# Supplementary material for: Repertoire and abundance of secreted virulence factors shape the pathogenic capacity of Pseudomonas syringae pv. aptata
Source: Front Microbiol. 2023 Jun 13;14:1205257. doi: 10.3389/fmicb.2023.1205257 (PMC10294431; doi:10.3389/fmicb.2023.1205257)
Supplement: Supplementary file 1 [file Data_Sheet_1.PDF]

Supplementary Information to

Repertoire and abundance of secreted virulence factors shape the pathogenic capacity of *Pseudomonas syringae* pv. aptata

Ivan Nikolić, Timo Glatter, Tamara Ranković, Tanja Berić, Slaviša Stanković & Andreas Diepold

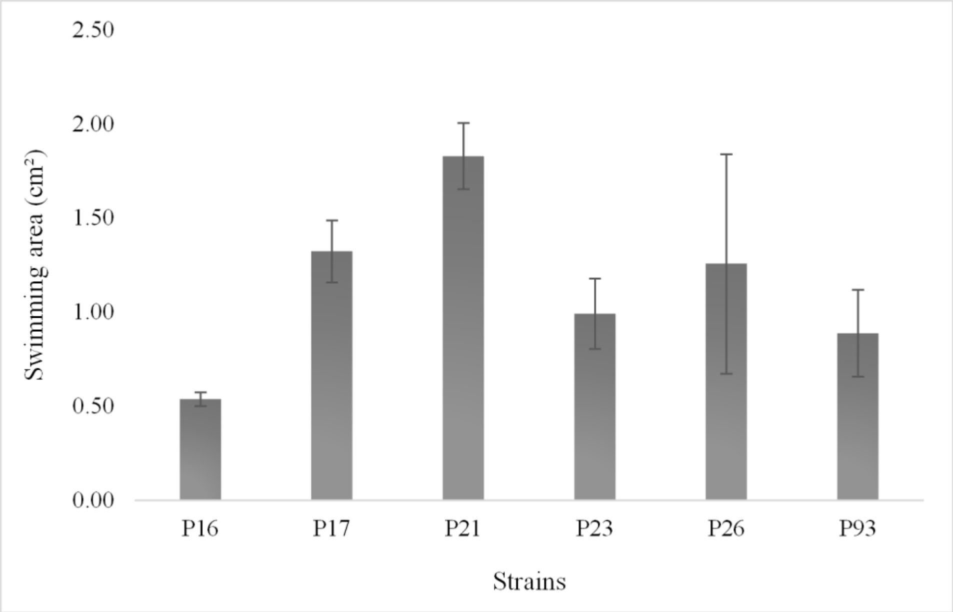

Suppl. Fig. 1: Swimming areas of strains used in this study

Swimming areas of *P. syringae* pv. aptata strain used in this study. $n = 3$; error bars denote standard deviation.

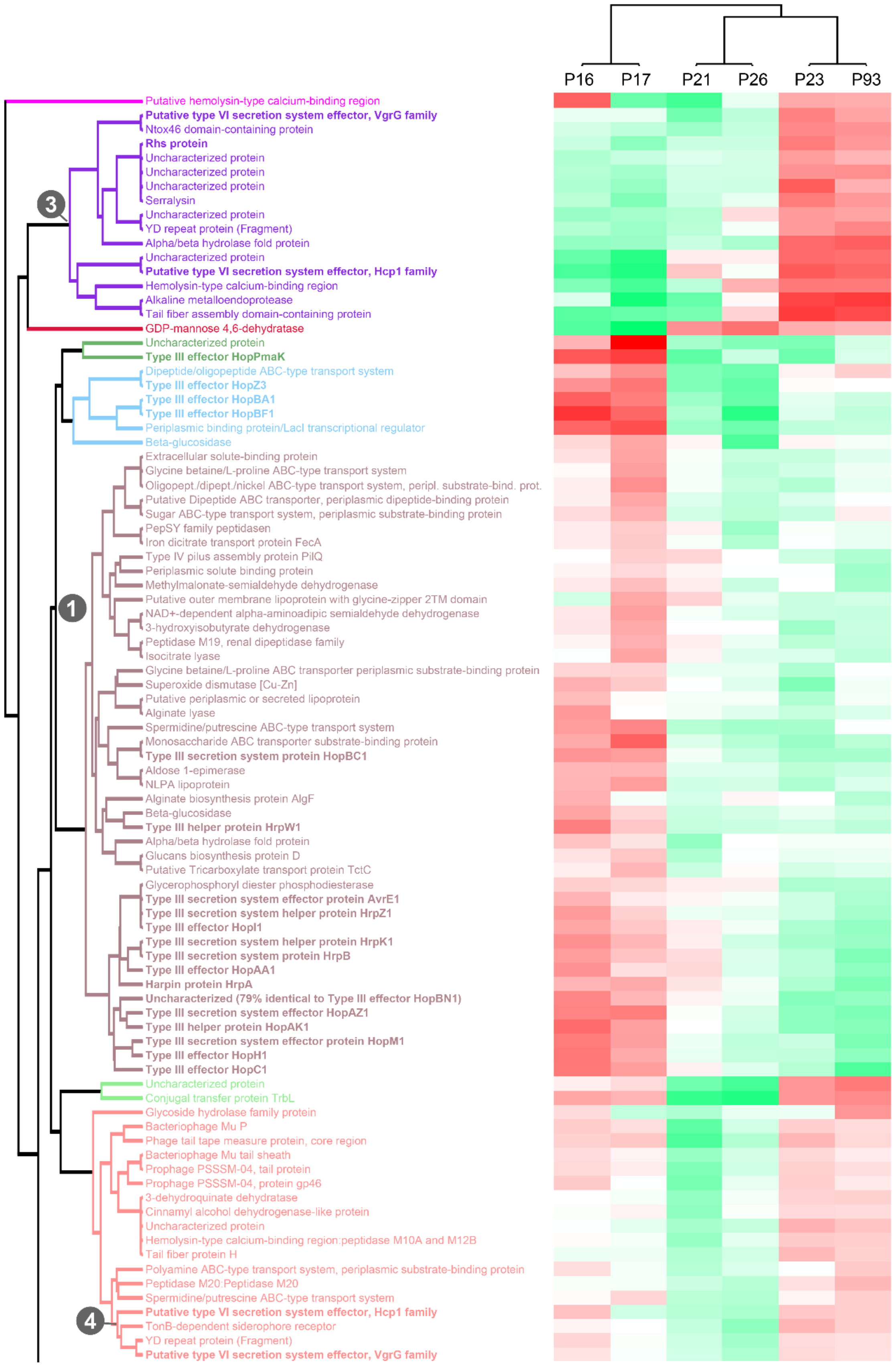

P16
P17
P21
P26
P23
P93
Putative hemolysin-type calcium-binding region
Putative type VI secretion system effector, VgrG family
Ntox46 domain-containing protein
Rhs protein
Uncharacterized protein
Uncharacterized protein
Uncharacterized protein
Serralysin
Uncharacterized protein
YD repeat protein (Fragment)
Alpha/beta hydrolase fold protein
Uncharacterized protein
Putative type VI secretion system effector, Hcp1 family
Hemolysin-type calcium-binding region
Alkaline metalloendoprotease
Tail fiber assembly domain-containing protein
GDP-mannose 4,6-dehydratase
Uncharacterized protein
Type III effector HopPmaK
Dipeptide/oligopeptide ABC-type transport system
Type III effector HopZ3
Type III effector HopBA1
Type III effector HopBF1
Periplasmic binding protein/LacI transcriptional regulator
Beta-glucosidase
Extracellular solute-binding protein
Glycine betaine/L-proline ABC-type transport system
Oligopept./dipept./nickel ABC-type transport system, peripl. substrate-bind. prot.
Putative Dipeptide ABC transporter, periplasmic dipeptide-binding protein
Sugar ABC-type transport system, periplasmic substrate-binding protein
PepSY family peptidasen
Iron dicitrate transport protein FecA
Type IV pilus assembly protein PilQ
Periplasmic solute binding protein
Methylmalonate-semialdehyde dehydrogenase
Putative outer membrane lipoprotein with glycine-zipper 2TM domain
NAD+-dependent alpha-aminoadipic semialdehyde dehydrogenase
3-hydroxyisobutyrate dehydrogenase
Peptidase M19, renal dipeptidase family
Isocitrate lyase
Glycine betaine/L-proline ABC transporter periplasmic substrate-binding protein
Superoxide dismutase [Cu-Zn]
Putative periplasmic or secreted lipoprotein
Alginate lyase
Spermidine/putrescine ABC-type transport system
Monosaccharide ABC transporter substrate-binding protein
Type III secretion system protein HopBC1
Aldose 1-epimerase
NLPA lipoprotein
Alginate biosynthesis protein AlgF
Beta-glucosidase
Type III helper protein HrpW1
Alpha/beta hydrolase fold protein
Glucans biosynthesis protein D
Putative Tricarboxylate transport protein TctC
Glycerophosphoryl diester phosphodiesterase
Type III secretion system effector protein AvrE1
Type III secretion system helper protein HrpZ1
Type III effector HopI1
Type III secretion system helper protein HrpK1
Type III secretion system protein HrpB
Type III effector HopAA1
Harpin protein HrpA
Uncharacterized (79% identical to Type III effector HopBN1)
Type III secretion system effector HopAZ1
Type III helper protein HopAK1
Type III secretion system effector protein HopM1
Type III effector HopH1
Type III effector HopC1
Uncharacterized protein
Conjugal transfer protein TrbL
Glycoside hydrolase family protein
Bacteriophage Mu P
Phage tail tape measure protein, core region
Bacteriophage Mu tail sheath
Prophage PSSSM-04, tail protein
Prophage PSSSM-04, protein gp46
3-dehydroquinate dehydratase
Cinnamyl alcohol dehydrogenase-like protein
Uncharacterized protein
Hemolysin-type calcium-binding region:peptidase M10A and M12B
Tail fiber protein H
Polyamine ABC-type transport system, periplasmic substrate-binding protein
Peptidase M20:Peptidase M20
Spermidine/putrescine ABC-type transport system
Putative type VI secretion system effector, Hcp1 family
TonB-dependent siderophore receptor
YD repeat protein (Fragment)
Putative type VI secretion system effector, VgrG family
1
3
4

Suppl. Fig. 2

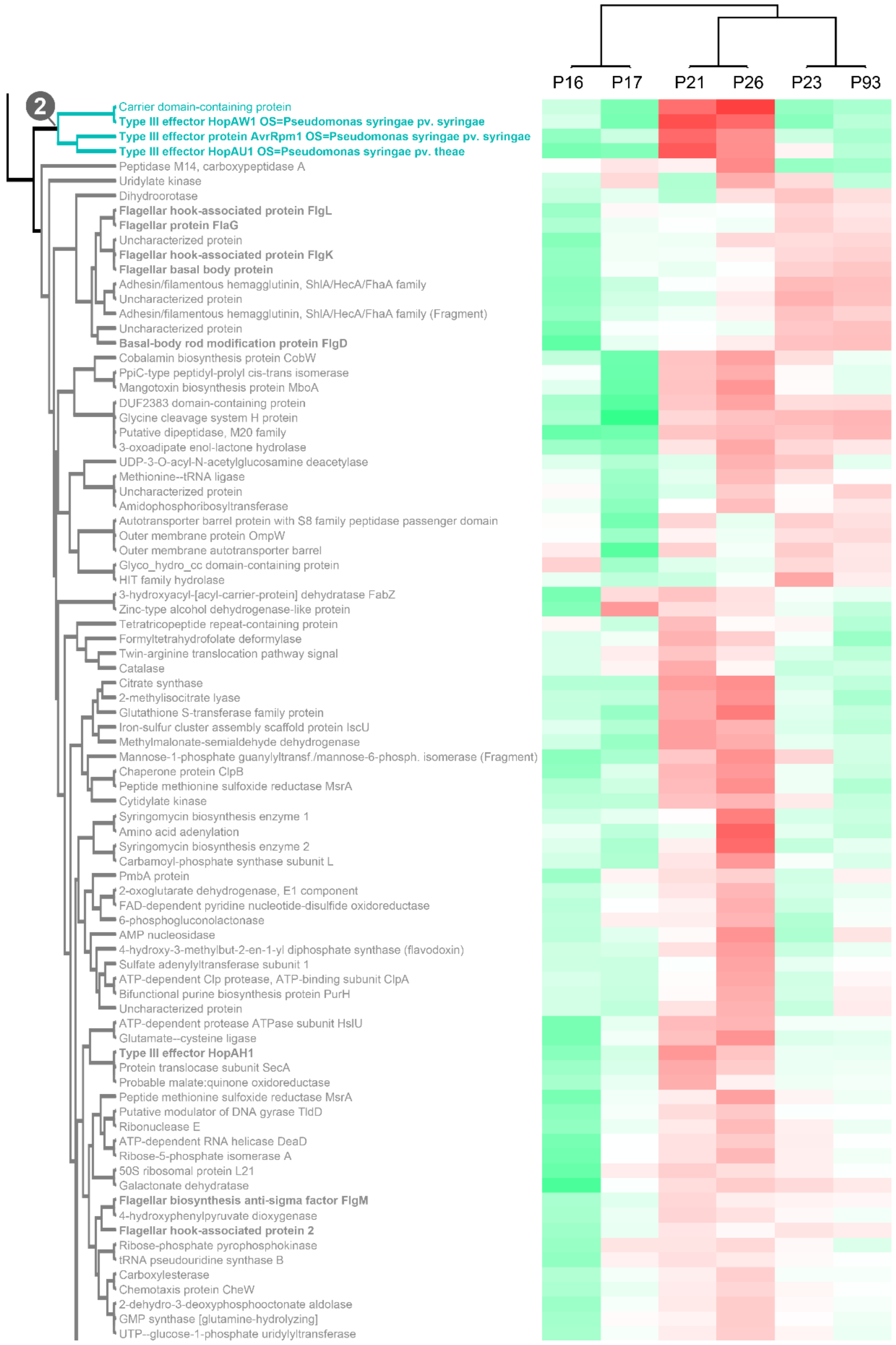

Suppl. Fig. 2, continued

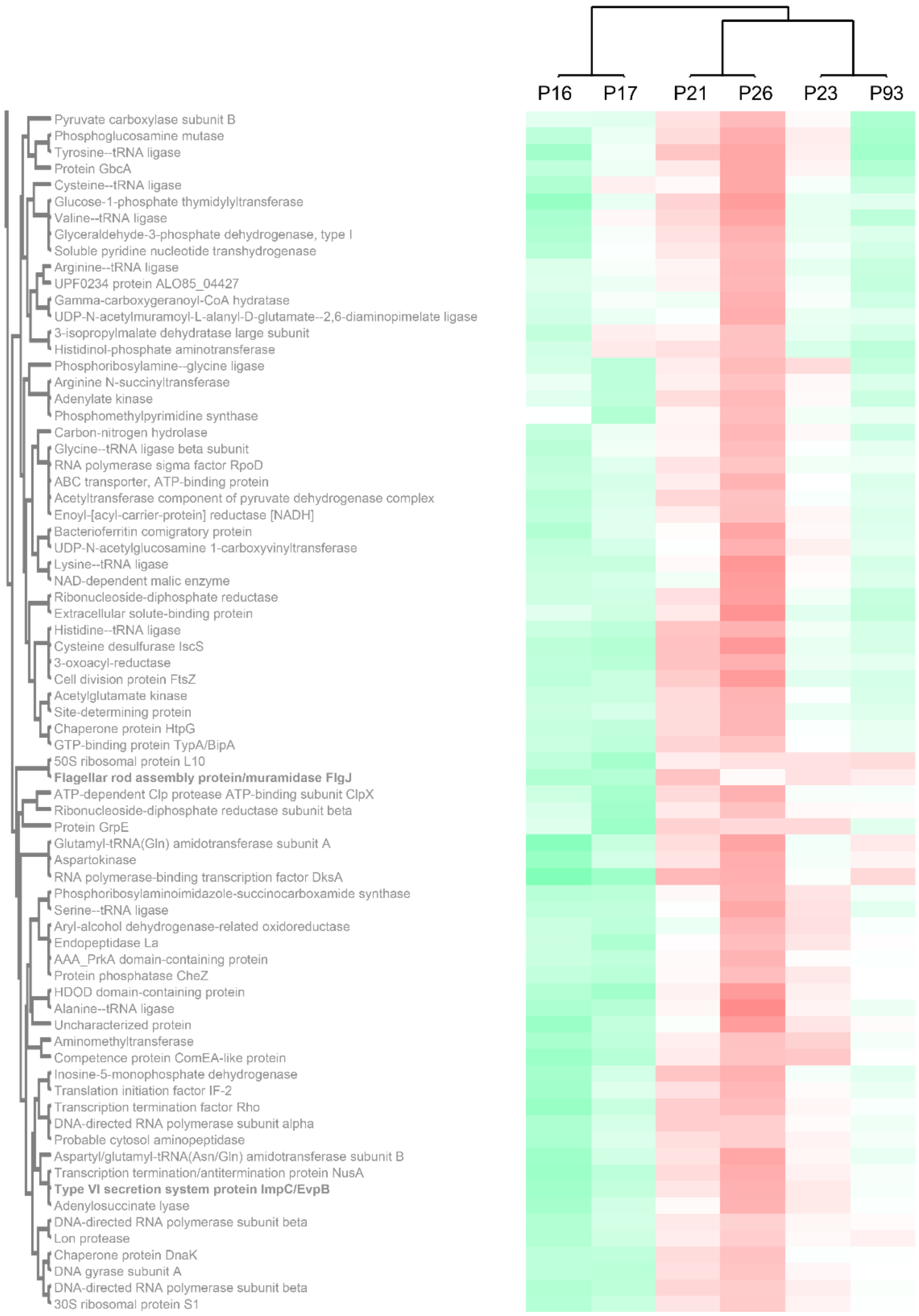

Suppl. Fig. 2, continued. **Clustering of strains and proteins in the culture supernatant indicates co-regulation of secretion of proteins in different strains.**

Full version of the clustering analysis displayed in Figure 3. Clustering of all strains and secreted proteins previously analyzed with a maximal pairwise $\log_2$ intensity difference of ≥3, based on the respective deviations from the average intensity. Color scale indicates average protein intensities. Different shades of red indicate increased protein intensity in the respective supernatant, whereas shades of green indicate lower intensities (median of 2 biological replicates). Known T3SS/T6SS-

secreted proteins in bold font; each of the 10 main clades is indicated by distinct font color. Numbers indicate the clade containing the majority of T3SS-secreted proteins (1), a distinct clade of T3SS effectors (2), and the two clades of T6SS-secreted proteins with distinct regulation (3, 4).

Suppl. Table 1: Distribution of measured diameters of T3SS pili and flagella identified in electron microscopy experiments

Diameters were measured manually using ImageJ (see material and methods for details), individual measurements are indicated.

Identified as	Individual diameter (nm)										**Average diameter (nm)**	**St.dev. (nm)**
**Pili**	8.20	7.11	8.12	6.82	8.17	6.85	7.02	5.70	7.11	6.82	**7.33**	0.83
**Flagella**	23.86	17.20	16.29	14.76	18.35	16.62	12.56	25.68	15.69	19.12	**18.01**	4.02

Suppl. Table 2: Additional information for Table 2, relative quantification of secreted proteins

Measured intensities for proteins with at least five detected peptides and a maximal $\log_2$ intensity difference between individual strains of ≤ 3. See Table 2 and main text for details.

Protein	Average protein intensity (log 2) in indicated strains						Overall avg. log 2 intens.	Max. diff. log 2 intens.
	P16	P17	P21	P23	P26	P93		
**Type III secretion system**								
HopAH2 protein	25.226	26.209	25.993	25.101	26.396	24.214	25.523	2.182
**Flagellum**								
Flagellin	34.499	35.046	35.588	36.593	35.820	35.922	35.578	2.094
Flagellar hook protein FlgE	29.056	29.485	29.506	31.566	30.591	31.278	30.247	2.511
Flagellar basal-body rod protein FlgG	26.871	27.631	27.577	29.343	28.457	29.406	28.214	2.536
Flagellar hook-length control protein	26.109	27.754	27.658	28.876	27.683	28.808	27.815	2.767

Suppl. Table 3: List of potential T6SS effectors as determined by Bastion6

Bastion6 T6SS effector prediction results for proteins analyzed in proteomics analysis. Single method-based models and ensemble results, as indicated for all proteins with a prediction score larger than 0.7. T6SS-related proteins detected in the *P. syringae* pv. aptata secretome analysis are marked by bold font.

Protein identifier	Protein annotation	Single Model Results									Ensemble Model Result Score
		AAC	DPC	QSO	BLOSUM	DPC-PSSM	S-FPSSM	Pse-PSSM	CTDC	CTDT	
A0A0Q0C9C0	Twin-arginine translocation pathway signal	0.994	0.967	0.981	0.985	0.938	0.992	0.909	0.968	0.955	0.966
A0A0Q0FP17	Insecticidal toxin protein	0.991	0.969	0.983	0.931	0.801	0.952	0.948	0.974	0.935	0.948
A0A0Q0FP42	DUF3274 domain-containing protein	0.987	0.987	0.971	0.852	0.945	0.983	0.988	0.970	0.869	0.948
A0A0Q0DDT3	LPS-assembly protein LptD	0.975	0.989	0.995	0.942	0.976	0.975	0.731	0.981	0.897	0.944
A0A0Q0C8Q1	CrtC domain-containing protein	0.988	0.995	0.972	0.882	0.981	0.841	0.971	0.931	0.918	0.943
A0A0Q0DLS3	Insecticidal toxin protein	0.992	0.965	0.984	0.855	0.987	0.838	0.972	0.948	0.897	0.939
A0A0Q0C096	Putative insecticidal toxin protein	0.981	0.977	0.983	0.714	0.861	0.986	0.977	0.966	0.925	0.937
A0A0Q0BX38	Alginate biosynthesis protein AlgE	0.969	0.987	0.995	0.935	0.963	0.966	0.876	0.885	0.902	0.937
A0A0Q0CEY7	Alginate lyase	0.938	0.933	0.959	0.746	0.951	0.994	0.985	0.962	0.932	0.936
A0A0Q0DLW9	Insecticidal toxin protein	0.976	0.953	0.956	0.919	0.844	0.980	0.968	0.946	0.892	0.936
A0A0Q0DSL3	Rhs family protein	0.986	0.978	0.942	0.728	0.852	0.969	0.947	0.944	0.930	0.927
A0A0Q0C688	**YD repeat protein**	**0.994**	**0.921**	**0.969**	**0.601**	**0.870**	**0.916**	**0.992**	**0.977**	**0.966**	**0.926**
A0A0Q0CIG8	Type III effector phosphothreonine lyase	0.994	0.946	0.965	0.894	0.921	0.805	0.967	0.907	0.920	0.926
A0A3M3EB84	Type III effector HopAI1	0.993	0.941	0.942	0.885	0.919	0.839	0.964	0.904	0.926	0.925
A0A0Q0D6F6	Putative type VI secretion system effector, VgrG family	0.953	0.946	0.893	0.904	0.926	0.973	0.967	0.936	0.866	0.925
A0A0Q0D543	Outer membrane porin OprE	0.955	0.966	0.950	0.755	0.967	0.996	0.732	0.968	0.945	0.925
A0A0Q0BXY6	Outer membrane porin	0.952	0.966	0.963	0.954	0.931	0.990	0.742	0.951	0.853	0.922
A0A0Q0C5E5	OprD family outer membrane porin	0.976	0.985	0.984	0.981	0.924	0.993	0.648	0.917	0.869	0.920
A0A0N8T848	Glycoside hydrolase family 18 protein	0.983	0.940	0.980	0.954	0.897	0.980	0.796	0.923	0.796	0.911
A0A0Q0DBI7	Outer membrane adhesin like proteiin	0.943	0.993	0.949	0.790	0.904	0.916	0.788	0.934	0.905	0.910
A0A0Q0DIJ7	Anaerobically-induced outer membrane porin OprE	0.908	0.981	0.980	0.977	0.954	0.997	0.694	0.811	0.918	0.909
A0A0N8T849	WW domain-containing protein	0.992	0.996	0.998	0.944	0.983	0.269	0.925	0.958	0.946	0.909
A0A0Q0DD34	Catalase-peroxidase	0.985	0.799	0.966	0.924	0.838	0.942	0.899	0.947	0.866	0.908
A0A0N8T9E7	Type VI secretion system effector, Hcp1 family	0.961	0.909	0.905	0.871	0.969	0.867	0.989	0.876	0.871	0.908
A0A0N8T980	Zona occludens toxin	0.951	0.967	0.933	0.853	0.938	0.786	0.792	0.921	0.933	0.907
A0A0Q0DZB3	Rhs protein	0.939	0.928	0.941	0.804	0.940	0.917	0.907	0.961	0.815	0.905
A0A0N8T7S5	Type III effector	0.978	0.971	0.981	0.769	0.957	0.850	0.955	0.829	0.885	0.905
A0A0Q0FVM6	RHS repeat-associated core domain-containing protein	0.949	0.873	0.820	0.914	0.983	0.975	0.893	0.864	0.912	0.903
A0A0Q0FN69	Nucleoside-specific channel-forming protein Tsx	0.914	0.994	0.978	0.670	0.888	0.921	0.904	0.879	0.916	0.902
A0A0Q0DI86	YD repeat-containing protein	0.985	0.960	0.986	0.931	0.812	0.481	0.861	0.972	0.930	0.900

A0A0Q0CV75	Putative type VI secretion system effector, VgrG family	0.857	0.913	0.905	0.945	0.922	0.945	0.896	0.909	0.840	0.898
A0A0Q0C7Z5	YD repeat-containing protein	0.952	0.974	0.955	0.900	0.517	0.868	0.900	0.979	0.894	0.898
A0A0Q0IAL8	Porin	0.994	0.991	0.987	0.815	0.913	0.969	0.649	0.970	0.756	0.897
A0A0N8T9B6	Type III helper protein HopAK1	0.868	0.989	0.984	0.976	0.952	0.992	0.915	0.653	0.904	0.895
A0A0Q0FJN8	Putative glycine-glutamate dipeptide porin OpdP	0.947	0.960	0.987	0.612	0.919	0.989	0.769	0.946	0.836	0.893
A0A0Q0D2R3	YD repeat-containing protein	0.991	0.985	0.993	0.941	0.816	0.427	0.832	0.972	0.895	0.892
A0A0Q0E0H9	Sucrose porin	0.951	0.962	0.950	0.769	0.928	0.932	0.946	0.947	0.709	0.892
A0A0Q0DL37	Myo-inositol catabolism protein IolB	0.987	0.974	0.904	0.675	0.866	0.838	0.810	0.931	0.918	0.892
A0A0Q0BGR4	Type III effector HopBB1	0.968	0.783	0.936	0.507	0.946	0.895	0.953	0.951	0.943	0.889
A0A0Q0FHJ9	Alkaline phosphatase	0.985	0.953	0.953	0.394	0.822	0.978	0.856	0.911	0.920	0.881
A0A0Q0C4L0	TIGR03756 family integrating conjugative element prot.	0.956	0.940	0.958	0.794	0.902	0.929	0.888	0.802	0.827	0.881
A0A0N0GH35	Type III effector HopF2	0.925	0.785	0.891	0.762	0.929	0.880	0.810	0.930	0.911	0.877
A0A0Q0C296	DUF1329 domain-containing protein	0.934	0.938	0.938	0.850	0.933	0.818	0.814	0.869	0.813	0.877
A0A0Q0CXH4	phospholipase C	0.982	0.874	0.983	0.652	0.788	0.995	0.782	0.980	0.774	0.876
A0A0Q0IJ36	DUF1329 domain-containing protein	0.948	0.959	0.899	0.767	0.955	0.703	0.827	0.938	0.812	0.874
A0A0Q0DAE5	Outer membrane porin	0.821	0.963	0.939	0.907	0.933	0.993	0.701	0.773	0.869	0.871
A0A0Q0D6E9	Pectate lyase/Amb allergen	0.940	0.897	0.893	0.896	0.921	0.998	0.908	0.666	0.866	0.869
A0A0Q0FUG0	Putative 3-carboxymuconate cye	0.918	0.934	0.947	0.794	0.602	0.748	0.846	0.962	0.893	0.869
A0A0N8T9D8	Outer membrane porin	0.947	0.977	0.970	0.961	0.912	0.995	0.676	0.739	0.770	0.868
A0A0Q0IAK6	Lipoprotein	0.984	0.906	0.814	0.594	0.916	0.819	0.955	0.837	0.902	0.864
A0A0N8TA35	50S ribosomal protein L15	0.981	0.912	0.915	0.775	0.867	0.716	0.966	0.873	0.779	0.864
A0A0Q0FW13	Type III effector HopAH1	0.924	0.987	0.926	0.953	0.857	0.935	0.965	0.552	0.876	0.862
A0A0Q0E0Z2	Non-heme catalase KatN	0.773	0.892	0.860	0.933	0.848	0.636	0.906	0.912	0.908	0.861
A0A0Q0DNS3	Outer membrane porin	0.900	0.974	0.931	0.670	0.944	0.999	0.709	0.837	0.768	0.856
A0A3M5WLB8	Type III effector HopX1	0.967	0.837	0.893	0.886	0.900	0.763	0.719	0.840	0.863	0.856
A0A0N8T9W4	DUF2778 domain-containing protein	0.948	0.871	0.761	0.660	0.842	0.960	0.917	0.902	0.816	0.855
A0A0Q0IF84	FlhE protein	0.988	0.617	0.828	0.568	0.917	0.882	0.818	0.936	0.953	0.851
A0A0Q0IFS3	HopAH2 protein	0.883	0.971	0.809	0.813	0.986	0.859	0.626	0.803	0.886	0.851
A0A0Q0IT12	Cupin domain-containing protein	0.986	0.865	0.889	0.847	0.960	0.630	0.838	0.772	0.856	0.849
A0A0Q0CG93	Fimbrial protein	0.968	0.763	0.901	0.860	0.950	0.845	0.991	0.844	0.675	0.849
A0A0Q0C1R6	Adhesin	0.954	0.978	0.974	0.860	0.906	0.820	0.532	0.805	0.792	0.849
A0A0Q0CVD4	Sushi domain-containing protein	0.600	0.856	0.868	0.812	0.864	0.817	0.817	0.974	0.914	0.849
A0A0Q0C5H9	Levansucrase LscA	0.867	0.975	0.640	0.793	0.967	0.973	0.973	0.836	0.747	0.848
A0A0N8T8T5	Fimbrial bioproteinsis outer membrane usher protein	0.937	0.973	0.870	0.754	0.844	0.841	0.583	0.869	0.854	0.848
A0A0N8T7Y0	Type III secretion system effector protein AvrE1	0.941	0.924	0.940	0.786	0.971	0.395	0.917	0.882	0.800	0.848
A0A0Q0BXZ7	TonB-dependent receptor	0.986	0.997	0.992	0.967	0.481	0.891	0.232	0.957	0.843	0.845
A0A0Q0BT79	YD repeat protein	0.985	0.778	0.739	0.797	0.786	0.973	0.986	0.921	0.698	0.843
A0A0Q0BVQ5	Hemolysin activator protein, HlyB family	0.979	0.918	0.983	0.731	0.742	0.783	0.456	0.955	0.822	0.842
A0A0Q0DT79	Catalase	0.945	0.940	0.908	0.675	0.663	0.682	0.554	0.970	0.927	0.841
A0A0Q0FST6	Glycoside hydrolase, family alpha amylase catal. subun.	0.942	0.922	0.932	0.777	0.629	0.960	0.743	0.933	0.696	0.841
A0A0Q0DST2	Outer membrane porin	0.880	0.929	0.919	0.652	0.951	0.991	0.700	0.807	0.771	0.840

A0A0Q0IJ17	Iron dicitrate transport protein FecA	0.972	0.961	0.956	0.944	0.372	0.881	0.292	0.931	0.933	0.839
A0A0Q0BVV7	Super protein	0.878	0.836	0.973	0.958	0.851	0.737	0.701	0.953	0.668	0.839
A0A0Q0BT59	DnaJ domain-containing protein	0.821	0.819	0.758	0.932	0.798	0.953	0.922	0.757	0.869	0.838
A0A0Q0CXV1	Peptidase C13 superfamily protein	0.978	0.990	0.939	0.812	0.279	0.942	0.601	0.873	0.895	0.837
A0A0Q0DQM4	Type VI secretion system effector, Hcp1 family	0.924	0.660	0.815	0.812	0.953	0.930	0.993	0.793	0.785	0.837
A0A0Q0BTQ0	PA14 domain-containing protein	0.908	0.987	0.919	0.538	0.972	0.728	0.714	0.870	0.790	0.836
A0A0Q0CAC5	Alginate lyase	0.904	0.813	0.944	0.511	0.970	0.987	0.987	0.892	0.619	0.835
A0A0Q0C453	Deferrochelatase	0.911	0.788	0.635	0.958	0.788	0.719	0.865	0.910	0.873	0.834
A0A0Q0IMU7	4-phytase	0.947	0.997	0.967	0.609	0.777	0.911	0.275	0.918	0.845	0.832
A0A0Q0BX48	Alginate biosynthesis protein AlgX	0.971	0.837	0.921	0.626	0.911	0.451	0.756	0.941	0.861	0.832
A0A0Q0C4H0	Cytochrome c domain-containing protein	0.959	0.876	0.882	0.610	0.847	0.730	0.882	0.879	0.760	0.831
A0A0Q0D8S3	Chitin-binding protein	0.728	0.775	0.641	0.860	0.902	0.980	0.963	0.809	0.885	0.829
A0A0Q0DEY0	Copper resistance protein B	0.940	0.983	0.963	0.709	0.907	0.535	0.775	0.737	0.845	0.828
A0A0Q0FN00	L-sorbosone dehydrogenase	0.857	0.916	0.968	0.903	0.631	0.918	0.628	0.688	0.879	0.822
A0A0Q0D919	Tannase/feruloyl esterase family protein	0.969	0.806	0.875	0.377	0.901	0.973	0.728	0.793	0.872	0.820
A0A0Q0BYM2	VCBS repeat-containing protein	0.906	0.814	0.773	0.648	0.931	0.875	0.926	0.724	0.843	0.820
A0A0Q0BWR0	Autotransporting lipase, GDSL family protein	0.961	0.992	0.970	0.761	0.844	0.776	0.317	0.785	0.826	0.818
A0A0Q0D9W4	Putative lipoprotein	0.976	0.842	0.655	0.868	0.981	0.539	0.834	0.920	0.726	0.818
A0A0N8T874	Secreted protein	0.921	0.880	0.859	0.673	0.941	0.856	0.706	0.659	0.884	0.817
A0A0Q0IIE1	Tail fiber protein H	0.781	0.984	0.971	0.929	0.981	0.986	0.736	0.567	0.683	0.815
A0A0Q0D451	DUF4329 domain-containing protein	0.957	0.878	0.750	0.743	0.632	0.564	0.783	0.893	0.913	0.815
A0A1S6YAB7	HopBD1 type III effector	0.891	0.581	0.364	0.871	0.918	0.879	0.939	0.931	0.913	0.812
A0A0Q0FVA2	Aldose 1-epimerase	0.938	0.967	0.977	0.585	0.571	0.443	0.688	0.952	0.853	0.812
A0A0Q0ICK8	Nucleoside-specific outer membrane channel protein Tsx	0.702	0.945	0.900	0.365	0.771	0.933	0.762	0.899	0.842	0.809
A0A0N8T952	30S ribosomal protein S19	0.912	0.832	0.913	0.219	0.863	0.742	0.909	0.857	0.861	0.809
A0A0Q0DMX1	ADP-ribosylating toxin	0.831	0.872	0.845	0.807	0.944	0.876	0.773	0.664	0.790	0.809
A0A0Q0BYJ4	FGE-sulfatase domain-containing protein	0.957	0.900	0.887	0.859	0.098	0.953	0.744	0.800	0.896	0.809
A0A0Q0DGE1	L-sorbosone dehydrogenase	0.795	0.883	0.959	0.837	0.641	0.935	0.699	0.728	0.806	0.808
A0A0Q0DHV7	Neutral zinc metallopeptidase	0.956	0.856	0.860	0.919	0.789	0.689	0.761	0.802	0.673	0.806
A0A0Q0C079	Superfamily protein	0.864	0.633	0.954	0.962	0.856	0.702	0.658	0.897	0.715	0.806
A0A0Q0IJF7	Flagellar biosynthesis protein FlgJ	0.852	0.822	0.890	0.370	0.829	0.847	0.763	0.806	0.907	0.805
A0A0Q0BRB6	Putative type VI secretion system effector, Hcp1 family	0.899	0.892	0.920	0.896	0.965	0.932	0.995	0.707	0.419	0.805
A0A0Q0BV33	Outer membrane autotransporter barrel	0.931	0.985	0.962	0.951	0.852	0.920	0.451	0.602	0.706	0.802
A0A3M2XX23	Type III secreted effector hopPmaA	0.964	0.912	0.796	0.872	0.896	0.231	0.579	0.883	0.854	0.801
A0A0Q0FGQ1	Rhs family protein with PAAR motif	0.812	0.825	0.850	0.676	0.239	0.758	0.852	0.934	0.942	0.799
A0A0Q0IL40	Homogentisate 1,2-dioxygenase	0.932	0.916	0.576	0.795	0.817	0.734	0.653	0.812	0.838	0.794
A0A0N8T7V9	Type III secretion system helper protein HrpK1	0.826	0.866	0.820	0.520	0.972	0.840	0.791	0.752	0.771	0.793
A0A0Q0FCB3	Pili bioproteinsis outer membrane usher protein FimD	0.772	0.928	0.941	0.837	0.860	0.941	0.528	0.739	0.662	0.791
A0A0Q0DMU9	**TonB-dependent siderophore receptor**	**0.953**	**0.987**	**0.969**	**0.907**	**0.112**	**0.721**	**0.312**	**0.910**	**0.869**	**0.791**
A0A0Q0CED4	**Serralysin**	**0.809**	**0.978**	**0.962**	**0.830**	**0.892**	**0.927**	**0.463**	**0.580**	**0.778**	**0.791**
A0A0Q0DN93	MltA-interacting MipA	0.958	0.829	0.852	0.650	0.824	0.633	0.424	0.870	0.844	0.790

A0A0Q0DMQ1	Peptidoglycan hydrolase FlgJ	0.707	0.723	0.689	0.421	0.911	0.834	0.897	0.866	0.925	0.789
A0A0Q0C7K3	Putative integral membrane protein	0.702	0.971	0.872	0.854	0.804	0.992	0.586	0.570	0.846	0.789
A0A0Q0C7G4	RHS repeat-associated core domain-containing protein	0.815	0.712	0.611	0.733	0.968	0.957	0.760	0.889	0.707	0.788
Q4ZV88	Type III effector HopAP1	0.890	0.851	0.704	0.719	0.512	0.773	0.943	0.819	0.800	0.787
A0A0Q0C227	RHS family protein	0.948	0.756	0.556	0.774	0.728	0.968	0.985	0.760	0.728	0.787
A0A0Q0E0I9	Sucrose-6-phosphate hydrolase	0.860	0.967	0.934	0.911	0.888	0.974	0.874	0.495	0.554	0.786
A0A0Q0DGY4	TonB-dependent siderophore receptor	0.956	0.961	0.973	0.848	0.134	0.727	0.225	0.940	0.885	0.786
A0A0N8T954	Putative Type IV pilus-associated protein	0.739	0.988	0.892	0.732	0.783	0.828	0.610	0.715	0.778	0.786
A0A0Q0DXX0	TonB-dependent siderophore receptor	0.936	0.961	0.984	0.744	0.108	0.785	0.303	0.939	0.884	0.786
A0A0Q0D5Y8	TonB-dependent siderophore receptor	0.954	0.994	0.994	0.701	0.123	0.817	0.285	0.900	0.870	0.782
A0A0Q0FE60	Penicillin amidase	0.960	0.867	0.934	0.366	0.666	0.826	0.476	0.919	0.759	0.781
A0A0Q0BV93	Cysteine dioxygenase	0.983	0.848	0.759	0.634	0.531	0.493	0.688	0.964	0.816	0.780
A0A0N1JBR5	Restriction endonuclease	0.886	0.728	0.786	0.475	0.859	0.610	0.795	0.906	0.795	0.779
A0A0Q0IKU0	TonB-dependent siderophore receptor	0.953	0.989	0.978	0.714	0.120	0.741	0.326	0.953	0.821	0.779
A0A0Q0CY49	Tat pathway signal:copper-resistance protein CopA	0.542	0.866	0.779	0.916	0.643	0.835	0.663	0.853	0.817	0.776
A0A0Q0FSL3	TonB-dependent receptor	0.783	0.979	0.960	0.575	0.734	0.960	0.289	0.769	0.795	0.776
A0A0Q0DMX0	Autotransp. barrel prot., S8 fam. Peptid. passenger dom.	0.944	0.977	0.920	0.970	0.832	0.910	0.403	0.577	0.630	0.776
A0A0Q0IC24	Penicillin amidase	0.960	0.618	0.944	0.306	0.647	0.859	0.561	0.926	0.861	0.776
A0A0Q0FV00	LPD7 domain-containing protein	0.836	0.870	0.786	0.714	0.548	0.834	0.688	0.744	0.851	0.775
A0A0Q0DPI0	Ig-like_bact domain-containing protein	0.693	0.885	0.919	0.708	0.953	0.928	0.534	0.603	0.818	0.774
A0A0Q0BYH0	BNR repeat-containing glycosyl hydrolase	0.864	0.947	0.939	0.893	0.784	0.914	0.923	0.530	0.520	0.773
A0A0Q0BTV2	Oligopeptidase B	0.975	0.754	0.958	0.644	0.473	0.963	0.396	0.787	0.819	0.772
A0A0Q0DPI5	Beta-glucosidase-related glycosyl hydrolase	0.889	0.928	0.934	0.688	0.586	0.884	0.267	0.808	0.764	0.770
A0A3M5WIF0	Type III effector protein AvrB3	0.969	0.905	0.530	0.390	0.827	0.515	0.931	0.863	0.820	0.770
A0A0Q0IH46	PhcA	0.824	0.655	0.864	0.599	0.761	0.374	0.918	0.907	0.819	0.769
A0A0Q0FUW7	Single-stranded DNA-binding protein	0.964	0.957	0.944	0.770	0.331	0.348	0.434	0.850	0.906	0.768
A0A0Q0DRB6	Glucose dehydrogenase	0.795	0.938	0.827	0.872	0.890	0.967	0.488	0.606	0.684	0.767
A0A0Q0FSP4	Ovule protein	0.692	0.785	0.715	0.670	0.612	0.772	0.675	0.931	0.840	0.766
A0A0Q0D665	S-formylglutathione hydrolase	0.970	0.940	0.748	0.364	0.611	0.799	0.419	0.785	0.937	0.765
A0A0Q0DM93	Esterified fatty acid cis/trans isomerase	0.911	0.951	0.906	0.800	0.774	0.540	0.896	0.645	0.593	0.765
A0A0Q0CVS2	pectate lyase	0.642	0.996	0.835	0.839	0.955	0.999	0.667	0.375	0.828	0.764
A0A0Q0C504	YD repeat-containing protein	0.872	0.707	0.373	0.342	0.968	0.868	0.899	0.928	0.805	0.762
A0A0Q0DS86	PAAR domain-containing protein	0.672	0.897	0.381	0.573	0.937	0.857	0.980	0.668	0.925	0.761
A0A0N0X6U3	Type III effector hopW1	0.935	0.870	0.614	0.923	0.932	0.120	0.475	0.857	0.863	0.760
A0A0Q0DMN3	TonB-dependent siderophore receptor	0.865	0.993	0.953	0.818	0.133	0.687	0.220	0.858	0.899	0.760
A0A0N8TA54	Allergen V5/Tpx-1 related protein	0.899	0.788	0.558	0.672	0.233	0.902	0.883	0.800	0.912	0.759
A0A0N8T9G5	6-phosphogluconolactonase	0.868	0.719	0.687	0.798	0.361	0.859	0.805	0.824	0.800	0.759
A0A0Q0C4Y3	TIGR03751 family conjugal transfer lipoprotein	0.876	0.684	0.839	0.747	0.701	0.677	0.631	0.827	0.744	0.758
A0A0Q0DMA8	Quinoprotein glucose dehydrogenase	0.899	0.918	0.914	0.907	0.836	0.987	0.476	0.626	0.497	0.758
A0A0Q0DGE7	Membrane protein involved in aromatic hydrocarbon degradation	0.926	0.970	0.925	0.516	0.564	0.913	0.407	0.740	0.727	0.758
A0A0Q0C4N1	Replication protein A	0.815	0.662	0.719	0.392	0.990	0.932	0.971	0.786	0.649	0.757

A0A0N8T8P5	**Alkaline metalloendoprotease**	**0.687**	**0.996**	**0.971**	**0.890**	**0.802**	**0.993**	**0.375**	**0.510**	**0.734**	**0.757**
A0A0Q0BZX0	DUF3034 domain-containing protein	0.959	0.506	0.893	0.643	0.757	0.961	0.446	0.774	0.785	0.756
A0A0Q0CFT2	Peptidase M14, carboxypeptidase A	0.972	0.884	0.912	0.715	0.341	0.662	0.110	0.923	0.855	0.756
A0A0Q0D672	ZnMc domain-containing protein	0.745	0.605	0.592	0.573	0.323	0.743	0.966	0.968	0.964	0.755
A0A0Q0D4S0	ABC transporter	0.768	0.878	0.791	0.790	0.880	0.524	0.877	0.822	0.543	0.754
A0A0N8T8I6	Flagellar hook protein FlgE	0.823	0.976	0.904	0.864	0.533	0.990	0.355	0.551	0.791	0.752
A0A0Q0C264	TonB system transport protein	0.969	0.937	0.980	0.793	0.398	0.575	0.130	0.741	0.894	0.751
A0A0Q0BSZ3	Peptidase S1, chymotrypsin	0.748	0.562	0.533	0.914	0.840	0.891	0.919	0.770	0.725	0.751
A0A0Q0BWQ0	Peptidase M23B	0.927	0.859	0.862	0.742	0.394	0.128	0.434	0.963	0.920	0.750
A0A0Q0FQV4	Glycosyltransferase sugar-binding dom.-containing prot.	0.794	0.769	0.869	0.406	0.893	0.947	0.894	0.696	0.613	0.750
A0A0Q0FIC0	Beta-glucosidase	0.954	0.824	0.967	0.530	0.531	0.831	0.219	0.951	0.658	0.749
A0A0N8T7T1	Lipoprotein	0.763	0.752	0.531	0.671	0.819	0.667	0.911	0.732	0.859	0.748
A0A0N8TAA6	Secreted protein	0.945	0.615	0.914	0.421	0.876	0.618	0.884	0.935	0.499	0.747
A0A0Q0DPF6	Putative lipoprotein	0.654	0.969	0.748	0.445	0.940	0.921	0.652	0.603	0.819	0.747
A0A0Q0C1H9	Rhs element Vgr protein	0.630	0.650	0.733	0.860	0.825	0.908	0.806	0.676	0.763	0.747
A0A0Q0FH11	Alpha-2-macroglobulin	0.841	0.846	0.904	0.849	0.945	0.521	0.497	0.748	0.603	0.747
A0A0N8TA31	Killer protein	0.715	0.846	0.798	0.865	0.574	0.638	0.393	0.829	0.841	0.746
A0A0Q0BZ89	Beta-glucosidase	0.861	0.848	0.859	0.684	0.560	0.851	0.236	0.893	0.708	0.746
A0A0Q0BUZ1	STN domain-containing protein	0.947	0.980	0.933	0.778	0.112	0.709	0.305	0.847	0.755	0.743
A0A0N8T920	Secreted protein	0.796	0.655	0.724	0.360	0.620	0.877	0.728	0.885	0.829	0.743
A0A0Q0DMU2	3-oxo-C12-homoserine lactone acylase PvdQ	0.942	0.849	0.829	0.750	0.563	0.875	0.623	0.665	0.643	0.743
Q08I86	Type III effector HopD	0.640	0.896	0.713	0.604	0.949	0.822	0.623	0.565	0.886	0.742
A0A0Q0C1J6	**Hemolysin-type calcium-binding region:peptidase M10A and M12B**	**0.790**	**0.972**	**0.934**	**0.933**	**0.553**	**0.952**	**0.311**	**0.556**	**0.726**	**0.742**
A0A0Q0CEH2	Outer membrane autotransporter barrel	0.869	0.991	0.930	0.870	0.866	0.854	0.263	0.435	0.730	0.742
A0A0N8T8D5	Rhs family protein with PAAR motif	0.708	0.734	0.433	0.439	0.784	0.951	0.991	0.798	0.822	0.742
A0A0Q0ILE3	Aldose-1-epimerase superfamily protein	0.963	0.798	0.937	0.482	0.608	0.296	0.540	0.943	0.734	0.740
A0A0Q0DM01	Peptide chain release factor RF-3	0.610	0.917	0.799	0.691	0.836	0.829	0.662	0.717	0.661	0.740
A0A0Q0ICD7	Outer membrane ferripyoverdine receptor	0.947	0.949	0.975	0.376	0.114	0.802	0.345	0.884	0.825	0.740
A0A0Q0BR59	Type III effector HopH1	0.717	0.778	0.701	0.489	0.778	0.856	0.986	0.778	0.636	0.739
A0A0Q0IPQ3	Outer membrane ligand receptor	0.909	0.865	0.928	0.284	0.777	0.944	0.249	0.806	0.698	0.739
A0A0Q0DNU1	TonB-dependent outer membrane receptor	0.928	0.982	0.978	0.784	0.115	0.665	0.181	0.920	0.716	0.739
A0A0Q0DAM4	DUF3828 domain-containing protein	0.758	0.662	0.779	0.414	0.921	0.585	0.818	0.722	0.876	0.739
A0A0Q0IK58	Rhs family protein	0.910	0.372	0.760	0.674	0.990	0.890	0.993	0.869	0.428	0.739
A0A0Q0BYS1	Phage capsid protein	0.842	0.749	0.609	0.638	0.757	0.939	0.919	0.724	0.617	0.739
A0A0N8TAD7	Peptidase S9, prolyl oligopeptidase active site region	0.977	0.878	0.960	0.673	0.595	0.571	0.517	0.594	0.779	0.738
A0A0Q0BWB9	Cbb3-type cytochrome c oxidase subunit	0.972	0.955	0.960	0.902	0.330	0.596	0.867	0.428	0.726	0.738
A0A0Q0DLM4	Tol-Pal system protein TolB	0.597	0.866	0.690	0.757	0.317	0.850	0.671	0.826	0.872	0.738
A0A0Q0BRG7	DUF4352 domain-containing protein	0.746	0.476	0.625	0.809	0.859	0.564	0.648	0.898	0.856	0.737
A0A0Q0DMZ9	Abhydrolase_10 domain-containing protein	0.736	0.685	0.790	0.867	0.813	0.450	0.502	0.767	0.862	0.737
A0A0Q0C0M9	DUF1989 domain-containing protein	0.943	0.813	0.721	0.493	0.701	0.749	0.596	0.833	0.668	0.737
A0A0Q0C4T2	Alpha-1,4-glucan:maltose-1-phosphate maltosyltransferase	0.810	0.822	0.865	0.709	0.436	0.777	0.431	0.852	0.723	0.736

A0A0Q0D8X1	DUF1852 domain-containing protein	0.959	0.581	0.545	0.245	0.791	0.648	0.888	0.938	0.806	0.736
A0A0Q0C7S9	Putative type VI secretion system effector, VgrG family	0.848	0.871	0.883	0.887	0.893	0.406	0.801	0.592	0.592	0.735
A0A0Q0IQW5	Syringomycin biosynthesis enzyme 2	0.941	0.765	0.902	0.452	0.379	0.006	0.795	0.939	0.908	0.734
A0A0Q0D1M6	TonB-dependent siderophore receptor	0.845	0.969	0.908	0.818	0.139	0.651	0.259	0.816	0.833	0.733
Q88BH0	Type III effector HopK1	0.885	0.776	0.727	0.801	0.766	0.380	0.639	0.918	0.584	0.731
A0A0Q0C6G4	VirK family protein	0.859	0.841	0.832	0.564	0.721	0.664	0.909	0.782	0.479	0.730
A0A3M5X1E1	Type III effector HopT1-1	0.907	0.592	0.353	0.433	0.848	0.565	0.813	0.921	0.890	0.729
A0A0Q0DIF8	Rhs family protein	0.884	0.783	0.624	0.317	0.502	0.913	0.903	0.761	0.763	0.728
A0A0Q0C763	Prepilin	0.748	0.958	0.751	0.667	0.767	0.934	0.690	0.498	0.702	0.728
A0A0Q0FN94	TonB-dependent siderophore receptor	0.951	0.945	0.942	0.801	0.137	0.624	0.193	0.797	0.797	0.727
A0A0Q0D786	Aconitase B	0.868	0.393	0.760	0.319	0.864	0.642	0.702	0.963	0.789	0.727
A0A0Q0D8W4	Putative signal peptide protein	0.884	0.913	0.971	0.331	0.878	0.790	0.325	0.782	0.573	0.727
A0A0Q0CVW3	Type III effector HopZ3	0.873	0.767	0.735	0.901	0.528	0.305	0.683	0.733	0.821	0.724
Q87Y16	Type III effector protein AvrPto1	0.583	0.811	0.699	0.498	0.912	0.806	0.649	0.743	0.768	0.723
A0A0Q0DXX3	DNA/RNA non-specific endonuclease	0.758	0.736	0.717	0.642	0.426	0.835	0.600	0.878	0.733	0.723
A0A0N8TAB7	Glucans biosynthesis protein D	0.850	0.818	0.859	0.546	0.455	0.498	0.535	0.765	0.859	0.721
A0A0Q0IP33	DNA-binding transcriptional activator OsmE	0.799	0.647	0.804	0.654	0.623	0.548	0.830	0.860	0.640	0.721
A0A0Q0DLQ9	N-acetylmuramoyl-L-alanine amidase	0.914	0.815	0.773	0.523	0.494	0.795	0.950	0.681	0.595	0.721
A0A0N8T9T9	Putative secreted protein	0.879	0.667	0.764	0.651	0.927	0.832	0.861	0.534	0.608	0.720
A0A0Q0E0W9	CigR	0.506	0.809	0.825	0.460	0.712	0.925	0.804	0.584	0.858	0.720
A0A0Q0DJZ6	Sugar ABC-type transport system, periplasmic substrate-binding protein	0.831	0.816	0.909	0.710	0.174	0.496	0.541	0.862	0.786	0.719
A0A0Q0DSY5	Putative periplasmic ligand-binding protein	0.698	0.967	0.688	0.765	0.686	0.851	0.435	0.549	0.827	0.719
A0A0Q0ID75	Autotransporter barrel protein with phosphatase-like passenger domain	0.719	0.962	0.903	0.828	0.828	0.800	0.380	0.555	0.618	0.719
A0A0N8T8C2	Glucans biosynthesis protein G	0.723	0.741	0.811	0.875	0.572	0.498	0.553	0.731	0.802	0.716
A0A2R3F5Q2	Type III effector avrA1	0.849	0.662	0.696	0.440	0.810	0.589	0.677	0.854	0.716	0.716
A0A0Q0FKW3	Peptidase aspartic, active site protein	0.839	0.825	0.976	0.820	0.101	0.418	0.355	0.941	0.751	0.716
A0A0Q0FGW7	Type III effector HopI1	0.832	0.732	0.724	0.718	0.337	0.596	0.726	0.731	0.845	0.715
A0A0Q0ILZ0	Imelysin, Metallo peptidase, MEROPS family M75	0.712	0.855	0.727	0.963	0.839	0.350	0.608	0.656	0.722	0.715
A0A0Q0DIF4	Oxidoreductase alpha	0.944	0.830	0.771	0.877	0.086	0.509	0.062	0.934	0.887	0.714
A0A0Q0DQV9	Pirin	0.694	0.812	0.477	0.833	0.812	0.704	0.377	0.891	0.706	0.714
A0A0Q0DP26	Phenol degradation meta-pathway protein	0.884	0.789	0.849	0.808	0.856	0.799	0.234	0.774	0.474	0.713
A0A0Q0C9U5	DUF4105 domain-containing protein	0.914	0.870	0.892	0.518	0.909	0.764	0.788	0.646	0.357	0.713
A0A0Q0DAI4	Conjugal transfer protein	0.336	0.686	0.716	0.623	0.740	0.833	0.751	0.759	0.879	0.712
A0A0Q0DMP2	Putative Membrane protein	0.822	0.853	0.891	0.536	0.939	0.968	0.932	0.400	0.464	0.711
A0A0Q0BXL6	RHS family protein	0.821	0.713	0.772	0.662	0.725	0.984	0.936	0.465	0.602	0.710
A0A0Q0DXG2	Alpha-ketoglutarate-dependent dioxygenase AlkB	0.916	0.516	0.813	0.594	0.483	0.478	0.707	0.860	0.763	0.709
A0A0Q0C803	Membrane-bound lytic murein transglycosylase A	0.800	0.797	0.910	0.765	0.597	0.645	0.522	0.461	0.858	0.709
A0A0Q0CVK2	Lipoprotein	0.538	0.603	0.606	0.663	0.757	0.821	0.719	0.726	0.882	0.709
A0A0Q0FEQ9	Thiamine pyrophosphate-binding protein	0.940	0.710	0.713	0.454	0.901	0.767	0.783	0.592	0.630	0.708
A0A0Q0DN10	Transcriptional regulator	0.867	0.538	0.682	0.491	0.591	0.652	0.584	0.856	0.842	0.708
A0A0Q0DZ24	Conjugal transfer protein	0.821	0.660	0.867	0.805	0.308	0.227	0.238	0.933	0.961	0.708

A0A0Q0DGR5	Twin-arginine translocation pathway signal	0.503	0.780	0.797	0.740	0.821	0.807	0.492	0.719	0.706	0.707
A0A0Q0FI54	GSDH domain-containing protein	0.885	0.861	0.823	0.822	0.494	0.730	0.503	0.639	0.614	0.707
A0A0Q0CIW2	DUF4105 domain-containing protein	0.916	0.777	0.833	0.264	0.870	0.752	0.785	0.615	0.602	0.706
A0A0Q0BRH4	ATPase	0.833	0.402	0.577	0.800	0.935	0.832	0.834	0.638	0.693	0.706
A0A0Q0BXK5	Putative cytoplasmic protein	0.931	0.860	0.684	0.680	0.631	0.457	0.340	0.653	0.871	0.705
A0A0Q0FCL0	SmpA/OmlA family outer membrane lipoprotein	0.655	0.709	0.611	0.421	0.625	0.836	0.602	0.809	0.865	0.705
A0A0Q0C4S4	Translocation and assembly module subunit TamA	0.972	0.841	0.894	0.426	0.443	0.539	0.188	0.925	0.704	0.705
A0A0Q0IBZ8	Allantoate amidinohydrolase	0.867	0.715	0.682	0.677	0.748	0.345	0.582	0.903	0.636	0.704
A0A0Q0DL55	Putative lipoprotein	0.721	0.854	0.859	0.111	0.366	0.826	0.391	0.842	0.909	0.704
A0A0Q0DLX5	Endolytic peptidoglycan transglycosylase RlpA	0.760	0.954	0.804	0.357	0.315	0.554	0.227	0.872	0.939	0.703
A0A0Q0C5C9	FGE-sulfatase domain-containing protein	0.821	0.795	0.576	0.552	0.824	0.986	0.926	0.562	0.545	0.702
A0A0N8T802	Protocatechuate 3,4-dioxygenase, alpha subunit	0.740	0.691	0.565	0.694	0.662	0.427	0.329	0.909	0.911	0.701
A0A0Q0FAW2	Putative TraI family relaxase/helicase	0.904	0.697	0.659	0.503	0.526	0.624	0.745	0.695	0.806	0.701
A0A0Q0BVG8	Peptide methionine sulfoxide reductase MsrA	0.963	0.492	0.762	0.495	0.604	0.618	0.148	0.969	0.830	0.701
A0A0Q0C2C2	Cupin-like domain protein	0.931	0.509	0.595	0.538	0.928	0.450	0.615	0.723	0.856	0.700
A0A0N8T9X7	Transporter	0.731	0.770	0.705	0.499	0.801	0.857	0.638	0.591	0.741	0.700
