# Supplementary material for: Repertoire and abundance of secreted virulence factors shape the pathogenic capacity of Pseudomonas syringae pv. aptata
Source: Front Microbiol. 2023 Jun 13;14:1205257. doi: 10.3389/fmicb.2023.1205257 (PMC10294431; doi:10.3389/fmicb.2023.1205257)
Supplement: Supplementary file 2 [file Data_Sheet_2.PDF]

>tr|A0A0Q0BTG7|A0A0Q0BTG7_PSEAP Pyrimidine/purine nucleoside phosphorylase OS=Pseudomonas syringae pv. aptata OX=83167 GN=ppnP PE=3 SV=1
MFKVNEYFDGTVKSIAFTQADGEATIGVMAAGEYEFGTAQREIMHVISGELSVKLPDSTD
WETFATGNQFNVPANSKFQIKVKVDTAYLCEYR
>tr|A0A0Q0DC31|A0A0Q0DC31_PSEAP Bifunctional protein GlmU OS=Pseudomonas syringae pv. aptata OX=83167 GN=glmU PE=3 SV=1
MRSALPKVLHPVAGNSMLGHVIHSARQLSPSGIHVVIGHGADAVREQLAADDLNFVMQDK
QLGTGHAVAQALPALTADTVLILYGDVPLIEVETLSRLLELVNPQQLGLLTVTLDDPTGY
GRIVRDDQNRVCAIVEHKDASDAQKAITEGNTGILAVPAAHLADWLGRLSNNNAQGEYYL
TDVIAMAANDGLVVATEQPHDAMEVQGANDRKQLSELERHYQMREARRLMAAGVTLRDPA
RFDVRGEVSVGRDVLIDINVILEGKVVIEDDVVIGPNCVIKDSTLRKGVVVKANSHIEGA
ILGEGSDAGPFARLRPGSVLGAKAHVGNFVELKNANLGEGAKVGHLTYLGDAEVGARTNI
GAGTITCNYDGANKHKTTLGADVFIGSNNSLVAPVDILDGATTAAGSTITQNVPAEQLGV
ARARQRNIEGWKRPVKIRKD
>tr|A0A0Q0D5T9|A0A0Q0D5T9_PSEAP Fatty acid oxidation complex subunit alpha OS=Pseudomonas syringae pv. aptata OX=83167 GN=fadB PE=3 SV=1
MMIYEGKAITVKALESGIVELNFDLKGESVNKFNRLTLNEFRQAVDAVKSDASIKGVIVT
SGKDSFIVGADITEFVDNFKLPEAELVAGNLEANRIFSDFEDLNVPTVVAINGIALGGGL
EMCLAADYRVIASSARVGLPEVKLGLYPGFGGTVRLPRIIGADNAIEWIASGKENAAEDA
LKVGAVDAVVAPDKLQAAALDLIQRAISGEFDYKAKRQPKLDKLKLNAIEQMMAFETAKG
FVAGQAGPNYPAPVEAIKTIQKAANFGRDKALEIEAAGFVKMAKTAAAQSLIGLFLNDQE
LKKKAKGYDKIAKDVKQAAVLGAGIMGGGIAYQSAVKGTPILMKDIREEAIQLGLNEASK
LLGGRLEKGRLTAAKMAEALNAIRPTLSYGDFGNVDLVVEAVVENPKVKQAVLAEVEANV
GENTILASNTSTISISLLAQALKRPENFVGMHFFNPVHMMPLVEVIRGEKSSEEAVATTV
AYAKKMGKNPIVVNDCPGFLVNRVLFPYFGGFARLVSAGVDFVRIDKVMEKFGWPMGPAY
LMDVVGIDTGHHGRDVMAEGFPDRMKDDRRSAIDALYDAKRLGQKNGKGFYAYETDKKGK
PKKVNDPAVLDVLKPIVYEQREVSDEDIVNWMMIPLCLETVRCLEDGIVETAAEADMGLI
YGIGFPPFRGGALRYIDSIGVAEFVALADQYAELGALYQPTTKLREMAAKGQSFFGQASS
EE
>tr|A0A0Q0FQS3|A0A0Q0FQS3_PSEAP Multifunctional fusion protein OS=Pseudomonas syringae pv. aptata OX=83167 GN=nnrD PE=3 SV=1
MFDTKPHLPDALYSAAQVRDLDARLIAAGTPGLELMLRAAQALWHTLLRRWPEANELTVL
TGRGNNAGDGYLVAAMAHKAGWQVRVLAVGDPAALTGDAAAAHAAAFGIDIQAWAGQPLA
GVVLDALLGTGLEGKVREPYVSAINAINDSGLPVVAVDIPSGLSADTGQTLGVAVRADVT
VTFIGLKVGLLTGDAADLVGELIFDDLRTDPALVAQAPFSATRLDKHNLPQLAARPRTAH
KGLFGRVLVIGGDHGFGGAALLSAESALRSGAGMLTLATRPEHVPAALTRMPEIMSAAIH
SANQLMGLIEPASVLVVGPGLGQAAWGRSLLSAAANADRPQVWDADALNMLAARLVSLPK
GSVITPHPGEAARLLGVGIKDVQADRAAAVRALARKFDTVCVLKGSGSLIANASGQLALC
DRGHPAMATAGLGDVLAGLTGALMAQHLKPFDAACLAVWLHASAGQKVGECGRGLAASDI
IPAIRQLLEELQPCLI
>tr|A0A0Q0DH31|A0A0Q0DH31_PSEAP Siroheme synthase OS=Pseudomonas syringae pv. aptata OX=83167 GN=cysG PE=3 SV=1
MEFLPLFHNLRGSRVLVVGGGEIALRKSRLIADAGAVLRVVAPEIEAQLSELVVQSGGEM
ILRGYSECDLDGCVLIIAATDDEPLNAQVSRDARLRCVPVNVVDAPALCTVIFPAIVDRS
PLVIAVSSGGDAPVLARLIRAKLETWIPSSYGQLAGLAARFRNQVKGLFPNVQQRRAFWE
DVFQGAIADRQLAGQGAEAERMLLAKIAGEPPSETGEVYLVGAGPGDPDLLTFRALRLMQ
QADVVLYDRLVAPTILDLCRRDAERVYVGKRRAEHAVPQEQINQQLVALAKQGKRVVRLK
GGDPFIFGRGGEEIEELAAHGIPFQVVPGITAASGCAAYAGIPLTHRDHAQSVRFITGHL
KNGTTDLPWSDLVAPAQTLVFYMGLIGLPVICEQLIRHGRSADTPAALVEQGTTVNQRVF
TGTLANLPQLVAEHDVHAPTLVIIGEVVKLREKLAWFEGAQATL
>tr|A0A0Q0C5R9|A0A0Q0C5R9_PSEAP UDP-N-acetylmuramoyl-L-alanyl-D-glutamate--2,6-diaminopimelate ligase OS=Pseudomonas syringae pv. aptata OX=83167 GN=murE PE=3 SV=1
MAFNLSKLFAHTDRDALIRELTLDSRNVRPGDLFLAVPGINVDGRAHIADALKRGAAAVA
YEVEGSIVLPITDVPLIPVKGLAAQLSAIAGRFYGDSSRSLNLVGVTGTNGKTSVTQLVA
QALDALGQRCGIVGTLGTGFYGSLQSGRHTTPDPIAVQATLTDLRQAGARAVAMEVSSHG
LDQGRAAALAFDVAVLTNLSRDHLDYHGTMEAYAAAKAKLFAWLDLKCRVINLDDDFGRE
LAAVKQESRLITYSQLDSSAYLYCRDAKFDDDGVRATLVTPQGEHFLRSSLLGRFNLSNV
LAAVGALLGLDYALDEILKALPKLEGPVGRMQRLGGADKPLVVVDYAHTPDALEKVLEAL

RPHAKGRLLCLFGCGGDRDRGKRPLMAEVVERLADGVWVTDDNPRSESPVSIFDDIRPGF
VAADKVRFIEGRGQAIAELIASANADDVVVLAGKGHEDYQEINGQRQPFSDLQEAASALA
VWEPANA
>tr|A0A0Q0D598|A0A0Q0D598_PSEAP Bifunctional polymyxin resistance protein ArnA OS=Pseudomonas syringae pv. aptata OX=83167 GN=arnA PE=3 SV=1
MSTKAVVFAYHDIGCVGLQALLDAGYEIAAVFTHADDPKEKTFFGSVAQMCARHGIAVHA
PEDPNHPLWVERIGKLAPDFIFSFYYRQLLGDSLLACAKKAALNLHGSLLPRYRGRAPAN
WVLVNGESETGVTLHQMVKRADAGPIVAQQRVSISATDTALTLHGKLRDAAADLLCETLP
LLAAQGQLPATPQDESRATYFGRRTPADGLIDWSLPATQLYNLIRAVTQPYPGAFCPVGD
NKLIVWAASVDTSSNGEAPGTVISHEPLRIACGDGSLVITAGQRGDNGLYLSGAQLAREF
GLVAGSQLLDKAKRRSVRRTRVLILGVNGFIGNHLSERLLQDDRYEIYGMDIGSDAIERL
RAKPNFHFIEGDISIHTEWIEYHIKKCDVVLPLVAIATPIEYTRNPLRVFELDFEENLKI
VRYCVKYNKRVIFPSTSEVYGMCQDANFNEDTSNLIVGPINKQRWIYSVSKQLLDRVIWA
YGQKGLQFTLFRPFNWMGPRLDRLDSARIGSSRAITQLILHLVEGTPIRLVDGGAQKRCF
TDVVDGIEALARIIENRDGRCNGQIINIGNPDNEASIRQLGEELLRQFEAHPLRGHFPPF
AGFREVESQSFYGKGYQDVSHRTPSIDNAKKLIGWTPGIELSETIGKTLDFFLREAMAEK
ADQC
>tr|A0A0Q0BTA9|A0A0Q0BTA9_PSEAP Dual-specificity RNA methyltransferase RlmN OS=Pseudomonas syringae pv. aptata OX=83167 GN=rlmN PE=3 SV=1
MKGEFMIASTGKTNLLGLTQQEMEKFFDSIGEKRFRAGQVMKWIHHFGVDDFDAMTNVSK
ALREKLKACAEVRGPEVVSEDISSDGTRKWVVRVESGSCVETVYIPQGKRGTLCVSSQAG
CALDCSFCSTGKQGFNSNLTAAEVIGQVWIANKSFGSVPATVDRAITNVVMMGMGEPLLN
FDNVIAAMHLMMDDLGYGISKRRVTLSTSGVVPMIDELSKHIDVSLALSLHAPNDALRNQ
LVPLNKKYPLKVLLESCRRYMSSLGEKRVLTIEYTMLKDINDKVEHAVEMIELLKDTPCK
INLIPFNPFPHSGYERPSNNAIRRFQDLLHQAGYNVTVRTTRGEDIDAACGQLVGQVMDR
TRRSERYIAVRELSAEADAAPVAVTRT
>tr|A0A0Q0CBL2|A0A0Q0CBL2_PSEAP Ribose-phosphate pyrophosphokinase OS=Pseudomonas syringae pv. aptata OX=83167 GN=prs PE=3 SV=1
MSKMMVFTGNANPDLARRVVRQLHIPLGDVSVGKFSDGEISTEINENVRGKDVFIIQPTC
APTNDNLMELVVMADAFRRSSASRITAVIPYFGYARQDRRPRSARVAISAKVVADMLTVV
GIDRVLTVDLHADQIQGFFDIPVDNIYGSPVLVDDIEDQRFENLMIVSPDIGGVVRARAV
AKSLGVDLGIIDKRREKANHSEVMHIIGDVEGRTCILVDDMVDTAGTLCHAAKALKEHGA
AKVFAYCTHPVLSGRAIENIENSVLDELVVTNTIPLSAAAQACGRIRQLDIAPVVAEAVR
RISNEESISAMFR
>tr|A0A0N8T8U3|A0A0N8T8U3_PSEAP Aspartate-semialdehyde dehydrogenase OS=Pseudomonas syringae pv. aptata OX=83167 GN=asd PE=3 SV=1
MKRVGLIGWRGMVGSVLMQRMREEQDFDLIEPVFFTTSNVGGQAPSVGKDVAPLKDAYSI
DELKTLDVVLTCQGGDYTNEVFPKLREAGWQGYWIDAASSLRMQDDAVIVLDPVNRKVID
QQLDAGTKNYIGGNCTVSLMLMGLGGLFDAGLVEWMNVMTYQAASGAGAQNMRELIRQMG
AVHASVADDLANPASAILDIDRKVAEAMRSESFPTENFGVPLAGSLIPWIDKALPNGQSR
EEWKGQAETNKILGRFKSPIPVDGICVRIGAMRCHSQALTIKLNKDVPIADIEGLISQHN
PWVKLVPNNREASMHELSPTAVTGTMSIPVGRLRKLNMGSQYLGAFTVGDQLLWGAAEPL
RRMLRILLER
>tr|A0A0Q0FNY0|A0A0Q0FNY0_PSEAP CTP synthase OS=Pseudomonas syringae pv. aptata OX=83167 GN=pyrG PE=3 SV=1
MTRYIFVTGGVVSSLGKGIASASLAAILEARGLKVTMLKLDPYINVDPGTMSPFQHGEVF
VTHDGAETDLDLGHYERFIRTTMTQNNNFTTGRVYEHVLRKERRGDYLGATIQVIPHITD
EIKRRIIKGAGDADVALVEIGGTVGDIESQPFLEAIRQLRFEVGARRAMLMHLTLVPYIA
TAGETKTKPTQHSVKELRSIGLQPDVLVCRSDHPIDVSSRRKIAQFTNVEERAVIALEDA
DTIYKIPGILHSQGLDDFVVERFGLQCGGADLSEWDKVVDAKLNPEHEVTIAMVGKYMEL
LDAYKSLIEAMSHAGITNRTKVNLRYIDSEDIENQGTGLLEGVDAILVPGGFGLRGVEGK
ITAVQFARENKVPYLGICLGMQVAVIEFARNVLGWKDANSTEFDRTSAHAVVGLITEWED
ATGAVETRTESSDLGGTMRLGAQECQLEAGSLVHDCYTKDVIVERHRHRYEVNNNLLPQL
IEAGLKISGRSGDGALVEVVEAPDHPWFVACQFHPEFTSTPRDGHPLFSGFVKAALAQHK
KNS
>tr|A0A0Q0DWR2|A0A0Q0DWR2_PSEAP tRNA sulfurtransferase OS=Pseudomonas syringae pv. aptata OX=83167 GN=thiI PE=3 SV=1
MVGTSRSVSMKLIVKVFPEITIKSPPVRKKFIRQLGKNIRTVLRELDADIVVGGVWDNLE
VETRLTDPKVLQGIRERLSCMPGIANFLQVAEYPLGDLDDVVAKCKLHYADLLPGKMFSV
RCKRAGRHDFSSMDVEKYVGSKLRMQCGAAGIELKKPDLVVRMEIRDQRLFVVHDQHQGM

GGYPLGALEQTLVLMSGGFDSTVAAYQIMRRGLMAHFCFFNLGGRAHELGVMEVAHFIWK
KYGSSQRVLFVSVPFEEVLGEILQKVDNSHMGVVLKRMMLRAASAVADRLEIDVLVTGEA
ISQVASQTLPNLSLIDAATEKLVLRPLVATHKQDIVDLATEIGTADFARHMPEYCGVISV
NPKTNAKRNRVEYEEKQFDMAILEQALERAKLVSIDRVIDDLSRNVDIEEVSQALAGQVI
LDIRHPDAQEDQPLQVPGVEIQTLPFYALNSRFKALDDTRQYLLYCDKGVMSRLHAHHLL
SEGHANVRVVYRPS
>tr|A0A0Q0C9M3|A0A0Q0C9M3_PSEAP Serine--tRNA ligase OS=Pseudomonas syringae pv. aptata OX=83167 GN=serS PE=3 SV=1
MLDSKLLRTQLQDVADRLASRGFTLDVARIESLEAQRKVVQTRTEQLQAERNARSKSIGQ
AKQRGEDIAPLMADVERMGNELSEGKVELDGIQAELDALVLSIPNLPHESVPVGADEEGN
VEVRRWGTPTSFDFEVKDHVALGEKFGWLDFETAAKLSGARFALLRGPIARLHRALAQFM
INLHINEHGYEETYTPYLVQAPALQGTGQLPKFEEDLFKISREGEADLYLIPTAEVSLTN
IVSGEILDAKQLPLKFVAHTPCFRSEAGASGRDTRGMIRQHQFDKVEMVQIVAPDDSMAA
LESLTGNAERVLQLLELPYRTLALCTGDMGFSAVKTYDLEVWIPSQDKYREISSCSNCGD
FQARRMQARWRNPETGKPELVHTLNGSGLAVGRTLVAVLENYQQADGSIRVPEVLKPYMG
GLEVIG
>tr|A0A0Q0CY88|A0A0Q0CY88_PSEAP Ketol-acid reductoisomerase (NADP(+)) OS=Pseudomonas syringae pv. aptata OX=83167 GN=ilvC PE=3 SV=1
MKVFYDKDCDLSIIQGKKVAIIGYGSQGHAQACNLKDSGVDVTVGLRKGSATVAKAEAHG
LKVTDVASAVAAADVVMILTPDEFQSQLYKNEVEPNLKKGATLAFSHGFAIHYNQVVPRA
DLDVIMIAPKAPGHTVRTEFVKGGGIPDLIAVYQDASGNAKNVALSYASGVGGGRTGIIE
TTFKDETETDLFGEQAVLCGGTVELVKAGFETLVEAGYAPEMAYFECLHELKLIVDLMYE
GGIANMNYSISNNAEFGEYVTGPEVINAESRQAMRNALKRIQDGEYAKMFISEGATGYPS
MTAKRRNNAAHGIEIIGEKLRSMMPWIAANKIVDKDKN
>tr|A0A0Q0CWN3|A0A0Q0CWN3_PSEAP ATP-dependent zinc metalloprotease FtsH OS=Pseudomonas syringae pv. aptata OX=83167 GN=ftsH PE=3 SV=1
MNDMAKNLILWLIIAAVLVTVMNNFSSPNEPQNLNYSEFIQQVKDGKVEKVSVDGYVITG
KRSDGDTFKTIRPNIPDNGLIGDLVSNNVVIEGKQPEQQSIWTQLLVASFPILVIIAVFM
FFMRQMQGGAGGKGGPMSFGKSKARLLSEDQVKTTLADVAGCDEAKEEVGELVEFLRDPG
KFQRLGGRIPRGVLMVGPPGTGKTLIAKAIAGEAKVPFFTISGSDFVEMFVGVGASRVRD
MFEQAKKHAPCIIFIDEIDAVGRHRGAGMGGGHDEREQTLNQLLVEMDGFEMNDGIIVIA
ATNRPDVLDPALLRPGRFDRQVVVGLPDIRGREQILKVHMRKVPMGEDVNPGVIARGTPG
FSGADLANLVNEASLFAARSGKRVVEMKEFELAKDKIMMGAERKSMVMSDKEKRNTAYHE
AGHAIVGRLVPEHDPVYKVSIIPRGRALGVTMFLPEEDRYSLSKRALISQICSLYGGRIA
EEMTLGFDGVTTGASNDIMRASQIARNMVTKWGLSEKLGPLMYSEDEDAGYLGRGGSQNS
SVSGDTAKLIDSEVRSIIDHCYGTAKQLLTDNRDKLEAMADALMKYETIDADQIDDIMTG
RTPREPRDWGGGSGTNGTPITPDPRPETPIGGPAAEH
>tr|A0A0Q0C8C8|A0A0Q0C8C8_PSEAP Enolase OS=Pseudomonas syringae pv. aptata OX=83167 GN=eno PE=3 SV=1
MKTQIQAIHAREILDSRGNPTVEVDVTLECGAMGRASVPSGASTGAHEAVELRDKDTQRY
SGKGVLKAVSNVNTEILESLRGMNAIDQEQIDHLMIKLDGTSDKSRLGGNAILGVSLAVA
RAAASALNLPLFQYLGGEQAARMPVPMFNILNGGVHANWQGPDFQEFMIAPTGAGSFKEA
LRWGSEVYHELKAVLKDAGYSTAVGDEGGFAPALKKNSDAIELIIKAIERAGYTPGSQIE
IAIDPASSGFYENGLYHLRSEGRKVDAQELINLYSSWVDKYPIAVLEDGLAEDDWSGWKL
LNAALGDRIELVGDDLFVTNVERIQRGITENVANAVLIKPNQIGTLTETKAAIEMAYGAN
WGAMVSHRSGETVDSSIADLTVAMGTGHLKTGAPCRGERVEKYNQFLRIEEDLGSRAFYA
GHDAFVR
>tr|A0A0Q0CE01|A0A0Q0CE01_PSEAP Enolase OS=Pseudomonas syringae pv. aptata OX=83167 GN=eno PE=3 SV=1
MAKIVDIKGREVLDSRGNPTVEADVLLDNGIIGSACAPSGASTGSREALELRDGDKSRYM
GKGVLKAVANINGPIRDLLLGKDPVDQKALDHAMIELDGTENKASLGANAILAVSLAAAK
AAAQDQDLPLYAHIANLNGTPGVYSMPVPMMNIINGGEHADNNIDIQEFMIQPVGAKSFA
EGLRWGTEIFHHLKAVLKARGLNTAVGDEGGFAPNLASNKEALDAIAEAVANAGYTLGTD
VTLALDCAASEFYKNGKYKLSEEGEYSSAEFAEYLAELTRKHPIISIEDGLDESDWDGWK
ILTDKIGEKTQLVGDDLFVTNTKILKEGIDKKIANSILIKFNQIGTLTETLEAIQMAKAA
GYTAIISHRSGETEDSTIADLAVGTSAGQIKTGSLCRSDRVSKYNQLLRIEEQLGSKAVY
RGRAEFRG
>tr|A0A0Q0C6R9|A0A0Q0C6R9_PSEAP tRNA-dihydrouridine(20/20a) synthase OS=Pseudomonas syringae pv. aptata OX=83167 GN=dusA PE=3 SV=1
MNIENNEASANIGDPLATVSRRFSVAPMMDWTDHHCRYFMRKLSKHALLYTEMVTTGALL

HGDRERFLRHDESEHPLALQLGGSTAADLAACSRLAEEAGYDEVNLNVGCPSDRVQNNMI
GACLMAHPELVADCVKAMRDAVGIPVTVKHRIGINGHDSYAELCDFVGKVQEAGCQSFTV
HARIAILEGLSPKENRDIPPLRYDVVAQLKSDFPDLEIVLNGGIKTLEQCSEHLQTFDGV
MLGREAYHNPYLLAHVDQQLFGSTAPVISRYDALESMRPYIAAHIASGGNMHHVTRHMLG
LGLGFPGARRFRQLLSVDIHKAENPMLLLDQAAAFLQGH
>tr|A0A0Q0BY73|A0A0Q0BY73_PSEAP S-adenosylmethionine synthase
OS=Pseudomonas syringae pv. aptata OX=83167 GN=metK PE=3 SV=1
MSEYSLFTSESVSEGHPDKIADQISDAVLDAIIAEDKYARVACETLVKTGVAIIAGEVST
SAWVDLEDIVRNVILDIGYNSSDVGFDGATCGVMNIIGKQSVDIAQGVDRSKPEDQGAGD
QGLMFGYASNETDVLMPAPITFSHQLVQRQAEARKSGLLPWLRPDAKSQVTCRYENGKVV
GVDAIVLSTQHNPDVSYKDLREGVMELIVKHVIPAHLLHKDTQFHINPTGNFIIGGPVGD
CGLTGRKIIVDTYGGMARHGGGAFSGKDPSKVDRSAAYAGRYVAKNIVAAGLAERCEIQV
SYAIGVAQPTSISLNTFGTGKLSDDKIIKLVRDNFDLRPYAITTMLDLLHPMYQATAAYG
HFGRTPVEMTVGDDTFTAFTWEKTDRADALRAAAGL
>tr|A0A0Q0C0Y9|A0A0Q0C0Y9_PSEAP Bifunctional protein HldE OS=Pseudomonas
syringae pv. aptata OX=83167 GN=hldE PE=3 SV=1
MKLSMPRFDQAPVLVVGDVMLDRYWHGGTSRISPEAPVPVVKVDQIEDRPGGAANVALNI
AALGAPASLVGVTGDDEAAESLTNSLKAAGVLARFQRIADQPTIVKLRVMSRHQQLLRID
FEEPFRTDPLALSAEVYSLLDGIKVLVLSDYGKGALKNHQALIQAARKRGIPVLADPKGK
DFAIYRGASLITPNLSEFEAIVGHCVDEAQLVTKGAQLMQELDLGALLVTRGEHGMTLLR
PDQQALHLPARAREVFDVTGAGDTVISTLAAAIAAGEELPHAVALANLAAGIVVGKLGTA
AISAPELRRAIQREEGSERGVLGLEQLLLAVDDARAHKERIVFTNGCFDILHAGHVTYLE
QARALGDRLIVAVNDDASVSRLKGPGRPINSVERRMAVLAGLGAVDWVISFPEGTPENLL
THVKPDVLVKGGDYGVDQVVGADIVQAYGGEVRVLGLVENSSTTAIVEKIRGQG
>tr|A0A0Q0DMV2|A0A0Q0DMV2_PSEAP dITP/XTP pyrophosphatase OS=Pseudomonas
syringae pv. aptata OX=83167 GN=ALO85_02941 PE=3 SV=1
MNLTQLVLASHNGGKLKELQAMLGGSVTLRSVSEFSLIEPEETGLSFVENAILKARNAAR
LSGLPALADDSGLAVDFLGGAPGIYSARYADGQGDAANNAKLLEALKDVPDDQRGAQFVC
VLALVRHADDPLPILCEGLWHGRILHAASGEHGFGYDPLFWVPERNSSSAELGPTEKNQL
SHRARAMVLLRQRLGLQ
>tr|A0A0Q0C403|A0A0Q0C403_PSEAP Beta-hexosaminidase OS=Pseudomonas
syringae pv. aptata OX=83167 GN=nagZ PE=3 SV=1
MSSGLQGSLMVDVAGTWLTSEDRQFLRQPEVGGLIIFARNIEHPRQVRELSAAIRAVRPD
LLLAVDQEGGRVQRLREGFVRLPPMRAIADKPDAELLAEQCGWLMATEVLAVGLDLSFAP
VLDLDYQRSAVVGTRSFEGDPQRAALLAGAFIRGMNAAGMAATGKHFPGHGWAEADSHVA
IPNDERSLEEIRASDLVPFARLSQQLAAVMPAHVIYPKVDSQPAGFSRRWLQDILRDELQ
FDGVIFSDDLSMAGAHVVGDAASRIEAALTAGCDMGLVCNDRAAAELALGAAQRLKVKPS
PRIARMRGQASASTDYRQHPRWQAALQALRAAQLID
>tr|A0A0Q0DKJ4|A0A0Q0DKJ4_PSEAP Multifunctional CCA protein
OS=Pseudomonas syringae pv. aptata OX=83167 GN=cca PE=3 SV=1
MLSRLRRLFMQIYKVGGAVRDRLLGQPVTDIDWVVVGASTEDMLIKGYRPVGTDFPVFLH
PLTNEEYALARTERKSGVGYGGFVFHASPEVTLEQDLIRRDLTINAMAEDKDGNLTDPYN
GQKDLEARILRHVSPAFAEDPLRVLRVARFAARYAGYGFTIAPETLGLMRQLSESGELKA
LTAERSWKEISRALMEEQPQVFIQVLHDCGALKELMPEVEALFGVPQPAAHHPEIDTGVH
VLSVLEQSARHKHPLTVRWACLLHDLGKGLTPEAEWPRHIAHEHTGLRLIKAVNERFRVP
RDCQELALLVGQYHTHGHRALELKPSTLLELLQSFDVYRRPQRFEEFIAACEMDARGRHG
FEQRSYPQADYLRAAAEVARAVSVQPLLEQGLKGKELGDALKNERLKALKTYKVEHLA
>tr|A0A0Q0FCY9|A0A0Q0FCY9_PSEAP Ribokinase OS=Pseudomonas syringae pv.
aptata OX=83167 GN=rbsK PE=3 SV=1
MQAKIVIVGSLNMDLVIRAQRLPRPGETLSGETFDTVPGGKGANQAVAAARLGASVAMIG
CVGADAYGEQLRAALLAEQIDCQAVTVVEGVSTGIASIVVDANSQNAIVIVAGGNGRLSP
ALIERFDTLLADSQIVICQLEVPTETVFHTLARARALGKTVILNPAPASEPLPANWYALI
DYLIPNESEAQTLTGVNVDSPAAAENAASSMLAAGARNVIITLGEHGTLFANASGMEHIP
ARRVQAVDTTAAGDTFVGGFAAALSAGHGELQAIRFGQAAAAISVTRAGAQPSIPTFEEV
QEFNSL
>tr|A0A0Q0DCR8|A0A0Q0DCR8_PSEAP 4-hydroxy-tetrahydrodipicolinate
reductase OS=Pseudomonas syringae pv. aptata OX=83167 GN=dapB PE=3 SV=1
MRRIAVVGAAGRMGKTLIEAVQQAPGAGLTAAIDRPDSTLVGADAGELAALGRIGVPLSG
DLAKVADEFDVLIDFTHPSVTLKNLAFCRKAGKAMIIGTTGFSAEEKQRLEEAGKDIPIV
FAANFSVGVNLCLKLLDTAARVLGDEVDIEITEAHHRHKVDAPSGTALRMGEVVANALGR

DLEKVAVYGREGQTGARDRQTIGFATIRAGDVVGDHTVLFAADGERVEITHKASSRMTFA
KGAVRAAMWLDGKAPGLYDMQDVLGLH
>tr|A0A0Q0CI03|A0A0Q0CI03_PSEAP Malate synthase G OS=Pseudomonas syringae pv. aptata OX=83167 GN=glcB PE=3 SV=1
MQRRFHATAAHVVSRIAHYMTPGSITERSPSVLDPAEQSEASAMTEYVQVGDLQVARVLF
DFVQNEATPGTGVDATAFWAGADKLIHDLAPKNKALLAKRDELQAKIDAWHQSHAGQAHD
ASAYKAFLQEIGYLLPEAADFQITTQNVDEEIATMAGPQLVVPVMNARFALNASNARWGS
LYDALYGTDAISEAGGAEKGKGYNKVRGDKVIAFARAFLDQAAPLSAGSHADSTAYKLID
GKLVVSLKGGSNTGLHDDAQLVGFQGDALAPFALLLKHNGLHFELQIDAASPVGQTDPAG
VKDILMEAALTTIMDCEDSIAAVDADDKVVVYRNWLGLMKGDLAESVSKGGETFTRTMNP
DRVYTTPEGGEVTLHGRSLLFIRNVGHLMTIDAILDKHGNEVPEGILDGLLTSLAAIHNL
NGNTSRSNSRNGSMYIVKPKMHGPEEAAFTNELFERIEQVLGLPRNTLKVGIMDEERRTT
VNLKACIQAASERVVFINTGFLDRTGDEIHTSMEAGPVVRKAQMKAEKWIAAYENSNVDI
GLKCGLQGRAQIGKGMWAMPDLMAAMLEQKIAHPLAGANTAWVPSPTAAALHALHYHKVD
VFARQAELAQREQASVDDILTIPLAPNTDWTPEEIQNELDNNAQGILGYVVRWIDQGVGC
SKVPDINDIGLMEDRATLRISSQHMANWLRHGVVSETQVLESLKRMAPVVDRQNANDPLY
HPLAPDFDNNIAFQAAVELVIEGTRQPNGYTEPVLHRRRREFKAINGL
>tr|A0A0Q0IB09|A0A0Q0IB09_PSEAP Regulatory protein, LysR:LysR, substrate-binding protein OS=Pseudomonas syringae pv. aptata OX=83167 GN=ALO85_00543 PE=3 SV=1
MNIDTRIKFRHLVCFLEVARQGSLARASDVLAISQPALSKSLKELETLLDTTLFVRSKSG
AALTEAGVAFMRFAGPSVQALREGVSSLRSGEHDTVTARLGVLSTAESLLVPEVVHRLHQ
QHPALIVSVMTGPSAYLLSQLRVGELDLVVGRMTDSPQIQGLTFEHLYNESMTLVVRSGH
PLLAAPLKPESLEQFPLVLPLAGTTIRKFADSLFVQCGIRMPRQRLETLSLTLSRRYVQC
SDAVWIAPLDAVSLELKGGTLVELDMGIREPGGSVGLCSNPALPLTRAAQWCVDELRRLG
EAYRNV
>tr|A0A0N8T8F5|A0A0N8T8F5_PSEAP Nuclease OS=Pseudomonas syringae pv. aptata OX=83167 GN=ALO85_02805 PE=4 SV=1
MGISRLLEKALLVGVFFMPAIWVSGAQAFCPAPASLPVAQVQRVVDGDTLKLTDGRNVRM
IGLNTPETGRKGRSAEPFAESAKKRLQTLVNESGGKVRLRIGLQGKDHYGRTLANVYDRR
GANLEAQLLSEGLGYLVAVAPNVTLVDCQQVAERQARQARLGVWRHSPVQSSARLSKSGF
AIVSGQVKRVQRNRGGIWIELQGSLVLRIAPAHLSAFDTAMLKRFEGRQVEARGWVVDRS
RRGGLKKGQSRWQLSLTHPAMLDLSRR
>tr|A0A0Q0C9T4|A0A0Q0C9T4_PSEAP Uncharacterized protein OS=Pseudomonas syringae pv. aptata OX=83167 GN=ALO85_100661 PE=4 SV=1
MKTGTQRVRVILEKQAHRRVAVFCKGLFRARSPVVWRREAYLREEGA
>tr|A0A0Q0DFJ5|A0A0Q0DFJ5_PSEAP Phosphoribosylamine--glycine ligase OS=Pseudomonas syringae pv. aptata OX=83167 GN=purD PE=3 SV=1
MNILIIGSGGREHALAWKVAQDPRVQTVFVAPGNAGTAIEAKCENVAIDVLALEQLADFA
EKNVALTIVGPEVPLVAGVVDLFRSRGLDCFGPTAGAAQLEGSKAFTKDFLARHKIPTAD
YQNFTEIEPALAYLREKGAPIVIKADGLAAGKGVIVAMSLAEAEDAVRDMLAGNAFGDAG
SRVVIEEFLDGEEASFIVMVDGKNVLPMATSQDHKRVGDGDSGPNTGGMGAYSPAPVVTA
EVHQRVMDLVIWPTVRGMADEGNVYTGFLYAGLMIDKAGNPKVIEFNCRFGDPETQPVML
RLQSSLVLLVEAALAQALDKIEAQWDPRPSLGIVLAAGGYPGDYAKGAVIEGLDAAAQLE
GKIFHAGTALQDERVVTSGGRVLCATALGDSVGNAQENAYALAAKVDWQGCFYRKDIGYR
AIARERGEDQE
>tr|A0A0Q0C4S6|A0A0Q0C4S6_PSEAP Type VI secretion system protein ImpC/EvpB OS=Pseudomonas syringae pv. aptata OX=83167 GN=ALO85_02260 PE=4 SV=1
MTDKQPVTQQDSALVEVENFAPQSSLLDSIISESRVARSETERTRTRDLIGELVAQVLEG
EMTPSKDLIAVLDARIAEIDSMLSEQMNEIMHAREFQQLEASWRGLKYQVDQTETSTTLK
IHLLNASKKDLVRDLKASSEFDQSALFKKIYEEEYGTFGGAPFGMLLGDYEFNRSPEDMY
LLEEISHVAAAAHAPFISAASAELFGWDSFTDMAGPRDLAKIFDTVEYAKWKSFRASEDS
RYVGLTLPHVLGRLPYGPDTTPVEEFNFVESVDGRDHNKYLWMNAAYALGTRVTDAFSRY
GWCVAIRGVEGGGLVEGLPTHTFKTDDGEIALKCPTEIAITDRREKELSDLGFIPLVHCK
GTDYAAFFGTQSTQKQKQYNTDIANANARLSAQLQYIFATSRIAHYMKAIMRDKIGSFAS
RKDVELFLNKWLSSYVLLDDTASQEAKAKFPLREARAEVFEVPGKPGVYKAVTYLRPHYQ
LDELTASLRLVAELPQSTRG
>tr|A0A0Q0CEU8|A0A0Q0CEU8_PSEAP Elongation factor G OS=Pseudomonas syringae pv. aptata OX=83167 GN=fusA PE=3 SV=1

MARTTPIGRYRNIGIVAHVDAGKTTTTERVLFYTGKSHKMGEVHDGAATTDWMVQEQERG
ITITSAAITAFWQGSEKQHKDQYRFNVIDTPGHVDFTIEVERSLRVLDGAVVVFCGTSGV
EPQSETVWRQANKYGVPRIVYVNKMDRAGANFLRVIAQIKQRLGHTPVPIQLAIGAEDNF
QGQIDLMSMEAVYWNDADKGMVPRREPIPAELQELADEWRSNMVEAAAEASEELMNKYLE
GEELTNEEIKAALRQRTIAGEIVLAVCGSSFKNKGVPLVLDAVIDYLPAPIDIPAIKGSD
PDNEEILMERHADDNEPFSALAFKIATDPFVGTLTFVRVYSGVLASGDGVINSVKGKKER
VGRMVQMHANAREEIKEVRAGDIAALIGMKDVTTGETLCNADKPIILVRMDFPEPVISVA
VEPKTKDDQEKMGIALGKLAQEDPSFRVKTDEETGQTIISGMGELHLDILVDRMRREFNV
EANIGKPQVSYRERITKNCEIEGKFVRQSGGRGQFGHCWIRFAPADEGQEGLQFVNEVVG
GVVPKEYIPAIQKGIEEQMKNGVVAGYPLIGLKATVFDGSYHDVDSNEMAFKVAASMATK
QLAQKGGGELLEPIMAVEVVTPEDYMGDVMGDLNRRRGMILGMEDTVSGKVIRAEVPLGE
MFGYATDVRSMSQGRASYSMEFKKYNTAPSHIVETVTKKQG
>tr|A0A0Q0DXY5|A0A0Q0DXY5_PSEAP Uncharacterized protein OS=Pseudomonas syringae pv. aptata OX=83167 GN=ALO85_101378 PE=4 SV=1
MNLAQRCVLVIWHGKSPGVLHTPQRERVQSSLTFRFGLSAG
>tr|A0A0Q0DG10|A0A0Q0DG10_PSEAP Type IV pilus assembly protein PilN OS=Pseudomonas syringae pv. aptata OX=83167 GN=ALO85_02809 PE=4 SV=1
MARINLLPWREELREERKKRFLTALVGVLVVSVGILFLIDRYVSNGIEHQMARNAFLQTQ
IAQLDIRIKEISDLKARRKQLLERMKIIQDLQGNRPITGRVFDQLARTLPDGVYYSQVKM
TDKLIAISGAAESNNRVSDLMRNLEASDWLEAPSLTEVKATTAGAVDQANVFQLTVRQTQ
PPVAAVPAAGAKP
>tr|A0A0Q0IJ22|A0A0Q0IJ22_PSEAP Molybdopterin converting factor subunit 1 OS=Pseudomonas syringae pv. aptata OX=83167 GN=ALO85_01815 PE=4 SV=1
MKVQVQYFARYRELLGLDNESVEGEFATLDALRQMLLQRGEAWQVLAEQNLMCARNQELC
KPSEPLSDGDEVAFFPPVTGG
>tr|A0A0Q0BYU9|A0A0Q0BYU9_PSEAP UPF0060 membrane protein ALO85_03223 OS=Pseudomonas syringae pv. aptata OX=83167 GN=ALO85_03223 PE=3 SV=1
MHISALTCEPFMLNYLWFFLAALFEIFGCYAFWLWLRQGKSALWVIPALVSLTVFALLLT
RVEAAYAGRAYAAYGGIYIVASIAWLGLVERVRPLGTDWLGLAFCVIGATIILLGPRWSA
A
>tr|A0A0Q0IR15|A0A0Q0IR15_PSEAP Rhomboid family protein OS=Pseudomonas syringae pv. aptata OX=83167 GN=ALO85_00610 PE=4 SV=1
MAFSRRLKVILVLSAVLIAVQAINSITGNSLVHFGIMPRSLIGLRGIVFAPFLHGSIQHL
LSNLLPFIVLSWLVATEGVRRYAWVAGLVCLLGGLLVWSFGRSNVHVGASGLIFGLWAYL
LARAWYQRSIASVLIALLVLAAYSGLVFGFVPVAGVSFESHIAGAFAGVCVAWLMHSRAL
LAQK
>tr|A0A0N8T8P9|A0A0N8T8P9_PSEAP Diaminopimelate decarboxylase OS=Pseudomonas syringae pv. aptata OX=83167 GN=ALO85_00394 PE=3 SV=1
MHKVPETVLAAIKEVRALETDPLAAFVYDLDALQQHVSEVMAALPAGVELYYAIKANSEA
LMLETIAPLVSGFEISSGGEIERVMACPTRKPYVFSGPGKLDSDLRSALLNKVEAIHLES
LNEIARLQQLAEQAGRVQPVFLRINPQLPAAQSSRLAMAGTATPFGIDEADLAEAVRRVD
NASHLMLKGFHVHAMSHQMSVERHEQLLDFYLQRWQEWKALARDPGQLTHFNVGGGIGVD
YLNSQQFDWQRLCRYLEKRLGEQHDAPILRFEPGRFISAYCGYYAIEVLDSKTSHGEHFL
VCRGGTHQFRLPVAQSHDHPVIHLPTAPQSTESSEQAYTIVGQLCTPKDVLSRRQPLKGV
NIGDLLVLPLAGAYGYNISHADFLCHPRPSQHFVRNGERVRQ
>tr|A0A0Q0BVB4|A0A0Q0BVB4_PSEAP NADH:ubiquinone oxidoreductase, subunit E OS=Pseudomonas syringae pv. aptata OX=83167 GN=ALO85_00741 PE=4 SV=1
MNSPLIQTDRFALSETERSAIEHEMHHYEDPRAASIEALKIVQKERGWVPDGAIYAIGDL
LGIPASDVEGVATFYSQIFRQPVGRHIIRVCNSMVCFIGGHENVVDEIKSSLGIGLGQTT
ADGRFTLLPVCCLGNCDKAPAVMVDDDTFGDVQPAGVAKMLEGYL
>tr|A0A0Q0BWR6|A0A0Q0BWR6_PSEAP Plasmid stabilization system family protein OS=Pseudomonas syringae pv. aptata OX=83167 GN=ALO85_01050 PE=4 SV=1
MPQIELSEKADSDLEAIHEHYAGLMGHQRADEVVNTILESIEQLATFTGMGRPSQTPDVR
ELVLTRYPFVVSYVVRTQVVFIVRVLHERNERLNTAKIQ
>tr|A0A0Q0FSW2|A0A0Q0FSW2_PSEAP Putative paraquat-inducible protein PqiB OS=Pseudomonas syringae pv. aptata OX=83167 GN=ALO85_03604 PE=4 SV=1
MSDSTLPPPASVPRPEVKRRRLRVSLIWLVPIVAAIIGISMALHDWMNIGPRITVSFLTA
EGLEANKTQVKYKNVVIGMVTDISLSDDRTHVLATIELNTSATPFTRVDSQFWVVRPRIG
AHGVSGVDTLLSGAFIGADAGSAEETKASFTGLETPPPVTFGEKGKRFTLHTDDLGSLDI

GSPIYFRRIQVGQVVAYDLSKDGRGVDIQIFINAPNDQYITTDTRFWNASGVDITVGASG
VKVNTQSLTSIISGGIAFREPNWSPDSKPADENAEFKIFDDQATAMAPPDGEPRYIRMRF
NQSLRGLTVNAAVDFLGVNIGKVVSVDLDYDPATKTFPGIVGAVIYPKRLGAAESKLKEL
GGSGDEEEQSARVLGAFVANGLRAQVRNGNLLTGQLYIAMEFDPKAPKVAFNAKARPLEI
PTVPGSFDKLQEQLQAFVEKLSNLPIDQLAGNLNGTLSELQKTLKQVNSSVLPQMRGTLQ
QAEKTLGTANDSFAEDSPARQQLGQAMDEVQRTARSVRVLTDFLSRHPESLIRGRTGDAA
PRSFNAPSSSRAIDPEPKQ
>tr|A0A0Q0CVN7|A0A0Q0CVN7_PSEAP Putative nuclease of the RecB family OS=Pseudomonas syringae pv. aptata OX=83167 GN=ALO85_04125 PE=4 SV=1
MGGPDGTHERFSMKDWCRQNPDEAPPLNPNETNSQALRRGFIRMGWVTEETDTQVLILHP
GTPKTTVEAIENEDLALDDVDTPDSDRETVFELEWQLRDFIAHNIETLRIDGKALRLYVD
SIGRDGVEYPTGVGPIDILALDSDDSFVIFELKRGRVADRAIGQISRYMGWIKKNLAKGR
MVKGVIVAKSISSNLRHAVIAVPNVSLFEYQVSFNLNQVLEADETI
>tr|A0A0N8T803|A0A0N8T803_PSEAP D-alanyl-D-alanine carboxypeptidase/D-alanyl-D-alanine-endopeptidase OS=Pseudomonas syringae pv. aptata OX=83167 GN=ALO85_00553 PE=4 SV=1
MIKSLRPLLLASLLLPLALPSYAAVINTTLPPKVQQAMKASKLGDDALSLVMLPLNGPGT
PTVFNADVSVNPASTMKLITTYAALEMLGPTHQWKTEFFTDGTLSNGVLRGNLYLKGGGD
PKLNMEKLWLLMRDLRANGVQQVTGDLVLDRSHFVQPQLPVFNDDGNDDNKPFLVKPDAL
MVNLKALRFVTRNDDGRILVSVEPPIASIRIDNQVKASASKQCTGDVRYNPVTQADGSVN
VTVTGQLGNGCNSQTYLSLLDHPTYAAGAVRAIWQELGGTIQGKDRVGVLPGNAKLLAKA
FSPDLVEVIRDINKFSNNTMAQQLFLSLGEEFRNEADGDDGKAAQRVIRQWLAKKGITAP
HLVMENGSGLSRAERVSAREMAVILQAAWRSPYAAEFMSSMPLAGLDGTMRKRLKRTPLL
GEAHIKTGTLNTVRAIAGFSRDSNGNTWAVVAILNDPRPFGASSILDEVLVDLYRQPKLT
NTTVSIQQ
>tr|A0A0Q0C646|A0A0Q0C646_PSEAP Uncharacterized protein OS=Pseudomonas syringae pv. aptata OX=83167 GN=ALO85_101344 PE=4 SV=1
MSLEALHLQGFFIFVTAALHLRTAGLCSGVITQYLSIRSGNSYT
>tr|A0A0Q0IQ76|A0A0Q0IQ76_PSEAP MerR family transcriptional regulator OS=Pseudomonas syringae pv. aptata OX=83167 GN=ALO85_00765 PE=4 SV=1
MLEPSHNDELPPIPGKRYFTIGEVSELCAVKPHVLRYWEQEFPQLNPVKRRGNRRYYQRQ
DVLMIRQIRGLLYDQGFTIGGARLRLTSGEPKDDTQQYKQMIREMIAELEDVLVMLKA
>tr|A0A0Q0FW61|A0A0Q0FW61_PSEAP Poly(A) polymerase I OS=Pseudomonas syringae pv. aptata OX=83167 GN=pcnB PE=3 SV=1
MLKKLFQSFRSPLRKPQQHTRTTPEVLNSSQHSLQRSQFSRYAVNIVERLQNAGYQAYLV
GGCVRDMMLNITPKDFDVATSATPEQVRAEFRNARIIGRRFKLVHIHFGREIIEVATFRA
NHPQDDEEEDSNQSSRNESGRILRDNVYGTLEEDAQRRDFTINALYYDPVSERVLDYANG
VHDIRNRLIRLIGDPEQRYKEDPVRMLRAVRFAAKLDFGIEKHSAQPIRALAPMLRDIPS
ARLFEEVLKLFLSGHAAPTFEMLVDLELFEPLFPASSKALEYNPTYTHTLISNALINTDL
RIKQNKPVTPAFLFAALLWPALPAKVLRAQERGMPPIAAMQEAAHELIIEQCQRIAIPKR
FTLPIREIWDMQERLPRRSGKRADLLLDNSRFRAGYDFLLLRETAGEQTDGLGQWWTDYQ
DCNDSERRDMIRDLSNKPEAAGTAPRKRRRNSGAKRKRTTGEAQSGE
>tr|A0A0Q0IJ31|A0A0Q0IJ31_PSEAP Ferrochelatase OS=Pseudomonas syringae pv. aptata OX=83167 GN=hemH PE=3 SV=1
MTDHALLLVNLGSPASTQVADVRSYLNQFLMDPYVIDLPWPVRRLLVSLILIKRPEQSAH
AYASIWWDEGSPLVVLSKRLQQAMKKEWSHGPVELAMRYGEPSIETVLTRLAEQGFKKVT
LAPLYPQFADSTVTTVIEEAKRVVRAKSLKMQFSVLQPFYDQPEYLSALVESVRPHLDQP
YDHLLLSFHGLPERHLHKLDPTGKHCLKDDCCMTAPAEVLATCYRAQCIQSAAAFAKRMG
IPDGKWSVSFQSRLGRAKWIEPYTEAHLDELAAKGVRKLLVMCPAFVADCIETLEEIGDR
GAEQFKEAGGEELILIPCLNDDPNWAKELNRLCERAPLML
>tr|A0A0Q0DN76|A0A0Q0DN76_PSEAP Putative permease OS=Pseudomonas syringae pv. aptata OX=83167 GN=ALO85_04646 PE=4 SV=1
MNETTLQYKTLILLLVLVTIAFIWILLPFYGAVFWAVILGIIFAPVQRKLQIKFNWSRNL
TSLCTLMICLVIAILPVIVISALLVQEGTTLYKNVETGQLDIAGYLAEFKDLLPHSMQAL
LDRLGMGDLEGLRDKIAKGAMQGSQYLATQAFSFGQGTFDFVVSVFIMLYLLYFFLRDGQ
ELVRKIRTAFPLGEQQKRRLQLKFTRVVRATVKGNVVVAVTQGALGGFIFWVLDIPSALL
WAVIMAFLSLLPAVGAGIVWAPVAVYFLLSGMIWQGVVLGLFGVFVIGLVDNVLRPILVG
KDTKMPDYLILISTLGGMSVFGLNGFVIGPLVAALFMSSWGLFSGAKKPVKLPG
>tr|A0A0Q0DAV0|A0A0Q0DAV0_PSEAP KAP NTPase domain-containing protein OS=Pseudomonas syringae pv. aptata OX=83167 GN=ALO85_03373 PE=4 SV=1

MNILNFQSENPSERDAFKGESHDRVAQAIHDYINSRENHRVIGLEGEFGSGKSSILQMLE
RKIKEKSSKSVLWIFDCEQNYQGSIKSNFIELFTDQITSLLTKIGDKAAVPEVLKTRDIA
LGRHFSYTKDTRSHISVWAVLLIASGVLSPTFIRDYLPQLRGNMELPWWQHLFYLGTSLA
PLIILILAYFFNGKEQVGKKRWNISSLLKGSSDDQITETIEISKEVSPLDLKRALRNHLH
AVKDHHFIIVIDNLDRLPRDVLRSVWSDLEIFTSVTGSENLSVIVPFCSTKVSQYLNGDS
EQSYDSRDFIAKKFPVVFRTPPIITSGWKDAFRTLWAETFGPESQSDADTCSIILQRHSP
MTAGLVTPRLQKRFINDIATTMLVTAGQPSLICIAAYIAICKYNNISIESLLKEPAAPST
EDAAATDNSGEKEQREKNSANLARTKRTLKTLLGEDMNRGWHIQVLQIHFQTSPDIAIAE
LIDQPLATAIESQDGKQLASLAEYFGFTDSFLRGLEGEVSPNNLFLALHKAANSENCDIS
VLIPHINKKFTENSLLTALVPEAGYFEATRGLISLGLSKAIFDGELKSARDAFLSINRRA
YEPDAKDAYQKVVNMYDSYLNALDEPFPETTMDSSEVLFHILVPDPDLLVIDVNRITLSQ
KGRSDAVRQIISNDTISFDLTPLPQEEWIYCLHQCFRGIKMSSVTHDYQLSDEKSTEIIN
AITYDPTEERSWVGLAFFEKYSNTIPALIKSHLPSTESNCIKVAMAIVYMRLGMGAELAE
IPEIESALTKESELLNSLTKALLHSGNLFTLTLEPESRSIVAPILAHAIKKRIIAKIYVF
HVYREYTQLCEALSNAGLAESEFLAWITSFSQLAEHLPTLDEIDPKFLNTILMGSDDSML
QLRAHILDRDFGSEIDKAGWLAIFNSPNARVANTLDRMVVEKLDFKGEKNAHAAFSTYIS
ALIKKSTFEDPSEQVIGNIKNVLDLFDKDTLFVIGTELRACTYDGTAPIEASIFILSTLN
SLLPSVTPRNKVEEERLLQILLFIHRNPESTSQASDFLDHQWEQLADWSISKDNKDTYAS
YVTKLRDRLPNVYADLSNKTGFKARMKKFASALMARPSDTED
>tr|A0A0Q0BUF7|A0A0Q0BUF7_PSEAP Type IV pilus assembly protein PilO
OS=Pseudomonas syringae pv. aptata OX=83167 GN=ALO85_02810 PE=4 SV=1
MNMSEWLEGLRKVDLSELDLNNLGSWPAAVKAIAGLLVAVTIFALGYFFFIQDLETQLES
AQSSEVTLKEQFSNKAFQAANLEPYKKQMEEMENTFGALLRQLPSDTEVPGLLEDITRTG
LGSGLEFEEIKLLPEAVQQFYIELPIQITVVGGYHDLATFVSGVASLPRIVTLHDFEIKP
VDPKVPTKLRMSILAKTYRYNDEGLKK
>tr|A0A0Q0C0Z1|A0A0Q0C0Z1_PSEAP Type IV pilus bioproteinsis regulatory
protein PilG OS=Pseudomonas syringae pv. aptata OX=83167 GN=ALO85_02927
PE=4 SV=1
MEQHASALKVMVIDDSKTIRRTAETLLKNAGCEVITAIDGFDALAKIADNHPRIIFVDIM
MPRLDGYQTCALIKNNRAFKSTPVIMLSSKDGLFDKAKGRIVGSDQFLTKPFSKEELLSA
IKAHVPGFVAAEQHIS
>tr|A0A0Q0FV25|A0A0Q0FV25_PSEAP Alpha/beta hydrolase fold protein
OS=Pseudomonas syringae pv. aptata OX=83167 GN=ALO85_03430 PE=4 SV=1
MIQLTAEHTPAGTSYLATGQGHPVVLIHGVGLNKEMWGGQIVGLATHYKVIAYDMLGHGA
SPRPAPETGLPGYAEQLHELLTHLQLPQAAVVGFSMGGLVARAFALEFPQYLSGLVVLNS
VFNRSAEQRASVIARTSQAAEHGPDANAGEALSRWFSREYQAASPAQIAAIRHNLASNDP
QGYLTTYKLFATQDMYRAEDLGDIRAPTLIATGELDPGSTPQMARELAARIPGADVAILP
DQRHMMPVESPRLVNQVLLGFFEKIGLDKPASAHHSIKGIVA
>tr|A0A0Q0DSH0|A0A0Q0DSH0_PSEAP IclR family transcriptional regulator
OS=Pseudomonas syringae pv. aptata OX=83167 GN=ALO85_03365 PE=4 SV=1
MDNSTDRNSEKAEVGVGAVSRLFAVLRCLGDCDEGGERVTQLAQRVGLSQPTTHRLLRSL
MDEGMVEQDLLSKRYRLSIEFFALAAKAGNTGNLRDVVRPSLLRLSASLGDSLFLLARSG
FDAICLDRSEGPYPIRTFTGDIGGRVALGVGQGSLAILAFLPEDERETVIAYNLPRLKDF
HLYDEVFLRSEVENVRRQGYAGRNTGALPGMAGLAVPILDRSGRAVAALSVATISDRLGP
DRLMTVVELLKREATAISARINPFDPSLRRPSQVFGQVD
>tr|A0A0Q0C487|A0A0Q0C487_PSEAP Peptide chain release factor 2
OS=Pseudomonas syringae pv. aptata OX=83167 GN=prfB PE=3 SV=1
MSSGLTDAKDLLLMSAEEEDQAAVDDVAAEVERLRESLEKLEFRRMFSGDMDPNNAYLDI
QAGSGGTEAQDWANILLRMYLRWADKRGFDATIMELSAGEVAGIKGATVHIKGEYAFGWL
RTEIGVHRLVRKSPFDSGNRRHTSFSAVFVSPEIDDNIEIDINPADLRIDTYRSSGAGGQ
HVNTTDSAVRITHVPTNTVVSCQNERSQHANKDTAMKMLRARLYEQEVQKRNAASQALED
TKSDIGWGHQIRSYVLDASRIKDLRTNIERSDCDKVLDGDIDEYLIASLKQGL
>tr|A0A0Q0CZJ5|A0A0Q0CZJ5_PSEAP CheC-like chemotactic response regulator
rotein OS=Pseudomonas syringae pv. aptata OX=83167 GN=ALO85_01109 PE=4
SV=1
MSTLALLICDDSNMARKQLLRALPEDWDVSVTLATQGQEGLEAIRKGQGQVVLLDLTMPV
MDGYQTLTAIRAENLDAKVIVVSGDVQDEAVRRVMELGALAFLKKPADPDELKSTLERLG
LLGKPAASPVALPALNNKGGVISFQDAFRETVNVAMGRAAALLAKVLGVFVHLPVPNVNM
LEVGELHMALADAHSDQRLTAVCQGYIGGGIAGEALLIFHDSEISGISQLMGGDHSDSSD
MEMLLDLSTILIGACLSGIAEQIDIAFSQGHPQLLGAQGGIDELIRINQQRWKKTLAVEI

SYSVEGHNLHFDLLMLFTEDSVELLTKKLAYMMS
>tr|A0A0Q0IJ08|A0A0Q0IJ08_PSEAP GTP cyclohydrolase 1 OS=Pseudomonas syringae pv. aptata OX=83167 GN=folE PE=3 SV=1
MSSSLPQHYRDILLGLGEDPDREGLLDTPKRASKAMQYLCHGYTQSVEEIVNGALFASDN
DEMVIVQNIELYSLCEHHLLPFIGKAHVAYIPTGKVLGLSKIARIVDMFARRLQIQENLT
KQIADAIQKVTGAAGVAVVIEAQHMCMMMRGVEKQNSTMNTSVMLGAFRDSSTTRMEFLQ
LIGRSRQ
>tr|A0A0Q0BZQ8|A0A0Q0BZQ8_PSEAP Helix-hairpin-helix DNA-binding motif-containing protein OS=Pseudomonas syringae pv. aptata OX=83167 GN=ALO85_100421 PE=4 SV=1
MSTSMHKENSQRPPVLQHVSQNVRRLRNSADLSQTALAEKSGVSRRMLVAIEAGEKNVSL
ATLDRVAEALEVAFSDLIQAPENHDYSRINELAWAGTIAGSKAVLLARAVARREVELWEF
CLQPGDAYVSEADPDGWSEQVYVVEGHLTIRFSDPAATQQIGAGEFFMFPSNQPHRYCND
ADVPLRFVRNVAL
>tr|A0A0Q0CEA9|A0A0Q0CEA9_PSEAP 3-beta hydroxysteroid dehydrogenase/isomerase family protein OS=Pseudomonas syringae pv. aptata OX=83167 GN=ALO85_02363 PE=4 SV=1
MKILVTGASGFIGGRFARFALEQGMSVRINGRRAEGVEHLVRRGAEFIQGDLADPYLVRA
LCDDVEAVVHCAGSVGTWGRRQDFMQGNVQLTENIVEGCLKQRVRRLVHLSSPSIYFDGH
SHRGIKEEQVPKRFHNHYAATKYLAEQKVFGAEEFGLEVIALRPRFVTGAGDNSIFPRLL
HMQRKRRLSIVGNGLNVVDFTSMQNLNEALLSSLLATGSALGKAYNISNGTPVPLWDTIN
YVMRQMHLPQATRYRSYGLAYSAAAINEAACMLWPGRPEPTLSRVGMQVMNTDFTLDISR
ARHYLDYQPQVSLWAALDEFCGWWQAQHAI
>tr|A0A0N8T894|A0A0N8T894_PSEAP 50S ribosomal protein L9 OS=Pseudomonas syringae pv. aptata OX=83167 GN=rplI PE=3 SV=1
MQLILLEKVANLGNLGDKVNVKAGYGRNYLLPYGKATAATAANVAAFEERRAELEKLAAD
KKASAETRAAQLAELEVTITATAGDEGKLFGSIGTHDIADALTASGVEVAKSEVRLPNGT
IRNVGEFDVAVHLHSDVEATVRVVVVAA
>tr|A0A0Q0IRW6|A0A0Q0IRW6_PSEAP Zinc-containing alcohol dehydrogenase super protein OS=Pseudomonas syringae pv. aptata OX=83167 GN=ALO85_03432 PE=4 SV=1
MKAIVYSGPRDVSVKNVPDAKIQKPTDALVRVTSTNICGSDLHMYEGRTSFETGRIFGHE
NLGQVIEVGAGVDRIKVGDWVCLPFNIGCGFCENCEKGLTGYCLTANPGSAGAAYGFADM
GDHEGGQAELLRVPYADFNCLLLPEDAAEREEDYVMLSDIFPTGWHATELAGLLPGESVA
IYGAGPVGLMAAHSAMIKGASQVFVVDNHPDRLALAAKMGATPINSLEQGAVDQILNLTD
GKGTDRGCECVGYQCCDRHGHEANHVTMNNLVASTKATGGIGVVGVFVPQDPGAKSDLAK
EGKMAFDFGSFWFKGQQIRTGQANVKAYNRRLAELIHHDRAKPSQIISHRLKLEEGPDAY
KHFDARDEGWTKVVLKPGA
>tr|A0A0Q0DTE1|A0A0Q0DTE1_PSEAP Putative Fe2+-dicitrate sensor, FecR/PupR family OS=Pseudomonas syringae pv. aptata OX=83167 GN=ALO85_03833 PE=4 SV=1
MNTRPVSARVLDAAIEWQLNMDSNGGNDAELARWLAADEEHARAWSQLSMVNQRFALPAG
PARKALQQSPGALRHSLRKLGGGLAGVFMALGLALFVGDRYLPIDYWLADQRTATGEQRD
LRLADNTLIRLNTHSAIDVRFDAQQRRVILQDGEILVQTGGHDDPRPFIVETADGKMRAL
GTRFLVRRESDGTLLSVLQSAVVAHPRNASGEQVLREGQQVLLSRNGLGPLLGMPSGADA
WTRGMLVVDNTRLADMLAELGRYRTGHLGVDPKVADLRVTGTFPLNDTDLALKALLPTLP
VQIEQHTAWWVSVLPRDQQPGNPPAKL
>tr|A0A0Q0D5Z5|A0A0Q0D5Z5_PSEAP Allergen V5/Tpx-1 related protein OS=Pseudomonas syringae pv. aptata OX=83167 GN=ALO85_02620 PE=4 SV=1
MPVIHYLLRLSLPRVSISFLAVLCSGVFASAALAGDETQLIESLNAYRSQAQRCGEQVSM
ELPPLTSDARLVLPASGNLDLQQALTRASYPMVTVQAISLSGPNNADAALKAVLESFCRV
VLDPQFVDIGVSREGRDWRIVLARSLVAARLGDWQAEGQKILDMINNARTQARQCGTQSF
AATTPLAWNLVLGTAAQRHSQAMANQNFFDHKDRDGRTPGDRAELAGYVGQQVGENIAAG
QDSARKVVDGWLLSPGHCANLMNPDFRELGAAYAMDPKSDAGIYWTAMFGTQQ
>tr|A0A0Q0FMN9|A0A0Q0FMN9_PSEAP Malonate/sodium simporter, MadM subunit OS=Pseudomonas syringae pv. aptata OX=83167 GN=ALO85_02960 PE=4 SV=1
MWDLIEKGLEHNGLITAFAFVGIIMWVSVVLSRRLTFGRVHGSAIAIVIGLILAWVGGTL
TGGQKGLADITLFSGIGLMGGAMLRDFAIVATAFEVQATEARKAGLIGVIALLLGTILPF
IVGASIAWVFGYRDAISMTTIGAGAVTYIVGPVTGAALGATSDVMALSIATGLIKAILVM
VGTPMAARWMGLDNPRSAMVFGGLAGTVSGVTAGLAATDRRLVPYGALTATFHTGLGCLL

GPSVLYFIVRAIVG
>tr|A0A0Q0IBU5|A0A0Q0IBU5_PSEAP Thiazole synthase OS=Pseudomonas syringae pv. aptata OX=83167 GN=thiG PE=3 SV=1
MSNVRSDKPFVLAGRTFESRLLVGTGKYIDMEQTRLAIEASGAEIVTVAVRRTNLGQNPG
EPNLLDVLPPDRYTILPNTAGCFDATEAVRTCRLSRELLDGRNLVKLEVLADQKTLFPNV
IETLKAAEILVKDGFDVMVYTSDDPIIARQLAEIGCIAIMPLAGLIGSGLGICNPYNLQI
ILEETKVPVLVDAGVGTASDATIAMELGCEAVLMNSAIAHAQQPVMMAEAMKHAVIAGRL
AYLAGRMPRKLYASASSPLDGLIK
>tr|A0A0N8TA28|A0A0N8TA28_PSEAP GntR family transcriptional regulator OS=Pseudomonas syringae pv. aptata OX=83167 GN=ALO85_04326 PE=4 SV=1
MRKSDREAFLGSVLGNEQPPAHLARTVIEEKLRNAIIDGSLPSGTALRQQELATLFGVSR
MPVREALRQLEAQSLLRVETHKGAVVAPLITEDAVDAYALRILLESEALRLSIPLLDADD
LATAKGYIEQLEVETDFGQIGRLNRMFHLSLYAKTHNKRLMRLVEEGLNEEERFLRFNLS
DMGLGKLSQDDHWQLLRLAEQKAIEPCVEALQHHLNRGVQAVTQYLTSKKATKAKPTRTA
KKNPA
>tr|A0A0Q0DIG2|A0A0Q0DIG2_PSEAP Uncharacterized protein OS=Pseudomonas syringae pv. aptata OX=83167 GN=ALO85_100447 PE=4 SV=1
MRNNIIMNIKALKEAAKTLRDKLEFYKQRDSDASILLLSLESLLCRAAQGEITEMVEPRD
IPGYKLFTETNLQAYGDLEKAYADFCLELSEVRETDAYEMLAAKMAKRRKSLQ
>tr|A0A0Q0D472|A0A0Q0D472_PSEAP Diguanylate cye/phosphodiesterase OS=Pseudomonas syringae pv. aptata OX=83167 GN=ALO85_100036 PE=4 SV=1
MKYGAEREIVQPFFILSETTMTITEQLSALSSILARGDLHSLFQPIVSLSEHRILGYEAL
TRGPSNSALHSPLNLFAIARQAGRLSELELSCRDSACRRFSQQKLPGKLFLNVSPESLLE
TSHPPGRTLEMLRRYQIAPKDVVIELTEQMPTDDFDLLYNALHHYRDMGFSIALDDLGAG
YSSLRLWSELRPDYVKIDRHFIDGIHQDAVKREFVGSMLQMAKASRATVIAEGIELPEEL
ATLKDMGVNLVQGYLLARPQERPPRDTRAMLPKTEITVVPLNEEAADLSALLNPQPSVSQ
STPTAEVLEAFRRQANLNSLAVLDDDARPCGIVHRHSLSEALLKPFGTELFARKPISRLM
SSDFLAVEVSQSLQQVSRLLTSRARQRIEEDFIITSNGAYLGLGRVIDVLKLITEMKIQQ
ARYANPLTLLPGNVPIQQCLTRLLQQGRASMICYVDIDSFKPFNDIYGYARGDEVLLCLA
QCLNDRIDPARDFVGHIGGDDFLMVLGFEDWERRLKTLLDDFQNQCRRFYRAEHLEAGCF
IALNRQGQRQEFPLLSLSIGVVHLHEESCTLVDASQLADLASQAKHFAKDVAGASIHVID
STRLDLLMQA
>tr|A0A0Q0IB01|A0A0Q0IB01_PSEAP Aminoglycoside phosphotransferase OS=Pseudomonas syringae pv. aptata OX=83167 GN=ALO85_00500 PE=4 SV=1
MALTDQSTEIRNGEELDASLIDAYLKAHVPDLHGTPTISQFPGGASNLTYLLQYPERELV
LRRPPFGHKARSAHDMGREYRILNQLKEAFPYCPEAYLHCTDESLIGSEFYVMQRVKGII
LRSDLPSELALDATQTEQLCKNFIDKMVDLHRVDYQACGLGDLGKPQGYVQRQISGWSER
YEKALTPDAPAWEQVKRWLVDKMPADSPTSSIVHNDYRFDNVILDPANPMQIIGVLDWEL
TTLGDPLMDLGNTLAYWVQADDPAPVQLMRRQPSNAPGMLTRQAFVDYYAERAGVRIDNF
DFYYTYGLFRLAGIVQQIYYRFFHGQTQDKRFAQFIHMNRLLEQMSLSVIEKSSL
>tr|A0A0N8T8G8|A0A0N8T8G8_PSEAP Histidine kinase, HAMP region: chemotaxis sensory transducer OS=Pseudomonas syringae pv. aptata OX=83167 GN=ALO85_01095 PE=4 SV=1
MKWFYNAKLSAKLFMAFALCALFTLGVGVIGSRGISALSVSLDNVFTNSLLSVTKTGEVK
ASVIAQNRDLYRLLAAMAGNAPQSVKDDILASMTVNRTQAEKSYATYRATPLDDDERAAG
DQMDRDWPGYQALIEQAKGVALSGDIAGARAIIDGDVRSGYLKVMDQLSTIVNSNNRQAG
EAAVASDQTQDAAQRNLYIGIGLSFFAAFVMGLFISRAISTPINTAVTNARRIASGDLTL
PIVSQYHDEAGMMLVALSDMQDNLKSTIGQISSAADQLASAAEELNAVTEESSRGLIRQN
DEIQQAATAVNEMTAAVEEVARNAMSTSEASRQTSTDAAAGRDQARDAVNAINTVSAEIS
SSTTMVEELAGRVREIGQVLDVIRGIAEQTNLLALNAAIEAARAGEQGRGFAVVADEVRA
LAARTQASTGEIETMIGSVQASADQAVRAMGNSRTLASNTQTLAQATGQSLERIAQSVAE
INDRNMLIATASEEQSHVAREVDRNLVNIQDLSAQTAAGANQTSASSQELSRLAISFNNL
VGKFKV
>tr|A0A0Q0BXP0|A0A0Q0BXP0_PSEAP Amidase family protein OS=Pseudomonas syringae pv. aptata OX=83167 GN=ALO85_02816 PE=4 SV=1
MNNQFGFCLALSIAPCTFASQAYANFDGPVFGSIIENKAVYFESSVELQRRMSAGSLTSA
GLVTDLLQRIEVLNKNGPALNALIEINPDALQIAAQLDGERSRGEQRGPLHGIPIVIKDN
LDTADRMQTTAGALAMVGAPAPHDAFVVQRLREAGAIIIGKANLSEWAHFRGYEVPSGWS
GRGGQTRHPYDLNADPLGSSSGSAVALAAGFSPLAVGTETNGSIIQPAATSGVVGLRPTL
GRLSRTGMIPLSSRQDTPGPMARTVTDTAILLTAMSGTDPLDEATARACADTVNYVDQLR

ADALNGKRLGYSSHTHDGMLMDDDPEFQKVKSRLSSAGAILVPVDVPSIDSTPEYRVLLH
DFKRELNAYLSTRTVLGVSTLDDIIAFNTASDGAQAYDQDLLIDSSGATLDQENYLSIAT
HLRTAHRQLIDGLLQQHSLDALIDWSEVSFKAVGAIAGYPGITVPVGLEENGLPRGLYFL
STAWDEADLLSYAYALEQALAASAASGHSGISQDN
>tr|A0A0Q0BUF1|A0A0Q0BUF1_PSEAP Glutathione-regulated potassium-efflux system protein KefB OS=Pseudomonas syringae pv. aptata OX=83167 GN=ALO85_00527 PE=3 SV=1
MCSIITKEFYMPHEGSLLQVAVVFLLAAVLTVPLAKRLKLGAVIGYLFAGVIIGPSVLGL
IGDTESVSHISELGVVLLLFIIGLELSPKRLWVMRKSVFGVGMAQVLLTGLIIGGVAFAA
FNQSLNTAVILGLGLALSSTAFGLQSLAERKQLNSPHGRMAFAILLFQDIAAIPLIAMVP
FLAGADHAMDTGESINHGLRVLGSIAIVVIGGRYLLRPVFRIVAKTKIQEVSTATALLVV
IGTAWLMELVGVSMALGAFLAGLLLADSEYRHELEAQIEPFKGLLLGLFFISVGMGANVG
LLLSSPLIVLGLTLLLIAIKLPMLFLIGRTLGELNNRSALQLGLVLAAGGEFAFVVFKIG
SAQGLFEAGLYDMLVLTITLSMAITPLLFIALARWIKPRIKPVEVPAEYRDIQTDKPRVV
IAGMGRMGQIVARILRAQKIPFIALDTAVESIEFSRSFGNVPVFYGDPLRPEILRAAKVD
EAEFFVIATDDPDTNIKTAELIRKLYPHMKIIARARNRQHVHRLMDLGAHAVRETFYSSL
EMSRQTLMGLGLSETQADARISRFKRHDEQVLAAQHEVYDDDAKVLQTAREARADLAQLF
ESDRLDEEAAKS
>tr|A0A0Q0FDG7|A0A0Q0FDG7_PSEAP Probable peptidoglycan glycosyltransferase FtsW OS=Pseudomonas syringae pv. aptata OX=83167 GN=ftsW PE=3 SV=1
MIFGVIKPYPSPLISGRGIDLDFPMLVGCLALLGLGLVMITSASSEVAAVQSGNTLYMMT
RHLVYLLIGLGACGVTMMIPVATWQRLGWMMLLGAFGLLLLVLVPGIGREVNGSMRWIGF
GAFNVQPSEIAKVFVVIFLAGYLIRQQQEVRESWMGFFKPFIVLLPMAGLLLMEPDFGAT
VVMMGAAAAMLFLGGVGLFRFSLMVVLAVASVVVLVQAQPYRMARLTNFTDPWADQFGSG
YQLTQALIAFGRGEWFGVGLGNSVQKQFYLPEAHTDFVFSVLAEELGVVGSLITVALFLF
VSIRGMYIGMWAERAKQFFGAYVAYGLSFLWIGQFLINIGVNVGLLPTKGLTLPFLSYGG
SSLVICCASLGLLLRIEWESRNNMGSEEAEFKESDFAEDTPNGR
>tr|A0A0Q0DSM2|A0A0Q0DSM2_PSEAP NUDIX hydrolase OS=Pseudomonas syringae pv. aptata OX=83167 GN=ALO85_03452 PE=3 SV=1
MKQRATVICKRDGQVLYVRKPKSRWALPGGKIEAGETPFQAAVRELCEETGLADLDLLYL
DVYEKDQVAHYVFTAQVPASSEPSPQNEIAACKWLAPQKLGDLKASSATKTIVKSYATES
EGVPGARSHVTG
>tr|A0A0Q0D3S1|A0A0Q0D3S1_PSEAP Alginate biosynthesis protein AlgK OS=Pseudomonas syringae pv. aptata OX=83167 GN=ALO85_01807 PE=4 SV=1
MNSPLRRLSTPTLLSLAVALGLSGCAGLPDQRLANEALKNGDTALAEQNYRQLADLGYSD
AQVGLADIQVATRDPAQLKQAEATYRAAAQTSPRAQSRLGRLLAAKPDATEAEHREAEGL
LKKAFANGESGTLIPLAMLYLQYPHTFPEVNAQQKISEWRAAGYPEAGLAQVLLYRTQGT
YAQHLDEVESICKQALSATDICYVELATVYQTRGQAEQRTALIEKLKSEYAAGRVNAQRV
DSVARVLGDSTIGTPDEKTAQQLLESVAPGYPVSWVTLAKLLYDFPELGDVNKMMEYLNN
GRAADQPRAELLLGRLYYEGKWVTPDAVKAEEHLKKATATEISAHYYLGQIYRRGYLGQV
YSQKAVDELLTAARGGQNSADFALAQLFSQGKGTMPDPVNAWVFAQLALASQTPQATELA
EALNTQLPPEKLATAKDLLAREQKVRGANATQNAMALQALQEEKDGEEAL
>tr|A0A0Q0BRF6|A0A0Q0BRF6_PSEAP Glycosyl transferase family protein OS=Pseudomonas syringae pv. aptata OX=83167 GN=ALO85_04258 PE=4 SV=1
MMSSESQNPTPSPLVSIIAPCYNAERFLEVAIQSIFAQDYENFEVIIVDDGSTDNSIAML
ESLQQRYPFQLYRQANQGVSAALNHGLRYAKGVYLSTPDLDDIMLPSSVRIRAEYLDNHP
AVGCVGALVSYMDGDGKEIKRQSRDHIERLTFDDILRDAVVVGAPVALYRMKAIKDADGY
DPQIKVQDFQATLKIAHLGYEIHVLPEIVTRYRRHPNNLSRKYKVLLEADLKSIEPYRDH
REYQSGRTQAINKALKYAAVADKRYAWSLLFSIPWRQINRTTLRRCKRLLLSYR
>tr|A0A0Q0C3H8|A0A0Q0C3H8_PSEAP HAD superfamily hydrolase OS=Pseudomonas syringae pv. aptata OX=83167 GN=ALO85_03304 PE=4 SV=1
MIEPDLETAGPDLEPVGPDLEIEVEQRLLAVFDFDGTITRHDSFVPFLKFAFGQRAFSVR
MARLVLPSIGYLAKRLTRDELKGRLIKAFLSGVEVQWLQQKAEAFCQSHWKRLMRPAALE
SIAAEIEKGAIVTLCSASPAMVLQPFADRLKVELIGTNLEVIDGKLTGLIEGSNCRCDSK
VARLESVYGPLTQFRVRAWGDTRGDHELLAAAQDAHWRLFHPTWRRGRYKGPVNAKLHNP
DGKDGPTR
>tr|A0A0Q0CIQ9|A0A0Q0CIQ9_PSEAP DNA-3-methyladenine glycosidase I OS=Pseudomonas syringae pv. aptata OX=83167 GN=ALO85_04886 PE=4 SV=1
MSPVLGCDSKSGSIPDSAARPLVGSPMTRCFWCNEDPLYIAYHDQEWGVPLRDAQKLFEL

LLLEGFQAGLSWITILKRRARYREVLHGFDVERLAQLSDAELEALMLDPSIIRNRLKLKA
VRTNARAWLALEDPVALLWSFVGGTPKINHFKDRSEVPAITAEAEAMSKALKKAGFTFVG
PTICYAYMQASGMVMDHTVDCDRYAILSR
>tr|A0A0Q0FN14|A0A0Q0FN14_PSEAP Histone deacetylase family protein
OS=Pseudomonas syringae pv. aptata OX=83167 GN=ALO85_03480 PE=4 SV=1
MSLPLIYHEDYSPEFPADHRFPMDKFRLLRDYLIDSGLTSDVQLMRPELCPADILALAHD
PSYISRYLSGDLSREDQRRLGLPWSEALARRTIRAVGGSLLTAEQALKHGLACHLAGGTH
HAHYDYPAGFCIFNDLAVISQYLLQSGRVDKVLIFDCDVHQGDGTARILADTEDAITVSL
HCEKNFPARKAQSDWDIPLPMGMGDADYLNVVDDLLNYLLPFYKPDLVLYDAGVDVHKDD
ALGYLQLTDQGLANRDEAVLRHCLGRDIPVMGVIGGGYSKDRQALARRHGILHHSAQRVW
NDMGL
>tr|A0A0Q0FFY5|A0A0Q0FFY5_PSEAP Beta-lactamase-like protein
OS=Pseudomonas syringae pv. aptata OX=83167 GN=ALO85_01048 PE=4 SV=1
MRFAVLGSGSRGNGTLVASNDTYVLVDCGFSLRDTERRLARLGVSASQLSAILVTHEHAD
HVHGVGLLSRRYNVPVYLSSGTLRGMRKPVEIAGLLAGGESLQIGALDIDVVSVAHDALE
PTQFVFSDGKRRFGLLTDLGSYCSNVLQRYQGLDALMIEANHCRDMLARGQYPVFLKQRV
GCETGHLNNHQAASLVSELGWQDLQHLVLAHLSSKNNLPHLARQCFVDTLGCDPDWLQLA
DQDSGLDWRHIA
>tr|A0A0Q0BWC6|A0A0Q0BWC6_PSEAP Uncharacterized protein OS=Pseudomonas
syringae pv. aptata OX=83167 GN=ALO85_101613 PE=4 SV=1
MFSEHRFTGSMCIRSGAPHQASSCVDCSAAPVVPGLGYHARLSSLRGYARVPTAQE
>tr|A0A0Q0BUW1|A0A0Q0BUW1_PSEAP 50S ribosomal protein L21 OS=Pseudomonas
syringae pv. aptata OX=83167 GN=ALO85_02139 PE=4 SV=1
MSDKGRPPLPPFTLETAVQKVRGAEDGWNSRDAAKVALAYTEDTYWRNRSEFVNGRAEAQ
AFLERKWRKELDYRLIKELWAFTDNRIAVRYAYEWHDDSGNWFRSYGNENWEFAANGLMQ
RRFACINDLPIAESERKYHWPLGRRPDDHPGLSELGL
>tr|A0A0Q0C9Y9|A0A0Q0C9Y9_PSEAP Uncharacterized protein OS=Pseudomonas
syringae pv. aptata OX=83167 GN=ALO85_01471 PE=4 SV=1
MKKLSSRQPLTPRDELDSALTDRVFVKGLRAFEAQALLARRGFQVVMATKGFGVGTLLEN
NLPRR
>tr|A0A0Q0BTQ2|A0A0Q0BTQ2_PSEAP Inorganic pyrophosphatase OS=Pseudomonas
syringae pv. aptata OX=83167 GN=ppa PE=3 SV=1
MSYSKIPAGKDLPNDIYVAIEIPANHAPIKYEIDKDSDCLFVDRFMATPMFYPANYGFIP
NTLADDGDPLDVLVVTPYPVTPGSVIRARPVGILHMTDDGGGDAKVIAVPHDKLSQLYVD
VKEYTDLPPLLLEQIKHFFENYKDLEKGKWVKIEGWGNADAARAEITKSVAAYKG
>tr|A0A0Q0DBX0|A0A0Q0DBX0_PSEAP Putative pterin-4-alpha-carbinolamine
dehydratase OS=Pseudomonas syringae pv. aptata OX=83167 GN=ALO85_01833
PE=3 SV=1
MTTLNQAHCEACRADAPQVSEAELPELLKQIPDWNIEVRDAVMQLEKVFLFKNFKWALAF
TNAVGEIAEAEGHHPGLLTEWGKVTVTWWSHSIKGLHRNDFIMAARTDEVAKGAEGRK
>tr|A0A0Q0DS61|A0A0Q0DS61_PSEAP Uncharacterized protein OS=Pseudomonas
syringae pv. aptata OX=83167 GN=ALO85_200155 PE=4 SV=1
MQTDRHYPKNPPTVGTVLLTSYDSFAHENEIPKSRAADALRMGKELADGFDDEAHHLGAL
MLMISDVPADPLLKASAAQKGSVLGLASLGYLLSYGSTGKKAKRIIESGGGVFLIRLSGD
IENPKADIKVFSSWSEYQKFLGPILKTGDFYPGETSSFS
>tr|A0A0Q0C1C2|A0A0Q0C1C2_PSEAP Uncharacterized protein OS=Pseudomonas
syringae pv. aptata OX=83167 GN=ALO85_04362 PE=4 SV=1
MTVLIRNVAKPLLLTLSLLGLSAQALAETRVDDAWVRATVPGQSATGAFMRITADTDSKL
VDVASPVAKTVQIHEMSMKGDVMSMQRVNSVDLPAGKPVMLDSDGYHVMFMGLSAQVKEG
DQVPLTLTVEDAKGTKQTIQVTAVAKSLTSDEHSSHGDHSGH
>tr|A0A0N8TA76|A0A0N8TA76_PSEAP Uncharacterized protein OS=Pseudomonas
syringae pv. aptata OX=83167 GN=ALO85_03852 PE=4 SV=1
MLEASLSQLEQLVGDLVQQNQALQETNAQLGAELAKAKDENENLQLSLMEQEEKHGSTAA
RIQALVDRATSASAVGA
>tr|A0A0Q0DI59|A0A0Q0DI59_PSEAP GCN5-related N-acetyltransferase
OS=Pseudomonas syringae pv. aptata OX=83167 GN=ALO85_05296 PE=4 SV=1
MSPTTHPPHRNGGAMPATSDAHYQYRPVTAADIPAAHALSVSLKWPHREQDWAMVQRTSE
GFVAEHDGQLVGVAFTCHQGDWSSIGLVIVRDDHQGKGIGRRLMKLCLDATAPRTPILNA
TELGAPLYQSLGFVDFARIQQHQGVADLSGMAPSKDDLPIRTLGATDHAELIRLANAGSG
LDRTAVLTDLLHDAEQAVGIEHAGRLQGIALLRRFGRGHIIGPVVARDVDQAQRLIEHLL

QQIPGLFVRFDILADCGLAPWLESLGLPCVDRAPRMVLGTPPASSPDVQQFALVTQAIG
>tr|A0A0Q0CD99|A0A0Q0CD99_PSEAP Aldo/keto reductase family oxidoreductase OS=Pseudomonas syringae pv. aptata OX=83167 GN=ALO85_02432 PE=4 SV=1
MRTLELAGRTVPVLGQGTWHMGEKGHQRSAEIAGLCLGIEQGLTLIDTAEMYADGAAEEI
VGQAIAGQRDKVFVVSKVYPHNASRAGIPTACEASLRRMNTDYIDLYLLHWPGQYPLTET
VEAFERLREAGKIGAWGVSNFDVPDMIELNNPRCATNQVLYNPEQRGIEFDLMLWSADRH
LPLMAYCPIGQAGDLLYHPALHEIAKRHDATPAQISLAWVLRQDNMIAIPKATNPEHIRL
NIAAEQIRLTERDLADIDLAWPAPTRKVPLAMV
>tr|A0A0Q0D7B9|A0A0Q0D7B9_PSEAP 50S ribosomal protein L4 OS=Pseudomonas syringae pv. aptata OX=83167 GN=rplD PE=3 SV=1
MQLNVNDAQAIEVSELTFGGEFNETLVHQAVVAYMAGGRQGSKQQKTRSDVRGGGKRPWR
QKGTGRARAGTIRSPIWRGGGTTFAARPQDHTQKLNKKMYRAALRSILAELVRTDRLVVV
QDFAVEAPKTKDLLNKLTGMGLTDVLIVSDAVDQNLYLAARNLPHVDVRDVQGSDPVSLI
AYDKVLITVSAVKKFEELLG
>tr|A0A0Q0DP70|A0A0Q0DP70_PSEAP Luciferase OS=Pseudomonas syringae pv. aptata OX=83167 GN=ALO85_05421 PE=4 SV=1
MQLPTWEVTMRAEKSYPIKTNPMRSSPNKIQLGVFSTNTEGGCTVTNAPERLRGDDWAGN
LEIARIADDAGFEAFIPVGRWKGFGGTNNTGGVCFETYTWAAGIAALTSQIAVFTTSHLP
TVHPLFAAKQAVTIDHISGGRFGLNILCGWYGAEMRMFSGHMMEHDKRYDYAEEWLHIAK
NAWEQQAPFDFKGEYFNICGAISQPQPLNKPYLINAGGSPRGKLFCAEHCDAAFLIVKHL
DGEQAVREQIASYKDLAKQKFGRDIKIWCYGYVVQKDTQEEADAYLDYYANQVGDDEGCD
IITGELGIQTGIFTPEDAVRFRFHFKAGFAGVPLVGTPERIVELFRKYSDWGIDGIALTW
LDYHEGIKDFVRETLPLMEDAGLRESRGVEAITSKVSAEVA
>tr|A0A0Q0BYQ5|A0A0Q0BYQ5_PSEAP Uncharacterized protein OS=Pseudomonas syringae pv. aptata OX=83167 GN=ALO85_03245 PE=4 SV=1
MKKTGTGLFAMLALLCAFTVQARETQVTDASIVKAIIQESIDGYSGICPCPYNSARNGSR
CGKRSAYSREGGEEPICYKEDITKEMINDYRRRLKD
>tr|A0A0Q0D4C1|A0A0Q0D4C1_PSEAP Uncharacterized protein OS=Pseudomonas syringae pv. aptata OX=83167 GN=ALO85_04015 PE=4 SV=1
MTRTSIYDRISERRLADEALAREAAEHAAAQQALERVEALIPVPKPLLDAPCSFNFGGFH
FGFPEGFQCNAIEATVQVGGEPVVVSIQRRDVPEEATLAEAFAEAVKALQPLHGEVQVIR
HYETLLAGNAALALDFHFRAGLEERHGRLIGAIVPCADHNERQWLSVGCVIDSEKPALKT
WLLDFDSMLEGLTAP
>tr|A0A0Q0C8G9|A0A0Q0C8G9_PSEAP Response regulator OS=Pseudomonas syringae pv. aptata OX=83167 GN=ALO85_02170 PE=4 SV=1
MQSAPKKLCILIADDSTSDRLLLSTIVARQGHRVLSAGNGVEAVAIFKAESPQLILMDAM
MPVMDGFEAARRIKAMAGESLVPIIFLTSLTEGEALARCLDAGGDDFMSKPYNPLVLAAK
INAMNRLRVLHETVRQQRDQIARHHEYLLNEQRVAKAVFDQIAHAGCLGASNIRYLQSPY
ALFNGDLMLAAYTPAGHMQVLLGDFTGHGLPAAVGAMPLAEVFYGMTAKGYGLVEILREM
NAKLKRILPVDMFCCATLLCINLPQRQVEVWSGGLPDGYLLRGVQGEHVPLASNHLPLGV
LSVDAFDARTDVYPLAPGDRIFLLSDGVLDTSNSQEELFGARRLREVLSANREPQLLIEE
ILEALNELGGKARDDVSLLEVSAVEAPLKSLPAMMYSDSGASSPLDWSASFEFRASTLKR
FNPLPYVLQLLLEVHGLRDQGAALHMVLGELYSNALEHGVLALDSSLKHDAAGFALYYQQ
RAERLDRLQSGFIRVTLQVETVAQGGRLTLSVEDSGQGFDVEKTRTPTSAGNELYGRGLH
LVCELSREARWSRDGRTACVEFSWKGVA
>tr|A0A0Q0ICC2|A0A0Q0ICC2_PSEAP Isochorismatase family protein OS=Pseudomonas syringae pv. aptata OX=83167 GN=ALO85_04770 PE=4 SV=1
MSDLKTMFQLSGRVHAPAHLSNATLIIIDAQKEYLRGPLALTGVREAMANIGRLVEKARA
VKCPIVHIRHLGTVGGPFDPHGERGELVPELLPQGDEHLVEKRLPNAFNGTGLHELLQSL
GHLDLIVCGFMSHSSISTTVRATKDYGYRCTLVDDACATRDLPSPHGVVSAEVVHRTEMA
IMADNFATLALTKDLV
>tr|A0A0Q0CEE1|A0A0Q0CEE1_PSEAP Uncharacterized protein OS=Pseudomonas syringae pv. aptata OX=83167 GN=ALO85_02371 PE=4 SV=1
MSTRRMFILRRPFSSLLLLIVAALAVLAWQYRVNLQAFPTIISAYTAKEYCSCRYVMNNS
EAYCQGYIKQYLPSTLVDDSSQKRVIASGLGRTSSASWQGERQGCRLLP
>tr|A0A0Q0BY62|A0A0Q0BY62_PSEAP Thioredoxin domain-containing protein OS=Pseudomonas syringae pv. aptata OX=83167 GN=ALO85_03518 PE=4 SV=1
MLSINAGPFALAISHLLLLGALITATWVEARLVRHTLTRKTAVFGLFILGIATARIAFVL
TYFAQYSDAPWKALDIRDGGFVVWPGVICALLGGLIIGWRRPGQRRALTFAMSIGLAIWV
SGGLILDHYQKGNSLPDTTLRTLTGEPVRASDYRGKPVVINLWATWCPPCRREMPVLRDM

QREREDITFLFVNQAESAGEVSDYLSKQQLVLSNSFLDPDGEFARQVGSAALPTTLFYSP
DGRLIDSHLGELSRASLTHYLDK
>tr|A0A0Q0C3P0|A0A0Q0C3P0_PSEAP Uroporphyrinogen-III C-methyltransferase OS=Pseudomonas syringae pv. aptata OX=83167 GN=ALO85_01577 PE=4 SV=1
MIDTCRLRYYAKMQRMGYVSETALPKDEESVLKTPASKPEPVRTERTGSRNSGLVFLALL
LAIAGIAVAGWGVWQLRMLQAGHQQQRGQVEDIAEQTLVLAQNDQQLSARIAQFPPANQL
EDSRRLVAQLQGDQQRLSQRLETVLGASRKDWRLAEAEHLLRLASLRLSALQDINSAQAL
VQGADEILREQDDPGSFAAREQLAKSLAALRSVEQPDRTGLFLQLAALREQAVQLNKLAP
EYEDKGESLMGLTADKTETSRWTQWWDEISHYFRIDFNADKDIRPVLAGQSLSQVRLALS
LALEQAQWAALNGEPEVYSKAITEAQSVLKANFDQDDPQGKALGQGLDAVASQPVSVKTP
DLAPTLSAVQAYLERRHAAGQPAEAQQGTSR
>tr|A0A0Q0IB22|A0A0Q0IB22_PSEAP Deoxyguanosinetriphosphate triphosphohydrolase-like protein OS=Pseudomonas syringae pv. aptata OX=83167 GN=ALO85_00549 PE=4 SV=1
MNALDWQTLLNRERLGKTLHSPEELGRSPFHKDHDRIIFSGAFRRLGRKTQVHPVSSNDH
IHTRLTHSLEVSCVGRSLGMRVGETLRSALPEWCDPSDLGMVVQSACLAHDIGNPPFGHS
GEDAIRNWFNQAAGRGWLDAMSDTERNDFLNFEGNAQGFRVLTQLEYHQFDGGTRLTYAT
LGTYLKYPWTARHADSLGYKKHKFGCYQSELPILEQIAGKLGLPQLEDQRWARHPLVYLM
EAADDICYALIDLEDGLEMDLLNYAEVESLLLGLVGDDLPETYRQLGPGDSRRRKLAILR
GKAIEHLTNAAARAFVEQQDALLAGTLPGDLVEHMHGPAKRCVLNAKDMARKKIFQDKRK
TLHEIGAYTTLEILLNAFCGAAVEQFGGRTPSFKHRRILDLLGNSAPDPKAPLHASFLRM
IDFIAGMTDSYASEMAREMTGRSGQI
>tr|A0A0Q0DUJ7|A0A0Q0DUJ7_PSEAP DNA helicase OS=Pseudomonas syringae pv. aptata OX=83167 GN=ALO85_00976 PE=3 SV=1
MPDDLPSALPHDLDVDLVPPRQLPFFRRLLARLMGRGLTGLRSQHAPSWSQGHADGYLNG
HIEGVREGYEEGYLDGQESGRQVLVINDTRPARHRGPKVDDHLFDDWRLALTPELKKRIK
ADVVEKLPAHAQPSAAQWKMIFSDTPSTYVIAGAGAGKSTSLVLRILLLHHYLGFELNAM
TVVTFTRESRKDFISKLIDVMALWGHVLEQKQARELVRTFHSRILPLVRSLPGFEQLRAF
ENLSSQSSGLEEVDSNPFDLRINDAQRQQLNLCYRDLYAGNERFREVMAPLYRHALQLKE
LDRDHPDVQKRVAVTELSSKRDEEWCDTVEDLWIRAGAWPIKGIEPMRQAVEINGFSFSF
HGYIAELDAWVVLGLDASEDQQLKRPGAKLPVWAEGVIKRTLFQAFCSKPLIWMDNYQSA
SEVLQSLAGEAAAGPGFDYRVKGELGAAPLLDCFVTAASFIENLGLDVPSAVSEMSFASD
DPDRFFFEALSLFWKAFETHLLAQSPPIMTYNRMFALFGETSPENLKHVSDPMLRPLAHL
MIDEFQDVSPQIVSWLRASLREIRRRGPALHTGRIAQHSSLLCVGDDWQSIYGWRGSSPK
YFMEFAKEFSSPATTRVMLSDNFRSHQHIIDAAEHIVRAAPAIPGKKARASGLLQELLPV
KVLDRDEDELAERVAEHYHDGDSILLLYRKSSEKARFSARLQALIDADSANGAQNRRIRT
LTYHSSKGLQADAVFLLGDCQHLTVSPYKNQVYRLAGLGDTNDLQPFDTAQKDEVLRLAY
VAITRSARHCYWYIDESATSGDAVQMPKASDRIARDKPFFEDLRNKARVG
>tr|A0A0N8T8U2|A0A0N8T8U2_PSEAP LuxR family transcriptional regulator OS=Pseudomonas syringae pv. aptata OX=83167 GN=ALO85_05074 PE=4 SV=1
MLRDNGKPPGTTPPSPAPPATVQLTVKERQVLEWVAIGKSAWEISRIQGCSEATVHFHTS
NIRRKFGVTSLKAALVMAIRQGVILVE
>tr|A0A0Q0BZR0|A0A0Q0BZR0_PSEAP Iron compound ABC transporter, periplasmic iron compound-binding protein OS=Pseudomonas syringae pv. aptata OX=83167 GN=ALO85_04619 PE=4 SV=1
MPLDRDPGRPEPGDDVSVRRPADPIQPTNRRGVREGHPHASAFPAVPGYLPGMCFLMCIA
PWHLLQRRPHSMSLPRLAALAASLTLSTLALAAPTQSPLTLENCGATLTFTHAPTKAVTI
GQAGTEMLYELGLGDRLVGTSLWFNNVLPRFKAQNDKVERLADNEPSFEAVIGKRPELVA
VQLEWMVGKQGVVGTREQFHDLKIPTYILPSDCEGKDNLVGADGTRLEPFRIETIYKSIE
QLAQIFDVQDRGDALVTDLKARLSAAIANVRQNKANDVSALFWFSSSDLASDPFVAGRKG
IPDFMLNSLGMRNIVQSDEEWPTVGWETIAKANPSILVIARMDRRRFPADDYLKKIEFLK
TDPVTRNMDAVKHDRIVIIDAEGMQAGLRLFTGFEELADAANRFNAAK
>tr|A0A0Q0DYG2|A0A0Q0DYG2_PSEAP Putative syringolin exporter SylE OS=Pseudomonas syringae pv. aptata OX=83167 GN=ALO85_00599 PE=4 SV=1
MPGGYSKGDTRPPMRFILLILGLDVLGIGLAIPVMPTLIATIWPSSAEHVSLALGVALTL
YSAMQFLCAPLLGALSDCHGRRPILLLALAGMCLGNLMAGFAGSLTVLLIGRAIAGITAA
NIATAMAYIADISEGEQRTHFYGAAGSVIAIALVFGPVIGGGLASYGPHLPFLVAGGLAA
INLLYGYMRLPESLAAEHRRAFEWRRTNPFGSLRGLWSTQGLRPYLLAATCSWFAYGIFQ
SCFVLANQMRYGWSMLEVSYALAALALGMAFAQRVLVRKLTPIMSNQRIIVTGYACCLLG
YGFYTAAVSVWLTVVGMCFHAVGLIAEPALRSELSRHARAGHQGELQGGLTSLLSLVGGL

APVIGALIFAGNLGSGQHVLWLGAPFVVSLLMYVLAIGCIQRGRTSAACNG
>tr|A0A0Q0IEF7|A0A0Q0IEF7_PSEAP Putative exonuclease of the beta-lactamase fold involved in RNA processing OS=Pseudomonas syringae pv. aptata OX=83167 GN=ALO85_03523 PE=4 SV=1
MSYPQIIHHGAVDTVTGSCHQLHIDVSSSLLIDCGSVQERGRQSESASFGFDPEAIRALL
ISHVHNDHVGRIPELLASGYKGPILCSEPSAHLLPLVMEDLLKIEFAHDPGQVARYLDVI
NKRIIALPFDDWFSLIDNESVHCRVRLQRAGHILGSAYIECDLQYPAQGRSVRVVFSGDL
GASHTPFLPAPKPPERADVLVLESTYGNRVHEDRNIRQLGLERIIDKALEDNGTVLIPAF
SIGRTQELLYELEDILRRKMPSNHCTESSEHRGESAPDWSRLPIILDSPLASRFTKVYQS
FEDYWDEDARLRLDVGRKPLGFEQLVTVDTHEEHLRTVNYLASTARPAIVIAGNGMCAGG
RIVNYLKAMLGDTRHNVVFVGYQGKGTPGEAIQLHGPSGGYVELDRERFDINAGVHTAKG
YSAHADQVELVEFVTGMKEWPTQIRLVHGEAAAKKVLGNILSRKYSLEKRPLELFIP
>tr|A0A0N8TAB9|A0A0N8TAB9_PSEAP Alginate biosynthesis sensory transduction protein kinase AlgZ/FimS OS=Pseudomonas syringae pv. aptata OX=83167 GN=ALO85_01581 PE=4 SV=1
MRIEPVKHDARPAPGKDFFLPELCLPQALLVLVVLAELLVLVLVLVEPMRSGFDWVRLAL
MSLFVQWIVLLSAALLCGLRPWLARLTPGLAGMLSCLLVVGLTLLCTAVTDVCQLTGRIS
VSGMVERYLRYSTIALIMSALMLRYFYLQSQWRKQQQGELRARIESLQARIRPHFLFNTL
NSIASLVASNPVKAEQAVLDLSDLFRASLAKPGSLVTWGEELALAKRYLSIEQYRLGERL
QLDWRVSAIPDDLPIPQLTLQPLLENALIYGIAPRVEGGVVTVEANYEGGEFILSVSNPY
EEVANRQTSNGTQQALTNIGARIAALFGPHASLSVERRDGRHYTCLRYPCARLTQEARAI
>tr|A0A0Q0FPC1|A0A0Q0FPC1_PSEAP OmpA/MotB OS=Pseudomonas syringae pv. aptata OX=83167 GN=ALO85_00946 PE=4 SV=1
MVTPMLRALLTAFILSSATLPAFAQAALPLPAGDIPALRIQGSNTINAQLGPALVEGLMR
QQGLQSVQILPGNTLNEQRVVGTTATGQPVSVEIAAHGSGTGFTALKAGNADIAASSRPI
KDQEAKDLTSLGDLKSAASEQTIAIDGVAVIVHPGNPLRQLDTLQLARIFSGEVRNWSEV
GGNPGTIHLYVRDEKSGTYETFKDLVLTKYGKTLLGSAARFESSEQLSDEVSKDINGIGF
TGLPSIRRAKAVAIADGDSRPMLPTISLIATEDYPLSRRLYFYVPTSTPQRWAQALVRFA
QSAEGQAIVAQSGFVAQTVQVLKVQPTAQMPADYQALARKAERLSVNFRFAQGSARLDNK
AQQDLKRVADYLKTSGKLDQHVTLVGFGDAKSDPERAALLSRLRAMAVRRELLKSGVTFR
DIVGLGDEMPVATNEIDDGRIKNRRVEVWVQ
>tr|A0A0Q0BRB0|A0A0Q0BRB0_PSEAP ClpB protein OS=Pseudomonas syringae pv. aptata OX=83167 GN=ALO85_05084 PE=3 SV=1
MNLKSLFAKLNETSRTATESAAALCLSENHYDVEVEHLLLQLLDNNDNDLPVILRHYDVV
PDRLQAQLVTALGTFKKGNTRTPALSPHITRMIEQAWVLASIEFGQSQIRTGHLVQALLD
DDALRRVVIGSAPELEKINADDLRLNMSALVEGSPETKLSKPLANADASTGATTKGNGKT
PALDQYTVNLTQSARDGRIDPVLGREFEVRQMVDILTRRRQNNPILTGEAGVGKTAVVEG
LALRIVQGDVPSVLKGVAIHTLDLGLLQAGAGVKGEFENRLKSVIEETKRSLHPIILFID
EAHTLIGSGGQAGQNDAANLLKPALARGELRTIAATTWAEYKKYFEKDAALARRFQVVKV
EEPDEDKAIHMLRGLLAKMQEHHKVTVMDEALVQAVRLSNRYITGRQLPDKAVSVLDTAC
ARVALGQSAQPGALEDCKRHIDNLNAEIAVLEQETAKGSDHARRLTALHEELKAEQETRE
SLEQQWKRELELVEKLGALSVPQSGEPDAGQIAAVRAELASVQGEQPLVHALVDGGTIGE
VISGWTGIPLGKMLRDEIDTVQSLPALLGERVLGQDHALEEIGKRIKISRARMEDPNKPI
GVFLLLGPSGVGKTETALALADTLYGGERNVITINMSEYQEAHTVSSLKGSPPGYVGYGE
GGVLTEAVRRKPYSVVLLDEVEKAHPDVLELFFQVFDKGVLDDGEGREINFRNTVIILTS
NTGTDRIMQWCLNTEQKPTPDDIVEGLRDELNQVFKPAFLGRLTIVPYYPVQDAILERIV
GLKLERIRKRFERNHQAVLRYDEALIKAIASRCTEVDSGARNIDNILSKTLMPELAQRVL
ERMAQDKPIQSLTIDLGSDGDFAYTLT
>tr|A0A0Q0D9Z5|A0A0Q0D9Z5_PSEAP Phosphoribosyl-AMP cyclohydrolase OS=Pseudomonas syringae pv. aptata OX=83167 GN=hisI PE=3 SV=1
MKDWLDEIHWNSDGLVPAIAQDHKTGRVLMMAWMNREALSLTASENRAIYWSRSRGKLWR
KGEESGHVQKLHELRLDCDADVIILMVEQIGGIACHTGRESCFYRVYENSGWKTVDPVLK
DPDAIYPAGH
>tr|A0A0Q0FUL8|A0A0Q0FUL8_PSEAP DNA-binding response regulator TctD OS=Pseudomonas syringae pv. aptata OX=83167 GN=ALO85_01990 PE=4 SV=1
MASRESFLPFTDRGRPVNSTATSLDTPINNNSDNQETAMRVLLVEDHLQLAESVAQALKS
AGLTVDVLHDGVAADLALNSEEYAVAILDVGLPRMDGFEVLARLRARSKNLPVLMLTARS
DVKDRVHGLNLGADDYLAKPFELSELEARVKALLRRSVLGGERQQRCGVLAYDLDTRRFT
LGEELLTLTSREQAVLEALIARPGRVMSKEQLAAQVFGLDEEASPDAIEIYVHRLRKKLD
GHSVAIVTFRGLGYLLESRDD

>tr|A0A0Q0CGY5|A0A0Q0CGY5_PSEAP Sigma E regulatory protein, MucB/RseB OS=Pseudomonas syringae pv. aptata OX=83167 GN=ALO85_01997 PE=4 SV=1
MRAVPLLPLLLGGWLAFPVQADDAQDAINRLAKVDQQQSYQGTFVYERNGSFSTHRIWHR
IADGHIQERLLQLDGSAQEVLRVDGLTQCVSGTLEAGVANPADSSARAFDVKRLSAWYDM
KIAGKSRVAGRQATIVALSPKDQHRYGLELHLDNETGLVLKSLLLSEKGQLLERFQFTDL
DTASVLSEQMLKTSADCKPVSVVKTKPEASISPVAWHSDWLPPGFDVSSSGVRKDPATQS
PVTHLMYSDGLARFSVFIETVKGGASSDIRTQLGPTVAVSRRLTTPQGDMMVTVVGEVPM
GTAERIALSMRNDESSANK
>tr|A0A0Q0DMJ3|A0A0Q0DMJ3_PSEAP BolA-like protein OS=Pseudomonas syringae pv. aptata OX=83167 GN=ALO85_03540 PE=3 SV=1
MTMQQRIESALALLQPHHLQVLDESHMHSRGQQTHYKAVLVSDQFQGLNSVKRHQKVYAT
LGPLMGEFHALALHTYTPEEWSKIGAAPASPTCAGGH
>tr|A0A0Q0FGY6|A0A0Q0FGY6_PSEAP Putative 3-methyladenine DNA glycosylase OS=Pseudomonas syringae pv. aptata OX=83167 GN=ALO85_01346 PE=3 SV=1
MPTALPDHFFHRDAQVLARALLGKVIRHKVGDLWLAARIIETEAYYCAEKGSHASLGYTE
KRKALFLDGGHIYMYYARGGDSLNFSAEGPGNAVLIKSAFPWTDATSDENALAQMQLNNP
DASGAMRPAQRLCAGQTLLCKALGLKVPVWDAERFDPEKLLVEDVGQTPERIIQTTRLGI
PAGRDEHLMYRFVDAGYARFCTRNPLRRGQVEGRDYLFLDQGN
>tr|A0A0Q0BXE7|A0A0Q0BXE7_PSEAP Cobalt-precorrin-5B C(1)-methyltransferase OS=Pseudomonas syringae pv. aptata OX=83167 GN=cbiD PE=3 SV=1
MREETAEQPAPLRSGLTTGSCATATSLAAARLLLCGQVSDAVGIVLPKGKQVQMRLEFCR
LVDNFAEAATLKDAGDDPDVTHGALVFARVRLQTAAGVRFVAGAGVGTVTRPGLVLAVGE
PAINPVPRRMMTEHLLQLAEEQGYSGGFEVTIGVEGGEALALKTMNPRLGILGGLSILGT
SGIVRPFSCAAYIASIHQGIDVATTNGYRHIAACTGNASEDTMRRVYKIPDIALIEMGDF
VGAVLKHLRKVPVDKLSVCGGFGKISKLAAGHMDLHSRHSSIDLPQLAQWAKDVGADAGL
QQQILAANTSQQALAISAAAGVPLGDEVCRHALNFARSIVPASVQVEVFAIDRQGGLVGQ
AGVHSQREAT
>tr|A0A0Q0C1N0|A0A0Q0C1N0_PSEAP Sigma-54 dependent transcriptional regulator OS=Pseudomonas syringae pv. aptata OX=83167 GN=ALO85_03635 PE=4 SV=1
MHGSAPTRRLLMLDGGDDCHPLIPVLIEAGWTVQESPSGSAIISDCDVGLIHLTSDHFEH
LPRLEALFVNSPVRWIAVLSASDLRREKIGDFVCRWFFDFHTLPFDAGRLHASLDRAFTA
GPPPVQGSPSPFSADPELLGDSRPVRELRRLLSKLAPTDSPVLIRGERGTGKELIARSLH
AQSLRRDKPFIVVDCAAPDGQSIHTVLFGHEDGGFDDAQARRVGLLEAADGGTLLLDEVG
ELPLDTQASLLRFLEDRQIERADGGEPIAVDVRVLAATREDLETAVRKKRFREDLYYQLN
VLQVGVAPLRERHGDLALLANHFAHVYSQETGRRARSFSQDALVALGKHDWPGNVRELAG
RVRRGLVLAEGRQIEAANLGLLGEEDSVGNMGTLEDYKSRAERQALCDVLTRHSDNLSVA
ARVLGISRPTFYRLLHKHQIR
>tr|A0A0Q0D609|A0A0Q0D609_PSEAP Uncharacterized protein OS=Pseudomonas syringae pv. aptata OX=83167 GN=ALO85_101674 PE=4 SV=1
MEFAGTDSLSVGQSLIKQPAGQVFRPHLKGFTVFAVQKSFNCVAEPASRP
>tr|A0A0Q0C2M9|A0A0Q0C2M9_PSEAP Uncharacterized protein OS=Pseudomonas syringae pv. aptata OX=83167 GN=ALO85_04317 PE=4 SV=1
MGKQSRSKRASPEPMVIYRSHVAAFRSRYLLSFLTPIFMGIFSLAITAGTMVDAYFPNEP
ERAGQSMMLMMGVSLVLSFGGIMMSRGHACGMWILAAILVSCFFAVLPTAPAHWRRFELM
LYVLGLLFPLLGLLSLNSERGRELRMQWRIIRSERKEIRQEMRRRKDLEVHRENLRKRKA
LRK
>tr|A0A0Q0DBS5|A0A0Q0DBS5_PSEAP Chemotaxis protein methyltransferase OS=Pseudomonas syringae pv. aptata OX=83167 GN=ALO85_03282 PE=4 SV=1
MPDTASLTDREFGQFQNWLYNAAGIKLTLAKKALVAGRLFKRLKHYELDNYGEYFKLIMN
DQRNGELQVALDLLTTNETYFFREPKHFDFLRQQVLTKATPGKMFRIWSAASSSGEEPYS
LAMTLAEQLGTTPWEVVGSDISTRVLSKARSGHYPLERTETLPQPLLFKYCLKGIGRQEG
TFLIDKSLRSRVSFVQVNLNDTLPDLGEFDVIFLRNVMIYFDQETKSKVVARLIPRLKPG
GYFIISHSESLNGINDMLKMVSPSIYRKP
>tr|A0A0Q0DP38|A0A0Q0DP38_PSEAP Putative copper-binding protein OS=Pseudomonas syringae pv. aptata OX=83167 GN=ALO85_01946 PE=4 SV=1
MLKKALLLAALLLPACVVHAHEYKAGSLLIGHPWSMELPPNAPTVAAYFTLENKGDSADR
LISVDTPIAGQAQLHEHVHADGLMKMQHVQAVDIPAGAKVAFAPMAWHVMLLDIKDRSKL
VVGQRFPMTLHFEKAGDVEVQVVVQKQAPEHQH

>tr|A0A0Q0C1G6|A0A0Q0C1G6_PSEAP Uncharacterized protein OS=Pseudomonas syringae pv. aptata OX=83167 GN=ALO85_03647 PE=4 SV=1
MSADEKRIRDFAYQIWESEGKPSGHDERHWEMARKLAEAEALAPNKSSVRKSTKPKLPEV
DSAKAPAKGAAKPAAKSAPAPKPAGEAKPKPAPKAAAKPAPKAKPPTAAATDAPAKPAPK
KPRKPSNS
>tr|A0A0Q0BUS2|A0A0Q0BUS2_PSEAP Transcriptional regulator MraZ OS=Pseudomonas syringae pv. aptata OX=83167 GN=mraZ PE=3 SV=1
MFRGANAINLDAKGRLAMPSRYRDELDSRSAGQLIVTIDAVDPCLCLYPLSEWELIEAKL
RDLATFREENRRLQRLLIGNAVDLELDGSGRFLVPPRLREYARLDKRVMLVGQLNKFQLW
DEDAWNALADADLAAIQKPGAMPDELRDLIL
>tr|A0A0N8T802|A0A0N8T802_PSEAP Protocatechuate 3,4-dioxygenase, alpha subunit OS=Pseudomonas syringae pv. aptata OX=83167 GN=ALO85_00541 PE=4 SV=1
MPVQLLQETPSQTAGPYVHIGLAPQVAGNPTRELEIWNEMARPEAPGEHIVLLGNVFDGN
GHLIRDAYLEFWQADHQGAYHSEFDPDRPFNGFGRTATTDDGQWILKTIKPGSAHNAAGV
PMAAHINVSLFARGINIHLQTRLYFDDEAQANAQDPVLNLIEQPPRRETLVARRCTHEGQ
TAYRFDIRVQGEGETVFFDF
>tr|A0A0Q0ICR3|A0A0Q0ICR3_PSEAP Uncharacterized protein OS=Pseudomonas syringae pv. aptata OX=83167 GN=ALO85_03108 PE=4 SV=1
MNVVSGLDEAAVEGLVRSQATFEVIGLRGNFKAGIKMVETKIESLGMKCRVKSDTKSALA
QGGAFGAVLGILSAPVTIAAGVIGTAACVGHALSTYDPDYEILKDYINKKLIVRYKK
>tr|A0A0Q0DL66|A0A0Q0DL66_PSEAP Uncharacterized protein OS=Pseudomonas syringae pv. aptata OX=83167 GN=ALO85_02665 PE=4 SV=1
MLSHNDWQHKHDQFLSLSQALLHKSEECLSHLELIPNDEDATGCLLTTLRKLAQEAEAVP
VPCIADFSRQLCQLLKTGGQANELSQEALLAVKNCLTLMYWQVELLDPHTGELTMDDNEQ
LELLEKLASVSAQSASPKDVTRQ
>tr|A0A0N8T8E3|A0A0N8T8E3_PSEAP Uncharacterized protein OS=Pseudomonas syringae pv. aptata OX=83167 GN=ALO85_01386 PE=4 SV=1
MTQEKTMNSTDNLDYIAACELAECPSSFGAYAAAANPGLLKARDVTDAKPGNEAAVVAGS
LLAFAAGMSAQSKQDVINSFLFATLVANKAFSAQTQGDQWYDKFNEVLSKLGWLSTQWNY
ARYRATHQRFTMDELGLEILGSAIAAAALPGPASLLMLKVAGDAIAALRAKKEPLRLFES
QTKAHRGGSFRIASCIESEDSIVSLAMAAVSFQADSDVTNVLFWEWQDTSVQTWKGEDSL
LLNTALYARHRDMIQQRLDDNARRAIADFEI
>tr|A0A0Q0IJL9|A0A0Q0IJL9_PSEAP Phosphoadenosine phosphosulfate reductase OS=Pseudomonas syringae pv. aptata OX=83167 GN=ALO85_02472 PE=3 SV=1
MSQPFDVAELATTYANKSAQDILKLAFEHFGDELWISFSGAEDVVLVDMAWKLNKNVKVF
SLDTGRLHAETYRFIEQVRGHYKIEIEIVSPDHSKLEPFVKEKGLFSFYRDGHGECCGIR
KIEPLRRKLSGVSAWATGQRRDQSPGTRSAVAAVEIDSAFSTAERTLYKFNPLAQMSSEE
IWGYIRMLELPYNSLHKRGFISIGCEPCTRPVLPNQHEREGRWWWEEATQKECGLHAGNL
IARD
>tr|A0A0Q0BVY1|A0A0Q0BVY1_PSEAP Small-conductance mechanosensitive channel family protein OS=Pseudomonas syringae pv. aptata OX=83167 GN=ALO85_01173 PE=4 SV=1
MEQDIIRFFTDSQFLGISLANWILAIFASTLSFILVRGVIGFVLRKMRSRADAPSTHMRY
IVVQVLSGTSNTLLLLASILIGIGMLDLPERWLGRVSSLWFVVAALQVGLWANRAIALAL
QRYFVRHNAGGTFQGSALATLSLWGAKVLLWATVVMAMLSNVGVNITAFVASLGVGGIAV
ALAVQNILGDVFASLSIAVDKPFEVGDFIVVGALSGTVEHVGLKTTRIRSLGGEQIVMAN
ADMINNTIQNYKRLQERRIVFEFRLTYDCSVDQIREVPKKVEAIINAQKLARFDRSHFRG
FGETSLEFETVFIVLDPSYNVYMDVQQAINLDIMEAFAELDVRFAFPSRTVYVASLPPVK
TARPTALEAADATT
>tr|A0A0Q0C6U6|A0A0Q0C6U6_PSEAP Sarcosine oxidase, gamma subunit OS=Pseudomonas syringae pv. aptata OX=83167 GN=ALO85_00787 PE=4 SV=1
MTSLIQNSPVGANARALCTLQDLTDRPRVGFRGTQSADYLTARGFSLPEAPNRAVTQADG
SHVARLSQTEYLLLGSPFDDGQRIADEEARWELDHSANYLLPREDSHAWLQLSGACIAEV
MAKLCGVDLRPNAFAPGSVAQTSAARINVIVINLGAQAEPCFQILFDRASLAYFKDAMLD
AMTEFGGRYL
>tr|A0A0Q0BRU9|A0A0Q0BRU9_PSEAP Putative 4-oxalocrotonate tautomerase OS=Pseudomonas syringae pv. aptata OX=83167 GN=ALO85_04086 PE=4 SV=1
MPLLKIDVIEGRSDQEIETLLDAIHDAMVNAFQVPERDRYQVLNEHKRNRLIIQDTGLGF
TRSDKVVVITAISRPRPAEMKKRFYALIAEGLERNCGIAAEDVMVSIVINSDEDWSFGLG

RAQFLTGEL
>tr|A0A0N8T9T9|A0A0N8T9T9_PSEAP Putative secreted protein OS=Pseudomonas syringae pv. aptata OX=83167 GN=ALO85_100636 PE=4 SV=1
MKGSVTLGLIVAALFSSVSQAADVKSCSDLSGRWETTAGDQHLVLNISPSAEACGTNCVT
LELKYNLNETRVNKAYCHEGVEGIKGQGPMVISFEGAYGGHSIGTYNRQLDVLWAGVIPK
DSKGNWETKMDNYWFKRVGN
>tr|A0A0N8T7V5|A0A0N8T7V5_PSEAP Uncharacterized protein OS=Pseudomonas syringae pv. aptata OX=83167 GN=ALO85_04157 PE=4 SV=1
MNNEFDEFDGYDDQYERPSLVDDRSHRNHWLNRASDLHASAGAIWYSMHGGNHREIIETL
GFSEGFSMSTACFPVYHMLCGLALEVIMKAVIVSRGEPAPEIHDLNELATLVGMKRNVNE
KRILSFYQASVVWAGRYPIPRKADDQKLREYWKLANKVLTKPKAMGKETTLTFYESSGAT
AWEKYNGLFGSYSSLFNHHYPAPLKK
>tr|A0A0Q0DPZ5|A0A0Q0DPZ5_PSEAP Uncharacterized protein OS=Pseudomonas syringae pv. aptata OX=83167 GN=ALO85_00766 PE=4 SV=1
MDALMLNDSDTLGSAFKRAFYRVDGITMYACWAIWVGVLIWDLLGSEGSGIHTVVLILIG
LLNPFLFLLLGLWRLPGLLTALIIIGINIKFLFAWL
>tr|A0A0Q0BWD1|A0A0Q0BWD1_PSEAP Phosphohistidine phosphatase SixA OS=Pseudomonas syringae pv. aptata OX=83167 GN=ALO85_02214 PE=4 SV=1
MKVWVLRHGEAQSRARSDAERELTAHGREEVLKSALQLTDKPVRRIIASPYVRAQQTAEL
VRQSLAFDDSIVTAPWLTPDSSPREVLAQLDKLGIDEVLLVSHQPLVGELIGVLAHGSPQ
QAEPMSTASLAELEGEFVVAGGMRLNSVRHV
>tr|A0A0Q0DJZ6|A0A0Q0DJZ6_PSEAP Sugar ABC-type transport system, periplasmic substrate-binding protein OS=Pseudomonas syringae pv. aptata OX=83167 GN=ALO85_04396 PE=4 SV=1
MGKAGLHESNLEMTMFNKKNKLQHSVTLATMIMLSGLSVSAWADKYEDAAKKWADSEFKP
STLSDDEQLKELKWFIEAAKPFRGMDIKVVSETLTTHEYESKTLAKAFTEITGIKVTHDL
LQEGDVIEKLQTAMQSDKSIYDGWVNDSDLIGTHFRYGKALSLTDLMASPEGSKVTSPTL
DLNDFIGTSFTTAPDGKLYQLPDQQFANLYWFRADWFERPDLKKKFKDKYGYELGVPVNW
SAYEDIAKFFSEDVKEIDGKKVFGHMDYGKKDPSLGWRFTDAWFSMAGSGDKGLPNGLPV
DEWGIRVEDCHPVGSSITRGGDTNGPAAVFATQKYIDWLKAYAPPEAAGMTFSESGPVPS
QGNVAQQIFWYTAFTADMTKPGLPVMNADGTPKWRMAPSPKGPYWKEGMKLGYQDVGSWT
FLKSSDEKKRLAAWLYAQFVTSKSVSLKKTIVGLTPIRESDINSKEMTELAPKLGGLVEF
YRSPARVQWSPTGTNVPDYPKLAQLWWSHVAEAVTGEKTAQEALDGLAKDQDAIMTRIER
SKVQEASKCAPKMNPETTAEAWYKKAEESNGKFLAPQRKLANEKPKGETIAYADLLKSWE
AAKK
>tr|A0A0Q0BT80|A0A0Q0BT80_PSEAP Trk system potassium uptake protein OS=Pseudomonas syringae pv. aptata OX=83167 GN=ALO85_03801 PE=3 SV=1
MSLPTLRIIGFIIGIFLITLAVAMIVPMATLVIYDRFEDLREFLWASAITFIAGLALVVP
GRPDNVQLRPRDMYMLTVSSWIVVCIFAALPFLLTQHISYTDSFFESMSGITATGSTVLS
HLDSMSPGILIWRSMLHWLGGIGFIGMAVAILPLLRIGGMRLFQTESSDRSEKVMPRSHM
VAKFIVLAYVGFTALGSLAFWAAGMSLFDAINHAMSAISTGGFSTSDQSLAKWPQPAVHW
VAIVVMILGSLPFTLYVATLRGNRKALIKDEQVQGLIGLLLITWLVMTVWYANTTDLPWT
EALRHVALNVTSVVTTTGFALGDYSLWGNFSLMLFFYLGFIGGCSGSTAGGVKIFRFQVA
YILLKTNLNQLIHPRAVMKQKYNGHRLDDEIVRSILTFSFFFTITICLIALMLSLLGLDW
MTALTGAASTVSGVGPGLGEVIGPAGNFSTLPDAAKWILSGGMLLGRLEIITVLVLCVPA
FWRH
>tr|A0A0Q0CG97|A0A0Q0CG97_PSEAP Bifunctional protein FolD OS=Pseudomonas syringae pv. aptata OX=83167 GN=folD PE=3 SV=1
MSAHIIDGKAAAARVLQQVKHDVNTLKAEGIEPALAVILVGNDAASEVYVRNKILRAEEA
GIRSLEHRLPADSSQARLLALIAELNADNTVNGILLQLPLPAHLEENRVLQAIDPGKDVD
GFHSENVGGLSQGRNVLTPCTPSGCLHLLEETCGDLSGKHAVVIGRSNIVGKPMAALLLQ
AHCSVTVVHSRSHDARALCQLADIVVAAVGRPRLIDASWLKRGAVVIDVGINRIEDHGRN
RLVGDVDFDSVIEVASAITPVPGGVGPMTIAFLMKNTVTAARQQAHAQRSQSEAVCLSTC
>tr|A0A0N8T9G8|A0A0N8T9G8_PSEAP Uncharacterized protein OS=Pseudomonas syringae pv. aptata OX=83167 GN=ALO85_00966 PE=4 SV=1
MTTIKITAGGYEFLAQAHPDAPQTVEAFLKLLPYRQKFIHVRWSGEGCWVPLDDYQLKLD
DTLIGFENATSHPSVGDILFYPGGYSETEIILAYGSCCFASKMGQLAGNHFLTITQGKEN
LRKLGVKTLWEGAQEVVFELA
>tr|A0A0Q0FJH7|A0A0Q0FJH7_PSEAP Uncharacterized protein OS=Pseudomonas syringae pv. aptata OX=83167 GN=ALO85_100987 PE=4 SV=1

MLYPESTLFFSKSPSPLREIGAVSPQHVDPRCIRAIHPDDK
>tr|A0A0Q0DAD2|A0A0Q0DAD2_PSEAP Enolase 2 OS=Pseudomonas syringae pv. aptata OX=83167 GN=ALO85_200133 PE=4 SV=1
MNDEKRMTLDDYDFSKVKVNPTRPTQDPAHILLRVVEGAGVALWRSSPAGQAELLPTRRD
LFQYEGGYSWGYKGEGCKNLAFAIIGRVYECDDLSSEDMYEKAMKLVDTLIPALQQQMNH
DLSVTVIRKVLGDGQRPIF
>tr|A0A0Q0DEM1|A0A0Q0DEM1_PSEAP Transport permease protein OS=Pseudomonas syringae pv. aptata OX=83167 GN=ALO85_03122 PE=3 SV=1
MPSFCSPGVMKWRDLIINLVSKEIKIRYMGATLGFVWSLGNPLVVTLTYYTVFTYILPSS
QDRFALHLVTGVVHWMLLAQIVSQSGEWLINNGNLIRKLRFPRLLLPVSGALAIVVFWAG
CMLVYASLFAMLGGVFSRALLFYPIVLIAFMALIMGFGLALSVIQVTVRDAKHFIDVFLP
LLFWLTPIVWVTSSLPDGIARIAAYNPIGLYFNTFTSILHTGVVPETRDLLLCVFMGAAS
LLVGLLMFRKVDNVVEYL
>tr|A0A0N8T9Q2|A0A0N8T9Q2_PSEAP AraC family transcriptional regulator OS=Pseudomonas syringae pv. aptata OX=83167 GN=ALO85_02076 PE=4 SV=1
MFKRPQRIHNDKKGHPVPDSRAALLEQRPAELEVILPQPDHCFRWYEHDYPYALARWNHH
PEFEIHLIRQGSGKLVAGDYIGEFAAGHVALIGPDLPHDWIGDLAPGEHLPGRDVVLQFD
GAALLALRGTLPEMGDLRRLFEQARRGLEFTGATAVQAARLLEEIGPAQGLERLILFLQL
VNTLMKAPAQEVRLLASTWYAPTLDARSSERINKAFDYLLTELTSDIRLSVIAQRLDMSD
PGFSRFFKRTTGHCFIDLMRKLRVQRACRLLLHSEMSVSDICFEVGYANLSNFNRHFRVE
MQQTPSEYRRAAASV
>tr|A0A0Q0DMM9|A0A0Q0DMM9_PSEAP Bacterioferritin OS=Pseudomonas syringae pv. aptata OX=83167 GN=ALO85_200100 PE=3 SV=1
MQGHPEVIDYLNTLLTGELAARDQYFVHSRMYEDWGFSKLYERINHEMEEEAQHADALMR
RILMLEGTPRMRPDDLDVGTTVPEMLASDLRLEYKVRAALCKGIELCELHSDYVTREILR
VQLADTEEDHTYWLEQQMGLIKSIGLQNYLQSQF
>tr|A0A0N8T8L9|A0A0N8T8L9_PSEAP Uncharacterized protein OS=Pseudomonas syringae pv. aptata OX=83167 GN=ALO85_00692 PE=4 SV=1
MKFSMMSAGVVLGLALSGAAFADDATDADATSGASDETVMTQTQDAKAAQKAQNQKTETT
KGGRPQNATPQKQSN
>tr|A0A0Q0DFN1|A0A0Q0DFN1_PSEAP Uncharacterized protein OS=Pseudomonas syringae pv. aptata OX=83167 GN=ALO85_01329 PE=4 SV=1
MTELQHVVSLTRLYERLIDRLGLALETASTSVRLRNESPAELELKGLSRAEFQLIEAYLE
KGSGGAGATVVDMSSGSALVAAEHGRAPRASATIVWLKDRRRAKASVRLGRNS
>tr|A0A0N8T9B7|A0A0N8T9B7_PSEAP Uncharacterized protein OS=Pseudomonas syringae pv. aptata OX=83167 GN=ALO85_02677 PE=4 SV=1
MPTSFLEIVELPDGRIELRRADDEGSLVTLDFSADAKAFMQGQHVEVAKAMLSVGVQMAG
RLAEGEIEKDDVPHVLH
>tr|A0A0Q0DYH6|A0A0Q0DYH6_PSEAP Uncharacterized protein OS=Pseudomonas syringae pv. aptata OX=83167 GN=ALO85_00585 PE=4 SV=1
MQIKTASLIANPCDDEYDDMALLCCHAENGMLFSLTRFPDENEVEITVSDDKSLNVSSLK
VTFSAKRLLVEIDAQDAKQLDGHHQYEILHATDAGELQDVHQTLQIILENVGEYTSTIS
>tr|A0A0Q0DJH7|A0A0Q0DJH7_PSEAP Methionine aminopeptidase OS=Pseudomonas syringae pv. aptata OX=83167 GN=map PE=3 SV=1
MSSAIGIKTEQDLVQLRIAGRLAADVLAMITPYVKAGVSTEALDDICNEYIVKELKVIPA
NVGYHGFTKTTCISPNAVVCHGIPSATDILKEGDIVNIDVAVIKDGWYGDTSRMYLVGEV
SPLARRLVETTYEATRAGIHAVRPGATLGDIGYAIQSVAHREGFSVVREYCGHGIGRKYH
EEPQVLHYGAQGKGLRLKPGMVFTIEPMINAGRAGTRTLPDGWTVLTSDLSLSAQWEHMV
AVTSTGFELLTPWPDGTGDYPAI
>tr|A0A0Q0CGL2|A0A0Q0CGL2_PSEAP Chemotaxis protein CheW OS=Pseudomonas syringae pv. aptata OX=83167 GN=ALO85_04018 PE=4 SV=1
MNKSSAQGSEDPILQWVTFRLDNESYGINVMQVQEVLRYTEIAPVPGAPSYVLGIINLRG
NVVTVIDTRQRFGLDPVEVSDNTRIVIIEADKQVVGILVDSVAEVVYLRQSEVETAPNVG
NDESAKFIQGVCNKNGELLILVELDKMMSEEEWSDLENI
>tr|A0A0Q0DY18|A0A0Q0DY18_PSEAP Uncharacterized protein OS=Pseudomonas syringae pv. aptata OX=83167 GN=ALO85_101103 PE=4 SV=1
MEISADDSLRKAGHFTRNRPFVKYAAPVLFRQATTLDQK
>tr|A0A0Q0E0P8|A0A0Q0E0P8_PSEAP Periplasmic binding protein/LacI transcriptional regulator OS=Pseudomonas syringae pv. aptata OX=83167 GN=ALO85_03276 PE=4 SV=1

MAMLRLSKRFTLTLGVVLALALSPIIQAQTGKRLALVQVNQQALYFTQITQGAQAAAKAA
GAELVVFNANDNPSAQNDAIEAYIQDKVDAILVCAIDVNGIKPAVTAAAQAGIPVVAIDA
VINGDNAVQVGVDNREAARQIGQYTGEYINRELAGKASIGVVGALGSYVQNLRLDGFREG
LAKTASQAKIVNTVDGNNVQDTAQAAAENLLTANPDLQIIYATGEPALIGSVAASVSQGA
GERVRIFGWDLSSQAVQGLDDGSVAVVVQQNTQAMGKTAVESALALLSGKTVAREQSIPV
TLVTKANLAAYRAEFK
>tr|A0A0Q0CCX5|A0A0Q0CCX5_PSEAP Uncharacterized protein OS=Pseudomonas syringae pv. aptata OX=83167 GN=ALO85_02968 PE=4 SV=1
MSFIVREGDLTTTGGFVLAASASEVIERRRVARMGDPVWCPACKQIGFIAQGNPTYIDER
VAVATQGHEVQCGCTPGSNTLITSQEHVQADMDATITIPEELANTARQHAEHLARSMKDG
SFTPDVLRPLT
>tr|A0A0Q0DFR0|A0A0Q0DFR0_PSEAP Uncharacterized protein OS=Pseudomonas syringae pv. aptata OX=83167 GN=ALO85_01384 PE=4 SV=1
MSDLDEALLTDAGLLSFARNVEPDLRQNIIDCLHHARLVADDHHSSRMNWRAWLEAFQRS
IAATGGLRSAGINDARLKIHSFRDIGKLQLPVVDNAGELRQLYRSSFDKLLSSDHATSFF
SSWFTSGRSESFQLMPCAMRSKDEVSILLCSFQMTTLALRPARYFWQIVVGEMQVHAVAT
AFRFSRQSFEPFRQTVQDALADRAAAEIISL
>tr|A0A0Q0C7F9|A0A0Q0C7F9_PSEAP Zinc import ATP-binding protein ZnuC OS=Pseudomonas syringae pv. aptata OX=83167 GN=znuC PE=3 SV=1
MSDALIRLDKVAVTLSGQNVLDDIQLSVKPGEIVTLIGPNGAGKTTLVRAVLGLLKPDSG
SVWRKPKLRVGYMPQKLHVDQTLPLSVLRFLRLVPGVDRTAAASALEEVGAGKVIDSPIQ
GISGGEMQRVLLARALLRKPELLVLDEPVQGVDVAGQAELYSLITRLRDRHQCGVLMVSH
DLHLVMSTTDQVVCLNRHVCCSGHPEQVSHDPAFVELFGKNAQSLAIYHHHHDHAHDLHG
AVVNDAATSHTHVHGDGCKHG
>tr|A0A0Q0FNH0|A0A0Q0FNH0_PSEAP ATP-dependent DNA helicase DinG OS=Pseudomonas syringae pv. aptata OX=83167 GN=dinG PE=3 SV=1
MISTELKTQIQGAYSRFLEAKSLKPRYGQRLMIAEVAKVLGDIDTDEEGRRSGDPAVVAV
EAGTGTGKTVAYAIASIPTAKAAGKRLVIATATVALQEQIVYKDLPDLMRNSGLNFTFAL
AKGRGRYMCLSKLDALLQESDATNATAQLFEEEGFKIEVDEASQKLFTSMLQKLAGNKWD
GDRDSWPQELADQDWARLTTDHSQCTNRHCPNFQQCAFYKAREGMGKVDVIVTNHDMVLA
DLALGGGAVLPDPRDTLYVFDEGHHLPDKAIGHFAHYTRLKSTADWLEQTAKNLAKLLAQ
HPLPGDLGKLIEQVPELAREIKGHQQFMFGACEQLADFRAGEDMQGRERPRHRFVGGVIP
EHIREMGIELKKGFARLDDLFTRLTELLKEGMDGEVNIGIASHQAEEWYPLFGSLLARAH
GNWELWTAFTAEDPEDSPPMARWLTLADSGAMFDIEVNASPILAAEMLRRNLWNIAYGAL
VTSATLTALGKFDRYRMRAGLPKGAVTAVVPSPFHHADAGVLRVPDLKADPRDSVAHTAA
IIRDLPGLVEGSRGTLVLYSSRKQMQDVFDGLDRDWRKQVFIQGNLSKQETLNKHKARVD
SGESSVLFGLASFAEGVDLPGAYCEHVVIAKIPFAVPDDPVEAALAEWIEARGGNPFMEI
AVPDASLRLVQACGRLLRTEEDRGTITLLDRRVVTQRYGKAILNALPPFRREIT
>tr|A0A0Q0DFA1|A0A0Q0DFA1_PSEAP Outer membrane lipoprotein Blc OS=Pseudomonas syringae pv. aptata OX=83167 GN=ALO85_01690 PE=3 SV=1
MIKVLVRFGICVMASFLAIGFAHSADNLEPKTVDSVDLKQYQGTWYEIARLPMFFQRKCA
QSEARYTLKDDGNIAVTNRCRTIEGKWEEATGTASPQVPGKTDKLWVVFDNWFSRLLPSV
AKGDYWILDVSEGYRTAVVGNPDRKYLWLLSRTPTVSASVREDMLSKARQQGYDTSRLIW
REDDSKIGKGEK
>tr|A0A0Q0FN69|A0A0Q0FN69_PSEAP Nucleoside-specific channel-forming protein Tsx OS=Pseudomonas syringae pv. aptata OX=83167 GN=ALO85_02735 PE=4 SV=1
MPQPPFTRPATPSRFLMPLLFAAAAALPCTSVLAQPAAAEDSAQGEALSPPASPKEGKGA
YFSDWLNQDLTLIGSKDISFGPKPNDDIYLEYEYFGRKGPFELYGYVDVPKILGIGNDND
KGVWDHGSPLFMEHEPRVSIDYLAGRSLAIGPFKEWYVAFDWIYDHGSNTANRANTLYSG
LGTDIDTHSRVNLSANFYGRYQWENYGASNEYSWDGYRAQLKYIVPISTFDNGASLTYIG
FTNFDFGSDLKNDPARTGNSTVATNVLLYAFTHLRFTLVGRYFHNGGNWQDGSELNFGDG
NFRARSDGWGYYAGVGYQF
>tr|A0A0Q0BTW3|A0A0Q0BTW3_PSEAP 6-phosphogluconolactonase OS=Pseudomonas syringae pv. aptata OX=83167 GN=pgl PE=3 SV=1
MATCDLKLPAGLVAHDFDTAQQLADALAETVAERLKQAISKNGLATLVVSGGRSPVAFFQ
RLAAQPLEWSKVVISLADERFVPTEHADSNAGLLHRHLLQGPVAKAKFLGMYSVASSVEE
AAQAADQALAELPPIDVLILGMGDDGHTASLFPNSPNLNEALDLQGERRCLPMLAPSVPH
QRLTLTRRLLASARSPILSVSGQAKLDTLRTALAGDDLAEMPVRAFLNPSLEIYWCP

>tr|A0A0N8T862|A0A0N8T862_PSEAP 4-hydroxythreonine-4-phosphate dehydrogenase OS=Pseudomonas syringae pv. aptata OX=83167 GN=pdxA PE=3 SV=1
MKPKRFALTPGEPAGIGPDLCLLLATQPQPYPLIAITSRDLLLERAAQLGVSVSLIKVTP
DAFPDLPAPAGSLYVWDTPLAAPVETGVLNKANAAFVLETLTRAGQGCLDGLFSGMITAP
VHKGVINDGGIAFSGHTEFLAELTHTEQVVMMLATGDLRVALVTTHLPLRDVADAITADR
LERVTRILHADLVNKFGIAHPRILVCGLNPHAGESGHLGREEIDIIEPALERLRSEGLDL
RGPLPADTLFTPKYLEHCDAVLAMYHDQGLPVLKYKGFGAAVNVTLGLPIIRTSVDHGTA
LDLAGTANIDTGSLHVALQTAYQMAETHS
>tr|A0A0Q0I9V8|A0A0Q0I9V8_PSEAP Orotate phosphoribosyltransferase OS=Pseudomonas syringae pv. aptata OX=83167 GN=pyrE PE=3 SV=1
MVFGAFFMQAYQRDFIRFAIDRGVLRFGEFTLKSGRTSPYFFNAGLFNTGSALAQLGRFY
AAAVVESGIAFDVLFGPAYKGIPLASATAVALAEHHDRDLPWCFNRKEAKAHGEGGSLVG
SPLAGNVLIIDDVITAGTAIREVMQIIKDQNATAAGVLIALNRQERGNGELSAIQEVERD
FGIPVVSIVSLNQVLEFLADDPQLKQHLPAVEAYRAQFGI
>tr|A0A0Q0DKQ0|A0A0Q0DKQ0_PSEAP Uncharacterized protein OS=Pseudomonas syringae pv. aptata OX=83167 GN=ALO85_01199 PE=4 SV=1
MKTSDGFDARRLRPRGQGNWGKRIGTLIALLLVVAGLLLAVAGASSLTVHPQMLGELNAS
PGGAAFVAIGGLLVLYIGVWLWRRCRRQRSGGGLSISSHLMKKHN
>tr|A0A0N8T8Y4|A0A0N8T8Y4_PSEAP SnoaL-like domain-containing protein OS=Pseudomonas syringae pv. aptata OX=83167 GN=ALO85_01826 PE=4 SV=1
MNYEEQSQANSALITRFYEAFAQLDAESMSACYTDDVLFSDPAFGELRGAQVGDMWRMLT
SRAKNFSVVFDQVRADDQTGSAHWVATYLFSQTGRTVVNDIQARFVFRDGKICEHRDHFD
MWRWSRQALGLKGLLLGWTPLVRNAVRAQALKGLKTFSESRRA
>tr|A0A0Q0IL68|A0A0Q0IL68_PSEAP AP endonuclease OS=Pseudomonas syringae pv. aptata OX=83167 GN=ALO85_02689 PE=4 SV=1
MPVSTSTPRIRIGINPISWSNDDLPALGGETPLSTALSEGKAIGYEGFELNGKFPKDAKG
VGDVLRPHDLALVSGWYSSRLARRSVAEEIEAITAHVQLLAENGASVLVYGEVADSIQGQ
RIPLVERPRFHTYAAWHEYAEKLTELARFTLSRGVRLAYHHHMGAYVESPQDIDRLMALT
GEEVGLLFDSGHCYMGGGEPLQVLSKHIQRICHVHFKDVRKPVVQLARNNLWSFPDCIIN
GTFTVPGDGDIDFSALLKVLLQADYNGWLVVEAEQDPAVAPSYVYAKKGYDTLRQLLADA
R
>tr|A0A0N8T956|A0A0N8T956_PSEAP Probable membrane transporter protein OS=Pseudomonas syringae pv. aptata OX=83167 GN=ALO85_02776 PE=3 SV=1
MHSILDFYQTLGWGLSALVIGTFLLAGTVKGVIGLGLPTVAMGLLGLAMLQAQAAALLII
PSTFTNVWQLVAGGNLRPLLKRLWPMLSMIFIGTLVGAFWLGMSGGHSMTRALGGALFLY
ALSGLFLPTFKVAAAAERWLGPLCGLITGVIASATGVFVIPVVPYLQALGLERDQLVQAL
GLSFTVSTLALAAGLSWNGALGGAELGASTLALIPALLGMLLGQWLRQRISAVLFKRVFF
IGMGLLGLHLLIKG
>tr|A0A0N8T9R6|A0A0N8T9R6_PSEAP Drug/metabolite transporter superfamily permease OS=Pseudomonas syringae pv. aptata OX=83167 GN=ALO85_01927 PE=4 SV=1
MQYAFPLITVLIWAINTAVTKASAGVIFPAEIGFYRWALAGLLFTPFMLGPVWANRAAIR
PVLGKIFILAVLGTALYQSLAYFAASLTTATNMGIIQSMVPMMALGLSIACLGTRLTSGA
LLGAALSFAGVVVVVSAGNPAGLIEQGVNRGDAMVLVAAASYAVYSTLLKKWQLRLPPLQ
LLYVQILLAIIVLLPPFMLSAKTGLNASNIPMVLYAAIPTSMLAPWLWMTSIMRLGPSRT
TLFFNLMPIATALIAAATLGEKLALYHLFGGALTLCGVILAERWTTPLRSKAD
>tr|A0A0Q0D0F2|A0A0Q0D0F2_PSEAP PepSY family peptidasen OS=Pseudomonas syringae pv. aptata OX=83167 GN=ALO85_03207 PE=4 SV=1
MKLMPAVFAAVALTAVAGAAQADVGPDEVIRLYKSQALGDFEQFNKNAVAKHQGFTVSDT
ELDKDRTGRLIYQIELKDAKGVEWNYDVDAKTGEVVRDAKDN
>tr|A0A0Q0CGF8|A0A0Q0CGF8_PSEAP Killer protein OS=Pseudomonas syringae pv. aptata OX=83167 GN=ALO85_200160 PE=4 SV=1
MTKGAIVVIDPVSSGRRYGLEILQKGYTSVALVTRTKFPGRLHRLFSSSDFQEVIFSENL
IDSIEKLSLQNVKAIVPGSDSALKFCDTLAEHLGLTGNPVRTQKARYSKLEMKRALKLNN
VPTTGVEALSLDTARKSDLSFTQFPVVVKPTQGTGSKNVKVCHNVGDIYRALSLIESTNE
SFNSGEKEALIEPYIVGKEYCVAIANFGKGNDKELLCIAEYEKIQINNNPSIYKNIRSVR
IDDDLSSRIFDYATRVNMALEADYGINDIELKVDGDDIQLIEQNGRLPGADLPRMIELCT
GRNLYQVNIDIYLGLAGVPRVPVEYKKHFCVCCLINFESGLIDRIEGVAEIEKLSSFQDM
NIIVSEAEIIEPTHDFLSTWGFVFLLHDDAETLMQHSKMVHEHMKIIYKV

>tr|A0A0Q0IR36|A0A0Q0IR36_PSEAP 50S ribosomal protein L31 type B OS=Pseudomonas syringae pv. aptata OX=83167 GN=rpmE2 PE=3 SV=1
MKPDIHPQYRTVLFHDTAADAYFLIGSTVDTDRTQQHTDGTTYPYVALDVSSASHPMYTG
QQRKTTTEGRIAGFNKRFAAFGSAGKKETAAAQ
>tr|A0A0Q0CFR6|A0A0Q0CFR6_PSEAP Phosphoglucomutase OS=Pseudomonas syringae pv. aptata OX=83167 GN=ALO85_05451 PE=3 SV=1
MPVNCGIDHLGSYSKSLFFLRISFLRGGCMSLSPFAGKLAPAQLLVDIPRLVTAYYTGQP
DASVPTQRVAFGTSGHRGTSFDLGFNEWHVLAISQAICLYRKANGIDGPLFLGADTHALS
TPAAATALEVLAANGVQVMISQGDEYTPTPAVSHAIICYNRGRTSGLADGIVITPSHNPP
QSGGFKYNPPNGGPADSDVTKWIENKANELLAEKVTGVSRISHEKALRADTTHRHDYINT
YVADLKNVIDMDAIRNSGLRLGVDPLGGAGVNYWSAIGEHYGLNLDVVNKFVDPTFRFMT
VDWDGQIRMDPSSSHAMQSLIGLKDRYQVAFACDPDHDRHGIVTPSGGLMTPNSYLAVSI
DYLFQNRPEWRADAAVGKTVVSSGMIDRVAKRLGRRLYEVPVGFKYFAQGLFEGSLGFGG
EESAGASFLRRDGTVWTTDKDGLIPALLAAEMTARTGRDPSQIYKTMTEQLGEPFSTRVD
AKANPQQKALLSKLSPEQVTSTELAGEPIQQVLSNAPGNDQAFGGVKVMTENGWFAARPS
GTEDIYKIYAESFVSEDHLKRLVAEAQVLVDGAISPN
>tr|A0A0Q0C553|A0A0Q0C553_PSEAP Membrane protein OS=Pseudomonas syringae pv. aptata OX=83167 GN=ALO85_02943 PE=4 SV=1
MDFVLDLLATVSRWSRSNLSEIALALVGCLLVLFGSDIKGWLEQRISGITGALRIPLIAL
LCAVGSGAALIYATPWVVRGLSQFNNYSLAPVLLVVLVLIGVIADRK
>tr|A0A0Q0FK41|A0A0Q0FK41_PSEAP Dihydrolipoyllysine-residue succinyltransferase component of 2-oxoglutarate dehydrogenase complex OS=Pseudomonas syringae pv. aptata OX=83167 GN=ALO85_02200 PE=3 SV=1
MAIEIKAPSFPESVADGTISKWYKKEGDAVKRDEMLVDIETDKVVLEVLAEADGVMGAIT
KEEGAIVLSNEVLGTLNDGATASAAPAPAAAPASAPASAPAAAPAASAGEEDPIAAPAAR
QLAEENGINLASVKGTGKDGRITKEDVVAAVEAKKSAPAAAPAAKPAAAAVPVVAAGDRT
EKRVPMTRVRATVAKRLVEAQSNMAMLTTFNEVDMTEVMALRSKYKDLFEKSHNGVRLGF
MSFFVKAATEALKRFPAVNASIDGSDIVYHGYADVGVAVSSDRGLVVPVLRNAEHMSLAE
IEGGIATFGKKARDGKLSIDEMTGGTFTITNGGTFGSMMSTPIVNPPQAAILGMHNILQR
PMAVNGQVVIRPMMYLALSYDHRLIDGKEAVTFLVTIKNLLEDPARLLLDI
>tr|A0A0N8T7W7|A0A0N8T7W7_PSEAP Cytochrome O ubiquinol oxidase, subunit I OS=Pseudomonas syringae pv. aptata OX=83167 GN=ALO85_04262 PE=3 SV=1
MFGKLSLEAVPFHEPIVMVTLAMIALGGIAVVGLITYFRKWTYLWSEWLTSVDHKKIGVM
YIVVAMVMLLRGFADAIMMRTQLAMAQNGAEGFLPPEHYDQIFTAHGVIMIIFMAMPFFT
GLMNIVLPLQIGARDVAFPFLNSLSFWLLVAGMLLINLSLGVGEFAKTGWVAYPPLSGLQ
YSPGVGVDYYIWALQLSGLGTTLTGVNFLVTVLKMRTPGMKLMDMPIFTWTCTWANILIV
ASFPILTATLAFLTLDRYLDFHIFTNEMGGNPMMYVNLFWAWGHPEVYILILPAFGVFSE
VISTFSGKRLFGHKSMIFASGAICILGFMVWLHHFFTMGAGANVNAFFGLATMLIAIPTG
VKLFNWLFTMYQGRLRFTAPVLWTLGFMVTFSIGGMTGVLLAIPGADFVLHNSLFVIAHF
HNVIIGGAVFGYIAGFAFWFPKAFGFTLNEKWGKAAFWFWIVGFFVAFMPLYALGFLGMT
RRLNATDMPEWNIYLDVALFGAVLIAMGIASQLIQLFVSIRDRDNNRDLTGDPWNGHTLE
WSTSSPPPFYNFAELPKADDVDPFTDAKRAGTAYKVPARYSAIHMPNNTATGLYMGMLLT
VFGFAFIWHIWWLVGASLVATIAVFVAHAMRDDQGYMVPAEDVARIEGEHHKVLAANGAY
TPVKSSLEQV
>tr|A0A0Q0FWX0|A0A0Q0FWX0_PSEAP 3-oxoadipate enol-lactone hydrolase OS=Pseudomonas syringae pv. aptata OX=83167 GN=ALO85_01551 PE=4 SV=1
MLKDQNRGSSEMPDLLIDGKTLHYSDQGTGPVVLLGHSYLWDKAMWSAQIDTLASQYRVI
VPDLWGHGDSSGFPEGTRNLDDLARHALALLDHLNIERCSIVGLSVGGMWGAIAALLAPE
RITGLVLMDTYLGKETEAKKAYYFSLLDKLEEVGSFPEPLLDIVVPIFFRPGIDPQSPVY
TSFRAALASMNTEQLRQSVVPLGRMIFGRDDRLGLIEQLNADTTLVMCGDADIPRPPEET
REMANLIGCPYVLVPEAGHIANLENPAFVSGALMTFLARVNQKQG
>tr|A0A0N8TAA6|A0A0N8TAA6_PSEAP Uncharacterized protein OS=Pseudomonas syringae pv. aptata OX=83167 GN=ALO85_03254 PE=4 SV=1
MKLITTVTTSLLMLMNGAHAEGLTTSVDGKELTVSFGQPTIKQVLHYRKMYDKKPFPDAE
IYYYDNGTYKLISQGENHYGVYSIEGNVDEETFTVRFISLPSSDWGNKTAFHQLTFVRGD
KQNIFIQNAIVGTGEALAQQNGTYTQEKNTVTNPVSTTWKNK
>tr|A0A0Q0C2V3|A0A0Q0C2V3_PSEAP CBS domain-containing protein OS=Pseudomonas syringae pv. aptata OX=83167 GN=ALO85_03391 PE=4 SV=1
MKTVAQLLKLKDLQNQQVHTIGPDQMVLEALKLMADKNIGALPVVEGNVVVGVISERDYA
RKVVLKGRSSVGTPVRDIMSSKVITVDSQRSVEACMGIMTDSHLRHLPVVEGGQLLGLLS

IGDLVKEAIAEQASLIQQLEQYIRGE
>tr|A0A0Q0D2Q0|A0A0Q0D2Q0_PSEAP Succinate dehydrogenase flavoprotein subunit OS=Pseudomonas syringae pv. aptata OX=83167 GN=ALO85_02197 PE=3 SV=1
MANINALSFDAIIIGGGGAGMRAALQLAQGGHKTAVVTKVFPTRSHTVSAQGGITCAIAS
ADPNDDWRWHMYDTVKGSDYIGDQDAIEYMCSVGPEAVFELEHMGLPFSRTEQGRIYQRP
FGGQSKDFGKGGQAARTCAAADRTGHALLHTLYQANLKAGTVFLNEYYAVDLVKNNDGAF
VGIIAICIETGETSYIRANATVLATGGAGRIYSSTTNALINTGDGIGMALRAGVPVQDIE
MWQFHPTGIAGAGVLVTEGCRGEGGYLINKHGERFMERYAPNAKDLAGRDVVARSMVKEI
IAGNGCGPDGDHVMLKLDHLGEEVLHSRLPGIMELSKTFAHVDPATAPIPVVPTCHYMMG
GVATNIHGQAITQDAAGVDQIIPGLFAVGEVACVSVHGANRLGGNSLLDLVVFGRAAGIH
LEQALREGVDYARASESDIDAALARLAGLNERTTGEDVATLRKELQSCMQNYFGVFRTGE
YMQKGIAQLADLRVRIANVKINDKSQAFNTARIEALELQNLLEVAEATAIAAEHRKESRG
AHAREDFEDRDDENWLCHTLYFPGDKSVTKRAVNFSPKTVPTFEPKIRTY
>tr|A0A0Q0IAK6|A0A0Q0IAK6_PSEAP Uncharacterized protein OS=Pseudomonas syringae pv. aptata OX=83167 GN=ALO85_100384 PE=4 SV=1
MTDVSAPYRPQSTSFETGLKFEENRYGKSAQRHPQTQRRPRGHPAKRFDIHIPKNQGPPF
MKSRFSLSSSFLILLSLTGCAANMDAPKHDQPGESALYGSWHVAGVAVSDSGVQALGDDD
PSFMGRRLSFSHDQLAWKASTDSTEDVCKGPVFHELPAMTGAELEPQLHKLGIEKAVRYS
VECKSGSWGPFDKETPAFFLAQDGALALSWYDGGLLKLVRD
>tr|A0A0Q0DHL0|A0A0Q0DHL0_PSEAP AraC family transcriptional regulator OS=Pseudomonas syringae pv. aptata OX=83167 GN=ALO85_00635 PE=4 SV=1
MTGTKSTAASSPATLKRKLHKHCTHFQAQHDLARKHVVLYLYQIKVMSNEAVADYLNFND
PANFRRSFKRWTGSTPTLIQRLFNVE
>tr|A0A0Q0FWT9|A0A0Q0FWT9_PSEAP CbsD/ElaB super protein OS=Pseudomonas syringae pv. aptata OX=83167 GN=ALO85_04899 PE=4 SV=1
MATTSLRKASLQSMEAEIESLLKSLEGLKADATDESRKTLKNLKANAESALSHSRSLLSD
VYEDVKEKTRENALATRDYAQEHPWTTAGVAVGAIGLIAAYMLFKRGN
>tr|A0A0N8T982|A0A0N8T982_PSEAP Putative GntR-family transcriptional regulator OS=Pseudomonas syringae pv. aptata OX=83167 GN=ALO85_00921 PE=4 SV=1
MILSSANIETSPARLSAVEALAAKLKARILNGDFEPGEFLRDVKMAEEYEVARNTFRSAA
QLLVSFGLLLKVPHRGFCIPEFGPDDVVDIARLRGVLETEAVRMIILNGTVPANALDAVE
RLRNAAPDTPRSDIVTADGDFHRAIIRASGSARLQRSYAMLESEIELLLVLQQPCYEDPK
QIVKDHEHLIACLRSRDFETARTAFVEHWEDLSAKLLRAQFERRQSL
>tr|A0A0Q0CBY9|A0A0Q0CBY9_PSEAP Ammonium transporter OS=Pseudomonas syringae pv. aptata OX=83167 GN=ALO85_03885 PE=3 SV=1
MTLRQFAGLGALLSLALPGLALAADPVAEPVLNSGDTAWMLTSTALVLFMTIPGLALFYG
GMVRSKNILSVMMQCFAITGLISILWVVYGYSIAFDTTGMEAGVVNFNSFFGGMGKAFLA
GITPASITGPAALFPEAVFVTFQMTFAIITPALIVGAFAERMKFSAMLVFMGIWFTLVYA
PIAHMVWGGVGGLLWDWGVLDFAGGTVVHINAGVAGLVACLVLGKRKGFPTTPMAPHNLG
YTLIGAAMLWVGWFGFNAGSAAAANGTAGMAMLVTQIATAAAALGWMFAEWLTHGKPSAL
GIASGVVAGLVAVTPAAGTVGPMGALIIGLAAGVICFFCATSLKRKLGYDDSLDAFGVHG
IGGIVGAILTGVFAAPALGGFGTVTDIGAQVWIQFKGVAFTVVYTAIVTFIILKVLDAVM
GLRVTDEEESVGLDLAQHNERGYNL
>tr|A0A0Q0IEM0|A0A0Q0IEM0_PSEAP Two-component DNA-binding response regulator QseB OS=Pseudomonas syringae pv. aptata OX=83167 GN=ALO85_02112 PE=4 SV=1
MRLLLVEDDRAIGQGIRVALNTEGYTLDWLEDGLSALHALRSEPFDLLLLDLGLPRMDGL
DLLRQLRAEALTLPVLILTARDGTADRIAGLDAGADDYLIKPFDVDELKARVRALLRRSQ
GRAQPLLEHAGISLDPASQQVSFKGSEVPMTPMEYQLLHQLMIRPGKVVTRERLSNTLYG
WQDKVESNTLEVLIHNLRKKLSTELIRTVRGVGYLLELKA
>tr|A0A0Q0C3U2|A0A0Q0C3U2_PSEAP Uncharacterized protein OS=Pseudomonas syringae pv. aptata OX=83167 GN=ALO85_01404 PE=4 SV=1
MKFRFLLWMLGLLMARASRNNPAFQQQLAGKDLTFQLQTADGKVARHFVVNDQRIRSASG
TVAEPAFAIVFRDAAFGFDTLQAKNKQLAFMKGIQDKDIQIKGNPALVIWFQGLVKYLKP
RKKTV
>tr|A0A0Q0D439|A0A0Q0D439_PSEAP Glutathione S-transferase OS=Pseudomonas syringae pv. aptata OX=83167 GN=ALO85_02523 PE=3 SV=1
MLKIWGRKNSSNVRKALWIAEEVGVPYETQDAGGAFGLVNEAAYRAKNPNGRIPMIEDGD

FVMWESNAIVRYLAARHAPDSDLYPADLQARANADKWMDWTTSTIAAPFSPVFWGVVRTP
AEKQDWPAIEQGIKTLHSLLLVADEALSRQPYLSGNAFGMGDIPLGCFAYGWFEMPIERP
PLPHLQAWYERLKMRPAYRKAVMTPLT
>tr|A0A0Q0C6D1|A0A0Q0C6D1_PSEAP Regulatory protein, DeoR OS=Pseudomonas syringae pv. aptata OX=83167 GN=ALO85_03596 PE=4 SV=1
MLNQPRLDEIMRLLVQQQRVKASDLAQLLFVSEETIRRDFKHLEEEGRLRRIHGGAILPR
SSEELPLQERSRLKPRAKAGIAVRAAQLVSEGMAIFLDTGTSTLALAQQLTRFSQLKIIT
NSLDIAQLISHQSDNQVLVAPGDVRRTDNALIGPHTLEFARQFHYDIAFMGIGGIDLDFG
LMDYQEPEAMLRRTLVRHCTRSVVLADDGKFGHRTFINTLPFAAITTLVTNRALSDEFAT
RLEKDHVDTLYS
>tr|A0A0Q0DIS7|A0A0Q0DIS7_PSEAP Isochorismatase hydrolase OS=Pseudomonas syringae pv. aptata OX=83167 GN=ALO85_04242 PE=4 SV=1
MIKVNARPDRFAFDTSRTAVVIIDMQRDFLEPGGFGAALGNDVAPLQAIVPSVQRLLALA
RDEGMAVIHTRESHRPDLADCPQAKRDHGSPGLRIGDPGPMGRILIRGEPGNQIIDTLAP
RAGEWIIDKPGKGMFFATDLQQRLTDAGIIHLIFAGVTTEVCVQTSMREANDRGYRCLLI
EDATESYFPAFKQATLEMITAQGGIVGRVASLTDLEQALLTRSTL
>tr|A0A0Q0FT54|A0A0Q0FT54_PSEAP Histidine kinase, HAMP region: chemotaxis sensory transducer OS=Pseudomonas syringae pv. aptata OX=83167 GN=ALO85_03765 PE=4 SV=1
MHYPSSSDREIKEGYTTMNLLRGTMLSTRLISAFVVCAVVTLGVGLVGYTGITGLKNSVD
GIVNNNLVSVYNTSNARSNAIAHYRDLHRALLYKFAKADQAKYDETLKSLADNQKEVEQL
FNAYRQTPLEDDERAAGDQFEKDWPAYINASSRVIELAAKGDLESANKIFAQEVDPAYKK
TNDELKIMVASNKRQSDDVAIEAAKTSSVAYGSLGIGVVIAFVAAIILGLLVTRSITRPL
LGALRTAKQVADGDLSQQIDVTGNDEISTLQSALSTMQVNLRNTVKEIANAADQLASASE
ELNAVTEESSKALIRQNDEIQQAATAVNEMTAAVEEVARNASGASESSEETSRSAIEGRD
QVKNAVGSVNTMAEQISQSTEKVTVLATRVNEITGVLAVIQSIAQQTNLLALNAAIEAAR
AGEQGRGFAVVADEVRALAHRTQASTADIEAMMAQIRAGADEAVISMNNSRSLAGETRDQ
AVQAGHALDRITEGVSMINEKNLVIASAAEEQAHVAREVDRNLVNIQDISTQTATGAHQT
NASSAELSRLAASFGVLVNKFKT
>tr|A0A0Q0DQ84|A0A0Q0DQ84_PSEAP Uncharacterized protein OS=Pseudomonas syringae pv. aptata OX=83167 GN=ALO85_200205 PE=4 SV=1
MRMENNCETNSSKQSNVDVLDTIPHQGRDFFWERHQFARHGYLKINHLMDAGVRDNIMAE
VKYLLANHAKRRDMLIESTGNTPRYLSNVTRDTIAEHGAYITKAYDSNYLKGLVAHIVKE
EIIPTPWIWDNYIINSQHKAGDTHGWHWGDYPFTIIWIVEAPRIEAGGLLECVPHTDWDK
KNPRIENLLVENNIESHYHQTGDVYLLKTDTTLHRVKPLTEDVTRIILNMAYERAKDVDR
YVEHETFVFRD
>tr|A0A0Q0CC10|A0A0Q0CC10_PSEAP Lipoprotein OS=Pseudomonas syringae pv. aptata OX=83167 GN=ALO85_02552 PE=4 SV=1
MSISFKALVPAAPEMARKLHPTTIRRFSVTGIWEGIMRSLVMLLALLALSGCMKVSDMGE
GVREQFSDAGLLDHSETRRTANWRVQPDSFIYIAQGAFVPPGNAYPRPNVVAEEAFKGFI
EYFPMVRRARGPLGLDEAMVEARAAGAHYLLYARFAKADDRIGNGDQWADQEAVDRLGVD
SGVIQLMLIETGTRYLIDSARIRSRGGLLTLYDKSPEDLLGPPLEDYARTLLGVER
>tr|A0A0N8T7V8|A0A0N8T7V8_PSEAP Gamma carbonic anhydrase-like protein OS=Pseudomonas syringae pv. aptata OX=83167 GN=ALO85_04078 PE=4 SV=1
MKFRLGESRVQTDPQSWVAPNATLIGNVRLEAGASVWFNAVLRGDNELIHIGENSNVQDG
TVMHTDMGSPLSIGKGVTIGHNAMLHGCSVDDYSLIGINAVILNGAKIGKYCIIGANSLI
GENKVIPDGSLVMGTPGKVVRELTDVQKKMLEASAAHYVHNAQRYARDLAVQEE
>tr|A0A0N8T7Y8|A0A0N8T7Y8_PSEAP Uncharacterized protein OS=Pseudomonas syringae pv. aptata OX=83167 GN=ALO85_01859 PE=4 SV=1
MDGSDLAILATMSLNMRGKISLFDIQDLEADLEALTGVAVDFDVYNNMPDDWRASIERDV
VML
>tr|A0A0Q0C0Z6|A0A0Q0C0Z6_PSEAP Uncharacterized protein OS=Pseudomonas syringae pv. aptata OX=83167 GN=ALO85_101269 PE=4 SV=1
MRMLGVKRLWQAPSNRARQASEVILNNDMLILGSVSKGGCVVRMQAGWTVNNVFGSIMAD
>tr|A0A0Q0D3M7|A0A0Q0D3M7_PSEAP Uncharacterized protein OS=Pseudomonas syringae pv. aptata OX=83167 GN=ALO85_01771 PE=4 SV=1
MQPDSLLTQAELDFIQSMQRNPQLNVRDSTRSLLVNGGIQIQDLLTKLAAHEQVTIHAQF
ENQQMNFPLHLVEDEFHALHLELGAPSIFEEGPKIRPWRLVLEEPIPLETEKGALTALWV
HELSFKGALLEYRKEGKLPRNFSAWFNPPGQAPIPMRGKLERMIGQNVGAYHFSKHGKDD
AERLRQYILQEHRRKHPSLHK

>tr|A0A0Q0D051|A0A0Q0D051_PSEAP Alpha/beta hydrolase fold protein OS=Pseudomonas syringae pv. aptata OX=83167 GN=ALO85_03202 PE=4 SV=1
MEPDMSVQQRNNVNISGDGPITLIFAHGFGCDQNMWRFMAPHFAARFKVVLFDLVGNGNS
DVSAWYPHKYSSLKGYATDLLEVVNEFAAEGPVVHVGHSVSCMIAVLAELQSPGRFDGHI
MIGPSPHYLNEGDYMGGFTRADVDSLLGTLESNYLGWSSTMAPALMGASDRPELSEELAN
SFCRTNAEIARQFARVTFLSDHRADVAQLKSRTLILQSSDDMVVPVEVGEYLHRVITDST
LRMIDNVGHYPHMSAAQECITAMNQFLASYESAAHDR
>tr|A0A0Q0DTC4|A0A0Q0DTC4_PSEAP Hexapeptide repeat-containing transferase OS=Pseudomonas syringae pv. aptata OX=83167 GN=ALO85_02654 PE=4 SV=1
MKHRMMHSHNLREALPDYARKMAVPLDELEAAYQWMLDNEVCFETRIKDRTLSFICYLNI
EPRIEHPLARRFYKLLAIETKGPLIPLYGINWPTLRDRMLRTWEQAYNILICKIPSHTLR
LLWLRIGGAKIGKGSTVWRNTEVLGVDSLRIGNDSTVGWHCQLDARGGLVIGDHVTIASH
VLIIAGGHDLNEPEFWAVGGPVFIGDYAWICSRALLSFGADIGEGAVVGGNSVVSKPVPP
YAIVSGPNAEIKGERARGLNYKVGGKGLFTLFH
>tr|A0A0Q0DT95|A0A0Q0DT95_PSEAP Formyltetrahydrofolate deformylase OS=Pseudomonas syringae pv. aptata OX=83167 GN=purU PE=3 SV=1
MSRAPDTWILTADCPSMLGTVDAVTRFLFEQGCYVTEHHSFDDRLSGRFFIRVEFRQPEA
FDEQGFRAGLSERGETFGMIFELTAPNYRPKVVIMVSKADHCLNDLLYRQRINQLSMDVV
AVVSNHPDLEPLAGWHGIAYYHFPLDPNDKPAQEAKVWQVIEESGAELVILARYMQVLSP
DLCRKLDGKAINIHHSLLPGFKGAKPYHQAYNKGVKLVGATAHYINNDLDEGPIIAQGVE
VVDHSHYPEDLIAKGRDIEGLTLARAVGYHIERRVFLNANRTVVL
>tr|A0A0Q0CAD5|A0A0Q0CAD5_PSEAP Pyrroloquinoline-quinone synthase OS=Pseudomonas syringae pv. aptata OX=83167 GN=pqqC PE=3 SV=1
MSDATALSPAEFEQALRAKGAYYHIYHPFHVAMYEGRATREQIQGWVANRFYYQVNIPLK
DAAILANCPDREIRREWIQRLLDHDGAPGEDGGIEAWLRLGQAVGLDPDQLRSQELVLPG
VRFAVDAYVNFARRANWQEAASSSLTELFAPQIHQSRLDSWPQHYPWIDPAGYEYFRTRL
GQARRDVEHGLAITLQHYTTYEGQQRMLEILQFKLDILWSMLDAMSMAYELNRPPYHSVT
DQRVWHKGITL
>tr|A0A0Q0BVZ7|A0A0Q0BVZ7_PSEAP BRO domain-containing protein OS=Pseudomonas syringae pv. aptata OX=83167 GN=ALO85_00057 PE=4 SV=1
MKTDPKLILCTDCVDPLIFIRHHRALRAVRLGYQCWFSLQDMARLMGKALDERSTRKLDS
DQHRHVWLHSHGEWQKCLMISDSGIYALLVHHYVPENRALRLWLSSEVIPTLCSEFTPPA
RHITVVWWRRMLGLPPAREKFAA
>tr|A0A0Q0CBC0|A0A0Q0CBC0_PSEAP Glutamyl-tRNA reductase OS=Pseudomonas syringae pv. aptata OX=83167 GN=hemA PE=3 SV=1
MAFLALGINHKTASVDVRERVAFTPEQLVEALQQLCQLTESREAAILSTCNRSELYIEHE
HLGADSILAWLANYHHLSLEELRASAYVHEDDAAVRHMMRVASGLDSLVLGEPQILGQMK
SAYAVAREAGTVGPLLGRLFQATFSAAKQVRTDTAIGENPVSVAFAAVSLAKQIFSDLQR
SQALLIGAGETITLVARHLHDLGVKRIVVANRTLERASMLAAEFGAHAVLLSDIPAELVN
SDIVISSTASQLPILGKGAVESALKLRKHKPIFMVDIAVPRDIEPEVGELDDVYLYSVDD
LHEVVAENLKSRQGAALAAEQLVSVGAEDFMSRLRELAAVDVLRAYRQQSERLRDEELSK
AQRMLANGSNAEDVLIQLARGLTNKLLHAPSVQLKKLSAEGRVDALAMAQELFALGEGST
DKTPQ
>tr|A0A0Q0BWE0|A0A0Q0BWE0_PSEAP Alpha/beta hydrolase family protein OS=Pseudomonas syringae pv. aptata OX=83167 GN=ALO85_02216 PE=4 SV=1
MSQPVFFAHANGFPSATYSKLFCALAPEFSVAHLEQHAHDPRFPVDDNWLNLVDELLHHL
REQPGPVWGVGHSLGGVLHLHAALRCPELYRGVVMLDSPVLGLADQLFIRAAKRLGFIDR
ITPAGRTLGRREAFDDLPSARAYFAGKTLFSRFDPDCLSAYLQHGLREDGGQWRLRFDPA
TEISIYRSIPHTSPVPSRQLKVPLAMVRGKHSRVIMPHHGYLARRMREGEYLSMPGGHMF
PLERPDETALLLKTLLARWDARSASRVTA
>tr|A0A0Q0DB48|A0A0Q0DB48_PSEAP Secretion protein HlyD OS=Pseudomonas syringae pv. aptata OX=83167 GN=ALO85_04274 PE=3 SV=1
MRRKTLTVTAVVLVIACAAIAGWKLTRPAILPKHAAVAVPVKVITVSLEDVPRFVTGIGS
VLSLQSVVIRPQVDGVLTRVLVKEGQQVKAGDLLATLDDRSIRAALEQARAQLAQSKAQL
DVAQLDLKRYRQLTQENGISRQTFDQQQALVRQLEATAKGNEASINASQVQLSYTQIRSP
VTGRVGIRNVDEGNFLRVSDAAGLFSVTQIDPIAVEFSLPQQMLPTLQGLIAERNAATVK
AYQGDGAANGLLLGEGTLSLIDNQVSAGTGTIRAKAQFKNPGEQLWPGQLVTVKIQTGIE
LNSLKVPPQVVQRGVDLHFVYRLTADNKVEVVPVKVLYQDSEQTLISGPRAGDLLVSDGQ
SRLRPGATVEVITAPAADVASAQAEPAR

>tr|A0A0Q0BZ01|A0A0Q0BZ01_PSEAP Probable 4-amino-4-deoxy-L-arabinose-phosphoundecaprenol flippase subunit ArnE OS=Pseudomonas syringae pv. aptata OX=83167 GN=arnE PE=3 SV=1
MTWLMLASACLLTCLGQIAQKYAVQDWRGAFPGAFAALRSIWLWLALACLGSGLLVWLLV
LQRLDVGIAYPMLGVNFVLITLAGRYVFNEPVDAQHWLGIALILVGVFQLGHQA
>tr|A0A0Q0C574|A0A0Q0C574_PSEAP Putative pre-16S rRNA nuclease OS=Pseudomonas syringae pv. aptata OX=83167 GN=ALO85_02931 PE=3 SV=1
MAALRLLLGIDYGTKQIGVAVGQAITGQARELCTLKAQNGVPDWDKVQALINEWKPDAIV
VGLPLNMDGTPSDMSARAEKFSRKLNGRFGVTVYTHDERLTTFEAKGERMARGGQKGSYR
DNPVDAIAAALLLQGWLDEHPELLNV
>tr|A0A0Q0C8M3|A0A0Q0C8M3_PSEAP Chemotaxis protein methyltransferase OS=Pseudomonas syringae pv. aptata OX=83167 GN=ALO85_02136 PE=4 SV=1
MSTGNLDFDQFRVFLEKACGILLGENKQYLVSSRLNKLMEQQSIKSLGELVQRIQTQPRS
GLREQVVDAMTTNETLWFRDTYPFEVLKNKVLPEQIKASPGQRLRIWSAACSSGQEPYSL
SMSIDEFERANQGQLKSGVQIVATDLSGLMLNNCKTGEYDSLAIGRGLSPDRLQRFFDVK
SPGRWVVKAPIKSRVEFRSFNLLDSYASLGKFDIVFCRNVLIYFSAEVKKDILLRIHGTL
KPGGYLFLGASEALNGLPDHYQMVQCSPGIIYKAK
>tr|A0A0Q0C466|A0A0Q0C466_PSEAP 4-hydroxyphenylpyruvate dioxygenase OS=Pseudomonas syringae pv. aptata OX=83167 GN=ALO85_02637 PE=4 SV=1
MADLYENPMGLMGFEFIEFASPTPNSLEPVFQMMGFTKVATHRSKDVSLFRQGAINLILN
NEPHSLASYFAAEHGPSVCGMAFRVKDAQKAYNRALELGAQPLEIATGPMELRLPAIKGI
GGAPLYLIDRFGEGTSIYDIDFNFIEGVDRHPVGAGLKFIDHLTHNVYRGRMAYWAGFYE
KLFNFRELRYFDIKGEYTGLTSKAMTAPDGMIRIPLNEESSKGAGQIEEFLMQFNGEGIQ
HVAFFTDDLIKTWDALKATGMRFMTAPPDTYYEMLQGRLPDHGEPEAELKSRGILLDGSS
EGGERRLLLQIFSATLMGPVFFEFIQRKGDDGFGEGNFKALFESIERDQIQRGVLTPD
>tr|A0A0Q0BRF0|A0A0Q0BRF0_PSEAP Uncharacterized protein OS=Pseudomonas syringae pv. aptata OX=83167 GN=ALO85_04154 PE=4 SV=1
MNEHDLTNNNDSLPNLNFKAVEPTAKFPDVDFGFFEREEEKKKILASSNKVIDALQNTLD
SVEEKAKTFSANLFASGFADQLRRLQGVETGLTKVEVGQQAVEQIDHYEASSSVTSRPLH
QPEASGEWTAGIGIDNELDEEERLEELVSSMMEVDTEDDSYFDTFDFSQSRAKVTEPEPL
PASEHEPEPEPEPVVGLDDEPEAVEAPVTHLVVSEESVEAWEEIAPDEVSEVGEVHVPDE
LTVPVQDDDEPVYESFTPPADVEVPAHVQAISDELAPFLAPSPVDDLQEPGGEPSKAFDA
AVFASGRSHKPVPSVLDRQIRLVEKARQARLAMTTKPEFMSETANARRNSLAERSYQAKI
ESLNIMKDLAQQTDSLKAFLENNDLEGASDIIRLLKRAEYLKAQAKHFDKLREELVLINF
KKTYKE
>tr|A0A0N8T7T8|A0A0N8T7T8_PSEAP Uncharacterized protein OS=Pseudomonas syringae pv. aptata OX=83167 GN=ALO85_02304 PE=4 SV=1
MKLPKLDGVSLDPKFFSKHIWNADLVYFEGPLLSLYRDEQGADLLYAWLDCTDKSNRWCV
IPISRKMLRDYLETQITLRDVFIESSWIAIFHTGSSAKRRNSATLTCWNKLPQEYHPDED
SFLSPDIATEAADKFAEEVSEAYFLGLDGDMYIDDIAAIPKTYQQLYSFHYGLEHLDRVA
VRDTLDRLGSSWTGGFSAVHLFTGLNQVTPSIHRPQVMEMLFQSPGHIKLDLLPSLAKRI
ETSANQIHDSDSFRALEEFYSATYRYFRENNIGGFDDERELQRRSLPDEIVEELSNQVTF
FFKLMHWEDYRAQFGSLHIDPLHQLRALLAYYRRLRKLKPYLDSGRLVLGRSMIEPLPPE
PST
>tr|A0A0N8T913|A0A0N8T913_PSEAP Putative Lipoprotein OS=Pseudomonas syringae pv. aptata OX=83167 GN=ALO85_03946 PE=4 SV=1
MLGRERAVRQFQKITSSTNPTSKVPPPETWVRVFPWSFTLKSFSKVVAAAVCGLVLAGCT
GTPMKTPQYDSSQYTVVGHSEASATGLMLFGLIPIRQNDRFVRAQNSAIQAKGGDALINT
QVQEKWFWAWVLNGYTTTVSGDVIKLKTAK
>tr|A0A0Q0C545|A0A0Q0C545_PSEAP Putative lipoprotein OS=Pseudomonas syringae pv. aptata OX=83167 GN=ALO85_02864 PE=4 SV=1
MSLDGHLDGMMNFLRLPLILLMTGVLGLAGCSVHQPTALYQLDSGEPGQPKQSSGLAVLL
GPVSVADYLQRETLLQRQPDGTLTASSDGRWAGNLSSDIDQLLLRQLAWKLDSQRVVMAP
ATASFTPDVQVVLSITRLDSGAKQPAVLDAQWRLVDRKGHVRDSRLVHLEQVHAGSSADQ
VKAQGMLLQRLADQVSTAIKPISWQALEEPKKTPVAKAKEPDKPRIPLASPIRTDLEVFR
F
>tr|A0A0Q0BXB6|A0A0Q0BXB6_PSEAP 3-dehydroquinate dehydratase OS=Pseudomonas syringae pv. aptata OX=83167 GN=aroQ PE=3 SV=1
MATILVLHGPNLNLLGTREPGVYGTVTLPQINQDLEQRARDAGHHLMYLQSNAEYELIDR
IHAARGEGVDFILINPAAFTHTSVAIRDALMGVSIPFIEVHLSNVHKREPFRHHSYFSDV

AVGVICGLGASGYRLALEAALEQLAASAKP
>tr|A0A0Q0IDQ4|A0A0Q0IDQ4_PSEAP Biotin carboxyl carrier protein of acetyl-CoA carboxylase OS=Pseudomonas syringae pv. aptata OX=83167 GN=ALO85_01373 PE=4 SV=1
MDIRKVKKLIELLEESGIDELEIREGEESVRISRHSKTPAQPYYAPAPVAAPVAAPAPAA
APAAPEVPSAPKLNGFVVKSPMVGTFYRTPAPTSPAFVEVGQTVKKGDTICIVEAMKMMN
HIQAEASGVIESILVENGQPVEFDQPLFTIV
>tr|A0A0N8TAA4|A0A0N8TAA4_PSEAP Phosphofructokinase OS=Pseudomonas syringae pv. aptata OX=83167 GN=ALO85_03319 PE=3 SV=1
MAKILTLTMNPALDLTVQLGQLELGQVNRSTAMLTHAAGKGLNVAQVLADLGHDLTVAGF
LGVDNQQAFEALFERRQFVDEFVRVPGETRSNIKLAEGSGRITDLNGPGPQVSEEAQHAL
FTRVEQIAQGFDVVVVAGSLPRGVTPEWLQKLLVMLKDLGLKVALDSSGLALRAGLKAGP
WLIKPNTEELADALDAPIISIAAQAEAAARLQAQGIEHVVISQGSEGVHWFSPSVALHSL
PPKVTVASTVGAGDSLLAGMVHGLITGQEPHKTLRTATAIAAMAVTQIGFGITDAAQLKR
LEGGVTVRSLSEQ
>tr|A0A0Q0C518|A0A0Q0C518_PSEAP Phage-related protein OS=Pseudomonas syringae pv. aptata OX=83167 GN=ALO85_04159 PE=4 SV=1
MNTFIRTHEFDNWLKGLKDVTARLRITNRIMQAQNGNFGDCEPVGDGISEMRIFVGPGYR
VYYTRRGEVVYLLLCGGSKGSQKRDIKLAKSILDEIEKEGRK
>tr|A0A0Q0DXH0|A0A0Q0DXH0_PSEAP Uncharacterized protein OS=Pseudomonas syringae pv. aptata OX=83167 GN=ALO85_03608 PE=4 SV=1
MRKTVMMIAVLALAGCDGGGPARDKVQGTAATAPVSTPQWFIQMNSREAVSDSSAWLLER
SYAPIIVDVDGKQQVLIGPYESQAKAEEERVALQAKVTKSHRFAEPSVVQHTR
>tr|A0A0Q0CY46|A0A0Q0CY46_PSEAP Uncharacterized protein OS=Pseudomonas syringae pv. aptata OX=83167 GN=ALO85_100685 PE=4 SV=1
MSIIEPVRRYFRRREAEMMIQVAHKVSLLVQEQAVPLGSFVFPGMDYVAMLEVDGKRVGH
IDYCINPLRDRLYIDKIEIYADYRRRGFALSALWQLWQKHYLPIVPLYQFGTSDGFWHKA
RTRFAAADAVIGDEIRGSMEMSAEMDRWQHLVPEPIHERLQRELMASDEWPAIKAKWDAE
YGPCRED
>tr|A0A0Q0IDB0|A0A0Q0IDB0_PSEAP Uncharacterized protein OS=Pseudomonas syringae pv. aptata OX=83167 GN=ALO85_01666 PE=4 SV=1
MINSTPSPPLPNSLEDSLMQVSDILRCASATAYETGDNLDGLKRDLAFSVVHLINMAKAE
LERSLECVQNP
>tr|A0A0Q0C283|A0A0Q0C283_PSEAP GCN5-related N-acetyltransferase OS=Pseudomonas syringae pv. aptata OX=83167 GN=ALO85_03904 PE=4 SV=1
MGVNGIQIRRFVPSDESGVSAMILPIQREEFGIPITAEDQPDLKAIPAFYQTGTGDFWVA
VQDDQVIGSIGLRDIGAGQAALRKMFVAAPFRGREFSVATRLLERLIEESTRKGVSEVFL
GTTDKFHAAHRFYEKHGFREITKEDLPASFPLIAVDSKFYVLGLKEQ
>tr|A0A0Q0C523|A0A0Q0C523_PSEAP Cyclohexadienyl dehydratase OS=Pseudomonas syringae pv. aptata OX=83167 GN=ALO85_04254 PE=4 SV=1
MMNLKYSALTALLLGITGMAQAQDYTSHLDKVLQTGQLAVCTTGDYKPYTLLREDGEYEG
IDITMARSLAKSLGVNVQWVPTTWKNLMPDMVAGKCDIGMGGISVSLERQKKAFFSNTLD
VDGKIPLVRCADQALYQTVEQINQPSVRLIEPAGGTNEAFARAHLSKASLAFHDNKTIFQ
ELLDKKADVMITDASEALYQQKRMPGLCAVNPTRYMQYGEKAYLLPRDDVTWKGYVDQWL
HLSKATGEYQQALGEWLAVPAE
>tr|A0A0Q0FNV2|A0A0Q0FNV2_PSEAP Probable potassium transport system protein kup OS=Pseudomonas syringae pv. aptata OX=83167 GN=kup PE=3 SV=1
MSQANSHAEAGGAAKPIGLLIAAVGVVYGDIGTSPLYTLKEVFQGGYGVDVTHDAILGVL
SLIFWSLIWVVSFKYMAFVLRADNQGEGGIMALMALARRASAKHPRLQMMMVVFGLFGAA
LFYGDSMITPAVSVLSAMEGLELAFDGLDHWIVPMALVVLVGLFLIQRHGTARIGVLFGP
VMVIWFLVLGALGVYGIMQSPEVLKAVNPAWGVNFFIIHPGIGVAILGAVVLALTGAEAL
YADMGHFGRKPISRAWFILVLPALLLNYFGQGALVLGNPEAVRNPFYLLAPGWALLPLIG
LSTMATIIASQAVISGAFSMTLQAIQLGYIPRMHIQHTSSDAQGQIYIGAVNWALMAGVI
LLVIGFESSGALASAYGVAVTGTMLCTTILVSTVMLMLWKWPPLLAVPLLICLLLVDGLF
FAANVPKIFQGGAFPVLAGAVLFILMTTWKRGKQLLAERIDEGGLPLPIFIGSIRVQPPH
RVQGTAVFLTARSDAVPHALLHNMLHNQVLHEQVVLLTVVYEDSPRVPSAQRFEVESYGE
GFYRVILHFGFIDEPDVPAALALCHLPELDFSPMRTTYFLSRETVIPSKMDGMARWREAL
FAFMLKNANGNLRFFKLPFNRVIELGTQVEM
>tr|A0A0Q0CC45|A0A0Q0CC45_PSEAP Acyl carrier protein OS=Pseudomonas syringae pv. aptata OX=83167 GN=acpP PE=3 SV=1

MSTIEERVKKIVAEQLGVKQEEVVNTASFVEDLGADSLDTVELVMALEEEFETEIPDEEA
EKITTVQAAIDYVTSHQA
>tr|A0A0Q0BU33|A0A0Q0BU33_PSEAP ABC transporter ATP-binding protein OS=Pseudomonas syringae pv. aptata OX=83167 GN=ALO85_01663 PE=4 SV=1
MNAVAQGHTASTSTQPQPAAQPTAEALLQVCGVSLEYRTPERVVRATHQVSFEVDPADRF
VLLGPSGCGKSTLLKAVAGFIEPSEGEIRLAGHQVTEPGPDRIVVFQEFDQLPPWKTVIE
NVMFPLLASRTLKRPEALERARYYLEKVGLSAFADAYPHTLSGGMKARVAIARALAMQPK
ILLMDEPFAALDALTRRKMQEELLELWDEVRFTLLFVTHSIEEALVVGNRILLLSPHPGR
VRAEINSHQYDLKSLGGVGFQQTAQRIHRLLFDEGEAGPVEQDLNFQDIRIAY
>tr|A0A0Q0E0N9|A0A0Q0E0N9_PSEAP Uncharacterized protein OS=Pseudomonas syringae pv. aptata OX=83167 GN=ALO85_03313 PE=4 SV=1
MPNDEKVVPLSKSFPLQGAGTPLARLPVVLLQVRDKAAQQLKDALQALFDNADDTLFEMA
DRARSNTEQNIFFEAMRDLRLKRKSIERGFLDKFYESFLVLGQYQIQEPVLPAAVSFDKL
SLVHNDDLEKTVAVDAMVSKVMSRDALALGQLTARLNVLIPQAIAEDSNPLGPAMLCRFF
LEAGRSLGVEIKVKLIILKLFEKYVLANTDDLYSEANQLLIATGVLPDMKALPARRSSDR
AAPRSRSAEMSDPALNEAADKLDKGVQEVFSALQELLLHVRGHLAPRHEPNAEVRPISSR
DLLRLLSHLQQYVPSQDTSDDFDLRNQLEQLLTRVNVKTGKSRIVGVGDEDVINLIAMLF
EFILDDRNLTPSLRALIGRLQIPMLKVAVIDKSFFSRGSHPARRLLNEIATAALGWGGRD
DYQRDSLYQRVEQIVQRLLNDFVDDPAIFSELLADFLAFTSDERRRSELLEQRTRDAEEG
RARAELARQQVQHELNQRLLGKTLPEVVVRLLQEAWSKVLLLTCLKHGEESSEWQAGLET
MDELIWSVELHDDPQALQRLLELVPGLLKSLRDGLSSAAFDPFATSEFFSQLESLHVKAF
QHFSRLQESEGDTAQSDATQADGQAVEGPPMMEVIEEIVLITAEEQMLNEPVVQLPDDDA
GLQMVDKLRVGCWVEIQEDEEHKLRCKLTAIVEPTGRYVFVNRTGMKVLEKTRTGLAIEF
RRGAVRILDDALLFDRALESVISNLRKLKGA
>tr|A0A0Q0BYH0|A0A0Q0BYH0_PSEAP BNR repeat-containing glycosyl hydrolase OS=Pseudomonas syringae pv. aptata OX=83167 GN=ALO85_02115 PE=4 SV=1
MRVISRNLTAWSAGLIVVAIFLSAWLSHPQHRIAPFALSPAPVGAESVAPKASYMSRFAS
SDLDDFVHSSAVTALPGGDLMSVWFAGSREGAGDVEIRTSRFDAASGEWGGEQVLATRES
TQSGTGKYIRKLGNPVIALAPDNRLWLFYVSVSVGGWAGSTVNAMVSSDMGASWSPPWQL
VTTPFLNISTLVRGAPVFHADGSIGLPVYHEFLGKFAEYLYLSADGEVIDKFRISRGNNS
LQPTVVPLDGQRAVALLRYAGETFHRVLASRTEDGGQSWSEPYPLTPSNPNSSLAAVGVP
GRGLLVALNDLREGRFKLSLYGTDEEMNVWRPLMDFDKSPDPLGTPFTLEAYKEVIGEGF
RSSSGALRQPMEEAFLSNLDQRVCSPQGCDFEYEYPYFIRSPDGLYHLVYSWNNTFIKHV
SFNEAWLAEQLL
>tr|A0A0Q0D2E0|A0A0Q0D2E0_PSEAP Transcriptional activator protein Anr OS=Pseudomonas syringae pv. aptata OX=83167 GN=ALO85_02998 PE=4 SV=1
MMSEPVKSRAQNQAHCKDCSLAPLCLPLSLNTEDMDCLDQIVKRGRPLKKGEFLFRQGDT
FESVYAVRSGALKTFNISDSGEEQLTGFHLPSELVGMSGMDAEAYPVSAQALETTSVCEI
PFERLDELSAKLPQLRRQLMRVMSREIRDDQQMMLLLSKKTADERIATFLINLSARFRAR
GFSANQFRLSMSRNEIGNHLGLAVETVSRVFTRFQQNQLISAEGKEIHILAPIELCALAG
GSMQS
>tr|A0A0Q0C6G4|A0A0Q0C6G4_PSEAP VirK family protein OS=Pseudomonas syringae pv. aptata OX=83167 GN=ALO85_03746 PE=4 SV=1
MKHSYAAALFALSLPGLAHAGDALPTYSAITQALNTGESVAVVIDLGQCKSSIAGAEPSK
TKGGKRIDAYRITPDGTLAFSDTHFSLDRNNKPIEQFIRYQIRSNGTATFSMTTLNVPGY
QQVGSPVSYECGVGKGLSFFVSQ
>tr|A0A0N8T7T6|A0A0N8T7T6_PSEAP Aliphatic sulfonates import ATP-binding protein SsuB OS=Pseudomonas syringae pv. aptata OX=83167 GN=ssuB PE=3 SV=1
MTSLKQQPPHLLRGIPLAVRKLKKAFGAREVLKDIDLHIPAGQFVAIVGRSGCGKSTLLR
LLAGLDKPTQGQLLAGSAPLDDAREDTRLMFQEARLLPWKKIIDNVGLGLSGDWRAQALE
ALEAVGLAERANEWPAALSGGQKQRVALARALIHKPRLLLLDEPLGALDALTRIEMQQLI
EKLWGQYGFTVLLVTHDVSEAVAIADRVILIEEGQIGLDLLVDLPRPRVRGSHRLAALEA
EVLNRVLALPGSPPDPEPFSPLPTQLRWAN
>tr|A0A0Q0BZ30|A0A0Q0BZ30_PSEAP FAD dependent oxidoreductase OS=Pseudomonas syringae pv. aptata OX=83167 GN=ALO85_00917 PE=4 SV=1
MDNQNMPAPLTPVHTSADLPAEADVVVIGGGIIGAFTAYYLARRGMKVALVEKGRIGAEQ
SSRNWGWCRQQNRDARELPMATKSLELWEQFAEQSGEQTGFTRCGLLYLSNNDQELEGWA
RWGEFARTVNVQTQMLNAEQAAERGRATGKPWKGGVFAPTDGIADPSRAAPAVARAIMAL
GSSVHQNCAVRGVETEGGRLSAVVTEKGTIRTRLAVLAAGAWASSFCRQYGIRFPQATIR

QTVLSVSAPSQEIPSALHTTGASMTRRFDGSYTLAISGRGRVDITPQLLRFSTQFLPMFQ
RRWRNLAPGGLEGWQAGHESLKRWRLDQPTPMERMRILDPSADASAVALTYKRAVELVPA
LQGTSIQASWAGYVDSTPDGVPGIGEMASLPGLVIAAGFSGHGFGIGPGAGHLIADIVSG
ASPIVDPRPYHPDRFQTSAWGKVADF
>tr|A0A0Q0DP21|A0A0Q0DP21_PSEAP Branched-chain amino acid ABC transporter, periplasmic amino acid-binding protein OS=Pseudomonas syringae pv. aptata OX=83167 GN=ALO85_04995 PE=4 SV=1
MAGAPANQPCTIAEPGAPAGQSRVPPWCTPSTNRDTFVSFCDVSTLAQTLLKLESAMKCT
AAKPSNNVLREQSMKRRSLIKAFTLSASIAAMGLTWTVQAAETIKVGILHSLSGTMAISE
TSLKDMALMTIDEINAKGGVNGMMLEPVVVDPASNWPLFAEKGRQLLTQDKVAVVFGCWT
SVSRKSVLPVFEELNGLLFYPVQYEGEEMSPNVFYTGAAPNQQAIPAVEYVMSEDGGSAK
RFFLLGTDYVYPRTTNKILRAFLHSKGVKDTDIEEVYTPFGHSDYQTIVANIKKFSAGGK
TAVISTVNGDSNVPFYKELANQGLNATDVPVVAFSVGEEELRGIDTKPLVGHLAAWNYFQ
SVENPVNKKFVADWTAYAKKKNLPGADKAVTNDPMEATYVGIHMWAQAVEKAKSTDVDKV
REAMAGQTFAAPSGFTLTMDKTNHHLHKPVMIGEIQDDGQFNVVWQTKEPIRAQPWSPYI
PGNDKKPDTPAKSN
>tr|A0A0N8T8I4|A0A0N8T8I4_PSEAP Flagellin OS=Pseudomonas syringae pv. aptata OX=83167 GN=ALO85_02156 PE=3 SV=1
MALTVNTNVASLNVQKNLGRASDALSTSMTRLSSGLKINSAKDDAAGLNIATKINSQIKG
QTMAIKNANDGTSIAQTAEGALQESTNILQRMRELAVQSRNDSNSATDRVALNKEFTQMS
SELTRIANSTNLNGKNLIDGSASTMTFQVGSNSGASNQITLTLSASFDANTLGVGSAITI
VGSDSAAAETNFSAAMAAIDSALQTINNTRSDLGAAQNRLTSTISNLQNINENASAALGR
IQDTDFAAETAQLTKQQTLQQASTSVLAQANQLPSAVLKLLQ
>tr|A0A0Q0C2P1|A0A0Q0C2P1_PSEAP Uncharacterized protein OS=Pseudomonas syringae pv. aptata OX=83167 GN=ALO85_200171 PE=4 SV=1
MSAVTLSIVFGLVMGRFFTGSVYEFAALFIVSVGVSLLTFHLLNQRRVRLQAQTAYCCSV
MVASNQMMELFQMAKRGQFMPLVGEIPELIIGGFLVPAIAVSAVQLFRILMES
>tr|A0A0N8T9Z1|A0A0N8T9Z1_PSEAP Flagellar motor rotation protein MotA OS=Pseudomonas syringae pv. aptata OX=83167 GN=ALO85_04022 PE=3 SV=1
MDVLSLIGLILAFVAIIGGNFLEGGHLGALLNGPAALIVLGGTLGASLLQSPMNAFMRAM
KIIHWIIFPPRIDLPGGVDRVIGWSMTARKEGLLGLETVADSEPDGYSRKGLQLLVDGAE
PAAIRSILEVDFITQETRDIQAAKVFESMGGYAPTVGIIGAVMGLIHVMGNLADPSQLGS
GIAVAFVATIYGVAMANLILLPVANKLKAIAHRQARYREMLLEGLLSIAEGENPRSIELK
LQGFME
>tr|A0A0Q0DUC7|A0A0Q0DUC7_PSEAP ATP-grasp domain-containing protein OS=Pseudomonas syringae pv. aptata OX=83167 GN=ALO85_03299 PE=4 SV=1
MIWFLEGQSSQREVIMGARDALPVSVQIIASHRQQRSEITGQADVALQEPVDNEERIDWV
LQTARSLGVKIIVAGRIGSFYEAQRARFVAEGLDLVTGGTSLQTFMDVDDKSRFTAAAEA
AGLACIPGIAVSNAEELQAAYDALAVSGEVCIKPVVGIYGRGFWRFKTDVDDFRCFANPD
ARETTFQAYLDAYRQADDVPAMLLMPYMPGSECSVDMVCEHGKAVAFVGRRKVGLNQTFE
RDSEAVKLAVRAAEHFACDGLINVQTRDDADGKPRLLEINPRYSGGIGYTRETGINLPGI
FAARRLGLKEPETHWLEDIRVKAITVAVRASV
>tr|A0A0Q0DE98|A0A0Q0DE98_PSEAP Uncharacterized protein OS=Pseudomonas syringae pv. aptata OX=83167 GN=ALO85_01260 PE=4 SV=1
MNEVLDTQERHNISRFLLASSLWFICVGIQSVFLPWVLISVVHASAFQFGIAQMCLMLPV
LLLVIYGGYVADRKPLRRLLVCVYLGGALTALVFAVWSYQSAITYANVLVYALAFGVLSA
FCAPARELYLNVLGKKQLQKIVTINIGIEFGIQTVGFAIGGIAPQVGVGLMFLLLSFLFG
MSSLAASYISVTTPSKPHSGLIAETRTALRFSIQHPVIFPVLLLNGLIGVFFLGSFFVML
PLHISRLPGYDASLLALFNIVFMVGLIASVIVLTVKGGIENKLRALISSCLLASLLLAVI
PSVLTEWILFLGVLSWGFLGGIALSMGQTLIQENAPSGNKAQVLALLMLFFMGGSPIGSF
LLGTLLEFVDAPVVGVISAVAMIVSIMAVSYTYTLWNAQHERIDTAL
>tr|A0A0Q0CE26|A0A0Q0CE26_PSEAP Holliday junction ATP-dependent DNA helicase RuvA OS=Pseudomonas syringae pv. aptata OX=83167 GN=ruvA PE=3 SV=1
MIGRLRGSLAEKQPPHLVLDVNGVGYEVEVPMTTLYRLPHVGETVTLHTHLVVREDAHLL
YGFYEKRERELFRELIRLNGVGPKLALALMSGLEVDELVRCVQAQDTSALTRIPGVGKKT
AERLLVELKDRFKAWESLPGTFTLVSNGPNQAEPVASAESDAVSALISLGYKPQEASKAV
SAIKEKDLSSADLIRRALKGMG
>tr|A0A0Q0DLC2|A0A0Q0DLC2_PSEAP Single-stranded DNA-binding protein OS=Pseudomonas syringae pv. aptata OX=83167 GN=ALO85_01037 PE=4 SV=1

MRVMIQGRAIIDNWTKDGKEFSALKVQASRVAILPHRVEAVQLAPSQSYNQPQSEQQHTD
RNSTQNDAAPHFDDDIPV
>tr|A0A0Q0DH40|A0A0Q0DH40_PSEAP Uncharacterized protein OS=Pseudomonas syringae pv. aptata OX=83167 GN=ALO85_00687 PE=4 SV=1
MKTSQSGFTLLEMLAALTVMAVCSGVLLVAFGQSARSLQQVSRSDRLSHAARTIMDQESA
GPLENGTRKGELAGIDWTLDIRQLPGANGQARMFRLDLMLSEHQRKAQFSTLKLRGAVSG
AGS
>tr|A0A0Q0FDF9|A0A0Q0FDF9_PSEAP 30S ribosomal protein S15 OS=Pseudomonas syringae pv. aptata OX=83167 GN=rpsO PE=3 SV=1
MALSVEEKAQIVTDYQQAVGDTGSPEVQVALLTANINKLQGHFKANGKDHHSRRGLIRMV
NQRRKLLDYLKGKDVSRYSALIGRLGLRR
>tr|A0A0Q0C9R1|A0A0Q0C9R1_PSEAP MFS transporter OS=Pseudomonas syringae pv. aptata OX=83167 GN=ALO85_03329 PE=4 SV=1
MYIYRLVLLLVVGIYLFSPAIMDWWIDATGAWYRPYMLWLILIVVTFILQSQRDADEL
>tr|A0A0Q0DR67|A0A0Q0DR67_PSEAP 2-dehydropantoate 2-reductase OS=Pseudomonas syringae pv. aptata OX=83167 GN=ALO85_02468 PE=3 SV=1
MSVDSRRPRVGIIGTGAIGGFYGLMLARAGYDVHFLLRSEFDTVAREGLQVKSAVHGELS
LKPVQAYRSAAEMPPCDWLLVGTKSTGNAALGPIIRQAAAPNASVLLLQNGLAVEDQLRN
VLPDSLHILGGLCFVCVNRVAPGVVAHEAFGAVSVGYHSGPAKDEASRMAVVEACAALFR
TAGIDAPVMANLQQARWQKLVWNVPYNGLSALLQTSTGRLMADPDSQALIRSLMDEVVQG
AEACGHALPPGFAQHLFTVTESMPDYRPSMYHDLAERRPLELDAIYARPLAAALAVGFDM
PRVRALYQALAFIDRGNHPASKE
>tr|A0A0Q0CXU4|A0A0Q0CXU4_PSEAP Ribonuclease R OS=Pseudomonas syringae pv. aptata OX=83167 GN=rnr PE=3 SV=1
MADWQSLDPEAAREAEKYENPIPSRELILQHLAERGSPASREQLVEEFGLTTEDQFEALR
RRLRAMERDAQLIYTRRGTYAPVDKLDLILGRISGHRDGFGFLVPDDGSDDLFMSPAQMR
LVFDGDRALARVSGLDRRGRREGVIVEVISRAHESIVGRYFEESGIGFVVPDNPKIQQEV
LVTPGRNNGAKIGQFVEVKITHWPTPRFQPQGDVIEVVGNYMAPGMEIDVALRTYDIPHV
WPEAVLKEAAKLKPEVEEKDKEHRVDLRHLPFVTIDGEDARDFDDAVYCEAKPGKLRLFS
GGWKLYVAIADVSSYVKIGSALDAESQVRGNSVYFPERVVPMLPEQLSNGLCSLNPHVDR
LAMVCEMTISKTGEMTDYVFYEGIIHSHARLTYNKVSTILEQPKTAEAKSLRGEYGDVVP
HLKQLYAMYKVLLGARHVRGAIDFETQETRIIFGSERKIAEIRPTTRNDAHKLIEECMLA
ANVATAQFLKKHEIPALYRVHDGPPPERLEKLRAFLGELGLTLHKGKEGPTPKDYQALLE
TVRDRPDYHLIQTVMLRSLSQAVYSSDNNGHFGLNYEAYTHFTSPIRRYPDLLTHRAIRS
VIRSKQDTPHVRRAGAATIPKARIYPYDDALLDQLGEQCSMSERRADEATRDVVNWLKCE
YMKDRVGESFPGVITAVTGFGLFVELTDIYVEGLVHVTALPGDYYHFDPLHHRLAGERTG
RNFRLGDTVEVRVMRVDLDERKIDFEISEAAAAAKPTARKRRGNDTAAPAATATAEKDKA
PAKSGRRSASTKDSSPEAYRPSDAAAKNAEVRKSRELKKALLADAKGGSKASSGKSDRSG
SAPSSGKPASKHRKGSSKTGSAPAAKSSGARKPKAKS
>tr|A0A0Q0BXW6|A0A0Q0BXW6_PSEAP Uncharacterized protein OS=Pseudomonas syringae pv. aptata OX=83167 GN=ALO85_03887 PE=4 SV=1
MSDDDDIKDDLEDDLEGEEDGEELAAASGDDPVEADDDVPAPAAKGKAKAAVSVDELPSV
EAKNKERDALARAMEEFLARGGKVQEVEANVVADPPKKPDNKYGSRPI
>tr|A0A0Q0C4B1|A0A0Q0C4B1_PSEAP Uridylate kinase OS=Pseudomonas syringae pv. aptata OX=83167 GN=pyrH PE=3 SV=1
MAQQGSGYQARYKRILLKLSGEALMGSEEFGIDPKVLDRMALEVGQLVGIGVQVGLVIGG
GNLFRGAALSAAGMDRVTGDHMGMLATVMNALAMRDALERANITAIVMSAISMVGVTDHY
DRRKAMRHLSAKEVVIFAAGTGNPFFTTDSAACLRAIEIDADVVLKATKVDGVYTADPFK
DPNAEKFDHLTYDEVLDRKLGVMDLTAICLCRDHKMPLRVFNMNKPGALLNIVHGGAEGT
LIEEAQ
>tr|A0A0Q0DMK8|A0A0Q0DMK8_PSEAP UPF0176 protein ALO85_05412 OS=Pseudomonas syringae pv. aptata OX=83167 GN=ALO85_05412 PE=3 SV=1
MTQEPMTQPIVVAALYKFVTLSDYVELREPLLQAMIDNGIKGTLLIADEGINGTVSGSRE
GIDGLMAWLKNDPRLVDIDHKESYCDEQPFYRTKVKLKKEIVTLGVEGVDPNKSVGTYVE
AKDWNDLISDPEVLLIDTRNDYEVSIGTFEGAIDPKTTSFREFPEYIKAHFDPAVHKKVA
MFCTGGIRCEKASSYMLGEGFEEVYHLKGGILKYLEEVPEQESHWRGECFVFDNRVTVRH
DLTEGDYDQCHACRTPISAEDRASEHYSPGVSCPHCWDSLSEKTRRSAIDRQKQIELAKA
RNQPHPIGRNYRLPSEA
>tr|A0A0Q0DP74|A0A0Q0DP74_PSEAP LuxR response regulator receiver OS=Pseudomonas syringae pv. aptata OX=83167 GN=ALO85_03730 PE=4 SV=1

MLADIDGLQVVGQADSGEESLKKARELKPDVVLMDVKMPGIGGLEATRKMLRSHPDIKVV
AVTVCEEDPFPTRLLQAGAAGYMTKGAGLAEMVQAIRLVFAGQRYISPQIAQQLALKSFQ
PQVNNSPFDLLSEREIQIALMIVGCQKVQTISDKLCLSPKTVNTYRYRIFEKLSISSDVE
LALLAVRHGMVDASA
>tr|A0A0Q0CGX7|A0A0Q0CGX7_PSEAP Inhibitor_I42 domain-containing protein OS=Pseudomonas syringae pv. aptata OX=83167 GN=ALO85_02007 PE=4 SV=1
MTSARFIAPLSLALLTACAQTPKNIVSIDTQSDCPLTLTPGQTLILTLPSNPTTGFRWLT
QNPAQNILRSLGPEVYANAESKEMVGNGGQSVWRFKATDAGTGRLMMVYQQPWAPEVAPE
QTFECAISVK
>tr|A0A0Q0IRW1|A0A0Q0IRW1_PSEAP Metal ion ABC-type transport system, permease protein OS=Pseudomonas syringae pv. aptata OX=83167 GN=ALO85_03396 PE=3 SV=1
MMLYSLVVEPFIEFGFMRRALVACLALGIGSGPVGVLLMLRRMSLVGDAMSHAVLPGAAV
GFMVAGLSLPAMGFGGLIAGLAVALLSGLVSRLTSLREDASFASFYLTSLAAGVLIVSLH
GSSVDLLHVLFGTILAIDDTAIYMVGSIASFTLILLAIIYRPLVLECFDPGFLRAVGGRG
SLYHVLFLLLVVLNLVAGFQALGTLMAVGMMMLPATAVRFWTNSLSGLMVISTLLATLSG
LIGLIVSYHLGVASGPAIVLTASAFYGISLLFGRTGIVRRLFPKPHLAH
>tr|A0A0Q0CX39|A0A0Q0CX39_PSEAP TetR family transcriptional regulator OS=Pseudomonas syringae pv. aptata OX=83167 GN=ALO85_00470 PE=4 SV=1
MAPRTKTRERIVQTSLELFNQQGERSVTTNHIAAHMEISPGNLYYHFANKQAIIAELFSE
YEVLIGSFLTLPADRTPTIEDKRDYFLAIIDAMWRYRFLHRDLEHLLTSDEELARRYRRF
SYRCLMQAMTIYRGFIKAGILKMDESQIEWTSLNTWIVLTSWVRFLCTNRENSTEINQSA
IRRGVYQVLMLESVFVAPQAREAFDALCETFHASLPEVLM
>tr|A0A0Q0BTZ2|A0A0Q0BTZ2_PSEAP Polar amino acid ABC-type transport system, permease protein I OS=Pseudomonas syringae pv. aptata OX=83167 GN=ALO85_01634 PE=3 SV=1
MDFTLWDIVRNLLIGLQWTLALSLVAFIGGGLIGLLIMGMRTSEKSGPRVTAKLYIELFQ
GTPLLMQLFLVFFGVALMGLNISPWAAAALALTLFTSAYLAEIWRGCVESISKGQWEASA
SLALTPFEQMRYVILPQALRIAVAPTVGFSVQVVKGTAVTSIIGFTELTKTGSMLANATF
EPFMVYGFVALGYFILCYPLSLCARHLERRLHASA
>tr|A0A0Q0C8L5|A0A0Q0C8L5_PSEAP LysR family transcriptional regulator OS=Pseudomonas syringae pv. aptata OX=83167 GN=ALO85_03380 PE=3 SV=1
MDLLQSMQVFVKLAELGSFTKVADATQVGRPHVTRIIQDLEASLDVRLFQRTTRSVKLTA
EGQRFYERVKNNLADLAETTSMFDRNGSTLRGRLRIDIPAAFSQRSFMESLKGFTRAFPD
IELALGVTDRTVDLVAEGIDCALRIGELPDSSLVVREIGRATMVTCAAPSYLQAFGTPET
LDELAGHCGVSFLSGQSNRPLPWHFSLNGDDFALPPRGGITVNESNAYVQCGLAGFGILQ
APGIAVESFLASGELVEVLEKFRPLPRPVSVLYPSRTHLAPQVQAFIAWLREHFPKLHPR
WFDVR
>tr|A0A0Q0DXG2|A0A0Q0DXG2_PSEAP Alpha-ketoglutarate-dependent dioxygenase AlkB OS=Pseudomonas syringae pv. aptata OX=83167 GN=ALO85_03658 PE=4 SV=1
MQRKGDTPATLDLFADQTAQPRSDERIGPGSWLFRGFALSAMPQLLSALEATLGLSPFRH
MQTPSGLSMSAALSSCGQLGWITDRHGYRYSATDPQTGQAWPAMPDVFMQLAQDAALAAG
YAGFVPDACLINRYIPGARMSLHQDRNEHDHRWPVVSVSLGIPAIFQFGGQLRSDKTQRI
SLFHGDVVVWGDEDRLRFHGILPIKQAEHPQLGEQRINLTFRKAGRDS
>tr|A0A0Q0CAX5|A0A0Q0CAX5_PSEAP HIT family hydrolase OS=Pseudomonas syringae pv. aptata OX=83167 GN=ALO85_00419 PE=4 SV=1
MDTLFTKIINREIPAKIIYEDDQVLAFHDIAPQAPVHFLVIPKKPIRTLNDLTEEDKGLA
GHILFTAQRLALELGCEEGFRVVMNCNELGGQTVYHIHMHVLGQRQMNWPPG
>tr|A0A0Q0BV93|A0A0Q0BV93_PSEAP Uncharacterized protein OS=Pseudomonas syringae pv. aptata OX=83167 GN=ALO85_03470 PE=4 SV=1
MDMTQNRHPERLGAFIDALTELLGSEPHEGDLLRRGGKLLAQLVSHDDWLAEEFTVPDAN
RYQQFLLHADPQQRFSVVSFVWGPGQRTPIHDHRVWGLIGMLRGAEDSQGYLRNAQGHLE
TSGPAIRLQPGQVEALSPHSNDVHQVSNAFNDQVSISIHVYGADIGTVKRAVYDLDGTEK
LFISGYSNAAATATAHQDQPTGSPTP
>tr|A0A0N8T8K3|A0A0N8T8K3_PSEAP YecA family protein OS=Pseudomonas syringae pv. aptata OX=83167 GN=ALO85_03236 PE=3 SV=1
MSFAEQLTRLQVFLDADELHEEALDYVAAHGYLTALSICAEEVPEREWIDALFSEPPQYS
SEAQQTEVEATLIALKAHIARQLASDEEFELPCDLDLGDDPDDSDLRGWCIGFMEGVFLR
ENAWFESAEEEVSEMLLPIMVGSGLFDEQPEFADIAQDANLMDDMIVQIPEALTALYLLC
QAPDEKPAILKPRHH

>tr|A0A0Q0CY39|A0A0Q0CY39_PSEAP PerM family protein OS=Pseudomonas syringae pv. aptata OX=83167 GN=ALO85_01044 PE=4 SV=1
MMFNVLRNWVQRYFSDEEAVVLAVLLFLAFTLVLTLGGMLAPVIAGLVLAFLMHGLVSVL
ERLRMPEVAAVGLVFTLFIGVLLVFLLVLVPLLWHQLITLFNEAPGMLAKWQSVLLLLPE
RYPHLVSDEQVLLAIEVARGEVGKIGQLALTFSISSLPLLVNLMIYLVLVPILVFFFLKD
RHMIGRWARGYLPRERALINRVADEMKRQIANYIRGKVIEIFICGGVTYIAFAALGLNYA
ALLAMLVGISVVVPYVGAVVVTVPVALIGLFQWGWGDQFIYLMIAHGIIQALDGNVLVPL
LFSEAVNLHPVAIICAVLLFGGLWGFWGIFFAIPLATLFKAVLDAWPRNEPTVAPLL
>tr|A0A0Q0FJG9|A0A0Q0FJG9_PSEAP Uncharacterized protein OS=Pseudomonas syringae pv. aptata OX=83167 GN=ALO85_00375 PE=4 SV=1
MAMYYVAIAMLTLLLVPGPTNSLLLQSGISRGLGMYSLKLVAAEWAAYLIQITSWGLSID
AMIEHYGWVVVATKVLAVMFLFYISLKLWFSIQQDTDGKPAPISIRALFLATLNNPKGLF
FATIIAPAGTFLHLESYGAFMTVFSLVVIPVGITWVALGAFFGRKLPSLLSGNRVNRFVS
LVIGLFAIIALYNVATTAIFA
>tr|A0A0Q0BUD9|A0A0Q0BUD9_PSEAP LuxR family DNA binding response regulator OS=Pseudomonas syringae pv. aptata OX=83167 GN=ALO85_01351 PE=4 SV=1
MELPVDNDPLTKVLVVDDQPLIVEELCEFLESSGYECVRCHSSLEAIERFSADSTIGIVL
CDLEMPGMNGIEMVEAMKMTGGKTHLFEAIMLTGQAEKKDVIKALRAGIADYYQKPVDLE
ELLEGVQLQVQALHERQKNRQQLGLLNEKLQFLAASIDDLYKNLDSVQNNPQPARKARDR
QERDGQMPVALAKLSPRQLDVARLVSTGLTNYQIACELGITENTVKLYVSQVLRLTHMHN
RTQLALAFSPGKSAERHRQIESQD
>tr|A0A0Q0D3G9|A0A0Q0D3G9_PSEAP Putative aminoacrylate peracid reductase RutC OS=Pseudomonas syringae pv. aptata OX=83167 GN=rutC PE=3 SV=1
MTKKAIIPAGTSKPIAPFVPGSMADGVLYVSGTLPFDKDNNVVHVGDATAQTRHVLEAIK
SVVETAGGTMDDVTFNMIMIRDWADYAKVNEVYAEYFAGEKPARYCIQCGLVKPEALIEI
ASIAHIG
>tr|A0A0Q0DTY2|A0A0Q0DTY2_PSEAP GNAT family acetyltransferase OS=Pseudomonas syringae pv. aptata OX=83167 GN=ALO85_00260 PE=4 SV=1
MPLYTPDTHDYPELTEVWERSVRATHDFLPDAYITRLKVLLPQYLGSVTLFCTRDEQLNI
TGFAGTRNSRLDILFIEPDHRGQKLGTQLLNHAIEQFNIRELDVNEQNTQALGFYRRHGF
EVVSRSEVDGLGQPYPMLRMRLTGRH
>tr|A0A0Q0BZZ0|A0A0Q0BZZ0_PSEAP Ribosomal RNA small subunit methyltransferase J OS=Pseudomonas syringae pv. aptata OX=83167 GN=rsmJ PE=3 SV=1
MIDQQAGSRIRVEALAVGEQEQAAQWAQRLGLPLSDACADFALQLTDHGLQLQQLGDDAP
GAVRVDFVEGAVAHRRLFGGGTGQMIAKAVGIQPGIRPSVLDATAGLGKDAFVLASLGCD
MSLIERQPIIAALLEDGLARGRGDRDVGSIIARMRLLTGNSIDIIRAWTGEPPQVIYLDP
MFPHREKTALVKKEMRLFRPLVGDDMDAPALLEAALALATHRVVVKRPRKAPCIDGVKPG
YALDGKSSRYDIYPKKALKPKAATDESGA
>tr|A0A0Q0C9T3|A0A0Q0C9T3_PSEAP IucA/IucC OS=Pseudomonas syringae pv. aptata OX=83167 GN=ALO85_00392 PE=4 SV=1
MNMATPLPLQRNAASTTTTTTTTGTGAWLADVSAERYQQVQRRVIGQLLQTLLYEAALPY
RCEPLDDHRHRFAVAVSGGVEYHCEGLLSTSFELIRLDHATLERVDSAGERSVPDLHLAL
TELLSPFKDSPHLTRFIQEIEQTQLKDLQARNQGYQPARPAHQLDVDALEQHFMDAHSYH
PCYKSRIGFSLADNRHYGPEFATPFAVVWLAVAKSSASVGHSRSMDFQAFIRQELGTQRW
QEITRDLAAQGKSIDDYQLMPVHPWQWDNVTVSTFYPELASGELIYLGTSTDSYKAQQSI
RTLANASQPQRPYVKLAMSMTNTSSTRILARHTVLNGPIITDWLHQLIATDSTARALDFV
ILGEVAGVSFDYRHLPESRSAQTYGTLGAIWRESLHQYLRDDEQAVPFNGLSHVENRYGD
GQQTPFIDAWVSQYGLKEWTRQLLQVTVPPIIHMLYAEGIGMESHGQNIVLIVKQGWPQR
IALKDFHDGVRYSPAHLGRPELCPELVPLPASHAKLNRNSFIITDDVNAVRDFSCDCFFF
ICLAEMAIFLRQQYQLDEALFWQMTADVILDYQRAHPQHRDRFGLFDVFAPSYEVEELTK
RRLLGDGERRFRSVPNPLHTYRPQ
>tr|A0A0Q0CBU1|A0A0Q0CBU1_PSEAP MutT/nudix protein OS=Pseudomonas syringae pv. aptata OX=83167 GN=ALO85_05524 PE=4 SV=1
MSRPMSNWTSRQSPVRPPKPLFSNVSPAVASPCISLCRLDEQKVCLGCFRHVEDIREWRS
ADDDRRRQIRHEADQRRARAEVDQAAG
>tr|A0A0Q0CIJ4|A0A0Q0CIJ4_PSEAP Uncharacterized protein OS=Pseudomonas syringae pv. aptata OX=83167 GN=ALO85_03292 PE=4 SV=1
MFAGAVFIENRPVKLLPILLLALLPLTANALQVGERLSSWTLLDQYDQPYTLNNQTQTLL

VARSMDAAKLVNAALQDKPKGFLESRHTVFVADIQKMPTIIANMFAIPKMRDYSYRVMLD
REARIVPQYAGDDDKVLWLQLRDGQLVSQQQFGSADALRAALDRK
>tr|A0A0Q0FSJ4|A0A0Q0FSJ4_PSEAP Ferric siderophore uptake system, MotA/TolQ/ExbB family protein OS=Pseudomonas syringae pv. aptata OX=83167 GN=ALO85_04360 PE=3 SV=1
MNLLASPFESIEHAVIWLLVLFSVATWGLALLKGVQFSRLKSQDRKFHKRFWAASSLDSA
AQLAQEQPGAAARVALAGYAAIQVPDGAQANDLSQSINHQDRLERALRQQIVRERRSLET
GLAILASIGSTSPFIGLFGTVWGIMSALKGISAAGSASLETVAGPIGAALVATGVGIAVA
VPAVLVYNYFLRRLKLTAADLDDFAHDFYSLAQKSAFRVLLHPVLKSGTAGVHAGQNVKE
AS
>tr|A0A0Q0DF16|A0A0Q0DF16_PSEAP Uncharacterized protein OS=Pseudomonas syringae pv. aptata OX=83167 GN=ALO85_04724 PE=4 SV=1
MASMKRPRLKEVQAQSGYRLALTFIDDQQFVLDMSADVQTFPGLRPLIAADAFAQAQVGD
DGWTVEWPEPDIQIGADTLYLDAQAQAATDENTRIFIGWRARTGLPLAQAAIALGVSPRS
ITRYSNSREPTPRTLALACLGWDALQQQAQAAEERGVYGKDKKAR
>tr|A0A0Q0C4P7|A0A0Q0C4P7_PSEAP Gamma-carboxygeranoyl-CoA hydratase OS=Pseudomonas syringae pv. aptata OX=83167 GN=ALO85_00939 PE=3 SV=1
MIMTSTSFATIELINDPRGFATLWLNRPDKNNAFNAQMIRELILALDQVQGDASLRFLLL
RGRGRNFSAGADLAWMQQAADLDYNTNLDDARELAELMYNLARLKIPTLAVVQGAAFGGA
LGLISCCDMAIAAADAQFCLSEVRIGLAPAVISPFVVKAIGERATRRYTLTAERFDGQRA
QQIGLVAECYPAAELQDQTQHWVDNLLLNSPQAMRVSKELLREVGNGELTPSLRRYCESA
IARIRTSAEGQEGLRAFLQKRTPGWQPVKDQQPGDPVTTVSAARHEPQKDSQS
>tr|A0A0Q0DRL0|A0A0Q0DRL0_PSEAP Uncharacterized protein OS=Pseudomonas syringae pv. aptata OX=83167 GN=ALO85_02023 PE=4 SV=1
MTTPNSDPLHGVTLEQILRALVEHYEWSGLAERVDIRCFKSDPSIKSSLTFLRKTPWARE
KVEALYVKLHRSKGW
>tr|A0A0Q0FIA2|A0A0Q0FIA2_PSEAP Phosphinothricin N-acetyltransferase OS=Pseudomonas syringae pv. aptata OX=83167 GN=ALO85_00695 PE=4 SV=1
MTSTFDLRDARDDDMPAVQAIYADHVLHGISSFELEPPDLAELLQRRRMVLAKGLPYLVA
ERGGEIVGYGYVTPYRPRPGYRFTVEDSVYVRDGLGGQGIGQALLSALVQHCEAGGWRQM
IAVIGNSQNIASLRLHERLGFRRVGVFESVGFKHGRWVDTVLMQRMLGEGWGSVPVERT
>tr|A0A0Q0C2A1|A0A0Q0C2A1_PSEAP Uncharacterized protein OS=Pseudomonas syringae pv. aptata OX=83167 GN=ALO85_102123 PE=4 SV=1
MGFSGSAIEKLKGLSVTCTGSRLPKVCFKQVYSDTQR
>tr|A0A0N8T9J1|A0A0N8T9J1_PSEAP 50S ribosomal protein L23 OS=Pseudomonas syringae pv. aptata OX=83167 GN=rplW PE=3 SV=1
MNQERVFKVLLGPHVSEKATVLADKKGQFVFKVATDATKLEIKKAVESLFSVKVERVTTL
NVLGKSKRTARGLGKRNDWKKAVISLQPGQDLDFSSSAE
>tr|A0A0Q0DD59|A0A0Q0DD59_PSEAP Arginine biosynthesis bifunctional protein ArgJ OS=Pseudomonas syringae pv. aptata OX=83167 GN=argJ PE=3 SV=1
MAVGLGPLPALHPVPGFELGISSAGIKRPGRKDVVVMRCAEGSSVAGVFTLNAFCAAPVI
LAKQRVQGTVRYLLTNTGNANAGTGEPGLVAARRTCEKLAQLTGVDASAVLPYSTGVIGE
PLPVEKIEGALQAALDDLSVDNWAAAATGIMTTDTLPKGASRQFSHDGVTVTVTGISKGA
GMIRPNMATMLGYIATDAKVAQSVLQDLIRDGANKSFNRITIDGDTSTNDCCMLIATGQA
DLPEITEAKGPLFEALKKAVFDVCMEVAQAIVRDGEGATKFVTVEVNGGGNHQECLDVGY
AVAHSPLIKTALFASDPNWGRILAAVGRAGVPDLDVSKIDVFLGGVCIASQGCRATTYTE
EQGSAVMAEEEITIRIELGRGDCSETIWTTDLSHEYVKINAEYRT
>tr|A0A0Q0C4L3|A0A0Q0C4L3_PSEAP Alpha-ketoglutarate MFS-type transporter KgtP OS=Pseudomonas syringae pv. aptata OX=83167 GN=ALO85_05115 PE=4 SV=1
METSNPASHGSAATAAGQKTTSSRLKSIFSGSVGNMVEWYDWYVYAAFSLYFAKAFFPKG
DTTAQLLNTAAIFAVGFLMRPIGGWLMGLYADYKGRKAALMASVLLMCFGSLIIALTPGY
ESIGVGAPILLVFARLLQGLSVGGEYGTSATYLSEMATKERRGFFSSFQYVTLISGQLIA
LGVLIVLQQTLTTEQLYAWGWRIPFVIGALCAIVALFLRRGMEETESFTRKKKEKPKESL
MRTLMRHPKELMTVVGLTMGGTLAFYTYTTYMQKYLVNTVGMSITDSTSISAATLFLFML
LQPVVGALSDKIGRRPILIAFGVLGTLCTVPILTTLHTIQTWWGAFFLIMAALIIVSGYT
SINAVVKAELFPTEIRALGVGLPYALTVSIFGGTAEYIALWFKSIGMETGYYWYVTACIA
CSLLVYVFMKDTRKHSRIETD
>tr|A0A0Q0D896|A0A0Q0D896_PSEAP Thymidylate synthase OS=Pseudomonas syringae pv. aptata OX=83167 GN=thyA PE=3 SV=1

MKQYLELVAHVIKHGTLQANRTGVNTISFPGAMLRYDLQEGFPAITTRRMAFKSAIGEMV
GFLRGVNNAAEFRELGCKVWDQNANENAQWLNNPFRKGEDDLGEIYGVQWRKWPAYKRID
AGNVAAIELALGQGYRQIAESEEDGQSFVVLYKAIDQIRQCVDTIINDPGSRRILFHGWN
CAQLDEMALPPCHLLYQLHPNPQTREISLTLYIRSNDLGLGTPFNLTEGAALLSLIGRLT
GYTPRWFTYFIGDAHVYENHLDMLNEQMTREPYPMPKLVISDRVPEFAKTGVYQPEWLEL
IEPSDFTLEGYQHHPAMTAPMAV
>tr|A0A0N8T8D2|A0A0N8T8D2_PSEAP Delta-aminolevulinic acid dehydratase
OS=Pseudomonas syringae pv. aptata OX=83167 GN=ALO85_01631 PE=3 SV=1
MSFTPANRLFPLTRLRRNRRDDFSRRLVRENVVTVDDLILPVFVLDGENRRESIASMPGV
ERLSVDLLLKEAEHWVALGIPALALFPVTPPEKKSLDGAEAWNPDGIAQRATRALRDRFP
ELGVITDVALDPFTTHGQDGILDEEGYVQNDITVDALVKQALSHADAGAQVVAPSDMMDG
RIQAIRESLELAGHVNVRIMAYSAKYASAYYGPFRDAVGSSLNLGKANKASYQMDPANSN
EALHEVAADLAEGADMVMVKPGMPYLDILYRVKDEFKVPTFVYQVSGEYAMHMAAIQNGW
LSEGVILESLTAFKRAGADGILTYFAVRAAQLLKGQ
>tr|A0A0Q0C5C0|A0A0Q0C5C0_PSEAP Uncharacterized protein OS=Pseudomonas
syringae pv. aptata OX=83167 GN=ALO85_05634 PE=4 SV=1
MVSMCPDRLLIGSKPAINVQSIDLERKCPLTQTPQSPSFSDRIGGGGAATSAGIRFEQQL
GALFSSWILSGERLNDTFRLGAASAEWIRFETEAPVDDILIKTSDGGYVAVQAKTSASLS
EDLGSPFGKTVSQFVRHWFAAQQGDASMGWNRPLDPVRDRLVLAVGPQASNQIKLDLPTA
LRLVAQPGAGALTKAQQHAYQVFSSLVEQSWTSITRSEYDRHLTQQLAELIAVFTFDGDA
GHALASGSIFKALGQNVDVVAVFTALTDISGQLMKQRGGGDLRAWRTLLAARGIALSAPP
EYRSDIEALQRHSKAIASALTSYERIDMGNGNPLSVRRECQVEVEAAVLNGPLLIIGEPG
AGKSGVLNALAKKLFDDGVDVLQLAVDRYSIESLEGLTRELGLAHPLLDVIEAWEGTGPA
CLIIDALDATRGGRGESVFRALIERVIERKGRWNIVASIRTFDLRMGQQLRSLFNGTPPH
APLADSAFPNVAHIEVPPWSESEFQQLLQQSPTLATCLDKSPARLRELAMVPFNTRLLGE
LISANAVKNLDTISSQADLLGLYWDHRVEGHGSPAKICLNRMVKRMVEARALRAPLTEVA
AENPAMIDTLCQQGVIVIVDNKRWMQFRHHLLFDYAAAKLFLDPDAISRGIHRFQKSDAL
GLMLSPALGFVLREIWESQENRKPFWASLNALINDEQGDPIIRSTAGRLGAEFPSKKEDC
LQLGKLAISGDKPAQDTLSHVTGALAVQLEDQKDVPLEPWVHLADCLASSPGSVANTFRF
LLHNLIGRTADESKPMIGRAARSLLRFALTQPEPGFMVRTAIDFVISTYSSDVVASKELL
AQLFTRDRLDRFGHEEVPAVCYKIQAVGEVDPEFVSTVVYPVVFDYQVTGDQQTTISHSQ
ILNLTSNARQDYGMARWSLGEYLPKFLQLHPKAAARAVIKAVEGYVAREHPIADNAVIVQ
LEFPDGTVHLREDRSYIWAHDPNSPHERDAGRLVGKLREYLITASEAEAVAVIEVLRTEA
SLAIFWSRMFLCAVERSDSLVDLLWQIASAEPFLVLPDTRKDAIDLVAKGFAVQPILDRQ
KLEERVLQFDFSKFKYAEARQNFLERFFGAIGRENLVTEAARRILDASLQDVAETNERLF
SVQTGWRDTEDFFWFRDLDKNLPENSLAIEAIKSAEKLLEPQAKSPPATDASLDELLAAL
NSIQARLAVNKVHVGLRRYAEGVIGQGCEAIVSRKLLASTSSPPTALQDSSFTTFLETAA
KSENPEMDEDVEVQFEKSAAWGSPAARVEAAQSYWDLFLQRPDLYPKLATQAEQLLADPH
PAVRLSGAIRLIRIWDIDRERFWHLLEERLRNETNLTVIEHVLGDVVSRLIYSDQGRTLA
LLQMLSQREVPGSERESRLQEAVADKLTILWITYESAEAKRVIDHWLGEPWRHSASANKI
MATLRDAIVAGLGDKPDKYLGLRNRSRALISDIVNAANTRLARYFTAADLDGPESEEVRE
CARLIDTAGMQMLFATGADSDRSQDDPSNPHQDLDVFLQENADLIRRIGEYAQPHTTYYL
LQLVERLIKVDAGTAFDLAVSILQSSTRSGYQNDTLGVDLLVKLVGVFLADHKEVFENPA
RRNALIDCLEIFMNAGWPAARRLLYRLPEFIQ
>tr|A0A0Q0BTC8|A0A0Q0BTC8_PSEAP Uncharacterized protein OS=Pseudomonas
syringae pv. aptata OX=83167 GN=ALO85_01256 PE=4 SV=1
MQERLDQLRLPKPVQGAISDLVRALDATSTCADVEAEAALQIEYIHGLETSRKLRPADAE
ALYIIFDDAVQARLQALAD
>tr|A0A0N8TA17|A0A0N8TA17_PSEAP ABC transporter ATP-binding protein
OS=Pseudomonas syringae pv. aptata OX=83167 GN=ALO85_01979 PE=4 SV=1
MTDSTTLYPVFAEEMLPGGGHRSFILKRGELLRLTDLHGNANVSLTLLNAHEKTERLNLP
DSLKCQHTARLSNGHCLYSDMGRVLAAIVTDTCGWSDSIGGVLNAQEVAEKYGQGRYQEL
RNGFFRNGVDNLLVELGKWGLGLSDLLMTLNLFSRVDVDEAGTLHFAPNNSKAGDYIELY
APMDTLVVLTALQHPMDPNPEYTPQPLKLSWMKADASVAEHCRTSRPENERGFINTDRLF
A
>tr|A0A0Q0C8Y1|A0A0Q0C8Y1_PSEAP Endoglucanase-like subfamily M42
peptidase OS=Pseudomonas syringae pv. aptata OX=83167 GN=ALO85_03226 PE=4
SV=1
MTPANIPDPDLKYLQKVLLEMLAIPSPTGFTDTIVRYVAERLEELGIPFELTRRGTIRAT
LKGKQNSPDRAVSAHLDTIGASVREVKDNGRLALAAVGCWSSRFAEGSRVSVFTDTGVIR

GSVLPLMASGHAFNTAVDEMPISWDHVELRLDAYCTTRADCESLGIGIGDFVAFDPLPEF
TESGHISARHLDDKAGVAALLAALKSIVESGAEPLIDCHPLFTITEETGTGAAGVLPWDV
SEFVGIDIAPVAPGQHSSEHAVSVAMQDSGGPYDYHLSRHLLRLGVDNELPVRRDLFRYY
YSDAHSAVTSGHDIRTALLAFGCDATHGYERTHIDSLAALSKLLGAYILSPPVFASDAKP
AQSSLERFSHQIEHDAQMESDTRVPPVDSLIGQNREES
>tr|A0A0Q0DXP9|A0A0Q0DXP9_PSEAP HMG-CoA lyase-like protein OS=Pseudomonas syringae pv. aptata OX=83167 GN=ALO85_03582 PE=4 SV=1
MADVKILDVTLRDGGYRNNFNFTEDQAQIVVAGLASAGVDLCEIGYCKGSFAPKGEHGLT
SDVGAGFIEKIAQAANGRIELAVMVHPRNIATDDFAMLKEQGVSMIRVCLRRDQLDEGLV
TLSLARDHGFSVSANVTHVTSQTPGSVLDTAVRAEGAGADIVCCADSNGHMLPADVDRLF
SRLSSRLLVPLGFHAHNILSLALANAALNTSTRPYAAWAKGPAIFT
>tr|A0A0Q0FEV2|A0A0Q0FEV2_PSEAP PilZ domain protein OS=Pseudomonas syringae pv. aptata OX=83167 GN=ALO85_01252 PE=4 SV=1
MSRNDRDYEEKRDYIRMRVDADVNLIHAGQVIPAVCIDLSSSGMQVQAPRSFQVGDKLDV
RIDSDHPALKGLEAETEVVWVNDEEGGGQKLGLSIVSMK
>tr|A0A0Q0DPT3|A0A0Q0DPT3_PSEAP Uncharacterized protein OS=Pseudomonas syringae pv. aptata OX=83167 GN=ALO85_03661 PE=4 SV=1
MKLAAVVVLALAMTGCATVNDIEHTPPTMNVMSGKNPQEYADCFAGKISSSRKPPLIEPR
HDGLRVIVAQKLSKAPAALVDIESRSGGSTIKVYERLSNVPIRFRDVQNAAEACISG
>tr|A0A0Q0DCG2|A0A0Q0DCG2_PSEAP Ribose ABC-type transport system, permease protein RbsC OS=Pseudomonas syringae pv. aptata OX=83167 GN=ALO85_00515 PE=3 SV=1
MKNTPSPTLTAPVRRGGNYLGLGTYIGLAGALLVMIVLFSLLSDHFLSYQTFSMLANQIP
DLMVLSVGMTLILIIGGIDLSVGSVLALAASAVSVAILGWGWSVFPAALLGIACATLAGT
ITGSITVAWRIPSFIVSLGVLEMARGAAYQMTNSRTAYIGDSFAWLSDPIAFGIAPSFII
ALLVIFVAQAVLTRTVFGRYLIGIGTNEEAVRLAGINPKPYKILVFSLMGLLAGVAALFQ
ISRLEAADPNAGAGLELQVIAAVVIGGTSLMGGRGSIISTFFGVLIISVLAAGLAQIGAT
EPTKRIITGAVIVIAVVLDTYRSHRARRQG
>tr|A0A0Q0CHI4|A0A0Q0CHI4_PSEAP Acetyltransferase OS=Pseudomonas syringae pv. aptata OX=83167 GN=ALO85_03377 PE=4 SV=1
MLNLDWLSNHMHHSDTMALWLHRQFDYEFASQSLADWQLDFSQGQANSHWKCLIAMEDGE
LLGGASLASDDLAERPDLGPWLACVLVKPEARGRGVAGQLIDGICSHARSVGITTLYLHT
HDQHSFYAKRSWRVLERIEAWGKEHYLMSQDL
>tr|A0A0Q0FHZ7|A0A0Q0FHZ7_PSEAP Flagellar biosynthesis chaperone FlgN family protein OS=Pseudomonas syringae pv. aptata OX=83167 GN=ALO85_02132 PE=4 SV=1
MQDTTLLQMITDDMVSARQLLELLQAESLILHGRDMGEMEQVLAQKQALVILLDQHGRKR
SQVLAGMGLPANRSGLQQLASQSDIGEQLLLAADELNTLINECQTLNERNGSLIQMQQVS
TAHQLRILNGGDTPTLYDSRGSTALRAKPRPLSQA
>tr|A0A0Q0IB70|A0A0Q0IB70_PSEAP D-alanine--D-alanine ligase OS=Pseudomonas syringae pv. aptata OX=83167 GN=ddl PE=3 SV=1
MTAVAQSSLRSTLEPKSFGRVAVLFGGKSAERAVSLNSGNAVLSALLEAGVDAFGIDVGD
DFLQRLVSEKIDRAFIVLHGRGGEDGTMQGLLECLEIPYTGSGVLASALAMDKLRTKQVW
QSLGLATPLHAVLENESDCICAATELGLPLIVKPAHEGSSIGMAKVNSVDELIAAWKAAS
TYDSQVLVEQWIQGPEFTVASLRGQVLPPIGLGTPHSFYDYDAKYLASDTQYRIPCGLGD
AQEQELKQLAARACEAIGIAGWARTDVMQDAQGKFWLLEVNTVPGMTDHSLVPMAARAAG
LDFQQLVLAILADSVQARG
>tr|A0A0Q0C7L5|A0A0Q0C7L5_PSEAP Syringolin siosynthesis protein SylB OS=Pseudomonas syringae pv. aptata OX=83167 GN=ALO85_00601 PE=4 SV=1
MQTVQRFHCKNALKLELTCLYKKNNWRWLLGWSGDVLVIVCAVALTQWSLYAYPLSVLII
GSRQRALASLVHEAAHLTLTQSRRLSYAIGHYLTGLPIFQDYEAYRHSHVHMHHHHLGDA
DKDPDYRYYIESGLQATEDRFDFMFNHLLRTVLFLNIFSYLRYLVIHRLGALLKSPRKAL
TLIAVHAVLFSLFYATTGVYGYLMFWLVPYLTAFQVIGWLSEVSEHFGLFGREQEALRIT
RNRFPSWWERLFIGMHGDNLHLTHHLFAGIPYWNLHKAHSILLQDPDYAEANLAKGGIIS
GRRDTQSVMRQILSIYRLQNRRHSPYGTAPQTLCEQSAREGTQTTNGDDR
>tr|A0A0Q0FQG0|A0A0Q0FQG0_PSEAP Extracellular solute-binding protein OS=Pseudomonas syringae pv. aptata OX=83167 GN=ALO85_200077 PE=4 SV=1
MSSSAPYNEQVIRDIKKVLNANFSFPTSITNIELLVKGMNYTLRVECKNTKYYLKIFSAS
RDEIDISFEMHVINRLSASGIGVATAINSINGDAYINLTLAGEDRLAVLYAHVGGRTLTS
DPRDIFLFSKALAEIHRTPTSFFLQYTSRIFSIESECYRIRSSIEKHSYIPKPLASKLLD

NCYSIQALASTDCAVLSHGDAWLGNAKYVNGVIYFIDLEDSKLANRNFDLGVMAYNLIVK
KYEVRENIQALLRGYNELNISITSGSIKPYIQLRSLFVLCFLLENKLVDQSLLQKVFERA
EYFTSSKFDSEMAHLP
>tr|A0A0Q0C2I1|A0A0Q0C2I1_PSEAP Cation efflux family protein
OS=Pseudomonas syringae pv. aptata OX=83167 GN=ALO85_04014 PE=3 SV=1
MFERLIQFAIEQRIVVMLAVLLMAGLGIASYQKLPIDAVPDITNVQVQINTSAPGFSPLE
TEQRITFAIETNMAGLPGLQQTRSLSRSGLSQVTVIFEDGTDLFFARQLVNERLQIAKDQ
LPEGVDAMMGPISTGLGEIFLWTVEAREGALKEDGTPYTPTDLRVIQDWIIKPQLRNVPG
VAEINTIGGFARQYQIAPDPKKLAAYKLTLNDLVAALERNNANVGAGYIERGGEQLLIRA
PGQLGTVDDIANIVIANVQGTPIRISSVAEVGIGKEMRSGAATENGREVVLGTVFMLIGE
NSRTVSQAVAAKLAEINRTLPAGVEAVTVYDRTTLMEKAIATVKKNLIEGAILVIVILFL
FLGNIRAALITAMVIPLAMLFTFTGMFTNKVSANLMSLGALDFGIIVDGAVVIVENAIRR
LAHAQQKHGRMLTRSERFHEVFAAAREARRPLIFGQLIIMVVYLPIFALTGVEGKMFHPM
AFTVVIALLGAMILSVTFVPAAIAMFVTGKVKEEEGLVMRTARQRYAPILSWVLGHRSIA
FGMALVLIVLSGFTASRMGSEFIPSLSEGDFALQALRVPGTSLTQSVDMQQRLEKAIIEK
VPEVQRVFARTGTAEIAADPMPPNISDSYVMLKPQSEWPDPDKSRETLIADLQKAAASVP
GSNYELSQPIQLRFNELVSGVRSDVAVKVFGDDMNVLNQTAAKIAATLQKVPGASEVKVE
QTTGLPVLTINIDRDKAARYGLNVADVQDAIAIALGGRQAGTLYEGDRRFDMVVRLSEQL
RTDVEGLSSLLIPVPASAGNQQISFIALSQVASLDLVLGPNQISRENGKRVVIVSANVRG
RDLGSFVEEAGTTIDNGVQIPAGYWTSWGGQFEQLQSAAKRLQIVVPVALLLVLALLFMM
FNNLKDGLLVFTGIPFALTGGVMALWLRDIPLSISAGVGFIALSGVAVLNGLVMIAFIRS
LREQGHSLHSAINEGALTRLRPVLMTALVASLGFIPMALATGTGAEVQRPLATVVIGGIL
SSTALTLLVLPALYQWAHRREDEEAVEALK
>tr|A0A0Q0BTV2|A0A0Q0BTV2_PSEAP Oligopeptidase B OS=Pseudomonas syringae
pv. aptata OX=83167 GN=ALO85_01068 PE=4 SV=1
MSFSNATSSAPIARKADGPDPYAWLQNRDTDEVLDYLKAENAWQEQQLAEQTGLRESLFE
EIKGRILETDLSLPSPWGPYLYYTRTTQGDEYARHYRCPRPADDSNTVDESAEELLLDPN
EMADGGFFSLGAFSISPDHQRLAYSLDTSGDEVYELYVKELSTGQISSLPFENCDGSMTW
ANDSQTLFFGELDETHRPGKLHRHTLGTASAEQVFDEPDGRFFLHCFRSSSERQLIILLN
SKTTSEAWVLDADHPQQAFTCLAPRSEGHEYYPDHGLLDGVWTWFIRSNQSGINFALYQA
AQHASGVPVREDWQTLVAHDDKVMLEGVSLNAKGISLSLREGGLPIIEVRPQALPTYRVQ
LPDAAYSLYVQDTLEFDSDKIRLRYQSLNRPAQVRQLTLASGEQVVLKETPVLGTFDADA
YVSQRLWATASDGTQVPISLVVKRELAGQATPLYLYGYGAYGESLDPWFSHARLSLLDRG
VAFAIAHVRGGGELGEAWYRNGKQEHKQNTFGDFIACAEHLVAQGLTSAEQLVISGGSAG
GLLIGAVLNQRPELFKAAIAEVPFVDVLNTMLDPDLPLTVTEYDEWGNPQEPEVYARIKA
YAPYENVTAQAYPAMLVIAGYNDSRVQYWEAAKWVAKLRATKTDDNPLLLKTELGAGHGG
MSGRYQGLRDVALEYAFIFKVLQIAE
>tr|A0A0N8T9C8|A0A0N8T9C8_PSEAP Thiol:disulfide interchange protein
OS=Pseudomonas syringae pv. aptata OX=83167 GN=ALO85_00358 PE=3 SV=1
MRLPQIFAAAALMLAGTFSMSALADAAPDQAIRKTLDSLELQLPVESISPSPLNGLYEVK
LKGGRVLYASADGQFVVQGYLFQVQNGKPVNLTEKTERLAISKTINAIPASEMVIYPAVG
ETKSHITVFTDTTCPYCHKLHEEVGQLNKMGVEVRYLAFPRQGLGSPGDEQLQAVWCSKD
RKGAMDRMVDGKDIKAAKCDNPVSKQYELGQSIGVNGTPAIVLADGQVIPGYQPAAQVAK
LALGAK
>tr|A0A0N8T8B6|A0A0N8T8B6_PSEAP Tetratricopeptide repeat-containing
protein OS=Pseudomonas syringae pv. aptata OX=83167 GN=ALO85_02990 PE=4
SV=1
MNKWWIPALTALALLNGCASVERGSIPVVDSSSKVSNGERAAASRRTNTAPVQQQPQAVP
QDSGVVVMVPGAGGADAGQSYSAPASQAPFSVNTPPVDAAPVNQAPVSQAPANNSYSTPA
ASAPTGIPSSGGGLSEDEQLDGPVLALLTTAQQQQTSGDLNGASSSLERAQRVAPREPQV
LYRLAQVRLAQGDAAQAEQLARRGLTYANGRTSLQASLWGLIAQSREKQGDAAGAALARQ
KAR
>tr|A0A0Q0DLN6|A0A0Q0DLN6_PSEAP CBS:transporter-associated region
OS=Pseudomonas syringae pv. aptata OX=83167 GN=ALO85_00366 PE=4 SV=1
MDNLPLGPLLGVLALLLLWAGLLTAVEAAHHQVKAMRAASRPEKSAPLPELAFNLNSLIL
GNTLIRVLITVIATLIAANYWFYNGPTVAWLATMSVMLVFAEYMPRRLANRHPVSTLLLG
NSVLRIPMKIVWPLAYVFNVLAKTLLRPFLSRAVQQQDQAFEDDTQNTPRNDAAQEYSPR
ASVLSGIRALDSITVNDILIPRNEVDGVNLDDPMEMIIERLIISRHTRLPVYHNDINQVQ
GVINTRDISHLLPKGTLTKEQLLAVCYEPYFVPESTPLQLQLLNFHKQQRRLGVVVDEYG
EVLGIVTLEDILEEIVGEFESEQRLDNPHVKQQDDGRLEVEGAASIRDLNKSLGWHLPSD

GPKTVNGLVTEALETIPDAPVCLKIGPYRLEILETEDNRVKRVAMWQNTLVRTFK
>tr|A0A0N8T878|A0A0N8T878_PSEAP Peptidase S24, S26A and S26B OS=Pseudomonas syringae pv. aptata OX=83167 GN=ALO85_04790 PE=3 SV=1
MSVTFLGPLSAGGIKLPFISLSSVGSRSPLVEKHVSIAELCEVREPHAYLARIEDQGMQG
GGMCSGDMLVVDRSQYAEHGDIVVASLNARQICRRLHMRGDVVILESQNPDFPPLHVTDN
DDLIILGVVTYSVKNLLEGRAASSAHR
>tr|A0A0N8T9U6|A0A0N8T9U6_PSEAP Xenobiotic compound monooxygenase OS=Pseudomonas syringae pv. aptata OX=83167 GN=ALO85_03755 PE=4 SV=1
MSRQIRLNAFDMNCVGHQSPGLWAHPRDRSWQYKDLEYWTDLAKVLERGKFDGLFIADVL
GIYDVYRGNGEAAIRQATQVPVNDPLQLIPPMALVTEHLGFGLTASLSFEHPYPFARRLS
TLDHLTKGRAGWNIVTSYLESGAKNLGQKTLTEHDARYDYAEEYLEVCYKLWEGSWEDGA
ILRDRERRVFSDPTKIHEIRHVGKHFQVPGIHLCEPSPQRTPVLYQAGASSRGKQFAAEH
AECVFVAAPSKVLLKKTVADIRRRTAEAGRDPSKVLIFNLQTVILGETDAKAKAKFEEYK
SWVSYEGAMALISGWTGIDFSQFKPDEPLRHVHTNAIQSAVETFSTADPNTIWTPQALAD
WVGIGGFGPLFVGSPETVADLLQEWVEETDVDGFNLAYALTHETFIDAVELLVPELQKRG
VYKTEYANGTLREKLFGDGSRLEAGHPGAGFRDLAALHRNRQTETA
>tr|A0A0Q0DKM0|A0A0Q0DKM0_PSEAP Lipoprotein-releasing system ATP-binding protein LolD OS=Pseudomonas syringae pv. aptata OX=83167 GN=lolD PE=3 SV=1
MSESGMSDKAVLSCRNLGKSYEEGPESVVVLSGLQLELHPGERVAIVGSSGSGKSTLLNL
LGGLDTPSEGSVWLAGEELSALGEKARGLLRNRALGFVYQFHHLLPEFTALENVCMPLLI
GRTPIPEARKRSTALLERVGLGHRLAHKPSELSGGERQRVAIARALINQPGLVMLDEPTG
NLDHHTAQGIQDLMRELSTSSRTAFLIVTHDMNLARQMDRVLRLEDGRLVEA
>tr|A0A0Q0DIK4|A0A0Q0DIK4_PSEAP Amino acid ABC transporter permease OS=Pseudomonas syringae pv. aptata OX=83167 GN=ALO85_02297 PE=3 SV=1
MNIDLHGFGPALAAGALMTVQLALSALCLGLILGLLGALAKTSPYKPLQWLGSTYSTLVR
GIPELLWVLLIYFGTVNGMRALGKLFDIPDLALSAFAAGVIALGICFGAYATEVFRGAIL
AIPKGHREAGLALGMSRSRILFKLVLPQMWRIALPGLGNLFMILMKDTALVSVIGLEEIM
RHAQIAVGFTKQAFTFYMVAAFMYLGLTVLAMAGMYFLEKRASRGFLRSAS
>tr|A0A0Q0BX62|A0A0Q0BX62_PSEAP Uncharacterized protein OS=Pseudomonas syringae pv. aptata OX=83167 GN=ALO85_01727 PE=4 SV=1
MKLLIPGAVALVLLSGLAVAAPAAPVSASPMHDQYLPPDDLSLREGVPEQQQLMRVTEYT
IVAGNQRQSSQQPIPVTSPLSLRLKGKSLNKGASISQVLVHFDGGDSKSLKKPTYDEPSS
TLTLYYPLSQYRVLVDLLRNEKVYCQFLSYANGHIWADLHTTSARAR
>tr|A0A0Q0DTX2|A0A0Q0DTX2_PSEAP Ferredoxin--NADP reductase OS=Pseudomonas syringae pv. aptata OX=83167 GN=ALO85_04461 PE=4 SV=1
MFSGVLTYMSNMNHERVLSVHHWNDTLFSFKCTRDPGLRFENGQFVMIGLQQPNGRPLMR
AYSIASPNWEEHLEFFSIKVPDGPLTSQLQHLKEGDEIIISKKPTGTLVLDDLKPGKHLY
LLSTGTGLAPFMSVIQDPETYERFEKVILCHGVRYVNEVAYREFITEHLPQNEFFGEALR
DKLIYYPTVTREPFENEGRLTDLMRSGKLFSDIGLPPINPQDDRAMLCGSPSMLDETSEV
LNSFGLTVSPRMREPGDYLIERAFVEK
>tr|A0A0Q0FC57|A0A0Q0FC57_PSEAP Putative methyltransferase OS=Pseudomonas syringae pv. aptata OX=83167 GN=ALO85_04138 PE=4 SV=1
MNDLKNQIVNLKKTIFKITKNVIDSDSKNQTLAFKCFVQLALHTSIHNLTKRFEHHGLSP
FMLDHHLVVGEALIQYEGRHEALTHIDESSLIDAVNLYNHIVKKNEPFTDVLSLLAEEIT
ISKKQAKSLGQYMTPPDLARAVASLLNPDDYPDKPTKVCDFCVGYGALILGRIEKGLKAN
PKSLRHVEVIVNDIDPFMSNVTALQIITNTVHNNLDIKELVVLCSDVIKDYRSGNASFVI
KYRTPQIVLDDIERIHKEKALAKHSKRIDVTDTGIASKSVGAV
>tr|A0A0N8T872|A0A0N8T872_PSEAP Uncharacterized protein OS=Pseudomonas syringae pv. aptata OX=83167 GN=ALO85_101741 PE=4 SV=1
MMSNLLSEAVRGLERHDYPSEKTSSACKPRSVSQPAPVAQGHKHGNWHEIRKTLNTAGRQ
FKNQRLFDRQMAEKLNFLTLKSETN
>tr|A0A0Q0IJC7|A0A0Q0IJC7_PSEAP Biopolymer transporter ExbD OS=Pseudomonas syringae pv. aptata OX=83167 GN=ALO85_03926 PE=3 SV=1
MSFSTQDSDEVLSEMNVTPLVDVMLVLLVVFIVTTPLMTNAIKVNLPKTDAVAPAEKKNP
VVVSVDQDGKFYLAKSEIAPELLEKSLQDEKAKDPEIRVQLQADTAVNYGQVAKAMASIE
RSGITKISVMTAK
>tr|A0A0Q0CET6|A0A0Q0CET6_PSEAP Epimerase domain-containing protein OS=Pseudomonas syringae pv. aptata OX=83167 GN=ALO85_200003 PE=4 SV=1
MSDAPVLITGGAGFIGSHLTDALLASGQTVRILDNLSAGKRSNLPLDNPRVELIEGDVAD

ADLVKRAAQGCKAVVHLAAVASVQASVDDPVRTHQSNFIGTLNVCEAMREAGIKRVIFAS
SAAVYGNNGEGEAITEDTTKAPLTPYASDKLASEYYLDFYRRQHGLEPVIFRFFNIFGPR
QDPSSPYSGVISIFAERIRKGLPISVFGDGEQTRDFYYVGDLLKLLLQALSRESAIEGAI
NVGLNKTTSLNELLAALAQVVGKQPEVTYQAPRSGDIKHSRASNQRLLEHFTLDEPTPLV
RGLELLMGQ
>tr|A0A0Q0BXA2|A0A0Q0BXA2_PSEAP YdcF-like superfamily protein
OS=Pseudomonas syringae pv. aptata OX=83167 GN=ALO85_04844 PE=4 SV=1
MPLRYLLKALLLPPGILFVLLILGWWLRRTRPRLAATFFAVGLGGLWLMSLPVAVEFAAR
KLEQVPPLPQQQWATLAQQADAIVVLGNGRERNSPTWGTDTPTGLGLERLRLAARLAKES
GLPILTSGGLHFDQPPSEASIMAQSLQDDFAVAVRWQEGLSRTTWENATMSAAILQPQGI
KRVVLVTQAWHMPRARWSFEQAGFKVVGAPVGFLGVDNARPFGGWLPESRVFTQSGVLLN
EAAGLLVYPWIYRKGQ
>tr|A0A0Q0DX78|A0A0Q0DX78_PSEAP Transcriptional regulator, AsnC family
OS=Pseudomonas syringae pv. aptata OX=83167 GN=ALO85_03624 PE=4 SV=1
MQLDCQQQRGFFFLNARKSADIQHAFFGQNKKQGQFMQPKLSPIDRKILRLLQHDASVSA
AEIAERVELSQSPCWRRIHRLQEDGVIERTVALLSTQKLGLSMTVFVEVKLSGHGRRYLA
EFEEAIIGHPEVLECYTMAGGMDFMLKVVAQDIASYERFLRDHLLQLPHVHEAHSNIAMS
TVKRTTELPLD
>tr|A0A0Q0ILK1|A0A0Q0ILK1_PSEAP Tol-Pal biopolymer translocation system
protein YbgC OS=Pseudomonas syringae pv. aptata OX=83167 GN=ALO85_00240
PE=4 SV=1
MRAQNGVQSFAHRCRVYYEDTDAGGIVYYVNYLKFMERARTERLRELGFAQSELAQENLL
FVVHSSEARYHKPARLDDELLVSAEVTELNRVSLRFKQQIRRASDATLLCEGQFLVACVR
ADSFKPRAIPEALRAAFADQGGAGIHSEQEIKRGS
>tr|A0A0N8T8H6|A0A0N8T8H6_PSEAP Uncharacterized protein OS=Pseudomonas
syringae pv. aptata OX=83167 GN=ALO85_03515 PE=4 SV=1
MNKRDVLQRIIDKLNIDLDIAERAAQTAYETATHEENIAENKYDTLGLEASYLAAGQARR
VEEIRQSLTLYRKWSLKPFDESRGIQTGDLVVVEDVSGQTHYLFLGPDAAGLKVSAGDQL
VTVITARAPLGQSLLGKFEDDAVQIVINGSRQSYEITQTL
>tr|A0A0Q0FWV8|A0A0Q0FWV8_PSEAP MTS domain-containing protein
OS=Pseudomonas syringae pv. aptata OX=83167 GN=ALO85_01474 PE=3 SV=1
MSLPLPDSVALIALAQRLETSGYRFITPTPLTHQHVNQRPENRTAASLRDVFGWSRLIPE
SMLPVEEAQGLLAAGILERKEDGLKSRVRFSSLDNLLLVHSAFPTTDEDSVFFGPDTYRF
AQSINRHLQGTSHPINRAVDIGCGTGAGAMLIAVARPQAQVHAVDINPKALHFAQTNATV
AGLKNMECCHSDILSGLSGSFDLIVANPPYMKDTKRRAYRHGGDALGADLSVRIVRESLD
RLTPGGSLVLYTGVAMVGEHDPFFEAVRGDIDHAALAWTYRELDPDVFGEELLEEGYEDV
DRIAAVELVVTRRSA
>tr|A0A0Q0DAK5|A0A0Q0DAK5_PSEAP Putative cytoplasmic protein
OS=Pseudomonas syringae pv. aptata OX=83167 GN=ALO85_100355 PE=4 SV=1
MSCRTRNSLMQRTSIFDRISEARIREQERLEEERLAQAIVDAIPVPERFHTNELSFIRPQ
GFKDKTFHVFTLTDIGPSPLSVVIGRTPVEGDKQLEDLSQRLIADLKKALSHLEWIEHLV
PVEVAGVEGRRAEFRWRQQGTPVHQVQFIFLHHDEHRHPVLIQVTGTSNHPGGMTIEEKT
AFYLLVDTLELRSYPDLDQAVDRV
>tr|A0A0Q0C4B6|A0A0Q0C4B6_PSEAP Succinyl-diaminopimelate desuccinylase
OS=Pseudomonas syringae pv. aptata OX=83167 GN=dapE PE=3 SV=1
MTAPADLSPTLQLACDLIRRPSVTPVDADCQTVMMQRLGDAGFKLEPMRIEDVDNFWATH
GTTDGPVLCFAGHTDVVPTGPLQNWQNDPFDALIDEHGMLCGRGAADMKGSLAAMLVAAE
RFVADHPDHKGSVAFLITSDEEGPAHHGTKAVVERLAARKERLDWCIVGEPSSTTLVGDV
VKNGRRGSLGATLTVRGKQGHVAYPHLAKNPIHLAAPALAELAAEHWDHGNDFFPPTSFQ
ISNLNAGTGATNVIPGDLVAVFNFRFSTESTVEGLQQRVADILDRHELDWHVDWALSGLP
FLTEPGALLDAVSSSIKSVTGRETKASTSGGTSDGRFIATLGTQVVELGPVNATIHQVNE
RILASDLDVLTEIYYQTLVKLLA
>tr|A0A0Q0BUM3|A0A0Q0BUM3_PSEAP Transcriptional activator ChrR
OS=Pseudomonas syringae pv. aptata OX=83167 GN=ALO85_04736 PE=4 SV=1
MGPQHHPDDATLVSYAAGALSQVISLVTAAHLERCAECRARLRQAEAIGGVLMQQHTSSV
VPLKGRSAMLARLDEQKAGLVPALHSMPAANQDPDLMPVCMQAHFGRHLSTLKWKTLIPG
VQRVRAEGIEQGNLMLLKIAPGVSMPVHSHESGEMTMVLKGAYYDVQGEFGLNDVADLDS
HIHHQPIAYPDRECICVLATESKLRFQGLLARMMQPFFGI
>tr|A0A0Q0D4C5|A0A0Q0D4C5_PSEAP D-erythrose-4-phosphate dehydrogenase
OS=Pseudomonas syringae pv. aptata OX=83167 GN=ALO85_04003 PE=3 SV=1

MPQPRPYKVALNGYGRIGRCVVRALCERGAKAGFEVVAINDLADMASLEYLTRFDSTHGR
FPGEVRVEGQYLHINDHRIKVLRSATPEGIDWAGLEVDLVLECSGVYNTREDGLRFLEAG
APRVLFSQPMASERDVDATVVFGINQHKLTGRELLVSAASCTTNCSVPLLRLLDQAIGLE
YMTITTIHSAMNDQPVIDAYHHEDLRRTRSAFQSIIPVSTGLARGIERLLPELAGRIQAK
AVRVPTVNVSCLDITLQMSRDTDAVEINRILREAATSGPLKGLLAYTELPHASCDFNHDP
HSAIVDASQTRVSGPRLVNLLAWFDNEWGFANRMLDVADHFLRVADQ
>tr|A0A0Q0BRX1|A0A0Q0BRX1_PSEAP Type III secretion system outer membrane protein HrcC OS=Pseudomonas syringae pv. aptata OX=83167 GN=ALO85_04194 PE=3 SV=1
MRKALMWLPLLLIGLSPATWAVTPEAWKHTAYAYDARQTELATALADFAKEFGMALDMPP
IPGVLDDRIRAQSPEEFLDRLGQEYHFQWFVYNDTLYVSPSSEHTSARIEVSSDAVDDLQ
TALTDVGLLDKRFGWGVLPNEGVVLVRGPAKYVELVRDYSKKVEAPEKGDKQDIIVFPLK
YASAADRTIRYRDQQLVVAGVASILQDLLDTRSHGGSINGMDLLGRGGRGNGLAGGGAPD
APSLPMSSSGLDTNALEQGLDQVLHYGGGGASKSGGKSRSGGRANIRVTADVRNNAVLIY
DLPSRKAMYEKLIKELDVSRNLIEIDAVILDIDRNELAELSSRWNFNAGSVNGGANMFDA
GTSSTLFIQNAGKFAAELHALEGNGSASVIGNPSILTLENQPAVIDFSRTEYLTATSERV
ANIEPITAGTSLQVTPRSLDHDGKPQVQLIVDIEDGQIDISDINDTQPSVRKGNVSTQAV
IAEHGSLVIGGFHGLEANDKIHKVPLLGDIPYVGKLLFQSRSRELSQRERLFILTPRLIG
DQVNPARYVQNGNPHDVDDQMKRIKERRDGGELPTRGDIQKVFTQMVDGAAPEGMHDGET
LPFETDSLCDPGQGLTLDGQRSQWYARKDWGVAVVVARNNTDKPVRIDESRCGGRWVIGV
AAWPHAWLQPGEESEVYIAVRQPQISKMAKESRPSLLRGAKP
>tr|A0A0Q0DV69|A0A0Q0DV69_PSEAP Putative S-adenosylmethionine-dependent methyltransferase OS=Pseudomonas syringae pv. aptata OX=83167 GN=ALO85_01587 PE=4 SV=1
MKQYVENYQPTEGFAPATGHVEGDSVSPKAMIFHILKDLSADVEVLDIGFGSGTLGALIK
ANPDTAHWSVDGIDGWEANCQNSQLHENATYRNIWHGLAQELSSERLAQYRIICLLDVVE
HLTADTARWLVRTLLSSLGPDAYLFISTPLWFYPQETQQTGDLEEHLIGVPASSMMALCP
HLYAVNQPLIGGFVFSRRSLDFVEFFQPTADKSFSYEKGMHVAKAVGMKLEPNVAFTMD
>tr|A0A0Q0BS61|A0A0Q0BS61_PSEAP Uncharacterized protein OS=Pseudomonas syringae pv. aptata OX=83167 GN=ALO85_01832 PE=4 SV=1
MPVFNTPGKPATGKQQIFMSDFTETQASAPIGTAEKMIEIWSRLSPEKQSALLARFGTQE
NALAALVTTHLVSPRDK
>tr|A0A0Q0I9Z8|A0A0Q0I9Z8_PSEAP DUF87 domain-containing protein OS=Pseudomonas syringae pv. aptata OX=83167 GN=ALO85_04044 PE=4 SV=1
MPVFNFRDEDALGKVASVDTTNVIVDVENVDQLKRLQVNHLAVLQSSRPGQHLIGLITQV
TRKRGIQDIANDGIIEQTSELNLCKIALIGTMLDRDGERENVFRRTLESVPEIDANCFSL
EGGNLTNFMRTLSSVSADGNALTLGKYTLDEHAVAYLNGNKFFQRHAFIGGSTGSGKSWT
TAKIIEQMAGLSTANAIVFDLHGEYSPLVGQGIQHFKVAGPADVETKRTIDDGVLYLPYW
LLSYDALVAMFVDRSDQNAPNQAMIMSREINQAKRKYLEDGDQKEILKHFTVDSPVPFDL
DVLMSRLNEINVEMVPGASAGKEKQGDFYGKLARMISRLENKISDRRLGFMFNGGGDVLD
FNWLEKFTTAVLGSSEENGKAGIKIINFSEVPSDVLPLIVSLVARVTFSVQQWTPSELRH
PIALLCDEAHLYMPQRNMAESADDISLDIFERIAKEGRKYGVSLVVISQRPSEVNKTMLS
QCSNFVSMRLTNAEDQGVIKRLLPDSLGGFSDILPTLDTGEALVVGDASLLPSRIRIDEP
INKPNSGTVNFWDEWQKPVKDKRLSIAVDNWRKQNIQ
>tr|A0A0N8TA44|A0A0N8TA44_PSEAP Acetylornithine aminotransferase OS=Pseudomonas syringae pv. aptata OX=83167 GN=argD PE=3 SV=1
MTATCLMSTYQPLSLSFTHGLGTRLWDQGGREYLDAVAGVAVTNVGHSHPLLVEAIRDQA
GLLLHTSNLYTIDWQQRLAQKLAAVSGLDAVFFNNSGAEANETALKLARLHGWHKDIERP
LVVVMENAFHGRTLGTLSASDGPAVRLGFSTLPGDYIKVPFGDLAAFDKVCTTYGHRICA
VLVEPIQGEGGAQVAPAGYLKALRERCTRRDWLLMLDEIQTGMGRTGKWFAFQHEGIVPD
VMTLAKGLGNGIPIGACLARGRAAQLFTPGSHGSTFGGNPLACRVGCTVIDIIEQQALVA
NAALQGRQLLAGLRNALGAHPNVLDVRGQGLMIGIELRQAIPELTRIAAEDHGLLINVTR
GKIIRLLPPLVLDAAEVAQIVQGLAASLDSACDRSFVRSA
>tr|A0A0Q0BWB9|A0A0Q0BWB9_PSEAP Cbb3-type cytochrome c oxidase subunit OS=Pseudomonas syringae pv. aptata OX=83167 GN=ALO85_100080 PE=3 SV=1
MSTFWSLYVAVLTLGTLIAMLWLLVSTRKGQPDEPTDETVGHTFDGIEEYDNPLPRWWFY
LFVATIVFALGYLLLYPGLGNWQGLLPGYEYRDQDSKTQFSDGQQGWTSIHEWEREVANA
EARYGPLFARYAAMPVEEVAKDPQALKIGARLFASNCSVCHGSDAKGAYGFPNLTDADWR
WGGDAQAIKTSILAGRMGVMPALGGVLGEQGVRDVAAYVLTQLDARALPQDAKADPVAGQ
KTFASLCVACHGPEGKGMPILGAPDLTHPNAFIYGSSFAQLQQTIRDGRQGQMPAQQALQ

GNDRVHILAAYVYSLSRQEPPAESR
>tr|A0A0Q0DLZ2|A0A0Q0DLZ2_PSEAP 3-oxoadipate:succinyl-CoA transferase, subunit B OS=Pseudomonas syringae pv. aptata OX=83167 GN=ALO85_02397 PE=4 SV=1
MTYSTSEMMTVAAARRLRNGSVCFVGIGLPSKAANLARLTSAPDVVLIYESGPIGAKPTV
LPLSIGDGELAETADTVVSTSEIFRYWLQGGRVDVGFLGAAQVDRFGNINTTVVGDYHHP
KVRLPGAGGAPEIAGSAKSVLIILKQSARSFVDKLDFITSVGHGEGGDSRKRLGLPGAGP
VGIITDLCIMEPEAGTHEFVVTTLHPGVTREQVIAATGWEIRFADTVVVSEEPTDVELTA
LRDLEARTAAAHGQVAGEA
>tr|A0A0Q0C5G2|A0A0Q0C5G2_PSEAP Enoyl-CoA hydratase OS=Pseudomonas syringae pv. aptata OX=83167 GN=ALO85_04549 PE=3 SV=1
MGCCCYSPAPEHPNLPEDHVVTDYQSFRVELADSIAHVQINRPEKLNAMNESFWTEIIDI
FDWIERTDEVRVVVLSGAGKHFSSGIDLALLASVASQMGKDAGRNARLLRRKIRDMQASF
TAVDICRKPVLAAIHGYCLGGAIDLISACDMRYAAADAQFAIREIDMGMAADVGTLQRLP
RIIGDGVLRELAYTGRTVGAEEAQRIGLVNRTFDDPDGLLQGVLAVAREIAGKSPIAIEG
TKQMISYMRDHRVDDGLEHVAIWNAAMLQSPDLKLAMAAQMSKQKPVFDN
>tr|A0A0Q0DHT5|A0A0Q0DHT5_PSEAP tRNA/tmRNA (uracil-C(5))-methyltransferase OS=Pseudomonas syringae pv. aptata OX=83167 GN=trmA PE=3 SV=1
MSVPFDPASYDRQLEEKTVRLRELLAPFDAPEPQVFDSPREHYRLRAEFRLWREDQKRYY
AMFAPGDNRTPILLEGLPIASERINALMPVLRERWEASPTLNHKLFQVDFLTTLAGDAMI
TLCYHRPLDAEWQAAAEQLAAELDVSLIGRSKGQKLVIGHDYVTEKLDVAGRTFSYRQPE
GAFTQPNGTVNGKMLNWAFDALGERQDDLLELYCGNGNFTLPLATRVRKVLATEISKTSV
NAALSNLDDNGVDNVTLVRLSAEELTEALNEVRPFRRLHGVDLKSYDFGSVFVDPPRAGM
DPDTCELTRRFERILYISCNPETLAANIAQLHDTHRVERCALFDQFPYTHHMESGVLLVR
R
>tr|A0A0Q0DMH4|A0A0Q0DMH4_PSEAP Organic hydroperoxide resistance transcriptional regulator OS=Pseudomonas syringae pv. aptata OX=83167 GN=ALO85_03510 PE=4 SV=1
MSADESPTRAAPADCDSLLLDNQLCFALYSTSLMMTKVYKPLLQALGLTYPQYLAMMVLW
ERDGLTVGDVSTRLLTDPGSLTPLLKRLEGEGFISRTRSKEDERVVLLHLTAQGRELQQK
ALTVPGCVLSSSGLTMEKLRDLQAQLLQLREHLHHSL
>tr|A0A0N8T914|A0A0N8T914_PSEAP Oxidoreductase, zinc-binding protein OS=Pseudomonas syringae pv. aptata OX=83167 GN=ALO85_02506 PE=4 SV=1
MKALQGVEGHVEWGEEPSPALDVGQVRIKVAAAGLNRADLLQREGKYPPPPGVTQTLGLE
CSGVIAEVGPGSSWVVGDRVCALLAGGAMAEEVVVDGRHVLPVPEGLSLHEAAAIPEVYA
TAWLNLFELAGLKPGEKVLLHAGASGVGSAGIQLCKAFGNPVWVSVGSAERLAYCVELGA
QGGVVRSESLEGLSDFAPFNVILDPVGASYAELNVKLLALDGRWVLIGIMSGREAQVDLA
HVLGKRIQLMGSTLRSRDDTFKADLLSDLGQHVWPLFTEGRLKPQLAKSFAIKDAEAAFE
ELATNTVSGKIVLVIDESLV
>tr|A0A0Q0FHD1|A0A0Q0FHD1_PSEAP Shikimate kinase OS=Pseudomonas syringae pv. aptata OX=83167 GN=aroK PE=3 SV=1
MGAGKSTIGRLLAKELRLPFKDSDKEIELRTGANIPWIFDKEGEPGFRDREQAMIAELCD
ADGVVLATGGGAVMRSENRQALRAGGRVVYLHASIEQQVGRTARDRNRPLLRTADPARVL
SELLAIRDPLYREIADIVIETDERPPRMVVLEILARLAELPPR
>tr|A0A0Q0DMI7|A0A0Q0DMI7_PSEAP Putative exported protein OS=Pseudomonas syringae pv. aptata OX=83167 GN=ALO85_100785 PE=4 SV=1
MSRFLKLADSQRAHINSLRIAIVLIALVAAGLGFGWWSAPKQLTVHVPPDLRSGSTRMWW
DVPPQSVYTFGLYIFQQMNRWPVDGEQDYQANITRLDAYITPSCKHYLQSDFELRRSSGE
LRKRVRGVYEIPGRGYGDSPEIRTVTNSIDDWTVTLDITADEYYGGQLVKRALARYPLHV
VRMDVDPETNPFGLAWDCYNGAPQRIEGNVEAPATPSKGVFK
>tr|A0A0N8T973|A0A0N8T973_PSEAP Biotin carboxyl carrier protein of acetyl-CoA carboxylase OS=Pseudomonas syringae pv. aptata OX=83167 GN=ALO85_00858 PE=4 SV=1
MKPERIKALIDLMAESDLSELSLCEGDAQLRLLREPAQVANRVNPAVATAQTTPAARPVS
PTQASTTAAVQQTQACASLYGVLHLTPAPDQPPFVEIGTSVEVGQTLAVVEAMKMFHPVK
AEAAGVVTAILVANGEEVQAGQALFSIA
>tr|A0A0Q0C777|A0A0Q0C777_PSEAP Uncharacterized protein OS=Pseudomonas syringae pv. aptata OX=83167 GN=ALO85_101348 PE=4 SV=1
MLYKPVIDRPVPPDQQHDHFEHPNYADSSTQPIRRKSAD

>tr|A0A0Q0BUP3|A0A0Q0BUP3_PSEAP Probable membrane transporter protein OS=Pseudomonas syringae pv. aptata OX=83167 GN=ALO85_00101 PE=3 SV=1
MDMEAGGLGFVVAGLVVGFIVGMTGVGGGSLMTPILLWFGINPATAVGTDLLYAAITKSG
GVLVHRKHDNIDWKVTGLLTLGSVPAVLMTLWFLSTLHTAPEALNAIIKQALGFVLLLTA
LAVLFKKKLLAFAHRRGDGYSFFSGTSLTVLTVITGLILGTMVALTSIGAGALGTVALFI
LYPLLPTRRLVGTEIAHAVPLTLVAGLGHASMGNMNWELLGWLLMGSLPGIYLGSHMAGR
VSDDLLRPFLAIMLGMIGFKLAF
>tr|A0A0Q0C808|A0A0Q0C808_PSEAP Uncharacterized protein OS=Pseudomonas syringae pv. aptata OX=83167 GN=ALO85_01085 PE=4 SV=1
MNDSRRPFGATEPEPIDDNEDRMGSMETLDFDEEEPGRIGDLIPEGELQHEIPDQRVREA
GLTGASTDDHHSTDDDLSPETLIHEDGARSPNEPGEDLPADLDVTVVDEDDIGGGSGLDE
EELALIDPLDGNQR
>tr|A0A0Q0DVH1|A0A0Q0DVH1_PSEAP TonB OS=Pseudomonas syringae pv. aptata OX=83167 GN=ALO85_02929 PE=4 SV=1
MANNDLPLELANAGVRPADRLGFTMMIAALIHLAVILGVGFTYVKPEQISQTLEITLASF
KSEEKPKQADFLAQDDQQGSGTLDKAETLKTTELAPYQDTKVNKVTPPPASKPVVKQEAP
KTAVATTAPSQQKTVAKRDEVKPEPTTKAAPTFDSSELSNEIASLEAELSTEQQLYAKRP
KIHRLNAASTMRDKGAWYKDDWRKKVERVGNLNYPEEARRKQIYGNLRLLVSINRDGSLY
EVLVLESSGQPLLDQAAQRIVRLAAPFAPFTGDLADIDRLEIIRTWKFARGDKLSSN
>tr|A0A0Q0DTY5|A0A0Q0DTY5_PSEAP Putative Xenobiotic reductase OS=Pseudomonas syringae pv. aptata OX=83167 GN=ALO85_00349 PE=4 SV=1
MNGTWTPEFSLREVAMSLLLEPYTLRQLTLRNRIAVSPMCQYSSVDGLANDWHLVHLGSR
AVGGAGLVITEAMAVTPDGRITPEDLGLWNDEQIEPLQRITRFINAQGAVAGVQLAHAGR
KASTWRPWLGKHGSISLNEGGWTPVGPSAIAFDPQHTTPVQLSETQIQELIKAFVDSARR
ALEAGFKVVEIHAAHGYLLHQFLSPLSNQRTDEYGGSFENRIRFTLQVTEAVRAVWPAEL
PVFVRVSATDWVEDGWNADETVELARRLKAIGTDLIDVSSGGTSANAEIPVGPGYQTRFA
ERVRKESDIATGTVGMITDPAQAEHILRTGQADIILLARELLRDPYWPLRADEDLGGRLA
TWPAQYQRATHRDQPIHESDLRD
>tr|A0A0Q0C432|A0A0Q0C432_PSEAP Pyruvate decarboxylase:pyruvate decarboxylase OS=Pseudomonas syringae pv. aptata OX=83167 GN=ALO85_05200 PE=3 SV=1
MSTTRLTMAQALVKFLDNQYVEVDGVQSKFVAGIFTIFGHGNVLGLGQALEQDSGDLVVH
QGRNEQGMCHAAIGFAKQHLRRKIYACSSSVGPGAANMVTAAATASANRIPLLLLPGDVY
ASRQPDPVLQQIEQFHDLSISTNDAFKAVSKYWDRINRPEQLMSAALNAMRVLTDPADTG
AVTLALPQDVQAEAYDYPDSFLQKRVHRIDRRPASKAMLDDALQLLVGKRKPLLICGGGV
RYSGAADALQAFAERFDIPFAETQAGKSAIVSAHPLNMGGIGETGTLAANRLAKEADLII
GVGTRYSDFTTASKWLFQNPDVQFLNLNVGAFDVQKLDGVQVLADAQMALQALTESLQAC
GYRAAWGDAPRSARAELDAEVDRLYAVEYQREDFVPEINDHLDPAVLRDFIELTGSCLTQ
SGVLGILNQSLPADAVIVAAAGSLPGDLQRAWRSTGVDTYHVEYGYSCMGYEVNAALGVK
LAAPHREVFALVGDGSYMMLHSELATSIQERRKINVVLLDNMTFGCINNLQMEHGMNSFG
TEFRFRNPETGKLDGDFVPVDFAMSAAAYGCKTYKVSTAEQLRQALADAQRQTVSTLIDI
KVLPKTMIHKYLSWWRVGVAEVSTTGTTAQVYEKLNRELLKARQY
>tr|A0A0Q0D2R3|A0A0Q0D2R3_PSEAP YD repeat-containing protein OS=Pseudomonas syringae pv. aptata OX=83167 GN=ALO85_05069 PE=4 SV=1
MVRHRCACHASISEVRSMAASTSVHSNALNFMSCLKSGVDPRTGLYNISISMPDLQSNDL
RGPGFRLDMSYSQLNTLDSGYGLGWNLQLSQYDPATQILSLSTGETFRVDGTSSTGQLTM
TEKKLDTFHFYKLDQESYRVVHKSGLVEILRLHSSGNKKMAWAVKIIAPSGHSIALKHTA
FNSSTYRLDSITDDLGQTLLEIARSSTSVELDLHPYAGTGGTPLARFLMTLAGSDKRVSR
ITLPTDNNASWRFEYGLENGNQLCVKHVETPTGSSEDVYYQDEGHTFPSSAARLPIPRVT
RHVIDPGLKLAKLDVRYTYKDGQQRSRNFLGAGLPIAWEDNGLDNLYKYLQDYDYVCTES
LWVDNKAVRSIERTFNRFHLQTIEVTTQNENQQTVTTAYNILQGEHYSRQPNDCQLPSKV
TTRWQRLDEPERTRTETVTDTYDSYGNQLVHTRADGMAEISSWYPADGDDGCPADAEGFV
SRLKEKVFKPALSSQPGAPTLSTRYRYRILPALTGSGSPDWIVPEGETLLQLESDGTSVE
LQRILLEYINQPDAPFQHGRTGKNIESLNGNDTVTVYEYSNSESESEQLKVPVQLIDIITIG
FDNARISKQQQQSLLTGQVLLTLEEGVETRYIHDALNRLIKETIAPNSTAGHEASREYLY
TLCSAPEQQAEQVLVDARKVKTRSVMDGLGRAILEERDHIDSNNPHAMHMIHTAHYNAWN
NVQYETDYDWFDSQQRFTTRNTYTYDDWNGQSVITTDQGVQTHQYYDPIGNEEHDGPIQR
RWVQSGEAEPLISGRSETWLNMFGKPVRIKSQNAAGMDLGSQSFLYDGLGRCTEQTDESN
HKTQFSYDAWSRMVSTRLPDSSVARRDYAPHSNTELPIGLEMIHPDGITKTPAGKQTFDG
LGRLTLAQAGQRIERYEYEDGHRQVKTRKTAKGDDISYTYNLALTDQIVSSTAPDETAGF

GYDKVSARLTSATNQQGKRGYAYDAHNQLTKETWKDRQDRTWITLYRTSLQGRTMSRTDV
EQKEVKSPVKGVVKGRLKGGVKHRVKGVETTYRYDGFGQLKSVVQGNVKVTIMYDPLGQP
SEMTTHDQAAGTSLITRMEYDDQGQEVLRTQIAGDQPVRTLEQQWQPDGLMQSRHLQEAG
NRLLLEEFRYDARSRLTGVSYSGSKLPVHASGRAILKQTFNFDELDNMTLNHTEFADEST
ETAIFRYGKEGDPDSRDRCQLLGITYRPARSTPDPTFRYDANGNQLNDEHGNQLDYDSQS
RLSRVEKPTGEPINTYAYDGHNQLVTTRNGSDSEILRFYQDQQLSSTVQDNRRTLFLHID
EQPVGQQTVGESAETLLLLTDANLSVLAEFQQNTLRTAVYSAYGERHSDDALLSVAGFNG
EVCEKDTGWYLLGNGYRAYNPGMMRFHSPDSLSPFGAGGLNPYTYCLGNPIAWRDPTGHD
ASSQSGRLRRPDEDAIPAEMPGGMGIESWAMVAVGVVFTVLGVYATVATLGLATPVSGPV
TVLGLSMSAQAAAYVTAGVLATGTTLSAASTAASAYGAATGNNTALQAGHYLAYAGGALE
LGGGFLKGAVKSAIKAGTQALGRAAYQTRLGSAFSSVNATSISPVNGVVRLPKLTGALSS
TQQSAVDALTTLLRNRNNVPLAGNLTSTRTASTPFANVPAGGVPSIPRAVAPTPVPPPPR
PQSFWQKFFPLRQQYLPRIGGVGDWRTVRI
>tr|A0A0Q0C1U8|A0A0Q0C1U8_PSEAP Uncharacterized protein OS=Pseudomonas syringae pv. aptata OX=83167 GN=ALO85_00797 PE=4 SV=1
MAKDIENPCISVCQLSGDLCVSCGRTKDDIRKWKRMKRPEKMAAVQRATQRLKSLQKKSI
>tr|A0A0Q0C148|A0A0Q0C148_PSEAP Binding-protein dependent transport system inner membrane protein OS=Pseudomonas syringae pv. aptata OX=83167 GN=ALO85_00450 PE=3 SV=1
MVIAVPLTEGASPNLKQRLARAERVNRWKAQALIAPLVIFLLLVFLVPIAALLYKSVGNL
EVVTALPTTVVEVQKWDGKGLPPESVYKAMSLDLAETRKNSTLGDLSKRLNIELAGYRSL
LSKTARALPFKTEPASYKEALEALDERWGDPAYWQAVRRNTSSITPYYLLAAVDHRIDDL
GEVAPATPDQAIYLDIFARTFWMGLVITAICLVLAYPLAYLLASLPARQSNLLMILVLLP
FWTSILVRVAAWIVLLQSSGLINSALLAMGIIDKPLELVFNRVGVYISMVHIMLPFMILP
IYSVMKNISPTYMRAAISLGCHPFASFWRVYFPQTYAGIGAGCLLVFILSIGYYITPALL
GSPNDQMVSYFVAFYTNTSINWGMATALGGLLLLATIVLYLIYSWLVGASRLRLS
>tr|A0A0Q0C8E2|A0A0Q0C8E2_PSEAP Thiopurine S-methyltransferase OS=Pseudomonas syringae pv. aptata OX=83167 GN=tpm PE=3 SV=1
MQADFWLQRWSAGQIGFHQSEVNKDLQQYWSSLNVVPGARVLVPLCGKSQDMSWLSGQGY
HVVGAELSEAAVERYFTERGEQPHITSQGDFKVYAVPGIEIWCGDFFALTVRDIGHCAAF
YDRAAMIALPADMRERYVQHLEALMPQTCSGLLITLEYDQALLEGPPFSVPQTWLHRVMS
GSWEVTNVGGQDTLHSSAKGLKAGLERMDEHVYVLERV
>tr|A0A0Q0IFC2|A0A0Q0IFC2_PSEAP Uncharacterized protein OS=Pseudomonas syringae pv. aptata OX=83167 GN=ALO85_03200 PE=4 SV=1
MTDHARGSRGTWQTCLALLACLCLDAMHPASAEEADDMALALVEQRNLGEGLAWLGYQVA
SRTATFAGIVQAVGKTEAQELVQKELQRLKPEYQAQWDRNLAAAYAHAFNAEELRSLNQG
ADSPALGNRFRSRNTQVSVDMKARSSELLGQFVSRALSNAEAALRR
>tr|A0A0Q0CFH0|A0A0Q0CFH0_PSEAP GTP-binding protein TypA/BipA OS=Pseudomonas syringae pv. aptata OX=83167 GN=ALO85_05037 PE=4 SV=1
MSLTSQQTESSDRDTQVIENLRNIAIIAHVDHGKTTLVDKLLRQSGTLERGELNDERVMD
SNDQEKERGITILAKNTAINWNGYHINIVDTPGHADFGGEVERVMSMVDSVLLLVDAQDG
PMPQTRFVTKKAFEAGLRPIVVINKVDRPGARPDWVLDQIFDLFDNLGATEEQLDFKVVY
ASALNGIAGLEHTDMAEDMTPLYQAIVDHVPAPKVDRDGPFQMQISALDYNSFLGVIGVG
RIARGRVKPNTPVVAIDVNGKKRNGRILKLMGHHGLHRIDVEEAAAGDIVCISGFDQLFI
SDTLCDPLNVEAMKPLTVDEPTVSMTFQVNDSPFCGKEGKFVTSRNIKERLDKELLYNVA
LRVEEGDTADKFKVSGRGELHLSVLIETMRREGFEMGVGRPEVIIRMVDGVKHEPYENVT
IDLPEEAQGAIMEQMGNRKGDLTNMVPDGKGRVRLEYNIPARGLIGFRNEFLTLTSGAGI
LTSIFDRYDVMKSGDMSGRQNGVLVSVATGKALTYSLETLQARGKLFLGHGEDVYEGQIV
GINSRDNDLGVNPTKGKKLDNMRASGKDETIALVPPIRFTLEQALEFVQEDELCEVTPKS
IRLRKKILGESERTRAAKKSGN
>tr|A0A0Q0DTL0|A0A0Q0DTL0_PSEAP Uncharacterized protein OS=Pseudomonas syringae pv. aptata OX=83167 GN=ALO85_100134 PE=4 SV=1
MRVRNSESRHGGESLSARDELSACLANPGPMLLSGFLMPAIQGQARALSSASIRCNTSAS
PRSAQYTATSCPSASTL
>tr|A0A0Q0CI23|A0A0Q0CI23_PSEAP Putative ATP-dependent zinc protease OS=Pseudomonas syringae pv. aptata OX=83167 GN=ALO85_03836 PE=4 SV=1
MKPILALLSLLALPVMAAEPTLYGRYENIKVAEIGQTFKAKMDTGALTASLSAKDIELFK
RDGDDWVRFRLATKGADDKVYEHKVSRISKIKGRAEGDDDDDEGINPAKRPVVDLELCLG
DKKRTVEVNLVDRSHFNFPLLIGAKALREFDAAVNPARRYTADEPDC

>tr|A0A0Q0DJN4|A0A0Q0DJN4_PSEAP AsnC family transcriptional regulator OS=Pseudomonas syringae pv. aptata OX=83167 GN=ALO85_05534 PE=4 SV=1
MDKFDRAILDILQTDCTRSVADIAERIGLGSTACWRRIQKLEEAGIIDRRVALLNPQRLN
VSVSVFAAIRTNQHNAAWLEQFHAAVGELPEVVECYRMAGDTDYMLRIVVADIAGYDEVY
KQLISIPGISDVSSSFAMEQIKFTTGLPLHYASFGG
>tr|A0A0Q0CFT0|A0A0Q0CFT0_PSEAP ABM domain-containing protein OS=Pseudomonas syringae pv. aptata OX=83167 GN=ALO85_03585 PE=4 SV=1
MEMMMKMELFDAQSVTDDNAVYLINVVQVNEGAQDLAIQILEETVSYVARTYSAFKWSRL
MRSIDGKTVINQAQWSDRSQFESLFTDAEFLSRYSRLKETGTWEYHLYNVAEYITPEIAL
QKAG
>tr|A0A0Q0ICS0|A0A0Q0ICS0_PSEAP Uncharacterized protein OS=Pseudomonas syringae pv. aptata OX=83167 GN=ALO85_03146 PE=4 SV=1
MRALAEFIMRGRMQATLVVAGCAALPLLFWLSAAAGCLVLLRRGFSDAVGVLSWALLPAL
VWWYFGEPRTAMVLAGSLSLAMVLRASESWVRVLLVSVALGVVYAVILGTVFREPLEAMS
QELQKHLPTMLAGLYEQLNVEERARLGALIAPVLNGLIAAVLQIVSVLCLMLGRYWQAMM
YNPGGFGREFRAVKLPLVPALVLVVCMLVGPNFGPQIAMLTPLCSVPLVFAGLALIHGLV
AEKRLSRFWLVGMYITLLVFMQLIYPLLVVIAIVDSLIDFRGRRSSKDSGNGPANGEG
>tr|A0A0Q0CX88|A0A0Q0CX88_PSEAP Filamentation induced by cAMP protein Fic OS=Pseudomonas syringae pv. aptata OX=83167 GN=ALO85_02486 PE=4 SV=1
MSEHLWIWQQPNWPRFHWREERLAPLIRACAKAQGRLLGMSGAVGGDAQMCEGLNALLQN
IVTSSAIEGERLNVNSVRSSLARRLGIADDGKHSPRSEGLAELMLDATRGHDQPLDLQRL
CTWHRWLFPEEDSFVSARILVGQLRSDEPMQVVSGRLDRPIVHFEAPPRAGLEQQLEDFL
DWFASSQSDPTLDPFVRAGIAHFWFVTLHPFDDGNGRLTRAITDLALTQGDQQAIRLFAM
SASILADRAGYYHVLETSQKSTTDITEWLEWFLRTLLKSIENALVHIERVLGKTRFWQLH
RDNDLSAEQIKVLNRLLDGGERGFENGLSAAQYQAVAKVSKATATRHLSDLLAKNCLVRL
PGGGRSTRYQINLPLP
>tr|A0A0Q0IPT8|A0A0Q0IPT8_PSEAP PpiC-type peptidyl-prolyl cis-trans isomerase OS=Pseudomonas syringae pv. aptata OX=83167 GN=ALO85_03726 PE=4 SV=1
MAKATARHILVSSEEKCNELKAQIEGGADFAEIAKANSSCPSSRQGGDLGSFGPGQMVKE
FDTVVFSAPVNTVQGPVKTQFGYHLLEVTSRQD
>tr|A0A0Q0DAG9|A0A0Q0DAG9_PSEAP Putative lipoprotein OS=Pseudomonas syringae pv. aptata OX=83167 GN=ALO85_02303 PE=4 SV=1
MLKLTASALILAATLGLAACDSKSEQKAQDAQAHQEKSQEKMESAQDKVNEAAKENTEAA
KDKAESKEAAAEESKTFVPTSEVTNTIQPEKK
>tr|A0A0Q0DJV8|A0A0Q0DJV8_PSEAP BON domain protein OS=Pseudomonas syringae pv. aptata OX=83167 GN=ALO85_00140 PE=4 SV=1
MKKFAIAAATATALTLTMANAAFAQTSAQAPMVLAANTTTQEVKEDTSDTWITTKVKADL
LTEKGIPGSDIKVETNKGVVSLSSMVKVTEAQKTTAVAIAKKIKGVKDVSAAGLKAE
>tr|A0A0Q0C815|A0A0Q0C815_PSEAP Transport permease protein OS=Pseudomonas syringae pv. aptata OX=83167 GN=ALO85_01131 PE=3 SV=1
MLELFRSLWGYRGFVFSSIRNEFSARFARSRLGGLWMIINPLAQVAIYALVLSNVLAAKL
PGIDNKYAYAVYLMAGMLAWSYFAEIIGRCLTLFIDQGNLMKKMRFPRITLPVIVTGSCL
LNYVLLFAAILLVFAALGLWPHWQMLWLIPLTLVVTALAVGLGLILGVLNVFVRDVGQVI
PILLQVWFWFTPIVYPVNIIPEQFKSVMGINPMFPIVSAYHDVLVYGRAPDLQSMAITAA
VAAGLMLLGLFMFRRAAPEMVDVL
>tr|A0A0Q0C8F1|A0A0Q0C8F1_PSEAP 30S ribosomal protein S8 OS=Pseudomonas syringae pv. aptata OX=83167 GN=ALO85_200017 PE=4 SV=1
MNGFQLTFFTEQSTNYQHLPLGEWLVEFAKREGALGATLVSGTEGLDHLGHLHTAHFLGG
ADHPVTVTISTDEIGCDRLLEALAKESFSLSYIKLPIEYGRVGQAKN
>tr|A0A0Q0D8P6|A0A0Q0D8P6_PSEAP Probable transcriptional regulatory protein ALO85_03618 OS=Pseudomonas syringae pv. aptata OX=83167 GN=ALO85_03618 PE=3 SV=1
MGAQWKVKHKEAAANAKGRTFGKLSKEIMIAARAGADPDMNSRLRLVVEQAKKASMPRET
LERAIKKGAGLLGESVNFERLTYEGFAPHRVPVIVECLTDNINRTVSEIRVLFRKGQLGA
AGSVSWDFLYQGMIEAVPAAADADPELAAIEAGAQDFEPGEEGATLFLTESTDMDAVCKA
LPEFGFTVQSAQLGYRPKSTVDGLTDEQMAEVEAFLEAIDNHDDVQNVYVGLAG
>tr|A0A0Q0DSJ4|A0A0Q0DSJ4_PSEAP Major facilitator transporter OS=Pseudomonas syringae pv. aptata OX=83167 GN=ALO85_102137 PE=4 SV=1
MNSLPHTEAAANEPLRAAYISARIDRLPAVATIWRLVALLSIAGFFELYDLFQTAYISPG

LIRDGIFATGSQGLFGFSDQAAFASATFLGLFFGASLVSPIADRYGRRAIFTFALIWYTV
ATVIMGLQTSALGVIGMRFVVGIGLGVELVTIDTYLSELVPKRIRSSAFAFAFFIQFLSV
PSVALMSWWLVPQDPFGFSGWRWVVISSAVFALFVWWLRSALPESPRWLAQQGRFDEAER
VLDGIEARCIKDHGKPLDEPEPDNVALTGKGRFADMWQPPYRRRALMLIVFHVFQAIGFF
GFGNWLPALLSGQGLSVTHSLAYAFVITLAYPLGPLLFVKFANRFENKWQIVGSALGAMI
FGSLFAFQTSAAGLIFCGIMITFCNAWLSFSYHSYQGELFPTNIRARAVGFCYSFSRLST
VFSSLLIGIFLDHFGTPGVLAFIVGSMLIVIVTIGRFGPRTRNLALEQIAHR
>tr|A0A0Q0C0S7|A0A0Q0C0S7_PSEAP FMN-dependent NADH-azoreductase
OS=Pseudomonas syringae pv. aptata OX=83167 GN=azoR PE=3 SV=1
MHILHIASSPRAERSVSLKLASRYLETFTSLHPESTVDALDVWTTDLLPFDGPALNAKYA
DLQGVQMSAEQQAVWKQIHALGERFHRADVILFSVPMWNFGIPYRLKHLIDAVSQRGVLF
EFDAQGMRGLLKGKKVVVFASRGVALGEDFPTEAYDHQVAYLRTWARMVGIPAIDAVLSE
ATLADPATAEANFNAALAEASKLAADHSDPQGARVT
>tr|A0A0N8T9K3|A0A0N8T9K3_PSEAP MutT/nudix family protein OS=Pseudomonas
syringae pv. aptata OX=83167 GN=ALO85_02871 PE=4 SV=1
MSNTAASEKPMTQTTRSAPSAFETIKRENCFKGFYKLDKLHVRHELFAGGMSKEISRELF
VRHDAVCVLPYDAKRDEVVLIEQFRVGAMKKTNNPWLVELVAGLIDKDEQPEEVAHREAE
EEAGLKFEALWPITKYFPSPGGSDEYVHLFLGRCTSEGAGGLHGLESEGEDIRVTVWSFD
DAMQAMKDGIIKNASTIIALQWLALNRAEIRGLWS
>tr|A0A0Q0DG45|A0A0Q0DG45_PSEAP Uncharacterized protein OS=Pseudomonas
syringae pv. aptata OX=83167 GN=ALO85_01098 PE=4 SV=1
MGAVMFNARLKKELQAQKAELSMYRQMQKGMDAQMVCLTLDANHHIVHANENFLNTLGYS
LEQLLGKDLDHLVPTYVKQLDCYRSLKVAVQKGESVIDRYRFLHADGRLVWIRALWQPVL
DEQGRLMTLQCYGSDITQIVETAAENSAFIQALLRSTAVIEFNLSGQVLTANDQFLRGMG
YSLAQIKGKHHSMFCDPAETSQASYREFWAMLNRGEFVAGRFKRIDSNGREVWLEATYNP
VHDAHGRLYKVVKFATMVTDQVAREEEVSQAASVAFEISQQTDVSAQRGADVVQNTVNTM
RKISEEMQSASSGIEALGKQSLLINSIVQTIGGIAQQTNLLALNAAIEAARAGEQGRGFA
VVADEVRQLAGRTSAATEEIVSVVQQNQALADEAVRGMANSRTQAEQGLVLANEAGAVIV
EIQEGAKQVVGAVGRFANQLK
>tr|A0A0N8T8D7|A0A0N8T8D7_PSEAP Uncharacterized protein OS=Pseudomonas
syringae pv. aptata OX=83167 GN=ALO85_01360 PE=4 SV=1
MQVEGFFEWLGQAIGAIIRFFVDGLAWLFNGFTHAGGNFVDGLSRTLGMDTSLISIIALV
IGLMLLYSAVRAFMRASIILGIIWLALGLWLLSWIIH
>tr|A0A0Q0FQA4|A0A0Q0FQA4_PSEAP Single-stranded DNA-binding protein
OS=Pseudomonas syringae pv. aptata OX=83167 GN=ALO85_200057 PE=3 SV=1
MARGVNKVILVGTCGQDPEVRYLPNGNAVTNLSLATSEQWTDKQSGQKVEKTEWHRVSMF
GKVAEIAGEYLRKGSQVYIEGKLQTREWEKDGIKRYTTEIVVDMQGTMQLLGGRPQGDAQ
QGQGGGNYNQSAPRPQQSRPQQSAPQQSAPQQNYNQQPPQQRDSRPAPQQQAPQPAADFD
SFDDDIPF
>tr|A0A0Q0IGR1|A0A0Q0IGR1_PSEAP Secretion protein HlyD OS=Pseudomonas
syringae pv. aptata OX=83167 GN=ALO85_00376 PE=3 SV=1
MSIVGFFRKLNFRKILLRLVLIDLTISLAGYAFYKGASGEEETAFLSAAAEVTDMEYAVM
AQGVLHGRNQVDVGAQVSGQLQSLKVKPGDMVKKNQLLAEIDPLPARNALRQAVVKIEQL
TAERDATLHKLKLAESNDRRYQALGSTTAVSKADQDKARFELEAQRANLVSLTAQLQTAR
VQEEAARIKLDYTRIFAPMDGQVLAIVTQEGQTVIADQMAPVILKMAQLDTMTVKAQVSE
ADVLKIKPGLAVYFTIPGDPDKRYNATLKAIEPGPVDAASQSGATGENSKAVFYNALFDV
PNLESRFYIDMTANVRIVLEAKEGVLTIPVAALGDRREDGTYSVRVLGEGDEVRTADVVP
GINNQVRVEVLSGLKAGDRVVIGERGGVEGA
>tr|A0A0Q0C479|A0A0Q0C479_PSEAP Uncharacterized protein OS=Pseudomonas
syringae pv. aptata OX=83167 GN=ALO85_102234 PE=4 SV=1
MDVVDDAAGIGVLVINATAMCERLNPVLRAHTLELTPQVANRVRQLFFNLLPLSYRYRRQ
DWQCDHRDRK
>tr|A0A0Q0DRZ5|A0A0Q0DRZ5_PSEAP Chemotaxis sensory transducer protein
OS=Pseudomonas syringae pv. aptata OX=83167 GN=ALO85_04322 PE=4 SV=1
MSLLSRFRAGTSRTEGAISVITSARELNAKLSALSFDPVYLTGFVSPHVDFDQVAKSVAA
RFPNAKISLCTTSGELCSGNDSLYCAADNQWDRIVLALFDSSVIKSAEVVHIPLHSEDIR
GAGKRLSMRERIARLTDSIKRTQINTPIDHRDTLAYVTFDGLSASESFFMEALYESGRFP
CLFVGGSAGGKGDFKKTLIHDGKRSYENHAQVVFLKSAKNVRFGVFKSQNFEPTPLSLSV
LTASLEERYISQVVNARGEITTMVAALCDALKCERHELEGKLARYSFAIRVGEELFVRSI
ARIDYESERVHLFCDVAPGEELVMVKRTSMIDTTRKDFARFLQGKSGKPLLGLLSDCILR

RLNNGSELGRMNDVFGATPVVGFSTFGEILGLNLNQTLTAVFFFKVPEGQPFRDDYIDNF
IAYYGEFKAFFLRRQIKKLAGLSHVVVKQIDQFKQRNFVQTIDPSGLDEQIRPVFGGLSD
LGDVLVKAHHDQEEIAAQLRHYSGELHVSMDELSQTITQQETVIDKAGNTVKSLVSQADE
VVLGSRELAKSSLRIQSVVQMIQQIAGQTNLLALNAAIEAARAGEMGRGFAVVADEVRQL
AQKTSQNANDIGTDIERLAEEIRKVAQHIEEQSLEVGTLTGLLSELEGSSDMTAGTSQRT
RQLADTLRGLTQ
>tr|A0A0Q0CA66|A0A0Q0CA66_PSEAP SdiA-regulated OS=Pseudomonas syringae pv. aptata OX=83167 GN=ALO85_01484 PE=4 SV=1
MNEAMQLETIKAKRLTWSNVARRQVSLAQLVLSAAALGLLILAYAVSTHFQVHQQLYQYG
RTWLKGNTAADKNIWLPDFHVVIDAKPLAPDVANISGITYDYDNDRLLAVTNKGPIQLLA
LNVNGDIIARYPLIGFDDTEGVAYLGNGRIVLCDEDLQQLDIITLPPQARPIHVEEAQFI
ELLINPSIHNKGFEGVTYDPANDRLFAIKERDPRQMFEVSGVLRSIDQGRLQIKIADRLD
WITKSVAARDLSDGYYDPRTGHLLLLSDQSHSVTELDSKGRFVSIRSLLGTFSDLKHSAP
QPEGLTMDRAGNLYVVSEPNLFYKFSKVPE
>tr|A0A0Q0D3V7|A0A0Q0D3V7_PSEAP Helix-turn-helix, AraC type:AraC-type transcriptional regulator OS=Pseudomonas syringae pv. aptata OX=83167 GN=ALO85_03881 PE=4 SV=1
MDTHNAASAPGTDVCRAEIIQLLTQRFAAPGVYETAIAPLHVVRCDAPSELIHALHRPAL
CVIVQGRKQVALGDEQYVYDPLSYLVVAVTVPVAGRVLEASGEAPYLCIRLDFEPAHIAQ
LIAEAPLNGVPDEPQRGLFLEPMDQPLLETVLRLVRLLDTPRDIGMLAPLALRELYYRLL
RGNNGRRLYEIAVGDSQTHRVTRAVDWLNSHYTETLRIDELARLANLGNSTLHHRFKALT
AMSPLQYQKKLRLQEARRLIINEGLDASSACYRVGYESPSQFSREYSRQFGCPPSTDLSR
VRQIG
>tr|A0A0Q0C1L0|A0A0Q0C1L0_PSEAP Xanthine/uracil permease family protein OS=Pseudomonas syringae pv. aptata OX=83167 GN=ALO85_01724 PE=4 SV=1
MTQQEFNDPLWRTILSGAQMLFVAFGALVLMPLITGLDPNVALFTAGLGTLLFQIVTGRQ
VPVFLASSFAFITPIILAKGQFGLAATMGGVMAAGFVYTFLGLAVKIKGTGFIDRLLPPV
VIGPVIISIGLAMAPIAANMAMGKSGDGAQLIPYQTAMLISMPALLTTLIVAVFGKGIFR
LVPIISGVLVGFALSFYFGVVDTKKIADAAWLALPHFTAPEFNWQAILFIVPVALAPAIE
HIGGVIAVGSVTGRDYLKKPGLHRTLFGDGIATTAAGLFGGPPNTTYAEVTGAVMLTKNY
NPQIMIWASFFAITLAFIGKFGALLQSIPVPVMGGILCLLFGSIAAVGMNTLIRHKIDLA
EARNLVIVSVTLVFGIGGVLIGTGNGPNDFGLKGIALCAVTAIVLNLILPGNDSWKNNKP
DDQLP
>tr|A0A0Q0D5H2|A0A0Q0D5H2_PSEAP Acetyltransferase OS=Pseudomonas syringae pv. aptata OX=83167 GN=ALO85_02444 PE=4 SV=1
MSPASMTLNYRRLTPADAPAYRALMLEAYERHPDAFTSDVKEREVLPAQWWEKRLDDSAG
ASEVVFAAVEDGHLLGVAGLCVETRKKARHKSTLFGMYVPLAHRNRGIGARLVRSVLDHA
GTRSELLVVQLTVTQGNTEAVGLYERMGFVTFGLEPLAVAVGNGFVAKVHMWKDLRG
>tr|A0A0Q0BU56|A0A0Q0BU56_PSEAP TonB domain protein OS=Pseudomonas syringae pv. aptata OX=83167 GN=ALO85_100679 PE=4 SV=1
MPKQSRSSVRRLSADADQGIDGRVGRASGGQRMSCVLGCMMLTGLLSAGGAQALPSYPVP
LHMPEPGYPTSMLRSFVKRSVSIRVFIQADGGVRFLEVLGAPDPRLISLTRSAVEQWVFE
PWQPSASHPEGEAVTVTFNFTERPHTNPPLAANVELRQVLCWQLNTEMFEGRKWRKDVKP
DALMRTELYLSSSPVIEHFLTLDERQALVLELLEATPDIVAQCQKNPMRRYVDYLPESVR
KAL
>tr|A0A0Q0DP35|A0A0Q0DP35_PSEAP Tabtoxin resistance protein OS=Pseudomonas syringae pv. aptata OX=83167 GN=ALO85_01937 PE=4 SV=1
MNPAQLRRVTAESFAHYRQGLAQLLFDTVHDGASVGFMADLDMAQAYGWCDGLRVDVAAG
TLLLWVVVENENVLASAQLSLCQKPNGLNRAEVQKLMVLPAARGRGLGRQLMEAVEQAAV
KHKRGLLHLDTEAGSTAEAFYRSLAYHRVGELPDYCATPDGRYRPTAIYFKTLGQPT
>tr|A0A0Q0DX85|A0A0Q0DX85_PSEAP Amino acid ABC transporter permease protein OS=Pseudomonas syringae pv. aptata OX=83167 GN=ALO85_100180 PE=3 SV=1
MTTQYCVRGTTMPIVAKPYDFIDEPSHYPLPQSRVAPPPIKALQVVPARHPGRWFGSIFA
ALVLLAIVHSLATNPRWEWGVFGQWFFSPSVLRGLAQTLLLTLLSTLFSIILGTALALAR
LSGSPLLAALAWGYIWFFRSMPALLVLIILYNFAYLYDHIVLGVPFTQLVFAEWSTVDVL
SQFTVAVLGLSLMQSAYTAEIIRGGLIGVDAGQHEAAAALGLPVSRRIFRIILPQALRSI
LPSGFNEIIGLVKGTSIVYVLALPELFYTVQVIYNRTQAVIPLLIVATVWYLIITTVLTS
AQYYVERHFARGSARVLPPTPLQRLRGWLKGSVSGEKHHG

>tr|A0A0Q0BXS4|A0A0Q0BXS4_PSEAP AAA ATPase OS=Pseudomonas syringae pv. aptata OX=83167 GN=ALO85_03953 PE=4 SV=1
MRVLIKLKKLSNPKPVEISTKNTLYVREMAKLRAYFKLNPTERARTRPPFDKDVLDAQSV
LQELKRIQHSKCAYCETILEHNPSRFVSHYRPLSNAFDPHQDAEHLECYAWFAYEWRNLM
LICKDCDNHKLNFFPIAGRTAIALSSWRAAQLREMPLLLNPFEDNPSDHLMFDTDGVACS
TSAAGKETINILNLNRPALCIKRKEQIGAGLKIILGNSNEHALREALREYTDHEVLHGGA
VRNCLGHVCRILQTQIRHGRYSAHTDLIKKIIRLRNEFSVHDWEEAISRCKSNDLAFVIQ
NNELGELQPRRYAYINRIEIGRFKGVEHFTLDLDAGPTGQSRSETSCTMLLGENATGKSS
ILQAIALALMPSGERQRLRLDQKSIIPYGIDDTFMPPLQPAVTLRFSNGEAVAISMNLQT
GRLKSVGSPHNVVFGYGSRRYFVLGKSTKAQTCPNRTLFNQAANLPDPTNWLLNLKEEKK
FDAVARALASLLSLRAGEFVGRDDHSIFIQRHTSPLPIEHLSDGYKSLFAMAVDIMREML
KHWDSLEFAQGIVLIDEIENHLHPRWKMRVMRALRDSFPRVQFITSTHDPLCLRGMLEGE
VQVLYRDRVGHLQRVEQLPNVANLRIEQILTSDYFGLATTEDPLRQHALEQLAKYAAHGD
SDLTQTERRHRDDLLENYGSLPMIGDTLDRQIIAQALTRHIRDADIATPFEHASAREASV
RAIIEVLERNRKKYGTR
>tr|A0A0N8T8X8|A0A0N8T8X8_PSEAP Uncharacterized protein OS=Pseudomonas syringae pv. aptata OX=83167 GN=ALO85_101483 PE=4 SV=1
MAQAQSSPVEASFLARHYAYNSLTGEGVDLSDYPVIRYCATGKIVTPESSAYFQKIGGCM
QKQRAALYEEEYLKGTPAARILEKILNFNDALPLAFRDMANW
>tr|A0A0Q0DGQ7|A0A0Q0DGQ7_PSEAP Aminotransferase OS=Pseudomonas syringae pv. aptata OX=83167 GN=ALO85_03825 PE=3 SV=1
MSLFSAVEMAPRDPILGINEAFNADTRSTKVNLGVGVYCDENGRIPLLRAVAEAEKVRVA
QHAPRGYLPIDGIAAYDQAVQKLLLGADSPLIASGRVLTTQSVGGTGALKIGADFLKQLL
PDAVVAISDPSWENHRALFETAGFAVQNYRYYDAATHDVNRAGMLEDLHNLPNNSVVVLH
ACCHNPTGVDLSLDDWKKVLEVVKSKGHVPFLDMAYQGFGQGIQEDALAVRLFAESGLTF
FASSSFSKSLSLYGERVGALSIITESKEETTRVLSQVKRVIRTNYSNPPTHGAIISAAVL
NDPTLRAMWEEELGEMRLRIQGMRKAMVERLADNPAGQDFSFVARQCGMFSYSGLTAEQA
QRLRSEFGIYALDTGRICVAALNQKNIDVVCDAIKQVL
>tr|A0A0Q0FDD5|A0A0Q0FDD5_PSEAP NADPH-dependent FMN reductase OS=Pseudomonas syringae pv. aptata OX=83167 GN=ALO85_100028 PE=4 SV=1
MPHRSSKPGFCSIPNLPLNQMTDISMSQVHTIAVLVGSLRKESINRKIALALAELAPASL
KLNIVEIGDLPLYNEDIDGDSPPPAYTTFREQLGAADGVLFVTPEYNRSVPGALKNAIDV
GSRPYGKSAFSGKPGAVISASPGAVGGFGANHHLRQSLVFLDVLCLQQPEAYLGGAGSFF
DESGALSDKTRPFLQKFIDAFAAWVDKNGRAADN
>tr|A0A0Q0DUU5|A0A0Q0DUU5_PSEAP Uncharacterized protein OS=Pseudomonas syringae pv. aptata OX=83167 GN=ALO85_01512 PE=4 SV=1
MPVLHYGQTTIEWYFQLDTNLKRHYVTVERGRPVLLRGPQVDDPEQEALVLRRARWIREK
LAQVNLPLANEAIVTGSRLRYAGRTYFTEVRHTPDILKPRLTFTASRFIVDNPDSASIQP
DALIPLLERFYRERAQDKLLVRVRHWQRETGLQATGARIRHFQSRWASCDANNTLEFHPR
VMELPASVQDYVIIHELCHTVEKNHTKAFWGLVASYMPEWQKQHQVLERATFGDAV
>tr|A0A0Q0DEJ9|A0A0Q0DEJ9_PSEAP Uncharacterized protein OS=Pseudomonas syringae pv. aptata OX=83167 GN=ALO85_03115 PE=4 SV=1
MKTTHLQHHPSLGYIAAGSLRSLPVGLLLPHDCSSLISFRAHPFRKPERVIRKLHAAQRP
LRIYGAGSYCFYGVQSDSPLAPLLLWDGAHFLNVGEKITVIEDTPIECYLDRDYFAGSLK
VIARSANSVTYKKVSRLTAESNDDLDSWTFGIPVGPGDATVLNAVVKRILEIDIPHKEIL
LCGTPGGNFAYFDKVRIVGEDITAPPVQICKKKNRLALEARHSNLVILHDRVFLPKHFGE
VVRRFGPRYPLMTLQSMFFDNRISMHPRRYSDYGMAMDEVAQGLLGLHRTSGNAQSIAPS
IFPDVDRTGFCFASPMRYNNDASYPTGSLYICRKDVWNACPLDESLHWVEYEDIEHALRA
SRAGIPNRVNPFGITQSVTSRALLGGNAPVESVDGCLGVSGPCYVSLLEKKPLLNLSAET
ALARLRQFADKYLANPSAVVIPTGLDHVSVRAWIELIDNVVQQSTFKNDSETVREFIGDF
ERLVLLDQLPSTRQEFLVNRFLTDPALAKQTLITQSCEVRNMLRQRGTQTWFARQQDDYF
HHNLLSLPGILISAVRAYRNNGKSFYFESLWAAVKAIYNSTPFMSYSKGAK
>tr|A0A0Q0IA06|A0A0Q0IA06_PSEAP Uncharacterized protein OS=Pseudomonas syringae pv. aptata OX=83167 GN=ALO85_04061 PE=4 SV=1
MRAMDALTQNLIDSLTKILQCPQEDSLLALKICAPVLIEHLQVVKHVAVDRREIQSQLHK
ALDQWLEHHPQPESAQKALLTLLDQELLAKQLLDKEASA
>tr|A0A0Q0DX55|A0A0Q0DX55_PSEAP Uncharacterized protein OS=Pseudomonas syringae pv. aptata OX=83167 GN=ALO85_03709 PE=4 SV=1
MPALTTYTTRVRPDWVDYNGHLRDAFYLLIFSYATDGLMDALGLDSDNREASGHSLFTLE
LHLNYLHEVKLGAEVEVHTQLIAHDAKRLHLYHSLHLAGDEMELAGNEQMLLHVDLAGPH

ATMFTKATKERLDAMSAGQSELPRPKLLGRVIGLHAGK
>tr|A0A0Q0DM79|A0A0Q0DM79_PSEAP Outer-membrane lipoprotein carrier protein OS=Pseudomonas syringae pv. aptata OX=83167 GN=ALO85_02833 PE=3 SV=1
MNRPLRTLILLGLLCLSSLAQAFDLQQLSDQLAKPAVVQGRFVQEKHLRALPQPLLSKGR
FVLARDFGLLWLLETPLKQDYRINADGIARRETDGNVSTWKPVPNKNAGAEQNRLFLAVL
QGDSSGLQRDFDLQLKGEANAWHLTLTPRSMLLQQVFKQINIDGGELVQRIELLETQGDS
TVLKMIDSSSAPTLTDAQRNDFKD
>tr|A0A0Q0C3K4|A0A0Q0C3K4_PSEAP Putative acyltransferase OS=Pseudomonas syringae pv. aptata OX=83167 GN=ALO85_01462 PE=4 SV=1
MDFRKDINGLRAIAVIAVLLFHFHPDWLPGGFAGVDVFFVISGYLITGIILRGLRSGKFR
LATFYTSRARRIVPALAALCFALLLLGWFSLLPLDYRALGTHVASSLGFVSNIVYWREAG
YFTASAHEKWLLHTWSLSVEWQFYLLYPIALLMLSRFVALHNLRWWVLGASLTGFVACVS
ASSQWPEAAFYLLPTRAWELLAGGVACLFPLQLKALQQRVLEHIGLTLIVAGFFLLSVQD
TWPGYLVLMPVLGTFAVIVAARNDSLLTCNPLAQWTGTRSYSLYLWHWPVVVWMNDAGLL
GETRAVLVGIGMAVTLGLVSWWLIEQPASRPSSDQRSRYATLGTLIALVFVCGALVSATG
GVVSPLRPVSVSDKARFIQEYADRQHNLYEPYWLKCDAFSALTQRGQSAIDEACTRKQGA
GGVFLWGDSHAQALSLGLRTLLTHSTPFYQVASASCRPALSDHQGRTSATSRACDYSNRT
ALQGIEKLRPDIVVIAQKDGHDKTDWTQVAIRLKGLGVKHIVLIGPVPQWNPSLPSVIAN
RHWGLSESHIRDPALDQSVMLVDQATRALAASAGIQFVSLIDKLCIADACRVRLEDNRSL
LQIDSGHLSAEGSLYVVRNYVLPQLVN
>tr|A0A0Q0C9V5|A0A0Q0C9V5_PSEAP Na/Pi co transporter family protein OS=Pseudomonas syringae pv. aptata OX=83167 GN=ALO85_01586 PE=4 SV=1
MGKPQGAFRMLTLLDLLSAVALLIWGTHIVRTGILRVYGTQLRRVLSQNMSKRPLAFIAG
ILVTALVQSSNATAMLVTSFVGQGLMTLTPALVIMLGADVGTALMSRVLTFDLSWLSPLL
IFLGVVFFLSRKQTRAGQLGRVGIGLGLIVLALQLIVEAAAPITQAAGVKVLFASLTGDL
LLDALVGAMFALISYSSLAAVLLTATLAGTEVIGLPVAIGLVIGANIGSGLLAFLSTSMQ
NVAGRQVALGSLLYKLLGLLLIFPVLDPLVAWIDTLGFSPQELVIGFHLLYNTVRCLIML
PTVAPMARLCSYLLPQQNEVGGLAKPRHLDLTALSTPSLALANAVRETLRMGDLIESMLA
SMLAVLRGTQTAVTQEVRRLNDDVEALYSAIKLYLAQMPREDLGEHDNRRWAEIIELTIN
LELASGLIERMLRKIQQQKTAHRRSFSEVGLEELADLQIQLQSNLRLGLSVFLSGDHESA
RQLLREKRQFRAEERRLAHAHVSRLRNKVVQSIETSSLHLELIADMKRLNSLFCSSAYVV
LETADTGALSSEGEADRSL
>tr|A0A0Q0IPB6|A0A0Q0IPB6_PSEAP Peptidase OS=Pseudomonas syringae pv. aptata OX=83167 GN=ALO85_03767 PE=4 SV=1
MPCDLSGSPLAAQTCCSRPKGLFFENTSGQSLRALGNYWRHPMLFPNPSGNGALINFELF
SCGLLGAEEGNEVLVNTVHAAPPYGNTHILDHRKTDLQGYGSARNTQRMEFKDRLKAARQ
HAKLNQAELAVRAGITQTSISDLERGKSKATAHVAKIADVCGVNALWLSDGKGDMTAFIT
SNEPSNVSMAEQSSRMYRYPLVSWVAAGEWSEAVEPYPPGAADEYDVSDYKAKGPAFWLV
VKGDSMTAPTAPSIPEGSQILIDTRAEVLPGKLVIAKLAGSNEATFKKLVEDGGVRYLKP
LNSAYPTVQCTEGCRVIGVVVRSLTKFA
>tr|A0A0Q0DKD0|A0A0Q0DKD0_PSEAP Putative Pillin OS=Pseudomonas syringae pv. aptata OX=83167 GN=ALO85_02790 PE=4 SV=1
MRHIAKGFSLIELLVTVSLVGILAAIAIPNFTSSIQSNKADTELSDLQRALNYARLEAIN
RGVAVRIAPTSGTAWTNELQVYLVSDTQATPTALRKVAAMSSGATLVADNNATAIDFNNL
GALIAPAAAVTMTYTRGTIIKTIKVCLTGRITLGGGC
>tr|A0A0Q0IBI2|A0A0Q0IBI2_PSEAP Nitrilase/cyanide hydratase and apolipoprotein N-acyltransferase OS=Pseudomonas syringae pv. aptata OX=83167 GN=ALO85_00157 PE=4 SV=1
MEYSGGHVIQERVAMSFAVIQMVSQSDVLANLASARRLLEQAAEGGARLAVLPENFVAMG
RRDVADIGRAEAQGHGPILPWLKLAARDLKLWIVAGTLPLPPDERPDGKVTACSLLMDEL
GEQVARYDKLHLFDVDVADNRGRYRESDDYAHGSRVVVADTPVGRLGLTVCYDLRFPELY
TALREAGAELITAPSAFTAVTGAAHWDILIRARAIETQCYVLAAAQGGVHPGPRETYGHA
AIVDPWGRVLAEQAQGEAVLLATRDSEEQASIRARMPVSSHRRFFSQGAMRPASE
>tr|A0A0Q0D8M9|A0A0Q0D8M9_PSEAP Putative type VI secretion system effector, Hcp1 family OS=Pseudomonas syringae pv. aptata OX=83167 GN=ALO85_100814 PE=4 SV=1
MSKYVDRATPKLFELCCRGSHIKNVTIRIHRAGTEKFKYLDIVLEEVLISLVSGQGADQS
GFPIEVVNLNYGRIKFEYSQQRRADGGSAGIVSGGWDRTANKPFA

>tr|A0A0Q0BY28|A0A0Q0BY28_PSEAP Phosphoserine aminotransferase OS=Pseudomonas syringae pv. aptata OX=83167 GN=serC PE=3 SV=1
MDMSKRAFNFCAGPAALPEAVLLRAQAELLDWHGKGLSVMEMSHRSDEFVSIATKAEQDL
RDLLSIPSNYKVLFLQGGASQQFAQIALNLLPENGKADYIDTGIWSQKAIDEASRYGAIN
VAASAKAYDYFAIPGQNEWKLSKDAAYVHYAPNETIGGLEFNWIPETGDVPLVADMSSDI
LSRPLDISRFGMIYAGAQKNIGPSGIVVVIIREDLLGRARSLCPTMLDYKVAADNGSMYN
TPPTLAWYLSGLVFEWLKEQGGVEAIGKRNEIKQRTLYDFIDASELYSNPINKPDRSWMN
VPFRLADDRLDKPFLAGADANGLLNLKGHRSVGGMRASIYNAIDINAINALVAYMKDFEK
EHG
>tr|A0A0Q0BX41|A0A0Q0BX41_PSEAP Cell division protein ZapA OS=Pseudomonas syringae pv. aptata OX=83167 GN=ALO85_01649 PE=4 SV=1
MSNGNSVTVQILDKEYSIICPQEERTNLVSAARYLDGKMREIRSSGKVIGADRIAVMAAL
NITHDLLHKEHLPDVQTSGSTREQVRDLLDKVDLVLATDTPGTPDSTRG
>tr|A0A0Q0CD58|A0A0Q0CD58_PSEAP Paraquat-inducible protein B OS=Pseudomonas syringae pv. aptata OX=83167 GN=ALO85_00871 PE=4 SV=1
MSFCIVGAVIYPDRLGMANEKIVKTLGVTDDARTAQLIADFVKRGLRAQPRSESLLTGQL
YISLGFIANATQVPFDVSAQPLIIPTVPGELEKMQEQVQQIIDKVGKVPVQEIAGNLNGS
LGEAQKTFRLFDAQVIPELHGVLLQSRSTMDAAGAALAEDSPVRQQIDRTTDEVQRMARS
VRVLTDYLSRNPEALIRGRTRQDPPGLYQRPAPAQRAD
>tr|A0A0Q0D5J3|A0A0Q0D5J3_PSEAP S-adenosyl-L-methionine-dependent methyltransferase OS=Pseudomonas syringae pv. aptata OX=83167 GN=ALO85_03491 PE=3 SV=1
MMLTSQKNIINTGIPSRTAFQVAELRAAHQLLDEPIIFNDPLALPILGEQAATDLRQYPF
QSNDLHARGIRAVVVTRSRLAEDELKKFVHSGVKQYVVLGAGLDTFALRNTYLEEGLHVF
EVDHPSTQEWKRSLLKEASIKVPDSLTFVAVDFESNTLAEELQKAGFRADQPAFFSWLGV
TIYISQEAILDTLTFVASLPKGSGVIFDYGVTPTLLDPIENAIGDHLVSLIGQLGEPWKT
WLDPAVIEHELQSIGFQSVRDYGSVELNELYLARRKDGLRMLGTFRLICAKT
>tr|A0A0N8T8C4|A0A0N8T8C4_PSEAP Polar amino acid ABC-type transport system, permease protein II OS=Pseudomonas syringae pv. aptata OX=83167 GN=ALO85_01635 PE=3 SV=1
MAYHFDFTPVLQSIDLLLRGALFTLELTAIGTLLGVSLGIVGAVVRAWKIQPFATIFAIY
VELIRNTPFLVQLFFIFFGLPSLGVQITEWQAAVLAMVINLGAYSTEIVRAGIQAIPKGQ
LEASAALAMSRYEAFRHVVLVPALGKVWPALSSQIIIVMLGSSVCSQIATEELSFYANFI
QSRNFRSFETYIVTTLIYLAMALLIRQLLAWIGRRYISRNR
>tr|A0A0Q0FU10|A0A0Q0FU10_PSEAP Response regulator receiver:transcriptional regulatory protein OS=Pseudomonas syringae pv. aptata OX=83167 GN=ALO85_01968 PE=4 SV=1
MKLLVVEDEALLRHHLRTRLTEAGHVVEAVANAEEALYQVAQFNHDLAVIDLGLPGIGGL
DLIRQLRALGKAFPILILTARGNWQDKVEGLAAGADDYVVKPFQFEELEARLNALLRRSS
GFIQSTITAGPLLLDLNRKHAALAEQPLALTAYEYRILEYLMLHHQQVVPKERLMEQLYP
DDDERDPNVIEVLVGRLRRKLDGSVAFKPIETVRGMGYLFNERCT
>tr|A0A0Q0DJA2|A0A0Q0DJA2_PSEAP Phenylalanine 4-monooxygenase OS=Pseudomonas syringae pv. aptata OX=83167 GN=ALO85_01834 PE=4 SV=1
MAQTLYVAREPDASGFIDYTADEHAVWNTLITRQMEIVEGRACQEYLDGIKQLGLPLDRI
PQLGEINKVLGATTGWQVERVPALIPFQTFFELLASKRFPVATFIRSAEELDYLQEPDIF
HEIFGHCPLLTNPWFAEFTHTYGKLGLSASKEQRVYLARLYWMTIEFGLVDTPQGRKIYG
GGILSSPKEALYSLSAAPEHQPFDPLEAMRTPYRIDILQPLYFVLPDLKHLFDLAQQDIM
ALVGQGMQLGLHAPKFPPKTKSHAA
>tr|A0A0Q0D9G0|A0A0Q0D9G0_PSEAP Arsenate reductase OS=Pseudomonas syringae pv. aptata OX=83167 GN=ALO85_01962 PE=3 SV=1
MQMTDLTLYHNPRCSKSRGALELLQARGLTPDIVLYLETPPDADRVRDLLGKLGIGARQL
LRTGEDDYKQLNLADPSLSDEQLIAAMAAHPKLIERPILVAGDKAVIGRPPENILELLP
>tr|A0A0Q0D1Z4|A0A0Q0D1Z4_PSEAP Binding protein OS=Pseudomonas syringae pv. aptata OX=83167 GN=ALO85_00053 PE=4 SV=1
MTAYREVSMSATTPNPQDFATHAISLTLNGQLRQLDVLPWTTLLDLLRDQLDLVGTKKGC
DHGQCGACTVLRDGTRINACLTLAIMCDGAELTTIEGLADGDQLHPMQQAFIKNDAFQCG
YCTPGQICSAVGLANEGRASTRAEIQELMSGNLCRCGAYSNILAAVEEALPQFHNAAPKP
EVNA
>tr|A0A0Q0BT42|A0A0Q0BT42_PSEAP Uncharacterized protein OS=Pseudomonas syringae pv. aptata OX=83167 GN=ALO85_02288 PE=4 SV=1

MQLTNETMAKHTFLKGMYQDEYFPDAVVKMCEDVLVNLCHEIEQQKPSDLDALYALTHKA
TEQINELEEVFEENGSEIETVGRETIAEDFVRIADAYGFTDADIEELIAPREW
>tr|A0A0Q0BUQ7|A0A0Q0BUQ7_PSEAP Uncharacterized protein OS=Pseudomonas syringae pv. aptata OX=83167 GN=ALO85_04428 PE=4 SV=1
MQTRFCQAVVGMYLVAGMVLLAGCTQESVSPCDELIGDYATKPKNQPRMRIEKSGEKYLF
THSESGAQVSEPLTPLPAAEVVEMFEGKIVPPRCILTADGMKIMKLPAGSGVNPTEDPDR
KLSQYPEPTPFLMGISAGVAAVLGLYPVPHQEKLKDIDEEDEDDDGYIYSK
>tr|A0A0Q0CVY2|A0A0Q0CVY2_PSEAP Glutathione S-transferase family protein OS=Pseudomonas syringae pv. aptata OX=83167 GN=ALO85_04222 PE=3 SV=1
MITVHHLNNSRSQRILWLLEELDLPYEIKRYQRDPKTNLAPPELKAVHPLGKSPVIEDGP
HVVIESGAIVDYLIRRHGDGRLQPDPASATYDEYVQWLHFAEGSAILPLMLNLYVGRLGD
AGAPLHPRIQSELANYMGYLNEALGLTPYLLGDELSGADIQMSFIGEFAKAQAMLQPYPN
VTAWVERLQGRPAYRKALEQGGEYSFAK
>tr|A0A0Q0C9X4|A0A0Q0C9X4_PSEAP Large-conductance mechanosensitive channel OS=Pseudomonas syringae pv. aptata OX=83167 GN=mscL PE=3 SV=1
MSVLKEFKAFAVKGNVVDMAVGIIIGAAFGKIVSSFVGDVIMPPLGLLIGGVDFSDLAVT
LRPAQGTAPAVLLAYGKFIQTVLDFIIVAFAIFMGVKAINRLKREEAKAPTLPPTPSKEE
VLLGEIRDLLKEQSKPAAPITVDPARTL
>tr|A0A0N8T9F6|A0A0N8T9F6_PSEAP Phosphate transporter OS=Pseudomonas syringae pv. aptata OX=83167 GN=ALO85_02393 PE=3 SV=1
MIDLFSGLDAWVLVSLLLALAFVLTFEFINGFHDTANAVATVIYTKAMPPHLAVLFSGVF
NFLGVLLGGVGVAYAIVHLLPVELLINVNTGHGLAMVFSLLAAAITWNLGTWYFGIPASS
SHTLIGSILGVGLANALLNDIPLADGVNWQKAIDIGASLVFSPLAGFIVAGLVLIALKWW
RPLSKMHKTPDQRRKLDDKKHPPFWNRLVLVISAMAVSFVHGSNDGQKGIGLIMLVLIGI
VPSQFVLDLTSTTYQIERTRDATQHLNQFYQRNSSTLGEYLAMGKAEKGDLPSSSACNPK
QTEPTIDALLDRLKGVSDYHALAPESRIEVRRYLLCLDDTARKVGKLPDLSAREKSDLEK
LRKDLTTTTEYAPFWVILAVALALGIGTMVGWKRVVLTIGEKIGKQGMTYAQGMSAQITT
ACAIGLANVFSLPVSTTHILSSGVAGTMVANKSGLQGGTVRTILLAWVLTLPATVALSAA
LFWLASKALA
>tr|A0A0N8T9L0|A0A0N8T9L0_PSEAP Carbamoyltransferase OS=Pseudomonas syringae pv. aptata OX=83167 GN=ALO85_02888 PE=4 SV=1
MALTILGLSGALSHDPSAALYIDGKLIAAAEEERFVRDKHAKNRMPYESAKFCLEQAGIK
PSDVDVVAIPFAPISLFGKARWHYAKRYWYAPDRALDALLMGNRRYKRYRNKIVWCLEQL
GFDPKKIKIEPVEHHLAHASSAYHCSGFTEKTAIMGIDGKGEYATTFFGYGENGKIHKIK
EFFDPDSLGGLYGAITEFLGFEMLDGEFKVMGMAPYGDASKYDFSRLATFENGELVINTE
LANVIGLRRYKENGKGFYFSPKLIEWLGPKRVGDVADEPYIHYAASMQALFEKLALQMMD
HYLGDILKETGKIAFAGGCALNVKLNQRIIARPEVKELFVQPASGDAGTAVGAAAYVSHA
RGVPVEKMEHVYLGPSYSNEDVIAACARHPSKPVWRQIDNTPERIAEIMVKGNPVAWFQG
RMEFGPRALGGRSIIGCPSVTGVADRINHQIKFRERWRPFCPSMLDTVAPQMIKIDHPAP
FMTFTFEVAEEWKTRVPEVVHEDGTSRAQVLKREYNPRYYDMMKALENLTGNGVSLNTSL
NRRGEPMICSPTDALNMFFGSDLEYLIMEDILVVKEGVEAYDTLG
>tr|A0A0Q0D457|A0A0Q0D457_PSEAP CvpA family protein OS=Pseudomonas syringae pv. aptata OX=83167 GN=ALO85_02561 PE=4 SV=1
MPFTPVDWAILGIVAISALISLKRGFVKEALSLTTWIIAGVVAWMFGAGLSQYLVNYIET
PSARVIASCTILFVATLLVGAMVNFLIGELIRVTGLSGTDRFLGMVFGAARGGLLVVVAV
GLLSLGPVQQDQWWQQSRLVPQFLMVADWSKNLILGMSSKWLASGISVPADMPFKEQILP
STMPQDVLGKSSSTK
>tr|A0A0Q0DQJ9|A0A0Q0DQJ9_PSEAP Short-chain dehydrogenase/reductase SDR OS=Pseudomonas syringae pv. aptata OX=83167 GN=ALO85_01773 PE=3 SV=1
MEPAMSESVRFDDKVVIVTGAGGGLGRAHALLFAKHGARVVVNDLGGSAHGEGASASAAD
RVVAEIRAAGGTAIANHDSVTEGGRIVQHALDAFGRIDVLVNNAGILRDKTFANMEDADW
DLVYRVHVEGAYKVTHAAWPYLREQNDGRVIFTSSTSGIYGNFGQANYATAKLGLYGLTR
TLALEGRKHRIFVNAIAPTGGTRMTEGLIPANVFELLKPELVSPLVVYLGSEQCQSSGEL
FEVGGGWIGKVRWQRSQGACFDPQTGFSPEDVAAQWQVIGDFANAAHPADTGEALKEMMA
NLQKYVK
>tr|A0A0N8T8F6|A0A0N8T8F6_PSEAP 4-hydroxybenzoyl-CoA thioesterase OS=Pseudomonas syringae pv. aptata OX=83167 GN=ALO85_02834 PE=4 SV=1
MRSKGFLHIDTEIVVPFFDVDMMNIVWHGHYVKYLEVARCALLDHIGHNYMQMLESGYGW
PVIDLQLRYVRSAVFGQTLNVRASLVEWENRLKINYLITDAHTGERLTRASTVQVAVDSQ
SREMQLASPQVFIDAVKNALESTIESASP

>tr|A0A0Q0C421|A0A0Q0C421_PSEAP Uncharacterized protein OS=Pseudomonas syringae pv. aptata OX=83167 GN=ALO85_02725 PE=4 SV=1
MTVGYSNRTPQQALAALLDRYAPQRLLLIGAQAFPALQAFQEAHPQTEVALAEPGALPAH
LAAQRFDLALVVDCLEHIPKRTGLELLGGIRNLNASRIAVLADLQACGWQETDFFSLALQ
SSERFARDEQVLNLFTYDLREYKQVPDWLNAKYWANPENFGKYWW
>tr|A0A0Q0IAB8|A0A0Q0IAB8_PSEAP RF_PROK_I domain-containing protein OS=Pseudomonas syringae pv. aptata OX=83167 GN=ALO85_03815 PE=4 SV=1
MLVISNNVHLPDAEIELTAIRAQGAGGQNVNKVSSAVHLRFDINASSLPPFYKERLLALS
DSRITSDGVIVLKAQQYRTQEQNRADALLRLSELIVNAARVEKKRRPTRPTLGSKTRRLE
SKSKRGSIKAGRGKVDF
>tr|A0A0N8T9R8|A0A0N8T9R8_PSEAP Transcription antitermination protein NusB OS=Pseudomonas syringae pv. aptata OX=83167 GN=nusB PE=3 SV=1
MISDDTDQFNPRDAKSPEAAKGKSAKRREARQMATQALYQWHMAGHSLNEIEAQFRVDND
FSNVDGTYFHELLHGVATNKTEIDTALSPCLDLTIEELDPVELAVLRLSTFELLKRIDVP
YRVVINEGIELAKVYGSTDGHKFVNGVLDKLAPRLREVEVKAHKR
>tr|A0A0N8TAD4|A0A0N8TAD4_PSEAP ATP synthase subunit alpha OS=Pseudomonas syringae pv. aptata OX=83167 GN=atpA PE=3 SV=1
MQQLNPSEISEIIKGRIDKLDVTSQARNEGTVVSVSDGIVRIHGLADVMYGEMIEFPGGV
YGMALNLEQDSVGAVVLGAYTTLAEGMSAKCTGRILEVPVGKELLGRVVDALGNPVDGKG
PLNNTETDAVEKVAPGVIWRKSVDQPVQTGYKAVDAMIPVGRGQRELIIGDRQIGKTALA
IDAIINQKNSGIFCVYVAIGQKQSTIANVVRKLEENGALANTIVVAASASESAALQFLAP
YSGCTMGEFFRDRGEDALIVYDDLSKQAVAYRQISLLLRRPPGREAYPGDVFYLHSRLLE
RASRVSEEYVEKFTNGAVTGKTGSLTALPIIETQAGDVSAFVPTNVISITDGQIFLESAM
FNSGIRPAVNAGVSVSRVGGAAQTKIIKKLSGGIRTALAQYRELAAFAQFASDLDEATRK
QLEHGQRVTELMKQKQYAPMSIADMALSLYAAERGFLTDVEITKIGSFEQALIAYFNRDH
ADLMAKINVKGDFNDEIDSGMKAGIEKFKATQTW
>tr|A0A0Q0BSL8|A0A0Q0BSL8_PSEAP Corrinoid adenosyltransferase OS=Pseudomonas syringae pv. aptata OX=83167 GN=ALO85_00219 PE=3 SV=1
MGFRLSKIYTRTGDAGETGLGDGRRVSKDHPRVEAIGEVDTLNSQLGLLLAGLIDEAQRV
PALKEVIEVLAPCQHRLFDLGGELAMPTYKALNEAEVERLEAAIDVWNDELGPLENFILP
GGSSLIAQAHICRSLARSAERRCQHLNAVEPLEGAGLAYINRLSDLLFVAARLIARRQGI
AEILWQPAPKPV
>tr|A0A0N8T902|A0A0N8T902_PSEAP NmrA family protein OS=Pseudomonas syringae pv. aptata OX=83167 GN=ALO85_03948 PE=4 SV=1
MNHATALPIVVAGATGDLGHRVVRALAERGAHVIALVRPGTESARLNGLRNNTTTITPVS
LDDPQGLRRAIAGSGCVVSTLNGLEEVIIGQQGRLLEAAVAAGVPRFIPSDYSLDYTRTR
PGDNRNLDLRRRFVTQLDAADISVTSILNGGFLELLEGDAPIVLPGRRVLHFGDAQQPLD
FTAKDDVAAFTADAALDPHTPRFLRIAGNSLSPAQIASLLTELTGQRYRTLRPGNIGTLS
TLIGVVRALTPASDKPFPAWQGMQYLRDMMSGRGKLLNLDNDRYGQREWASVRDTLARTH
VQGSNQ
>tr|A0A0Q0FG94|A0A0Q0FG94_PSEAP DszA family monooxygenase OS=Pseudomonas syringae pv. aptata OX=83167 GN=ALO85_04932 PE=4 SV=1
MMARKKILLNAFNMNCIGHINHGLWTHPRDTSTQFNSLEYWTDLAKLLERGLFDGLFIAD
IVGVYDVYQNSVDVTLKEAIQLPVNDPLLLVSAMAGVTRHLGFGLTANLTYDAPYLFARR
MSTLDHLSRGRVGWNIVTGYLDSAARAMGLSEQNEHDRRYDQADEYLEVLYKLWEGSWED
DAVINDREQRVYAQPGKVHKVRHQGEFYQVEGYHLCEPSPQRTPVLFQAGSSERGLQFAG
QNAECVFISGQNKAATREQVDKVRASAVQAGRNPEDIKVFMGLNVIVAATEALAREKHAE
YRRHASAEAGVAHFAASTGIDFSRYELDEPIQYVKNNAIQSATKNLKNNDWTRQKLLDQH
ALGGRYITLIGSPEQVADELESWIEETGLDGFNLTRIVTPESYEDFIDLVIPELQRRGSY
KTAYQDGSLRKKLFPQGTDRLPQRHAGAAHRHI
>tr|A0A0Q0DIA5|A0A0Q0DIA5_PSEAP START domain-containing protein OS=Pseudomonas syringae pv. aptata OX=83167 GN=ALO85_02194 PE=4 SV=1
MGPLYRMAVACGFTVMLAGTAQAEEWKEVKNQDGIKVSLSDVPGSDYKAYQGIALINASV
SKLRALQEDVTGACAWIHECKLQKVLKHEGNKTWTYSQFNTPWPVTPRDSVLLITTQEGP
DGSVTRNLEEQPSYIPEEKGYVRVTEVKGFWKMVPKGPNQTEVTYQVHTEPGGSVPSMLA
NKFVVDAPYNTLKALRERAAQTK
>tr|A0A0Q0FMB4|A0A0Q0FMB4_PSEAP Phosphotransferase OS=Pseudomonas syringae pv. aptata OX=83167 GN=ALO85_200037 PE=4 SV=1
MDWNSLIYFPVLKTKDAELRAISAVDVLYRRKMLPVYEITKSRITRKNSFGDILKRLDQI
KNIQGDMPYILDVTTDEKQKNEQTESILSPANGYESWRTWLDLNCGPNIIPVIHINYELD

EVLNEAKSFVSSVTNKYKKMALRLPAHLEAEEYEEIISIVVSELKGSRLYVLLDEGCIRE
KVKDDGLAAVAGAYQRAFDTITRMPNSQEWLERVVCVAGSFPYQVAQEGKGDAYGKFEIY
EHSLFVSLKHNRPGLQFGDFASINVKQIEMRGGTFVPRIDFGTDTTFYYHRKRRDAGSYI
LCAKQVLVDPMYSTNHSWGDAEIDSASKNTPSGISPSFWISVRACNYMIRRVKLLSA
>tr|A0A0Q0DF05|A0A0Q0DF05_PSEAP Uncharacterized protein OS=Pseudomonas syringae pv. aptata OX=83167 GN=ALO85_100289 PE=4 SV=1
MVTRTWRTTMSTAINHLPSTLLKLPVVLTPSAWNESVHLEAPSHIAEVGTRLGDVVLEAY
RELHLQPDETQIDFGIYRFPPNGDRSGREWLELKLHRIDAVHGNSYLCISLRDEKPLYLC
>tr|A0A0Q0DCZ6|A0A0Q0DCZ6_PSEAP Insecticidal toxin complex protein TcdA1 OS=Pseudomonas syringae pv. aptata OX=83167 GN=ALO85_00113 PE=4 SV=1
MCEFNYKDISMGLNRLSKIKRLSARAGLDSLAAQALTSVAAFRQKLSTRKLLFSEVDELY
EAAREEREAALIYEKCLLARSSPLLKNAVRLGINPPGESLRDYEEQFGNRASAYTSPGSV
SSMFSPAAYLTALYRNARGLYPEESPYHIDKRRPDLKGLLLSQSNMSKEVSALSLSNEVL
MTLAGKEMAVDDQNAVLEALAEFRLSASTPYHHPHARLRQSRIQKDPKFKQLAANPRVTG
LFSGATMAGMAFDMPPELYTILTEEVTSENAAALYAKNFGDLPEEYLLNPQSLRRYYGLS
DEEVTLFTTIDWEGEQDGGGEGEYVDNVLTTMIDGAVYRLQCGQHYTLDFAWLFPKGNGA
YELRFSYNDVHQAFKAFRVHLNDGGTLFDNPDWTPPDAGTTCVVQIASGVPEGSFTLYLE
RYRQDGLFVRAPIAYDVIISRSAVAYLLKLNKAIRLWRATGMHPRALETIVNSVNSNNIT
DETLQLLFQVQRCVQRYGVEPEEALVLCGGLLSQSGYDDNQSLFDQVFNSPPLNGESFAP
STTQINLLPDNAADHSFEKAVLKRAFNVDDVGLFTLLSVFDNSVSTGTFTLNLKNLSAMY
ALSRWARLHGLSVAELRQLLKAADLPRLTSELENTQLWSGWLQKVDSLTQWLNARKLSVA
SVELLTRPTFIQVASTEISALLDEVKRVIDANGDADTLAKRISLLAPVLVSSLALPSAAV
AESVLAWANGLQPAEWTVDQFWDGAATNDVKAVAFCYGLAQLALIYHATGINPQAFSLFV
ASPARLLGPVPETVVLPRALATLQALCNFSAWLKSLGDGASTLLAAFVADTLTPADLAIA
MNDDAARFEQATEQAFSQAQAASDTQLSAWSEIDAVLQWAALSAAFGVTPVNIGELLALS
YTAGNQPSWDDWVRVADAFSAGLSQNETKGMEAALASGLSAALCGYLLKSGLIAQVANKS
REGLYQYLLLDNLNGPQVMASRVAEAIVSLQTFIQRTLSAAESQGLVDKAAVTGQFFTDW
ERYNQRYSTWAGAAKLVYYPENYVDPTVRLGQSGMMNTMLQTLGQAQLNTDTVGDAFNTY
LNSFEEVANLRVISGYHDNLDVHEGKTYFIGTNQSEVREFYWRSADEGRRGEDGQLAANA
WTDWRKIECAAQPWGDCIRPVIFKSRLYLCWLERKDVTPPDAQGMPGTTKMFDYAINISY
LRYDGNWTSPKIIDVTDKIKSFAGPIESDDKPVIEPPGLYCTSFDAETTMAVLIYKKQES
YTNGGDNVADKHLIYVYEDVTYTHIAADNAKTFIEYMWFELNTVSKIIVSNRYAQGLAIG
SALTFLKSEGEVFEISGGASNITISDGSSGDPALVSLVARAVVREPYSYYFFLRSFHPEI
VNENEYYVFEAEYYLGTLWFVRVSGLIFFIISRGNGNASSPTLVIRYSDDTGPEYQSEMA
FYDDPNKQFYARVFYDYHPERIKDYVSFYMNIDGESVMGGGACQATSAPKPLSVNSLKME
CHDEEDAKTKQEYTASDMVESESGGVAEYIFSFPMVDDIVFTKEWENITHYLTVTFKVDG
LAYSSYYVTVYRAVGSASNVIQIKTTSEKAQYIEVDAYRTRLNTLFARKLVERAASGIDT
ILSYETQEIQEPQLGAGFFVTLNLPVYDETKHGDEKWVRIYYHCFKVDDDSYLAWSGSLS
DQSITSVELFVPYPDDGWFLEQDIHLRIQYQCADFNQANSQSVWIGYLPGSRSVDIARPG
RHALAPHIVQSVIGRDNSTVPMDFSGANALYFWELFYYTPMMSAQRFLQEQQFTLADQWL
RYVWSPSGYVVRGEHVDRNWNVRPLQEDISWNDAPLKAVDPDAVAQNDPMHYKVATFMRA
LDLLIARGDSAYRKLERDTLTEAKVWYSQALNLLGEQPYIRANAQWTEPSLGDASSQALA
EQHLTVLSLLRKGRAETLKAMASTNTATASSLFLPEVNEVMQGYWLTLRQRMYNLRHNLT
LDGQPLLLPLFAKPADPKALLNAAVAAESSGGSELPVTSLPLWRFDPMLESARGLVFQLI
QFGNAVQGVLERQDAESLNALLQNQGTELMASSIRVQEGMLRDLEAEKAVLSKAKDSAQK
RFDSYSRMHDENINARERLSMEMQVASQSVAAGAKVAHMTAAALGLAPNIFGLANGGMKY
EGVGNAVGIGITMASDVLMITSLRIAQEEMYRRRREEWEIQRNNAEGDIHQIEAQLAALD
VRIESAELQKTHLGMQQGHAQAQLDFLQTKFSNSALYSWLRGRLATIYFQFYDLAVSRCL
MTEKAWHWESGKSDTYIRGGGWQGTWAGLTCGEGLMLNLAQLETARMKWSKRALEVTRTV
SLADFYRSTLAESDPFELSAAVSALLNGDTPPEGSAERVRLDESGALTASITLADLNIVD
DYPSGLGDQRRIKQVSVSLPALLGPYQDVQAVLNYTGGVNELPPGCDNMAISRGVNDSGQ
FQPDFNDPRWLPFEGADIREGSMIISFPQAETKQKALLESLTDIILHISYTIRSS
>tr|A0A0Q0IMG2|A0A0Q0IMG2_PSEAP Uncharacterized protein OS=Pseudomonas syringae pv. aptata OX=83167 GN=ALO85_101690 PE=4 SV=1
MCERKDELQIAVRMIDLRGAKLDEGGHMRFFCKAR
>tr|A0A0Q0DPN4|A0A0Q0DPN4_PSEAP Uncharacterized protein OS=Pseudomonas syringae pv. aptata OX=83167 GN=ALO85_102168 PE=4 SV=1
MQTIDIDFDFIRTIALRIGSNPRIGHRNIGGIFRPSATYMCLNTTSTILIWRHQFAIFIY
VRLRFTVTPDLRREIIIAAHYDIII

>tr|A0A0Q0FFS6|A0A0Q0FFS6_PSEAP Uncharacterized protein OS=Pseudomonas syringae pv. aptata OX=83167 GN=ALO85_01032 PE=4 SV=1
MKSSIALYQALISIDVPEDRAAAVVDALESDMQTQLATKADIDTLESRLELKLTIRMAVM
LTAAVGVMLTAFRFMH
>tr|A0A0Q0CV75|A0A0Q0CV75_PSEAP Putative type VI secretion system effector, VgrG family OS=Pseudomonas syringae pv. aptata OX=83167 GN=ALO85_02242 PE=4 SV=1
MLMDLAAMLSPQNRRLFKFKNLANPEQELLLESFRGTEGLSRAYQFDLLLVCQDSGVELK
SMMGQHVVIEIELADGSPRYIAGYLTRFASGGSDGGMAKYTATLNPWFSMLKNRFDTRIF
QGNTVEEVVTQVFAMCTAFSKHEFRLSKPLKRYTYITQYRESDFNFVQRLLEEEGMFYYF
EHTAEGHTMIICDDSTTLVPLPEQPQIRFHSASVTETADSITDWNGDRRLQSGKMAVQTF
DYRQPNNRLPVTMNSLNQQGDVENFEVYDFPGQYTHGTYDEGEALLRLRVEALELRGKSF
RGASNCRAMKPGYTFELLQHYDHDQGSPEDRQFLLVLIDSEGHNNYLNGQQASYRNTFSC
VRKKIVFRPQLTTNRSVISGPHTAIVVGPPGEEIFTDELGRVKIQFHWDRKGEHNDKSSC
WVRVAQSGASGGFGSIQIPRVGDEVVVVFLDGNPDRPLVMGSLYNSQNTPPWSLPANKTQ
SGFLTRSAKGDGGTANFFRFEDKAGAEQVIMHAERNMDTEIELDEKHEVGNNRKVTVGGT
NTEIIQKDTVTNVQQGSFTLKVDNQFIQVDAKQYILLKVGDSSISITPEGIQIKGKVINV
LGESTFVKGDRVDINK
>tr|A0A0Q0C0U3|A0A0Q0C0U3_PSEAP 50S ribosomal protein L6 OS=Pseudomonas syringae pv. aptata OX=83167 GN=rplF PE=3 SV=1
MSRVAKNPVKLPSGVEVKLVGQLLSVKGAKGTLELNIHSSVEIVEEAGELRFAARNGDQQ
TRAMAGTTRALVNNMVQGVSQGFERKLQLVGVGYKAQAKGTVLNLALGFSHPVDYELPNG
ITAETPSQTDILIRGIDKQLVGQVAAEIRDFRRPEPYKGKGVRYADEVVRRKEAKKK
>tr|A0A0Q0DPK1|A0A0Q0DPK1_PSEAP Glycosyl transferase, group 2 family protein OS=Pseudomonas syringae pv. aptata OX=83167 GN=ALO85_03643 PE=4 SV=1
MIGILIPVHNEEQLLDLCLETIKQAAAHPDLKGECVEVLVVLDSCTDRSAEIARAHGVMI
LEVSARNVGQARAQGAEFLLDRGARWLACTDGDSTVASDWLAEQLALDADAVCGTVTPGD
WSKDISAAAQAAYLQHYQHRDGHRHIHGANLGVSSIAYISAGGFPALPCHEDVHLIQQLE
LIGARIAWSCRPKVTTSTRLDSKARGGFGDFLSSLNA
>tr|A0A0Q0CEJ9|A0A0Q0CEJ9_PSEAP Uncharacterized protein OS=Pseudomonas syringae pv. aptata OX=83167 GN=ALO85_00955 PE=4 SV=1
MLIGSYIPSLVVISILVAILAAYTALDLVGRIVSARGRAVHLWTAGGAIAMGVGTWSTHF
IGMLAFVLPIDLGYDVPLVLLSLLIAIGFSGFALWLATQPRLPALQLSLGAVLLGLGISA
MHYTGMAAMRMQPGIQYTPWLFVLSVVIAIAASAAALCIAFHLRQQRPRVYLLRASAALL
LGLAVIGMHYTGMAAANFADGSFCGALPQGLSSNGLDRIVLVASLSILGIALFSCILDSH
LETRTAVLADSLTEANLELTHLALHDNLTGLPNRALLTERIDRAMKRAVETGGCFALMFM
DLDGFKPVNDAFGHHIGDLLLRQVALRLRNSLHRRDTLARVGGDEFVLLVELQHAEDVLA
VARRQVSEVGTPFNIGEHQLQISLSIGICMYPGNGSTQHELLINADAAMYHTKAAGKNGY
SFFDASMNSNARNQLQMSQDLHRAIKHRQFCIYYQPKFDAVTGLPVGAEALLRWNHPEQG
ILGPDLFISMAEKTGLIIQIGEWVLDEACRQMREWYTQGYSHWRIAVNLSALQFCHSGLV
TAVADTLARHQLPANCLTLEITETTAMHDADASLAVLRRLSEMGVDLSIDDFGTGYSSLM
YLKRLPANEIKIDRGFVRDLEHDTDDAAIVSAIVAVGQALNLRIVAEGVETAVQQRFLTH
LGCHSLQGFLLGYPLPAQQFLEEIRAAEAKAAVDASDTGQAAF
>tr|A0A0Q0DV45|A0A0Q0DV45_PSEAP Putative glucose-6-phosphate 1-epimerase OS=Pseudomonas syringae pv. aptata OX=83167 GN=ALO85_01422 PE=3 SV=1
MPTSPVEPTVESILYDELNCWRICHGEAELLVAQQGAHILSYQVAGQPPLVWLNEEAVFK
RGKPIRAGMPICWPWFGNLERNPQSVQAMRDSSEPAKAHGEVRALDWQLLGIGADGDALL
VEFVLPEAEGHLPGWPHNVALKLSIRLDHALNVSLVSYNCGTEPVVISQALHTYFAVSDV
RQVSVEGLDGLRYIETLANWEEREQSGDLTFTGETDRIYKDTPGVLSIVDPEWQRRIHIR
STGSNSAILWNPWIEKTARFTDMAADGWQRMVCVETANVLDDVVTLAPDQMHVLGVSIWS
EAL
>tr|A0A0Q0FEI6|A0A0Q0FEI6_PSEAP Nucleoid-associated protein ALO85_01177 OS=Pseudomonas syringae pv. aptata OX=83167 GN=ALO85_01177 PE=3 SV=1
MMKGGMAGLMKQAQQMQEKMAKMQEELANAEVTGQSGAGMVSVVMTGRHDVKRINLDDSL
MQEDKEVLEDLIAAAVNDAVRKIEQASQDKTASMTAGMQLPPGMKLPF
>tr|A0A0Q0FPB5|A0A0Q0FPB5_PSEAP Uncharacterized protein OS=Pseudomonas syringae pv. aptata OX=83167 GN=ALO85_00982 PE=4 SV=1
MNMPKMPSLTRLQHRLAWCFFPSVLVASALVLFTMGKGVHAEPKNGTQTLVFLRHAEKPA
MGLGQLNCQGLNRAIELSEVLPREFGKADFIFAANPSRHVEEGEGDQSYSYVRPLMTVGP

SAIKLGLPVNLDFGANDTADLADELMQDKYHNAVIYTAWSHGYLPELINKVAEEASGKSI
NLLDDWTNDDFDSVVVVTLKWVDGKATLDYKNQKQGLDNGTQTCPEAT
>tr|A0A0Q0IDN1|A0A0Q0IDN1_PSEAP Uncharacterized protein OS=Pseudomonas syringae pv. aptata OX=83167 GN=ALO85_04840 PE=4 SV=1
MKSGIWQFAGVSLLCLSIGAQALADERDEPPRNPLYGSSGENAQQRQQQQRAQEQINQQ
RQIQQQQNQLRQVERQQQQQINQQQTNQQRQIQEQQNQQRQVERQQQQQIQQQQLQQRNV
ERQQQQQIEQQQRDQQRQIQQQQNQQRQQLREAQDNLPIQSGPKEAWQTQPPRQGYYQDI
PRGNNRNWQAGNGRPNDRWEGRPDGHGNGWGPGPQYRPGYAIDRVPGGYSRIPYRGQDYY
YSGGYWYRPQGPRYVVVTPPYGVRVSRLPSYSQEVWIGSSLLFLAAGTYYAYQPDSQDYV
VVNPPQNEAVYQAPPAEQQGNGYDPVAYPANGQSPQQVELDKYQCYRWAAEQSGFDPANV
TGQPPPDVVDLYRRSMGACLAGRGYSVN
>tr|A0A0Q0C5Q6|A0A0Q0C5Q6_PSEAP Glutamyl-tRNA(Gln) amidotransferase subunit A OS=Pseudomonas syringae pv. aptata OX=83167 GN=gatA PE=3 SV=1
MHQMTLAEIARGLADKKFSSEELTRVLLSRIATLDPQLNSFISLTEDLAITQAQAADARR
AAGENGPLLGAPLAHKDLFCTQGIRTSCGSLMLDNFKAPYDATVVSRLASAGTVTLGKTN
MDEFAMGSANESSHYGAVKNPWNLECVPGGSSGGSAAAVAARLLPAATGTDTGGSIRQPA
ALTNLTGLKPTYGRVSRWGMIAYASSLDQAGPMARTAEDCALLLQGMAGFDPQDSTSIDE
PVPDYSASLNTSLKGLRIGVPKEYFSAGLDPRIAQLVHESVKELEKLGAIVKEVSLPNLQ
HAIPAYYVIAPAEASSNLSRFDGVRFGYRCEDPKDLTDLYKRSRAEGFGPEVQRRIMVGA
YALSAGYYDAYYLQAQKIRRLIKNDFMSAFADVDVILGPTTPNPAWKIGAKTNDPIAEYL
EDFYTITANLAGLPGLSMPAGFVDGLPVGVQLLAPYFQEGRLLNVAHQYQQVTDWHTRAP
EGF
>tr|A0A0Q0IAY0|A0A0Q0IAY0_PSEAP Glutathione S-transferase family protein OS=Pseudomonas syringae pv. aptata OX=83167 GN=ALO85_01868 PE=4 SV=1
MTSKLRLFTSPSAFPNPQRLRLFIHEKGIADQFEETIYDMTPGGEQRGWRHLNMNPWGET
PTLELADGSFISETAAIARYLDQAYPGRKIMGEGALEQGLDNMWDNRIWVHILYRIVTAF
HVLHTGLGFKLELTKNEAWGEHCRKEALSHAALVNRHLSDGREWLLGGDAPTFADITLAT
AIAFSKFPVNATPLDERFEFLDLYWQRWQKRPAFQAAYADRSSGIPELDSPAE
>tr|A0A0N8T8H8|A0A0N8T8H8_PSEAP DnaA protein OS=Pseudomonas syringae pv. aptata OX=83167 GN=ALO85_03565 PE=3 SV=1
MCDQRHGYKLCALYTEALDGLHEPLVSMKPIQLPLSVRLRDDATFVNYYPGANAAALGYV
ERLCEADAGWTESLIYLWGKDGVGRTHLLQAACLRFEQLGEPAVYLPLAEVIDEGIELFD
HLEQYELVCLDDLQAIVGKPDWEEALFHLFNRLRDSGRRLLIAASKSPRELPVKLPDLKS
RLTMALVFQMRGLSDEDKLRALQLRASRRGLHLTDDVGHFILTRGTRSMSALFELLERLD
QASLQEKRKLTIPFLKETLGW
>tr|A0A0Q0DHV7|A0A0Q0DHV7_PSEAP Uncharacterized protein OS=Pseudomonas syringae pv. aptata OX=83167 GN=ALO85_00059 PE=4 SV=1
MLWKKGRRSDNVVDARDGGSSGGGGGGMRIGGKGLGIGGIVIIVAIGLLTGQDPMQILGQ
LTGQVTEQSAPPSAQTREAPPANDQQAEFVRSILGDTEDTWRAVFAQNGREYKDPTLVLF
SGQVNSACGRATSATGPFYCPADQQVYLDMEFFREMAQRFSAAGDFAQAYVIAHEVGHHV
QTLLGISAKVDKARQAGQKMEGANGLLVRQELQADCFAGVWAYNAQNRLNWLEPGDIEEA
LNAANAIGDDRLQQQSSGRVAPDSFTHGTSAQRVRWFKAGFAQGQINQCDTFAAKSL
>tr|A0A0Q0BTR1|A0A0Q0BTR1_PSEAP Putative malonic semialdehyde reductase OS=Pseudomonas syringae pv. aptata OX=83167 GN=ALO85_03130 PE=3 SV=1
MTSTVFITGATSGFGEACARRFAEAGWSLVLTGRRKDRLDALSAELSKQTKVHTLVLDVR
DRKAMESGIAGLPEEFGSIRGLINNAGLALGIDPAPKCDLDDWDTMIDTNVKGLVYTTRL
LLPRLIAHGRGASIVNLGSVAGNYPYPGGNVYGGTKAFVGQFSLNLRNDLIGTGVRVTNL
EPGLCESEFSLVRFGGDQAKYDATYAGAEPIQPQDIADTIFWIMNTPAHVNINSLELMPV
SQTWAGFAIDRNRG
>tr|A0A0Q0DMG1|A0A0Q0DMG1_PSEAP Uncharacterized protein OS=Pseudomonas syringae pv. aptata OX=83167 GN=ALO85_03514 PE=4 SV=1
MSFPTRRRDLYVSLKLIVCIALGIWLGALAVFLTALLYYKSLPPAQTQALDNAAAQLTTP
PAQKPASPSAPETEMFRKYEQSLRESEARQAREQTQEQQQRTFNRSKCDFWIQQDRTAPS
EKSRASINQYCG
>tr|A0A0Q0FCX6|A0A0Q0FCX6_PSEAP Chemotaxis protein methyltransferase CheR OS=Pseudomonas syringae pv. aptata OX=83167 GN=ALO85_00509 PE=4 SV=1
MPVPGATMPIDRSMDIELRLLIEAIYLQYSYDFRDYSGASVKRRVIHALRQFDCATISAL
QERVLHDPAAFMQLLQILTIPVSEMFRDPSHFLAIRNEVVPVLRTYPSLKVWIAGCSTGE
EVYSTAILLREEGLLERTIIYATDINPSSLEKAKQGIFSMDNVRAYAENYRKAGGQRDFA
EYYTSAYDYAIFDKTLCENVTFADHSLATDSVFSETQFISCRNVLIYFNKKLQDRAFGLF

HDSLCHRGFLALGSKETLEFSAYGGAFEALVKPERIYRKS
>tr|A0A0Q0DVB5|A0A0Q0DVB5_PSEAP 50S ribosomal protein L2 OS=Pseudomonas syringae pv. aptata OX=83167 GN=rplB PE=3 SV=1
MAIVKCKPTSPGRRFVVKVVNQELHKGAPHAPLLEKKSKSGGRNNNGRITTRHIGGGHKQ
HYRLVDFRRNDKDGIAATVERIEYDPNRTAHIALLLYADGERRYIIAPKGVSAGDQLIAG
ALAPIKPGNALQLRNIPVGSTVHGIELKPGKGAQIARSAGASAQLIAREGVYVTLRLRSG
EMRKVLSECRATLGEVSNSEHSLRSLGKAGAKRWRGVRPTVRGVAMNPVDHPHGGGEGRT
SGGRHPVSPWGFPTKGAKTRGNKRTDKMIVRRRK
>tr|A0A0Q0C4X6|A0A0Q0C4X6_PSEAP Protein-export membrane protein SecF OS=Pseudomonas syringae pv. aptata OX=83167 GN=secF PE=3 SV=1
MLRTINFMGVRNVAFAITMLLTALALFSWFHKGLNFGLDFTGGTLIELTYERPADLGKVR
QELVSAGYHEAVVQSFGATTDLLVRMPGEDPQLGTQVAEALRKAGADNPAVVKRVEFVGP
QVGEELRDQGGLGMLMALGGVLIYLAFRFQWKFAVGAIVSLIHDVVVTMGILSFFQITFD
LTVLAAVLAIIGYSLNDTIVVFDRVRENFRLLRKASLIENINISTTQTLLRTIATSVSTL
LAIVALWVFGGDSLEGFSIALFIGVLAGTYSSIYIANVVLIWLNLTTEDLIPPVVVEKAD
DLP
>tr|A0A0Q0C3N4|A0A0Q0C3N4_PSEAP GCN5-related N-acetyltransferase OS=Pseudomonas syringae pv. aptata OX=83167 GN=ALO85_02448 PE=4 SV=1
MLELHTERLYLRTLLASDWPLFFRLHSEPETMQYVFGEIEEAQIRKGFDHRLPEWSPASD
HWLCLVVMDAQSHAELGVSGFRILSPGHAEVGYLLLPEHQGKGFGTESCRAIIDYAAAIG
LDSLESTVTDGNIASCKVMEKCGFTFERRVPQAYQIGDQWFDDLIYHLPLR
>tr|A0A0N8TA21|A0A0N8TA21_PSEAP Uncharacterized protein OS=Pseudomonas syringae pv. aptata OX=83167 GN=ALO85_02009 PE=4 SV=1
MSGGACQVEKSGLISFTALPLWVESGMTNVFISALLAGLCVASAASASASTFTAQVMDKE
GKPLADAVVTLKGPPDPSPVALKASMDQRDQEFAPHVLAIHTGTQVKFPNSDNIRHQVYS
FSPAKRFELRLYEGTPSDPLLFDKPGVVVLGCNIHDWMVGYIYVTDEPWFGVTDSNGQLK
LDQMPAGHYNATLWHPQIADMQPVSGGEFEVPAAGLTQRFNLAVEMQAEDKPGKPAPGGF
GDAFHKATHE
>tr|A0A0Q0DQK7|A0A0Q0DQK7_PSEAP Iron uptake protein OS=Pseudomonas syringae pv. aptata OX=83167 GN=ALO85_100226 PE=4 SV=1
MMLLAALLCYVGFTCLCLSMARHYGELLKGPLTEQRRRALTLAGWSALLLSSWAAVDDHG
WMMGTVHWFAALMGSAVLLVALLSFRPRWVLALAGCGALLSPLAAVSQLLA
>tr|A0A0Q0CIF7|A0A0Q0CIF7_PSEAP Peptidase M20:peptidase M20 OS=Pseudomonas syringae pv. aptata OX=83167 GN=ALO85_03271 PE=4 SV=1
MLDDPIALTRALLAFQTLNPPGDEEACAAFLAEQLTRHGFVCELQRFGERRFNLVAWLEG
DGPGKPLGFTGHLDTVPLGNATWSHSPFAGEIVDGRLYGRGASDMKAGIAAFIVACQRSR
DSIRRGPGVRLILTGGEETGCDGAKALCSDAPHLLGELGALLIGEPTANYPILGHKGALW
LRCASHGLTAHGAMPEEGVNAIYVAAEHIGRAQTFEVGPAHPLMRKPTLNVGTISGGLNI
NSVPDYAAFTLDLRTAPNLDHDEIRGRLAAHLGSRAELSTLIDLPGICASLDEPWVQQVF
ARCQALHDTPLEEKAVPYFTDAAVLLPAIGYPPTLILGPGEPAMAHKVDEYCEVDKLHQC
VELYAGLIEDWTAMHAPMND
>tr|A0A0N8TA10|A0A0N8TA10_PSEAP Ion-translocating oxidoreductase complex subunit B OS=Pseudomonas syringae pv. aptata OX=83167 GN=ALO85_02058 PE=3 SV=1
MTELIRLLPDVDLIRSIDALLPQTQCGKCGHPGCKPYAEGIAGGEAINKCPPGGDETIAQ
LAELLSVPALTLDMQRGTAPAQVAFIREAECIGCTKCIQACPVDAILGASRLMHTVIIDE
CTGCDLCVAPCPVDCIEMHPLPLATVTPIVGGLAPTPELQEARQRKRSHARMRFEARNAR
LHREQQKRLAERLARSQRVESARDVLPRSAVALARNDKPAGPDEALKKARIALAMSRAQL
NKSLKAFGHPPIAEQAARLVELQREYEAAEQTLAALLPPPAQANSEEKLRP
>tr|A0A0Q0FR32|A0A0Q0FR32_PSEAP 4-hydroxybenzoyl-CoA thioesterase OS=Pseudomonas syringae pv. aptata OX=83167 GN=ALO85_02952 PE=4 SV=1
MNFHTRKWVKPEDLNPNGTLFGGSLLRWIDEEAAIYAIVQLGNQRVVTKYISEINFVSAS
RQGDIIELGITATDFGRTSITLKCQVRNKITRKSILTVDKIVFVNLDEDGQPAPHGRTEI
RYIKDQFDVED
>tr|A0A0Q0D9D0|A0A0Q0D9D0_PSEAP Major facilitator transporter OS=Pseudomonas syringae pv. aptata OX=83167 GN=ALO85_04652 PE=4 SV=1
MLGAETSLKRSGSVLPVTRLPGVGRITGTSLLVSHADRRNTLSLDSLNFFLADVRDGLGP
YLAIYLLAVHKWDPASIGVVMTIAGIAGLLTQTPAGALIDRTPYKRAMIGVAALLVTLSC
LILPFTSSFSVVALTQALSSIAASVFAPAIAAISLGITGPKAFTRRTGRNETFNHAGNAC
AALLAGGFAYLFGPIAVFYLMAAMALASIVAVSFVSADAIDHDVARGFDASHEVSGHQPS

GLSALLSNKPLLMFGICCALFHLANAAMLPLVSQKLSQINMQMATPLTSACIVAAQLVMV
PAALLVGMKADVWGRKPLLLAGFLFLPIRGVLYTLSDDPYWLVAVQMLDGIGAGLFGALF
PLMVKDLTQGSGRFNVSLGALSTLFGLGAALSNSLAGFVVHAAGYSAAFLTLAGVAAVAF
CLLWLTVPETLSRSHNETGLKPDNAVGA
>tr|A0A0Q0DM61|A0A0Q0DM61_PSEAP Uncharacterized MFS-type transporter ALO85_00959 OS=Pseudomonas syringae pv. aptata OX=83167 GN=ALO85_00959 PE=3 SV=1
MTITLQIVSIVFYTFIAFLCIGLPIAVLPGYVHDQLGFSPLIAGLAIASQYLATLLSRPF
AGRATDTLGSKRSIVFGLWGIVISGSMTLLATLLHDFATLSLSILIVARLFLGVSQGLIG
VGTISWCIGKIGPEHTARSISWNGIASYGAIAIGAPLGVVVIDGIGFNSLGIAMMVMAGL
ALRLIRDKPSVPIIVGERLPYWSVFGRIAPYGLGLTLSSIGYGTLTTFITLFYLDRGWEG
AAYCLSMFGVCFILARLLFIGAINRLGGYNAAILCMAVEICGLLMLWLSPSSGVALAGAG
LTGVGLSLVYPALGVEAIKSVPTPSRGAGLSAYAVFFDLALAIAGPAMGAIALGQGYDWI
FFYAALLSVCALALTIWLSRRAAQAQ
>tr|A0A0Q0BSB2|A0A0Q0BSB2_PSEAP Shikimate dehydrogenase (NADP(+)) OS=Pseudomonas syringae pv. aptata OX=83167 GN=aroE PE=3 SV=1
MTQKQEMYFMTHAEKPSILAGLIGSGIQASRTPAMHEREGDAQGMRYLYRLIDIDALQLD
VDALPRLLEGARQCGFTGLNITFPCKQAVIPLLDELSDEARGIGAVNTVVFKDGKRIGHN
TDCLGFAEGFQRGLDKAPRQQVVQMGAGGAGAAVAHALLSAGVERLSVFEVEPQRAQRLV
DNLNQHFGTGRAQVGIDLPAAMAEADGLVNTTPVGMSKLPGTPLPIELLRPELWVAEIIY
FPLETELLKHARVLGCRTLDGSTMAVFQAVKAFELFSGQSADAERMMAHFHTL
>tr|A0A0Q0IDB8|A0A0Q0IDB8_PSEAP Ferric uptake regulation protein OS=Pseudomonas syringae pv. aptata OX=83167 GN=fur PE=3 SV=1
MPNTPLASRPHDHSHCVHSALAEADALCAKQGLRLTTLRRRVLELVWQSHKPLGAYDILA
VLSDEDGRRAAPPTVYRALDFLLENGLVHRIASLNAFTGCNHPTHAHQGQFLICRLCHAA
IELQHPAISNAVVDAAAGVGFAVEGQTVEIVGVCAGCKAA
>tr|A0A0Q0DE08|A0A0Q0DE08_PSEAP Cystatin-like fold protein OS=Pseudomonas syringae pv. aptata OX=83167 GN=ALO85_01272 PE=4 SV=1
MKTKLTALALLLAVSAPMVHAAAEPSGYTYRMVAETPNNVNDKEIAGLFDRWNKALQTGN
STTVASLYASDAVLQPTVSNKVRATPAEIKDYFDKFLALKPVGEINYREIRRLGPDAAAD
NGVYTFTLTAPDGKKTKVQARYSFLYHRVGNEWKILNHHSSAMPEVQPEYQVSR
>tr|A0A0Q0IR67|A0A0Q0IR67_PSEAP Carboxy-S-adenosyl-L-methionine synthase OS=Pseudomonas syringae pv. aptata OX=83167 GN=cmoA PE=3 SV=1
MSKETDRIFAQPLTQVPDFAFNEDVVRVFPDMIKRSVPGYPTIVENLGVLAAQFAQPNTV
LYDLGSSLGAVTQALRRHVRGEGCEVIAIDNSSAMVERCREYLNAQDSMFQELLPVQVLE
GDILALTFKPASVVALNFTLQFIAPEQRLALLGRIRDALVPGGALILSEKLRFDDQQEHA
LLTDLHIAFKRANGYSDLEIAQKRSAIENVMKPDSLEEHRQRLLAAGFSKVVPWFQCLNF
TSLIALP
>tr|A0A0Q0BS38|A0A0Q0BS38_PSEAP Uncharacterized protein OS=Pseudomonas syringae pv. aptata OX=83167 GN=ALO85_01846 PE=4 SV=1
MSDALQLILEDTDGTQLETSCTRVAVIWQGKELWIQQDGRGQLLIGVDVEEDDAEYANLL
LRPLATNLVSLQLEMEPADMGVEEDGHVHGPDCNH
>tr|A0A0Q0CWQ1|A0A0Q0CWQ1_PSEAP ABC-type nickel/dipeptide/oligopeptide-type transport system OS=Pseudomonas syringae pv. aptata OX=83167 GN=ALO85_00072 PE=4 SV=1
MNPLHSLRSQAAGLLLASLACTVQAAPQHGVTLYDEAPKYPADFKHFDYVNPDAPKGGTL
RQAGFGGFDSLNPFINKGVPADDIGLIYDTLARSSLDEPFTEYGLVAGKIEKSPDNSWVR
FYLRPEARFHDGHPIRADDVEFSFKTLMEHGSPMYKGYYADVDQVIVEDPLRVLFKFKHK
GSRELPLILGQLPVLPKHFWADRDFSKGNLDFPLGSGPYKVADVKAGRSVSYERVKDYWA
KDLPINKGFYNFDVLTTDYYRDNTVALEAGKAGQFDYWIETSAKNWATAYDTPAVRDGRL
IKEELPNGNPTGMQGFVFNLRKPVFQDVRVREALTLLLDFEWTNKQLFNGSYSRTKSYFD
NSEMAASGLPSAEELKLLEPLRGKIPDRVFSEPFTLPVTDGSGMIRPQQRRAFQLLQEAG
WKIVDDKMVDAQGNPIKIEFLIAQTEFERILLPYKRNLSDLGIELVIRRADVSQYINRLR
SRDYDMIVTGYPQSSSPGNEQRVYFDSSSADNPGSRNFMGLKDPAVDSLVNGLINADSRQ
SLITHTKALDRVLLWGFYVVPNWHIKTWRVAYWNHLDHPKAKALSDIGLMTWWARPDVKP
ATATPSAPDQAKPANAEQ
>tr|A0A0Q0IPK0|A0A0Q0IPK0_PSEAP Putative lipoprotein OS=Pseudomonas syringae pv. aptata OX=83167 GN=ALO85_03758 PE=4 SV=1
MKPSFVEQKPSGRFHLKTHGMLLLALALTGCGTINTTFRPDSVAGEKLTDWKSNCSSIPR
VYSGVMLDFCELNAEPKQSSAYQKTNAPEWVLLDMGLSGIVDTLLLPYTIYQQNQYGYIN

ASRFK
>tr|A0A0Q0DLN8|A0A0Q0DLN8_PSEAP Uncharacterized protein OS=Pseudomonas syringae pv. aptata OX=83167 GN=ALO85_101330 PE=4 SV=1
MEHSDMTCFTPYAKRPSSVAHFYNLSVTLTNSGFRVRSKHRRLKAIQSAQE
>tr|A0A0Q0FNS4|A0A0Q0FNS4_PSEAP Tol-Pal system protein TolR OS=Pseudomonas syringae pv. aptata OX=83167 GN=tolR PE=3 SV=1
MALIARDRRRKRKPVAEMNVVPYIDVMLVLLVIFMVTAPMINQGVKVDLPKVSSEALPQD
NNNQVLTISIKADKTYYWNLGSEVDTDKQMDKAMTLPQLTAAVTKIVAAGRDAGKQTQVF
IRGDKTVDYGSVMGTMGGLQKAGVGNVGLITEAP
>tr|A0A0Q0BW17|A0A0Q0BW17_PSEAP Uncharacterized protein OS=Pseudomonas syringae pv. aptata OX=83167 GN=ALO85_100853 PE=4 SV=1
MSDKYAVALESGGVLPERENLILQAWGDVPVSYVQDEELLSACSFLDLKINKWEVNKQWS
TQQESEPWIRMEILRGQALEDYFQHNGKTLRNAGQYKALLILELDEGGVEVDVFLFMRLI
SVFSESFKVYRFCDGVQEVDEKALASISARNTVMERLVGCW
>tr|A0A0Q0DEY5|A0A0Q0DEY5_PSEAP Putative Type IV pilus protein PilM OS=Pseudomonas syringae pv. aptata OX=83167 GN=ALO85_100437 PE=4 SV=1
MNIYWLAMVVITAVLGMVYEKSHQAEQSSQTVEISLIAHNVLVYRNALSEYAYAHKAASG
TVADNQLALPTWYARYPGVEGVIDAGRSYAFVGSPPPGLVSEMINLTGGSLAIGTASSGS
LVTPSSGNVGVALPATVPNGAAVAYQ
>tr|A0A0Q0FW94|A0A0Q0FW94_PSEAP Uncharacterized protein OS=Pseudomonas syringae pv. aptata OX=83167 GN=ALO85_04905 PE=4 SV=1
MIMAAESTSFVLNRWITMPSALWSFTLDFYARPGVEQACLTLQANGANVCMVLCGVWLGT
RDVACDAQRVAQIKQLATPWHDDVVRPLRDLRNQWRNAALEDASLTPLRMKVKALELEAE
QGLMMKLEALTGDWPAGEARNAEEWLLELAEGDAEKNRDALHVLRIAVGLAVDQT
>tr|A0A0N8T8B2|A0A0N8T8B2_PSEAP Uncharacterized protein OS=Pseudomonas syringae pv. aptata OX=83167 GN=ALO85_101829 PE=4 SV=1
MFSSGIEKTILLTLLAAVGGAFKLGLETQHRWLVGSEQEVVAKGSYIKLKELEDGYYSKK
YVDENYLSKNSVLEGFVTKKQYNEIAAELVEYRSKAQLAAKALKSETKQLSYGEVWHPQN
PEFVIRFDSYSVDVDSVFTGIVTTILPESERSTYRMSPLSNSRVYKFRYEGQSYSLKLAF
KNVRDEIFLEATLTQIL
>tr|A0A0Q0BZZ6|A0A0Q0BZZ6_PSEAP Ribosome maturation factor RimM OS=Pseudomonas syringae pv. aptata OX=83167 GN=rimM PE=3 SV=1
MSATPASADDLIVIGKIYSVHGVRGEVKVYSFTDPIGNLLDYKTWTLRREGSVDKQVELV
SGRLQSKFLVTKLKGLDDREEARLLSGYEICVPRNLFPDLDDGEYYWYQLEGLKVIDQLG
QLLGKIDHLLETGSNDVMVVKPCAGSLDDRERLLPYTEQCVLAIDMAAGEMKVDWDADF
>tr|A0A0Q0FLJ1|A0A0Q0FLJ1_PSEAP Diaminopimelate decarboxylase OS=Pseudomonas syringae pv. aptata OX=83167 GN=lysA PE=3 SV=1
MDAFNYRDGELFAEGVALSAIAERFGTPTYVYSRAHIEAQYRAYADALSGMPHMVCFAVK
ANSNLGVLNVLARLGAGFDIVSRGELERVLAAGGKAEKIVFSGVGKTREDMRRALEVGVH
CFNVESTDELERLQEVAAELNVRAPISLRVNPDVDAGTHPYISTGLKENKFGIAIAAAED
VYIRASQLPNLEVIGVDCHIGSQLTTLEPFIDALDRLLDLVDRLGDCGIHLHHIDLGGGL
GVRYRDEEPPLAADYIKAVRERLAGRDLGLLFEPGRFIVANAGALLTRVEYLKHTEHKDF
AIVDAAMNDLIRPALYQAWMDVTAVRPRDSEPRAYDIVGPICETGDFLAKGRELALAEGD
LLAVHSAGAYGFVMSSNYNTRGRAAEVLVDGSQAFEVRRRETVAELFAGESLLPE
>tr|A0A0Q0CCN1|A0A0Q0CCN1_PSEAP Type IV pilus bioproteinsis protein FimT OS=Pseudomonas syringae pv. aptata OX=83167 GN=ALO85_02789 PE=4 SV=1
MKHAGFTLIELLIVVALVAILANVATPSFKELIDSSRGLATARELASGIRSARAAAITRN
QIVTLHAIENDWSNGWRIILDADGKGPDENDPVLIERANSGTARVVDNRKRVEEISFNGL
GTLRRRTARTLHVCAADQPVSNYRVIVAPAGRVRVEDTKIDAALCG
>tr|A0A0Q0BZ00|A0A0Q0BZ00_PSEAP NADH-quinone oxidoreductase subunit J OS=Pseudomonas syringae pv. aptata OX=83167 GN=ALO85_00746 PE=3 SV=1
MEFAFYFASGIAVVSTLRVITNTNPVHALLYLIISLIAVAMTFFSLGAPFAGVLEVIAYA
GAIMVLFVFVVMMLNLGPASVQQERAWLKPGIWIGPVILGALLLAELLYVLFANPTGAGV
GHTTVDAKAVGISLFGPYLLVVELASMLLLAAAITAFHLGRNEAKEPSQ
>tr|A0A0Q0DKR8|A0A0Q0DKR8_PSEAP Signal transduction histidine kinase OS=Pseudomonas syringae pv. aptata OX=83167 GN=ALO85_00886 PE=4 SV=1
MSENKARVDNAATGDIKHQGKDIFFAAVETTRMPMIVTDPNRPDNPIIFANQAFLEMTGY
ASEEIIGSNCRFLQGPDTDRAAVQSIRDAIEERVDISTEILNYRKDGSSFWNALFISPVY
NDAGDLIYFFASQLDISRRRDAEEALRQAQKMEALGQLTGGIAHDFNNLLQVMGGYIDLI
GSAAEKPTIDVQRVQRSVHHAKSAVERASTLTKQLLAFARKQKLQGRVLNLNGLVSTTEP

LIERTFGPEVVIETDLDPALKNCRIDPTQAEVALLNIFINARDALIGRPDPKIFIETRNL
VVDELANMSYDGLLPGRYVSIAVTDNGIGMPASIRDRVMDPFFTTKEEGKGSGLGLSMVY
GFAKQSGGAARIYTEEGVGTTLRLYFPVDEAVLSKNDPPKASERRIGSCERILIVEDRPD
VAELAKMVLDDYGYVSEIVLNAREALKRFEAGATYDLLFTDLIMPGGMNGVMLAREVRRR
YPKVKVLLTTGYAESSIERTDIGGSEFEVVSKPCMPQDLARKVRQVLDGPNGIA
>tr|A0A0N8T8I3|A0A0N8T8I3_PSEAP Flagellar L-ring protein OS=Pseudomonas syringae pv. aptata OX=83167 GN=flgH PE=3 SV=1
MNRLSVPRFSVLIASLCGITLLSGCVAPTAKPNDPYYAPVLPRTPMSAAANNGAIYQAGF
EQNLYGDRKAFRIGDIITITLSERMAASKAATSAMSKNSNNSIGLTSLFGSGLTTNNPIG
GNDLSLNAGYNGARTTKGDGKAAQSNSLTGSVTVTVADVLPNGILAVRGEKWMTLNTGDE
LVRIAGLVRADDIATDNTVSSTRIADARITYSGTGAFADTSQPGWFDRFFLSPLFPF
>tr|A0A0N8T8V0|A0A0N8T8V0_PSEAP Lysine exporter protein LysE/YggA OS=Pseudomonas syringae pv. aptata OX=83167 GN=ALO85_04353 PE=4 SV=1
MFLSFDLLLAFTLFAFVTSITPGPNNMMLLASGVNFGFSRTLPHMLGISVGFFVLVLAVG
FGLGSVFKAWPVLYTILRYVGAAYLLYLAWKIATSGPASDSVDSQGKPLSFMSAALFQWV
NPKAWIMAIGAISTYTPMQGYFYNVLVISAVFALINLPSVGVWAGFGSLLRNVLRDPLGL
RIFNGVMAALLVASLYPLFIEH
>tr|A0A0Q0C5P7|A0A0Q0C5P7_PSEAP MFS transporter, phthalate permease family OS=Pseudomonas syringae pv. aptata OX=83167 GN=ALO85_00096 PE=4 SV=1
MQKSKPTHVRYLILLMLFLVTTINYADRATIAIAGSSIQKDLGISAVTLGYIFSAFGWAY
VAGQIPGGWLLDRFGSKKVYAMSIFTWSLFTLLQGYVGEFGVSTAIVALFMLRFLVGLAE
APSFPGNARIVAAWFPTAERGTASAIFNSAQYFATVLFAPLMGWIVYTYGWQHVFVVMGA
FGIVFSMIWMKVIYGPRNHPMINEAELEHISSNGALVDLDQDKGKGTQKTASSGPKWDYI
RQLLTNRMMLGIYLSQYCINGITYFFLTWFPVYLVQERGMTILKAGIIASLPAICGFIGG
VLGGIISDYLLRKGHSLTFARKAPIIGGLLLSTSIVTCNYVDIEWVVVGFMALAFFGKGV
GALGWAVMSDVSPKQIAGLSGGLFNTFGNIASITTPIVIGYIISSTGSFKWALVFVGANA
LLAVISYIFIVGEIKRVELKEPPAKGPLLNDSVSDLSEAKS
>tr|A0A0Q0ILP9|A0A0Q0ILP9_PSEAP Tol-Pal biopolymer translocation system protein TolA OS=Pseudomonas syringae pv. aptata OX=83167 GN=ALO85_00237 PE=4 SV=1
MQPIREPSASESYFWPSVWAVALHILIFGMLFVSFAMTPDLPEAKPIVQATLYQLKSKSQ
ATTQTNQKIAGEAKKTAARQTEVEQLEQKKIEQQKQEAVKAAEQKKEESAQKAEEQKAAD
EAKKAEQKAEEAKKADDAKKADEAKKVADAKKLEDKQQADIAKKKAEEEAKKKAEEDAKK
AAAEEAKKQAADEAKKKAAEDAKKKAAEDAKKKAAADSAKKAQEAARKSAEDKKAQALAD
LLSDKPERQQALADERGDETAGSFDDLIRVRASEGWSRPPSARNNMSVTLQIGMLPDGTI
ASVSIAKSSGDGPFDSSAVAAVKNIGRLTEMQGLKPADFAPYRSFKMTFTPGDLAL
>tr|A0A0Q0CXL7|A0A0Q0CXL7_PSEAP Cupin_2 domain-containing protein OS=Pseudomonas syringae pv. aptata OX=83167 GN=ALO85_01264 PE=4 SV=1
MSMNNPTFSSGDFVHVPASLAAGEDVGTLLHEGVRTTTLDAFSKTRKHEVHVVDLPSRSI
SMTFGALQPLQGSGLHRHNYETIIYIISGTGFSMVGETLVAWTAGDAVYIPVWRWHKHVN
TSEDAEVTYIACENTPLLQALGVALREEAS
>tr|A0A0Q0IL84|A0A0Q0IL84_PSEAP Uncharacterized protein OS=Pseudomonas syringae pv. aptata OX=83167 GN=ALO85_100104 PE=4 SV=1
MSDKRSFQQSAALSDVVTRRVTARVGIFFDGTGNNRVNSQIGADCQALMEINEGQHIKAC
AGRHVQPGSSYSNDPSNIARLVDIYRLQSVAENDGEGLKVYYPIYVSGAGTTSGGRDSVW
PGQSFGRGATGVISKVENAFKKLESVLKAFPRDNPDCVLEALELDVFGFSRGAASARHLV
NEILKQRSGMLEPILESRKARLSDSFSWSNGSVRLKVIGLFDTVAAIGSFKDMGNTRDAS
NRRVNLYLPPGCAQQVLHLVARDESRRNFALNSVRPVWPREIVLPGAHSDIGGGYPPQME
ESVLLTRPRSSVVSRGSPCEEAPSWKEAQAELNRMNRDHWIDPLDSQAWLGVQACESKCY
PCRKSLNGMSTVVAAVGMGRQVFGHLSRVSLLVMHTLACDEGVPFNPVPLTPEFQLPPEL
ENISSRLIDYARGGPYLLDSHEEALLRWRYIHQSAHWNAVVGRVGTLGDAVFVHAPQSGG
RILHPNIGQPGYPQ
>tr|A0A0Q0C595|A0A0Q0C595_PSEAP HAMP domain protein OS=Pseudomonas syringae pv. aptata OX=83167 GN=ALO85_02863 PE=4 SV=1
MNRPTPVKTDNFFLLIFRALRHRRVPIALRIASHNVILVALALVIYAGVMGLQFKQAMHE
QADALGQALTTQTATSATELLVSNDILSLNVLLGNLVKNPLVAHAAIYSVDNRILAEAGQ
RPKNGLLGEAQGLYEIKITFQDVMAGHLRISLDMSQFQQPLTISLQSMGILAGILLALAL
ALSLRLGRHISTPLMQMRIWLRNIDPYTPATDRQDEIGDLARQLRTSFAPPVPEPEPLPE
PEYEGEEDDSEDPYLEPEEKPAPKVSARSSEAKPRLPAPPVQRRIVAEEDEDDDDEDPFA

DLRDQSDNSPAVRPVAASAAPVEPHNSAVLAVQLGAQDQLRRLPRARLTELLQRYRDCLD
QAASLYESELHTLNDGSTLMLFHSHESGDDYLTNAICCGELLRALSHALQIEVADSGITL
QLQLGLTLGEGLSGLSQIDLLLTETAQDALALSQHSRNLLLVERRINDDPLIRQRARIRP
IASPEGACCVERLMEPYPSMLERQLARMHESNKA
>tr|A0A0Q0DKV9|A0A0Q0DKV9_PSEAP Uncharacterized protein OS=Pseudomonas syringae pv. aptata OX=83167 GN=ALO85_01197 PE=4 SV=1
MSMSHDQVIKRMREDLEQLGNEVRNMPAHEPRNVLVSAASGAVFALVFSWLAFKVWF
>tr|A0A0Q0DJG4|A0A0Q0DJG4_PSEAP Molybdenum cofactor biosynthesis protein B OS=Pseudomonas syringae pv. aptata OX=83167 GN=ALO85_00530 PE=3 SV=1
MKAKADTPFVPLNIAVLTVSDTRTRETDTSGQMFVDRLTDAGHGLIERVLLKDDLYKIRA
QVATWIADDQVQVVLITGGTGFTGRDSTPEAVACLLDKQVDGFGELFRQISVPDIGTSTI
QSRALAGLSNGTLVCCLPGSTNAVRTAWDGILAQQLDSRFRPCNFVPHLKQAEPCATRG
>tr|A0A0Q0BUN3|A0A0Q0BUN3_PSEAP Sugar ABC-type transport system, ATP-binding protein OS=Pseudomonas syringae pv. aptata OX=83167 GN=ALO85_00089 PE=3 SV=1
MAEIRLQNLAHSYSAKPGPDDYAIREMSHVWEQGGAYALLGPSGCGKSTLLNIISGLLSP
SQGQVLFDSKVVNELPPERRNIAQVFQFPVIYDTMTVFDNLAFPLRNQGMDEARILIKVN
EIADVLDLQPLLKKKARNLSADEKQKVSMGRGLVRDDVSAILFDEPLTVIDPHLKWKLRR
KLKQIHEQFNITMVYVTHDQLEASTFADKIAVMYGGQIVQFGTPRELFEKPSHTFVGYFI
GSPGMNLIDVTPQPGGVGFADIHLPLPESLQQKLANTPWKTLKVGIRPEFVHVWDGPYDD
AMCADVTYVEDLGTYKIITLKLAGQLLKVRLQEDKPVPQGQAWISFPGQWLMLYADDYLI
EAGPASEVSHA
>tr|A0A0Q0D579|A0A0Q0D579_PSEAP Uncharacterized protein OS=Pseudomonas syringae pv. aptata OX=83167 GN=ALO85_00852 PE=4 SV=1
MHRTKISAAIAMISAATLATSLAYGATAIPLPASAPADKPIGKLEQVHAFYDAMPTGVAV
TETGRIFVNFPRWGDKVPFTVGEIRDGKVVAYPDLAVNKENPKDPGDGLISVQSVVADGK
GRVWLLDTAAPGFASPRRGGAKLVAVDLASNRIVKRLVFPDNVILPGTYVNDMRFDFRKG
AEGTVYVTDSSLSGPGAIIVMDIASGHAVRRLNGAQSTSVDPAFVPVVEGVAVLGDGPNG
TRKPVGVASDGIALSADGKTLYFSPLSSRHLYAVATELLNDSKVSEQQLAAAVQDLGEKG
ASDGLEADAKGAVYAGDYEHNSIRKRLPDGTWQTVAHDPRMLWPDTLSIGPDGYLYFIVN
QLHRQANFNAGHDKREKPYSLLRLKIDAAPAPTH
>tr|A0A0Q0IBZ1|A0A0Q0IBZ1_PSEAP Bacterial mobilization protein OS=Pseudomonas syringae pv. aptata OX=83167 GN=ALO85_02494 PE=4 SV=1
MSEIEALGESNYQLLAIGRNLNQIARKLNEGDPYPIQKELIKGLSAIIDRHTHVVSSAIR
ASLEHWDLE
>tr|A0A0N8TA05|A0A0N8TA05_PSEAP Putative membrane protein OS=Pseudomonas syringae pv. aptata OX=83167 GN=ALO85_00608 PE=4 SV=1
MIDALRMSLNATAVFGFVMYILVAAILFALFQFIYSRITPHKEFALIRENNSAAAVALGG
SLIGFALPASNIISYSISILDVIVWAVIAAVVQLLAFGVTSLVLKGLSTRIAKGEMAAAI
YSASVAISVGLLNAACMTPSV
>tr|A0A0Q0BYY9|A0A0Q0BYY9_PSEAP TonB-dependent siderophore receptor OS=Pseudomonas syringae pv. aptata OX=83167 GN=ALO85_04628 PE=3 SV=1
MSMRRTLVSLCVIQAISQAAWADQVAGVQPAMELQNIDIQGAANAETAQGPVDGYRATRS
ASATRTDTSLHETPQSVSVVTREAVEDIGSTRLQDALDYAGGVGRANNFGGQGLTTFTVR
GFTTGEFYRNGFPINRGYPNMPDANTIERLEVLRGPATTLYGRGDPGGTFNVVSKQPLAE
PSVTLGSQLNDQGMKRGTLDASGPLDDEGRFAYRLNVVGEGGDTFRDHVETERYGVAPVL
SWQVNDATRITFEGDFMRNNAPLDRGLTRYANQTGTASRDTFFGEKSVGKLHNDNNMAQL
RFEHDLNPDWTLGGGVQILDGSLQGDAIEANGLATDGRTLGRNFNYRKLEWTDRDVQLNL
TGHFSAGGFEHTLLTGVEYEDYDYKSIIQRSSGAVSAYPIDLFNPVYGQARPALTRTTTH
DKENLKTWAAFVQDQMALTERLKLLGGVRLERFEHDYDSFLPGTSSWTASDNAVTPRLGL
SYDLTDDLAVYATTARSFKPNSGASRLGGGFKPEEGKSYEVGTKWEALDGQLSVDTAIYQ
IEKRNVLTTDPLDSTFSVAAGEVRSRGLDLNVVGNLTPQWRVMGSYAYVDAEVTKDNTLR
SGTRLMNIPEQTFSLLNVYEFQDGALRGLGLGGGGRYVDQRAGRTANTSFSMDSYTVFDL
LAYYKVNPHVKLNLDLKNVFDTDYEEGSFGNVYAYPGAPRTVQVGIAYTL
>tr|A0A0Q0C6S5|A0A0Q0C6S5_PSEAP ABC-type transporter, permease protein OS=Pseudomonas syringae pv. aptata OX=83167 GN=ALO85_01279 PE=3 SV=1
MDYETFRLFIQGLASSGYLPEALAYGFVVNALLAGLLIGPVLGGLGTLVVVKRFAFFSEA
VGHAALTGVAIGILLGEPYTGPYGALFGYCLLFGIVLNYLRNRTGLAPDTLIGVFLSVSL
ALGASLLLVLAGKINVHILENVLFGSVLTVNGNDLLVLLIVGSLVMGLSLPLYNRIMLAS
FNPQLAAVRGVAVKTLDYLFVILVTLITVAAVKVIGAILVGALLVIPAAAARLLSQSLKG

FFWISVAIATLSTLCGILLPIVFDLPVPSGAAIILVAGMAFALAAIARGTLPSLKGNIG
>tr|A0A0Q0DE80|A0A0Q0DE80_PSEAP Circularly permuted ATPgrasp domain protein OS=Pseudomonas syringae pv. aptata OX=83167 GN=ALO85_01218 PE=4 SV=1
MIRTFYDEMYDAAGVVRPHYREFARWLGDTPPELLAQRRREADLLFHRAGITFTLYGDEQ
GTERLIPFDTIPRSIPASEWRVVERGCIQRVKALNMFLADLYHDQRIIKAGIIPAEQVLA
NEQYQLAMQGLNLHRDLYSHISGVDLVRDGDGTYYVLEDNLRTPSGVSYMLEDRKMMMRL
FPELFSAQRIAPIDHYPNLLLDTLKSSSHLDNPSVVVLTPGRFNSAFFEHAFLAREMGVE
LVEGADLFVRDDRVFMRTTDGPKAVDVIYRRLDDAFLDPLAFNPDSMLGVPGLLSAYRSG
NVVLANAIGTGVADDKSVYPYVTDMIRFYLDEEPILKNVPTWQCRKPEELSHVLANLGDL
VVKETQGSGGYGMLVGPAATAAEIEAFRARLKAKPHAYIAQPTLCLSTCPTFVENGIAPR
HIDLRPFVLSGRETRVVPGGLTRVALREGSLVVNSSQGGGTKDTWVVED
>tr|A0A0N8T8Y7|A0A0N8T8Y7_PSEAP Protein YebG OS=Pseudomonas syringae pv. aptata OX=83167 GN=ALO85_01780 PE=4 SV=1
MAVETLYRSTRDLETTFVDRKLADAHDQMLELAELLTDVLIKNVPGISEKHAEDASIYMA
KNRAVFAAAFKNNATALSELSEPAEAKD
>tr|A0A0Q0DCT8|A0A0Q0DCT8_PSEAP Lipopolysaccharide export system protein LptA OS=Pseudomonas syringae pv. aptata OX=83167 GN=lptA PE=3 SV=1
MRLVKTLPLLLGLGAALGSASAWSLPTDRDQPIHIQSDDAQLDDKKGVATYKGNVIITQG
SMKITGNTVTITRNAQGEVDVFTSVGNLAYYEQKPAVDKPIVQAYAVTIQYYAGQDRIVL
IDKAKVINDGNTSEGEKIVYDTVRQVVTAGRANGGAKVTTPRPRIDMVIQPKKKTDQPQK
AQ
>tr|A0A0Q0DPE5|A0A0Q0DPE5_PSEAP Two-component DNA-binding heavy metal response regulator OS=Pseudomonas syringae pv. aptata OX=83167 GN=ALO85_03049 PE=4 SV=1
MHILLIEDDTKTGEYLKKGLGESGYKVDWTQHGADGLHLALENRYDLIVLDVMLPGIDGW
QIIEVLRARQDVPVLFLTARDQLQDRIRGLELGADDYLVKPFSFTELLLRIRTILRRGVV
READHFHLADLELDLLRRRVTRQQQVIVLTNKEFALLHLLLRREGDVLSRAQIASEVWDM
NFDSDTNVVDVAIKRLRSKVDLPYPVKLIHTVRGIGYVCEVRPCDASLP
>tr|A0A0Q0DJU9|A0A0Q0DJU9_PSEAP Uncharacterized protein OS=Pseudomonas syringae pv. aptata OX=83167 GN=ALO85_100458 PE=4 SV=1
MRPETRLTAEQTARGQCGQFMASSQSAVAFRRYERMLREQRLKP
>tr|A0A0N8T8V9|A0A0N8T8V9_PSEAP Prophage PSSSM-04, protein gp46 OS=Pseudomonas syringae pv. aptata OX=83167 GN=ALO85_00433 PE=4 SV=1
MIIEGSLQASLLRSVVISLFTWRRAEADDPFDDAERYGWWGDTYPAQANDRIGSRLWLLR
RVRLTAQTQRDAEFYAREALDWLIEDGQVKHINILTEQVQSNRLNLGVELVVSDGQLVRF
NPSEQWQVIYAV
>tr|A0A0N8T993|A0A0N8T993_PSEAP ATP-dependent RNA helicase rhlE OS=Pseudomonas syringae pv. aptata OX=83167 GN=ALO85_03481 PE=3 SV=1
MNLSYGISMTFASLGLIEPLLRALEALGYQTPTPVQTQAIPPVLAGRDLMAAAQTGTGKT
AGFALPLLQRLTMEGPKVAPNSIRALVLAPTRELADQVHESIRQYAEHLPLTTYAAYGGV
SINPQMMKLRRGVDVLVATPGRLLDLHRQNAVKFSQLQTLILDEADRMLDLGFAEELRGI
YAVLPKQRQTLLFSATFSDEIRLLAAQMLNDPLTIEVSPRNVAASSVKQWVVTVDKKRKA
DLFIHLMKKHRWGQVLVFAKTRVGVDQLVDRLQGLGMNADGIHGDKPQATRQRALDRFKS
NEVKILVATDVAARGLDIDDLPTVVNLDLPIVAEDYIHRIGRTGRAGLTGEAISLVCADE
VELLSAIEVLTRQTLERKEEQDFEPEHRVPSTDASGQILKKPKKPKKPKVSGSKRNLGKW
VDSGESEPAPVVKPVRKVPVFNTGPRKKKP
>tr|A0A0Q0BXW7|A0A0Q0BXW7_PSEAP Pectate lyase OS=Pseudomonas syringae pv. aptata OX=83167 GN=ALO85_01080 PE=4 SV=1
MRTILLTVLLVVAATAQATDYYVVAPNGDDHAAGTKGAPLRTIMRAQQAAKAGDTVYFRGG
VYTYTAGINRCATRTDTVNAITLNNSGSENKPIRYWAYPGETPVFDFSAMKDDCRVKGFN
VTGSWLHLKGLEVKGVPQQPENHLNHESWGIWNSGSHNTFEQLNLHHNMGPGLFIQNGGY
NLVLNTDSHHNYDPYTSNGAGQSADGFGAHIKAGHPGNVFRGCRAWANSDDGFDLINAFS
PVTIESSWAWQQGYLPGTRTKLEAGNGNGIKAGGYGGKYVPDGVKHIVRNSVAFDNKSAG
FYANHHTLALDFINNTAFANGVNYNMAGIAPDGSLIPLGNLSNNIAYKGRLTVNTEGLDM
AHNSWTLPTPITDADFEDVSDTGWDAPRQPDGSLPVLRSFHLRAGGRLAGMGAFH
>tr|A0A0N8TA75|A0A0N8TA75_PSEAP Uncharacterized protein OS=Pseudomonas syringae pv. aptata OX=83167 GN=ALO85_03860 PE=4 SV=1
MSNLPPTLTVKFFRTETGNEPVREWLIDLPRDDRKAVGTDIKTVQFGWPIGMPVVRKMEP
DLWEVRIDLKEKIARVLFTVEARTMVLLHGFIKKSEKTPASDLETARQRKAALKRKR

>tr|A0A0Q0DUB1|A0A0Q0DUB1_PSEAP Uncharacterized protein OS=Pseudomonas syringae pv. aptata OX=83167 GN=ALO85_03267 PE=4 SV=1
MFESSANQGIPMTDPRNVAHDKKVQQEKKNHGEEIAPNAEHAPQPGMKPALKHDDDEQEE
QA
>tr|A0A0Q0DUF9|A0A0Q0DUF9_PSEAP Monosaccharide ABC transporter membrane protein OS=Pseudomonas syringae pv. aptata OX=83167 GN=ALO85_01020 PE=3 SV=1
MNAKTIIAPASTSPRSRLRLSLDRFGLPLVFILLCLVLAFASEYFMTWRNWMDILRQTSI
NGILAVGMTYVILTKGIDLSVGSILAFAGLCSALVATQGYGLLAAVSAGMFAGAMLGVVN
GFMVANLSIPPFVATLGMLSIARGMTFILNDGSPVTDLPEEYLALGIGRIGPIGVPIIIF
AVVALIFWMVLRYTTYGRYVYAVGGNEKSARTSGIGVRKVMFSVYVVSGLLAGLAGVVLS
ARTTSALPQAGMSYELDAIAAVVIGGTSLSGGTGSIVGTLFGALLIGVINNGLNLLGVSS
YYQQVAKGLIIVFAVLIDVWRKKKR
>tr|A0A0Q0DKR1|A0A0Q0DKR1_PSEAP Dipeptide/oligopeptide/nickel ABC-type transport system OS=Pseudomonas syringae pv. aptata OX=83167 GN=ALO85_00902 PE=4 SV=1
MKIARVVTGTLLLLAANAAFAESTLRIGIQDDPDVLDPHRSRTYSGRLVYTALCDKLVDV
NPDLTYSPQLATAWNWSEDGKTLTMTLREGVTYHDGEPFDAASVKFNLDRARTLPDSLRK
SELASVDSVEVIDAKTVAIKLKQADATLVSQLSDRAGMMLAPKAAQGEFASNPVCSGPYK
FVQRVQQDRIVLERFANYWNKQAYHFDKVVFLPIPDTSVRLANLRSGDLDIIERVAPTDV
KTVKADSKLAIYNTPGLGYMQLMFNIGNGEKANSPMGKDKRVRKAFELSIDRDAINQVVF
EGLYAPSAQPFPKNSPYYDKELPIPARDVEKSKALLAQAGVKLPLAVDLKVANNPIAQQV
GQIIQAMAEEAGFKVNLIATEYATMLSEQASGNFQIGMSAWSGRPDPDGDIHQFVTCKGG
QNDGKYCNARLDELLNKARTVNDVAQRQALYNDALRLLADEVPTAYLYFDPRIIAMRNNV
TGFVPNPDGLIRLNNVAFKP
>tr|A0A0Q0IHQ8|A0A0Q0IHQ8_PSEAP Autotransporter barrel protein with pertacin-like passenger domain OS=Pseudomonas syringae pv. aptata OX=83167 GN=ALO85_02223 PE=4 SV=1
MNAPFVLRPLSWTLKTVIFLSPLLTGSHALAQILVDKPQTIDATGPLDSYRVVSTGNLTA
NGATTLQISTITGAKLTLTGSQVSAGTSSSAVSLTGADALIVGSVLTGGADGLGMGNESS
QLVGSTATVIGSTITATNRGINAGSLSNLTLEGTSVTATGTNGRGMEMWDSTVKASGSTI
TGQQYGVRLRADPAVPSSNLLVLEGTRVEGITGSALIVGTPTGAPATADIQVNNSSTLTG
GNGKILELINGSTAHMTVDNSRLQGDVSAGEGSTASLSLQNNATLTGRLENVSSLSLSSQ
GQWVMVENGQVNELSMNGGSVRFGDAAAFYTLSLASLSGSGTFMMDVDFGGQATDFLDIT
GSATGSHTVLIGSTGVDPLSDTSLHVVHAAAGDASFSLAGGAVDLGAWSYDLIKQGDNDW
YLDTATRTISPGAQTVMALFNTAPTVWYGELTTLRSRMGELRMDQARSGGWIRTYGNKFN
VADASGFGYQQVQSGVALGADGKLPMGAGQWLAGVMIGQSTSDLSLDHGASGKVDSYSLG
AYSTWLDSESGYYVDGVIKLNQFKNKARVNLSDGSRTRGNYDNLGVGASLELGRHITLDN
GYFLEPYTQLAGLIVQGKDYGLDNGMRAEGDRSRSLLGKVGTTTGRSFDLGKGRTLQPYV
RVAVAHEFVNRNEVKVNDNVFNNDLSGSRGELGAGVSVSLSDNLQLHADFDYSNGDAIEQ
PWGASAGLRYSW
>tr|A0A0Q0C0K4|A0A0Q0C0K4_PSEAP Isovaleryl-CoA dehydrogenase OS=Pseudomonas syringae pv. aptata OX=83167 GN=ALO85_00941 PE=3 SV=1
MSYPSLNFALGETIDMLRDQLQSFVATELAPRAAQIDKDNLFPADMWRKFGEMGVLGITV
SEEYGGAGLGYLAHVVAMEEISRGSASVALSYGAHSNLCVNQINRNGSSEQKARYLPRLI
SGEHVGALAMSEPNAGSDVVSMKLRADKRGDHYVLNGSKTWITNGPDADTYVIYAKTDLE
KAAHGISAFIVERDWKGFSRGSKFDKLGMRGSNTCELFFDDVEVPEENLLGVLDGGVRVL
MSGLDYERVVLSGGPTGIMQACMDVVVPYIHDRKQFGQSIGEFQLIQGKVADMYTQLNAS
RAYLYAVAQACDRGETTRKDAAGVILYSAERATQMALETIQILGGNGYINEFPAGRLLRD
AKLYEIGAGTSEIRRMLIGRELFNETR
>tr|A0A0Q0DFY9|A0A0Q0DFY9_PSEAP Uroporphyrinogen decarboxylase OS=Pseudomonas syringae pv. aptata OX=83167 GN=hemE PE=3 SV=1
MTALKNDRFLRALLKQPVDVTPVWMMRQAGRYLPEYRASRASAGDFMSLCKNPQFACEVT
MQPLDRYPLDAAILFSDILTIPDAMGQGLYFETGEGPRFRKTVSTLADIEALPIPDAQQD
LGYVMDAVSTIRRELNGRVPLIGFAGSPWTLATYMVEGGSSKDFRKSKAMLYDNPQAMHL
LLDKLAQSVTSYLNGQILAGAQAVQIFDSWGGSLSSAAYQEFSLAYMRKIVNGLIRENDG
RKVPVIVFTKGGGLWLESIADIGADTLGLDWTCDIGEARQRVGSKVSLQGNMDPTVLYAR
PEAIRQEVARILASYGSGTGHVFNLGHGITPEVDPANAGAFINAVHELSAQYHQ
>tr|A0A0Q0IJG1|A0A0Q0IJG1_PSEAP Recombination-associated protein RdgC OS=Pseudomonas syringae pv. aptata OX=83167 GN=rdgC PE=3 SV=1

MWFKNLLVYRLTQDVPFDAEALETALATKPARACASQEVATYGFVAPFGKGEDAPLVHIS
QDFMLIAARKEERILPGSVVRDALKEKVDEIEAEQMRKVYKKERDQLKDEIIQAFLPRAF
IRRSATFAAIAPKQGLILVNASSPKRAEDLLSTLREVIGSLPVRPLTVKVSPSATMTDWV
KTQKAADNFFVLDECELRDTHEDGGIVRCKRQDLTGDEIQLHLSTGKVVTQLSLAWQDKL
SFVLDDKLVVKRLKFEDLLQDQAEQDGGDEALGQLDASFTLMMLTFGEFLPELFEALGGE
EIPQGI
>tr|A0A0Q0C8W0|A0A0Q0C8W0_PSEAP Uncharacterized protein OS=Pseudomonas syringae pv. aptata OX=83167 GN=ALO85_03410 PE=4 SV=1
MSKHTDKPSSQTPEQSAPTTDQSGKPVDVVRKDIAQENPETEGVDKVITPTSIKEKEQDA
ETLRAKNRELERKLNGQG
>tr|A0A0N8T9Z9|A0A0N8T9Z9_PSEAP Uncharacterized protein OS=Pseudomonas syringae pv. aptata OX=83167 GN=ALO85_00592 PE=4 SV=1
MTDMNFNLFSIIAASAISASVALPASASVEVNDKKAHAAQTYTATYTTKYLQQSANFYAA
LDHKSAQ
>tr|A0A0Q0FUC5|A0A0Q0FUC5_PSEAP Protein phosphatase CheZ OS=Pseudomonas syringae pv. aptata OX=83167 GN=ALO85_04025 PE=3 SV=1
MDNNDSMADFESTLKRHAQELVTSLEKGRFGDAVQLIHELNQTRDRGLYQEVGKLTRELH
SAIVNFHIDPHMPQAEEVSQITDATERLSYVVRLTENAANRTMDLVEESTPVMNGLSSDA
KALSDDWGRFMRREIGAEEFRELAKRVDGFLTRTEKETHQVSAHLNDILLAQDYQDLTGQ
VIKRVTQLVTEVESNLLKLVLMASHVDRFAGIEHDEQSILDEKDPKKHLAQGEGPQIHAD
KREDVVSGQDDVDDLLSSLGF
>tr|A0A0Q0BXE0|A0A0Q0BXE0_PSEAP TPR repeat-containing protein OS=Pseudomonas syringae pv. aptata OX=83167 GN=ALO85_01369 PE=4 SV=1
MKPVIILFGLLLLGGCASDGRPLWTQTTAQPGQCPKLTSDQEFSLNLAQNMADEGRLHAS
LANLESLPDSLGEVRLRKARVLRLLGSNEAEPLYRSLLGTCRAAEGEHGLGQLAAARGDN
ALAQQHLLMASKLQPTDEKIRNDLGVVYLNQLKLEQARFQFLTAMELKQSDSLAAVNLAT
LLIYQNNWTQAAELASRAGLTPEQVTDAQARAEQLRKRPAAPQASARPIASLTEQNDRQS
QEVRP
>tr|A0A0Q0C7E4|A0A0Q0C7E4_PSEAP DNA polymerase I OS=Pseudomonas syringae pv. aptata OX=83167 GN=polA PE=3 SV=1
MLAPPPPKRLILLMTQAPLVLVDGSSYLYRAFHALPPLATSKGLPTGAVKGVLNMLKSLR
RQYPDSPLAVVFDAKGGTFRDALYNDYKANRPSMPDDLRVQVDLLHACVKGMGYPFLCVE
GVEADDVIGTLARSSAAADRPVVISTGDKDMAQLVDGHITLVNTMTGSVLDVAGVKEKFG
VGPEHIIDYLALMGDKVDNIPGVPGVGEKTAVGLLVGIGGGIKELYENLDKVASLPIRGA
KTLAAKLEEHREMAFLSYELATIKIDVPLDIELDQLHCGEPDRDTLMELYAELEFKSWIE
DLQRDAKRAGQELTVEEPTVEDREAAYEVILEQGQFDAWLKKLQAAPLFAFVTQSNGTDA
QRAQLVGLSFAIQTHEAAYIPLTHSYMGVPQQLDRDTVLKALKPLLEDPDKTKVGQHAKF
AINLLANCAIDGDQAQGIDLQGVRFDTILESYVLDSTATRHDRDSLVAKYLTHTPINFQE
IAGKGAKQLSFDQIALEQAGNYAAEEADLTLRLHEVFDARLAAIPTLQPVLNDIEMPLVP
VLARIERQGALVDANLLGIQSVELGDKMTALEREAFAIAGEEFNLGSPKQLGVILYEKLG
MPILSKTATGQASTAEAVLAELAEQDFPLPKVLMQYRSMSKLKSTYTDRLPEQINPRTGR
IHTSYHQAVAVTGRLSSSDPNLQNIPIRTAEGRRIRQAFVAPKGYKLLAADYSQIELRIM
AHLAQDEGLLHAFRNDLDVHRATAAEVFGVELENVTTDMRRSAKAINFGLIYGMSAFGLA
KQIGVDRKQSQAYVDRYFARYPGVLNYMERTRAQAAEQGFVETIFGRRLYLPDINAKNQS
LRKGAERMAINAPMQGTAADIIKKAMVAVNGWLDESGLDARVILQVHDELVLEVREDLVD
QVSEQIRPHMSGAAELAVPLLVEVGVGHNWDEAH
>tr|A0A0Q0BS48|A0A0Q0BS48_PSEAP Uncharacterized protein OS=Pseudomonas syringae pv. aptata OX=83167 GN=ALO85_01856 PE=4 SV=1
MNKTLSVLNAAALVALVAFHFQDSGIKDAQAATPAPVRHQISQAPKLAIMTERVASAAML
ANDDDESLQFPRAEQRWVF
>tr|A0A0Q0DCS8|A0A0Q0DCS8_PSEAP Protein GrpE OS=Pseudomonas syringae pv. aptata OX=83167 GN=grpE PE=3 SV=1
MADEQNLDAQAQDQAAEAGAGDELTTRVQVLEEQLAAAQDQSLRVAADLQNVRRRAEQDV
EKAHKFALEKFAGDLLPIIDSLERGLDLSSPDDESIRPMREGIELTLKMFQDTLKRYQLE
AIDPHGQPFSADQHQAMAMQESADVEPNTVLKVFQKGYQLNGRLLRPAMVVVSKAPSPAT
PSINEQA
>tr|A0A0N8T8K9|A0A0N8T8K9_PSEAP NADH-quinone oxidoreductase subunit K OS=Pseudomonas syringae pv. aptata OX=83167 GN=nuoK PE=3 SV=1
MNAIPLEHGLAVAGVLFCLGLVGLMVRRNILFVLMSLEIMMNAAALAFVVAGSRWGQPDG
QVMFILVISLAAAEASIGLAILMQLYRRFHTLDIDAASEMRG

>tr|A0A0Q0ID06|A0A0Q0ID06_PSEAP RHH_1 domain-containing protein OS=Pseudomonas syringae pv. aptata OX=83167 GN=ALO85_101905 PE=4 SV=1
MEANMASPVLSFRVEEGLVEMLDQLALATDRDRQYHLKRALSRYVEAEAWHLKAIDEGLA
DIDAGKTIDLETVKAKWVARAANRVK
>tr|A0A0N8T8F1|A0A0N8T8F1_PSEAP UPF0056 membrane protein OS=Pseudomonas syringae pv. aptata OX=83167 GN=ALO85_01382 PE=3 SV=1
MLHVLFSVYLKMLVLYSPFFVLSCFISLTRGHSRKEQRRLAWKVALATLVSSVLLYLFGR
VIFDVFGITVDAFRIGAGSVLFISALGMAQGKSAVQTDNIQQDVTIVPLTIPLTVGPGTI
GALLVMGVSQPHWDDKLTAILSIALASLTVGVVLYLSNRIERILGDQGLQIVSRLMGLFV
CALAAQIIFTGIRGYLLP
>tr|A0A0Q0C794|A0A0Q0C794_PSEAP Uncharacterized protein OS=Pseudomonas syringae pv. aptata OX=83167 GN=ALO85_04017 PE=4 SV=1
MIFEVAVVFLGILWVVTLFMFLAHTRRQRKLDAERDEAAALRDQRIKELARRLDNYQNGT
VRMGEALHELRAVVAPLPDKLTALEQRDPSTLSFAQAARLVGMGASIDELTQSCGLTQAE
AQLMTKLHGNTAS
>tr|A0A0Q0BYC2|A0A0Q0BYC2_PSEAP Flagellar P-ring protein OS=Pseudomonas syringae pv. aptata OX=83167 GN=flgI PE=3 SV=1
MIKLKQLIAATLLLSTAFGVHAERLKDIASISGVRANQLIGYGLVVGLNGTGDQTTQTPF
TLQTFNNMLSQFGIKVPAGSGTVQLKNVAAVAVYADLPAFAKPGQTVDITVSSIGNSKSL
RGGALLMTPMKGVDGNVYAIAQGNLVVGGFDAEGRDGSKITVNVPSSGRIPGGASVERSV
PSGFNQGNTLTLNLNRSDFTTAKRVVDKINELLGPGVAQALDGGSVRVTAPLDPGQRVDY
LSILENLEVDPGQTAAKVIINSRTGTIVIGQNVKVSPAAVTHGSLTVTITEDPIVSQPGA
LSGGQTAVVPRSRVNAQQELHPMFKFGPGTTLDEIVRAVNQVGAAPGDLMAILEALKQAG
ALQADLIVI
>tr|A0A0Q0CVB5|A0A0Q0CVB5_PSEAP Type VI secretion system protein ImpK OS=Pseudomonas syringae pv. aptata OX=83167 GN=ALO85_05082 PE=4 SV=1
MTEAVMQGAATAATEKPTLKDLVRDFITMALIVRRGRQAISVTAFESSVDTFFANLERQA
RSANYSVEQVKDAQYALCAFLDESVLRSGDNDMRRHFEMEPLQFRYFGVHLAGEGFFEKI
ELLRADVKKNLDVLEVYHLCLALGFEGKFGLGQKDQLRYLANTLGQDIARYRKTPKALSP
DWALPDQVSQMLRHEVPVWLYLVLIALVCLGVYLTLDWLLGKDVAALAEQISQLFNA
>tr|A0A0Q0DN37|A0A0Q0DN37_PSEAP McrBC 5-methylcytosine restriction system component-like protein OS=Pseudomonas syringae pv. aptata OX=83167 GN=ALO85_03247 PE=4 SV=1
MPSLNQIFFGPPGTGKTYATVEATLQILDQPFLAKNAGNRSALKARFDELLAAGDVRFVT
FHQSFSYEDFVEGLRATTDEQGQIRYEVVSGVFKSLCESIASELSGKYRAFKVGDRYGTG
YKVTRATPDVVEIEKPQGKHLPIGMSLLNTLASYVDAGTFTIEELGNGRWDKKVPGSILD
PFLVNGYKNFLPSMVEHMLGKSEEGLFEPAPIQHSDAKVLIIDEINRGNVSRIFGELITL
IEPSKRAGASEALEVTLPYSKERFSIPSNIHLIGTMNTSDRSLAALDIALRRRFTFIEVP
PNPELLEDIEVDGIAIDELLSVMNQRIAVLLDQDHCLGHAYFMPLESDPTLERLAGIFQE
QILPLLQEYFFEDWQRIQWVLNDQRKAPENRFLIQPSQDLSVLFGDAVTLGQNNERWELN
LPAFKNIESYLGVIDHNLSVSAPLEAKSVRTEGVDIRQAANGSIEVYRGGQQVKPAKPHL
REIASKHGLSITSASGIKLNTRSLGRKIIKFLSEQQR
>tr|A0A0Q0FET0|A0A0Q0FET0_PSEAP ABC transporter, transmembrane region:ABC transporter OS=Pseudomonas syringae pv. aptata OX=83167 GN=ALO85_01181 PE=4 SV=1
MISRITRRHRQALRLAASFIAPYRWQALGALLALVITAGITLSIGQGIKLMVDQGFMTRS
PQLLERSIGFFMLLTVGLAVGTFARFYLVSWIGERVVADLRKKVFDHLIELHPGFYENNR
SSEIQSRLTADTTLLQSVIGSSLSMFLRNALMVIGGIVLLFITNPKLTSIVVVALPLIIA
PILMFGRRVRNLSRESQDRVANVGSYVAEALGQIKTVQAYNHQQQDKQRFSHTVEQAFET
ARKRILQRSWLITLVIVLVLGAVAVMLWVGGMDVISGRISSGELAAFVFYALMVGMAFGT
LSEVIGDLQRAAGAAERIGELLQARSEISVPTTDLQSLPQRISGRLALENVTFAYPSRPE
RNALDDLTLSIEPGETLALVGPSGAGKSTILDLLLRFYDPIKGRILIEGVPIAQLDPHDL
RRCFALVSQSPALFFGSVEDNIRYGNATASLEQVEAAARIAHAHEFVLDMADGYRTHLGE
GGVGLSGGQRQRLAIARALLVDAPILLLDEATSALDAQSEHLIQQALPSLMQGRTTLVIA
HRLATVQNADRIAVMDQGRLVAVGTHQQLIASNPLYARLAALQFNAGVE
>tr|A0A0Q0C2G5|A0A0Q0C2G5_PSEAP Uncharacterized protein OS=Pseudomonas syringae pv. aptata OX=83167 GN=ALO85_02476 PE=4 SV=1
MRSLTLHLKILITALVTLGIAITAYQILALGIPVSEDETDDLWNIDAKVEFVANPKDSVK
VQMFVPPLAHDYISLNESFISNNYGVSVNRADGNRKVTWSARRATGNQTLYYRLVLTKRY
SGEKPKVKGPIFRDSMTVEGPEKIAAEALLAPIRQHSADVETFITEAIKRVNNLNDDNVK

LLLAGDTSASNKARVTELLLSIAHVPLEKAHTIRLDAEQQTPELWLRSFNGKEWLYFNPD
TGEAGLPDDRLLWWTGEEGLVSVEGGKKVQVTFSLNNSEMNAMRLAKLTDASTDSDFLAY
SLYGLPLQTQQTFMIMVMIPIGVLVILILRNLIGLQTLGTFTPVLIALAFRETQLGFGIA
LFTVITALGLSLRSYLEHLKLQMLPRLSVVLTFVVVLIAAISLFSHKLGLERGLSVALFP
MVILTMTIERLSITWEERGGSHAMKVAIGTLFAASLAHLIMSVPELTYFVFTFPAVLLVL
VGFMLAMGRYRGYRLTELVRFKAFLKEEKAK
>tr|A0A0Q0FFI7|A0A0Q0FFI7_PSEAP Ethanolamine ammonia-lyase heavy chain OS=Pseudomonas syringae pv. aptata OX=83167 GN=ALO85_03099 PE=4 SV=1
MASFSHSVGAQTYRFDSLKEVMAKASPARSGDYLAEVAAQNDGERVAAQMALANIPLKHF
LEEALIPYEQDEVTRLIIDTHDKLAFAPVSHLTVGGFRDWLLSDKADEASLRALAPGLTP
EMAAAVSKIMRVQDLVLVAQKIRVVTRFRNTMGLRGRLSTRLQPNHPTDDPSGIAASVLD
GLLYGNGDAMIGINPATDSTSSIVALLEMLDAIVQRYEIPTQSCVLTHVTTSIEVINRGV
PVDLVFQSITGTEAANASFGISLKLLQEGYEAGLSLNRGTLGQNLMYFETGQGSALSANA
HHGVDQQTCETRAYAVARHFNPFLVNTVVGFIGPEYLYNGKQIIRAGLEDHFCGKLLGVP
MGCDICYTNHAEADQDDMDTLLTLLGVAGINFIMGIPGSDDIMLNYQTTSFHDALYARQS
LGLRPAPEYEAWLEKMGIFTQAEGRVRFGDSLPPAFRQALAHLA
>tr|A0A0N8TAD2|A0A0N8TAD2_PSEAP ATP-dependent DNA helicase Rep OS=Pseudomonas syringae pv. aptata OX=83167 GN=rep PE=3 SV=1
MQTTLHTAMSRLNPRQQEAVNYVGGPLLVLAGAGSGKTSVITRKIAHLIQNCGIRAQYIV
AMTFTNKAAREMKERVGTLLRGSEGRGLTVSTFHNLGLNIIRKEHTRLGYKPGFSIFDET
DVKALMTDIMQKEYSGDDGVDEIKNMIGSWKNDLILPAEALENARNPKEQTAAIVYTHYQ
RTLKAYNAVDFDDLILLPVKLFQEHADILEKWQNKVRYLLVDEYQDTNASQYLLVKLLIG
TRHQFTVVGDDDQSIYAWRGARPENLMLLKDDYPSLKVVMLEQNYRSTSRILRCANVLIS
NNPHAFEKQLWSEMGHGDEIRVIRCRNEDAEAERVAMEILTLHLRTDRPYSDFAILYRGN
YQAKLIELKLQHHQVPYRLSGGNSFFGRQEVKDLMAYFRLLVNPDDDNAFLRVINVPRRE
IGSTTLEKLGNYATERKVSMYAATDEIGLGEHLDSRFTDRLQRFKRWMDGVRQQCAQNDP
IAALRSMVMDIDYENWLRQNSSSEKAADYRMSNVWFLIEALKNTLEKDEEGGMTIEEAIG
KLVLRDMLERQQEEEDGAEGVQMMTLHASKGLEFPYVFIMGMEEEILPHRSSIEADTIEE
ERRLAYVGITRARQTLAFTFAAKRKQYGEIIDCAPSRFLDELPPDDLAWEGNDDTPTEVK
AVRGNTALADIRAMLKR
>tr|A0A0Q0CIT0|A0A0Q0CIT0_PSEAP Hydrolase, haloacid dehalogenase-like family OS=Pseudomonas syringae pv. aptata OX=83167 GN=ALO85_01523 PE=4 SV=1
MSHVQAVIFDAFGTITRIENARHPFRQLLKLGIAQGRRPKPDDASMLMRHPWGLAQAADH
FGIDVTPAELARIQAELDAEVASIQPFADALACIEALQAHGLKVAVCSNLARPYGAAVKR
IFPGLESYGFSYEIGTLKPDRRIYEAALTELAVDACKVWMIGDSQRCDRDGPIALGIKGH
YLNRTGAGSPSDFRDLMAFKHSVFESRSP
>tr|A0A0Q0IQ55|A0A0Q0IQ55_PSEAP Uncharacterized protein OS=Pseudomonas syringae pv. aptata OX=83167 GN=ALO85_00768 PE=4 SV=1
MLMPKKRPVIITCAVTGAIHTPSMSPHLPITPREIADAAIGAAEAGASIVHLHARDPHDG
RPSQDVDLFRQFLPQIKAKSDVVINITTGGAPTMGVEERLQPVAQLKPELASLNMGSMNF
GLYEMLDRYSEFKHDWERPYLADSDDRIFRNTFRDIAHILNTCAENRTRFEIECYDIGHL
YTAAHFLQRGLLKAPIFIQSVFGLRGGIGGHPEDLAHMRRTADRLFGDAYQWSILGAGRN
QIPLGTMGLSMGSHVRVGLEDSLWDGPGKLAASNADQVKRIRTVIEALGGQVATPDQARE
MLDLKGQDKVNF
>tr|A0A0Q0CD77|A0A0Q0CD77_PSEAP Putative transcriptional regulator OS=Pseudomonas syringae pv. aptata OX=83167 GN=ALO85_00882 PE=4 SV=1
MDTGARLKLVRESYKLSQRELARRSGVTNATISLIEQNRVSPSISSLKKLLEGIPMTLAD
FFTFDQPPGQDQYVFRAGDQPDLGRNGARLLLVGATLPSRQMRFLREQYAPGADSGDEPI
VHSEGEECGLVTRGTVELTIDGQVNILGPGDGYYFPTTLPHRFRNIGQDEAEIISANTPA
NF
>tr|A0A0Q0BU60|A0A0Q0BU60_PSEAP Uncharacterized protein OS=Pseudomonas syringae pv. aptata OX=83167 GN=ALO85_101189 PE=4 SV=1
MPGRAEKPKHGEAPDYCCFCECEYYIFITEKSSAFGLILCRKCRQSAVVLRTLRKCLQVF
QVFGMGPTGVSEVVC
>tr|A0A0Q0CFK9|A0A0Q0CFK9_PSEAP Putative phosphate starvation-inducible protein psiF OS=Pseudomonas syringae pv. aptata OX=83167 GN=ALO85_01928 PE=4 SV=1
MDLESGSMNKLGIPVLLVGLMLSAQGFAATAQQNKMTTCNADASAKSLKGDERKAFMSTC
LKAAPPPAATQQEKMKTCNATASTQALKGDERKSFMSDCLKKK

>tr|A0A0Q0CC92|A0A0Q0CC92_PSEAP GntR family transcriptional regulator OS=Pseudomonas syringae pv. aptata OX=83167 GN=ALO85_02477 PE=4 SV=1
MLKVVEKTMTVADDSETLSENVFRRIQSAIVKGEIAPGSKISEPELARTYGISRGPLREA
IHRLEGQRLVVRVPHVGARVVSLSHAELIELYEIRESLEGMACRLAAERMTQAEIDELRG
VLDTHERDAAFQAGIGYYQQEGDFDFHYRIIQGSGNRTLSQMLCGELYQLVRMYRIQYST
TPNRPRQAFAEHHRILDAIADRDGELAELLMRRHIGASRRNIERHYQAASQTTSDRGKQ
>tr|A0A0Q0II02|A0A0Q0II02_PSEAP Uncharacterized protein OS=Pseudomonas syringae pv. aptata OX=83167 GN=ALO85_200118 PE=4 SV=1
MDKELFRHMFENEVKAKFGDDASFASYDYVHHRNYRDLRVGGRMLGGRPESVSQLYVIVN
DLITEEEKKAVFPGAFDLGNFKQLPENLQTYKALEDEIRKESKVEAHDRLKKIKILALPL
LGRSFSEFFSEDKTLTLKVLKTLYRFQTEQNKIFTLLAPPRISKKPSYEVRDCYGIEGST
EEVLLISDLKSHASFEMPRERLAFINSTYGKMRGLLDNVDWKLSQEAGVECGGSLQRFTQ
IVHHLATRIQSVLHVEVQEPVRLPIDEQLYTYLNRMELDHHTEGNLQLLSKVTEHEPPFG
EVRDLIAELAKYIKLPLSTPQHLLIEAETCESQFRDSANSFKWFYSKLLDREIDNNTFEK
ALPLFGKLCKVLNFAEFKKHISMSLALGAMALVLSELHKSTPYQPYWYGKGTISGGLQEY
LNAVRFEHWNLDAPEGSHRYWTGRVDYFSYTMLGQGNVFKGRVHLSNTINQSFMKILDTQ
DLELIDNKLTLFEAIFGSVEQ
>tr|A0A0Q0DBC0|A0A0Q0DBC0_PSEAP Uncharacterized protein OS=Pseudomonas syringae pv. aptata OX=83167 GN=ALO85_04172 PE=4 SV=1
MSCIDNPKGEFEKMIAALGADYYRILAVPFNPARSTYTVKHVAEMRKPSAGFFPDEVIKP
EVFEKLKRINSSNCTLKIICKSTKYHYVTLYDVSHEELYALSANGVKPCIVSLVRVSKQG
DKFYSVVLRFGQKRIPDESTYARKVSGSFQINVGSKVTTSLEEGIVLCGFVDKKSGMWIN
ANRAVNQNCPTCEDRFDIFLKNRSVDENPEKMPELDRSGSTFLYFESCRSQIIYKAKDAG
DKFDDTEIQCRLFYRLQKEHYTTAEIAQYIEERKKPQESSDF
>tr|A0A0Q0DVK5|A0A0Q0DVK5_PSEAP Glycosyl transferase, group 1 OS=Pseudomonas syringae pv. aptata OX=83167 GN=ALO85_02887 PE=4 SV=1
MTRSVERHVLQFCHGYDGPFLDCARQYASLFAGKGYRVTTVFLTGAADPDVVADCGSDEV
LFMEYSSSAIRGLKLGAIRDLRKIAATRSFSFCIAHRFKPVYIALLATKLPVIGVHHAFG
DYHRRSRKLFANLFRKRLSLLGVSDAVRDDMRSSLLKWPAERIQTLYNRIDVEQLQGSQF
SAEDARAELGLSASAWIVANVGRLHPDKDQATLLRGFAEALPDLPENSQLVILGKGRLEE
DLKALALELGIGPQVLFLGQVPDARRYFKAFDVFALSSDHEPFGMVLLEAMVAGVPLVAT
SCGGAKEVVEDVGLLFPLGDVHSLAHGLVHMAGLDAEQRQDCAERMLLRLRERFSDHAVR
NVFWRLPQVSSLTAGA
>tr|A0A0Q0DI97|A0A0Q0DI97_PSEAP Ferric siderophore uptake system, ExbD/TolR family protein OS=Pseudomonas syringae pv. aptata OX=83167 GN=ALO85_03979 PE=3 SV=1
MGLHLNEGGDDLAENHEINVTPFIDVMLVLLIIFMVAAPLATVDIKVDLPASTATPQPRP
EKPIFLSVKTDKSLYLGEEKVAREQIGQVLDARTKGKKDTTIFFQADKGVDYGDLMEVMN
TLRGAGYLKVGLVGLETAGKK
>tr|A0A0N8T9T5|A0A0N8T9T5_PSEAP Magnesium/cobalt chelatase, subunit BchD OS=Pseudomonas syringae pv. aptata OX=83167 GN=ALO85_03633 PE=4 SV=1
MQWLPTFLRGQPKSHKDLVRQPRSSRPSELLLVIVDASASTRRHQALSQAKGLLSQMFDD
AYRRRARLALLTASGTAPRWQHQGLKASSALIPWLDALGAGGGTPLPEALQQAAEWLAQR
QKRYPAEQQRVLVLTDGRIKSLPALPAFDCASLLIDIEKGPIRLGRARELASSLGAEYRH
IDELKRV
>tr|A0A0Q0BY72|A0A0Q0BY72_PSEAP Glutathione peroxidase OS=Pseudomonas syringae pv. aptata OX=83167 GN=ALO85_03549 PE=3 SV=1
MMNIRFFSIPLLLLAMSGAAMAADCPPLLQGELPKLRAKENIDLCKRFAGKPLVVVNTAS
FCGFAPQFKGLEALSQRYKGQGLEVLGVPSNDFKQEAKDGEETAKVCYVNYGVTFTMTEP
QAVRGDDATHLFKVLAEQSSAPRWNFYKYVVDRQGKVIANFSSMTKPDDPELIAAIEKAI
ASKP
>tr|A0A0N8TAB8|A0A0N8TAB8_PSEAP Phosphate-specific transport system accessory protein PhoU OS=Pseudomonas syringae pv. aptata OX=83167 GN=ALO85_01416 PE=3 SV=1
MIHKDSHTHHISQQFNAELEDVRSHLLEMGGLVEKQVNDSVTALIEADSGLAQRVREVDD
RINQMERSIDEECLRILARRQPAASDLRLIISISKSVIDLERIGDEATKIARRAIKLCEE
GEAPRGYVEVRHIGDQVRNMVRDALDSFARFDAELALSVAQYDKTIDREYKTALRELATY
MMEDPRSISRVLDVIWVLRSLERIGDHARNISELVIYLVRGTDVRHMGLKRMKEEVQGIS
SETANVPGNADDK

>tr|A0A0Q0C8P5|A0A0Q0C8P5_PSEAP Uncharacterized protein OS=Pseudomonas syringae pv. aptata OX=83167 GN=ALO85_03244 PE=4 SV=1
MDLLANVPKWDDIERNLEDKFSHLNQSFDQGWESASDGWHGFTRRVSNEATLAFGSIGGN
RIDSVKLAMAKSYPIIQLDLMRKWASIDITEILPVLLQLVKEVAMIMGGSVAVGTIMGAA
AGSLAFGAGSVPGAIVGSGIGAQVGNLILLGMGLSAIATYFYEGLPACLTTIYEGMVTAW
NAEEGVKPAGLDPSGASAWLIDERIDAAAQKLAKGQEQLIMLLLTAIVTYITRGQIKSGI
TGSLDSIAARSAKLQADMTNKQIAGWLARNEQKLLAHPDLRPSEAATVAKKESEILTPNK
SETSTPKEPEKRDRIDKEDEFPYNRNPVLGVLENSVFAQKISKPTKTFSKVGQNLYSRAA
GHPIDTVADLTSALEKGTIKPSQVPLDYVIIDGQNVIANTRSSTALINAGIPKSQWYGAN
KTGVTAYDQITFDDLVRDQLRKNYGGSVLNARK
>tr|A0A0Q0IT12|A0A0Q0IT12_PSEAP Uncharacterized protein OS=Pseudomonas syringae pv. aptata OX=83167 GN=ALO85_03265 PE=4 SV=1
MKSLYTLALACAACAGMASANADVQSVPKESTQMTHSNGQLQPPVSYATLWADEKGATHV
GHCRLEGLEFKSYAPPAAPQWVGVSPDDIESIAYAVLPVGYVGNWHHAPGPQWVITLQGQ
WSVETTDGSVLVQGPGEMQFNADTRSRPRPDDERVGHLTRTVGDQPNVQLIIKLKPSAAD
KRTNGSCAY
>tr|A0A0Q0DMQ3|A0A0Q0DMQ3_PSEAP 30S ribosomal protein S14 OS=Pseudomonas syringae pv. aptata OX=83167 GN=rpsN PE=3 SV=1
MAKKSMKNRELKRQLTVAKYAKKRAELKAIIVDLNASPEARWEATVALQKQPRDASAARM
RNRCRITGRPHGVYRKFGLGRNKLREAAMRGDVPGLVKASW
>tr|A0A0Q0C491|A0A0Q0C491_PSEAP YaeQ OS=Pseudomonas syringae pv. aptata OX=83167 GN=ALO85_00351 PE=4 SV=1
MAQPSTTYKFELNLTDLDRGVYESVKQTIARHPSETEERMTVRLLAYAFWYNEQLSFGRG
LSDVDEPALWEKSLDDRVLHWIEVGQPDADRLTWCSRRTERTSLLAYGSLRVWEGKVIPA
IKNLKNVNIAAVPQEVLEVLAKDMPRVIKWDVMISEGTVFVTDDRGQHEVQLQWLAGERG
>tr|A0A0Q0IGL1|A0A0Q0IGL1_PSEAP NADH-quinone oxidoreductase subunit I OS=Pseudomonas syringae pv. aptata OX=83167 GN=nuoI PE=3 SV=1
MFKYIGDIVKGTGTQLRSLVMVFGHGFRKRDTLQYPEEQVYLPPRYRGRIVLTRDPDGEE
RCVACNLCAVACPVGCISLQKAETEDGRWYPDFFRINFSRCIFCGLCEEACPTTAIQLTP
DFEMADFKRQDLVYEKEDLLISGPGKNPDYNFYRVAGMAIGGKPKGSAQNEAEPINVKSL
LP
>tr|A0A0Q0FSQ0|A0A0Q0FSQ0_PSEAP 6,7-dimethyl-8-ribityllumazine synthase OS=Pseudomonas syringae pv. aptata OX=83167 GN=ribH PE=3 SV=1
MTLKTIEGTFIAPQGRYALVVGRFNSFVVESLVSGAVDALVRHGVSESDITIIRAPGAFE
IPLVVQKVAQRSEFAAIVALGAVIRGGTPHFEYVAGECVKGLSQVSMEFGVPVAFGVLTV
DSIEQAIERSGTKAGNKGAEAALSAIEMVSLLSQLEAK
>tr|A0A0Q0C9R5|A0A0Q0C9R5_PSEAP Alcohol dehydrogenase OS=Pseudomonas syringae pv. aptata OX=83167 GN=ALO85_00378 PE=4 SV=1
MFMKAFQIGSQQGLDALTATTRPEPIAGPGQAMVAPRLVSLISRDVQILRGTYGPRQLPE
RIPVSEGVGEVIAVGEGVSQVKPGDRVICGHFPNWLQGEFRSSVFAHDVGVTHDGWLAEK
VVLPAAALIHVPDALADKDVAALASAGLTAWNALMEVCKVKPGELVLCLGTGGVALAALK
LARLHGARVAITSSSDEKLEIARQLGADITINYRTHPDWAAQVMALSDNAGADIIIETGG
QDTLGQSIAAAAVNGRIAVIGVTPGQHSPIPDYLSLILKNVTIRGIANGSREMFVDLIRA
IEANGIETVVARTFKFAEAPEAYAYFAAAQHIGKVLIEFEQG
>tr|A0A0Q0DGV6|A0A0Q0DGV6_PSEAP Uncharacterized protein OS=Pseudomonas syringae pv. aptata OX=83167 GN=ALO85_03221 PE=4 SV=1
MMVYRVAADAVVVFHLLFIVFVLFGGLLVLRRPWLALLHVPAAAWGTAVEFLHLYCPLTP
LENTLRSNAGGQGYTGGFVEHYLIPLIYPAGLTPGMQLWLGGIVVLLNVSVYGLLLLRYA
ARSRRS
>tr|A0A0Q0FAS8|A0A0Q0FAS8_PSEAP Ferric siderophore uptake system, MotA/TolQ/ExbB family protein OS=Pseudomonas syringae pv. aptata OX=83167 GN=ALO85_03980 PE=3 SV=1
MKRIQSIASPTQMPRLPRAWRSIAALVLSLMFVPMALADQSAPTAAPATSTAPAATAPAA
PDAAAPVQAMEPAAEDNSLGMAHDLSPWGMYQNADVVVKAVMIGLAIASIITWTIWISKG
FELLGAKRRLRGEIANLKKARSLSEASATASTEGTLAHLLVHDALEEMRLSANSRERGI
KERVSFRLERLVAACGRNMSMGTGVLATIGSTAPFVGLFGTVWGIMNSFIGIAKTQTTNL
AVVAPGIAEALLATALGLVAAIPAVVIYNVFARSIAGYKAQVSDASAHVLLLVSRDLDHL
PEPSERNQQQPHMVKVG
>tr|A0A0Q0IDA2|A0A0Q0IDA2_PSEAP TonB OS=Pseudomonas syringae pv. aptata OX=83167 GN=ALO85_01643 PE=4 SV=1

MRITAFMIAAALAPPVGAAEPFLVPIYTPTPVFPPELVKTHYAGKVRAQLWIKSNGQVRE
ARAIESGHPQLAAAVEQALRQWRYKPWVGTVGAPPMTTITVPVIFGSHGYRRFNTEVTVG
LGNIRCGYLNHEVKSARQDYPKEPLSKVDVFWYTGRALFGSHVAHLRNEPQRQVLLEQLG
AAIPMMVSNCRSNPDRLYGDYLPTPIKALMVGLAEQAEGSE
>tr|A0A0Q0C1K3|A0A0Q0C1K3_PSEAP Regulatory protein, GntR OS=Pseudomonas syringae pv. aptata OX=83167 GN=ALO85_03717 PE=4 SV=1
MKNNTDFAYQAVYRYLVRLVDQQQGEPALKMPSLRQLAQRLNVSISTVQSAYSLLEKEGR
VYSLPKSGYYSVARSLSAQGHVLPDSDGDLLQALYDNARLPGMALLGCDEPALLHAQDSP
LPAMERELGRHYPRQRDPVFQPFGEPELRTALAARYTRDAAHCWHADNVYVAADFHGALK
AVLGTLRLRGGTVLVESPCAWGVLRLLQSFDIRVIELPLDETGSLDAARLDGLLRGHDIG
LVVLPSLLNPVRGSVRPQNNSQAVAEVLNRHQVWVLENDSHAGLQFVGGQAPLRHWIDPQ
RLVIIGGFEKSMGPEAPYAYLLCKSLDTRWQQYFCLRAFELPPLRQRAIARLCSSGRLDR
QLLALRETLGRRMQSMTRQLDEHTGGALRYQLPEGGCGVWAESAYPVDMRQVFIALLAQS
IVVSPGELFTLQNRYSQHLWVSYATDWTREPARLLGALGEAVSQARLP
>tr|A0A0Q0DUY2|A0A0Q0DUY2_PSEAP Glycosyl transferase family protein OS=Pseudomonas syringae pv. aptata OX=83167 GN=ALO85_01481 PE=4 SV=1
MTTPTFLSVVIPAKNEADNLPSLIKEICTALDGERYEILVVDDGSTDDTLKVLTHLKDSG
LTVLRILRHERSLGQSTSVYHAALAANGTWLATLDGDGQNDPADIPGMLALVRAHATGSG
AIQLVAGHRVNRRDTASKRWASRFANGLRSRLLKDATPDTGCGLKLIERAAFLRLPYFDH
MHRFIPALIQRHNGRMVTHPVNHRHRQAGVSKYGNLDRALVGILDLVGVWWLIRRTRLDV
QAQELRP
>tr|A0A0Q0IDC6|A0A0Q0IDC6_PSEAP Methyltransf_25 domain-containing protein OS=Pseudomonas syringae pv. aptata OX=83167 GN=ALO85_01672 PE=4 SV=1
MMKLDPQTLAQITAATLGNYNSVADDFREGTRDHDVSQNIDALLRHIEGPAPWQILDFGC
GPGRDLKAFTAMGHVAVGLDGSERFAEMARAETGCEVLQQNFLELDLPHGRFDGLFANAV
LFHIPKQELPRVLRQLHATLKPGGVLFSSNPRGENQEGWNGERYGAYHDLESWRELLTEA
GFVELEHYYRPAGLPREQQPWLASVWRR
>tr|A0A0Q0CZ96|A0A0Q0CZ96_PSEAP 3-dehydroquinate synthase OS=Pseudomonas syringae pv. aptata OX=83167 GN=aroB PE=3 SV=1
MWGHMQTLKVELGERSYPIHIGEGLLDQPELLAPHIVGRQVAIVSNTTVAPLYLERLTQT
LAGYNVLPIVLPDGEAFKNWETLQTIFDGLLTARHDRRTTVIALGGGVIGDMAGFAAACY
QRGVNFIQIPTTLLSQVDSSVGGKTGINHPLGKNMVGAFYQPSVVLIDTTSLNTLPEREL
SAGLAEVIKYGLICDEPFLTWLEEHVDALRGLDQAALTVAIERSCAAKALVVGADERESG
VRATLNLGHTFGHAIETHMGYGVWLHGEAVAAGTVMALEMSSRLGWISTQERDRGIRLFQ
RAGLPVVPPQEMTEDNFLEHMAIDKKVIDGRLRLVLLRRMGEAVITDEYPKEVLQATLVA
DYRALVDQLRG
>tr|A0A0Q0FBQ5|A0A0Q0FBQ5_PSEAP Type III secretion system protein HrpB OS=Pseudomonas syringae pv. aptata OX=83167 GN=ALO85_04200 PE=4 SV=1
MTISHLGNVKSISPELGQDVPQGLVSEPAQADVDIFTAATQPDGVSSGAPLSEHIASAIS
GGLGETEKMSQQAMRSMKKASGTGDALDIAAMTRTLSQCSLQTALTTKVVSKTAQALDKL
TNLQ
>tr|A0A0Q0C5L6|A0A0Q0C5L6_PSEAP SsrA-binding protein OS=Pseudomonas syringae pv. aptata OX=83167 GN=smpB PE=3 SV=1
MAKLKKHPTGTIAQNKKARHDYFIEHKFEAGLVLSGWEVKSLRATKVQLVDSYVLLKDGE
AWLMGCHITPLKTASTHVIADPTRTRKLLLNKRELEKLTSSVQQKGYACVALSVYWKEHL
VKCEIALGKGKKEYDKRHTERERDSDRELQRAVRSKGKED
>tr|A0A0Q0C259|A0A0Q0C259_PSEAP Uncharacterized protein OS=Pseudomonas syringae pv. aptata OX=83167 GN=ALO85_100267 PE=4 SV=1
MAMVFCRGCAKEIHETALNCPQCGASQVSATPAKQLQQTGSPWMAIVSLVLGILCSLALF
DDGEWDLDTVVGLGMCSIAGLVLGVISINKKLPGNGIAIAGTVLSAVSLLIFFGLIAN
>tr|A0A0Q0D5X8|A0A0Q0D5X8_PSEAP Uncharacterized protein OS=Pseudomonas syringae pv. aptata OX=83167 GN=ALO85_02647 PE=4 SV=1
MADESKREDLDHKPENAGKVGEKNKQLNQQGEQARPATSPDPQKDKSGN
>tr|A0A0Q0DLJ9|A0A0Q0DLJ9_PSEAP Homoserine dehydrogenase OS=Pseudomonas syringae pv. aptata OX=83167 GN=ALO85_00357 PE=3 SV=1
MKPVKVGICGLGTVGGGTFNVLQRNAEEIARRAGRGIEVAQIAVRTPNPNCQITGTPTTS
DVFAIATNPEIDIVVELIGGYTVARDLVLKAIENGKHVVTANKALIAVHGNEIFAKAREK
GVIVAFEAAVAGGIPVIKAIREGLSANRINWVAGIINGTGNFILTEMREKGRTFPDVLAE
AQALGYAEADPTFDVEGIDAAHKLTILASIAFGIPLQFDKAYTEGITKLTTADVNYAEAL
GYRIKHLGVARSTSQGIELRVHPTLIPADRLIANVNGVMNAVMVNGDASGSTLFYGAGAG

MEPTASSVVADLVDVVRAMTSDPENRVPHLAFQPDSLSAHPILPIEACESAYYLRIQAKD
HPGVLAQVASILSERGINIESIMQKDVEEHDGLVPMILLTHRVVEQRINDAIQALEALQD
VVGPVVRIRVEHLN
>tr|A0A0Q0FQY8|A0A0Q0FQY8_PSEAP YjeE family ATPase OS=Pseudomonas syringae pv. aptata OX=83167 GN=ALO85_02851 PE=4 SV=1
MSDLTLHVVGEEAMMDFGARLAKVTEGLGVIFLDGDLGAGKTTLSRGLIRGFGHVGAVKS
PTFTLVEPYEIGAIKVFHFDLYRLVDPEELEYMGVRDYFDGDALCLIEWPQRGAGFLPKP
DLTITIGPHGEGRSVILSPLGSRGERWCATLALEFK
>tr|A0A0Q0CXZ1|A0A0Q0CXZ1_PSEAP 3-ketoacyl-reductase OS=Pseudomonas syringae pv. aptata OX=83167 GN=ALO85_03084 PE=4 SV=1
MPSDRYIDFANSDMGRRLVKAVGLPAPTHLERWQAGRLRPVEGTLLISAGPLGDQVRQFA
SRLTDSLYSFGSDMPGATTWVSNQGPRLKAVVFDASQILRTDQLRQLRDFFQPLLRNLDH
CAHVVILGKSPETLADPLAASTQQAIEGFSRSLAKEARNGATVKLLQVDDDAQDQLEGAL
RFFLTPKAAFISGQFVHLSACPSKVQDWTRPLAGRKAVVTGAARGIGASIAETLTRDGAQ
VILLDVPQTKNELEALASRLGGQALALDICAADAPAQLLEHLPDGVDILVHNAGITRDKT
LVNMPEDFWDSVLAVNLNAPQVLTQVLLDAGALKDNARIVLMASISGIAGNRGQTNYTTS
KAGLIGFARAMAPGLKSRGISINAVAPGFIETKMTAHMPFTLREAGRRMSSLGQGGSPQD
VAEAVAWFSQPGSGAVSGQVLRVCGQNVIGA
>tr|A0A0Q0FK62|A0A0Q0FK62_PSEAP Succinate--CoA ligase [ADP-forming] subunit alpha OS=Pseudomonas syringae pv. aptata OX=83167 GN=sucD PE=3 SV=1
MSVLINKDTKVICQGFTGSQGTFHSEQAIAYGTKMVGGVTPGKGGTTHLNLPVFNTVKEA
VDTTGATASVIYVPAPFCKDSILEAAFGGIKLIVCITEGIPTIDMLEAKVKCDELGVVLI
GPNCPGVITPGECKIGIMPGHIHLPGKVGIVSRSGTLTYEAVKQTTDAGFGQSTCVGIGG
DPIPGSNFIDILKLFQEDPKTEAIVMIGEIGGSAEEEAAAYIKAHVTKPVVSYIAGVTAP
PGKRMGHAGAIISGGKGTADEKFAALQDAGVKTVRSLADIGKALAELTGWPTK
>tr|A0A0Q0FUM4|A0A0Q0FUM4_PSEAP ABC transporter periplasmic substrate-binding protein OS=Pseudomonas syringae pv. aptata OX=83167 GN=ALO85_01982 PE=4 SV=1
MLLAAFFHYWRFLNMQKSRLFGLLTAGLLAALSLSANAAQKDHFNVCWTIYAGWMPWEYA
GSQGIVDKWAKKYGIKIDVTQLNDYVESINQYTAGQFDGCTMTNMDALTIPAAGGVESTA
LIVSDFSNGNDGIVLKGEGKKVSDLKGMDVNLVELSVSHYLLARALDSVKLSEKDLKVVN
TSDADISAAFNTDDVQAVTTWNPMLSDIKAKPGVTEVFNSSQIPGEIMDMMVVNTQTLKD
NPALGKALTGAWFEVVELMNAKNAQGKAALEHMAKASGTDLAGFQAQLDTTKLFATPKEA
LEFATSKQLPDTQRKVADFSFAHGLLGEGARDADAVGMSFANGVTLGDKGNLKLHFDPGY
VQMAVDGKL
>tr|A0A0Q0CED1|A0A0Q0CED1_PSEAP Arginine exporter protein ArgO OS=Pseudomonas syringae pv. aptata OX=83167 GN=ALO85_02361 PE=4 SV=1
MWQSYLNGLLIAAGLIMAIGTQNAFVLAQGLRREHHVAVAMLCIVCDAILVAAGVFGLAN
VLAQNPTLLAVARWGGVIFLSWYGLQALRRACSRQSLEHSAAVGRKSLRTVLLSALAVTL
LNPHVYLDTVLLIGSLGAQQGVPGAYVAGAASASLLWFSSLALGAAWLAPWLARPATWRM
LDLMIAVMMFSVAFQLIRSA
>tr|A0A0Q0IPC4|A0A0Q0IPC4_PSEAP Uncharacterized protein OS=Pseudomonas syringae pv. aptata OX=83167 GN=ALO85_101113 PE=4 SV=1
MDRLLLVGDRLKTLNTLDPEHCTARAENYFPLKAVSCEATLMNGRTLQRKMFERCCWPNS
GALAAHPAQTQVFQGS
>tr|A0A0Q0C4Y3|A0A0Q0C4Y3_PSEAP Uncharacterized protein OS=Pseudomonas syringae pv. aptata OX=83167 GN=ALO85_03993 PE=4 SV=1
MPMPTWIDHLGSARSALLYCALTAGLLAGCSTNKEQMLPHGQNTMMDVWNQGTTGSAGSA
SGRQLLDARSELRRPSDVRATQPLQDSAAYTRTAQNEIYSQFKRLPNPDLVMYVFPHLAG
SDPAPVPGYTTVFPLYQRVQYAMPGERVEDY
>tr|A0A0Q0BTR9|A0A0Q0BTR9_PSEAP Uncharacterized protein OS=Pseudomonas syringae pv. aptata OX=83167 GN=ALO85_04243 PE=4 SV=1
MDINLPHVVAEVTHAFHDYERALLANELSMLDAYFWNSEQTVRYGVAENLLGADTIARYR
RQCQPVGPGRTLLRTVVSTFGEDFATVSTEFHDGVTQRLGRQMQTWARLEGGWKVVAAHV
SIDLSSLEPRP
>tr|A0A0Q0DVS5|A0A0Q0DVS5_PSEAP Uncharacterized protein OS=Pseudomonas syringae pv. aptata OX=83167 GN=ALO85_101455 PE=4 SV=1
MAGTVSSVDDRFETFGSIAVLAILPKAFCRFKGHPITIFL

>tr|A0A0Q0DB22|A0A0Q0DB22_PSEAP Type III secretion protein HrpD OS=Pseudomonas syringae pv. aptata OX=83167 GN=ALO85_04198 PE=4 SV=1
MDLTAEDYWTQWWCNPWPWAHAGWQSRFAERCGLTVSDCEALMVSRHSVFLQSVGITPSQ
PPMPAAPVLSWLALTTVQRDQALDLAQRICFSRNESDGHDGQWCWSLTKALRPGVWLDLE
HDDVRLLLGAWLGPEYWPRLRLAWAPDEVADSPCSAPENKLQTLWQAVLWRVTAA
>tr|A0A0Q0BWP4|A0A0Q0BWP4_PSEAP Uncharacterized protein OS=Pseudomonas syringae pv. aptata OX=83167 GN=ALO85_01072 PE=4 SV=1
MNETLGIMQPYFFPYIGYFQLIAAVQRGLVFDIVKYKRKSWMNRNRVLGSKGDWQYINVP
VCVSEGALIKDATIIDLACAHRRITNQLEHYRSKAPYFRETLQVVEQTFGTPATHLCELN
TRALKVVCEYLGISFNWESCAAMNLDLPPIEHAGQWALEISTVLKARQYINATGGREIFI
PGEWQERGIELRFLEPASFSYSTGPMNFVENLSIIDVLMWNAPETVLTYLRNETRAVI
>tr|A0A0Q0BRW3|A0A0Q0BRW3_PSEAP Uncharacterized protein OS=Pseudomonas syringae pv. aptata OX=83167 GN=ALO85_04166 PE=4 SV=1
MNLYTERQAYEKAIRDSESSAHLFEHVAEPSDQLFQKGMESLLRKIRNRTTSFNISLLGK
LTNENSSKYLYCYYSNFADEDGYTTFEIFEFYLNPITGKTIAFNGKLFDVFNVIDIELFK
INNTICDFYDMYKMYATFKYQPEHNFVEFLNSPNFQESWQYLKLDEY
>tr|A0A0Q0C0V6|A0A0Q0C0V6_PSEAP Uncharacterized protein OS=Pseudomonas syringae pv. aptata OX=83167 GN=ALO85_02904 PE=4 SV=1
MTASSHAPLKYLQAYPQALQDQVQQLIAEDRLADYLQQRYPARHQVQSDKALYAYALALK
QDHLRNAPAIKKVLFDNRLDLTHRALGLHTKISRVQGGKLKASNEIRVAALFKEAPPEFL
KMIVVHELAHFRESDHNKAFYQLCEYMLPGYHQLEFDLRVYLTYRDLQAARAPC
>tr|A0A0Q0C0M6|A0A0Q0C0M6_PSEAP LysR family transcriptional regulator OS=Pseudomonas syringae pv. aptata OX=83167 GN=ALO85_00944 PE=3 SV=1
MNLNKVDLNLFIVFDAIYTEANLTRAGQIVGITQPAVSNALARLRETFNDPLFVRTAQGM
VPTPMAQNIIGPVRNALSLLRVSVQESRIFNPLQANKNYRISMTDLTEAVILPALFQRLR
RLAPAVTIESFLSKRRETTKELAAGRLDFAVDAPLNTDPQVRHVKLMEDRYVCGMRKGHP
LANKATLTLDDYLSQSHIHISSRRSGLGHVDLALGKMGIQRRIALRSQHYMMATQVLQQT
DMVMTVPERFARRNDLHYVYLPVSDVPSVETHLYWHESTDQDPANRWMREQIIELSQRVV
AQEQNLENA
>tr|A0A0Q0D9J1|A0A0Q0D9J1_PSEAP Filamentation induced by cAMP protein Fic OS=Pseudomonas syringae pv. aptata OX=83167 GN=ALO85_200060 PE=3 SV=1
MQTSFTSRHNASKITIITSDNCAYLTASPFMSKFQRASLATAARPCFVCNGYFMRRDQID
ILPYMAAFAIVVEVGSFIEASDVLGITASAVSRQVSKLERALSLRLLERSTRHLRLNNDG
ERVYDFCKKILESSSQIFELKEQLHNEPKGIIRIAAPRLLIPALSEVIPSFLTQYPLIDV
QSFFSGFSESDNNTDTEMDIILKLSAVPPSNFIARKLTDVRRSLYASSQYLECHGLPTSV
DKLALHSFIKINNRVDEKEWHFKSDDRTCSFEVSSRYSANDYEAALNAAIGHLGIVALPR
LYARAAVREGLVVPVLVDWQLIDGGEKEFVWFLYNQNKYGSLKVKAAVEYLIKHLRLNLE
ID
>tr|A0A0Q0DBB5|A0A0Q0DBB5_PSEAP Choline ABC-type transport system, permease protein OS=Pseudomonas syringae pv. aptata OX=83167 GN=ALO85_03847 PE=3 SV=1
MLTDHKIPLGEYIAAFVDWLTANGADYFDAIASTLEMMIHGFTFALTWFNPFVLIGLIAA
LAHFIQRKWGLTVFVILSFLLILNLHYWQETMETLAQVLFATLVCVVIGVPLGIIAAHKP
LFYTIIRPILDLMQTVPTFVYLIPTLTLFGLGVVPGLISTVVFAIAAPIRLTYLGIRDVP
QELLDAGKAFGSSRRQLLTRIELPYAMPSIAAGITQCIMLSLSMVVIAALVGADGLGKPV
VNALNTADIALGFEAGLAIVLLAIMLDRICKQPEAKVGSDA
>tr|A0A0Q0BRI1|A0A0Q0BRI1_PSEAP Lipoprotein OS=Pseudomonas syringae pv. aptata OX=83167 GN=ALO85_04199 PE=3 SV=1
MKFLSAGLLLICMVLLGGCSDETDLFTGLSEQDSNEVVARLADQHIDARKRLEKTGVVVT
VATSDMNRAVRVLNAAGLPRQSRASLGDIFKKEGVISTPLEERARYIYALSQELEATLSQ
IDGVIVARVHVVLPERIAPGEPVQPASAAVFIKHSAALDPDSVRGRIQQMVASSIPGMST
QSAESKKFSIVFVPATEFQETTQWVSFGPFKLDSANLPFWNLMLWLVPVGLSVLLLIIAL
LLRSDWRASVLRRIGFAGRSRSTVPARA
>tr|A0A0Q0C7M0|A0A0Q0C7M0_PSEAP Putative manganese efflux pump MntP OS=Pseudomonas syringae pv. aptata OX=83167 GN=mntP PE=3 SV=1
MNPISLLFLALAMSTDAFAAALGKGASLHKPRFLEALRTGLIFGAIETITPVIGWGIGQV
AARFAESWDHWIAFTLLLVLGLHMIYNGIKHDDDEEQEKPGQHSFWILAVTAFATSIDAL
AVGVGLAFVDVNIVVAALAIGLATTVMVTIGVMLGRVLGTMVGKRAEIIGGIVLIVVGAT
ILYEHLSAAQ

>tr|A0A0Q0C858|A0A0Q0C858_PSEAP Glycosyl transferase family protein OS=Pseudomonas syringae pv. aptata OX=83167 GN=ALO85_04756 PE=4 SV=1
MISEHPQVNSDPTAQPPGNTRFNGAITGLYGDVLQGWALDTQHVDERLVVEVYVDGACVS
LVRADQFHPRAGADDPFHGFGVQLRQGWLDNAKHISARVANSDCWLAGDLQLPTTPSAEP
APISSQVWHSGGLRLGGWSWDPAAPQRHVQITVREGSRVLGQATCNVHHQALVYRATSDH
GFSFDLPWELADGKPHTLDIENDLGHTLSGSPITLCCWHDGLEGLLKQHERTPTEQNLTL
LTRIAADQAMRLPKSAGFHHYAEWFERFRPAAPKDDQLQHCRTGVLLISDGDSTLEAISL
ASLNSQRTAPDQLAKADAAHLLPALKQLLEAGCDAVVPLMAGDHLGMHALEHLSTLLEDG
CAWGYADCDRDGPQGERSLPWLKPVWDIDLFMGADIFTPGAIFSAAIIDKALALIATTDG
QQTLDWHHLTAAIALTTETSQAVVAHLPHVLYHRSHLAAASPEQAEPSAQRLQAIGWLSE
SLASGAIVTQLPKFPTLLRTQWPLPATLPRVSLIVPTRDQLGLLRTCIEGLLTATDYPDL
EIIVVDNQSTDPHTLVYLQQLSGRGVKVLPYPHPFNYSAINNYAATHASVELIGLVNNDI
EIIAADWLKEMVSQLLRPNVGAVGAKLLWPNRMVQHGGVVVGVNGLAAHTGNHLEQRDPG
YLGMNQITRRQSAVTAACLLLRKSVFDTLQGLDEQAFPVAFNDVDLCLRIRQQGLNIIWT
PFAELIHAESASRGKDQTPEKRARGQREQQGFIERWSQSGQSDPYYHPALSLDYLSGPYG
GLAIPPRHRQLRRVGT
>tr|A0A0Q0DJT4|A0A0Q0DJT4_PSEAP Short-chain dehydrogenase OS=Pseudomonas syringae pv. aptata OX=83167 GN=ALO85_03880 PE=3 SV=1
MSNIQGKVVLITGASSGIGEAAARLIAAKGAHVVLGARRIERLQTLAAGIEAQGASARFR
ALDVTDALDMQAFADFAKHAFGKIDVIINNAGVMPLSPLAALNIAEWNQMLDVNVRGVLH
GIAAVLPSMQSQGHGQVINISSIGGLAVSPTAAVYCATKFAVRAISDGLRQETDKIRVTV
VSPGVVESELADSISDETAREAMKAFRKVALEPDAIARALVYAIEQPDGVDVSEIVVRPT
GSAY
>tr|A0A0Q0FMF0|A0A0Q0FMF0_PSEAP GTPase Obg OS=Pseudomonas syringae pv. aptata OX=83167 GN=obg PE=3 SV=1
MKFVDEVSIRVKAGDGGNGCMSFRREKFIENGGPNGGDGGDGGSIFMVADVNLNTLVDYR
YTRHFDAERGSNGGSADCTGRKGEELVLRVPVGTTIIDATTQEIIGDLTKDGQRLMVAQG
GWHGLGNTRFKSSTNRAPRQTTPGKPGDQRDLKLELKVLADVGLLGLPNAGKSTFIRSVS
AAKPKVADYPFTTLVPNLGVVSVDRWKSFVVADIPGLIEGASDGAGLGIRFLKHLARTRL
LLHLVDMAPLDESSAPDAAEVIVNELEKFSPSLAERDRWLVLNKCDQILEEEQEARKQEI
VDRLEWTGPVYVISAIAKEGTEQLTRDIMRYLEERSQRIAEEPGYAEELAELDQRIEDEA
RAQLQALDDQRALRRSGVKSVHDIGDDDWDEEDVDDEDGPEIIYVRD
>tr|A0A0Q0CB15|A0A0Q0CB15_PSEAP Uncharacterized protein OS=Pseudomonas syringae pv. aptata OX=83167 GN=ALO85_00443 PE=4 SV=1
MIEKIEAVMQHWGEQRMRIGLGGGLSSPMAGIMEWGAYIPRRTPGSRALMGNGSGLDYIS
SEVEAAIAQLSRSPAKSRGPELAQLATLRYVESLPVREQMRLVGINEGADRTYRNWINKL
HQQVLAILAERSASRSNNAVADKAAQG
>tr|A0A0N8T818|A0A0N8T818_PSEAP D-galactarate dehydratase OS=Pseudomonas syringae pv. aptata OX=83167 GN=ALO85_00097 PE=4 SV=1
MMNLIQHADSPRYIRLHPLDNVVVVVNDQGVPAGTEFDNGLVTVDNVPQSHKVSTVDLAV
GEAVIRYGHTIGYALQPIPRGSWVREDQLRMPSAPALDSLPMSDAVPEKQAPLEGYTFEG
YRNADGTVGTRNILGITTTVQCVTGVLDHAVKRIRDELLPKYPNVDDVVALTHSYGCGVA
ITATDAYIPIRTVRNLARNPNLGGEALVISLGCEKLQAGQVMHEGDSSVDLSEPWLYRLQ
DSSHGFNEMIEQIMELAETRLKKLDLRRRETVPASELILGMQCGGSDAFSGITANPALGY
ASDLLLRAGATVMFSEVTEVRDAIYLLTSRAQDQDVAQALVREMDWYDRYLAKGEADRSA
NTTPGNKKGGLSNIVEKSLGSIVKSGSSAINGVLGPGERVSSKGLIFCATPASDFVCGTL
QLAAGMNLHVFTTGRGTPYGLAMAPVVKVSTRTELAQRWPDLIDIDAGRIATGRASIEDL
GWELFHFYLDVASGKKKTWTEHYRLHNDITLFNPAPIT
>tr|A0A0Q0DCE2|A0A0Q0DCE2_PSEAP Response regulator receiver OS=Pseudomonas syringae pv. aptata OX=83167 GN=ALO85_04563 PE=4 SV=1
MEDHPFQLIATQVLLNNHGYFLLTPVLTAAEAMAAMQRSAEPYGLVLCDQCLPDMSGLDL
IDEAARHGWLRQAILLSGLPDTQLENLQQLALQRDLPLLGCLSKPLHGPDLSRLLGHLID
>tr|A0A0Q0CWA9|A0A0Q0CWA9_PSEAP Arc-like DNA binding domain protein OS=Pseudomonas syringae pv. aptata OX=83167 GN=ALO85_01854 PE=4 SV=1
MKQATYSSRTADKFVVRLPDGMRNRVQEVAKNHHRSMNSEIIARLEQSLIQEGALGDEPS
LRLDSPELSLHERELLQRFRQLSHRQQNALVSLIAHDTELASEES
>tr|A0A0Q0CG81|A0A0Q0CG81_PSEAP Cold shock DNA-binding protein OS=Pseudomonas syringae pv. aptata OX=83167 GN=ALO85_00757 PE=4 SV=1
MSNRQTGTVKWFNDEKGFGFITPQGGGDDLFVHFKAIQSDGFKSLKEGQTVTFVAAKGQK
GMQAEEVQVA

>tr|A0A0N8T8M1|A0A0N8T8M1_PSEAP Citrate/protein symporter, MFS family OS=Pseudomonas syringae pv. aptata OX=83167 GN=ALO85_00709 PE=4 SV=1
MHGSSPLRKESSRSKAGAVFRVTSGNFLEQFDFFLFGFYATQIASAFFPASSEFASLMMT
FAVFGAGFLMRPLGAVILGAYIDDVGRRKGLIVTLSIMASGTLLIVLVPGYASIGLWAPA
LVLVGRLLQGFSAGAELGGVSVYLAEMATPGRKGFYTSWQSGSQQVAIVVAAALGYGLNQ
WMAPAAVADWGWRIPFAIGCLIIPFIFLLRRSLQETEEFATRSHRPTMRQVFATLLQNWR
VVIAGMLMVAMTTTTFYLITIYAPTFGKTVLNLSTSDALLVTLLVGVSNFIWLPVGGMLS
DRFGRKPLLVAMTLLTIVSAYPALSFLALAPSFGHMLEVLLWFSFLYGLYNGAMIPALTE
IMPVEVRVAGFSLAYSLATAVFGGFTPAISTWLIHLTGDKAAPAYWMTFAAACALGATLV
LYRHISTRPQLAV
>tr|A0A0Q0DD09|A0A0Q0DD09_PSEAP Histidine kinase, HAMP region: chemotaxis sensory transducer OS=Pseudomonas syringae pv. aptata OX=83167 GN=ALO85_00105 PE=4 SV=1
MPLEATMNLRSLSISRRASLCFGVITLLLIGLGAFSYVQIGHLRIAEQNIEENSLPSIQV
VDDIQIALLHARLESIRMLASNDPSVHATAEAKVREAIEALRANSDFYQKHLLSGEADRA
QFEEANNKMGVYIDGLKQVVALDSADHDKAVSLANGEQAQKAAAYQEKLTVLRGHNAEEA
VVSGKDATAVYDHSVNVLMIVLIAAFLLTVVLAIALTRSIVDPISVSLKLAEDIAAGDLT
RQLSVSGSDEASRLMTALNTMSGNLRTTIHEISGASAQLSTAAVEMTSITESADRTLQQQ
NSEIEQAATAVNEMSAAVEEVARNATSTSQAAQQSSLSADLGNQRVAETLTAMHNLTGLV
EGSSAQVTALAGQAQNISKVLGVIRGIAEQTNLLALNAAIEAARAGEQGRGFAVVADEVR
ALAHRTQTSTQEVEQMISAIQAGSSATVESMQRSTQEVHSTRKTAEDAGESLRQITDSVL
EINNRNLQIAAASEQQAHVARDVDRSLVSIRDLAVQSSEGTRQTLIASTELSQLAVNLND
LVLRFKT
>tr|A0A0Q0DEB5|A0A0Q0DEB5_PSEAP Uncharacterized protein OS=Pseudomonas syringae pv. aptata OX=83167 GN=ALO85_01185 PE=4 SV=1
MPWYAWLILLVAIGSVVGGLMMLRDTAKKLPLTEEQLKRVHERNAEMDAKDANDR
>tr|A0A0Q0DSL8|A0A0Q0DSL8_PSEAP Uncharacterized protein OS=Pseudomonas syringae pv. aptata OX=83167 GN=ALO85_00875 PE=4 SV=1
MRIFNLLTFAAVFSTSIVFTAPVNAAVSPVSEQSCHFMPVVDSAASLQHTQSVGVLYSEN
TLSNLQYLNNYHSVALRSGSNGALDARIRNAFINSSDPALATDWLVGSLKKEFASVTVYD
TLDALMQARPDVVVMLDTYNRLVSKRNSQVEARFMARFYDNDLQYIGKAEGEVVREMTSV
WVQGKAAPEIAAQIDQQRDLQVNALKQFDASLKALVMQADRAQVAAN
>tr|A0A0Q0BS18|A0A0Q0BS18_PSEAP Aspartokinase OS=Pseudomonas syringae pv. aptata OX=83167 GN=ALO85_01851 PE=3 SV=1
MALIVQKFGGTSVGSVERIEQVADKVKKFREAGDDLVVVLSAMSGETNRLIELARQISDQ
PVPRELDVIVSTGEQVTIALLAMALMKRGVPAVSYTGNQVRILTDSAHNKARILQIDDQK
IRSDLKAGRVVVVAGFQGVDEHGNITTLGRGGSDTTGVALAAALKADECQIYTDVDGVYT
TDPRVVSQAQRLDKITFEEMLEMASLGSKVLQIRAVEFAGKYNVPLRVLHSFKEGPGTLI
TIDEEESMEQPIISGIAFNRDEAKLTIRGVPDTPGVAFKILGPISAANIEVDMIVQNVSH
DNTTDFTFTIHRNDYQAALQVLEATAREISAREVSGDTKIAKVSIVGVGMRSHAGVASRM
FEALAKESINIQMISTSEIKVSVVIEEKYLELAVRALHTAFELDAPRQGE
>tr|A0A0Q0FKZ9|A0A0Q0FKZ9_PSEAP Putative aminoacrylate hydrolase RutD OS=Pseudomonas syringae pv. aptata OX=83167 GN=rutD PE=3 SV=1
MFHEFHACQHADAPTLVLSSGLGGSGRYWADDLALLTRDYRVLVYDHAGTGRSPAVLPGD
YSIRHMAVELLALLDSLDIQRCHFMGHALGGLVGLELALLRPELLQSLILINAWSSPNPH
SARCFSVRKKLLLNSGPEAYVQAQALFLYPADWIAANGPRLADDEAHALAHFPDTDNLLR
RIHALETFDVEADLTRIHTPTLLIANRDDMLVPWQQSRHLANALPNATLVVLDYGGHASN
ITDPQPFQRALLAFLSTQP
>tr|A0A0Q0C524|A0A0Q0C524_PSEAP 50S ribosomal protein L5 OS=Pseudomonas syringae pv. aptata OX=83167 GN=rplE PE=3 SV=1
MARLKEIYRKEIAPKLKEELKLSNVMEVPRVTKITLNMGLGEAIGDKKVIEHAVADLEKI
TGQKVVVTYARKSIAGFKVREGWPIGVKVTLRRDRMYEFLDRLLSISLPRVRDFRGLNAK
SFDGRGNYSMGVKEQIIFPEIDYDKIDALRGLDITLTTTAKNDDEGRALLRAFKFPFRN
>tr|A0A0Q0C3D7|A0A0Q0C3D7_PSEAP Oligopeptide/dipeptide ABC transporter, ATP-binding protein OS=Pseudomonas syringae pv. aptata OX=83167 GN=ALO85_00903 PE=3 SV=1
MNLAKPDITGRDSPDSNAQKPILEGIGLSKHFAVESGFLQPKKPPVQAVNEVNLSVRKGE
TLALVGESGSGKSTLGRLLLNLLQPTAGDVIYEGRNLANLTPEKLRQVRRDLQIIFQDPF
ASLNPRMTVESIVGEPIWLHSQASRSDRQAKVAELLRTVGLAPEHGGRHPHEFSGGQRQR
IGIARALASEPRLILGDEPVSALDVSVQAQVVNLLEDLKHKFGLTLVIVAHGLAVIRHMS

DRVAVMYLGEIVELAPVDALFENPLHPYTQALMAAVPVSHPDLRQPRALLGGDMPSPSRP
PSGCRFHTRCPHARALCKEAAPVMETVEAERQVACHFWREIANAGSATLIIPTPSAAYTQ
RLNLFKHHQSLAVESQP
>tr|A0A0Q0DKL2|A0A0Q0DKL2_PSEAP EthD OS=Pseudomonas syringae pv. aptata OX=83167 GN=ALO85_01227 PE=4 SV=1
MIKISVLYAYEEGARFDHDYYRDRHMPLVQELMGKYCKGYSVDRGIGGATPGSDPVYIGM
CHIYCDSIEDFEAGMGPHSKQINADIINYTDLIPEMQISEVRADVKTAQ
>tr|A0A0Q0DSR5|A0A0Q0DSR5_PSEAP D-Glucarate dehydratase OS=Pseudomonas syringae pv. aptata OX=83167 GN=ALO85_00872 PE=3 SV=1
MKIIRVTVTPIAFRDPPLLNASGIHEPFALRSIIEVESDNGYIGLGESYGDAPALAIQQQ
VQNQLIGLDPFNLNQLRSIVKATVAAHKPASLAGAELAPGSHASKAVSNAYSAFEVAFLD
LQARSMNLPLVDLLGGAIRDQIPFSAYLFFKYAQHADTPYAPDSWGEALSEEQIVAQARR
MIEAYGFKSIKLKAGALQPEHEVSCIKALKKAFPGYPLRIDPNGNWSLETSIRMAELLGD
DLQYYEDPTPGLDGMSELHKRTGLPLATNMVVTDFDEFRRSVALDSVQIVLADHHYWGGL
RDTQALAKMCDTFGLGVSMHSNSHLGISLMAMAHVAASVPNLDYACDTHYPWQEPDEEVI
KGGKLPIVDGCVSITRAPGLGLELDYDQLGKLNDQYHSCGIRQRDDVKQMQKYKPDWKAV
KPRY
>tr|A0A0Q0BWW1|A0A0Q0BWW1_PSEAP Adenylate cye OS=Pseudomonas syringae pv. aptata OX=83167 GN=ALO85_01594 PE=4 SV=1
MQKETEIKLRVSRETLAALREHPLLKKRNKSGWERLELSNQYFDTPEGELAQAKVALRIR
RDGDQIIQTMKTRGQSVAGLSERNEYNWDLSKAKLDLKKLEGDCWPEQLADLDKKTIKPL
FTTDFVREKAEIAWGRGKAKVVIEAALDLGQVVAGKQKEEICELELELREGEPAALLELA
AELAATLPLMPCDISKAERGYRLLDASSYSLSLQAPALTAETPLDDAYSALAWHLLGSSQ
RLAEQYRFNGHWRLLQDWVEMLAELRALTSSLGQAAPRTSTAQLRTALDALLEDWRPLVQ
AGQEDADVRGAAHEQFLEELQDTRWGEFSLNTSRWLLSRSWTAERNTRGNRQGAALLSSW
LPRLLGEEATSLQLSRYQQQPEDLAEQLPRIERIQSWLHWARGALDLPELDRLYGELRKL
EELAHLDISDEVLDARVQQAITVFQSRAWKTLLRL
>tr|A0A0Q0C005|A0A0Q0C005_PSEAP 30S ribosomal protein S16 OS=Pseudomonas syringae pv. aptata OX=83167 GN=rpsP PE=3 SV=1
MLTIRLALGGSKKRPFYHLTVTDSRNARDGSHKEQVGFFNPVARGQEIRLSVNEERVNYW
LSVGAQTSERVAQLLKEHNKTKAAA
>tr|A0A0Q0DU91|A0A0Q0DU91_PSEAP Putative membrane protein OS=Pseudomonas syringae pv. aptata OX=83167 GN=ALO85_03269 PE=4 SV=1
MDAVLRAAAIYFVLMVLFRVAGRRSLTDLTTFDFVLLLIIGEATQQALLGDDFSVTNAIL
IISTLIAIDVGFSLAKRRSKRLAKFLDGGPTVIVEDGKPLTKRMREARISESDVMEAART
TQGIVEMKDIRYAIIERNGEISVIPFK
>tr|A0A0N8T8E8|A0A0N8T8E8_PSEAP Ribosomal silencing factor RsfS OS=Pseudomonas syringae pv. aptata OX=83167 GN=rsfS PE=3 SV=1
MTKQKMSSEDVINVAIAALEEVKGADILTIDVRDKTSIADYMLICTGTSNRQLNALVDNV
RDKVKAAGLKSLSEEGKGDSDWVLLDLGDVIVHVMTAAARQFYDLERLWQGAEQSRAASA
AHHTPGNE
>tr|A0A0Q0IBC1|A0A0Q0IBC1_PSEAP Translation initiation factor IF-2 OS=Pseudomonas syringae pv. aptata OX=83167 GN=infB PE=3 SV=1
MTQVTVKELAKVVDTPVERLLQQMREAGLPHTAAEQVVTDNEKQALLTHLKSGHKAKVEE
PRKITLQRKTTSTLRVAGSKSISVEVRKKKVFVQRSPEEIEAERKREMDERRAVENAARQ
KAEEEAKRRAEEDARNQPAASQPASAAAEPVAAAEPVREASPAAAAAPAPASAAPSADAR
KRDEQRRPDKPRADDRNARGGDGERKNAPHRASVKEKAPAPRVAPRTTDEESDSFRRGGR
GKGKLKKRNAHGFQSPTGPVIRDVAIGETITVGELSAQMSVKAAEVIKFMFKMGTPVTIN
QVLDQETAQLIAEELGHKVTLVSDNALEDSLAESLKFEGESFSRAPVVTVMGHVDHGKTS
LLDYIRRAKVAAGEAGGITQHIGAYHVETERGMVTFLDTPGHAAFTAMRARGAKATDIVI
LVVAADDGVMPQTIEAVQHAVAAGVPLVVAVNKIDKPGADLDRIRSELSVHGVTSEEWGG
DTPFVSVSAKMGTGVDELLEAVLLQAEVLELKATPSAPGRGVVVESRLDKGRGPVATVLV
QDGTLRQGDMVLVGSNFGRIRAMLDENGKPVKEAGPSIPVEILGLDGTPDAGDEMSVLSD
EKKAREVALFRQGKFREVKLARAHAGKLENIFENMGQAEKKTLNIVLKSDVRGSLEALNG
ALNGLGNDEVQVRVVGGGVGGITESDANLALASNAVLFGFNVRADAGARKIVEQEGLDMR
YYNVIYDIIEDVKKALTGMLGSDVRENILGIAEVRDVFRSPKFGAIAGCMVLEGTVYRNR
PIRVLREDIVIFEGELESLRRFKDDAAEVRAGMECGIGVKSYNDVKVGDKIEVFEKVQVA
RSL

>tr|A0A0Q0CII1|A0A0Q0CII1_PSEAP Type 4 prepilin-like proteins leader peptide-processing enzyme OS=Pseudomonas syringae pv. aptata OX=83167 GN=ALO85_03295 PE=3 SV=1
MPLLDLLASSPLAFVTTCCILGLIIGSFLNVVVYRLPIMMERDWKAQSRELLGLPAEPDQ
PVFNLNRPRSSCPHCAHKIRPWENLPVISYLLLRGKCSQCKAPISKRYPLVELTCAVLSA
YVAWHFGFGWQAAAMLVLSWGLLAMSLIDADHQLLPDSLVLPLLWLGLIVNAFGLFTSLN
NALWGAVAGYLALWSVFWLFKLVTGKEGMGYGDFKLLAMLGAWGGWQILPLTILLSSLVG
AVLGVIMMRVRRVESGTPIPFGPYLAIAGWIALLWGGQITDSYMQFAGFR
>tr|A0A0Q0CAG6|A0A0Q0CAG6_PSEAP Uncharacterized protein OS=Pseudomonas syringae pv. aptata OX=83167 GN=ALO85_02213 PE=4 SV=1
MNESKIQHNESILLRILWMLLFLGVWHVAQIILAGVVLVQLVYRLIYGAPNGGLMNFGDS
ISQYLAQIGRFGTFHSDHKPWPFGDWPAPRAPEGEAPHHVPPAAQPVRDEELKP
>tr|A0A0Q0BW91|A0A0Q0BW91_PSEAP SNARE associated protein OS=Pseudomonas syringae pv. aptata OX=83167 GN=ALO85_03101 PE=4 SV=1
MEFNPLDLILHLDVYLDLLVTNYGTWVYAILFLVIFCETGLVIMPFLPGDSLLFIAGAVA
AGGGMDPVLLAGLLMLAAILGDSTNYVIGRTVGEKLFSNSSSRIFRRDYLLRTQDFYARH
GGKTVTLARFLPIIRTFAPFVAGVGRMSYPRFVGFSVLGSVLWVGSLVTLGFFFGNVPFI
KHNLTFLVLAIILLSLVPMVIGVVRSRTARPFDAR
>tr|A0A0Q0D0A9|A0A0Q0D0A9_PSEAP C4-dicarboxylate transport protein OS=Pseudomonas syringae pv. aptata OX=83167 GN=ALO85_03164 PE=3 SV=1
MEISKTRWYSQLYVQVLIGIVLGAAIGYFEPDVGAKLQPFADGFIKLIKMLLAPIIFGTV
VVGIAKMGSIKEVGRIGVKALVYFEILSTIALVIGLIVVNIVKPGAGMNINAGTLDGSAV
SKYSQAASEQGGTIEFFLNIIPTTFLGAFSNGVMLQVILLSVLMGVALVQMGETSKPLIN
TIDLFLQGLFRIVAMVMRLAPLGAGAGMAFTIGKYGIGTLLSLGQLLVALYITTLIFIVV
VLGSVARWSGMPLLQFIRYFKDEILITLGTCSTEAVLPRMMVKLEKLGCKKSVVGMVLPT
GYTFNADGTCIYLTMAAIFIAQATNTPLTFVDQMILLGVFLLTSKGSAGVAGAGFVTLTA
TLTTIHSIPLVGLVLLLGIDRFLNEARAVTNLIGNGIGTLAIAKWDNSFDAEACESEIAA
MNNAKAARKALLAQK
>tr|A0A0Q0CWK7|A0A0Q0CWK7_PSEAP Glutathione S-transferase OS=Pseudomonas syringae pv. aptata OX=83167 GN=ALO85_00217 PE=4 SV=1
MSLHLIIGDKRYSSWSLRPWLVLEMTGAPFTDQVIRLNQPDTRANILKYSPTGKVPALQC
EHGTIIDSLAICEYLVERFPDAGLWPSDTAARAQARSACAQMHSGFVSLRSNMPMDLRQD
QALEVIPVDTQNDIDRIVELWAECRTAAAESGPFLFGKPSIADAFFAPVAIRLHSYRVEL
PEEALKYIETIYQWPAFKRWQQAGLEES
>tr|A0A0Q0CZY4|A0A0Q0CZY4_PSEAP Basal-body rod modification protein FlgD OS=Pseudomonas syringae pv. aptata OX=83167 GN=ALO85_02142 PE=3 SV=1
MAVDSTTANSVSSTFLNTLQNPTPTATNSTGTLGKDSFLQLLVTQMKNQNPLDPQDNTQF
VAQLAQFSSLESMQNLTSTVDTIASSYKSSQALQASSLVGRSVIIDASSTSVDTTKGLTG
SIVVPSESSLTTVKVYDSQSNLVDSVDLGTQKAGTTSFAWDGTDSSGTQLPSGTYTFKAE
GSVEGKNTQLSTYLPATVNSVTMGVNGAETTLNLASGSVALSKVQTIGL
>tr|A0A0N8T7W6|A0A0N8T7W6_PSEAP Bacilysin biosynthesis protein BacA OS=Pseudomonas syringae pv. aptata OX=83167 GN=ALO85_04228 PE=4 SV=1
MDSKFLNLSVGDWREVMIHTLGPSGTSSEAAAGFFTEWLGQRFPESQVQINLSDSYEHAC
AAMDERIPGVLIVANAYPQIHNFYMNPRLSLVATFVFDTPEYGLVSKGPLTTRKLTIATH
PAPLMLIQELLPDGLEVDSVIFSLSTSAAAAAVARGEVDVALTTEVAAHIHGLQFISKTR
PIRMLWSVFAPIQWSTQPVEVSARWR
>tr|A0A0Q0FAW2|A0A0Q0FAW2_PSEAP Putative TraI family relaxase/helicase OS=Pseudomonas syringae pv. aptata OX=83167 GN=ALO85_04039 PE=4 SV=1
MLSFFTRRKASPISSNAAAGFFKPESPDALLSTSRRRQLIENIWQRTSLPREQFETLYMQ
AFKSYAALVQHLPASENHHHAYHGGMLDHGLEIVAYALKIRQMYLLPIGAPPESQAAQSE
AWSAASAYGALVHDLGKIAVDVKVELADGTIWHPWHGPLDQPYRFKYVKGRDYRLHGAAS
SLIYSNVIPAKALDWLSGFPELWSQLVFAFAGQYEHADILGEIVSQADQASVAQELGGNP
GRAMSAPKQSIQRQLAEGLRMLVADKFKLNQPDGPSDGWLTQEGLWLVSKPAVDQLRALL
LSQGTSLSQPRQLVGNKAENHTAPVEEPAKVLPEGSSSHGLNFLTWLRGGVISHRIIIND
AKARVHTVDGTAFLVSPDIFKRYALEHPDIEREAKERDLEAWQVVQRSFEKLKKHRKTGA
GLNIWTCLVKGPRKSKQLRGYLLIEPTDVFSEVPYDNPVISLAELVDKDTSE
>tr|A0A0Q0FHM0|A0A0Q0FHM0_PSEAP Uncharacterized protein OS=Pseudomonas syringae pv. aptata OX=83167 GN=ALO85_01143 PE=4 SV=1
MARQFDLNYEKDTVTKPKIKKNSIKKHENLDESATSSTSVKMASAEKNDADMDALEKIRN
SAFYDEAWYRSNHRDLKNLDIDFALHYFNYGWRQGKSPSFGFSGDMYLAAYPDVKTIDMN

PLLHYETYGKKEKRYVIPYAVEAIYNSTYFDDAYYQKNHNGKSTSRLASALDYITCKDNL
RNPSEHFDADAYLESYPDVKEDGVMPLLHFELHGKKENRTAGLSNPLAKFLTYENNSNIS
HATKRVCLFACYMGDGAIPDETIFLLEKVRSVCDAIVLIGDCGIQPDEFRKIEHLVCHAH
FVRHREYDFGSYKRAFAYADENGLLKNADEILICNDSVVGPCGDINDFFKCREEDGNPEF
YGTTINNFGFRNIQSHGNSLYSPHIQSYFLSIAKSIFNATFWRDFIYSVKHEEHKTDIII
NYEMGMSKLLSSHGYPPHSMYKSIAGLNPAARECMDVLNTALFIKKSMLPGLSPERAGIV
NGIFKSKNFPFQLKDNKIVSNDNQNTHTPIIKNKKLRIVDCATSGNMVSLLTVSEHSYVN
LELIISNDSEFTSTSATSRTEAGKDTYTGLADSYLTQGLHLFIFEFDKKHIEKNAGLTFS
ERGNPVDLQYVYGDIPCYNLLNHKNSGLYPRIEKNTLHLQNKQQSIISIMLSNNYSQTDK
SLYASIINNEIQAKYSLYSERASLTGDNAYEAFKYALKSDDSCFYITSKEVVDRETDEHI
KKHLAILGSAQHKELFLNAKSLFCSFGFPGIIFPGLKDIHISALKYKLYLMWHGVSAGDK
DSYEIASYNGNRNDGIFACSTYEERNFKLLGHDTVYLGGYPRMDKWCNDAPLDVNAAVIF
FTWRRSLYEATLAEFLSSDYVTTIVELVKAIAKSRPKLELYYFIHNSIPAQHVECLAAIL
RAHSSNIRFVNNNDTPTFNHIFNTSQYLITDYSSVGYDFAYYKKRAPIFFMPQKFIKGHY
VPTSLFEKIIPGPKCLDIKSVIKSLRPEQYKNHKSSTKAFFRFMDSENCKRAFDVTHPES
ESAS
>tr|A0A0Q0BXV8|A0A0Q0BXV8_PSEAP Alkylphosphonate utilization operon protein PhnA OS=Pseudomonas syringae pv. aptata OX=83167 GN=ALO85_01090 PE=4 SV=1
MVLLLLDARTIVSLPSCPKCNSEYTYEDGSLLICPECAHEWSADAVGADASDDVKVIKDS
TGNVLQDGDTITVIKDLKVKGSSLVVKVGTKVKNIRLVDGDHDIDCKIDGIGAMKLKSEF
VRKV
>tr|A0A0Q0FNE2|A0A0Q0FNE2_PSEAP 3-oxoacyl-synthase III OS=Pseudomonas syringae pv. aptata OX=83167 GN=ALO85_02759 PE=4 SV=1
MHNVVISGTGLFTPANSISNEELVQSFNAYVAQFNSDNAAAIERGDVQALSESSAAFIEK
ASGIKSRFVMDKEGILDPQRMKPNLPERSNDEWSILCEMGVAAARQALQRAGKTAADIDG
VIVACSNLQRAYPAISIEIQQALGVAGYGFDMNVACSSATFGIQTACNSVQLGQARALLV
ISPEICTAHLNFRDRDSHFIFGDGATAVVIERADLATSPYQFDIVSTRLLTQFSNNIRNN
FGFLNRTSDEGQSAPDKLFVQEGRKVFREVCPMVAELVAAHLQDNGINITDVRRFWLHQA
NLSMNHLIVKKLLGRDASVEEAPVILDTYGNTSSAGSVIAFHTYQDDLPQGALAVLSSFG
AGYSIGSVILRKR
>tr|A0A0Q0DSW9|A0A0Q0DSW9_PSEAP TetR family transcriptional regulator OS=Pseudomonas syringae pv. aptata OX=83167 GN=ALO85_02433 PE=4 SV=1
MSSIRERNKELILRAASEEFADKGFAASKTSDIAAKAGVPKPNVYYYFKSKENLYREVLE
SIIEPILRASTPFNPEGVPAEVLSRYIRSKIQISRDLPFASKVFASEIMHGAPHLTAQQI
EQLNGQARHNIECIQAWIDSGQIAPLDPHHLMFTIWAATQTYADFDWQIATVTGKAKLQD
ADYEAATQTILRLVLKGCEPDR
>tr|A0A0Q0IGM1|A0A0Q0IGM1_PSEAP Glucarate dehydratase OS=Pseudomonas syringae pv. aptata OX=83167 GN=ALO85_00663 PE=3 SV=1
MNIQQSNITSKAPVITEMQVVPVAGHDDMLLNLSGAHGPYFTRNVIILKDNAGHVGVGEV
PGGELIRQTLEDARSLVVGSSIGTYQKILNEARKAFADRDSGGRGLQTFDLRIAIHAVTA
LEAALLDLLGQHLEVPVAALLGEGQQRDQVEMLGYLFYVGDQRKTDLAYRSEPEADNDWF
RIRHEEALTPEAVVRLAEAAHQRYGFQDFKLKGGVLSGDQEIEAVTALAERFPDARITLD
PNGAWSLKEAIRLCRDQHHVLAYAEDPCGAENGYSGREVMAEFRRATGLKTATNMIATDW
REMGHAIQLQSVDIPLADPHFWTMQGSVRVAQMCNEWGLTWGSHSNNHFDISLAMFTHVA
AAAPGTITAIDTHWIWQDGQRLTKAPLQIIGGKVEVPKKPGLGVELDMDQLAKAHELYKG
MGLGARNDAVAMQFLIPDWKFNNKQPCLVR
>tr|A0A0Q0C2X7|A0A0Q0C2X7_PSEAP Uncharacterized protein OS=Pseudomonas syringae pv. aptata OX=83167 GN=ALO85_100988 PE=4 SV=1
MVVAGMGVINSAATRVHPSGPCSSTRSVRVADMSETLFPVTFLL
>tr|A0A0Q0C3M1|A0A0Q0C3M1_PSEAP L-carnitine dehydratase/bile acid-inducible protein F OS=Pseudomonas syringae pv. aptata OX=83167 GN=ALO85_100077 PE=3 SV=1
MGALSHIRVLDLSRVLAGPWAGQILADLGADVIKVERPGCGDDTRSWGPPFLQDAAGQNT
SEAAYYLSANRNKQSVTIDFTRPEGQRLVRELAAKSDIVIENFKVDGLAAYGLDYESLKA
VNPRLIYCSITGFGQSGPYARRPGYDFMIQALGGLMSFTGLPDGEEGAGPVKVGVALTDI
LTGLYSTTAILAALAHRDQSDIGQYIDMALLDVQVACLANQGMNYLTTGVAPKRLGNAHP
NIVPYQDFPTADGDLILTVGNDSQFRKFAAVAGQPQWAEDPRFLTNKLRVAHRAELIPLI
RQVTVFKATAQWVSELEAAGVPCGPVNDLAQVFADPHVVARGLAIELPHALAGRVPQVAS
PIRLSETPVEYTRAPPLLGEHTAQVLQALLGIGEEELTLLREAGVL

>tr|A0A0Q0BRQ6|A0A0Q0BRQ6_PSEAP DNA-binding protein OS=Pseudomonas syringae pv. aptata OX=83167 GN=ALO85_04135 PE=4 SV=1
MKNLQLGQTIRRLRGVCGLSQAELGLRTGFDSNTISRFELGTVTPSVDALYKLAVQLDCS
VRDFFLDFDDDAQKRAYLFNMICDANSEELNRYVELVSTPVKKA
>tr|A0A0N8T8I8|A0A0N8T8I8_PSEAP Flagellar basal-body rod protein FlgG OS=Pseudomonas syringae pv. aptata OX=83167 GN=ALO85_02147 PE=3 SV=1
MLPALYVAKTGLAAQDTNLTTISNNLANVSTTGFKSDRAEFQDLLYQIKRQPGAQSTQDS
ELPSGLQLGTGVRIVGTQKNFTAGSLQTTNNPLDLAVNGRGFFQVTQPDGTIAYSRDGTF
HLDANGQIVTANGYALEPAIVVPQNAQTFTVGQDGTVSVTLAGATATPQIIGNIQTADFI
NPAGLQAMGGNLYLETGSSGAPQIGTPGLNGLGPTLQNTLENSNVSTVEELVNMITTQRA
YEMNSKVISTADQMLQNLTQNL
>tr|A0A0Q0CBS6|A0A0Q0CBS6_PSEAP Tyrosine recombinase XerC OS=Pseudomonas syringae pv. aptata OX=83167 GN=xerC PE=3 SV=1
MDQHLDAYCMHLRSERQVSPHTLEAYRRDLGKVLAYCQKAQLSSWSDLDIQHLRSFTARQ
HQQGQSSRSLARMLSAVRGFYKYLNREGICQHDPANGLSPPKGERRLPKTLDTDRTAQLL
DGGVEDDFLAHRDQAILELLYSSGLRLSELTGLNLDQLDLRDGLVQVLGKGSKTRVLPVG
SKARQALEVWLPLRALTHPQDDAVFVSQQGKRLGPRAIQVRLKAAGERELGQNLHPHMLR
HSFASHLLESSQDLRAVQELLGHADIKTTQIYTHLDFQHLATVYDSAHPRAKRKGAADD
>tr|A0A0Q0DMI2|A0A0Q0DMI2_PSEAP FrnE-like thiol oxidoreductase OS=Pseudomonas syringae pv. aptata OX=83167 GN=ALO85_03539 PE=4 SV=1
MSARLIYVMDPMCSWCWGFAPVADALVQQARAAGVPLHLVMGGLRSEGAALEPAKRRYIL
EHWQAVEEATGQPFCREGALPEGFVYDTTPACLAVTAARQLDPDRAWELVGLIQRAFYSE
ARDVTRPSLLAELAEQTGLSRQAFADEFESPQRQAATAADFAWAQDLGIAGFPTLLAERN
GQLALLTNGYQPLSSLSPLLGRWLERAASA
>tr|A0A0Q0DTZ8|A0A0Q0DTZ8_PSEAP Lipid-A-disaccharide synthase OS=Pseudomonas syringae pv. aptata OX=83167 GN=lpxB PE=3 SV=1
MSSPLCVALVAGEASGDILGSGLMRALKVRHPDIRFIGVGGPLMEAEGMQSSFPMERLSV
MGLVEVLGRLRELLARRKLLVQTLINEKPDVFIGIDAPDFTLNIELQLRRAGIKTVHYVS
PSVWAWRQKRVLKIREGCDLMLTLLPFEARFYEEQGVPVRFVGHPLADTIPLESDRAGAR
AGLGFAQDTPVVALMPGSRGGEVGRLGGLFFDTAELLLARRPDLRFVLPCASPQRRAQVE
QLLQGRDLPVTLLDGQSHVALAACDAVLIASGTATLEALLYKRPMVVAYRLAPLTFWILK
RMVKSPYVSLPNLLAQRLLVPELLQDDATPEALARTLLPLIEDGREQTAGFDAIHRILRR
DASNQAADAVLSLLGRSPSL
>tr|A0A0Q0C2D2|A0A0Q0C2D2_PSEAP Signal peptidase I OS=Pseudomonas syringae pv. aptata OX=83167 GN=ALO85_02000 PE=3 SV=1
MSLNFPLLLVIAVFVCGVLALIDLVILAPRRRAAISNYQGSVGEPDIAVVERLNKEPLLV
EYGKSFFPVLFIVLVLRSFLVEPFQIPSGSMKPTLDVGDFILVNKFAYGIRLPVLDQKVI
QIGDPQRGDVMVFRYPSDPSVNYIKRVVGLPGDRIRYTSDKRLFINGELVAKKLIGTEPG
TLGSAELYEEQLGEVEHQIRQEMSRYRAPPDSEWTVPAAHYFMMGDNRDNSNDSRYWDDP
NIPKDELGMVPDKNIVGKAFAVWMSWPEPKLSHFPNFARVGLIK
>tr|A0A0Q0DZ33|A0A0Q0DZ33_PSEAP Energy transducer TonB OS=Pseudomonas syringae pv. aptata OX=83167 GN=ALO85_200016 PE=4 SV=1
MYRVYNLRPQPAPANWAHDNRQLLVGLVFWSLFLAGIADNQKISSEGWYVVTPLEGALVT
MRMPSEKSCKADVAQTCVPGYELRAQQH
>tr|A0A0Q0BX29|A0A0Q0BX29_PSEAP Secretion protein HlyD OS=Pseudomonas syringae pv. aptata OX=83167 GN=ALO85_01670 PE=3 SV=1
MKHMSCLPLGGLLLSGFLLSGCGKDETPPEPVRPVVFVEAKPESNQDFGRFAGNIQARYQ
SVLGFRVAGRIARRDVDVGAEVKKGDLLATLDPTDQQNAVRARQGDLANVQAQYINAQAN
ARRQQELFDRGVGAQAQLDIALTDLKTASSSQEQARAAAQQARDQLSYSELRSDHDAVVT
EWKVEAGQVVTAGQEVVTLARPDIKEAVIDLPDTLADQLLSDVVFTVASQLNPEVNTTAT
IREIEPQADRTTRTRRARLSLAQTPAAFRLGTAISVTLSSTITPRMRLPINALQEVDGKR
QVWIVDSQSQTVNPRVVTIASRGSDSFVLTDGVKAGEKVVSAGVNSLKPGQKVKVDEESP
R
>tr|A0A0Q0IJD3|A0A0Q0IJD3_PSEAP FAD-dependent pyridine nucleotide-disulfide oxidoreductase:BFD-like-binding region OS=Pseudomonas syringae pv. aptata OX=83167 GN=ALO85_100079 PE=4 SV=1
MATSSRLDVRNPDSPRVVIIGAGPAGTRCAETLLAAGIKPILIDENRRDGGQIYRRQPEG
FKRDYSALYGTEAAKARDLHESFDRLRPQIDYRPDTLAWNLTHGELCCASQGIHTVVEYD
ALILCAGATDRLMPIKGWQLAGTYSLGGSQIALKSQSVSIGNRVVFMGSGPLLYLVASQY
VKAGADVAAVLDTSPFGKRVHAMPKLLARPGLLFNGMKLLGQLYSAGVPVHLGVQPEEII

GSEQDGVTGVRVKLANGTALNVDCDALALGYHLRPETQLADLAGCQLRFDEASGQWLLAV
DEEGRTSVKGFYAAGDGAKIRGADAAEQAGRLVAMALLEDLDLPVDNSRQAEVRKSLVAM
DRFRVGLAEAFPWPAAQAAALPDDAIVCRCEMISAGELRGVVREKGACEVNRAKAFSRVG
MGRCQGRYCSQAGAEVIAAEAGVPVEQVGRQRGQAPVKPLSMLIDEVTS
>tr|A0A0Q0DC74|A0A0Q0DC74_PSEAP Uncharacterized protein OS=Pseudomonas syringae pv. aptata OX=83167 GN=ALO85_04880 PE=4 SV=1
MGARKITLNFPVLGHSLIYAAQKNRKSRMTASIVMFNCRGTIFMTSLQVMTGDAVVKTVV
EEARSKTVYQSWMNERAPEHQHDAVMFLNHCLALAEREHCDLPAQPEALEQWMEQNVGVV
ADQYALYLEQRRAGKPRRFFKSKAHAMYFIQQVAPTKMVDGAWLYGLLPRWADYRFHGLI
RTYLEELGDGEQAQNHVSLYRKLLADLDCDTSAPLTDDAYLQGAIQLSLGQLSEQYLPEV
IGYNLGYEQLPLHLLITSFELNELGIDPYYFTLHVTIDNASTGHARKAAQSVLELMPVGQ
ERDEFYRRVASGYRLNELGMGSTQVIQSFDLEQEVIAMLERKRTFGQHMHSDYCRLDGKT
VNEWLAEPDQIPEFLAVLENRGWIKRHEDPLHSRFWKLFEGAGAPMFGVFNGYEKQLVHD
WIAGEWLSDGSAQPTVGKRLPEAFRARFRNLQDGAQPSPALAVPASADADPDVRELHRKL
ESAPASEKMSTLIESMSPARHATAAGLQATRLFVSSMTERMTGAHA
>tr|A0A0Q0DLM1|A0A0Q0DLM1_PSEAP Methionine ABC-type transport system, permease protein OS=Pseudomonas syringae pv. aptata OX=83167 GN=ALO85_01621 PE=3 SV=1
MDAFLNLFSNVDWYEIWVATGDTLIMLGVSLGFTILLGLPLGVLMFLTSPRQLLEHKQLY
ATVGVIVNMLRALPFIILLIVMMPLTEIITGTSLGILGTLPPLIAGATPFFARLVETALR
EVDRGIIEATQAMGATTRQIIVKALLPEARPGILAAITVTAIALVSFTAMAGAVGAGGLG
DLAIRYGYQRFQNDVMFVTVVLLLVLVQILQTIGDRLVAHFTHR
>tr|A0A0Q0DKU3|A0A0Q0DKU3_PSEAP Type III secretion system effector HopAZ1 OS=Pseudomonas syringae pv. aptata OX=83167 GN=ALO85_01172 PE=4 SV=1
MTFSIRGGAGPSTYYTQPQPQPKPFDELKLEPEKIQQKTAKLEKEGYFSVRMSEHTKSYL
LEQSRLSNPAWRNETVGKVVDLRTTEHERSVTAVAKDIAASLTGRKQQLRPHEFQLRRAD
NPRSEQWHQDRAPLKVICISAIEGRGTEFVTSPDSKAVFTNGEYAEMNPVSAEVVAQKTK
EAKQDRFYFFAGKGITEESIPKLVHRSPSETGRSIFMARWK
>tr|A0A0Q0C1E5|A0A0Q0C1E5_PSEAP Unsaturated fatty acid biosynthesis transcriptional regulator DesT OS=Pseudomonas syringae pv. aptata OX=83167 GN=ALO85_01924 PE=4 SV=1
MLPRAEQKQQTRRALLDAAHQLMESGRGFGSLSLREVARTAGIVPTGFYRHFEDMDQLGL
ALVSEVGQTFRETIRLVRHNEFAMGGLIRASVKIFLERVAANRSQFLFLAREQYGGSLKV
RQALGALREGISADLTADLAKMPKWQHLNADALSIIADLVVKSVFAMLPELIDPPPASLA
PHLTPQAKITQQLRFIFIGARHWRGLGSHD
>tr|A0A0Q0IFX5|A0A0Q0IFX5_PSEAP Uncharacterized protein OS=Pseudomonas syringae pv. aptata OX=83167 GN=ALO85_00664 PE=4 SV=1
MRHLARTRPRLTLAALVGLTAGIAWSWLIPHSTLTQSLLTGWDTGVWLYLIMIFIRTLRS
DADDVRRVAMREDENAGVILVTVCVAALASLAAIIFEIANSKGLSNDAKMLHHAFTGLTV
IGSWLMIGVIFSLHYARMFYTWKGKEPALAFVGGEKNPDYWDFLYFSFTLSVAVQTSDVG
VATREMRKVVLGQSLICFVFNTAILGFSINIAASLFN
>tr|A0A0N8T831|A0A0N8T831_PSEAP tRNA pseudouridine synthase B OS=Pseudomonas syringae pv. aptata OX=83167 GN=truB PE=3 SV=1
MAQVKRIRRNVSGIILLDKPLGFTSNAALQKVRWLLNAEKAGHTGSLDPLATGVLPLCFG
EATKFSQYLLDSDKSYETLAQLGKTTTTADSEGEVLLTRPVTVGRDDIEAVLPHFRGQIS
QIPPMYSALKRDGQPLYKLARAGEVVEREPRSVTIARLELLACEGDTARLSVDCSKGTYI
RTLVEDIGEKLGCGAYVAELRRTQAGPFTLAQTVTLEELEQVHADGGNEAVDRFLMPSDS
GLLDWPLLKFSEHSSFYWLHGQPVRAPDAPKFGMVRVQDHEGRFIGIGEVAEDGRIAPRR
LIRSE
>tr|A0A0Q0D6Z8|A0A0Q0D6Z8_PSEAP Polar amino acid ABC-type transport system, permease protein OS=Pseudomonas syringae pv. aptata OX=83167 GN=ALO85_00971 PE=3 SV=1
MYQPPGWLQELWNAREVLWSGFLTSIQCSALAIAAGTLIGMLAGLVLTYGGFFARLPIRL
YVDLIRGTPVFVLVLAVFYMVPALGWQISAFQAGAIGLTLFCGSHVSEIVRGALQAIPRG
QLEAGKAIGLRFNQSLRYVLLPQAMRQILPTWVNSSTEIVKASTLLSVIGVAELLLSTQQ
VIARTFMTLEFYLFAGFLFFLINYAIELLGRQIEKRVALP
>tr|A0A0N8T983|A0A0N8T983_PSEAP Putative 4-aminobutyrate aminotransferase and related aminotransferase OS=Pseudomonas syringae pv. aptata OX=83167 GN=ALO85_00919 PE=3 SV=1
MSNSNIQAIRGDMPNGFNPANVDRLDAAAREHIQRRIDLLGPAYRLFYNEPVDVVRGKGA

YLYDKDGNEYLDAYNNVVSVGHCHPRVIAAITEQMNKLCSHTRYMQDGILDYAEQLRATA
DGEMGADGHLMFTCSGSEANDLAIRIARHHTGNTGVIVTAEAYHGNSATVAAFSPSLGLN
AKLDPFVRRVPAPDSYRIPREALGRYMADQVAAQIRDIQRHGGGVAAFIADCLFSSDGVF
CDPVDVFGPIAEVVRKAGGLFIADEVQSGFGRSGTHFWGHQRHAVEPDIITMGKPMGNGY
PVAGLMVRPEVVASFGRDMRYFNTFGGNSVAIAAAQATLDVITEEQLMANANRVGSLIMA
GLKDLAARYEHIGDVRGTGMYFAVELVKDRASKTPDMQTALRLVNHLREKRVLISATGPD
ASILKIRPPLIWTAGEAGRLLDALESAFAEL
>tr|A0A0N8TA81|A0A0N8TA81_PSEAP Putative chloramphenical resistance permease RarD OS=Pseudomonas syringae pv. aptata OX=83167 GN=ALO85_05519 PE=4 SV=1
MQAANPRRGYILGLSAYIIWGLFPIYFKAIAAVPAIEIIIHRALWSALFGSIVLMFWKHP
GWWRDLRNNPQRLAVLALSGTLIAANWIVYVWAVNNGRMLEASLGYYINPLVNVLLAMLL
LGERLRRLQMLAVAFAAIGVAQHVWHVGSLPWVSLALALTFAFYGLIRKKAPVAALPGLV
VETWMLVPLALIWLTLNPTAVSAQPEFWTTTQAIWLAAAGPVTLVPLVCFNAAARHLPFT
TLGFLQYIAPTLVLLLAVLLYGEHLTTSTIITFAFIWTGLAIYSVDIWLKSRSRH
>tr|A0A0N8TA11|A0A0N8TA11_PSEAP tRNA U34 carboxymethyltransferase OS=Pseudomonas syringae pv. aptata OX=83167 GN=cmoB PE=3 SV=1
MIDLAPLVRRLAGTPLADWANGLQAQLDTKMAKGHGDLQRWQSALDALPDLQPERIDLID
SFTLEAECNGETRTVLRKALLGLSPWRKGPFNVFGVHIDTEWRSDWKWSRVSPHLDLKGK
RVLDVGCGNGYYQWRMLGAGADSVIGVDPNWLFFCQFQAMQRYLPDLPAWHLPFALEDLP
ANLEGFDTVFSMGVLYHRKSPIDHLLALKDCLVKGGELVMETLVVPGDVHQVLVPEDRYA
QMRNVWFLPSVPALELWMRRAGFTDVRCVDVSHTTVDEQRSTEWMRFQSLSDYLDPTDHS
KTVEGLPAPMRAVIVGRKP
>tr|A0A0Q0DZ02|A0A0Q0DZ02_PSEAP Orf12 OS=Pseudomonas syringae pv. aptata OX=83167 GN=ALO85_200012 PE=4 SV=1
MAVKKASETANRRGQVSEEASERLARELADKPYDSTPLAKAPDKRKPVPVSISLPPGILE
KIEDDVRENKRSGKAHRTVSAIVRHALEAQGYKLD
>tr|A0A0Q0BX44|A0A0Q0BX44_PSEAP Outer-membrane lipoprotein LolB OS=Pseudomonas syringae pv. aptata OX=83167 GN=lolB PE=3 SV=1
MFLRHVLVFSLIALLTGCAGLTSREAVQGKGDPAQWREHKQQLSSLDGWQINGKVGIRAP
KDSGSGTLFWLQRQDYYDIRLSGPLGRGAARLTGRPGAVVLEVANQGRYEATSPESLLQD
QLGWKLPVSHLVWWVRGLPAPDSKSNLTLDGDSRLASLEQDGWQVEYLSYVEQNGYWLPE
RVKLHGQDLDVTLVIKDWQPRKLGQ
>tr|A0A0Q0CEI2|A0A0Q0CEI2_PSEAP Polar amino acid ABC-type transport system, permease protein OS=Pseudomonas syringae pv. aptata OX=83167 GN=ALO85_00970 PE=3 SV=1
MNYQLNFDAVWRDFPSLLAGLGLGLELALISIAIGCVIGLANAFALLSRYKALRIVASIY
VTVVRNTPILVLILLIYFALPGLGIRLDKLASFIVTLSLYAGAYLTEVFRAGLLSIHKGQ
REAGLAIGLGEWQVRAYIIVPVMLRNVLPALSNNFISLFKDTSLAAAIAVPELTYYARKI
NVESYRVIETWLVTTALYVVACYVIALLLRTLEQRLAIRR
>tr|A0A0Q0BWX7|A0A0Q0BWX7_PSEAP Methionine ABC-type transport system, permease protein OS=Pseudomonas syringae pv. aptata OX=83167 GN=ALO85_01673 PE=3 SV=1
MWVDRLLQGTLDTFLMVGVSSLIALLLGIPLAVILVTSSKGGIYEAPGINRVLGGFVNLF
RSIPFLILMVALIPFTRMIVGTTYGVWAAVVPLTIAATPFFARIAEVSLREVDHGLVEAA
QAMGCRRWHIIWHVLLPEARPGIVGGFTITLVTMINSSAMAGAIGAGGLGDIAYRYGYQR
FDTQVMLTVIVLLVAVVALVQLGGDRLARSFDRR
>tr|A0A0Q0CVJ6|A0A0Q0CVJ6_PSEAP Uncharacterized protein OS=Pseudomonas syringae pv. aptata OX=83167 GN=ALO85_04177 PE=4 SV=1
MAIEEVEIRSLGDLVTLSLGCELKNIKLPEDLLVRLKISKKEKAEYLDASAVDRFRNNLL
DQVSEMSNGAPLNTLSLEALQDINAELRVRDLRTFLRQS
>tr|A0A0Q0DQC2|A0A0Q0DQC2_PSEAP Homoserine/lysine/threonine efflux protein, LysE/YggA family OS=Pseudomonas syringae pv. aptata OX=83167 GN=ALO85_01742 PE=4 SV=1
MYLAEFLTVALIHLLAVASPGPDFAVVVRESVAHGRKAGTWAALGVGSAIFLHVGYSLLG
IGIIVSQSIVLFNALKWAAAAYLLYIGIKALRAKPAAATADVAIKATPCERTARGAYISG
FVTNGLNPKATLFFLSLFTVVINPHTPLLVQGGYGVYLAVATAAWFCLVARLFSQARVRA
GFARMGHWFDRAMGGVLIALGIKLALTEVH
>tr|A0A0Q0CEC7|A0A0Q0CEC7_PSEAP Integral membrane protein OS=Pseudomonas syringae pv. aptata OX=83167 GN=ALO85_100220 PE=4 SV=1

MKQSLFETRHQQEWQRFEQRLTALEQGKGDVSGGANFPGDYRRLCHHLALAQERGYSSHL
VDPLHQLAMRGHQQLYRHRSTAGVRLLSFVLSGFPRRVRAEWRFVLVASLLLFGSLIGMA
LLVYGFPQLIYSVVSPEQLADMQSMYDPSASRVGQPVERGSDTDWMMFGYYIMNNIGIAF
QTYASGLLFGLGSLFFLLFNGLMTGAIAGHLTDIGYGQTFWPFVIGHGAFELTAIALAGA
AGLKLGWALLAPGRLTRSAALRIAARASMQLVGGVILFLLIAAFIEAYWSSMTWPGPLLK
YLTGAVLWVLVAAYLTLAGRTPHAPD
>tr|A0A0Q0ISI5|A0A0Q0ISI5_PSEAP NAD/NADP-dependent betaine aldehyde dehydrogenase OS=Pseudomonas syringae pv. aptata OX=83167 GN=betB PE=3 SV=1
MARFELQKLYIDGGYVDASNPETFDAINPANGEVLAQIQRAGKDDVERAVVAAEKGQKIW
AAMTAVERSRILRRAVDILRERNDELAALETLDTGKAISETRYVDIVTGADVLEYYAGLV
PAIEGEQIPLRDSSFVYTRREPLGVVAGIGAWNYPIQIALWKSAPALAAGNAMIFKPSEV
TSLTTLKLAEIYTEAGVPNGVFNVLTGSGREVGTWITEHPRIEKVSFTGGTDTGKKVMAS
ASSSSLKEVTMELGGKSPLIVFDDADLDRAADIAMMANFYSSGQVCTNGTRVFVPNALKA
EFEAKILERVKRIRAGNPEDENINFGPLVSFEHMESVLGYIAKGKEQGARLLCGGDRLTG
GVFDKGAFVAPTVFTDCTDEMTIVREEIFGPVMSILGYDTEDEVVRRANDTDFGLAAGIV
TRDLNRAHRVIHLLEAGICWINAWGESAAQMPVGGYKQSGVGRENGISSLAQYTRIKSVQ
IELGDYASVF
>tr|A0A0N8T9B5|A0A0N8T9B5_PSEAP TetR family virulence regulator TvrR OS=Pseudomonas syringae pv. aptata OX=83167 GN=ALO85_02622 PE=4 SV=1
MKVRTEARREAIIDAAASVFLEMGYERASMNEVTKRMGGSKATIYSYFPSKEELFIAVVD
RLATAHLADAVSELAIQDDRPIDLRTLFTRFGERMLMVLINDDKALAVYRMVVAESGHSD
IGMMFYESGPSQCLQTVTSMMALAMRRGQLRDTDPHIAALQLTALLTAETEIRLYQQAPM
PLSIEEIRALVKRAVDTFMLGMEKR
>tr|A0A0Q0DC06|A0A0Q0DC06_PSEAP Uncharacterized protein OS=Pseudomonas syringae pv. aptata OX=83167 GN=ALO85_01528 PE=4 SV=1
MSFFQNVALQPGESLNSLLMRKAELNGYGCAQALLGERGLKLTAAYSSPELAQICECFEL
NEEQLSTTLIGQTHYLGQPSYLRNGHSPVCPECLASDGYAKDAWSHLLVTACPTHGTVLL
GNCPDCEKPISYHRRTLELCDCGYDLRLAVSVQASEFAMKLSALMADAGVGEHKGFTEIC
RRQGFEPLLPDFLTLLAKHQAQRSGLPMAKPRFSSSSPLQDSLVMVGCLESLLSDWPARF
DAAMQNQLRSGHGVGLAERIGSWYRELFSTYSSSSFDFVRDQFRAQVAEHFDGRLGLNTR
AMMFGPDNAEAMQWFSAAEAARLLGVAPDILANLVIKQAVIGRVHQEGKSRFVAIHRSTL
DQIASARAEYLSATEARRRLNVSKVFFERVVQAGGLRRYKRDERPVLVAGEFLATEIDAV
VAQLIKRVRKKARVSLPIGIQDISVRHGISNAKIVAVLQDILQGTICPTGHVPALPGLAG
LQFDQAEIEQRVRDNNPDVALSVDHLAQISGWKPAVIKKWIQGGYLNAVEEKHGKAKRDV
VPVSALVRFLLTYVPTAEISKQLETKTPYLLQSLRPAKIECIVPPQEAGGAHRGLLLRSA
DLARGAQLRKPTIRDLANQMAQMDFIQC
>tr|A0A0Q0BR59|A0A0Q0BR59_PSEAP Type III effector HopH1 OS=Pseudomonas syringae pv. aptata OX=83167 GN=ALO85_100599 PE=4 SV=1
MSSNHILVRSTQLAFRITMITPSRYPGIYIAPLSNEPTAAHAFKEQAEEALDHISAAPSG
DKLLRKISTLASQKDRKVTLKEIEINNQCYTEAVLSRRQLEKYEPENFNENRHIASQLSR
KGTFTKGEGSNAIIGWSPDKASIRLNQNGSPLHLGMDNDDKITTLAHELVHARHVLGGSS
LADGGDRYNPRTGSGKEELRAVGLDKYRYSLTKKPSENSIRAEHGLPLRMKYRAHQ
>tr|A0A0Q0CIN6|A0A0Q0CIN6_PSEAP Rubredoxin OS=Pseudomonas syringae pv. aptata OX=83167 GN=ALO85_01408 PE=3 SV=1
MKKWQCIVCGLIYNEADGWPDDGIAPGTLWQDVPEDWMCPDCGVGKMDFEMIEIG
>tr|A0A0Q0DQ16|A0A0Q0DQ16_PSEAP Ribose 1,5-bisphosphate phosphokinase PhnN OS=Pseudomonas syringae pv. aptata OX=83167 GN=phnN PE=3 SV=1
MVGRLIYLIGPSGSGKDSLLDAARDTLAQRDCRIVRRVITRSAEAVGEAAQAVTVEQFEQ
MRERGAFALSWQANGLHYGIPVEIDQWLLEGNDVLINGSRAHLQKAQARYPTLLAVLLTV
NQEVLRKRLLARNRESLAEIEERLARNSRFAGDLLASNPHVFPLDNSADLQHTVATLLGL
LDKRHACA
>tr|A0A0Q0BUU3|A0A0Q0BUU3_PSEAP Aminotransferase s-III OS=Pseudomonas syringae pv. aptata OX=83167 GN=ALO85_02108 PE=3 SV=1
MSEHISESISFVHNITLSHGRNAEVWDTAGKRYIDFVGGIGVLNLGHCNPQVVTAIQDQA
AKLTHYCFNATPHDPYIRFMEQLTQFVPVSYPLSGMLTNSGAEAAENALKIVRGATGRTA
VIAFDGGFHGRTLATLNLNGKVAPYKQKVGVLPGPVFHVPFPSKDNGVSTEQALMAMDRL
FSVEIDVNDVGCIIFEPVQGEGGFLAMQPDFAQALRAFCDKHGIVLIADEIQSGFGRTGQ
RFAFSRLGIEPDLILLGKSIAGGIPLGAVVGRKPLLDNLPKGGLGGTYSGNPIGCAAGLA
SLAQMTDANLSSWGEQQEHAIVSRYEAWQASRLSPYLGRLTGVGCMRGIELITAEGEPGT

RQLAELLSSARDAGLLLMPSGKSRHIIRLLIPLTIEPDVLHEGLDIFERCLAALA
>tr|A0A0Q0CAE3|A0A0Q0CAE3_PSEAP Ribosomal protein alanine acetyltransferase OS=Pseudomonas syringae pv. aptata OX=83167 GN=ALO85_03041 PE=4 SV=1
MQYAGEGFSLRILAFDDAELINRYENTHRQHLQPWEPLRDDDYFILENARSRVQQQWESM
QAGSAVFFLITAPGGELLGRCSYTNIVRGAFQACNLGFSLAETSQGQGLMKRTLEVTNRY
CFEHMGLHRIMANHLPHNLKSQRLLQSLGFEQEGYARAYLKIAGVWEDHVLRALINPNHA
>tr|A0A0Q0DAU3|A0A0Q0DAU3_PSEAP Uncharacterized protein OS=Pseudomonas syringae pv. aptata OX=83167 GN=ALO85_03447 PE=4 SV=1
MNMLSVSFFTGTPGALRSWLIAALLMGLAGCSSMVTPEMKRLPDRVELTSIPFFRGNAYQ
SGPMVLASMLANQQVQTTPGLLDKPLQLPGAEDRLEQNMQKVAREYGFMVYPLDGQLQDL
LTQVSAGYPVMLRFAQGSVLWKGPRYAVLIGYNRVKETVLLNAGMDRRYSMSFSSFASAW
KDAGSWAVLIQSPRQLPANVDAQRWLQAAEALSTSGQEQAAGEAKRTLARGVK
>tr|A0A0Q0C5Q1|A0A0Q0C5Q1_PSEAP Ribosome-binding factor A OS=Pseudomonas syringae pv. aptata OX=83167 GN=rbfA PE=3 SV=1
MAKEYSRTQRIGDQMQRELAQLIRREIKDPRVGLVTITAVDVSRDVGHAKIFMTVMGQDS
AEEIAQTIKVLNAAAGFLRMQLAREMKLRSVPQLHFHYDESVVRGAHLSALIERAVAEDG
QHQEGPASPDAKPESTEE
>tr|A0A0N8T8G7|A0A0N8T8G7_PSEAP dTDP-4-dehydrorhamnose reductase OS=Pseudomonas syringae pv. aptata OX=83167 GN=ALO85_01140 PE=3 SV=1
MKILLLGKNGQVGWELQRALAPLGEVIALDRQGADGRCGDLADLERLAATVRALAPDVIV
NAAAYTAVDKAESEPDLAMLINGEAPGVLAREAAALGAWLIHYSTDYVFDGSGEQQWRED
AATGPLSVYGRSKLKGEQAIQASGAKALILRTSWVYAARGHNFAKTMLRLATERDSLNVV
ADQYGAPTGAELIADVTAQILHRVRADQNPAALAGIYHLAAAGETSWHGFAQFVLEHAAL
NGVALKVAPDQIGAIPTEAYPVPAPRPRNSRLALSKLETAFQLKMPSWQQGAQRMLDEIQ
R
>tr|A0A0N8T9J0|A0A0N8T9J0_PSEAP 50S ribosomal protein L3 OS=Pseudomonas syringae pv. aptata OX=83167 GN=rplC PE=3 SV=1
MTIGVVGRKCGMTRIFTEEGVSIPVTVIEIEPNRVTQFKTEETDGYRAVQVTVGERRASR
VTAAQAGHFAKANVAAGRTVMEFRLEEGDYQAGDQINAEIFAAGQLVDVTGQSKGKGFQG
TIKRWNFRGQDNTHGNSVSHRVPGSIGQCQTPGRVFKGKKMSGHMGAERVTVQSLEVVRV
DAERNLLLVKGAVPGATGGNLVVRPAAKARG
>tr|A0A0Q0BRG7|A0A0Q0BRG7_PSEAP Uncharacterized protein OS=Pseudomonas syringae pv. aptata OX=83167 GN=ALO85_04162 PE=4 SV=1
MHRRRLAIALICLSAPCFASAATAKKVAKKVELPDCATTAYDGSNLSQAESDYMFSMQKN
LMTSEALGFAIGNDTILGDYYKMRKGADFNADVMRLRELAKQPTLAKLMYRDFKTANDSV
GHQIDDHKWSKETYDILFCRIAAIGDTGTNAYNTYATSYLKELQAKQDAMHGQVGYFDVT
VNDCTVSKSIDTGNRYTTVKPDPDARFLTVNATFKNTDNEGRLPLEGSLFINMDGKDYKF
DTTESIMSDGYGIRMRSLNPLIKLNTKIVYKLPNELAGDVYWKPGRNAEDKKLWCTYLPS
A
>tr|A0A0Q0FDH2|A0A0Q0FDH2_PSEAP Arabinose 5-phosphate isomerase OS=Pseudomonas syringae pv. aptata OX=83167 GN=ALO85_00171 PE=3 SV=1
MNQSSDLIQSAQRTIRLEIEAIQGLLERLDGDFVRACEMILASKGRVVVVGMGKSGHIGN
KIAATLASTGTTSFFVHPAEASHGDMGMITRDDIILALSNSGSTNEIVTLLPLIKRLGIK
MISLTGDSESILAKAADINLNAHVVHEACPLNLAPTSSTTAALVMGDALAVALLDARGFT
AEDFAFSHPGGALGRRLLLKVENVMHSGESLPSVQRGTLLRDALLEMTRKGLGMTAIVEA
DGTLAGIFTDGDLRRTLDRPVDIRQTIIDEVMTLHGKTAHAEMLAAEALKIMEDNKISAL
VVVDQNDRPVGAFNLQDLLRAGVM
>tr|A0A0N8T9K2|A0A0N8T9K2_PSEAP Methylenetetrahydrofolate reductase OS=Pseudomonas syringae pv. aptata OX=83167 GN=ALO85_02954 PE=3 SV=1
MSQERRFSFEFFPTKTDAGHEKLLTVARQLATYNPDFFSCTYGAGGSTRDRTLNTVLQLE
NEVKVPAAPHLSCVGDSTEDLRKLLAQYKDAGIKRIVALRGDLPSGMGMASGELRHANDL
VKFIREETGSHFHIEVAAYPEMHPQARNFEDDLQHFVNKANAGADSAITQYFFNADSYFT
FVERVEKLGVSIPIVPGIMPITNYSKLARFSDACGAEIPRWIRKQLEAYGDDVQSIQAFG
EEVITQMCERLLQGGAPGLHFYTLNQADPSLAVWNNLQLPR
>tr|A0A0Q0BRU2|A0A0Q0BRU2_PSEAP Aminomethyltransferase OS=Pseudomonas syringae pv. aptata OX=83167 GN=ALO85_04302 PE=3 SV=1
MPDRAARTGNAIGTARSNKESNMSTESLLTTPLHALHRELGAKMVPFAGYDMPVQYPAGV
MKEHLHTRAQAGLFDVSHMGQIRLTGADAAKALEALVPVDIIDLPVGMQRYAMFTDENGG
ILDDLMVANLGNDQLMLVVNAACKNQDLAHLCKHLAGHCKIEPLFEERALLALQGPAAVT

VLARLAPEVAKMTFMQFASVTLLGAKCYVSRSGYTGEDGYEISVPAEQAEALARRLLEEP
EVAPIGLGARDSLRLEAGLCLYGHDMDTQTSPIEASLLWAISKVRRADGARAGGFPGAER
IFAQQQNGVSKKRVGLLPQERTPVREGTEIVDEQGTVIGTVCSGGFGPSLAGPLAMGYLP
NDYTALNTPVWALVRGKRVPMLVAKMPFVAQRYFRG
>tr|A0A0Q0BTU5|A0A0Q0BTU5_PSEAP Uncharacterized protein OS=Pseudomonas syringae pv. aptata OX=83167 GN=ALO85_01062 PE=4 SV=1
MRDTPIIERIDTPELLQRNPEQNVNGWERIGSLAGGVLLMGKGVRRGGFLGLIQLAIGGA
ALARGISGHCSAKSLMEKGRSDLQSARSTIERAGAQLNNLKDDAEDAVKTAAAKGVEALT
EPKPVI
>tr|A0A0Q0IS07|A0A0Q0IS07_PSEAP Flavin reductase domain protein OS=Pseudomonas syringae pv. aptata OX=83167 GN=ALO85_03428 PE=4 SV=1
MIEPGIYKEVMASFPSGVTIVTTLDPEGNLFGITASAFSALSIDPALVLFCPNYESDTYP
VLRDSKKFAIHLLSADQQTEAYAFASKGKDKTKGIEWQLSELGNPLLSNATAIIECELWR
EYDGGDHAIIVGQVKNLILPATPVTPMVYHRGKMGALPAIV
>tr|A0A0Q0CAA7|A0A0Q0CAA7_PSEAP Phosphate ABC-type transport system, periplasmic substrate-binding protein PstS-2 OS=Pseudomonas syringae pv. aptata OX=83167 GN=ALO85_01419 PE=4 SV=1
MTLKRLMTALTLVAAGVTAANTLAAVDPAIQAYTKTSGVSGNLSSVGSDTLANLMTLWAE
AYKKEYPSVNIQIQAAGSSTAPPALTEGTANLGPMSRKMKDGELSAFEQKHGYKPTAIPV
AVDALAVFVHKDNPIKGLTLQQVDAIFSATRLCGAKADAKTWGDVGVTGDLAGKPIQLFG
RNSVSGTYGYFKEEALCKGDFKANVNEQPGSASVVSAISNSLNGIGYSGIGYKTSNVRTV
PLAKKEGGEFVEDSEANTLNGSYPLSRFLYVYVNKAPNQPLAPLEAEFVKLVLSRQGQEV
VMKDGYIPLPARVVEKTLADLGLQGASGVALK
>tr|A0A0N8T7W1|A0A0N8T7W1_PSEAP Type III secretion protein HrcU OS=Pseudomonas syringae pv. aptata OX=83167 GN=ALO85_04191 PE=4 SV=1
MSEKTEKATPKQLRDAREKGQVGQSQDLGKLLVLMAVSEITLALADESVSRLEALLSLSF
KGIDRSFAASVELIASEGFTVLLSFTLCSVGIAMLMRLISSWMQIGFLFAPKALKIDPNK
INPFSHAKQMFSGQNLLNLLLSVLKAIAIGATLYVQVKPVLGTLVLLANSDLTTYWHALV
ELFRHILRVILGLLLAIAMIDFAMQKYFHAKKLRMSHEDIKKEYKQSEGDPHVKGHRRQL
AQEILNQEPSAAPKPVEDADMLLVNPTHYAVALYYRPGETPLPLIHCKGEDEEALALIAR
AKKAGIPVVQSIWLTRTLYRSKVGKYIPRPTLQAVGHIYKVVRQLDEVTDEVIQVEVEL
>tr|A0A0Q0DL82|A0A0Q0DL82_PSEAP Uncharacterized protein OS=Pseudomonas syringae pv. aptata OX=83167 GN=ALO85_03112 PE=4 SV=1
MSRHKACGSSQRRHASRYLPICKKCRALQRLRSRIGVLADASPKPKPSTGPQIVSAMYAY
LMTSWEELPEQNKRALGFDSIVGGEEEEAALNRLATLFMEYADVSLRRALVARRRRLGEG
GG
>tr|A0A0Q0CDA8|A0A0Q0CDA8_PSEAP Transcriptional regulator, TetR family OS=Pseudomonas syringae pv. aptata OX=83167 GN=ALO85_02435 PE=4 SV=1
MSIDANSSGTPAPAKQPAAKRRLPKGEVRKAEIIKAAMTLFARDGYAGASLTNIAKVAGL
SQVGLLHHFPTKLVLLQAVLEHRDQYIAGRLQDADQVASLQGFLSFLKQVMSFSIEDAAV
SQALMIINTESLSVTHPAHRWFSERFAIVHGHLQAHLKLLVEEGEVRADIDARQISLEIA
AMMDGMQIQWLRSPGDVQIEEGFARFLERLARDLAAR
>tr|A0A0Q0D8T5|A0A0Q0D8T5_PSEAP Uncharacterized protein OS=Pseudomonas syringae pv. aptata OX=83167 GN=ALO85_03693 PE=4 SV=1
MSNNIKAVPTNDYNAVIATASKYVEGLRTGSVATIEEAFHKDAVMYGFTNGQLLGGPISN
LYTFVETNGTAPDITTRLDVLAITPTTAVVRVDMEKDAIGADYNDYLTLIKIDDSWKVIA
KVYHQFEG
>tr|A0A0Q0BUY3|A0A0Q0BUY3_PSEAP Uncharacterized protein OS=Pseudomonas syringae pv. aptata OX=83167 GN=ALO85_100836 PE=4 SV=1
MGSGFWSGLAHRILGHTCAARVGRLNQSAYHSRSCQVPDNRLRI
>tr|A0A0N8T9L6|A0A0N8T9L6_PSEAP Putative esterase OS=Pseudomonas syringae pv. aptata OX=83167 GN=ALO85_02868 PE=4 SV=1
MTSDTPSLLYIHGLNSSALSRKACQLSALMKSLGLADRLQVPELHHHPRQAMLQLEAAIT
ALGRPLLVGSSLGGYYATHLAQRHGLRAVLINPAVNPHQLFDGFLGVQQNLYTGEQWQLT
EDHIRALAELEVPAPQDPQQFQVWLQTGDETLDYRRAEKFYRACALRIQAGGDHSFQGFA
ERMPALLTFAGFAPDLLQKIDLSAL
>tr|A0A0Q0DB75|A0A0Q0DB75_PSEAP TPR repeat-containing protein OS=Pseudomonas syringae pv. aptata OX=83167 GN=ALO85_04151 PE=4 SV=1
MPKLDRWGKGMSTRTEKMLEEIRRRFSLIATDIEMDNKSSLFDRNSHVERYFKQILNAIY
ETNLVSTNLGISNYPAIDLKDATRRIAYQVTSTNTKQKITNTIKTFFEHELEKEIDTLNF

LILKNIEGPKISTGKEGSVDYEVITIFDLSRIISDLDDEKQIEEIHQIVMVEYIVEAVGT
IGVKGGAPSNLTTFPRLLEQIGFDAEEHPNDIEIFGDEMEAFVKALADLTVEQRTVLYEL
LIRCNTISRDHKTVYISSKIVTVQFTAKENFVIGSLIDLDLIRVDEEFTADYRDRPKTAL
LLRFPSDVDVNIFAELKRFAGNNLDMLQKMIISLNFKCLTL
>tr|A0A0Q0DBQ5|A0A0Q0DBQ5_PSEAP Methyl-accepting chemotaxis protein OS=Pseudomonas syringae pv. aptata OX=83167 GN=ALO85_03256 PE=4 SV=1
MRNLKVSARAALSFMFIALLLVALGGISVWKMGDIRETARDLEDDALASVVVADRINASM
LRLRLEVRRMLSQTNPQDMATTTGMIASLSEELKKQVALYAPFVASDKEGQLFKAVSENT
DKALVAFDQVTALIRKNAANEALAYLSSNVTPVTRLLDDAVNGLVQMNIDEAANSGKESE
ASYQSGLTFVIFIIAAATLATIVLAVLFTKSIVAPLRDMLRVNDTIAKGDLRSVITVTGK
DEFSDLMRATQVMQNNLRDTIRLIGDSSTQLASAAEEMNAVTEESSRGLLRQNNEIDQAA
TAVNQMTAAVDEVARNAAAASDAAKASDQSTRTGAARVTSTVDAIEKLSKTVQSTSVDVE
RLAVQSKDISKVLEVIRTIAEQTNLLALNAAIEAARAGEQGRGFAVVADEVRALAHRTQT
STQEIEKMIGDILTGTAQATKAMSESCDQADGTLTIAHEAGAALTLIAKAINEINEMNLM
IATASEQQAQVARSVDGNLMSIRDLSIQSATGANQTAASSELSVLAADMNKLVARFSL
>tr|A0A0Q0FGA4|A0A0Q0FGA4_PSEAP Transcriptional regulator, GntR family OS=Pseudomonas syringae pv. aptata OX=83167 GN=ALO85_01637 PE=4 SV=1
MPHTSLAVPESALRAIRKLIAEQGYKPGESLPSQRDLAVLLGVSRASLREALSSLSALGV
VSVQPGKGVFVQSISEPEQRTGGFSWPYEAQVSPADTFQLRYALEGFAAGMAAVTLTADE
RDVLEDNVEAMRLELRAGDFEAAARLDFEFHRHILVASGNQAMLGIVTAGADIFLESQKM
PFIRAARVMETWQEHRKILRALARHASGPAQKAMQDHIRGAALRTGIVFVSPAN
>tr|A0A0Q0DM00|A0A0Q0DM00_PSEAP Uncharacterized protein OS=Pseudomonas syringae pv. aptata OX=83167 GN=ALO85_01385 PE=4 SV=1
MKQELDAVVVNAGCLMFFPEPQRVSLKNDVLDSMLYVQLAASKQHSKFAEPEKWNETRLA
AAQRFGWLPSANEHTSQPLPEESVETVWSCMTRALAPFVPAETLRHAEAFMRKVGNQSFT
NEALHLLRSQTIQSGLNHAGKLQVSVLLQLAFVDTRQNLKLAQFQFATRQPLSSGFLFEV
IEPKSVYGNIAVTVYAMQLFEQVYSQFRDGIDTALSSKRAGLIMSLGGSSDV
>tr|A0A0Q0IP38|A0A0Q0IP38_PSEAP Two-component sensor histidine kinase OS=Pseudomonas syringae pv. aptata OX=83167 GN=ALO85_01932 PE=4 SV=1
MRLSEFIVIHVDRIVDEWEDFARTLKPAAETMTRAELRDHASSILLAAARDMKTAQSKSE
EIAKARGEELNKTPSLDEAASSHGELRHEVGFDLVQMTSEFRHLRASVIRLWVESLAAPQ
LADFQDVIRFNEAIDEALAESTAAYAERVGRSRDIFLAILGHDLRAPLQAVSMSTELLAR
KITLDDKTQSYVSRIQTSTRHMGSMVSDLLEFVRSRLGAGLPVERKYMDLASACREAIEE
ACAGRPDSVPVLNIEGNTKGHWDAGRISQMLQNLIGNALQHGAASHEITVTVTGAEKAVI
LVVHNEGKPIAEDAIGTIFDPLVRSTEENSETRGTSTSLGLGLFIVKEVVNAHGGSITVT
STIGEGTTFNVVLPKN
>tr|A0A0Q0DR10|A0A0Q0DR10_PSEAP tRNA--hydroxylase OS=Pseudomonas syringae pv. aptata OX=83167 GN=ALO85_00580 PE=4 SV=1
MRGRASPSFCEPMYPIPEIEAFLLCRTPDSWVQAALRNLDILLIDHANNEKKAAGAAFQF
MFQYNDKFDLLSKMSRLAREELRHFEQVISIIRKRQIPMANISSARYAGALRKLVRNHNP
YRLTDALIVGAIVEARSCERFAALVPHLDEELAKFYGGLLKSEARHFQDYLKLAYSYGDK
ADVDAKIEEIRLAERELIESPDEEFRFHSGVPAAA
>tr|A0A0Q0CX65|A0A0Q0CX65_PSEAP Uncharacterized protein OS=Pseudomonas syringae pv. aptata OX=83167 GN=ALO85_03800 PE=4 SV=1
MKTYSIREVILNISEMPDSWFYLPDTPWTLDTKGAFSLDSRDFPPDSTDYLPPQVLSEGW
KETLETPIIEDIISNVNEQLSQPSVENYFDAFKFYYENDAFKIFKI
>tr|A0A0N8T804|A0A0N8T804_PSEAP ABC transporter OS=Pseudomonas syringae pv. aptata OX=83167 GN=ALO85_00516 PE=4 SV=1
MSASAQTAVLSVSGIGKTYAQPVLGDVDLTLMRGEVLALTGENGAGKSTLSKIIGGLVTP
TTGNMEFQGQPYQPASRTQAEKLGIRMVMQELNLLPTLTVAENLFLHDLPRRAGWIDRKR
LRENAIEAMAQVGLDAIDPDTLVGELGIGHQQMVEIARNLIGDCHVLILDEPTAMLTSRE
VEMLFEQITRLQARGVSIIYISHRLEELARVSQRIAVLRDGKLVCVEPISRYNSEQLVTL
MVGRELGEHIDMGARQIGEVALSVKGLSRAGKVQDVSFDVRRGEIFGISGLIGAGRTELL
RLIYGADVADSGHIEVGRPLQKVSIRSPSDAVEQGIALITEDRKSEGLLMSQSISANIAL
GNMHSISSAGLVNSDDELKLAQRQVAAMRIRSSSPGQLVSELSGGNQQKVVIGRWLERDC
TVMLFDEPTRGIDIGAKFDIYALLAELTRQGRALVVVSSDLRELMLICDRIGVLSAGRLI
DTFERDSWTQDQLLAAAFAGYQKRDALLNDAAPRNDS
>tr|A0A0Q0FEQ2|A0A0Q0FEQ2_PSEAP Peptidase U61, LD-carboxypeptidase A OS=Pseudomonas syringae pv. aptata OX=83167 GN=ALO85_01189 PE=4 SV=1
MPATSSNTLHPRALRAGDAVALVSPAGPVDPGKIQAAVSVLEGWGLRPRVYPHALGSYSF

YSGTDEERLADLNAALADPEIRAVICTRGGYGVQRIVQQVDMDAVRRDPKLVTGFSDITA
LHGALWNHARLATIHGPVASQLERGGLFVSGMRHVLMSSEPVLLKADPASPTARVRTGGS
AKGLLLGGNLCILDTSVGTPFMPDLSGAILLIEEVNEPAYRVDRMLTHLGNCGILASLAG
IAVGEFTPAPNTGRTISPADVLLERLGSLGIPVLGGLPVGHGDLNQAVPLGTQAILDADA
GTLLVAAAARS
>tr|A0A0Q0CES8|A0A0Q0CES8_PSEAP Type III pantothenate kinase
OS=Pseudomonas syringae pv. aptata OX=83167 GN=coaX PE=3 SV=1
MILELDCGNSFIKWRITANSDAAVVSVGVVDSDAALLEHLRNLPDTTFSDCRLVSVRSAE
ETARLVSVLADAFSVAPVCAVPARELGGVINGYDDFERLGLDRWLAFVGAYHLVKRACLV
IDLGTAVTSDFVDADGAHLGGFICPGMPLMRNQLRTHTRRIRYDDTEAERALVRLVPGRA
TAEAVERGCSLMLRGFAVTQVEIARGYWGEDFAIFVTGGDAALVADVLPGARIVPDLVFV
GLALACPLR
>tr|A0A0N8T7S4|A0A0N8T7S4_PSEAP Uncharacterized protein OS=Pseudomonas
syringae pv. aptata OX=83167 GN=ALO85_04042 PE=4 SV=1
MNMLMTLGLSIESPLAVPGASNYRPESWPPPPDWPVVIDAQGKVISHWGDPVWRLDPWAK
RALTLNFGDGPATKTAAPISSANANLLRIITGWWLYGPYGARGYRSLKTRFDQMRRLFVL
CEEAGILASELSQFPRVCERIPAFLHTSRKTEFLSLMHELYASRSALGFTLLDSGSLTRL
AALFPTHQTAQTPYIPPRIWRYQATRLRECLDDFQSHREQVEACYRFCFAAYEHNQRISE
RTACINAPPFSVHKMQLSGSKTRLRFLGAFADTAKRFGIFELLERWVGQGNDLQVRALTS
YLKLVTGAGLAYILNFSLMRVEEAWNLRVGCLEVEHDKSFGDIFLLRGQTTKTLSDSYAL
WVTSPSVAVAVEAMEFISMFRSLHAPHEAARVLAGRYLTDFSYEPWSPLRNKVNHSLRPG
IQPYAGLLGQYRKLFDLERLRITSEDLKLARLATPTLPEEYQVGAVWPLAWHQLRRTGAV
NMQASGLVSDASLQYQLKHVTRSMSLYYGQNHSHVRLEEKAHTLYVRTMYETLGRELQQL
TSERFVSPHGDKRKAEIVRLISPEDAKKMIGLAKKGAVACRPIILGVCSSREPCPYGGID
NIAHCGGGDSVDAKPCPDVLYDSERLGAVDDLEHVLKERLTSAQNGSPLMESLMAQQRSV
ESFRRVVGSANGR
>tr|A0A0Q0BWE5|A0A0Q0BWE5_PSEAP Putative lipoprotein OS=Pseudomonas
syringae pv. aptata OX=83167 GN=ALO85_02232 PE=4 SV=1
MNALNKTLAYLALAGLAGCTLVPSHRPVPKPPAEPVRLIMLAETSDGGGKNCTFVAKDDV
VRLNKGDHECTNDQMSFIKLDNVRSAMKILLESRDCDDPGGWVFGLETYIDPVTTAWISI
DQLRSKPVESIITRGVVLTKAYSGGENIKGKLSCVRVDVSD
>tr|A0A0Q0D8M2|A0A0Q0D8M2_PSEAP Uncharacterized protein OS=Pseudomonas
syringae pv. aptata OX=83167 GN=ALO85_101257 PE=4 SV=1
MGAGCSGYSAAQAKHREKASSGAVEACSLHPDHFVEVASSQGFELLEMLSPDKQLERYCQ
RREEGLSLTPNIYLVYSVKA
>tr|A0A0Q0CDR0|A0A0Q0CDR0_PSEAP 3-ketoacyl-CoA thiolase OS=Pseudomonas
syringae pv. aptata OX=83167 GN=fadA PE=3 SV=1
MSLNPRDVVIVDFGRTPMGRSKGGMHRNTRAEDMSAHLISKLLERNNKVDPAEVEDVIWG
CVNQTLEQGWNIARMASLLTQIPHTSAAQTVSRLCGSSMSALHTAAQAIMTNNGDVFVIG
GVEHMGHVSMMHGVDPNPHMSLHAAKASGMMGLTAEMLGKMHGITREQQDAFGLRSHQLA
HKATLEGKFKDEIIPMQGYDENGFLKVFDYDETIRPDTTLESLAALKPAFNPKGGTVTAG
TSSQITDGASCMIVMSAQRAQDLGIQPMAVIRSMAVAGVDPAIMGYGPVPATQKALKRAG
LSIADIDFFELNEAFAAQALPVLKDLKVLDKMNEKVNLHGGAIALGHPFGCSGARISGTL
LNVMKQNGGTFGVSTMCIGLGQGIATVFERV
>tr|A0A0N8T9D4|A0A0N8T9D4_PSEAP Ribosome-recycling factor OS=Pseudomonas
syringae pv. aptata OX=83167 GN=frr PE=3 SV=1
MINEIKKDAQTRMQKSLESLTHAFTRIRTGKAHPSILGGVMVPYYGADTPLAQVANVTVK
DSRTLQVVAFERNMLAAVDKAIQSSGLGFNPTNLGELLLISMPALTEETRKGFTKQARDA
AEDARVAVRNIRRDALSQLKDLVKEKEISEDEERRAADDVQKLTDKFVAEIEVAVKQKEA
DLMAV
>tr|A0A0Q0DQT8|A0A0Q0DQT8_PSEAP Uncharacterized protein OS=Pseudomonas
syringae pv. aptata OX=83167 GN=ALO85_100475 PE=4 SV=1
MPNQESDMIAIPRSIALLAALDLIIDLPRAFARYPGTTYEEAMR
>tr|A0A0Q0D9Z6|A0A0Q0D9Z6_PSEAP Putative Tricarboxylate transport protein
TctC OS=Pseudomonas syringae pv. aptata OX=83167 GN=ALO85_01989 PE=4 SV=1
MKFSLRHIAATAGCMLIASQLLAEPKRPECIAPASPGGGFDLTCKLVQSALINEKILTSP
MRVTYMPGGVGAVAYNAVVAQRPADAGTLVAWSSGSLLNLAQGKFGRFDENAVRWLAAVG
TSYGAIAVKSDSPYKNLDDLVQALKADPSKVVIGSGGTVGSQDWMQTALIAKAAGINPRA
LRYVALEGGGEIATALLGGHIQVGSTDISDSMPHILSGDMRLLAVFAEKRIDEPEMKDIP
TAKEQGYDIVWPVVRGFYLGPKVSDEDYNWWKASFDKILASEDFAKLRDQRELFPFAMSG

TELDTYVKKQVADYKVMAKEFGLIQ
>tr|A0A0Q0FEJ6|A0A0Q0FEJ6_PSEAP Transcriptional regulator GntR OS=Pseudomonas syringae pv. aptata OX=83167 GN=ALO85_01162 PE=4 SV=1
MPMNDQSLPLKKQPRRLGLSRNVTQDDIVYEHVFDAILEQRLAPGTKLSEEALGEIFGVS
RTIIRRALSRLGHEGVVLLRPNRGAVVASPSVEEARQVFFARRMVETAITQLAVEHATAE
QLAELRQMVSDERDSFARGDRGAGIRLSGEFHLQLAVAARNAPLISFQRSLVSQTSLIIA
QYETGNRTHCSYDEHSQLIDAIEARNAALAVELMMHHMDHIDSKLNLDEGSASEDLHAVF
SHVMGRKKTER
>tr|A0A0Q0CZF6|A0A0Q0CZF6_PSEAP Uncharacterized protein OS=Pseudomonas syringae pv. aptata OX=83167 GN=ALO85_01114 PE=4 SV=1
MDTHVTHASFSQVRLASLLLLVIVCTGLFFAPERSQAEVANLATCPVGTHTATWSPGVTN
TAKTTDVVTTSNWGPCVLPSFPLVVSASSIQSFQAPLSCQSLLLQTSNVVWIITWSDGVT
STYTFNTSFNTVNGLITAVLGVGTITDGRFKDATALSTFELVNFQAALNNSCGTTTGVTG
VSGLSTLVITP
>tr|A0A0Q0FNF8|A0A0Q0FNF8_PSEAP GlcG protein OS=Pseudomonas syringae pv. aptata OX=83167 GN=ALO85_02633 PE=4 SV=1
MKIKAVLTQTEVSLMLGAAREEAQANGWAVSIAVVDDGGHLLAFERLDDAAPISSYIAIE
KARTSALGKRESKGYEEMVNGGRTAFLSAPLLTSLEGGVPVIVDGQVIGALGVSGVKAEQ
DAQVAKAGVAALDAD
>tr|A0A0Q0CVT5|A0A0Q0CVT5_PSEAP Uncharacterized protein OS=Pseudomonas syringae pv. aptata OX=83167 GN=ALO85_04141 PE=4 SV=1
MKIWACAKGVNGFINREDGRYKLYFIWQILKMKRENPSEFSALTFFSTHSREDKRREMFA
VKNGYFRYKSAEAGSSRSGDSDGESLPHAYLIAILSQLPTINFKIGEEVVPFTFTRLIAE
ETRLRFGNGKGYVPDLYGEFTSDNPYYKKWGGKLAIEVCYSNPCSTQKIVDFRDHGIPII
EIKIARSINIETYIDKNDIEGSIERGFQKITDIASNQLFCRVLSDPVSVQFHQEYVRQAD
KEKEGLVEKNKRETKFLFNKLREAESLAANLGKERDVFADAKSLVEKDKAGLEAATTELT
QIVADLKVELGSCKKAYTNKVHEVDSLKAKLEKTKSGWWAKLKESFNVKRTQKKP
>tr|A0A0Q0C3G8|A0A0Q0C3G8_PSEAP MEKHLA domain protein OS=Pseudomonas syringae pv. aptata OX=83167 GN=ALO85_03255 PE=4 SV=1
MSQEQFIQMLDESYRHWTGHGLPSPRQLDSQQRLTWLHTQAPYSLLAHDGATDPRFTYVN
ECALQCFKYPRDSFIGMPSRFSASELDRAQRQVLLEQVTANGIAEGYSGWRVDANNQPFM
IYAGVVWTLLNSQGQACGQAALFWPDEQRIGVVD
>tr|A0A0Q0CHG9|A0A0Q0CHG9_PSEAP Putative amino acid synthesis-associated protein OS=Pseudomonas syringae pv. aptata OX=83167 GN=ALO85_03431 PE=4 SV=1
MSFEIRKIVTYTEETFIEGGKPTDKPVTMIGLAAVIKNPWLGRGFVEDLKPEIRANCSAL
GELMVKRLTDAIGGAEKIEAYGKAAVVGAEGEIEHASAVIHTLRFGNHYRDAVKAKSYLS
FTNKRGGPGTSIQIPMMHKDDEGLRSHYITLEMHIEDSPRADEIIVVLGAANGGRLHPRI
GNRYIDLEELAAEKAQ
>tr|A0A0Q0DJL6|A0A0Q0DJL6_PSEAP Cell shape-determining protein MreB OS=Pseudomonas syringae pv. aptata OX=83167 GN=mreB PE=3 SV=1
MFKKLRGMFSSDLSIDLGTANTLIYVRERGIVLNEPSVVAIRTHGNQKSVVAVGTEAKRM
LGRTPGNIAAIRPMKDGVIADFSVCEKMLQYFINKVHENSFLQPSPRVLICVPCKSTQVE
RRAIRESALGAGAREVFLIEEPMAAAIGAGLPVEEARGSMVVDIGGGTTEIALISLNGVV
YAESVRVGGDRFDEAIITYVRRNYGSLIGESTAERIKQEIGTAYPGGEVREVDVRGRNLA
EGVPRAFTLNSNEVLEALQESLATIVQAVKSALEQSPPELASDIAERGLVLTGGGALLRD
LDKLLAQETGLPVIVAEDPLTCVARGGGRALEMMDKHTMDLLSSE
>tr|A0A0Q0IJ76|A0A0Q0IJ76_PSEAP HAD family hydrolase OS=Pseudomonas syringae pv. aptata OX=83167 GN=ALO85_03888 PE=4 SV=1
MIKLVTFDLDDTLWDTAPAIAGAEVTLRDWLGENAPRLGAVPVEHLWEIRSRLVAEDPSF
KHRISALRRRVLFHALEDAGYDPEEAQDLADRGFEVFLQGRHQVQIFPEVQPMLEILAKT
FTLGVITNGNADVRRLGLADYFAFALCAEDLGIGKPDPAPFVEALRRAKVDAGSAVHVGD
HPRDDIAGAQQAGMRAIWYNPQGKAWDADRLPDAEIHNLSQLPEVLARWA
>tr|A0A0Q0BVH5|A0A0Q0BVH5_PSEAP Helix-turn-helix XRE-family protein OS=Pseudomonas syringae pv. aptata OX=83167 GN=ALO85_02484 PE=4 SV=1
MKAFFPVQCTDTLLRIALLVKQTRLQQGIRQVDLAERLGISLRTFRRIEAGNAEGVSLRD
FMLVVWGLGVSERLFQGLRDDESFSVEQLEAADRKRVRLPRHKPEDF
>tr|A0A0Q0DUS9|A0A0Q0DUS9_PSEAP Uncharacterized protein OS=Pseudomonas syringae pv. aptata OX=83167 GN=ALO85_04884 PE=4 SV=1
MLAGQPVNSFPSKQNGVGMGRKVDTTWYGTYLEAIAFENLSGDKSVGTPELADHLGVKPK

TLARIRSAGRFIHEVLPGVKPEQIQCGYASLELLSKLWGADPSGAQSRLESVLANRTKLP
ELEEAIRRLKLGENKSSTESNLVGPSQLGFMARMDVWIASSDLVHFDSYRGTAFRLKPCL
GSCPGYLINTENGQPSALVLCKQGSGWRDPAGVARELYEHAIARRHTAPAIWYVFEKDSA
VLQHLAELSIWWGGSPTSDDPWLLLAYLTESGKLEVLFEEYFYNLIGSMTKGEGALRPND
LIATGEAMDGSKACITIPLRNIQPISAATKHRPYSEVLRERLLAIAGQGHATSDQIDRLA
AIDLGL
>tr|A0A0Q0FGJ9|A0A0Q0FGJ9_PSEAP Polar amino acid ABC-type transport system, periplasmic substrate-binding protein OS=Pseudomonas syringae pv. aptata OX=83167 GN=ALO85_01636 PE=4 SV=1
MTKRYSALLTALFASLMLSQTPAHANGLDDVVARGTLKVAVPQDFPPFGSVGPDMKPRGL
DIDTAQLLADKLKVKLELTPVNSTNRIPFLTTGKVDLVISSLGKNPDREKVIDFSRAYAP
FYLAVFGPPDSPVKDIADLKGKTISVTRGAIEDIELSKVAPEGAVIKRFEDNNSTIAAYL
AGQTDLIASGNVVMVAISERNPKRIPALKVKLKDSPVYVGVNKGQPELLGKVDEILNAAK
ADGSLEKASQTWLKQPLPADL
>tr|A0A0N8T9Q3|A0A0N8T9Q3_PSEAP DUF4124 domain-containing protein OS=Pseudomonas syringae pv. aptata OX=83167 GN=ALO85_02084 PE=4 SV=1
MRQSLICLLLLISLPALAQIYKYTDANGNTAFSNQPPNGAKTEVVELPPLNSIETQTPSR
PVVNSAPQPAPAAPQSQPQTAYDVLELADLPTDEALRANNGTFIIGVKIQPRLQPTHSLQ
LLLDGNLYGQPSNLPRFQVVNVDRGEHSFAVVVKDGERIIQQSETITLTVQRVHLGKP
>tr|A0A0Q0BSY8|A0A0Q0BSY8_PSEAP tRNA (cytidine(34)-2'-O)-methyltransferase OS=Pseudomonas syringae pv. aptata OX=83167 GN=trmL PE=3 SV=1
MSNLSWPVHGCPDASTLRTTAPSTVAPRYFGSSLNQSARPAGNSSICSSSTGSGCCRRPI
MVPTVNPTIAMEPRTRGHSFGLGSSSRVECILSFRSEPCMFHVILFQPEIPPNTGNVIRL
CANSGCTLHLIEPLGFELDDKRLRRAGLDYHEYATLQTHADLASCLEKIGNPRLFAFTTK
GSRPFHDASFEPGDAFLFGPESRGLPADILDSLSSEHRLRLPMREGCRSLNLSNTVAVAV
YEAWRQHGFA
>tr|A0A0Q0IEE3|A0A0Q0IEE3_PSEAP Corrinoid adenosyltransferase OS=Pseudomonas syringae pv. aptata OX=83167 GN=ALO85_03560 PE=3 SV=1
MQGVAVLSSAVLPSVVACPERRTQMNESPERDERHLARMQRKKAFMDQRIASSPNECGLL
LVLTGNGKGKSSSAFGMLARAMGHDMQCGVVQFIKGRNSTGEEMFFRRFPEQVRYHVMGE
GFTWETQDRQRDIAAAEAAWAVSQEMLRDPAIGLVVLDELNIALKHGYLDLEQVLSDLQA
RPPMQHVLVTGRGAKAELIDLADTVSEIGVVKHAFQAGIRAQKGIEL
>tr|A0A0Q0D7G6|A0A0Q0D7G6_PSEAP ADP-heptose:LPS heptosyltransferase II OS=Pseudomonas syringae pv. aptata OX=83167 GN=ALO85_02896 PE=4 SV=1
MKILIVGPSWVGDMVMAQTLFQCLKQRHPECEIDVLAPEWSRPILERMPEVRAALSFPLG
HGALELATRRRIGKSLAGQYDQAILLPNSLKSALVPFFAGIPKRTGWRGEFRYGLLNDVR
TLDKQRYPLMIERFMALAFDKGAELPRPYPQPSLRIDPTTRDAALSKFGLTLDRPVLALC
PGAEFGESKRWPAEHYAQVADASIREGWQVWLFGSKKDHPVGESIRQELIPGLREESVNL
SGETSLAEAIDLLSCADAVVSNDSGLMHVAAALNRPLVAVYGSTSPGFTPPLADEVEVVR
LGIECSPCFERTCRFGHYNCMRLLEPDAVIQALSRLSSMPVEVA
>tr|A0A0Q0FVC0|A0A0Q0FVC0_PSEAP Uncharacterized protein OS=Pseudomonas syringae pv. aptata OX=83167 GN=ALO85_03368 PE=4 SV=1
MALNKSTQELKRHLKGTATNLENTAEEILKLASQMNDVDVTAVLQMVNRLYSDADQLKAY
ADEVKAKRIVRAKPL
>tr|A0A0Q0CZX6|A0A0Q0CZX6_PSEAP Methyltransf_11 domain-containing protein OS=Pseudomonas syringae pv. aptata OX=83167 GN=ALO85_02128 PE=4 SV=1
MLLAKKYCQGHGIELGAAAHNAFNLAHCLNVAPCNGVDFLHPRDMEDYQQYFVEQMKVSG
DVSKVDMLGDFQCIHTADSSVDYLISSHVIEHVPNLLSAYVESFRVLKNGGVFFCIFPKR
TAAKTDRARRLTTLAQMIEDYENKVDMTTVTEGEWRGHYQVFSLQSMLRAVNYVNSSGLG
CWYIECVEETDSKVGNGHTVVLRKVEGLSAEKWQDVGDFNTQFNQLLMAGKLENALTLTK
IMLSFNFFDATRLHLAGALSRQLGNVAEGVEFLRQALVVDPENEDYRKEFIEVTGTPFHN
PVL
>tr|A0A0Q0C288|A0A0Q0C288_PSEAP Putative DNA-binding protein with peptidase domain OS=Pseudomonas syringae pv. aptata OX=83167 GN=ALO85_03931 PE=4 SV=1
MSQAAFVNPSILTWSRERAGLSAAQVARKLPVKPERVVEWEAGEAKPTFLQAQKWASVAH
VPFGFLFLLQPPVELLPLPDLRTVGNLAPLRPSLELLDTVKDAIRKQDWYLEYLHNHEQK
PLSFVGRFDSRSTVKAVVDDIRRTLGVDPEKSRLDHDRYSRALIDAAEAAGVLVMRSGIA
LGNTHRKLEVSEFRGFAISNPLAPLVFINSSDAPTARLFTLMHELAHIWIGSSGVSDAGT

ANGREDERFCNAVAGEFLVPEALFRTLRDTAVEWESNLAQLATRFHVSKLVIGRRALDLG
FVTQEQYGTYYQRVLKAFQEEKGGAGNYYRNATAKNSARLSRAVLIEAMSGRMLLREAGN
LLGIQPVKLRTLAQTVTL
>tr|A0A0Q0BUQ6|A0A0Q0BUQ6_PSEAP Uncharacterized protein OS=Pseudomonas syringae pv. aptata OX=83167 GN=ALO85_05399 PE=4 SV=1
MIKRSSARTQGLPPLKNGATFRKKRLTKVRISGIVRAGL
>tr|A0A0Q0DZ29|A0A0Q0DZ29_PSEAP DUF190 domain-containing protein OS=Pseudomonas syringae pv. aptata OX=83167 GN=ALO85_200168 PE=4 SV=1
MKGFLVTFFTQQNRRYQGKMLGDWVVDLAKEMGLRGATLSTGIEGFGHTGRLHSSHFFEL
ADQPTEIRMAITEDESEQLFKRLELEDISLFYIKTPVELGFVGTEVT
>tr|A0A0Q0DU34|A0A0Q0DU34_PSEAP Uncharacterized protein OS=Pseudomonas syringae pv. aptata OX=83167 GN=ALO85_02364 PE=4 SV=1
MRNDPYDDVDDVPSLSAKDDDDDFAPSNGARERTTVYSRTAPVVKVKAPSTGPLWALVGA
LFIAFCGLGWWSFQQVSLMEQQLVATQESFARISEEAAGRLQDISGKVVATEALSSDGEA
LKQRIKLLEAQLEDQDKQREGVEGQQGSLDKRLEQMAAQTAQQQSENAQLQEQLKSVVAE
LTTLKAALPDLKTAQADQGKLDAQIKSVAADVATLKKQGNPSAAVERLEQDLMVLKSEQE
NRPAPSAQANTAEFDAFRAQVTRNINTLTSQIQNLSQQLNARR
>tr|A0A0Q0IE38|A0A0Q0IE38_PSEAP Glucose-1-phosphate thymidylyltransferase OS=Pseudomonas syringae pv. aptata OX=83167 GN=ALO85_01139 PE=3 SV=1
MARKGIILAGGSGTRLHPATLSVSKQLLPVYDKPMIYYPLCTLLLAGIRDILIISTPQDT
PRFSQLLGDGSQWGLNLTYAVQPSPDGLAQAFTIGADFIGNDASALVLGDNIFYGHDFQS
LLLNASNRESGASVFAYHVQDPERYGVAEFDASGRVLSLEEKPQVAKSNYAVTGLYFYDN
QVVDLARQLKPSPRGELEITDLNTLYLEQKQLHVEIMGRGYAWLDTGTHDSLLEAGQYIA
TLERRQGLKVACPEEICYRAGWIDAAQLEKLAQPLIKNGYGQYLKNVLKEKVF
>tr|A0A0Q0DII5|A0A0Q0DII5_PSEAP Putative polysaccharide deacetylase OS=Pseudomonas syringae pv. aptata OX=83167 GN=ALO85_02318 PE=4 SV=1
MRFLALLLLTLSITGVAHAAPAENSGKPPKAYLSLIIDDLGQNQDRDSRTLALPGPVTLA
IMPDTPHATEFARQAHRAGKTVMLHMPMDPATGPYAWHPELPLPELESRLNAALLKVPYA
AGINNHMGSRMTAEPVAMTWLMAELQRRHLFFVDSRTSAKTVAAAEAQRIGLASVSRDVF
LDDERTAEAITRQLQTAIKIARKYGSAVVIGHPYPVTLDVLERELPNLKAQGVEWIDLRS
MISERGNQASAAHGKNGIYR
>tr|A0A0Q0DLI4|A0A0Q0DLI4_PSEAP Alginate biosynthesis transcriptional regulator AlgB OS=Pseudomonas syringae pv. aptata OX=83167 GN=ALO85_01605 PE=4 SV=1
MEAATENQGRILLVDDESAILRTFRYCLEDEGYSVATANSAAQADTLMQRQVFDLCFLDL
RLGEDNGLDVLAQMRIQAPWMRVVIVTAHSAVDTAVDAIQAGAADYLVKPCSPDQLRLAT
AKQLEVRQLSARLEALEGEVRKPKDGLDSHSPSMMAILETARQVAVTDANILILGESGTG
KGELARAIHGWSKRAKKSCVTINCPSLTAELMESELFGHSRGAFTGASESTLGRVNQADG
GTLFLDEIGDFPLTLQPKLLRFIQDKEYERVGDPVTRRADVRILAATNLNLEDMVRSGRF
REDLLYRLNVITLNLPALRERSEDILTLADRFLARFVKEYARPARGFSEEARAALLNYRW
PGNIRELRNVIERASIICPQERVEVSHLGMAEQPTNNAPRVGAALSLDELEKAHIGAVLA
TSETLDQAAKTLGIDASTLYRKRKQYNL
>tr|A0A0Q0IEX5|A0A0Q0IEX5_PSEAP Uncharacterized protein OS=Pseudomonas syringae pv. aptata OX=83167 GN=ALO85_03824 PE=4 SV=1
MSIEPQRQKEPTPNQDAPASPGPDRNDPAIEPQVGDVKPETEKGNGQSQGQTPSPSQSQT
QSQSQSNSDSAYVPDHEPEEKTEDLRNHQPTTGIDADIDTPGG
>tr|A0A0Q0BZ84|A0A0Q0BZ84_PSEAP Methyl-accepting chemotaxis protein OS=Pseudomonas syringae pv. aptata OX=83167 GN=ALO85_00924 PE=4 SV=1
MNLINRTSFAKKLMAALTVCAVITLAVGGVGMLGINRLSDALELTFSNNLVSVANTNETM
TALTTHNRGLYRLLDAKDTATAEKMRQSINAELERAQKVYAIYRATPLEDDERAAGDQYD
ALMPAYMSASQNIFDLQQAGKLEDARIRLNALADGEFNKARGYLQIMIDSNKRQIKEGAE
AADRLQSTSVFMLTAGVVIAFVVAIMLGILITRMISGPLRSAIEVAQRIASGDLTQSVSS
TRGDEAGQLLNAIGTMQGNLKRTIQEISSASDQLASAAEELGAVTEESTRGLTRQNDEIQ
QAATAVNEMTAAVEEVARNAVSTSEESKSLATDAANGRGQVDNTVKGIGTMVSEITESTG
SVTTLAGHVRDISKVLEVIRSIAEQTNLLALNAAIEAARAGEQGRGFAVVADEVRALAHR
TQASTVEIEGMIGTVQSGADGAVAAMSKSLATATNTQELAQRAGSALEKITSGVGMINER
NMVIASASEEQAQVAREVDRNLVNIQELSAQSAAGANQTSASSQELSRLATSFNTMVAQF
KL
>tr|A0A0Q0FB75|A0A0Q0FB75_PSEAP Ntox46 domain-containing protein OS=Pseudomonas syringae pv. aptata OX=83167 GN=ALO85_02243 PE=4 SV=1

MDLLANVPSWDDIERNLDDKFNTLNQGYSDGLQSAHDSWNGFTRRVSTAATQAFGYMGSN
RIDSVKAAMRLSRDIVESDLKRKWASIEIEQILPVLLQLVKEVAMILGGSVAVGTIAGGV
AGSFFFGVGAVPVAAAGAGIGLQVGNLILLTLGLASIAEYFYQGLPACLATLQEGFATAW
HADEGLKPEGLDPTGGSAYVMQERIDRAAQQLAKGQEQLVLLLFTAIVTYWTRGQVKAGV
MGSMDSIAARSTKLQAEISNKQFANWLARNEQKLLAQPELRGTEPVSFKQTDAQQYDKQE
QPAARPQAKPAEKKVLEVQSRRDYLNKRFGRTGDLVQDIYIRGNRETAVDFFKSQGLKPV
DYEKYMSGLDFTKPVYVETINRGKNLWQYQVPDGVQGVWFSPTPNVAPGQLGINPLGKIY
KTEIVVPKVLNIYQSTDKVTLLRSTSAPVVDTWSIRHQPYDAIGGARQMTALQKELFKLI
APGQP
>tr|A0A0Q0FI22|A0A0Q0FI22_PSEAP STAS domain protein OS=Pseudomonas syringae pv. aptata OX=83167 GN=ALO85_02169 PE=4 SV=1
MTQSDPQQSLWLVYAINVSVMKHLEVPMTVTSKLSDDGRTLILEIEGRFDFNSHQAFRAA
YEDHPQSLNYVVDLRGTHYLDSSALGMLLLLRDHAGGDKALVRLVNCTPDVLKILAISNF
SKLFELSIPSAVSPEKTVHSDR
>tr|A0A0Q0D6E6|A0A0Q0D6E6_PSEAP Uncharacterized protein OS=Pseudomonas syringae pv. aptata OX=83167 GN=ALO85_02404 PE=4 SV=1
MFLPNLAPMCPEIITSYADVYSGRQGHGYPMSGDISGVTDTCEGHAGTRKNLTPATDGRA
GHVGHCN
>tr|A0A0Q0DAA1|A0A0Q0DAA1_PSEAP Mangotoxin biosynthesis-involved protein MgoB OS=Pseudomonas syringae pv. aptata OX=83167 GN=ALO85_04330 PE=4 SV=1
MENDMNTLHSNLKPDTLIKNPVACRVVSTVDVSGDAASVWAVVGNFGGFQAFIPALESIE
VTGEGPGSVRNKLFKDGNVAIEQLNSRDDQALYMTWSLIHTSLPVSNLWAAMTVEASGDG
DTCIASWTIVADPVEGGPEGDAFEAFLQGFADGAMSNVRSLFG
>tr|A0A0Q0CXE2|A0A0Q0CXE2_PSEAP Ureidoglycolate lyase OS=Pseudomonas syringae pv. aptata OX=83167 GN=allA PE=3 SV=1
MRKLIIEPLTKEAFAPFGDVIETDGSDHFMINNGSTMRFHRLADVQTAQPEDKAIISIFR
AEALPMPLTIGMLERHPLGSQAFVPLLGNPFLIVVAPVGDEPESEATRAFVSNGRQGVNY
HRGVWHHPVLTIEKRDDFLVVDRSGEGNNCDEHYFAESQLLVLDPHPLEG
>tr|A0A0Q0FAZ8|A0A0Q0FAZ8_PSEAP Uncharacterized protein OS=Pseudomonas syringae pv. aptata OX=83167 GN=ALO85_200108 PE=4 SV=1
MSKEFSVFTDTATISIFDIDAIKHRVSDSPDWWSIVEDEILETNKGNIAFLGLGDDGDYT
IKLLDNIENETGALNLHFPSGQVFIGAGEDTSGGDLEPDGSDVIQGKTLKFAPGDYSMKF
ARAGNTIELCFTPTLEKYNSIKEPIRI
>tr|A0A0Q0BV02|A0A0Q0BV02_PSEAP Short-chain dehydrogenase OS=Pseudomonas syringae pv. aptata OX=83167 GN=ALO85_03155 PE=4 SV=1
MSDKKKLLLTGASRGIGHATVKHFNAAGWEVFTASRQNWVADCPWAEGLLNHIHLDLENI
DSVSENMAAIKDKLGGRLDALVNNAGVSPKTEDGGRMGVLESDYSTWIKVFNVNLFSTAL
LGRGLFDELKAAKGSIINVTSIAGSKVHPFAGVAYATSKAALSALTREMAFDFGPHGIRV
NAIAPGEIDTSILSPGTAEIVERLVPMHRLGKPEEVASLIYFLCTAGASYVNGAEIHVNG
GQHV
>tr|A0A0Q0DAI5|A0A0Q0DAI5_PSEAP ADP-ribosylation/crystallin J1 OS=Pseudomonas syringae pv. aptata OX=83167 GN=ALO85_02245 PE=4 SV=1
MIDLRSPNDMLDHYVERYDHLLPAPSAQLLQRMDYMRISSQLLTYIASQGI
>tr|A0A0Q0BVU6|A0A0Q0BVU6_PSEAP Uncharacterized protein OS=Pseudomonas syringae pv. aptata OX=83167 GN=ALO85_00401 PE=4 SV=1
MDTVHTLPYSNKKVLYIFASVGVLIALSEAVYDLAFANLAYTLTGSTLSVMTTYAIGYSA
EILVTLLGAGFIDRFDKWKLFIATQIINIIVFTVAVTTLSTPGSSVEQVWLFAFLVDLVH
QYVRLIMFSFIPFLFSREEIIRVNGFIAIINGVARAVGPAIGALVIFQVGLSMALTASIV
FMMGALFLALSLWSVAAGTSVTRPDVESSSFKARFQESVTGASMATVNLLRSSQWRNFLA
SYSTCVLVISVLALLWIPFLRDFHAFSPEQTGYLYALGTTGAVLGGFTMSSFQDKSLITT
ILSAHALMFTGIAMTLWFRGSFLWVAVGMFLFQFGTTVYFRSTASAIQLTLPKEIIGSWY
GAIDFMSRFAGLVGVILAGWSYDWIGAYWVYSVLLGLLVLSGLAWKAGNQVAWLSPS
>tr|A0A0Q0FNI7|A0A0Q0FNI7_PSEAP Uncharacterized protein OS=Pseudomonas syringae pv. aptata OX=83167 GN=ALO85_102023 PE=4 SV=1
MEAEQYPPDLNVRRGYDVAVDEHAKSHAYRRDVVVGILSELSDDQTLISYARLIWVRTPS
GITTRLSIRQNT
>tr|A0A0Q0DMY9|A0A0Q0DMY9_PSEAP Short chain dehydrogenase OS=Pseudomonas syringae pv. aptata OX=83167 GN=ALO85_03222 PE=3 SV=1
MQNRIMITGAGSGLGREIALRWAREGWRLALSDINDAGLQETLALVREAGGDGFVQRCDV
RDYSQLTAFAQACEQRFGGIDVIVNNAGVASGGFFNELSLEDWDWQIAINLMGVVKGCKA

FLPMLLASNGRIINIASMAALMQAPAMSNYNVAKAGVVALSESLLVELHDQGIGVHVVCP
SFFQTNLLDSFRGPTPAMKAQIGRLLEKSPITAGDIADCIFRQVGEGEFMILPHEEGRMA
WEMKRKQPQAMYDEMTVMCAKMRAKAQKGHA
>tr|A0A0Q0CVZ7|A0A0Q0CVZ7_PSEAP Uncharacterized protein OS=Pseudomonas syringae pv. aptata OX=83167 GN=ALO85_04171 PE=4 SV=1
MNLPDNVFENPYKADQFLQFSMNVNEIIPNLVSTLEAYQAFTISENLEYAQYLLIEKGIH
IEDKALAEFFSIHDVLACLFGQYGDLDVEDVWNSYVKDFNEHVAHAECKSAGFTIFKAYC
FRAHKLEAVQEQWSDAEDI
>tr|A0A0Q0DQV5|A0A0Q0DQV5_PSEAP Uncharacterized protein OS=Pseudomonas syringae pv. aptata OX=83167 GN=ALO85_03896 PE=4 SV=1
MTMTDDIDNDEEDFAASTLIEAIENQIEAGNPPAARAVFNMLTLVNYEREDILEMMAHVL
AIEIDALLEQDRPFDTQWYEAALRALPDLPPEK
>tr|A0A0Q0C1N3|A0A0Q0C1N3_PSEAP Uncharacterized protein OS=Pseudomonas syringae pv. aptata OX=83167 GN=ALO85_03659 PE=4 SV=1
MMQGVTVNNPVPRTTKFAVSYKLNGERRFEFAQLQSASTEEAEAALKKMHGESDDLITDV
KVSKAL
>tr|A0A0Q0C3K8|A0A0Q0C3K8_PSEAP Cardiolipin synthase A OS=Pseudomonas syringae pv. aptata OX=83167 GN=clsA PE=3 SV=1
MFHMDYHDPYFFGYVLGFIHLLGTGAAIHALLTVRTSQGAIAWAMPLLFIPYFTLLPYLV
FGRSSFDAYIKARRQANQEMRIAIGDLNWRPWMEEAVAARRSDAYAALRAMPKLGNMPAL
ANNKVKLLINGEETFGAIFQAIREAKKTILIQFFIIHDDKLGRELQSLLLEKAAEGVAIF
VLYDRIGSHALPGAYIDKLRDGGVQIKAFATRGGWLNRFQINFRNHRKIVVVDGLKGYIG
GHNVGDEYMGLKPPLAPWRDTHVQVIGPVVACLQESFAEDWFWATRELPPLSLPDEFPED
GVLCQLLTSGPADAQETCSLFFVEAIHAAEERVWITSPYFIPDEAVTAALTLAVLRGVDV
RLLLPSRPDHYVVYAASSLYAFDAVRAGVRVFRYEPGFLHQKVVLVDNEITAIGSANLDN
RSFRLNFELMLLTVDSDFSSQVESMLTADFNLAREISVQESHETRRLHQLGMRVARLISP
IL
>tr|A0A0Q0DM52|A0A0Q0DM52_PSEAP Iron-sulfur cluster-binding protein, Rieske family OS=Pseudomonas syringae pv. aptata OX=83167 GN=ALO85_01014 PE=4 SV=1
MPSTLISETDTRDDGTLEQWYVACTQKQLRKGKPHRARILGLNIVLFRQRDGQPAALLDQ
CVHRGTRLSAGKITDDCLVCPYHGWRYDANGQVVHIPSVDARQGAAKSPDYRYRQRHFAV
CEQDGLVWVYTGTRDARETPIFRLPAYGERPWQSYFMVNTFDADVGSLVQNFLDVPHTVF
VHEGIFRSSSGRAMEATLSCKAQSIEVVYHDEVDEIGMMQWLTNPDAEPLVHTDRFLAPN
VTFCDYLWGARSGFLIVSQITPVDDRQSRVYTYIAYRFPVPRWVLRILRPFIHLYTHIVI
QQDVRIIQAHREGLDNAAGFKPCNVPADAIHVGVEQLLAAVRRGEALPEAQRRERRIRFE
L
>tr|A0A0Q0FUN7|A0A0Q0FUN7_PSEAP Glycerol-3-phosphate regulon repressor OS=Pseudomonas syringae pv. aptata OX=83167 GN=ALO85_02042 PE=4 SV=1
MNLPPRQQQILELVRDRGYVSIEEMATLFVVTPQTIRRDINQLAEMNLLRRYHGGAAYDS
SIENTAYAMRADQMRDEKQRIAEAIAAQIPDNASLFINIGTTTESIARALLNHNNLKIIT
NNLHVASILSTKDDFEVLIAGGNVRRDGGVVGQGSIDFITQFKVDFALVGISGIDEDGSL
LDFDYQEVRVSQAIIANARKVLLAADSSKFGRNAMVRLGPITLIDCLVTDQPPVPALQQL
LNQHKIRLEVV
>tr|A0A0Q0DE84|A0A0Q0DE84_PSEAP Glycerophosphoryl diester phosphodiesterase OS=Pseudomonas syringae pv. aptata OX=83167 GN=ALO85_01243 PE=4 SV=1
MTLIYGHRGAKGEAPENTLTSFQECLKHGVRRCELDLHLSKDGELMVIHDPTLKRTTDRR
GKVNEHLADDLVTYDARKGGPGWVSPCPIPRLEELFEQCDFEHWQLEVKSASRTRAANTV
LAIREMAQRHGLLDKVTITSSSREVLRAALDLTPDIARGLVAEYAWLDPLKVAQSYGCEI
LALNWTLCTPERLQKAQRQGLHVSVWTVNEPALMRRLADFGVDSLITDFPGLATATLGNH
>tr|A0A0Q0C2Z8|A0A0Q0C2Z8_PSEAP Type IV pilus bioproteinsis protein OS=Pseudomonas syringae pv. aptata OX=83167 GN=ALO85_02794 PE=4 SV=1
MRATSRGFTLIELMIVVAIVGILAAIAYPSYTEYVKRTQRSAIASLLSEQTQALERFYSR
TAPQPTYMGATVSLGNTYYKITPTLNATDFTLTADPIGGTLMAGDKCGSFVITSTGARTN
TGATSGVTVKDCWGR
>tr|A0A0Q0BWG8|A0A0Q0BWG8_PSEAP LexA-like transcriptional regulator OS=Pseudomonas syringae pv. aptata OX=83167 GN=ALO85_03104 PE=3 SV=1
MNTSGDRLRILLRECHLTATDFAANRKITPQHVNNWFKRGVPMARIDEVAELLTVNARWL
RTGEGPKHPNECANENTGGDTRIIIHQARNVSRGDVEIQIFMEAESTHGVGKTVLAEAPG

QKIRLPLQVLQNMGIDPKNCMCVAMVGNSMADKIQDGSILGVDRELTQVIDGEIYALEHG
GILRVRYLYRLPNGGLRLRSHNDAEYPDELFSAEDIDREKIRILGWIFWWSTLNSRRNAM
LLL
>tr|A0A0Q0D665|A0A0Q0D665_PSEAP S-formylglutathione hydrolase
OS=Pseudomonas syringae pv. aptata OX=83167 GN=ALO85_00282 PE=3 SV=1
MPLENISCQKSFGGWHKRYKHHSQVLGCDMVFAVYLPPQAEQGGKLPVLYWLSGLTCTDE
NFMQKAAAHRLAAELGIIIVAPDTSPRGADVADDPDGAWDFGQGAGFYLNATEQPYARHY
QMHDYVVKELPALIEGHFPASQVRSISGHSMGGHGALVCALRNPGRYRSVSAFSPISNPI
DCPWGQKAFSRYLGEDRSRWREWDASVLIAQASEKLPTLVDQGDRDDFLVNQLKPEVLVQ
AAKTADYPLTLRMQPGYDHSYFFIASFIEDHLRHHAAALNS
>tr|A0A0Q0C6A9|A0A0Q0C6A9_PSEAP DNA helicase OS=Pseudomonas syringae pv.
aptata OX=83167 GN=ALO85_02491 PE=4 SV=1
MVTIIWGATKKPVSSLRLKEFFEANEKYDGFLYIGYPVIGTPDGPYPIDAIWLNSNKGLI
AFNLVEGKDLSNYEELQDDSANKLEAKLKNHKNLMKGRNLQIPLSIVTFAPIGTTPEHGV
DPEYPLCKGENLGEFLDGLEDSDIAAYNEALSVIQSISTIRKGRKKRELKRADSRGAILK
SLEDSIANLDNLQGRAVIETVDGVQRIRGLAGSGKTIVLALKAAYLHAQHPDWKIAVTFN
TRSLKGQFKRLINTFFIEQTNEEPDWDNLSIINSWGAPGGTDRNGIYYNFCQAHGLEYLD
LTAAKHKFRQNLEFEGVCGKALSEAQSIKPYYDAILIDEAQDFSPEFLKLCYAFLKEEKR
LVYAYDELQNLSNRSLPSPDEIFGKKPDGTPNVKFGKQRPGEPQQDIILEKCYRNSRPML
ATAHALGFGIYRRPPTGASTGLIQMFDNKELWIDVGYSVTSGSLEENHQVRLSRTEESSP
RFLETHSPLDDLIQFHKFDSLEDQAAWLSKEIEKNITEDELQHGDIVVINPDPLTTRNAV
APARKILFQRGISSHLAGVDTSPDIFFDSDGSSIAFTGIFRAKGNEAGMVYIINAQDCFS
SFGNISRIRNRLFTAITRSKAWVRVLGVGPNMEQLQEEFNQVRAHQFELDFRYPTEEERK
QLNIVNREMTTEEKQSVKSGAENLTKLVSDLQQGLVYLEDLPESSVHLLREILERGKK
>tr|A0A0Q0ICK0|A0A0Q0ICK0_PSEAP Erythronate-4-phosphate dehydrogenase
OS=Pseudomonas syringae pv. aptata OX=83167 GN=pdxB PE=3 SV=1
MRIVADENIPLLDAFFAHFGEIHRLPGRAMDRAAVADADILLVRSVTAVTRELLEGSPVR
FVGTCTIGTDHLDLDWFQQAGIQWASAPGCNARGVVDYVLGSLLTLAEIEGVDLAQRTYG
VVGAGQVGGRLISVLKALGWNVLVCDPPRQASEGGDFVSLDEILQRCDVISLHTPLSKTG
TSPTWHLLDDARLRQLRQGAWLINASRGAVVDNAALHDVLLEREDLQAVLDVWEGEPQVN
VALAELCVLGTPHIAGYSLDGRQRGTAQIYQALCGFLDQPATIELDDLLPRPWLAQVSLD
AACDPVWALNMLCRGVYDPRRDDADFRRSLTGDTASQRLAFDALRKQYPPRREIEGLKVH
LEGESEALAQWVRALGAVLV
>tr|A0A0Q0C0X9|A0A0Q0C0X9_PSEAP Glycosyl transferase, group 1
OS=Pseudomonas syringae pv. aptata OX=83167 GN=ALO85_04348 PE=4 SV=1
MIFINARFLTQDISGVQRYAEQMCRALKRLRDDLVFVAPHNIRLHDCASALEAHCIGRST
GHAWEQIDLPLYLKRRGSPLLISISNTGPMLYGNQIATHHDINYVRYPQSYTRLFRLAYR
TITPILLSRARTLITGSHFSRKEISSFYGYAEDKVLVVPAAVSDDFIPGSTAVNHPKYLL
AVSSPAVHKNFRRMIQAFLSLRGHTDLQLHIVGAASALFADPNLQHLACRDPRIRFLGRL
SDAELIAQYQGATAFVFPSLYEGFGIPPLEAQACGCPVLAANAASIPEVLQASALYFDPL
DVSHMAAAMQRILLDAPLRKALRVQGLQNVQRFSWELSAQRISQRIDTLLAADPAQQSKL
HVAAESSTGKP
>tr|A0A0Q0DQW9|A0A0Q0DQW9_PSEAP Probable septum site-determining protein
MinC OS=Pseudomonas syringae pv. aptata OX=83167 GN=minC PE=3 SV=1
MGFRQNKGRAFYNGSGRLRLAGVLAISLGTFSDKLLCISTMSQIESQNPEPVFQLKGSML
AITVMELARTNLEALDRQLAAKVAQAPNFFSNTPLILALDKLAPHEGPVDLPGLVRICRQ
HGLRTLAIRANRIEDIAAAIAVDLPVLPPSGARERVIEPVEAEAPKKIPEKPPEPLIKPT
RVITAPVRGGQQIYAQGGDLVVVAPVSPGAELLADGNIHVYGPMRGRALAGIKGDTKARI
FCQQLSAELISIAGQYKVSEDLRRDPLWGSPVQISLSGDVLNIIRL
>tr|A0A0N8T8G4|A0A0N8T8G4_PSEAP RES domain protein OS=Pseudomonas
syringae pv. aptata OX=83167 GN=ALO85_01107 PE=4 SV=1
MIAVYRLVKRKWLAQAFDGEGAKLYGGRWNSKGNACVYCAGSESLALLEVLVHLNNATVA
QHYTMLELQIAEEHVLNARPDTLPRDWREEPAPPATASFGDDWLASAQSLALAVPSVIIP
RESNYLLNVQHPEFRAVVATAKELEFVVDPRLG
>tr|A0A0Q0ITJ7|A0A0Q0ITJ7_PSEAP Uncharacterized protein OS=Pseudomonas
syringae pv. aptata OX=83167 GN=ALO85_101649 PE=4 SV=1
MRMAVFVDHAGCPWNVRLIAKTQRWFRADEARTTRNDR
>tr|A0A0Q0DJ51|A0A0Q0DJ51_PSEAP Putative addiction module antidote
protein OS=Pseudomonas syringae pv. aptata OX=83167 GN=ALO85_04160 PE=4
SV=1

MRELKVFEASDYLNNDAEIAEFLSISLEDPNPDVFLQALKEAAKARGMTQLAKDSGMNRE
SLYKALAPGAKPRYDTVLKVMNALGVKLTAQHV
>tr|A0A0Q0FNL2|A0A0Q0FNL2_PSEAP Uncharacterized protein OS=Pseudomonas syringae pv. aptata OX=83167 GN=ALO85_02676 PE=4 SV=1
MESVAMAHELYTRTNQKIYFAGLSLEALGRAEKGQAVNSPALLQAERESALFHLYGALLG
LCHEIAGYYRLPQASARRAEELLTQEVLNAIAIPEMAELVELAQNRQTWLAQLLAAYNAL
YEPPRAPKKLKGDVTQPMIQAVNLDAEPESELSREELENWRQQLKGLAIRFREGLSEC
>tr|A0A0Q0DSS4|A0A0Q0DSS4_PSEAP Uncharacterized protein OS=Pseudomonas syringae pv. aptata OX=83167 GN=ALO85_00846 PE=4 SV=1
MRALAELSFDFVWHLMFTDDKQLDQDLAVRSLENLSDYFSLMSDEEKRAFIAVAHERKAR
LLAVPDADGYSPIRLATEEQAQFLDHVVSGQFFQQFEGPLAADASDRVVPIRR
>tr|A0A0Q0DA28|A0A0Q0DA28_PSEAP Integrase/recombinase XerC OS=Pseudomonas syringae pv. aptata OX=83167 GN=ALO85_100235 PE=3 SV=1
MAMASTASYEHVLHRYPSVQEWLELLGNLGRAPATLEAYGRGLAHYLLHCEASSLEAESI
TFEQVTLYIRRLLPGQENAVANSTLHQRLTAIRLWYDHLVFQGRCAQNPVPRGQHGRLCQ
IPGHSGFVRGLVPRLIKLPDIPTDEQWRYFLSIAARSSIRDRLMLSLAYCGALRRAELVG
LRIEDLDLAHRLISVRAETTKGRRSRVVCYSPDIAPILGTHLHALRLAGWSKGALFRSES
DRNQGSALTRWTWSKTVERWARDSNLSCLSTHTFRHLRLTHLARAGWKLHELTAYAGHRD
PKTTLIYVHLSGADLTARLAHSVGSLDARLFAELFKSE
>tr|A0A0Q0FAX8|A0A0Q0FAX8_PSEAP Uncharacterized protein OS=Pseudomonas syringae pv. aptata OX=83167 GN=ALO85_04053 PE=4 SV=1
MTNLCEALRATANKWRKINQDHRGGIVMIWQGTVYGWKDSLRDASHERPGVFAVDETGHI
FIAAGGDDYNGAKGWVAADLDKQ
>tr|A0A0Q0CVW9|A0A0Q0CVW9_PSEAP Uncharacterized protein OS=Pseudomonas syringae pv. aptata OX=83167 GN=ALO85_04223 PE=4 SV=1
MNLAEQYRAYIACLNDRRWQDLGNFVDDGVHYNGERIRLAGYRAMLENDVRIIPDLRFNI
DLLVVEAPQVAARLLFNCSPQGRFLGLEALGKRIAFSENVFYTFGDGKIVGVWSIIDKAA
VERELRGEAASVSSDRAT
>tr|A0A0Q0DA78|A0A0Q0DA78_PSEAP Uncharacterized protein OS=Pseudomonas syringae pv. aptata OX=83167 GN=ALO85_02287 PE=4 SV=1
MNEPLDYKLMDSIVVCINDTAGFGRKAFESFSLATDVARDMGVDGDDANDLMVEFFERFS
IDLNDYDPYRYFLEEGFNFFSFRRAKDRRGNIPLRVGMLYSALKARRWDTQAFEQATFSD
APLYERTEDIPIDGYKIKSR
>tr|A0A0Q0D7M9|A0A0Q0D7M9_PSEAP Putative potassium efflux system component, mechanosensitive ion channel family OS=Pseudomonas syringae pv. aptata OX=83167 GN=ALO85_02914 PE=4 SV=1
MFSLRSFCAAALFALCLSSFPVLAADPPSSDAVQQSLDKIADRKLPDAEQKALQQVLEQT
LAFLASKKDSEQKLEALKQQLAQAPKQTSENQRELARLKESKVVPVAQRYGGLDVPQLEQ
LLSQRSTQQSDLQSELNDANSLAITAQTRPERAQTEISANQTRIQQINAILKNGKDNGKP
LSADQRNLLNAELASINALNLLRRQELAGNSQLQDLGNSQHDLLTEKVARQEQEIQDLQT
LINDKRRAQSQKTVADLSLEAQKSGGSSLLATESAANLKLSDYLLRGTDRLNELTQQNLK
TKQQLDNLTQTDQALSEQINVLSGSLLLSKILYKQKQALPHLELDKGLADEIANIRLYQF
DINQQREQMSTPTAYVERLLATQPPENVTPQLRRTLLDLAITRSDLLERLNRELSALLNE
SITLQLNQKQLTSTAQGLRATLDEQMFWIPSNKPLDVEWFQNIWPRLQKQVSTLPWTSSL
SELSDGLTQRPLLFLPLLLLIGILTWRRKALYQKLNRLHADIGHFKRDSQWKTPLALLFN
VLLAMPVALGLALCGYALQIDARGQNANLGEALLQIALAWLVFYTAYRVLAPQGVAQLHF
RWETAQVEFLRGWVRRLGLVVLALVAVVAVAEHQPAALADDVLGIGVVLTCYALMTWLLA
RLLISSPTHHNASLFRKTVGVVFTALPVALFLAVCFGYYYTALKLSDRLIDTLYLMMIWL
MVEATFVRGLSVAARRLAYQRALAKRQAARENGDSDIPVEEPKLDIEQVNQQSLRLIRLA
LLAGFVGALYLVWAELITVFAYLDNIILYEYTSGTGANMSMVPISLSDFLGAGVIIVITF
VLAGNLPGLLEVLVLSRMNLAQGSAYATTTLLSYTIAGVGFVTTLSTLGVSWDKLQWLVA
ALSVGLGFGMQEIFANFISGIMILFERPVRIGDTITIGALSGTVSKIRIRATTITDFDRK
DIIVPNKTFITGQLINWSLTDTVTRVTLKLGVDYGSDLDLVRSLLLQAARENPRVLKEPE
PIVYFLNFGESTLDHELRMHVRDLGDRNPVLDEINRFINREFKKQHINISFRQMEIYLKN
TQGLEYKLVPAEPGDKHGAPAGQTTLQPVNTQVTPATKDAPEPPELRLD
>tr|A0A0Q0IIY6|A0A0Q0IIY6_PSEAP Peptide chain release factor 1 OS=Pseudomonas syringae pv. aptata OX=83167 GN=prfA PE=3 SV=1
MKASLLNKLDVLSDRFEELTALLGDAEVISDQTRFRAYSREYAEVEPVVTLYAQLLRVQG
DLEGAQALLKDSDPDMREMAVEEVRETRQQLVELEAQLQRMLLPKDPNDGRNVFLEIRAG
TGGDEAAIFSGDLFRMYSRYAERRGWRVEILSENEGEHGGYKEVIARVEGDSVYGKLKFE

SGAHRVQRVPETESQGRIHTSACTVAVLPEPDEQQAIEINPADLRVDTYRSSGAGGQHVN
KTDSAIRITHLPSGIVVECQEERSQHKNRARAMSWLSAKLNDQQTSAAANAIASERKLLV
GSGDRSERIRTYNFPQGRVTDHRVNLTLYSLDEVLAGGVDAVIEPLLAEYQADQLAALGE
>tr|A0A0Q0DKC3|A0A0Q0DKC3_PSEAP Phosphonate ABC transporter, inner membrane subunit OS=Pseudomonas syringae pv. aptata OX=83167 GN=ALO85_02771 PE=3 SV=1
MKRAGNLLLVLALIAAVIASFVYLGLDLLTLGSHDNLTQMGRYALRFMSPDLSGPHLQAI
GQGALETLAMSALGTLLAAIAGLLLALPAAGRFGWFLRSLSRLLLNALRAIPELVWAVLM
VLAAGLGPNAGTLALALHTTGVLGRLFAEALENTAPQPAAAIRLQGGSAWQAFSYGTLPN
LWPQLLAYTLYRWENNIRMASVLGFVGAGGLGQMLYVSLSLFQEAQASTVILAMLILVFA
VDALSSWSRQRWVRN
>tr|A0A0Q0IKP5|A0A0Q0IKP5_PSEAP Phage_X domain-containing protein OS=Pseudomonas syringae pv. aptata OX=83167 GN=ALO85_00911 PE=4 SV=1
MFIDWLSISQEHDHDLPVVCDVQFLTVDTLSGDVISTKQPKFKHTESYSTSVTIHVQGRK
IRIDGNPSRIGRLDNLFGFTNIDQCVSVYNALLAQYGLPAFTRCTKVYLRDGASGAKSGD
LVADGAKIERIDLTTNVSLGEGNCLAYLRGISSQRIGHSIGFLYPNGRTVTWTPKGNGQG
GRLQYRKAYDKSFEMDQNSLPKIKRAFGENSQEYAYVKQVRDYCASNGVVRMEQELKNEY
LKREGLAYWGMFNESRLTTIHNEFLGLDQRMKVTAMDLMSIADKLIEEGVCKGRASANAT
ASQAILWMSGSPHGISQRAFETHAARLNRIGINIRNACDTSRYAPVFVRQCREVTKSALS
IPAWYRRPNHLQLAA
>tr|A0A0Q0IFT0|A0A0Q0IFT0_PSEAP Major Facilitator superfamily transporter OS=Pseudomonas syringae pv. aptata OX=83167 GN=ALO85_00676 PE=4 SV=1
MFNWYRDVTPKERKTFWACFGGWSLDALEVQMFGLAIPALIAAFALTKGDAGLISAVTLV
TSALGGWVGGTLSDRYGRVRTLQWMILWFSFFTFSSAFVTGFNQLLIVKALQGFGIGGEW
AAGAVLMAETIQSRYRGKVMATVQGAWAVGWGLAVVLFTLIYSFVPEDIAWRVMFFVGLL
PALMIIWVRRNVEEPDSFQRMQKSAAPKDNFFKSMAGIFRPELLRVTLLGGLLGLGAHGG
YHAVMTWLPTFLKTERNLSVLSSGGYLAVIIVAFWCGCVCSGLLIDRIGRRKNIMLFALC
CVVTVQCYLMLPLSNTQMLFLGFPLGFFAAGIPASLGSFFNELYPAEVRGAGVGFCYNFG
RVLSAVFPFLVGHMSQSMSLGSAIGIDAGIAYGVAMIAAFCLPETRGRNLEATLPVAEPT
GQGRRAAGLKTFHSRCCGTALTLYRRTHD
>tr|A0A0Q0BZQ1|A0A0Q0BZQ1_PSEAP Major facilitator transporter OS=Pseudomonas syringae pv. aptata OX=83167 GN=ALO85_02645 PE=4 SV=1
MTINSDGDGMQMRGSAAQNMGLMISVLLVALNLRPSMAAVGPLLVAIRLDVPLGFGTASL
LTMLPVMAMGLAMFFGMGIARYLGSYRSIMLSLALIGLATVARLFLDSAAELILSAVLAG
LGIALIQALMPALIKLRFKANVSLYMGFYVTAIMGGAALAASFSPLVMDSSGSWRVGLAV
WSLLALLAMCFWFAQRHALQSDASTAAGHRQTFFHTPRAWMLGIFFGLGTASYTCVLAWL
APYYLENGWSEQSAGLLLGFLTLIEVVSGLLAPALANRSRDKRMALVVLLTFMMIGFAGL
ILMPQQLGLLWTCLLGLGIGGLFPLSLIVSMDHFDDPQQAGGLTAFVQGVGYLIASLSPL
LAGVIRDLTGSFEGAWWALMALVAIMLLMVVRLDPRRYADHLRNALDEPVITGREAA
>tr|A0A0N8T898|A0A0N8T898_PSEAP Branched-chain amino acid ABC-type transport system OS=Pseudomonas syringae pv. aptata OX=83167 GN=ALO85_03132 PE=4 SV=1
MSTEVILSVEHLMMHFGGIKALSDVSLKVRRNSIFALIGPNGAGKTTVFNCLTGFYKATG
GRIELHTRGKTTNVIRLLGEPFQATDFVSPKSFLSRVYYKMFGGTHLVNRAGLARTFQNI
RLFKEMSVVENLLVAQHMWVNRSLISGILNTRGYRKAEEDALNTAFYWLEVVDLVDCANR
LAGELSYGQQRRLEIARAMCTRPQVICLDEPAAGLNPQETEALSGMIRLLRDEHDMTIVL
IEHDMGMVMGISDDIVVLDHGNVIAMGGPEQIRNDPKVIAAYLGADEEELV
>tr|A0A0Q0FT91|A0A0Q0FT91_PSEAP Xylose ABC-type transport system, periplasmic substrate-binding protein OS=Pseudomonas syringae pv. aptata OX=83167 GN=ALO85_03741 PE=4 SV=1
MNTLKRTLLASALALLSLPVMADSAHPKIGFSIDDLRLERWSRDRDYFVAAAEKLDAKVF
VQSADANEQKQISQIENLISRGVDVIVIVPFNATVLTNAVAEAKKAGIKVVSYDRLILNA
DIDAYISFDNEKVGEMQANGVLKAAPKGNYFLLGGAPTDNNAKILREGQMKALQPAIDRG
DIKLVGQQWVKEWSPTEALSIVENALTRNNNKIDGIVASNDATAGGAIQALAAQKLAGTV
PISGQDADLAAVKRVIDGTQTMTVYKPLKLIATEAAKLSVQLARKEKPEFTSKYDNGSKK
VDTILLTPTALTKDNIDLLEKDGFYTKAQIDGQ
>tr|A0A0Q0CDI9|A0A0Q0CDI9_PSEAP Putative biotin carboxylase OS=Pseudomonas syringae pv. aptata OX=83167 GN=ALO85_02729 PE=4 SV=1
MKHVLIINRYDDELSDYRNYIDHSQVDVSYISLAGHSRLIDPQVSANVVEVSALDADLIL
QEARAIHRRQPVDFVIAFSEYDLDAAALVRTEFNIPGAKVADNLLCRNKASMKEALTGSS

VRYPQYRNVASRGGVEAFCHEHGFPVILKPQVGAASDGVVKIERLEDIPDLPDFNGYEVE
EYIEGDIYHVDAILAADSMPYFKVSKYLNTCLDFRNGLPLGSVTVDDRAFIARVRAFTEE
VCLRLNLHNQAIHLEIIERRGELVFLEIGGRVGGGEIPFVALRSEGIDLYELWTRAALEV
AIAPVDTRITGFLMMPNPFPGGFTFDPLMELSHPLLTYSSVQSQGDSNAFSYEDIPARLH
FAADSQAAIEQAIVQCMSILQTSIRAT
>tr|A0A0Q0DAN3|A0A0Q0DAN3_PSEAP Putative aldehyde oxidoreductase, FAD-binding subunit OS=Pseudomonas syringae pv. aptata OX=83167 GN=ALO85_03413 PE=4 SV=1
MRTFDYARAASPAQAFSTASGEGQRFYLAGGTTLLDLVKLDVMQPQQLVDINHLALKQVE
SLPDGRLRIGALVSNTDLASHPLVQQRYPVLSEAILAGASTQLRNKATTAGNLMQRVRCP
YFRDGISACNKRQPGSGCAAIGGMNRSVHAVLGTSEHCIATHPSDMCVGMAAIGGQVTVQ
GANGSRDIPFADFHLLPGDTPEREHALAPHELITHVTLDAPLSGSRSSYLKLRDRTSYQF
ALASSAVILVMDGKRITDARIALGGVGTKPWRALDAERALIGQRADLNTFAQVAALAMKG
SRAYEHNAFKIPLGQQVIVRNLRDLTA
>tr|A0A0Q0FCJ0|A0A0Q0FCJ0_PSEAP Topoisomerase II OS=Pseudomonas syringae pv. aptata OX=83167 GN=ALO85_100032 PE=4 SV=1
MKALLVALALCGISSFAMAEESQTKVAAQQARVEQYTYGTHLDIARVISISQIPNVCAVV
PARMVYEDSQGARHTMGYQVMGNGCSNG
>tr|A0A0Q0IDS2|A0A0Q0IDS2_PSEAP UPF0250 protein ALO85_01337 OS=Pseudomonas syringae pv. aptata OX=83167 GN=ALO85_01337 PE=3 SV=1
MSVVTACETFTAMTDTDIKSHKIEFPCNDYPIKVIGDTSVGFTAAVMEVLEKHATVDLKT
LAERQSSNGKYTTVQLHIVATGEDQLRDINSALRATGFVHMVL
>tr|A0A0N8T830|A0A0N8T830_PSEAP PTS IIA-like nitrogen-regulatory protein PtsN OS=Pseudomonas syringae pv. aptata OX=83167 GN=ALO85_00164 PE=4 SV=1
MIRIENILTPGRSLVDVPGDSKKRVLEDIANLIAREVPGMDFETVLGSLVAREKLGSTGF
GNGIAIPHCRLPGCEDPVSAVIHLTNPINFDAIDGAPVDLLFVLLVPQAATDAHLELLRQ
IASMLDRADVRERLRSAKSGEALYQVVLDVQNGH
>tr|A0A0Q0FGW7|A0A0Q0FGW7_PSEAP Type III effector HopI1 OS=Pseudomonas syringae pv. aptata OX=83167 GN=ALO85_01306 PE=4 SV=1
MPPRRYEFPEKQMINLTNLASSLARVALSDSTKPTIDRAMNVVSHIAGKVALQVNSSLLE
QKGRLNERQQRGLEVILDALLGKEPVTHVETHEGGGRFNLARAAFDVASVVWERDQSMRN
VMSFLGVSDSQGKMLFSLGKTLADAMASLEPEHGKHNSEANYAFFSSNLKLNKLMNDIAD
QVINEIHQQSTDRVRRPTPGPSWRPESTQQQARPQTPPNARPQANSAPPPPKAQPNSGAQ
RPSTERPHKTPAADASAKVDDSAPAKPPVKPLYEHLGLSDMTADLSAVKKAYKKASLKNH
PDKNVGNEAEATERFKVISNAFRILSDPELRKKYDNGLIDENGNEFKT
>tr|A0A0Q0DKS0|A0A0Q0DKS0_PSEAP Methylthioribulose-1-phosphate dehydratase OS=Pseudomonas syringae pv. aptata OX=83167 GN=mtnB PE=3 SV=1
MSREQLSLEIIEAGRFLYGRGWSPATSSNYSARLSASEALLTVSGKHKGQLGPDDVLATD
LAGNSLEPGKKPSAETLLHTQLYSCRPQIGAVLHTHSVNATVLSRLTTADHLVFEDYELQ
KAFSGVVTHESQVVVPIFDNDQDIARLAAKVQPWLDTHPECAGYLIRGHGLYTWGAKMSD
ALRQIEAFEFLFECELKMRAVLNR
>tr|A0A0Q0DBB1|A0A0Q0DBB1_PSEAP GTPase Der OS=Pseudomonas syringae pv. aptata OX=83167 GN=der PE=3 SV=1
MESRMVPVIALVGRPNVGKSTMFNRLTRTRDAIVGDLSGLTRDRQYGEAKWQGRSYILID
TGGISGDEHGMDEKMAEQSLLAIEEADVVLFLVDARAGYTAADQMIGEHLRKRNKRSYVV
ANKIDNIDENLARAEFSPMGLGDAIPVAGAHGRGISQMLEIALREFPRDEDELEEGAEVE
EVAEGQEAKRIPGPSEKDGIKIAIIGRPNVGKSTLVNRMLGEDRVIVYDEPGTTRDSIYI
PFERNEEKYTLIDTAGVRKRGKIHEEVEKFSVVKTLQAIKDANVVIFVMDAREGVVDHDL
NLLGFALEAGRALVIALNKWDGMTPGERDFVKIELERRLFFVDFADIHFISALHGTGVGN
LYQSVQNSFKSAVTRWPTSRLTQILEDAVSEHAPPMVGSRRIKLRYAHLGGANPPLIVIH
GNQVEKVPKSYVRYLENTYRRVLKLVGTPIRIEFKGGENPYEGNKNTLTDRQVNKKRRMM
SHHKKADKKRRDKR
>tr|A0A0Q0FWL0|A0A0Q0FWL0_PSEAP Uncharacterized protein OS=Pseudomonas syringae pv. aptata OX=83167 GN=ALO85_01427 PE=4 SV=1
MLKKYTSGLLLLCALALSHTAWANDNASRQPLTLYLVLHDDLSHHNTLELRQDYFAWLIK
DLESFTDRYVRLAFIRDVPGLTDFAYQNDDVDETYYAWKNTMDRYVIDNDMERNATTKYL
LLTHNKINSKTLGASEDKSYTGIASLETFAIPAHELGHMLGGTHEAAEVLYKGGWWCQTN
IIESHEKIRSNCYVYSDANKQIMAEHLNRFP
>tr|A0A0Q0D2Q4|A0A0Q0D2Q4_PSEAP Putative lipoprotein OS=Pseudomonas syringae pv. aptata OX=83167 GN=ALO85_02206 PE=4 SV=1

MRKSLAYALMLSATLGLAACDKKSENTQQDANKAAQQSQESMEKAQEKVNDAAKESQEAA
QKNAEAAQQRTNENADKAPEAAPATK
>tr|A0A0Q0IJF7|A0A0Q0IJF7_PSEAP Uncharacterized protein OS=Pseudomonas syringae pv. aptata OX=83167 GN=ALO85_03905 PE=4 SV=1
MFDISHITALTSSPTLTTVAKEVSPTSSTSAISKLSSERSADSATLSTLSSQLNSSAMRA
QIRDSRLGFKELGAVATSINDKITGTHYYANRALHDAEVPDTDDPALLERARQATLFKNG
GGANPFSGLSQDQLKLIMYDEGGEFTINERSAASSENHLQQEAWNRAMGKRYVDEYNETG
KSTQTLVMLFAHYDQLPPIEKAQYPANYAANITSGDSSAMDIFKTLKSQSGIQGT
>tr|A0A0Q0C2U1|A0A0Q0C2U1_PSEAP Uncharacterized protein OS=Pseudomonas syringae pv. aptata OX=83167 GN=ALO85_03404 PE=4 SV=1
MSHSSQQQFRSVWATLQSLRKQVADLQLSELERAESLRGHQTVDDREVIEQSFVALEQAI
DDMEVTLASIGEAAGEIGKL
>tr|A0A0Q0D8B4|A0A0Q0D8B4_PSEAP Uncharacterized protein OS=Pseudomonas syringae pv. aptata OX=83167 GN=ALO85_100819 PE=4 SV=1
MRVLVGLHQQEQGHQYGAMSVKLQKTNQGSEKKRFICKIGPFL
>tr|A0A0Q0D3P5|A0A0Q0D3P5_PSEAP Alginate biosynthesis protein AlgJ OS=Pseudomonas syringae pv. aptata OX=83167 GN=ALO85_01801 PE=4 SV=1
MTRSLRILYIAIFLGILLALGIWSLRSFSTFSTSAETTVLNGKWTKAAETHYDDEFPIKR
LGTNLWAALDFKLFNEGRPGVVLGKDQWLYTDEEFDAVANGEQNEADNLAIIQGVRDALE
KQGTKLVLAIVPAKTRLYPEHIGDSKPASLHTDLYQQFHAQVAKAGIFAPDLLAPLQAAK
QQGQVFLRTDTHWTPMGAEVAAQQLSAAIAQKTPLEGEPEPFVTQAKGTEAYKGDLTTFL
PLDPLFSNMLPKPDELQKRSTDPVAGEAAGGDALFADSDIPVGLVGTSYSANPNWNFVGA
LKEALRSDVVNYAEDGHGPILPMLKYLQTDAFKNTPPQVVIWEFPERYLPAHNDLGEFDP
KWIAELKKSRDTQENVALNAKQSESPNRAQN
>tr|A0A0Q0BUA3|A0A0Q0BUA3_PSEAP Phosphoglycerate/bisphosphoglycerate mutase OS=Pseudomonas syringae pv. aptata OX=83167 GN=ALO85_00498 PE=4 SV=1
MGSIYLIRHGQASFGAENYDVLSPMGIRQSQVLGAYLAELGLSFDRCVSGELMRQRDTAQ
HVLSQYTEAGLDTPDVQLDSAFDEFDAEGVIRALIPAMLEEEPQALDILRDAAANPAGFQ
RLFNLITRRWLSGNHDTPGLQSWQSFVTRVEAGLKRILQAAGPHERIAVFTSGGTITALL
HLITGMPASKALELHWHIVNTSLHQLKFKGNDVTLASFNGYTHLQLLKAPELITYR
>tr|A0A0Q0D3S8|A0A0Q0D3S8_PSEAP Probable alginate O-acetylase OS=Pseudomonas syringae pv. aptata OX=83167 GN=ALO85_01802 PE=3 SV=1
MVFSSNVFLFLFMPIFLGLYYLSGQRYRNLLLLIASYVFYAWWRVDFLALFVGVTVWNYW
IGLKVGAAGVRTKPAQKWLLLGVIVDLCILGYFKYANFGVDSINAAMTSMGLEPFILTHV
LLPIGISFYVFESISYIIDVYRGDTPATRNLIDFAAFVAIFPHLIAGPVLRFRDLADQFN
NRTHTLDKFSEGATRFMQGFIKKVFIADTLAVVADHCFALQNPTTGDAWLGALAYTAQLY
FDFSGYSDMAIGLGLMMGFRFMENFKQPYISQSITEFWRRWHISLSTWLRDYLYITLGGN
RGGKVATYRNLFLTMLLGGLWHGANVTYIIWGAWHGMWLAIEKAVGINTKPYSFNPIRWA
LTFLLVIIGWVIFRAENLHVAGRMYGAMFSFGEWQLSELNRASLTGLQVATLVVAYLTLA
FFGLRDFYQNREKDTGKSTKADGPATAQPGTIKAVPGDAPGSLHMPGYTVGSEAQVQPAY
WEADWPRYAMRALILLLFVASILKLSAQSFSPFLYFQF
>tr|A0A0Q0ID24|A0A0Q0ID24_PSEAP Phage integrase:Phage integrase, N-terminal SAM-like protein OS=Pseudomonas syringae pv. aptata OX=83167 GN=ALO85_04716 PE=4 SV=1
MLAVLLYHGLRREEGAQLKTSDLQERRGIKHLRVNGKGGKLRWLPLHPVAAERIYAYMEK
DLERGAGNGSLFRSLRGRASGTGLSADGIYKIVCHWAMSAGIKVDGLGVHGLRATAATNA
LEHDADIAKVQIWLGHANISTTRLYDRRGQRPEDSPTFKVKY
>tr|A0A0Q0DAS4|A0A0Q0DAS4_PSEAP Rhs family protein OS=Pseudomonas syringae pv. aptata OX=83167 GN=ALO85_03375 PE=4 SV=1
MSAAARVNDPIEHTGSLTGLLAGLAIGAIGAALVVGTGGLAAVAIVGASAATGAGVGQLI
GSLSCCNHQTGQIVSGSSNVYINGEPAARAHADQAKCDEHSSRPQVIAQGSSNVYINGHP
AARVGDRTACDAKIVVGSSNVFIGGGTETTDPINPEVPELLERGILLVGLASAFVLASPV
IVIAGLVGGIAGGTVGSMGGAQLFGEGTDGQKLMAFGGALLGGGLGAKGGKWFDTRYDIK
VQGVGSNLGNLKITPKGAAKVSNIAESEAALGRASQARADLPQSKELKVKTVSSNDKKTL
SGWGNKKPEGYERISAEQVKAKSEEIGHEVKSHPYDRDYKGQYFSSHAEKQMSIASPNHP
LGVSKPMCTDCQGYFSQLAKYSKVEQTVADPKAIRIFKTDGSVETIMRSE
>tr|A0A0Q0C3J1|A0A0Q0C3J1_PSEAP N-acetyl-anhydromuranmyl-L-alanine amidase OS=Pseudomonas syringae pv. aptata OX=83167 GN=ALO85_03314 PE=4 SV=1

MQLDSASGWCDGVQHCPSPNFNARPEGEISLLVIHNISLPPAQFKTGKVQAFFQNQLDID
EHPYFVGIADLRVSAHFLIERDGEVVQFVSCLDRAWHAGVSSFQDREGCNDFSVGIELEG
TDDQPFTDAQYDTLIDLARQLRQAFEAITPERICGHSDVAPGRKTDPGPCFDWARFRAAL
LD
>tr|A0A0Q0FNR6|A0A0Q0FNR6_PSEAP LysR family transcriptional regulator OS=Pseudomonas syringae pv. aptata OX=83167 GN=ALO85_04462 PE=3 SV=1
MGYIDMRFTLRQLQVFVAVARQESVSKAAVLLSLSQSAASTSITELERQSSCQLFDRAGK
RLSLNATGRQLLPQAVALLDQAKEIEDLLNGKSGFGSLAVGATLTIGNYLATLLIGGYMQ
RHPESQVKLHVQNTAHIVQQVAHYEIDLGLIEGDCSHPDIEVQSWVEDELVVFCAPQHPL
AKRAQVTLEELTHEAWILREQGSGTRLTFDQAMRHHRNGLNVRLELEHTEAIKRAVESGL
GIGCISRLALRDAFRRGNLVELATPELDLARQFYFIWHKQKYQTSAMREFLELCRAFTAG
VQRSDEIVLPSLY
>tr|A0A0Q0C1I2|A0A0Q0C1I2_PSEAP GDT1 family protein OS=Pseudomonas syringae pv. aptata OX=83167 GN=ALO85_01740 PE=3 SV=1
MLESFLVPTAVVALAEIGDKTQLLALVLAARFRKPWPIIAGIVAATLANHAAAGAVGAWF
SSYLSDAVLHWILAASFTATALWTLVPDKMDDDEASTARKFGPFMTTLITFFIAEIGDKT
QIATVMLAAQYSYLWLVILGTTVGMLLANVPVVLAGNFAAEKLPLTLIRRLAACAFFVLA
LVAVYKAMQVSGWV
>tr|A0A0Q0IRZ2|A0A0Q0IRZ2_PSEAP Spermidine/putrescine ABC-type transport system OS=Pseudomonas syringae pv. aptata OX=83167 GN=ALO85_03360 PE=4 SV=1
MKSTKRLALTGLSCLPLAAALLSTPAMAETTLYLGMNGGTMERLYADQVLPAFEKANNVK
VVIVPGTSADILAKVQASKDNPQMHVIFLDDGIMYRAISMGLCDTLQPSASLADLPAKAK
IKEQAAAVSLGVTGLAYNTRMFKEQGWSAPTSWMDLADKRFKDKVVFQSLASSTFGLHGF
LMFNRIQGGSEKDVEPGFKAWPKTIGPNVLEYIASSAKISEMVQTDEAALFPLTPTQVTA
LKIKGVPVEYASPKEGGVVLNVAECAIANNNQPELAQKLAAYLLTPEAQAPALEFGDQIP
SNPKTPTTDKTRSQVEAMEKYLETAVTIDWDQVNQIRPEWNARWSRSIER
>tr|A0A0Q0INX4|A0A0Q0INX4_PSEAP Uncharacterized protein OS=Pseudomonas syringae pv. aptata OX=83167 GN=ALO85_05046 PE=4 SV=1
MAFLLLLPLLVSGFLVCLKDPTVYCRLHRYEGQMLYFLVGRYGIYCFLAAMFVTAALAGL
FSHDWGVFCPKWAQTTDATSPVCFAVNTDFMGALGQMGQDFDITGRNFSQVGVFLALSGL
LTLAMPSLGAFLTVRILKWQLGAGKKEEIAVYLLGQSVDHSPICSTLFDAFVDKEEVMIT
MADRKVYVGYIMDVGAPTEVTGVNQEILLIPTVSGYRDKDTLKVVYTTDYPSDTPLRPIG
FRQENIVSISIFSEEVREAFKRVDSERAGEETAKEKAAKDQLVKAITELVAVVQAAQR
>tr|A0A0N8T958|A0A0N8T958_PSEAP Gamma-glutamyltranspeptidase OS=Pseudomonas syringae pv. aptata OX=83167 GN=ALO85_02770 PE=4 SV=1
MKFEPLARSLIATALIVAYSPTFAASQAPVAAENGMVVTAQHLATHVGVDVLKAGGNAVD
AAVAVGYALAVVYPAAGNLGGGGFMTIQLADGRKTFLDFREKAPLAATANMYLDKDGKVI
PDLSAKGHLAVGVPGTVSGMEMALSKYGTRKREEVIAPAIKLAEEGFVLGQGDVDMLSSA
TDVFKADMADSGSIFLNKGEPMQVGQKLVQKDLAKTLKEVSEKGSDGFYKGWVAKALVDS
SQAGKGIITQADLDHYKTRELAPIECDYRGYHVVSAPPPSSGGVVICQILNILEGYPMKE
LGFRSAQGMHYQIEAMRHAYADRNSYLGDPDFVKNPVEHLLDVKYAAKLRAAIEPQKAGD
SSKIKPGVAPHEGSNTTHYSIVDKWGNAVSVTYTLNNWFGAGVMASKTGVILNDEMDDFT
VKVGVPNMYGLVQGEANAIEPGKTPLSSMSPTIVTKDGKAVMVVGTPGGSRIITATLLTM
LNVIDYGMNIQEAVDAPRFHQQWQPEETNIDTFAVSPDTLKILESWGQKFAGPQPLNHVA
AILVGAPSLGGKPVGNNRFYGANDPRRNTGLSLGY
>tr|A0A0Q0C0W0|A0A0Q0C0W0_PSEAP GNAT family acetyltransferase OS=Pseudomonas syringae pv. aptata OX=83167 GN=ALO85_02902 PE=4 SV=1
MSIKWICKHHTELSIEQLYAVLQLRAEVFVVEQQCVYLDVDGQDLTGDTCHLMAWQEDKL
VAYLRLLDPIQQGGDVTIGRVVTAPSIRSRGIGHELMEQALENAERKWPDQPIYLSAQAH
LQGYYSRYGFNPVGEVYLEDDIPHIGMRRDLD
>tr|A0A0Q0FM01|A0A0Q0FM01_PSEAP Fructose-1,6-bisphosphate aldolase OS=Pseudomonas syringae pv. aptata OX=83167 GN=ALO85_03999 PE=3 SV=1
MALISMRQMLDHAAEFGYGVPAFNVNNLEQMRAIMEAADKTDSPVIVQASAGARKYAGAP
FLRHLILAAIEEFPHIPVVMHQDHGTSPDICQRSIQLGFSSVMMDGSLREDGKSPADYDY
NVDVTRRVVSFAHACGVSVEGELGVLGSLETGMAGEEDGVGAEGVLDHSQMLTDPEEAAD
FVKRTQVDALAIAIGTSHGAYKFTKPPTGDILAIERIKEIHKRIPNTHLVMHGSSSVPQE
WLKIINEFGGDIKETYGVPVEEIVEGIKHGVRKVNIDTDLRLASTGAIREFMAKNPSEFD
PRKYLAKTVSAMRDVCIARYEAFGTAGNASKIKPISLDAMFERYARGELDAKVN

>tr|A0A0Q0C0K7|A0A0Q0C0K7_PSEAP Cytochrome c oxidase, cbb3-type, subunit IV OS=Pseudomonas syringae pv. aptata OX=83167 GN=ALO85_03008 PE=4 SV=1
MMDIGMIRGLGTLVVMVAFIGLMLWVFSPARKADFDDATLLPFADDPEAIGKVENAKAER
EGRS
>tr|A0A0Q0D7B0|A0A0Q0D7B0_PSEAP 30S ribosomal protein S8 OS=Pseudomonas syringae pv. aptata OX=83167 GN=rpsH PE=3 SV=1
MSMQDPLADMLTRIRNAQMAEKPVVSMPSSTLKVAVAKVLKDEGYIAGYQISSEVKSSLS
IELKYFEGRPVIEEVKRVSRPGLRQYKSSDDLPKVRGGLGVSIVSTSKGVMTDRAARAAG
VGGEVLCTVF
>tr|A0A0Q0DE17|A0A0Q0DE17_PSEAP Uncharacterized protein OS=Pseudomonas syringae pv. aptata OX=83167 GN=ALO85_01180 PE=4 SV=1
MNPQERSNEVQVVRATPNLPVGGAVLDQHGEEVLITEEMVQAACQECDKNWVTPEK
>tr|A0A0Q0BTV4|A0A0Q0BTV4_PSEAP Uncharacterized protein OS=Pseudomonas syringae pv. aptata OX=83167 GN=ALO85_101932 PE=4 SV=1
MFKVGTQGWPAGAAKGRSFMMFLHYWVKQGMHDTTKGIVHTLRCG
>tr|A0A0Q0FQT5|A0A0Q0FQT5_PSEAP Protein adenylyltransferase SelO OS=Pseudomonas syringae pv. aptata OX=83167 GN=selO PE=3 SV=1
MKALDELIFDNRFAHLGDAFSTSVLPEPIDAPQLVVASQSALALLDLAPGQADLPLFAEI
FSGHKLWSEAEPRAMVYSGHQFGSYNPRLGDGRGLLLGEVYNDAGEHWDLHLKGAGRTPY
SRMGDGRAVLRSSIREFLASEALHALGIPSSRAGCVVSSSTPVWRETQEHAAMVLRLAQS
HVRFGSLEYFFYTKQPEQLKTLAEHVLTMHYPHCQEQPEPYLAMFREIVERNAELIAKWQ
AYGFCHGVMNTDNMSILGITFDFGPFAFLDDFDEHFICNHSDHEGRYSFSNQVPIAQWNL
SALAQALTPFISVEALREAIGLFLPLYQAHYLDLMRRRLGLTVAQEQDEQLVSQLLKLMQ
NSGVDYTLFFRRLGDQPAAEALRTLRDDFVDIKGFDGWAQAYQARIALEDNGTEQERQTR
MHAVNPLYILRNYLAQNAIAAAEKGDYEEVRRLHQVLCTPFTEQPGMQGYAQRPPDWGKH
LEISCSS
>tr|A0A0Q0ID68|A0A0Q0ID68_PSEAP Cystine transporter subunit OS=Pseudomonas syringae pv. aptata OX=83167 GN=ALO85_01680 PE=4 SV=1
MNISAIRGTFLFSAMGLMLGAGIAGQAMAGEQLQKIKDSGTLNVGLEGTYPPFSFVDESG
KLTGFEVEFSEALAKELGVKAKIQPSKWDGILAALDSKRLDVVINQVTISDERKKKYDFS
EPYTVSGIQALVLKKNEDAIKSAKDLTGKKVGVGLGTNYEEWLKANVPGAIVKTYDDDPS
KIQDLSVGRIDAILVDRLAALEMAAKTKNKLVPAGDAFSRQESGIALRKGEPELLAAIDK
AIDKLRADGTLKKLSEKYFNADVTK
>tr|A0A0Q0IAI7|A0A0Q0IAI7_PSEAP D12 s N6 adenine-specific DNA methyltransferase OS=Pseudomonas syringae pv. aptata OX=83167 GN=ALO85_04164 PE=4 SV=1
MKYLGSKTKLLGFIDSVVTECVSNIGKPSDEIVLCDLFSGSGKVANHFKDRFKLIANDLE
YYSYATLENLLNNDSSTVEECQPILDYMNHNMAGEEGFIFQNYSEAGGRTYFTNDNALLI
DAGISLVYGMYGRGELTDQQFYYCLCSVLEAADRASNTTGLYSAYLKKLSTTALKPIEFK
PFDLKESVASNEVYLGDANDLIQEIRGDVLYLDPPYTPMQYSNAYHLLNTIAQNEQPTIH
GISGRPEGRNVSPWSSKKLVEGEFRRLVESANFEYLVMSYSNESIMPVELIGDVMSSYGK
YEMKKMQYKKFKSRTEASNQAYVTEYLHVLHKAS
>tr|A0A0Q0DS65|A0A0Q0DS65_PSEAP 50S ribosomal protein L27 OS=Pseudomonas syringae pv. aptata OX=83167 GN=rpmA PE=3 SV=1
MAHKKAGGSTRNGRDSEAKRLGVKMYGGQAIIPGNIIVRQRGTQFHAGYGVGMGKDHTLF
AKVEGVIKFQVKGAFGRRYVSIVPKTEVSAA
>tr|A0A0Q0BUU2|A0A0Q0BUU2_PSEAP GCN5-related N-acetyltransferase OS=Pseudomonas syringae pv. aptata OX=83167 GN=ALO85_00111 PE=4 SV=1
MITIRSMTLDDYDAVIELMRSTPGISLRDADSREATARYLERNPGMSFVAEADGALCGCV
MCGHDGRRGYLQHLIVLPEYRRRGTAHELVERCLECLDALGIYKCHLDVMKSNEAAGRYW
QGQGWTLREDIDRYSKVRRDGNV
>tr|A0A0Q0DJQ9|A0A0Q0DJQ9_PSEAP UPF0125 protein ALO85_00117 OS=Pseudomonas syringae pv. aptata OX=83167 GN=ALO85_00117 PE=3 SV=1
MADASIQIEVVYASVQHQVLKTVDVPAGSSVRQALALSGMDKEFPELDLSQCAVGIFGKV
VADPAARVLEAGERIEIYRPLVADPMEIRRLRAAKAREKRTPTG
>tr|A0A0Q0C8P4|A0A0Q0C8P4_PSEAP Spermidine/putrescine ABC-type transport system OS=Pseudomonas syringae pv. aptata OX=83167 GN=ALO85_03362 PE=3 SV=1
MSKNGPLALSFHALVVIFMMAPLVVVCLVAFTPENTLSLPTTHFSLRWFKAVFERADFID
SFYNSLILAFVSATLATLIAVPAALAITRHTFPGRNFFNALFLSPIIIPHLVLGVAMLRL

FALMGVNGSFTWLIFAHVLVITPYVLRLVLAAAIGIDRSAEHAAESLGADRFTLFRQITL
PMILPGVAGGWLLAFINSFDEVTLSIFVTSPATQTLPVRMYVYATESIDPMMAAVSALVI
ALTAATMILLDRVYGLDRVLVGKH
>tr|A0A0Q0DA05|A0A0Q0DA05_PSEAP Uncharacterized protein OS=Pseudomonas syringae pv. aptata OX=83167 GN=ALO85_04057 PE=4 SV=1
MIRIDSIWLATEPMDMRAGTETALARVVAVFGAAKPHCAYLFANRRANRMKVLVHDGLGI
WLAARRLNQGRFFWPGVRHGSEVELDAEQLQALVLGLPWQRVGSGAAITVL
>tr|A0A0Q0IHK0|A0A0Q0IHK0_PSEAP Sulfite oxidase, molibdopterin-binding subunit OS=Pseudomonas syringae pv. aptata OX=83167 GN=ALO85_100559 PE=4 SV=1
MNEPRKRITQRIRLEPAQERQLVDIQRRSFLRAGLTVGAMSMLTGCNLQDGDQVDKVLWA
MSRWNDRVQAWLFNGQKLAPTYSKAQLTNPFPFNAFYPEYNVPELDLSDYQLAVSGLVRD
KQPWTLEALRKLPQRTDITRLICIEGWSAIGQWGGVPLKTFLEYIGADTTARFVGFKCAD
RYYSSLDMPTALHPQTLLALDFGEVALPPDYGYPLRVRVPTKLGFKNPKHIVEIFVSNEN
PGGYWEDQGYNWFSGI
>tr|A0A0Q0BWK9|A0A0Q0BWK9_PSEAP 3-oxoacid CoA-transferase OS=Pseudomonas syringae pv. aptata OX=83167 GN=ALO85_04356 PE=4 SV=1
MAGLDKRVATYEEALAGLTDDMTVLCGGFGLCGIPENLIAQIKRMGIKGLTVVSNNCGVD
GFGLGILLEDRQIRKMISSYVGENALFEKQLLSGELEVELTPQGTLAEKLRAGGAGIPAF
YTATGYGTPVAEGKETRQFNGRNVILEEAITGDFALIKGWKADHFGNVIYRHTAQNFNPV
VATAGRITVVEVEEIVEPGVLLPTQIHTPGIYVDRVIQGTFEKRIEQRTVRKS
>tr|A0A0Q0IE81|A0A0Q0IE81_PSEAP GCN5-related N-acetyltransferase OS=Pseudomonas syringae pv. aptata OX=83167 GN=ALO85_01096 PE=4 SV=1
MSVHIDVNTNPTEEDRLAILEPLKRFNAEQAGDGKSEKVAMFIRDDQTREVLGGLHARIF
YEWLFIELLVVPEQARGQGMGSRLMQMAEDLAVEKGCTGIWLDTFDFQAPDFYRRHGYTE
FGQIDDYPPGNKRFFFQKRLPNTTA
>tr|A0A0Q0C0U4|A0A0Q0C0U4_PSEAP Uncharacterized protein OS=Pseudomonas syringae pv. aptata OX=83167 GN=ALO85_101663 PE=4 SV=1
MTKFSVQVYLDFTCAVTFITTQQSMSRLPDRVLAKLARQDWRSGQWRHNLPHKIIEELA
>tr|A0A0Q0IBZ8|A0A0Q0IBZ8_PSEAP Uncharacterized protein OS=Pseudomonas syringae pv. aptata OX=83167 GN=ALO85_02490 PE=4 SV=1
MPFGSQSAFDTYARNIHTAARSVFSISRSKLNEALSCGYGFRTYASLRAALKHAPLSDAT
TFDHAVFQSTLATLEDWSKVPVLAVLVEGNAFDIEIEKWPSGPGQRNGPRDLDVSYHIVM
NVSNADGKAVQGGQPFTLPVFAETAHDEKFRVDSGHGYRVTEGHSVTRFRTGTQTLRTSL
RDGRWGGEAFIYSSAEQQDDLLTLGTIKSAMVKSLLPTTSNRVICGIYRPDHYDENARRI
EITLGGPVLDFLGSSPFHFEIPKMEKRFFVMDDGRSNTEGVGVIVNGSWGAAVNSNGIDE
TDNPTSLDEVRVRMQIAVEKRLSLLGFNSNRR
>tr|A0A0Q0FSG3|A0A0Q0FSG3_PSEAP Uncharacterized protein OS=Pseudomonas syringae pv. aptata OX=83167 GN=ALO85_100192 PE=4 SV=1
MFFNRIGHRSDTKKAEDAALMQAIKSIDTLVVRGGRMSMDAKEIEGLVRSSRDKAKKLVT
PQAES
>tr|A0A0Q0CD14|A0A0Q0CD14_PSEAP 5-oxoprolinase subunit A OS=Pseudomonas syringae pv. aptata OX=83167 GN=pxpA PE=3 SV=1
MRIDLNADLGEGFGPWTMGEDEALMSLISSANVACGFHAGDPLIMDSTVRRAKALGIDLG
AHVGFPDLQGFGRRRMNIELKELCAIVVYQLGALAGLAAAAGYRVTHMSFHGALGNMAAA
DAELAGPLVRAVAAFDPQLIISSSSSAAIESAAEACNLRVATTFLADRAYDENCLLMPRG
IAGAVVKNPEEVRARVAQLLQDGTVTTLSGKRVAVNAQSILLHGDTPGAVELARSIRRSI
EEQGGVITPVSQLLGS
>tr|A0A0N8T952|A0A0N8T952_PSEAP 30S ribosomal protein S19 OS=Pseudomonas syringae pv. aptata OX=83167 GN=ALO85_200051 PE=4 SV=1
MAGTSTSHKSKKPVSIILPPNGGNMNAISNALTSASVTDNARSKKQTSKAKAVPTRYFEE
GTALNRILTESPYLARCSDNKTAALVRPREHAIRFPYMQINRREMVSWLIFDLDHANSLI
WDDLGLPQPNLIVRNRVNGHSHLYYAISPVCTSEKARDKPIQYMKAVYSAFALLLKADPD
YNSGPVAKTPGHPWWLTTELHDSVYELADLADYVDTQVSSWSKASRLEDLPESRHVILFE
KLRYYAYSVVGQQREEGNFESFCRLLDTYAANINNFSRQGFQTGNLPLSSIRATVRSVSR
WTWTKYWGNSSCCRGVMALDRSLPLKQRQRLAAKRTHDVRKRATEAKIREACHALQKRGE
QLRLITVAKEAGVTRQTVASHIHIFSELATCPTATDLQPQRGIEPIERSERISLSADLTV
STRPGSKYLHFGSQRENYKNVNHAVHQITACSAVMVAWPLEDSQGHDSSTVLLRSTSPPL
HKSSASLDLGSLLEPYSARADA

>tr|A0A0Q0DBX2|A0A0Q0DBX2_PSEAP TatD family deoxyribonuclease OS=Pseudomonas syringae pv. aptata OX=83167 GN=ALO85_03316 PE=4 SV=1
MTLIDTHTHLDFADFDADRAQVLDNCLALGVQRMVVLGVYQRNWQRLWELTEANPSLYAA
FGMHPVYIDEHRTAHLTELGDWLTRLQGHPQLCAVGEIGLDYYVDQPDRARQQALFDAQL
QLANDFNLPALLHVRRSHADVIATLKRHRPKRSGIIHAFAGSREEAREYIRLGFKLGLGG
AATWPQALRMHRVIAELPLDSVVLETDSPDMAPAMHPYQRNSPQHLPDICEALAGLMKIS
PERLAQVSTDNACELFDWPRLSHS
>tr|A0A0N8T8A1|A0A0N8T8A1_PSEAP TpRbsC-like sugar ABC-type transport system, permease protein OS=Pseudomonas syringae pv. aptata OX=83167 GN=ALO85_03059 PE=3 SV=1
MLLSLEPRGQQSRAMLWCSPLLAAVLTLVCGSLLFIGLGLNPVVTLHTLLIAPVSDWYGL
SELMVKTLPILLCALGLAVAYQARIWNIGAEGQLLLGALAGSAVAVNIIEMESRWALVMV
LLAGTLAGAAWAGVTAWLRTHFNANEILTSIMLNYIALNLLLYFVHGPLKDPAGMNFPES
ATFGDASRLPLLMEDGRAHIGVYFALFALVAVWVLLQRSFVGFQIKVLGLDKRAAGFVGF
REKRLVWLALLISGALAGLAGVSEVTGPIGQLVPQVSPGYGYAAITVAFLGRLNPIGILF
SSLLIALLYLGGESAQMTLNLPLAITQLFQGMMLFFLLACDVLILYRPRLKLHWAKRQMT
PVTGAA
>tr|A0A0Q0D7F7|A0A0Q0D7F7_PSEAP Lipopolysaccharide kinase OS=Pseudomonas syringae pv. aptata OX=83167 GN=ALO85_02892 PE=4 SV=1
MAGWKLEPEYAFLEPEFGSLDAVFALQGQQLTRDPLSDVIRIERAGVYYYVKRYVGAGKG
LRRYMGKPRVKSEWQNLKRFAKWGIPTAEVVAWGLERNGAAYDRGALITRGLPNTEDLSA
LAQRKDPRLADRHWVDALSRQLAAFTRIMHDNHFTHNDLKWRNLLVDNEGKLFFIDCPNG
AFWWSFMLRYRITKDLACLDKVAKYHLSATQRLRFYLQYRQRTRLNASDKKRIRHIVSFF
EGRE
>tr|A0A0Q0IBW6|A0A0Q0IBW6_PSEAP Uncharacterized protein OS=Pseudomonas syringae pv. aptata OX=83167 GN=ALO85_101218 PE=4 SV=1
MGHTRSPRPTVGLSCCGFCVTPEAAIYLSTTFTYLPRPAPSNKACRVGNGCGHVAHG
>tr|A0A0Q0CAM5|A0A0Q0CAM5_PSEAP Succinate dehydrogenase, iron-sulfur protein OS=Pseudomonas syringae pv. aptata OX=83167 GN=ALO85_02198 PE=4 SV=1
MTDMLQVSVYRYNPDQDAAPFMQEFQVDTGGKDLMVLDVLALVKEQDEGFSYRRSCREGV
CGSDGMNINGKNGLACITPLSAVVAKNKLIVRPLPGLPVIRDLVVDMSIFYKQYEKVKPF
LQNDTPAPAIERLQTPEEREKLDGLYECILCACCSTSCPSFWWNPDKFLGPAALLQAYRF
LADSRDTRTSERLASLDDPFSVFRCRGIMNCVNVCPKGLNPTKAIGHVRNMLLQNGV
>tr|A0A0Q0DH19|A0A0Q0DH19_PSEAP Alpha/beta hydrolase fold protein OS=Pseudomonas syringae pv. aptata OX=83167 GN=ALO85_00728 PE=4 SV=1
MNIDDAFRVTFHTQQVGDVNVFYREVGAKDAPVLLLLHGFPSSSHMFRDLMPLLASQYRL
IAPDLPGFGNTKAPPRGQFDYTFENLYKVIERFTEALGLKKYALYLFDYGAPTGLRLAAA
NPEKVTAIISQNGNAYLEGFSDQWGPWQAYWREPSAANREACRASLSPQVIRDWQYGTGA
DPEKLSPDGYNLDIMYMARPGAEEIQLDLILDYRSNVAAYPSFQEYLRKYQPPLLAVWGK
HDPAFIPPGAHAYRKDVPAAEVHLLDAGHFALETHAPEVAEYIRDFLSRTLKS
>tr|A0A0Q0DIS4|A0A0Q0DIS4_PSEAP Phage baseplate assembly protein V OS=Pseudomonas syringae pv. aptata OX=83167 GN=ALO85_00434 PE=4 SV=1
MSLLNRMLVRGTVVLARASSKMQALQMRLTAGEVKDDMEHFEPYGFTSNPLAGAEGIAAF
IGGDRSHGLLLVVADRRYRLKGLESGEVAIYTDEGDKIHLKRGKVIDIETDTLNIKAAVA
VNFDTPQITQTGKIVSQGDQLAAGISQISHLHGNVQGGNGQSGPPVGGAG
>tr|A0A0Q0DJ46|A0A0Q0DJ46_PSEAP Uncharacterized protein OS=Pseudomonas syringae pv. aptata OX=83167 GN=ALO85_04169 PE=4 SV=1
MHWTFKDRYQPHLTLNVDYDMPETLKQLGTTIDEFKAYEKLEGEKAERFLNRSENFTILM
IHIALSSVYAVYDENYLFDYSAYVERIRKNLIDVHPAFAAKAFADCFCKIRYEQSILTNC
NDELDEGFVFTEKE
>tr|A0A0Q0BVV0|A0A0Q0BVV0_PSEAP Uncharacterized protein OS=Pseudomonas syringae pv. aptata OX=83167 GN=ALO85_00400 PE=4 SV=1
MQEVITTHSMSWKTELAALVASAKDETECVALEAFQKALTPYLDTPDSLRTLFGKIQMLA
PAAEALDLKSNFASLDELKSAIPEHFEIIAA
>tr|A0A0Q0D8X5|A0A0Q0D8X5_PSEAP Uncharacterized protein OS=Pseudomonas syringae pv. aptata OX=83167 GN=ALO85_101557 PE=4 SV=1
MRFCARRAACVAPELTSAGVVRAGLLAFRALHLREFLQASFRHVAA
>tr|A0A0Q0IJA0|A0A0Q0IJA0_PSEAP Uncharacterized protein OS=Pseudomonas syringae pv. aptata OX=83167 GN=ALO85_03955 PE=4 SV=1

MPSTYNIYKIKHHKIKELTDKLKSVGLIEQKTLPILNYSKTFYFSENIKGNDVWWWKTYR
EFFNDDVKEPKNTFNFGVLLCQNLENKEQVYAVSLGKSHFYLSKFIHLDFGIDLAIRVAD
ENSILLKKSRYFTGTKRQDVSSYQRFQIDSYEAGESVDHLKLKAANKAIWGDRNIIFADS
IQMDMDKKPLELSAIFQQIDESSRGEKIIHLPKLESVGAELASELDELLLKHLKNADGQV
LIEEFQVHGIAICFSFHDYDYEIKAKVDKVNHRKALGNTIEIQAVSDFLSDHPDITDINN
VTIQFKSEDTGRFTRTLKEIIDCPIEYEEQQYFLKGGEWFVFNQIFMDYLKRSIGGIEIK
LEEPLIESEFIAWQAEKKRNAKPDDDKVDYREAYFNQKICSERNFKLLDRILTDIKSIEQ
KRRNYRVEVADIYKSGEIISVKISKKKPELIYNIEQSRDAIILIKNKAIKFDGELHSAAL
WFVFDEDVEKITDVNSIQFLLAVESWHKLVRSHGLIPRIYISKHEKDVKKKT
>tr|A0A0Q0BZL6|A0A0Q0BZL6_PSEAP Putative monovalent cation/H+ antiporter subunit E OS=Pseudomonas syringae pv. aptata OX=83167 GN=ALO85_02705 PE=4 SV=1
MKRLFPAPLLSLALWLLWLVLNLSISPGHLLLGAVLGFLAPLLMAPLRPLPVRIRKPGTI
LKFLWRVGRDVVVSNVQVGLSVWRLESRPPRSAFVRIPLDLHNAHGLAALAMVTTVVPGT
VWSELALDRSVLLMHVFDLEDEAQFIEHFKTTYERPLMEIFQ
>tr|A0A0Q0DJP5|A0A0Q0DJP5_PSEAP Superoxide dismutase OS=Pseudomonas syringae pv. aptata OX=83167 GN=ALO85_00161 PE=3 SV=1
MPYTLPALPYAYDALEPHIDAQTMEIHYTKHHQTYINNLNAAVEGTEFADWSVEKLVASV
QQLPENLRPAVINQGGGHANHSLFWEVMVPGGGGLPSGAVASAIDEQLGGYESFKEAFTK
AALTRFGSGWAWLSVTPDKKLVVESSGNQDSPLMNGNTPILGLDVWEHAYYLRYQNRRPE
YINAFYNVINWDEVSRRYQAALA
>tr|A0A0Q0IE01|A0A0Q0IE01_PSEAP FtsN-like cell division protein OS=Pseudomonas syringae pv. aptata OX=83167 GN=ALO85_02801 PE=4 SV=1
MAVAKKKPAPKRGASRYQAPAKKPIPGWLWLAIGLTVGAFVVFLMKLEPAGDDVKRVRAD
AKAAKIAEANKTPPSPTAPVKPKYDFYTLLPESEVIVPNEAVPEKTPPPVAPTAPVSPEQ
AAKIDTARAQAALSGLTPPPAPAVATTKPAAVTTFFLQAGSFRKQADAEKVRAQIILLGQ
TSTVESGTVKDETWYRVLVGPFSNREQLTTAQKQLASGGFSNLLLQQRQSR
>tr|A0A0Q0C035|A0A0Q0C035_PSEAP Extracellular solute-binding protein OS=Pseudomonas syringae pv. aptata OX=83167 GN=ALO85_00006 PE=4 SV=1
MGHTLFKPLLLTTALLACAPLAQAASTLVYCSEASPAGFDPSQYTSGTDFDASAETVFNR
LTQFKRGGTEVQPGLATRWDVSPDGLTYTFHLRDGVKFHTTDYFTPTRTFNADDVLFTFN
RLLDANHPFRKAYPSESPYFTDMGLNTTIKSVEKVDPLTVKFTLNNIDAAFVQNLAMSFA
SIQSAEYADKLLKEGKAEDLNQKPIGTGPFVFKRYQKDSQIRYVGNTEYWKPEDVKIDNL
IFSISSDAAVRLQKLKANECQVSGYPRPQDIEEMKKDPKLKVLSQPGFNLGFVAYNVTHP
PLDQLKVRQALDMAIDKPAIIKAVYQSAGQLAENALPPGQWSFDPTIKDAPHDLTKARQL
LKEAGVAPGTKIDLWAMTVQRASNPNARMSAQMIQSDWEKIGIKANIVSYEWGEYIKRAK
AGEHDAMIYGWTGDNGDPDNWLGVLYSCAAVKGSNYAKWCDPAYDKLVQQAKLTSDRDER
IKLYQQAQHILKQQVPITPIANSKVFQPMRQEVQDFKISPFGLTPFYGVSLGTVK
>tr|A0A0Q0DV84|A0A0Q0DV84_PSEAP 50S ribosomal protein L14 OS=Pseudomonas syringae pv. aptata OX=83167 GN=rplN PE=3 SV=1
MIQTQSMLDVADNSGARRVMCIKVLGGSHRRYAGIGDIIKVTVKEAIPRGKVKKGQVMTA
VVVRTRHGVRRADGSIIRFDGNAAVLLNNKQEPIGTRIFGPVTRELRTEKFMKIVSLAPE
VL
>tr|A0A0Q0CDM5|A0A0Q0CDM5_PSEAP Uncharacterized protein OS=Pseudomonas syringae pv. aptata OX=83167 GN=ALO85_02623 PE=4 SV=1
MTIIDFNKSVKRSFRGNTATAPQLEKMLTVILKTDAPVLMGICSDGLRSNSTVLGGEFYG
ENFSGSVLSGGSNTLVELSSELAFLETRFTLVTQLGEMINVESRGALLVDEMGQHELQNG
VWPISEGHYQCTCTPRFQVSLGPNEWLEKSAFIGRFEYLYGNEALISFYRIKF
>tr|A0A0Q0C0U0|A0A0Q0C0U0_PSEAP 30S ribosomal protein S12 OS=Pseudomonas syringae pv. aptata OX=83167 GN=rpsL PE=3 SV=1
MATINQLVRQPRKRIVEKSDVPALQNCPQRRGVCTRVYTTTPKKPNSALRKVCRVRLTNG
FEVSSYIGGEGHNLQEHSVVLIRGGRVKDLPGVRYHTVRGSLDTSGVKGRNQGRSKYGTK
KPK
>tr|A0A0Q0DL49|A0A0Q0DL49_PSEAP Zinc-type alcohol dehydrogenase-like protein OS=Pseudomonas syringae pv. aptata OX=83167 GN=ALO85_03073 PE=3 SV=1
MKAIAYYQSLPISDVKSLQDIELPEPTAGDRDLLVEVKAISVNPVDTKIRQNVQPEDGAG
KVLGWDVAGVVKAVGSQVTLFKPGDKVFYAGSLTRPGANSELHVVDERIVGHMPKTLSFA
HAAALPLTAITAWELLFERLQIPQQKGEGAHSLLIVGAAGGVGSILTQLARKLTSLKVIG
TASRPETESWVNELGAHHVLDHSKPLSEELKRVGLKHVTHVASLTQTEQHLDQLVEALKP

QGKLALIDDPKSLDVSKLKRKSLSLHWEFMYTRSMFETEDMIEQHNLLNRVAELIDAGTL
KTTFGEHFGTINAENLRRAHALLESGKAKGKIVLEGF
>tr|A0A0N8T850|A0A0N8T850_PSEAP Uncharacterized protein OS=Pseudomonas syringae pv. aptata OX=83167 GN=ALO85_101948 PE=4 SV=1
MKAKVRQRPEKGQMTPFAPLFATVKPKTTLLLQFFVSGRNCSRNC
>tr|A0A0Q0DNT1|A0A0Q0DNT1_PSEAP Phosphatidylglycerol--prolipoprotein diacylglyceryl transferase OS=Pseudomonas syringae pv. aptata OX=83167 GN=lgt PE=3 SV=1
MLPYPQIDPVAVAIGPLQIHWYGLMYLVGIGGAWLLASRRLNKFDPTWTKEKLSDLIFWL
AMGVIVGGRLGYVLFYDLSAYIANPLLIFEVWKGGMAFHGGFVGVMIAAWWFGKRNGKSF
FQLMDFVAPLVPIGLGAGRIGNFINAELWGKPTDVPWAMVFPPFSDPAQLARHPSQLYQF
ALEGVALFIILNLYARKPRPTMAVSGMFALFYGIFRFVVEFVRVPDAQLGYLAWGWVTMG
QILSLPMIIAGLFLIWLAYKRDPSASKAAV
>tr|A0A0Q0D036|A0A0Q0D036_PSEAP Uncharacterized protein OS=Pseudomonas syringae pv. aptata OX=83167 GN=ALO85_101712 PE=4 SV=1
MNWLFPVSIGKEIGSVVQVCAVGSSRRPGRRELLFYGALRERWLGAQFAVNENESE
>tr|A0A0Q0BT72|A0A0Q0BT72_PSEAP Response regulator receiver:transcriptional regulatory protein OS=Pseudomonas syringae pv. aptata OX=83167 GN=ALO85_03805 PE=4 SV=1
MSELLLIDDDQELCELLSSWLSQEGFQVRACHDGTSARQALADAAPAAVVLDVMLPDGSG
LELLKQLRADHPDLPVVMLSARGEPLDRILGLELGADDYLAKPCDPRELTARLRAVLRRS
HPVASSSQLELGDLTFSPMRGVVSVDEKELTLTVSESRLLEALMRQPGEPLDKQELAQLA
LGRKLTLYDRSLDMHVSNLRKKIGPHSDGRPRIVALRSRGYYYAL
>tr|A0A0Q0C0N1|A0A0Q0C0N1_PSEAP 2-dehydro-3-deoxygalactonokinase OS=Pseudomonas syringae pv. aptata OX=83167 GN=ALO85_02181 PE=4 SV=1
MQAQLIALDWGTTSLRAYRLGEHGQVLEQRALSAGIMQLPTTPRLIAGQFCSDGFELAFE
QACGDWLDAQPDLQVIACGMVGSAQGWREAAYRETPASVNELSAALQTVRSVRGVTVHII
PGVLQRSTLPNVMRGEETQVLGVLAGLEEGQGSQPLLIGLPGSHSKWVQVERGRIVHFDT
FMTGEVYAALCAHTILGRTMQPSQAFDEQAFDRGLSIALSDAGSAGPLSTIFSTRTLGLT
GQLAASAQPDYLSGLLIGHELGAIARLHLHNHEQLPAVILIGSDSLCTRYARGLAVCGFP
GVTLAEQATERGLWQVAVQAGLIARRSSQHIREV
>tr|A0A0Q0IJF3|A0A0Q0IJF3_PSEAP Uncharacterized protein OS=Pseudomonas syringae pv. aptata OX=83167 GN=ALO85_03936 PE=4 SV=1
MNSDQLNQYDAERLHQRVAAELGITAEELTTWMINDIERVTEGGKDVGHMVVFRESTPAQ
VLDRVQHKQSHFTAMTGVIDLS
>tr|A0A0Q0C810|A0A0Q0C810_PSEAP Binding-protein dependent transport system inner membrane protein OS=Pseudomonas syringae pv. aptata OX=83167 GN=ALO85_01981 PE=3 SV=1
MRLINRHPDRAGRLILVILPFALLLFAYFMGSATRLAENPSDKLLPSAVQMADAVKRMAF
VADPRSGDYLLWQDSASSLQRLAIGLGISALLGLCLGIAAGILPLCGAPLSPLLTVLSMV
PPLAILPILFIVFGLGELSKVMLIVIGITPILARDLEQRAREIPVELLIKAQTLGASTWT
LILRVILPQLLPRLLIALRLVLGSAWLFLIAAEAIASTDGLGYRIFLVRRYLAMDVILPY
VVWITLLAWLMDWSLKALTRRAFPWYEGARA
>tr|A0A0Q0FB27|A0A0Q0FB27_PSEAP Type VI secretion system protein ImpM OS=Pseudomonas syringae pv. aptata OX=83167 GN=ALO85_02251 PE=4 SV=1
MIGCFGKVPASADFVSLHGASDDVCEFDAWLQGALADMQHREDWRTLFDRLPVCFFSYRA
RSGNWLVGGLISSRDSSARRYPFFIFQTVKSSDAGLFVNPFTLSELFAGQIKPLLHMAAQ
GESTAVLFERIRALRPLQGQDFELFRRVHEKFLVNFTLRDIATSLESSYPEFISNAVLTR
LQALSRPSSKAPVGISLPLPAERGLKNPTADLWVNWLTRIEPNRAVPQISILADDFMRPR
LFCFASRNTSGVYRVVTGVSEHSESYDVLAPFDAFDEHHRGHVFPDIDRPLYDVIDRFVD
VLDLKPV
>tr|A0A0Q0BXD2|A0A0Q0BXD2_PSEAP RNA polymerase-binding transcription factor DksA OS=Pseudomonas syringae pv. aptata OX=83167 GN=dksA PE=3 SV=1
MPTPEKQQNHLISGFEPYVQTKGEEYMGEPMRAHFTKILNKWKQDLMQEVDRTVDHMKDE
AANFPDPADRASQEEEFSLELRARDRERKLIKKIDKTLQLIKDEEYGWCESCGVEIGIRR
LEARPTADQCVDCKTLAEIKEKQVGK
>tr|A0A0N8T8L3|A0A0N8T8L3_PSEAP Nucleoside 2-deoxyribosyltransferase OS=Pseudomonas syringae pv. aptata OX=83167 GN=ALO85_00653 PE=4 SV=1
MHAPLVYLAGFDVFRTDAVEHGRYLKALCAAHGLEGLYPFDNEVTHGRTAHETAQQIYSM
NVAMIHRCTAMLVNLNVFRGLEPDSGTAFEVGMAVALNKPVWAYFEPVASLREL VPHDEN

GLDANGFMVEDFDLPRNLMLACSWAGTSSTVELGAEALAKYLSGFQALPASG
>tr|A0A0Q0D922|A0A0Q0D922_PSEAP Undecaprenyl-diphosphatase OS=Pseudomonas syringae pv. aptata OX=83167 GN=uppP PE=3 SV=1
MMDLWTAAQALILGVVEGLTEFLPISSTGHQIIVADLIDFGGERAMAFNIIIQLGAILAV
VWEFRRKILDVVVGLPKQQQAQRFTLNLLIAFMPAVVLGVIFADTIHHYLFNAITVATAL
VIGGVIMLWAERREHTVRTETVDDMSWSDALKIGLVQCLAMIPGTSRSGSTIIGGLLFGL
SRKAATEFSFFLAMPTMVGAAVYSGYKYRDMFRPDDFAVFAIGFITSFIFAMIAVRGLLK
FIATHSYAVFAWYRIAFGLLILATWQFGWIDWASAKA
>tr|A0A0Q0C5E9|A0A0Q0C5E9_PSEAP Uncharacterized protein OS=Pseudomonas syringae pv. aptata OX=83167 GN=ALO85_04574 PE=4 SV=1
MAHSPQICIPATYMRGGTSKGVFFRLTDLPEAAQVPGAARDALLLRVIGSPDPYEKQIDG
MGGATSSTSKSVIVSRSSRPDHDVDYLFGQVSIDKPFVDWSGNCGNLSAAVGPFAITSGL
VDPGRVPQDGVAVVRIWQANIGKTIIAHVPISNGVVQETGDFELDGVTFPAAEVPLEFLD
PAADEEGAGGSMFPTGNLVDELEVPGIGILKATMINAGIPTIFVNAADIGYSGTELQDDI
NSDPQALARLEAIRAYGALRMGLIGNIDEAAARQHTPKVAFVAAPADYVASSGKPVAAQD
IELLVRAVSMGKLHHAMMGTAAVAIGTAAAIPGTLVNLAAGGQARSAVNFGHPSGTLRVG
AKAVQEGGDWKVTKALMSRSARVLMEGWVRVPQPGE
>tr|A0A0Q0CC38|A0A0Q0CC38_PSEAP Lysine exporter protein LysE/YggA OS=Pseudomonas syringae pv. aptata OX=83167 GN=ALO85_02550 PE=4 SV=1
MEFSNGFLLSLSLCLDIGLANIAMITLSMQRGFSRGLWLGLGTCVGDLAYAILAMLGMAV
LLQYEAVRYVLWIGGTAVLAWFCFRMIMSATTVSNGLDSQAVESEKSSLALFLRGIFLAM
SSPSAILWFAAVGGALIARQGSDLVSASTFLAGFGAAGVVVWSISLCALAHQGGRFMGERM
LRWSYIASALIFAYFTLYVVITGYLEFVVASPALPAH
>tr|A0A0Q0C828|A0A0Q0C828_PSEAP Plasmid stabilization system OS=Pseudomonas syringae pv. aptata OX=83167 GN=ALO85_102222 PE=4 SV=1
MGRECCQQRRQSHRLDIRPGLRITHYRKRAVIAFAVDAVQVKILGIFYGGQDFEAVFQAD
LDE
>tr|A0A0Q0BZX0|A0A0Q0BZX0_PSEAP Uncharacterized protein OS=Pseudomonas syringae pv. aptata OX=83167 GN=ALO85_00407 PE=4 SV=1
MRTYTTRYVMAAALCSAAGPTLAIADEISSYAVDVTAKVSSDIRTRGISDSLNRPGAKVT
LQVAHETGLVALAEFTTVSKKQFLDGDGAGVLLAGGYRFGDPDAWHYGVGLAAEMFPGAQ
FKAPNRFDFETFTPGEEHSTNYNSQFAVLEIGYGALEGRIARVLSKNYRGANSGGVCGAQ
LQFRDDPTKGLDCYAKGDRNSRGSMLYDLDYKYALGINTNLKLHAGYQQIENFSEADFAD
YGAGLVHHWWGVDWGLDWVTTKTRARELYMAEDDGHVRTTDGSRWVASISRTF
>tr|A0A0Q0IRN0|A0A0Q0IRN0_PSEAP Uncharacterized protein OS=Pseudomonas syringae pv. aptata OX=83167 GN=ALO85_200087 PE=4 SV=1
MPEEQQPKGAQWPAGETMTAHCPNCETPATVDIVNVKAWEMIWRPVDCDNCFAEFELSAD
GSTALLLGPAEQSTARGRELLSTIFVFDPNEDTP
>tr|A0A0Q0CW08|A0A0Q0CW08_PSEAP Uncharacterized protein OS=Pseudomonas syringae pv. aptata OX=83167 GN=ALO85_04221 PE=4 SV=1
MIRACASELKNMNRRKKIQQLLKAHAKKASAKLAPQSKEKYISKADRVKLAAEADQDSNS
PSEG
>tr|A0A0Q0C676|A0A0Q0C676_PSEAP Uncharacterized protein OS=Pseudomonas syringae pv. aptata OX=83167 GN=ALO85_00485 PE=4 SV=1
MLKIVPDPPLFTANPDVSPEDALMHASDLLRCAATSACEFSDSMTGTQRDMTLSIMHLVE
MAKVMVDRTIDNLQTE
>tr|A0A0Q0BTT7|A0A0Q0BTT7_PSEAP Uncharacterized protein OS=Pseudomonas syringae pv. aptata OX=83167 GN=ALO85_100809 PE=4 SV=1
MLRSSILGINMSVLKGVLDWMITKLASKQSPVRPETVENLAAQAALELAAILRVGGALEH
DEIPMITRRMQSVIDTTNAVVAACEPSQAEGRYCRREL
>tr|A0A0Q0FUG5|A0A0Q0FUG5_PSEAP Putative Fe-S protein OS=Pseudomonas syringae pv. aptata OX=83167 GN=ALO85_00581 PE=4 SV=1
MPDYIIKTPCVGLCSTVYGDLVCRGCKRFHHEVIHWNGYNEDEKRAVWLRLEQLLVQVMT
AKLEVFDADKLRAQLTQRKIRFVPHQSEYCWAYQLIARGARVISQVEAYGFVLLPEFRDW
TLPELRDAIDREFFLLSEAHYQRYIAPGFLRDAFGA
>tr|A0A0Q0DFD2|A0A0Q0DFD2_PSEAP Uncharacterized protein OS=Pseudomonas syringae pv. aptata OX=83167 GN=ALO85_01625 PE=4 SV=1
MPAAMNTAALREQIQLAHQHEAQTGHLLQQLEQKLPHLHPAIHLPEVDAKDVLTRFVTAY
IDLVPDLLDAAHEVALQAGIEGQIKPVLKIAEHFFAAPPSIMDGHEGLESLLDEAYLAHR
LVEEVNDLYIKHFGQPLIPSNTTVASVIAHQLIGEQFANLLDEAVHHAVDELLDEESFAL

DSVEAYREKLTNPETGAAWQRWPSLSRQLGVGLELERSAV
>tr|A0A0Q0BYP8|A0A0Q0BYP8_PSEAP NUDIX hydrolase OS=Pseudomonas syringae pv. aptata OX=83167 GN=ALO85_02765 PE=4 SV=1
MAADKACAVVLRDRGVLEILAFEHPLAGLQLVKGSVEPGESSGAAAVRELMEEAGIQATA
KRNLGEWHSTITGHIWAFHECEVAQDLPDNWVHFAEDDGGHDFRFFWHPLMSEPSDRWHH
IFKDALSFLRETVT
>tr|A0A0Q0C5W2|A0A0Q0C5W2_PSEAP ABC transporter ATP-binding protein OS=Pseudomonas syringae pv. aptata OX=83167 GN=ALO85_00090 PE=3 SV=1
MSLTLEHVSRAVDGQVWIDDASLSFEPGSFNVLLGRTLSGKTSLMRLMAGLDKPDSGRVL
MNGVDVTNRPVRLRNVSMVYQQFINYPSMTVFENIASPLRQAGLSRDVIHSRVQETAKML
RIEKFLQRHPLELSGGQQQRTAMARALVKDAELILFDEPLVNLDYKLREELRQEMRELFA
ARHTIAIYATTEPNEALALGGTTTILHEGRVVQSGKTPEVYHRPATVLAAELFSEPPINL
MPGRIEGNEVSFANFVHFPLNVDLRAVGDGEYRFGVRPSHLSLVPSNDDDLELAVTVELA
EISGSETFLHVRNELFSLVLHLPGVHAYDVDAAIRVYIPTHKLFVFDQQGGLIQAPGLRM
ARVA
>tr|A0A0Q0DCF3|A0A0Q0DCF3_PSEAP Short-chain dehydrogenase/reductase family oxidoreductase OS=Pseudomonas syringae pv. aptata OX=83167 GN=ALO85_00501 PE=4 SV=1
MSRTQLFDLDGKVAFVSGASRGIGEAIARLLAQQGAHVIVSSRRLEGCQAVADAIVSEGG
KATAIACHIGELEQISSAFAQIRQQFGRLDVLVNNAATNPQFCNVLDTDPGAFQKTIDVN
IRGYFFMSVEAGKLMRENGGGSIINVASINAISPGAYQGVYSMTKAAVVNMTKVFAKECA
EFGIRCNALLPGLTDTKFASALVKNDAILNMALSQIPLKRVAAPSEMAGAVLYLASEASS
YTTGVALNVDGGFLS
>tr|A0A0Q0DI79|A0A0Q0DI79_PSEAP LysR family transcriptional regulator OS=Pseudomonas syringae pv. aptata OX=83167 GN=ALO85_03025 PE=3 SV=1
MIAIEDLRLAVTLSRCESLSAAARILNVSPPALSMRLRKLETQLGITLASRDARRLSLTA
DGERFARESAQLLEQLEALPESFRQRDERLVGTLRLAAPFGYGRQRIAPLLARFARLHPQ
LCLQLDLRETPWPDRHDSDAVIHIGSLNDSQWIARPLAQNHRWLCASPAYIEQHGVPSTP
DQLAGHRCICIRENDEDVTLWHLSKGQTKKTLRIEPALLSNDGSVARRWAEQGLGIVLRS
QWDVSDAIASGRLVRVLADWQLASAPINLLVPVRKHRSARVQALTEFLETALKA
>tr|A0A0Q0IQS7|A0A0Q0IQS7_PSEAP Flagellar motor switch protein FliN OS=Pseudomonas syringae pv. aptata OX=83167 GN=ALO85_04036 PE=3 SV=1
MADENDMTSAEDQALADEWAAALGEAGDSQADIDALLAADAGNSGSRMAMEEFGSVPKST
GPVSLDGPNLDVILDIPVSISMEVGSTDINIRNLLQLNQGSVIELDRLAGEPLDVLVNGT
LIAHGEVVVVNEKFGIRLTDVISPSERIKKLR
>tr|A0A0Q0CVZ1|A0A0Q0CVZ1_PSEAP Type III secretion protein HrpG OS=Pseudomonas syringae pv. aptata OX=83167 GN=ALO85_04195 PE=4 SV=1
MDFSEFEEVVGLWRDQRPASPLDCWVDDANARLEMVGGGVRCSIELLDPYDANDPQRIEA
LLSHGGASLACACDGAFAIDPQTRCMVLVSWMPGPCSLADLLNRLESLANQRAALLSLMH
TTLVNSVPALAGRTTLNHRQPGV
>tr|A0A0Q0DHW4|A0A0Q0DHW4_PSEAP Transcription elongation factor GreB OS=Pseudomonas syringae pv. aptata OX=83167 GN=ALO85_00034 PE=4 SV=1
MSRAFVNEDNAAAEAEQPVERLVSTQINYVTAEGLTMLKKHVSSLQAQHSTQTALGEAAD
KQRLADLERDLRYFNQRLQSAQVVAPAVSTEKVQIGSWVTFADEQDNQQRVHLVGEDQAD
AAKGQINWGSPLGRALIGAQKGDEVLWQRPAGDQSIEVLLIEPDA
>tr|A0A0Q0BZ42|A0A0Q0BZ42_PSEAP NADH-quinone oxidoreductase subunit C/D OS=Pseudomonas syringae pv. aptata OX=83167 GN=nuoC PE=3 SV=1
MTADNALYIPPYKADDQDVVVELNNRFGAEAFTAQPTRTGMPVLWVERARLFEILTFLRN
VPKPYSMLYDLHGVDERLRTNRRGLPGADFTVFYHLLSIERNSDVMIKVALSESDLSVPT
ITSIWPNANWYEREVWDMFGIDFPGHPHLSRIMMPPTWEGHPLRKDFPARATEFDPYSLT
LAKQQLEEEAARFRPEDWGMKRSGANEDYMFLNLGPNHPSAHGAFRIILQLDGEEIVDCV
PDIGYHHRGAEKMAERQSWHSFIPYTDRIDYLGGVMNNLPYVLSVEKLAGIKVPEKVDVI
RIMMAEFFRITSHLLFLGTYIQDVGAMTPVFFTFTDRQKAYTVIEAITGFRLHPAWYRIG
GVAHDLPRGWEKLVKDFVDWLPKRLDEYTKAALQNSILKGRTVGVAAYNTKEALEWGVTG
AGLRSTGCDFDLRKARPYSGYENFEFEVPLAANGDAYDRCMVRVEEMRQSIKIIDQCMRN
MPEGPYKADHPLTTPPPKERTLQHIETLITHFLQVSWGPVMPANESFQMIEATKGINSYY
LTSDGGTMSYRTRIRTPSYPHLQQIPSVIKGSMVADLIAYLGSIDFVMADVDR
>tr|A0A0Q0IAG1|A0A0Q0IAG1_PSEAP Type IV pilus bioproteinsis/stability protein PilF OS=Pseudomonas syringae pv. aptata OX=83167 GN=ALO85_04105 PE=4 SV=1

MPVRASLLLCVVVLLTACVSTGNVNPLTTAQGREEARKAYVQLGLGYFQQGQTERAKVPL
KKALELDNSDAQANAALALVFQTEMEPELAEQYFRKALAASNNETRIVNNYGSFLFEQKR
YKEAYERFEQAAADNLYPERSRVFESLGMTSLKLGNREQAREHFTKALRLDRQLPLSLLE
MAQLSYEDKQYVPARDYYDRFSQLSEQNARSLLLGTRLAKIYDDRNKAASFGLLLKRLYP
GTPEYQQYLSEQ
>tr|A0A0Q0CWU8|A0A0Q0CWU8_PSEAP Putative auxin efflux pump OS=Pseudomonas syringae pv. aptata OX=83167 GN=ALO85_00145 PE=4 SV=1
MLAIFLETLNITAPVFAMLFLGVFLRRIGAINDGFIVAASGLVFNVTMPALLFLGIIHAD
LRTALQPKLLIFFSIATLLSFAFAWCWAIWRCPHEERGIYVQGAFRGNNGVIGLALAASM
YGPDGISLGAILAALVIVFYNTLSTVVLAVYSPVIKSDPWSIFKSVLANPLIISVIIASP
FAYFAIGLPKWLETSGSYLAQMTLPLALICIGGTLSLASLRKSGKLAISASVMKMITLPV
LCTLAAWLAGFRGAELGILFLYFGSPTAAASYIMARTANGNYELAAAIIVITTLAAAVTT
NVGIFILQWGGWI
>tr|A0A0Q0FUP1|A0A0Q0FUP1_PSEAP Cys-tRNA(Pro)/Cys-tRNA(Cys) deacylase OS=Pseudomonas syringae pv. aptata OX=83167 GN=ALO85_02045 PE=3 SV=1
MERCMTPALDLLKKNRAEHRIHSYEHDPKAPSYGLEAAEKLGLEPAQVFKTLLASTEKGE
LLVAVVPVVGTLDLKALAQAAGAKKTEMADPAAAQRSTGYLLGGISPLGQKKRLRTFIDE
TAQRFASIYVSAGRRGLEVELSPAVLAEHTQARFAQIGRE
>tr|A0A0Q0DMS0|A0A0Q0DMS0_PSEAP Flagellar two component sensor histidine kinase FleS OS=Pseudomonas syringae pv. aptata OX=83167 GN=ALO85_02162 PE=4 SV=1
MSQAAQLTSALSVQAPYSPELESRQGLEQAFSLFNQMSAQLTNSYGVLEARVTELKGELA
HAGAQRLQELAEKERLANRLQNLLDLLPGGVIVIDGMGVVREANPAAIDLLGQPLLGMLW
RHVISRCFAPREDDGHEVSLKDGRRLSIATRSLDAEPGQLVLLNDLTETRHLQEQLARHE
RLSSLGRMVASLAHQIRTPLSAAMIYASHLTERELPVETQQRFAARLKDRLHELEHQVRD
MLVFARGELPLTDRLTPGALFQALQNAAATHVQGLAVRWQCDSIDGELLCNRDTLVGALL
NLIENAVQASAGRTLLKVHAYSRGNELRICFSDNGSGIDKAGLARIGEPFFTTKTTGTGL
GLAVVTAVSRAHQGRVHYLSRVGRGTCAIISLPLIPASSSTGTN
>tr|A0A0Q0DKL4|A0A0Q0DKL4_PSEAP Putative homoserine kinase type II OS=Pseudomonas syringae pv. aptata OX=83167 GN=ALO85_00920 PE=4 SV=1
MQVIGKDQFSESLIALQGDLAAPFTPVSLEEGRRIVLEQFGISAQLTRFATEKDDTFRCD
CSSGQSYVLKIANPQESPIELSLQIEVMQHIGRRAPQLPIPRVYPALDGGYLTTVVTASG
ESRQVRLLSFLPGTPLDKTTLNAQGREQIGQLLAHLRLATADFAHPAESRQVCWDVQHLR
VLEPLLNGVVEPARRHSLERALERFGEVEELIAGCRQQVLHNDFNTSNIVIDAHRPQCVG
AIIDFGDTVKTAIAIDVSTAMMNQLLAVHPGQDLDIFAPAKDLLRGYLQVADLTPEELAL
IPHLAMARLVARALITTWRSQLFPQNSAYILRNAAPGWGQLDWLLNRSPSQMSALLMHFA
H
>tr|A0A0Q0DWS3|A0A0Q0DWS3_PSEAP Heavy metal--translocating P-type ATPase OS=Pseudomonas syringae pv. aptata OX=83167 GN=ALO85_02102 PE=3 SV=1
MGTTIKNSPAHDHDHDHDHDEHAHEQKPVEHAHSCCSSDAQPAVVTFGEASAAGGRLSSF
RIEAMDCPTEQTLIQNKLGKLAGVQKLEFNLINRMLGVWHDLPSTDPIREAISSLGMQAE
PVEAGAASTESAPVVKKHWWPLALSGVAALGAEVVHFASLGPTWVVALLALVSIFSCGLT
TYKKGWIALKNFNLNINALMSIAVTGAILIGQWPEAAMVMFLFTIAELIEAKSLDRARNA
ISGLMQLTPELATVKQADGSWQEVEAKNVELEAIVRIKPGERVGLDGEVVSGSSTIDQAS
ITGESLPVEKTVGDKVFAGTINQAGSLEYRVTAAANNSTLARIIHAVEAAQGSRAPTQRF
VDSFSRIYTPVVFVVALALALVAPLFFGGEWYDWIYRALVLLVVACPCALVISTPVTIVS
GLAAAARKGILIKGGVYLEMGEKLDYLALDKTGTLTHGKPVQTDYVPLNPAVSDSAPGIA
ASLAGRSDHPVSQAIAKAADGSLTLYEVSAFEALGGRGVKGEINGQMYHLGNHRLVEELG
LCSPELEARLDALEMQGKTVVLLLDASGPIALFAVADTVRETSREAIAQLHELGIKTVML
TGDNPHTAKAIADQVGIDEAQGNLLPADKLSAIEALYSRNHRVGMVGDGINDAPALARAE
IGFAMAAAGTDTAIETADVALMDDDLRKIPTFIALSRRTSAVLKQNIVLAIVTKVLFIGI
TFAGLATMWMAVFADMGVSLLVVFNGLRLLKK
>tr|A0A0Q0FSX2|A0A0Q0FSX2_PSEAP Regulatory protein, LysR OS=Pseudomonas syringae pv. aptata OX=83167 GN=ALO85_03792 PE=3 SV=1
MQNQSAGHMKTHFFKVVQTVAQHGSFSEAAAILGCSQSNISYAIKEVEDFFEKRLFIRSR
TGCTLTPEGKVINQTLGNIMATLEQLKKMGSVAASPFITTRTSAKPA
>tr|A0A0Q0D7E0|A0A0Q0D7E0_PSEAP Putative integral membrane protein, YGGT family OS=Pseudomonas syringae pv. aptata OX=83167 GN=ALO85_02938 PE=4 SV=1
MIGLNTAAIYVLQTLGSLYLLIVLLRFVLQLVRANFYNPLCQFIVRATQPLLKPLRRIIP

SLFGLDMSSLVLAIIVQMILMALTLLLMFGTTGDPLHLLLWSIISVTALFLKIFFFALII
SVILSWVAPGSHNPGAELVNQICEPALAPFRKIVPNLGGLDISPILAFLVLKLLDMLVIN
NLAAMSGMPDVLRLLM
>tr|A0A0Q0FWU8|A0A0Q0FWU8_PSEAP Methyl-accepting chemotaxis protein OS=Pseudomonas syringae pv. aptata OX=83167 GN=ALO85_01468 PE=4 SV=1
MNILSPGIYLTNRLRFPAKFAVLAIIIVIPLIVLGLRVFNSLNASIDTVAQERVGREYLQ
LTTPVLRLSMLQRAVSNRLLAGDASAAQDMTSNRAQLETALANLADMDARQGQQLETENR
VQRLRESTRSLMDSIKPGLSQAEVFAQWNEQLAQTLNFIYYVSATSGMVLDEDYASLFLI
DLSTIRMPREINVAGQIRGITAGLITGQGLSVSMRGSLESLLKIELQFRAELEQSIRLLK
RRSPELAARISDPITASTAAMDSFRGDLHAYVKGTEFSVQQGQALSARGNVVVSGLYKAQ
DEIQTALQDELNTRYDALVLQREVVIAMCVIMGLLLLYAFCSIYRALRLTIDSLLGVTRR
LGEGDLSARVAVVSKDEVADIANGLNLMADAFASSISHMDRTSYELTDVASRLGASIGLA
KQSMNAQQAETEQVATAINEMTASVADVAQNTEGAALAADEANTASRNGLRIMHQAHSTI
QALAEEVELSAQKVQALALHSQSIGGVIQVISTIADQTNLLALNAAIEAARAGEQGRGFA
VVADEVRTLASRTQASTEEIRGIIQQLQSATDAAVQQMQAGQQKAHACISAASDASGSLS
SISQGVERIVEMNTQIASAAVQQHAVSEDINRNVMEIRNSSGTLMRGIDNNAVTADELAR
VASDMRNVVARFKLTA
>tr|A0A0Q0DA04|A0A0Q0DA04_PSEAP Transcriptional activator MetR OS=Pseudomonas syringae pv. aptata OX=83167 GN=ALO85_02032 PE=3 SV=1
MLITNTAHGALTVLEIRHLKTLHALREADSLVEAAERLHLTQSALSHQFKELEERMGMPL
FVRKTKPVRFTSAGLRLLQLADSMLPQLRSAERDIARLAGGTAGRLHMAIECHSCFQWLM
PTIDQFRDAWPEVELDLSSGFSFAPLPALARGDLDLVVTSDPLELPGITYVPLFTYEAML
AVANQHALATRPYVVPEDLLAETLITYPVERDRLDIFTRFLEPADIEPAQVRTSELTVMM
MQLVASGRGVCGMPHWALHEYSSRGYVKAKRLGEKGLFATLYAGIRADMLDAPYMRDFLL
TAKDTSFSTLDGVSVVR
>tr|A0A0Q0DKQ5|A0A0Q0DKQ5_PSEAP ABC-type transporter in pyoverdin protein cluster OS=Pseudomonas syringae pv. aptata OX=83167 GN=ALO85_01280 PE=4 SV=1
MTAAQQLDIVSQGPGIEFNQVSLTLGRTTILDQVHFNVRAGSVHALVGPNGGGKSSLIKT
LLGQTPHQGQLSLMWPAVPGLIGYVPQALEFDRGLPMTVDDFMAAMCQRRPAFLGLSKHY
AAAIGEALERVGMQDKRKRRMGALSGGERQRVLLAQGLIPAPQLLVLDEPMSALDEAGIQ
VFERLLGDWRRSGVTVLWIEHDLDAVRRLADQVTGLNRRILFDEPAATALTPKRLLTLFS
AHPRTTDSSAGRIA
>tr|A0A0Q0C7X8|A0A0Q0C7X8_PSEAP Uncharacterized protein OS=Pseudomonas syringae pv. aptata OX=83167 GN=ALO85_02026 PE=4 SV=1
MDTIIYQIERNAPKREEERKRCLIFTSDQASATAVHVWLQWD
>tr|A0A0Q0IKN3|A0A0Q0IKN3_PSEAP Transposase OS=Pseudomonas syringae pv. aptata OX=83167 GN=ALO85_04667 PE=4 SV=1
MASFTDHVFALMHLLDFRFTPSIRGLGETKLYVP
>tr|A0A0Q0IA97|A0A0Q0IA97_PSEAP Aminotransferase OS=Pseudomonas syringae pv. aptata OX=83167 GN=ALO85_02310 PE=3 SV=1
MLAHVESYAGDPILSLMETFGKDSRADKVNLSIGLYYDAQGRIPQLACVATAQQQLAEGE
QAASVYLPMEGLAAYRQAVQTLLFGADHPLVQAGRVATIQTVGGSGALKIGADFLKYAFP
DSQVWVSDPTWENHNALFGGAGFKVNAYPYFDAQTGGVRFEAMLDTVQGLPAQSILLLHP
CCHNPTGADLTVAQWDRLIDVIKDRQLIAFLDIAYQGFGKGIDEDVYAIRAMANAGITTL
VSNSFSKIFSLYGERVGGLSVVCEDAASAANVFGQLKATVRRNYSSPPAHGAFLVSKVLN
TPHLRDQWLGEVEAMRLRIIDMRTRLVEVLAQKVSGHDFSFILRQNGMFSYTGLTPQQVD
ELKDVHGIYMLRSGRVCMAGLNEGNIDKVCDAIASLFAR
>tr|A0A0Q0C979|A0A0Q0C979_PSEAP Putative fluoride ion transporter CrcB OS=Pseudomonas syringae pv. aptata OX=83167 GN=crcB PE=3 SV=1
MIQTILAVSIAGIAGTLLRFATGTWVSANWPRYFYAATLAVNIVGCLIIGVLYGLFLLRP
EVPIEIRAGLLVGFVGGLTTFSSFSLDTLRLLESGQVPLALGYAGISVFGGLLATWAGLS
LTRL
>tr|A0A0Q0CX84|A0A0Q0CX84_PSEAP AsnC family transcriptional regulator OS=Pseudomonas syringae pv. aptata OX=83167 GN=ALO85_02482 PE=4 SV=1
MPDTRPPVLDEIDRQLIAALQINARESVAMLARQLGIARTTVTSRLARLESSKVITGYGV
RLGQRVVSGGLQAYVGITVQPRSGKEVVRRLSAMAQVQQLCAVSGEFDYVAWLRTDSPEQ
LDQLLDLIGSVDGVEKTTTSIILSSKIDRGQPV
>tr|A0A0Q0FHP0|A0A0Q0FHP0_PSEAP Phosphoglycerol transferase I OS=Pseudomonas syringae pv. aptata OX=83167 GN=ALO85_01137 PE=4 SV=1

MGSGMQFKNGIKLGAPAAVACIMVMTLIVLLVRSIGLYPGVLGDEYTYSSMARLMPLSAA
YIPDYLYLAIYKSTNLCGRGFMECSKLFNTVFFVAAAPFIYMTARRFCGSRLSVLIVFLS
MLGPINSYTAYFMPEPLFFLSFWVGISYFLSLNAQSSLKEWALFGFIMGCSSLVKPHAMF
MIPAFCLCIIFFAYKSMSTWLVTGLKSSAVFVVVMLATKFVVSFIIAGTSGLTLFGNFYT
STFESSASSLQRYLDIFSTAPRIIEGHLLAIVMMFGTSVAIIIFGSIKALAKKSISSEDK
VVFCTLALLLNLIVIVGLFSASVAGTNVVETAFRLHMRYYDFMLPLLFIAAATQINAVHT
ASLKYCRFIIAILLLAMIGYAAMTLMHPFTPSYVDSAELRGYTLNVNWFIALAVFSALAV
VVWCLSASSGTIVFLLFYLPLSVGVTAVHSNAEVRQRMVIDAYDRAGLFAKSYVPANELS
SLVVVGNSVAATLRSLVYIDDLGASRDLSFVEGVPYTAAQTPSDKKWILAVGDIEFAKDE
FEVMRFNGFSLAKKMTNVYPLNINFRHSSLPDTVIKASGLSHPESWGAWSDRGDIVFRFA
KPLPSKFQLVLTAKAFGPNVDKPFEVLVGHRSYPVKLTADNASYTLDIVNVDSADVLTIK
VPLPTSPKQLGMSKDDRTLGIGLDQLEIRKVE
>tr|A0A0Q0DH05|A0A0Q0DH05_PSEAP Putative lipoprotein OS=Pseudomonas syringae pv. aptata OX=83167 GN=ALO85_03216 PE=4 SV=1
MLRFLRLATVIGGLCLSASALATTVDSATYGYPLTNPFEATIATTPPALRPDLPDDEDID
QDVYTLNLHPEREFTLPDNFWAVKKLHYRLAKQDHAAPLIFLIAGTGAPYNSTINEFLKK
LYYGAGYHVVQLSSPTSYDFMTSASRFATPGVSTDDAEDIYRVMQAIRAQQAQLPVTDYY
LTGYSLGALNAAFVSKLDETRRSFNFKKVLLLNPPVNLYTSISNLDKLVQTNVKGINNTT
TFYELVLAKLTRYFRQKGYIDLNDALLFDFQQSKQHLTNEQMAMLIGTSFRFSSADIAFT
SDLINRRGLITPPKFPITEGTSLTPFLKRALQCDFDCYLTEQVIPMWRARTDGGSLLQLV
DQVSLYALKDYLHSNTKIAVMHNADDVILGSGDLGFLRKTFGDRLTVYPYGGHCGNLNYR
VNTDAMLEFFRG
>tr|A0A0Q0DNW3|A0A0Q0DNW3_PSEAP Iron dicitrate transport system permease protein OS=Pseudomonas syringae pv. aptata OX=83167 GN=ALO85_00389 PE=3 SV=1
MTAAVKLGLGLLILVACMGLSLATGATWISPAAIVRSVWQADALSAIEHVLLDTRLTRTL
MAVAVGSSLAVAGALMQALTRNPLASPGLFGINAGATFAIIACSSLLALSSMSQWLWCAF
IGAAVAGTIVWVIGNRGQGSLNPLRIVLAGAAITALFTAFSQALLVVNQDGLDTVLFWLA
GSLSERELSTAAPLLVCVVMGLVGALVLAGQVNVLNTGEAIATALGQRTGLIRLMMSVVI
IALAGSAVALAGSIGFIGLLVPHMVRKTLSIDHRWLLPGCAVLGATLLLLADTLARVVIV
PQEVPVGVMTALFGAPFFIALVRRGARYG
>tr|A0A0Q0E0Z2|A0A0Q0E0Z2_PSEAP Non-heme catalase KatN OS=Pseudomonas syringae pv. aptata OX=83167 GN=ALO85_01470 PE=4 SV=1
MFMYNKRLQYTVRVARPNPGLANLLLEQFGGAQGELAAAGRYFTQGLSEEDPGRKDLLMD
IATEELSHLEIVGSIIVMLNKGAKGQLAEGIEEEGELYRSINGNGNDSHITSLLYGSGAP
LTNSAGVPFTAAYIDTIGEPTADFRSNIAAESRAKIVYERLMNVTDDPGVKEALGFLMTR
EIAHQLSFEKALHAIQPNFPQGKLPGMPEFTNKYFNMSGEPNVRGAWNQGDDWEYVESPQ
AAVDGGDGTASVTLDEKDTEVLEMMKERTQSDPAANPITGADLGSGLVQGKDA
>tr|A0A0Q0C8M7|A0A0Q0C8M7_PSEAP Xanthine dehydrogenase, iron-sulfur subunit OS=Pseudomonas syringae pv. aptata OX=83167 GN=ALO85_03412 PE=4 SV=1
MILMTEPKKHKETAVDSSAVQPSRRNFLKFGASGIAAATLSTWIPSDGLLQAAPLAPAAP
VVEDSDAPAAGEQRIQLKVNGQVHTLNVPANAVLLDVLRDRLQLTGTKKGCDHGQCGACT
LLVNGLAINSCLSIAVQHQGDSITTIEGLAENGKLHPVQEAFWEHDAYQCGYCTSGQIMS
AVAILKDPNIAFDDASVREAMSGNICRCGAYKNILSAVQSARSKMGGAA
>tr|A0A0Q0C4C1|A0A0Q0C4C1_PSEAP Outer membrane protein assembly factor BamA OS=Pseudomonas syringae pv. aptata OX=83167 GN=bamA PE=3 SV=1
MGAWSSPFGKGSRLGGADRYQSGSGRNVARAGQRSGPTVKRCRVANLPHFAAVCLLPVGI
RKDFMKRLLLTAVLSALMIAEVHAESFTISDIRVNGLQRVSAGSVFGALPLNVGEQADDG
RLVDATRSLFKTGFFQDIQLGRDGNVLVINVVERPSVASIEIEGNKAISTEDLMKGLKQS
GLAEGEIFQRATLEGVRNELQRQYVAQGRYSAEVDAEVVPQPRNRVGLKININEGTVASI
QHINVVGNKVFSDEDLTDLFELKTSNWLSFFKNDDKYAREKLSGDLERLRSYYLDRGYIN
MDIASTQVSITPDKKNVYITVNINEGEKYSVKSVKLSGDLKVPEDQVKSLLLVQPGQVFS
RKVMTTTSELITRRLGNEGYTFANVNGVPTPNNEDHTVDITFVVDPGKRAYVNRINYRGN
TKSEDEVLRREMRQMEGGWASTYLIDQSKTRLDRLGFFKEVNVETPPVPGTDDQVDVNYA
VEEQASGSITASVGFAQSAGLILGGSISQNNFLGTGNKVSIGLTRSEYQSRYNFSYVDPY
YTPDGVSLGYSAFYRTTNYDDLDVDVASYAVDSLGAGINLGYPISETSRLTYGLTVQQDE
IKTGKYTVDEIFDFVQKEGDKYLNFKGSVGWSESTLNKGVLATRGHSQSLVFESTLPGSD
LSFFKLDYRAQYFHPITDNYTLRLHTELGYGDGYGSTSGLPFYENYYAGGFNSVRGFKDS
TLGPRSTPSRGAGVTGNQGTLEDPDQDPLPFGGNALVQGGVEVMFPLPFVKDQRSLRTSV

FWDVGNVFDTNCDSKKTTASNKTVECNNIDLSGMASSVGIGVTWITALGPLSFALAMPIK
KPDDEAETQVFQFSLGQTF
>tr|A0A0Q0CH33|A0A0Q0CH33_PSEAP Tricarboxylate transport sensor protein TctE OS=Pseudomonas syringae pv. aptata OX=83167 GN=ALO85_100487 PE=4 SV=1
MINDSLRWRLLWNLALLLVVLMLASGMSAYWNGREVADTAYDRTLLASARTIAAGLTQRD
GTLSADIPYVALDTFAYDSEGRIYYQVNDIHQKLISGYENLPSPPPGTLRTDDYPALAKF
YNGIYQGQDVRVVSLLKPVTDPGMNGTAEIRVAETQEARKGMARSLMADTLLRLGMLGGG
ALLLVWFTVSAALRPLERLRTAVEERQPDDLRALPMVEVQHELRPLVGALNHFTERLRVQ
FERQAQFIADAAHELRTPLAALKARVELGLREHDPQHWRSTLESAALGTDRLTHLANQLL
SLARIENGARAIAEGGAQQLDLSQLARELGMAMAPLAHSRGVALALEADEPVWLRGEPTL
LNELLSNLIDNALAHTAPGGNVVLRVYAPGVLEVEDDGPGIPADERERVFERFYRRSQQG
SGAGLGLAIVGEICRAHLARITLHEGAERGLKVRVSFSST
>tr|A0A0N8T9F1|A0A0N8T9F1_PSEAP MoxR-like ATPase OS=Pseudomonas syringae pv. aptata OX=83167 GN=ALO85_02385 PE=4 SV=1
MSDQTPAPSGSSTESLAEPAAESTTQLQQRQRASQLAQALRNELQKAVIGQNAVIDDVLT
ALIGGGHVLLEGVPGLGKTLLVRALASCIGCEFARIQFTPDLMPSDVTGHAVYDLQTEQF
KLRKGPLFTNLLLADEINRAPAKTQAALLEAMQERQVTLEGRALPIPQPFMVLATQNPIE
QEGTYPLPEAELDRFMLKLRMDYPEAQEELNMVRQVTRSSRADMLDVRPLRVIMQAREVQ
ALQRIASELPIDEQVLDYAVRLARSTRTWPGLSQGAGPRASIALVRGGRARAVLRGGEFV
TPDDIKGCALAVLRHRVRLSPELDIEGLSVDQVLRQILDQVSAPRL
>tr|A0A0N8T9D9|A0A0N8T9D9_PSEAP Inactive-like protein of metal-dependent protease OS=Pseudomonas syringae pv. aptata OX=83167 GN=ALO85_00328 PE=4 SV=1
MSFILLHFFTLTTTETPMTTLLALDTATEACSVALLHDGKVLSHYEVIPRLHAQRLLPMI
KTLMAEAGIAMSALDAIAFGRGPGAFTGVRIAIGVVQGLAFALERPVLPVSNLAVLAQRA
WREHGVSQVASAIDARMDEVYWGCYHETAGEMRLLGDEAVMAPELATLPAQTSGQWFGAG
TGWGYAERIPVSLAGHDAAMLPHAQDLLTLATFAWHRGEALPADDAQPVYLRDKVATPKA
R
>tr|A0A0Q0DJY1|A0A0Q0DJY1_PSEAP GNAT family N-acetyltransferase OS=Pseudomonas syringae pv. aptata OX=83167 GN=ALO85_101410 PE=4 SV=1
MEKIIYLARVDGIPVAPGLIHLKSRELEAIFVLPVFMDQGGSAHAVYT
>tr|A0A0Q0C510|A0A0Q0C510_PSEAP Uncharacterized protein OS=Pseudomonas syringae pv. aptata OX=83167 GN=ALO85_04108 PE=4 SV=1
MLMSLKWVDVQEIAIQLAESHPEVNPLTVNFVKLRNLVIALPEFDDVPDRGGEKVLEAIQ
GLWIEEAD
>tr|A0A0Q0C7F0|A0A0Q0C7F0_PSEAP Sigma-54 factor, interaction region OS=Pseudomonas syringae pv. aptata OX=83167 GN=ALO85_01657 PE=4 SV=1
MSLHENFGQPLLTFPDAEKSPLSIRAKALVFIDPRSRRLREEMEQLAPRSLPVLIRGESG
TGKELLARHIHRGSDRSGLFVSVNCGAISPTYADAELFGYAAGAHSGTVSSRAGWFGSAN
GGTLYLDEIGDLPLPIQVKLLAALENHEVTRVGAQQPSPVDVRLVAATSIDLAQAVAAGK
FHERLYHYLSEGRLDLPALREQPGNILPLAEYFVGIYSQRLNLPVQLISEDAQRTLEAHS
WPGNTRELENVIHFALLVSSGDEIRAHDINLPDIADPLTLIESQTQLLLDGGDRRQLSAL
RQLLESVTSELARRPA
>tr|A0A0Q0DLW3|A0A0Q0DLW3_PSEAP Precorrin-2 C(20)-methyltransferase OS=Pseudomonas syringae pv. aptata OX=83167 GN=ALO85_01388 PE=3 SV=1
MMQARGLLIGLGVGPGDPELITVKALRLLRESPVVAYFVAKGKKGNAFGIIEAHLQDVQT
LMPLVYPVTTEALPATLSYEKLISDFYDESSRHIAAHLDAGRDVAVICEGDPFFYGSYMY
LHDRLAEHYDAEVIPGVCSMLGGASVLGAPLVYRNQSLSVLSGVLSHDDLKRKLADADAA
VIMKLGRNFHKVREVLIELGMAGRALYVERATMSNQKIVPLDQVDPTSSPYFSLIIVPGD
RWQG
>tr|A0A0N8T9Z0|A0A0N8T9Z0_PSEAP Flagellar biosynthesis protein FlhA OS=Pseudomonas syringae pv. aptata OX=83167 GN=flhA PE=3 SV=1
MDRSQLLSSARSNITGLGRGQLGVPLLLLIMLAMMMLPMPPFLLDVLFTFNIALSIVVLL
VCVYALRPLDFSVFPTILLVATLLRLALNVASTRVVMLHGQDGHAAAGKVIQAFGEVVIG
GNYVVGIVVFAILMIINFVVITKGAGRISEVSARFTLDAMPGKQMAIDADLNAGLIDQPE
AKRRRAEVAQEAEFYGSMDGASKFVRGDAIAGLLILFINLIGGMLVGIFQHNMTFADAGR
VYTLLTIGDGLVAQLPSLLLSTAAAIMVTRASGSEEMGKLINRQMFASPKALAVSAAIMI
VMGLVPGMPHFSFISLGLVAAGGAYLLWKKENQVKVEAIAEVQRQQDLLPSPTRVQDAKE
LGWDDVTPIDIIGLEVGYRLIPLVDRNQGGQLLARIKGVRKKLSQELGFLMPTVHIRDNL

DLAPSAYRLTLMGVILAEAEIYPDRELAINPGQVFGTLNGITARDPAFGLEAVWIEISQR
SQAQSLGYTVVDASTVVATHLNQILYKHSHELIGHEEVQQLMQLLAKSSPKLAEELVPGV
LSLSSLLNVLQALLAEHVPVRDIRSIAEAIANNAGKSQDTAALVAAVRVGLSRAIVQSIV
GVEPELPVITLEPRLEQILLNSLQKAGQGQEEGVLLEPSMAEKLQRSLIEAAQRQEMQGL
PVILLVAGPVRAMLSRFGRLAVPNMHVLAYQEIPDNKQVTIVATVGPNG
>tr|A0A0Q0DCJ0|A0A0Q0DCJ0_PSEAP Cell division protein FtsA OS=Pseudomonas syringae pv. aptata OX=83167 GN=ftsA PE=3 SV=1
MANVQSGKMIVGLDIGTSKVVALVGEVAADGSLEIVGIGTHPSRGLKKGVVVNIESTVQS
IQRAIEEAQLMAGCRIHSAFVGVAGSHIRSLNSHGIVAIRDREVSAADLERVLDAAQAVA
IPADQRVLHTLPQDYVIDNQEGVREPLGMSGVRLEAKVHVVTCAVNAAQNIEKCVRRCGL
EVDDIILEQLASAYSVLTDDEKELGVCLVDIGGGTTDIAIFTEGAIRHTAVIPIAGDQVT
NDIAMALRTPTQYAEEIKIRYACALAKLAGAGETIKVPSVGDRPPRELSRQALAEVVEPR
YDELFTLIQAELRRSGYEDLIPAGIVLTGGTAKMEGAVELAEEIFHMPVRLGVPHSVKGL
ADVVRNPIYSTGVGLLMYGLQKQSDGISLSGIVGNSSYSDETKAPVLERIKRWVQGNF
>tr|A0A0Q0DM75|A0A0Q0DM75_PSEAP 3-oxoacyl-ACP synthase OS=Pseudomonas syringae pv. aptata OX=83167 GN=ALO85_02829 PE=3 SV=1
MTAYLNALGVICSLGDSREEVSRRLFAGDSSGMVFESGWVPERALPVGAVKSALPPIPPA
VHLHRSRNNQLLLAAALQIEEDISQAITRFGAGRIGVIIGTSTSGIDEASESMAVWLRER
TFPDDYDYRQQELGAPANFLAAWLQLSGPAYVISTACTSSSRALLSARRALDMGLCDAVL
CGGVDSLCKLTLQGFSALEAMSPQLCNPFSNNRNGINIGEAAALFLMTREADSRHSIALL
GAGASCDAHHISAPEPSGRGARDAMLQALRNARIEAEQIGYLNLHGTATQHNDAMESLAV
QGVFPNGVPCSSTKPLSGHTLGAAGALEAAFCWLSLAPQNTEQALPPHLWDGEADPLLPA
LQWTDAGSRLTPENARYMMSNSFAFGGNNISLIIGDAP
>tr|A0A0Q0D3L2|A0A0Q0D3L2_PSEAP Uncharacterized protein OS=Pseudomonas syringae pv. aptata OX=83167 GN=ALO85_01764 PE=4 SV=1
MNTQVNPAALAADNATVQEKIRAFLVSELAEWSINPDEVYINGVNDPEERIVIGSTSLTA
EAANRVFEKDIPAYSTRTAGLFTVAYSYADEHRLAAPDLAKVGEVIGQLVRDLG
>tr|A0A0Q0FUI9|A0A0Q0FUI9_PSEAP Uncharacterized protein OS=Pseudomonas syringae pv. aptata OX=83167 GN=ALO85_00595 PE=4 SV=1
MRLVQFELSDGQRRVGLVDGDQVREVQGVESVRELALAAIEAGSALAHQVEQRGVGETHD
YSQLLEELRILPPLDHPDPAHLLVSGTGLTHLGSASARDKMHQQSGDEASMTDTMRIFKW
GVEGGKPAAGQAGVQPEWFYKGDGSIVVRPGHSFSLPPFAEDGGEEPELSGLYVIGHDRK
PYRLGFAIGNEYSDHVMERRNYLYLAHSKLRACSFGPELRVGDLPQSLSGTSRVWRDGKV
LWEKEFLSGEANMCHSLENLEYHHFKYAQFLRPGDVHVHFFGTATLSFADGVRTQPGDRF
EISQAEFGKPLVNGLAPSEPVFQPGGIGKL
>tr|A0A0Q0C6P9|A0A0Q0C6P9_PSEAP Carboxyphosphonoenolpyruvate phosphonomutase-like protein OS=Pseudomonas syringae pv. aptata OX=83167 GN=ALO85_04793 PE=4 SV=1
MAIQGGAMNTSQAFRKLHERNELFVMPNAWCEGSARLIQQLGYASLGTTSAGIAYAQGYR
DSSPSMDHEDRFAAIERIVRAVDIPVTADLENGLAKTLGKVEEHFRRALTVGCAGASIED
IADYHDPNTPLYFSIEEACDRVRAASEAAHALDADFIVTARTDYLLGAHEYSLTEVIKRL
VAFEEAGADCLFAPGVKSIEDIEAIQRALSKPLNVLPVFGMTIDRLRTVGVRRISLGSSL
FRNAYRQISTVLLGLEEPEGLDFLQSGLSLNQLDEVMR
>tr|A0A0Q0IM06|A0A0Q0IM06_PSEAP ArsR family transcriptional regulator OS=Pseudomonas syringae pv. aptata OX=83167 GN=ALO85_02367 PE=4 SV=1
MPIDLDDIIKALAHPVRREILTWLKNPRASFPAQEYSIEHGVCAGQIDQRAGLSQSTVSA
HLATLQRAGLISSKKVGQWHFFRRNEEVIQAFVQALVEELNNPT
>tr|A0A0Q0FI75|A0A0Q0FI75_PSEAP Thermostable hemolysin delta-VPH OS=Pseudomonas syringae pv. aptata OX=83167 GN=ALO85_03184 PE=4 SV=1
MPDIDSNIRLPLSFGNTGAPTLTLFRALAHEQQREPLELFVQQQFRKAHDADVRQFMPEL
FGLQDGSGALCAVAGIRHASAGTLFLENYLDAPIEQQVNAAAGRLIERQGIVEVGNLAAN
SLGSARLSIITVTWLLAMSGFEWVAFTGNAALVNSFNRLGLNPVTLCAADPARLGEGRHT
WGSYYDSRPSVHVGDIRAGFLHLSSMGIFERFGLPQTLEPDCHVA
>tr|A0A0Q0D6Z0|A0A0Q0D6Z0_PSEAP Uncharacterized protein OS=Pseudomonas syringae pv. aptata OX=83167 GN=ALO85_01006 PE=4 SV=1
MRECNHSIAGPFMTSTTEHSTIPDVLDSLLGIAPGSPLHAVRHARDKVALATQGSQDLFF
DPALAHNLSLNERLWVAYYATLLSAQPTLSGYYLSQLQAAGAEANVLADVDASQIDLLED
QRLAAILRFTRILIESPVHGDQSALQALQHQGLSTAEIVVLAQLIAFLSYQVRLAAGLGA
LKSAGAA

>tr|A0A0Q0IRP5|A0A0Q0IRP5_PSEAP Uncharacterized protein OS=Pseudomonas syringae pv. aptata OX=83167 GN=ALO85_200028 PE=4 SV=1
MQLYEVDEIKELFATGEVNDALTSGWRIVAVVSSVAPGGDLPVACYVMGRYLPKEDRL
>tr|A0A0Q0CVQ9|A0A0Q0CVQ9_PSEAP Tetratricopeptide repeat protein OS=Pseudomonas syringae pv. aptata OX=83167 GN=ALO85_05581 PE=4 SV=1
MFKRILSALSGKKSQASADNAPAATTSPEAHDGELITAYDSYGREIKITRSEWRDKVFLP
NLKQQWGDADELYNLIVSGLNDGFATDLMPAAERLVEIDANPERSYVVNGIVLMHNARLD
AAEATLREGLAKVGVSGIILTNLAKVFAERGDQSVAEATLWQAVEADPNQENGLLWWLSI
QQERGGEAAYLDALRTVAALPDSWRAQLWMARHYLQQQNVAQARVLYEEVLAGGHFDRSA
LQMISGDLGNNGHIPLIIELVGPAYDEHKHDSTAGLNLLRAYQELGRVDEGEALLTRLYA
LGFAPIKSHLDQFAHAFEDVRRQEDKGIPIDPASVTISTLALNKPVWHYGLRNADWLFAQ
KPEGAPEIGFFALSKIMEKAEPAESQREDDVGRYTRAIPLYMAESVHYWNDYVANCYVQV
AEGAGPVVSGVEVDGNTLFDIVPPTTKYFVTGEVGCSGEGDQAQWRISLSLWNCATRSRQ
TVENGSAGKAELGALVLDLQQRLLAGIGLKREQPLDVFYQQPAAEVLPVYLTQLGQSFML
TLLANDHLPKSSMWGERAMLEWPLNMALQWPEMETAKLMYISGLGKAFGYKSETVAEHQQ
RCLQVLSELERASSPAWRLAPLVWKVFGMQAELEDYRANLPPDTEPAYIEWLERVSQA
>tr|A0A0Q0C1W4|A0A0Q0C1W4_PSEAP DNA topoisomerase OS=Pseudomonas syringae pv. aptata OX=83167 GN=ALO85_00809 PE=4 SV=1
MRLFLCEKPSQAKDIAAALGATRRGDGCWVGANATVTWCIGHLLETAPPDAYDARYKRWV
LEDLPIVPEKWKMLVKPRTASQFKAVKRLLGEARELVIATDADREGEMIARELIEHCRYR
GPIQRLWLSALDDASIRKALAALKPGADTFSLYHSALGRSRADWLIGMNMSRLFTLLGRQ
SGYQGVLPVGRVQTPTLRLVVDRDRSIANFVPAPYWAIDVRLLHDNQAFIAQWRAPSDAC
DDQDRCLNQALAQQAAHDMHATTSARLAKLRTERIREAAPLPFDLGTLQEICSKKLGLGA
QETLDIAQSLYETHKVITYPRSDCGYLPNSQHGEAAGIIAALCKADPALAGLSPHLDPQR
RSRAWNDSKVTAHHGIIPTAAVRGVERLTGKPRAVYTLIRARYLAQFLPNHEYDRTQADF
DCAGHALRAVGKQIVEPGWKRAMPEALAPAKGREAPAPQPLPALFQGCECAIAEVILKDL
WTQPPKPFTEGDLIKAMKNVAKLVEDPLLKQKLKDTTGIGTEATRAGIIQGLLDRGYLTK
QGKSLCAAPAAFSLIDAVPRAIADPGTTAIWEQALDMVQSGEMSLEEFVAKQAAWMSKQV
SRCVGMRMSISGPASPAGVAPPWKKKRKTTKRVAKTSTATADTAAKKPRRAARPAAKA
>tr|A0A0Q0C6W4|A0A0Q0C6W4_PSEAP Isopenicillin N synthase-like dioxygenase OS=Pseudomonas syringae pv. aptata OX=83167 GN=ALO85_03069 PE=3 SV=1
MDQLPVIDIAPLYGTDTQAWQDVARQIDSACRAWGFFYIKGHPISAQRIEQVQSAAKEFF
ARPAAEKLLIDITQSTHHRGYGAIATEQLDPSLPSDLKETFDMGLHLDARHPDVLAGKPL
RGPNRHPDIPGWEALMEQHYLDMQALAQTLLRAMTLALGIERDFFDQRFKDPVSVLRMIH
YPPRGTATSAEQQGAGAHTDYGCITLLYQDMAGGLQVRDVRGEWIDAPPLDGTFVVNLGD
MMARWSNDRYLSTPHRVISPLGVDRYSMPFFAEPHPDTRIECLPGCQSDDHPARYPVTTC
AEFLLSRFADTYAYRREQEAS
>tr|A0A0N8T9X5|A0A0N8T9X5_PSEAP Lipoprotein OS=Pseudomonas syringae pv. aptata OX=83167 GN=ALO85_01960 PE=4 SV=1
MKQFALLSLIGGALLVGCTADPLPIQQDHSYVTEWIGERPLIDNSRLTMTLGADGRAYGN
AGCNHWFAPYTLNDHSISFGPVGKTRKMCAPALMEQEQRFIKAMSSVQRWDISPIEQLRL
WPTQGKPLRFWLEDS
>tr|A0A0Q0CYK4|A0A0Q0CYK4_PSEAP Proline iminopeptidase OS=Pseudomonas syringae pv. aptata OX=83167 GN=ALO85_01696 PE=3 SV=1
MQTLYPQIKPYARHDLAVEQPHVLYVDESGSPEGLPVVFIHGGPGSGCDAHSRCYFDPNL
YRIVTFDQRGCGRSTPHASLENNTTWKLVEDLEAIREHLGIDKWVLFGGSWGSTLALAYA
QTHPDRVHALILRGVFLARQQEIDWFYQAGASRLFPDYWQDYVAPIPLDERDNILAAFHK
RLTGPDQIAQMHAAKAWSTWEGRCATLRPNPQVVDRFAEPHRALSIARIECHYFMNNAFL
EDNQLIRDMPKIAHLPAIIVHGRYDVICPLDNAWELHQNWPGSELQVIREAGHSAAEPGI
ADALVRAAAEVARNLLDLPPEEA
>tr|A0A0Q0IT73|A0A0Q0IT73_PSEAP SMI1/KNR4 family protein OS=Pseudomonas syringae pv. aptata OX=83167 GN=ALO85_01442 PE=4 SV=1
MEEVIEQLREANEPVPVPLELPDEDQLVEIEEQLFINIPFVFKEFLLTVSDVVYGSLEPV
TVTDPQSHTYLPDVAATAWDLGVPRELIPICQDGDDYYCVEEDGTVLLWSAEEELVTEDS
WESVWHWARDVWLES
>tr|A0A0N8T9S6|A0A0N8T9S6_PSEAP 6-phosphogluconate dehydrogenase-like protein OS=Pseudomonas syringae pv. aptata OX=83167 GN=ALO85_03654 PE=4 SV=1
MTKQHARNLSMRWTASDAPFYDLRSTFMQLGIIGLGRMGGNIARRLMLNGHTTVVYDRDE
ASRNALANDGSTAVADLKELVGTLAKPRAVWVMLPAGAPTEDTIQTLSELLDADDVIIDG

GNTFYKDDIRRAQELTEKGLHYVDVGTSGGVWGLERGYCMMIGGEAAVVERLDPLFATLA
PGLGSIERTRDRTSTDDRAERGYIHSGPAGSGHFVKMIHNGIEYGMMQAFAEGFDLLKQK
NSELLPPEQRFDLNTADIAEVWRRGSVVSSWLLDLTADALATDPHLDAFSGSVADSGEGR
WTIEAAIEQAVPVPVLSSALFARFRSRQTSSYADKMLSAMRFGFGGHKEPK
>tr|A0A0Q0DIX1|A0A0Q0DIX1_PSEAP Uncharacterized protein OS=Pseudomonas syringae pv. aptata OX=83167 GN=ALO85_04077 PE=4 SV=1
MSTDTPIEKPVRSPCVSICALDEQDICTGCQRTVTEITRWSRMSNDERRVVLGLCDERAR
ASGLMWTVGEAR
>tr|A0A0Q0C662|A0A0Q0C662_PSEAP Uncharacterized protein OS=Pseudomonas syringae pv. aptata OX=83167 GN=ALO85_100592 PE=4 SV=1
MAASCLQRVEITTPRRLPPGRFLGRCGLLAQELNAPSRFMRLRSCQPGPGTEPE
>tr|A0A0Q0C9Q4|A0A0Q0C9Q4_PSEAP Uncharacterized protein OS=Pseudomonas syringae pv. aptata OX=83167 GN=ALO85_00379 PE=4 SV=1
MTIGFRVLEAARKVSAEWVSRYRDVPVANVSDSMNRMTAGGARLRPMHREGLLAGPALTV
KARPGDNLMLHYAIDIAEPGDVIVVDAGGDLSNALIGEMMVAYAVKRGVAGIVINGAIRD
AGSIAAGTFPMFAAGISHRGPYKDGPGEINVAIAIDGMVIEPGDLVIGDEDGLLCVPYDQ
VADVYDRASAKHHAEQKQLEQIASGENDRSWVLESLKKKGCQLPQ
>tr|A0A0Q0D022|A0A0Q0D022_PSEAP Uncharacterized protein OS=Pseudomonas syringae pv. aptata OX=83167 GN=ALO85_101187 PE=4 SV=1
MGVIACHGSVLLVEQQTPSFNLQAAIFKHRASRCRPAARRVREPGYGKVSRSLR
>tr|A0A0Q0DZB3|A0A0Q0DZB3_PSEAP Rhs protein OS=Pseudomonas syringae pv. aptata OX=83167 GN=ALO85_03374 PE=4 SV=1
MIATPPLAFSKEIQMLMDLAAMFSPQNRRLFKFRNLANPTQDLLLESFSGTEGLSYAYSF
ELTLLCQDSGIKLKSMMGQHSVIDIELADGPARHVAGYITRFASIGSDGGIARYTATLNP
WFSMLENRIDSRIFQDKTVEEVVAQVFASCSAYSRHEFRLSKPAKRYGYITQYRESDFNF
VQRLLEQEGMFYYFEHTSEGHTMIICDDSTRLESLPNQPKIRFHTASVTETADSITDWQS
SRQLQASRISVQTFDYSQPRNRLPVTINSMNMQGDVENFEIYDYPGQFSHSTYDEGGALL
RLRVEALELRGKTFQGSSNCRAMQPGYTFELLQHYNHDYGPAEDRQFLLMSVRSEGNNNY
SNGHPASYFNTFSCVRRKIVFRPQSVTPRPTICGPQTAIVVGAPGEEIFTDRLGRIKVQF
HWDREGNQDDKSSCWVRVAQSGASSGFGSIQIPRVDDEVVVVFLDGNPDRPLVMGSLYNS
RNIPPWSLPANKTQSGFLTRSIKGQASNANFFRFEDKPGAEQVSLHAERNLDTDVEVDES
HTVGGDRTITVKGKHSETIKLETSIAVEEGSYFVTVDKGEVKIKSAESITLEVGSSKLVM
NRDGTITLSGITVNVEGTTKINLNKDQ
>tr|A0A0Q0FEQ9|A0A0Q0FEQ9_PSEAP Uncharacterized protein OS=Pseudomonas syringae pv. aptata OX=83167 GN=ALO85_01284 PE=4 SV=1
MSKAAAAAQPASSLSRFWHKWRFHINVLLVLIPLGFMPKYFSDVSLNRGDQGLGQREVGE
IQVGPWSLRLAEDRNEAPRLSGPSGYMKSFNAALCNACIDRVKATYLRIGKPRSLRTAGV
IFFGGGYRMSAFMQIAQNTDPDAELWITMEGWDGAVHQTSIPLQQASPSTVAWLKKQGAQ
P
>tr|A0A0N8T816|A0A0N8T816_PSEAP Ribosomal RNA large subunit methyltransferase E OS=Pseudomonas syringae pv. aptata OX=83167 GN=rlmE PE=3 SV=1
MARSKTSHNWLKEHFDDKYVKMAQKDGYRSRASYKLLEIQEKDKLIRPGMTVIDLGAAPG
GWSQVTSRLIGGQGRLIASDILEMDSIPDVTFIQGDFTEDAILEQILEAVGNTQVDLVIS
DMAPNMSGLSAVDMPRAMFLCELALDLAGRVLRPGGDFLIKVFQGEGFDVYHKDIRKLFD
KVQMRKPSSSRDRSREQYLLARGFRGIDGAASIERF
>tr|A0A0N8TAC5|A0A0N8TAC5_PSEAP Uncharacterized protein OS=Pseudomonas syringae pv. aptata OX=83167 GN=ALO85_101554 PE=4 SV=1
MVTVLYGKGTNERRILGSCSTKHKTINPMLSMTKTHLKLFSAFAVK
>tr|A0A0Q0CH38|A0A0Q0CH38_PSEAP Uncharacterized protein OS=Pseudomonas syringae pv. aptata OX=83167 GN=ALO85_02048 PE=4 SV=1
MDSKEHQLTELLGLTARTLTHLTASMTSMSFELLRSEDEVTRTAGRRMIDRMATISSGLD
EHWRLIGDLTGVHLAQEQVETVAEIQQQRKVVTPIGG
>tr|A0A0Q0CH26|A0A0Q0CH26_PSEAP Uncharacterized protein OS=Pseudomonas syringae pv. aptata OX=83167 GN=ALO85_02019 PE=4 SV=1
MCGLCGLLGEDVHWSDPLAAELPRRRERLRRIAAINKVVAPFRLKVEDFQGVSYLLLGAT
GKQELATGLEQLWQKAELLIGRPLDPLDSGLLDHLQRSS
>tr|A0A0Q0C8J3|A0A0Q0C8J3_PSEAP TPR repeat-containing response regulator OS=Pseudomonas syringae pv. aptata OX=83167 GN=ALO85_02125 PE=4 SV=1
MLAYNQKSFLIVDDFSDFRSSVRSMLRELGVKEVDTADSGEQALRMCSQKRYDFVLHDFN

LGDGRKNGQQVLEDLMIERLLSYESVFIMVTAENSQAMVMSALEWEPDGYLTKPFNRAGL
AQRLEKLVQRKTLLKPILQALDRRKPAEVLAACDKLIEQDPRYAPLCLRYKADALRDLKQ
NEPLEAFLKTILADRATPWAYGALGSLLLKRGKTAEAQAVYEQAIKAFPTMPALFDGLAD
VLVALGDGKRAQSVLESAVRLSPLAVRRQKLLGKLALGNDDFESASKAYRQAVSQGQHSR
FKDPETNLGLAHALISKGGDQGLDARTRVEINNALVDVAKEHGDDEGLQVRTRLMKAASL
QHSDPEAAAKLTEQAMARLDGMEQVLSADAALMVAAQLKKLGQEEAGAGVLKSCAGAYGD
DPAVMKSIASMTDDPAILGASKAAVDFNLQGVRSYKAGNLTEAQAFFRSALGLQPKNISI
ALNMVQSLLHPGQNLGQAGIDECRASLTMLGKIPESDPRYERYHKLRERAFSA
>tr|A0A0Q0C7S9|A0A0Q0C7S9_PSEAP Putative type VI secretion system effector, VgrG family OS=Pseudomonas syringae pv. aptata OX=83167 GN=ALO85_01357 PE=4 SV=1
MPDPDSAPFFYLEIARLQRRFTVVSFSATEAISQPYAIELEILGDGFDLDLTSLMYKPAW
LSCGSEQGFPGQSFQGQGFHGQIHGATRKHYHPGPACYTLLMGPRLACLGLRQHSRIFQH
MTATQIIAQVLEEHGLKNAFRFYLQTECIERESCVQYQESDLQLVQRLCAAEGIHYHFVH
SRRRHLLVFGASPWETGFTAPAPAPRPAIAPLQRGWIIGSDGEPARTDASGRVHVRLEWD
WQRPEGPDAGCWLHLAAGLDVECRGGMAVIVSFCADDKRPPQITGCLSTLADDGGHRCDQ
APADNVEMHLDWQLITGPDRQLQLADGTRVELAEGSTLTVDAGASTVQIDDSGLTLSSPR
ISFASPPSDPYATE
>tr|A0A0Q0FMR8|A0A0Q0FMR8_PSEAP UDP-glucose 4-epimerase OS=Pseudomonas syringae pv. aptata OX=83167 GN=ALO85_04674 PE=3 SV=1
MILVTGGAGYIGAHIALELLEDGRDVVVLDNLCNSSREPLRRVEELCGRQVVFIHGDVRS
KATLHRLFAQHPVKAVVHCAGLKAVGESVREPLRYFETNVSGSVNLCQAMAEAGVFDLLF
SSSATVYGECEQMPLDENCPLGLPTNPYGHSKLMAEHVMQSVARSDPRWSIGLLRYFNPI
GAHPSGLLGESPCNTPNNLLPFLLQVANRLRPALHIFGSDYPTPDGTGVRDYLHVMDLAE
GHLKALDRIHDQRGVSVWNLGTGQGYSVLEVVQAFERISGKAVPLIFEPRRPGDIAACWS
DPGKAARELDWRARFNLDSMLTDAWRWQCMNPQGYRPTALVI
>tr|A0A0Q0ICW1|A0A0Q0ICW1_PSEAP HAD family hydrolase OS=Pseudomonas syringae pv. aptata OX=83167 GN=ALO85_03118 PE=4 SV=1
MIKAVIFDMDGVLIDAKEWHYDALNKALSLFGYNISRHEHLTAYDGLPTSRKLDMLSVER
DLPVALHAFINEMKQQYTMEIVYAQCKPTFVHQYALSSLKTLGYKLAVASNSIRNTVEVM
MNRADLDRYLDLRLSNEDVRHAKPAPDIYTKAISQLGLRPEECLIVEDNENGIKAARDSG
AHVLVVAETCEVNLNNILGKVEQINFEKAAVL
>tr|A0A0Q0CI37|A0A0Q0CI37_PSEAP Sarcosine oxidase, gamma subunit, heterotetrameric OS=Pseudomonas syringae pv. aptata OX=83167 GN=ALO85_03841 PE=4 SV=1
MSTANVYQQRPTTDVKAESPLFHANLDSLIGKGRASPGVFLREKKLLGHLTIRGNAHDPA
FAAGVHKALGMELPAALMLVINGESSLQWLGPDEWLLIVPGGEEFAAEQKLREELSGQHI
AIVNVSGGQSILELSGPKVRQVLMKSTSYDVHPDNFPVGKAVGTVFAKSQLVIRRTGEET
WELLVRRSFSDYWWLWLQDASAEYGLSIQA
>tr|A0A0Q0CDP6|A0A0Q0CDP6_PSEAP Putative chloramphenical resistance permease RarD OS=Pseudomonas syringae pv. aptata OX=83167 GN=ALO85_02609 PE=4 SV=1
MYKGVVLSVLASTLFGVMYFYTSLMAPLDGEEIFGWRMLLTVPCATLFMLVSGDWRRVRE
VAVRLRQQPLLIFALLLSSVLLGAQLLIFMWAPLHGRSLEVSLGYFLLPLSMILTGRVVY
GEHLSYLQKVAAVFAMIGVAHELWRLGSFSWETLLVAGGYPLYFVLRRTLKTDHLGGLWF
DMLLMLPIGLWFIAHGHPQAIQQTPLLYLLIPLLGVISASALVSYILASRLLPFSLFGLL
SYVEPILLVGVALLLGESIGRDEWLTYLPIWMAVVVLMIEGAKHMRAQRRRDAS
>tr|A0A0Q0DQU9|A0A0Q0DQU9_PSEAP Oligopeptide/dipeptide/nickel ABC-type transport system OS=Pseudomonas syringae pv. aptata OX=83167 GN=ALO85_03911 PE=3 SV=1
MNSNTLWLIVQRFGAAIVTLLIVSIVVFAITAVLPGDAAQQALGQFATPEQVAALRIKLG
LDQPGVVRYLHWLTNLLSGNLGESVSNAMPVSELIAGRFPKTLMLSATTALVSVPVALAL
GIGAAMYRGSRLDSALNFITLSLVAVPEFLVATLAVLIFAVNLGWLSALSYGADVSSPWQ
FMRTYALPVMTLCCVIVAQMARMTRAAVIDQLDSAYVEMARLKGVSPVRIVLRHALPNAI
GPIVNAVALSLSYLLGGVVIVETIFNYPGIASLMVDAVTNRDMALVQGCTMLFCSAYLGL
VLMADLCAILSNPRLRNQ
>tr|A0A0Q0FQJ6|A0A0Q0FQJ6_PSEAP Ferric siderophore transport system protein, ExbD/TolR family OS=Pseudomonas syringae pv. aptata OX=83167 GN=ALO85_200165 PE=3 SV=1
MASGLLGGDEQEEDGFNPEINTTPLVDVMLVLLVIFIITVPAIQHSVKIDLPKAAAQQDN

KPPPSVDLALDNEGHLHWDDRDISDSDLPGLIAEAAARQPVPELHLRAERNTPYEKVVQV
MTAAQSGGLDKIGFVTKAVTP
>tr|A0A0Q0CG93|A0A0Q0CG93_PSEAP Uncharacterized protein OS=Pseudomonas syringae pv. aptata OX=83167 GN=ALO85_00799 PE=4 SV=1
MTFDTYMQVEGIPGESLDAVFKDWIELSDFDVGASQSASATTTSAGGASNGRASMSDFFF
RKAVDKSTPKLHEACCSGKHFKEVTIAVNRAGTEKIKYLEIKLEEVIISSVSLNGNGSIE
SGFPTETVRLNYGRIKMLYTQQKRSDGQGGGQVAGGWDGIKNRVYA
>tr|A0A0Q0CGZ8|A0A0Q0CGZ8_PSEAP Uncharacterized protein OS=Pseudomonas syringae pv. aptata OX=83167 GN=ALO85_101971 PE=4 SV=1
MIISIRSETARPVTRTRDLLPLNPRPQPGKMPDIFFTSLFRTPP
>tr|A0A0Q0IAU5|A0A0Q0IAU5_PSEAP Uncharacterized protein OS=Pseudomonas syringae pv. aptata OX=83167 GN=ALO85_101425 PE=4 SV=1
MREYERYQLDSIASEYRSRGYVVDVEAQLSDSGLRFDAIARRGDDKELVFVEIVNPRLSD
DEIAARRLAIADAALRFPYALIDFRYIDIKQSAFLEFNTRDDNSRDQQFRELLKARFPVF
NKKPKDAARQMLSLWAGYASLLRGLGRLCRHPESEEASILDLYNSFLQRRILVSAEITDD
SVSHDLYQMHEVVIAATQGALVDIEYVKQLRGHYQALRKQATDYSKKGWPIDTTRW
>tr|A0A0Q0DTK3|A0A0Q0DTK3_PSEAP Mangotoxin biosynthesis protein MboB OS=Pseudomonas syringae pv. aptata OX=83167 GN=ALO85_02727 PE=4 SV=1
MAIYSVRIGDQTYTAESQQPLTDALPHEALVRGCLKGVCRVCKCTLVSGRVLEQGKVVAL
NEAFLPCVSRAETDIDIRVAISTFHKARLKSKRMLSGQIMEVVLEVKKVFYNAKSVISLK
YPEGSAVRSYSVVTLGGKDYDSLTCHVKLREGGLFSGLLLQWPVGEPLEYSIASPALPVY
EQSLSRLNVVSGGSGMGAALSRAQELVAKYGISEVAIYAVNRSGLSDYHARCIEMFRKTV
ECDVHVTNFPFSEWTHPDFAIGEHLMHDTLTIGVGSDGVIGKLKNLPLCELESFG
>tr|A0A0Q0FLN9|A0A0Q0FLN9_PSEAP MotA/TolQ/ExbB proton channel OS=Pseudomonas syringae pv. aptata OX=83167 GN=ALO85_02527 PE=3 SV=1
MWELVKSGGWMMLPIILSSIAAAGIIIERLWTLRASRITPPHLLGQVWQWIQEKKLDGEK
LKQLRADSPLGEILAAGLANSRHGREIMKECIEEAAARVIHELERYLSALGSIAAMAPLL
GLLGTVLGMIDIFGSFNSSGATANAGVLAGGISKALICTASGLIVAIPAIFFHRFLQSRV
DELVVGMEQQAIRLVEVVQGDRDVDLIDAKIDLKSLARAGGGKKK
>tr|A0A0Q0DJY0|A0A0Q0DJY0_PSEAP 3-oxoacyl-reductase OS=Pseudomonas syringae pv. aptata OX=83167 GN=ALO85_02541 PE=4 SV=1
MSLQGKVALVTGASRGIGQAIALELGRNGAVVVGTATSESGAERISATFKENGIEGFGLM
LDVCDAESVNSVLSTIQERVGAPLILVNNAGITRDNLMMRMKDDEWYDVVNTNLNSLFRL
SKGVLRGMTKARWGRIISIGSVVGAMGNAGQVNYASAKAGLEGFSRALAREVGSRAVTVN
SVAPGFIDTDMTRELPEAQRESLITQIPLGRLGQAEEIAHVVAFLASEGAGYVTGATIPV
NGGMYMS
>tr|A0A0Q0C482|A0A0Q0C482_PSEAP Uncharacterized protein OS=Pseudomonas syringae pv. aptata OX=83167 GN=ALO85_00347 PE=4 SV=1
MNTRGLLDQLLKSGQSMLQDKNKGKSGKQSSGSDSLINGLGSLLGGSKGQGVSQNGLGSL
LSGAGGGALAAGAMSLLRGKRSRGMGGKVLTYGGLAALGVLAYKAYNNWQASQGLTAQRE
PQTIDRLPEAEAEQHSQAILRALVAAAKADGHVDERERELIEGEFTKLNNDQTLQQWLHA
ELNKPLDPAEVARAATTPEMAAEMYIASVMLVDEEHFMERAYLDELARQLKLDPGLKTEL
EGQVRNAQ
>tr|A0A0N8T9J3|A0A0N8T9J3_PSEAP 30S ribosomal protein S4 OS=Pseudomonas syringae pv. aptata OX=83167 GN=rpsD PE=3 SV=1
MARYIGPKCKLARREGTDLFLKSGVRAIESKCNIEAAPGIHGQRRGRQSDYGTQLREKQK
VRRIYGVLERQFSGYYKEAAGKKGATGENLLQLLECRLDNVVYRMGFGSTRAESRQLVSH
KSVSVNGKTVNVPSYQVRAGDVVAIREKAKNQLRIVQALDLCAQRGRVEWVEVDTEKKSG
VFKNVPARSDLSADINESLIVELYSK
>tr|A0A0Q0CD44|A0A0Q0CD44_PSEAP 5-oxoprolinase subunit A OS=Pseudomonas syringae pv. aptata OX=83167 GN=pxpA PE=3 SV=1
MQVDFNSDMGESFGAWTIGDGVDTELMTFISSANVATGFHAGDPGTMRRTIEQAKRLGVA
IGAHPGFRDLVGFGRRHINSTPQELVDDMLYQLGALRELARVQGVALQHFKPHGALYMHL
ARDEAAARLLVENLQRLEPDLLLYCMPGSVICKIAEELGQPVIREFYADRAYDLSGSIVF
TRHVRALDPTEVAARVVRACLQGLVRTVEGEDLSIEFDSICLHSDTPGALDLAEATRLAL
DAAGIKVRAPR
>tr|A0A0Q0IN60|A0A0Q0IN60_PSEAP Uncharacterized protein OS=Pseudomonas syringae pv. aptata OX=83167 GN=ALO85_02903 PE=4 SV=1
MLSQALKKDSKMQVSKTKSSFYRRLYVAYLIDSQIASSVPELMAATGMPRRTAQDTISAL
ADLDIVCDFEQLEGGRNHAGSYRIRDWGAVDKGWIADNLPRIKAVLEYP

>tr|A0A0Q0BY66|A0A0Q0BY66_PSEAP Phosphoglycerate kinase OS=Pseudomonas syringae pv. aptata OX=83167 GN=pgk PE=3 SV=1
MLMTVLKMTDLDLQGKRVLIREDLNVPVKDGVVSSDARILASLPTIRLALEKGAAVMVCS
HLGRPTEGEFSAENSLKPVADYLSKALGRDVPLVADYLDGVDVKAGDVVLFENVRFNKGE
KKNTDELAQQYAALCDVFVMDAFGTAHRAEGSTHGVAKFAKVAAAGPLLAAELEALGKAL
GAPAQPMTAIVAGSKVSTKLDVLNSLSGICNQLIVGGGIANTFLAAAGHKVGKSLYEPDL
LDTARAIAAKVSVPLPTDVVVAKEFAESAAATVKLIGEVADDDMILDIGPQTAAHFAELL
KSSGTILWNGPVGVFEFDQFGEGTKTLAKAIAESQAFSIAGGGDTLAAIDKYGVAQKISY
ISTGGGAFLEFVEGKVLPAVEVLEQRAKA
>tr|A0A0Q0FW28|A0A0Q0FW28_PSEAP NADH dehydrogenase OS=Pseudomonas syringae pv. aptata OX=83167 GN=ALO85_03289 PE=4 SV=1
MTHRIVIVGGGAGGLELATSLGKTLGKKGTASVTLVDANLTHIWKPLLHEVAAGSLNSYE
DELNYVAQAKWNNFQFQLGRMTGLDRASRQIHLAETLDENGAELVPARSLGYDSLVIAVG
STTNDFGTKGAAEHCLFLDSRKQAERFHQQLLNHYLRAHAGQADSAQEITVAIVGAGATG
VELAAELHNAAHELAAYGLGQIKPENLRITVIEAGPRVLPALPERIGAPVHKTLEKLGVT
VLTNAAVSEVTADGLITATGQVIPASLKVWAAGIRAPAFLHELDGLESNRINQLQVLPTL
QTTRDENIFAFGDCAACPQKGTDRNVPPRAQAAHQQASLLVKSLRLRIEGKTLPEYTYKD
YGSLISLSSFSAVGNLMGNLMGSVMLEGWLARMFYVSLYRMHQMALYGTFRTLMLMLGSK
IGKGTEPRMKLH
>tr|A0A0Q0CFG2|A0A0Q0CFG2_PSEAP Sorbitol and other polyol-specific dehydrogenase OS=Pseudomonas syringae pv. aptata OX=83167 GN=ALO85_02078 PE=4 SV=1
MKRLEGKSALITGSARGIGRAFAQAYIHEGARVAIADIDLQRAQATATELGPNAYAVRMD
VTDQSSIDQAIAAVVAQVGKLDILINNAALFDLAPIVDITRDSYEQLFSINVGGTLFTLQ
AAARQMIAQGHGGKIINMASQAGRRGEALVAVYCATKAAVISLTQSAGLDLIRHGINVNA
IAPGVVDGEHWDGVDALFARHENRPLGEKKRLVGEQVPFGRMGTADDLTGMAIFLASADS
EYVVAQTYNVDGGNWMS
>tr|A0A0N8T9E7|A0A0N8T9E7_PSEAP Uncharacterized protein OS=Pseudomonas syringae pv. aptata OX=83167 GN=ALO85_02374 PE=4 SV=1
MAFDAYIKIEGIPGEALDDRYKDCIEITGYGFGMHQSTSATASSSGGASSGRTSLSDFTF
TKSLDKSSCKLMESLCAGKHLKEVVLSLHRAGGDKVKYFEVLLEEVIISNYTQHANDGIP
SETISLNYGKIKTTYTQQKRSDGAGGGNVSGGWNRINNKVYS
>tr|A0A0N8T909|A0A0N8T909_PSEAP Sulfate/thiosulfate import ATP-binding protein CysA OS=Pseudomonas syringae pv. aptata OX=83167 GN=cysA PE=3 SV=1
MSIEVRNVSKNFNAFKALNNISLDIQSGELVALLGPSGCGKTTLLRIIAGLETPDDGSIV
FHGEDVSGHDVRDRNVGFVFQHYALFRHMTVFDNVAFGLRMKPKRERPNETRIAEKVHEL
LNMVQLDWLADRYPEQLSGGQRQRIALARALAVEPKVLLLDEPFGALDAKVRKELRRWLA
RLHEDINLTSVFVTHDQEEAMEVADRIVVMNKGVIEQIGSPGEVYEKPSNDFVYHFLGDS
NRLSLGEEGHLLFRPHEVSLSRQEIEDHHAAEVRDIRPLGATTRVTLKVEGQDELIEAEV
VKDHDSLVGLAKGETLFFKPKVWQKL
>tr|A0A0Q0C252|A0A0Q0C252_PSEAP Flagellar biosynthesis protein FlhF OS=Pseudomonas syringae pv. aptata OX=83167 GN=ALO85_05567 PE=4 SV=1
MQVKRFFAADMRQAMKLVRDELGAEAAIIGNRRIAGGVELTAALDYKLSALAPRVPNMEL
EDELRKTQSRIVSAQAELNNRSDSDASINLQLFSGKPANPADTHIEPTLEEPPRPFAYAP
PQAAEPAIGQRAYDSMRSELNGLRELLEVQLGSLAWNQLQGSRPQQANLWRRLQRVGLSG
PLSRDLLSMIPEIDEPRQAWRMLLAHLARMIAVPEVEPLEEGGVIAMVGPAGMGKTTTLA
KLAARYVLKYGPQNIALVSMDSFRIGAQEQLKTLGRILNVPVTHIDPGQSLVQALEPLLR
KRVVLIDTAGLQASDPALRMQLESLAGRGIKSRNYLVLATTSQKQVLTAAYHSYKRCGLA
GCILTKLDETASLGEVLSLAISHELPVAYLTDGPRIPDDLHTPRRHQLVSRAVSVQMQDE
PSEEAMADMFADLYHTPGKRVG
>tr|A0A0Q0FLT7|A0A0Q0FLT7_PSEAP HAD family hydrolase OS=Pseudomonas syringae pv. aptata OX=83167 GN=ALO85_02535 PE=4 SV=1
MRRDYDLLIFDWDGTLADSVGRIILSMRTAAIETDLEIRDDTAIKGIIGLGLPEAIRTLY
PQISGNQLIDFRQRYADSYMAMDNVPSPLFDGVVESMQAFREDGYRLAVATGKARRGLDR
VLKANGWQDYFDATRAADETASKPDPLMLNEIMAHCEVAPQRSLMIGDASFDLLMARNAG
MDSVAVGYGAQPLESLRQFEPRLAIEHFSELRTWLNGRGV
>tr|A0A0Q0CE18|A0A0Q0CE18_PSEAP Uncharacterized protein OS=Pseudomonas syringae pv. aptata OX=83167 GN=ALO85_101727 PE=4 SV=1
MGVGRVVPDVWIFELAVDFGQTIMFVIVVKDTPEWFRSARTNP

>tr|A0A0Q0IE88|A0A0Q0IE88_PSEAP Uncharacterized protein OS=Pseudomonas syringae pv. aptata OX=83167 GN=ALO85_01091 PE=4 SV=1
MNTVLNTPAPYLKPDECVVLFDGVCKLCNGVVKFLIRHDPHRRLRLAAVQSEQGQALLKW
AGLPLDDFHTIAVIVNNRVFVRSDAFLHIMGLLPAPWPLLKVLGIFPRFLRDWAYNRIAL
NRYRLFGRYDHCLLPSPEDRQRFLDG
>tr|A0A0Q0CV89|A0A0Q0CV89_PSEAP Agmatine deiminase OS=Pseudomonas syringae pv. aptata OX=83167 GN=aguA PE=3 SV=1
MTTLNSTPRADGFHMPAEWAPQTQVWMVWPERPDNWRLGGKPAQAAHVAIAKAIARFEPV
TVAVSAAQYDNARARLDMPNIRVVEMSSNDAWVRDSGPTFVINDRGEVRGVNWEFNAWGG
FDGGLYAPWNLDSQLGSKVLEIERCPRYVTEGFVLEGGSIHVDGEGTLITTEECLLNRNR
NPHLTREQIETILGDYLAVDKVIWLPDGLFNDETDGHVDNFCCYIRPGEVLLAWTDDPED
PNYSRCHAALSILENTLDAKGRAFIVHKMPIPGPLFATEEECAGVDQVHGSQERNPSVRL
AGSYVNFLIVNGGIIAPSFDDPMDGKAREILQKLFPEHEVVMAPGRELLLGGGNIHCLTQ
QQPAPHNR
>tr|A0A0Q0BTC7|A0A0Q0BTC7_PSEAP Uncharacterized protein OS=Pseudomonas syringae pv. aptata OX=83167 GN=ALO85_100953 PE=4 SV=1
MFHPQIRLNASGATDSYQPHGSNGIRWVIASCPNGAMSV
>tr|A0A0N8T7W4|A0A0N8T7W4_PSEAP ErfK/YbiS/YcfS/YnhG OS=Pseudomonas syringae pv. aptata OX=83167 GN=ALO85_04081 PE=4 SV=1
MRWLLALFCMSFMPLSQAAFTQTIVPKGSEQKIINNKVINTESIASKPIDKVLVLKSARQ
LQLISGGEALKTYRISLGKNPKGTKLQEGDQRTPEGFYWLDWRKPSNNYNLSMHISYPNV
SDSARARSEGVKPGSMIMLHGTPVDEEYPEWYFHTLDWTNGCIALKNNDMREIWDLVKDG
TMIEIRP
>tr|A0A0Q0CHB1|A0A0Q0CHB1_PSEAP Beta-lactamase OS=Pseudomonas syringae pv. aptata OX=83167 GN=ALO85_200064 PE=4 SV=1
MPVTYTPINKCAPGMWPMNYAASLIKGATEVMFAIGTDDPKGARSRLDDLAE
>tr|A0A0Q0D965|A0A0Q0D965_PSEAP Short chain dehydrogenase OS=Pseudomonas syringae pv. aptata OX=83167 GN=ALO85_100233 PE=4 SV=1
MSVLNRLVPYAGLRVLISGGAAGIGEVLAAAYLEAGAKVHVCDVSEAAIAAFLDRHPGSV
ATHADVADPAQIEAVFRVQRARFDGLDVLINNAGIAGPTSGIDAISEEEWQRTIDINLTG
QYRFAHHAVPLLKASPNAHLIHIASVAGRLGYAWRTPYAATKWAIVGLMKSLASELGESD
IRVNALLPGIVEGPRMDGVIRARAQQMNVPEEAMREEYLKKISLKRMVTAEDVAAMALFL
CSPAARNVTGQAISVDGNVEYL
>tr|A0A0Q0BZA6|A0A0Q0BZA6_PSEAP HAD family hydrolase OS=Pseudomonas syringae pv. aptata OX=83167 GN=ALO85_02443 PE=4 SV=1
MARLEQIGFLGFDVFGTVVDWRNGVARAAAPFLQQHGVNVDPLDFADQWRGLYQPSMQRV
RAGERPYVTLDVLNRESLETVLARHEVDFGSISDAELAELNKAWERLDPWPDSVAGLTRL
KRRFSIGTLSNGHIAGMLNLAKFGGLPWDVIVGAEIAGTYKPMPQAYLKSAKAVGLAPHC
VAMVAAHNADLTAARANGFKTVFVRRPAEHGPGQTSDLTAEQDWDVIVDSLTEAADALDC
>tr|A0A0Q0IS15|A0A0Q0IS15_PSEAP Pyruvate dehydrogenase OS=Pseudomonas syringae pv. aptata OX=83167 GN=ALO85_05360 PE=3 SV=1
MSRMHRVDHNRRGRALRAFYRTHYHCLDKEPRMSKTIADHLAQTLAAAGVSHIWGVSGDS
LNGLTDSLERTDSIRWMHTRHEEVAAFAAGAQAASSGKLAVCAGSCGPGNLHLINGLYDC
HRNRVPVLAIAAHIPSSEIGLDYFQETHPQELFKECSHFVELVSNPEQFPRVLERAMRAA
ISQKGVAVIVLPGDVALSDAPDVAAKWIEATAPKVVPAENDLQSMADMLNASKAVTLLCG
AGCEGAHEQLLALADTLGAPIVHALRGKQHVEYDNPFDVGMTGLIGFSSGYHAMLSCDTL
VILGSSFPYRNFYPEKADIIQIDLDPTQLGRRTPVALGLVGGVRETLEALQPKLKPHTDR
RFLDKALKHYAKAREELDELATPTPDGTPIHPQYLTRLVDEQADADAIFTVDVGTPTLWA
ARYLHMNGKRSLLGSFNHGSMANALPQALGAKAEHPDRQVIALCGDGGLSMLLGDLLSIR
QLNLPIKMVVFNNSSLGFVDMEMKASGYVPHGTDLHETNFAGIALGAGILGLRVENAEEL
PAALRKAFDHPGPVLIDVVTAKQELGIPPKIKLAQAKGFSLYMMRAIMSGRGSEVVELAK
TNLR
>tr|A0A0Q0C0H3|A0A0Q0C0H3_PSEAP Putative Kef-type K+ transport system, NAD-binding subunitt OS=Pseudomonas syringae pv. aptata OX=83167 GN=ALO85_05298 PE=4 SV=1
MFDSGTTMDSSLSRRERLHIVIFEANTPAGRTFDKAVLVAILLSLLVTIIDSIESIHRDY
AMLFAWIEWGFTLMFAIEYMLRLYCSPRPLKYAFSFYGLVDLLAIVPGVLSIYYSDAQYL
LIVRIIRMLRIFRVLKLGTYLRQANYLLAALRGSKQKIIVFLVTVSTLVTVFGTLMYVIE
GPEHGFTSIPKGIYWAIVTLTTVGFGDIVPKTPLGQMLSSLVMITGYSIIAVPTGIFTAE
LANAMRGEQLKHDCPVCSKNLHEHGAAFCSRCGNQLFKKTEHTA

>tr|A0A0Q0FMS8|A0A0Q0FMS8_PSEAP HAD family hydrolase OS=Pseudomonas syringae pv. aptata OX=83167 GN=ALO85_00918 PE=4 SV=1
MSDLRPKYITFDCYGTLTNFKTAALTRDLFADRIPAERMEQFIKDYAAYRLDQVMGDWLP
YDQILKIALARTCKRWGIEYRDEGQVYYDAVPTWGPHPDVPAGLEKIAGKIPLVIFSNAM
DEQIMSNVDKLGAPFYKVFTAQQAQAYKPRLAAFEFMLDNLGCGPEDVLHVSSSFRYDLM
SAHDMKIKHKAFVARGHEQPANAAFGYHQIADIGGLAGLVGL
>tr|A0A0Q0FLQ4|A0A0Q0FLQ4_PSEAP Mg-dependent DNase, TatD family OS=Pseudomonas syringae pv. aptata OX=83167 GN=ALO85_02549 PE=4 SV=1
MLVDSHCHLDRLDLAQHDGSLDAALDAARGRGVGHFLCIGVSADNAGAVKALAERYADVD
CSVGIHPLDVKPGEMPPLDWLLKELDHPRVVAIGETGLDYHYEPEAAELQQASFRVHLEA
ARITEKPVVIHTRGARADTLSLLREAALPHAGVLHCFTEDWEMAKAALDLGYYISLSGIV
TFRNADALRDVARQVPADRLLVETDSPYLAPIPYRGKPNLPQYVREVAEFLAMLRGESYE
RFAEQTTANFARLFPLAHVQR
>tr|A0A0Q0D433|A0A0Q0D433_PSEAP PsiE super protein OS=Pseudomonas syringae pv. aptata OX=83167 GN=ALO85_03895 PE=4 SV=1
MSYTPELVAELEILALFNLGNTQEGLKVHHVAAPTAVAAAQRLFDKGLTTQVDGGYLTSL
GLEAAQHAQSLLTILNVSRQAA
>tr|A0A0N8T9E4|A0A0N8T9E4_PSEAP 3-oxoadipate:succinyl-CoA transferase subunit A OS=Pseudomonas syringae pv. aptata OX=83167 GN=ALO85_02396 PE=4 SV=1
MNVMLGSEHLMADILSLRDAVKQLVNDGDTVALEGFTHLIPTAAGHEIIRQGKKDLTLVR
MTPDLIYDQLIGAGCAKRLIFSWGGNPGVGSLHRLRDAVEKQWPRAMEIEEHSHADLANA
YVAGASGLPFAVLRAYAGSDLPKVNPLIKTVTCPFTGEVLAAVPSVRPDVTVIHAQKADR
KGNVLLWGILGAQKEAALAAKRCIVTVEEIVDDLKAPMNSCVLPTWALSAVCLVPGGAHP
SYAHGYYERDNPFYQAWDPIARDRETFTAWIDEYIHGTADFSEFQSKLARASEAK
>tr|A0A0Q0CA28|A0A0Q0CA28_PSEAP Uncharacterized protein OS=Pseudomonas syringae pv. aptata OX=83167 GN=ALO85_102127 PE=4 SV=1
MQKVESLMQESVDTTRCAKLPECCVQGKRYGPMGLALCHN
>tr|A0A0N8T9T3|A0A0N8T9T3_PSEAP Cobalamin biosynthesis protein CobW OS=Pseudomonas syringae pv. aptata OX=83167 GN=ALO85_03630 PE=4 SV=1
MIRWRSDLRVLHAVLVLFARRTAKGHLMKTLAKLPVTIVTGFLGSGKTTLLRHMLDNAEG
RRIAVIVNEFGELGIDGEILKQCTIGCTEEEANGRVYELANGCLCCTVQEEFFPVMRELV
ARRGDLDHILIETSGLALPKPLVQAFQWPEIRNACTVDAVITVVDSPAVLAGTFAAFPDQ
VDAQRKLDPNLDHESPLHELFADQLASADLVILNKADMIDAEGLATVRAEVAEELPPAVK
VIEASSGRLPISVLLGVGAESELHIDARKTHHDHHDDDDHDDHDHDAFDSISLTLPQADE
KVLLDALNQLVVQHGILRVKGFAAIPGKPMRLLIQGVGTRFDKHFDRAWRADEERATHLV
LIGQELDAAALQAQLNAAIVG
>tr|A0A0Q0E130|A0A0Q0E130_PSEAP NAD(P) transhydrogenase subunit beta OS=Pseudomonas syringae pv. aptata OX=83167 GN=ALO85_100087 PE=3 SV=1
MSMNLVTLLYLIASICFIQALKGLSHPTTSRRGNLFGMLGMGLAVITTIGLVFKLAALST
AEGTSAGIGYIVVGLLVGGTAGSIMAKRVEMTKMPELVAFMHSMIGLAAVFIAIAAVVEP
QSLGIVRNLGDAIPAGNRLELFLGAAIGAITFSGSVIAFGKLSGKYKFRLFQGAPVQFAG
QHTLNLVLGLATLFFGLTFMFTGSLTAFAIMLALAFVIGVLLIIPIGGADMPVVVSMLNS
YSGWAAAGIGFSLNNSMLIIAGSLVGSSGAILSYIMCKAMNRSFFNVIGGGFGGATDSAG
PAGAREARPVKSGSAEDATFLLTNADTVIIVPGYGLAVARAQHALKELTEKLVHHGVTVK
YAIHPVAGRMPGHMNVLLAEAEVPYDQVFEMDDINSEFGQADVVLVLGANDVVNPAAKND
PKSPIAGMPILEAFKAKTIIVNKRSMASGYAGLDNELFYLDKTMMVFGDAKKVIEDMVKA
VENA
>tr|A0A0Q0FV46|A0A0Q0FV46_PSEAP Spermidine/putrescine ABC-type transport system OS=Pseudomonas syringae pv. aptata OX=83167 GN=ALO85_03423 PE=4 SV=1
MVLNKRATAVLAAGVLAFSSVAAIAAESVNFVSWGGSTQDFQKEAWAAPFSKASGITVVQ
DGPTDYGKLKAMVESGNVQWDVVDVEADFALRAASEGLLEPLDFTTIKRDEIDKRFVTDH
GAGSFFFSFVLGYNESKVGGKAPGDWAAMFDTKTYPGKRALYKWPSPGVLELALLADGVA
PDKLYPLDLDRAFKKLDTIKKDIVWWGGGAQSQQLLASGEVSMGQMWNGRVYALQQDGAP
VGVSWKQNLVMADFLVVPKGAKNKDAAMKFIANATSAKGQADFSNLSAYAPVNTQSVARL
DSTLAPNLPTAHVADQITLDFAYWAKNGADIATRWNEWLVK
>tr|A0A0Q0IIE1|A0A0Q0IIE1_PSEAP Tail fiber protein H OS=Pseudomonas syringae pv. aptata OX=83167 GN=ALO85_00430 PE=4 SV=1
MDYPKSVPGVGLASGKFVDENPATGTPGSLIPAQWGNSVTQEILNVILGAGLVPNEEDVT

QLHRAILGLAASDYKKSVRCATTVSIGLSGLQTIDDVTLVAGDRVLVKNQDTASQNWIYV
AAAGAWARAQDANESTECTPGHMVPVQAGTKNAGTVWQLVNTTVPVLGTTDLAFERLLGR
SGVAAGDYTRVKVNKYGQVEAGSNPTTLSGNGISDAYTKAEVYAKSEVDTRLDSRALADA
ISYVGIAGGVLGQPYMRRSSDSATCWLQTKLLYAPVQQGTGVGQLNNVVKIGWSDNGLKA
TVDATDMGTLWYANNFDPGSKANWGSTLAAYGITNAYTKAESDARDLQRVMADSITYVGF
ASNDVNFPYMRRASDGQVYFLQPRLGFPPIEQGGGPNMSTNKIRLGYNSAGSLRLQVDVT
DFGDLTNDYNLPTKLAGLGMSAIGSYAFARVISSQGQVNQGGMIAGSNLIYSSTNGGDGA
GNNSGLIGVGTWRAHGAFSSSERTLFQRVS
>tr|A0A0Q0DLS3|A0A0Q0DLS3_PSEAP Insecticidal toxin protein OS=Pseudomonas syringae pv. aptata OX=83167 GN=ALO85_05110 PE=4 SV=1
MGASSMEAQSQNQSPPSLQPAVATPSLPKGGGAIQSIGKGWGAVGTNGAASLEIALPISQ
GRGYAPALSLSYQSTSGNGVFGLGWNLNTSKVARRASKGVPNYTDDDLIFGPGGDVCLPE
RDDSGALVSSQVSRYNGHDLNETYQVVRYFSRVEGAFARVEHWRVDIADPGFWLIHGADG
SLNLYGRRTSSRIADPADMNRVTEWLLDESMNAVGEHILYEYKPEDHQGLAEDHPRNFRA
QRYLSRVRYGNAKAHPVLYLWQEDSLDGLLWHFDLIFDYDQRDTRSDPPPEYDEQFTWPV
RSDPHSSFAYGFELGNLRLCRQVLMFHHFPDELGEAPLLTRRLLLEHYQTALGYNLLSAA
HSQAWDGTDWRRVDQQPPVQFQYTDFSLESGIYTPLEPMAGLNDGQQYQLVDLYGDGLPG
ILYRDDKAWLYREPVRDTAGTADAVAYGTCQPLPRIPTADSATPVRQVLTDLTGDGRLDW
VVAQPGMAGFFTLNPDRSWSNYATFSAFPTEFFHPQGQMADLVGDGLSDLALIGPRSVRL
YANRRADGFAAAVDIPHDEDRLPLLSDSSTELVAFSDLLGTGQQHLIRIRHNEIRVWPNL
GRGRFGKGQLFATLPYTYEVFDSSQVRLADLDGSGASDVLYLQADGFQVFMNRGGNGLAA
PFDQHWPEGVRYDRFCQFSAVDLLGLGFSSLVLTVPHMAPRHWSLYYAEDRAGSVRKPYL
LKALDNNLGAAGEVSYRSSAQEWLDEKNELRTAGSVAVSELPFPVHVVVKQTMQDKVTGN
TLTQLFRYRQGFYDPREREFRGFGLLLQTDTETSSQDQEDFTAAVLNKTWFHTGRYPARP
CTDHDRSDPLARLPGEHVLSRLDAATQTELPITDADDATLQDMARALSGSVLRSEVFGLD
ASQRPTVLYSTWSCRYLVRQLQALSAHRPYASMLPLSLEVITYRYEAEELEDPMCEHSLN
LAWDRYGSTLHSVSVNYARRKKPGDAPPFADPHQQQWWEASHDDAQQQFYLNEMHAEAIH
LDSPQSWRLGLPYRTRGDAMLIPASALTPEQISYEQFADPSGPFATLPRTLTSQSVQRYI
GCGDGEATFQALADAVETAELDDHALSAYERVMDSVTLAEKLVKIGYQQMPSFLPADSLN
LWSVKRGFATYAGQEHFFHPTQFRPTRSHGWSLVEYDAYHLFATRITDPAGCVTTAEYDY
RVLQPKRIIDPNQNSQEADYDAFGRIWATSFYGTELGEAVGFPPLNRAAHYWASAGEVAL
QPEYALGRQASALYYDGNTALGQVHIPLATAALVADRYPEDLDRQIRISMASIDGFGRTL
QTRQKVEDGNAYSVDEWGNLELVDGKPKIVHASPRWRVSERVEYNNKGLAVRVYRPYFAN
GHLYVNDASIRSQNIVDKQFYDPLGRPTITITAKGWMRRQTYRVWYTISEDENDTAEEVL
AARKAAERG
>tr|A0A0Q0IB83|A0A0Q0IB83_PSEAP Enoyl-[acyl-carrier-protein] reductase [NADH] OS=Pseudomonas syringae pv. aptata OX=83167 GN=ALO85_00076 PE=3 SV=1
MGFLAGKRVLIVGVASKLSIASGIAAAMHREGAELAFTYQNDKLKGRVEEFAAGWGSGPE
LCFPCDVASDEEINKVFEELSKKWDGLDVIVHSVGFAPGDQLDGDFTEATTREGFRIAHD
ISAYSFVALAKAGREMMKGRNGSLLTLSYLGAERTMPNYNVMGMAKASLEAGVRYLAGSL
GPEGTRVNAVSAGPIRTLAASGIKNFRKMLAANEAQTPLRRNVTIDEVGNAGAFLCSDLA
SGISGEIMYVDGGFNTTAMGSMDE
>tr|A0A0Q0CXA2|A0A0Q0CXA2_PSEAP Ribose-5-phosphate isomerase A OS=Pseudomonas syringae pv. aptata OX=83167 GN=rpiA PE=3 SV=1
MTQDQLKQAVAQAAVDFILPKLDDKSIVGVGTGSTANCFIDALAKHKAAFDGAVASSQAT
AARLKAHGIPVYELNTVSDLEFYVDGADESDEHLNLIKGGGAALTREKIVAAVAKTFICI
ADGSKLVPVLGAFPLPVEVIPMARSHVARQLVKLGGDPVYREGVLTDNGNIIIDVHNMSI
TNPVELEAQINAIVGVVTNGLFAARPADLLLLGTNDGVKTLTR
>tr|A0A0N8TA98|A0A0N8TA98_PSEAP Tellurium resistance protein TerZ OS=Pseudomonas syringae pv. aptata OX=83167 GN=ALO85_03305 PE=4 SV=1
MALTLAKNQTISLEKTAGTGLKKVSMGLGWDPEKASGFFGKLLGGGGGDIDLDASCIMLD
ADKKPLDLVWFRQLQSRDGAIQHSGDNRTGEGAGDDETISVDLEKLPATVKYLVFTVNSF
TGQNFEKVANAYCRIVDLGSRNELGRFDLSEKGQHTGVVMSYLARTPSGWDFTAVGHVTN
GRTADDLVELAIGAVRV
>tr|A0A0Q0FDF1|A0A0Q0FDF1_PSEAP Stringent starvation protein A OS=Pseudomonas syringae pv. aptata OX=83167 GN=ALO85_00191 PE=4 SV=1
MGVTNRLACYSDPADHYSHRVRIVLAEKGVSAEIIEVVAGRHPPQLIEVNPYGSVPTLVD
RDLALYESTVVMEYLDERYPHPPLLPVYPVARANSRLLIHRIQRDWCGLVDLILDTRSKE
PARVQARKELRESLAGVSPLFAEKAFFMSDELSLVDCCLLPILWRLPILGIELPRPAKPL

LDYMERQFAREAFQTSLSDAEREMR
>tr|A0A0Q0CW01|A0A0Q0CW01_PSEAP Uncharacterized protein OS=Pseudomonas syringae pv. aptata OX=83167 GN=ALO85_100787 PE=4 SV=1
MKGILSGLLMVSCFAGAVVANADERYKFEAYPASAVSQKAVSKIDWKSNPVYAKDLKSKD
AVNWTVGKKADFAGHFVVNSFSCGTGCQSTVFVDVNDGKIYKSPMQFPSEGTDLPKVAGG
KAYQVSSNLIFMSSLGYAGTDMVQSDGVVVFDEKLKQFQIVAKSKPYNMKF
>tr|A0A0N8T869|A0A0N8T869_PSEAP Phosphoglycerol transferase, alkaline phosphatase superfamily OS=Pseudomonas syringae pv. aptata OX=83167 GN=ALO85_01215 PE=4 SV=1
MAKTDAQIHRQARLQNPTVKSHLAYILLSGFALMVMYTLLRIGLLVYNREMIGDTPASTF
LEALFNGTRFDLRLTVYLLIPLVLSLFSARAMAARGFFRFWLTLVGSITLFFGLMEMDFY
REFHQRLNGLVFQYVKEDPKTVLSMLWYGFPVVRYLLAWALVTWLLSLVFKGIDRATRPR
VAAKTSPEATRSIAPWYVRAGVFVLVLLVMVVCIRGTLRQGPPLRWGDAYTTDSNFANQL
GLNGTLTLITAAKSRMSEDRDNIWKATLPQNEAQQTVRDMLLTPNDKLVEPDIAAVRRDF
TPPAENTLPIRNVVVILMESFAGHSVGALGNDANITPYFDKLSKEGLLFDHFFSNGTHTH
QGMFATMACFPNLPGFEYLMQTPEGSHKLSGLPQLLSTGRNYDDVYVYNGNFAWDNQSGF
FSNQGMTNFVGREDFVNPVFSDPTWGVSDQDMFDRGAQELKARQDGKPFYALLQTLSNHT
PYALPDPLPVERVTGHGSLDEHLTAMRYADWALGQFFEKAKKEPYYKNTLFVVLGDHGFG
NDKQLTEMDLGRFNVPLLLIGPGVQEKFGQRSSIVGTQVDVVPTIMGRLGGLNRNQCWGR
DLLNLPEGDKGFGVIKPSGSEQVVAIISGNRILIEPTEMPAKLLTYTLGAKPHAEEVPDA
PDTQELKRKLESFLQTATKSLLDNTAGVEASKNRN
>tr|A0A0Q0IAG6|A0A0Q0IAG6_PSEAP HTH-type transcriptional regulator IscR OS=Pseudomonas syringae pv. aptata OX=83167 GN=ALO85_04115 PE=4 SV=1
MRLTTKGRYAVTAMLDLALHAQNGPVSLADISERQGISLSYLEQLFAKLRRGNLVSSVRG
PGGGYQLSRDMKGIQVAQVVDAVNESVDATRCQGLGDCHAGDTCLTHHLWCDLSQQIHEF
LSGISLADLVTRREVQEVAQRQDMRRGHNHMSQLGKIETSAVE
>tr|A0A0Q0BVA0|A0A0Q0BVA0_PSEAP GNAT family acetyltransferase OS=Pseudomonas syringae pv. aptata OX=83167 GN=ALO85_00735 PE=4 SV=1
MNKVTVAVAQWQKNNADIRRIRDSVFIAEQSVPPELEWDAEDAGAVHFLAYEGEYAVGTA
RLLPDGEIGRLSVLRDWRGLKVGEALLGAVIHEAEQRGLKPLKLSAQVQAVEFYQRFGFS
VISDEFLEAGLPHVDMLREA
>tr|A0A0Q0DKP5|A0A0Q0DKP5_PSEAP Transcriptional regulator GntR OS=Pseudomonas syringae pv. aptata OX=83167 GN=ALO85_00877 PE=4 SV=1
MMSKPGQTVLVALRKMIASGELAAGERLMEVPTAELFGVSRMPVRMAFRTLEQEGLLVPF
GGRGFQVRSISPIEIAGAVDVRGVLEGLAARQMAERGVTQEARDELEACLVQGDALFEKG
HVTEDDLEVYHDMNMRLHRVIVEGSGNRAIADALSRNDHLPFASVTALAVDRDNLIREYR
RFNFAHMQHHAVVDALVNGQGARAEAIMREHANATLRYAEIFGTGQNERMKVIQRPD
>tr|A0A0N8T9J2|A0A0N8T9J2_PSEAP 30S ribosomal protein S7 OS=Pseudomonas syringae pv. aptata OX=83167 GN=rpsG PE=3 SV=1
MPRRRVAAKREVLDDPKYGSQILAKFMNHVMESGKKAVAERIVYGALEKVKERKNSDPLE
IFEKALDAIAPLVEVKSRRVGGATYQVPVEVRPSRRNALAMRWLVDFARKRGEKSMALRL
AGELLDAAEGKGAAVKKREDVHRMAEANKAFSHYRF
>tr|A0A0Q0DDJ9|A0A0Q0DDJ9_PSEAP Putative GbcB protein, subunit beta OS=Pseudomonas syringae pv. aptata OX=83167 GN=ALO85_00486 PE=4 SV=1
MSQSFLSPVTTQTWANGRHLVRVVKVIQETWDVRTFCFMADQPIMFFFKPGQFVTLELEI
DGLPIMRSYTISSSPSVPYSFSITVKRVPGGRVSNYLHDTLIEGQELAVHGPVGLFNAID
FPNPKILYLSGGVGITPVMSMARWFYDTNANVDMVFVHSARSPKDIIYHRELEHMASRID
NFNLHLVCEKHGLGEPWAGYRGYLNQKMLELMAPDFMEREVFCCGPTPYMTAVKRLLQEN
GFNMAQYHEESFGATPPEARADAVEQAEIAADAPDVDAADLHQVEFTDTGKSIRVGPGET
VHAAAAKLGLMIPKACGMGICGTCKVMKLSGEVEMEHNGGITDEDVAEGYILSCCSVPKG
DVRIEY
>tr|A0A0Q0CVW3|A0A0Q0CVW3_PSEAP Type III effector HopZ3 OS=Pseudomonas syringae pv. aptata OX=83167 GN=ALO85_100535 PE=4 SV=1
MAGKVTAWPFCSARLLNQADESRFNANHGGDYSMNISGPNRRQGTQAENTESASSSSVTN
PPLQRGEGRRLRRQDALPTDTRYNANQTAASPQNARAAGRYESGASSSGANDTPQAEGSM
PSSSAFLQFRLPGGRNHSELEDFHTMMLNSPKASRGDAIPEKPEAIPKRLLEKMEPINLA
QLALRDKDLHEYAVMVCNQVKKGEGPNSNITQGDIKLLPLFAKAENTRNPGLNLHTFKSH
KDCYQAIKEQNRDIQKNKQSLSMRVVYPPFKKMPDHHIALDIQLRYGHRPSIVGFESAPR
NIIDAAEREIFSALGNVKLKMVGNFLQYSKTDCTMFALNNALKAFKHHEEYTSRLHNGEK
QVPIPATFLKHAQSKSFVENHPKKDTTVTKDQGGLHMETLLHRNRAYRAQRSAGQHVTSI

EGFRMQEIKRAGDFLAANRVRAKP
>tr|A0A0N8T8C1|A0A0N8T8C1_PSEAP Histidine utilization repressor HutC OS=Pseudomonas syringae pv. aptata OX=83167 GN=ALO85_01688 PE=4 SV=1
MVSVDALQRLNALENTVSNNKDSHVSTPPAASSLAAQMGESPAPLYARVKQMIALQIQNG
TWPPHHRVPSESELVTQLGFSRMTINRALRELTAEGLLVRMQGVGTFVAEPKSQSALFEV
HNIADEIAARGHRHTCKVMVLKEEAAGSERALALDMREGQRVFHSLIVHYENDIPVQIED
RFVNAQVAPDYLRQDFTLQTPYAYLSQVAPLTEGEHVVEAILAEADECRLLQIEAGEPCL
LIRRRTWSGRQPVTAARLIHPGSRHRLEGRFTK
>tr|A0A0Q0D6A2|A0A0Q0D6A2_PSEAP Putative Lipoprotein OS=Pseudomonas syringae pv. aptata OX=83167 GN=ALO85_00253 PE=4 SV=1
MLKRSTLSLGSAVLCGSFLVGGCANQLSQRSEHEERVERKLLEHTLQVDIGEPKTLELPQ
RRVRIHEHKSFEVTDFEVTRHYDRYTPYQPWREVYEIPLGAVAVVAGVGANVVNVFALGN
LPESMTKDWIHYGVAGLNPFMNAPSHGRAEQNLASIDEVQKDKRIENSTLPWNERPVMVK
AGNETHEMSTDRNGILRLNLLDSPFAEQDLSRITRIYINVEDDQDSAHTSASLPISHSLR
GKLLEAHALIYDDLEDDEVSQWVHRVKRLSDMGLEEEASELEQNLIELTRNDPQLQGEFL
KSLARDAGRLVAVPAQSQ
>tr|A0A0Q0C945|A0A0Q0C945_PSEAP Uncharacterized protein OS=Pseudomonas syringae pv. aptata OX=83167 GN=ALO85_00637 PE=4 SV=1
MTNTIINKNVLPRSGISQRYKLHRHIVSMSVTVVLMAICAWFYSSFSGVHVVDLGVGRNL
SVSGLREHWRQGNVVVMIRHAERCDRSSNPCMGDADGITSNGRDTALAVGAGLQKMGMDH
ARMIASPKTRTQQTAQLLAGHAVVAEDWINDCDSGFMKAVQAHKVSGENMVLVTHSGCID
QFERKAGVRGGERSSEYTQAFFVKVDGSHPPKILGSLNAGQWANLNSEQFN
>tr|A0A0Q0DPD6|A0A0Q0DPD6_PSEAP Adenine phosphoribosyltransferase OS=Pseudomonas syringae pv. aptata OX=83167 GN=apt PE=3 SV=1
MTFDQSNFKSLIRPVVDFPKPGVVFRDITPLFQSPKATRQVIDSFVQRYIDAEFSHIGVM
DARGFLIGSVVAYQLNKPLVLFRKQGKLPADVLSEAYQTEYGEAYLEVHADSLCEGDSVI
MFDDLIATGGTLIAAANLIRRMGAEVYEAAAIIDLPELGGSQRLNDLKIPTFCLTEFALD
EQ
>tr|A0A0Q0FJN1|A0A0Q0FJN1_PSEAP Transcriptional regulator, LuxR family OS=Pseudomonas syringae pv. aptata OX=83167 GN=ALO85_00037 PE=4 SV=1
MISDTTSLDSVSCQVVEALNKVILALQTEVSSASISTFHQQALERACALVPFDKAWWGRS
TWRDNWPVEHSSFLLGLPTGYPAEWEQLKTSDASVGRMHQTQGVCQVIHCTAADAPPALA
ELGRQYDLCFALGIVLTDPHTQQGVHLTLFRSADHTPFSPLDKRLIEWLMPHLIAAEQAS
YLRTLTTLRETQGAYDETAMAVSDRYGVLLSMEPAFSSMLGSEWPDRPSNRLPRQCNPQE
GYSSQNLHITSQPVGDLVLLTARRRSSLELLSTRELQVARGFAVGQTYKEVARNIGMSPC
TVRHHLRKIYNKLGVTRKAQIAQLLHLTDG
>tr|A0A0N8T8C2|A0A0N8T8C2_PSEAP Glucans biosynthesis protein G OS=Pseudomonas syringae pv. aptata OX=83167 GN=opgG PE=3 SV=1
MIVSPCDAPKTPVKRLRSALLASSALVCLFSAGQLWAFNLDDVAAKAKEMAGQKFEAPRS
NLPNELRDMKFADYQKIRFRDDKAEWAGEKTPFKVSFYHQGMHFDTPVKINEVTATSVDE
IKYDPNRFDFGDLKIDPKSTEKLGYAGFRVLYPINKDDKQDEIMTMLGASYFRVIGKGQV
YGLSARGMAIDTASPSGEEFPRFKEFWIEKPGPDDNHLVIFALLDSPRATGAYQLTLRPG
TNTLVDVKSRMFLRDKVNKLGVAPLTSMFLFGANQPSRVPNYRRELHDSSGLSIQAANGE
WLWRPLNNPKHLSISSFSVENPRGFGLLQRGRDFSQYEDLDDRYDKRPSAWIEPKGDWGK
GTVELVEIPTADETNDNIVAYWKPETLAEPGQEMAFDYRLHWTMQENSIHSPDLGWVKQT
QRSIGDVRQSNLIRQPDGSLAFLVDFVGPVLAALPEDKTIRSQVTTDDNVELVENNLRYN
PVTKGYRLTLRVKVKDSSKPTEMRAYLLREIPAEPGKEPALLVADKAEEKKAAAKEAAKP
AVAKESANDQVEIAKADAPKPEAAKPETAKSEAGKADAAKGKGEVAKADAGKADASKADV
AKDKDGKEIQQPETEAAPTHPEPAKTLQVMTETWSYQLPSDE
>tr|A0A0Q0CXM4|A0A0Q0CXM4_PSEAP RsbW family anti-anti-sigma factor OS=Pseudomonas syringae pv. aptata OX=83167 GN=ALO85_01253 PE=4 SV=1
MSTGRIQFAEQEGTFVLKFVGEVRLTLCSALDATIERIFTALNFSAVVIDLTETRSIDST
TLGLLAKLSILSRQKVGLLPTLVTTHEDITRLLQSMGFDQVFNIVDRPLPRPECLTDLPS
QDQCEEVVKAKVLEAHKILMGLNDSNREAFHDLVNALEGQR
>tr|A0A0Q0DMM6|A0A0Q0DMM6_PSEAP Flagellar biosynthesis regulatory protein FleR OS=Pseudomonas syringae pv. aptata OX=83167 GN=ALO85_02163 PE=4 SV=1
MPINVLLVEDDLSLREALGETLELAGYDYQAVGSAEEALVAADAQPFSLVISDVNMPGMD
GHQLLSLLRRSRHPQLPVLLMTAHGAVERAVDAMRQGAADYLVKPFEPKALIALVARHALG
RLEPAERDGPIAVEPASIQLLNLASRVAKSDSTVLISGESGTGKEVLARFIHQNSPRADK
PFIAINCAAIPDNMLEATLFGHEKGSFTGAIAAQAGKFEQADGGTILLDEISEMPLGLQA

KLLRVLQEREVERVGARKPIVLDIRVVATTNRDLAGEVAAGRFREDLFYRLSVFPLAWQA
LRQRTADILPLAERLLAKHVNKMKHAPVRLSAEAQQCLVSYPWPGNVRELDNAVQRALIL
QQGGVIQAQDFCLSGPVTSLPVAASVEAAVAVTVTTVAENAGAGVSPESVGALGDDLRRH
EFQMIIDTLRSERGRRKEAAERLGISPRTLRYKLAQMRDAGMDVEAYLFAT
>tr|A0A0Q0BXY0|A0A0Q0BXY0_PSEAP UPF0434 protein ALO85_02530
OS=Pseudomonas syringae pv. aptata OX=83167 GN=ALO85_02530 PE=3 SV=1
MDTKLLDILACPVCKGPLKLSADKTELISKGAGLAYPVRDGIPVMLESEARTLSTDERLE
K
>tr|A0A0Q0FF88|A0A0Q0FF88_PSEAP Putative ABC-type polysaccharide/polyol
phosphate export system OS=Pseudomonas syringae pv. aptata OX=83167
GN=ALO85_03121 PE=4 SV=1
MNRVVIKAENLTKQFVLRHNRGGLKKKFLGLFDAQYRERAETFTAVSDVSFTLHKGESLA
LVGHNGSGKSTLLQLVSRILVPTSGRLTSVGRVAPLIEIGVGFHPELTGEENIYLNASLF
GLSNKEVRSRFNEIVDFSGLGNFIDTPVKNYSSGMYMRLGFSVAVHVEPQTLLADEVLAV
GDEEFKRKCHERIRMMQDDGMALILVTHAVEEGKEFCQRYLTLERGRMTEHGYY
>tr|A0A0Q0BTP5|A0A0Q0BTP5_PSEAP UPF0313 protein ALO85_03143
OS=Pseudomonas syringae pv. aptata OX=83167 GN=ALO85_03143 PE=3 SV=1
MQSAKPLFDYPKYWAECFGPAPFLPMSREEMDLLGWDSCDIIIVTGDAYVDHPSFGMAII
GRLLESQGFRVGIIAQPNWQSKDDFMKLGEPNLFFGVAAGNMDSMINRYTADKKIRSDDA
YTPGGMAGKRPDRASLVYSQRCKEAYKHVPIVLGGIEASLRRIAHYDYWQDKVRNSILID
ACADILLYGNAERAIVEVAQRLSYGHKIEDITDVRGTAFIRRDTPQGWYEVDSTRIDRPG
KVDKIINPYVNTQDTEACAIEQEKGNVEDPNEAKVVQILASPRMTRDKTVIRLPSVEKVR
NDAVLYAHANRVLHLETNPGNARALVQKHGEVDVWFNPPPIPMTTEEMDYVFGMPYARVP
HPAYGKEKIPAYDMIRFSVNIMRGCFGGCTFCSITEHEGRIIQNRSEESIIREIEEIRDK
VPGFTGVISDLGGPTANMYRIACKSPEIESACRKPSCVFPGICPNLNTDHSSLIQLYRSA
RALPGVKKILIASGLRYDLAVESPEYVKELVTHHVGGYLKIAPEHTEEGPLNQMMKPGIG
SYDKFKRMFEKYTKEAGKEQYLIPYFIAAHPGTTDEDMMNLALWLKGNGFRADQVQAFYP
SPMATATAMYHSGKNPLRKVTYKSDAVTIVKSEAQRRLHKAFLRYHDPKGWPMLREALER
MGRADLIGPGKDQLIPLHQPVTDSYQSARRKNSTPAGSHKVAKTAKTTLIQTQHTGLPPR
GSDGSKPWDKREEAKAAAMARNKQAAKERMDAAKGKGTKPPKRKPVVPR
>tr|A0A0Q0C6G9|A0A0Q0C6G9_PSEAP Uncharacterized protein OS=Pseudomonas
syringae pv. aptata OX=83167 GN=ALO85_01249 PE=4 SV=1
MPARELQEQLNTLREQLEHNPPMSESERENLHELMQQIETKIQLEQATHEQDSSLADGVN
LAVERFELEHPTIAGTLRNIVQTLGNIGV
>tr|A0A0Q0CH17|A0A0Q0CH17_PSEAP Aspartate/glutamate ABC-type transport
system OS=Pseudomonas syringae pv. aptata OX=83167 GN=ALO85_02040 PE=3
SV=1
MNYNWDWGVFFKSTGVGSETYLDWFISGLGWTIAIAVIAWIIALILGSVLGVMRTVPNRL
VSGIATVYVEIFRNVPLLVQLFIWYFLVPDLLPENLQEWYKQDLNPTTSAFLSVVVCLGL
FTAARVCEQVRTGIEALPKGQESAARAMGFKLPQIYWNVLLPQAYRIIIPPLTSEFLNVF
KNSSVASLIGLMELLAQTKQTAEFSANLFEAFTLATLIYFTLNMSLMLLMRAIEKKVAVP
GLISLGGK
>tr|A0A0Q0BXD8|A0A0Q0BXD8_PSEAP Alginate biosynthesis protein Alg8
OS=Pseudomonas syringae pv. aptata OX=83167 GN=ALO85_01809 PE=4 SV=1
MQRLKHGLLQAAGWLFYLSLLMGLAAALPTSIFDSQSKNFIFLIGAVGIWRYSMGITHFV
RGMIFLYIVYPHLRRKVRKLGSAADPSHVFLMVTSFRIDALTTAQVYNSVIREAIDCGLP
TTIVCSLVEMSDELLVKSMWAKFNPPERVKLDFVRIPGTGKRDGLAYGFRAISRHLPDSR
AVVAVIDGDTVLNEGVVAKTVPWFQLFDNVGGLTTNEFCEVRGGYIMSEWHKLRFAQRHI
NMCSMALSKRVLTMTGRMSVFRATVVTDPEFIADVESDSLNHWRLGTFRFLTGDDKSSWF
SLMRLGYDTFYVPDAAINTVEHPPEKSFLKASRKLMYRWYGNNLRQNSRALGLGVRRLGI
FTSIVLFDQRVSMWTSILGLTVALIASFKYGGAFLLMYLLWIGMTRLILTLLLSLSGHRI
GPAYPIILYYNQIVGALMKIYVFFRLDRQSWTRQDTKLSRDMASFQGWFNTWSSRTMTFS
AGTIFVAVLLTMV
>tr|A0A0Q0BSN8|A0A0Q0BSN8_PSEAP Sec-independent protein translocase
protein TatB OS=Pseudomonas syringae pv. aptata OX=83167 GN=tatB PE=3
SV=1
MFGISFSELLLVGLVALLVLGPERLPGAARTAGLWIGRLKRSFNAIKQEVEREIGADEIR
RQLHNEHILSLEDEARKMFAPNQPPENSSEPVNPSPAPAAAEAASPAHDEAAHHEIGPAQ
PAAPKTALSLEKTAKPADAGTPVPTPSDHDSSLPPRAP

>tr|A0A0N8TAA0|A0A0N8TAA0_PSEAP Gluconolactonase OS=Pseudomonas syringae pv. aptata OX=83167 GN=ALO85_03264 PE=4 SV=1
MKPTRKKHFLRPSSWLLGGALLAAGSAGAADAVAPVRQVVIDPAFSQLVAPQARVELLTD
QAKWAEGPLCLADGRLIWSDIKANKVSSWQAKGGVSTWLEKADFQNGHTLDGQGRVVAAS
HGKRGIVRQEPNGEWHTLVDTYQGKRLNSPNDVVADKSGALWFSDPTFGVLSKSEGYGGK
PEQGGEFIYRYVPDTGEITRLDTSEVHSPNGLAFSPDQQLLYVADSQMAHDFTNKKLAHR
IMVYQVRDGRLINGKVFADIEPGIPDGIAVDALGNLWSSSKEGIQVFSAAGKPLGKLLVS
AADTSNLTFCSSEQTSWAYITAANKVLRVEVTKSVAGQ
>tr|A0A0Q0C069|A0A0Q0C069_PSEAP Uncharacterized protein OS=Pseudomonas syringae pv. aptata OX=83167 GN=ALO85_00056 PE=4 SV=1
MLFFHMLPSWSELRQRLNHDMGYQPGGHFRNHRNEPAPSRSLIIRRFDCVRRAFVLAEWE
AGRILVQRFSDLDIASIIKDLINVVAQTAMIVVGSAFTGGLIGAGVGSAFFGVGAYPGGA
LGTALGLQTSTFILGVLGLKSIAEYVVDGFPHIVGYYLSGIKTAWQGPREQGLNPFMSDD
PYAQNSATQDIAKGHVEVVLLLLGAMVEYLTRGRGDARLLAQEMRASPRGERLGQWMLKH
EDALKKRPDLQRPGSSKSTLDAQDAFTPVPYSNREVEKNLENAKGIIKASETIGQPVEAV
INGRKRLLRVDIEPNGKLQIQSGGGKDSIVDFRPDLSKPLAPQIDKAFKRLPQSVRDELI
RNAEKGLKRLQETGNM
>tr|A0A0Q0ICH5|A0A0Q0ICH5_PSEAP DNA-binding response regulator, LuxR family OS=Pseudomonas syringae pv. aptata OX=83167 GN=ALO85_01270 PE=4 SV=1
MTDTVRLVLADDHEVTRTGFVSLLAGHPEFEVVGQAADGQQAIDLCLELQPDIAILDIRM
PVLNGLGAARILQQRMPGLKVVIFTMDDSTDHLEAAISAGAVGYLLKDASRDEVIDGLQR
VARGEEALNSAVSARLLRRMTERNTSGAALPETLTPRERQVLGLVAGGFSNREIGEKLGI
TTGTAKAHVEKVIGKLGASDRTQAAVRGVALGLVSQSTSDWQ
>tr|A0A0Q0C0T9|A0A0Q0C0T9_PSEAP PBPb domain-containing protein OS=Pseudomonas syringae pv. aptata OX=83167 GN=ALO85_02190 PE=4 SV=1
MVHSRLLSSLLFGLIALPSLAFAAGKCERLIVTGSPDAPPYLWRDPQDPRHLMGANADLL
TQAAGELGIKVEFLYGGKRSQALEEVRTGRMDLLADAPLNAAQLEALDYIHPPIVQNEIM
VWTRHDHSVPFNAVSELQGHPGAMSEKTRLTPSFDALARQSLTLERVPALTPAFQKLALG
EVDYVLAGRYAGLVMVQTLGLSKDLTAQPLPVDTPGFYLALSFNSACNEPWLRGQLAKKM
TESAASGLAGDVIRHNLELWKAQLLQPASASAPNK
>tr|A0A0Q0DRF8|A0A0Q0DRF8_PSEAP Uncharacterized protein OS=Pseudomonas syringae pv. aptata OX=83167 GN=ALO85_04008 PE=4 SV=1
MQFLSPLILFTSCTLLATTVFADAQAPSPVQSECQIKSQEISSKIQYAKTEGNKAEQSDL
EKALAEVNANCTETSLIKQRKQKVLDAKQEVSRRQTDLNKAMEKGDPEKINKRKDKLADS
RKELQEAQTELEKVQSDD
>tr|A0A0Q0IQ98|A0A0Q0IQ98_PSEAP Phosphonate metabolism PhnG OS=Pseudomonas syringae pv. aptata OX=83167 GN=ALO85_00816 PE=4 SV=1
MQATPETTARQHWMGVLARAHADQPSREQLNRHEAALRDTDYQMIRAPEIGMTLVRGRMG
GTGSAFNLGEMSVTRCVVRLADGRTGYSYLAVRDKRHAELAALADAHLQGAQQAWWLSEL
IEPLARAQAERRARKDAEAAATKVEFFTLVRGED
>tr|A0A0Q0D8V9|A0A0Q0D8V9_PSEAP Cupin_2 domain-containing protein OS=Pseudomonas syringae pv. aptata OX=83167 GN=ALO85_03780 PE=4 SV=1
MPHLKRTGLSMEKFDIHRKTRFDSLVYEYGLDGSRLLPWEGYPMPFAGGWCVVRPGTCSE
AHTQIDQEIFIAIKGSARLVVGDRQMDFTVGDIAAIPKHTNHYVINDSAEDFHFYVVWWD
MNYVNAFVAQHSETTGCLDE
>tr|A0A0Q0BUJ3|A0A0Q0BUJ3_PSEAP Membrane fusion protein (MFP) family protein OS=Pseudomonas syringae pv. aptata OX=83167 GN=ALO85_01148 PE=3 SV=1
MSIKTISTLEADADDMPTSDRGVRRIGVAIVLVTFGLFGTWAAFAPLGSAVYGSGVVMVQ
SYRKTVQHLEGGIVKELLVRDGDTVHKGDPLIVLDDSQLRSQYESTRNQLISTQAREARL
RAERDELPSIPPLTISGTDSVRAKEAIAGEQQVFSTRKNSRLTEISVQRERIGQLKQQIS
GLRDMIRTKISLEKSYSSEITELKDLLSQGFVDKQRLLEQERKLDMLKSEVADHESTITK
TQLQISETELQIIQLNKNFSSDVAKELSDVQAKVFDLQETATALQDRLSRVVIRAPEDGM
VLDMKVHTVGGVVSAGTPLLDIVPESSELVVEAHVAITDIDRISIGKLTDVRFSAFNSAT
TPVIEGEVTRISADRLKDDAGEPYYLVRVKLTEKGMKRLGDHKLQPGMPAEVLINAGERT
MLQYLLKPASNVFIKSMIEE
>tr|A0A0N8T9S1|A0A0N8T9S1_PSEAP Cytochrome C family protein OS=Pseudomonas syringae pv. aptata OX=83167 GN=ALO85_03769 PE=4 SV=1
MKALILFGALLLSGQLSAAQLDMQLGQISRTWQTEDLLTHPQLQVITVKSDVAYKKDMTY

YAVPLSALLTGIKPSDHLQAVALDGFAAELAAAPLLNDKGARAWLAIEDPAHPWPPLAAG
KPSAGPFYLVWTDPQVGNISPEQWPFQVASIKSMTSVAQRFPALLPDPVLKTDDPVNKGF
ALFQKNCLACHRLNGAGDAQFGPDLNIPYNPTEYLGMDLLKLYIRNPQDLRQWPQAKMPA
FSEAVLPDADLQMLIAYLHHMAGRKIKP
>tr|A0A0N8TAA2|A0A0N8TAA2_PSEAP Chemotaxis protein CheY OS=Pseudomonas syringae pv. aptata OX=83167 GN=ALO85_03287 PE=4 SV=1
MAKSVLVVDDSSSVRQVVGIALKSAGYDVIEACDGKDALGKLTGQKVHLIISDVNMPNMD
GITFVKEVKKLASYKFTPIIMLTTESQESKKAEGQAAGAKAWVVKPFQPAQMLAAVSKLI
LP
>tr|A0A0N8T975|A0A0N8T975_PSEAP Nitrogen assimilation regulatory protein Nac OS=Pseudomonas syringae pv. aptata OX=83167 GN=ALO85_00857 PE=3 SV=1
MNLRRLKYFVKIVDIGSLTQAADVLHIAQPALSQQLVTLEAELQQQLLIRTKRGVTPTEA
GKVLYGHAQVILRQCEQAQSDVNASGLALSGPVSVGLAPGTAASTLSLPLLKRVRERHPG
ILLYLNENFGTTLSELIMNGRMDMAVLYGGREVHGLSFVPLIREQLYLVSAEEALQGREE
IPLSELADIDLLLPRTYNTVRRLVDEAFVRIERSARVVAEVESSTTLAAAVADRFGSTIL
PESAARVLADTHAVNLSRIVEPEIEAPLALCLSGHLPLSVPAQAVKAILLELVAEMRGNR
RTQ
>tr|A0A0Q0CZN4|A0A0Q0CZN4_PSEAP Uncharacterized protein OS=Pseudomonas syringae pv. aptata OX=83167 GN=ALO85_01142 PE=4 SV=1
MQKLQKYFLNTEVLMKTRHSLMDIYAGHEGYVSDKWDIYLNTYDRLFKNLETSAVSLLEI
GIQNGGSLEIWSKLFSHFKIIVGCDINPDCAQLNYESEKIKLLIGNINDSNIQRQLDAIS
NEFDIIIDDGSHTSSDIITSFFLLFPKLNTGGIYIIEDLHCSYWQSFEGGLFNKSSSMNF
LKSLTDIINYEHWGVPINKSQLLAEFNIPADVDTEGLLSEIHSIEFINSMCIVTKNQPQN
NVLGVRHVVGSQELVAKNKFANGGFLVPEPQQSHVQSSSNFESEKDKKIATLTLEISELK
RLLNEAQPKNG
>tr|A0A0Q0BYV8|A0A0Q0BYV8_PSEAP Uncharacterized protein OS=Pseudomonas syringae pv. aptata OX=83167 GN=ALO85_03174 PE=4 SV=1
MNKAVFTQHYLARTALYIPSLNTACTTHQVRSRYMSREVRQITPDVQEIIQHALRSLLGK
GFVIALFGSEDATGAMHYHLRIDHDATGLGIEHHDNVEDGFIDDIFMLATRMKAMLKQRE
TLSRMHGGSQATGQVRLLTWITEDNSQTVMQTAEAAGRECLSALRERRLRA
>tr|A0A0N8T912|A0A0N8T912_PSEAP Uncharacterized protein OS=Pseudomonas syringae pv. aptata OX=83167 GN=ALO85_101769 PE=4 SV=1
MTQYWCIKQSDLQVMIFERVCSSRGSLFACFFVRRFIHLCGFEVNEVGNR
>tr|A0A0N8T918|A0A0N8T918_PSEAP Glyoxalase/bleomycin resistance protein/dioxygenase family protein OS=Pseudomonas syringae pv. aptata OX=83167 GN=ALO85_02498 PE=4 SV=1
MIDHLDHLVLTTADIDACKDFYVRVLGMTPERFRQGRLAFRFGNQKINVHERGKEFEPKA
HLPVPGALDLCFIATIPLEQVIVHLQEQDWPIVEGPVERTGATGPIWSVYVRDPDLNLIE
ISEPAADASM
>tr|A0A0Q0BTL6|A0A0Q0BTL6_PSEAP Beta-alanine--pyruvate aminotransferase OS=Pseudomonas syringae pv. aptata OX=83167 GN=ALO85_03053 PE=3 SV=1
MNMFEVSDVPVTRQLKLDAHWMPYTANRNFQRDPRMVVAAHGSYLTDDKGRKIYDALSGL
WTCGAGHTRKEIQEAVSRQLGVLDYSPGFQFGHPLSFQLAEQITDLTPGNLNHVFYTNSG
SECADTAVKMVRAYWRLKGQATKTKMIGRARGYHGVNVAGTSLGGVNGNRKMFGQLMDVD
HLPHTLLASNAYSKGMPEAGGIALADEMLKLIELHDASNIAAVIVEPMSGSAGVIVPPQG
YLKRLREICDQHNILLIFDEVITGFGRTGSMFGADSFGVTPDLMCIAKQITNGAIPMGAV
IASSEIYQTFMNQPTPEYAVEFPHGYTYSAHPVACAAGLAALELLQRENLVQQAAAVAPH
FERALHGIKGAKNVVDIRNYGLAGAIQLAPRDGDAIVRPFEAGMKLWKAGFYVRFGGDTL
QFGPTFNSQAQDLDRLFDAVGETLNLID
>tr|A0A0N8T8R0|A0A0N8T8R0_PSEAP Binding-protein dependent transport system inner membrane protein OS=Pseudomonas syringae pv. aptata OX=83167 GN=ALO85_00024 PE=3 SV=1
MANRYGKGLLGGAVAIALLALLIHWIGVSTIKQYQDDLLFYLQAHLILVVVSMLAALVVG
IPAGIALSRPSMVGRAERFMQIFNIGNTVPPLAVLAIALGILGIGSGPAIFALFLASLLP
IVRNTYEGLKNVQGSLKEAAVGIGMTPRQVLWRVELPNAVPIIIGGVRVALAINVGTAPL
AFLIGANSLGSLIFPGIALNNQPQLVLGAACTALLALLLDGLVILASRLWLERGLAR
>tr|A0A0Q0INV8|A0A0Q0INV8_PSEAP Imidazolonepropionase OS=Pseudomonas syringae pv. aptata OX=83167 GN=hutI PE=3 SV=1
MKTLWKNCHIASMAHGKYSIIEHAAIVTSGALIEWIGPQAELAEPEHDNCIDLGGAWVTP
GLIDCHTHTVFGGNRSGEFEQRLQGVSYAEIAATGGGIASTVRATRDASEDELYASAERR

LRHLLKDGVTTVEMKSGYGLDLENERKILRVIRRLGNTQPVTVRATCLAAHALPPEYADR
ADDYINHICNDMLPALAAEGLVDAVDAFCEYLAFSPAQVEQVFITAGQLALPVKLHAEQL
SSLGGSSLAARYKALSADHLEFMTEDDAIAMAAAGTVAVLLPGAFYFLRETQLPPMDALR
KHGVPIAISTDLNPGTSPGLSLRLMLNMACTLFRMTPEEALAGVTFNAAKALGMSATHGS
LEVGKVADFVAWNIERPADLAYWLGGDLDKRIVRHGVESSI
>tr|A0A0Q0C3K0|A0A0Q0C3K0_PSEAP Regulatory protein, LysR:LysR, substrate-binding protein OS=Pseudomonas syringae pv. aptata OX=83167 GN=ALO85_01589 PE=3 SV=1
MTRSLPPLYALRAFEAAARHTSFTRAAEELSITQSAVSRHIRTLEEHFACRLFQRNGRNL
QLTESARALLPGVRDGFLALERACSTLQTDEGILRMKAPSTLTMRWLLARLSRFRHLQVG
NEVQLTSAWMEIDAVDFTQEPFDCAILLSQGHFPSDWEATLLFPELLIPVGAPTLLQDPP
WDVERLAKAELLHPTPDRRDWRTWLNRMGLADQVSLKGGQVFDTLELGMIAAARGYGVSM
GDLLMVAEDVAHGGVQWRKLLSGLAAYEARRRAVATPQRFSSGGGGRHAVAVS
>tr|A0A0Q0C4K9|A0A0Q0C4K9_PSEAP Imelysin-like peptidase, M75 superfamily OS=Pseudomonas syringae pv. aptata OX=83167 GN=ALO85_02356 PE=4 SV=1
MFRPKLLLTSLAALALGACSPSDPQAVTSAAIAKQVILPTYSRWVEADRQLAASALAFCQ
GKTDLATARADFLKAQKTWAELQPLLIGPLAEGNRAWQIQFWPDKKNLVGRQVEQLVTAQ
PQITAQELSKASVVVQGLSAYEYILFDADIDMANAEQKARYCPLLMAIGERQKLLAEEIL
NSWNSTDGMLAQLSKFPNQRYADSHEAIAELLRVQVTALDSLKKKLGTPLGRQSKGQPQP
FQADAWRSKSSLSSLEASLISAETVWTGVDNKGLRSLMPAEQKPLADKIDAAYANSRKLL
SELKPPLADLLATDAGRQQLNAFYDSLNAVHRLHEGELAKALGIQLGFNANDGD
>tr|A0A0N8T989|A0A0N8T989_PSEAP Putative aldo/keto reductase OS=Pseudomonas syringae pv. aptata OX=83167 GN=ALO85_02442 PE=4 SV=1
MDYVKLGNTGLDISRLCLGCMTFGEPDAGTHPWTLGEEASRPIIKHAVEQGINFFDTANS
YSAGTSETIVGKLLKEFTRRDETVIATKVFFPANMWEGSTRPNEKGLSRKAIMSSIDASL
TRLGTDYVDLYQIHRWDYDTPIEETMEALHDVVKAGKARYIGASSMYAWQFAKAQQVATA
HGWSRFVSMQNYLNLVYREEEREMIPLCIDQGVGLMPWSPMARGRLTRPHGQQTERTRTD
VSGQSFYEATEKEDGRVIDTVEQLAAERGVPMAQIALAWVLAKRGVSAPIVGASKATQLD
DAIAALQLVLSDEDIKRLEAPYVPHAVTGFV
>tr|A0A0Q0DGM8|A0A0Q0DGM8_PSEAP DUF1338 domain-containing protein OS=Pseudomonas syringae pv. aptata OX=83167 GN=ALO85_02107 PE=4 SV=1
MSTARFADPDQIRAGFSRAMSQMYKHEVPLYGTLMALVSEVNEQVMSHDSSVLDSLRQTG
EIQRLDMERHGAIRVGMAQELATLARLFAVMGMQPVGYYDLSTAGVPVHSTAFRAVHEQA
LQVSPFRVFTSLLRLELIESDGLREFASQVLGKRSIFTPGALALIDKHEADGGLSEADAA
EFIQQALETFRWHNTATVSLDDYQKLNTQHRLIADVVAFRGPHINHLTPRTLDIDAVQAG
MPGKGITPKAVVEGPPRRQCPILLRQTSFKALEEPITFIGQGSIQSGSHSARFGEIEQRG
AALTPKGRELYDRLLNDAREALGEFPSEANAVRYAGLLEETFKAFPDSHAEMRRQGLAYF
RYFPTESGIAARASGSLEQLVEAGHVRFEPLVYEDFLPVSAAGIFQSNLGDNAQAHYATN
SNQQDFERALGRGTLNELDLYADTQRRSLEKCARELELSSPAC
>tr|A0A0Q0DPC7|A0A0Q0DPC7_PSEAP Regulatory protein LysR OS=Pseudomonas syringae pv. aptata OX=83167 GN=ALO85_03690 PE=3 SV=1
MQISDIEVFAAIASSRSLSEAARRLGLSPMAISRRLAALENDLGVRLVHRTTRSVSLTPE
GEVFLPHAKTMLHASETARATLKADAGTASGVLRVTAPSVFGQTVIMPLLPALMQENPAL
SIDLTLSDSIVDIAGLGIDVAIRIATIRDSALVARSLAPNPRVVCASPDYLARHGQPSIL
DELRQHPCITLHAMPYWPFIREGEPVAIRAQGAFSANSVEAVRTACKQGLGLAMLTYWDV
QQEIREGSLLQIHFEDVIPEQLSVTAVLPTRQRVPHRVRLFVDRLEAELESKAVAGGATL
>tr|A0A0Q0C416|A0A0Q0C416_PSEAP Acetyltransferase OS=Pseudomonas syringae pv. aptata OX=83167 GN=ALO85_02625 PE=4 SV=1
MNCIVEYSDPHHRLAVIQLWKTVFNDDMPHNRPDLSIDKKRAVDDHLFFVALADDQVVGT
LLAGYDGHRGWLYSVAVDPQQRGNGLGTRLVRHAEKALADLGCVKINLQIHTFNESVQAF
YQTLGYTPELIISMGKRIDANVTAQS
>tr|A0A0Q0CH06|A0A0Q0CH06_PSEAP Transgly domain-containing protein OS=Pseudomonas syringae pv. aptata OX=83167 GN=ALO85_05018 PE=4 SV=1
MTGQSTMGAFRHSDSKQETLSADREDDPRTPKYPRRKRYGWRVFWVVVVLAGVALGAAVA
VESRTSRLQAREFSRFAASLGYSMQPGPSHDVVYPGDGPFDRRLGYSSMDEFLSRLLKRD
YVITRQTRFSPELLRYVRHGFFVPYEEKTQAGLSITDCRSEPLYEFKYPQQFYPTFAAIP
PMLVDSLLFIENRDLLDPQQPMANPAVDWPRFVKAAWSQLAKMFALPGQSAGGSTLATQL
EKYRHSPDGLTLSGSEKLRQMISASVRAYQPGAETLAVRQRVVRDYLNSVPLSAVPGHGE
VHGLAEGLRVWFGADFGKTNEILASNPGNKQALADKALALREVLSLVIAQRRPSHYLAKG
RSELAELTDSHIRLLAQANVIDRPLAEAALAAQVTYRDWAQQPTLQPIETNKGISVARTR

LSNLLNRPLYDLDRLDLSATSTLHGELQRSVSQYLRDLANPEFAAKTGLMGERLLTPAST
TQVRYSFTLFERGVDGSRVRVQTDSTDQPFDINEGSKLELGSTAKMRVLTTYLEIIAELH
GRYAGLSPAQLRKVPVEEPDRLTRWAVDYLLINKDRDLAKMLSAALDRTYSASPAEAFFT
GGGMHRFNNFRREDNARIPTLRESLRESINLPFIRLMRDVVRYSTYQAPNNSAELLKDDD
NPRRQEYLAQFADREGTVFLLRFWKRYKEKTTQERLDTFLDGIHPTAIRLAAVHRYLLPG
ADQGTFNAFIRAHLDEPRASAKLTDKRLADLYQSYGPGSYDLPDQGYIARVHPLELWLVG
YLLKHPEAQFKDAVAASRFERQEVYGWLFKSRHKGARDSRVRTMMEVEAFLDIEQRWQRV
GYPFDHLVPSLATAIGSSGDRPAALAELIGIIQNDGIRLPAVRIDSLHFAADTPYDTQLS
INPELGKRVLPSEVAAMREALSQVVDGGTAKRVQGTFKMQDGSVLAMGGKTGTGDNRIE
SVGAGGRILSSRAINRTATFVFYIGDNHFGALTAFVPGRAAEGFRFTSALPVQVLKGMAP
ILTPYLENHGQAMCNGPLADAPKGV
>tr|A0A0N8T916|A0A0N8T916_PSEAP Uncharacterized protein OS=Pseudomonas syringae pv. aptata OX=83167 GN=ALO85_02521 PE=4 SV=1
MADATLDHQLGLLAHLRSILVALGEAEQVPEESHALFMERFDELVELLPQDPIESQYLGQ
DILCQVIQRYPQIAHLVPRDLLWFFAGDCLHFMPDDEIELYQALEERRYEAEQNEEPFDW
NQEKQLLSMSTQGSKH
>tr|A0A0Q0CYH3|A0A0Q0CYH3_PSEAP Catalase OS=Pseudomonas syringae pv. aptata OX=83167 GN=ALO85_04921 PE=3 SV=1
MGRDKESLMASKNAPPVTETPKSEMAGTDTLDRGNTNEKLESLEQFRSDATQQALRTNHG
VKISDNQNTLKVGSRGPSLLEDFIMREKITHFDHERIPERIVHARGTAAHGYFQTYEDHG
ALSKAGFLRDPGKKTPVFVRFSTVQGPRGSGDTVRDVRGFAVKLYTDEGNFDLVGNNMPV
FFIQDAIKFPDFVHAVKPEPHNEIPTGGSAHDTFWDFVSLVPESAHMVLWTMSDRAIPKS
LRTMQGFGIHTFRFINTEGKSSFVKFHWKPKFGVCSLVWDEAQKLAGKDTDFHRRDLWES
IEMGDYPEWELGVQIVAEEDEHKFDFDLLDPTKIIPEELIPVTPLGKMVLNRNPDNYFAE
TEQVAFCPGHIVPGIDFSNDPLLQGRLFSYTDTQISRLGGPNFHEIPINRPIAPNHNGQR
DAQHRTTIDKGRASYEPNSIDGGWPKETPAGPVDGGFETYPERVEAHKVRERSESFGDHF
SQATLFFQSMSHHEKEHIIAAYSFELGKVEREYIRARQVNEILANIDLELAKRVAANLGL
PAPTAGTVPVRQTSVKESPALSQVNLLSGDIVSRKVAILVADGVDSKAVEAMKAALTAEG
AHAKVLGPTSAPVKTADGKSLPVDASAEGLPSVAFDAVFVPGGKASIEALKGDGVALHFI
LEAYKHLKAISFAGEAKELLSLLRLEEDAGLLEVSDSKSFKAFFHAIAQHRVWDREVKAK
AVPA
>tr|A0A0Q0IJH0|A0A0Q0IJH0_PSEAP Polyamine ABC-type transport system, periplasmic substrate-binding protein OS=Pseudomonas syringae pv. aptata OX=83167 GN=ALO85_02512 PE=4 SV=1
MKHLLLASLLGSAMAMSSLVMAADTDLKTLEAAAKAEGAVNSLGMPDDWANWGGIWKDLE
AKYGLKHMDTDMSSAQEIAKFEAEKDNASGDIGDVGAAFGPIATAKGVTQPYKPTTWEQI
PTWAKDKDGHWALAYTGTIAFIVNKNLLHGSEIPKSWADLKTGKYKVSIGDVSTAAQAAN
GVLAAAIAMKGDETNIAPGLALFTDLAKQKRLSLANPTIQTIEKGEIEVGVVWDFNGLSY
KAKMTKPDDYVVLIPSDGSVISGYTTIINKYAKHPNAAKLAREYTFSDAGQIRLAEGNAR
PIRVEHIKLPEDVQAKLLPQDQYKNAKPIEKADAWEKTSKALPQMWNEQVIIEMN
>tr|A0A0N8T9A5|A0A0N8T9A5_PSEAP Uncharacterized protein OS=Pseudomonas syringae pv. aptata OX=83167 GN=ALO85_02686 PE=4 SV=1
MTQATQAAPHSAQGTKWKWAKRVFNLFFFIAVPVLLFMLVKKLDWQEVKQALASYKASTL
AIAAAVAFASYATYCGFDVLARYYTRHKLSIKQIVPVTFVCYAFNLNLSSWVGGIALRYR
LYSRLGLDVSTITRILSLSLITNWLGYMLLAGFVFSMRFLELPKEWSIGTTTLQLIGFGL
LAVCFAYLLACRFSKKRSWTIRDQEFNLPSMKQALMQASLGALNWSLMAAVIYTLLPDKA
FYPAILGVLLISSIAGVITHIPAGLGVLETVFITLLAHQFSKGSLLAALIGYRAIYFLLP
LLLALVVYLVLEKRAKKMGEKNQQRMGEEAKS
>tr|A0A0Q0DS87|A0A0Q0DS87_PSEAP Glycerol-3-phosphate ABC transporter, permease OS=Pseudomonas syringae pv. aptata OX=83167 GN=ALO85_200056 PE=4 SV=1
MARAKSKPISPTNPQKRIDEHPAFLLGKHPTEDSFLASYGQQFVMLAAPPGTGKGVGAVI
PNLLSYPDSMVVNDPKFENWEITSGFRASAGHKVYRFSPERLETHRWNPLSAINRDPLYR
LGEIRTIARVLFVSDNPKNQEWYNKAGNVFTAMLLYLMETPDMPFTLPQAYEIGSLGTGM
GTWAQQIIELRSTGPNALSFEALRELNGVFEASKNKSSGWSTTVDIVRDVLSVYAEKTVA
WAVSGDDIDLSKAREEKMTAYFSVTEGSLKKYGPLMNLFFTQAIRLNSKVIPEQGGHCAD
GTLRYKYQLALLMDELAIMGRIEILETAPALTRGAGLRFFFIFQGKDQLRAIYGEEAANG
IMKAIHNEIVFAPGDIKLAEEYSRRLGNTTVRVHNQSLNRQKHEIGARGQTDSYSEQPRP
LMLPQEVNELPYDKQLIFVQGTQTTPALKILARKIYYYEEEVFKSREKLPPPPLPVGDAS
KIDALTVPVRTIESKVAVADTKPMQAEQRQRWNPKDKASEVAQAEADKAQPVEVEPDPEP

VQADDTSEPM
>tr|A0A0Q0CFZ4|A0A0Q0CFZ4_PSEAP Chromosome segregation protein SMC OS=Pseudomonas syringae pv. aptata OX=83167 GN=ALO85_101379 PE=4 SV=1
MVAGLQRQVAQAASIALVDEVEHGLEPHRLIRFMDSLGTKNTPETLQVFLTTHSPVALRE
LSDANGR
>tr|A0A0Q0IAN4|A0A0Q0IAN4_PSEAP Type III transcriptional regulator HrpS OS=Pseudomonas syringae pv. aptata OX=83167 GN=ALO85_04203 PE=4 SV=1
MNLDDEFDDHLDAERVPNLGIVAESISQLGIDVLLSGETGTGKDTIARRIHNMSGRQGRF
VPMNCAAIPESLAESELFGVVSGAYTGADRSRMGYIEAAQGGTLYLDEIDSMPLALQAKL
LRVLETRALERLGSTSTINLDICVIASAQACLDDAVEEGKFRRDLYFRLNVLTLKLPPLR
DQPERILPLFTRFVAASAKELSVPIPDVCPLLQQVLTGHRWPGNIRELKAAAKRHVLGFP
LLGADSQTEEHLACGLKFQLRAIEKALIQQALKRHCNCIDAASLELDIPRRTLYRRIKEL
SI
>tr|A0A0Q0CCM7|A0A0Q0CCM7_PSEAP Type IV pilus assembly protein PilV OS=Pseudomonas syringae pv. aptata OX=83167 GN=ALO85_02791 PE=4 SV=1
MAGETRYRQTGMTLIEVLVSVLILAIGLLGAAAIQLNALKYTDSSAMTSQASFIAYDMMD
RIRANVDGNASANGSTNVLATYALANLDAAPAANLNDARAQDLSDFRSNIINFAGQSGTG
SITVSNAPEVTIIIGWSDNRAAGASNQATGSPAATPVPRSFQLVSRIGVNP
>tr|A0A0Q0FN22|A0A0Q0FN22_PSEAP AsnC family transcriptional regulator OS=Pseudomonas syringae pv. aptata OX=83167 GN=ALO85_03483 PE=4 SV=1
MTLDKYDRILLDELLKNGRASFAQLAKLVNLSAPAVAERVAKLEASGVITGYEAKVDLSK
VGLPIQCIIELRMGSHGNQQAYKDLWKVPELIQCYRVTGDPCVIMRAAVDSMPHLEDLIN
RIAKFGFSKTSIVLSTAVERSLPADYLISNGKQEH
>tr|A0A0Q0C8S5|A0A0Q0C8S5_PSEAP Phytochrome:GAF:ATP-binding region OS=Pseudomonas syringae pv. aptata OX=83167 GN=ALO85_05373 PE=4 SV=1
MQAFPRHSQLKLRVQRAPVMVTHFLISLACISRARHPSLHAALSRQPRIFLRCPSGPLAA
QIPAAADKNPSESRLASNDCTSYQAVAGCSRLKHPVEFTQLIKDMLDAKPDAALEAALAE
CAREPIRVPGAIQPHGILLSVAGAPLCIEQVSANCATALGMTADELLGKPLSWLLSPEHS
ALIDHAYSHPAAPNLDPIKLTLGTAHYSASLQRIDDVLIIELEDFVETDHGQSRILARVL
RNLQAATTLETLFDISVHEIQALTGYDRVMIYRFEPEGHGKVVAQALTGPLPSYSGLNFP
GSDIPAQARELYRLNWIRVIPDATYVPVPLIPTLRPATGQPLDLGFSTLRSVSPVHCEYL
KNMGVRSSMSISLLDGGELWGLVACGHPEPLRVSRELRDACAMIGQLLSVKISAIVATDV
QREREAKVALLGQLADAMSRANHEVLDGLVSRPELLMSLTLADGAAVLIDDQVHLFGTCP
AIEQVRELYTWIRDTGLAHQRTRQRATGLQGLGVFHTNSMQRELAASAVYRDVASGVIAF
TLPKPIDNAVMWFRAQLTSTMNWSGDPTHHVSTRPESSASHRLHPRQSFDVWEQEVTGIA
RPWSRADLYGAEDIRRSALESDLERQVQREQEAVRLRDELVAVVSHDLRNPMSIIIMQCG
MMQRWALGDTHFENRNIRRALGTIEKATTRMNSLLEDLLDTAQIEAGRYQLSRLPLSVTS
LLEEACSLLVMLTTEKNIELNCTAAQGLVVDADPERIFQVLSNLVGNAIKFTPKGGRINI
DAVADQHEVLFRVSDDGIGIPVEHLPFIFQRYWSVKEGNPRGNGLGLYICQGIVTAHGGR
LWADSSLDSGSVFSFTLPMHQGLDTVGETTFLKQSGTTHRLAQSISSKLERQQLEDRLTR
AGLLNELNHRVKNTLATVQAIASLTVNSSASLESFHKSFGARLFALSQAHDALARAEWIS
TELVDLIEQLQEVDSGAHRICFEGDPVTLEPRFSLTLSMVLHELMANAVQHGALSSAAGQ
VTVTSTLTAALSPPTLKIEWKETGGPPVVATTVKGFGLRLIRRSIERELKGQADIQFART
GIIWSMLIPRPDKPESHL
>tr|A0A0N8T8T9|A0A0N8T8T9_PSEAP 3-isopropylmalate dehydratase large subunit OS=Pseudomonas syringae pv. aptata OX=83167 GN=leuC PE=3 SV=1
MAGKTLYDKLWDSHLVKQRDDGSALIYIDRHIIHEVTSPQAFEGLRLAKRKPWRIDSIIA
TPDHNVPTTAERKGGIGAIEDQVSRLQVQTLDDNCDEYGITEFKMNDPRQGIVHVIGPEQ
GATLPGMSVVCGDSHTSTHGAFGALAHGIGTSEVEHVLATQCLVAKKMKNMLVSVEGQLP
FGVTAKDIVLAVIGKIGTAGGNGYAIEFAGSAIRDLSIEGRMTICNMSIEAGARVGMVAT
DEKTVEYVKGRPFAPKGAEWDLAVEAWKDLVSDPDAVFDTVVKLDAAQIKPQVSWGTSPE
MVLAVDQNVPDPAQEPDLVKRGSIERALKYMGLKANQPITDIQLDRVFIGSCTNSRIEDL
RAAADVAKGRKVASTIKQAIVVPGSGLIKAQAEKEGLDKVFIEAGFEWREPGCSMCLAMN
PDRLGSGEHCASTSNRNFEGRQGAGGRTHLVSPAMAAAAAVNGRFIDVRDLIQH
>tr|A0A0Q0CHH5|A0A0Q0CHH5_PSEAP ABC transporter permease OS=Pseudomonas syringae pv. aptata OX=83167 GN=ALO85_03363 PE=3 SV=1
MSLLSEMRQGGRGYWLSAPALALYIGLLVLPLGLTLVLSFNVFDYQVGVKSDSYTLANYM
AVVTDSYFYEIFLRTFWISALVTLLCVLIGVPEAYILRRMGTPWRSIFLILILTPLLISV
VVRAFGWSLLLGADGLINQVIQFMGGRPVKLLYTPFAVVIALVHVMLPFMIIPVWTSLQK
LDPTAEQAALSLGASQAKVMRLIVLPQVMPGVLSGSLIVFGLAASSFAIPGLLGGRRLKM

VATVIYDQYLSELNWPMGATLAVALLLVNLLVMLSWNRMIEGRYKKTLGE
>tr|A0A0Q0CFV9|A0A0Q0CFV9_PSEAP UvrABC system protein C OS=Pseudomonas syringae pv. aptata OX=83167 GN=uvrC PE=3 SV=1
MTQTFDPSAFLATCSGRPGVYRMFDAEATLLYVGKAKNLKKRLASYFRKTGHAPKTGALV
SRIAQIETTITGNETEALLLEQTLIKEWRPPYNILLRDDKSYPYVFLSDNAFPRLSIHRG
TKKAKGRYFGPYPSAGAIRESLSLLQKTFQVRQCEDSYFKNRTRPCLQYQIKRCKGPCVG
LVEPEVYAEDVRHSVMFLEGRSNALSDELNASMEKAAMALDFERAAELRDQVALLRRVQD
QQSMDGGTGDVDVVAAFVNPGGACVHLISVRGGRVLGSKNFFPQVGIEEEVGEVMSAFLA
QYFLGGIDRELPGEVIVNVINDDFPAFVDAVEELRGVEMVISHRVRGTRARWQQMAVTNA
EQALTARLANRQHVASRFEALAKVLGLEDPPMRLECYDISHSSGEATVASCVVFGPEGPI
KSDYRRFNIEGVTAGDDYAAMHQALTRRYSRIKAGEGKLPDVLLVDGGKGQMSMARDVLN
ELQVPDLILLGVAKGTTRKAGFETLYLNDSAHEFTLPGDSPALHLIQQIRDEAHRFAITG
HRARRGKTRRTSTLEGVAGVGPTRRRDLLKHFGGLQELSRASIDEIAKAPGISKKLAESI
YANLHSE
>tr|A0A0Q0BUB8|A0A0Q0BUB8_PSEAP CrfX protein OS=Pseudomonas syringae pv. aptata OX=83167 GN=ALO85_00570 PE=4 SV=1
MHDPFEESLRDMLNSSSSSRDDDACLGRVLKTANRQVGAGVLFGLLGRWSEALMIAVNNG
SAHVSPVSRRTSTGARSIDKAD
>tr|A0A0Q0CI31|A0A0Q0CI31_PSEAP Uncharacterized protein OS=Pseudomonas syringae pv. aptata OX=83167 GN=ALO85_05511 PE=4 SV=1
MRSLQTSALGVPGFAGQTIDPITGYYLLGNYLKTAAPPWLARPSIQRVR
>tr|A0A0Q0BTK6|A0A0Q0BTK6_PSEAP PAS:GGDEF protein OS=Pseudomonas syringae pv. aptata OX=83167 GN=ALO85_04299 PE=4 SV=1
MTQSAATILIIDDDVHVRDLLEVLLQNQGYRTLTAESGELALAMIKRQAPDLILLDIMMP
GMDGYEVASQLKAEKNTANIPIIMLSALDDQSSRLSGLEAGAEEYLNKPVDSAELWLRVR
NMLRLKAFGDYLKNHSLILEDQLQQRTIDLERFRTVMDASEDAIFLINRNTMSLIEFNRR
ACQLLGYTAEELSHKTPAELGETSMEKLEVVYDRIIAGKGPSEPLETQIRDKSGRDVEVE
IHRQAYRTGEDWVIVGIVRDITRRKESDQRLLTMAHYDTLTSLPNRDLFFTSLQMGMTQA
AISRWKLAALTINLDDFKNINETWGHVLGDEVLLEVSRRLSNCLNASDTLGRVDGDQFAM
ILMLRDGQAGTRETLERIRNALRVPFHIEGQSLVMTASIGIALYPEDGEDSRELIRHAYT
AMNSARKIGPDNYRFYTPQMNADVSARLDLEAALREAVRNQAFEIVYQPKLDLNNDRICG
LEALLRWPRPGHPGVSPAVFVPVLESLGLIGEVGNWVVDSVCAQIARWQRSGLGSFQVAV
NISGQQISNSSLVADIRQALEKHNVAPHWLEVELTESSLMENTAHTIATLGALRASRVSI
SIDDFGTGYSSLAYLRRFPIDKLKIDIAFIREVTSNPQDAAIARAIIELAHSLDLLVIAE
GVETPEQLAFLRENHCDQIQGYLISKPLPLDELETFLRSPASRVG
>tr|A0A0Q0C706|A0A0Q0C706_PSEAP Uncharacterized protein OS=Pseudomonas syringae pv. aptata OX=83167 GN=ALO85_01951 PE=4 SV=1
MRRALLFAFALLALPAVQAADLQPFSASYTADWKQLPMSGTAERSLEAGANGTWTLSFKA
SMMIASLTEVSTLKVDKDTLLPQTYSFERSGLGKSKKVDLAFDWNTKFVTGTDRGDAIKL
PLNRGILDKSTYQLALQHDIAEGKKSMSYQVVDGDEVDTYDFRVLGSEKVTTKTGQVDAI
KVERVRDPTQSKRTTVLWFAKDWDYLLVRLQQVETDGKEYNIVLLDGTVNGKAVKGS
>tr|A0A0Q0C7I1|A0A0Q0C7I1_PSEAP PspA/IM30 OS=Pseudomonas syringae pv. aptata OX=83167 GN=ALO85_00611 PE=4 SV=1
MTTSIWKKLVTAVRGGASEVGESIADANAIRILDQEIRDAENALVKARDGLITIKAKHKL
SAQRLEEHATSIANWEGRAMQALNKGEEELAVECANKIAEIEALRDQEQELADQFSKQVN
LLQDQVIKAESRIKSLKQQVEMAKARETVQKARVATATATGGASGAVETAVGSLARLKQR
QDEQDAKLEAAEEIASQSSGSDLERRLQDAGIGAKSGGAQDVLARLKAKSQTPAQ
>tr|A0A0Q0DK28|A0A0Q0DK28_PSEAP Putative membrane-bound lysozyme-inhibitor of c-type lysozyme OS=Pseudomonas syringae pv. aptata OX=83167 GN=ALO85_04000 PE=4 SV=1
MKGVIALGVLAVLGGCSSMSSPQQNDPWNRWVCDSNAEVNWRYIDSAKKEVDVRLNQSDQ
VFRLKAEPGAASGTLYSNNVLAFVNKGSEGLIYWDATNDLIGRGCKAR
>tr|A0A0Q0DL90|A0A0Q0DL90_PSEAP GCN5-related N-acetyltransferase OS=Pseudomonas syringae pv. aptata OX=83167 GN=ALO85_02617 PE=4 SV=1
MPTIMLLHTPPPEFIRSQIQQMVVDYVTDISLVAIAPSNPLYNLYQYGVGYEVHLYLNAM
DGSQQLTVELIVALDDEDPETVLGFLLYLPVQDDPQACAVAYMAVRDSHRRQGIARSMLE
KMTSRYPHAELYCVVGKVAYFEAMGFQVLGARGPQVIMNTRDHGTDGLLAVMDIAPIYNS
VEVRQIHAYLLKQHGNSAMVDAEKQRDRHLDRMTLEARTFAEQHLGNNQLADGASKTSRL
H

>tr|A0A0Q0DC40|A0A0Q0DC40_PSEAP Porphobilinogen deaminase OS=Pseudomonas syringae pv. aptata OX=83167 GN=hemC PE=3 SV=1
MSSREIRIATRKSALALWQAEYVKARLEQAHPGLIVTLVPMVSRGDKLLDSPLSKIGGKG
LFVKELETALLENNADIAVHSMKDVPMDFPQGLGLFCICEREDPRDAFVSNTFASLDQLP
AGSIVGTSSLRRQAQLLARRPDLQIRFLRGNVNTRLAKLDAGEYDAIILAAAGLIRLGFE
DRITSAISVDDSLPAGGQGAVGIECRSVDAEIHALLAPLHHEDTAVRVIAERSLNKHLNG
GCQVPIACYAVLEGDDVWLRGLVGDPSGSVLLHADARAPQTSAQALGVQVAEALLEQGAA
DILKAVYGEANNE
>tr|A0A0Q0BZ23|A0A0Q0BZ23_PSEAP AcrB/AcrD/AcrF family RND-type efflux transporter OS=Pseudomonas syringae pv. aptata OX=83167 GN=ALO85_04616 PE=3 SV=1
MNLTSRAMGASRLTLFAALLILLAGVATFLSFPSQEEPSVTVRDALVSVSLDGLSAERSE
ELLARPLEERIRELAEIKNVLTTVQPGRVIVQVTAYDNVQDLGLLWQRLRAKVSETSGGF
PPGTQGPFVDDDFGRVAVASVALTAPGFTMSEMRGPIKRLRNQLYALKGVDQVQLYGLQD
ERIYLDVEHQTLAAAGLSPQQLLQQLQARNLIATGGNPVIGQQSTTLSISGEVLSLEQLR
QFPVQLPNGQKVALQNLSRVSVSPVDPPETEAIYQGQDAVVLAVSMQPGLNVQTFGDALR
KRLGELEQQLPTGFTLHVVTFQSSVVEHEMSKMYHVMAETVVIVMCVVMLFLGWRTGLVV
GVIVPLTILSTLLVMRALGIELQTVSIAAIILALGLLVDNGIVIAEDIERRLHAGEDRRS
ACEEAGRTLMIPLLTSSLVIVLAFSPFFLGQTSTNEYLRSLAVVLATTLISSWLLSVTVT
PLLCFYFARMPAVKASADEADEYASVFYRGYRKVIEGLLNHSLIFIACMMGLLALAVVVL
TRVPYDFLPKSDRMQFQIPLVLEPGSSSRDTSRAVRDISQWLSSDQDVEHSIGYVASGGP
RIVLGLNPPLPAPNIAYFTVSVREGADLDAVIERARQFVLTHHPEIEAQPKRFSMGTTEA
GIAVYRVIGPDEEVLRGIARQIEQALSALPGTLDVHDDWRTRLLRYDVVVDPYKALQAGV
ATQDIAQALQLRNSGLSVSSLQDGDIAVAIVAREQSTLSKPGNDLDSTLIYPASGGAPLM
LSAIATVEPRAEASTLQRRNLQRAITVVGHNPSMTATSIVEQLAPQIAAISLPAGYHIEP
GGEIEDSAEANQALLQYMPHALVAMLLLFVWQFNSFRKLLIVISSVPFVLIGVALALVLT
GYPFGFMATFGLLSLAGIIVNNAVLLLERIEVEREQGLAVHDAVVNAAIKRLRPIVMTKL
TCIIGLVPLMLFAGPLWEGMAITIMGGLALGTLVTLGLIPILYALLFDRFERWRKAS
>tr|A0A0Q0FC15|A0A0Q0FC15_PSEAP Dihydroneopterin triphosphate pyrophosphohydrolase, putative Actinobacterial type OS=Pseudomonas syringae pv. aptata OX=83167 GN=ALO85_04245 PE=3 SV=1
MSNFQVDKPLRERKSSRLLIVSPSMRILLFRFQHTKGALAGSNYWATPGGGVEDGETYEA
AAIRELREETGIRVNSVAEHIANRRFPMLLPSGESVLAVERYYVVYAESQVLSRSEWTLQ
ETQVMTEHRWWSAHELNSTTEIIKPDDLVRTLEKAGVFKAER
>tr|A0A0Q0IAP6|A0A0Q0IAP6_PSEAP Type III secretion system protein HrpE OS=Pseudomonas syringae pv. aptata OX=83167 GN=ALO85_04197 PE=4 SV=1
MLAKRSITLTADAVLPEPVLRREDIANSLLARDILMDAQRQAEQLLVLEQAKADRRHQEA
LAQFWERANAFLDELHVQREALQQQAMTAVEELLTEGLRQLLDETTLAERARALVRNLAA
SQLNEAVATLSVHPEMAEPVAEWLAEGRFAEHWELKRDATLATESLRLSDANGAFEIDWA
TLRNGLAGTEPAA
>tr|A0A0Q0C1P0|A0A0Q0C1P0_PSEAP ISPsy24, transposase orfA OS=Pseudomonas syringae pv. aptata OX=83167 GN=ALO85_03580 PE=4 SV=1
MTKQRRTYSTEFKRDAAGLVLDQGYSHIEAFRLLGVVESTLRRWVSQLQQKRNVVTPQSK
H
>tr|A0A0Q0ILD7|A0A0Q0ILD7_PSEAP Glycosyl transferase, group 1 family protein PslI OS=Pseudomonas syringae pv. aptata OX=83167 GN=ALO85_02656 PE=4 SV=1
MRIGLDYRAAAGYPTSGIGRQNLALEQAFREHPDVQLQLFGVAPYDHPVRRLMHAPRWAA
PLNSVHRLPDRLRFEGQFLPAALRDAGIQIYVANINMGLPLGRKPAGMRYVLQLHDLFQL
TQHNSHGSQLKARLYRMTDYLSIAWSLKVADQIWVPSQFTANEAARMFPRIKAKLRVIPL
LVEAFKGEPADIAQLRLPLRYWLCVGTREPRKNIKWFVDAWQTARMQFVDTPELVLVGGD
DPLTEAQRQLPGLHVLSGLSDAELHAVYKNAERLWQPSKSEGFGLPVIEALNTGTPVAVA
TGSSLDEVAPPDSPRFSPVDSGNLIRLMGSLSQAPEEDPAIAKDWTQRYQLEAFSERVHA
ALEELR
>tr|A0A0Q0ILI2|A0A0Q0ILI2_PSEAP Decarboxylase family protein OS=Pseudomonas syringae pv. aptata OX=83167 GN=ALO85_00270 PE=4 SV=1
MILQEEKSRMPYYVPDDLLSRHFQSDGLDLTSKIEEHINEVAPGTKNLPLYRDMMLTVLRM
AQDDRNRWDVKITLQTLRELDKAFRVLERFKGRRKVTVFGSARTPMEHPLYAQATDLGEK
LARADMMVITGAGGGIMAAAHAGAGLKHSLGFNITLPFEQHANPTVEGSENLLPFHFFFT
RKLFFVKEANALVLCPGGFGTLDEALEVLTLIQTGKSPLVPVVLLDTPGGSFWQGALDFI

KHQLQDNHYILPADMKLMRLVYSADEAVEEINRFYANFHSSRWLKNKFVIRMHHALNEQA
LEHMQEAFADLCINENFHQHSYQGEEHDEAQFSHLTRLAFTFTGRNQGRLRELVDYINQQ
ENWEDASATRDAARNRTPQ
>tr|A0A0Q0FVL5|A0A0Q0FVL5_PSEAP Glutathione S-transferase family protein OS=Pseudomonas syringae pv. aptata OX=83167 GN=ALO85_03835 PE=4 SV=1
MSAPTMTLYFNAASPFARKVMVMLHETAQLDRVELQTTVLTPVSPSAELNEDNPAGKLPA
LRLADGNVIHDSRVILDYLDHQHVGLPLIPREGSARWRRLTLASLADALLDAAVLIRYET
ALRPQEKHWDQWLDNQQQKIERSLHYFDSDAITELSTSFDVASISVAVALGYLDFRQPDL
NWRSTYPRLSAWYLDVSQRPSMIATQPPV
>tr|A0A0Q0IJD8|A0A0Q0IJD8_PSEAP Delta-9 fatty acid desaturase OS=Pseudomonas syringae pv. aptata OX=83167 GN=ALO85_03963 PE=4 SV=1
MWYNGLLDLSVWQVIAVTLAMTHITIVGVTVYLHRYSAHRSLELNAGLKHFFRFWLWLTT
AQNTREWTAIHRKHHAKCETVDDPHSPVVKGLSTVLRKGAELYREEAQNQDTLRIYGKNC
PEDWIERNLYSRYKLLGIALMAVIDVALFGALGITVWAVQMMWIPFWAAGVVNGLGHAVG
YRNFECRDAATNLVPWGIVIGGEELHNNHHTYPNSAKLSVKPWEFDMGWAWIKLFSFLGL
AKVQRVAPIAHRVEGKGHMDMDTAMAILNNRFQIMAQYRKLVIAPLVAQELAKADESVRH
QFRRAKRLLSRETSLLDDRHHLRIQTMLEHSHALKVIYEKRLALQQIWVKTSSNGHDMLS
AMKEWIHEAEASGIHSLREFADQLKTYSLRPAHA
>tr|A0A0Q0ICQ5|A0A0Q0ICQ5_PSEAP Uncharacterized protein OS=Pseudomonas syringae pv. aptata OX=83167 GN=ALO85_03061 PE=4 SV=1
MISMKEIFCVVGTLQCTVFMLLRLASVFFLKPKLG
>tr|A0A0Q0BSL3|A0A0Q0BSL3_PSEAP Uncharacterized protein OS=Pseudomonas syringae pv. aptata OX=83167 GN=ALO85_00114 PE=4 SV=1
MKRTALTGLFLSAALLASPVFAADDLCAANIKTIENANTSSSTNLSPENKTMLETTVKNA
KAAQAQNDEKKCVEITTKTLTALKNTGSGSEGTK
>tr|A0A0Q0DVA1|A0A0Q0DVA1_PSEAP Putative Recombinase OS=Pseudomonas syringae pv. aptata OX=83167 GN=ALO85_01530 PE=4 SV=1
MSSPSMRKRLEDYSDGLIAAAEYVRMSTDHQRYSTENQSAAIHAYALSHGMDIVKTYRDE
GKSGLDIGGRDALRRLLDDVQAGNVQFQIILVYDVSRWGRFQNTDESAHYEYLCTSSGIQ
VVYCAEPFENDGSPLATIYKGIKRSMAGEYSRELSQKVFAGQCRLVEKGFHQGGPAGYGL
RRALIDEKNEFKAELSRGQQKSIQTDRVILIPGPEAEIEIVQHIYHRFIHNGMNEREIAN
ALNADGVLTDFDRPWSRSSVHEVLTNEKYIGNNVYNKTSSKLRKRSSRNPPDKWIRCDGA
FQGIVSLEVFTCAREIILQRSHRLDDTQMLELLRALLQQAGSLSGMLIDEQDNMPSSTVY
ISRFGGLLRAYTLIGYAPDRDYRYLDINRSLRQLHPQVFEDVIKHLENAGASVEISAQND
VLTVNGEWTASVVIARCHSTPAGTLRWKLRFDISLAPDITIAVRMARANLQVRDYYLVPL
IDMGAWPQKMAEENSPLIDSYCFQALDVLDGLAARCSLKEACQ
>tr|A0A0N8T9H1|A0A0N8T9H1_PSEAP Lipopolysaccharide core biosynthesis domain protein OS=Pseudomonas syringae pv. aptata OX=83167 GN=ALO85_100135 PE=4 SV=1
MTSQSINSTRYVRNAVDEHTPAPTRVLDFSQCKGTKKGPVFLIGSGKSAKDFPIEEFSHI
PMITMNGAFSMFAGTNIKPLFYVCSDRDFPNQQPELFAAAMRSSENVGLWEDQFRGDIPR
PSGRAYALKKSPRVSRVAALCSREDALVRKLSLWSSRSRDIGFSKDLEFGFFDARTVMYL
ALQLSYHLGFDNVFLVGFDMNQSVGRFYESSSDNCSPCGLDQHYESRILPSLELMSKQVV
GSDFQVFNVSDSSRVPHEVIPKLSLDEVRHKVSCALQQ
>tr|A0A0Q0BXA4|A0A0Q0BXA4_PSEAP Alginate biosynthesis protein AlgF OS=Pseudomonas syringae pv. aptata OX=83167 GN=ALO85_01800 PE=4 SV=1
MTFNTTANRSAAKRLFKTCAMAAGLGLMSLQAFAGDSALYGPTAPKGSTFVRVYNASNAE
ISATVGNTNLNEVAPLGSTAFSFMPQGDYTAKLGSQSLPVKLAGDHYYTIVNNASGKPQL
VEEPPFKNKQKSLVRVQNLSDKSLTLKTADGKTDVVKAVAAKGTGEREINPVKVSLALYD
GDKKVTDVKPVALERGEAAVLYITGSGSSLSPVWVKPPVATR
>tr|A0A0Q0C519|A0A0Q0C519_PSEAP 50S ribosomal protein L7/L12 OS=Pseudomonas syringae pv. aptata OX=83167 GN=ALO85_200055 PE=4 SV=1
MNQAIRVGAYGAAESSIKEVIQPQVPLRLPCYDFTPVMNHTVVTVPPKVRLATSGATHSH
GVTGGVYKARERIHRDILIRDY
>tr|A0A0Q0FAX0|A0A0Q0FAX0_PSEAP Uncharacterized protein OS=Pseudomonas syringae pv. aptata OX=83167 GN=ALO85_04051 PE=4 SV=1
MATHKELVAELINVLNSDGSSEVRAGAAKGLGAAGGADALRALRAALKHDSKILVRAASA
EAVGLILGRGNLQDMMDQ
>tr|A0A0N8T903|A0A0N8T903_PSEAP Uncharacterized protein OS=Pseudomonas syringae pv. aptata OX=83167 GN=ALO85_101029 PE=4 SV=1

MDSDNRLYKLAVTPTGRRLWTYMAAILEVTEMSQGKSFPLKRFMVNFQTHLDGGRIESGP
DGYRLTRIGHEYFQGRYHAESPQRVERAAVQQMIISIRSGVGEGEWIALP
>tr|A0A0Q0CZ83|A0A0Q0CZ83_PSEAP FabA-like domain protein OS=Pseudomonas syringae pv. aptata OX=83167 GN=ALO85_02828 PE=4 SV=1
MTEWPLAELIPHAGDMILIDQVLSVDEEQIRTRLTVRADGLFNQADGSLPAWLGIELMAQ
SVAAYAGCKARNKGEAVKLGFLLGSRKFDCNVTRFPLGSELHIHAVRSLQDDNGMGMFEC
TLTGPGIEAFARLNVYCPPNTADYLAEGAPA
>tr|A0A0Q0CAJ1|A0A0Q0CAJ1_PSEAP Uncharacterized protein OS=Pseudomonas syringae pv. aptata OX=83167 GN=ALO85_02193 PE=4 SV=1
MDCRSGCGACCIAPSITSPIPGMPDGKPAGERCLHLSVEFLCALFGRAERPAVCSQFKAA
DDVCGADQADAIRLIGWWEKATAVA
>tr|A0A0Q0DLN0|A0A0Q0DLN0_PSEAP Uncharacterized protein OS=Pseudomonas syringae pv. aptata OX=83167 GN=ALO85_00319 PE=4 SV=1
MRQPDIEIYLKDEDVDHKAIAQWLGDALGSCSDWKQKGQTWKCTAGTVAVTWLPRAVGKW
NSLHLDSDQTPWEDDIACARAAFKALNVEVRCAPGTWVEEESDETADRWIRVSADGEEEI
TWRTA
>tr|A0A0N8T9T0|A0A0N8T9T0_PSEAP Cointegrate resolution protein T OS=Pseudomonas syringae pv. aptata OX=83167 GN=ALO85_03666 PE=4 SV=1
MARGGVNKALVQKARASLLSRGENPSIDAVRIEMGNTGSKTTIHRYLKELESTQAPAPDI
NEELSDLVAHLAQRLQEQAQERIDQAHDQYQAERDILQRRLAATQEQISQLEKHAQQRAE
TLEQQAAALRHTQGTLQDVQTEHARLLQANQDLEARLHDKDGQIHSLEEKHQHAREALEH
YRNSIREQRVQEQRRHESQVQQLQMELRQAQQGMAMHQENITQLNRDNERLLAENRTTLR
ELHSQTDQLKQANTQNVAFAEQQQHAHTQCALLEERLRSAQDENAALKQGMTDAQQQSRM
LELLLTKKEAALENLQIRLDQPDKPKAAKKKPSDQT
>tr|A0A0Q0CEX9|A0A0Q0CEX9_PSEAP Mig-14 OS=Pseudomonas syringae pv. aptata OX=83167 GN=ALO85_02886 PE=4 SV=1
MLNRFKGWRERGWVQIDAAAYEQAWQRFGGSVATHPLVVARLSAFSGIAVRYLAWEQGGE
VKAAIATWGRSLALSKDELKRHGKKGLFDLGNAELILPVADDIQVPVRHRARYMSALNEG
RISTFKPQTESLAMARTPEELSKKFRYNQRRELRLLEEAGGTVRPVSEFSSAELASMYCD
LFQRRWGFEATGARHKAEVIELLRELLIGSVVFLNDAPIAIQLVYRVEAPQWVSIEYVNG
GVDPQTRDFSPGSVLSFVNTQSAWENARAQGKSLRFSFGRADREYKDRWCNPVPVFQI
>tr|A0A0Q0C2R6|A0A0Q0C2R6_PSEAP Orf10 OS=Pseudomonas syringae pv. aptata OX=83167 GN=ALO85_200211 PE=4 SV=1
MKIELPDIPEQQRLIFEMATREAIKQLEANLHAPSIPGPKDLDEALFPRTHLLRKHEGWE
APHAEIVRSYFRHFQDHFDAYATDKKLAGLLRIASDRRIRKFKEGSQDVPYEIWRNFLIL
TGRVPQDIVPILAFMG
>tr|A0A0Q0DS71|A0A0Q0DS71_PSEAP Uncharacterized protein OS=Pseudomonas syringae pv. aptata OX=83167 GN=ALO85_101657 PE=4 SV=1
MAIPAPKVRAMMVSRTKPKTRETMVIELTAASDLSRFIERVVRLGKGWAIKPAIYAILRA
AKTPEAKAASLQVTGRK
>tr|A0A0Q0CYJ3|A0A0Q0CYJ3_PSEAP ABC transporter periplasmic substrate-binding protein OS=Pseudomonas syringae pv. aptata OX=83167 GN=ALO85_01665 PE=4 SV=1
MNNQGENHMNTAFQRPALTVLAATLGLFGALLSGGAQAEGKISIAQQFGIGYLILDVVRD
QKLIEKHGKEQGLDIEVDWNSISGATAMNEALLAGALDVVSAGVPPMLTVWDRTKGKQNV
KAIASLGSMPNYLLTNNPNVKTLKDFTDKDRIAVPAAGVGFQSRTLQIETAKLFGNDNYK
KFDNISVSLAHPDATSALIAGGSEINSHFSSPPFQYQELENPNVHKVLSSYDVLGGQATF
NVLYTTEKFHDKNPKTYKAFYDALAEAEKIIKADKPAAAQTYIRVEQSKLPLPLVEKIVA
DPEIDFTVTPQRTFIYAEKLHELGVLKNKAASWKDFFFEEAHGTEGS
>tr|A0A0Q0IAJ5|A0A0Q0IAJ5_PSEAP Uncharacterized protein OS=Pseudomonas syringae pv. aptata OX=83167 GN=ALO85_04149 PE=4 SV=1
MDKPEIIKCECRCSQEFRQKLVELGYLSGFIKKQKIEDPNNKDFLIDVSEFDTPVRTAFL
SRTKGVSEMLMSIVKNNALIISGADKSDMRDIERKFNKTNSNISQLARLTEKQSFNLKGK
SYDLEKLFHEFIREKTALGEQVNRRLSIKTYPAVTSGKIFDAKMDLASHRDKEGNYDDRF
YFAWDKQTKDALRPAGSELKPMIIQLMNEKPIQKEGAPVNNPLILEALEIYQRLNSDLEH
IHTLKLEGKNYQIELYKSLYSRKNECNALHKRLLEENINALRKT
>tr|A0A0Q0DE29|A0A0Q0DE29_PSEAP Glutamine amidotransferase type-1 domain-containing protein OS=Pseudomonas syringae pv. aptata OX=83167 GN=ALO85_01223 PE=4 SV=1
MSLRICILETDNLRPELVEQYHGYGRMFETLFARQPIAAELTVYNVVQGDYPPADEQFDA

YLVTGSKADSFGTNPWIQTLKVYLLELYQRGEKLLGICFGHQLLALLLGGRTERAVQGWG
VGIHHYQLAPARPWMTPAMDTLTLLISHQDQVTALPENATVLASSEFCPFAAYHINDQVL
CFQGHPEFIHDFSRTLLDLRQEALGEQIYQQGVASLDHDHHGATVAEWMMRFVAHKPEKQ
SV
>tr|A0A0Q0BZK8|A0A0Q0BZK8_PSEAP Uncharacterized protein OS=Pseudomonas syringae pv. aptata OX=83167 GN=ALO85_02752 PE=4 SV=1
MLQPMLPLSVVPVTSQLDPAKRMPDIPPVAPVQQSSSETSIDLRNEDTERSALLLREEQQ
RQQQQQQAHGGGEAEEYVGLVVPGDSLNADNTVPVVPLIDDEPRQGLWVDIQI
>tr|A0A0Q0BY20|A0A0Q0BY20_PSEAP Uncharacterized protein OS=Pseudomonas syringae pv. aptata OX=83167 GN=ALO85_03547 PE=4 SV=1
MNTQTQHAIHTLTNAFAPMNCLIMAARKGCFSFTIVNEHGIARHSERLYPDQYASAEPLQ
AVIERTRQALIA
>tr|A0A0N8T9D8|A0A0N8T9D8_PSEAP Outer membrane porin OS=Pseudomonas syringae pv. aptata OX=83167 GN=ALO85_00251 PE=4 SV=1
MRVMKWSAIALAVTAASAQMATAAPFVSDQADAKGFVEGSTLDLKLRNYYFNRDNKNGNS
DLRDWTQGLWANFSTGYTQGTVGVGAEAFGYFGMQLWAPDAYSGSTNLAFNRNTGENENS
LGKLGGAVKFRISKTELKIGDMQPTSPVFAVGGSRLLPQTASGLSLQSSEISGLDLEAGH
FYSGTSQNDTSHDGGIFANYAGVEANSADFAGGKYAITDSLGVAFYAAKLEDIWDQYYGN
VNYALPLGGDQSLAFDANIYRTVDEGSAKAGSISNTAFSGSAAYSFLAAHTVTLAFQKIN
GDTPFDYIGIGDNNRGGDSIFLNNSIQYSDFNGPGEKSWQVRYDLNMTPYGVPGLSFMTR
YIRGTDIDGTNISANSAYRKNDGSPLYGADGKHHETNLEAKYVVQTGPAKDLSFRMRQAW
HRANADQGEGDVNEFRLIVDYPISIL
>tr|A0A0Q0FSF7|A0A0Q0FSF7_PSEAP Inducible histidine transporter OS=Pseudomonas syringae pv. aptata OX=83167 GN=ALO85_02099 PE=4 SV=1
MTSQDGLKRGLSARHIRFMALGSAIGTGLFYGSASAIQQAGPAVLLAYLIGGAAVYMVMR
ALGEMAVHDPVSGSFSHYATRYMGPLAGFVLGWTYAFEMIIVCLADVTAFGIYMGFWFPE
VPRWIWVLGIVFLIGALNLCNVKVFGETEFWLSILKVSAIVAMIVAGFGIMIFGIGSSAS
GSEIGISNLWAHGGFMPNGVTGLIASFAVVMFAFGGIEIIGITAGEAKDPQRSLPQAINA
VPLRILLFYVLTLFVLMCIYPWPQIGTQGSPFVQIFDNLGIASAATILNIVVISAAVSAI
NSDIFGAGRMMYGLAREGQAPASFARVSRHGVPWMTVLVMGIALLGGVLLNYLIPKDVFL
LIASLATFATVWVWLMILLTQVAMRRSMNREEAAQLKFAVPFWPYGPAAAIAFMLFIFGV
LGYFPDNRAALIVGAIWIVLLLIAYAVWVKPKALNNTH
>tr|A0A0Q0IBD3|A0A0Q0IBD3_PSEAP Chaperone protein DnaJ OS=Pseudomonas syringae pv. aptata OX=83167 GN=dnaJ PE=3 SV=1
MAKRDYYEVLGVERGSSEADLKKAYRRLAMKHHPDRNPDDKASEELFKEANEAYEVLSDA
SKRAAYDQYGHAGVDPSMGGGGGFGGGAGGANFSDIFGDVFSDFFGGGRAGGGGGRGGAQ
RGSDLRYTLELNLEEAVRGTNVNIRVPTLVNCKPCDGSGAKKGSSPVTCPTCGGIGQVRM
QQGFFSVQQTCPRCHGHGKIISDPCDSCHGEGRVEESKTLSVKVPPGVDTGDRIRLSGEG
EAGTQGGPTGDLYVVINVREHAIFQRDGKHLFCEVPISFTDAALGGELEVPTLDGRVKLK
IPEGTQTGKQFRLRGKGVAPVRGGGAGDLMCRVAVETPVNLSKRQRELLEEFRTSLENDE
SHSPKASGWFEGVKRFFGDL
>tr|A0A0Q0DNW7|A0A0Q0DNW7_PSEAP Homoserine/lysine/threonine efflux protein, LysE/YggA family OS=Pseudomonas syringae pv. aptata OX=83167 GN=ALO85_100825 PE=4 SV=1
MNWHTWWVFAVTVFLLSGTPGPNMLHVLSRAVSLGFARSIPAMIGCLVAVVAVLSISAAG
LGALLAASPRAFEALRYLGVAYLFWLGYKAWRGSAQSPQDAPVSSPAVSSWRVFINGLIV
GFGNPKLLLFAAAFLPQFIVPSQPQMTQLIILVMTFAACEMFWYMAYALGGTQLRRYLTR
PAMRRAFDRFTGVLFAGFGVLLLRFRPQ
>tr|A0A0Q0IBG5|A0A0Q0IBG5_PSEAP ABC-type nickel/dipeptide/oligopeptide-type transport system OS=Pseudomonas syringae pv. aptata OX=83167 GN=ALO85_00074 PE=3 SV=1
MKLSPLNRRRFERFKANRRGWWSLWLFLILFGLSLGAELIANDKPLAVRYDGSWYFPVFE
RYPETTFGGEFPLEANYKSPYIKELLAAKDGWTLWAPIPFSYQSINYDLKVPAPAPPSRE
NLLGTDDQGRDVLARVIYGFRISVLFALTLTILSSIIGVIAGALQGFYGGWVDLLGQRFL
EVWSGLPVLYLLIIILASFVQPNFWWLLGIMLLFSWMSLVDVVRAEFLRGRNLEYVRAARA
LGMENGAIMFRHILPNAMVSTMTFLPFILTGAIGTLTALDFLGFGLPAGSPSLGELVAQG
KSNLQAPWLGISAFAVLAIMLSLLVFIGESARDAFDPRK
>tr|A0A0N8T847|A0A0N8T847_PSEAP Protease PfpI OS=Pseudomonas syringae pv. aptata OX=83167 GN=ALO85_100145 PE=4 SV=1
MRHPSSLTLEDPMSKALTGKRIAILVTDGFEQVELTGPKEALEQAGATVEILSAEEGKVK

GWNHDKPADDFKVDRTFKAANASDYHGVVLPGGVQNSDTIRVDTDAQQIVKDIDASGKPL
AVICHGGWLLISAGLVKGKTLTSFNSLKDDLVNAGAKWVDQEVVTDGKLISSRQPDDIPA
FNSKLIEALSA
>tr|A0A0Q0C2D6|A0A0Q0C2D6_PSEAP N-(5'-phosphoribosyl)anthranilate isomerase OS=Pseudomonas syringae pv. aptata OX=83167 GN=trpF PE=3 SV=1
MSAVRSKICGITRIEDALAAAEAGADAIGLVFYPKSPRAVTVLQARAIIAALPPFITTVG
LFVNASRCELNETLDAVALDMLQFHGDETPEECDGYHRPYIKALRVKAGDDIAGVCRTYR
NARGVLLDTYVEGVPGGTGETFDWALIPDDLDKPVILAGGLTSANVAQAIAQVRPYAVDV
SGGVEKSKGIKDREKILAFMSAVHGT
>tr|A0A0Q0C7T2|A0A0Q0C7T2_PSEAP Putative signal transduction protein OS=Pseudomonas syringae pv. aptata OX=83167 GN=ALO85_01991 PE=4 SV=1
MSKMADEVQRNLIKAIDRDALFLPTLPEVALRIRLAAEDTEISISALSKVIGSDTALSAR
LIKVANSPLLRPNFEVSDVNTAIRRLGVNYTCNLAIGLVVEQMFHAKSPVIEQKMRDIWK
QSLQVAGISYTLCQRYTNLKPDQATLAGLIHLIGILPILTYAEDHYELLSDPISLNHVID
SIHPVIGERLLRSWDFPEPLACVPGQFQNFARVSKSPDYTDLVQIATVHIHRNTDHPFGL
IEMVDLPAYSQLGLARAEGVLSAQMDEAKSMLY
>tr|A0A0Q0DJK8|A0A0Q0DJK8_PSEAP Ferric siderophore uptake system, MotA/TolQ/ExbB family protein OS=Pseudomonas syringae pv. aptata OX=83167 GN=ALO85_03927 PE=3 SV=1
MNASLSSMIVPAVLWALILFSVLSWALLLIKSAQYVRQKSQNKQFTKVFWSAPDLLTAAE
HSAQYPGALARIANSGFEAMAVDESPRTTQQLAHTINRSDRLERNLRQQIQKERRALESG
QAILASIGSTAPFIGLFGTVWGIMEALQSIGVTGSASLEAVAGPIGHALVATGVGIAVAV
PAVLIYNFFLRRLKLAVADMDDFAHDFDALAQRSAFAVTRQPIASKNGHAVREAS
>tr|A0A0N8T9S8|A0A0N8T9S8_PSEAP Nickel/dipeptide/oligopeptide ABC-type transport system OS=Pseudomonas syringae pv. aptata OX=83167 GN=ALO85_03724 PE=3 SV=1
MLGYILRRLLLIIPTLLCILLVNFFIVQAAPGGPVEQAIARLQGIGGATVGAGASESAGG
HSKASRGLDPALIEQIERQYGFDKPMHERLWLMLKNYARLDFGSSFFRGATVTDLILQKM
PVSISLGLWATLLTYLVSIPLGIRKAVKHGSQFDIWSSTAIIIGYAMPAFLFAMLLVVVF
CGGTSLNWFPVRGLVSDNFEQLTTIGKIADYFWHLALPVSALVIGGFATLTLLTKHSFLN
EITRQYVTTARAKGMSERRVLYGHVFRNAMLLVVSGIPQAFISVFFAGSLLIEVIFSLDG
LGRMSYEAAVSRDYPVVFGSLFIFTVFGLLIKLIGDICYTLVDPRIDFSARNA
>tr|A0A0Q0BTN2|A0A0Q0BTN2_PSEAP Uncharacterized protein OS=Pseudomonas syringae pv. aptata OX=83167 GN=ALO85_04153 PE=4 SV=1
MSKLERLAEEAAIKQKRLDDEAAKTRNDNTPIEIEIRLREAKAELENLKPYLKTLARKPY
WDEWQKSLRKKNSA
>tr|A0A0N8T8U4|A0A0N8T8U4_PSEAP Fimbrial protein OS=Pseudomonas syringae pv. aptata OX=83167 GN=ALO85_02225 PE=4 SV=1
MNTALNALLPSLILLGASYSAAAYSDVQDDTPSNDCYWVSSPGIKDYRRDIGTLYVPKDA
AVGTFIGSIDMLHATTDQAGLEAACKNDGTALLEFNATATAPIFSGTVEPINGEDLTGKI
LQTNIPGVGVRVRLEYPYDRNCPNCFTPVGGSASVPFIGINDHQKMLTSLRFLHLNNYLT
LVKTGPIPPGPNVLNGSELFSGSVTTLGKIMKFGLTGTILQAQCSVGADPVSADPVQLGE
WDSTDFTGPGFTTPAVPFSIALSNCETYTGAGFVATAHIQLDGVEGSVPEGPIGSGVFSL
TSDSDAKGMGIQVLKGDGVTPMELQTEVPLIAITPGNVVLNFYARFYQTQASSAIRPGKA
KGALSFTMTYK
>tr|A0A0Q0DZ74|A0A0Q0DZ74_PSEAP Two component response regulator reciever-domain protein OS=Pseudomonas syringae pv. aptata OX=83167 GN=ALO85_03408 PE=4 SV=1
MSSESDDQTSLPKVLLVEDETMLAMLMEMLLEDLGFATAYHASTLNEGIEYARNGEYDLA
ILDINIIGGNSFPIAAAIADRGIPFMFCSGYGRLGIPDTWLDRRCVAKPFSAEQLSEALS
ELLQV
>tr|A0A0Q0FQW1|A0A0Q0FQW1_PSEAP Adenosylhomocysteinase OS=Pseudomonas syringae pv. aptata OX=83167 GN=ahcY PE=3 SV=1
MIPFPPPVRGALQQVFPVRLGWGVVCQDDPRPANLKRTTAPIRIFMEWRLSMSAVMTPAE
FNDYKVADISLAAWGRRETIIAESEMPALMGLRRKYAGDQPLKGAKILGCIHMTIQTAVL
IETLVALGAEVRWSSCNIFSTQDQAAAAIAAAGIPVFAWKGETEEEYEWCIEQTILKDGQ
PWDANMILDDGGDLTEIIHKKYPAMLDKIHGVTEETTTGVHRLLDMLAKGELKIPAINVN
DSVTKSKNDNKYGCRHSLNDAIKRGTDHLLSGKQALVIGYGDVGKGSAQSLRQEGMIVKV
TEVDPICAMQACMDGFELVSPFIDGENDGTEASIDKALLGKIDLIVTTTGNVNVCDSNML
KALKKRAVVCNIGHFDNEIDTAFMRKNWAWEEVKPQVHKIHRTGPGSFDAQNDDYLILLA

EGRLVNLGNATGHPSRIMDGSFANQVLAQIFLFEQKYADLAPAKKAERLTVEVLPKKLDE
EVALEMVRGFGGVVTKLTKTQADYIGVTVEGPFKPDAYRY
>tr|A0A0Q0BVB0|A0A0Q0BVB0_PSEAP Regulatory protein, LysR:LysR, substrate-binding protein OS=Pseudomonas syringae pv. aptata OX=83167 GN=ALO85_00654 PE=3 SV=1
MDLRDLTYFETIAELGHLGRAAEKLNRSQPALTKSIQRLEESFGTRLFQRDGRRIKLTPV
GELLQARGKELQQSIAQTQREIRDFASGMVGNIRVGCAATMAEYLMPKLTSALLQRTPDV
TLKLVIGQDDLLRESLRSGQLDMIICALAPDSEDVTSFAILQDEAVVVASKNHPIFKPAL
KMSDLCAYRWVLPPVSVSSRKWLDATFAAHGLPLPTVQIEANSISLLPGLISRSNLLSFI
ARESLEFGKTMQHLREVPLEQTTLKRTIGVTLRKGGYLSPAAEGMLQMLRDNGGDFLGWA
>tr|A0A0Q0FEW4|A0A0Q0FEW4_PSEAP 50S ribosomal protein L3 glutamine methyltransferase OS=Pseudomonas syringae pv. aptata OX=83167 GN=prmB PE=3 SV=1
MSMITSRLRTVRDHIRWAVSRFHEEDVFFGHGTDNAWDEARQLVLGALHLPWEIADSYLD
CRLEVDEISDLQRLIKRRIDERVPTPYLLGEAWFCGLSFIVDDRVLIPRSPIAELIEERF
APWLADEPARILDLCTGSGCIGIACAEVFPDAEVALADLSYDALEVANQNIERHGMEDRV
YTVQGDGFDGLPGQRFDLIVSNPPYVDAEDFADMPDEYQHEPELALACGSDGLNLVRRML
AQAADHLNEKGLLIVEVGNSQVHVQALYPEVDFAWLDFQRGGHGVFMLTAEQCRDHQALF
QSKV
>tr|A0A0Q0D349|A0A0Q0D349_PSEAP Baseplate J-like protein OS=Pseudomonas syringae pv. aptata OX=83167 GN=ALO85_00432 PE=4 SV=1
MPFETPTLPALINRTQVDLADEALRQSDARVLSRAHSGAAYGLYGYQDWIADQILPDTAD
EETLERQAILRLRQPRKVAQAATGTVRFTAAAGAVLDADTVLQFSDGRFYRVTKGVTTVA
GNNTTTVEAVDAGVLGNADAGLVMTAVQPVEGIDSTFTVIADGLSGGISQESIESLRARV
VRSYRVIPHGGNQDDYVTWALEVPGVTRAWCVRRFMGPGTVAVFFMRDDQADPIPDAEQL
AAVAAYIEPLRPVTADVYVLAPVQKPVVYTIRLTPDTSAVRAAVEAQLLDLHNREGGLGE
TLLLTHIAEAISRATGETDHVLVSPVANVTAAANQLLTFGGIQWSS
>tr|A0A0Q0DIL7|A0A0Q0DIL7_PSEAP Integrase family protein OS=Pseudomonas syringae pv. aptata OX=83167 GN=ALO85_02292 PE=4 SV=1
MSRLAIPLSDLQCRTAKTRERAYKLFGGEGMYLFVKPSGVKTWRLKYTKPSGKEGTLIIG
KIGQRAVVDSNPGSQNLKATLDRGGSPKVRLASGGSHGHSDVMDAYFYQCAHCGNSSPFI
AALQQPIRLAISCANLRPSLSSGRRRAFLPLLQPRSYKALRHSPS
>tr|A0A0Q0BX35|A0A0Q0BX35_PSEAP Homoserine kinase OS=Pseudomonas syringae pv. aptata OX=83167 GN=thrB PE=3 SV=1
MSVFTPLARPELETFLAPYGLGRLLDYQGIAAGSENTNYFISLEQGEFVLTLVERGPVQE
MPFFIELLDVLHEADLPVPYALRTTDGQALRELADKPALLQLRLPGKHISEPNTQHCVQI
GELLANLHLATRGQIVERKTDRGLDWMLNEGRNFLSHLGETQRALLEKSLQEIEAFKPQI
MALPRANLHADLFRDNVLFEGTHLTGLIDFYNACSGPMLYDLAIALNDWCSRENGQLDAV
RARALLGAYAGLRPFTAAESKLWAIMLRIACVRFWLSRLIAAETFAGQDVLIHDPAEFER
RLAERQEAHIALPFAL
>tr|A0A0Q0BV06|A0A0Q0BV06_PSEAP Sulfate adenylyltransferase subunit 2 OS=Pseudomonas syringae pv. aptata OX=83167 GN=cysD PE=3 SV=1
MVDKLTHLKQLEAESIHIIREVAAEFDNPVMLYSIGKDSAVMLHLARKAFFPGKLPFPVM
HVDTRWKFQEMYRFRDQMVEEMGLDLITHINPDGVAQGINPFTHGSAKHTDIMKTEGLKQ
ALDKHGFDAAFGGARRDEEKSRAKERVYSFRDSKHRWDPKNQRPELWNVYNGNVNKGESI
RVFPLSNWTELDIWQYIYLEGIPIVPLYFAAERDVIEKNGTLIMIDDERILEHLTDEEKS
RIVKKKVRFRTLGCYPLTGAVESEATSLTDIIQEMLLTRTSERQGRVIDHDGAGSMEEKK
RQGYF
>tr|A0A0Q0DGS7|A0A0Q0DGS7_PSEAP Asparagine synthetase OS=Pseudomonas syringae pv. aptata OX=83167 GN=ALO85_03228 PE=4 SV=1
MCGLAGELRFDHQPADLAAVERITHELAPRGPDAWGFHSQGPVALGHRRLKIMDLSDGSA
QPMIDSNLGLSLAFNGAIYNFPELRAELESLGYQFHSGGDTEVLLKGYHAWGADMLPKLN
GMFAFAIWERESKQLFIARDRLGVKPLYLSKTDKRLRFASSLPALLKGGDISGMLDPVAL
NHYLNFHAVVPAPRTLLAGVEKLPPASWMRIDASGKVEQKVWWTLPYGPHEDEKNLTLED
WRDRVLDSTREAVAIRQRAAVDVGVLLSGGVDSSMLVGLLREVGVEDLSTFSIGFQDAGG
ERGDEFQYSDLIAKHYGTRHHQLRIQEREIIEQLPAAFRAMSEPMVSHDCIAFYLLSREV
SKHCKVVQSGQGADELFAGYHWYPQVDGASDPVAAYRDAFVDRSYAEYAETVQPKWRTAN
DAAGDFVRDHFAQPGASASVDKALRLDSTVMLVDDPVKRVDNMTMAWGLEARTPFLDYRL
VELSARIPGKFKLPDGGKQVLKEAARMVIPSEVIDRKKGYFPVPGLKHLQGDTLNWVREL
LTDSSQDRGLFNPAMVDKLLTNPEGQLTPLRGSKLWQLAALNLWLSEQGL

>tr|A0A0Q0FQI1|A0A0Q0FQI1_PSEAP 30S ribosomal protein S13 OS=Pseudomonas syringae pv. aptata OX=83167 GN=rpsM PE=3 SV=1
MARIAGVNIPDNKHTVISLTYIYGVGRTTAQKICATTGVNPAVKIKDLSDEQIEQLRGEV
AKFTTEGDLRREINMKIKRLMDLGCYRGLRHRRGLPVRGQRTKTNARTRKGPRKPIRK
>tr|A0A0Q0C7R5|A0A0Q0C7R5_PSEAP Uncharacterized protein OS=Pseudomonas syringae pv. aptata OX=83167 GN=ALO85_02011 PE=4 SV=1
MLHKTSLLLGSLLVGMAGATPTSALGDNGRLIATGGATSIEGSAGGGITPWAVITGYSEQ
GEWGATAFATHVNLPDYNLDVAGLAFAYGNRVELSYAHQRFDISNLQHRLSLPDDNLSQD
VFGVKVRLFGDLIYESLPQVSLGVQYKHQNDFLIPSLVGARRDSDVEGYLTASRLFMGAA
FGYNVVVNGGVRYSRANETGLLGFGGDRRDSRSLLKEGSVAVLFNPRWALGVEYREKPDN
LSFAGESDWADVFLGYFPNKHVSVVLAYARLGEIATLDNQNGTYLSVQGSF
>tr|A0A0N8T895|A0A0N8T895_PSEAP Uncharacterized protein OS=Pseudomonas syringae pv. aptata OX=83167 GN=ALO85_03111 PE=4 SV=1
MPKGQYIDSIIRALMPIERNIARIAKLDDQLTQFRQLWKWATYFWAFFQSSELSLNRLGS
SPSYLRTFLDKKLTTELQPLCSTFGDNYSWHLGSSASASVPQVLSQARVSWPVRCRPVS
>tr|A0A0Q0FLP5|A0A0Q0FLP5_PSEAP Uncharacterized protein OS=Pseudomonas syringae pv. aptata OX=83167 GN=ALO85_02511 PE=4 SV=1
MKHNVILIVLDGLNYEVARHAMGHLQAWHAAGRAALYKMECELPALSRPLYECILTGVAP
IDSGIVHNNVSRLSNQRSIFHYARDAGLSTAAAAYHWVSELYNRTPFDPARDRHTEAADL
PIQHGLFYWADHYPDSHLFADAESLRLSHAPNFLLIHPMNIDDAGHKHGLDTAQYRNSAR
SADIILADYLQRWLDTGYQVLVTADHGMNNDRSHNGLLPEEREVPLFVIGDAFSLNVDAA
PRQTDLCGTVCELLGIPHDKPVCREIFN
>tr|A0A0Q0FKX2|A0A0Q0FKX2_PSEAP Long-chain-fatty-acid--CoA ligase OS=Pseudomonas syringae pv. aptata OX=83167 GN=ALO85_01743 PE=4 SV=1
MLQTRVLAPADGAYQCPLLIKRLLLSGGRYEKTREIIYRDSVRYTYATLNERICRLANVL
TAAGVKAGDTVAVMDWDSHRYLECMFAIPMIGAVIHTVNVRLSPEQIAYTINHADDRLVL
VNSEFTALYQAMSGHLNTVEKTLLLTDLPDKSADLPNLVGEYETLLTAASPEYVFEDFDE
NSVATTFYTTGTTGNPKGVYFTHRQLVLHTMGVATIMGCIDSTRLLGTDDVYMPITPMFH
VHAWGIPYAATMLGLKQVYPGRYDPELLVELWRREKVTFSHCVPTILQMLLNAKSAQDVD
FGGWKIIIGGSSLTRSLYQAAKSRGIQLTAAYGMSETGPLISVAHINEELKAGSEDERIT
YRIKAGVPGMLVEAAIIDQQGNSLPADGETQGELVLRAPWLTGSYFREPEKGAELWAGGW
LHTGDVATLDGMGFIDIRDRIKDVIKTGGEWVSSLELEDLCSRHPAVREVAVVGIADPQW
GERPFALLVIRDGHQLDAKGLKEHLKPFVEQGHINKWAIPSQIALVTEIPKTSVGKLDKK
RMRRDIVEWQSSNSAFLSTL
>tr|A0A0Q0DCL8|A0A0Q0DCL8_PSEAP Uncharacterized protein OS=Pseudomonas syringae pv. aptata OX=83167 GN=ALO85_101782 PE=4 SV=1
MEKMHCTGCGPAERFARQDADKKGMKTCSDPSKKRGAPLELNDLAAR
>tr|A0A0Q0C3W7|A0A0Q0C3W7_PSEAP Uncharacterized protein OS=Pseudomonas syringae pv. aptata OX=83167 GN=ALO85_101093 PE=4 SV=1
MSQSEKYAPLPSLGEAGGLSASPCAESSAAAGCARTLVQGSAAAAANSSGIRNLEGVAGR
VNGGCGIEAPFRGRVLLIASVCTAHAKLFQDVECLGYQAQKMTDRAGKRPQAVRGVTASA
PRCSVLPQRPGRAAASMTTARPIIRLMPTRRRILPNAAAPSAHCSASTARRSSGSCNDCI
RKNSATMTTYRPIMICT
>tr|A0A0Q0BWF4|A0A0Q0BWF4_PSEAP AraC family transcriptional regulator OS=Pseudomonas syringae pv. aptata OX=83167 GN=ALO85_03079 PE=4 SV=1
MPSNGQINQRQPAGERQIPALSGLPRPLYARAESLSAGSWTPPHRHDWVQFSYAISGVLG
VHTAEGSFFAPPQWGVWVPAGLEHEVVTSMRAEMRSLYIRTADSGWAPQRCRVLEVTPLA
RELIKSFCLLPVDYPEQDSHEARLVQVLLDQLAQLPEVGFSLPLPRHPRLLGLCNELIEE
PGRLITLHDWAARLGTSEKTLMRMFQRETGLSFRGWRQRARLLSSLSALEEGASVTHTAL
ACGYDSTSAFIAAFKGLFGHTPGDLFK
>tr|A0A0Q0CEZ4|A0A0Q0CEZ4_PSEAP CheW-like protein OS=Pseudomonas syringae pv. aptata OX=83167 GN=ALO85_02925 PE=4 SV=1
MAEPRTAFELLLDMDRRCRSLAAGLPLQEAHQQDWVGIGFRMGEQRFVAPIGEIAEILHE
PHHAVMPGVKFWVAGIANLRGRLLPLMDLCGFFGHELAPVRKQRRVLVVEHHDVFAGLLV
DEVFGMQRFSQLSLIPQTPQDMDQRMVPFLRGQFIREQAWQIFSPWALVQSADFMDLAS
>tr|A0A0Q0DPB9|A0A0Q0DPB9_PSEAP Extracellular solute-binding protein OS=Pseudomonas syringae pv. aptata OX=83167 GN=ALO85_03689 PE=4 SV=1
MSFKTALVLPLIAFTLMAGCDKPAEQKPVAKAAPSPSSSYLETIKARDKLIVGVFSDKPP
FGFVNEQGQYVGFDTDLGKRFAKDLLGDEKKIEFVVVEPASRIPFLQSDKDVDLILANMTV
TPERREAVDFTNPNLRVAVQALVPQASPVKTLDDLASKTTIVTTGTTADLWLTKNHPDWK

VLKFEKNSESLQALANGRGDAYAQDNLVLFSWAKKNPGYRVLEQKLGDEAPIAPAVKKGN
IELRDWVNTELARLGEEKYLLKLYDQYVRPELAEGTDPNAVIVEGGSWKP
>tr|A0A0Q0DIV7|A0A0Q0DIV7_PSEAP Uncharacterized protein OS=Pseudomonas syringae pv. aptata OX=83167 GN=ALO85_04156 PE=4 SV=1
MANTQKAPSAAKNGFDEAQTAGLYADQAKELGNAIFKKITSMSSNNALAEIHAKIATAAS
QEDDALLESLFADLKKAKNDQKNVADQYKTIRNSNSFETILQAYSEEFRELAYSVATEVI
KGTHVALKNVKGRKSSSNDAGEGKGKRGPRTSVDHTITNGKGEKTVLVSRAGRAAANLTQ
DAETFAFLGFKIEKDEEGKEVLNPSTIKLNDGNEVPATRPNIVRAITDKTGLSDFTISA
>tr|A0A0Q0CWJ3|A0A0Q0CWJ3_PSEAP Uncharacterized protein OS=Pseudomonas syringae pv. aptata OX=83167 GN=ALO85_100602 PE=4 SV=1
MTGDTACLEGYGRLTELKLFDYRVHGFLPFFVALGELVSCEFLGSVGNRPLPQPR
>tr|A0A0Q0DYS9|A0A0Q0DYS9_PSEAP D-alanine--D-alanine ligase OS=Pseudomonas syringae pv. aptata OX=83167 GN=ddl PE=3 SV=1
MKKSVAIVFGGQSSEHEVSLQSARNVINAIDRERYALTLIGVDKLGRWLHFDEADYLLNA
TDPARIKLSASGKPLSLLPGSVGGQFVEVESGQPLAAIDVVFPLIHGAFGEDGALQGLLR
MLAVPFVGADVLSSAVCMDKDVTKRLLRDAGITVAPFRVLSRGESLSFTEAADQLGLPMF
IKPANQGSSVGVSKVSDEAGFAAALALGFEFDHKLLIEQGIVGREVECAVLGNVDVQVSV
CGEVIANDEFYAYDTKYLNGDQARIAIPAELPADLSDQVREVALQAYKVLGCAGLSRVDF
FVTEGRDIIINEVNTLPGFTSISMYPKLWQASGLSYAELIHRLIDLALERADQARLLKTE
IFA
>tr|A0A0Q0IJP2|A0A0Q0IJP2_PSEAP Bifunctional phosphoserine phosphatase/homoserine phosphotransferase OS=Pseudomonas syringae pv. aptata OX=83167 GN=ALO85_02473 PE=4 SV=1
MEIACLDLEGVLVPEIWIAFAEKTGIESLRATTRDIPDYDVLMKQRLRILDEHGLKLADI
QEVIGTLKPLEGAVEFVNWLRERFQVVILSDTFYEFSQPLMRQLGFPTLLCHRLITDETD
RVVSYQLRQKDPKRQSVLAFKSLYYRIIAAGDSYNDTTMLGEADAGILFHAPDNVIREFP
QFPAVHTFDDLKKEFIKASNRDLML
>tr|A0A0Q0CVM9|A0A0Q0CVM9_PSEAP Anaerobic nitric oxide reductase transcription regulator OS=Pseudomonas syringae pv. aptata OX=83167 GN=ALO85_04089 PE=4 SV=1
MKKDVISTHQLKALHALLPLLGDLAVELPAEHRYYRVLDALRELVPSDAVALLRLEQGEL
IPVAAHGLASDAMARRYRVASNPRLEAIIEAQGAVLFPPNCDLPDPYDGLIGSSALPVHD
CMGCSLRINDKTWGVLTLDALTVGQFSEQDLQTVETFAMFAAATVVAASRFDDLLRTVAG
ERKRAEAYRLSARRPKPEMIGNSDSFRQLLSEIDLVASSDLTVLITGETGVGKELVAQRL
HDLSARADKPFITVNCAALPEHLIESELFGHVKGAFSGAIENRLGKFEVASTGTLFLDEI
GELPLAAQATLLRVLQGGQLQRVGSDKLHVVDIRLIAATNRDLIEEVRAGRFRADLYHRL
SVYPLRVPSLKERRDDIMLLAGAFAEENRWRVGVPSLRFSDSARQALVGYSWPGNIRELE
HLMARAALKAKKRAASLKRQSVLSLELQDLDLLESPSERVAEKSGSILAQQLPSHDFKSS
VDGYKKAILEEALRLSEGNVSAAARGLNLDRANLTRLAGRLGVSILR
>tr|A0A0N8T8T5|A0A0N8T8T5_PSEAP Fimbrial bioproteinsis outer membrane usher protein OS=Pseudomonas syringae pv. aptata OX=83167 GN=ALO85_05064 PE=3 SV=1
MTETALARFIPRTLNMTLLPDSGFIPVRLRFMQVLIVCGSVTVPLELTKAAPPVNFQPGF
LRQGQDYDSEAAASVLNHLSVVESLGPGDHWVEIHVNMRYFGQRQIRFDADPQGNGLLPC
LSRELLEQIGVRLDSLADPALLQVACVALGQLIPDARVVLDGGRLQLSISIPQIAMRRDA
NGRVEPALWDYGINAAFINYQTSAQQTTHRETGTSSSADLYLNTGINLGSWRLRSNQSVR
QDAQGHREWTRAYAYAQRDLPGTHANLTLGETYTGGDVFRSMPIKGGLIKTDQEMLPDSL
QGYAPVIRGVAQSRAKLEVLQNGYPIYSTYVSAGPYEIDDLNTAGSGELEIVLTEADGQV
RRFTQPYSTMSNLLREGVWKYSAALGRFNGAYATEHPWLWQGTLAVGTGWNSTLYGGLMT
SDFYHAAALGVSRDMGVLGAMAFDVTRSRASIDQPGQSSVQGMSYAIKYGKAFTTHTNLR
FAGYRYSTEGYRDFDEAVSQRSDDDTFTGSRRSRLEASVHQRIGARSSVGLTLSQQDYWG
SDIEQRQFQFNFNTHRAGITYNFYASQSLSVASNRGNDRQFGLSISMPLDTGHSSNATFD
LQNSANRHSQRGSLSGSLYENRVNYHASLSNDDGKQQSASLAAGYQAPFASLGAGVTQGN
DYRSTSVNASGALLLHADGIEFGPNLGDTIALVEVPDTPGVGIQNATGVRTNSRGYALMP
YLRPYRYNPIALQTDQLGPEVEIDNASTQVVPARGAVIKTTFAARTVTRLVINATTQSGK
PLPFGAQVSDAQGNILGIAGQGGQVLLSTGMQAQTLDVHWGEKSDPQCRLYIDPAGMPLT
KGYRMQDMTCAQ
>tr|A0A0N8T9U2|A0A0N8T9U2_PSEAP NADH:flavin oxidoreductase/NADH oxidase OS=Pseudomonas syringae pv. aptata OX=83167 GN=ALO85_03694 PE=4 SV=1
MEYLMNDTHNTTDLFSPVTMGSMELANRIVMAPVTRSRYGEDGVPNELHATYYAQRATAG

LIVAEATNISAQGRGYAATPGIWSDQQVAGWRAVTDAVHAAGGKIVSQLWHVGRFSSVDL
QPNGEAPVAPSAIKAEGNTYTVDGFVPVSMPRALETGEIPGIIEQYKHAAENAKRAGFDG
VEVHSANSYLLDQFLRDSTNQRTDQYGGSIENRARLTLEVTQAIVDIWGSDRVGIRLSPV
TPDAGNTPPDSNVMGLHGYLIQQLNTFNLAYLHFVEGATATSREVPEGVDMDALSARFNG
PFIGNNNYDLEMAIERRAQGKIDAVAFGRLFISNPDLVARLRHGAELTIAPRESYYGGGA
KGYIDWPMGNY
>tr|A0A0Q0D4A0|A0A0Q0D4A0_PSEAP 5'-nucleotidase OS=Pseudomonas syringae pv. aptata OX=83167 GN=ALO85_02469 PE=4 SV=1
MTEAEDHKLVLAISSRALFDLRDSHEVYMADGVEAYRKYQIEHEDEILAQGDAFPLVEKL
LSLNASLQKARVEVVLVSRNSADTGLRVFNSIQHYGLDISRAAFAGGRSPYPYLAAFGCH
LFLSTHAEDVRSALDAGFAAATILSGGARRAASAELRIAFDGDAVLFSDESERVYQSGGL
AAFQASEREWAREPLRGGPFKPFLAALNQLQREFPDASCPIRTALVTARSAPAHERVIRT
LREWDIRLDESLFLGGLQKSAFLEAFAADVFFDDQPGHCEKARDVVATGHVPHGISNEIV
PLADGG
>tr|A0A0Q0IBY1|A0A0Q0IBY1_PSEAP Ferrichrome-iron receptor OS=Pseudomonas syringae pv. aptata OX=83167 GN=ALO85_100169 PE=3 SV=1
MSRSLDTLLRPSLLAVAIALCTPMTSPLSMAAQQASLVAYDLPAGPMATTLNQISSQGGL
ALSLDPALVAGKTSSAVKGNFDAATALREALRGSGLQLVQSSAGTYTLMAMEQNALNLSD
VNINANSTPLEGSEAAGYRSENVSSVGALGGMRLQDTPYSISVTPQALLKNIQATSLDDV
IKHNPFTQMYSPTSAGYASAVNIRGFSSAGSLNIANDGLRFTNGADGSNYMEEMEQLEVI
TGLTGFLYGPASPGGLVNYVLKRPTYQRYNSVTLGNAGGENYYLHGDFGGPIDSEGKFAY
RLNVLTQDGETAVDLNKRRREMISLALDWNVSDDLLVQFDASHKKTEIRGLTSYWYFGDQ
SLRPSAASLDNDKLYSQKWSFSDYESDKAGVRAKWRLNDTFTLRAAFAAQQYTSENTYTG
PTVYSAGLYSQPLYAFAPIETEEKTGSLFLDAAFDTGFIGHKVTVGYQGNTSRQKQYEDH
IPYTPNQPGPQYTSPVGTIPLDQTPQVGKPAYSIGHGDQRLANSSESNNVLIGDVITFNE
QWSAILGVTNTQIKTYANEFVYAKAFGSAETETTYNESKTSPNISLVYKPLPWLTTYATY
IEGLQSGGVAPSGTANAGQAFAPEISEQYEIGAKATLGETLLTLALFNIEKPNGYTTASN
IYVVDGRQENNGLEFSVTGKVIPELTVVGGFTLLDPKIKKTGVAADEGNVPTNVAKQLAK
VYAEYDINPIRGLAMTGGAFYTGKQYTDQSNENDLPSFTTFDAGARYRLRLAENDLTLRV
NVSNLTNKEYWLNSSYLGDPRTVAFSAQLEF
>tr|A0A0Q0C4J0|A0A0Q0C4J0_PSEAP Superoxide dismutase OS=Pseudomonas syringae pv. aptata OX=83167 GN=ALO85_02360 PE=3 SV=1
MAFELPPLPYAKDALVPHISAETLEYHHDKHHNTYVVNLNNLVPGTEFEGKTLEEIIKTS
SGGIFNNAAQVWNHTFYWNCLAPNAGGEPTGALAEAINKAFGSFDKFKEEFSKTSIGTFG
SGWGWLVKKADGSLALASTIGAGNPLTSGDTPLLTCDVWEHAYYIDYRNLRPKYVEAFWN
LVNWDFVAKQFAA
>tr|A0A0Q0FUJ5|A0A0Q0FUJ5_PSEAP Fosmidomycin resistance protein OS=Pseudomonas syringae pv. aptata OX=83167 GN=ALO85_00622 PE=4 SV=1
MATSAPPATAKASTTPQASPLVMRIIGAVALAHLVNDLIQAILPSIYPMLKASYDLSFTQ
IGLITLTFQITASLLQPWVGYYTDRHPNPLVLPVGSICTLIGIVMMSMVGSFPLILLAAA
LIGIGSSTFHPEASRIARLASGGRYGLAQSTFQVGGNTGTAFGPLLAAAIIIPFGQGNVA
WIGLFALFSLGLLYAISRWYRAHLNLFKLKAGQAATHGLSRKRVIASLAVLAFLVFSKFF
YMTSLTSYFTFYLIEKFDLSVASSQLHLFLFLGAVAAGTFFGGPIGDRIGRKAVIWFSIL
GAAPFTLALPYADLFWTSVLSVIIGFVLASAFSAIVVYAQELVPGNVGMIAGLFFGLMFG
FGGIGAALLGYLADSHGILFVYTLCSYLPLLGILAILLPRTK
>tr|A0A0Q0D461|A0A0Q0D461_PSEAP Uncharacterized protein OS=Pseudomonas syringae pv. aptata OX=83167 GN=ALO85_02553 PE=4 SV=1
MTDSNKAGDLFAQIPKTKGLPPVHLWNPDFCGDIDMRIARDGTWYYLGTPIGRKPMVRLF
STIMRRDGDDYFLVTPVEKVGITVDDAPFVAVSLEVLGEGEGQVLRFLTNVEDQSEAGPE
HLLRVVTDPDTQEPAPYIHVRTNLEALIHRNVFYQLVELAVSRDIDGQRWLGVWSHGVFF
PIGLEP
>tr|A0A0Q0DL25|A0A0Q0DL25_PSEAP Putative membrane protein OS=Pseudomonas syringae pv. aptata OX=83167 GN=ALO85_02743 PE=4 SV=1
MFAARSVLVFALLPLFAGCQLLGKQTEEPKVSTAGMLRMQGDLTGENGQLLFKPCNEQRR
YVVKDRGNTGILQEAASLADSKGTVFADLRGSFAASKAANSDGQLDLHQLYRVERPGQAC
EDLNFKRLTLHVNGNKPAWNVNVSGKGMVLEREGVAPLALPYVEEKLPDGSFSVSSEANN
QRIEIWVAPQRCVDSVDGSVQHLTAELRINGQAQRGCGYYGGSRDD
>tr|A0A0Q0BY11|A0A0Q0BY11_PSEAP Uncharacterized protein OS=Pseudomonas syringae pv. aptata OX=83167 GN=ALO85_02504 PE=4 SV=1
MSVRISRQHWDGLLNELDLARRQRHLLTYRALVERLQLPSPAMQTLTAALEHLAAMDARA

EQPLRSSLVISQGASRLPRPGFFECAERLGRFTGPSDGVAAASWHASEVVRVFEYSYPEV
EAQ
>tr|A0A0Q0CA05|A0A0Q0CA05_PSEAP Regulatory protein, LysR:LysR, substrate-binding protein OS=Pseudomonas syringae pv. aptata OX=83167 GN=ALO85_04895 PE=3 SV=1
MFESFGDVSLDLLRAFEVAARLRSFTAAALELGTTQPAVSQQIKRLEEQLATRLFDRIYR
GIELTDAGEVLFSHVQAGLQSIDSGLIAITEHHQHEVLQVATDFAFAAYWLMPRLHRFHK
VNPDVDVSLVTSERTHSMLRADIDVAVLFGDGRFKQGQSHWLFSEEVFPVCSPQLLAGRQ
TPLPNDALRDFPLLHLRGESINNWFDWAGVFRALDIPQAPAPGQLRFDNYTLLIQAAIGG
QGIAIGWRHLVDDLLDQGLLCRPIAGAAISGQGYYVVLPQRKRRVQIVQQFVDWLASEQA
LSGVSLAGRPLPSIAV
>tr|A0A0Q0DPE9|A0A0Q0DPE9_PSEAP Metall resistance MreA-like protein OS=Pseudomonas syringae pv. aptata OX=83167 GN=ALO85_03696 PE=4 SV=1
MSEHDHPHGHTHQSHEAVIKRLKRADGHLRGIITMIEEGRECVDIAQQLHAVEKAVCQAK
RTLIQDHIDHCLEDTLGSLARGERAPLEAFKQITKYL
>tr|A0A0N8TA14|A0A0N8TA14_PSEAP DNA helicase OS=Pseudomonas syringae pv. aptata OX=83167 GN=ALO85_01984 PE=4 SV=1
MHPEHFDLSSPVYLPITFESCSRLLNAEGAEMLGSLTDPELAAVLVIQNLAQAAPKDLTS
DAIERVLAKLIAWAVDTSISINVGLSSEAQRLFDSRKLYFGLNIVKKLNLRFLLADEVSA
REYLLHDGKWDVEYKIRHHRGNNPFREQMVTPIHREHWLSTEQDKLVRTFRANLDEDLHV
QGYAGIGKSHLLGALIECLRPEKTLLIAQTPAKLETLRSRMGGARYGKTGFTFKDFALLL
LHGPKFWSAGKLSTMLSKQALSTELHIIGFRAYDGRATLNICIKVLEQYCKSKDHSLSAL
HIPAFKLPLSSLESKVLLEYSIRLWTYFDKHPEHISSIGIETLFLIKKAALAGCVVPPRF
THILIDESQDIPSSLLRIIERGTQAVVTLGDEYQQVKSAIIKRKSDVRQTDITYSIRSGR
NIERLVNPLIAQHSKKIKIPFEGARNADVDIEFYPENFLPPEGCVVMTASNWDIMKWAIL
IHDANSAFSFPAGEEQDFKKFMSTAIALFRPDFYSVEHHTDGLHPYFTDMPDWQHVRDAY
KYDDSFLWIEAELEKGFKISDMNRLCRKIGPIGRTCMLMKAEAAGGMEFDRVLLTPELMT
NVKFRTPYELDERICAVYIAISRAKKQLYIPYDVEEWITYQKQEGYRESPGY
>tr|A0A0Q0BUZ9|A0A0Q0BUZ9_PSEAP CRM domain-containing protein OS=Pseudomonas syringae pv. aptata OX=83167 GN=ALO85_00128 PE=4 SV=1
MPLTPEHKKQYKSIGHHLKPVLIVADNGLTEGVLAELERALSDHELIKIKLNILDRESRL
GAIAELCKAGSADLVQVIGKMALIYRKNPKVNKQLSNVHRAHG
>tr|A0A0Q0C7U4|A0A0Q0C7U4_PSEAP Arginine/lysine/ornithine ABC-type transport system OS=Pseudomonas syringae pv. aptata OX=83167 GN=ALO85_02069 PE=3 SV=1
MLDALMQNLGLSAFSLKGFGPLLLEGTWMTVKLAVLSLALSILLGLIGASAKLSSSALLR
VPAQIYTTLIRGVPDLVLMLLIFYSLQTWLTMLTDAMEWEYIEINPFGAGVITLGFIYGA
YFTETFRGAILSVPRGQVEAATAYGLKRGQRFRYVVFPQMMRYALPGIGNNWQVLLKATA
LVSIIGLADLVKASQDAGKSTYQLFYFLVLAALIYLLITSASNFALRWAERYYAAGSREA
QR
>tr|A0A0Q0BXZ3|A0A0Q0BXZ3_PSEAP GntR family transcriptional regulator OS=Pseudomonas syringae pv. aptata OX=83167 GN=ALO85_02513 PE=4 SV=1
MDTGMREDVPRAVTTICHALQEQIEHGLLPHGSKLPAERKLSEVFATTRITLREALLQLE
AQGLIYREERRGWFISPPRLAYDLIRRSHFHAMVCAQGRVPHTQLLSARLQPASALICEM
LELPALSSVIQICRARRIDQRLVLYVEHYLRPELFPGILEHDLNESLTELYARSYDVHYG
QVRFDMVPTALHADAAAALKVSLGSPGLRIARVNYDRKDRLIDCDLEYWRHDAIHVRAEV
HGD
>tr|A0A0Q0DJA6|A0A0Q0DJA6_PSEAP Uracil phosphoribosyltransferase OS=Pseudomonas syringae pv. aptata OX=83167 GN=upp PE=3 SV=1
MPIREIRHPLIRHKLGLMRRADISTKNFRELAQEVGALLTYEATADLTLESYDIQGWAGT
VSVEKIAGKKITVVPILRAGIGMLDGVLSLIPGAKVSAVGVARNEETLQAHTYLEKLVPE
IDERLAMIIDPMLATGSSMVATIDLLKKAGCKEIRAMVLVAAPEGIAAVEQAHPDVMIYT
ASIDERLNEHGYIIPGLGDAGDKIFGTKQKDA
>tr|A0A0Q0BZ64|A0A0Q0BZ64_PSEAP LysR family transcriptional regulator OS=Pseudomonas syringae pv. aptata OX=83167 GN=ALO85_00708 PE=3 SV=1
MPINFDLNDLQAFRAVVENGSFRKAAETVQITQPALSRRIEKLESALNVKLFERTTRRVS
LTTVGRAFLPQVERILDDLDSALMTIEGVGSTRMGSVTIACVPSAAYYFMPQVISAFHRL
FPKIRIRVMDASANEVNSAVALGEADFGVSFSGNLAPEVDFELLLQERYVMACRRDHPLA
TCDSVSWEEMYRHDYIGLDITSGNRLVLDQALAPSRPHKESICETRHVTTMIGLVEAGLG
VAAVPSIAMPMTAHPFLVSVPLVAPEVIRNVGLIKRRGRTLTPAALELEQLVRKMPSRSA

GN
>tr|A0A0Q0D9U5|A0A0Q0D9U5_PSEAP HopBC1 OS=Pseudomonas syringae pv. aptata OX=83167 GN=ALO85_101372 PE=4 SV=1
MFRINTGSSSYNVQSSASSEGSEGVKSYGLHDNLNVRANTTKVTDYVGTKAANQIPCLLS
NATFQGDVISVANSMAVLAAMGQGHGNVIVADLDKAHLAGVQSVLAIAQSSGSQEAWLRE
VKRQHKSGYADHLELLIQRNQEHLSFDSLKQRLHRGGVSLVRADMASDSATELAGLAPHG
VSGMYVSNIEMYLGGFLDKANTSINERQQALNTFKNNIVSLMGAEAFLIRGESVGMQVHN
KNAAINDWIPQVR
>tr|A0A0Q0D6G6|A0A0Q0D6G6_PSEAP Phospholipid/glycerol acyltransferase OS=Pseudomonas syringae pv. aptata OX=83167 GN=ALO85_02368 PE=4 SV=1
MSRLRGYARVVRVLLVVALGLTIASTFAVLERTKVGGSMERRQRWSRWFMARLTNALPFR
VTVTGQLPTQPMLWVSNHVSWTDIALLGMLAPLSFLSKAEVRTWPVAGWLALKAGTLFIR
RGSGDSRLIQKQMCNHLQQGNALLIFPEGTTTDGKSLRTFHGRLLSSAIDAGVPIQPVAI
GYSRDGKPDPIAPFIGDDDLLSHLRRLFANEQSDVHIHLLTPIPTADQERAALAFKAQQA
VQVALFGEPPAQPSEVVSRPARAA
>tr|A0A0Q0BTB0|A0A0Q0BTB0_PSEAP Polyamine aminopropyltransferase OS=Pseudomonas syringae pv. aptata OX=83167 GN=speE PE=3 SV=1
MSDYQETLYEGYGQRFRIEKMLHEVRTDHQHLVIFQNPRMGRVMALDGVIQTTEADEFIY
HEMLTHVPILAHGAAKRVLIIGGGDGGMLREVAKHMSVEHITMVEIDATVVEMCKEYLPN
HSSGAFDDSRLNLVIDDGMRFVATTEEKFDVIISDSTDPIGPGEVLFSENFYQACHRCLN
EGGILVTQNGTPFMQLSGVQTTAGRMNGLFADWHFYHAAIPTYIGGAMTFAWGSTNKSYR
KLPLETLRQRFSGSGIVTRYYNPEVHIGAFALPQYVLQAVGKASND
>tr|A0A0Q0FB05|A0A0Q0FB05_PSEAP Putative carboxyl-terminal protease OS=Pseudomonas syringae pv. aptata OX=83167 GN=ALO85_02319 PE=3 SV=1
MLHLSRLTSLALAIAIVIGAPLAQAAEKTAPATPAAVSPAKTNATAKPPLPLDELRTFAE
VMDRVKAAYVEPVDDKTLLENAIKGMLSNLDPHSAYLGPEDFQELQESTSGEFGGLGIEV
GVEDGLVKVVSPIDDTPASKAGIEAGDLIVKINGTPTQGQNMQEAVDKMRGKIGEKITLT
LVRDGGTPFDVTLARATIQVKSVKAQMLENGYGYIRITQFQVKTGDEVGKALAKFRKDNG
KKMSGLILDLRNNPGGVLQSAVQVADHFLTKGLIVYTKGRIANSELRFSADPADASEGVP
LVVLINGGSASASEIVAGALQDQKRGILMGTDTFGKGSVQTVLPLNNDRALKITTALYYT
PNGRSIQAQGINPDIVVRRAKVTNEADGENYKEADLMGHLGNGNGGADKPTVKSGAAAKA
RPQDDDFQLSQALSLLKGLSITRGN
>tr|A0A0Q0FUZ2|A0A0Q0FUZ2_PSEAP 50S ribosomal protein L36 OS=Pseudomonas syringae pv. aptata OX=83167 GN=ALO85_200008 PE=4 SV=1
MSEHETIPSTARDALIIELLSDVGRLHDDVKRIPQLLELSMRDSLDIVADAVEDAEDTAL
LLQKTTKEVIQATAAKAGVDVALEMSTAIHQSLERVFEPALQRAAIKIDGLEKRVSALSG
NFRDTQASRFNYIVLAGFVAVSIAVVCAMGWVAIKAQDVNETNRWFYHEYKKQQAVIDTL
PPAVKKQFAQ
>tr|A0A0Q0C7G4|A0A0Q0C7G4_PSEAP Uncharacterized protein OS=Pseudomonas syringae pv. aptata OX=83167 GN=ALO85_01668 PE=4 SV=1
MAILCKYTYDPLDRVSTLTPLAQAVSNRFYNDGQLMSELQGGRQRTCIRAGGQLLAQQSR
EGDEVVTTMIAGDRSNSVLHVSEDGRPVDIAYTPFGHHQAGQTIADLPGFNGEQPDPVTG
HYLLGNGYRAYNPVLMRFNSPDSMSPFGRGGLNAYAYCVGDPVNRVDPTGHISIGLTFLS
GILFSAVGAGAIGASVGLKESDSGLSTALAAVGSVSLLVGFTGFATTIHKWRGAKFQIKG
VEKKYQKKLDTAELARKKLIARYDKKVSRLQTELRRHTDGPENARVFLGNLRNADALLVD
GVTFYSDRRLPGIEQFHLDYLFRDTGRGFTAFRVDAQGRLDVRNIRSRTTLSQNTYRIRR
SQSDA
>tr|A0A0Q0IFE0|A0A0Q0IFE0_PSEAP TetR family transcriptional regulator OS=Pseudomonas syringae pv. aptata OX=83167 GN=ALO85_03251 PE=4 SV=1
MQQQRRTRAGMIEQTRAKLVAAARQAFASQGFAHTSMDDFTAEVSLTRGAIYHHFGNKEG
LLLAVIEQIETEVGERLQAISDAAPSPWEAFRRRCRAYLELALEPEIRRIILQDARAIFG
DVPQAAQSLGIAALQAALDGLIAEGVVAPLHTTVTARMIYGAVTEASFWIAEPDGDIEAR
LAQALEGLERLLSGLLNS
>tr|A0A0Q0DLK6|A0A0Q0DLK6_PSEAP Cysteine desulfurase, main protein CsdA OS=Pseudomonas syringae pv. aptata OX=83167 GN=ALO85_00315 PE=3 SV=1
MPLFSPWRGDFPAIAALQRQGQTYLDNAATTQKPQALLDALTHYYANGAANVHRAQHLPG
AHATQAFEASRGKVARWLNAGENGQIIFTHGATSALNLLAYGLEHEFAAGDEIAISALEH
HANLLPWQQLAQRRGLKLVVLPLDIDGVIDCDAAASLIGPRTRLLAVTQLSNVLGTWQPL
ARLIALAKSQGALTVVDGSQGVVHGRHDVQALGCDFYVFSSHKLYGPEGLGVLYGRNEAL
PSLAHWQFGGEMVLTADYQHAKFRPAPLGFEAGTPPIASVIGLGATLSYLDSLDHQAVAE

HEARLHRRLLEGLQQRDGIQVLGSPDLALVSFVVDGVHNADVAHLLTEQGIAVRAGHHCA
MPLFKTLGLPGAIRVSLALYNDSDDLLQFFSALDQALELLR
>tr|A0A0Q0DHL7|A0A0Q0DHL7_PSEAP NADH-quinone oxidoreductase subunit B OS=Pseudomonas syringae pv. aptata OX=83167 GN=nuoB PE=3 SV=1
MAMQYNLTRIDPDAPNEQYPIGKRETVSDPLEDQVHKNIYMGKLEDVLNGAVNWGRKNSL
WPYNFGLSCCYVEMTTAFTAPHDIARFGAEVIRASPRQADFMVIAGTCFIKMAPIIQRLY
EQMLEPKWVISMGSCANSGGMYDIYSVVQGVDKFLPVDVYVPGCPPRPEAFLQGLMLLQE
SIGKERRPLSWVVGDQGVYRAEMPSQKEQRREQRIQVTNLRSPDEV
>tr|A0A0Q0C8K1|A0A0Q0C8K1_PSEAP Flagellar export protein FliJ OS=Pseudomonas syringae pv. aptata OX=83167 GN=ALO85_02168 PE=4 SV=1
MAQSRAARLAPVVEMAEAAERTAAQRLGHFQGQVNLANNKLQELDQFRQDYQQQWLQRGS
AGVSGQWLLGYQRFLSQLDVAVAQQYKSLEWHKANLDRARSAWQDCYARVEGLRKLVQRY
MDEARRLEDKREQKLLDELSQRLPRHEQF
>tr|A0A0Q0DTF9|A0A0Q0DTF9_PSEAP Undecaprenyl-phosphate galactosephosphotransferase OS=Pseudomonas syringae pv. aptata OX=83167 GN=ALO85_02663 PE=4 SV=1
MRLQPVDSLSLTRTSLVEYFLFGIRLAHGIACILPGLVIMLLIKDGGTDAHQSYLTMLLF
FGCVGVLVFQAMGVYSEALFSNDLRMGAMALAWTAAFGLLLFMQQAMGLFGYLPPEELLI
WYLTSLALFGIIRLLLLTLFKHQMRKGVFLQHAVILGATENGQRLAEYLLEHQDIRSGVT
GFIDDRIGRMPKTVANLPLLGNTSDLERLIREEKVTQVLVALPWTAENRMDYIIRELRRL
PVNVLLVPDMIAFRHTHNRITEVARLPMFNASDLPLSGWSPFIKRAEDIVLSSLALLALS
PLMLLVALAIKLDSPGPVFFRQKRYGYNNRLIEVFKFRSMHQHQADATAERQTTRGDARI
TRIGRFIRKTSLDELPQLFNVVAGSMSMVGPRPHATATKAAGILFEQAVKEYTSRHRVKP
GITGLAQINGYRGETDTVEKIEKRVEFDLEYIENWSVWFDLYILMRTVPAVLFTREAY
>tr|A0A0Q0DRI8|A0A0Q0DRI8_PSEAP Putative polysaccharide deacetylase OS=Pseudomonas syringae pv. aptata OX=83167 GN=ALO85_02015 PE=4 SV=1
MAKEILCAFGVDVDAVAGWLGSYGGEDSPDDISRGLFAGEVGAPRLLKLFERYGLRTTWF
IPGHSMETFPEQMKAVADAGHEIGVHGYSHENPIAMTPEQEEIVLDKSIDLITQMTGKRP
TGYVAPWWEFSNVTNELLLKKGIKYDHSLMHNDFHPYYVRVGDKWTKIDYSQHPDTWMKP
LVRGEETDLVEIPANWYLDDLPPMMFIKKAPNSHGFVNPRHLEEMWRDQFDWVYREHEHA
VFTMTIHPDVSGRPQVLLMLERLIEHIQSHAGVKFVTFDEIADDFVRRNPRTR
>tr|A0A0Q0DH37|A0A0Q0DH37_PSEAP tRNA 5-methylaminomethyl-2-thiouridine synthase TusD OS=Pseudomonas syringae pv. aptata OX=83167 GN=ALO85_00710 PE=3 SV=1
MQVNTRQTHTPALEFFIIAHVLLAEPIMKFAISLFSAAHAPSSRRALQFARAVLAGGHEI
VRLFFYQEGVHSASANVVTPQDEQDMALQWRDFIVEHKLDGVVCIAAALRRGVLDTQEAE
RYQRPAANLREPWELSGLGQLHDAVQSADRLICFGGP
>tr|A0A0Q0CBJ9|A0A0Q0CBJ9_PSEAP Glutaredoxin NrdH OS=Pseudomonas syringae pv. aptata OX=83167 GN=ALO85_01829 PE=4 SV=1
MLLRTLKKFALIMLVVVIYQNWGRIENFINPPPADVAQSYSQAKVVMYATDWCGYCKQTR
RFLDSKGIAYQEFDIEKSEEGRKAYEALGGRGIPLIDVNGTLIRGFDPDQILAALK
>tr|A0A0Q0DIT3|A0A0Q0DIT3_PSEAP Oxaloacetate decarboxylase OS=Pseudomonas syringae pv. aptata OX=83167 GN=ALO85_04096 PE=3 SV=1
MPKASHQDLRRSFRALTSSNACFHTASVFDPMSARIAADLGFEVGILGGSVASLQVLAAP
DFALITLSEFVEQATRIGRVAQLPVIADADHGYGNALNVMRTVVELERAGISALTIEDTL
LPAQFGRKSTDLISTAEGVGKIRAALEARVDPEMSIFARTNAAIIPVQEAISRVQQYQAA
GADGITIVGIRDFDHLAQISEGVTVPLMLVTYGNPELHDNARLAEMGVRVCVHGHAAYFA
AIKATYDCLREQRQILGSESNMSATELTHTYTQPEDYVEWARKFMNVNE
>tr|A0A0Q0CWL9|A0A0Q0CWL9_PSEAP Hydroxyacylglutathione hydrolase OS=Pseudomonas syringae pv. aptata OX=83167 GN=gloB PE=3 SV=1
MIQIHALAAFNDNYIWLLQDLSSQQCAVVDPGDAKPVLGWLAQNPDYRLTDILITHHHND
HVGGVAELKQLTAARVLGPAAETIPARDIALVDNDRLTVLGLEFVVHAVPGHTLGHIAFY
HEDATTPLLFSGDTLFAAGCGRLFEGTPQQMHDSLGRLAELPDSTLIYCAHEYTLSNLRF
AQAVEPDNVDIGERLAEVTRWRSENRISLPSNLALEKRTNPFLRTGETSVKEKADERSGG
QNTSQSAVFASLRAWKDKF
>tr|A0A0Q0FFH3|A0A0Q0FFH3_PSEAP Uncharacterized protein OS=Pseudomonas syringae pv. aptata OX=83167 GN=ALO85_03102 PE=3 SV=1
MWSFSNWRRQRTLARHPVAEELWQNVCARLPILDGLSNGLRAQLRDACVLFLHDKHLSAL
PGVDLDDEQRLFLAAQAQLPLLNLGELNWYQGFHEIVLYPDDFVSPQRHRDASGVEHEWD
GEHSGEAWLQSPVILAWPGVLSSGGWDGYNLVIHELAHKLDMLNGDANGLPPLHSDMQVT

EWARVMQSAFDDLNRQLDHNLDAEPAIDPYAAEDPAEFFAVTSEYFFSAPDLLHGSYPAV
YEQLKAFYRQDTLARLDLLRQQDPAYRDS
>tr|A0A0Q0DK05|A0A0Q0DK05_PSEAP Peptidoglycan lytic exotransglycosylase OS=Pseudomonas syringae pv. aptata OX=83167 GN=ALO85_02455 PE=3 SV=1
MIRSALVSLMCLLPLLPLQVSARQTGPEVVQHTVQVRDLPAIRSSKVLRVLVNQSRNSSG
DVKGQEIGVEYHRLQAFEQYLNSHSRAGQKITIKVIPKAKNQLLTALQRGEGDMIAPGEL
LDVSDAKGIQASAPIVHDVPLVLVGVRGQRSVRRVDQLSGKTLSLPTGSAADEALHQVNR
QLELRKLPLAKIEWVDPSLAVEDVLEMVQAGIYPMTLVEQPIAERWARIMPKLRIERGLT
LQTHGDISWFVRDNATQLRASVDDFLKDYKPSAQQDQVFANAYKNTYKVNNPLVRNNLQR
LEQLRPMLQRHADAQGMDWLDLAALAFKESKLDPSAKGSGGATGLLQITPSAAKSVGVPS
IQSADDNIRAGSRYMALIQRKYFSSKRVNERERMAFVMAAYNLGPERVQGMREEARRRGL
NPNQWFFQTERVAMEQGGANVVAFVNSVNKYYLAFDRERDSLEKSGPKPAVKR
>tr|A0A0Q0DU95|A0A0Q0DU95_PSEAP Pyruvate kinase OS=Pseudomonas syringae pv. aptata OX=83167 GN=ALO85_02381 PE=3 SV=1
MCMTVRRTKIVATLGPASNSPEVIEQLILSGLDVARLNFSHGTPDEHKARARLIREIAAR
HGRFVALLGDLQGPKIRIAKFTDKRIELKVGDKFTFSTAHPLTSGNQQIVGIDYPDLVKD
CGVGDELLLDDGRVVMRVDTQTANELHCTVLIGGPLSDHKGINRRGGGLTAPALTEKDKQ
DIKLAAEMDLDYLAVSFPRDAADMEYARQLRDESGGTAWLVAKIERAEAVANDEVLDELI
RASDAVMVARGDLGVEIGDAELVGVQKRIILHARRHNKAVIVATQMMESMISSPMPTRAE
VSDVANAVLDYTDAVMLSAESAAGSYPIEAVQAMARICIGAEKHPTGKTSSHRIGHSFTR
CDESIALAAMYTANHFPGVKAIIALTESGYTPLIMSRIRSSVPIYAFSPHRGTQARAAMF
RGVYTVPFDPAALPPGQVSQAAVDELLKRGLVEQGDWVILTKGDSYHTIGGTNGMKILHV
GDPLV
>tr|A0A0Q0DGE7|A0A0Q0DGE7_PSEAP Membrane protein involved in aromatic hydrocarbon degradation OS=Pseudomonas syringae pv. aptata OX=83167 GN=ALO85_03548 PE=4 SV=1
MKIMTKALNRTTLGLALGLASSQLLAAGFALNEQSISGMGTGFAGRSSSADDASTVFGNP
AGMSRIKREQISVGAAAVIAKTDIDNGRGTFGGSNDGDMVPVVGVPMGYYVKPIDDHWAF
GLGVYVPFGLVTDYEKGFAGRYWGDKSHVQVVTFQPTVSYAFNDKVSIGFGPTINRIDGE
LTSATLNAATPGRNDGKVKIEGDDTAIGFNAGILVQATDTTRLGLTYHSKVDYKLKGKTK
IEGSGFGPFGGQKYDASLDISTPESVDFSITQQIDENWTVYAGSTWTRWSRLQDITVNND
GVPALLGGSAGPIGTISEEQNWHDTWAHAIGASYKVNKEWTLRTGFSVDQSPTNNVNRSP
RIPTGDRKIISFGAGWSPTDDLTIDVAYSYLREDETKIRDSSATKGSYSADYRNTAHGLG
TSVTYRF
>tr|A0A0Q0FBL0|A0A0Q0FBL0_PSEAP tRNA (cytidine/uridine-2'-O-)-methyltransferase TrmJ OS=Pseudomonas syringae pv. aptata OX=83167 GN=trmJ PE=4 SV=1
MLQNIRVVLVGTTHPGNIGGAARAMKNMGLSRLVLVDPRIFPSPDADARASGATDILEGA
QVVATLEEALVGCRLVLGTSARDRSLPWPMLDPRASGEKVIEQAGEGAEVALVFGREHAG
LTNEELQRCHFHVHIPSDPAFSSLNLAAAVQVLSYEVRVAWLAATEQGEAFRPASAHNAE
LATMDEMEGFYIHLEATLVAIGFLDPEKPRHLMARLRRLYGRSEVERSELSILRGVLTET
QKAARGEPYKRKDQ
>tr|A0A0Q0FH28|A0A0Q0FH28_PSEAP Biosynthetic arginine decarboxylase OS=Pseudomonas syringae pv. aptata OX=83167 GN=speA PE=3 SV=1
MSVRRTRKDDGSQWTVADSRSVYGIRHWGAGYFAINEAGRVEVRPNGPDSSPIDLYEQVD
NLRKSGLSLPLLVRFPDILQDRVRQLTGAFDSNIARLEYQSQYTALYPIKVNQQEAVVEN
IIATQNVSIGLEAGSKPELMAVLALAPKGGTIVCNGYKDREFIRLALMGQKLGHNVFIVI
EKESEVELVIEEAAELKVAPQVGLRVRLSSLASSKWADTGGEKSKFGLSAAQLLSVVERF
RKAGLDQGIRLLHFHMGSQIANLADYQHGFKEAIRYYGELRKLGLPVDYIDVGGGLGVDY
DGTHSRNASSINYDMDDYAGVVVGMLKEFCDAQSLPHPHIFSESGRSLTAHHAMLVVQVT
DVEKHNDEVPEIADKASLPETVQWLVDLLGPTDIEMVTETYWRATHYMSDIAAQYADGKI
SLSEKALGEQCYFAVCRRLYNSLKARQRSHRQVLDELNDKLADKYICNFSVFQSLPDTWA
IGQVLPILPLHRLNEEPVRRAVLQDLTCDSDGKIKQYVDEQSIETSMPVHSLNEGEDYLL
GIFLVGAYQEILGDMHNLFGDTDSVNIYQNPDGSVYHAGIETHDTIEDMLRYVHLSPEEL
MTHYRDKVASAKITPRERTYFLDALRLGLTRSSYLSS
>tr|A0A0Q0FI12|A0A0Q0FI12_PSEAP Major facilitator transporter OS=Pseudomonas syringae pv. aptata OX=83167 GN=ALO85_02130 PE=4 SV=1
MMRQIWKSFRALYFASLMMLIGSGLLSTYLALRLAADQVDSLWVGALMAANYFGLVLGGK
IGHRLIGRVGHIRAYATCAGIVGAAVLGHGLISWLPAWLVLRVIVGLGMMCQYMVIESWL
NEQAAAKQRGMVFSGYMIASYLGLVLGQLVLVVHPQLGPELLMLVAMCFALCLVPVAMTR

AIHPAPLHPAPLEPRFFMKRVPQSLTAVLGAGLIIGSFYGLAPVYAAEQGLSTEQVGLFM
GCCILAGFLVQWPLGLLSDRYDRSVLMRIFAAALVVASLPLAVFPSVPVEVLFGVGFLAS
LMQFCLYPLAVAFANDHVEAERRVSLTAMLLMTYGIGASIGPLAAGVVMKLFGGHMLYAF
VCGAGVILVLLIRPKAVTHLHQVENAPLHHVAMPDSMSSSPLVVALDPRVDEQVVQEQMQ
ATVEPEPEPDSEVVSEAEVDTESEVLVQAGPAAEAETAEADEHERERESETWKA
>tr|A0A0Q0IJE1|A0A0Q0IJE1_PSEAP Epoxide hydrolase OS=Pseudomonas syringae pv. aptata OX=83167 GN=ALO85_03947 PE=4 SV=1
MTYLIEPFPLQIPQVQLDDLAQRLAQTRWPDPETVSDNSQGPRLERLRALVERWRNGYDW
RATEAQLNQWNSSRVEIDGLGIHFLHIRSPHPDALPLLMTHGWPGSILEFRKMIGPLTDP
VAHGGQASDAFHLVIPALPGFGFSDKPRSTGWGVGKTAAAWAQLMERLGYGQRWAAQGGD
WGAAVTTALGHMRPPGLIGIHLNMVMYQATEAEIAQATPEEQRMLADAHRYDRELSGYMK
VHSTRPQSVGFALSDSPVGLAAWVYALFQDASDSGGEPERVIELDALIDDIMLYWLPNAG
PSSIRFYWEGMREMQQQGPAPAMPIPAGVSMFPGELLRLSRRWAQARFDDLRFFAEAEHG
GHFAAMENPKTLTEHVRETFRLLR
>tr|A0A0Q0CWT3|A0A0Q0CWT3_PSEAP Putative oligoketide cye/lipid transport protein OS=Pseudomonas syringae pv. aptata OX=83167 GN=ALO85_00116 PE=4 SV=1
MTTHIQRSALLPYPARFLYDLVNDVARYPEFLPWCSSATVVEASEAQMRASLEIAKGGLS
QKFMTRNTLVPGESIVMDLVEGPFEQFHGVWTFKPLGEKACKISLDLSFDYAGTIVRATL
GPLFNQAANTLVDAFCQRAKELHG
>tr|A0A0Q0BXR1|A0A0Q0BXR1_PSEAP LysR family transcriptional regulator OS=Pseudomonas syringae pv. aptata OX=83167 GN=ALO85_02825 PE=3 SV=1
MELRHLRYFIAVAEELHFGRAAQALGISQPPLSQQIQTLEQELGTRLFERTNRRVELSES
GRLFLDEARLVLAQVDKATDVARRAQLGEIGELKIGFTSSAPFTSSIPQAIFAFRQAYPD
VHLTLTEITSQEVAAGLEDKTIQVGIMRPLALPDSLVVFELLSEPLVAIMRADHPLAAES
EEGIYMAALAAEPFVFFPRTYGSGIYAQVLSLARAAGFSPLITQEAGEVMTIIGLVAAGL
GVTVLPASYRRMRIDGVVYRNVLDPGATSAVWLVQRKDEQSPMAKAFTELLTRNVG
>tr|A0A0Q0CZX0|A0A0Q0CZX0_PSEAP Flagellar hook-basal body complex protein FliE OS=Pseudomonas syringae pv. aptata OX=83167 GN=fliE PE=3 SV=1
MSQGVEFNRLMLDMRAMQMDAMSAPKPVSGAQEAGASSFADMLGQAVNKVAQTQQASSQL
ASAFEVGKSGVDLTDVMISSQKASVSFQALTQVRNKLVQAYQDIMQMPV
>tr|A0A0Q0D3H6|A0A0Q0D3H6_PSEAP Peptidoglycan-associated protein OS=Pseudomonas syringae pv. aptata OX=83167 GN=ALO85_01758 PE=4 SV=1
MFTSRRLIIVATAVAMLSGCATSNPYDNQGQAQSSGGMSKTAKYGGLGALAGAVAGAAID
HNHRGKGALIGAAVAGAASAGYGYYADKQEAALRESMANTGVEVQRQGDQIKLIMPGNIT
FATDSSAIASSFYSPLNNLASSLKQFNQNNIEIVGYTDSTGSRQHNMDLSQQRAQSVATY
LTSQGVDQAHLSVRGAGPDQPIASNADVNGRAQNRRVEVNLKPIPGQQY
>tr|A0A0Q0BTK7|A0A0Q0BTK7_PSEAP 30S ribosomal protein S18 OS=Pseudomonas syringae pv. aptata OX=83167 GN=rpsR PE=3 SV=1
MARFFRRRKFCRFTAEDVKEIDYKDLNTLKAYVSETGKIVPSRITGTKARYQRQLATAIK
RARFLALLAYTDSHGR
>tr|A0A0N8T9C7|A0A0N8T9C7_PSEAP 50S ribosomal protein L19 OS=Pseudomonas syringae pv. aptata OX=83167 GN=rplS PE=3 SV=1
MTNKIILALEAEQMTKEIPTFAPGDTIVVQVKVKEGDRARLQAFEGVVIAKRNRGVNSAF
TVRKISNGVGVERTFQTYSPQIDSMAVKRRGDVRKAKLYYLRDLSGKAARIKEKLS
>tr|A0A0Q0BT77|A0A0Q0BT77_PSEAP Uncharacterized protein OS=Pseudomonas syringae pv. aptata OX=83167 GN=ALO85_01899 PE=4 SV=1
MASTAATPPRTKKRFAYLRNMRWYSWFLVVALLGYGLAFIMHWDDRALLWFQERFESKAG
RHASIWLPDYHVDIDAKVLPGMEKDEASDLSYNPVTKTLFAVMGKNAFLVELTLTGDVLR
KIPLVGWSNPEGVAVMNDGLIAITDERQHKLTIVKVNADTTTLNIADFPQYELGKSANQN
KGYEGIAWDPQRQQLILGEERPPALYTWKSDGSDVLTGDKQPLSSRSLDMRNLSSLSIDP
RTGHMLALSADSHMLLEVDEQGEQVSFMTLLGGMNGLKDTIPRAEGVALDEAGTLYMVSE
PNLFYSFHRALHKH
>tr|A0A0Q0DPZ9|A0A0Q0DPZ9_PSEAP Inosine/uridine-preferring nucleoside hydrolase OS=Pseudomonas syringae pv. aptata OX=83167 GN=ALO85_00756 PE=3 SV=1
MTLIQFSRQFIRGALLLSILGTAAVQAAEKRDLIIDTDPGADDVVALLLALASPEELNVM
AITTVAGNVRLDKTSRNARLAREWAGREEVPVYAGAPKPLVRTPIYAENVHGQEGLPGVP
VHEPAKGLAEGNAVDYLIRTLSKAKPHSITIAMLGPQTNLALALVQAPEITQGIKEVVVM
GGAHFNGGNITPVAEFNLFADPHAAQIVLASGVKLTYVPLDVTHKILTSEQRLKQIAALG

NNAGKLVDGILNEYVKLDMEHYGLPGGPVHDASVIAWLLKPELFSGRQINVAVDTREGIG
FGQTVADWYGTLKQPQNVFWVENGDAQGFFDLLTERLARLK
>tr|A0A0Q0BRI4|A0A0Q0BRI4_PSEAP Twin-arginine translocation pathway signal OS=Pseudomonas syringae pv. aptata OX=83167 GN=ALO85_04236 PE=4 SV=1
MSNVDRRSFIKLAGIIGLSAALPFGRAYSADAPLVVGFIYVGARDDFGYNQAHAQAAAII
KTLPNVKVIEEENVPETVAVQKTMEAMIRQDGATLIFPTSFGYFEPHVVKVAKKYPDVRF
AHCGGLWQKGRDPMNAGSFFGYIDEAQYLNGVIAGHMSKSKKLGFVAAKPVPQVLRNLNA
FAMGARSVDPSIVTTVIFTGDWSLPVKEAEAANGLIDQGCDVLTCHVDGPKVIVETAEKR
GVMTCGYHASQAALAPKGYLTGAEWNWETPYRAHVAAAQSGAPMINFLRGGLKEGFVKTS
AYGPAVTADARQKADAIKAQMMAGQFVIFKGPLKDNKGNVVIADGVAQGQTDIALESMNY
LVEGVLGQL
>tr|A0A0Q0DU22|A0A0Q0DU22_PSEAP Beta-ketoadipate pathway transcriptional regulator PcaR OS=Pseudomonas syringae pv. aptata OX=83167 GN=ALO85_02395 PE=4 SV=1
MNDELRKSFASLAPPIVASPAKRIQALTGDPDFMTSLARGLAVIHAFQERKRHLTIAQIS
HRTEIPRAAVRRCLHTLIKLGYATTDGRTYSLLPKVLTLGHAYLSSTPLATSSQPYLDRM
SDQLHEACNMATLEGDDILYIARSATTQRLISVDLSVGGRLPAYCTSMGRILLAALDDVS
LHEYLDHVELQPKTSRTIRTPEALLECLQLVRQQGWCIVDQELEQGLRSIAVPVYDASGQ
VLAALNVSTSAGRVARSELEQRFLPIMLDASRDLSTQLFT
>tr|A0A0Q0IAH1|A0A0Q0IAH1_PSEAP Uncharacterized protein OS=Pseudomonas syringae pv. aptata OX=83167 GN=ALO85_04249 PE=4 SV=1
MIKLLMRLKARFDRKSKGQQSDRLPPAQPADQQESMSRVQENLTWLPVYREPEPLRLQPP
ACPPLRQQDEPLRARYHAYVAQTEQADPASQDESCRETGRQLARELQSAAIDIVTSKAES
SPDVYWLTQSAAIASLFADGAQSDAFQRYQEYVQHYKDQRLTAGQVWAFDIYVAEHTPRQ
VRTFLPHPSSETRLPDEPSPGADDIDQLLSYLPLLYPDGVAIKSYIIKENTYWPDYFPVV
EAFYRAVAKDCWCDIDYLNHGAADMLNDDIYIAQANLADMQTLLTYCIRGERFCDGHHGA
MIEKGYVLKILRRLAVLRED
>tr|A0A0N8T999|A0A0N8T999_PSEAP Translational regulator CsrA OS=Pseudomonas syringae pv. aptata OX=83167 GN=csrA PE=3 SV=1
MLILTRKVGESINIGDEITVTILGVQGLQVRLGINAPKNVSVHREEIYKRIQAELAPNQD
PQ
>tr|A0A0Q0DA22|A0A0Q0DA22_PSEAP Dihydroorotase OS=Pseudomonas syringae pv. aptata OX=83167 GN=pyrC PE=3 SV=1
MSDRLTLLRPDDWHIHLRDGAVLPHTVADVARTFGRAIIMPNLVPPVRNAQQADAYRQRI
LAARPASSRFEPLMVLYLTDQTTPDDIRTAKASGFVYAAKLYPAGATTNSDSGVTSIDKI
FPALEAMAEVGMLLLVHGEVTRGEIDVFDREKVFIDEHLRRVVERFPTLKVVFEHITTGE
AVQFVNEASANVAATITAHHLLYNRNHMLVGGIRPHFYCLPILKRNTHQTALLDAATSGS
GKFFLGTDSAPHAQHAKENACGCAGCYTAYAAIELYAEAFEQRNALDKLEGFASLHGPAF
YGLPANQDTITLVRDEWTAPASLPFGELTVIPLRAGETLRWRLEEHA
>tr|A0A0N8T7V3|A0A0N8T7V3_PSEAP Type III secretion system chaperone protein ShcM OS=Pseudomonas syringae pv. aptata OX=83167 GN=ALO85_04208 PE=4 SV=1
MNKNDEYQTLINEICTLSLISEPERFYESANFKISDVDFTLHYQDRDEGRAILIYGDMGA
LPARNRDSALLALMDINFHMFAGAHSPAFSWNAQTGRVLLMGSVGLERATAEGVLLLMKS
FADLAREWREHGFMGPVGTASASVSTPASKATMRDTPASSGRFQ
>tr|A0A0Q0BYE6|A0A0Q0BYE6_PSEAP Uncharacterized protein OS=Pseudomonas syringae pv. aptata OX=83167 GN=ALO85_02116 PE=4 SV=1
MIALGLAHLAFAWTLFVLLAPLTASLWWRCGLLAAVSLLSVISIDGLSMASYARSLTDDL
AISSLVLLGWLTLQRLGVLKPLAVSRRAVVLLVFAALALTLYPATLGLTYFDPYRWGYNP
RPMIMIVAVIALGLVFMRNALAAVMLSAATLAFTFRIKPSENYWDYLIDPLLGLYCCGAL
LILAVRFVYRRAMETRRPAALSVGNV
>tr|A0A0Q0BW22|A0A0Q0BW22_PSEAP Sulfur carrier protein TusA OS=Pseudomonas syringae pv. aptata OX=83167 GN=tusA PE=3 SV=1
MSEPIENLTVDAELDAVGLFCPEPVMMLHQKVRDLPAGGLLKVTATDPSTRRDIPKFCVF
LGHELVAEQAEEGTFLYWIRKKSD
>tr|A0A0Q0DV93|A0A0Q0DV93_PSEAP Glycerophosphoryl diester phosphodiesterase OS=Pseudomonas syringae pv. aptata OX=83167 GN=ALO85_200099 PE=4 SV=1
MLKPAPLVRSSLIAHRGAKAYVPENTLLALEKAAACGAEWVEIDVKLTRDGQPVVIHDDL

LDRTTNGRGAVVLHELKAIRMLDAGSWFAPEFAGLQVPTFEEIVACALRLNLGLQVELKP
TIGDDVETAEVVMPILKNLWPADNDRLFVSSFSVRSLTAARRLWNDVPLAIASVVAPADP
VALLAEYDCRILHVLDDMLDDHHLVRLKSSGVEFAVATINSPKRAHYLLEHGAQSILSDY
PDLLNLPNGGCLQ
>tr|A0A0Q0BSG4|A0A0Q0BSG4_PSEAP Diguanylate cye domain protein
OS=Pseudomonas syringae pv. aptata OX=83167 GN=ALO85_00112 PE=4 SV=1
MPQRRNLKNSIFVPLKLSQLIGLFSWVISWCLNPSIDRSLDIFICCALAGAALSIIVTSN
SRSMLAWRLFGIIYMASLTFLFNEQVERMGVEASMWGLVVTSLLITGMSVFFVKFIDYIA
AAALIWLIMWQMDLTAVGVNHAPLFYVFLISSTLLGGCLNATFLSLVMQTMAARDRYQEL
SETDTLTHAPNRRALVASLNTALACAEKSSLWFAMIDLDHFKSINDTHGHDVGDNVLVGF
ATLLKGTKGLIAFGRLGGEEFGVILSAANAIDAVHALEELLRRAQKDEAAQVPYSFSAGV
ASLSRVETVSDLLKEADENLYHAKRNGRKCIAFEGRIVSSSAASTMTQNQAAQSVDAPTH
A
>tr|A0A0Q0C6Q4|A0A0Q0C6Q4_PSEAP Periplasmic substrate-binding protein
OS=Pseudomonas syringae pv. aptata OX=83167 GN=ALO85_00803 PE=4 SV=1
MKQWLSISLSASLLFAGLAQAATPLIGVGMAKYNDNFQTILRHGVEKQVALLDADIFMEN
GQDDVELQMRQFRNLVNSKVDAIVVAMVSGKSAPEMMRLANEAKVPLVFVNRNPEPAKWP
ALTAFVGSDELESGTLQMEELARRANYKGNVVILVGDPSNKSSVMRTEDVEKVVAKYPDM
KVVQKKIGNWERSQAATIVIEWVKQGVDFSIIAANNDEMAIGAIMGLEKTGKKAGDYLVG
GIDGTPDGLKLMAEGKMAVSVFQDALGQADGAVDAAMRMARGETLASPVIWVPFKLITPE
NRQQFE
>tr|A0A0N8T9W7|A0A0N8T9W7_PSEAP HDOD domain-containing protein
OS=Pseudomonas syringae pv. aptata OX=83167 GN=ALO85_00810 PE=4 SV=1
MVEIIPRPAQDGFAMPSIEQLFDGLHSLPSIPKVAQDLMLQFDNPSSNLESIARNIEKDP
VIAAKVLRLANSARFRGSRESSSIEDAAMRLGFNTLRTLVMASAVTGAFKAGPSFDLKGF
WLKSFQVAGICRMLAKQTGADTEIAFTCGVMHNIGELLIQTGAPDVAERLNNAAKAGTPG
RAASETLQLGFSYPEVGAELASRWHLPQVIRDAIAYQATPLKAPKDASLPRIVAEAITIS
DALEAHGGATPQAQQASGGPLMEGIDLDALFAGLPAVLEADKAFSELLS
>tr|A0A0Q0FND5|A0A0Q0FND5_PSEAP Chemotaxis protein CheV OS=Pseudomonas
syringae pv. aptata OX=83167 GN=ALO85_02666 PE=4 SV=1
MAGILDTVDQRTQLVGENRLEILMFRLAGRQLFAINVFKVQEVLQLPKLTLMPQRHSFVC
GVVNLRGQTLPVIDLSQAIGMRPLVPGPGSTIIVTEYNRSVQAFLVGGVDRIVNMNWDAI
MPPPVSAGRQHYLTAISKVDDQLVEIIDVEKVLAEIVPYNAKVSREKLEDPVLENARGRE
VLLVDDSKVALAQLRDTLSQLGLKLHVASDGLKALNMLKAWADAGENVHEKLLMVFTDAE
MPEMDGYRLTTEIRNDARLRGLYIVLHTSLSGSFNESMVKKVGCDNFLSKFQPDKLVEVV
RERLLMIV
>tr|A0A0Q0IFR2|A0A0Q0IFR2_PSEAP Transcriptional regulator OS=Pseudomonas
syringae pv. aptata OX=83167 GN=ALO85_04644 PE=4 SV=1
MPTCSNDPISELLLGMRLFGVQFRRLEVVPPLGVGFSNAIGRAQFHFVGRGPVWLRSAEN
VLCRLDSGDAVLIPRGGHHAILSAEDTAASDIQLFDPSTAIVDGVHNGAYDEEGGVSDHA
IIFSCCMELELGGMQPLVAAMPDVMQVSTLLETCPELRPMLAAMERETLMLQAGHAGILA
RLAEVVAALLVRGWVAGGCGNATGWLGALQDPRLSRAIVMMHQRPGRRWTVATLAREARH
SRSVFAQRFLEATGMPPLQYLTELRMRLALHSLSREHCSLEAVASQLGYGSLAAFSRAFK
RTVGLSPGAVRAAKSVAG
>tr|A0A0Q0D6Y2|A0A0Q0D6Y2_PSEAP Uncharacterized protein OS=Pseudomonas
syringae pv. aptata OX=83167 GN=ALO85_00985 PE=4 SV=1
MMGAVVSLDALLDERRVWKGRQQSAPQVSPQPSGHALLDAALPTGGWPAAALTEILIPAN
GSGELRLLWPSLARLSAIAERIVLVAPPYIPYPQAWLSAGVDLRQLVVIEASPRDALWAA
EQCLRSGSCGAVVCWPGMVDDRALRRLQVAAETGQTLAFACRPQQAAANPSPAALRIALD
TRPAQLRVLKCRGGLAPPFPIPFPTDA
>tr|A0A0N8T7W8|A0A0N8T7W8_PSEAP Uncharacterized protein OS=Pseudomonas
syringae pv. aptata OX=83167 GN=ALO85_04158 PE=4 SV=1
MSKSNGDDSFRAFLTACTENQDEVVAQNSPYVAMMDALDSFLLTHITNPTKTPSDLVMHA
LRINARFMLMTGFRIGLTGHAAGVYPTLRTALENACYAFLMSQDEALSDVWMKRSLSVAN
TKAFKKTFNKAIAEARDLMDELHPNNLGTWMYELYQASMEFGAHPNALTILLHTRFSDDE
ATGWTKYENIALYTVGNFEFDRTLLACVEMGLAVAIVLSMTFEKPSQEIIEAVNKLNKMK
NELEDMIRSKFGIAAVSI
>tr|A0A0Q0IAM8|A0A0Q0IAM8_PSEAP Cytochrome O ubiquinol oxidase, subunit
III OS=Pseudomonas syringae pv. aptata OX=83167 GN=ALO85_04261 PE=3 SV=1
MSNIAINSGAHDHGHDHDHGHDDHHDSGGMTVYGFWLYLMTDCVLFASFFAVYAVMVNSV

AGGPSGQDIFLLPFVAVETAFLLVSSITYGFAMLALYKGNKSQVLGWLALTFLCGAAFIG
MEVYEFHHLIEEGFGPSRSGFLSAFFALVGLHGVHVTSGLIWMAIMMFQVQKKGLTNTNK
TRLSCLSLFWHFLDVVWIGVFTVVYLMGAL
>tr|A0A0Q0IRR4|A0A0Q0IRR4_PSEAP Putative lipoprotein OS=Pseudomonas syringae pv. aptata OX=83167 GN=ALO85_03349 PE=4 SV=1
MNSKSLIACMTLLGCFSAVTLPVHAAETADPKVTESKDNVHELEVGSRVPEKYRRSDLEV
KDWKSKGLETPAKESEWVKINDKYVRFQKVNGQIMEIVPVKK
>tr|A0A0N8T9W5|A0A0N8T9W5_PSEAP Glutamine synthetase, type III OS=Pseudomonas syringae pv. aptata OX=83167 GN=ALO85_00836 PE=3 SV=1
MLPPETQRLIEQHGIKYVLAQFVDIHGSAKTKSVPVTGLEMVAEDGAGFAGFAICGMGME
PHGPDFMARGDLSSLTPVPWQPGYGRVVCIGHVDGKPWPYDSRYVLQQQVERLGQRGWTL
NTGLEPEFSLFKRDVTGSLQMVDASDNLDKPCYDYKGLSRSREFLERLTEALQPVGFDIY
QIDHEDANGQFEINYTYSEAMESADRFTFFRMAAGEIANDMGMICSFMPKPDPKRAGNGM
HFHLSIASASNKNLFHDASDPSGMGLSKLAYHFAAGLLAHGPALCAFSAPTVNSYKRLVV
GNSLSGATWAPAFIAFGANNRSAMVRVPYGRLEFRLPDAGCNPYLVSAAIIAAGLDGIDR
QLEIDHVCNENLYTLSLEEIAARGIKTLPQSLNEACDALQADPLFGEVLGSEIVDEFIRL
KRMEWVEYSRHVSDWEVKRYIEFF
>tr|A0A0N8T8R4|A0A0N8T8R4_PSEAP ABC transporter, ATP-binding protein OS=Pseudomonas syringae pv. aptata OX=83167 GN=ALO85_00035 PE=4 SV=1
MAQYVYTMHRLSKVVPPKREILKNISLSFFPGAKIGVLGLNGSGKSTLLKIMAGVDTEFD
GEARPMPELNVGYLPQEPQLDPNKTVREVVEEAVSVIKDAQARLDQVYAEYAEPDADFDK
LAAEQAKLESILQASDGHNLERQLEVAADALRLPAWDAKVSVLSGGEKRRVALCRLLLSA
PDMLLLDEPTNHLDADSVAWLEHFLHDFPGTVVAITHDRYFLDNVAGWILELDRGAGIPY
EGNYSGWLEAKSDRLAQESKQQTAHEKAMKDELEWVRKGAKARQSKSKARLQRFEEMQSQ
EFQKRSETNEIYIPAGPRLGDKVIEFKNVTKGYGDRVLIDNLSFSMPKGAIVGVIGGNGA
GKSTLFRMLMGKEQPDSGSIEVGETVQLACVDQSRDDLDGSKSVFQAVSDGSDVIRIGNY
EIPSRTYVGRFNFKGGDQQKFVKDLSGGERGRLHLALTLKEGGNVLLLDEPSNDLDVETL
RSLEEALLDFPGAAIVISHDRWFLDRVATHILAYEDDSQAIFFEGNYTEYEADRKKRLGE
AAAQPHRVRHKKLA
>tr|A0A0Q0DTV8|A0A0Q0DTV8_PSEAP 1-deoxy-D-xylulose 5-phosphate reductoisomerase OS=Pseudomonas syringae pv. aptata OX=83167 GN=dxr PE=3 SV=1
MGCHVSGPQQITILGATGSIGLSTLDVVARHPALYQVFALTGFSRLDELLALCIRHTPQY
AVVPNQVVARKLQDDLAAAGLDTRVLVGEGGLCEVAADPRVDAVMAAIVGAAGLRPTLAA
VEAGKKVLLANKEALVMSGALFMQAVRQSGAVLLPIDSEHNAIFQCLPGDFARGLGAVGV
RRIMLTASGGPFRETPLEQLHNVTPEQACAHPVWSMGRKISVDSATMMNKGLELIEACWL
FDARPDQVEVVIHPQSVIHSLVDYVDGSVLAQLGNPDMRTPIANALAWPARVDSGVAPLD
LFRIGQLDFQAPDEERFPCLRLARQAAEAGGSAPAMLNAANEVAVAAFLDGRIRYLEIAG
IIEEVLDHEPVTAVEGLDAVFAADAKARLLAGQWLERNGR
>tr|A0A0Q0BTJ5|A0A0Q0BTJ5_PSEAP Uncharacterized protein OS=Pseudomonas syringae pv. aptata OX=83167 GN=ALO85_04140 PE=4 SV=1
MKIKIEHTTQEDKAAIKVFCPYDDQFIKGAGNSSGKFSNSQNCWIFPARSEAKTRALLIE
IFGTDDTATSPKVDVRVTFPNMYYANKDAIRLAGRMLARATSRDSGAILGDDVELVSGWV
RSDGSAKNWETRTSEGSVYEIFDFEASKLEELRALDFIEVEVIGGEEIEDTITIKELVKF
TGNVKKDEKATFIEYPFLVVVMNHDTKTIDVAGRDLLMTNKQWKNAYSIFSEIVEK
>tr|A0A0Q0C2Y7|A0A0Q0C2Y7_PSEAP GCN5-related N-acetyltransferase OS=Pseudomonas syringae pv. aptata OX=83167 GN=ALO85_03372 PE=4 SV=1
MIQIRPMTPEDFERFWPTFQAVVIAQETYAYDPALTQEQARDLWMTAPLHTLIAEEDGQL
LGSYYLKANAAGPGNHVCNCGYMVTDAARGRGVARLMCEHSQQLARDSGFLAMQFNSVVA
SNEVAVGLWSKLGFETVGRLPKAYRHARLGLVDCLVMFKWLADEPVAKEPAQPKLIGRKN
IESVVSRPRRK
>tr|A0A0Q0CEJ6|A0A0Q0CEJ6_PSEAP Glutathione S-transferase OS=Pseudomonas syringae pv. aptata OX=83167 GN=ALO85_00965 PE=4 SV=1
MLRILGKTSSINVRKVLWTCEELKIPFTQEDWGSDFKPADTEAFVALNPNAMVPVIVDDD
FVLWESNSIVRYLANRYAGDPVYPLDPRARAHTDQWIDWQGSDLNRSWSYAFMSLVRRST
EHQAPAAIAVSCAQWTRHMQILDRQLEKTGAYVCGERFTLADIPIGLSVNRWFETPLEHP
DLPAVNAYYERLSHRSGYLLYGRNGTP
>tr|A0A0Q0C4P3|A0A0Q0C4P3_PSEAP ImpA, N-terminal OS=Pseudomonas syringae pv. aptata OX=83167 GN=ALO85_02248 PE=4 SV=1
MSEFLNDLSLADLTAPINGGSGEDLSFSTLFDQVKEARRADPDYLTQGDWQTDLKSSDWE

QTITLAAQGLAEQSKDLMLVAWLSEALAHKYHFVGITFGLTVAERILQNFWDDLYPSLED
GVEERAARLAWLKTTLTEVVGGLPITQGQNFGLLRYDESRHVENLALQNPKAMQSAVEEG
KINAEIFQRSVVLTDSDHLRLKASEIAASLYACQQLQATADTLFGRDAPSLVTLSDILQR
AGQLTEKLLKDRGIELSPAPAAAQAAPETVVDQPAGAVMTQASQDSASAPLRTTPTTRDE
AFTMLAGIAQFFKNTEPQSPVPYLIERAIKWGNMPLEGWLNDVIKDSNVVDSIRDVLGTK
EPKQ
>tr|A0A0Q0DPI7|A0A0Q0DPI7_PSEAP Thioesterase super protein OS=Pseudomonas syringae pv. aptata OX=83167 GN=ALO85_02173 PE=4 SV=1
MVWDRATPFVIDLNVAAEDIDGLGHANNAVYVSWLERCAWRHSQFLGLDLTEYRRLDRAM
AVVRHEIDYLASAYENDELQLATWIVDWDRRLKMTRCFQLKRPADNLTLLRAQTTFVCIE
LSTGKPKRMPAEFIEGYGAAISDNAVPNPYVGQ
>tr|A0A0N8T9E0|A0A0N8T9E0_PSEAP Uncharacterized protein OS=Pseudomonas syringae pv. aptata OX=83167 GN=ALO85_00312 PE=4 SV=1
MSRWLLRAIITRHLKFGCVMSEQEKKRQEALVRQRYYRERQRAEGFKQSTLWIHGEAEAQ
GRLAAREGKPLLPMQSHDPVSWAVGWVAEKLRTRE
>tr|A0A0Q0FWD0|A0A0Q0FWD0_PSEAP Heme biosynthesis protein HemY OS=Pseudomonas syringae pv. aptata OX=83167 GN=ALO85_01576 PE=4 SV=1
MKRFYVLLFIAIAAAALIGVAVAEHSGYVLIAYQNFRYESSLWATLALLVVALLVIALLR
LLISLLTTSGRVVNPWSRRNRRRRVQIAIEQGQMDLAEGRWSSAQRHLQRAAEADTHPLL
YYIGAARAANEQGRYEDCDALLERALERQPQAELAIALNHAQLQQDRGDTDGALTTLLAM
KERHPHNPQVLRQLQRLHQQRGDWSDVVRLMPELRKDKVLPPKELAELERRAWGENLSLA
AYSEGIEGAPTGLPSLESAWQGLGSAQRQEPQLVLAYADQLRRLGAEAQAEEVLRSALKR
EYNSHLARLYGLVRGTDPLKQLQAAEGWLKHHPADPSLLLSLGRICLQGRLWGKARDYLE
SSLRMERNPETCAELARLLGQLGETDRSNQLFQEGLGLLDERLLSRPLPVLTKA
>tr|A0A0Q0DGD2|A0A0Q0DGD2_PSEAP NlpC/P60 family lipoprotein OS=Pseudomonas syringae pv. aptata OX=83167 GN=ALO85_03562 PE=4 SV=1
MSIALRLLVISVAALLGACASAPPPPKAPRIVQRPVVTAPPQILSPAAEDVLFRALGLVG
TPYRWGGNTPDSGFDCSGLIGYVYRDAAGISLPRSTREMIVMGAPNIRREQLQSGDLVFF
ATSGGSQVSHAGIYVGEGRFVHAPATGGTVKLDSLDKPYWQRAYLNAKRVIQPASLAQNR
P
>tr|A0A0Q0FLY7|A0A0Q0FLY7_PSEAP Outer membrane lipoprotein I OS=Pseudomonas syringae pv. aptata OX=83167 GN=ALO85_02465 PE=4 SV=1
MKKNSLKVNTMNNVLKFSALALAAVLATGCSSVSKETEARLTATEDAAARSQARADEAYR
KADEAMAAAQKAQQTADEANERALRMLDKASRK
>tr|A0A0Q0DKX2|A0A0Q0DKX2_PSEAP Transglutaminase OS=Pseudomonas syringae pv. aptata OX=83167 GN=ALO85_01220 PE=4 SV=1
MRLSISHETTYHYDDQVRSSIQYLRLTPHDSERQQVLSWQLTLPRPVRAQIDPFGNILHV
LSLEEPHESIIIGARGQVEIDETREAEHESQSALPFLRVTRLTEPDEAIRSFAAMECRKR
ADRTGLIDLMQALNQHMTYTQGATEVDTSAAQAFAGRSGVCQDHTHAFLACARSLGVPAR
YVSGYLCSEDSEHLSSHAWAEAWIDDAWYSFDVTNQLAIPERHLKLAVGLDYLDACPVRG
MRRGGGSEHMHAKVVVSPMTGTPKVKTRVQVQAQHQ
>tr|A0A0Q0IHZ5|A0A0Q0IHZ5_PSEAP Oxidoreductase, zinc-binding protein OS=Pseudomonas syringae pv. aptata OX=83167 GN=ALO85_100107 PE=3 SV=1
MYTAIGYAAQSATAPLAPMTFERRALRADDVAIEILYCGVCHSDIHQARNEWGIAVYPLM
PGHEIVGRVTATGANATKYKVGDMVGVGCMVDSCRECSACKSDLEQYCLEGPTMTYATPD
RVDGSNTMGGYSNSIVVAEHFVVRVPEKLDPAAAAPILCAGITTYSPLKHYGVKAGDKVG
ILGMGGLGHMGIKFAKAMGAEVTLFTRSAAKAQEAHRQGADHVIVSTDAEQMKAAAGHFD
FLLDTIPVQHDLNPYLETLRFDGAHILVGLIEPIEPPVHAANLVMSRRVLAGSLIGGIAE
TQEVLDFCAENDITCDIEMLDIRNINEAYERMLAGDVKYRFVIDMATLKA
>tr|A0A0Q0CGA6|A0A0Q0CGA6_PSEAP Putative carbonic anhydrase OS=Pseudomonas syringae pv. aptata OX=83167 GN=ALO85_00833 PE=4 SV=1
MIRKNPSGHLPVIAESAYIDKTAIICGKVIIKDNVFVGPYAVIRADEVDATGDMEPIVIG
ANSNIQDGVVIHSKSGAAVTIGEFTSIAHRSIVHGPCIVGDRVFIGFNSVLFNCQVGNGS
VVRHNSVVDGRDLPEHFYVPSTTRIGPGTDLSQFPPVSISASEFSEDVAHTNIDLVRGYK
ALQNEF
>tr|A0A0Q0ICB7|A0A0Q0ICB7_PSEAP Uncharacterized protein OS=Pseudomonas syringae pv. aptata OX=83167 GN=ALO85_01212 PE=4 SV=1
MPTPASSRTPWAAWLNQLQGSPLVSLGFALALLIVVGLGGISLYNAFYGTSQVNFNHALL
GGIAGFLATALGAVMAVALRNVSQRVQDIMLGFAAGMMLAASSFSLILPGLEAARDITDS
GPWAAATVVAGLGLGVLLMLGLDKFTPHEHESVGRQGPHSERISRVWLFVLAITLHNLPE

GMAIGVSFASGDMNVGLPLTTAIAIQDIPEGLAIALALRATGLSAFKAMLVAIGSGLMEP
LGSLVGLGISSGFAIAYPLSMGLAAGAMIFVVSHEVIPETHRNGHQTSATLGLMGGFAVM
MFLDTALG
>tr|A0A0Q0CWV2|A0A0Q0CWV2_PSEAP Putative modulator of DNA gyrase TldD OS=Pseudomonas syringae pv. aptata OX=83167 GN=ALO85_00158 PE=4 SV=1
MSDLSSVSEHLLAPGGLSLDSLQSVLGDLAGPGIDAADLYFQGMISESWSLEDGIVKEGS
FNLDQGVGVRAQSGEKTGFAYSNAITPEALRTAAQAARSISRAGQNGRVQAFTSQDVAQL
YAPDNPLEVLSRAEKVELLKRIDVATRALDSRIQQVSVSMAGVWERILVAAADGSLAADV
RPLVRFNVSVIVEQNGRRERGGHGGGRTDYRYFLTDDRAMGYAREALRQALVNLEAIPA
PAGTLPVVLGSGWSGVLLHEAVGHGLEGDFNRKGSSAYSGRMGEMVASKLCTIVDDGTLA
GRRGSLSVDDEGTPTECTTLIENGVLKGYMQDKLNARLMGVARTGNGRRESYAHLPMPRM
TNTYMLGGQSDPQEIIASVKRGIYCANLGGGQVDITSGKFVFSTSEAYLIEDGKITAPVK
GATLIGNGPEAMSRVSMVGNDLSLDSGVGTCGKDGQSLPVGVGQPTLKIDAITVGGTGS
>tr|A0A0Q0DSV7|A0A0Q0DSV7_PSEAP Uncharacterized protein OS=Pseudomonas syringae pv. aptata OX=83167 GN=ALO85_05131 PE=4 SV=1
MSITIQSLPRLNSLPFLTAPQWLCQWTPADESVFDGSGI
>tr|A0A0Q0CAD2|A0A0Q0CAD2_PSEAP Uncharacterized protein OS=Pseudomonas syringae pv. aptata OX=83167 GN=ALO85_05293 PE=4 SV=1
MENLSTRYLSTPVSVPGNRRRNLNLVLTAPDIAVAFHDQDTPGLIPVAEASQPLTVDLVV
WPAALPGYTYQLLFNSIATGPQKLILPSHAPGDPLQLEIPSELLLEGFHDVAYVVVNPIN
QTEQKSEIFRIQIDRTAPGAPELAAIQFPAEIQNGLTAAELDQLGGQLDVQIAGYTGMAK
HDRILTRWGDIVGPTAVVNETDMGLDKVSVTFTRAFLQTLGEEEKQVCYQVIDRAGNPSI
LSNTVPVKLRLKDLPSDYPAPVIDQAVGDLVDYLEAQAGVVVDIPHYPGAAALDSIQLFW
GINNPLTPVFVTPGDENEAVILTLKLPFEAIAQGPQGDTAVHYEVFRNGELTGTSLDTHI
DVFTTLPFPTPLNNVTVQGTSVQNPNTQDNFIDEDDYELNGRGVVAWNTGFEINDDLNLY
WGNQVKPQWYQIRSSDVAAARDLIIPIDNELMKLQGTGEAIPVYFTVTRTGNPNSMTSPM
QPVTVRSREEQPGGENGLTGPTFNLTPNGVLGPNENPDGADVKVSPYLNIAEGQKITFTF
KGFDDSNNPIEAATYVATRKLDEVDVVQGHVFTVPQINTLLICTGFAEASYTVDPVEGSN
QSAANSTVTRVVVHMLKPTDFTCLSR
>tr|A0A0Q0FLG1|A0A0Q0FLG1_PSEAP DUF262 domain-containing protein OS=Pseudomonas syringae pv. aptata OX=83167 GN=ALO85_03952 PE=4 SV=1
MSAQKYSVNQHLIETLLSWVKSGEIAIPEIQRPFVWDASKVRDLMDSLYQGFPVGYIIAW
RNPTVKLKDGSLADAAEVLRVPRLMPDFIR
>tr|A0A0Q0C0A9|A0A0Q0C0A9_PSEAP RDD domain-containing protein OS=Pseudomonas syringae pv. aptata OX=83167 GN=ALO85_02388 PE=4 SV=1
MSSRPASPRSAALPTPLDTRIEIETPEGIDMLLRPAGLVSRALAFGIDFAIRAALLGVFF
VLLQLFDKLGMGLAALVFFLVNWWYMVLFEVLNQGRTPGKRLLGLRVVHDDGTPIDWSSS
LIRNLLRFVDMLPLGYGLGAITCLNHPLFKRLGDLAAGTLVIYSDRPVARPTMPQAEPVM
VPFALRLDEQRAVLGLAERQAELSSARTQELAAILAEPLRIPADTAVAHVNGIARNLLGP
T
>tr|A0A0N8TAC3|A0A0N8TAC3_PSEAP Cobalamin synthesis protein/P47K:cobalamin synthesis protein/P47K OS=Pseudomonas syringae pv. aptata OX=83167 GN=ALO85_01450 PE=4 SV=1
MAARGIRRSRHEQGSNLAAAWRILMANLLPVTVLSGFLGAGKSTLLNYVLRNRENLRVAV
IVNDMSEINIDGSEVQRDVSLNRAEEKLIEMSNGCICCTLREDLLEEVSRLAQDGRFDYL
LIESTGISEPLPVAETFTFRDEAGKSLMDLARLDTMVTVVDGVNFLLDYEAADGLSTRGE
TMGEDDDRSITDLLIEQIEFADVILLSKIDLISSSEREELMAILKRLNPEAEIVPMVMGQ
VPLATILNTGRFDFQRASQAPGWLKEMRGEHLPETEEYGIASTAYRARRPFHPERFFDFI
NRPWSNGKLLRSKGFFWLASKYKDAGSWSQAGGLMRHGFAGRWWRFVNRDSWPDDKESVS
SILENWTTETGDCRQELVFIGQNIDFAQLTAELDNCLLTDAEMEAGADRWRALADPFGDW
HEEVAA
>tr|A0A0Q0DQS9|A0A0Q0DQS9_PSEAP Diaminopimelate epimerase OS=Pseudomonas syringae pv. aptata OX=83167 GN=dapF PE=3 SV=1
MLLRFTKMHGLGNDFMVLDLVSQHAHILPKHAKQWGDRHTGIGFDQLLLVEAPNNPDVDF
RYRIFNSDGSEVEQCGNGARCFARFVLDKRLTAKKQIRVETKSGIIELDIRSDGQISVDM
GPPRFVPEEIPFEAAEQAISYTVDVDGQNIEMAAVSMGNPHAVLRVDDINNAPVHELGPK
IEHHPRFPARVNVGFLHVVDRQRAQLRVWERGAGETQACGTGACAAAVAAISQGWMDSPL
LIDLPGGRLSIEWAGPGHSVMMTGPAVRVYEGQVRL
>tr|A0A0Q0DDR9|A0A0Q0DDR9_PSEAP D-2-hydroxyglutarate dehydrogenase OS=Pseudomonas syringae pv. aptata OX=83167 GN=ALO85_100263 PE=4 SV=1

MPMNRSRVDSMTDRVLIDELQTLVDPGKVLTDAGSLETYGKDWTKQFTPAPLAIVFPKTT
EQVQAIVRWANERLVALVPSGGRTGLSAAAVAANGEVVVSFDYMNQVLDLNLTDRTAVCQ
PGVITRQLQALAEENGLYYPVDFASSGSSQIGGNIGTNAGGIKVIRYGMTRNWVAGLKVV
TGKGDLLELNKDLIKNATGYDLRQLFIGAEGTLGFVVEATMRLDRAPKNLTAMVLGTSDF
NSIMPVLHAFHGKLDLTAFEFFSDKSFARVMARGDVPSPFDTPCPFYVLLEFEATTEDLA
DQALATFEHCVEQGWVLDGVMSQSEQQLRNLWKLREYISETISHFTPYKNDISVTVSKVP
EFLAEIDAIVAEHYPDFEVLWYGHIGDGNLHLNILKPESLDKDEFFVKCARVNKWVFETV
EKYNGSISAEHGVGMTKRDYLTYSRSPVEIEYMKALKAVFDPNWIMNPGKIFAV
>tr|A0A0N8T8T0|A0A0N8T8T0_PSEAP Cytochrome c-type biogenesis protein CcmE OS=Pseudomonas syringae pv. aptata OX=83167 GN=ccmE PE=3 SV=1
MNPLRKKRLLIIVALLAGVGLAVTLALGALQENINLFYTPSQIANGEAPLDRRIRAGGMV
EKGSLQRSADSLDVRFVVTDFNKSVTITYRGILPDLFREGQGIVALGKLNAQGVVVADEV
LAKHDEKYMPPEVTKALRDSGQAAPAVPSTPVKQG
>tr|A0A0N8T874|A0A0N8T874_PSEAP Uncharacterized protein OS=Pseudomonas syringae pv. aptata OX=83167 GN=ALO85_04773 PE=4 SV=1
MPWGDVHLKISPYAAPLPSKANTDSVIESLFMRLSLLDASMAQGYHLLFVAAISLCQARS
SDTSHGCGHRLSEYQVLNLKTEAHMTGTQPGSNSGNKNDPGFDEKSPNTQDPQIDPTEPA
KTPATDKGSKDDYPPYTPDK
>tr|A0A0N8TAA5|A0A0N8TAA5_PSEAP Tellurium resistance protein TerD OS=Pseudomonas syringae pv. aptata OX=83167 GN=ALO85_03309 PE=4 SV=1
MALTLQKGGNLSLSKTDPTLTKVLIGLGWDPRATDGQDFDLDASAFLLAANGKVRSEADF
IFYNQLKSADGSVEHTGDNRTGAGDGDDEVVKVDLARVPADVEKVVFVVTIHDADSRKQN
FGQVGGSFIRVLNEQSGSEVVRYDLAEDASTETAMIFAELYRNNGEWKFRAVGSGFAGGL
KAVANSFGMNF
>tr|A0A0Q0FIB3|A0A0Q0FIB3_PSEAP GntR family transcriptional regulator OS=Pseudomonas syringae pv. aptata OX=83167 GN=ALO85_00661 PE=4 SV=1
MQEPASTAPKRRQHSLATDLVTELSQRILLGKIAPGQKLPSENQIVREHGVSRTVVREAI
SKLQASGLVVTHHGIGTFVLERSDQTGLRLKVETVSRVRDIIELRIGLETQAVALAAVRR
TDEQLAAMRQALDDYQDLLANDDSCVEADKRFHMLIAEATGNPYFLEIMQHLGSAVIPRS
RIASSERAGNNLAHQGYLANLEHEALLSAIRRKDPDAARAAMWTHLSNSRERLVPLE
>tr|A0A0Q0BZ97|A0A0Q0BZ97_PSEAP Taurine dioxygenase OS=Pseudomonas syringae pv. aptata OX=83167 GN=ALO85_02447 PE=4 SV=1
MDQMALKAIEGIAGAVRAPYQSITVNRLTPIIGAEVGGVDLSQPLSAEQLTEIRRAFLEN
HVLVFRDQHLTVEQHKAFGRLFGPLRALPVDSIDGDDPELVVVRANAQSRFAAGELWHTD
GTADMEPSMGSMLYVKETPAIGTGGDTLFANMHLAIEMLSPAMQQFLGGLTAIHDGEIPW
KGYQPPPGLPKSEHPLVVRHPETGRRSLFVNSGFTSHIVQLSPGESHAVLTMLFDLIARE
PSLSCRVRWEPNTLVFWDNRCTQHHAVWDYFPHSRYGERVTILGGRPQA
>tr|A0A0Q0IF98|A0A0Q0IF98_PSEAP Putative cytochrome OS=Pseudomonas syringae pv. aptata OX=83167 GN=ALO85_03252 PE=4 SV=1
MQWRNSATCFGGLAKFFHWTSAASFIAAYIVVYYVIWFMDDTSPESLPVLNIHWVLGLLV
GFLVLPRLLWRLLDMQPDNPPGSALEHILAHVAHGGLYGLMIAMPLTGYLGTGAPTDFGL
FSVTGFNETKLFAWFSVTFSLSYEAFEAPIDTVHHFLGKWVAWVVVSLHVLAALFHHLVR
RDSVLRRMLPWSSLPKAS
>tr|A0A0Q0BTX7|A0A0Q0BTX7_PSEAP Penicillin-binding protein 1B OS=Pseudomonas syringae pv. aptata OX=83167 GN=ALO85_100041 PE=3 SV=1
MTRSRTSRSAKKKPPRSLNKWLGWALKLGIVGLVLLGGLAIYLDAIVQEKFSGKRWTIPA
KVYARPLELFVGQKLSRDDFLIELDALGYRRESVANGPGAAAVNGNTVDLNTRGFQFYEG
TDAAQQVRVRFSGDYVADLSSGNGAKLAVARLEPLLIGGLYPKNLEDRILIKLDQAPPYL
LDGLVAVEDRDFYHHFGVSPKSIARAIWVNTSQGQMRQGGSTLTQQLVKNFYLTNERSLT
RKLTEAMMAVLLEIHYSKQEILEAYLNEVFVGQDGQRAVHGFGLASQYFFSQPLSELKIH
QVAMLVGLVKGPSFYNPRRNPERALERRNLVLDLFEQQGVATPEVVAAAKKMPLGVTKTG
TLADSSFPAFLDLVKRQLREDYRDEDLTEEGLRIFTSFDPILQMKSQAAMDDTFKKLAGR
KGSDEVESAMVVTNPETGEVQALIGGREAGFSGFNRAIDAVRPIGSLVKPAIYLTALERP
SQYTLTSWIADELFQVKGADGQIWKPQNYDHKSHGNIFLYQGLAHSYNLSTAKLGLELGI
PNVFKTLAKLGVTREWPTYPSMLLGAGGLSPMEVATMYQTIASGGFNTPMRGIRSVLTAE
GEPLKRYPFQIEQRFDPGAIYLVQNAMQRVMREGTGKSVYNVLPSSLNLAGKTGTSNDSR
DSWFAGFSQDLLAVVWMGRDDNGKTPFTGATGALQVWTSFMRKAGPLPLDMAMPDNVVQA
WVDAQTGQGSDASCPNAVQMPYIRGSEPQPGATCGGAPAPATEVMDWVKGWLN
>tr|A0A0Q0C496|A0A0Q0C496_PSEAP TsaA-like domain-containing protein OS=Pseudomonas syringae pv. aptata OX=83167 GN=ALO85_00263 PE=4 SV=1

MSYNVSPVGFVRSCFKEKFAIPRQPQLAPAARGVLELLAPFDQGEAVQGLEQVSHVWLLF
LFHLALEDKPRLKVRPPRLGGNQSMGVFATRATHRPNGIGQSVVKLDKVEAGRLWLSGID
LLDGTPVLDIKPYVPYADAIGDATNHIASAAPALIPVHWQDAALQQAREHALRLNEPLVE
LIEQCLAQDPRPAYQLPTPERRYGAQFWDLDVRWHYPQAGVICVLEVLIA
>tr|A0A0Q0IHS9|A0A0Q0IHS9_PSEAP Phenylacetic acid degradation-related protein OS=Pseudomonas syringae pv. aptata OX=83167 GN=ALO85_02215 PE=4 SV=1
MSIWRQAPDIEALMAAQKSTIGEVLDIRFESFTDDSLTASMVVDQRTHQPFGLLHGGASV
VLAESLGSMASYLIVDPSKYFCVGLEVNANHLRGVRSGRVTGVVRPVHIGRTTHVWDIRI
SNDEGKPSCISRLTVAVVAHGKEPPVN
>tr|A0A0Q0DVU1|A0A0Q0DVU1_PSEAP Homoserine O-succinyltransferase OS=Pseudomonas syringae pv. aptata OX=83167 GN=metXS PE=3 SV=1
MPTVFPHDSVGLVTPQTAHFSEPLALACGRSLPAYDLIYETYGQLNAARSNAVLICHALS
GHHHAAGFHSADDRKPGWWDSCIGPGKPIDTQKFFVVSLNNLGGCNGSTGPSSIDPDTGK
PFGANFPVVTVEDWVNSQARLADLLDIDTWAAVIGGSLGGMQALQWTISYPHRVRHCLAI
ASAPKLSAQNIAFNEVARQAILTDPEFHGGSFQERGVIPKRGLMLARMVGHITYLSDDSM
GEKFGRGLKSEKLNYDFHSVEFQVESYLRYQGEEFSGRFDANTYLLMTKALDYFDPAANF
NDDLAKTFANATAKFCVMSFTTDWRFSPARSRELVDALMAARKDVCYLEIDAPQGHDAFL
IPIPRYLQAFGNYMNRISL
>tr|A0A0Q0IBA2|A0A0Q0IBA2_PSEAP PLDc_N domain-containing protein OS=Pseudomonas syringae pv. aptata OX=83167 GN=ALO85_00098 PE=4 SV=1
MGWISENHLWLFAALTALIVISDIWAVLRIRKSKTQSSNKLMWIIVVVAVPIGGVLALLI
AGPRHVDTPARPEQQSTKY
>tr|A0A0Q0BT04|A0A0Q0BT04_PSEAP tRNA (guanine-N(7)-)-methyltransferase OS=Pseudomonas syringae pv. aptata OX=83167 GN=trmB PE=3 SV=1
MMTDSHVPHPESPTVEEGEERPHRRIKSFVMRAGRMTEGQQRGLDQGLPLYGLSLTDTPV
DFDQVFGRVAPRTLEIGFGMGHSLLEMAAAAPEHDFIGVEVHSPGVGALLNGVLTQGLTN
VRVYDCDAIEVLSRCVADNSLDRLMLFFPDPWHKSRHHKRRIVQPEFAALVRSKLKVGGV
FHMATDWGPYAEYMLEVMSVAPGYRNQAEDNQYVPRPAERPITKFERRGEKLGHGVWDLK
FEKVD
>tr|A0A0Q0FNH8|A0A0Q0FNH8_PSEAP Drug/metabolite transporter superfamily permease OS=Pseudomonas syringae pv. aptata OX=83167 GN=ALO85_02709 PE=4 SV=1
MKSISRTPRISKAEAVLILITMLWGGTFLLVQHAMTVSGPMFFVGLRFAAAALIVALFSM
KVLRGLTFLELKAGVFIGIAIMLGYGLQTIGLQTIPSSQSAFITALYVPCVPLLQWLVLG
RRPGLMPTLGIILAFTGLMLVAGPQGASLQLSSGEIVTLISTVAIAAEIIMISAYAGEVD
VRRVTVVQLATASALAFLMIVPTEEHLPDFSWLLVFSAVGLGTMSAAIQIAMNWAQKSVS
PTRATVIYAGEPVWAGIVGRLAGERLPGVALLGAALIVAGVIVSEMKRRSASDDQSIVDE
EGNEQAVRKAE
>tr|A0A0Q0C556|A0A0Q0C556_PSEAP Outer membrane protein assembly factor BamB OS=Pseudomonas syringae pv. aptata OX=83167 GN=bamB PE=3 SV=1
MWRKGMRDVIRWKHAALLALAILAAGCSSNSKKELPPAELTDFKEEVVLHKQWSRSVGDG
QGKTYNMLVPAIDGDRIYAADVTGEVVSLDRLTGDVIWKKDLDLPVSGAVGVGYGLVMIG
TLKGEVIAMDATSGEEKWRARVTSEVLAPPATNGSVVVVQTQDDRIVGFDASTGSQLWIY
ESTPAVLTLRGTGAPLATNRLAVAGLSTGKVVALDITNGVPVWEQRVAIPQGRSELDRVV
DIDGGLLLSGGTLYVATYQGRVAGLDLESGRVLWQRDASSYSGVAQGFGSVYATLASGTV
EGIDERSSSALWSNESLARRQLGAPEVYSSYVAVGDMEGYLHLLSQVDGRFVGRERIDSD
GLRARPLVVGDMIYVYGNSGKLEALTIKQ
>tr|A0A0Q0D8B0|A0A0Q0D8B0_PSEAP GntR family transcriptional regulator OS=Pseudomonas syringae pv. aptata OX=83167 GN=ALO85_02091 PE=4 SV=1
MADEKPLIRKRRGAGRVTITAVARQAGVSAITVSRYFNQPEQVSSELRERIASTVSALGY
VPNLVAGGLASARSRVVAMVIPNISGPIFANTIQGFSDTLSRHGFQLLLASSYFSAEQEE
SAVRAFLGWSPAALVLTSRFHSPATENMIEQTDVPVIETWDHQPDRSPIQIGFSHYDVGV
MAARYLHDKGYRRIAFVQNSIAGDFSALERRDGYAATVEGLGITPWVFVPDADRAPFEAG
KQAMDTLMNQAPRPDAIFFANDNLAAGALLASQRAGLSIPQDCAVMGFGDYAFAEMMLPS
LTTIKPPALQIGVMAATRVLESLGVLPVEGEIQRLNLLECSLVEREST
>tr|A0A0Q0C6L1|A0A0Q0C6L1_PSEAP Cytochrome c2 OS=Pseudomonas syringae pv. aptata OX=83167 GN=ALO85_01230 PE=4 SV=1
MKKTALLLMALTLPALAQGAGDAEAGKAVFTRLCSGCHKIGPSARSAFGPPLNGIIGRQA
GQQEGYVYTDAMKNSGLVWTREVLRTYIKDPSEVVPGTRMKLWWMGNDERMEDLLEYLDA

NK
>tr|A0A0Q0ILJ7|A0A0Q0ILJ7_PSEAP DUF4398 domain-containing protein OS=Pseudomonas syringae pv. aptata OX=83167 GN=ALO85_00331 PE=4 SV=1
MELINMNTLTAKTKFKGLRGLKLAALALGSTFILAGCAGNPPSEQYAVSQSAVNSAVSAG
GTEFAAVEMKSAQDKLKAADLAMQDHKYEEARRLSEQAEWDARVAERKSQAAKAAKAVQD
ARQGVNELREEGLRQVQ
>tr|A0A0Q0IFW1|A0A0Q0IFW1_PSEAP Two-component DNA-binding response regulator OS=Pseudomonas syringae pv. aptata OX=83167 GN=ALO85_00636 PE=4 SV=1
MNILVIEDHRDIHDNLLEFFELRGHAVEGALDGLSGLHLAASKRFDAIILDIMLPGIDGN
QICHSLRQYSKSEVAIVMLSARDELDDRLTGFKVGADDYITKPFAMSEVLARVEAIVSRR
QRQDNRMMVVADLQFDLDTLEVTRKGVPLKLNPTNMKLLELLMRKSPHIVKRSELEEALW
GRDAPNSSSLRSNIHVLRRVLDGGHDTPLLHTAHGLGYKLCVQR
>tr|A0A0Q0D8H5|A0A0Q0D8H5_PSEAP Polyketide cye SnoaL-like domain protein OS=Pseudomonas syringae pv. aptata OX=83167 GN=ALO85_01906 PE=4 SV=1
MNKLHSNREVLRAVYKDMTRITEFADADIVLHKADQGAGGGLSIAIGREAVLSHQINLIR
RTHQTLYMDVHDIIANDHFGSVLGEMRASCEGRKIIMPFCDLWRFRDGRIIECWENVYDV
RALGNFINGKEPVLNQWRYG
>tr|A0A0N8T959|A0A0N8T959_PSEAP Lipoprotein signal peptidase OS=Pseudomonas syringae pv. aptata OX=83167 GN=lspA PE=3 SV=1
MPNASFGRLAWLWLSVLVLVIDQASKYYFENALSLYQQIIVIPDLFSWTLAYNTGAAFSF
LADGAGWQRWLFALIAVVVSAVLVVWLKRLGRDDTWLAVALALVLGGALGNLYDRIVLGH
VIDFILVHWQNRWYFPAFNVADSAITVGAIMLALDMFKSKKTGETVND
>tr|A0A0Q0BUT3|A0A0Q0BUT3_PSEAP Uncharacterized protein OS=Pseudomonas syringae pv. aptata OX=83167 GN=ALO85_03516 PE=4 SV=1
MTNVIAFPDAHERDRLKSSQDPALRYLNKKERQLIDQLRSTSHAGRQYVYDYASIMHLSR
PLYPEPVD
>tr|A0A0Q0DJ83|A0A0Q0DJ83_PSEAP Short-chain dehydrogenase/reductase SDR OS=Pseudomonas syringae pv. aptata OX=83167 GN=ALO85_01874 PE=4 SV=1
MKLEGKIAVVTGASKGIGAGIAKALGAEGATVIVNYATGKADADAVVAWIAEHGGSAFAV
QADMSQSADVIRLFETVGTKYGALDILVNNAGVAVFQMIDDLTEEAFHTQFNLNVLGYLL
AVREAVKLLGPTGSIINISSILSTDPYLASSVYAATKGAVDTLTFALARELGARGIRVNS
ILPGHTNTPATHGHFAGELGEKILAGTPLGRFGEPEDIAPLAVFLASQDSHWITGESIRA
SGGVRGVGY
>tr|A0A0N8T882|A0A0N8T882_PSEAP Short-chain dehydrogenase/reductase SDR OS=Pseudomonas syringae pv. aptata OX=83167 GN=ALO85_01235 PE=4 SV=1
MIEHLKLAGKVALVQGGSRGIGAAIVQRLAKEGAAVAFTYVSSEVSALEIQDSIVANGGR
ALAIRADSADEKAIRQAVQTTAETLGRLDILVNNAGILAIAPLNEFSMQDFDKTLAINVR
SVFIASQEAARHMEEGGRIINIGSTNADRMPFAGGATYAMSKSALIGLTKGMARDLGPQG
ITVNNVQPGPVDTDMNPAQGEFAETLKALMALPRYGTSEEIASFVAYLAGPEAGYITGAS
LTIDGGFSA
>tr|A0A0N8T829|A0A0N8T829_PSEAP Uncharacterized protein OS=Pseudomonas syringae pv. aptata OX=83167 GN=ALO85_00142 PE=4 SV=1
MPYSKGFQKRETPLPMSRGLKRTLGSLLAVIALYSLLGFFILPGVALRIANQQLANYATV
PAKLERLEFNPFTLELTLWGLNIGTPGKEQVAFEKLYANLQINSLWTRALHLQRVELIKP
RTELLFDKQGKLNLAQLFKLPASEPAKDEPATKPFPLRIDEIKLAGGYVHFQDLRPSQPI
EFLYDALDFELKNLSTLPEDNADMTLVAAGPDGGQIDWVGRISLIPIASEGTLKVTDSKM
KLWWPYVRDALPLALEDGVLNFSTAYTLNLSKETELKLTNVSASVAPFVLNAPDGRPLVR
LQRLDVSETSVDLAKQLVTVGKVRSQKLETWAAREADGQLDWQKLFASQARKPAAAHTPA
VDEKAAEPAPATETTAKASAQPVAQSKPWQVLLRDVQLRNYMVHLADRVPQEPVALDVGP
LNLDIQNFDSLNKAPFTLKLDTGLGKQGNLTAAGDVNLNPVSARLNVTTRDIDLRLAQAY
ISPFIRLELRSGMLGSDLAVDLKSTEPLAFNVTGKAQVNQLHTLDTLKQRDLLKWQQLAL
EGLDYQHGKGLSIAKVNMDQPYARFMINEDRTTNIDDLLIPQPADAATGKTAAQPKAART
QAAGKKENPLAIYVGEVNINNGSANFADMTLTPNFATAVQQLNGRIGTIDNRKPTPAPVD
IEGKVDRYAPVTIKGSLNPFDPMASLDITTSFKRVELTNLTPYSGKFAGFRIRKGRLNLD
LHYLITKGQLKAQNKVLVEQLQLGERVDSPDALDLPIRLAVALLKDTKGRISLELPIEGD
LNNPQFSVMPIVWQTLRNLVLRAAQAPFKMLGGLVAGGSSEDLGSVSFAPGASDLSAEAQ
KALDKLSVALKERPDLKLEIEGTSAASSDGPLIAQQRLEREYQYTYYKILQRRGDKVPAK
AGLIEVPEEEKAPMLEGIYRTRLKQQPPAEWTSLGKEQRAAQMRAAVLKFWSSNEVLLRE
LGQSRASSIKDYLVDKGKLEDARVYFVDAQLGQAQPDGKVISPLHLDSE

>tr|A0A0Q0FHS6|A0A0Q0FHS6_PSEAP Uncharacterized protein OS=Pseudomonas syringae pv. aptata OX=83167 GN=ALO85_03536 PE=4 SV=1
MIDAFERTGPLMEASSYPAWAQHLIKDCSAAKARVVEHELYQQMRDAKLSPQIMRHYLIG
GWPVVEQFAVYMAKNLTKTRFGRHPGEDMARRWLMRNIRVELNHADYWVNWCAAHEVTLE
DLHDQRVAPELHALSHWCWQTSSSDSLAVAMAATNYAIEGATGEWSAVVCSSGVYAEAFP
EETRKKAMKWLKMHAQYDDAHPWEALEIICTLVGNKPSVQLQAELRQAVTKSYDYMYLFL
ERCMQLDRVKPRGRVAALEA
>tr|A0A0Q0DL53|A0A0Q0DL53_PSEAP Putative regulator of the myo-inositol utilization operon IolR OS=Pseudomonas syringae pv. aptata OX=83167 GN=ALO85_02687 PE=4 SV=1
MTSTDQPTLPGTSVDMPPNSAEGLLRLITDEYEGLPRQLKRIASYMSQQSDRIMVDRISD
IARECEVHPSAIVRFSQRFGFSGFSEMQALFREAYTHKTTPVQNYQQRIRSLIANKSQKA
NAGDLARECVSATLSGLERLSVELDDDEFEKAVDLVVNADNIYVVGVRRSFAVADYLVYN
LQHTNKRIHLISGLGGSYREQMRSVRANDLVIAISFTPYGKETQHCLRIAQHHQAKTLII
TDSNLSPLAKRANSVLLVNEGSSFAFRSLSATLCLCQALFIAVAYRLELKVDEIHEQVGF
DD
>tr|A0A0Q0BTL0|A0A0Q0BTL0_PSEAP Putative lipoprotein OS=Pseudomonas syringae pv. aptata OX=83167 GN=ALO85_03095 PE=4 SV=1
MNKLLLVALALALTGCATARTLNAAKPGAPVVYAGTRLNMYALQGGCCAADRFGAEAPSY
PGLDLPGSALLDTLLLPLSLLTAAGLGVQATGGL
>tr|A0A0Q0CIY5|A0A0Q0CIY5_PSEAP Uncharacterized protein OS=Pseudomonas syringae pv. aptata OX=83167 GN=ALO85_01516 PE=4 SV=1
MNAREANLIAHRYQARAQAFNDLHALLAPFFRRTPLAASMNEISECVSEALHANTLCGWL
PDFGDFDELEALVGEIRRDGGRKRFTSLNDIPTHLREHFDDTDEAFTKFANEIREECRDG
YDSLLEQQEILNEHLESVRFDQVFAFDEDSLEVETTRLINQVFDHLHTQWLAYEKLARSL
VGMAHLIDEPDPDKGLTEALLFD
>tr|A0A0Q0C7C7|A0A0Q0C7C7_PSEAP Dioxygenase, TauD/TfdA family OS=Pseudomonas syringae pv. aptata OX=83167 GN=ALO85_04931 PE=4 SV=1
MPAASLASSSAQPASQSFEVRPFTEKVGAEIVGLDLSRPLNDADFARVHQAHLDHHVLVF
RDQQITPQQQIDFSRRFGVLQIHVLKQFLLANHPEILIVSNIVENDKPVGLGDAGKYWHS
DLSYKELPSLGSMLYAQELPSEGGDTLFADMHLAWETLPQHLRDAVEGRSAVHSYTARYA
EGHNAANWRPTLSAEQLAQVVEVSHPIVRTHPENGRKALFVSEGFTTRILGLPEDESRQI
LNEIYAHSVKPEHIYRHQWQANDMVFWDNRSLIHLAAGCPAHLRRKLHRTTIQGTAPF
>tr|A0A0Q0FLY1|A0A0Q0FLY1_PSEAP Uncharacterized protein OS=Pseudomonas syringae pv. aptata OX=83167 GN=ALO85_02463 PE=4 SV=1
MPRPAWSLYAYQMLEPDEQLDLFACQEVRVHLIARQLELGGSIDRTLCGTLLPAQPQMRA
VELQAMRDERLCPLCKAIMDAQRRGMNPIWPEL
>tr|A0A0Q0BUR3|A0A0Q0BUR3_PSEAP Putative small protein OS=Pseudomonas syringae pv. aptata OX=83167 GN=ALO85_03508 PE=4 SV=1
MMKGQKQCVGVERADGCLQAGAVFTVLKGHLQRWLELHRQRHLLAQMSDGTLKDLGLSRA
DIQQEAERPFWDDPLKH
>tr|A0A0Q0BSS5|A0A0Q0BSS5_PSEAP ABC transporter permease OS=Pseudomonas syringae pv. aptata OX=83167 GN=ALO85_00073 PE=3 SV=1
MLAYILRRLLLIIPTLLGILIINFVIIQAAPGGPVEQMIAKIEGFDGATSRIAGGGAEVS
VAGSNYRGAQGLDPALVKEIERMYGFDKSAPERLWIMIKNYATLDFGDSFFRDAKVIDLI
VEKMPVSISLGLWSTLIMYLVSIPLGIAKATRHGSQFDVWTSSAIIVGYAIPAFLFAILL
IVVFAGGSYFNWFPLRGLTSNNYDELSTLGKLFDYFWHLVLPVTALVIGNFATMTLLTKN
SFLDEISKQYVTTAKAKGLSNHKVLYGHVFRNAMLLVIAGFPSAFIGIFFTGSLLVEVIF
SLDGLGLMSFEAAINRDYPVVFGTLFIFTLLGLVVKLIGDITYTLVDPRIDFESRKH
>tr|A0A0Q0CWP4|A0A0Q0CWP4_PSEAP Uncharacterized protein OS=Pseudomonas syringae pv. aptata OX=83167 GN=ALO85_100416 PE=4 SV=1
MRSELLMLSNVRLFRLAVGSVLLLAGMALLIVHGLNWIRVQGWW
>tr|A0A0Q0FEM2|A0A0Q0FEM2_PSEAP PilZ domain protein OS=Pseudomonas syringae pv. aptata OX=83167 GN=ALO85_01244 PE=4 SV=1
MSTLDEAGRREYYRIDDSVALEINPLSAADQASQDAMQDTSTLFDLLSELHVSEFESQHL
MRQLDERDRVLNSFLKSLSKRIDLLGEVVAHTALGKLGAPQPVKLSEGGIQFNSQQAFAV
GEQLSIKMVLMPQAAGLMLRARVCQCDTLADGNFDISTEFVNLPDAQRQLLARHVLQRQA
QQRRQALEQGQPSGN
>tr|A0A0Q0II46|A0A0Q0II46_PSEAP Sugar transferase OS=Pseudomonas syringae pv. aptata OX=83167 GN=ALO85_04350 PE=4 SV=1

MRTSVRGILRAHQSALSLAHRLLDLTVIVLIGYWQTGLTVTDSTEAWSHIFLAVLVFHWV
SEYHQLYGSWRGERILRELTKVFSYWAMTFITLLSLNSLLLNQTQLPDNGQMSWFALALA
VLCGYRLLIRLALHTLRRRGFNTRRVAIVGTGQVGERLARSIALAPWMGLILLGFYDAHP
QQMNLQAHKRRPPVLGDLQQLIEDARAGRIDKVYITLAFSAEPQLRELITGLSDTTASVY
LIPDVFMFDLLHARSESINGLASISIFDSPMDGAWSLAKRAEDIVLSSLILLLIAVPLLT
IAAAIKLTSAGPVLFRQRRYGLDGRSIMVWKFRSMSVQENTDVVLQATRNDARITPLGRF
LRRTSLDELPQFFNVLHGDMSIVGPRPHAIAHNEQYRRQVSGYMLRHKVKPGITGWAQIN
GWRGETDTLDKMRMRVEFDLEYIEHWSIWLDLKIILLTLLKGFVNKNAF
>tr|A0A0Q0D659|A0A0Q0D659_PSEAP Methionine aminopeptidase OS=Pseudomonas syringae pv. aptata OX=83167 GN=map PE=3 SV=1
MTITLKTPEDIEKMRIAGRLAADVLEMIAPHVKPGVTTEELDRLCHDYIVNEQKAIPAPL
NYKGFPKSICTSINQVVCHGIPSDKKLKDGDSLNIDVTVIKDGYHGDTSRMFHVGTVPVW
AERLSKVTQECLYKAIEIVKPGTRLGDIGAVIQKHAEKNGFSVVREFCGHGIGKVFHEEP
QVMHFGEAGTGIELQEGMTFTIEPMINQGRPETKILGDGWTAITKDRKLSAQWEHTLLVT
ADGYEIFTLRSDDTIAQKAP
>tr|A0A0Q0DGW1|A0A0Q0DGW1_PSEAP Probable RNA 2'-phosphotransferase OS=Pseudomonas syringae pv. aptata OX=83167 GN=kptA PE=3 SV=1
MSKQKHDELSKFLSYVLRHEPEAIGLELSRDGWADIDMLIACAAKNEQVFDRDFLERVVA
ESEKKRFLISEDRRYIRAAQGHSNSSVSIQYTQVVPPEFLYHGTATRFLESIFKEGLVAG
ARHYVHLSESKETARSVGLRYGKPIVLEIEALHMHNLGFKFFQAENNVWLIDKVPASWLV
LKG
>tr|A0A0Q0C142|A0A0Q0C142_PSEAP OsmC/Ohr family protein OS=Pseudomonas syringae pv. aptata OX=83167 GN=ALO85_00422 PE=4 SV=1
MAMKARIQWAGEAMFLGESGSGHVVVMDGPPESGGRNLGVRPMEMVLIGLGGCSNFDVVS
ILKKSRQPVESCEAFLDAERADEDPKVFTKIHLHFVVKGRGLKEAQVKRAIELSAEKYCS
ASIMLGNAGVKITHDYEIVELG
>tr|A0A0N8T7U4|A0A0N8T7U4_PSEAP Uncharacterized protein OS=Pseudomonas syringae pv. aptata OX=83167 GN=ALO85_100921 PE=4 SV=1
MKFNDTFFNRDLMLSLGTEETSGRFYLSLPVSNGLIDYEEYYEIDKASFDLFHTDLIAAD
AFATTCRRRERDDFLIQKPGAKRGTAIWPLTSGIDSGTR
>tr|A0A0Q0DH52|A0A0Q0DH52_PSEAP Outer-membrane lipoprotein carrier protein OS=Pseudomonas syringae pv. aptata OX=83167 GN=lolA PE=3 SV=1
MRLIRMLLATALTFSMIPAHADSKDVARLTQLLEKSQTLTARFSQLTLDGGGTQLQETTG
EMALQRPGLFNWHTDAPQEQLMVSDGKKVSLWDPDLEQVTIKKLDQRLTQTPALLLSGDV
SKISESFDITAKEAGGVIDFVLKPKTKDTLFDSLRLSFRNGIINDMQLIDSVGQRTNILF
TGVKANESIAASKFQFQIPKGADVIQE
>tr|A0A0Q0CEB1|A0A0Q0CEB1_PSEAP Chromosome replication initiation inhibitor protein OS=Pseudomonas syringae pv. aptata OX=83167 GN=ALO85_02362 PE=3 SV=1
MFDYKLLSALAAVIEQAGFERAAQVLGLSQSAVSQRIKLLEARIGLPVLVRATPPSPTDI
GRRLLNHVQQVRLLERDLQSQVPALDEEGMPERLRIALNADSLATWWAPAIGAFCTQHRL
LLDLVVEDQDVGLKRMRAGEVAACLCGSERPVAGARSQLLGAMRYRALASPAFIARHFPD
GVSPEALVRSAALVYGPDDLLQHRYLALLGVQDGFEHHLCPSSEGFIRMIEAGMGWGLAP
ELQVRDQLRNGTLLELLPDRCIDVPLYWHHWRNGGQLLTRLTEHLAQHSAHWLVPLSED
>tr|A0A0Q0IL49|A0A0Q0IL49_PSEAP Putative integral membrane protein, CopD superfamily OS=Pseudomonas syringae pv. aptata OX=83167 GN=ALO85_02736 PE=4 SV=1
MTAFSTVYTLHLLAALVWVGGMFFAWMVLRPAVIAALEGPSRLKVWVQVFPRFFAWVWAA
VVLLPITGIGMIQLNFTGFETAPRYVQIMMGLYVVMVALFLRIQSLQLPELRRAVEAEQW
AEGAAALGNIRRLVGINLIIGLAVVMLAAARPGL
>tr|A0A0N8T8G3|A0A0N8T8G3_PSEAP dTDP-glucose 4,6-dehydratase OS=Pseudomonas syringae pv. aptata OX=83167 GN=ALO85_01141 PE=3 SV=1
MILVTGGAGFIGSNFVLQWCARNGEPVLNLDALTYAGNLANLQSLEGNEQHRFVHGNIGD
AALLDRLFTEHRPRAVVHFAAESHVDRSITGPEAFVETNVMGTFRLLEAARAYWNGLETA
DKAAFRFLHVSTDEVYGTLGANDPAFTETTPYQPNSPYSASKAASDHLVRSYHHTYGMPV
LTTNCSNNYGPFHFPEKLIPLMIVNALAGKALPVYGDGQQIRDWLYVEDHCSGIRRVLEA
GALGETYNIGGWNEKANIDIVQTLCTLLDELAPAAARQVINQKTGQPVSAYAELITYVTD
RPGHDRRYAIDARKIERELGWKPAETFETGIRKTVEWYLTNQKWVNGVMDGSYRDWIVQQ
YEVSDA

>tr|A0A0N8T8Z1|A0A0N8T8Z1_PSEAP HU family DNA-binding protein HU-nu OS=Pseudomonas syringae pv. aptata OX=83167 GN=ALO85_01831 PE=3 SV=1
MALTKDQLIADIAEAIDAPKTTARNALEQLGQIVADQLENGVEITLPGIGKLKVAERPAR
TGRNPSTGAAIEIAAKKVIKFVPAKVLTDSINK
>tr|A0A0N8T832|A0A0N8T832_PSEAP Triosephosphate isomerase OS=Pseudomonas syringae pv. aptata OX=83167 GN=tpiA PE=3 SV=1
MRRPMVAGNWKMHGTRASVAELIEGLCKQSLPGSVDIAVMPASLFTCQVVEGLKATSIIV
GAQDAAIQAEQGALTGEVASSQLADAGCKLVLVGHSERRQLIGEQEDVLNKKFAAIQAKG
LTPVLCVGETLEERKAGQTLEVVGRQLNSVIAEFGVNALVNAVIAYEPVWAIGTGLTASP
QEAQEVHAAIRAQLAQENAEVAQGVRLLYGGSVKAANAVELFSMPDIDGGLIGGASLNAD
EFGAICRAAGN
>tr|A0A0Q0CDJ6|A0A0Q0CDJ6_PSEAP High-affinity leucine/isoleucine/valine ABC-type transport system OS=Pseudomonas syringae pv. aptata OX=83167 GN=ALO85_02747 PE=3 SV=1
MPELFHYMQQLVNGLTIGSTYALIAIGYTMVYGIIGMINFAHGEVYMIGSYVAFMALAGL
AMLGIHSLPLLMIAGFAAAIIVTSAYGYSIERVAYRPLRNSNRLIPLISAIGMSIFLQNS
VQLSQGSGNFAIPNLLPGSLSFGPGGAEEVLISYMQIVVFVVTLVAMTGLTLFISRSRMG
RACRACAEDMKMANLLGINTNNIIALTFVVGAALAAVAAMLLSMQYGVINPNAGFLVGLK
AFTAAVLGGIGSIPGAVLGGLVLGVAEAFGADIFGDQYKDVVAFGLLVLVLLFRPTGILG
RPEVEKV
>tr|A0A0Q0FMW5|A0A0Q0FMW5_PSEAP TspO/MBR family protein OS=Pseudomonas syringae pv. aptata OX=83167 GN=ALO85_00922 PE=4 SV=1
MTFFIFLLACCAAGATGIIFKPGAWYESLEKPSFTPPDWAFPVAWTTIYLLLAWAGYRLT
LIPGSEIVLALWAAQIALNTLWTPVFFGANRLFAAMVVLVLLWIVVAAMIVMALQLDIIT
GLILFPYLVWLSVAAALNFSILRHNR
>tr|A0A0Q0D366|A0A0Q0D366_PSEAP Anhydro-N-acetylmuramic acid kinase OS=Pseudomonas syringae pv. aptata OX=83167 GN=anmK PE=3 SV=1
MDFFYIGVMSGSSLDGIDIALLKQDDRSRLLATHYTPMPEDLYAELLGLCSSGADELARA
AIAEQKWCRLVAQGVQTLLEDQNMVPAQIRAIGSHGQTIRHEPARGYSIQIGNPALLAEL
TEITVVSDFRRRDIAAGGQGAPLVPAFHEALFDDNKDHRAVLNIGGFSNLSLIESDRPVE
GFDCGPGNVLLDAWIQSQRHESYDKDGAWAASGKVDSALLKKLLSDQFFLTKGPKSTGRE
VFNLGWVHHHLFQLPTLAPEDVQATLLELTALTITESLQSAQAITRELLVCGGGAHNKAL
MKRLAELLPGTEVSSTEKFGVDPDWVEAMAFAWLAHCCLEGVPANRPTVTGAKGRRVLGA
IYPA
>tr|A0A0Q0CB04|A0A0Q0CB04_PSEAP Anthranilate phosphoribosyltransferase OS=Pseudomonas syringae pv. aptata OX=83167 GN=trpD PE=3 SV=1
MSTPMDIKSALNRVVNQLDLTTEEMSDVMREIMTGQCTEAQIGAFLMGMRMKSETIDEIV
GAVSVMRELAGKVELKTLDGVVDIVGTGGDGANIFNVSTAAAFVIAAAGCTVAKHGNRAV
SGKSGSADLLEAAGVYLNLTPVQVARCIDSVGIGFMFAQSHHTAMKHTAGPRRELGLRTL
FNMLGPLTNPAGVRHQIVGVFNQALCRPLAEVLLRLGSKHVLVVHSQDGLDEFSLAAPTF
VAELKNGEVTEYWVQPEDLGIKSQSLYGLAVESPAASLELIRDALGRRKTENGQKAAEMI
VLNAGAALYAADHATSLKEGVALAHDALHTGLAREKLEELGAFTAVFKQENEA
>tr|A0A0Q0ILT9|A0A0Q0ILT9_PSEAP Uncharacterized protein OS=Pseudomonas syringae pv. aptata OX=83167 GN=ALO85_00353 PE=4 SV=1
MVIRMENISLLLSEALAPYQASIGPAGSRGERLVTLKDKSGTSVIERAFGADQLSDKRQL
TDVVDGLRRDLMVAEGRIEPCVIAAMRNAAISRTFVGTPSFA
>tr|A0A0Q0BX96|A0A0Q0BX96_PSEAP RNA polymerase sigma-70 family protein OS=Pseudomonas syringae pv. aptata OX=83167 GN=ALO85_01786 PE=3 SV=1
MNRNINDKYSHTQKSLPESTPPMSPRGLFPVIPVAHSALNHTVEGLYRAHNAWLTTWLRR
RLGCPHSAADLAQDTFIKVLGARDTAQIVEPRAFLTTIAKRVLCNHYRRQDLEHAYHQAL
AELPERVAPSEEERAIILETLVELDQLLDGLPPLVKRAFLLSQVDGLSHGRIAAELDISI
ATVKRHLHKAALRCYFAL
>tr|A0A0Q0DK15|A0A0Q0DK15_PSEAP Uncharacterized protein OS=Pseudomonas syringae pv. aptata OX=83167 GN=ALO85_02459 PE=4 SV=1
MGLIALLRGELLDTWQNQLPKDAPNYFVLNVLPADKENFAQTLSKLSTHAAPLYPVVPGR
LTSINGEPVSNFVTKDSRGENATRRDLSLTWAAELPEGNTLTAGNWWKALPDSGQSDAMP
GVSIEAKLADSLKIKLGDRLSFIVGGLTREAMVTSLRDVNWDTFQPNFFMIFQPGTLADL
PTTYLTSFYLPPGQDKQIVELSRAYPSISILGVEALLAQVRSILDQVTLAVQFVLLFVLA
AGIAVLFSGLQATLDERIRQGALLRALGAERALLIKSRRIEFGLLGAASGVLAALGCELV
SFVLYRYAFSLEWQPHLWLLLLPVIGALLVGGAGVFGTRRALNVSPLTVLREG

>tr|A0A0Q0E0H9|A0A0Q0E0H9_PSEAP Sucrose porin OS=Pseudomonas syringae pv. aptata OX=83167 GN=ALO85_03263 PE=3 SV=1
MWHPFFFDLHNFSLKRKGSANVTKTIKTKQEVHMQNASRWLLAGVLGTSTTVAQAATLEE
RMAAFEARASAAEKRAAAAEQQTQALARELQQLKLATPAVQAAAPTSTPTVAASPQPALD
DRVAKLEARQQSMEKPSGAGHLTDGFSFKGYARSGLLINDGLGGGRGGPYTTPAGSVGGA
VGRLGNEDDTYMRFDLSKEIYARNGTRSKFTVSIADGVESYNDWTATESKLNVRQVFTEL
DHIAAFKGNSVFENSTLWAGKRFDRDNFDIHWLDSDVVFLAGTGGGIYDVQMTKDWRSNY
SLIGRNYGDFSEGGVDADVESYILTSNQFFDNGQWQWMFNAIGAKKNDVGTRTNQAGFAP
ADSGLHTMLANHQKDFFGREGFFKTALLYGQGLGAQVKDIGSDGELLDDARALRLAMYGE
TPIAPGWRIGPSLLAEQSKDRYVKGDDYRWLTLNMRLANEINSNFEMAYELSWQTMDLNP
QGYLQRNAVDGNFWKFTVAPTFKPDMGDLLTRPELRVFASLMNWSSDLDRYSTTGNFGKS
DFNAGGVWQFGIQMETWF
>tr|A0A0N8T815|A0A0N8T815_PSEAP Aspartyl/glutamyl-tRNA(Asn/Gln) amidotransferase subunit B OS=Pseudomonas syringae pv. aptata OX=83167 GN=gatB PE=3 SV=1
MQWEVVIGLEIHTQLSTQSKIFSGSATTFGSEPNTQASLVDLGMPGVLPVLNKEAVRMAV
KFGLAVNAEIGQHNVFARKNYFYPDLPKGYQISQMELPIVGKGHLDIPLEDGTVKRIGIT
RAHLEEDAGKSLHEDFQGMTGIDLNRAGTPLLEIVSEPDMRNAKEAVAYVKAIHAIVRYL
GICDGNMAEGSLRCDCNVSIRPKGQVEFGTRCEIKNVNSFRFIEKAINSEIQRQIDLIED
GGKVIQQTRLYDPNTNETRAMRSKEEANDYRYFPDPDLLPVIIEDSFLEETRATLPELPP
QKRERFQSQFGLSTYDASVLASSREQADYFEQVVSISGDAKLAANWVMVELGSLLNKQGL
EIDQSPVSAEQLGGMLKRITDNTISGKIAKMVFEAMANGEGSADEVIEKRGLKQVTDSGA
IESMLDEVLAANAEQVEQYRAADEAKRGKMFGFFVGQAMKASKGKANPQQVNELLKAKLE
G
>tr|A0A0Q0BXG1|A0A0Q0BXG1_PSEAP Octanoyltransferase OS=Pseudomonas syringae pv. aptata OX=83167 GN=lipB PE=3 SV=1
MAATLGFRDLGLIDYETAWHAMKRFTDERGREAADEVWLVQHPPVFTQGQSGKAEHLLLP
GNIPVVQVDRGGQVTYHGPGQLVAYLMLDVRRLGFGVRDLVTRIENTLIALLADYGVKAA
AKADAPGVYVDGAKIASLGLRIRNGCSFHGLALNVDMDLEPFRRINPCGYAGLAMTQLSD
QAGQIEFSEVSARLRAQLVKHLDYAEQATLTGGINHYD
>tr|A0A0Q0CDX0|A0A0Q0CDX0_PSEAP PAS/PAC and GAF sensor-containing diguanylate cyclase/phosphodiesterase OS=Pseudomonas syringae pv. aptata OX=83167 GN=ALO85_05191 PE=4 SV=1
MNSYPVGDDDEERIRILNAFSLMDTPPEHEYDQIVQMASRIFDVPIVLISLVHRDRQFFK
ARVGLDVCETGRDVSFCNFALMGRNVFLVPDALQDERFSSNALVVGAPHIRFYAGAPLIT
ASGHVLGSLCLIDNKPRETFSERDQRVLQDLAVMVIERMELRRLTLENEDSHTRFMNITS
TSPDAIICSDSKNRIISWNNSAELIFGHRSEDAMGKPLDIIIPPEMRSRHHAGLSRVAAG
GPASIIGKSINLTAIHRDGHTLPIELSLSQWTYAGEPQFGAIIRDITQRVEADKLLKHAA
EYDHLTGLANRSTLKRRIKEACDAQLSAAILLIDLDGFKDVNDTLGHAAGDFVLKVTSHR
LREHVPACHMVSRLGGDEFVVFLSDTADPDKAIKLGLALIAVIEEPIEFEDNSIYIGASI
GVSVRSGADYDDEQMLGNADLALYQAKSDGRSLVRTFTSEHRQTASRRGALSSSMRQAWA
NKEFELYYQPQVRLADGRITGAEALIRWNHPTLGVVSPAVFLPALEAGLLAVPVGEWILR
SACEQAAKWRNTGCEEFRIGVNLFAAQFRMRDFAEMVESALEDFSLPAHSLELEITENII
LRNEQRIMEPLRHLRSLGVGIAFDDFGTGYASLSLLKDYPVTRLKIDRSFVSGVERSKKD
EVIVEAVVRLANGFNLQVTAEGIETREQESLMRHYVCDEGQGYLYGRPMPAHDFTERYIS
SHNRQADVLKHK
>tr|A0A0Q0C9S8|A0A0Q0C9S8_PSEAP Tellurium resistance protein TerE OS=Pseudomonas syringae pv. aptata OX=83167 GN=ALO85_03310 PE=4 SV=1
MAVSLSKGGNVSLSKEAPGLTEITVGLGWDPRVTDGTEFDLDASIFIVGENGKVLDDNSF
IFYNNKKSADGSVEHLGDNRSGAGEGDDESVTVKLTGLAAAVKKLVFAVTIHSAEERKQS
FGQVSNAYIRVVNKADGKEIARYDLSEDASTETAMIFGELYRNGEEFKFKAIGQGFAGGL
KPLAEAHGVAIG
>tr|A0A0Q0C0T1|A0A0Q0C0T1_PSEAP Alpha/beta hydrolase fold protein OS=Pseudomonas syringae pv. aptata OX=83167 GN=ALO85_200176 PE=4 SV=1
MSTFNTKDGTEIYYKDWGTGKPVLFSHGWPLDADMWEYQMEYLSSRGYRTIAFDRRGFGR
SGQPWTGYDYDTFADDIADLIEHLDLRDVTLVGFSMGGGDVTRYIANYGSERVAKLALLG
SVTPFFLKTADNPEGVEQSVFDGITEGLLKDRAQFISDFASTFYGINHGQKVSEGVQTQT
LNIALLASLKGTLDCVTAFSATDFRPDMAKIDVPTLVIHGDDDQVVPFEASGKRAAAMIK
GAELKVYPGAPHGFAVTHAETLNKDLLAFLQG

>tr|A0A0Q0C5U2|A0A0Q0C5U2_PSEAP Uncharacterized protein OS=Pseudomonas syringae pv. aptata OX=83167 GN=ALO85_04422 PE=4 SV=1
MEHSLLVWLLPTLALVAGVVIGFLVARLLPNTVPNSTQRQLDDIQERFDAYQNEVVTHFN
STANLVQKLSQSYQDVQEHLAEGANRLALDELTRQRLLAALHPEANHAHRDHRDRLTPPR
DTETPKDYAPKSPNSPGMLDEHYGLKK
>tr|A0A0Q0DTJ5|A0A0Q0DTJ5_PSEAP C4-dicarboxylate transporter/malic acid transport protein OS=Pseudomonas syringae pv. aptata OX=83167 GN=ALO85_02652 PE=4 SV=1
MSRLAILIKGNAPFAALTSPREAIRQFTPNWFAVTMGTGILSLALAQLPGSPAPLRALGE
ALWFLNIALFTLFSVMYASRWLLFFDEARQIFGHSTVSMFFGTIPMGLATIINGLLVFGP
ARWGSAIVPVAEALWWLDVAMALTCGVLIPFMMFTRQEHSIEKMTAVWLLPVVAAEVAAA
CGGSLTTHLAGADSQFTVLITSYILWAYSVPVALSILVILLLRLALHKLPHESMAASSWL
ALGPIGTGALGMLVMGSDAPAVFAAHGLASIGTVAAGIGVLVGMLFWGLGLWWMALAVLI
TARYVKQGIGFNLGWWAFTFPLGVYAVATLKLGATLHLGFFDVFGTWLVALLALMWSVVS
IRTLAGAYRGYLFVSPCIAAQGCVRR
>tr|A0A0N8T9F2|A0A0N8T9F2_PSEAP Uncharacterized protein OS=Pseudomonas syringae pv. aptata OX=83167 GN=ALO85_02392 PE=4 SV=1
MTDPLDKATSTAPATLGEGCLSRYDPAELTAENGTDFDGAAALWRELQQAQALGERLEEE
GGQEDE
>tr|A0A0Q0IJM6|A0A0Q0IJM6_PSEAP Universal stress protein family, UspA-like protein OS=Pseudomonas syringae pv. aptata OX=83167 GN=ALO85_02470 PE=4 SV=1
MVRSMLYAADLGLYAPYVTQHALALARTFNAELYVIHVVEPLGLFAESVLQSYLGEDALK
ELRGKGVNAFLSGIEQRMLDGFREELGEGHSDLSLIRTVRVVQGEPASVILEQAHRLNVD
LLVVGSHSRRMEEGTSVGRTAARVLQFSDVPVYMVPMMLKKGRG
>tr|A0A0Q0DAD4|A0A0Q0DAD4_PSEAP OmpA domain-containing protein OS=Pseudomonas syringae pv. aptata OX=83167 GN=ALO85_05078 PE=4 SV=1
MNKIKYYFLRYQPYVLGVCFFLAIFIVWSIGRALGFSSMNSLLTGIGLFLLLSAAYVLLL
YRGVSQHQNLEGLLRDDADQAVLNASPADREEVSLLRERLLQSLERLQSNKPNGSNSKDA
LYALPWYLVVGQPAAGKSTMLYQSGLNFPYAEREGARVAGLGGTRNCDWFFSSEAVLLDT
AGRYMNDQEEAGKWRAFLQLLRQHRQRRPLNGLIVTVSIHDILQSSVEDQERIAKRLRER
IQETHALLEVRLPVYLVFTKCDLVPGFVPFYRQLDDVARGEVMGKTFPHKGFEQADWGQR
FGVAMEELINYWKQIANQQLVLQDIQVTRQDTAVYRFPLELEALKPRLLLFVDALLRANP
YQSAEMLRGFYFTSALDADQPEMGGHTRQVTERFQLESEQEAVDGNVQPHPLFINSLFRK
VIIPDQHLVALYTTNHRERRRKAAWIGGASLVALLLCSLWGWSYWNNKDNIQSISTELAQ
AAAEDSKATGQYTEWQTLDRMRFWTSLYYDRHHDEGVPLRLRLGLYRGYDVEPLLRTRYF
NRLETVMLKPTADNLTRSLYLLTTIKVYQRNAKDLMPVTGVDSVEPKALPPDNRAQSIAA
FGKATLDTYVMLSRAQREKADPAFLKAKMPDYWYPAIARQTGKTITANAEAGSNQDYEYA
SRQINFYSDQIHELDVPRILDNAFLTSSSRNYINSLLTQSLKAIETITLESDTLFAFGRA
DFQSLKTEGQNQLSAIAGKLLNTPNIGKIVIAGHADQLGDPQSNMQVSKQRAQTIKTYLV
GKGVPAELVDAVGEGSEKPLVKCDMQQPRAQLIQCLEPNRRVEIEVRALN
>tr|A0A0Q0C0R0|A0A0Q0C0R0_PSEAP DNA helicase OS=Pseudomonas syringae pv. aptata OX=83167 GN=ALO85_05557 PE=4 SV=1
MPSMQWTEEQLPAIHSFAKKLLVQAFAGTGKTTTLVGYATHNSSVKMLYLCYNKAVEIAA
KNRFPRNVTCKTAHGLAYAVYGSQYKHKQAGNLRLTDIARTINTQDWELAKDIVSTLNAF
MASKDLELLEDHFVRFQSNRTLTSVQQQYMSKALNLTRDVWQKMVDINDRSVPMTHDGYL
KLYQMSQPDLSQRFGAILLDEGQDVNPVIANLVQIQTITQVTVGDRHQQLYRFRGAVDAL
NSPAMKDAEKHFLTQSFRFGPAVAYVANVILSFKGEKIPLQGLGQPTLVKRALPDDLPHR
TYLHRTVSGVIENALRLVNQNHRMQWIGGIDSYSLRDLEDLFYFSRHMNDQVQQRKLLTD
YADYDQYVVIAKATQDPEMLRSIKIIENYADLPQRIEQLRAASVTSELDATVTLTTAHRA
KGLEWDFVGLYDDFSADPLSPDIDAGKRDDELNLLYVAVTRAMKILAVNSLVIDIMQRFK
DNRSVIAATP
>tr|A0A0Q0FCU5|A0A0Q0FCU5_PSEAP D-ribose pyranase OS=Pseudomonas syringae pv. aptata OX=83167 GN=rbsD PE=3 SV=1
MKKTPLLNIALSRVIASLGHGDILMIVDAGMPVPAGVELIDLALTRGVPDFISVLDVVLS
EMQVESHVLASEMAEVKPPALQVIESLNLDDQLGQQRWISHEDLKVLSRKAKAIIRTGEC
QPYSNVALVSGVVF
>tr|A0A0Q0BRB1|A0A0Q0BRB1_PSEAP Protein-export protein SecB OS=Pseudomonas syringae pv. aptata OX=83167 GN=secB PE=3 SV=1
MTDQPNNDAVANEENGPQFSLQRIYVRDLSFEAPKSPAIFRQEWTPTVSLDLNTRQKQLE

GDFYEVVLTLSVTVNNGDEVAFIVEVQQAGIFLIKGLDDGAMSHTLGAFCPNILFPYARE
AIDNLVVRGSFPALMLAPVNFDALYAQELQRMQEAGETPTVQ
>tr|A0A0Q0C9F0|A0A0Q0C9F0_PSEAP DNA-binding transcriptional regulator OS=Pseudomonas syringae pv. aptata OX=83167 GN=ALO85_02576 PE=4 SV=1
MMAFRPTRPDKTWGNIALDVGERLQSIRKLKGLSQRELAKRAGVTNSTISMIEKNSVSPS
ISSLRKVLGGIPMSMVEFFSEESVPENAAQVVYKASELIDISDGAVTMKLVGKSHQGRAI
AFLTETYPPGADTGEEMLVHEGEETGILVEGRLELVVGLDTYVLEAGDSYYFESTRPHRF
RNPFDVPARLISAATPANF
>tr|A0A0Q0DMX5|A0A0Q0DMX5_PSEAP Major facilitator superfamily OS=Pseudomonas syringae pv. aptata OX=83167 GN=ALO85_03158 PE=4 SV=1
MQAGQALVLFAFFFVVESRLASAVCVLLMAGFGFASVSPIQRLVMDKARAAGAPNLAASV
NIGLFNLGNAIGAWLGGWVIAAGFGYAAPNWVGGMLSTCALLLAIASGWLTVSHVAKRKG
AIACNAS
>tr|A0A0Q0C3L4|A0A0Q0C3L4_PSEAP Glutamine ABC transporter, permease protein OS=Pseudomonas syringae pv. aptata OX=83167 GN=ALO85_00865 PE=3 SV=1
MFDVFTIVRDNWTLLLVGQYPHGPMGGLISTLMLAALALLLAFPLGLLLSLARVSPWRWL
RYPATVWVYVMRGIPLLMVIFWTYFLVPLLIGHNITGFTTMLCTLVVYQSAYLCEIIRGA
IQALPTGQYEASRALGMGYLRTTIWVILPQALYNALPSLISQFISIIKETSLGYVINVQE
VTFAANQINNQLLTKPFQVFFILAITYYVLCYGLTRLAAVVEQRIARKREGDVSPVPVPR
PLTTDN
>tr|A0A0Q0BXR3|A0A0Q0BXR3_PSEAP Peptidase S1 and S6 chymotrypsin/Hap OS=Pseudomonas syringae pv. aptata OX=83167 GN=ALO85_02826 PE=4 SV=1
MRKQIWLMALLSLSLTVAADEKIIGGTPAPAGAYPFFTSLTKEDGQHLCGGALIAAQWVL
TAAHCIRGQSPAFVQIAIEKYGPPVVSRDKIRIAEAFIPADFKGWQIYSKSGDEKESGLY
DIALLKLERPTESKDILKLDGIDQVVAHEVGMPVTLAGFGMRETGTMPNELYHVDGSILE
DRKCIDVPPGYPPTYFDPLLNVCSSKISGGGDSGGPLLFKTQSGYVGVALVSRGLLDASQ
MTRISFYKDWITNIIANDKCGQREKPEGTVSVRSVGSSGC
>tr|A0A0Q0DLV3|A0A0Q0DLV3_PSEAP Pyridoxamine 5'-phosphate oxidase-related protein OS=Pseudomonas syringae pv. aptata OX=83167 GN=ALO85_05101 PE=4 SV=1
MHTPTASRDYPMNNPDNSAFVTTIQALEAIYGPIAPPSLLKEVDHIHEVYRPFIEQAPFA
VLASAGPDGLDASPRGDQAGLVHIEDSKTLYLPDRRGNNRIDTLRNIVNDPRVALLFLIP
GVGETLRVNGTAQISVEPELLSRFAVKGQLPRSVLRISVTSVYFQCSRATIRSGLWDPAK
HVERSALPTAGQILKAVSAEQIDGEAYDKALPGRIADTLY
>tr|A0A0Q0FNQ1|A0A0Q0FNQ1_PSEAP tRNA(Ile)-lysidine synthase OS=Pseudomonas syringae pv. aptata OX=83167 GN=tilS PE=3 SV=1
MTTPSRTGHDLSARLLQVLAPWRNAPVWHVGFSGGLDSTVLLHLLAELASRETLPALNAI
HVHHGLQTAADGWPEHCQQVCKTLGVAFESVRVQVEPAASLEQAARQARYSAFSERLVEG
EVLLTGQHRDDQAETLLFRLLRGAGVRGLAAMPDQRVLGRGHLLRPLLDVSRKQLEDYAH
RHGLNWIEDPSNDDQQFSRNFLRSQVLPLLTSRWPQAAASLARTARHLGEAQQLLDELAL
QDIANAQAATSFTWVGLPVLSLGPIAALSAARQRNALRHWLAPLTRLPDTDHWAGWEALR
DAAHDAGPLWRLADGTLHRAQNCIWWVPADWQPLCRQAMDWPDPGVPLDLPGNGCVVLEG
EAPSGCLQVRYRQGGEVLRLNGRGSRDLKRLLNEQSVPAFLRGRLPLLYLDNVLLAVANL
PGLDGSPNENWRLRWVTPTGDQSLS
>tr|A0A0Q0IN06|A0A0Q0IN06_PSEAP Oxidoreductase, FAD-binding protein OS=Pseudomonas syringae pv. aptata OX=83167 GN=ALO85_02876 PE=4 SV=1
MLMPSVISTDVLIVGAGVAGLWLNARLRRQGFSTVLVESASLGGGQSLKSQGIIHGGAKY
ALHGALSGASEAIADMPRRWREALDGKGELDLSGVRLLSEAHYLWSPGTIAGNLTSFFAS
KAVRGRVDQVKGEQLPPALQNPKFKGKVYRLAELVVDVPSLIGRLAELAGDSLLAGQHIE
PLFEGDELIGLRVDGRDIHAQRIVLSAGGGNADLLNSLGVSQPQMQRRPLHMVLVKGPTL
KPLYAHCLGGGPKPRITVTTHPAADGQWVWYLGGDLAEADGVARQPDAQIAVARKELEAL
LPWVDLSQAQWATLRVDRAEPAQSGLVRPDNAFLDSQQRLMIGWPTKLALAPDFADRVLS
QLSRDGIHPTPQAALSDVPRPPMAIPVWDELLP
>tr|A0A0Q0C0S1|A0A0Q0C0S1_PSEAP Phosphate/phosphonate ABC transporter periplasmic protein OS=Pseudomonas syringae pv. aptata OX=83167 GN=ALO85_200117 PE=4 SV=1
MKLRLSCKAIMIALSLVLSNVAASEELKVYNFSPVNQYNINLSASFWNPIIKYVSEKSGV
NLTLKLGRTSSDTTSYVLAQEVDFAFTNHLFSPERDKMGWKVFGRRDAPALEGQIVVPAD
SPIHSLSELEGKEVVYPGPEAFIAYKVTSSELVKKKINTSTVFAGNMDGAFSQLLSGKAQ

AMGANSQLVSGYTEREGKSFRVLWSSASFNDLALMASPRVSKKERDAVANAFFNMQNDPD
GSRVLREATELVHAPTPITFIPATEADYASYRDFYNSLPANLK
>tr|A0A0Q0IFN4|A0A0Q0IFN4_PSEAP ATP-dependent Clp protease, ATP-binding subunit ClpA OS=Pseudomonas syringae pv. aptata OX=83167 GN=ALO85_04641 PE=3 SV=1
MLNRELEVTLNLAFKEARSKRHEFMTVEHLLLALLDNEAAATVLRACGANLDKLKHDLQE
FIDSTTPLIPVHDEDRETQPTLGFQRVLQRAVFHVQSSGKREVTGANVLVAIFSEQESQA
VFLLKQQSVARIDVVNYIAHGISKVPGHGEHSSEGEQDMQDDEGGEASSSGNPLDAYASN
LNELARQGRIDPLVGRESEVERVAQILARRRKNNPLLVGEAGVGKTAIAEGLAKRIVDNQ
VPDLLANSVVYSLDLGALLAGTKYRGDFEKRFKALLNELRKRPQAILFIDEIHTIIGAGA
ASGGVMDASNLLKPLLSSGEIRCIGSTTFQEFRGIFEKDRALARRFQKVDVSEPSVEDTI
GILRGLKSRFEQHHSIEYSDEALRAAAELASRYINDRHMPDKAIDVIDEAGAYQRLQPVE
KRAKRIDVPEVEDIVAKIARIPPKHVNSSDKELLRNLERDLKLTVFGQDAAIDSLSTAIK
LSRAGLKSPDKPVGSFLFAGPTGVGKTEAARQLAKALGVELIRFDMSEYMERHTVSRLIG
APPGYVGFDQGGLLTEAITRQPHCVLLLDEIEKAHPEVFNLLLQVMDHGTLTDNNGRKAD
FRNVIVIMTTNAGAESAARASIGFTHQDHSSDAMEVIKKSFTPEFRNRLDTIIQFGRLSH
EVIKSVVDKFLTELQAQLEDKRVLLEVSEAARSWLAQGGYDAAMGARPMARLIQDKIKRP
LAEEILFGELSEHGGVVHIDIKDGEITFDFETTAEMA
>tr|A0A0Q0C7K3|A0A0Q0C7K3_PSEAP Putative integral membrane protein OS=Pseudomonas syringae pv. aptata OX=83167 GN=ALO85_00607 PE=4 SV=1
MRRSSVKLVLAGALPLALAGCGPSEQKDTVNAKANFSSVQECADAKVPVNICSDAYMQAL
SDHRRIAPTYDDKASCDADFVPDYCQVTSDGKYMPKLGGFELGFSGQVPKEALDRANQEV
NQSASAGMSGTTGLMAGLLIGNLLSNGFGNNRYYSQPIYQTRDDRGGYYSSSLPSQIDRG
KTFGRSTQAQSSPDRSYGRSTLGRNLGSSGSGSSSVSSTISRGGFGSQASARSGWGGKSS
SGSGSFGG
>tr|A0A0Q0IQH1|A0A0Q0IQH1_PSEAP Uncharacterized protein OS=Pseudomonas syringae pv. aptata OX=83167 GN=ALO85_01952 PE=4 SV=1
MTVRVTERDDAHLSHEAVADGIQIWDVHQQDQLVGMFHVESDALRYKEELEALESQGNH
>tr|A0A0Q0FTU2|A0A0Q0FTU2_PSEAP D-isomer specific 2-hydroxyacid dehydrogenase OS=Pseudomonas syringae pv. aptata OX=83167 GN=ALO85_00779 PE=4 SV=1
MTRLVIASQLDDDFNQVISERLAVTHPEARVIGVPAGVPSDLPGEVSILLARPINVRGYE
APASPPPGWPYGLKWIQVVSSGIDFYPGWLFDGPPVSTSRGSAAENIAEFSLAAIFAAAK
HLPDIWVHDSQWNFTALTPLKGTTLGILGFGAIGRSLADKALALGIKVIALRQSQTPFEV
DGVESARDIHELFSRADHLVLAAPLTPATRHIVNADVLNSAKPGLHLINIARGGLLDHEA
LLKALDRGTIGLASLDVTEPEPLPDGHPLYAHPRVRLSPHTSAISSNSRHEIADSFLANL
DRFLSGQALQNQADRQRGY
>tr|A0A0Q0D413|A0A0Q0D413_PSEAP Aminotransferase, class V OS=Pseudomonas syringae pv. aptata OX=83167 GN=ALO85_05535 PE=3 SV=1
MILTRLQSELPMTTSALYLDYAATTPVDEQVIEAMVACMGREGHFGNPASSGHGYGQVAR
QAVEQARAQVAGAVGAPVDTIVWTSGATESNNLAIKGIAQDASAQRRHIITSVLEHKAVL
DTVSTLERQGFPVTWLKPDAQGLIQPHAVQAALREDTLLVSLMLVNNELGTLTDIASIGA
LVREHGALFHVDAAQGVGKVKVDLASLAVDLMSFSAHKAYGPKGIGALYVGPRAHSLLKA
QIHGGGHEGGFRSGTLATHQIVGMGTAFALAISQADAENRRIEALSRHLREGLLSLPDVQ
LNGCPQQRIPHTLNLCITRSGFTSAQLSHSLALSSTSACNSASNAPSHVLLALGMDSAKA
LASVRVSLGRYTTQADVERAIEVFSKALSAPAAFW
>tr|A0A0Q0CYG3|A0A0Q0CYG3_PSEAP Putative lipoprotein OS=Pseudomonas syringae pv. aptata OX=83167 GN=ALO85_01618 PE=4 SV=1
MKPFAPRYLLLVAFSILLGACQSAPTVEAPGSEAQPDAFAQLEQNIAGNELATAEDQLAA
LQAANPADIRLEPFQRRLAEAYLSRSQISLQKGDMNAAASALSRARALMPKAPALTSGVN
GAIAHARKAELEKAEADLKAAEARRVAKLIDPSAESTTIELKIGDMRALRRQLNDIAADT
VAFNCEVLMVVPRTEDYPWLATLLTKRVVKTNPDFELQLRRQIERNEKAQIILTPAKS
>tr|A0A0N8T821|A0A0N8T821_PSEAP Uncharacterized protein OS=Pseudomonas syringae pv. aptata OX=83167 GN=ALO85_04414 PE=3 SV=1
MRTAPMVDSYDDSLDGEKSKTQVKRELHALVDLGERLTTLKADVLAKLPLTDALRKALAE
APKHTANIARKRHILFIGKLMRDQDQEAILVLLDQLDASTRQYNERFHNLERWRDRLIAG
DDADLEKFVVEYPDADRQQLRSLIRQAQHEVARNKPPATSRKIFKYIRELDELQRGLR
>tr|A0A0Q0DI90|A0A0Q0DI90_PSEAP K+-transporting ATPase, F subunit OS=Pseudomonas syringae pv. aptata OX=83167 GN=ALO85_05073 PE=4 SV=1
MKALRRQGCSAFLQLLYAPASLSISRLYTLPVDNHPTRPSPYRWCLMGEISMSVMDGISL

LMGVGLFIYLLVALLRADQSQE
>tr|A0A0Q0DMZ9|A0A0Q0DMZ9_PSEAP Uncharacterized protein OS=Pseudomonas syringae pv. aptata OX=83167 GN=ALO85_05326 PE=4 SV=1
MGVRMNRLLHAAAFAGSMMIVAAPVQARITQLEILRTEPAFGGESFGNTGAYERVFARAH
GELDPGAPENKIIQDIELAPRNGRGMVEYTTDVAILRPADVAKSNDILLFDVPNRGSKRA
MLLFNADMQGSPAAFNNFEVAGDGFLQRQGTTLIAFAWQGDVAPGDNRMMVQLPTAINTD
GSPVTGVVRSELTTLSPTVSLNLSAGWFTSTPPHTSYKSVETDNTKVLADGFRPTLTVRS
RANAPRQEIPVNDWRFGDCNTPSETQICLPAGFQPGKLYELIYQAKEPAVMGIGFAIARD
VGAFFQQAEHDDTGVPNPVVHGPKVKSIITGSSQAGRFIRSMIALGFNRSEEGRRVYDGA
MPHIGGGLMPLNIRFAQPGRAWGQQVDHDSPAYDFPFTYGRETDPVTGRSQGLLDRCNDN
NTCPLIFHMATALELWEGRQSLGLTDPLGLRDAPEPANVRTFIMASTQHAPPSLPLPAKP
PFGLCQVQSNPNPHTWTMRAAFINLVGWVRDETTPPASIKPSVADGTLVPADRVSFPLIP
ATNYGGVSRPELRYTGAVNSLQVLDFGPGFRPEDSSGILTNEPPKSGVGSYGLLVPQVDA
DGIDVGGVRDVYLGAPIGTYTGWNTFRPEFFDGGFCNFQGSFLPFAATKKERVAAGDPRL
SLEERYPTKEAYVSAVSKAADSLVEARMLLPEDRERLVKEAQEKGVRAGP
>tr|A0A0Q0DBA4|A0A0Q0DBA4_PSEAP GNAT family acetyltransferase OS=Pseudomonas syringae pv. aptata OX=83167 GN=ALO85_04266 PE=4 SV=1
MPDRGRTYGGRPRALIPERTATQRPNMTDASAAHYWLMRRDLAVPVLIPAWPPGIQPHPL
TLERAAEIHSLLTLGYGSAAGCVADYPDWLAAFEHDPECDPSLCFLAMRNETVVGVIVCW
TSAFIRDLVVHPDVRHSGIGHALLNHLFTHLRTHNEASVDLHVMENNLAARRLYEKSAMV
YLKRFDIPAA
>tr|A0A0Q0IA81|A0A0Q0IA81_PSEAP Uncharacterized protein OS=Pseudomonas syringae pv. aptata OX=83167 GN=ALO85_02280 PE=4 SV=1
MNAGMNQQRVRGFSASLSNEARQRVADWLRTTRPVKARPDVRAMLLKRYPAGLFNDAEFE
ALARVLTD
>tr|A0A0Q0CAC0|A0A0Q0CAC0_PSEAP Homoserine/lysine/threonine efflux protein, LysE/YggA family OS=Pseudomonas syringae pv. aptata OX=83167 GN=ALO85_01570 PE=4 SV=1
MALETWLGFFAACWIISLSPGAGAIASMSCGLQYGFWRGYWNALGLQIALVAQIAIVAAG
LGAVLAASDMAFTLIKWFGVAYLVYLGIKQWRALPADLADESAVRPVGKPMTLVLRGFLV
NISNPKALIFILAILPQFIDTAAPLLIQYAIIAATMVVVDLIVMAGYTGLASKVLRLLKT
PRQQRRMNRTFASLFVGAAGFLATLHRGTA
>tr|A0A0Q0CAB7|A0A0Q0CAB7_PSEAP Cbb3-type cytochrome c oxidase subunit I OS=Pseudomonas syringae pv. aptata OX=83167 GN=ALO85_03010 PE=3 SV=1
MSTTISPTTYNYTVVRQFAIMTVVWGVVGMSLGVFIASQLVWPGLNLDLEWTTFGRLRPL
HTNLVIFAFGGCALFATSYYVVQRTCQTRLISDGLAAFTFWGWQAVIVGAIVTLPMGYTT
TKEYAELEWPVAILLAIVWAAYAVVFFGTIVKRRTRHIYVANWFYGAFIVVTGMLHIVNH
ISLPVSLFKSYSAYAGATDAMIQWWYGHNAVGFFLTTGFLGMMYYFVPKQAERPVYSYRL
SIVHFWALITLYIWAGPHHLHYTALPDWAQSLGMVMSIILLVPSWGGMINGMMTLSGAWH
KLRTDSILRFLVVSLAFYGMSTFEGPMMAIKTVNSLSHYTDWTIGHVHAGALGWVAMITI
GSVYHMIPKLYARPHMYSVSLINTHFWLATVGTVLYITSMWVNGITQGLMWRAVNDDGTL
TYSFIETMQASHLGYIVRAIGGAIFTSGMLLMAYNVVRTIRASDPQAAAAATRIAVAGAH
>tr|A0A0Q0BW03|A0A0Q0BW03_PSEAP Uncharacterized protein OS=Pseudomonas syringae pv. aptata OX=83167 GN=ALO85_00026 PE=4 SV=1
MTYLIDAWLDRPHPYLRILHRETGEVCAVLEEEALEELRDQGDLDVCSLSSSEPLVLKEL
VRNLFLFCYARALRPMGELH
>tr|A0A0N8T859|A0A0N8T859_PSEAP Fumarylacetoacetate hydrolase family protein OS=Pseudomonas syringae pv. aptata OX=83167 GN=ALO85_01898 PE=4 SV=1
MSYQHKYVDGTNIHFPLGKVVCIGRNYAEHAKELGNAVPTEPLLFMKPGSAVVALEGGFT
IPADRGSVHYEAEIVVLIGKPLSKSPSREEVLDAISGFAPGLDLTLRDVQSRLREKGLPW
ELAKCFDGAAVIPPFVPAETFPDLEDIGIRLTLNGKVVQDGNSNMMLNPIVPMIQHMAAN
FSLQAGDLIMTGTPAGVGPFEAGDQIALELVGQTPFESVVR
>tr|A0A0Q0IDT1|A0A0Q0IDT1_PSEAP Uncharacterized protein OS=Pseudomonas syringae pv. aptata OX=83167 GN=ALO85_01333 PE=4 SV=1
MSKPKRPNKAKSIIAQPLFRSRQEQPAKGKGSYRREAFQSKSWEASCFMAA
>tr|A0A0Q0BYP3|A0A0Q0BYP3_PSEAP Phosphonate ABC transporter, periplasmic phosphonate-binding protein OS=Pseudomonas syringae pv. aptata OX=83167 GN=ALO85_02773 PE=4 SV=1
MLKRTLALAAGLALSFSALTSQAADTLRVSAIPDEAPTELLRKFKPLGAYLEQRLGMKVE

FVPVADYPAVVEALATDRLDMAWLGGFTFVQVNLKTGNAVPLVQREQDAKFTSKFITSDP
NVKTLADLKGKTFAFGSVSSTSGSLMPRYFMLKENIKPETFFSRVAYSGAHDATAAWVQA
GKVDAGVLNASVWDKLVASGKVDTNKVHVFETTPAYFDYNWTVRGSLDPALAAKIKQAFL
DLDPANPEQKAILDLQAASRFIETKPENYKGIEEAARAADLLK
>tr|A0A0Q0D1P9|A0A0Q0D1P9_PSEAP Methyl-accepting chemotaxis protein OS=Pseudomonas syringae pv. aptata OX=83167 GN=ALO85_00384 PE=4 SV=1
MFNSHLKNTNKALERRLAMLEQAGQILNLETVAVVLDGTGKIQTVNALFETEMSYAQAEL
VGRNLSDLSPPELNGDVHQKRALTAIRDGKHFSGTLRLVSKTGSRVWLRTVVVPYKNETG
GVEQITLYSSVLTRTIEASRENEALVNALQRSTAVIDFTLDGIVIGANDNFLRAMGYELN
QIKGKHHKMFCVPEESASDAYTRFWERLRRGEFIVDRFRRIDKGGRDVWLEASYNPITDA
NGKLYKIAKFATVITEQVEREKAVAEASEMAYQTSIDTDSSAQQGRKVIKDTLQMLGQLT
ISMEEATRGIEALDSQSQVIGTIIKTISGIAEQTNLLALNAAIEAARAGEQGRGFAVVAD
EVRQLASRTTKAAEEIVSVVKTNQSLASDAVGVIVRSTEQASEALVLANDADRVIQEIQH
GANTVVRAVSRFVDQRSS
>tr|A0A0Q0D786|A0A0Q0D786_PSEAP Aconitase B OS=Pseudomonas syringae pv. aptata OX=83167 GN=ALO85_100317 PE=4 SV=1
MTTFEYTQTFVPLPYKTVTSGVLMFKSTDDTTEPDIHGYLSNPETLAALNRHGREGWELV
SVQQINRGHEQIGNQNAQGWAFGYAISTGFLFFFKRSTASLTSLDKPPQT
>tr|A0A0Q0BYZ4|A0A0Q0BYZ4_PSEAP Uncharacterized protein OS=Pseudomonas syringae pv. aptata OX=83167 GN=ALO85_100586 PE=4 SV=1
MKTVSLQGIQLSPGQRRMLEQQRHVREFMNPVLTQQVAETLAEIEVRKEQGTKPEKIWFH
DRESSWQGTISVAEWMGY
>tr|A0A0Q0DV73|A0A0Q0DV73_PSEAP 50S ribosomal protein L29 OS=Pseudomonas syringae pv. aptata OX=83167 GN=rpmC PE=3 SV=1
MKANELREKSAQQLNEQLLGLLRDQFNLRMQKATGQLGQSHLLSQVKRDIARVKTVLNQQ
AGK
>tr|A0A0Q0DID7|A0A0Q0DID7_PSEAP Uncharacterized protein OS=Pseudomonas syringae pv. aptata OX=83167 GN=ALO85_04052 PE=4 SV=1
MKDSDLIAQILERARQRIEQVAIAGDREVMFHSAAEAQGWIGALQAENLLGNEQCEMLDA
ELKVAVSKWDGGPE
>tr|A0A0Q0CIX5|A0A0Q0CIX5_PSEAP ATP synthase subunit c OS=Pseudomonas syringae pv. aptata OX=83167 GN=atpE PE=3 SV=1
METVVGLTAIAVALLIGLGALGTAIGFGLLGGKFLEGAARQPEMVPMLQVKMFIVAGLLD
AVTMIGVGIALFFTFANPFVGQLAG
>tr|A0A0Q0C1G4|A0A0Q0C1G4_PSEAP PhoH family protein OS=Pseudomonas syringae pv. aptata OX=83167 GN=ALO85_01813 PE=4 SV=1
MDDHGRTPSTNQPILYVLDTNVLIHDPNALLNFEEHHVAIPMTVLEELDKLKAGKHSVAA
ECRQAIRLIDKTLGEASPEEVEKGVPIDRGTGVLKGYLSILMSKREEPNSLLPEHLNDNI
IINQLIDLHARNKDLSVVLVTKDINMRLKARACGIAAEDYSTDQLVDDVSLLSRGYHDMT
GSFWDRVSKVDTRQERGRTWHRVQLTEMLPAVHINEFIVDEQGFVGWIKGVKNDELLILD
MHQEPLLHQEAWGLKPRDIYQGLALFALLDPDIHLVNLSGAAGSGKTILALAAAIEQTMV
TKRYRRIIATRSVQGLDQEIGFLPGTEAEKMEPWLGAITDNLEALHMDDENTHGSVDYIL
SKVPLQFKSLNYIRGRSFQQSLILIDECQNLTPHQMKTIITRAGAGSKVVCLGNLAQIDT
PYLSATSSGLTYLTERFKDFPNGVHITLQGVPRSILAEYAESHL
>tr|A0A0Q0FJN8|A0A0Q0FJN8_PSEAP Putative glycine-glutamate dipeptide porin OpdP OS=Pseudomonas syringae pv. aptata OX=83167 GN=ALO85_00009 PE=4 SV=1
MKTTSTALLALSLSSFGALVQAEPASQAFVPTELSSKNAQADANGFIEDQHLTGTTRNWY
ANELKRRGEVFRYNDNGVAEQTSRRINWQQGTIVNYTSGYTQGVVGFSTELALYNAIALD
RDTDDFAGNSNRTLAHSDGDAVGQWSKMGLGNVKARVSNTVLTMGRQSVNTPVIAFIGNR
ALPSSFQGVAVQSDELNNLSFQAGSFDRVSPRMEQSLDKFRSEYGDRSQTADRLDMFGAD
YKPTDSLTTSFYASNLEDFWHQYYFGFTHELGDSKALALSTNLNYYKTKDSGQSKQGPID
NDTYSLSFTGTHNAHSISLAYQQVSGDEYFDYAHDTNAIFLANSLLSDFNGPNEKSLQIA
YVLNMAEYGVPGLKFNIYQARGWDIDGTKYKGNVYGDVQTINGVRTASGVNAMDGETHYE
YGIGSSYSVQSGPLKATAIRATYTTHRASENQADGNINEFRLVTTVPFNIL
>tr|A0A0Q0C3Y1|A0A0Q0C3Y1_PSEAP Long-chain-fatty-acid--CoA ligase OS=Pseudomonas syringae pv. aptata OX=83167 GN=ALO85_02757 PE=4 SV=1
MIDNFWKDKYPSGIPADINPDEYPNVQAVLKQSCQRFADKPAFSNLGKTITYGELYELSG
AFAAWIQQHTDLQPGDRIAVQLPNVLQYPIAVFGAIRAGLIVVNTNPLYTAREMEHQFND
SGAKALVCLANMAHLAEKVVPKTQIRQVIVTEVADMLSPFKRLLINSVIKYVKKMVPAYH

LPKAVKFNDVLGKGRGQPVTEVSPGSADVAVLQYTGGTTGVAKGAMLTHRNLIANMLQCR
ALMASNLGEGCEIIITPLPLYHIYAFTFHCMAMMLLGNHNILISNPRDLPAMVKELSKWK
FSGFVGLNTLFVALCNSEGFRNLDFSALKVTLSGGMALQQAAAERWKQVTGCQVCEGYGM
TETSPVATVNPSQYVQMGSIGIPVPSTLCKVIDDAGNELAFGETGELCIKGPQVMKGYWQ
RQEATDEMIDSEGWLKTGDIAIIQPDGYIRIVDRKKDMILISGFNVYPNELEDVLATLPG
VLQCAAIGVPDEKSGETIKVFVVAKPGVTLTKDKVMEHMRANLTGYKVPRSVEFRDVLPT
TNVGKILRRELRDEELKKLGVKK
>tr|A0A0Q0IDE3|A0A0Q0IDE3_PSEAP PAS protein OS=Pseudomonas syringae pv. aptata OX=83167 GN=ALO85_01606 PE=4 SV=1
MKLAMKLRTRLFLSISALITVALLGLLLGLVSVMQMARTQESLIQHNFAILDLGLKLRQN
MGDQLVLMTGSNRNEPELQRMQKQFEELLEEGSAQDTQQDVRNGFEKTRGDYQRFIAAWQ
QYGNDPRGIRGNSQLSESFDTLRNGLMDVHRKALENISNAEINSRDRALWIAGLLGLVGL
AVLCIGFVTAHGIARRFGAPIEALAKAADRIGEGDYEVTLPISSAAEMNLLTRRFGIMAE
ALRQHQATNVDELLAGQQRLQAVLDSIDDGLLMIDRQGHLEHLNPVAQRQLGWDESRLGH
SLGEALGRPELDEQLHLVLRGGTLERAPEDLAIEIDGESRLLTYSMTPVSHTKGHILGAV
MVLHDVTEQRAFERVRSEFVLRASHELRTPVTGMHMAFGLLQERLHFAPESREADLLNTV
TEEMQRLMQLINDLLNFSRYQNGLQKLKLAPCSIETLLQEAKVRFEDQAQEQDIVLMLDM
QEPMPRLHADQSQLERVLDNLLDNALRHTPPNGLIRLQARRHGERAIISVEDNGEGIAYG
QQGRIFEPFVQVGRKKGGAGLGLALCKEIVQLHGGRMGVYSRPGQGTQFYMALPL
>tr|A0A0Q0C296|A0A0Q0C296_PSEAP Uncharacterized protein OS=Pseudomonas syringae pv. aptata OX=83167 GN=ALO85_00605 PE=4 SV=1
MLMKQRLGSALALLFTINTCQAVDTTLLGSRLTPLGGEMAGNATGTIPPWTGGITRPPSG
YKGSGTRHVDPYAADKPLFTITHANMQHYRTRLTPGQVAMFETYPDSYRMPVYPTRRSGS
APQWVYDNTRRNAQKARLIGGGNGFVDAYGGIPFPLPEKGIQAVWNHIVRYRGHYAVRRT
SEVAIKRDGSFKLNTSRQEVLFRFYDPAGSFASLDNVLFYYLSFIQCPTRMAGSAVLVHE
TLDQIQEPRQAWAYSVRQRRVRRAPSLAYDMPIPASEGLRTADDTDMFNGSPDRYDWTLL
GKREIYIPYNNYRISSPDITYRQLLQVGHLNPAYTRHELHRVWVVEGTLKPTARHVYSRR
TLYLDEDSWQAAIVDQYDARGQLWRVSMAYLKNYYELPTTWSALDVFHDLQARRYHVQNL
DNQEPGTSDFSQPVPSVNAFKPAALRRRSIR
>tr|A0A0Q0CVQ1|A0A0Q0CVQ1_PSEAP Type III secretion system ATPase HrcN OS=Pseudomonas syringae pv. aptata OX=83167 GN=ALO85_04183 PE=4 SV=1
MNAALNLWKDAHAKRLSQYCAVRVIGRVSAVRGILLECKIPSAKVGDLCEVSKADGSLLL
AEIVGFTQECTLLSALGPPDGIQVGAPIRPLGVAHRIGVDDSLLGCVLDGFGRPLMGDCL
GAFAGPEDRRTTLPVIADALPPTQRPRITRALPTGIRAIDSAILLGEGQRVGLFAGAGCG
KTTLMAELARNMDCDVIVFGLIGERGRELREFLDHELDETLRRRSVLVCATSDRSSMERA
RAAFTATAIAEAFRARGQKVLLLLDSLTRFARAQREIGIASGEPLGRGGLPPSVYTLLPR
LVERAGMSENGSITALYTVLIEQDSMNDPVADEVRSLLDGHIVLSRKLAERGHYPAIDVS
ASISRILSNVTGREHQRANNRLRQLLAAYKQVEMLLRLGEYQAGADPVTDCAVQLNEAIN
AFLRQDLREPVPLQETLDRLLQLTSQLPE
>tr|A0A0Q0BZR6|A0A0Q0BZR6_PSEAP Maleylacetoacetate isomerase OS=Pseudomonas syringae pv. aptata OX=83167 GN=ALO85_100553 PE=4 SV=1
MYAAHRYRVDPTAFVRIQRVEHLARQPPAFYQAHPDAQSDKPE
>tr|A0A0Q0IMS9|A0A0Q0IMS9_PSEAP 50S ribosomal protein L30 OS=Pseudomonas syringae pv. aptata OX=83167 GN=rpmD PE=3 SV=1
MATVKVTLIKSMTGRIPNHRLCIKGLGLRRIGHTVEVLDTPENRGMINKAYYMLRVEG
>tr|A0A0Q0DIC0|A0A0Q0DIC0_PSEAP Sterol-binding domain-containing protein OS=Pseudomonas syringae pv. aptata OX=83167 GN=ALO85_04068 PE=4 SV=1
MLLRGLLASIEHGINRVLRLDSTALPRMARLSGRVIAVDCRDPSLKLFILPSDEGLLLAA
DWAAEADCTLRAPASSLVRLALSKDKTAVLHGPDVELDGDSGALLELVGILQDLELDWEH
ELSNWIGPVASQLFSSHLRNSGRWTRESFNSLSQNIADYLSEESRTLVGKHEAEARFAEL
DQIKLDLERLEARFARLAHSLNTSDNA
>tr|A0A0Q0BYU0|A0A0Q0BYU0_PSEAP Substrate-binding region of ABC-type glycine betaine transport system OS=Pseudomonas syringae pv. aptata OX=83167 GN=ALO85_03199 PE=4 SV=1
MSMKSITRLLGCSLLALGLLGQTATAAEKTKPIQFGALTWESGSLITEVLRTIVEKGYGY
PTDTLPGSTVSLETALAKNDIQVIAEEWTGRSPVWVKAEEEGKVLGLGNIVKGATEGWWV
PEYVIKGDAAKGIKPLAPDLKSVADLARYKDVFKDPESPDKGRFLNSPSGWTSEIVNTQK
LKAYGLADSFVNFRTGSGAALDAEVASSIRRGKPVLFYYWSPTPLLGKYKLIKLDEPAFD
AEAWKTLTDASNPNPRGTSSLAATLSIGVSAEFSKQYPELVTVFKGVDFPIDLLNKTLGE
MSDKHEDAKVVAARFLKQHPDVWKAWLPADVAERVSSSL

>tr|A0A0Q0CHP4|A0A0Q0CHP4_PSEAP Tartrate dehydrogenase OS=Pseudomonas syringae pv. aptata OX=83167 GN=ALO85_03418 PE=4 SV=1
MDRFRENAAMNNKKLRIAAIAGDGIGLEVLPEGVRVVQAAAARHGLELEFEYFEWASCDY
YLKHGKMMPDDWFEQLNGFDSIFFGAVGWPEKVPDHISLWGSLLKFRREFDQYANIRPVR
LFPGVPCPLANRKPGDIDFVVVRENTEGEYSSLGGIMFENTDNEFVLQESVFTRRGVDRI
LKFAFEMASKRERKHVTSATKSNGMAISMPYWDKRTEAMANQYPGISWDKQHIDILCARF
VLQPERFDVVVASNLFGDILSDLGPACAGTIGIAPSANLNPERHFPSLFEPVHGSAPDIF
GQNIANPIAMIWSGALMLEFLGQGDQRFTAAHDEIITAIEQVIANGDVTPDLGGMRSTQQ
VGAAIAERVSAAR
>tr|A0A0Q0DYG8|A0A0Q0DYG8_PSEAP Nitrilase/cyanide hydratase and apolipoprotein N-acyltransferase OS=Pseudomonas syringae pv. aptata OX=83167 GN=ALO85_04603 PE=4 SV=1
MALGKRPTPPYPSRQFPDGRHSILFMRKLFAICLVLLSVASLVSYAIWTDQRPAGHYLSD
LRIRLAINEGQPGERGNLLGIEPELFPTDYQNIERLHRKLAAYLQQARDKGLINPRTVVV
LPEHIGTWLFASGEKDQLYQAATLDDAMDWLSWSNPLQFIKAMLEAEGRGRLDDAHLRIK
AQSMARDYQTLFGGLAREFGVTLVAGSIVLPDPSVEGGQLKVGSGALYNSSLTFGSDGLP
LGQPQRQLYPERYQKRYVRTASDAPLNVIDTPAGRLGVLIGSDSWYPENYARLNQKGAQL
IAVPAFVIGKAAWSEAWRKPRHSDIDVPTDTPSEGEAWHHLTLTGRAPQSTAQAGLSVFM
RGQFWNQGVAGQSFASHAGQTIAEPQQENAAGGARLINLWL
>tr|A0A0Q0DN32|A0A0Q0DN32_PSEAP WbcX-like glycosyl transferase, family A OS=Pseudomonas syringae pv. aptata OX=83167 GN=ALO85_02883 PE=4 SV=1
MVFSENSDITLVVTSCGRFDLLKRTLETFDHFNTAPIRHVLITEDSGDDRVQACIPEHWH
PHTRFIINNPKLGQMRSVDAAYSLIETSWVFHCEDDWDFYRPHFIEESMTLLKHDPQALQ
VWLRSYNHDLRIHSPYVYLNEREVVEGIAFYKLGSHKPEWQGFSFNPGLRRLADYLQHAP
YAGHGGEKKLSQLFAAQNRYALILENDAVLHTGFGEHVELPQERLDKQRRKRRDRVKLIV
AVLAGLVAGWLLNNVS
>tr|A0A0N8T8J5|A0A0N8T8J5_PSEAP Uncharacterized protein OS=Pseudomonas syringae pv. aptata OX=83167 GN=ALO85_03157 PE=4 SV=1
MKKLLISAAMATSLLGMSLASQASENCPAVAKIEQVSPGVYRASGNDGEWTGVLQGVVTK
KMTVQSFDLAIAIQEGADAPQMLQHCTYNIGGREALDMRFIAKNNKDFTIKTAGSAWKKE
DGPFGLVYNVCEKTSAENCKFTVVQ
>tr|A0A0Q0BTF8|A0A0Q0BTF8_PSEAP Putative glucose-6-phosphate 1-epimerase OS=Pseudomonas syringae pv. aptata OX=83167 GN=ALO85_04285 PE=3 SV=1
MAMTTHDAQLPEHPLQRFFRSRRARPTFEWERHQLRDVLVIDHPQCQAVFSRQGAQLLHF
QPQGQKPWLWCAAQWPQVGAIRGGVPVCWPWYGRHPGESGWPAHGWGRLLDWKLIDSSEC
EEGVSLHWRLQLWDWQVDLHAELGQGMELRLSTCHEDSEPCHFSHALHAYWRIGDVAEVA
LEGLDGAQGYDELSRQACQQQGELRVAGACQRVFEHAGAVQLQDPIWQRRLNIDTGDSTN
TVVWHPGSRPLLGVCGSETTGFVRVEAASGSGDTFSLRPGEQVQLKLQASLAG
>tr|A0A0Q0DV81|A0A0Q0DV81_PSEAP Glyoxalase/bleomycin resistance protein/dioxygenase OS=Pseudomonas syringae pv. aptata OX=83167 GN=ALO85_01475 PE=4 SV=1
MPRLNRIIETALYVEDLAHASEFYSGTLELEVMFESATLVAFNVGGVSTLLLFKRGASLQ
TQHLSGGEIPPHDAQGRIHVCFAINADEMQPWADRLARASVAIEGRTEWPKGGSSIYFRD
PDENLVELLTPGCWAIY
>tr|A0A0Q0C6D6|A0A0Q0C6D6_PSEAP Substrate-binding region of ABC-type glycine betaine transport system OS=Pseudomonas syringae pv. aptata OX=83167 GN=ALO85_03712 PE=4 SV=1
MNRLISCSVLALSASALLSSNVMAADAASCQNVRMGVVNWTDVIATSAMTQVLLDGLGYK
TKQTSASQQIIFAGIRDQRLDLFLGYWNPLMTQTITPFVEARKVKVLDKPSLEDARATLA
VPTYLADKGLKSFADIARFEKELGGKIYGIEPGSGANTQIKAMIAKNQFGLGKFQLVESS
EAGMLAAVDRAVRRKEAVVFFGWTPHPMNVNVAMTYLNGSDDALGPNEGMATVWTVTSPT
YAEQCPNVHKLLTNLTFTAADESRMMQPLLDHKDALESARQWLKDHPQDQARWLKGVTTF
DGKPAAANLQLSSQ
>tr|A0A0Q0DC56|A0A0Q0DC56_PSEAP ATP synthase gamma chain OS=Pseudomonas syringae pv. aptata OX=83167 GN=atpG PE=3 SV=1
MAGAKEIRSKIASIKSTQKITSAMEKVAVSKMRKAQMRMAASRPYAERIRQVIGHLANAN
PEYRHPFMIERPVKRVGYVVVSSDRGLCGGLNTNLFKTLVKDMAVNRENGVEIDLCVVGS
KGAAFFRNFGGNVVAAISHLGEEPSINDLIGSVKVMLDAYLDGRIDRLSVVSNKFINTMT
QQPTVEQLIPLVATPDQGLKHHWDYLYEPDAKELLDGLMVRYVESQVYQAVVENNAAEQA
ARMIAMKNATDNAGDLISDLQLIYNKARQAAITQEISEIVGGAAAV

>tr|A0A0Q0BTB8|A0A0Q0BTB8_PSEAP MutT/NUDIX family protein OS=Pseudomonas syringae pv. aptata OX=83167 GN=ALO85_04080 PE=4 SV=1
MKFCSQCGNPVIQRIPEGDSRLRYVCEHCHTVHYQNPNIVAGCLVTLGGKVLLCRRAIEP
RLGFWTLPAGFMENGETIEQAARRETMEEACATLSELHLYTLIDVPHINQVHVFYRGEMA
NEAFAAGIESLEVQLFDEADIPWSDLAFMTVGRTLEYFFADRRRQAYPVHTSALPPSRPL
LDT
>tr|A0A0Q0CBL5|A0A0Q0CBL5_PSEAP D-erythro-7,8-dihydroneopterin triphosphate 2'-epimerase OS=Pseudomonas syringae pv. aptata OX=83167 GN=ALO85_01767 PE=4 SV=1
MARLEPGTARIRVKDLRLRTFIGINEDEILNKQDVLINLTILYAAQEAVRDNDIEHALNY
RTITKAIIQHVEGNRFALLERLTQEVLDLVMSHEAVTYAEVEVDKPHALRFAESVSITLA
GQR
>tr|A0A0Q0DM84|A0A0Q0DM84_PSEAP 3-hydroxy-3-methylglutaryl-CoA lyase OS=Pseudomonas syringae pv. aptata OX=83167 GN=ALO85_00942 PE=4 SV=1
MSLPQKVNLVEVGPRDGLQNEAQPISVDAKVRLVDDLTAAGLSHIEVGSFVSAKWVPQMA
GSAQVFERIQRREGVIYSALAPNLRGFEDALAAGVREVAVFAAATEGFSQRNLNCSIGES
LERFAPVMEAARMHGVRVRGYVSCVLGCPYEGSVSPEQVATVANQLHAMGCYEISLGDTI
GTGTPGATRALLNAVAAQIPRGKLAGHFHDTYGQALVNIYASLEEGIQVFDSSVAGLGGC
PYAKGASGNVATEDVLYMLQGLGIETGVDLDQVIAAGQRICDVLQRSNGSRVAKARLSA
>tr|A0A0Q0FVN2|A0A0Q0FVN2_PSEAP Glycine betaine/L-proline ABC transporter periplasmic substrate-binding protein OS=Pseudomonas syringae pv. aptata OX=83167 GN=ALO85_03848 PE=4 SV=1
MKGSKSLLLAATLCMPVLAQAAEPEACHTVNFSDVGWTDITVTTAVTSAVLESLGYKTKT
TMISVPVTYKSLADGKNMDVFLGNWMPTMENDIKPYREAGTVETVRANLENAKYTLAVPE
ALYDKGLHDFADITKFKKELGGKIYGIEPGNDGNRLIQSMIDKNAFGLKDAGFKVVESSE
AAMLSQVDRAVKRKNDVVFLGWEPHPMNTRFKMKYLTGGDDFFGPNYGQATIYTNTRKGY
SQECSNVGQLLKNLSFTLNMESTLMGNVLDDKMKPDAAAKAWIKKNPQVLDTWLAGVTTV
DGKPGLEAAKAKLTQ
>tr|A0A0Q0DJC8|A0A0Q0DJC8_PSEAP Protein sirB1 OS=Pseudomonas syringae pv. aptata OX=83167 GN=ALO85_04957 PE=4 SV=1
MALDEIRAQGYKESSHWISSRTEWSMTPRQHCLACLQQTPPSVFEAALWVSTEHDANFAW
HEVMSEMDRLQRQINAALPVLPAPELAQPLLRQLNALGFQQDDWNPPKPDSALVHKIVEQ
RRGQPLGLAMIALEIARRLDIAMEGVNFPGHFLLRVPGADHLLDPCSGRRLYPADCRELL
IRQFGPTMQLRAEHMTRANPTSMMQRLSRNLRHLHQINDNVLAALKDADRIVELGQATSS
DHLARASLYQYLECPQAERFDLERALLLSEDPLQRLLLAERLSHLPVNHRVH
>tr|A0A0Q0DKS9|A0A0Q0DKS9_PSEAP Regulatory protein, LysR:LysR, substrate-binding protein OS=Pseudomonas syringae pv. aptata OX=83167 GN=ALO85_01152 PE=3 SV=1
MTAILDIELVRTFHAVARIGKFSAAAEQLHKSPAAVSVHVQRLEAVAGGRLLNRDNQAVS
LTALGKRLLLSTTELLSTHDRVLADLQGTHLAGRITLGVPDEYAVHVIRDILPVFAAAWP
NVVLELKSAPSYALREQVARGKLQTAVIAQPKGQLGADTQFLVSTKPVWVGPVSVALASA
DPVPLAIHAAQCPYREAMLASLKDNGRKVRVVLESPSNQAIKACVEAGLAISLIDRSGVT
DAMQIIDDLPEIAEHEIVFLRSPSSQNDEAVSLLAQALQKYFRV
>tr|A0A0N8T7V7|A0A0N8T7V7_PSEAP AAA_31 domain-containing protein OS=Pseudomonas syringae pv. aptata OX=83167 GN=ALO85_04127 PE=4 SV=1
MNAKIPSFKINELASAIRTFIIVFAQQKSSGKTTKAINMAFKLILEGKKVAYVDLDSIRA
AEDFAILSAENKSERINHYTNMSLEYSKDQKTKGLAARFSNKVIEITKEPVLKIHGSSLS
SFSMKAVKERFHGYDFIIIDLPANLYNNSDEIKPEISQLFIDSDLVVFPVKAEPQELKTA
PILSRYVSSLKAFCESNKEIKTNVKFCTAMCFDITKYKGKNGTIQMADTIVEYNKVYGAT
MPAFENPMVFRAIYPTQLGQGMTIYSSDTTETRSAQKEFNLVVQDIINQLN
>tr|A0A0Q0BZC4|A0A0Q0BZC4_PSEAP Drug/metabolite transporter superfamily permease OS=Pseudomonas syringae pv. aptata OX=83167 GN=ALO85_03482 PE=4 SV=1
MPQSVRVSPLLIGAFLALYLIWGSTYLVIRIGVESWPPLMMAGVRFLIAGCLMYGFLRFR
GVPAPTWAEWKAAFVIGFLLLACGNGGVTLAEHAGVASGVAALAVATMPLFTLLFGLFWG
NRTTNLEWAGIVLGLIGIGLLNLGSNLQASPYGAAVVIFAAAAWAFGSVLSKHLPLPKGP
MASAAEMITAGATLLIGSALSGERLTHMPTAAGWGALLYLVVFGSIVAFSAYMYLLKNVR
PAAATSYAYVNPAVAVMLGVVFAGETIGVEECMAMVVIISAVVLIGLPQWRRSPPQEAVS
TRTS

>tr|A0A0Q0D6U5|A0A0Q0D6U5_PSEAP Methylcrotonyl-CoA carboxylase, beta subunit OS=Pseudomonas syringae pv. aptata OX=83167 GN=ALO85_00940 PE=4 SV=1
MTILQTRINTRSPEFTLNRDALLKQVEALRTLLSNVQQGGGPKAQERHTSRGKLLPRERI
NRLLDTGSPFLEIGQLAAHEVYGEEVPAAGVIAGIGRVEGVECMIIANDATVKGGSYYPL
TVKKHLRAQTIAQQNRLPCIYLVDSGGANLPRQDEVFPDREHFGRIFFNQANMSAQGIAQ
IAVVMGSCTAGGAYVPAMADEAIMVRQQATIFLAGPPLVKAATGEVVSAEELGGADVHCR
TSGVADHYADNDDHALALARRSIANLNWRKQGQLNTVAPIAPLYPGDDLYGIIPADTKQP
FDVREVIARLVDGSVLDEFKALFGTTLVCGFARLHGYPIAILANNGILFAEAAQKGAHFI
ELACQRGIPLLFLQNITGFMVGQKYEAGGIAKHGAKLVTAVACAKVPKFTVIIGGSFGAG
NYGMCGRAYDPRFLWMWPNARIGVMGGEQAAGVLVQVKCEQAERAGQAFSAEQQAELKQP
ILDQYEHQGHPYYSSARLWDDGVIDPAQTRDILALALSASLNAPIEPTTFGLFRM
>tr|A0A0Q0DXX9|A0A0Q0DXX9_PSEAP GTP cyclohydrolase FolE2 OS=Pseudomonas syringae pv. aptata OX=83167 GN=folE2 PE=3 SV=1
MNKPLPDVALTEISPALVSLDWVGMQGVEVPIRLAEASIRHPVHAHVDLQVDLADPSVKG
IHMSRLYRLLDGYAERQIVSPDTLAALLEAMVESHLDCHSSHARLKLSFNLLCRRPALIT
EGLSGWKSYPVTLDATWHAGRLCLDSSVDVTYSSTCPCSAALSRQLLEEAFAARFGRQSF
VDPMQVAAWLRENASFATPHSQRSVATVQVRVAEQATELGLMTLIDLVEQALGTPVQTAV
KRADEQAFARLNGQNLMYVEDAARKVQQALEGRYAASSVSVRHFESLHPHDAAAQTSNYL
S
>tr|A0A0Q0CG03|A0A0Q0CG03_PSEAP Ectoine/hydroxyectoine ABC-type transport system OS=Pseudomonas syringae pv. aptata OX=83167 GN=ALO85_03678 PE=4 SV=1
MSDLAHTTIPSVITAQIQLKHVVKDFGRNRIIDHLDLSIPQGQKVALIGPSGSGKSTVLR
LIKGLENYQQGSIEVAGETVPARRASGWRWPGARKAPVEHRVGMVFQQFNLFPHLTVQEN
VTEAPLRVLRLTREEALARAHQYLGLVGLAHKADAYPSQLSGGQQQRVAIARALAMRPQV
MLFDEVTSALDPELVGEVLAVIRSIAHEFQMTMLLVTHEMKFAQDIADRVLFMEGGRIVA
DGTPSEILLNPQNERTRRFLQRVEQR
>tr|A0A0Q0C8W8|A0A0Q0C8W8_PSEAP GCN5-related N-acetyltransferase OS=Pseudomonas syringae pv. aptata OX=83167 GN=ALO85_03450 PE=4 SV=1
MTESNLLPAAIQTERLLIRVAKPGDGIVFNQAVAESAEHLQKWLAWATPTPGIDQSELIC
RHAYARFLLNEDLRALLFLKNNGALVGSSGLHNANWQLRSFEVGYWGRTQYLGAGLISEG
VAALVSYALDNLMANRVFLTMDERNLASQRLAERVGFEYEGTLRQDRMSIQGGLRNTRVY
SIIGN
>tr|A0A0Q0DJL5|A0A0Q0DJL5_PSEAP Integral membrane protein OS=Pseudomonas syringae pv. aptata OX=83167 GN=ALO85_100290 PE=4 SV=1
MVFCRGCAKEISDDAGTCPHCGAPQLVISPASASTDNPPAWSWYTEVLKKYAVFTGRARR
KEYWMFFLFNILISIVLGFIGGLIGDGGIIANLYSLAVLVPGIAVGVRRLHDTDRSGWWL
LVPIANLVFLIQEGHPGPNRFGPSPK
>tr|A0A0Q0DKK4|A0A0Q0DKK4_PSEAP Cytochrome OS=Pseudomonas syringae pv. aptata OX=83167 GN=ALO85_101206 PE=4 SV=1
MQLRNSSSRYGLVSIVLHWGVAAVVFGMFALGLWMVGLDYYDTWRKAGPDLHKSIGITLF
AVMLIRVVWRLLSPPPPPLTSYSKLTRIGAAFGHLFLYVALFAVMFAGYLISTADGVGIP
IFGLFDVPALVSGLPDQAETAGWIHLYLAWVIVIFAGLHGLAALKHHFIDRDVTLKRMLG
RN
>tr|A0A0Q0C783|A0A0Q0C783_PSEAP Nitroreductase-like family II protein OS=Pseudomonas syringae pv. aptata OX=83167 GN=ALO85_01060 PE=4 SV=1
MSTNPRIADHPIDPQFTERWSPRAFSGESIDQETLLSFFEAARWAPSAYNSQPWRFLYAR
RDTPNWERYLGLLNEFNRSWAQHAAALVIVISKTTFVAPGASEESPALWHTFDTGSAWGY
LALQASLSGWHTHGMAGFDQDLTRKELNIPEGYAVHAAIAIGKLGDKSTLPDYLQGREVP
SPRKPLAELAAEGDFSL
>tr|A0A0Q0CIM0|A0A0Q0CIM0_PSEAP Uroporphyrinogen-III synthase OS=Pseudomonas syringae pv. aptata OX=83167 GN=ALO85_01578 PE=3 SV=1
MSGWRLLLTRPAEESTALARVLAEDGIFSSSLPLLETEPLPLTPAQRSIIFELLNYCAVI
VVSKPAARLAIELIDEVWPQPPLQPWFSVGSATGQILLDYGLDASWPEQGDDSEALLELP
RLKQAIAIPGSRVLIMRGDEGRELLAEQLRGLGVGVDYLPLYRRYLPQHAPGALAQRVAV
ERLNGLVVSSGQGFEHLLQLAGDSWPDLADLPLFVPSPRVASIARAAGARTVIDCRGASA
AALLAALREQPQPAVKA

>tr|A0A0N8T930|A0A0N8T930_PSEAP Phospho-2-dehydro-3-deoxyheptonate aldolase OS=Pseudomonas syringae pv. aptata OX=83167 GN=ALO85_05143 PE=3 SV=1
MAASTRDLMSNTMADLPIDDLNVASNETLITPDQLKRDIPLTDAALHTVSQGREMIRNIL
DGHDHRLFIVIGPCSIHDLKAAKEYAERLKALAAEVSDTLYLVMRVYFEKPRTTVGWKGL
INDPYLDDSFKIQDGLHIGRQLLLDLAEMGLPTATEALDPISPQYLQDLISWSAIGARTT
ESQTHREMASGLSSAVGFKNGTDGGLTVAINALQSVSSPHRFLGINQEGGVSIVTTKGNA
YGHVVLRGGNGKPNYDSVSVALCEQALNKSGIRPNIMVDCSHANSNKDPALQPLVMENVA
NQILEGNESIIGLMVESHLNWGCQAIPKDLSELQYGVSITDACIDWESTEKTLRSMHAKL
KDVLPARKRK
>tr|A0A0Q0BYQ2|A0A0Q0BYQ2_PSEAP Pseudouridine synthase OS=Pseudomonas syringae pv. aptata OX=83167 GN=ALO85_02799 PE=3 SV=1
MSENIELRAEVPSELGGQRLDQVAAQLFAEHSRSRLSAWIKDGRLTVDGGVLRPRDIVHG
GAVLELIAEQEAQGEWVAQDIELDIIYEDDQILVINKPAGLVVHPAAGHADGTLLNALLH
HVPDIINVPRAGIVHRLDKDTTGLMVVAKTIQAQTQLVTQLQSRSVSRIYECIVIGVVTA
GGKIDAPIGRHGQQRQRMAVMEGGKQAVSHYRVLERFRSHTHVRVKLETGRTHQIRVHMA
HINYPLVGDPAYGGRFRIPPAASLTMVDSLKTFPRQALHARFLELDHPTTGKRISWESPL
PDDFVWLLSLLKQDREAFIG
>tr|A0A0Q0CIZ3|A0A0Q0CIZ3_PSEAP Protein product OS=Pseudomonas syringae pv. aptata OX=83167 GN=ALO85_03573 PE=4 SV=1
MDIIRIIFAILLPPVGVFMQVGFAGAFWLNILLTLLGYIPGIIHAIYIIVSRK
>tr|A0A0Q0D5V6|A0A0Q0D5V6_PSEAP Uncharacterized protein OS=Pseudomonas syringae pv. aptata OX=83167 GN=ALO85_02732 PE=4 SV=1
MKVFWGLGKILMLGFWLVVLVNAAIEAPSPFGVMIDMAGAVLLLTHLLELILFNGSLRGR
RHPWRDRGKIILFGIFHVQSIGRSVRGHTHA
>tr|A0A0Q0BXM5|A0A0Q0BXM5_PSEAP Uncharacterized protein OS=Pseudomonas syringae pv. aptata OX=83167 GN=ALO85_02817 PE=4 SV=1
MLNAAPMGKLARSTEQQSLGPDRFTPSSVAVREHRNRERADRIIDQFVERFTEKDFFPDD
GDEALFALLVQLPEWPADLSIRIHNENDEVLAIFLKGNDESVVQNSVVLMQNADAYIVPD
NIPVADDEPLLRWVFSQLPVGSRVGMGGNFPGSHSDSGRIVTLREQIAGLANDQRPLLFD
ALVAAGEGASKVQLDTRTRNPFLPLWIRQQTLLSAALSELCALYPQVPVGRLEELLEQLP
LTVQDDADFLDNGVLPDSFDEALNISVQEWSSSLAIDGLSRTRSFNACTDELAREITSEL
LAAESGRALLIFEPGDSESFPADPDDTRIVLRHDGYGNYSARDAENGEFVSFKPGTDSFY
LAIGSQLKTEERSALGMQFEQDVKGIRDVLTRRAIEKNRGWFDPEQPTEIESDCLPDWFA
NATDVEKQRWKVAVQDYSQALLEAQAPDLLDPSIYGQPDRLRHYARARLQERILLDHGMT
VDPDLITVHTISVEIDPGVGIDIDYEYVGPSELEPHYETQERSLTDLSLENIAFTNINFL
LTSRAFDDQGQLVRFLNAGYLFGLIRDLNIGESYSRYLSTVLSTSAAGQWHCERYARVMR
AQMRLDAIEAKMAGDFLGDGGLPPELANRGYKWVAAVLDHPTDSDDRAMVEGHRIQVSQL
LINGVSLSGVLVIGTESRSSVATLMVFTPQAPDGICFREMSNSQEFRQRILLEPDFLDYL
VGKAPLAAQADVRHVLTAGRDTLFMDLQPCTGNFLEAVYESEVARVISAVDEQTNTNWET
YWESAWEITKVVGDIVLIFTPFKVQLPIAALRSFYAIWQGVGKKSSEQGIAPLYFVQAAL
LLADALTLSKGRRVRTSSAAKTGRSVLDPKTAVSKTPVGLKMRDDGIYRGIHEKAQEGVP
SRFYAVQQEKAYAVTYDSDSAAWRVIDSRRPDAYYQLPIQLDDQGVWVHASTGLRGGGRH
RITKLPMESAGGAGTPGKSAGKLRYKIHMNDFFDTAEFKAAYEHIQAKDLEASVKNAVEK
YVLDGAAHLHKWKPTAKNKLDKNGKFYFLDLTSIGDSTGRGDWRLGLKRVEKGMLEPFDI
LNHKNM
>tr|A0A0Q0CIT9|A0A0Q0CIT9_PSEAP ATP synthase subunit a OS=Pseudomonas syringae pv. aptata OX=83167 GN=atpB PE=3 SV=1
MAEQTASGYIQHHLQNLTFGHLPNGEWGFAHTAAEAKEMGFWAFHVDTLGWSVALGLIFV
LIFRMAAKKATSGQPGALQNFVEVLVEFVDGSVKDSFHGRSAVIAPLALTIFVWVFLMNA
VDLVPVDWIPQLAMMISGDSHIPFRAVSTTDPNATLGMALSVFALIIFYSIKVKGIGGFI
GELTLHPFGSKNLFVQALLIPVNFLLEFVTLIAKPISLALRLFGNMYAGELVFILIAVMF
GSGLLWLSGLGVVLQWAWAVFHILIITLQAFIFMMLTIVYLSMAHEDNH
>tr|A0A0Q0BVU3|A0A0Q0BVU3_PSEAP Uncharacterized protein OS=Pseudomonas syringae pv. aptata OX=83167 GN=ALO85_101969 PE=4 SV=1
MRSARSDGTGAQTCRSWRSVIEVAYGSANGLRDLNGDILDIARIESGRFSLNPERANLRR
LVELVLWVFDGQVTMLTKLRNEGFRRCEATTTSAGDVGLIVEPQLAFVGEVIVPIFHEHP
QKLVLCRKGNGLASGQTEKLVGLEKGVQVSRMYAHPQPSAEGKR
>tr|A0A0Q0DNE0|A0A0Q0DNE0_PSEAP Phosphate import ATP-binding protein PstB OS=Pseudomonas syringae pv. aptata OX=83167 GN=pstB PE=3 SV=1

MNNLSLANEKTKIQVRGLEFFYNNQKSLKSIDMTIPEKRITAIIGPSGCGKSTLLRVFNR
IYAMYPKQEARGEVLLNGENILAPGYSMNRLRSHVGMVFQKPVPFPMSIYDNISYAIKHH
EKLSRREMEDRVEQALRGAALWDEVKDKLKQSATGLSGGQQQRLCIARTIALRPQVLLLD
EPTSALDPISTGRIEQLITELKEQFTVIIVTHNMQQAARCSDYTAFMFMGELIEHGDTDT
IFTKPSKTQTEDYITGRFG
>tr|A0A0Q0DUM9|A0A0Q0DUM9_PSEAP Regulatory protein, LysR:LysR, substrate-binding protein OS=Pseudomonas syringae pv. aptata OX=83167 GN=ALO85_01395 PE=3 SV=1
MRRKIPSTTALVSFEAAARHESFTKAANELSLTQGAVCRQIGGLEEFLGVELFRRSRRGV
KLTEAGLSYSRRVSQQLDAVERDTLSVMGQQGANAIELAVVPTFGTQWLLPRLKDFQHKH
PDVTVHLTNRTRPFLFADTDFDAAIYFGDADWSGTQSHRLMGENSMPVCSPSFLEGRDSL
TARELAELPLLQQTTRPYAWRQWFNAQDLDVARDMTGPRYELFSMLAQAAMHDMGVALIP
PFLIQRELQEKRLVIANPRSLPGSKAYHLMIPERKVESASLTAFRDWLVGQACAYQLPQE
TPEPAIR
>tr|A0A0Q0IAN9|A0A0Q0IAN9_PSEAP DNA polymerase III, chi subunit OS=Pseudomonas syringae pv. aptata OX=83167 GN=ALO85_04307 PE=4 SV=1
MTQVDFYILPSADPAARLDFACKLTEKAWRLGHKVYLHCSDAAQREDLDARLWRFRGEVF
LPHGDAESDHDAAVVLGLGDDSGEHSDLLVNLDLSIPAFFKRFARVAEVVVEDPAIRLAA
RESFRSYREQGYPPQDHRLQRL
>tr|A0A0N8TAB0|A0A0N8TAB0_PSEAP Sodium/alanine symporter OS=Pseudomonas syringae pv. aptata OX=83167 GN=ALO85_100112 PE=3 SV=1
MLEMINDFLSGKVLIVLIVGLGSYFTIRSRFVQFRHFFHMFSVFRDSLRSSAGQLSSFQA
LMLSLAGRVGAGNIAGVGIAVTLGGPGAVFWMWVTALVGMASSFIECSLAQLYKRKDSEG
QFRGGPAFYIQYGLGKRWLGMVMAVLLLVTFGFAFNGLQSHAVTDSLKSAFNVDTRTTGI
CLVVLLGLAFIGGIKRIAAISDLLVPVKTLIYIAVTIYVIALQIDHVPGMLMTIVRSAFG
LDPVFGGLIGSAIVMGVKRGVFANEAGLGSAPNVAAVAQVEHPVAQGVVQAFSVFLDTFV
ICTCTALLILLSGIYDIGYDGNGIVLAQNSLAAVVGDWGRVFISVALALFVFTSILYNYY
LGENSLRFLFGEKIQTIIIYRIAVLVLIMWGAVVDLKDVLAFADITMTMLAFVNLIALAM
LFKVVKRILNDYDAQRRAGVKTPVFDSSQFPDLDLDRNAWPANPTRQSTQDAEAAAKSVP
EAR
>tr|A0A0Q0CZM0|A0A0Q0CZM0_PSEAP Uncharacterized protein OS=Pseudomonas syringae pv. aptata OX=83167 GN=ALO85_01083 PE=4 SV=1
MIEPKRVLRALAEHWALLEPLCEHFDQGTLSLSELRLQLGAQQQDSTPQDITNLLDVWIR
LDILVPVAKSPNRFELNAQIHDFLSYLRREHRLGLCLEIEAYLRHLERLAGYIQDAFEVR
DANDLARQLRLLDMRVRDVLKKLANDEQALVAVADRAKTSDRQIPLRQRYAEVLATWDEY
VEPMIQLVNADGAFEQGVRKVENVLLRLLTEQQRLGHLVDDDMLLRTHARILEMQTSAQL
TLRHARELLLPLREEARRHNAVTRGAALALSAIRRKGLDAVPQAAMPMFTRPQSTFLGSA
SQVEAYVYALARFEPKPARFPKASGARKGQAPRAPRTVKEMLERCEDALPMPDLMVWLLE
QEPTGETDELLYWFSRLSRDKRFARERLERREYFTREHRVSLRSFALLAAKDEQAEHSES
TVHAS
>tr|A0A0Q0DG90|A0A0Q0DG90_PSEAP Hemolysin-type calcium-binding region OS=Pseudomonas syringae pv. aptata OX=83167 GN=ALO85_04758 PE=4 SV=1
MQYDAPVTATEFSSFLTATSLTDSTTAAISTLLALDSASTVNLASWDGVNAIEIPTGQTG
TTDVITGTIAGALGDLVSLNVTPDVAAAKAIILDSQANLHVNITPTVATDAAADVSSQAR
IAVSADASAITQFVLTTGTGDDLIIVSGDQNNFIDAGAGNDTIITGNGNNTVIAGAGNNN
VITGSGNDTIVLSGTNHADVVNAGAGYDVVQLDGSVADYTFVTGNNFNVNLTGAQTAAIT
GAEFLTFVGTAGTETVVLAQSEAEASALRLYDGLLGRDADLGGAQNFANQVNAGTSLTDI
ANEFLNSDEFVNTSTLAPINTLYNELLGRTTGADGGGLQTWQTLLANGGTLGDVAAGIAG
SAEAQRFDQSNAEFVQDLYAAALGRNADQAGLDNWVNNLFNGSTRADVAKAIVNSDEAAL
KADSDFIDNLYLTATGRASDTAGKATFTDILANGGTQADVAIGIVGSVEAVAHNDNVIVL
HGAV
>tr|A0A0Q0C1P5|A0A0Q0C1P5_PSEAP Uncharacterized protein OS=Pseudomonas syringae pv. aptata OX=83167 GN=ALO85_101903 PE=4 SV=1
MKPTDENSTNYLMVSAAAKNLGEQACTLGIKHIKNGTLRLQFNREVAYYAKSIVNDISEG
KKSPEQGLKELKDEQNSLFTQSWEVAKKGVGAVAGAMQFVAGAGICYGSVGTLCLVAGVP
IMAHGANNVYENGRNLWEGRSDTEGPVRKGYQAAAKAVGYGAQEGNMAYGSADLAMSVYG
VTKLVLKPDSWRLFRYIRTDYVRAYRNTSIPVLVIDRAADAITVDGMQKQ
>tr|A0A0Q0DV53|A0A0Q0DV53_PSEAP DNA-directed RNA polymerase subunit alpha OS=Pseudomonas syringae pv. aptata OX=83167 GN=rpoA PE=3 SV=1
MQISVNEFLTPRHIDVQVVSPTRAKITLEPLERGFGHTLGNALRRILLSSMPGCAVVEAE

IDGVLHEYSAIEGVQEDVIEILLNLKGLAIKLHGRDEVTLTLSKKGSGVVTAADIQLDHD
VEIVNPDHVIANLASNGVLNMKLTVARGRGYEPADSRQSDEDESRSIGRLQLDSSFSPVR
RIAYVVENARVEQRTNLDKLVIDLETNGTLDPEEAIRRAATILQQQLAAFVDLKGDSEPV
VIEQEDEIDPILLRPVDDLELTVRSANCLKAENIYYIGDLIQRTEVELLKTPNLGKKSLT
EIKDVLASRGLSLGMRLDNWPPASLKKDDKATA
>tr|A0A0Q0FVL0|A0A0Q0FVL0_PSEAP HTH_37 domain-containing protein
OS=Pseudomonas syringae pv. aptata OX=83167 GN=ALO85_03859 PE=4 SV=1
MNEHIGSDFDDFLAEQGFAEEVSAAALKRVISWQIAEVMKQQKVTKKALAERMHTSRTAV
DRALDQNDPGMTLATLASAARALGQRVEVRLVPEREAVHN
>tr|A0A0Q0FGL4|A0A0Q0FGL4_PSEAP Acetylornithine deacetylase
OS=Pseudomonas syringae pv. aptata OX=83167 GN=ALO85_01595 PE=3 SV=1
MPLPSMKDQFAALIAVPSVSCTQPSLDQSNRPVIDLLAGWLGDLGFACDIQQVSPGKFNL
LATYGTGPGGLVLAGHSDTVPFDEALWKTDPLKLTEVDGRWVGLGSCDMKGFFALVIEAV
RGLVDQPFKQPLLILATCDEESSMAGARALADAGRPLGRAAVIGEPTGLKPIRLHKGVMM
ERIDILGRSGHSSDPSLGHSALEAMHDAISELKGLRTQWQAKYRNPQFTVPQPTLNLGCI
HGGDNPNRICGQCSLEFDLRPLPGMDPEMLRSAIRQKLQPLAELHQVRIDYAPLFPECAP
FEQVADAELVRVAERLTGHTAAAVAFGTEAPYLQRLGCETLVLGPGDIACAHQPGEYLEM
SRLDPTVRLLRQLIEHYCLTPQ
>tr|A0A0Q0DED1|A0A0Q0DED1_PSEAP GTP cyclohydrolase 1 OS=Pseudomonas
syringae pv. aptata OX=83167 GN=folE PE=3 SV=1
MTLEQNYTAILGQLGEDVSREGLLDTPKRAAKAMQYLCRGYTQTLEEVTNGALFSSDASE
MVMVKDIELYSLCEHHLLPFIGKAHVAYIPQGKVLGLSKVARIVDMYARRLQIQENLSRQ
IAEAIQQVTGALGVAVVIEAKHMCMMMRGVEKQNSAMITSVMLGEFRENAATRSEFLSLI
K
>tr|A0A0Q0FI88|A0A0Q0FI88_PSEAP MarR family transcriptional regulator
OS=Pseudomonas syringae pv. aptata OX=83167 GN=ALO85_03238 PE=4 SV=1
MPMTDEHRFGMQLGHLTRGWRAELDRRLADLGLSQARWLVLLHLARFTEPPTQRELAQSV
GVEGPTLARLLDSLEKQGLVQRQAVLEDRRAKKILLSDTALPLIEKIENIANVLRIELFE
GVSEEDLRVSMRVHSQILANLERS
>tr|A0A0Q0DFM1|A0A0Q0DFM1_PSEAP AsnC family transcriptional regulator
OS=Pseudomonas syringae pv. aptata OX=83167 GN=ALO85_01313 PE=4 SV=1
MGCIVSKLDRYDLSILAELQRDARISNQELAERIGLSPSPCSRRVKQLEDDGYIVRQVAL
LDRKKLGLNLTAYVLIGMDRHTPERFENFEQQIRNLPQVLECSLVTGMDADYQLKVVVPD
MDHYQKLLLGHLTRIEGVTSVKSSFVLNQVLASTEMPLTHLRN
>tr|A0A0Q0D6A5|A0A0Q0D6A5_PSEAP Cell division coordinator CpoB
OS=Pseudomonas syringae pv. aptata OX=83167 GN=cpoB PE=3 SV=1
MGAAPVVDNNSGSGSSSYPPAGYGTSGAYAGGGVTAPASAQGQLFMQLQQMQDEIARLRG
VVEVQQNDIQRMKQEALERYQELDQRIASGAAAPATNNSQPAGGAIDAGGTPSASAAQQA
PAAGTEPPDPAKEKLYYEAAFDLIKAKDFDKASQAFTAFLRKYPNSSYAGNAQYWLGEVN
LAKGDLQGAGQAFAKVSQQYPKHAKVPDSLYKLADVERRLGHTDKVKGILQQVVAQYPGT
SAAQLAQRDLQRL
>tr|A0A0Q0BWL5|A0A0Q0BWL5_PSEAP UPF0260 protein ALO85_01061
OS=Pseudomonas syringae pv. aptata OX=83167 GN=ALO85_01061 PE=3 SV=1
MAAKVEPFWIRKTLDQLDTQEWESLCDGCGLCCLQKLEDEEDNAVYYTRIACKLLDLKTC
QCSDYTNRRASVPDCIQLTPGQADEFKWLPPTCGYRLVSEGKDLPLWHHLVCGDRTAVHH
ERISQSGRMLSENTVAEDDWEDYLIFRAG
>tr|A0A0Q0IRD5|A0A0Q0IRD5_PSEAP Iron-sulfur cluster carrier protein
OS=Pseudomonas syringae pv. aptata OX=83167 GN=ALO85_02060 PE=3 SV=1
MSAVNRAAVETILRQYTDPYLNQDPVTAGCVRSIDIQGAQVSVQLELGYAADLFRNGWAQ
VLKTAIENLDGVSSATVAISSVISAHKAQSQIPGLANVKNIVAVASGKGGVGKSTTAANL
ALALSLEGARVGILDADIYGPSQGVMFGIPEGTRPKIKDQKWFVPVEAHGIEVMSMAFLT
DDNTPMVWRGPMVSGALLQLVTQTAWNDLDYLVIDMPPGTGDIQLTLAQKVPVAGAVIVT
TPQDLALLDAKKGVEMFRKVNIPVLGVVENMAVHICSNCGHAEHLFGEGGGAKLATQYDV
ELLASLPLSMLIREQADGGKPTAIAEPESQIAMVYQELARHVGARIVLQEAASPAMPTIS
VSDD
>tr|A0A0Q0FDD0|A0A0Q0FDD0_PSEAP 50S ribosomal protein L13 OS=Pseudomonas
syringae pv. aptata OX=83167 GN=rplM PE=3 SV=1
MKTFTAKPETVKRDWFVVDAAGQTLGRLATEIASRLRGKHKPEYTPHVDTGDYIVVINAE
QIRVTGAKTTDKIYYSHSGFPGGIKSINFEKLIAKAPERVIETAVKGMLPKNPLGRDMYR
KLKVYAGAVHPHTAQQPQELKF

>tr|A0A0Q0DGM1|A0A0Q0DGM1_PSEAP Hpt domain protein OS=Pseudomonas syringae pv. aptata OX=83167 GN=ALO85_02171 PE=4 SV=1
MSIHLDYSVLNALQEVMEDEYPTLLDVFLKDSEQRIAQLRLAVGSHDPDFQELSLTAHSF
KGSSSNMGALQLSELCRQLEDQARQQQFAGLAELIRTIDSEYMTIRGLFNSERQLFVS
>tr|A0A0Q0C622|A0A0Q0C622_PSEAP Uncharacterized protein OS=Pseudomonas syringae pv. aptata OX=83167 GN=ALO85_01905 PE=4 SV=1
MRRLLLALPFALLPLVAAHAAEEHDHDHGDEHGSLGAHEHGVGRLDVVLEGKTLEFEFDS
PAMNIVGFEHVATSDEDKAKVAKARELLLKPNALFSIADAANCSATSVKLESPLFGDKDD
DHDEHADAGDAEHHEHSEIHGHYKFVCDAPAILKKLDLSQIFTTFPDTKKLQVQLISPGG
QSGAEVIAANPTLKF
>tr|A0A0Q0DLK4|A0A0Q0DLK4_PSEAP Electron transport protein SCO1/SenC OS=Pseudomonas syringae pv. aptata OX=83167 GN=ALO85_01624 PE=4 SV=1
MTRTQKAVFIAAAIVALLLGVILSQVLPGRSSQDQAALNDAGIVLLPKSRQLPALSMTDQ
NGAAVALDGLKDQWTLMFFGYTFCPDICPTTLAQIKQIRAELPEEAVKHMRVVLVSVDPN
RDTPQQLKQYLGYFDPQYIGLTAPVADIQTLANAVSIPFIPADTSKPNYFVDHSGNLALI
GPDGTQRGFIRAPLNNQKLVKQLPGLLERD
>tr|A0A0Q0DAG7|A0A0Q0DAG7_PSEAP Conjugal transfer protein OS=Pseudomonas syringae pv. aptata OX=83167 GN=ALO85_200137 PE=4 SV=1
MRKFRKSKEDVIEQERQLAARTSIDAATAQEISLVEQAWIHAARYFEKSIAADEKAKTKR
ATRLNYVFGTIAVMSVAAVMGLTPLKTVQLGLVRVDRNSGYTDVVWADDKGKPQEQIDDE
FWLSAYTRFRESYNFSSNDANYSMVKLMSYDETFTEYRNFQLSSKGYLEVLGTNRQIRTD
INNINFLDRKDREGTAQVRITKTVLDRNGVPDPQLHPVTWVATVTYDYKNPAKKAGDQWL
NPRGFGIKAYTMTQEVGVSNGK
>tr|A0A0Q0IH46|A0A0Q0IH46_PSEAP Uncharacterized protein OS=Pseudomonas syringae pv. aptata OX=83167 GN=ALO85_101045 PE=4 SV=1
MPSHFRTLSPWLSDPAKARIAIKESRMNPPHNASRNPAAVIDQNMNTYREPLPIPLRFEA
GEGDEHTHTHGAIESVLESAGFRPEEIRAIYFGNWLRDYSQLLDPKIVRATSMPKSFPDL
LSRDALTRIVDVLAVKEFTDLMKTDRSRFVVTAERLGVYRPAEHIDNPRTPDPKPANPKE
RDADFDAWVLPDDPLLKVDPDTSMKRYIQRSVQVMAAGLESAVNGGPGSTDGLRDMGAAL
HILEDFFAHSNFAELSLIKLGHERILPWTSDADGKHRLPLVTGTFGGSDIIASLAEPLGR
IMFSTDEKPFEAIKAGERYERDQIIQIVLSEHPDPRLLAGYEAFLEARDQWASQPFSEQV
EQFYAFIGTPGRLLGNAFGVVMQSLAIWLGNSVDDLQTLLDEDPNSSGSTDPSHSQLAKD
HAQHPLHTLAARLARTAVLQVGQAVLGQWHGKEGAEHPVQVATRFFTHPMDCDWQDSIVR
EWAEANPEQVERATLKSELDQSRLQLQRSAQNDLQRFSQDGSDFLSVFFESVSLADLWAR
ITGK
>tr|A0A0Q0DCX0|A0A0Q0DCX0_PSEAP Cell division protein ZapE OS=Pseudomonas syringae pv. aptata OX=83167 GN=zapE PE=3 SV=1
MTPLERYQADLKRPDFFHDAAQENAVRHLQRLYDDLIAAHDSKPGLFGKLFGKKEQTLVK
GLYFWGGVGRGKTYLVDTFFDALPFQDKVRTHFHRFMKRVHEEMKTLKGEKNPLTIIARR
FSDEARVICFDEFFVSDITDAMILGTLMEELFKNGVTLVATSNIVPDGLYKDGLQRARFL
PAIALIKQNTDIVNVDSGVDYRLRHLEQAELFHFPLNEVAQESLRKSFKALTPDCAETIE
NDVLMIENREIRALRTCDDVAWFDFRELCDGPRSQNDYIELGKIFHAVLISGVEQMDVAT
DDIARRFINMVDEFYDRNVKLIISAEVELKDLYTGGRLMFEFQRTLSRLLEMQSHEFLSR
AHKP
>tr|A0A0Q0IRL1|A0A0Q0IRL1_PSEAP Conjugal transfer protein TrbL OS=Pseudomonas syringae pv. aptata OX=83167 GN=ALO85_200173 PE=4 SV=1
MFKKMILTVAVAASCMSGLALADAPVKLAETDKGKVLVDAKGMTLYTFGKDTSGKSVCNG
PCASNWPPLPVSADAKAGHDYSVVTRDDGTKQWAYKGMPLYSWVKDTKPGDTTGDGVNQV
WHVAKP
>tr|A0A0Q0CF32|A0A0Q0CF32_PSEAP DNA topoisomerase 4 subunit B OS=Pseudomonas syringae pv. aptata OX=83167 GN=parE PE=3 SV=1
MATPSASSYNADAIEVLSGLDPVRKRPGMYTDTSRPNHLAQEVIDNSVDEALAGHARSVQ
VILHADNSLEVSDDGRGMPVDIHPEEGVSGVELILTKLHAGGKFSNKNYQFSGGLHGVGI
SVVNALSTQVRVRVKRDGNEYEMTFADGYKATELAVVGTVGKRNTGTSVYFAPDPKYFDT
PKFSVSRLKHVLKAKAVLCPGLLVSFEDKSSGEKVEWHYEDGLRSYLQDSVTEFLRLPDE
PFCGSFTGNKEAVDWALLWLPEGGDSVQESYVNLIPTAQGGTHVNGLRQGLLDAMREFCE
FRNLLPRGVKLAPEDVWERIAFVLSMKMQEPQFSGQTKERLSSREAAAFVSGVVKDAFSL
WLNTHSELGLQLADLAINNAGRRLKASKKVERKRITQGPALPGKLADCAGQDPARSELFL
VEGDSAGGSAKQARDKEFQAILPLRGKILNTWEVDGGEVLASQEVHNIAVAIGVDPGAAD
ISQLRYGKICILADADSDGLHIATLLCALFVQHFRPLVDAGHVYVAMPPLYRIDLGKEIY

YALDEAERDGILDRLVAEKKRGKPQVTRFKGLGEMNPPQLRETTMDPNTRRLVQLTLDDF
AATSEMMDMLLAKKRAGDRKSWLESKGNLAEVLT
>tr|A0A0Q0IR07|A0A0Q0IR07_PSEAP BNR repeat-containing glycosyl hydrolase OS=Pseudomonas syringae pv. aptata OX=83167 GN=ALO85_04596 PE=4 SV=1
MDMSCPAPAPDNSHLIIIRAEKSMRESFHRRTRLCAGMFLAVVASSLFALPAMAGSEALA
TAVAADAPVYAIESAKASRTLLLDVAHAGARLVVVGDHGHILLSDDQGNTWSQARVPTRQ
LLTAVFFVDEQHGWAVGHDAQVLASSDGGKTWNKQFEDLKREAPLLDVWFKDLDNGLAIG
AYGLLLSTADGGQHWEDVSDRLDNEDQYHLNGIAQVKDAGLFIVGEAGSMFRSRDEGRTW
EKIAGPYQGSLFGVIGTAQPSTLLAYGLRGNLFRSSDFGDSWQPIELKGARGPLEFGLAS
ATLLADDSLVLVGNGGSVMRSTDDGETFAVFNRPDRISLAGVTGNPQGNLILVGQGGVRI
ASPTGAETTQQ
>tr|A0A0N8TAC9|A0A0N8TAC9_PSEAP Chemotaxis protein CheY OS=Pseudomonas syringae pv. aptata OX=83167 GN=ALO85_01411 PE=4 SV=1
MAGRSILIVDDEAPIREMIAVALEMAGYDCIEAENSQQAHAIIVDRKPDLILLDWMLPGT
SGIELARRLKRDELTGDIPIIMLTAKGEEDNKIQGLEVGADDYITKPFSPRELVARLKAV
LRRAGPTDGEAPIEVGGLLLDPISHRVTIDGKPAEMGPTEYRLLQFFMTHQERAYTRGQL
LDQVWGGNVYVEERTVDVHIRRLRKALGDAYENLVQTVRGTGYRFSTKS
>tr|A0A0N8T9Y1|A0A0N8T9Y1_PSEAP VOC domain-containing protein OS=Pseudomonas syringae pv. aptata OX=83167 GN=ALO85_200091 PE=4 SV=1
MFGFNRGKKVRFSHTNIVSADWKRLSKFYRNVFGCHQVGPVRKLSGDAVSGGTGVPDAII
EGIHLSLPGCEDSGITLEIFQYMEGVNREVAYANSRGITHLAFEVEDLVDICAKVVRNGG
WMLGKVVKQNVKGVGVCEFLYVRDPERNIIEIQKWTKPEKRT
>tr|A0A0Q0DP52|A0A0Q0DP52_PSEAP Outer membrane protein OmpW OS=Pseudomonas syringae pv. aptata OX=83167 GN=ALO85_01902 PE=4 SV=1
MHKHLLRASLVALTLTAPVAAHAYQSGDFIVRAGVAHVQPNEDSGEVRLDGAKVSGTKAT
VDGENQLGLTFAYMLTQHVGVELLAATPFSHTVSVKGLGPGLDGKLADIKQLPPTLSLQY
YPMEPSSKFQPYAGVGINYTTFFDTDLTGNRKNQGFSNLKLKDSVGLAGQLGMDYMITDN
VLLNASVWYVDIDTKATVDGPSALAVGKTKVDVDIDPWVYMVGVGYKF
>tr|A0A0Q0DC05|A0A0Q0DC05_PSEAP Arginine N-succinyltransferase OS=Pseudomonas syringae pv. aptata OX=83167 GN=ALO85_01842 PE=4 SV=1
MLVMRPAQMADLAEVQRLAADSPIGVTSLPDDAGRLTDKIGSSEASFAAEVSFNGEETYF
FVLEDSESGKLVGCSGIVASAGYSEPFYSFRNETFVHASRELKIHNKIHVLSQCHDLTGN
SLLTSFYVVPELVGTAWSELNSRGRLLFVASHPERFADSVVTEIVGYSDEQGDSPFWDAI
GRNFFDINYAAAERLCGLKSRTFLAELMPHYPIYVPLLPDAAQEAMGQVHPRAQITFDIL
MREGFETDHYIDIFDGGPTLHARVSGIRSIAQSRLVPVKVEPGQGSDVGKGGRLYLIANG
LLQDYRAVLLELDWAPGRPVVLSLQAADALGVGEGASVRIVAV
>tr|A0A0Q0CDW3|A0A0Q0CDW3_PSEAP RNA polymerase-associated protein RapA OS=Pseudomonas syringae pv. aptata OX=83167 GN=rapA PE=3 SV=1
MTMSFFTLPLAFGARSVNILSFFKRETGSMAQQYQPGQRWISDSEAELGLGTVLAQDGRL
LTVLYPATGETRQYALRNAPLTRVRFSPGDVITHFENWKMTVREVDDVDGLLVYHGLNAQ
NELVTLPETQLSNFIQFRLATDRLFAGQIDQLSWFSLRYNTLEHTSRQLQSSLWGLGGVR
AQPIAHQLHIAREVADRIAPRVLLADEVGLGKTIEAGLVIHRQLLSGRANRVLILVPENL
QHQWLVEMRRRFNLQVALFDAERFMESDAGNPFEDTQLALVALEWLVEDEKAQDALFAAG
WDLMVVDEAHHLVWHEDKASREYSLVEQLAEVIAGVLLLTATPEQLGQDSHFARLRLLDP
NRFHDLKAFRAESENYRPVAQAVQELLDKGKLSAAAQETIHGFLGAEGDSLLAAVNTGDE
EAKSRLIRELLDRHGTGRVLFRNTRAAVQGFPERKLHQYPLPCPVEYLELPVGEHADLYP
EVSFQSQSEVSEEERWWRFDPRVDWLIDTLKMLKRVKVLVICAHAETAMDLEDALRVRSG
IPATVFHEGMNILERDRAAAYFADEEFGAQVLICSEIGSEGRNFQFSHHLVLFDLPSHPD
LLEQRIGRLDRIGQKHTIELHVPFLETSPQARLFQWYHEALNAFLNTCPTGNALQHQFGP
RLLPLLESGDDDEWQTLIDEARSERERLESELHTGRDRLLELNSGGAGEGEALVEAILDQ
DDQFSLPIYMETLFDAFGIDSEDHSENALILKPSEKMLDASFPLGDDEGVTITYDRNQAL
SREDMQFITWEHPMVQGGMDLVLSGSMGNTAVALIKNKALKPGTVLLELIYVSEVVAPRS
LQLGRYLPPAALRCLLDPNGNDLASRVSFNTLNDQLESVPRASANKFIQAQRDQLTPRIN
AGEEKITPKHAERVAEAQRRLAADTEEELARLTALQAVNPTVRDSELVALRAQREQGLAM
LEKAALRLEAIRVLVAG
>tr|A0A0Q0C3I1|A0A0Q0C3I1_PSEAP Nicotinate-nucleotide pyrophosphorylase OS=Pseudomonas syringae pv. aptata OX=83167 GN=ALO85_03312 PE=3 SV=1
MPNLRIADLTAEIEANVRRALLEDVGSGDITAQLIPAERLAKATIISRDAAVIAGTAWVD
AVFRQLDPRVAVHWQVTDGDRVSPNQALFHLEGPARSLLTGERSALNFLQMLSGVATRAQ
YFADMVAGTQVKLLDTRKTLPGLRMAQKYAVTCGGCHNHRIGLYDAFLIKENHIAACGGI

AQAVEAAHRIAPGKPVEVEVESLGELQQALDAGADIIMLDELSLDDMREAVRLTAGRASL
EASGGINEDTLRVIAETGVDYISIGAMTKDVKAVDLSMRLSL
>tr|A0A0Q0ID16|A0A0Q0ID16_PSEAP Cyclic nucleotide-binding protein OS=Pseudomonas syringae pv. aptata OX=83167 GN=ALO85_01067 PE=4 SV=1
MSEPLSNLNNAIRDMLLDCGLFDTLLPGDFFTVAGYFSITDIDKGETIFAEGDAGTFMCI
IHQGTVSVQKLAADGQQVEIAVLRRGRAFGEMAVLDGERRSASCIAATSCQLLNLGRDSL
DKMLEDAPKIAAKIIRALAISLSKRLRMVDGQLLSQQV
>tr|A0A0Q0BTI1|A0A0Q0BTI1_PSEAP RraB domain-containing protein OS=Pseudomonas syringae pv. aptata OX=83167 GN=ALO85_01217 PE=4 SV=1
MSTAYQEDISSNVLRRMKEGGFDFARIYPIEFYAIFPDEERARQAAEQFRGESLNTQVNV
RDDGHWHLQLSKVMYATYDGIGDFEQDFQTVISPMDGEVEGWGVTQEIRRLHA
>tr|A0A0Q0IGU4|A0A0Q0IGU4_PSEAP RNA polymerase sigma factor, FecI/PupI family OS=Pseudomonas syringae pv. aptata OX=83167 GN=ALO85_00398 PE=3 SV=1
MTQAATLSQQTLHSLYRDHHGWLESWLRRRMGNAWDAADLSQDTFLRVLSSSQQIADMQE
PRAYLLTVGKRLLSNFYTRRSLEQAYLEALAQLPEESVPSPEQRWLLLETLQALDELLDG
LSAVVRRAFLWSQLEGLGYREIAERLSVSERTVKRYMAQAYEHCLLVDW
>tr|A0A0Q0D0W7|A0A0Q0D0W7_PSEAP tRNA-specific 2-thiouridylase MnmA OS=Pseudomonas syringae pv. aptata OX=83167 GN=mnmA PE=3 SV=1
MRDPAPSNSEMKRVIVGMSGGVDSSVSAVLLMEQGYQVEGLFMKNWEEDDGTEYCTARED
LADAQAVCDKIGIKLHTANFAAEYWDNVFEHFLEEYKAGRTPNPDILCNREIKFKAFLDY
ALMLGADLIATGHYVRRRDIDGRTELLKGLDPNKDQSYFLHAVGGEQIARTLFPVGELEK
PEVRAIAEKHGLATAKKKDSTGICFIGERRFTDFLRQYLPAQPGEIKTTEGEVIGRHSGL
MYHTIGQRQGLGIGGLKDASDDPWYVLVKDLDNNELIVGQGNDHPWLFSRALVSSEIYWV
NPIDLSSPRRLTAKVRYRQGDQPCTLEKTADGYRATFDDPQRAVTPGQSVVFYDGEICLG
GGVIEIAEPWTSKDKR
>tr|A0A0Q0DQU7|A0A0Q0DQU7_PSEAP AsnC family transcriptional regulator OS=Pseudomonas syringae pv. aptata OX=83167 GN=ALO85_03919 PE=4 SV=1
MEGLVKLDRIDINILVELQKDGRMTNVSLADAVGLSASPCLQRVKRLESAGYISSYKAHL
NLAKITESVTVFTEISLSDHKREDFAKFEANIRHVDEVLECHLISGGYDYLVRFMTRSIQ
HYQEVIEELLDKNIGISKYFSYIVIKSPVMKDGVPLRKLLRH
>tr|A0A0Q0D5A8|A0A0Q0D5A8_PSEAP UDP-4-amino-4-deoxy-L-arabinose--oxoglutarate aminotransferase OS=Pseudomonas syringae pv. aptata OX=83167 GN=arnB PE=3 SV=1
MSQTFLAFSRPSIGDEEIAAVTRVLRSGWITTGPECQKLEEEFAARVGAQHAVALSSATG
AMHVALLALGVGPGDEVITPSQTWVSTANMICLQGATPVFVDVDRDTLMTSAALIAQAIT
PRTKAIVPVHYAGAAFDLDPLYALADRHGITVIEDAAHAAGTGYQGRPVGQQGTAIFSFH
AIKNMTCAEGAMLVTDNARLADRVRQLKFHGLGVDAYDRLTLGRKPQAEVMEPGFKYNLA
DINASIARVQLQRLDAINAQRQALASHYLERLASSPVLPLALPRHAQQHAWHLFILRIDP
ERCGLDRDAFMKALQARNIGTGIHFIATHLHSYYRKRFPDVRLPDTEWNSSKLCSIPLFP
DMTLDDVERVVGAIESTVESSH
>tr|A0A0Q0C8V2|A0A0Q0C8V2_PSEAP Putative membrane protein OS=Pseudomonas syringae pv. aptata OX=83167 GN=ALO85_03367 PE=4 SV=1
MLPQPATSSVSPRHLSLQKWRGRVGMGLVASLSVLAGMTDAIGFMATGDFVSFMSGNTTR
LAVAISDGDLSVMVRLTLAILAFIAGNALGVVVVRLGGRRALPLLLGIATLLCAAAAWPL
ESNMLALIWAILAMGMLNAAVEQVNGLPVGLTYVTGALSRFGRGLGRWMLGERRDGWRVQ
LVPWVGMFIGAVIGALLEHRLGLNALLISGSLSALLGLVSLKIPHRWQRQYMPR
>tr|A0A0Q0D681|A0A0Q0D681_PSEAP tRNA pseudouridine synthase D OS=Pseudomonas syringae pv. aptata OX=83167 GN=truD PE=3 SV=1
MSERELLGPRAYGEALGRAVLKATAEDFQVDEVLDIPLSGDGEHLWLWVEKRGLNTEEAA
RRLARAAGVQLRTVSYAGLKDRQALTRQWFSIQLPGKADPDLSAAQDDTLQILKSGRHKR
KLQRGAHAANGFTLRLTQLDADKDALNQRLETIARQGIPNYFGTQRFGYQGGNLGEARDY
AARKALPEQRAVRSRLLSTARSYLFNRVLAARVVDGSWQKAQVGDLLAFTDSRSFFPAGV
DECSDPRLAILDLHPTGPQWGEGPSPAGGATAALENTVADDESVLRDWLVRAGMEHERRI
LRLPIGRLTWHYPESDILQLEFVLPPGCFATVLVRELIDLVPVGQTDSSCVF
>tr|A0A0N8T8T4|A0A0N8T8T4_PSEAP RND-type multidrug efflux system, membrane fusion protein OS=Pseudomonas syringae pv. aptata OX=83167 GN=ALO85_03043 PE=3 SV=1
MKKPILTLGRVVLTLLVVTLAAVVVWRMVMYYMYAPWTRDGHIRADIVQIAPDVSGLIQK
VEVTDNQPVHKGQVLFTIDQDRFRLALRQAQATVAERQETWEQARRENKRNRGLGNLVAR

EQLEESQSREARALSALGESKVAVDAAQLNLDRSVIRSPVDGYLNDRAPREHEFVTAGRP
VLSVVDSTSFHIDGYFEETKLDGIHVGQGVDIRVIGDNARLTGHVVSIVAGIEDRDRSSG
SNLLPNVNPAFSWVRLAQRIPVRIAFDDVPADFRMIAGRTATVSIIDDTQERSR
>tr|A0A0Q0DQH2|A0A0Q0DQH2_PSEAP Release factor glutamine methyltransferase OS=Pseudomonas syringae pv. aptata OX=83167 GN=prmC PE=3 SV=1
MTIIASVLRSAELPDSPTARLDAELLLAAALGKPRSFLHTWPERIVSTEAAVAFAGYMER
RRKGEPVAYILGQQGFWKLDLEVAPHTLIPRPETEMLVEAALELVPAFAPAQVLDLGTGT
GAIALALANERQQWKVTAVDRVPEAVALAERNRQRLQLNNAEVFESHWFGGLQGRQFDLI
ISNPPYISDADPHLSAGDVRFEPSSALVAGSDGLDDLRTIIEQAPAHLNADGWLLLEHGY
DQGPAVRELLIRQGFERIQTRRDLGEHERITFGCKPC
>tr|A0A0Q0IHY0|A0A0Q0IHY0_PSEAP Histidine kinase OS=Pseudomonas syringae pv. aptata OX=83167 GN=ALO85_02211 PE=4 SV=1
MKFRHWDINTRTQIISLGPALLLTALLISFFTFVRIQDLRQELNHTGQLIANQLATAVEP
GVTTGDIEGLKALMRATLSMPSVRYVEIQDSSNTAVIYIDQPRDNEQQARQMEVFLAPVQ
PQRTTIHGNPGTPAAGKSQPAQGASQRYLGRVLVGMSSEAFSRRQQEILLKAGVLAMVAL
LFAFLLARSLALSLSRPISDMGEALKAIQQGDYHTPMPVSDDAELGALARHINNLAENLH
RASLEQQRSVAQLIQTREEAEQANRAKSDFLAMMSHELRTPMNGVLGMLQLLETTHMTHE
QSEYATLATESTEHLLRVINDILDFSRIERDALQMEGIPFNLAELVSASTHAFNHSAAQR
TLLLELHIQPGLETLNVIGDPTRIRQILVNLIGNALKFTEQGKVCVDARWAMLDNELLHF
TCAVQDSGIGIDAERLESMFDAFKQADSSISRRYGGTGLGLPIARTLAERMGGTLHAESE
EGSGSLFTLQIPLPVSRDASVAIAKPLSTLDSHGRERSILLVEDNPVNRTVVQAMLRNLG
CEVDIAVDGAEAVAKASAGNHALILMDCRLPVMDGYEATRRIRQLSGLERIPIIALTANA
LQGDRDACLQAGMDDYLPKPFKHADLQQVLQRWLSFEAPVTGDKC
>tr|A0A0Q0DJJ8|A0A0Q0DJJ8_PSEAP Two component transcriptional regulator, LuxR family OS=Pseudomonas syringae pv. aptata OX=83167 GN=ALO85_00550 PE=4 SV=1
MEWNMNSVFIIDDHPVIRMAVRMLLENENYEVVGETDNGVDAMQMVRECMPDLIILDISI
PKLDGLEVLARFNTMGLPSKILVLTSQTPNLFAIRCMQSGASGYVCKQEDLSELLSSVKA
VLSGYNYFPSQALTCAVREDGQTNELELFKLVNDRELMVLQLFAQGRSNKEIAKGMFLSN
KTVSTYKTRLMQKLKTRTLVELIEMAKRNALV
>tr|A0A0N8T8C5|A0A0N8T8C5_PSEAP Two-component system osmolarity response regulator OmpR OS=Pseudomonas syringae pv. aptata OX=83167 GN=ALO85_01599 PE=4 SV=1
MSSTAQNAEGEKILIVDDDPGLSSLLERFFTSKGYRARAVANVEQMDRLLAREVFNLVVL
DLMLPGEDGLSACRRLRAANNQVPIIMLTAKGDELSRIKGLELGADDYLAKPFNPDELMA
RVKAVLRRQAAPVPGAPGSEDESVSFGDYVLSLATRELKRGEEVHMLTTGEFAVLKALVM
HAREPLTRDKLMNLARGREWDALERSIDVQISRLRRLIEPDPSKPRYIQTVWGVGYVFVP
DGAGSR
>tr|A0A0Q0IQ70|A0A0Q0IQ70_PSEAP Glutamate synthase-like protein, small subunit OS=Pseudomonas syringae pv. aptata OX=83167 GN=ALO85_00838 PE=4 SV=1
MKTVDLSSATVRDLNQALHDQVKEVQDREWLVTHPDGAHNLAVGVNEAISIDIKGHAGYY
CAGMNQKASITVHGNVGVGCAENMMSGAVRVKGSASQAAGATAHGGLLVIEGDAGARCGI
SMKGIDIVVGGSIGHMSCFMGQAGRLVVCGDAGDALGDSLYETRIYVKGKVESLGSDCIA
KEMREEHLQELQELLNRAGFNEKAADFKRYGSARQLYNFKVDNASAY
>tr|A0A0Q0FQQ0|A0A0Q0FQQ0_PSEAP Protein HflK OS=Pseudomonas syringae pv. aptata OX=83167 GN=ALO85_02846 PE=3 SV=1
MLYQMESAMAWNEPGGNSNNQDPWGGKRRGGDRKGPPDLDEAFRKLQESLKGLFGGGNKR
GSDGGGSGSGGGSGKGGGFGLLGIGLVVLVAFWLYSAIYVVDEQEQAVVLRFGQYHETVG
PGLNIYFPPFDRKYMENVTRERAYSKQGQMLTEDENIVEVPLTVQYKISDLQAFVLNVDQ
PEISLQHATESALRHVVGSTAMDQVLTEGRELMASEIKERLQRFLDTYRTGITVTQVNVQ
SAAAPREVQEAFDDVIRAREDEQRARNQAESYANGVIPEARGQAQRILEDANGYRDEVVS
RAKGEADRFTKLVAEYRKAPEVTRQRLYLDTMQEVFSNTSKVLVTGDKGQNNLLYLPLDK
MIESSRSSNGNGSNSGTPSQSANDAAAAAAAARANEAQQRGEVRTRETR
>tr|A0A0Q0DJP9|A0A0Q0DJP9_PSEAP Chaperone protein DnaK OS=Pseudomonas syringae pv. aptata OX=83167 GN=dnaK PE=2 SV=1
MGRIIGIDLGTTNSCVSILENGNVKVIENAEGTRTTPSIIAYANDGEILVGQSAKRQAVT
NPHNTLYAVKRLIGRKFEEDVVQKDIQMVPYKIVKADNGDAWVEVNGQKMAPPQISAEIL
KKMKKTAEDYLGEAVTEAVITVPAYFNDSQRQATKDAGRIAGLDVKRIINEPTAAALAYG

MDKAKGDHTVIVYDLGGGTFDVSVIEIAEVDGEHQFEVLATNGDTFLGGEDFDIRLIDYF
VDEFKKESGMNLKGDPLAMQRLKEAAEKAKIELSSSTQTEVNLPYITADATGPKHLVVKI
SRSKLESLVEDLVQRTIAPCEMALKDAGIDRSKINDVILVGGQTRMPLVQKLVTEFFGKE
ARKDVNPDEAVAMGAAIQGAVLAGDVKDVLLLDVSPLTLGIETMGGVMTALIEKNTTIPT
KKSQVFSTADDNQNAVTIHVLQGERKQAGQNKSLGKFDLAEIPPAPRGVPQIEVTFDIDA
NGILHVGAKDKATGKQQSIVIKANSGLSEEEIQQMVRDAEVNSEEDRKFEELASARNQGD
ALVHSTRKMIADAGDKVTAEEKTAVEAALVALEAAIKGDDKAAIEAKVEELSKVSAPIAQ
KMYAEQAENPDAAAKPAEETAKADDVVDAEFEEVKDHK
>tr|A0A0Q0FQE0|A0A0Q0FQE0_PSEAP 50S ribosomal protein L7/L12
OS=Pseudomonas syringae pv. aptata OX=83167 GN=rplL PE=3 SV=1
MSISQDDILNAVAEMSVLQVVELIKAFEEKFGVTAAAGSAGPAAAAAVVEEQTEFNVMLL
EAGEKKVNVIKAVRELTGLGLKEAKAVVDGAPAQVLEAVSKDAADKAKATLEEAGAKVEL
K
>tr|A0A0Q0FI71|A0A0Q0FI71_PSEAP Peptide deformylase OS=Pseudomonas
syringae pv. aptata OX=83167 GN=def PE=3 SV=1
MIHKILKMGDERLLRIAPPVPAEMFGSSELDTLIADMFETMHSVGGVGLAAPQIGIDLQL
VIFGFERNERYPQAEAVPQTILINPLITPLSPTLEEGWEGCLSVPGLRGMVDRYQSIRYE
GFDPQGQPIERVAHGFHARVVQHECDHLIGRLYPSRITDFSKFGFMDVMFPDMDPNADE
>tr|A0A0Q0DZE1|A0A0Q0DZE1_PSEAP Uncharacterized protein OS=Pseudomonas
syringae pv. aptata OX=83167 GN=ALO85_03385 PE=4 SV=1
MCRPKEPIAGIFLLLVLAAGGWVAQEPFPHFGIDLPVEALLDSDHPATPVPPRHRMQLDE
GQFSSALLESMPLYALAEPPGSP
>tr|A0A0Q0BU71|A0A0Q0BU71_PSEAP Periplasmic solute binding protein
OS=Pseudomonas syringae pv. aptata OX=83167 GN=ALO85_100968 PE=4 SV=1
MIRVRRLLNLAAVFWVSLLIIAPAQAEVRVLTSIKPLQLIAAAVQDGVSNPEVLLPPGAS
PHNYALRPSDVRRVQDVELLYWIGPDMETFLPRVLKGRKLPTVAVQTLPGMHLRHFGEDN
ASHDDHEHDHDNDADEHDHDHRPGSLDSHLWLSTVNARVIAAKMAADLSAADPANATRYT
SNAEAFSKRLDTLDARIKQRVSAVAGKPYFVFHEAFDYFEEAYGLKHAGVFAIAAEIQPG
AQHVAAMRERLKAAGKTCVFSEPPLRPRLAETLSTGLPVKLAELDGLGGYIPATAQGYEQ
VLQKLADDLAGCLSSL
>tr|A0A0Q0DCD8|A0A0Q0DCD8_PSEAP GCN5-related N-acetyltransferase
OS=Pseudomonas syringae pv. aptata OX=83167 GN=ALO85_01434 PE=4 SV=1
MTDVQIRPVNVDDHHAWLPLWQAYLRFYNTELAEGVSDITWQRLVDANEPTHSALAWRGD
EAVGMVNFIYHRSNWSISNACYLQDLIVAPEQRGTGIGRQLIEFVYATAKADGCDKVHWL
THETNATAIQLYERIAERPGLIQFRKAL
>tr|A0A0Q0DF72|A0A0Q0DF72_PSEAP Uncharacterized protein OS=Pseudomonas
syringae pv. aptata OX=83167 GN=ALO85_01650 PE=3 SV=1
MPIQNSPYKAFATLLNSGGHQVSPAELHGLLLGRSCAGAGFDNEGWFADASVLLETEPQD
NIRQALVGLQEMVKGELTGDDMTVVLLLPGDDEPLTERAAALGQWCQGFLAGFGLAIGDK
PLGTEAKAVLEDLAAIAQVQDALEESEDGETDYMEVMEYMRVAPLLLFTEFNEPSAPQPK
PSLH
>tr|A0A0Q0FJY9|A0A0Q0FJY9_PSEAP Uncharacterized protein OS=Pseudomonas
syringae pv. aptata OX=83167 GN=ALO85_02999 PE=4 SV=1
MSTTLPLISSITIRTATSQTTYSNAEQITTAASASETKGVQVNLSAEGKAAASSSSRNAD
IEQSGLPGSVQKILKAIRELQRRIEETMEQIQKALNDPSLSPEERRTKAAALQTVLSTLQ
AQVSNSTADLSSLMNSLGSSDADKTKAGMLVLAKM
>tr|A0A0Q0DUP1|A0A0Q0DUP1_PSEAP Uncharacterized protein OS=Pseudomonas
syringae pv. aptata OX=83167 GN=ALO85_01519 PE=4 SV=1
MLSPTAPQSCARPGQCRHGVGMEMDWESLLDELWSYAASDPMELETKIRSSLKGHSSEGL
LQAISLLITQGQHATARTLLSEFLKNHQAAEDIRRRLELILRFKKHRAQQTLSIKSKQRS
KELLLKDVNSLVASGKLQAAESLLLEAIATFEEPDYLNLLSRVYMRQRRPIDGAKAMQRA
LVLRRQQQAFVEIATEESTDDLPTASDLAFLNDSAESLTTLESPPPPINKALTETADDSL
NFIQHTASKYPSLKTDGWWEGQLTPSEAPMCLGKPEAGDAPDMASAARADSTEEHAAEDE
TAPSAPIKNILKLTHPRKETEAGEGLTKVKVFNKSGRSLTSIQPTRLDTPEPLDSDGPPS
HVIPSLVMKASAPTPPIQDQISDENDEDEDEDGEDLALTDLELDLPTTPQPNLGLEALED
DLDLDHDLYDLTIIELGTGLEEEIRTDLDDFRDIDDDYAAYAFDPDEVFDGEEAVAAEPN
DWLADKLSREERALQKAAELIGKANWPLSTLSLVQQIFVMSGWGQTRLALEREIEKGLVP
QELILAAHIKVIWSENDIYWIAFDKNGSSSLSYYALSWPTALLIVRSFESLPQVEEIEVF
LESLFASWYENTVLRRAFKAFSRYLWFRFANLEGCLPANQPFDFCDPHELPAEEYSDLGL
WDVLEIEKTEILRAYGVFKTKHPQEPGCYLSDKPLVEEKPPLNAEKRNKKQHPEDTNTPQ

ETEGEDETLDDCAYEAPSQWSAVSLTSALHAQRTDLTLPNIEP
>tr|A0A0Q0FSS6|A0A0Q0FSS6_PSEAP Uncharacterized protein OS=Pseudomonas syringae pv. aptata OX=83167 GN=ALO85_04991 PE=4 SV=1
MSDKELYEIEYTLLGEHHVDIIENLDVPIIEVVHELALKHHVELDELPMGEAGLPLAAGI
TDVSIHSLEGSEGSATV
>tr|A0A0Q0DZZ8|A0A0Q0DZZ8_PSEAP RNA polymerase sigma factor OS=Pseudomonas syringae pv. aptata OX=83167 GN=ALO85_03832 PE=3 SV=1
MSSVPSTHSEIVGALYRDHRGWLLAWLRRNVACPQRAQDLSQDTFVRLLGRHELLAPKEP
RAFLATVAKGLMFDHFRRAALEQAYLGELMLVPEAEQPSPEKQMLILEDLKAIDRMLGTL
SSKARAAFLYSRLDGMSHAEIAEKLGVSVSRVRQYLAQAMRQCYVALYGEPT
>tr|A0A0N8T9A0|A0A0N8T9A0_PSEAP LacI transcriptional regulator OS=Pseudomonas syringae pv. aptata OX=83167 GN=ALO85_02631 PE=4 SV=1
MIRIGSRSTGRPTLNEVAKLAGVSPITASRALRGINTVAPELVEKVRAAAQSLGYVANPA
ARALASSCSQTVVVLVPSLSNQLFIETLEAIQDVLRARGLDVLIGNYHYSPEEEEKLLRN
HLVNRPRGILLTGFDHTAASRQMLETSGIPCVHMMELDTRLGAYCVGFSQQQAGAEAARH
LLGRGRHRLAYMAAQLDPRVLQRGTGFRRVLEDAGLFDPALQVSTRQASSIGLGGELFAR
LLDQHPDVDGVFFCNDDLAQGAALEALRLGVAIPERVSLVGFNDLPGSAHMVPRLTSIRT
PREEVGQRAAQVLLGLLDGVTQHPQVDLGFELVVRESS
>tr|A0A0Q0C7D5|A0A0Q0C7D5_PSEAP UPF0391 membrane protein ALO85_01602 OS=Pseudomonas syringae pv. aptata OX=83167 GN=ALO85_01602 PE=3 SV=1
MLSWAITFLIIAIVAAVLGFGGIAGTATGIAKILFVVFLVMFVVSFFFGRRGRG
>tr|A0A0Q0FCQ8|A0A0Q0FCQ8_PSEAP Transporter OS=Pseudomonas syringae pv. aptata OX=83167 GN=ALO85_00535 PE=4 SV=1
MLNTQTNPVAQAARAGMGEKIRGALAVGKTRWGMLALVFFATTLNYIDRAALGVMQPILA
KEMSWTAMDYANINFWFQVGYAVGFVLQGRFIDKVGVKRAFFLAVLLWSLATGAHGLATS
AVGFMVCRFILGLTEAANYPACVKTTRLWFPAGERAVATGIFNAGTNVGAMLTPALLPVI
LTVWGWQAAFVAMGSLGLLWAITWRLKYYNPEEHPTVSKTELDYINQEVEPAPVKVPFTG
ILKMRGTWAFALAYAITAPVFWFYLYWLPPFLNQQYNLGISVTQMGIPLILIWLTADFGS
VGGGILSSWLIGRGMRATTARLLSMLIFAITICSVVFAANASGLWVAVLAISLAVGAHQA
WTANIWSLVMDYTPKHMMSTVFGFGGMCAAIGGMFMTQIVGAVLTATNNNYNVLFTMIPA
MYFIALIWMYFMAPRKVPTLSE
>tr|A0A0Q0CYX4|A0A0Q0CYX4_PSEAP YjgF OS=Pseudomonas syringae pv. aptata OX=83167 GN=ALO85_01307 PE=4 SV=1
MANADITFTPDPDPESISSDVADFNGVLVSTQIPTHPDGSLELGGITEQSECTLRALKDA
LERAGSSMDRVMHLTIYLTDMADRAAFNEVYKRFFKKPWPVRAAVGVAALAVEGMRVEVT
AMAAKAG
>tr|A0A0Q0CHJ7|A0A0Q0CHJ7_PSEAP Uncharacterized protein OS=Pseudomonas syringae pv. aptata OX=83167 GN=ALO85_03370 PE=4 SV=1
MLRIVVVHLFQWLKRLSSALFFRSAEDNYSRSFTREDDDEGRWTFVSIVIVLTLSLYCLA
GLGYYAKTNIWDGFTQEQKENIAQAMVVSSQNL
>tr|A0A0Q0DIP2|A0A0Q0DIP2_PSEAP Indole-3-glycerol phosphate synthase OS=Pseudomonas syringae pv. aptata OX=83167 GN=trpC PE=3 SV=1
MSVPTVLEKILARKAEEVAARRAIVSLTELEQQAARADAPRGFANALITQAKAKQPAVIA
EIKKASPSKGVIREHFLPGEIARSYQAGGATCLSVLTDVDFFQGADAYLQEARAACNLPV
IRKDFMIDPYQVVEARALGADCILLIVSALDDVQLAELAAVAKGLKLDVLVEVHDGDELE
RALKTLDTPLVGINNRNLHTFEVSLETTLDLLPRIPRDRLVVTESGILNRADVELMEISD
VYSFLVGEAFMRAEHPGAELQRLFFPERKMAVSTSSSD
>tr|A0A0Q0C2R2|A0A0Q0C2R2_PSEAP NAD-dependent epimerase/dehydratase OS=Pseudomonas syringae pv. aptata OX=83167 GN=ALO85_200036 PE=4 SV=1
MTSYALTGAVIDDEGVTTIINEFTTSAARAAEWIRFYTGNSFTGTLILITTDIDGIKAKR
RFVLDR
>tr|A0A0Q0CHA5|A0A0Q0CHA5_PSEAP Conjugal transfer protein OS=Pseudomonas syringae pv. aptata OX=83167 GN=ALO85_200183 PE=4 SV=1
MIKRTLVPNKGELGILLVALAYLPGIASADWLTTLNQWGSNIRLGLYTLAGTLGLICLIW
SGAQWLIARSTGDRSHTFMDYLEQVGVLLVVGGSVLLGTAAWQVFGTGVPG
>tr|A0A0N8TAC6|A0A0N8TAC6_PSEAP Quinone oxidoreductase OS=Pseudomonas syringae pv. aptata OX=83167 GN=ALO85_04891 PE=4 SV=1
MAKRIQFSAVGGPEVLEYVDFEPEAPGPQAVVVRNKAIGLNFIDTYYRSGLYPTPLPSGL
GAEGAGVVEAVGAEVTRFKVGDRVAYGTGPLGAYSEVHVLPEANLVKLADSISFEQAAAL
MLKGLTVQYLLRQTYQVKPGEIILFHAAAGGVGSLACQWVKALGAKMIGTVSSPEKAAHA

KALGAWETVDYSHEDVAKRVLELTDGKKCPVVYDGVGQDTWLTSLDCVAPRGLVVSFGNA
SGPVSGVNLGILAQKGSVYVTRPTLGSYANNAQNLQAMADELFDMLASGKLKVDGIQQYA
LKDAAKAQTELSARRTTGSTILIP
>tr|A0A0Q0D2T5|A0A0Q0D2T5_PSEAP Rhs family protein OS=Pseudomonas syringae pv. aptata OX=83167 GN=ALO85_200162 PE=4 SV=1
MPPANQHLVPVFPELKRLVRLGITPSATTILIRDDSTMPSMRPSQYAMQQRTDGGPFLRF
DLEDTGALSEEELSAIESDEEYESIGDEIYGISRVYYSDVALAEHEPGWHLLLEVAPSYL
TIYPINPRAEHPEYGQPRYHGIRSIIITRPVYTPYSHPATPYEVDELLDGLPKGLYTDWR
YGLGFKYEYRCIVQALSSLKGVDTILIHGGTGHNDAKLKGNEFYLGVARLEQLRKSLDRL
TQRHQRETASDKKLACYTSLLHEAAPELYPPRARKLPPDLLANLVSLGSLSPRLSSKDQQ
QVAKLTEQNVSALAKSAPNSLFNLKAEIELVTLGELIEIYRKMMDNKVSEPRWQRFLSEH
PFVLDMAFGYPVKKIADQPYIGGKGFSGRGGQFSDFLMAARATGNLALIEIKHPQTELIG
QSYRQTFVPSYELSGAVGQIISQRSVLQREVFGLSQELDERVHAHAIAAIVIVGQTPEDL
AKQRAFEQYRSGLKDVLVVTFDELQVRLESIHQALTPKPPVEPEPISEEDLPF
>tr|A0A0Q0DAA8|A0A0Q0DAA8_PSEAP Polar amino acid ABC-type transport system, periplasmic substrate-binding protein OS=Pseudomonas syringae pv. aptata OX=83167 GN=ALO85_02298 PE=4 SV=1
MQSYKKILLIAAATLAFSGSAFAEKMKMGIEGAYPPFNNKDASGQVVGFDYEIGQALCAK
MKVECEAVTSDWDGIIPALMASKFDFLISSLSITEERQQVVDFTNPYYSNKLQFIAPKTS
SIDVSSIEKAKESLAGKSVGAQRSTLAATWLEDKLEIMDPKLYDTQENAFLDLSSGRVDA
MLADKYVSFEWLKSEAGQNFEFKGEPVVENDKIGIAVRKGDDALRERLNVALKEIIADGT
YKKINDKYFPFSIL
>tr|A0A0Q0DVC4|A0A0Q0DVC4_PSEAP Nucleotidyltransferase domain protein OS=Pseudomonas syringae pv. aptata OX=83167 GN=ALO85_200033 PE=4 SV=1
MISLDKVTHQFMMYGKDVEAIYLYGSAARGEQDEFSDLDLLIVSELDPFHLICRVKDDLG
ICIENEVEIISKKQLTTMFETGSDLAWTIYQEAKLLFCQSNHVQCPQPNKHIITKEFLAS
QYQKLKSSASALNNNDAVGFECIQIYSAIRRVYISLHLQLNLYGSDKAVRGLNSFSNFDH
LYQDLRSFVRQGKAGLRKKLPSREWIFNTIRISTNWISNVAKDYGVSNGELQNLITYNER
AFKARNHYRQVLEFYSKEFQKLPPPFTLSNTAIDAWCKDAYDAGYDRSIELYRFSVGDLI
FRQSDTLSRIYDTLSECGIPTGFDRYINLVLDY
>tr|A0A0Q0FHR5|A0A0Q0FHR5_PSEAP Uncharacterized protein OS=Pseudomonas syringae pv. aptata OX=83167 GN=ALO85_03506 PE=4 SV=1
MTEERERVERILAEVHDDFGMIRVLEVEDYRFLEFGDAIEQSCTFTADPSWLEYDYTRAM
LIGALCHDAPESALFLGLGAGTLTQACMKFLPLEDVEVIELRPDVPRLAMEFMGLDDDPR
LYIRIGDALDLLDSAEPADLIFVDLYTDVGPGVGHLAWNFLQSCQQRLNPGGWLIINQWA
TDDGKPLGAALLRGLYHRHYWELPVKEGNVILLVPADLDQTLDIDALSGRAEALAPHLGY
SLASLIRAIRSAT
>tr|A0A0Q0FNP4|A0A0Q0FNP4_PSEAP Uncharacterized protein OS=Pseudomonas syringae pv. aptata OX=83167 GN=ALO85_00322 PE=4 SV=1
MPRFSFTLAGLCAGLLLSANVFALSLGDLSQSDATGGLKDALTQGAQVAVKQLGVPGGFS
KNEEVRIELPGKLGQIAKKMKMLGMGAQVDQLETSMNQAAEAAVPQAQALLVDAVKKMSV
TDAKAILSGGKDSATQYLSSTSREQIRAKFLPIVKKSTDQVGLAQKYNAFAGKAAALGAL
DSKSANLEGYVTEQALNGLFEMIAKQEESIRANPAAAATGLAKKVFGAL
>tr|A0A0Q0CC34|A0A0Q0CC34_PSEAP Malonyl CoA-acyl carrier protein transacylase OS=Pseudomonas syringae pv. aptata OX=83167 GN=ALO85_02540 PE=3 SV=1
MSASLAFVFPGQGSQSLGMLAEQGAQHPLILDTFEQASEALGYDLWALTQNGPAELLNQT
DKTQPAILTASVALWHVWLAEGGAVPAFVAGHSLGEYSALVAAQSLSLADAVKLVERRGQ
LMQEAVPAGQGGMAAILGLDDADVIAACAEAAQGEVVSAVNFNSPGQVVIAGSAEAVKRA
MELCKARGAKRALPLPVSVPSHCELMRPAAERFAEAVEAIEWQAPEIALVQNVSASAVSD
LATLKRDLLEQLYKPVRWVESIQCLASRGATQLVECGPGKVLAGLNKRCADGVMTYNLDT
PDAFAAARAALA
>tr|A0A0Q0IAV9|A0A0Q0IAV9_PSEAP N-succinylarginine dihydrolase OS=Pseudomonas syringae pv. aptata OX=83167 GN=astB PE=3 SV=1
MPMKSCEVNFDGLVGPTHNYGGLSYGNVASQSNSQQSANPREAALQGLAKMKALMDLGFT
QGVLAPQERPDVSGLRRLGFTGSDEQVIEKAARQDMPLLVASCSASSMWVANAATVSPSA
DTADGRVHFTAANLNCKYHRSIEHPTTTRVLGAMFADAKHFAHHPALPPVAQFGDEGAAN
HTRFCRDYGEAGVEFFVFGRSAFDTRYPAPQKYPARQTLEASRAVARLHGLSEAGVVYSQ
QNPAVIDQGVFHNDVIAVGNGEVLFYHQDAFLNTEPMLDELRDKLSRVGGQLRAICVPHA
EVSVQDAVRSYLFNSQLLSRSDGSMLLIVPQECQANASVWAYLQRLIADDSPVAEVKVFD

LKQSMQNGGGPACLRLRVALNDTELAAVNPGVIMTAPLYETLTQWVDRHYRDRMSESDLA
DPRLLDECRAALDELTQILKLGAVYPFQLN
>tr|A0A0Q0BTD6|A0A0Q0BTD6_PSEAP Soluble pyridine nucleotide transhydrogenase OS=Pseudomonas syringae pv. aptata OX=83167 GN=sthA PE=3 SV=1
MAVYNYDVVVLGSGPAGEGAAMNAAKAGRKVAMVDSRRQVGGNCTHLGTIPSKALRHSVK
QIIQFNTNPMFRAIGEPRWFSFPDVLKNAELVISKQVASRTGYYARNRVDVFFGTGSFAD
ETSVNVVCTNGVVEKLVANQIIIATGSRPYRPADIDFSHKRIYDSDTILSLGHTPRKLII
YGAGVIGCEYASIFSGLGVLVELVDNRDQLLSFLDSEISQALSYHFSNNNVMVRHNEEYE
KVEGLDNGVILHLKSGKKIKADALLWCNGRTGNTDKLGLENIGLKANGRGQIEVDETYRT
SVSNVYGAGDVIGWPSLASAAYDQGRSAAGSMVDNGSWRYVNDVPTGIYTIPEISSIGKN
EHELTQAKVPYEVGKAFFKGMARAQISGERVGMLKILFHRETLEVLGVHCFGDQASEIVH
IGQAIMSQPGEANTMKYFVNTTFNYPTMAEAYRVAAYDGLNRLF
>tr|A0A0Q0IBB0|A0A0Q0IBB0_PSEAP Lipoprotein OS=Pseudomonas syringae pv. aptata OX=83167 GN=ALO85_00083 PE=4 SV=1
MYKMIAAFSFLAIASATMTGCNDKEGSQGSAATTKPNYYSSDKCNIDSIAGQISAGTGAS
IYVPRGVAVFNGWAIDTANQDAPKEMRLRLTGYKGTPTVFNAPAIVDRIDLVKTYNNEKL
LKSGFSFTADLSSMEPGGYSVAIEIPGANSSLLCQGKVLLVIE
>tr|A0A0Q0DMK1|A0A0Q0DMK1_PSEAP Glutathione peroxidase OS=Pseudomonas syringae pv. aptata OX=83167 GN=ALO85_03499 PE=3 SV=1
MMSENLLSIPVTTIKGEQKTLADFSGKALLVVNTASKCGFTPQYEGLEKLWQDYRDQGLV
VLGFPCNQFGKQEPGDEGAISGFCELNYGVSFPLFKKIDVNGEQAHPLFVQLKQQAPGLL
GTERIKWNFTKFLIGQDGRLVKRFAPLTKPEELTGEIEALLR
>tr|A0A0Q0C5F7|A0A0Q0C5F7_PSEAP Uncharacterized protein OS=Pseudomonas syringae pv. aptata OX=83167 GN=ALO85_101781 PE=4 SV=1
MIIVRANGAESMPGIAAQTFARSELWKKLQPLSIHRKLNLP
>tr|A0A0Q0DRF2|A0A0Q0DRF2_PSEAP Aspartate/glutamate ABC-type transport system OS=Pseudomonas syringae pv. aptata OX=83167 GN=ALO85_02039 PE=3 SV=1
MDFTGIVPAIPGLWNGMVMTLKLMAMGVVGGLVLGTLLALMRLSSNKLLANVAGAYVNYF
RSIPLLLVITWFYLAVPFVLRWITGEDTPIGAFASCVVAFMMFEAAYFCEIVRAGVQSIS
KGQMGAAQALGMNYSQMMRLIILPQAFRKMTPLLLQQSIILFQDTSLVYTVGLVDFLNAS
RSSGDIIGRANEFLIIAGLVYFTISFAASLLVKRLQKRFAV
>tr|A0A0Q0DLD8|A0A0Q0DLD8_PSEAP Uncharacterized protein OS=Pseudomonas syringae pv. aptata OX=83167 GN=ALO85_101787 PE=4 SV=1
MSMLPLGLLAQGIEAMGFTRLNLSVFAARVRNIL
>tr|A0A0Q0C1B0|A0A0Q0C1B0_PSEAP Uncharacterized protein OS=Pseudomonas syringae pv. aptata OX=83167 GN=ALO85_02074 PE=4 SV=1
MRTAKSLLLALVILSPVSAFAYTSDEVKATTVIKEHQASVQKYAALHNKPMPEIKEYTYG
MKLDVAKLVRKSPDLQTCSVMPKLMTYEDSKGKLNTVQYQVLSGCRNSQ
>tr|A0A0Q0BUU6|A0A0Q0BUU6_PSEAP Peptidylprolyl isomerase OS=Pseudomonas syringae pv. aptata OX=83167 GN=ALO85_04391 PE=4 SV=1
MRLSSIRFLPTLRGWLVSTTVLGDAMLQNIRDNSQGWIAKTIIGLIIALMAFTGIEAMFT
ATSNKQNAAEVNGEDISQNELSQAVDMQRRQLAQQLSQQLGKDFDPAMLDEKLLRESALK
GLIDRKLLLQGAADAKFSFSDAALDQQLLQTPEFQVDGKFSADRFDQVIRQLGYSRLQFR
QMLGQEMLIGQVRAGVAGSAFVTDAQVEAFARLEKQTRDFASLTLPADTSAVKVTDDEVK
AHYDEHAKEFMSPEQVVLDYIELKKSSFFDKVQVKDEDLQAAYQKEIANLSEQRRAAHIL
IEVNDKLNDEQAKAKIEEIQQRLAKGEDFAALAKEYSQDPGSSNKGGDLGYAGKGVYDPA
FEEALYALNKDQVSQPVRTDFGWHLIKLLGVEAPSVPTFASLKGKLTNDLKSQLVEQKFV
EVTKQLEDSAFESSDLSQPAQDLGLKVQTTAPFGREGGEGLTANRAVIQAAFSPEVLEEG
ANSNTLELDPETVVVVRSKEHLQPQQLPLESVASSIRAQLVKEHASAAAKAKGEALLAGL
RDGKIPLAAKQEGRDWKMMEAVTRSQEGVDPQVLQTLFRMPKPTGKDKPEFASITASDGS
FVIVRLNGVNQAAAPTDAEKAQYRRFLASREGQQDFAAYRAQLESKAKIEKF
>tr|A0A0Q0DUH9|A0A0Q0DUH9_PSEAP Fatty acid desaturase OS=Pseudomonas syringae pv. aptata OX=83167 GN=ALO85_04706 PE=4 SV=1
MHPHQCATSSTDRPVTWLLAYLLVTGLLYFLVTHVPMGPARLVEPGIVDAHMPLLPFTLP
LYLSYTLVMPVLVYMGRQSSWLLPVFFVGALAAGLCLVSHLFWPTMILRPESGSAWLDWL
YQLDAPLAASPSGHVALPVAISVVMGGLRLRSAWVFALWSAVLMLTVMTTGQHVFTDMAC
GVFIGLACGLTTLVLSRCAVDMRTLSALLLEWLCILVTIRVAIYLADWRFYLLAVLIVAA
RQHALFVLYHDATHYHLTRQRSTNDFLINLAIGVPGLVPIEFYRPLHLDHHQHAGTEQDP

ERRFLYYRQPWQFRPLTAKLLARQLLGDLLLINTLRNIAAYKAAGGAPPALTRPLISAAL
VWLMIVAALIWQCSAQTFGLVAMLWFLPLITVGTLLQKIRSMAEHSGGPGVTPGWEEWTY
AWRVGWLGRFFIWPYHINLHLQHHRTASIPWHALPSAVRAEEQLMASRSLASLMWSRLKQ
KY
>tr|A0A0Q0CE84|A0A0Q0CE84_PSEAP Tol-Pal system protein TolQ OS=Pseudomonas syringae pv. aptata OX=83167 GN=tolQ PE=3 SV=1
MEANVVDHSSMWSLVSNASIVVQLVMLILVAASVTSWIVIFQRSNMLRAGRRALDSFEER
FWSGIDLSKLYRQAGSNPDPDSGVEQIFRAGFKEFSRLRQQSGVDPDAVMEGVARAMRVA
ISREEEKLEAGLPYLATVGSTSPYVGLFGTVWGIMNSFRGLATAQQATLATVAPGIAEAL
IATAIGLFAAIPAVIAYNRFAARSETLISRYYTFADEFQAILHRKVHTSEE
>tr|A0A0Q0DCE8|A0A0Q0DCE8_PSEAP Lipid A biosynthesis lauroyl acyltransferase OS=Pseudomonas syringae pv. aptata OX=83167 GN=ALO85_01535 PE=4 SV=1
MDKFKGAVMVGALRLFALLPWRAVQWTGTAIGWLMWKLPNRSREVARINLSKCFPELSEP
ELEKLVGRSLMDIGKTLTESACAWIWPAQKSIDLVREVEGLDVLKDALASGKGVVGITSH
LGNWEVLNHFYCSQCKPIIFYRPPKLKAVDDLLRKQRVQLGNRVAASTKEGILSVIKEVR
KGGSVGIPADPEPAESAGIFVPFCGTQALTSKFVPNMLAGGKAVGVFLHAMRLPDGSGYK
VVLEAAPEAMYSTDTETSAAAMSKVVEKYVRAYPSQYMWTMKRFKKRPPGEARWY
>tr|A0A0Q0IFL3|A0A0Q0IFL3_PSEAP 3-hydroxyacyl-CoA-acyl carrier protein transferase OS=Pseudomonas syringae pv. aptata OX=83167 GN=ALO85_00671 PE=4 SV=1
MGAQSKILTIGKYKVYAETYWKGSDAKTILLVNGALSTTGSFGQTVKYLQPHYNLVLFDL
PFSGQSRAHNPDDMIVSKEDEVNILLQLIDLYQVNHLLSVSWGGVSALLALSACPPSVEK
AIISSFSPVLNEAMLDYIDNAQVYLAAREKRKIGSLLNDTVGKYLPRLFKLYNYKHLISL
AEHEYRQIDFHISQIRQLSASNYVERFSSINIPVLFINGEKDEYTTVEDARMFSNYIAKS
SFNVVPDTGHFLDLESKAAWHHSQRNVLTFLGTEANQEVWPSVTAMA
>tr|A0A0Q0FSU3|A0A0Q0FSU3_PSEAP Glutamine/glutamate ABC transporter permease OS=Pseudomonas syringae pv. aptata OX=83167 GN=ALO85_03687 PE=3 SV=1
MASSGLELLWVSAPQLITGAGRTLGISLLAIVFSSLGGLCYGVLRSLGKRWLEVPLQIYL
ELFRAIPVLVWLYLFFFGLPIFLGLSIPAFWCAVLVLSLWGASEVGEVVRGGLRSIPRGQ
REAGLAIGLGLGQLYGRVLLPQALKRLTPPVINVYTRLIKTSSLAVLIGVVDVTKVGQQI
IERTYESVLIYGFLFLFFFIVCYPLSAASRVLERRWNHA
>tr|A0A0Q0DBN5|A0A0Q0DBN5_PSEAP Iron-binding protein IscA OS=Pseudomonas syringae pv. aptata OX=83167 GN=ALO85_04112 PE=3 SV=1
MAISMTEAAANHVRRSLEGRGKGDGVRLGVRTTGCSGLAYVLEFVDEAASEDTVFELHGV
KVIIDPKSLVYLDGTELDFVREGLNEGFKFNNPNSRGECGCGESFNV
>tr|A0A0N8T8S8|A0A0N8T8S8_PSEAP Cytochrome c-type biogenesis protein OS=Pseudomonas syringae pv. aptata OX=83167 GN=ALO85_03036 PE=3 SV=1
MKRCLMIVLFSVLCSGIAHAAIDAYAFRDDAERARYSELTRELRCPKCQNQDIADSNAPI
AADLRKEIYRMLGEGQSNQQIIDFMVDRYGDFVRYKPSLNARTWLLWFGPAGLLVGGFVV
IGLIVGRRRKAQSAQADELCEDEKQRLATLLDEHRP
>tr|A0A0Q0BXZ1|A0A0Q0BXZ1_PSEAP O-succinylhomoserine sulfhydrylase OS=Pseudomonas syringae pv. aptata OX=83167 GN=metZ PE=3 SV=1
MGGMTQEWDAGRLDSDLEGVGFDTLAVRAGHHRTPEGEHGEPMFFTSSYVFRTAADAAAR
FSGEVPGNVYSRYTNPTVRSFEERIAALEGAEQAVATATGMAATLAVVMSLCSAGDHVLV
SRSVFGSTISLFDKYFKRFGVEVDYVPLADLSGWDAAIKPNTKMLFVESPSNPLAELVDI
TALAEIAHAKGAMLIVDNCFCTPALQQPLLLGADIVVHSATKFIDGQGRCMGGVVAGRSE
QMKEVVGFLRTAGPTLSPFNAWIFLKGLETLSLRMRAHCANAQALAEWLEQQDGIEKVHY
AGLKSHPQHELAQRQQKGFGAVVSFEVKGGKDGAWRFIDATRLISITANLGDSKTTITHP
STTSHGRLAPQEREAAGIRDSLIRVAVGLEDVADLQADLARGLAAL
>tr|A0A0N8T8E1|A0A0N8T8E1_PSEAP Rieske region OS=Pseudomonas syringae pv. aptata OX=83167 GN=ALO85_01292 PE=4 SV=1
MIFLCTSSHLPEAHSKGFALREHALFAVRRQGQVFVYKNRCPHRGIPLEWQPDQFLDSSA
SLIQCATHGALFLIENGECVAGPCEGQSLTVIKSREDAEGIWVSLHLEA
>tr|A0A0Q0C5X1|A0A0Q0C5X1_PSEAP Uncharacterized protein OS=Pseudomonas syringae pv. aptata OX=83167 GN=ALO85_04367 PE=4 SV=1
MKLHAFFLALMIPALSAHAEGKSISIPHDRTGQHALVEKSGNAKERVVITRREGMLGVIY
SKRVYNCANHTVNLVGTGSTLEIMEQAKAVSGMGPVIRDSTAEYIESEACS

>tr|A0A0Q0DTH2|A0A0Q0DTH2_PSEAP Alpha/beta hydrolase OS=Pseudomonas syringae pv. aptata OX=83167 GN=ALO85_100250 PE=4 SV=1
MCGEGAQYPVTRRSSAGLASRMQILHLFHFVDRTIPLMSISPLARFKARPLRWISMIAVI
LGLPVGCAVLEHKERELVFRIEPGTASWYSGLPADVQEIELSTPAFGTAQNIHAWWWPAA
DKNAPAVLYLHGSRWNLTGQLFRIQQLKAQGYSILAIDYRGFGQSMGQLPSERSVYEDAR
IAWERLKQLQPDPQRRLIYGHSLGGAVAVDLAAELGEDAEKGNAPIQARGLIIESTFTNL
ADVATALANTSLPVRWLLSQKFDSLDKIADIHMPVLIVHGTEDRYVPARFSEQLFAAAQE
PKKLLLVPGGTHNNSMQLGQPAYSRAIRALLDTPASLPQVTKQGKDSKNAG
>tr|A0A0Q0IPV9|A0A0Q0IPV9_PSEAP Extracellular solute-binding protein OS=Pseudomonas syringae pv. aptata OX=83167 GN=ALO85_03685 PE=4 SV=1
MLDLKRTFYAPYTAFDFLKWIRDPERSAMTGRPFLSRLLFAAVTAITLLGCSPSGQDGQT
AKTLKVAFFRDNTTLVSLDPFQVYWLEHRVVLRNIAESLTDQDPQTGKIIPWLAERWEIS
DNALEYTFHLRKDVTFSNGTRFDAQAVKTAYDADKSFAAQLPATFGATYLKGYEHAEVLD
DFTIKLVLAQPNAGFLQATSTTNLAILAPESYALSVKQRSLGAIIGTGPFVLERYTPESG
LRLTKRKGYAWPSANAQNKGEAHLDAVEVSYVPEESVRNGQFVQGQVDILWPRNPFSEVD
LNLFKSRGATIQSRSLPGPALNLYPNTRGNRILADRSVRLALQKAIDRKTYAATVYNPQF
PVVSGIYDVTTPFYKSQADKLAYDPQGAERLLDAAGWQKNADGYRYKEGKRLKLAYNLSP
AETAGDVLIQDQLRKVGIELKLNVVTTAEWAAANAAGNYDLTSTYMTRADPIILQTIIDP
RSANSSTLATNLYAPETLPKALALFDAGLTATQSEQRARAYGELQDLLVDEGSAFPVYER
VWQAATAKNVRNFHWTAEGFALFNDIELATP
>tr|A0A0Q0D8U6|A0A0Q0D8U6_PSEAP XRE family transcriptional regulator OS=Pseudomonas syringae pv. aptata OX=83167 GN=ALO85_03729 PE=4 SV=1
MSGIGCRLRKERERLRMSQRTFGEIGGVEANAQGKYENGDRAPKADYLAAVAAKGVDVLY
VLTGTRTPVPIDNLSLIEEKILGNYRVLGKEDQDAIRRLTTTIAELSGLSSGAAKLPSAK
>tr|A0A0Q0ISX5|A0A0Q0ISX5_PSEAP Dephospho-CoA kinase OS=Pseudomonas syringae pv. aptata OX=83167 GN=coaE PE=3 SV=1
MTHPDEKPWILGLTGGIGSGKSAAAKCFSDLGVDTVDADHAARWVVEPGRPALEQIAAHF
GTGVLQTCGELNRGALRKLIFENPEQRRWLEALLHPLINQEIVSHLAKAKSPYAILVSPL
LVESGQYRMVQRLLVIDAPAHLQIERTMLRDSSSQEQVEAILKVQIQREDRLRHANDVLV
NDRDHAWLSSEVERLHHFYLTLRGGQS
>tr|A0A0Q0IPG2|A0A0Q0IPG2_PSEAP Uncharacterized protein OS=Pseudomonas syringae pv. aptata OX=83167 GN=ALO85_03718 PE=4 SV=1
MTPNAPVNPDPEPVLASEPPARYVVAVRALCEFTAKVGDLDLRFTPSPTAQEGIAGHRTV
ATRRGADYQAELSLSGDYRELTVRGRADGYDASRNQLEEVKTYRGELDAMPANHRQLHWA
QVKVYGWLLCQRLQLESVTLALVYFNIVSEQETLIREPFSAAALQAFFERQCTIFLGWAQ
QELAHTQALHATLGRLKFPHASFRTGQRVLAESVYKAVSTGCCLMAQAPTGIGKTVGTLF
PLLKAAPGQKLDKIFFLTAKTPGRRLALDALKVINHSAPELKLRVLELVARDKACEHPDK
ACNGDSCPLASGFYDRLPAARSAALTSPWLNQAGVRDVALQHQVCPYYLSQELARWADVV
IADYNYYFDLSALLFGLGQLNQWRVAVLVDEAHNMVERARQMYSASLDQSQLKALIQTAP
EPVKKALQRLDRQWNALHKVQPGAYQAYSAAPEKFIGSLNQCISTIGDHFNEHPQAVDGT
LQGFYLEAIGFARIAELFDEHFIFDITRREAGGKRILSRLSLRNVVPARFIRPRLSAARS
TVLFSATLNPRHYYADLLGLPANTAWIDVESPFHHDQLDVRIVSRISTRFTHRQASLAPI
VELMAEQFDTRPGNYLAFFSSFDYLQQVAELMALTHPHISLWQQARGMAESERHAFLDRF
TLSSQGIGFAVLGGAFGEGIDLPGARLIGAFIATLGLPQLNPVNEQFKQRMAALFGAGYD
YTYLYPGVQKVIQAAGRVIRSQSDSGVVMLIDDRFAEHKVKQLFPAWWRPETSMA
>tr|A0A0Q0CWP0|A0A0Q0CWP0_PSEAP Uncharacterized protein OS=Pseudomonas syringae pv. aptata OX=83167 GN=ALO85_101791 PE=4 SV=1
MGSARSNLMIAVLRICRAGLIVERTAGMNPVLLSSVITVGGPTSGLDGRMQMTKRCIKHV
N
>tr|A0A0N8T8W0|A0A0N8T8W0_PSEAP N-acetyl-gamma-glutamyl-phosphate reductase OS=Pseudomonas syringae pv. aptata OX=83167 GN=argC PE=3 SV=1
MVKVGIVGGTGYTGVELLRLLAQHPQAEVVVITSRSEAGMPVADMYPNLRGHYDGLAFSV
PDVKTLGACDVVFFATPHGVAHALAGELLAAGTKVIDLSADFRLQDPVEWAKWYGQPHGA
PQLLEDAVYGLPEVNREQIRSARLIAVPGCYPTATQLGFLPLLEAGIADNTRLIADCKSG
VSGAGRGLNIGSLYSEANESFKAYAVKGHRHLPEITQGLRRAAGGDIGLTFVPHLVPMIR
GIHSTLYATVTDRSVDLQALFEKRYADEPFVDVMPAGSHPETRSVRGANVCRIAVHRPQG
GDLVVVVLSVIDNLVKGASGQAVQNMNILFGLDERAGLSHAGMMP
>tr|A0A0Q0C502|A0A0Q0C502_PSEAP 4-phytase OS=Pseudomonas syringae pv. aptata OX=83167 GN=ALO85_200167 PE=4 SV=1
MMKSTLFKYAGLALSVCLVLPSVAAPLDTTGLRLDKVVLVMRHGIRPATNTAELQRWSAK

TWPAFGVRDGQLTDHGRAATVLLGQWQRRTLDSLGLFKAGQCPQAGDAYVWSSPVARTQA
TSAALVKGVFPGCNVAIHHVRESEDRLFHGGENGLAPLDPARTRAAMLAAMGGSVDAARE
RFAAPLLAMQQLVGVPTTCGKKTCALSKQPWKLKEKEGVTKLSGPLSVGAAMSETFRMQY
AEGLPLDQVAFGEGRTAADVSRLMALRSGKYALSNHVPYIAQRGASQLLGQILLALQPTS
VGSPPGSKWLAYVGHDSNIAQLRTLLGFDWKIAEYPENDAAPGGTIVFERWVNDRTGAQF
VSVAYVAQSLDQLRNLSSEPPYQAQYPGYAGEELMPLNGFVADMEKRIDRSATEIQHYDN
P
>tr|A0A0N8T8U0|A0A0N8T8U0_PSEAP 3-hydroxydecanoyl-[acyl-carrier-protein] dehydratase OS=Pseudomonas syringae pv. aptata OX=83167 GN=fabA PE=3 SV=1
MTKQHAFTREDLLRCSRGELFGPGNAQLPAPNMLMVDRITHISDEGGKFGKGELVAELDI
NPDLWFFACHFEGDPVMPGCLGLDAMWQLVGFYLGWQGNPGRGRALGSGEVKFFGQVLPT
AKKVTYNIHIKRVLKGKLNLAIADGSVSVDGREIYTAEGLRVGVFTSTENF
>tr|A0A0N8T8H4|A0A0N8T8H4_PSEAP Threonine/serine transporter OS=Pseudomonas syringae pv. aptata OX=83167 GN=ALO85_03517 PE=4 SV=1
MNDQANGVVERLDVAPESIASWNRNDTTWMLGLFGTAIGAGTLFLPINAGIGGFWPLMVL
ALLAFPMTFYAHRGLTRFVLSGREGADITDVVEEHFGKSAGAMITLLYFFAIFPILLIYS
VALTNTVGSFLEHQLHITPPPRAALAFLLIMGLLAVVRCGERFIVKAMSLMVYPFIVALL
FLAIFLIPHWTGGILSTATTFPELSAFIPTLWLAIPVMVFSFNHTPIISAFAVDQKRQYG
ENAEVRSSQILARAHGLMVVMVLFFVFSCVLTLSPVQLAEAKAQNISILSYLANHFNNPT
IAFVAPLIAFVAISKSFLGHYIGASEGLKGLVLKAGRRPAPKALDRMTAAFMLVVCWLVA
TLNPSILGMIETLGGPVISALLFLMPMYAIHKVPAMRKYAGAWSNYFVVAAGVVAISALI
FSLIR
>tr|A0A0Q0BUY6|A0A0Q0BUY6_PSEAP Cell division protein FtsQ OS=Pseudomonas syringae pv. aptata OX=83167 GN=ftsQ PE=3 SV=1
MYGPSTRPQPPAPGRNKPVPRGASRMVAKEPLSARLPKANFSFLKRLLWPVLLVVLGFGT
YEAAQRLLPYADRPITRINVQGDLSYISQQAVQQRIAPYVASSFFKIDLAAMRTELEQMP
WIAHAEVRRVWPDQVVIRLEEQLPVARWGDEALLNNQGQAFTPRELSNYEHLPQLFGPQR
AQQQVMQQYQVLSQMLRPLGFSIVRLELRERGSWFLTTGAGSAGPGIELLLGRDHLVEKM
RRFIAIYDKTLKDQITNIARVDLRYSNGLAVGWREQAAPATEKPAVAKN
>tr|A0A0Q0C5A1|A0A0Q0C5A1_PSEAP Transposase OS=Pseudomonas syringae pv. aptata OX=83167 GN=ALO85_01866 PE=4 SV=1
MMTRGTGVVGYNVQTAVDAQHHLIVAHEVTNVGSDRDQLSSMAKQAREAMASDSLSVVAD
RGYFKGEEILACHNAEITAYAPKPYAVSFGIYKKLRLGQDQWSPWVTSA
>tr|A0A0Q0IMV4|A0A0Q0IMV4_PSEAP dTDP-4-dehydrorhamnose reductase OS=Pseudomonas syringae pv. aptata OX=83167 GN=ALO85_200158 PE=3 SV=1
MRMRLMLLGGGNALGQALIRLGAEEDIGFLAPRPPQDGWDVASLTQLLDDTRPDALINLA
YYFDWFQAETVSEARLTHQERSVERLAELCQHHNITLVQPSSYRVFDGSRATAYSEKDEP
VPLGLRGQALWRIEQSVRATCPQHVLVRFGWLLDDSTDGVLGRFLSRAKEPGELLLADDR
RGNPTPVDDAARVIISVIKQLDCEAPLWGTYHYAGHEATTPLALGQAVLTEARLLHALAI
ESPTAQAHAARPDAAEEPQHAVLACKKILHTFGIKPRAWRAGLPSLLDRYYRHV
>tr|A0A0N8T9U0|A0A0N8T9U0_PSEAP Binding-protein dependent transport system inner membrane protein OS=Pseudomonas syringae pv. aptata OX=83167 GN=ALO85_03683 PE=3 SV=1
MTIFDNRLARLARLVASGETIPGKTTPAALWRRRSSLSRIVEAARPLLRRPGFLLAVAIV
AFALLAALAPHLLSSYAPYATSPADKLTAPNAAHWFGTDELGRDLYTRVVHGSSLSVQAA
LLAVGIAMAGGLSLGVISGFAGGRIDAVIMRLIDVLLALPGLLLALAIVTAIGFGTLPVA
IAVGVGIIPGFARTTRAEVLRVKTLPYVEAARLGGASWTRTLLRHILPNAWGPVAVLATL
DFGAAILATAGLSFLGFGAEPPAAEWGTLIANGRHFLMTAPWVSLLPGLFVVAIVFSFNH
IARTLEEIQR
>tr|A0A0Q0C6D9|A0A0Q0C6D9_PSEAP Nucleotidyl transferase OS=Pseudomonas syringae pv. aptata OX=83167 GN=ALO85_100006 PE=4 SV=1
MKHGHFGKDLDCVEEDCMKAMILAAGKGERMRPLTLHTPKPLVRVGDVPLIEYHLNALRD
AGFHQLVINHAWLGQQIEDYLGDGQRFDLSIRYSPEGLPLETGGGIQRALPLLGLEPFVV
INGDIWTDYDFSALRVPLAGLAHLVLVDNPAHHPTGDFSLVDGQVRDDDSAGQRLTYSGI
AILHPQLFANCEPGAFKLAPLLREAMHNGLVTGEHYRGRWVDVGTHERLAEAEQMLVETR
>tr|A0A0Q0C570|A0A0Q0C570_PSEAP tRNA dimethylallyltransferase OS=Pseudomonas syringae pv. aptata OX=83167 GN=miaA PE=3 SV=1
MNALPPAIFLMGPTAAGKTDLAIELTKVLPCELISVDSALVYRGMDIGTAKPSKTQLAEH
PHRLIDILDPAQSYSAADFRSDALAAMAEITARGNIPLLVGGTMLYFKALLDGLADMPAA
DAQVRAQLEADAQAFGWQALHDQLAVVDPVSAARIHPNDPQRLIRALEVYRVSGMSMTAH

REQQTAQSTEAAASGRQQLPYTVANLAIAPADRKVLHQRIALRFEQMLDQGFLDEVLALR
SRGDLHSGLPSIRAVGYRQVWDHLDGKLTRDEMQERGIIATRQLAKRQFTWLRSWEDLHW
LDSLASDNLSRALKYLGSVSILS
>tr|A0A0Q0IPH4|A0A0Q0IPH4_PSEAP 3-alpha-hydroxysteroid dehydrogenase OS=Pseudomonas syringae pv. aptata OX=83167 GN=ALO85_03797 PE=4 SV=1
MNLNNKTLIVTGVSSGIGAELARVARFQGARVIGIDRHEPQLTVDSFFQADLADPGSIDA
LIERLPKQIDCLCNIAGVPGTAPVQAVAQVNYLGLRHLTQRVLPRIVSGGSIVNVASILG
SQWPERLELHKALAATESYGEGQEWLAANPVAHETCYQYFKEALIVWSFKQSQEWFRDHS
VRVNCVAPGPVFTPILGDFVSMVGQERVARDGLHMKRPALADEVAEVIAFLCSDASRWIN
GVNLPVDGGLAASYV
>tr|A0A0Q0DPR5|A0A0Q0DPR5_PSEAP Putative Lipoprotein OS=Pseudomonas syringae pv. aptata OX=83167 GN=ALO85_04354 PE=4 SV=1
MNRMLALALVLTFTVALVSGCSTHHAVEQRAFSAEESHQLALEDLNRRGLSFDEYQARKA
QLMADPQIQQARDFDEKGETSVDLGVTGQQPQG
>tr|A0A0Q0IA83|A0A0Q0IA83_PSEAP Type VI secretion system protein ImpJ-1/VasE-1 OS=Pseudomonas syringae pv. aptata OX=83167 GN=ALO85_02254 PE=4 SV=1
MSKQSRVMWSEGMFLLPQHFQYQDEFHQHQLAESTLRSTPFHWGVQTLEVDVEALATGSL
QLKRLKLVFPDGSLYDAPQHDPLPAARDLKNLTKGGEIKVYAALKLPEPFGLNYVEDGQE
QKSARRFRKQFDTLPDLNEGDLENEITSLRLNVVMLVDGDSLDGYSYCPIARLARNSIGG
FNLDAHFVHPTLHVGTHETLVGLGRRLISVLQAKSKALSGRRRERADQIAEFGSSDVTLF
WLLNTVNRAYPQLAHLLAHPRLHPERLYLFLAELAGGLLTFSLDTQLTDIPDYDHQDPAA
SLVKLDELVRLLLENVIPNQCIVINLSQVRPSYWQGQLLDPRLTEADFYISVHADMPGSS
LLELVPRAFKVGSPEDIEVVVNSAMPGVTLNHSTRLPNAIPVRLDNHYFSIEPHGRVYER
MMEAQAISFYAPSAFTNLKLELLAVLK
>tr|A0A0Q0FEC5|A0A0Q0FEC5_PSEAP 2-amino-4-hydroxy-6-hydroxymethyldihydropteridine pyrophosphokinase OS=Pseudomonas syringae pv. aptata OX=83167 GN=ALO85_100056 PE=4 SV=1
MSLTRIFLGLGSNIERERHLVAGLDALDSFLTDLSCSPVFESHAVGIKSGPFFNLVVSAL
TDLPLMELDRRLKFIEADNGRYAPDRKGLPLDIDVLLYGEQVGNFDGLILPRAEILKNAF
VLRPLALIAPERLHPGVGISFSKLWADAQIDQQLWPVAFEWRGLALTPEDLLRR
>tr|A0A0N8T867|A0A0N8T867_PSEAP Putative auxin-binding protein OS=Pseudomonas syringae pv. aptata OX=83167 GN=ALO85_01232 PE=4 SV=1
MNERARELASRLIRNFNEAPLEHEVREPLYESSAARLGTGTAAQKLGASIDVVAPGKRSC
PYHFHHVQEEMFVIIEGEGSLRVAGEMLPIKTGDVLFIPAGADYPHQIINTSQAPLKYLS
ISTRETPEVCEYPDSGKYQAMVSVQGTRVFTANQRTTENLDYWDGEP
>tr|A0A0Q0DKN4|A0A0Q0DKN4_PSEAP Magnesium/cobalt transport protein CorA OS=Pseudomonas syringae pv. aptata OX=83167 GN=ALO85_01224 PE=3 SV=1
MGRVVAAAVYTKGKKVRDITLDEGAAWAAKPDHFVWIGLEQPTNAELTSLKEQFNLHELA
LEDALEVHSRPKLETFGDALFIVTYAPVRDNGKLLFIETHIFAGKGYIITSRNGHSKSYG
LVRQRCEARPLLLEHGEDFVLYALLDFVTENYQPVTEAIHAELEQLEQSVIGGSLRESDI
QHIHALRRDLLRMRRYVAPMVEVGEELQKLSFPFIDKNMRPYFRDVEIHVKRQMEDLGNL
CDIASQTIEIGLLLESSRQSIVQRKFAGWAAILAFPTAIAGIYGMNFENMPELKWEYGYF
IVLGVILGGCTALFASFKRSGWL
>tr|A0A0Q0CEL8|A0A0Q0CEL8_PSEAP Ribose/galactose isomerase OS=Pseudomonas syringae pv. aptata OX=83167 GN=ALO85_01022 PE=4 SV=1
MNTPFPVAIGCDEAGYELKELLKRHIESLGYPVTDFGTHSTAPVLYPDIALAVATAINAG
QQRLGVLVCGTGIGMAISANKVFGIRAAQAHDTYSAERARKSNDAQILSIGARVIGTELA
KSIVKSFLESEFEAARSGAKVERINAIERDGHQ
>tr|A0A0Q0FWS0|A0A0Q0FWS0_PSEAP Uncharacterized protein OS=Pseudomonas syringae pv. aptata OX=83167 GN=ALO85_01402 PE=4 SV=1
MRTPLIAASLGLLLLADFAQAQTVVATSNIIVRAFGRTIDFTSDTTTSIRDSKVVLQAKD
DAASYVATGGDIHGAQLDAAFDTLRTRVPEARGASDQTLAEAILAL
>tr|A0A0Q0IAK2|A0A0Q0IAK2_PSEAP DNA-binding heavy metal response regulator OS=Pseudomonas syringae pv. aptata OX=83167 GN=ALO85_04276 PE=4 SV=1
MRVLIIEDEEKTADYLRRGLTEQGYAVDVARDGIEGLHLALENDHAIVILDVMLPGLDGF
GVLRALRARKQTPVIMLTAREQVDDRIRGLREGADDYLGKPFSFLELVARLQALTRRSGG
HEPVQITIADLWIDLISRKATRAGLRLDLTAKEFSLLSSVLARRQGDILSKTSIAEMVWDI
NFDSDTNVVEVAIKRLRAKLDGPYEHKLLHTIRGMGYVLENRSAG

>tr|A0A0Q0D6E9|A0A0Q0D6E9_PSEAP Pectate lyase/Amb allergen OS=Pseudomonas syringae pv. aptata OX=83167 GN=ALO85_02423 PE=3 SV=1
MTPLQSLLQSGYLKIAATLVLLFSGAQCVRAADIPQGFAKDVTGGGSAAAVHPSTLDELR
SALCASHDSHGACTDDTPRVIVLDHTFDFRASVIANGNTTTTEPGCVVKPCPQGGEQLAL
NGANNFCQSRPRVMVTYDNAGLKPLKVGSNKTLIGLGDKAGIQGAGLFIGDGAHNVIVRN
LTLSDINPRVVWGGDALTLNKADGVWIDHNTFARIGRQMIVTGWGTASHVTISSNEFDGR
TPYSSTCDGHHYWVWLFLGSQDTLTLSRNYVHDTSGRAPHSGGMNNAQVRAQLVNNVFQR
MTYQGAIMSRTSSSQLLVEGNDFENVAHPLYNDVDQPGTAFALFDPVSKAANDACMSAIG
RTCVVNQQRSSGEDYRPRDASALEAFREYRQYLVMPVSAQEAKARVPGEAGVGKVSAGLL
Q
>tr|A0A0N8T848|A0A0N8T848_PSEAP Glycoside hydrolase family 18 protein OS=Pseudomonas syringae pv. aptata OX=83167 GN=ALO85_00488 PE=4 SV=1
MLNPKIINQYKNKKVDAASTMTDISGKNILMGFWHNWPSESGQGYQQGMFEEMALTGIHE
AYNVVAVAFMKGSGIPTFKPYNLSDEAFRAEVAALNAQGRAVLISLGGADAHIELHAGQE
EALAYEIIRLVEVYGFDGLDIDLEQAAITFADNRTVLPAALRMVREHYKTEGKHFIISMA
PEFPYLRASKKLSVLKEITTYFPFLKDFVTFLESLTSGGAYLAYINALEDIYDFIAPQYY
NQGADGIWADEANNGNGLWIPQNDDTVKKEFLYYLTDSLIHGTRGFTTIPADRLAIGLPT
NKDAAGSGYVINPQDVKDALRELEHAGNPIRGLMTWSVNWDNGISKDDHAYNWEFAKRYS
YLTGGETPVPGKPTVPADLRSIEQTPTSVKLVWSLSVGVPPVLRYELRRDNSVSLFADSP
SYDDIGLQPGTTYSYQVRAIDIMGNTSAFSAAISVSTQAESGGGEKPTTPVLSLSGVTDK
SVSLKWTLSSSDIGNGNGNGIDKYQIGRDGWPIAAVLADQPPEYTDTGLTAGKKYTYYVV
AKDTQSQWSEVSNLLEVSTDPAPEKPSVPVDFRSTARTTTSLTLDWSVSANANGPYHYEL
FRNDISIGTDKVPPFVDEGLWQSRLYGYRIIATDGLGNSSERSEELLATTDSQPSVDRPS
PPQNLRHTGSTTTSISLAWDEPASHGDLASYQIHTFNAPTVYVDSTVLAYTVEGLSPGKN
YQHLVGARDANAELSGPSNVVQASTSAALPEWKTDTDYAKGDKVMHAGASYICLNPHHSQ
IDWYPGGAPTLWAPWTEQAGGRGFRDWPKEWQ
>tr|A0A0Q0CFK0|A0A0Q0CFK0_PSEAP Urease accessory protein UreE OS=Pseudomonas syringae pv. aptata OX=83167 GN=ureE PE=3 SV=1
MAGSYLMLVIHDRIEPQAEWAAELHLNFEARSKSRLRCFSAENEDVGLFLQRGQSPLRDG
EFLQAKDGRVVRVCARPEKLMHVTCSSTFELTRAAYHLGNRHVALQVGDGWLRLLDDYVL
KAMLDQLGATVDTIEAPFQPEHGAYGGGHHHSRAGEEDFNYPPRMHQFGVRK
>tr|A0A0Q0BX74|A0A0Q0BX74_PSEAP DEDD_Tnp_IS110 domain-containing protein OS=Pseudomonas syringae pv. aptata OX=83167 GN=ALO85_200146 PE=4 SV=1
MRVTLEKPIIGVDVAKNELVIYHDQYDRLEAIPNTKVAITQWLKALASSCAIAIEATNVY
HVLFADAAHEAGCDVYMVDGYQLSHYRKGVNIRAKTDAQDARLLARYLKNELDELRPWIP
ASPLYRQLLSLFRRRAALVQARTGLVQSWTNEPFLRTAFANQVNSMKRFEALVEKKIRDV
LQEAGLMRQVNRCMKVEGIGFLTAARLVTSFQRGEFSGANAFIAFLGLDLRVSKSGQSDG
PRRLTKRGDPEARRLLHNAAMSGSRTPAWKPFYEEQRKRGFSTTQALVMLSRKLARVVFA
LLKNQSEYQTKVA
>tr|A0A0Q0BU24|A0A0Q0BU24_PSEAP Taurine ABC-type transport system, permease protein TauC OS=Pseudomonas syringae pv. aptata OX=83167 GN=ALO85_01664 PE=3 SV=1
MSLAPPAREEYEITLEPFTQEDLARDLPLAQRIWQLSWVRKCVIMIALAVIWELAARLQN
NDLLLPSFLQTAAAFYDGLISGELPGKVWISLTVLIQGYLIGIVLAFALTTLAVSTQLGR
DLLSTLTAMFNPLPAIALLPLALLWFGLGQNSLIFVLVHSVLWALALNTYAGFLGVSETQ
RMAGRNYGLKGLRFVWHILIPAALPSILAGLKIGWAFAWRTLIAAELVFGASSGKGGLGW
YIFQNRNELYTDKVFAGLAAVILIGLLVENLLFANIERLTVKRWGMQR
>tr|A0A0Q0DTN9|A0A0Q0DTN9_PSEAP Adenylate kinase OS=Pseudomonas syringae pv. aptata OX=83167 GN=adk PE=3 SV=1
MRVILLGAPGAGKGTQAKFITEKFGIPQVSTGDMLRAAVKAETELGLKAKSVMDSGGLVS
DDLIIGLIKDRLAEPDCANGVLFDGFPRTIPQAEALLKAGLEIDHVLEIAVDDEEIVKRM
SGRRVHEGSGRIYHTIFNPPKVEGIDDVTGEPLLQRKDDVEETVRHRLSVYHAQTKPLVE
FYSKLEAKNGKPKCSHIPGVGSVEDITAKVLEALK
>tr|A0A0N8T888|A0A0N8T888_PSEAP Putative nucleoside-diphosphate-sugar epimerase OS=Pseudomonas syringae pv. aptata OX=83167 GN=ALO85_03094 PE=4 SV=1
MYLTPQHILLAGATGLTGEHLLDRLLSEPTVTRVLAPTRKPLAEHSHLENPVGELATLLP
TLSGRVDIAFCCLGTTIKQAGSQDAFKAVDLDMVTAFASRARELGARHLLVISAVGADPA
SSTFYNRVKGEMELALRAQGWPQLTIARPSLLVGSRPETRWVEQLAAPIAKLIPGKYGAI
EACHLARALWRLALEEQNGERIVESDELRKLGK

>tr|A0A0Q0BSX4|A0A0Q0BSX4_PSEAP Acetyltransferase OS=Pseudomonas syringae pv. aptata OX=83167 GN=ALO85_02284 PE=4 SV=1
MILLNKQGATRMPLTRNTEPDITLVTVQPEDFETLVAIRIEAMRESLERVGRFDPVRARE
RFREGFSASCTHYIQVAGSKVGFVVVKPTSDGLLLDHLYIKPVVQGQGIGAAVLRQVLAH
ADATASTVRVGALKESDSNHFYARHGFQLVESGEFDNYYLRSQRLPMC
>tr|A0A0Q0DK10|A0A0Q0DK10_PSEAP Arylesterase OS=Pseudomonas syringae pv. aptata OX=83167 GN=ALO85_02462 PE=4 SV=1
MENPMRALFLSAGLGLLLLSQGALAGTVLIVGDSISAAFGLDTRVGWVSLLEQRLSKEGF
DDKVVNASISGDTSAGGQARLPALLAEHKPELVILELGGNDGLRGQQPAQLQQNLSSMID
RSQAAGAKVLLLGMRIPPNYGPRYTKAFEEVYSNLAKEKKVPFVPFFLEGVGGVPELMQA
DGLHPAAAAQSKLLENVWPSLKPLL
>tr|A0A0Q0CWR9|A0A0Q0CWR9_PSEAP ATP-dependent Clp protease ATP-binding subunit ClpX OS=Pseudomonas syringae pv. aptata OX=83167 GN=clpX PE=3 SV=1
MTDTRNGEDNGKLLYCSFCGKSQHEVRKLIAGPSVFICDECVDLCNDIIREEVQEAQAES
SAHKLPSPKEISGILDQYVIGQERAKKVLAVAVYNHYKRLNQRDKKNDDVELGKSNILLI
GPTGSGKTLLAETLARLLNVPFTIADATTLTEAGYVGEDVENIIQKLLQKCDYDVEKAQM
GIVYIDEIDKISRKSDNPSITRDVSGEGVQQALLKLIEGTVASVPPQGGRKHPQQEFLQV
DTRNILFICGGAFSGLEKVIQNRSTRGGIGFNAEVRSKEEGKKVGESLREVEPDDLVKFG
LIPEFVGRLPVLATLDELDEAALIQILTEPKNALTKQYAKLFEMEGVDLEFRTDALKSVA
RRALERKTGARGLRSILEGVLLDTMYEIPSQSDVSKVVIDESVIDGTSKPLLIYENSEPP
AKVAPDA
>tr|A0A0Q0C2K3|A0A0Q0C2K3_PSEAP Uncharacterized protein OS=Pseudomonas syringae pv. aptata OX=83167 GN=ALO85_02028 PE=4 SV=1
MNNGRDHRIDFFRGLALIFIFWDHVPDNPLAQLTVRNFGFSDAAEIFVFLAGYASILAYG
RIARRDGMLVAGVRILRRTWVLYVVHIFLLTLLMGIVFVANNHVETRDMVQQMGLEYFVG
NPQQALADELLLRFKPNLTDPLPLYIVLLLTLPLTLPLMLRKLEVAVGLSIALYLMVPLF
GWNLRAYEGGGVWYFNPVAWQLLFVLGGACALRSETATTAARPPLRQQPLFMVAAVYVMV
AGILTFSEKWPGLHAALVSSLHLESLYPINKTDLAPVRLLHFLALVYVVARLLPTSSTWL
ENWPARQTCRMGRYSLEVFCLGVLLAPLADMANALAGDTWPMQVTTAIVGLGLMMLMANG
LELNKRLEKSPHLLPT
>tr|A0A0Q0E0S2|A0A0Q0E0S2_PSEAP Uncharacterized protein OS=Pseudomonas syringae pv. aptata OX=83167 GN=ALO85_01557 PE=4 SV=1
MKKFLLAVGLLSIAGTALAAGKPCEELKSELDARLQAKGVTSYTLEVVEKGSAADKQVVG
TCEGGTKEIVYQRG
>tr|A0A0Q0IET6|A0A0Q0IET6_PSEAP GAF:ATP-binding region, ATPase-like:histidine kinase A OS=Pseudomonas syringae pv. aptata OX=83167 GN=ALO85_02118 PE=4 SV=1
MSQLDKDAFEVLLANCADEPIQFPGAIQPHGLLFTLTEPELTILQVSANVQTVLGHVPEE
VLGKGLDCVLGAGWADVIRSASAHDSFIDAQRLLMSINGIEFEALLHRHQGVLVLELEIQ
GKDAQSVSYSERTGNMGRMLRQLHAASDLQTLYEVSVREIQKMTGYDRVLIYRFEEEGHG
QVIAEASAPSMELFNGLFFPASDIPEQARELYRRNWLRIIPDADYTPVPLVPQLRPDTLQ
QLDLSFSTLRSVSPIHCQYMKNMGVLSSMSVSLIQGGKLWGLISCGNRTPLYVSHELRSA
CQAIGQVLSLQISAMEALEISRQREAKVRALEQLNLAMAGSEENVFDGLAQQPQLLMDMV
GATGVAIIEDRQTHCFGICPEPSDIRALHAWMIAGGEPVYASHHLSSVYPPAEAYQPIAS
GVLAMSLPKPVDNGVIWFRPEVKETVQWSGNPKKPLDMESSAGGMRLRPRTSFEIWKVEM
TGIATKWSYGDVFAANDLRRSALENDLARQVRREQQAVRARDELVAVVSHDLRNPMTVIS
MLCGMMQKSFSSDGPHTSRRISTAIDTMQQAASRMNVLLEDLLDTSKIEAGRYTITPQPL
EVSQIFEEAYTLLAPLAMDKSVEISFSAEPDIKVNADPERLFQVLSNLVGNAIKFTPKLG
KIDVSAMSNGSEIVFTVRDSGEGIPPEQLPHIFERYWTVKEGNPTGTGLGLYISQGIIKA
HGGELAAQSRVGEGSEFRFTVPMAV
>tr|A0A0Q0BTU7|A0A0Q0BTU7_PSEAP Type III secretion system protein HrpF OS=Pseudomonas syringae pv. aptata OX=83167 GN=ALO85_04196 PE=4 SV=1
MINFKSLQNNLDSTLTRALSDVDDVLDGGNGHFTADDIDAFSEASQQAAVSSNIADQCQQ
AGYKMTKNVIDGFQ
>tr|A0A0Q0BYW7|A0A0Q0BYW7_PSEAP ATP-dependent DNA helicase RecQ OS=Pseudomonas syringae pv. aptata OX=83167 GN=ALO85_03237 PE=4 SV=1
MSQSSIAPERSGILPRFAVQQDIPGFFMLEQAQRVLKDIFGYDSFRGRQGAIIERVASGG
DALVLMPTGGGKSLCFQVPGLLRDGLCVVVSPLIALMDDQVATLDELGVSAAALNSTLSA
EQQRELANRIRLGEVKMLYLAPERLVQPRMLSFLQNLKIALFAIDEAHCVSQWGHDFRPE

YLQLGQLAELFPDVPRIALTATADKRTREEIVTRLHLQNAERFLSSFDRPNIFYRIVPKE
QPRKQLLAFLSERRSDAGIVYCLSRKKVDEVAVFLSDNGYPALPYHAGLPSETRAANQKR
FLNEEGLIMVATIAFGMGIDKPNVRFVAHMDLPKSLEAYYQETGRAGRDGLPADAWMAYG
LQDVLMLKQMLQNSEGDERHKRLEQHKLDAMLALCEETRCRRQTLLAYFDEDMPNPCGHC
DNCVDGVQTWDATEPARQALSAIYRTGQRYGVGHLVDVLLGKSNDKVESFGHQHLSVFGV
GKARTESEWRSLFRQLVARGLADIDLEGYGGLRLSETCRPLLRGEVSLELRQDLKPQTTS
RSSSGSPASQLVRGEEREQWEALRALRRKLAEEHGVPPYVIFPDSTLLEMLRSKPGSMAE
MAKVSGVGARKLERYGEAFLEVLSGKAEAPRVVADVRHELISLARAGMTPTQIAGQLQCS
EKNVYTLLAEAIGKQQLSIEQALDLPEDLLGEVQDAFLDGEGELPPVSAIAEQFAGRVPE
GVLYCVRAALQSEFEV
>tr|A0A0Q0BX54|A0A0Q0BX54_PSEAP Uncharacterized protein OS=Pseudomonas syringae pv. aptata OX=83167 GN=ALO85_01613 PE=4 SV=1
MREPLTTKGQGYHAQGKSTDIDRPVGLLAFCPLFVVAAEDPPSAAPDVTIRTDGDRTIQE
YRQNGFLYAVKITPKHGKPYFLVRADGTSPNFIRSDQPDMLIPQWEIFSW
>tr|A0A0Q0DIX5|A0A0Q0DIX5_PSEAP Type III secretion system protein HrpO OS=Pseudomonas syringae pv. aptata OX=83167 GN=ALO85_04184 PE=4 SV=1
MDEPLEDDPQQVALQQVIGLLTPLRQHRQASAERAHRQAQLELKSMLDHLAETRASLNQE
RDNHKRRRESLSHAHLQKTLSLTDVDGWHEKERTMLDRLAYIRQDVQQQQMRVAEQQALL
EQKRLQAKASQRAVEKLACMEETLNEEG
>tr|A0A0Q0IJE5|A0A0Q0IJE5_PSEAP Phosphoenolpyruvate carboxykinase (ATP) OS=Pseudomonas syringae pv. aptata OX=83167 GN=pckA PE=3 SV=1
MTQTNNAVYTDLSTDELVKEALARKEGVLSDTGALVVETGHRTGRSPVDRFIVEEPSTQD
AIAWGPINRKFPEDKFNALWDLVGAYLAERERFVSHVHVGSDPAHYLPVKMTTETAWHNL
FGRCLFINPEQYNPAGKDEWEIQNAPWFVCDPERDGTNSDGTVIINFAARKVLIAGMRYA
GEMKKAMFSVQNFLLPASDVLPMHCAANMGEEGDVTLFFGLSGTGKTTLSADESRYLIGD
DEHGWGEGVVFNIEGGCYAKCIDLSEKNEPVIWKAIQHGAVLENVVLDPVTKKADYADSS
LTQNSRAAYPRELIEKRAPKNLGGEPNAVIFLTCDLTGVLPPVSILSEEQAAYHFLSGYT
ALVGSTEMGSGSGIKSTFSTCFGAPFFPRPAGEYAELLIKRIRGFKSKVYLVNTGWTGGG
YGVGKRFNIPTTRGVIAAIQSGALIGAETEHLDTINLDVPKSVPGVDTGLLNPRNTWADK
AAYDEAAKALAGLFVENFKKFDVSDAIKAAGPKL
>tr|A0A0Q0DJN7|A0A0Q0DJN7_PSEAP Periplasmic serine endoprotease DegP-like OS=Pseudomonas syringae pv. aptata OX=83167 GN=ALO85_04416 PE=3 SV=1
MFKALRFFGWPLLAGVLIAMLIIQRYPQWVGLPSLDVNLQQAPQTTNVMQGPSSYADAVI
AAAPAVVNLYTTKMVNKGNNPLFEDPQFRRFFGDNTPKQKRMESSLGSGVMMSPEGYILT
NNHVTTGADQIVVALKDGRETIARVIGNDPETDLAVLKIDLKNLPAITIARSDSIRIGDV
ALAIGNPFGVGQTVTMGIISATGRNQLGLNTYEDFIQTDAAINPGNSGGALVDASGNLIG
INTAIFSKSGGSQGIGFAIPTKLAMDVMKSIIEHGQVIRGWLGIEVQPLTQELAESFGLK
DRPGIVVAGIFRDGPAQKAGLQLGDVILSINGEPAGDGRRSMNQVARTKPKDKIAIDVMR
NGKEMRLSAEVGLRPPPAPTPVAAPE
>tr|A0A0Q0CA60|A0A0Q0CA60_PSEAP M23 family peptidase OS=Pseudomonas syringae pv. aptata OX=83167 GN=ALO85_01414 PE=4 SV=1
MLERLLILFALMLTVQSAGAVTIYKFTDADGVVSFSDRPTPGASVMVFRDRMVERIDGQV
HLSVRREKGVHSLYVRNDLYAPVEVELKLSSVTNVLGVSGSSATLRRTIPARSNQRVVVL
SPKVGSKPMNYASVLSSTLGDPKGSVQAYKYPLPWIGGPFRLTQGPNGRYSHYGVKGRYA
MDIAMPEGTPIIAARGGTVVKIENSQSGRGSNASGNFVRILHEDGTMGVYLHLMRGSVSV
REGQRVSVGTALARSGNTGNSTGPHLHFVVQRNSGNELISIPYEFATPVQSLPNFAVGGN
>tr|A0A0Q0DKS7|A0A0Q0DKS7_PSEAP Uncharacterized protein OS=Pseudomonas syringae pv. aptata OX=83167 GN=ALO85_00887 PE=4 SV=1
MIRDDRIAPGAPVYHETHAAAPLLKRISWSAILAGIVLAMVVSLLLNLLGTAIGSASIDP
MQEANPLSGIGTGAGIWVVVSSVISLFVGGWAAGRLAQREGAFHGLLVWASVSLITVYLV
SSAVTGVVRGGLNLAGSGMSALGSGIAQVAPAVGSKIQDQLRAQGIDFNLDDIQGEIETA
MRQTGKPELNPDNVKQEAQATQQDAQNTAKQSAQNPQQADQQLSGLMDRIKAKGDQAWDA
ADRQALVNLIKARGNKTDAEANQIVDQAQASYRQAYAKYQELKAQAEQKAREAAEVTAKR
VSQGAWILLITLVISGLVAAGAGVLGRRTQPPAKVVAAV
>tr|A0A0Q0BTW1|A0A0Q0BTW1_PSEAP Uncharacterized protein OS=Pseudomonas syringae pv. aptata OX=83167 GN=ALO85_01075 PE=4 SV=1
MKQETHVMKRRYSWPLWTLAALIVLLIAVNIALPYLVRDYLNDKLADMGDYRGEITDVDL
ALWRGAYRIKGLKIVKTDGKVPVPFFDAPMIELAVSWHSLWYDHAVVAKALFTGPQINFV
DGGSKQDSQTGQGTDWRTQMNKLLPITLNELRIDDGRITFNNFNSTPKVKIEADQVNASI
YNLTNVVDVEGNRDAEFKGKARLLGHADLESSARFDPFSNFEDFDFKLRATGIQLKKLND

FSSAYGKFDFNAGDGDVVIEAQADKGQLKGYIKPLLRNVDVFNWHQDVENKDKGVFRSIW
EALVGTSETVLKNQRENQFATRVDLSGSVHQQDISGFQAFLQILRNAFVQAFNARYESSK
GS
>tr|A0A0N8T9A1|A0A0N8T9A1_PSEAP Lactamase_B domain-containing protein
OS=Pseudomonas syringae pv. aptata OX=83167 GN=ALO85_02644 PE=4 SV=1
MASTHQKKDSSSLPVLSRHEGRYRNHATMKPSSLLRTLGIFWDQAFNKPKDTRPAGDIPV
QPLSRQQLLAAPNNTVYRLGHSTVLLKLRDQFWLTDPVFAERASPVQWAGPQRFHQPPIS
LEELPPITAVILSHNHYDHLDRMAIKALTEKTEHFLAPLGVGDTLIEWGVPAHKVRQLDW
WQSTEVAGIEFVATPSQHFSGRTLLDSNRTLWASWVMIDGDQRIFFSGDSGYFDGFKTIG
EQYGPFDLTLMETGAYNVEWPDVHMQPEQTLQAHLDLRGRWLLPIHNGTFDLSMHAWHEP
FDRILALAWEQNVSITTPMMGQPFYVQYPCRGLTWWLAVDEVVESEAAQPEAAESCSCRR
QNA
>tr|A0A0Q0FJL3|A0A0Q0FJL3_PSEAP Dipeptide/oligopeptide ABC-type transport system OS=Pseudomonas syringae pv. aptata OX=83167 GN=ALO85_00010 PE=4 SV=1
MKLLSLRNSIALALLSVAVSVSAKPLVVCTEASPEGFDMVQYTTAVTADAVAETMFNRLA
DFKPGTTEVIPALADSWEISDDGLTYTFHLRKGVKFHTTDYFKPTRDMNADDVLWSFQRQ
LDPKHPWHDKSSVGFPYFESMGFKELLKSVEKTDDYTVKFTLTRREAPFLADLAMAFASI
YPAEYADQLLKANKTGDLNSKPIGTGPFIFQRYAKDAQVRFKANPDYFRGKPPADALILA
IATDNNVRLQKLKANECQIALYPKPDDVPSIKKDPNLKIDELEAMTTGYIAINTTHKYLS
DVRVRKAIDMTFDKNAYVNAVFGKGNALVAVNPYPPTLLGYNKDLKNPPIDLDKARALLK
EAGVPEGTTFTLFTRNGGGPTNPNPMLGAQMMQSDLAKIGIKIDIRVMEWGEMLKRAKNG
EHDMVSAGWAGDNGDPDNFLTPMLSCEAAKNGENYARWCNADFQKLIDKARETVNPDERA
ELYEQAQAIFNKDQPWISMAHTRMFTAMRKNVEGYQISPLTTNNFATTQVK
>tr|A0A0N8T7X6|A0A0N8T7X6_PSEAP Cold acclimation protein B OS=Pseudomonas syringae pv. aptata OX=83167 GN=ALO85_04303 PE=4 SV=1
MAERQSGTVKWFNDEKGFGFITPESGPDLFVHFRAIQGNGFKSLKEGQKVTFIAVQGQKG
MQADEVQAEG
>tr|A0A0Q0BSY1|A0A0Q0BSY1_PSEAP YbbF/LpxH-related metallophosphatase
OS=Pseudomonas syringae pv. aptata OX=83167 GN=ALO85_03465 PE=4 SV=1
MTSAQLATPSRKQRVRTLWISDVHLGTRDCQAEHLSAFLKRYQADKVYLVGDIIDGWKLR
GGMYWPQAHTNVIRRLLTMSKRGTEVIYVTGNHDEFLRRYSKLVLGNIQLVDEVEHVTAD
GRRFLVIHGDQFDVITRYHRWLAFLGDSAYEFTLTLNRWLNHWRAKYGYGYWSLSAYLKH
KVKSAVNFISDFEEAIAHECVKRGLDGVVCGHIHHAEIRQVGGVEYLNCGDWVESCTALI
EHLDGNIELFRLADAHLKAQQVAVEEPASETLA
>tr|A0A0N8T908|A0A0N8T908_PSEAP Marine sediment meta DNA OS=Pseudomonas syringae pv. aptata OX=83167 GN=ALO85_03884 PE=3 SV=1
MKLVTAIIKPFKLDDVRESLSEIGVQGITVTEVKGFGRQKGHTELYRGAEYVVDFLPKVK
IDVAIDDKDLDRVIEAITKAANTGKIGDGKIFVVNLEQAIRIRTGETDTDAI
>tr|A0A0N8T896|A0A0N8T896_PSEAP Transglutaminase-like protein
OS=Pseudomonas syringae pv. aptata OX=83167 GN=ALO85_03140 PE=4 SV=1
MSAFYQIFHDTHYKYDSPVSLAQQLAHLWPRSSPWQRCTEQALEILPEPTTRRDELDVFG
NPLTRLAFERPHDELQVNARLRIEVMEKPFLDFNLSPEWEVTRSALTYSAKPMTEDILEA
CRYRFESPYVHLKRSFVEFSQVCFPPGRPLLLCVQALMEKIFEEFTFDEEATQVATPLVE
VLERRRGVCQDFAHLMLACLRSRGLAARYVSGYLLTQPPPGQPRLIGADASHAWVSVFCP
ASGWVDFDPTNNVQPALEHISLAWGRDFSDVSPLRGVILGGGTHDPDVRVTVMPVSG
>tr|A0A0Q0DHV2|A0A0Q0DHV2_PSEAP Uncharacterized protein OS=Pseudomonas syringae pv. aptata OX=83167 GN=ALO85_00060 PE=4 SV=1
MIKPLLVLLLSTAFVSLGAHAEDQAVNSISPDRLAINGAEATLGLSQEWVHPKPKIERVV
IVLHGRLRNAQTYLRSIERAANQSRERGKTLLIAPQFLDERDIVAHHLPDSILRWRQNSW
MEGATSTDPKPVSSFLVLDHILKRLSDSKLFPNLKEIVIAGHSGGAQVVQRYAMIGGKED
ALLQREGVKLRYVIANPSSYAYFDAERPEPVTAQSCPNFNDWKYGLNKMPFYSGKEKPAD
IEKNYVKRDITYLMGELDTDRNHPALDKTCAAEAQGAYRLIRGQNYFNYLQKRHPEGLNQ
RLVIVPKVGHNGDGIFTSPQGQAVLFKPF
>tr|A0A0Q0C4P6|A0A0Q0C4P6_PSEAP Peptidoglycan-binding LysM:Transport-associated OS=Pseudomonas syringae pv. aptata OX=83167 GN=ALO85_02270 PE=4 SV=1
MSLISFIKEAGEKILDALLPDKANADELLRKHISEVGLGNPNVRTRIDGGTVIVEGKVSS
QEEREKIVLILGNIHGVEKVDDQMTVEGSAGAESQYVEVKSGDTLSAIS KRVYGDANQYQ
KIFEANKPMLKSADKIYPGQKLRIPK

>tr|A0A0Q0C9W0|A0A0Q0C9W0_PSEAP GNAT family acetyltransferase OS=Pseudomonas syringae pv. aptata OX=83167 GN=ALO85_01435 PE=4 SV=1
MSDTLLDWQAAALPSAHTLTGRFIRLEKLDAARHADDLWAALEGPNADPKLWDYLPYGPF
SDRSAFDRWLAGHQAATDPWFYSVVDQQTDKAEGVISLMSIVAAHGRIEIGHVTFSAAMQ
RTPKGTEAIYLLAREIFALGYRRLEWKCNANNARSKRAAERFGFSYEGTFRQHMVVKGQS
RDTEWYSILDSEWPERQNAFERWLAVDNFKEGQQIKGLEAFR
>tr|A0A0Q0DJ05|A0A0Q0DJ05_PSEAP Amidase OS=Pseudomonas syringae pv. aptata OX=83167 GN=ALO85_04235 PE=4 SV=1
MNTTMTALDIPELQSALRSGDTTLTTLVHTLTAHIDADDRAEVWIHRVPLAQLVERAKAL
ESIAEQLGDALYDRLPLFGVPFAVKDNFDVAHMPTTAACPAFAYVPEDSAHVVQLLLDSG
AILMGKTNLDQFATGLVGVRSPYGAVRNAHDPAYVSGGSSSGSAVAVARGYVSFALGTDT
AGSGRVPAGFNGIVGLKPSLGLFSSRGVVPACRTLDCPSIFAKDVMQAWQVAQVIAAYDP
LDASSVAVQALPVQRRVRRVAVARHCEFFGDEQAKAAYLKTLEALQNDPLVTVSTIEFDV
FAEAAALLYQGPWVAERRAAIGEFFASNAADIHPVVRGIVQSADSFDAVDTFNARYRLAE
LTRAAQQLLTDIDVLVVPTAPCMPTIEAVLGNPVALNSQLGYYTNFVNLMNMCAIAIPTD
RRADGLPAGITLIGPAGADQRLAEIAAAWQPLFGQADKRDAVAMAPLPYNSPTVQVAVVG
AHLVGQPLNWQLLEGGARLLRSTTTSADYRLYALAATSPPKPGLVRIAEQGASIEIEVWE
MPLSQFGAFVAAIPAPLGIGSLQLADGQWVKGFICEPGGLEGALDITDYKGWRAYRAAHT
SSIAQ
>tr|A0A0Q0FFV4|A0A0Q0FFV4_PSEAP SirA-like protein OS=Pseudomonas syringae pv. aptata OX=83167 GN=ALO85_01043 PE=3 SV=1
MSDAVERPEACDAELDASGLNCPLPLLKAKMELNRLASGAVLKVIATDAGSQRDFRTFAR
LAGHELLHEEVDAGIYRYWLRKA
>tr|A0A0Q0D7C7|A0A0Q0D7C7_PSEAP 50S ribosomal protein L17 OS=Pseudomonas syringae pv. aptata OX=83167 GN=rplQ PE=3 SV=1
MRHRKSGRHLSRTSSHRKAMFQNMAVSLFEHELIKTTLPKAKELRRVAEPLITLAKEDSV
ANRRLAFDRTRSKEIVGKLFNDLGKRYATRQGGYLRILKCGFRAGDNAPMAYVELVDRPI
GGSVEAAE
>tr|A0A0Q0DRA1|A0A0Q0DRA1_PSEAP Transcription elongation factor GreB OS=Pseudomonas syringae pv. aptata OX=83167 GN=greB PE=3 SV=1
MSTKIITRDGHEALKKELDYLWREHRPDITQKVAWAASLGDRSENADYQYNKKLLREIDR
RVRYLRKRLEDMRVVQYSPEQEGRVFFGAWVEIENEAGDLKKFRIVGYDEIYGRNDYISI
DSPMARALLKKEVGDEVLVNTPEGEKLWFVNSIDYEK
>tr|A0A0Q0BU66|A0A0Q0BU66_PSEAP Cytochrome oxidase bioproteinsis protein Cox11-CtaG OS=Pseudomonas syringae pv. aptata OX=83167 GN=ALO85_100318 PE=4 SV=1
MSDIKSEPRMTQPLLSKRLVLRLTCLMVIMLMCGLALPSLYAVLTGHDRAGMQGEDVSRQ
VEVQFTAGNAAGMTWAFYPQSRQMQVHPGAVNEMIFIAQNPTDRPMQAQAVPGISPVKAA
AWFHKTECFCFTQQTLQPGERIEMPVRFIVDRDLPDDVKSLTLAYTLFDVTTR
>tr|A0A0Q0C6R6|A0A0Q0C6R6_PSEAP DEDDh 3'-5' exonuclease domain protein OS=Pseudomonas syringae pv. aptata OX=83167 GN=ALO85_01183 PE=4 SV=1
MMMPHWLVIDLEATTEEGGWPVAEMEVIEIGATLVNQDGRELDHFERFVRPARRPLLTHF
CRELTHINQSSIDSAAPLTTVWPQFERWLSHHRARIVGWASWGDYDRQQLEEEWRHHHLD
SALSSMPHVNLKQRFAQARHLQKPMGLNSALQLAGMQFSGQQHRALVDARNTARLLPLIL
PN
>tr|A0A0Q0D627|A0A0Q0D627_PSEAP Beta-lactamase OS=Pseudomonas syringae pv. aptata OX=83167 GN=ALO85_02606 PE=3 SV=1
MQWNGALLCTGLLLFNAVAVADDDTASVDAFVQAEASKVMQENHIAGLSIAVTRQGKQQF
YTYGVASKATGQPVTRDTLFELGSISKAFTATLATWAQANGQLSLTQSIDSYIPQLQATR
LGKVPVFHLGTHTAGGFPLQVPDKVQNTRQLMEYFKAWQPDYLPGTHRTYANPSVGLLGM
VAARSMKLPYEQALQQRLFPALGLSNTYINVPDEKQALYAQGYNKLDEPVRVNPGPLAAE
AYGVKSSSSDLLRFVEANIGLGQNDAALQKALRDTRTGYFKVGGMTQDLIWEQYPLPVHL
DTLLEGNASAMLNTVKTEMIEPPQAAQSSVWVNKTGSTNGFGGYVAFIPEQQLGIAILAN
KNYPNEERVKLAYRILQKMGCCSTP
>tr|A0A0Q0DWR6|A0A0Q0DWR6_PSEAP Putative nitrilotriacetate monooxygenase, subunit A OS=Pseudomonas syringae pv. aptata OX=83167 GN=ALO85_05041 PE=4 SV=1
MSTTARQMKLGAFLMATGHHVAAWRHPEVPADAGLDFKHYRHVARVAEAAKFDALFVADS
VAAATGDIASRMARSDHFEPLTLLSALSAVTEHIGLIATATTTYNEPYHVARKFASLDHL
SGGRAGWNLVTSDAAAEAQNFGRAEHVGHAERYSRAREFHQVVTGLWDSWADDAFTRDKA

SGEYYDPGRLHVLNHQGEHFSVKGPLNVARSPQGQPVVVQAGSSEVGRDLAAQTAEVVFT
AQTSLASAQAFYADLKGRLSAYGRAVDSLKIMPGVFIVVAETEALAKAKFESFQELVEPQ
VGVALLGRMLGNFDLSGYPLDGPLPELPLTDSGQRSRQKLLTELADQENLTLAQLGRRIA
GGRGHYSLIGTPEQIADELQRWFEQGAADGFNVLVPHLPGGLEDVARLLVPELQRRGLFR
TEYEGTTLRENLGLQRPAYRF
>tr|A0A0Q0IKQ1|A0A0Q0IKQ1_PSEAP AHS1 domain-containing protein
OS=Pseudomonas syringae pv. aptata OX=83167 GN=ALO85_00849 PE=4 SV=1
MLLIFCLPFYNDFKKGTDMADPIRYSFGADEHLFAEVSDSMSLEAFFKGLAVTRAVERLA
LDGVLDVCLANASFQIRFDPDRIAPHVLLEAVRAAEAGAVAERTLQTRIIEIPVLYNDPW
THETLMRFRDRHQDPGSTDLEYAARINNLADVDAFIAAHSGAPWFVSMVGFVAGLPFMFQ
MVERERQLQVPKYLRPRTDTPKHTLGHGGCFGCIYSVRGAGGYQMFGVTPAPIYDPQQKL
AYLKEHMVFFRPGDIVQFKPLDRQAYDEAVAEVEAGRFDLLIRPVEFSLDAFLADPVGYP
KSLQEVLA
>tr|A0A0Q0DND6|A0A0Q0DND6_PSEAP Putative ammonia monooxygenase
OS=Pseudomonas syringae pv. aptata OX=83167 GN=ALO85_00655 PE=4 SV=1
MSNASRFSLSSLSPLTQWAGLILMAGIAGQLLKLFDMPAAMFLGPMLVAIGFGVSGASIR
MPKRAFQLGQGTVGVLIAHAMSVSVLLTALHSWHVMLLATILTVVLSAAVGLVLVRFAGI
PSNTAAWGTSPGAASAMVAMSEDYGADSRIVATMQYVRVVCVVTIGALVSHFIGAPDSAA
ALHTSAAVLPSLNLVDLALSLAVIAAGVTLGSRLPAGALLVPLMLGGALQLSGVMQITVP
AWLLPIAYGAIGCYVGLRFDRPTVQYVWRRLPMMILASLLLIVLCAVSAWLIAVMLGKDY
LSVYLATSPGGLDTMAIIAIDTHADVGFVLAMQTLRLFGVILTGSFLARQIIRLTDKRIP
AL
>tr|A0A0Q0C210|A0A0Q0C210_PSEAP TlpA-like protein-disulfide reductase
OS=Pseudomonas syringae pv. aptata OX=83167 GN=ALO85_01961 PE=4 SV=1
MARRLTAAVILFGSLLLSGCGVDLGNDQNGQKVASERIKGHWLVVNYWAEWCGPCRTEVP
EFNALSEQLKDQKVTVLGVNFDNLQGDELKSAANALGIKFTVLAQDPAEQYSLPPSEALP
VTYIIDDKGKMREQLLGEQSAATVIQKLKTLRGEG
>tr|A0A0Q0IB48|A0A0Q0IB48_PSEAP Response regulator receiver:ATP-binding
region OS=Pseudomonas syringae pv. aptata OX=83167 GN=ALO85_00507 PE=4
SV=1
MLSEIQAKLLIVDDLPENLLALEALIKRGDRIVYKALSADEALSLLLQHEFAMAILDVQM
PGMNGFELAEMMRSTEKTKSIPIVFVSAAGRELNYAFKGYESGAVDFLHKPLDIHAVKSK
VNVFVDLYRQRKAMKIQVEELERSRQEQEALLQRLQSTQGELEHAIRMRDDFMSIVSHEV
RTPLNGLILETQLRKLHLAKDNAAAFTMEKLHAMVDRDERQIQSLIRLIEDMLDVSRIRT
GKLSIRPSPFDLSELVTQLLESFAAQIAAADSSITQFAEQPVIGVWDEFRIEQVVANLLT
NALRYGARKPIEVHVYSKDSEAFIEVRDQGIGITAENQKRIFQQFERVSSNHAVAGLGLG
LFISDQIVMAHGGRIEVESEEGKGSVFRICLPL
>tr|A0A0Q0BSZ3|A0A0Q0BSZ3_PSEAP Peptidase S1, chymotrypsin OS=Pseudomonas
syringae pv. aptata OX=83167 GN=ALO85_02317 PE=4 SV=1
MMMKPTMMTGACCIAALLPFSIHATPEDLGEGLMALAPSTALLNADGSRDHWNGIGRINS
RSGSNCTATLIDSRSVDSPSDAPAYVITSGHCINRQNGVIITDGEVEGSVQFNFFTDSKA
RSYPLKRVSWSSMQGVDLAVVELQPTLETLVNDGIQPIRLAREMPEQGRDILWVGAPLDK
DTGHLRMAACVHQTSEVIMEPPWVWRHTVRNQCRDVGTGASGSPLLTRDNGELYAVMNLT
NQASSDTPIEDLSDEIPGFPPMVADSNYGNPVTLLKKCFVSGTLSTDPAVCDLFPAFSVD
FETLGRQPAQHARVQLDAEGRNVYPSWDLRFMVDTPLYRYKKVDSAMQCEDPVGYSHTIS
SQDAAIDDPVDKQIGINWLCMVGVSSAEQRSSIGLMRNALTLGIELQPAGPTPAPQMEIG
KNRSGATSVVWSHEHRLIDYYTVKMGKPDTTDCSAPEGFKPQFRNLVLRAQSLPLKICTY
AHDINGQPSALREDIIPSPAAG
>tr|A0A0Q0DC96|A0A0Q0DC96_PSEAP CorA-like Mg2+ transporter protein
OS=Pseudomonas syringae pv. aptata OX=83167 GN=ALO85_00571 PE=3 SV=1
MFDEANAQWGLVHALVLDGKGGARFIARTELQDLALQPEESLWLHWDRSHPQTHAWLRTG
SGLSEFACDLLLEENTRPRLLPLPEQELLLFLRGVNLNPGAEPEDMVSVRIFAAAQRVIS
LRLRPLRATEELIELLGQGKGPKGASELILHLAQHLTDKFQDLVGELTESVDEEEEKNDA
DERYNPEHGKLLHIRRRAAGLRRFLGPQRDIYGQLSRIKLSWFAHDDADYWNELNNSLTR
YLEELELTRERVGLLLESEDRRLREHMNRTMYRFGIITCIFLPMSFLTGLLGINVGGIPG
SASSYGFLVACLLIVALGAGQWWLFRRLRWV
>tr|A0A0Q0DKJ9|A0A0Q0DKJ9_PSEAP Malonate utilization transcriptional
regulator OS=Pseudomonas syringae pv. aptata OX=83167 GN=ALO85_02959 PE=3
SV=1
MTDILSSYRRMSCAQSAHLNHKRHDMQIDDELTLKKLEIFLAFMRTGNLARAAEELQTSN

VSVHRAIHSLESALRCPLFKHEGRNLTPLESAYVLEERAQKLVQDVLATVELTRQAAGFS
AARFRLGALYSLTVKTVPQLIMGLKIRRSELNIDLILGSNMDLLYKLKNMEVDAMLVSLD
ESTHDPDCEHLALFSDDIFLAVPTDSPFAEHPEVDLGDLREATFITLTQGFATHRDGIRV
FQQAGFEPKVAMQVNDIFTLLSMVSSGVGYALLPGRIAAVYENRVRLIPLQSRYRMQQHI
GIVFLKSKQRDPNLLALLAECRMYTNRLLEQTT
>tr|A0A0Q0DJ99|A0A0Q0DJ99_PSEAP NADH:flavin oxidoreductase, Old Yellow Enzyme family OS=Pseudomonas syringae pv. aptata OX=83167 GN=ALO85_01763 PE=4 SV=1
MRMSAEALFTPFRLGSLELSSRVVMAPMTRSFSPGHVPNSKVVEYYRRRAAAGVGLIITE
GTTVNHKASNGYPNVPQFFGEAPLAGWRKVVEAVHAEGGRIVPQLWHVGAVRRPGTEPDG
SVPAYGPMEKVKDAQVLVHGMSKQDIDEIVAAFAQAAVDAKAMGMDGVEIHGAHGYLIDQ
FFWEGSNQRSDEYGGSLANRSRFALELIKAVRAAVGPDYPIIFRFSQWKQQDYTARLVQT
PEALGEFLQPLADAGVDIFHCSTRRFWEPEFEGSDLNLAGWTRKLTGKPTITVGSVGLDG
EFLQFMVNTDKVAQPASLENLLKRLGNDEFDLVAVGRALLVDPDWALKVREGREQDILPF
SRDALATLA
>tr|A0A0Q0C8T2|A0A0Q0C8T2_PSEAP LuxR family transcriptional regulator OS=Pseudomonas syringae pv. aptata OX=83167 GN=ALO85_100256 PE=4 SV=1
MCRSWKTNVRNILRICRLGKMMARGSIMWVTMREQEVLGLLLQGCTNKEIARQLSISDYT
VRDHVSSLLRKNQVRTRAELMARCVSLLLPGNTVQPPTSVGLRSEPAHT
>tr|A0A0Q0IBE1|A0A0Q0IBE1_PSEAP Uncharacterized protein OS=Pseudomonas syringae pv. aptata OX=83167 GN=ALO85_00099 PE=4 SV=1
MSIMTKWALQAAALFVLGWMAVSVASWSNDVANGTGSDRLAELNAGVLSSPVLAVSSSPA
AQGE
>tr|A0A0Q0FBB8|A0A0Q0FBB8_PSEAP LysR family transcriptional regulator OS=Pseudomonas syringae pv. aptata OX=83167 GN=ALO85_02269 PE=3 SV=1
MNMDIKQLKFLIALDETRHFGQAAARCNITQPTLSMRLRNLEEELGLPLVNRGQRFEGFT
APGERVLAWARTVLAAYDGLQAEAAACRGHLVGTLRLGVVPLSSFDPLPLLHRLHQLHPN
LRFELSSLSSEQVLEQLASNRIDLGVSYLERLDNERFDSLPLDDTRMGLLYDRRHFSFGD
DPLSWADLVELPLGMLTSGMHFRQSIDHNFHSRGLQPNPLIQTDAVHQLLQAVHGGFCCA
IMPLDGGLETLTEHLRLHPIADSRTLARLGLIMRRSAPRSALAEACFALVAEIPR
>tr|A0A0Q0FMQ0|A0A0Q0FMQ0_PSEAP p-hydroxycinnamoyl CoA hydratase/lyase OS=Pseudomonas syringae pv. aptata OX=83167 GN=ALO85_00843 PE=3 SV=1
MSKYEGRWSTIKVEIEHGIAWVILNRPEKRNAMSPTLNREMIDVLETLEQDPDAGVLVLT
GAGEAWTAGMDLKEYFREVDAGPEILQEKIRREASQWQWKMLRMYAKPTIAMVNGWCFGG
GFSPLVACDLAICADEATFGLSEINWGIPPGNLVSKAMADTVGHRQSLYYIMTGKTFNGQ
KAAEMGLVNESVPLAQLRQVTIDLANNLLEKNPVVLRAAKHGFKRCRELTWEQNEDYLYA
KLDQSRLLDKEGGREQGMKQFLDDKSIKPGLEAYKR
>tr|A0A0Q0IEA6|A0A0Q0IEA6_PSEAP NAD-dependent deacetylase OS=Pseudomonas syringae pv. aptata OX=83167 GN=ALO85_03564 PE=4 SV=1
MMQTAAALRHAQRILVITGAGLSADSGLPTYRGVGGLYNGKTDDGLPIEMALSGPMLRRD
PELCWKYIAELGKACLGGEPNVAHYAIAQLQRIKPECWVLTQNVDGYHRAAGSPPERLIE
IHGQLSPLFCQSCGAEDSQLSEHLQRPLPPLCPACSGILRPSVVLFQEMLPEKALETLHE
ELAKGYDAVLSIGTTASFPYIHEPVIRTRVSGGFTAEINPQPTDHSAQMDVFLPYRAAHV
MAELISHI
>tr|A0A0N8T8Z3|A0A0N8T8Z3_PSEAP Putative membrane protein, PsiE superfamily OS=Pseudomonas syringae pv. aptata OX=83167 GN=ALO85_01781 PE=3 SV=1
MGNKWAVKIRHRVHAQAESLGNLCVESFHFLALFAIGAITAWASVVAFLGMVEKGNVTVD
DILLLFIYLELGAMTGIYFKTNHMPVRFLIYVAITALTRLLISDVSHHNPPDIGIIYLCG
GILLLAFAILVVRYASYKYPSAKIPDSSSTVKGAVTEEKGEI
>tr|A0A0Q0BUZ6|A0A0Q0BUZ6_PSEAP Putative membrane protein OS=Pseudomonas syringae pv. aptata OX=83167 GN=ALO85_03218 PE=4 SV=1
MNDSIYLSIQNSPRFKELVSKRERFAWILSAIMLGLYAAFILLIAYGPHILGAKLSPTST
ITWGMPIGVGLILSAFILTAIYVRRANGEFDDLNNAILKEAQQ
>tr|A0A0Q0DCG3|A0A0Q0DCG3_PSEAP DNA gyrase subunit B OS=Pseudomonas syringae pv. aptata OX=83167 GN=gyrB PE=3 SV=1
MSENQTYDSSSIKVLKGLDAVRKRPGMYIGDTDDGSGLHHMVFEVVDNSIDEALAGHCDD
ISIIIHPDESITVRDNGRGIPVDVHKEEGVSAAEVIMTVLHAGGKFDDNSYKVSGGLHGV
GVSVVNALSELLLLTVRRSGKIWEQTYVHGVPQEPMKIVGESDSTGTQIHFKPSAETFKN
IHFSWDILAKRIRELSFLNSGVGIVLKDERSGKEELFKYEGGLRAFVEYLNTNKTPVNEV

FHFNIQRDDGIGVEIALQWNDSFNENLLCFTNNIPQRDGGTHLVGFRSALTRNLNNYIEQ
EGLAKKHKVATTGDDAREGLTAIISVKVPDPKFSSQTKDKLVSSEVKTAVEQEMGKYFSD
FLLENPNEAKAVVGKMIDAARAREAARKAREMTRRKGALDIAGLPGKLADCQEKDPALSE
LYLVEGDSAGGSAKQGRNRRTQAILPLKGKILNVEKARFDKMISSQEVGTLITALGCGIG
REEYNIDKLRYHNIIIMTDADVDGSHIRTLLLTFFFRQLPELIERGYIYIAQPPLYKVKK
GKQEQYIKDDEAMEEYMTQSALEDASLHLSESAPGISGTALEKLVNDFRMVMKTLKRLSR
LYPQELTEHFVYLPPITLEQLSDHEGMQAWLALFDARLRTGEKSGLVYKASLREDRERNV
WLPEVELISHGLSNYVTFNRDFFGSNDYKTVTALGAQISTLLEEGAYVQRGERKKPVNEF
KEALAWLMAESTKRHTIQRYKGLGEMNPDQLWETTMDPSVRRMLKVTIEDAIGADQIFNT
LMGDAVEPRRDFIESNALAVSNLDF
>tr|A0A0Q0DVF3|A0A0Q0DVF3_PSEAP Uncharacterized protein OS=Pseudomonas syringae pv. aptata OX=83167 GN=ALO85_02870 PE=4 SV=1
MVVSPVRERYRVDLAGLQAACEANYARLMRLLPDMRNEQRSRRVAVTQGDQMLGVLAVEV
LLDCPYTTTLQVCQEHSLPWLPVPKLEVQVYHDARMAEVIGAEHARRFRGIYPYPNADMH
QPDEKAQLNLFLGEWLSHCLACGHEFEAVR
>tr|A0A0Q0FTB9|A0A0Q0FTB9_PSEAP UTP--glucose-1-phosphate uridylyltransferase OS=Pseudomonas syringae pv. aptata OX=83167 GN=ALO85_03663 PE=3 SV=1
MIKKCLFPAAGYGTRFLPATKAMPKEMLPVVNKPLIQYGVEEALAAGLTEISIVTGRGKR
SLEDHFDISYELEHQIKGTDKEKYLVGIRKLIDECSFSYTRQTEMKGLGHAILSGRPLIG
NEAFAVVLADDLCVNLEGDGVLAQMVKLHKHYGCSIIAIQEVDPSETNKYGVIAGEEIKP
GLFRVTDMVEKPKPEDAPSNLAIIGRYILTPDIFEKIEQTEPGKGGEIQITDALMKQAAE
GNVLAYKFKGTRFDCGGAEGYIEATNFCFEHFYKAGKAH
>tr|A0A0Q0DZ96|A0A0Q0DZ96_PSEAP Uncharacterized protein OS=Pseudomonas syringae pv. aptata OX=83167 GN=ALO85_03442 PE=4 SV=1
MRLSQSGYVMSISLYAASIPVFQQMLNALSDVLTKAEAYATEKKIQPPALLQARLYPDML
PFTRQVQIAVDFAKGASARLAGVEIPQYDDTETTFAELQALLAKTLAFIGSITPDQVDGK
EGIEIVLRPGTEKEKRLNGQAYLLSYALPQFFFHVTTAYDLLRHNGVEIGKRDFMGKF
>tr|A0A0Q0FMF8|A0A0Q0FMF8_PSEAP Outer membrane protein assembly factor BamD OS=Pseudomonas syringae pv. aptata OX=83167 GN=bamD PE=3 SV=1
MQVKHLLLIAILALTAACSSKEVIDENLSEVELYQQAQADLGNNSYNSATEKLKALESRY
PFGRYADQAQLELIYSNYKNGEPEAAKSAAERFIRLHPQHPNVDYAYYMKGLTSFDQDVG
LLARFLPLDQTKRDPGAARDSFNEFAQLTSRFPNSRYAPDAKQRMIYLRNLLASYEIHVA
DYYLTRQAYVAAANRGRYVVENFQETPSVGDGLAVMVESYQRLHLDDLAATSLEVLKTNY
PNHPQLADGKFTPREAEADNRSWLSKATLGLIDGSAPLPPGQTRADQDMKKQYQDAKDSI
PKELLPENQAELDEQAAKDAEAKGTKSRSWFSYMTLGLFD
>tr|A0A0Q0C8K7|A0A0Q0C8K7_PSEAP Uncharacterized protein OS=Pseudomonas syringae pv. aptata OX=83167 GN=ALO85_03438 PE=4 SV=1
MTKVHIMSVVGSAVPAPLRERGLLACWYLIQDGEPVSGPLASLPVAEALSRKMQCQPLNS
>tr|A0A0Q0FHX0|A0A0Q0FHX0_PSEAP PepSY super protein OS=Pseudomonas syringae pv. aptata OX=83167 GN=ALO85_02113 PE=4 SV=1
MRKVLLISLCLASPLALAGPQCTTADKSQWQDQAKFQEQLKAQGYEISKFKVTDGNCYEI
YGFDKDKRKVEIYHDPVTGKAVKTETK
>tr|A0A0N8T8C9|A0A0N8T8C9_PSEAP Uncharacterized protein OS=Pseudomonas syringae pv. aptata OX=83167 GN=ALO85_01667 PE=4 SV=1
MRQPTPLCRYRYDALDRLAARTPAIGTIARSFYQSDTLVSEVQGAEHLRFLHRDRQLLAM
QSALATLLIGSDQQHSVLHTVSAGLPGPIAYTPYGHRQALNQLPGFNGERPDPLTGHYLL
GNGYRAYNPALMRFNSPDSMSPFGKGGMNAYAYCAGDPVNRSDPTGHEVDVGQVLSFVWI
GLGLFGAFVGVKTAVPAIKAIAKGGEPLSTKLIAASAVGQLAASSVFTVSRVINAVDPDT
PAANILLATAIGLLIPVLAVRTVSPRIKRWEDAGTNIKLVKQRPSNGVVTTAAVNIRRPS
VGGDQANDPGTAMSY
>tr|A0A0Q0FLD0|A0A0Q0FLD0_PSEAP Uncharacterized protein OS=Pseudomonas syringae pv. aptata OX=83167 GN=ALO85_03956 PE=4 SV=1
MAYCHPRLLVCETIMVIPNAPKPADRQGDIEIKALKNNQYNGLYTPRAAMAEAAGLKLGV
SAKMLHLDLAQQSLTIYPLITWPDHEEFLQPKYKKIQKIWFPIDEKTSDFYKKKTPLTSE
DLLELLRETLLPGFTLDPDYGLGIARELKDIIDAVELHSDCSEISFSESEQTCISNNKKI
FTFSTEDYYEATKNIRSISSLGQKAAAEVKDGTIYNLLAGKLDKPLKTIAIGRSPVRHLI
TAAALGKPPLSSGDQDSVITILKTHSKQIAEAKPEALAKLQDEIELVTLEVLIKKYEEKL
KRTTIESEWQDFFDSNKFILTMAFGYPIVQILGQASVGGGQLIGGGSKFADFISRNSQTN
NAAIFEIKTPGTPLLNKTPFRGKVYSATKEFTGAMVQILDQKYQLAKNLPILKSNDRDLM

LEAYAVHCCLIIGSTPKGLDEQKSFELFRRNSREVEVITFDELLEKLKQLHAFLSSNT
>tr|A0A0Q0DIJ0|A0A0Q0DIJ0_PSEAP Rhodanese-like proteiny domain protein OS=Pseudomonas syringae pv. aptata OX=83167 GN=ALO85_02321 PE=4 SV=1
MVAHLLEFATNHYLITGAFVILLGLLIAYEMSKGGASLSTRELTALVNSDQGVVIDVRSK
KDYTAGHIVGSLNFPQDKVLTRTAELEKYKDKTLIIVDAMGQHAGTTARELLKSGFKAAK
LSGGISSWRGDNLPLVK
>tr|A0A0Q0FHI9|A0A0Q0FHI9_PSEAP Uncharacterized protein OS=Pseudomonas syringae pv. aptata OX=83167 GN=ALO85_101062 PE=4 SV=1
MRSHDVPERQRKNRDPSTSGIKPAVPVSFIAKPGRGSAFR
>tr|A0A0Q0BSQ9|A0A0Q0BSQ9_PSEAP Peptidase S24, S26A and S26B OS=Pseudomonas syringae pv. aptata OX=83167 GN=ALO85_100222 PE=3 SV=1
MMSVVILGPLSEGGIKLPLYSFQVPAGFASPAVDYIEKHVSLDELAEVRAPHVYLAKILG
DSMIGAGIFDKDLIVVDRSRTAEHGEIVVAALNNSEPICKRLFMMDGVVKLQSENSAYPS
KHILEGDNLVIWGVVNYSMRRHGKA
>tr|A0A0Q0DUC1|A0A0Q0DUC1_PSEAP Uncharacterized protein OS=Pseudomonas syringae pv. aptata OX=83167 GN=ALO85_03301 PE=4 SV=1
MDSSAAFSNPTKLRADLLRGRLDVSVDSSTIAPDSLFGFAERRNPKRAFLFVSRVLGRHI
PARPSVMLKSFQDLAHKIPADLPGPVLVIGMAETAVGLGAGVHRAYSATRPDALYMVSTR
HPTGSALFARFEEEHSHASAHLIHLPVDPALREMMLGARSLVLVDDEASTGKTFINLHQA
LVDAGLSKIERVVTCVLTDWSAGAVSTSMGAVAEQVSLLQGSYSFDEDLSAPLPEMPEVG
TVAMGDWPLVTANDWGRMGVLSVADTLAPGITVKKGERILVVGTSEFVWRPFLLAERLEK
AGADVHFSSTSRSPIALGHAIDHALSFSDNYGLGIPNFLYNVRPGQFDRVLICTETPRQA
VPAELIEALNAEVICDE
>tr|A0A0Q0CW15|A0A0Q0CW15_PSEAP Glucokinase OS=Pseudomonas syringae pv. aptata OX=83167 GN=glk PE=3 SV=1
MKLALVGDIGGTNARFAIWEDDTLHSVRVFPTIDYAGPEKAIEVYLQDLELQRGDIGHVC
LAVAGPVDGDFFQFTNSHWQLSRKAFCADLQVDELLLINDFTAMALGMTRLKDDEYLTVC
HGVGKPDRPRVVVGPGTGLGVGTLIKLEGNRWMALPGEGGHADLPIGTAREALLWTRLMA
EHEHVSAEVVLSGAGLLLLYQVSCALDDIEPVLKSPAAITTAALSGDPVAAAVLEQFCVF
LGRVVGNHVLALGSLGGVYIVGGVVPRFTEFFINSGFKRAMAEKGVMSDYFNGLPVWLVT
AEYPGLMGSGVALQQAFGAQIRAIWHNNKNLQPDKDAST
>tr|A0A0Q0FNM3|A0A0Q0FNM3_PSEAP Protein-L-isoaspartate O-methyltransferase OS=Pseudomonas syringae pv. aptata OX=83167 GN=pcm PE=3 SV=1
MSREQDDLLRRGIGMTSQRTRERLIQRLCEEGISNQRVLDVIRKTPRHLFVDEALAHRAY
EDTALPIGHNQTISQPYMVARMSELLLAAGPLDKVMEIGTGSGYQTAVLAQLVERVFSVE
RIKVLQDRAKERLVELNLRNVVFRWGDGWEGWPALAPYNGIIVTAVATDVPQALLDQLAP
GGRLVIPVGSGEVQQLMLIIREENGFSRHVLGAVRFVPLLNGPIA
>tr|A0A0Q0C077|A0A0Q0C077_PSEAP Uncharacterized protein OS=Pseudomonas syringae pv. aptata OX=83167 GN=ALO85_00048 PE=4 SV=1
MSGKNSMHDPDPALSKPLWQRLAWLVGIWAGSVFALFIVASLMRMFMSAAGLTTH
>tr|A0A0Q0DEE1|A0A0Q0DEE1_PSEAP Universal stress family protein OS=Pseudomonas syringae pv. aptata OX=83167 GN=ALO85_01267 PE=4 SV=1
MHKVLVPFDGSEHAMRALGYVIELSGELTKSLEVHILNVQASPIDYSLYLAPDMIDGVKA
GLTNEGKRVLADAVALLTAAGVPFQAHVDLGNVAEQVEAEVKRLGCDAVVMGTRGLGNFG
GLLLGSVATRVIHEASVPVTLIK
>tr|A0A0Q0D9N8|A0A0Q0D9N8_PSEAP Flagellar biosynthetic protein FliR OS=Pseudomonas syringae pv. aptata OX=83167 GN=ALO85_04032 PE=3 SV=1
MQPMLALTDTQISTWVASFMLPLFRIIALLMTMPIIGTTLVPRRVRMYLAVAITVVVAPA
LPAMPPVQALDLSALLLIGEQIIIGAGMGLSLQLFFHIFVIAGQIISTQMGMGFASMVDP
TNGVSSATIGQFFTMLVTLLFLAMNGHLVVLEVLVESFTTMPVGSGLLVNNFWELANGLG
WALASGLRLVLPAITALLIINIAFGVMTRAAPQLNIFSIGFPLTLVLGMVILWMTMGDML
NQYQPIATQALQALRDMVRAR
>tr|A0A0Q0ITG0|A0A0Q0ITG0_PSEAP RpiR family transcriptional regulator OS=Pseudomonas syringae pv. aptata OX=83167 GN=ALO85_01439 PE=4 SV=1
MNLLQHIAQSRNLLRKSELKVADHVLLDPAAVMHSSMADLAHSVGISEPTIVRFCRAIGC
SGFQDLKLKLAQSLAAGASFGQFAIHEDDSVADYSLKIFDTTLHTLMEVRENLDPHALQL
AVTAMAGANRVEFYGFGASGAVAADAQHKFFRLLLTAAAYSDPHMQAMSAVTLKPTDVAV
CISQSGRSKDLLITANLVRESGATLITLCPSQTPLAELSSVNLAIDVHEDTEIYTPLTSR
IAHLVVIDVLAMGVAMARGPGLVNHLKSVKRSLRGLRLSPKSIKTHED

>tr|A0A0Q0CC87|A0A0Q0CC87_PSEAP Putative phage terminase, small subunit OS=Pseudomonas syringae pv. aptata OX=83167 GN=ALO85_02452 PE=4 SV=1
MPEEDRIMNKPLSDLQLNQWRDSNDSQEHSTSLALYLIAVPLFILAALLILNGLFSESLS
SVAIGVIGLVAALGFQRQNAARAARKP
>tr|A0A0Q0DCV8|A0A0Q0DCV8_PSEAP Stringent starvation protein B OS=Pseudomonas syringae pv. aptata OX=83167 GN=ALO85_00192 PE=4 SV=1
MNSSRPYLVRALYEWIVDNDCTPHVLVNADYPAVQVPQGFANDGQIVLNVSPSAVRHLHM
DNEAVSFEGRFGGVPHTLYVPVAAILGIYARENGQGMVFDLEPSMEEGEEIEIEIEDDTP
PPDSEPPRPSGRPSLKVVK
>tr|A0A0Q0CVM4|A0A0Q0CVM4_PSEAP Uncharacterized protein OS=Pseudomonas syringae pv. aptata OX=83167 GN=ALO85_04268 PE=4 SV=1
MTTPEQPPVQDAAADSVADTENKAVIAPFSFPFKPSEFAQAKKEPAWHQKNNKHGHHKTP
GVAPAGTRRSMGKR
>tr|A0A0Q0BU91|A0A0Q0BU91_PSEAP Endoribonuclease YbeY OS=Pseudomonas syringae pv. aptata OX=83167 GN=ybeY PE=3 SV=1
MLELDLQIASETSAPDEARFRLWCEMGLRQRSADSELTIRLVDETEGRELNNTWRHKNYA
TNVLSFPADVPDDMLDIPLLGDLVICVPVVNREAVEQGKSIDAHWAHMVIHGCLHLLGYD
HIDDEEAEEMEALERTLLEELGYPDPYADDESADLPHSDTPSKDHE
>tr|A0A0Q0DU50|A0A0Q0DU50_PSEAP Formyltetrahydrofolate deformylase OS=Pseudomonas syringae pv. aptata OX=83167 GN=purU PE=3 SV=1
MRTFRLVIACPDRVGIVAKVSNFLASHNGWITEASHHSDNLSGWFFMRHEIRADTLPFDL
DGFREAFTPIAEEFSMDWRITDSAQKKRVVLMASRESHCLADLLHRWHSDELDCDIACVI
SNHQDLRSMVEWHNIPYYHVPVDPKDKEPAFAEVSRLVGHHQADVVVLARYMQILPPQLC
REYAHQVINIHHSFLPSFVGAKPYHQASLRGVKLIGATCHYVTEELDAGPIIEQDVVRVS
HRDSIENMVRFGRDVEKMVLARGLRAHLEDRVLVHDNKTVVFG
>tr|A0A0Q0DU38|A0A0Q0DU38_PSEAP Multidrug resistance operon repressor MexR OS=Pseudomonas syringae pv. aptata OX=83167 GN=ALO85_02403 PE=4 SV=1
MVRRTKEEAQITRSQILEAAEQAFYERGVARTTLADIATLAGVTRGAIYWHFNNKADLVQ
AMLDSLQEPLDEMAEASQSEDEEDPLGCMRNLLIHLFHELALDPKTRRINEILFHKCEFT
DEMCDFRRQRQDNAIQCHDRITLGLNNAVRQGQLPKGLDTARAAVALFAYVNGIIYQWLL
VPDSFSLPAEAEQLVDVCLDMLRFSPTLLIDSNRSTVGA
>tr|A0A0N8T826|A0A0N8T826_PSEAP Histidinol dehydrogenase OS=Pseudomonas syringae pv. aptata OX=83167 GN=hisD PE=3 SV=1
MTTPTAIRRLDAADPDFARHLDHLLSWESVSDDSVNQRVLDIIKAVRERGDAALVEFTQK
FDGLQVASMADLILPRERLELALTRITPVQREALEKAAERVRSYHEKQKQDSWSYTEADG
TVLGQKVTPLDRAGLYVPGGKASYPSSVLMNAIPAKVAGVTEVVMVVPTPRGEINELVLA
AACIAGVDRVFTIGGAQAVAALAYGTESVPKVDKVVGPGNIYVATAKRHVFGQVGIDMIA
GPSEILVVCDGQTDPDWIAMDLFSQAEHDEDAQAILVSPDAEFLDKVAASITRLLPTMER
AAIVETSINGRGALIKVSDMAQAIEVANRIAPEHLELSVADPEAWLPQIRHAGAIFMGRH
TSEALGDYCAGPNHVLPTSGTARFSSPLGVYDFQKRSSIIYCSPQGASELGKTASVLARG
ESLSGHARSAEYRITDPDWKAGNTEDGK
>tr|A0A0Q0FBH0|A0A0Q0FBH0_PSEAP Putative aromatic amino acid transporter OS=Pseudomonas syringae pv. aptata OX=83167 GN=ALO85_03814 PE=4 SV=1
MSETTSPSGELKRGLKNRHIQLIALGGAIGTGLFLGSAGVLKSAGPSMILGYSICGFIAF
MIMRQLGEMIVEEPVAGSFSHFAHNYWGGFAGFLSGWNCWLLYILVGMSELTAVGKYIHY
WLPDIPGWVSAAAFFVLINAINLTNVKVFGETEFWFAIIKVVAIIGMIALGSYMLVSGSG
GPQASVSNLWEHGGFFPNGVSGLVMALAIIMFSFGGLEMLGFTAAEAEQPKTVIPKAINQ
VIYRILIFYIGALVVLLSLTPWDSLLASLNASGDSYSGSPFVQVFSMLGSDTAAHILNFV
VLTAALSVYNSGTYCNSRMLVGMAEQGDAPKALAQVDKRGVPVKAIFASAAVTLVAVLLN
YFVPQQALELLMSLVVATLVINWAMISYSHLKFRQHMNRTAQVPLFKALWYPYGNYVCLA
FVVFILGIMLMIPGIRISVYAIPVWLAVMWVCYRAKNRRSVVLSAQSGK
>tr|A0A0Q0C438|A0A0Q0C438_PSEAP Ribose import ATP-binding protein RbsA OS=Pseudomonas syringae pv. aptata OX=83167 GN=rbsA PE=3 SV=1
MIMFGSATANPPAQRDLPSGDGDGGAPDAQAPYLLEISHVTKGFPGVVALNDVQLRVRPG
SVLALMGENGAGKSTLMKIIAGIYQPDAGEIRLRGKPVRFETPLSALQAGIAMIHQELNL
MPFMSIAENIWIGREQLNGLHMVDHREMHRCTAELLERLRIKLDPEELVGTLSIAERQMV
EIAKAVSYNSDVLIMDEPTSAITETEVAHLFSIISDLRAQGKGIIYITHKMNEVFEIADE
VAVFRDGAYIGLQRAESMDGDSLITMMVGRELTQLFPEREKPAGDVLLSVNRLSLNGIFK
DVSFDLRAGEVLGIAGLMGSGRTNVAETLFGITPSDSGEVRFDGKTVHIGDPHQAIELGF
ALLTEDRKLTGLFPCLSVMENMEMAVLDNYAGNGFVQQKALRALCEDMCKKLRVKTPSLE

QCIDTLSGGNQQKALLARWLMTNPKVLILDEPTRGIDVGAKVEIYRLISLLASEGMAVIM
ISSELPEVLGMSDRVMVMHEGEMMGILDRSEATQEKVMHLASGHKVH
>tr|A0A0Q0BWW9|A0A0Q0BWW9_PSEAP Alpha-1,4 glucan phosphorylase OS=Pseudomonas syringae pv. aptata OX=83167 GN=ALO85_01695 PE=3 SV=1
MSQEPLVRDADVAAFRAAVLAKLTYSVGKDPDHAFEHDWFEAVALAARDHMVEHWMDHTR
QIYRKVQKRVYYLSLEFLIGRLLYDSLSNLGLLEVAREALTELGVDIERIRLLEPDAALG
NGGLGRLAACFMESMSTLGIAGHGYGIRYEHGLFRQGIVDGWQQEQTENWLDFGNPWEFE
RPEVVYSIGFGGSVDTVPTEAGDSRQVWRPGETVRAIAYDTPVVGWRGKSVNTLRLWRAR
AVEDLHLERFNAGDHFGAVAEVVRAESISRVLYPNDATEAGQELRLRQEYFFVSASLQDL
LRRHLNQHATLTDLSEHAAIQMNDTHPSIAVAELMRQLIDNHNIPWDTAWKITVGTLGYT
NHTLLPEALETWSVGLMERMLPRHMQIIYLINAQHIDTLRAKGVDDVNVLRAVSLIEEDN
GRRVRMGNLAFLGSHSVNGVSALHTQLMRKTVFAELHKIYPERINNKTNGITFRRWLFQA
NPKLTEMLVESLGEDVLDNAETRLKELEPFAEKSSFRKQMADQRLHSKRALAAIIHERLG
IAVNPAAMFDVQVKRIHEYKRQLLNLFHTVALYQAIRAEPGTDWVPRVKIFAGKAAASYH
SAKLIIKLTNDIARTVNNDPTVRGLLKVVFMPNYNVSLAESIIPAADLSEQISTAGLEAS
GTSNMKFGLNGALTIGTLDGANVEMSEQVGLEHMFIFGMSSQQVEARKQAGEFSAHDDVA
ASGRLNDVLQAIRGGVFSPDDPNRYVGLIDQLLDYDRFLVCADFDSYWAAQAKVEERWHD
SKEWWRSAVLNTARMGWFSSDRTIREYAGDIWKALD
>tr|A0A0Q0DBD1|A0A0Q0DBD1_PSEAP Sec translocon accessory complex subunit YajC OS=Pseudomonas syringae pv. aptata OX=83167 GN=yajC PE=3 SV=1
MSFFISPAFADAAAPAAGPAGSGFEWIFLVGFLVIFYLMIWRPQAKRAKEQKNLLGNLQK
GDEVVTSGGIAGKINKVTDDFVVIEVSDTVELKIQKGAIAATLPKGTLKAI
>tr|A0A0Q0FR56|A0A0Q0FR56_PSEAP Flavoprotein, family OS=Pseudomonas syringae pv. aptata OX=83167 GN=ALO85_02901 PE=4 SV=1
MPVTDVVIIGAGAAGLMCAFTAAARGRKVMLIDHANKPGKKILMSGGGRCNFTNMYTEPA
NFLSQNPHFCKSALARYTQWDFIAMVAKHGVPYHEKKLGQLFCDNKSSDILEMLLEECRQ
AGVSLHMDTSVQQIEKTDAGYSLQTTLGTLNCQSLVIATGGLSIPTLGATGFGYQVGKQF
GHTLLPTRAGLVPFTITDQLKELCTELSGTSVDCLVSCNDTSFRENILFTHRGLSGPAIL
QISSFWQPGDTVEINLLPDHDVPAWLNQQQAERPNSELKTLLGEIFTKKMANLIAEHWFV
SKPMKQYTHAELAEIAEKLASWQVVPAGTEGYRTAEVTLGGIDTREVSSKTMESLKSPGL
YFVGEVLDVSGHLGGFNFQWAWASAYAAAHYV
>tr|A0A0Q0DR95|A0A0Q0DR95_PSEAP Ornithine carbamoyltransferase OS=Pseudomonas syringae pv. aptata OX=83167 GN=ALO85_02047 PE=3 SV=1
MNARHFLSMMDYTPDELLGLIRRGVELKDLRNRGVLFEPLKNRVLGMIFEKSSTRTRLSF
EAGMIQLGGQAIFLSHRDTQLGRGEPIADSAKVMSRMLDAVMIRTYAHSNLTEFAANSRV
PVINGLSDDLHPCQLLADMQTFLEHRGSIQGKTVAWIGDGNNMCNSYIEAAIQFDFQLRV
ACPAGYEPNAEFLALAGERVTVVRDPKAAVEGAHLVSTDVWTSMGQEEETARRKKLFAPF
QVTRALLDLADKDVLFMHCLPAHRGEEISVDLLDDARSVAWDQAENRLHAQKALLEFLVA
PACKPA
>tr|A0A0Q0C0S9|A0A0Q0C0S9_PSEAP 50S ribosomal protein L16 OS=Pseudomonas syringae pv. aptata OX=83167 GN=rplP PE=3 SV=1
MLQPKRTKFRKQMTGHNRGLALRGSKVSFGEFALKSVARGRLTARQIESARRALTRHVKR
GGKIWIRVFPDKPVTKKPLEVRMGKGKGNVEYWVAQIQPGKVLYEIEGVTEELAREAFAL
AAAKLPLATSFVKRTVM
>tr|A0A0Q0CGU7|A0A0Q0CGU7_PSEAP Putative serine/threonine protein phosphatase OS=Pseudomonas syringae pv. aptata OX=83167 GN=ALO85_00617 PE=4 SV=1
MLDPARGYDLIGDVHGCAHTLEHLLELLGYRLQAGVWRHPERIVIFLGDIIDRGPRIRES
LHIVRDMVQAGQALCIMGNHEFNALGWVTPALSESGHQFVREHTPRHARLIGETLAQFEP
YPDEWSEFVNWFYELPLYLDAGRFRVVHACWDATLIDPLRGLHGTGCIDQHFVQASAVPG
SFASNVFNRLLRGTDMRLPHGLTLTGGDGLTREFFRTKFWEDDPQTYGDVVFQPDALPEG
VAKTPLSTSEKNALLRYGINEPLLFVGHYWRSGIPAPIRPNLACLDYSAVLYGKLVAYRL
DQETQIDPDKFVWVDVQRPEV
>tr|A0A0Q0C0K9|A0A0Q0C0K9_PSEAP Exodeoxyribonuclease III OS=Pseudomonas syringae pv. aptata OX=83167 GN=ALO85_00947 PE=4 SV=1
MKIVSFNINGLRARPHQLAALIDKHQPDVIGLQETKVSDEQFPQAEVEALGYHVHFHGQK
GHYGVALLSRNAPLALHKGFEGDDEESQKRFIWGTYADSNGQPVTIMNGYFPQGESRDHP
TKFPAKQRFYENLQTLLEGQFRNDQPLIVMGDVNISPQDCDIGIGADNAKRWLKTGKCSF
LPEEREWMERLKNWGLVDSFRHLYPEVVDRFSWFDYRSRGFEDEPKRGLRIDLIMTSTGL
QPRIKEAGVDYDLRAMEKPSDHAPIWLELS

>tr|A0A0N8TAC1|A0A0N8TAC1_PSEAP Acyl-CoA dehydrogenase OS=Pseudomonas syringae pv. aptata OX=83167 GN=ALO85_04856 PE=3 SV=1
MSGKASFNWIDPLLLDQQLTEEERMVRDTAEQFAQSKLAPRVLEAFRHEKTDPAIFREMG
EVGLLGATIPEEFGGSGLNYVCYGLIAREVERIDSGYRSMMSVQSSLVMVPINEFGTQAQ
KEKYLPKLASGEWIGCFGLTEPDHGSDPGAMITRARSVEGGYRLSGSKMWITNSPIADVF
VVWAKDDAGDIRGFVLEKGWAGLSAPAIHGKVGLRASITGEIVMDNVFVPEENIFPDVRG
LKGPFTCLNSARYGISWGALGAAEFCWHTARQYTLDRQQFGRPLAANQLIQKKLADMQTE
ITLALQGCLRLGRMKDEGTAAVEITSIMKRNSCGKSLDIARLARDMLGGNGISDEFGIAR
HLVNLEVVNTYEGTHDVHALILGRAQTGIQAFY
>tr|A0A0Q0CFS1|A0A0Q0CFS1_PSEAP Ectoine/hydroxyectoine ABC-type transport system OS=Pseudomonas syringae pv. aptata OX=83167 GN=ALO85_03680 PE=3 SV=1
MPVDAATLETAGFIARQLAHGAWITLQITVLAELLVLVLSFVIALMRLSPFRALRWFATV
YVEVLRGISALVLLFYLFFILPLFGIRLSPMTTGVLGLGLTFSAYGAEIIRSALINVSQG
QRDAVRALDFGPISAFRRIILPQALPFMIPPMGNQLVELLKTTSLVSLITLTDLTFTGSQ
LITTLGQQTLIWSIVLVCYFVMAWPLSWLVKRYEQHATAWRTARG
>tr|A0A0Q0DBP3|A0A0Q0DBP3_PSEAP Superoxide dismutase [Cu-Zn] OS=Pseudomonas syringae pv. aptata OX=83167 GN=ALO85_04251 PE=3 SV=1
MKNVRAVQPPRCLVPRSIRLSDQGRIEIMKPHLLLGLIGVFSFGVQAASLDVPINLVSAD
GAPKPIGSVTVSETQYGLLFTPNLKELPAGIHGFHVHENGSCEAGTKDGKKVAALAAGGH
FDPAKTGKHLGPYADGHLGDLPALYVAADGTASYPVLAPRLKKLSKVKGHALMVHAGGDN
HSDHPAPLGGGGDRAACGVI
>tr|A0A0Q0FUW7|A0A0Q0FUW7_PSEAP Single-stranded DNA-binding protein OS=Pseudomonas syringae pv. aptata OX=83167 GN=ALO85_200208 PE=3 SV=1
MARGINKAILVGTCGQDPDCRYLPNGTAVTNLSLATSEQWTDKQSGQKVEKTEWHRVSLF
GKVAEIAGEYLRKGSQVYIEGKLQTREWEKDGIKRYTTEIVVDMQGTMQLLGGRPQGDSQ
HSQNGQGSGDSDHQEPPRQQAPQQAAPEKPSGKGKAAPKPPRASGKQAQAKAPAPQPAGD
FDGGDDNIPFMDPYRFNWMLV
>tr|A0A0Q0BW14|A0A0Q0BW14_PSEAP Uncharacterized protein OS=Pseudomonas syringae pv. aptata OX=83167 GN=ALO85_101331 PE=4 SV=1
MSSKPKMPADFKQTSLCATRLSGFFLTTAYEPGPETEPADAERIPNQPVVV
>tr|A0A0Q0C2B4|A0A0Q0C2B4_PSEAP Uncharacterized protein OS=Pseudomonas syringae pv. aptata OX=83167 GN=ALO85_100723 PE=4 SV=1
MRQKGKRVKPRAYQAAAINGYRHERQKTPISSGIQRFSRICTALFSCSSVCDSHRQGAGS
LHRAQATGLITPASGQRSLVCSTRRSPARATRRTKSCTLRLPASSCA
>tr|A0A0N8TA77|A0A0N8TA77_PSEAP Phosphoribosyltransferase OS=Pseudomonas syringae pv. aptata OX=83167 GN=ALO85_03870 PE=4 SV=1
MRCQPSYPHEVYIWLKNKQSCLLCDERSEVPTPICVPCEAELPWLGNACVCCALPLPRSG
MSCAQCCKQPPGFTQVIAPWLYDFPVDGLITRFKHNGKWPMGRLLAELCAHYLQHRFEED
LPRPDCLIPVPLAIKRLRQRGYNQAAMLAGWLGKQLQLPIDEEHVLRSRETTAQQGLDAK
ARKRNLRGAFTLIDPDWVQGRHLALVDDVLTTGSTADVIARLLSNAGARRVDVYCLARTP
KPGD
>tr|A0A0Q0DLP6|A0A0Q0DLP6_PSEAP Putative Membrane protein OS=Pseudomonas syringae pv. aptata OX=83167 GN=ALO85_01659 PE=4 SV=1
MRKDYLAFFVSLFLSRLADQILLFIVPLVVFQTTNSASWAGLAFFVESLPRFLAFPLCGA
LCDKYPPVKILHISQIYRASLCVLAMLLYAIFGGIGWLVVLSALCGVLTTQGIMAREVLM
PHIFQHYSYTRTLSYSQIADQSGLVLGPLVAALMLEV
>tr|A0A0Q0BU37|A0A0Q0BU37_PSEAP Uncharacterized protein OS=Pseudomonas syringae pv. aptata OX=83167 GN=ALO85_100357 PE=4 SV=1
MRATRSPGRRFELEHKHRSPCHSSESIQWKTMEDTDLQALMKRVELLLKYAEQLKGQNAL
LLAQEKTWREERAQLIEKNEIARQKVESMISRLKALEQDS
>tr|A0A0Q0DIF4|A0A0Q0DIF4_PSEAP Oxidoreductase alpha OS=Pseudomonas syringae pv. aptata OX=83167 GN=ALO85_05089 PE=3 SV=1
MSTHHQADKTPTPRYKPYKGAAGGWGALISVTQHWLGSDNALKNLRMMLKTNQNGGFDCP
GCAWGDSPESGMVKFCENGAKAVNWEATKRRVDPAFFARYSVSSLMEQSDYWLEYQGRLT
EPMTYDAETDRYKPISWDNAFALIAKHLKNLPSPNMAEFYTSGRASNEAAYLYQLFVRAY
GTNNFPDCSNMCHEASGVALSQSVGVGKGTVTFEDFEHADAIFVLGQNPGTNHPRMLEPL
REAVQRGAQVVCVNPLKERGLERFQHPQHPVEMLTNGDRPTNTAYFRPALGGDMALLRGM
AKFLLQWEREAQANNAPSVFDHAFLNEHTEGVLEYLAAIDDTSWDAIVEQSGLPLSDIEQ
SARMYAKGKNVIMCWAMGITQHRHSVPTIQEIANLMLLRGNIGRPGAGLCPVRGHSNVQG

DRTMGINERPPVFLLDALEKRFQFKVPRENGHNVVEAIHAMAEGRAKVFIALGGNFAQAT
PDSHRTAEALSNCDLTVQISTKLNRSHLFHGKDALILPCFGRTDIDIQANGPQAVTVEDS
FSMVHASNGQLKPSSKQMRSEPAIIAGIANATLGKAPVDWLWLVEDYNRIRDLIADTIPG
FKDFNERVKHPGGFYLGNAAGARRWNTASTRANFKSNALPLTLINEHVSSTGQIPDLIMQ
SMRSHDQYNTTIYGLDDRYRGVKGQRDVLFVNEADIIRLGFQPGQKADLISIWSDNRERR
VKGFTLLPFDIPAGQAAAYYPEVNPLVPLESVGDGSSTPTSKFVAIRLERSAESARIL
>tr|A0A0Q0FT06|A0A0Q0FT06_PSEAP Xylose import ATP-binding protein XylG OS=Pseudomonas syringae pv. aptata OX=83167 GN=xylG PE=3 SV=1
MSDYLLQMNGIVKSFGGVKALNGIDITVKPGECVGLCGENGAGKSTLMKVLSAVYPYGTW
EGEILWDGQPLKAQSISETEAAGIVIIHQELTLVPDLSVAENIFMGHELTLPGGRMNYPA
MIHRAESLMRELKVPDMNVALPVSQYGGGYQQLVEIAKALNKKARLLILDEPSSALTRSE
IEVLLDIIRDLKAKGVACVYISHKLDEVAAVCDTISVIRDGKHIATTAMADMDIAKIITQ
MVGREMSNLYPTEPHDVGEVIFEARHITCYDVDNPGRKRVDDVSFVLRRGEILGIAGLVG
AGRTELVSALFGAYPGRYSGEVWLDGQAIDTRTPLKSIRAGLCMVPEDRKRQGIIPDLGV
GQNITLAVLDSYSNMTRIDAEAELGSIDREISRMHLKTASPFLPITSLSGGNQQKAVLAK
MLLTRPRVLILDEPTRGVDVGAKYEIYKLMGALAAEGVSIIMVSSELAEVLGVSDRVLVI
GDGQLRGDFINHELTQEQVLAAALSHSDAPHNNARKTA
>tr|A0A0N8T9V0|A0A0N8T9V0_PSEAP Glutamine/glutamate ABC transporter permease OS=Pseudomonas syringae pv. aptata OX=83167 GN=ALO85_03686 PE=3 SV=1
MTFDYAFAISTLPAFLKAVGVTLQVGLIAILTSLAVAVVNAAIAMFKVPVLHRLVPVYVE
LARNTPLLIQLFFIYFALPTLGIRVSGFASAVIAMTFLGGAYLTEILRAGIEAVPKAQIE
SGLSIGLSRWQLLRHVILPQAGILSLPALFANFVFLLKETTVVSAVAVPEILYTTKSYIA
LYYKTYEMLAVMTGLCVLLFLPLSLLLGLLERRLQHGQFGS
>tr|A0A0Q0FBE0|A0A0Q0FBE0_PSEAP Putative toxin OS=Pseudomonas syringae pv. aptata OX=83167 GN=ALO85_101354 PE=4 SV=1
MKLDFGPGFRVYYAIQGQKILFLLGGGSKDKQQNDIDQAKALWKWHKVKKK
>tr|A0A0Q0CXD2|A0A0Q0CXD2_PSEAP Regulatory protein, AsnC/Lrp OS=Pseudomonas syringae pv. aptata OX=83167 GN=ALO85_100085 PE=4 SV=1
MNFIMKAINERTKKSGADVTPPLDRIDRAILKTLQRDASISNVALAEKVKLSPPACLRRV
ERLKEAGVIKGVVALLNGDVLDAGMVVLIGVVLDRSTPDSFAQFEAAAQKVSGCMEFHVV
TGEFDYFMLLRTRDSQSFNRLHAEQLLYLPGVRQIRSFMGLRQVVSTTQLPL
>tr|A0A0Q0FTW9|A0A0Q0FTW9_PSEAP Sarcosine oxidase, beta subunit OS=Pseudomonas syringae pv. aptata OX=83167 GN=ALO85_00790 PE=4 SV=1
MPFNLLKYGLSSEYPVEVDLPAPKELKSSYDVVIIGGGGHGLATAYYLSKYHGITNIAVL
EKGYLGGGNTARNTAVIRSNYLTSEGVKFYAESVKMFQGLSNEFDFNIMYSERGQLTLAH
TDATVRSFRQRAEVNKHFGGRTEMIDRQQIRELVPSLNLDPGHLPVLAGLWHIDGGTARH
DAVAWGYAKQAAKRGVEIHQLTEVQELVIENGTIKAVKTNRGTIQCGCAVQAVAGMSSQM
MKKAGIRSPIQTFPLQAMVTQPFKPFLDPLVSSSALHCYVQQTSRGEVVFGGGSDPYPLF
NSRSTLDLKESLLAHAIEMFPFLANAKLMRQWAGITDMTPDYSPIMGLSPVRNYYLDAGW
GTWGFKATPICGKTMAELVASGGKVPELIKPFALERFSTFQQVNEMGATAASH
>tr|A0A0Q0BRY0|A0A0Q0BRY0_PSEAP Uncharacterized protein OS=Pseudomonas syringae pv. aptata OX=83167 GN=ALO85_04295 PE=4 SV=1
MYEPGHLHLTHVALQPSDISYDIHLRYNVEEDPKQGTSMHFTMQGEINGKSFEEQFQLPR
DLAFNFAHDASRIAIRHGLPNSAALPIAQHKDYDRMFADIRDKLHAKSGDPVKPEHLE
>tr|A0A0N8T9H2|A0A0N8T9H2_PSEAP Type III Pyridoxal 5-phosphate-dependent bacterial cryptic D-serine dehydratase OS=Pseudomonas syringae pv. aptata OX=83167 GN=ALO85_00974 PE=4 SV=1
MSTAVMDKGTAQPGDSLVSDVSLPALVIHNHALQHNIRWMQQFVSDSGAELAPHGKTSMV
PALFRRQLDAGAWGMTLATAVQTQAAYAHGVRRVLMANQLVGAPNMAIVADMLADPMFEF
HCMVDHPDNVAALGEFFAARGLRLNVMIEYGVPGGRCGCRSEEQVLALADAVLAQPALAL
TGIEGYEGVIHGDHAIQGIKAFAASLVRLAVDLQDKGAFALDRPIVTASGSAWYDLIAEA
FDAQAVRERFLSVLRPGSYVVHDHGIYKDAQCCVLDRRAELSEALRPALEVWAHVQSMPE
PGFAVIAMGKRDVAYDAGLPNPLLRYKPGVQSAGDDVSACKVTAVMDQHAFMTVAPGCEL
KVGDIISFGTSHPCLTFDKWRSGCLVDNDLRVIEPFSTYF
>tr|A0A0Q0C7J1|A0A0Q0C7J1_PSEAP Uncharacterized protein OS=Pseudomonas syringae pv. aptata OX=83167 GN=ALO85_00612 PE=4 SV=1
MPRKTQGDNAAVPAKVSKSSTDYMREMRQRLKEAGLVKREFWIRPENVDALRGIEKALRQ
PFLGDRIKLEDFMTENTHWTISSLQKALSELELVTNGKIELDVTEGDASGIRLTMADYGD
LPIYIAVEGEQILADATLIEVNKVKNVQAFNDVVLRSRDLFPLSSVGIEKLGGGQEVYCL

FGALSAASSLTVVVQEILTLADNVIRAADAFEDHFIY
>tr|A0A0Q0BTB9|A0A0Q0BTB9_PSEAP Uncharacterized protein OS=Pseudomonas syringae pv. aptata OX=83167 GN=ALO85_101135 PE=4 SV=1
MNLGVRFGHGCQDPVDSGVGSECSAALLLKETARDRAQILLDCGSPGASSADEDACLFSF
ARNFVTERVKFLSRIKRL
>tr|A0A0Q0C560|A0A0Q0C560_PSEAP Uncharacterized protein OS=Pseudomonas syringae pv. aptata OX=83167 GN=ALO85_04267 PE=4 SV=1
MDTKFSCVGCGGCCTDHHVPLTLDEAAQWAADGGNVIVLTEAFLSNGYGVSETQLTHASR
RSAEVSTGSTTAFVAITFAAYNVGRCRNLDEKNLCRIYERRPLVCRIYPMEINPHIPLRP
ENKGCPPESWEQGPDLIVNDRLVDRQLIDLIEQSRQADRDEIETKQLICQKLGIRTTALK
GNGFVAYLPDMGAFAAAIEEVANPAQDERTQGSKWAFHVAGQHVLDTLQQEGAEVTDRQP
VSYLFIPLQAA
>tr|A0A0Q0E0V9|A0A0Q0E0V9_PSEAP Transposition helper protein OS=Pseudomonas syringae pv. aptata OX=83167 GN=ALO85_01527 PE=4 SV=1
MMANEVIKVIRSEGFFHGRMLRSYQRAFQVIEASLAGERQILPMFGPSRIGKGEVAQALM
ADFPTQEVNGKICKPLIRVTAPTEPNQRALTLSIIRGLGGRVLSKCSTPDLYDQALRQLE
IAKVRAIIVDEVQHLAELHSPQKVRALADFFKVLSDELNISLVLLGLPAAERLLGLNEQL
RGRSLATELIYPYSWISAADRQDFAAGIALVAAAYSEQGWIFELSGDVAIKSLYASSLGR
FGMLVDLFSHAETNNANKIIDVRCLAKAYRNAVNDQPFSGNPFTPGTVISDHDLNAAYVK
VLREAHLPIPRL
>tr|A0A0Q0FLK1|A0A0Q0FLK1_PSEAP Uncharacterized protein OS=Pseudomonas syringae pv. aptata OX=83167 GN=ALO85_03944 PE=4 SV=1
MRGYMELISFMEALSDGLLDYLPEDQRAGQLTVEEVIEQWMSEKSYYSSLSLRKDIVTYI
RLQESGDFSVDEILSWYDLCFIPERFGVEEHVFFSGILKSIDSHIEKKKKSFLVKYFSWA
GCK
>tr|A0A0Q0BXT6|A0A0Q0BXT6_PSEAP Uncharacterized protein OS=Pseudomonas syringae pv. aptata OX=83167 GN=ALO85_100641 PE=4 SV=1
MNKESVDYELLNDIVKCIQEVFGFSNDAFETLSLATDINRELGIEGDDANELMLEFFERF
SINIDKYDPYRYFVPEGYDFFSFRRSKDRRGKIPITLGMLYLAAKAKVWNTQELESVEFS
PAQLYNCTSEIPLEGYKIHDK
>tr|A0A0Q0DN04|A0A0Q0DN04_PSEAP Uncharacterized protein OS=Pseudomonas syringae pv. aptata OX=83167 GN=ALO85_100433 PE=4 SV=1
MGQHISADDTCENDNDADDFSHRKKYFTESGSGTVRKLLNLFFSLSA
>tr|A0A0Q0DTP9|A0A0Q0DTP9_PSEAP Tyrosine recombinase XerD OS=Pseudomonas syringae pv. aptata OX=83167 GN=xerD PE=3 SV=1
MNMAAIDHPLIDRFLDALWLEKGLSDNTRDSYRSDLALFNGWLQERNVDLISAGREVILD
HLAWRVENAYKPRSTARFLSGARGFYRYLLREKLISVDPTLQIDMPQLGKPLPKSLSEAD
VEALLAAPDLSEPIGERDRAMLEVLYACGLRVTELISLTLEQVNLRQGVLRVMGKGSKER
LVPMGEESIVWVERYLRGARDELLGGKPSDVLFPSTRGDQMTRQTFWHRIKHQATVAGIG
KSLSPHTLRHAFATHLLNHGADLRVVQMLLGHSDLSTTQIYTHVARARLQELHAKHHPRG
>tr|A0A0N8T9D6|A0A0N8T9D6_PSEAP 2-C-methyl-D-erythritol 2,4-cyclodiphosphate synthase OS=Pseudomonas syringae pv. aptata OX=83167 GN=ispF PE=3 SV=1
MRIGHGFDVHRFAEGDFITLGGVRIAHGFGLLAHSDGDVLLHALSDALLGAAALGDIGKH
FPDTDPQFKGADSRVLLRHVLTLIHGKGWKVGNVDATIVAQAPKMAPHIDAMRALIAADL
QVELDQVNVKATTTEKLGFTGREEGIAVHAVALLLRA
>tr|A0A0Q0FSD8|A0A0Q0FSD8_PSEAP Uncharacterized protein OS=Pseudomonas syringae pv. aptata OX=83167 GN=ALO85_05033 PE=4 SV=1
MNAKDIIEKVDFKSYVSSNLCDPITRELFSKSKISDISFLLPPEVSAYDDQNKNTLKSII
EFRANNLAAQGRQLNGADLCAASLEGSRDEALRSWVPLEVENHLVIFIINSESFEIDGCM
SLI
>tr|A0A0Q0ILG0|A0A0Q0ILG0_PSEAP ISPsy9, transposase OrfB OS=Pseudomonas syringae pv. aptata OX=83167 GN=ALO85_02723 PE=4 SV=1
MNHKTVQKLMGQLGLKSLVRVKKYRSYKGEVGKAAPNILKRDFKAQHLNEKWATDVTEFK
VGGQKLYLSPIMDLYNGEIISYAIARRPLYSMVDEMLEGAFKKLEPHEKPILHSDQGWQY
RMPVYQRLLNEHSITCSMSRKGNCYDNAAMESFFGTLKSEFFYLNKFNNLDELHAGIDEY
IEYYNQSRIKLKLNGLSPVEFRMQAAQAA
>tr|A0A0Q0C8U7|A0A0Q0C8U7_PSEAP Putative S1-domain RNA-bindining protein OS=Pseudomonas syringae pv. aptata OX=83167 GN=ALO85_03191 PE=3 SV=1
MALIGRYNSLQIVKHTNFGLYLDGAQDGEILLPNRYIPKDVPTEDEDWLNVFVYLDSEDK

LLATTEKPKVQVGEFASLKVVEVNSIGVFLDWGLPKDLLLPYSEEKRTLQAGDYCVVHVY
LDKHTRRITATARLDRYLDKTPANYSVGQEVDLLVAEATDMGFKAIINNKHWGLIHKNEV
FKFMRAGKQEKGYIKEIRSDGNISLSLQPVGAEAASSLNSKILAKLRENDGTLPVSDKSD
PQVISGLFGVSKGNFKKAIGALYKQGQIVIHADRIELV
>tr|A0A0Q0BSP6|A0A0Q0BSP6_PSEAP Ribosomal RNA small subunit methyltransferase H OS=Pseudomonas syringae pv. aptata OX=83167 GN=rsmH PE=3 SV=1
MNSGFTHITVLLEEAVEALAVRADGCYLDGTFGRGGHSRLILSQLGPDGRLLGFDKDPQA
IATGQALAAEDGRFVIVQRSFAELGSETQERGLAGKVSGILLDLGVSSPQLDDPERGFSF
MNDGPLDMRMDPTRGVSAAEFIATAPAEEIARVFKEYGEERFAKRMANAVVARREVQPFE
RTADLAEVLKVANPAWEKGKNPATRAFQGLRIHVNNELGDLEAGLEAALDALEVGGRLVV
ISFHSLEDRIVKLFMRKLAKGEADNMPRNLPIQYKPFEPKIKIHGKAQFASDVETKANPR
SRSAVMRVAEKLR
>tr|A0A0Q0DXI3|A0A0Q0DXI3_PSEAP Pirin-like protein OS=Pseudomonas syringae pv. aptata OX=83167 GN=ALO85_03600 PE=3 SV=1
MNSIVSIQPRAIVHRTTGRTRGPITRLVSPGDLGQLLKPFVFLDRICFTSEPGKTGFGMH
PHSGIATLTYMIEGELAYEDTTGKSGVLLSGGVEWMSAGNGVWHDAQPVNGSAVRGFQLW
VALPAAQENGPAQSVYLSAAQVAQQGPANVLLGRYGSAKSSLPAPEGMNYLAVQLKDGEH
WRYTPPAGHSVGWLAVNTGQLDAGGPVGAGELVIFEESDRPIDIVAKGDTHFVLGSAVKH
PHDLVTGYYSVHTSKAALIQGELEIERIGALLREEGRL
>tr|A0A0Q0C2H6|A0A0Q0C2H6_PSEAP MAPEG superfamily putative membrane protein OS=Pseudomonas syringae pv. aptata OX=83167 GN=ALO85_04009 PE=4 SV=1
MSGYCPDASDEAEPYKDGRMTVAFWCVLVAILLPIICAGIAKFGSGKFGSGHNHDPRAFL
DKLEGFPRRAHAAQMNSFEVTPAFAAAVIIAHIAGNAQLVTIDVLAVLFITSRLLYIIFY
LADLAALRSLVWLVGMGLIIALFGVSAFPAVS
>tr|A0A0Q0DMI6|A0A0Q0DMI6_PSEAP Major facilitator transporter OS=Pseudomonas syringae pv. aptata OX=83167 GN=ALO85_03550 PE=4 SV=1
MTSIWRTSGWVLLGSALILALSLGTRHGFGLFLAPMSAEFGWGREVFAFAIALQNLMWGL
AQPFAGALADRFGAAKVVFVGGVLYAAGLLCMSTADSSLSLSLSAGLLIGIGLSGTSFSV
ILGVVGRALPAEKRSMGMGIASAAGSFGQFAMLPGTLGLISWLGWSGALLVLGVMVALIL
PLVGMLKDTPGVSTSVELTLGEALREACSHSGFWLLALGFFVCGFQVVFIGVHLPAYLVD
QHLPAKVGTTVLALIGLFNIFGTYTAGWLGGRMSKPRLLTVLYLLRAVVIVLFLWIPLSQ
TTAYLFGVAMGLLWLSTVPLTNGTVATLFGVRNLSMLGGIVFLFHQLGAFLGGWLGGLVY
DRTGSYDLIWQVSILLSLLAAALNWPVRERPVARLQAQGGLA
>tr|A0A0Q0DC92|A0A0Q0DC92_PSEAP Putative hemin ABC transporter, permease protein OS=Pseudomonas syringae pv. aptata OX=83167 GN=ALO85_01478 PE=3 SV=1
MTLAQWLTERPRRYLGLLIALTLLLLMSCLMCITFGAAPVPLSRVMAILMFKVSGTAEHP
PLWTAGQETIVWLIRTPRVLLGALAGAGLALIGCVLQAATRNPLADPHLLGATSGATLGA
VIVVMHVGEVFGMLTLPLAAFVGSLVSLCLVLWLASHQGRLDSQRLLLGGVAVSFVMMAI
ANLSLFLGDHRASSSVLFWMLGGLGLARWELLAVPFITVLCGWILLQGLGRSLNALMGGD
QTAVSLGLQVRNVRLLVFLIASLMTGVLVALCGSIGFVGLMVPHMARRLVGSEHRRLLPV
ATLLGALLMVWVDVISRTLIAPEDLPIGITTALLGGLFFIFMMKRGQ
>tr|A0A0Q0D2I4|A0A0Q0D2I4_PSEAP FlhB-like domain protein OS=Pseudomonas syringae pv. aptata OX=83167 GN=ALO85_03027 PE=4 SV=1
MSLPEHTPRQALALSYDGQHAPTLSAKGDDQLAEAILAIAREYEVPIYENAELVKLLARM
ELGDSIPEPLYRTIAEIIAFAWHLKGKFPAGQDPNAPPVERDITPRY
>tr|A0A0Q0E0W9|A0A0Q0E0W9_PSEAP Uncharacterized protein OS=Pseudomonas syringae pv. aptata OX=83167 GN=ALO85_01552 PE=4 SV=1
MKLPNRFITGLGILMISASPLLHAAPPDQRGNGPDDNRGVQQPGPQNDGGPNQRGNDRGP
GNDNGRGPDKGPASQPGRQAHQDNRGGNRPPQDFGPVRETFQQHRDVIGRGQPLPPGVHI
AKGRPLPPGYGKRLDSRSLHYLPQYDGYEWRRLGTDVVLIAVGSGIVYAILDGVLN
>tr|A0A0Q0BUE0|A0A0Q0BUE0_PSEAP 4-carboxymuconolactone decarboxylase OS=Pseudomonas syringae pv. aptata OX=83167 GN=ALO85_00538 PE=4 SV=1
MTEDERYEAGMQVRRAVLGDAHVDNSLSKLTPFNEEFQEMITRHAWGDIWTRPGLPRHTR
SLITIAMLIGMNREGELRLHLKAAKNNGVTREEIKEVLMQSAIYCGIPAANATFHLAEAV
WDEMGVESLQDN
>tr|A0A0Q0IKW6|A0A0Q0IKW6_PSEAP Uncharacterized protein OS=Pseudomonas syringae pv. aptata OX=83167 GN=ALO85_03487 PE=4 SV=1

MNPLNIIQDSLYFFRRNLGSIALLCLPVVILEVLAKQALGNAMSADTSPAYALVIGLFFY
PVYTAALILFLDTRSRGEDIHTRDVLAMAVRLWPTFAVLSAMSTLLIMFGLSLFVVPGIW
VMIKLAFSEYLLVLRKLTPFMAMRESMQMTTGHFTRILVCVLSVYIPLWLLEGASLYLFP
EPQSAAVSVITDSIGSFLQLFTTIVTFRLFMLISEPAHRA
>tr|A0A0Q0DP32|A0A0Q0DP32_PSEAP Peptide chain release factor 3
OS=Pseudomonas syringae pv. aptata OX=83167 GN=prfC PE=3 SV=1
MCALFQSHPRRFPMTQQAAEVAKRRTFAIISHPDAGKTTITEKLLLMGKAISVAGTVKSR
KSDRHATSDWMEMEKQRGISITTSVMQFPYRDHMINLLDTPGHEDFSEDTYRTLTAVDSA
LMVLDGGKGVEPRTIALMDVCRLRDTPIVSFINKLDRDIRDPIELLDEIEAVLKIKAAPI
TWPIGCYRDFKGVYHLADDYIIVYTAGHGHERTETKIIEKLDSDEARAHLGDEYERFVEQ
LELVQGACHEFNQQEFIDGQLTPVFFGTALGNFGVDHVLDAVVNWAPKPLARVANERTVE
PAEEKFSGFVFKIQANMDPKHRDRIAFMRICSGRYDKGMKMRHVRLGKDVRIGDALTFFS
SEREQLEEAYAGDIIGLHNHGTIQIGDTFTEGEALGFTGIPHFAPELFRRVRLKDPLKSK
QLRQGLQQLAEEGATQVFFPQRSNDIILGAVGVLQFDVVASRLKEEYKVECAYEPITVWS
ARWIDCDDKKKLEEFENKAVENLAVDGGGHLTYLAPTRVNLALMEERWPDVKFRATREHH
>tr|A0A0Q0DH24|A0A0Q0DH24_PSEAP Adenylosuccinate lyase OS=Pseudomonas
syringae pv. aptata OX=83167 GN=ALO85_00733 PE=3 SV=1
MQLSSLTAVSPVDGRYAGKTQALRPIFSEYGLIRFRVMVEVRWLQRLAAHPQITEVPAFS
AQANAILDTLAEDFSVEHAERVKEIERTTNHDVKAVEYLLKEQAAKLPELEKVSEFIHFA
CTSEDINNLSHALMLREGRDTVVLPLMRQIADAIRELAIRFADVPMLSRTHGQPASPTTL
GKELANVVYRLERQISQIAAVPLLGKINGAVGNYNAHLSAYADIDWEANARAFIEDELGL
GFNPYTTQIEPHDYIAELFDAIARFNTILIDFDRDIWGYISLGYFKQRTIAGEIGSSTMP
HKVNPIDFENSEGNLGIANALFQHLASKLPVSRWQRDLTDSTVLRNLGVGFAHSVIAYEA
SLKGISKLELNEQRIAADLDACWEVLAEPIQTVMRRYNIENPYEKLKELTRGKGIGPEAL
QTFIDGLDMPAEAKAELKKLTPANYIGNAAAQAKRI
>tr|A0A0Q0DQK3|A0A0Q0DQK3_PSEAP Uncharacterized protein OS=Pseudomonas
syringae pv. aptata OX=83167 GN=ALO85_01794 PE=4 SV=1
MPPREIAFHGLYMPTLTLIFLLAVVLAWALDRLLAGFDLYRYFWHPALLRLSLFICIFGA
LALPLYR
>tr|A0A0Q0DPZ0|A0A0Q0DPZ0_PSEAP TetR family transcriptional regulator
OS=Pseudomonas syringae pv. aptata OX=83167 GN=ALO85_00774 PE=4 SV=1
MTNQIKTSPTRGPSDHSVRDQVVEAATQHFGHYGYEKTTVSDLAKAIGFSKAYIYKFFDS
KQAIGEVICSNRLAMIMALVNSAISDAPTASERLRRLFRSLVEAGSDLFFHDRKLYDIAA
VAGRDQWPSAAAHDERIRQLIQQIVLEGRESGEFERKTPLDEAVQAIHLVMRPYINPVQL
QYNLDIVSTAPVHLSALILRSLAP
>tr|A0A0N8T9A2|A0A0N8T9A2_PSEAP Phosphatidylserine synthase
OS=Pseudomonas syringae pv. aptata OX=83167 GN=ALO85_02715 PE=4 SV=1
MQSTFRRSTLAALRGFALQSDAISIVPSAADYRRCLLEKIASATRRIYIIALYLQQDEAG
QEILDALYAAKSARPELDVVVLVDWFRAQRGLIGAGRQPGNSTWYQAQNLEYDEEVPIYG
VPVQTRELFGVLHLKGSVIDDCVIYSGASINNVYLHKLDKYRLDRYHLIENAALADAFQN
LVQREILSSPAVHRLDLQSPPSSRSLRSEIRAFRGDLKRATYDTSAGEKENGDMRVIPLL
GVGPRNNLNRAICDLIASSKIQLTICTPYFNLPVAVTREINRALKRGVQVDIIIGDKTAN
DFFIDPDEPFKVISALPYLYEISLRRFAHKHQKAIAQHRLNVHLWKHGDNTFHLKGIWVD
QRYTLLTGNNLNPRAFNLDLENALLIDDPKGQWSEPREAEIAHLMRNTQRVNQYDELDTL
ANYPEGVRKFLKRVSRVRVERLLYRIL
>tr|A0A0Q0BZV8|A0A0Q0BZV8_PSEAP Phosphoglycerate dehydrogenase family
protein OS=Pseudomonas syringae pv. aptata OX=83167 GN=ALO85_00380 PE=3
SV=1
MTRKILLTGPALAHDAMSYANDLGIQIFPSTPYLPAEELTSLIRDVQPDAIIVRQGKLTR
EMIEASPRLKAVAKHGVGYDTIDIQAAAEKGVPVMIALGANAQSVAEHAFALMFSVARQI
AWLDARTRQGHWDKATANGIELFGKTLGLVGLGAIGSVLMDLVAPLQMKVKVFDPYLKTL
PERSHVERETDFDRLIESSDIISLHCPLTDENRNLISTAQLNRMRSNCILINTARGELVD
TQALIQALQTNRIAGAGLDTFNPEPPAADSPLWQLPNLVATPHTGANTTESRDRVGLLAV
QQIVKVWDGDALDPRCIVNRQLLDS
>tr|A0A0Q0IIG2|A0A0Q0IIG2_PSEAP Bacteriophage Mu P OS=Pseudomonas
syringae pv. aptata OX=83167 GN=ALO85_00435 PE=4 SV=1
MIDPNVVTLTVDQHDYAGWKSVEISAGIERQARSFEVSITWQWPGTEISHPITPGAACEV
RIGGELILTGWVFAAPISYDGKQVTLKISGRSKTADLIDCSAINRPSQWKEVGVLKIVEA
LAAPYGLSVISEIPETSKMADHTIEPAETVFKSIDRLLTLFRIFSTDDEYGNVVLARPGS
RGQSADALELGKNVLSAVIARDFSGLFSEYRVIGQQTGNDQTFGKESSEVSAEVTDDRHD

DPAHKKRLRVLVVHEDAPITPKLALSRANWERGQRAGKALLTTYKVQGWRQSNGALWRHN
TMVRVIDPVIGFTSRNMLISAVTYSLSDQGTITTLVVGPPEGFQAEPGDPNKRSKVQVNQ
DAYSWLLPIDEETTS
>tr|A0A0Q0BTT8|A0A0Q0BTT8_PSEAP Type III secretion system protein HrcS OS=Pseudomonas syringae pv. aptata OX=83167 GN=ALO85_04189 PE=4 SV=1
MEALALFKQGMFLVVILTAPPLAVAVLVGVVTSLLQALMQIQDQTLPFGIKLGAVGLTLA
MTGRWIGVELIQFINMAFDLIARSGVSH
>tr|A0A0Q0FV50|A0A0Q0FV50_PSEAP GAF domain protein OS=Pseudomonas syringae pv. aptata OX=83167 GN=ALO85_03386 PE=4 SV=1
MGILEKCMSLELIASKSSLLTIEERAAIAEIEATTNVLRLVTRLTGMRFAGIAKFTDADW
IACSVYDPTDLGMDDGDVFELETTLCNEFCTNPQALFVPQISTNGRYANLPVVKRYAIES
YAGVPIYLPDGQLYGALCALDSRTTLFDDPDLAETLTLFARLIGCIFFANINEGS
>tr|A0A0Q0CD25|A0A0Q0CD25_PSEAP Undecaprenyl phosphate-alpha-4-amino-4-deoxy-L-arabinose arabinosyl transferase OS=Pseudomonas syringae pv. aptata OX=83167 GN=arnT PE=3 SV=1
MHVRHRHLALLLLAFVLAYLLPLGFHGLWIPDETRYAQISQEMLHSGNWIAPHFMGLRYF
EKPAAGYWLIALGQQVFGENLFGVRIVSALASGLSVLLAYLLAGKIWNDPRKQFASALLL
MSFGFVAGQAGYANLDPQFTFWTNLTLLAFWYAVHSIGRARLAAWAAVGVACAMGFMTKG
FLAWALPVIIALPYMLCQRRLAELLRFGPLAVLIAVTVCLPWALAIHQQEPDYWRYFFWH
EHIRRFAGDNAQHAQPWWFYIPLLVAACVPWALLLPVTLKQAWQEKSHPDIAFLLLWLLL
PLAFLSLSKGKLPTYILPCLLPLALLMANTLVERLDRGHSTALRANGIFNATVTFLGLVA
LIYLQLKQPVYENEPMHLSLAVIVLLGWTLANALQGLRPLTFWATPALGGWLLIALLPAA
LPNDVINNKTPDPFVVRYQAELADCTHLLSNDLGAASALAWRLKRPDVALFNTWGELEYG
LGYPDVQGREVRLQDIDAWMKNARSQGRVGVIMRGKSDEELKELESLPKDGQRYDEGNLA
ILVYEKSES
>tr|A0A0Q0FI83|A0A0Q0FI83_PSEAP AraC family transcriptional regulator OS=Pseudomonas syringae pv. aptata OX=83167 GN=ALO85_03159 PE=4 SV=1
MQDAGLATAPVLRRAQLPGDLFTRQNVRLDSANFFMLWRAIEAEAKSINAEVPAPLQIAR
VMSSDWFDPELFAALCSANLSGALDRIAKYVRLIAPMVIRVVPTKAHTTVTMDFLDAAEP
PAVFLAFKLVFFVQLARLATRSAVQPLQVNWPSPSTLGADLEAYQAYFGVAVTHAPQASV
VFAAADVGRPFLTENHKMWLFFEPGLRQRLSDLDRTSGMVERVRSALLESLPAGAVSMQE
ISRKLGVSTRTLQRRLQDEGTTFQQTLDTLRESLARHYLRSTAMSSAEISFLLGFEDANS
FARAFQSWTGSTPRKVRAEMDNSRHPSTAHSEE
>tr|A0A0Q0CC51|A0A0Q0CC51_PSEAP Helix-turn-helix motif protein OS=Pseudomonas syringae pv. aptata OX=83167 GN=ALO85_02503 PE=4 SV=1
MDLQVISRDGEPEYAVLPWAQYQALLKAAGLAGKPSSEPAASTSGTAASELPGFDQLRAL
REAKGLEAASLARTVGISPSYLELIENGTREPDGAIKRSLAWELGVPGWRGES
>tr|A0A0Q0FR05|A0A0Q0FR05_PSEAP RNA-binding protein Hfq OS=Pseudomonas syringae pv. aptata OX=83167 GN=hfq PE=3 SV=1
MSKGHSLQDPYLNTLRKEKVGVSIYLVNGIKLQGTIESFDQFVILLKNTVSQMVYKHAIS
TVVPVRPIRLPSATDADGADAEPGNA
>tr|A0A0Q0D5V0|A0A0Q0D5V0_PSEAP Sulfate transporter/antisigma-factor antagonist STAS:sulfate transporter OS=Pseudomonas syringae pv. aptata OX=83167 GN=ALO85_02607 PE=4 SV=1
MKTIRIRAEVLAGLTTSFALVPECIAFALVAHLNPLMGLYGAFIICTLTALFGGRPGMVS
GAAGSMAVVIVALVVQQGVQYLLATVLLGGLIMILFGLLRLGKLVRLVPYPVMLGFVNGL
AIVIAMAQLEHFKVGESWLSGTPLYVMLGLVALTMAIVYLLPRLTRIVPPALVAILSVGL
AVYLLGLPTRTLGDMAHIAGGLPVFAMPDIPWNLETLRIIAPYAILMALVGLLETLLTLN
LTDEITETRGYPDRECVALGAANVVSGAFGGMGGCAMIGQTVINLSSGGRGRLSGVVAGL
MILMFVLFLSPLIERIPLAALVGVMFVVAQQTFAWASLRVLRKVPVNDVLAIIAVTVVTV
LTDLATAVLFGIIIAAINFAWQQARELYADSHVEADGSKVYHLHGTLFFASTTPFLDQFN
PAEDPAQVTLDCRHLSFVDYSAIAALKTLRERYAKADKHLRVVHLSERCKRLLKRAGVQH
A
>tr|A0A0N8TA04|A0A0N8TA04_PSEAP NAD kinase OS=Pseudomonas syringae pv. aptata OX=83167 GN=nadK PE=3 SV=1
MEQFRNIGIIGRLGSVQVLETVRRLKRFLLDRHLHVILEETIAEVLPGHGLQTSSRKMLG
EVCDMVIVVGGDGSLLGAARALARHNVPVLGINRGSLGFLTDIRPDELEVKCAEVLDGHY
LVENRFLLQAEVRRHGEAIGQGDALNDVVLHPGKSTRMIEFEIYIDGQFVCSQKADGLIV
ATPTGSTAYALSAGGPIMHPKLDAIVIVPMYPHTLSGRPIVVDGNSELKIVVSKDMTIYP
QVSCDGQNHFTCAPGDTITVSKKPQKLRLIHPLDHNYYEVCRTKLGWGSKLGGGGD

>tr|A0A0Q0BR09|A0A0Q0BR09_PSEAP Uncharacterized protein OS=Pseudomonas syringae pv. aptata OX=83167 GN=ALO85_03989 PE=4 SV=1
MSDSLQPFSKPPQSARQWAMFCHLSAFAGLMFPFGNLLGPLIIWQLKKDSDPFIDAQGKE
ALNFQITVAIAATVSMLLVLLIIGFALLTLVGIGAAVLAIIAGIKANDGVDYRYPFTLRL
IK
>tr|A0A0Q0IP17|A0A0Q0IP17_PSEAP Uncharacterized protein OS=Pseudomonas syringae pv. aptata OX=83167 GN=ALO85_101887 PE=4 SV=1
MSAYLHLSYLGFRAASCRPQTVMCSKRQAASRKSLVVICFSLEA
>tr|A0A0Q0IRL7|A0A0Q0IRL7_PSEAP Integrase OS=Pseudomonas syringae pv. aptata OX=83167 GN=ALO85_200094 PE=3 SV=1
MNDLIDRAAGELSGKRERRLTAAEFQQLAHVPAAAEWFANIDNPRTRRAYQNDLQDFCSF
VGLAGAEEFRAVTRSHVLAWRADLEKRGLAGATIRRKLAALASLFDHLLENNAVAGGNPV
HGVKRPRIESNEGKTPALGDLQAKQLLDAPDPETLKGLRDRAILAVLLYHGLRREEAAQL
KTGDLQERRGIKHLRVHGKGSKIRFLPLHPVAAERIYAYLEQDVERDAAPGPLFRSMRGT
TTGAGVTANGLYTIVAQWASVAGIQVERLGVHGLRATAATNALEHDADIAKVQMWLGHAN
ISTTRLYDRRGHRPEDSPTFKVKY
>tr|A0A0Q0BWQ0|A0A0Q0BWQ0_PSEAP Peptidase M23B OS=Pseudomonas syringae pv. aptata OX=83167 GN=ALO85_00412 PE=4 SV=1
MKDTPPKAPPLYPKSHLLAASGIAALLSLALLVFPSSEVEAKRTNIPLDLEAPDQDVQDQ
DAQQATQATPETTESPFAQIDNADDQAQDTASAQPQAEKPAPAAAKDPAHREVTVSKGDT
LSTLFEKVGLPAATVHEVLASDKQAKQFSKLQNGQTLQFELTPDGQLKQLHTKLSEVESI
SLSKTATGGYAFNREISKPNVRNAYVHGVINSSLSQSAQRAGLTHSMTMDMANIFGYDID
FAQDIRKGDEFDVVYEQKVVNGKSVGAGNILSARFTNRGKTYTAVRYTNKQGNTNYYTAD
GNSMRKAFIRTPVDFARISSMFSMGRKHPILNKIRAHNGVDYAAPRGTPIKATGDGKVLL
AGRRGGYGNTVIIQHGDTYRTLYGHMQGFAKGIQTGGTVKQGQVIGYIGTTGLSTGPHLH
YEFQVNGVHVDPLGQKLPMADPIAKSEKQRFMQQSQPLMARMDQEKATTLASNKR
>tr|A0A0N8T806|A0A0N8T806_PSEAP Uncharacterized protein OS=Pseudomonas syringae pv. aptata OX=83167 GN=ALO85_00526 PE=4 SV=1
MSIQETVMTNKQRLIYSILIALGVLAIMLGLSHLQNSGAITEKTFQYIAISVAVLVVILN
GIMRRKVKR
>tr|A0A0Q0FEU1|A0A0Q0FEU1_PSEAP Putative iron-regulated membrane peptidase OS=Pseudomonas syringae pv. aptata OX=83167 GN=ALO85_01285 PE=4 SV=1
MSKKSRSKIWFLVHSWLALPIWFFVLIVCVTGTLAVVSKEIMWLANPEMRDNRPSDDAQR
LNFEQIRATIERNEPDLIVGTIMQPDGEYFALSVFITYPDGRSVPAYVNPYTGVIQGITP
SFDFRRFTRALHGWWLVPFTNGYSWGWYLVSILGLPLLASLVTGLVVYKKFWRGFFKPVR
FSQGPRIFWGDLHRLSGVWSIWFIAVISITGTWFLIQAILGDNQISISSKSQVASIIPHS
AVPLTADGSPAPTISLERAVEIARERIPGLEASFITPPFNAYSNMQVGGRSWYPLMFQTA
EINPYSGEIAASHLLSDRNKLEFVTESMRPLHTGDFGGIWIKLIWAFFGLLLSMMVLSGL
LIWSKRTAIATINALKRDARARPVAYTPEINTSPLATRTPENSL
>tr|A0A0Q0C7Q5|A0A0Q0C7Q5_PSEAP Regulatory protein, LysR:LysR, substrate-binding OS=Pseudomonas syringae pv. aptata OX=83167 GN=ALO85_01304 PE=3 SV=1
MLANMFSKIPLTSLRSFESAARLSSFKAAAIELFVTPSAISHQVKSLEHWLGLLLFERVA
KGVTLTTEGLDLYKNIHQHFSSINQSLSQLRSQTDENAITVTTTHAFASLWLIPRLGDFY
RRHPTMQVNIITSKTQADLHRDASIDVAISSVFEEVPDTYQLHLMDESFQAYTPYRTPLS
GETRSPLINVRWEADGHNPFDWTTWCQAAGHDDWLANATFMEYDDEHHALQAATSGYGMV
LASDVLAVDSVTRGFLTPYKPEVKLTGTHYVSACVPGRERNPPVRDFLDWLSKEASKHLV
LQRLLTESIDFSAHKNPLRNDRNQSMLKLQIY
>tr|A0A0Q0DQL3|A0A0Q0DQL3_PSEAP Protein product OS=Pseudomonas syringae pv. aptata OX=83167 GN=ALO85_04955 PE=4 SV=1
MKFNDNKLKWARSASGIALALLAFSNLALAGNYQSSVALPVIHSKGYVHTAELPVSRHAP
VTATIKSVSWNWNVVGWPQGLEVYLCQGVSVCLNVSRQRTGSTTFFGNQSASRKFYYTMK
VGDAGRVPVAGQQGRITVDW
>tr|A0A0Q0CCA9|A0A0Q0CCA9_PSEAP Two component DNA-binding heavy metal response regulator OS=Pseudomonas syringae pv. aptata OX=83167 GN=ALO85_04011 PE=4 SV=1
MRILVVEDEPKTAEYMHQGLTESGYIVDIAATGLDGLYLAQHQAYDVVILDVNLPEMDGW
EVLSRLRKTVSTRIMMVTARGRLEEKVKGLEMGADDYLVKPFEFPELLARVRTLMRRSEQ
TSTSKVLQVGDLELDQGRHRAFRGSQRIDLTTKEFALLHLLMRHSGEVMSRTQIISLVWD

MNFDCDTNVVEVSIRRLRAKIDDPFETKLIHTLRGVGYVLEVRE
>tr|A0A0N8T971|A0A0N8T971_PSEAP Malonate decarboxylase subunit beta OS=Pseudomonas syringae pv. aptata OX=83167 GN=ALO85_02964 PE=4 SV=1
MTDNARLLNQHSFIELGARARARALLDAGSFRELLGPFDRVMSPWLAMQGVVPQADDGVV
VAKGTVDGRPVVIAAIEGSFQGGSMGEVGGAKMAGALELAAEDNRNGIPTAAIVLLETGG
VRLQEANLGLAAIAEIHAAIVDLRQYQPVIGVVAGSVGCFGGMSIAAGLCSYLLVTQEAR
LGLNGPQVIEQEAGIEEYDSRDRPFIWSLTGGEQRFASALVDGFAADDVADVRQQVSGWL
KQGVPDTHRSSQYPLFLQRLASLDTEPQIDPQSVRTLYQGARS
>tr|A0A0Q0FT71|A0A0Q0FT71_PSEAP Peptidylprolyl isomerase OS=Pseudomonas syringae pv. aptata OX=83167 GN=ALO85_03720 PE=4 SV=1
MKAQARHILVKTAEEAEQLKLRIAKGEAFDVLAKKHSSCPSGKRGGDLGEVRPGQMVGAI
DQVIFKKPLRVVHGPIKSKFGYHLVQVFYRD
>tr|A0A0Q0CAB3|A0A0Q0CAB3_PSEAP Ribosomal RNA small subunit methyltransferase G OS=Pseudomonas syringae pv. aptata OX=83167 GN=rsmG PE=3 SV=1
MSSMVTPQHAQELSTGARELGIDLSPAQHEQLLAYLALLIKWNKAYNLTAVRNPDEMVSR
HLLDSLSVVPFIEGTRWIDVGSGGGMPGIPMAILFPERKVALLDSNGKKTRFQTQVKLEL
KLDNLEVIHSRAESYQPEVPFDGIISRAFSSLEDFTGWTRHMGDVNTRWLAMKGLHPDDE
LVALPSDFHLDSAHALTVPGCQGQRHLLILRRTA
>tr|A0A0Q0DE74|A0A0Q0DE74_PSEAP Uncharacterized protein OS=Pseudomonas syringae pv. aptata OX=83167 GN=ALO85_101788 PE=4 SV=1
MCAARGCRTSRWPRNKADRTASGSPACSYRNRPAVGADVKVAMRVIETHGVLLRYSLAAT
VVGPVTNENGWRLQIIRRMHAAGTGTKKTPF
>tr|A0A0Q0IK88|A0A0Q0IK88_PSEAP 50S ribosomal protein L21 OS=Pseudomonas syringae pv. aptata OX=83167 GN=rplU PE=3 SV=1
MYAVIVTGGKQYKVAPGEYLKIEKLEIATGESVTFDRVLLVGNGDDVNIGAPVVAGATVV
AEVVSQGRHDKVRIIKFRRRKHHMKRMGHRQWYTEIKITGIQA
>tr|A0A0Q0IJH3|A0A0Q0IJH3_PSEAP Probable M18 family aminopeptidase 2 OS=Pseudomonas syringae pv. aptata OX=83167 GN=apeB PE=3 SV=1
MCIAMSATQGSTSSNTVNSRHCCLELFMRAELNKGLIDFLKASPTPFHATATLVQHFEAA
GFQRLDERDMWAVETGGRYFVTRNDSSIVAFRMGRQSPLTGGIRMVGAHTDSPCLRVKPQ
PELQRQGFWQLGVEVYGGALLAPWFDRDLSLAGRVTFRRDGKVESQLIDFKLPIAVIPNL
AIHLNRTANEGWAINAQNELPPILAQVAGDERADFRALLTDQLAREHGLNADVVLDYELS
FYDTQGAAVVGLNGDFLAGARLDNLLSCFAGMQALLNTESDETALLVCTDHEEVGSSSAC
GADGAMLEQIVQRLLPSSEDYVRTIQKSLLISADNAHGIHPNYADKHDANHGPKLNAGPV
IKVNSNQRYATNSETAGFFRHLCMAEEVPVQSFVVRSDMGCGSTIGPITASHLGIRTVDI
GLPTFAMHSIRELAGSHDLAHLVKVLSAFYASHELP
>tr|A0A0Q0DUK6|A0A0Q0DUK6_PSEAP Uncharacterized protein OS=Pseudomonas syringae pv. aptata OX=83167 GN=ALO85_04865 PE=4 SV=1
MARHYDDLPTSTVKTRRQQEDQRRMEFRRAIESYSEARQLNQELCDYMDGMDNAVWQTIK
LPAVDRRNARQAG
>tr|A0A0Q0BWY7|A0A0Q0BWY7_PSEAP Glyoxalase OS=Pseudomonas syringae pv. aptata OX=83167 GN=ALO85_01629 PE=3 SV=1
MIEHKEPKSMQKKVAVILSGCGVYDGAEIHESVLTLLRLDQRGAQVQCFAPNIAQHHVIN
HLNGEEMSESRNVLVESARIARGEVKDIREANAEDFDALIIPGGFGSTKNLSDLALQGHA
CQVEAGLLKLAEAFAEAGKPVGLICITPALAAKIYGPGVTCTIGSDPETAAAITKMGGSH
AECAVDDIVEDEARKLVSTPAYMVAKSISEAASGINKLVDRVLELTHEGDA
>tr|A0A0Q0D1W9|A0A0Q0D1W9_PSEAP Dipeptide ABC-type transport system, ATP-binding protein OS=Pseudomonas syringae pv. aptata OX=83167 GN=ALO85_00013 PE=3 SV=1
MSLLEIKNLNVRFGDTKAVPVVDGLSLSVEKGEVLAIVGESGSGKSVTMMALMGLIDSPG
IITADALNFDGNDLLKLNSRQRRRIIGKDLAMVFQDPMTALNPSYTVGFQIEEVLRQHLN
MSGKAARQRALELLEKVEIPGAAARLNAYPHQLSGGMSQRVAIAMAIAGEPKLLIADEPT
TALDVTIQAQIMELLLSLQKEQDMALVLITHDLAVVAETAQRVCVMYAGQAVEVGQVPGL
FDVPAHPYSEALLAAIPEHSMGAERLATLPGMVPGRYDRPQGCLLSPRCPYVRDNCRTER
PALDPKAHSLARCFYPLNQEVA
>tr|A0A0Q0C8D8|A0A0Q0C8D8_PSEAP ParA OS=Pseudomonas syringae pv. aptata OX=83167 GN=ALO85_200141 PE=4 SV=1
MGTIVVVGGNKGGVGKTTTAVNVSTGLAMEGYDVCLVDADAQRSASKWYAEREAAQLEPK
ITLVEKRDNITVTLRTLSEKFDYVIVDVAGRNSRELITGAVVADIIIAPNQCSQLDLDTL

GELQEQVLRCRDLNPNLQAYAYQSMASPNPAVRITERREFLEFLSEYPEIKPLDSVGFYR
KAYKDSIPSGMSVLEGKNREAANEIKSLLKEVFHGS
>tr|A0A0Q0FCM2|A0A0Q0FCM2_PSEAP Uncharacterized protein OS=Pseudomonas syringae pv. aptata OX=83167 GN=ALO85_01858 PE=4 SV=1
MTISIILLIIQIFMMTAWGEGNLRKTPEGQKVLATLSSLGLFFLRWRANADYKKEMAFLG
KAMGWLAIASVVAFFILPASAKPVVSVYCVTFMGLWLSIRVGMDIESQLMDMLGWAALFF
CIPFIFLACDHFHVLPGSLLRATAAPLSLMLGHELLDYQLALAMAIIGGTGGLFMGLVNI
VVFSVFPLLLLFFIVVVSKASKFLLGRSVNAGRNFVALYVLVVGPTLLVLHSLQVF
>tr|A0A0Q0DP49|A0A0Q0DP49_PSEAP LuxR family regulatory protein OS=Pseudomonas syringae pv. aptata OX=83167 GN=ALO85_00046 PE=4 SV=1
MNTMDMNFSQPSPAKNLTQREIEVLKWSAEGKTAGDIAMILCLKERTIHFHIASAIQKMG
VCNKTAAAVQAALSGMF
>tr|A0A0Q0C6H7|A0A0Q0C6H7_PSEAP Ectoine/hydroxyectoine ABC-type transport system OS=Pseudomonas syringae pv. aptata OX=83167 GN=ALO85_03679 PE=3 SV=1
MEFDIAFAWSILPELFQGLLVTVQVVVLGFLLAVLLGLILALTLRSQVTIVHRLIKGYLS
FFRNTPLMVQLYVLFFALPLAGITFPAITTGVIGLGCYYAAYVAEAYRGAIDDIPAGQWE
AARALDFNRGDTWRRIILPQALKPMLPVLGNYLIGMFKETPLLAVITIPELFQAAKQIAG
MTYRYNEPYTVMALMFLAISVPTSLLFKYLERRSHV
>tr|A0A0Q0DQ18|A0A0Q0DQ18_PSEAP Histidine kinase OS=Pseudomonas syringae pv. aptata OX=83167 GN=ALO85_00770 PE=4 SV=1
MFRIDIMFSKLSISQKLYFSFAAIIVFIAVLVASAYRGFEQVEEATNSNVHTYQVLSEAQ
LALEQLINIETGMRGFVIASKDAFLEPLVAGQKRFTEELDSLKRLTADNAEQQRRLAALG
DTQKRWLDEDVNPIIALRRDLTARNMPDDELDARITSGADKAKMDSMRALLAEISGAEQK
LLVQRSQDMTDAKHLAILILMCGGLAAAVLAMILAAVLGRSTTARLQLAIDAATAIANGK
LDTVIDTSSHDELPKAFDRMQNRLREMIQQISHAANQLVVAVQQISGASEQLSGAIQEQS
TSASAMAATIEELTVSIHHVSENADEAHELASRSGQQSKDGAQVIENTLSSMNGIARTVQ
LSSTQVAGLGQHSEHISSIVSVIQGIADQTNLLALNAAIEAARAGEQGRGFAVVADEVRL
LAQNTGKSTKEIAGMIEKIQAGVRETVESMRSGVQEVNEGVEMAGTAGQAIIEIRDSSGK
VLQVVDQISFALREQTAASQDVARNVERSAQMAEQNNMSVQELLKTSGGLKTLATSLQTE
VGKFRL
>tr|A0A0Q0CC96|A0A0Q0CC96_PSEAP Uncharacterized protein OS=Pseudomonas syringae pv. aptata OX=83167 GN=ALO85_02457 PE=4 SV=1
MRLVYCALACLTLSGLAFSVQARNATENERYLCRWGSAIAGGAQASKLSGVTRYGARQKL
QARKFAKQWMRPMALRITEQTYDSESRLKPDAVTKVYYDGCIQHEVARR
>tr|A0A0Q0CF24|A0A0Q0CF24_PSEAP 3-hydroxyisobutyrate dehydrogenase OS=Pseudomonas syringae pv. aptata OX=83167 GN=ALO85_02947 PE=4 SV=1
MTGKNVGVIGLGAMGLGIARSLLRSGFTVHACDVRTSVTEAFAQEGGVACDSPARMAAAC
DVIITVVVNAEQTETVLFGENGAIAALRPGCLVIGCATVAPTFAVELGERLAAQNLLYLD
APISGGAAKAAAGQMTMMTSGPAESYAKAEAILNGMAGKVYRLGDVHGLGSKVKIINQLL
AGVHIAASAEAMALGLREGVDADALYEVITNSAGNSWMFENRVPHILNADYTPLSAVDIF
VKDLGLVLDTARSSKFPLPLSATAHQMFMQASSAGFGREDDSAVIKIFPGIELPKAKPEK
A
>tr|A0A0Q0C2A7|A0A0Q0C2A7_PSEAP Tail-specific protease OS=Pseudomonas syringae pv. aptata OX=83167 GN=ALO85_02505 PE=3 SV=1
MRYQLSSRRISMKQLFPSTALALFVGLSVLPMSASTFAANSWDNLQPDRDEVIASLNVVE
LLKRHHYSKPPLDDKRSAIIYQSYIKQLDPSRSYFMASDIADFDKWQFQFDDFLKSGDLN
AGFIIYKRYLDRVNARLNFALGELGKGVDKLDFNTKETLLVDRKDAAWPKDTAELDELWR
KRIKDEVLRLKIAGKDPAKIQETLTKRYKNQQARLNQTRAEDIFQAYINTFAMSYDPHTN
YLSPDSAENFDINMSLSLEGIGAVLQSDNDNVKIVRLVPAGPAAKTKQVAPADKIVAVAQ
GDKEMVDVIGWRLDEVVKLIRGPKGSVVRLEIIPASNAPNDQTSKIVAITREAVKLEEQA
AKKSILHIKQDGKDYKLGVIDIPAFYLDFKAYRAGDPEYKSTTRDVKKLLTELQAEKVDG
VVLDLRNNGGGSLQEATELTSLFIDKGPTVLVRNADGKVDVLEDEAKGAFYKGPMALLVN
RLSASASEIFAGAMQDYHRALVIGGQTFGKGTVQTIQPLNHGELKLTLAKFYRVSGQSTQ
HQGVVPDITYPSLIDTKEIGESALPEAMPWDSIKPAIKPAIDPFKPFLAQLQARHEARSA
KDAEFVFIEDRLALAKKLMNEKTVSLNEADRRAEHASIESKQLALENTRRKAKGEEPLKE
LKKEDEDALPVEDEKTKPEDDAYLAETGRILIDYLGLSAAVAKK
>tr|A0A0N8T8B4|A0A0N8T8B4_PSEAP YcgL domain-containing protein ALO85_01058 OS=Pseudomonas syringae pv. aptata OX=83167 GN=ALO85_01058 PE=3 SV=1

MKRICSIYRSPKRNEMYLYVLKSDVLKRVPPELMVAFGKPVHAFDLVLSPERALSREDIN
VVLENLDSQGYHLQMPPAEDDYIEHLPEELLRRNDPM
>tr|A0A0N8T8K6|A0A0N8T8K6_PSEAP Major facilitator superfamily transporter OS=Pseudomonas syringae pv. aptata OX=83167 GN=ALO85_05332 PE=4 SV=1
MPSLETTASGARSESSTGAGLSIAILALAGFVIVTTEFLIIGLLPSMARDLGISISTAGL
LVTLFAFTVMLFGPPLTAMLSHLDRKRTFIVILLIFASSNALAAVSSNIWVLALARFIPA
LALPVFWGTASETASLLAGPKHAGKAVAQVYLGISAAMLFGIPLGTVFADAVGWRGAFWA
LTLLSALMALLLALSMPRIEPTAKVGLAEQAKILRDPFFLANLLLSILLFTAMFGAYTYL
ADTLERIAGIESAQVGWWLMGFGAVGLIGNALGGRYVDRSPLGSTMAFALLLALGMTASV
PAAGSMPLLAVVLVIWGIAHTALFPICQIRVMKAAPQAQALAGTLNVSAANAGIGLGSII
GGLTIEHLGLGAVGYVAAVVALLSIAVAWMTSTQQNKATL
>tr|A0A0Q0FF78|A0A0Q0FF78_PSEAP Uncharacterized protein OS=Pseudomonas syringae pv. aptata OX=83167 GN=ALO85_102122 PE=4 SV=1
MTPEKAPSANPSGPKVEPMAAPNWPPAKAALAPRATPPTAPAVAPIFIAVLSDAILVEWH
AGHCDDIGKLLVGFVKAMM
>tr|A0A0Q0D620|A0A0Q0D620_PSEAP Uncharacterized protein OS=Pseudomonas syringae pv. aptata OX=83167 GN=ALO85_02701 PE=4 SV=1
MEQTTTTINDLFAQLALDSDDDSIEKFIASHPLPDDVKLIDAEFWTPRQSDFLKEQLHED
AEWAMVVDELNVRLHKKPE
>tr|A0A0Q0DJ25|A0A0Q0DJ25_PSEAP Uncharacterized protein OS=Pseudomonas syringae pv. aptata OX=83167 GN=ALO85_05603 PE=4 SV=1
MNPEKLKLLVTQEMPFGKYKGRLIADLPGHYLNWFAREGFPKGELGGLLALMQEIDHNGL
GALLDPLRTRPRQSFKDKG
>tr|A0A0N8T929|A0A0N8T929_PSEAP CHAD domain protein OS=Pseudomonas syringae pv. aptata OX=83167 GN=ALO85_02451 PE=4 SV=1
MSSMVDHLVAEVLALDVKLLACQARLAVSTDSEALHDLRTTVRRLRSVLRPLRENPSAAE
LEDAAKAVGQLTTPLRDMQVLAGFLEEQGLNEAAFTRNRYLESACPKVASSPELTRLLKL
IDRFPELLRLQQRQGMLRGLRKTIEKRMDKQWHKLRVAIAEPGHDRHDLRLLIKRVRYAA
EAYPLLSHQPKNMQARLKSAQGELGDWHDHLQWLAQAAEQPDLAPCVPGWQIGIVRAERK
AEASLKRLAKACF
>tr|A0A0Q0DTA2|A0A0Q0DTA2_PSEAP Zinc-containing alcohol dehydrogenase super protein OS=Pseudomonas syringae pv. aptata OX=83167 GN=ALO85_100189 PE=4 SV=1
MQLFFSALNLFHHNKSEVTCMSGNRGVVYLGAGKVEVQSIPFPKMEDPRGKRIEHGVILR
VVSTNICGSDQHMVRGRTTAQTGLVLGHEITGEVLEAGRDVEHLKVGDLVSVPFNVACGR
CRSCKEQNTGVCLTVNPARPGGAYGYVDMGDWVGGQAEYVLVPYADFNLLKLPDRDAAME
KIRDLTCLSDILPTGYHGAVTAGVGPGSTVYVAGAGPVGLAAAASARLLGAAVVIVGDVN
PTRLVHAKAQGFEIADLSQDTPLHEQIAALLGEPEVDCAIDAVGFEARGHGHAGAQAEAP
ATVLNSLMGVVRVAGKIGIPGLYVTEDPGAVDAAAKMGSLSIRFGLGWAKSHSFHTGQTP
VMKYNRQLMQAIMWDRIKIADIVGVEVISLDDAPRGYGEFDSGVPKKFVIDPHGLFSRA
>tr|A0A0Q0CFS3|A0A0Q0CFS3_PSEAP Putative Methyltransferase OS=Pseudomonas syringae pv. aptata OX=83167 GN=ALO85_03644 PE=4 SV=1
MSVEDSYFNELFLNNEDPWAFKERWYERRKRALTLAALPRERYRSIFEPGCANGELSADL
AGRCDSLVCCDTSNLAVDLARKRLAGLPHAKVLQARLPQQWPQGEFDLIVFSEMGYYLDA
DDLHGLIDRALAALAPDGQLLACHWRPDIEGCPLNAQAVHDILAARLSMHRLFSHHEQDF
LLDLWSRDGTSVAEQEFSDDRHTDPGAQ
>tr|A0A0Q0BVQ2|A0A0Q0BVQ2_PSEAP Formate/nitrite transporter family protein OS=Pseudomonas syringae pv. aptata OX=83167 GN=ALO85_00669 PE=4 SV=1
MDHEVDGKTPGLSAEEEREIDENQPPRAAVLHETIRMQGDHELERSVAALWWSALAAGLT
MGLSLMAMGLLNSRLEGIPGSHVISSLGYSAGFLAVILARQQLFTENTITAVLPVMSKLN
LANIGRLLRLWGVVLTGNLVGTLTVAYVMLNLPIFDTSTDKAFLEIGHKVMENDMAQMFS
KGIVSGWMIATMVWMIASMENAKIAIIVLITYLMALGDFTHIVVGSAEVSYLVFAGELGW
KDFWFAFAGPTLAGNIIGGSFIFALISHAQIRSEKDTTEKMERDRKNKEEKLRLEKAQKL
KEADFTAKREI
>tr|A0A0Q0C6I7|A0A0Q0C6I7_PSEAP Uncharacterized protein OS=Pseudomonas syringae pv. aptata OX=83167 GN=ALO85_101277 PE=4 SV=1
MGPFRGFVTPRSSTHLPFSPGTQVLEPPRNFYPQQRL
>tr|A0A0Q0CHE9|A0A0Q0CHE9_PSEAP Uncharacterized protein OS=Pseudomonas syringae pv. aptata OX=83167 GN=ALO85_03346 PE=4 SV=1

MQIEQLQDMQAYIRRTADDLELVSANLAGHLLYLERTSRAHEAQEVSERIIGLQASVDSL
RGIFR
>tr|A0A0Q0FEH7|A0A0Q0FEH7_PSEAP OHCU_decarbox domain-containing protein OS=Pseudomonas syringae pv. aptata OX=83167 GN=ALO85_01159 PE=4 SV=1
MSTFKTLTPSSLGRDAFIAAFADIYEHSPWVAQQAFDQSTGAQLDQVETLHARMSEILLG
ATHEQQLALINAHPDLAGKAAVQGELTQASTDEQAGAGIHHCTPEEFQRFTELNEAYKAR
FGFPFIMAVKGSDRHKILAAFEQRIHHSPEVEFACALAEINKIALFRLQAL
>tr|A0A0Q0IA09|A0A0Q0IA09_PSEAP Phage integrase family site specific recombinase OS=Pseudomonas syringae pv. aptata OX=83167 GN=ALO85_100627 PE=4 SV=1
MVKGALLTMSYVPFDVDHYERQEELSDLERTILSNRRYRSDWAYLQSSVPRLVIPLIDLV
AHAGVSDRLAVSSVSVILWHVSRTDIPYWSWSEMQWLALLDTQAGSRPYLAAVAYHMGGF
RTPKRITKFRQSAIYASFIFGHKIFKDELTRLSTVLKSLGYTARHLEKFLSGVLAALMLE
NGDPRLETFTEVLLIKGQGHRSVGIARLVGKVSHGLAALGILDKPLRKRGYADWREKSIE
GIDPVWVSWCRRWRDTSTLRPRTRESNYSFMLRTGIWLTREQPWVSSPVDWNTSTCAAVI
AAIDRMTVGEWALESALGTKLKGLGQPIAPNSKRAFLHALRRFFIDFELWGWGRLKFSPR
HHLATPRTVAFNSGINPRVIDDSSWLKLIWASLNLERKDLLSETHYPLAMVQAASVVWTH
TGLRSNEIMRLSVGCAHAQPHEVVHEDGTTIPPGTLCYLDIPASKTFKAFVKPVAVVVKE
RIDVWLQERPVNQAPLVDERTGEKVSYLFQFRGKRMGAGVINRTIIPMLCAKAGVPLDDS
RGRITSHRGRASVVTALASVPQGMSLMELMQWSGHSSPSSTLHYIRIRPTKLAASFVRAD
QMSHMVSVLIDHDVIARHSSDPYTFYDLGDSYCSNPFWSSCPHRMACAGCDFNVPKASAR
AQALESKASIGHYLEAVPLTADERAIVEGDLAKLDGLIRKLDDVPTLDGRTPSQIEAKKN
R
>tr|A0A0Q0CBF8|A0A0Q0CBF8_PSEAP LysR family transcriptional regulator OS=Pseudomonas syringae pv. aptata OX=83167 GN=ALO85_01791 PE=3 SV=1
MDTLQNMRAFSCVAQAGSFTAAAAVLDTTTANVSRAVSNLEAHLQTRLLNRTTRRIALTE
AGKRYLLRCEQILASVEEAEAEASDAHARPAGQLKVHSMPGVGQHYVIDAIARYRRNHPD
VAFDLTMTHRVPDLLEEGYDVSIVLASELADSGFVSQRLGITYSIVCASPEYVKTFGMAH
KPADLLNHACLRLVSPVFPLDKWLFDGPEGQEMVTINSSPLLVNSADAMKTAISSGMGIG
ILPIYSAIEGLRNGTLVRVLSDYRSQELNLYAIYPSRQYLDAKIRTWVEYLRGSLPEILA
ADEADLHVQALKSSF
>tr|A0A0Q0CWF1|A0A0Q0CWF1_PSEAP Uncharacterized protein OS=Pseudomonas syringae pv. aptata OX=83167 GN=ALO85_101678 PE=4 SV=1
MFCVAEKRVAHGRSGLYLRRILPFDFRQREHLQAIAHQGAAYFLVNASGSH
>tr|A0A0Q0C1V9|A0A0Q0C1V9_PSEAP Phosphonate metabolism protein PhnH OS=Pseudomonas syringae pv. aptata OX=83167 GN=ALO85_00817 PE=4 SV=1
MNAVLLQPAFTDPVLDAQRSFRVALKALSGPGVIQTLPCAHQPPALQGLDSATHALCLAL
LDLDTPVWLAPAFDTPAIRANIAFHCGSPIVTERHAARFALLDASTALQLHDFDTGNDRY
PDQSCTLMIQLPDLQGGRMLAWSGPGIERRHSVALPLDEGFWAAREARNVFPCGLDVFFV
SGNRLLGLPRSTRVQECA
>tr|A0A0Q0FC37|A0A0Q0FC37_PSEAP 3-oxoacyl-reductase OS=Pseudomonas syringae pv. aptata OX=83167 GN=ALO85_04213 PE=4 SV=1
MLLQGKIAVITGAASARGIGRATAITFANHGARVVIIDLDESAARDAAAALGEGHLGLAA
NVADEHQVHEAVSKIIAHYGRIDILVNNAGITQPIKTLDIRPSDYDKVLDVSLRGTLLMS
QAVIPTMRAQSSGSIVCMSSVSAQRGGGIFGGPHYSAAKAGVLGLGKAMAREFGPDQVRV
NSIAPGLIHTDITGGLMQDERRHAIIDGIPLGRLGAAQDVANAALFLASDLSSYLTGITL
DVNGGMLIH
>tr|A0A0Q0C584|A0A0Q0C584_PSEAP Uncharacterized protein OS=Pseudomonas syringae pv. aptata OX=83167 GN=ALO85_02909 PE=4 SV=1
MDSKVIVMDISPATIQAGWRGEAVPSVDLPVPEKLFNGQGEVVEWGAVSALLDTVLLDKF
KWSDNDEVTIMYVVPSLMPKAGKEKLLQLSFDDDKYRLKGVFLKSAASAILFSLDRTKTT
LEGETVPNVTGVAIRTDKKTAIITVDPVFQGVLLPSAALKLTKNNWEGSFDPQFALSDDS
INILADTIVLAVQRTNVDVRSLLYKNLVLSGSRVNESGFYEKLKKSITEKAPKGALIGMT
LDPKPESAAWRGAAMYAATEGVVGGAVTKQDYDHYSAGS
>tr|A0A0N8T828|A0A0N8T828_PSEAP Putative GTP cyclohydrolase 1 type 2 OS=Pseudomonas syringae pv. aptata OX=83167 GN=ALO85_04418 PE=4 SV=1
MAVALSTLVEEADRYLNSARIQDYCPNGLQVEGRPQVMRIVSGVTASQALLDAAVDAQAD
LVLVHHGYFWKGENPCVVGMKQRRLKTLLKHDISLLAYHLPLDVHPEVGNNVQLARQLDI
TVEGPLDPENPRVVGLIGSLSEPMTARDFARRVQDALGREPLLIEGSQMIRRVGWCTGGG
QNYIDQAVLEGVDLFLSGEASEQTFHSARENDISFIAAGHHATERYGVQALGDYLARRFA

LEHIFIDCPNPI
>tr|A0A0Q0DMY7|A0A0Q0DMY7_PSEAP Putative aspartate protease, retropepsin-like protease family OS=Pseudomonas syringae pv. aptata OX=83167 GN=ALO85_02866 PE=4 SV=1
MSDVQPGKRAGKVLMIVAWAAGLFLATRFFGDWEDRQQNPNAVVTSQHGDGYIEVQLAGN
RQGHFVTTGQINGRTVEFMIDTGATDVAVPGDMADRLGLKRGFPVTVSTANGNSQGFRTT
LDRLQLGDIVLTNVRALVAPGLDGEQVLLGMSAMKQLEFTQRGGNLLLRQSTK
>tr|A0A0Q0DQY7|A0A0Q0DQY7_PSEAP Lipid A biosynthesis lauroyltransferase OS=Pseudomonas syringae pv. aptata OX=83167 GN=lpxL PE=3 SV=1
MTEAQMDRPRFKASFLHPRFWPLWLGLGLLWLIVQLPFRVLLVIGRALGAIMYRVATDRK
KIASRNLELCFPHLSAAERKRLLKENFASTGIAFFEMAMSWWWSKKRLARLAHVEGLEHL
QNAQEKGEGVILMALHFTTLEIGAALLGQQHTIDGMYREHKSPLFDFVQRRGRERHNLDS
LAVERDDVRGMLKLLRAGRAIWYAPDQDYGAKQSIFVPLFGIQAATVTATSKFARLGRAQ
VVPFTQRRLADGSGYRLVIHPPLDNFPGESDEADCLRINQWVEKAVTECPEQYLWAHRRF
KSRPAGEQKLYEKHR
>tr|A0A0Q0CYY5|A0A0Q0CYY5_PSEAP D-alanyl-D-alanine carboxypeptidase OS=Pseudomonas syringae pv. aptata OX=83167 GN=ALO85_01338 PE=3 SV=1
MNITTFAKRLCLLVPLIITPTVWAAEQMTPAAPQLAAKAYVLMDATSGNVLVENNGDQRL
APASLTKLMTAYIATLEIRRGQIGENDPVTISENAWRTGGSRMFIKVGSQVTVSDLLHGI
IIQSGNDASVAISEHIAGSEDAFADMMNKTAADLGMTNSHFMNPTGLPNPEHYSSAHDMA
ILARAIIHEDPAHYAIYSQKEFFWNGIKQPNRNLLLWRDKTVDGLKTGHTDEAGYCMVSS
AVRDGMRLIAVVFGTNSEQARAAETQKLLTYGFRFFETQNFYQKGTELAQATVWKGTERQ
VKAGLAEDLSMTMPKGDMKKLSASMTMNPQLVAPIAKGDVIGKVEVKKDDQVVHTANLIA
LDGVEEGGIFRRVWDSIRLFFYGLFN
>tr|A0A0N8T9B2|A0A0N8T9B2_PSEAP Inositol ABC-type transport system, permease protein OS=Pseudomonas syringae pv. aptata OX=83167 GN=ALO85_02698 PE=3 SV=1
MSDAILQNKPAMTPTRSKRRLPTELSIFLVLIGIGLIFELFGWIVRDQSFLLNSQRLVLM
ILQVSIIGLLAIGVTQVIITTGIDLSSGSVLALSAMIAASLAQTSDFSRAVFPSLTDLPV
WIPVVVGLGVGLLAGAINGSIIAITGIPPFIATLGMMVSARGLARFYTEGQPVSMLSDSY
TAIGQGAMPVIIFLVVAVIFHIALRYTKYGKYTYAIGGNMQAARTSGINVKRHLVIVYSI
AGLLAGLAGVVASARAATGQAGMGMSYELDAIAAAVIGGTSLSGGVGRITGTVIGALILG
VMASGFTFVGVDAYVQDIIKGLIIVVAVVIDQYRNKRKTKR
>tr|A0A0Q0D569|A0A0Q0D569_PSEAP AHS1 domain-containing protein OS=Pseudomonas syringae pv. aptata OX=83167 GN=ALO85_00860 PE=4 SV=1
MSLVQAQNAPRITLLGTTALLFEAPGELDLALQQRIWALARLSKDWPQVREAVPGMNNLM
LTFAAPPRDLAGLKTRLLETWEQCQPLPLQGRIIELPVVYGGDGGPHMADVIAHTGLDIE
TIANLHCEPLYPVYALGSHPGYCYLGGMDQRLATPRRKVPVLDIGAGSVSIGGVQTGISA
SAGPSGWNTIGRTEMVFFDAGQQPPALLQPGDQLRLRIERIIR
>tr|A0A0Q0DJ98|A0A0Q0DJ98_PSEAP LabA-like protein OS=Pseudomonas syringae pv. aptata OX=83167 GN=ALO85_01880 PE=4 SV=1
MAVQNPNSSQKHLAVLIDADNAPAAIVEGLFEEIAKYGVASVKRIYGDWTGPQLGGWKKV
LLDYSIQPIQQFAYTKGKNATDSSLIIDAMDLLYTRRFDGFCLVSSDSDFTRLASRLREE
GLTVYGFGEEKTPKPFVAACDKFIYIELLREEITTPATGDSTPDAAATPSNVKSVATEPS
KKPKAPVGFIAKIMDDIADEDGWAPLGAIGTNITKLRPEFDPRTHGYKKLSDLIKGYPQT
FELQARPASGGTAVLYARHKLQGK
>tr|A0A0Q0BYN1|A0A0Q0BYN1_PSEAP Mandelate racemase/muconate lactonizing protein OS=Pseudomonas syringae pv. aptata OX=83167 GN=ALO85_05325 PE=3 SV=1
MRIVDIREKTVSIASPIANAYIDFSKMTCSVVAVITDVIRDGKPVIGYGFNSNGRYGQGA
LMRDRFLARITEADPDTLVDHENNNLDPFAIWKTLMTNEKPGGHGERSVAVGTIDMAVWD
AVAKIEGKPLYRLLADRYRDGVADDKVWVYAAGGYYYPGKDQTKLKAEMQSYLDRGYDVV
KMKIGAVPLDEDIRRIEAVLEVVGDGRRLAVDANGRFDLQTGIAYAEAIKKYNLFWYEEV
GDPLDYALQAELANHYELPMATGENLFSHQDARNLLRHGGMRPDRDYLQFDCALSYGLVE
YMRTLKVMEEMGWSSRRVVPHGGHQMSLNIAAGLHLGGNESYPDVFQPFGGFADGIKVEN
GYVGLPDIPGVGFEAKSALYAVMCELGEG
>tr|A0A0Q0BU26|A0A0Q0BU26_PSEAP Permease OS=Pseudomonas syringae pv. aptata OX=83167 GN=ALO85_04975 PE=4 SV=1
MPYQREIVWHMFCVHDRIQKNHRSYSDPVTTHRGSQAMTDQHPAGYSPRLHNHDLGPVPQ
KWTWYNILAFWMSDVHSVGGYVFAASLFALGLASWQVLIALLVGICIIQVIANLVAKPSQ

QAAVPYPVICRLAFGVFGANIPAIIRGLIAVAWYGIQTYLASSALVIVVLRFFPELAAYQ
SVTFLGLSWLGWFGFLALWVVQAAVFWTGMESIRRFIDWAGPAVYAVMFALAGWIVWRAG
WDNISFTLAEKELSGWAAFGQVVMAIALVVSYFSGPTLNFGDFSRYCRSMADVRRGNFWG
LPVNFLAFSLVTVVIVSGTLPIFGEMIHDPIATVARIDNITAVLLGAFTFVTATVGINIV
ANFVSPAFDFANVAPSRISWRAGGMIAAVASIFITPWNLFNNPEVIHYTLDVLAACIGPL
FGILLVDYYLIKKQQIDVDALFNDTPSGRYWYTNGVNWIAVKALLLTALVGLCFTFVEWF
KPIANFAWFTGCILGGAVFYALTRWSPVQAANLPAPVGQH
>tr|A0A0Q0DMY3|A0A0Q0DMY3_PSEAP 5-oxoprolinase subunit A OS=Pseudomonas syringae pv. aptata OX=83167 GN=pxpA PE=3 SV=1
MPAIDLNSDLGESFGAWSMGDDEAILDVVSSANVACGFHAGDPAGILRTLEAAAARGVAI
GAHVAYPDLVGFGRRNMDIPSDQLTADVIYQIGALQGLARSAGTSVSYVKPHGALYNTIA
GDQRQAAAVIQALLRIDPELKLVCLANSPLLDWAREAGLSCVAEAFADRAYTAEGTLVSR
SRPGAVLHDAERIAERMLRLVREGVIEAEDGREISLQADSICVHGDSPGAVNIARILKDR
LHDAGVTVRTFNRG
>tr|A0A0Q0C4P9|A0A0Q0C4P9_PSEAP Putrescine ABC transporter permease OS=Pseudomonas syringae pv. aptata OX=83167 GN=ALO85_02341 PE=3 SV=1
MNRFRFSNLMLVLGLLFIYAPMVILVIYSFNASQLVTVWGGWSVKWYVGLLDNSQLMGSV
MRSLEIACYTAIAAVALGTMAAFVLTRISRFKGRTFFGGLVTAPLVMPEVITGLSLLLLF
VAMAQLIGWPQERGIMTIWIAHTTFCAAYVAVVVSARLRELDLSIEEAAMDLGARPWKVF
LLITIPMIAPSLAAGGMMSFALSLDDLVLASFVSGPGSTTLPMEVFSAVRLGVKPEINAV
ASLILLAVSLATFLVWFFTRRAENNRKRAIQQAIEESAADAWKQPDPRRAPAA
>tr|A0A0Q0C4I1|A0A0Q0C4I1_PSEAP 10 kDa chaperonin OS=Pseudomonas syringae pv. aptata OX=83167 GN=groS PE=3 SV=1
MKLRPLHDRVVIRRSEEETKTAGGIVLPGSAAEKPNRGEIVAVGTGRVLDNGEVRALAVK
VGDKVVFGPYSGSNTVKVDGEDLLVMSENEILAVVEG
>tr|Q6L8X0|Q6L8X0_PSEAP Harpin protein HrpA OS=Pseudomonas syringae pv. aptata OX=83167 GN=hrpA PE=4 SV=1
MTIMSSLAGAGRGVVNTIGGAAQGINSVKSSADRNAALVSNTGSTDSIDATRSSISKGDA
KSAELDGTANEENGLLRESSMLAGFEDKKEALSNQIVASKIRNSVVQF
>tr|A0A0Q0D009|A0A0Q0D009_PSEAP Flagellar assembly protein FliH OS=Pseudomonas syringae pv. aptata OX=83167 GN=ALO85_02167 PE=4 SV=1
MSSSNKESASDLIRAKDAGLLDIWALPSFDPHVEPEPEPEPEQLDEPAEMEEVPLDEVQP
LTLEELESIRQEAWNEGFATGEKEGFHSTQLKVRQEAEVVLAAKVASLEQLMSNLLTPIA
EQDTQIEKAVIHLVEHIARQVIQRELVTDSAQIASVLRDALKLLPMGAQNVRIFINPQDF
LLVKAMRERHEESWKIVEDEDLLPGGCRIETEHSRIDASVETRIALAISKMHDQLHEQVT
HPAAADLSIDLQASPGAVPTADESDADTLDAP
>tr|A0A0Q0DL55|A0A0Q0DL55_PSEAP Putative lipoprotein OS=Pseudomonas syringae pv. aptata OX=83167 GN=ALO85_03087 PE=4 SV=1
MKHRSALFAVLLGTALLGISACQSDNPYVASSLPMPPAPAQAAKTLDMSAYPAPPRDYGR
YRSWGWLNGQLPPGTAWVDSAQVVEAVSNGLDQRGLRPARGNQPADLYVTTTTRQETRLR
QVREDYDPYYGGGMGYGRYDGYRPGYGGYASVPVVRTYQEKVMVVQINLIDARSGQPVWS
ASADARASNNLADRSSALRQAVQQALAAYPPS
>tr|A0A0N8T9H6|A0A0N8T9H6_PSEAP Phosphate ABC transporter ATP-binding protein OS=Pseudomonas syringae pv. aptata OX=83167 GN=ALO85_100069 PE=4 SV=1
MTQTHNPGPALLSIEGLHKSYGAVEVLKGVDLRMQKGNVVTLIGSSGSGKTTLLRCVNLL
EEFQGGHIRLEGQDIGYSNVDGKRTRHPERLIAQHRAMTGMAFQQFNLFPHLSALQNVTL
GLLKVKKMPKDQAVALAEKWLDRVGLLERRNHFPGQLSGGQQQRVAIARAIAMNPSLMLF
DEVTSALDPELVGEVLSVIKDLAEEGMTMLLVTHEMRFAYEVSDQIVFMNQGRIEEQGPP
KALFEQPKSARLSEFLKNIRF
>tr|A0A0Q0DX63|A0A0Q0DX63_PSEAP Molybdenum ABC transporter, periplasmic molybdate-binding protein OS=Pseudomonas syringae pv. aptata OX=83167 GN=ALO85_03774 PE=4 SV=1
MRTTTLRAILKFSVLAFTASAAAHASADEVQVAVAANFTAPIQAIATDFEKDTGHKLVTA
FGATGQIYTQIKNGAPFEVFLSADDTTPAKLEQEGDTVKGSRFTYAVGTLALWSAKDGYV
DSKGQVLKANQYQHLSIANPKTAPYGLAATQVLSKLGLTEATKAKIVEGQSITQAYQFVS
TGNAELGFVALSQVYKDGKLTSGSAWIVPDDLHDPIKQDAVILNKGKDSAAAKALVEYLK
GPKAAAIIKSFGYQL
>tr|A0A0N8T9E6|A0A0N8T9E6_PSEAP Beta-D-glucoside glucohydrolase OS=Pseudomonas syringae pv. aptata OX=83167 GN=ALO85_05121 PE=3 SV=1

MNKLCLLGLLVGMASQSVMAQNAAATDNTTLQAKNAFIGKLMKQMTLDEKIGQLRLISIS
SEMPQPQILKEIAAGRIGGTFNSITRSENRPLQEAAVAKSRLKIPMFFAYDVVHGHRTIF
PISLGMAASWDMDAIALMGRVSAKEASADSIDMTFAPMVDISRDPRWGRSSEGFGEDTYL
VSRISDVMVRSFQGKNVAANDSIMAAVKHFALYGAVEGGRDYNTVDMSMTRMYQDYLPPY
KAGIDAGAGGIMVALNSINGVPSTSNKWLMQDLLRKDWGFKGVTISDHGALKELIDHGVA
KDFREAAKLAIKAGVDLSMNDAAYGEQLPGLVKDGEVSMKEIDSAVREVLGAKYDMGLFA
SPYGRIGVAAEDPADTYSDDRLHRAEARDVARKTLVLLKNQNETLPLKKQGTIAVIGGLA
QSHLDMLGSWSAAGRPNQSVTVYEGLANAVGDKAKLVYARGANVSDNEHVLNYLNFIEKE
VEIDPRPAQEMIDEAVKVAEQADVIVAVVGESRGMSHESASRSSLNIPGKQRDLIKALKA
TGKPLVLVLMNGRPLVLVDEQEQADAMLETWFPGTEGGNAVADVLFGDYNPSGKLAMTFP
RSIGQLPVYYAHLNTGRPYHEGKPGNYTSHYFEEPNGPLYPFGYGLSYTQFDVSDITLSD
ANMTRKGKLTASVTVKNTGKVAGATVVQLYLHDVAASISRPVKELKNFEKVMLEPGEEKV
VTFTLSEDDLKFYNTELKHVAEPGEFKVMIGLDSQAVKEATFNLL
>tr|A0A0Q0FK17|A0A0Q0FK17_PSEAP Succinate dehydrogenase hydrophobic membrane anchor subunit OS=Pseudomonas syringae pv. aptata OX=83167 GN=ALO85_100058 PE=4 SV=1
MDMVTNVTNLSRSGLYDWMAQRVSAVVLAAYFIFLIGYMVFHPGLSYAQWHGLFAHNGMR
IFSLLALVALGAHAWVGMWTIATDYLTPMALGKSATAVRFLFQAVCGVLMFAYFVWGVQI
LWGI
>tr|A0A0Q0FCT4|A0A0Q0FCT4_PSEAP Molybdenum cofactor guanylyltransferase OS=Pseudomonas syringae pv. aptata OX=83167 GN=mobA PE=3 SV=1
MNVPTPLPPCSILLLAGGRGQRMGGRDKGLIEWQGTALIEHLHRLTRPLTDDLIISCNRN
IDHYARYADRLVQDDDTDFNGPLAGIRAALPLARHRWLLILPCDAPLVDKALLHALREKA
AEYPERPIMVREGQHWQPLLGMIPVAHAATLEAAWQAGERSPRRALEPLQPVALQLEAGD
PRLANLNTPCLLTGISENRNK
>tr|A0A0Q0C3I9|A0A0Q0C3I9_PSEAP Uncharacterized protein OS=Pseudomonas syringae pv. aptata OX=83167 GN=ALO85_00914 PE=4 SV=1
MNALPSTSLPKHVSLLGYGGLLPFISLALLIPFYSDYRPLFAIALVNYGAVILSFVGALH
WGFAMTAQDMKAEQRSGRFIWSVIPALIAWIATLLPMPLGCLLLVIGFVVHLWQDRQLSR
VISLPAWYLPMRLRLTVVASVCLLLAAIVEVLHL
>tr|A0A0Q0CGD5|A0A0Q0CGD5_PSEAP Uncharacterized protein OS=Pseudomonas syringae pv. aptata OX=83167 GN=ALO85_01953 PE=4 SV=1
MSLSLRDQLLKAGLVNQKQAKQVGKEKQKEQRLVHKGQAQADDSQKRAAQEAMAEKAKRD
QELNRQQQEKVEQKARTAQVKQLIEVSRLPKLITEDYYNFVDDKKVKRIPVNAMVRNKLS
SGSLAIVHHGGGYEIIPREAALKIQERDPRRIVLLNTPTEAPDADDPYAAYQVPDDLMW
>tr|A0A0Q0BZ98|A0A0Q0BZ98_PSEAP Phosphate transport system permease protein PstA OS=Pseudomonas syringae pv. aptata OX=83167 GN=ALO85_00648 PE=3 SV=1
MSKDVTGSEHLYRVRSFKNKIAMVLSCGATAFGLLWLVWILLTTIIKGIDALNLQLFTGM
TPPPGTEGGLANAFYGSVLMSGIGLLIGTPIGLMAGIWLAEFARYTKLGNTVRFINDILL
SAPSIVLGLFVYTVVVLPLNAVTGHQVGFSAIAGALALALLVIPVVVRTTDEMMQLQPST
MREAALALGVPQWKLTLQIVLRAAKAGVVTGVLLALARITGETAPLLFTAFGNQFWSSDL
LKPIASVPVVVFQYAMSPFDDWHSLAWAGALVMTLFVLILSLLSRLILLRNRAS
>tr|A0A0Q0DUL9|A0A0Q0DUL9_PSEAP Chromosomal replication initiator protein DnaA OS=Pseudomonas syringae pv. aptata OX=83167 GN=dnaA PE=3 SV=1
MSVELWQQCVELLRDELPAQQFNTWIRPLQVEAEGDELRVYAPNRFVLDWVNEKYLGRLL
ELLGEHGQGMAPALSLLIGSKRSSAPRAAPNAPLAAAASQALSANSVSSVSSPAPATAAP
AAAVATPAPVQNVAAHNEPSRDSFDPMAGASSQQAPARAEQRTVQVEGALKHTSYLNRTF
TFENFVEGKSNQLARAAAWQVADNPKHGYNPLFLYGGVGLGKTHLMHAVGNHLLKKNPNA
KVVYLHSERFVADMVKALQLNAINEFKRFYRSVDALLIDDIQFFARKERSQEEFFHTFNA
LLEGGQQVILTSDRYPKEIEGLEERLKSRFGWGLTVAVEPPELETRVAILMKKADQAKVD
LPHDAAFFIAQRIRSNVRELEGALKRVIAHSHFMGRDITIELIRESLKDLLALQDKLVSV
DNIQRTVAEYYKIKISDLLSKRRSRSVARPRQVAMALSKELTNHSLPEIGDVFGGRDHTT
VLHACRKINELKESDADIREDYKNLLRTLTT
>tr|A0A0Q0C609|A0A0Q0C609_PSEAP YegD-like heat shock protein/molecular chaperone OS=Pseudomonas syringae pv. aptata OX=83167 GN=ALO85_01930 PE=3 SV=1
MNDQSPARACGIDFGTSNSTVGWQRPGMESLIALEDDKITLPSVVFFNMEERRPVYGRLA
LHEYLEGYEGRLMRSLKSLLGSKLIKHDTSVLGTAMPFKDLLALFIGELKKRAEQTAGRE
FEQVVLGRPVHFVDDDAQADQEAEDTLAEVARKIGFKDVSFQFEPIAAAFDYESTIQDEE

LVLIVDIGGGTSDFSLVRLSPERRQHDDRQQDILATGGVHIGGTDFDKQLSLQGVMPLFG
YGSRMKSGAYMPTSHHMNLATWHTINAVYSQKSQLALGSMRYDIEDTGGIDRLFKLIEQR
AGHWLAMEVEETKIQLTHADSRHLLMDRVEAGLSVDLSRVMFEAAIDAQLERVRNSVTNL
LNDAGVSVEQVSTVFFTGGSSGIPALRNSVSNMLPNARHVEGNIFGSIGSGLAIEAKKRY
G
>tr|A0A0Q0CIT4|A0A0Q0CIT4_PSEAP Negative transcriptional regulator
OS=Pseudomonas syringae pv. aptata OX=83167 GN=ALO85_01433 PE=4 SV=1
MYIPTAFKNNDTPSLHEQIEQTRLAILVTHGAEGLQATHLPLLLRRDEGSYGTLYGHLAK
ANPQWQQLSSGAEALVIFPGGDAYVSPSFYPSKIEHGKVVPTWNYVAVHAYGQAEVFTDA
AQLHQLLADLTHRHESDRAQPWSIDDAPADYIAKMLGAIVGFSIPVQRLQGKRKLSQNRS
AADMAGVRDGLASSQDANDQHVARLMSEGSSS
>tr|A0A0Q0IN75|A0A0Q0IN75_PSEAP Bifunctional protein PyrR OS=Pseudomonas
syringae pv. aptata OX=83167 GN=pyrR PE=3 SV=1
MSLPDPAELIRQMATDLNAHLSKRGISDPRFIGIRTGGVWVAQALLKALNNPAPLGTLDV
SFYRDDFSQNGLHPQVRPSELPFEIEGQHLVLIDDVLMSGRTVRAALNELFDYGRPASVT
LVCLLDLDAGELPVRPNVVGATLALAPNERIKLSGPEPLALELQDLSTAL
>tr|A0A0Q0II69|A0A0Q0II69_PSEAP Putative lipoprotein OS=Pseudomonas
syringae pv. aptata OX=83167 GN=ALO85_05617 PE=4 SV=1
MKYIFVVLAILAVAGCAATSKPVTQSKRGIHINCSGLTSSWAKCYELATNSCASRTFKVI
AKSGDATEEPEDYPFGLNPAGYTSRSMIIVCRKAAPLADPSPQD
>tr|A0A0N8T9Y0|A0A0N8T9Y0_PSEAP 30S ribosomal protein S11 OS=Pseudomonas
syringae pv. aptata OX=83167 GN=ALO85_200011 PE=4 SV=1
MKNTQRQNQQLLADYYRKNLGDPAGMAGQSKSLHEMRARAESYEDSWNIQEENLLGDFRP
PRTKDEFLQWYLSCEKKINDDIAFFVDFLKNESTLEQVAFYICMEEMVDGSFDDLMAVTQ
LGMPIQCKMVAGNNYWDEMGNGNFSLVHSTMFRQSSDYMRVVLSNAGVSIDVPPTECLMN
GNILLMWASRREYNVRLIGAMGLVEGSAPTRFGATTFAMERLGLPKDVIAYHKAHIMIDT
YHSEAWYETVLNHYSSCGEEIIKELSIGVMVRYNVAVRYYYTYMYNKLRSIGL
>tr|A0A0Q0IMK5|A0A0Q0IMK5_PSEAP Aliphatic sulfonates import ATP-binding
protein SsuB OS=Pseudomonas syringae pv. aptata OX=83167 GN=ssuB PE=3
SV=1
MPESLMDIRVEHKAFAGNTVLHGIDLSLQSGEIVSLLGPSGCGKSTLLRIVAGLEQDFRG
SVQRIQGEVAFVFQEPRLMPWLTVAQNIGFSDDDRYDRHWVGQLIEEVGLSGFADALPKA
LSGGMAQRVAIARGLYSHPAVLLLDEPFSAVDAFTRMKLQDLLLQLAARHAITLLLVTHD
VDEALYLSDRVLVMGSRPGTITHQLPVGLQTPRDRRDPLLARLKAQALTELQQTHVI
>tr|A0A0Q0IMT5|A0A0Q0IMT5_PSEAP Transcription termination/antitermination
protein NusG OS=Pseudomonas syringae pv. aptata OX=83167 GN=nusG PE=3
SV=1
MAKRWYVVHAYSGYEKHVMRSLVERVKLAGMEDGFGEILVPTEEVVEMRNGQKRKSERKF
FPGYVLVQMDMNEGTWHLVKDTPRVMGFIGGTADKPAPITDKEAEAILRRVADGSDKPKP
KTLFEPGEVVRVTDGPFADFNGTVEEVNYEKSRIQVAVLIFGRSTPVELEFSQVEKA
>tr|A0A0Q0IBJ0|A0A0Q0IBJ0_PSEAP Putative BON superfamily
periplasmic/secreted lipoprotein OS=Pseudomonas syringae pv. aptata
OX=83167 GN=ALO85_00193 PE=4 SV=1
MTPNRLSLLVLTLCLSITGCSSVITATREKPIEDDRGTRTFGSKIDDSLIETKAAVNISK
ASPDLAEGSHIVVTSFNGIVLLAGQTPRADLKAMAEQAASAVQRVKKVNNELQVMAPSSL
LARNNDAWLTTKVKSQMIADASIPGSRIKVVTENGIVFLLGLVTQDEANRATNLVQGVSG
VQKIVKLFEYID
>tr|A0A0Q0C8V5|A0A0Q0C8V5_PSEAP DLH domain-containing protein
OS=Pseudomonas syringae pv. aptata OX=83167 GN=ALO85_03171 PE=4 SV=1
MSHGADNFYTSDKVVAQKVLFNNQYKMKVAGNLFIPKGSNPNVKAPAIIVGHPMGAVKEQ
SANLYAQKLAEQGFVTLSLDLSFWGGSEGQPRNAVSPDLYAEDFSAAVDFLGTQTFVDKK
RIGVLGICGSGSFVISAAKIDPRMKAIATVSMYDMGAANRNGLKHSQTLAQRKTIIEQAA
LQRDVEYSGGDTAYTSGTVHKLTDTSGPIEREFYDFYRTPRGEFTPEGQSPELTTHPTLT
SNIKFMNFYPFNDIATISPRPMLFIAGSKAHSLEFSEEAYKLAGQPKQLIIVPDAGHVDL
YDRVDLIPFDKLGEFFKNNLK
>tr|A0A0Q0FE82|A0A0Q0FE82_PSEAP Uncharacterized protein OS=Pseudomonas
syringae pv. aptata OX=83167 GN=ALO85_02485 PE=4 SV=1
MAQAYIYMECPVSGETLTLGKLTIQAGVGTFQYAPDAVQENIWVPDPFRYPLSTRSYTVT
KNGGVPGFIDDAMPDGWGERLLHRVEKGPLDSIQLLLKSPNSDRAGNIMAGLSRVAQDGL
GQTPPKAFRAKGLDHFIDACEAIYDSQLSAEQLEVLKVRDQRSSAGGARPKRTYLGDRKL

ILAKPRDKFDHYDLPSVEHACMTFAGLKGLQAANTSLYRGERINTLLVERFDRAFDEQTH
RFRRLPMLSGLTLLNAEWKARTHPDWQYAALADELYRRGAPDVDRQALYRRMAYNVLVGN
ADDHPRNHAVIWKEGGWRLSPMYDVLPILDEGPAQALAMSVGIEGSRLNRGNLLSQHAHF
ALSKEQAEAALDEVAGWEAELHDYYSQFLSGAELDAAVDATSGARLKR
>tr|A0A0Q0BSE1|A0A0Q0BSE1_PSEAP Endonuclease I OS=Pseudomonas syringae pv. aptata OX=83167 GN=ALO85_00520 PE=4 SV=1
MTFRFSSLLCTTLLITASFAAQAQAPRTFSEAKKVAWGLYAPQSTEFYCGCKYTGNRVDI
AGCGYVPRKSAKRASRIEWEHIVPAWQIGHLRQCWQNGGRKNCTKTDPVYKRAEADLHNL
VPSIGEVNGDRSNFSFGWIPEQKGQYGSCLTQVDFKAKKVMPRPSIRGMIARTYFYMSKQ
YNLRLSRQDQQLYQAWNKTYPPQAWERQRNQQVACVMGRGNDFVGPVDLKSCK
>tr|A0A0Q0C4R0|A0A0Q0C4R0_PSEAP Uncharacterized protein OS=Pseudomonas syringae pv. aptata OX=83167 GN=ALO85_00962 PE=4 SV=1
MFAHEGLGLILVSIVFLLGAIAMFIHPFHALGSWFRRKKQERKSD
>tr|A0A0Q0DR76|A0A0Q0DR76_PSEAP LuxR family transcriptional regulator OS=Pseudomonas syringae pv. aptata OX=83167 GN=ALO85_02055 PE=4 SV=1
MLALKIRIAKNGLPMNKVLIVDDHPVIRLAVRMLMERHGYEVIAETDNGVDALQLAREHL
PDIVILDIGIPKLDGLEVIARLSAMTLPFKVLILTSQAPGHFSMRCMQAGAAGYVCKQQD
LTELLSAIKAVLSGYSYFPNQALHTVRSSMGNASESDMVDRLSGREMMVLQQLARGKTNK
EIADGMFLSNKTVSTYKTRLLLKLNAHSLVDLIELAQRNGLV
>tr|A0A0Q0C6I2|A0A0Q0C6I2_PSEAP PLDc_N domain-containing protein OS=Pseudomonas syringae pv. aptata OX=83167 GN=ALO85_01213 PE=4 SV=1
MGSSFNGLVGLIILALDIWAIINVLKSGAETGKKILWVLLIVLLPVVGLIIWAILGPRGN
VRM
>tr|A0A0Q0IRJ1|A0A0Q0IRJ1_PSEAP Integrase OS=Pseudomonas syringae pv. aptata OX=83167 GN=ALO85_200170 PE=4 SV=1
MTTSVAVLEKPHRDEIKELVKLVRMDEKYAALVADGFLPLDVQSSIYNFQRKSRIEELSQ
KYGLI
>tr|A0A0Q0CAP9|A0A0Q0CAP9_PSEAP Polysaccharide export protein OS=Pseudomonas syringae pv. aptata OX=83167 GN=ALO85_04351 PE=4 SV=1
MNRSVTALLLVSLALQGCAFAPGQHMTPDDVTLDKPDEPRAQLIEVTPKTLQQQLRATA
EAKALPQVLLDYRTPEYVIGAGDTLLVTVFEHPELTAPGSQDQLDANTREVLNDGTLYFP
YVGRIRASGRTVSEVREQLRMGLAPQYTEVKVDVKVLRYNSQRILLSGSFKVPGPQPITN
IPLSLVQAVSVAGVDLTDANLAGLTLRREGKDYLIDIDSLNRRDSQLSRIFLKDGDYLHL
NSNSRNKIYVLGEVRNPQVISFGTTRLTLLEALGNSGGLSPDSADGDAVYVIRGAENFAQ
APSTVYHLNAKKPTAYLLAGQFELKAQDVVFVGPADITRWSRFISQLLGSASVIQTGAAF
RN
>tr|A0A0Q0ICP8|A0A0Q0ICP8_PSEAP WavE lipopolysaccharide synthesi OS=Pseudomonas syringae pv. aptata OX=83167 GN=ALO85_03113 PE=4 SV=1
MPHLVAVDSRKISVVIQGPLYRNLSSKRNIFACIASIRTHLPQAEIIVSTWRHEDTSDVT
ADQIVMSDDPGAFVDDAGNQININRMLLSTLCGIQSASRPYVMKMRADHNLTSAALAVIG
QSDDTEPGETQLFDTPITTTTLYIRDPERLPMLFHISDLVQFGTRDAMLAMWEQPLFERD
ALFNARPSRNPFGNFIGYTSARMVSEQALMLGVMRRKGIEARLARPCEVGLSNLKLWDNI
LRRNFRVLNHCEAGVDFPERFLTSRRVLTTLYKASAIEQLQRLDPQGYRLRIARIWLNQY
LLTCLRPGWWVSFATIVLFSTSPTLARRIRSYWRTLRKVAHAESYRV
>tr|A0A0Q0DPI0|A0A0Q0DPI0_PSEAP Uncharacterized protein OS=Pseudomonas syringae pv. aptata OX=83167 GN=ALO85_02222 PE=4 SV=1
MLMMVDSKTTATLDDKKKARIAYRLARSQRGSGIDADLAPAQVAGIVDPADGTLNKDVLA
GEFLEVTLPQWAGLVTDPGDSDTFFIDWAIGAAADNDAFKEVFSDAVTAPVDPDTFPMVI
DIPLSSLMQGLAKPADGAYSLRYRIRQPNDQETISPSIDLIVDTTPPGEHLEPEQMVLAT
DQLTQAFLDANPGGLVGTLPDYDNWKPGDKAAFYWVGTPLPESAEELPDPVGVVDLASPT
NNTVTFPVSAIEASRDEPYYAIYILVDKATNRTSLSSPKRIDVALGLLPDHLKEPVVPLA
QPPERLINLPDARLGVEVLIESFDNWKPADRIEVTWGTEVLRPDEVGSSPAFPISISVPD
LVLQGQYNQANGGDQPTEVSYRILRGVVASEVKSISVNVNFATIGPDPITGPGWPDPVND
ALRPVEVRGQTSNLVNILTAADYNQPAKVSFNLYQKLQAGEVIDFYWGTSHVSEAQYTVT
VSDTAGSAREAEIPWSYIDHEGNRDDLPVHYRIHAPDSENTQHSPDTLVQVNAIVIIPEA
PVFEGTSPNGWLNCDSLFADGIENPGEGEPGIRVRVPDLSKYLSDNETVTLHWYGSEGSN
GDAPISGTEKDEVIVLGGNHPATGFIWLVTPYDKHILPIYNPSGSSPNGSARIRYSFSMG
GETITSLETTARVGMYDATGPCPIVLKTGQPTKN
>tr|A0A0Q0FFA9|A0A0Q0FFA9_PSEAP SMC domain-containing protein OS=Pseudomonas syringae pv. aptata OX=83167 GN=ALO85_03110 PE=4 SV=1

MVQFNSAEFAGISIMTPELKARQQIDPKLEKAGWVIQDMKELNLSAGVGVAVRECPTDTG
PADYMLFVNRVAVGVIEAKKDSAGENLTVTEIQTERYATSSLKWRKDDTPLRFLFEATGQ
IIRFTDNADPVPRSREIFNFFKPEALATWLAQPETLRRRLAEQMPALPELNLRDCQISAV
TGLEQSLALNKPRALVHMATGAGKTFTAITSVYRLLKFGGAKRILFLVDMYEVFSPYLES
YDSKLRNGIDPGLKRLFYAMPVHSQFVLLAFMIQQSDVVRAFLDDHLGIDPDDGIESVLF
VLRQPPWKSKAPDGDPRFWNARGVVRDFLSRLHDIALAPIEISRQVSTSIWNKTKLQFKY
LYVKDIAALRRLVGNQAPAQFFRDLESTYVSELIEEVRIRIRLKKNDGSVTFRELSEGEQ
QLLTVLGLLRFTAEDESLFLLDEPDTHLNPRWSVDYISYLKQFIANGTKQEETSHILLTT
HNPLAVAELDREQVQILRMSKQEGQRQILACYPEVAPRGMGYAAIVTSDMFGIASSMDAS
TQLQLEKQRALGAKPDLSVTEQGALDEINDDLNRLGFRFFHPDDEFSRYLRLRNEALKAR
FGVIDPLDLAEKAVTMTRQEREDLATDLITKLVEDENEELGGPSR
>tr|A0A0Q0DLH0|A0A0Q0DLH0_PSEAP DUF446 domain-containing protein
OS=Pseudomonas syringae pv. aptata OX=83167 GN=ALO85_02991 PE=4 SV=1
MDGRLPEVAEQLLLIERELRLLGWWDATPPSEQALASQEPFSVDTLEFAQWLQWIFLPRM
KVILERDLPLPNASGILEMAEMVYAGCMSETRQLQAHLARFDQLITAAR
>tr|A0A0Q0IGT5|A0A0Q0IGT5_PSEAP HpcH/HpaI aldolase OS=Pseudomonas
syringae pv. aptata OX=83167 GN=ALO85_00391 PE=3 SV=1
MMLRTNTLKSRIADGQPAHGVISSIPAPAAIELIAEAGFDFVIIDMEHVLINPETVENMI
RVAESYDLTPLVRVADLNPKTLLRLLDGGAQGIVLPMIESPEQLADAIAACKYHPLGRRS
LNAGRPGSFGKHSLAQYVEAANQQIMVVAMIESAEGVRRAAEIASVPGLDMILEGAADLS
QSLGMPWQIDQPDVQNALIATWQATHAAGVAYCAIPRQPGDAARWQARGVNTFVLGDERG
IAFRALQARLAQHSAEGK
>tr|A0A0Q0DZ87|A0A0Q0DZ87_PSEAP DUF4174 domain-containing protein
OS=Pseudomonas syringae pv. aptata OX=83167 GN=ALO85_03390 PE=4 SV=1
MLIRSLTLATLFVAAGTALAADNDGPLAQERGKSRPLIIIAPSTIDPVLVKLKKSLDEPA
NREAFAQRNMVLFTVVNTMGERDGKSMDPQSTMALIRELKLGAGAGTKVILVGKDGEKKV
DKTLKNPESLDPKEIFATIDQMPMREQEAAAPAPEPPAKPAPAEKPGKPVKGGAPGKTLD
D
>tr|A0A0Q0FKI5|A0A0Q0FKI5_PSEAP Ribulose-phosphate 3-epimerase
OS=Pseudomonas syringae pv. aptata OX=83167 GN=ALO85_00448 PE=3 SV=1
MQPFVIAPSILSADFARLGEEVDNVLSSGADFVHFDVMDNHYVPNLTIGPMVCSALRKYG
VTAPIDVHLMVSPVDRIIGDFIEAGATYITFHPEATQHIDRSLQLIREGGCKAGLVFNPA
TPLNLLEYVMDKVDMILLMSVNPGFGGQKFIPGTLNKLREARALIDASGRDIRLEIDGGV
NVNNIREIAAAGADTFVAGSAIFNAPDYREVIDKMRTELASSRA
>tr|A0A0Q0ICV3|A0A0Q0ICV3_PSEAP TetR family transcriptional regulator
OS=Pseudomonas syringae pv. aptata OX=83167 GN=ALO85_03056 PE=4 SV=1
MPMTLEVPAHSAHVTKPASRIRQKNEEAIIQAAEDEFARHGFKGTSMNTIAIKAGLPKAN
LHYYFTNKLGLYVAVLSNIIDLWDSTFNHLSVEDDPAEALARYIRAKMEFSRRQPQASRI
FAMEIISGGECMSEYFSQDYRLWFQGRAAVFQAWIDAGKMDPVDPVHLIFLLWGSTQHYA
DFASQICRVTGRSRLTRQDMDQASNNLIRIILKGCGLTPPAL
>tr|A0A0Q0CBB6|A0A0Q0CBB6_PSEAP Thioredoxin reductase OS=Pseudomonas
syringae pv. aptata OX=83167 GN=ALO85_01765 PE=3 SV=1
MSDVRHSRVIILGSGPAGYSAAVYAARANLKPLLITGMQAGGQLTTTTEVDNWPGDPHGL
TGPALMERMREHAERFETEIVFDHINSVDLAGKPFSLQGDSATYTCDALIIATGASARYL
GLPSEEAFMGKGVSACATCDGFFYRNREVAVVGGGNTAVEEALYLANIASKVTLVHRRET
FRAEKILIDKLHARVAEGKIELKLNATLDEVLGDNMGVTGARLKNNDGSSSELKVDGVFI
AIGHTPNTSLFDGQLALKDGYMVVQGGREGNATATSVEGVFAAGDVADHVYRQAITSAGA
GCMAALDVERYLDGLADVSF
>tr|A0A0Q0DPL6|A0A0Q0DPL6_PSEAP TetR family transcriptional regulator
OS=Pseudomonas syringae pv. aptata OX=83167 GN=ALO85_03612 PE=4 SV=1
MVIEESGNESAETAPPKRRRAPKGDMRRKALLDAATVVFARDGYSSASMREVALLAGITT
VGLLHHFPNKEALLAALLERRDQRVTSRFQDLVTEPTLEGFLKFLKLSMSFSIESAEECQ
AALLMNTESLSKAHPAWAWHLERFHLTHEHARGHLQALIEAGEVRGDINVKALAQEIFSV
MDGLQIQWLRSPQDVDVMAVFDIYVQRLGRDIKAHP
>tr|A0A0Q0FWJ9|A0A0Q0FWJ9_PSEAP Sodium/hydrogen exchanger family protein
OS=Pseudomonas syringae pv. aptata OX=83167 GN=ALO85_04867 PE=4 SV=1
MLVTKRIAVAQKGTAISACNAIITRSFAIMHAINFIQDLAVIMLVAGVVTILFHRLRQPV
VLGYIVAGFIIGPHTPPFSLIHDEDTIKILAELGVIFLMFCLGLEFSLRKLFKVGATAFI
AAFLEIALMIWIGYEIGQFFGWKTMDSLFLGAILAISSTTIIVKALNDLKMKNQRFAQLI
FGVLIVEDILGIGIIALLSGIAVSGSVSSGEVFSTVGKLSLFMIVALVVGILVVPRLLSY

VAKFESNEMLLITVLGLCFGFCLLVVKLEYSMVLGAFLIGAIMAESRELLKIERLIEPVR
DMFSAIFFVAIGLMIDPGILLQYAWPIAVITVAVVLGKMLSCGLGAFIAGNDGRTSLRVG
MGLSQIGEFSFIIAALGMTLQVTSDFLYPVAVAVSAITTLLTPYLIRGADPLSLKLAAIM
PRRVARVFGMYGEWLRSIQPQGQSAVLAGMIRRILLQVGVNLALVMAIFFSGGYFAERLG
GYMSEWVGDVSHQKAWICGAALLLSLPFLIAAYRKLKALSMLLAEMGVKPEMAGRHTVRV
RKVVAEVIPLLSLMVIFVMLAALSASILPANELLLVVALVAALVAALLWRWLIRVHTRMQ
IALLETLGNHQENSGH
>tr|A0A0Q0IC41|A0A0Q0IC41_PSEAP Putative phosphotransferase
OS=Pseudomonas syringae pv. aptata OX=83167 GN=ALO85_02985 PE=4 SV=1
MSDQDIRLQSLKVWLDEQLPALFSAHGWGAVPPATLTAASSDASFRRYFRWEGADRTFIV
MDAPPPQENCKPFVDIAHLLEKSGINVPKIYAEDLTQGFLLLNDLGRQTYLDVIDADNAD
ALFADAIQALLAYQQLPMDAPLPSYDVALLRRELELFPEWYVKRHLGVEMDEAQLSDWQQ
VSELLINSALAQPKVLVHRDYMPRNLMISEPNPGVLDFQDAVYGPVTYDVTCLFKDAFLS
WPQERVSDWLRTYWDQARAIGIPVQEDFAAFERASDLMGVQRHLKVIGIFARICHRDGKP
RYLEDVPRFFAYIEAVLSRRPELAQLGQLLTSLQPVDAAV
>tr|A0A0Q0IAX0|A0A0Q0IAX0_PSEAP Arginine N-succinyltransferase
OS=Pseudomonas syringae pv. aptata OX=83167 GN=ALO85_01843 PE=4 SV=1
MIVRPVRSSDLPALIELARSTGAGLTTLPANEQRLAHRVGWAEKTFRGEAERGDADYLFV
LEDDDGRVVGISAIAGAVGLREPWYNYRVGLTVSASQELNIYREIPTLFLANDLTGNSEL
CSLFLHSDSRNGLNGRLLSKARMLFIAEFPRLFGNKIIAEMRGMSDEQGRSPFWESLGRH
FFKMEFSQADYLTGVGNKAFIAELMPKFPLYSCFLSEDARNVIGRVHADTEPALTMLKSE
GFSYQGYVDIFDAGPAIECETGKIRAIKDSQALVLAIGTPGDDAPQFLIYNRKREDCRVT
VGAARFAAGTLVVAPQTAKRLRMSAGDNVRAVPLSAAREGV
>tr|A0A0Q0ISW1|A0A0Q0ISW1_PSEAP Glucose-6-phosphate isomerase
OS=Pseudomonas syringae pv. aptata OX=83167 GN=pgi PE=3 SV=1
MAYYRTPSDVTALPAWQALNKHRQTMQNFSMREAFNTDPQRFSQFTLSSAGLFLDYSKNL
ITTETRDLLVSLAGEVGLKDAIKAQYDGELVNSSEGRPALHTALRRPVGDKLKVNGVDVM
PDVHRVLNQMTELVGRIHDGLWRGYTEKPITDVVNIGIGGSFLGPELVSEALVAYAHKGV
RCHYLANIDGSEFHELSMKIRAETTLFIVSSKSFNTLETLKNAQAARAWYLAQGGSEVEL
HRHFIAVSSNNAAAVAFGIREENIFPMWDWVGGRYSLWSAIGLPIALAIGMSNFKELLSG
AYTMDQHFQSAPFEQNMPVLLGLLGVWYGNFWGAQSHAILPYDHYLRNITKHLQQLDMES
NGKSVRQDGTPTSTDTGPVIWGGVGANGQHAYHQLLHQGTQMIPADFIVPIVSFNPVADH
HQWLYANCLSQSQALMMGKTRAEAEAELREKGMDEQEVQKLAPHKVIPGNRPSNTLVVER
ISPRRLGALVAMYEHKVFVQSVIWGTNAFDQWGVELGKEMGKAVYQRLTGGTEEPADDAS
TQGLINYFRGRHRG
>tr|A0A0Q0FBT1|A0A0Q0FBT1_PSEAP Uncharacterized protein OS=Pseudomonas
syringae pv. aptata OX=83167 GN=ALO85_04175 PE=4 SV=1
MGMPEGKALILDTTTAMSITKPETLPKPIQRALNQIAHSRPLLYQAACRDQIRKEIDTLL
ARGMSHQDAIEPLRACPPTLDPDY
>tr|A0A0Q0DIE9|A0A0Q0DIE9_PSEAP 30S ribosomal protein S14 OS=Pseudomonas
syringae pv. aptata OX=83167 GN=ALO85_200005 PE=4 SV=1
MDKAVEYEKRLTLFLDFLGFSEKVKATEKNAELLIDLVDALKDAAETANIGSAPDFQATQ
FSDCLVISYKFESPDALFDIVNKLSLIVVSLAGRGYLIRGGLTYGWLLHTDEIVVGPAMI
RAHSLESKVAKAPRVILDPEIFNAALKKPKDEVLIVIKKFLKRDKDKDGNVIWFFDYFSW
QTVVATVGGENDLFPKYFKTLSGLIKAGLKSNDPGVLEKYVWMQRQYKIARDSLLSTPSN
NPFRKYHPGYLESIEKLPPLRRAAARAKELIAEARKKENEKKK
>tr|A0A0Q0DQG1|A0A0Q0DQG1_PSEAP Lipid A deacylase OS=Pseudomonas syringae
pv. aptata OX=83167 GN=ALO85_01710 PE=3 SV=1
MKRLFCLAVIAAALAGQASIAQADGVEFSVGQTGESTMTYRLGVQFDWDKTWLQSDIGRL
TGYWDGAYTYWDGKDYKDNHSLSFSPVLVYEFGNGNVKPYVEAGIGVSVFSNTQVEDRKF
GSAFNFEDRIGFGLRFAGGHEVGIRATHYSNAGIKEPNDGIESYALHYKMPF
>tr|A0A0N8T824|A0A0N8T824_PSEAP RNAse G OS=Pseudomonas syringae pv.
aptata OX=83167 GN=ALO85_00155 PE=4 SV=1
MSEEILINITPMESRVAVVENGVLQEVHVERTQRRGIVGNIYKGKVVRVLPGMQAAFIDI
GLDRAAFIHASEISMREGPAVESIASLVHDGQSLVVQVTKDPIGSKGARLTTQLSIPSRY
LVYMPRTAHVGISLKIEDEAERERLKKVVSDCVAQEGIKEAGGFILRTAAEGAGADEILM
DIRYLRRLWDQIGEQIKTVSPPTVIYEDLGLALRTLRDLVSPRIEKIRIDSRETFQKTTQ
FVAELMPEIADRLEHYPGERPIFDLYGVEDEIQKALERKVPLKSGGYLVIDPAEAMSTID
VNTGAFVGHRNLEETIFKTNLEAATAIARQLRLRNLGGIIIIDFIDMEDGEHQRQVLRTL
EKQLERDHAKTNIIGITELGLVQMTRKRTRESLEQVLCEPCHCCAGRGKLKTPETICYEI

FREILREARAYQAVGYRVLANQRVVDRLLDEESGNVAELESFIGRTIRFQVETMYSQEQY
DVVLL
>tr|A0A0Q0BWU0|A0A0Q0BWU0_PSEAP DUF4124 domain-containing protein
OS=Pseudomonas syringae pv. aptata OX=83167 GN=ALO85_02992 PE=4 SV=1
MHWMILAAALAISATSQAASIYKWVDAQGVTHFDAQPPTGQPVQEINVQTPPPAAAPGMP
ADDGAAQQREIDQKVKSQVKAQEAKRAENCETLRTNLAQLQNNPRVREQVGGESRRLTDE
NRKTRIDETNRAISEYCR
>tr|A0A0Q0BW41|A0A0Q0BW41_PSEAP Alpha/beta hydrolase fold protein
OS=Pseudomonas syringae pv. aptata OX=83167 GN=ALO85_04792 PE=4 SV=1
MLLSTLSQGRRLMKRIASAALFIASCTTALMVSAAPLPASSAANIDGVEVHTVTLKTDAM
KMALWRDSPKGTHTQTMKESQVVLFIHGATISGNLSAGYAIDGYSWAQDVANSGREAWVV
DLPGYGRSDDYSEMREASPHANGESVGNAKSLVPVIDAAVDYVLKFTGAKKLTIIATSRG
AIPVGYYLSAHGEKVDKVVFNSPIVRRDTTSPEIVRGLFGSAERPDKSFYEIPVDKRLSM
IEEDRPAGTETQLEQGFIRNWPGDAALERAHQKNMIKVPGGFAQDIYDAWHGVYWDPATI
TVPVLITRGDYDHVLTTAADIDWLYTHLSNASSKRYVLIDKGTHAMLFEKKRFELYREVR
VFLEGSYNRLSLACSFCGGRN
>tr|A0A0Q0FGU1|A0A0Q0FGU1_PSEAP Probable nicotinate-nucleotide
adenylyltransferase OS=Pseudomonas syringae pv. aptata OX=83167 GN=nadD
PE=3 SV=1
MTALPRRIGMLGGTFDPVHIGHLRGALEVAELLELDELRLTPSARPPHRDMPSVTAEDRL
AMVQSAVAGVSPLTVDDRELKRDKPSYTLDTLESMRAELAPRDQLFLLLGWDAFCGLPTW
HRWEELLEHCHIVVLQRPDADSESPDAMRNLLAARAVSDPKALKGPGGQITFVWQTPLSV
SATQIRQLLASGKSVRFLVPDAVLAYIDARGLYRAPNTDGSS
>tr|A0A0Q0FDC3|A0A0Q0FDC3_PSEAP GAF domain protein OS=Pseudomonas
syringae pv. aptata OX=83167 GN=ALO85_00104 PE=4 SV=1
MNNNLTSAELAVISEIEATSSLLRLVTRLTGLRFAAIAKVTEASWTACAVYDEIQFGLEA
GHQLKLETTFCNELRLHRQPIVINEVATDPVYAEHPITKMYGFQSYFSLPIIFPNGDFFG
TLCGLDTKPAPLDDANTLDTLKVFCTLIASTLYAHYQLQEMQEIQEVTA
>tr|A0A0Q0BYT1|A0A0Q0BYT1_PSEAP Uncharacterized protein OS=Pseudomonas
syringae pv. aptata OX=83167 GN=ALO85_03190 PE=4 SV=1
MAAMRIKASHSKAKPAPAVETSDSINAQIAAFLKSGGEIQQIAKGVSGQTFAPSKQINLG
KK
>tr|A0A0Q0E117|A0A0Q0E117_PSEAP Uncharacterized protein OS=Pseudomonas
syringae pv. aptata OX=83167 GN=ALO85_01400 PE=4 SV=1
MSLLRNLGAVFLLTAAAGANASSFIVTTDAVVDAVNAATRATSNVSSSLKDDKIVLAARD
DAASFVASSGDIRSARLEGALQHIRQVLPELQASDSQLAQAILAI
>tr|A0A0Q0DLE9|A0A0Q0DLE9_PSEAP Pyridoxine/pyridoxamine 5'-phosphate
oxidase OS=Pseudomonas syringae pv. aptata OX=83167 GN=pdxH PE=3 SV=1
MTQTLADMRRDYTRDGLTEAQSPDEPFALFHAWFADAVNTEQPPVEANAMTLATVDEQGR
PHCRVLLLKGLDTQGFTFFTNYESAKGQQIAARPFAAMTFFWPTLERQVRIEGRVEKVSA
QESDAYYQVRPLGSRLGAWASPQSRVIADRDELEGLIRQTEQRFADTQPHCPEHWGGYRL
LPERIEFWQGRSSRLHDRLNYRLIDDRWTRERLAP
>tr|A0A0Q0DPZ2|A0A0Q0DPZ2_PSEAP Phage repressor protein, Serine
peptidase, MEROPS family S24 OS=Pseudomonas syringae pv. aptata OX=83167
GN=ALO85_100128 PE=4 SV=1
MRNGRFHFAAYIVQTAAIAHQRARAGCATRNVRAPFHDPRHRAAQSAAQADAHALFTPVL
HHGFYFLDHRVSPVGPFRNAAQRLSLHRTKALQYILYFYDTTEHYIMYIAQHPTACMIRR
MNKKWYEVARQVMETQEISQEEMAERMGVTPGAVGHWLNGKREPKIEVINRFLTELGLPI
LTTSIPASEPGMHNVEPTVQPSRFYRYPVISWVEAGGWSEAVEPYPAGYSDTFEISDYKA
KGRAFWLVVRGDSMTAPAGQSIPEGMLILVDTGIEPTAGKLVIAKLPESNEATFKKLVED
AGRYFLKPLNPAYPTLAVTEECKLIGVIRQMTMRL
>tr|A0A0Q0FIM6|A0A0Q0FIM6_PSEAP Type II secretion system protein
OS=Pseudomonas syringae pv. aptata OX=83167 GN=ALO85_00690 PE=4 SV=1
MSLFKYRALDAQGAPQNGTLEARDQDAAIAALQKRGLMVLQVDAAGLGGLRRALGSGLLN
GAALVSFTQQLATLLGAGQPLERSLGILLKQPGQSQTKALIERIREQVKAGKPLSVALEE
EGSQFSPLYISMVRAGEAGGALESTLRQLSDYLERSQLLRGEVINALIYPAFLVVGVLGS
LALLLAYVVPQFVPIFKDLGVPIPLITEVILNLGEFLSDYGLAVLAGLIALIWGMAIRIR
DPQRRERRDRRLLSIRVIGPLLQRIEAARLTRTLGTLLTNGVALLQALVIARQVCTNRAL
QAQVEQAAESVKGGGTLASAFGAQPLLPDLALQMIEVGEQAGELDTMLMKVADVFDVEAK
RGIDRMLAALVPALTVVMAGMVAVIMLAIMLPLMSLTSNI

>tr|A0A0Q0ITA6|A0A0Q0ITA6_PSEAP Type I site-specific deoxyribonuclease, HsdR family OS=Pseudomonas syringae pv. aptata OX=83167 GN=ALO85_01511 PE=4 SV=1
MSQSPEKVGVELPAINWLVKLGYTHLSSNQVQVEYRHLAPVLDDVLQARLLILNPWLESV
PGGIPTALIELRKRLNDELLPANKAFWEQVVHRSDIQVKDVEGRPRSVRFFDASDAANND
FHVVDQYVGRNADGDFFRPDLLLFVNGLPLAIIECKASHHRLDEALGQLDGYQNCFPTQF
VFNQVCVGLNRREGAYGAILTKPAYYARYRLQAQESAAVAALLGDVLSEQESLLWALFEP
SRFLQLITHFALFETRDGKTMKKLPRYQQWRAVRKTLDRLCAPKPMGGVVWHTQGSGKSL
TMALLARMLRADSTGLNNPTVLVLTDRKDLDKQIFDTFHAVGISAIQAVSVEGLLKMLSN
DYGSVFTSTVQKFQENEQPGEQPAVTPDEEDNALQATRHRRIREEKDFFILHERNVRHRQ
VDENGVLVKAKWEEISREKVHFRVLSTKSNFYVLVDEAHRSQYDFLAAFMRASLPNAKFI
AFTGTPLLQEDKHTLGEFGGGEYIDEYRLHEAVADGATLSIKYQDAWVALSANAELDAAF
KEQFSGQSEARQHELKRELLRRWRQAGNRMEQVAEHLVEHFLSNVKAKGLKAMLVCDGRD
MAVGYKDLLDAIMAERVQKGLPTFDSKVVISLASITASRTGASAMEEASEYEVDLDKIQS
IEERVKAEVKAGNTPVAMPSEQIGNFVSKLFPLPYGEESSGLDGKPQANNVGLIIVSDML
LTGWDAPIVGTMYLDKPLKEHTLLQAIARVNRTLPGKNAGYIVDYHGVVEHLDHALKIYG
GEVKPSQVWEGVETELPKLQATLDRILKLLPKKHDPVAQREGYKTDAERFLDPATRLDMV
EEFLDLVKQFNRSVDIILPDVRGVPFKPYFTLLGEIRLMLRDKLPGNTYKERITKLESVL
LQQLLDEYVAASPAKSLLGHEVSILDASDMARLKQLASPGSRALVMKNQLKHTIVTGKDK
DPAFFDKLAEELQKLLEEEKAGRITQAKFLEQLELFGQRIQDKDNTGFIKPAHSAVYHYL
TALLAEDTARVATTKLFEDGELSRTVATDNWKKMPDLHPDIRDHLRGLLMPLAGWERSVA
REHAKRILDILLKN
>tr|A0A0N8T8P7|A0A0N8T8P7_PSEAP Uncharacterized protein OS=Pseudomonas syringae pv. aptata OX=83167 GN=ALO85_100903 PE=4 SV=1
MDFFKTLYFLRNSANISLIDQKRLSDGTDCSRMDRERRQRESLSTDA
>tr|A0A0Q0BR94|A0A0Q0BR94_PSEAP dTDP-4-dehydrorhamnose 3,5-epimerase OS=Pseudomonas syringae pv. aptata OX=83167 GN=ALO85_02276 PE=3 SV=1
MNVVATKLPEVLILEPKVFGDERGFFYESFNAKAFQAATGLTREFVQDNHSRSQRGVLRG
LHYQIENAQGKLVRVTVGKVLDVAVDIRRSSPNFGQWVGVELSAENARQLWVPEGFAHGF
VVLSEHAEFLYKTTDYYTPSAERCIAWDDADLAIDWQFDGQPNLSAKDQQGKRLKEADLF
P
>tr|A0A0Q0IBF4|A0A0Q0IBF4_PSEAP Toluene tolerance protein Ttg2B OS=Pseudomonas syringae pv. aptata OX=83167 GN=ALO85_00173 PE=3 SV=1
MRNKSLMDRVRLLGRSAIDIVTVLGRSGLFLVHALLGRGGAGSSFQLLVRQLYSVGVMSL
VIIVVSGMFIGMVLALQGFSILTKYGSEQAVGQMVALTLLRELGPVVTALLFAGRAGSAL
TAEIGNMKSTEQLSSLEMIGVDPLKYIVAPRLWAGFISLPILAMIFSVVGIWGASWVAID
WLGVYDGSFWSNMQNSVSFTNDVLNGVIKSVVFAFVVTWIAVFQGYDCEPTSEGISRATT
RTVVYASLAVLGLDFILTALMFGDF
>tr|A0A0Q0DPX9|A0A0Q0DPX9_PSEAP Uncharacterized protein OS=Pseudomonas syringae pv. aptata OX=83167 GN=ALO85_00416 PE=4 SV=1
MLYLWIKALHVISMVCWFAALFYLPRLFVYHSMSEDTVSRERFIVMERKLYRGIMTPAMI
ATLVFGGWLISFDPSGYFSQGWMHAKLALVVLLIGYHHVCGAQMKRFARGENGRSHVFYR
WFNEIPVLILIAIVILVIVKPF
>tr|A0A0Q0C6Z9|A0A0Q0C6Z9_PSEAP TqsA superfamily permease, member of the PurR regulon OS=Pseudomonas syringae pv. aptata OX=83167 GN=ALO85_01947 PE=4 SV=1
MMTDMRRWFWIGGLALLVAFVVLLHPILTPFLVALLLAYMGDPLVDRLERAGLSRTLSVV
VVFGLFTLVLMALLLILVPMLAKQLFRLYELAPQILDWLQHAALPWVQAKFGLADGFWKF
DKIKAAISEHMGQAGDIVSIVLSQATASSLALIGWLTNLVLIPVVCFYLLRDWDIMTGKI
RGLLPRQREAQIVKLAGECHEVLGAFIRGQLLVMVGLGVIYAAGLMLVGLELGLLIGVIA
GLAAIVPYMGFVIGIGSALVAGLFQFGGDLYPMMGIVAVFMIGQALEGMVLTPLLVGDRI
GLHPVAVIFAILAGGELFGFTGILLALPVAAVIMVLVRHMHDLYKDSEIYAGAEDPEL
>tr|A0A0N8T8U8|A0A0N8T8U8_PSEAP Glycosyl transferase, group 1 OS=Pseudomonas syringae pv. aptata OX=83167 GN=ALO85_04347 PE=4 SV=1
MKILFINSLYAPDIGGGAEIILQRTVEGLQQRGHSVAVLATTDRPGLQLAEVNRVKVYRA
GLLNHYWHFIAQRPGRLARFAWHWRDRYNAGMREYVERVMELEQPELVVCHNLTGWSVSA
WDQITQANCPIVQVLHDFYLLCPASTMFSKGKNCQRRCTTCTRLRSHHAQQSEQVSAVVG
VSRFMLDTLRAQDYFKHARGYVVHNASPFTPALREQPKALVDNAPLRFGYLGTLSTNKGL
EWMIEQFQRLPFKATLQIAGRGQPSDEKRFRAMVTSPDISFVGFQRPEHFYRQIDVAVVP
SLWNEPFGMVAVEACAHSLPVIASRMGGLTEIIQEPLNGLLCSPDDPDSLGLAMLRLHQQ

PELLARLGSQARSSVASLMNLDLMLDQYESIFAQTLQDRST
>tr|A0A0Q0FIL2|A0A0Q0FIL2_PSEAP NADH:ubiquinone oxidoreductase, subunit L OS=Pseudomonas syringae pv. aptata OX=83167 GN=ALO85_00748 PE=4 SV=1
MNLLFLTFVFPLIGFLLLSFSRGRLSENLAALIGVGSIGLSAIVTAWVIWQFNVAPPEGG
HYTQVLWRWMDVDGFQPNFALYLDGLSVTMLGVVVGVGFLIHLFASWYMRGEAGYSRFFS
YTNLFIASMLFLILGDNLLFIYFGWEGVGLCSYLLIGFYYSNRNNGNAALKAFIVTRIGD
VFMAIGLFILFQQLGTLNVQELLVRAPEHFKVGDFWIVLATLMLLGGAVGKSAQLPLQTW
LADAMAGPTPVSALIHAATMVTAGVYLIARTHGLFALAPDILHLVGIVGGVTLVLAGFAA
LVQTDIKRILAYSTMSQIGYMFLALGVGAWEGAIFHLMTHAFFKALLFLASGAVIVACHH
EQNIFKMGGLWKKLPLAYASFIVGGAALSALPLLTAGFYSKDEILWEAFASGNNGLLYAG
LVGAFMTSLYTFRLIFIAFHGEAKTEAHAGHGIAHWLPLSVLIVLSTFVGALITPPLAGV
LPQSVGHAGGEAKHSLEIASGAIALAGILLAALLFLGKRRLATAIANSGPGRFLSAWWFA
AWGFDWIYDKLFVKPYLAISHVLRSDPFDRTIGLIPRLVKGGHDTMSRTETGQLRWYAAS
IAVGAVLVLGAVVLVAI
>tr|A0A0Q0IF84|A0A0Q0IF84_PSEAP Uncharacterized protein OS=Pseudomonas syringae pv. aptata OX=83167 GN=ALO85_05331 PE=4 SV=1
MNLIYKYWSTPMKKIAPLLVLLVSAASQAGDYSVTTTRTPTFINGPGHSAFSRHQLRLAD
YPRGAANNSRLQKISWTATSFPESTGEQAKICFRRGGVHESSCIQIDPGMPGDTDQFNSL
GFSYASDVLIKHQAVNGPWQSKPAGPETVTFHLTY
>tr|A0A0Q0C0T0|A0A0Q0C0T0_PSEAP OmpA family protein OS=Pseudomonas syringae pv. aptata OX=83167 GN=ALO85_100094 PE=4 SV=1
MNRIPRVLSVCLLLGSAGLYGCAGHKDSGQALQQASADFQKVKEDTDVLRSAPKDVIRAG
ESLARAERLSSYLGSGADVAHYAYLSSRYSEIAREHSNLMLSQERLAKMDMERQRMQLAL
REAKLASAQQQGRWLEDQILSLATTETDRGLVMTLGDVLFDAGHAELKNSASRTILKVVQ
FLQINPRRVVRIEGYTDSTGDRQDNLKLSKDRAQAVADVLMDLGVDEKRIHVEGYGQEFP
VNANTSERGRAQNRRVEIVFSDEKGQLGAAR
>tr|A0A0Q0DL43|A0A0Q0DL43_PSEAP Ethanolamine ammonia-lyase light chain OS=Pseudomonas syringae pv. aptata OX=83167 GN=eutC PE=3 SV=1
MNELQDSAAPANPWLELRRLTPARIALGRTGTSLPTSAQLDFQAAHAQARDAVHLAFDHA
AISAQLTEKGRETLLLHSAAADRDSYLQRPDLGRRLNDESAQTLRDYAAAHPGGLDLAVV
VADGLSALAVHRHAVPFLIRLEEQASAEGWSLSPVIMVEQGRVAVADEVGELLGAKMVVI
LIGERPGLSSPDSLGLYFTYAPKVGLNDAHRNCISNVRLEGLSYGMAAHRLLYLMREACR
RQISGVNLKDEAELQTLESDGTDEHSDKPIGNFLLDGPAAPH
>tr|A0A0N8T9F5|A0A0N8T9F5_PSEAP Uncharacterized protein OS=Pseudomonas syringae pv. aptata OX=83167 GN=ALO85_02407 PE=4 SV=1
MKAITRKCMVVAGALAVLAVYSAGAYRNELARQAPYVSSCTFGHCVPTDATFSALR
>tr|A0A0N8T9B0|A0A0N8T9B0_PSEAP Glycerophosphoryl diester phosphodiesterase, periplasmic OS=Pseudomonas syringae pv. aptata OX=83167 GN=ALO85_101200 PE=4 SV=1
MSCALPFDQQNADLNATRPTCAAPLTLELKKAFPNTPGVHKVDAFAGQTNAQADLAVSRV
RYGCVQ
>tr|A0A0Q0DI46|A0A0Q0DI46_PSEAP Uncharacterized protein OS=Pseudomonas syringae pv. aptata OX=83167 GN=ALO85_03004 PE=4 SV=1
MPAATAASPWYRHLWPWIIIAILTGSVTLSLSMVFIAVTHPDPLVTDNYYEAGKGINRSL
NREVLAQTLKLHARLNMDELTGEVELRLSGNSNPERLELNLISPTRPDSDRRVFLTRSPS
EPGRYIGQLQDKVNGRRFVELLGVEGEQTWRLFEEEQIGTNDVTLGDEPLQAAQRRKD
>tr|A0A0Q0FC79|A0A0Q0FC79_PSEAP Disulfide bond formation protein B OS=Pseudomonas syringae pv. aptata OX=83167 GN=dsbB PE=3 SV=1
MSDNTLYLRREKRFLVLLGIICLALIGGALYMQIALGEAPCPLCILQRYALLFIAIFAFI
GAAMPGRRSVTVFETLVTLSALGGIAAAGRHVWILAHPSDSCGIDVLQPIVDGLPLATLF
PTGFQVSGFCTTPYPPVLGLSLAQWALTAFVLTAVLVPACIIRNRRNPY
>tr|A0A0Q0C3Z7|A0A0Q0C3Z7_PSEAP Ankyrin OS=Pseudomonas syringae pv. aptata OX=83167 GN=ALO85_02615 PE=4 SV=1
MKHLIYGLLLFAAAANATPPASTPTPTSPPSELSAEQTASQLRTLFFDASREGNNPMLDT
FIEAHYDLNIRDEQGYTGLILAAYHGHEDSVIRLIDAGADPCAKDNRGNTALMGAIFKGE
LSIAKRLVQADCGANLTNNAGQTAAMYAALFKRTEVLKELTDKGADLSIRDSMGNDVEGL
SKGEFQAPPVR
>tr|A0A0N8TA47|A0A0N8TA47_PSEAP Uncharacterized protein OS=Pseudomonas syringae pv. aptata OX=83167 GN=ALO85_101089 PE=4 SV=1
MAGIGMGHCRPYTMAERQAWRCSVRTRRSAGIHAVQEVK

>tr|A0A0Q0IQ48|A0A0Q0IQ48_PSEAP Uncharacterized protein OS=Pseudomonas syringae pv. aptata OX=83167 GN=ALO85_100323 PE=4 SV=1
MHIHANQMELTWRLNWQASWASENNETPYVFSSNKENTMKFSRLTSLLVCSAGLIPLGAH
AAMDGATRTTFTQECVAAAKKQGLNDKTAATHCECGAKQVDSHFTDKEIASLKDTKAAPP
AALTAKLQKLVAENCTATK
>tr|A0A0Q0DTR8|A0A0Q0DTR8_PSEAP Uncharacterized protein OS=Pseudomonas syringae pv. aptata OX=83167 GN=ALO85_101797 PE=4 SV=1
MRFTKNYDECCVMLRAIAPRHVNAWLNLNCYAL
>tr|A0A0Q0BUP8|A0A0Q0BUP8_PSEAP Carbamoyl-phosphate synthase small chain OS=Pseudomonas syringae pv. aptata OX=83167 GN=carA PE=3 SV=1
MTKPAILALADGSIFRGEAIGADGQTVGEVVFNTAMTGYQEILTDPSYAQQIVTLTYPHI
GNTGTTPEDAESNRVWSAGLVIRDLPLVASNWRNKLSLGDYLKANNVVAIAGIDTRRLTR
ILREKGAQNGCILAGDNITEEAAIAAARSFPGLKGMDLAKEVCTKDTYEWRSSVWDLKTD
SHPEIAASELPYHVVAYDYGVKVNILRMLVERGCRVTVVPAQTPASEVLAYKPDGVFLSN
GPGDPEPCDYAIKAIREVLETDIPVFGICLGHQLLALAAGAKTVKMGHGHHGANHPVQDL
DTGVVMITSQNHGFAVDEATLPSNVRAIHKSLFDGTLQGIELTDKSAFSFQGHPEASPGP
NDVAPLFDRFIEAMAKRR
>tr|A0A0N8T891|A0A0N8T891_PSEAP Adenine deaminase OS=Pseudomonas syringae pv. aptata OX=83167 GN=ALO85_03070 PE=3 SV=1
MYDWLNALPKAELHLHLEGSLEPELLFALAERNRIALPWDDVETLRKAYAFNNLQEFLDL
YYRGADVLRTEQDFYDLTWAYLLRCKAQNVIHTEPFFDPQTHTDRGIPFEVVLAGITGAL
KDGKSKLGVDSGLILSFLRHLSQEEAEKTLDQALPFRDAFVAVGLDSSEMGHPPSKFQRV
FDRARNEGFLTVAHAGEEGPPEYIWEALDLLKIERIDHGVRAIEDERLMQRIIDEQIPLT
VCPLSNTKLCVFDDMAQHNILDMLERGVKVTVNSDDPAYFGGYVTENFHALYTHLGMTED
QARRLAQNSLDARLVKP
>tr|A0A0Q0BVC7|A0A0Q0BVC7_PSEAP Uncharacterized protein OS=Pseudomonas syringae pv. aptata OX=83167 GN=ALO85_04623 PE=4 SV=1
MIRSRLGAISLAAFLALSGTSAFAGSTGPTNPVSDSPKVPGTGAEKNVDGTSPGAPGTSP
GTKGIDPLPGKNGSADDKPHDSKQPHDADHQGSQDGHKAP
>tr|A0A0N8T8A9|A0A0N8T8A9_PSEAP Uncharacterized protein OS=Pseudomonas syringae pv. aptata OX=83167 GN=ALO85_100523 PE=4 SV=1
MAGAAQDVGQLHGVCAKGTDDKELFANCGGGRHAAFLDDERSVLFRTCGAVSTIDGARLL
AGSLGAWLAWIYHDWAGVRWGGASGDKFDRLSEVRR
>tr|A0A0Q0DG68|A0A0Q0DG68_PSEAP Glycosyl transferase, group 1 OS=Pseudomonas syringae pv. aptata OX=83167 GN=ALO85_01150 PE=4 SV=1
MDIGLSCTVWANGERAGHLDGIGIYSRALWQGMDRLSQAEDINVKPYAFGKHLPVLACGK
PQRLSSDYRIHAVLSGVLKLPLGCSASIAKDVQLFHATDHHIPRIKGVPVVATIMDVIPV
LYPEWIKQDLKRLKSWLFTTSIERADHIITISEYSKQDMVSHLGIEPERISVTPLGVDPV
YFERIDIAERNRVLATHGLKPGFFLVVGTLQPRKNLQRVLEAFQQLPAQVRKEHPLVIVG
RDGWSNEELLPQLEALQQRGEGRWLSYLPQNEVLALLQSAGALVFASLYEGFGLPAIEAF
AAQCPLIASNNSSLPEVTGDAAWAVDPHSAESISTAMLAVLEQPEERERKVESGLGRARH
FSWDACAQNTLDIYRKVLAARR
>tr|A0A0Q0DUM1|A0A0Q0DUM1_PSEAP Zinc-containing alcohol dehydrogenase super protein OS=Pseudomonas syringae pv. aptata OX=83167 GN=ALO85_01017 PE=4 SV=1
MRFIVMSTATERTAEQLSPVIPKTMQAVVCHGPEDYRLETVDVPTPGPDEILTKVELCGI
CMGDIKTYRGAPSFWGDAEQPRYVKPPMIPGHEFVCRVVALGPGAEKRGVQIGDRVISEQ
IVPCWGCRFCNHGQYWMCQKHDLYGFQNNVQGAMAQYMIFTKEGIIHKVPDSIAPDEAIL
IEPLACSLHAAERADVDFDDIVVVAGAGTLGLGIIGAVRMRNPKKLIVLDMKPERAALAL
RMGADEVWNPAEVDVLAKIREITDGYGCDIYIEATGHHKAVNQGLAMLRKLGRFVEFSVF
NDEATVDWSIIGDRKELDVLGSHLGPYMYPRAIDFIGNRKIDMRDVVTHKFPLAEFKEAF
AVMERGDKSLKVVLEP
>tr|A0A0Q0DPJ2|A0A0Q0DPJ2_PSEAP Uncharacterized protein OS=Pseudomonas syringae pv. aptata OX=83167 GN=ALO85_03728 PE=4 SV=1
MPGIRTAAQAKAWLDQQGKSVQEFARENSIDPATTYQVLAGRKKGRRGEAHKVAVLLGMK
IGTIPNTEALHEEARE
>tr|A0A0Q0INZ3|A0A0Q0INZ3_PSEAP Sco1/SenC/PrrC family protein OS=Pseudomonas syringae pv. aptata OX=83167 GN=ALO85_04363 PE=4 SV=1
MTQHLTRREVVAGLSLLSLGLLTGCGSSNALPFKHGKDMSNEIVGRNFRLKDSQGNVKTL
SSFRGLMPMIFFGFTQCPAICPTALARAAQIRKLMGEEGKTLQVVFITLDPERDTPEVID

AYVKAFDPTFVALSGTLEETAATAKEFGVFFEKVPLGDTYTISHTATSYVYDTRGVLRLG
LSHRLSAQQCTEDLLTLMEVC
>tr|A0A0Q0C516|A0A0Q0C516_PSEAP Elongation factor G OS=Pseudomonas syringae pv. aptata OX=83167 GN=ALO85_200074 PE=4 SV=1
MTPNAELYNPSTEYADKLISRIGQTPSWIAKRIGVTDKRIRYILDGERTVKGETTPIQMT
YTEQFALECLVAEAIALRM
>tr|A0A0Q0D1L4|A0A0Q0D1L4_PSEAP Uncharacterized protein OS=Pseudomonas syringae pv. aptata OX=83167 GN=ALO85_00399 PE=4 SV=1
MFKKVDDGSLSLAFSIEGLQFEPNLTSLAKSPTSFCHKKLISSPGPLISDFVTHEKNFHY
STYGIHVGQDDRLTFMGDPIVEIDGFFVDCREGSATLHRIVRLRFKPSLERRLVIPRGVA
HTFDNLESIVTRDEPVWYVDHDNPAWNLDNDLVSVPRSSALDEFPIIRPNRYTLPDEAHL
FLSKISQSLLENPKSYLARFSVQIAGAKKFVMLEPKQWANDDRSLAAVVEKAKIPGVEVR
RNRYALTGGKSFTLVPNTNACVSDVLLLKSDYAESAAYHWHARTRKIYTFLNNEGAEITL
SFIDLRENSETFGQMTNHTIISDPRINIRIEQGIAYRITSTQDILIRCEHEVFVDKNEPR
TDIPMFGQDLVPLSDTLPYPRISLPTLQCPHSVVYKMAKFEQHNFT
>tr|A0A0Q0CBJ2|A0A0Q0CBJ2_PSEAP Dihydromonapterin reductase OS=Pseudomonas syringae pv. aptata OX=83167 GN=ALO85_01769 PE=4 SV=1
MAMSSAPILITGASQRVGLHCALRLLEHGQRVIISYRTEHESVTELRQAGAVAIHGDFSC
EAGIMAFVELLKTQTSSLRAIVHNASEWLAETPGDEAENFTRMFSVHMLAPYLINLHCES
LLTASEIADIVHISDDVTRKGSSKHIAYCATKAGLESLTLSFAARFAPLVKVNGIAPALL
MFQPKDDAAYRANALAKSALGIEPGAEVIYQSLRYLLDSTYVTGTTLTVNGGRHVK
>tr|A0A0Q0C824|A0A0Q0C824_PSEAP 3-ketoacyl-reductase OS=Pseudomonas syringae pv. aptata OX=83167 GN=ALO85_02018 PE=3 SV=1
MQQLKQKRAVITGAGSGIGAAIARAYAAEGARLVLGDRDADSLAKIAAECRQLGAQVQEC
VADVGSVDGAQASVDACVEQFGGIDILVNNAGMLTQARCVDLTLDMWNDMLRIDLTSVFV
ASQRALPHMIAQRWGRIINVASQLGIKGGAELTHYAAAKAGVIGFSKSLALEVAKDNVLV
NAIAPGPIETPLVAGISSAWKTAKAAELPLGRFGLAEEVAPVAVLLASEPGGNLFVGQTL
GPNSGDVMP
>tr|A0A0Q0BSR5|A0A0Q0BSR5_PSEAP Uncharacterized protein OS=Pseudomonas syringae pv. aptata OX=83167 GN=ALO85_00065 PE=4 SV=1
MASVLVGQFHARDAEGRVYPVHEFQESQPNEAQGGQPVITYRLAIGDRVKHLGGEDFELV
QSGIKITRTPT
>tr|A0A0Q0C1T6|A0A0Q0C1T6_PSEAP Uncharacterized protein OS=Pseudomonas syringae pv. aptata OX=83167 GN=ALO85_01783 PE=4 SV=1
MQTIEQVIDMDNPFQMITDTFHPDYRVNLSIQGLDGSIMLTLSNECGVVAKRLISAAQRN
DPQRLRKLIESVQFGIAIERGDSAIKILAALTDGATLKKAPVPANGLWPQSQATASAGV
>tr|A0A0Q0BTA2|A0A0Q0BTA2_PSEAP Uncharacterized protein OS=Pseudomonas syringae pv. aptata OX=83167 GN=ALO85_04163 PE=4 SV=1
MSTRLTLDEKNDLINEAMKSSIRHNAEVRCESIDGGSGLYMEGYYLRYKNDKDKIIKAAG
IDASRVTPEENIENILIGKEIISEILNKPEFSRLQSQIKDGHLSIDISTPVTSLKFSDEI
ANHVGKSKALAISERVNADGRLIGLDYFIPALEGSRSTLGTALKDTSDYVKDVLGEIKNK
AVVKQEEKPVVRAKLKM
>tr|A0A0Q0IC99|A0A0Q0IC99_PSEAP Plasmid stabilization system family protein OS=Pseudomonas syringae pv. aptata OX=83167 GN=ALO85_100501 PE=4 SV=1
MQVEWLKTALKNLDEEAAYIALDNPAAAAAFVKAIQSSVTQLASFPAMGREGRIAGTREW
PLPDLPYLIPYRIRSGRLQVLRIFHTGRQSPPVW
>tr|A0A0Q0C1A2|A0A0Q0C1A2_PSEAP Histidine ABC-type transport system, permease protein OS=Pseudomonas syringae pv. aptata OX=83167 GN=ALO85_02095 PE=3 SV=1
MFPESFTFSIANWVNDGVDSLVTQYGDVFRHISDTLLWAIVNLEGLLRMAPWWLMLMIVG
GIAWHATRKIVTTLVIVGLLFLVGAVGLWDKLMQTLALMLVATIIAVLIGIPLGILSARS
NRLRSVLMPLLDIMQTMPSFVYLIPVLMLFGLGKVPAIFATVIYAAPPLIRLTDLGIRQV
DGEVMEAINAFGANRWQQLFGVQLPLAMPSIMAGINQTTMMALSMVVIASMIGARGLGED
VLVGIQTLNVGRGLEAGLAIVILAVVIDRITQAYGRPRHEAHK
>tr|A0A0Q0DUK8|A0A0Q0DUK8_PSEAP GNAT family acetyltransferase OS=Pseudomonas syringae pv. aptata OX=83167 GN=ALO85_00990 PE=4 SV=1
MQMDFIFRDAVETDVDDLLLLENQCFDGDRLTARSFHWMIRRANASLIVAEQAGQLTGYA
LVLFHRGTSLGRLYSLAIADAARGKGLGRQLLQRAEQQAVGRDCAYLRLEVRPDNGAAIA
LYERSGYRRFAQVDDYYQDHAPALRYEKRIVEHARLDSRSVPYYQQTLEFTCGPACLLMA

MKALQPARSMGRGEELQIWREATTVFMTSGHGGCSPQGLALAALQRGFAVKLLVSVSGPL
FLGGVRSDNKKQVIRLVHEQFCDALQTAGVQTLPNRSLNLPGLLADGGKPLVLISSYRLT
RSKAPHWVMVTDCDSDFVYLHDPDIDHSLHRQAIDCQHMPVSHADFNRMSCFGADKLRAA
VIVFAAPVDDHACASADRPQDSLP
>tr|A0A0Q0E1D9|A0A0Q0E1D9_PSEAP Uncharacterized protein OS=Pseudomonas syringae pv. aptata OX=83167 GN=ALO85_01405 PE=4 SV=1
MKKPWKFAAVAALIATTSGCVSYNVSQPSAPLEGTVKTDLKADVKVGGTISGESSTNVLF
NFLSFGGDNQFADGVTYGGASGGGGLGLALPDPVSTTKAAAAFKAVKSSGADLIVAPRYE
VSVKDYFIFKKVDVKVTGNKGSISSIR
>tr|A0A0Q0C803|A0A0Q0C803_PSEAP Membrane-bound lytic murein transglycosylase A OS=Pseudomonas syringae pv. aptata OX=83167 GN=ALO85_01106 PE=4 SV=1
MVLLMMLSFKPWLRGLALVLPLAALLTACDKSDKSDKPVTPPAPPHTTYSQAGWDALPAV
SDADLQAGFASWRSACVRLKSDAVWGPTCAAAASLAAAPDAAQIRTFLQEQLQVYSLRAD
GGSADGLITGYYEPVYPGSLTQTATATVPVYGIPDDLIVVNLDSIYPELKGKRLRGRLEG
RVLKPYDDAATISAQGLNAPVIAWLTDPMDLQFLQIQGSGRVSLEDGSQLRLGYADQNGR
PYRAIGRWLVEQGQLKKEDVTMGSIAAWAKAHPERISELLASNPSYVFFARNPDSNEGPR
GSLNVPLTPGYSVAIDRKVIPLGSLLWLSTTRPDGSSVVRPVAAQDTGGAIAGEVRADLF
WGTGDEAGKLAGDMKQKGNIWLLWPKGMPLPQSAETAPAATPAAASH
>tr|A0A0Q0DJX3|A0A0Q0DJX3_PSEAP Uncharacterized protein OS=Pseudomonas syringae pv. aptata OX=83167 GN=ALO85_00141 PE=4 SV=1
MKPLAHTRIKRLINLTLAAMAGSVVASAYASSTLRCGSQLVSTGDRAFEVQQKCGEPVSQ
EVLGTQETFNSNYRRSEAVRIEEWIYGPDNGMYQYLRFEGGRLVGIESKRRN
>tr|A0A0Q0CFX6|A0A0Q0CFX6_PSEAP Helix-turn-helix, Fis-type OS=Pseudomonas syringae pv. aptata OX=83167 GN=ALO85_03795 PE=4 SV=1
MNNPHCELPDHSVPTEHFSPRGDSSQTHAGGSPTAEELTSCLFFSPDDGRIWLNDQRMLL
LHTSSFGTLRREIIERLGQEQARGMFTRAGYLSGARDARLIRERWPQADASSAFRAGTHL
HTLEGMTKVEPLHFKFDAESGFYEGEFLWHHSCEADEHVAAYGIGQDPVCWTEIGYATGF
VSGLFGQMVVFREVECRGMGHASCRVVGKTSEQWGDVERDLSYLRAVNSPAPVHSAPTPA
ASYEASPPTATDQPLVGASAAFNAASQALQRVAMTPATVLISGESGVGKEMFARQLHQLS
RNREGPFVALNCAAIPDNLIEAELFGVERGAYTGATHSRPGRFERANGGTLFLDEITSLS
LAGQSKLLRALQEREIERVGGVHGIKVNVRVVAATNVDLRKAVADGDFREDLFYRLNVYP
IALPPLRERRDDIPLLINFFLKRFCQEYGRTPAGLTMRALKTLLGYDFAGNVRELQNLIE
RGLIASDEGQAIDLVHIFRNESLPRDAYSLNHHGALRQASAPAGQQAPAASLLDSLNQPD
QAFSIDGLEQRLIQEALDKSEGNLAAASRLLGLSRAQFAYRLKKHQP
>tr|A0A0Q0DMC3|A0A0Q0DMC3_PSEAP Putrescine-binding periplasmic protein OS=Pseudomonas syringae pv. aptata OX=83167 GN=ALO85_00978 PE=4 SV=1
MLRNSLAALMLLASTSHAAETVNIYNWTDYIAPDTLKNFEVATGFKTTYETFETNEALNA
KLLAGHSGHDVVFPSIHFMGRQIQNGLLKTLDKRQLPNWKNLNPVLLKALEGSDPGNQHG
FPYLWGSTGIGYDTTKVKAILGKDAPLDSWDLVLKPENMKKLAQCGVVFLDSAPALLPIT
LNYLGLPPHSEVPDDYRQAKAVLDSVRPYIRNFSSADYIADLAKGKICVAVGYSGDISQA
QEQAQKEGNGFTIDYVVPKEGAPMWFDMAAIPADVTDTKGAYAFLNYLLRPEVIANITNT
VHYANGNEKADALIGPGLWTDTTVYPDADMLSRLFVMSQVPETIDTLREKLWDTFRADK
>tr|A0A0Q0FWI6|A0A0Q0FWI6_PSEAP Uncharacterized protein OS=Pseudomonas syringae pv. aptata OX=83167 GN=ALO85_01567 PE=4 SV=1
MANYEVRLSSAELEGDATPEVLVEFWDSEAVNERTGRKGDVAFTAFVTASGNGDGYDTVK
SKADVDGVEGIDGKDDAILIELAKAFTKMNLSIK
>tr|A0A0Q0DCK9|A0A0Q0DCK9_PSEAP UPF0234 protein ALO85_04427 OS=Pseudomonas syringae pv. aptata OX=83167 GN=ALO85_04427 PE=3 SV=1
MSVLSHRQVMRARIVRTPFIWRTYMPSFDVVSELDKHEVTNAVDNAIKELDRRYDLKGKG
TFEFKELTVTLTAEADFQLEAMIEILKLALVKRKIDAKCLEIKDAYASGKLMKQEVILRE
GIDKELAKKIVAHIKEAKLKVQAAIQGEQVRVTGKKRDDLQEAIAALRAYDSGMPLQFNN
FRD
>tr|A0A0Q0INW3|A0A0Q0INW3_PSEAP Histidine ABC transporter, ATP-binding protein OS=Pseudomonas syringae pv. aptata OX=83167 GN=ALO85_02096 PE=4 SV=1
MSSVEPNKIVVKNVFKIFGNRSKEALQLIKQNQSKDQVLAQTGCVVGVNDLSLSIGSGEI
FVIMGLSGSGKSTLVRHFNRLIDPTSGEILVDGEDILRYDMEALRQFRRHKISMVFQSFG
LLPHKTVLANVAYGLTVRGESKALCQERAQHWITTVGLKGYENKYPHQLSGGMRQRVGLA
RALAADTDIILMDEAFSALDPLIRAEMQDQLLELQASLHKTIVFITHDLDEAVRIGNRIA

ILKDGRLIQVGTPKEILHSPADDYVDRFVQRRVATL
>tr|A0A0Q0D8Q9|A0A0Q0D8Q9_PSEAP Acyl-CoA dehydrogenase family protein OS=Pseudomonas syringae pv. aptata OX=83167 GN=ALO85_03752 PE=4 SV=1
MTQTTGSNPLSLGTDYETLANRFRPIFREIGAGSVEREQARALPHEAIAWLQKAGFGAVR
VPTEYGGAGASISQLFQLLIELAEADSNIPQALRAHFAFVEDRLNAPPGADRDQWFARFV
AGDLVGNGWTEVGAVKIGDVITKVSPQGDGFVLNGTKFYSTGSIFADWIDVYAQRSDTGA
DVIAVVDARHAGVRHSDDWDGFGQRTTGSGTSVYDNVPLPATHVIPFEQRFKYQTAFYQL
VLLAVLAGIGRAVERDIAQEVRDRKRVFSHGNAGSVSQDSQVQQVVGQIAAQVYAAEAAT
LRSAEPLQRAYVARFGNDPQREKDANIAAEIETAKAQVIVSELVLRAATELFNALGASGV
SVNKALDRHWRNARTAASHNPLIYKARIVGDWRINGTEPPFVWQIGSGKGNA
>tr|A0A0Q0D9A6|A0A0Q0D9A6_PSEAP Integration host factor subunit alpha OS=Pseudomonas syringae pv. aptata OX=83167 GN=ihfA PE=3 SV=1
MGALTKAEMAERLYEELGLNKREAKELVELFFEEIRHALEDNEQVKLSGFGNFDLRDKRQ
RPGRNPKTGEEIPITARRVVTFRPGQKLKARVEAYAGTKS
>tr|A0A0Q0CE09|A0A0Q0CE09_PSEAP Uncharacterized protein OS=Pseudomonas syringae pv. aptata OX=83167 GN=ALO85_00352 PE=4 SV=1
MPLPASYCTTARLMLALSLAAMPCVSQARETLTWLMRDIPPSTIFSGSMRGQGAIDQLMP
LLTAQMPEYDHVLMRVNRARALQMLSDDTSLSCDPTMLWTAERARTLLFSIPIGAILSSG
LIVRKEEMSKFEPYVEDGKIDVAALMASRTIKLGIVAERSYGEMIDHTLHDVDEGTLVPH
YGNDAVGSLLQMERLGRLQAFISFWPEARYHAMQQGIRPEELAFLPIKGNPAYQFVYVSC
SSSPEGEQAIALVNRVMRVLRETSLMGFYAQWLEPSQRAGYLEDVKALLDKE
>tr|A0A0N8T9C9|A0A0N8T9C9_PSEAP Cell division protein FtsB OS=Pseudomonas syringae pv. aptata OX=83167 GN=ftsB PE=3 SV=1
MRSPNWLFLVLLLLLAGLQYRLWVGNGSLAQVASLTQQIADQHAENQVLLERNRVLDAEV
MELKKGLETVEERARHELGMVKDGETLYQLAQ
>tr|A0A0Q0C0K1|A0A0Q0C0K1_PSEAP MFS superfamily metabolite-proton symporter OS=Pseudomonas syringae pv. aptata OX=83167 GN=ALO85_00993 PE=4 SV=1
MSKPQSPTSRTHEQKARRAVVATVIGNGLEWFDFTVYSFFSVIIAKVFFPTGSELTSYLL
ALATFGVGFFMRPVGGIVLGIYSDKRGRKAALSLTILLMALGTLIIGLTPGFAQIGYLAP
LLIVLARLLQGFSAGGEMGSATAFLTEHAPEDKKAFYSSWIQASIGVAVLLGSALGAALS
SYLTQAQLESWGWRVPFLIGTLIGPVGFYIRSRVDETPAYKAAKPVESPLREVIKTYPRQ
TLISFSLVVLWTVCTYAILFYIPSYAQRVLGLPSWMGFSAGMMGGAVLIVATPIVGAMAD
RSGHRPWLLGSAIAIFLSAYPMFLFINQIPGLFSLLIFELVLGLLISGYIGSILAAFGEL
LPTHVLSTGLSVAYNFAVTIFGGFATFTITWLIASTGSNLAPAFYIMFAAIVSGIGALLY
HDRAPAAHQPLSGAYQ
>tr|A0A0Q0DFC0|A0A0Q0DFC0_PSEAP 5-formyltetrahydrofolate cyclo-ligase OS=Pseudomonas syringae pv. aptata OX=83167 GN=ALO85_01648 PE=3 SV=1
MISAQSLTRPQLRRQLRKARKALSQSQQREAARDIYRQLAQHPLFRRARHVSLYLPMDGE
IDPRLLLREAQRRGKATYLPVLNAWPRTRMVFQRVRPGETFIPNRFRIPEPRIDRAKQRT
IWALDLILMPLVGFDDEGGRLGMGGGFYDRSLAYLARRKTWRKPVLLGLAHECQKVDRLA
QASWDVPLQGTVSDKGWYVTQTL
>tr|A0A0Q0DH65|A0A0Q0DH65_PSEAP Isocitrate dehydrogenase [NADP] OS=Pseudomonas syringae pv. aptata OX=83167 GN=ALO85_00727 PE=3 SV=1
MSNRSKIIYTFTDEAPALATYSLLPIIEAFTATSDIDVETRDISLAGRVLSSFPELLADK
KVPDHLAELGKLATTPEANIIKLPNISASVPQLKATIKELQNQGYNIPDYPENPATDAEK
ETRARYDKTKGSAVNPVLREGNSDRRAPLSVKNYARKHPHKMGAWSADSKAHVAHMSNGD
FYGSEKAAEIAADNTVKIELVAQDGTTTVLKEKTSVKAGEVVDCSVMSAKALRSFIAAEI
EDARKQGVLFSVHLKATMMKISDPIMFGQIVDEFYKDALTKHADTLKQIGFSLNNGIGDL
YERITALPADKQAEIKADIEAVYAVRPQLAMVNSDKGITNLHVPSDVIVDASMPAMIRDS
GKMWGTDGQLHDAKAVIPDRCYATIYQAVIEDCKKNGAFDPTTMGSVPNVGLMAQKAEEY
GSHDKTFHIQTNGVVRVTDSQGNLLMEQNVEAGDIWRMCQAKDAPIQDWVKLAVNRARAS
NTPAIFWLDSSRAHDSVMIEKVRKYLGDHDTSGLDIQILSPVDAMKLTLERTRAGKDTIS
VTGNVLRDYLTDLFPIMELGTSAKMLSIVPLMNGGGLFETGAGGSAPKHVQQFVEENFLR
WDSLGEFLALAASLEHLGHAYNNPKALVLSKTLDQATGEFLDRNKSPSRKVGGIDNRGSH
FYLTLFWAQALAAQNDDADLKAQFAPLAKTLTDNEEKIVAELNAVQGKPVDIGGYYFPNP
EVTSKAMRPSATLNAAIAAL
>tr|A0A0Q0DCJ8|A0A0Q0DCJ8_PSEAP Insulinase-like:peptidase M16 OS=Pseudomonas syringae pv. aptata OX=83167 GN=ALO85_03578 PE=3 SV=1
MPAAHATDTQRLTLANGLNVVLCHEPRLKRCAASLRVAAGSHDAPRAWPGLAHFLEHLFF

LGTERFPAGDNLMTFVQRHGGQVNASTRERTTDFFFELPQAAFAQGLERLCDMLAKPRMD
IADQLREREVLHAEFIAWLGDSASRDQARLLTAINPQHPLRGFHAGNRYSLPVPNPAFQQ
ALKDFYRGFYQAGQMTLCLTGPLPIAELQALATNHGTVFASGMKLKQRPPPALMASPRQA
GEQNHLLFAVEDLPDKADEAVAFFCHWLNAAQPGGLVAELVRKGLGTSLHAAPLYQFGGQ
LLLDIEFTGDLANATPISSLLFSWLGFFKAHWPTRIEEYHRLEQRRLQMCGALALANHHC
RQTPAQLSEQGQKALSALLSKLTADVDNLDPEPVTWHLPAPNPFLGTAADDPAEAALYLR
WQLPSPQPAFWRRLDAALKPLVEDARQAGVTLTFTAYGPYWQLQLNGLREPMVAVLQQSL
QRLQHPDAHTLEHDEPVLIPIRQLLKQLADHYLRSDPEVAISDLPDVWAASRWSSLTSGF
DPARQSMLDAVLNAAPGVRQTSPPDLPTIRAGKHWATQVSSSSEDAVLVFCPAPTTSIED
EAAWRLLAHLAQAPFYQRLRVELQLGYAVFSGLRQIDGRTGLLFGVQSPTSSAQQLFAHI
GAFIGKLPQLVRDADLPEQANALAAQFEPSSLPQQQRADLQWQAQLAGHRGDHAQTLQGA
LSNLDTHSLLASADQLISATGGWLIVANRPASAAVPQSLPEQ
>tr|A0A0Q0C8B6|A0A0Q0C8B6_PSEAP Hydrogenobyrinate a,c-diamide synthase OS=Pseudomonas syringae pv. aptata OX=83167 GN=cobB PE=3 SV=1
MRAPRDCPAVLIAAPASGQGKTTVTAALARLHRNQGRKVRVFKCGPDFLDPMILERASGA
PVYQLDMWMVGADESRRLLWEAADEADLILIEGVMGLFDGTPSSADLARHFGVPVLGVID
GTAMAQTFGALALGLARYQPDLPFAGVLANRVGTVRHAQLLENSLTEGLRWYGALSREVG
FELPSRHLGLVQASELNDLDVRLDMAAAALASTCEVALPPAVTFTAPDPVQVEPLLRGVR
IAIARDEAFAFLYGASLDLLRNMGAELSFFSPIHDRELPDADSLYLPGGYPELHHVALAA
NGAMQASIRAHHIAGKPILAECGGMLYLLEALTDVEGVRAELVGLLSGEAVMQKRLAALA
LQAVELPEGTLHGHTYHHSLTSTELQPIARGVSPNGGRGAEAVYRLGRLTASYVHFYFPS
CPRAIAALFKP
>tr|A0A0Q0D9E3|A0A0Q0D9E3_PSEAP Endoribonuclease L-PSP OS=Pseudomonas syringae pv. aptata OX=83167 GN=ALO85_00808 PE=4 SV=1
MSDRELIIPPTMQSIMDRAGYAPAVRVGDTLYCAGQVGRTRDMEVILNPEAQFIACWENL
RSVLAEGGCTFDDIVDMTTYHVAMSEHMAVFREVKNRIFPRGQCAWTCIGVAELAHPGLL
LEIKCVAVRRNST
>tr|A0A0Q0BT50|A0A0Q0BT50_PSEAP Binding-protein dependent transport system inner membrane protein OS=Pseudomonas syringae pv. aptata OX=83167 GN=ALO85_02340 PE=3 SV=1
MKIRKIKRAFERIKPNGRQMVIGVPFLWLFLFFALPFLIVLKISFAEADVAIPPYTEIFS
YADEKLQMVLNFANYTLLSGDDLYVSAYLGSLKMAFISTLLCLLIGYPMAYAIASARKDL
QTVLLLLIMMPTWTAILIRVYAWMGILSNNGLLNAFLIWLGLIDAPLQILNTNLAVYIGV
VYSYLPFMILPLYANLVKHDTSLLEAASDLGSSTFNSFWKITVPLSKNGVIAGCMLVFIP
VVGEFVIPELLGGPETLMIGRVLWQEFFNNRDWPVASALAVIMLLVLIVPIILFNRSQAK
ELEGKA
>tr|A0A0Q0E048|A0A0Q0E048_PSEAP Uncharacterized protein OS=Pseudomonas syringae pv. aptata OX=83167 GN=ALO85_02567 PE=4 SV=1
MWSISMPNLEAVTLPSQRPDSWSSSNAQCCARYLICAEAEPALLCQVLNLFAMQYLIPDQ
LSAVQQESLLLIDLQVCGLSWHRAQVIGEKMRNLIDVCSVEVEQVDVQNTAQSRVALATG
>tr|A0A0Q0DMW4|A0A0Q0DMW4_PSEAP Twitching motility protein OS=Pseudomonas syringae pv. aptata OX=83167 GN=ALO85_05264 PE=4 SV=1
MRSCMDITELLAFSAKQGASDLHLSAGLPPMIRVDGDVRRINLPPLDAKEVKALIYDIMN
DKQRQDFEERLETDFSFEVPGVARFRVNAFNQNRGAGAVFRTIPSKILSMEDLGMGNVFR
KITDVARGLILVTGPTGSGKSTTLAAMIDYLNCNKHHHILTIEDPIEFVHESKKCLVNQR
EVHRDTLGFSEALRSALREDPDVILVGEMRDLETIRLALTAAETGHLVFGTLHTTSAAKT
IDRIVDVFPAQEKSMIRSMLSESLHAVVSQALLKKVGGGRVAAHEIMMGTPAIRNLIRED
KVAQMYSSIQTGGSMGMQTLDMCLADLVKKGLITRESARERAKVPDNF
>tr|A0A0Q0DLM6|A0A0Q0DLM6_PSEAP Putative lipoprotein OS=Pseudomonas syringae pv. aptata OX=83167 GN=ALO85_01691 PE=4 SV=1
MSLRSVALISFCVLLAACSKITQENYSKLSAGMPKAQVESLLGSPTECSGALGMSSCTWG
DQNTFISVQYAADKVVLFSGQGLK
>tr|A0A0Q0D5U5|A0A0Q0D5U5_PSEAP Transcriptional regulator PsrA OS=Pseudomonas syringae pv. aptata OX=83167 GN=ALO85_02680 PE=4 SV=1
MAQSETVERILDAAEQLFAEKGFAETSLRLITSKASVNLAAVNYHFGSKKALIQAVFSRF
LGPFCISLDRELERRQAKPEHKPSLEELLEILVEQALVVQPRSGNDLSIFMRLLGLAFSQ
SQGHLRRYLEDMYGKVFRRYMTLVNEAAPRIPPIELFWRVHFMLGAAAFSMSGIKALRAI
AETDFGVNTSIEQVMRLMVPFLAAGMRADTGVSDPAMIAAQLRPRTKSSTSAPAVAKA
>tr|A0A0Q0CZU7|A0A0Q0CZU7_PSEAP Cobalamin biosynthesis protein CobD OS=Pseudomonas syringae pv. aptata OX=83167 GN=cobD PE=3 SV=1

MSVALLSVAGVALDALLGEPKRWHPLVAFGNFASRVEQRFNSGGRGWRSHGVTAWVITVV
PLTLLATLLSWLPGVGWLFDILALYCALGMRSLGEHVQPVAQALRSDDLVEARQRVGYLV
SRQTTELDATEVARAATESVLENGSDAVFAALFWFVVAGAPGVVLYRLSNTLDAMWGYRN
ERFERFGWAAAKIDDLLNYIPARLVALTYALLGKTQLALRCWRTQAPAWDSPNAGPVMAA
GAGALGVELGGAAIYHGELHQRPQLGEGTPADADSIDRGWQLVQRGVWLWLLVICVAAEF
YA
>tr|A0A0Q0DE38|A0A0Q0DE38_PSEAP Uncharacterized protein OS=Pseudomonas syringae pv. aptata OX=83167 GN=ALO85_102193 PE=4 SV=1
MEGDFSQRMGLGYASDVMQLSDMNKDLRTGLLNAIIECYLEKYTSSFPNTGMIQHSNLES
FAKIAYTRFFKLRRDQIPLIFRYFLETICELFEKGAWHRVYSFIEFIITHAPNLNSKHDE
LTSSEDFINECNTTLRAENSAYRIVGGRVMQITSAEEVSEINSAIAANGFAGVNTHIRTA
LTMLTDRENPDYRNSIKESISAVESLAKQLTGNPRATLTPALSELERSHSLHPALKNAFS
SLYGWTSDAHGIRHALMDVSNLTYADARFMLITCSAFINFAIDSTKG
>tr|A0A0Q0CZK8|A0A0Q0CZK8_PSEAP MotA/TolQ/ExbB proton channel OS=Pseudomonas syringae pv. aptata OX=83167 GN=ALO85_01104 PE=3 SV=1
MDMNQLHDITFYVMYAAIAIAIFVAIERGIYFAYVRRQSRALQTALGASVHSEKDLPETL
TRRTSLPLEMVLPVLAQKNAQGSRRDLDDVIETQYLSTRAPLARSLWLIETITTAAPLLG
LLGTILGIIDTFKALASAGVSDPGQISAGIGTALFATGLGIAIALFCVVFHNYFQDSLER
INDQLKILLIRASSGACVHDEAEHPVASAQPKYRTA
>tr|A0A0Q0D5P7|A0A0Q0D5P7_PSEAP Cytochrome OS=Pseudomonas syringae pv. aptata OX=83167 GN=ALO85_02683 PE=4 SV=1
MDASTSQRYTARARWLHWSMAILIVLAYTLILSRTQFSKGSEYRLLVVQSHYWVGILILI
MAFFRAAERRRHRPPGITPPLEGALRIVATLTHYALYAFLFAQPLLGLLTVMVEKGALPI
PLTDLQIPWPLATSDHLAETFEDVHKLLGSIFYYVIGLHVIAALWHHLVRKDDTLKRML
>tr|A0A0N8T820|A0A0N8T820_PSEAP Uncharacterized protein OS=Pseudomonas syringae pv. aptata OX=83167 GN=ALO85_100972 PE=4 SV=1
MQEVIVSYLIVAYMKYFVVRRQLSNDFNAPVNVAGFFK
>tr|A0A0Q0FGE5|A0A0Q0FGE5_PSEAP Amino-acid ABC transporter ATP-binding protein YecC OS=Pseudomonas syringae pv. aptata OX=83167 GN=ALO85_01678 PE=4 SV=1
MIVVEKLTKAFKGNEVLKGIDLRIEAGEVVAIIGPSGSGKTTLLRCLNLLETPDSGSIKV
GDIEIDGTRSMNQQQGLIRQLRQQVGFVFQNFNLFPHRTALENVIEGPVVVKKVAREAAE
ALGRKLLAKVGLSGKEDAYPRRLSGGQQQRVAIARALAMEPQVILFDEPTSALDPELVGE
VLTTIRALAEENRTMVIVTHEMSFARDVANRVIFIDKGVIVEQGEAKALFANPKEERTRQ
FLERTRS
>tr|A0A0Q0C5S6|A0A0Q0C5S6_PSEAP Glutamine synthetase OS=Pseudomonas syringae pv. aptata OX=83167 GN=ALO85_02082 PE=3 SV=1
MSKSVQLIKDHDVKWIDLRFTDTKGKQQHVTMPARDALEDDFFEVGKMFDGSSIAGWKGI
EASDMILLPDDETALLDPFTEEPTLVLVCDVIEPSTMQGYDRDPRAIAHRAEEYLKSTGI
GDTVFVGPEPEFFIFDEVKFKSDISGSMFKIFSEQASWMTDGDVDGGNKGHRPGVKGGYF
PLPPVDHDHEIRTAMCNALEEMGQTVEVHHHEVATAGQNEIGVKFNTLVKKADEVQTLKY
CVHNVADAYGRTATFMPKPLYGDNGSGMHVHMSISKDGKNTFAGEGYAGLSDTALYFIGG
IIKHGKALNGFTNPSTNSYKRLVPGFEAPVMLAYSARNRSASIRIPYVNSPRGRRIEARF
PDPAANPYLAFAALLMAGLDGIQNKIHPGDAADKNLYDLPPEEAKEIPQVCGSLKEALEE
LDKGRAFLTKGGVFSDDFIDAYIELKSEEEIKVRTFVHPLEYELYYSC
>tr|A0A0Q0FV70|A0A0Q0FV70_PSEAP Spermidine/putrescine ABC transporter permease OS=Pseudomonas syringae pv. aptata OX=83167 GN=ALO85_03421 PE=3 SV=1
MLLTPNAMSPGLRTGLYLTTALIALFLLLPILFIVLLSFGSSQWLVFPPPGWTLKWYQQF
FSNPDWMAAAMSSFKVAILTTLASVALGLPTAFALVRGRFPGRDLLYGVFTLPMIVPLVI
IAVAVYALFLKLGYTGTLFSFVVSHVIVALPFTIISIINSLKLFDQSIEDAAVICGASRL
QAIYKVTFPGIRPGMMAGALFAFLVSWDEVVLSVMMASPTLQTLPVKMWTTLRQDLTPVI
AVASTLLIALSVLIMFIAATLRRRNEARSTS
>tr|A0A0N8T8Q8|A0A0N8T8Q8_PSEAP Dipeptide ABC-type transport system, permease protein OS=Pseudomonas syringae pv. aptata OX=83167 GN=ALO85_00011 PE=3 SV=1
MFSFIARRVGLLIPTFFGITLLTFALIRLIPGDPVEVMMGERRVDPEMHAQAMERLGLNK
PLYAQYIDYIGKLAQGDLGESLRTRTSVWSEFTSLFPATLELSLAALIFAGILGLLAGVI
AALKRGSLFDHGVMGISLAGYSMPIFWWGLILIMFFSVTLGWTPVSGRIDLLYDIPPVTG
FMLIDTLLSDEQGAFLDALHHMILPAIVLGTIPLAVIARMTRSSMLEVLREDYVRTARAK

GLSPARVVFVHGLRNALIPVLTVFGLQIGTLLAGAVLTETIFSWPGIGKWLIEAIGARDY
PVVQNGILLIACLVILVNFVVDILYGFANPRIRHQR
>tr|A0A0N8T811|A0A0N8T811_PSEAP Ribonuclease H OS=Pseudomonas syringae pv. aptata OX=83167 GN=rnhA PE=3 SV=1
MSDSVELFTDGACKGNPGPGGWGALLICKGVEKELWGGEANTTNNRMELTGAIRGLEELK
RPCEVTLVTDSQYVMKGITEWMVNWKKRGWKTAAKEPVKNADLWQLLDEQVSRHTVKWQW
VRGHIGHPGNERADQLANRGVDEVRGIRHG
>tr|A0A0Q0C4D8|A0A0Q0C4D8_PSEAP Chemotaxis signal transduction protein WspD OS=Pseudomonas syringae pv. aptata OX=83167 GN=ALO85_00342 PE=4 SV=1
MTGVSGFSSLTHDQAQDIDDCWNRIGIHGDRSCPLLAEHIHCRNCAVYSAAATRLLDRYS
LAQESHEHFQGSVLLRDLETRSILVFRLGEEWLGLASRCLSEVAPSQTIHSLPHQRSRAL
LGVANVRGALVACISLVELLGLDSTAVSTPSTRVVPRMLILSAEGGPVVVPVDEVDGIHA
IDERELEAASASGSHANARFTRGVLQWKGRSLRWLDEDELLSAVYRSLT
>tr|A0A0Q0IFM5|A0A0Q0IFM5_PSEAP Type I secretion outer membrane protein, TolC OS=Pseudomonas syringae pv. aptata OX=83167 GN=ALO85_00699 PE=4 SV=1
MFRHPLKAALLTAILLGVAPLSQAMGPFQVYELALRNDPVYLGALKERDAGLENRVIGRA
GLLPRVSYSYNKGRNDSKATLKGSTQDTTDHRHYNSFGSSLTVQQPLIDYEAYANYRKGL
AQALFADETFRSKSQELLVRVLTLYTQALFAQDQINIAHAKQQAFERQFAQNRQMFEQGE
GTRTDILEAESRNELAIAEQIEASDEQDAALRELAALLGEPSIDVAQLEPLRDSFQALVL
APSSFTEWHALAMANSPILASQRQSLEVARYEVERNRAGHLPTVNAYASIRQNESDSGNT
YNQRYDTNTIGIELSLPLYAGGGTSASVRQANSKREQAEYELEGKTRETLIELRRQFNAC
ASGVGKLRAYQKALISAQALVESTRQSILGGERISLDALNAEQQLYSTRRDLAKARYDYL
MAWIKLHYYAGTLRDTDLARIDEAFVVAR
>tr|A0A0Q0C7S3|A0A0Q0C7S3_PSEAP MutT/nudix protein OS=Pseudomonas syringae pv. aptata OX=83167 GN=ALO85_02031 PE=3 SV=1
MKIIRIAAALLIGADGRTLLVRKRGTQAFMQPGGKIEPGEPAPQALARELEEELGLIVDP
AQAVFLGEFTAPAANEPGFEVNCQLYEVRTDAQAVPAAEIEEVLWVDADSHAGLQLAPLT
RDLILPLYLKRQAARS
>tr|A0A0Q0DL16|A0A0Q0DL16_PSEAP Exodeoxyribonuclease 7 small subunit OS=Pseudomonas syringae pv. aptata OX=83167 GN=xseB PE=3 SV=1
MARKKAALDFEQSLADLQALVERLENGELSLEDSLTAFEQGVRLTRDCQSALTQAEQKVQ
VLLERDGELSEEPFDDAELPE
>tr|A0A0Q0IDT9|A0A0Q0IDT9_PSEAP tRNA-dihydrouridine synthase B OS=Pseudomonas syringae pv. aptata OX=83167 GN=dusB PE=3 SV=1
MSAVRIGPYTVQNGLILAPMAGVTDQPFRQLCRRLGAGLVVSEMVTSDMSLWNSRKSRLR
MIHEGDPEPRSVQIAGGDPQMLADAARANVELGAQIIDINMGCPAKKVCNKAAGSALLKD
EQLVNDILQAVVAAVDVPVTLKIRTGWDRDNRNGLTVAKIAEQAGIQALAVHGRTRADLY
TGEAEYDTIAQIKQAVSIPVFANGDIDSPQKARHVLKATGADGLLIGRAAQGRPWIFREI
EHFLRTGETLPAPQSSEVERILLEHLAALHVFYGDVMGVRIARKHVSWYLATLPGAREFR
AHFNRLEDTEAQCANVREFFSQGCKGPDNENDKEVAA
>tr|A0A0Q0DBU3|A0A0Q0DBU3_PSEAP LacI transcriptional regulator OS=Pseudomonas syringae pv. aptata OX=83167 GN=ALO85_03277 PE=4 SV=1
MTRPVTLKDVANHAGLSKAAVSRYLNRSISLPPETAKRIDTAIKALDYRGNSLARRLSKG
GSETIGLVLPDIANPFFAELADAAEEEASAHGYNLVLCVTRNLLERESTFVRWLDSRNVD
GLLLVSNRPDDGTLASQLMSYRNIILLDEDVPGTSQPKVFADNHQGGRLATDYLLRHGHR
RIAHVSGPSALMSARERYAGYREALALAGIAEVPEYLCFGDYSREFGRIATAQLLALPHP
PEAIFAASDFIALGVLDVLRERNISVPDAMSLVGFDDAVYASLLTPPLCTIRQSSRELGR
LGVAQLLQRMTGGDCAQDVVRVPVELVCRGTVATRSD
>tr|A0A0Q0DVN3|A0A0Q0DVN3_PSEAP Putative s II aldolase OS=Pseudomonas syringae pv. aptata OX=83167 GN=ALO85_02949 PE=4 SV=1
MIDENALRQEICDVGRLLYGRGYTVGSAGNISARLDDGWLITPTDACLGRMDPADIAKVN
LAGEWVSGSKPSKTLALHREVYDRNPQVGGVVHTHSTHLVALTLAGVWQEDDILPPLTPY
QVMKVGHIPLIPYERPGSPKVAERVAELANSVRGVMLERLGPVVWESSVAKACYALEELE
ETARLWLMSNPKPAPLDPAALEELRQVFGARW
>tr|A0A0Q0IB12|A0A0Q0IB12_PSEAP Putative lipoprotein OS=Pseudomonas syringae pv. aptata OX=83167 GN=ALO85_00518 PE=4 SV=1
MNIKSLAFPLLASAVVLLAGCSTPSVVTLQNGTQYVTKDMPKTKTRDGFYEFEDISGKTI
RIKADDVATVKPEE
>tr|A0A0Q0FER8|A0A0Q0FER8_PSEAP Putative lipoprotein OS=Pseudomonas syringae pv. aptata OX=83167 GN=ALO85_01194 PE=4 SV=1

MRVPLVLLGAFIALGIGCSAQAEDCDANQASMNRCAANEQAALDADLNKQYNAQMAWLKT
PAKKQALKDAQRKWIALRDADCLYQAGKPEDSGSIWPLLQSQCLAEQTRVRLKQLQAYVA
CREEGCPR
>tr|A0A0Q0IK49|A0A0Q0IK49_PSEAP TrbL/VirB6 plasmid conjugal transfer protein OS=Pseudomonas syringae pv. aptata OX=83167 GN=ALO85_200092 PE=4 SV=1
MESNFYEWVGGSIDVVLNDYVQLMAQTVIGDFSVLLALGGALFFMTMGLLSVGGFIEQPL
PHLFKIAIKWAFIGALALNANTYLGWVVEAIRGLEVGLADAFSPNGKGATATSVYQVIDR
AMTDGFKIASDLLARAGRRGVTEIGMMCYDLLMAGTMLVATFLITLPAGAMIITAKVLMS
FLLGCGPIFIAMLLFGQMTGKWFDQWFAQVMNYIMQSAAITSVLTLGMKFFTTMLTDIQP
TAGNGPATDMLEVLALSGIVLYMVYQASSLGSQLAGGLSSSAITLRQMAQAAINPAKAVY
NAVNPMTTRRDLQSGMMTSARAANHWVAGNTAWNPAYRQHVMQNLGRNWGKARGGKVSS
>tr|A0A0N8T7Z1|A0A0N8T7Z1_PSEAP Arginine/lysine/ornithine ABC-type transport system OS=Pseudomonas syringae pv. aptata OX=83167 GN=ALO85_01837 PE=4 SV=1
MESLEMKKLMLIGAMALSVLAQPIFADEKPLKIGIEAAYPPFASKAPDGSIVGFDYDIGN
ALCEEMKVKCTWVEQEFDGLIPALKVRKIDAILSSMSITDDRKKSVDFTGKYYNSPARLV
MKDGTTISDSLAELKGKKIGVQRGSIHERFAREVLAPKGVEVTPYGSQNEIYLDIGAGRL
DGTVADATLLQDGFLKTDAGKGYAFVGPSFVDPKYFGDGIGIAVRKGDKADLDRLNAAIA
AIRANGKYKAIQDKYFDFDIYGK
>tr|A0A0Q0BRP7|A0A0Q0BRP7_PSEAP Nitrilase/cyanide hydratase and apolipoprotein N-acyltransferase OS=Pseudomonas syringae pv. aptata OX=83167 GN=ALO85_04097 PE=4 SV=1
MRDISGLPNLNLALIQTTLAWHDREANLEHFEPLLDQARGADLVILPEMFTTGFSMESEA
LSEPEAGPTSVWLLAQAKRIDAVVTGSVMMRAADGSHRNRLLWARPDGELLHYDKRHLFR
MAGEHQHYTPGDRQVMFELNGWRVRPLICYDLRFPVWSRDAQDTDLLLYTANWPGARRLH
WNRLLPARAIENLCYVAAVNRVGSDGKGFAYTGDSQVLDFQGESLLSVGEADGVFKATLG
AADLHAYRTRFPAGLDADSFNIE
>tr|A0A0Q0DX23|A0A0Q0DX23_PSEAP Transcriptional repressor NrdR OS=Pseudomonas syringae pv. aptata OX=83167 GN=nrdR PE=3 SV=1
MALLILLLPATMHCPFCGANDTKVIDSRLVAEGEQVRRRRECLACGERFTTFETAELVLP
RLIKQDGSRQPFDEEKLRAGMQRALEKRPVSVERLEAALVHIKHKLRATGEREVKSLVVG
ELVMTELQKLDEVAYIRFASVYRRFQDLNEFREEIDRLAREPGKE
>tr|A0A0N8T9Q4|A0A0N8T9Q4_PSEAP DUF4124 domain-containing protein OS=Pseudomonas syringae pv. aptata OX=83167 GN=ALO85_02085 PE=4 SV=1
MRRLLSCLLLLLTLPAVADVYTYIDAQGNRVFTDQPRKNATRVDIAPSNNMTGTPPTRTL
EAHPAKPAVQPMFHYQLLRILVPEPDATLVNPSGDLIVTVTSEPVLQAGHSYRLLLDNVA
VGQAGRSPVFPLSNVDRGTHQLSVEIFDELGRVLEKTPNQPFHVQRISLAQKRATHPCKE
DDYGVRPECPLKDKPEPKSSILPFF
>tr|A0A0Q0FFJ9|A0A0Q0FFJ9_PSEAP Branched-chain amino acid ABC-type transport system OS=Pseudomonas syringae pv. aptata OX=83167 GN=ALO85_03134 PE=3 SV=1
MMDGIFLQQMVNGLTLGSVYGLIAIGYTMVYGIIGMINFAHGDVYMISAYLAAIGLAVLS
FFGVESFPFLILGTLIFTIVVTGVYGFVIERVAYKPLRNSTRLAPLISAIGISLILQNYA
QISQGARQQGVPTLLEGAWRFEVGSGFVQITYTKIFILIAAFVGMGVLTYIIKYTKLGRM
CRATQQDRKMASILGINTDRVISYVFIIGAAMAALAGVLITMNYGTFDFYAGFIIGIKAF
TAAVLGGIGSLPGAMLGGLILGVAESQFSGLINSDYKDVFSFGLLVLILIFRPQGLLGRP
LVAKV
>tr|A0A0Q0BYR0|A0A0Q0BYR0_PSEAP 4-hydroxy-3-methylbut-2-enyl diphosphate reductase OS=Pseudomonas syringae pv. aptata OX=83167 GN=ispH PE=3 SV=1
MQIKLANPRGFCAGVDRAIEIVNRALEVFGPPIYVRHEVVHNKFVVEDLRSRGAIFVEEL
DQVPDDVIVIFSAHGVSQAVRTEAAGRGLKVFDATCPLVTKVHIEVARYSRDGRECILIG
HAGHPEVEGTMGQYDAANGGAIYLVEDEEDVASLQVRNPEALAFVTQTTLSMDDTSRVID
ALRKRFPAIGGPRKDDICYATQNRQDAVKQLADECDVVLVVGSPNSSNSNRLRELAERMA
TPAYLIDGAEDMQQGWFDGVERIGITAGASAPEVLVRGVIQQLQAWGATGADELAGREEN
ITFSMPKELRVKSLL
>tr|A0A0Q0C461|A0A0Q0C461_PSEAP Myo-inositol catabolism protein IolH OS=Pseudomonas syringae pv. aptata OX=83167 GN=ALO85_02694 PE=4 SV=1
MKGIKGGIMRIALDPYMYRHLSLGAMVDKTAELGYDYIELSPREDFMPFYKYPRVDRARI
REFRKALSDAGVKLSSLLPLYHWAAPDEDLRIAAVRNWKRAIQVAVEMDCDLMNTEFSGQ

SDHALVCENQFMKSMDELMPHFEREGIKLDIQAHPYDFCERNNESVDIIRGLDRDWINYL
YAAPHTFFYDDGVGDIASMLRYAGDKLTHLIIADTYNHRASSNLRYIVNPPGVTATVHQH
LDIGQGEVNWEAFFSTLRDIKFDGIATVAVFAWEERADESSRFMLERVKSELLR
>tr|A0A0Q0DLT8|A0A0Q0DLT8_PSEAP Putative immunity protein OS=Pseudomonas syringae pv. aptata OX=83167 GN=ALO85_100441 PE=4 SV=1
MEPYAMKSLFTTVLACCSLTALTIHASAQPSSPEGMYRTHAQNYKDMVLATCIASAYKFS
ANVGTDAGSSVTALREWANYDWEKSPEKPREFVDNYLARDYTNPLVESEIKGVKFDLLKC
LDLYHSKELDAQTKKAVINPTHTDVQDYKRP
>tr|A0A0Q0DJM7|A0A0Q0DJM7_PSEAP Transporter OS=Pseudomonas syringae pv. aptata OX=83167 GN=ALO85_100582 PE=3 SV=1
MSTKVESRTMRDLFWLGVSIMSTDKVSVHGSWASRWVFILAATGSAVGLGSIWKFPYMVG
AYGGGAFVLVFLACIALIGIPVMIAETLIGRRSRLSPANALKTLAQEAGHSPRWSWGAFA
GMITALLILSFYSVVGGWSLDYIIDMGRGDFQGVSPDQVGAYFGAVIADPWRLILWHTIF
MLLSAVVIGKGVEAGLEKSLRIMMPMLFLLMLALLGYSLTTGHFMEGVHFMFDFNPEKVL
DGLLPAMGHAFFSLSVGVGSIMVYGAYMTKGASISSTVVGIALLDTFVSLLAGLALFPIV
FAAGLNPSEGPGLMFVTLPYAFGNVAFGQLMGIVFFVLVAVAAWSSAISLLEPMVAYLVE
RTRIRRAWVTFWLAFTCWFVGLGTVFSFNIWQKAKFFVNDGGAFHLYQWGASTGLDFFGV
IDFFTSRIMLPLGGLCFVVFAGWVMGRETVRDELSIRSPLLFNLTYFLMRYVAPLGILVV
FAAQLWK
>tr|A0A0Q0BZG6|A0A0Q0BZG6_PSEAP Fumarylacetoacetase OS=Pseudomonas syringae pv. aptata OX=83167 GN=ALO85_02642 PE=4 SV=1
MTTALNRRSCVESANGHADFPLQNLPIGVFSHGQTAPRGGVAIGDRIFDLRVATESGMFQ
GQAAEVAEIASRDQLNDFLALGAAARRALRAALLQLLDEDSPQRDRLQAVGGLLLPMSEC
TLHMPARVGDYSDFYVGIHHALNVGKLFRPDNPLLPNYKYVPIGYHGRASTLCPSGTTVR
RPNGQTLVPGQEVPVFGPCKRLDYELELGVWIGPGNAQGEAIGIDQAAEHIAGLCLLNDW
SARDIQAWEYQPLGPFLSKSFATTISPWIVTAEALEPFRRAQPPRPEGDPQPLAYLLDDH
DQQGGALDIELEVLLLTAQMKADGLAPHRLGLSNALNMYWTVAQMVAHHSVNGCKLQPGD
LLGTGTLSGPQEGQYGSLLEMTAGGKQPVNLPNGETRMFLQRGDEVILRARCHREGHVSI
GFGECRGVVLD
>tr|A0A0Q0ICF0|A0A0Q0ICF0_PSEAP Isochorismatase hydrolase OS=Pseudomonas syringae pv. aptata OX=83167 GN=ALO85_01236 PE=4 SV=1
MIMDIQDNSALILIDLQQGIHHPKLGRRNNPLAESHVSALLDAWRQSGRPVIHVRHFSTS
PESVFWPQQSGVEYQPAFLPQADERELSKQVPDAFCGSFLEMWLRSDGIRQLVIAGVVTN
NSVESTARSGGNLGFDVLVAHDACFTFDQQDFFGTPRSAEEVHAMSLANLHGEYATVLST
AQILQQVAVE
>tr|A0A0Q0E022|A0A0Q0E022_PSEAP PepSY-associated TM helix OS=Pseudomonas syringae pv. aptata OX=83167 GN=ALO85_03834 PE=4 SV=1
MKSQTVRRWSIVHTWSSLICTLFLLMLAVTGLPLIFHHEIDHLLGDAPHYKEMPADTPRL
DLEQLARAAEAHRPGEVMQYFGWDDEDPNGVMAITAATAGTEPNSSHTFALDARTGEALE
MPSANGGFMMVMLRLHVDLYANLPGKLLLAFMGLLFVVAIVSGTVLYAPFMRKLEFGQVR
VNKSRRTRWLDLHNLIGVVTLTWALVVGVTGVISACADLLIASWRNDALATMIAPYKDAP
PLSQRAPATRLLEIAESAAPGMQADFIAFPGTRFSSEHHYAVFLKGNTHLTAHLATPVLI
DARTLQVTAVVERPWYMDALGMSQPLHFGDYGGMPMKILWAVLDVLTIIVLGSGVYLWWV
RRRAARSVSTVQAQVAQ
>tr|A0A0Q0C4N9|A0A0Q0C4N9_PSEAP GABA permease OS=Pseudomonas syringae pv. aptata OX=83167 GN=ALO85_02300 PE=4 SV=1
MSSTANSSELEQGLKPRHITMLSIAGVIGAGLFVGSGHAIAEAGPAVLLAYAAAGTLVVL
VMRMLAEMAVASPDTGSFSTYADRAIGHWAGFTIGWLYWWFWVLVIPLEANAAATILHAW
FPNIAIWVFTLVITLLLTATNLFSVKNYGEFEFWFALIKVVAIIAFVVLGVAAIFGLLPT
SNVSGVSHLFDSGGFLPNGMGAVLAAMLTTMFSFMGTEIVTIAAAESKNPGKQITKATNS
VIWRIFLFYLVSIFIVVSLVPWNDPRLAEVGSYQTVLDAMGIPNAKLIVDLVVLVAVTSC
LNSALYTASRMLFSLGKRGDAPALSKRTSKGGTPHWAVIMSTAAAFLTVFANYIAPAAVF
DFLLASSGAIALLVYLVIAVSQLRMRQKRIAAGEAIDFKMWLFPWLTWAVIAFIVAVLTI
MLLQEEHRLEIIATGVLSLLVIGAGLIVSRRRKDGRVGAAALG
>tr|A0A0Q0CZW1|A0A0Q0CZW1_PSEAP Catalytic LigB subunit of aromatic ring-opening dioxygenase OS=Pseudomonas syringae pv. aptata OX=83167 GN=ALO85_03504 PE=4 SV=1
MFPSLFISHGSPMLALEPGESGPALKRLAASLPRPRAIVMVSAHWESHELIVNGNPQPET
WHDFGGFPPQLFAVQYPAQGLPELTRKVVELLAASDLPARIDSRRPFDHGVWVPLSLMYP
DADIPVVQVSLPSRQGPELQTRVGRALASLREQGVLIIGSGSITHNLRDLDWNAGPDSIE

PWAAAFRDWMIDKLQNDDEAALHHYRSLAPHAARAHPSDEHLLPLYFARGAGGVFGIAHQ
GFTMGALGMDIYRFG
>tr|A0A0Q0CIL4|A0A0Q0CIL4_PSEAP Thioesterase superfamily OS=Pseudomonas syringae pv. aptata OX=83167 GN=ALO85_04868 PE=4 SV=1
MEPGNAQLSMTVLMTPDMANFSGNVHGGTLLKYLDEVAYACASRYAGRYVVTLSVDQVIF
REPVHVGELVTFLASVNYTGNTSMEVGIKVVTENIRERSVRHSNSCFFTMVAVDDNRKPA
SVPPLEPESADAKRRFIQAQQRRQIRQELEKRYQDIKEEAL
>tr|A0A0Q0DLC0|A0A0Q0DLC0_PSEAP Glycolate oxidase iron-sulfur subunit OS=Pseudomonas syringae pv. aptata OX=83167 GN=ALO85_02634 PE=4 SV=1
MQTTLSEHARTLPRAEEAERILRSCVHCGFCNATCPTYQLLGDELDGPRGRIYLIKQVLE
GQPVTEKTRVHLDRCLSCRNCETTCPSGVDYHNLLDIGRAAVEALVPRPLAERVLREGLR
RVIPHPGLFKSLTGMGLKFRPLLPQTLATKLPATVHPAGLRPHTGHARQVLLLEGCVQPG
LSPNTNSATARVLDRLGIGITAVRQAGCCGAVDYHLNAQEAGLDRARRNIDAWWPALQGS
AESLIQTASGCGAFVKDYGHLLRHDPAYADKAGQISARTKDLVELLRDEPLETLCIKTDQ
RLAFHCPCTLQHAQKLGGEVERVLTRLGFNLTTVPDGHLCCGSAGTYSITQPVIARQLRD
NRMNALESGKPQVIATANIGCQTHLASAGRTPVRHWIELIDAALGTPESH
>tr|A0A0Q0DQD3|A0A0Q0DQD3_PSEAP Fusaric acid resistance protein region OS=Pseudomonas syringae pv. aptata OX=83167 GN=ALO85_01793 PE=4 SV=1
MIASLQRITDLPWRRELHSWARSDGVTWVYIFKVLLAAFLTYWLALRLELPQPHTAMITV
FIVMQPQSGQVFAKSFYRLLGTLAGSAMMVLLMALFAQSPLPFLGSLALWVGLCSAGAAR
YRNFRAYGFVLAGYTAAMVGLPALAHPEGSFMSAVWRVVEISLGILCSTAISAAVLPQSS
SAAMRNALYLRFGGFARFVVNGLRGDISRAEFETGNVRFVAEAIGLEGLRSVTVFEDPHM
RRRNGRLNRLNSEFMAVTTRFNALHQLLERLRLEQADQVLKHIDPGLEHLAELLDGFADR
ALTNDDAALLATRLESCRAYFPARVRSLRTELERTLPSDAERLDFHTSFELLYRLLSELL
DYARTHASLADHSHEREQWKGSFVPRTHWMTALAAGARATCVVGLMSVYWVLTAWPSGAS
MVLASAATVALSSTTHNPRRMSLQMAGGTLLGALSGFIETFFLFPHIDGFPLLCLLLAPV
FVLGAYLGSRPQWTGYGLGLLIFFSLGSVPDNLTVYSPYTFINDYIAMVVGMLICAAAGA
IILPPNSRWMWQRLEQALRNQVVFAISAPLKRLGSSFESQPRDLMHQAYGLAIGKPLVQR
ELLRWMFVVLEIGHAIIELRHEQALLPIHPAYAEYQPWRIALRVMGRALVRLFIQPDAVN
LQRCLSAVDQAIKRVQEADEPFASHFDTSVLRRVKSYLHFIRSSLLDPQSPLAAYGVART
VSGVVHAAA
>tr|A0A0Q0C1Z5|A0A0Q0C1Z5_PSEAP GCN5-related N-acetyltransferase OS=Pseudomonas syringae pv. aptata OX=83167 GN=ALO85_03907 PE=4 SV=1
MLAQQRLDFEFRPMTAADVGNAHELSIALKWPHRLEDWAMLQRVAQGFVAVHNGRLIGSA
FACHQGEFSTIGLVIVSDDYQGKGIGRKLMELAVGSVAPRTAILNATLAGAPLYAKMGFV
SFGEIQQRQGQAQPPGTTELKAQERCRGLSKADHPRVLELANAGSGMDRSVTLGDVLNNL
EHAVGIERDGQLQAFALLRPFGRGLCIGPVVAQSVDQARHMIGELLARVPYAFVRIDIPT
DSGLADWLEEAGLKRVDSVTCMSMGAVPQATQGVRQFALITQAIG
>tr|A0A0Q0DV56|A0A0Q0DV56_PSEAP Xanthine phosphoribosyltransferase OS=Pseudomonas syringae pv. aptata OX=83167 GN=xpt PE=3 SV=1
MEALHKKIREEGIVLSDQVLKVDAFLNHQIDPALMKEIGDEFARLFADAGVTKIVTIEAS
GIAPAVMAGLNMGVPVIFARKHQSLTLTENLLSATVYSFTKQVESTVAISPRHLSSDDKV
LIIDDFLANGKASQALISIIKQAGATVAGLGIVIEKSFQGGRAELDAQGYRVESLARVES
LAGGVVTFK
>tr|A0A0Q0IIX5|A0A0Q0IIX5_PSEAP Cyclopropane-fatty-acyl-phospholipid synthase OS=Pseudomonas syringae pv. aptata OX=83167 GN=ALO85_100030 PE=4 SV=1
MGRPCACWSNAYRSFPIRPLTASTVLPPCNPRTHSMKSSSSSFSANLGTNGLTANLLRRG
VLRQLEGLRNGQLVIVERGERHVFGKNGALIQAEIHILDSAAWGMVASNGSIGAGEAFIH
GYWTTPDLTAVVRVFVSNLDVLDAMEGGMARLTRPLIHALHWLNRNTREGSQKNIAAHYD
LGNTLFEQFLDPTMMYSAAQFLSEYDTLEQAQLNKLERICQKLALKPTDHLLEIGTGWGS
MAIYAAQHYGCQVTTTTLSKEQFAYTERRLIELGLQDRVTLMLTDYRDLTGEYDKLVSIE
MIEAVGHRFLPTYFKQCANLLKSNGMMLLQAITIREQRYAQAKSNVDFIQRYIFPGGALP
SVAKMLDIVGSDTDMNLLHMEDFGLHYARTLRLWHDNFKDAHGKLAELGYDEHFLRLWEF
YLCYCEGGFLERTIGTAQLLLAKPEALREPLLGRFNA
>tr|A0A0Q0DUN3|A0A0Q0DUN3_PSEAP Short-chain dehydrogenase/reductase family protein OS=Pseudomonas syringae pv. aptata OX=83167 GN=ALO85_00956 PE=4 SV=1
MEKVLIITGGSRGIGAATARLAASQGYRICINYLSDHTAAEKTAGQVRAMGAKAITVQAD
VSNEDEIMRLFARVDSELGRVTHLVNNAGTLAQASRVEDMSEFRLLKMMMNNVVGPMLCS

KHALLRMLPAHGGQGGSIVNVSSLAARLGSAGEYVDYAASKGALDTFTIGLSREVAAENV
RVNAVRPGFIFTDFHALSGDPFRVSKLEGALPMGRGGTAEEVAEAILWLLSDNASYATGT
FIDVAGGR
>tr|A0A0Q0FNQ9|A0A0Q0FNQ9_PSEAP tRNA (guanine-N(1)-)-methyltransferase
OS=Pseudomonas syringae pv. aptata OX=83167 GN=trmD PE=3 SV=1
MASLRIEVISLFPEMFSAISEYGITSRAVKQGLLQLTCWNPRDYTTDRHHTVDDRPFGGG
PGMVMKIKPLEDALVQARQAAGDAAKVIYLSPQGRQLNQSAVRELAQEEAIILIAGRYEG
IDERFIDAHVDEEWSIGDYVLSGGELPAMVLIDAVTRLLPGALGHVDSAEEDSFTDGLLD
CPHYTRPEVYADQRVPDVLLSGNHAHIRRWRLQQSLGRTYERRADLLESRSLSGEEKKLL
AEYIRERDDS
>tr|A0A0N8T8L7|A0A0N8T8L7_PSEAP Two-component DNA-binding response
regulator OS=Pseudomonas syringae pv. aptata OX=83167 GN=ALO85_00631 PE=4
SV=1
MSGKRILIVEDDADSASILEAYLRRDGFNVGLAENGQRGIDMHRQWKPDLILLDVMLPLV
SGTDVLSAVRRCSDTPVIMVTAMGDEPEKLGALRYGADDYVVKPYNPREVVARVHAVLRR
SQQNGSNERHLRYQNLLVELDAVTAIIEGQDGAETVLDLTPTEFKILSTLLKTPSKAFTR
EELLEICLPDSEALARVVDAHVHNLRRKLEIHGVDNVLVTVRSVGYRFR
>tr|A0A0Q0BUV5|A0A0Q0BUV5_PSEAP Flagellar motor switch protein FliG
OS=Pseudomonas syringae pv. aptata OX=83167 GN=ALO85_02166 PE=3 SV=1
MNERAMVAKLSKVEKAAVLLLSLGETDAAQVLRHMGPKEVQKVGVAMAQMRNVHREQVEE
VMSEFVDIVGDQTSLGVGSDGYIRKMLTQALGEDKANGLIDRILLGGNTSGLDSLKWMEP
RAVADVIRFEHPQIQAIVVAYLDADQAGEVLGHFDHKVRLDIILRVSSLNTVQPAALKEL
NQILEKQFSGNANTSRTTLGGIKRAADIMNFLDSSIEGSLMDSIREVDEDLSVQIEDLMF
VFNNLSDVDDRGIQALLREVSSDVLVLALKGSDEAIKEKIFKNMSKRASELLRDDLEAKG
PVRVSDVETAQKEILTIARRMAEAGEIVLGGKGGEEMI
>tr|A0A0Q0DJM8|A0A0Q0DJM8_PSEAP Heat shock protein 15 OS=Pseudomonas
syringae pv. aptata OX=83167 GN=ALO85_03901 PE=3 SV=1
MAQKMEEDDKVRLDKWLWAARFFKTRALAKAAIESGKVHCRGERCKPGKEPRIGDEFQIR
MGFDDRTVVVEALSIVRRGAPEAQTLYHETPESIARREHAAAQRKAGNLGVTTDGKPTKK
QRRELFGFRASQNND
>tr|A0A0Q0BWR0|A0A0Q0BWR0_PSEAP Autotransporting lipase, GDSL family
protein OS=Pseudomonas syringae pv. aptata OX=83167 GN=ALO85_100502 PE=4
SV=1
MKNLATFPVQNKNNRDPFMTKTSRRWPFAACLLSLACGTAAAAPYSTMVVFGDSLADAGQ
FPDTAGPRGSTLRFTNRVGPTYQDGSGEAFNLNSSTLIGRMLGVPAGDLAASTSPVNAAL
GAPDGNNWAVGGYRTDQILDSINSQSTVVDPNTGTLLRSRTGYLPANSFRADPNALYYLT
GGGNDFLQGRVLSAGSAAQAANQLADSAQALQQAGARYIMVWLLPDIGKTPALSGSPLAS
ATSALSAGFNQQLVSRLAQINAQIIPLNVPLLINEILAQPARFGFDPNENLVSTCFSGDN
CRESTTNGRSSATPNPSRLFFNDRVHPTEAGQRLLADYAYSLLSAPWEISLLPEMANGTL
RMHQDEIRAQWLSDWGNWQGVGQWQSMLSAGGQKMDFDAQDSSADADGRGYNLTIGGSYR
FAENWRTGVVAGAYRQSLEAGPRDSDYKLNSYIATAFLQYQANHWWGDLSVSGGKLDYEN
AERKFALGVSEGQEKGDTDGEMWAVSGRVGFDIAGPTSRWHLSPFVSADYAHIDVDGYSE
KGDRSTALTFSDQTRKSRRAGVGVQGKFQVTPSTQVWGEVAHEREFETDQQDVTMALNSV
QSVGFTLQGYTPQRDLNRATLGVSQKLTQDLTLRGNYNWRKNDDVTQQGVNVALSLSF
>tr|A0A0Q0CDR8|A0A0Q0CDR8_PSEAP Polysaccharide export protein
OS=Pseudomonas syringae pv. aptata OX=83167 GN=ALO85_02661 PE=4 SV=1
MKSMMLIASLLLLSACNTPSRVGLPDDRAQIEAGQAAGRALAGKPLPPERIRPGDTLRIV
RNSGEAPSISAFTANSIYELTLFQVLNDGTFSYPYIGTVKAAGLTLQQLNDLLESKLQTV
YRETALTINISQSPGNTVFVGGAVRNSVTLPVNVATNLDQAIVGAGGVALDADAGQVALL
RQEENGLYKTYFFDYSKFLWAGGVGGPTAPVLLRRGDIVFVPKSSVGNKVDGVSLYFNQL
IPFAKSIGLGLNYELRNNN
>tr|A0A0Q0DP85|A0A0Q0DP85_PSEAP Methionyl-tRNA synthetase OS=Pseudomonas
syringae pv. aptata OX=83167 GN=ALO85_03781 PE=3 SV=1
MSKFIVTITPPTPNGDLHIGHIAGPFLAADVFTRVQRQRGHECVLVSYSDDYQSYMLRKG
LEQDVDPVELARRNSDRIEASLAAINIQPDNWMRPHDNPWFAEAVGEVFGTLRDAGAIEC
RDSSEPYCPDCDVWGYEAFARGNCNYCGAESDASQCENCAQAPDAELMTDLRCKLCGQPS
QWKNVHRAFLKLAGFKPQLRDRLLGQSWRKPLDSWLRDSLEHLHDWGVTRPGDGGLDLQA
DGSCRVHTWFMGLAGYMAAFREHADRVGRPELFRQYWQSGQGTLVHFLGFDCVFSHAVVY
PAQLTALRDIKVRQRFMPNQFLKLDGLNLSTSRNHAIWVGDLAREACVDSARLYLASIAP
EESEGDFRLAQFHAWRTEVFSEFFPALLEAGSNRDDHWWSGLCGADAGLLEALRVQWSKA

SEPAQFSMKRMAQVLLDVIAITRARLAEGRPISHLAAFIAVLGKALIPDTSAQIISAYGL
PEARVHATLMNGPAAEYSI
>tr|A0A0N8T8D9|A0A0N8T8D9_PSEAP Translation initiation factor Sui1
OS=Pseudomonas syringae pv. aptata OX=83167 GN=ALO85_01355 PE=4 SV=1
MAKKAASLAALGGLVFSTDAGRHCPDCRQPVAECTCKQTAIPEGDGIARVRRESKGRGGK
TVTTISGVPLAEEPLKELAKALKQRCGTGGSLKEGVIEIQGDHVELLVAELIKKGFKAKK
SGG
>tr|A0A0Q0CH65|A0A0Q0CH65_PSEAP Small-conductance mechanosensitive
channel, MscS family OS=Pseudomonas syringae pv. aptata OX=83167
GN=ALO85_04321 PE=4 SV=1
MMDALNVINIGTDGINSLFTIVLQYGATVVLRLVIAALLWIVGRWLIGVLVRMAQRSLTR
QRFDPTVLRYVGSFITVTLNIILVIAILSYCGIETTSFAALLAAVALAIGMAWSGLLANL
AAGGFIIVLRPFKVGDLISAAGVTGTVVEIGLFVTSINTPENVLNLVGNNKIFSDTIVNF
SSNAFRSVELTAILPGSADEQKIIPRLKDQIARLPNVLSSPAVDVHLSSATADSITLSVR
PYCHNDHYDSVHRQTLALIRALMLHLS
>tr|A0A0Q0C9Z5|A0A0Q0C9Z5_PSEAP Uncharacterized protein OS=Pseudomonas
syringae pv. aptata OX=83167 GN=ALO85_100676 PE=4 SV=1
MGHGKSPGMGLKKGADYSSTRGNNRTVQTINDGQIMSRVGTFQVVETSTPPSRLATKPTQ
GADSSACWFDEAALMACVFAEVKPLPATHPLLFANVIAGVLPGRPGQRVRRLK
>tr|A0A0Q0FPJ9|A0A0Q0FPJ9_PSEAP Thiol peroxidase OS=Pseudomonas syringae
pv. aptata OX=83167 GN=tpx PE=3 SV=1
MAQVTLKGSPVQVSGNLPEVGSKAHDFTLVGAGLADKTLASFAGKRKVLNIFPSVDTPTC
ATSVRKFNTQANDLSNAVVLCISADLPFAQARFCGSEGLENVQNLSSFRSADFAQNYGVA
VADGPLAGLTARAVVVLDENDKVLHSELVAEIGSEPNYDAALAVLK
>tr|A0A0Q0CW22|A0A0Q0CW22_PSEAP Type III secretion protein HrcV
OS=Pseudomonas syringae pv. aptata OX=83167 GN=ALO85_04181 PE=4 SV=1
MNRVINFLNMVALSAMRRSELVGAFFVIAIVFMMITPLPTGLIDVLIAVNICISCLLIML
AMHLPRPLAFSTFPAVLLLTTMFRLALSVSTTRLILLNQDAGHIVEAFGQFVVGGNLAVG
LVIFLILTVVNFLVITKGSERVAEVGARFTLDAMPGKQMSIDSDLRANLITVHEARKRRA
ELNKESQLFGAMDGAMKFVNGDAIASLIIVAINMIGGISIGVLQHNMAAGDALQLYTVLT
IGDGLIAQIPALLISVTSGMIITRVPNTEAGAEANIGREIAEQITSQPKAWIIASVAMLG
FAALPGMPTGVFITIAIICGAGGLLQLQRAKPKADEQRTAAVAPEMNGKEDLRTFSPSRQ
FVLQFHPGQDSAQIEALVSEIRKRRNRLVVQYGLTLPSFIIEHVDDIAPDEFRFTVYDVP
MLKATFTQSHVAVEARQLAGENLPAAIPGNTDRQEDQWVWLPAEQSGDLNPVSSTTLIIE
RMERTLQSCAPQFIGLQETKAILSWLESEQPELAQEMQRVLTLTRFSAVLQRLASERVPL
RAIRVIAETLIEHCQHERDTNVLTDYVRIALKSQIYHQYCGAEGLQVWLLTPESEGLLRD
GLRQTQTETFFALSNEISQMLVQQLHIAFPVRAPEQAVLLVAQDLRSPLRTLLREEFYHV
PVLSFAEISNAAKVKVMGRFDLEDDLEPLDNEHAA
>tr|A0A0Q0FEK7|A0A0Q0FEK7_PSEAP Enoyl-CoA hydratase OS=Pseudomonas
syringae pv. aptata OX=83167 GN=ALO85_01225 PE=4 SV=1
MSDLVSYHLDDGVATLTLNNGKVNAISPDVIVAFNAALDQAEKDRAIVILTGQPGILSGG
YDLKVMTSSAEAAIDLVAQGSTLARRMLSHPFPIIVACTGHAVAKGAFLLLSADYRIGVV
GPFSIGLNEVQIGMTMHHAGIELARDRLRKSAFNRSVINAEMFDPEAAMAAGFLDRVVSV
EELQSTALAVAGQLKKINMNAHKKTKLKVRKALLDTLEAAIEKDRQHML
>tr|A0A0N8T7Z2|A0A0N8T7Z2_PSEAP HTH OST-type domain-containing protein
OS=Pseudomonas syringae pv. aptata OX=83167 GN=ALO85_102178 PE=4 SV=1
MSNQLQNLNVAMFIDADNAPSKKLGSVLAELASYGAVSIRRAYGNWKSPSLDPWGKVLHE
HAIQPVQQFDLVKGKNATDMAMAVDAMDVLFNKPVDVFCLVSSDCDFTPLVMRLRAEGKQ
VVGFGERKAPEPFVNACSRFLYFDQYLMSDDQQVSATVLAGKKAISDSAVQSGALVRKTA
NELKGDTKLLNLLRNAVAYAENEDGWAQLSVVGSRIGNQASFDARNYGYPRLVDLLAAID
LFEMKRVNNHPQIRHKRRVAIAGG
>tr|A0A0Q0C030|A0A0Q0C030_PSEAP Uncharacterized protein OS=Pseudomonas
syringae pv. aptata OX=83167 GN=ALO85_00326 PE=4 SV=1
MSLLPYYVGCPSWSENAWRDSLYPADARPNDFLNLYTQVFNAVEGNTTFYARPAATTVQR
WAETMPDDFRFTAKFHKDISHNGDLREQVGAAEEFIRLLAPLGGRIAPFWLQLPASFTPQ
RLAELAGFLDELNVPLAVEVRNMAFFMKGDEERMLNRLLLDRGVERICLDSRALFSCVST
DPAVLHAQSKKPKVPPRPAALTLFPQVRFIGGPDLEANDQFLQHWVEKVAVWIEEGRTPY
VFLHTPDNIRAAEQAMRFHQQLMARLPGLPALFELDRGPQVEQLGLL

>tr|A0A0Q0IRI0|A0A0Q0IRI0_PSEAP Beta-ketoacyl-acyl-carrier-protein synthase I OS=Pseudomonas syringae pv. aptata OX=83167 GN=ALO85_04314 PE=4 SV=1
MTAPSSPAALLPLKIIATGVAVPANRVDSSALDTLLNKPAGYVEKRSGVHCRFHATLDAS
QAELAAQALQDALRREQIPAGSIDLLISASAVPMQALPCTAALILKAAHLPSGTPGFDIN
SSCVGFITALQVAAGLLNAGIYKRIAVVSADLVSRGIDWDDEESSLILGDGAACAIVERG
DGSSGILSSLSETYPQGSDLCEIQAGGTRRNPRAGMTDSDFLFHMKGKQLFRQAAALIEG
YLARLLERSGLTLAEIATVVPHQASHLSLEHMRKRLGVRPEVLVDIYRYHGNQVSASIPT
ALHTAFTTGRFTPGKPVMLIGTAAGLTLAGMVLLP
>tr|A0A0Q0DLH4|A0A0Q0DLH4_PSEAP Uncharacterized protein OS=Pseudomonas syringae pv. aptata OX=83167 GN=ALO85_102097 PE=4 SV=1
MRSWVRMRSESTSAFGQPSETNPTLGADGVVTVDMANLGVKGARARSAGADWALAAPLIK
KCAILAASGQDDQRLHENYDVRERQPGPARAWPIASPPTISRLHAVYRGGWPMLQRVTVT
F
>tr|A0A0N8T8R1|A0A0N8T8R1_PSEAP Uncharacterized protein OS=Pseudomonas syringae pv. aptata OX=83167 GN=ALO85_00005 PE=4 SV=1
MFNLRPAATLVALAAALSSLPAMAEEARYNQISLRAEVNQEVQRDLMLVTLYTEAQDSDP
AKLAAQITETLNKALGQARQVKDVKIRQGSRNSYPVYDDKGQKITGWRERAELRLESADF
AVLSKLTGELLTDLKMGGMDFSISPSTRKTSEDALLKDAVTAFKARAQLVTEALGGTGYK
LVNLNLNTSGYPQPYLRAPVMMMAKSLREDAAPTPDVEAGTSQVSVAADGVIEVAIP
>tr|A0A0Q0C6P7|A0A0Q0C6P7_PSEAP Metallo-beta-lactamase super protein OS=Pseudomonas syringae pv. aptata OX=83167 GN=ALO85_00784 PE=4 SV=1
MLRKSLLALSFAALFPLAAQAAAPLAIEVYNPADKAVFPVSSELITGKHDAVLIDAQFQR
NDAEALVQKIKASGKQLTTVYISHSDPDYYFGLDVIKAAFPKAKIVASAPTVKAIKASMQ
GKLAYWGPILKDNAPASLVVPEVLKGDHLTLEGQPLQIKGLNGPAPERSYVWIPSLKAVV
GGVVVSSGIHVWVADTQSTKSRHDWLATLKGIEALKPATVIPGHYLGEVPSGTQAVTFTA
DYLKSFEEQAAKAKDSAALIDAMQKSWPQLAEPASLEMSAKVIKGEMKWPN
>tr|A0A0Q0BR03|A0A0Q0BR03_PSEAP Atypical short-chain dehydrogenase/reductase family protein OS=Pseudomonas syringae pv. aptata OX=83167 GN=ALO85_03981 PE=4 SV=1
MTAPSLLIAGCGDIGSRLATRLLPHGWTVHGLRRTVSELPAGVHGVAGDLFKTPKPAQWP
DAALDYVVYCATPSQRDEAGYRMAYVEGLRNVLSWLEQTGQRPKRLIFVSSSGVYGQQNG
EWVDETSATEPGNYTGTVMLEAEQVALNSGFPATAVRLTGIYGPGRSDLSNRVRQGHSVR
IDPPVYANRIHADDAAGLLEHVLLADQRGVALESCYLGVDDDPAALADVVAWIREYLGVT
EWSEEVSVQRVGSKRCSNARARALGWSPIYPDYKAGYAALLG
>tr|A0A0N8T9L1|A0A0N8T9L1_PSEAP Flagellar motor rotation protein MotA OS=Pseudomonas syringae pv. aptata OX=83167 GN=ALO85_02858 PE=4 SV=1
MAKIIGIIVVLASVIGGYVLSHGKVMALFQPYEVLIIGGAALGAFLQANPGYMFMHVFKK
SLKMFGSRFTHAYYLEVLGLVYEILNKSRREGMMAIEGDIEDAASSPIFAKYPGVLKDER
MTAYICDYLRIMSSGNMAPHELEGLFDMELLSMKEDLEHPSHAITGIADGMPGFGIVAAV
LGIVVTMASLGSGDKAAIGMHVGAALVGTFFGILAAYGFFGPLATSLAHDAKEEMNVYEA
IKASLVASASGMPPSLAVEFGRKVLYPLHRPSFSELEQAVRGR
>tr|A0A0Q0DHQ1|A0A0Q0DHQ1_PSEAP Achromobactin ABC-type transport system, ATPase protein OS=Pseudomonas syringae pv. aptata OX=83167 GN=ALO85_00387 PE=4 SV=1
MASIATHDLTLSYQRQVIIDSLDLQLPAGQVTVLIGSNGCGKSTLLKSLARLLKPQQGTV
VLNGADIHQKSTATVARELAILPQMPSAPEGISVRQLVALGRYPYQNWMQQWSAEDEAMV
ARALAQTGMQDLADRPVDALSGGQRQRAWIAMTLAQDTDIVLLDEPTTFLDLAHQIEVLD
LLRDLNRQEGKTIVMVLHDLNLACRYADHMVAVHERSAFAQGRPADILSEALVKQVFGLN
CRIIADPFFGTPLCIPFGRELPQ
>tr|A0A0Q0DHU5|A0A0Q0DHU5_PSEAP L-proline/glycine betaine ABC-type transport system OS=Pseudomonas syringae pv. aptata OX=83167 GN=ALO85_00022 PE=3 SV=1
MSFLSAFSHLDWAQVLHLTWQHITLVGIAVTLAIVVGVPLGVLMTRFPVLAGPLQASATV
LLTVPSIALFGLLLPFYSKFGQGLGPMPAITAVFLYSLLPIMRNTYLALTGVEPGIREAA
KGIGMTFGQRLRMVELPIAVPVILAGVRTAVVMNIGVMTIAATIGAGGLGVLILASISRS
DMSMLIVGAVLVSILAIIADLLLQWLQRTLTPKGLLK
>tr|A0A0Q0DDG4|A0A0Q0DDG4_PSEAP Alpha/beta fold family hydrolase OS=Pseudomonas syringae pv. aptata OX=83167 GN=ALO85_00468 PE=4 SV=1
MSKHVSTRPTFLSHANPFVPAVGMGNPHLQTLWGPLLRKPTLLARTRERLWLQDGDFLDM

DWHGPDESDKPLVLVLHGLTGSSNSPYVAGLQKAMAALGWPSVALNWRGCSGEPNLLSRS
YHSGASEDLAEVIAHLRSLRPLAPIYAAGYSLGGNVLLKYLGESGAGSDLQGAVAVSVPF
RLDECANRIGQGFSRVYQRHFMREMLAYIRDKQHRFQHEGISEGLAELAALGSLENMRTF
WDFDGRVTAPLHGFTDATDYYRRASSRYYLGQIQTPTLIIQSSDDPFVFPHSLPEPSELS
SCTEFELHTKGGHVGFVEGSLRNPRYYLERRIPLWLCATQERDGSPTS
>tr|A0A0Q0DGY4|A0A0Q0DGY4_PSEAP TonB-dependent siderophore receptor OS=Pseudomonas syringae pv. aptata OX=83167 GN=ALO85_03208 PE=3 SV=1
MRTPYRPAMRHAWRLLPLGMAVASPLLAAEVLSLDAVNVTGFSEPESTGDYKAERASIFG
LDQASLLDTPASVSVFNAALIKDRQAKLLSEVLRNDASVGDGYAPVGYYENFVVRGFSLN
AANSYRINGRSIAGEQNVALENKQQVELLKGLSGLQSGVAEPGGVINYQTKRAQDVRSVT
VSTNEHGERYIATDVGGWFGSEQQFGLRANLAHEDIRSYVEHADGQRDFASLAFDWNISE
RALLQLDVEYQTKEQRSVPGYQLLGGTALPHDASPRNLLGHQNWSNPVGIDSLNMNGRFE
YRFNDSWKGSLSASRSRVVIDDYSAFAWGCYGSASCAGQAVPNHFSAEGGYDIYDFRSPD
DTRRNDEVEAAMSGTFATGSLGHELTFGSSAYRRTVDTRGTFNEFVGTGNINEAPEQVAP
STLALAHTERRLDSRQYGLFATDRISFNEHWQTVLGGRGVRLDEQAFNEDGSDARHTERY
VFLPQAALIYKPVDNVSLYTSYSKGLSLGGTAAWFTTNASEILAPTVSRQLEAGIKYDWQ
RMSLTAAVFQARQAYQYSRPNDDGTFTYVQQGEQKNTGLELGASGWVTDRLQISASAAAI
RARVEDSGTEAYDDHQALNVPRYRGTLQADYSLPVPGLALLGGVQYSASKYADREGTVQV
NDYALFNIGSRYSTRISGYDTVLRLTVDNLFDKRYWRDAGEYLGDDYLFMGAPRTARLSA
SVNF
>tr|A0A0Q0CYC9|A0A0Q0CYC9_PSEAP RNA binding S1 OS=Pseudomonas syringae pv. aptata OX=83167 GN=ALO85_04916 PE=4 SV=1
MLTTPGGFMDSINSRIAEELGVRPQQVAAAVALLDEGSTVPFISRYRKEVTGSLDDTQLR
HLEERLRYLRELDERRVSILASIEEQGKLTPELARDIKLADTKTRLEDLYLPYKQKRRTK
GQIALEAGLGELADGLFNDPSLAPEAEAARFVDADKGVADVKAALEGAKYILMERFAEDA
SLLDKLRSFLKQEAVISARVVPGKEEEGAKFRDYFEHDEPLKSMPSHRALAIFRGRNEGF
LSSALKVGEELPGAMHPCELMIGERFGIQNQNRPADKWLAEVVRWTWKVKLYSHLETDLL
GELRDGAETEAINVFAHNLHDLLLAAPAGQRATLGLDPGLRTGCKVAVVDATGKLLDYAT
VYPHVPKNQWDQTIAVLAALCAKHSVDLIAIGNGTASRETDKLAADLIKKYPGLKMTKVM
VSEAGASVYSASELAAKEFPDLDVSIRGAVSIARRLQDPLAELVKIDPKSIGVGQYQHDV
SQLKLARGLDAVVEDCVNAVGVDVNTASVALLARISGLNTTLAQNIVAHRDENGAFKTRA
ALKKVSRLGEKTFEQAAGFLRVMTGDNPLDASAVHPEAYPLVQRIAAETDRDIRSLIGDA
SFLKRLDPKKFTDETFGLPTVTDILQELEKPGRDPRPEFKTAEFQDGVEDLKDLQLGMIL
EGVVTNVTNFGAFVDIGVHQDGLVHISALSEKFIKDPREAVKAGDVVKVKVMEVDIPRKR
VGLSMRMSDTPGEKIDGARGARPGSSPRQNNAPRKETTAPAPANNAMASLFANAKQLKKR
>tr|A0A0N8T8E6|A0A0N8T8E6_PSEAP Uncharacterized protein OS=Pseudomonas syringae pv. aptata OX=83167 GN=ALO85_100726 PE=4 SV=1
MHELLKVAIESAVLLFVPGPTNTLLLAAGVSKGFLKSAGLPLFELSGYVIAITFYSILIE
YVSGNTVCLFFMKVFCAGYISYMSCRTWKLSGDFLKATETILGFNIFITTLFNPKAFIVS
SIIFSVTLIDDPLLIAQEMLVFSGVMLLASLFWLSVGTFVNLHSQEGGGTRQVILKASAS
VLMMFSCAILYSGVSSLLS
>tr|A0A0Q0FMT9|A0A0Q0FMT9_PSEAP HTH cro/C1-type domain-containing protein OS=Pseudomonas syringae pv. aptata OX=83167 GN=ALO85_101636 PE=4 SV=1
MTTTTEQGLTDMSVAENIKKARENKGLKIEEAASICGIPLGTYRKYEAGSSLPTADPIRA
MAKGFGVSTDEILMEPDERSVKAELRKLFSVVATLPECQQAEVKRAIKGLVMVFQQEDLS
Q
>tr|A0A0Q0BTL5|A0A0Q0BTL5_PSEAP Histidine--tRNA ligase OS=Pseudomonas syringae pv. aptata OX=83167 GN=hisS PE=3 SV=1
MSKSLQAIRGMNDILPEQTPLWRHFEGTVARLLDNYGYRQIRMPIVEFTELFKRSIGEVT
DIVEKEMYTFADRNGDSLTLRPEGTAACVRAVLEHGITGGGQVQKLWYIGPMFRHERPQK
GRYRQFHQIGVEVFNLDGPDIDAELIVMTWRLWGLLGIRNAVKLELNSLGTSEARARYRD
ALVEYLSARLDQLDEDSQRRLKTNPLRVLDTKHPETQAVLVDAPKLADYLDDESRVHFEG
LKTRLDAAGIPYVINPKLVRGLDYYSKTVFEWVTDQLGAQGTVCAGGRYDGLVEQMGGKP
TAGVGFAMGIERLVLLLETLEQVPEEIARQVDVYLCAFGEAAELAALALTEKVRDQLPTL
RLQVNAGAGSFKSQFKKADKSGALYALILGEEELAAKVIGVKPLRGQGEQQNIAWDALSE
HLASCVVQG
>tr|A0A0Q0ISK3|A0A0Q0ISK3_PSEAP Acetylglutamate kinase OS=Pseudomonas syringae pv. aptata OX=83167 GN=argB PE=3 SV=1
MTLERDAASNVAKVLSEALPYIRRFVGKTLVIKYGGNAMESEELKTGFARDIVLMKAVGI
NPVVVHGGGPQIGDLLKRLSIESHFIDGMRVTDAQTMDVVEMVLGGQVNKDIVNLINRHG

GSAIGLTGKDAELIRAKKLTVTRQTPEMTKPEIIDIGQVGEVVGVNIGLLNMLVKGDFIP
VIAPIGVGPDGESYNINADLVAGKVAEALKAEKLILLTNIAGLMNKQGEVLTGLTTEQVD
GLIADGTIYGGMLPKIRCALEAVQGGVNSSHIIDGRVPNAVLLEIFTDSGVGTQITNRKR
H
>tr|A0A0Q0D096|A0A0Q0D096_PSEAP Short-chain dehydrogenase/reductase SDR OS=Pseudomonas syringae pv. aptata OX=83167 GN=ALO85_03162 PE=3 SV=1
MNGTGNTILVTGGTSGIGLGLALRLHKAGNTVIIAGRRKALLDKIVSEHPGIESVVLDVS
DPQSIQRNSEALAISHPHLNVLINNAGIMHWEDLTDPQYLSTAEDIVTTNLLGTIRMVYA
FTPQLIKQPSATIVNVSSALAFVPLPATPTYSATKAAVHSFTQSLRVQLEGSPIEVIELA
PPGVRTTLLGQENDEHAMPLEEFLDQIFDLFKISPTPQELVVERAKPLRFAEASGAHDEV
LKMLAGYKPPAA
>tr|A0A0N8T7X1|A0A0N8T7X1_PSEAP Uncharacterized protein OS=Pseudomonas syringae pv. aptata OX=83167 GN=ALO85_04211 PE=4 SV=1
MQRGVLIDRAKPLPTQQRSAAMPGPFEKKWRCISRTVTYVGWSLFWLLLWDVAVTMDFML
IQGAGIDLPLMPLTLLCSALIVLISFRNTSAYNRWWEARTLWGAMVNSSRSYGRQVLTLI
EGNANEPGNPVKEVLFNRHVAYLRVLRAHLKGDVSTAKLEGLLPVSEIQRAKESNNFPND
ILNGSAAMISEEFAEGRIDSIRLARLESTMVELSNCQGGMERIANTPLPYPYVYFPRLFS
TLFCIIMPLSMVTTLGWFTPAISTVVGCMLLAMDRIGTDLQAPFGASQHRIRMEDLCNTI
EKNLRSMFDAPARQSLVIDAQDQQGSAWQPHRLVV
>tr|A0A0Q0BUI8|A0A0Q0BUI8_PSEAP Acyltransferase 3 OS=Pseudomonas syringae pv. aptata OX=83167 GN=ALO85_04732 PE=4 SV=1
MQRAFTVSCHLLHRAHHMGIPHNPALGYRPEIDGLRALAVIPVVLFHAGLPLFSGGFVGV
DIFFVISGYLITSIIIAEKIRGRFSLINFYERRARRILPALFVMMLICLPVAWLTLDPSD
LKYFAKSLVAVPTFSSNVLFWLESGYFDATAELKPLLHTWTLAVEEQYYLFFPLLLMLAW
GLGRKWLIVLLIVAALASLALAQLGAHQATSNAFYLLPARAWELLAGSFIAFYFAWRPRS
IGRASVVDQAATLLGILLIGYAVVGFDSSTPFPGLNALVPVLGAVLIIVFAHGKTWVGSA
LSSRAPFAIGMLSYSAYLWHQPVFAFARQYNLNEPGLPLMLALTLVSLALAWLSWRFVEQ
PFRKAGTFNRRQIFTTAGAASTLFIALGLVGYVNNGFPQRFAVDPELHQAFADPLIRDKC
DQPLDGKSGNVDFCLFGLADKEAVPDMALFGDSHSEALLSTFDAAARDQGRTLAHIGLGG
CLPLLGVDVANGNYAAGVCEALAEREFEYVKQQRIKKVVLVARWTLYTGDDYSERTMSTY
FLTSRADPEKSRETSRRVFSQALEHTIEAYRSIGAEVFIIAQAPQQMINPESLYYRLARD
ASEDDVQALQRVSELSVPVEKHDLLQRFTRQLFLRASQSKHISLITLDDAFCKDRQCLIG
DLHSYYKDFNHLNARGAGLLAGQISHILDQ
>tr|A0A0Q0FCG4|A0A0Q0FCG4_PSEAP 6,7-dimethyl-8-ribityllumazine synthase OS=Pseudomonas syringae pv. aptata OX=83167 GN=ribH PE=3 SV=1
MQPTAIDSKSKSHANERVAFIQACWHKDIVDQSRKGFIAEMANQGYAESDIDIFEVGGAF
EIPLHAKLLANTGRYAGIVGAALVVDGGIYRHEFVAQSVVSALMQVQLETEVPVFSVVLT
PHHFHAGEEHQKFFFDHFVHKGEEAAKTCADTLNKVRSLRRLDAQQKAAC
>tr|A0A0Q0CFP1|A0A0Q0CFP1_PSEAP Efflux pump membrane transporter OS=Pseudomonas syringae pv. aptata OX=83167 GN=ALO85_03675 PE=3 SV=1
MNFSKFFISRPIFAAVLSLLILIAGAISLFQLPISEYPEVVPPTVVVRANFPGANPKVIG
ETVASPLEQAITGVEGMLYMSSQATADGKLTLTITFALGTDLDNAQVQVQNRVTRSEPKL
PEEVTRIGITVDKASPDLTMVVHLTSPDKRYDMLYLSNYAVLNIKDELARLGGVGDVQLF
GMGDYSLRVWLDPNKTASRNLTATDVVNAIREQNRQVAAGQLGSPPSPNATSFQMSINTQ
GRLVSEEEFENVVVRAGADGEITRLKDIARIELGSSQYALRSLLNNQPAVAIPIFQRPGS
NAIDISNDVRARMAELKKSFPEGMDYSIVYDPTIFVRGSIEAVIHTLFEALILVVLVVIL
FLQTWRASIIPLVAVPVSLIGTFAVMHMFGFSLNALSLFGLVLAIGIVVDDAIVVVENVE
RNIELGLEPVAATHKAMAEVTGPIIATALVLCAVFVPAAFISGLSGQFYKQFALTIAIST
VISAFNSLTLSPALAAVLLKGHDAPKDRFSRFLDRMLGSWLFRPFNRFFEKASNGYVGTV
ARVIRSSGIALLVYAGLMVLTWMGFASTPTGFVPSQDKQYLVAFAQLPDAASLDRTEDVI
KRMSELALKQPGVQDAIAFPGLSINGFTNSPNNGVVFVTLKPFDERKDPSLSANAIAGAL
NGQFASIQEAYMAIFPPPPVQGLGTIGGFRLQIEDRGNLGYDELYKETQNIIAKSRSVPE
LAGLFTSYTVNVPQVDAAIDREKAKTHGVAVSDIFDTLQVYLGSLYANDFNRFGRTYQVN
VQAEQQFRQDADQIGQLKVRNNLGEMIPLATFVKVSDTAGPDRVMHYNGFITAEINGAAA
PGYSSGQAQAAVEKLLREELPTGMIYEWTDLTYQQILSGNTALFVFPLCVLLAFLVLAAQ
YESWSLPLAVILIVPMTLLSAIAGVMIAGSDNNIFTQIGLIVLVGLACKNAILIVEFAKD
KQAEGMSPLDAVLEACRLRLRPILMTSFAFIMGVVPLVLSSGAGAEMRHAMGVAVFSGML
GVTFFGLLLTPVFYVLIRNYVERQEARKAARVNSQQNLPAEMH
>tr|A0A0Q0DR85|A0A0Q0DR85_PSEAP ErfK/YbiS/YcfS/YnhG OS=Pseudomonas syringae pv. aptata OX=83167 GN=ALO85_02464 PE=4 SV=1

MLSRIPAVTRCLSLAALCAASSVHALEFPLPPPGEDIVGQVQVIKAKYEDTFADLGTQYD
LGYLEMIAANPGVDPWLPGAGKDIVLPTRFILPSGPREGIVINLAEYRMYYYPKGQNVVH
TYPLGVGREGWGSPIGVTKVTAKTPNPTWTPPASIKAEHAADGDPLPDVVPAGPDNPLGP
FKFGLGLSGYLIHGSNKKFGIGMRTSHGCFRMYNNNVLELADMAPVGTTVRIISEPYKFG
ISGGKVYLEAHTPVDDLGNPSVVDKHTAVINALLKRDDLANNLRMNWDVVRDVVAAEDGM
PVEIAEPGKAPANAEEPVIFQ
>tr|A0A0Q0ISG7|A0A0Q0ISG7_PSEAP Oxygen-dependent choline dehydrogenase OS=Pseudomonas syringae pv. aptata OX=83167 GN=betA PE=3 SV=1
MTTQSEYDYIIIGAGSAGNTLAARLTEDAGVTVLLLEAGGPDYRLDFRTQMPAALAFPLQ
GRRYNWAYETEPEPHMNNRRMECGRGKGLGGSSLINGMCYIRGNAMDYDGWAKEPGLEDW
SYLDCLPYFRKAETRDIGPNDYHGGEGPVSVATPKAGNNPLFHAMVEAGVQAGFPRTDDL
NGYQQEGFGPMDRTVTPNGRRASTARGYLDEAKKRSTLTIVTHALTDRILFEGKRAVGVA
YLVGDSDTRIQARARKEVLLCGGAIASPQILQRSGVGPAEVLNKLDIPVVHDLPGVGQNL
QDHLEMYLQYACTQPVSLYPSLKWWNQPAIGAEWMFLGTGIGASNQFEAGGFIRSSEAFE
WPNIQYHFLPVAINYNGTKGVQEHGFQAHVGSMRSPSRGRVQVKSKDPREYPSILFNYMA
SEQDWQEFRDGIRLTREIMQQPALDPYRGREISPGIDVQSDEALDQFVREHAETAYHPSC
SCKMGTDEMAVVDGQGRVHGLQSLRVVDASIMPIITTGNLNAPTIMIAEKIADKIRGRQP
LPRSTADYFVAGDKPARGKPLREISHQA
>tr|A0A0N8T9K4|A0A0N8T9K4_PSEAP Oligoribonuclease OS=Pseudomonas syringae pv. aptata OX=83167 GN=orn PE=3 SV=1
MQNKQNLIWIDLEMTGLDPDTDVIIEMATIITDSELNTLAEGPVIAVHQSDETLAKMDEW
NTRQHGGSGLTQRVRESTVSMAEAEAQTLEFIKLWVPERSSPICGNSICQDRRFLYRHMP
TLENYFHYRNLDVSTLKELAARWSPELKFKKGSTHLALDDIRESIAELRFYREHFIKA
>tr|A0A0Q0BV61|A0A0Q0BV61_PSEAP Short-chain dehydrogenase/reductase family oxidoreductase OS=Pseudomonas syringae pv. aptata OX=83167 GN=ALO85_03160 PE=3 SV=1
MNIQGLSLGRQPRHDNGEWRQKNFSKTGAFRGQVGALGECHLFIVWAMYALSEVLNGQST
FEDKTAFAPLLPRQGSRMKKTVLITGASSGFGLMLATHLHQQGFNVVGTSRYPERYASSV
PFKLLRLDVDDDGSIQSFTEALFKNITTLDVLVNNAGYMVTGLAEETPIETGRQQFETNF
WGTVKVTNAVLPHLRQQKAGQIITVSSMVGLIGPPNLSYYSASKHAVEGYFKSLRFELSQ
FNIHVSVIEPGWFKTNLGGNAISVAGSQIADYDNYRKKVNVVTQKGIDEAESPQAVVNAI
VNVIATKKPPFSHPVAKMAGMILFLQRYLPSLFESAIFKSIASGKKL
>tr|A0A0Q0CEV3|A0A0Q0CEV3_PSEAP UvrABC system protein A OS=Pseudomonas syringae pv. aptata OX=83167 GN=uvrA PE=3 SV=1
MDKILIRGARTHNLKNIDLTLPRDKLIVITGLSGSGKSSLAFDTLYAEGQRRYVESLSAY
ARQFLSMMEKPDVDTIEGLSPAISIEQKSTSHNPRSTVGTITEIYDYLRLLYARVGQPRC
PDHDIPLEAQTVSQMVDLVLTQPEGSKLMLLAPVIRERKGEHLSVFEELRAQGFVRARVN
GKLCELDELPKLDKQKKHSIDVVVDRFKVRADLQQRLAESFETALKLADGIALVAPMDDE
PGEEVIFSARFACPICGHAISELEPKLFSFNNPAGACPTCDGLGVKQFFDIKRLVNAELT
LAEGAIRGWDRRNVYYFQMLGSLSKHYGFSLEMPFKQIPADMQKILLNGSGSQSVDFRYL
NDRGDIVKRAHPFEGIVPNLERRYRETESATVREELAKFLGTQPCPDCRGTRLRREARHV
WVGEKTLPAVTNLPIGDATHYFETLKLTGRRGEIADKILKEIRERLQFLVNVGLDYLTLD
RSADTLSGGEAQRIRLASQIGAGLVGVMYILDEPSIGLHQRDNDRLLGTLKHLRDIGNTV
IVVEHDEDAIRLADYVVDIGPGAGVHGGHIVAEGTPDEVMSHPDSLTGKYLSGRVKIAVP
AKRTPRNKKLSLTLKGARGNNLQNVNLEIPIGLLTCVTGVSGSGKSTLINNTLFPLSATA
LNGATTLEAATHDSINGLQHLDKVVDIDQSPIGRTPRSNPATYTGLFTPIRELFAGVPES
RSRGYGPGRFSFNVKGGRCEACQGDGLIKVEMHFLPDIYVPCDVCKSKRYNRETLEVKYK
GKNIHEVLEMTIEEAREFFDAVPALARKLQTLMDVGLSYIKLGQSATTLSGGEAQRVKLS
RELSKRDTGKTLYILDEPTTGLHFADIQQLLDVLHRLRDHGNTVVVIEHNLDVIKTADWL
VDLGPEGGSRGGQIIATGTPEQVAEMEQSYTGHYLKPLLTRDKA
>tr|A0A0Q0DJT9|A0A0Q0DJT9_PSEAP K+-dependent Na+/Ca+ exchanger related-protein OS=Pseudomonas syringae pv. aptata OX=83167 GN=ALO85_00146 PE=4 SV=1
MPAPALIQMLSGLLLLLIGAELSVRAAVHLAAIFKVRPLIVGLTVVAMGTSAPQMAVSLQ
AAFADNTDIAVGSVIGGNIFNVLVILGLCALIIPLRVARQVLHVDIPLMIGACLLAIGLS
WSGEFSKLDGAVLLAGLLFCLIVIIRQGGHAPRHGQAETTEKPRTFTRILMLASGLLLLT
AGGHLLVDASVVIAIHLGLSERIVGLTIIAIGTSLPALMTSLIAAFRGERDIAVGNVIGS
NLFNLLGVLGLTALVAPVPLTISPNALVFDLPIMLGVSLLCVPLFYSGYRIDRLEGLFLL
SLYLTYGLHILSISTGMALAERLESKMLTLVLPVLGAVVVWGTIRAWRRQH

>tr|A0A0Q0CDY5|A0A0Q0CDY5_PSEAP Putative outer membrane lipoprotein, OmpA family OS=Pseudomonas syringae pv. aptata OX=83167 GN=ALO85_00332 PE=4 SV=1
MRKQLMIPALLAMSVALAACATKPNPNLEQARSNFTALQTNPQATQVAALETKDASEWLT
KAEQAFRNDDDVQKVDQLSYLTNQRIELAKQTIALRTSEAALQNASADRAKARLEARDAQ
IAALKNSLNAKQTERGTLVTFGDVLFDYNKAELKPTAQGDIGKLAGFLQENPDRKVIVEG
YTDSTGSASYNQSLSERRANSVRMALVRMGVDPARIVTMGYGKEYPVADNSSNSGRAMNR
RVEVTISNDNQPVAPRSSMK
>tr|A0A0Q0DPG5|A0A0Q0DPG5_PSEAP Uncharacterized protein OS=Pseudomonas syringae pv. aptata OX=83167 GN=ALO85_101284 PE=4 SV=1
MDHKTNFLLSNIPEGADYAADAGDLDLEDIPQERMEAVIDLLRNTDDEVVKFLAAKLLAS
WGVFEGFSVLKEFVEKKSAIITNIYPHRLHGYDDTFRQVLMAVVMFYANMAGAGKKDVAR
AEVYPLLSKIIELAASNPFEITDVLSFIKREQYFEYLPLIKGYLMSIIDKPEVHRWKIYD
AIEFLVGFDSQFVVSLLEERRKSIEDFKPSIPG
>tr|A0A0Q0BUY8|A0A0Q0BUY8_PSEAP Pyoverdine-specific efflux macA-like protein OS=Pseudomonas syringae pv. aptata OX=83167 GN=ALO85_100174 PE=3 SV=1
MKRSRHPRRALLVALCLFPVIAVCAWQILPDSKGSAATVTVTRGTIENSVTALGTLQPRS
YVDVGSQASGQIMKIHAQVGDQVKEGDLLVEIDPSTQKAKLDAARYAIDNLKAQLQEQRA
LHELAEQKQQRQKRLAAGGATRAEDVQAAESEFRATQARVDMFKAQILEAQAALRSDEAA
LGYTRIFAPMSGTVVALDAREGQTLNAQQQTPLILRIARLSPMTVWAEVSEADIGHVKPG
MTAYFTTLSGGNRRWSSTVRQILPVPPRPLDQANQGGGSPSSSRKNGGGRVVLYTVLLDV
DNSDQALMAEMTAQIFFVAEKAENTLTAPLAALRNGAQTDRQTAQVLSANGEVQNRQVRT
GISDRLRVQVLDGLNEGERLLVPGAECARR
>tr|A0A0Q0BS96|A0A0Q0BS96_PSEAP Glutaredoxin-like domain protein OS=Pseudomonas syringae pv. aptata OX=83167 GN=ALO85_00544 PE=4 SV=1
MPPECQLFSTLGCPLCEVAEAVLLPFAIEHGLLVELVDICEDEQLLERYELRVPVLRRVD
TGDELDWPFDAPQVASFLSR
>tr|A0A0Q0CVF4|A0A0Q0CVF4_PSEAP Thiol-specific antioxidant protein LsfA OS=Pseudomonas syringae pv. aptata OX=83167 GN=ALO85_02327 PE=4 SV=1
MTLRLGDIAPDFEQESSEGRIRFHEWLGDSWGVLFSHPADFTPVCTTELGFTAKLKDEFA
KRGVKAIALSVDPVDSHIKWIDDINTTQNTLVNFPILADADRKVSDLYDLIHPNANDTLT
VRSLFVIDPNKKVRLTITYPASTGRNFHEILRVIDSLQLTDNYKVATPANWVDGDDVVIV
PSIKDEAEIKQRFPKGYKAVTPYLRLTPQPNR
>tr|A0A0Q0DJQ4|A0A0Q0DJQ4_PSEAP LysR family transcriptional regulator OS=Pseudomonas syringae pv. aptata OX=83167 GN=ALO85_03935 PE=3 SV=1
MPQVEQALSGVVIFVTAARAGSFTSAADRLGITKSAVGKSIAKLEERLGCKLFHRSTRSL
GLTVDGEAYFASCSLAVDEVLAAEAFLTSNQKTPGGRLRIDLPAAYGRSVVLPVLLDILE
ANPGLKLTVTFNDSVIDLIEEGVDLSIRFGALKDTSGLVARRLAVQKQIICAAPRYIQKH
GYPESPEDLQQHRCIVNYRRGQRVSWTVLDESGELARITPPGTHEVGDGDAIIAMAAAGQ
GICQMPGSLIRDHLKDGRLVPVLEKYHPDDVAIHAVWPQTRHLQPKVRRVVDELVARAER
GDLS
>tr|A0A0Q0DBM1|A0A0Q0DBM1_PSEAP Stress responsive A/B barrel domain protein OS=Pseudomonas syringae pv. aptata OX=83167 GN=ALO85_04232 PE=4 SV=1
MILHLVFFKFHEPFQWCDSEVLEAEAATRGHPHRISEIAGWACGRNISVRKRAMDFVAMG
LFESQEELSSFLIHPDHQDGVLKWQKIASWQVVDIDINSDTTQWSGLLDGWTDVVSAPLD
AVSVSR
>tr|A0A0Q0CBF1|A0A0Q0CBF1_PSEAP Alginate lyase OS=Pseudomonas syringae pv. aptata OX=83167 GN=algL PE=3 SV=1
MQTPKLIRPTLLSMAILSSMAWATGASAALVPPKGYDAPIEKMKTGDHNFSCEAIPKPYT
DKLVFRSKYEGSDKARATLNAVSEEAFRDATKDITTLERGVSKVVMQYMRDGRPEQLDCA
LNMMTTWAKADALESREFNHTGKSMRKWALGSMSSAYLRLKFSESHPLANRQQDAKIIET
WFSKLADQVVSDWSNLPLEKINNHSYWAAWSVMATAVATNRQDLFDWAVKEYKVAANQVD
KDGFLPNEMKRRQRALSYHNYALPPLAMIASFAQANGVDLRPENNGALKRLGDRVLAGVK
DPSIFADHNGEKQDMTDLKKDPKFAWLEPYCSLYTCSPDVLEEKHEKQPFKTFRLGGDLT
KVFDPSHEKGDKGDNVSE
>tr|A0A0Q0FVM6|A0A0Q0FVM6_PSEAP Uncharacterized protein OS=Pseudomonas syringae pv. aptata OX=83167 GN=ALO85_03840 PE=4 SV=1
MASSGQSVLCVYRYDPLDRLADCAPAGQGSTRLFYQKTHLAMQIQGQVQHTLMRTDEHLL

ACLSAENNQRDGALLVTDQQQSVTAAQGLEFAYTPYGHRHPSGPASLPGFTGQRVDPVTG
HYLLGNGYRAFNPVLMRFNSPDSLSPFGEGGVNAYGYCGGDPVNGVDPSGQAGARILAQI
FRSRRALVTAKQVTGHRNSVLVPGRPASLGLTRTGSASGAKEPVAVPDFINKLAQPENVP
AHLKTRRRLPAVPSDARLHTTWAAPESSIAPRNIYHLPSGVVRRPKRIELPATPFQRVLT
GDLPMDTIYEYPRLSHSQARALEVIRRGKMSQRDHSYSISVSVNFRQSDPTSSVNIAVRF
>tr|A0A0Q0DMP7|A0A0Q0DMP7_PSEAP 30S ribosomal protein S10 OS=Pseudomonas syringae pv. aptata OX=83167 GN=rpsJ PE=3 SV=1
MQNQQIRIRLKAFDHRLIDQSTQEIVETAKRTGAQVRGPIPLPTRKERFTVLVSPHVNKD
ARDQYEIRTHKRVLDIVQPTEKTVDALMKLDLAAGVEVQISLG
>tr|A0A0Q0FV59|A0A0Q0FV59_PSEAP Putative Lipoprotein OS=Pseudomonas syringae pv. aptata OX=83167 GN=ALO85_03357 PE=4 SV=1
MLRKWMLMLCVGLVLSGCGGVPVTRYSQEAPKLDLRQYFTGRVEAWGMFQKRSGEVTKRF
TVVIDGHSEGEVLVMHEAFSYSDGTKQVREWRLRPDGPGRWKGTAGDVVGEAYGEVSGNS
FHWNYVLRLPVDGTEYDVSLDDWMYLIDEKTMANRSSMTKLGVEVGQITLFFRKVGK
>tr|A0A0Q0BUL1|A0A0Q0BUL1_PSEAP Short-chain dehydrogenase/reductase SDR OS=Pseudomonas syringae pv. aptata OX=83167 GN=ALO85_01123 PE=4 SV=1
MNRFTQKVIVVTGAGSGIGEATAKRFAEEGASVVLVGRNRDKLAKVAAQLKGEGHLIRAT
DVADPSDVEALFKEVATRFGRLDVLVNNAGIVKSGKVTELGIEDWKELMSVDLDGVFYCT
RTAMPALIASKGNIINVSSVSGLGGDWGMSFYNAAKGAITNFTRSLAMDHGADGVRVNAV
CPSLTRSELTEDMLGDKALMAKFMERIPLGRPGEAEDVGDVIAFLASDDARFVTGVNLPV
DGGLSASNGQPPQA
>tr|A0A0Q0E0T2|A0A0Q0E0T2_PSEAP Uncharacterized protein OS=Pseudomonas syringae pv. aptata OX=83167 GN=ALO85_01517 PE=4 SV=1
MKERAMNNSRTVSTTKELKKAIDDKIGDIIITDAKLATCVKVASNIPYKTLAAIIAVAGA
SAAVFWNPVGWFGAAAGSAAGVGVVASAAAAAGITTGTAAIIGGVLVLLAALGVSALAMV
KDYDFDAGISASSSGLEGGIPKGKTSAHLKIKRNV
>tr|A0A0Q0DAM1|A0A0Q0DAM1_PSEAP Type VI secretion system protein ImpH/VasB OS=Pseudomonas syringae pv. aptata OX=83167 GN=ALO85_02257 PE=4 SV=1
MDLETRPHEDPLRFRSQLSLNFPASEVSDLQFEREGKLSAAGLPLSEVQVTFMGLVGPSG
VLPRPYTELLIDRHIQYRDDAAHAFLDIFSHRMTTLFYEAWQKYKFYIEYERNGTSNFDR
YLLNLVGFGPEGLKQKFDKGESPLRRELFSYFSGMFAQKPRNALNLEVMLSFYFSLPFKI
QQFAGRWLKLDSSQCTQLGRKNAVLGQSAVAGNRVWDYQSCVRIEMGPLELAEYQRFQPG
TEDYQKLVELVRFYIGAELDFQIAPKLKREAVPVARLGRQGNVSLGWLGWLKRPGVDVEP
SRCAVFHIPFDGVSL
>tr|A0A0Q0DPF8|A0A0Q0DPF8_PSEAP Uncharacterized protein OS=Pseudomonas syringae pv. aptata OX=83167 GN=ALO85_03611 PE=4 SV=1
MMAQASARFTQGSVARHIVVTASASALSLFAVFLVDILTLVYVSMLHDPVLLAAVGIAKV
LMFFNGALATGLIIAGASVLSARIGHRAARSIPRLTGSLMLMVITVSGLAAAAQLTFIVP
IMRWLGAEAATYEAARSFIWLTLPFTALQAVMQMSAQMLRTIGDNRRAMGVVLSAAASLA
VADPFFIFGLGLGLDGAGIAFAVSAAVSASIGLYWVRSRIGMVFTRHLKLLRLHARHTFR
LALPAMAANLATPVGLAYLVASLSAFGTSALAAMTVLDRVIQFSYCAFFALPGALAPVLG
QNIGASRDDRVRSAVIFTRRLVVCYGLAVWLVLLLCGGMIADLYQLGDEARAVFMAFCLW
GGGLWVLIGLDFVAIAVFLTMNRSWWVAVFAWLRATAGTVPFVFAGTHWFGSSGALPGMF
TGNALIALVSITTASLTAKRFFTSRAPVMSVGVH
>tr|A0A0Q0CGU0|A0A0Q0CGU0_PSEAP Rhomboid family protease GlpG OS=Pseudomonas syringae pv. aptata OX=83167 GN=ALO85_00616 PE=4 SV=1
MSPVAVLRLPLTTDLSGFVRLLLRLQIPHRVSEEAGEQVLWVPDAPRLVEEVRELYTRFP
EGDEHVQLTQDTGSATYKPPGLIEQLRSSPVTALVLLVTLLVAGLTLLGDNLEAVRWLTF
QDFRINGEYATFLPLSESLASGQWWRIISPMLIHFGILHLAMNGMWFWELGRRIEIRQGS
LSLLLLTLLFSSVSNYVQYLFSGPSLFGGLSGVLYGLLGHCWIFQMLAPNPVYRLPRGVL
IMMLVWLALCLSGLVSMLGFGEIANGAHVGGLIIGCVTGLLGGAIARRKA
>tr|A0A0Q0DVG1|A0A0Q0DVG1_PSEAP Epoxyqueuosine reductase OS=Pseudomonas syringae pv. aptata OX=83167 GN=queG PE=3 SV=1
MGSDVWQNYTHLGPGFSLRMSAITAVVPTSDDLAALAVSIKAWGRELGFQEVGISGLDLA
EHEQHLQRWLDAGYHGEMDYMAAHGSKRSHPDELVPGTLRVVSLRMDYLPGDTEMAQRLT
EPEKAYVSRYALGRDYHKLIRKRLQQLAERIQQAIGPFGFRAFVDSAPVLEKAIAEQAGL
GWIGKNTLVLNRKAGSFFFLGELFVDIPLPVDAPHATEHCGRCTACLDICPTAAFIGPYV
LDARRCISYLTIELKTAIPEELRPLIGNRVFGCDDCQIVCPWNRFARPTEQSDFQPRHNL
DNAGLAELFLWDEEKFLGSTEGSPLRRAGYERWLRNLAVGLGNAPSTIPVIEALQSRRDY

PSELVREHVEWALQRHGVAPR
>tr|A0A0Q0DFS6|A0A0Q0DFS6_PSEAP Uncharacterized protein OS=Pseudomonas syringae pv. aptata OX=83167 GN=ALO85_101253 PE=4 SV=1
MRRFFVTVAGVAGGHAQAGVTHGLNGPSSFGHVQKQQSA
>tr|A0A0Q0CBU7|A0A0Q0CBU7_PSEAP Nucleoside diphosphate kinase regulator OS=Pseudomonas syringae pv. aptata OX=83167 GN=ALO85_100264 PE=4 SV=1
MYLLRCVSVRPCLCRQEARQTPPTGGVLFYSWRIKESAQTMTTAPSIILTRLDVQRLEKF
IADQNEDTPGIQALQAELDRADQVVGHDEVPAGVVTMNSRVHCREEVSGKDYHLKLVYPQ
DAGADGTVSVLAPMGSALLGLQIGQHIDWPAPGGKTLKLTLLAVEYQPEAAGEYE
>tr|A0A0N8TA30|A0A0N8TA30_PSEAP Sodium/hydrogen exchanger OS=Pseudomonas syringae pv. aptata OX=83167 GN=ALO85_200191 PE=4 SV=1
MPNQLQGQIWHRSIAKLIEKLNRPEFWNSLVRLLKEYAPVDNWVVLMFGSEGVQAICYPD
TADEEEIDGLLNRYVKGLYMLDPFYIANRENQQSGFFHLLDIAPTHFLETEYYHLYFEQF
VSVDEVQYNVQLDSERTLCLSMGSKSRFTQEHIAIFDLIKPWVLALMKQRIASDVEEKES
SRPQQWQDKIIELAPQLTGREIEVLKLALSGFSNSEIAGKLSVSPETVKVHRRNFYSKLN
IKSQSELFAHFFQSTIS
>tr|A0A0Q0ILQ7|A0A0Q0ILQ7_PSEAP Holliday junction ATP-dependent DNA helicase RuvB OS=Pseudomonas syringae pv. aptata OX=83167 GN=ruvB PE=3 SV=1
MIDADRLITATGGRDRDEQIDRAIRPLSLADYIGQPTVREQMELFIQAARGRSEALDHTL
IFGPPGLGKTTLANIIAQEMGVSIKSTSGPVLERPGDLAAILTNLEPNDVLFIDEIHRLS
PIVEEVLYPAMEDFQLDIMIGEGPAARSIKLDLPPFTLVGATTRAGMLTNPLRDRFGIVQ
RLEFYNIADLSTIVSRSAGILGLVIEPQGAFEIARRARGTPRIANRLLRRVRDFAEVRGN
GQITRQTADKALNLLDVDEHGFDHQDRRLLLTMIEKFDGGPVGVDSLAAAISEERHTIED
VLEPYLIQQGYIMRTPRGRVVTRHAYLHFGLNIPSRMGEIPVPDDFGDGPVDL
>tr|A0A0Q0C289|A0A0Q0C289_PSEAP DUF58 domain-containing protein OS=Pseudomonas syringae pv. aptata OX=83167 GN=ALO85_00588 PE=4 SV=1
MQPYLIPQPGIRVSLSELIEMRHRVREVQLFSTPSQRSPLIGLHHSKLRGRGVDFDQVRV
YQAGDDVRSIDWRVTARTQEPHTKLFHEERERPIFILVEQSRRLFFGSGLMFKSVLAAQA
AALIGWAALEHNDRVGGLVFGDNEHYEIKPRRSKQSLLQLLNRLVRVNQSLHTEIPPDRD
AFGTALRRAREVLRPGSLVIVLCDERALSDAAEQQLSLLSRHSDLLLLPLSDPLDRALPA
AGLLRFAERGAQLEIDTLNPELRRAYRQQGEERTARWELLAQKLRVLLMPLSTQAELVEQ
LREYLNARRPVKRK
>tr|A0A0Q0DM55|A0A0Q0DM55_PSEAP GNAT family acetyltransferase OS=Pseudomonas syringae pv. aptata OX=83167 GN=ALO85_00948 PE=4 SV=1
MSLPAAITGEIRQLDSGYSRETRSLLFRAYRHDPTFAYLFNAEREGYEQRVLATIRQLVK
QHFLQNQPALGLFLQDRLVGVALIAPPQRRLGVTESWAWRLRMVMDTGLGCTQRYLAYYN
AVLACVPSETVHVLPLIGLDPEFQGQKLGQDLSEQLLAALHDWCAVDEHSQGIVVDTGNP
RYLEFYKRQGYEEIGEIAVGPVREHVFFHPNPQVSLPVPDVMA
>tr|A0A0Q0CHJ0|A0A0Q0CHJ0_PSEAP Spermidine/putrescine ABC transporter permease OS=Pseudomonas syringae pv. aptata OX=83167 GN=ALO85_03422 PE=3 SV=1
MPAGGASSTAGAASQKAPGMLPSPSLAQRWRGSRNLLPALIFLGLFFFAPLIALLLRGVL
EPVPGLGNYEQLFANSAYSRVLLNTFSVAGLVTLFSLLLGFPLAWVITLVPRGWGRWILN
IVLLSMWTSLLARTYSWLVLLQASGVVNKALMAMGIIDQPLEMVHNLTGVVIGMSYIMIP
FIVLPLQATMQAIDPMVLQAGSICGASPWSNFFRVFLPLCRPGLFSGGLMVFVMSLGYYV
TPALLGGAQNMMLPEFIVQQVQSFLNWGLASAAAALLVLITLVLFYFYLKLQPESPVGAS
NAR
>tr|A0A0Q0FJ62|A0A0Q0FJ62_PSEAP Glutamate carboxypeptidase OS=Pseudomonas syringae pv. aptata OX=83167 GN=ALO85_00629 PE=4 SV=1
MKHMRSFTRSPLAAHLALALALTALAPAAMAEPHKAIQADAEQYKDEALKLLERLVNIDS
GSGYVPGLTKVSDIAIEELKKLGATIELVPNTPEASNHVIATLKGTGKAKILLMAHMDTV
FKEGSAAERPFHIKDGRAYGPGVMDDKGGIVAAIYALKVLHNLKFTDYAQITVLLDASEE
TGSGVGTELIKKTAKEHDVTLNLEPGRPADGLVVWRKGSATALVEVKGKASHAGFAPELG
RNAATEVAHQILQLGKLGDEEKKTTINFTVLKAGDRTNVIPDQASAKADVRAAVPEEFDR
VEQDLAKVSANKLVPDTEVKTSLVRGLPPMPQTAQSDALVAMAQGIYGELGRTLTIEGSG
GAADSSLSASVGTPTLDGFGIVGGNIHTPEEYAEVGSVAPRIYLLSRMIMKLSGQQ
>tr|A0A0Q0DJ59|A0A0Q0DJ59_PSEAP Chaperone protein HscA homolog OS=Pseudomonas syringae pv. aptata OX=83167 GN=hscA PE=3 SV=1
MIILNSMALLQIAEPGLSPQPHQRRLAVGIDLGTTNSLVAAVRSGLSEPLADAEGQVILP

SAVRYHADRVEVGQSAKIAASQDPFNTVLSVKRLMGRGLTDVKQLGEQLPYRFVGGESHM
PFIDTVQGPKSPVEVSADILKVLRQRAEASLGGELVGAVITVPAYFDDAQRQATKDAARL
AGLNVLRLLNEPTAAAVAYGLDQKAEGVVAIYDLGGGTFDISILRLTGGVFEVLATGGDT
ALGGDDFDHAIASWIVADAGLSADIDPSAQRSLLQAACSAKEALTDAESVEVAYGEWRGT
LTREALNALIEPMVARSLKACRRAVRDTGIELEEVEAVVMVGGSTRVPRVREAVAELFGR
QPLTEIDPDQVVAIGAAIQADTLAGNKRDGGELLLLDVIPLSLGLETMGGLMEKVIPRNT
TIPVARGQEFTTYKDGQTAMKIHVLQGERELISDCRSLARFELRGIPPMVAGAAKIRVTF
QVDADGLLSVSAREMGSGIESSIQVKPSYGLTDDEVTRMLKDSFEYAGDDKVARVLREHQ
VDAERLLEAVQGALDADGERLLDEEERLVINLQMDELRELMQGTDGYAIEQQTKRLSQVT
DAFAARRLDSTVKAALAGRNLNEIEE
>tr|A0A0Q0C789|A0A0Q0C789_PSEAP Short-chain dehydrogenase/reductase SDR
OS=Pseudomonas syringae pv. aptata OX=83167 GN=ALO85_01041 PE=3 SV=1
MASESTNTPVWLITGCSTGFGRELAKLVIARGWRLVATARDKSRVADLAEGTQGQVLAVD
LDVSKADQIEAAVAAAKSQFGKIDVLVNNAGYGYQSSIEEGEDAEIRAQFDANVFGLFAI
TRAVLPIMREQRSGHIINITSVAGLIGFPGSGYYAASKHAVEGFSDALLAEVKPLGISVT
CVEPGPFRTDWAGRSLRQTPSRIDDYKDTATKRLESTRQSSGNQAGDPVRAAEAMIALTQ
KANAPRHLVLGSWGYDAVVADRQARLKQIEQVRDISIGADFPKE
>tr|A0A0Q0C9Z7|A0A0Q0C9Z7_PSEAP Oxidoreductase alpha subunit
OS=Pseudomonas syringae pv. aptata OX=83167 GN=ALO85_01536 PE=3 SV=1
MSESENSQAPLQGSQTDGTPAGGWPALSAVNKHLLHQHVLIKGNRTLMSMNKPGGFDCPS
CSWPDPKKPHTFEYCENGAKALAWESTKKRVTPEFFARHTVSELATWSDHDLEAEGRITE
PMRYDAATDKYLPVTWDEAFAEIGEELRGLSDPWKLELYTSGRTSNEAAFLYQLFGRLYG
MANFPDCSNMCHETTTAALPESIGVGKGTTTLEDFENADAIFIIGQNPGTNSPRMMSELH
AAARRGASIISFNPLRERALVRFAAPQDPRDMLSLHGVEISSQYHQVRIGGDMIALQGVC
KAVIEADDVAQRENLPRVLDVAFIEEHTHGFEQYADYCRQLPWDIIERHSGLTRQAIEEV
ATVYKRSKGVICCWGMGVTQHKRGGDAMQQIINLLLLRGNIGKLGAGACPVRGHSNVQGD
RTMGIYEKAGEPFLASMSKVFGFEASREKGHAVAETCEALLRGEVEALISMGGNFFRAIP
DTDVICPTVSRLKMTVLVNTKLNRSATVHGQKAFLLPCVARSERDLQNGVAQTITVEDSM
SMVHGSTGMLEPASKHLISEIAIIAGIAKATLAPNPKVDWDSYVGDYARIRDKIEEVLPE
QFYDFNQRILEPGGFRLPNAASERKWDTESGKANFLFPEGIYDEDDTPPGAEHLQLMTIR
SHDQFNTTVYSNDDRYRDIFGDRMVVMLNPQDIERLGLKAGDYIEFQTALDPTTARRAAG
FKVIPYDVPQGCCAAYYPETNGLLPLANRDKHGNTPAAKSIPVNLIRMSPEGTQVALGRR
GLIAAAS
>tr|A0A0Q0CDV9|A0A0Q0CDV9_PSEAP ThiJ/PfpI OS=Pseudomonas syringae pv.
aptata OX=83167 GN=ALO85_02648 PE=4 SV=1
MHMKILMVLTSHDQLGDTGKKTGFWLEEFVAPYYAFKEAGADITLVSPKGGQPPLDPKSD
EPDAQTEDTKRFSKDADAQKALANTGLLSGVNAADFDAVFYPGGHGPLWDLAEDKHSIAL
IEAFAKADKPHGMVCHAPGVLRHVKAPDGQPLVKGRRVTGFTNSEEEAVGLTKVVPFLVE
DTLKQLGGDYSKGDDWGVYVLTDGKLVTGQNPASSAQAAKDLITLLS
>tr|A0A0Q0DJE4|A0A0Q0DJE4_PSEAP Pressure-regulated protein OS=Pseudomonas
syringae pv. aptata OX=83167 GN=ALO85_100307 PE=4 SV=1
MLKIEERASLTKQSPESERNSDGRPALKGSIVIDLRQDEHGHWFALLSCGHTQHLRHDPP
WQSRPWVLDPLQRSQMRGQAFCCGWCANAADNDSLAAEKSR
>tr|A0A0Q0DNY0|A0A0Q0DNY0_PSEAP Diguanylate cye OS=Pseudomonas syringae
pv. aptata OX=83167 GN=ALO85_100045 PE=4 SV=1
MACFYRCQAFQVWPSTCRQWHLIAKGLSMAPEQSGPVFRKTRCTGSHLLWRQVLKRDLRL
ASWLIFVVCACVVMLMAWQAWTARRNTLENIQTSTINLAGALSTYTDGIFKQSELMLIGL
TERVEKDGAGPEQIERLRWVIAREMGALPQIDTVSLYNADGICIFSSNPKVIGINSGQRQ
FFQHHLQMPDKAPYIGPTIRSRASGEWVISVSRRINNPDASFAGLMVVTIGVDHLLKFYS
DIKVGRTGIISLTSTTGQLRVRYPHLEQEIGRDLSSTPIFTSKRGQVSGTAQFVSRIDGV
PRFYAFKRSDVYPIVTVVALGQKEAMLDWLGQTRQSILVMLVLLGLVIGLGVRLIGHIHS
RIRAEDQLLDSQAALIQLNQHLELIASEDKLTGLANRRRFDQFLEVEFKRARRERSLLSL
ILIDADHFKRYNDHYGHLAGDECLIALARVVEQFIRRPSDVAARYGGEEIAVVLPGTDES
NARQVAEKILQAIRDEQIEHPASPFGIVTISLGVATFVGTDRQLDQRGLIELADRALYAA
KSQGRNRVNVASENATGTLPAWTRVKASADARADTEPRSGQRD
>tr|A0A0Q0DL13|A0A0Q0DL13_PSEAP Inositol 2-dehydrogenase OS=Pseudomonas
syringae pv. aptata OX=83167 GN=iolG PE=3 SV=1
MKMALKLGVIGTGAIGQDHIRRCSKTLVGSQVVAVTDINLEQAAKVVRDLDLGAEVYADG
HALIAAPDVEAVLVCSWGPSHEEYVLAAIAAGKPVFCEKPLAVTAEGCRHIVEAEIASGR
RLVQVGFMRPYDQGYRALKAVIDSGRIGEPLMLHCAHRNPSVGENYKTDMAITDTLIHEL

NVLRWLLDDDYVSVQVVFPRKTSKALAHLKDPQIVMLETVKGTRIDVEVFVNCQYGYDIQ
CEVVGETGIARLPEPSQVQLRSEAKLSNAILMDWKDRFIAAYDVELQDFIDGVKAGTLYG
PSAWDGYAAAVAADACVLAQNTGAVVPITLAMRPVFYS
>tr|A0A0Q0C087|A0A0Q0C087_PSEAP H-NS histone OS=Pseudomonas syringae pv. aptata OX=83167 GN=ALO85_02390 PE=4 SV=1
MSLINEYRATEEAIKELQARLKNLSQDDKLQTELEFEGKLRTLMGEYQKSLRDIIALLDP
DAKVNKAPRGGAVKPAGTKRARKVKQYKNPHNGEVIETKGGNHKTLKEWKAKWGSDDVES
WATLLG
>tr|A0A0Q0DMP2|A0A0Q0DMP2_PSEAP Putative Membrane protein OS=Pseudomonas syringae pv. aptata OX=83167 GN=ALO85_05050 PE=4 SV=1
MHTAGDIARFCAEIPCMIEKSGTHTQRRAATFAKTPATATSLCPFRGPDVAIVPVRYALD
RSRYDAAPQKLKPLLTGSRWAVMPKLKTRSYTLRQLYDGYVYVYDETAETLHEYAVSAAT
GNLSRIVWTDAQLGSNQRSGGSDAKPFLLYPRSNLLRIAFSAQQWTWRICEHLRSNPESR
SSWMKALDLKRYCMTMAEPDTLPLNRIAEAVADIDKEHVVDDDRFADSAIPTHESSSEET
QPLFSPIGADVFWQGSVEDQDSSLLIALDDPLAIFNDLGMQLAGDQAAYRNWQAEHEHKL
QIAQTVTTLCGAESEPEKLPASVRDNAALTHQYLSDVEAYFEQCTFEQEQIGNNTAPGGV
LLLPDIFKSPEMRRSIEMRYGRAPSDDALQAWNDRHKWRREVDLSGARQYLQQHLPTGDT
LLLQVRDTQSDFQQWAIHLGTEPLKLFVDPTHPESLLYLQTLMLNLQIIYAQDNAASAWL
AEQEANDSSLFGTLRYGFSPALKHALHQEANALLNGLGDVTNLATRIGELNGTLNHQGFA
DKPWMKALKQPVQDSFKALSQLASSAGKATFESILLAWVPIDSRLAVGKQQNIVALIRTL
LIGQILLDSKARIAIDHTMLAKLKNWISEWQVLNKQISDTRRSWLYPTAYNPRKSIASHL
RALEQKLRLHELSMPALLDYQNNQYAKQLQDEIRLHFQSGKAITKEWLSTVRRWLDGLGG
IAGSITWGVIMLNFINTAFTYRDLTRDGDFSTKDIGKVTYGLGYSFNLLMAVFVEAPWAV
IRDAKPLSIGGYNIGILDKSAAYWKARGNAVWGDAIRGFRGSMVAMGAFGIAAVTLEIFD
VADDFYAAKTPEEKNVIIVKGISVFTMGVGSTFQLMGGLSPASAFATVAMSSWFSVALLI
IGSIYLFTTMTLNYFKQDSVGWWLRKCCWSRTLDYRYAETAQGEHEEVRALMEIKLSPQV
YVKSTVYYENRYLGKGDHYSVAVQNGAGVQVHLPNLIRGQSVHFNIVSSKRPWGVLPVEK
IDDPLHQTFLDRGQFRKAEQFGKLTNTPPSKASENFTYPLMPPDNEDLIWETWVPLDKDA
TYLELQIWYPASLLDPGEEDRSYLFQMQLSPKGDTAIDGLAAVELEVKTSNRAGTLTLEV
AEGTPL
>tr|A0A0Q0E139|A0A0Q0E139_PSEAP Tryptophan synthase alpha chain OS=Pseudomonas syringae pv. aptata OX=83167 GN=trpA PE=3 SV=1
MSRLEHRFAQLKTEGRAALVTFITAGDPGYDTSLKVLKGLPAAGADVIELGMPFTDPMAD
GVAIQLATLRALDAGQTLQKTLQMVSEFRVDDQTTPIVLMGYYNPIHRFGVEAFVAQAKE
AGVDGLIIVDLPPEHDAELATPAQASGIDFIRLTTPTTDDARLPRVLERSSGFVYYVSVA
GVTGAGSATTEHVTEAIARLRRHTDLPISVGFGIRTPEQAAAIARLADGVVVGSAFVDKI
ASAESPEHAIDGVLTLCAALAEGVRSARR
>tr|A0A0Q0DQE4|A0A0Q0DQE4_PSEAP Flagellar protein OS=Pseudomonas syringae pv. aptata OX=83167 GN=ALO85_04035 PE=3 SV=1
MRRWLSVLLTLPSLAWAAEPAAQAAPAQVANTVSGGMGGQVVQLLFGLILVIGLIFVLAW
LMRRVQRVGPNSNQVIELVSSRALGPRDRLVLVQIGNEQVLLGITPGRITPLHVLKEPVQ
VPVREQATPEFAQRLMELMGKDKDKK
>tr|A0A0Q0DPP4|A0A0Q0DPP4_PSEAP Dimethylmenaquinone methyltransferase OS=Pseudomonas syringae pv. aptata OX=83167 GN=ALO85_03584 PE=4 SV=1
MSAHFEAVPIAQAVLEACEHLDTASLSDALDSLGVDGGLSGIASQVPGTRCVGIAFTVQY
EPVDASALFRGAANYIDHVPAGAVIVSSNNGRQDCTVWGDIMTHFALANGIKGTVIDGVA
RDIDTVIECSYPLFSRGRFMQSAKNRAQLQAVQVPLLIDGVTVRPGDLLVCDGSGCVVIP
QQLAVEVVRRAQAVEQTERSIIEAISAGWTLEKARSTYRYDQPWLTDAEKISVAMPSQA
>tr|A0A0Q0C540|A0A0Q0C540_PSEAP Queuine tRNA-ribosyltransferase OS=Pseudomonas syringae pv. aptata OX=83167 GN=tgt PE=3 SV=1
MSRTCRMSFELLATDGKARRGRLTFPRGVVETPAFMPVGTYGTVKGMLPRDIEATGAQMI
LGNTFHLWLRPGTEVIKGHGDLHDFMQWKGPILTDSGGFQVFSLGAMRKIKEEGVTFASP
VDGAKVFMGPEESMQVQRDLGSDVVMIFDECTPYPADEDVARVSMELSLRWAKRSKIAHG
ENTAALFGIVQGGMHENLRKRSLEGLDEIGFDGLAIGGLSVGEPKHEMIKVLDYLPGLMP
ADKPRYLMGVGKPEDLVEGVRRGVDMFDCVMPTRNARNGHLFTDTGVLKIRNAFHRHDNS
PLDPTCDCYTCQNFSRAYLHHLDKCGEMLGSMLNTIHNLRHYQLLMAGLREAIQQGTLAA
FVDAFYAKRGLPTPPLG
>tr|A0A0Q0D8A3|A0A0Q0D8A3_PSEAP Histidine ammonia-lyase OS=Pseudomonas syringae pv. aptata OX=83167 GN=hutH PE=3 SV=1
MTTLSAKTLNLIPGQLTLAQLRDIYLQPVSLTLDSSADQQIDDSVACVERILAENRTAYG

INTGFGLLASTRIASEDLENLQRSLVLSHAAGVGQPISDELVRLIMVLKVNSLSRGFSGI
RRVVIDALIALINAQVYPHIPLKGSVGASGDLAPLAHMSLVLLGEGKARYKGEWLNGVDA
LAAAGLQPLTLAAKEGLALLNGTQVSSAYALRGLFEGEDLFAAALTCGSLTVEAVLGSRS
PFDARIHAARGQRGQIDAAACYRDLLGESSGVSESHRNCDKVQDPYSLRCQPQVMGACLT
QLRQAAEVLEIEANAVSDNPLVFAAENDVISGGNFHAEPVAMAADNLALAIAEIGSLSER
RISLMMDKHMSQLPPFLVANGGVNSGFMIAQVTAAALASENKALAHPHSVDSLPTSANQE
DHVSMAPAAGKRLWEMAENVRGILAVEWLAACQGLDLREGLKTSPKLEQARSLLRSKVPF
YEKDRFFAPDIEAAIQLLSSTCLNPLVPARLLPSL
>tr|A0A0Q0IKU0|A0A0Q0IKU0_PSEAP TonB-dependent siderophore receptor
OS=Pseudomonas syringae pv. aptata OX=83167 GN=ALO85_02438 PE=3 SV=1
MSGQQSQSRNRFTPGLLAMGIWAATTQAAFAAGEQVGEAQPATTMELGATQISSDQLGST
TEGSQSYTTGPMQTATKLPLTIRETPQAVTVVTRQRMDDQAMTSINDVVRYTPGLFLDQS
SGPGRQTYTSRGFDIDNIMYDGLPASYSGYTAGVQPNLAMFDHVEVIRGATGLATGSGNP
SAAINMVRKRPTAVPQVTLTGTVGSWDDYRGVFDASGPLNDSRTVRGRIVGSYQDANSFR
DKEKSDHGVFYGVVEADLSDATTATLGLSRQEDQTNYFWGGLPLGVDGHHLNLPRSTYSG
SDWENKKIQIDTVFGELEHRFDNDWKLRVAGSSSSLDGLFSGTYLSRYNGPLETTAYQSK
PEEQQTAFDAYASGPVEAFGRTHEVVVGTSRRVYDQTSNEYSPYDTGLPIGAPKPDFVRN
GKTHSIATQDAVYLTTRLSLADPLTLILGGRLDWYDYDDRVGDEDYKVTRNLTRYAGLIY
KLDEHHSLYTSYTDVFQPQSAQDLSGKVLKPVVGENYEVGIKGEYFNGALNTSLAVFQID
QKNLKTQSPTQAGCAVLTCYDAAGLVRSQGIEMELQGALTENWQVGAGYTYARTHYLKDA
DPANKNQQFNTDTPEHLFKVSTVYRLQGALEKMRVGGNVYWQSRMYNDIALTDGSYRLEQ
GSYAVADLMAGYQVSKNLDVQLNANNIFDRTYYTAIGASSVWGSTDVYGNPRSYALTAKY
SF
>tr|A0A0Q0DNU9|A0A0Q0DNU9_PSEAP Na+/H+ antiporter NhaP OS=Pseudomonas
syringae pv. aptata OX=83167 GN=ALO85_02075 PE=4 SV=1
MLELAAGFICLTALLTYVNYRFIGLPPTIGVMVTALMFSLLLQGLSFLGYPGLEDRVQQL
IGQIDFGDLLMNWMLSFLLFAGALHVNLGDLRSYRWPIGLLATFGVLIATTVIGALAYWI
FALFGWHVSPLYCLLFGALISPTDPIAVLGVLRTANASKPLKTTIVGESLFNDGTAVVVF
TVLLGIAQLGETPTVGETALLFVHEAVGGVLFGGLIGYAVYLMIKSIEQYQIEVMLTLAL
VIGGSAMASELHVSGPIAMVVAGLIIGNLGRNLAMNDMTRRYMDGFWELLDDMLNALLFA
LIGMELLLLPFSWQHLIAASLLAVAILLSRFVTVAPAILLLRRWRKVPHGTIRILTWGGL
RGGVSVALALALPTGPERDLLLSITYIVVLLSILLQGLTIGKLVNAVTRAPQAKEPAEH
>tr|A0A0Q0DBJ9|A0A0Q0DBJ9_PSEAP Transcriptional regulator of aromatic
amino acids metabolism TyrR OS=Pseudomonas syringae pv. aptata OX=83167
GN=ALO85_04296 PE=4 SV=1
MRIHVSFIDRVGITQEVLAILGGRNLNLDAVEMVPPNVYIDAPTLSHQMLEELKDALFRV
RGVEAITVVDILPGQRRHLQLDALLAAMTDPVLALDSAGHVLLANPALIALIGREPAGES
IGELFSDPSLHVALLENGFRLPLREITLNGEALLLDATPITEAGALITLYLPSRIGERLS
ALHHDHAEGFDALLGESAAIRTLKARAQRVAALDAPLLIQGETGTGKELVARACHASSAR
HGEPFLALNCAALPENLAESELFGYAPGAFTGAQRGGKPGLMELANQGSVFLDEIGEMSP
YLQAKLLRFLNDGSFRRVGGDREVRVNVRILSATHRDLEKMVSEGTFREDLFYRLNVLNL
EVPPLRERGQDILLLARYFMEQACAQIQRPVCRLAPGTYPALLGNRWPGNVRQLQNVIFR
AAAICEHTQVDIGDLDIAGTAVARQNDSSIDSLENAVEAFERNLLEKLYADYPSTRQLAN
RLHTSHTAIAHRLRKYGIPDKR
>tr|A0A0Q0BZS2|A0A0Q0BZS2_PSEAP Phospholipase/carboxylesterase
OS=Pseudomonas syringae pv. aptata OX=83167 GN=ALO85_00680 PE=4 SV=1
MNMLKFFAALLFAFVGMAQAQDTLHTDLPLDYLAQTNVDKPDQPLVIFIHGYGSNAADLF
GLKEELPADYNYVSVQAPMELRADSYKWFTQKPGVADYDGVTEDLKSSGTRLAAFIGKAT
EKFHTQPGKVFLIGFSQGAMMSYEVALRQPKLVGGFAALSGRLLPVVKSEVKASDDLKAL
SVFIGHGTQDRQVAYASAPQAEATLKSLGLTPQLHAYEGMGHSINEAEVMDLAAWLKQSN
RQ
>tr|A0A0Q0DGN5|A0A0Q0DGN5_PSEAP SH3 domain protein OS=Pseudomonas
syringae pv. aptata OX=83167 GN=ALO85_05055 PE=4 SV=1
MKGIIRCFFNAPDRQTFRPGRSCKTVDHLPLPSLAGPLNEALFSFQIKIIIAMSRHFSAL
LSRAPGLFAVSRRLLGAGLVGAALTVVMPGSAQAAGSDRWVSDSLTTYVRSGPTDDHRIV
GTLKSGQKVELLSASGKFSQVRGEGGSTVWIPSSDLQEVPGQAERVPQLTQQVADLTEQL
AGIDNTWKTRVQGMQETLDARKKLVDELEARTKTLNDQLADSQAELRATQARLGDENKQV
MMRYMVYGGSIAGAGLLVGLILPALTKGRKKNDGWV
>tr|A0A0Q0C6E5|A0A0Q0C6E5_PSEAP DNA primase OS=Pseudomonas syringae pv.
aptata OX=83167 GN=dnaG PE=3 SV=1

MNDENAMAGLIPQSFIDDLLNRTDIVDVVSSRVQLKKAGKNYTACCPFHKEKTPSFSVSP
DKQFYYCFGCGAGGNALGFIMDHDNLDFPQAVEDLAKAAGMEVPREQSVKGQKPRQPTDS
PLYPLLTAAAEFYRQALKSHPSRKAAVDYLKGRGLSGEIARDFGLGFAPPGWDNLYKHLS
SDSLQQKVMIDAGLLIENAETGKRYDRFRDRVMFPIRDSRGRVIAFGGRVLGDDKPKYLN
SPETPVFHKGQELYGLFEARKFNRNLDEIIVVEGYMDVIALAQQGLRNAVATLGTATSEE
HLKRLFRVVPNVLFCFDGDQAGRNAAWRALEATLSNLQDGRRARFLFLPEGEDPDTLVRS
EGTDAFKARINQHAQPLADYFFEQLTKESDPRSLEGKAHMATLAAPLIDKVPGANLRILM
RQRLTEITGLTGEAVSQLVQSAPAEAPPSYDPYVDYDAMPDFADYQPQGGYEPQQEWTQK
KTGGPNQKKWDNKSWGKKGKRQDFEPRTPRVPTAVEPPMQAALRTLLHHPELAEKVENAS
HFAAEDQSNAQLLVALIEARQKNPNLRSLQLIARWHGTEQGRLLRALAEKEWLIEADNLE
QQFFDTITSLSARQRERSLEHLISKARQTELSAEEKIQLRELLNRNVPAQTPTSTGA
>tr|A0A0Q0BZS8|A0A0Q0BZS8_PSEAP Uncharacterized protein OS=Pseudomonas syringae pv. aptata OX=83167 GN=ALO85_02753 PE=4 SV=1
MALISRVREPQAMSDDAQLIDLGAERAKRIHDLNDKRLNEVRNAFEQAMPLSKQKKSGKK
PKKR
>tr|A0A0Q0IPN6|A0A0Q0IPN6_PSEAP Cell division inhibitor OS=Pseudomonas syringae pv. aptata OX=83167 GN=ALO85_100933 PE=4 SV=1
MYKKFEWPLTLYFDGDCPLCAREVRTLRAHAKPERLLFVDIRDDTFDATSLGFTLEDMES
LLHARFNDGTWVKGLDATLWSWRAAGLGAWVAPLSWRFMRPVFNLGYRLFCRSRPYLAWL
PHPDGSRRCSSNSCAVPDAKPASAKRNSSTER
>tr|A0A0N8T880|A0A0N8T880_PSEAP Uncharacterized protein OS=Pseudomonas syringae pv. aptata OX=83167 GN=ALO85_01216 PE=4 SV=1
MMKQWAISYLDKDGVQQHREAGFDERPSEEEAARFLRKDLYPVTDELNLNDLDGRTEDPT
VKTLKDHNSVQIISITEVA
>tr|A0A0Q0DLD9|A0A0Q0DLD9_PSEAP Uncharacterized protein OS=Pseudomonas syringae pv. aptata OX=83167 GN=ALO85_04712 PE=4 SV=1
MALMFSRLARNFARNGYYPTDELTLERTLQALLPATSGRMRILDPCSGEGVALAEVAHRL
ERDRTETYAVEYDKERADHSKTLLDRVLQGDLMDTMISRQSFGLLWLNPPYGDLVADHSG
ASQYQGSGRKRLEKAFYQRSLPLLQYGGVMVFIVPHYVLDDELCGWLTNHFTGLRICAAV
DRTFKQVVIFGIRVRRQDLARSRELAAMREYLRAIGSGEEAADLLPATWPWEQYAVLPIA
NDLEHFYRITLEPEQFSEEVLRLRGLWPDFTLHFGQTGAQPRAPVKALSRWHLALALAAG
AMTGVVTSRSGRVLVLKGDTYKDKVPKTEFTEDEDGNVFETRILTDRFVPRIRAWDMTPG
SSYLGCALTISSDATTPAAYETTSNTEARSQDALFELGRVVLTHSVQSLLENNSLDVMQY
LKRHATGDWGEISNDDWDSNQQALSAEGRLFSGYDIDAEDETRLWIITESDRSATTVMLP
SDY
>tr|A0A0Q0C6L7|A0A0Q0C6L7_PSEAP Enolase-phosphatase E1 OS=Pseudomonas syringae pv. aptata OX=83167 GN=mtnC PE=3 SV=1
MPIKAILTDIEGTTSAVSFVFDVLFPFARKHLPAFVRENAEQPAVAQQLQAVRDQAGEPD
AEVERVIAILLEWIAEDRKATPLKALQGMVWEQGYNAGQLKGHVYPDAVDALKHWQQQGY
RLYVYSSGSIQAQQLIFGCSEAGDLSGLFSGYFDTTSGPKREAQSYQTIAQAMDCPAEDI
LFLSDIVEELDAAKAAGMATCGLARDGGALAGHTYVASFALIDPASF
>tr|A0A0Q0BXT2|A0A0Q0BXT2_PSEAP LysR family transcriptional regulator OS=Pseudomonas syringae pv. aptata OX=83167 GN=ALO85_01127 PE=3 SV=1
MNLRTLRTFVEVVRQGSFSQAADVVSLTQSTVSKAVKTLEDELGAPLLSRIGHRSELTAA
GEIVYRRALTLLAERSDLLTELNDLRGLKGGVLRIGLPPVGSGALFAAMFTQYRARYPDI
DIQLTEHGASKLHECLASGEVELAALLKMNSFSEEFDYQDVRIEPVVVVMAANHPLAKRK
RVVIEDLAETPFILFEAGFALNTMIKLACDRKGVTPKVTARSGQVDFIVDLVSAGLGVAF
LPRMLAQKHYHDGIRLVALNEPDTDWHMVLAWRKNAHLSPAAEAWLTLARETRLPTTS
>tr|A0A0Q0DH60|A0A0Q0DH60_PSEAP LysR family transcriptional regulator OS=Pseudomonas syringae pv. aptata OX=83167 GN=ALO85_00646 PE=3 SV=1
MNRNELRKADINLMVVFETLMQERNVTRAAEKLFLGQPTISAALNRLRSLLNDPLFIRVG
HRMEPTARAHEILKHLTPALDAMSTALSLTTDFDPSVSKMTFRIGLTDDVEFGLLPAMLK
AIRLEAPGVVIVVKHVDYLNISEVLMSGDITVGVCLTRELPANAKRKTLRNVQPRLVRAD
KPASPMSLDEYCSRPHVVVSHVASVNSFSDEWLTALGRKRQVVLSVPQFATLPALMAGTD
MISGLSDYAAKAMSALGLLYDEPLPFPTPGLDLSMTWLSVMDSDPAERWLRSRIEEFMGE
RQEAPALAG
>tr|A0A0Q0CEB7|A0A0Q0CEB7_PSEAP Uncharacterized protein OS=Pseudomonas syringae pv. aptata OX=83167 GN=ALO85_02369 PE=4 SV=1
MEIRMTQIARIRDNTSERRLQAERLIGARALQEAQALRFSVFSEEFNARLNGAEQGLDID
DYDVHCSHIGVRDLNTLVNLQGPILELGRTCVDPTYRNGGTIAVLWSELAEVLNEGGYSY

LMGCASIPMQDGGIQAHAIMQRLRERYLCNEHLRAEPKNPLPTLDIPSNVICEMPPLLKA
YMRLGAKICGEPCWDEDFQVADVFILLKRDDLCPRYARHFKAAV
>tr|A0A0N8T8I2|A0A0N8T8I2_PSEAP Cyn operon transcriptional activator OS=Pseudomonas syringae pv. aptata OX=83167 GN=ALO85_02137 PE=3 SV=1
MIIAIILTYGDAVLLRHIRYLLAIADHSNFTRAAEALHVSQPALSQQIRQLESSLGVQLF
DRTRRSVVPTDAGRVYLDHARRCLLELEAGKRALDDVSDLSRGQLRLGVTPTFSEYLIAP
LIDRFSALYPGVAIILTELPLEQITEALISDALDLAIGFAGSHAVEIDSQALFDEQLCLV
MAKPGPEKQAALTLEDLQALRFALLAPGFATRQLLDAWCQMQNFKPTVGLEANSIAILLK
VVAQGRMATILPDAIVREQPGLREVQTIPALPGRTVALLRRKNGYQSAAANAFAKLVSGS
STQT
>tr|A0A0Q0DM01|A0A0Q0DM01_PSEAP Uncharacterized protein OS=Pseudomonas syringae pv. aptata OX=83167 GN=ALO85_02345 PE=4 SV=1
MLSRTLLCLAITAVSTPLLADTVWLKNGDRLTGTIKVFDGGKLLLQTEYGGAIALDWKQV
KTLKSDQPLLVKQDEHGGEVSNSLQAADDGKVVLANGDAPKTVELASIHQIIKPKPVITD
LVWKGNIDAALDFQQAENDTNDYNVAFKTSARHGDWRHNAKGDYNRETTDDVVGTDNWSA
EYSLDRFLTEKFFWDGRVSYKRDKVEDLSRQRVVGTGPGYQLWDDELGAFKVGALLNRTD
FEFSNDEKENFYSVAGTWDYNRYLIGKKVEFFTNGELGKPLSAVADYSLDVEAGLRYKVT
DWASLNLKAEKNVISGSSNGDVDKTRYTAGFGVSW
>tr|A0A0Q0C4X1|A0A0Q0C4X1_PSEAP Bleomycin resistance protein/glyoxalase family protein OS=Pseudomonas syringae pv. aptata OX=83167 GN=ALO85_04234 PE=4 SV=1
MPATEPATVTFTSAIPILRIFDARKAREFYIDFLGFSIEFEHRFEADLPLYLGISRNGLN
LHLSEHHGDACPGAAVFIPTQNIEQLRDELIGKQHGYARPDIIQQGWGKTLEVCDPFGNR
LRFCES
>tr|A0A0Q0CAH5|A0A0Q0CAH5_PSEAP 2-dehydro-3-deoxy-6-phosphogalactonate aldolase OS=Pseudomonas syringae pv. aptata OX=83167 GN=ALO85_02182 PE=4 SV=1
MLKQALKENGLVAILRGLRPEEAPAVGDALYEAGFRVIEVPLNSPQPFDSIRLLRQQLPA
DCLIGAGTVLTPEQVTQVKDAGGQVIVMPHSDAKVLRAAKAAGLFLSPGVATPTEAFAAL
AEGADVLKLFPAEQMSPAVVKAWLAVLPKGTILLPVGGISPDNMKVFLDAGVKGFGLGSG
LFKPGMSVADVTERAQAYVAAWKLHSAALAD
>tr|A0A0N8T813|A0A0N8T813_PSEAP 3-deoxy-D-manno-octulosonate 8-phosphate phosphatase KdsC OS=Pseudomonas syringae pv. aptata OX=83167 GN=ALO85_00170 PE=3 SV=1
METEIMTQDLMQRGKAIKLAVFDVDGVLTDGRLYFMEDGSEIKTFNTLDGQGIKMLIASG
VTTAIISGRKTAIVERRAKSLGIEHLFQGREDKLVVLDKLLAELNLSYEQVAYLGDDLPD
LPVIRRVGLGMAVANAAGFVREHAHGITRARGGEGAAREFCELILSAQGNLEAAHSAYL
>tr|A0A0Q0C018|A0A0Q0C018_PSEAP Extensin-like_C domain-containing protein OS=Pseudomonas syringae pv. aptata OX=83167 GN=ALO85_00325 PE=4 SV=1
MSMRVLLVFLLLCAMAAFAVWRGWVDVPERWNPWAPLDVRAEPNFLTSYKLSRLRNDPAL
CDQVLSSSGLRFSRQADSDPSARCPLQNTLRIQGGDVALSSSFLASCPLAVAYALFDIHT
LQPAAQAVFGQRVARIDHLGSFACRNIYNRANARLSQHATANALDMAGFRLADGQRIGVL
KDWGDEGDKGRFLRLLRDGACKNFSTVLGPDYNAAHRDHFHVDMGRWSVCK
>tr|A0A0Q0FCA2|A0A0Q0FCA2_PSEAP Protein translocase subunit SecD OS=Pseudomonas syringae pv. aptata OX=83167 GN=secD PE=3 SV=1
MLNKYPLWKYVLILAVLVVGFIYSAPNLYPDDSAIQISGASTALQVNQADVDRAAKALAD
AGISVKASSLAENGKGGLLRLSKQEDQLPAKDVVRKALGDDYVVALNLARTTPTWLRNLG
ASPMKLGLDLSGGVHFLLEVDMDKAIDARLKVYEGEVKSLLRKEKVRYRSLPQLGNVIQL
GFTDDAEREQARALVRKTFTDFEITPAELNGIPVLRMALTPAKLAEIREYSIKQNLTTVR
NRVNELGVAEPLVQRQGANRIVVELPGVQDTAEAKRILGKTANLEFRLGAGPDDSKGTTE
MFEFREGGRPAAAVERGLIITGDQVTDAKASFDEHGRPQVNINLDGHGGELMSRATRSNV
GRSMAVIFIEQRPTTTYTKQMVNGVEKDVPVQTFKEDKKIISLATIQSPLGSQFRITGLN
GQGESSELALLLRAGGLAAPMYFAEERTIGPSLGADNITKGIDASLWGMLFVSLFIMAIY
RFFGLIATVALALNMVMLLALMSLLGATLTLPGIAGIVLTMGMAVDANVLIFSRIREEIA
NGMTVQRAINEGFDRAYTAILDANLTTLLVGGILFAMGTGPVKGFAVTMSLGIFTSMFTA
IMVTRAMVNLIYGGRDFKKLWI
>tr|A0A0Q0DDI1|A0A0Q0DDI1_PSEAP Uncharacterized protein OS=Pseudomonas syringae pv. aptata OX=83167 GN=ALO85_00496 PE=4 SV=1
MIICGVEIKGSEAIFALATRRNGDIEHLPLATKKLALEDDDEAANVKAFSTQLAGFVREN
GISHVVIKKRSKKGEFAGGPTTFKIETIFQLMSDCNVVLISPQTVNAQNKKHTFVLPDTL

NKYQHEAYKAACAGVMKSV
>tr|A0A0Q0BRD4|A0A0Q0BRD4_PSEAP Uncharacterized protein OS=Pseudomonas syringae pv. aptata OX=83167 GN=ALO85_101162 PE=4 SV=1
MRKPAQGRMMADPRRSSVARAFKYFALPLSTARRAQVLPVLGEP
>tr|A0A0Q0DQG4|A0A0Q0DQG4_PSEAP Cysteine-rich CPXCG family protein OS=Pseudomonas syringae pv. aptata OX=83167 GN=ALO85_01736 PE=4 SV=1
MMQELQSYDCPYCGEPVEAVLDLSGGDQQYIEDCPVCCRPIVFDLQTDGEDWNLDVRSEN
E
>tr|A0A0Q0C380|A0A0Q0C380_PSEAP Deoxyuridine 5'-triphosphate nucleotidohydrolase OS=Pseudomonas syringae pv. aptata OX=83167 GN=dut PE=3 SV=1
MHALQAKILDPRIGNEFPLPAYATVGSAGLDLRAMLKEDTLLEPGQTLLIPTGLSIYIGD
PGLAALILPRSGLGHKHGIVLGNLVGLIDSDYQGELMVSCWNRGQTAFNIAVGERIAQLV
LVPVVQAHFELVETFDESQRGAGGFGHSGSH
>tr|A0A0Q0FKG7|A0A0Q0FKG7_PSEAP Uncharacterized protein OS=Pseudomonas syringae pv. aptata OX=83167 GN=ALO85_00417 PE=4 SV=1
MADSHFKIVFEGQVREGVELDTAKANLAGLFKSEPSAVQKLFSGQTVALKRGITYQDAER
YLKALNDAGVEARIEPDIEISLSLDEVVSQPSPKPAHLEISPYAPPKAQVGADQPGHSTL
KVLSINGRIGRLRYLAWSMVLSAATLMLVGVCLLVMNGSLVAGGLLMAVVLVAFAVIGAQ
IGVQRLHDLGWSGWLLLLTMVPFVGSVFPFLMILLPGTKGPNAFGPPSPPNSRGVKTLAV
LWVLLIPIVVIGLTAGGGGMLRDELELQTDEYEQSLPYDDEESRDSALTAPADVNIESEE
QSDK
>tr|A0A0Q0DXK5|A0A0Q0DXK5_PSEAP Na+/solute symporter OS=Pseudomonas syringae pv. aptata OX=83167 GN=ALO85_03599 PE=3 SV=1
MISTTMVSDLYITFGLIGVFLAGMIAVLYATNRKSESFSDYAVGGRSYGPWFIAMCYTNS
WWPGATFTAFFALSVSGALGFYGMVYATLGVTAMYLMANRAWTWGKRFNLTTQPDLLGMR
FNSPVVKRIASIIGIISVFPWVVMGIQALATLFQFASFGRWGVTTCLLVGVAVILIRQYW
TVSMGMRGLIMTDMYQGLIAYVLCAALCIFLLFGAQASFSNLSQLPASMLVIPGGAGSTY
GPMYMFSLIFTGVIGSMCWPMSFQRIYTASGVRSVKKGTLLTILLVGGFYGILMLFAAAI
SQDPNVAAHPQHGWFLSLFDIGGPWLLGVAIMIVLAASIGHVDGCVQVCGTQFANDLATW
SKPRSDREKTILAKVGMVVFIAAASLLAYLTFDYARLQLLAQISYQGIIQLAVPLFFGVF
SRHGNKQGAIAGMLVGIVIAIVLTTVYPDDIPALGSLTSGIVGLIFNAGIFVACAVAIKP
SADEVRRVDELFAMAAPARRGVGPVVAMS
>tr|A0A0Q0C8G3|A0A0Q0C8G3_PSEAP Prevent-host-death family protein OS=Pseudomonas syringae pv. aptata OX=83167 GN=ALO85_200096 PE=3 SV=1
MNVKILGRLSESGKVIPFYSFKIPAGFPNPAADHIEQDFSFDRLMDLRAPHIYVAKIDGD
SMEGAKIFHDSLVVVDRSRTPSSGSIVIAALNNEPLCKILILQGDHVVLKSANPAYPARH
ILEGDELSIWGVVRHGVTSFE
>tr|A0A0Q0DBY2|A0A0Q0DBY2_PSEAP Arginine/ornithine ABC-type transport system, permease protein AotQ OS=Pseudomonas syringae pv. aptata OX=83167 GN=ALO85_01838 PE=3 SV=1
MLKGYGAVILEGAWLTLQLALSSMALAIVLGLIGVALRLSPVRWIAWLGDLYSTVVRGIP
DLVLILLIFYGGQDLLNRVAPLLGFDDYIDLDPLMAGICTLGFIFGAYLSETFRGAFMAI
PKGQAEAGMAYGLSSGKVFFRILVPQMIRLAIPGFTNNWLVLTKATALISVVGLQDMMFK
AKQAADATREPFTFFLAVAAMYLVITSVSLLALRYLEKRYSVGVRAADL
>tr|A0A0Q0CVP6|A0A0Q0CVP6_PSEAP Branched-chain-amino-acid aminotransferase OS=Pseudomonas syringae pv. aptata OX=83167 GN=ilvE PE=3 SV=1
MADVPLNTTSTARKPHPWVYLDDRFVAAADALVPITTQAFNYGTAVFEGIRAYVADNGEL
NVFRLDDHLKRMENSARLLQLDSLPGRAELKEIILDLLRRNDALTDSYIRPMAYKKALMP
GVGFGVKLTGVSSGLVINTLAMGAYMPAAGIRCAVSSWRRIPDVSLPSRAKITGSYANSA
LAMESAQRNGFDDALMLNIHGNLAESTTSNVFVVKDGSLITPPLSAHILEGITRDSIMTL
AREILGLNVVERDISFSELYSVDECFLAGTGVEIVPVTQIDHYVLQNHKVGELTAALRSL
YLQTVKGSHSRYAHWLSPL
>tr|A0A0Q0DLW1|A0A0Q0DLW1_PSEAP RND efflux system, outer membrane lipoprotein OS=Pseudomonas syringae pv. aptata OX=83167 GN=ALO85_02400 PE=3 SV=1
MSKSLISLAVTAFILGGCSLIPDYKQPEAPVAAQYPQGPAYSPVEGAKMAAAEQGWRHFF
NDPALQQLIQTALLNNRDLRVAALNIDAYRAQYRIQRSDLFPAVSATGSGSRQRVPGDQS
QTGQPGITSSYSATLGVSSYELDLFGRVRSLSEQALETYFASEEARRSTQISLVASVANA

YMTWQADKELLKLTQDTLKTYEESFRLTSRASEVGVSSALDLSQSRTAVESARAKLAQYQ
RLVAQDQNSLVLLLGTSLPDNLPASHLLDSDMLTEMPAGLPSDLLQRRPDILEAEHSLKA
ANANIGAARAAFFPSISLTASAGTSSSELSGLFKGGSGAWLFQPSINIPIFNAGNLRASL
DYSKIQKEINVANYEKAIQTAFQEVSDGLASRKTYDEQLQAQRDFVAANQDYYRLAERRY
RIGIDSNLTFLDAQRSLFSAQQTLITDRLSQLTSEVNLYKALGGGWYERTGQTETISKEK
PSVQLF
>tr|A0A0Q0DXN8|A0A0Q0DXN8_PSEAP Putative cobalt transporter subunit CbtB
OS=Pseudomonas syringae pv. aptata OX=83167 GN=ALO85_03629 PE=4 SV=1
MSTISSHAAAPSSTRTQRLTAAISAALLGACLVYFAGFSHIAAVHNAAHDTRHSAAFPCH
>tr|A0A0Q0C727|A0A0Q0C727_PSEAP 50S ribosomal protein L6 OS=Pseudomonas
syringae pv. aptata OX=83167 GN=ALO85_200046 PE=4 SV=1
MTHWMLSLFVVLITALIVGSIAERVGQSRVIGEIASGLLLGASVLGGVSPELFNYLFVGS
PGQHLKELAELGVIILMFEVAWHAPASLENIAKKHAPIVIAVAGIVFSFSIGCVLAYVAK
PVMAPEKPFFSFILFCGLALSITALPVLVRIIRENTFINKSSGSIALASAVCSDMVAWAG
VAVISAMYASGYTSTKQITINGILFLTFIVTSLYVVSPIIRKFKWLDGDRFPAFAQITIA
FSYCLLCSEISDSLGFNRVTGAIVAGYTLSRVPGLESNWKKIVGGFSSLALTPVFFAYSG
IQASLSLSGSLVVWLLLFFVGSLIGKLGGCYLGARIVGLPKSTSVEVGVLMNTKGLVELV
VLNIGLEIGVVNQATYSVLLILALVSTGMTIPVLNWWYKRAGKSQSAPAEAVT
>tr|A0A0Q0D8Y1|A0A0Q0D8Y1_PSEAP Nicotinamide mononucleotide transporter
OS=Pseudomonas syringae pv. aptata OX=83167 GN=ALO85_03636 PE=4 SV=1
MSGLELFAAAIGVIAVWLTVKQNPWCWPIGLVMVVIYIWIFFDVKLYSDMLLQVIYAGLQ
VYGWLQWTRHGDGLPVKAVTTLQNGSVLKGLAVGVLISVALGAGMAHFTDAAQPWLDAAL
TGFSLVAQVWMAQKRVQCWPLWILLDVIFVGLFIYKDLYLTAALYGLFTLLAVQGWREWR
NDLALAR
>tr|A0A0N8T953|A0A0N8T953_PSEAP Single-stranded DNA-binding protein
OS=Pseudomonas syringae pv. aptata OX=83167 GN=ALO85_200119 PE=4 SV=1
MTFAGSASDSLILEFEKEMGLKFPYSYTLFLKKFGALSFAGDTYYGATKSGLAATSIPSV
FFATMKARSQGDADSKMVVIQSSGYGPIYSIDTSINSGPNENPIIETELSFKREPNKKII
FENYELFLLSTIRKAIEEL
>tr|A0A0Q0IPA8|A0A0Q0IPA8_PSEAP Regulatory protein, TetR OS=Pseudomonas
syringae pv. aptata OX=83167 GN=ALO85_03701 PE=4 SV=1
MAQLGRPRTFDRDAAITQALHLFWEHGYDATSLSQLKATIGGGITAPSFYAAFGSKQALF
NEVMEHYLDTYGRVTDSLFDTTLPPRDAIELTLRRSAKMQCEPGHPTGCLVALGLMSACS
DESKAISEPLARARDLNRAGIVACIQRAVAAGELPATVIPETLAAVFDSFLLGLSTLARD
GVPHATLDAAVTQVMRLWDSLGNGADKH
>tr|A0A0Q0FV86|A0A0Q0FV86_PSEAP Mannitol/polyol ABC-type transport
system, permease protein OS=Pseudomonas syringae pv. aptata OX=83167
GN=ALO85_03459 PE=3 SV=1
MMTLKQSRRLQSLLLGALSWIIAALVFFPIFWMVLTSFKTEIDAFATPPQLFFTPTLENY
LHIQERSDYFHFAWNSVLISFSATVLAMLIAIPAAYSMAFFETKRTKSTLLWMLSTKMLP
PVGVLMPIYLLAKTFGLLDSRLALIIIYTLINLPIVVWMIYTYFKDIPGEILEASRLDGA
STRQEIFRVLMPICKGGLASTALLSLILCWNEAFWSLNLTSSAAAPLTALIASYSSPEGL
FWAKLSAVSTLACAPILIFGWISQKQLVRGLSFGAVK
>tr|A0A0Q0BWG7|A0A0Q0BWG7_PSEAP Succinate dehydrogenase, cytochrome
subunit OS=Pseudomonas syringae pv. aptata OX=83167 GN=ALO85_02196 PE=4
SV=1
MLYAMDKSLASEEGFGEVKAYMTSPLAKLIIWGLLSALLYHLVAGIRHLIMDSGVGETLE
GGKLGSKIVIAVSVILILLAGVWIW
>tr|A0A0Q0DN21|A0A0Q0DN21_PSEAP DNA topoisomerase 4 subunit A
OS=Pseudomonas syringae pv. aptata OX=83167 GN=parC PE=3 SV=1
MAAICCCASQRNNEARMSDSLDLSLDGVERRSLADFTEQAYLNYSMYVIMDRALPHIGDG
LKPVQRRIIYAMSELGLGADAKHKKSARTVGDVLGKFHPHGDSACYEAMVLMAQPFSYRY
TLVDGQGNWGAPDDPKSFAAMRYTEARLSRYSEVLLSELGQGTADWVPNFDGTLDEPAVL
PARLPNILLNGTTGIAVGMATDVPPHNLREVASACVRLLDDPKATVAELCEHILGPDYPT
EAEIITPRADLLKIYETGRGSVRMRAVYRIEDGDIIVTSLPHQVSGAKVLEQIAAQMQAK
KLPMVADLRDESDHENPCRIVIIPRSNRVELDELMQHLFATTELESSYRVNINIIGLDGK
PQLKNLKTLLGEWLTFRIGTVRRRLQFRLDKVEHRLHLLDGLLTAYLNLDEVIHIIRTAE
HPKAELIARFDLSEIQADYILDTRLRQLARLEEMKLRSEQDALRKEQAKLQALLGSESKL
RKLVRAELIADAETYGDDRRSPIVARAEAKALSENELMPTEPVTVVLSEKGWVRCAKGHD
IDATGLSYKAGDGFKTSSIGRSNQFAVFIDSTGRSYSVAAHTLPSARGQGEPLTGKLQPP

PGATFECVLLPEDDALYVIASDAGYGFVVKGEDLQAKNKAGKALLSLPAGAKVILPRPVP
DREQNWLAAVTTEGRLLVFKISDLPQLGKGKGNKIIGIPGERVASREEYVTDLAVIGQGA
TLVLQAGKRTLSLKPEDLEHYKGERGRRGNKLPRGFQRVDALLVENS
>tr|A0A0Q0C6S4|A0A0Q0C6S4_PSEAP GntR family transcriptional regulator OS=Pseudomonas syringae pv. aptata OX=83167 GN=ALO85_00767 PE=4 SV=1
MTDRPILLPAMRQVSRDTLQDQVYRQIREALMSGRFQPGQKLTIRGLAEALGSSPMPVRE
ALNRLSAENAFEVTETARLRVPMMTPERLREIRDARVALEGLLAEKAVVLINDADLADIS
QLCGQMQKAADNVDVALYLWTNFAFHRRIYAVAKAELIVAAVENFWLHIGPCFALVAPDR
AHLQRSMEAHNRIVEALAARDGAAARAAVTDDIMQAADSLTRLIANSDRSRSRVSGVKKA
>tr|A0A0N8T7T9|A0A0N8T7T9_PSEAP Uncharacterized protein OS=Pseudomonas syringae pv. aptata OX=83167 GN=ALO85_02247 PE=4 SV=1
MNTSDPVNASGHYSDKWKERFAFFEAHGGPSAPGFRPALKQLPFLKKVKINFNFFAFFFG
PVYLFIMGLWKKNLCIIAIMIVVSVALNIVMDMFEFRYAKEASSALGFAFNSLYGQLTNY
AYYLKEVKGEQGWNPFEGLRW
>tr|A0A0Q0DC61|A0A0Q0DC61_PSEAP Protocatechuate 3,4-dioxygenase, beta subunit OS=Pseudomonas syringae pv. aptata OX=83167 GN=ALO85_00542 PE=4 SV=1
MRAHKKNEENAMSAADNSRFVIRDRNWHPKALTPDYKTSILRSPRQALVSIPQSISETTG
PDFSHLKFGQHDNDLLLNFNNGGLPIGERILLAGRVCDQYGKPIPHTLVEIWQANAGGRY
RHKRDAYLAPIDANFGGVGRALTDSEGNYSFRTVKPGPYPWRNGPNDWRPAHIHVSISGP
SIATRLITQLYFEGDPLIPICPIVKAIANPDAVQSLIARLDLGLGNPMDCLAYRFDIVLR
GQRKTHFENC
>tr|A0A0Q0C6X2|A0A0Q0C6X2_PSEAP Branched-chain amino acid ABC-type transport system OS=Pseudomonas syringae pv. aptata OX=83167 GN=ALO85_03133 PE=3 SV=1
MSAPVAVPVSKPVEIKKSLIDAVVAGLLALIVFGPIVGIVLDGYSFNLQPTRVAWLVGVV
MIGRFLISLFLQTPRGLRVSQSFESSDSGVHVLKPDHRSRLYWIIPMLIVIAIVFPIFAN
KYILTVVILGLIYVLLGLGLNIVVGLAGLLDLGYVAFYAIGAYGLALGYQYLGLGFWSAL
PLAAIAAALAGCILGFPVLRMHGDYLAIVTLGFGEIIRLVLNNWLSFTGGPNGVPVPAPT
FLGLEFGRRAKDGGVPIHEYFGFDYNPDLKFIFIYAVLFLVVLAVLFIKHRLTRMPIGRA
WEALREDEIACRSMGLNHVLVKLSAFTIGASTAGLAGVFFASYQGFVNPTSFTFFESALI
LAIVVLGGMGSTVGVVIAAFVLTVAPELLRSFSEYRVLLFGILMVLMMIWRPRGLIRISR
TGVKPRKGALVTEGAAR
>tr|A0A0N8T861|A0A0N8T861_PSEAP tRNA N6-adenosine threonylcarbamoyltransferase OS=Pseudomonas syringae pv. aptata OX=83167 GN=tsaD PE=3 SV=1
MLVLGLETSCDETGVALYDSERGLLADALFSQIDLHRAYGGVVPELASRDHVKRMLPLIR
QTLAEADCVATDIDAIAYTAGPGLVGALLVGASCAQALAFAWDIPALGVHHMEGHLLAPM
LEENPPQFPFVALLVSGGHTQLVRVDGIGQYELLGETLDDAAGEAFDKTAKMMGMQYPGG
PEISKAAMQGVAGRFVFPRPMTDRPGLEFSFSGLKTSALNTWQQCRNAGDDSEQTRCDIA
LAFQQAVVETLTIKCKRALKQTGLKSLVIAGGVSANKALRASLESMLGDLRGHVYYARPE
FCTDNGAMIAFAGCQRLQVGQKEDLSISVQARWPMEQLSGL
>tr|A0A0Q0C205|A0A0Q0C205_PSEAP Ribonucleoside-diphosphate reductase subunit beta OS=Pseudomonas syringae pv. aptata OX=83167 GN=ALO85_01976 PE=4 SV=1
MRATPEKQATHPLDPTGQSTRLTLKQEKYTMLSWDEFDKEDGEVVAKATNAGHANEANMD
RLDSAGGLAAQEARAVTAADSAALIRAKKALDALDVAEGLAELEGASARVAVDEKMMINC
RADLNQLVPFKYDWAWQKYLDGCANHWMPQEVNMTADIALWKNPEGLTDDERRIVMRNLG
FFSTADSLVANNLVLAVYRLITNPECRQYILRQAFEEAIHTHAYQYCIESLAMDEGEIFN
MYHEIPSVAKKAAWGLKYTRSISDPKFETGTVDTDKELLRNLIAYYCVLEGIFFYCGFTQ
ILSMGRRNKMTGVAEQFQYILRDESMHLNFGIDVINQIKIENPHLWDAEMKEEATQMILQ
GTQLEIEYARDTMPRGVLGMNAAMMEDYLKFIANRRLSQIGLKEEYPGTTNPFPWMSEIM
DLKKEKNFFETRVIEYQTGGALSWD
>tr|A0A0Q0IAY5|A0A0Q0IAY5_PSEAP Uncharacterized protein OS=Pseudomonas syringae pv. aptata OX=83167 GN=ALO85_01861 PE=4 SV=1
MQPISGDLVVVIDDQIQSLIEEINDAPQQHQQTRFIDEFQNMMIDQLMGPFGLTRAMFDD
RDGGAITTLQNFEKGIAANDADDARHASWKQANEKSFERKDYDAALNAAHDDMRSADGKF
YDGYKVASEIPEGPRMDARDHVVSASSIERSAKGQLGQTREERVITATLDDNVVLTAFNM
NCSKGEKDLLEWAALPSSKDPSKTNAEYFEVDEQTLLDKYKKATKAVGSTQNRALFSKQS
GEFLVEGAKASGKLVVRQILGLLLKDLIDGLVQDVRYLVSKGLAEAKPLMQLVKERIQAT

YERIKLKWADYLKEGASAGISGFLSTFVTLIINSFITTAKNLVRMIREAVLSIVRAVKLI
MAPSPGTESKDIAVEVAKILASSIALCVGIALEEVIQKALEAIPVLLPFAATIAPVITGV
VTGALTLFTVMAFDRLRDSFAFQNKQLADVHRGQAVGLLKIKQSMFMLDSAYRHVIVTTE
MLRVTFAEDWFEVQAMKAVTTTKAADYRKAVDSLDDLLELF
>tr|A0A0Q0FP42|A0A0Q0FP42_PSEAP Uncharacterized protein OS=Pseudomonas syringae pv. aptata OX=83167 GN=ALO85_02342 PE=4 SV=1
MNGADEYAVAQGNTRLIPNLNTTCKMEVPADLPGVVIFLHGVNDPGASYSMVESGLCQGV
NERLDRPDLVPGRYGARYKAAEKTAPEKRSSDQKVIVDDSDTYLYRRDTNDPKTHSLLIP
FYWGYRAASEDIKRDDAGDPLRMRNQFQDINGNRLDRHFAKGGGFFANATNNLQEMYGEG
FEPNFKTHSVEFLSPDNSLYFGKAPSRQYFVLAAHRLAMLVREIRRLSPDETITIIGHSQ
GTLITLLAQALLVDEGQRCADTVIMVDSPYSVLPDVTPKDHDTLSTLINIVSAVTKNPYT
QPPLSDLRNPATYAGRSGPKWSPAQGTRKNKLGNLTVFPERDNRGKVYLYFCPDDTTVAL
DDVQGIGTYGVPDALPDGRMAMMVLQTRRFYQRMWTKRHRDGEPVLVGKTPQPEFLRAKD
ESRYPGGSFFVGLVSQAPVSEGQERLINAEALNPAHEPQMFGGEAIKGTPTTSGRDRPDD
VGKSVALGKDAATFLWVQMPSEYNPYEIDKEQALARYNSLSNDPEKHTRSVRISNNRRSF
QREETPFEVRTRMEHDSSTWGYNSYHSGLLRSPENHRWVTAMDIAIGQSKSLDDPAMCEL
LVAIGNWRMDAKQFLNTTESPAWGRLSPRAQALVKASHQYYQKGIFPPYGVVPMSPPALV
KNALNKGAEQ
>tr|A0A0Q0DNT5|A0A0Q0DNT5_PSEAP Major facilitator family transporter OS=Pseudomonas syringae pv. aptata OX=83167 GN=ALO85_04630 PE=4 SV=1
MIKPLACAASIKNRLTDTRPASGSPCSSETFMPHSSQSAHNAMTMTPAMVMLFAFCCGAI
VANIYYAQPIIELIAPDIGLSSTAASLIVSLTQIGYALGLFFLVPLGDLLENRRLMLVTT
VVAILSLLGAAFAEQPNVFLLVSLLVGFSSVSVQMLIPLAAHLAPEASRGRVVGGIMGGL
LLGILLARPIASLVADHFGWRAVFGSAAVVMIGISVVLATTMPKRLPDHRASYGQLLFSL
WTLLRTQPVLRQRAFYQACMFATFSLFWTAVPLELSRNHGLSQSQIAIFALIGAIGAIAA
PISGRLADAGYTRIASLGALLFGALSFLPGLVHPAYSVIGLAITGVVLDFCVQTSMVLGQ
RTVYALDAASRSRLNALYMTSIFIGGAIGSAVASPLFDHGGWTWVLIAGTALPLIALLAL
LRDRSRQNA
>tr|A0A0Q0C903|A0A0Q0C903_PSEAP Sulfurtransferase OS=Pseudomonas syringae pv. aptata OX=83167 GN=ALO85_00713 PE=3 SV=1
MKQLNIDGRSIELDKDGFLQDLSDWSLDVAHALSAEEGIELSAEHLEILELLRGFYAEFQ
LSPATRPLIKYTALKLGAEKGNSMHLNRLFNGTPAKLAAKLAGLPKPTNCI
>tr|A0A0Q0DSC4|A0A0Q0DSC4_PSEAP Mannitol/polyol ABC-type transport system, permease protein OS=Pseudomonas syringae pv. aptata OX=83167 GN=ALO85_03460 PE=3 SV=1
MNTSASPERTDLSGEQQPLKSRRFKPGWFLVSPSVALLLVWMIVPLGMTIYFSLIRYNLL
SPGENEFVGLENFQYFVTDSGFLPGAGNTLLLVGSVLAISVILGVLISALLEASEFFGRG
IVRVLLISPFFIMPTVSALIWKNLIFHPVSGVLAAVWKFFGAEPIDWFAHYPMLSIIIIV
SWQWLPFAILILMTAMQSLDQEQKEAARLDGAGPLAIFWHLTLPHLARPIAVVVMIETIF
LLSVFAEIFTTTNGGPGFASTNLAYLIYIQALLQFDVGMASAGGLIAVVIANIAAIILIR
MIGKNLTDKS
>tr|A0A0Q0C4R6|A0A0Q0C4R6_PSEAP Putrescine-pyruvate aminotransferase OS=Pseudomonas syringae pv. aptata OX=83167 GN=ALO85_02336 PE=3 SV=1
MSANNPQTLEWQALSSEHHLAPFSDYKQLKEKGPRIITRAEGVYLWDSEGNKILDGMSGL
WCVAIGYGREELADAASKQMRELPYYNLFFQTAHPPVLELAKAISEIAPQGMNHVFFTGS
GSEGNDTMLRMVRHYWALKGQPNKKTIISRVNGYHGSTVAGASLGGMTYMHEQGDLPIPG
VVHIPQPYWFGEGGDMTPDEFGVWAAEQLEKKILELGVENVGAFIAEPIQGAGGVIVPPD
SYWPKIKEILSRYDILFAADEVICGFGRTSEWFGSDFYGLKPDMMTIAKGLTSGYVPMGG
LIVRDEIVAVLNEGGDFNHGFTYSGHPVAAAVALENIRILREEKIVERVRSETAPYLQKR
LRELSDHPLVGEVRGVGLLGAIELVKDKTTRERYTDKGAGMICRTFCFDNGLIMRAVGDT
MIIAPPLVISFAQIDELVEKARKCLDLTLAVLQG
>tr|A0A0Q0BSI1|A0A0Q0BSI1_PSEAP Carbamoyl-phosphate synthase large chain OS=Pseudomonas syringae pv. aptata OX=83167 GN=carB PE=3 SV=1
MPKRTDIKSILILGAGPIVIGQACEFDYSGAQACKALREEGYRVILVNSNPATIMTDPAM
ADATYIEPIKWQTVAKIIEKERPDALLPTMGGQTALNCALDLEREGVLEKFGVEMIGANA
DTIDKAEDRSRFDKAMKSIGLACPRSGIAHSMEEANAVLEKLGFPCIIRPSFTMGGTGGG
IAYNREEFEEICARGLDLSPTKELLIDESLIGWKEYEMEVVRDKKDNCIIVCSIENFDPM
GVHTGDSITVAPAQTLTDKEYQILRNASLAVLREIGVETGGSNVQFGICPNTGRMVVIEM
NPRVSRSSALASKATGFPIARVAAKLAVGYTLDELSNEITGGKTPASFEPSIDYVVTKLP
RFAFEKFAKADARLTTQMKSVGEVMAIGRTFQESLQKALRGLEVGVCGLDPKLDLSHPES

MSTLKRELTVPGAERIWYVADAFRAGMTVEEIFAMNMIDPWFLVQIEDLIKDEEKIKTLG
LSAIDRDVMFRLKRKGFSDARLAKLLGVTEKNLRTHRHKLEVFPVYKRVDTCAAEFATDT
AYLYSTYEEECEANPSTRDKIMILGGGPNRIGQGIEFDYCCVHAALALREDGYETIMVNC
NPETVSTDYDTSDRLYFEPVTLEDVLEIVRVEKPKGVIVQYGGQTPLKLARALEAAGVPI
IGTSPDAIDRAEDRERFQHMVERLNLRQPPNATVRSEDEAIRAAAKIGYPLVVRPSYVLG
GRAMEIVYQEDELKRYLREAVQVSNDSPVLLDHFLNCAIEMDVDAVCDGTDVVIGAIMQH
IEQAGVHSGDSACSLPPYSLPAHIQDEMREQVKKMALELGVVGLMNVQLALQGEDIYVIE
VNPRASRTVPFVSKCIGVSLAMIAARVMAGKTLKELNFTKEIIPNFYSVKEAVFPFAKFP
GVDPILGPEMKSTGEVMGVGDTFGEAFAKAQMGASEVLPTGGTAFISVRDDDKPLVEAVA
RDLINLGFEIVATAGTAKLIEAAGLKVRRVNKVTEGRPHVVDMIKNDEVTLIINTTEGRQ
SIADSYSIRRNALQHKIYCTTTIAAGEAICEALKFGPEKTVRRLQDLHAGLKA
>tr|A0A0Q0DI96|A0A0Q0DI96_PSEAP Succinate--CoA ligase [ADP-forming] subunit beta OS=Pseudomonas syringae pv. aptata OX=83167 GN=sucC PE=3 SV=1
MNLHEYQGKQLFAEYGLPVSKGYAVDTPEAAAEACDKIGGTEWVVKAQVHAGGRGKAGGV
KLVRSKEDAAAFAQQWLGKRLVTYQTDANGQPVTKILVESCTDIAKELYLGAVVDRSSRR
IVFMASTEGGVDIEKIAHDTPEKILKATIDPLVGAQPFQGRDLAFQLGLEGKQVTQFAKI
FTGLAKLFQDHDLALLEVNPLVIKADGDLHCLDAKINIDANAMYRQPKLKGFHDPSQDDP
REAHAAKFELNYVALEGNIGCMVNGAGLAMGTMDIVNLHGGKPANFLDVGGGATKERVTE
AFKIILSDANVAAVLVNIFGGIVRCDMIAEGIIGAVKEVGVKIPVVVRLEGNNAELGAKV
LAESGLNIIAATSLTDAAQQVVKAAEGK
>tr|A0A0Q0FNM7|A0A0Q0FNM7_PSEAP Diacylglycerol kinase OS=Pseudomonas syringae pv. aptata OX=83167 GN=ALO85_00266 PE=3 SV=1
MSPFKGQTGLKRILNAAGYSLDGMRAAFKGEAAFRQLVLLNVVLIPLSFFLHVSKGEHAL
LVAVCLLALIVELLNSAVEAAIDRISLDLHPLSKNAKDMGSAAQFIALSMIGLVWAIILL
G
>tr|A0A0Q0D1G5|A0A0Q0D1G5_PSEAP Response regulator receiver:transcriptional regulatory protein OS=Pseudomonas syringae pv. aptata OX=83167 GN=ALO85_00754 PE=4 SV=1
MRLLLVEDHVPLADELLAALGRQGYAVDWLADGRDAVHQGATEPYDLIVLDLGLPGMPGL
EVLQQWRSKGLATPVLILTARGSWSERIEGLKAGADDYLTKPFHPEELQLRIQALLRRSH
GLANQPTLESAGLNLDEGRQCVSRDGVDIQLTSAEFRLLRYFMLHPGQILSKSHLSEHLY
DGENERDSNVIEVHVNHLRRKLGRAVVETRRGQGYLYGGAGG
>tr|A0A0Q0D6H6|A0A0Q0D6H6_PSEAP Two-component DNA-binding response regulator ColR OS=Pseudomonas syringae pv. aptata OX=83167 GN=ALO85_02351 PE=4 SV=1
MRILLVEDNRDILANMADYLGMKGYTVDCAQDGLSGLHLAATEHYDLIVLDIMLPGIDGY
TLCKRLREDARRDTPVIMLTARDQLDDRLQGFRSGADDYLLKPFALSELAARIEAVLRRA
QGGGRRTLQVADLVYDLDTLEVTREGRMLKLNPVGLKLLAVLMQKSPHVLRREILEEALW
GDDCPDSDSLRSHVHQLRQVIDKPFGKPLLQTVHGVGYRLAEGRDGV
>tr|A0A0Q0CIG8|A0A0Q0CIG8_PSEAP Type III effector phosphothreonine lyase OS=Pseudomonas syringae pv. aptata OX=83167 GN=ALO85_03279 PE=4 SV=1
MPINRPAFNLKLNTAIAQPTLKKDAGAELRRLNQSEVRANTQTRFAVNHRAPTYDVAQSA
LGENHGGWTAANHFKMTGSEVFIHMDRLEPNCKGEFAGDKIHLSVAPEDVPDAFNAIGKI
LQASDSPVDSWKVTDMKCLQAEMPAAKQRVALGAQFTIYAKPDREDNTYSPEYMGKMRGI
SSIEQELSAAGVRQSSHRPDSDVSPGHWSYASYRNEHKSNRSGTSNQHRNLEAEPFFQLV
SFSDGASGSSRSSADHQALLPPPWAR
>tr|A0A0Q0BRJ1|A0A0Q0BRJ1_PSEAP Branched-chain-amino-acid aminotransferase OS=Pseudomonas syringae pv. aptata OX=83167 GN=ALO85_04256 PE=3 SV=1
MSQESINWDTLGFDYIKTDKRYLSHWRDGAWDQGSLTEDNTLHISEGSTALHYGQQCFEG
LKAYRCKDGSINLFRPDQNAQRMQRSCSRLLMPHVPTDVFIEACKQVVKANERFIPPYGS
GGALYLRPFVIGVGDNIGVRTAPEFIFSIFCIPVGAYFKGGLKPNNFVISGYDRAAPNGT
GAAKVGGNYAASLMPGSEAKKHSFADCIYLDPQTHSKIEEVGSANFFAITKDDVFLTPKS
PSVLPGITRLSLIELAKSRLGLTVEEGDVFIDQLGEFKEAGACGTAAVITPIGGISYKDN
LHVFHSQTEVGPVTQRLYKELTGVQSGDIEAPAGWIVKV
>tr|A0A0Q0DP18|A0A0Q0DP18_PSEAP GntR family transcriptional regulator OS=Pseudomonas syringae pv. aptata OX=83167 GN=ALO85_00001 PE=4 SV=1
MENQSALPRARRKHRSLAQELVTELSERIRSGQLKRGDKLPTESAIMEEQGVSRTVVREA
ISRLQASGLVETRHGIGTFVLDTPSPSGFRIDPATIVTLRDVLAILELRISLEVESAGLA

AQRRSDEQLAAMRSALDALNESAAHATDAVASDFQFHLQIALSTGNRYFTDIMTHLGTSI
IPRTRLNSARLAHDDQQHYMDRLSREHEEIFDAIARQDSDAARAAMRLHLTNSRERLRHA
HEEAESQRA
>tr|A0A0Q0CW89|A0A0Q0CW89_PSEAP Uncharacterized protein OS=Pseudomonas syringae pv. aptata OX=83167 GN=ALO85_01863 PE=4 SV=1
MCMNSKETVLQFDLSNKFVSTKDYARGLVEAINDLAPYLSVAERESLCFSKLGLKSDNVS
EQAYIQAAVEVTVCAHFARFFPNGFVYEEKVNPPKDVDCSVRVEDFKYNIEVKCADFSKK
HEVDDSEGFKIGSLGRLGDYDSIVADLEDMFSRGGHTLSRQRHMDNNLKSFLISAHEKFA
SITTKNELNILVVGCDDAMDMQKWYSYLYGAQGLFTLESYSDTSAYNRVDLVLLTNLYHR
HKDPKLKDKLSGHWRLSEAFCILCENPKSLKPDETFIEFSKTIRHHNNELHSHTVEGEAP
DFILKGLAIPSYVGSELQAKGIYYFHPPEPETEISQEQH
>tr|A0A0Q0C5U4|A0A0Q0C5U4_PSEAP Extracellular solute-binding protein OS=Pseudomonas syringae pv. aptata OX=83167 GN=ALO85_02077 PE=4 SV=1
MNIRKKLLLSAGVSAALFNLTVQAAETVTIATVNNGDMIRMQRLSKTFEQQHPDIKLNWV
VLEENVLRQRLTTDIATQGGQFDVLTIGTYETPLWGAKQWLEPVTDLAPDYDLEDIFPSV
RQGLSVKDTLYALPFYGESTITYYRTDLFKQAGLSMPEHPTWTQLGEFAAKLHQPDKGVY
GMCLRGKAGWGENIALLSTMANAFGARWFDEQWKPQLTSPEWSKAANFYVNTLKNYGPPG
VSSNGFNETLALFNSGKCALWVDASVAGSFTTDKAQSKVVDSVGFAPAPVEVTDKGSSWL
YAWSLAIPTTSKHKDAAKAFITWATSKAYIQLVAETDGISNVPPGTRTSTYSEAYLKAAP
FAKVTLQMMQHADPAHPSAKPVPYVGIQYVTIPEFQAIGTSVGKLFSAAVTGQTTTEQAL
TAAQAVTEREMKRAGYPK
>tr|A0A0Q0C5T4|A0A0Q0C5T4_PSEAP ATP-dependent Clp protease proteolytic subunit OS=Pseudomonas syringae pv. aptata OX=83167 GN=clpP PE=3 SV=1
MQDMSRNSYIQQNSDIQAAGGLVPMVIEQSARGERAYDIYSRLLKERVIFMVGPVEDYMA
NLIAAQLLFLEAENPDKDIHLYINSPGGSVTAGMSIYDTMQFIKPDVSTICIGQACSMGA
FLLAGGAEGKRHCLPNSRMMIHQPLGGFQGQASDIDIHAKEILHIRHRLNSLLAHHTGQS
LETIERDTERDNFMSAERAAEYGLIDSVINKRQMPA
>tr|A0A0Q0CYI6|A0A0Q0CYI6_PSEAP Uncharacterized protein OS=Pseudomonas syringae pv. aptata OX=83167 GN=ALO85_100409 PE=4 SV=1
MNQSTCNANPLSLPIPSVPLASRHGIGVRNASQLVVHVDPYGSMEEGDLIELFWDGCYVA
STILKASDVGKPVILRVPASFLQNGKARTYYRVMKIGGVPVTSPCRKLWVKLNAPGGQLV
SANTEENQGLAPLYVAPSVIRHGLSRRHLEGGLPMTIEPYLNMAVHDEITIRWGDLRMDL
PPLTADDVGEPVDLVVPPELIREGGDEQRLEVSYCVIDRVGNNSLWAPPRIIKIQPLGH
>tr|A0A0Q0CBG8|A0A0Q0CBG8_PSEAP Peptidase M48, Ste24p OS=Pseudomonas syringae pv. aptata OX=83167 GN=ALO85_01739 PE=3 SV=1
MRPSFAFCALSAALLLGGCQAVNTTSGDSVGVERKQYMFSMLSTDEVNKMYAQSYQQTVG
EATSKGVLDTTSANAKRVHAIADRLIAQAPKLRPDSAQWQWEVNLIKSDELNANCGPGGK
IIVYSGLIDTLKLTDDEIAAVMGHEIAHALREHGREAMSKAYGVSMAKQGAGALLGLGQD
SLALADTVVNYSLTLPNSRSNENEADLLGLELAARAGYNPNAAITLWQKMTQNSGGSQPE
FMSTHPASESRIASLQAAIPKVMPLYQKAAKS
>tr|A0A0Q0DJH1|A0A0Q0DJH1_PSEAP Membrane protein OS=Pseudomonas syringae pv. aptata OX=83167 GN=ALO85_00546 PE=4 SV=1
MARETAQNAQEILRADFQVLVADTEKLLAHTASLAGDQADELRAQIRESLLRAKETLKTT
EDSLRERGEAAVVATEDYVQNNPWQSVGIAAGVGFLIGLLATRR
>tr|A0A0Q0C7L8|A0A0Q0C7L8_PSEAP Uncharacterized protein OS=Pseudomonas syringae pv. aptata OX=83167 GN=ALO85_101136 PE=4 SV=1
MVLQAISARVFRVGHIQTTVVLLSDTADGAMLAPPLQQNSVVSHLFDNILMAHSQYAKSI
DA
>tr|A0A0Q0BSA8|A0A0Q0BSA8_PSEAP 4-hydroxy-4-methyl-2-oxoglutarate aldolase OS=Pseudomonas syringae pv. aptata OX=83167 GN=ALO85_00572 PE=3 SV=1
MHYLTPDLCDAYPDLIQVVEPMFSNFGGRDSFGGQIVTLKCFEDNSKVREQVELDGKGKV
LVVDGGGSLRCALLGDMLADKAAKNGWEGMLIYGCVRDVDVIAQTDLGVQALASHPKKTE
KRGIGELNVPVTFGGVTFRPGEYLYADNNGVIISPSPLTMPE
>tr|A0A0Q0IA87|A0A0Q0IA87_PSEAP Transporter OS=Pseudomonas syringae pv. aptata OX=83167 GN=ALO85_02331 PE=4 SV=1
MTIKAINVRNQFKGTIKEIVHGDVLSEIDVQTASGVVTSVITTRSVKELELVVGSEVIAF
VKSTEVSIAKL
>tr|A0A0Q0DAE2|A0A0Q0DAE2_PSEAP 50S ribosomal protein L5 OS=Pseudomonas syringae pv. aptata OX=83167 GN=ALO85_200038 PE=4 SV=1

MNMTEEDMRRALFGSGESGIGSAVQHALPPAVQTAAKRNGTRNFVPKLVVTLSVGNEFEG
AMELITHEADTLSHLQAEVEAMKIARKKYKYVELVSVLPAK
>tr|A0A0Q0IED2|A0A0Q0IED2_PSEAP Organic hydroperoxide resistance protein OS=Pseudomonas syringae pv. aptata OX=83167 GN=ALO85_03511 PE=4 SV=1
MDTLYTAVATATGGRDGRAVSDDKILDVKLATPKELGGAGGAATNPEQLFAAGYSACFIG
ALKFVAGQSKRSIPADTSITAHVGIGQIPGGFGLDIDLHVSLPGLEQADAQQLVDAAHVV
CPYSNATRGNVDVRLHVTV
>tr|A0A0Q0C658|A0A0Q0C658_PSEAP Uncharacterized protein OS=Pseudomonas syringae pv. aptata OX=83167 GN=ALO85_101760 PE=4 SV=1
MQVTCISSIICAFRNIAKQKICFMVHNKHLLIDMFYISGWNATFDQTLTIANSITQIRLC
PR
>tr|A0A0N8T981|A0A0N8T981_PSEAP Uncharacterized protein OS=Pseudomonas syringae pv. aptata OX=83167 GN=ALO85_04676 PE=4 SV=1
MLRANTWRTRRLSILRRAADPLTPFCLVPARDVKLAAMNFFRTHCSPIAWILYAVVLFNG
LACSIGHGQMMAAFAGTAMASVSHADHGMSDMHAGHHTMHMAMHDSSKKMDMSGSMKAQS
GECSFAGTLTLAMIFFVALGWLIRVRRTRFVLPELWLGNLPRYTLPGLNPQAP
>tr|A0A0Q0FDY8|A0A0Q0FDY8_PSEAP M16 family peptidase OS=Pseudomonas syringae pv. aptata OX=83167 GN=ALO85_00465 PE=4 SV=1
MNALARRAAGLLLSTICLPLTALAADPQPTHEFTLDNGLKVVVREDHRAPVVVSQIWYKV
GSSYETPGQTGLSHALEHMMFKGSSKTGPGESSLILRDLGAEENAFTSDDYTAYYQVLAR
DRLSVALELEADRMATLKLPADEFSREIEVIKEERRLRTDDQPMGKAFERFKAMAYPASG
YHTPTIGWMADLERMKVEELRHWYESWYAPNNATLVVVGDVQPGDVKALAERFFGSIPRR
DVPPSKKPLELAEPGERKITLHVKTQLPSLIYGFNVPSVATAEDSRSANALRLIAALLDG
GYSARISSRLERGEELVSGASSRYDAFARGDSLFMISATPNLQKKKTLADVEAGIWRLLD
ELKTKAPSAEELERVRAQVIASVVYERDSITSQATMIGELETVGLSWKLMDNELEALQSV
TPQDIQKAANTYFTRERLSVAHVLPEEKAK
>tr|A0A0Q0ITI9|A0A0Q0ITI9_PSEAP TrpBA operon transcriptional activator OS=Pseudomonas syringae pv. aptata OX=83167 GN=ALO85_04897 PE=3 SV=1
MKTDKIAVTCQENSQIMSRDLPPLNALRAFESTARLGSVSQAAEQLNVTHGAVSRQLKLL
EEHLGVSLFSKDGRGLKLTDAGIRLRDVSGDAFDRLRGVCAELSKGSADAPFVLGCSGSL
LARWFIPRLSRLNAELPDLRLHLSAGEGDLDPRRPGLDALLIFAEPPWPADMQVFELASE
RIGPVLSPRFARFERLCDAPVEALLEESLLQTTSRPQAWPSWAQQNGIDPQSLRYGQGFE
HLYYLLEAAVAGLGIAIAPEPLVIDDLKAGRLAAPWGFSETPAQLALWVPRRAADGRAQQ
LAQWLKNELKRSGEPIRN
>tr|A0A0Q0D9W4|A0A0Q0D9W4_PSEAP Putative lipoprotein OS=Pseudomonas syringae pv. aptata OX=83167 GN=ALO85_03982 PE=4 SV=1
MRSALAISLLAVILGGCASHADRNPDGTWINQTAIDAAVKQGNLRQALLANGPNLEWKIN
SKANQAIYSNGFELGEGKIVSAAEGKLHVDFYGNFFEDLSVKGDELVQAASESGPEQHFQ
KPEDPAPDGAQPGSSFEKALYGAYMGGKWTVVEGDGQGSTVQFMPDGSVQGLPENDRYAL
CLAGDCAAMSGEYDSMWLEKAEKGNPWIFARKGKQLEIFQAVNNAGADQMPELRPGPRRW
LLEQQ
>tr|A0A0Q0C4T2|A0A0Q0C4T2_PSEAP Alpha-1,4-glucan:maltose-1-phosphate maltosyltransferase OS=Pseudomonas syringae pv. aptata OX=83167 GN=glgE PE=3 SV=1
MNEQPYGPDSLATDVPQATLPLSLTQALQLPRIVIEDTLPVIDGGLFAAKAIIGQPVTVT
SKVYADGHDKMAVNIRWRAADEEHWHSAPMRELGNDSWVGEFTPTSVSRHVFRLEAWIDQ
FGSYRYELEKKFGAGVPVDLELEEGRIHLVHAAERSQNEQRQQIESLLGQLEQGDKDEKV
ALLLHSETASLMKLADNQAFLSRSVEFPMDVERELAQFASWYELFPRSMTDDPARHGTFN
DVHSRLPTIRDMGFDVLYFPPIHPIGRAHRKGPNNSLTAGPDDPGSPYAIGSEDGGHEAI
HPQLGSREDFRNLVKAAADHGLEIALDFAIQCSQDHPWLKQHPGWFSWRPDGTIRYAENP
PKKYQDIVNVDFYAPDAIPGLWTELRDIVLGWVNEGVKIFRVDNPHTKPLPFWQWMIGEV
RSQHPEVMFLAEAFTKPAMMARLGKVGYSQSYTYFTWRNTKAELSEYFTQLNEVPWRDCY
RPNFFVNTPDINPRFLHESGRPGFLIRAALATMGSGLWGMYSGFELCEAAPIPGKEEYLD
SEKYEIRVRDYAAPGNIIAEIAQLNRIRRQNPALQTHLGLKLYNAWNDNILYFGKRSADG
SNFILVAINLDPYNAQEADFELPLWEMGLPDDAATSGEDLMTGHRWTWYGKYQHTRLDPA
QPFGIWRIQAAQ
>tr|A0A0N8T8G9|A0A0N8T8G9_PSEAP Sensory box domain protein OS=Pseudomonas syringae pv. aptata OX=83167 GN=ALO85_01110 PE=4 SV=1
MSNAIEINELHWLLAVTQNIDVGIVVLDLNYRVTVWNTFMENRSGVVPYVAVDKTFFEIF
PEVNAKWFSKKIDNVVTLGTPAFTIWEQRPYLVRFKNYQPITGQEEFMYQNTTLFPLRST

TGAISHVCLVIYDVTDVAANRNQLLAANDKLQQLSGSAKS
>tr|A0A0Q0DJV9|A0A0Q0DJV9_PSEAP DedD-like protein OS=Pseudomonas syringae pv. aptata OX=83167 GN=ALO85_02560 PE=4 SV=1
MALLDNVFKQRMVGALVLIAVAVIFLPMLFTREDETRQVQVEAPAAPQAPAAAQVRVDPV
PVPEPQVLPQEPVPGDDDMSSNQPPAMPIAPAPTAQTAPAAPASKPAAKPAPAPAPATAA
APVATPAPAKSAAPAGVDANGLSISWSVQLASMSNRANADNLQKTLRTQGYNAYIRTADG
VNRVFVGPLIERAEADRLRDQLDKQQKLKGIVVRFQPQGG
>tr|A0A0Q0FID0|A0A0Q0FID0_PSEAP Alpha/beta hydrolase fold protein OS=Pseudomonas syringae pv. aptata OX=83167 GN=ALO85_04621 PE=4 SV=1
MPLMRAAKIAGMLMSLTLSGVALAETPGYGQQLEGFTYPHALHKFSFQSQGQTLEMGYMD
VAPGGKANGRTAVLLHGKNFCAATWEDTIKGLSAAGYRVVAPDQIGFCTSTKPEHYQYSF
QQLSMNTHALLEKLGIDKASVIGHSTGGMLATRYALMYPKQTEKLVMVNPIGLKDWKALG
VPYRSVDQWYERELKTTAEGIRAYEQKTYYDGRWKPEYDKWVDMLAGLNKGPGHKIVAWN
SALIYDMIFTQPVFYEFPKLQVPTVLMIGDADTTAIGSDIAPPEVKAKIGNYKVLGKQVA
QMIPGARLVEFKGKGHAPQMEDPQSFNKALVSELQ
>tr|A0A0Q0DYU5|A0A0Q0DYU5_PSEAP Two-component DNA-binding response regulator, sigma-54 dependent OS=Pseudomonas syringae pv. aptata OX=83167 GN=ALO85_02036 PE=4 SV=1
MSINSDLTVLIVEDDPHVLLGCQQALALEDIASEGVSNAEEALKRIGDNFAGIVISDIRL
PGIDGLELLSRLKARDSSLPVVLITGHGDITMAVNAMRDGAYDFMEKPFSPERLVDVTRR
ALEQRGLAREVWALRKQLAERDSLEGRIIGRSPAMQNLRALIANVADTSANVLIEGETGT
GKELVARCLHDFSRRKDSQFVALNCGGLAESLFESEIFGHEANAFTGAGKRRIGKIEHAH
NGTLFLDEVESMPVNLQIKLLRVLQERTLERLGSNQSVAVDCRVIAATKSDLNALGKTNQ
FRSDLYYRLNVVTLELPPLRERREDILQLFEHFLQLSSLRFDREPPVLDRQTSSNLMSHD
WPGNVRELRNVAERFALGLPAFKQSGTVSENQSLGFAEAVEAFERSLLGDALQRSGGNLS
QASQELGMAKTTLFDKVKKYGL
>tr|A0A0Q0DV31|A0A0Q0DV31_PSEAP Uncharacterized protein OS=Pseudomonas syringae pv. aptata OX=83167 GN=ALO85_03994 PE=4 SV=1
MKGNPLLKYLVIPFLLIVIFVGIKSLGSAPAPSTETPAPKLTAEQAKALGVEGDTPSDTL
RTVVAEGRELKQQITDVIAQNNAYKQDNETLKQRLANVDQNVEQRLKNAQEQFKLDSQQQ
QQSVLEGLRKQMDELTRMGQNSNSNSDLPIGLGVQPGDGQQFKSETNGSDIVWIDPQDAT
ALDSNGKPITAGNSVPASAYSFPTAFGESVDRGQKALTTGAQSVRDDMGGQQQERKKVRR
AYTLPQNSTLMGSVAMSALIGRVPVDGTVNDPYPFKVLIGPDNLTANGIDLPDVAGAVAS
GTASGDWTLSCVRGQIKSLTFVFTDGTVRTLPAPVQGGNQQNNNQNNSGDQQPIQGGLGW
ISDPYGIPCIAGERRSNAQQYIGSQALITAAGAGAASLIKTEGSTSSYVSNASGTLGSTG
LSGNEAMGKILTQGVTDVSSWVNKLYGQAFAAIYVLPGAQVSLHIDQELEIDYELKGRKV
KYASGANHANANLD
>tr|A0A0Q0BSI8|A0A0Q0BSI8_PSEAP Peptidoglycan D,D-transpeptidase FtsI OS=Pseudomonas syringae pv. aptata OX=83167 GN=ftsI PE=3 SV=1
MMKLDGALYPWRFRVVVGLLVSLVLAICWRIVDLQVIDHTFLKEQGDARSLRHIPIPAHR
GLITDRNGEPLAVSTPVTTLWANPREMQQDKSKWPMLAAALGQDLKALTERLDSQANKEF
IYLVRGLTPEQGQSVIDLKVPGVYGIEEFRRFYPAGDVTAHMVGFTDLDDHGREGVELAY
DDWLAGVPGKRQVIKDRRGRLIKDVQVTKNAKAGKTLALSIDLRLQYLATRELRNAIVEN
EAKAGSMVIMDVKTGEVLAMVNQPTYNPNNRRTMVPAAMRNRAIIDVFEPGSTMKPISMT
AALDSGRWKPTDKVEVYPGTLQIGKYTIRDVTKTEGPILDLTGILINSSNVGMSKVAFDV
GGEAIFRAMQRVGFGQYTGLGFPGERVGNLPNYREWRKAETATLSYGYGLSVTALQLVHA
YGALANNGRIVPLTILKTDKEPESTQAIPEKTAKTLQGMLQQVIEDPRGVYRARVPSYHV
GGKSGTARKTSIGTKGYAENSYRSLFAGFGPMSNPRYAIVVVIDEPSKGGYYGGLVSAPV
FSKVMSGTLRLMNVTPDNLAPPEQVNAAPAAKGGRG
>tr|A0A0Q0DBH7|A0A0Q0DBH7_PSEAP TPR_21 domain-containing protein OS=Pseudomonas syringae pv. aptata OX=83167 GN=ALO85_04101 PE=4 SV=1
MSGTEDEELAVMKDWWQRNGKPLLTGGLLALVVVLGWQFWHRYQASQSQGASMLYQQLLE
TALTPSGQADTARVAEISGKLKSEFGGTTYAQFGSLFVAKVAVDAGKLDDAAAELKPVAD
KPANDTLGEIARQRLARVLAAQNKTDDALKLLDGDADKAFLASREELKGDLLVQLGRTDE
AYAAYQKAKSALSEDAAVGGLQMKLDDLAKGDA
>tr|A0A0Q0BVX0|A0A0Q0BVX0_PSEAP Putative allantoinase/urate catabolism protein OS=Pseudomonas syringae pv. aptata OX=83167 GN=ALO85_01160 PE=4 SV=1
MSADYPRDLIGYGNNPPHPRWPGNARIALSFVLNYEEGGERNILHGDKESEAFLSEMVAA
QPLQGQRNMSMESLYEYGSRAGVWRILKLFRQFDIPLTIFAVAMAAQRHPEVIRAMVAEG

HEICSHGYRWIDYQNMDEAQEREHMRQAIQILTEISGERPLGWYTGRTGPNTRRLVMEEG
GFLYDCDTYDDDLPYWEPNNPTGKPHLVIPYTLDTNDMRFTQVQGFNNGEQFFQYLKDAF
DVLYAEGEDAPKMLSIGLHCRLIGRPARLAALKRFIEYAKGHEQVWFTRRVDIARHWHTE
HPFTRKAEQ
>tr|A0A0Q0DUE4|A0A0Q0DUE4_PSEAP FA_desaturase domain-containing protein
OS=Pseudomonas syringae pv. aptata OX=83167 GN=ALO85_04707 PE=4 SV=1
MVIQAQLYMDIHPARRFDNANLAVLVLQTLFWGATLTWLSHTGAGPWKWLILIPFCLVMQ
GVFSMMHEAFHSLAHSDKTVNYLMMCWASTLFGASATLIHINHLGHHSRNRTRAELADFA
MPDESLLRKRLEYYFAVLGGIWLAAFVGSLLLPLLPPRLADRWSQSGEVNTYAAAFKDFS
ISDFRRIRKEVITGIIVWLTCAYLLGWSWEVVLIAYIAFGFSWSSLQWVYHMRTPIDVVE
GAYNMRAPRVVRWAFLNFNYNLTHHRHSQMHWQHMYAASNPKETRPLWFGWLQVFMPPQR
LPDDLTQLDKTYF
>tr|A0A0Q0CES2|A0A0Q0CES2_PSEAP 30S ribosomal protein S13 OS=Pseudomonas
syringae pv. aptata OX=83167 GN=ALO85_200039 PE=4 SV=1
MQQPIERAGYSWLINALDLRCVALDHECVIGSRPALSLPAGGVKIEVFTKGYAREHRPVD
QMLFALSYEGVNIGVLERAFQNPAVRNDLTAAIVEKPASGYLRRLWYICEHIAGHLLPLP
DAAQNIPYEHLLPPDKYFTGPEQMSRRHRVRMNLLGTPKLSALVTRQSWADDNLLNAQIV
ERMHDQMVDFSVSEIARAAQYLLTTETRSSYEIEHERPAPDRILRFVRVLEQAGQRSLDE
EFLFDVHRIALATGRPDPSIGAYRYMQNWLGRGDLIHLIPPAPENVPEMMEEWFLMRERV
MRSNAPALVKLNAIAHSFIYIHPFMDGNGRLSRFIMQDILANEGVKLQGIIMPISAGLLV
NLETYIGALDALSRRILGATQYHRTDLASDPELGEAHDHLFAHLDFTDLQGEVNKSAARV
LDGLMRDEVNHLVRRDRFKEQMNRELDLSNRDLELLIAVIYENSGHVSKRKRTGLFQHLT
DEDIALAESIYADLHAPQATGSK
>tr|A0A0Q0C0P8|A0A0Q0C0P8_PSEAP Citrate synthase OS=Pseudomonas syringae
pv. aptata OX=83167 GN=ALO85_05059 PE=3 SV=1
MADKKAQLIIEGAAPVELPILTGTVGPDVIDVRGLTATGRFTFDPGFMSTASCDSKITYI
DGDNGILLHRGYPIEQLAEQSDYLETCYLLLNGELPTAEQKAQFVAVVKNHTMVHEQLKT
FFNGFRRDAHPMAVMCGVVGALSAFYHDSLDINNPQHREISAVRLVAKMPTLAAMVYKYS
MGQPMMYPRNDLSYAENFLHMMFNTPCEIKPISPVLAKAMDKIFILHADHEQNASTSTVR
MAGSSGANPFACIAAGIAALWGPAHGGANEAVLTMLDEIGDVSNIDKFIAKAKDKNDPFK
LMGFGHRVYKNRDPRATVMKQTCDEVLKELGITNDPQLELAMRLEEIALTDPYFIERSLY
PNVDFYSGIILKAIGIPTSMFTVIFALARTVGWISHWKEMLSSPYKIGRPRQLYTGNVQR
DIVALQDRK
>tr|A0A0Q0IHP7|A0A0Q0IHP7_PSEAP Recombination protein RecR OS=Pseudomonas
syringae pv. aptata OX=83167 GN=recR PE=3 SV=1
MSFSPLIRQLIDAFRVLPGVGQKTAQRMALQLLERDRSGGSRLAQALGQAMDGVGHCRSC
RTLTEEELCPQCADPRRDDTLLCVVEGPTDVYAVEQTGYRGRYFVLKGHLSPLDGLGPEA
IGIPQLMERVSQQGTFTEVILATNPTVEGEATAHYIAQLLHDKGLVASRIAHGVPLGGEL
DLVDGGTLAHSFAGRKPIAM
>tr|A0A0Q0IAD4|A0A0Q0IAD4_PSEAP Transketolase, TPP-binding subunit
OS=Pseudomonas syringae pv. aptata OX=83167 GN=ALO85_04215 PE=4 SV=1
MTATTLSAPSTSLAQRAHNIRRHALRMGQVQGQGYVGQALGAADLLAVSYFHALNYRPED
PEWEQRDRFYLSIGHYAIALYAALIEAEIIPFDELETYGSDDSRLPMSGMATYTPGMEIT
GGSLGHGLGIAVGACLGLKRKNSSAFVYNLLSDGELNEGSTWEAAMSASHWKLDNLIAII
DVNNQQADGHSSEVLAFEPIVDRWQAFGWFTQRVDGNDLDALVAAFDNARQHAGTQPRVI
ICDTKMGKGVAFLETREKTHFIRVDEHEWDVALNNLDEGKTV
>tr|A0A0Q0BWS3|A0A0Q0BWS3_PSEAP NlpB/DapX family lipoprotein
OS=Pseudomonas syringae pv. aptata OX=83167 GN=ALO85_01047 PE=4 SV=1
MKRLAGLSALALIISSTSGCGWLWGEDGYFRDRGSDYLQATQTPPMQLPADVTGVKRLDP
LLPIPRNVADSTQKDGEYIVPRPQPLGTTSESSDFSLQKSGDAHWILAQRQPAEVWPVAH
QYFEDNGFRIAEERPQTGEFSSSWQRLDELSASVAKRLSAAGVSADSETRVRVRIEPGVQ
RNTSEVYVVSVERPAGSTADVAFPTRTTNLGLDAALTDDLLASMGRNAEKGGSISLLAAR
DYDTPSRVALSEDGSGNPVLNLGADLDRAWSSVGRALDQGDWRVEDINRSLGLYYINLAE
KAKTPENEPGFFGRMFGSKSTKEEIDARAERYQVRLSKVGDNVQVTVEKNINTVAPADIA
RRVLSVIQDNLG
>tr|A0A0Q0E016|A0A0Q0E016_PSEAP Binding-protein dependent transport
system inner membrane protein OS=Pseudomonas syringae pv. aptata OX=83167
GN=ALO85_03865 PE=3 SV=1
MSASPEQALPLRKGGFFRLRLRPTAAVALAPAMAVLLAFWLLPLTHLIMLGTQGSDGNGS
GYWQVLSSAQYLGSLLQTCLLALVVTLAALLVGGISGVFLARNHFFGRSALVALLTFPLA

FPGVVVGFLVILLAGRQGIFAALGLHLAGERWVFAYSLTGLFIGYLYFSIPRVILTVMAA
CESLDRSLEEAAHSLGAGHWRVVCDVIIPGLAPALVSCGAICFATSMGAFGTAFTLGTRL
NVTPVAIYNVFTNYANFTVAAALSVILGLVTWAVLLLARRMVGKSGVVL
>tr|A0A0N8T8Z4|A0A0N8T8Z4_PSEAP Uncharacterized protein OS=Pseudomonas syringae pv. aptata OX=83167 GN=ALO85_01797 PE=4 SV=1
MPSLETRMLIGILLILTWLILLLRYPAKALPVSLAAAVALGAVAAIVIWQDSRETRQLEH
LDIRLSYNPQGCPADRPLQVSITNTNQVTLQELRWRIAAYAPGDSVNLTDNTYASARYRG
PGELQAKGTWQDCVPVPALRNGYRPQTLEFRAEHLQGSFSD
>tr|A0A0Q0DNA8|A0A0Q0DNA8_PSEAP NolW-like protein OS=Pseudomonas syringae pv. aptata OX=83167 GN=ALO85_00736 PE=4 SV=1
MNMSVRNLLNAALTSMLIVFSAHVFAATEVVPLNYRTSADLLPVAKSFLGDEGTVSAYGN
QLIVNAESRKIEELRALISQLDTAPKRLLISVDTSDSATRNSSGSPVIEYGTASREGGVQ
QVQTSEGTPALIQVGQSVPLTSSSTDAYGYSRSDTEYRNVTQGFYVTASVTGDTVHLSIS
TNHDRMSQERPDAVKVQSTDSQVSGRLGEWITLASNSNESVADQSGLAQRYSTQGRDDMI
LRVKVETLE
>tr|A0A0Q0DZG4|A0A0Q0DZG4_PSEAP Peptidyl-prolyl cis-trans isomerase OS=Pseudomonas syringae pv. aptata OX=83167 GN=ALO85_03369 PE=3 SV=1
MAMSKELQITDLHPGEGKAAVKGALITTHYTGTLEDGTVFDSSHERGKPFQCVIGTGRVI
KGWDQGLIGMKVGGKRQLFVPAHLAYGDRSMGAHIKPGADLTFEIELLEVLTRDD
>tr|A0A0Q0C5U6|A0A0Q0C5U6_PSEAP Toluene tolerance protein Ttg2E OS=Pseudomonas syringae pv. aptata OX=83167 GN=ALO85_00176 PE=4 SV=1
MTESAVRLVGPGELRLIGVLDYRTGPQLRKQGAALIKSGDLADLTLDCSGVEKSSSVGLA
LLLAFMRDARDAGKSMTLRSLPEDMRQIARVSGLTELLDAH
>tr|A0A0Q0IPQ3|A0A0Q0IPQ3_PSEAP Outer membrane ligand receptor OS=Pseudomonas syringae pv. aptata OX=83167 GN=ALO85_05458 PE=3 SV=1
MVGREFNVCCLFNPAIAFTSLALSLTGASPCHCCAPTVRLTASSFRPLSAKPGRRLSCMS
LITFKTVHAAPSSKQRECPISRFGCVAAMTAMSGVGMKQALQRARVSTAAMGYCPLALAI
GGVLLSGTIRAQENNTDDPRTLSTVVVTGNRGAEQRTVTSSPVPIDVVSARQLQSTGKPG
LMEALSAVIPSLTLPEKTGWDASGIARAPNLRGLSAAEVLVLVNGKRRHTSATLNINGIN
TGAAPADLDLIPISAIDHVEVLRDGAAAQYGSDAIAGVINVILKADTSGTSVSNAGLGYD
GKKQTVQQSLNKGFEIGDGGIVQLALDARSQNDDNKASANGYSHEQAYDLAGKSTYGGYG
TPKTNLLTLGYNAELPINDSLSLYSFTTYSRRKAEQGQNFRLPTITNTITTGPNGYPAGY
TPTWYIDEDDFQAAFGGKGTVGEWDWDLSSTYGRNEAEQGTTHNQNPSLGEDTPNRFTSG
TWISTELTTNLDFKRGFDIGLQKPLDLSYGLEHRRETYEVQAGDYESYANGGYCITPGNC
ASSGAQVTNGISPDEESSASRNSVASYVDVGFNPVPDWYVGSAFRYEHYNQGVGATRSGK
LTTRYDFTRQFAIRATVSNGFRAPSLANSLFSARSTTYGVVDGVYQSINYGVLPTGSGAA
KALGAEDLKPERSTNFSLGFTLTPTYRLTLTADAYVINLRDRITLTGTLLGNEVTQVLLN
NGIDSTSGGQYFINGADTRTKGLDLVSNYSQDLGQYGSLKWTAAFNWNQTKILGYKESTS
ILGTSYELMDREARNLITGVQPRTKLILGGDWSIERFNLNLALTRYGAYKEVNSSANRDL
DRVYGAKWITDLDLGYNLSKNLNVAIGAKNLFDVYPKKQGIPSSTMVSSYGTYSPYGFTG
GYYYTRLTYAF
>tr|A0A0Q0BVA5|A0A0Q0BVA5_PSEAP Translation initiation factor IF-1 OS=Pseudomonas syringae pv. aptata OX=83167 GN=infA PE=3 SV=1
MSKEDSFEMEGTVVDTLPNTMFRVELENGHVVTAHISGKMRKNYIRILTGDKVRVELTPY
DLSKGRITYRAR
>tr|A0A0Q0C6V1|A0A0Q0C6V1_PSEAP 30S ribosomal protein S6 OS=Pseudomonas syringae pv. aptata OX=83167 GN=rpsF PE=3 SV=1
MRHYEIIFLVHPDQSEQVGGMVERYTKLIEEDGGKIHRLEDWGRRQLAYAINNVHKAHYV
MLNVECTGKALAELEDNFRYNDAVIRNLVIRRDEAVTGQSEMLKAEENRSERRERRDRPE
HSDSADGDDGDNSDVSDNADE
>tr|A0A0Q0DCK1|A0A0Q0DCK1_PSEAP Putative small integral membrane protein OS=Pseudomonas syringae pv. aptata OX=83167 GN=ALO85_00086 PE=4 SV=1
MEWMSWTLPTAAFFISIALLLAGMTVWELRSASIERRGFLPIATTRGDRLFIGLLGSAYL
HLLVIGATDWNIWIASGASLVWLLVVMRWG
>tr|A0A0N8TA78|A0A0N8TA78_PSEAP Transcriptional regulator GbdR OS=Pseudomonas syringae pv. aptata OX=83167 GN=ALO85_03849 PE=4 SV=1
MMTSFNQGTQPQNRAPQSIGFLLLDNFTLISLASAVEPLRMANQLSGRELYRWTTLSVDG
GQVWASDGLQITPDAAMHKAPLLDTVIVCGGVGIQRSVTREHVSWLQGQARQSRRLGAVC
TGSWALACAGLLDGFECSVHWECLASMQEAFPRVSMSTRLFTLDRNRFTSSGGTAPLDMM
LHLISRDHGRELSAAISEMFVYERIRNEQDHQRVPLKHMLGTNQPKLQEIVALMEANLEE

PIDLDELAVYVAVSRRQLERLFQKYLHCSPSRYYLKLRLIRARQLLKQTPMSIIEVASVC
GFVSTPHFSKCYREYFGIPPRDERVGSNTAQQVAMMPIPQALVLAPLSGPMSALSQARNE
STFASVRL
>tr|A0A0Q0C8Z8|A0A0Q0C8Z8_PSEAP Proteinral secretion pathway protein G OS=Pseudomonas syringae pv. aptata OX=83167 GN=ALO85_00689 PE=4 SV=1
MKQETIPMTFSRTRFKPARRQGGFTLLEMLAVIVLLGIVATIVVRQVGGNVDKGKYGAGK
AQLASLGMKIESYALDVGSPPKTLQQLTEKPGNASNWNGPYAKPSDLKDPFGHAFGYRFP
GQHGSFDLIFYGQDGQPGGEGYSADLGNWE
>tr|A0A0Q0C0L3|A0A0Q0C0L3_PSEAP DeoR family transcriptional regulator with sugar-binding domain OS=Pseudomonas syringae pv. aptata OX=83167 GN=ALO85_01023 PE=4 SV=1
MSDSTQRMASDIDLMTEVAMLYYLENVTQEAIAKRFDLSRAKVSRLLKRARDEGIVEVRV
LQHPAMNNELELALVERFQLDRALIAVDHSDPDTQRSAVASLVANYLNKTLGDGMIVAVG
MGRNVGAVADNVFLPVTRNCTFVCAIGGSLKAGEYMNPDHICRRLALRFGGESESLYAPA
LVANPELRSVLISNDTVRSTLDRARRADMALIGIGDMSENSNMVRMGWFSPQEIAQARLS
GTVGDMMGYDFIDIHGQPAVNAIQGRVIGLTVQELFRIPDVVAIASENTKAAATLGALRS
GVINTLATTVTNAHTILALDDATRKN
>tr|A0A0Q0IJG7|A0A0Q0IJG7_PSEAP 3-oxoacyl-[acyl-carrier-protein] synthase 2 OS=Pseudomonas syringae pv. aptata OX=83167 GN=ALO85_02543 PE=3 SV=1
MSRRRVVVTGMGMLSPLGNDVPSSWQGILAGRSGIGLIEHTDLSAFTTRFGGSVKGFNVE
EYLAPKEARRLDLFIQYGLAASFQAVRNAGLEVTDANRERIGVAMGSGIGGLTNIENSSR
LLHEQGPGRISPFFVPGSIINMISGFLSIHLGAQGPNYSIATACTTATHCIGMAARNIAY
DEADVMIAGGAEMAACGLGLGGFGAARALSTRNDDPTRASRPWDKGRDGFVLSDGAGAMV
LEELEHAKARGATIYAELVGFGMSGDAYHMTSPPDDGAGAARCIVNALRDAKVNPDQVQY
INAHGTSTPAGDLAEASAIKSVFGDHAYKLAVSSTKSMTGHLLGAAGAVEAIFSVLAMVD
QVAPPTINLDEPSEGCDLDFVPHEARRMPIDVVLSNSFGFGGTNGSLVFRRFAE
>tr|A0A0Q0DYV1|A0A0Q0DYV1_PSEAP Putative G3E family GTPase OS=Pseudomonas syringae pv. aptata OX=83167 GN=ALO85_02020 PE=4 SV=1
MSIALNVITGFLGSGKTTLLKRLLADENMGDTALLINEFGDVGIDHLLVEEVAPDTVLLP
SGCVCCTVRGELKDALLGLLERRNRGEIPAFRRVILETTGLADPAPILTTLSNDPQLRGR
FHIGLIVTLVDACHATLQERLHPEWLAQVAAADRLLLSKTDLVDESVLDGLREHLQALNF
SAPLLDTADVHSGDQLLLGEGMRSEEPAAEVTRWQLHRVLSTSARHGDAQVCCLTFERPL
DWVGFGVWLSMLLRCHGERILRVKGLLNVNDNQAPIVIHGVQHCLHAPVHLAAWPGEDRT
SRLVFILRGLDPDLLRRSFEVFSSCFAPPLNESAA
>tr|A0A0Q0D205|A0A0Q0D205_PSEAP Binary cytotoxin component OS=Pseudomonas syringae pv. aptata OX=83167 GN=ALO85_00018 PE=4 SV=1
MVTTVEPQFTDGEAHSVEEVSRVSKDLMDTLTSRGAEVARAPGLLITNEDVRRIRRYVNT
GLELPTTLDEVSQLTGGQDNGIPGLGAEAIVELYLGIQAHARSWSAIETAMQKVGSDLYV
FSGNLIAIATDVVDFIKGLDSYQTLQVGDLTPSQIDAMPPVALMDQDSRRLPGLLSLVDE
LKVHIKDHSASTTRTRVGVTDFKRRLKDEIAPGVALKIRLAGSGDTDDEIARLTADVDQL
NGRINHKLAEYEEYCEYKWIGFWWGLVGGAVSLSIFGPKASKALDEKDRLIEEKRQIEKQ
IKQSNALLSDLLAFETSLQDLKARVDGAASSVSNIESLWVLLEELVDASYDRIKNTNNAL
YLVSFVSRFQTLMSNWREIQVQAFDLLTAFNNALDEPVA
>tr|A0A0N8TA37|A0A0N8TA37_PSEAP 5-oxopent-3-ene-1,2,5-tricarboxylate decarboxylase OS=Pseudomonas syringae pv. aptata OX=83167 GN=ALO85_200022 PE=4 SV=1
MRVETISYLKRNAADLPLEEPLVVTQNGVPAYVVESYADRKRRDESIALVKLLAISSREY
SQGKHCSTDELKARLSRRFAQK
>tr|A0A0Q0C3V6|A0A0Q0C3V6_PSEAP Inosose isomerase OS=Pseudomonas syringae pv. aptata OX=83167 GN=ALO85_02692 PE=4 SV=1
MNHPLRFALNRMVAPRLSLESFVDLAVELGADAIEIRNDLKGIEIEDGTAPEVVRDLCAA
RGVEVLSINALYPFDVWNQERAAQATKLAQYARDCGARGLVLCPLNDRADPRNGAQRAAG
LRTALTALAPILREHGILGFVEPLGFEECSLRLKRQAVDAIKAVGGLDVYRLVHDTFHHH
LAGEVELFPELTGLVHISGVEDAQTPLNSIRDGHRVLVGEGDILGNASQIERLLDSGYEG
HLSFEPFAESVHVLDDIPQAIAASMTHLSRAVSQRH
>tr|A0A0Q0BVK6|A0A0Q0BVK6_PSEAP Heat shock protein DnaJ OS=Pseudomonas syringae pv. aptata OX=83167 GN=ALO85_02986 PE=4 SV=1
MLWPGTLIGAGVGYAIASIPGAMLGALLGQALDRRLKLQSWAHLRERLGGRATVHKDDLL
FVLLGRLAKSDGRVLASHIHQARTEMRRLNLNEADQLRAINAFKRGRDGTDGLRSYLRDL
QGQPDIAEDMLRACWRMAWADGKASRVERELIGVWGMWLGWTGPQIEALAADHDPMKRSP

ISSSGGDYKSAMTLLGIKSDTDPLSIKRAYRRLLSRHHPDKIAGSGANPQQVRVATERTS
ELHNAYRVVKARRGFN
>tr|A0A0Q0C4I2|A0A0Q0C4I2_PSEAP Reactive intermediate/imine deaminase OS=Pseudomonas syringae pv. aptata OX=83167 GN=ALO85_03983 PE=4 SV=1
MTKTVITSDKAPAAIGPYSQAIKAGNTVYMSGQIPLDPSTMELVEGIEAQITQVFENLKS
VAEAAGGSFKDIVKLNIFLTDLGHFAKVNELMSAYFTQPYPARAAIGVAALPRGAQVEMD
AILVIE
>tr|A0A0Q0DJ39|A0A0Q0DJ39_PSEAP PAS:GGDEF protein OS=Pseudomonas syringae pv. aptata OX=83167 GN=ALO85_04246 PE=4 SV=1
MNPQGYEHDHVIPTNIEDVAKVLQAAAHSASCTAGLLMVSSATGWEQLVSSGQSLPKADE
AWLRMISSYGDVVLVGQDSPANTMETNSSGSTLIYVAIRGFRHELLGAMLLRNSDQAMVL
SAAQRYALQAQAAHIRTYLQPGRDPEANNPLVTSFERLRLLESVVINANDAILITEAEPI
DLPGPRIVYCNPAFLAITGFTEEEVIGRTPRILQCEETSRATLDVIREALSHWRAVEVEL
LNTRKDGSKFWVELSIVPVANEKGWFTHWVSVQRDITERKESQQLAQRARLEWEEKLALQ
SRLEERERISEELTYIAFHDELTSLFNRAYLMNELTTAFEIRDPRYERATVLFLDLDGFK
VVNDSMGHLAGDKLLKAVANRLQTCVRSNDVLARIGGDEFAILLMGKDQPETAVKMAERI
VSQLHTAIQIENQDIFISCSIGIVTAGDSHTKPEELLRDADVAMYSAKKQGRGRWSIFNT
SMREAAIDTLVIQNALRQALEDKDFLVFYQPIYSGTCEHLVGVEALVRWNHATMGIISPD
AFIPIAEDLGIIHELGAWVMHQACSEVQAWRKEFSHLDLRLNVNVSGKELSHPGFVDQVT
RIIATTGLPAGQLQIEVTESVFLYQPDAIAEVLRQIRQLGVRVALDDFGTGYSSLGYIDR
YPIDAVKIDRSFVSRMMTYERSKAIVSSILSLGRALNLDITAEGVETAEQYNLLRKMGCP
YFQGYLLNRPMRSEALLHIIKSAAECTA
>tr|A0A0Q0BV45|A0A0Q0BV45_PSEAP Inner membrane protein OS=Pseudomonas syringae pv. aptata OX=83167 GN=ALO85_03235 PE=4 SV=1
MAQGSPRTSRHRSVRYLLVAIGWLSVTLGVVGIFLPVLPTTPFLLLAAACFARSSPRFYD
WLVNHPQLGPWIRDYLEGNGIPLKGKVYAIGLMWLSISLSCYLVPSPWARVFMLTSAVLV
TIYILRQKTRTPGCCKR
>tr|A0A0Q0DY38|A0A0Q0DY38_PSEAP Uncharacterized protein OS=Pseudomonas syringae pv. aptata OX=83167 GN=ALO85_01959 PE=4 SV=1
MVEHDFRYSMLNPQHTLTECRTLAPGRYQVTGNGGSIHDNDQLLVTIKGSKSLHMRLTVE
KVRHLINPPGQWIAVAKGPVFDELAIHQWKVNCDSCAAELNFEFMVESKLGVKAQKPAAT
ARIAELGWKTDGEKHFCKKCQEKAA
>tr|A0A0Q0DP40|A0A0Q0DP40_PSEAP Permease OS=Pseudomonas syringae pv. aptata OX=83167 GN=ALO85_01903 PE=4 SV=1
MYLFRLAMASLANRRFTAFLTAFAIALSVCLLLAVERVRTEARSSFASTISGTDLIVGAR
SGSINLLLYSVFRIGNATNNIRWDSFEHFANSKQVKWAIPVSLGDSHRGYRVMGTNEAYF
EHYQFGRQQHLEIAEGREFKTDPFEVVLGSEVAKALHYKLGDKLVLAHGVAAISLVKHDD
KPFTVVGILKPTGTPVDRTLHISLGGMEAIHIDWHNGAPARGNERISADQARNMDLTPTA
ITAFMLGLNSKISTFSLQREINEYRGEPMLAILPGVALQELWSLMGTAEKALFVISLFVV
LTGLIGMLTAILTSLNERRREMAILRSVGARPWHIASLLILEAFALALAGVISGLALLYI
GIFVARDYVLDNYGLYLSSMPPGQYEWTLLGAILGCALLMGTVPAWRAYRQSLADGLSIR
L
>tr|A0A0Q0CA40|A0A0Q0CA40_PSEAP CBS domain protein OS=Pseudomonas syringae pv. aptata OX=83167 GN=ALO85_01413 PE=4 SV=1
MDPSPSFNLSSLFADFGMILFALFLVLLNGFFVAAEFAMVKLRSTKVEAIADKNGWRGHI
LRKVHGQLDAYLSACQLGITLASLGLGWVGEPAFAHLLEPLLAAVGVDTPELIKGVSFFT
AFFIISYLHIVIGELAPKSWAIRKPELLSLWTAAPLYFFYWLMYPAIYLLNASANAILRI
AGQGEPGPHHEHSYSRDELKLILHSSRGQDPSDQGMRVLASAVEMGELEVVDWANSREDM
VSLDFNAPLKEILALFRRHKFSRYPLYDAERGEFVGLLHIKDLLLELAALDHIPESFNLA
ELTRPLERVSRHMPLSQLLEQFRKGGAHFAVVEEADGKIVGYLTMEDVLEVLVGDIQDEH
RKVERGILAYQPGKLLVRGDTPLFKIERLLGIDLDHIEAETLAGLIYDTLKRVPEEEEQL
EVEGLRIIIKKMKGPKIVLAKVLKLD
>tr|A0A0N8T854|A0A0N8T854_PSEAP Conserved secreted protein OS=Pseudomonas syringae pv. aptata OX=83167 GN=ALO85_100242 PE=4 SV=1
MPLNVELVDSGVKGSTSMFFQAHITRIISLALILSGLSGCSLLSFDRTQEPEVHLLKVQV
VKARLTQQDFKLYFEVDNPNEDSLLVRGLKYKITLNDMVLVDDTFSDWFVVDGNSRKTFV
VPIRTNLWRYARYIAQLLKKPDEPVHYQLDGKLKTGIVFRHNVRIGTSGDVLPEDLIQRR
>tr|A0A0Q0C4R7|A0A0Q0C4R7_PSEAP Uncharacterized protein OS=Pseudomonas syringae pv. aptata OX=83167 GN=ALO85_04700 PE=4 SV=1
MLRHKKFFQNGAGKCYFQCSSDRSAILLESNLTDR

>tr|A0A0Q0IKJ9|A0A0Q0IKJ9_PSEAP Bacteriophage protein OS=Pseudomonas syringae pv. aptata OX=83167 GN=ALO85_101451 PE=4 SV=1
MDLSWLAAWLDSANTFFQYIWDFMASGIYQFFKDALVIITKALIYSYLQFKVIMLDIAYT
VVKEISEESGVVALVKSAWGSIPGDIQSTLAFFKIPQGLTMIFSAIPARWAMKFIPGAN
>tr|A0A0Q0C563|A0A0Q0C563_PSEAP Uncharacterized protein OS=Pseudomonas syringae pv. aptata OX=83167 GN=ALO85_102188 PE=4 SV=1
MPRRSAADRTKGIDLAPWRTVLPVASGVSG
>tr|A0A0Q0C4S8|A0A0Q0C4S8_PSEAP Uncharacterized protein OS=Pseudomonas syringae pv. aptata OX=83167 GN=ALO85_100762 PE=4 SV=1
MLGRVRLFVTYLNNLPIKQTWPLGTESVADQRTICELHTLG
>tr|A0A0Q0DJ72|A0A0Q0DJ72_PSEAP Molybdenum co factor biosynthesis protein E OS=Pseudomonas syringae pv. aptata OX=83167 GN=ALO85_01816 PE=4 SV=1
MSVRVQAAAFDPGTEVNALHAANLGIGAVVSFVGYVRDFNEGREVSGMFLEHYPGMTEKA
LAKIIAEAEQRWPLLRVDVLHRVGALEPGEPIVFVGVASAHRQAAFEACDFVMDYLKTRA
PFWKRENTSEGTHWVEGRHSDHQAADRWK
>tr|A0A0Q0CAL0|A0A0Q0CAL0_PSEAP Uncharacterized protein OS=Pseudomonas syringae pv. aptata OX=83167 GN=ALO85_05060 PE=4 SV=1
MFRKGSAVDFADGLLPFTPFVAATQSAIGGKSARCH
>tr|A0A0Q0BYP6|A0A0Q0BYP6_PSEAP Uncharacterized protein OS=Pseudomonas syringae pv. aptata OX=83167 GN=ALO85_101123 PE=4 SV=1
MGQEYVVTNLKVVLSARRLAHLAGGVELAAQIPLLLFDTLIVETKAAFDEVVERIKKAAH
HPFSQGCVETL
>tr|A0A0N8T8J7|A0A0N8T8J7_PSEAP Addiction module antitoxin OS=Pseudomonas syringae pv. aptata OX=83167 GN=ALO85_101942 PE=4 SV=1
MLVEWRPAARMNLKQILSYIADRNIRAASELGMAIEVATSALPQHPYLYRHGRVHGTREI
VVHPNYLVVYKVTDRIEVLAVLHARQAYPTDAG
>tr|A0A0Q0FLK7|A0A0Q0FLK7_PSEAP Poly C5 epimerase 3 OS=Pseudomonas syringae pv. aptata OX=83167 GN=ALO85_03934 PE=4 SV=1
MIGVSALGFTGLGNGYDGTLKVVLNLAGDATALKSLEADANGNRFEILLSGNHANELNAS
TEGNAVDLVN
>tr|A0A0Q0FDI1|A0A0Q0FDI1_PSEAP Uncharacterized protein OS=Pseudomonas syringae pv. aptata OX=83167 GN=ALO85_101862 PE=4 SV=1
MRLLVTDEAVGLEPFGVATECTTAPLLDQDRPDPAGKGIERLS
>tr|A0A0Q0C651|A0A0Q0C651_PSEAP Dihydrofolate reductase OS=Pseudomonas syringae pv. aptata OX=83167 GN=ALO85_00455 PE=3 SV=1
MKTHLPLSLIAALGENRVIGVDNSMPWHLPGDFKYFKATTLGKPIIMGRKTWDSLGRPLP
GRLNLVVSRQTDLQLEGAEVFASLDAAVVRAEQWAQEQGVDEVMLIGGAQLYAQGLPQAD
RLYLTRVALSPDGDAWFPEFDTTQWALVSNAENAAVDDKPAYNFEVWERV
>tr|A0A0Q0BSH2|A0A0Q0BSH2_PSEAP Transcription termination/antitermination protein NusA OS=Pseudomonas syringae pv. aptata OX=83167 GN=nusA PE=3 SV=1
MSKEVLLVVESVSNEKGVPASVIFEALELALATATKKRFEDEVELRVEINRQTGNYETFR
RWTVVEDEDLDDPAYELAVDQAQAKKPGAVAGDLIEEKIDSIEFGRIAAQTAKQVIVQKV
REAERAQVVDAYRERLGEIISGTVKKVTRDNVIVDLGNNAEALLAREDIISRETFRVGVR
VRALLKEIRTENRGPQLILSRTAPEMLIELFRIEVPEIAEGLIEVMAASRDPGSRAKIAV
RSKDKRIDPQGACIGMRGSRVQAVSGELGGERVDIVLWDDNPAQFVINAMSPAEVAAIIV
DEDAHAMDIAVGADNLAQAIGRGGQNVRLASQLTGWTLNVMTESDIQAKQQAETGDILRN
FIEELEVDEELAQVLVDEGFTSLEEIAYVPLEEMLNIDGFDEDIVNELRARAKDRLLTKA
IATEEKLADAHPAEDLLSLEGMDKDLAMELAVRGVITREDLAEQSIDDLLDIDGIDDDRA
GKLIMAARAHWFE
>tr|A0A0Q0DMA0|A0A0Q0DMA0_PSEAP Uncharacterized protein OS=Pseudomonas syringae pv. aptata OX=83167 GN=ALO85_04693 PE=4 SV=1
MTLPNPVEVPDPNIDDPALPNPVPEKEPPPSTPPPNEKPVGDPPANAPPITV
>tr|A0A0Q0CIH3|A0A0Q0CIH3_PSEAP Probable chemoreceptor glutamine deamidase CheD OS=Pseudomonas syringae pv. aptata OX=83167 GN=cheD PE=3 SV=1
MNTPVGVAEIVLGPGEVVFQTRPTRLRTLLGSCVAITFWHPWRRIGGMCHFMLPGRIRRH
QPLDGRYADEAMEILIRHALANGTLPEEYQVKLFGGGEMFPAHRHDPHMQNVADSNVHAA
LALAEQNRLKLMAQDLGSTGHRSIIFDLWDGNVWVRHQPMEAMEKDAKQKNQRTAGR

>tr|A0A0Q0DTX6|A0A0Q0DTX6_PSEAP N-succinyl-L,L-diaminopimelate transaminase OS=Pseudomonas syringae pv. aptata OX=83167 GN=ALO85_00310 PE=4 SV=1
MNNAMQLLQPYPFEKLRALLAGVTPNPEKRPVALSIGEPKHRSPDFVAKALADNLDQMAV
YPTTLGIPALREAIAGWCSRRFGVPQGWIDPARNVLPVNGTREALFAFTQTVVNRSDDGL
VISPNPFYQIYEGAAFLAGAQPHYLPCLSENGFNPDFDAVSPDIWKRCQILFLCSPGNPT
GALIPVETLKKLIALADEHDFVIAADECYSELYFDEQTPPPGLLSACVELGRKDFKRCVV
FHSLSKRSNLPGLRSGFVAGDAEILKAFLLYRTYHGCAMPVQTQLASIAAWNDEEHVRAN
RDLYREKFAAVLDILSPVLDVQRPDGGFYLWPNVGTDDAAFCRDLFVDQHVTAVPGSYLS
REVDGVNPGAGRVRLALVAPLAECIEAAERIRAFLSK
>tr|A0A0N8TA15|A0A0N8TA15_PSEAP Methionine--tRNA ligase OS=Pseudomonas syringae pv. aptata OX=83167 GN=metG PE=3 SV=1
MSEPRKILVTSALPYANGSIHLGHMLEYIQTDMWVRFQKHRGNQCIYVCADDAHGSAIML
RAEKEGITPEQLIANVKAEHSADFADFLVDFDNFHSTHSDENRELSSMIYKRLRDAGHIA
TRSVTQYFDPEKKMFLADRFIKGTCPKCAAEDQYGDNCEKCGATYAPTDLKDPKSAISGA
TPVLKDSKHFFFDLPAFDVMLKRWTRSGTLQDAVANKIAEWLDSGLQQWDISRDAPYFGF
EIPDEPGKYFYVWLDAPIGYMASFKNLCARRPDLDFDAYWGKGATTELYHFIGKDIVNFH
ALFWPAMLEGADLRTPTGINVHGYLTVNGQKMSKSRGTFIKARTYLDHLPPEYLRYYYAS
KLGRGVDDLDLNLEDFVQKVNSDLIGKVVNIASRCAGFIHKGNAGVMVEANAAPELTDAF
LAAAPSIADAYEARDFARAMRETMALADRANAYIAEKAPWALAKQEGRQDEVQAVCALGI
NLFRQLVIFLKPVLPNLAADAEKFLNVEPLTWEDHKILLTNHQLNPFSALMTRIDPVKVE
AMATASKEDLTATDSSADTAPAGNGELAKDPLSAEIDFDAFAAIDLRVALILKAEHVEGA
DKLLRLTLDIGDEQRNVFSGIKSAYPNPSELEGRLTMMIANLKPRKMRFGISQGMVMAAG
PGGEEIYLLSPDSGAKPGQRIK
>tr|A0A0Q0DEA7|A0A0Q0DEA7_PSEAP Glutathione S-transferase, N-terminal:Glutathione S-transferase OS=Pseudomonas syringae pv. aptata OX=83167 GN=ALO85_01193 PE=3 SV=1
MYKVYGDYNSGNCYKVKLMLNLLGSEYEWVPVDILNGETQTPAFLEKNPNGKIPVLELED
GTCLWESNAILNFLADGSSYLPSEPRLRTQVLQWQFFEQYSHEPSVAVARFIQFYLGLPA
ERMTEYRAMQKAGYRALAVMEQQLDRTPFLVGENFSIADIALYAYTHVAHQGGFDLAPYT
GIRRWLDRVKAQPGYIGMLD
>tr|A0A0Q0BTH8|A0A0Q0BTH8_PSEAP AsnC family transcriptional regulator OS=Pseudomonas syringae pv. aptata OX=83167 GN=ALO85_04233 PE=4 SV=1
MTYQTLNPTDVRILTALQQDGRITNQTLADQIGMSASPCWRRVRQLEEHRYIQGYRAVLD
RRKIGLGVMVFIRISIDSHSEAEARKFEKEVMQLEDVVACYSIGGDADFLLQVVAPDLDS
FADFAMTVVRRLPGIKEMQSMFVLKEIKPFVSFPVKQPA
>tr|A0A0N8T979|A0A0N8T979_PSEAP LysR family transcriptional regulator OS=Pseudomonas syringae pv. aptata OX=83167 GN=ALO85_100121 PE=3 SV=1
MTGGARMDRFDAMQAFARVVETGSFTKAAATLHMSKTSVTQLVQQLEARLRVKLLNRTTR
RVNVTADGAVFYERVVRLLADMDDAETSLSSAASLPRGRLRVDVPSPLASMLLMPALPGF
HARYPDIQIDMGVSDRMVDLIGENVDCVVRGGEIADQSLMARRVGDLQLGVYAAPDYLQR
MGTPVHPQELEDSAHRIVGYLWARTGKALPYAMQRNGEKLHVQGRYTLAVDDGNAYVAAG
LAGMGILWLPDYMAKPYRERGALVPLFEDWQLDSMPMYVAFPPNRHISIKVRVFIDWITE
LMAEHAPVTRPPAHPAQ
>tr|A0A0Q0DIJ7|A0A0Q0DIJ7_PSEAP Anaerobically-induced outer membrane porin OprE OS=Pseudomonas syringae pv. aptata OX=83167 GN=ALO85_02326 PE=4 SV=1
MKKSTLALAVTVGVLAQQASAAGFLEDSKASVSSRTLYFDNDFREGTTHNNRETATGLKF
DYLSGFTQGTVGFGLDVQGLVGVHLDGGRGNHGPAANGGILPTDSDGSAVSEWSRLGANG
KVRFSKTELKVGNALAPNLPILVSNDGRLLPQAFQGGILTSKDLDNVTFTAGQLNKSIGR
ASSNWTDLSIAGASETGDQFRFAGADWKVTKDLTLQYYYANLEDFYKQHFLGLVHVYQID
ANQSFKTDLRYFNSSSDGKNSDAATGYRFNNNNGYAKNAGEVDNSTWSAMFTYALGGHAF
MVGHQQIGDDGGMVFLNQGNVTKNGTSTSSLEGNGGSSFYLFTDSMINGFNRAGENTTFG
QYSYDFAKVGVPGLKAAIAYLHADDIRDRTTGKEEYSEWETDMRVDYVIQSGPMKGFGTT
LRHGTYRADGDLNNSTNTDQTRLIFNYTYNFM
>tr|A0A0Q0C158|A0A0Q0C158_PSEAP Anthranilate synthase, component II OS=Pseudomonas syringae pv. aptata OX=83167 GN=ALO85_00427 PE=4 SV=1
MLLMIDNYDSFTYNVVQYLGELGADVKVIRNDELSIAEIEALNPEHIVVSPGPCTPNEAG
VSLDVIKHFAGKLPILGVCLGHQSIGQAFGGDVVRARQVMHGKTSPVIHEDGGVFDGLNH
PLVVTRYHSLVVKQDTLPDCLEVTAWTALEDGSVDEIMGLRHKTLNVEGVQFHPESILTE

QGHELFANFLKQSGGHRQG
>tr|A0A0Q0DTZ2|A0A0Q0DTZ2_PSEAP 7-carboxy-7-deazaguanine synthase OS=Pseudomonas syringae pv. aptata OX=83167 GN=queE PE=3 SV=1
MADMQDTLRITEIFHSLQGETRTAGLPTVFVRLTGCPLRCQYCDSAYAFSGGTIQTLDDI
LGQVASYRPRYVCVTGGEPLAQPNAIPLLKRLCDAGYEVSLETSGALDISAVDPRVSRVV
DLKTPGSKEVSRNRYENMELLTANDQVKFVICSREDYDWAVSKLIQYGLDRRAGEVLFSA
SHHELKGRDLADWIVADNLPVRLQMQLHKILWDDEPGR
>tr|A0A0Q0CER8|A0A0Q0CER8_PSEAP 50S ribosomal protein L11 OS=Pseudomonas syringae pv. aptata OX=83167 GN=rplK PE=3 SV=1
MAKKITAYIKLQVKAAQANPSPPVGPALGQHGVNIMEFCKAFNARTQGIEPGLPTPVIIT
VYSDRSFTFETKSTPASVLLKKAAGLTSGSARPNTVKVGTVTRAQLEDIAKAKNADLTAA
DMEAAVRTIAGSARSMGLNVEGV
>tr|A0A0Q0C7P2|A0A0Q0C7P2_PSEAP Putative transposase OS=Pseudomonas syringae pv. aptata OX=83167 GN=ALO85_102075 PE=4 SV=1
MKLLGIQPVTTPVRSPQNNGMAKSFMKTIKGGRVA
>tr|A0A0Q0FCH0|A0A0Q0FCH0_PSEAP Short chain dehydrogenase OS=Pseudomonas syringae pv. aptata OX=83167 GN=ALO85_01875 PE=3 SV=1
MEYQMNTSSERTAIVTGASSGIGRATTEALARSGYTVFGTSRKAGDSEAQVSMLTCDVTD
DDSVRALVATVLAQTGRIDLLVNNAGIGMLGGAEEFSIPQVQALFDVNLFGVMRMTNAVL
PSMRQRGQGRIINIGSVLGLIPAPYSAHYSAVKHALEGYSESLDHEIRAFNVRVSVIEPA
YVRTVFDQNGIEPDSQLQAYDQARAGVRALLADVMPTADLPEVVAAVVLKAASDVRPRHR
YTAGKTARQISLLRRFAPAGAFDKSLRKQFRLPE
>tr|A0A0Q0DQD6|A0A0Q0DQD6_PSEAP Flagellar motor rotation protein MotB OS=Pseudomonas syringae pv. aptata OX=83167 GN=ALO85_04021 PE=4 SV=1
MARRRQVEEHENHERWLVSYADFITLLFAFFVVMYSISSVNEGKYKILSQALVGVFNDTE
RTMKPIPIGEQRPVSVKPAEPLVKDSEQTDAGIGQASDDPLQTIAQDVRDAFGDLIKSDQ
MTVRGNELWIEIELNSSMLFGSGDAMPSDKAFTIIEKVAGIVKRFDNPIHVEGFTDDQPI
STAQFPTNWELSSARSASIVRMLAMDGVNPARLASVGYGEFQPIVPNTSTAGRAKNRRVV
LVISRNLDVRRSLTSAGSANVQPDAALRRAGTQTAPVPAKPPVRANAVNSPSPAP
>tr|A0A0Q0FKW3|A0A0Q0FKW3_PSEAP Peptidase aspartic, active site protein OS=Pseudomonas syringae pv. aptata OX=83167 GN=ALO85_01776 PE=4 SV=1
MKRPFILSLLVSGLGLVAPLSQAETLPLPLTGPAYAIANEAYMAYNRKDYDLAIAKANEA
LRQRGDAQQLRDLITLAERDKYRRDHPQRAYKTRPKPGYLEGNQALRAYARHDYDGSASH
ARKAIAQAPKNLDYRMMLIEALQRQQRLDEAQAAINDAEQALGPQQVLTRRRQAIQEQVA
VDKAATGYKALARGDNDTAVSEAREAVRSFPKQMAYRKLLVSALIAQGQYAEARSAATEA
LALNGNDATLLVQRGQMRQRLGDQDGARQDFAQAMAVGNLPLREQASLYAAMGQPSEALQ
RLQQARDAGQLQPGDEVQIAYFLSQAGDDQGALDTFKRVDRQSGLKPREVQDAAYSAMRT
SNDAQAIAYFKRVLDYQQTGDLQMPAQQVFDTRRAVSDLSREWGLTNTTTYRGASTSSGL
SGAPGGSNDSVQNSTEVFWRPFGYRNARFVELYGRVTDTLWSKEGDSDTGADALQGALGI
RVKPFSAVNVIGAFERTFPLGNSNADGDWLVRLGYGSSIGTDLRVDVPSWWTSQLYAEGG
RYLQDKRNYFNSEWQVGRSFRLDSISPRLVVFPHVVAAVDYDSKMRSEVDSLGRSSTSSG
NAGGLGVGTGVRYWFREDKYKAPQSYVDVSVQYREKIFGDDRAEGVFARMTFSW
>tr|A0A0Q0C650|A0A0Q0C650_PSEAP Ribose ABC-type transport system, permease protein OS=Pseudomonas syringae pv. aptata OX=83167 GN=ALO85_03750 PE=3 SV=1
MSALDLSVAQAGDPAQRWLVQLAQRGSLLVFLAILLGFAVSAPNFISLGNISNVFAQSAT
LGILALGLTCVVIGGGSNVVSGGLDLSLAANLGLCAAVYSSLNNAGFDAWQAIGLTLGCG
LLVGAFNGLAVVFLRLPPLLATLASMNLIAGLELVLTQNTVLATDSALLDSLASGVWLGI
PALAWVLLGVAAVLWLVIQHSAFGLRLYAIGEYPQAAEAAGLNLRRYVFASYLIAGGCAA
LAAFCSAAFFSGSTTGSGDMLLSVVAIAFLGVVFSRRLVANIPGTLLATLLLGFLINGFQ
LLNISSFWVNGVQGVLILLVVAFSGAFGRREGEQ
>tr|A0A0Q0FCW8|A0A0Q0FCW8_PSEAP Ribose operon regulator OS=Pseudomonas syringae pv. aptata OX=83167 GN=ALO85_00514 PE=4 SV=1
MATIKDVAALAGISYTTVSHVLNKTRPVSEPVRLKVEAAIAQLDYVPSAVARSLKAKTTS
TIGLLIPNGMNPYFAELARGIEDYCERNGFCVILCNSDDNPEKQRGYLRVLLEKRVDGLI
VSSVGGDVGVAGGLAEVRTPLVIVDRELDGIDADMIRIDHEQGAYLATRHLLDLGHRAIA
CIGGPVHSTVAEMRLAGYRRALQQASVQVRAEWSLHSEFTSSAGHEAACQLLAERPPTAI
FAGNDVIAIGVLRAAAERLIRVPQDLSVIGFDDIQMSRYVYPALTTVGQSIMQLGETAAE
MLLSRIATPHGLPAEKRLVTPSVVVRESTAAPNTLSNE

>tr|A0A0Q0IT85|A0A0Q0IT85_PSEAP Phosphate transport system permease protein PstA OS=Pseudomonas syringae pv. aptata OX=83167 GN=ALO85_01417 PE=3 SV=1
MVKRNSLRNWFKSGAPGVWMSAGAVSIAVIMTIGLLAVIAVRGLGHFWPADLVSAQYAVP
NQESSVLVGERVEQEQVPRARLKSAGLPVPDQGPEFMTRELFKVGNRDLNPSDFNWVVGE
WLTGQSRPADLMTLERREWGNFYGYLVNVKEGGRVVAEGDAAWPVLQERIKRVEKLADEL
YTLEKKDIGAINHAIERLRLQARKLELNGRLDAAAQADMAAERAELDARYKVIEARLDGL
HQAFDRDSLTARDGNGKEIELSLGKVVRAYQPNAMGTVTKLGFYFEKLWEFLSADPREAN
TEGGIFPAIFGTVMMTLIMAVIVTPFGVLAAVYLREYARQGPVTRLIRIAVNNLAGVPAI
VYGVFGLGFFVYVLGGSVDRLFFPEALPAPTFGTPGLLWASLTLALLAVPVVIVATEEGL
ARIPLTLREGSLALGATKAETLWKIVLPMASPAMMTGLILAVARAAGEVAPLMLVGVVKL
APSLPLDGNYPYLHLDQKIMHLGFHIYDVGFQSPNVEAARPLVYATALLLVLVIALLNLS
AVSIRNHLREKYKALDN
>tr|A0A0Q0CHC1|A0A0Q0CHC1_PSEAP 50S ribosomal protein L23 OS=Pseudomonas syringae pv. aptata OX=83167 GN=ALO85_200050 PE=4 SV=1
MSETRLAAIMGGSHPLAALSNPREAIRQFTPNWFAATMGTGILALALGQLPFVAQWPKAL
GEALWFFNIVLFCLFTLMYASRWVLFFDEAKQIFGHSTVSMFFGTIPMGLATIINGFLLY
GLPRWGGGVVALAEVLWWIDVTMALACGVLIPYMMFTRQQHSIDQMTAVWLLPVVAAEVA
AASGGLLAPHLADASAQFTMLITSYVLWAYSVPVALSILVILLLRMALHKLPHESMAASS
WLSLGPIGTGALGMLLLGNDAPAIFAAHGMASIGGVAEGIGLIGGVLFWGLGLWWMVLAL
LITGRYFREGIPFNLGWWGFTFPLGVYAVTTLKLGSMLHLTFFSFFGVVLVLMLTVMWMI
VAAKTLAGAYRGNLFVSPCIHSLSKASKT
>tr|A0A0Q0C0W2|A0A0Q0C0W2_PSEAP Bifunctional protein PutA OS=Pseudomonas syringae pv. aptata OX=83167 GN=ALO85_02908 PE=3 SV=1
MATTTLGVKLDDPTRERLKAAAHSIDRTPHWLIKQAIFNYLEKLESGATLLELDGAAGKE
NEDRGELAEDAGLQCFLDFAESIQPQSVLRAAITSAYRRPEQEVVPMLLEQARLSAPVAE
ATNKMAASIAEKLRNQKSAGGRAGIVQGLLQEFSLSSQEGVALMCLAEALLRIPDKGTRD
ALIRDKISNGNWQPHLGNSPSLFVNAATWGLLLTGRLVATHNEAGLTSSLSRIIGKSGEP
MIRKGVDMAMRLMGEQFVTGETIGEALANASRFEAKGFRYSYDMLGEAALTEHDAQKYLA
SYEQAIHSIGKASHGRGIYEGPGISIKLSALHPRYSRAQYERVMDELYPRLLSLTLLAKQ
YDIGLNIDAEEADRLELSLDLLERLCFEPQLTGWNGIGFVIQAYQKRCPYVIDYVIDLAR
RSRHRLMIRLVKGAYWDSEIKRAQVEGLEGYPVYTRKVYTDVSYIACARKLLAAPEVIYP
QFATHNAHTLAAIYQIAGQNYYPGQYEFQCLHGMGEPLYEQVVGKVADGKLNRPCRVYAP
VGTHETLLAYLVRRLLENGANTSFVNRIADHSISIQELVADPVSQIERMATQEGGFGLPH
PRIPLPRDLYGTERANSSGIDMANEHRLASLSSALLATAHNDWKAAPMLGCPASAGTLSA
ALNPSDLRDVVGHVQEASLPDVDNAIQCALSAGPIWQATPPGERAAILERAADLMEAEIQ
PLMGLLVREAGKTFANAIAEVREAVDFLRYYAVQARNDFTNDGHRPLGPVVCISPWNFPL
AIFSGQVAAALAAGNPVLAKPAEQTPLIAAQAVRLLLEAGIPEGVVQLLPGRGETVGAGL
VGDERVKGVMFTGSTEVARLLQRNVAGRLDSQGRPIPLIAETGGQNAMIVDSSALTEQVV
IDVVSSAFDSAGQRCSALRVLCLQEDSADRVIEMLKGAMAENRLGNPERLSVDIGPVIDA
EAKAGIEKHIQAMRDKGRTVYQVAIADSAELKRGTYVMPTLIELESFDELQREIFGPVLH
VVRYKRKELGLLIDQINASGYGLTLGVHTRIDETIAKVIDNVNAGNVYVNRNIVGAVVGV
QPFGGEGLSGTGPKAGGPLYLYRLLSTRPQDAIEKSFVRSDALSAPDTRLREVLGKPLQA
LKAWAASNQQSDLDALCSQYAEQSQSGITRQLAGPTGERNSYAILPREHVLCLADDENDL
LIQLAAVLAVGSSAVWPETDISKPLRARLPKDVQARIKLVPDWAKDEVTFDAVLHHGDSD
QLRAICQQIAQRSGAIVGVNGLSHGETNVPLERLVIERALSVNTAAAGGNASLMTIG
>tr|A0A0N8T9D3|A0A0N8T9D3_PSEAP Crossover junction endodeoxyribonuclease RuvC OS=Pseudomonas syringae pv. aptata OX=83167 GN=ruvC PE=3 SV=1
MTLILGIDPGSRITGYGVVRDTGRGCVYVASGCIRTGSGELHERLQIVYRGVREVIKTYG
PVTMGIEKVFMARNADSALKLGQARGAAIVAGAEEALEIAEYTATQVKQAVAGTGGANKE
QVMMMVMHLLKLTQKPQIDASDALAIALCHAHTRSSLIPHGLATARSRGGRLRL
>tr|A0A0Q0C4A1|A0A0Q0C4A1_PSEAP DNA-binding stress protein, Dps family OS=Pseudomonas syringae pv. aptata OX=83167 GN=ALO85_00247 PE=3 SV=1
MAIDIGISEEDRKSIVDGLSHLLSDTYVLYLKTHNFHWNVSGPMFRTLHLMFEEQYNELA
LAVDSIAERIRALGFPAPGTYSTYARLSSIKEEEGVPSAEDMIKSLVQGQEAVVRTARSI
FPLLDKVSDEPTADLLTQRMQVHEKTAWMLRSMLESK
>tr|A0A0Q0DQX8|A0A0Q0DQX8_PSEAP DUF1232 domain-containing protein OS=Pseudomonas syringae pv. aptata OX=83167 GN=ALO85_02502 PE=4 SV=1
MGMKAPWNFIRYLPGARRLLAAGRLPGLLFAVARKGNSEGSRLGKLKEDLRLLQALCLAY
WRGEYRAISPKAILSIVAGLMYFLSPLDAIPDWIPGLGMLDDIAVLAWVTKHLSNELDAF

RAWRAQQSPEKLIVVEQLPKTQALLEQERGKV
>tr|A0A0Q0C693|A0A0Q0C693_PSEAP Glycoside hydrolase family protein OS=Pseudomonas syringae pv. aptata OX=83167 GN=ALO85_03657 PE=4 SV=1
MAALIEDYALLGNCETAALVARDGSLDWLCFPRFDSTACFAALLGGDEHGRWKIAPTAEV
AAVERRYRDGTLILETVFETRDGRAMLIDFMPMKTSGHVVRMVVGLSGRVEFAVDLAIRF
DYGSSVPWVERKDAHTLTAVAGPEMLVLRSPVALHPQDHHTASLFHVDEGERKVFTLAYQ
ASFAPLLDQIDADRALETTAAYWREFSDRCPDVGPWTAQVKRSLITLKAMTYAPTGGIVA
AVTTSLPEQLGGERNWDYRYCWLRDATMTLLAFMNLGYFEEAQAWRDWLLRSVAGNPEQI
QIMYGVGGERRLQEYELPWLSGYENSLPVRVGNGAATQVQLDVYGEVADAMIQALRGGMT
QHPRSLAISKVVMPYLEKIWHLPDAGIWEIRSEPRHFTHSKVMAWVAFDRNATQIAQAAE
SDEDRALAVHYRKVADEIHADVCRHGFDAELGSFVQTYGSTEVDASLLQIVLTGFLAPED
PRVIGTVAQIERTLMQDGLLLRYDNERSVDGVAGREGTFLVCSFWLADAYVLLGRADDAA
RLFERLVGLCNDVGLLAEQYDPKGGRMLGNFPQAFSHIGIINTALNLHRAVCPALARTSG
ALEEA
>tr|A0A0Q0DQH7|A0A0Q0DQH7_PSEAP Uncharacterized protein OS=Pseudomonas syringae pv. aptata OX=83167 GN=ALO85_101499 PE=4 SV=1
MQDAQCNARMRLMAPDGLVFASSECLLGVFCAISIKT
>tr|A0A0Q0IEM9|A0A0Q0IEM9_PSEAP NAD+-dependent alpha-aminoadipic semialdehyde dehydrogenase OS=Pseudomonas syringae pv. aptata OX=83167 GN=ALO85_02109 PE=3 SV=1
MVAELLARLGVANSAHTQGDYPVYTPIDGSRIASVTLEGKAQVVARIDSAHNAFLKWRTV
PAPRRGELVRIFGEVLREHKADLGELVSVEAGKITQEGLGEVQEMIDICDFAVGLSRQLY
GLTIASERPGHHMRETWHPLGVVGVISAFNFPVAVWAWNTTLALVCGNPVIWKPSEKTPL
TALACQALFDKALKIFGDAPAALAQLVIGDRDAGEALVDDPRVPLISATGSTRMGREVGP
RVAARFGRSILELGGNNAMILAPSADLDLAVRGILFSAVGTAGQRCTTLRRLIVHRSIKD
EVVARVKAAYAKVRVGDPREGNLIGPLIDQQAFSAMQNALTKARDEGGQVFGGERQLQDK
YPNGYYVTPAIAEMPGQSDVVRHETFAPILYVLAYDDFEEALRLNNEVPQGLSSCIFTTD
LREAEAFQSAAGSDCGIANVNIGTSGAEIGGAFGGEKETGGGRESGSDSWRAYMRRQTNT
VNYSRELPLAQGIVFD
>tr|A0A0Q0DHN1|A0A0Q0DHN1_PSEAP Regulatory protein, LuxR OS=Pseudomonas syringae pv. aptata OX=83167 GN=ALO85_00373 PE=4 SV=1
MNRQVNAKDEVKTHIYLELGKLVSSVGDDMFLSNMYQLINTSLAVSRVELSEWTVDDSQA
TVTDVQLLGYAGAELNPQMQTVCPTRARHRSDHPLVKRVLEVEDDLLIHLNARMKNQQGN
DCLGTSHQCSMISRKANRCCVISLHRPQADKDYSLQELSFLKYLSEALLPLSERHARAHR
QSGMKSAGGPLSHGPLSLEHKQLQNEFNERLRACDVSLSAREKEACLEFLAGSTVPEIAE
KLCVKSSSVETYLKRSAAKLGISGRHGLAKWLVCAE
>tr|A0A0Q0C6C6|A0A0Q0C6C6_PSEAP DUF1883 domain-containing protein OS=Pseudomonas syringae pv. aptata OX=83167 GN=ALO85_03660 PE=4 SV=1
MKFVHQREHLNEDDLVVIECSQTCNIRLMNDANFRSFKNGGRHTYHGGAFDRFPAKIAAP
STGFWNITIDTAGRKADTNAGRKLPFTHKIKIVRRSTTRLG
>tr|A0A0Q0C4Y4|A0A0Q0C4Y4_PSEAP Uncharacterized protein OS=Pseudomonas syringae pv. aptata OX=83167 GN=ALO85_04231 PE=4 SV=1
MNFDAALHKCVIVVDKALGLGHAANAVSVIGVSMGRSVEGLVGPDMQSSDSVNYPGVIYA
PLPILLSSNESLHELQEKALADDEIFSIPFSALAQSCKTYQEYESRISEADSSSIELVAI
GLIGPKKKISKLSGNFPLFK
>tr|A0A0Q0BR55|A0A0Q0BR55_PSEAP DSBA oxidoreductase OS=Pseudomonas syringae pv. aptata OX=83167 GN=ALO85_04060 PE=4 SV=1
MKRPTRRQILYCIGAVALAISAFLLTEVMQNEPGVTGDPVAPAADQKRHSGGWVYGSRGA
RFTIVEYADLECPYCKDYFPHLKAWIDQHPDVNLQWHHLPLPMHEPAASYEARWAECAGI
EGGNDAFWLAVELIYQRTRSNGAGTAGNPQIPGLEDRQHFIDNCAARNPSVQQAVISQAH
KASQDGITATPTLVIKDKQSGRSIKLQGAPDGDVLLSAMDWLASTRDR
>tr|A0A0N8T9R4|A0A0N8T9R4_PSEAP Thioredoxin domain-containing protein OS=Pseudomonas syringae pv. aptata OX=83167 GN=ALO85_01934 PE=4 SV=1
MTMSNTELTAFVEERVTRAQQTLADGKVSKLDDVATGKLSVFLPLQRALQGTGTPQDLGV
LDAINDTLQSLKILDSKETLLNYQNKAAQVRVVKNDTDFSSQWTAISPLVVLEFSNPGVN
EHGEANNSTRTGAILDELAPEFAGRVVMLRVPVDTSPATAQNYNVTVTPTVIFFKNGKKI
EETADFHLKSWFRAKLNELLSLKE
>tr|A0A0N8T8B0|A0A0N8T8B0_PSEAP Uncharacterized protein OS=Pseudomonas syringae pv. aptata OX=83167 GN=ALO85_01035 PE=4 SV=1
MTAEPICETTFVQTLLDIAKFPERHRAVANTWADHFNVPAEARDEFILHYLTHTSSTRCW

CVALHNDGSVARPTVARIGRQLQYFDGQLISAVRFDEKRKVPGHAPSPSQALKLAHQLIT
HDGADALLTSFCKPARDLARGEAELSIRPLVKYNMGALSSEGRNKRFYAPRGRFYITCIG
AALKRFCQNLDQELLHAVRSVQCPSAKLYNWLAQGDRTRRLQALKAQPVLVPVLIVGVGM
PWPMIAGGLLVECPWFELQGFCCSWEGETIMDGAGFVGKAVDTGLPLNRVLAWLFSVPTS
SIRFLGQQRVYDTGSALSRLNSEGLEAGWEHLIAGSLLGNRRPRTKAEWRFFYSFRSAIP
WELLRSLRDMNNLLAGCPTDWADPAWSGIATKLVDFRELFDNLDRAGSCEARNTKRRLYA
FLSGLNFRQISNVVDAFHGALADIRARLERDHPPEPSDCFTRWPGLLLGSDPITCSTTGL
QIVELRCPADLDQEHRSLGHCIDTYDFRAYSGNCRLLSIRSEGLPLASVELTLRTGRNER
VTGDFTPQHLHIAQIRDHENKTPDAHSVVMNAFELFMAAVRSGRMPVLLEWPNMAMKMAR
YADEKSVFNIRFGEEIVGWANSLLDKGL
>tr|A0A0Q0C1R4|A0A0Q0C1R4_PSEAP Uncharacterized protein OS=Pseudomonas syringae pv. aptata OX=83167 GN=ALO85_03609 PE=4 SV=1
MARKTPAQQAADQIKDQAFSDLQALIEESDKLLKESAALVGEEAETLRAQLSAKLKQALD
SMASVRDRTKPVVEATETYIGGHPWQTVAISAGFGLVVGLLLGRR
>tr|A0A0Q0ILN6|A0A0Q0ILN6_PSEAP Peptidoglycan-binding LysM:peptidase M23B OS=Pseudomonas syringae pv. aptata OX=83167 GN=ALO85_00277 PE=4 SV=1
MSLTVIQQLRSSRSYQRLVIGIALSVLLVGCSSSPSGGVRVVDRNGNAVAQRPTVTTGQY
VVRRGDTLFSIAFRYGWDWKALAARNQIPEPFTIKPGQTIRFDGRSSSTPSVASTSGAGR
PVTSTSTTTSSSGSVKTTVMSKPVAVVPVVIPPSATTGTGPAGKSPTGWTWPSSGILIGK
FSSNGSLNKGIDIAGDLGQPVLAASDGSVVYAGSGLRGYGELIIIKHSDTYVSAYGHNRR
LLVREGQQVKAGQTIAEMGSTGTDRVKLHFEIRRQGKPVDPLGFLPRR
>tr|A0A0Q0C599|A0A0Q0C599_PSEAP Glycosyl transferase Ssg OS=Pseudomonas syringae pv. aptata OX=83167 GN=ALO85_02884 PE=4 SV=1
MKVLLLVQKEQRAILDRLYDGIAAHCECEVRWLSSAEQRNLRGYFRREVDVSRYDRIVFF
LRFKQEVRQVGFIRTIPNLVILEHDAYQNYIPCKYTGKFSAHYRKLPWARVICSGFMVSE
RLRQEGFDAVFVPKGYDQSLLQLQARERDIELAFVGSTNSVAYSGRKALLDELGQVENLL
VTRTKSGEEYCDTLNRIRFFVSADVGMGEYMIKNFEAMACGCVLLAFDQGAEENRALGFV
DMHNIVLYRDIPELREKLAKLRDNVTLAGEISCNGQTLVEERFTFHALGKAIVEAMHAPL
RSMPAASWIDRLRSRLGW
>tr|A0A0Q0DMZ6|A0A0Q0DMZ6_PSEAP HopAC1 OS=Pseudomonas syringae pv. aptata OX=83167 GN=ALO85_02890 PE=4 SV=1
MSKTLLHDLKTLDRGHHTDPDGIYTFERLIIGARQQGLEIRAIDCASSYHLKGIAGEQSI
TRQQMMNYFASRTIRRHQEVMGSHKWIALVGNSHSNTYQGVVPGVAELQGGIGLRVVDVT
PGHSRGITLDPGELVSGGIADDKVYIKADYRVEMEVLSSRPVRLPSIENRLSRPGMFLVQ
EGEGNLPTIIHRARDTWTHHTPVLRNAEGKLYIERLRWPRIHLKPFDDIDALVAALETMN
LTRIT
>tr|A0A0Q0D6W3|A0A0Q0D6W3_PSEAP Aldo/keto reductase OS=Pseudomonas syringae pv. aptata OX=83167 GN=ALO85_00983 PE=4 SV=1
MITRQLGNHGPQVSALGLGCMGMSDFYTTGIDEKESIATLQRALELGVSFFDTADMYGPY
TNEALLGRALEGKREGIYLASKFGIVRGDDPHARGVNGSPAYIRQSIDASLKRLNTDYLD
LYYQHRVDPNVPIEDTIGAMAELVKAGKVRHIGICEASAATIEKAHAVHPLAAVQSEYSL
WSRDPEQDGVLATCRRLGIAFVAYSPLGRGFLTGELRTPDDFAADDYRRFTPRFQGENFN
RNLQLVEKVKTLATARGISASQLALAWVLAQGEDIIPIPGTKQRKYLESNVAAASLTLSA
DDLVQLEAIFPAQGSASGERYNAASMKSLNG
>tr|A0A0Q0ISG0|A0A0Q0ISG0_PSEAP Uncharacterized protein OS=Pseudomonas syringae pv. aptata OX=83167 GN=ALO85_101116 PE=4 SV=1
MTISSFLSRVGRKPLKRRGVGFFRDGSLPIACAHLSQFQTV
>tr|A0A0Q0C793|A0A0Q0C793_PSEAP SpoU family RNA methyltransferase OS=Pseudomonas syringae pv. aptata OX=83167 GN=ALO85_01063 PE=4 SV=1
MANKRYACIGLYNPKSPENVGSVMRAAGCYGVASVFYTGKRYERARDFITDTKKIHQDIP
LIGIEDLKSILPLGCIPVAVELVDGARALPEYTHPDRALYIFGPEDGSLDQEIRDWCEDV
VYIPTEGCMNLAATVNVVLYDRMAKGINTRSGPQFGREKIDPASDR
>tr|A0A0Q0D0N1|A0A0Q0D0N1_PSEAP Uncharacterized protein OS=Pseudomonas syringae pv. aptata OX=83167 GN=ALO85_101134 PE=4 SV=1
MFIAFSLDRGLLLAEAALTNDAPRAEAAPNADACKTLRRLTPDAAFSASAEPKFFIEESD
RAG
>tr|A0A0Q0DUG8|A0A0Q0DUG8_PSEAP 4-hydroxybenzoate octaprenyltransferase OS=Pseudomonas syringae pv. aptata OX=83167 GN=ubiA PE=3 SV=1
MYLSLLKSLNRLNPRAWDFIQLTRIDKPIGVYLLLWPTLWAVWIAGKGAPSLQTVFIFVL
GVFLMRAAGCVINDFADRKVDGHVKRTEQRPLVSGKVSSREALVLFAVLVGLSFVLVLFT

NAATIWLSFGGLALAASYPFMKRYTYYPQVVLGAAFSWGMPMAFTAETGELPAAAWLLYI
ANLLWTVGYDTYYAMVDRDDDLKIGVKSTAVLFGDADRVIILTLQGLALGCLMLAGARFE
LGACFYIGLLAAAGCFAWEFWSTRERERDACFKAFLHNHWAGLAIFLGIVADYAVR
>tr|A0A0N8TAD3|A0A0N8TAD3_PSEAP Thioesterase superfamily OS=Pseudomonas syringae pv. aptata OX=83167 GN=ALO85_01421 PE=4 SV=1
MIELEQEDPTPQGDLALQITALPRETNGFGDIFGGWLVSQMDLAGTAMASKVAGGRVATV
AIDRMAFLVPVAVGEQLSFYTRTLEVGRTSIKMMVEVWSDDPLTSEWRKVTEAAFVFVAI
DASGRTRSVPARR
>tr|A0A0Q0BYM2|A0A0Q0BYM2_PSEAP Uncharacterized protein OS=Pseudomonas syringae pv. aptata OX=83167 GN=ALO85_03168 PE=4 SV=1
MHLSIVQRCASRLGLQALWVLVGLASSTLVSAAGALPTYGEMDAAGFAGSPRENYALQDF
SDKYRATLDISAKDDVFRPGVINVYDKASGAALIRVQSDELVLGTDPKTGKVKTNVHELP
YGEQSVLIYQDFNFDGIKDLALMDGQNSCYHGPSYQVFLGTADGFRHSDSFTKLAQNNCG
LFSVDEKARKIETMTKDGCCWHQTSTYSIRNGEPVLETQTVLDHTGGSGLPTETVSRNQN
GKMTHTTSIVWEEDQQREILLSFRLAPSGKRIVLFRSGDASPVFYAAVDSKNQVGLLFPQ
ADGEHLKYDAASHVLSFVRGDTVYRIVGDAKGAPTAMQVVVRGKTTELKLLAEPAQGSLN
KVADALKAAQ
>tr|A0A0Q0DMX1|A0A0Q0DMX1_PSEAP Uncharacterized protein OS=Pseudomonas syringae pv. aptata OX=83167 GN=ALO85_05253 PE=4 SV=1
MASFQQCQQQSAHVYRLAAFRSAGAVDDLDRAAHAALQRHDQEASDELSLYGGFYTDGPD
HGVFDERNEVPMLPQDVLKDMWILDFSTLYTRRMERFWSLHSENFCTLAKARYLVAASHC
LRKGQLSAVDFKHVTSVVTADPLQPPTLKALLAPLPATPGVTVHTLDIDGIKAHNMLRIV
TAAGREIIYWPDAEQPFMAFDSERAVYRWLKARFMGEHADKAITRQFLRGETSRKKDGER
FFRAVSQLLAHDWRADIPLVNLGQAPIVGDPFVYLRDVVRQGMTADARERLTSNADLRKQ
MWIGYLGAFMQAFGGLAALGWPIALTLVGASLANTGLNIDQAVNGKTAAQRKAGVLGAIN
NIIYLLFNLPLLMSLRAVPQPVPVAATEGLPISSEVLESSSAPAHPLENMEGNLLLDSLT
PSAAEGRFRGIYTLGNGDTWIKLAQLPYRVMFNEQQQCWLIVNPDNPFAFAGSKPVRLST
DGEWALLDSPGLQGGAPMDVPMPSSSASTATGKPYVVTVHSAFWDRYLQTDLFSEQHYAEV
ALARQKGVMSIWQPDPEEAPVSDSSPDGDEVYKDPWHGKHRVFRLGEDDYYGRNIMLYTQ
DDAEFNQFLRTGESKRANQVQLIERLADDIHVVGYNNDVELYRGGSGDRGTSGAVFRSGQ
IKVGDVLVNTDVTSFSENPYVVSAFASSRAGAPINAMIGPVTFDDTSVVFVLPKGRYLRA
TPIAPFSASPEEAETIFLPGCYFQIDSIEEVSGEFYRIMKIQMQEVDRPANGRGLYDMRT
GEPFSRDQYALKLGNNAKPLVDRFFPQEPLADLFGFH
>tr|A0A0Q0BT96|A0A0Q0BT96_PSEAP Fimbrial biosynthesis protein FimA OS=Pseudomonas syringae pv. aptata OX=83167 GN=ALO85_04272 PE=4 SV=1
MKKLSLTLAVLGVMGVGMAGTASAANNGTMVFNGTLTNISCDVGPGTGVSAGTNPGEINI
DLGNVSFSDIGVSSESRFETAAPIQLLVNCSAGADQYNMVKMRLIARNGSGLDNNDAKLL
RTTGAADGVGIGLLNASSVLMDLSGDETVDTTLIKDGANGATAEINFGAVYVLNGTPTNP
GNADGFLPFVMDYE
>tr|A0A0Q0DID9|A0A0Q0DID9_PSEAP C-5 cytosine-specific DNA methylase OS=Pseudomonas syringae pv. aptata OX=83167 GN=ALO85_04054 PE=3 SV=1
MNTFSLLITPKIGEARGVPRIWLEGQKLLNAGIEIGTRFSLIRPEGQTRLELVPATSPEL
NTGDVTVSRRVKNGITTPLIEIRTALLRSLFKTAEKVRVVIRLGRIVITPLDNDKRIEER
LARMKRKLDAQEPLAVCSLFHGGGVLDRAIHAGLARSGIDTFVRIGIEVEDQYLDASLRN
NPMLWRDTSFAICADARDVQRGSGTPECDLVVAGIPCTGASRAGKAKNKLNSAEQHPSAG
SLFIDFLDFVRYSNPVVAIVENVPDYQTSTSMEVIRSVLKSLGYRLFECILDGAVFGAFE
SRSRMALVAYTDGAFEPLRLDQITPLRTKERSLKDLMDVVPETDKSWKCYDYLGRKEERD
IASGKGFRRQLLTPEATSCGCIGRGYAKARSTEPFIRHPRNPALSRLLTVSEHARVKTIP
VEIVEGVSNTTAHEILGQSVIYAAFEAIAATVGKLITKLHRTARPAGVMAA
>tr|A0A0Q0CXK1|A0A0Q0CXK1_PSEAP MbtH-like protein OS=Pseudomonas syringae pv. aptata OX=83167 GN=ALO85_01277 PE=4 SV=1
MTSVFDREDIVFQVVVNHEEQYSIWPDYKAIPNGWRTVGKSGFKKECLAYIEEVWTDMRP
LSLRQKMEAQTA
>tr|A0A0N8T9J9|A0A0N8T9J9_PSEAP 30S ribosomal protein S11 OS=Pseudomonas syringae pv. aptata OX=83167 GN=rpsK PE=3 SV=1
MAKPAARPRKKVKKTVVDGIAHIHASFNNTIVTITDRQGNALSWATSGGSGFRGSRKSTP
FAAQVAAERAGQAALEYGLKNLDVNVKGPGPGRESAVRALNGCGYKIASITDVTPIPHNG
CRPPKKRRV
>tr|A0A0Q0DKI8|A0A0Q0DKI8_PSEAP RNA polymerase sigma factor RpoD OS=Pseudomonas syringae pv. aptata OX=83167 GN=rpoD PE=3 SV=1

MSGKAQQQSRIKELITLGREQKYLTYAEVNDHLPEDISDPEQVEDIIRMINDMGIPVHES
APDADALMLADADTDEAAAEEAAAALAAVETDIGRTTDPVRMYMREMGTVELLTREGEIE
IAKRIEEGIREVMGAIAHFPGTVDHILSEYTRVTSEGGRLSDVLSGYIDPDDGIAPPAEV
PPPVDPKAAKAEGADDDEEESADASDEEDEVESGPDPVIAQQRFGAVSDQMEITRKALKK
FGRDDKHAIAEMVALAELFMPIKLVPKQFEGLVERVRGALERLRAQERAIMQLCVRDARM
PRADFLRQFPGNEVDESWTDALAKGKSKYAEAIGRLQPDIQRCQQKLSALEEETGLKIAE
IKDINRRMSIGEAKARRAKKEMVEANLRLVISIAKKYTNRGLQFLDLIQEGNIGLMKAVD
KFEYRRGYKFSTYATWWIRQAITRSIADQARTIRIPVHMIETINKLNRISRQMLQEMGRE
PTPEELGERMEMPEDKIRKVLKIAKEPISMETPIGDDEDSHLGDFIEDSTMQSPIDVATV
ESLKEATREVLSGLTAREAKVLRMRFGIDMNTDHTLEEVGKQFDVTRERIRQIEAKALRK
LRHPTRSEHLRSFLDE
>tr|A0A0Q0BWB1|A0A0Q0BWB1_PSEAP tRNA 5-carboxymethoxyuridine methyltransferase OS=Pseudomonas syringae pv. aptata OX=83167 GN=cmoM PE=3 SV=1
MSDRHFDQLATRFAEKIYGGAKGAIRLAVLQADLTETLPVRPLRVLDIGAGLGHMSLWLA
EQGHQVTLAEPAEPMLEGARQRFADAGQTATFIQAPWQALPDLLSDTYDLVLCHAVLEWL
AEPHSILPVLRQLTKDDGWLSLAFYNRDALIYRNLLKGHFRKMRKNDMAGEKQSLTPQQP
LDPRELATQLEGLWQVETRSGVRVFHDYMPVEFQARAELTDLLEMELAHRRHPAFAGLGR
YLHWMCRPV
>tr|A0A0Q0DHM5|A0A0Q0DHM5_PSEAP High frequency lysogenization protein HflD homolog OS=Pseudomonas syringae pv. aptata OX=83167 GN=hflD PE=3 SV=1
MSPTQEQLIALGGVFQAAVLVDRIAKTGQISEAALGCMLGSLLVVDPKDTLDVYGGDDLN
LHEGYRAMASALERDPATLQREPLRYALSMLGLERQLAKRDDLLEVIGKRIPVIQSQVEH
FGIAHENVIAATGALYQDTLSTLRQRIQVQGDMRNLQQPNNASKIRGILLAGIRSARLWR
QVGGHRWQLVFSRRKLLKELYPLLHG
>tr|A0A0Q0C5S8|A0A0Q0C5S8_PSEAP Ribosome maturation factor RimP OS=Pseudomonas syringae pv. aptata OX=83167 GN=rimP PE=3 SV=1
MHEGVQVSSKLEQLQDLLAPVVVALGYQCWGIDFSSQGKHSVLRIYIDKEGGVLVDDCAI
VSRQISGVLDVEDPISTEYTLEVSSPGMERPLFTIEQFASYAGEQVKIKLRSPFEGRRNF
QGLLRGVEEQDVVVQVEDHEFLLPIDMIDKANIIPTFD
>tr|A0A0Q0FGD2|A0A0Q0FGD2_PSEAP Zinc ABC-type transport system, permease protein ZnuB OS=Pseudomonas syringae pv. aptata OX=83167 GN=ALO85_01617 PE=3 SV=1
MADFLLYALLAGVALAVVAGPLGSFVVWRRMAYFGDTLSHAALLGVALGFLLDISPTIAV
TVGCLLLAVLLVTLQQRQPLASDTLLGILAPSTLSLGLVVLSFMHEVRIDLMAYLFGDLL
AISPADLGWILGGSTLVLVLIVALWRPLLAMTVHEELARVEGLPVATLRMTLMLLIAVVI
AVAMKIVGVLLITSLLIIPAAAAQRHARSPEQMALGASLLGVIAVCAGLSLSWFKDTPAG
PSIVVSAAALFLLSFVLPRRAV
>tr|A0A0Q0IEK3|A0A0Q0IEK3_PSEAP Glutamate--ammonia ligase OS=Pseudomonas syringae pv. aptata OX=83167 GN=ALO85_02129 PE=3 SV=1
MTRNDPHPQQEMLMGIDEVECVTPDLNGVPRGKGMTAEGFLEGRRLQLARGVLLQCIMGG
YPPAHFYGSDDGDLALISDPQQLHRLPWSTSPRALAICDAQDLDGQPCMLSTRNQLKAVI
ARYAAQDLVPVVATELEFFIFAPNPEPLYPFEPPTGLDGRREQGHSAFSVSSNNGLRPFF
AEVYACMAALGLPRDTVMHEMGVSQFEINLLHGDPLLLADQTFLFKHLLKEVALKHGLIV
VCMAKPLAHTAGSSMHIHQSVVAAETAVNVFSDAQGEPTDAFRHFIAGQQACMADFTALF
APNVNSYQRLCHPYASPNNACWSYDNRAAGLRIPSSAAAARRVENRLPGADANPYLAIAA
SLAAGLHGIEQRLQPTPPLQGELEVPEELLLPCTLHAALERLKSSQLAKELFGHEFIEGY
IASKTLELTSFFDEITPWERRVLAAQV
>tr|A0A0Q0CFF5|A0A0Q0CFF5_PSEAP MerR family transcriptional regulator OS=Pseudomonas syringae pv. aptata OX=83167 GN=ALO85_02103 PE=4 SV=1
MKIGELAKLTDTQVETIRYYEREGLLPAPARSDGNYRLYTQAHMERLSFIRNCRSLDMTL
EEIRNLLNLRDSPQDQCENVNALIDEHIEHVNARITSLQALQEQLLDLRRRCSDGTTEHC
AILERLEVTGAVAAPEGEPSHVGRSHGH
>tr|A0A0Q0DKC8|A0A0Q0DKC8_PSEAP Glutamate 5-kinase OS=Pseudomonas syringae pv. aptata OX=83167 GN=proB PE=3 SV=1
MRSKVTGAQRWVVKIGSALLTADGKGLDRNAMGVWVEQMVALHEAGVELVLVSSGAVAAG
MSRLGWTARPSAMHELQAAAAIGQMGLVQAWESSFAEHGRHTAQILLTHDDLSDRKRYLN
ARSTLRTLVELGVVPVINENDTVVTDEIRFGDNDTLAALVANLVEADLLVILTDRDGMFD
ADPRNNPEAQLIYEARADDPALDAVAGGTGGALGRGGMQTKLRAARLAARSGAHTVIVGG

RIERVLARLKGGERLGTLLSPERGMLAARKQWLAGHLQTRGTLVLDDGAVAALAKDQKSL
LPVGVKLVQGSFRRGEMVVCVAPDGREIARGLSNYSAIEAQKIIGHSSESIVRELGYMAE
PELIHRDNLILV
>tr|A0A0Q0FI44|A0A0Q0FI44_PSEAP Uncharacterized protein OS=Pseudomonas syringae pv. aptata OX=83167 GN=ALO85_03188 PE=4 SV=1
MLSHRLHVLSFSAMLFCQSVSAQVLERDLGDFDLKLATTPTRSMAQGLVTPTGGSGSFHG
GMDLTHESGFYFGQWAPNMGLASDTGMELDSYMGFKKPFDNTLGYELGMIRYSYPDTSQI
DSHEFYAGLRMRDSRIGAAFSNDVGTRDSTVFVDLGAIEQLGVGVRMQYANHQFDTPQSA
ADGGLINGFNDWSLNLSRPWLGIDMNLIYSGSSLSGGDCSVYSGHNARCDGTFTLKAVRS
FF
>tr|A0A0N8T905|A0A0N8T905_PSEAP Chorismate mutase OS=Pseudomonas syringae pv. aptata OX=83167 GN=ALO85_03943 PE=4 SV=1
MEVMYTTIEEVRAHIDTIDRDVVALLAKRGKLVNQAAAFKKTTDDVRAPNRVEQVIAKVR
AVANETGASPEVVEQVYRAMIAAFINEELKAHAALANA
>tr|A0A0Q0BRV6|A0A0Q0BRV6_PSEAP Uncharacterized protein OS=Pseudomonas syringae pv. aptata OX=83167 GN=ALO85_04155 PE=4 SV=1
MTDSTDTNHETLTAKEQYSIVIEKSDHLPAAKVVGEFVLDALINSSGESQKIRELREEIK
SAISEADDDKAHSLMVELKTIKDAEQENANALAEISKKFSISQILASYSKDPAFEEIAYG
LALQVLSKTQQALTSPSGKSKPAREKTPPKPALVYVISKGDESAELVMRIGKGAANLAQD
AEAFTLLGFAVEKDTDGKDTLVPATYTDKMGVEHQASRRTIATAIEEKTAFEGYTIVTKE
>tr|A0A0N8T9I9|A0A0N8T9I9_PSEAP Beta-xylanase OS=Pseudomonas syringae pv. aptata OX=83167 GN=ALO85_200065 PE=3 SV=1
MRRASRRSFLTAFVRLLACLPFVNSRLLAAETFTALRQPAAEKGIRFGFAVDPAKLNDDA
AYRQLVALQASIVVPENALKWQTVHPEPERYNFAPADAIAAFAKAHDQRMRGHTFCWHRS
LPDWVHHTVTPMNAEAVLTTHISTVASHYRGLISAWDVVNEAIQLEDGQPDGLRNSFWYQ
MLGPRYLDIAFKAAHKADPDALLCYNDYGLEKDTHYGESRRTAVLALLRGLKQRGIPIHG
LGIQSHLRAGDTFGPGLSRFILAVRDMGLSIHITELDVDDSHLTGSIADRDGSVAATYKR
YLDVVLATRSVSTVITWGVWDSPHVAGATASNGPLAQRALAFGPRGEVKPASWVIAHCFE
NAQMQKDARI
>tr|A0A0Q0C9B3|A0A0Q0C9B3_PSEAP 3',5'-cyclic adenosine monophosphate phosphodiesterase CpdA OS=Pseudomonas syringae pv. aptata OX=83167 GN=cpdA PE=3 SV=1
MNRPFLIAQISDLHLKADGRLTYGVVDTLGALRRAVAHINASKQRPDIVVISGDLVDFGR
EDEYAVLKPELERLQMPFYLVPGNHDDRHNLLAAFAEQVYLPLSASGPLDWVVEKYPVRL
IGMDTTIPGEHGGQLDYCQLDWLHAQLSRRPYVPTVIVMHHPPFITGIGHMDREPFGNAS
ALERVIAQHPQVERLLCGHLHRPMQRRFGGSLASICPGTSHQIVLDLDEAAPAHFNLEPA
GYVLHRWHSEQGFVSHNAVFGDYEGPYPFYDVNGLID
>tr|A0A0N8TA02|A0A0N8TA02_PSEAP AraC family transcriptional regulator OS=Pseudomonas syringae pv. aptata OX=83167 GN=ALO85_00626 PE=4 SV=1
MPTLTLRLGDLSVGFIQSLTDAVNSFDRNPDALLEQYGLDPIRLSQARARLSIPRYMRLG
HAAIQLTGQPGLGLRMGQLSRFAQAGLAGITAAQAPNVREAARTLIRFEPLYGSNYRGQS
SFHEDSAGAWMRFYSISPYNAYNRFVVDSILAGWLAQLSTLAGTELRAQRVEIEFEAPDY
AAQYAVLSDNPVVFGAATNQLRLDQTSLALRNPDHCPSTWRHLLQLCEGELEQQTRTRSL
RERIIQLLGPLLNGGSEPDLEEVAARLKLPTWTLRRKLAEEGTCFRAVLNDTRRDLAMTY
IRDTELAFGEIAYLLGFASAEAFQRAFKRWNGQTPGEFRRSQRQSA
>tr|A0A0Q0IHX0|A0A0Q0IHX0_PSEAP Putative permease OS=Pseudomonas syringae pv. aptata OX=83167 GN=ALO85_02219 PE=4 SV=1
MFNNDRLLVQILLLALFGACLWVMAPFWSALVWGAVLAFASWPLMRLLTRWFKGRESLAA
LVMCLCWMLLVAAPLVWLGFNLADHVRDATGLIKDIQVDGLPDPPQWLAGIPLVGERLVA
LWTTIDQQGAAFMATLKPHLGQVGNWILARSAQIGSGILELTLSIVFAFFFYRDGPRLAA
FVLSLLERLIGERAQYYLDLVAGTVQRVVNGVIGTAAAQAVLALIGFLIAGIPGALVLGI
LTFLFSLIPMGPPLVWLPATAWLVWQGEYGMAIFLGIWGMFIISGVDNVLKPYLISRGGN
LPLVIVLLGVFGGLLAFGFIGLFIGPTLLAVAYSLLTDWVGSERARPR
>tr|A0A0Q0C849|A0A0Q0C849_PSEAP Glycosyl transferase, group 1 OS=Pseudomonas syringae pv. aptata OX=83167 GN=ALO85_04755 PE=4 SV=1
MRIVIDLQGAQSESRFRGIGRYSLSLAQAIARNAGSHEIWLALNNCMSESIPDIRAAFAG
LIPESRIRIFDVPGDANPTPWVARASEVIRESFLSTLKPDAVVVSSLFEGYWANAVTSVG
AVPATHQTAVILYDLIPFMNEDVYLPTAELKDYYHRKIDWLKKADTLLAISDSSRQEAVT
HLGIDQAKIANISAAIGEQFIPDEVNLSKSSAILERLGVKSEFILYAPGGFDPRKNFARL
LEAYSTLPSALKARHQLVIASKLHAQQRMELLAFAEQYGVKASELVLTGYVNDADLIALY

SLTKLFVFPSTHEGFGLPVLEAMACGAAVIGSNCSSVPEVIGFDEALFDPFSVGSIAAKM
LEGLESDGFRSRLLTNAGEQCKKFSWDESAKKAIEALESRHSSVAADQPDLPDPGAALLE
RLISLSDTAPTDIELFQVAKSIAFDFAGADHRQLLLDVSSIVHSDAKSGIQRVVRSLLHE
FLNDQPAGLTVRPIYFADGQYYYANAFASRIYDQFSETTDAIIDFAQDDIYLSLDLNMHL
ASQLYPLHSQMRQMGVQVSYIVYDLLLFHRPDWWVAPNAELFNDWLQKISMVASSLICIS
GAVADELKQWLADNVDKCAGNKPSVKSFHLGADIDNSLPSAGLPDDAGELLARLAQKPSF
LMVSTVEPRKGHAQTLSAFESLWEQGQDINLVIVGKRGWLVDELVARLENHPEKGNRLFW
LEGISDEYLEKIYSASTCLIAASEGEGFGLPLIEAAQYNLPMIIRDLPVFKEIAGTHAFY
FSGLHPTDMSEAIAAWLELHKDSMHPDSSDMPWLTWEQSARQLQAALILSN
>tr|A0A0Q0CVL1|A0A0Q0CVL1_PSEAP 30S ribosomal protein S1 OS=Pseudomonas syringae pv. aptata OX=83167 GN=ALO85_04130 PE=4 SV=1
MVMDYGVFIDLGPMDGLLHNSKIIERNGEVNEIFTQGREVDLYVFSINREKCTLGLSQYP
RQITKLELPTNEVTGKEKTYTVGHQLSGYAKLITETGIHFEDEYGFKGFISNRAISCDPT
KINDIKKQAAEGEELQVKIINLNGKNIYYLEMVEYLRPNLEAYFHFQLERDKAFDGEIVR
VDKDEIFLKIEKDIYAFMMKRNLPEFYMDKNLKEEFEIGQVLSVKSKGYSSKLLNVYADE
I
>tr|A0A0Q0IN26|A0A0Q0IN26_PSEAP Type I secretion outer membrane protein, TolC OS=Pseudomonas syringae pv. aptata OX=83167 GN=ALO85_02873 PE=4 SV=1
MLRKLSLVVAVSFASSGLTWAAELPRPTQTGLLSVYQQAVDNNADLAASRADYDARKEAV
PQARAGLLPNISGSAQNSSTRTSIDQPRAVATRSGNLYQATLSQPIFRADRWFQLQAAEA
SNEQAALELSATEQNLILQSAQSYFSVLRAQDNLASTKAEEAAFKRQLDQANERFDVGLS
DKTDVLQAQASFDTSRASRLIAQRQVDDAFQALVTLTNREYNSIEGIVHTLPVLAPTPND
AKAWVDTAAQQNLNLLASNYAVNAAEETLRQRKAGHAPTLDAVATYQRGDNDALGFSNPN
YTGRNYGGDVEQRSIGVQLNIPIYSGGLTSSQVREAYARLSQSEQRRESLRRQVVENTRN
LHRAVNTDVEQVQARKQSIISNQSALEATEIGYQVGTRNIVDVLDAQRQLYASVRDYNNT
RYDYILDNLRLKQAAGTLNPGDLQDLSRYLKADYNPDKDFLPPDLATAAQKNFERPAKR
>tr|A0A0Q0CYS2|A0A0Q0CYS2_PSEAP Uncharacterized protein OS=Pseudomonas syringae pv. aptata OX=83167 GN=ALO85_01334 PE=4 SV=1
MDSSMPSRFTRRPQLRQLIAASSLVALVACAEKPTAADATPLQSSKVQTNAPAVAPTPAL
VVDDNLTIQPAVSFSEWQAGFRAQALKAGIRADVFDLAFAGVTPDMSVVKADRSQPEFSR
PVWEYLDGAISAARVRKGQALLSQYADDLQKIEQQYGVDRQALVAVWGMESNFGSFQGTQ
SVIRSLATLAYEGRRPGFAQSQLLAALEIIQHGDITPDKMLGSWAGAMGQTQFIPTTYNT
HAVDFDGDGRRDIWNTPADALASTAHYLQSSGWQRGQPWGFEAVLGSGFDYSLADSTTRK
SLAEWQQLGLKQPDGSAIPVAASQQQAALLLPAGYRGPAFLVLDNFRAILKYNNSTSYAL
AISLLSDRFKGAGYVVGSWPRGDLPLSRSERIELQTLLSARQYDAGAPDGIIGANTRKAI
RSAQQSFGWPADGYPTHELLENLRKPVGQ
>tr|A0A0N8T883|A0A0N8T883_PSEAP RsbU family regulator of sigma subunit OS=Pseudomonas syringae pv. aptata OX=83167 GN=ALO85_04785 PE=4 SV=1
MKHPSDGSTVCALKGTSETRQTYQVVACYCGEARSESSKWLPVTPGRLRQPDLAPFAPMP
KTSAKLLIIDDDDVVRASLAAYLEDSGFSVLQASNGLQGIQIFEQENPDLVVCDLRMPQM
GGLELIRQVTAIAPQTPVIVVSGAGVMSDAVEALRLGAADYLIKPLEDLAVLEHSVRRAL
DRARLLTENQVYREKLEKANRELEASLHLLQEDQDAGRQVQMNMLPITPWSIDAFKFAHQ
IIPSLYLSGDFVDYFRVDERRVAFYLADVSGHGASSAFITVLLKFMTTRLLFESKRGGTL
PEFKPSDVLGHINRGLISCKLGKHVTMVGGVIDEESGKLTYAVGGHLPLPVLYSEGQAHY
LEGRGLPVGLFNEATYQDHEIDLPESFSLTMLSDGILDLLPGDTLKEKEAVLPELVKTAG
GSLDGLQQVFELATLGEMPDDIALLVLSRNLE
>tr|A0A0Q0ILY2|A0A0Q0ILY2_PSEAP 60 kDa chaperonin OS=Pseudomonas syringae pv. aptata OX=83167 GN=groL PE=3 SV=1
MIMAAKEVKFGDSGRKKMLAGVNVLADAVKATLGPKGRNVIIEKSFGAPLITKDGVSVAK
EIELKDRFENMGAQLVKDVASRANDDAGDGTTTATVLAQAIVNEGLKAVAAGMNPMDLKR
GIDKATIAIVAELKKLSKPCTDTKAIAQVGTISANSDHSIGDIIAEAMEKVTKDGVITVE
EGSGLENELSVVEGMQFDRGYLSPYFINKPDTMVAELDSPLLLLVDKKISNIREMLPVLE
AVAKAGRPLLIVAEDVEGEALATLVVNNMRGIVKVAAVKAPGFGDRRKAMLQDIAVLTGG
TVISEEIGLSLETTTLEHLGNAKRVILNKENTTIIDGAGVKTDIDSRISQIRQQIGDTSS
DYDKEKLQERLAKLSGGVAVIKVGAGSEVEMKEKKARVEDALHATRAAVEEGVVPGGGVA
LVRSLQAIEGLKGDNADQDVGIALLRRAVEAPLRQIVANSGDEPSVVVDKVKQGSGNYGY
NAASGEYGDMIEMGILDPAKVTRSALQAASSIASLMITTEAMIADVPDDKPAGGGMPDMG
GMGGMGGMM
>tr|A0A0Q0DCH0|A0A0Q0DCH0_PSEAP Putative lipoprotein OS=Pseudomonas syringae pv. aptata OX=83167 GN=ALO85_01561 PE=4 SV=1

MPNRLLLILITAFTLATLAGCAGPLPAVNPDMAWVDMRTITGQLIMGDKLDGKNTYDGRYF
QVTPGSHELQVRYDYEYRSGGMGMIGDEYTEITCYVSVRYDHFAAGQRYVLEVRSLANSV
DAWLYDAERKVVAEEEEEGGVHCI
>tr|A0A0Q0DJF9|A0A0Q0DJF9_PSEAP Uncharacterized protein OS=Pseudomonas syringae pv. aptata OX=83167 GN=ALO85_00548 PE=4 SV=1
MSEPKSHQSNTRRELRKALIRLRMEMHRQEIRHESTQLMQPLQRFRGIRHDWKSALGFRH
APLWGVGSVALLGFLSGKRAKTGRTSLLGRLLKLGITLTPVVKVAMQARALQNRNRKV
>tr|A0A0Q0CZ71|A0A0Q0CZ71_PSEAP Acyl carrier protein OS=Pseudomonas syringae pv. aptata OX=83167 GN=ALO85_02840 PE=4 SV=1
MSDLHQDIKLLIIDALGLEDITPEDIGNDMTLFGEGLGLDSVDALELGLAIQKRYGIRID
ADAKDTRNHFASVDSLAAFVSARQPA
>tr|A0A0Q0CA74|A0A0Q0CA74_PSEAP ParB-like nuclease OS=Pseudomonas syringae pv. aptata OX=83167 GN=ALO85_100431 PE=4 SV=1
MSNRKLSPDKTQPIYVPNNAFLDECVRVEIINILPLKVLRPAVKESTKYHQILASIQDVG
LVEPPAVTPAPGTSGQYFLLDGHLRIEVLKDLGELKVDCLVATDDDTYSYNKRTNRLSAV
QSNKMIVRAMERGVSAERLGKALGLSPQTIRQRFRLLNGICAEVVALLGDTDCPANVFNL
LRQMKPLRQIEAAELMIGNKNLSVLLAKALLAATPPDQLVMIKKPSQDHQVSAESMARLE
RELAALQMQIKTVEEDYGPDVLHLTVIKGYLTKLLANAAVVRWLARHQPEYLKEFQSIAE
LTELPWADNGPEDDSQSTTAEG
>tr|A0A0Q0DCN5|A0A0Q0DCN5_PSEAP Cell division protein FtsL OS=Pseudomonas syringae pv. aptata OX=83167 GN=ftsL PE=3 SV=1
MSRLFLKPLPGGSFIMLLLFIAVLISAIGVSYSAHWNRQLLNSLYSEMSVRDKAQAEWGR
LILEQSTWTAHSRIETLATEQLKMRIPSAAEVRMVAP
>tr|A0A0Q0BVU1|A0A0Q0BVU1_PSEAP Putative Transposase OS=Pseudomonas syringae pv. aptata OX=83167 GN=ALO85_00374 PE=4 SV=1
MSYRGIVLSEDEEVELNRRFESITVSQREGRRAQVILLAAQGCSRNEIARLTGLSVVSVT
RWCKRFRELRLQGLVDLPGRGRKSSLPAEALRKALDQVTQPCVGHARWSCRSMAKLVGIS
PASVQRIWAANNIRPHLAEVSELHSGSDVEEKSSKGGGGISETPE
>tr|A0A0Q0IIX8|A0A0Q0IIX8_PSEAP Carboxylesterase OS=Pseudomonas syringae pv. aptata OX=83167 GN=ALO85_01818 PE=4 SV=1
MSDSLIIEPSSPADACVIWLHGLGADRYDFLPVAEALQESLRSSRFVLPQAPTRAVTVNG
GYAMPSWYDIKSMSSEARAIDHDQMEASAQKVLDLIEQQRDSGIDPARIFLAGFSQGGAV
VLHAGYRRWQGPLGGVLALSTYAPTFSSEMTLSASQQRIPAYCLHGKHDAVVPYPMGRAV
YDHLTAQGVTVEWQEYPMEHQVLPEEIRDIGVWLAERLR
>tr|A0A0Q0CC16|A0A0Q0CC16_PSEAP Biopolymer transport protein ExbD/TolR family OS=Pseudomonas syringae pv. aptata OX=83167 GN=ALO85_02528 PE=3 SV=1
MKFRRRRPRENIDINLVSLIDVVFILLLFFIVTTTFTRETQLRVDLPQAVTGTPDVDASA
KQLDIAINADGIFSLNNQLLPKNDLATLIEALQRESGGDTSLPLSISADGKTPHQSVITA
MDAAGKLGFSHLRMTTVEATPAN
>tr|A0A0Q0DUE6|A0A0Q0DUE6_PSEAP Type IV pilus bioproteinsis protein PilB OS=Pseudomonas syringae pv. aptata OX=83167 GN=ALO85_03297 PE=4 SV=1
MSGGFMTDAVLTGLAKQLVLAELLTEPVALQAYQQARRDKISLVSYLVQNGLVKSLTLAE
MASDQFGVPFMDLASLDKESQPKGLVSEKLVRQHHALPLWRRGNKLFIGISDPTNHQAVT
DIQFSTGLNTEAILVEDDKLTTAIDRFFDNDSGLGNLEDVDLGLDIEPADGKETSLATQN
DADDAPVVRFVNKMLMDAIRLGSSDLHFEPYEKIFRVRLRTDGILHEVARPPIHLANRIA
ARLKVMASLDISERRKPQDGRVKLRVSKTKAIDFRMNTLPTLWGEKIVMRILDPTSAQMG
IDALGYEPEQKALYLEALKQPQGMILVTGPTGSGKTVSLYTGLNILNTVDINISTAEDPV
EINLEGINQVNVNPRQGLDFSQALRAFLRQDPDVIMVGEIRDLETAEIAIKASQTGHMVL
STLHTNSAAETLTRLHHMGVAAFNIATAINLIIAQRLARKLCSHCKKELDIPRDTLIQEG
FPEAKIGTFKIFGPVGCEHCNGGYRGRVGIYEVVKKTPELERIIMEEGNSLEISRQMRKD
GFNDLRTSGLSKAMQGITSLEEVNRVTKD
>tr|A0A0Q0C917|A0A0Q0C917_PSEAP Protease inhibitor Inh OS=Pseudomonas syringae pv. aptata OX=83167 GN=ALO85_00702 PE=4 SV=1
MKLNRFFQFAPAALALLVSLSGACMAMSLKLPSPAELSGKWHLFIHSQADRACELQLNTE
APQLGGDTACATKWLNEAPVGWFPTPDGLALTDNEGNRLIHFNHMGEQRYEARLPSGQVL
RLERAAE
>tr|A0A0N8T823|A0A0N8T823_PSEAP Transcription elongation factor GreA OS=Pseudomonas syringae pv. aptata OX=83167 GN=greA PE=3 SV=1
MIKYPMTVQGARALEEELTHLTKVIRPKLSQDIGTARELGDLKENAEYHAAREQQGMVEA

RIRDIEGRMQNAVIIDVTTIPHTGKVIFGTTVEIANVETDESVVYQIVGEDEADIKKGKI
SVGSPIARALIAKEEGDVVVVKTPSGVIEYEIVEVRHI
>tr|A0A0Q0CD32|A0A0Q0CD32_PSEAP Dipeptide/oligopeptide/nickel ABC-type transport system OS=Pseudomonas syringae pv. aptata OX=83167 GN=ALO85_00900 PE=3 SV=1
MTAIASLSHSAILPRSRSPFLKKFLANKGAVLGAAVLLLFVLAAVLAPWIAPHDPLKANF
LAVRKAPSLIYWMGTDELGRDLFSRLLYGARSSLLAGGVSVAIAMFIGVPLGLLAGYFGG
KLDMIISQVMEALLSCPFLVLAIALGAFLGPSLTNAMIAIGLSAMPIFARLTRGQVLSIK
HEDYLEGARAIGLPDRWIILRYVLPNVMSPLVVQATLTIASAILAEASLSFLGLGQQPPS
PSWGSMLNTAKNFMEQAPWMSIAPGVAIYITVLCFNLLGDGLRDALDPKG
>tr|A0A0Q0BTU2|A0A0Q0BTU2_PSEAP Phospholipid/glycerol acyltransferase:phospholipid/glycerol acyltransferase OS=Pseudomonas syringae pv. aptata OX=83167 GN=ALO85_01079 PE=4 SV=1
MSQQSQFSLLGKRRFLPFFITQSLGAFNDNIFKQSLILAILYKLSIDGDRSIYVNLCALL
FILPFFLFSALAGQFGEKYPKDKLIRIIKFGEIVIMVVGAAGFLFNHLELMLAALFAMGT
HSALFGPVKYSILPQHLRESELVGGNALVEMGTFLAILAGTISAGVMMSSSHYVWVVAAA
IVLVACLGFLASFGIPRADAASPEMKLNWNIFTQSWATLRMGLGQTPAVSRSIVGNSWFW
FVGAIYLTQIPAYAKEWMYGDETVVTLILAVFSIGIALGSLLCERLSGHKVEIGLVPFGS
MGLTIFGLLLWWHSGGFPQNVQANDWLAVLSSGQGWLVLFDILGIGVFGGFYIVPLYALI
QSRTPLKERSRVIAANNILNALFMVVSAIVSILLLSVAKLSIPQLFLAVSVMNIAVNIYI
FKIVPEFTMRFMIWLLGHSMYRVEHRNLDRIPDEGAALLVCNHVSFVDALLIAGAVRRPI
RFVMYYKIYQLPVLNFIFRTAGTIPIAGRNEDMDIYEKSFKRIAQYLAEGELVCIFPEGK
LTTDGEINGFKNGMSRIIQETPVPVIPMALQGLWGSFFSRDPSKTLFRRLWSRVVLVAGS
PIAADVATPVDIREEVKALRGKVQ
>tr|A0A0Q0FV76|A0A0Q0FV76_PSEAP 2Fe-2S iron-sulfur cluster binding protein OS=Pseudomonas syringae pv. aptata OX=83167 GN=ALO85_101044 PE=4 SV=1
MALLKRLTEHDRPVLPFTLDGQPASGLLGDTLLTAVLTASDHLRGSDFSAEPRAGFCMMG
ACQDCWVRLGDGQRVRACSTLLEAGQAVIREPGRCV
>tr|A0A0Q0DRE2|A0A0Q0DRE2_PSEAP Argininosuccinate synthase OS=Pseudomonas syringae pv. aptata OX=83167 GN=argG PE=3 SV=1
MADVNKVVLAYSGGLDTSVILKWLQDTYNCEVVTFTADLGQGEEVEPARAKAQAMGVKEI
YIDDLREEFVRDFVFPMFRANTVYEGEYLLGTSIARPLIAKRLIEIANETGADAISHGAT
GKGNDQVRFELGAYALKPGVKVIAPWREWDLLSREKLMDYAEKHNIPIERHGKKKSPYSM
DANLLHISYEGGVLEDTWTEHEEDMWRWTKSPEDAPNVATYLELTYRNGDIVALDGVEMT
PATVLATLNRIGGENGIGRLDIVENRYVGMKSRGCYETPGGTIMLRAHRAIESITLDREV
AHLKDELMAKYASLIYTGYWWSPERLMLQQMIDASQAHVNGVVRLKLYKGNVIVTGRKSD
DSLFDANIATFEDDAGAYDQADAAGFIKLNALRMRIAANKGRKLF
>tr|A0A0N8T923|A0A0N8T923_PSEAP Amidophosphoribosyltransferase OS=Pseudomonas syringae pv. aptata OX=83167 GN=purF PE=3 SV=1
MCGIVGIVGKSNVNQALYDALTVLQHRGQDAAGIVTSHDGRLFLRKDNGLVRDVFHQRHM
QRLVGHMGIGHVRYPTAGSSTSAEAQPFYVNSPYGITLAHNGNLTNVEQLAKEIYESDLR
HVNTNSDSEVLLNVFAHELAVRGKLQPTEEDIFAAVTDVHNRCRGGYAVVAMITGYGIVG
FRDPDAIRPIVFGQRHTDEGVEYMIASESVSLDVLGFTLIRDLAPGEAVYITEDGKLHTR
QCAANPKYAPCIFEHVYLARPDSIIDGISVYKARLRMGEKLADKILRERPDHDIDVVIPI
PDTSRTAALELANHLGVKFREGFVKNRYIGRTFIMPGQAARKKSVRQKLNAIELEFRGKN
VMLVDDSIVRGTTCKQIIQMAREAGAKNVYFCSAAPAVRYPNVYGIDMPSAHELIAHNRT
TQDVADLIGADWLVYQDLNDLIEAVSGSKKIKIDNFDCSVFDGKYVTGDIDEHYLNRIES
ARNDASKIKTQAVSAIIDLYNN
>tr|A0A0Q0BV91|A0A0Q0BV91_PSEAP ATP-binding region, ATPase-like:Histidine kinase, HAMP region:Histidine kinase A OS=Pseudomonas syringae pv. aptata OX=83167 GN=ALO85_04629 PE=4 SV=1
MSLPNPSDGWRSSTSRLLALYSSLFVAWSVILLGVLYWEVTSYLDTLARQSLTHRQQLFS
RFEGQGLVDALATSMTFDTRSVDAYGLFDENFRPVAGPINSVPKNLPIDGQIHVLNDCIE
SDDPGMPQGSCDAIVNRTSDGRWLVLVRENGSLFAVSTLILHALLWGISLTIIPGVAGWH
LLRRRPLRRIRAIQASAESIIAGDMAGRLPVSNRRDELDMLAVIVNAMLDRIEKLMNEVK
GVCDNIAHDLRTPLTRLRAQLYRIQQQSAHDSTHATQIAQVISEADTLMARFRGLLRISE
LEDRQRRSAFVELELRPLLQELHDFHLPLAEEDQITLILDLHETLPTLVGDRALLFEALT
NLLGNAIKFTPTGGVVKVSAYPAPGGTTPCIEVEDSGPGIPVAERAAVFQRFYRSEAGTE
QSGFGLGLSVVAAIVNLHGFTLQIGTSEYGGASLLLECRTVNTLA

>tr|A0A0Q0BYG2|A0A0Q0BYG2_PSEAP Flagellar hook-associated protein 2 OS=Pseudomonas syringae pv. aptata OX=83167 GN=ALO85_02158 PE=3 SV=1
MASPISASTGLGSGLAIGNIVTVLVDAEKTPKQNQIDKQSASTTASLSGVSQLTSALAAF
QKTLDTLASSTTPAFQGFAATSSNEAVVKATAGNTAVNGTYAINISQLATPSKVATAALS
STQASAIPSGTLKITQNGTDYNVAIDKTSTLQEVRDKVNSTLQGKGITANIINDNNGSRL
VFSSTTTGKGSDISVTGSSGQEALNIDGTKLMSASGTGAGAITDVAKDAAFTVDGLSLTS
KTNTVSTAISGLTFELVAPSASADPSASTKVTVATNTDGLKTSLQSFVDSYNTLVSLVSS
LTKGTVDSQGKYTAALTGDSTPRALLATIRDQIATATSNAGLSSLSQLGIKTQQSDGKL
TLSTTELTTALSDKKLGSQIQAMFTGVTNADGTSSGGLIARMNTALEPYTKADGVLAGKT
TSLNKVQTRLANDQEALNRRITTLTATLTKKYNAMDLVVGQLKATASSITSIFEAMNAQK
NAS
>tr|A0A0Q0D9H6|A0A0Q0D9H6_PSEAP tRNA-cytidine(32) 2-sulfurtransferase OS=Pseudomonas syringae pv. aptata OX=83167 GN=ttcA PE=3 SV=1
MGTLSVNQNKLQKRLRRLAGEAVADFNMIEEGDKVMVCLSGGKDSYTMLDVLMHLQKVAP
IKFDIVAVNMDQKQPGFPEHVLPAYLKELGIEYHIVEKDTYSVVKELVPEGKTTCSLCSR
LRRGTLYTFADEIGATKMALGHHRDDIVETFFLNMFFNGSLKAMPPKLRADDGRNVVIRP
LAYCHEKDIQAYSDLKQFPIIPCNLCGSQENLQRQVVKEMLQDWERKTPGRTESIFRALQ
NVQPSQLADRNLFDFSNLRIDETAASRFVNVVNI
>tr|A0A0Q0DC73|A0A0Q0DC73_PSEAP FecR protein OS=Pseudomonas syringae pv. aptata OX=83167 GN=ALO85_00525 PE=4 SV=1
MSDPLFSNAEHDAITDAAAHWCMRLHAEDCTAAERKAFEQWLKAHPLHAIEYNAMLEVWD
IAGQIEPLNPAPVLQAHAARPDQVRPRRGRRWAKLAAAAAVVAMALPIAGYVGWHQGWLP
DAYETYNADTATRLVMLEDGSRAELNLGTELTYANYRDRRSVTLTKGEAFFEVSHDSTHP
FVVDAGAGSIRVTGTRFNVWMYQDQVRVTLVEGSVQINSDRAHPSSSHALAPGMQAIYKT
GDDAPLISQTEARDTSLAWRNGKLIFNDLPLSQALPLINRYLQTPILLADSATGAMRLGG
SYNTHDLSSLLGSLPKVLPVYVTQNQNGNPVLNRRTSDTPKG
>tr|A0A0Q0D4A4|A0A0Q0D4A4_PSEAP MoxR-like ATPase OS=Pseudomonas syringae pv. aptata OX=83167 GN=ALO85_02450 PE=4 SV=1
MGRKLDACLSAINEVVLGKEAQVRLAMTCLVGGGHLLIEDLPGMGKTTLSHALAKILGLS
YQRIQFTSDLLPGDILGTSVFDRDSGQFTFHPGPIFAELVLADEINRATPKSQSALLEAM
EEGQVSIEGATRLLPDPFFVIATQNPFSSGGTFALPESQLDRFMMRISMGYPARAAEKAL
LLGESRRELLPRLQPILDHAQLGQLQAQARQVRASDALVDYVLRLVEATRSQPQFAYGLS
PRASLAILAAARAWAMLLGRDYVIPEDVQAVLPSVIGHRLRERSDPTGHGGGALVQWLLR
EVPVL
>tr|A0A0Q0CZS6|A0A0Q0CZS6_PSEAP DTW protein OS=Pseudomonas syringae pv. aptata OX=83167 GN=ALO85_100155 PE=4 SV=1
MSRARCERCLRPATHCLCALIPQLDSRTRVLILQHPDEVSHALNTARLAALGLLNAQLLV
GEVFDDLQRILNPPGYQPRLLFPGEAAQTLTPYAQDSLPMLLVVPDGTWRKARKLLHLNP
LLAELPRVTPGDVPVSRYRLRKAPGPEALSTLEAIVHTLQTLEAPTSFEALLKPFDALIE
GQIQAMGSETYQRNHGGQG
>tr|A0A0Q0C7W4|A0A0Q0C7W4_PSEAP RpiR family transcriptional regulator OS=Pseudomonas syringae pv. aptata OX=83167 GN=ALO85_02821 PE=4 SV=1
MDILYQIRARQDSLSAGEGRIARLLLSDIAFAASASLDELASRAEVSSATLSRFARSIGC
RDLRDLRLQLAQASGVGSRFLHPEPAPEQSAFFTRIVDDIEATLRQHLAGFEATRFAAAI
ACVADARMIHTFGMGGCSSLCSEELQTRLVRLGYPVAACRDPVMMRMVAATLGPQHSLIV
CSLSGRTPELLDAVTLARSYGARVLAITLPDSPLAQLAEVVLPLQIAETNFIYKPTAARY
GMLLTIDVLATELALASPEDNQERLRRIKLALDEYRGGEDGLPLGD
>tr|A0A0Q0CAA3|A0A0Q0CAA3_PSEAP ATP synthase subunit delta OS=Pseudomonas syringae pv. aptata OX=83167 GN=atpH PE=3 SV=1
MAELTTLARPYAKAAFEHAQAHQQLANWSAMLGLAAAVSQDDTMQRMLKAPRLTSAQKAT
TFIEVCGEKFDAKAQNFIHVVAENDRLPLLPEIAELFDLYKAEQEKSVDVDVTSAFALNQ
EQQDKLAKVLSARLGREVRLHAAEDASLIGGVVIRAGDLVIDGSVRGKIAKLAEALKS
>tr|A0A0N8T8C8|A0A0N8T8C8_PSEAP Cystine ABC-type transport system, permease protein OS=Pseudomonas syringae pv. aptata OX=83167 GN=ALO85_01679 PE=3 SV=1
MIEESLQLALDSAPFLLKGAYYTVFLSIGGMFFGLLLGFGLALMRLSRIKPVNWIARIYV
SFFRGTPLLVQLFVIYYGLPELGIQLEPLSAALIGFSLNMAAYACEILRAAISSVERGQW
EAAASIGMTRWQTMRRAILPQAARTALPPLGNSFISLVKDTALAATIQVPELFRQAQLIT
ARTFEIFTMYLAAALIYWILATALSHLQNKLEARVNRHDLET

>tr|A0A0Q0C580|A0A0Q0C580_PSEAP Hydroxypyruvate isomerase OS=Pseudomonas syringae pv. aptata OX=83167 GN=ALO85_02951 PE=3 SV=1
MPRFAANLSMLYPQHDFLERFAAAAADGFGAVEYLFPYAYSTQELKQRLSDNGLVQALFN
APPGDWEAGERGIATLPGRETEFRSGIERALEYAQVLGNDRVHVMAGLLPNESLRERHQA
VYLENLAFAAEQAKGAGVTILIEPINTRDMPGFFLNRQDQAQDICKLVGADNLKVQFDCY
HCQIVEGDLTSKLRRDFAGIGHIQIAGVPDRHEPDLGELNYEHLFNVIDELGYSGWIGCE
YRPKGDTSAGLAWLRALQARG
>tr|A0A0Q0I9Z4|A0A0Q0I9Z4_PSEAP Uncharacterized protein OS=Pseudomonas syringae pv. aptata OX=83167 GN=ALO85_04050 PE=4 SV=1
MKLALAQLIAVLASIGLGEAGQRTADLAYNEAGIIALLLGTILMMAAFAREVFEVLREKS
LI
>tr|A0A0Q0DEL0|A0A0Q0DEL0_PSEAP Nicotinate phosphoribosyltransferase OS=Pseudomonas syringae pv. aptata OX=83167 GN=pncB PE=3 SV=1
MSESAFSNRIVQSLLDTDFYKLTMMQAVLHNYPNVDVEWEFRCRNGEDLRPYLDEIKHQI
ELLCELSLSPEHLAFLERITFIKPDFLRFLGLFRFNTRYVKTTIENDELCIRLHGPWLHV
ILFEVPLLAIVSEVRNRHRYPDTLLSQARNRLYDKFEWLTTHATTDELAELKVADFGTRR
RFSYRVQEEMLGVLKNDFPGQFVGTSNVHLARQLDLKPLGTMAHEWIMAHQQLGPRLIDS
QIAALDCWVREYRGLLGIALTDCITTDAFLNDFDLYFAKLFDGLRHDSGDPVKWAEKCIS
HYQKLGIDPMSKTLVFSDGLNLPKALDIFRALRGRINVSFGIGTNLTADIPGIAPMNMVL
KMTACAGQAVAKISDEPGKTQCKDPNFVAYLRHVFKVPNLPSPEKPA
>tr|A0A0Q0FFL9|A0A0Q0FFL9_PSEAP MaoC-like dehydratase OS=Pseudomonas syringae pv. aptata OX=83167 GN=ALO85_03085 PE=4 SV=1
MMTAHWRYLNSLQPLSNLFIQAALRRKVTGMQLPDLGLRSWIAVDTDKLEAYRKVCGFEE
SSLLPPTYPHVLAFPLQMQLMTSEEFPFPLLGLIHLANRTRTHRPLGGVSQLYISVQATD
LRPHAKGATFTLVTQAEDGMGLLWEEESTMLCTAVHLEDSPVSYPEAAPLPLSELQGWRA
TAQIGREYAKVSGDYNPIHLSAPSARLFGFPRAIAHGMWLKARTLAALGEHLPASNVEIS
VQFQKPVRLPADVTLSASAAGSQGQFRVEGQEGIVHMVGSWQPLSE
>tr|A0A0N8T7X5|A0A0N8T7X5_PSEAP Co-chaperone protein HscB homolog OS=Pseudomonas syringae pv. aptata OX=83167 GN=hscB PE=3 SV=1
MGTPCHFALFDLKPEFQLDLDQLATRYRELARNVHPDRFADAPEREQRVALERSASLNEA
YQTLKSPPKRARYLLAMNGNEVPLEVTVHDPEFLLQQMQLREDLEDLQDEADLAGVATFK
RQLKIAQDELNQSFAACWNDAAQREHAEKLMRRMQFLDKLSHEVRQLEERLDD
>tr|A0A0Q0C4Z7|A0A0Q0C4Z7_PSEAP Glycerol-3-phosphate ABC transporter, ATP-binding protein OS=Pseudomonas syringae pv. aptata OX=83167 GN=ALO85_200207 PE=3 SV=1
MNALTPITCAPDIVLKGLHKTYGTEPILHGLDLVFKAGELNVILGPSGCGKSTLLRMVAG
LEEASQGQILMGGRDVTRLPPKERGCAMVFQNYALYPHMSVADNIGYALKLARVARKERE
QRIRECAASLGLEDLLQRKPGELSGGQRQRVAIGRALIRKLPVLLFDEPLCNLDSALRHE
MRLLIRSLHRQTGTTILYVTHDQTEAMTLAERVVILDKGRVEQIGTPAEIYDQPASTFVA
SFIGTPPMNLLPVHCLDDRVLMLADGQRLPIELGIASNRTGKWLMGLRPETFMLDQEGVT
ATVESSENLGSHTLLYCQLAGTRCVVSLAGRQHQIPGTQVLLAINSTPLLFDIETGQRQL
VTFSHTTK
>tr|A0A0Q0BVX6|A0A0Q0BVX6_PSEAP Diaminopimelate decarboxylase OS=Pseudomonas syringae pv. aptata OX=83167 GN=ALO85_04373 PE=3 SV=1
MPINVEDYYSAETFKKMKAFADKQETPFVVIDTDIISKAYDDLRAGFEFAKVYYAVKANP
AVEVINLLKEKGSSFDIASIYELDKVLSCGVGPERMSYGNTIKKSKDIRYFYEKGVRLFA
TDSEADLRNIAKAAPGSKVYVRILTEGSTTADWPLSRKFGCQTDMAMDLLILARDLGLVP
YGISFHVGSQQRDISVWDAAIAKVKVIFERLKEEDGIVLKLINMGGGFPANYITRTNSLE
TYAEEIIRFLKEDFGDDLPEIILEPGRSLISNAGILVSEVVLVARKSRTAVERWVYTDVG
KFSGLIETMDEAIKFPIWTEKKGEMEEVVIAGPTCDSADIMYENYKYGLPLNLAIGDRMY
WLSTGAYTTSYSAVEFNGFPPLKSFYL
>tr|A0A0Q0DJW3|A0A0Q0DJW3_PSEAP Thymidylate kinase OS=Pseudomonas syringae pv. aptata OX=83167 GN=tmk PE=3 SV=1
MTGLFITLEGPEGAGKSTNRDYLAAQLRAQGVQVLLTREPGGTPLAERIRELLLAPSDEA
MSADTELLLVFAARAQHLAEVIRPALARGEVVLCDRFTDATYAYQGGGRGLSHERIAALE
QFVQGDLRPDLTLVFDLPVEIGLSRAAARGRLDRFEQEGRAFFDAVRSTYLERAKAEPTR
YRLVDAGQTLADVQASLDTLLPQLLELQRG
>tr|A0A0Q0D1K1|A0A0Q0D1K1_PSEAP Putative transporter-like membrane protein OS=Pseudomonas syringae pv. aptata OX=83167 GN=ALO85_00381 PE=4 SV=1

MARTQPTQPVTELERTTMRRVAWRLLPFLILCYLIAIIDRGNIGMASLQMNHDLGLSPAI
FGFASSLFFVSYFLVEVPSNLALQKFGARIWIARIMITWGLISAGTAFVQGANSLYVMRF
LLGAAEAGFFPGVLLYMTYWLPSAYRARMVAIFMVAIPGANFLGSPLSGYLLTLDGWMGM
RGWHWLFILEGIPAVLLGIACLFILTDRPAQARWLDDEQRTWLVNKLDEERKLKTNIGHI
SLWKLLKHKDIWVMALIYSGASAAGSTMSVWAPQLLKSFGLSNLEIGFVNAIPYGIASVA
MIIWGRSSDRSNERRWHTSATLFLITAGLLLALVTSALPATVVMLTMVLIGAYSMKGPFW
ALVSNWLSSSTAAAGLAAIGALANLIGGGIMVNAYGAIHQATGSYAIALLPLAALCTAGA
IAVVVMGRSRKREAHEDAKSVEVN
>tr|A0A0Q0ISL0|A0A0Q0ISL0_PSEAP Ribonucleotide reductase, alpha subunit
OS=Pseudomonas syringae pv. aptata OX=83167 GN=ALO85_02569 PE=4 SV=1
MRLKFAVATVALLSIPMGSAMADTFWRNVLSSGATTGSTYLTFKDHKLIVAAQDDAGSFV
ASDGGIRGPFLEAAIEQVRKDNPGLKATDMELANAILDKNLVAENQ
>tr|A0A0Q0C373|A0A0Q0C373_PSEAP Extracellular solute-binding protein
OS=Pseudomonas syringae pv. aptata OX=83167 GN=ALO85_05520 PE=4 SV=1
MLAQYLFLIVCRATSGPAKAPSPTRRCFMLSLSKTLAALLLCGAASLAQAAETAICYNCP
PEWADWGTQLKAIADSTGVQVPLDNKNSGQALAQIVAEQAAPVADVVYYGVTFGLQAQKA
GVVDTYKPKHWDEIPAGLKDPEGHWFAIHSGTLGIMVNVDALGGLPVPQSWADLLKPEYK
GMVGYLDPSSAFVGYVSAVAINQAMGGTLDNFGPAIDYFQKLAKNSPIVPKQTAYARVLS
GELPILVDYDFNAYRARYKDKANVAFVIPKEGTIGVPYVMSLVAKAPHRANAEKVLDFVL
SDEGQALWAKAYLRPVRSKMPAEVAAQFLPDSDYARSGVVDYQHMAAVQEAFAARYLREV
K
>tr|A0A0N8T805|A0A0N8T805_PSEAP Putative lipoprotein OS=Pseudomonas
syringae pv. aptata OX=83167 GN=ALO85_00532 PE=4 SV=1
MKQRTLPAAFLLALSIASLAGCASPTVITLNDGREIQAVDTPKYDEDSGFYEFKQLDGKQ
TRINKDQVRTVKDL
>tr|A0A0N8T7W0|A0A0N8T7W0_PSEAP Sensory box domain protein OS=Pseudomonas
syringae pv. aptata OX=83167 GN=ALO85_04247 PE=4 SV=1
MTSSSQRLALLEAVVEQSFNAVLITDANLANGGPFIVYVNPAFCSMTGYTAEALIGASPR
ILQGPDTDPQVIERMRECLSESLFFEGSTVNYRADGSPYIVEWKISPVRDDAGTVCNFVS
VQQNISPRIRAEREQHLLAQALNAALDPIIITDCNSTIVFANEAFQQITGYSSSEILGEN
PRMLSSGKHDEAFYAQFKEALKSGAPLRTTFINKRKDGSLFYAEHSISPLCNLEGAVTHY
VSISQDVTTRLGREQKLLEIAHSDPLTGLDNRRSAEHTLERQIPAVSMSGKPFSLIICDI
DHFKAVNDRYGHPAGDSVITSVAAILKQRIRLLDVAARWGGEEFLILVPDSSLKQAAELA
ERIRKSVESLVIPETGSVTISLGVAELSAGETAASLIQRADKALYQAKRLGRNRVECA
>tr|A0A0Q0IB20|A0A0Q0IB20_PSEAP Two-component response regulator
OS=Pseudomonas syringae pv. aptata OX=83167 GN=ALO85_00511 PE=4 SV=1
MPDTARTILVVEDDAIVRMLIVDVLEELEYRVLEAEDATSALTFVADDSNHIDLLMTDQG
LPDMKGTALAKKVIELRPQLPVLFASGYSENIDVPPGMHSIGKPFSIDQLRDKVKSILGN
SLD
>tr|A0A0Q0C3I7|A0A0Q0C3I7_PSEAP Uncharacterized protein OS=Pseudomonas
syringae pv. aptata OX=83167 GN=ALO85_03298 PE=4 SV=1
MKWLRRSIWLVVFVALGIGALSLYYVLPRHDVVMITGVEVKRMDADGVINAENPADGPTR
DVYFINTEDPDSKKVVVYRNEDTGWSFPWYFKFDSADIQAKAQGYSRDSQQLALIRYYGW
RITILSMFPNVTEVEAVTSRDQPFPIFNTIFFVVVGLLVVMVVVGVRRRFKGRARVDGVV
R
>tr|A0A0Q0D3K4|A0A0Q0D3K4_PSEAP Cyclic pyranopterin monophosphate
synthase OS=Pseudomonas syringae pv. aptata OX=83167 GN=moaC PE=3 SV=1
MLTHLDSQGRANMVDVTDKAVTSREAVAEAFVRMLPTTLQMIVSGGHPKGDVFAVARIAG
IQAAKKTSDLIPLCHPLMLTSIKVHLAAEGDDAVRITASCKLAGQTGVEMEALTAASVAA
LTIYDMCKAVDRGMVIESVRLLEKLGGKSGHFIADQAQVSS
>tr|A0A0N8T7Y5|A0A0N8T7Y5_PSEAP Uncharacterized protein OS=Pseudomonas
syringae pv. aptata OX=83167 GN=ALO85_101339 PE=4 SV=1
MMIPLPAEWPFSGLINRYSVFQVGAVKRQGSVEKSMKSWRPLSGQVFFMRSECDPDPGCF
EPLQGDHRVRYLKG
>tr|A0A0Q0FBY1|A0A0Q0FBY1_PSEAP Putative Membrane protein OS=Pseudomonas
syringae pv. aptata OX=83167 GN=ALO85_04165 PE=4 SV=1
MKRLATFLLFLSMILSGSAFAATDPFANCGLTPDGKKIDVWNSGVCPQNITQRIHYNFLG
VGAFGKYETQNLITTGDDIDSTLSEGQKNLNASNQNLMQLIIMFSYGIALFICIFLSAPY
AFAYVKAMINGEFHQRGNEQTRYHGYGLLSIFATAAGVLAFMPGLMGVKGSLSFGGYAGS
SAGNMGLLGENFIIGSFLNHLEDGGLEVKTDNGTLKDYREKQQTSQTYYSSQATVAGFIS

TSLVIANTSAFNNAIENLKSNDKSWKIRDKTADVWQPNDKDGIDFIKKFSEDPNQTMWST
DSITFAQNPTEISETLPYFKAINYNKSYRDFSDIEGLVNQAAKLASDIQTSSFSDSPSGF
DQIKQSAVAYFYEDAQANILRKNFLQWMEKSDAIADLINNYACSKNYSARIAAQNFIDAH
SEKPTHSGGNTSCVSNDWKVMGEGKTEDYVTQINEKTKALIDEYYDVRIKINTAMNTSFQ
SDTLHKKIVEIYQKGFLHFVFFLPQLMDDLNFSSTVQNEFQNSTPMMTIQTQAMGSYIVD
DWSIAHGYGDANGVDLQIGEAVRELNLIPNSTGSSSLKDGSAILQKLYSTQATASAQQRD
YDQAVGTGLNNPKVELTACLNTTTYPVTCMQKYGQKVSETMTDIMYIAATVKLVSVITTH
YGDKKKDKVMSEQAKEDEKAGISKKQTAKNGKKINSKPNFLQIVGAVTNGLASAAFGWAL
FGYLFSTALKFASIVLMTTPFAVLYILNILYFLIMIATMPLAIISFYRMNDMNNLRNIGR
TLFNIFLSLVAFGPIIVSMYLLNYQMTAVFQKIIIPAATNGLGVAFASGSMQIFQSALEA
AVALICFMVVGYTCTRYAFKILTEALEIFELNMPFLEHGEAFLGRVGAIQTVASGGIFRL
LNIAIANKVDNVAFSAMRKIFRRKKR
>tr|A0A0Q0DN93|A0A0Q0DN93_PSEAP MltA-interacting MipA OS=Pseudomonas syringae pv. aptata OX=83167 GN=ALO85_100137 PE=4 SV=1
MSPAISRTHLRSALLKPVLTVLITSAAMTVAGHACAQDSTAESQSMLGDRFNVNFGLGMA
VLPRYMGADEYRSQVVPMLTVQRGIFFADTTRGLGLQWQSDSGFGASAALNYDFGRTEKN
STNRPGSNELRGMGTVKGATVGDFMVSQQLMPWLSVSAEAELRVAGEQRGNRYRLGFEGI
AYHSQTDTVTLDIDAHAGDGRYNQTYFGVSPIQSQRNGFSRFNADSGIYAYSAALAWQHT
FDAHWSTVASVGVTHYTDQTRGSPLVETDAEGSGLIALNYAF
>tr|A0A0Q0BT26|A0A0Q0BT26_PSEAP Imidazole glycerol phosphate synthase subunit HisH OS=Pseudomonas syringae pv. aptata OX=83167 GN=hisH PE=3 SV=1
MQTVAVIDYGMGNLHSVAKALEHVGAGRVLITSDAKVIREADRIVFPGVGAIRDCMAEIR
RLGFDSLVQEVSQDRPFLGICVGMQALLDSSEENGGVDCIGMFPGQVKFFGKDLHEEGEH
LKVPHMGWNQVAQAVDHPLWHDIPDQARFYFVHSFYIDAANQRQVVGRGHYGLDFAAALA
DGSRFAVQFHPEKSHTHGLQLLQNFAAWDGRW
>tr|A0A0Q0C067|A0A0Q0C067_PSEAP YceI-like family protein OS=Pseudomonas syringae pv. aptata OX=83167 GN=ALO85_02372 PE=3 SV=1
MLTMNRLLLVLLAAVALPAHANWYLDNESSRLSFTSTKNADIAEVHRFLVLHGKVDAKGL
AEVEVETDSVSTGIPLRDERLREQVFQVRKFPTAQINAQLDMRPINDLAPGAQLELRLPL
TVSLRGKTHSYNAELLATRLDERRFQVVTLEPLVIHAQDFDMAPDFKALRNAAGLSAVSL
SVPVGAVLIFTAR
>tr|A0A0Q0CYL9|A0A0Q0CYL9_PSEAP Transcription termination factor Rho OS=Pseudomonas syringae pv. aptata OX=83167 GN=rho PE=3 SV=1
MNLTELKQKPITELLEMAEQAGIENMARSRKQDVIFSLLKRHAKSGEEISGDGVLEILQD
GFGFLRSADASYLAGPDDIYVSPSQIRRFNLRTGDTIVGKIRPPKEGERYFALLKVDTIN
YDRPENAKNKILFENLTPLFPTIRMKMEAGNGSTEDLTGRVIDLCAPIGKGQRGLIVAPP
KAGKTIMLQNIASNITRNNPEVHLIVLLIDERPEEVTEMQRTVRGEVVASTFDEPPTRHV
QVAEMVIEKAKRLVEHKKDVVILLDSITRLARAYNTVIPSSGKVLTGGVDAHALEKPKRF
FGAARNIEEGGSLTIIATALVETGSKMDEVIYEEFKGTGNMELPLDRKIAEKRVFPAINI
NRSGTRREELLTADDELQRMWILRKLLHPMDEVAAIEFLIDKLKQTKTNDEFFLSMKRK
>tr|A0A0Q0D3W6|A0A0Q0D3W6_PSEAP L-fucose permease OS=Pseudomonas syringae pv. aptata OX=83167 GN=ALO85_03940 PE=4 SV=1
MNKPALQQTPDGFYLNRTPWFAFILLCSIFALWAAAASMNDVLIAHFKKAFLLSDFQTAF
VQSAFYLGYFFVAIPAALVVRRFSYKTTILIGLMLYLFGCALFFPAASTAKYGMFLMALF
VIAAGLSFLETACNTYSTLMGPRETGTRRLNISQTFHPFGAMTGVYVGSFVMFKDTDATR
EQLAQMSASEAAAQQLQMIQSTLLPYKWMIAVLILMFILIAITRFPACKGNGTVSESRSS
IGQSLGRLRRNPRFCFGVLAQFLYVGAQVGVWSFTIRLAMQMGGMNERSASWFLLTTFAA
YFIGKMIANLLMRKMHPAKVLAVYGVLCIVLLAYTILVPNITAVYAAVGVSVFLGPCWPT
IYGLTIDGLGEDTGVGGSLLVMSIVGGGVMPIFQGLLSDANGGNMQIAYSVPLLCFVAIV
MYAVYCMRQPQPVLGGSATAVAQ
>tr|A0A0Q0C5B6|A0A0Q0C5B6_PSEAP Transcriptional regulator GntR OS=Pseudomonas syringae pv. aptata OX=83167 GN=ALO85_01884 PE=4 SV=1
MIRPDSAPLFLTPSTDEKGRDEQIYQHVLEAIVEHRLAPGTRLPEDALADVFEISRTGIR
KVLQRLALERLVTLRPKRGAEVAQPSAQEAQDVLGARQLIEPALMADIATRVGGQRLEAL
RALCDQEHDAQHAGRHSEAIQLSARFHVQLSALAGNQVLTEQVAQLTTRSSLIVAVYGSR
RSVGCDCGQHVDLLALLESGQGEQARDWMAEHLRRIRASLNVDAPLSETVDFHSIFKRR
>tr|A0A0Q0FP55|A0A0Q0FP55_PSEAP Uncharacterized protein OS=Pseudomonas syringae pv. aptata OX=83167 GN=ALO85_101446 PE=4 SV=1
MRHASRYDRAAFVSPCPELAPGSLNTGRMRTAGVYKQPRM

>tr|A0A0Q0D8V2|A0A0Q0D8V2_PSEAP L-carnitine dehydrogenase OS=Pseudomonas syringae pv. aptata OX=83167 GN=lcdH PE=3 SV=1
MSFITEIKTFAALGSGVIGSGWVSRALAHGLDVVAWDPAPGAEAALRNRVANCWGALEQQ
GLVPGASQNRLRFVATVEECVRDADFIQESAPERLELKLNLHGRISAAAGPNVLIGSSTS
GLLPSDFYESSAHPERCVVGHPFNPVYLLPLVEVVGGKNTAPEAIQAAIKVYESLGMRPL
HVRKEVPGFIADRLLEALWREALHLVNDGVATTGEIDDAIRFGAGLRWSFMGTFLTYTLA
GGDAGMRHFMTQFGPALQLPWTYLPAPELTGKLIDDVVDGTTDQLGKHSISALERYRDDC
LLAVMEAVKTTKARHGMSFAE
>tr|A0A0Q0DU56|A0A0Q0DU56_PSEAP PilZ-domain protein OS=Pseudomonas syringae pv. aptata OX=83167 GN=ALO85_02383 PE=4 SV=1
MYKERKIERHQLQDYLQVFNRHTGKLVGCLGNVSGEGLMLISSLPVLVGARFDLCLKLPN
GNVGQPIDVSARCLWCHEDETPGSYDSGFELCQVSTEYLEFVQLLRQYFCFYPSYEASA
>tr|A0A0Q0C591|A0A0Q0C591_PSEAP Putative Toluene tolerance protein OS=Pseudomonas syringae pv. aptata OX=83167 GN=ALO85_02881 PE=4 SV=1
MQALDHAHYLEQVQGAEVLEADGTGDKVLRLRDGSMLKLFRRKRLVSSAAWYPYAQRFAD
NCETLAKRDIPCPRIRSVSRIAEIERDAVHYDPLAGFTLRQLLGSAVADDKLRRQLGSFI
AGLHEKGIYFRSAHLGNVILTPEGSLGLIDIADMKTYRRALRKSLRLRNFKHMLRYQEDR
QWLLDNGNPAFLDGYMSAQTLCDTSELAQAVQ
>tr|A0A0Q0BX70|A0A0Q0BX70_PSEAP Uncharacterized protein OS=Pseudomonas syringae pv. aptata OX=83167 GN=ALO85_101016 PE=4 SV=1
MNKASADMRNSCEMVDCHQARRISPGVSGGPVLLIWLI
>tr|A0A0Q0C5W7|A0A0Q0C5W7_PSEAP ATP phosphoribosyltransferase OS=Pseudomonas syringae pv. aptata OX=83167 GN=hisG PE=3 SV=1
MLTIALSKGRILDDTLPLLAEAGIVPTENPDKSRKLIIPTTQADVRLLIVRATDVPTYVE
HGAADLGVAGKDVLMEYTGQGLYEPLDLQIAKCKLMTAGAIGAVEPKGRLRVATKFVNVA
KRYYAEQGRQVDIIKLYGSMELAPLIGLADKIIDVVDTGNTLRANGLEPQELIATISSRL
VVNKASMKMQHARIQALIDTLRKAVESRHRS
>tr|A0A0N8T7T7|A0A0N8T7T7_PSEAP Probable Fe(2+)-trafficking protein OS=Pseudomonas syringae pv. aptata OX=83167 GN=ALO85_02306 PE=3 SV=1
MTRTVMCRKYKEELPGLERAPYPGAKGEDIFNHVSQKAWADWQKHQTLLINERRLNMMNA
EDRKFLQTEMDKFLSGEEYAQAEGYVPPEK
>tr|A0A0Q0BZJ0|A0A0Q0BZJ0_PSEAP Multisubunit potassium/proton antiporter, PhaF subunit OS=Pseudomonas syringae pv. aptata OX=83167 GN=ALO85_02704 PE=4 SV=1
MSALLDNAILISLLIFALAMVITLLRLLKGPSAQDRVLALDYLYIIAMLMMLVLGIRYAS
DTYFEAAMLIALFGFVGSFALAKFLLRGEVIE
>tr|A0A0Q0DQJ3|A0A0Q0DQJ3_PSEAP Putative lipoprotein OS=Pseudomonas syringae pv. aptata OX=83167 GN=ALO85_01734 PE=4 SV=1
MSMLRRILFGLLAVSSLTLVGCAHSPQQLSPTPKLNAQLAAVGRGQPVVVRVVDGRPGPT
LGTRGGMYPETSAISVTGADIVPKLQAQAEAAVRLLGFTPTQGGSAPQLTVTLADLKYQS
PKEGLYVTEATISSTFRADVRNNGRTYSGRYAASLDQRFGMAPNQETNTKLVSDVLSDAL
TRVFQDPTIGSTLSQ
>tr|A0A0Q0IN12|A0A0Q0IN12_PSEAP Pyridoxal phosphate homeostasis protein OS=Pseudomonas syringae pv. aptata OX=83167 GN=ALO85_02936 PE=3 SV=1
MSTIAANISTLEQRIRDAALAAERDPASVGLLAVSKTKPASDLREAYTAGLRDFGENYLQ
EALGKQLELSDLPLCWHFIGPIQSNKTRAIAENFDWVHSVDRLKIAQRLSEQRPEALEPL
NICIQVNVSGEASKSGCTPQDLSALAAAIIALPRLRLRGLMAIPVPTDDPAAQDASFAAV
RTLQEQLNLPLDTLSMGMSHDLEAAIAQGATWVRIGTALFGARDYGQP
>tr|A0A0Q0BRT4|A0A0Q0BRT4_PSEAP Uncharacterized protein OS=Pseudomonas syringae pv. aptata OX=83167 GN=ALO85_04092 PE=4 SV=1
MNTQSIIVPQISTFPGHEARARLILRWLVKLNVVEPELTTCGRTYNKMAYAVAPGAKRVV
KNPDALPFGQVVNGLEIVTKRCIYTPLNDFAEEAGCPECRREVGEALFDSLEDWMPGHTD
NFTCPECRHEDDINGFLFLDACGFSNLGFIFNNWLDASFTQTFLDDFAERLDRPVSCVNV
RL
>tr|A0A0Q0D9N4|A0A0Q0D9N4_PSEAP Site-determining protein OS=Pseudomonas syringae pv. aptata OX=83167 GN=ALO85_04028 PE=3 SV=1
MGSMHPVQVIAVTGGKGGVGKTNVSVNLSLALAELGRRVMLLDADLGLANVDVLLGLTPK
RTLADVMEGRCELRDVLLQGPGGVRIVPAASGTQSMVHLSPAQHAGLIQAFSEIGDNLDV
LVIDTAAGIGESVVSFVRAAQEVLLVVCDEPTSITDAYALIKLLNRDYGMNRFRVLANMA
QSPQEGRNLFAKLTKVTDRFLDVALQYVGAVPYDECVRKAVQKQRAVYEAFPRSKCALAF

KAIAQKVDTWPLPANPRGHLEFFVERLVHQTSAGPVQ
>tr|A0A0Q0IJ85|A0A0Q0IJ85_PSEAP Uncharacterized protein OS=Pseudomonas syringae pv. aptata OX=83167 GN=ALO85_03930 PE=4 SV=1
MTTDHILTDSVPKEDPASPQLGQYDREADPVLAMNANRPGVGPKKGDEVKKRTEHDETYD
EGEPEVDDADSVSDEGGGILPG
>tr|A0A0Q0E1H4|A0A0Q0E1H4_PSEAP Diguanylate cye domain protein OS=Pseudomonas syringae pv. aptata OX=83167 GN=ALO85_03571 PE=4 SV=1
MLRPSAARLSHFLPSLGLLVAGLAAAYVKDLNVFFTSLFNVLPTLVLLLGGAYCFVYRRQ
RELFLMLTVYIAYYLLDTQTDYYRDHGKVREDAAVIFHLVCLLLPVLFGLYAAWEERTHL
FQDMVARLAVLVALGGVAVGLEQSYPVELQVWLADIRWPALHGAWMSLIQLSYAMFLVSF
AVLIWQYLEKPRPLHAAQIVGLLGLFWALPKTFILPFTLNILCSQVMLMIAAAVAHEAYQ
MAFRDELTGLPGRRALNERMQRLGRNYVLAMSDVDHFKKFNDTHGHDVGDQVLRLVASKL
SKITNGGGKAYRYGGEEFAIVFAAKSIDECMPHLEAIRETIASYEIQLRNKNDRPQDDQQ
GRQCRSGAQAGSVSVTVSIGVAERQPEHRTPEEVLKSADQALYAAKGAGRNCVVAYGQLN
RRGAVRTTEAAT
>tr|A0A0Q0BUI3|A0A0Q0BUI3_PSEAP Diguanylate cye OS=Pseudomonas syringae pv. aptata OX=83167 GN=ALO85_04734 PE=4 SV=1
MIRDVLCFSMLMSRCHMLVSALERPSLEDAQLLAEVEQTITSGVGSLRFSRVLEKRYQEE
TRLPRREFLTAVGIGGCLIYNLFLISDWLILRDVFFYVAVGRLCLITPMFIALLMLAQRV
STRWALETIAAFATVACSLMPLLVMIHSNSPYQIFYQLGMLLIMVYCTMIQQLPLRHAAV
AMSCMLIIQLATTYIASFADFVIWQANAVLFVSTVALLLMASYFLERAGRMSYLFALRGR
LLQVQLMEFARTDGLTRLFNRRYQDEVMTSVWEHARNTPANVAIILLDIDHFKSYNDNYG
HPQGDTCLRLLCQKVQQSAHEHGAVAFRFGGEEVLVLMIGNPVQAREVAEALQTSVAELK
LPHPVLGENALVTVSLGVACATAPMTSAENLIGAADNALYAAKRAGRNCVCFA
>tr|A0A0Q0IMR2|A0A0Q0IMR2_PSEAP Uncharacterized protein OS=Pseudomonas syringae pv. aptata OX=83167 GN=ALO85_03996 PE=4 SV=1
MTMSSTQISAFQAAAGFSPANSNTLWTGIAMGILTLWGVWVFLSIYRGWATQNLDRMVAA
ASAARWAVLFMIMTFMLLS
>tr|A0A0Q0E108|A0A0Q0E108_PSEAP Uncharacterized protein OS=Pseudomonas syringae pv. aptata OX=83167 GN=ALO85_101271 PE=4 SV=1
MRRVNGECPSDIKILIYDTFISMESALCDAGAKARGRLKA
>tr|A0A0Q0FBR9|A0A0Q0FBR9_PSEAP Ferredoxin, 2Fe-2S type, ISC system OS=Pseudomonas syringae pv. aptata OX=83167 GN=ALO85_04109 PE=4 SV=1
MPQVIFLPHAEHCPDGLVVEVEPGTSILEIAHEHHIEIESACGGVCACTTCHCVIREGFN
SLDEADELEEDMLDKAWGLEPQSRLSCQAIVGNEDLTVEIPKYSLNHAAEAPH
>tr|A0A0Q0DYZ0|A0A0Q0DYZ0_PSEAP Uncharacterized protein OS=Pseudomonas syringae pv. aptata OX=83167 GN=ALO85_04323 PE=4 SV=1
MIVSRGFQPVLPVPVRLSHHQNGYLYVTMNKSAGIAIGVIVVAGALATAGAWYTGNQLEG
ALNDSISKTNQELAKSFKGTDTSVKLEMLSLDKHFFSSTAHYRVDIQNLSDSTQGGEFLF
VDHIEHGPIPLSHLKTLNLLPVMALSNFQMEKSPSAEKWFAMSKDVPPLTGQASIGYDRA
TKGTLQMAPLEMTDVDGTFRFSGLDLNAESSANAEKFTLAGNMDNLQLNVASPDGPVSIE
IKGMTFDTGGTKGKSGFYLGHTNMKIQNAGFQVVGKPQVQIRDFTNTNLVQEDAGNLAAQ
INYDAGMIAYAGKDVGSAHMGFKIANFDVLATQALYQLYQDKILPQQQQAAQEKRPFRLE
LSPADQELMNSQMKTLLAAKPHIELEKLSLKTSNGESHMRIAVDLADPGPLDQPSDALLM
KSLGEVNAKVVLSKPMIRDLATQQAMREGQTDLKVIADQAKAAGDMASAMAEMMQLAKVD
GDNIVSDLHYANQMVDFNGQKMTVQQFMSNVMGKVGVLGNQ
>tr|A0A0Q0BZQ2|A0A0Q0BZQ2_PSEAP Sulfur transfer complex subunit, TusC/DsrF OS=Pseudomonas syringae pv. aptata OX=83167 GN=ALO85_00711 PE=3 SV=1
MPRSLLVVSRQSPWSGPGAREALDIVLAGGAFDLLVGLLFLDDGVFQLLPQQSPDALQQK
DLTANLKALPMFGVEDLYVCGQSLAERGISPGALSEELSQLTTRAELSALFDRYDEVITI
>tr|A0A0Q0CIL1|A0A0Q0CIL1_PSEAP Uncharacterized protein OS=Pseudomonas syringae pv. aptata OX=83167 GN=ALO85_01568 PE=4 SV=1
MMAKYEIRLWAEDIEEHDHPEIAIEFYENDDLQEHAWVRLHPRGKHKIRGELGRDEEVLT
VLALAFRKKVAELADW
>tr|A0A0Q0IE73|A0A0Q0IE73_PSEAP Uncharacterized protein OS=Pseudomonas syringae pv. aptata OX=83167 GN=ALO85_01082 PE=4 SV=1
MHLDLSEMSQLAPIFRELFKGYHISRRDPELYAQLSNCQDQYRTLFKALGFELVCDTRGF
YYFVPELAAAQVNKTAQRLALFTFILVEHLADQGRDPMSVLDGGSLGRDELPSMLEKYRD
LFLQAEVQTPEELEEKIMRRMTQLGFASEEVGIYRFLPPMHRFLDVCLSVQQDRDLAASL

HSALPLPTPVLIDDDSDEKLLETDDPLDLAEFGEETEEEALARAIADEQRQEMDT
>tr|A0A0Q0DBI7|A0A0Q0DBI7_PSEAP Outer membrane adhesin like proteiin OS=Pseudomonas syringae pv. aptata OX=83167 GN=ALO85_04167 PE=4 SV=1
MSWYNDITANIIHMNNKYKKNKSMFCSYLLLMFFTLVFTAQANAALTSQGSHPELLSNAT
CSGGVTGSYALYGNRFGLSIRDSALIGYHPADLNSRNLGTAGIVYIEDAPGASVNGFGSD
GMGLYNYNGKTYFVGPYYNTRRAYFEFQICYINVSTPPAPPAAPTFISSGYEDQNASCPA
DRPSGYLVQRRTFENWTDGRRNESGWSTIADYCSPIRSSIQTDARYYGCPSGQSGQIVQS
RTYEIWTDGSARNYSAWNVAGNTCVSAPMTANPTQRRELCAEGYTGAITYHWVVYYTNDS
YSAYDADGKLISYTLSTPHQQELLLSNTCALIPSQGVASVPGHESMTCNAYYNVVKGTYL
GEVVKYGNYVSSYDSAKKQTNTVFKVTSIDITQCVPDPEKTYSNESITAACAAGQSGIIT
KIRTVATAPNGSKSYPSGTDYTVSSNTCAGTSVDNTPVVLATAAKTGLIENLSLTSSMLS
DSTYSTKLVDSLKSQSIKAGETHRLNIVVNDLSPGKYNAQNVTNIVKAFKIAVGSGAEFK
MTLPRSIDKYAGNGEIQGGKGLTLTSSELNGNKLTVKYMDAGDKKTLSMPVENSADVQLF
NGDMTGIFFKTD
>tr|A0A0Q0FBW9|A0A0Q0FBW9_PSEAP Uncharacterized protein OS=Pseudomonas syringae pv. aptata OX=83167 GN=ALO85_04150 PE=4 SV=1
MIIDIQNEETTQATPATDAKPAKVSKGSPELRGKVSLKKCSHYIDSPKMENGKDLHAGRQ
FYDSNIPNDMIHPSIMEKTLTAKEMKDYRMRYAESDAKFLYTHSTDCKNNAPRSKGKQMD
GFRFILSCKQEELYPYETDEQQAEFLCTLARTVLQKMSGSKEINFYYKIVPHLKDGNPHA
HIMMSSYTIDGQYYSFFRGKQDHGLIKLFDDVKHDLEAKYPHLLQMEHEKREKNRLMVDV
NKETNEVTGEHAEIFNRLNTILMSHQGAGYSHPKLKKELEDAGFELRYTKAKSRSKFKDI
QIFHKDAGEAFRSYANLPYQVKRVMENFETYQHFSKGLKIRSDVSATVAKATILVNAFPS
KNVTELNKVLYEELGVMLVPSFKKDLDTGRKYVNSWSFYLSRENVKFPAIKAGVEEPKKL
IITAKEAEDLHEQVEQIIHTQTITQITAGIRYTQKNREEFPQFRFQKNETNEEFLARMLA
SKAYKQSLTSQIAVGNTLISSYNKRTMLDTNGVDSVKVYQANMSSAKSAIQWYVAQGFAS
IVFKGTENPEIQRMMYIQGILHDVKIDNYIPSEELKKEAQDLLDAERDKVIKSNKEKIKA
HLDAVKAEPNGEKQYLYLRSNRKLDIDVDQRPKLYGFLFGIKMGLEQDAFKHMDKLTLSK
EEIKNVIKHFASEEKLTTFELQDVLRRAGFLDDTDPISTARQKVPETAQTTNTVSQTIAP
EPDTAPSQSSDTKPEQEKKTQRIEPPGSGKIKP
>tr|A0A0Q0DXI8|A0A0Q0DXI8_PSEAP TetR family transcriptional regulator OS=Pseudomonas syringae pv. aptata OX=83167 GN=ALO85_03620 PE=4 SV=1
MAIKEAIRTGGRSARVQESIHRAVRDLLQEHDRSALSVPMIATRAGVTPSTIYRRWGDLT
TLLADAALERLRPDVPIDQGSLHQDLLTWSEMYLDEMSSAPGRALMRDVAASTSGCVGKC
AAMVREQLQVMIDRAMARGEASPSADDMIDAVIAPMIYRILYAESAPTPERVRQWVDRCL
S
>tr|A0A0Q0DAC1|A0A0Q0DAC1_PSEAP Deoxyguanosinetriphosphate triphosphohydrolase OS=Pseudomonas syringae pv. aptata OX=83167 GN=ALO85_02291 PE=4 SV=1
MALTRSLYFSRRYELTSMQEALIPATAEPRSVWTSLLSASRRKKNDAATVVPAASSSSGR
VELRTEIERDYDRILFCAPVRRMADKTQVFPLDRNDSVRNRLTHSHEVSNLARSFGTTLV
YSNSIASEVPTAVRDIPSLLAAVGLVHDLGNPPFGHQGEAAIQSWFFDNISSVAGKKFSV
LTERQYRDFLKFEGNAQAFRLVTRLQVLNDDFGLDLTVATLAAMMKYPVGAMGVNKSNVC
TKKHGFFLSEESIAKHVLESVGLSIGKRHPMSYIMEACDDIAYAVLDIEDAVKKGLASFS
DLIAYLDHHAPQDFVINELVEKSRAKHVEYRILDLSPAELNDVSMQRFRVYAIGMMINSC
VDAFVDNQNSYIEGLAPKALLDISSVSRLRELLGDFSFNHTFQHRSVLEVELTGYNTIRG
LMDIFWGAILETNPDNGVCRPSHPYNRYVFMRISENYRRVYNSPPGDLKGLPLRYRQCLL
LTDMISGMTDSYAVNLLQELRSYQARG
>tr|A0A0Q0FGZ9|A0A0Q0FGZ9_PSEAP Uncharacterized protein OS=Pseudomonas syringae pv. aptata OX=83167 GN=ALO85_01349 PE=4 SV=1
MKTVLQHTLPRRQQGAAAIEFSAVFVIFFAVFYGMVSYSLPLLMVQSFNAAASEAVRRSV
ALSPTVTGYNDLLKSQAQSVVMNQLSWIPPALGFNINHTSVTYSAGILTVTIQYPKTRLA
QVLPLLTLPGIGEVPRLPNNLTAQASLQLVP
>tr|A0A0Q0C0A3|A0A0Q0C0A3_PSEAP von Willebrand factor type A domain protein, family OS=Pseudomonas syringae pv. aptata OX=83167 GN=ALO85_02384 PE=4 SV=1
MKQVLKPSITLLRWLGGLMGVALLLGTFHALGIEYPAKIDDLYWGALLALVVVSVLDAIW
LLRSPSPRVQRELAGSLTLDRWNEVRLDVQHDNCRPVIVRLFDHVPHGLEHENLPQSLTL
AARGKGSIGYRLRPSSRGHFTFEQCEISLPGTLRLWTTRRYLAVPGTTRVYPDFARLCGG
QLMAVDNWLSQLGIRQLQRRGLMEFNQLREFREGDSLRQIDWKATARQRAPIAREYQDE
RDQQIVFMLDCGRRMRSQDGDLSHFDHALNACLLLSYVALRQGDSVGIATFAGEQDRYLA

PVKGPGQLNRVLNTVYDLQTTRRPADYSAAVRHLMVRHKRRSLVVLMTNLRDEDDEELLA
AARQLGKQHRVLIASLREEVLDGLRHSPVHTYDQALSYCGTIDFLNARASLHDRLSAQGI
VIMDARPGELGPQLVNRYMSWKKAGTL
>tr|A0A0Q0DQB4|A0A0Q0DQB4_PSEAP Ribosomal RNA small subunit methyltransferase C OS=Pseudomonas syringae pv. aptata OX=83167 GN=rsmC PE=3 SV=1
MDPRSEVLLRQAELFQGSLLLVGLPADDLLGKLPDARGWCWHAGDQAALDARFEGRVDFG
VEAPQTDFKAAVLFLPKARDLTDYLLNALASRLAGRELFLVGEKRGGIEAAAKQLSPFGR
ARKLDSARHCQLWQVTVEHAPQAVSLESLAKPYQIELQDGPLTVVSLPGVFSHGRLDRGS
ALLLENIDKLPSGNLLDFGCGAGVLGAAIKRRYPHNEVIMLDVDAFATASSRLTLAANGL
QAQVLTGDGIDAAPMGLNTILSNPPFHVGVHTDYMATENLLRKARQHLKSGGELRLVANN
FLRYQPLIEEHVGQCEVRAQGNGFKIYSAKRP
>tr|A0A0Q0BRA3|A0A0Q0BRA3_PSEAP Polar amino acid ABC-type transport system, ATP-binding protein OS=Pseudomonas syringae pv. aptata OX=83167 GN=ALO85_02299 PE=4 SV=1
MAEATPALEIRNLHKRYDQLEVLKGISLTARDGDVISILGSSGSGKSTLLRCINLLENPH
QGQILVAGEELKLKAARNGDLIAADNRQINRLRSQIGFVFQNFNLWPHMSVLDNIIEAPR
RVLGQSKAEAIAVAEELLAKVGIADKRHAYPAQLSGGQQQRAAIARTLAMQPKVILFDEP
TSALDPEMVQEVLGVIRALAEEGRTMLLVTHEMNFARQVSSEVVFLHQGMVEEQGTPQQV
FENPLSARCKQFMSSHR
>tr|A0A0Q0CWR3|A0A0Q0CWR3_PSEAP Extracellular solute-binding protein OS=Pseudomonas syringae pv. aptata OX=83167 GN=ALO85_00107 PE=4 SV=1
MAEFRCKVSVDHLINHDAGVNWIDLYHSPYINVSRKNIEHLGQNPFGRFTLEETVKK
>tr|A0A0Q0ILR5|A0A0Q0ILR5_PSEAP DTW domain protein OS=Pseudomonas syringae pv. aptata OX=83167 GN=ALO85_00268 PE=4 SV=1
MSHAVSRLRDERLARSTRPFIARGSRAPRCPECRVISSYCLCEWRPSVAADSGICLLMYD
TEPLKPTNTGWLIADVIQDTHAFLWSRIEVDEQLLALLDDPQWQPYIVFPGEFVAEERVV
NKVSRLDGKRPLFILLDATWTEARKMFRKSPYLERFPVLSLEPEQISRYRLRRSRRDDHF
CTAEVAALCLELAGDASASGVLDAYLDVFSAHYLGAKYQNPIDPDDVAHTHLKAFI
>tr|A0A0Q0C5H5|A0A0Q0C5H5_PSEAP Protein-glutamate methylesterase OS=Pseudomonas syringae pv. aptata OX=83167 GN=ALO85_00508 PE=4 SV=1
MKTTFGADAPSHSLPVVDAIVVGASAGGVEALLKIFSGLRKGFSLPILMVLHLPDDRHSQ
LANVFQNRLAIPVKEADDKESIVPGTLYVAPAGYHVSVESDFSLSLSQEDKVFYSRPSID
ILFGSAADAYAPRLAGVLLTGANNDGANGLLQIKKYGGFTVIQDPAQAQASTMPEAGLAL
HSPDYLLSLNDIGRLLVELERTAC
>tr|A0A0Q0BSZ6|A0A0Q0BSZ6_PSEAP GAF/PAS/GGDEF/EAL domain protein OS=Pseudomonas syringae pv. aptata OX=83167 GN=ALO85_00482 PE=4 SV=1
MFMKSQTDAAGRSAAEVVTQLPVPSRLGMLRFERLNEANWALLFLDPNCERQFGLAAVDL
CALIGSPYASLMEPEARYQLHDDIQQQLASSPNYLIRYTLHTPKGPLGLLEIGEAYKQHN
RHLLRGYFLIVEGLVTTGEPVTDSDLETRNLRLQIALELNQRAQRDQFAHLERVRAQQDL
ILRLTRHRYTTANTLLEAAELITKSACDIYDVDHVSIWNLNDKRLEPITDYSRETGDYQS
RTPVDISPYPTYLQALNTSRAIDASNIQTDPRTCEMAKILNPGETRAVLDASIRIDGQVI
GVLCLEQSGSTREWQSDEIAFAGELADQFAQVINNHNRRAATNALHLFQRAVEQSANAFL
LVNCNGVVEYVNPSFTAITQYSSEEVSGHKLSELPALENLNQLLLEANSSLTNSNSWQGE
FKSRRKNLEPYWGQLSISKVYGDNRELTHYIGIYEDITQSKLAQQRIERLAYTDNLTNLG
NRPAFIRNLDERFARDTDTPMSLLLVDIDNFKRINDSLGHQTGDKLLISLARRLRNTLSP
SDVLARFASNEFAVLIDNTDQEAGQATATQVLATLDKPMFVDNQLISVTGSVGLACAPLH
GRDPQTLMKNAGLALHKAKANGKHQVQVFTEALNAEASYKLFVENNLRRALTQNELEVFY
QPKLCLLTGRLLGMEALLRWNHPEKGMIRPDQFISVAEETGLIIPIGKWVARQSCRMSKD
LTAAGFGNLQVAINVSPKQFSDPELVSSIAAILKEEELDPSLLELELTEGLLLEATEDTR
QQLDSLKKLGLSLAMDDFGTGYSSFSYLKKFPIDVIKIDRSFIRDIPDDEDDMEITSAVI
AMAHNLKLKVVAEGIETAAQLAFLRRHRCDVGQGYLFDRPIPGEELIEKLKRYPRRPSA
>tr|A0A0Q0D426|A0A0Q0D426_PSEAP D-isomer specific 2-hydroxyacid dehydrogenase OS=Pseudomonas syringae pv. aptata OX=83167 GN=ALO85_03916 PE=4 SV=1
MIYAELRPSKVHIDCFSHCKRQIPSCPQITMQSAGQRRFSDIREAWKQAFRQILLCFYGM
MGLIFHAQETPMPALLYKADPARAERWRVLFAEHAPDIEWRVWPDIGDPADIHYLGAWLA
PDDLQELLPNLKVLFALSAGVDQLDLSRIPKALPVVRLLDPGISHAMCEYATFAVLSLHR
EMLRYRQQQVEGVWKAHPLIPANQRRVGVMGLGLQAQHILSSLKPFGFQLSGWARSAHRI
DAVQCYAGEEQLDAFLGQCDILLCVLPLTGQTEGILNGRLFEQMPEGAALINMGRGGHLV

EADLIEALDSGQLSAAVLDVLQQEPAAADHPFWNHPKILLTPHVAAMTQPESAFPGLLDN
IRRFERGEAMQGQVDRGQGY
>tr|A0A0Q0DLL1|A0A0Q0DLL1_PSEAP EVE domain-containing protein
OS=Pseudomonas syringae pv. aptata OX=83167 GN=ALO85_01646 PE=4 SV=1
MAYWLMKSEPDEFSISDLQRLGNARWDGVRNYQARNFLRAMAEGDAFFFYHSSCPEPGIA
GIGKIARSAYPDPTALDPESHYHDAKATEEKNPWSAIDVEFVETFKRVLGLGYLKQQSQL
EQLPLVQKGSRLSVMPVTAEQWAAILTLR
>tr|A0A0Q0C531|A0A0Q0C531_PSEAP Type III secretion system protein HrpT
OS=Pseudomonas syringae pv. aptata OX=83167 GN=ALO85_04193 PE=4 SV=1
MKISSVAVVLVVFATLTGCATHGCSGTACKRPDSTSRELVIWWPPDMRDGLDDQDHERDY
TVVKLRD
>tr|A0A0Q0FUX4|A0A0Q0FUX4_PSEAP Glycerophosphoryl diester
phosphodiesterase OS=Pseudomonas syringae pv. aptata OX=83167
GN=ALO85_200049 PE=3 SV=1
MKLSLRQLQIFCAVAQHGSTTSAAISVSLSQSATSAALNELESALDTRLFDRVGKRLVLN
DNGHALLPQARRMLENAQQIEASFLPGHAGLKTRLRVGCSTTIGNYVLPLILGELRQSTP
HLRVDVDMANSMAIARRVADFEIDMGLIEGPCHLPELSAEPWLIDELLIAAGQRHPLALK
QQVTLDDLRTAEWLLREPGSGTREEVEQLLLRHLHDLAETRQIGSSEAIKHTLAQGIGIS
CLSRWVVRDLLASEVLVELPHALPALTRRFFLIRHRDKYISPGLERFWEKCSQFVSQE
>tr|A0A0Q0CA49|A0A0Q0CA49_PSEAP Sulfatase OS=Pseudomonas syringae pv.
aptata OX=83167 GN=ALO85_01549 PE=4 SV=1
MGRSDYMKRKNILFIMADQMAAPMLPFYARSPILMPNLSRMAANGVVFDSAYCNSPLCAP
SRFTLVSGQLPSRIGAYDNAADFPADIPTYAHYLRALGYKTALAGKMHFCGPDQLHGYEE
RLTSDIYPADYGWSVNWDEPDVRPSWYHNMSSVLQAGPCIRTNQLDFDEEVLFKSQQYLY
DHVRQDGDAPFCLTVSMTHPHDPYTIPLPFWDLYADDEIPMPTPHANQAALDPHSQRLLK
VYDLWDKPMPTDKIRDARRAYFGACSYIDLNVGKLMQTLDEVGLAEDTIVVFSGDHGDML
GEKGLWYKMHWFEMAARVPLVVYAPGQFKPGRVSASVSTADLLPTFVEMAKGKLDAGLPL
DGRSLMPHLKRKGGHDEVFGEYMAEGTTSPLMMIRRGAYKFIYSEQDPCLLFDVKKDPKE
QKDLSQSPTHEKLFNDFLAEARAKWDIPAIHQQVLASQRRRRFVAKSLATGKLKSWDHQP
LVDASQQYMRNHIDLDDLERKARFPQP
>tr|A0A0Q0IDZ3|A0A0Q0IDZ3_PSEAP Ketoacyl_synth_N domain-containing
protein OS=Pseudomonas syringae pv. aptata OX=83167 GN=ALO85_02841 PE=4
SV=1
MITFNIARWQAWAPGLASAEDWQRWSLDPTLLETSDAAPDVSFLPAMQRRRLGRMARMAF
AVGWPLTEGLERVPLVFVSRHGETPRTFEILRDLAANEPLSPTQFSLSVHNAVIGLWSIM
RGETSEMTALAATGDGLEYGAFEAAALLAEGAEAVLLVVTEEQPPQAYAQWIDDVPFPYA
VGLLLTPGNEWQLSLNTDTQGTKRTQWPHALNLLRALHTDQSVCLHPWNNRLWNWQRSH
>tr|A0A0Q0BW97|A0A0Q0BW97_PSEAP Uncharacterized protein OS=Pseudomonas
syringae pv. aptata OX=83167 GN=ALO85_03050 PE=4 SV=1
MKTALSTTFGLLLLSAAVMTQAADMKVVKPMRGTVDSVSADTLDFTTRSGQHESIKLTDQ
TGIRLVSEADFKQIKGDSFVGSAAIPQADGTLKALEVTVFEASLKGSGEGHYGWENADGS
TGTMTNGTVGTLAGTDGRTLTVKYEGGEKKLVVPQDVPIAYVEPGKVDQLTKGAKVVVFP
ADDGKSARGVAVGKGGFTPPM
>tr|A0A0Q0DGJ9|A0A0Q0DGJ9_PSEAP Flagellar basal-body rod protein FlgC
OS=Pseudomonas syringae pv. aptata OX=83167 GN=ALO85_02141 PE=3 SV=1
MSLANVFNIAGSAMSAQTTRLNTTASNIANAETVSSSMDQTYRARHPVFATVMQGQQSTG
GSLFQDQGEAGQGVQVNGIIEDSSNLEARYEPNHPSADKDGYVYYPNVNVVEEMADMISA
SRSFQTNAEIMNTAKSMMQKVLTLGQ
>tr|A0A0N8T7Y4|A0A0N8T7Y4_PSEAP AraC family transcriptional regulator
OS=Pseudomonas syringae pv. aptata OX=83167 GN=ALO85_01881 PE=4 SV=1
MIARRKFSDAMQGKNLRFLKEEQPESTRQTCAGFLLLEHFSLPAFTQVLDAIVTANLLRR
DLFSTITLGLGEGEVVSDLGLVIRPDELFGRDSLAGLDLLVICGGYRTELKASDNLIRLL
REAAAQGVALAGLWNGAWFLGRAGVLDGYRCAIHPEHRPALAEIASVGQVTSEAHVADRD
RLTASSPTGAYHMALEWIKGLHGKALVEGIEDILSFEESRYRRIKTSPLLSVSAPLREVI
GLMESNLEEPLEMEELAVYGGRSRRQLERLFKEQLGTTPQRYYLELRITEARRLLQHTEL
SQTEVLVACGFVSPSHFSKCYSSYFGYRPSAEKRRVK
>tr|A0A0Q0IPT2|A0A0Q0IPT2_PSEAP FMN-dependent NADH-azoreductase
OS=Pseudomonas syringae pv. aptata OX=83167 GN=azoR PE=3 SV=1
MNLLHLDSSILGDHSASRQLTREVVQAYLNVHADSQVTYRDLASDALGHFSAASLAAAGT
PVDVRDAAQQQEVAGNEATLQQFLDSDVLVIGAPMYNFTIPTQLKAWFDRILIAGRTFRY

SEAGPEGLCGGKKVIIVSTSGGLHAGQPTGTGHEELLKTLFAFIGITDLQFVRAHGLAYG
EEPRASAMSAAQQYIQNELFAA
>tr|A0A0Q0FH19|A0A0Q0FH19_PSEAP Pentapeptide repeat-containing protein OS=Pseudomonas syringae pv. aptata OX=83167 GN=ALO85_01294 PE=4 SV=1
MYEAIMSHPKQIDSPLYMLLRKDDVEGFNQEKPRTGTIDMVGGDFRGLDLRELDTAGIDF
TDAYFRSADLRGLDLRNTPMEGASIAHAQISGVFFPAELSADEILMSVNFGTRLRYRTH
>tr|A0A0Q0ITV5|A0A0Q0ITV5_PSEAP PqqA binding protein OS=Pseudomonas syringae pv. aptata OX=83167 GN=pqqD PE=3 SV=1
MKHDPQFRALTPKWHQGYRFQYEPAQKAHVVLYPEGMIKLNESAALIGGLIDGKRTIAAV
IDELHQQFPNVPELGMDVDEFMEGAKKKNWIDLV
>tr|A0A0Q0BUW3|A0A0Q0BUW3_PSEAP Putative SAM-dependent methyltransferase OS=Pseudomonas syringae pv. aptata OX=83167 GN=ALO85_00143 PE=3 SV=1
MSSLNQALSVALDHRQALLDQLHGQGTDCYRLFHGSQEGASGLTIDRYGPQLLVQSFHTR
LDRDALLALHEQVNTCLGLDTLLVYNDRSQGNSRVDRHDTVYKAEEAALADHVGHEWGLN
YRVRGRHAGQDPLLFLDLRNTRGWVKQHSAGKSVLNLFAYTCGVGLSAAAGGASEVCNLD
FAEGNLAVGRENGALNPQLPPMQFIQSDYFPAIRQLAGLPIAARRSQQLPSYVKLQQRQY
DLVLLDPPAWAKSAFGTVDLLRDYQSLLKPALLATADDGVLICCNNLAKVSMQDWREQVL
RCATKAGRPVRDCQEMRPAADFPSQDNQPPLKTLILQL
>tr|A0A0Q0DLA4|A0A0Q0DLA4_PSEAP Cardiolipin synthase B OS=Pseudomonas syringae pv. aptata OX=83167 GN=clsB PE=3 SV=1
MSDTTGQWRDGNSVELLINGEDFYARVFECIRAARKEVLIETFIIFEDRIGESLQQALLE
AAANGAKVVVVTVDDYGTSDLSSTFVRTMIDAGIQIQLFDPRPRFMGMRTNLFRRLHRKVV
VIDGELGFIGGINYSVDHMTDTGVTAKQDYAVLVRGPIVGDIHHSALNMLSKVVRDKLPP
IELKLDRAGDASMLLAERDNDEHSTDIEEQYLEAIRAAKQRITLANAYFYPSYRFLRELR
NASRRGVKVTLILQGQPDMPFVRVCSRLTYTYLLRDGVAIHEYKQRALHGKVALIDQDWS
TVGSSNLDPLSLALNLEANLFIRDRALNEHLQNHLMELAAAHSKQMSLKGAARGQWWRAP
MIVLSFFFLRRFPAIAGLFPVHGVRLKPLRAGDVVPEAKVIEQQQNNHPMDQEKTL
>tr|A0A0Q0DKV1|A0A0Q0DKV1_PSEAP 1-aminocyclopropane-1-carboxylate deaminase OS=Pseudomonas syringae pv. aptata OX=83167 GN=acdS PE=3 SV=1
MNLSKFKRYPLTFGPSPITPLKRLSEHLGGKVELYAKREDCNSGLAFGGNKTRKLEYLVP
EAIDGGYDTLVSIGGIQSNQTRQVAAVAAHLGMKCVLVQENWVNYSDALYDRVGNIEMSR
IMGADVRLDSAGFDIGIRPSWEKAMADVEEGGGKPFPIPAGCSEHPYGGLGFVRFADEVR
QQEEELGFKFDYIVVCSVTGSTHAGMLVGFAADDRAQRVIGIDASAKPEQTRAQVLRIAQ
NTAKLVELGREISAEDVVLDTRYAYPEYGLPNDGTLEAIRLCARLEGVLTDPVYEGKSMH
GMIDMVRNGEFPEGSKVLYAHLGGVPALNAYSFLFKDG
>tr|A0A0N8TA31|A0A0N8TA31_PSEAP Killer protein OS=Pseudomonas syringae pv. aptata OX=83167 GN=ALO85_200174 PE=4 SV=1
MNTYSKTADRQGHAAGNAVPGRGKTRVCALFAVCLLGSGSAFAGDPCKSVLCLYGKFAGN
SGSSECRSAEQDYFSILVKKHGNIKWSETASARQDYLNSCPDADQSYTQKINDKFGKVQG
>tr|A0A0N8T9C5|A0A0N8T9C5_PSEAP Outer membrane protein Skp, OmpH family OS=Pseudomonas syringae pv. aptata OX=83167 GN=ALO85_00298 PE=4 SV=1
MRKLTQLVMLATVLVASPAFADMKIAVLNYQMALLESDAAKRYAVDAEKKFGPQLTKLKS
LESSAKGIQDRLVSGGDKMAQPERERLELEFKQKARDFQFQSKELNEAKAVADREMLKQL
KPKLDQAVEEVIKKGGFDLVFERGAVIDVKPQYDVTRQVIERMNQLK
>tr|A0A0Q0DEN7|A0A0Q0DEN7_PSEAP MaoC-like acyl dehydratase OS=Pseudomonas syringae pv. aptata OX=83167 GN=ALO85_03092 PE=4 SV=1
MPFVPVAELKQHIGKDAGYSDWLTIDQQRINLFADATGDFQFIHVDPVKAAQTPFGATIA
HGFLSLSLIPQLMEGIMVLPEGLKMAVNYGIDSVRFVQPVTVNSRVRLKVQLTDVSEKKT
GQWLLKATATLEIEGQEKPAFVAEPLTLCFV
>tr|A0A0Q0C2R9|A0A0Q0C2R9_PSEAP Lytic transglycosylase OS=Pseudomonas syringae pv. aptata OX=83167 GN=ALO85_200113 PE=4 SV=1
MLTTSAFLALAMQCAPSIHPSTLTPIVKTESSFNPYAIGVVGKVLPRQPQSLDEAVLVVK
QLVEEGANFSIGLGQINRQHFDVSRPEPVFEPCTNLRMAARELQACYVKASKADPDVQSA
LHKAISCYYSGNPKRGFKAEAEFGGSSHVQRVLANAGGTTVTVPALEGGSAEPSQLQRAQ
APVSAVEPTYESWDVLRQYPRYLPPAPPSVSAPPAAPAPPAVSPEEPSTLPKEDQ
>tr|A0A0Q0DLQ1|A0A0Q0DLQ1_PSEAP Hexuronate transporter OS=Pseudomonas syringae pv. aptata OX=83167 GN=ALO85_01655 PE=4 SV=1
MKIKGIRWWMVGLVTAGLVVNYLARNTLSVAAPTLMSELSISTEQYAHIVVAWQLCYAVM
QPVAGYIIDLIGTKMGFAIFAIAWSAACAAAAFATGWQSLAIFRGMLGLTEAAGLPAGVK
ATTEWFPAKERSVAIGWFNIGSSFGALLAPPLVVWAILQSGWELAFLIVGGLGIVWSGLW

LLLYKHPRDQKRLGDAERDYILSGQEARLKDAPAQKGSWKRLFKNRNFYAIASARILSEP
AWQTFNAWIPLYLMTERHMNIKEVAMFAWLPFLAADIGCVLGGYLSPLFHKYCKVSLFTS
RKMVMLFGCSCMIGPACIGLVESPYTAIALLCIGGFAHQTLSGALYSITSDSFGKNEVAT
ATGMGGMFGFFGAAAFTMVFGVLVTKIGYSPLFVVLAIFDIVAAIVVWNVAREVPQHDEP
LPLAAGEQGPRTVPAT
>tr|A0A0Q0D690|A0A0Q0D690_PSEAP Protein SlyX homolog OS=Pseudomonas syringae pv. aptata OX=83167 GN=slyX PE=3 SV=1
MSLEERVMELESRMAFQDDTIQALNDVLVKQRRELDHLQLQMAAMLKRQEEMGSQFETFE
EDAPPPHY
>tr|A0A0Q0CH80|A0A0Q0CH80_PSEAP Uncharacterized protein OS=Pseudomonas syringae pv. aptata OX=83167 GN=ALO85_04312 PE=4 SV=1
MITPQPELIKKGLYSSFALITFFVTISTFKSSVCWLVALGLFILFIRTTYLVYLSESFTA
ISIHSFTGLFSSLLFMNASVIYLIAKSEYGTSTTDALSWAMIPALLMLVTFLFIYFTKAT
SSQLYLEIKNNKVCITHSYVSTRSGNLLCGAILAVGIAAMIWGHVQHIIVVSVWIALINL
YLLYWYRNSIRMLKKILALEKKHKRSYTFEYIDEIRKARSRWWLGRLLKWATRR
>tr|A0A0Q0C2C2|A0A0Q0C2C2_PSEAP Uncharacterized protein OS=Pseudomonas syringae pv. aptata OX=83167 GN=ALO85_02499 PE=4 SV=1
MQSTTRFRSKRTTARQLASLAVIAAAAMLPVSAAYAAKKPPLVVTEGIKAYRLCTGSDKL
SHVIEGSIDQQAMTDVSALHFKETPAHSTFDWHPAPEEQYVITLSGTLEFSTTGGENFVL
HPGEVLLAQDTTGPGHRWKLIDDQPWRRAYVITKPGDKGAFVPKDGASAKGC
>tr|A0A0Q0CI57|A0A0Q0CI57_PSEAP Putative leucine-responsive regulatory protein OS=Pseudomonas syringae pv. aptata OX=83167 GN=ALO85_02580 PE=4 SV=1
MRTQHQSNRELDKIDRNILRILQADGRISFTELGERVGLSTTPCTERVRRLEREGIIMGY
TARLNPQSLKASLLVFVEISLDYKSGDTFEEFRRAVLKLPHVLECHLVSGDFDYLVKARI
SEMASYRKLLGDILLKLPHVRESKSYIVMEEVKESLSLPIPD
>tr|A0A0Q0DGH7|A0A0Q0DGH7_PSEAP LysR family transcriptional regulator OS=Pseudomonas syringae pv. aptata OX=83167 GN=ALO85_03509 PE=3 SV=1
MFVMPSIWEIDVLATYPSIDTELLRTFVAIADHGGFTRAGEAVNRTQSAVSMQMKRLEED
VVQRPLFQREGRVLNLTAEGQVLLGYARRILKLHSEVFNTLREPHMIGVVKIGSPDDYVM
RFLPGILAQFAQAYPLVQVEVHCEPSDLLLQRHDLDLTIVTRKPGTEIGTLLRQERLMWM
EAIGFAPHEQTPIPLAMFNTHCFCRDWACNALDGVGRDYRIAYSSSSMAAITAVVSAGLA
VTAQFQSLATADLRILGEAEQLPQLPTASIVLLRKPENPSPITECMADYIIAGFKP
>tr|A0A0Q0CXB0|A0A0Q0CXB0_PSEAP 30S ribosomal protein S21 OS=Pseudomonas syringae pv. aptata OX=83167 GN=rpsU PE=3 SV=1
MPAVKVKENEPFDVALRRFKRSCEKAGVLAEVRSREFYEKPTSERKRKAAAAVKRHAKKV
QREQRRAVRLY
>tr|A0A0Q0BXI0|A0A0Q0BXI0_PSEAP Acyl-CoA dehydrogenase domain-containing protein OS=Pseudomonas syringae pv. aptata OX=83167 GN=ALO85_01316 PE=3 SV=1
MNLNQFAETHEVTNQPPPLDGANLYRIDVPLQEWSSRFGAGWAQPRIDAYGALAGGPLMA
AGFLANRHKPEFASHDRYGHRIDLVEFHPAYHQLMSAAIEHGIPSLPWTYPQPGAHVARA
AMSYLHTQADPGSGCPLTMTFASVPALRLQPDLAEIWLPKVLSTEYDPSNVGIAHKNGAT
IGMAMTEKQGGTDVRANTTRAFPVGAGGPGQAYELVGHKWFCSAPMCDAFLTLAQTEKGL
SCFLLPRHRPDDTRNEFYIQRLKNKLGNWSNASSEVEFRGALAWMIGEEGRGVPTIIEMV
AMTRFDCMIGSSALMRQALTQATHHCAHRAVSGRLLAEQPLMQNVLADLALESEAALALT
LRMGRALDHQDDDHEKRFVRLVTAVGKYWICKRAPAMINEAAECLGGAGYVEDSILPRLY
REAPVNSTWEGSGNVQCLDVLRTLSKEPGALDALFDELGDGHGDRHLAAHIGTLKAAFQD
TGDIQYRARQLTEDIAIALQGKLLLEAGNAAVSDGFIAGRLVSSGRVYGALPKGVDVETL
LQRASPQVA
>tr|A0A0Q0FCR9|A0A0Q0FCR9_PSEAP 4-hydroxyphenylpyruvate dioxygenase OS=Pseudomonas syringae pv. aptata OX=83167 GN=ALO85_100029 PE=4 SV=1
MFSQANTLLAPLNKAGKGQECGAVQSRPKQVAFIQELKSMQRSIATVSLSGTLPEKLEAI
AAAGFDGVEIFENDLLYYDGSPRNIRQMCADLGIAITLFQPFRDFEGCRRDRLQRNIDRA
ERKFDLMQELGTDLVLVCSNASADSVGDENILLDDLGLLAERAGARNLRVGYEALAWGKH
VNTWQQVWNLVRKVDHPALGVLLDSFHTLSLKGDPSAIAQIPGDKIFFVQMADAPLLAMD
VLEWSRHFRCFPGQGEFDLAGFLAPILRSGYTGPLSLEVFNDGFRAAPTRANAADGLRSL
LYLEEKTRQLMARDEPAAVPEILFNPPAASTYNGVEFLEFAVDESHGARLSGWLQRLGFA
KLGQHRSKAVSLLGQGDIRIVLNAEPYSFAHSFFEAHGPSLCATALRVDDGHQSLERARV
FKGQPYRGLVGPNEREIPSVRAPDGSLIYLVDNAAPGESIYDSDFVVDPQAVAKGGLQRI

DHMAMALPADSLDSWVLFYKSILDFEADDEVVLPDPYGLVKSRALRSRCSTVRLPLNISE
NRNTAISHALSSYRGSGVHHIAFSCEDIFAEVSRAKEAGVPLLDIPLNYYDDLAARFDFD
EEFLSELAYYNVLYDRDAQGGELFHVYTDAFDGRFFFEILQRKNGYVGYGAANVPVRLAA
MAKARNVAARQARL
>tr|A0A0Q0CC24|A0A0Q0CC24_PSEAP Endolytic murein transglycosylase
OS=Pseudomonas syringae pv. aptata OX=83167 GN=mltG PE=3 SV=1
MIRKILVLLETAVVLAGLLLGFAFWQQYQALNQPLEVAQEQLLDVPAGSTPTGVLNRLQA
DGVIKDAFWLRLYWRFNLSGQALHSGEYRMVPGMNVKGLFDVWKRKEVVQYSLTLVEGWN
FRQVRAALAKQPKLDQTLAGLSDSELMAKIGHPDVFPEGRFFPDTYRYVRGMTDVELLKQ
AYSRLEEVLDEEWNARSSEAPYSNPYQALIMASLVEKETGVPQERGQIAGVFVRRLKLGM
QLQTDPTVIYGMGERYNGKLTRANLKEATPYNTYVIAGLPPTPISLVGREAIHAALNPVD
GSSLYFVAKGDGSHVFSDDLDAHNAAVRDYQLKRRADYRSSPAPATPAPATDSPSSEAVP
ADVPDAPQSEPNAQ
>tr|A0A0Q0ITL2|A0A0Q0ITL2_PSEAP DUF892 domain-containing protein
OS=Pseudomonas syringae pv. aptata OX=83167 GN=ALO85_01472 PE=4 SV=1
MTRTTIEDLFIHELSDVYSAEKQITKALPRLARASTNPLLAEAFKAHLEETQGQIDRIDE
LVEGAGLKLKRMKCAAMEGLIEESKELLEEIEKGAVLDAALIGACQKVEHYEIASYGTLI
AMAKHLKMC
>tr|A0A0Q0DCA2|A0A0Q0DCA2_PSEAP N5-carboxyaminoimidazole ribonucleotide
mutase OS=Pseudomonas syringae pv. aptata OX=83167 GN=purE PE=3 SV=1
MSALVGVIMGSKSDWSTLSHTADMLEKLGIPFEVKVVSAHRTPDLLFQYADEAEGRGIEV
IIAGAGGAAHLPGMCAAKTHLPVLGVPVQSSMLSGVDSLLSIVQMPAGIPVATLAIGKAG
AINAALLSASILGAKHPQFHAALKKFRTEQTDSVLDNPDPRHA
>tr|A0A0Q0FIG7|A0A0Q0FIG7_PSEAP Leucyl/phenylalanyl-tRNA--protein
transferase OS=Pseudomonas syringae pv. aptata OX=83167 GN=aat PE=3 SV=1
MLTWLTRNSLDFPPLEKALREPNGLLAAGGDLSADRLISAYRHGCFPWFQDGQPILWWSP
DPRTVLFPEELHVSRSLGKVLRQSRYRVTFDQDFASVIKACAAPRSYANETWITGSMQAA
YVELHRRGHAHSVEVWDQDELVGGLYGLAMGQLFFGESMFSRADNASKVGFATLVEHLTD
WGFVLIDCQMPTQHLHSFGARAIPRQTFADYLSRHLDQPTDANWSARRV
>tr|A0A0Q0BRE4|A0A0Q0BRE4_PSEAP Type III secretion protein HrcQb
OS=Pseudomonas syringae pv. aptata OX=83167 GN=ALO85_04187 PE=4 SV=1
MSTEDLYQDDVEMLDDYEEPVPEQADQQLRDDEHTEHAFGYADSEAEHEEASGEHNESPM
LDSLELALTLRCGELRLTLAELRRLDAGTILEVSGIAPGHATLCHGEQVVAEGELVDVEG
RLGLQITRLVARS
>tr|A0A0Q0IAL8|A0A0Q0IAL8_PSEAP Porin OS=Pseudomonas syringae pv. aptata
OX=83167 GN=ALO85_04286 PE=3 SV=1
MKKINKGKSLSRLQLVCKLSALGAMGLAGSVHAAEPFAADSPWMTGDWGGKRTELLDKGY
DFSLEYVSEMASNLKGGYNDDTTGRYSDQFALGMKVDLQKVLGWQDAEFKLAITERSGRN
ISNDRIGDPRAGTLSSSQEVWGRGQTWRLTQMWIKQKYFDGALDVKAGRFGPGEDFNSFP
CDFQNLSFCGSQVGNYVSTWYNWPISQWALRIKYNITPEVYAQVGVYEQNPSNLETGNGF
KLSGSGTEGMILPVELVWTPSFNSLPGEYRVGYYKSTPNANDVYEDVNGQPQALTGAAFK
SHSSKHGWWVVAQQQLTTHDGDASRGLSIFANATVHDKATNFVDNYQQIGFTYKGPFNSR
PKDDIGIGIARIHVNDDVQDRVRLANQISGINDYDNPGYLPVQSTEYNSEIYYGFHVTNW
LTVRPNLQYIKHPGGVDQVDDALVAGIKIQSKF
>tr|A0A0Q0DTC2|A0A0Q0DTC2_PSEAP Y1_Tnp domain-containing protein
OS=Pseudomonas syringae pv. aptata OX=83167 GN=ALO85_03850 PE=4 SV=1
MPDCATTRHLRKGRYSAVGQIYMVTSVTKGRVPVFAEMQLGRLLVREMKRCEEQGLVKSM
AWVVMPDHLHWLFELKAGDLPSLMKQLKARSSIAVGKRCVRPHALWQSGYHDRAIRDEKD
IAPIARYIVANPLRAGLVKKLGDYPLWDAIWV
>tr|A0A0Q0C0L9|A0A0Q0C0L9_PSEAP Putative metal-dependent hydrolase
OS=Pseudomonas syringae pv. aptata OX=83167 GN=ALO85_01011 PE=4 SV=1
MSLFKRILFRRSAAMLSPQAIPHRSIRFTFDDSIPRYWHGGRTHTTRFFDCLSFMFPQGE
KIFIESVVHFRHLLDRGSALDKAVDDFVYQEASHIREHRAYNLLLGTQGAPVEKLERMLL
RRRKESERLPASFRLALTASLEHITAILSEQILRNPAYMAGADPTMARLWRWHAMEEIEH
KAVAYDVLCKVERNPLRRYLLRWVAMTSLSVYFTFDLTYFTYHLVRGDRQHRNWREWLRL
QWWLFVNPGLLSRIVPAGLFWFAPGFHPDRIDTRELLDNARQALDEQR
>tr|A0A0N8T866|A0A0N8T866_PSEAP Uncharacterized protein OS=Pseudomonas
syringae pv. aptata OX=83167 GN=ALO85_100310 PE=4 SV=1
MKDSLVELISKVSSGCMGGDEIVHIADEAAQAYADPQAFLAANPDINYDDTFPIPLGEWV
VVGSLPETVLFQADSYMDLFEQIVQSFGKDVTFNIKPKQLAKVEPLVALNRIQIQLSSMN

KEMGGYVLMNFSQPLDDELQAVLVYGCDEARVVELAASAGIHAAPALQALRG
>tr|A0A0Q0IKH5|A0A0Q0IKH5_PSEAP HylII OS=Pseudomonas syringae pv. aptata OX=83167 GN=ALO85_02958 PE=4 SV=1
MYYGEKFNAWSHLVGAVLAAVGAIWLLVMACLHGDVWKVVSIAIYGVCLVTLYSVSTVYH
SIKGRSKSIMQKVDHFSIYLMIAGSYTPFCLVTLRGAWGWTLFGIVWGLALIGILQEIKP
RSEARIMSIVIYAVMGWIVLVAVKPLLAALGVSGFIWLAGGGVLYTVGILFFAYDQIRHF
HGIWHLFVIGGSLMHFVAICFYVL
>tr|A0A0Q0DES2|A0A0Q0DES2_PSEAP Putative phosphodiesterase OS=Pseudomonas syringae pv. aptata OX=83167 GN=ALO85_03119 PE=4 SV=1
MQIISHRGYWLQRPERNLPEAFHRSFDLGFGTETDVRDVAGQLVISHDMPAGGELTLDGL
LDIMAGRNLPLAINVKADGLAQALAETFARYGHTNWFAFDMAVPDMRSYLNANLITYTRL
SDVEPSPAWLEQAAGVWLDGFEGEWFSSQVIGDLLSLGKRICVVSPELHGRGHDALWQQL
LEFRSQDRLMLCTDLPADAATFFT
>tr|A0A0Q0C222|A0A0Q0C222_PSEAP Haloacid dehalogenase OS=Pseudomonas syringae pv. aptata OX=83167 GN=ALO85_03908 PE=4 SV=1
MSFLRPKFITFDCYGTLTNFHMGTMTRELFADRVPAEQMDQFVRDFSAYRLDQVMGDWMP
YDEILKVALARTCKRWNVEYREEGQLYYDAVPTWGPHADVPAGLAKIADKIPLVIYSNAS
DSQIMSNVEKLGAPFHKVFTADQAKVYKPRLAAFEFMLDNLGCGPEDILHVSSSFRYDLF
SAHDMKIKNKAFVARGHEQPANSFYEYHQIPDIGGLAGLVGL
>tr|A0A0N8T904|A0A0N8T904_PSEAP Putative phosphatase OS=Pseudomonas syringae pv. aptata OX=83167 GN=ALO85_03932 PE=4 SV=1
MRPQTSFIFDLDGTLTDSVYQNVAAWKEALDAEKIPLAMWRIHRKIGMSGGLMLKSLSRE
TGLNISEEQAERLSEKHAQAYERLQGQIIALPGAVELLETLDKENLKWCIATSGGIDTAT
INLKALKLDINKINIVTRDDVSYGKPDPDLFLAAAKKINAPIDECLVIGDAIWDMLAARR
CKATGVGLLSGGYDIGELERAGALRVYEDPLDLLNHLDEIASRP
>tr|A0A0Q0IF02|A0A0Q0IF02_PSEAP Uncharacterized protein OS=Pseudomonas syringae pv. aptata OX=83167 GN=ALO85_03249 PE=4 SV=1
MKTPTLVLASFLSLCCCAAFAQDGADMLQKRQETRTLERQQAREKLAGSEDKKHNEKSAV
TSSIKHEDVSKSEEKPIY
>tr|A0A0Q0CDM0|A0A0Q0CDM0_PSEAP Uncharacterized protein OS=Pseudomonas syringae pv. aptata OX=83167 GN=ALO85_02731 PE=4 SV=1
MKVGNKAFLPVLALIISTIGVASAIAFVKLSEVDATSTLMLRMFMAGGMASALAAWPAGK
NTETGTVGTARSRRAMLWLLVLSGVVSCVDLLSNHWAVKLTSMANTSVLMNLSPVFVALL
SYLFMKEKIGVYQVLALALSIGGACLLVFDGSASVAFSSQSVTGDLLALNSALFYAIYLI
LIKSLRDCFTSRKIIIWNSFTCAVLLLPIALLTSPKILPDTVQGWVVIALLALISQLLGH
GLMAYALRHVDVVLASVSALARPVVAILIGFFFFDEQLTLVQWLGVAAVLVGVWWYKRPV
PVVRPVVQAKTPSEA
>tr|A0A0Q0DKN7|A0A0Q0DKN7_PSEAP Uncharacterized protein OS=Pseudomonas syringae pv. aptata OX=83167 GN=ALO85_00910 PE=4 SV=1
MNWILAIAIAGSSTASIMRPYSTEAECRKALEEFFDKSTGKTQYSGSCFEKNSPVLKLLK
G
>tr|A0A0Q0BVB8|A0A0Q0BVB8_PSEAP Uncharacterized protein OS=Pseudomonas syringae pv. aptata OX=83167 GN=ALO85_00651 PE=4 SV=1
MIKTKLTALTLAGLMAVASGAAFAESTTTPETPINKNATKLPGTNTQPVDRTSSETPGVQ
TESKEAAAERSMKHDKATHGTHDKSTVTEKTKTQ
>tr|A0A0Q0DMB6|A0A0Q0DMB6_PSEAP Diguanylate cye domain protein OS=Pseudomonas syringae pv. aptata OX=83167 GN=ALO85_00998 PE=4 SV=1
MMMSLPAEDRTQLFKDTATQFWKHITPIVYVALGIHCVLLTVFLGLGMKIMWIANIVSTL
VYVSCIYLIRRGRHRQAGQLMSLEIIGHAVLATWQLGWESNFSFYIFCVIPVIAFTFQLN
VVRRIVYSVAILMSLVGCFAFRRYTGQGTGLSQNVLDAFGIVNVHTATTLSIYATALSVR
FTSSMQLSLFHIANRDSLTNLYTRRRVLHRIRELEARRQGLSASLILLDIDHFKQINDIH
GHETGDVVLQRVAEIIGSSVRSSDMAARWGGEEFLVLMPDTTPEDALRAAERIWRRIAEE
AGRINERIIAVSATVAVATIATGEAFQLALNRADTSLYMGKQQGRNRVMIAG
>tr|A0A0Q0IK81|A0A0Q0IK81_PSEAP Type IV pilus expression regulatory protein PilS OS=Pseudomonas syringae pv. aptata OX=83167 GN=ALO85_100060 PE=4 SV=1
MTADSPSLVSPQAQRILRLYHLYRLTIGVVLVLLISSSLETELLQLANPNLFRSGCWLYL
VFNILVVVLLERPSRQAQFFGLAMTDAIMLSALFYAAGGPSSGIGNLLIASVAISNVLLR
GRIGLLIAAVSAIGIIYLTFYISFGTPSVSNDYVQAGSLGALCFAAALLVQALTARMQVS
EVLAEQRAADVDSLEALNALILQRMRTGILVLDANHQVLLANQSALHLLSQEQLVGQMID

TCSPQLVERLRQWLVNPIMVPRSITTAPVGLVLQPSFATLNRGDQRQTLVFLEDLSQIAH
QAQQLKLAALGRLTAGIAHEIRNPLGAVSHAAQLLNESEELSAADRRLGQIIHEQSGRID
RVIENVLQLSRRREAQPELLDLKTWLEHFVAEFRSSTAPNQAIHVDISPGTLTTRMDSGQ
LTQIMTNLVQNGLRYSGKNHPSAQVWLKLHNEPRSDLPTLEVLDDGPGVSEQHLSKIFEP
FFTTESKGTGLGLYLSRELCESNQAHLEYAPRPTGGSCLRITFAHPFKTS
>tr|A0A0Q0ID32|A0A0Q0ID32_PSEAP Aspartyl/Asparaginyl beta-hydroxylase
OS=Pseudomonas syringae pv. aptata OX=83167 GN=ALO85_01077 PE=4 SV=1
MALSARFKESFMTLSFVAKASILMLFLGSTLYVHLRGRARLPLLRQFVNHSALFAPYNAL
MYLFSRVPSEPYLDRSKFPELDILKDNWETIRDEAMHLFDEGYIRAAEKNNDAGFGSFFK
KGWKRFYLKWYDKALPSAEALCPKTVELVNSIPNVKGAMFALLPGGSHLNPHRDPFAGSL
RYHLGLSTPNSDDCRIFVDGKEYAWRDGQDVMFDETYVHWVKNETDQTRVILFCDIERPL
TNRFMTRMNRSVSAFLGRATAPQNLDDERVGGINQAYAFSKRFSDGFSGKVKQFKRANPK
AYRILRPVLAVAVLTLLGYWLFG
>tr|A0A0Q0DPW8|A0A0Q0DPW8_PSEAP Putative phosphoserine phosphatase
OS=Pseudomonas syringae pv. aptata OX=83167 GN=ALO85_04333 PE=4 SV=1
MRLALFDLDNTLLGGDSDHAWGDYLCRRGILDAATYKTRNDEFYQDYLAGTLNMTDYLNF
TLEILGNTDMAQLEQWHREFMRDCIEPMMLPKALELIAKHREAGDKLVVITATNRFVTAP
IVAQLGIDTLLATECEMADGRYTGRTTGVPCFREGKVTRLNQWLEDNAFSLEDSYFYSDS
MNDLPLLEQVANPVAVDPDDKLRAEAERRGWPVITLRH
>tr|A0A0Q0FWG3|A0A0Q0FWG3_PSEAP Alginate biosynthesis regulatory protein
AlgR OS=Pseudomonas syringae pv. aptata OX=83167 GN=ALO85_01580 PE=4 SV=1
MNVLIVDDEPLARERLSRMVNEIEGYRVLEPSASNGEEALALIETHKPDVVLLDIRMPGL
DGLQVAARLCEREAPPAVVFCTAHDEFALEAFQVSAVGYLVKPVRPEHLIDALKKAERPN
RVQLAALTRPAAESGSGPRSHISARTRKGIELIPLDQVIYFIADHKYVTLRHEGGEVLLD
EPLKALEDEFGDRFVRIHRNALVARERIERLQRTPLGHFQLFLRGLNGDALIVSRRHVAG
VRKMMQQL
>tr|A0A0Q0C6F9|A0A0Q0C6F9_PSEAP Uncharacterized protein OS=Pseudomonas
syringae pv. aptata OX=83167 GN=ALO85_01233 PE=4 SV=1
MFSKPDIQRVLETAFLPSKCECVVAPNETFSVKLLHPESGDIQLYVTGLSLSEVDSSRSI
ARLVLSLREQRDLMGQMNLSMRRMA
>tr|A0A0Q0BY34|A0A0Q0BY34_PSEAP Cys regulon transcriptional activator
CysB OS=Pseudomonas syringae pv. aptata OX=83167 GN=ALO85_02471 PE=3 SV=1
MKLQQLRYIWEVAHHDLNVSATAQSLYTSQPGISKQIRLLEDELGVEVFARSGKHLTRVT
PAGERIITTAGEILRKVESIKQIAQEFSNEKKGTLSIATTHTQARYALPPVISNFIKQYP
DVALHMHQGSPMQIAEMAADGTVDFAIATEALEQFSDLVMMPCYRWNRCIVVPQGHPLTK
APKLTLELLAEYPIVTYVFGFTGRSKLDEAFSHRGLVPKVVFTAADADVIKTYVRLNLGV
GIVARMAIDEAVDSDLVVLDASDLFESSVTKIAFRRGTFLRGFMCDFIEKFAPHLTREVM
AKAIQCHNKQEMEELFANVELPVH
>tr|A0A0Q0FC45|A0A0Q0FC45_PSEAP Ornithine cyclodeaminase OS=Pseudomonas
syringae pv. aptata OX=83167 GN=ALO85_04226 PE=4 SV=1
MMLHLDGSTVKSLINVQLSLHLSEAAFRLHSSGQVEQPLRTVIKGGDNALMGVMPAYIKS
GPYQGFGLKSVVVRFGEAAGGPSHMGTILIYDEAQVTPVVAVDAGAVTEIRTAAASAHAT
RLLAAPDASRLAILGTGLQARAHLQAMQAVRPIRYVSVWGRSNEACREFAAWSAQHCDLP
VSICTSAAEAVRDADIICTTTAARSPILFHADLPVRCHINAIGASAPGFQELHEDVYQDI
TLFTDCNRSVLAASDCVIKAVQKGLLPQSGCATEIGTLCKDETLQKNRGITVFKSVGLAV
QDLVFSREIIRQWVASRCNGSSLT
>tr|A0A0Q0D374|A0A0Q0D374_PSEAP Iron-sulfur cluster insertion protein
ErpA OS=Pseudomonas syringae pv. aptata OX=83167 GN=erpA PE=3 SV=1
MSVESFTPTALEFTPNAAHKVKTLVDEEGNDRLKLRVFVTGGGCSGFQYGFTFDEDVADD
DTIVEREGVSLVVDPMSFQYLAGAEVDYQEGLEGSRFVIKNPNASTTCGCGSSFSI
>tr|A0A0Q0CE92|A0A0Q0CE92_PSEAP Uncharacterized protein OS=Pseudomonas
syringae pv. aptata OX=83167 GN=ALO85_101875 PE=4 SV=1
MRFIRMMTREYRDQGKMRAVHTAPFFVCRKAYPTKKALLGAFCLRGI
>tr|A0A0Q0DKY1|A0A0Q0DKY1_PSEAP Uncharacterized protein OS=Pseudomonas
syringae pv. aptata OX=83167 GN=ALO85_101373 PE=4 SV=1
MHEHPSCALHFAVSCTIGIGQKGDCIASGDQEQALLIGIDADRTAGES
>tr|A0A0Q0BVG8|A0A0Q0BVG8_PSEAP Peptide methionine sulfoxide reductase
MsrA OS=Pseudomonas syringae pv. aptata OX=83167 GN=msrA PE=3 SV=1
MTNPTETAILAGGCFWGMQDLLRSYPGVLHTRVGYTGGDVPNATYRNHGNHAEAIEIVFD
PAVISYRQILEFFFQIHDPSTANRQGNDLGPSYRSAIYYLSEQQRDIAEDTAADVDASKL

WPGRVVTQIEPASEFWEAEPEHQDYLERIPNGYTCHFIRPNWKLPKRA
>tr|A0A0Q0DCI6|A0A0Q0DCI6_PSEAP Uncharacterized protein OS=Pseudomonas syringae pv. aptata OX=83167 GN=ALO85_01449 PE=4 SV=1
MLAYRFKPEAAIRQAQGDTPDVLGDILQDGVNLALWQRQLPAHIEDFGALLLSMGEPLAE
SMTLEVQGGEVEPDLSALASGYSDLLGYQGFIADVSWLVGAYACLLGAECVGLRLRVLDK
AMCPRFHVDHVPVRLITTYGGVGSQWLHEGVMDRKQLGRLDAEPTDAAHIQQINSGDVAL
LKGERWHGNEGFGLIHRSPQLLRNERRLILTLDWLA
>tr|A0A0Q0CYF8|A0A0Q0CYF8_PSEAP Serine O-acetyltransferase OS=Pseudomonas syringae pv. aptata OX=83167 GN=ALO85_01682 PE=4 SV=1
MSDIPEASADGRSSHWQLQRIVSQLRGAREDWRARNRRVSGEHGGRELPSRAAMGEILEA
LSGALFPMRLGPVDLREESEDFYVGHTLDVALNALLAQARLELRYVAHQQGGDLNSIDAR
ALELIQGFAMALPGIRLLLDTDVLAAYHGDPAARSVDEVLLCYPGILAVIHHRLAHYLYR
AGLPLLARISSEIAHSATGIDIHPGAQIGPSFFIDHGTGVVIGETAIIGERVRIYQAVTL
GAKRFPSDEDGQLQKGHARHPIVEDDVVIYAGATILGRITIGKGSTIGGNVWLTRSVPAD
CNLTQANLLQQDDCSRK
>tr|A0A0N8T8U5|A0A0N8T8U5_PSEAP 30S ribosomal protein S12 OS=Pseudomonas syringae pv. aptata OX=83167 GN=ALO85_200044 PE=4 SV=1
MTNCGANYTLAATPSAVTENMLRLVGSSLDITSAINDFRPSYHQDFANVQSVVCRYLNAP
SGSANAALLAQALSTALLNWGACRRRSPILRPIPQIVSALKDKRLHERLLKLSQQSLAAF
SLYLQEHRLLDSDAPLNDVGAFDQEILGILNTLASVLFFNNTNVTYPMKALLLITGLIPA
LDSQVRGGLTRAGKAGFTGQQLLPRNVQQAAGRRICELPYYLGHCWNLHREVFEEGILKS
DHKGLQDTPGRVFDILLFMQNQATRNQLLAFKAERV
>tr|A0A0N8T9L4|A0A0N8T9L4_PSEAP Methionine biosynthesis protein MetW OS=Pseudomonas syringae pv. aptata OX=83167 GN=ALO85_02940 PE=4 SV=1
MRADLEIIQEWIPAGSRVLDLGCGDGELLTWLRDHKQVTGYGLENDAANIAECVAKGINV
IEQDLDKGLGNFASNSFDVVVMTQALQAVHYPDRILDEMLRVGRQCIITFPNFGHWRCRW
YLASKGRMPVSEFLPYTWYNTPNIHFCTFGDFEELCRERDAQVLDRLAVDQQHRHGWASK
LWPNLLGEIGIYRVTSPGLTDHRIAV
>tr|A0A0Q0E0K8|A0A0Q0E0K8_PSEAP Chemotaxis protein CheW OS=Pseudomonas syringae pv. aptata OX=83167 GN=ALO85_03283 PE=4 SV=1
MGSLVTTRQTAATVEEEAQYLTFMLGGEMFAIGILGIKEIIEYGSLTVVPMMPAFVRGVI
NLRGAVVPVVDLSARFGRQNSAITRRSCVIIIEANSEDGQPQDIGLLVDNVSAVLEIPAS
QIEPPPNFGARIRADFISGMAKVDGKFVIVLEVDRVLSIDEMSSLAEASQSLPTDVDAT
>tr|A0A0Q0C2U8|A0A0Q0C2U8_PSEAP GTP 3',8-cyclase OS=Pseudomonas syringae pv. aptata OX=83167 GN=moaA PE=3 SV=1
MSEPVLMDRFARKVDYLRMSVTDRCDFRCVYCMAEEMTFLPRQQILSLEEILQVAERFVA
LGTRKIRLTGGEPLVRAGVVGLCEKIAALPGLRELCMTTNGSQLDKLAAPLFKAGVTRLN
ISLDSLDPQRFRELTRTGDLHKVIAGIDAANAAGFVHTKLNCVVMHGRNDDEINDLLAFA
IDRNLDVSFIEEMPLGIISEHSRAESFYSSDQVRERIAERYTLVPSTDSTQGPSRYWRLA
EAPGIRIGFISPHSHNFCGTCNRVRMTVEGRLLLCLGNEHSVDLKAVLRANPGQPEKLEK
AIIDAMQLKPWSHNFTHDDGVQVVRFMNMTGG
>tr|A0A0Q0ICD7|A0A0Q0ICD7_PSEAP Outer membrane ferripyoverdine receptor OS=Pseudomonas syringae pv. aptata OX=83167 GN=ALO85_04786 PE=3 SV=1
MKKLLVSPRRCPSPRSRGASSARCITTSSAIAGMTLMLRPFPPSLTGLVVLGLSAAAVAA
PVQLDLPAQPLSKSLTQLSQESGLQISADDYLIASKQAPAVRGSLEPAQALSRLLLGSGL
DAQVQGNAVKVFQPASNGPMTLGNVEISAQTGIAGMATTEGSGSYTTGSMSSATRLPLSI
RETPQSVRVITRQRIEDQGMNDLNDVVKYAPGVTLRKFGSDRQQFLARGFAIDNLMYDGL
PTIVSTFTLDTISGADLALYDRVEVVRGATGLMTGAGSPSATLNLIRKRPTTTPQVSVTT
SAGSWDRYRTEVDASNRLNDSGTLRGRVVAAYETGNSFVDERDKERQTFYGVLEADLSES
TTWTIGASRQRDDATSDWGSLPSGPNGENLDLPRSTFLSNDWAYWDRHNVSLFTDLTHHF
DNGWSSKLAATRIWAESDMYSNYLTANGTGYGQAAGQYDTTDVQTSLDGSLSGPFQLFGR
EHELMLGVSRREEKFDQKGGFYGSTPIDIHHFNPNVIAKPDLATRTAYVSQNTATEEGVY
AAARFNPIDPLHLIVGSRVSWYDYQNRSANNGDYKVIREVTPYAGVVYDLNDTYSAYASY
TEIFKPQNQLDVGGGVLDPMTGESYEIGLKGEYFDGSLNASLAVFDMTQENRAYAIVNPP
AACQQQNRVCYEAAGEVRSRGIDAEITGQLTPDWQASASYTLVLSQYVKDRTNDKGDLFA
PNQPKHLFKAATSYHLPGNLNKWRVGADVLAQSETYNRVGNGHSVQDAYAVVGLMAGYRF
DEHWDARVNLNNVFDEKYWQGIPLATGSGVYGDPRNLMVSLKWSL
>tr|A0A0Q0DX02|A0A0Q0DX02_PSEAP Riboflavin synthase, alpha subunit OS=Pseudomonas syringae pv. aptata OX=83167 GN=ALO85_01913 PE=4 SV=1
MFTGIIESIGSIRALTPKGGDVRVYVETGKLDLTDVKLGDSIAVNGVCLTAVELPGDGFW

ADVSRETLTVTAFVDLKSGSRVNLEKALTPTTRLGGHLVSGHVDGVGEIVARADNARAIQ
FTVRAPRELAKYIAHKGSITVDGTSLTVNSVNGAEFELTIVPHTWSETIMADYQPGRKVN
LEVDLLARYLERLLLGDKAAEPGHGTITESFLAENGYLKS
>tr|A0A0Q0CGV9|A0A0Q0CGV9_PSEAP Uncharacterized protein OS=Pseudomonas syringae pv. aptata OX=83167 GN=ALO85_00594 PE=4 SV=1
MNFMTTTPTTLLAALEASTMLEIDGLHAWDFSLGEELNVECMDGRERKVWRFTKAQVEAA
TFDESLQSWVINDGNADHRIICLDAFTPSDEDDSAQD
>tr|A0A0Q0DJC0|A0A0Q0DJC0_PSEAP Glutathione peroxidase OS=Pseudomonas syringae pv. aptata OX=83167 GN=ALO85_01762 PE=3 SV=1
MSAFHDIKLKALGGQELPLANFRNKVVLVVNVASKCGLTPQYAALENLYQQYKDKGLEIL
GLPCNQFAGQEPGTEQEIAEFCQLNYGVTFTLGEKLDVNGPNRHSLYRLLAGEGAEFPGD
ITWNFEKFLVGKNGGVSARFAPRTTPDDPAVIQAIEKALAQS
>tr|A0A0Q0CXF0|A0A0Q0CXF0_PSEAP Uncharacterized protein OS=Pseudomonas syringae pv. aptata OX=83167 GN=ALO85_01222 PE=4 SV=1
MPAVDYLFCRSRLLLSLRNNNKRRSAMFKYKHSKVRQAGLILFTTALLLIVPNLNRLFG
>tr|A0A0N8T9C6|A0A0N8T9C6_PSEAP Putative lipoprotein OS=Pseudomonas syringae pv. aptata OX=83167 GN=ALO85_00333 PE=4 SV=1
MSRERNLNGMILSAVGAVIGTVGCLWFYGYLHFAKPEDALLLGEFTLLKTVPGEDYKVAA
TPAAEVAQCVDGVLVLFDTSRKGLSGVLIDDRRQAVRCLGQQTPRQ
>tr|A0A0Q0BRZ6|A0A0Q0BRZ6_PSEAP Acetyltransferase OS=Pseudomonas syringae pv. aptata OX=83167 GN=ALO85_04257 PE=4 SV=1
MIEDMKFIELLKNRKIRKQLRKLDKLERLAQKIRLQYPRAEVGVGTYGAPDIVEFGDDSV
LRVGSYTSIAEGVRILLGGEHRTDWITTYPFPAMVEQLADIKDYSPSKGDVTIGSDCWIC
ANALIVSGVTIGHGAIVGAGAMVVRDVAPYSVVGGNPCKFIRWRFEEDVRQLLLQTAWWD
WPMEEVKSVARTLCSSDMDAFLSYVLQRQATSRPLGN
>tr|A0A0Q0DJF0|A0A0Q0DJF0_PSEAP FAD-dependent pyridine nucleotide-disulfide oxidoreductase OS=Pseudomonas syringae pv. aptata OX=83167 GN=ALO85_00564 PE=4 SV=1
MTKLKLVMIGNGMAGVRTLEELLKLSEELYHITVFGAEPHPNYNRILLSPVLAGEQNFED
IVLNDLDWYERNGIQLLLNRRVVKIDRIRRLVIADDGTQAHYDRLLIATGSRPFILPIPG
NTLNGVIGYRDIGHTQQMIDTAVTHKRAVVIGGGLLGLEAANGLSLRGMHVTVVHNGQTL
LERQLDKTSGRMLQAALEKRGLSFRLDEQTNALLGDDQGRVTAVQFNNGDRIDADLVVMA
AGIRPNTELAEQAGLPCNRGILVNDTLQTYDPRIYAIGECVSHRGIAYGLVAPLFEQARV
CANHLAQLGFARYPGSVTSTKLKVTGIDLFSAGDFLGGEGTESITLSDPIGGVYKKLVIK
NDVLVGACLYGDTADGNWYLQQIRDGQGIDAIRDHLMFGKPAAPEAACA
>tr|A0A0N8T8Y0|A0A0N8T8Y0_PSEAP SnoaL-like domain protein OS=Pseudomonas syringae pv. aptata OX=83167 GN=ALO85_01716 PE=4 SV=1
MADFLHRFAEGFAALNKDNLDQVAELYSEDVSFSDPMHDIHGLAAMRRYFGELYANVEDL
RFDFHAFDEVRPGEGYLVWTMSYSHPRLAAGRTIQVEGCSHLKWRDKVYRHRDYFDAGAL
LYEHLPIMGRVIHWLKKRLG
>tr|A0A0Q0DR02|A0A0Q0DR02_PSEAP Site-determining protein OS=Pseudomonas syringae pv. aptata OX=83167 GN=ALO85_02517 PE=3 SV=1
MAKILVVTSGKGGVGKTTTSAAIGTGLALRGHKTVIVDFDVGLRNLDLIMGCERRVVYDF
VNVVNGEANLQQALIKDKKIEGLFVLAASQTRDKEALTKEGVEKVLMELKESFEYIVCDS
PAGIETGAHLAMYFADEAIVVTNPEVSSVRDSDRMLGLLASKSRRAERGEDPIKEHLLLT
RYNPERVSKGEMLGVEDVKEILAVTLLGVIPESQAVLKASNQGVPVILDDQSDAGQAYSD
AVDRLLGKTLEHRFLDVHKKGFFERLFGGN
>tr|A0A0Q0DU17|A0A0Q0DU17_PSEAP Pseudouridine synthase OS=Pseudomonas syringae pv. aptata OX=83167 GN=ALO85_02426 PE=3 SV=1
MRLDRFLSNLPRFNRQQVRLALASGRVQVDGQATRDPHHEVREFSCVALDEEILQAGKVP
RYFMLHKPEGCVSATSDAQHPTVLDLLDEPDKHELHIAGRLDFNTTGLMLITNDGQWSRR
LTQPQTKMPKVYYVETEQPIDERYAAKFAEGLYFAFENLTTQPAQLTLLGPQSARLSIVE
GRYHQVKRMFGHFDNKVLRLHRETIGALALDPNLRPGSYRALNAGEIGLF
>tr|A0A0Q0C6Y5|A0A0Q0C6Y5_PSEAP GT2/BcbE-like lipopolysaccharide biosynthesis protein OS=Pseudomonas syringae pv. aptata OX=83167 GN=ALO85_03117 PE=4 SV=1
MSALNIILLTGRQSEAPHDEGEYPHYLCEYNGKPLIHTLIDNCAVLQPRRMICTFSNHDI
ARLHLRNMVPQMHPSASVLPVHNITRGAACTALLASEEIDNDDELLILSVSDFLDIELQD
VVRAFRESHEDAGVVIFNSLHPRYSFARLDSDCRVIEAAEKNPISSNAIAGVYWFKSGSL
FVSAAKEMIRKDARVNDNFYIAPALNELVLLQKRIGAYRIEPNQYRPLKTDSQLHAFEAG

EMR
>tr|A0A0Q0C7A8|A0A0Q0C7A8_PSEAP Flagellar protein FliL OS=Pseudomonas syringae pv. aptata OX=83167 GN=ALO85_04038 PE=3 SV=1
MAQSEEVKDPATTGKGKGKGKLKLIIIAVVALLLAVGLSVGATWFLMHKSESKVDPAAAAAA
ANVKPVAVFEPMTPAFVVNFNANGRQRYMQVSITMLGRNAADMEALKVHMPLIRNNLVML
FAAIPFESLASPIGQEMLRQKATASIQEVAQKELGKTVIEQLLFTNFVLQ
>tr|A0A0Q0D8Y8|A0A0Q0D8Y8_PSEAP Isochorismatase hydrolase OS=Pseudomonas syringae pv. aptata OX=83167 GN=ALO85_03759 PE=4 SV=1
MSKPLVRWPIDPRRTAVIVVDMQKVFCEPSGALYVKSTADIIQPIQKLLQTARAAQVPVV
YLRHIVRGDGSDTGRMRDLYPNVDQILARHDPDVEVIEALAPRPGDVIVDKLFYSGFHNT
DLDTVLRARDVDTIIVCGTVTNVCCETTIRDGVHREYKVIALSDANAAMDYPDVGFGAVS
AEEVQRISLTTIAYEFGEVTTTADAIERIASA
>tr|A0A0Q0D0J8|A0A0Q0D0J8_PSEAP Alpha/beta fold family hydrolase OS=Pseudomonas syringae pv. aptata OX=83167 GN=ALO85_04643 PE=4 SV=1
MRHRPTVRPSLLPQGNRLMNHIRFVRLLRNTWLVALLSGLSSIALAIEKNYTVTAPDGVK
LAVQESGDPSGPALIFIHGLLGSHLNWQKQIGSPQLQRYRMITYDLRGHGLSDKPENIEA
YRNGRHYADDLAAVVEATASKRPVLVGWSVGGVVMSNYLAAYGDAKIGGIVYVDGVIELN
AALITAHPQVYAGLSSEDLKTHLDAVRTFLGLCFQTKPDALTFERLLSSAAMASWAMTRA
TPAMTVDAAKGLPRAKVPVLMLYGGKDELVNVQPSIARARQLNARIQSKVYENSGHAPFL
EEASRFNTDLTAFMDSAAIAAKGLE
>tr|A0A0Q0D493|A0A0Q0D493_PSEAP DoxD-like family protein OS=Pseudomonas syringae pv. aptata OX=83167 GN=ALO85_02456 PE=4 SV=1
MNTLIKSVLTTRAGYGLTVLRVIVGIAFIAHGSQKLFGAFGGYGLEGTAQYMESLGLTPG
YLMALMSGSAEFFGGLGLLLGLLARPAAVVVILLLLVAIFTVHIHNGFFMANNGYEYALA
LLGGAVAVLIEGAGKLSLDRIIAR
>tr|A0A0N8T870|A0A0N8T870_PSEAP Cation ABC transporter periplasmic cation-binding protein OS=Pseudomonas syringae pv. aptata OX=83167 GN=ALO85_01281 PE=3 SV=1
MKEDRLPMPISFKRRPLLCGLLTGLMVALLAPSSFAAEPAKRLRIGITLHPYYSYVSNIV
GDKADVVPLIPAGFNPHAYEPRAEDIKRIGSLDVIVLNGVGHDDFADRMIAASETPNIKT
IEANADVPLLAATGVAARGAGKVVNPHTFLSISASIAQVNNIARELGKLDPDNAKTYTAN
ARAYGKRLRQMRADALAKLTKAPNADLRVATVHAAYDYLLREFGLEVTAVVEPAHGIEPS
PSQLKKTIDQLRELDVKVIFSEMDFPSTYVETIQRESGVKLYPLSHISYGEYTADKYEKE
MAGNLDTVVRAIQESGA
>tr|A0A0Q0DF54|A0A0Q0DF54_PSEAP Probable GTP-binding protein EngB OS=Pseudomonas syringae pv. aptata OX=83167 GN=engB PE=3 SV=1
MQLKNPILGLCQQATFMLSAAKVDQCPDDEGFEVAFAGRSNAGKSSALNTLTHASLARTS
KTPGRTQLLNFFGLDEDRRLVDLPGYGYAKVPIPLKLHWQRHLEAYLGSRESLKGLILMM
DIRHPMTDFDVLMLDWAIASNMPMHILLTKADKLTYGAAKNTLLKVQAEIRKGWGDAVSI
QLFSAPKRMGLEEAYTVLADWMELEDKAPAE
>tr|A0A0Q0DBF1|A0A0Q0DBF1_PSEAP Type III secretino system transcriptional regulator HrpR OS=Pseudomonas syringae pv. aptata OX=83167 GN=ALO85_04204 PE=4 SV=1
MSTDIDNDVRTCWNVTALSAGHQIAMNSALLDMDLLLCGETGTGKDTLASRIHELSSRTG
PFVGMNCAAIPESLAESQLFGVVNGAFTGVCRAREGYIEASNGGTLYLDEIDSMPLSLQA
KLLRVLESRGVERLGSTDFIPLDLRVIASAQRPLDELVEQGLFRRDLFFRLNVLTLQLPA
LRKRREQILPLFDQFTQDVAAESGRSVPTLDNRRVQILLSHDWPGNVRELKSAAKRFVLG
LPLLGAEPVEARDPVTGLRMQMRVIEKMLIQDALKRHRHNFDAVLEELELPRRTLYHRMK
ELGVASHIDLVAES
>tr|A0A0Q0BU93|A0A0Q0BU93_PSEAP Sensor y box histidine kinase/response regulator OS=Pseudomonas syringae pv. aptata OX=83167 GN=ALO85_04551 PE=4 SV=1
MSTPGLLSERAIILAPRGRDSQIATRILDEAGYPSTVAHDVSELVRELSAGAGLAIIADE
ALRNADMTPLLELLGRQPPWSDLPIVLLTHHGGPDHNPSARMGNLLGNVTFLERPFHPVT
LVSLVMTAVRGRRRQYEARARMEDLHESEGRLQNALKAGRLGSWVLEAEHLKLTCSDITK
SHYGRDEQNTLDYQEWLAAVYFEDQPRMQSALQRSLDTGVDLIIEYRNVWPDGSLNWVDV
RARATRSKNGLIGSLAGVTSDITERKQAESQLRRLNETLEQQVEERTSQLRHKEEILRQS
QKMEAVGQLTGGIAHDFNNMLTGIIGSLELIRRRLARGRIEDLNGLIDLGVTSANRAAAL
THRLLAFSRRQSLDSKPVEMNHLVNAMDELLQRSVNESIHLQLRLAHDLWTAEADPNQLE
SALLNLVLNARDAMPDGGTLVVETSNQQLDRSFTNAYENLLPGDYVVLSVSDNGCGMPET

VISRAFDPFFTTKPIGQGTGLGLSMIYGFSKQSHGHVLIKSEVGVGTTVQLFLPRFKGLA
SEAEQTFQSNAVYAENGETVLIVEDDPAVRTLVSEVLGELGYTFIEAGEASDAVPILESG
QRIDLLISDVGLPGMNGRQLAEIARQLRPELKVLFITGYAEHAAVRGGFLDTGMQLITKP
FAFDHLTSKVREMIEA
>tr|A0A0Q0BUL0|A0A0Q0BUL0_PSEAP Small-conductance mechanosensitive ion channel OS=Pseudomonas syringae pv. aptata OX=83167 GN=ALO85_00223 PE=4 SV=1
MDLNAEVGHLWQASQAWIPMIMEYGSRLLLAVVTLCVGWWLINRLTGKLGALLALRHADL
ALQGFVSNLANVILKILLVVSVASMIGVETTSFVAAIGAAGLAIGLALQGSLANFAGGVL
ILLFRPFRIGDWIEAQGVSGTVDNIQIFHTVLRTGDNRTVIIPNGNLSNGIITNTNRQPT
RKITFDIGVSHDADLKKALQVLLDMADDPRVLKDPAPQAVVAALGENAITLSLRVWTSSG
DMGDVTSMFNIEARDRLKDAGIEIPLPQRIVRVVQE
>tr|A0A0Q0DNF2|A0A0Q0DNF2_PSEAP Peptidase OS=Pseudomonas syringae pv. aptata OX=83167 GN=ALO85_00755 PE=4 SV=1
MKTLTTLLAALTFVLTSCFIQARGVDPQEVLRLSEAGTLHSAEKLDAKALSKHPDASIIG
TQFKNIYGRYVYNVELRDKQGVEWVLEMDAATGQVYRNHQDN
>tr|A0A0N8TA41|A0A0N8TA41_PSEAP Short chain dehydrogenase/reductase oxidoreductase OS=Pseudomonas syringae pv. aptata OX=83167 GN=ALO85_03433 PE=4 SV=1
MSYPPQAIDKGISTSVTTFYSERFFQMNEEYPKPPFASQPQDVPGLQGKMDPYPDCGEKS
YKGSGRLQGKIALITGADSGIGRAVAIAFAREGAQVAISYLNEHEDAQETKRWVEEAGRK
CLLLPGDLAQKQQCEDIVSKTVAEFGRIDVLVNNAAFQMTHETLDEISDEEWVKTFDINI
TAMFRICKAAVPHMPRGGSIINTSSVNSDMPKPTLLAYATTKGAIANFTGGLAQLLGEKG
IRVNSVAPGPIWTPLIPATMTDEDVKSFGSETPLGRPGQPVEVSPIYVLLASDEASYISG
SRYAVTGGKPIL
>tr|A0A0Q0D7E9|A0A0Q0D7E9_PSEAP ACT domain protein/phosphoserine phosphatase SerB OS=Pseudomonas syringae pv. aptata OX=83167 GN=ALO85_02862 PE=4 SV=1
MWLECGFFPLRGCALREIVLINITGVDRPGLTAAITGVLAQGGVNILDIGQAVIHDTLSF
GILVEIPDTVQGSSVLKDILFTAYELDQQVRFTAVSETDYQHWVDGQGKARHIVTLLTRK
VTAEQLQCVSAITAKYGLNIDQIDRLSGRMPLDTPADKGKGCIEFTVRGEPADPKAMQAE
FLAVAQDLNVDIAFQQDSLFRRNRRLAVFDMDSTLIEAEVIDELAKAAGVGEQVSEITER
AMRGELDFSESFKERLALLKGLDVSVLDEIGASLRLTEGAETLFSELKRLGYKTAILSGG
FTYFAKQLQAKLGIDYVFANELEVVDGKVTGVAVEPIVNAQRKADLLRELAHKEGLSLEQ
TIAVGDGANDLPMLAIAGLGVAFRAKPLVKQSAKQAISTLGLDGVLYLLGFRDREGQH
>tr|A0A0Q0ILL8|A0A0Q0ILL8_PSEAP Histidine triad protein OS=Pseudomonas syringae pv. aptata OX=83167 GN=ALO85_00250 PE=4 SV=1
MHSPGNQELFIVFVLDSRLQQDTLPIGDFPLCRLLLSNDSHYPWFILVPRRADITEVFQL
SAADQIQLWQETSALAKVLNELFDADKINVAALGNVVSQLHMHVIVRKRDDAAWPAPVWG
KQAAIAYTDEQLSAIRRQLKPVLTNDFRFEEA
>tr|A0A0Q0DIR2|A0A0Q0DIR2_PSEAP Putative Membrane protein OS=Pseudomonas syringae pv. aptata OX=83167 GN=ALO85_04131 PE=4 SV=1
MKYIHVYLFALLMLFASASFADEATLTGTDCNRPATTTELNRCMEKAIENTPYVGSLVGM
LKTPISVVMVNFFGNKLAFWGDNSSFQLTYEEVEKDFITNKKDTLLWGVRFSFLFIYVGW
TCLLIFKTQSKGFLGNSKKDIVYYTLTSTGVLILLGFGTIFDFYAGSILTAVAVGLSFIV
TFISNFFVATQADIGHVTTEANANSRQESTLLISNMIKQHATNILYGSQIYNKYNFNKTN
ETGMRIFEENEYSTCMAQSADSSRFLSTLLLDGETQKTEKCLRTIGYKTFSMGNVSYSGK
EDSTRLALLEMNDMAHMIALDSIKLMCGNALNVENRRVDWETSHDVYNQCIERITTGEVG
KGANGEIKFFEQNDGITEASIQSQIEQMGQIYIKASTQFVANHAKDAVTAGKTPLVDIAS
FALYFATAQNSSDKLEQMYEDEFGKFVTSVYDPTILGTADMAGLSENTNIDSDRAISQSL
KLTRVFDINQTIDNMVIGSLKSQVNAGFKNSVEYTANVLFGDLYKIAGMTFEDCTKEINS
CIAPVLNHSAAEFSNGIKIVKYYWTAYIVVKVGALTAGEGSGQSIKSTGRSLDFLAFIFA
IAVAFLTSTLVLSGLVTPIIFIGRYAQSLFMLPANFVKMIVRLFSLIIPHDESEDQEKSF
HVFMDLILSLFWIMLEPLVILFLMVVTFSIEGIISVFVGWLVYFICMPLIDTSGMIGMAL
SLIFMMLVYAALTCYVKVKLASTFVQTVDSIQGLFKAEVVADATGSAISKYQEISNKLEK
ISRDVIR
>tr|A0A0N8T849|A0A0N8T849_PSEAP WW domain-containing protein OS=Pseudomonas syringae pv. aptata OX=83167 GN=ALO85_04545 PE=4 SV=1
MRMDAITMKKYQRTVWSVAFVTGLLVAWLVSAAPDNGPSSTASPAVSVAQVQAVGVEVNA
GTFEQPRLAGEPAFKGAVFFAQTGQFAAFYIAAFDSQPEQLDPALPDTPHSTEFWTYAGQ

HSGLGPDLKPFDEPTREGALHLAMIEGKNALLSSKINGAPGPQTPYPVTAQSNDFWDFIG
FFDKPGTPEAMKTDQDLVWPGVFVYFEQSKTVYLAQHTGRPSVDRWPAPDTEQGAGYWKL
AGTRSNIPAALTKPWTDMSVSQGSYYRNPLTPLRSRYYASTFIGFAPLLNAEFPSGPQSN
QYWDYRGVHEGSNIDQKAFGENTAREKLHRAKMQGKWVSFAAHFSGPVEQPYPTSPTNTT
YWGVRHITQNAGTWWDPRTDLDTDTWVGAIHSTPLGQGKALYRALKSWTAPETGWDYSQT
DEFQSLGTSLHAGTVADPKTQQEFTWPGAVHEMLIDHNPVYFVSLAEGFPSEHDWPLPVT
PESNEHWSYIPEIKPLGTFEAPRRFDDYSQAGSVYVDDTVPGSRTFYVSNYEGYPANDLD
SFSTVGTVDPYWIYAGRHAGTWSDFKPFDDITRVGALHEASIKGQRVALRSLFDGMPSAA
TPYPSDLASNTHWEFVTVFKHAGTADDFKGRDDATWPGALHLDEDQHLIFAARQQAQPVA
GGWVYPVGETSDDQWSYVSSLNGGTAETPRERNDINTPGAIFADQLGLTRQKLFRSRFSG
FAQDAGAIYPSGEESNDFYEYAGEHAGTFSDPKPFTDPTWPDAVHVATINGQRAFLKSKM
TGKPSSQTPYPEGLTSTDFWEFMFTSDQAGTYADPKSLSGQTWIGAVHEYSSSGRSQLYI
AQQSGNPIADNWPLPTAGDTEYWKVMGLVRHKGTFADPKGLDEMTSQGLIHATTIEGQDV
YYRSLARGVPQANNWSYPAPGTDNEHWESLGANVPEGTWADPKGSSGFTSPGRIHAVQAR
DRTLYLLSKVDGLFAENDWPIPLDGENDYWTVVGASRHSGEIIDPKDQQEVTWTGAIHMR
QDENTRRYYSSKIVGNLATIGADHPLPAAETDNAWWSFIGRAAHAGTQSDPVQSGEVIRP
GETVRVTRTADEYYQARFAGVFNAERALPDSQKDNEDWSYIGKSAFAGTLQSPKDAYEIT
WPGAIHRFEVDGKAYFAKSLVEGAPGQDGWHYPAPPDSNEQWSYLDMGIHAGNWLDPKPD
FDATWPGALHVVKIPTGIGEGFTRWFYRSKIWGYVMDDEYGYLNPNNFEHVGYSIFQGTL
NSPKMFDQPTWVGAIHQDLETQFLFEAKKSGQMGVEVGEHPTTPTDNESWHFLGISKHSG
VLRDPKEWDEYTWPGRLHRYEHGGKTLYFHAQMTGTPSDHYWYYPTDESSTEQWAYYGTT
LHAGTFADPHTADEVTWGGAIHRVEKDGIRFYFKARIAGIPNEQNWAYPTSATSNEYYIY
IATARHAGTISDPKEVNEPVIPGDYVIKARKDGDYYFIAKNSGVPSANNWPFPAGQVDTE
NWVFYGISRNPGTLDDPKEWDEVSWRGAVHVRKVIGMRLLFSVNSDREGIPSQDKWTLPP
SQPLDADEVKPPAPDPKYPAWKFLQVTHLNGTRDQPKSLSDWTQNGLIHQTSIAYQNMLF
TSKFTGKKDYPQDKPAKEDPVDDKSSTWWTFFSKGQGTFKEPKTWYDYAYPDDIYSYDYQ
GERLLFRAEKEGRPAAHGRYFPTSEYSTADWTYLYRNEGNYADPKTWNEFTRKGEIHRHE
VNGQALFFQALHEGSPAKLNWYYPAGGTNNMYWTYVGKHAGTWQDPKEWDEPTYPGAIHA
QNNITFYRSKFSGNAAAHNWYYPTSGKENAQWFIVNKDAYAAPGINDFTKTFDQYTWVTA
HNAYLDAITPQLERGIRGFMLDIHLDRGDENGKKQVRVCHLPADYGCWSSAPLLKDVLKE
FIAYLKKDRNAVISLLFESTLTSDQLRPVLEQVPEIADYSHVSNGDSWPVLGDMIDTNKR
LVMLSQGEVAKRYTLAGKQAEVLWAPKTQVENTYDLGATSLVHDWQCKSRFTSMDLSLRR
RDGRLPRLFVLNQFHSWGSTTLHAGDMDNNLTWLQRRVENYCGEATGWRKPNYLAIDFNQ
VGDALPYAATLSQGGLYFYEDNRANRAEDTSCVVPVNQSGGTSGVQYDLKLASRGCENDE
LKSMELEGVRAGTRIELYDNPNGDRQDDFTIIDVKQSIPMGKRVRIDSFEGTSDTFYYRK
LASRNNGLDGKVSRIKVFNKPDDNDISDASIVFYEGNGATENIVCTVPFNVDRQFKMGSG
NNSYGCDNDEMRSAKILKAGKGSWFSVTGKPDGTFGQGFTEVRFKRAILVPITIPSFNRS
YENADVKVAVSHGGGLDGSVSYAYFGPASEQKGKPPIKEASTGP
>tr|A0A0Q0D044|A0A0Q0D044_PSEAP Putative prophage antirepressor, BRO-domain protein OS=Pseudomonas syringae pv. aptata OX=83167 GN=ALO85_100821 PE=4 SV=1
MFTATPCVTPTPHFEAILFLRHRRTLRTIFTESQAWFCLADLARLMGRALDERATLKLDA
DQRREVWLEAHGECQRQLMISESGVLALLVHHYVPENRALRQWLTHEVLTTLHDQQNVTL
DNPRMSQLQWPGTSLSVLHWRSETWVRLRDMPNVVAEAAPRPKRPLWRRVLKRINTMRTA
A
>tr|A0A0Q0IJI5|A0A0Q0IJI5_PSEAP Cell division topological specificity factor OS=Pseudomonas syringae pv. aptata OX=83167 GN=minE PE=3 SV=1
MNIFDFFRDRKKGSTASVAKERLQIIVAHERGQRSTPDYLPALQKELVEVIRKYVNIESD
QVQVALESQGSCSILELNITLPDR
>tr|A0A0Q0CWE6|A0A0Q0CWE6_PSEAP Uncharacterized protein OS=Pseudomonas syringae pv. aptata OX=83167 GN=ALO85_00523 PE=4 SV=1
MGLVKISENMHANLRSASVALSRSINAQAEHWMRVGMLAELHPALDYSEICQMLIRLENA
GETVLTASNFNISEPSASSLSKAAS
>tr|A0A0Q0ILT1|A0A0Q0ILT1_PSEAP Uncharacterized protein OS=Pseudomonas syringae pv. aptata OX=83167 GN=ALO85_00327 PE=4 SV=1
MRIDGTSSRSYPIKRKPQKTPSIVEGSFEEVVVDAELPPPPARARRETGDSTIGSTANVP
ARPQDIIFPRSMSSRVAHALASYMATASFVDWDLEVSGLDLHV
>tr|A0A0Q0DQU3|A0A0Q0DQU3_PSEAP Uncharacterized protein OS=Pseudomonas syringae pv. aptata OX=83167 GN=ALO85_100647 PE=4 SV=1
MAALHQGFVSLAHVFLIRRSSVGVGQGSSGPDQLRQHTMIDCCSLQATRKLRYNKSR

>tr|A0A0Q0C802|A0A0Q0C802_PSEAP Bacterioferritin OS=Pseudomonas syringae pv. aptata OX=83167 GN=ALO85_02050 PE=3 SV=1
MKGDITVIQHLNKILGNELVAINQYFLHARMYEDWGLKKLGGHEYKESIHAMKYADKLIK
RTLFLEGLPNLQDLGKILIGEHTKEMLECDLKIEQKGLADLKAAIAHFETVGDFGSRELL
EDILEPKEEQIDWLETQLGLIVKVGIENYLQSQMGD
>tr|A0A0Q0C699|A0A0Q0C699_PSEAP GAF:ATP-binding region, ATPase-like:histidine kinase A OS=Pseudomonas syringae pv. aptata OX=83167 GN=ALO85_03593 PE=4 SV=1
MHAELIYAAYTLLHSMDHRHHKTQFHPIAVRPMTHHPAPLPASEEERVLSLEHLKILDSA
PEQDFDDIVLLATTLCDAPIALVSLVDRERQWFKACIGLDVQETHRDLAFCAHAILQPSD
VLVVEDATTDPRFRESPLVLGPPYIRFYAGAPIRTEAGHAMGTVCVIDIWPRVLSEQQRR
ALLALARQTAALMQYRLVSEQRDQHAAELALGLESSQSQSLALERNLRQSQRVSSLGMLT
ASIAHDFNNLLQALSASLQLIRMRSRRPADVETLSDTGLRAVDHGRQLVTRLLNSVRQDG
SELICIDVSERIDAARDLLLRSAGDTLELSFDLSARGWGVLCAEAQLHTTLLNLLSNARD
AISGPGKVHISTRLESVKDDPQFPEGDYLVLSLKDNGPGMAAELKEQMFEPFFTTRSSDP
GAGLGLVQVQEFAVNAGGGARVETAPGSGTTVYLYLRILGPISATNAPAHITEQ
>tr|A0A0N8T7X4|A0A0N8T7X4_PSEAP 2-isopropylmalate synthase OS=Pseudomonas syringae pv. aptata OX=83167 GN=leuA PE=3 SV=1
MTMLKDPSKKYRAFPTIDLPDRTWPSKTIDAVPIWCSSDLRDGNQSLIEPMDAAKKLRFW
KALVSVGVKEIEASFPSASQTDFDFVRTLIEDGHIPDDTTIQVLTQAREDLIARTFESLR
GAKKAIVHLYNATCPSFRRIVFNQDKAGVKEIAVNAAKLFVKYAAQQPETQWTFQYSPET
FSATELEFAKEVCDAVIEVWNPTPENKVILNLPATVEVATPNIYADQIEWFGRNITRRDS
VLISLHTHNDRGTGVAATELGLMAGADRVEGCLFGNGERTGNVDLVTVALNLYTQGINPG
LDFSDIDGVRKVVEECNQIPVHPRHPYVGDLVHTAFSGSHQDAIRKGFTQQKEGELWEVP
YLPIDPADIGRSYEAVIRVNSQSGKGGIAYLLEQEYGISLPRRMQIEFSQVVQGETDRLG
LEMTAEQIHSLLRREYLQANVPYALISHKLQEENGNSSVDAEVHVDGETQHWRGKGKGAL
EALVAGLPLAVEIMDYNEHAIGSGTTAKAAAYIELRVNGERAVHGVGIDENITTASFRAL
FSALNRSISQTQAKAA
>tr|A0A0Q0C3P2|A0A0Q0C3P2_PSEAP Ser/Thr protein phosphatase family protein OS=Pseudomonas syringae pv. aptata OX=83167 GN=ALO85_01529 PE=4 SV=1
MRIHLLSDLHNEFEPYLPSKLDVDLVILAGDIDIKTRGVAWAGKAFTCPVLYVPGNHEYY
GGHLTKTLEKMRLACDSHVQILDLEQVVIEGVRFLGATAWTDFSATGNAPLASISAQNLM
NDFRQIRTENYRRIRPTDLVERSVNAREWLWARLKEPYDGATVVITHHAPSLRSLQDNPH
AGGHMDAAYANRWEHLMGSDRANLWVHGHSHTAVDYDVEGTRVVCNPRGYPNEDTGFDAG
LVVTV
>tr|A0A0Q0DPB3|A0A0Q0DPB3_PSEAP PAS protein OS=Pseudomonas syringae pv. aptata OX=83167 GN=ALO85_100406 PE=4 SV=1
MFSRALRHCNSRPFLSCKPSIEIQSMNRSMLSVFSAVLLCAQSALTFAADSTSSTPANGS
DQQIRTQVEAKRDQLSGADKINPAQQQSSSSLLDAPVDTDDAPAQAQRP
>tr|A0A0Q0DEJ2|A0A0Q0DEJ2_PSEAP UDP-N-acetylmuramate--L-alanyl-gamma-D-glutamyl-meso-2,6-diaminoheptandioate ligase OS=Pseudomonas syringae pv. aptata OX=83167 GN=mpl PE=3 SV=1
MVMHIHILGICGTFMGSLAVLAKELGHRVTGSDANVYPPMSTQLQAQGIELTQGYDPVQL
IPAPDLVVVGNALSRGNPAVEYVLNTGLPYVSGPQWLADHVLQGRWVLAVAGTHGKTTTS
SMLAWVLEHAGMSPGFLIGGIPQNFAVSARLGETPFFVVEADEYDSAFFDKRSKFVHYRP
RTAILNNLEFDHADIFPDLPAIERQFHHLVRTIPSEGLVIHPTTEPALSRVIEMGCWTPV
QTTGQNGQWQARLLSEDGSRFEVLFEGELQGTVEWDMTGQHNVANALATLAAARHVGVVP
LLGVQALSAFKSVKRRMEKVADVRGITIYDDFAHHPTAIATTLDGLRKKVGDASIIAVVE
PRSNSMKLGAHRDGLRESVSQADQVVWYAPPNLGWDLAATVAGGTVPALVCDSLEAIIAQ
VKSQAQPGTHIVIMSNGGFGGLHGKLAEALK
>tr|A0A0Q0CA83|A0A0Q0CA83_PSEAP Uncharacterized protein OS=Pseudomonas syringae pv. aptata OX=83167 GN=ALO85_01514 PE=4 SV=1
MDREYHLVRELLEPGRRQRDEARARIRSMLAMESIAVEEVEVSERDIDRIEKAVRAGQGI
KEVFPRLSTVGTRTDGEGVNLLVHFTKKEGAPIRYIGGDDPAEAAAVRELDLQKKFYLGA
ADLAQKVGLTLPKASALRAHLGVDEDVACRDVFEFGSTRIPRFSDNAFRRMREALATVDI
EDVWRARQR
>tr|A0A0Q0IMJ5|A0A0Q0IMJ5_PSEAP Uncharacterized protein OS=Pseudomonas syringae pv. aptata OX=83167 GN=ALO85_04705 PE=4 SV=1
MPSAARQFRIRSLCFHTSKASMSLCATAVGRPDLRSVDIDRMFEIYSACYQDTCRARFEQ

DLDDKTHCVVLRDTHDTIMGFTTLKLFEMSWDNQPCKFIFSGDTIITPEHWGSQQLAFAW
SRLVGDLWRQAPDVPLYWFLICKGHRTYRYLRAMVLDYAPRAGHSTAPRVQALMDHLAYT
RFGDAYDPATGVLSFKTPQGRLSAELALISENHAKLPEVEYFLQKNPGYAEGDELVCLCR
LAPKNLRPLARRLFMTEQYSAC
>tr|A0A0Q0C0R8|A0A0Q0C0R8_PSEAP Orf50 OS=Pseudomonas syringae pv. aptata OX=83167 GN=ALO85_200089 PE=4 SV=1
MDATLTPLLHLQPLPAGSASRLLRRGCAVTLVVAVHVLVLSMVTRSSPPALAPQVIRTIY
ASVINEPTPAPAASAAPTPPPTVAPVAPAPSPAPVAVKPVARPKPATKPQVMQHPVVAAA
TSAPATISTAAAAPVVAQIPVPPPQPKTLTRGVEYVHEPQPEYPDSAREEGHEGTVILRV
LVDEHGKPGAVDIVRSSGFGNLDEAGRTAVRGALFKPHVEEGHAVSVYVIVPLRFQLDS
>tr|A0A0Q0E197|A0A0Q0E197_PSEAP Uncharacterized protein OS=Pseudomonas syringae pv. aptata OX=83167 GN=ALO85_01423 PE=4 SV=1
MPSFIMRRFLLMTLLLLSALSGPAHAQLPETDWLELMPTSDQKALEAMPEIDHNSPEAMG
TFDSKGGLKQSKGLPAVMYSTKTVPAMNGKTIRLGGYPVPLETDAKGRSTLFFIVPYPGA
CIHVPPPPPNQLVLVRYPKGLKLDDIYTPLWVEGTLKIEKVSNDLADAAYAMDASKVRVV
VESDL
>tr|A0A0Q0C3Q4|A0A0Q0C3Q4_PSEAP Acyl-CoA thioesterase II OS=Pseudomonas syringae pv. aptata OX=83167 GN=ALO85_03478 PE=4 SV=1
MSQVLDDLVELLTLEAIEENLFRGHSQDLGFRQLFGGQVLGQSLSAASQTVEEDRHVHSL
HGYFLRPGDAGLPVVYQVDRVRDGGSFSTRHVNAIQKGKPIFTCSASFQYDEEGFEHQAT
MPQIVGPEDLPSELELLTRRAHLIPEAMHDKFLRPKPIQFRPVTSEDPYDPKPGEPVKYV
WFRADGSLSDVQALHRYMLAYASDFNLLTTSLLPHGKTVWQRDMQVASLDHSLWFHNDLR
TDDWLLYAMDSPWAGNSRGFSRGSIFNRDGQLVASVSQEGLIRHRKDWA
>tr|A0A0Q0ISH4|A0A0Q0ISH4_PSEAP ZapA-like cell division protein OS=Pseudomonas syringae pv. aptata OX=83167 GN=ALO85_03853 PE=4 SV=1
MNVDGSGVTVLTILGNEYSVKAPAGEDLTLMKAAAMLNASLADTKRKYPTLIGDRLLVLA
ALNLCSQQIELEQLHKVELKRYREQVEATVDVIAKTIAPN
>tr|A0A0Q0IPG6|A0A0Q0IPG6_PSEAP Uncharacterized protein OS=Pseudomonas syringae pv. aptata OX=83167 GN=ALO85_03757 PE=4 SV=1
MVGRISDSELHEMRIRKLQNDIADSERLGMPVKFMHLSALTPTSREQHIERHGELFTGQQ
MLDWWAEGDNRVRCRCACTPVLLDRQGRPLTPDLIASAKQALKAFKLS
>tr|A0A0Q0DU44|A0A0Q0DU44_PSEAP DIM6/NTAB family flavoprotein oxygenase OS=Pseudomonas syringae pv. aptata OX=83167 GN=ALO85_02409 PE=4 SV=1
MSDAIHSYEPSKGHGLPHDPFNAIVGPRPIGWISSQDANGKLNLAPYSFFNAFNYVPPII
GFCSVGRKDSLNNIEQTGEFVWNLATRSLAEAMNQSCAPVAPDVSEFELANLTPLASQIV
SVPRVLETPVSFECKVTQIIQLQRADKELVPSWLILGEVVAVHIDKSLLKDGVYDTAAAE
PILRGGGPADYFQLGPEALFKMFRPGA
>tr|A0A0Q0BUM2|A0A0Q0BUM2_PSEAP UDP-N-acetylmuramate--L-alanine ligase OS=Pseudomonas syringae pv. aptata OX=83167 GN=murC PE=3 SV=1
MVENQRAMPQPEMRRIRRIHFVGIGGVGMCGIAEVLLNLGYEVSGSDLKGSAVTERLESF
GAQIFIGHRAENTIGADVLVVSSAVNTSNPEVATALERRIPVVPRAEMLAELMRYRHGIA
VAGTHGKTTTTSLIASVFAAGGLDPTFVIGGRLNAAGTNAQLGTSRYLIAEADESDASFL
HLQPLVAVVTNIDADHMATYEGDFNKLKKTFVEFLHNLPFYGLAVMCIDDPVVREILPLV
KRPTLTYGFSESADIRAINVRQDGMLTFFTVLRRDREPLDVSVNMPGNHNVLNSLATIAI
ATDEGVSDEAIVQGLSGFQGVGRRFQVYGELPVDGGNVMLVDDYGHHPREVAAVISAVRG
GWPDRRLVMVYQPHRFSRTRDLYDDFVQVLADANVLLLMEVYPAGEEPVPGADSRNLCHS
IRQRGQLDPIYIERGVELAPLVKPLLRAGDILLCQGAGDIAGLAPQLLKSPLFAGAKVAS
TEGKLK
>tr|A0A0Q0DPF2|A0A0Q0DPF2_PSEAP Uncharacterized protein OS=Pseudomonas syringae pv. aptata OX=83167 GN=ALO85_03015 PE=4 SV=1
MSIKEFTMDKEQSTDVAARTQKEKNNPNSAITVQEAPPHESLYRHASPLHLPSDISDTLK
APVLPDIDPDSISHEQAHAGITIHIPGSIKMCWGDEIRFYWGKNLSSTTLFHRVGENSVI
RVLCISYTFTPYIQYGLVDLYYELYRDHRLIGTSPVLRVNVNYAVPVTSRQRQRKRRFTD
H
>tr|A0A0Q0ISZ2|A0A0Q0ISZ2_PSEAP Protein-glutamate methylesterase/protein-glutamine glutaminase OS=Pseudomonas syringae pv. aptata OX=83167 GN=cheB PE=3 SV=1
MPNKKISVLLVDDSAVVRQVLVAILNDTPDIHVMGAASDPIFAMSKLAQEWPDVIVLDVE
MPRMDGITFLKKIMSERPTPVVICSSLTQKGAETSLQALSAGAVEIITKPTTGLKNFLID
SAAELVAAVRAAANSNVRNLGKRTAAPVLTPASKLTADAILPAASGHAMAQTTERIVAIG

TSTGGTQALEAVLTALPRVCPGMVIVQHMPEKFTASFAERLNSLSQIEVREARNNDRILP
GLALIAPGGKHMMVTRSGAYYHVQVIDGPLVNRHRPSVDVLFRSVAKFAGKNATGIIMTG
MGDDGARGLKEMLDAGSATVAQDEASCVVFGMPKEAIKLNAAQRIIPLQEIHQVILHR
>tr|A0A0Q0IS23|A0A0Q0IS23_PSEAP Probable malate:quinone oxidoreductase
OS=Pseudomonas syringae pv. aptata OX=83167 GN=mqo PE=3 SV=1
MDDRMFKKVNTALLGLAISMGLMPVQAAETKKVDVLLVGGGIMSATLAVWLNELEPSWSM
EMVERLDGVAEESSNGWNNAGTGHSALAELNYTPEDKNGNVDISKAIEINEAFQISRQFW
SWQVKTGVLKDPRSFINSTPHMSFVWGDNNIQFLRKRYAALQASPLFSGMQYSEDHEQIK
KWVPLMMEGRDPAQKLAVTWSPIGTDVNFGEITRQFVSNLKAKQNFNLQLSSEVEDINHN
DDGTWRVKYKNLKDGTVTETDTKFLFIGAGGAALHLLQESGIPEAKEYGGFPVGGSWLVT
DNQTLAMQHMGKAYGIASTGAPPMSVPHLDTRVLDGKRVILFGPFATFSTKFLKNGSYFD
LLTSTTTHNVWPMTRVGIEQYPLIEYLAGQVMMSDDDRFAALKEYFPNAKKEDWRLMQAG
QRVQIIKRDEEKGGVLKLGTEIVASKDGSIAGLLGASPGASTAAPIMLGVLEKVFKDKVA
TPEWQAKLHQIVPSYGTKLNNNPDRVAEEWAYTAEVLQLTPPPPVNKTGNAPTAAPAAQP
AKSNPASDMAL
>tr|A0A0Q0C8H8|A0A0Q0C8H8_PSEAP Flagellar basal-body P-ring formation
protein FlgA OS=Pseudomonas syringae pv. aptata OX=83167 GN=ALO85_02134
PE=4 SV=1
MNAKTTISRHLALETRRLLCAIALLCLSSTVTLARAENFTLPEQLIGVTQGFLEFTVEDY
LATNQIDGRHEIQVKQLDPRLRMGACDKDLTATLESPRPVGRVTVRVRCEGSSPWTVFVP
AQVLLYRNVVTVMRPLKRDAIVTEEDVAMRERDVSSLGQGFLASLDQAVGQKVVRQMVID
QVITPVALEQPQMVHKGDQVVIIARSGSLAVRMPGEAMSDGGFNEQIRVKNLNSQRVIKA
SVTGPGQVEVAM
>tr|A0A0Q0BX55|A0A0Q0BX55_PSEAP Isoflavone reductase OS=Pseudomonas
syringae pv. aptata OX=83167 GN=ALO85_04968 PE=4 SV=1
MERLSKGVTMQADNANVKQRILVIGAGELGLAVLRGLVEKAGAHGLSIAVLLRQSSLSTQ
TPAKRVEIEEVRALDIAIETADLADATVDELAAVMARYDTVISCVGFAAGRGTQRKLTDA
ALKAGIKRYLPWQFGVDYDLIGRGSPQDLFDEQLDVREMLRTQQRTEWVIVSTGMFTSFL
FEPAFGVVDLQGGRINALGSLDTAVTVTTAQDIGRLTAAIVMHEPRIVNQVVYTAGDTLT
YAGLADLVERVTGRDIERHVWSVAQLQAELTEMPDDNLRKYRAVFAMGRGVAWDVASTYN
AKSGLSVTRAEQWALANLTQT
>tr|A0A0Q0DZ20|A0A0Q0DZ20_PSEAP SOS response-associated protein
OS=Pseudomonas syringae pv. aptata OX=83167 GN=ALO85_200018 PE=3 SV=1
MCSHYEAPSPHLVAEAFGVAPFEQGRLDLWPGYIGPFLRSPDGRAEDEESPAAMEVLTGS
FGLIPSWSKDSKIARRTYNARSETVAEKPSFRHAWRHAQHCIIPAVAIYEPDWRSGKAVA
TRIVREDAELLGIAGLWEQWRDPSTDQILHSYTMLTVNADDHDFMKAYHKPQDEKRMVVI
LPKGSYADWLTARPEQSAAFMNRYPADRLAVAM
>tr|A0A0Q0C7X0|A0A0Q0C7X0_PSEAP Siderophore-interacting protein
OS=Pseudomonas syringae pv. aptata OX=83167 GN=ALO85_02025 PE=4 SV=1
MNSPQSIHRVMHEIKRRKLQVVRVTELTPLMRRITLQGPELAGFISLGTDDHVKLFFPQT
AEEQAALENLNPAGDKDTPRPPMRDYTPRRYDEKTGELDIDFVLHGEGPAATWAAQAAPG
QFLHIAGPKGSMVVPDMFDSYLLIGDETAIPAISRRLESLPANRAALVVVEVANPQEQQE
FDSAAQVDVIWIVRGEQNLLDVTRRLEMPEGKLYAWVATESALSRKLRRVLLDEFGLEED
FVKAAGYWKLDSTAE
>tr|A0A0Q0DDF3|A0A0Q0DDF3_PSEAP Aliphatic sulfonates family ABC
transporter periplasmic substrate-binding protein OS=Pseudomonas syringae
pv. aptata OX=83167 GN=ALO85_03471 PE=4 SV=1
MLPFFKSPLPFKSLLLGALLSVMAGQQVLAAEQAPLLVANQKSLLKTLLQVSGELKDVPY
EIQFSEFPAAAPLGEALNAGAVDIGALGDAPYVFALGAGAPLKVVSITHAQGRFTTAVLV
PKNSPIKTVADLKGKRIVTGRGSIGHYLAIRALHEAGLKTSDVTFINLLPSESRLLLDSG
DADAWATWDPYTTISITQSDARVLVSGSELLSNHLYLAATSKAIEGKRAQLDDFVARVER
AFNWSNAHPEEYAAAQAKVTGLPLAVHLAVAKATKMQRTPIDDQIVQGLQTTADTYLAEG
ILSKRIDVSTGFDKSFNASRAQIAQATAQ
>tr|A0A0Q0DV91|A0A0Q0DV91_PSEAP ATP synthase subunit beta OS=Pseudomonas
syringae pv. aptata OX=83167 GN=atpD PE=3 SV=1
MSSGRIVQIIGAVIDVEFPRDSVPSIYNALEVQSAAGTTLEVQQQLGDGVVRTIAMGSTE
GLKRGLEVTDSGAAISVPVGKATLGRIMDVLGNPIDEAGPIATEERWGIHRPAPSFAEQA
GGNDLLETGIKVIDLVCPFAKGGKVGLFGGAGVGKTVNMMELIRNIAIEHSGYSVFAGVG
ERTREGNDFYHEMKDSNVLDKVALVYGQMNEPPGNRLRVALTGLTMAEKFRDEGNDVLLF
VDNIYRYTLAGTEVSALLGRMPSAVGYQPTLAEEMGTLQERITSTKNGSITSIQAVYVPA

DDLTDPSPATTFAHLDATVVLSRDIASLGIYPAVDPLDSTSRQLDPNVIGQEHYDTARGV
QYVLQRYKELKDIIAILGMDELSETDKQLVNRARKIQRFLSQPFFVAEVFTGASGKYVSL
KDTIAGFKGILNGDYDHLPEQAFYMVGGIEEAIEKAKKL
>tr|A0A0Q0BTN4|A0A0Q0BTN4_PSEAP Uncharacterized protein OS=Pseudomonas syringae pv. aptata OX=83167 GN=ALO85_03082 PE=4 SV=1
MGFIIGVSVLLILIGTVLYLFDKGAQTRWLRPYIGLMLIGLLIGGMGLLVGLVSDEIEVY
FLLAAKWVIAVTGITFCLRLIVLIAKRFIARN
>tr|A0A0Q0FP34|A0A0Q0FP34_PSEAP Uncharacterized protein OS=Pseudomonas syringae pv. aptata OX=83167 GN=ALO85_100905 PE=4 SV=1
MRCSEPNITFNDQREDLRRNENEVMPEQEGKQSDNRLNVR
>tr|A0A0Q0BUB0|A0A0Q0BUB0_PSEAP Uncharacterized protein OS=Pseudomonas syringae pv. aptata OX=83167 GN=ALO85_01353 PE=4 SV=1
MLDWKNRAGSAGARTADTSSTKRRGYLGNLFYSRALGALILIYLLVALAVGWYWSKEPAL
FPVQQNAQAAAEREGKQMVIGYTTVETLKTVAGTLLSKPGGYLSNDRMPPGLWLDNIPSW
EYGVLVQVRDLSRALRKDFARSQSQSAEDGDLARAEPLFNFNNRSWLLPSSESQYAEGIN
SLSRYQARLSDPNQKGALFYARADNLNNWLGDVGIRLGSLSQRLSASVGRVKLNSTLKTE
AAVSVKPGEVPQVDEEIVETPWLEIDNVFYEARGQAWALSHLLRAIEVDFADVLAKKNAT
VSVRQIIRELEASQEPMWSPMILNGSPFGVFANHSLVMANYISRANAAVIDLRQLLNQG
>tr|A0A0Q0ITQ3|A0A0Q0ITQ3_PSEAP DNA-binding protein OS=Pseudomonas syringae pv. aptata OX=83167 GN=ALO85_01520 PE=4 SV=1
MIFTSRGTTLAVMQNTLHIESLRAALVQRGWTQKDLADQLGVSAQSVTNWFKGKDFPRPD
KLLKLATTLQLSFSQLVAPPEDQPVVAFRKKAGTKTTDEHILKAMGIGGLLKPLVDFLPK
LPTLRTKISSPSISYARLQSDASQVRSKLGLGERSVLGYEALIAEFKACGAVLVPVLWGQ
KQQHKNALHILLPASDVTFVFVNLDTKLEDFKFWMAHELAHVYTPELAGSDEGEDFADAF
AGALLFPEACVQLAYAEAAQAPSAAGEVSVLQQHARHHQISLNTVFQQAQGYAAENNLPS
LRVPERTIHAVRNSSTPQLVSTILFDPTPPKPAQYIAAASNVFQSEFFLALKRMIREHGT
GPSYVQQIMDVSLSDASALYGELAR
>tr|A0A0Q0IC32|A0A0Q0IC32_PSEAP 7,8-dihydroneopterin aldolase OS=Pseudomonas syringae pv. aptata OX=83167 GN=ALO85_02975 PE=3 SV=1
MDRVFIEGLEVDTVIGAYDWERGIRQCLRLDLSFAWDNRPAAAGDDLSKALDYASVSTRI
QAFAEQAQFQLVETFAERLAEVLMGEFNIPWLRLKLTKPGAVAAAAGVGVEIERGCR
>tr|A0A0Q0C1M2|A0A0Q0C1M2_PSEAP C1 Peptidase family protein OS=Pseudomonas syringae pv. aptata OX=83167 GN=ALO85_05462 PE=4 SV=1
MTYTVKQYGWIRDLPDHRDHLYAAPPTALAALPHRVDLRPHCPPVYDQGQLGSCTANGIA
GAIQFDRMKQKLTPAFEPSRLFIYYNERVIEHTVDSDSGAMIRHGIKSVVKQGDCPEKEW
PYDIEKFAVKPPAACYKDARKYKAVSYQKVAQNLNQMKGCLAAGYPFVIGFSVYESFESK
KVAKTGHAPMPGPHEKMLGGHCVLAVGYNDAHQHFILRNSWGAGWGMEGYFTLPYSYLLD
ENLSSDFWTIRVVAA
>tr|A0A0Q0DJ88|A0A0Q0DJ88_PSEAP Ribosome-binding ATPase YchF OS=Pseudomonas syringae pv. aptata OX=83167 GN=ychF PE=3 SV=1
MGFNCGIVGLPNVGKSTLFNALTKSGIAAENFPFCTIEPNSGIVPMPDPRLAALAAIVNP
KRILPTTMEFVDIAGLVAGASKGEGLGNKFLANIRETDAIAHVVRCFEDENVIHVSNSVD
PKRDIEIIDLELIFADLDSCEKQLQKVTRNAKGGDKEAVVQKGLLEQLIAHFSEGKPARS
LMKSMSNDEKATIRGFHLLTTKPVMYIANVAEDGFENNPLLDVVRAIAEEEGAMLVPVCN
KIEAEIAELDDGEEKDMFLEALGLEEPGLNRVIRAGYEMLHLQTYFTAGVEEVRAWTVRV
GATAPQAAGVIHTDFEKGFIRAEVVAYDDFIQFKGESGAKEAGKWRLEGKEYIVKDGDVM
HFRFNV
>tr|A0A0Q0C6F8|A0A0Q0C6F8_PSEAP Allantoate amidohydrolase OS=Pseudomonas syringae pv. aptata OX=83167 GN=ALO85_100163 PE=4 SV=1
MSTPSIPELIEPSATDLAAFEAVFRQSSAIGATPAGGLHRLAASAEDGQVRDLFRDWLEA
HGFEVRIDAVGNLFGLMIFDPDAPYLLCGSHLDSQPSAGRFDGVYGVLAGAVAVSSLVRQ
LLERGEVPRCNLAVVNWTNEEGARFQPSLIGSSVFTGALTLDDAWESRDGDGIRLKDALQ
AIGYLGNDRVDLNIAGYVEIHVEQGAGLESSQTAIGVVRETWAALKRRVRFDGEQNHTGP
TPMAARRDALLAAAHTITAVHDEALQHGVQMHSSVGRIEVYPNSPNVVPSRVSLLIEYRS
RDVDLLSAASERLDATLRAIVDKTMTSFEVESSVLRSPARLHDGFAALAHGVGAELGLST
TDSMTVAGHDAISLNRRYPVCLLFIPSSNGVSHNEAEYTSDQDMHNGLRMLTGLLHRACT
SSASFG
>tr|A0A0Q0CEW8|A0A0Q0CEW8_PSEAP Putative lipoprotein OS=Pseudomonas syringae pv. aptata OX=83167 GN=ALO85_05237 PE=4 SV=1
MSLLRITSLACLALLLGACQSLFTPNMRTPLQVQRDASELIKPGCTTADCPLVNIDTVHF

PDEPRLDAIVQKTLLQLTVADSSASPPVSIKAYQEQFLSQAQGRNASYLQAKVREQHDGI
VVVELSSYLETGGAHGNPGRAFINYSRQQQKVLTLADMIIPGKESEFWQKAQLAHQGWLV
STKMNEDSEFMKTWPFKRTPHIALTYGAVILKYSVDTIAPYSMGHIELKIPYPQLNGILK
PELFPGRG
>tr|A0A0N8T7X9|A0A0N8T7X9_PSEAP Glycine cleavage system H protein OS=Pseudomonas syringae pv. aptata OX=83167 GN=gcvH PE=3 SV=1
MSDLRFTVDHEWLRAETDGSVTVGITPYAQESLGDVVFVQLPELQAYTQHAEVSVVESVK
AASSINMPLDGEVVEVNSALDATPELVNEDALGAGWFFRFIPQDANAIHGLLDQDAYDRL
IKANAEA
>tr|A0A0N8T892|A0A0N8T892_PSEAP Cystathionine beta-lyase OS=Pseudomonas syringae pv. aptata OX=83167 GN=ALO85_03125 PE=3 SV=1
MPQDKDPRAHTALSQSGRSASSGPGIAVNPPVVRLSTVLFDSLESLRQAEIRTAGPERSL
TYGANGNPTAFALQDLISELEGAHGTCLYPTGLAAVAQMFQSFLRPGDHVLMTESVYGPV
RRLAKTMLTAFDIQFDFYAADGSNVESLIKANTRMIYAEVPGSLTFDMCDLPALSQLCKA
RNLLLAVDNSWGSGVLFKPLELGADISLMALTKYVAGHSDVMMGSVSTTEAHWHALKTMN
TAVGNTVSPDDAYLVLRGARSLAARMAMHERHALQIAQWLQAQPQVARVLYLALPDDPGH
AIWKRDFHGCNGLLSFEFNTTDRQVLDRFTGALKLFGVGYSWGGFESLITEVEQHGPDRG
AGPMLRLQVGLESPEDLIADLRNGFAAG
>tr|A0A0Q0BY17|A0A0Q0BY17_PSEAP Peptidase S49, SppA OS=Pseudomonas syringae pv. aptata OX=83167 GN=ALO85_02536 PE=4 SV=1
MSDEWKAPVSQSADEGINGREEAKSWKLLEKTLLASVQEQRRARRWGIFFKLLTFAFLFV
AVIVPMLDFEGGTSRRSSHTALIDVQGVIADKEAASAENIVNALQKAFEDEKTKGVILRI
NSPGGSPVQSGYVYDEIRRLRATKPDIKVYAVITDLGASGAYYIASAADQIYADKASLVG
SIGVTAAGFGFVGAMDKLGVDRRTYTSGEHKAFLDPFQPQKADETQFWQGVLDTTHRQFI
ASVKQGRGDRLKDKEHPELFSGLIWTGEQAVALGLVDGLGSASYVARDVIKEKDIVEYTV
EESPFDRFSKKLGTSIAERIAMLVGFNGPSLR
>tr|A0A0Q0CEY7|A0A0Q0CEY7_PSEAP Alginate lyase OS=Pseudomonas syringae pv. aptata OX=83167 GN=ALO85_02906 PE=4 SV=1
MVDLGSWNLSIPVGSPPKTIETAQLVKGYDGKYFKSKTDAIVFWSPVTGSKTENAKYSRS
ELRETWADGSLHNWVYPDADNYLNAKLEVDQVPSSGKIVIGQIHAYESTEPLLKVEFQYK
EDTKTGDIVAKVRNRYDDDESKVYTIVEGVKLNETFNYVINLSKGGKLSVSAKDKDWSTR
ISETWRDKPLYFKAGVYTQDNTGYTSEGGKATFYNLDIDHNQS
>tr|A0A0Q0FHN3|A0A0Q0FHN3_PSEAP Uncharacterized protein OS=Pseudomonas syringae pv. aptata OX=83167 GN=ALO85_04728 PE=4 SV=1
MSQERYGIRRFALLNTAGYSLGLFPLENPLSVYGANNLGKSASINALQFPILARMSDMSF
GKYSLEQSRRFYFASDTSYILVEVSLPHGPHVIGVVGRGPGGGFGHQFFAYAGELDLGHY
QKNDTCLRQKELFSNLESQGLKAYELKPDELRRLLVGGHTSIPLDLTLIPLRSTSEQSLK
TFRALFINLLHMREITAAKLKQLFLDAFEHSLRSGSVDYIAACEEAFRDVRRMEQDYNSL
VLAGPLVEALAAGVKQRDVLRGKLHRLSPILDSLLGTWSDYSGARKEELVIQAEHYRAQQ
DEMQNDQRSSTQELMRLEREISSIQRWVGELSVLKNRFALVDDVKVLEQQLLAAKDAHDE
LAGALAQSRQFSAEDLDERLRDLEKRLKSVKQQLDHADNNSYARLREEFSQPDVERLMRL
FNSALFSLPLGEQGIALDDGDAWVKSLETILDGFKGDQFAVPGLTINLSSIEPPALQALA
DRAALRDQKERLEKELKQLKTQQAVAADRSASKTQTEALYQQVLDAQKALEDFRRCQTLS
AEESTKLEELAQMEAAQDELKRSSDAFTERVQQLSAKLQLIARQIGDMESKQRTLDDALR
RRQLLPADLPFGTPFMDPIDDSMDNLLPLLNDYQDSWQGLLRSDGQIEALYAQVRLKGVA
KFDSEDDMERRLQLLINAYAHRTDEALTLGKARRAAVTDIARTLRNIRSDYDSLEHQLAL
FNREINKRQVSNLASFRIVLAPNKEALKHIDQIIHSAGQYEEGETLSVFDLSQSAEQDNK
NEEAKEYLARLVAANHNQLGLKDLFELAFEITKVNGQPVIHTDIDGAASNGTTMTIKALT
NMYLLLHLMDRDQAGRVRLPYYLDEAADIDEKNQAALLETSLQLGFVPILASVKPQVSAH
VAIDLEGGSGPNGIYIDEADWKYIQRRDDVKPEVPELAS
>tr|A0A0N8TA56|A0A0N8TA56_PSEAP Chalcone_isomerase domain-containing protein OS=Pseudomonas syringae pv. aptata OX=83167 GN=ALO85_03356 PE=4 SV=1
MRAPRWLWLVMLLSAGASADWREVLPNAQVVGGGELSLFGFRVYTARLLSPTKPFVADAP
LALELTYHRDIDREDLVDASIDEIKRISGSKVTEQQLTEWRQQMNQSFVDVQPGMKITGV
YLPGREARFYVGDKLQHVVPDSQFAKAFFSIWLDPKTRNPELREQLLGSTGS
>tr|A0A0Q0DFY0|A0A0Q0DFY0_PSEAP Type IV pilus assembly protein PilM OS=Pseudomonas syringae pv. aptata OX=83167 GN=ALO85_02808 PE=4 SV=1
MFELFSKKANTLLGIDISSTSVKLLELSRSGTRYKVESYAVEPLPANAVVEKNIAELEGV
GQALSRVLVKAKTSVKIVAVAVAGSAVITKTIEMDAGLSDDDMENQLKLEADQYIPYPLE

EVAIDFEVQGYSVRNPERVEVLLAACRKENVEVREAALALAGLTARVVDVEAYALERSFG
LLAAQLGNGHDELTVAVVDIGATMTTLSVLHHGRIIYTREQLFGGRQLTDEIQRRYGLSM
EEAGLAKKQGGLPDDYVSEVLEPFKEALVQQVSRSLQFFFAAGQYNSVDHIMLAGGTASI
SGLEHLIQRRIGTPTMVANPFADMALSSKVNAGALASDAPALMIACGLALRSFD
>tr|A0A0Q0D1N5|A0A0Q0D1N5_PSEAP TetR family transcriptional regulator
OS=Pseudomonas syringae pv. aptata OX=83167 GN=ALO85_00408 PE=4 SV=1
MARLKTEILPEQIAGKSVERKKLPAQQRATETYERILVATANTLCEVGIERLSTNLVCER
AGLTPPALYRYFPNKYALLTVLGERLLEKQNERIDHWITPELIAGTPQALEQALAELILD
TYRVTDATLGGMWTLKALRAVPALEHVRLDSHRRVSQAQAAIIGSAFPDADPERIAMVAR
VAVEMIHATIELLFDEPLEPAKTCAMVAAMIVSHLDRLAPEPGHGKR
>tr|A0A0Q0DSI0|A0A0Q0DSI0_PSEAP Malonate transporter MadL subunit
OS=Pseudomonas syringae pv. aptata OX=83167 GN=ALO85_02961 PE=4 SV=1
MIIYGVALLAICTLAGVILGDMLGVLLGVKSNVGGVGIAMILLICARLWMEKHGGMSKDC
EMGVGFWGALYIPVVVAMAAQQNVVTALKGGPVAVLAAVGSVVLCACTIAVISRTNRGEP
LPKEEPLLTPEIAPAGGR
>tr|A0A0Q0BUP6|A0A0Q0BUP6_PSEAP Integration host factor subunit beta
OS=Pseudomonas syringae pv. aptata OX=83167 GN=ihfB PE=3 SV=1
MTKSELIERIVTHQGLLSSKDVELAIKTMLEQMSQCLATGDRIEIRGFGSFSLHYRAPRV
GRNPKTGQSVSLDGKFVPHFKPGKELRDRVNEDEEEGF
>tr|A0A0N8T922|A0A0N8T922_PSEAP 50S ribosomal protein L32 OS=Pseudomonas
syringae pv. aptata OX=83167 GN=rpmF PE=3 SV=1
MAVQQNKKSRSARDMRRSHDALEASTLSVEKTTGEIHLRHHVSPEGVYRGRKVIDKGADE
>tr|A0A0Q0IC89|A0A0Q0IC89_PSEAP Smr domain protein OS=Pseudomonas
syringae pv. aptata OX=83167 GN=ALO85_04769 PE=4 SV=1
MQDDDFSLFRSETRGVKPLKHEHADVGKPKTDRKMLARLRQSATVRTDSVTVDGLSDQFV
IDVGPEDVLSWSRDGVQEGQMRKLKLGQISFEGSLDLHGMSVEVARETLWEFLAEATRLE
IRCVRVTHGKAVRLDGKRPMIKSHVNTWLRQHSQVLGFCSCLAKHGGAGAVYVILRRTMM
EGRDE
>tr|A0A0Q0D0L1|A0A0Q0D0L1_PSEAP Aspartate/glutamate leucyltransferase
OS=Pseudomonas syringae pv. aptata OX=83167 GN=bpt PE=3 SV=1
MTELARLKFYATQPHTCSYLPEEQATTLFLDPSQPMDVQVYADLSDMGFRRSGDHLYRPH
CQNCSACVPARIPVSLFVPDRQQKRILKRNADIQVQSAKPVFTQEYFDLYRRYIEQRHAD
GDMFPPSREQFSTFLVRDLPFSRFYEFRVDQRLLAVAVTDLLPNGLSAVYTFYEPDEERR
SLGRYAILWQIAEAARLQLQAVYLGYWIKNCKKMNYKTQYRPIELLTNQRWVTLY
>tr|A0A0Q0DFB2|A0A0Q0DFB2_PSEAP Uncharacterized protein OS=Pseudomonas
syringae pv. aptata OX=83167 GN=ALO85_01647 PE=4 SV=1
MKRKPDLLWILVILFGLGVVTTGYAQSLWSTKDDAPVEITQQQQKVPGRH
>tr|A0A0Q0DGB0|A0A0Q0DGB0_PSEAP Ribonuclease VapC OS=Pseudomonas syringae
pv. aptata OX=83167 GN=vapC PE=3 SV=1
MLKYMLDTNICIFTIKNKPTSVREAFNLHHGQLCISAITLMELVYGAEKSLSPERNLAVV
EGFTARLEVLPYDSDAAAHTGMIRAELARSGTPIGPYDQMIAGHARSLGLVVITNNQREF
RRVEGLRVEDWVSQ
>tr|A0A0N8TAD0|A0A0N8TAD0_PSEAP Peptidyl-prolyl cis-trans isomerase
OS=Pseudomonas syringae pv. aptata OX=83167 GN=ALO85_01573 PE=3 SV=1
MSRYLLTSLFLLLPLAQAAEAPPASSDGHDLAYSLGASLGERLHQEVPDLDLKALVEGLQ
QAYQGKPLALKQDRIDQILREHDAAIAQAETAGTDAPTEAALKAERTFMDSEKAKPGVKV
MADGILMTELTPGTGPKPSADGRVEVRYVGRLPDGTIFDQSTQPQWFRLDSVISGWTSAL
QSMPTGAKWRLVIPSDQAYGAEGAGDLIDPFTPLVFEIELVAVSQ
>tr|A0A0Q0C092|A0A0Q0C092_PSEAP Uncharacterized protein OS=Pseudomonas
syringae pv. aptata OX=83167 GN=ALO85_02344 PE=4 SV=1
MDTENKARAFNPVPLILISLGIWLPIIGTLLSSYLSQDPATPSGPMAFGGAVVLLAQVEI
FAIIFLLVYAFAVKPWRLSTTLRTVFLINAIAAVVAAIGIIVTLANISLQ
>tr|A0A0Q0IJA9|A0A0Q0IJA9_PSEAP Uncharacterized protein OS=Pseudomonas
syringae pv. aptata OX=83167 GN=ALO85_101149 PE=4 SV=1
MRQTTVTLAIHTLGITCLKRCLHGCFTLVPICALLFDGPARLDLRHFACALSVLMMNQDL
AWPCA
>tr|A0A0Q0DXV5|A0A0Q0DXV5_PSEAP 50S ribosomal protein L20 OS=Pseudomonas
syringae pv. aptata OX=83167 GN=rplT PE=3 SV=1
MARVKRGVIARKRHKKILKLAKGYYGARSRVFRVAKQAVIKAGQYAYRDRRQKKRQFRAL
WIARINAGARVNGLSYSRFIAGLKKASIEIDRKVLADLAVNEKAAFAAIVEKAKATLA

>tr|A0A0Q0BVV2|A0A0Q0BVV2_PSEAP Uncharacterized protein OS=Pseudomonas syringae pv. aptata OX=83167 GN=ALO85_01238 PE=4 SV=1
MLLFRSLILLLTLIAPAAFADGLYQVEILLFRQNGEPASTRQLVPENWDKGAQKIDANDE
RSPALNDLASKLQSSGHYEVLMQRAWQQSIGSEPGKVAITSGEERLGHYPIEGTVSLAMA
RFTDIGAQFWVNQLDPHGVVISSERLKQTARVKNGELTYLDNGNLALLIKVSPL
>tr|A0A0Q0BUX3|A0A0Q0BUX3_PSEAP Sugar ABC-type transport system, permease protein OS=Pseudomonas syringae pv. aptata OX=83167 GN=ALO85_00087 PE=3 SV=1
MSLRKIIPLWIYLLFLLVPIYWLINMSFKTNTEILGGLTLWPQDFTLANYKVIFTDESWY
SGYLNSLYYVCLNTVISLSVALPAAYAFSRYRFLGDKHLFFWLLTNRMAPPAVFLLPFFQ
LYSSIGLFDTHIAVALAHCLFNVPLAVWILEGFMSGVPKEIDETAYIDGYSFPKFFVKIF
IPLIGSGIGVTAFFCFMFSWVELLLARTLTSVNAKPIAAVMTRTVSASGIDWGVLAAAGV
LTILPGMLVIWFVRNHVVKGFALGRV
>tr|A0A0Q0ITM2|A0A0Q0ITM2_PSEAP Peptide deformylase OS=Pseudomonas syringae pv. aptata OX=83167 GN=def PE=3 SV=1
MAILNILEFPDSRLRTIAKPVAMVDEGIRQLVDDMFETMYEAPGIGLAATQVNVHKRVVV
MDLSEDRSEPMVLINPEIEKLTDEMDQYQEGCLSVPGFYENVDRPQKVRVKALDRDGKPY
ELVAEGLLAICIQHECDHLNGKLFVDYLSNLKRDRIKKKLEKQHKLNA
>tr|A0A0Q0C719|A0A0Q0C719_PSEAP Regulatory protein, LysR:LysR, substrate-binding protein OS=Pseudomonas syringae pv. aptata OX=83167 GN=ALO85_03054 PE=4 SV=1
MPATLTDEGREVYQSSLQLLSALESFRTEVNGLHQNLRGELNIGLTDNLVTLPHMRITHA
LARLKERGPGVHINIRMIAPAEVERGVLDGGLHVGVVPQASALPGLEYHSLYSERSLLYC
AVGHPLFYADNAALDDVRLAQQEAIAPTFRLPNDVQDHYQALTCTASASDREGIAFLILT
GRYIGYLPDHYANAWVQEGRLRALKPDSKFYDLNLVTVTRKGRRPHLVLESFLETLAATR
>tr|A0A0Q0CBC4|A0A0Q0CBC4_PSEAP Uncharacterized protein OS=Pseudomonas syringae pv. aptata OX=83167 GN=ALO85_01775 PE=4 SV=1
MKALAALLRQATLIKTSLAALLVGAIALGTSELYVLIQPKGPGVVGIVWQPDNATVGISG
NWDRLGARQLLVQWTAVDDQSFIPGTSMASVPVLPDWTRIGKEPWAQEVIFGLAGYFSET
RSRDNIEQLAVLSAQLAKVKTPLNVTGWYFPAEVDPSWSRAKELPALLAKLPRPLWISVY
DGANIGPAATADWLKTWLPDDIGVFFQDGVGVYARTAPVARTYADALRNRLGKNRVRIIV
EAFRPQVGGGFRPATAAELKPQIAAFDGYPLYLFDGPHYVTPDLIEALNK
>tr|A0A0N8T879|A0A0N8T879_PSEAP Uncharacterized protein OS=Pseudomonas syringae pv. aptata OX=83167 GN=ALO85_01210 PE=4 SV=1
MTFIQEKFASVFSDYTVTTQPRPDGGVLLSLRTQDGKQIRRSVSYAQLHTPVQLEWVISA
IRRDLAAQASELPAISMLQSQHRFDLPTYHTR
>tr|A0A0Q0FDV8|A0A0Q0FDV8_PSEAP Peptidase U62, modulator of DNA gyrase OS=Pseudomonas syringae pv. aptata OX=83167 GN=ALO85_00479 PE=4 SV=1
MFDFSARLKQHFAALQSEAQFFSVRYVHRTGQRLCVRKNVAEPPSLISDEGAMLTVRLNG
VETYAATNDISLKGLQAALQQAETLARRLAPHALLDLSDQAVSTAKADYLSPNMDRAFPP
LSDCIGLLMAECSAVPVDERLVNWQAILGLETVEQIYLNSAGAQLRQAQRFVFPGMSVTA
YDGRDSQTRTLGGHNFGQQGGIEVLSSCGLIGSAANVADEALQLILAPNTPSGPRDLLLM
PDQMVLQIHESIGHPLELDRILGDERNYAGTSFVKTTDFGTLQYGSSLLNVTFDPEIPEQ
LASYAHDDDGTSASKQFLIRDGLLQRPLGGALSQYRSGLAGVANSRASSWNRAPIDRMAN
LNIEPGDQSLDSLIGNIEHGILMSSNRSWSIDDARNKFQFGCEWGQLIENGELKGVVKNP
NYRAISAQFWKNLSAVGDASTFQVLGTPNCGKGEPNQVIRVGHASPACVFANVDVFGGDA
>tr|A0A0Q0BU16|A0A0Q0BU16_PSEAP Metal ion ABC-type transport system, periplasmic substrate-binding protein OS=Pseudomonas syringae pv. aptata OX=83167 GN=ALO85_01658 PE=4 SV=1
MKKTFLLTALAAVFSIGLAHAGEKLVVGATPVPHAEILELIKPTLAKEGVDLEIKVFTDY
VQPNVQLNEKRLDANYFQTKPYLDGFNKAKGDNLVTGVGVHVEPFGGYSSKYKTLADLPE
GATIAIPNEGSNAGRALLLLQKGGLITLKDPTNALSTPKDIATNPKKFKFRELESAVLPR
ALKQVDLALINTNYALEAKLNPKKDALIIEGADSPYVNFLVTRKDNAHTDAIEKLSKALT
SQEVKDFINKKYDGAVLPAF
>tr|A0A0Q0DLL9|A0A0Q0DLL9_PSEAP LuxR family transcriptional regulator OS=Pseudomonas syringae pv. aptata OX=83167 GN=ALO85_00267 PE=4 SV=1
MTYDILIADDHPLFRSALHQALSIGLGPDARLVEAESIAQLESRLGEKADWDLVLLDLNM
PGAYGFSGLVLLRGQYPQIPVVMVSAQEEASIVVRSREFGASGFIPKSSSLEVIQQAVRT
VLDGDVWWPPQVNEVISVSDEAKAASAGLASLTPQQFRVLTMVCEGLLNKQIASELSVSE
ATIKAHVTAIFRKLGVRTRTQAALLLQQLESISSS

>tr|A0A0Q0CW84|A0A0Q0CW84_PSEAP Putative hydantoin racemase OS=Pseudomonas syringae pv. aptata OX=83167 GN=ALO85_01885 PE=4 SV=1
MRIQVINPNTSEAMTHKIGLAAQAVARPGTQIIACSPADGPVSIEGHFDEAIATLGMLEE
VRKGREQQVDAHIIACFGDPGLLAAREYASAPVIGIAEAAFHMASLISTRFAVVTTLTRT
RIIAEHLLQRYGFSELCTSVRCINLPVLALEESGPELIECMAEQARRVRDEEGAGAIVLG
CGGMADLGRQLSEAIGLPVIDGVAAAVKLAESLVDLGLTTSKHGDLADPIGKPFKGRFAY
LSR
>tr|A0A0Q0DIT9|A0A0Q0DIT9_PSEAP Response regulator receiver OS=Pseudomonas syringae pv. aptata OX=83167 GN=ALO85_04298 PE=4 SV=1
MAQILIIEDNAANMRLAELLLTSAGHAVLSAVDAESGLKLARERQPDLILMDIHLPEMDG
LTATSLLKKDASTAAIPVIALTAMAMKEDKEKIRLAGCNAYVIKPLRYKELYRVIDTLLE
KNLPQQPIT
>tr|A0A0Q0FMU7|A0A0Q0FMU7_PSEAP Lipoyl-binding domain-containing protein OS=Pseudomonas syringae pv. aptata OX=83167 GN=ALO85_00862 PE=4 SV=1
MNIKEIRQLAAWIRDAGLTVLELKRPGFELLIRRGEPVQACAAPSAPQVAAEQSALTAHV
VSADNPGVFVSSHPDERNSYVKPGDAVAAGQLLGLLRIGLLYLPVRSHQAGRVLRFLAAE
NERVGYGQPLVELEEHDHAH
>tr|A0A0N8T801|A0A0N8T801_PSEAP Uncharacterized protein OS=Pseudomonas syringae pv. aptata OX=83167 GN=ALO85_101350 PE=4 SV=1
MLLCGAEDGLGHAGDSNSCKPEAAGRGLSLISAITAQHRCVVIDFCLRHY
>tr|A0A0N8T9B1|A0A0N8T9B1_PSEAP Monovalent cation/H+ antiporter, subunit A/B OS=Pseudomonas syringae pv. aptata OX=83167 GN=ALO85_05201 PE=4 SV=1
MSLIVLLLLPFIGSCLAAFLPHNARNAESFLAGLIALIGAVQMALWFPQIADGGVIREEY
FWLPSLGMNFVLRIDGFAWLFSMLVLGIGALVSLYARYYMSPDDPVPRFFAFFLAFMGAM
LGLVMSGNLIQIVFFWELTSLFSFLLIGYWHHRADARRGAYMALMVTGAGGLALLAGMMI
LGHVAGSYDLDRVLASGDAIRAHALYPVLLTLILIGALTKSAQFPFHFWLPHAMAAPTPV
SAYLHSATMVKAGVFLMARMWPALSGTEQWFWIVSGAGACTLILGAYAAIFQNDLKGLLA
YSTISHLGLITLLLGLNSPLAAVAAVFHILNHATFKASLFMAAGIIDHESGTRDIRRLSG
LVRLLPYTATLAMVASASMAGVPLLNGFLSKEMFFAETVFISATAWVEIALPVIATIAGI
FSVAYSLRFTVDVFFGPAAKDLPHLPHEPPRWMRAPVELLVLTCLVVGIFPAQSVAPLLA
AAARPVVGGTLPEYSLAIWHGWNLPMVMSLVAMAGGIILYLLLRKPLKHERITAPPLVGR
LNGKRFFERSQVVMMHWARRFERKVSTRRLQPQLFLLVLAAVLGGFIPMYFSGLTWGDRP
KIPGSGVFVTLWLIAIACAIGAAWQGKYHRLAALVMVSVCGLMTCITFVWFSAPDLALTQ
LVVEVVTTVLILLGLRWLPRRNEDVAPLSARLRARTRRIRDFGLAVLVGLGMAILSYAML
TRQTPNAISSFYLSRALPQGGGTNVVNVMLVDFRGFDTFGEITVLAAVAMTVFALLRRFR
PPKESILLPAQQRLLARDVVTDLVNPRSASDTALGFMMVPAALVRLLLPIAFIISMYLFV
RGHNQPGGGFVAGLVMSVAFILQYMVAGTQWVEAQMSLRPLRWMGTGLLCAVLTGAGSMA
LGYPFMTTHTAHVDLPILGDIHIASALFFDVGVYAVVVGSTLLILTALAHQSVRSHRPTQ
LPKPVANPQGIL
>tr|A0A0N8T897|A0A0N8T897_PSEAP LrgA protein OS=Pseudomonas syringae pv. aptata OX=83167 GN=ALO85_03091 PE=4 SV=1
MLLRGLTWLVLFQLLGTALNHLFLSILPGPIIGLVLLMAYLMWRGEVSEPISMAASSLLR
YLPLLLVPPAVGVMVYASAIAEDFWAIFGTLTLSLMISMVFVGWLMQTLIKHQAQRRELP
>tr|A0A0Q0DXE3|A0A0Q0DXE3_PSEAP 2-nitropropane dioxygenase, NPD OS=Pseudomonas syringae pv. aptata OX=83167 GN=ALO85_03775 PE=4 SV=1
MSQWPDSRITELFAIELPLLQAPMAGASGSQMAIAVAQAGGLGALPCAMLTPEKIDQEVA
TFRQRTGNAPLNLNFFCHQPPAYDAECAERWKQSLKPYYEELGADLDAPTPVSDRAPFDN
DSCALIERLRPEVVSFHFGLPQPSLLDRVRATGAKIISSATTVEEAIWLEQHGCDAVIAM
GYEAGGHRGLFLSDQLHTQVGTFALVPQIADATSIPVIAAGGIADGRGVAAAFVLGASAV
QIGTAYLFCPEASVSALHRQALHDATGSQTALTNLFTGRPARGIVNRLMREAGPISPLAP
AFPLAGGALMPLRAQAEKLGSSDFVNLWAGQAVGIKHRLGATELTRQLAENALKILSPR
>tr|A0A0Q0IF72|A0A0Q0IF72_PSEAP Response regulator receiver:transcriptional regulatory protein OS=Pseudomonas syringae pv. aptata OX=83167 GN=ALO85_03176 PE=4 SV=1
MTSVLLVDDDRELTEMLSQYLTRESFDVTCTNSAEEGEVEALSGRHDIVVLDIMMPRISG
IEVLRRIRAQSQVPVILLTARGDNIDRISGLELGADDYVPKPSSPGELVARLRAIMRRVR
AVPVIETVRDVIQAGELMLWPGKREARWQGVTLELTGNEFCLLEELARHAGQLVSKQNLS
MNALGRPLARYDRSIDVHISSIRHKLGPRNDNQSWIQSVRNLGYMLITP
>tr|A0A0Q0D543|A0A0Q0D543_PSEAP Outer membrane porin OprE OS=Pseudomonas syringae pv. aptata OX=83167 GN=ALO85_04663 PE=4 SV=1

MPPDSASQKTLNYSMRMNPMERINRAAPFVAGFCGLLIAQQSYAEGFLEDSKASILARNF
FSNADNRSGAADPNYTQEWGQGFMLNYQSGYTPGTVGFGVDALGQLGIRLDSGKGHHYNP
QSTAFNGNIFPTDSDGRAVDEFSSLGATLKARVSRTELKYGTLVPMLPVVMSTDRMLQQT
FRGGQIQSGDLKDFTFTAGQLEHVMGRGSTNQMGMSIPGANNPLTGEFVNKFYYAGVDYK
PTKDLTLQYYYGNLENFYKQNFLGLKYDMQLGPGILRTDLRHFDNRSDGANGHDPAFYTM
GYYGNGVTKGEVDSRLSSAQFTYLINGHTFAAGYQAVTGNSDAVWLNQGDGSTAYFMTES
MIGKFQRAGERTWQVRYGYDFARLGVPGLNFLAMYESGSNIQTSDGDKKEWERNVTLSYV
VQSGPAKNLSVALRHAQLRTEFASQRDSDEHRVIVSYPINIF
>tr|A0A0Q0BYH8|A0A0Q0BYH8_PSEAP Flagellar basal body rod protein FlgB OS=Pseudomonas syringae pv. aptata OX=83167 GN=ALO85_02140 PE=3 SV=1
MSISFDKALGIHEKALNFRAQRAEVLANNISNADTPNFKARDLEFSSVLAAETDKAQNGH
FALNKTNSRHIEAEGMGNDDGTLMYRTPSQPSLDQNTVDSQVEQASYTENAMGFQASFTL
LNSKFKGLVAALRGE
>tr|A0A0Q0DGB7|A0A0Q0DGB7_PSEAP TRAP dicarboxylate transporter, DctM subunit OS=Pseudomonas syringae pv. aptata OX=83167 GN=ALO85_04737 PE=4 SV=1
MDAFILLGSFIALILIGMPVAYALGLSALIGAWWIDIPADALMIQIAGGVNKFSLLAIPF
FVLAGAIMAEGGMSRRLVAFAAVLVGFVRGGLSLVNIVASTFFGAISGSSVADTASVGSV
LIPEMERNGYPRDFSTAVTVSGSVQALLTPPSHNSVLYSLAAGGTVSIASLFMAGILPGL
LLSAVMMGLCMIFARKRNYPKGEVIPLRQALKIAGEALWGMMAMVIILGGILSGIFTATE
SAAIAVLWAFFVTMFIYRDYKWRDLPKLMHRAARTISIVMILIGFAASFGYILTLMEIPM
KITTAFLTLSDNRYVILMCINVMLLLLGTVMDMAPLILILTPILLPVIVGVGIDPVHFGM
IMLVNLGIGLITPPVGAVLFVGAAVGKVTIEATVKALVPFYVALFLVLMLVTYIPAISLW
LPSVVL
>tr|A0A0Q0DIH9|A0A0Q0DIH9_PSEAP Glutaredoxin OS=Pseudomonas syringae pv. aptata OX=83167 GN=ALO85_02322 PE=3 SV=1
MAQVIVYSSDYCPYCIRAKQLLQSKSVAFEEIRVDGKPQLRAEMTKKAGRTSVPQIWIGS
THVGGCDDLFALERAGKLDALLA
>tr|A0A0Q0DBR6|A0A0Q0DBR6_PSEAP Inner membrane protein AmpE OS=Pseudomonas syringae pv. aptata OX=83167 GN=ALO85_03315 PE=4 SV=1
MSFLVLLLAVLIEKFSALRQRVQRDGPWLKELAKLEASPRTSTRPWVMLALLVLVPVLIL
QLALTIIGSVAYGWLVLPIHLLVLIYSLGRVDLIAVLGPFRDACRRGDAQAAVHVAERDM
GVQADNSEQLLQGVQGYLLWQAFQSFFAVIFWYFVLGPVAALAYRLLALAAEHGKTPALV
ERAGQLRHAFDWVPVRLLAASFALVGNFVAVTRVMLHELLNWNISAAQLITRVGCAASEV
PPPMMGAQGVTGLDMLWELLVRAAVVWYAGFALWTLLL
>tr|A0A0Q0BXJ4|A0A0Q0BXJ4_PSEAP Short-chain dehydrogenase/reductase SDR OS=Pseudomonas syringae pv. aptata OX=83167 GN=ALO85_05537 PE=4 SV=1
MAPKSKTLQPCSSRCLMSLIPELNSEANVLICGASRGIGLALCAALLARDDVAQVWAVAR
QASTSTELAMLAEQYGQRIKRVDCDARNEQSLEALVSETLDGCDHLHLVISTLGILHQDG
AKAEKGLVQLTLASLQASFATNTFAPILLLKHLLPLLRKQPSTFAALSARVGSIGDNRLG
GWYSYRASKAALNQLLHTASIELKRLNPASTVLAIHPGTTDTELSQPFQANVPEGQLFEP
AFSADRIIEVVGAHGPADSGTFWDWDDKPIVW
>tr|A0A0Q0CWK2|A0A0Q0CWK2_PSEAP Ribosomal subunit interface protein RaiA/YfiA OS=Pseudomonas syringae pv. aptata OX=83167 GN=ALO85_00165 PE=4 SV=1
MQVNISGHQLEVTKPLREYVELKLKKLEGHFDKITNVQVTMTVEKLKQKIEATLHIHGGE
VVANAEHDDMYAAIDLLTDKLDRQLLKHKEKQQSILKGAAAR
>tr|A0A0Q0C1K7|A0A0Q0C1K7_PSEAP Uncharacterized protein OS=Pseudomonas syringae pv. aptata OX=83167 GN=ALO85_03588 PE=4 SV=1
MSQVDALNFIDVHYHANPDAFIRRHGAIEAGRCYARAQGRVVLKNHLGCTAAQAWEARDQ
GFPVSGSVVLNEIAGGIDYRVVERSLCVRGDEPGRFIVHLPTVTGRTHASTLARNLSHPL
LRDKPIKPARVTSENGRLTPQALDILRMARDYPLVISTGHADADEVRTLIDEALRIGVPR
LMLNQPANPLTGLDAAELALIGTEPSVYIEQTALTYLLGYQDRQDFSDVLSHVGNVVYSS
DLGQTSQIDVHEWLSLSGQWFDAFGLSGERRIEVTLLNPQRMLAL
>tr|A0A0Q0CY43|A0A0Q0CY43_PSEAP Uncharacterized protein OS=Pseudomonas syringae pv. aptata OX=83167 GN=ALO85_101235 PE=4 SV=1
MMLAGAEAALKGIPLHECPYRHPAMRASWLKGFAQEQQQSFKF
>tr|A0A0Q0CFN7|A0A0Q0CFN7_PSEAP Uncharacterized protein OS=Pseudomonas syringae pv. aptata OX=83167 GN=ALO85_03716 PE=4 SV=1
MSSIHEQAMNYVYQQVLQRLLGYFSRAERTALQLLIQRLIVAAGGIERIAGFKVLVAFGG

GKDSAYTLAFLRAAQLSIACRSPGTFNLRVANRRHAGMTPAVMDNINRTYSALFLYDDPR
VEMLVIDNQYTQAFEPDLPFSSAGREQNRLDMLLGGHLSAGDARTTFCNTCYLGLAEFLG
RALSWGSGVDAVVSGDSRREQRQYATWIMRLAQRTGQYSGSWGKQTLASVLKVIDTIGQA
YYHELYGEDDDSPPASRAMVLPEKSSTPAFITIADLVSCKADEHWNLLTEFLDFRFDDLS
FSFSESDCANPLLMAHMRGLTAQYLQARTYADGIAEYLELATSLMRRKQMPPRLIDQTLS
AYAGRARIEARRELASSFAQEGFGLNETQLVCMLFSPFVDHGHGLESFLRSCHSGMLVAL
PDLHKALSGSTAPDQVMQWLVDISGLSLKSLHNLYGKQRVNFDDPQSIIARIRAADPDKR
RIMTVDPATGQAVAEMLSGR
>tr|A0A0Q0DIX9|A0A0Q0DIX9_PSEAP Type III secretion system protein HrcT
OS=Pseudomonas syringae pv. aptata OX=83167 GN=ALO85_04190 PE=3 SV=1
MPFDAHSAFQFMLGMGLAMARLMPCMLLVPAFCFKYLKGPLRYAVVAVMAMIPAPAISKA
LESLDDNWFAIGGLLIKEAVLGTLLGLLLYAPFWMFASVGALLDSQRGALSGGQLNPALG
PDATPLGELFQETLIMLVILTGGLSLMTQIIWDSYSVWPPTAWMPGMNAGGLDVFLEQLN
QTMQHMLLYAAPFIALLLLIEAAFAIIGLYAQQLNVSILAMPAKSMAGLAFLLIYLPTLL
ELGTGQLLKLVDLKSLLTLLVQVP
>tr|A0A0N8T7W3|A0A0N8T7W3_PSEAP Transcriptional regulator, ArsR family
OS=Pseudomonas syringae pv. aptata OX=83167 GN=ALO85_04253 PE=4 SV=1
MILEQIKAVGNDTRMLIMEWLKNPQAHFPPQDHGDPAIGVCVSHIQAKANLSASTASAHL
AILQRAGLVQATRIGKWTYFRRDEQAIDRFADRLKKEL
>tr|Q2UZV7|Q2UZV7_PSEAP RNA polymerase sigma factor OS=Pseudomonas
syringae pv. aptata OX=83167 GN=hrpL PE=3 SV=1
MLPNLVILDATEPRQPSSSAGIRQLTADQVQMLRAFIQKRVKNADDVDDILQCVFLEALR
NEHKFQHASKPQTWLCGIALNLIRNHFRKMYRQPYQESWEDEVHTDLESNGDITHQVDGH
RQLARVIEAIDCLPTNMQKVLEVSLEMDGNYQETANMLGVPIGTVRSRLSRARVQLKQHI
DPFA
>tr|A0A0Q0CBD4|A0A0Q0CBD4_PSEAP Amino acid ABC transporter permease
OS=Pseudomonas syringae pv. aptata OX=83167 GN=ALO85_04944 PE=3 SV=1
MTSFQPQRPPEQAEQSLLKRVFGFRTRLYLTWLVMFVLFAGFFLSFDLKLSIILDKLPNL
IGLHLAPNGFLQGAALTLFVSVCSIVVSVALGFVTALARLSSSAVAFGIASFYASFFRGT
PLLIQILLIYLGLPQLGIVPGAITAGVIALSLNYGAYLSEIFRAGIIGVSAGQREAALAL
ALRPAQIFWRVTLPQAMRTIIPPTTNQFISMLKDSSLISVMGVWEVMFLAQSYGRSSYRY
IEMLTTAAVLYWIMSIGLELLQSRLERHYGKAYQARK
>tr|A0A0Q0C647|A0A0Q0C647_PSEAP Rdx family selenoprotein OS=Pseudomonas
syringae pv. aptata OX=83167 GN=ALO85_03466 PE=4 SV=1
MSVEKPEVIITYCIQCQWLLRAAWLAQELLSTFSDDLGKVSLQPGTGGVFRITCDGVQLW
ERKADGGFPEAKVLKQRLRDQIDPARDLGHNDRHTPADPQ
>tr|A0A0Q0BW05|A0A0Q0BW05_PSEAP Phosphatase/phosphohexomutase-like
protein OS=Pseudomonas syringae pv. aptata OX=83167 GN=ALO85_01250 PE=4
SV=1
MSQKIGSVSRYFREILMLAPVLTPLPTFPALLFGLSGCLVDFGAQAANSRTPGDEHTQFT
PGAKAILQTLRDQSMPCAWLDELPESVSATLSVPVSDWMIPAPRPTAGWPAPDACWLALM
ALKVSHLDGCVLISGDPRLLQSGLNAGLWTIGLASCGPLCGLSPSQWQALSNAEREQRRA
QATLKLYSLGVHSVIDHLGELESCLADIALRRSKGEKP
>tr|A0A0Q0DLB6|A0A0Q0DLB6_PSEAP Gas vesicle protein OS=Pseudomonas
syringae pv. aptata OX=83167 GN=ALO85_01054 PE=4 SV=1
MSDADQVQKSQPSPSQSHDDVIAQFKWQGKQIDWLLQWLVKFVANTKVEMGVTLSVGGNL
VSGHLISHDTYFEQLADDISAPFSSFANGTDATMKEMILSFKPGESSEDTPAFHFLHLKD
CRTYSTDGNPICDAGVLWRGRISAVDGFTIGLIAEKPDAA
>tr|A0A0Q0C7N1|A0A0Q0C7N1_PSEAP Asparagine synthetase OS=Pseudomonas
syringae pv. aptata OX=83167 GN=ALO85_04833 PE=4 SV=1
MGLSTFAKAVGRYSSVWGANLNFGGFIFMCGITGFFGVKNKNLDETTVIKNMLSMIEHRG
PDQAGYYVNNSMGLGTMRLSIIDLAAGIQPLSDSTGQYWISYNGELYNYIELRRRLQVLG
HHFSTESDTEVVLRSWMEWGDEALEKFNGAFAFAIYDTVKDEVFIARDRFGKRPLFYTQT
DGGYLFASEMKCFFGFPGFEFKFDEMSLSSILAHWTPLPSQTPFQGIRQLPPGSIMTLRE
AQCEIRPYYALDFRAVDFDGSENEAKEEIRNTLEQAVKLRLRSDVPMGLYLSGGVDSSVL
TSIAARIIKQKPLRTFSVEFEDKNLDESSDQQLVSALFSTKHERLIINHKDIVDNFSATV
FHAEMPLFRTAPVPMYMLSKHVRSTGIKAILSGEGADEVFLGYNIFKETLLRSQWDTTEH
DEKKRLLAKLYPYMKSFSEDNDSNLMGFYNAYAKEKIPGLFSHEIRFQNGRFASRLLKTK
NDPFAGIQAWVAKDSVYSALSPTQKAQWLEFNTLLSGYLLSSQGERMGLAHGIENRCPFL
DINVQKLASSINLRFDDGFTEKYMLRMAYDGILPDRIIHKAKTPYRAPDSGPFVEHALDY

LNHLCESNALDAIELINPVFAKALIKKVVERTLAGGEDITVRDNQAFVYLMSLIELDKWF
VKRQGAAYINPCRVDKLLVQAVDARH
>tr|A0A0Q0BUZ0|A0A0Q0BUZ0_PSEAP Sugar ABC-type transport system, permease protein OS=Pseudomonas syringae pv. aptata OX=83167 GN=ALO85_00088 PE=3 SV=1
MRKVQNNKAWWLVMPVFLLVAFSAIIPMMTVVNYSVQDIFDQSSRYFVGTDWFRQVLQDP
RLHDSLLRQFIYSGCVLLIEIPLGIAIALTMPTKGRWSSLCLIVMTIPLLIPWNVVGTIW
QIFGRGDIGLLGYTLNNVLGINYNYAANASDAWVTVLVIDVWHWTSLVALLCYSGLRAIP
DVYYQAARIDRASGWAIFRHIQLPKMKSVLLIAVMLRFMDSFMIYTEPFVLTGGGPGNST
TFLSQTLTTMALGQFDLGPAAAFSLVYFLIILLVSWVFYTAMTHSEKN
>tr|A0A0Q0DMA3|A0A0Q0DMA3_PSEAP Sigma-70 region 2:Sigma-70 region 4 OS=Pseudomonas syringae pv. aptata OX=83167 GN=ALO85_01113 PE=3 SV=1
MRITASLRTFCHLSPSHSESTITRLWIDEVTAVARQRDRDSFMRIYDHFAPRLLRYLTGL
NVPEGQAEELVQEVLLKLWHKADSFDPAKASLGTWLFRIARNLYIDSVRKDRGWVQVQNS
LEQLERLEAPVDRTLDYSQRQEQQLNIAIQNLPADQARVLRMSYFEALSHREISERLGMP
LGTVKSCLRLAFQKLRSRIEES
>tr|A0A0Q0C3V1|A0A0Q0C3V1_PSEAP Putative zink aminopeptidase OS=Pseudomonas syringae pv. aptata OX=83167 GN=ALO85_01562 PE=4 SV=1
MATHPLPRHRHLLALTAVTLLAGCSSVSYYGQLAQGQWQLLQAREPVEKIVADPTRDAGL
REHLARSQLARTFASEHLHLPDNKSYRLYADLGRPYVVWNVFATDEFSLEPVTHCFPIAG
CVAYRGYYSPGGARGEAALQRQAGKDVYLSGVEAYSTLGWFNDPILSSMMGWGDERLATL
IFHELAHQRFYVKDDTEFNESYASFVEQEGTRQWRAARGLPPESVSQSARRDQFTRLVLD
TRERLKALYRQPLSAE
>tr|A0A0Q0DJN1|A0A0Q0DJN1_PSEAP Peptidyl-prolyl cis-trans isomerase OS=Pseudomonas syringae pv. aptata OX=83167 GN=ALO85_00094 PE=3 SV=1
MPNVKLTTNHGDIVLQLNEEKAPQTVANFIEYVKAGHYENTVFHRVIGNFMVQGGGFEPG
MKEKKDKRPSIQNEADNGLPNKKYSVAMARTMEPHSASAQFFINVADNAFLNHSAKTVQG
WGYAVFGEVIEGTDVVDKIKGVATSSKAGHQDVPVEDVIIEKAEIVE
>tr|A0A0Q0BUX0|A0A0Q0BUX0_PSEAP Flagellar hook-associated protein FlgL OS=Pseudomonas syringae pv. aptata OX=83167 GN=ALO85_02152 PE=4 SV=1
MRISTTQFFESTNTNYQRNYSNLNKTSEEVSSGIKLNTAGDDPVGAARVLQLAQQNSMLT
QYETNIGTINTNVVTTETTLTSIIDTMQAAREQIVSAGSGAFTDSDRLAKASALKQYQSQ
ILGLMNSQDPNGQYIFSGSKASTPPYAQNADGSYSYKGDQTSVNLAVGDGLVMASNTTGF
EAFEQSVNTTRTSATLLSPATDDGKIGLSGGLVTSTPTYNASYQGGEPYTLTFLSGTQFK
ITDASGTDVSSDTSSGGKFSHGSFDAQTFTFRGVEMTLNVNLPAADRVSDATADAALTNR
SYQLASTPDSVSTARSAGNTSTATVSSSAVGNTAADRAAFNNTFPTEGAILKFTSPTDYD
LYAAPLTSSSKPVSSGTMTGSTANASGVNFNISGTPAAGDQFIVESGTHQTENILNTLTA
AIKALSTPTDGNLVASQNMTAALNSALGNMSSAIEQASTARSSGGARQLAATAQGTTNDL
LKGNNTTEQGTYVNADIVEATTRLTLQKTMLDASQQVFTMLSKLNLFSQL
>tr|A0A0Q0DLI0|A0A0Q0DLI0_PSEAP Phosphatidate cytidylyltransferase OS=Pseudomonas syringae pv. aptata OX=83167 GN=ALO85_00302 PE=3 SV=1
MLKQRIITALILLPIALCGFFLLTGMYFALFIGVVVVLGAWEWARLAGFAAQSMRIGYAA
VVAVLLFLMYLLPGLEPWVLVASVIWWSVATFLVLTYPDSSSHWASAACKLVIGLLILLP
AWQGLVLIKQWPLGNWLILSVMVLVWAADIGAYFSGKAFGKRKLAPKVSPGKSWEGVYGG
LVVSLGITAAVGVSRDWTVAQFIAALFGAAVIVFISVIGDLTESMFKRQSGVKDSSNLLP
GHGGVLDRIDSLTAAIPVFAVLLWAADWGVM
>tr|A0A0Q0DML6|A0A0Q0DML6_PSEAP 50S ribosomal protein L24 OS=Pseudomonas syringae pv. aptata OX=83167 GN=rplX PE=3 SV=1
MQKIRRDDEIIVIAGKDKGKRGKVLKVLADDRLVVGGINLVKRHTKPNPMSGVQGGIVEK
EAPMHASNVAIFNGATNKADRVGFKVEDGKKIRVFKSTQKAVDA
>tr|A0A0Q0BSD3|A0A0Q0BSD3_PSEAP 4-hydroxybenzoate transporter OS=Pseudomonas syringae pv. aptata OX=83167 GN=ALO85_00540 PE=4 SV=1
MTLSAGSPASSTLDVQAFINAQPLSGYQWRVVALCFLIVFLDGLDTAAMGFIAPALSQDW
GIDRASLGPVMSAALIGMVFGALGSGPLADRFGRKVVLVVAVLLFGLFSLASAYSSNIDQ
LLVLRLLTGLGLGAAMPNATTLLSEYTPERLKSLLVTSMFCGFNLGMACGGFVSAKLIPS
MGWHSLLMLGGILPLLLAVVLMVWLPESARFLVVRNRGAERIRKVLSPIAPKALAGITDF
SVPEQKTVSSRNVLKVIFSGNYSAGTLLLWLTYFMGLVIVYLLTSWLPTLMRDSGASMEQ
SAFIGALFQFGGVLSAVGVGWAMDRYNPHKVIGCAYALAGVFAWLVGQSLGNVALLATLV
LLAGMCINGAQSAMPSLAARFYPTQGRATGVSWMLGIGRFGAILGAWAGATLLGLGWTFE
QVLTALVVPAALAAIAVLIKGWVSHSDAT

>tr|A0A0Q0FNU3|A0A0Q0FNU3_PSEAP Lysine--tRNA ligase OS=Pseudomonas syringae pv. aptata OX=83167 GN=lysS PE=3 SV=1
MSDQQLDPQALQQEENTLIALRKEKLAAGRAKGQAFPNDFRRDSYCNDLQKQYVDKTKEE
LAEAAIPVKVAGRIMLNRGSFMVIQDMTGRIQVYVNRKTLPEETLAEVKTWDLGDIIAAE
GTLARSGKGDLYVEMTSVRLLTKSLRPLPDKHHGLTDTEQRYRQRYVDLIVNEEVRETFR
VRSQVIAHIRSFLMKRDFLEVETPMLQTIPGGAAAKPFETHHNALDMEMFLRIAPELYLK
RLVVGGFEKVFEINRNFRNEGVSTRHNPEFTMLEFYQAYADYEDNMDLTEELFRELAQLV
LGTTDVPYGDKVFHFGEPFVRLSVFDSILKYNPELTAADLQDIDKARAIAKKAGAKVLGF
EGLGKLQVMIFEELVEHKLEQPHFITQYPFEVSPLARRNDENPNVTDRFELFIGGREIAN
AYSELNDAEDQAERFQAQVADKDAGDDEAMHYDADFVRALEYGMPPTAGEGIGIDRLVML
LTDSPSIRDVILFPHMRPQA
>tr|A0A0Q0ILP2|A0A0Q0ILP2_PSEAP Pseudouridylate synthase, RsuA family OS=Pseudomonas syringae pv. aptata OX=83167 GN=ALO85_00261 PE=4 SV=1
MTDPIRLSKRLIELVGCSRREAELFIEGGWVTVDGEVIEEPHFKVSTQKIELSPDAKADS
PEPVTIILHKPASASAEEALQMITPGTLSVEHSYSKRPLKGHFMRLEAISSLQANASGLM
VFSQDWKILRKLTDDRSKIEQEYVVEISGEMVAHGLNRLNHGLTYKGKELPAVKASWQNE
NRLRFAMKNPQPGIIALLCEAVGLKIVAIRRIRIGGVSMGKLPEGQWRYMTAKEKF
>tr|A0A0Q0CVU9|A0A0Q0CVU9_PSEAP Putative Coenzyme A pyrophosphatase OS=Pseudomonas syringae pv. aptata OX=83167 GN=ALO85_04079 PE=4 SV=1
MLDELLRRVVRHTPETLDSDRQFPEAAVLVPITRSEQPELILTLRASGLSTHGGEVAFPG
GRRDPGDPDLIFTALREAEEEIGLPPGLVEVIGPLSPLISKHGIKVTPYVGVIPDFVEYQ
PNDGEIAAVFSVPLEFFRQDTREHTHRIDYEGRSWYVPSYRYGEYKIWGLTAIMIVELVN
VLYDTRISLHHPPERSTI
>tr|A0A0Q0FHA0|A0A0Q0FHA0_PSEAP Type IV pilus assembly protein PilQ OS=Pseudomonas syringae pv. aptata OX=83167 GN=ALO85_100023 PE=3 SV=1
MAGKAAHDRPQRTLMKWARPSMHSSAQRKLIMTRILSIIGVSLCIAMLSPVLQAANLKTL
DVAALPGDRIELKLAFDAPVPAPRGYTTSQPARIALDLPGVTSQLATKSRDLGSGNARSV
VVVQAQDRTRVIINLTTLSPYSSRVDGNNLFVVIGQGAGAAQASQPTTAMGAQRVAVPPP
TPASARPFIPAGVKAIRNVDFQRGELGEGNVVIDLSNPSIAPDIQEQGGKIRVDFAKTQL
PEPLRVRLDVKDFATPVQFVSASASGDKASILIEPSGAFDYSAFQTENKLTISVRPLTNE
DVDRRAADKPAYTGEKLSLNFQDIDVRSVLQLIADFTNLNLVASDTVQGGITLRLQNVPW
DQALDLVLKTKGLDKRKVGNVLLVAPADEIAARERQELESLKQIAELAPLRRELLQVNYA
KAADIAKLFQSVTSAESKADERGSITVDDRTNNIIAYQTQERLDELRRIVSQLDIPVRQV
MIEARIVEANVDYNKQLGVRWGGSTNTSGNRKWTTYGLDNNGDEAGNTSGNLTPNVPFVD
LGAAGATSGIGLGFVTNNTLLDLELSAMEKTGNGEIVSQPKVVTSDKETAKILKGTEIPY
QESSSSGATTVSFKEASLSLEVTPQITPDNRIIMEVKVTKDEPDYLNAVLGVPPIKKNEV
NAKVLISDGETIVIGGVFSNTQSKVVDKVPFLGDVPYLGRLFRRDVVSESKSELLVFLTP
RIMNNQAISVSR
>tr|A0A0Q0FLL2|A0A0Q0FLL2_PSEAP Uncharacterized protein OS=Pseudomonas syringae pv. aptata OX=83167 GN=ALO85_101278 PE=4 SV=1
MKLLEIRCVGFFDFFLARQSGIRTGALLFFRGVSGVRMKPSG
>tr|A0A0Q0DMU0|A0A0Q0DMU0_PSEAP UPF0301 protein ALO85_02930 OS=Pseudomonas syringae pv. aptata OX=83167 GN=ALO85_02930 PE=3 SV=1
MSAGQGNAKLGLMKKVSPSYLKHHFLIAMPHMHDENFAQTLTYIVEHNANGAMGLVINRP
QSLTLADVLEQLRPELPAPRHCQDIVIHTGGPVQTDRGFVLHPSGQTFQATVNLPGGISL
STSQDVLFSIADGYGPDQNVITLGYAGWDAGQLDAEMADNAWLTCSFDPAILFDVDSEQR
LDAAARRLGINLNLISTQAGHA
>tr|A0A0Q0BWD7|A0A0Q0BWD7_PSEAP Short chain dehydrogenase OS=Pseudomonas syringae pv. aptata OX=83167 GN=ALO85_05299 PE=4 SV=1
MESVSQSAKESFMTTTKKTALIIGASRGLGLGLVQRLTEQGWQVTATVRDPQNAENLKAV
EGVRIEAVDLDETASLEVLVQKLRGEVFDVLFVNAGISGAAHQSAAKSTAAELGQLFLTN
AVAPIRLAERFVDQIRPGTGVLAFMSSWLGSVTCPDGANLALYKASKAALNSMTNTFVTE
LGENRPTVLSMHPGWVKTDMGGENAAIDVMTSTTGLVEQLNAYAGKGGHHFIDYQGKTIA
W
>tr|A0A0Q0BS43|A0A0Q0BS43_PSEAP Uncharacterized protein OS=Pseudomonas syringae pv. aptata OX=83167 GN=ALO85_01855 PE=4 SV=1
MSKSALSFLVLAIMAVVTDLLLPVNAQAMSIAANIAAGVFASLFVVALFVGRRFKFDPVL
R
>tr|A0A0Q0IN82|A0A0Q0IN82_PSEAP Uncharacterized protein OS=Pseudomonas syringae pv. aptata OX=83167 GN=ALO85_02878 PE=4 SV=1

MPRTLIRKNPSNFKTLPLFVEATAESLSYQSVGMPMNFAQTLQRRRKIDVPDTEHFATEL
ANLGVSVRLTVSWQNRDYWVLVRQRRQDRGDVVLKLISGYVPAHELTLPLHTAIQEVAEE
CLIETPQGWLSGLFKETWLPAPYAAALHYREAMPFRLSPLSGAARPVRTGSLTLLERPRA
YVHLPTASLQLIYDMRLEIPKEARPVSLFHVDEALENDQLVARLNRSKPDLYLMPLENGA
PLPELYTLKRDKLIPAGTRGLYLAESFAAQDGWVVREERIRWKDWLRQQGMAPPAKKSGL
KRLTGKARELLHSISGKL
>tr|A0A0Q0CH95|A0A0Q0CH95_PSEAP Uncharacterized protein OS=Pseudomonas syringae pv. aptata OX=83167 GN=ALO85_200120 PE=4 SV=1
MSPSDIAGNESYSSEQAAFDMLSPQQRDTFAILLADIGKAMALDRLCERRGKAMGYALGL
LSCQAIDGNQLAGMEREANTAFKLRFEAVQPL
>tr|A0A0N8T899|A0A0N8T899_PSEAP Extracellular ligand-binding receptor OS=Pseudomonas syringae pv. aptata OX=83167 GN=ALO85_03135 PE=4 SV=1
MSHTFYRKGFLAIAVATALGVSSFAQADVKIGVAGPMTGANASFGQQYMKGAQAAADAIN
AAGGVNGEKIVLVAGDDACEPKQAVAVANRLVDQDKVIGVVGHFCSSSTIPASDIYSDAG
VIAITPGSTNPTVTERGMKEMFRMCGRDDQQGIVAGDYIVDVLKGKKVAVINDKDTYGKG
LADATAKQLTARGVKPILEEGLTRGEKDFSALVTKIRSVGADVVYFGGLHPEAGPLVRQM
REQGLKDVKFMSDDGIVTDELVTTAGGAQYVDGVYMTFGADPRALPDSKAVVDQFRKAGY
EPEGYTLYAYASVQALAAGFNGAKSNKGDAAAVWLKANPVKTVMGEKAWDAKGDLKVSDY
VVYQWDAAGKYKQLEKQK
>tr|A0A0Q0DAB0|A0A0Q0DAB0_PSEAP Putative adenylate-forming enzyme OS=Pseudomonas syringae pv. aptata OX=83167 GN=ALO85_100314 PE=4 SV=1
MIPVMTLWHYWRARRLRFSSRQALEHFQDRQLKRFAHRVLAKSPYFRAYSALPFNEWPLM
DKALMMTQFDQMNTAGLQRDQLLACARRGEAERDFTPRIGRFSVGLSSGTSGQRSVFVVS
PQEQQIWAGGMLAKMLPDGIFAGERVALFLRADNNLYHSVDNRWLSLAFYDLFAPFTEQF
PRLQAQAPTIIVAPAQVLRALALAVLDGQIQLEVKKVISVAEVLDAQDRQLLNTVFREVG
EVYQATEGFLAATCSHGTLHLNEEFVHIEPQWLDEQRFTPLITDFTRSTQPIVRYRLDDV
LVRQSEPCPCGQHSMAISRIEGRRDDQLSLPDQHGEMQIIFADLCSRAIANALPLTSDYR
LIQLSKTRLQLIADCTQTELEHSRRQLVALFTQQGIATDKLEWQLTVQNVMPNFDRKRRR
IVRQAKA
>tr|A0A0N8T8E4|A0A0N8T8E4_PSEAP Magnesium and cobalt efflux protein CorC OS=Pseudomonas syringae pv. aptata OX=83167 GN=ALO85_01326 PE=4 SV=1
MSEDRSSNGQKSWLGKLTQAFVHEPKNRQELLELLREAHQNKLLDSEALAIVEGAIQVAD
LQVRDIMVPRSQMISIKATQTPREFLPAVIDAAHSRYPVIGESHDDVLGVLLAKDLLPLI
LKADGDSDDVKKLLRPATFVPESKRLNVLLREFRANHNHMAIVIDEYGGVAGLVTIEDVL
EQIVGDIEDEHDVEEDSYIKPLPSGDFLVKALTPVENFNEFFDSTFSDDEFDTVGGLVMN
AFGHLPKRNEITEIGAYRFRILSADSRRIHLLRVTPISRP
>tr|A0A0Q0D8U1|A0A0Q0D8U1_PSEAP Uncharacterized protein OS=Pseudomonas syringae pv. aptata OX=83167 GN=ALO85_03634 PE=4 SV=1
MTELIWALEETTMNAPLRFNDALLIAGHAFEPFQCVAWAPQDGNGELSLTVIDRTSNRIG
RKQIPSSTYSDKRQLASALEQARAEISNEGYDLEPWTMPA
>tr|A0A0Q0FK22|A0A0Q0FK22_PSEAP GntR family transcriptional regulator with aspartate aminotransferase domain OS=Pseudomonas syringae pv. aptata OX=83167 GN=ALO85_05058 PE=4 SV=1
MTNLLLYQRIAQQLAEDIRRGVYQPGERVPSVRKMSSQLNVSHATVLQAYANLEDQGLIR
ARPQSGYYVHQTPALTASTPDIARVERPGLVTRSSIIQQVLEESRREGVFALGAAVPHVD
YLPVRALHQQLAKVTRFHSPRAFSYMFSPGFEPLRRQVAIRMRDAGVVVDPSEVVITHGC
VDALQMALRVLTRPGDLIAAESPAYYGLLQLADLLGLKVIEIPSDPATGISLEALQLAAN
QWSIKALVLTSRLSNPLGGTMPEERQKNLLRLTSDFDIQVVEDDVYGELMFEVGRTKALK
AFDRLGRVIYCSSFSKTLSPGVRIGWMIAGKYQAEIQRLQTFSTHSACSVTQMAVAAYLE
NGGYDRHLRFIRLEYRKNLSAYQLAVQQFFPEGTQMSRPAGGFILWVSLPGRVNTQELHV
RALEQGISIAPGLIFSNTEQFNHCIRLNCGMKWNKEAERALMTLGILSKQLCQEAVQAY
>tr|A0A0Q0D071|A0A0Q0D071_PSEAP Two-component system response regulator QseB OS=Pseudomonas syringae pv. aptata OX=83167 GN=ALO85_03179 PE=4 SV=1
MRLLLIEDDVALGEGIQQALSREGYTVDWIKDGSSALHALLSESFDLAILDVGLPTLSGF
EVLRRLRHSGSTLPVMILTARDATEDRIIGLDSGADDYLVKPFDVSELKARLRALLRRSA
GRAKVLIEHNGIRLDPSTQQVSYHDAAVALTPKEYQLLYELLSPPGRVMTRERLMQLLYG
WNEEAESNTLEVHIHHLRKKFSSTLIRTIRGVGYLVEERS
>tr|A0A0Q0FC20|A0A0Q0FC20_PSEAP Type III secretion system negative regulator HrpV OS=Pseudomonas syringae pv. aptata OX=83167 GN=ALO85_04192 PE=4 SV=1

MIEVEEKTAFYYNVAAQSPAVWPVANGVSFVSRREHHDWGIALHIEGRALRPEQLREALQ
LRFSEAERFNDYFLFLDMRRDFVVWHAVSDAPHAVTNLDDIRRNELILAGLDHLV
>tr|A0A0N8T9T7|A0A0N8T9T7_PSEAP HAD superfamily Cof subfamily hydrolase OS=Pseudomonas syringae pv. aptata OX=83167 GN=ALO85_03656 PE=4 SV=1
MSELPVQFLLSDIDGTLLRRDHSLSQANIDAIKRLRIAGVHFTLASSRPPRAMRQVIEAL
DVDLPTVAFNGGTITHPDGSLLVAHRIDPRAVRICLELFAGQNVAIWVFADDQWLLIDPD
AAYVDHELDALGYDYVQVESFEPYLDRVDKIVAASGDFELLKTLEARLNPQIEGLALAAR
SQKYYLDVTALEANKGSALVALADYLGVDLSRTAAIGDGGNDVAMFHKAGLSIAMGQGEQ
TVKGQADHVTDSNEQDGVANAIERYILNV
>tr|A0A0Q0BWN8|A0A0Q0BWN8_PSEAP Uncharacterized protein OS=Pseudomonas syringae pv. aptata OX=83167 GN=ALO85_01069 PE=4 SV=1
MTENDYLIAWSVYAFAALGCLLVWFLMTRWMWRYLKEPLLVAVAVLLFTPTMVDPAKDAM
APAVAITALDLMFKVGSNIWTAVSDLAMYGMFAFAAYLLFVLIRWPIERSRKARQPKPAA
QPVETDPADEPFAEDDHYARKNAAPLAPTRAARVEPRL
>tr|A0A0Q0BTE9|A0A0Q0BTE9_PSEAP Uncharacterized protein OS=Pseudomonas syringae pv. aptata OX=83167 GN=ALO85_04273 PE=4 SV=1
MEYAMSSQKMNGKVMWKLRTLDKRLWVVILSALWLTAPAVSWAKDGAITLRGSIVNSSCK
VSSTEMTAPGVQSRTLEVAPGVTIHVSTERNVCGEQAMPFVTQYQVLPAAVVIDGEALQA
RAAVVTLSYQ
>tr|A0A0Q0CZT1|A0A0Q0CZT1_PSEAP Uncharacterized protein OS=Pseudomonas syringae pv. aptata OX=83167 GN=ALO85_100612 PE=4 SV=1
MPVEEFRLGTQQNSFRKRSRTCAEVKGSLAHIHSRQIRWVCTRLFLVACHEQTDAAATGS
SLRES
>tr|A0A0Q0BVD6|A0A0Q0BVD6_PSEAP N-acylglucosamine 2-epimerase OS=Pseudomonas syringae pv. aptata OX=83167 GN=ALO85_00656 PE=4 SV=1
MPSVEMTSGTVWAQPYRRGRHRFMNNNNQTFSSWLRSPAHHQWLALEGNRLLTFAKASRL
DNGFGSLDDYGRLMVGATAGTMNTARMTHCFAMAHAQGIPGCAALVDHGIAALRGPLHDA
EHGGWFSAALEDGGKTEKQAYLHAFVALAASSAVVAGRPGAQALLSDAIQVIQTHFWSEQ
EGAMRESFARDWSHEESYRGANSNMHSTEAFLALADVTGDAQWLDRALSIVERVIHEHAG
ANNFQVIEHFTQDWQPLPDYNRENPADGFRPFGTTPGHAFEWARLVLHLEAARRQAGRAN
PEWLLDDARQLFANACQYGWDVDGAPGIVYTLDWQNQPVVRHRLHWTHCEAAAAAAALLQ
RTGEQQYEDWYRCFWEFNETLFIDHEHGSWRHELNERNEPSADIWPGKPDLYHAYQATLL
PVLPLAPSLASALAGLE
>tr|A0A0Q0C1L4|A0A0Q0C1L4_PSEAP ABC transporter binding protein-like protein AabH OS=Pseudomonas syringae pv. aptata OX=83167 GN=ALO85_03681 PE=4 SV=1
MKGITPLSFPALMGSALLLNAVISFPALAGLLEKGRTDGLTAGIANEQPYAYIGTDGKAT
GANVEILRAILEPLGIKQIETPITDFGSLVPGLAAGRFDLIGAGLFINPNRCKVIAFSNP
VTRSGGAFIVKTGNPLNLHSLKDVAGNTKVRLATQPGSNQVQEAKDSGIANGNVVLFDKD
TEALAALQAGRVDVVYFPDAEVISLIKKANSADIERALPFEQIPDASGKPGWNYHAYGLP
KNDPAFEQAFNEQLAKLRASGELLKILQKYGYTENELADPAITAVQRCNP
>tr|A0A0Q0DTD1|A0A0Q0DTD1_PSEAP Biotin synthesis protein BioH OS=Pseudomonas syringae pv. aptata OX=83167 GN=ALO85_03873 PE=4 SV=1
MRDRLILLPGWGLGVSPLEPLAAALRGLDEHLRVEVEPLPDIDSCDLPDWLDELDTNLPD
DAWLGGWSLGGMLAAELAARRGDRCCGLLTLASNACFVTQGAWPNAMPAQDFEAFLAGCA
DDPDLTLKRFSLLCTQGAEDPRGLARLLKAGAPHLSKTALIDGLKILRKLDTRSALQTYR
GPQLHLFAGLDAFVPAEAAGDLLALLPDVEVGLIEQASHAFLLENPHGVAAAIHAFLHEL
DDD
>tr|A0A0Q0FGF4|A0A0Q0FGF4_PSEAP RNA polymerase sigma factor RpoT OS=Pseudomonas syringae pv. aptata OX=83167 GN=ALO85_01684 PE=3 SV=1
MSSHESLFDYEATLQACARGEKQALQRLYQQESARLLGVAQRLVRDRALAEDIVHDAFLK
IWSHAASFDASRGSARGWIFSVTRHLALNTLRNTAREVRVEDDGSEDHQEQATLEGWQEI
GDAFDWRVNPGRITSCMEQLEPVRRNCIFHAYVDGYSHQEIAQKIGAPLGTVKAWIKRSL
TALRECMG
>tr|A0A0N8T7X3|A0A0N8T7X3_PSEAP Putative lipopolysaccharide ABC-type transport system OS=Pseudomonas syringae pv. aptata OX=83167 GN=ALO85_04305 PE=4 SV=1
MIVFRYLSREVLLTLSAVSAVLLVFIMSGRFIKYLAQAASGALDPGVLFLIMGFRLPGFL
QVILPLGLFLGILMAYGRLYLESEMTVLAATGMSQQRLLAITMGPATLVGLLVAWLSFSL
APQGATQFSLLINQQDAMTEFDTLVPGRFQALRDGTRITYTKELSEDRSQLAGVFISEKR

MSSDKSKDNGITVLVAEKGHQEVRPNGSRFLILENGYRYDGNPGAADYRVIKYDTYGAML
PKPEISEEITDREAIPTSELIGNPAPRSVAELQWRISLPLSVFIVTLMAIPLSRVNPRQG
RYLKLLPAILLYMSYLAILISVRSSLEKGKLPLSLGMWWVHAIYLSIGLLLFYWEPLRLK
MASRRSVTEVTRGQA
>tr|A0A0Q0IIZ1|A0A0Q0IIZ1_PSEAP Peptidyl-prolyl cis-trans isomerase OS=Pseudomonas syringae pv. aptata OX=83167 GN=ALO85_01761 PE=3 SV=1
MPIAANKAVSIDYTLTNDAGEVIDSSTGAAPLVYLHGAGNIIPGLEKALEGKEVGDQLNV
AVEPEDAYGEYSAELVSTLSSSMFEGVDKLEVGMQFHASGPDGGMQIVTIRDLEGDDVTV
DGNHPLAGQRLNFDVKVVAIRDASEEEMAHGHVHGEGGHQH
>tr|A0A0Q0DLD3|A0A0Q0DLD3_PSEAP Uncharacterized protein OS=Pseudomonas syringae pv. aptata OX=83167 GN=ALO85_01031 PE=4 SV=1
MNTQNWRYIMSNSTNETKYFDLHTVGIGYLNRIREVKPRKGAPFMAVTVAALKGTSEKPE
YAYIDCNVVGAEADKLIRRCQEAVAAEKKVLVSFRIGDIWADVFTYSSGAKQGQAGVSLK
GRLLYLAWIKVEGEYVYQAPQKEEAPASSESSESEATPVVQSEASASSEPTDVATPAATP
PKPTRAKPARAGRGEKQAA
>tr|A0A0Q0DZ49|A0A0Q0DZ49_PSEAP LysR family transcriptional regulator OS=Pseudomonas syringae pv. aptata OX=83167 GN=ALO85_03419 PE=3 SV=1
MNKYPSLNDLNVFVHVARRSSFVGAADELGMSAAYVSKRIRLLEQGMGVVLLHRSTRQVS
ITEDGERVYEWARGMLDSVQQMGEEVAALHGEPSGLLRVVSSQGFGRRFVAPALSELASR
YPKLDIRLDIQDRLVDLIEEGVDLDIRVGNEIAPHLRARHLASNWRVLCASPGYLNLHGT
PLNLGELANHDCLVIKERDRPFGIWHLNGPQGAETVKVTGSLSTNHGEIARQWCLDGRGV
LLRSLWDVRTDLAEGRLVQVLPDYRQDADIWAVHTSPLMSSAKVRVTVEFLRDYLALHPS
PVNH
>tr|A0A0Q0C961|A0A0Q0C961_PSEAP Glutathione S-transferase domain-containing protein OS=Pseudomonas syringae pv. aptata OX=83167 GN=ALO85_00715 PE=4 SV=1
MGLLVEGHWQDQWYESSKDGAFQRENAQRRNWVTPDGQPGPSGAGGFAAQAGRYHLYVSL
ACPWAHRTLILRKLKGLEGLIDVSVVSWLMLEDGWTFDQQKGSSGDALDDLQFLHQRYTA
DDPRYTGRVTVPVLWDKQQQCIVSNESAEIIRMFNSAFDELTGNTLDFYPQALRASIDEL
NEQIYPKVNNGVYRAGFATSQGAYEEAFDDVFAELDVLENLLGEKRYLTGKHLTEADIRL
FTTIVRFDAVYYSHFKCNLRRIADYPNLSNWLRELYQWPGIAETVSFEHIKGHYYGSHRT
INPTGIIPKGPALDLQADHDRERLSGEGVWNN
>tr|A0A0Q0FCL0|A0A0Q0FCL0_PSEAP SmpA/OmlA family outer membrane lipoprotein OS=Pseudomonas syringae pv. aptata OX=83167 GN=ALO85_100486 PE=4 SV=1
MGMRPANHLLLWSALLCLPATATASNVHRCEDASGKITFTTLGCPDGHSTRLQRAFNAPP
GTRIDVLPPASTAQTAREKPADQTVVVVGTRDDGCGNRLSAEQRRAAIINQRTPPGMTQR
DVESLLGRPDKVTNRNGELHYVYNQKKGRSNKVTFDEHGCVKGKR
>tr|A0A0Q0BUG2|A0A0Q0BUG2_PSEAP AMP-dependent synthetase and ligase OS=Pseudomonas syringae pv. aptata OX=83167 GN=ALO85_02837 PE=4 SV=1
MDRMNWLNLEHLLLDALPDRLLTLAPSLDHAQFRDQALSVAAGLQARGITHLAVHLEDAA
DLAIALFAAWRAGVHVLLPADLQGQTRERWANQVDLWLTDLPGDTHLSDLRAAPLPAAAL
DLDQCRLSLCTSGSSGEPKLIEKRLRQLANEVCGLEQLWGAELGSACMIGSVATQHIYGL
LFRLLWPLCAARPFVRRQLPFAEDLQRASREYPSFAWVASPALLKRMGDNLDWPGLAAVR
RVFSSGGALPAEAAQSLQQRLGQWPTEILGSSETGGIAWRQGGQRWQAFDGVELSQDAQG
ALRISSPYLPPGHVEQTADAALIGADGRFELLGRLDRIVKLEEKRVSLPLIEQALAAHEW
VSEARLGVVQANRASLGALLVLSDAGLLALRNQGRRALTEALRNYLQPHCETIALPRRWR
LLRQMPLNAQGKLPQADVEALLLAPRSKQPEVLEQQNIEGELHLQLSVPPDLAFFSGHFP
KAPILPGVVQVDWAISLGQRLLDLPSGFAGMEVLKFQQLVRPGDRLTLTLRFDAARSKLH
FAFRNADNAPCSSGRILLEDDHA
>tr|A0A0Q0DLU8|A0A0Q0DLU8_PSEAP Two component hybrid sensor histidine kinase/response regulator RetS OS=Pseudomonas syringae pv. aptata OX=83167 GN=ALO85_01381 PE=4 SV=1
MRGLRIAIASTVGLLTLLFMLSAQAEHGAGWSTLLDEKAHLTLDEIRSARYQNQFSPTEL
ERVTAADRGGAVWLHYRLQPTQHEQLLRIFAPDLSSADLYVMDGDQQIDHVRTGNDVPIE
DQRLPSNDFLLPVPQSSAPLDIYLKLVSAQKMRPSITLEPAIKSAANETQPLLFGLLFGA
LSMLIVQNLTRYGHTRSRSNLWLAACESLLGLSALLLLNLLRPIDPWNIAQTPSAHLALL
LAAVAGLIYTYYFFVHRNARKLDRLLLGNAVLMGIGASLVLFDEQLPINILTFGLVSLTT
LSILAVSMWHWQKGYRSARLFVLGMITFNIGYMVVLPGLLWLSLVPPQWLILALLSVFCI
SGLLMSLALSERHRSITEDRFSISRDQAASTAEINAKAEFLAKISHEIRTPMNGVLGMTE

LLLGTPLSVKQRDYVQTIHSAGNELLTLINEILDITKLESGQIELDDVQFDIGALVEDCL
NIFRAKAEQQQVELISQIQPQVPRVINGDPTRLRQTLLSLLENALKKTDEGEILLAVALE
SSKSGNTRLRISVQDSGESLSDEEREELLHAELHSRNFLASSRLGGHLGLVIARQLIVLM
DGEFGIQNTGAQGNTLWLNLPLDAKLLEQPTIDLDSPLKDARVLVVDDNDTCRKVLVQQC
SAWGLNVSAVASGKEALALLRTKAHLRDYFDVVLLDQNMPGMTGMQLAAKIKEDPSLNHD
ILLIMLTGISNAPSKIIARNSGIKRILAKPVAGYTLKTTLADELTQLNKGVVSNKPGPVV
TPPVNVPGDFRILVAEDNSISTKVIRGMLGKLNLNPDTASNGEEALRAMKAQRYDLVLMD
CEMPILDGFSATEQLRAWEVGNQRVRTPVVALTAHILTEHKDRARQAGMDGHMAKPIELS
QLRELVEHWVAHRDKARGLTADDERYRQN
>tr|A0A0N8T7U3|A0A0N8T7U3_PSEAP Organosulfonate ABC-type transport system, periplasmic substrate-binding protein OS=Pseudomonas syringae pv. aptata OX=83167 GN=ALO85_02328 PE=4 SV=1
MRTVILRRSLVALFAAAVALGAVTQAQAEDLRIGYQKYGTLVLLKAKGSLEKRLAEQGVK
VQWTEFPGGPQLLEGLNVGSIDFGVTGETPPVFAQAAGADLLYVAYEPPAPTSEAILVPK
DSPITSVKDLKGKKVVLNKGSNVHYLLVKALEDAGLKYTDIQTVFLPPADARAAFERGSV
DAWVIWDPYQAAAEKQLQARTLKDGTGIVDNHQFYLATKPYAQKNPKVIQALVEEVRAVG
EWSKANPDEVTQQVAPLLGLPADITLTSVKRQGYGAQFLTPPVVAAQQKIADTFYQLKLI
PKPLSIADVVWTPPAAVAQAQ
>tr|A0A0Q0C1Q1|A0A0Q0C1Q1_PSEAP Transcriptional regulator MexT OS=Pseudomonas syringae pv. aptata OX=83167 GN=ALO85_03714 PE=3 SV=1
MNRNDLRRVDLNLLIVFETLMHERSVTRAAEKLFLGQPAISAALSRLRGLFDDPLFVRTG
RSMEPTARATEIFALLSPALDSISTAVSRASEFDPATSTSVFRIGLSDDVEFALLPTLLK
RLRSEAPGIVLVVRRVNYILMPPLLASGEISVGVSYTQDLPANAKRKVLRRSKPQLLRAD
SIPGPLSLDDFCARPHALVSFAGDLSGFIDEHLEKLDRKRHVVLAVPQFNGLATLLTGTD
IIATVPDYAAQVLTAAGGVRSEDLPIETRTFELHMAWRGAQDNDPGERWLRSRIQMFFGD
PDSL
>tr|A0A0Q0BSL2|A0A0Q0BSL2_PSEAP Guanylate kinase OS=Pseudomonas syringae pv. aptata OX=83167 GN=gmk PE=3 SV=1
MTHITGTLYIISAPSGAGKTSLVKALMDAQQEPQHGAQAKIRVSVSHTTRAMRPGEVDGV
NYNFVDRATFLSMIEHGDFLEQAEVFGNLYGTSQSHLQQTLDEGHDLILEIDWQGARQVR
AQMPQARSIFILPPTQQALRQRLTNRGQDSDEIIEARMREAVSEMSHYSEYEYVVVNDDF
AGALEDLKAIFRANRLTQQHQQEQYSELFQELLA
>tr|A0A0Q0IP42|A0A0Q0IP42_PSEAP Bacterioferritin OS=Pseudomonas syringae pv. aptata OX=83167 GN=ALO85_01926 PE=4 SV=1
MMNSAPQSQLSDVNTLRQRARQNVEDGAVTQGYSADRETVLRLLNESLATELVCVLRYKR
HYYMASGLKASVAAAEFLEHAEQEAQHADKLAERIVQLGGEPEFNPDTLSKNSHAQYVAG
NTLKEMVYEDLVAERIAVDSYREIIQYLGDSDPTTRRIFEEILAQEEEHADDMADILNDL
>tr|A0A0Q0BXL3|A0A0Q0BXL3_PSEAP Putative ATP-dependent zinc protease OS=Pseudomonas syringae pv. aptata OX=83167 GN=ALO85_03900 PE=4 SV=1
MKTFDHLTVIGLREWVALPDLGVAGLRAKIDTGASTSSLHATEIEPFERNGQKWVRFNAH
LGTVVQLRHRRCEAQLVAMKTIKSSNGQAQVRYVIRTTLALGDRIWPVEFTLACRKSMRY
RLLLGSKALIDGHLVVNPGITYVQDKPVFPASTSSGVA
>tr|A0A0Q0BSQ0|A0A0Q0BSQ0_PSEAP UDP-N-acetylmuramoylalanine--D-glutamate ligase OS=Pseudomonas syringae pv. aptata OX=83167 GN=murD PE=3 SV=1
MSLIVSDRFRIVVGLGKSGMSLVRFLANQGVSFAVADTRENPPELATLRRDYPQVEVRCG
ELDVDFLCRADELYVSPGLALATPALQQAHARGVKLSGDIELFARYAKAPVIAITGSNAK
STVTTLVGEMAAAAGKRVAVGGNLGTPALDLLSDEVELYVMELSSFQLETTDQLNAEVAT
VLNISEDHMDRYNGLPAYHLAKHRIFRGARQVVVNRQDALSRPLIGEGLPCWTFGLNKPD
FNGFGLREENGEKYLAFQFENLMSVRELKVRGAHNQANALAALALGHAVGLPFDAMLASL
REFTGLEHRCQWLREHDGVHYYNDSKATNVGAALAAIEGLGSDIDGKLVLIAGGDGKGAD
FSGLRAPVAKYCRAAVLLGRDAELIAQALGDAVPLVRVDTVQAAVEHSAKLALRGDAVLL
SPACASLDMFKNYEERGRVFAQAVECLS
>tr|A0A0Q0BTE0|A0A0Q0BTE0_PSEAP Sensor histidine kinase OS=Pseudomonas syringae pv. aptata OX=83167 GN=ALO85_01257 PE=4 SV=1
MAMRLSGFILENIEAIVREWTDFARTLSALGEPLDTTELRDHAEQMLRAIATDLQTDQSS
QQQVDKSQGHGVSNEQTAAKSHAITRLMSGFTIDQVVSEFRALRASVIKQWMSRETQDAQ
QQIEDMIRFNEAIDQALAESITSYTGAVQASRNIFLGILGHDLRTPLSAILLGADVLLRT
HDLGARPTKVASRIYSSVKRANQIVGDLLDFTRSQIGPGIPLKKTEVDMQPICTRIVDES
STVHPEADIRLTSDEPVIGRFDGDRLEQVFSNLIGNAIQHGNSSDPVEVTLAVSESGLRF
TVHNTGSPIPEDVLPFIFNPMDRYSPEKMTDNGPYSSLGLGLFIVDRIVDAHGGRIEVTS

KADLGTTFAVVIPLNAE
>tr|A0A0Q0C664|A0A0Q0C664_PSEAP EmrB/QacA family drug resistance transporter OS=Pseudomonas syringae pv. aptata OX=83167 GN=ALO85_00481 PE=3 SV=1
MQNSAAPIMDEKTLRWMPWVIAIAFFMQSLDGTILNTALPSMARSLAEDPLRMQSVVIAY
MLTIALLIPASGWIADRFGIKRIFFSAILLFSFGSLLCALSWSLNVLVAARVIQGLGGAL
MLPIGRLIVLRAYPRSELVRIMGFITVPGLLGPLLGPTLGGWMVEYLSWHWIFLINIPVG
ILGCYAVKHFIPDLPGGGRTRFDGVGFILFGAAMVLITIALEGLGELHLPHMRVVLLLFA
GMGCLAAYWLRAGHIESPLFAPSLFRTRTFAVGIMGNLFARLGSGALPFLVPLLLQVALG
YSPSQAGMSMLPLAAAGMFAKTVARWLIERLGYRTILTGNTLLLGILLASLALVDVQTPY
WVLLVHLGLLGAVNSLQFTAMNTVTLIDLDDASAASGNSLLSVVAQLSLSLGVASAAALL
GGFSEEVATGEVSSMLGAFQLTFLSVGVLAMFAAGIFLQLPAKEHRVAKA
>tr|A0A0Q0C1J6|A0A0Q0C1J6_PSEAP Hemolysin-type calcium-binding region:peptidase M10A and M12B OS=Pseudomonas syringae pv. aptata OX=83167 GN=ALO85_03594 PE=4 SV=1
MPGPNESSPAELLPDGATDDRVTSLLWGPFWLGDSSGTHLTYSFHRADSVYATDYSRSQE
PGDAYSLTDAQAAAAKSALDAWSAVADIKFTEVQDTPDNVGDIRFGGFKGLQGTEFGQAY
APGTLGRSGDVWIGPNVNAADPAKGTDDYLTFMHETGHALGLKHSFEASQYNDVLLDAKF
EDARYTIMSYTNNYSFKPTTPMLLDVAAMQFIYGANTHYHTGDDVYKWAPGQSVFETIWD
AGGKDTIDASNQEASVKINLNEGEFSTIGKAFLDYNANPDAPTSMNSGLAIAYGAHIENA
IGSAFNDTLIGNDLDNVLNGRGGLDTMIGGLGNDTYVIDQAGELDLVQEKADQGVDTLKI
TYDNTSPIAQVINLNAGTLANFENVHLKGEGAFTVLGNDRNNTLTGNDADNVLFGGAGND
KLVGGQGADIMTGGSGADRFVFNNLSEMGKGHNSDVITDFNSQQGDKLSFLKMDANVDTK
ALDAFSFIGSGEFTGAGQLRFADHVLSGNVNADLHADFEIQLVGVTEFHAHDLAV
>tr|A0A0Q0CXJ4|A0A0Q0CXJ4_PSEAP Ankyrin OS=Pseudomonas syringae pv. aptata OX=83167 GN=ALO85_01211 PE=4 SV=1
MTDTDISASKTMTEDEAAEFAEQVFDVARQGNAVMLDRLLEKGVPADLRNHKGDTLLMLA
SYHCQADAVRVLLNHKADPEIRNDNGQSPIAGAAFKGDLAVVRLLVEAGADIDGASADGR
TALMMAAMFNRNEIVDYLISQGADPHATDAKGITALGAAQAMGATVTIEQLTKMGVQPAR
S
>tr|A0A0N8T844|A0A0N8T844_PSEAP Membrane protein-like protein OS=Pseudomonas syringae pv. aptata OX=83167 GN=ALO85_03464 PE=4 SV=1
MDTIRRFSSFAEFYPFYLAEHSSATSRRLHFVGTSLVIFLLAFGVGSGRWGLLWLLPVAG
YGFAWAGHFFFEKNRPATFKHPFYSLLGDFVMYRDMLLGKVPF
>tr|A0A0Q0CXC0|A0A0Q0CXC0_PSEAP Thiosulfate sulfurtransferase GlpE OS=Pseudomonas syringae pv. aptata OX=83167 GN=glpE PE=3 SV=1
MTEFKRIPPEQAQALREQGAVLVDVRDPQAFESNHIPDSVHLDNHSIADFIREADLDKPL
VVVCYHGNSSQSAAAYLVGQGFSDVYSVDGGFELWRATYPQETVQG
>tr|A0A0Q0D685|A0A0Q0D685_PSEAP Acyl-[acyl-carrier-protein]--UDP-N-acetylglucosamine O-acyltransferase OS=Pseudomonas syringae pv. aptata OX=83167 GN=lpxA PE=3 SV=1
MSLIDPRAIIDPTAVLADNVEVGPWSIIGPGVEIGEGTVVGPHVVLKGPTRIGKHNRIYQ
FSSVGEDTPDLKYKGEETRLVIGDHNVIREGVTIHRGTVQDRAETTLGDHNLIMAYAHIG
HDSVIGNHVILVNNTALAGHVHVDDWAILSGFTLVHQFCHIGAHSFSGMGTAIGKDVPAF
VTVFGNPAEARSMNFEGMRRRGFSEEAIHALRRAYKTVYRQGLTIGQALADLAEPAAQFP
EVAVFLQSIQTSTRGIIR
>tr|A0A0Q0CA32|A0A0Q0CA32_PSEAP Uncharacterized protein OS=Pseudomonas syringae pv. aptata OX=83167 GN=ALO85_01401 PE=4 SV=1
MRLVHLLYIAPFASLMYVSQVQALDTTTQGLVKTGYATSQVSTGPFDNKLIMAARDDAAA
FIASGGQMRGARLESALHALRQADAKLHASDLELAQAILVQ
>tr|A0A0Q0D5B6|A0A0Q0D5B6_PSEAP UDP-glucose 6-dehydrogenase OS=Pseudomonas syringae pv. aptata OX=83167 GN=ALO85_00889 PE=3 SV=1
MKISVFGSGYVGLVQATVLAEVGHDVVCMDIDQAKIDQLRQGQVHIYEPGLANLVRENLD
QQRLCFTTDEQLAVEHAEVLFIAVGTPSRADGSADLSGFFAVGEAIARHRREPLIIVEKS
TVPVGSGDALRNHVDQALRQAGRLLQFDIVSNPEFLKEGSAVNDCRRPDRIVIGCENEAV
RNVMRELYAPFNRNHERIMFMDLRSAELTKYAANCMLATKISFINQIAELAEHLGADVES
VRLGIGADSRIGYDFIYPGCGYGGSCFGKDIRALIHSAREAHCSNDLLSVVEAINERQKN
KLFERVNAFYQGDLRGKTFALWGLAFKPNTDDMRDAPSRVLMEALWEAGASVRAFDPEAM
QETQRIYGHRKDLMLMGTPESALNEADALIVCTEWQQFKAPDFELIQQRLNAPIIFDGRN
LYDTERLAKRGFHYFPIGRGESCDLPIPQKQWMACNSLVSSQVV

>tr|A0A0Q0D002|A0A0Q0D002_PSEAP Decarboxylase OS=Pseudomonas syringae pv. aptata OX=83167 GN=ALO85_02124 PE=4 SV=1
MFSIFAILMLDCIALMENRMIPRQVINATVSPKGSLETLSQREVQQLSAAGSGSTYTLFR
QCALAILNTGAHVDNAKTILEAYESFEVRIHQQDRGVRLELLNAPADAFVDGEMIASTRE
MLFSALRDIVYTESELDSQRIDLSNSQGITDYVFHLLRNARTLRAGVEPKMVVCWGGHSI
NTEEYKYTKKVGHELGLRSLDICTGCGPGVMKGPMKGATIAHAKQRIVGGRYLGLTEPGI
IAAEAPNPIVNELVILPDIEKRLEAFVRVGHGIIIFPGGAGTAEEFLYLLGILMHPDNKD
VPFPVILTGPKNTEPYLQQLHAFVGATLGEEAQRHYQIIIDNPADVARQMTQGLKEVKQF
RRERNDAFHFNWLLKIEESFQHPFDPTHENMSRLQLNHDVPTHELAANLRRAFSGIVAGN
VKDKGIRLIEQHGPYQIHGDPSIMGPLDKLLQAFVDQHRMKLPGGAAYVPCYQVVT
>tr|A0A0Q0IRS6|A0A0Q0IRS6_PSEAP Putative DNA modification/repair radical SAM protein OS=Pseudomonas syringae pv. aptata OX=83167 GN=ALO85_03394 PE=4 SV=1
MQLIDKLSILADAAKYDASCASSGAPKRSSQDKSGLGSTNGMGICHSYTPDGRCVSLLKI
LLTNFCLYDCQYCVNRRSSDVPRARFTPEEVVTLTLDFYRRNCVSGLFLSSGIIRSADYT
MEQLVEVARLLREVHEFRGYIHLKTIPDADPALIEKAGRYADRLSVNIELPTDLSLQTLA
PEKDVASIKQAMQTIYTGEQTVRNEPRSPRFAPAGQSTQMIVGADATDDSTILHSAQSLY
SNFMLRRVYYSAFSPIPNSPNSVPLAAPPLMREHRLYQADFLLRGYGFTASELLDGPGDL
ALDIDPKLAWALGNRQVFPLDLNQADPALIARVPGIGIRTTQRLVELRRQRRIRYEDLTR
MRCILAKAKPFIITSDYHPPHAETTSEFLHHQLRDRPQPQQMGLWG
>tr|A0A0Q0FGM8|A0A0Q0FGM8_PSEAP Radical SAM domain protein OS=Pseudomonas syringae pv. aptata OX=83167 GN=ALO85_04924 PE=4 SV=1
MSSLYPVRGRGTASNPHNRFAPSQSVVEDDGWYQEVPLTQGTQVTSETAKSIITRNSSPD
IPFDRSINPYRGCEHGCIYCFARPSHAYWDMSPGLDFETKLIAKTNAAALLEQQLSKPGY
RCAPITLGANTDPYQPIEREYRITRATLEVLLRYRHPVSIITKGSLILRDLDLLAEMARL
RLVSVFISLTTLDDELKCILEPRAAAPKARLRAIRVLRQAGVPVGVLCAPMIPMINDSEL
EALLSEAKAAGALSASYVMLRLPLEVAPLFDEWLKTHYPQRADHVMSLIRQSRGGALYDS
TFGSRMRGEGPFADLLAQRYALAIKRLGLNKRGRFALDCDAFCPPGGQMSLL
>tr|A0A0Q0C550|A0A0Q0C550_PSEAP Uncharacterized protein OS=Pseudomonas syringae pv. aptata OX=83167 GN=ALO85_04147 PE=4 SV=1
MTNNYEIDEQKRKADEESDGGILNDIKEFFEDDESEDFEEDAKTKTEPKKKLIGEEYLTG
LPMNVIRYLFIPCVLAFLIYRNSSAIYASGFGISSLLYVIPLVFFSCIAVYLSFVFLKAQ
IASRASTPDERVRISGITLKAVLLRGGSIGSPYFLKRQGLIFEFFAGLFVPTSVFILVFL
VMNMLNIFAFKVVYVVVAFVLLLALMNYPVSIMRSKAADEIQRLG
>tr|A0A0Q0C4Q6|A0A0Q0C4Q6_PSEAP Fe/S biogenesis protein NfuA OS=Pseudomonas syringae pv. aptata OX=83167 GN=nfuA PE=3 SV=1
MCEAVDWRKLHARLRGIFMTAITITDAAHDYLADLLEKQNTPGIGIRVFITQPGTQYAET
CIAYCKPGEEKPEDKAIGLKSFTAWIDGFSEAFLDDAVVDYATDRMGGQLTIKAPNAKVP
MVNADSPINERINYYLQTEINPGLASHGGQVTLIDVVEEEAKNIAVLQFGGGCQGCGQAD
VTLKEGIERTLLERIPELSGVRDVTDHTQKENAYY
>tr|A0A0Q0C4J7|A0A0Q0C4J7_PSEAP Sec-independent protein translocase protein TatC OS=Pseudomonas syringae pv. aptata OX=83167 GN=tatC PE=3 SV=1
MSADIPENDQQMPLISHLTELRTRLLRCVAAVFLIFAGLFYFTQKIYTLVSAPLRVYLPE
GATMIATDVASPFITPFKLTMMVALFLSMPVILHQIWGFIAPGLYKHEKRVAVPLLVSSI
ILFYAGMAFAYFLVFPLIFHFFASVTPEGVSMMTDIASYLDFVMTLFFAFGVAFEIPVAV
VLLVWIGIVDVKYLKKIRPYVIIACFVVGMILTPPDIFSQTLLAVPMWLLFELGVLCSTM
IKKRGEHPDDQAEDADKQDQPPATTP
>tr|A0A0Q0CV81|A0A0Q0CV81_PSEAP Uncharacterized protein OS=Pseudomonas syringae pv. aptata OX=83167 GN=ALO85_02290 PE=4 SV=1
MEMAQGEKEMTTRTRSHDESVLEMLREDEAFAIEYLSVALEEIDEDGGEDVFLTAIRRLI
EARGGMGNLSKNTGLARPNLYRSIAAGGDPKLSTILKVLQALGVGMSKVVSHRADVGSQS
PDQ
>tr|A0A0Q0FBD1|A0A0Q0FBD1_PSEAP Uncharacterized protein OS=Pseudomonas syringae pv. aptata OX=83167 GN=ALO85_02255 PE=4 SV=1
MNKRLQGNCSSKPALARCLAVLAMMMALSACGVTDRVAKRMDDTWAGDMMFGDNEKVILT
TDGGNQLNPDEYGKPLSVVVRVYQLSSLERFESINADTLWDDPQKALGNTLVESRELILL
PGMGQTDQWPLNPNASYVGIAAFFRTDVDSRWKVAFDANTLRKDGIWFSSDGVRVLVDNN
YILAVRGVDVLNKLKTEEQFNKSNSIPEQPAGKSMTERAQDAAVQQVQDSAGSAAKKAAD
SQFNSLLEGAK

>tr|A0A0N8T855|A0A0N8T855_PSEAP Tryptophan 2-monooxygenase OS=Pseudomonas syringae pv. aptata OX=83167 GN=ALO85_02481 PE=4 SV=1
MKNNRHPANGKKPITMFGPDFPFAFDDWIEHPKGLGSIPVENHGAEVAIIGAGIAGLVAA
YELMKMGLKPVVYEASKMGGRLRSQEFEGAKGIVAELGGMRFPVSSTAFFHYVDKLGLES
RPFPNPLTAASGSTVIDLEGTTYYAQMLSDLPALFQEVADAWADALESGSQFGDIQQAIR
DRDVPRLKELWNKLVPLWDDRTFYDFVATSKAFAKLSFFHREVFGQVGFGTGGWDSDFPN
SMLEIFRVVMTNCDEHQHLIVGGVQQVPVGLWSHVPEHCAHWPKGTSLSSLHRGAPRPGV
KRIARAEDGSFAVTDNWGDTRQYAAVLTTCQSWLLTTQIECEESLFSQKMWMALDRTRYM
QSSKTFVMVDRPFWKDKDPETGRDLMSMTLTDRLTRGTYLFDNGDDKPGVICLSYSWMSD
ALKMLPQPIEKRVKLALDALKKIYPKVDIAARIIGDPITVSWEADPHFLGAFKGALPGHY
RYNQRMYAHFMQQDMPSEQRGMFIAGDDVSWTPAWVEGAVQTSLNAVWGIMNHFGGKTHA
ENPGPGDVFHEIGPIALGD
>tr|A0A0N8T9G7|A0A0N8T9G7_PSEAP Putative lipoprotein OS=Pseudomonas syringae pv. aptata OX=83167 GN=ALO85_00963 PE=4 SV=1
MKSLFRPALLLTVALPLFLAGCGDKEPEQRTAFTQFLQTRIVDKPGVHVPKLTDEEKKTF
GDYTSHYAVISDFGAGMDSAVQPLAGLMQKGSFHSVSDVIQRRADLAAVQTGLDEVGEKL
TIEQGKADAARAKLKQPEDLKVVYDKAYDRTVSVPANTFREVLPQIKGTFSSGLKVADYV
DAHKSQIDISGSTITVKDPVVQTELNKLLQELNEQGKNAQQAQSKLQSLMTGR
>tr|A0A0Q0BVC2|A0A0Q0BVC2_PSEAP Uncharacterized protein OS=Pseudomonas syringae pv. aptata OX=83167 GN=ALO85_101725 PE=4 SV=1
MFHGVSPEHVEACEINQWRVHIRLSAADIIAPILRRTVCSRYMPLIGWNTNSRRLRWNLY
G
>tr|A0A0Q0IFJ0|A0A0Q0IFJ0_PSEAP GCN5-related N-acetyltransferase OS=Pseudomonas syringae pv. aptata OX=83167 GN=ALO85_00642 PE=4 SV=1
MLMNITITEVHPSEIPHTVDFVMRARAAIFPLLDTVTVPPDLAGFEQVYLNGENGKFLIA
RCEGQIIAAVGYLPYDHRFPQFDYRGRRTVEIVRLFVTPEFRGDGLASRLCQALWAHAEA
GGIEVLYLHTHPFLPGAIRFWEKQGFAVTDVESDPVWNTTHMERVL
>tr|A0A0Q0BVP4|A0A0Q0BVP4_PSEAP Probable allantoicase OS=Pseudomonas syringae pv. aptata OX=83167 GN=alc PE=3 SV=1
MKVYAAPFEKFVNLADARLGTKILSVTDDWFADANRLFQPTPAVWKEGVFDDNGKWMDGW
ESRRKRFEGYDSAVIRLGVAGTIKGVDIDTSFFTGNFPPSASLEACFLASGEPDENTAWT
EVLPSVELQGNSHHYHEISNDQAFSHLRFNIYPDGGVARLRVYGVPHRDWSKVSEDEQID
LVAALNGGRSIACSDEHYGSMSNILNPGRGVNMGDGWETARRRTPGNDWVIVALGHKGEV
EKVIVDTLHFKGNYPDSCSIQGALVKGGTDSQIETQSLFWRELLPSQKLTMHAEHEFAEQ
IKAIGPITHIRLNVFPDGGVSRLRVLGKVAR
>tr|A0A0Q0IBH0|A0A0Q0IBH0_PSEAP Phosphoheptose isomerase OS=Pseudomonas syringae pv. aptata OX=83167 GN=gmhA PE=3 SV=1
MMDMQSRIRRLFQASIETKQQAMEVLAPFIEQASQVMVNALLNEGKMLACGNGGSAGDAQ
HFSSELLNRFERERPSLPAIALTTDSSTITSIANDYSYNEIFSKQIRALGQPGDVLLAIS
TSGNSANIIQAIQAAHDREMIVVALTGRDGGGMASLLLPEDVEIRVPANVTARIQEVHLL
AIHCLCDLIDSQLFGSEE
>tr|A0A0Q0D7B1|A0A0Q0D7B1_PSEAP 50S ribosomal protein L1 OS=Pseudomonas syringae pv. aptata OX=83167 GN=rplA PE=3 SV=1
MAKLTKRQKAIAEKIEAGKSYNFVDAAALLTELSTVKFSESIDVAVNLGVDPRKSDQVVR
SATVLPHGTGKTVRVAVFTQGPAAEAALAAGADRVGMDDLAAEMKAGDLNYDVVIASPDA
MRVVGQLGQVLGPRGLMPNPKVGTVTPDVATAVKNAKAGQVRYRTDKNGIIHTSVGKVGF
DAVKLKENVEALIADLKRIKPASSKGIYVKRITLSTTMGPGLVIDQGSLEA
>tr|A0A0Q0DBY1|A0A0Q0DBY1_PSEAP Tellurite resistance TerB OS=Pseudomonas syringae pv. aptata OX=83167 GN=ALO85_03307 PE=4 SV=1
MLDWLKTNATAARDKLASEVSKFKNREFMEAVVSGCALVSAADGDISSSEKQKMAGFIQN
SQELKVFDMKDVIQGFQEACSKFEFDFEIGRAEALKTIGKIKKKEDAARLLVRVCCAIGG
ADGSFDEKEREVCRTICRELGLNPSDFDL
>tr|A0A0N8T8Y2|A0A0N8T8Y2_PSEAP Uncharacterized protein OS=Pseudomonas syringae pv. aptata OX=83167 GN=ALO85_01713 PE=4 SV=1
MNSALYSGWISHRRFAPRAHQFTYRIGLLYLDLDEQDSVLGLSPLAGRGRFAPFSFRERD
YLPELTGQGLTLIEAVRERVGKALGRTPSGRVCLLTQARSWGLSFNPVSFFYCHEADGTL
AGILCEVTNTPWGERYSYVLPATGEGHQHFAVAKAFHVSPFLPRDLEYRMSFSQPAERIG
VHMADWQGELKMFDATLNLTRQNLNRKTLHRYLIAYPWMTAKTCLAIYWQAMRLLVKRIP
IFSHQAADGEYRAAAVQSKDSLHEKQ

>tr|A0A0Q0C670|A0A0Q0C670_PSEAP Uncharacterized protein OS=Pseudomonas syringae pv. aptata OX=83167 GN=ALO85_00480 PE=4 SV=1
MSHQQRFEALVAVLKAAISPTEHFTLAYDAEASDFIRFNHGQVRQAGSVQQVIVTFKLID
DGRHANLELTLSGDAETDQQRLVDALQQLRETLPSLARDPYLLPNTNAWQSSNVQDLPLP
DSARVVEQISELAGDLDLVGFYAAGPLYRGFASSWGALGWHQANSFNFDWSLFHENGQAV
KANYAGHHWSDEAFAQRFQQAREQLEFLGRPLHALKPGQYRAYLAPAALDEVIGMLTWGG
FSAQSLASKRSPLQRLYDGETLFSPLVNFDEQVSGSLSPAFSREGYPRKDLKLIVDGQAR
DRLVDSSSAAEYDLPANGADDGEGPSALSIAGGSLELDQILARLGTGLYISNLWYLNFSD
RAAARLTGMTRFATFWVEEGKIVAPVSTMRFDDSAYSLLGSALEDLTKERELILQSSTYS
QRQTGSIQLPGALISRLTLTL
>tr|A0A0Q0E040|A0A0Q0E040_PSEAP AsnC family transcriptional regulator OS=Pseudomonas syringae pv. aptata OX=83167 GN=ALO85_02566 PE=4 SV=1
MQIELNSYDRRILALLQEDASLSSAQIAEQVGLSQSPCWRRIQRLKDEGVIRRQVALLDR
KKIGLNTQIFAEVKLNAHGRSNFTEFTDAIRGFPEVLECYVLMGSVDFLLRIVTPDIEAY
ERFFFEKLSLVPGIQEVNSTVALSEIKSTTSLPLLR
>tr|A0A0Q0CX25|A0A0Q0CX25_PSEAP Transcriptional regulator, LysR family OS=Pseudomonas syringae pv. aptata OX=83167 GN=ALO85_03469 PE=3 SV=1
MSACWYYAFCLCLISFLCAVRMKIDDIDAFVAVIRCQSISHAAESLDLTQPAITRRVQNF
EQALGVELFDRNTKPLKPTPMGLQVYQKCLAILREMDSLRELVASDTPPSGLLRLGVPQT
IGDVVLLDALKQLRGEFPDLRAQVSTGWGSHLIGKIENGELDAAAALFPAGKIFPDNIIG
QSIGKMELVVVCAKGLAPKKPGKLADCYEQGWVLNPDGCGFRAGLQRTLTDQGLSMKVNL
ETFGTELQLGLVADGMGLGLVPRPLLERSAHREQLVALPLKDFKPVMDLWLFYPRFLGNL
QAPVEAFGTLVARSLKPVSAAA
>tr|A0A0Q0C5C9|A0A0Q0C5C9_PSEAP Uncharacterized protein OS=Pseudomonas syringae pv. aptata OX=83167 GN=ALO85_01860 PE=4 SV=1
MLCAVVFGEIKSMLEFLRATRDTYVLTDRPGDAPSDVNRKWSIRIGGLHQDHLRDAVNRL
RQQHLPRADKPSKKRSLKPTALLDSLAKALGAKSYDHWLTIEQPKIIALLNEHGMSRPVD
LIKWATPAFTPLAAQQVADRLFNSGMDIPQRMFTGVGSPLFAASSYGRIDIDTLAGKYFV
NDKQRYNFCEQYSEQVVLRAVHMSEKSALSYLDLTGRMLMLNSVREHVDGMFNLLGSNLM
QPSTGEPVMRVYNTTESDREWDLCLFQLFRAEIESSSAGWVEVIPVPGNVNVIFLKGQDG
TFDWVIRDQRDEALSTNSLHPFFVQSEVPTAMDQGKLLGHLYYSTAIWRERLEHDAESRH
YANGGTAANWPGYQALIERELVAAQGFQMPRQPSGRVHGDFFAHRINERCLMVSPLITIA
EFQAFSSRTGWRSLRAEKAHLAHFDHFDDLEALNSCDPDELPIALTWLDAVAYCRDFETH
SGLPVRLLTIEEWRQITPPPSINFRAINTVRSFKVKSGERPNDPIYEQMGWGVVGSDGEL
GTNSSDVERVDGVMRFGASLNWVINSEGLPFLSTAGFAEWLADHRHQQAPAGCTATHQSI
RGGGVERALCPVHSSMSYKGAKVGFRLCYVANADA
>tr|A0A0Q0C8L7|A0A0Q0C8L7_PSEAP Glycosyltransferase, subfamily GT2 OS=Pseudomonas syringae pv. aptata OX=83167 GN=ALO85_02127 PE=4 SV=1
MINGQKVGCGIVTYNRPALLKKLYDSLPAEIIDCVVIINDGEVYPEFSAYDSEVFFNNET
NLGVGKSKNRALSRLMDAGCEHLFLIEDDIYIKSPEVFELYILTSTLTGIQHLNYSQHGL
MNKTPSGEANAVYRIDYGNGINLPLYRHCVGAFSYYSRRSLEAAGLMDETYHNAFEHVDH
TLAIINQNMHPPFWFFADIDNSQLYLGDEPWTLEGSTISSDTLHRKNALAAIEHFTLKHG
CEPVRHPLASDQELMDSLNSIKQNFGLNI
>tr|A0A0N8TAB3|A0A0N8TAB3_PSEAP Uncharacterized protein OS=Pseudomonas syringae pv. aptata OX=83167 GN=ALO85_01518 PE=4 SV=1
MKPLRLTHVPGCWVSQKLPSGQEERRGIVKMAIAKESEDQLQVHWFSPEKKLAYVDASAV
HSGFQNGMDVVDETPGSGVLSLGQGVIMQQRTLAGSEQVLVDFPERGERHWLPYQYLRQV
KGVKHRFVTASKGEPIDAERMRMRMLAHAIKVWNENTGALSHLEIEPLPHQIHLVHHILA
SGNFNWLIADDVGLGKTIETGMLLHALQQRDLAKRILLITPAGLTKQWQEELYHKFKLED
FEIYGEDFFINEPRQWKMHDCVIGSMDRLKQVDHLESLLQAEPWDLVIFDEGHRLSRRQY
GQKLDSSERYDLAKNLRSQTKHMLLLSATPHQGMQDKFVALLELLRPERRTELMMLNLNP
EILHDMVFRNHKADVTDAEGNFIFQGKITSALQVPSSHESIEFDKTLQAYLRKGYDAGEA
LGRTGNAIGFVMTVYRKLAASSAAAIHRALSNRLQRLLDDEAKGTSKDEPSDERYLGEWE
EQFTSDAREFFAGEVQLLKDLIDEAAALKANDLKLKRFIDEIIGKIHTANPDEKVLIFTE
YRTTQNYLRDALANHYGSDQVDLINGSMQHAERREAIKRFEEQGRFLISTEAGGEGINLQ
SKCHVMVNYDLPWNPMRLVQRIGRLYRYGQKKKVVVFNIQQADSLDQNIVDLMYERIDSV
VNDLAEVQQHEFNDGLKDEILGQVAELIDVEDILQQATKLGIDRTRERIDEALKQARTAA
AKQRELFAHAATSDPNELRDELEITVDHLYSFVLGMFDQLGIEVTERSHKERLLRIRLSD
QVMREMGLSPKASQRMDVTLDRLLASNRPDTHMLDLNSKLMQYLLGKACEYDFGGLAAML
QAPELGEGALLGAMLRWQGPQGKRMRQEFVAIQVDDGKAKLNHAKVSQWLMRPAEYSVLS

PDGQTSKLLFKAAEEMANQRLADASNRYLIPENLDWAAAGWTH
>tr|A0A0Q0FWF3|A0A0Q0FWF3_PSEAP Uncharacterized protein OS=Pseudomonas syringae pv. aptata OX=83167 GN=ALO85_01399 PE=4 SV=1
MERTISSDLFSESTVNTAKTSLPLRAFANLMLWQRRLSSRHQLARLDARLLADAGISESQ
RYEELSKPFWR
>tr|A0A0Q0DIW9|A0A0Q0DIW9_PSEAP Uncharacterized protein OS=Pseudomonas syringae pv. aptata OX=83167 GN=ALO85_04263 PE=4 SV=1
MAASKASARSFGQFQQLKKQESIAKPGLVDTPLCVLAALVQFKKISRHWQGEVLAVETQL
HRASCF
>tr|A0A0N8T8R5|A0A0N8T8R5_PSEAP Binary cytotoxin component OS=Pseudomonas syringae pv. aptata OX=83167 GN=ALO85_00017 PE=4 SV=1
MNIHVLAMPAQLPKPDMEIIIASREKLKREADRLGDIYLPIMNEALHSLLSEVGHVDKEA
LDTLTLVPHMYNSEEMLPFLEAVEKLRGDPEDAKSSAAIADFNEEISLLLDTREASLSSQ
AKALDRALINLEAVRVDGVEHLTPALEQEIAVLEARLETEHARLMEVIRQGAAVNDLIRD
VESLSFFDKLKPLVASLERLADVDPLNPLIGSVKAGIAGVSNILDLLDAAVDYDHLIALR
ERLQTQMTGLQETTDATRAALETEVSKRGQLSGLASVEVCKTDYVREMSKLLEALKRVLA
SSRLPETAVIEKRVEHFSRQADALNNYLIDLRRSWRS
>tr|A0A0N8T9H0|A0A0N8T9H0_PSEAP ABC-type transporter, ChvD family ATP-binding protein OS=Pseudomonas syringae pv. aptata OX=83167 GN=ALO85_00961 PE=4 SV=1
MISTANITMQFGPKPLFENVSVKFGAGNRYGLIGANGCGKSTFMKILGGDLEPSGGQVML
EPNVRLGKLRQDQFAYEEFTVLDTVIMGHEELWKVKAERDRIYSLPEMSEDDGMAVAELE
TEFAEMDGYTAESRAGELLLGLGIGIEQHNGPMSEVSPGWKLRVLLAQALFSDPEVLLLD
EPTNHLDINTIRWLENILTQRNSLMIIISHDRHFLNSVCTHMADLDYGELRLFPGNYDEY
MTVATQSREQLLSDNAKKKAQISELQSFVSRFSANASKAKQATSRAKAIDKIQLAEVKPS
SRVSPFIRFEQTKKLHRQAVIVDRMAKGFDGKTLFKDFSFQVEAGERVAIIGPNGIGKTT
LLRTLVNELQPDAGTVKWTDAAEIGYYAQDHAHDFEDDSTLFDWMGQWTQGGEQLVRGTL
GRMLFSNDEILKSVKVISGGEQGRMLFGKLILQKPNVLVMDEPTNHLDMESIESLNLALE
NYPGTLIFVSHDREFVSSLATRIIELSPSGVVDFSGTYDDYLRSQGVTF
>tr|A0A0Q0D5W9|A0A0Q0D5W9_PSEAP Mangotoxin biosynthesis protein MboA OS=Pseudomonas syringae pv. aptata OX=83167 GN=ALO85_02726 PE=4 SV=1
MISTAEMLKTLESTVDTCLEAEFKKDTVMGEFHKGDWFNESYYKRHILECVIRIHMNNEL
DARAVQVAAVDNISAAKQLSYYLYDEFGHDEMFGQDLITYGYSPSDVQAQFAFPETWKLM
GYLNFCVSKFGPLSSITWDWFLEYYGDKYSSFITQKASASMGQPAVSGAASHVAFDEAED
HSGMMNNMLASVIKDEKDLQKAVVHIKAFIPMVGEYFQALRAATL
>tr|A0A0Q0FJY5|A0A0Q0FJY5_PSEAP Cbb3-type cytochrome oxidase maturation protein OS=Pseudomonas syringae pv. aptata OX=83167 GN=ALO85_03002 PE=4 SV=1
MPALYIMIPAALLLVGVAIYVFFWAVDSGQYDDMDGPAHSILFDDTPPVEHAPPESPQPG
PDEKRDV
>tr|A0A0Q0DPH4|A0A0Q0DPH4_PSEAP 3-isopropylmalate dehydratase small subunit OS=Pseudomonas syringae pv. aptata OX=83167 GN=leuD PE=3 SV=1
MKAFTQHHGLVAPLDRANVDTDQIIPKQFLKSIKRTGFGPNLFDEWRYLDVGQPYQDNSK
RPLNTDFVLNHERYQGASVLLARENFGCGSSREHAPWALEEYGFCAIIAPSYADIFFNNS
FKNGLLPIILSEEDVDQLFKQVEATPGYQLSIDLQAQTVTRPDGKVLSFEIDAFRKHCLL
NGLDDIGLTLMDAEAIAGFESKHRASQPWLFRD
>tr|A0A0Q0C8N8|A0A0Q0C8N8_PSEAP Uncharacterized protein OS=Pseudomonas syringae pv. aptata OX=83167 GN=ALO85_101890 PE=4 SV=1
MAKDNAFAVELVVCRCLLSVPAPSVPLAQTLMGQYVVQKCIV
>tr|A0A0Q0ILW1|A0A0Q0ILW1_PSEAP Uncharacterized protein OS=Pseudomonas syringae pv. aptata OX=83167 GN=ALO85_02394 PE=4 SV=1
MTKDELRAELERQEQRYKDVYGGEITTYAAQPEPERKPWRKRASLLDQAFKQELQKMEEG
LKEEP
>tr|A0A0Q0BZU1|A0A0Q0BZU1_PSEAP DUF2147 domain-containing protein OS=Pseudomonas syringae pv. aptata OX=83167 GN=ALO85_100313 PE=4 SV=1
MKRRYCDDLSKQKNKKGDAMNVRQWLFLSSVLMCSTGLHAAAGPLEAKGLWLTAEKDAVV
RLDDCADKAGAICGQIVWVKDAASTSSDCGVQILQLTRYDQDAWRDGWVYDPRDQKKYKG
AVRVKDGRLNVRAFVGTEVLGKTEQFERIATLPPAPVCKS
>tr|A0A0Q0IAH6|A0A0Q0IAH6_PSEAP Glucose-6-phosphate 1-dehydrogenase OS=Pseudomonas syringae pv. aptata OX=83167 GN=zwf PE=3 SV=1

MMPLITVEPCTFALFGALGDLAVRKLFPALYQLDRAGLLHEDTKILALAREPGDEESHLA
YIEKSMRRFIPETELEPDNLGRFLARLSYLHVDFLKAEDYVALAERVGTAETLIAYFATP
ASVYGAICENLASVNLAERTRAVLEKPIGHDLASSRRVNDAVAQFFPENRVYRIDHYLGK
DTVQNLIALRFANSLFETQWNQNHISHVEITVAEKVGIEGRWGYFDQAGQLRDMIQNHLL
QLLCLIAMDPPSDLSADSIRDEKVKVLKALAPFTPERLATQVVRGQYTSGYSEGKAVPGY
LEEENSNTQSDTETFVALRADIRNWRWSGTPFYLRTGKRMPQKLSQIVIHFKEPPHYIFA
PEQRLQISNRLIIRLQPDEGISLQVMTKDQGLDKGMQLRSGPLQLNFSDTYRSARVPDAY
ERLLLEVMRGNQNLFVRKDEIEYAWQWCDQLIAGWKKAGDAPKPYPAGTWGPMSSIALIT
RDGRSWYGDM
>tr|A0A0Q0BTS9|A0A0Q0BTS9_PSEAP Uncharacterized protein OS=Pseudomonas
syringae pv. aptata OX=83167 GN=ALO85_04129 PE=4 SV=1
MNNKNITSAEFFLNQFNDYANELSFNGETLHAVTDKSLIMKKSDGKLINFSKSDLEKDIS
FQMEMGIFDEEEITKATAQRKFVQVRSLLPA
>tr|A0A0Q0C2W8|A0A0Q0C2W8_PSEAP Uncharacterized protein OS=Pseudomonas
syringae pv. aptata OX=83167 GN=ALO85_03347 PE=4 SV=1
MNTFEQSIIEAAQAATPNNTDAEREAARVAATQAVAEIMSTRKGLDRTLALAELLDSYSE
HDDQEDE
>tr|A0A0N8T9A9|A0A0N8T9A9_PSEAP RNA polymerase-binding protein DksA
OS=Pseudomonas syringae pv. aptata OX=83167 GN=ALO85_02699 PE=4 SV=1
MTKEKLLAMPADDYMNAEQHAFFVELLQGMKVEIHERIEQSRIAIESLDTPADPADAASV
EEERHWLVNVIDRDQRMLPQLEMALSRIADDTFGWCDDSGEPIGLKRLLISPTTKYCIEA
QERHEQIDKHQRQA
>tr|A0A0Q0DB68|A0A0Q0DB68_PSEAP Uncharacterized protein OS=Pseudomonas
syringae pv. aptata OX=83167 GN=ALO85_04308 PE=4 SV=1
MDTSKKAESAHLLNDLESIRQLLGDDPAHSPQSNDAARPEGQIPLLFDVVGARPDAAYQQ
DFDEDDIPTLTPDVHTAPAPQVQQADQPQALPNPDALLHLDSELRAAAQLIMQDVIDDFA
PHIENEIKRRLEARLERLLAQQQK
>tr|A0A0Q0DK90|A0A0Q0DK90_PSEAP Phosphopantetheine adenylyltransferase
OS=Pseudomonas syringae pv. aptata OX=83167 GN=coaD PE=3 SV=1
MNRVLYPGTFDPITKGHGDLVERASRLFDQVVIAVAASPKKNPLFPLEQRVELAREVTKH
LPNVEVVGFSTLLAHFAKEQNANVFLRGLRAVSDFEYEFQLANMNRQLAPDVESLFLTPS
ERYSFISSTLVREIAALGGDITKFVHPAVAQALTERFKR
>tr|A0A0N8T9F0|A0A0N8T9F0_PSEAP DUF4350 domain-containing protein
OS=Pseudomonas syringae pv. aptata OX=83167 GN=ALO85_02386 PE=4 SV=1
MSRRTGLLIGLLVLLLLASGALFLGTQLERYEKTVDQGPSPEAKANPWLAAEHFLRGLSV
TVNTVDTLAQLPDPSQSPQTLLLLDDREDMTPAQTQKLLDWAEAGGHLLFVAEQLWDEQK
GRSGDLLLDSLQIHQYLTKDINAKEQQLTDAQPPLPIPLMTPAPKHKKMPWPELTRLYLE
NESAPAYMSFDPAFHLEDPEDHAQSWANSDDATHLLQISRGNGLITVITDANLWKTRSIG
HYDNAWLLWYLTQDSQVTLVLQTEHDNLLSLLLRNFPQALLTLALLIGLGLWHIGLREGP
LRRPAPLARRQLTEHLRASAEFLRKHTGQQTLIKRLQQDILRRARQLHPGFERLVVAEQW
QVLARLTRKPTSAISDALRPRPPQRLSHSEFTRQVAHLQTIRNAL
>tr|A0A0N8T7V1|A0A0N8T7V1_PSEAP Nucleoside diphosphate kinase
OS=Pseudomonas syringae pv. aptata OX=83167 GN=ndk PE=3 SV=1
MAVQRTFSIIKPDAVAKNVIGEITTRFEKAGLRVVASKLKQLSKAEAEGFYAEHSARGFF
GDLVAFMISGPVVVQVLEGENAIALNRELMGATNPKEAAAGTIRADFADSIDANAVHGSD
SEAAAAREISYFFAATEVTAR
>tr|A0A0Q0D998|A0A0Q0D998_PSEAP Uncharacterized protein OS=Pseudomonas
syringae pv. aptata OX=83167 GN=ALO85_00783 PE=4 SV=1
MNAPLKIRNPCPPGACDCKRELLDAEDADLRILLLSRDAEKTLLDRLERIDSLEDLEHMQ
HKIFQQLGVRVDVAPGFNEVRTMRGISIVVEEKVGLCRKTRQSIPAAIRRALEARPQIAY
QLLNANDLLRDA
>tr|A0A0Q0C6I9|A0A0Q0C6I9_PSEAP Molybdenum import ATP-binding protein
ModC OS=Pseudomonas syringae pv. aptata OX=83167 GN=modC PE=3 SV=1
MASPIEVRLQMAYPDFTVRTDLSLPGSGITALFGPSGSGKTTCLRCIAGLEKADQGFIRV
HDEVWQNTEKGIFLAPHKRAIGYVFQEASLFAHLSVRDNLEFGMKRIPRQQRRIQLPQAS
ELLGIDHLLQRNPDKLSGGERQRVGIARALLTSPRLMLLDEPLAALDTRRKSEILPYLER
LHRELDIPMLYVSHAQDEVARLADHLVLLNAGNVLASGPIQETLARLDLPLAMGSDAGVV
IEGTVSAYDQHYQLLTVTLPDSKLSMRVAHAQMQVGTLLRIKVQARDVSLNLQPDYQSSI
LNRLPVTVIEEALADNSAHVLVKLDAGGTPLLARITRYSSDQLNLHRGQSLWAQIKAVAV
LA

>tr|A0A0Q0CGZ1|A0A0Q0CGZ1_PSEAP Cold-shock protein OS=Pseudomonas syringae pv. aptata OX=83167 GN=ALO85_02061 PE=4 SV=1
MSNRQTGTVKWFNDEKGFGFITPQSGDDLFVHFKAIQSDGFKSLKEGQQVSFIATRGQKG
MQAEEVQVI
>tr|A0A0Q0CIU9|A0A0Q0CIU9_PSEAP Uncharacterized protein OS=Pseudomonas syringae pv. aptata OX=83167 GN=ALO85_01583 PE=4 SV=1
MLNGLWLGFFVVATISALVQWLVGGNAGIFAAMVESIFAMAKLSVEVMVLLFGTLTLWLG
FLRIAEKAGIVDWLAKVLGPLFLRLMPEVPPGHPALGLITLNFAANALGLDNAATPIGLK
AMRSLQELNPSKTAASNAQILFLVLNASSLTLLPVTIFMYRAQQGAPDPTLVFLPILLAT
SVSTIVGLLSVAFMQRLRLWDPVVLAYLIPGALLLGTFMAFLGTLSAAALAGLSSILGNL
TLFGLIMMFLIIGTLRKVLVYEAFVEGAKEGFDVAKSLLPYLVAMLCAVGVLRASGALDF
GLEGIRHLVQWLGLDTRFVDALPTAMVKPFSGSAARALLIETMQTQGVDSFAALAAATIQ
GSTETTFYVLAVYFGAVGIQRARHAVGCALLAEFSGVVAAIFVCYWFFGATAT
>tr|A0A0Q0DM31|A0A0Q0DM31_PSEAP Carrier domain-containing protein OS=Pseudomonas syringae pv. aptata OX=83167 GN=ALO85_01302 PE=4 SV=1
MKEKIKSYLTQNFLFEFDEDITEQDDLIKLGLIDSVGYIQLISFLKKEFGIVFTQEDMMG
NVLVSIQNMVEFVEKRATATR
>tr|A0A0Q0D7D8|A0A0Q0D7D8_PSEAP GTPase HflX OS=Pseudomonas syringae pv. aptata OX=83167 GN=hflX PE=3 SV=1
MFFERHSGGERAILVHLDGQDPEAREDPQEFQELAISAGADTVAFINVPRHRPSAKYLIG
SGKVEELRDQVKAEQADLVIFNHTLTPSQERNLERVFECRVLDRTGLILDIFAQRARTHE
GKLQVELAQLEHMSTRLVRGWTHLERQKGGIGLRGPGETQLETDRRLLRVRLRQIKGRLE
KVRSQRDQARRGRRRADIPSVSLVGYTNAGKSTLFNSVTDSDVFAADQLFATLDPTLRRL
QLDDLGPIVLADTVGFIRHLPHKLVEAFRATLEESSNSDLLLHVIDSHEPDRMSQIEQVM
AVLGEIGAEGLPILEVYNKLDLLEGVEPQIQRDADGKPQRVWVSARDGRGLDLLKQAIAE
LLGDDLFVGTLLLPQSLARLRAQFFELGAVQSETHDEEGASVLAVRLPRVELNRLVSREG
LQPLEFIEQHTLQ
>tr|A0A0Q0BTD9|A0A0Q0BTD9_PSEAP Uncharacterized protein OS=Pseudomonas syringae pv. aptata OX=83167 GN=ALO85_04142 PE=4 SV=1
MIKLCVAGMVGLVMCGSVLAASNEDEAAALASLTEVQKMYEIRPQGTPNDAGTRTLSKQD
INDCVTQMTEAKNKLEAVKQQYGTTQAYRSMQTRMLTGQVRGRLATCKRTKDTLGY
>tr|A0A0Q0FQN5|A0A0Q0FQN5_PSEAP Uncharacterized protein OS=Pseudomonas syringae pv. aptata OX=83167 GN=ALO85_02946 PE=4 SV=1
MKPTLIAAAELDRIDTWAKYSSHMCGGCVSSCCTLPVEVKIKDLIRIGIVDEFERGDPAK
NIAKRLQKEGIVERYNQKSEIFTLQRMSNNDCLYLDRKSRLCTIYDKRPDTCRNHPKVGP
RPGYCAYKPKEVVRQESGSLRNATSAPVPKF
>tr|A0A0Q0IBF9|A0A0Q0IBF9_PSEAP Tryptophan--tRNA ligase OS=Pseudomonas syringae pv. aptata OX=83167 GN=trpS PE=3 SV=1
MTTRILTGITTTGTPHLGNYAGAIRPAIVASRQSDFDSFYFLADYHALIKCDDPLRIQRS
RLEIAATWLAAGLDVDRVTFYRQSDIPEIPELTWLLTCVAAKGLLNRAHAYKASVDKNVE
GGEDPDAGITMGLYSYPVLMAADILMFNAHQVPVGRDQIQHVEMARDIGQRFNHLFGNGK
EFFVMPEALIEESVATLPGLDGRKMSKSYDNTIPLFSGAKDMKSAISRIVTDSRAPGEAK
DPDTSHLFTLYQAFSTAEQCAEFRSDLLQGLGWGEAKNRLFTLLDAQLSEPREKYLGLIE
RPADLEDILLAGAQKARRVATPFLEELREAVGLRSFRTVVQNADTGKKKASKGARFVSFR
EDDGSFRFRLLAADGEQLLLSRNFADGKAAGLVSKQLQQGGELDLRSEDDRFTLWLDGAC
VADSPVFADAAARDSAIETLKLALAPQQD
>tr|A0A0Q0D5W4|A0A0Q0D5W4_PSEAP Uncharacterized protein OS=Pseudomonas syringae pv. aptata OX=83167 GN=ALO85_101238 PE=4 SV=1
MPRLILLRHAACLPLEKAEYVNGTRPEGQRQREKTHGQRRNLSNFWFHDTYFTERSQKRS
YKPSLAISARFKLPRARPTPR
>tr|A0A0N8T7X8|A0A0N8T7X8_PSEAP Phenylacetate-coenzyme A ligase OS=Pseudomonas syringae pv. aptata OX=83167 GN=ALO85_04230 PE=4 SV=1
MYALFKPDLLELARVTYQQHALFESDGISVEELNAIQLDRLRQTLRYVNAHSPFYRDLLS
ASDAALSTLDWHAFRQLPFTTKDDLRTAGHFMLSAPLSDAWIYYETTGTTGAATPCPRNE
RDSIFNNTPLILRYEALFQAHGERHIVGVMGPTELHSTGDTFEDVCRSLGHTVVKMWPRS
PVVGMERVVTLIRELKISALVCTPAVAISLARHLINKGQNPSALGVKLIFTLGELTTPGL
LRNIGDVWGSAVYNCMYASQESSILAACDKQGHLCTVPLNVVYEVVNPITGEVLEPKDGQ
MTGELVITHLYQGQKPLIRYRTGDTVRARVLSQGKWQIIPIGRLRDTLVINGRNHCAYDV
EAALFEHLTGCLDYHIQVETVSGIDKLSVIIELQPNSEARNDLNTVASYMETALGVPVSI
TVGPTDAVTGTAAMVSWKAARLHDKRTGGDNLERDTALKIAARRA

>tr|A0A0Q0DYQ0|A0A0Q0DYQ0_PSEAP Uncharacterized protein OS=Pseudomonas syringae pv. aptata OX=83167 GN=ALO85_100330 PE=4 SV=1
MIFLGGIRREAQRSKQACRQQTEETRLLHVEKSPVVKKGGQEQRDTTGVPVSRAFVPPCN
LYWRSPTLGPATCLAASRNPSRLLQIVVPRTCVDSCTSLIKRELCQLVLRLVFQGLAESS
TASCRRNCLVLVRDRTVMRQRWMKCFVACL
>tr|A0A0Q0C2V7|A0A0Q0C2V7_PSEAP Glucose 1-dehydrogenase OS=Pseudomonas syringae pv. aptata OX=83167 GN=ALO85_03337 PE=4 SV=1
MMHISLKQQVAIVTGASSGLGAGAARALAEAGAAVVINYNSKPEPAEKLAEEIRAAGGKA
LAVGADVSKEEDVERLFAQTIEHFGALDILVANSGLQKDASIVDMSLEDWNTVINVNLTG
QFLCARAALRQFIKQGMRPEVSRAMGKIIHMSSVHQLIPWAGHVNYAASKGGVDLLMRSI
AQEVGELKIRVNSVAPGAIRTPINADARKGDAEKEMLKLIPYGRIGEPEDVANAVLWLAS
DASDYVHGTTLFIDGGMTLYPEFRGNG
>tr|A0A0Q0IPU6|A0A0Q0IPU6_PSEAP Uncharacterized protein OS=Pseudomonas syringae pv. aptata OX=83167 GN=ALO85_03704 PE=4 SV=1
MLEQVNVIYEGWGERWQWGTLVSTTALTGRPLIVFEYSNEARQRGLELSSYTLPLDGGRL
RRDFPDHQLYLPGPVYDSLPDGWGMLLMDRLFRRRGLTTARIGPLERLAYIGSSAMGAMT
FEPVAPEGQESDVHIPLEQLAADVKQVLRGNGGEFLQALLLVGGSPQGARPKALVYRDPE
TGGFTTAATPGFEAWLIKFPAKEEHAEVCAIEMVYAECLRMCGIETPDTQYFNLPDGLAA
FASKRFDRRDGLRIPMQSLAAFTGANYQSPGVLDYVNFLRATQMCTNDVREMARAFERAV
FNVAFNNRDDHPKNFAYIMSQDGQWKLSPAYDVTFCEGAGGYHQMDVMGEALEISRAQML
RLAEEAEMSPDAATGVIDGMCDVASRFTAIAENLCPQVITRDTLRMIQSRIDQNVARLHH
SQADGGHGRFS
>tr|A0A0Q0CIP8|A0A0Q0CIP8_PSEAP Putative membrane protein OS=Pseudomonas syringae pv. aptata OX=83167 GN=ALO85_01430 PE=4 SV=1
MVLLQQTIISKIRFLGKTIGYVGWSLFWLLIWDVIVTVDFMLYLERKITLPSMPLTLLGS
ALVVLTSFRNSSAYNRWWEARTLWGALVNSSRSFARQVLTLVEDDDGINPVKATLLRRHV
AYVKCLSAHLKGAKCGDEVQMLIPREEFERRHDTNNFPNDLLNTSAALLAKEYQAGRLDS
IRLARLESTMVDISNCQGGMERIANTPLPYPYVAFPRLFITLFCLIVPIGLVETLGWFTP
LASTVVGFMLLAIEKIGTDLQSPFRASEHEIQMTALCENIERNLDSMLRGAQEECKAS
>tr|A0A0Q0ID00|A0A0Q0ID00_PSEAP Uncharacterized protein OS=Pseudomonas syringae pv. aptata OX=83167 GN=ALO85_01036 PE=4 SV=1
MSDINNFVTYVIRMPNDTVSRTALTTELNEAVISNGGVITGTSVEDEMTLNELFEARMND
LDVQEARRAAKELATTEYLEE
>tr|A0A0Q0FSV0|A0A0Q0FSV0_PSEAP Acetyl-CoA acetyltransferase OS=Pseudomonas syringae pv. aptata OX=83167 GN=ALO85_03616 PE=3 SV=1
MSIERTPPRQDPVVIVSAVRTPMGGFQGYLQSLSATALGSIAIRAAVERAGIEPEDVEHV
LFGCVLPAGLGQAPARQAALGAGLSHATLCSTVNRMCGSGMQTAIMAHDLLLADSTAVVV
AGGMESMSNAPYLLDRARSGYRMGHGKVLDHMFLDGLEDAYEPGRLMGTFAEDCAGLNGF
SREAQDAFALASLARAQQAIAGGHFDAEIVPVQVTVGKESRQITHDEQPPKARPDKIPTL
KPAFREGGTVTAANSSSISDGAAALLLMRQSEAQRRGLKPLAVLHAHAAFADRPSLFPTA
PIGAIQRLLGKTGWALDDVDLFEINEAFAVVPLVAMHELGLEHEKVNVNGGACALGHPIG
ASGARIIVTLLAALQHRQLKRGIAAICIGGGEATAIAVEILN
>tr|A0A0Q0C1I4|A0A0Q0C1I4_PSEAP Uncharacterized protein OS=Pseudomonas syringae pv. aptata OX=83167 GN=ALO85_03607 PE=4 SV=1
MKVCLVQSGGFAGAVKRCEVDTATLEQPEAEQLQRLVSDSGLNQSSSAFNDEARDLNQYE
ITIEDDAKAICLTFDEHNVPASARQLLGFLKQRVKPGKL
>tr|A0A0Q0C3E2|A0A0Q0C3E2_PSEAP Uncharacterized protein OS=Pseudomonas syringae pv. aptata OX=83167 GN=ALO85_101714 PE=4 SV=1
MVSVDRRNGLFMASLIVVFVVEKSLFPSANRGVVTGSYQGNKNN
>tr|A0A0Q0DPY7|A0A0Q0DPY7_PSEAP S-adenosylmethionine decarboxylase proenzyme OS=Pseudomonas syringae pv. aptata OX=83167 GN=speD PE=3 SV=1
MKSKLKLHGFNNLTKTLSFNIYDICYAETPQDQQAYVEYINSVYDAERLTRILTDVVDII
GANILNIARQDYDPQGASVTILISEQPVTPTESQIEESPGPLPETILAHLDKSHITVHTY
PEIHPVDGIATFRVDIDVSTCGVISPLKALNYLIHKFESDIVTVDYRVRGFTRDVEGKKH
FIDHEINSIQNYLSEDTRNGYQMTDVNVYQENLFHTKMLLKQFELDNYLFGDATSNLSPE
QREQVTAKVKHEMLEIFYGRNVAV
>tr|A0A0Q0IAQ7|A0A0Q0IAQ7_PSEAP Keto-hydroxyglutarate-aldolase/keto-deoxy-phosphogluconate aldolase OS=Pseudomonas syringae pv. aptata OX=83167 GN=ALO85_04281 PE=4 SV=1
MTQNENNQPLTSMANKIAQIDELCAKAKILPVITIARDQDVLPLADALAAGGMTALEVTL

RSAFGLSAIRILREQRPELCVGAGTILDRKMLADAEAAGSQFIVTPGSTQDLLQAAVDSP
LPLLPGVSSASEIMIGYAMGYRRFKLFPAEISGGVAAIKALGGPFNEVRFCPTGGVNEQN
LKSYMALPNVMCVGGTWMIDNAWVKNGDWGRIQEATAQAMALFD
>tr|A0A0Q0DJ09|A0A0Q0DJ09_PSEAP Uncharacterized protein OS=Pseudomonas syringae pv. aptata OX=83167 GN=ALO85_04152 PE=4 SV=1
MKEKIKTFDQLPDLYGTIIELALRFGYWKVGNKFNDRKGECVIVLDDLTVFMHSDVPLIV
VKEIGLAVRKSKRREVSLFLDGAVVTPKQIKMLVEMERQTA
>tr|A0A0Q0C0S4|A0A0Q0C0S4_PSEAP 30S ribosomal protein S5 OS=Pseudomonas syringae pv. aptata OX=83167 GN=rpsE PE=3 SV=1
MSNHDQKRDEGYIEKLVQVNRVAKTVKGGRIFTFTALTVVGDGKGRVGFGRGKSREVPAA
IQKAMEAARRNMIQVDLNGTTLQYAMKSAHGASKVYMQPASEGTGIIAGGAMRAVLEVAG
VQNVLAKCYGSTNPVNVVHATFKGLKGMQSPESIAAKRGKRVEEII
>tr|A0A0Q0DUG6|A0A0Q0DUG6_PSEAP Alkyl hydroperoxide reductase AhpD OS=Pseudomonas syringae pv. aptata OX=83167 GN=ALO85_01007 PE=3 SV=1
MSELIEIEGFTNQVLGWKAWLPTVDLDSASAEQVAVLEESHPQAKTSDYYLTLAHHPDIL
RQRSQAFNAIMYAPGGLSRAERELASTVVSRINRCVYCASVHAQRFEQLAKRNDVIAQVF
ADPATAGTTAREKAIVQFAIDLTLEPASLNAGHIQALYEQGLDETQVLDLIHAISIFAWA
NRLMLNLGEPVFPEA
>tr|A0A0Q0ID13|A0A0Q0ID13_PSEAP Uncharacterized protein OS=Pseudomonas syringae pv. aptata OX=83167 GN=ALO85_100938 PE=4 SV=1
MGVSNADGCTLNASLTQRGIIPLSGGVLRNFPWRNIMSTASFRFGVMVGTALRECIRAVT
HSSNPEPGRVAPQQLRMMPEPAMSNEQSEQYRLPALARCKHVDLAGWYDHHMQEIQAKPV
KKPRKSRASKALQTSQKQDAEPPVKAPVMGPLDQLIA
>tr|A0A0Q0CYL3|A0A0Q0CYL3_PSEAP Na(+)/H(+) antiporter NhaA OS=Pseudomonas syringae pv. aptata OX=83167 GN=nhaA PE=3 SV=1
MTQVPNRETPRGIAILAGFLASESAGGIVLMAAALAALIVANSPLSAGYFSILHSVWLGL
SVELWINDGLMAIFFLMVGLEIKREVLAGGLATWGQRALPGFAAAGGMLVPALIYIAINW
GNPQTLSGWAIPAATDIAFALGVLSLLGNRVPTSLKVFLAALAILDDLGAVTIIAFFYSS
GLNLPMLAAAFVTLAVLIVMNRLNVRRLLPYLLLGALLWFFVLQSGVHATLAGVALALCI
PMGKPEEEARSPLLFLEEKMHYWVAFAVVPIFGFANAGVSLAGITLGNLIDPVPLGVALG
LFVGKQIGVFLAAVLAIRAGLAVLPEGSNWVQLYGVAILCGIGFTMSLFIGNLAFPGSQH
LIDEVKVGVLIGSGLAAIAGIVLLRSRFSRA
>tr|A0A0Q0FFX3|A0A0Q0FFX3_PSEAP Luciferase OS=Pseudomonas syringae pv. aptata OX=83167 GN=ALO85_01053 PE=4 SV=1
MTQNNQSLGLENHTVFSGTFQPDQLTIGFISPARGYPDGPFPSLEDQAEIVQRLDQSDAA
ALWLRDVPFYDPSFGDTGQVLDPLVYAGWLAALTHRIAIGTAGIVSPLREPVITAKQIAT
ADQLLGGRFLAGMASGDRATEYPAFGVDFASRAERYREALTLIKTLISESFPRLKTQHFG
QLRGDIDLVPKPAAQRVPIIAIGRAGQSLEWLAANVDAWIWHGDALRMPEAVPHWREATA
FRGFVPFGYGAMFDLDANPDAPLQTGRVLKGGRKALKDFFSRQREAGINHVALNLKPTRR
PSVEVIDELQEHILPEFSITSG
>tr|A0A0Q0FI66|A0A0Q0FI66_PSEAP Uncharacterized protein OS=Pseudomonas syringae pv. aptata OX=83167 GN=ALO85_03205 PE=4 SV=1
MMNSFVRNLVGGVLVASCSIGAVQAQTLPNSTLNNGASSNPYNSPIHRANPNSRQGTESS
VPPSRGPNIAPPVRKPTLENGGIGNGYPTRQNTPRPSTTPITDPKRGN
>tr|A0A0Q0IRM4|A0A0Q0IRM4_PSEAP Integrase OS=Pseudomonas syringae pv. aptata OX=83167 GN=ALO85_200152 PE=4 SV=1
MHDPNLCLSCALPFASVEWTEDLSGIGDDSTEWMTFVDDEVCLCTDEDRNE
>tr|A0A0Q0FDB3|A0A0Q0FDB3_PSEAP UDP-3-O-acyl-N-acetylglucosamine deacetylase OS=Pseudomonas syringae pv. aptata OX=83167 GN=lpxC PE=3 SV=1
MIKQRTLKNIIRATGVGLHSGEKVYLTLKPAPVNTGIVFCRADLDPVVQIPARAENVGDT
TLSTTLVNGDVKVDTVEHLLSAMAGLGIDNAYVELSASEVPIMDGSAGPFVFLIQSAGLE
EQDEPKKFIRILREVTVEEGGKRATFVPFEGFKVSFEIDFDHPVFRDRTQSASVDFSSTS
FVKEVSRARTFGFMSDIEYLRKHNLALGGSVENAIVVDKDGVLNEDGLRYEDEFVKHKIL
DAIGDLYLLGNSLIGEFRGFKSGHALNNRLLRTLIEQKDAWEVVTFEDASTAPISYMRPV
AAV
>tr|A0A0Q0FLL8|A0A0Q0FLL8_PSEAP Putative 2Fe-2S ferredoxin domain protein OS=Pseudomonas syringae pv. aptata OX=83167 GN=ALO85_03913 PE=4 SV=1
MNGRFVRLGERDRPVVSLMVDGAPIEALQGDTLMVALLTRKATLRQSEFDPGRRAGFCLM
GACQDCWVWTRSGERLRACSNEVRDGLDIVTTQPEAKWPLLHG

>tr|A0A0Q0C3Q6|A0A0Q0C3Q6_PSEAP NAD transhydrogenase, alpha subunit II OS=Pseudomonas syringae pv. aptata OX=83167 GN=ALO85_01397 PE=4 SV=1
MEELISPGVYNLIIFVLAIYVGYHVVWNVTPALHTPLMAVTNAISAIVIVGAMLAAALTV
TPLGKTMGTLAVALAAVNVFGGFLVTRRMLEMFRKKAPKAKDEAPKS
>tr|A0A0Q0FNK1|A0A0Q0FNK1_PSEAP LexA repressor OS=Pseudomonas syringae pv. aptata OX=83167 GN=lexA PE=3 SV=1
MIKLTPRQAEILGFIKRCLEDNGFPPTRAEIAQELGFKSPNAAEEHLKALARKGAIEMTP
GASRGIRIPGFEARPDDSSLPVIGRVAAGAPILAQQHIEESCNINPSFFHPSANYLLRVH
GMSMKDVGILDGDLLAVHTTREARNGQIVVARIGDEVTVKRFKREGSKVWLLAENPDFAP
IEVDLKDQELVIEGLSVGVIRR
>tr|A0A0Q0BXY4|A0A0Q0BXY4_PSEAP Phosphate acyltransferase OS=Pseudomonas syringae pv. aptata OX=83167 GN=plsX PE=3 SV=1
MSAPIIAIDAMGGDFGPRNIVQASLACLTATPSLHLALVGQASLIEELVSAHEAVDRSRL
RVIHASEAIAMDERPSQALRGKPDSSMRVALELVASGQAQACVSAGNTGALMALSRFVLK
TLPGIDRPAMIAAIPTRSGHCQLLDLGANVDCSAEALYQFAVMGSVLAEILGVATPRVAL
LNVGTEDIKGNQQVKRAAGLLQSASGLNYIGYVEGDGLYRGEADVVVCDGFVGNVLLKSS
EGLATMIAARIETLFQRNLLSRAVGALALPLLKRLQTDLAPARHNGASLLGLQGVVVKSH
GSASVSGFQSAIHRALVESRENLPQRLKGRLETMFQDDRS
>tr|A0A0Q0C1N7|A0A0Q0C1N7_PSEAP Periplasmic binding protein/LacI transcriptional regulator OS=Pseudomonas syringae pv. aptata OX=83167 GN=ALO85_05463 PE=4 SV=1
MPDAVPTPKTNRLHAIGRTLVLTLLATSLVATAYAEDSDVPSLAGKRIAISMTGTSHYFD
IKAFQAQVDEVKRLGGTPITLDAGRNDKNLVNQLQTVVTQKPDAVIQTLGTLSVIDPWLK
RISKAGIPLFTIDAPSQYSLNNTTSDNVATGKALAGQLIKDAGGQGNVLVFNGFYGVPVC
AIRYDQLKLALKDHPELKIIEPELRDVIPNTVQDAYSQVSALLNKYPKGSVSAIWAAWDI
PQLGASKALIDAGRTEIKTYGVDGTPEVLELMRREDSPVGAVVAQQPALIGKTAVQNVAR
YLAGQHDLPKETQVPTLLTTAANLPDVQKLRGDN
>tr|A0A0Q0FNB9|A0A0Q0FNB9_PSEAP Gluconate permease OS=Pseudomonas syringae pv. aptata OX=83167 GN=ALO85_02630 PE=4 SV=1
MFGMTHETFLLVDALVTIVGLVLLITTFKVHPFVALTLAAGFLGLTSGMPVEKVMKSFQD
GFGGVLGFVGIILGLGTMLGKLMADSGGADQIAQTLIRTFGKQKVHWAMMFSAFLVGIPL
FFEIGFVLLIPLVFIVARRTGVPIVKIGIPLLAGLSAVHGLVPPHPGPLLAIGIFGADIG
KTIFYGLIVALPTAIIAGPIFGNWISKSIPGTPSKELMDQIAKESSTENLPGFGITLVTI
LLPVFLMLLKTFADVVLPENNMFRIWMDLIGHPITALLAALLLAFYTFGAARGFDRSKIM
KLLDQSLAPVAAIVLIVGAGGGFKQMLVASGVGDVIGHMAVQAQINPIMLAWLVAAVIRI
ATGSATVATITGAGIVAPVVGLIPGVNRELLVLATGAGSLILSHVNDAGFWLVKQYFNMT
VAETFKTWTVMETILSVVGLIFIMLLSMAV
>tr|A0A0Q0DL47|A0A0Q0DL47_PSEAP Tripartite ferrous iron transport system, permease protein EfeU OS=Pseudomonas syringae pv. aptata OX=83167 GN=ALO85_101825 PE=4 SV=1
MLVPFLIMLREGIEAALIVGIIASYLKQTGRGEWMPAVWIGVFLAAALSLFVGGGLELMS
AEFPQKQQELFEGIVGLIAVGILSSMVFWMRKVARSIKNALHESLDAALTGSKNQTYALI
AMVFFAVAREGLETVFFLLAVFQQSDGSGAPLGALLGLLLAVAFGVAIYSGSMRLNLSLF
FRWTGLFILVVAAGILANSVQALHEAGVWNHLQEVVFDISALLPMDGPAGSVLAGMFGYQ
DAPTVSTLTAYLIYLIGALLLFFKPHTPAAGKVMQPNTSSVTNE
>tr|A0A0Q0IL33|A0A0Q0IL33_PSEAP Probable S-methyl-5'-thioinosine phosphorylase OS=Pseudomonas syringae pv. aptata OX=83167 GN=ALO85_02682 PE=3 SV=1
MTVYAIIGGTGLTQLEGLNIRQSLPMNTPYGAPSGEIQIGDYAGREVMFLARHGHPHRLP
PHQVNYRANIWALKQAGAEAILAVNAVGGIHPAMGTGHFCVPHDLVDYTSGRQHTFFADD
LEEVTHIDFSYPYSEPLRARLIAALAAEGCAFSDRGVYACTQGPRLETVAEIIRLERDGC
DIVGMTGMPEAALARELELEYACLALVVNPAAGKSTTVITMAEIEQALREGMGKVKATLA
RVLSAS
>tr|A0A0Q0ISI9|A0A0Q0ISI9_PSEAP ATP-dependent dethiobiotin synthetase BioD OS=Pseudomonas syringae pv. aptata OX=83167 GN=bioD PE=3 SV=1
MTAAYFITGTDTDAGKTTVAAGLLHAARSSGLSTAAGKPVASGCFVTPHGLRNPDALALL
AECSVELDYAEVNPLAFEPAIAPHLAAREAGVALTVDSVLQPMRHMLARQADFTLIEGAG
GWRVPLSGQSTLSDLAIALDLPVILVVGVRLGCINHAVLTAEAIARDGLRLAGWVANIVD
ADTSRLEENLATLVERLPAPCLGQVPRLNEASAENVAQYLDLKVLS

>tr|A0A0Q0FNF0|A0A0Q0FNF0_PSEAP Tripartite ferrous iron transport system, periplasmic protein EfeO OS=Pseudomonas syringae pv. aptata OX=83167 GN=ALO85_02604 PE=4 SV=1
MSNRPSGPSGKARPPRVLRLAVAGSVILMIAAGGLFYYASQVAAKKRAANAGTETVVNIH
AHNCEPNALTVAAGKNAFRIVNRSERAVEWEILDGVLVIEERENIAPGLSQVINANLTPG
DYTITCGLLSNPRGTLHVTPTAESDAKAKARPSMTAFIGPLSEYRVYLSLQSSALIKAVT
ALQQAIDAGDLSAAQAAYLPARAVYQRIAPAAQRLAELDNAINARADYYEKREQDPGFGG
FHRIEYGLYEQHSVEGLAPVAQRLQTDVTQLKQQLMAQSLAPEQLAAIATRTLRSLAEVR
SNGEEERYSHSDLNGFAANLDGTRKIVDVLRPLLARSAGDLLQKIDAAMADLDTTLDALS
SADGGVRPYDQVDKAQRQQIAAKAGALADALNGIDPALGLSGL
>tr|A0A0Q0C4Y7|A0A0Q0C4Y7_PSEAP DUF2383 domain-containing protein OS=Pseudomonas syringae pv. aptata OX=83167 GN=ALO85_04279 PE=4 SV=1
MLILNNYFHIKNWHGVCNYLYIEHFRINLPLGNYQMTDINKETISILNDLVETSIDGKEG
FETSAKHAIGSDVKAFFTSRAIEVGQAVTELQAHVTRLGGKPEDSSSVSADIHQAWVKLK
TAFTSNDDLAVLEEAERGEDVAKKAYTKALEKSREKGVDPAVIAVIEKQYQGVLANHDKV
KKLRDAARAAAKHNA
>tr|A0A0Q0CG48|A0A0Q0CG48_PSEAP Uncharacterized protein OS=Pseudomonas syringae pv. aptata OX=83167 GN=ALO85_04332 PE=4 SV=1
MEPITTLNTLHIVATALLLISALVLASGVVKVRLEGDTTVQNRLLKRPLLFFWVLMAVCL
ATLPFSGWWLVHLKGLSLGQTWVLGSSVLYTLGLCSWVWLVARLNRLRLGAKGGKRGFTL
ALTVISVVCFVVMAGLTGFKPA
>tr|A0A0Q0BVE2|A0A0Q0BVE2_PSEAP NADH-quinone oxidoreductase subunit A OS=Pseudomonas syringae pv. aptata OX=83167 GN=nuoA PE=3 SV=1
MPESTGLIAHNWGFAIFLLGVVGLCAFMLGLSSLLGSKAWGRSKNEPFESGMLPTGSARL
RLSAKFYLVAMLFVIFDIEALFLFAWSVSVRESGWTGFVEALVFIAILLAGLVYLWRVGA
LDWAPEGRRNRQAKLKQ
>tr|A0A0Q0DLM4|A0A0Q0DLM4_PSEAP Tol-Pal system protein TolB OS=Pseudomonas syringae pv. aptata OX=83167 GN=tolB PE=3 SV=1
MQDDIHTWRSSLVINLFRGLLVVLCFASAMVSAEEKNILVTSGSDRAAPIAVVPFGWQGG
NVLPEDMAEIISNDLRNSGYYAPIPKQNMISLPTQASEVIFRDWKALGAQYVMVGNITPA
GGRLQIQYALFNVATEQQVLTGNVSGTNDQLRDMAHYIADQSFEKLTGIKGAFSTRMLYV
TAERFSENNTRYTLQRSDYDGARAVTLLQSREPILSPRFAPDGKRIAYVSFEQKRPRIFV
QHIDTGRREQITNFEGLNGAPAWSPDGSKLAFVLSKDGNPEIYVINLASRQLSRVTNDSS
IDTEPFFGKDGSTLYFTSDRGGKPQIYKTNINGGGAERVTFVGNYNANPKLSADEKTLVM
IHRQDGFTNFKVAVQDLARGSVKILTDSNLDESPTVAPNGTMVIYATRQQGRGVLMLVSI
NGRVRLPLPTAQGEVREPSWSPYLN
>tr|A0A0Q0DKX8|A0A0Q0DKX8_PSEAP HAD family hydrolase OS=Pseudomonas syringae pv. aptata OX=83167 GN=ALO85_03477 PE=4 SV=1
MTLEGIRHWVFDMDGTLTVPIHDFPAIKRELGIPQDDDILGHLAALPAEESAAKHAWLLE
HERELALASQPAEGAVELVRELSARGYQLGILTRNAQELAYITLKAIGLDDCFAVEDVLG
RDEATPKPDPAGLLQLATRWSVEPEQMVMIGDYMHDLNCGRAAGAKTILVNLKDNPWPEL
TDWHAVDCAELRGMLG
>tr|A0A0Q0BT64|A0A0Q0BT64_PSEAP Imidazole glycerol phosphate synthase subunit HisF OS=Pseudomonas syringae pv. aptata OX=83167 GN=hisF PE=3 SV=1
MWPKLRRFATANNAEQLEIDPVALAKRIIPCLDVDNGRVVKGVKFENIRDAGDPVEIARR
YDEQGADEITFLDITASVDGRDTTLHTVERMASQVFIPLTVGGGVRTVQDIRNLLNAGAD
KVSINTAAVFNPEFVGEAAARFGSQCIVVAIDAKKVSGPGETPRWEIFTHGGRKPTGLDA
VLWAKKMEDLGAGEILLTSMDQDGMKNGFDLGVTRAISDALGIPVIASGGVGNLEHLAAG
VIEGHASAVLAASIFHFGEYTVPEAKAYMASRGIVVR
>tr|A0A0Q0D3R2|A0A0Q0D3R2_PSEAP GDP-mannose 6-dehydrogenase OS=Pseudomonas syringae pv. aptata OX=83167 GN=ALO85_01810 PE=3 SV=1
MRISIFGLGYVGAVCAGCLSARGHEVVGVDISSTKIDLINNGKSPIVEPGLEELLQKGIS
TGKLRGTTDFAEAIRATDLSMICVGTPSKKNGDLELDYIESVCREIGYVLRDKATRHTIV
VRSTVLPGTVANVVIPILEDCSGKKAGVDFGVAVNPEFLRESTAIKDYDLPPMTVIGEFD
TASGDVLQSLYEELDAPIIRKDIAVAEMIKYTCNVWHATKVTFANEIGNIAKAVGVDGRE
VMDVVCQDKALNLSQYYMRPGFAFGGSCLPKDVRALTYRASSLDVEAPLLNSLMRSNTSQ
VQNAFDMVASYDTRKVALLGLSFKAGTDDLRESPLVELAEMLIGKGFDLSIFDSNVEYAR
VHGANKDYIESKIPHVSSLLNSDFDQVINDSDVIILGNRDERFRALANKTPEGKRVIDLV
GFMANATSEDGRAEGICW

>tr|A0A0Q0FUQ9|A0A0Q0FUQ9_PSEAP GTPase Era OS=Pseudomonas syringae pv. aptata OX=83167 GN=era PE=3 SV=1
MNDTTATRCGYVAIVGRPNVGKSTLLNHILGQKLAITSRKPQTTRHNMLGIKTEGAVQAI
YVDTPGMHKNGEKALNRYMNKTASAALKDVDVVIFVVDRTRWTDEDQMVLERVQYVQGPV
ILAINKTDRIEDKSDLMPHLEWLQGQLPNASIVPISAQHGHNLEALESLIASHLPENDHF
FPEDQITDRSSRFLAAELVREKIMRQLGAELPYQITVEIEEFKQQGRTLHIHALILVERD
GQKKIIIGDKGDRIKRIGSDARRDMELLFDSKVMLNLWVKVKGGWSDDERALRSLGYGDL
>tr|A0A0Q0CVC7|A0A0Q0CVC7_PSEAP Type VI secretion system protein ImpB/EvpA OS=Pseudomonas syringae pv. aptata OX=83167 GN=ALO85_02261 PE=4 SV=1
MAKKESTQHKLDRVRAPRVHITYDVDIGDAQEKKELPFVVGVLGDFSGNPLEPLPKLKDR
KFIFIDRDNFNGVLKGIKPRLAYRVDNTLAKDGTQLGVELNFNALEDFEPQNVVRQVEPL
RKLLEVRNKLADLRNKMGGNDKLEELLMDVLQNTEKLKTLGKEFGREAAAPATDSKE
>tr|A0A0Q0IQ39|A0A0Q0IQ39_PSEAP Phosphoenolpyruvate-protein phosphotransferase OS=Pseudomonas syringae pv. aptata OX=83167 GN=ALO85_04335 PE=3 SV=1
MLNTLRKIVQEVNSAKDLKAALGIIVLRVKEAMGSQVCSVYLLDAEVNRFVLMASEGLNK
RSIGKVSMAPNEGLVGLVGTREEPLNLENAADHPRYRYFAETGEERYASFLGAPIIHHRR
VVGVLVIQQKERRQFDEGEEAFLVTMSAQLAGVIAHAEATGSIRGLGRQGKGIQEAKFVG
VAGSPGAAVGVAVVMLPPADLEVVPDKTVTDIAAELALFQNALEGVRGDMRTLSAKLATQ
LRPEERALFDVYLMMLDDASLGSEVTDVIKTGQWAQGALRSVVSEHVKRFELMDDAYLRE
RASDVKDLGRRLLAYLQEARQQALVYPDNTILVSEELTPAMLGEVPEGKLVGLVSVQGSG
NSHVAILARAMGIPTVMGLVDFPYSKVDGINLVVDGYHGEVFTNPSEIMRKQFGKVVEEE
RQLSQGLDALRELPCVTLDGHRMPLWVNTGLLADVARAQQRGAEGVGLYRTEVPFMINQR
FPSEKEQLAIYREQLAAFHPLPVTMRSLDIGGDKSLSYFPIKEDNPFLGWRGIRVTLDHP
EIFLVQTRAMLKASEGLNNLRILLPMISSTHEVEEALHLIHRAWGEVRDEGTDVPMPPVG
VMIEVPAAVYQTRDLARQVDFLSVGSNDLTQYLLAVDRNNPRVADLYDYLHPAVLQALQS
VVRDAHAEGKPVSICGEMAGDPAAAVLLMAMGFDSLSMNATNLPKVKWMLRQINLSKAKE
LLAQLMTNDNPQVISSSLQLALKNLGLSRMINPSSVKGH
>tr|A0A0Q0IRA0|A0A0Q0IRA0_PSEAP Uncharacterized protein OS=Pseudomonas syringae pv. aptata OX=83167 GN=ALO85_02001 PE=4 SV=1
MTFSTSQKGFSFVGWLLVLALVAFATSTAFKLVPHYLDYMTMKKIIEAVDTNKTLDITTV
DDFYGYVGKNMQVNSIRDIDLNKALKVTVENNAFQAHLNYEQREPWFQNIDLVLKFDKQF
SVGKP
>tr|A0A0Q0CHF5|A0A0Q0CHF5_PSEAP Transcriptional activator MltR OS=Pseudomonas syringae pv. aptata OX=83167 GN=ALO85_03461 PE=4 SV=1
MNMTSPMNKTTRIADPSYELMDDHNGLSIIYREHGFPCPLVRWHFHKEYELHLIVASSGK
VFVGDYIGNFNPASLFLTGPNLPHNWISQIDDGEVVPKRDMLVNFTDELLESGNQVFAEL
KTLTPLLERARYGIEFRCKRTIRQAMKQMQKIADSRGITRLGHFFILLELLAASEDYQLL
SGTAVAHTADEHSVDRTNRAVDYIFAHYGRELPLEEVAEYLGMKPTYFSRVFKQATGRCF
VEFVNRLRISKSCELLADGDKPVTDVCFESGFNNISNFNRRFQQLKGMTPSHYRRLAVQR
LTEQNLY
>tr|A0A0Q0C3I3|A0A0Q0C3I3_PSEAP Short chain dehydrogenase/reductase oxidoreductase OS=Pseudomonas syringae pv. aptata OX=83167 GN=ALO85_00863 PE=3 SV=1
MPFSDYKTALVTGASSGIGEAVVERLCAEGLQVHALARSADKLAALAQRTGCIAHAIDVT
DLAGLTALFQAHHFDVVVNNAGVDRPGSLLKADAEGIDLLVDVNLRAVLQIARLSLPGMV
ERDCGHIINISSIAAAYNFGGNSTYHATKAAVSMLSRQLRIDAFGKRVRVTEICPGRVAT
DIFAHVHGDSEEVRKRFIEGYELPVAKDIADAIAYVIAAPIAVNIGHMEITPTLQVPGGL
STARPQDYEG
>tr|A0A0Q0C074|A0A0Q0C074_PSEAP 3-hydroxyacyl-CoA-acyl carrier protein transferase OS=Pseudomonas syringae pv. aptata OX=83167 GN=ALO85_02425 PE=4 SV=1
MSPEKAVLDIQGQFRVYTELYRTDAAAKTIILVNGSLATTASFTQTVRNLYPTFNVVLYD
QPYSGKSKPHNLHGAPLTREEEGQLLLELIDHFNAEHVLSFSWGGAATLVALAHRPKRIE
SAVISSFSPVINEKMHDYLCHGITHLGAHNRYDMGHLINNTVGKHLPTLFKRFNFRHISS
LDAHEYDQMHYHFSQVMELDPDNYLTAGKNITVPILFINGALDEYTCAHDAAHFSRYLRD
CSFSTIDATGHFLDMEHKAACRDSKQVLMNFLQPAQVSRDVHNRVQDYPSLTF
>tr|A0A0Q0C7H1|A0A0Q0C7H1_PSEAP Gamma-glutamylcyclotransferase OS=Pseudomonas syringae pv. aptata OX=83167 GN=ALO85_01642 PE=3 SV=1

MENPINELAAAVHDGLGLTYPPVIDMGPQLTREQLLASMNTTMRRHKGGPVWLFAYGSLI
WRPECTAVESQRARVHGYHRGLYLWSHEHRGTPEVPGLVFGLDRGGSCTGFAYRLPDECL
EKSLLALWEREMPYPSYRPHWLNCRLEDGRKVQALGFVLERHLPSYAGNLPDSVLSQVLA
SARGRYGTTREYVEQTAKALRSHAMPDLNLEARLKRCKSETA
>tr|A0A0Q0FC91|A0A0Q0FC91_PSEAP Homoserine/lysine/threonine efflux protein, LysE/YggA family OS=Pseudomonas syringae pv. aptata OX=83167 GN=ALO85_04225 PE=4 SV=1
MLDLTQVLTYSAALGIAAAIPGPGMAALVARSVSGGAVSGFCLLSGLILGDLTYLSFAVF
GLAMIAEHFNALFQVVRWGAAIYLCYLAWQFWFADHQAIEIGKSAKKKELLSAAASGLTI
TLGNPKTIAFYLALLPLVINLETVSLQTWGLVLVPLTVLVLLAVGAVFIFAALRIRHLLS
SESAQRKLFRGAAAIMVAAAASMLVR
>tr|A0A0Q0CYJ6|A0A0Q0CYJ6_PSEAP Metal-dependent hydrolase OS=Pseudomonas syringae pv. aptata OX=83167 GN=ALO85_01609 PE=4 SV=1
MRRWGKGSTRIVGLHDPQVNEHHLLQNGLPEDGRLRLLSFNIQVGISTQRYHHYLTRSWQ
HLLPHGGRAGNLQKIGDLINEFDLVALQEVDGGSMRSGFVNQVEHLAQLGGFPYWYQQLN
RNLGRLAQHSNGVLSRLRPTKIEDHPLPGPAGRGAILVRFGEGEDALVVVMMHLALGTRT
RTRQLAYIRELIGGYRHQVLMGDMNTHANDLLEHSPLRDLGLLAPQIEATFPSWRPQRCL
DHILLSPTLTLERVQVLAQPISDHLPVAVEIRLPDSLRGDSLPVPSGGSLA
>tr|A0A0Q0FUB8|A0A0Q0FUB8_PSEAP Flagellar biosynthetic protein FlhB OS=Pseudomonas syringae pv. aptata OX=83167 GN=flhB PE=3 SV=1
MAENENGQDKTEDPTEKKVKDSRADGQIARSKELTTLVVMLMGAGGLLMFGSDIALMMSE
LMRDNFTISRETLMDQSYMGKALLSSGMHALVVVLPFLIAMLVAALVGPIMLGGWLFATK
SLMPKFSRMNPAAGLKRMFSPHALVELLKSFGKFLITLAVALVVLNNERKDLVAIAHEPL
EQAMIHSLVVVGWSSFWMACGLIFIAAADVPFVLYEAHKKLLMTKQEVRDEHKNSEGSPE
VKQRIRQLQREMSQRRMMASVPEADVIITNPTHFAVALKYDPEQGGAPMLLAKGTDLVAL
KIREIGAHNEILILESAALARSIYYSTELDQEIPAGLYLAVAQVLAYVYQIRQFRAGQGK
RPDPLGDIRIPPDLQRDA
>tr|A0A0Q0BUV3|A0A0Q0BUV3_PSEAP Toluene tolerance protein Ttg2C OS=Pseudomonas syringae pv. aptata OX=83167 GN=ALO85_00174 PE=4 SV=1
MQNRAIETGVGLFLLAGILALLLLALRVSGLSTSASTDTYKLYAYFDNIAGLTVRAKVSM
AGVTIGKVTAIDLDRDTFTGRVTLEIQKKVDNLPSDSTASILTAGLLGEKYIGLSVGGDD
KLLKDGGTIHDTQSSLVLEDLIGKFLLNTVSKDAK
>tr|A0A0Q0DAR8|A0A0Q0DAR8_PSEAP Uncharacterized protein OS=Pseudomonas syringae pv. aptata OX=83167 GN=ALO85_03334 PE=4 SV=1
MKNRKLIMKDAYNLQRFVDAQQPLFQQVLTELGAGQKRSHWMWFVFPQIQGLGRSDTARH
YAITDLDEARAYLQHPVLGPRLEQCAAIIAPQVERSARQIFSSPDDMKLHSSMTLFSIAA
PERPVFQKVLDTFFGGERDAMTVKLLSR
>tr|A0A0Q0DXF3|A0A0Q0DXF3_PSEAP Uncharacterized protein OS=Pseudomonas syringae pv. aptata OX=83167 GN=ALO85_03610 PE=4 SV=1
MMSFNLANRPLPERTALEDEKSRLFELWQSNLGKAKGEAARLMGERAKRKGKWSEWVRSE
LDTMSPPEYANMVRSEVNRLMAASR
>tr|A0A0Q0BSW5|A0A0Q0BSW5_PSEAP Uncharacterized protein OS=Pseudomonas syringae pv. aptata OX=83167 GN=ALO85_02262 PE=4 SV=1
MSRCAVEKFATFPRPRSDPGNGREGFKDDVPMTQTFDIEALIKLRKQTRAISDALKVQAS
DYLSTLALLIRPQTFFGEYLQGAQRSSGRETQHHFKELKELYDRIASAEPFKLVNELEVP
LNLISTTPELFPLEYDMVLSQSGQTIRITSPVRWVVGFNSFDLAQFRKVIKDPNRSSAEL
YRYVVHYLVLFYCLSKSPGMSRLFEGLRFPVSFERLKDFGDLPFCVISSPVRSELPDESV
IRNSTQIAGNTSFEELVGHENILEMNDEIRQRLLLTIEGL
>tr|A0A0Q0DYQ8|A0A0Q0DYQ8_PSEAP Elongation factor 4 OS=Pseudomonas syringae pv. aptata OX=83167 GN=lepA PE=3 SV=1
MSDLSHIRNFSIIAHIDHGKSTLADRFIQMCGGLTEREMEAQVLDSMDLERERGITIKAH
SVTLYYKAKDGITYQLNFIDTPGHVDFTYEVSRSLAACEGALLVVDAGQGVEAQSVANCY
TAIEQGLEVMPVLNKMDLPQADPDRVKEEIEKIIGIDATDAVACSAKSGMGVDEVLERLV
ATIPAPTGNIEDPLQALIIDSWFDNYLGVVSLVRVRHGRVKKGDKILVKSTGKLHLVDSV
GVFNPKHSATVDLKAGEVGFIIAGIKDIHGAPVGDTLTLSTTPDVDVLPGFKRIQPQVYA
GLFPVSSDDFEDFREALQKLTLNDSSLQYLPESSDALGFGFRCGFLGMLHMEIIQERLER
EYNLDLITTAPTVIFELLLKTGETIYVDNPSKLPDLSAIEDMREPIVRANILVPQEHLGN
VITLCIEKRGVQHDMLFLGTQVQVSYDLPMNEVVLDFFDRLKSVSRGYASLDYHFDRYQS
ANLVKLDVLINAEKVDALALIVHRDNAHYKGRALTEKMKELIPRQMFDVAIQAAIGGQIV
ARTTVKALRKNVLAKCYGGDVSRKRKLLEKQKAGKKRMKQVGNVEIPQEAFLAVLRLE

>tr|A0A0Q0C952|A0A0Q0C952_PSEAP Alpha-ketoglutaric semialdehyde dehydrogenase OS=Pseudomonas syringae pv. aptata OX=83167 GN=ALO85_00697 PE=4 SV=1
MPNILGQNFIAGGRSALGQSLQKSLDATTGEELPYSFHQATDGEIDAAALAAKAAFPEFR
QLSPARRADFLDAIADELDGLGDDFVAIVCQETALPQARIQGERGRTSGQMRLFSKVLRR
GDFVGARIDLALPDRKPLPRVDLRQYRIGVGPVAVFGASNFPLAFSTAGGDTAAALAAGC
PVVFKAHSGHMATADLVASAIIRAAERTQMPKGVFNMIFGNGVGEGLVKHPAIQAVGFTG
SLNGGNALCKMAAERPQPIPVFAEMSSINPVVLLPGALQARGETVAKELAGSVVMGAGQF
CTNPGVVIGLRSPAFSTFIEQLTEQMGSQAPQTMLNAGGLRSYSKGVEHLLSHPGVTHLA
GKPQEGKQAQAQLFKADVSLLLNGDQLLQEEVFGPTTLLIEVADDAQLKDALQALRGQLT
ATVIGEPADLSQYSWLQPILEEKVGRILFNGYPTGVEVCEAMVHGGPYPATSDARGTSVG
TLAIDRFLRPVCYQNLADSQLPPALQNANPLGLRRLVNGEWSDQPVA
>tr|A0A0Q0BV57|A0A0Q0BV57_PSEAP Short chain dehydrogenase OS=Pseudomonas syringae pv. aptata OX=83167 GN=ALO85_03180 PE=4 SV=1
MKRIAFVVMSLISLPLWALDAPDQQRLAGIQKEWAHIQYGLPEKQRVDAFEKLATQATAF
TQDRPDAAEAWIWSGIVTSSWAGAQGGLGALGKAKQAKADLEKAMTLDANALQGSAYTSL
AALYDKVPGWPIGFGDSAKAGQLLEQALKINPNGIDTLFFSAQHQFDQGNYAQAQQALNK
ALQAPPRPGREEADAGRRKDIQSLLTSVNKKLN
>tr|A0A0N8T7U8|A0A0N8T7U8_PSEAP Putative PHP family metal-dependent phosphoesterase OS=Pseudomonas syringae pv. aptata OX=83167 GN=ALO85_03808 PE=4 SV=1
MNVDLHCHSTASDGALSPTALVTRAHENGVRVLSLTDHDTLEGLEEARAAAHSLGMQLVN
GVELSCTWGGATIHILGYGFDTQAPALTEAISRLHEGRWLRAEEISRKLAIKGMPGALEG
ARAIQQELGDSGNAPARPHFADFMVRSGFVKDRAEAFRKWLGAGKLGDVKQHWPTLEDTV
ATLRASGAWVSLAHPSHYDFTRSKRRKLVGDFVQAGGHAIEVVNGMQPADQVGTLAILAR
EFGLLVSAGSDFHGPGTWGEIGTYRPVPEDLPPLWCRFKHEQPTASV
>tr|A0A0N8T7Y6|A0A0N8T7Y6_PSEAP Uncharacterized protein OS=Pseudomonas syringae pv. aptata OX=83167 GN=ALO85_01864 PE=4 SV=1
MNRLAEKNRFKARLNDLLRELERELERPNLDPYAERDPDRRPHEHDTRLLFVNELLSLLD
WKLGVRGNVLQEARLQANTTKFMDYVGVVEATQKPLLLVEAKAWDKPQVSPRGDGTYASE
AALVAAAIKHIRDGKPAGTSPIISEWDSYLRQVHGYLETLKTRYKHTLPRAIIISGEWIA
VFRAPDETFLGIVLPDDIVIFYRPEFRERAEELFELLHRSVLTEEPPVPLRPAQLTQYLE
LNDVSGTFMGVHVHYERSGSTLFTPRPRILIYPAIFVVRTDGAVYTVIRSTGHCELDYQT
DPNGADTLALHLDEVRQHTEALIELCGMELGGALLAAEISQFPGFPSNEFPQKAVTAIGT
VGDDWLIATGDVVHFLLLEPRVGDCRYHSWQQCGDDATLQSAISIRSVNPPSFFVDNQRH
HCAHQVVQDRRENRCLIKGIDSRTCCQACVFYESCWTEEEKAALPCGR
>tr|A0A0Q0ILX4|A0A0Q0ILX4_PSEAP Uncharacterized protein OS=Pseudomonas syringae pv. aptata OX=83167 GN=ALO85_05108 PE=4 SV=1
MPDKIVPDPPTALFTVNASVSQEEALNYASDLLRCIITTAYESGDSTQGTSRDLAFSVLH
MAEMAKPWLTVHWNASFDQMTSRGHDRPRANLSYADPASSPPAGRSSPTSAA
>tr|A0A0Q0ILZ9|A0A0Q0ILZ9_PSEAP Phage T7 F-exclusion suppressor FxsA-like protein OS=Pseudomonas syringae pv. aptata OX=83167 GN=ALO85_02347 PE=4 SV=1
MMRVFLLLFLLFPVLELFLLVRVGMSIGFLWTFLLVVATSMLGLFVMRVAGFATALRARE
SLARGELPAQEMLEGLMVAVGGGLLLLPGFISDILGVICLLPITRRLLINKVRQRAQDQA
MRQRAFADDLQTPANQGASSHRPTAIHQPTREPDVIEGEFEHRDK
>tr|A0A0Q0C1F7|A0A0Q0C1F7_PSEAP Urease accessory protein UreD OS=Pseudomonas syringae pv. aptata OX=83167 GN=ureD PE=3 SV=1
MRQANEDTMNLPANTALFTPSWHAELELGYGRFDDSTRPTLRRHKGPLRVQKHLYAEGPE
VCQHIIVHPPGGIAGGDRLDISATVGADAWAQLTSPGAAKWYRAASPAFQQLELHVQPGA
TLEWLPQETIVFSNAQAELSTRIELHGDAKLCYWDVVALGRPASGERFEHGHFQSHLDIR
RDGTLLWHERQRIIGGDGLLGSPIGLDGRTVFATLLMTGEVGSELLEACRSLSMPNPVRG
ELTQLPGLLVARCLADEALHARAWLIELWKRLRPALLGREAVTPRIWNT
>tr|A0A0Q0IHQ3|A0A0Q0IHQ3_PSEAP Cytochrome c-type bioproteinsis protein OS=Pseudomonas syringae pv. aptata OX=83167 GN=ALO85_03037 PE=4 SV=1
MIDFWMATWLLLMVALSFLLIPLLRGHHAQREEDRTALNVALYQERLSELQVQQERGVLS
VAQLQSARAEAARELLADTEGAEPARTSRLGKPLLVLAALLVPVLGLAGYLQLGASDRVE
LSREFARPPTSLADMTQRLERSVQAQPDSAENLYFLARSYMAQNRPGDAAQMFERSVALA
GRQPELLGQWAQALYFASDKHFTPQVQVLTDEALLADPKEVTSLGLLGIAAFETQRYQAA
VDYWTRLLAALPADDASRSALEGGIARARENLAKRVANAAPAVKTKSIKIHVELAAALQG

KVRPNDSVFIFARAINGPAAPLAVKRITVADLPADVELSDADAMMPQLNLSNFAQVQLVV
RVSRTGQPTTGEWVGRSQPLASDSGVQQALIIDSPDN
>tr|A0A0Q0BTQ8|A0A0Q0BTQ8_PSEAP DNA-binding transcriptional regulator HexR OS=Pseudomonas syringae pv. aptata OX=83167 GN=ALO85_04284 PE=4 SV=1
MDRVRNLLEQIQGRLDELNKAERKVAEVILLNPQMATRLSIAALAQAAKVSEPTVNRFCR
SFSVSGYPELKLQLAQSLASGAAYVSRAVEADDNPEAYTQKIFGSAIASLDSACQQLDPA
LISKSVDMLIQARQIHFFGLGASAPVALDAQHKFFRFNLAVTAHADVLMQRMIASVAHTG
ELFVIISYTGRTRELVEVARIARANGASVLGLTAAGSPLAQASTVSLNIPLPEDTDIYMP
MTSRIIQLTVLDVLATGMTLRRGVDFQPHLRKIKESLNDSRYPIEDQG
>tr|A0A0Q0CX76|A0A0Q0CX76_PSEAP ATP-dependent hsl protease ATP-binding subunit hslU OS=Pseudomonas syringae pv. aptata OX=83167 GN=ALO85_03567 PE=4 SV=1
MAGKDTGSTGRSSYEQIGKRIQRLVSAPNVQKIQWVIVARRDDEPVDSWNVVLQEIEETE
GIEIDRQPDGSVRIGWQRYIDN
>tr|A0A0Q0DF92|A0A0Q0DF92_PSEAP Glutamate--cysteine ligase OS=Pseudomonas syringae pv. aptata OX=83167 GN=gshA PE=3 SV=1
MKDYTLSELLNRRLALLGERNNLSLLEQCLHGIERECLRVTATAELARTPHPQALGAALT
NGQVTTDYSESLLEFITPALKNPAETIDNLDRVHRFVYSKLGDELLWSPSMPCPLPDEEH
IPIAYYGTSNIGKLKYVYRKGLALRYGKTMQCIAGIHYNFSLPEDAWALLKQTEDFAGDA
RDYQSHSYIALIRNFRRYSWLLMYLFGASPALDAGFLRGRKHQLEQHFDADTLYLPYATS
LRMSDLGYQSDAQADLTPCYNDLVSYTDSLRKAVATPYKPYVEVGTHDQNGEWVQLNTNV
LQIENEYYSNIRPKRVTYSGERPIQALVARGVQYVEVRCLDINPFLPTGISLEQSRFIDA
FVLYCALEESQQLASHECSNASSNFLSVVKEGRRPGLSLMRDNRPVDLKTWATELVEKIT
PIARLLDQAQGTDEHLKSIAVQQAKIDDTALTPSAQVLASMEAHNEGFTAFSLRQSQVHA
EYFRTHPLSAQEQADFEAQAKTSIEEQVELEATEEVVDFDTFVGSYQASILSISN
>tr|A0A0Q0FUG0|A0A0Q0FUG0_PSEAP Putative 3-carboxymuconate cye OS=Pseudomonas syringae pv. aptata OX=83167 GN=ALO85_00593 PE=4 SV=1
MPHKFLPWLAIASAMSAFTLQAQAMPDTELLIGSYTQGKSEGIYRFAFDSKTGMIDAKPL
QVVKAENPSWLTVSRDQRYLFAVNENGPGQKDAVGKVSSFAIDPKTRQITPINQVQSRGE
EPTHSSLSYDGRYLFVANYAVNPDPGGALTVVPVSKDGTLSEAVQVLPLGPGSKVNSERQ
MSSHVHLAVPTPDDKYVVTADLGADKLFVYHYDASQAKPLQPAKVPFVQLPGGSGPRHTL
FSGDGKHAWLVLEMTAQVAVFDYHDGAFKQTQLVDMKNKGVDEKNGGGALHTSPDGKFLY
VTNRGDANQVVVFRIDQASGKLEEIQRRSLEGKEPREFAFDPTGQFMLFANQKSNQIVTV
RRDPQSGKIGETVQKFDADAPSYLRFLTDK
>tr|A0A0Q0C3G6|A0A0Q0C3G6_PSEAP Extracellular solute-binding protein OS=Pseudomonas syringae pv. aptata OX=83167 GN=ALO85_03262 PE=4 SV=1
MKQLKSLLPAALLTLSAGFSALSNAADLTISCGAVGAELQLCKEAVDSWSKQTGNNVQVV
STPNSATERLSFYQQILSAQSTDIDIIQIDMVWPGMLAKHLTDLRELLPATATQGYFQAQ
VDNATVDGRLVSMPWFTDSGLLYYRKDLLDKYQQPVPQTWEDMTATAKKVQKAERDAGNA
TMWGYVFQGRAYEGLTCNALEWISSQPDGGLVNPRGDIVVNSQASRVALTLAKSWVGDIS
PPGVLNYTEEEGRGVFQSGNALFMRNWPYVWALVQSKDSAIKDKVGVAPLPSGGANGTHA
STLGGWGLAVSRYSRNPKLAAELVAYLTSAQQQKQRALAGAYNPVIESLYADPELLAAMP
YYPQLHSILSNGVMRPAAITANGYPRVSNAFFDRVHSVLAGDIPVDQALVELERELTRIK
RRNW
>tr|A0A0Q0DHS8|A0A0Q0DHS8_PSEAP L-proline/glycine betaine ABC-type transport system OS=Pseudomonas syringae pv. aptata OX=83167 GN=ALO85_00023 PE=4 SV=1
MKRICLFLGFALLFPALAQAAEKPLLRVGARVFTEQTILAELTAQYLRTKNYDVQITGGL
GSNLARSAQESGQLDLLWEYTGVSLVSYNHIDEKLDSAQTYARVKEVDAKKGLIWLSPSK
FNNTYALALPQKIADKYPQVNTMSDLAKVFKDEAKEGHLVALDTEFANRSDGLVGMVKHY
DMNLGRENTRQMDSGLVYTALQNGQVFAGLVYTTDGRLNAFKLKVLQDDKHYFPDYTAAP
VIRQEYLDKHPDIETLLKPLADLLDNQTMIDLNARVDVGHESPSAVAADFLRQHPLN
>tr|A0A0Q0D4V9|A0A0Q0D4V9_PSEAP Uncharacterized protein OS=Pseudomonas syringae pv. aptata OX=83167 GN=ALO85_100370 PE=4 SV=1
MSVAIPIRNQRPHGSTSQQPAQTVLQETHLPLYLYKQKSPVARQGS
>tr|A0A0Q0DSQ5|A0A0Q0DSQ5_PSEAP H+/citrate symporter, CitM family OS=Pseudomonas syringae pv. aptata OX=83167 GN=ALO85_00907 PE=4 SV=1
MLATLGVITILAMLVSIMSKRISPLVALIALPIIAALLAGFGLQTSGFIITGIKNVAPVV
GMFVFAILFFGIMTDAGMLDPIIDRILKRVGTRPTRIVMGTALLALLVHLDGSGAVTFLV
TIPAMLPLYTRLGMDKRILACVTAMAAGVNFLPWTGPVLRSSAALHVPVSDLFQPLIPVQ

IVGLIFVFTCAFFLGRREERRLGLGPDNLDVKPHQRVLSDAERELRKPRLFWVNLLLTLV
VMGVMIAGVVDPVVMFMLGTVIALCINYPAVDAQRARIDAHAKTALTMASILLAAGVFTG
IMQGTGMLKAMAEVAVGQIPAGHGKLIPVVVGFISMPLSLLFDPDSFYFGIMPVVAEVGK
ALGVDPMQVAQASLLGVHTTGFPVSPLTPATFLLVGLCKIELADHQRFTIPFLFAASVIM
TLTAMVIGVF
>tr|A0A0Q0IRC7|A0A0Q0IRC7_PSEAP RNA polymerase sigma factor
OS=Pseudomonas syringae pv. aptata OX=83167 GN=ALO85_01995 PE=3 SV=1
MFMLTQEEDQQLVERVQRGDTRAFDLLVLKYQHKILGLIVRFVHDTHEAQDVAQEAFIKA
YRALGNFRGDSAFYTWLYRIAINTAKNYLVSRGRRPPDSDVRSEDAEFYDGDHGLKDIES
PERALLRDEIEGTVHRTIQLLPEDLRTALTLREFDGLSYEDIASVMQCPVGTVRSRIFRA
REAIDKALQPLLQES
>tr|A0A0Q0BWM0|A0A0Q0BWM0_PSEAP NADPH-dependent FMN reductase
OS=Pseudomonas syringae pv. aptata OX=83167 GN=ALO85_04352 PE=4 SV=1
MRVVSLSGSPSPKSRSGVVLAHAGRWLQSHGVEVTTLRIRDFNAEDLLFARFDSPQVLEL
IEAVSQADGLLIGTPVYKASFSGALKTLLDLLPERALHGKVVLPLATGGSIAHMLAVDYA
LKPVLSALKSQEVLHGIFAIDTQVSYDDNELGGTLDEILTERLHEGLEHFHHGLQRRLQA
RHQQAGGHLQLAL
>tr|A0A0N8T9H4|A0A0N8T9H4_PSEAP LysR family transcriptional regulator
OS=Pseudomonas syringae pv. aptata OX=83167 GN=ALO85_00951 PE=3 SV=1
MPAIMDRFNAMRVFTRIVELGGFARAAESLHMPRASVTVLIKQLEAHLGVQLLQRTTRQV
SATPDGKAYYQRCVSLLADLDDAEASFSATGAEPKGLLRVDLPVSLGRMIIIPALPEFSR
RYPQIRLEIGMSDRPVDLIREGVDCVLRAGAALDETLVARPLGSLTQITCASREYLAQHG
TPLNLQELAQHQVIEYFSTSTGKRYGLEFMADGMGVEHASACSIAVNSADGYVAACEAGF
GLIQVPRYHVAAQLRSGVLVEVLRLHRPPPLPLTALYPPHRQLSRRVRVFVDWLVELCAH
TDIRDRLVS
>tr|A0A0Q0C1X7|A0A0Q0C1X7_PSEAP Regulatory protein, LysR:LysR, substrate-binding protein OS=Pseudomonas syringae pv. aptata OX=83167
GN=ALO85_00831 PE=3 SV=1
MQLYGVQSTALRYFLEVARCGSISEASIRLNVASSAVSRQISKLERELDAVLFERQARGM
MLSEAGVRLAAYARKSQLEAEQVVLEITELQGLQRGHVRIACSEGFALDFLPQCIARFRR
QYQGIHFSMEVCAPAQATERVRSGDADLCLTFSLTPQNEIKVEHIHSGAICAVVSHNHPL
AARKEVTLAELQPYAIALTNTDTTLRQLFDICCGVQGLLLDPVLTSNYIGALLRFVREEG
GISLSSEMTLDKRAHEKQLKSLPISDDGMKARRIELQSMAGRNLPAAVAAFRDFLIEELA
KT
>tr|A0A0Q0C2F1|A0A0Q0C2F1_PSEAP Periplasmic serine endoprotease DegP-like
OS=Pseudomonas syringae pv. aptata OX=83167 GN=ALO85_01998 PE=3 SV=1
MSIPRMKSYFSLIAAVLMLGQVATAQAENLPDFTGLVEQASPAVVNISTRQKLPDRAVAN
QQMPDLEGLPPMLREFLERSMPPGSRPPGSGAGKGDRQREAQSLGSGFIISPDGYVLTNN
HVIDGADEILVRLSDRSELKAKLVGTDPRTDVAVLKIDGKDLPTAKLGNSNTLKVGEWVL
AIGSPFGFDHSVTKGIVSAKGRSLPNDTYVPFIQTDVAINPGNSGGPLFNMAGEVVGINS
QIFTRSGGFMGLSFAIPIDVAMDVANQLKANGKVSRGWLGVVIQEVNKDLAESFGLDKPA
GALVAQVLEDGPAAKGGVQVGDVILSANGQPIVMSADLPHLIGNLKDGSKAELEVIRDGK
RQKLTVTVGALPDEGQEMGDVGGTGAERSSNRLGVSVIELTAEQKKSLDLKGGVAIKEVT
GGPASLIGLQAGDVITHLNNQAITSSKQFTEVAKSLPKDRSVSMRVLRQGRATFITFKLS
E
>tr|A0A0Q0DSE2|A0A0Q0DSE2_PSEAP Integrase catalytic subunit
OS=Pseudomonas syringae pv. aptata OX=83167 GN=ALO85_03449 PE=4 SV=1
MTDGYDCYPNALAERVSGTLETEFLLPRSKDFAEAIRMVCASVQIYNNGWPHLSLKYKTP
YAEHRACRG
>tr|A0A0Q0FHX9|A0A0Q0FHX9_PSEAP 3-oxoacyl-synthase III OS=Pseudomonas syringae pv. aptata OX=83167 GN=ALO85_02155 PE=4 SV=1
MMIGIKSIASYVPVAGVDNYAQGARFGKDEEFILGKIGSTFLPRKDAGQETSDLCVEAAN
TLFANNPQLKRESIDVLIVVTQNGDEEGLPHTAAIVQDKLGLSTNVAAFDISLGCSGYVY
GIYAIKGFMEAAGLKNGLLITADPYSKIVDQEDRNTTMLFGDAATATWMGEGAEWQLGKA
KFGTDGSGAPHLKVSNGIFFMNGRQVFNFALLKVPAHLHELLAESDLTPDDIDAFCIHQG
SAAIVDAVARRFEDKPEKFLKDMVETGNTVSSSIPLLIEKHVLGSSWKRVALSGFGVGLS
WGSAIIYKG
>tr|A0A0Q0ILB2|A0A0Q0ILB2_PSEAP Helix-turn-helix, Fis-type OS=Pseudomonas syringae pv. aptata OX=83167 GN=ALO85_02646 PE=3 SV=1
MRVDLTARSQRQRDTLMLNSNALRKLDMQDLMVFVSVYEQRNLTLVAEALHVSQSTVSYC

LKKLRANFEDDLFICTRNGMRPTRKALAMHDHVQHILRSVNLCHNGLDLFDPTRAQTTFT
VCAPEYFELLVLPHLLRDFAAAGCSVTVNVQKLEKQLPAEQLNDGLFDLALCFGPGFHRV
PDSLISQVLMEDDLVCVMDSRFAPLSALIDLQTFIQHRHVYPTPWTSDTNMVDGWLQRQG
LERNIVARANSYRAALQLLEGTDFMLVLPRRIQTLLGNELWISVFELPPDLPGFSLDMLW
SAQADRDEASGWLREQVVKVCAERGLL
>tr|A0A0Q0C876|A0A0Q0C876_PSEAP C-5 sterol desaturase OS=Pseudomonas syringae pv. aptata OX=83167 GN=ALO85_04315 PE=4 SV=1
MSDLAIPIIIMLLAVTGEAIILQWMQRKPVDWHDVVFNLNSGHLMLWLFRGLEVICYGLV
ATYLSLGLLDTWPPLWVWAFALLAWDFCFYWLHRLHHHVGVLWAVHVVHHQGEHFNLSLG
VRNSWYSSLTSIPFFIPLAVLGVPLHVFVAVSILHYSMQFFNHNALTPRLGVLEKIFVTP
AHHRVHHVKNRAYSDKNFGGTFIFWDKLFGTFSRLPDVPYTFGIGGPRSSANPFWASNVP
FLHYLRLSIKSNEPTRSRVSGLSIFSGALLLFALVVGYIYQYGYGYSDITWPQVVLFVFL
VAGSVALGGMTEARTWSVPVWLIVSLGLASLFLGYYGWSQVYWQLPMVALALHGAWVAVS
SRGYNLSAANPVEKHHG
>tr|A0A0N8T9B9|A0A0N8T9B9_PSEAP Universal stress protein OS=Pseudomonas syringae pv. aptata OX=83167 GN=ALO85_02670 PE=3 SV=1
MPYKHILVAIDLTDECDPVIRRASGLAKASGAKLSVVHIVEPIAMAFGGDVPMDLSQLQQ
QQIDQARQKLAKLEDKYPELENSKSHMVFGQPRQEIHKLAKSEECDLIVVGSHGRHGLAL
LLGSTANDVLHGAPCDVLAVSLKKPE
>tr|A0A0Q0D695|A0A0Q0D695_PSEAP Threonine synthase OS=Pseudomonas syringae pv. aptata OX=83167 GN=ALO85_00356 PE=4 SV=1
MRYISTRGQAPALNFEDVLLTGLASDGGLYVPENLPRFTQEEIASWAGLPYHELAFRVMR
PFVAGSIPDADFKKILEETYGVFAHSAVAPLRQLHGNEWVLELFHGPTLAFKDFALQLLG
RLLDYILAKRGQRVVIIGATSGDTGSAAIEGCRRCDNVDIFILHPNNRVSDVQRRQMTTI
FGDNIHNIAVEGNFDDCQEMVKASFADQSFLKGTPLVAVNSINWARIMAQIVYYFHASLQ
LGGPARSVSFSVPTGNFGDIFAGYLARNMGLPINQLIVATNRNDILHRFMSGNQYVKETL
HATLSPSMDIMVSSNFERLLFDMHGRNGAAIAELMSTFRQGGGFSVEEERWTETRKLFDS
LAVSDEDTCTTIAEVFASTGELLDPHTAIGVKAARECRRSLDTPMVILGTAHPVKFPEAV
EKAGVGKALELPAHLSDLFERDERCTVLPNDLKAIQAFVSQHGNRGKPL
>tr|A0A0Q0FWM3|A0A0Q0FWM3_PSEAP ATP synthase subunit b OS=Pseudomonas syringae pv. aptata OX=83167 GN=atpF PE=3 SV=1
MNINATLIGQSVAFFIFVIFCMKFVWPPVIAALHERQKKIADGLDAASRAARDLELAQEK
AGQQLREAKAQAAEIIEQAKKRGTQIVDEARETARVEADRVKAQAHAEIEQELNGVKDAL
RAQLGSLAVNGAEKILGATIDQNAHAELVNKLAAEI
>tr|A0A0Q0C1B2|A0A0Q0C1B2_PSEAP Cytosine-specific methyltransferase OS=Pseudomonas syringae pv. aptata OX=83167 GN=ALO85_03495 PE=3 SV=1
MRLLTKCLELHPDIPVFVIFWAVTSITAPKGYRLGGLGEILTMHQIDAVDLFCGAGGLTA
GLLKTGINVRAGYDIDHNCEYAYRKNNGAEFITESVELTKIEDIAAWYRPKRIKLLAGCA
PCQPFSTYNQGRDTSTDRKWPLLYSFEKLIKGVNPELVTMENVPDVTKHKVYHDFVKSLE
DAKYHIWSGTIHCVDYGLPQQRKRHVLLASKLGPITMIPKTHPEPVSVAQAIGHLPRLAA
GETDPDDPLHRAATLSPVNLQRIMLSKPGGTWKDWPEYLRADCHRKPSGKTYASVYGRMR
GDKPGPTMTTLCYGFGNGRFGHYDSEQNRAISLREAATLQAFPEDYQFMPSDEITFKAVG
RMIGNAVPVRLGEIIGLSIQRHLDELNQEKTPC
>tr|A0A0Q0BYN7|A0A0Q0BYN7_PSEAP LysR family transcriptional regulator OS=Pseudomonas syringae pv. aptata OX=83167 GN=ALO85_100325 PE=3 SV=1
MRRHPPNAINAAPYAQEAWALELVWLEDLSALAEYGSFVRAAEARHVTQPAFSRRVRSLE
NWMGVELFVRTPQGATLTEAGRQILPSAQEAARHLYRMRSEAQEVAGVAARSLRFAATHS
LSFTFFPKWLKTAENGAPIEAVQLHSDSMAACEQMLIHGQVQFLLCHRHPDVPPLLAPDQ
FVSKKVGEDVLVPLASASANVGTSPAALPYLAYTHESGLGRIVAHRLRGKEDYLHLKPLF
SSHLAAVLMSMALESKGVAWLPKSLTEQEVLDGRLVRALDESWDIPLEIHLTRPKALSSQ
SVEMFWGRAGQ
>tr|A0A0Q0C475|A0A0Q0C475_PSEAP Ditrans,polycis-undecaprenyl-diphosphate synthase ((2E,6E)-farnesyl-diphosphate specific) OS=Pseudomonas syringae pv. aptata OX=83167 GN=uppS PE=3 SV=1
MEKIKPAVPSSVPRHVAIIMDGNNRWAKKRLLPGVAGHKAGVDAVRAVIEVCAEARVEVL
TLFAFSSENWQRPAEEVGALMELFFTALRRETKRLNENEISLRIIGDRSRFHPELQAAMR
EAEVRTSGNSRFVLQIAANYGGQWDIAQAAQRLAREVQAGHLQPEDITPQLLQTCLATGD
LPLPDLCIRTGGEHRISNFLLWQLAYAELYFSDLFWPDFKHDAMRAALADFASRQRRFGK
TSEQVEAGARA

>tr|A0A0Q0CFW3|A0A0Q0CFW3_PSEAP Putative cytosine/uracil/thiamine/allantoin permease OS=Pseudomonas syringae pv. aptata OX=83167 GN=ALO85_03706 PE=3 SV=1
MTTSSPSQSAGQLETRGIEPVPENECNGHPLQLFWVWFAANISILGLPLGATLVAFQGLA
IWQAIIVAILGAAGSFAVVGVISIAGRRGRAPSLTLSRAIFGVRGNIGPTLVSLMSRLGW
ETVNTTTAAFVLLSLFAILFGTPVEAKNSPGLTLVFIALFVLLTLTVSGLGHATLLVIQK
WATYLFGALNLVVGGFLLTHIDWSLVFDAPAAPLSAMIIGVGTMAAGTGIGWANAGADMS
RYQHASVKATRLVASAAFGAGIPLVLLITLGGLLSVGNDHLASATDPIIAIREMLPTWMA
VPYLITAFGGLLLSNNLSVYSAGLTTLTLGLKVKRVHAVIVDIIAIFAGSIYFMLIADSF
YGPFITFISMLAVPITAWVGIFVVDLLHRHIYSPKDLLDQTPRSAYWYRGGVEWRALGSW
ALAIVLGFSFTTVATTPEHVLFKGFLADSWLGHNGLGWIVTFIVAGGLYALLGGARDRRI
APKEAAHAR
>tr|A0A0Q0CA96|A0A0Q0CA96_PSEAP Beta sliding clamp OS=Pseudomonas syringae pv. aptata OX=83167 GN=ALO85_01505 PE=3 SV=1
MHFTIQREALLKPLQLVAGVVERRQTLPVLSNVLLVVEGQQLSLTGTDLEVELVGRVQLE
EPAEPGEITVPARKLMDICKSLSNDALIDIKLDDSKLIVKAGRSRFTLSTLPANDFPTVE
EGPGSLTFTLVQSKLRRLIERTSFAMAQQDVRYYLNGMLLEVSAGILRAVATDGHRLAMC
SMSADIEHADRHQVIVPRKGILEMARLLTEQDGTVSIVLGQHHIRATTGEFTFTSKLVDG
KFPDYERVLPKGGDKLVLGDRQALREAFSRTAILSNEKYRGIRLQLASGQLKIQANNPEQ
EEAEEEISVEYNGDSLEIGFNVSYLLDVLGVMTTEQVRLILSDSNSSALVQESDNDDSAY
VVMPMRL
>tr|A0A0Q0BY81|A0A0Q0BY81_PSEAP Ubiquinone biosynthesis O-methyltransferase OS=Pseudomonas syringae pv. aptata OX=83167 GN=ubiG PE=3 SV=1
MSNVDRAEIAKFEALAHRWWDRESEFKPLHDINPLRVNWIDERVGLAGKKVLDVGCGGGI
LSEAMALRGATVTGIDMGEAPLAVAQLHQLESGVSVEYRQITAEDMAEEMPEQYDVVTCL
EMLEHVPDPSSVIRACYRMVKPGGQVFFSTINRNPKAYLFAVVGAEYILNLLPRGTHDFK
KFIRPSELGAWSRDAGLQVKDVIGLTYNPLTKHYKLTSDVGVNYMIQTLREA
>tr|A0A0N8T9H3|A0A0N8T9H3_PSEAP Binding-protein dependent transport system inner membrane protein OS=Pseudomonas syringae pv. aptata OX=83167 GN=ALO85_01001 PE=3 SV=1
MIGWALRRLTQSLLVVLLMTLVVFVGLNAIGNPMDILVGEDLNQAERLQAIAHLGLDQPL
WQQYLIFLKGAVHGNLGQSFVYHEDAMRLILQRLPATFELAFSALFLAVVIGVPLGMFAG
MYPEHPLSRLMMAASIVGFSLPAFWVALMMIMLFSITLGWLPASGRGETREWLGVQWSWL
TLDGLQHLLLPALNLALFKISLVLRLTRAGVREVLPQEFVKFARAKGLSPMRVMCMHVMR
NTMIPVVTVLAMELGSTIAYAVVTESIFAWPGAGKLILDSINMLDRPVVVAYLMVVVVIF
VVLNLIVDGLYYLLDPRVRIEASR
>tr|A0A0Q0ISJ7|A0A0Q0ISJ7_PSEAP Stress response kinase A OS=Pseudomonas syringae pv. aptata OX=83167 GN=srkA PE=3 SV=1
MAHPFATLTPDLVLDAVESIGFLSDARILALNSYENRVYQVGIEDGQPLIAKFYRPQRWS
NEAILEEHNFTAELVEVEVPVVAPMVHNGATLFEHAGFRFTLFPRRGGRAPEPGNLDQLY
RLGQLLGRLHAVGSTKPFAHREALGVKNFGHDSVNFLLENDCIPRSLLPAYESVARDLLK
RVEDVYAATPHTNIRMHGDCHPGNMMCRDEMFHIVDLDDCRLGPAVQDIWMMLAGDRQER
LGQLSELMDGYNEFHDFDPRELALIEPLRALRLMHYSAWLARRWDDPAFPHSFPWFGTER
YWGDHILALREQMAALNEEPLKLF
>tr|A0A0Q0DG36|A0A0Q0DG36_PSEAP GCN5-related N-acetyltransferase OS=Pseudomonas syringae pv. aptata OX=83167 GN=ALO85_01100 PE=4 SV=1
MSRPLPTIRTSRLLLAALQPEQALTLSRLADEPTIAAMTAALPSPYTLEHAQVFIAQTHD
QYAAGQTVSLGVHIQATGELTGIVSLRLSMSHHSGHLGYWTGTRYQNQGYACEAAKGLLK
HGFTDMNLNRIAGQCFSDNPASARVLEKCGLSYEGCTREAFLKNGVFKNMLLFSLLRSEY
ASSEATS
>tr|A0A0Q0ICS5|A0A0Q0ICS5_PSEAP LysR family transcriptional regulator OS=Pseudomonas syringae pv. aptata OX=83167 GN=ALO85_03136 PE=3 SV=1
MLNKRHLPSIAALQCFEAVTRHLSFTRAAEELNLTQSAVSKQVAQLEELVQHLLFQRVRR
RLQLTPAGALYLAEVRKILTQIEMSTHFLRSYGGETEVLRVSTPSTFGARWLVPRLKGWR
LRHPHIHLDLINEQESDDLIKGRCDVSFYFGQAAMPGAESVKLFGEELVPVCAPESLPDE
PLGDPTQLSELVLLQNAFRPEAWHEWFESQGYHTDHSYHGPRFETFYMCIRAAQVGCGVA
LLPRFLVEEELAEGKLVIAWDYALPSTNSAYYLSYPEHTADVPKIRVFLQWMLEQLDQPP
A

>tr|A0A0Q0CFH3|A0A0Q0CFH3_PSEAP Soluble lytic murein transglycosylase-related regulatory protein OS=Pseudomonas syringae pv. aptata OX=83167 GN=ALO85_100213 PE=4 SV=1
MASSVTWAGLALLCFALTSHAAELPPPAYQLAAHAANIPSEVLYSVALQESGTPLRGQLV
PWPWTLNVAGASYRFATRADACTALIIALAQAGPKRVDVGLGQVNMGWNGQRFKSSNPCD
ALNPYKNLDVAAQMLAELRALGGDWITVAGRYHRPAGGAPAANYRKAFAKHLSRVTGIQM
LVTNP
>tr|A0A0N8T7R6|A0A0N8T7R6_PSEAP H+/citrate symporter, CitMHS family OS=Pseudomonas syringae pv. aptata OX=83167 GN=ALO85_03975 PE=4 SV=1
MLTFLGFAMVIAFMYLIMSKRLTALIALILVPIIFALFGGFASQIGPMMLAGITKLAPTG
VMLMFAILYFALMIDSGLFDPAVRKILKMVKGDPMRISVGTAVLALVVSLDGDGATTYMI
CVAAMLPLYSRIGMSPRIMAGLIIILAGGVMNMTPWGGPTARAASALHVDASDIFVPMIPA
MLAGVIAILAIAYFYGKRERARLGELHLQGDEVDHSEISVSQFPDARRPKLIWFNAALTL
TLMVALIMGLLPLPVLFMIAFSIAMIINYPNLQDQKDRVSAHAGSVLAVVGLIFAAGIFT
GILSGTGMVDAMSKSLLAVIPDALGPYLAVITALVSMPFTFFMSNDAFYYGVLPVLSEAA
AHYGISPVEMARASIVGQPVHLLSPLVPSTYLLVALAGIEFGDHQRFTLKWAILVCMCIL
VAALLMGIFPFYSSM
>tr|A0A0Q0DIP1|A0A0Q0DIP1_PSEAP Uncharacterized protein OS=Pseudomonas syringae pv. aptata OX=83167 GN=ALO85_03812 PE=4 SV=1
MSSTKNPCISLCKFDDDICLGCGRSKREIKSWKKLDKDDKRTVLDESAKRLAKLKTAGRK
KKK
>tr|A0A0Q0IMB7|A0A0Q0IMB7_PSEAP Acyl-CoA dehydrogenase domain-containing protein OS=Pseudomonas syringae pv. aptata OX=83167 GN=ALO85_00945 PE=3 SV=1
MDFAYSPKVQALRERVTAFMDAYVYPAEPVFERQVAEGDRWQPTAIVEELKARAKSEGLW
NLFLPDSGLGAGLSNLEYAPLAEIMGRSLLGPEPFNCSAPDTGNMEVLVRYASEEQKARW
LEPLLRGEIRSAFAMTEPDVASSDATNMAARAERDGDQWVINGRKWWTSGACDPRCKILI
FMGLSSPEGPRHQQHSMILVPVDTPGVKIVRPLPVFGYDDAPHGHAEVLFENVRVPFENV
LLGEGRGFEIAQGRLGPGRIHHCMRSIGMAERALELMCRRSIDRVAFGKPLARLGGNIDL
IADSRMEIDMARLLTLKAAYMMDTVGNKVARSEIAQIKVVAPNVALKVIDRAIQMHGAAG
ISSDFPLAYMYAMQRTLRLADGPDEVHRAAVGKYELGKYMPAGTTRRSS
>tr|A0A0Q0BWP7|A0A0Q0BWP7_PSEAP Uncharacterized protein OS=Pseudomonas syringae pv. aptata OX=83167 GN=ALO85_00444 PE=4 SV=1
MLKQFPDTSQHDAYLALAQRIQDAITSDKAQIEHQVLLIREPGESMAHWERIMDQISEAE
GISVTRNPENGTARVSWYIDSL
>tr|A0A0N8T8X7|A0A0N8T8X7_PSEAP Bacteriophage N4 adsorption protein B OS=Pseudomonas syringae pv. aptata OX=83167 GN=ALO85_01777 PE=4 SV=1
MTSLYWPYWLAHYYSVLEVATIVVGLIILVSSIDDLFIDIWYWSRRLYRKFTAERRYRPL
TAEQLIARDEQPLAIMVPAWLEYDVIAPMIENMVSTLDYQNYVVFVGTYINDQRTIDEVE
RMRRRYKQLHRVEVPHAGPTCKADCLNWVIQAIFLYEKTHSVQFAGTILHDSEDVLHPLE
LRLFNYLLPRKDMIQLPVVSLERNWYEWVAGTYMDEFAEWHGKDLVVRESMTDTVPSAGV
GTCFSRRALMVLADENQNQPFNTESLTEDYDVGARLAKYGMQAIFVRFPVQFRVLRKSWF
RKPYESTLEMPLCVREFFPDTFRTAFRQKARWTLGIGLQGWEQMGWTGSLANRYLLFRDR
KGVVTSFVSILAYAILIQLLALIVLRSSGVWNTSFPTPFETTGLIQYLLVANGIALLWRI
LHRCYFTTVLYGWQHGLLSIPRMVVGNFVNFMAAARAWRMFLVGKVLNRKLVWDKTMHDF
PSSDLIAVAPRRLGSVLLSWQAINDEKLQTALAEQQTRQVPLGRILLSHGWLDDETLAEA
IAFQNDLPRVFDIASKRADSRVLADEFCLRWRVVPLQMNALGRQEIAVASPLPPDGLQQI
TQQLGAEPVQLIARESDIVAQLRQLQVVEGQPLPARAPLLGDLLIEQGLLDRDVFQKAML
GYRPHVHGRIGDYLVDIGVLPRETIEQAVARQHNHYRSDDQTEQPL
>tr|A0A0Q0BVC8|A0A0Q0BVC8_PSEAP Sulfur carrier protein ThiS OS=Pseudomonas syringae pv. aptata OX=83167 GN=ALO85_00459 PE=4 SV=1
MHIQLNGEPHELPDGETVAALLTRLDLAGRRVAVELNLDIVPRSQHATTVLSEGDQVEVV
HAIGGG
>tr|A0A0N8T876|A0A0N8T876_PSEAP Uncharacterized protein OS=Pseudomonas syringae pv. aptata OX=83167 GN=ALO85_01245 PE=4 SV=1
MFRPLFVFIGTRYTRAKRRNHFVSFISLTSMIGLALGVVVMIVVLSVMNGFDHEMRTRVL
GMVPHATIESADPITDWRGLAAKVQENPQVLAVAPFTQMQGLLTHDGKVQKVLLNGIDPL
EERKVSIIDHFIKQGQLDSLSPGSFGIMIGDKAAAKLGVGIGDKLTFVAPEVTVTPAGMF
PRMKRFEVTGIFHVGAGEIDGFLGLTNLDDLGRLHRWKPNQVQGLRLKFDDLFAAPRTSW
EIAQKLGENNFYSRDWTRTHGNLYQAIRMEKAMIGLLLLLIVAVAAFNIISTLVMVVNDK

KGDIAILRTLGATPRQIMAIFMVQGTVIGVVGTLIGAALGILAALNVSAAIAALEGLIGH
KFLNADVYFIDYLPSQLMAQDVFQVCGAALVLSFLATLYPAWRAARTQPAEALRYE
>tr|A0A0Q0C212|A0A0Q0C212_PSEAP Deoxyribose operon repressor DeoR
OS=Pseudomonas syringae pv. aptata OX=83167 GN=ALO85_03938 PE=4 SV=1
MDVKKTDRIKQIQQALQDQKAIHLREMAALLEVSEMTLRRDLSRHPEQLRLLGGYITRAH
DDPEPGDYRVSEQGTRHVEEKRRIGKLAATFIRPGDTVFFDCGTTIPFVVDFIPDELEFT
AVCNSLNVLLKLQQKPNCSIVLCGGIFHRKNQVFESHAETSILDGVRLTWAFVSAAGVSL
DCGVTCFNFHEVEVKQKVMRQARQSLLLADHSKFDAVRTAHFGALSDFHCVVSDKKIPRS
YREAIEAGGAQLVL
>tr|A0A0Q0IH30|A0A0Q0IH30_PSEAP Uncharacterized protein OS=Pseudomonas
syringae pv. aptata OX=83167 GN=ALO85_00030 PE=4 SV=1
MNTVADQERIMQRSLCLTRECMGLMTRIECVIRPLRSDSGQWMVLFAAGMAAEQPSAIKS
QGPFRGLPEAQSVLTSVIESLSLHGYQCADDVPIWALHVQAELRRIDSDRMVCQSSSLF
>tr|A0A0Q0CWM8|A0A0Q0CWM8_PSEAP Lipopolysaccharide export system protein
LptC OS=Pseudomonas syringae pv. aptata OX=83167 GN=lptC PE=3 SV=1
MFTKKIRKFLLLGVLALLLAAVGYWNISPESFMDQPDASLDDTAIDYYAINTRSVQYLPD
GTLQYDMTSDKVEHVKATDVSLLTTPNLNMYRGGAFPWHVQSKRGEVSSDGDQVELMDSV
RVERTDEKQRTTIITSSRMTVFPQKEYAQTDQDVRIDGAGGVTTAKGMKAYLKDSRMDLL
SNVRGQYEAR
>tr|A0A0Q0IRS3|A0A0Q0IRS3_PSEAP Uncharacterized protein OS=Pseudomonas
syringae pv. aptata OX=83167 GN=ALO85_03454 PE=4 SV=1
MIIGTSALEQLVSRAVVGRKTVDEVSQDAFSSVAAAGLTSGSAPVTAQTSSSTSQDSKQE
NSGYNESFARMMINLKSAGDTSLSEKTADRDNGVIGSVAGTTSTTDTSSTDSVQQKFMDS
LKQTAEEKVREKLTGITQKDYDAMAPQDQLAIDQKVQDALKDKQNDVADDINTRIRGIKA
GMVA
>tr|A0A0Q0FL84|A0A0Q0FL84_PSEAP Transcriptional regulator, TetR family
OS=Pseudomonas syringae pv. aptata OX=83167 GN=ALO85_01753 PE=4 SV=1
MFVSDHLVRIRALGLCRAHADGAGRTAQATRIDKTAAPVKMPAHFGITGWMCVNKKPPLA
DPANKRVRKAKPETVVKTTREPSATSLKRRMRLMEGKRTLILDAALEIFSRYGVHGSSLD
QVASLADVSKTNLLYYFSSKDDLYLNVLRQLLAVWLSPLLHFTADKDPVQAISAYIKAKL
EMSRDHPAESRLFCMEVMQGAPLIQGELQHPLRDTVQAKVAVIQHWIDSGQLAPVNPHHL
IFTLWATTQHYADFRTQVEAVTGKTLDDPAFFEEVLASLRSMVLDGILPRKA
>tr|A0A0Q0C8R6|A0A0Q0C8R6_PSEAP Uncharacterized protein OS=Pseudomonas
syringae pv. aptata OX=83167 GN=ALO85_03193 PE=4 SV=1
MVGHTLKRAFPALKLHQGRAMKRILIAYFGTLLAFLILDGLWLGVLMGPTYREWLGPLML
DTPVLGPAIAFYLLYGAGVVFFGVMPALRVQRLSHATLCSGLLGLIAYGTYDLTNWATLQ
GWPAQLALVDHAWGTIVSALAGTAGYLAVRRFA
>tr|A0A0N8T8D8|A0A0N8T8D8_PSEAP Flp pilus assembly protein TadB
OS=Pseudomonas syringae pv. aptata OX=83167 GN=ALO85_01367 PE=4 SV=1
MSASLVLGLISLLLFIASIRMFYLALNQGASERVRQRLMAGQVRPVAEKTGWSYLDRAFL
RAGLGQPTKRMGLALSLYALLILIGYATAGVVGALSVLLAVPLTLRVFVSWRYEVRVRRM
IQQLPQLLDHTVRSLKSGRTLADAVLHGIDATDQPLKDGMSRIERNVQLGVSLPDAARDF
AELYERDEFHLFAIGLRVNHRYGGNASELMENLIKLIRDREQAGRQLRAMTGETRMTAVV
LALLPISTAGYFLAVNPNYLLHMWDDESGKIMLSAAFALQATGCVVLWRMLRSI
>tr|A0A0Q0IFP7|A0A0Q0IFP7_PSEAP Regulatory protein, TetR OS=Pseudomonas
syringae pv. aptata OX=83167 GN=ALO85_00694 PE=4 SV=1
MTRHDPTETGTAVTRQRRAPKGEKRREELLDAALQVFSLEGYTGASVAKVAAIVGISVAG
LLHHFPSKISLLMGVLERRDEVNGRIAAEVRTDNTLTGLLGGLRAINRSNATAPGVVRAF
SILNAESLLENQPAYEWFQTRYERIHAHLLGQFSALVERGEVRADVDLDKIIRQLLAMMD
GLQIQWLRFPDQVDLVECFDTYIAQVDAAVRARP
>tr|A0A0N8TA12|A0A0N8TA12_PSEAP Arginine/lysine/ornithine ABC-type
transport system OS=Pseudomonas syringae pv. aptata OX=83167
GN=ALO85_02070 PE=3 SV=1
MKKAWLTLSALALCIAAGNTMAKEYKELRFGVDPSYAPFESKAADGSLVGFDIDLGNAIC
KELKVTCKWVESDFDGMIPGLKARKFDGVISSMTVTPAREKVIDFSNELFSGPTSLVFKK
GAGFTADPASLKGKTVGYEQGTIQEAYAKAVLDKAGISTKAYANQDQVYSDLTSGRLDAS
IQDMLQAELGFLKSPAGADYEVSKPIDSELLPAKTAIGIAQGNKELKALLDKGIKAMHDD
GTYAEIQKKHFGDLNLYSGK
>tr|A0A0N8TA48|A0A0N8TA48_PSEAP Glutaminase OS=Pseudomonas syringae pv.
aptata OX=83167 GN=glsA PE=3 SV=1

MQDLLNEILDEVRPLLGQGKVADYIPALGEVRPDQLGIAVYGNDGQVFSAGDAETAFSIQ
SISKVFSLVQAIGHSGEEIWQRLGHEPSGQPFNSLVQLEFEQGRPRNPFINAGALVICDI
NQARFAAPSLSMRDFVRRLCGNRAITSDSKVADSEYQHRSRNAAAAYLMKSFDNFHGDVE
EVLRSYFHHCALSMNCIDLAKAFGFLAHQGFCAHSGEQIVTARQATQVNSIMATSGLYDE
AGNFAYRVGLPGKSGVGGGIVAIVPERASVCVWSPELNAAGNSLVGMAALEKLSARVDWS
VF
>tr|A0A0Q0DSN8|A0A0Q0DSN8_PSEAP Biotin carboxylase OS=Pseudomonas syringae pv. aptata OX=83167 GN=ALO85_00859 PE=4 SV=1
MMFDKVLIANRGEIALRIQRACKGLGLRTVAVYSEADRDAGYVQQADEALCIGPAAAGQS
YLNQAAVLYAAQLTGAQAIHPGYGFLSENAAFAERIEQAGLTFIGPSAACIRVMGDKVAA
KRAMREAGVPCVPGPDTSMPDDPHSILQIARDIGYPVIVKAAGGGGGRGMRVVQEESQLL
DAIALTREEARHAFGNPELYIEKFLGQPRHVEIQVLCDAYGNAVWLGSRDCSMQRRHQKV
LEEAPAPGIDAALMARVGERCAQACRQMGYQGVGTFEFLFEKDEFYFIEMNTRLQVEHPV
TEMTSGIDIVQQQIRMAMGERLSFSQADVRVSGHSLECRINAEDPRSFMPTPGLVTRWQI
PGGFGVRVDSHVSHGYRVPPYYDSMVAKVITHGASRDEALARMRLALSEMHVEGISTNIA
LHRDILQDPVFCKGGMDIHHLERWLQTRSQP
>tr|A0A0Q0FHE4|A0A0Q0FHE4_PSEAP Intracellular septation protein A OS=Pseudomonas syringae pv. aptata OX=83167 GN=ALO85_02838 PE=4 SV=1
MKRLIGLILVLAGVLYPFAVYWGTEHFAPWQFALLLGSLWLARALTGERKQGSLAMAIVA
LIFCVVLGALDSHMMLRWYPVLVSTFMLGLFGSSLVYGPPIVEKMARLNRPDLPEAGIRY
TRKVTQIWCLFFLVNGLTSAILTLWAPLSWWTLYTGLISYGLMGVLFAVEWIVRPPSAGG
K
>tr|A0A0Q0IEP7|A0A0Q0IEP7_PSEAP Flagellar hook protein FlgE OS=Pseudomonas syringae pv. aptata OX=83167 GN=ALO85_02143 PE=3 SV=1
MSFNIGLSGLYAANKSLDVTGNNIANVATTGFKSSRAEFADQYAQSIRGTSGNTNVGSGV
RTAAVSQQFSQGSLTTGTANSLDLAINGDGFFMMSNNGEKLYTRAGAFHTDKEGYVVNSS
NMKLQGYNVDANGSVVTGALSDLRVNASNLDPKATSTITNSANLNSTTPLPTVATFDATD
TKSYNNKYSTPTYDTQGNAHTLDQYFVKTGTNTWSMYSLMDGRSISDPTSTAPDKNDLTF
DSSGNLVTTAGAAVPTDSANIKFNADGTFAVNNWVPGVQVGTGTTATWAANGAAGAASIK
LDMSSTTQTASVSGLLKQDQNGYATGQLSGMNVDSSGNLFATYTNGKSQVIGQTSLTSFA
NVQGLAQAGGTNWRETFASGVPVSGAPQSGTLGYITGQALEESNVDLTMELVNLIKAQSN
YQANAKTISTQSTIMQTTIQMT
>tr|A0A0Q0CYR4|A0A0Q0CYR4_PSEAP Taurine catabolism dioxygenase OS=Pseudomonas syringae pv. aptata OX=83167 GN=ALO85_01303 PE=4 SV=1
MGLKLSSVPFEISYPAQSFGVEIENLKLHKNLSFDTIAAIKKCLAYRGFVLFRNQNLTRR
EQIAVTRLLGNPNLKLHSWAPQIETATFGDDEVIPNGLPLINHGEILYFVNGPDFCDKTQ
DEHAIWDEKDNHTKGKGTSCWHTGDSEAINVETINCLYAELAAAQGGATLFCDTVAAYND
LEDSLKKRIDNLRVVHYFVDPQRTEPVNQPLVKTNPITRQKYLYLNYNTMERVEGLSKSE
SYRLLKFLFDHQIKDQYVYEHFWKQDDFLIWNCNGTMHKRGALISNHNRIMRRTQAIIPE
FTSYYERDKSLAVTSHDNEDWHPYPFERAELESAGSA
>tr|A0A0Q0INX1|A0A0Q0INX1_PSEAP Uncharacterized protein OS=Pseudomonas syringae pv. aptata OX=83167 GN=ALO85_02073 PE=4 SV=1
MMMKLVKSMVFAVAALSATAAFADDGNEYASPAADKMRLAQEMRFKEQNSSKAERYVNAD
EKAEAERSTKSEG
>tr|A0A0Q0IHN6|A0A0Q0IHN6_PSEAP Heme exporter protein D OS=Pseudomonas syringae pv. aptata OX=83167 GN=ALO85_03032 PE=3 SV=1
MSFASFSDFLAMGRHGVFIWSAYGLCLVVLLINVALPLWARQRFLKQQARRLKRENRP
>tr|A0A0N8T856|A0A0N8T856_PSEAP Putative phosphopantetheinyl transferase OS=Pseudomonas syringae pv. aptata OX=83167 GN=ALO85_101821 PE=3 SV=1
MKRGASWAFDEDGRLAPPKPFPRQNVLLVSCVTRPGGARDEARNRIRACVRTAVEQWLEL
PSGAITFISAPGVAPRLLIDGLPEPGFSISHEAGCSLAAINLHGAVGVDLMQVQAVPDWH
AVALDYLGAEVATGLSGLPESIRTIAFAKAWCRREAFLKLHGLALEEWTAVGGLQGVGVE
VELGEGMVGVVALSQVLMEYPNQTERRTAP
>tr|A0A0Q0IDD4|A0A0Q0IDD4_PSEAP Flagellar protein FliL OS=Pseudomonas syringae pv. aptata OX=83167 GN=ALO85_01645 PE=3 SV=1
MKAWILMLLALSLPVMAQEEAKEEGGAPKAAYVSLTPPFVGNYALDGGPKLRVYKADISL
RVTGADAEAAVKRNDALIRNQLVSLFTQQTVDTMSSAEAKENIRQEALKQVQRVMNDEEG
KPIVEDLLFNNFIVQ

>tr|A0A0Q0BWM9|A0A0Q0BWM9_PSEAP D-isomer specific 2-hydroxyacid dehydrogenase OS=Pseudomonas syringae pv. aptata OX=83167 GN=ALO85_01059 PE=4 SV=1
MRVLIAEHDYHVYTQLLRKAAPDLEVFSTGDSAELSRMASDCPVWLGQPDLMANLLRQGH
TPQWLQSTWAGITPLLADSLSRDYRLTRAVGIFGQVMAEFVLTYMLGHEREVLARLMSQV
ERKWDNRTGQSLSGRKALIVGAGDIGQRVAEFLLPFGVQVYGVASSARTQEPFIEVTALS
DMTRLLGDMDYVINLLPNTPETHDLYDAKLFASFKPTALFINVGRGVAVVDADLVEALKE
GHLAGAVIDVCRQEPLPQRHPFWTAYGLLLTGHSSAPTSPVAMTELFVHNLNAFQAGEAL
RGEVDFSRGY
>tr|A0A0Q0D0E3|A0A0Q0D0E3_PSEAP Oxidoreductase, short-chain dehydrogenase/reductase family OS=Pseudomonas syringae pv. aptata OX=83167 GN=ALO85_03181 PE=3 SV=1
MRLPDAHVVLTGASGGIGLAIAEALCSQGAKVLAVSRHVHPLRQLLELYPANLHWVEADL
CTAQGRQQVVRAVSAVPGVNLLINAAGTNHFAMLEQLSASDIDSMLTLNVHAPIQLTSLM
LPVLKQASEAMVVNVGSTYGSIGHAGYATYCASKFALRGFSEALRRELADTRVGVLYVAP
RATRTSMNSAAAQALNDALSSAVDDPQDVAQAVLQAIAGNRRELYLGWPERFFVRLNSLL
PTLVDRGLRKHLPLIRHHSSPIAQEKHIP
>tr|A0A0Q0C4A8|A0A0Q0C4A8_PSEAP Putative secreted protein OS=Pseudomonas syringae pv. aptata OX=83167 GN=ALO85_00269 PE=4 SV=1
MLRLIVPTLTLLLVAPLCAQAASKQEFELSKMLEKVAKESSVGTPRAINEDILDQGYTVS
GNQLINHLSVREGQAQQMRANPDVMRNQLGNSVCHNNGFRQLMTKGAVLKYQFTEYKTNR
PVVTQTFQASDCTVKPKQ
>tr|A0A0Q0IDM0|A0A0Q0IDM0_PSEAP DNA-binding protein Fis OS=Pseudomonas syringae pv. aptata OX=83167 GN=ALO85_01378 PE=3 SV=1
MTMMTETLVSGTTPVSDNVNLKQHLNTPSEEGQTLRGSVEKALHNYFAHLEGASVTDVYN
LVLSEVEAPLLESVMNYVKGNQTKASELLGLNRGTLRKKLKQYDLL
>tr|A0A0Q0E0Y2|A0A0Q0E0Y2_PSEAP DNA helicase OS=Pseudomonas syringae pv. aptata OX=83167 GN=ALO85_04866 PE=3 SV=1
MMRDDLSLLLNSLNDAQRQAVAASLGRQLVLAGAGSGKTRVLVHRIAWLMQVEQASPHSV
LSVTFTNKAAAEMRHRIEQLMGISPAGMWVGTFHGLAHRLLRAHWQEAGLVQTFQILDSD
DQQRLVKRVMRELGLDEQRWPARQAQWFINGQKDEGLRPKHIQASGDLFLTTMKSVYEAY
EIACQRAGVIDFSELLLRALDLWRDNPGLLAHYQRRFRHVLVDEFQDTNAVQYAWLRLLA
QGGDSLMVVGDDDQSIYGWRGAKIENIHQYSSDFPDTEVIRLEQNYRSTASILKAANGLI
INNSGRLGKELWTDVGDGELINLYAAFNEHDEARYVVETIESALKTGISRNDIAILYRSN
AQSRVLEEALLRERIPYRIYGGQRFFERAEIKNAMAYMRLLEGRGNDAALERVINVPARG
IGEKTVEAIREHARHADVSMWEAMRLLVANKGLTGRAATALGGFIELIENLAAKVMEMPL
HLMTQTVIEQSGLITYHEQEKGEKGQARVENLEELVSAARAFENHESEEDLTPLAAFLGH
ASLEAGDTQAQEHEDSIQLMTLHSAKGLEFPHVFLVGMEEGLFPHKMSLEEPGRLEEERR
LAYVGITRAMKQLVMTYAETRRLYGSETYNKVSRFVREIPPALIQEVRLSNSVNRPFGGT
PKFNSSSLFNGTGIPETEFTMGQRVQHAVFGEGVILNFEGAGAQARVQVNFPEGSKWLMM
GYAKLVPL
>tr|A0A0Q0IL99|A0A0Q0IL99_PSEAP Monovalent cation/H+ antiporter, subunit C OS=Pseudomonas syringae pv. aptata OX=83167 GN=ALO85_02707 PE=4 SV=1
MEEVIAIAIGVLAASGVWLILRPRTFQVVMGLCLLSYGVNLFIFSMGSLFIGREPIIKDG
IPQDLLNYTDPLPQALVLTAIVISFAMTALFLVVLLASRGLTGTDHVDGREPKA
>tr|A0A0Q0CWD6|A0A0Q0CWD6_PSEAP Ribosome modulation factor OS=Pseudomonas syringae pv. aptata OX=83167 GN=rmf PE=3 SV=1
MRRLKRDPLERAFLRGYQYGVHGKSRELCPFTLPSVRQAWINGWREGRGDNWDGMTGTAG
IHRLNELHAVG
>tr|A0A0Q0C1H6|A0A0Q0C1H6_PSEAP GNAT family acetyltransferase OS=Pseudomonas syringae pv. aptata OX=83167 GN=ALO85_03652 PE=4 SV=1
MEMEAPEVLVLQASYTNPVHADAIGFVLNEYAMDAMGTGRPLSSDTRRQLAIELAKRPYA
FSVLAFISGEPVGLVNCFEGFSTFACRPLVNIHDVAVLSQYRGLGISQKMLLKVEEIARQ
RGCCKLTLEVLEGNEVAQGAYRKLGFDNYQLSPEMGRAMFWQKAL
>tr|A0A0Q0FVA2|A0A0Q0FVA2_PSEAP Aldose 1-epimerase OS=Pseudomonas syringae pv. aptata OX=83167 GN=ALO85_03398 PE=3 SV=1
MKKHALLSTLGLSLMISTLTAQAAALSSEHSTFGQLPDGSAVEKYTLRNSHGVQASIITY
GATLQSLLVPDKAGKVEDIVLGFDDVQGYQNNGTVYFGATIGRFGNRLAGGKFSLDGKTY
QVPLNDKTNALHGGTQGFDKRLWKAEETKTGDSVGVKLTYLSPDGEMGFPGALLTEVTYS
LNEKNELKIDYRATTDKPTVLNLTNHSYFNLAGAGSGDVLKQAATLHASHYTPVNDKLIP

TGELPAVAGTPMDFLKPTAFGKHIKDDHQQLKYAEPKQGGFDFNWVLDTKGDVKKLAAEV
SDPQSGRRLQLFTTEPGVQLYTGNFLDGSIHGKAGKVYPHWGAFTLETQHYPDAPNQPKF
PSTRLDPGKAYTQTTVFKFLND
>tr|A0A0N8T907|A0A0N8T907_PSEAP PAS:GGDEF protein OS=Pseudomonas syringae pv. aptata OX=83167 GN=ALO85_05544 PE=4 SV=1
MQTFRKARFFMSDYSRNALRTAVLYGLLSILWFATGSALLSFFVKDPARLAVGQQMIGYG
WALLSTVMIFIARDRLKAIVGGEALFERQQQDYDRLLLAAVVFDSTREGVLVSDAEGRIV
HVNRAFVDITGYQADEVLGFNPSKFKSGRHGPEFYRQMYHSLQSAGQWSGEIWNRRKSGE
IYPQWQTIRSIKGEDGQARHFVAVFSDITAIKRSEQELAQLAHYDALTELPNRLLLTDRI
SQALTSARSSKRGCALLVVDLDNFRNINDSLGHNIGDEVLKAAAERLKALADSGMTVARL
GGDEFALLVKNCTHMVHAAALAQQVINAFKAPYPIAGHQLFVSASIGISLFPGDALTAEQ
LLRNADSALFKAKTDGREGFALYTEELTAQAQQRVQLASELRQAIEQDQLRVFYQPVYDL
VTGRICAVEALVRWQHPTRGMVSPGDFIPIAEQNGLIADIDAWVLNTACRQMQAWLAEGV
SIASVAVNVSSRLFSRGDVDRRVAEVLNETGLDPAFLELEVTESAVMEDPEQAIEQLHRL
RELGLSLAIDDFGTGYSSLLRLKSMPVQKLKIDQGFVAGLPGDVDDIAIVRAVIALASSL
GMRVLAEGIEHANQASFLLSNGCELGQGYWFARPMAAQSIDWTHAPAFN
>tr|A0A0Q0BRJ9|A0A0Q0BRJ9_PSEAP Serine acetyltransferase OS=Pseudomonas syringae pv. aptata OX=83167 GN=ALO85_04116 PE=4 SV=1
MFERLREDIQSVFHRDPAARNAFEVLTCYPGMHAIWLHRFAHILWRTGWKWPARVVSNFG
RWMTGIEIHPGARIGRRFFIDHGMGIVIGETAEIGNDVTLYQGVTLGGTSWNAGKRHPTL
EDGVVVGAGAKVLGPFTVGAGAKIGSNAVVTKAVPAGATAVGIPGRIIVKSDDEVEARRK
AMAEKLGFDAYGVSADMPDPVARAIGQLLDHLQAVDGRLEGMCDALGRLGSDYRAKELPE
LRDEVFDCVKDRQDDKVG
>tr|A0A0Q0DIH4|A0A0Q0DIH4_PSEAP Acyl CoA:acetate/3-ketoacid CoA transferase, subunit B OS=Pseudomonas syringae pv. aptata OX=83167 GN=ALO85_04357 PE=4 SV=1
MALSREQMAQRVARELKDGYYVNLGIGIPTLVANYVPDDIDVMLQSENGLLGMGAFPTED
TIDADMINAGKQTVTARIGASIFSSAESFAMIRGGHVDLTVLGAFEVDIEGNIASWMIPG
KLIKGMGGAMDLVAGADNIIVTMTHASKDGESKLLTRCSLPLTGAGCIRKVLTDLAYLEI
ENGAFILRERAPGVSVEEIVAKTAGKLIVPDDVIEMSF
>tr|A0A0Q0DA51|A0A0Q0DA51_PSEAP dCTP deaminase OS=Pseudomonas syringae pv. aptata OX=83167 GN=dcd PE=3 SV=1
MSIKSDKWIRRMAQEHGMIEPFVERQVRGEGADRVISFGVSSYGYDVRCADEFKVFTNIN
SATVDPKNFDEKSFVDIKSDVCIIPPNSFALARTVEYFRIPRNVLTICLGKSTYARCGII
VNVTPLEPEWEGHVTLEFSNTTTLPAKIYANEGVAQMLFLESDEECEVSYKDRGGKYQGQ
RGVTLPRT
>tr|A0A0Q0DIB0|A0A0Q0DIB0_PSEAP Heat shock protein Hsp20 OS=Pseudomonas syringae pv. aptata OX=83167 GN=ALO85_02175 PE=3 SV=1
MATAFSLAPLFRNSVGFDRFNDLFESAARNETTSGYPPYNVERHTDDHYRIVIAAAGMQE
QDLELQVEKGVLTVVGNKREQEAENVTYLHQGIARREFKLSFRLADNIEVKGADLSHGLL
NIDLVRNVPEEAKPKRIPLNSQKALEN
>tr|A0A0Q0ITR6|A0A0Q0ITR6_PSEAP Threonylcarbamoyl-AMP synthase OS=Pseudomonas syringae pv. aptata OX=83167 GN=tsaC PE=3 SV=1
MVSSWRVQQAAQDIRAGAVIAYPTEAVWGLGCDPWDEEAVYRLLAIKSRPVEKGLILIAD
NIRQFDFLFEDFPELWLDRMASTWPGPNTWLVPHQNLLPEWITGIHETVALRVTDHPTVR
ELCALVGPLISTSANPAGRPAARSRLRVEQYFRGQIDGVLGGSLGGRRNPSVIRDIATAQ
IVRAG
>tr|A0A0N8TA00|A0A0N8TA00_PSEAP Uncharacterized protein OS=Pseudomonas syringae pv. aptata OX=83167 GN=ALO85_00614 PE=4 SV=1
MSARQDDTSCVQYAFRLGETEIAVNPLIGKTLRLEFLGAIHCSHCGRKTKSSYSQGYCYP
CMLKLAQCDLCIMSPEKCHHDQGTCRDPAWGEQFCMTDHIVYLANSSGVKVGITRSTQLP
TRWLDQGASQALPILRVATRQQSGFVEDLLRSQVADRTNWRALLKGDAQPVDLKAIRQAL
FDSCGAGLQGLQERFGLQAIQLLHDAEPVEFRYPVEAYPTKIVSFNLDKNPIAEGTLLGI
KGQYLIFDTGVINIRKYTAYQLAVHQ
>tr|A0A0N8T954|A0A0N8T954_PSEAP Putative Type IV pilus-associated protein OS=Pseudomonas syringae pv. aptata OX=83167 GN=ALO85_02793 PE=4 SV=1
MPTAKALRGGLKYLYGALLALYLASPVYAFTPAQVPLLSSPAVPPNLMLLVDNSGSMYNI
IWDSGFDASVNRPNISYYSTQCLSGVITVVCPFITTLSDDDTLSVGDINAYSIFTLSLMC
PFGGRPVFRNNTQYCLNLPDPAGNGLTRYTADYLSYLIDLVPPNFFGVRDYTNGVIPNDY
RMNVAKTVATNLVTNNTSLRIGLATFNDPNNFDLGPGGKIARVVSDLSPVAISTYQPTGV

SQQKATQNITDLKTAISNLTPSANTPLAETYYEITRYFRGMTPFYQSGSTYVSPIQYRCQ
KNYGVVITDGLPTYDRTFPTNDPDDVLDTTRSLPNWDLNAANDGANLSGDGEGDTLYLDD
LAKFAYDIDLRRDAVTTGGDLTGKSWDTAGFVKQNMSTYTIGFTASNQMLNDAANDTHGH
GKYFQANDSSGLSSALDLALSDIYAKVGSGAGGAANSSTLQTGTQFYQTLYDPTDWHGTI
KAFNLDQITGALSAPVWTTDTAIVTGSTPPVFESWSTLSTPTKIPLEYANFSPAQQTALN
ATLPTNATGAELIAWAKGTANAKFRARPRLLGDIVNSSLGIALPSQKTASDLTGDTTYTT
YLATKANSMTPSILANANDGFFNVINTSDGSRRYAYMPSTALTSLATIASANYGSGVHKF
TVDGQIAVFDTQLSAGSAWRTVAYSGTGSGGKAFFAIKLFEGTSNTVSALWEVKAPDTSN
ANNKFNNLGYAYSKPDVARMADGTGIVVVGNGYGSFTGRASLYVLNANTGAFIAEIQTPL
LNNETDNGLSSVKLRVNAQNVVQAAYAGDLKGRLWKFDMSGATAASWKVAFGGAPLFTAP
RGANQPITVQPFVLDHPLNGKIVYFGTGKFIEVADKQTTTLQDFYAIWDADSGAGSTVEA
NLQAQAVNGSVVSNGTTYFTSTTNDVDWSVKKGWYMPLSAVTPYLGERIIYPAQTSHGRI
IFSTASVNSADPCESTGTGRVFELNAATGSMLNYQVLDTNGDSAINRSDLLVAGLGYIGI
PVVSAIVAGTRDGNEVKIVNNSTGAPPDGLPEKGGIGNQRIMWRQIQ
>tr|A0A0Q0BUC9|A0A0Q0BUC9_PSEAP Two-component response regulator OS=Pseudomonas syringae pv. aptata OX=83167 GN=ALO85_01362 PE=4 SV=1
MAVHPIRQQLLLVDDEEDANEELAELLEGEGFCCFTASSVKMALHQLTLHPDIALVITDL
RMPEESGIQLIRHLRDHTSRQHLPVIVTSGHADMDDVSDLLRLHVLDLFRKPIYHVRLLE
TLNNLFPEPKVFQVQ
>tr|A0A0Q0ITK4|A0A0Q0ITK4_PSEAP Type I restriction-modification system, S subunit OS=Pseudomonas syringae pv. aptata OX=83167 GN=ALO85_01509 PE=4 SV=1
MSQCEADSLRETAIGDVASDWEMGVIGDFVTALQAGVSVNAFETDSGCTEGELGVLKTSA
VLRGRFFAEQYKVVVAEDMGRVATSVVGDRIIISRMNTPALVGESGYVTKDEPNLFLPDR
LWQTEPSDRPHSQRWLSYWLQHPGVRRLIAASATGTSNSMKNISKETVLSLPVPRTPLPE
QQKIAAILTAVDDKLDVIFRQIKATQALKQGLMQALFSRGVGTQDTTGRWIQHTEFKDSE
LGTIPALWDVGVIADYVSALRSGVSVNAEDRMHGDDEIGVLKVSCVLRGGFYPNCHKTVV
PEERERVAEPVLQGRIIVSRANTPALVGESAYVNSAWPNLFLPDKLWQIEPSESPHSIKW
LSFYLQSPFVRQEISKAATGTSGSMKNISKPAFLSIRMPLVPLAEQEHIAAILSDVTSKI
EALNSKQNHFQTLKRGLMQKLLTGEWRVKIDSPTGTV
>tr|A0A0Q0BXU8|A0A0Q0BXU8_PSEAP Acyltransferase 3 OS=Pseudomonas syringae pv. aptata OX=83167 GN=ALO85_01138 PE=4 SV=1
MSEQIGQSERIYTLDVLRGIAALSVVFWHWQHFFYVGDQPALFDVARQPFFAYLTILYKY
GALAVELFFCISGFVFFYLFFNDISSRRVSASRFFMNRFSRLYPLHIFSFAAVALFQFIY
FQQNAVYFIYQFNDVYHAILNILMIPAWGLEKGWSFNGPVWSVSVEIFLYGLFFTVCLMG
RCRYLLVPLLIFISWLAYPEYHKLSAGVFSFFSGGLAYLIFARVRSLTGRNTSCIAALIA
MLAAWSAVWLSPTLNIYLLMGVAFPASVMFIASVSYVQPTLMKRFGVIGDVSYSSYLLHF
PLQILFAMAFDALGYNREIFYNTWMLLLFMAVLIPLSFASHRLYEVPLQHWLRRRFNVWS
TNRREIT
>tr|A0A0Q0C061|A0A0Q0C061_PSEAP Oxidoreductase FAD-binding OS=Pseudomonas syringae pv. aptata OX=83167 GN=ALO85_04371 PE=4 SV=1
MRVASVLKKVLFQLHWFFGITAGLVLTPMGMTGALYSFEDEILDALNPDVLLVEPRGATL
PPVELVRRLEAATGLTVAILRVETTGNRAAQVFFTPKTGERRGPKRNFDPYTGELKGNAV
GEGFFDFVLKLHRYLAMGEAGKQVTAACTLILVFFCLSGLYLRWPRAARNWRVWLAVDWA
KKGRSFNWDLHSIFGTWCLLFYLLFALTGLTWSYDWVSNGLNKLIGDSPLEEGVAPPAKA
AGRLPLAVDYVAVWDSIQKAAGPDLLAYNLRLPAAGGQPATVFYLLKDSPHPRALNTITL
DPANGQVSSVVRYAERSFGAQLLASNYALHVGSYFGLVGRVIMTGASLLMPLFFITGWLL
YLDRRRKKRDIERARGEVQGDDLDDDASWLVCFASQSGFAEQLAWQTAGQLQASGLSVRV
KRLGDLSEDELYRSHNALFVVSTFGDGEAPDSARRFERKLLGRPLSLNQLDYAVLALGDR
QYPHFCGFAHRLHGWLTERGGRSLFPPVEVDSADPAALQHWQQQLAQLTGCVPAAHWQTP
VFDNWTLARRVLLNAGSSGSNVYLLDLTPPTSAHWQAGDLVEVMPRHTLRVIEPFLAGLG
VDPGAWVSVDGLQEPLAQALESRQLPHSRSHLVGLHAQALIDALVPVSVREYSIASIAQD
GCLQLIVRQEVHADGSLGLGSGWLTEHCAVGGAVSLRVRRNSSFHLPAEPLPLILLGNGT
GLAGLRSLLKARIAQGQARNWLLFGERNRAHDFHCSAQLQGWLQAGHLTRLDLAFSRDQA
EKIYVQDRLREAADELRVWLDEGAAIYICGSLLGMAAGVDQVLNEVLGEQAVSELIEEGR
YRRDVY
>tr|A0A0Q0FWX9|A0A0Q0FWX9_PSEAP DNA replication and repair protein RecF OS=Pseudomonas syringae pv. aptata OX=83167 GN=recF PE=3 SV=1
MSLSRVSVTGVRNLHPVTLSPSPRINILYGANGSGKTSVLEAIHLLGIARSFRSSRLLPV
IQYEQPSCTVFGQVDLAQGGHSNLGVSRDRQGEFQIRIDGQNARSAAQLAEILPLQLINP

DSFRLLEGAPKIRRQFLDWGVFHVEPRFMATWQRLQKALKQRNSWLRHGTLDAASQAAWD
RELCSASDEIDEFRRAYIKALKPVFEQTLSELVELEGLTLSYYRGWDKEKELSTVLASSL
HRDQQMGHTQAGPQRADLRLRLGAHNAADILSRGQQKLVVCALRIAQGHLVSQVRRGQCI
YLVDDLPSELDDNHRRALCRLLEELRCQVFITCVDQEFLREGWQTETPVALFHVEQGRIT
QTHDHRE
>tr|A0A0N8T7T3|A0A0N8T7T3_PSEAP HSDR_N_2 domain-containing protein
OS=Pseudomonas syringae pv. aptata OX=83167 GN=ALO85_02286 PE=4 SV=1
MDFLEALNSLSAKVRQQAASIHTEEATKTALVMPFIHTVLGYDVFDPSEVVPEYTCDVGT
KKGEKIDYAIVKEGNIQILIETKKIGEPLNINHASQLFRYFHVTTARISILTNGRVYKFF
TDLDAPNKMDEKPFLELDLLAIDDHAIPELQKLTKSSFDVESILNAAGELKYVGQIKRVI
ATQFSQPDDDFVRYFASRIYEGAITQKVREQFTILTKKATSQFLNDQVNDRLKLAMSGVQ
APTVAETIIENNTQPEKEEPIDDRIVTTVEEMEGFHIVKSIIRTVVEAKRIAHRDTQSYF
GILLDDNNRKPITRLHFNRAQKYIGIFEKDKGETRHPIASLDDIYGFADALKDTVLSYAE
S
>tr|A0A0Q0C0X0|A0A0Q0C0X0_PSEAP SMR family multidrug efflux transporter
OS=Pseudomonas syringae pv. aptata OX=83167 GN=ALO85_02875 PE=3 SV=1
MNAYYLLAIAVCAEVIATTSMKAVKGFSTPLPLVLLITGYAIAFWMLILVMRTIPVGITY
AIWSGMGIVLVSIAAFFIYGQKLDLPALLGMGMIVAGVAVIQLFSKTAGH
>tr|A0A0Q0DTA4|A0A0Q0DTA4_PSEAP Histidine kinase, HAMP region: chemotaxis
sensory transducer OS=Pseudomonas syringae pv. aptata OX=83167
GN=ALO85_02618 PE=4 SV=1
MTSSLFDHLISALTPLLIQQQVSPMNVLRQSKFSTKLLSAFIICALITLAVGALGMVGVT
RLANALELTFSNNLVSVGNTNEALTSFTAHNRGLYRLLDAQDGGVSETDKDRVRQALGDD
LARAQKVFAIYRATPLEDDERAAGDQLELMLPAYVSGSQQVST
>tr|A0A0Q0IEC6|A0A0Q0IEC6_PSEAP Elongation factor P OS=Pseudomonas
syringae pv. aptata OX=83167 GN=efp PE=3 SV=1
MKTGKELKPGTVIRLENDPWLVQKAEFTKSGRNSAIMKTKLKNLLTGYKTEIVYSADDKL
DDVILDRKEATLSFISGDTYTFMDTTDYTMYELNAEDIESVLPFVEEGMTDVCEAVFFED
RLVSVELPTTIVRQVDYTEGSARGDTSGKVMKPAKLKNGTELSVADFIEIGDMIEIDTRE
GGSYKGRAK
>tr|A0A0Q0DP57|A0A0Q0DP57_PSEAP Two-component DNA-binding response
regulator RoxR OS=Pseudomonas syringae pv. aptata OX=83167 GN=ALO85_00003
PE=4 SV=1
MSDDDIQVEGEELPHLLLVDDDATFTRVMARAMSRRGFRVSTAGSAEEGLALAQQDIPEY
AALDLKMDGDSGLVLLPKLLEMDPDMRVVILTGYSSIATAVEAIKRGACNYLCKPADADD
VLAALLSEHANLDTLVPENPMSVDRLQWEHIQRVLTEHEGNISATARALGMHRRTLQRKL
QKRPVRR
>tr|A0A0N8T873|A0A0N8T873_PSEAP Uncharacterized protein OS=Pseudomonas
syringae pv. aptata OX=83167 GN=ALO85_101199 PE=4 SV=1
MVSDCLVESTLNLFVLFAVFQRDYSCKFCAIVNGVTVNTCTDFPAKKERQRWAVP
>tr|A0A0Q0BTM1|A0A0Q0BTM1_PSEAP Uncharacterized protein OS=Pseudomonas
syringae pv. aptata OX=83167 GN=ALO85_102229 PE=4 SV=1
MQEHSSMGVKVLRMKQLCACLGLSRSTIYDRINPESKRYDETFPKPFKMGGSAVGWLEAD
VQKWLSDWLSK
>tr|A0A0Q0DI73|A0A0Q0DI73_PSEAP Uncharacterized protein OS=Pseudomonas
syringae pv. aptata OX=83167 GN=ALO85_101118 PE=4 SV=1
MCLSVPAPAFSVCFDAISSTASPWPDYVCGNPLNRGSLQ
>tr|A0A0Q0FFT7|A0A0Q0FFT7_PSEAP Uncharacterized protein OS=Pseudomonas
syringae pv. aptata OX=83167 GN=ALO85_01030 PE=4 SV=1
MINLPGYLAIRTISGRNGAFNVGRLSTSIGEFVIKDALLDQYHEGKYRGDFVITEIRPSY
YTNGGRLVVEVRARLDSMSLDDVANLSADEAERLSNNETDPLDEEASGGSSSTKPRPTIQ
TTPRRSVSAGSDASFGMSRSEPEKQVTAREVDAELFGTIWPIGPSVKLDTTVDRKRLRQQ
CVRLGELGYELDFKLQIWNLSTY
>tr|A0A0Q0DIZ0|A0A0Q0DIZ0_PSEAP Multicopper oxidase OS=Pseudomonas
syringae pv. aptata OX=83167 GN=ALO85_04084 PE=4 SV=1
MPFMSFTRRQILTGIAGLAVVGLGAGGASRYWMGRRESEAGTDFVLIAAPLDVELVPGHK
TPAWAFGGSAPGTELRVRQGEWLRLRFINQLPVETTIHWHGIRLPLEMDGVPYVSQLPVK
PGEYFDYKFRVPDAGSYWYHPHVSSSEELGRGLVGPLIVEEREPTGFAHERTVSLKSWHV
DEVGAFTEFSVLREAAREGTAGRLSTLNGIAQAVIELPAGQITRVRLLNLDNTVTYRLNL
PDAEAQIYALDGNPVKPRPFGDEYWLGPGMRICLAIKAPPAGEEISLRNGPVRLGTFRSV

ANNDAPGQWPPELPANPVAEPDMENAEKINFNFEWVGSMSVNTDNGKPPSLWQINGEAWD
ITDKTCADRPIARLKLGKSYIFELKNMTQYQHPIHLHGMSFKVIASNRRKIIPYFTDTFL
LGRNERARVALVADNPGVWMFHCHVIDHMETGLMAAIEVA
>tr|A0A0Q0CBX1|A0A0Q0CBX1_PSEAP Iron-sulfur cluster assembly protein CyaY OS=Pseudomonas syringae pv. aptata OX=83167 GN=cyaY PE=3 SV=1
MSLTEARFHDLVDATQQNIEDVFDESGLDVDLENSAGVLTVKFENGSQLIFSRQEPLRQL
WLAARSGGFHFDYDEENSRWVCDTSDELLSEMLVRMTLEQADVELDFSPVTRSTAQAAFQ
QCQPGSSLAVYQPVSAR
>tr|A0A0Q0DF82|A0A0Q0DF82_PSEAP Methionine ABC-type transport system, periplasmic substrate-binding protein OS=Pseudomonas syringae pv. aptata OX=83167 GN=ALO85_01622 PE=4 SV=1
MKKLLAIFAAVTALSAQANETLTVAASAVPHAEILEFVKPTLAKEGVDLNIKVFNDYIQP
NVQVSQNRMDANFFQHQPYLDEFNKGKGTDLVAVAKVHIEPFGAYSDKLKTLADLPNGAN
VALPNDATNEGRALLLLAKAGLITLKDPGNILSKPSDVVNNPKDLKFRELEAATLPRVLT
QVDLALINTNYALSAKLDPTKDALIIEGADSPYANILVARPDNKDSDAMKKLVAALQSPE
VKTFLAEKYKGAVVPAF
>tr|A0A0Q0FEY3|A0A0Q0FEY3_PSEAP Uncharacterized protein OS=Pseudomonas syringae pv. aptata OX=83167 GN=ALO85_04772 PE=4 SV=1
MLASSRDNLMKRLSITLSVFALLGSGAAYGEIFKCTSPQGKVSYASIPCDESAQSALQRE
GPAYMRNADEPLDYVHKTNLKAIEYLKVGGRTRVQVIETERYKAFQRNRPPPPNIPSQCQ
SPRYDSQCFDPSGGMSSKKQAAFRNTAR
>tr|A0A0N8T9J6|A0A0N8T9J6_PSEAP Conjugal transfer protein OS=Pseudomonas syringae pv. aptata OX=83167 GN=ALO85_200109 PE=4 SV=1
MNELSDFNPNRLILARQRRSYTKKLLADYAGLNSKLITLHETGAQDPTPESLGMLFRVLK
FPVNFFLGRDIEAPTDENASFRSFSRMTAGKRDAALAAGGIVYLLADWMDQKINLPSADI
PDCSGMDPEAAAIEGIVREYGHV
>tr|A0A0Q0FW76|A0A0Q0FW76_PSEAP LacI transcriptional regulator OS=Pseudomonas syringae pv. aptata OX=83167 GN=ALO85_05345 PE=4 SV=1
MTSVKDVAKLAGVSLMTVSRALNDPGKLSPETYQRVRNAIDELQFVPSLSARRIRGDNLQ
TRTIGVFALDTATTPFAVELLLSIEQTAQQAGWNVFILNLLSNPPTDQNIDLMLSHRPDG
LIFSAMGLRKVNIPQRLKSKPLVLANCVADDSSLVSYVPDDETGQYRALHHAFSKGYRRP
LFINLPKQSLAWEIRQAGMQRACEAFGLAGDELLQYNLSEHDAYWETATILARHMLDGRP
QFDILICGNDRIAFCAYQLLLGQGLKIPGDVAVLGYDNMIGIAELFIPALTTVQLPYYEI
GRNAARHLIDSLDVSGSQPVDCPLVVRESL
>tr|A0A0Q0IJH7|A0A0Q0IJH7_PSEAP Polyamine ABC-type transport system, permease protein OS=Pseudomonas syringae pv. aptata OX=83167 GN=ALO85_02509 PE=3 SV=1
MSRVERGPASIYHKTVVYLLFLILLLPLAGTFLYSIATSWSATILPSGLTFKWYVALWSD
PRFLTAFGQSLLVCIGALVLSVVLILPLLFVVHYYFPRLDGLMNILILLPFAVPPVVSSV
GLLQLYGSGPMAMVGTPWILIGCYFTVALPFMYRAITNNLQAINLTDLMDAAQLLGASTW
QAAFMVVLPNLRKGLMIALLLSFSFLFGEFVFANLLVGTRYETLQVYLNNMRNSSGHFNS
ALVISYFLFVLVLTWAANRLNKDKD
>tr|A0A0Q0DCC0|A0A0Q0DCC0_PSEAP Response regulator NasT OS=Pseudomonas syringae pv. aptata OX=83167 GN=ALO85_00560 PE=4 SV=1
MLRILLINDTAKKVGRLRSALIEAGFDVIDESGLVIDLPARVEAVRPDVILIDTESPGRD
VMEQVVLVSRDQPRPIVMFTDEHDPGVMRQAIKSGVSAYIVEGIQAQRLQPILDVAMARF
ESDQALRAQLHARDQQLAERKRIELAKGLLMKMKDCNEEDAYTLMRRQAMSRQQKLIQVA
EQIIAMSELLG
>tr|A0A0N8T911|A0A0N8T911_PSEAP Succinate-semialdehyde dehydrogenase OS=Pseudomonas syringae pv. aptata OX=83167 GN=ALO85_03959 PE=3 SV=1
MQLKDSTLFRQQAYINGQWLDADGGQSIKVNNPANNEILGTVPKMGAAETRRAIEAADKA
LPAWRALTAKERGNKLRRWFELMIENQDDLGLLMTLEQGKPLAEAKGEITYAASFIEWFA
EEAKRVYGDVIPGHQPDKRLIVLKQPIGVTAAITPWNFPAAMITRKAGPALAAGCTMVLK
PASQTPFSALALAELAERAGIPAGVFSVVTGSAGDIGAELTGNPIVRKLSFTGSTDIGRQ
LMAECAKDIKKVSLELGGNAPFIVFDDADLDKAVEGAMISKYRNNGQTCVCANRIYVQDA
VYDAFAEKLKAAVGKLKIGNGLEEGVTTGPLIDDKAVAKVKEHIADAVSKGATVLTGGNS
LEGSFFEPTILVNVSKDAAVAREETFGPLAPLFRFKDEAEVIAMANDTEFGLASYFYAQN
MSRVFRVAEALEYGMVGINTGLISNELAPFGGIKSSGLGREGSKYGIEDYLEIKYLCLSV
>tr|A0A0Q0DTB3|A0A0Q0DTB3_PSEAP DNA topoisomerase 1 OS=Pseudomonas syringae pv. aptata OX=83167 GN=topA PE=3 SV=1

MGKSLVIVESPAKAKTINKYLGNQYVVKSSIGHIRDLPTSGSASAAKDPAAKRGKAAAGE
GPVLTPKEKAQRQLVSRMGVDPEHGWKAKYEILPGKEKVIEELRKLAKDADIIYLATDLD
REGEAIAWHLREAIGGDDSRYKRVVFNEITKKAIQEAFSKPGELDIARVNAQQARRFLDR
VVGYMVSPLLWQKVARGLSAGRVQSVAVKLVVEREREIRAFNPEEYWEIHADLGTAKGAN
VRFEVARENGEAFKPLNESHAMAALEKLKASSYSIVKREDKPTSSKPSAPFITSTLQQAA
SNRLGYGVKKTMMMAQRLYEAGYITYMRTDSTNLSADAVSMARDYIETEFGKKYLPESPN
VYSSKEGAQEAHEAIRPSDVNTHPSKLTGMERDAERLYELIWRQFVACQMLPAQYLSTTV
TVAASDFELRAKGRILKFDGYTRALPQMAKPGDDDVLPDMAQGETLKLNKLDPSQHFTKP
PARYSEASLVKEMEKRGIGRPSTYAAIISTIQDRGYVALHNRRFYSEKMGDIVTGRLSES
FSNLMDYGFTAGMEENLDDVAQGERDWKNVLDEFYGDFRKKLEVAEAADGGMRANQPVMT
DIPCKVCGRPMQIRTASTGVFLGCSGYSLPPKERCKATVNLTPGDEIADDDEGESESRVL
RGKHRCPICSTAMDAYLLDEKHKLHICGNNPDCTGYEIEEGTYRIKGYEGPSLECDKCGS
EMQLKTGRFGKFFGCTNPDCKNTRKLLKSGDAAPPKMDPVKMPELKCDKVDDTYILRDGA
SGLFLAASQFPKNRETRAPLVIEIAPHKDEIDPKYHFLCEAPKKDPDGRPAVIRYSRKTK
EQYVQTEVEGKPTGWRAFYDGSAWKVEDKR
>tr|A0A0Q0C3L3|A0A0Q0C3L3_PSEAP Substrate-binding region of ABC-type glycine betaine transport system OS=Pseudomonas syringae pv. aptata OX=83167 GN=ALO85_01548 PE=4 SV=1
MKKLSTAVAVAVMTLGSLAAQAEDASCSTVKMADPGWSDIAATNAITSVVLEGLGYTPKV
DTLAVPIAYGGLKDGKLDVFLGNWMPAQQGFYDKFVATGDVTQLAKNLEGTEFTLAVPDY
VWDAGVHNFADLNKFADKFDKKIYGIGSGAPANLSLQEIIKKNEFDLGKWKLVESSETAM
LAEVNRNVKKKAFVVFLGWTPHPMNVQIKGMHYLKGGEKYFGDTGSVFTLTRKGYAQACP
NVGKLLTNLSFTLDMENSIMAEVVNKKVSNSAAAKAWIKANPAVLEKWLEGVQTADGKDG
LATVKARL
>tr|A0A0N8T9X7|A0A0N8T9X7_PSEAP Uncharacterized protein OS=Pseudomonas syringae pv. aptata OX=83167 GN=ALO85_01969 PE=4 SV=1
MLNLNKFFLALAFLGGSAAVQAGDGRLDVARIEFSKTSEKLDDTNSWGHALGEQAHSNVS
VGTLYSPPVYGSLTGTHNGAPVSREILLGSYDVTQHVPVSARLLSTDATMSFHSLS
>tr|A0A0N8TAB6|A0A0N8TAB6_PSEAP Pyruvate carboxylase, subunit A OS=Pseudomonas syringae pv. aptata OX=83167 GN=ALO85_01437 PE=4 SV=1
MITKILIANRGEIAVRIVRACAEMGIRSVAIFSEADRHALHVKRADEAYSIGAEPLAGYL
NPRKLVNLAVETGCDALHPGYGFLSENAELADICAERGIKFIGPSAEVIRRMGDKTEARR
SMIKAGVPVTPGTEGNVADIDEALAEGERIGYPVMLKATSGGGGRGIRRCNSREELEQAF
PRVISEATKAFGSAEVFLEKCIVNPKHIEAQILGDSFGNVVHLFERDCSIQRRNQKLIEI
APSPQLTPEQRAYIGDLSVRAAKAVGYENAGTVEFLLAEGEVYFMEMNTRVQVEHTITEE
ITGIDIVREQIRIASGLPLSVKQEDIIHRGFALQFRINAEDPKNNFLPSFGKITRYYAPG
GPGVRTDTAIYTGYTIPPFYDSMCLKLIVWALTWEEAMDRGLRALDDMRLQGVKTTAAYY
QEILRNPEFRSGEFNTSFVESHPELTNYSIKRKPEELALAIAAAIAAHAGL
>tr|A0A0Q0CA13|A0A0Q0CA13_PSEAP SMF protein OS=Pseudomonas syringae pv. aptata OX=83167 GN=ALO85_01542 PE=4 SV=1
MPLFENAPSSPAELEARLRMHRLPEIGPKRFSRLIEAFGSASSALSAPASAWRALGIPGA
CAEARRDPLVRDGASAALAWLERPAQHLLMWDDPGYPALLAQIADPPPLIFIAGDPSILE
RPQLGMVGSRRASRPGLDTASAFARSLAAAGFVITSGLARGIDGAAHQGALDVGGLTIGV
LGTGLEKLYPQQHRALAAQMAAQGGAVISEFPLDAEPQASNFPRRNRIISGLSLGILVVE
ASVASGSLITARLAAEHGREVYAIPGSIHHPGAKGCHQLIRDGATLVETVEHILEGLQHW
KRVAPSVDPVTPAKPGPCDHPLLALLHAAPHTSEGLSVSCGWPLPKVLAGLTELELDGRI
SCEAGRWFARAP
>tr|A0A0Q0DKB7|A0A0Q0DKB7_PSEAP Cell division protein FtsX OS=Pseudomonas syringae pv. aptata OX=83167 GN=ALO85_00462 PE=3 SV=1
MSATRSPKVSERVAPKAADPVPPKQKKQRHDHDDDGPDFSALLSAWLESHRSSLLDSLRR
LGKQPIGSFFTCLVMAIALSLPMGLSLLLSNVERLGGSWQRAAQISIYLNLDASTADGTR
LRDEIKAMPGVADAEYISSEQALGEFKQQSGLGEALKELPENPLPGVVLVTPDEVDKPAL
EALRTRLAELPKVEQAQLDLVWVERLAAILKLGDRFVFGLTVLLVSALLLVIGNTIRLHI
ENRRIEIEVIKLVGGTDSYVRRPFLYMGALYGFGAGILSWGVLAYGLNWLNGAVVGLAGL
YGSDFALAGVPMGDGLSLLLGAVLLGYIGAWIAVARHLRELAPR
>tr|A0A0Q0ITU4|A0A0Q0ITU4_PSEAP Nitrate/sulfonate/bicarbonate ABC-type transport system OS=Pseudomonas syringae pv. aptata OX=83167 GN=ALO85_01465 PE=4 SV=1
MMAVETRCLVDVQKVRHVYGKGSKERLVLDDVTLTLNENEIVGLLGRSGSGKSTLLRSIA
GLVVPTEGEVNFPRRAQGRAMSVGMVFQSFALFPWLTVLENVEVGLEAQGVPAAVRRKRA

LAAIDLIGLDGFESAYPKELSGGMRQRVGMARALVIDPDVLLMDEPFSALDVLTAETLRT
DLLDMWSEGRMPIKSILMVTHNIAEAVLMCDRILLFSSNPGRVISEIKVDLPQPRNRQDP
IFRALVEEIYVLMTQDNDAGEIRQGVFPGTGLGMVLPQVSTNALAGLIEAIHAPPYSLKA
DLPELAAALHYNADELFPIAEVLQLLRFAELKGGDIRLLPAANRYALADVDERKQLFAQH
LLSFVPLVAHIRRVLDDRPTHTAPARRFRDQLEDFMSESDAMQTLNAVIQWGRYAELFAY
DELADQFSLDNPS
>tr|A0A0N8T7Z7|A0A0N8T7Z7_PSEAP Dihydroorotate dehydrogenase (quinone) OS=Pseudomonas syringae pv. aptata OX=83167 GN=pyrD PE=3 SV=1
MPIAVSSKAYAIAGGGRATSLSRASPDRPAHVYNRHPEFSCSRAHPAMYNLARQLLFKLS
PETSHDLSLDLIGAGGRLGLNGLLSKSPAKLPVSVMGLEFPNPVGLAAGLDKNGAAIDGF
AQLGFGFVEIGTVTPRPQPGNPKPRIFRLPNAEAIINRMGFNNLGVDNLVSRVEAAKYRG
VLGINIGKNFDTPVERAVDDYLICLDKVYAHASYVTVNVSSPNTPGLRSLQFGDSLKQLL
QALSLRQQELTQRHGRRVPLAIKIAPDMTDEETVLVAAALIESGMDAVIATNTTLSRQGV
EGLPHADQAGGLSGAPVREKSTHIVKVLAGELAGRLPIIAAGGITEGRHAAEKIAAGASL
VQIYSGFIYKGPALIRESVDAIAAMPRAAR
>tr|A0A0N8T921|A0A0N8T921_PSEAP Putative metal-binding protein OS=Pseudomonas syringae pv. aptata OX=83167 GN=ALO85_02538 PE=4 SV=1
MLNDPIPSHVDPRKLSDRGTTLQGEVLLGDLKRLCDPLADTVGTVQAKFVFERDERRSVV
IHSSIDVPVKMVCQRCLELVTLPIHSECSYVVVKEGANTQSLPKGYDVLELGEDPLDLLA
LIEEELLLALPIVPAHHPEECQQPAGLDEPEPSVDEVTRSNPFSVLAQLKRDPNV
>tr|A0A0Q0C6B2|A0A0Q0C6B2_PSEAP Glutathione reductase OS=Pseudomonas syringae pv. aptata OX=83167 GN=ALO85_03664 PE=3 SV=1
MAFDFDLFVIGAGSGGVRAARFAAGFGAKVAVAESRYLGGTCVNVGCVPKKLLVYGAHFS
EDFDHAKGFGWSLGEASFDWCTLIANKDSEISRLNGIYRKLLVDSGVTLLEGHAKIVGPQ
QVEINGQTYRVERILIATGGWPQVPEVPGREHAITSNEAFHLKTLPKRVVVVGGGYIAVE
FASIFNGLGADTTLVYRRELFLRGFDGSVRTHLHEELLKRDMTIRFSSDIERIDKQADGS
LLLSMKGGGTLETDCVFYATGRRPMLDNLGLDSVDVKLDEHGYIKVDEHYQSSEPSILAI
GDVIGGVQLTPVALAEGMAVARRLFKPEQYRPVDYNHIPTAVFSLPNIGTVGLTEEDAIK
AGHDVQIFESRFRPMKLTLTDDQERTLMKLVVDAKTDRVLGCHMVGPDAGEIVQSLAIAI
KAGATKQVFDDTIGVHPTAAEEFVTMRTPVKR
>tr|A0A0Q0BU18|A0A0Q0BU18_PSEAP Regulatory protein, TetR OS=Pseudomonas syringae pv. aptata OX=83167 GN=ALO85_01878 PE=4 SV=1
MSTSDAPRTYHHGNLPAALRQAAWDIVGEAGVRGMSLRECARRANVSHAAPAHHFGSLEN
LLAEVVADGYERMADVIIAVQQELDDTLLGCGIGYVSFAKSYPEHFRLMFGAGVNRTLPR
LLKSGERALQVLRQSIRNAWIAKYGSEPEPELLEQRTFLAWSTAHGYASLTIDRKTNNPP
IPSAESVLQLMTCAVLMPSG
>tr|A0A0Q0DJE5|A0A0Q0DJE5_PSEAP Two-component hybrid sensor histidine kinase/response regulator OS=Pseudomonas syringae pv. aptata OX=83167 GN=ALO85_00510 PE=4 SV=1
MNRTSVVDEQRFRKLLGRNVGLPLGVGVLSAVFFILLISYLLTAIQWVEHTDRVINNTNR
SMKLSIDMETGMRGFLLTGDQHFLDPYEVAKPLLLAELQGLEKLVDDNPTQLDRFRRLST
LQQEWNAFAQEMITLRRTNGDYQTPVLAGRGKRITDQIRTEYDQAVEMEQQLRLNRNAEV
TRSTILSVTLYLIFVVIVSAILAYIGRRDLLSLSNTYSENLRLQEINSERLQKVAWLRNG
QSELAEQVLGQLTLSMLGRNILQFLTQYLGASVAAVYIRQDHGGLTRAASYGFSREQEQQ
DQTIHSDEGIVGQAAQQDRIITLDELPDNYFKVASGLGDGSPRSVVVVPTSNDDQVNGVI
ELGFLRALTERDIEFLELVADNVGTSIEAARYRQRLQEVLAETQQLNEELQVQQEELRTA
NEELEEQSRILKESQAHLETQQAELEQTNEQLADQSQALAEQRDALDRNNEELNIAQAEL
QARADELQRASKYKSEFLANMSHELRTPLNSSLILAKLLAENPGENLTAEQVKFAESIYS
AGNDLLNLINDILDISKVEAGKLEVRPENSSVPRLVEGLRTLFQPLAAEKKLAFTVDIQT
GAPTMLFTDRQRLEQILKNLLSNAIKFTEAGSVSMVVSRQPGAGIAFIVRDSGIGIAEEH
QQGIFEAFRQADGTTNRRYGGTGLGLSISRDLAALLGGSISVTSAPGQGSIFSLVLPEHY
VEQDDREPGSDLPIVPAPAALQPYAPVLPVIPQPVPAPVPMDTPVPVFADDRDKAPFQRR
CILVIEDEVRFAQILYDLAHELGYDCLVAHAADDGFNLASRYTPDAILLDMRLPDHSGLT
VLQRLKELAPTRHIPVHVISVEDRQEAALHMGAIGYAVKPTTREELKDVFAKLEAKLTQK
VKRILLVEDDALQRDSIARLIGDDDIEITAVGFAQEALDLLRDHVYDCMIIDLKLPDMLG
NELLKRMSMEDICAFPPVIVYTGRNMTRDEEAELMKYSRSIIIKGARSPERLLDEVTLFL
HKVESQLSNERQKMLKTARSRDKVFEARKILVVDDDVRNIFALTSALEHKGAVVEIARNG
LEAIARLNEVEDIDLVLMDVMMPEMDGFEATIEIRKDPRWRKLPIIAVTAKAMKDDQERC
LQAGSNDYLAKPIDLDRLFSLIRVWLPKMERI

>tr|A0A0Q0IHV3|A0A0Q0IHV3_PSEAP Regulatory protein, IclR OS=Pseudomonas syringae pv. aptata OX=83167 GN=ALO85_02185 PE=4 SV=1
MHNDSITPEKITGKDSVPTGTQTLLRGLGVVQAVAGGARDLKEIARLIGTTRSTTHRLAS
CLVEERYLRIVPQVGYLLGPKLIELGFQAREEVPLAMLARPYLDRLSELTGDTVHLAVRE
GDDVLYLHKNPGRNGPEMRSRVGHRMPLVRTGIGKALLLDSAQSEWQRLYEISMPSTGRN
PLWPAHEQQTWEQLKARREEYVEGGYAFDLEDNEPSIRCVAAPVRDASKQIVAGISVAST
VPYMPLEKMAELVPLIKTIAAELSADLGG
>tr|A0A0Q0FJJ7|A0A0Q0FJJ7_PSEAP Putative small protein OS=Pseudomonas syringae pv. aptata OX=83167 GN=ALO85_00042 PE=4 SV=1
MFNDLSRLGKYLGQAARLMVGMPDYDTYVEHMQNTHPDKPVMTYEQFFRERQEARYGGKA
GPKCC
>tr|A0A0Q0FBM6|A0A0Q0FBM6_PSEAP Inositol-1-monophosphatase OS=Pseudomonas syringae pv. aptata OX=83167 GN=ALO85_04118 PE=3 SV=1
MQPMLNIALRAARSASELIFRSIERLDTIKVDEKEAKDYVTEIDRAAEQSIITALRKAYP
THGILGEESGLHEGSGEGTDYLWIIDPLDGTTNFVRGIPHFAVSIACKYRGRLEHAVVLD
PVRQEEFTASRGRGAALNGRRLRVSQRKSLEGALLGTGFPFRDNQMDNIENYLGMFRSLV
GQTAGIRRAGAASLDLAYVAAGRFDAFWESGLSEWDMAAGALLIQEAGGLVSDFTGGHDF
LEKGHIVAGNTKCFKAVLTAIAPHLPASLKR
>tr|A0A0Q0DQY3|A0A0Q0DQY3_PSEAP Peptidyl-prolyl cis-trans isomerase OS=Pseudomonas syringae pv. aptata OX=83167 GN=ALO85_02501 PE=3 SV=1
MPVAIDRGGLISSRLYSFALLAHEQGISMKQHRLAAAIALVGLVLAGCDKQASTVELKTP
AQKASYGIGLNMGKSLAQEGMDDLDSKAVALGIEDAVGKKDQKLKDEELVEAFSALQKRS
EERLAKMSEEASAAGKKFLEENGKKDGVVTTASGLQYQIIKKADGAQPKPTDVVTVHYEG
KLIDGKVFDSSVERGSPIDLPVGGVIPGWVEGLQLMHVGEKIKLFIPSDLAYGAQSPSPA
IPANSVLVFDLELLGIKDPAAAPTPGADADEEEAAPAASAPAKK
>tr|A0A0N8TA50|A0A0N8TA50_PSEAP LysR family transcriptional regulator OS=Pseudomonas syringae pv. aptata OX=83167 GN=ALO85_05382 PE=3 SV=1
MLNYRQLHYFWVVAKTGSIVRACEQLNLTAQTVSGQISLLEASLGVDLFQRVGRQLEMTE
AGRLALPYAEQMFQLGNDLENLLRSQPDEQQLLLRIGVADVVPKSIVYRLIAPTMTLDES
IRITCREDKLERLLADLAIQRLDLVISDSPMPPHLDIKGYSQKLGECGVSFFATREIVER
IGTDFPQCMHGAPLLIPGQETVVRGRLMRWFAEQGIQPRIIGEFDDSALMKAFGKSGSGV
FVAPSVIADEVEDQYGVQVIGHTDAVTESFYAITVERKVKHPGVVAIAESARNQLFTDAG
>tr|A0A0Q0C2A8|A0A0Q0C2A8_PSEAP Uncharacterized protein OS=Pseudomonas syringae pv. aptata OX=83167 GN=ALO85_00609 PE=4 SV=1
MSWFKRAMGLEAPKASGRDSTQPVSTVSPLGLASGRMLCLDSSLKLLLDGHSEVVVPGDE
KVWSVGHIDLGQSMALHRFYLDNEDYFLQVVSDGPNPEDVQDIILFGYYSAEPITSKDEL
LRLTGPSSKIGLPTYEHDGEVFERQWGTSPGQTELTPLDEDIVSPDATYRVKHLSMLYAR
ETGLINRREFLLFSVEEDEEGSITLTTAVGITLQSTDINVL
>tr|A0A0Q0C686|A0A0Q0C686_PSEAP NAD-dependent benzaldehyde dehydrogenase II OS=Pseudomonas syringae pv. aptata OX=83167 GN=ALO85_03796 PE=3 SV=1
MSASDGTPLLQRAIEAECVFNGDWIPSSSPSLPVIEPATGELLMNTAMADAADIAVACRE
AALAQPAWAALGPREKADIFLLAADHAVCAFDELALYVARETGGSLQKGQHEVNETIVLL
RQAAGMLSQAHGHGLILPSQAGRLSYARRVAHGVVGVISPFNFPLVLSLRSVAPALAAGN
AVVLKPDPQTPISGGFLIARLFEEAGLPKGLLHVLPGLVDAGEALCRDTNVQMITFTGST
GAGRKVAEAAGRNLKKVSLELGGKNPLVILEDADLDLAASNAAFGAWLHQGQICMATGLI
LVHESIVADLTRKLADKARALTVGNAARGEAALGPLINKRQLQHVHQVVSDSLQAGARLE
TGGEYEGLFYRPTVLSGVKPGMRAFDEEIFGPVAVVVSFSTDEEAIELANRSEYGLAAAV
ISPDIGRATALGDRLRCGMLHINDQTVADECVNSFGGRGASGNGSSAGSPSDWDEYSQWQ
WVTVKNQAPAYPF
>tr|A0A0Q0C5V4|A0A0Q0C5V4_PSEAP Uncharacterized protein OS=Pseudomonas syringae pv. aptata OX=83167 GN=ALO85_00100 PE=4 SV=1
MLIRIEEGVYGDAWVVILDDYPVKCRSWQEAREFADRMKSRIDAPHSLPHMARRAAAEIS
VQAAAR
>tr|A0A0Q0FB15|A0A0Q0FB15_PSEAP Putrescine-binding periplasmic protein OS=Pseudomonas syringae pv. aptata OX=83167 GN=ALO85_02337 PE=4 SV=1
MELRMKKFGKTLLALSLMGVVATAAQADDKVLHVYNWSDYIAPDTVAKFEKESGIKVVYD
VFDSNETLEAKLLAGKSGYDIVVPSNNFLAKQIKAGVYQELDKSKLPNWKNLNESLLKAV
SVSDPDNKHAFPYMWGSIGIGINPDKVKAALGADAPVNSWDLLFKPENAAKLKSCGISFL
DSPTEMLPIALHYLGYPTDSQDKKQLAEAEALFMKIRPSIGYFHSSKYISDLANGNICVA
VGYSGDIYQAKSRAAEAGGKVKVAYNIPKEGAGSFYDMVAIPKDAENVEGAYKFMTFLMK

PEIMAEITNAVRFPNGNAAATALVDKEITSDPGVYPPADVQAKLYAIADLPAATQRIMTR
SWTKIKSGK
>tr|A0A0N8T9F4|A0A0N8T9F4_PSEAP Putative lipoprotein OS=Pseudomonas syringae pv. aptata OX=83167 GN=ALO85_02382 PE=4 SV=1
MRIVTIVVLLTALSGCTRWAMNSNLDHAYRAYQEGDCDRVMLDLSKVERQSRSRRYVQPE
VSMLRGQCLERQKLYVDAAQTYQFLMAQYPESEYAFRARARLDTLAQTGLYPRAQPARPI
PVPVRNAPVSK
>tr|A0A0Q0CCL8|A0A0Q0CCL8_PSEAP 30S ribosomal protein S5 OS=Pseudomonas syringae pv. aptata OX=83167 GN=ALO85_200015 PE=4 SV=1
MNIDAELKKIKESHFFSNMGVSFSSEKRIICIENVRKVFVEPLNLEFKGCYAEVEWLPTS
PTQDDPFYKLPKPSTKLIELRVKVNKSVMAATKNLGKDPFLCGPHDFSVAARNGVCFAFR
QYISECYYGHGVQWKEVVDIYYKGHWPVGFTKDRLVVI
>tr|A0A0N8T8G6|A0A0N8T8G6_PSEAP Uncharacterized protein OS=Pseudomonas syringae pv. aptata OX=83167 GN=ALO85_100652 PE=4 SV=1
MIVSLTLMVSKSQIKQKAFFTLLSEMTANGRKARSPGLIVATHNLREFQRTDGLRLDDRA
TR
>tr|A0A0N8T9W4|A0A0N8T9W4_PSEAP Uncharacterized protein OS=Pseudomonas syringae pv. aptata OX=83167 GN=ALO85_100924 PE=4 SV=1
MSENTNNSNVAYCSFSLNSESVSTLRCGQASYEAFSGMGAPFVNNPQYQSVQGKGAIPAG
RYFIVDRQSGGRFGWVREPLQDFLAGTDRGEWFGLYRDDGEVDDQTTVDNIRRGAFRLHP
VGPRGLSEGCITLNSQIGFDELKKYLRSQRGEVISGTNLKYYGVIDVVGAGDKNSK
>tr|A0A0Q0FTE7|A0A0Q0FTE7_PSEAP Transcriptional regulator, LysR family OS=Pseudomonas syringae pv. aptata OX=83167 GN=ALO85_03640 PE=4 SV=1
MTITRTAWPCWSEAILRSKSEPQGRVVISCPPALAAMGFNETIARFAAEHPKVQIQVEST
NRRVDPLTENIDIALRVRFPPLEDDGLVVKKLGHSAQRLVARADLAARLSATPSLDALST
LPSLDMPSSNHEHRWLLETQPGQYAEIRHTPRLISSDLDMMLQAALQGVGVAQLPELIVR
EALQDGRLIELFSEHRPKCGIVHAVFASRRGLLPAVRGLLDVFETSFKELSLSPDSLYCS
LNRTNSRIQDH
>tr|A0A0Q0BS30|A0A0Q0BS30_PSEAP Uncharacterized protein OS=Pseudomonas syringae pv. aptata OX=83167 GN=ALO85_01892 PE=4 SV=1
MIRPEKGHSAGKGIIMQNSHAAVSGDNQAVSSTVKLYVWAAVILTIAEMIGAVSIPLGPG
KVVLLPMVWALLLGAMIGLASRRMPGTIGIDHGTQLRSASILQPALLLFIAKLGLVVGGS
LPVVVFASGWALMFQEFGHFVGTVMLGLPVALMLGIKREAIGATFSVGREPSLAIIGERYG
MDSPEGRGVLAEYLTGTLFGALFIAIVAGFIASLGIFHPNSLAMGSGIGSGSMMAAAAGA
IAAQQTPEVAKEVMTLAAASNLITTTIGTYFTLFISLPLAVWGYRVLEPLIGRTTKASMS
EEGLRHSDVSLEVPELGWSGKISAWLAAGALALIANYVGYKTLSGDAFTGMGIMIFCAFV
GEVFCNLLRRKIPAVCMVSLVAMFLTSPACPWAAEIARITSSINLLAVITPMLTFAGLSI
AKDLPAFRRLGWRIVLVSFLANFGTFIGAVLIAELFH
>tr|A0A0Q0FTT3|A0A0Q0FTT3_PSEAP Uncharacterized protein OS=Pseudomonas syringae pv. aptata OX=83167 GN=ALO85_102102 PE=4 SV=1
MVNVCRYQSFNAFAFSGGGKTSTTQLTVLFAPQSTAELGNLISQLKAIDRL
>tr|A0A0Q0BYK3|A0A0Q0BYK3_PSEAP Pyoverdine biosynthesis protein PvdM, putative dipeptidase OS=Pseudomonas syringae pv. aptata OX=83167 GN=ALO85_03820 PE=4 SV=1
MTRPRWKKALFIGLPLALAISAGTGFLAWNYWSPAGYPVKVMQQADDLQERIISFDSHIT
VPMKFGSEDNEADKDGSGQFDLVKAARGRLSGAALTVFGWPEIWNGDNAPHRPTPGFVDE
ARHQQEVRYKIITGMVRDFPNQVGIAYTPDDMRRLHGEGKFAIFISMLNAYPLGSDLSLL
DHWTARGMRMFGFSYVGNNSWADSSRPLPFLNDTPDALGGLSEIGRQAVRRLNDLGVIID
VSQMSSKALQQVSELSRTPMVASHSAPRALVDIPRNLSDEELQLIKRSGGVVQIVAFPAY
LKPLSQKTQDKLNALRKDFDLPPLPNLAMALMPGDPIITVWPEQRFGAYASKLYAILEEE
PKATVKDFVDAIDYTVRKIGIDHVGISSDFNDGGGVKGFNDVSEIRNVTAELIERGYAEA
DITKLWGGNFLRVWEQVQAAARPDVKHMNLSNAATRPSGADAHD
>tr|A0A0Q0DL22|A0A0Q0DL22_PSEAP Azurin OS=Pseudomonas syringae pv. aptata OX=83167 GN=ALO85_03139 PE=4 SV=1
MIRKLLAISLLSLASGQLLAAECSVTVDSTDQMMYDTKAIGIDKSCKEFTVNLTHSGSLP
KNVMGHNWVLGKEADAQAITTDGMSVGIDKDYVKPDDTRVIAHTKVIGAGEKDSVTFDVS
KLDPAEKYQFFCTFPGHISMMKGAVTLK
>tr|A0A0Q0C5N5|A0A0Q0C5N5_PSEAP Outer membrane protein assembly factor BamE OS=Pseudomonas syringae pv. aptata OX=83167 GN=bamE PE=3 SV=1
MQNTKLLLTSFTFVGLLALAGCSFPGVYKIDIQQGNVVTQDMIDQLRPGMTRRQVRFIMG

NPLLTDTFHSDRWDYLYSLQPGGGERQQERVSLIFNGNDQLVSLSGDFMPGVSRDEAILG
KGSDTTAPAENQPVQQPEEPAKPGSLLDTIQKDVDSVETVPVPTQEPLDTNTR
>tr|A0A0Q0CX45|A0A0Q0CX45_PSEAP HNOB domain protein OS=Pseudomonas syringae pv. aptata OX=83167 GN=ALO85_00493 PE=4 SV=1
MTAMAKIAPQLPIEVDSETGVWTSDALPMLYVPRHFFVNNHRGIEEVLGADAYADILYKA
GYKSAWHWCEKEAECHGLEGVAVFEHYMKRLSQRGWGLFKIQDIDIEKGTASVKLEHSCF
VYVYGKVGRKVDYMFTGWFAGAMDQILAASGSAVRTVAEQVYGGSEEGHDDGLFVVKPL
>tr|A0A0N8TA03|A0A0N8TA03_PSEAP Uncharacterized protein OS=Pseudomonas syringae pv. aptata OX=83167 GN=ALO85_00620 PE=4 SV=1
MLSVGGDSRYPRRMTLFPGLIDLPRQLRHPEVRDLAWVILAPPMLETAPWPQRHPLAGSD
WVKAPQQLTDFLQQLDRDSRPLEDWLAQASTRRLGRYYERLWQFAVQHAPGVEIIAANLP
IRLGNQTLGELDMLLRDGDGVHHVELAIKLYLGPQDGDGRDPAHWLGPGCQDRLDRKLTH
LSQHQLPMSARPESRATLAALGVEAFSARLWLGGYLLYPWPGSCQSPAGVHPQHLKGRWL
HQRDWKSFQAQSHPGRWQPLPRHAWLAPALYNQAWSDQQLHEWLSELDPLAPAQLLVRLR
ENAEGHWEEAERLFLVSDMWPNLAQGV
>tr|A0A0Q0DMB7|A0A0Q0DMB7_PSEAP GDP-mannose 4,6-dehydratase OS=Pseudomonas syringae pv. aptata OX=83167 GN=gmd PE=3 SV=1
MNKLGHPIFDGVKKSSMKAIITGITGQDGAYLAELLLEKGYTVYGTYRRTSSVNFWRIEE
LGIQNNPNLHLVEYDLTDLSASIRLLQTTGATEVYNLAAQSFVGVSFEQPLTTAEITGIG
AVNLLEAIRIVNTKIRFYQASTSEMFGKVQAIPQVESTPFYPRSPYGVAKLYAHWMTINY
RESYDIFATSGILFNHESPLRGREFVTRKITDSVAKIKLGLIESFELGNMDAKRDWGFAK
EYVEGMWRMLQADEPDTFVLATNRTETVRDFVSMAFKATGVTIKWEGEAESEKGICADTG
KVLVSVNPKFYRPTEVELLIGNPAKAKEVLGWEPKTNLEELCRMMVEADLRRNEKGFSF
>tr|A0A0Q0DBE4|A0A0Q0DBE4_PSEAP Uncharacterized protein OS=Pseudomonas syringae pv. aptata OX=83167 GN=ALO85_102257 PE=4 SV=1
MQSFTHVQCDIPPGFVRSSRSERHHGNSVRYLQHFDRHLTHCGLEHRELNQSSE
>tr|A0A0N8T9U5|A0A0N8T9U5_PSEAP Phage integrase OS=Pseudomonas syringae pv. aptata OX=83167 GN=ALO85_05468 PE=4 SV=1
MRDLRAKTGTDKAESSGDILQARDQLGHTTVAMTENYIRKRIGKKVTPTK
>tr|A0A0Q0FJZ7|A0A0Q0FJZ7_PSEAP Heme exporter protein C OS=Pseudomonas syringae pv. aptata OX=83167 GN=ccmC PE=3 SV=1
MKSNAMKSSIGLAWLHTLGSPKGFYRVSARLLPWLSVAAVLLLVIGMVWGLAFAPPDYQQ
GNSFRIIYIHVPAAMLAQSCYVMLAVCGVVGLVWRIKLADVALHCAAPIGAWMTALALAT
GAIWGKPTWGAWWVWDARLTSMLILLFLYFGLIALGNAISNRDSAAKACAVLAVVGVVNI
PIIKYSVEWWNTLHQGSTFSLTEKPAMPAEMWLPLLFTTLGFYCLFGVLLMMRMRLEVLR
REARTQWAKAEILRSLGHTTAQSENTL
>tr|A0A0Q0IF63|A0A0Q0IF63_PSEAP Uncharacterized protein OS=Pseudomonas syringae pv. aptata OX=83167 GN=ALO85_102095 PE=4 SV=1
MRSGLNDGRHEAPETWLKRAPDAHRARRIAKLAGALIRS
>tr|A0A0Q0DBM7|A0A0Q0DBM7_PSEAP Uncharacterized protein OS=Pseudomonas syringae pv. aptata OX=83167 GN=ALO85_04144 PE=4 SV=1
MSNGLIDLEDEMYRLYTVFYPNGKAARIGFDALPSHTISLISRFPEATAHLLASSAYRLT
RRVFSQPFTIEQHTPRSMLRLRPARSHTYTYTSHQDLALAIQTTITSNTNLQILDKLAQL
TFRTINQPSLLGDLDDLRTLTEFVALSVHEHTRSHVQTPSPNVFRYFFCKQ
>tr|A0A0Q0DS40|A0A0Q0DS40_PSEAP Conjugal transfer protein OS=Pseudomonas syringae pv. aptata OX=83167 GN=ALO85_200144 PE=4 SV=1
MEQSKESKPLLFLGFPLPIALVVVFVIAAWGIYIALAKLDEISASLRQIQANQQVAIALQ
QSLQVQSNEAQTIERQAGVR
>tr|A0A0N8T8G5|A0A0N8T8G5_PSEAP Ferric siderophore uptake system protein, ExbD/TolR family OS=Pseudomonas syringae pv. aptata OX=83167 GN=ALO85_01101 PE=3 SV=1
MRTWDEPKKRKAHIELIPMIDVMMFLLVFFVLVSLNVIPALGMKTQLPSASSSQQLKPQN
KSILTLGLNGELQLDGKATTVDALVPALKALEKPDSKTTIIVNSDKGVEVARLVEIMDTL
RLGGFTSVSIATRKS
>tr|A0A0Q0C747|A0A0Q0C747_PSEAP Histidine kinase, HAMP region: chemotaxis sensory transducer OS=Pseudomonas syringae pv. aptata OX=83167 GN=ALO85_01038 PE=4 SV=1
MSSATSGVLSNMTVRAKLLVGFGLLILMILLMAYTGKDATDTLKRRADLSGDIAQFSSIA
RDMRIERLVYFLKADDAQASKWLEALERTERQLVSISPRFKTPNNIALLKEAQTTMQLYR
GFYTRSVEATREREQLRTLAGASAETINGLLLKIADAANDESGNAADRQKLPALFISVQK

MRTAFRSYTASPSKSGEDTVRQAIAQVVASIDTLKNTRLPSADVQALAAGMATYSGQLET
LVAAQAKVDEAQGGITTSIATILGITDKMTAIQNELRASDAEKAQEKILLWLALSALLGV
LAAWLITRSIVHPLKETVEIVEVVAEGDFTYRTEVTRNDELGALQGSMLRMTSGLRSLIG
EMKDGIVQVASAAEQLSAVTEQTSAGVNAQKLETEQIATAMQQMTATTHEVSRNAAQAVN
TAQIASQLAQKGGQVVDRTRTQIETLAREMNMTRTAMAALRGNTQSIGGVLDVIKTVADQ
TNLLALNAAIEAARAGEAGRGFAVVADEVRGLAVRTRTSTDEIALLINELQTSTDHMGQV
LEQNLALTDSSVELSTQASEVLQSITASVHEIEVMNEQIAVATEQQTNVGEEIGRGVTNV
RDISDQTASASEETATSSVELARVSARLQEMTNRFKV
>tr|A0A0Q0CX17|A0A0Q0CX17_PSEAP Alkanesulfonate monooxygenase
OS=Pseudomonas syringae pv. aptata OX=83167 GN=ALO85_100108 PE=4 SV=1
MAGVFMSIEFIGYIATQPGSEIHPRSGAAIQPDYVKTVAKAHEDAGFDRALIAYHSNSPD
STLVAAHAASVTTKLKFLVAHRPGFIAPTVAARQFATLDVFNGGRTAVHIITGGDDKELR
ADGSHIGKDDRYARSDEYLSVVRQEWTSDKPFDHNGTYYQLEGAHSLVKSPQQPHIPLYF
GGSSAAAIAVAGKHADVYALWGETYEQVRDVVKQVRAEAAKHGRTIRFSLSLRPILAETE
EQAWARADSILEKAKALADRNGFVRREPPNEGSRRLLAAAAQGSRLDKRLWTGIAGLLGA
QGNSTSLVGTPEQVAEALLDYYDLGITTFLIRGFDPLEDAIDYGKKLIPLTRALVAQREQ
QAREQVA
>tr|A0A0Q0CHG2|A0A0Q0CHG2_PSEAP Putative alcohol dehydrogenase
OS=Pseudomonas syringae pv. aptata OX=83167 GN=ALO85_03379 PE=4 SV=1
MKAWLLKDFGLENLVLGDAETPVPKAGELLIKVGAVSLNFRDKAIVDGIYEPHHVPKPLI
PVSDAAGTVVAVGEGVSRFAVGDRVNSHLYSRWIDGPPGPDEPDYCFGSPLPGGLAAYMI
IHEQSAVRAPDAMSDEEAATLPIAALTAWYALVDFGQVEAGQTVLVQGSGGVSIFAAQIA
TALGANVIATSSKDENLHAMKALGTVAGVNYRTHPEWADEVLKLTDGKGVDLLLDVAGGD
GINQSIAATKAGGRIAQIGFLTGQTSALNLMPMIFRQTTFRGIAVAPRSSFERMNAFLNE
HKIRPVIDHVYPFEQAREAYQHLARGAFGKVVIKVDQA
>tr|A0A0Q0FSE9|A0A0Q0FSE9_PSEAP Urocanate hydratase OS=Pseudomonas
syringae pv. aptata OX=83167 GN=hutU PE=3 SV=1
MIAEDFFVTENNQDLKQDWTRHREGVVRAARGTQLTAKSWLTEAPLRMLMNNLDPEVAEN
PNELVVYGGIGRAARNWECYDKIVESLTQLNDDETLLVQSGKPVGVFKTHSNAPRVLIAN
SNLVPHWASWEHFNELDAKGLAMYGQMTAGSWIYIGSQGIVQGTYETFVEAGRQHYDGNL
KGRWVLTAGLGGMGGAQPLAATLAGACSLNIECQQSRIDFRIKTRYVDEQAADLDDALAR
IAKYTAEGKAISIALCGNAADILPEMVRRGVRPDMVTDQTSAHDPLNGYLPKGWTWDEYR
ARSVSEPASVVKAAKQSMAEHVEAMLAFQQAGIPTFDYGNNIRQMAKEVGVTNAFDFPGF
VPAYIRPLFCRGIGPFRWAALSGDPQDIYKTDAKVKELIPDDEHLHNWLDMARERISFQG
LPARICWVGLGQRAKLGLAFNEMVRSGELSAPVVIGRDHLDSGSVASPNRETESMRDGSD
AVSDWPLLNALLNTASGATWVSLHHGGGVGMGFSQHSGMVIVCDGTDEAAERIARVLHND
PATGVMRHADAGYEIAIDCAKEQGLNLPMIGR
>tr|A0A0N8TAB2|A0A0N8TAB2_PSEAP HTH OST-type domain-containing protein
OS=Pseudomonas syringae pv. aptata OX=83167 GN=ALO85_01510 PE=4 SV=1
MDEIIPLPSDPLPDLQRTVQRKLGRCILQSQQYERLLKAMVAHGELSGPPERLQAIRDEK
VACAHKKTLGTLVGMLTESYLKLPDLSDEPEQAEPIDRVWVSFRCQMELTEERYAETKAA
LKELVDLRNELVHHFLQRFDLWGVDGCIAAESYLDESYETIDGHYLTLRDWAKSMDEARQ
LMVSFMQTQEYRDAVINGIWLDGIVHWPSSGITACLREAETKLAHAGWTPLFEAIRWIAK
TYPELTPKRYGCGSWRHVIHESQQFEIRKQSQADNGPTVVWYRSRPRETSKELE
>tr|A0A0Q0BU86|A0A0Q0BU86_PSEAP Putative membrane protein OS=Pseudomonas
syringae pv. aptata OX=83167 GN=ALO85_00547 PE=4 SV=1
MTLGTESAPPEGTPGASPRRLGAAFLGLLHTHVELFGIELQEQKARTVSLLLFAGLALVF
GLLLLTGLSALLLIVLWDSYRLAGIVGLCTFYLLAAIFCGLRLKAAIFDESSPFHATLEE
LANDRERLMP
>tr|A0A0Q0CEX4|A0A0Q0CEX4_PSEAP Pyruvate dehydrogenase E1 component
OS=Pseudomonas syringae pv. aptata OX=83167 GN=ALO85_05246 PE=4 SV=1
MSRFSVVLLLGPILGGLKSSSICSKTTRRRIPPSGNPRIYVVCPQGQSQTQATDSGSLSA
LEQAMQDLDPVETQEWLDALESVLDKEGEDRAHYLMTRLGELATRSGSQLPYAITTPYRN
TIPVTHEARMPGDLFMERRIRSLVRWNALAMVVKTNIGDPDLGGHISSFASSATLYDIGF
NYFFQAPTDEHGGDLIYFQGHASPGVYARAFMEGRITEEQMTNFRQEVDGNGLSSYPHPW
LMKDFWQFPTVSMGLGPIQAIYQARFMKYLEHRGYIPEGKQKVWCFLGDGETDEPESLGA
IALAGREKLDNLIFVVNCNLQRLDGPVRGNAKIIQELEGVFRGAQWNVTKVIWGRFWDPL
LAKDVDGILQRRMDEVIDGEYQNYKAKDGAFVREHFFNSPELKAMVADLSDDEIWKLNRG
GHDPYKVYAAYHEAVNHKGQPTVVLAKTIKGYGTGAGEAKNTAHNTKKVDVDSLRHFRDR
FDIPVKDSELEALPFYKPEEGSAEARYLSERRAALGGFVPQRRAQSFSLPTPPLETLKAI

LDGSGDREISTTMAFVRILTQLVKDKEIGQRIVPIIPDEARTFGMEGMFRQLGIYSSVGQ
LYEPVDKEQVMFYREDKKGQILEEGINEAGAMSSFIAAGTSYSSHNQPMIPFYIFYSMFG
FQRIGDLAWAAGDSRTRGFLVGGTAGRTTLNGEGLQHEDGHSHILASTIPNCRTFDPTYG
YELAVIIQDGMRRMFEEQQDVFYYLTVMNESYAQPAMPAGVEEGIVKGMYLLEEDTKEAA
HHVQLLGSGTILREVREAAKILRDEFNIGADVWSVTSFNELRRDGLAAERNNRLHPGQKP
QLSYVEECLNGRKGPVIASTDYMKLFADQIRQWVPTKEYKVLGTDGFGRSDSRKKLRHFF
EVDRHFVVLAALEALADRGDIEPKVVAEAIAKFGIDPEKRNPLDC
>tr|A0A0Q0CYV1|A0A0Q0CYV1_PSEAP Ribosomal protein L11 methyltransferase
OS=Pseudomonas syringae pv. aptata OX=83167 GN=prmA PE=3 SV=1
MPWLQVRLAISPEQAETYEDALLEVGAVSVTFMDAEDQPIFEPELNTTPLWAHTHLLALF
EADTNAEMALAHLSLLTGEELPEHSAEVIEDQDWERIWMDNFQPMCFGKRLWIVPSWHAA
PQPDAVNLLLDPGLAFGTGTHPTTALCLEWLDGQDLQGCNVLDFGCGSGILAIAALLLGA
EQAVGTDIDVQALEASRDNAGRNNIAAERFPLYLPEDLPHQQADVLVANILAGPLVSLAP
QLATLIRAGGRLALSGILAEQGEEVAAAYADSFDLDPIANRDGWVRITGRRR
>tr|A0A0Q0BTI6|A0A0Q0BTI6_PSEAP Transketolase, C-terminal subunit
OS=Pseudomonas syringae pv. aptata OX=83167 GN=ALO85_04216 PE=4 SV=1
MSSANHTPAPSGTTKKKLTTSAMIASIASEGQATRPAPFGHALAALAEQRQDIVGLSADL
SKYTDLHIFAKAHPERFYQMGMAEQLLMSAAAGMAREGFVPFATTYAVFASRRAYDFICM
AIAEDNLNVKIVCGLPGLTTGYGPSHQATDDLAIFRAMPNLMIVDPCDALEIEQAVPAIA
AHQGPVYMRLLRGNVPLVLDEYGYTFEIGKAKTLRTGNDVLIIATGLMTMRALEAAKQLQ
ADGIDVAVLHVPTIKPLDEQTILAEARKPGRLVVTAENHSVIGGLGEAVATVLLRNGVTP
TFRQIALPDAFLDAGALPTLHDRYGISTPAVCTQIKAWL
>tr|A0A0Q0CE67|A0A0Q0CE67_PSEAP 30S ribosomal protein S2 OS=Pseudomonas
syringae pv. aptata OX=83167 GN=rpsB PE=3 SV=1
MSQVNMRDMLKAGCHFGHQTRYWNPKMGKYIFGARNKIHIINLEKTLPMFNEALTFVERL
ASGKNKILFVGTKRSAGKIVAEEAARCGSPYVDHRWLGGMLTNFKTIRQSIKRLRELEVQ
AEDGTFAKLTKKEALMRTRDLEKLDRSLGGIKDMGGLPDALFVIDVDHERIAITEANKLG
IPVIGVVDTNSSPEGVDYIIPGNDDAIRAIQLYMGSMADAVIRGRNNVAGGTDVFVEEAP
AAAAVEG
>tr|A0A0Q0DCQ8|A0A0Q0DCQ8_PSEAP Nucleoid protein HU beta subunit
OS=Pseudomonas syringae pv. aptata OX=83167 GN=ALO85_00078 PE=3 SV=1
MNKSELIDAIAASADIPKAAAGRALDAVIESVTGALKAGDSVVLVGFGTFSVTDRPARIG
RNPQTGKTLEIAAAKKPGFKAGKALKEAVN
>tr|A0A0Q0C0J1|A0A0Q0C0J1_PSEAP Uncharacterized protein OS=Pseudomonas
syringae pv. aptata OX=83167 GN=ALO85_00934 PE=4 SV=1
MSKHPKVTKPGRSVDTWAILFIVILAVGAAMFWASSR
>tr|A0A0Q0BTN0|A0A0Q0BTN0_PSEAP Protease La-like domain protein
OS=Pseudomonas syringae pv. aptata OX=83167 GN=ALO85_03089 PE=4 SV=1
MTLPLFPLNAVLFPGCVLDLQLFEARYLDMIGRCMKQGEGFGVVCITEGSEVGSVPGGYS
MIGCEALVTDFQQQENGLLGIRVVGGRRFRVVAAEVQRDQLLVAEVEWLEEPVERPLQEE
DADLVALLEALAEHPMVASLNMGVSAGGQYALSNQLAYLLPFTEKDKVELLEIDDPEERL
DAIQELLDEMQGDLQA
>tr|A0A0Q0DL08|A0A0Q0DL08_PSEAP Uncharacterized protein OS=Pseudomonas
syringae pv. aptata OX=83167 GN=ALO85_03106 PE=4 SV=1
MIVMEFDVQLSLEQMLEYEAFIYAENDGVPVHIVTDHGEHRADLRRIVNVVESVVKYGKP
LFLPRLSTVKKSARCAALEAAVLSVIGIDPGLVKSVFPHHQLNPVVNAFFTVEKGVVDQL
STRRYLDLSKDDNAILHEFVENLLAEVKGESVQMKLNGFKRAANKAYKGLRDLIDALFVR
YSRLLVIRLDLGYRNKYCAPAGLLSVCYEEAKKHREDLLKYFRLKMSPAIVGYAWKLEYG
LDKTYHYHFLLFFDGAKSHQDIVIAKAVGDHWSNCVTDGKGIYYNCNANKAEYKARGILG
IGMIGHDELDLRNGLTTAAAYLTKVDLFVRMVISEGGRCFGKSGKPKPKKVSGGRPRRNS
PKCRVQSESLT
>tr|A0A0Q0ITP7|A0A0Q0ITP7_PSEAP Iron-siderophore/hemincobalamin ABC-type
transport system OS=Pseudomonas syringae pv. aptata OX=83167
GN=ALO85_01476 PE=4 SV=1
MMLALESLAWAPEQTSVCTHEHFALKDIDLQIAAGEFVGLIGPNGSGKTSLLRCAFRYSR
PHSGKVAINARDIWSCSPRWCARHVAVVAQEFPDAFGLTLLDVANMGRAPHQGFFAADSD
HDRAIVQNALNAVGLAGYEQHRFDTLSGGEKQRCLLARALVQEPRLMVLDEPTNHLDPRH
QLELLGLVKQLGMATLASIHDLNLAAAFCDRLCVIHHGRLIAQGTPREVLTPEMLLNVFG
AKALVDSHPLRDYPRITWIP

>tr|A0A0Q0DPX1|A0A0Q0DPX1_PSEAP Short chain dehydrogenase/reductase family oxidoreductase OS=Pseudomonas syringae pv. aptata OX=83167 GN=ALO85_00418 PE=3 SV=1
MENCMTRYALITGASSGIGLAMAEALARRGRNLILVARQRDLLETIALELTQRFGVEVLF
RACDLGEPLRLSGFLLELEDGAHQIDLLVNAAGIGTCGPFLAQDWSAEQDLIDLNILALT
RLCHTVGNLMAIQGGGQILNIASVAAFQPGPWMSTYYASKAYVLHFSEGLREEVKKAGVK
VSVLCPGPTRTAFFRTAQLQVNVDSLMSPEEVALYTVRALAKNRAVITPGFSNRLWTLSP
RLVSRWLARQISGYINKKYCPR
>tr|Q8G8R2|Q8G8R2_PSEAP RNA polymerase sigma factor RpoS OS=Pseudomonas syringae pv. aptata OX=83167 GN=rpoS PE=3 SV=1
MALGNEAPEFDIDDEVLLMEAGIVLKEASNEKQPAAVSGRTKVKHSTSLKQHKYIDYTRA
LDATQLYLNEIGFSPLLSPEEEVHFARLSQSGDPAGRKRMIESNLRLVVKIARRYVNRGL
SLLDLIEEGNLGLIRAVEKFDPERGFRFSTYATWWIRQTIERAIMNQTRTIRLPIHVVKE
LNVYLRAARELTQKLDHEPSPEEIANLLEKPVGEVKRMLGLNERVSSVDVSLGPDSDKTL
LDTLTDDRPTDPCELLQDDDLSQSIDQWLSELTEKQREVVIRRFGLRGHESSTLEDVGLE
IGLTRERVRQIQVEGLKRLREILEKNGLSSESLFQ
>tr|A0A0Q0D6I1|A0A0Q0D6I1_PSEAP Putative immunity protein OS=Pseudomonas syringae pv. aptata OX=83167 GN=ALO85_100718 PE=4 SV=1
MEDWANYDWEEGPDEIRALVKKYLARDYTNPLAESQIKGIKFDLLKCLDMYHSKELDALT
KKVVTDPNHTYMKNIKKP
>tr|A0A0Q0CXR9|A0A0Q0CXR9_PSEAP Uncharacterized protein OS=Pseudomonas syringae pv. aptata OX=83167 GN=ALO85_03124 PE=4 SV=1
MSTPNTPHTPVHDRFDEVLAMIRSARQQAAQAVNTQLIELYWQVGAYISRKLENAEWGDA
VVTQLAEHLATTQPGLRGFTRRNLFRMRQFFEVYRDDEVVSAVLTQLPWTHHLIIFSQSK
RPEEREFYLRMAIREKWSSRELERQFKNALFERTVTQPAKASAMLKETRPAALDIFRDAY
MVEFLELPPGHAEADLHGGLLKRLRDFLIELGRDFCFVGSEYALQVGGQDFALDLLFFHR
GLNCLVAIELKVGRFEPEYLGKLDFYLEALDRNERKPHENPAIGVLLCASKNDEVVEYAL
NRSLSPALIAEYQTRLPDRQLLQAKLHEFYASAMLNDQ
>tr|A0A0Q0DSK3|A0A0Q0DSK3_PSEAP Flavin reductase-like protein OS=Pseudomonas syringae pv. aptata OX=83167 GN=ALO85_03416 PE=4 SV=1
MDNHIRPVELEKAYRLLNHGPTVLVSSSHEGTHNVMAAAWACALDFSPPKVTLVIDKMTK
TRELVEKSGYFALQVPNLDQLQMTFDVGTQSKVFTPDKLDKANVDLFRIDGHDSPLVAGC
SAWLICQVIPEPHNQQAYDLFIGEVVGAWADTRVFKDGHWEFEKADPKWRSLHYVAGGHF
YTIGEPAVVETDDE
>tr|A0A0Q0DJQ0|A0A0Q0DJQ0_PSEAP FAD dependent oxidoreductase OS=Pseudomonas syringae pv. aptata OX=83167 GN=ALO85_03909 PE=4 SV=1
MSDKGNDMRSESYWLDTAPDFTGAQDGAVEGQADVVVVGGGFTGLAAARALALKGASVVV
LEAGRVIGEASGRNGGQCNTGVAQDYASLSATLGANQAREYYKAYESAVQSVVTVIDEEH
IACDVKRNGKLKLAAKPGHYEGLARTCELIRREVDADVELLSAQDVRAEIDSNEFHGGLL
QRNGIQMHVGRFGLGLADAAVRHGARVYQNATVKDWKANRGGFVVNTSKGSIQAGQLMLA
TGACQHGGLGWYRRRIVPVGSFVIVTEVLPQALIDQLFPHQRAYVTSRLIGNYFRLTPDN
RLLFGGRARFAMSNSSSDAKSGKVLQAAMVQMFPQLAHVKVDYCWGGLVDMTSDRLPRAG
EHNGVFYSMGYSGHGVQMSVHMGRMMAEVMEGKAQANPWKDLSWPAIPGHFGKPWFLPLV
GAYYRYQDSRH
>tr|A0A0Q0BSJ9|A0A0Q0BSJ9_PSEAP Trigger factor OS=Pseudomonas syringae pv. aptata OX=83167 GN=tig PE=3 SV=1
MHALDSGVGNGRLTSVQRGISMQVSVENTSALERRMTIGVPAERIETEVNKRLQQTARKA
KIPGFRPGKVPMSVIRQRYEDGARQEALGDLIQATFYEAVVEQKLNPAGAPAVEPKSFEK
GKDLEYVATFEVFPEFTVAGFDSISVERLSADVADSDLDNMLEVLRKQNVRFEVADRAAQ
NEDQLNIDFVGKVDGEVFAGGSATGTQLVLGSGRMIPGFEDGLVGAKAGEERVLNVTFPE
DYQNLELAGKAAEFTVTVNTVSEPKLPELNEEFFKQFGIKETGIEGFRTEVRKNMERELR
QAIKSKVKNQVMDGLLAANPIEVPKALLENEVNRLRVQAVQQFGGNIKPDQLPAELFEEQ
AKRRVELGLIVAEVVKQFDLKPDDARVREMIQEMASAYQEPEQVVAWYYKSEQQMNEVRS
VVLEEQVVDTVLQKASVTDKSVSYEEAVKPVEAPKAD
>tr|A0A0Q0IMG9|A0A0Q0IMG9_PSEAP Uncharacterized protein OS=Pseudomonas syringae pv. aptata OX=83167 GN=ALO85_00950 PE=4 SV=1
MNKANITRGAKIVGLGLVALLLLVVISVALLLGTHMGSQWALARVPGVQLENFQGRLGGQ
WSAERLVWQKGADRVEVQSIDFAWTPACLLKMTLCIDRLHAQQVLMHFPPSDTPASDGPI
QLPTLRLPLALQLGDIQLGGVQLDGVEQLRDLKLAAHWTAEGLKIDSAHLQRDALVLDLN
GLLKPEGDWPLSVQGQLQLPPQDGQPWTLALDIQGDLLKTLQLKADSSGYLPGLLTGELQ

PLAEHLPARIKLTAEHFKPSAALPDTLQLDQLLLTAEGNLDSGYLIDGSASLPAEKGPVA
LALKGRVDANGATIAGLDLAADDQQRLAINGKMNWQSGLSAEASIDWLDFPWQRLYPLAS
EPQVALHAFKGDISYTDGHYLGNFNASLKGPAGAFTLVSPFSGNLQEIHLPQLELVAGQG
KASGFLNLQFADGIGWDTALDLSALDPSFWVAELPGTLAGPLRSKGQMRNEKLDLSADLD
LKGRLRGQPALLQAKADGRDQQWNVSSLSIRLGDNRIQGTGSLQERLKGQLDLDLPRLAQ
LWPRLQGQVKGRLDLAGTLQAPQGQLALQGSQLVLQDNRLQTLTLNARLDQAQRAIINLK
GAGIQAGDTQLGTLLVDGQGTLKNQQLKLDLQGPLLKLSIGLDGGLDKGDWRGRLASGTV
QSGGQNWRLQQPAKIERLADGRLNFGAHCWLSGVASLCGGDQRLMPEPHLRYQLKNFPLD
SLAQWMPKDFAWKGALNADVQLDIPAAGPSGKVTIDAGGGTLRVRNNNQWLDFPYQTLKL
ATTLAPKRIDTRLDFDGGKLGRLLLNAQIDPLAKDKTLAGDFNLSGLDISVARPFAPMVQ
KLNGRLNGNGRLSGTLLAPLVNGNVTLEGGEVAGAELPMELHDLKVQALIAGESVQLNGG
WKSGSTGQGSLGGQVAWGQSLNVDVSLKGSRLPINVEPYAAVEAAPDLKISMQGERLAIS
GKVLLPKGAITIRELPPSTVKLSGDTVIVGQQTEQGAPPLAVVMDIDVDVGQEKLSFSGF
GLTANLVGHVHIGDNLDTRGELNLNDGRYRAYGQRLTIRKARLLFAGPVDQPYLDIEAIR
QTDDVIAGIRLTGSAEQPTTEVFSEPAMSQEQALSYLVMGRPLSTSGEDNNMVAQAALAL
GVAGSSSTTGKLANNLGIKDFELDTSGTGDKTNVVASGKITDKLSLRYGVGVFEPANTIA
LRYLLSKRVYLEAASGVASSLDIFYKRDF
>tr|A0A0Q0IAS9|A0A0Q0IAS9_PSEAP Type III secretion system protein HrcR OS=Pseudomonas syringae pv. aptata OX=83167 GN=ALO85_04188 PE=3 SV=1
MIMEGVNPIMLALFLGSLSLIPFLLIVCTAFLKIAMTLLITRNAIGVQQVPPNMALYGIA
LAATMFVMAPVAHDIQQRVHEHPLELSNADKLQSSLKVVIEPLQRFMTRNTDPDVVAHLL
ENTQRMWPKEMADQANKNDLLLAIPAFVLSELQAGFEIGFLIYIPFIVIDLIVSNLLLAL
GMQMVSPMTLSLPLKLLLFVLVSGWSRLLDSLFYSYM
>tr|A0A0Q0DCJ2|A0A0Q0DCJ2_PSEAP Protein HflC OS=Pseudomonas syringae pv. aptata OX=83167 GN=ALO85_01458 PE=3 SV=1
MSLFHHHHDHADHDSHDSHGHGGHGHHHHHHGASGPAAFPWRRASLAALLIAFAIAAASL
VQVRSGEATVVTRFGNPSRVLLEPGLNWRWPAPFEATIPVDLRLRTTSSGLQDVGTRDGL
RIIVQAYVAWQVQGDADNVQRFMRAVQNQPDEAARQIRTFVGSALETTASSFDLSSLVNT
DASKVNITAFENQLRQQIDQQLLATYGVRVLQVGVERLTLPSVTLNATVDRMRAERETIA
TERTAVGKREAAQIRSAAERDARIVEADATVKAADIEAQSRVEAAQIYGRAYAGSPQLYN
LLRSLDTLGTIVTPGTRLILRTDAAPFRVLVDGPPTLDGKSGTQP
>tr|A0A0Q0C2W5|A0A0Q0C2W5_PSEAP TerC family integral membrane protein OS=Pseudomonas syringae pv. aptata OX=83167 GN=ALO85_03445 PE=4 SV=1
MEYLLLELATSPAAWIALATLVVMEVVLGIDNLIFISILTNKLPEHQREKARKLGIGMALV
MRLGLLSTVAYIVQLTEPVFEVFGQAFSWKDMILIAGGLFLVWKATTEIHHSMDVKTEEE
KALGSVVMLSMSAAIVQILMLDLVFSIDSIITAVGMTEHLPIMVIAVISAVVVMLVAANP
LAKFINDNPTVVMLALGFLIMIGMTLIAEGFGAHVPKGYIYAAMAFSAAVEALNMLVRRA
RRKKAVNQVSTH
>tr|A0A0Q0CAY4|A0A0Q0CAY4_PSEAP cAMP-regulatory protein OS=Pseudomonas syringae pv. aptata OX=83167 GN=ALO85_00423 PE=4 SV=1
MVAITFTPKANSLDKLLPHAERRRLPAKTNIINAGDRSASLFLILKGSVTVLIEDDERRE
MIVAYLNSGDFFGELGLFEQPGKESVRSAWVRAKTECEVAEISYDTFRELTRQEPEILYA
LCSQMAERLRNTTRKVGDLAFLDVTGRVARTLLELCKQPDAMTHPDGMQIKITRQEIGRI
VGCSREMVGRVLKALEEQSLVNVKGKTMVVYGTR
>tr|A0A0Q0DA60|A0A0Q0DA60_PSEAP Glutaredoxin OS=Pseudomonas syringae pv. aptata OX=83167 GN=ALO85_02049 PE=3 SV=1
MDIIETIKEQIANNTILLYMKGAPNAPQCGFSAKASQALMACGEKFAYVDILQNPEIRAN
LPKYANWPTFPQLWVAGELVGGSDIITEMMADGSLQTLVKEASAAKAE
>tr|A0A0Q0E057|A0A0Q0E057_PSEAP 50S ribosomal protein L28 OS=Pseudomonas syringae pv. aptata OX=83167 GN=rpmB PE=3 SV=1
MSRVCQVTGKGPVTGNNISHANNKTRRRFLPNLQHHRFWVEGEKRFVRLRVSAKGMRIID
KRGIEVVLAELRRDGKI
>tr|A0A0N8T863|A0A0N8T863_PSEAP Two-component DNA-binding response regulator OS=Pseudomonas syringae pv. aptata OX=83167 GN=ALO85_02989 PE=4 SV=1
MIRVLVAEDHTIVREGIKQLIGLARDLQVVGEASNGEQLLETLRHVACEVVLLDISMPGV
NGLEAIPRILALTNPPAILVLSMHNEAQMAARALKVGAAGYATKDSDPALLLTAIRRVAA
GGRYIDPDLADRMVFEVGLTDSRPLHSLLSEREFSVFERLAQGANVNDIAQQLALSSKTI
STHKARLMQKMKLNSLADLVKYAMEHKLL

>tr|A0A0Q0DQR7|A0A0Q0DQR7_PSEAP Glutathionylspermidine synthase OS=Pseudomonas syringae pv. aptata OX=83167 GN=ALO85_00606 PE=4 SV=1
MKKISIEERPDWRTTAEREGFNFHTIDGERYWDERGYYLFSEQQITRDLEAPTQELHQMC
MDAVARVVDSEALLHQLAIPEAFFDLIQTSWKEGHPHLYGRFDFAYDGTGPAKMYEINAD
TPTSLMEAGAVQLLWLEEQMARGVLPAHATQFNSIAEDLVRAFAEFPGTTPFYFSAMSGS
IEDRGTTDFLRRMAAHVGIDARHIDIEDIGLGADGRFVDLDGRWIERLFKLHAWEHIFHE
EFGQHVSAAGTQFVEPAWKAILSNKGILPLLWQFNEGHPNLLPAHVDQDPNKAVPKGWVR
KPYFSREGANIEMRTPGDQVISVDGPYTDAPYILQAYSPLPRFGDSYTLIGSWVIGDLAS
GIGIREDDSLITKDTSRFLPHVVID
>tr|A0A0Q0CXX2|A0A0Q0CXX2_PSEAP High-affinity branched-chain amino acid transport ATP-binding protein OS=Pseudomonas syringae pv. aptata OX=83167 GN=ALO85_03131 PE=3 SV=1
MSKPILELKEIDVFYGPIQALKKVSLHINEGETVSLIGSNGAGKSTLLMSIFGQPRAASG
QIIYQGTDITQKSSHYIASNGIAQSPEGRRVFPDMSVEENLMMGTIPIGDKHSAEDMQRM
FELFPRLKERRNQRAMTMSGGEQQMLAIARALMSRPKLLLLDEPSLGLAPIIVKQIFSTL
RELAATGMTIFLVEQNANHALKLSDRAYVMVNGEIRLSGTGKELLVNEEVRNAYLGGH
>tr|A0A0Q0IT35|A0A0Q0IT35_PSEAP Uncharacterized protein OS=Pseudomonas syringae pv. aptata OX=83167 GN=ALO85_01469 PE=4 SV=1
MADEKHYRIDYLLHGSYKTFYIRAGAMDNKEAWHWASVDAGFGAIPKYRSDPVPKLSKPQ
AEKLGLSNVEWARA
>tr|A0A0Q0IA64|A0A0Q0IA64_PSEAP ADP-ribose diphosphatase NudE OS=Pseudomonas syringae pv. aptata OX=83167 GN=ALO85_02271 PE=4 SV=1
MRQKPTVLARAIVASSRLFRVEELQLRFANGVERTYERLASKGTGPGAVMIVAMLDADHA
VLIEEYCGGTDAYELSLPKGLIEPGEDVLAAANRELKEEAGFGARELELLTELSLSPGYM
SQKIQVVLATDLYEERLEGDEPEPIRVDRINLRELSSLAQNAQFSEGRALAALYLTRDLL
TQRGLFQP
>tr|A0A0Q0IQY6|A0A0Q0IQY6_PSEAP Secretion protein HlyD OS=Pseudomonas syringae pv. aptata OX=83167 GN=ALO85_00578 PE=4 SV=1
MATAAAENTSAPAPEQEPKSGKRKFMLIGLAIVVVILALGIWAWYEIYGQWSEETDDAYV
NGNVVEITPLVTGTVISIGADDGDLVHAGQVLLKFDPSDAEVSLQSAEANLGKVVRQVRG
LYSNVDGMKAQLAAQRAAVQTAQDNYNRRRSLAAGGAISQEELSHARDSLTSAQSALNNI
QQQLSTSVALVDDTVVSSHPDVKAAAAQLRQAYLANARSTLVAPVTGYVAKRTVQLGQRI
QPGTATMAVIPLDQLWIDANFKETQLGKMRIGQPVEISSDLYGSDVKYNGTIDSLGAGTG
SAFALLPAQNATGNWIKIVQRVPVRVHINPEELAKYPLRIGLSTTVEVNLHDQSGPVLAQ
QPRKQAAFSTQVYTEQLADADALIARLIHENSAPGGKTAVQR
>tr|A0A0Q0C880|A0A0Q0C880_PSEAP Sensor histidine kinase/response regulator fusion protein OS=Pseudomonas syringae pv. aptata OX=83167 GN=ALO85_03498 PE=4 SV=1
MAVLMDISLKNRLSFKQARLAVLIGFVLGTLLSLFQIAIDYASEDASINREIKSLLEIIQ
NPASRIAYNIDSELAQELTLGLLRSPAVVSARLTDNNDAVLSSVERPMATGRYRVLSDWL
FGESRQFQEKLHLNHLPNETIGTLYLTVDTFAFGSHFLQRAEITLLNGFVRSLALAGILT
ILFYATLTKPLVRVIRELSSRDPRNPDQGKITCPPYHAQDEIGVLVETFNRQFETMSSEF
SRRRAAEDRLTDHLNELENIVSARTNELKASNLRLRESNEELQIARSTALDMAHARSAFL
AHMSHEIRTPLNGLLGMLALSLDSPLGAEQRQQLMIAHDSGKVLVELLNDILDMSKFDAG
HLEIERIPFDLGMLIEDTANLLSQNAAPSVELTCLIAPDFPAMVTGDPTRVRQIVSNLLS
NALKFTRFGRVDVRLTHHRDASSGENRVRIEVRDTGIGIAQDAQTRIFQPFTQAETAITR
QYGGTGLGLALTNNLCEAMNGTLSIQSQSGFGSQFCAELPLPVHVPAVTQTPLKGRVTVV
SSAGSGLVELLGSLLPFWGVDLKRRSIDDRPEAVDLDGSPDLVITDCPECLFAIRPTTPV
PILLVTAYGNFMPGEQVAALAPLQQQARPLARNALYHTLRRILGQEETGLKDTQALEHTA
PRLARVLLVEDNPVNQLVAKGILGKLGCEVTISSHGGEALEQLEQGHFDLVLMDCNMPVM
DGYEASRQIRSSGRWPNLPIVALTANAMPDERERCKAAGMNDYLAKPFRRAELVSILDQW
IPITGID
>tr|A0A0Q0DC67|A0A0Q0DC67_PSEAP Regulatory protein, LysR:LysR, substrate-binding protein OS=Pseudomonas syringae pv. aptata OX=83167 GN=ALO85_01438 PE=3 SV=1
MRKSLMRMTLRQLRIFNEVCDLRSYSRAAEEMSLTQPAVSLQIRQLEELIGQPLFEYVSK
KLYLTEAAQALQQASRDIFGRMESLDMQLSDMQGSLQGQLKLAVESSCKYFIPHLFAAFK
RQHPEVSLSLMVVNRAQVIRRLSDNRDDLVVMSAVPLDMGLEFLPFLNNPIVAVAPPDHP
LSSRATLNLKDLEPWPLLTRETGSGTRRACEEFFKEKRVHFTQTLEVSSSEAQRECVVAG
LGLAMLTRHAVCQELATGALVELPVAELPLYRSWCVAQARDKRLSPVAHAFLAFIRTERA

QISQLVERFDGQPRAT
>tr|A0A0Q0IJR1|A0A0Q0IJR1_PSEAP Uncharacterized protein OS=Pseudomonas syringae pv. aptata OX=83167 GN=ALO85_04001 PE=4 SV=1
MKIAALALMSCIVLAGCTGSGSRAKTCQIFSPAAVNVPTTENSQRVEAHVTGEPADDRNQ
EQNCP
>tr|A0A0Q0FGH0|A0A0Q0FGH0_PSEAP Sulfur acquisition oxidoreductase, SfnB family OS=Pseudomonas syringae pv. aptata OX=83167 GN=ALO85_01677 PE=4 SV=1
MVPFTTTPVDKPRVVSIDGERRESDPATVATHRVEAPQEPDVAPRLLKATVLQNDAQAIA
AAHDLAAAAQINAANRDRQRALPWAEIEQFTRSGLGSISIPRTYGGPQVSFVTVAEVFRI
ISAADPALGQIPQNQFGILHLLQSSATEQQKKTLFQSVLNGWRIGNAGPERGTKNTLELK
ARITTNGDRLSITGQKFYSTGALFAHWVAVKALDDQGRQIMAFVQRGTPGLRIVDDWSGF
GQRTTASGTVLLDNVPVDAALVVDNWRLSLTPNIQGAVSQLIQAAIDAGIARGAIDDAIA
FVREKSRPWIDANVERASDDLYVIADIGKLKLELHAAEALLRKAGQELDEISAAPIDEHS
AARASIAVARAKVLTTEISLLASEKLFELAGSRATLAEFNLDRHWRNARVHTLHDPVRWK
YHAVGTWHLNGTLPARHSWI
>tr|A0A0Q0C2D7|A0A0Q0C2D7_PSEAP Acetyl-coenzyme A carboxylase carboxyl transferase subunit beta OS=Pseudomonas syringae pv. aptata OX=83167 GN=accD PE=3 SV=1
MSNWLVDKLIPSIMRSEVKKSSVPEGLWHKCPSCEAVLYRPELEKTLDVCPKCNHHMRIG
ARARLNIFLDVEGREELGADLEPVDRLKFRDGKKYKDRLTAAQKQTGEKDALISMSGTLL
GMPVVASAFEFSFMGGSMGAIVGERFVRAANYALENRCPFVCFAASGGARMQEALISLMQ
MAKTSAVLARLREEGLPFISVLTDPVYGGVSASLAMLGDVIVAEPKALIGFAGPRVIEQT
VREKLPEGFQRSEFLLDHGAIDMIVSRSELRPRLGNLLAQMMNLPTPRFVAPVIEPIVVP
PAPATI
>tr|A0A0Q0BR97|A0A0Q0BR97_PSEAP Uncharacterized protein OS=Pseudomonas syringae pv. aptata OX=83167 GN=ALO85_102258 PE=4 SV=1
MESTGGFEGYLVGSGDDDYETLCDLLSYRSPKALLVSDGLTLRDRQNLDQTIAEELAKAS
LPMPLPDTPFHRYIQEAFLAQHLELSEIAYKRHYGNYSAILELQRL
>tr|A0A0Q0DMN5|A0A0Q0DMN5_PSEAP Uncharacterized protein OS=Pseudomonas syringae pv. aptata OX=83167 GN=ALO85_02160 PE=4 SV=1
MSAALKRIEETREALVGALADRDWEAIVKLDLACRECVDAVVGEAPDDEPALRSNLEELL
GVYRQLIDVATGERQAVVEEMTQIQNAKSATKVYHLFG
>tr|A0A0Q0FSX9|A0A0Q0FSX9_PSEAP Glyoxalase/bleomycin resistance protein/dioxygenase family protein OS=Pseudomonas syringae pv. aptata OX=83167 GN=ALO85_03761 PE=4 SV=1
MNSLSYVNVFAEDIVRLSTFYSNLFGFREIEAIRSPIFRGLDTGKSCIGFNALDAYELLG
LEAFAGSKGCRFLLNIDVSDQAEVDRLVPLAVAQGAILVKEPYLTYYNWYQAVLLDPEQN
VFRINVML
>tr|A0A0Q0DJD6|A0A0Q0DJD6_PSEAP Ribosomal subunit interface protein, RaiA superfamily OS=Pseudomonas syringae pv. aptata OX=83167 GN=ALO85_01784 PE=4 SV=1
MQIQVNSDNHIESSIRLEEWVRTTVESTLEHYDENLTRVEVHIRDENGDKPGPHDIKCQM
EARPKGHQPISVSHKADTVDQAVDGAAVKLDHALEHLFGKLRGKRGAAPIIAEQDEDPVS
PDALLEEEFLEKEQEKEAARLT
>tr|A0A0Q0FW44|A0A0Q0FW44_PSEAP Uncharacterized protein OS=Pseudomonas syringae pv. aptata OX=83167 GN=ALO85_03291 PE=4 SV=1
MDDSDYLRLLTIQAEQANAFLSNARKWERERWVCQRLLQGLNITHRNEDFMPASQEPPDV
LFRDGRFEVFFVLDEGRRLNDEWREELARRRSAFSLSQLVRREAKPKRIPASELLHRLAP
TLRKKSTNYRERGIDLGGLDIIAFASLKREVLDLNSHFPPPTEYLRQGWRSLSLVGPTFA
RVLFAHPGAPDFLRTNLGRSVVFDVGISL
>tr|A0A0Q0DL65|A0A0Q0DL65_PSEAP Transporter OS=Pseudomonas syringae pv. aptata OX=83167 GN=ALO85_03080 PE=4 SV=1
MQSLKHFKRVATDWFLWGMVLATVLAYFFPRFGATGGNMHAEYVIKVGVFVVFFLHGVNL
SSEQIKKGLTNWRLHVMIQVFTFVVFPLIWLACNKLLGSQVPALLMLGFLYLCALPSTIS
SSVALTGSAGGNVPAAILNASMSSVIGIFITPWLVSLVVGTGAGGIDLGDTLLDLCAMLL
LPLVLGQLMRPLLGKFFARHKKYTNLIDKLVILLLVYAAFCNSMISGMWQSQGNSVLLTA
FVGTALLLAVILLMTTRTARVLKFDHADKVAAVFCATKKSLAAGAPMAALIFGSNPGLGL
ILLPIMIYHPMQLIVCSIIAESYASRHRQQLSAAALEEAQAA

>tr|A0A0Q0C385|A0A0Q0C385_PSEAP Cytochrome c5 OS=Pseudomonas syringae pv. aptata OX=83167 GN=ALO85_02575 PE=4 SV=1
MKLTWKILSSAGVLTLWAFNVQAADSARAPDDIIAAHCVACHSVGLLNAPKIGDTAAWEQ
RAQQKGGLDGLLASTIKGLGIMPAKGTCGDCTNDELKSAIQSMSGLK
>tr|A0A0Q0DL30|A0A0Q0DL30_PSEAP NH(3)-dependent NAD(+) synthetase OS=Pseudomonas syringae pv. aptata OX=83167 GN=nadE PE=3 SV=1
MHAVQRQIAEQLKVQPPFADQNALQAEVARRVNFIKECLQNARLKTLVLGISGGVDSLTA
GLLAQRAVKELRESTGDNAYRFIAVRLPYVVQADEHEAQASVDFIEPDERHTINIGSSVK
ALAAEVKAFDGLPATSVDFVLGNTKARMRMVAQYTVAGAYHGLVIGTDHAAEAVMGFFTK
FGDGACDLAPLSGLVKNQVRAIARHFGAPESLVEKVPTADLEDLSPGKPDEASHGVTYAE
IDAFLHGEPVREEAFRIICETYAKTQHKRELPYAP
>tr|A0A0Q0DG55|A0A0Q0DG55_PSEAP Uncharacterized protein OS=Pseudomonas syringae pv. aptata OX=83167 GN=ALO85_01146 PE=4 SV=1
MQSPELDGLKISSRVTLLEPGMYIFRYASQPPVDKPVCIALQQAPLGKGSIDFFPAEGVS
KNMLTRLGDTIVARVKGGITTVLITEYHMFDAVIDPIDLRIDRIDTSTAIMRNFTIAATA
EPSAAISSTLTPVALKLVAHLQGEGDVMSTEGWTGEPDGTRRLEGFSILWDDKPAGVDLV
YTSTVAGSGPSPNVATGAFTGTRGKSLPLVSAGFSLVGPDRHFYELSGHLVFRGAAPQVI
VANQMMYGPTGTEPLVALHICIAPATKMNAARYKSPWENSALTQKISSTGQMA
>tr|A0A0Q0BUB7|A0A0Q0BUB7_PSEAP Uncharacterized protein OS=Pseudomonas syringae pv. aptata OX=83167 GN=ALO85_101119 PE=4 SV=1
MSNRRLCQPGLQPGATSRTEAFRFAGLFSFARKGMFAH
>tr|A0A0Q0D7K3|A0A0Q0D7K3_PSEAP HopAN1 protein OS=Pseudomonas syringae pv. aptata OX=83167 GN=ALO85_02948 PE=4 SV=1
MSLNNARPLLGCIADDFTGATDLANMLVRGGMRTVQSIGIPSAEMAAGLDADAIVIALKS
RTTPSADAVAESLAALEWLRERGCEQIFFKYCSTFDSTAAGNIGQVSEALLEQLGSDFTL
ACPAFPENGRTIFRGHLFVQDQLLSESGMQNHPLTPMTDANLVRVLQAQTRHKVGLLRYD
SIAQGVEGVRNRIAELRAEGVSMAIADALSDADLYTLGEACADLPLLTGGSGLALGLPGN
FRKAGKLRDIDAAKQVAISGGEVVLAGSASVATNGQVAAWLEADRPALRINPLDLAAGKP
VVEQALAFAKDAGQTVLIYATSTPGEVKAVQKELGVERSGAMVEAALGEIAKGLLDAGVR
RFVVAGGETSGAVVQALGVQLLQIGAQIDPGVPATVSSGAQPLALALKSGNFGARDFFTK
ALKQLAGAA
>tr|A0A0Q0DSH7|A0A0Q0DSH7_PSEAP Epimerase domain-containing protein OS=Pseudomonas syringae pv. aptata OX=83167 GN=ALO85_03451 PE=4 SV=1
MTQTALVVGASGIVGSAITQLLLENDWQVAALSRSPSARPGVIPVAADLQNPESVHAALA
DVKPTHIFITTWSRQATEAENIRVNAAMVRNVLDAVRPAGSVKHVALVTGLKHYLGPFEA
YGKGTLPQTPFRESQARLDIENFYYAQEDEVFAAAEKDGFTWSVHRPHTVTGVAVGNAMN
MATTLAVYASICKATGRPFVFPGSRVQWDSLTDMTDARQLARQQLWAATTPAAANQAFNI
TNGDVFRWSWMWGQIAEYFGLQPADFPSEPAPLETQMANDQAVWDDIVREHQLKESDINR
LISPWHSDADLGRPIEVVTDMSKSRKLGFTAFQASDDAFFDVFEKLRRDRLIP
>tr|A0A0Q0D3Z4|A0A0Q0D3Z4_PSEAP Uncharacterized protein OS=Pseudomonas syringae pv. aptata OX=83167 GN=ALO85_03965 PE=4 SV=1
MSASLRSVDGQDEAAILREIQSALRDLRFGAVEITVHNAQVVQIERKEKFRLQNPAKQS
>tr|A0A0Q0BTB6|A0A0Q0BTB6_PSEAP UBA/THIF-type NAD/FAD binding fold protein OS=Pseudomonas syringae pv. aptata OX=83167 GN=ALO85_01259 PE=4 SV=1
MLKKARIVKFPGVFDEQVYNERVSRSGCFLGNSTLEQMANQGKLSNAVVGVAGAGGIGGA
VALGLARLGVRHIKIADPDSFDISNINRQFGASVETVGRNKALVVAELVHRLAGDVSVDV
FPEGIQKHTAEAFVEDCDLVLDQMDFYSIAERYALHRAYRHSARCKGLLASSVVGWGANI
YKFDKAGLTLEDFYDIPEEAQMTPELVERLVMLQASYQPRFPSIPDIFSWMKETGNVPIL
SVAPTASHYLILCRSALMLCDLEGAPYSTELPPMPKYYWFDGSTFDNGIYEFDGTWANPE
EHRKHFNVN
>tr|A0A0Q0DLA9|A0A0Q0DLA9_PSEAP Cell division inhibitor SulA OS=Pseudomonas syringae pv. aptata OX=83167 GN=ALO85_02678 PE=3 SV=1
MQFHQAPHSQLPLFEAFLAQPIAPLLTDAVETPWGDEPEPFSELSLRGAAGNCLNLLAPI
LRELSEELDARWLTLIAPPSSLTQTWLRDAGLNRERILLLQPRGTQSVLELTREALRLGR
SHTVVSWINPLGAIARQQLISAAKIGEAQSLNIRLG
>tr|A0A0Q0IQB1|A0A0Q0IQB1_PSEAP Uncharacterized protein OS=Pseudomonas syringae pv. aptata OX=83167 GN=ALO85_00807 PE=4 SV=1
MNIQDLTKHVKDQTVRELDLISMEGGSYVLHAKLDGKSVPVEDSSGKTLHVASVEEARKV
LSSVSDVKLFMTQAVAHDEMVGLDDVRAQSSRHEIPLRSSL

>tr|A0A0Q0FI35|A0A0Q0FI35_PSEAP Major facilitator transporter OS=Pseudomonas syringae pv. aptata OX=83167 GN=ALO85_03209 PE=4 SV=1
MTAIVQSTPQPFTKGDYKTLSLAALGGALEIYDFIIFVFFALALSQLFFPPDMPEWLRLL
QSFGIFVTGYLARPLGGILMAHFADRLGRKRVFSLSILMMALPCLLIGVMPTYAQIGYWA
PLVLLALRILQGAAVGGEVPSAWVFVAEHAPKGHRGYALGVLQAGLTFGYLLGALTATWL
ARVFSPGEILDWAWRIPFLLGGVFGVVGVWLRRWLNETPVFIALHAQREHLPALPLGQVL
RDHHQSLLPAALLTFVLTSAVVVLVVVTPTVMQQRFGISASDTFALSAVGIVFLNIGCVL
AGMLVDRIGAWRGILFYSLLMPMGIGVLYTSLIEQWMPLGIAYAIAGLACGIVGVVPSVM
IGLFPANIRVSGISFTYNIAYAFWASTTPLLLIALVPWNIWVCVLYSLIMGATGLMTAAV
FGLRRGMVVDDLASGKAG
>tr|A0A0Q0DAB8|A0A0Q0DAB8_PSEAP Uncharacterized protein OS=Pseudomonas syringae pv. aptata OX=83167 GN=ALO85_04313 PE=4 SV=1
MSTADQTRSTGTYSAKWQERFNFFETYGAPNDPRFKAGLKSLPGFRKKMRINFNVIAYFF
GPIYFFVLGLWKKGIALIGIMLATNALILLVCTLLGTEVPYALGGGLNVAFSLMYALTVN
YSYYLKEVKGEQGWNPFKGMRL
>tr|A0A0Q0DM24|A0A0Q0DM24_PSEAP Uncharacterized protein OS=Pseudomonas syringae pv. aptata OX=83167 GN=ALO85_01311 PE=4 SV=1
MRIPSRLIGGVLVATLLTQISACGSIFYPDRRGQIDGKVDPAIAALDAFALLFYIVPGVI
AFAVDFATGAIYYGPGEHAQIDPQKLQKALKADGSVDNTKLQAIIETELGRSLPLNDPRL
IQHKGSVEQLAALGLVPAA
>tr|A0A0Q0FTB0|A0A0Q0FTB0_PSEAP Lipoprotein OS=Pseudomonas syringae pv. aptata OX=83167 GN=ALO85_03615 PE=4 SV=1
MSLRLIRALLSGLLILGLSACALIPHRDPLTISVVGIEPIPGQGLELRMAVTLRVQNPNE
TEINYNGVALDLDVNDRLLASGVSNQKGTVGRFSEAVLVVPVSVSAFAALRQALGLSQSQ
RLDNLPYTLRGKLAGGLFGTMRFSDSGTLDLRQAEGDPW
>tr|A0A0Q0IBC7|A0A0Q0IBC7_PSEAP Chemotaxis sensory transducer OS=Pseudomonas syringae pv. aptata OX=83167 GN=ALO85_00109 PE=4 SV=1
MLHLFSPRRDKKALQQLISSLKGGTEPSVGGCAEKSAVLECWSSLSAQRRLDEQRIAALE
QELETARHLLGEASASAAAYETRFDLVNRASSEGLWDMEVVAGDPVNPNNRFWWSQQLRK
LLGFNDERDFPNVLASWADRLHPQDKQATLGAFAKHLNDRSGNTPYRVKNRLAMKDGTYR
WFYAQGETLRDARGTPLRVAGSLRDIHDELERDLEHDVIITRFELAREMLSDGLWDMEVI
AGDPVNARNPFWWSPQFRRLLGFETVEEFPDVLDSWASRLHPEDKERSLTAFVAHLNDRT
GKTPFDIEYRLKMKTGEYRWFRARGQTRRNPEGVPLRVVGALVDVHLKHEQDALRDTEAQ
HQKNLEENIAQLTQIVGTIQSIASQTNLLALNAAIEAARAGEAGRGFAVVADEVRKLATR
TTEATEQAASMMSR
>tr|A0A0Q0CDC4|A0A0Q0CDC4_PSEAP Uncharacterized protein OS=Pseudomonas syringae pv. aptata OX=83167 GN=ALO85_03490 PE=4 SV=1
MKTNFALLLLAVISISGCVQTAYSKSIAVTKDASGRVVQTVETETVTQPGQGYEMRLEKI
QGVQR
>tr|A0A0N8T9V8|A0A0N8T9V8_PSEAP RNA pyrophosphohydrolase OS=Pseudomonas syringae pv. aptata OX=83167 GN=rppH PE=3 SV=1
MIDPDGFRPNVGIILTNDAGQVLWARRINQDAWQFPQGGINPQETPEDALYRELNEEVGL
ERHDVQILACTRGWLRYRLPQRLVRTHSQPLCIGQKQKWFLLRLISNEQRVRMDLTGKPE
FDGWRWVSYWYPLGQVVTFKREVYRRALKELAPRLLSRD
>tr|A0A0Q0C4R9|A0A0Q0C4R9_PSEAP Hemerythrin OS=Pseudomonas syringae pv. aptata OX=83167 GN=ALO85_00967 PE=4 SV=1
MTLNIFEALRESHDRQRSYADALIQTSGDSAEREKAYKQLKEELQAHETAEERFFYIPLM
AHDNGVDLSRHAISEHHEMDEMMEELDETEMSSPAWLATAKKLSEKVHHHLKEEEQKFFQ
MAGKLLDEKQKQSLAGEYVKEYEEQLAEG
>tr|A0A0N8T827|A0A0N8T827_PSEAP Toluene tolerance protein Ttg2F OS=Pseudomonas syringae pv. aptata OX=83167 GN=ALO85_00177 PE=3 SV=1
MQAVEVKSFLEGKLPEIKVEVEGEGCNFQLNIISDELAGLSPVKRQQQIYTHLNPWIADG
SIHAVTMKFFSRAAWAERS
>tr|A0A0Q0CY49|A0A0Q0CY49_PSEAP Twin-arginine translocation pathway signal:copper-resistance protein CopA OS=Pseudomonas syringae pv. aptata OX=83167 GN=ALO85_01065 PE=4 SV=1
MPTRTSRRTFVKGLAASGLLGGLGLWRSPAWAVANPGQPNGLSGTEFDLYIGETQVNITG
NAKTAQTINGGIPGPLLRWREGDNVTLRVKNRLDEATSIHWHGIILPANMDGVPGLSFDG
IAPDGLYVYRFKVRQHGTYWYHSHSGFQEQSGVYGPLVIDAKEPEPFEYDREHVIMLSDW
TDEEPVQLMKTLKKQSDYYNNNQRTVGDFINDVGEKGWSETTRDRWMWAKMRMDPTDLAD

VSGATYTYLMNGQAPNMNWTGLFKPGERIRLRLINGSAMTYFDVRIPGLKMAVVASDGQH
VKPVTVDELRIAVAETFDVIVEPAEGAYTLFAQSMDRTGYARGTLASRPGLQAVVPPLDP
RPLLSMDDMGMAGMEHGSMNHAAAPATHDMSGMDHSKMGHDAMPAMDHAQMPARKTPTPQ
SHPESENNNPLVDMQAMSTSPKLDDPGIGLRGNGRRVLTYADLRSTFEDPDGREPSRTIE
LHLTGHMEKYAWSFDGVKFSDAKPLMLKYGERVRVVLVNDTMMTHPIHLHGMWSDLEDEN
GRFMVRKHTIDMPPGSRRSYRVTADALGRWAYHCHMLYHMEMGMFREVRVEE
>tr|A0A0Q0C4Q4|A0A0Q0C4Q4_PSEAP L-glutamine synthetase OS=Pseudomonas syringae pv. aptata OX=83167 GN=ALO85_02335 PE=3 SV=1
MSTNLDQLTDWLKEHKITEVECMMSDLTGITRGKISPTNKFIAEKGMRLPESVLLQTVTG
DYVEDDIYYELLDPADIDMICRPDQNAVYLVPWAVEPTAQVIHDTYDKQGNPIELSPRNV
LKKVLKLYADQGWQPIVAPEMEFYLTKRSDDPDYPLQPPIGRSGRPETGRQSFSIEAANE
FDPLFEDVYDWCELQNLDLDTLIHEDGTAQMEINFRHGDALSLADQILVFKRTMREAALK
HDVAATFMAKPMTGEPGSAMHLHQSIIDIETGKNIFSNEDGTMSELFLNHVGGLQKFIPE
LLPLFAPNVNSFRRFLPDTSAPVNVEWGEENRTVGLRVPDAVPQNRRVENRLPGADANPY
LAIAASLLCGFIGMVEGINPSAPVVGRGYERRNLRLPLTIEDALERMENSKTIEKYLGKK
FIIGYVAVKRAEHENFKRVISSWEREFLLFAV
>tr|A0A0Q0CCW9|A0A0Q0CCW9_PSEAP Malonate decarboxylase, gamma subunit OS=Pseudomonas syringae pv. aptata OX=83167 GN=ALO85_02963 PE=4 SV=1
MSLSQRGLNWFNALSAGAREVAGLPASLKVADGFLGEQAVRFVAVIADTDNRFPRARQGE
VGLLEGWGLAKAVDEAIEQDRDRAAKRALIAIVDVPSQAYGRREEALGIHQALAGAVDSY
ARARLAGHPVIGLLVGKAMSGAFLAHGYQANRLIALRDPGVMVHAMGKASAARVTLRSVE
DLEKLAASIPPMAYDIDSYASLGLLWETLAVSQIEQPAADDLTQVRDVLNRAIKDVQARG
VDLSSRLGASNRTASTNVRQLLRAQW
>tr|A0A0Q0BRB2|A0A0Q0BRB2_PSEAP Putative transmembrane protein OS=Pseudomonas syringae pv. aptata OX=83167 GN=ALO85_101073 PE=4 SV=1
MIQMGGGSMSPALFRFGAAVFVFFMTLVGVFSVYWTWEYVLPLYGRIYRNAPVVETPYLA
FCLLMVPPAVLLIIIGGSIAVWTGKKFDPPNNSFLGAFQSLMLKISVKTIIYVVPSIAVL
TTIVLLARDYTPCPKLLISGSAWQLFWVNDERVCFKPTRYINDGWSCKMVGDQEYCIQVD
GK
>tr|A0A0Q0DNU1|A0A0Q0DNU1_PSEAP TonB-dependent outer membrane receptor OS=Pseudomonas syringae pv. aptata OX=83167 GN=ALO85_05042 PE=3 SV=1
MRIALSDQGIFQVGTQMHTTGGDGVRHTLLAQAIDQAHTARGRRCGWLLMGLTALPCVPA
MAETAAGQNTETVALESVTVTATRREESLQKVPVAVSVIEGEQLERDNRNGVASIVQQVP
SLNFRTGASNKDTSLFVRGVGTISTSPGVEPTVATVIDGVVYARPGQATLDLLDLERIEV
LRGPQGTLFGKNASAGVLNVTTKAPTEETHGYIDQSYYSGNESRTRFGIGGSLIPQTLKG
SIMTLFGSYDGNVDNKLNGQEVNGYNRKGARGKLEFTPNDDITFTLAADYMQSHDDAPNG
VVTKALTPSFASALAPVRADSNNRNVVTDYRSHVEDVNKGLSGQLDWQLGDYTLTSITAW
RGWDNTQYQDGDRLGTVTAAFPGTEDKGDLAFNQYSQELRLASPKGQFVEYVGGLFYMHG
KSDETYQRTLITPTTQDRGIADYSTTTDSYSVFGETTFNFTPDLRAIVGARWTHDDLEYD
HRRVSTSATTVSGIQPATSSSGSVDEDGKSGRLGLQYDLSDSVMTYITYSRGYKGPAYNV
FFNMQPRDTEALKPETSNTWEVGLKATTWNNRLTTNLAVFHSEYDNYQANFFDSVAGQVV
TRLINAGSVSTEGVELDYALQATRNLKFSGALSYTRARIDSFACPAGAAASCNVDGKTLP
YSPDWKSYVRADYTLPLDNGLDIELGTDYSWQSEVQYDISQNPDTKQGAYGIWNASVALA
DYNSGWRVALLGKNLADKSYSPMLATGGNYVYRSVPRDDERYFGVQLRKDF
>tr|A0A0Q0CWF4|A0A0Q0CWF4_PSEAP Molybdopterin molybdenumtransferase OS=Pseudomonas syringae pv. aptata OX=83167 GN=ALO85_00529 PE=3 SV=1
MLMSNQPKTLLPVEDAIARLLKMAEATPITERQRVSLADAEGRVLAVDLVSTLDLPPWPN
SAMDGYALRAADWHGEPLTVSQRIFAGQAPEALAPGTCARIFTGAPMPEGADCVEMQENA
EVLADQRVRFNEPLGVGQNIRPQGQETRVGDTVLAAGTRLGPIELGLAASLGLAELEVIR
RVRVAALSTGDELIEPGQPLGPGQIYNSNRVLLCSWLKRLECEVVDAGILPDDLEKTRAA
LANLQGVDLILSTGGVSVGEADFLGHALREEGELTLWKLAIKPGKPLTFGHFRGVPVIGL
PGNPASTLVTFALLARAYLLRRQGVIDVAPLQFPVPAGFVWTRPGNRREYLRGRLEQGRA
VAYRNQSSGVLRSAAWADGLIEVREGSTVAEGDWVNFIPLSEVLG
>tr|A0A0Q0D0D0|A0A0Q0D0D0_PSEAP Putative nucleic acid-binding protein OS=Pseudomonas syringae pv. aptata OX=83167 GN=ALO85_03229 PE=4 SV=1
MKYPMCIEWGNETTAFGIQIPDIPGAVTAGDTFEEAHAAAVEIAHIMLQEIAASGRTIPK
AGNVAEHARNPDFAGMGWGMIEIDVTPYLGKTEKVNVTLPGFVIRQIDRYVRDHSIKSRS
TFLADAALEKLGRA
>tr|A0A0N8T8T1|A0A0N8T8T1_PSEAP Flg_hook domain-containing protein OS=Pseudomonas syringae pv. aptata OX=83167 GN=ALO85_03028 PE=4 SV=1

MTGDITSVPATNATLTLLRAGISPAQVLTMLQSAENLIPEGETANAEVLTLKQVNQNFQL
LLRLVLANGSLTNLPVTSSVPFTPGSLLQVAQASANELTLTLQQLNGALKNSMTSIDTQQ
LPVGTLLQGKVMTSQVIAQAVNQLATQNPAAATPIYRSIVMLLNTALAGSSLTIESPQPL
TVGSLLSAQVQGNQALNFVALPGRFDQLAVAQQLSAQQNRQGSLDTLINALQNLQSGSPT
STSPAISAPLLASINQLLADLPDVEQMTTPKGVAQALNASGVFLEARLLAGLNPAQQLPD
MKANLMRLIAQILPGLPDNMSYGAAAASNTLARTMPNAIRNALGTLGLVAARTQPSIFPL
PSRTVSGGEKEDDLEILLKLAAAAVSRLQSHQLGGLEQTRTNADGTQVTTWQLEVPMRNA
HDIVPLQVKVQREDTPDQDAAEDRDGIEIKDTREKLWKVDLAFDLEPLGPLQVHAQLLRG
TLSSQLWAERPDSAALIEHELGYLRERLIACGLAVGELACSHGAPPSGPRTALEQRWIDE
NA
>tr|A0A0Q0CI15|A0A0Q0CI15_PSEAP HTH-type transcriptional regulator BetI OS=Pseudomonas syringae pv. aptata OX=83167 GN=betI PE=3 SV=1
MPKVGMQPIRRQQLIQATLTAVDQVGMGDASIALIARLAGVSNGIISHYFQDKNGLIAAT
MRHLMNALIQNVRERRQALTEDSPRAHLQVIIEGNFDASQVSGPAMKTWLAFWATSMHHP
SLHRLQRINDHRLYSNLCCQFRRTLPLEQARSAARGLAALIDGLWLRGALSGDAFDTEQA
QRIAYEYMDFQLAKSAS
>tr|A0A0Q0E0U1|A0A0Q0E0U1_PSEAP Rubredoxin reductase OS=Pseudomonas syringae pv. aptata OX=83167 GN=ALO85_01407 PE=3 SV=1
MSAPVVIIGTGLAGYNLAREFRKLDADTPLLLITADDGRSYSKPMLSTGFGKNKEADGLS
MAEAGAMADQLKAEVRTHTRISGIDPGHKRLWIGEEAVYYRDLVLAWGAETIRVSVEGDA
GDAIFPINDLQDYARFRAAAAGKRRVLILGAGLIGCEFANDLILGGYEVDLVAPCEQVMP
TLLPPEAAAAVKSGLESLGARFHLGPVLTRLNRNGEGLQAHLSDGQVMDCDLVVSAIGLR
PRIELAAAAGLQTGRGILVDRLLQTSHANIHALGDCAEVDGLNLLYVMPLMSCARALAQT
LAGNPTSVKYGPMPITVKTPACPLVVSPPPRGSDGHWSVEGQGADIKALCHDADGHLLGY
ALTGAAVMEKLALNKVLPALLA
>tr|A0A0Q0IMC2|A0A0Q0IMC2_PSEAP Mg2+ transporter protein, CorA-like protein OS=Pseudomonas syringae pv. aptata OX=83167 GN=ALO85_00988 PE=3 SV=1
MIQGLKLDNNQLLACDPQKAQVLWFSKPDAGERRLLLERFHLDEHAVASALDPDEISRIE
FHPDALFVIWKRPENYSGKDSFSFDVSSFGVLLSRDQLVLICDDEQPLSALGAHRPLNQP
LDILLELLFNNIHHYLGHLKVIKMVARELQQKFNSSLENRHLLQMFNLSESLVYYINAIH
SNGAVLTRLRNHAQGRQYAADSLALIDDMIIENDQCYKQAEIYSTVFAGLMDARGNLVNN
STNTLLRKLTLINVVFLPLNLIAGIGGMSEFSMMTAPLHWWVAYPALLLAMLGMGAVMVW
GLRRMARAA
>tr|A0A0Q0BXT0|A0A0Q0BXT0_PSEAP Probable membrane transporter protein OS=Pseudomonas syringae pv. aptata OX=83167 GN=ALO85_03970 PE=3 SV=1
MDEHQLLGVGLGTVIGMVLALTGAGGGILAVPLLVFGLGLSIVEAAPVGLLAVGLAAGIG
AVLGLRQGIVRYRAAGYIASIGVLMAPLGLWLAHRLPNTPLALVFSVVLLYACGRMFIRA
SRELRHGKPAPRAEILPCVLNPLQGRLRWTMPCLRALTLTGVGSGLLSGLLGVGGGFVII
PALTRYSDLDMKSVVATSLAVIALVSMGSVITASLSGVMHWAVGAPFALGAVIGLVIGRQ
VARYLAGPRLQQLFAVCGIVAAFMLALSVR
>tr|A0A0Q0C0D4|A0A0Q0C0D4_PSEAP Uncharacterized protein OS=Pseudomonas syringae pv. aptata OX=83167 GN=ALO85_05127 PE=4 SV=1
MVWRAPATSAISAAGARTCNRSEQGGMLGHAVSLFQRDGKLQYIVMTFPIALAEIMGIHS
FRPHVMPPIWSPLRQDTLFHPSSLLLEQTSVLRPEHSSSRHDTLNASMPFILPDGFSVPE
PTPLPAVTEESEPKDAAPHIERQLRAMRNSAGFRLIADSTLLLQACQLDMDFPPTRQDYV
RQQLTTLLTQTSDKALDPDALQITFSNDDRPAVDDNGHEHYSVSLSLTDVALASFEPAHF
QALSRSTLADISLCEDTPALATARMFKRIGELPLKRNYNAVLQTFRTRHEVTWRTLSRLA
FLDNLARQLNHRHISRDGYWLALDALGLDRFPDATDTLSMSGRGEKSEVRALMLNGKQVP
EIFQISSKTTSHCFIHILGAKGNVIEYISDDPRQMSRRLLAAMNASGLYGQVFLSLEQHA
GIVVEAPLIEGDLFTAVTQASFAGLLADSHARSTLDLLRPVARGLALAGAADLWQTQSSI
LEQIPVASKMAGQVMASYLQENHTLTINPEHVFIAYRPGYSSTPLGNPRLPPTSVHTPDP
RPISLSEALVGNYRVDHPAGYIDHGGSTVVFFDPTGQGDSEQGQVLDISPQALEDYITAF
DFLTWMTQRIHDFWDQQRTTIQQAFRTTFINQALISLKRGSLTRGTFDLLVDTLAHPAPH
NWRSLGFFVQGALIDAMQQQYTGLLVLEQPGSPNVLYQAGHADAFIEFADDAALKLHLSR
ATASPEWRETVMRYVPRRHHQRLDYLLKLWGGTQAPNPPVSILRPWIDALYNPDTRQAMN
HSLCEKRLEDTPFAFLHRLLKQNALDDAEDQIVTSTQVTLADWTGRLQQLQRLLVPMSLL
LTPALVASLATEIGITSLAIAATHLPGSRYAEKNQALLATLTLGLLQLAPHTPRMLRSLS
RIVKPAVSSSQTVAITLDRAFGTRPLRRQFVTPRQTRLEKFFHTDALLKRWTVSTAVSSN
LLQVHAWKLGKRFLLWTSDRGQARTLVVSSHGYYTPWSRTVKIPNGTEIRTYAPHGYELV

DPKLHRVVNKNARPFALSNTAVHSVMQPTPLEPLLLTAKLIAGTSLPGRLKNYSLSKFQT
TTSESYDEIASVVGNSRASPLRGWLPPTPMDVLTVRNRFGMPPPSLADLFESLSIQGIHY
DRILLVHCRCAAISALMNRSPVYHAPTASSVIANMA
>tr|A0A0Q0BXZ7|A0A0Q0BXZ7_PSEAP TonB-dependent receptor:TonB-dependent receptor OS=Pseudomonas syringae pv. aptata OX=83167 GN=ALO85_01103 PE=3 SV=1
MMKFSRIHLALVAAGMSHGMVMADSSASDVGTIGVQGRATVGGGYMVQEESIKARSTVTK
EALDKQTATGNAIDKLKYTPGLNISSEDATGLSGFKFTMRGLNSDQVGMSVDGMPINDSG
NYALYSNLLGDPENIDQIFVTQGSAESDGPHIGSSGGNIGIVTIRPTKETGAFVKQVAGS
NGTYKTFARLNTGEINGLSNWLSMSHTEGQKWRGDGAVRADKVEWNSFFDMGSGNTANLI
LKYHEQDNNSYSQLTKSQFQQNGRKFDPYPSTPALGSNGKLSSYYELAQNPFQTFTGVLN
TQFKLADNLSLSVIPYYFWGNGSGVNSSSYALNRGSNQNGVFDLSNLPTAAAYNADGSST
TGVYYRPSRTQTWRPGITTKLNWDVNDEHSVQVGYWYERARQSQTQPFIRLKSNGKPESI
QPDSDYMVDANGNEIQGRDRYTVTPAQKVWAQDTWYFAPDWTLVGGLAFMNVEREGTNHG
SLTERPEKRDQTYNKLLPNFGLKYQLDERDQLFYSLSRNMRVPQNYVLYDPGTGSIDSKP
ETSWNHELGWRYTEQDMTLAATLFYLQFKDRQVSSKDVNGDFADINAGAVDNSGLELEWS
GLLPHHFNYYTSYTYTRAKQKDDLTVFNNGTAIELPTSGKQFTNVPKNMLAANIGYDDGS
YYGTFGAKLTSKLYGDLTNDESIPGRTIFNLGAGIYLPVDKKIVKDATLRLNVDNLFDKE
YLDGVYTTKTNAGTYSGFRDGDPAYIVGVERTVTVSLEANF
>tr|A0A0Q0IH37|A0A0Q0IH37_PSEAP Uncharacterized protein OS=Pseudomonas syringae pv. aptata OX=83167 GN=ALO85_00033 PE=4 SV=1
MKRYWLLLSLAIVLAGCQSTRDQMLAEGYPPGFADGYQDGCSSGRDAAGATTGQFRKNVP
RYLKDKLYAQGWTDGFRQCETSQDNRDRLDPGQVFNERDRAWEREKTRSAAKAYRPN
>tr|A0A0Q0CYP5|A0A0Q0CYP5_PSEAP Uncharacterized protein OS=Pseudomonas syringae pv. aptata OX=83167 GN=ALO85_04834 PE=4 SV=1
MQPTRRSYSKPFKAQVIPECAQPGASIASVALSHSLNANLVHKWIRLQAQKSPALPPAFI
PLAIPPAGANSHGDAVHPQIPLVHASHQCFE
>tr|A0A0Q0ICP0|A0A0Q0ICP0_PSEAP Xanthine/uracil permease family protein OS=Pseudomonas syringae pv. aptata OX=83167 GN=ALO85_03055 PE=4 SV=1
MQPETLDNDDLIYGLNDRPKPWTALLAAFQHVLASFVGIITPPLIIGSTLGLTQYMPYLI
SMALMVSGTGTFIQARRPFGIGAGMICLQGTSFAFLGAVLSAGFLVKQRGGSPEDIMAMI
FGVCFFGALVQIALSRCISQLRRVITPLVTGIVITLIGVSLIKVGVTDLGGGFNAPDFGA
PVNLALGAFVLAVIIVLNRSTTPWVRLSAIIIGLAVGSLAAWFSGKLVPQSLHDLPLISV
PMPFRFGFNFDWSAFLPVALIYLISTIETVGDLTANCMLARQPISGPSYLARLKGGVLGD
GVSCMIAATFSAFPNTTFAQNNGVIQLTGVASRYVGLYIGAVLFVLGLFPHIGAIVQQIP
KPVLGGATLVMFGSVAAAGVRILAQSPLDRRSMLIIATSLGVGLGIASQPALLHQLPKLV
QNLFDSAITSGGITAIVMCLLIPESKVAAATTDDVPEPEPVEQPR
>tr|A0A0Q0DEZ2|A0A0Q0DEZ2_PSEAP Uncharacterized protein OS=Pseudomonas syringae pv. aptata OX=83167 GN=ALO85_101921 PE=4 SV=1
MWRGNQKDNDGSSLGEGGCQGMTGDASAAAQRSLRSPLATRQFAQQGCCERHHQD
>tr|A0A0Q0CV33|A0A0Q0CV33_PSEAP Putative Membrane protein OS=Pseudomonas syringae pv. aptata OX=83167 GN=ALO85_04047 PE=4 SV=1
MILHTNDYLEYYLTLVGWLINGGIWNMIEDSGLFAAPFAAIVISEWLRARGEGADEGNKG
VLSLARVENRFYTAILVIILACMPLVNVSIDTIQFDRSRSDQCQYSIPNPADTGWETSFS
TLNGKSATVPVWWLFVHAMSKAATAASVAAIPCGVDLQQVRMDVNKARINDPLLAQEVAD
FTNDCYARARAKLFMTQPTLGNDQLNDVNWIGSHFFLQTSGYYSDGFSGFRSHAPRTKWP
YDATRDAGLPQTTGGGGFPTCTQWWSDGSVGLRARLLEQVSPDLLSKLAQWAKFMTQTEV
SDSVIRDLVSPRKQQLTQGQVYSDYGGQVGGSVLNDFTRLTATVGQAAGALVMYPAMDSV
RQAAPMVMAFLKMALIICIPFVLVIGMFDLKVVMTITFAAFALIFVDFWFQLARWVDSTI
LDALYGNGIGSNVPHSNFDPVFGASNAQGDLLLDFVMGAMFLVLPSFWVVALGWTGMKLG
SLMNGLSEGTKSAQAAGGKGAGEIINQVKK
>tr|A0A0Q0FKV4|A0A0Q0FKV4_PSEAP Uncharacterized protein OS=Pseudomonas syringae pv. aptata OX=83167 GN=ALO85_01719 PE=4 SV=1
MAASVIEKPKLGISACLMGAEVRFNGGHKESHLCTQALSKYFDFVQACPEVAIGMGIPRE
PIRLVGDAENPKALGTVNRDLDVTEALADYGIQMAAELGDICGYIFMQKSPSCGLERVKV
YRENGAPVDGGGRGIYAQAFCERHPDLPVEEDGRLNDAVLRENFVTRVFAYAAWKQLLKE
GVTRRALTEFHSRYKYQLMANDPVQYKALGKMLGSMGRTDPNEIAPMYFSQLMAALKKCA
TRSTHTNVLQHLCGYLKQTITSEDKQEIQQVIAQYHQGIVPLIVPLTLLKHHFRQHPDPY
VALQVYMQPHPENLSLRNAI

>tr|A0A0Q0BYD7|A0A0Q0BYD7_PSEAP Permease OS=Pseudomonas syringae pv. aptata OX=83167 GN=ALO85_02119 PE=4 SV=1
MTQKEKTPTFPQGRDVLNWKIKTLARSPREMMITQSIFIAVYLTGCTLYMWVGWIMFSDS
LYSLVGTVLALAGILAYFVGLLIRQKTIFNYTLKTDGATVEYYLHYPDFASSFFKGIAIF
VMLVFGLVAVLTGSLLFLVGPVAMAFIAAIKLLNWENPVHHRQTAPWALHEFVTVDHKRL
MVIIHCDDITTGFAARFPSKALMDKYLAFLREVLPANVEYIEKATNWYQG
>tr|A0A0Q0ICJ5|A0A0Q0ICJ5_PSEAP zinc-ribbon_6 domain-containing protein OS=Pseudomonas syringae pv. aptata OX=83167 GN=ALO85_01171 PE=4 SV=1
MQGQREIAMYRFFEQLSSRITAPFVGESKRNSKVWQCTCGQSVFFPNTQCLACSAALGYL
PEQGRVATLEAGPEPTTWRLSDEPGAGLYRRCANLGTPAACNWLFPEHNAGEFCVACSLN
HTIPDLSVVENGERWRKVETAKRRLVAQLISLGLQVIPKTVDEETGLAFDFVGVDLEGNL
PTTGHANGLITLNIEEADDAHREAMRVQMHEPYRTLLGHFRHEVGHYYWDRLIANTHWQD
SYRNLFGDERASYADALDHHYKNGAPDNWQESFVSAYATMHSWEDWAETWAHYLHMMDAV
DTALGFGMSARDMELDYQPFPLEVLYDPQHPGGPAFLSFVNAWIELASMLNELSRSMGQP
DFYPFVLPPAVIAKLHFIHLVIQDAGGKADEVLQAQ
>tr|A0A0Q0BZV0|A0A0Q0BZV0_PSEAP Uncharacterized protein OS=Pseudomonas syringae pv. aptata OX=83167 GN=ALO85_100415 PE=4 SV=1
MGTFEPGAHVRLRSGGKIMVVKHEAAITPLPDTLLVMCEYRSKNRLVQGFYSERDLILAR
REPMPNHD
>tr|A0A0Q0BST0|A0A0Q0BST0_PSEAP Aspartyl/glutamyl-tRNA(Asn/Gln) amidotransferase subunit C OS=Pseudomonas syringae pv. aptata OX=83167 GN=gatC PE=3 SV=1
MALERSDVEKIAHLARLGLNDADIPRTTEALNSILGLVDQMQAVDTTGIEPLAHPLEATQ
RLREDAVTERNRRDTYQAIAPAVQDGLYLVPKVIE
>tr|A0A0Q0FSE4|A0A0Q0FSE4_PSEAP DNA-binding transcriptional regulator NtrC OS=Pseudomonas syringae pv. aptata OX=83167 GN=ntrC PE=4 SV=1
MSRSETVWIVDDDRSIRWVLEKALQQEGMTTQSFDSADGVMSRLARQQPDVIISDIRMPG
ASGLDLLARIREQHPRLPVIIMTAHSDLDSAVASYQGGAFEYLPKPFDVDEAVSLVKRAN
QHAQEQQGLDVAPTLTRTPEIIGEAPAMQEVFRAIGRLSHSNITVLINGESGTGKELVAH
ALHRHSPRSASPFIALNMAAIPKDLMESELFGHEKGAFTGAANLRRGRFEQADGGTLFLD
EIGDMPADTQTRLLRVLADGEFYRVGGHTPVKVDVRIIAATHQNLETLVQAGKFREDLFH
RLNVIRIHIPRMSDRREDIPTLAKHFLSRAAQELAVEPKLLKAETEEYLKHLPWPGNVRQ
LENTCRWITVMASGREVHISDLPPELLSLPQDAAPVTNWEQALRQWADQALSRGQSSLLD
SAVPTFERIMIETALKHTAGRRRDAAVLLGWGRNTLTRKIKELGMKIDGGDDDEADEG
>tr|A0A0Q0FFR6|A0A0Q0FFR6_PSEAP Uncharacterized protein OS=Pseudomonas syringae pv. aptata OX=83167 GN=ALO85_01073 PE=4 SV=1
MLLNLFNEMRAAKVPVSLRELLDLINALKQRVVFADIDEFYFLARTILVKDERHFDKFDR
AFGAYFNGLENLDEHLQALIPEDWLRKEFERSLTDEERAQIQSLGGLDKLIEEFKKRLEE
QKERHAGGNKWIGTGGTSPFGSGGYNPEGIRVGDAGERKGKAVKVWDQREYKNLDDQVEL
GTRNIKVALRRLRKFARQGAADELDIDSTIDHTARDAGLLNIQMRPERRNTIKLLLLFDI
GGSMDSHIKVCEELFSACKTEFKHMEYFYFHNFIYESVWKNNQRRSSERTATQDLLHKYG
EDYKVIFIGDASMAPYEITQPGGSVEHWNEEAGYVWMQRFMAKFKKIIWINPYPKDAWDY
TTSTHIVRDLIEGQMYPLTLSGLEEGMRYLAK
>tr|A0A0Q0C7N5|A0A0Q0C7N5_PSEAP Erythronolide synthase OS=Pseudomonas syringae pv. aptata OX=83167 GN=ALO85_04815 PE=4 SV=1
MLPLGRRFDMSNAVHNIYAVNRWRPIAGSPSLATDESVLVIIPVTSAAGVSPNRQRLLSL
VGGQAHCSFTALDTLERMTATLADYIPGTIKSILWIGGSLDVSVHAEDQGLQYGIEEKIF
FRFFKALLALKADEVRLSITVVTKNNFNVNNEGTNTAVNAGLHGFVGAAAKEYSHWTISL
LDVDDAFVARATSNPSLVRSSQGIEKRKVKGQTYCCRDDQWYAEHLEVLPVENGSEPVKP
IYRDNGVYLIIGGAGGLGQAWTESLLQRHDSQVIWAGRKAINDEIRHSLRAMERLGRAPL
YVSVDATQPHQMEQLRHTILRQYGRLDGIIHSAIVLEDKTVANMSETTFSSALAAKVDIC
RNIENYFADLDLDFIAFYSSIQSFARQAGQSNYAAGCTFKDSFAHYLQAKCATTHVRAMN
WGYWGTVGIVSDASYQKVLEKSGMASINPTIGMLAFEALIAGDHLQASFIAAYDPEKITL
IDKVTLHQNTTEFGFVQSTTQLNNDGVEGCMMEYVYRNEHYMALREEGVFYLEDMDSYLC
RIVAADLETLGVFSGTEFSVEDVLRKTDIRAGFSRWLTETLMSLVRQGYLRHDAGQFSKT
AEFPESDASAAWLSWNTAKVEWLLDDNKSAQVKLVEALLIHTKEILTGKIKATDVMFPDS
SMRMVEGIYKNNRVADYFNLVLSRTVVDEINTLLAADPQRKIRILEVGAGTGGTTAVLLK
HLAPFHANIAEYCYTDLSKSFLFHAQREYGPGNPFLRYEIFNVEKSVDDQPLQRDYYDIA
VATNVLHATRDIAASIRRVWEVLKDQGSLIVNELSRNTLFNHLTFGFLDGWWLYNDDHIR
VPGSPVLEPATWHNTALQVGYGSTQFPAELAHDMGQFVMLMRAQKHAASLAPKVVESGSS

HSSGNESSMHTPSNTPSAVNVAMLKAKSQAFFTDLIADVVQLPVDEIALDLSLEKLGFDS
ILVVTLTNKLREHFEQVSSSLLFDIDSVEGIIDYFIEQDKQALAALLGIDFSDAQPPSAA
PVAVASHTQAPASAPAVTEFDVIDLGAFSLQEVDAPSASLPQVFDFQETYPEPRRSGDIA
IIGLNGTYPGSRTLDEFWENLSQGRDLVTSVPKARWEQTGFNRVENPENFKGGFIDGFDE
FDAEFFDFSETDALLTDPQERKFLECAFSVLEDAGYTKESLAESDMTTGVYVGAMYQEYQ
LHSETAKGLYEGQAISNSFASIANRVSYILNLEGPSVAIDSMCSSSLSAVHLACSALKLG
EIDYAIAGGVNLTLHPNKYGVLKQGGDLSQTGKCASFSESADGYVPGEGVGAVLLKRYED
ALRDGDHIYGVITGSLIVHAGKTNGYTVPSPKAHSKLISKVVQQAGIRSADISYIEAHGT
GTNLGDAIEIHGLRQAFSIDQDSAQQGKFCSVGSVKSNIGHCESAAGMAGLTKILLQLKY
QQIVPSLHSKKINKNLQIEKTSFSIPQLLSHWRLPNKEVKKRIAGLSSFGAGGANAHFII
EEHEEVSNQVNYTMDYPELIVLSAKSKEALHQRAAQLLGYIHYNAQQNTSLNNQPRYWLG
NIAYTLQTGRTQMPYRLSFLADSIEHLEDNLDAFLNDKKSKSPLHVCYAGEMDATAKLLY
EDNSLELILSEWLKQGKYQNIVALWAKGVVINWGGLLGKRGQRISLPTYPFSRNRHWLPM
GNHTAGVSQKVEAAPVQQRVKTVAAMVEPVVEKVAGKIVQKTVEPVVGEPVITAPVAVEK
VTAEPKSLFIHRWNKVDVSPHPKPAQTDTKICVVGLDTLDYFQRDLETCGLDAGSSEWQQ
VLPNGFAQSFADLAKSVSRQLAKKVKAWLADSTQPLHLQIIMPFDRQYQLYAALTGALEQ
HVLGAANVKLTIIYCEALCSFANISTLINRAADTQTHIAEYLCAHDSIYAGSVQKVSQPE
SGSLLNDNGVYVLANGLNEVGRSVIRAIDDETSGCHIAILGDALSAPDIDEQIGLLQNSL
DNTKVGYYAAELDDRKSVFNALLLIKDQLGDVQGVHYIVPADDKVQQHKEPLKDKALIRH
LAALINLDEATYSFDSQLQSFSIVMTVEEHDSKQLNTLAKTFARYRAALQGSGYREGKTV
ALEVEGNAANDIATAFIHALDKNHSDEQFAFIWKAATLADNTPVIQPQVTVEPPVASPAP
LAAVAQKQPAYSFDGASDELQATVSWLVSISSELLDKESASFSPVATFTENGYESISIVG
LCELINSRLGLAISVVVFFEHDTIQSLAQYLVAEHGDQIEEVLETDVED
>tr|A0A0Q0DX71|A0A0Q0DX71_PSEAP Uncharacterized protein OS=Pseudomonas syringae pv. aptata OX=83167 GN=ALO85_03745 PE=4 SV=1
METMKLHEVLTARLRIDKPQALCNPQRGQRSSGEILGGEFNGDGLQGQVLPGGSLFLVPQ
DDSLARFSLYYTLLTDDGIKIDVVGEGLVAFDEADRAPLDESRCRFTCSKQFSVASGAHD
DLQRNLYVGRVNLHAGDDRMRVSIFQVNEI
>tr|A0A0Q0CIG1|A0A0Q0CIG1_PSEAP Glutamyl-Q tRNA(Asp) synthetase OS=Pseudomonas syringae pv. aptata OX=83167 GN=gluQ PE=3 SV=1
MSGGLEFDSGHLFSPLRSTCPPKPVTRNIPPMKKPAYIGRFAPTPSGYLHFGSLVAALAS
YLDARAADGQWLLRMEDLDPPREVAGAQAGILETLERYGFEWDGQMVRQSERHDAYAEIL
QRWFNHGLAYACTCSRKQLEDFQGVYPGFCRNAGHSPDNAAIRIRVPELEYRFEDRVQGT
FKMHLGRESGDFVIRRRDGLYAYQLAVVIDDAWQGVTDIVRGADLLDSTPRQLYLQELLG
LPQPRYLHVPLITQPDGHKLGKSYRSPPLPADQATPLLLRALRALGQPLDAHMTDGTAQE
VLAWGIRHWNADLIPRLRNIEEARID
>tr|A0A0N8T7V6|A0A0N8T7V6_PSEAP Putative outer membrane lipoprotein with glycine-zipper 2TM domain OS=Pseudomonas syringae pv. aptata OX=83167 GN=ALO85_04119 PE=4 SV=1
MNKSMLVGAVLGAAVVTAGGAVATYSLVKGGPEYADVLAVEPINQQIKTPREVCKDVTVT
RQAPVKDQHQIVGSVIGAVAGGLLGNQVGGGNGKKIATVAGALGGGYAGNKVQEGMQERD
TYTTTQNRCTTVNDVSDKVVGYNVKYKLNEKVGQVRMERDPGSQIPVDKNGQLILGQAQ
>tr|A0A0Q0FWB4|A0A0Q0FWB4_PSEAP Osmotically inducible protein C OS=Pseudomonas syringae pv. aptata OX=83167 GN=ALO85_01559 PE=4 SV=1
MSIVKKASAHWEGDLKTGIGSISTETGVLREAPYGFKARFEGGKGTNPEELIGAAHAGCF
SMALSMILGGAGLTAESIDTQAEVSLDQVEGGFAISAVHLTLKAKVPGATQEKFDELTKQ
AKEGCPVSKVLNAKITLDATLLS
>tr|A0A0Q0DJF2|A0A0Q0DJF2_PSEAP Uncharacterized protein OS=Pseudomonas syringae pv. aptata OX=83167 GN=ALO85_01733 PE=4 SV=1
MNLFRSLFTARPQHRHYARIDQAGVCRAFKHCAQRPTGVEWVEVTEQRLNWLNQPLPASA
RMTQRSERSTRGQLLTA
>tr|A0A0Q0IA68|A0A0Q0IA68_PSEAP Imidazoleglycerol-phosphate dehydratase OS=Pseudomonas syringae pv. aptata OX=83167 GN=hisB PE=3 SV=1
MLAFFSPNDFAELVMAERKAYVERNTLETQIKASINLDGTGKARFDIGVPFLEHMLDQIA
RHGLIDLDIECKGDLAIDDHHTVEDVGITVGQAFSQAIGDKKGIRRYGHAYVPLDEALSR
VVIDFSGRPGLQMHVPYTRATVGGFDVDLFQEFFQGFVNHANVTLHIDNLRGTNTHHQIE
TVFKAFGRALRMAVELDERMAGQMPSTKGVL
>tr|A0A0Q0BT34|A0A0Q0BT34_PSEAP Uncharacterized protein OS=Pseudomonas syringae pv. aptata OX=83167 GN=ALO85_100520 PE=4 SV=1
MHYKIIAHSAGWAYTAAEPGPIACRTVVTDGSIVSTEDQVF

>tr|A0A0Q0CI43|A0A0Q0CI43_PSEAP Glycine cleavage system H protein OS=Pseudomonas syringae pv. aptata OX=83167 GN=gcvH PE=3 SV=1
MSNIPAELRFAESHEWARLESDGTVTVGISDHAQEALGDVVFVELPEVGKVFAAGDTAGV
VESVKAASDIYSPVAGEVIKVNTALGDSPESLNSEPYSAWIFKLKPSDAEGDLAKLLDPA
GYKSAIGE
>tr|A0A0N8T9J7|A0A0N8T9J7_PSEAP Glyoxalase/bleomycin resistance protein/dioxygenase OS=Pseudomonas syringae pv. aptata OX=83167 GN=ALO85_200186 PE=4 SV=1
MRWLFLLLLMLNAFYYIWHQQEAPLRAKEVMPLSLHRASQQDIRLLSESDAGRVSHDRDA
QCLYVGGFARQEDARLVEQRLNSLDIQSQFQPLQTSGATAYWLKIAPESRRLVEAGLISQ
LGQDFPQLKNQIMSCEGIATAD
>tr|A0A0Q0IDU9|A0A0Q0IDU9_PSEAP Silent information regulator protein Sir2 OS=Pseudomonas syringae pv. aptata OX=83167 GN=ALO85_04835 PE=4 SV=1
MPEFGSAAKALSHAPEIVIFTGAGISAESGIPTYNDPLTGIWAPYDPRTMETAKAFREIL
RWYGDGTCGRANRSLKQLPMMHISR
>tr|A0A0Q0FUR3|A0A0Q0FUR3_PSEAP Uncharacterized protein OS=Pseudomonas syringae pv. aptata OX=83167 GN=ALO85_101614 PE=4 SV=1
MEEIDDLIRGKIREWQSRRSEIKSLMQSNPERTLELSHVLDLMDEEHTAILSGSTERRAV
DNDRQSATLQATNIKHSLRPIAAKCDLQLRITASSPEDLRKLLETAVYELQGRIEAKGAV
AGEHRNYPGSMSGTLGNYHFELDIEGESGNE
>tr|A0A0Q0C1M8|A0A0Q0C1M8_PSEAP Putative Fe-S-cluster oxidoreductase OS=Pseudomonas syringae pv. aptata OX=83167 GN=ALO85_01711 PE=4 SV=1
MMLITDTGVPERYIDTDEWGGEVMLRLDDGWCAALDRNTMMCTIYEKRPLICREFEAGAE
DCLNERKGIATAYL
>tr|A0A0Q0BXJ3|A0A0Q0BXJ3_PSEAP Uncharacterized protein OS=Pseudomonas syringae pv. aptata OX=83167 GN=ALO85_100422 PE=4 SV=1
MGVGEQCFGTGHDRVSALKRGIRVYAGVSRGGQLQGGLLYWVA
>tr|A0A0Q0C2J2|A0A0Q0C2J2_PSEAP Glycerol-3-phosphate dehydrogenase OS=Pseudomonas syringae pv. aptata OX=83167 GN=ALO85_05022 PE=3 SV=1
MELLFSKVNINVQIRLSSTAHELSRRPPMPHTNVPTPPLSEVYDIAVIGGGINGVGIAAD
ASGRGLSVFLCEKDDLASHTSSASSKLIHGGLRYLEHYEFRLVREALAEREVLLAKAPHI
VKPMRFVLPHRPHLRPAWMIRAGMFLYDNLGKREKLPASRTIHFGADSPLNSSITRGFEY
SDCWVDDARLVVLNAMSARENGAHIHTQTRCVSARRIKGLWHLHLERSDGSLFSIHAKAL
VNAAGPWVAKFIREDLKLDSAYGIRLIQGSHLIVPKLYEGEHAFILQNEDKRIVFTIPYL
EHFTIVGTTDREYQGDPNKVDITEGEMDYVLKVANDHFKKQLSRADVLHTFSGVRPLCND
ESDNPSAITRDYTLSLSGGADEAPILSVFGGKLTTYRKLAESAMAQLAPFFKQMKPSWTA
KAALPGGEDMTTAEALATEMTRRCSWLPDPIAKRWSITYGSRSWRLMEGAQSLEDLGQHL
GAGLYTREVDYLCDQEWATSIEDILWRRTKLGLFTTPEEQAAVSSYLETVVRNKASFEAA
>tr|A0A0Q0CBE2|A0A0Q0CBE2_PSEAP Putative membrane protein OS=Pseudomonas syringae pv. aptata OX=83167 GN=ALO85_01770 PE=4 SV=1
MSTSPVTLMVARRVAQGRYHEMMAWLHEGEQLATDFPGYLGSGVLAPPPGDDEFQIIFRF
TDEHTMHAWEHSASRRSWLVRGSGLFAQPSEHRVSGIDGWFGAIDQRPPRWKQAVAIWLA
FFPVSLIFNFLLGPLLNHLDLLPRIMISTAVLTPLMVYLFIPLSTRLLAPWLQSTPAPAR
PTQSPSPR
>tr|A0A0Q0D2S5|A0A0Q0D2S5_PSEAP PAS domain S-box/diguanylate cye domain-containing protein OS=Pseudomonas syringae pv. aptata OX=83167 GN=ALO85_02176 PE=4 SV=1
MPNLPDYAVPKATAQSASARLPLTDAERALQETRDRLELALDLAQMGTWDLDIIRNRLQA
SARAALLHGMPALPFDESGGQFFGSLPGEERRRMRAIYAVALETGQDCLQVTYRFKLKNG
ASRLLEGRARIYRDEQGQPVRLSGTLMDITQQCAQIALEASEEKFAKAFHSSPDSITITE
IDSGRYVEVNDGFTRLTGFSAQEVLGRTAHEVGIWTDTLQREHMLEELRQKGRVHHREMD
GRDKHGTPLVLDISVEFITLNNTACLLMNARDIGALKNAQAQIQHLAYHDPLTGLPNRAL
LLDRLSQQVSLLQHQNLRGALLFLDLDHFKHINDSLGHPVGDSVLSIIAARLEASVRQED
TVARLGGDEFVVLICGLEGSQDEVTQQVRELADNLRNLLAEPMFLDGHRLQVTPSIGVVL
IPDDGLTPADLLKRADIALYRAKDCGRNASQFFHVSMQQAVSQRLRLENDLRLALTRNEF
HLHFQAQVDCRNEYITGAEALLRWQHPEYGLMAPDQFVQILEESGMILEVGSHILNEACK
TGGLLLRQGLIPENRFLMGVNISPRQFLQHDFIERVERSLTLHRLPASMLKLEITEGIVI
QNLDDTILKMLSLKALGVSFAMDDFGTGYSSLTYLKRLPVDTLKIDQSFVQDATNDPNDA
EIIRAIVAMAQSLNLNVIAEGVETSEQLAFLEKVGCYTYQGYLFSEPLPGPQFESLLQLK
RPAQQTAQQD

>tr|A0A0Q0DA68|A0A0Q0DA68_PSEAP Ribonuclease 3 OS=Pseudomonas syringae pv. aptata OX=83167 GN=rnc PE=3 SV=1
MSVSLSRLERQLGYTFKDQELMVLALTHRSFAGRNNERLEFLGDAILNFVAGEALFERFP
QAREGQLSRLRARLVKGETLALLARGFDLGEYLRLGSGELKSGGFRRESILADALEALIG
AIYLDAGMEAARERVLAWLTTEFDSLTLVDTNKDPKTRLQEFLQSRACELPRYEVVDIQG
EPHCRTFFVECEINLLNEKSRGQGVSRRIAEQVAAAAALIALGVENGHE
>tr|A0A0Q0DC26|A0A0Q0DC26_PSEAP Transcriptional regulator, LysR family OS=Pseudomonas syringae pv. aptata OX=83167 GN=ALO85_04978 PE=3 SV=1
MTLRALSACNALMIRVWTALSPLSNNQKIEFMLPLAQHWKPMSLVQDRRILYFFEAVRLG
SVRAAADFLNVAPSAVSRQIAQLEQELGSPLLERHRRGVKPTEAGDKVLAYYRQRLTQQE
VLLDSIQALKGLHSGSVTLVSGEGFLESLAQPLARFSELYPRIELIVNVCGSNEVIRQVV
EDEAHIGLVFNPAADPKIRSHAHQRQPVCVITAPDHPLAEEPGPIQLRALEDYRVALPAV
SYGIRQILLQAEHQLGITVQPTLTCSTFAMLKHFAMQGGVTLLPTFVVEEEIKAGRLRAL
PLQHEVFSSPQTHLISRLGRQLSVGANRLMGMLLQDMTAFRN
>tr|A0A0Q0BT69|A0A0Q0BT69_PSEAP Dead/deah box helicase domain-containing protein OS=Pseudomonas syringae pv. aptata OX=83167 GN=ALO85_02493 PE=4 SV=1
MNSFANELSNKLSSHEIFWGLFEMLQSAGAVSTLPDGVYEEFDSLSENDIAKLLYCARVF
AQSDLEKFKSISQSIALSCMLISQEPLVLEKCSRILTELGNFPGLGYAEQHYGIGSESLI
GTLQRRLAQEINTVQVGGQRLPLTDYQKNVWDQLAGTGSLAISAPTSAGKSFLVIEHMCR
LASENSSFCAVYIAPTRALLSEVLQTVQVRLLDMENIRVSSVPSLDPENDNKQVFVLTQE
RLQVLLAISDMSFDLVIVDEAQNLSDGSRGMILQECLEQVLQRSKITKIIMLAPGAEGFF
ETAKVIGLNDLRSLTTSVSPVQQNRIVVHKVRGKNELKLELLTGRRILPLGQLATERGMD
IPATRLAAVALELGSSGGSLVYSTGPSDAEDIALQISLARKNVEDIRLLELADFIEKHIH
PDYKLALQVRNGVAFHYGRMPSLLRESLEEGFKSGALKFLVCTTTLFQGVNLPARNVFID
TPTRGRGTSLDPALLWNFAGRAGRMRKDIVGNVFLVDYDEWPEKPMSEFVKFKIKPAIQE
TLTTSHFQVLDALSGNMPKRSRFDESSGKVTASAGLLIARAAKGDVGEFIARILPDVEKE
RVGSLVTAAIAAAESLQLPSIILTSNWMVDPFGQKRLYDKIVSKINADECDDLIPINPHE
PGASSLYGGIFHRILKSVYGQEGNFGGYVSSLAIPWMKGMPYPVILNTAIKKEVKRIDKK
TAEYFEKKILDPKSRVRQPKALDSSQVIRKTFDLIEDVVRFQFVQLGKVYIDLLSLALCN
EGYPELVSDIFDFSLALELGISTQSGRSYVELGLSRIAASALDQIYPNSKLTISEARAWL
WDLDVQNLKVGRVILEEIQKLGLIRSEA
>tr|A0A0N8T9U7|A0A0N8T9U7_PSEAP Putative paraquat-inducible protein PqiA OS=Pseudomonas syringae pv. aptata OX=83167 GN=ALO85_03606 PE=4 SV=1
MASMSTVHPIEHSSGLIICEHCDSLYEAHPLQPGEAAFCLRCEALLGRGHRVTIEQLLAL
TIAAAILFVFANLFPVISINMKGLSNEVTLWQSVEALAQGRITIIALVAGLSIIFAPLLQ
IVLLFWVLVHAHKGIVAPGFRTCMRALEHLRPWSMLEVCMLGILVAIIKLSGMLDVHPGV
GLWAMAMLMVLILLIANKDIRRLWDELGVQPQ
>tr|A0A0Q0C4G5|A0A0Q0C4G5_PSEAP Secretion protein HlyD OS=Pseudomonas syringae pv. aptata OX=83167 GN=ALO85_02402 PE=3 SV=1
MQFKPAVTVLVTAIALATLLSGCSKKEQAAPPAQTPQIGVVTIKAQPYTLTTELPGRTTA
FRVAEVRPQVNGIILKRLFTEGGDVKAGQQLYQIDPAVYEANANSAKATLQSAKSMSDRY
KQLVSEQAVSRQEYDTALASTQEAQATLQTAQINLRYTKVLAPISGRIGRSAVTEGALVS
SAQTDAMATIQQLDPIYVDVVQSSADMLKLRSDLDSGKLQKAGDNAAKVKLTLEDNSVYA
QEGKLEFSEVSVDQTTGSVTLRAVFPNPEHRLLPGMFVHAQLQAGVNSKAILAPQQGVTR
DLKGTPTAMVVNQDNKVEMRTLVANRTVGSDWLIEDGLKEGDRVITEGLQYVKPGAEVKV
AEATNVKGANPAPAPATDKAAGSKGE
>tr|A0A0Q0C3H4|A0A0Q0C3H4_PSEAP DNA gyrase inhibitor YacG OS=Pseudomonas syringae pv. aptata OX=83167 GN=yacG PE=3 SV=1
MSQPMTVQCPTCDAPVEWSAASPSRPFCSERCKLIDLGAWASEEHAIPVSPDAEDELFSG
DLEAPHRGH
>tr|A0A0N8T8J9|A0A0N8T8J9_PSEAP Peptidyl-prolyl cis-trans isomerase OS=Pseudomonas syringae pv. aptata OX=83167 GN=ALO85_03151 PE=3 SV=1
MLKKIALAACSVLFAANLMAAPDKATHVVLDTSFGQIEIELADTKAPVSTQNFLGYVDSG
FYTNTIFHRVIPGFMVQGGGFTPQMVQKQTKDPIKNEAQNGLHNVRGTIAMARTSDVNSA
TSQFFINTNDNAMLDNGARDFGYAVFGKVVKGMDVVDNIVNARTTTQKGMQNVPIDPILI
KSAKRVN
>tr|A0A0Q0FP03|A0A0Q0FP03_PSEAP Putative phosphatidylserine/phosphatidylglycerophosphate/cardiolipi n synthase OS=Pseudomonas syringae pv. aptata OX=83167 GN=ALO85_02373 PE=4 SV=1

MNGAIFPWRENNRFQLLIDGPAFFPRMIAAIDRAEQQVDLELYLVEAGACADAIVRALVE
AGRRGVIVRCLFDDFGSKAFDAGLRKQLTDAGVILRFYNPIHWRRGVRNFYRDHRKLLLV
DKALAVVGGTGVTDEFWEPDKDASQWHEVMVEITGPLVIDWQALFNRQFHANARRFAWRP
KENFGLDHLPKAPTTGEGLGRVAYADSRQHRDILQSLVRALNTGQKRIWLATPYFLPTWK
VRRALRRAAARGVDVRLLLTGPRTDHPSVRYAGQRYYPRLLRAGVKVFEYQPCFLHLKMV
LIDDWVSLGSCNFDHWNLRFNLEANLEALDPGLTRDVVASFEKDFALSAETTLADWKARP
LWRRVKQRLWGWIDRLVVNLLDQRN
>tr|A0A0N8TA29|A0A0N8TA29_PSEAP Orf10 OS=Pseudomonas syringae pv. aptata OX=83167 GN=ALO85_200126 PE=4 SV=1
MWWKILLSIVFMMAVCFVIGLYAGGAMYLHLTQGHFAGLAWDTLWETRKLAWNDRRMLYV
PWSWCVTAALTFLPVGITLMALFVRLKPKTSLHGDARFANNRELRQFEYQGEYKNTSK
>tr|A0A0Q0C1M0|A0A0Q0C1M0_PSEAP Amino acid ABC transporter permease OS=Pseudomonas syringae pv. aptata OX=83167 GN=ALO85_01820 PE=3 SV=1
MEKQIVAPKQKLSLSDPKVRAWLFQIITVVAVIALGWFIFDNTQTNLQHRGITSGFGFLE
NSAGFGIAQHLIDYSEADSYARVFVIGLLNTLLVSVIGIVLATLLGFVIGVARLSPNWMI
SKLATVYVEVFRNIPPLLQILFWYTAVFLTLPGPRQAHGYLDMFFVSSRGLNMPRALPAE
GAWAFLASLVVAVIAIVMMVRWANKRFEATGQPFHKFWVGLALLLVIPGLSVLIFGSPVH
WELPQLKGFNFSGGWVLIPELISLTLALTIYTAAFIAEIVRSGIKSVSHGQTEAARSLGL
RPGPTLRKVIIPQALRVIIPPLTSQFLNLAKNSSLAAAIGYPEMVSLFAGTVLNQTGQAI
EVIAITMSVYLAISISISLLMNWYNKRIALIER
>tr|A0A0Q0C6J1|A0A0Q0C6J1_PSEAP Uncharacterized protein OS=Pseudomonas syringae pv. aptata OX=83167 GN=ALO85_01231 PE=4 SV=1
MKNTLKKLLLAVACLAAGPAFAACQMTPVAYDMPSQRLDEALQQLAHRSGCPVKVNLGAD
SSRKVKKFKGTFTPDQALWLVLKKTGLEGYVENDGLTVDRRGQDFVNQRATELRTAIDEA
GARMEARKKKRFLHQLDTIESGAKKVVLEQSFVSAAEMASYKRDFDELSSQIPASK
>tr|A0A0Q0DTE2|A0A0Q0DTE2_PSEAP LysR family transcriptional regulator OS=Pseudomonas syringae pv. aptata OX=83167 GN=ALO85_02612 PE=3 SV=1
MSYSPEALEAFAQIAALGSFSAAARRLGKSQSTISEAIARLEIDLGLELFDRSSRQPVLT
EAGRVMLGRVDDLLCASDRLRRAAARLAAGVEPRLTLVLSDANQFADFERLMTELDQRFP
ELEMECVFAEHGDAISLVQSGRASLGLLSAQASYPPEIGHATIEESADFGLFVSHKHPLA
ALERVDYQQLARYRALRLNTLIEHSIPTDDLPTSGHRHWSAPNYLLLMDMAAFGFGWSAL
PRWLVSRYSGGLLKELQVSGWPRRSAVDVIWSFQRSLGPAGAWLLESLVPFAGE
>tr|A0A0Q0DK33|A0A0Q0DK33_PSEAP Sensor protein OS=Pseudomonas syringae pv. aptata OX=83167 GN=ALO85_04010 PE=4 SV=1
MKLTRLSTRLGLTVAALIGCLVLMMEMLAYAAISRQLDIRAEDALNEKFRQIEHSMSEGF
LGVVDIVQYPHTLRDQIVGHDSFSLTIFDVGNPRQELMSVGSQEGRSLPVPDTDALPQGF
QELKSEQGHKILLGYRTVRLKTGQDVLVRLSMDRESDTVLLHAYVRSTCLALPVILLLVG
FGVWWVVRRGLRPLGAFRKVTALISARDLSHRMKVKGLPDELRDLAHAVNFMLHRLDGDV
QQLAQFSDDLAHELRSPMNNLMGRAQVTLSRPRPSEEYKQALESCTEELERMSRMISQML
FLASVSQPAAPLPVEVIDLRQEADKVAELFSSSAEDRDITLQVQGHARASGDRLMIQRAI
SNLLSNAIRHGLSGSVITLTLATHEDEVSLAVRNAGDGIDAEHLPRLFDRFYRVHVSRAR
QQGGTGLGLAIVRSIMSLHEGQVTVQSEPGQFTSFSLIFPKLV
>tr|A0A0Q0DSA8|A0A0Q0DSA8_PSEAP Conjugal transfer protein OS=Pseudomonas syringae pv. aptata OX=83167 GN=ALO85_200014 PE=4 SV=1
MTQEQGLTEDTLVLDFLDQAGVTERLNRQGTKDVAINRPYELWVDGPNGWEYEEAPWLTY
NLCWRLANALCALNYRVLAPHSPIHTVELPGGERGQIVMAPACEKGTLSTTFRKPSLDRF
THMDYVNSGRYDRARAIASPILALKDWQRDMQEAHAAGHWHRFMEIAIAHRQNIIIFGGP
GSGKTTYGKSLIDMFPVNRRMITIQEILEDPMPFHPNHVHLLYGHVVTPKALVASALRMK
PDHLFLAELTGDEVWHFIEILNTGTKGTVTTAHANDSEAGYARVCGLVKQSPIGLGLDYA
YIERLVRTSFDVVVYMENTYIEEVHYEPEHKLALLNGQRQRAA
>tr|A0A0Q0CY66|A0A0Q0CY66_PSEAP Quinolinate synthase A OS=Pseudomonas syringae pv. aptata OX=83167 GN=nadA PE=3 SV=1
MVQMAQPLSGFFYCLYFGCKLAFRALALSGPVNMTQISERFLVQAHLDAKQPRTLSPAEQ
ARYRADIAEELKKQDAVLVAHYYCDPVIQALAEETGGCVADSLEMARFSNNHAASTVLVA
GVRFMGETAKILNPEKRVFMPTLEATCSLDVGCPVDEFSAFCDQHPERTVVVYANTSAAV
KARADWVVTSGCALEIVESLMDNGEKIIWAPDKHLGRYIQRETGADMLLWDGACIVHEEF
KSKQLEDMKALYPEAAILVHPESPEAVIELADVVGSTSQMIAAAQRLPNKTFIVATDRGI
FYKMQQLCPDKIFIEAPTAGNGAACRSCAHCPWMAMNTLERTLQCLREGSNEIAVDPALI
PNAVRPLQRMLDFTQAARLRQAGNA

>tr|A0A0N8T8Q1|A0A0N8T8Q1_PSEAP IucA/IucC OS=Pseudomonas syringae pv. aptata OX=83167 GN=ALO85_04509 PE=4 SV=1
MTLTSRLKHYPHTTFGWANLSDLDSSIVVPQAINQRLLRRHLNGQRKGDCGADMTNTDRT
VLSNMVSELATTRALLNCLIKEFALPEECLHYTWPEGMQGIAPGSFVDGGQWKGIPLTIS
LPNQQQFFVLVDRRDHLGSHRYLSDVYARQGQSTWRCLAFAEFARQLLTACEHMTRASND
ELLDQVLQSQHLTAAIVAHNMTGQHPEPLSGYLASEQGLWFGHPNHPAPKARLWPEHLAQ
ETYAPEFQAQTALHLFEVPLEGLRITSNGLSDSEVMAGFADQSRARPGHALICMHPVQAQ
LFMQDRRVQRLLELGDISDLGASGLLASPTASMRTWYIEGHDYFIKGSLNVRITNCVRKN
AWYELESTLIIDELFQRLQQTRPETLGGLSTVAEPGSMSWAPKGVSEADGHWFREQTGAI
LRENFCRRSGADCSVMAGTLFARDLRSRPLVHDFLERFNGRELEDQHLLDWFDEYQSLLL
SPVMALFFNHGIVMEPHLQNAVLIHDNGRPQQLLLRDFEGVKLTEELGIKAIQVGLHPRI
RQSLLYTREQGWNRITYCLLINNLSEAVLALSWERPHLAPLMWQRVERQLQRIRDELMLP
APELDALIAGQSIACKTNLKVRLAAKADREANYVRLASPWATEARYA
>tr|A0A0Q0C2M5|A0A0Q0C2M5_PSEAP Delta 5 fatty acid desaturase OS=Pseudomonas syringae pv. aptata OX=83167 GN=ALO85_04316 PE=4 SV=1
MAESLAPLSFPADGEQAFHHALKQAAHAYLADDHRYASIRQKVTALLLIVLCGGFYASSL
MQHNAWAFVGCYFLFVLTGMLLNVMVFHDATHNAFFRATWANRLVGRLMTLPLGIDPDYW
RVRHVQFHHAYANVEHYDLDTEENGVLRQTPFQGWRKHMRYQHLYWPMVAALSLPYISWI
FDWSDRLGKTPLKAKSILPGNKGWAVFLASKACHLLLVLIVPLVIFEGSWTTVLIAYFVS
QMLASLWVVFLLLGTHWAEAEFYQPSDSPLMPHGWYRHNFATTCDWQTSPRWLHHAVGGL
NFHLTHHLFPGWSHRHYPALANIIADLAPRHGLHYRCIDYRELLRQQRKFLRSMGRDTQV
TNIT
>tr|A0A0N8T800|A0A0N8T800_PSEAP Uncharacterized protein OS=Pseudomonas syringae pv. aptata OX=83167 GN=ALO85_00519 PE=4 SV=1
MAKQSTATPSTPGPYERMGMRVQKIINSPTAQKSRAALLFRQPDEAEDDWAQLLEEIAEN
DNVTLAWRDDGGVQIFWTLPKED
>tr|A0A0Q0DXL5|A0A0Q0DXL5_PSEAP Nickel ABC-type transport system, permease protein NikB OS=Pseudomonas syringae pv. aptata OX=83167 GN=ALO85_03684 PE=3 SV=1
MSRYILSRIAQALLVLWGAYSVTYFILYLLPGDTLSIMLSASGVEIDSLSAQDLAQAKLY
YGLDRGLFEQYVDLLWGAMHGDFGNSLTLNRPVSELLIERLPQTLSLAGLAIVLSLIGGI
GLAYLTAYVRWQPLKVALSRLPSLGFSVPVFWMGLLFIQLFAFTLGWFPATGNQGFSSLI
LPAVTLAIPSAAVYAQVLQRGFQGVWKEPYIITAYAKGLTRAQVQARHAFKNAALPLLTL
IGLQVGNTVSGAVLVETIFARSGVGRLAQEAVLRQDIPVVLAIVAVSAAAFVVVNLIVDL
LYPALDPRIAHMPKVS
>tr|A0A0N8TA26|A0A0N8TA26_PSEAP Mangotoxin biosynthesis-involved protein MgoC OS=Pseudomonas syringae pv. aptata OX=83167 GN=ALO85_04328 PE=4 SV=1
MKAEDYMSFIDAWEGRATIRTRPRRMVENDDKLIYPLSRQPLVLSDTFTRECAHLRDYAL
VQSLYKFINDVVIFETEIVDRTARSIAKDHFTIRFPFACRYDAMTVVVDEDYHALVAMDF
MQQTIALTGIEPIQLPDEIELSRAIPAALALAPEHLRSAVELICVAIAENTVTNDVAAFA
KDESVKQSVKGLMADHLLDEGRHSGFWARLVRIYWHTAPQQDRESIARIMPVFIAQYLTN
DIQNGFDFTLIDHLQVPDTIKHALREETRAVSFPVNRHHPLVANIVRFFKNSSMLDDPCV
QRALADYLPAQGTLQ
>tr|A0A0Q0DFE9|A0A0Q0DFE9_PSEAP Uncharacterized protein OS=Pseudomonas syringae pv. aptata OX=83167 GN=ALO85_01656 PE=4 SV=1
MHNESIRYLIVPGWQGSPDDHWQTHWQNSLPNSTRVEQADWLKPRREDWVGELQRTIAAH
DTPVILIAHSLGCVTVAHWARLAPLETLRQVQGALLVAPADVDRPNCPPALRNFAPIPTD
LLPFPTQIVSSDNDPAISSQRAMEMARNWGAELGFLSQAGHINVKSGHQRWEQGFAYLYR
LQNRLEQHARRRA
>tr|A0A0Q0C2T7|A0A0Q0C2T7_PSEAP Uncharacterized protein OS=Pseudomonas syringae pv. aptata OX=83167 GN=ALO85_03407 PE=4 SV=1
MMPGLYIGCAGWSLAREHWPDFPDEGSHLQRYASRFNAVEINSSFYRPHRAETYARWAES
VPAGFRFSVKMPKLITHELRLRECEAVLATFLSQCTAMDDKLGCLLVQLPPSLDYAPQIT
EGFFTALRNQYAGRVVLEPRHQSWRQAQALLIEHRIAQVAADPSVISDGHLPGGWQGAHY
WRLHGAPKIYHSAYTVEHLEGLAGRASASVAQGIETWCIFDNTASGAAVGDALALQALVK
>tr|A0A0Q0DGW7|A0A0Q0DGW7_PSEAP Glycine betaine/L-proline ABC-type transport system OS=Pseudomonas syringae pv. aptata OX=83167 GN=ALO85_03217 PE=4 SV=1
MMMRKILGAGAALVLAVSSTLAIAETKTLTIGYVEGWSDSVATTFVASEVIKQKLGYDVK
LQSVAAGIMWQAVATDKLDLMLSAWLPGTHGEYYAKYKDQVVNYGPNFKDAKIGLIVPEY

VKAKTIEDLKTDASFNKKITGIDAGSGVMLKTDQAIKDYGLDGYKLQASSGAGMIAELTR
AEKAKKSIVVTGWVPHWMFAKWKLRFLDDPKGVYGEAETVDNVGSLGLEKKAPEVVAFLK
KFQWASKDEIGEVMLAIQDGAKPEAAAKDWVAKHPDRVAAWTAK
>tr|A0A0N8T868|A0A0N8T868_PSEAP Xanthine dehydrogenase, molybdopterin-binding subunit OS=Pseudomonas syringae pv. aptata OX=83167 GN=ALO85_01165 PE=4 SV=1
MLAVFQQGLNTGVGRSVKHDSADKHVSGEAVYIDDRLEFPNQLHVYARLSDRAHARIVSI
DTSPCYAFEGVRIAITHQDIPGLKDIGPLLPGDPLLAIDKVEFVGQPVLAVAARDLDIAR
QAAMAAIIEYEDLQPVLDVVQALRQKHFVLDSHTHKRGDSAAALARATHRLQGNLHIGGQ
EHFYLETQISSVMPTEDGGMIVYCSTQNPTEIQKLVAEVLGVPMNRIVVDMRRMGGGFGG
KETQAASPACLCAVIARLTGQPTKMRLQRFEDMQMTGKRHPFYIEYDVGFDDDGRLQGIQ
LDLAGNCGYSPDLSASIVDRAMFHADNAYYLGEATVNGHRCKTHTASNTAYRGFGGPQGM
VAIEEVMDCIARFLGKDPLHVRKANYYGKTERNVTHYYQTVEHNLLEEMTTDLEQSSQYA
ERREAIRAFNAGSPVLKKGLALTPVKFGISFTASFLNQAGALIHIYTDGSIHLNHGGTEM
GQGLNTKVAQVVAEVFQVDIERIQITATNTDKVPNTSPTAASSGTDLNGKAAQNAAEILK
QRLVEFAARHHQVSEAEVEFRNGHVRIGELYLPFAELAQLAWMGQVSLSSTGYYKTPKIF
YDRSQARGRPFYYYAFGAACVEVIVDTLTGEYKMLRTDILHDVGASLNPAIDIGQVEGGY
IQGAGWLTTEELVWNDKGKLMTSGPASYKIPAVADMPLDLRIKLVENRKNPEDTVFHSKA
VGEPPFMLGIAAWCAIKDAVASLGDYRHQPDIDAPATPEKVLWGCEQMRQHSAARRAPSE
GAEPIVSAASRANEEAPGSPSPIQDAPVGASLLAKAPGLDHSVLEQK
>tr|A0A0Q0IGQ2|A0A0Q0IGQ2_PSEAP Putative metallophosphoesterase OS=Pseudomonas syringae pv. aptata OX=83167 GN=ALO85_00382 PE=4 SV=1
MFHVMFSMPCLYVIARFLWPMPWALSAKIAVAVVLVLASQYHLYCRFSSGSVFSPEFPRY
VVMLFNWAFGAILLLAVLQIIVDIGTLLTMLVRRQTLSIPANLRYFIGVAALLLAAVGVQ
QAVRVPPVKDIQIAIKNLPPQFEGYQVLQLTDMHISRLFDAPWTQAVVQQSNALGVNLIV
ITGDLIDGSLGNRKQDIEALRDLRAPDGVYVIPGNHEYFFDNQAWMQHFVSLGMIPLANS
HTMIEHDGARIALAGVTDVTAPKTGFPAPDLQKAIAGIAKDTPIILLDHQPRNAKETATH
GVALQLSGHTHGGMIFGLHWLLALANGGFVSGLYDVGGMQLYVNNGTALWPGFAIRLGKP
SELTRITLRRAP
>tr|A0A0Q0BY56|A0A0Q0BY56_PSEAP Bifunctional chorismate mutase I / prephenate dehydratase OS=Pseudomonas syringae pv. aptata OX=83167 GN=ALO85_03528 PE=4 SV=1
MSEQELKALRVRIDSLDEKVLELISERARCAQEVARVKMSSLAEGEVPVFYRPEREAAVL
RRVMERNRGPLSNEEMARLFREIMSSCLALEQPLKVAYLGPEGTFTQAAAMKHFGHAVIS
LPMAAIDEVFREVVAGAVNFGVVPVENSTEGAVNHTLDSFLEHDMVICGEVELRIHHHLL
VGESTKTESITRIYSHAQSLAQCRKWLDAHYPNVERVAVASNAEAAKRVKGEWNSAAIAG
DMAAGLYGLTRLAEKIEDRPDNSTRFLIIGNQEVPPTGDDKTSIIVSMSNKPGALHELLV
PFHENGLDLTRIETRPSRSGKWTYVFFIDFVGHHKDPLVKAVLERISSEAVALKVLGSYP
KAVL
>tr|A0A0Q0ILF6|A0A0Q0ILF6_PSEAP UPF0225 protein ALO85_05206 OS=Pseudomonas syringae pv. aptata OX=83167 GN=ALO85_05206 PE=3 SV=1
MSSAICPCGSGDLLLACCGRYHAGQPAPGAEKLMRSRYSAYVLGLTDYLVQTTLPVQQGG
LDREAIAQWSAQSTWLGLEVESSEVFGGKPEHAFVTFTARWHDGNGEHSHKERSSFVQNQ
GRWYFIDSTVPLKAGRNDACPCGSEQKFKKCCSAYVD
>tr|A0A0N8TA53|A0A0N8TA53_PSEAP FAD dependent oxidoreductase OS=Pseudomonas syringae pv. aptata OX=83167 GN=ALO85_05383 PE=4 SV=1
MACCWQSFYISGTIAAIVKNIKRSLRMANTPYPQSYYAASANAAPERPALQGEVETDVCV
IGAGYTGLSSALFLLENGFRVTVLEAAKVGFGASGRNGGQIVNSYSRDIDVIERTVGPRQ
AQLLGQMAFEGASIIRDRVSRYGIQCDLKDGGVFAALTSKQLAHLEAQQRLWERYGHTGL
ELMDKRRINEVVACDQYIGGLLDMTGGHIHPLNLALGEAAAVETLGGTIYEQSAAVRIER
GANPVVHTAQGRVRAKFVIVAGNAYLGNLVPELAAKSMPCGTQVITTEPLSDELAKTLLP
QDYCVEDCNYLLDYYRLSADKRLIFGGGVVYGARDPANIEAIIRPKMLKAFPQLKNVKID
YAWTGNFLLTLSRLPQAGRLGDNIYYSQGCSGHGVTYTHLAGKVLAEALRGQAERFDAFA
DLPHYPFPGGQVLRTPLTALGAWYYSLRDRLGF
>tr|A0A0N8TAD1|A0A0N8TAD1_PSEAP Import inner membrane translocase, subunit Tim44 OS=Pseudomonas syringae pv. aptata OX=83167 GN=ALO85_01441 PE=4 SV=1
MQRFLSIAMALCIGLTMSLDVNAARFGGGKSMGSAPTHQTRQAAPSTPAAAPNAAGRPAA
APSGASRWLGPLAGIAAGGLLASMFMGDGFNGLQLFDILIMGLIAFLIFRFIARRRQQQQ
PKMAAAGASPYQRETSQPAQNPIFGSSSPASTAPVINAPAWFNEQRFLDAARGHFQSLQQ

HWDANEMDKIAEFVTPQMLQFLKKERADLGDGFQSTYIENLQVQLDGVDDRADKTIATLT
FSGVSKTSRFDQGEVFSESWNMERPQGDNQPWLVAGIRQNG
>tr|A0A0Q0C4T3|A0A0Q0C4T3_PSEAP Uncharacterized protein OS=Pseudomonas syringae pv. aptata OX=83167 GN=ALO85_101028 PE=4 SV=1
MFKNSPRVYRSDEPTGLRIPLLLSSLRFGTLAPCATTFGSR
>tr|A0A0Q0IL22|A0A0Q0IL22_PSEAP Uncharacterized protein OS=Pseudomonas syringae pv. aptata OX=83167 GN=ALO85_02702 PE=4 SV=1
MAVSNLDTHALFVLGDLRAQLVKQFQSRFVYITEQSAEGIYIAEIDTESALVVDDKPRLE
LKVGDHFRASVLPSREGGKLELKFREIKTTVYGLGEYAFVDVPEGHGIVFKEGQTVFMVF
AAKEQLQIGMSKLLKGAVGKAAKWRKGELTFKASE
>tr|A0A0Q0C318|A0A0Q0C318_PSEAP Trans-hexaprenyltranstransferase OS=Pseudomonas syringae pv. aptata OX=83167 GN=ALO85_02777 PE=3 SV=1
MQPQAFYRAVADDFSAVDLIIKKQLTSRVPLVSKIGDYITSAGGKRLRPLLVLLCGKALG
REGDDVRLLAATIEFLHTATLLHDDVVDMSGMRRGRSTANALWGNAPSVLVGDFLYSRSF
EMMVELGSMEVMKILSKATRIIAEGEVLQLSKIRDASTTEETYMEVIRGKTAMLFEASTH
SAAALCNAPQEQSEALRTFGDHLGVAFQLVDDLLDYRGDAETLGKNVGDDLAEGKPTLPL
IYTMREGSPEQAALVRQAIQKGGLEDLESIRNAVESAGALDYTAQLARDYAARAIACLDA
LPPSEYRDALVELSEFAVARTH
>tr|A0A0Q0DFV6|A0A0Q0DFV6_PSEAP Uncharacterized protein OS=Pseudomonas syringae pv. aptata OX=83167 GN=ALO85_01383 PE=4 SV=1
MSADTENAAYLGNIPSMVAFLPGLASEERTDILNVLLDAQLFAGRQFDFKTQWGNWMHYY
RSRLKTRGIQQKGLVLGDSLVVSSVDDLLEATFKVSHPADRKRLSDMVQRAVAAMGVNQA
AESYFQNGLDQGRLGSFQVVPCEKYEPGRMLLLLCSLHLSVDDHAPGRRRLLFHFKGGSY
IFDSKVYATHRDEVMRYLDGRALGLVRSASI
>tr|A0A0Q0DKF2|A0A0Q0DKF2_PSEAP Nitrilase/cyanide hydratase and apolipoprotein N-acyltransferase OS=Pseudomonas syringae pv. aptata OX=83167 GN=ALO85_02480 PE=4 SV=1
MRVALYQCPPLPLDVSGNLTRLEQQAQAAAEQGAQLLICPEMFLSGYNIGAQAVGELAQA
QDGPAATRIAAIAQASGIAILYGYPERAAYRQIYNAVQLIDSQGTRLCNYRKTHLFSDLD
KSMFAAGDDHYPVVELNGWRLGLLICYDVEFPENTRRLALAGAELILVPTANMLPYDFVC
DVTVRARAFENHCYVVYANYCGSEGAIRYCGLSSLCAPDGSRPLLAGQDEALLVGTLDKS
LLAQARTANDYFMDRRPALYTSLT
>tr|A0A0Q0BXU4|A0A0Q0BXU4_PSEAP 5'-nucleotidase OS=Pseudomonas syringae pv. aptata OX=83167 GN=ALO85_03951 PE=4 SV=1
MAFDLSNTLVVGISATALFDMSESDQMFKAGLEADPERVIDNYRRFMQEREDEPLEPGTG
YHLVEALLDLNRHMESGDLSPLVEVVVMSRNSPDTGIRVLKTIRRDQLAITRSAFTGGES
VADYMDAFDVDLFLTTNVDDAQKVIDSRQCAAAILKAPPANAPQIPKGQVRIAFDGDAVL
FSDASELVYKTQGLDAFQAQEDALQHTPMEEGPYASLLIKLAKLQERLPTGSKDSPIRIA
IVTARNSPSEMRVINTLRAWGVYVDEAFFLGGVGKPKVLTAFNPHIFFDDQDVHLEAAAN
LVPSGKVPYLTRPNIDSGVVSSAE
>tr|A0A0Q0C1V8|A0A0Q0C1V8_PSEAP Sarcosine oxidase, delta subunit OS=Pseudomonas syringae pv. aptata OX=83167 GN=ALO85_00789 PE=4 SV=1
MKIMTCPLNGPRNISEFTYGGEFKHMPDPQTCSDAEWADYVFNSEDTLGVVREWWMHNPS
SYWFLAERHTGSDEIIRTFDPRELFTARIDFAPLASKEIAG
>tr|A0A0Q0DTI4|A0A0Q0DTI4_PSEAP Uncharacterized protein OS=Pseudomonas syringae pv. aptata OX=83167 GN=ALO85_02651 PE=4 SV=1
MPNSAFSASHFSGNHAARDENIVINSYTTVLRKTGVPHELFATYWRDVHGPLCARLEGLG
WYVQHHLTREKDGHLWPVIEGVTALPDYVLDGGVEIGFLSAEAQKQFKDASALLFSDEQN
MFEETIAYDVPDGSSTLVDRLPDPIPNETATLDKMHLHFHLASADLPAARKAIEQLASDM
ASSDAVLKLRLHLAQPYDNAKPAPPAPDVNHEVEASRLNIVMMELVFESAWTRRTYYASE
QFKTITQGISKHVRYITPFGVSGVYTYVRDALMTTAGVRGSRQAELIRQLGAINQTRPEI
ESLFGAPSTF
>tr|A0A0N8T9J5|A0A0N8T9J5_PSEAP Bifunctional ligase/repressor BirA OS=Pseudomonas syringae pv. aptata OX=83167 GN=birA PE=3 SV=1
MLTLLKLLADGAFHSGQVLGDALGISRSAVWKQLQQLEADLGIDVHKVRGRGYRLATPIS
LLSPYGIEQSGFPANWSVRTYDSIDSTNAEAARLIVQGVPMPVLVVAEQQTSGRGRRGRK
WVSPFAENLYYSLVLRIDGGMRQLEGLSLLVGLAVMNVLRDMGVQGAGLKWPNDVLVGRQ
KIAGILLELIGDPADVCHVIIGIGVNVNMRSTSEVDQLWTSVRLETGGLVDRNQVAARIS
TQLEALMTVHRKEGFAAFQKEWERGHLWQGAAVKLLSGVGVVEGVVLGVDSLGALRLEVN
GVEKSFSGGELSLRLRDDS

>tr|A0A0Q0C3Q3|A0A0Q0C3Q3_PSEAP Regulatory protein, GntR OS=Pseudomonas syringae pv. aptata OX=83167 GN=ALO85_01432 PE=4 SV=1
MPDLSVAFPPTLTGIALDPNQGLSKQLYALLRQQVLDGRLSSGTRLPASRDLAHSLGISR
NSVIRAYDQLYAEGFIEGRVGDGTYVAQLSESSAAPRRTGRKKLSTNLPTGVSTGLHTGL
STIGSEIPSDSSCEVIHSPVLALLEKHHLHEPPTGRPRAFRVGVPAFDLFPFALWGKLYA
AFWRKPDLAQLGYGSAAGDSRLRELIAVYLRSSRGLHCSAEQIVITSGAQHAISLCAQLL
LAPGEGVAVENPGYRAAGHAFALAGARLHGVAVDNEGMRCADLEQADCRLAYVTPSHQYP
TGVVMSLARRLELLAWAEKHQGWIVEDDYDGEYRYSGSPLAPLAALDRNARVLYVGTFGK
IAFPALRLGYLVLPEPLVPVFSQRKAVDMRHTEIGVQAVMAQFIASGHFQRHIRRMRRAA
LSRRNVLLDNWPADIPGCAAMPRVVAGLHVMVKVDSVAREQALIAKANGVGVEMNALSDY
WLPDSTEPVDNRAGLVLGFAAVPEDEMVEALNRLRGVWSE
>tr|A0A0Q0FHA8|A0A0Q0FHA8_PSEAP Malic enzyme OS=Pseudomonas syringae pv. aptata OX=83167 GN=ALO85_02806 PE=4 SV=1
MSDLKTAALEYHANPRPGKLSVELTKPTATARDLALAYSPGVAEPVREIARDPELAYKYT
GKGNLVAVISDGTAILGLGDLGPLASKPVMEGKGVLFKRFAGIDVFDIEVDSESPQAFID
TVKRISITFGGINLEDIKAPECFEIEKALIEQCDIPVFHDDQHGTAIVTAAGMINALEIV
GKTLDSAKIVCLGAGAAAISCMKLLVSMGAKIENIFMVDRTGVVHSGRTDLNQYKAAFAH
ATEKRSLTDALDGADVFVGLSGPNLLSAENLKLMAKDPIVFACSNPDPEISPELAHATRS
DVIMATGRSDYPNQVNNVLGFPFIFRGALDVRAKRINEEMKIAAANALRELAKLPVPQEV
SDAYGGIKLEFGREYIIPKPMDTRLLGLISDAVAKAAIETGVATLPYPKHYPLTSVDDVF
KG
>tr|A0A0Q0DS78|A0A0Q0DS78_PSEAP Type IV pilus assembly protein PilX OS=Pseudomonas syringae pv. aptata OX=83167 GN=ALO85_02792 PE=4 SV=1
MNKIVRFNSIQTFPARQRGMVLLVSLVFLLLLTLLGISSMQNATLQEKMAGSVVVRNVSF
QAAEAQLRLGESKIMESGFSMVPCTPPAACAPPSDSTTVVRPGLGTSGVTWIGTANALFG
IQNLGTTPTPIKRPANCTGSVTMYRVTAIAIQGTSRTVLESIYANC
>tr|A0A0Q0BTA5|A0A0Q0BTA5_PSEAP Diaminobutyrate--2-oxoglutarate transaminase OS=Pseudomonas syringae pv. aptata OX=83167 GN=ALO85_01262 PE=3 SV=1
MNPTMAIYERESNVRSYCRDCDVVFATAQGAVIEDVEGRQFIDFFAGAGSLNYGHNNPRL
KKELLDYIQQDGITTTLDFHSAAKSRFIEQFERIILTPRGMNYKLQFTGPTGTNAIEAAL
KLARKITGRTSIAAFTHAFHGVTLGSLALTANADKRAVSGVELSNVVRLPYDGFLGRDID
SAHIIETMFSSAGAGFEPPAAIILEVVQGEGGLNCARPQWLQRIQRLCQASGALLIIDDI
QAGCGRTGTFFSFESADISPDIICLSKSISGYGLPMSLVLIKPEHDQWAPGEHNGTFRGN
NTAFVTAAAALSYWQDNAFERSIAQRSAVLRLALEKIIAHSPPEAVRIVGRGLFMGLRFG
NSQRASRLSAECLSQGLLVETCGPLNEVLKLMPPLTIDLPVLERGLDILKRAYLSTLHNE
SSRGIA
>tr|A0A0N8T8I0|A0A0N8T8I0_PSEAP Nucleotide sugar epimerase/dehydratase WbpM OS=Pseudomonas syringae pv. aptata OX=83167 GN=ALO85_03520 PE=4 SV=1
MMSRLRTRLLALPRRKKRIIQVATDVFLVWLALWMAFVVRLGIDELINPLEKHTWLFISA
PVAAIPLFIRFGMYRAVMRYFGNDALIAIIKAVTMSSLILGCMVYVYSNHQQLVPRSVIF
NYWWLSMVMIGGLRLAMRQYFLGDWFAAAQHVPFAKSDNGLQKVAIYGAGAAGNQLVAAL
RMGRLMHPVAFIDDDESISNRVISGLQVYKPRNIQRMIDSTGAQEILLAIPSSSRGRRRE
ILGFLEGFPLHVRSVPGFMDLAAGRVKVDDLQEVDIADLLGRDAVPARDNLLEHCIKGKT
VLVTGAGGSIGSELCRQILLLQPTQLLLLDHSEFNLYSILSELEQRSARESLSVKLLPIL
GSVRNHPKLLSIMKTWKVDTVYHAAAYKHVPMVEHNIAEGVINNVVGTLNTAQAALQAGV
SNFVLISTDKAVRPTNVMGSTKRLAELILQALSRETAPVIFGDKANVYQVNKTRFTMVRF
GNVLGSSGSVIPLFHKQIQSGGPLTVTHPKITRYFMTIPEAAQLVIQAGSMGHGGDVFVL
DMGEPVKIVELAEKMIHLSGLAIRSEKNPHGDISIEFTGLRPGEKLYEELLIGDNVVATE
HPMIMSANEDCLPWDVLKGRLTKLLDAVDRDDYTSVRQLLRETVNGYTPEGEIVDWMYQE
RSGET
>tr|A0A0Q0BZ83|A0A0Q0BZ83_PSEAP Proteinral secretion pathway protein K OS=Pseudomonas syringae pv. aptata OX=83167 GN=ALO85_00685 PE=4 SV=1
MNVTGRSQRGVALLVVLWVLALLSLLLGGLAGWVQLESRQSLWLRQNTQALMAAEAGMNM
AVQGLLDAEQRKRWIADGRVVSLRMDDTQLLVSIRSERGKLDLNSAPVADISRLLQACGA
AKNQASGIAQVLESQRNGGQSPLRVVEEVRQLPGMNQALYTRLLPEITLWSGLDRPDPAF
ASALLRRALNLPNQSAVGADPGEVLAIDTRAERPGGVTALLQTTVLLSPSEGGAQPYRVL
RWQE
>tr|A0A0Q0DRQ0|A0A0Q0DRQ0_PSEAP Sigma factor AlgU regulatory protein MucA OS=Pseudomonas syringae pv. aptata OX=83167 GN=ALO85_01996 PE=4 SV=1

MSREALQESLSAVLDNEADELELRRVLNAIDDADTRATWSRYQVARAAMHKELLIPHLDI
SAAVSAAIADEVSPLKAARGPWRTLGRLAVAASVTVAVLAGVRLYNQDDIAGAQLAQQTQ
QPANLTVPQVKGPAVLAGYTESAEQAPGPMANGVLQGGGQGDQRLPGYLRQHAQEAALKG
TESALPYARAASLENR
>tr|A0A0Q0DL37|A0A0Q0DL37_PSEAP Myo-inositol catabolism protein IolB
OS=Pseudomonas syringae pv. aptata OX=83167 GN=ALO85_02690 PE=4 SV=1
MNLLTKSKSTGRDVVALAPGSLEYVNFAAYRLESGETLPLSAGENELCVVLLTGHASVSG
EAPGQGAFNWENIGDRQSVFEEKSPFAVYLPPHSQAQFVARGAVQLAVCTAPGDAGKHFP
ARLIMPGSMKRSVRGKGANTRYVCDILPDSEAAHSLLVVEVRTPSGHSSSYPPHKHDRDN
LPHESFLEETYYHQVNPPQGFVFQRVYTDDRSIDQAMAVENNDLVTVPKGYHPVSVPYGY
ESWYLNVMAGPTRAWQFHNDPQHSWLLDL
>tr|A0A0N8TA45|A0A0N8TA45_PSEAP Aldolase II super protein OS=Pseudomonas
syringae pv. aptata OX=83167 GN=ALO85_03332 PE=4 SV=1
MLISQKSSRSNTMSNVSALPVQSGTTPSGSGSVRDRVSPEEWEVRVKLAAAYRLAALKRW
TDHIYTHFSARVPGPDEHFLINAFGLLFDEITASNLVKVDIDGTIVDDPTGLGINYAGYV
IHSAIHAARHDLQAVLHTHTRDGIAVSAQKDGLLPISQHSIAFSGRVAYHGYEGIALDLS
ERERLVADLGDKSVMILRNHGLLTGGVSVEHAFQQLHALEYACNIQIAAQSAGNAELVFP
PREVIAKVEQQAKAIKDGHAPGVARHWNALIRELERNDTDYKT
>tr|A0A0N8T9J4|A0A0N8T9J4_PSEAP 50S ribosomal protein L15 OS=Pseudomonas
syringae pv. aptata OX=83167 GN=rplO PE=3 SV=1
MKLNDLSPAPGSRREKHRPGRGIGSGLGKTGGRGHKGQSSRSGGTIAPGFEGGQQPLHRR
LPKFGFVSLKAMDRAEVRLSELAKVEGDIVTVQSLKDANVINQNVQRVKIMLSGEVTRAV
TIKGIAATKGARAAIEAAGGKFEE
>tr|A0A0Q0C1E1|A0A0Q0C1E1_PSEAP 3,4-dihydroxy-2-butanone 4-phosphate
synthase OS=Pseudomonas syringae pv. aptata OX=83167 GN=ribB PE=3 SV=1
MALNSIEELVEDIRQGKMVILMDDEDRENEGDLIMAAECVKPEHINFMARFARGLICMPM
TRERCETLKLPLMAARNGSGFGTKFTVSIEAAEGVTTGISAADRARTVQAAAAKDAKAED
IVSPGHIFPLMAQAGGTLARAGHTEAACDLARMAGFEATGLICEVMNDDGTMSRRAELEA
FAQQHDIKIGTIADLIHYRMIHERTVQRIAEQPMDSELGSFNLVTYRDSVEGDVHLALTL
GTICAEEPTLVRVHNMDPLRDLLMVNQPGRWSLRAAMRAVNEAGSGVVLLLGHPLDGDVL
LAHIRETAGQQPIKTPTTYSTVGAGSQILRDLGVRKMRLMSSPMKFNAISGFDLEVVEYV
PSE
>tr|A0A0Q0DY27|A0A0Q0DY27_PSEAP Response regulator
receiver:transcriptional regulatory protein OS=Pseudomonas syringae pv.
aptata OX=83167 GN=ALO85_01974 PE=4 SV=1
MEPHTWHVLIVEDDQRLAELTRDYLQSHGLRVNIEGDGALASARIIAEQPDLVILDLMLP
GEDGFSICRSVRDRYDGPILMLTARTDDTDHIEGLDTGADDFVCKPVHPRVLLARIKALL
RRSEAPQVPAAELRRLVFGPLVVDNALREAWLREQSIELTGAEFDLLWLLVANAGRTLSR
EEIFTALRGVGYDGQDRSIDVRISRIRPKIGDDPIHPRLIKTVRSKGYLFVPEAAQDMSG
HVFSG
>tr|A0A0Q0IQZ3|A0A0Q0IQZ3_PSEAP ATPase AAA OS=Pseudomonas syringae pv.
aptata OX=83167 GN=ALO85_00587 PE=4 SV=1
MEHREALIALRTFLSTQILGQEKLIERLLIALLADGHMLVEGAPGLAKTKAIKELAEGIE
AQFHRIQFTPDLLPADITGTEIYRPETGSFVFQQGPIFHNLVLADEINRAPAKVQSALLE
AMAERQVSVGRSTYDLSPLFLVMATQNPIEQEGTYPLPEAQLDRFLMHVKIGFPDAAVER
KILQQARGEALNGETKPERRVSQQAIFAARKEILGLYMADAVEEYLVQLVMATRTPAKFD
PELAEWIAYGASPRGSISLDRCARAHAWLAGRDFVSPEDIQAVLFDVLRHRVILSFEAEA
AGIDQDRVIQRILDVVAVA
>tr|A0A0Q0DQ05|A0A0Q0DQ05_PSEAP Spermidine/putrescine import ATP-binding
protein PotA OS=Pseudomonas syringae pv. aptata OX=83167 GN=potA PE=3
SV=1
MWVMSDVDTSAGANDVLVSFRGVQKSYDGEALIVKDLNLDIRKGEFLTLLGPSGSGKTTS
LMMLAGFETPTAGEILLGGRAINNVPPHKRDIGMVFQNYALFPHMTVAENLAFPLSVRGM
SKTDVGEKVKKALGMVQLDTFAHRYPAQLSGGQQQRVALARALVFEPQLVLMDEPLGALD
KQLREHMQMEIKHLHQRLGVTVVYVTHDQGEALTMSDRVAVFHQGEIQQIAPPRSLYEEP
KNTFVANFIGENNRLNGRLVSQDGDRCVVELERGEKVHALAINVGQPGGPVTLSIRPERI
NLNGRSESCANRFSGRVAEFIYLGDHVRVRLEVAGKNDFFVKQPIAELDPGLSVGDVVPI
GWQVEHVRALDPMQQVH
>tr|A0A0Q0DSN4|A0A0Q0DSN4_PSEAP ANK_REP_REGION domain-containing protein
OS=Pseudomonas syringae pv. aptata OX=83167 GN=ALO85_03383 PE=4 SV=1

MNEGRDTAILFALIKQGMTLPMTEQKIYGVEGESEDFRVAVASAQRTFRFFWREMSWERR
RIVKALDLAAVKVSFTTDSADPDSPSVENMWVTDVDFDGLTLSGILMNEPVWVSSINAGD
PVSVSLDRLNDWVYVFGGRAFGGFTIDALRSGMSAAERVEHDEAWGLDFGEAGTVMLVPP
AEGKSPVCFTRALDSASDKRALNKLERLEHPMGLNAQGAVEEGLRDDPGLATDYDDSGWQ
MIHRETLAGNCNFVATLLYMGADSAATNSNGHDVLTLARIAGWPRTIELLEGDRSNLEKH
VQRRGFPAWPIGLTMAVIGVVGLYFAALSQSTGSLIVRNDSLLSTGLFIALVWFLGQGLI
LCTGPWYFRLRERTPIWGKARALDLLAMLIGVLLAFFQHDHLGNYLHSL
>tr|A0A0Q0C7Y9|A0A0Q0C7Y9_PSEAP Filamentation induced by cAMP protein Fic
OS=Pseudomonas syringae pv. aptata OX=83167 GN=ALO85_02819 PE=4 SV=1
MAFTPYIPQDKAPDELQDQVVQIMRLDAALHAKIPGPLRLPMMNLLRVVNSYYSNKIEGN
STIPADILRAEDAPQEFKGAKDLQEIKRHIEAQRRLTDEPINQVDVCTKSAISRMHREFY
VGVPEDMLDIQLNEKGDTVRMVPGEFRHLGVRVGKHIPPTVEQMPTYLNDFERMYRLDWI
HGLSGFFAAAASHHRLMWIHPFMDGNGRTGRLFTDQYLKAAGFGGYGLWSMSRGFARNVT
AYYEMLGAADHPRKGELDGRGELSDSGLLRWTRFFTETALDQVQYFSAQLEPERLGERID
VYFEMRSRRALSDSKGETLPELRIEARDVYKALLYQGDQQRADIQARLGVGERTTRSLLS
QMAKEGLITLDGRKPVALNLSRHSIEFLFPYLW
>tr|A0A0N8T919|A0A0N8T919_PSEAP UDP-N-acetylenolpyruvoylglucosamine
reductase OS=Pseudomonas syringae pv. aptata OX=83167 GN=murB PE=3 SV=1
MTLQVQSAISLKPFNTFGVDVQARLFAEAHSDDDVREALAYSAEHDVPLLVIGGGSNLLL
SADVQSLVVRMASRGIRIVHEDCLESIVEAEAGEPWHPFVQSCLELGLAGLENLSLIPGT
VGAAPMQNIGAYGVEIKDVFHSLTALDRETGELHEFSLQDCAFGYRDSVFKHQVARWLIL
RVRFKLSRVARLHLEYGPVRQRLDEQGIGQPTPFDVSRAICAIRSEKLPDPAVLGNAGSF
FKNPIIAAELYATIKQQHPGVVGYPQDDGRVKLAAGWLIEQAGWKGYRDGDAGVHKLQSL
VLVNYGQASGLQLLSLARRIQTDIAERFGIELEMEPNLY
>tr|A0A0Q0CG12|A0A0Q0CG12_PSEAP Regulatory protein, LysR:LysR, substrate-
binding protein OS=Pseudomonas syringae pv. aptata OX=83167
GN=ALO85_03581 PE=3 SV=1
MTMTPVDGSATFDVNNLFRKPRICFDWEDLRYFVMFSTVSSLSGAARILGVEHATVSRRI
ASLERSLNVKLVDRRKRLYELTDEGRRVAAIGRQIEAQTQAVGRLAVAAQVVQGVEVTIS
APPSVAVEKLVPQLGAFRAMHPGIMLRLLGDHQYSSLSSCQSDLCIRFSKPVESGIVARR
IGTASFSFYCARFYLEQRPPQQYEFIAYDNELMSDSQNGWLRDIVGDRPFVLEAKTVELQ
IRAAETGVGIALLPSFAVEDNDRLCRVELSAPLEVDVWLGVHEDLRAVASIQAAMNYVES
CYR
>tr|A0A0Q0ISY4|A0A0Q0ISY4_PSEAP Uncharacterized protein OS=Pseudomonas
syringae pv. aptata OX=83167 GN=ALO85_03303 PE=4 SV=1
MALFSPYALGATLYMPATRDDIVDVVFGEKIPELRSLVVCLEDAVALTDVDTALINLRQV
LTRIRDRGGRPANGPLLFVRPRDAAMARILNDWPLMAHVDGFVVPKLSLKSLTSWEQAVT
NPSLALMPTLETPEVFNPTAMVELGEALKASLYERIIALRIGGNDLMGCLGLRRNPAMTL
YSTPMGYVIPMLAGVMGSQGFALTAPVFEQLATPDILQHELALDITNGLVGKTAIHPSQV
NIIQNALRVSLEDMNSARMILNSVAPAVFKYNDAMCEPATHYKWATHIMERAKWHGVLSA
PASIMDASIRLAEAVS
>tr|A0A0Q0IR46|A0A0Q0IR46_PSEAP Group 1 truncated hemoglobin
OS=Pseudomonas syringae pv. aptata OX=83167 GN=ALO85_02012 PE=3 SV=1
MRRLLIAMMLTLLAGCAQQQQPPKDDSLYHDLGQRAGIQRIVEGMLINVAKDQRIVEHFK
KVDIVRLRDKLVEQLCVESGGPCRYSGDSMAEAHKGQNLTPSDFNALVEDLIASMDTEKV
PVPVQNRLIARLAPMRGQVIGK
>tr|A0A0Q0D0I7|A0A0Q0D0I7_PSEAP YccA-like protein OS=Pseudomonas syringae
pv. aptata OX=83167 GN=ALO85_00704 PE=3 SV=1
MQLNISTARSFAMREQDYSLKTGMQADQLEVSRVLRNTYGLLALTLAFSGVMAFVAQQMR
VGYPNIFVVLIGFYGLFFLTNKLRDSVWGLVSTFALTGFMGFLLGPILNRYLGMAGGAEV
VSSAFAMTALVFGGLSAYVLITRKDMSFLGGFITAGFFVLLGAVVAGMFFQISGLQLAIS
AGFVLFSSVCILFQTSAIIQGGERNYIMATVSLYVSIYNLFISLLQIFGIMSRDD
>tr|A0A0Q0DV21|A0A0Q0DV21_PSEAP Disulfide bond formation protein B
OS=Pseudomonas syringae pv. aptata OX=83167 GN=dsbB PE=3 SV=1
MYLARTRFLFFLASLACASIIGVAFYLQQAVGLDPCALCIVQRAAFIVCGVLALCAACHA
PGPTGTRRYSLALLLVALAGLATAGTQVWLQTASADQLIPVITRLEHVLSLLSLDMCIDR
LRSDALFCAEITWTLFGISLPEWSLLGFTGLALLPLYPLFSEFSHWLATRDRGSY
>tr|A0A0Q0D0C2|A0A0Q0D0C2_PSEAP Uncharacterized protein OS=Pseudomonas
syringae pv. aptata OX=83167 GN=ALO85_03214 PE=4 SV=1
MRFFGFKAMAVCVALAACQSMVSVQAATKPQAAAPAAAAVQNASLLGGKLAFTIPAGYVQ

GEMPEIDAKAKAQGVTGSLYTNAAEKRVLIVTETPMPMGVQANDNDGAVLDGLVTGILAQ
QSTGYKDFKKLGDKKIVKKNGLGVRQLDTSATMSDAQVLSTSIVAASGTRAAVLNVVSNA
KNPKEHAALVKTVIGQ
>tr|A0A0N8TA38|A0A0N8TA38_PSEAP RulB protein OS=Pseudomonas syringae pv. aptata OX=83167 GN=ALO85_200115 PE=4 SV=1
MLLNQKSAEQEISLKVREDFLLLPKTAELTRWVMAQSTGERTLPYFDPLDGGVFARVLIL
LESPARTVSLPRYVSRDNPGPAQRNLKGFIEQVGLARHKTVLWNLYPWLPDLDKPKGPLR
RAQIEEGIEMLITLLTLFPDLVTIVFAGRVAQKAIPRVRLAFPNFSLLEMPHPSPLSVCT
HPNVRQRILSTLRHAKQSIK
>tr|A0A0Q0DLX7|A0A0Q0DLX7_PSEAP Putative biofilm formation regulator BifA OS=Pseudomonas syringae pv. aptata OX=83167 GN=ALO85_05103 PE=4 SV=1
MRVWFFEGHEAICPATRTKERPLKLELKNSLSVKLLRVVLLSALLVGILLSCAQIVFDAY
KTRQAVASDAERILGMFRDPSTQAVYSLDREMGMQVIEGLFQDDSVRMASIGHPNESLLA
EKNRPLKTSSTRWLTDIILGQERSFTTQLVGRNPYSEYYGDLRITLDTANYGESFLVNAG
IIFIAGVLRALAMGLVLYLVYHWLLTRPLSKIIEHLIAINPDRPSEHQLPLLKGHEKNEL
GIWVNTANQLLASIERHTYLRHEAENNLQRMAQYDFLTGLPNRLQLQTGLDRILEDAGRQ
QHRVAVLCVGLDDFKGINEQFSYQSGDQLLLALADRLRAHSVRLGALARLGGDQFALVQS
NIEQPYEAAELAQNILDELEAPFIVGDQQIRLRATIGITLFPEDGDNTEKLLQKAEQTMT
LAKARSRNRYQFYIASVDSEMRRRRELEKDLREALPLNQLYLVYQPQISYRDHRIVGVEA
LIRWQHPEHGLVPPDVFIPLAEQNGTIIAIGEWVLDQACRQLREWLDQGFTDLRMAVNLS
TVQLHHAELPRVVNNLLQIYRLPPRSLELEVTETGLMEDINTAAQHLLSLRRSGALIAID
DFGTGYSSLSYLKSLPLDKIKIDRSFVQDLIDDDDDATIVRAIIQLGKSLGMQVIAEGVE
TVEQEAYVMTEGCHEGQGYLYSKPLPGRELLAYLKQSRQDHAASL
>tr|A0A0Q0FNA4|A0A0Q0FNA4_PSEAP DNA-binding response regulator, LuxR family OS=Pseudomonas syringae pv. aptata OX=83167 GN=ALO85_02664 PE=4 SV=1
MTCNLLLVDDHSLIRAGVRALVADMPGYTILGETADGSQLASLAQRLKPDIILLDICMKS
IDGLVALEHLLKVQPHCKVLILSAQNDPDMIMRTLDIGAQGYLLKDTTAAELEQALEALR
GNERYLSPAIAQMVIECALRGTPSDRAQAAKERFRLTARQLEILRLIVRGKSTREIADGL
GLSVKTIETHRAQVMKRLQIFDVAGLVLFCVRERIISLDD
>tr|A0A0Q0C2D0|A0A0Q0C2D0_PSEAP 3-deoxy-manno-octulosonate cytidylyltransferase OS=Pseudomonas syringae pv. aptata OX=83167 GN=kdsB PE=3 SV=1
MTAAFTVVIPARYGSSRFPGKPLKTIAGKPMVQLVWEQACKSSAERVVIATDDARIVEAC
QAFGAEVLLTRDDHNSGTDRLAEVAAQLGLAADAIVVNVQGDEPMIPPAVIDQVASNLAA
HPEAGMSTLAEPIDDVAALFNPNIVKVATDINGLALTFSRAPLPWARDALAANRDQLPAG
VPYRRHIGIYAYRAGFLHDFVSWGPCWLENTESLEQLRALWNGVRIHVADALEAPPGGVD
TPEDLERVRRLLEG
>tr|A0A0Q0D7A2|A0A0Q0D7A2_PSEAP 50S ribosomal protein L10 OS=Pseudomonas syringae pv. aptata OX=83167 GN=rplJ PE=3 SV=1
MAIKLEDKKAIVAEVNEAAKAGLSAVVADARGVTVGAMTGLRKEAREAGVYVRVVRNTLL
KRAVADTEFSVLNDVFTGPTLIAFSNEHPGAAARLFKEFAKGQDKFEIKAAAFEGKFLAA
NQIDVLASLPTRNEAISQLMSVIQGATSKLARTLAAVRDQKEAAAA
>tr|A0A0Q0FQ48|A0A0Q0FQ48_PSEAP Ferric siderophore uptake system, MotA/TolQ/ExbB protein OS=Pseudomonas syringae pv. aptata OX=83167 GN=ALO85_200198 PE=3 SV=1
MDTGTLGLNVLWQQADVVTRGVALTLLVMSIASWYVMVDKLVRLALHRLRSPKLLEAFWS
APTLKDGVQQLGHHSAYAELTQAGVSAARQHRDALDGDMHPPAFSEWLNRALRQATLTVG
GQLQGGMAILATVGSSAPFVGLLGTVWGIYHALMKIGLTGDASIDKVAGPVGETLIMTAL
GLAVAIPATLGYNLLVRSNKQTLAELGKFAFELHDLLVIGARATPRGGQPARSTFNAQAE
IV
>tr|A0A0Q0D5R2|A0A0Q0D5R2_PSEAP 2-hydroxyacid dehydrogenase OS=Pseudomonas syringae pv. aptata OX=83167 GN=ALO85_05204 PE=3 SV=1
MTTVLVLVENLDTYLPILESSGFQLIRAPTAELRAEAIATHGPSIIAVVTRGPLGFFAEE
MDALPQLRIICVSGAGYEKVDLPAAQARGIVVTNGAGVNAATVADHTLALLLSLVRDIPQ
ADASVRRSEWRKVVRPSLAGKRLGIVGLGAVGLAIAKRAAAFDIAIGYHNRKPRSDCDYT
WHATAQALAAASDFLVIATPGGSGTQHLIDARVLEALGPDGFLVNIARASVVDTQALVSA
LQHEQIAGAALDVFDDEPTVPDVLKTLDNVVLTPHVAGLSPEASRDSVQMVNDNLLAFFA
GQPVLTPVTE

>tr|A0A0Q0C1Y4|A0A0Q0C1Y4_PSEAP SMP-30/Gluconolaconase/LRE protein OS=Pseudomonas syringae pv. aptata OX=83167 GN=ALO85_00769 PE=4 SV=1
MRIEIVVDVKTTLGEGPGWDVEQQRLYWIDSFDGRVLRCTDDGRELRAWDVGQKIGSMAL
RKHGDAALVALQTGIYNLDLPSGDLELIVDPEPGLPNNRLNDGKVDRQGRFIVGSMDTQE
DQASAKLYRLDPDLSLHTLDEGIIVSNGPCWSPGGETFYFADTWSGEIWTYDYDNASGAV
ANRRTFTKVDTSAGGAADGCTVDAEGCLWQALVYAGKLVRYTPDGQVDRIIDMPVKKVTS
LTFGGPNLDTLFVTSMAKPPLPRFPQDGQQRGALFAITGLGVKGIAERRFAS
>tr|A0A0Q0IAS6|A0A0Q0IAS6_PSEAP 4-hydroxy-3-methylbut-2-en-1-yl diphosphate synthase (flavodoxin) OS=Pseudomonas syringae pv. aptata OX=83167 GN=ispG PE=3 SV=1
MHGESPIKRRESRKIWVGSVPVGGDAPIAVQSMTNSDTNDVAATVAQINRLEAAGVDIVR
VSVPDMDAAEAFGRIKQLVKVPLVADIHFDHRIALRVAELGVDCLRINPGNIGREDRVRA
VVDAARDRGIPIRIGVNAGSLEKDLQKKYGEPTPEALVESALRHVEHLERLNFQDFKVSV
KASDVFMAVAAYRLLAKEIVQPLHLGITEAGGLRSGTVKSAVGLGMLLAEGIGDTIRISL
AADPVEEVKVGYDILKSLRLRSRGINFIACPSCSRQNFDVVKTMNELEGRLEDLLVPLDV
AVIGCVVNGPGEAKEAHIGLTGGTPNLIYIDGKPAQKLTNDNLVNELERLIREKAAEKAE
ADASVIVRG
>tr|A0A0Q0DB56|A0A0Q0DB56_PSEAP Uncharacterized protein OS=Pseudomonas syringae pv. aptata OX=83167 GN=ALO85_04137 PE=4 SV=1
MRILTALTFCVLASTAQGKCQDRYYYYEAKPLPAKVKNWQIYEDNNVQFSREIKDFLSMT
AACSPATDGEAYHFTAYFNYIVEPNLWKKLSNPLYKNMAIRLPNGVYAQGANASTMDVVE
FNQTNRKTFTQYRAELSYAGKTSVIYVYLVRKGIDQMYTPMLNVSFSKTYQRDGYYFTEY
KP
>tr|A0A0Q0FI50|A0A0Q0FI50_PSEAP GCN5-related N-acetyltransferase OS=Pseudomonas syringae pv. aptata OX=83167 GN=ALO85_03198 PE=4 SV=1
MSEIQFYPLPDECRPLLGKFYREHQSSMRAASKGQAWVAKQAEIIGALCLTPVAEGHWLT
GLFVAPALRGQAVARGLIEAALQQLGGPIWLFCHPDLLVFYQRSGFETAQRLPQALGERF
MRYSRSKPLIALCREA
>tr|A0A0N8TAD6|A0A0N8TAD6_PSEAP Coenzyme PQQ synthesis protein B OS=Pseudomonas syringae pv. aptata OX=83167 GN=pqqB PE=3 SV=1
MYIQILGSAAGGGFPQWNCNCVNCAGFRDGSLRAHARTQSSIALSDDGINWVLCNASPDI
RAQLQGFAPMQPGRTLRDTGISAIVLMDSQIDHTTGLLSLREGCPHQVWCTDMVHEDLST
GFPLFEMLKHWNGGLTWNRIELQGSFVIPACPNLRFTPFPLRSAAPPYSPHRFDPHPGDN
IGLLVEDTRTGGKLFYAPGLGKVDEALAEKMRDADCLLVDGTMWDDDEMQRRGVGTRTGR
EMGHLAQNGPGGMLEVLEGFPEQRKVLIHINNTNPILDEDSPERAELVRRNVEVAFDGMS
IEL
>tr|A0A0Q0IDF2|A0A0Q0IDF2_PSEAP D-aminoacyl-tRNA deacylase OS=Pseudomonas syringae pv. aptata OX=83167 GN=dtd PE=3 SV=1
MKGLLQRVRSARVEVGTEVVGAIEQGILVLVGIEPQDTRASADKLLHKLLNYRVFSDAEG
KMNLSLREVGGGLLLVSQFTLAADTKSGLRAGFSKAAPPALGAELFDYLLSQARIAHPTV
AAGQFGADMQVHLINDGPVTFLFDT
>tr|A0A0Q0DYZ8|A0A0Q0DYZ8_PSEAP 50S ribosomal protein L18 OS=Pseudomonas syringae pv. aptata OX=83167 GN=ALO85_200013 PE=4 SV=1
MTDSRKRSFVEMPKDWPLPDHYLLELGRIAATWAVLEGSLNLYIKKLLGINDPDDARPQI
ITTHMNFKQKQDAFGTLCDVLQDSHPGLKNFDAVMTAMNKASKARNRYVHGSLHYDPDTA
TLLLSSVSARGSFKVTFVPTTVENLKDVSVLIHKASLALHNLVTGSKHQGLFPTT
>tr|A0A0N8T7S6|A0A0N8T7S6_PSEAP Uncharacterized protein OS=Pseudomonas syringae pv. aptata OX=83167 GN=ALO85_04045 PE=4 SV=1
MESPHEVAIRISKMDSGDRKFNEADTRHKIIDQILHGVLSWPVSQTMHESFISPGFADYR
LKRGNGDDLLFLEAKKEGVYFTIPKSFNSAKTHQFITVQTLLTDANIRAAIEQVRAYCME
EGCSFAGITNGHEWILFKTFESGKNWRNLKALVIHGNRYFAECFTEATNILGYSSIKEGS
LKTSLGSINRNNREIFYPKDKISSYDHEVTSNGHARELRPLANKYFGSIDVSEPVFMDAC
YVSQRDYENAFSNFRLIIKDSLTPYMADRGIQQSDDNEKGGLFGNRIAKSLKDKKSGEVI
VLFGGKGAGKSTFLRKLLYHNPPQYLSKYSRVAIADLLNTPEEETKIYDKIWSEIIRCLD
VDGLLETDRASLLQLFEDRFELAKKQVLFGLAETSEAFNIALNNLVEAWLCDKKYVAKRL
VFYWKLKHKGCVVAIDNTDQLPHNLQDFCFTTSQEISDQLGCLVIISMREERFHASRIHG
TLDAYQNSGFHISSPVAQAVFEKRIKFVQLMLEDREFCIELFGEQFDEKARVFKTLFRIF
ANEFRQGNDSPLNEFLSACAHGNIRLALELFRDFLKSGYTGVDEMVSAKGGLWKLKIHQV
LKPVMIPYRFFYEESQSSIPNLFQIRSKSNGSHFTGMRILQKLADGIDPSNPFYISISEL
KDYFSENFNMVEDLEKNLDVFLKWDLIESNNRVDYYCEAVDSLKITTYGLYIFNVLSSFF

TYIELVSTDCGIFNESKAHEMVELSNEDFRLFNARKRLERIRTRIAKAEAFISYLEAEEE
QELNFFGPNLQRFTPKIRASFEIEKQEVLNSAQKKRNR
>tr|A0A0Q0DRX5|A0A0Q0DRX5_PSEAP Uncharacterized protein OS=Pseudomonas syringae pv. aptata OX=83167 GN=ALO85_101816 PE=4 SV=1
MSDSTKPIVLDGYMNAGILESTQTNNISAVMPEVTKLLALS
>tr|A0A0Q0DAR3|A0A0Q0DAR3_PSEAP Short chain dehydrogenase OS=Pseudomonas syringae pv. aptata OX=83167 GN=ALO85_03341 PE=4 SV=1
MKKILIIGATSTIAHACARRWAAQGCDFFLVARNTDKLAGNAADLRARGAGHVDTYQMDV
THFSAHPAMLADCLAALGQIDIVLLAHGTLPDQKACEQNAGLAVQEFITNGASMIALLTV
LAKHFEVQRCGTLAVISSVAGDRGRPSNYLYGSAKAAVSTFCDGLQARMFKLGVHVVTIK
PGFVDTPMTRGLPLPAWLVAQPEDVAERIVNGIARQSTSLYAPGFWAWIMWVIRSIPQPL
FKRLNL
>tr|A0A0Q0DTT0|A0A0Q0DTT0_PSEAP Leucine-rich repeat protein OS=Pseudomonas syringae pv. aptata OX=83167 GN=ALO85_00346 PE=4 SV=1
MLDLSLTGKAPEPPHLQLIKDKSPEWLLHAAPATHATLRKALRRPLRWLAGARKSSPDQL
AELQRLYAEHREYEQQVRPTLDSLSTLENFARPLLTAAIKDRFGLEVDVANTWLFHASRA
RVDQSFNTASRDPITQANIALRASTQSLLKAALQSFEAWETATGAMDSSTGIKAQVFSSF
DIIGQQISGTSLPIPPTGFAALCRELDLGGQYQAHIQAAFSRPSTPDETADAAASRLRQS
FMQLEASSIRLQLQITSLQQLISRGLQGALLELLDGKQHVRLDNRPVSCSVLCLGDIELT
GILVIGKDREMATQTERIVVYIPDDPVAPLKEYDSVEVFINALRDRMFINGYLNFFMRFI
PARHRSALFEKLSERLHPKVKKGGIFERQWLEREADRNARLDLRETVLQGPLLDNLYERK
RAALRDDALFHGVPTAAQDQKTFDERVQYFMDTAFNVLNIAGFVVPVLGEVMMAVTAIQL
AHEVYEGVESWARDEKQQAFAYLFDVVENVALISALGAAATSAAGIPAVQAPEFVNSLKP
VDLPGGMTRLWKPDLTPFAHDIVLPKGLQPDATGLYTWQGKQWLPLEGRTYSVSSAANGD
GYLIEHPTRADSYRPALRHNGAGAWLHELDQPLAMEGLNLFRRLGYSSDTFSDSTARRIV
NVSNTPESVMREALTDQRRPPALLEDTARRFQLDQEIERCIEQLEANDVNAAAPLQLELL
SQDRGWPGNRALVLVDAEGQKLQTFAPAYQPVAVDTLDITVHADQPDALRQVLEKLSNNE
IRTLLNEEFGAGQLGMSPRLITLRAQLAARARATRGWLFESRYRALNVAEADGAQTLQKA
FPGLPPLVAQELASHASSAERLQLTTERRVPLPIAEEASAYLQQVRLARAYEGLYLTSVS
SADSDCLALHSLEALPQWPAQVRLEVHNRYFGGRLIDSIGPTDAPVRKVLIKDGNRYETR
DADDRHLHGLDDLYSSVLHALPDAERNQLGFAHPGQGQALAALIQENPLPRRHLAPLLNM
QAIKPGTKSPMRLADGRLGYPLSGGARVDWHMTDESLLDKIRMLELEDAFPEDILSRLRH
TGWDNRTIDARLNDLLGEQMLLRASLDAWAYESVAMPAMSQTHIDSRRRLNDAIWSHWRL
NNLPEIGRTLEPLRLQQVSLSDFPRHLPDFFFSRVTALHLENIAVEPDAIDLAPAGMNLP
RQLNNSAELGVFLQRFPHTRALNLVSEPSAGLAPETSVFFNLPQQVSRVLPQLTELGLIN
QRIFLDQAQMNHLRNMPGLRMLDLSGNRLISVLPMDLSWLHLDRLVLERVGMHRWPSWLT
DMIPNNIRELSVAHNNLTELPGWILDNPPSAEHRTVIDLRGNALSRHTAIHARINEAVAG
SSFSFVMDTPLAVRAAVNRQLTQGAELFAAVDQWTHASSSMAAPSEQLIESRRQIGRILI
NHWRTFSLGQIHSPLRLADIALTDFPRQLPDFFYRQVRYLHLSRITGTAADLDQLLRRMT
DLTSLELNGHVAPLLQLPPALLELRSLRSLSLMDQGMLVDQQHIDFFGRIATLERLELDG
NRIGAINNLNSLGGTALNWLSLNNAGLVEWPTWVKSMVPAQIRTLLLEGNLISELPEHIL
ANPGSEFAHTEISLLSNPLSEDTMRRAHLSERYGRSFTFDMDLPPELSAMDWTEHHDSDS
SISDYESSEDSRAATPEPVTVEPWLDDSNPLSTGRREIWEQLEAREHTRHLLDLIDSLRH
SADYRNIASRAALRERVWRVLATVSEDPQLALTLNAIAEEPLRLFHHNDTCSDGILLEFN
QMEVLVFIRQSLQDVVTEQRGALLYRLTVRLYRLSELDVAAREQAGNRDEAEVRLAYRTH
WASTLDLPVPPEGMMYPAHAAIRPGEFETALLRVQIGERGEPFLRFAGQQDYWINYLRET
HSGRFEALERNYRTELNRLTDEFEQRNISLDDPEYEGRILEFEATFKEQQTALIRELTNA
EGMEHH
>tr|A0A0N8T9C0|A0A0N8T9C0_PSEAP DNA-3-methyladenine glycosylase II OS=Pseudomonas syringae pv. aptata OX=83167 GN=ALO85_02605 PE=4 SV=1
MFFIGFAFLAGRSGQVQVIGHGVLNVPRSSQISDHFRLCVNPVCPPGRFNLEVIMSSKSH
SADREHAEAVAALRSIDVQWQSLIDHVGPCLHPVSAAQDPFQALVKAVAYQQLHARAGDA
MVRRLRALFLDDSLPEVSFPGAQALVDLDDQALRSCGFSASKCRAIKAIAEARLDGLVPE
VSAALAMGNEALVERLIQLPGVGRWTVEMMLIYGLGQLDVMPASDFGVCEGYRRLYALQL
KPSHRQMARLAERFAPYRTIAAWYLWRVPVDFSELGSSRI
>tr|A0A0Q0C872|A0A0Q0C872_PSEAP Cytidylate kinase OS=Pseudomonas syringae pv. aptata OX=83167 GN=cmk PE=3 SV=1
MKIKAPVITIDGPSGSGKGTVAGLLARKLGWCLLDSGALYRLLAFAARNHGVDLTNEEAL
KVLAAHLDVQFEATAAGQGQRIILEGEDVTHAIRNEQVGSGASQVASLPAVRDALLLRQR
AFQEEPGLVADGRDMGTVVFPDAPLKIFLTASAEERARRRYLQLKAKGDDVSLSSLLDEI

CARDERDTQRAVAPLKPAHDAIQLDSTELSIEQVLERILSEIALRGIAG
>tr|A0A0N8T8I9|A0A0N8T8I9_PSEAP Uncharacterized protein OS=Pseudomonas syringae pv. aptata OX=83167 GN=ALO85_02145 PE=4 SV=1
MPFRACIIGIASASLMTFALLSQAAPAHYYKWQGDSRIVCAQASPGPGWKRLKGHFIKAD
CSM
>tr|A0A0Q0IAR9|A0A0Q0IAR9_PSEAP Putative Permease OS=Pseudomonas syringae pv. aptata OX=83167 GN=ALO85_04304 PE=4 SV=1
MVKLDRYIGKSVLLAILAVLGIILGLVSLFAFIDEMGDISDTYTLADALSYVMLTAPRRV
YDTLPMAALIGCLIGLGTLASSSELTIMRAAGVSIGRIVWAVMKPMLVLMIAGVLVGEYV
APYAENQAQAARSLAQGSGDAQSARHGLWHRQGDEFIHINTVQPNGLMLGVTRYRFDDQR
HMLTSSFAKEAHFKTDYWELKDVATTHFNDGSTEVVNTPVERWDVALSPQLLSTVIMPPE
SLSISGLWSYAHYLADQGLSNGRYWLAFWTKVLQPAVTVALVLMAISFIFGPLRSVTLGQ
RVFTGVVVGFTFRIIQDLLGPSSLLFGFPPLLAVLLPAAACALAGILLLRRAG
>tr|A0A0Q0IMU7|A0A0Q0IMU7_PSEAP 4-phytase OS=Pseudomonas syringae pv. aptata OX=83167 GN=ALO85_200047 PE=3 SV=1
MTRRKRGTLTPQENLSMNNRSISRAPRFPVTRLALLISLNSLCLISPFIQADDDVPTSAT
APASDNSAAMTLGTVSVIGQGETRQVQRVTQKDVKAYSAGTSPMKVLQRLPGVNFQSGDP
LGREEGSQRISLRGFDMHHLGYTLDGVTLGNMSFGNFNGLSITRAIIAENIASSEVAPGI
GSLGTASNSDLGGTIQFTSSNPDKEFGARLSQTLGSYDTSRTFMRVDTGEYNGLSAYVSG
EKYDADAWKGHNNPQKSDAVNAKVNYNFGDNRVSFFHSTSSHDEANLPNMSKSIIQRLGY
NWSYYTPDWTRAVNAANGIYSGGVTSANDATYNASSLRDDELDILNGNFSLTDDLVLDAT
AYHHHDSGRGNSYYPFVYTSGSTTVPSNSIRSTKYGIDRTGFQSSLTYYLGQHEIQGGFW
IQSNKNDISRYLFDTTNATPQNHIVKTDGLPLAATVLDQSYNDLTRQFYLRDTYTLLDDR
LKLEFGAKNTVTTSTAEGKGGGYASGSLQARDRFLPQVGATYKLDEDDEVFTSYSENMAA
FPSGGYSPFFTTQSAVDAQNGFKDLKPETSKTVELGVRRSTKLYSASAAIYNTKFDNRLV
AITNCTGIVICQNGVANVGSVTSRGLELSFGLTPNENWRWSNSMSYNHSTYDDDYASGGN
TVHVKGKTVVDTPKLMYSSNLDWNWEHWNAGLQGSYISKRYYTYTNDSSVSGYWLANANL
GYDFGKLGALKDTTLSLNMVNLFDKRYISTLNTDASAATDPAGNLQILQVGTPRSAFVTL
GVKL
>tr|A0A0Q0FL70|A0A0Q0FL70_PSEAP Probable malate:quinone oxidoreductase OS=Pseudomonas syringae pv. aptata OX=83167 GN=mqo PE=3 SV=1
MAHNEAVDVVLVGAGIMSATLAVLLKELDPGITLEVVELMDSGAAESSNPWNNAGTGHAG
LCELNYTPPAADGSIDIKKAVHINTQFEVSKQFWAYLARKGTFGSARSFINPVPHLSFVQ
GEKGVSFLKTRFEAMSKHHAFSSMEYTEDKATLAEWMPLMMPGRPADEKIAATRMMNGTD
VNFGALTNQLLGHLTSAPGTQIKYRSRVTNLTRNGAGWTVSVKDVNGGGTREIDAKFVFL
GAGGAALPLLQLSGIEESKGFGGFPVSGQWLRCDNPEVVKHHQAKVYSQAAVGSPPMSVP
HLDTRVVDGKKSLLFGPYAGFTTKFLKHGSYLDLPLSVRVANIKPMLAVARDNMDLTKYL
VSEVMQSMEQRLESLRRFYPEAKAEDWRLEIAGQRVQIIKKDAKKGGVLQFGTELVSAQD
GSLAALLGASPGASVTVSIMLELLERCFPEKTRTEWATKLEEIFPAREKILETDAPLYER
ISAQNDEALELVEKSSQEHSFA
>tr|A0A0Q0FL17|A0A0Q0FL17_PSEAP Putative glutathione S-transferase-related protein OS=Pseudomonas syringae pv. aptata OX=83167 GN=ALO85_01828 PE=4 SV=1
MSELILHHYPTSLFAEKARLMLGFKGLAWRSVTIPSIMPKPDLTALTGGYRRTPVLQAGA
DIYCDTALMARRLEQEKASPAFYPKGQEFTVAGLAAWADSVLFMHAVSLVFQPESMAVRF
AKLPPDAAKAFVADRSTLFNGGTASRPPVEQVKHQWPTLMSRLELQLSHGGDFLFGVPSI
ADFSVAHTLWFLKQTPVTAPFVDDYPSVRAWLDRVLGFGHGTFSDLSSAEAIEIARNATP
APLPAEALVDPNGFKVGEKVAVSAVDYGVEAVEGELVFGGREELILRREDERAGVVHVHF
PRMGYRIEKR
>tr|A0A0Q0DJR9|A0A0Q0DJR9_PSEAP 4-aminobutyrate aminotransferase OS=Pseudomonas syringae pv. aptata OX=83167 GN=ALO85_03960 PE=3 SV=1
MSKTNESLMQRRHAAVPRGVGQIHPIFAASAKNATVTDVEGREFIDFAGGIAVLNTGHLH
PKIIAAVQEQLTKLSHTCFQVLAYEPYVELCEKINAKVPGDFDKKTLLVTTGSEAVENAV
KIARAATGRAGVIAFTGAYHGRTMMTLGLTGKVVPYSAGMGLMPGGIFRALYPCELHGVS
VDDSIASIERIFKNDAEPKDIAAIIIEPVQGEGGFYVAPKAFMLRLRELCDKHGILLIAD
EVQTGAGRTGTFFAMEQMGVAADLTTFAKSIAGGFPLAGVCGKAEYMDAIAPGGLGGTYA
GSPVACAAALAVLDIFEEEHLLERCKAVGEQLVTSLKAMQAKYPVIGEVRALGAMIAVEL
FEGGDSHKPNAAAVAQVVAKARDKGLILLSCGTYGNVLRVLVPLTAEDELLNRGLAILDE
CFAEIA

>tr|A0A0Q0BWA4|A0A0Q0BWA4_PSEAP Transcriptional regulator, CopG family OS=Pseudomonas syringae pv. aptata OX=83167 GN=ALO85_101096 PE=4 SV=1
MATSIKIDDELKNRIQHLAGLRQRSSHWIMREAIAQYVEREEARESFKQEALASWAAYQE
TGRHLTGQETRAWLNTWGTEAEAELPKCHE
>tr|A0A0Q0CAL7|A0A0Q0CAL7_PSEAP Response regulator receiver:transcriptional regulatory protein OS=Pseudomonas syringae pv. aptata OX=83167 GN=ALO85_02239 PE=4 SV=1
MSQATTILVIDDEPQIRKFLRISLVSQGYKVLEAATGAEGLTQAALNKPDLLVLDLGLPD
MDGQQVLSEFREWSAVPVLVLSVRASEAQKVQALDAGANDYVTKPFGIQEFLARIRALLR
QASGSEKPDSALEFGPLTVDLAYRRVLLDGQEVALTRKEYAVLAQLARHPGRVITQQQLL
KDIWGPTHTEDSHYLRIVVGHLRQKLADDPTAPRFILTEPGIGYRLLADH
>tr|A0A0Q0DFE1|A0A0Q0DFE1_PSEAP Uncharacterized protein OS=Pseudomonas syringae pv. aptata OX=83167 GN=ALO85_01687 PE=4 SV=1
MSQTRILRAIDYPRMPWKNGGGSTEEIARDAGQDLDGFGWRLSIADIETSGGFSVFAGYQ
RIITVLQGAGMTLDVDGLSSRPLLPSDPFAFSGDSQVNCTLLDGPIRDFNLIYAPHRYTA
RLHWIDVRHPQRLFSSASTLVLFSMAEQVAISVESQPWEVLGKHDCAQVDNAGGLLEVEL
QSPRASRCCLIELTATGQ
>tr|A0A0Q0DAA0|A0A0Q0DAA0_PSEAP Putative oxalate/formate antiporter OS=Pseudomonas syringae pv. aptata OX=83167 GN=ALO85_02309 PE=4 SV=1
MSTSTALGGDAVQPAFLSKERIIAKPGFNRWLVPPAALAIHLCIGMAYGFSVFWLPLSKA
IGVTAPVACTPDMGFFAQMFASTCDWQISMLGWIYTLFFIFLGCSAAIWGGWLEHAGPRK
AGVVSALCWCGGLLISALGIYTHQIWLMWLGSGVIGGIGLGLGYISPVSTLIKWFPDKRG
MATGMAIMGFGGGAMVGAPLATALMGHFASAESVGVWQSFVVMAVIYFIFMIGGALGYRV
PPTGWKPEGWTAPAAKAKNSMITNRHVHVSVAWKTPQFRLVWLVLCLNVSAGIGILGMAS
PLLQEVFAGKLLGLDLTFSQLNADQLKAIAAIAAGFTGLLSLFNIGGRFFWASCSDFIGR
KATYFVFFALGFLLYASVPTLSHMGSIALFVAAFCIVLSMYGGGFATVPAYLADLFGTQM
VGAIHGRLLTAWAAAGVLGPVLVNYLREYQLAHGVARADAYDITLYILSGLLVLGFICNL
LIRPVADKYFMTDAELAAEQAIGHDKGANATTSLEWKASAGSKPLVIAAWLAVGIPLAWG
VWITLQKTAVLFH
>tr|A0A0Q0IPX7|A0A0Q0IPX7_PSEAP Aminoglycoside phosphotransferase OS=Pseudomonas syringae pv. aptata OX=83167 GN=ALO85_03597 PE=4 SV=1
MSYADDLTKRLRSALTSCGFAADAEIENVTQGVASPSRLSTEWAGFRVSSVQGGCYAKVL
HADMAPLIDFDNVAQASESAGRSAAAPRLLGADQANGVLVFEDLGVDWRAARLDDLLAPG
RLQALWALKRQVHAGPVPDVVRSPMADIQRLRTLCEHDNVALPAEHQWIDRCVDMAWQAL
QSRSVQAAPLHGDGVASNVMVSSEGQLRLVDFDYGGCMDPWYDVAITLNELYSFERQWRE
GISAWAGQCLEIDYAVCRLYALINDWYWTLWGLWAGTTSSRPLEFSKVGQWTLLRCRQCV
QDPRLEGWMRQVQEGRA
>tr|A0A0Q0D6H1|A0A0Q0D6H1_PSEAP Alcohol dehydrogenase II OS=Pseudomonas syringae pv. aptata OX=83167 GN=ALO85_02421 PE=4 SV=1
MISTFFIPAVNIIGTGCIDEAMLAIRKYGFRKALIVTDAGLAKAGVASQIAGLLVEQGID
SVVYDGARPNPTIANVENGLALLQERQCDFVISLGGGSPHDCAKGIALCASNGGHISDYE
GVDRSAKPQLPLIAINTTAGTASEMTRFCIITDEARHVKMAIIDRNVTPLLSVNDPDMMV
AMPRSLTAATGMDALTHAIEAYVSTAANPITDACALQAMALIANNLRDAFNNGANITARE
NMAYAQFLAGMAFNNASLGYVHAMAHQLGGFYDLPHGVCNAVLLPHVQRFNASASVSAAR
LTDVAHAMGSNIRGLSPEEGAQAAIDAIHALSAAVEIPAGLAALGVKEEDFPLLATNALK
DACGLTNPRPADLEQIQDIFRQAL
>tr|A0A0Q0IPR2|A0A0Q0IPR2_PSEAP Ribose ABC-type transport system, permease protein OS=Pseudomonas syringae pv. aptata OX=83167 GN=ALO85_03751 PE=3 SV=1
MSTLTLSTSSTLVKVLPRLLPAVLPLLVSVILLFFAINAPGFLSWGNLSSLVLNNFVLLA
IVAIGMTLAVAAGGIDLSVGTALDFAGLGFVVTLNGGHGLGAAIAVALLAGGIAGAFNAM
LIAGLRISPFLATLGTLFIGTSVQQLLSDGGQPIYIAQGALPGISAVLILGVPLPLLVVG
LLAGLYGLILARGRLGREILALGTQPQLAYYSGLPTRRIAVQVFFASALACAVAGILLSS
TVNAYVPMSGNAYLINAIGAVFIGTTLSRQGRPNIPGTLLGVLFINVIANGLLLIGWNFY
WQQVATGALIFLVLAFSFTSRHLLRNAV
>tr|A0A0Q0D0V9|A0A0Q0D0V9_PSEAP ATP-dependent Clp protease adapter protein ClpS OS=Pseudomonas syringae pv. aptata OX=83167 GN=clpS PE=3 SV=1
MHAFSKIRLTFNQDDPQSHEDDSAGIAVQDAKPALQAPPMYKVVLFNDDYTPMDFVVEVL
EVFFNLNRELATKVMLAVHTEGRAVCGLFTRDIAETKAMQVNQYARESQHPLLCEIEKDG

>tr|A0A0N8T8D1|A0A0N8T8D1_PSEAP D-cysteine desulfhydrase OS=Pseudomonas syringae pv. aptata OX=83167 GN=ALO85_01681 PE=3 SV=1
MIKNQLARFNRLDLISAPTALEKLERLSVWADRDIYIKRDDTTTLALGGNKVRKLEYLAA
DALAQGADTLITAGAIQSNHVRQTAALAARLGLGCVALLENPIGTEDPSYLKNGNRLLLE
LFDAKVELVENLDNADEQLQALAARLSSSGKKPYLVPIGGSSPVGALGYVRAGLELAEQI
KQTGIDFAAVVLASGSAGTHSGLALALAHELPQLPVIGVTVSRSEEAQLPKVQGLAERTA
ELLGVALPEHFKVELWDEYFAPRYGEPNAGTLSAIKLVASHEGLLLDPVYTGKAMSGLLD
GIGRQRFNDGPLIFLHTGGAPALFAYPDAFDQ
>tr|A0A0Q0FV05|A0A0Q0FV05_PSEAP Lytic transglycosylase OS=Pseudomonas syringae pv. aptata OX=83167 GN=ALO85_200105 PE=4 SV=1
MNTFALRFTDVAQQSLEDQVEHLAVYQGFSSAAQRIDSLIDAIQDKLLSTPLGYPVSPQL
SELGVLHYRELNTDGYRIFYEVMDSDGITDISVLLVLGSKQSVEQALIRYCLLQPM
>tr|A0A0Q0DVP4|A0A0Q0DVP4_PSEAP Uncharacterized protein OS=Pseudomonas syringae pv. aptata OX=83167 GN=ALO85_02882 PE=4 SV=1
MPGSKVQQYFTAAIVIFLLSFILNPSAKITNNLFYAFTALPGLFFLIKYRGAGVFAKPLG
IAWAVFMACFLVPALQAQEFQYYKHIIYVSLFVFVVAGITDNGFFRRGVFVRSMFWALCL
YILLSSIHSWVTGQFEFGQRVAILPGRMENVIYASAWLVCSLALAMPLWAKERRWVEAVC
AVLVTLVITAFIVQTRTALVGCAVLFAVCTAYALYRFPLRTTVVLLALGVVGAAAFWFVK
DEQWVALLMVRGDSYRIELFEIMTGEWRRCGWMLGCGVDFYTTQTLTGGIPIQHPHNIFV
SLGLYTGAASLVMFLVVMAITLWDSVRNGDAWGLYLVCALVMLNFDGAQLVGNPDELWVL
VLLPASMVLGRVVQTHRLRQQQ
>tr|A0A0N8T8C3|A0A0N8T8C3_PSEAP Uncharacterized protein OS=Pseudomonas syringae pv. aptata OX=83167 GN=ALO85_01685 PE=4 SV=1
MLSAVKPSGDFCMNAKRLLLCLAGTTLAFTCLSSVAMAQTDLPESVRVPAGNKVGMQTTGV
GEITYECRAKANMPNEMEWAFVGPKAVLNDKSGKQVGTYYGPPATWEAKDGSKITGTQVA
VAPSSTGNLPYQLVKANPAEGKGAMTGVTYIQRVALKGGVAPAKACAESNKGAKEVVKYQ
ADYLFWTAS
>tr|A0A0Q0C841|A0A0Q0C841_PSEAP TRAP dicarboxylate transporter, DctP subunit OS=Pseudomonas syringae pv. aptata OX=83167 GN=ALO85_01118 PE=4 SV=1
MDFKRKLLIAALPLAFCVSGMAHADIKIKFAEVHPAGYAPVVAEQKMGKKLEDQSKGEIS
FKMYAGGVLGSEKEVVEQVQSGAVQMTRVSLGIVGPVVPDVNVFNLPFVFRDQAHMRTII
DGEIGQEILDKITNSEFNMVALAWMDGGTRNLYTKKPVRQISDLKGMKIRVQGNPVFIET
INDMGGNGIAMATGEIFSALQTGVIDGAENNPPTYFQHNHYQNAKFFTMTEHLILPEPIV
MSKTTWQKLNPEQQALVKKLAREAQMEERVLWDKSSAEAETKLKAAGVEFITLTPEQKKA
FYDATQPVRDKFGAPYKDLISRIEAVQTNPALAASPPQAAQ
>tr|A0A0Q0C966|A0A0Q0C966_PSEAP N-methyltryptophan oxidase OS=Pseudomonas syringae pv. aptata OX=83167 GN=ALO85_00698 PE=4 SV=1
MQQRCNVAVLGLGAMGAATAYQLAKAGVDVIGIDRYAPPHTQGSSHGDTRITRLSAGEGP
QYLPLVRSSQRIWRELEALSGESLFEQCGVLVMTSSPAYDPEDVNDFTHQTIALARAYGV
RHEVLAAAAIRERFPQFAPVLDSAIGYFEPEGGFVRPERCIAVQLKLARQHGARLLTHET
VTQLQAHGDQVQITTDKGSIIADKVVVSAGMWSAGLLGAPFSDLLRVCRQKLFWFELQHE
AAFAAPSPSFILIHGPGDADVNYGFPPLPGENSMKVATEQYLETSAADRLDRTITAREEQ
EMFRTQVQGRIAGLTPKVVKSSVCAYTVTPDCHFIIDEHPHLKNVTVVSACSGHGFKHSA
GLGLALAQRCIDGESDVDLSAFSLQRFARQA
>tr|A0A0Q0FHT7|A0A0Q0FHT7_PSEAP Oxoacyl-reductase OS=Pseudomonas syringae pv. aptata OX=83167 GN=ALO85_03534 PE=4 SV=1
MFDYSAHPELLKGRIILVTGAARGIGAAAAKAYAAHGATVLLLGRTEASLAQVHDEINAA
GQPQPLVIPFDLQSADPAQYHALATMIERTVGRLDGLLHNASIIGPRTSVEHLPGEDFME
VMHVNVNATFMLTQALLPLLQRSEDASIAFTSSSVGRKGRAQWGAYGVSKFATEGLMQTL
ADELEGVSTVRANSINPGATRTGMRAQAYPDENPQNNPAPEEIMPVYLYLMGPDSKGING
QALNAQ
>tr|A0A0Q0DBA6|A0A0Q0DBA6_PSEAP Uncharacterized protein OS=Pseudomonas syringae pv. aptata OX=83167 GN=ALO85_03838 PE=4 SV=1
MKTARAMALATLMAVGASPAFAFNLNDAANAVSNATNGNQKATAAPEVAGLLNTLGTQLN
VTPEQAVGGTGALLGLAKNKLTSTDYSQLAKSVPGLEQMSGTSALESLGGAKGLGSLLGG
KSGSSSMLNSALGNVQSMGDVNNAFKALGMDSTMVSQFAPLILQYLGQQGASGSALQSLS
GLWGAGS
>tr|A0A0Q0CEZ9|A0A0Q0CEZ9_PSEAP Chemotaxis protein CheY OS=Pseudomonas syringae pv. aptata OX=83167 GN=ALO85_02926 PE=4 SV=1

MARILIVDDSPTEMYKLTGMLEKHGHEVLKAENGADGVALARQEKPDAVLMDIVMPGLNG
FQATRQLTKDADTSMIPVIMITTKDQETDKVWGKRQGARDYLTKPVDEETLMKTLNAVLA
G
>tr|A0A0Q0DPX8|A0A0Q0DPX8_PSEAP Phosphonate ABC transporter ATP-binding protein OS=Pseudomonas syringae pv. aptata OX=83167 GN=ALO85_00819 PE=4 SV=1
MISARSLDAAGERLPEAVQPLLKVRGLTRLYGVQKGCQDVSFDLYPGEVLGIVGESGSGK
STLLSLLSGRCPPDRGSIDYRSKEGDWFDLYSASEAQRRTLLRTEWGFVEQNPRDGLRMG
VSAGANIGERLMAQGVRNYQQLRGAALDWLTQVEIDPQRIDDLPRTFSGGMQQRLQIARN
LVSSPRLVFMDEPTGGLDVSVQARLLDLLRGLVRELDLAVVIVTHDLAVARLLADRLMVM
RRSEVVESGLTDQILDDPQHPYSQLLVSSVLQP
>tr|A0A0Q0BR36|A0A0Q0BR36_PSEAP Probable protein kinase UbiB OS=Pseudomonas syringae pv. aptata OX=83167 GN=ubiB PE=3 SV=1
MKLLAVRRLFRIQRVVIRYRLDDLLFALPLPWWMLALRFVLPWRWLPRRKSELSRGVRFR
LALQDLGPIFIKFGQLLSTRRDLLPEDIADELMLLQDRVPPFDQQLAIKLIEEQLGARIC
DVFSRFDEKPLASASVAQVHAACLKTGEEVVVKVVRPGLKPIIGQDLAWLFILARTAERV
SADARLLHPVQVVMDYEKTIYDELDLLREAANASQLRRNFEGSDLLYVPQVYWDWCRPKV
LVMERIYGLQVTDMAGLADQRTDMKLLAERGVEIFFTQIFRDSFFHADMHPGNIFVSTVN
PWAPKYIAIDCGIVGSLTPEDQDYLARNLFAFFKRDYRRVAQLHIDSGWVPAETKLNEFE
AAIRTVCEPIFEKPLKDISFGQVLMRLFQTARRFNMEVQPQLVLLQKTLLNIEGLGRQLY
PELDLWSTAQPYLERWMRERVSPKTLLGNLQSQVEQLPHIAGMTRDLLERMSRPHASDPP
RPWHGRKDEPVLRLIGAALLVGGAIQGWVMSEAATQLLTLTAWPAAIMLIAGLYLIVRR
>tr|A0A0N8T8F9|A0A0N8T8F9_PSEAP FAD_binding_3 domain-containing protein OS=Pseudomonas syringae pv. aptata OX=83167 GN=ALO85_02831 PE=4 SV=1
MPITDREIRQVVVIGAGPAGSIAAALLKRQGHDVLIIERQLFPRFSIGESLLCHCLDFVE
EAGMLEAVQAAGFQPKNGAAFARGEQYSDFDFSDTFSNGKPTTFQVQRSEFDKLLADQAA
LQGVDIRYQEEIVSADFDGPQPVLRVRREDGSEYSVQASFVLDASGYGRVLPRLLDLEAP
SGFPVRQAVFTHIEDRIESPPFDRRKILVSIHPQHSDVWFWSIPFSEGRCSIGVVASAER
FKDKPEDLDACLRAFIDETPQLASVLKNAIWDTPARTIGGYSANVKTLHGKGFALLGNAA
EFLDPVFSSGVTIAMRSASMAAGVLSRQLQGENVDWESEFAVPLKRGVDTFRAYVEGWYD
GTFQNVIFYPGSAPDIRRMISSILAGYAWDERNPFVSEPKRRLRTLSEICADSGP
>tr|A0A0Q0FJX6|A0A0Q0FJX6_PSEAP Periplasmic protein thiol:disulfide oxidoreductase DsbE OS=Pseudomonas syringae pv. aptata OX=83167 GN=ALO85_03035 PE=4 SV=1
MRRWVMVLPLVLFLGVAAFLYRGLYLDPAELPSALIGKPFPAFSLPAVQGERMLSRADLL
GKPALVNVWGTWCVACRVEHPVLTRLAQQGVLIYGVNYKDVNADALKWLKEFHDPYQLNI
RDQDGSLGLNLGVYGAPETFLIDAQGVIRYKHVGVIDDAVWREKLAARYQQLIDEARP
>tr|A0A0Q0D794|A0A0Q0D794_PSEAP 50S ribosomal protein L17 OS=Pseudomonas syringae pv. aptata OX=83167 GN=ALO85_200041 PE=4 SV=1
MPSIRKMLSALALLPLATHAFAAPAEFWYSHSGAAGSAIADLCRSFNSQREESDRLHCVS
QGSYEQTLQKTVAAYRAGIGPALVEIYDVATPDMLLGGATRPVEAIMADHHRAYPEDTFL
PALRRYYADDHGTLAAQPFAASTAVLYTHRNALAAAGISAPPATWEAFADALRALKKSGQ
KCPVVSAFAPWIWLEQASAAQGTDVAIRSAGSDRYQFDKGAHLRLMKDLAQWTREGLVVH
EDSTRSGQQALAFATDDCAMLIDSTGAWNVVHSTLRSDIQVTALPIYAGTQRRANVPGGS
SLWVMRGHSVRDYRLVSEFLAFVLQPDNQLMFSARTGYLPVTQAGATRLQSAASEPSAIT
VGLTVLNDIDGQPSAPLRCGFITLMRLIWSQEMENALAGRQSIDLALRQTTLRANELLGL
FQQMHQAQASNESNEATP
>tr|A0A0Q0FPE3|A0A0Q0FPE3_PSEAP LexA repressor OS=Pseudomonas syringae pv. aptata OX=83167 GN=lexA PE=3 SV=1
MYSMSTLSPRRSAILTFIRDRIAQQGQSPSLAEISEAFGFASRSVARKHIVALTEAGLIE
VVPHQARGIRLLNTEPRPELLEIPVLGRVAAGVPIGPDLDIHTTLHLDRSTFTRVPDYLL
RVQGDSMIEDGILDGDLVGVHRNPQASDGQIVVARLDGEVTIKRLQRRADQIRLLPRNPA
YEPIIVTPDQDFAIEGVFCGLVRRE
>tr|A0A0Q0BWG0|A0A0Q0BWG0_PSEAP Putative Membrane protein OS=Pseudomonas syringae pv. aptata OX=83167 GN=ALO85_03090 PE=4 SV=1
MNLDWHGAWLSVIHHPLFGIAITLGAYQLAMAAYERTRWLFLQPVLVSMVVVIGVLLFCD
LEYTEYRKSTEILVTLLGPATVALAVPLYLNMRRIRQLFWPVLTTLVVGGVFATGLCVGF
GVLFGADHMILMTMAPKSVTSPIAMLVAEQIGGVAALAAVFVLITGVFGAIFGSGLLSLI
GVDNPAARGMALGLTAHAVGTSVALQEGEESGAFAALAMSLMGVATALFLPLAMSLAI

>tr|A0A0Q0FPJ3|A0A0Q0FPJ3_PSEAP Monosaccharide ABC transporter substrate-binding protein OS=Pseudomonas syringae pv. aptata OX=83167 GN=ALO85_01019 PE=4 SV=1
MKFGTSLAAVAAFSLLASSISMAADGKTYKIGAAVYGLKGQFMQNWVRELKEHPAVKDGT
VKITVFDGNYDALTQNNQIETMITQHYDAIIFVPIDTKAGVGTVARAMENDIPVIASNTR
VAGNKVPYVGNDDVEGGRLQAQAMVDKLKGKGNVVIIQGPIGQSAQIDREKGELEVLSKH
PDIKIIEKKTANWSRAEAQTLTEDWLNAHQNGGISGIISQNDDMALGALQAVMSRNLKPA
DVPITSIDGMPDAIQAAKKNEITTFLQDAQAQSQGALDVALRTLAGKDYKPQSVIWERYA
KDVKWGDGTDKNYILPWVPVTDANADALYKQVSGGK
>tr|A0A0Q0D6V3|A0A0Q0D6V3_PSEAP Carbohydrate kinase, PfkB OS=Pseudomonas syringae pv. aptata OX=83167 GN=ALO85_00975 PE=4 SV=1
MQEYRHMSLPRIALIGECMIELQQHADGSLRQSFGGDTLNTAVYLSRLLGERAKVDYVTA
LGDDSFSDAMCRIWADEGIGLGKVQRLPGRLPGLYCIQTDANGERRFLYWRNEAAVRDCF
MTPAAEPILAALASYDVLYFSGITLAVLGEQGRARLLEALARARQRGVRVAFDNNYRPRL
WASVEQAREAYLACLQHVDLALLTEDDEQALYGYADSEQLLAAYRGRGIGEIVVKRGAQS
CLVEAGGERFEIAAQKVERVVDTTAAGDSFSAGYLAARLTGQHPLQAAEAGHRLASIVIQ
HPGALVPKSVMPV
>tr|A0A0Q0BYL5|A0A0Q0BYL5_PSEAP Sensor histidine kinase OS=Pseudomonas syringae pv. aptata OX=83167 GN=ALO85_03177 PE=4 SV=1
MRPSRLFWKLFLAFWLATSLTFMVGAGLFMFTQSGRGDPSFSTVLDNEVRILQRSGIAAG
QALLDAWQPEGHSKVGLYDSQGMLLAGDLLENPRFELAVRTREGALVHIRSTQRPDHDDQ
PSHLNPLITGTVMSALFSWFLSSYLVAPLIKLREAMGKVARGRFDTRVKPDMGRRRDEIV
DLAEDCDRMANQLKVMADSQQQLLHDISHELRSPLTRMSAAIGLLRQEPTQLDMLERIER
ESERMDSLIEELLTLARMQSDVESLTRDQVDVISLLAAIVEDAEFEAVLKQCRVRLQAPG
SFVAQVDSELLYRAFENVIRNAVRHTAVGTDVVVVANVQAQPLCLMVNVIDQGPGVDEAC
LQRIFQPFERGPENAAQGFGLGLAIALRAIEMHGGQIAARNRPGGGLVVDVLLPVS
>tr|A0A0Q0C8X7|A0A0Q0C8X7_PSEAP Senescence marker protein-30 OS=Pseudomonas syringae pv. aptata OX=83167 GN=ALO85_03399 PE=4 SV=1
MNWLPVSEHRFKLAEGSFWDAEEQALYWVDISGFLVCRLVAGQIRQWRMPEPVSAFIPTE
QGDALVTLASGVYRLDLESDKTSLLPFCVADPTPGNRANEARCDARGHLWLGSMQNNIDA
EGGDVPLVRRSGGLFRVEPDARVTRLLDGQGIVNTLLWNEEGSVLYSADTLDDVIYRYPV
LADGALGPRQVWAAKHDRGSPDGSAMDAEGYVWNARWGGNCLIRFAPDGSVDRVVELPVS
HPTSCVFGGPDLQTLYVTSAAPADANGRFDGALLMAQVGVKGTPCQRFAG
>tr|A0A0Q0BW90|A0A0Q0BW90_PSEAP Coproporphyrinogen-III oxidase OS=Pseudomonas syringae pv. aptata OX=83167 GN=ALO85_05288 PE=3 SV=1
MRMLAISPAGPGNARMLDAIRWDSDLIHRYDVAGPRYTSYPTAVQFHTQVSAFDLLHALR
ESRKASRPLSLYVHLPFCANICYYCACNKVITKDRGRAQAYLQRLEHEIQMLACHLAPGQ
VVEQLHLGGGTPTFLSHDELRRLMAQLRLHFNLLDDDSGDYGIEIDPREADWSTMGLLRE
LGFNRVSLGVQDLDPTVQRAINRMQSLEETRAIVEAARTLQFRSVNIDLIYGLPTQNPQT
FSHTVDKVIDLQPDRLSVFNYAHLPERFMPQRRIDVADLPDAESKLLMLERTVEQLGNAG
YRYIGMDHFALPDDELATAQEDLTLQRNFQGYTTHGHCDLIGLGVSAISQVGELYSQNSS
DLNEYLRLLDSDQPATRRGLICNDDDRIRRAIIQQLICHFTLDFGEIEKTFCIDFRDYFS
EAWPQLLGMASDGLITLSETGIEVRPAGRLLVRAVCMVFDTYLTRQNRQQFSQVI
>tr|A0A0Q0C1G0|A0A0Q0C1G0_PSEAP Inner-membrane translocator OS=Pseudomonas syringae pv. aptata OX=83167 GN=ALO85_01942 PE=3 SV=1
MSQPLIVTAAQKVGPRGTLTAGCVILAVLLALPLLSLLPADNSFQVSAYTLTLVGKILCY
AIVALALDLVWGYAGLLSLGHGLFFALGGYAMGMYLMRQAAGDGLPAFMTFLSWTELPWY
WAGTDHFLWAMCLVVLAPGLLALVFGFFAFRSRIKGVYFSIMTQALTFAGMLLFFRNETG
FGGNNGFTNFRSILGFSISAQGTRAALFMATVALLVASLYIGWRLAQSKFGRVLTALRDA
ENRLMFCGYDPRGFKLFVWVLSAVLCGLAGALYVPQVGIINPSEMSPTNSIEAAVWVALG
GRGTLIGPLLGAGLVNGMKSWFTMAFPEYWLFFLGALFIIVTLYLPKGVIGMLKKRSEQ
>tr|A0A0Q0BSM9|A0A0Q0BSM9_PSEAP Cell division protein FtsZ OS=Pseudomonas syringae pv. aptata OX=83167 GN=ftsZ PE=3 SV=1
MFELVDNVPQSPVIKVIGVGGGGGNAVNHMVKSNIEGVEFICANTDAQALKNIGARTILQ
LGTGVTKGLGAGANPEVGRQAALEDRERIAEVLQGTNMVFITTGMGGGTGTGAAPIIAEV
AKEMGILTVAVVTRPFPFEGRKRMQIADEGIRMLSESVDSLITIPNEKLLTILGKDASLL
SAFAKADDVLAGAVRGISDIIKRPGMINVDFADVRTVMSEMGMAMMGTGCASGPNRAREA
TEAAIRNPLLEDVNLQGARGILVNITAGPDLSLGEYSDVGSIIEAFASEHAMVKVGTVID
PDMRDELHVTVVATGLGAKIEKPVKVIDNTLQTTQQAPAQQASRQEAPSVNYRDLDRPTV
MRNQAHAGATAAAKMNPNDDLDYLDIPAFLRRQAD

>tr|A0A0Q0C2G8|A0A0Q0C2G8_PSEAP Uncharacterized protein OS=Pseudomonas syringae pv. aptata OX=83167 GN=ALO85_02056 PE=4 SV=1
MSTDNEQLDVDDDLVSVDTDDTEPVTVEPAKTNLSKRRTIDNLLEERRLQKQLADYDFDF
>tr|A0A0N8TA54|A0A0N8TA54_PSEAP Allergen V5/Tpx-1 related protein OS=Pseudomonas syringae pv. aptata OX=83167 GN=ALO85_03405 PE=4 SV=1
MPALPRCSWRIWLYLVPLLPLYASPVSAAEEQRLVQAINDFRGQPQRCEARTTNISRALA
LNSSVALPIGFSGNLRDALKASGYQAVIVRTLRLAGAQSTDAALEMLRSRYCGALLDPQY
ADIGITRQGGDWRVVLAKPLIDESLEDARSAGRALLAQVNAARAKPRMCGKQPFPSARPL
SWNITLETAAQGHSQSMASENYFTHRGFDNDSPADRARAAGYGGRQIGENIAAGQSTASK
AMASWLASPGHCANLMNPMFTEVGAAYATATNADYGVYWTMLFGAP
>tr|A0A0Q0CZP6|A0A0Q0CZP6_PSEAP LapA_dom domain-containing protein OS=Pseudomonas syringae pv. aptata OX=83167 GN=ALO85_100122 PE=4 SV=1
MRIFKRVILIVAVLLVVLATTVFVLENRQSVAVTFFGWSAPQLPLALPVVLALLVGMVIG
PILAWIASLRKKRAPSPRSV
>tr|A0A0Q0DC48|A0A0Q0DC48_PSEAP Transcriptional regulator, LysR family OS=Pseudomonas syringae pv. aptata OX=83167 GN=ALO85_04862 PE=3 SV=1
MGMRNCLSKPSSIVREDLMNLESKWLEDFSALAATRSFSQAAERRFVTQPAFSRRIRSLE
SALGLTLVNRSRTPIELTAAGQLFLVTARTVVEQLGEVVRHLHHLEGGQGEVMQVAAAHS
LALGFFPRWIAQLRNEGLNIATRLVATNVGDAVHALREGGCDLMLAFYDPDAALQMDAEI
FPSLHLGHTEMLPVCAADAEGRPLFDMEGEGSVPLLAYSTGAFLGRSVNLLLRQRNLRFT
TVYETAMADSLKSMALEGLGIAWVPQLSVRAELARGELVVCGGPQWHVPLEIRLYRCALV
RKANVRLLWRKLEGAAAPDSR
>tr|A0A0Q0DSZ3|A0A0Q0DSZ3_PSEAP DNA polymerase III, epsilon subunit OS=Pseudomonas syringae pv. aptata OX=83167 GN=ALO85_03484 PE=4 SV=1
MLERIAVIDFETTGISPGHSCRATEIAVVIMEQGRIVERFQSLMNAGVRIPAFIEGLTGI
SNNMIRTAPSAERVMGDVADFVGSTPLVAHNASFDQKFWDYELSRIERTREQSFACSLLL
ARRLMPGAPNHKLGTLTRYARLPDTGKAHRAMADAEMAANLTAYLTNELRQTHGISAISH
KMLCSLQKVPAAKISETLKRQRGV
>tr|A0A0N8TA84|A0A0N8TA84_PSEAP D-amino acid dehydrogenase OS=Pseudomonas syringae pv. aptata OX=83167 GN=dadA PE=3 SV=1
MRVLVLGSGVIGTTSAYYLARAGFQVTVVDRQPAAAMETSFANAGQVSPGYASPWAAPGV
PLKALKWLLQRHAPLAIKATADIDQYLWMAQMLRNCTASRYAINKERMVRLSEYSRDCLD
ELRLETGIAYEGRSLGTTQLFRTQAQLDNAAKDIAVLEQSGVPYELLDREGIARVEPALA
GVTGILSGALRLPNDQTGDCQLFTTRLAEMAVALGVEFRYGQNIERLDHAGDSINGVWID
GKLETADRYVLALGSYSPQLLKPLGIKAPVYPLKGYSLTVPITNPDMAPTSTILDETYKV
AITRFDNRIRVGGMAEIAGFDLSLNPRRRETLEMIVGDLYPQGGDLTQADFWTGLRPTTP
DGTPIVGATPFRNLFLNTGHGTLGWTMACGSGRLLADLIARKTPRISAEGLDISRYGNTQ
ENAQHVHPAPAHQ
>tr|A0A0Q0IKP1|A0A0Q0IKP1_PSEAP Macrolide export system, MacA-like subunit OS=Pseudomonas syringae pv. aptata OX=83167 GN=ALO85_00930 PE=3 SV=1
MKKLKFRKVLIVVVLLVLAAGIAYSIQSPEKPPEYLTAKVERTEIENSVLASGVLQGIKQ
VDVGAQVSGQLKSLKVNLGDKVKQGQWLAEIDPVVLQNTLRQSQVNEQNLIAQKDAAVAQ
LKDAKAIYQRYKQLRADDAISQKDFDTAQSDFEVRSANLRSLEAQVRDARIQIETARINL
GYTRIVAPISGDVVGIVTQEGQTVIASQLTPVILKLADLDTMTVKAQVSEADVIHITPGQ
EVYFTILGDAEKRYYAKLKGTEPAPQNFLDSQSSNASKPNSAVFYNALFDVPNPDHRLRI
SMTAQVHIVREKAKDVLTVPVAALGDKNGDGTFPVRVMDAQGQAQSRNVQTGINNNVRVE
IKSGLAEGDQVVIGEPSATPAAAGA
>tr|A0A0Q0CEU3|A0A0Q0CEU3_PSEAP DEAD/DEAH box helicase OS=Pseudomonas syringae pv. aptata OX=83167 GN=ALO85_200188 PE=4 SV=1
MHDTHSERMSSGETRAAGGLALVFAFRMLGMFMVLPVLATYGMDLAGASPALIGLAIGAY
GLTQAVLQIPFGIISDRIGRRPVIYFGLIIFAIGSVVAANADSIWGIIAGRILQGAGAIS
AAVMALLSDLTREQHRTKAMAMIGMTIGLSFAIAMVVGPVITGMFGLSGLFLATGGMALI
GVLIVAYVVPKASGALMHRESGVAKQALGATLRHPDLLRLDLGIFVLHAMLMSSFVALPL
ALVEKAGLPKEQHWWVYLTALLVSFFAMIPFIIYGEKKRQMKRVLLGAVTVLMLAELFFW
AYGDTLRALVIGTVVFFTAFNLLEASLPSLISKVSPAGGKGTAMGVYSTSQFLGSAAGGI
LGGWLFQHGGLDVVFLGGAAMAAVWLAFAVTMREPPYVTSLRLPLSPQAQREAGLAERLM
SVAGVTDAVVVAEEAAIYIKLDTKLLDRASLEKLVNPASEACEA
>tr|A0A0Q0IMS2|A0A0Q0IMS2_PSEAP 50S ribosomal protein L18 OS=Pseudomonas syringae pv. aptata OX=83167 GN=rplR PE=3 SV=1

MTVKKVTRLRRARKARLKMHELEVVRLCVHRSSQHIYAQVISADGSKVLASASTLDKELR
DGATGNIDAATKVGKLVAERAKAAGVSQVAFDRSGFKYHGRVKALADAAREGGLEF
>tr|A0A0Q0FGH8|A0A0Q0FGH8_PSEAP DedA family protein OS=Pseudomonas syringae pv. aptata OX=83167 GN=ALO85_01630 PE=4 SV=1
MLQNFLNEFGYFALFLGTFFEGETILVLAGFLAFRGYMDLNIVILVAFCGSYAGDQLWYF
MGRKHGRKLLARKPRWQLMGDKALRLVRKHPDIWVLGFRFVYGLRTVMPVAIGLSGYPPG
RYLLLNGIGAAVWAAALGSAAYHFGAVLEGLLGNVKKYELWVLGALVLVGLALWIRRRVK
NARIAREEREEKSRFCASAAASDLETPVTVPVDLTKKSDE
>tr|A0A0Q0DY03|A0A0Q0DY03_PSEAP Uncharacterized protein OS=Pseudomonas syringae pv. aptata OX=83167 GN=ALO85_100748 PE=4 SV=1
MKKIMKLIVMGVLVLVLAALLTKWVVASGFIGWLLRTETGLDMYASTVSALGIIGGEDGE
FMLLALMVFVMLVPSAFLVGAAAKRLHTFRAKRQSCSK
>tr|A0A0Q0BR83|A0A0Q0BR83_PSEAP Carbon-nitrogen hydrolase OS=Pseudomonas syringae pv. aptata OX=83167 GN=ALO85_02278 PE=4 SV=1
MSRIVSVAATQMACSWDLEANIETAEKLVREAAAKGAQIILIQELFETPYFCQKPNPDYL
QLATTLEANVAIKHFQKIAKELQVVLPISFFELAGRARFNSIAIIDADGSNLGIYRKSHI
PDGPGYHEKYYFNPGDTGFKVWQTRYAKIGVGICWDQWFPECARSMALQGAEILFYPTAI
GSEPHDKTISSRDHWQRVQQGHAGANLMPLIASNRIGNEEQDGYDITFYGSSFIANQFGE
KVAELNETEEGVLVHSFDLDELEHTRSAWGTFRDRRPNLYGAVKTLDGSLES
>tr|A0A0Q0DJG2|A0A0Q0DJG2_PSEAP Flavodoxin OS=Pseudomonas syringae pv. aptata OX=83167 GN=ALO85_01772 PE=4 SV=1
MKVAIVCGTVYGSAEEVARHAAALLRAAGHETLVNPRLMLPELLAFEPQALLAVTSTTGM
GELPDNLMPLYAALRDSLPGQLRGLPGGVIALGDASYGDTFCAGGEQMRELFVELGVSEV
QDMLRLDGSESVTPETDAEPWLEAFIKHLG
>tr|A0A0Q0DQY9|A0A0Q0DQY9_PSEAP Diguanylate cye domain protein OS=Pseudomonas syringae pv. aptata OX=83167 GN=ALO85_04600 PE=4 SV=1
MGHAARRIARAVRNSGPVFVCWPARASLRAGGCRGQVAGENPSVAAGLLPSRCPPVDGAA
AKARFQAWGARGSPRQGRALRDAAIGGGDRSGRGIESCAEDSVSRRRQTACLASGIFALV
FLLQRQRMTYDVRQDVHRITKGYRKLSVAFSLCKRSFISSILFASNNSGTAQLCSLAGLS
QLWSPCGERHSISRSTLGLLNYRWSSGGGYLTRQVGCLEFSMNGDMQTRIKQLLSPAIAR
VQCIAIVCWLVVLSVTPYLQFGLPEILLTLGLLGACAWQYRAVDFRVWRCVGMAFVIIVA
LCFSRAGHLRTGTDINWSLSVAVMIILCSTLLVVYTRDYLVVAILAWCILSPAQGVDTDS
VAAVFMTLFFITSVSLGSVMNHTYTRTLRTVLSLEHQFRELSLTDDLTAILNRRALMQAL
DNHVLNNSAGYFLMLDIDDFKTINDQFGHDAGDQALRVMAGCLQKTVGSLAFGRMGGEEF
GVILPMCSEDDARDYVLNLLECIRKSLAVSPLTCSAGLADIPAAADSSEVLKTADINLYQ
AKRGGKDRAFWRGAQIGGHQAGGISVECDKTRVLTR
>tr|A0A0Q0BYM3|A0A0Q0BYM3_PSEAP Uncharacterized protein OS=Pseudomonas syringae pv. aptata OX=83167 GN=ALO85_200112 PE=4 SV=1
MPSFYSERTAEYALAPKFLKLLEPLGPAVPIFFSGRREDTLIAFDSLSGESFHLVAFFAR
RPKINETNSLTINGKINKRLLRVADCASKLGIKTICGISLTNNIFDQSRAESLWFDISHM
AAEQDIQFACEVTRDLELKSFHGHIQPVTPSTIISSIANSRIVDWAEATNIMRELPKLAD
DFQPRQSIFFSQTWRMRPLYFVIRRS
>tr|A0A0Q0DB97|A0A0Q0DB97_PSEAP Putative acyltransferase OS=Pseudomonas syringae pv. aptata OX=83167 GN=ALO85_03837 PE=4 SV=1
MRRLLTGCFVALLLLINTLVLIGPLLVFALLKLLFRGRMRDRCSAAVMWIAETWAEIDKV
IFATCIPTQWDIRGDEGLRGDTSYLVISNHQSWVDIPALVQALNRRTPFFKFFLKKELIW
VPLLGLAWWGLDYPFMKRYSKAFLAKHPQLKGKDLEITKAACELFKRQPVTIVNYLEGTR
FTAAKHAAQGSPYTHLLKPKAGGVAFVLAAMGEQLDAILDVTVVYPESGIPGFWDMLCGR
VSNVIVDIRTRELDPALWQGDYENDPVFREKVQGWVNQLWVEKDARIAALRLELPGH
>tr|A0A0Q0C0S3|A0A0Q0C0S3_PSEAP Chaperone protein PapD OS=Pseudomonas syringae pv. aptata OX=83167 GN=ALO85_02227 PE=4 SV=1
MICQQAFKPPAIAGCMLLFMCAAYIPASQAALTVNATRIVFDGDKRSTSVVISNPSDRPF
AVQTWVNTEADDTVTVVPFAPSPPLFRLNPGKEQQVQINGLPHDLPSDRESLFYFNVQEI
PQADPSQGNVLNIALRTRIKLFYRPALLTDNPIARLKDLQWSVRQIAGKAELTAMNPTPF
HVSFIRLDVSGGGSTEKVKEVAMLGPFSSRHYELSETKAVPGLQVVFSAINDYGGYSKPL
TATATLTD
>tr|A0A0Q0DBF3|A0A0Q0DBF3_PSEAP DNA repair protein RadC OS=Pseudomonas syringae pv. aptata OX=83167 GN=ALO85_05166 PE=3 SV=1
MSIRSWPAAERPRERLLELGAASLSDAELLAIFLRTGVAGKSAVDLARHLLKQFDGLRPL
LDADLSVFTSQLGLGPAKFAQLQAVMEMARRHMAESLRRESALENPTQVRGYLKALLRHE

PHEVFGCLFLDNKHRVMTFEILFRGTINASYVHPRQVVKRAMAHNAASLILCHNHPSGIT
TPSRSDIDLTKRLKEALMLVDVHVLDHVIVGDGEPLSMVERGLM
>tr|A0A0Q0D9T0|A0A0Q0D9T0_PSEAP Uncharacterized protein OS=Pseudomonas syringae pv. aptata OX=83167 GN=ALO85_00615 PE=4 SV=1
MSSFIEMIENITPDIYESLKLAVEIGKWSDGRKLTQEQRELSLQALIAWEVQNLPEDQRT
GYMGPQECASKSAPVPNILFKSDAIH
>tr|A0A0Q0BZW4|A0A0Q0BZW4_PSEAP 2,3,4,5-tetrahydropyridine-2,6-dicarboxylate N-succinyltransferase OS=Pseudomonas syringae pv. aptata OX=83167 GN=dapD PE=3 SV=1
MIKPRGIYMSTTLFSLAFGVGTQNRQGAWLEVFYAQPLINPSAELVAAIAPVLGYTGGNQ
AITFNVDMAYKLAEAAKSVDATQAALLTRLAESQKPLVATLLAEDATLTSTPEAYLKLHL
LSHRLVKPHGLNLAGIFPLLPNVAWTSQGAVDLVELAERQLEARLKGELLEVFSVDKFPK
MTDYVVPSGVRIADSARVRLGAYIGEGTTVMHEGFVNFNGGTEGPGMIEGRVSAGVFVGK
GSDLGGGCSTMGTLSGGGNIIIKVGEGCLIGANAGIGIPLGDRNTVESGLYVTAGTKVAL
LDENNELVKIVKARELAGQTDLLFRRNSQTGAVECKTHKSAVELNEALHAHN
>tr|A0A0Q0C7F3|A0A0Q0C7F3_PSEAP Methionine import ATP-binding protein MetN OS=Pseudomonas syringae pv. aptata OX=83167 GN=metN PE=3 SV=1
MIEFHNVHKTYRVAGKEIPALHPTSLRVDSGQVFGIIGHSGAGKSTLLRLINRLETPSGG
QIVVDGEDVTALDANGLRRFRQQVGMIFQHFNLLASKTVADNVAMPLTLAGDMPRKQIDH
RVAELLERVGLSDHAKKYPAQLSGGQKQRVGIARALATKPKILLCDEATSALDPQTTASV
LQLLAEINRELKLTIVLITHEMDVIRRVCDQVAVMDAGVIVEHGKVADVFLHPQHPTTRR
FVQEDEQVDENEQRDDFAHVQGRIVRLTFQGEATYAPLLGTVARETGVDYSILAGRIDRI
KDTPYGQLTLAITGGDMDAAFARFTAADVHMEVLR
>tr|A0A0Q0IMW6|A0A0Q0IMW6_PSEAP Glycerol-3-phosphate ABC transporter, permease protein OS=Pseudomonas syringae pv. aptata OX=83167 GN=ALO85_200129 PE=3 SV=1
MKPRAHFPQPRLALLFALPQLLALGLFFYWPAVQAIWWSFHLVPPFGGNEIFVGLSNYWR
VLKDPGVLASLGSTLVFSLSSVVLSIVIALVLALCLELKLRGNAVFRNLLIWPYAVSGAT
IGIVFHVLANPVMGIFAWLNAIAPGTWAPYSSPIQSMLLLIVAYAWCLVPFNFVMLVASL
KSVPDDYLAAAALDGAGPLRRMLDIQLPLIAPYLLFVFVIDLLESLSNSFGLVDTLTQGG
PGDTTNVLAYKIYTDGFMGLDLAGSSTLSVLMLLAVVALSVAQFKLLSRFQRRGVKA
>tr|A0A0N8TA80|A0A0N8TA80_PSEAP Uncharacterized protein OS=Pseudomonas syringae pv. aptata OX=83167 GN=ALO85_03876 PE=4 SV=1
MQITMNNTLYPGLSAMQVGQSRVDQAATQIASSNIEVSGRSQSSDFQLENLRSVDRSQRS
DLPSNIVELEAGKDQFAAGLAAQKASNEMLGTLIDTYA
>tr|A0A0Q0C4E6|A0A0Q0C4E6_PSEAP Catalase domain-containing protein OS=Pseudomonas syringae pv. aptata OX=83167 GN=ALO85_00258 PE=4 SV=1
MTTQNLTVQPLPFQPSFEVVPDDEAQTSKELVEAMHSILETTYQDTGHAIRSVHAKAHGL
LHGQIQVYDDLPEPLAQGAFARPGNFPVVMRFSTNPGDILDDKVSTPRGLAIKLVGVEGP
RLPGSAEATTQDFVMQNAKAFTAKDPKAFLKTLKLLAKTTDKAPSMKRALSAVLRGIESV
IEAAGGESGTLRSLGGHPMTHILGETFYTVVPFLYGPYYAKLSVVPVSAELIALTDKAVD
LKDNPDGLREAVKAFFIEHDASWEVRVQLATDLEKMPIEDASAIWPEDLSPYVTVARIDV
PRQSSWSEQNIQEIDEAMAFSPWHGLEAHRPLGGVMRVRKPAYEHSAGFRSQHNGCPMHE
PRR
>tr|A0A0Q0FBP6|A0A0Q0FBP6_PSEAP Uncharacterized protein OS=Pseudomonas syringae pv. aptata OX=83167 GN=ALO85_04128 PE=4 SV=1
MSKIDFSKLDSRMTRTQPESSANQLGGFALVVEEKTPAQLAQEALQELNELEPGNNNRRS
TFAMNSHAKRSIELVEAVTNTKRYKNSIFAAVGLDVEMMNRLRLKAVNNPAIQEILDELK
AETDRRDD
>tr|A0A0Q0CIQ3|A0A0Q0CIQ3_PSEAP Chromosome partitioning protein ParB OS=Pseudomonas syringae pv. aptata OX=83167 GN=ALO85_01497 PE=3 SV=1
MAVKKRGLGRGLDALLSSPTVSSLEEQAVKAPPSELQHVPLDLIQRGKYQPRRDMDPQAL
EELAQSIKSQGVMQPIVVRPIGDNRFEIIAGERRWRASQQAGVETIPAMVRDVPDETAIA
MALIENIQREDLNPIEEAMALQRLQQEFQLTQQQVADAVGKSRVSVANLLRLIALPEAIK
TMLSHGDLEMGHARALLGLGEDRQVEGARHVVARGLTVRQTEALVRQWLSGKAEAAEPVK
TDPDISRLEQRLAERLGSAVQIRHGQKGKGQLVIRYNSLDELQGVLAHIR
>tr|A0A0Q0DJR3|A0A0Q0DJR3_PSEAP Uncharacterized protein OS=Pseudomonas syringae pv. aptata OX=83167 GN=ALO85_100218 PE=4 SV=1
MKYFLDSNTLIEAKNRYYGMRICPAYWQWLLLQNQAFSLASIEPVKDELTKGHDELAAWA
IDNSSFFWGVSDEDTQTTFVKVVTLVAQAQGMKTGAMEEFLRGADPWLIAKALTSGATVV

THEVRNMDARRKFIIPNLCEQLNVPYMNTFELLHHLSAIFVLPQPGRMPPPSSETLSASS
TSGSPSS
>tr|A0A0Q0CER1|A0A0Q0CER1_PSEAP Uncharacterized protein OS=Pseudomonas syringae pv. aptata OX=83167 GN=ALO85_03995 PE=4 SV=1
MSVLRVITCSTLLAVVAGNAQVASAVEILRWERLPLAIPLRINQERIVFVDQNVRVGLPR
SLTEKLRVQSTGGAIYLFAKEAIEPTRLQLQNAKTGEIILVDIAATEAPTGQPALEPVKI
VEGETTSARYGGAATARSAKATTKSTDSSSRSSDEDDEPEEVVQRETPVPVVLTRYAAQM
LYAPLRTVEPVDGIAQVKVERNLDLATLLPTLPVKSTPLGAWRLDDYWVTAVKLQNQTTQ
RITLDPRELMGEFVTAAFQHPYLGSRGDASDTTTLYLVTRGHGLTQAAVFSATQADPRAA
QGAKHER
>tr|A0A0Q0BZ18|A0A0Q0BZ18_PSEAP Uncharacterized protein OS=Pseudomonas syringae pv. aptata OX=83167 GN=ALO85_100777 PE=4 SV=1
MPPPSIRLCPDCTGGHFDRGHNARPVDRRQADTTCINVGFYTLKRETTWLRSAARRDNSW
LDALIWLAP
>tr|A0A0N8T951|A0A0N8T951_PSEAP Periplasmic spermidine putrescine-binding protein OS=Pseudomonas syringae pv. aptata OX=83167 GN=ALO85_200157 PE=4 SV=1
MAFIRKNVKPKLVASSSVSDDSNSAETFTVAQLLELARRKGVETSPLDVQKLLSVLGVEL
LSVPMKDEISGVLSRLENKDGWVVKVNALHHPNRQRFTIAHEIGHYCRHRWQQKEFQDLN
FFRNGDSNPMEAEANRFASELLMPEQTFKEKVRLFAGSIEAIAQYFKVSTLAVRVRAKNL
GMKGHGLE
>tr|A0A0N8T8Z5|A0A0N8T8Z5_PSEAP Pyrimidine monooxygenase RutA OS=Pseudomonas syringae pv. aptata OX=83167 GN=rutA PE=3 SV=1
MDIGIFIPIGNNGWLISSNAPQYMPTFELNKQIVQKAEGYGFDFALSMIKLRGFGGKTQF
WEHNLESFTLMAGLAAVTSRIQLFATVATLTIPPAIAARMASTIDSISNGRFGINLVTGW
QKPEYEQMGLWPGDEFFHTRYEYLAEYAQVLRDLWATGSSDFKGEHFSMQDCRVSPRPQG
DMKLICAGQSEAGMAFSAQYADYNFCFGKGVNTPTAFAPTAQKLLEANEKTGRNVTSCVL
FMIIADDTDEAARARWEHIKDGADEEAIAWLSEKGSADKSAGSNLRQMADPTSAVNINMG
TLVGSWATVARMLDEVASVPGTQGVMLTFDDFVKGVEDFGEKIQPLMTSRKHIAQLKEVV
>tr|A0A0Q0IPA2|A0A0Q0IPA2_PSEAP 3-isopropylmalate dehydrogenase OS=Pseudomonas syringae pv. aptata OX=83167 GN=ALO85_03583 PE=4 SV=1
MTTQALRKRICVLPGDGIGIEVMEAALPVIAALNLPFDLIAADIGWECWRAEGETVPART
WEIMASCDATLLGAITSKPLAEAEAELPLALQGQKRRYVSPVIQLRQNLDLYANVRPVTD
MTGENRFRFVVIRENTEGLYAGLDFGCIPDALVPLLEEREALGAAWQREGQDDATVTLRL
QTRSGLTRIFEYAFEYARKNHFTRVTFVDKPMVLRHSSAFARGIFEATAQRYPDVTGVIE
NVDAVALWMVQRPERFGVIVAENMFGDILSDLGAGVMGGLGFAPSGNFGNSGAYFEPVHG
SAPAHAGLNKANPGAMFMAIALMLDHLGFPAESACISQAVSLVSRTSTRTYDLGGSHTTA
QVGEAIVQQCIELQSRTFGYGNEQVAAQGERHVSAL
>tr|A0A0Q0IJ92|A0A0Q0IJ92_PSEAP MvaT-like transcriptional regulator TurD OS=Pseudomonas syringae pv. aptata OX=83167 GN=ALO85_03933 PE=4 SV=1
MSKLAEFRQLEKHLAEQLQALEAMKGDQGLKKEIEFETKLRKLLEQYGFSLKHIINLLDP
QSSSRGQTVEKPAGKRKPRELKVYKNPKTGEVIETKGGNHKILKEWKAEHGSDVVEGWLK
K
>tr|A0A0Q0ITF4|A0A0Q0ITF4_PSEAP Two-component DNA-binding response regulator OS=Pseudomonas syringae pv. aptata OX=83167 GN=ALO85_01415 PE=4 SV=1
MSKVSVLVVDDATFIRDLVKKGLRNYFPGIHTEDAVNGRKAQTLLGKEAFDLILCDWEMP
EMSGLELLTWCREQDNYLRTVPFIMVTSRGDKENVVQAIQAGVTDFVGKPFTNEQLLTKV
KKALAKVGKLDAVMSTAPARMNSPLNDSLSALTGGKAEVVRAAPAAAAAAAPKPAPAPVR
SAQPTGRGQGQLRLPNGTQSCVIKALTLKDATLVIRVSENLPQVLDSAVLDLEQGEDNEV
ARLNGYLHSVAAFEQKPDSEWLQVTFRFVDHDAQKLDYLSRLIARGTAQKHFIPGA
>tr|A0A0Q0DKD4|A0A0Q0DKD4_PSEAP Uncharacterized protein OS=Pseudomonas syringae pv. aptata OX=83167 GN=ALO85_02797 PE=4 SV=1
MIRLLMWVALIAAVVWFVKRLINPPKPKSRAEPPELASTPMVQCAQCGVHLPQDRALSQA
QQWYCSEAHRLQGPAARDR
>tr|A0A0Q0IQZ9|A0A0Q0IQZ9_PSEAP Major facilitator transporter OS=Pseudomonas syringae pv. aptata OX=83167 GN=ALO85_00596 PE=4 SV=1
MSQELRLIRRITFKLIPFLILLYLIAYVDRSAVGFAKLHMGADIGIGDAAYGLGAGLFFI
GYFLLEIPSNLMLERFGARRWFARIMITWGAITIGMAFVQGPHSFYVMRFLLGAAEAGFF
PGVLYYITQWFPVRHRGKILGLFILSQPIAMMITGPVSGGLLGMDGILGLHGWQWLFIVI

GTPAILLTWPVLRWLPDGPQQVKWMDQAEKDWLTGELKKDLEEYGQTRHGNPLHALKDKR
VLLLALFYLPVTLSIYGLGLWLPTLIKQFGGSDLTTGFVSSVPYIFGIIGLLIVPRSSDR
LNDRYGHLAVLYVLGAIGLFCSAWLTMPVAQLAALCVVAFALFSCTAVFWTLPGRFFAGA
SAAAGIALINSVGNLGGYIGPFVIGALKEITGSLASGLYFLSGVMVFGLLLTGIVYHVLE
RKHVLPVSDFAASAKAR
>tr|A0A0Q0DAL1|A0A0Q0DAL1_PSEAP Phosphoesterase OS=Pseudomonas syringae pv. aptata OX=83167 GN=ALO85_03378 PE=3 SV=1
MKVGVISDTHGLLRPEAITALQGCAQIIHAGDIGSQDIVEQLTAIAPLHIVRGNNDMDAD
WATPIADHLRFEIEGWQILLVHDIADVPALLNDSVKLVVTGHSHKPLIDWRGDTLYLNPG
SAGRRRFKLPVTLALLEVSADAIEPSLVQLVD
>tr|A0A0Q0D8W4|A0A0Q0D8W4_PSEAP Putative signal peptide protein OS=Pseudomonas syringae pv. aptata OX=83167 GN=ALO85_03798 PE=4 SV=1
MPKSVLRTVATVSFIALSSTAYAYDLPGLNLGNTSFYDGSPAPAGPGWYLEEYLNYAKAN
RFNDVNGDKLQLPKQDVDVLAVTTQIIYVGQPMANGAMPGITAINTSLAHVDVDDGLGNT
ALSSRAGFGDLVIGPFLQLPTITHADGSPLLTQRIEADIAIPVGAYDRNRSINPGSNFWS
FNPYYAATYWFSPKWSASGRFMYLWNGKNDDPQAGFGNVSDTQAGQALHANLTLQYAVNE
QLSLGLNGYWLKQFTDTQVDGHDVSGRKEKVWAIGPGFLYAFNKENVLTVNSYFEQGAEN
RTEGNKVVLNFLHKL
>tr|A0A0Q0C1N4|A0A0Q0C1N4_PSEAP Uncharacterized protein OS=Pseudomonas syringae pv. aptata OX=83167 GN=ALO85_01787 PE=4 SV=1
MDVTSASRPLPQKRTAKARARGVTAAYRLAIASRALAAVFAGYLLASLVSTCIAQWLPIT
RAEAVVTGMMFSFLAYLGAVLWCFACRTALQAWIGVLTPCTLLGAAYTCGRWLL
>tr|A0A0Q0BYN0|A0A0Q0BYN0_PSEAP dTDP-4-dehydrorhamnose reductase OS=Pseudomonas syringae pv. aptata OX=83167 GN=ALO85_200069 PE=4 SV=1
MPMSPNRLVTALLSRMNDLQAKHILAPTLLATCIGLLAALAILHFAPISEMIISHSQYVS
TKDITELDPYEQLVIARMVKEKALITVDSLWSMQVSFYQTMISVLIALNAAIVGGAFVII
RSSSRAEVVKESKSHFDEYCKAGEFTKIIEKRAKKEIVKINATYGDILDSLSDHEARINA
HKDAILEVSNRLAEMDETENCSGGDGKLSE
>tr|A0A0Q0DPS0|A0A0Q0DPS0_PSEAP Choline/ethanolamine kinase OS=Pseudomonas syringae pv. aptata OX=83167 GN=ALO85_03598 PE=4 SV=1
MKTLGQSVSTQERQLEAAVRSIDSWQGRRVGYEPVSGGISNTNWRVEVEGADTAYFFKVP
GVGTEMFIDRHTAHEASVKAAQTGYGAPVFAFLEAFGVEVFEFMEGWRASSNHDFLQRDI
RHSALHGLKAFNDQPLLQQTKTVFDMIAEHQSQVAELKGIKPQDDDWLCLQYQRAKAALQ
ASGIDLAPCMNDTLAGNFMLNADRQIRLVDFEYASNNDRHYELALWFGEMFFSDDMELAL
IEDYFGQVSAQTVARIKLNKALADIKWSTWAMVQHAVSQLDFDFYKYGTWKHMRARSIIN
DSQWETWLRQV
>tr|A0A0Q0CXW5|A0A0Q0CXW5_PSEAP Type I restriction enzyme subunit M OS=Pseudomonas syringae pv. aptata OX=83167 GN=ALO85_03109 PE=4 SV=1
MNTSSLVQKVWNFCHTLRDDGVGYGDYLEQLTYLLFLKLAHEYAQEPYSRDTHIPKGYDW
AGLTSKVGEPLEAHYLATLHKLGQQSGMLGAIFFKAQNKIQDPAKLSRLVQLIDAESWIS
LGADTKGDLYEGLLQKNAEDTKSGAGQYFTPRALIETIVACVRPEPMKIIADPACGTGGF
FLGAYNWLTRPGATLNKAQKEFLRDKTFHGNEIVSNTRRMCLMNLFLHNVGELDGEPLVA
RSDALITEPKLKVDYVLANPPFGKKSSMTISNEEGDEDKEALTYERQDFWETTSNKQLNF
LQHIVSMLKVDGKAAVVLPDNVLFEGGAGEKIRRKLLDNCDVHTVLRLPTGIFYAQGVKA
NVVFFDNAPKDGRVHTKGVWFYDLRTNKHFTLKTRTLKPDDLQDFVTCYNPENRNERTAT
ERFKFFSYEDLMARDKASLDIFWLKDDSLDNLDDLPPPDVLQLEIIDHLEAALIAFREVA
LNLPRSEIVD
>tr|A0A0Q0BYL0|A0A0Q0BYL0_PSEAP Uncharacterized protein OS=Pseudomonas syringae pv. aptata OX=83167 GN=ALO85_200166 PE=4 SV=1
MSHDDLIAFKNLTLEHLENNDFQKAFSFNTNLDYKVSWSKGPACSIIPLDLEMSGVKPAE
LLAHEPKNKKNVHKNYFLDNTLTRVESFDRMGLLSEIESTKTDSGIRYSIRKNNFGEVNW
LKAVEFEKGLPIRACRIDSDSEFWSYRYKWENMKIVEITTFSSNSIPGIRLFVDYSGDAV
NSIFFDNKGSKIVIYNKND
>tr|A0A0Q0IL57|A0A0Q0IL57_PSEAP Mannose-1-phosphate guanylyltransferase/mannose-6-phosphate isomerase PslB OS=Pseudomonas syringae pv. aptata OX=83167 GN=ALO85_02662 PE=3 SV=1
MSTLIPCIIAGGSGTRLWPVSREAMPKPFMRLPDGQSLLQKTFLRAVGLPGVERLLTVTN
REVYFRTVDDYRQLNKGNARLDFILEPFGRNTAAAVAAAALHVSQLYGDEAQLLVLPADH
LITDVGEFSRAVGAAQKLAAEGWLVTFGILPTAPETGFGYIEKGPALNADACQVARFVEK
PDAITAQGYLDSGQHFWNAGMFCMRADAVLRELRTHAPDVLSAVSACLESSPRKEGNREL

QVELDGESFALTPDISIDYALMERSEKVAVVPCQLGWSDIGSWQAVRELSPVDAQGNYCN
GEAVLHDVSNCYIDSPKRLVGAIGLDNLIIVDTPDALLIADGRRSQEVKIIAQELKRQGH
DAYRLHRTVTRPWGTYTVLEEGKRFKIKRIVVRPQASLSLQMHHHRSEHWIVVSGMATVT
NGEREFMLDTNESTFIKPGHTHRLVNPGVIDLVMIEVQSGEYLGEDDIVRFTDIYGRVPD
KAVKR
>tr|A0A0N8T8V7|A0A0N8T8V7_PSEAP Uncharacterized protein OS=Pseudomonas syringae pv. aptata OX=83167 GN=ALO85_00439 PE=4 SV=1
MTQRITVVPAEGRTVPDPEAGDLLPVEGRQVTFNAWWQRRQNDGDITIKTEQSTTTHQAF
TA
>tr|A0A0Q0BUE2|A0A0Q0BUE2_PSEAP D-galactose 1-dehydrogenase OS=Pseudomonas syringae pv. aptata OX=83167 GN=ALO85_01310 PE=4 SV=1
MQPIRLGLVGYGKIAQDQHVPAIHANTAFKLVAVATQGQPCAGVGNFKSLGELLASGPQV
DAIAFCTPPQGRFALVQEALAAGKHVLVEKPPCATLGEAMALVTQVQEQGVSGLFAWHSR
YAQGIEAAREWLSTRTLQSVQIDWKEDVRKWHPGQAWIWQPGGLGVFDPGINALSIVTHL
LPLPLFVESAELRVPSNCQSPIAASIKMSDTRHLDIRAEFDFDHGHDELWSIEIRCAEGT
LRLDNGGALLSIDGVPQSVSEEGEYAAVYRHFQQMIGDKASDLDLQPLRLVADSFFVGSR
ASVEPFYD
>tr|A0A0Q0FAU8|A0A0Q0FAU8_PSEAP Phosphoribosyl-ATP pyrophosphatase OS=Pseudomonas syringae pv. aptata OX=83167 GN=hisE PE=3 SV=1
MTDTLSRLAEVLESRKDAAADSSYVASLYHKGLNKILEKLGEESIETIIAAKDAAVSGDC
SDVIYETADLWFHSMVMLAALGQHPQAVLDELDRRFGLSGHAEKAARTAE
>tr|A0A0Q0DXD3|A0A0Q0DXD3_PSEAP Cointegrate resolution protein S OS=Pseudomonas syringae pv. aptata OX=83167 GN=ALO85_03667 PE=3 SV=1
MNDVDRYIEAATRDNTRRSYRAAIEHFEVTWGGFLPATSESVARYLASHAGTLSVNTLKL
RLSALAQWHISQGFVDPTKAPMVRKVIKGIRALHPAQEKQAEPLQLQDLEKVIAWLEIEI
REASAQHDQPRLLRGRRDSALILLGFWRGFRSDELCRLQVQDVKAIADSGISLYLPRSKG
DRDNLGRTYQTPALQRLCPVQAYIEWINCAALVRGPVFRAVDRWGNLKEEGLHANSVIPL
LRQALQRAGIAAEHYTSHSLRRGFASWAHSNGWDLKSLMSYVGWRDIKSAMRYIDAAPFL
SPPEAGSGPKVSSINKLPS
>tr|A0A0Q0DL58|A0A0Q0DL58_PSEAP Lipoprotein OS=Pseudomonas syringae pv. aptata OX=83167 GN=ALO85_03088 PE=4 SV=1
MFRRIVLLTCAVLLTACQSNSVNRDFDAQRDFGGYRSWSWKEPAVQYQPDNDPRLKSDLT
EQRLRQSIGEQLDQRGLRMATGGARPDLKVQAWLIVENRQQTVSTNYGGGWSPWGGYGYW
NGPMNTETRSVDYKVGTVQIDMFDGKDGKLVWRGSTERILNDNAGSPSERDQAMRTTVAK
ILEQYPPR
>tr|A0A0Q0C769|A0A0Q0C769_PSEAP Uncharacterized protein OS=Pseudomonas syringae pv. aptata OX=83167 GN=ALO85_01071 PE=4 SV=1
MLRSYLRLVLFTMGLLFGVQIPGFISDYSKRVEAHLIEAQQAVKGYTATAQQFFKGDIQA
LIQHYRSSEDPVFRSDADTIDTLMNRTHILERQWLGLQGPWYSKAIFVATSSDPDIRRET
FNGYTWQVLLAPEVIAWGIISALLLALVIESFVLLLGWVVHGGRRKPQLERDWN
>tr|A0A0Q0CIX9|A0A0Q0CIX9_PSEAP Lipid A biosynthesis, N-terminal OS=Pseudomonas syringae pv. aptata OX=83167 GN=ALO85_01482 PE=4 SV=1
MKPDNETFWLILGFVGQAVFTGRFVLQWLYSEFKKRSIIPVGFWYLSMLGSTILLAYAIY
RQDPVFIAGQAFGLLVYMRNLQLISRQAKTPEEQ
>tr|A0A0Q0DLD4|A0A0Q0DLD4_PSEAP IclR family transcriptional regulator OS=Pseudomonas syringae pv. aptata OX=83167 GN=ALO85_02640 PE=4 SV=1
MSLPAGQAIKLPFPIGAQHPMTQQTESPAGGRQQKVQAAEVGLGVLKALAELSPATSLSR
LAEHLGMPASKVHRYLQALIASGFAEQDAVNNHYGLGREALQVGLASLGKLDVLKVSAPW
LASLRDELNQTCFLAVWGNKGPTVVYVEPSMGAVTLVTQIGSVLPLLGSSTGLVFDSFLN
GRETAALREQEVPLLSASQLREVEQHVEQIRTTGVHQIQGLLMPGINAASSPLFAMGNKL
VGVITVVGPGSVLNDETQSLAARRLLQTAVTISERMGGSRPSD
>tr|A0A0Q0C5M0|A0A0Q0C5M0_PSEAP Endolytic peptidoglycan transglycosylase RlpA OS=Pseudomonas syringae pv. aptata OX=83167 GN=rlpA PE=3 SV=1
MRRLIFACSLLTLLAGCANHIIDPHGYDETGTAAYYGARHHGNRTASGEPFNQNALTAAH
RQLPFGTRVKVTNLDNDRSVVVRINDRGPHTRGRLIDVSREAAEQLGMLRSGTAPVRVQA
LD
>tr|A0A0Q0C9Y2|A0A0Q0C9Y2_PSEAP Uncharacterized protein OS=Pseudomonas syringae pv. aptata OX=83167 GN=ALO85_01446 PE=4 SV=1
MLTLGNIFVLMLFASAAAWWWHAHGLREKALARVKQHCERLELQLLDDAVALRRLTFARD
AQGRKRLARVYGFEFTVTGEQRHPGTITMFGAHTAQIELAPYPFEIKTPLPSAEVIQMSE

WRQSHQKWKQ
>tr|A0A0Q0IQL0|A0A0Q0IQL0_PSEAP Pantothenate kinase OS=Pseudomonas syringae pv. aptata OX=83167 GN=ALO85_200034 PE=4 SV=1
MDVLMGLLAALMWGGTDFLVGLNARAVGVKRAVFFGQMVGFTITTALLLFFPSVIINAIS
VSVDVWLFGFLASSFTVLGALAISKAFALGKASIVAPVVTSYGVVTTLLSWASGEYLTVF
QLVFIVVCVFGVILSSVQPKSDAQPGNHTRKSILFAILAAMLYGTSFWLQGRFTLQYLGP
IAMLWLSYAVGITILLFMMINVKNGLRIPRPRSCFLLIATSLLNLGGFSAFSWGTALGSV
SIVTVISTLSGGVAAILGFLFFKERLGMIQISGVLLVILGAVMLHS
>tr|A0A0Q0DBL1|A0A0Q0DBL1_PSEAP Protoheme IX farnesyltransferase OS=Pseudomonas syringae pv. aptata OX=83167 GN=cyoE PE=3 SV=1
MSLKHFIQITKPGIIFGNVLSVAGGFFLASKGNIDFGVFLAAVIGTSLVVASGCVFNNCI
DRDIDQRMERTRNRVLVQGLVSLKLALLYATLLGIAGVGLLYTEANPLAALFAVIGFVIY
VGLYSLYLKRRSVHGTLVGSLSGAMPPVIGYCAVSNSFDFAALTLLVMFSLWQMPHSYAI
AIFRFNDYRAAKIPVLPVKRGILVTKRHIMLYILAFLVATLMLTVGGYAGLNYLAVAAGM
GMYWLYMAWKGYKAVDDTVWARKLFVFSIFTITALSVMMSVDFQVTKELLVTYAF
>tr|A0A0Q0FHM7|A0A0Q0FHM7_PSEAP L-lactate dehydrogenase OS=Pseudomonas syringae pv. aptata OX=83167 GN=lldD PE=3 SV=1
MIISSASDYRAAAKRKLPRFLFDYIDGGAYAEHTLRANGSDLADISLRQRVLKNVDNVSL
ETRLFGETLAMPIVLSPVGLSGMYARRGEVQAAKAAANKRIPFCLSTVSVCSIEEVASQS
KQAIWFQLYVLKDGGFMKNALERARAAGVTTLVFTVDMPTPGARYRDAHSGMSGPYAAPR
RILQAMTKPDWALNVGLLGRPHDLGNISRYLGKATTLEDYVGWLANNFDPSISWKDLEWI
REFWQGPMIIKGILDPQDARDALSFGADGIVVSNHGGRQLDGALSTAKALPPIVQAVGSD
LTVLADSGIRSGLDVVRMLALGAKGVLLGRSMAYALGADGQRGVENMLDIFAREMHVAMT
LTGVTSIEQIDESILVKSAV
>tr|A0A0Q0DJI9|A0A0Q0DJI9_PSEAP Sterol-binding protein OS=Pseudomonas syringae pv. aptata OX=83167 GN=ALO85_00499 PE=4 SV=1
MTSVADAVQTMKNKFNPAAAAGLDLIFGFNITDEDKHYALIVKDSTCELQEGENPDANVT
LVMDSQTLKGIVSGETDGMQAFMGGKLRAEGDMMLAMKLSELFPV
>tr|A0A0Q0FLR2|A0A0Q0FLR2_PSEAP Regulatory protein, TetR OS=Pseudomonas syringae pv. aptata OX=83167 GN=ALO85_02551 PE=4 SV=1
MQKEPRKVREFRRREQEILDTALKLFLEQGEDSVTVEMIADAVGIGKGTIYKHFKSKAEI
YLRLMLDYERDLNELLHSADVDKDKESLSRAYFEFRMRDPQRYRLFDRLEEKVVKGNQVP
ELVEELHKIRASNFEHLTLLIKGRISEGKLEDVPPYYHYCAAWALVHGAVALYHSPFWSN
VLEDQEGFFHFLMDIGVRMGNKRKRDGEVNAEPTPDPAAE
>tr|A0A0Q0C535|A0A0Q0C535_PSEAP Iron-sulfur cluster assembly scaffold protein IscU OS=Pseudomonas syringae pv. aptata OX=83167 GN=ALO85_04113 PE=3 SV=1
MAYSEKVIDHYENPRNVGKMNAEDPDVGTGMVGAPACGDVMRLQIKVNEQGVIEDAKFKT
YGCGSAIASSSLATEWMKGKTLDEAETIKNTQLAEELALPPVKIHCSVLAEDAIKAAVRD
YKQKKGLL
>tr|A0A0Q0IQ90|A0A0Q0IQ90_PSEAP Regulatory protein, ArsR OS=Pseudomonas syringae pv. aptata OX=83167 GN=ALO85_00771 PE=4 SV=1
MAINESLDIILSMRTFQHPDPEDLTLERLLYALSDPVRMGIVRCLGGVAEATCGELDGGR
PKSSMSHHFRVLRDAGLVQTRNVGTTHMNSLRKQMLESRFPGLLDSILAQH
>tr|A0A0Q0BU12|A0A0Q0BU12_PSEAP LysR family transcriptional activator OS=Pseudomonas syringae pv. aptata OX=83167 GN=ALO85_01891 PE=3 SV=1
MSRIFNAQMHAWLQVFACAARHLSFTRCAEELHVTPGAISQQMRQLEERLGFALFHRVGR
GLELTAEGQRLAVVANEVQSRISEELRLLYSGRIGGVFKLRCIPSFLSKWLMPRLPRLQA
AFPDIQLRIIAEDSSGSLRDDDFDLAIDLNDGSYPGLSTTPLLEEELFAVCSPDLLVGKP
PLDTPSQLVHFPLLHDITAWRGSYEYAEWEFYLTAIGADGIDVRRGHTFNRNHLTIDAAR
MGMGVAIARKALITDELEQGLLIVPFGHPIKARKKYVLAYREGALSTPSRRAVHDWLVNE
ALG
>tr|A0A0Q0CAF1|A0A0Q0CAF1_PSEAP PilT protein OS=Pseudomonas syringae pv. aptata OX=83167 GN=ALO85_03006 PE=4 SV=1
MFLLDTNVVSELRKRTADANVLRWSRTQMASSLFISSITILELETGILRIERRDPTQGAV
LRMWLDQHVLKAFAGRILTVDTQVARRCAQLHVPDPRSECDALIAATALVHGMTVVTRNT
MDFQSCGVALLNPWVSQLNEEAAVYSRASR
>tr|A0A0Q0CW73|A0A0Q0CW73_PSEAP Zinc-containing alcohol dehydrogenase super protein OS=Pseudomonas syringae pv. aptata OX=83167 GN=ALO85_01886 PE=3 SV=1

MRALTYHGANSVKVDTVPDPVIEQADDIILRVTATAICGSDLHLYRGKIPTVEHGDIFGH
EFMGVVEETGSAVTAVAKGDRVVIPFVIACGSCFFCNLDQFAACETTNTGRGAIINKKSI
PPGAALFGFSHLYGGVPGGQAEYVRVPKANVGPFKVPSTLADEKVLFLSDILPTAWQAVL
NAGIGQGSTVAIYGAGPVGLMSAACAKMLGAEKIFMVDHHPYRLAYAQKTYGVIPINFDD
DDDPADTIIRQTAGMRGVDGVVDAVGFEAKGSTTETILATLKLEGSSGKALRQCIAAVRR
GGVVSVPGVYAGFIHGFMFGDAFDKGLTFKMGQTHVHRFMPELLEHIEAGRLEPEAIITH
RMSLEDAAKGYKLFDKKEEDCRKVILTPGNNTIVVPDDTTEALVGGGVPAM
>tr|A0A0Q0D3L9|A0A0Q0D3L9_PSEAP GIY-YIG nuclease super protein OS=Pseudomonas syringae pv. aptata OX=83167 GN=ALO85_01827 PE=4 SV=1
MPEVASTSSASTAEQVAGKPWFVYLVRAANGALYCGISDDPQRRFAAHQSGKGARFFSSS
PAMALVYVEQWPSKGEALRHERLIKKLRKSAKEALAASYATSSIHERDAVPSV
>tr|A0A0Q0C4L4|A0A0Q0C4L4_PSEAP DNA-directed DNA polymerase OS=Pseudomonas syringae pv. aptata OX=83167 GN=ALO85_04040 PE=4 SV=1
MEKHEAVFALIDCNCFYASCERVFRPDLEKTPIVVLSNNDGCVIARSYDAKPFVKMGAPY
FQIKEVLRRNGIKVFSSNYAL
>tr|A0A0Q0DI52|A0A0Q0DI52_PSEAP Abortive infection protein OS=Pseudomonas syringae pv. aptata OX=83167 GN=ALO85_03017 PE=4 SV=1
MTTRHWLTFCLLSSGYALALFYGNLDPSAALSFGALFIAWFCVARSSYSSIRLCGHGLFI
VTGLALAFHLAPGFNNANVIEATRFSADAALFSMDLSLDKPLIGFWLILACPWILPKVDV
AHSLQTGLLALIVTSAFCMTAAVMLNVVGWTPKWPDQGLIWLLNNLLLVTLTEELFFRAY
LQGSLMRLFKGSRYATALSITLAAGLFGLAHAGAGPQWVALASMAGLGYGIAFRFGGLQA
AVISHLGLNLVHFGLFTYPMLARQG
>tr|A0A0Q0C5V1|A0A0Q0C5V1_PSEAP Uncharacterized protein (Fragment) OS=Pseudomonas syringae pv. aptata OX=83167 GN=ALO85_05395 PE=4 SV=1
MAHDYAIESLLRPAVELYTVYVCAAGAFLCLFAPWAFALTPLFGIVTSAGFLALGLVRLK
QAWQVLRYRRNIRRLPHYTMTSKEVPVSNQRLFIGLGFRWQQRHTQRLMDTYLPKYASYV
EATSLFRAARRFEERAEFAPYPVRLLARATSWDVPINPVRPLPPVGGLPRLHGIEPYEEN
VSLPLGERVGHSIVLGTTRVGKTRLAELFITQDIRRKKHGQHEVVIVFDPKGDADLLKRM
YLEAKRAGRLNEFYVFHLGWPDHSARYNAVGRFGRISEVATRIAGQLSGEGNSAAFREFA
WRFVNIIARALVALGRRPDYLQIQQHVINIEGIFQEYASKYFDEYDPKAWEAIVAIEGKL
NEKNVPFNMKGRPFRVVAIDQYLSQTRVADPVMDGLRSAVRYDKTYFDKIVASLLPLLEK
LTTGRMAELISPDYQDVNDPRPIFDWMQVVRKKAVVYIGLDALSDTEVAAAVGNSMFSDL
VSVAGHI
>tr|A0A0Q0BY40|A0A0Q0BY40_PSEAP 2-methylisocitrate lyase OS=Pseudomonas syringae pv. aptata OX=83167 GN=prpB PE=3 SV=1
MSHNNSTPGQRFRDAVASEQPLQVVGAINANHALLARRAGFKAIYLSGGGVAAGSLGIPD
LGITGLEDVLIDVRRITDVCDLPLLVDIDTGFGSSAFNVARTVRSMIKAGAAAIHIEDQV
GAKRCGHRPNKEIVSQQEMVDRIKAAVDARTDDSFVIMARTDALAVEGLESALERAAACI
EAGADMVFPEAITELAMYKQFANRAGVPILANITEFGATPLFTVDELREADVSLVLYPLS
AFRAMNKAAENVYGAIRRDGSQKNVIDSMQTRMELYDAIDYHTFEQKLDALFAQKKG
>tr|A0A0Q0C4J9|A0A0Q0C4J9_PSEAP Methyltranfer_dom domain-containing protein OS=Pseudomonas syringae pv. aptata OX=83167 GN=ALO85_02420 PE=4 SV=1
MSVITSTSPIAATDHLAQFLGLLETSLSQNSLIKLVLAKYVGSEADLQRIIIKPLTVKEQ
PCLSFVYRYKTRDITKNFSLAEAVAVIASLIPASFKNAHLLSLTDEVQLEFSKKGKTTLF
KSKAQQERQAPSAGHDREKKRYLELTRPFLTDLGVTNRQHELIPAMSRKWKQINKFIEVF
SHALSSSPLKLDQPIKVADFGSGKGYLTFAIHDYLCNTLQAQGQVTGVELREDMVSLCNE
AAAKLDHPGLTFAQGDVRTVAPSALDVMIALHACDIATDYAIHMGIRSGASIIMCSPCCH
KQIRLQIQSPTLLKPMLQYGLHMGQQAEMVTDSLRALLLEACGYETKVFEFISLEHTNKN
KMILAVKRTEPVDPAQLLARIQELKTFYGLSEHCLETLLKSDGFIG
>tr|A0A0Q0IAF6|A0A0Q0IAF6_PSEAP Bifunctional nitric oxide dioxygenase/dihydropteridine reductase 2 OS=Pseudomonas syringae pv. aptata OX=83167 GN=ALO85_04088 PE=3 SV=1
MLTSTQRDIIKATVPLLETGGEALITYFYQLMLDTHPEVRPLFNEAHQANGSQPRALANG
VLMYAKNIDRLENLGGLASRIINKHVALQILPEHYPIVGSCLLQAIREVLGAETATDEVI
AAWAAAYQQLADILIAGEQSVYDAIAAAPGGWRGGRQFTVLRKVAESAEITSFYLAPTDH
QPVIRHKAGQYIGLRLMIDGKEVRRNYSLSEQADGVRYRISVKRELGGLASTYLHDYVEE
GSTLDVFPPSGDFTLNAHTKPLVLISGGVGITPTLAMAEQAMESGERQVTFIHYARNGQV
HAFSDLLRDWQHRYPLFKAHVVYAERTDHEAYTPDAIGYPSLTHLEQWLPADRDVDAYFL
GPKPFMAFMKRALKDLGVPENQARYEFFGPAEALA

>tr|A0A0Q0DJZ1|A0A0Q0DJZ1_PSEAP Membrane-bound lytic murein transglycosylase D OS=Pseudomonas syringae pv. aptata OX=83167 GN=ALO85_04389 PE=4 SV=1
MSSSTSKPSHSDALTRLAQAMAVTASALLAGCQSTASVDPASNHRAPNLAAGIKQKPIFL
SHKPATPLAPPQDVWERMRQGFQLQQGNDQNPRIDQQRLWFANNPSFLENAGERGSLYMH
YIVERLEERNMPLELALLPVIESAYNPMAVSRSQAVGLWQFIPSTGRYFNLRQTSFYDGR
RDIQASTVAALDYLTRLHDMFNGDWLLALAAYNAGEGTVSRAIERNDKLNLPTDYWNLPL
PQETRDYVPKLLALSQVVLSPEAYGVNLNPIANQPYFEVVELNKRVDLSKVASAADIDED
ELIQLNPAYKKRLTVDGPQHLLVPTSKAQLLTASLLTMKQEELVAWQPYRVRKGDTLESL
ASRYQVTVSSLKGNNKLSGNRLKVGQSLSIPVKPGMQNSQPVFEALASNDKPSRTRSYKV
RSGDNLTNIAQANKVDVEDLQRWNKLTGKKLKVGQTLVMQDTSKPAGKAASKAVASTGGK
DNEKKSMQYKIQKGDSMYLVAKRFNVEMQHLKRWNPRSGQALKPGQTLTVYLPH
>tr|A0A0Q0FHY6|A0A0Q0FHY6_PSEAP Chemotaxis protein CheV OS=Pseudomonas syringae pv. aptata OX=83167 GN=ALO85_02135 PE=4 SV=1
MLIWCLGGGHFVRGFGMAGVMDSVNQRTQLVGQNRLELLLFRLDGKQLYGINVFKVKEVL
QCPKLTIMPKSSKIVRGVANIRGGTIPIMDLAMATGSTGMISLVNSFVIITEYNTKVQGF
LVHSVERIVNMNWEEIHPPPKGTGRDHYLTAVTRVDNQLVEIIDVEKILAEVAPVSEEIS
VGVIDAEVQHKAVSLRVLTVDDSSVARKQVSRCLETVGVEVVALNDGRQALDYLLKMVAE
GKKPEEEFLMMISDIEMPEMDGYTLTAAIRNDPRMQKMHITLHTSLSGVFNQAMVKKVGA
DDFLAKFRPDDLAARVVARIKAAE
>tr|A0A0Q0DAM4|A0A0Q0DAM4_PSEAP DUF3828 domain-containing protein OS=Pseudomonas syringae pv. aptata OX=83167 GN=ALO85_03348 PE=4 SV=1
MSRTVAIAFACLLSTTAHAACPAATPVDTARWIYEKHQDFYLNGKGSADYLSKPLLSLLK
KDWACQNGDQCAVSANPWTDAQDGDVQKPIDWKLVSTSDKQAVVEMTYNLGYKDAPQQPV
ASQTTRLLLAKNANSCWVLENLQGPQGVALMQSLEEFPYEGD
>tr|A0A0Q0DGP3|A0A0Q0DGP3_PSEAP Flagellar secretion chaperone FliS OS=Pseudomonas syringae pv. aptata OX=83167 GN=ALO85_02159 PE=3 SV=1
MNPMLALRQYQKIGSQAQTSEASPHRLVQMLMEGGLDRIAQAKGAMARRDIASKGVLISK
AIDIVGGLREGLDLENSPSDALVRQDSLYHYMMTRLAEANARNDQKMLDEVAGLLITVKE
GWDAIGTLQ
>tr|A0A0Q0D2G1|A0A0Q0D2G1_PSEAP Cytochrome c-type bioproteinsis protein CcmF OS=Pseudomonas syringae pv. aptata OX=83167 GN=ALO85_03034 PE=4 SV=1
MIPELGQLAMILAFCFALVQAIIPLIGAWRGDRLWMSLARPAAWGQFSFLIFAFGCLTYA
FMIDDFSVAYVAQNSNTALPWYYKFSAVWGAHEGSLLLWALILGGWTFAVSVFSRQLPQV
MLARVLAVMGMISLGFLSFLILTSNPFARLLPQMPANGRDLNPLLQDIGLIVHPPMLYMG
YVGFSVAFAFAIAALLGGRLDAAWARWSRPWTLVAWAFLGIGISLGSWWAYYELGWGGWW
FWDPVENASFMPWLVGTALIHSLAVTEKRGVFKSWTVLLAIAAFSLSLLGTFLVRSGVLT
SVHAFASDPARGVFILMFLLVVVGGSLTLFAIRAPVVKSQVGFGLWSRETLLLGNNLLLV
VAASMILLGTLYPLVIDAMSGAKMSVGPPYFNTLFVPLMGLLLVVMAVGVLVRWKDTPLK
WLLGMLAPVLIGSVVLAVLAGLLLGDFQWAVLATLMLAAWVLLAGVRDLLDKTRHKGLLS
GARGLTRSYWGMQLAHLGIVVCALGVVLSSQNSAERDLRMAPGESTELGGYTFVFEGARH
YEGPNFTSDRGTVRVLRDGVQLTELHPEKRLYTVQQSVMTEAGIDAGFSRDLYVALGEPL
GDGAWAVRVHVKPFVRWIWLGGLLTGLGGVLAALDRRYRTRVKNKVREALGLSGAAQ
>tr|A0A0Q0C559|A0A0Q0C559_PSEAP Response regulator/sensory box/diguanylate cye domain/EAL domain protein OS=Pseudomonas syringae pv. aptata OX=83167 GN=ALO85_02905 PE=4 SV=1
MERVPNQPDDGSSVLLVVDDYPENLVTMCAVLQRTDWHIVTASSGIEALTVLLEQEVDLV
LLDVQMPGMDGFEVARLMRGSQRTRLTPIIFLTANEQTQDAVQKGYANGATDYLFKPFDP
NVLKPKVQTLLEQQRNRRALQQLSLELESARAFNASVLANVAEGILVVDEAGTISFANPA
ICQLLNTSVDHLRGTQVLDYIIEPKVGHWLDSGFYQHYRKSDTYRVHDAILRTAQGTQLP
VALSCAALPAEQKAMVLTVLDMSVVRDLYQQLEKQAVTDALTGLLNRRGLYQAVESMLLR
NERADKYLVVLFMDLDGFKQINDSLGHEAGDQVLLWVAEQFKATMRPYDVLARIGGDEFT
VVIDGLDYPEQAAKIAEKLIERVSGRRQVDGVEITLGASVGIATFPDCGSNLDGLLRAAD
IAMYEAKRAGRQQYRFYDQNMNGRARSRLMLEESVRTAIDGRDFSVVYQPQIHVADGRLR
GFEALLRWQHPAVGEVPPALFIPLLEETRLINRLGSWIFDQGAEQRQAWSHIFAPDMVLS
VCVSPTQFCLPNLASELKRAMDRFELKPGQIEVEITETALVNNLTHSHKQLKLLHEVGVR
IALDDFGTGECSLSHLRNLQLDTLKLDRRFVANIVGSKREAAMASSIIDLSRNLDMLVIA
EGVETAEQYQWLADNGCQVIQGFLIAHPMVPEDALRFPGHFDVAGLRQSGLL

>tr|A0A0Q0C7S0|A0A0Q0C7S0_PSEAP Ribosomal RNA large subunit methyltransferase H OS=Pseudomonas syringae pv. aptata OX=83167 GN=rlmH PE=3 SV=1
MRLRLIAVGSRMPKWVEEGWHEYAKRMPSELALELVEIPLNTRGKNADVARFIRQEGEAM
LAKVQPGERIVTLEVQGKPWSTEQLAVELDRWRLDARTVNLMVGGPEGLAPEVCARSEQR
WSLSPLTLPHPLVRILIGEQMYRAWTVLSGHPYHK
>tr|A0A0Q0DEN0|A0A0Q0DEN0_PSEAP LysR family transcriptional regulator OS=Pseudomonas syringae pv. aptata OX=83167 GN=ALO85_03072 PE=3 SV=1
MMLRFDDLQLFVRAADLGSLSAAARVMDMSAAVASAALKRIERQLGTRLLARSTRSLRLT
AEGEGFLEYARAALGSLEEGRRLLASSQDRCEGVLQLSAPSDLGRNVLLPWLDEFQQQHP
QLTVRLLLGDRIADLFRQPVDVALRYGEPEDSSLVALPVLPDNRRVLCAAPSYLQRHGEP
HQVEQLAQHNCLLFMLGDRVHDRWTLNDGKREISLTVSGNRFSDDADVVRRWAVAGEGVA
YKSWLDVAADVRAGHLKVLMPDLIGERAPLNLLCAHRAQLSKPVILLLEMLKSRCKQHAV
SLSSASVF
>tr|A0A0Q0C1V4|A0A0Q0C1V4_PSEAP Histidine kinase, HAMP region: chemotaxis sensory transducer OS=Pseudomonas syringae pv. aptata OX=83167 GN=ALO85_00806 PE=4 SV=1
MQTLKALYESVEKQFFNTLTKKLSSLFLLVLVSALLYWVALNIRSDIMLQLRGTQTDPAA
LGVIQSQLDLLSNAILLSTLFTLVMVSFMVWYFRHLIVRPVMSMTRALEEVASGEGDLSK
DLPLLTHDEIRGLASTCNLFLAKQREIISSIQGLTVQIAVESARSLKNISDSSDSATDQA
RFAREVMDQSNMAVGSIEDVSQQTQGISSTTAQNLSMARDSYAELLEVTGNISQISSSLN
EFGGLVSGLNERSSSIKSIVGLIQQISSQTNLLALNAAIEAARAGESGRGFAVVADEVRT
LAQNVSKATDDISRNIDAMLQEVSSTHEQTIQISHSARETQLVVERASGHFESMIGDFES
TNDKLADIAAHIQQFATTNTGINERVTRIYSDSQAIDQRMQHSATATRDLSSVAEQVQAL
LGRFVLGHGELDAAITRASQCRDILQARLVELHKQGVNLFDQSYKLIPGTDPKQYTTGYT
ERFAQVCQEECDKLTKGTRGGKVTFIVDSKGYCPVNNSWVSQKPTGNREVDLPVCRNKRM
FSDPIGLRAAGNKQRFLLQTYLRDTGEIMTEIDVPFFFEGRHWGNLRMGFDAALLLGK
>tr|A0A0Q0C9X7|A0A0Q0C9X7_PSEAP ATP synthase epsilon chain OS=Pseudomonas syringae pv. aptata OX=83167 GN=atpC PE=3 SV=1
MAMTVHCDIVSAEGEIFSGLVEMVIAHGNLGDIGIAPGHAPLITDLKPGPIRLIKQGGEA
EVFYISGGFLEVQPNMVKVLADTVQRAADLDEASAQAAVLAAEKALNEKGADFDYGSATA
RLAEAAAQLRTVQQIRKKFGG
>tr|A0A0Q0FBL8|A0A0Q0FBL8_PSEAP Uncharacterized protein OS=Pseudomonas syringae pv. aptata OX=83167 GN=ALO85_04126 PE=4 SV=1
MKKEIEPIKAALKQLIDKKNEDSLYYPAAFISAMKIVAGEAAVKNLQEPPIHHEYYIAKK
EGDTPTRKNMYFCANSGLDVFAQSQLNLKVLAVEIRDSAPDLYRALERFNLKAHQANQNL
KVRGGYDDLTLATAENELYSAIRHQYPALRIESSDAKESPPKTKLKSLTPQ
>tr|A0A0Q0IAL3|A0A0Q0IAL3_PSEAP Fimbrial domain-containing protein OS=Pseudomonas syringae pv. aptata OX=83167 GN=ALO85_04269 PE=4 SV=1
MKHLSARGWLRSLAVLLLLQPASTFAVVCTTQGTGETEIHDDLSSTVAIPESMPDGEVVW
RSEPLTVQIECAREGWQSEAEEVFIYLNPDNRSIGQGIRAGMTLQGIDHLHGSGRVGTGS
YVPVCREGESTLENCPKVRFSLAFSVFIQKFGATPSSGVASDLLDYRFFQLGGATDPSLL
SGRSLSYVINNLRGLRFVACDADLQVLPETVEFGDVAIHQVAIGKVTATQTFSLLTSRSC
DSPFSIDARFRPVTGNLSGELLIPVGNDSLGIRITSAVNGQLLRYNEPFHLAELLGETNT
ARADFNAELLWNTNIPRPGPFNAEVMVDLFYK
>tr|A0A0Q0FNT8|A0A0Q0FNT8_PSEAP 5'-nucleotidase SurE OS=Pseudomonas syringae pv. aptata OX=83167 GN=surE PE=3 SV=1
MRILISNDDGVNAPGLVALHAALANYADCVVIAPDQDKSGASSSLTLDRPLHPHTLENGF
ISVNGTPTDCVHLGIHGLLERQPDMVVSGINLGANLGDDVLYSGTVAAALEGRFLQRPSF
AFSFLSRQPDNLATAAHYARLLVEAHEQLDLPPRTVLNVNIPNLPLEHIRGIQLTRLGHR
ARAAAPIRVVDPRGRAGYWIAAAGDVEDGGAGTDFHAVVQGYVSITPLQLDRTYQDGFSS
LNTWLEGLR
>tr|A0A0Q0DDJ0|A0A0Q0DDJ0_PSEAP Cell division ATP-binding protein FtsE OS=Pseudomonas syringae pv. aptata OX=83167 GN=ftsE PE=3 SV=1
MIRFEQVGKRYPNGHVGLHELSFRVRRGEFLFVTGHSGAGKSTLLRLLLAMERPTTGKLM
LAGQDLGQISNAQIPFLRRQIGVVFQNHQLLFDRTVFNNIALPLQILGLSKPEIAKRVDS
ALERVALSDKAELYPGDLSTGQQQRVGIARAIVHRPALLLADEPTGNLDPRLAAEIMGVF
EDINRLGTSVLIASHDLALIARMRHRMLTLQRGRLIGDGEAGV
>tr|A0A0Q0DJR6|A0A0Q0DJR6_PSEAP Uncharacterized protein OS=Pseudomonas syringae pv. aptata OX=83167 GN=ALO85_100369 PE=4 SV=1

MRQFVNHGYSRIAFVMDRETPGRPAGRIRAGHFNMRDIANQRRN
>tr|A0A0Q0C6V3|A0A0Q0C6V3_PSEAP NADH:flavin oxidoreductase/NADH oxidase OS=Pseudomonas syringae pv. aptata OX=83167 GN=ALO85_00772 PE=4 SV=1
MSALFEPFKLKDVTLRNRIAIPPMCQYSATEGVINDWHQVHLASMARGGAGLLVVEATAV
APEGRITPGCTGIWNDEQAQAFVPVVKAIKAAGCVPGIQIAHAGRKASANRPWEGDDHIA
ASDSRGWDTIAPSAIAFGANLPKVPRAMTLEDIARVRQNFVDAARRARDAGFEWIELHFA
HGYLGQSFFSEHSNQRTDEYGGSFDNRSRFLLETLAAVREVWPENLPLTARFGVIEYDGR
DEQTLAESIELARRFKAGGLDLLSVSVGFSTPDANIPWGPAFMGPVAERVRREADIPVTS
AWGFGEPKLAEEAVKSGQLDLVSIGRAHLADPHWAYFAAKELGVEKSAWTLPAPYAHWLE
RYR
>tr|A0A0Q0FNC5|A0A0Q0FNC5_PSEAP ABC-type transporter, ATP-binding protein OS=Pseudomonas syringae pv. aptata OX=83167 GN=ALO85_02669 PE=4 SV=1
MEKLMTLLKFSDVSLAFGAMPLLDKVSWQIARGERVCIIGRNGTGKSSMLKLVKGVQKPD
DGAVWRAPGLKIGELPQELPVADGRTVFDVVAEGLDGVGALLAEYHHLAQNCVTEEDLNK
LMHVQQDLEARDGWRLQQLVDSTLSRLQLPADKTLAELSGGWRRRVLLAQALVSEPDLLL
LDEPTNHLDIGAIAWLEEALKDFQGAVLFITHDRAFLQNLATRILELDRGGLIDWDGDYA
SFLVHKEAALAAEETANALFDKRLAQEEVWIRQGIKARRTRNEGRVRALKELRVERSERR
ERTGKANIQLDTAEKSGKQVMILENVSFAHPGGPTLIKDFSMVLQREDRIGLLGANGTGK
TTLLKLMLDNLQPTAGKVEVGTRLDVAYFDQLRHQLDLEKTVIDNVAEGRDFIDIDGQSR
HVLSYLGDFLFSPQRARTPVKALSGGERARLLLAKLFSKPANLLVLDEPTNDLDVETLEL
LEEVLLTFKGTVLMVSHDRAFLDNVVTSTLVFEGEGKVREYVGGYQDWLRQGGSPRLLGV
ADTKSGKAELASAVVQAAPVAAQDMPVAKKKLSYKLQRELEALPAQIDEVEGKMAALNAE
MAEPGFYQRSAEQTAAVLAQLENLQAELDKLLERWAELDE
>tr|A0A0Q0C2Y5|A0A0Q0C2Y5_PSEAP 30S ribosomal protein S20 OS=Pseudomonas syringae pv. aptata OX=83167 GN=rpsT PE=3 SV=1
MANTPSAKKRAKQAEKRRSHNASLRSMVRTYIKNVVKAIDAKDAEKAQAAYVLAVPVIDR
MADKGIIHKNKAARHKGRLNGHIKALSLAAAA
>tr|A0A0Q0CHQ1|A0A0Q0CHQ1_PSEAP Divalent metal cation transporter MntH OS=Pseudomonas syringae pv. aptata OX=83167 GN=mntH PE=3 SV=1
MTYKLPTTATAPFCPSATTDSVAIPPNASLTRKMMLFVGPGLLVSIGYMDPGNWATAIEA
GSRFGYSLLFVVVLASLSGMVLQNLCSRLGIATGRDLAQLSAARYSRKVAKGQWLLAELS
IIATDLAEVLGAALAFHLLLGVSITTGVALTAFDTLIVLALQGANFRRLEAIVLGLIATI
ASCFAVELFLIKPFWPDVVAGLKPSWDVLSSQEPLYIAIGILGATVMPHNLYLHSSVVQT
RVNGSDQASKASAIRYARLDTIGSLSLALLINAAILILAAAAFHKTGHTEVVEIQDAYHL
LDPLVGGAIASVLFGIALLAAGQSSTFTGTIAGQVVLEGFLQAKIPCWQRRLITRALALI
PALIGVVWLGDSSVGQMLVLSQVVLSLQLPFALWPLIRFTSDPQLMGPFVNSRLVKTAAW
GLFGLISAANLTLLWFWIS
>tr|A0A0Q0CFN2|A0A0Q0CFN2_PSEAP Putative membrane protein OS=Pseudomonas syringae pv. aptata OX=83167 GN=ALO85_03638 PE=4 SV=1
MTANRESSRPASRGRPPVRQLRLKVLVLLLLCALPVYGTVSLWLRQVTPIPALVLIVMSL
LAFVLYRHDKRQAGNAGQRTPENVLHGVELLGGWPGALLAQQVFRHKTRKVSFQVVFWLI
VLVHQALWIDWLFLGKRLVQLLPL
>tr|A0A0Q0ILM9|A0A0Q0ILM9_PSEAP 3-hydroxyacyl-[acyl-carrier-protein] dehydratase FabZ OS=Pseudomonas syringae pv. aptata OX=83167 GN=fabZ PE=3 SV=1
MMDIKEIREYLPHRYPFLLVDRVTELDIENKNIRAYKNVSVNEPFFNGHFPEHPIMPGVL
IIEAMAQAAGILAFKMLDSKPSDGTLYYFVGSDKLRFRQPVLPGDQLVLEAKFLSSKRQI
WKFECKATVDGKAVCSAEIICAERKL
>tr|A0A0Q0C1E6|A0A0Q0C1E6_PSEAP Uncharacterized protein OS=Pseudomonas syringae pv. aptata OX=83167 GN=ALO85_04997 PE=4 SV=1
MPLADHPSCRRIGTMTLPRTASTQALNASLASDTYPPDFSKTTQELARTLLHPLKPELDV
ETTWLTYRDSEVEFDHGSNIHRVPLIPGLIEYFTGHLQWIDNDVVALHPPLDTPSPTPDT
QQPDVKPPAIQPLGRAATVTFFTEVSEQLEQCYTQNLADYWSTATLDGETRSSRFVAEQI
ECLTSECAVLIALGKMTTGHYSLLSAALGPCATNATEGFADLQPHSVYSLSARDGDGPSL
SLYGAFVIARRLRDVPVQPDLEELEDVLLFTPHDGLEAFASLQQLNDALVQRVTEPEYRG
RLGDGVTAGQASPTNPTPVLQWQYTLMDGNFLSLLLARQVVAQQSVFAQTVQLARSQKMD
AVCFEQMIANLLKPRIYFDNHWRLDGFDSDLVQNQMPDWWKTMTLTQRQAWHRQAQRFGE
QVVNMRKASKEHFGQAENDSRTYPTRFIDQQLAQLLNRLSIAATAQQINISLTYRTGIEV
LDIPGAPVLPETKTESVSLRQLAHTQALHTKAKDAVLLRAVDAEGVSLAHLDKQAVTELI
ATLEDPRHLSDYLDLHLKTSAYAQQLKRSEKAMLRAQMKMALLEIEQQAFAHPGREWIKA

VLDSPGIQGRRTINEQVIEVRFFSVNQFKMTNVMLIAPAGTFEKGPLVLCTLDAADGVVF
RWFNSMYHLTTSFLEEAPFQQYLIQQIPVSRRIETLHAMQYEKEAKHWRLPEVFTQLTLL
PIPSRLLRPVVFVSQSKDIYEENHETKINHLINEAKRQMSLTTGTGQSGRGFDLIASIAI
LFLPGPIMMPVSLGAGLYKTWSAFSKIDENDLEGAAEEFLSALSYLAIAFVGHLALALKP
AGIAAKTVRRPHLVRRVGRDGQAQIGYLLSHSKAPRFADSKLIAAMDPKRFVAIEVEGQT
CFISRRANVFGHSRLYRVNPMDTTQLVHGQEFALRSTTGVWKIVGTQIPRMSQSAIRNAQ
AQLTSLTTLWPASLEEASSAERLSFETDYLALAKTSNAENFSQITAYVEGGSMDINPLLR
SGVRNATTRKFLSQFHKLKAWEGTAFRATYVSSDGVACLEREVGAVFTDNGVQSASMSRA
NASRWSQDGFVSRNASAENHPVFFIFAPAVPKKNMFTGFLGDHVAIAPGTYVQLGATKRI
NGQLFAWFDAPEQLVDQTYDLYTGEQELWV
>tr|A0A0Q0CEH2|A0A0Q0CEH2_PSEAP Outer membrane autotransporter barrel OS=Pseudomonas syringae pv. aptata OX=83167 GN=ALO85_04697 PE=4 SV=1
MPLDCLPAASLDGSAVMKIRCVHFYQTCNVLPVVILAIIPSWAEGACSLSSLPSAGTQVC
DGGGSGPFSALSGDHVLIFPQGGTGTVSGNVLFGPGMDSVDMQSGRILGNVIQGAGIDRF
TLSAGEVSGDLNQGDDPDDFVMSGGTLGALAQGDGRDTFLMTGGTLTRAFEDGDVAVMTG
GSIGRVDMKLDNNVFEMSGGTIINNLVTGFGRDTITLSGGIVGGNVSVSGGDDLVTLSGG
EIRGEVRTSVGNDNFNWLNGGYARSNVLMADGNDTARLYNLSEYYLSANPLLDGGPGNDL
LTFDNTVSSQPGRYANWETVNVSNGSQLNLAGTLVLGDSVSDTGVLNIDSASALTSTSGS
IAPFSAGSLVSLENAGTLDVSDAGRSLTGTLTVTGNYNGQNGRLLLLSALGDDASASDRL
VVAQGRISGSTRMIVSNSGGLGALTRGNGIEVVQAINGATSESSAFSLQNPLSAGAYQYY
LFKGGATAGSENSWFLRSAVIAPPTPAPAPAEPTPEPAQPTPLPTPVPVPVSVPEPTPTP
TPQPEPEPAPEPAPQPVPDPPPNPAPEPEPEPNSPPPVTPAVTLPVPATGTPPLPEPVRG
AAPIALYRPEVANYSVVAPTAAVLALSSLGTFHERQGDQALLTQNGTTPAGWARVFGSDF
RQRWSGDVSPGLDASFKGYQIGHDLYVWQQDSGQTQRVGLFVAHNRLDGKVKGFAEGFSD
YRTGHLKLQGESLGAYWTLTTPNGGYVDAVAMGTRLDGRSRSVRGIGVDTEGHVMSLSVE
TGYPLPLSQRWVAEPQLQIIYQRVDLQDQDDGIAHLGFDSQAYITGRIGVRFKGYYQVSS
VPIEPYLRVNLWHTKDGHDTLTFDHAEQIRTAHRSTTGSIGAGMVATLSSNASLHWSADY
LGDLGEHGAEGVNTSLGFRLVW
>tr|A0A0Q0DIY3|A0A0Q0DIY3_PSEAP Uncharacterized protein OS=Pseudomonas syringae pv. aptata OX=83167 GN=ALO85_04132 PE=4 SV=1
MGSTKEWMMEQENEAMIQWMRDSYNIPDDVDIDEDYPGYSGMVAVYEDELQREEYESSMQ
WYDDHPYHEIYNAFCGRIRQLEGMVTQDHNPFTDKMILQMVYAHSVTLFEAMVGDIIKAC
VRKFPHMMEKLVLGIAEVTKEKYTIKDIVKYGGVENIALSILNDSTFHNITVVNQYVNML
CDTRLNNRYESQMHKISMMRHDFVHRNGANKAGEYHQLTSLMVFEALEMISLYSVDVFQA
IKGSAEAPAAVDF
>tr|A0A0Q0DIH2|A0A0Q0DIH2_PSEAP Uncharacterized protein OS=Pseudomonas syringae pv. aptata OX=83167 GN=ALO85_02241 PE=4 SV=1
MDYELHEGSIKLPEGFQDRTVNMFVMGSTLPAPLSITVSRDTLLPSEHLKTYVDRQVKML
SSKLRGYTLLNRKAVSLSTVAPLEGIQIDAYYMTEGRPYYQRQAAFLIEPERAIVFSTTS
QTDFSAEQTQDWENLLASFQPRVADTSPANGSEQE
>tr|A0A0Q0IR74|A0A0Q0IR74_PSEAP Uncharacterized protein OS=Pseudomonas syringae pv. aptata OX=83167 GN=ALO85_05019 PE=4 SV=1
MNINGVSNSVNIPQQMGLVNGAKHVDPTEDVSLQEAIASEVKTTEKNVFYEYQKEPWLLE
PINEHITNRLDYVAHKKRLDVLELALVNVTYDSFRKELQETHPDIARKNFGFTLTESASI
KIIDYDNSLTENDTSVLTHAINGFREFKEQLQHHAKTIMTLVDHDHETFGGRYKLDISNF
QDVIDYGKIMNTSVQKMQDEWIQQVQSNAEKRDFSYISLAV
>tr|A0A0Q0BVZ1|A0A0Q0BVZ1_PSEAP Cell division protein ZipA OS=Pseudomonas syringae pv. aptata OX=83167 GN=zipA PE=3 SV=1
MEIGLREWLIVIGIIVIAGILFDGWRRMRGSKGKLKFRLDRSFSNLPDEEETTSAEVLGP
PRVLDTHKEPQLDEHDLPSMSASPREGKRGNSDKRSDKKRKDEPQQGDLNLDLDGPSLFT
GRDDDFPDDKPAQRITEDKDLLPVEEVLVISVISRSEGGFKGPALLQNILESGLRFGEMD
IFHRHESMAGNGEVLFSMANAVKPGVFDLDDIDHFSTRAVSFFLGLPGPRHPKQAFDVMV
AAARKLAHELDGELKDDQRSVMTAQTIEHYRQRIVEFERRALTQRRG
>tr|A0A0Q0CWG0|A0A0Q0CWG0_PSEAP Uncharacterized protein OS=Pseudomonas syringae pv. aptata OX=83167 GN=ALO85_00552 PE=4 SV=1
MATNRSRRLRKKLCVDEFQELGFELNLDFKQELDDKAIDAFLDAFLKEAMEANGLGYVGG
DDFGLVCLSKRGSVSEEQRAAVEAWLKGRSELTEVTVSPLIDVWYPEKEINPAA
>tr|A0A0Q0C8U0|A0A0Q0C8U0_PSEAP Uncharacterized protein OS=Pseudomonas syringae pv. aptata OX=83167 GN=ALO85_101525 PE=4 SV=1
MVNVVLQTAPAPDLGARGALHVFIAATRNQTHRNATLIIDLHDRQACSVCDTGAMGNDNN

EPSTTTHSEKRLKLAIRGRGTPLCPEENALPHTIVV
>tr|A0A0Q0C5I6|A0A0Q0C5I6_PSEAP Uncharacterized protein OS=Pseudomonas syringae pv. aptata OX=83167 GN=ALO85_100524 PE=4 SV=1
MIIKQSHTWGQLSGKSTRPCQFVRDICTVLSFGDSRKFLS
>tr|A0A0Q0DDM9|A0A0Q0DDM9_PSEAP Putative prophage repressor protein OS=Pseudomonas syringae pv. aptata OX=83167 GN=ALO85_03568 PE=4 SV=1
MTRQERIARAIAASGLKKGEIAAQCGVANSAVTQWISGESKSLRPENLYALARATGFRAE
WLAIGEGPEQAPAFDANVALIDQPKMSFRYPVISWVSAGSWEEAVQPYPDGFSDRYEISD
YDSKGPAFWLEVKGDSMTAPTGVSVPEGMMILVDTEADVQPGKLVIAKLPASNEATFKKL
VEDGGTRYLKPLNPAYKMIECGADCRIIGVAVRMTGKL
>tr|A0A0N8TA85|A0A0N8TA85_PSEAP Uncharacterized protein OS=Pseudomonas syringae pv. aptata OX=83167 GN=ALO85_100385 PE=4 SV=1
MLYTRKPWAQQQAKHFDPNDHASRSGMLKHCGGTGKSTARRSMTIVLRQYMV
>tr|A0A0Q0IM14|A0A0Q0IM14_PSEAP XaxB-like binary cytotoxin component OS=Pseudomonas syringae pv. aptata OX=83167 GN=ALO85_02418 PE=4 SV=1
MSTERLDELLILTPPDNEVMETARQNILAQVTALKLDFLPVMKEKMLPLQAALMSADKVY
GQELATVTVQLNNIDLKPIDQKQQLIEADARLSDTQKQQAISLLNGQRIRQVSNLTDVVR
RSAQAIAEHSDDVAQINLTLESNRLLETLQQQIDSMTQRSATQESAMALIAADRRLLDTT
IKTFEKYNIADQFKEMLPTPEELKLVTMTSPELALINAGIARLGKLLDKVSSALNYLDLV
EERDRLRSRYNALLDDSRTALREAQATREKLDELTALAGVAQSKTLWVQEAKKVYQSLYH
FLDQITSPADTSTSISQQVEHLQTYIKSFYNVKRIV
>tr|A0A0Q0C581|A0A0Q0C581_PSEAP Uncharacterized protein OS=Pseudomonas syringae pv. aptata OX=83167 GN=ALO85_04219 PE=4 SV=1
MMLATRLHLLCGKIASGKSTLAKSIATEHSAILLSEDQWLSRLYPDEITSVADYVRLAHR
IRDIIGPLVIDMLNSGMCVVLDFPANTLADRRWLRSLADDAQVKHRLHYLDVEDDICLAR
LHARNETAEHDFAATDAQFRLITRYFQIPHTDEGLDILVHGL
>tr|A0A0Q0DKM7|A0A0Q0DKM7_PSEAP Antibiotic biosynthesis monooxygenase family protein OS=Pseudomonas syringae pv. aptata OX=83167 GN=ALO85_00853 PE=4 SV=1
MNTPAKPLALMAVLKASAGKESALKTALSALVEPTRAEPGNIEYTLFEARDERGTFYMRE
CFKDQAALDAHVATPHYQQFAAVSGELLAEPPQLIFLDQVSS
>tr|A0A0Q0D6E1|A0A0Q0D6E1_PSEAP NADH:flavin oxidoreductase/NADH oxidase OS=Pseudomonas syringae pv. aptata OX=83167 GN=ALO85_05106 PE=4 SV=1
MTRIRHMTTIFDPITLGDLQLPNRIIMAPLTRCRADEGRVPNAMMAEYYVQRASAGLILT
EATSVTPMGVGYPDTPGIWSNDQVRGWSNITKAVHNAGGRIALQLWHVGRISHPSYLNGE
TPVAPSAIAAEGHVSLMRPITPLPTPRALELAEIGDIVEAYRIGAENAKAAGFDGVEVHG
ANGYLLEQFLLTGSNQRTDEYGGSVENRARLLLEVTDAVIDVWGAGRVGVHLSPRFDMHD
MSDANRTETFSYVAKELGKRGIAFICAREHDAEDSLGPQLKKDFGGVYIANEKFTKDTAN
AWLAEGKADAIAFGVPYIANPDLPERLASDAPLNEAHPETFYAKGPVGYIDYPRM
>tr|A0A0Q0CDQ3|A0A0Q0CDQ3_PSEAP Putative metal-dependent hydrolase OS=Pseudomonas syringae pv. aptata OX=83167 GN=ALO85_02684 PE=4 SV=1
MTSANPPVGPATPVSASVSLNVLTMNMHMGFGIFNRRFILPELREAVRTVSADIVFLQEV
HGEQQSHARKVKDWPTISQYEFLADSMWSDFAYGRNAVYPDGDHGNALLSKYPIIQHENL
DISIHGTEQRGLLHCILEVPHAGRVHAVCVHLGLRENHRRQQLKLLNELMARIPEGEPVI
VAGDFNDWRRRADASLQGSGLHEVFVERFGAPAKSFPARWPLLCLDRIYVRNATSHRPKV
LSSRPWSHLSDHAPLAVELTL
>tr|A0A0Q0CFF8|A0A0Q0CFF8_PSEAP Formimidoylglutamase OS=Pseudomonas syringae pv. aptata OX=83167 GN=ALO85_02101 PE=4 SV=1
MDNVLTFKRGRIPLLISMPHAGLKLTPVVEAALVDEALSLPDTDWHIPRLYDFAAELGAS
TLAAEYSRFVIDLNRPSDDKPMYAGATTGLFPSILFDGVPLFKDGQTPSVEERARYLQQI
WTPYHQTLEQELQRMRDEFGYALLFDAHSIRGHIPHLFDGRLPDFNLGTFNGASCDPELA
SRMEQVCAAAKDYSHVLNGRFKGGHITRHYGNPANNIHAVQLELAQSTYMDEFVPFHYRP
DLAEPTRAVLKPLLETFIAWGQERFG
>tr|A0A0Q0BV08|A0A0Q0BV08_PSEAP UPF0270 protein ALO85_03225 OS=Pseudomonas syringae pv. aptata OX=83167 GN=ALO85_03225 PE=3 SV=1
MLIPYDQLEPDTLTRLIEDFVTREGTDNGDETPLQTRVLRVRHALTKGQAVIFFDLESQQ
CQLMLKHDVPKEFFE
>tr|A0A0N8T9D2|A0A0N8T9D2_PSEAP Probable transcriptional regulatory protein ALO85_00244 OS=Pseudomonas syringae pv. aptata OX=83167 GN=ALO85_00244 PE=3 SV=1

MAGHSKWANIKHRKERQDAKKGKIFTKWIRELTVAARQGGGDPGSNPRLRLALDKALGAN
MTRDTIDRAVARGVGASDGDDVEELGYEGYGPGGVAIMVETMTDNRNRTAAAVRHAFTKC
GGNLGTDGSVAYLFDRKGQISFAAGVDEDSLIEAAMEADADDVVTNEDGSIDVFTSFSGF
YAVRNALEAAGFKAADAEIVMLPTTSAVLDLETAEKVLKLIDMLEDLDDVQNVYSNAEIP
DEVMEQLG
>tr|A0A0Q0DG79|A0A0Q0DG79_PSEAP Nucleoside-diphosphate-sugar epimerase
OS=Pseudomonas syringae pv. aptata OX=83167 GN=ALO85_01120 PE=4 SV=1
MASAHTTQTPFNRLLLTGAAGGLGKVLRETLRPYTNVLRLSDIAEMAPAVDDREEVQVCD
LADKDAVHQLVEGVDAIVHFGGVSVERPFEEILGANICGVFHIYEAARRHGVKRVIFASS
NHVIGFYKQTETIDAHSPRRPDSYYGLSKSYGEDMASFYFDRYGIETVSIRIGSSFAEPQ
NRRMMSTWLSFADLTQLIERSLYTPDVGHTVVYGVSDNKTVWWDNRLASKLDYTPKDSSE
VFREKVEAQPLPAADDPAMLYQGGAFVASGPFGDQ
>tr|A0A0Q0BSN4|A0A0Q0BSN4_PSEAP Phospho-N-acetylmuramoyl-pentapeptide-
transferase OS=Pseudomonas syringae pv. aptata OX=83167 GN=mraY PE=3 SV=1
MLLLLAEFLQQFYKGFAVFQYLSLRGILGVLTALTLSLCLGPWMIRTLQMRQIGQSVRND
GPQSHLSKSGTPTMGGALILSSIGISTLLWADLSNRYVWVVLLVTFLFGAIGWVDDYRKV
IEKNSRGLPSRWKYFWQSVFGLCAAIFLYTTAPSATETTLIVPMLKDVRIPLGIGFIVLT
YFVIVGSSNAVNLTDGLDGLAIMPTVMVGGALGIFCYLSGNVKFAEYLLIPYVPGAGELI
VFSGALIGAGLGFLWFNTYPAQVFMGDVGALALGAALGTMAVIVRQEMVLFIMGGVFVME
TLSVVIQVASFKLTGRRVFRMAPIHHHFELKGWPEPRVIVRFWIITVILVLIGLATLKLR
>tr|A0A0Q0FJX0|A0A0Q0FJX0_PSEAP Regulatory protein, MarR OS=Pseudomonas
syringae pv. aptata OX=83167 GN=ALO85_03046 PE=4 SV=1
MSVESLHRTISSGLVAASRQWRRICQNTLVTYGISEACAGPLLAIARLGDGAHQVKVAQA
AGMESPTLVRLLDQLCQADIVCRTEDPNDRRAKALSLTRKGRALACSIEAELTRLRAEVL
HGLQPADLEATLRVLQAFADAAQREHGGQA
>tr|A0A0Q0DKW5|A0A0Q0DKW5_PSEAP Putative citrate-proton symporter
OS=Pseudomonas syringae pv. aptata OX=83167 GN=ALO85_01241 PE=4 SV=1
MASTTSKGKAIFRVVSGNFLEMFDFMVYGFYATAIAKTFFPSDSAFASLMLSLATFGAGF
LMRPLGAIFLGAYIDRHGRRKGLIITLAMMAMGTLLIACVPGYATLGVIAPLLVLLGRLL
QGFSAGVELGGVSVYLAEISTPGRKGFFVSWQSASQQAAVVFAGLLGVGLNHWLSPEQMG
EWGWRVPFLIGCLIVPAIFIIRRSLEESPEFEARTHRPTLREVVRSISQNFGLVLGGMAL
VVMTTVSFYLITAYTPTFGKNELNLTDLESLLVTVCVGVSNFIWLPIMGSFSDRIGRKPL
LIAATVLAIATAYPALSWLVAHPSFGNLLMVELWFSFLYGSYNGAMVVALTEIMPVEVRT
TGFSLAYSLATATFGGFTPAACTYLIHELGNKAAPGIWLTGAAVLGLVATLVLFRKGGQK
LEAPVAASAS
>tr|A0A0Q0C6U0|A0A0Q0C6U0_PSEAP Glutamate synthase, large subunit
OS=Pseudomonas syringae pv. aptata OX=83167 GN=ALO85_00839 PE=3 SV=1
MSDPISHKPAPVLRESATFDRLTIQEIQRAAETGIYDIRGGGTKRKLPHFDDLLLLGASV
SRYPLEGYREKCGTDVVLGNRFAKKPLYLKIPVTIAGMSFGALSANAKEALGRGATIAGT
STTTGDGGMTPEERGQSQHLVYQYLPSRYGMNPDDLRKADAIEIVLGQGAKPGGGGMLLG
MKVTERVAGMRTLPVGVDQRSACRHPDWTGPDDLAIKIAEIREITDWEKPIYVKIGASRP
YYDVKLAVKAGADVIVLDGMQGGTAATQEVFIEHVGIPILPAIPQAVQALQEMGMHRKVQ
LIVSGGIRNGADVAKAMALGADAVAIGTAALIALGDNHPRLDEELKKIGSAAGYYDDWQN
GRDPAGITTQDPELSKRLDPVEGGRRLANYLRVLVLEAQTMARACGKSHLHNLDPEDLVA
LTVESAAMARVPLAGTSWIPGSGSGY
>tr|A0A0Q0CFL4|A0A0Q0CFL4_PSEAP Uncharacterized protein OS=Pseudomonas
syringae pv. aptata OX=83167 GN=ALO85_01919 PE=4 SV=1
MTRSEVRQKLEMQWWRQLGLTLAPLLVVCLFFGASEPLIPVLAIPLFIAGIGSMFVSLKP
FGAYKRALAVTQAALDTPEEPAAWLKLAAIRRLAFLAAGLPAWIAAIAVLFGLHPLPVCL
LAFASAVLLYLYRIPAPSR
>tr|A0A0Q0DPH1|A0A0Q0DPH1_PSEAP LysR_substrate domain-containing protein
OS=Pseudomonas syringae pv. aptata OX=83167 GN=ALO85_03793 PE=4 SV=1
MLHRSEQNPSLDALIKGVADILILPGTVLDEHYLEHHCWSDHYLLVAARSKQGKAQCFAE
LAQVSRYVAWRHPGVERLHQQLASAQVRLSHRGELSCLDTLLDLVGKRHCISIVPSALLS
GRLRELESIPLPLTVRRQISVVARPASLLSNAANAVVQALKKPPASNTRPD
>tr|A0A0Q0CGK6|A0A0Q0CGK6_PSEAP Flagellar biosynthetic protein FliP
OS=Pseudomonas syringae pv. aptata OX=83167 GN=fliP PE=3 SV=1
MGALRFLILLLLVMVTPAALAADPLSIPAITLSNGADGQQEYSVSLQILLIMTALSFIPA
FVMLMTSFTRIIIVFSILRQALGLQQAPSNQILTGMALFLTMFIMAPVFDRVNQDALQPY
LAEKLSAQDAVAKAQVPIKDFMLAQTRTSDLELFMRLSKRTDIPTPDAAPLTILVPAFVI

SELKTAFQIGFMIFIPFLIIDLVVASVLMAMGMMMLSPLIISLPFKIMLFVLVDGWALIV
GTLAGSFGGV
>tr|A0A0Q0C2J7|A0A0Q0C2J7_PSEAP Bacterioferritin-associated ferredoxin
OS=Pseudomonas syringae pv. aptata OX=83167 GN=ALO85_100119 PE=4 SV=1
MYVCLCQGVTDGQIRDAILEGCCSYREVRETLGVASKCGKCACLAKEVVRDTLTELQTSH
ALLAYPAKFSAA
>tr|A0A0Q0C1F5|A0A0Q0C1F5_PSEAP GCN5-related N-acetyltransferase
OS=Pseudomonas syringae pv. aptata OX=83167 GN=ALO85_01938 PE=4 SV=1
MIMPDVIRDALPDDLPGILDIYNDAVLNTTAIWNEQPVDLANRQAWYAARQSQAYPILVA
VDNAGDVLGYSSFGDWRPFEGFRHTVEHSVYVRADQRGKRLGPRLMDALIERARDCDKHM
MVAAIESGNAASIALHNRLGFKTTGQMPQVGTKFGRWLDLTFMQLDLSPGAQPPVSQAPS
AL
>tr|A0A0Q0BXC4|A0A0Q0BXC4_PSEAP Peptidase A24A, prepilin type IV
OS=Pseudomonas syringae pv. aptata OX=83167 GN=ALO85_01350 PE=4 SV=1
MMKLFFLLIWFAICAEQDARCKQISNGLTLGAAALAIIYLALTGMTWLGSPALEALLAVT
LALALTLPGYALGKLGAADVKLMTALALASNSAYLLGTFIGAGVAMATWFIVAKRAWPPT
QQWVTQRYRYMNPTAPDKYPFSPFLFAGLLLSTALLH
>tr|A0A0Q0C1Q6|A0A0Q0C1Q6_PSEAP UPF0246 protein ALO85_01811
OS=Pseudomonas syringae pv. aptata OX=83167 GN=ALO85_01811 PE=3 SV=1
MLMVISPAKTLDFETPPTTGRFTQPQYLEHSQELIDQLRELTPAQISELMHLSDKLAGLN
AARFGSWNPEFTLDNAKQALLAFKGDVYTGLEAETLSEAQLDYAQGHLRMLSGLYGLLRP
LDLMQPYRLEMGTRLANARGKDLYAFWGTRISEWLNEALADQGDDLLLNLASTEYFSAVK
RSSLKARIIDTEFKDFKNGQYKIISFYA
>tr|A0A0Q0BUV0|A0A0Q0BUV0_PSEAP Transcriptional regulator PobR
OS=Pseudomonas syringae pv. aptata OX=83167 GN=ALO85_02123 PE=4 SV=1
MARPATAAIPVFKLYGETTAWPTPDLIHCESIPERSRIHEWEIKPHRHGDLVQLLYVQSG
KAMLKVEDRVSEVEMPALQVVPALSVHTFRFSRDIQGHILSLARPLVEQLEAALDVSALS
ASRCYVLGADSDYVDTLFNAIADEYRQPRAGRDLMLHSLVNVLAVWLSRRSSEHAGEETH
AMDRGREHLQVFMHQLEASFREHWSIDQHAQAMGISAAHLNALCRRLCNQSALQIISERL
LLEAKRNLIYTTLTINQVSDSLGFSEPAYFSRFFKRATGQSPKAFRQQK
>tr|A0A0Q0DIR6|A0A0Q0DIR6_PSEAP Mu-like prophage, tail tube protein
OS=Pseudomonas syringae pv. aptata OX=83167 GN=ALO85_00437 PE=4 SV=1
MGQKVAGTCYIKVDGTQLTISGGGEAPLMNIKRETVVPGYYKETEKAAWLKFTAVHTADL
PLKLLTTGVDMTITCEFKNGKTYVLSGAYLVDEPSSKADDGTIELQFDGNQGSWQ
>tr|A0A0Q0DTJ0|A0A0Q0DTJ0_PSEAP Uncharacterized protein OS=Pseudomonas
syringae pv. aptata OX=83167 GN=ALO85_02627 PE=4 SV=1
MIASGLSELELRSMVECALLPLRCTCTIVDEQMNVQISHPVSGRTQRQKKLPLSRIKTVR
DIAELVAELREVPVTARVAKAYYSAA
>tr|A0A0Q0C4K1|A0A0Q0C4K1_PSEAP Sec-independent protein translocase
protein TatA OS=Pseudomonas syringae pv. aptata OX=83167 GN=tatA PE=3
SV=1
MGIFDWKHWIVILIVVVLVFGTKKLKGLGSDVGESIKGFRKAMNDDDKPAEQPAPQPQQA
QPAPQGSPLNQPHTIDAQAHKVDEPIRKDQV
>tr|A0A0Q0FD90|A0A0Q0FD90_PSEAP GCN5-related N-acetyltransferase
OS=Pseudomonas syringae pv. aptata OX=83167 GN=ALO85_00103 PE=4 SV=1
MNIEVRPAGMADADAISRVVLAALRTSNAKDYPSSVIERVQLSFSPAAIEQLMQQRQVFV
ASEGHVVRGTASLEGEVVRSVFVDPDRHRQGIGRMLMAELEVVARKAGAARMVVPSSLTA
REFYLALGFSVVREHLEGEERTLIMERQLRV
>tr|A0A0Q0CV12|A0A0Q0CV12_PSEAP Putative polyhydroxyalkanoic acid system
protein OS=Pseudomonas syringae pv. aptata OX=83167 GN=ALO85_04066 PE=4
SV=1
MAKITVERPHALGLEKAREKAEQLVEKLSTRYGLAHEWAGDTVKLEGKGAKGEVEVEEAL
IRVNIELNFILSAMSGSIKTEVERVLDKALAA
>tr|A0A0Q0D9I7|A0A0Q0D9I7_PSEAP Voltage-gated chloride channel family
protein OS=Pseudomonas syringae pv. aptata OX=83167 GN=ALO85_200128 PE=4
SV=1
MDQARKKNLAQFDLVHARYPFDDPKMVGFTESVNYVNSLAESQPGFIWREKYEDKKLVQE
LWGEGFAYTLSLWQDTESLKSFLYRTPHAEFIRRGKEWFLPIKHPRVVLWWVPEGHIPTL
SEAHERLELLYEHGASDLAFDLRSTEPPAVAY

>tr|A0A0Q0II83|A0A0Q0II83_PSEAP Transcription antitermination protein RfaH OS=Pseudomonas syringae pv. aptata OX=83167 GN=rfaH PE=3 SV=1
MSHSDQDVRWYLIQTKPRQEARAEEHLRRQFECYRPLQADAKKTGAKAQAGEPLFPGYL
FIHMDQVQDNWYPIRSTRGVSRIVTFGGHPVPVRDHLIEQIRQRLATPAPKAQFTQGDAV
LITHASWGDVEAIFLSEDGAQRAVILLNMLQRQQKVVLPISSLSRIEASA
>tr|A0A0Q0CE72|A0A0Q0CE72_PSEAP Ferredoxin OS=Pseudomonas syringae pv. aptata OX=83167 GN=ALO85_00275 PE=4 SV=1
MTFVVTDNCIKCKYTDCVEVCPVDCFYEGPNFLVIHPDECIDCALCEPECPAVAIYSEDE
IPAGMENFIELNAELAEVWPNITEKKDAMPDAAEWDGKTGKIAELER
>tr|A0A0Q0ITC7|A0A0Q0ITC7_PSEAP Uncharacterized protein OS=Pseudomonas syringae pv. aptata OX=83167 GN=ALO85_101774 PE=4 SV=1
MNITFTAPSYICRHAQWDQPGFNSREEFFFTSLNQINNHVDP
>tr|A0A0Q0CXG9|A0A0Q0CXG9_PSEAP Ethanolamine permease family protein OS=Pseudomonas syringae pv. aptata OX=83167 GN=ALO85_04767 PE=4 SV=1
MHWHGLCNRMFQRLSNPREKTMSTQLKPTLGTIHLWGIAVGLVISGEYFGWSYGWGVAGT
LGFLVTALMVATMYTCFIFSFTELTTAIPHAGGPFAYSRRAFGEKGGLIAGMATLIEFVF
APPAIAMAIGAYLNVQYPGLDPKHAAVGAYIVFMALNIVGVKLAATFELVVCILAVAELL
VFMGVVAPAFSFSNFALNGWAGSQTFGPEAIAGMFAAIPFAIWFFLAIEGAAMAAEEAKD
PKRTIPKAYISGILTLVILAIGVMLFAGGVGDWRTLANINDPLPQAMKAVVGDSSGWLHM
LVWIGLFGLVASFHGIIMGYSRQFFALARAGYLPASLARLSRFQTPHRAIIAGGLIGIAA
IYSDGLINLSGMTLTAAMITMAVFGAIVMYIMSMLSLFKLRKTEPDLERTFRAPGYPVVP
GIALALAVVCLVAMAWFNALIGLIFLGLMAIGFIYFSLTAKSRADAPTDAMLTGK
>tr|A0A0Q0FL29|A0A0Q0FL29_PSEAP Molybdopterin biosynthesis protein MoeB OS=Pseudomonas syringae pv. aptata OX=83167 GN=ALO85_01709 PE=4 SV=1
MQAVLSDQELLRYSRQILLQHVDIDGQLRLKQSRALIVGVGGLGSPVALYLAAAGVGELH
LADFDHVDLTNLQRQIIHDTQSIGQAKVDSAIARLTAINPEITLVAHRTALDTDSLGAAV
QAVDLVLDCSDNFATREAVNAACVAAGKPLVSGAAIRLEGQLSVFDPRRADSPCYHCLYG
HGSEAELSCSEAGVIGPLVGLVGSLQALEALKLLAGFGEPMVGRLLLIDALSTRFRELKV
KRDPACSVCGSASVQGEHA
>tr|A0A0Q0BWH4|A0A0Q0BWH4_PSEAP Electron-transferring-flavoprotein dehydrogenase OS=Pseudomonas syringae pv. aptata OX=83167 GN=ALO85_02187 PE=4 SV=1
MEREYMEFDVVIVGAGPSGLSAACRLKQKAAEAGQEISVCVVEKGSEVGAHILSGAVFEP
RALNELFPNWQELGAPLNTPVKRDDIYVLRDAEKAQKIPDFFVPKTMHNHGNYIISLGNL
CRWLAQQAENLGVEIYPGFAAQEALFDENGVVRGIVTGDLGVDREGNPKEGLYTPGMELR
AKYTLFAEGCRGHIGKQLLKRFKLDDEADVQHYAIGLKEIWEVDPAKHEQGLVVHTAGWP
LDDDNPGGSFLYHLENNQVVVGLIIDLSYSNPYLSPFDEFQRYKHHPVVKQYLEGGKRIS
YGARAIAKGGLNSLPKMVFPGGALIGCDLGTLNFAKIKGSHTAMKSGMLAADSVADALLA
GKEGGDVLNAYVDAFKASWLHEELFASRNFGVAIHKYGAIKGGAFNFIDQNIFGGKLPFT
LHDTKPDYACLKLAADSKKIDYPKPDGKLSFDKLSSVFLSSTNHEEEQPCHLKLTDPSIP
ISKNLPLYDEPAQRYCPAGVYEVITKEDGEKRFQINAQNCVHCKTCDIKDPSQNITWVAP
EGAGGPTYPNM
>tr|A0A0Q0D972|A0A0Q0D972_PSEAP Gluconolactonase OS=Pseudomonas syringae pv. aptata OX=83167 GN=ALO85_00800 PE=4 SV=1
MLIMDFSSSGNTPDHSRRGFLKQSLAVSATVAAIGALPRLSSAQPLTQRYPDPLVSVLDD
SFTHIRIFNASVEKLASGMRWAEGPVWIGDGRYLLVSDIANNRIMRWDEVTGELSVYREH
SNFSNGMCRDRQGRLLVCEGSSTTNEGRRVTRTEYNGRITVLADSFEGKPFNSPNDIACK
RDGSIWFTDPTFQAESNYEGQKVKQEQPFGVYRIDPSNGKVSRVIDDLAGPNGLCFSPDE
KTLYVVEGRAKPHGLIWAIAVNEDGTLGERRKLIEGLDYAAIDGIKCDDGGNLWCGWGGN
GDPKADMEKLDGVRVFNPQGKAIGHISLPERCANICFGGREGNRLFMASSHSLYSVFVNA
RGATFA
>tr|A0A0Q0D2X3|A0A0Q0D2X3_PSEAP Glycosyl transferase family protein OS=Pseudomonas syringae pv. aptata OX=83167 GN=ALO85_04346 PE=4 SV=1
MPRQTTLVTLTYGDRFSYLHTLISRSLESPLIGRVIVVSNASTAPLQALCDKWPDRVQVI
WLPHNTGSANGYSVGINAALQTGDDCIWLMDDDNAPTAHAIEALHRSLRERQLLDGQDNC
AVLGFRPTHQADIAAGVPLQYAIQRRSSFFGFHIAQLPYKIWRRLPWGQPDATRLMPERV
QLPFATYGGLLAHRSLYQKIGLPLDALKLYSDDSEYTWRITDGGGKLFLVPDARLDDLED
SWNIKARTRNVYESYLLGGSDLRAYYAARNQAWFDKHVWAASTLSYRLNRRMFLCLLSLY
AWRHDAAQRLDLLNDAIREGEAGRLGLNRAYPL

>tr|A0A0Q0BUG6|A0A0Q0BUG6_PSEAP 50S ribosomal protein L31 OS=Pseudomonas syringae pv. aptata OX=83167 GN=rpmE PE=3 SV=1
MKADIHPVYEAIDATCSCGNVIKTRSTLAAPLSLDVCNECHPFYTGKQKTLDVGGRVDKF
KSRFGAFGATKAK
>tr|A0A0N8T9K7|A0A0N8T9K7_PSEAP Chemosensory protein ChpC OS=Pseudomonas syringae pv. aptata OX=83167 GN=ALO85_02922 PE=4 SV=1
MSEGTFQTSRLTSLTGLLVPLSDRHLLLPNVAVAELIDYQDCSAEPDAPEWYLGPISWRE
LTLPLLSFEAACGGRTRVGGRARIVVLNALGGRNDVRFIALLTQGIPRSCKVDSQLSYVD
VPLAELELAAVQIGETVARIPDLEGLEQWLVDAGLS
>tr|A0A0Q0DBV7|A0A0Q0DBV7_PSEAP Helix-turn-helix, AraC type:AraC-type transcriptional regulator OS=Pseudomonas syringae pv. aptata OX=83167 GN=ALO85_04972 PE=4 SV=1
MGWPSDVVAACDFDSLRIDPAALFGQQHGDDACHILRLANPAQGRLRRQHRLELIIEPTA
QFGTNGAGRHSVGPDASTGKLGRHIAGQDFKPALERRIGSNPRSGYASGGTGQIDDAGVF
RHQRQQCLGEEKRAFEMHVEQAIELRFADRLQRIEQPVASVVDQAAQCCDAPDVLQDGAH
RRSKRLEAADVSDIQPERNSLATGLRDTLDHRCGGLRVGVVGKDDPHPASSHVFGCTCAD
ATAAASDYDDAHVMFLCGLNVSPQYPAPSPGAYPLLLETCLFLLLACAAPCPALSMRGMD
NQLNELRLLAARAENRRTETGIPRVAMVQGKIPEHMLAAVYDPMINVILQGSKHMTVGDS
TLEYDPATYFVMSIDLPAVGSVHPARSGEPYLAVSLTLDPAVLSTLFADLPKPASRLENK
PGFSVAPVTTELMDAWVRMLRLMGDPNAIAALAPVYEREIIFRVLQGPHGWMLREIAAPD
TAMARVNMSIQWIRRDFAEPLGVERLAERASMSVSAFHRHFKAVTNLSPLQYQKRVRLLH
ARTLMVANAKSVMAAAFEVGYESATQFSRDYSRVFGLPPAQDAGRILEATRGSSS
>tr|A0A0Q0CFU2|A0A0Q0CFU2_PSEAP Regulatory protein, LysR:LysR, substrate-binding protein OS=Pseudomonas syringae pv. aptata OX=83167 GN=ALO85_05445 PE=3 SV=1
MPMIKELKTFIAVAREGTFAAAGSKIGLTQAAVSAQMQRLEAELGVELFDRKGRSAQLNR
MGQQVLLQGQDLVRQFSSLGTTTVGLPASVLVTIGAIASVQRSFLPEALARFHQQFPACR
TRVIPGLSMDLVNQVDAGEIDMAAIIRPPFSLQSDLRWTTLVREPYRLIVPVDMPGDDWA
ELVSAHPFIRYDRSSFGGRQVDRFLRQTHVSLREVCELDELDAIIKLVANGVGVALVPET
ATLQTWHAGVRSIDLKAHTFHRDIGLVHRARRSLTEPVSTLVQLINDHVMTG
>tr|A0A0Q0DJ30|A0A0Q0DJ30_PSEAP Cysteine desulfurase IscS OS=Pseudomonas syringae pv. aptata OX=83167 GN=iscS PE=3 SV=1
MRLPDRRASMKLPIYLDYSATTPVDPRVAQKMIDCLTVEGNFGNPASRSHVFGWKAEEAV
ENARRQVADLVSADPREIVWTSGATESNNLAIKGVAHFYASKGKHLITSKVEHKAVLDTT
RQLEREGFEVTYIEPGEDGLITPSMIEAALRDDTILVSIMHVNNEIGTINDIAAIGELTR
SRGVLFHVDGAQSTGKVEIDLASLKVDLMSFSAHKTYGPKGIGALYVSRKPRVRLEATMH
GGGHERGMRSGTLATHQIVGMGEAFRIAKEEMAAENVRIKALSDRFFKQVENLEELYVNG
SLTARVPHNLNLSFNYVEGESLIMALKDLAVSSGSACTSASLEPSYVLRALGRNDELAHS
SIRFTFGRFSTEEEIDYAAQKVCEAVTRLRTLSPLWDMFKDGVDISKIEWAAH
>tr|A0A0N8T992|A0A0N8T992_PSEAP Endo/exonuclease/phosphatase domain-containing protein OS=Pseudomonas syringae pv. aptata OX=83167 GN=ALO85_05394 PE=4 SV=1
MLNTESLTERQALQVEPMPRLLLRLTLYGLLLVAVLLLLVFQLTWRPPARELLTASCNPA
VEAPKLVPGQALKVMTWNIQYLAGKRYVFWYDMADGSGPDERPTHEDLAYNLDEVARVIR
DEQPDVVLLQGVDDGAKNSDYQDQLALIKERVADLYPCSTQAFYWKSEFVPNPHIWGSVG
RKLATLSRFHIDSAERIQLPVPDANIISRQFQPKDALLVSYLPLRDGGKLAVINTSLTTA
RHAGDTAQKQVAATETQLDKLESGGTPWLIGGDFNLLPLGQYQRLPEQQRLGYAADSELH
ELWDKYPMIPDNAESSGIDRSKWLTRFPNDSRINGPDRTVDYLFYSPSLKRVSARVRRDD
TLLISDHLPVIGRFLLPVLP
>tr|A0A0N8T7X0|A0A0N8T7X0_PSEAP Putative peroxidase-related enzyme OS=Pseudomonas syringae pv. aptata OX=83167 GN=ALO85_04227 PE=4 SV=1
MIEVIMTVLKLHTIDSAPLKSKALLQSSIDNFGWIPNQSAYMAESPSLLGAYQRAHDLVI
ESSLSEEEKAVVWMTTGVENGCSYTIQAHAFIALSKGVDQSVVEALAHQPGRLNTRLQAL
REFTLEVINCRGRLSVTAVQDVLEAGFSKQNMLDVVLAVAQKNMSTILNSIAGTEIDARF
KWDAFQGLNTVSEA
>tr|A0A0Q0FTC6|A0A0Q0FTC6_PSEAP Regulatory protein, TetR OS=Pseudomonas syringae pv. aptata OX=83167 GN=ALO85_05470 PE=4 SV=1
MTQPTMPLPTPSLARPRRPGRPPKVDRDNLETREALLRRGLEVLTEQSFSATGLDYILKE
VGIPKGSFYHYFPSKEAFGRAVLDEYARYFAQRLDHWLLDEALSPLERLVGFVRSAKGGM
ARHDYRRGCMVGNLGQEISLLPDGFREVLEQTLLDWQSKLAGCLRAAQSMGELAADADCD

ELAAFFWIGWEGAVLRARLVKSATPLNTFIAGYLRGLPQ
>tr|A0A0Q0FHL2|A0A0Q0FHL2_PSEAP TonB OS=Pseudomonas syringae pv. aptata OX=83167 GN=ALO85_01102 PE=4 SV=1
MYFLFRVRQWLGGLPALVALIAIVIGVQTRPLKMEPVYDESAVELALIEPEPPAPEPVVQ
PEAPAVQPDEPPPPPPVVESEEAEPAPPPPKPAPKPKPKPEIKPEPRPKPLPKPAVAKPA
EPVQAPSKPIASAPVAPPSPPAPPAAPKVDTQGMEGGYLKGLRSELDGYKQYPTGRQASL
ERPSGEVVVWLLVDRQGRVLDSGIQTQASSMLLNRAAANSLRRIKQVKPFPEQAFGGRSE
QRFTATFNYSVQ
>tr|A0A0Q0CD65|A0A0Q0CD65_PSEAP Binding-protein dependent transport system inner membrane protein OS=Pseudomonas syringae pv. aptata OX=83167 GN=ALO85_00901 PE=3 SV=1
MLMFILRRLLSSIPTLILVSLFVFTLQKLLPGDPVLAMAGEERDPAVMEYLRDKYRLDDP
IPLQYLNWVSNVLTGDLGTSLRTEQPVTTLLASKLPVTLELAVLALLIALLIGIPTGIIS
AVRKGTAVDYGANVVALSGISIPHFWLGILLIMVFAVKLQWLPASGFVPMGEDFGQNLKT
LILPAFVLGAGLSGILMRHTRSAMLEVLRADYVRTARAKGLFPRTVILKHALRNALMPII
TLTTLLFGELLGGAVLTEQVFSIPGFGKMIVDAVFNRDYAVVQGVVLCVAIGFLMLNLLA
DVLYRLINPRLRTA
>tr|A0A0Q0IPD2|A0A0Q0IPD2_PSEAP Magnesium/cobalt chelatase, subunit BchI OS=Pseudomonas syringae pv. aptata OX=83167 GN=ALO85_03632 PE=4 SV=1
MTVSDSTMSETPHFPLAAVVGADDLKLALCLTAIDPRIGGVLIEGPRGMAKSTLARGLAD
VLASGQFVTLPLGATEERLVGTLDLDAALSEGRARFSPGVLAKADGGVLYVDEVNLLADH
LVDLLLDVAASGVNLVERDGISHRHAARFVLIGTMNPEEGELRPQLLDRFGLNVALSGHT
LPAERSQIIRRRLDFDSDPQSFCRQWQTQQDALKRRCEQARQLLSGIELDDQSLAMITER
CFAAGVDGMRADLVWLRAARAHAAWRGAGHIEEQDIEAVAEFALRHRRREPSPPPQNSEA
PPPPPGSSSKQAEPESGQGSWGELPAQAVTTGSRREVPSWPKKP
>tr|A0A0Q0DN42|A0A0Q0DN42_PSEAP YCII domain-containing protein OS=Pseudomonas syringae pv. aptata OX=83167 GN=ALO85_03161 PE=4 SV=1
MKYYLCKYIPPRADFLTSMTADERQWMSQHGTYLNELLEQGLIVAHGPVIDPAGSYGVSL
YQIEDDQDIAAITSQDPIVKNGSGYYEHYAMLHLKTRG
>tr|A0A0Q0DNE8|A0A0Q0DNE8_PSEAP Uncharacterized protein OS=Pseudomonas syringae pv. aptata OX=83167 GN=ALO85_100561 PE=4 SV=1
MLSDSRATKRAHIIHEKSVGPESADQRRQRRARGGEGHWRAAGQPCSNG
>tr|A0A0Q0IMD1|A0A0Q0IMD1_PSEAP Regulatory protein, LuxR OS=Pseudomonas syringae pv. aptata OX=83167 GN=ALO85_01025 PE=4 SV=1
MNTAQHFEYHDDPRFYLQLAKLVSSTGDDTFAGHMLQLVDSLVPIHGLGLSEWTLDLRES
SISRINPLGSAGLKHDPPGSEATGHPLLPSILHMQDPLLIQLKTPLSAQHPQHNTHQCNL
VSSHGERRLIICFYRLAALRAFSLTELSLLKSLSDTLLPLVEHHAQSLAQSSNNATRVEP
EPGSLDQAFSGRLAQDSVTLSAREQEVCLGLLTGGTVAEMAQRLKVKNSSVETYLKRATA
KLGVSGRHGLARWMAGD
>tr|A0A0Q0C0Y2|A0A0Q0C0Y2_PSEAP HopAC1 protein OS=Pseudomonas syringae pv. aptata OX=83167 GN=ALO85_02889 PE=4 SV=1
MPMTPGDTWPDASAALKRLDELRTLLARELNALPQAGEALLSALTGADVSERELEIFSLL
QQIDDYWTDPGETGESRRDRLVPALQRAMLDEARVRVHERDLDSGYLACLPESPEQAQGP
ALAYSTLWVQLHDDEQIEMAGVLVISQDQGRTLLMLPGLGITGFATQAMLLETLAQWLNA
PTLRDTLLGNAQRQHQERLAEIVQDADLYLEPFTAADVQLQPVTTAPFKHAFDRLLNKQR
NDIRYACEQPGTADRLKRQSLIQQAIDMPGLLGPAAMLELRELSNRQRQYQRDLPEWMKI
ASAADLQTYALHLQRYDAAHAAMLSVLGGAASPEQFAEMQLRTRLANDLGEDLDPRALTI
DTRRTLPATSETYRVTLPLTELALYGLHPGDETAGSDFLDQTLITLDGQPLDAAYSALTP
AYLAGVIDELNLRAVFATFQREAYQQEHNQQMLRALARTRLTTLGWAAKMQGHIQPEDFA
IVAALTSTPVSAPDPTMRVQQIKLNDRNVMARLLVFRKQDAQGQTQRLIMFASEAPGHQY
FKAFDTQTQLLHEVIGWTASPTMTTWLLDQVEVTARPELDAQLTALREKPQPAKEFLQFI
DHPDCETALRSFTDEQTRVLLSEQARHTPDWYLRASRAQRRELLAVEHAIEGALGNYQAQ
PHTRVQPFQDYVHQRASQQIGKLLGVPAGTVDPDLIVITSERETLTYTDMLLKGYNDSID
PLRTSAATDATFSGPEGIDLSALSPAAVAGSVRGQWLADEYIALIRNTLLNSENDGYAYR
RQYSVMITQLQMKAAALRSLLKGHVEPAQYVWLKQSLDNAHLSDSASRERFPLYPLQIHV
DKPLIASGLTDVDQLVIPSPLLTHIETVQGCLIILPTQIRHAALLYTPQAPDGIEFRLFS
DFVSSLDSEGMIDYYKDRCRIKARRTLSFFLRDMQKGNANKPPAIPRAFISDVADTCFNR
PLERRLRDVEETNSGRHDMLAKLIWVSVEIIATALTLPFPPASFAVGSLVSLHDSGQALA
ALSAGDRERATNYMLSALFNGLGAGSDLLVGLKGLGVLHQLENGQHSTPVLRSFQRQPSL
PRYEELYPVELQEQVFLLGKPNVHGHAAVFQAPHVASAPPLATGQFAARGGDGAWQPLLP

LPPVAQQAPPGLRIDLAVDISLENLPRIADGHAKGVHAINGKHYIQLSDRAFEVQYDAYW
RCWQIIDPANPFAFFGKQPVRLNDQGQWLLVERQRLRGGGLDTPGTYRPLPEEASASSSS
LNTLSDYEMPSGMRAHLDIVINKEVFDPTGAGLEVYFETYFTEVRQTFTARRENLYQDAQ
AFFARFTPPPRPPLPTYSLPGSVDTLIKHIFSHNNGLVFSEAPKSVASKRLLLLNMPLLA
EQRVEVLYIEHLLTDKHLRKLARYRQLGKKSRSGSHELKYYLQEANRGALNNASSEFDYY
HLIKAAHLYGIEVRPFSSSISYPFLGHSVLAAADDPTAAMKMSNFFGHRLISHDIEPASA
KRWVALLDQKLATTHDQVPGIAEMQGAVSVHVKDIPAGRPTRIRQGAGGAHEHTPTRCDF
SIAFADPTLPAKPLPPATALDNLLIRELGDPAAVAEGERWAGEYGFVLDENDTWLRVDPD
DWSVDRPMTAIQQSLTDATYEMPLETRKTLHNLANFEKKGLDMEYFFEDIELDTVRNTFD
LLRKNLQQDAARISSAQLPPRPTLPAIAPQTSTAGLLETLYRHAEGLVIGESHASVASKK
LIIDNLPLLSQQNVKTLYMEHLLSDLHQADLDRFLETG
>tr|A0A0Q0D478|A0A0Q0D478_PSEAP Polyamine ABC transporter permease
OS=Pseudomonas syringae pv. aptata OX=83167 GN=ALO85_02510 PE=3 SV=1
MTTFSRGKWLGLLCLLPFAVFFIVFQIAPLLWVAIHSLQSDAGWGWENFIRAFSSKFYRQ
AIQYSLEISFWSSLFGIIISVLGAYSLRKVDSRLRDFVNSFANMTSNFAGVPLAFAFIIL
LGFNGALTLILKQTGIIDDFNLYSKTGLIILYTYFQIPLGVLLLYPAFDAVREDWNESAA
LLGASSYQFWRYIGLPVLTPALLGTFVILLANALGAYATVYALTTGNFNVLPIRIAAMVA
GDITLDPNMASALAMILVGLMTLVTVVHQWLLRRSYHVTR
>tr|A0A0Q0D9P1|A0A0Q0D9P1_PSEAP Chemotaxis protein CheY OS=Pseudomonas
syringae pv. aptata OX=83167 GN=ALO85_04026 PE=4 SV=1
MDKNMKILIVDDFSTMRRIIKNLLRDLGFTNTSEADDGLTALPMLQSGAFDFLVTDWNMP
GMSGIDLLREVRKDERLKSLPVLMVTAEAKREQIIEAAQAGVNGYVVKPFTAQALKDKID
KIFERVNS
>tr|A0A0Q0C5T2|A0A0Q0C5T2_PSEAP Chorismate mutase OS=Pseudomonas syringae
pv. aptata OX=83167 GN=ALO85_02083 PE=4 SV=1
MRLILATSLLTMTFMSTCFAFTAEQKPSALEPLLQAISERLTIADQVALSKWDSGKAVED
PPRELQVISAAQARAAEFKLNPDDVQRLFRAQIEANKQVQNALLAQWHAAGKAPDTVRLS
LVDDIRPKLDRLQTQLLQAYADFQPLRKAETCQMQLDAALKRYQTDPIHDQALVLATADL
CPAKL
>tr|A0A0Q0C387|A0A0Q0C387_PSEAP 50S ribosomal protein L33 OS=Pseudomonas
syringae pv. aptata OX=83167 GN=rpmG PE=3 SV=1
MRELIRLVSSAGTGHFYTTDKNKRTTPDKIEIKKFDPVVRKHVIYKEGKIK
>tr|A0A0Q0IE30|A0A0Q0IE30_PSEAP Putative toxin-antitoxin system antitoxin
component OS=Pseudomonas syringae pv. aptata OX=83167 GN=ALO85_01108 PE=4
SV=1
MSLPISDYHPAVPRQDDFWQHLGIPARGARLYSALHDGLPYEVFERLAHYTDLNRSTLAE
HLGIAPATLQRRLKVRRFNAEESDRLFRLAAVYKATLDLFENDAEATRLWLANPVYGLGN
RRPLEMLATSAEAQAVLDLIGRLEHGVVA
>tr|A0A0Q0FME1|A0A0Q0FME1_PSEAP CreA protein OS=Pseudomonas syringae pv.
aptata OX=83167 GN=ALO85_02782 PE=4 SV=1
MMRFMKGLLAAALLMPVLVSAEEIGQVSTVFKMVGPNDRIVVEAFDDPRVDGVTCYLSRA
KTGGVRGGLGLAEDRAEASIACRQVGPIRFKETLKDGDEVFKERTSLVFKTMQVVRFFDK
KRNALVYLVYSDRVIEGSPQNAVTAIPILPWTTAPAP
>tr|A0A0Q0DYB2|A0A0Q0DYB2_PSEAP Flagellar motor switch protein FliM
OS=Pseudomonas syringae pv. aptata OX=83167 GN=ALO85_04037 PE=3 SV=1
MAVQDLLSQDEIDALLHGVDDGMVQTDNNSEPGSVKSYDLTSQDRIVRGRMPTLEMINER
FARYTRISMFNLLRRSADVAVGGVQVMKFGEYVHSLYVPTSLNLAKIKPLRGTALFILDA
KLVFKLVDNFFGGDGRHAKIEGREFTPTELRVVRMVLDQAFVDLKEAWQAIMEVNFEYIN
SEVNPAMANIVGPSEAVVISTFHIELDGGGGDLHVTMPYSMIEPIREMLDAGFQSDLDDQ
DERWVNALKEDVLDVNVPLTTTIAQRQLPLRDILHMRPGDVIPVELSDSLVMRANGVPSF
KVKLGSHKGKMALQVIEPIARR
>tr|A0A0Q0FWH5|A0A0Q0FWH5_PSEAP Glycosyl transferase family protein
OS=Pseudomonas syringae pv. aptata OX=83167 GN=ALO85_01483 PE=4 SV=1
MRTHLSPRLEGWLLVALAVLLVGGGLGLRLPQNVDEERFLGVALEMLQNGSWFVPHRAAQ
IYADKPPLFMWATAFFIWLTGSPNIALYLPGLLSAGATTAVLYDLGTRLWNRRIGRYAAL
LFLATYQTYSILRTGQIDSFLCLWIALGFYGLVRHVLLGPAWGWFYFSCAAMGLGIITKG
VGFVPALMLIPYAYAARKGWQGVVAMPGQAARWALGLLVLLLACCLWLVPMIISVVCDGG
PEGFAYVQEILLHQTANRYASAWDHREPFWYFFVKVIPQYWLPLVLVLPWLIPAWRRQLS
KRDGRVLVLLGWVLLVLLFFSLSSGKRKIYIFPALPGLVLVAAPLLPWLLKRWLHDRVRA
RRIVPVVVVVWLGLWFARGFVEPVIEGQNPHKELMEQAAYVTQGADLVLVNWREGHWLYA

RQPIVHFGFAKPSATEQAARWLREHPGTFALVPGEQLANCFLPEKARPLGQTSRADWFIV
DADADNGLCKPESPMQAYRFAWQQNVD
>tr|A0A0Q0DL42|A0A0Q0DL42_PSEAP NEL domain-containing protein OS=Pseudomonas syringae pv. aptata OX=83167 GN=ALO85_02760 PE=4 SV=1
MFTLRYNPFETIESSVAHASVTPPPQDAAPFEAEHANTEFIRLNLPDWYVGAPTALRQAL
HAGQQSARRCAQALEPMRNRLLSAQQFAAPLLSKAFVERFKLDLDVEAFQLMTWRYDSTW
SPAPLEQTLLQAALQNFAPSNRSRFDPYSAILRTGGLRYWLIDSAQRRYKVEYRDRQAID
LEQFADFCHELDLGRQYQTHLDSVFKPPGPAAQAVASAFMDSERAAVEVLAHIAVMKGDI
TEAAYQTLLDMVKSVDQPRWDGKGVRYCQLHMLDTYTFPGSLLQGALLIQQDGARPDDGP
CLVYLPSEPSHPIKQFASLRAFNVWLVTALGSEHYRRYFSRFVSLGQASAFFTKLDARLY
PARDRKLNLDVDLVVQAQPFSKPPFERLYDHLLAKTYDDSKAIAVPSAQVDQQAHDALIE
SLENNGMNLLNVAGFFVPVLGEVMSVVALYQLASEAFVAYEDWKHDEVEDAMQHVYDIGE
NVAQMLVAGGVVAAVNGLQPSMFIESLVQRRVDGAVRLGKPSIDAYAHTVSLPDNLSSNA
LGLYEYEGKTWLPLDGKLYRVESDAAGTQWRVRHPVNERSYAPKLKHNGAGAWRHEWENP
MGWDEVTAFRRLNPTYHAFPEEDIQKVLRITGTHEALLRQVHVENLQPPALLKDAIQRVE
TERQLHACIDALQAADVADVHVSHLEPWLKLLVSSPRWHEARGLLLIDAQGALLEAWNAG
SQMTLSSHVTGPTGQLTEVLGQLLENLSVDEAARLSGSDSADRAVQLRGLKRYLADYAQT
HVWRLLDDVQALKGRSDDPHVQLIQRDFESLPSSVALELIGMASDADKARMTTEKRIPLG
LAEHAREYQQQLRINRANEGFYRAVTDNPDTRAAGLGMLQYVPGWRGDVSIDLLKDSLEG
DEIASLDSDQASSHRLLVNTEQGVQCFEPSGESLGEVDQQFFRALLLALPEQVRLDIQLP
ADADELQLRSLLRNTAVERRERMAAVLQLQLIKPGIKWPQRLPHGRIGYPLSGRLRRFFR
RLGIGASRYSPELAVKSLYPDFSDAEVSGFLNALRAEHTGLARELSTFVRQRLSSLADEL
RTLQVTLDTWVAETPFSSMRRPREVAATRIHDCWKRLSVQCRNFQGDFLGYALDLDNLRI
GELPDITANFDHVAVLKARAMQLTDPQADALLKNFNRLRSLSLDFNDLRSLPTSIGQMPQ
LAELSISHNPLIWTESANATLQHLNRLEILDLNFCSLGEDVPINALNSLRLLFLRGSGIS
RLPAWNWLRSELIRIDVRDNRISEISIDELDNIERSLSSSRLHLHLNGNPLNSPTLERIR
LFREGRLRPRWGVSQSVNPVEAGPDSAPWLLGLNREQANTRTRLWQDLRACAGSTDFFQM
LSDLTHSADFSSNRDELIARVWSMIESASVNAELRAQLFDVAAHPQTCGDGLALVFGDME
VRVQIFSIMSSTPASAQPRKLFRMTRSLDRLDQVEKIALREIAFRQASGESVDEAEVRLA
YRVGLQARLELPGQPRTMWFRAIARVSEADLQTAYNEIIDRETTPEFFQSMIAREFWMSY
LEIRYAPEFEPVKQPFNQRLVALDELPPDEQSDQQYLEQIGVISRERERAINEFAVTLSR
QIAEAVNITAQ
>tr|A0A0Q0BWB4|A0A0Q0BWB4_PSEAP Ni_hydr_CYTB domain-containing protein OS=Pseudomonas syringae pv. aptata OX=83167 GN=ALO85_03051 PE=4 SV=1
MKTRPIHPWPVRLTHWVNAVGMVCMFMSGWAIYNASPLMPFTFPKFLTLGGWLGGSIAWH
FAVMWLLVINGLIYVLYGVFSRHFKRDLLPVRPSEVTRDMSDALRFRLVHVKGRYNAVQR
LMYWLVLAMGVLVVLSGLAIWKPVQFQGLVSLLGGFDFARWVHFGAMTAIGAFVIVHLLL
VVLVPSTLLPMITGGRQPNEDGTAQS
>tr|A0A0Q0BTX0|A0A0Q0BTX0_PSEAP Sensor protein OS=Pseudomonas syringae pv. aptata OX=83167 GN=ALO85_04277 PE=4 SV=1
MRVDSIALRLSGLFALVALVVFLLIGWALYLQVDKSLDLLPRAEVDARYSVLESAVNRFG
NPEHWGKIKTKLTLLSEEDKRLRFWVVSDNPLYEFGNPGADIRRFAQGPLGMHDLKLADH
PFSFKVLVSEFPAKEQRPALRFLTAIDTETFWHTQHSLLVALISLATLGILLASALGYWV
ARIGLRPLTSLSQEVQKLAPPRLSGRLQLSPLPPELEQFVASFNSTLERVEQAYSRLESF
NADVAHELRSPLTNLIGQTQVALTRGRSAEHYFEVLQSNLEELERLRSIINDMLFLASAD
QGTKVKAQTCTSLAEEVATTLDYLDFILEDAHVQVRVNGDASAPIEKPHLRRALINLLHN
AVQHTRAGEVIEVNIESRDAHVTVSVTNPGVQIAQEHLPRLFERFYRVDASRSNSGANHG
LGLAIVKAIALMHGGTVFVNSAKGANTFGIQLPR
>tr|A0A0Q0BRB6|A0A0Q0BRB6_PSEAP Putative type VI secretion system effector, Hcp1 family OS=Pseudomonas syringae pv. aptata OX=83167 GN=ALO85_02249 PE=4 SV=1
MKKEKQKMAFDAYIQIDGIPGEVLDDKHKDWIEVLGYEFGATQATSATASSSGGASSERV
ALSDFSIRKSVDKASAKLFEACCKGTHIAKIQLNVNRAGGDKLTYLTINLEEVVVSSVKA
VAGGSQKGEDNAVSDLPIEEISFNFARIKTTYTQQSRSDGQGGGNVTGGWDRTANKVFA
>tr|A0A0Q0C7J6|A0A0Q0C7J6_PSEAP GntR family transcriptional regulator OS=Pseudomonas syringae pv. aptata OX=83167 GN=ALO85_00598 PE=4 SV=1
MDYRKPSDRKSMHARIVQELGMQIVSGRFKPDEKLPAEALLCEEYAVSRPVLREATRVLV
AKGLVYSRPRVGTVVKARREWHLLDPDVLHWVMQSSPQNEFFHLLTSVRAVIEPAVAALA
AQHATDAEIESIHEAYLRMEAAPTPEDLLQPDLDFHSRIADATHNDLLAHLCNMLSLALR
EALKYSNKRPNLHELAMPRHKAILTAIQNRDALGARHASLVQLDDAHNALSVVLGGG

>tr|A0A0Q0D646|A0A0Q0D646_PSEAP 2-dehydro-3-deoxyphosphooctonate aldolase OS=Pseudomonas syringae pv. aptata OX=83167 GN=kdsA PE=3 SV=1
MAQKIIRVGSIEIANDKPMVLFGGMNVLESRDMAMQVCEEYVKVTEKLGIPYVFKASFDK
ANRSSVNSYRGPGLEEGMRIFEEIKRTFNVPLITDVHEPHQAALVAEVCDIIQLPAFLSR
QTDLVVAMAKTGAVINIKKAQFLAPQEMKHILTKCEEAGNDQLILCERGSSFGYNNLVVD
MLGFGIMKQFEYPILFDVTHALQMPGGRSDSAGGRRAQVLDLAKAGISQNLAGLFLEAHP
DPDNAKCDGPCALRLDKLEPFLAQLKSLDELVKSFPIVETA
>tr|A0A0Q0E168|A0A0Q0E168_PSEAP F0F1 ATP synthase, F0 subunit I OS=Pseudomonas syringae pv. aptata OX=83167 GN=ALO85_01496 PE=4 SV=1
MIMATDIKGAVAMESRMPDRLPFYRLAVFPLLLAQLIVLLMAALVLWQWQGVVAGYSGLC
GGLIAWLPNLYFAHKAFRFSGARAAQAIVRSFYAGEAGKLIFTAVLFALTFAGVKPLAPL
AVFGVFMLIQVVNWFAPLLTRTRLSRP
>tr|A0A0Q0CGL8|A0A0Q0CGL8_PSEAP RNA polymerase sigma factor FliA OS=Pseudomonas syringae pv. aptata OX=83167 GN=fliA PE=3 SV=1
MTASGYQMYSKTSRNSQYELIERYAPLVKRIAYHLLARLPASVQVEDLIQAGMIGLLEVS
TKYDSTKGASFETYAGIRIRGAMLDEVRKGDWAPRSVHRNTRMVSDAIRSIEAKTGRDAK
DHEVAAELQLSLDDYYGILNDTLGSRLFSFDDLLQDGDHEGLHEDGASGSLEPSRDLEDE
RFQAALADAIANLPERERLVLALYYDEELNLKEIGEVLGVSESRVSQLHSQCAARLRGRL
GEWRAR
>tr|A0A0Q0CWS6|A0A0Q0CWS6_PSEAP Toluene tolerance protein Ttg2A OS=Pseudomonas syringae pv. aptata OX=83167 GN=ALO85_00172 PE=4 SV=1
MSPDDAYAVELKGVSFKRGARSIFNNVDIRIPRGKVTGIMGPSGCGKTTLLRLMGAQLRP
SEGQVWVNGQNLPELSRSDLFDARKQMGVLFQSGALFTDLDVFENVAFPLRVHTELPEEM
IRDIVLLKLQAVGLRGAVELMPDELSGGMKRRVALARAIALDPQILMYDEPFVGQDPIAM
GVLVRLIRLLNDALGITSIVVSHDLSETASIADYLYVVGDGQVLGQGTPQELMSSDNPRI
RQFMTGEPDGPVPFHYPAPDFREDLLGKR
>tr|A0A0Q0BTD2|A0A0Q0BTD2_PSEAP GntR family transcriptional regulator OS=Pseudomonas syringae pv. aptata OX=83167 GN=ALO85_04244 PE=4 SV=1
MNSPNTTNALAEDVYRRLKQEIFDFYLLPYDRFTETQLADRYQVSRTPVRDALYRLQREG
YLDVEFRRGWSVKPLDFNQIDQLYDLRIVLECAAVERICASAEVHPELTVLRQHWLIDRD
SWQIDMQVVADLDEQFHTQLVAASGNLEMARIHQEVTERIRIVRRLDFFKSARIEHTYLE
HAAILNALQARKRDEALLLLRSHVEISKLEVRKLTISMLSDARRRYEA
>tr|A0A0N8T901|A0A0N8T901_PSEAP Uncharacterized protein OS=Pseudomonas syringae pv. aptata OX=83167 GN=ALO85_03882 PE=4 SV=1
MTDKRPLTHCPLAATLWLCVGTSVFAQDTALHSGKYEQLMLAVTPEHQVEGYYSETRGEA
FSCAFYLQGQVEAGKGAAVSSWLDDVYPGTLKASADGVVLTIEQGRQHPGCMNVMAPDIA
TGMDLTRTASKQWVGLVTVTADKAWLQKTPNAKATRGAYIVKDDVVGVLAFKDGAAQVEF
INADDRSFTGWISQDQYARLAAPGR
>tr|A0A0N8T7S3|A0A0N8T7S3_PSEAP Ultraviolet light resistance protein B OS=Pseudomonas syringae pv. aptata OX=83167 GN=ALO85_04046 PE=4 SV=1
MGVGIGPTKTLAKLANHTAKRLLSHTGGVVDICDLHNRNWVLRNTAVSEVWGVGKKMNAH
LQAMNIRTAMDLATADPRTLRDRFSVVIEKTARELAGTSCLELGEAAPPKQEICCSRMFG
KRLTAIQPIKEAVVTYTQRAAEKLRSQNSRCKKIRVSIRTGMFNPEEAKYANGALVELPY
PTNDVRLMTRAATEAVNRLFRPGFKYSKAEVLLLDLRQPGEFTDDMFAASQPASSEKVMG
VLDEINTRWGRGTLRAGSVPSNPEWAMRRELMSQSYTTRLDQLWEVKSL
>tr|A0A0Q0DJG9|A0A0Q0DJG9_PSEAP Uncharacterized protein OS=Pseudomonas syringae pv. aptata OX=83167 GN=ALO85_01728 PE=4 SV=1
MRKDKKQLIGDEIGDEQIKLFLNFEPYDATSPSLHKLIKAYRGLRINDFERFLVFFKEAG
HDFDGKDEHGNDFIALIKDQRNADEYIELIEKART
>tr|A0A0Q0FCK1|A0A0Q0FCK1_PSEAP Uncharacterized MFS-type transporter ALO85_100305 OS=Pseudomonas syringae pv. aptata OX=83167 GN=ALO85_100305 PE=3 SV=1
MHRYDKHFESLLRVFPRPLERHARKRMKARPMSETQQNTPQTPMAVTLTVVSIVMFTFIG
YLNIGIPLAVLPGYVHSDLGFGAVIAGLVISVQYLATLISRPWSSRIIDNLGSKKAVQIG
LTGCGLSGVFMLVPVWLDSWPMASLVILLIGRVILGAAESLVGSGSIGWGIGRVGAQNTA
KVISWNGIASYGALAIGAPLGVWMVDELGLWTVGVSIMLLCALGLYLTWHKEPAPIVQGE
RLPFMHVLGRVFPHGMGLALGGIGFGTIATFITLYYASNHWPNAALCLTLFGGSFIAARL
LFGSLINRLGGFRVAIVCMSVESLGLLLLWLAPTPDLALAGAALTGFGFSLVFPALGVEA
VNLVPASSRGAAVGAYSLFIDLSLGITGPLAGAIAAGFGFVSIFLFAALASLTGLALSVY
LYKQA

>tr|A0A0Q0D4S6|A0A0Q0D4S6_PSEAP Major facilitator transporter OS=Pseudomonas syringae pv. aptata OX=83167 GN=ALO85_200032 PE=4 SV=1
MSLENRKIFENELLKELKAITPSKLIGTDKIKILASKNKLKFKFEKAYVAELYLEHLTNA
NGYYLGWHVFGGPIFECTANFIAPYKSNLLNKACSSYTTLKIEDKKLTLVSGGVLKTPTP
EDAVAISKHIRQVIEEDYIPKIAGCIIASERTIQDVNDAPNIYAYPAIFIHCAIMQNPKI
LKKDLFKKVISNKKIVKDNNFDLELLESYLDSQLTD
>tr|A0A0Q0IMZ9|A0A0Q0IMZ9_PSEAP Lipopolysaccharide core heptose(I) kinase OS=Pseudomonas syringae pv. aptata OX=83167 GN=ALO85_02893 PE=3 SV=1
MKLFLAEPFKSLWAGRDAFVEVEGLSGEVYRELEGRRTLRTEVDGRGYFVKIHRGIGWGE
IAKNLATAKLPVLGAGKEWDAIERLHEVGVPTMTAVAYGERGSNPAAQHSFIVTEELAPT
TSLEDVSLNWRNEPPEPRLKRAFIAEVARLVGMMHRAGVNHRDCYICHFLLHTDKPVTAD
DFKLSVIDLHRAQTRRAITPRWRNKDLAALYFSALDIGLTRRDKLRFLQGYFQKPLREIL
LKEATLLTWLDKKADKLYQRKVRYGDAL
>tr|A0A0N8TAB1|A0A0N8TAB1_PSEAP Uncharacterized protein OS=Pseudomonas syringae pv. aptata OX=83167 GN=ALO85_01515 PE=4 SV=1
MKRAIEQETLAALVETGAAREFRVLREGETWRLELRLGAKWLPIRSRREPVRYWRSLTAV
GRFCEGVGIKVLTVQF
>tr|A0A0N8TA43|A0A0N8TA43_PSEAP Ubiquitin OS=Pseudomonas syringae pv. aptata OX=83167 GN=ALO85_101854 PE=4 SV=1
MKIYIQSLTTALLIPGGKLELDVEPSDTIGNVKSKIQDNLLLAPPPENQRLLFAGQRLDD
DRTLTDYNIQKETTLLLTLKI
>tr|A0A0Q0BZ36|A0A0Q0BZ36_PSEAP Sulfur transfer complex subunit, TusB/DsrH OS=Pseudomonas syringae pv. aptata OX=83167 GN=ALO85_00712 PE=4 SV=1
MATLHVLSHSPFADTRLDSCLRLLGNDDGVLLCGDATYALMPSSAQLKALQSSVDSSQLF
ALDEDLSARNLPLPAGVSSIDYPAFVELSLRFDKVNTWL
>tr|A0A0Q0DSG5|A0A0Q0DSG5_PSEAP Histidine kinase, HAMP region: chemotaxis sensory transducer OS=Pseudomonas syringae pv. aptata OX=83167 GN=ALO85_03387 PE=4 SV=1
MINTLRNISVRSKLVLGFALVIVLTVLIALTGWTGITSLSERSERISDIGKLSSLTRDVR
IARLAYSVNYDAERASNWLKAFESLENHVKYAQKVFDSPLNVPLVNTAADALKDYRIHYD
NLMQATAAREATRSVFGQYADAGADDLQKLNAIARSDDGTPAQRDAIVQAMTLFQKMRFD
LRGYTYSLKPENRAPAETSMNTVIESFKNLQGFDGQSAIIKHLVDSVISYQNTLNQFTAA
QARIDLAQAGITKDIGILFECADKLSENQVNLRVEDVSQAKMLLSVWLIAALIMSALAAW
VITNLIVGPLRDTLKLAERVADGDLTNNQVITRKDELGQLQGSMMRMTGNLRELIGGLRD
GVTQIASAAEQLSAVTEQTSAGVNSQKSETDQVATAMHEMSATVQEVARNAEQASHAAVN
ASKEAREGDGVVSKAVAQIEKLATEVTHSKSAMDELKNESNKIGGVLDVIKAVAEQTNLL
ALNAAIEAARAGEAGRGFAVVADEVRSLAQRTQTSTEEIAALISGLHTRTAQVATILDNS
QALTANSVELTRNAGVSINNMTQAISTIETMNHQIAAAAEEQSAVAEEINRSVLNVRDIS
EQTASASEETAASSVELARLGVHLQSLVSRFRV
>tr|A0A0N8T8K2|A0A0N8T8K2_PSEAP YihY/virulence factor BrkB family inner membrane protein OS=Pseudomonas syringae pv. aptata OX=83167 GN=ALO85_03196 PE=3 SV=1
MIFPHLRKLKVGKVLVRTVNEFLDDEMSTYASALAYQMLFSLFPFLLFLIALIGFLHLPD
FFSWLRLQSEFLLPPQALEQVNPVIDQLQQSKGGLLSIGIVIALWTASAGVRLMMSAMNA
AYDVVEGRPIWKRLPLSILYTVGIAGMLLAAAAFMVLGPQVMNWIAAQIGMEDFIVTLWT
ILRWPAIIILLMVAVALIYYVMPDVKQEFRFITPGSVLAVVVWIFASLAFGFYVKTFADY
NAMYGSIGAIIVLLLYFYISAAVLLLGAEMNAVIEHMSAEGKDAGEKEAGDGGEESSETD
KQHVSGLGRDHSVPLQKADEA
>tr|A0A0Q0IF33|A0A0Q0IF33_PSEAP Cytosine deaminase OS=Pseudomonas syringae pv. aptata OX=83167 GN=ALO85_03201 PE=4 SV=1
MDAFMQAAFDEAQLGLKEGGIPIGSVIVHGGKIIGRGHNRRVQEGSATKHGEMDALENAG
RQPASVYRDSVLYTTLSPCAMCSGAILLYGIRKVIIGENESFMGEEELLRSRGVQIDVLD
NPECTHMMKDFIASKPELWNEDIGE
>tr|A0A0N8T810|A0A0N8T810_PSEAP Carboxymuconolactone decarboxylase OS=Pseudomonas syringae pv. aptata OX=83167 GN=ALO85_100091 PE=4 SV=1
MDTRSKAMTDKTISEGEQIRRQVMGDVYVDRALGNATEFSQPLQDFVNEHAWGSVWSREG
LPLKTRSLITLATLTALKCPQELKGHVRGALNNGCTVEEIREALLHCAVYAGVPAAIDAF
RAAQEVIDSYVKAD

>tr|A0A0N8T8T7|A0A0N8T8T7_PSEAP Dihydrolipoyl dehydrogenase OS=Pseudomonas syringae pv. aptata OX=83167 GN=ALO85_02201 PE=3 SV=1
MSQKFDVVVIGAGPGGYVAAIKAAQLGLKTACIEKYQDKEGKLALGGTCLNVGCIPSKAL
LDSSWKFYEAKNGFSVHGISTSELNIDIPAMIGRKSTIVKGLTGGVASLFKANGVTTLQG
HGKLLAGKKVELTAADGTVEIIEADHVILASGSRPIDIPPAPVDQKVIVDSTGALEFQQV
PQRLGVIGAGVIGLELGSVWARLGAQVTVLEALEKFIPAADEAVSKEALKTFNKQGLDIK
LGARVTGSKVEGEQVVVSYTDAAGEQSITFDRLIVAVGRRPVTTDLLASDSGVDLDERGF
IYVDDYCTTSVPGVYAIGDVVRGLMLAHKASEEGIMVVERIKGHKAQMNYNLIPSVIYTH
PEIAWVGKTEQTLKAEGVEVNVGTFPFAASGRAMAANDTGGFVKIIADAKTDRVLGVHVI
GPSAAELVQQGAIAMEFGSSAEDIGMMVFSHPTLSEALHEAALAVNGGAIHIQNRKKR
>tr|A0A0Q0FL62|A0A0Q0FL62_PSEAP Putative nucleoside-diphosphate sugar epimerase/dehydratase OS=Pseudomonas syringae pv. aptata OX=83167 GN=ALO85_01722 PE=4 SV=1
MHILLTGGTGLIGRALCRSWSAQGHELTVWSRKPADVAELCGPSVRGIASLDELGAQPVD
AIINLAGAPIADRPWTRKRRLLLWESRVGLTEQLLKWLGTREQKPALMISGSAVGWYGDS
GEREIDETSLPAKEDFASQLCNAWEETAQRAEALGVRVVLIRTGLVLSDRAGFLQRLLPP
FKFGMGGPIGNGRQWMPWVHIQDQIAAIDFLLNLNEAKGPYNVCAPSPVRNRQFAKSLAG
ILHRPAFMPMPALALKVLLGELSVLLLGGQRARPERLQEAGFTFKYTELDIALQDLLGRH
>tr|A0A0Q0D710|A0A0Q0D710_PSEAP Amino acid ABC transporter, periplasmic amino acid-binding protein OS=Pseudomonas syringae pv. aptata OX=83167 GN=ALO85_00969 PE=4 SV=1
MHRGPSFVKACAFVLSASFMLANTVQAAEGSKLDAVLKRGNLVVGTGSTNAPWHFQGADG
KLQGFDIDIGRIIAKGLFNDPSKVEFVVQSSDARIPNLLTDKVDISCQFITVTASRAQQV
AFTLPYYREGVALLLPANSKYKEIDDLKAAGDDVTVAVLQNVYAEELVHQALPKAKVDQY
DSVDLMYQAINSGRADTAATDQSSVKYLMVQNPGRYRSPAFAWSPQTYACAVKRGDQDWL
NFVNTALHEAMTGVEFPAYAASFKQWFGVDLPVPAIGFPMEYK
>tr|A0A0Q0FGI5|A0A0Q0FGI5_PSEAP Uncharacterized protein OS=Pseudomonas syringae pv. aptata OX=83167 GN=ALO85_01660 PE=4 SV=1
MTAGLFLLADLSMLAWQRLSRITLEVFEQHQDIWLQPLRIAFGHIRSLAELKKIITLAVG
VNLIVGVTLATSAAMVIGEYSAGKDSYAGLQAAGAVTTIVILFFLARVALPMRVLGSVGF
SMIATGAFISAPSPNLIGYALGFLLIVGFDKMFNVYMRTLRQRVIPPQDFGKTVGVITLL
NNLSQPLAGLLVALLAAPLGIRQVILMLAVLTSVIGAGALWWFAKARGSFPTPIVEADRE
AGK
>tr|A0A0Q0FD62|A0A0Q0FD62_PSEAP Probable 5-dehydro-4-deoxyglucarate dehydratase OS=Pseudomonas syringae pv. aptata OX=83167 GN=ALO85_00095 PE=3 SV=1
MNPQELKSILSSGLLSFPVTDFNAQGDFHRAGYIKRLEWLAPYGASALFAAGGTGEFFSL
AASEYSEIIKTAVDTCATSVPILAGVGGATRQAIEYAQEAERLGAKGLLLLPHYLTEASQ
DGVAAHVEAVCKSVKIGVVVYNRNVCRLTPTLLEQLAERCPNLIGYKDGLGDIELMVSIR
RRLGDRFSYLGGLPTAEVYAAAYKALGVPVYSSAVFNFVPKLAMDFYHAIARDDHQAVGK
YIDDFFLPYLEIRNRKAGYAVSIVKAGAKIAGYDAGPVRAPLTDLTPDECDMLAALMDKQ
GKQ
>tr|A0A0Q0IIW3|A0A0Q0IIW3_PSEAP Polar amino acid ABC-type transport system, periplasmic substrate-binding protein AapJ OS=Pseudomonas syringae pv. aptata OX=83167 GN=ALO85_01819 PE=4 SV=1
MKMLKSTLAVVTALAAFGVIGAANAGTTLDAIKKKGFIQCGVSDGLPGFSVPDSTGKITG
IDADVCRAVAAAVFGDATKVKFSQLNAKERFTALQSGEIDILSRNTTWTSSRDAGMGLVF
TGVTYYDGIGFLVNNKLGVNSAKELDGATICIQAGTTTELNVSDYFRSNGLKYTPITFDT
SDESAKSLEGGRCDVLTSDQSQLYAQRSKLAKPDDYIVLPEVISKEPLGPVVRKGDEEWF
SIVRWTLFAMLNAEEAKITSKNVEAEAKSTKNPDVARMLGADGTYGQDLKLPKDWVVQIV
KQVGNYGEVFERNLGEGTPLKIKRGQNALWNAGGIQYAPPIR
>tr|A0A0Q0CIU4|A0A0Q0CIU4_PSEAP PAS:ATP-binding region, ATPase-like:histidine kinase A OS=Pseudomonas syringae pv. aptata OX=83167 GN=ALO85_01412 PE=4 SV=1
MLLLVTACLLVGLISGQYAWSLAAGLGLYLAWTLKQLLRLHDWLSSHKADEPPPDGYGLW
GEVFDSIYHLQRRDQRVRGRLQAVIDRVQESTAALRDAVIMLDSDGNLEWWNRAAETLLG
LKTPQDSGQPVTNLVRHPRFKEYFALGDYSEPLEIPSPTNDRLRIQLLITRYGNNEHLML
VRDVTRIHQLEQMRKDFVANVSHELRTPLTVIFGYLETLLDNVDEVNPRWVRALQQMHQQ
GGRMQTLLNDLLLLAKLEATDYPSDNHPVIVNSLLKTITADANALSGSKNQQIHLSLESN
MRLKGSESELRSAFSNLIFNAVKYTPAEGVIRVRWWADERGAHLSVQDSGIGIETKHLPR

LTERFYRVDTSRASNTGGTGLGLAIVKHVLLRHRGNLEINSVLGKGSVFTCHFASGQLSK
PASDDSDY
>tr|A0A0Q0C614|A0A0Q0C614_PSEAP Putative lipoprotein OS=Pseudomonas syringae pv. aptata OX=83167 GN=ALO85_01910 PE=4 SV=1
MMTLRPAFLLCFIGLLAACSSNDAPKPAAPPPVAPSIKLPAGPGPLQPYQRELSGQLLGV
PAGAEVELAMLVIDERGRPQKLLTNTLLKGNGQSLPFQLRFNPEAFPVGGRVELRGRASK
SGQLILHLPSMRIDQPATQALGQLQFVKAP
>tr|A0A0Q0IRY1|A0A0Q0IRY1_PSEAP Transporter OS=Pseudomonas syringae pv. aptata OX=83167 GN=ALO85_03371 PE=4 SV=1
MEWIADPTAWLGLLTLIVLELVLGIDNLVFIAILADKLPPEQRDKARVIGLSLALIMRLG
LLASISWMVTLTEPLFEVFGKTFSGRDLIMLFGGVFLLFKATMELHERLEGHVAQRAGNA
GYALFWPIVAQIVVLDAVFSLDAVITAVGMVEHLSVMMIAVIFSIGLMMIASKPLTKFVN
SRPTVIMLCLGFLMMIGFSLTAEGLGFHIPKGYLYAAIGFSILIEVFNQIARKRSKKSSH
GHLPRRERAAHAVMRLLGGRKLESGDVDEEITDMLDGESSEPVFDRRERVMISGVLQLAE
RPIRTVMTPRAEVDCIDLNDSAEKIRLKLMHSSYSRLPLIGERGIDEPLGFVHKKELFKE
LLSGNEPDLKLMSRKAINLLESFTILNALEQMRKESTHIAFVVNEFGDFIGVLSMTDILE
SIAGQLPDASEVEGPDIVEQGEDFVVSGALNLSLIRERTGFQAKATEDYQTLAGLVMSLL
DRLPSTGDSLSWQGWNLHVVGVEERRVTRVLLQKQPDMGAGK
>tr|A0A0Q0DLL4|A0A0Q0DLL4_PSEAP CheW domain-containing protein WspB OS=Pseudomonas syringae pv. aptata OX=83167 GN=ALO85_00344 PE=4 SV=1
MSDQSPRRNGTPLTASKLFLLFCIGEDRYALDATEIAEILPRVKLKAIAQAPHWVAGIFA
HRGEIVPVIDISALNSGQPARSRTSTRMVLVHYRYDDAHPAQLLGLILEQATETLRCPVA
QFKAYGLDNRLSPYLGPVREDEQGLLQWIHVHELLSEPVRELLYPVPPIDLELLEDAQ
>tr|A0A0Q0CBK5|A0A0Q0CBK5_PSEAP Short chain dehydrogenase OS=Pseudomonas syringae pv. aptata OX=83167 GN=ALO85_01796 PE=3 SV=1
MPIALITGCSSGIGRALADAFKATGYEVWATARKADDVAALSAAGFIAVQLDVNDSLALE
QLAAGLEHSGLDVLINNAGYGAMGPLLDGGVQALQRQFETNVFSVIGVTRALFPALRRNK
GLVVNIGSVSGVLVTPFAGAYCASKAAVHALSDALRLELAPFGVQVMEVQPGAIASSFAK
NASHEAEQLISEQSPWWPIREGIRARARASLDSPTPVTEFARDLLKAVQHTRPPRLLRLG
NGSRLLPLMAWLLPKRLLDMALRKRFGLNADL
>tr|A0A0Q0BXI8|A0A0Q0BXI8_PSEAP Leucine--tRNA ligase OS=Pseudomonas syringae pv. aptata OX=83167 GN=leuS PE=3 SV=1
MHELYQPREIEAAAQTFWDEQKSFEVSEQPGKDTFYCLSMFPYPSGKLHMGHVRNYTIGD
VISRYQRMLGKNVLQPLGWDAFGMPAENAAIDNNVAPAKWTYENIDYMRTQLKSLGLAVD
WSREVTTCKPDYYRWEQWLFTRLFEKGVIYRKNGTVNWDPIDQTVLANEQVIDGRGWRSG
ALIEKREIPMYYFKITAYADELLESLDELPGWPEQVKTMQRNWIGRSRGMEVQFPYDQAS
IGEAGALKVFTTRPDTLMGATYVAVAAEHPLATLAAQGNPALQAFIDECKGGSVAEADVA
TQEKKGQATSLFVEHPLTGEKLPVWVANYVLMHYGDGAVMAVPAHDERDFEFATQYGLPI
KPVVRTSAGDQTPAPWQPAYGEHGELINSGEFTGLTFQDAFDAIEAALVKKSLGQSRTQF
RLRDWGISRQRYWGCPIPIVHCDTCGDVPVPEDQLPVVLPEDVVPDGAGSPLARMPEFYE
CSCPKCGAPAKRETDTMDTFVESSWYYARYASPHYEGGLVEPNAANHWLPVDQYIGGIEH
AILHLLYARFFHKLMRDEGLVTSNEPFKNLLTQGMVNAETYFRMETSGKKTWINPADVTL
ERDAKAKVISATLTSDGLPVEIGGTEKMSKSKKNGIDPQTMIDQYGADTCRLFMMFASPP
DMSLEWSDSGVEGSHRFLRRVWRLAQAHVAQGPSTGLDVAALSDEQKAVRRAIHQAIKQA
SQDIGQNQKFNTAVAQVMTLMNVLEKAPQNTPQDRALMQEGVETVALLLAPITPHISHEL
WTQLGHNEPVIDAGWPAFDAHALVQDSLQLVIQVNGKLRGHIEMPASASREEVEAAARVN
ENVLRFTDGLTIRKVIVVPGKLVNIVAS
>tr|A0A0Q0D4W2|A0A0Q0D4W2_PSEAP Chaperone protein ClpB OS=Pseudomonas syringae pv. aptata OX=83167 GN=clpB PE=3 SV=1
MRIDRLTSKLQLALSDSQSLAVGLDHPAIEPAHLMQALLEQQGGSIKPLLLQVGFDINSL
RKELSAELDRLPKIQNPTGDVNMSQDLARLLNQADRLAQQKGDQFISSELVLLAAMDESS
KLGKLLLGQGVSKKALENAINNLRGEGAVNDPNVEESRQALDKYTVDLTKRAEEGKLDPV
IGRDDEIRRTIQVLQRRTKNNPVLIGEPGVGKTAIAEGLAQRIINGEVPDGLRGKRLLSL
DMGALIAGAKYRGEFEERLKSLLNELSKQEGQIILFIDELHTMVGAGKGEGSMDAGNMLK
PALARGELHCVGATTLNEYRQYIEKDAALERRFQKVLVDEPSEEDTIAILRGLKERYEVH
HKVAITDGAIIAAAKLSHRYITDRQLPDKAIDLIDEAASRIRMEIDSKPEVLDRLERRLI
QLKVEAQALKKEKDEAAIKRLEKLQGEIERLELEYADLEEIWTSEKAEVTGSAQIQQKIE
QSRQELEAARRRGDLNRMAELQYGIIPDLERSLQMVDQHGKPENQLLRSKVTEEEIAEVV
SKWTGIPVSKMLEGEREKLLRMETLLHNRVIGQEEAVVAVSNAVRRSRAGLSDPNRPSGS
FMFLGPTGVGKTELCKALAEFLFDTEEAMVRIDMSEFMEKHSVARLIGAPPGYVGYEEGG

YLTEAVRRKPYSLILLDEVEKAHSDVFNILLQVLEDGRLTDSHGRTVDFRNTVIVMTSNL
GSAQIQELVGDREAQRAAVMDAVGTHFRPEFVNRIDEVVIFEPLARDQIAGITDIQLGRL
RKRLAERELSMTLSPEALDKLIAVGYDPVYGARPLKRAIQRWIENPLAQMILSGSFMPGT
TITGKVVDDEITFV
>tr|A0A0Q0C8W2|A0A0Q0C8W2_PSEAP Histidine kinase OS=Pseudomonas syringae pv. aptata OX=83167 GN=ALO85_03248 PE=4 SV=1
MLFMSLRLRLSLILGVAFVAVWTLAATWMFQDVRSQMMYSLDQRLVASARMVAGLVAQLP
QPLSSKDHGAHFSADQLGIPDGMACEVSSLRGEILASNRSNTGTVIASPSGGFYDQVIDG
EHWRGFTLVQDDVRITTADREQEREQLNHSVLLSASMPVLVALLSCVATLWLGIGKGLAP
LNRMRDELRKRNINSLEPLHLQMLPCELKPLLETQNHLLMRISRAIERERRLTGDAAHEL
RSPLTAIKTHLQVAQMTSGKAQCLALSRAEQGTDRLHSTLEQLLLLARVEGSLSFDDGSQ
CGVEQVVSLAVQDATSSTERIVQTFQFVRPLACLDMPAVLAVAALRNLLDNALRHSPANT
QVVVSVTDDSGFALVTVKNVSVEMSQDNIHLLTERFWRGSSSTGCGLGLAIVQAIVERCG
CTIKFTTTSQYFEVTLGMPLVPAKGESYEKTCKCSTPDIKHPVSISNGTTHNRSPSENSK
PGNAS
>tr|A0A0Q0E0M9|A0A0Q0E0M9_PSEAP Haloacid dehalogenase I OS=Pseudomonas syringae pv. aptata OX=83167 GN=ALO85_03268 PE=4 SV=1
MLKHIIFDVNETLLDLAALDAFFAGLFGDSRLRTEWFLTLQECWMTNTITGQYQAFGDLA
QGALRMVAARHQVEVGDDQCHALAAAIKSLPAHKDVHQALQLLNDNGFVLIALSNGALEA
LQQQLEFAGLTGCFEHIMAGSEISQFKPAAATYQMVAQRLGIATDQMVMVAAHAWDIAGA
AQAGCRTAFVERPGKVLNPIGTQPDLIGKDVQDVAIKLCAGKL
>tr|A0A0Q0DSL3|A0A0Q0DSL3_PSEAP Rhs family protein OS=Pseudomonas syringae pv. aptata OX=83167 GN=ALO85_03434 PE=4 SV=1
MIMNVQSAALHHHTPRLNVVDPRGLEIRAIEFWRNQATDTPQRLVNRVAHDAAGHPVNCW
DARLWESQAAVNLATVFSLSGQALLSDSVDAGWRLMLAGDSGAVVAGWDGRGTERSVQYD
ALLRPVAIIENGRCIERRQYGGPDTKGHNQCGQCIRHDDPAGSRMDDEFALAGGVLEQTR
HFLFNPENVDWPEPLTERDALLEPGPGATTRWAHSPLGDVISQTDAQRNVQTFAHTVAGH
VEAISLGLPGQTERVLVHSIDYDAQGYVTSETAGNGVVTKALHDAANGRLIELKGTRADG
QLLQHLLYDYDPLGNVLRINDRAQPTRCCAGQRIEPVSTYQYDTLYQLIQATGREAKKVN
RGPVFPSFQTPLDPTQLANYTQTYRYDASGNLLQLTHTGTQSHSRTLVTSQTSNRSLPVI
NDRPPDEAAIAAAFDANGNLNELQAGQAMSWDWRNQLQQVRPVVREAGDDDKERYVYDAS
GQRLRKIHTTKAKAVVHNAEVRYLPGLEVHSNSATAETLHVIVTQAGRNEVRVLHWQAGQ
PEGLENDQVRYSFADHLGSGTLELDKNAHIISQESYYPFGGTSWWAGRSTVEASYKTIRY
SGKERDATGLYYYGLRYYAPWLQRWINPDPAGAVDGMNLYRFVRNSPLRFADQQGAAPHD
APLKVVADDLSEFEPEQLSKMYEARDVAVSLLTFTRSELLKASPGEDVKEAFDATFGALA
TSARAATSIDVEDSLRQMQELIEGIGSPESDLTLFLFNGPENTLASTDFQGEFQEAVERI
GVSASLLANYDVLKVARALIHEASHVRLNTVDAFYYPTDAGNPLLDGADTAQVEAWSSGI
LKSLREISTNGPDEEQFDPADYIAAMQALTKSARTPAQRKQEFLSNTTTRTLLLQMNADT
LSSLVMATGQPTRYAQTRMNQPGN
>tr|A0A0Q0C659|A0A0Q0C659_PSEAP Ribosomal RNA small subunit methyltransferase D OS=Pseudomonas syringae pv. aptata OX=83167 GN=ALO85_00467 PE=3 SV=1
MANPRPKGHNGLGQLRIIGGEWGSRRLTFPDAPGLRPTPDRVRETLFNWLAPYIAGARVL
DVFTGSGALYFEALSRGASMGLALDSNAAAIASLRQNLNALNCTSGQVSQTDALRHLETA
TASPFDVVFLDPPFHQGLLASACNLLESHGWLADTAWIYTESETPPSTTGLPGSWRLHRE
KKAGQVYYALWQRGQG
>tr|A0A0Q0BU03|A0A0Q0BU03_PSEAP Translational regulator CsrA OS=Pseudomonas syringae pv. aptata OX=83167 GN=csrA PE=3 SV=1
MLILTRRCAESLIIGDGEITVTVLGVKGNQVRIGVNAPKEVAVHREEIYLRIKKEKDEEP
SH
>tr|A0A0Q0FNZ7|A0A0Q0FNZ7_PSEAP Uncharacterized protein OS=Pseudomonas syringae pv. aptata OX=83167 GN=ALO85_101516 PE=4 SV=1
MIVVQFLKSLVIKALDKLKVQFIQAVIAKSWLNSAPPCNTLLQVLHVMDCFVAVDVSMRA
MILIVVSEPE
>tr|A0A0Q0DVR6|A0A0Q0DVR6_PSEAP Group 1 glycosyl transferase OS=Pseudomonas syringae pv. aptata OX=83167 GN=ALO85_02894 PE=4 SV=1
MQLAFVLYKYFPFGGLQRDFMRIALECQQRGHQIRVYTLIWEGDVPPGFEVLVAPVKALF
NHRRNEKLSAWMEADLAKRPVDRLIGFNKMPGLDVYYAADGCFEDKAQNLRSPLYRKWGR
YRHFADYERAVFAKDSKTQVLMISEVQQPLFIKHYDTPPSRFHLLPPGISLDRRAPPDAP
EIRAGFRKEFGLADDDLLLVQIGSGFKTKGVDRSLKALAALPAGLKKRTRLSVIGQDDPK

VFQLQSSALGLGDQVTFMKGRSDIPRFLLGADLLIHPAYNENTGTVLLEALVAGLPVLVS
AVCGYAHYIAEADCGRVLDEPFEQAQLNRYLAQMLQDDAARSAWSRNGLTFADTADLYSM
PQHAADVILAEHY
>tr|A0A0Q0C8C4|A0A0Q0C8C4_PSEAP Glycosyl transferase, group 4 protein OS=Pseudomonas syringae pv. aptata OX=83167 GN=ALO85_03521 PE=4 SV=1
MIEWLSVIAIAFLSLILTALLRKYALAKSLIDIPNARSSHSIPTPRGGGVAIVITFILAI
AVLGWAGYLPPSALAAIVGSGGLVAVIGFMDDHGHIAARWRLLGHFAAAAWALFWLGGLA
PLDVMGWTVDLGVLWQVLAAFYLVWMLNLYNFMDGIDGIAGIEAVTVCLGMSIIYAFGGQ
AGLSWEVLMLGAAAAGFLYWNFPPAKIFMGDAGSGFLGIVLGVLSIQGSWASPQLFWSWL
ILLGVFVVDATVTLVRRLLRKERLYEAHRSHAYQFASRHYGRHVPVTLAVGLINLVWLLP
IALWVALGDGEGLIWAVIAYCPLILLALRFGAGEAE
>tr|A0A0Q0C705|A0A0Q0C705_PSEAP Uncharacterized protein OS=Pseudomonas syringae pv. aptata OX=83167 GN=ALO85_101972 PE=4 SV=1
MRYIDQDELALPEGWLAKATRASAAVVAGAKPDEDESIWKELKDALAALFPEKKCWYCES
PVDRADNAVDHFRPKGRVADAANPHAGYRWLAFDHRNYRYACTFCNSRRKGIENDTVGGK
ADRFPLLDEATRLYTPGPLGQEKPILLDPCELNDWELIGCRRENGKPCATSQDAVERRRA
EESIAIYHLNYEPTCKQRHAAAVRLMADVEQAKLFFGQPGMNDHFTATAKKVRRAIDRKA
PYSGDMIFLLKGQRHADHPWIQKLLEA
>tr|A0A0Q0CIR5|A0A0Q0CIR5_PSEAP Uncharacterized protein OS=Pseudomonas syringae pv. aptata OX=83167 GN=ALO85_01461 PE=4 SV=1
MALNTANGVDARSSLMSFDKSTVVVWVAISLMLVETFSGALRFYFDQAGLSPLLYLPKAA
CILLFALELRTFRAGRLFWTFMILWLLSGLLAMLHRASVHNLAFSLFALSPLVFGLVCGR
HLLYRRKLLYRAIGFCLLASLLGIALDKLTSVPWKGYSYSVGETQLSANTSWSADEVDRI
AGFARVSNVLSIMIAFYTLYLIMFLRSRLMMLLLSAVALYAIVLTTSKAPAAAFALTIVL
LLLRRMSWTCRTVCTVVVGIGLLLPALGLIVSPDAYAVSSGGSLASLYDRMINTWPGLIN
AMSREGWMISGAGFGMVGSTMGLFPVDGAGVFLGMDSSALYLWAMLGVLGLLLYTLQIPL
LFRLIDDQTRVGHMLLAISFCWCLISWTTDMFEVAVANLFIGLAIGHVIAGREPSASHAP
GLSSTAR
>tr|A0A0N8T7R7|A0A0N8T7R7_PSEAP Catabolite repression control protein OS=Pseudomonas syringae pv. aptata OX=83167 GN=ALO85_03990 PE=4 SV=1
MRIISVNVNGIQAAVERGLLSWLQAQNADVICLQDTRASTFELDDPAFQLDGYFLYACEA
EVPAQGGVALYSRLQPKAVISGLGFETADRYGRYLQADFDKVSIATLLFPSGMNGDEDLN
QKFKLMDDFGKYMDKQRRKRREYIYCGSLYVAQQKLDIKNWRDSQQSPGFLAPERAWMDE
IVGTMGYVDALREVSREGDQYSWWPDNEQAEMLNLGWRFDYQILTPGLRRFVRSARLPRQ
PRFSQHAPLIVDYDWTLTI
>tr|A0A0Q0BY45|A0A0Q0BY45_PSEAP N-acetyltransferase domain-containing protein OS=Pseudomonas syringae pv. aptata OX=83167 GN=ALO85_02466 PE=4 SV=1
MSEALSIHHDEVGHHFEIIIDGHRAYLTYMDLGKQTLDFYRTFVPDALRGRGIAAALTKE
ALDYADSMGYSVIPSCSYVERYMELHDLQSQAAKL
>tr|A0A0N8T9R9|A0A0N8T9R9_PSEAP Thiamine-monophosphate kinase OS=Pseudomonas syringae pv. aptata OX=83167 GN=thiL PE=3 SV=1
MGEFELIRNYFAAAPCAQVGEEVALGIGDDCALLALAPGEQLAISTDTLVAGVHFPDACD
PFLLGQRALAVSASDLAAMGARPVAFTLALTLPQVDAAWLQTFARGLDQMAQGCSLRLIG
GDTTRGPLSLTLTVFGAVQAGSALTRAGAQAGDLLCVGGTLGDGAGALPLVLKQRSAEPS
ITEALLARYWSPQPQLALGQALRGRATSALDISDGLLADCGHIARASGVRLVVERDRLPM
SDQLLTLFDLPSARQAALSGGDDYILAFTLPSEHLPTLLKEGWPVHVIGRVEQGQGVILV
DDTGQDVTPDTRGYQHFGGEQ
>tr|A0A0Q0DT66|A0A0Q0DT66_PSEAP Uncharacterized protein OS=Pseudomonas syringae pv. aptata OX=83167 GN=ALO85_02742 PE=4 SV=1
MLRITELKLPIDHPEEDLRVALLKHLGIDSGELLDFTLFKRSYDARKKSSELCFIYTIDF
QVKDEEALLRKLADDRHVGPAPDVSYKVVGHAPEGLTERPIVVGFGPCGIFAGLLLAQMG
FKPIILERGTEVRQRTKDTWALWRKNVLSPESNVQFGEGGAGTFSDGKLYSQIKDPKFIG
RKVLHEFVKAGAPEEILYVSKPHIGTFRLTGVVENMRHQIEALGGEVRFQQRVTDVMIED
GQLLGVQLDSGEQLESRHVILALGHSARDTFRMLHGRGVFMEAKPFSVGFRIEHPQSLID
RARLGKYAGHPKLGAADYKLVHHASNGRSVYSFCMCPGGTVVAATSEPGRVVTNGMSQYS
RNERNANSGIVVGISPEQDYPGSPLAGIELQERLESHAYLLGGSSYEAPAQLVGDFIAGR
ASTALGSVEPSYKPGVKLVDLAEALPAFAIEAIREALPAFDKQIKGFSLHDAVLTGIETR
TSAPLRITRGPTMQSLNTKGLYPAGEGAGYAGGILSAGVDGIRVAEALVRDMLGIEG

>tr|A0A0Q0FT35|A0A0Q0FT35_PSEAP Cobalamine-independent methonine synthase MetE OS=Pseudomonas syringae pv. aptata OX=83167 GN=ALO85_03763 PE=3 SV=1
MKKLLPTSTAGSLPKPSWLAQPETLWSPWKLQGEELVEGKQDALRLSLQEQQLANIDIVS
DGEQTRQHFVTTFIEHLDGVDFEQRETVRIRDRYDASVPTVVGAVARRKPVFVEDARFLR
QQTRQPIKWALPGPMTMIDTLYDAHYKNREKLAWEFAKILNQEAKELEAAGVDIIQFDEP
AFNVFFDEVNDWGVATLERAVEGLTCETAVHICYGYGIKANTDWKKTLGSEWRQYEEAFP
RLQNSSIDIISLECHNSHVPMDLLELIRGKKVMVGAIDVASHAIETPEQVANTLRKALKF
VDADKLYPCTNCGMAPLPRHVASGKLRALSAGADIVRKELLSQ
>tr|A0A0Q0FLJ7|A0A0Q0FLJ7_PSEAP 4-aminobutyrate aminotransferase OS=Pseudomonas syringae pv. aptata OX=83167 GN=ALO85_03917 PE=3 SV=1
MIGKTMNSKVDDTPHLIRQRDQFVPRGIVTAHPLVIDRAQGSELWDVDGKRYLDFVGGIG
VLNIGHNHPSVVAAIQAQLTKVTHACFQVASYQPYIDLAKRLSLLIAGQSGIDHKAVLFT
SGAEAVENAVKIARAHTNRPAIISFRGGFHGRTLLGTTLTGMSQPYKQNFGPMAPEVFHT
PYPNEYRGVTTEVALAALHELLATQVAPERVAAILIEPMQGDGGFLTAPVEFMKALRALT
EQHGIVLIADEIQSGFGRTGKWFGFEHSGIQPDLVTVAKSLAGGMPLSGVVGRAEIMDAP
LPGGLGGTYGGNALSCAAALAVIDTYEQENLLARGEQLGKHLRAGLLALKERYACIGDVR
GTGFMLAMEMVKNDAARSPDADLNQKIIDQARLGGLLVIKCGVYRNVLRFLAPLVTTEQQ
IDEALGILDAALARVLKSS
>tr|A0A0Q0FK02|A0A0Q0FK02_PSEAP Uncharacterized protein OS=Pseudomonas syringae pv. aptata OX=83167 GN=ALO85_03044 PE=4 SV=1
MIGDLDISGIFIPTFLAMMGIAYLLFLVVHAVLSRAHFYRLVWHRALFNVGLYALLLGAV
DTLSRYLMT
>tr|A0A0Q0FD01|A0A0Q0FD01_PSEAP TM helix protein OS=Pseudomonas syringae pv. aptata OX=83167 GN=ALO85_00569 PE=4 SV=1
MELNLWTQSLLAAMTALWTKVANFIPNLFGALVVVLLGFVVAKLLDALLSKLLAKLGLDR
LVGGTGLTKIIGRAGVKVPVSTLIGKVVYWFVLLIFLVSAAESLGLQRVSATLDMLALYL
PKVFGAALVLLVGVLLAQLVNGLVRGAAEGVGIDYSAGLGRIAQGLVIIISISVAISQLE
VKTDLLNHVIVIALITVGLTVALALGLGSREIAGQIIAGIYVRELFQVGQQVQIGDTEGQ
IEEIGTVKTTLLTGEGELVSFSNRILLEQRVSSR
>tr|A0A0N8T9A7|A0A0N8T9A7_PSEAP Glycerophosphoryl diester phosphodiesterase OS=Pseudomonas syringae pv. aptata OX=83167 GN=ALO85_02717 PE=4 SV=1
MQGFPTTLLKKTLLAAAVMSGLMLSSGMAAAADAPGKVLSEKYGLPWPALIAHRGASFDA
PEETIPAYTLARDLGADYLEMDIQRTKDGVLIALHDDVLERTTNIAQVFPTRVKDPVSTF
TLAELKQLDAGSWFNKAYPDRARDSYNGLQILTLDEVIDIAEGGANKPGLYIETKVPNKF
PGVEADLKKLLAKRGWLDQRPAAAAGHVNVAHMPGRVVLQTFEKQSLELLQKEMPKVPKV
MLLWIGEGSIEPKSSVAFKDSGAKDKASYYATQEVKSAQEFEQWIDWAKAHGAIGTGPSS
QLAKGGDQSYMDLVKPWMNNLTHEKGMVIHPYTVDDAEDFKRISHDGADGFFTNRTAELL
KFYERPATESIETILKRNGY
>tr|A0A0Q0E0H1|A0A0Q0E0H1_PSEAP Tellurium resistance protein TerC OS=Pseudomonas syringae pv. aptata OX=83167 GN=ALO85_03308 PE=4 SV=1
MESTSLGFPPLTMAVFVGLAVTAMAIDMFSHRGNKPITLAQASAWSIFWVAISLAFAGFL
YVQHGSEVATLFVTGYALEKVLSVDNLFVFMALFSWFKIPDGLRHRVLYWGIIGAIVFRG
IFVAIGTGLLALGPWVEVVFAVIVAWTAIMMLRAGDDDDEEVDYSQHMAYRFAKKLFPVW
PKLHGSNFFVSRSVLEQEVKKPENAGITLAAKGALFATPLFLCLVVAEISDVLFAFDSVP
AIIAVSREPLIVFSAMLFAILGLRTLYFVLEALKRYLVHLEKAVIALLFFIALKLGLNAT
NHLFHHGYEISANTSLLVVVVVLIIGIVASLLFPGKDESAEEKA
>tr|A0A0Q0DVA4|A0A0Q0DVA4_PSEAP 30S ribosomal protein S17 OS=Pseudomonas syringae pv. aptata OX=83167 GN=rpsQ PE=3 SV=1
MAEAEKTVRTLTGRVVSDKMDKTITVLIERRVKHPIYGKYVKRSTKLHAHDETNQCHIGD
KVTIRETRPVAKTKSWALVDILERAVEV
>tr|A0A0Q0BUH4|A0A0Q0BUH4_PSEAP CHASE/PAS/GGDEF domain protein OS=Pseudomonas syringae pv. aptata OX=83167 GN=ALO85_04564 PE=4 SV=1
MANSYNRSHSQPDASVCWRLEFVCMTVKRFKLRILSFINGQLSAWLIAVLALLVGAALTI
MLALADNELYQRQLRQRFDIIAAERFSRLQERLDRQVTRLDTLARFFIYSQQVEPSEFDG
FVAPLLIGTQAYAWNPKVLQAERAAFERQSREDGSPGYAIREMDENGALKVAAVRNEYFP
VRFIQTLSGIPTPAGFDIASEPVRRAALERARLLKRTVATPRIRLLSLEPSDTYGILLVA
PVFSIGQPVAQLRGYVTAVISLTQLMNVGTADQDNLAVTMLDLSSPETPEQVYQSPLPAI
GSPLYASSLLSLGDRDYLVEVRPTQIFSLNNQIMMPGRVLWLGGLLSLMLSALLYSLISQ
RQRALQRVAQRTRELRQREQQLRAAHGQLRSVLDAATEVAIIATDLDGLIKTFNVGAQKM

LGYAQEDVLGRFRLMDFYQSADLEARASRLSKSRGQPISASQLMFLDAVQDGNHPSQEWT
LQRSDGSTLVVNMLVTAVRDDQGLWTGYLAVCIDVTEHKRVNQALAEKGQLLKKLGSQVP
GGIYQYHLAVDGSARFRYASAGMCELFEVDEEHLLGDSETILRRIQAVDLVRVRSSILIS
AEHLTPWSEEYRVELPRKGLRWLRAASTPERLADGSVLWHGFVSDITDLKRVEQELRALS
VTDVLTGAYNRRYFQDRMQAELKRIDRHGGNLSIIMLDIDHFKRINDRFGHAAGDQVLKA
ISEKISQRLRSDDVFCRLGGEEFMVICPGTRGAQAYQLALSLREELRKQVIEGVDQVVTG
SFGIASWREGEGIDAMLLRADSGVYAAKQAGRDRVEPEQG
>tr|A0A0Q0FJL9|A0A0Q0FJL9_PSEAP Cyclic diguanosine monophosphate-binding protein OS=Pseudomonas syringae pv. aptata OX=83167 GN=ALO85_100270 PE=4 SV=1
MASTDGVPITKQRCRTVLQLLSGLSCMDNRIDQITTCPLTPVAVTKRVHSMTDSPEDRRR
FKRIAFDAKTDLKQGDKSWKVQLVDLSLKGLLIDRPDPWNGDQTQPFEVDIILSNDAHVK
MDVEITHDNNRQLGFVCRHIGLESISHLRRLVELNLGDPEELDRELAALIELQE
>tr|A0A0Q0DJV3|A0A0Q0DJV3_PSEAP ABC-type multidrug efflux system, ATP-bindig protein OS=Pseudomonas syringae pv. aptata OX=83167 GN=ALO85_02524 PE=4 SV=1
MSTFPDMSSALSIRQLTKTYGNGFQALNGIDLDVAEGDFFALLGPNGAGKSTTIGIISTL
VNKTSGTVNIFGHDLDREPAALKRCIGVVPQEFNFNQFEKTLDIVVTQAGYYGIPAKIAK
ERAEQYLTQLGLWEKRDVPSRSLSGGMKRRLMIARALIHEPRLLILDEPTAGVDIELRRS
MWTFLTELNSKGITIILTTHYLEEAEQLCRNIGIIDHGVIVQNTGMKQLLSTLTVETFVL
DLKASWQTAPQLPGFPCRLLDSHTLEVQVDKNVGITALFSQLAFQNIEVLSLRNKSNRLE
ELFVSLVEKNLAKVAV
>tr|A0A0Q0BUD4|A0A0Q0BUD4_PSEAP Sugar fermentation stimulation protein homolog OS=Pseudomonas syringae pv. aptata OX=83167 GN=sfsA PE=3 SV=1
MRFSPQLEQGRLLVRYKRFLADIETDSGELLTIHCPNTGSMLNCMMPGGRVWFSRSNDPK
RKLPGTWEISETPQGRLACINTGRANTLVDEALRAGVISELTGFTALKREVAYGQEKSRV
DFRLEYPDGYLYLEVKSVTLGFDGSSVAAFPDAVTQRGARHLRELATLAREGVRAVLLYC
VNLTGIDAVRPAQEIDPAYAAALREAIDAGVQILAYGAQLTSEEIFIDRRLQVHGLD
>tr|A0A0Q0C2U4|A0A0Q0C2U4_PSEAP HTH hxlR-type domain-containing protein OS=Pseudomonas syringae pv. aptata OX=83167 GN=ALO85_03351 PE=4 SV=1
MSESASQPAAPRSDTVQQGLQPEVAETLLALVRQGQVLASDCPSRVVLNHVCSRWGVLVL
VVLRGGMHRFSEIRRKIGGVSEKMLAQTLQHLEHDGFVSRKSLPVVPPHVQYRLTPMGEE
VALQVETLVTWIETNLPRIMHAREASNTLHTTPE
>tr|A0A0Q0CG17|A0A0Q0CG17_PSEAP NAD(P)-bd_dom domain-containing protein OS=Pseudomonas syringae pv. aptata OX=83167 GN=ALO85_03692 PE=4 SV=1
MSKVFVIGAAGKVGQRLLKNLGGGGHEVIALHRKEEQSAAIKATGAIPLLGNLTELDASR
LSAAMTGSDVVVFTAGAGAGIELTNAIDGKGLETAVEAAVLAGVSRFLLVSAFPEAARGT
DTSAGFENYMRVKKQADVYLASSALDWVILRPGTLVDSAGTGEVSAGPAVAYSEIPRDDV
AAFLAALVDRIEISRQIIELTQGATPVDVALRTLSRR
>tr|A0A0Q0BV40|A0A0Q0BV40_PSEAP Putative AdrA-like diguanylate cye OS=Pseudomonas syringae pv. aptata OX=83167 GN=ALO85_03212 PE=4 SV=1
MSTYHRRGMAFAKRIYAPRCFGVSVGFVTVAVSLYYVNTAHWAWLLALLYSLVWPHVAYQ
LARTSREPYQAEWRNLLFDSMMGGFWVGAMGFSAVPGITVLAMMAMHNMAAAGPRLMLQG
LCMQALGVLISLAALDPVVNLHGNMAQIYACLPVLVTYPIFIGWLSHQVTLKLWEHRNIL
RKVSRTDSLTGLLNHGAWKDLLDLEYASSQNTYQECVIALIDIDHFKVINDTYGHLMGDT
VLQSISEALTENLRDSDLIGRCGGDEFCVILPDTCLLQAKEILERLRLAIDEMTYILHCN
LKVSLSIGIAAYSPEMPDASSWLHEADKALYLAKSTGRNRVASHQDAQPELQSLGAQA
>tr|A0A0Q0FUU7|A0A0Q0FUU7_PSEAP Uncharacterized protein OS=Pseudomonas syringae pv. aptata OX=83167 GN=ALO85_04318 PE=4 SV=1
MSTTDQTQSTGQHSAKWQERFNFFETYGAPNDPRFKPAVNALPDFKKKLLVCANIIAFFF
GPIYFFVLGLWKKNLALIGVIIAVNIVIALLFGILGMEVPAALGTGLNVVFSLMYALTAN
YSYYLKEVKGEQGWNPFKGMRL
>tr|A0A0Q0DJ60|A0A0Q0DJ60_PSEAP Putative lipoprotein OS=Pseudomonas syringae pv. aptata OX=83167 GN=ALO85_01782 PE=4 SV=1
MYLRYGVLIASLAIASGCVEERVVHERSHAHHEYVEEVIAPQPPPERVVEVEPAPRPGYI
WARGYWHWNGQRFVPVHGHWETERPGYHYVHPHWESAGDGWHWRAGVWLN
>tr|A0A0Q0BUN4|A0A0Q0BUN4_PSEAP L-threonine 3-O-phosphate decarboxylase OS=Pseudomonas syringae pv. aptata OX=83167 GN=ALO85_03556 PE=4 SV=1
MLEHGGRLRAAAQHYGIPLADWLDLSTGIAPWSWPIPEIPTRAWARLPETDDGLEAAACT
YYGVSRLLPVSGSQAAIQALPRVRSGGRVGVLSPCYAEHAHAWRKNGFVVREVGEQEVDY

FLDSLDVLVVVNPNNPTGLHLSAERLLEWHARLAERGGWLVVDEAFMDNTPGMSLAAETW
RSGLIVLRSFGKFFGLAGVRLGFVMAEPVLLRMLAQEIGPWSVSGPTRIIGQVCLSDQQG
QTRQRERCEQARERLVALLDRYRLSPQGGCALFQWLVTPEAQTLYEFCAQRGVLLRLFKG
DSSESSSLRFGLPCDEADWLRLHDVLLEYRKEYP
>tr|A0A0Q0CHN2|A0A0Q0CHN2_PSEAP Aryl-alcohol dehydrogenase-related oxidoreductase OS=Pseudomonas syringae pv. aptata OX=83167 GN=ALO85_03336 PE=4 SV=1
MQFRKLGKTDLSVSAICLGTMTWGEQNTQDEAFEQIRMAKDAKVNFLDTAEMYPVPPRGE
TYTKTESIIGEYFKKYGDRNDWVLASKVAGPGRMDHIRDGNPRLDRKNITAALEASLKRL
NTDYLDLYQLHWPDRKTNFFGVLGYTHDPDDQAVEIEETLSVLGDLVKAGKIRHFGLSNE
TPWGTQRFLHLAETLDLPRAVSIQNPYNLLNRTFEVGLAEIAIREQIGLLAYSPLAFGVL
AGKYLDGARPADGRLTLFERFQRYNNPQAVTATAQYVALAREHGLDPSQMALAYVTSRPF
VTSNIIGATKLDQLKINLESIDVTLSDEVIAGIEAIHTSQPNPAP
>tr|A0A0Q0FEE5|A0A0Q0FEE5_PSEAP Bis(5'-nucleosyl)-tetraphosphatase, symmetrical OS=Pseudomonas syringae pv. aptata OX=83167 GN=apaH PE=3 SV=1
MAVYAVGDLQGCLEPLQCLLEHVHFNPEQDRLWLVGDLVNRGPQSLETLRYLYNIRESLV
CVLGNHDLHLLAVARKKELLKKGDTLLEILEAPDRDDLLGWVRRQKLMHYDAERNVAMVH
AGIAPQWTLKKALKHAAEVEHALQDDTLYGAFLDGMYGNEPAKWDNDLQGVTRLRVITNY
FTRMRFCTSDGKLDLKSKEGVGTAIPGYAPWFSHQSRKTRDVKIIFGHWAALEGRCDEPD
VFALDSGCVWGGSMTLLNVDTLERHQCNCDAIGNAADGLVTRVQPGATPLP
>tr|A0A0Q0DBZ3|A0A0Q0DBZ3_PSEAP Helix-turn-helix, Fis-type OS=Pseudomonas syringae pv. aptata OX=83167 GN=ALO85_01835 PE=4 SV=1
MRIKVHCQNRIGILRDILDLLVAYGVNVARGEVGGEHGDAIYLHCPNLINLQFQALRPKF
EAITGVFGVKRVGLMPSERRHMELNALLGALEFPVLSIDMGGAIVAANRAAAQLLGVRVD
EVPGMPLSRYAEDFDLPELVRASQSRINGLRVKVCGDVFLADIAPLQSEHDDSEAMAGAV
LTLHRADRVGERIYHVRKQELRGFDSIFQSSKVMAAVVREARRMAPLDAPLLIEGETGTG
KELLARACHLASPRGQSPLMALNCAGLSESMAEIELFGYDPGAFEGARAEGRLGLLELTA
GGTLFLDGVGELSARMQAKLLRFIQDGSFRRVGSDEDMFLDVRVMCSTQVDLSELCAKGM
FRQDLYHRLNVLSLHIPPLRDCLDGLEPLVEHFIDQASRQIGCPLPGILPAAIKRLSQYH
WPGNVRQLENVLFQAVSLCDGGKVQAEHIRLPDYGVRQGLSEVSLEGGLGAIIGRFEKSV
LEQLYAQYPSSRQLGKRLGVSHTTIANKLRDYELGRESDPHPVDK
>tr|A0A0Q0IHU6|A0A0Q0IHU6_PSEAP Uncharacterized protein OS=Pseudomonas syringae pv. aptata OX=83167 GN=ALO85_02240 PE=4 SV=1
MKALICLPLLVLVLAGCAGKTGYRDSCATQLDAAWHELDLAKAEGFAGTVSYSKALSLLT
GAKTQQQFEAFEGCTEKSEKARFYIRESRAGR
>tr|A0A0Q0CC28|A0A0Q0CC28_PSEAP Type IV pilus bioproteinsis protein PilZ OS=Pseudomonas syringae pv. aptata OX=83167 GN=ALO85_02548 PE=4 SV=1
MNLPPSTGPRNGILSLTIKDKSVLYAAYMPFIRNGGLFIPTSKSYKLGDEVFMLLNLMDE
PEKVPVAGRVAWITPKGAQGNRTAGVGVQFNDGDNTARNLIETHLVGALKSDRPTHTM
>tr|A0A0Q0DAI4|A0A0Q0DAI4_PSEAP Conjugal transfer protein OS=Pseudomonas syringae pv. aptata OX=83167 GN=ALO85_200122 PE=4 SV=1
MAPRLPQRTPTLPTDMDTRYACVNSDCPCSELEELDLDDHVNRSTWTCIECNCPVSVDMA
NDWGEKCTVYRYQAQQLKKRDYIYKGKNLVAVEVTGSSATDVEGRWYFALAGHKYEFVEP
DRYYNCMPTGHHVR
>tr|A0A0Q0BSP0|A0A0Q0BSP0_PSEAP Polyribonucleotide nucleotidyltransferase OS=Pseudomonas syringae pv. aptata OX=83167 GN=pnp PE=3 SV=1
MNPVIKKFQFGQSTVTLETGRIARQASGAVLVTVDDDVSVLVTVVGAKQADAGKGFFPLS
VHYQEKTYAAGKIPGGFFKREGRPSEKETLTSRLIDRPIRPLFPEGFMNEVQVVCTVVST
SKKIDPDIAAMIGTSAALAISGIPFDGPVGAARVAFHESTGYLLNPTYEQLQASSLDMVV
AGTSEAVLMVESEAKELTEDQMLGAVLFAHDEFQVVINAIKELAAEAAKPTWAWQPKPEA
TELLSAIRSEFGEAISQAYTITVKADRYARLGELKDQIVAKLAVEEGSPSASEVKAAFGE
IEYRTVRENIVNGKPRIDGRDTRTVRPLNIEVGVLPKTHGSALFTRGETQALVVATLGTA
RDAQLLDTLEGEKKDPFMLHYNFPPFSVGECGRMGGAGRREIGHGRLARRSVQAMLPGAD
VFPYTIRVVSEITESNGSSSMASVCGASLALMDAGVPMKAPVAGIAMGLVKEGEKFAILT
DILGDEDHLGDMDFKVAGTSKGVTALQMDIKIKGITEEIMEIALGQALEARLNILGQMNQ
IIGQSRNELSANAPTMIAMKIDTDKIRDVIGKGGATIRAICEETKASIDIEDDGSIKIFG
ESKEAAEAARQRVLGITAEAEIGKIYVGKVERIVDFGAFVNILPGKDGLVHISMLSDARV
EKVTDILKEGQEVEVLVLDVDNRGRIKLSIKDVAAAKASGV
>tr|A0A0Q0DXK8|A0A0Q0DXK8_PSEAP Uncharacterized protein OS=Pseudomonas syringae pv. aptata OX=83167 GN=ALO85_03711 PE=4 SV=1

MNHDVIITCALTGAGDTTARSPHVPVTPKQIAAAAVEAAKAGATVVHCHVRDPHTGKFSR
DVALYREVMERIREADIDIIVNLTAGMGGDLEIGDGEHPMEFGANTDLVGPMTRLAHIEE
LLPEICTLDCGTLNFGDGDTIYVSTPSQLRAGARRIQALGVKAELEIFDTGHLWFAKQMI
KEGLLDNPLFQLCLGIPWGAPADTTTMKAMVDNLPADAVWAGFGIGRMQMPMAAQAVLLG
GNVRVGLEDNLWLDKGVLATNGQLVERASEILSRLGARVMTPAEGRIKMGLTKRG
>tr|A0A0Q0C6Y6|A0A0Q0C6Y6_PSEAP Uncharacterized protein OS=Pseudomonas syringae pv. aptata OX=83167 GN=ALO85_101878 PE=4 SV=1
MSRAVSCDPREGCYTGLRRGAATPALQKQKTVDNDKERWLWNHTPGTC
>tr|A0A0Q0C3B8|A0A0Q0C3B8_PSEAP UPF0312 protein ALO85_02956 OS=Pseudomonas syringae pv. aptata OX=83167 GN=ALO85_02956 PE=3 SV=1
MLKKSLAALALGTALLSAGQAMAADYVIDKEGQHAFVDFKISHLGYSFIHGTFKDWDGTF
SFDAAKPEASKINVDLKTASLFTNHAERDKHISSKDFLDVAKYPEAKFVSTAVKSTGEKT
ADVTGDLTLHGVTKPIVIKATFNGEGKDPWGGYRAGFNGTSTLNLNDFGIKGPGPTSQTL
DLDISFEGVQKK
>tr|A0A0Q0BWR8|A0A0Q0BWR8_PSEAP Anthranilate synthase component 1 OS=Pseudomonas syringae pv. aptata OX=83167 GN=trpE PE=3 SV=1
MLMNREEFLRLAAEGYNRIPLARETLADFDTPLSIYLKLADQPNSYLLESVQGGEKWGRY
SIIGLPCRTVMRVHGHHVSVTHDGVEIESLDVEDPLDFVETFKARYNVPTIAGLPRFNGG
LVGYFGYDCVRYVEKRLANCPNPDPLGVPDILLMVSDAVVVFDNLAGKMHAIVLIDPAQQ
DAYASGVAQLDELMHKLRQPITPRRGLDLDRPPAADPVFRSSFTQDDYEAAVDTIKQYIL
AGDVMQVVPSQRMSIDFSAAPIDLYRALRCFNPTPYMYFFNFGDFHVVGSSPEVLVRVED
NLITVRPIAGTRPRGATEEADRALEEDLLSDHKEIAEHLMLIDLGRNDTGRVSEIGTVKL
TEKMVIERYSNVMHIVSNVTGQLKSGLTAMDALRAILPAGTLSGAPKIRAMEIIDELEPV
KRGVYGGAVGYMAWNGNMDTAIAIRTAVIKDGELHVQAGGGIVADSVPVLEWEETLNKRR
AMFRAVALASQTAED
>tr|A0A0Q0C3J6|A0A0Q0C3J6_PSEAP Putative two-component sensor CbrA OS=Pseudomonas syringae pv. aptata OX=83167 GN=ALO85_03328 PE=4 SV=1
MPMSFSLTQMLLVSAAYLLVLFGVAWVSEHGLIPRWIIRHPLTYTLSLGVYASAWAFYGT
VGLAYQYGYGFLSSYLGVSGAFLLAPVLLYPILRITRTYQLSSLADLFAFRFRSTWAGAL
TTVFMLIGVLPLLALQIQAVADSIGILTREPVQDRVAVAFCALITLFTIFFGSRHIATRE
KHEGLVFAIAFESLIKLIALGGVGLYALYGVFDGPQQLELWLLQNQTALASLHTPLQEGP
WRTLLLVFFASAIVMPHMYHMTFTENLNPRSLVSASWGLPLFLLLISLAVPLILWAGLKL
GATTSPEYFTLGIGIAANSKSLALLAYIGGLSAASGLIIVTTLALSGMALNHLVLPLYQP
PAEGNIYRWLKWTRRGLIVAIIALGYGFYLMLGAQQDLANLGIVAFVATLQFLPGVLSVL
YWPTANRRGFIAGLLAGISVWAVSMLLPLIGNLQGFYIPWLNMIYVLDDTSWHMAAIASL
AANVLLFTLISLFTNASPEEAGAAEACAVDNVRRPQRRELHAVSPQEFATQLAKPLGAKA
AQKEVEQALRDLYLPFDERRPYALRRLRDRIEANLSGLMGPSVAQDMVETFLPYKSGSEK
YVTEDIHFIESRLEDYHSRLTGLAAELDALRRYHRQTLQELPMGVCSLAEDQEILMWNRA
MEELTGIVAQRVVGSRLETIAEPWKGLLTGFTSLPDEHLHKQKLGLDGQTRWLNLHKAAI
NEPLAPGNSGLVVLVEDLTDTQMLEDKLVHSERLASIGRLAAGVAHEIGNPITGIACLAQ
NLREEREEDGELKEISSQILEQTKRVSRIVQSLMSFAHAGAHQHSDEAVCLAVVAQDAIG
LLALNRRNFEVHFYNLCNPEHWAVGDSQRLAQVLINLLSNARDASPAGSAVRVRSEVSEH
TVDLIVEDEGSGISKAIMDRLFEPFFTTKDPGEGTGLGLALVYSIVEEHYGQITIDSPAD
PEQQRGTRIRVTLPRHVDATSTAN
>tr|A0A0Q0BVR4|A0A0Q0BVR4_PSEAP Phosphate transport system permease protein OS=Pseudomonas syringae pv. aptata OX=83167 GN=ALO85_00649 PE=3 SV=1
MTENTQYLSANVSAGESEGEKRAKRDHRHDLWFKRSLQAAAMLVLALLGCIALSTLWGGS
LAFQTFGFSFLTSTEWDVNAGKFGALVPIYGTLVTSFLALLIAVPVSFGIAIFLTEVAPP
WLRMPIASAIELLAGIPSIIYGMWGLFVFGPFMAEHLSPWITENLGALPLIGPMFQGPPL
GIGMLTAGIVLAIMITPFITSVMQEVFRTVPVALKESAYALGGTTWEVVWDIVLPYTRSA
VVGGVFLGLGRALGETMAVTFVLGNAHQFSASLMMPSSSIASVIANEFNEAYTDLHRSAL
IALGFLLFVVTFIVLALARLMLSRLSRKEGL
>tr|A0A0Q0BWA2|A0A0Q0BWA2_PSEAP Aerotaxis receptor OS=Pseudomonas syringae pv. aptata OX=83167 GN=ALO85_03016 PE=4 SV=1
MRNNQPVTQHERTFPAEQRLISTTDTRGTITYCNDAFVDISGYSEAELVGAAHNTVRHPD
VPPAVFEHMWTTLKSGQPWMGIVKNRCKNGDHYWVNAYVTPVLENRHVVGFESVRIKPTA
EQIRRAEALYTRLNKGKSAVPNRDKWLPVLQDWLPFILVSQLSFLIGVWLNSHWGFALAA
ALSVPLGLLGLSWQQRGIKRLLRLAEQTTSDPLIAQMYTDSRGPQARLEMSILSQEARMK
TCLTRLQDTAEHLNSQARQSNSLANASSIGLERQRVETEQVAAAINQMAATTQEVASHVN

RAADATQQANELTRRGRDIAGETREAIQRLSTSVGETGLTVTRLAKDSDEIGGVVDVIKG
IADQTNLLALNAAIEAARAGEMGRGFAVVADEVRQLAQRTAESTGQIHGLIAKLQQTATF
AVHTMDTGRRQAEEGVARVLEADQALVGISEAVANITDMTTQIATATEEQSAVAEEINRN
IATIASLADQTSDEARRSAVLSGELTDTANSQYSLVERFNR
>tr|A0A0Q0IQG5|A0A0Q0IQG5_PSEAP Trp repressor binding protein WrbA OS=Pseudomonas syringae pv. aptata OX=83167 GN=ALO85_01963 PE=3 SV=1
MSTPYILVLYYSRNGSTSEMARQVARGIELGGMEARLRTVPAVSADCEATAPEIPESGAL
YATLDDLKNCSGLALGSPTRFGNMAAPLKYFLDGTSNLWLTGALVGKPAGVFTSTASLHG
GQETTLMSMLLPLLHHGMLIMGLPYSESALLETTGGGTPYGPSHHAGADGKRALDRHETD
LCRALGQRLARTAVQLDAPRS
>tr|A0A0Q0D6B6|A0A0Q0D6B6_PSEAP Response regulator receiver:CheW-like protein:ATP-binding region OS=Pseudomonas syringae pv. aptata OX=83167 GN=ALO85_04477 PE=4 SV=1
MTPDQMRDASLFELFTLEADAQTQVLSAGLLALERNPTQADQLEACMRAAHSLKGAARIV
GVDFGVSVAHVMEDCLVSAQEGRLLLQAEHIDALLSGTDLLMRIATPGSDSIGQPDIDSY
VERLNTLLTSGAGAARTVSTPLPDPASDALLASAMLQLETSSGAAVPEPLVAASPAPAHP
AVMPEPEAPFTPLRERRVAEGGERVLRVTAERLNGLLDMSSKSLVETQRLKPLLAGMQRL
KRLQSSSDRALEVLGASFGEEGPSADVQKALEDARNLLSQAQQVLARHTAELDEFGWQSA
QRAQLLYDTALACRMRPFADVLNGQARMVRDLGRELGKQVRLQIEGEKTQVDRDVLEKLD
APLTHLLRNAVDHGIESPEQRVASGKDPEGLIRLQASHQAGLLVVELSDDGGGVDLERVR
QSIVDRKLSPAETAAQLSEEELLSFLFLPGFSMRDKVTQISGRGVGLDAVQHMVRQMHGS
VELQQWAGEGSRFRIEMPLTLSVVRSLVVEVGGEAYAFPLAHIERMRDLQPDDILQLEGR
QHFWDEERHVGLVSASQLLNRPPAQKAGETLKVVVIRERDAVYGVAVERFIGERTLVVMP
LDTRLGKVQDVSAGALLDDGSVVLIIDVEDMLRSVEKLLNTGRLERIDRRNRQVDQVSRK
RVLVVDDSLTVRELERKLLLSRGYQVSVAVDGMDGWNALRAEDFDLLITDIDMPRMDGIE
LVTLLRRDTRLQSLPVMVVSYKDREEDRRRGLDAGADYYLAKASFHDDALLDAVVELIGD
AQG
>tr|A0A0N8TAD5|A0A0N8TAD5_PSEAP Putative zink aminopeptidase OS=Pseudomonas syringae pv. aptata OX=83167 GN=ALO85_01563 PE=4 SV=1
MRARKAEAFERMRRDYRTLRDEQWAGDKRFDAWINSPMNNAKLLPFGLYDQWVPAFETLF
RQVNGDWQAFYHAVDKLGAMPVEARKAALRALMP
>tr|A0A0N8T9B8|A0A0N8T9B8_PSEAP Uncharacterized protein OS=Pseudomonas syringae pv. aptata OX=83167 GN=ALO85_101050 PE=4 SV=1
MVGALCGLDASGLECPTVLSCTVVDTLIRTVSTHFDCYF
>tr|A0A0Q0DIV1|A0A0Q0DIV1_PSEAP Site-specific recombinase, phage integrase family protein OS=Pseudomonas syringae pv. aptata OX=83167 GN=ALO85_04174 PE=4 SV=1
MAVDNLKNIDGTWYVRVAIPKDVRSEFGNRTEFIKSLKTGRKSEAMVLRTKDLHEFKNLI
AKARKKAVAEQEMGLIAGQPLNVDFAELMGNLEEIKQGLAEQKEARKKAFEASDYEDVQA
AREITSHHNLLNQRAEEKESIGEDLDPAMASLLSDLNDFQAQPTKSFKKSEELLERLETT
MQLGSFKGYAENADEKQQVSTLIAEPTKFIKKHPLNEKNFVDFHKYEVKRKVTLRSIDRH
VNRLNMLKDFLNKQKFEMDYESTRVFLESLECGDKTKLQYLCSFNAFYKFMFNNVDFRQE
YPINPFNNHAVNKIRRGARKEEVRKAFTKDQVKSLYSNALSQEKQRLADLIRLGCYTGAR
IEEICQIKLEDLIEVDGVYCIDIKQSKTNAGERLVPIHSELLPLVKRLEAESKDGYLLKT
NRGGKYGTKSKEMSSEFSAFKIALGYSRELVFHSFRHTMVTELERADIKNILVMSIVGHE
VGGSLSMTFDRYSDGPTPVAKKEAIEKVKFDI
>tr|A0A0Q0C2F6|A0A0Q0C2F6_PSEAP Arginine/lysine/ornithine ABC-type transport system OS=Pseudomonas syringae pv. aptata OX=83167 GN=ALO85_02068 PE=3 SV=1
MIELLQEYWKPFLYTDGVNVTGLAMTMWLLSASIVIGFCVSIPLSIARVSGKRWVRWPVQ
FYTYLFRGTPLYIQLLICYTGIYSLAAVREQPMLDAFFRDAMNCTILAFTLNTCAYTTEI
FAGAIRSMAHGEVEAAKAYGLTGWKLYAYVIMPSVLRRSLPYYSNEVILMLHSTTVAFTA
TVPDILKVARDANSATFMTFQSFGIAAVMYLTVTFILVGLFRLAERRWLAFLGPAH
>tr|A0A0Q0C7Y8|A0A0Q0C7Y8_PSEAP Urea carboxylase-associated protein 1 OS=Pseudomonas syringae pv. aptata OX=83167 GN=ALO85_01978 PE=4 SV=1
MNSPTHLCCDATIPAGEPFLAEVKAGQTVRILDLQGNQAVDTLFFSLSNPRERYDVQRTL
RRQNSVYLTTGSVLFSNLGQPMLTIVDDTCGRHDTLGGACAQESNTVRYALEKRYMHSCR
DNYLRACMHDGRLSKADIGPNINFFMNVPVTAEGGLTFEDGISAPGKYVELRAEMDVIVL
ISNCPQLNNPCNGYNPTPAQLLIRD

>tr|A0A0Q0C2G1|A0A0Q0C2G1_PSEAP Endonuclease III OS=Pseudomonas syringae pv. aptata OX=83167 GN=nth PE=3 SV=1
MNAAKRQEIFRRLHEDNPDPKTELAYTTPFELLIAVILSAQATDVSVNKATARLYPVANT
PQAIYELGVEGLSEYIKTIGLYNSKAKNVIETCRMLVELHNGEVPQTREALEALPGVGRK
TANVVLNTAFRQIAMAVDTHIFRVSNRTGIAPGKNVVEVERQLMKFVPKNYLLDAHHWLI
LHGRYVCQARKPRCGSCRIEDLCDYKAKTSDD
>tr|A0A0Q0D3N5|A0A0Q0D3N5_PSEAP Mannose-6-phosphate isomerase, type 2 / mannose-1-phosphate guanylyltransferase OS=Pseudomonas syringae pv. aptata OX=83167 GN=ALO85_01799 PE=3 SV=1
MIPVILSGGSGSRLWPLSRKQFPKQFLALTGEHTLFQQTLERLVFEGMQEPIVVCNKDHR
FIVNEQLAALNLETQAILMEPFGRNTAPAVALTAMKLVNEGNDGLMLVLPADHVIEDQKA
LQRALALATVAAERGEMVLFGVPANKPETGYGYIKSTADALLPEGVSRVSQFVEKPDEKR
AKEFVEAGGYYWNSGMFLFRASRFLEELKKHDPDIYDTCLLTLERSVQDGDALEIDSSTF
ACCPDNSIDYAVMEKTQRACVVPLSAGWSDVGCWSSLWEVNAKDANGNVTKGDVVIQDSR
NCMIHGNGKLVSVIGLDNIVVVETKDAMMIAHKDKVQGVKQMVATLNEQGRTETQNHLEV
YRPWGSYDSVDMGGRFQVKRISVKPGACLSLQMHHHRAEHWIVVSGTAQVTCDENVFLLT
ENQSTYIPIASVHRLRNPGKIPLEIIEVQSGSYLGEDDIERFEDIYGRSNALEAGVKTQT
IAR
>tr|A0A0Q0BZ74|A0A0Q0BZ74_PSEAP Putative transcriptional regulator FerR OS=Pseudomonas syringae pv. aptata OX=83167 GN=ALO85_00842 PE=4 SV=1
MAKPSDIADVCQAPVQAAEAPLPTDSALDDLIGYAMRRAQLKLFQNLIGRLSTHDLRPAQ
FSALAIIEQNPGLMQADLAKALAIEPPQVVPLLNKLEERALAVRVRCKPDKRSYGIFLSK
SGETLLRELKQIAVDSDLESTAALDQKERQDLLRLLKKVYQS
>tr|A0A0Q0C3U9|A0A0Q0C3U9_PSEAP High-affinity branched-chain amino acid transport ATP-binding protein OS=Pseudomonas syringae pv. aptata OX=83167 GN=ALO85_02744 PE=3 SV=1
MLQFENVSTFYGKIQALHSVNVEVRQGEIVTLIGANGAGKSTLLMTLCGSPRAHSGSIRY
MGEELVGLESSIIMRKSIAVVPEGRRVFARLTVEENLAMGGFFTEKEDYQEQMDKVLHLF
PRLKERFNQRGGTMSGGEQQMLAIGRALMSKPKLLLLDEPSLGLAPIIIQQIFEIVEQLR
RDGVTVFLVEQNANQALKVADRAYVLENGRVVMQGTGEELLVDPKVRDAYLGG
>tr|A0A0Q0BW09|A0A0Q0BW09_PSEAP UPF0114 protein ALO85_00029 OS=Pseudomonas syringae pv. aptata OX=83167 GN=ALO85_00029 PE=3 SV=1
MERFFENAMYASRWLLAPIYFGLSLGLLALCLKFFQEIFHVIPNIFSLAEADLILVLLSL
IDMALVGGLLVMVMISGYENFVSQLDIDEDKEKLNWLGTMDSSSLKMKVAASIVAISSIH
LLRVFMDATNIKPEYLMWYVIIHMTFVISAFAMGYLDKVTKH
>tr|A0A0Q0DZ39|A0A0Q0DZ39_PSEAP Glycoside hydrolase family protein OS=Pseudomonas syringae pv. aptata OX=83167 GN=ALO85_03338 PE=4 SV=1
MADFPAEAQNRIEDHGIIGDMRSAALIANTGSIDFCCWPDFDSPSIFTALLDTPDAGIFQ
LAPDLPDARRLQIYLPDTNVLQTRWISEDAVVEVTDLMPIGEDQDDLPRIVRRVQVRFGE
ATLRMRCRVRMDYSRADTTARVDGQDICFEAAGQPGMRLSATTPLRLEQHDAVAEFHMHK
GQTIEFILGGIDDSNVAAGRCSRYFDDTLKFWRRWSGQSHYTGRWREMVTRSALALKLLT
SRKHGGIVAAATFGLPETEGGERNWDYRYTWIRDASFTVYAFMRLGYTDEANAFMNWVRG
RLDDCCEQTEKLGILYALDGHAELPEEHLDHLSGYGGAQPVRIGNEAYKQVQLDIYGELM
DAVYLANKYGDAISHDGWQHVVRQVDYVCEHWNDRDVGIWEMRGGDQHFLHSRLMCWVAL
DRALRLAFKRSLSGPFERWTKVREEIHDDIWKNFWNDDLGHFVQHKGSCNLDASMLLMPL
VRFVGANDPKWLATLDAIEGALVRDGMVFRYRNDDDHDDGLAGEEGAFVACSFWYVECLA
RAGRVEQAHLEFEQLLRYANPLGLYAEEFDAHAHHLGNTPQALSHLALISAASFLDRKLG
GGQALWQP
>tr|A0A0N8T980|A0A0N8T980_PSEAP Zona occludens toxin OS=Pseudomonas syringae pv. aptata OX=83167 GN=ALO85_00912 PE=4 SV=1
MAIKIHHGPNGSYKTSGAVWDDAVPAAKAGRMIVTNIRGMSSEKFHSLFPDLPDTFDLLY
IDHESQEGMERIRTWFHWVPRNAFMIFDEAQTLFPQKWTDKVLDRFDFPGGMDAAKAADR
PMNFLDAWTRHRHWNWDIILTTPNIKYVHTDIRQTSEAAYQHSNLMLLGKWLKFLVAKDY
KEAMHSAQENRAPTDGSNIVALRKIDKRVFKLYDSTATGQHRDTMAGKNALASPRVVILL
GVLVAIFGTIYWRNGASAFSNPLSVGSPKPVAPVSQAPVPQGPAKAPNVASDILPNKQVV
PLPSVTSDPFGSYEIVIKGSITSETRGTIFVFELSKGDRSFTQTSRDMLSAGYAIFPHGG
CVAELHYRGEQRTVACLGSSSSGGGEKWLGAERRAAAAGPNPSSVHSSTSTPGTQTPASK
GASFTVVADSSRTPRTIN
>tr|A0A0Q0CXB4|A0A0Q0CXB4_PSEAP UPF0229 protein ALO85_02978 OS=Pseudomonas syringae pv. aptata OX=83167 GN=ALO85_02978 PE=3 SV=1

MSYVIDRRLNGKNKSTVNRQRFLRRYRDHIKKAVEEAVSRRSITDMEHGEQISIPGRDID
EPVLHHGRGGKQTVVHPGNKEFTTGEHIARPQGGAGGKGPGKAGNSGEGMDEFSFQITQE
EFLEFMFEDLELPNLVKRNLTGTDTFKTVRAGISNEGNPSRINIIRTLRSAHARRIALSG
SSRAKLREATAEFERMKREEPDNFGDLQELEVEIDRLKARIRRVPYLDTFDLKYNLLVKQ
PNPSSKAVMFCLMDVSGSMTQATKDIAKRFFILLYLFLKRNYDKIDVVFIRHHTSAREVD
EEEFFYSRETGGTIVSSALKLMQEIMAARYPSSDWNIYAAQASDGDNWNDDSPICREILT
KQIMPFVQYYTYVEITPREHQALWYEYERIGEDFADTFAQQQLVSAGDIYPVFRELFQRR
LVS
>tr|A0A0Q0DL34|A0A0Q0DL34_PSEAP Uncharacterized protein OS=Pseudomonas syringae pv. aptata OX=83167 GN=ALO85_03114 PE=4 SV=1
MKVFVVSKLSSSTASYQLICQLARIQHEHRKTVPVLVIDDRYNLSELPVSDSSTEHALFC
LDDFLHDNGVSGIVANRIAQHMDKVESDWVLIDFGQHDSATCMDFIQKIARYNYLSARLR
KRNLVIMVPAMHLGIYSRTLNASFNNLPASTTASVRSVQIGRLLMVTRLERVLMSIPRLA
GVLRRMRRVALRVFRRVPRSH
>tr|A0A0Q0C3E4|A0A0Q0C3E4_PSEAP Putative integral membrane protein OS=Pseudomonas syringae pv. aptata OX=83167 GN=ALO85_03266 PE=4 SV=1
MNTRAYGVAALAGFVGGNVSSFIKWGTEIPFPPRTPDRPVPPVEMLDSLGINAQQLTYVY
SEHVVNYGSALVHHGFSIFFAMFYCLLVLRYPRTALWQGLGFGLLMTLGFHGVILPAFHW
APPLWELPASELLSETFGHLLWMWVIEVIRRDLQQRWLPHTRPA
>tr|A0A0Q0FB56|A0A0Q0FB56_PSEAP Type VI secretion system protein EvpE OS=Pseudomonas syringae pv. aptata OX=83167 GN=ALO85_02259 PE=4 SV=1
MSSQHKLLPSLLDRLLDDRPHQSVESSSQRLSSLADYKASIVRDLEILVNTRQSLVAGEL
EGFANLSGTILDYGMPDFTSRSVLDPQDRLLIQRQLEKAISVGDRRFRSVKVQLLAQQTG
QRMLTFRVDAVLRLQDISRQVSFDAVLQVNTQEYKVQNLN
>tr|A0A0Q0BZH8|A0A0Q0BZH8_PSEAP Alkanesulfonate monooxygenase OS=Pseudomonas syringae pv. aptata OX=83167 GN=ssuD PE=3 SV=1
MNVFWFLPTHGDGHYLGTTKGARPVTLNYLKQVAQAADDLGYYGVLIPTGRSCEDSWVIA
SALVPLTERLRYLVAIRPGIISPTVSARMAATLDRLSGGRLLINVVTGGDPDENRGDGSF
LDHSERYEVTDEFLKIWRRVLQGEAVDFEGKHLRVQNAKALYPPIQKPYPPLYFGGSSDA
AHDLAADQVDVYLTWGEPPAAVAQKLADVRERAARKGRTVKFGIRLHVIVRETSEEAWKA
ASTLIEHISDDTIAAAQKSFSRFDSEGQRRMAALHDGRRDNLEIAPNLWAGVGLVRGGAG
TALVGNPEEVAARIKEYADLGIESFIFSGYPHLEEAYRFAELVFPLLPEPYASLAGRGIT
NLTGPFGEMIANDLPPQAK
>tr|A0A0Q0CWA1|A0A0Q0CWA1_PSEAP Short-chain dehydrogenase/reductase SDR OS=Pseudomonas syringae pv. aptata OX=83167 GN=ALO85_01877 PE=3 SV=1
MTTPSTVLITGASSGIGAVYAERFARRGHNLVMVARDKARLDALATRLREENGVAVEVIQ
ADLTQSADLTALETRLREDASIGVLINNAGIAQSGGFVQQSAQSIDKLVALNIVALTRLA
AAVAPRFAQSGSGSIVNLGSVVGFAPEFGMSVYGATKAYVLFLSQGMNVELAPKGVYVQA
VLPAATRTEIWERAGIDLNSIAEVMDVEELVDAALVGFDRREQVTIPPLHVAGRWDSLDE
ARLGLMSDIRQAQAAERYRPQA
>tr|A0A0Q0D4T9|A0A0Q0D4T9_PSEAP FAD dependent oxidoreductase OS=Pseudomonas syringae pv. aptata OX=83167 GN=ALO85_02795 PE=4 SV=1
MSKQKIVIVGGGVIGLLAAFNLASEQVSVVLLDRSGVGQESSWAGGGIVSPLYPWRYSLA
VTALAHWSQDFYPHLAERLLAQTGIDPEVHKTGLYWLDLDDEQSALEWAAREKRSLNRVD
ISAVNDAVPVLGEGYSRAIYMADVANVRNPRLVKSLKAALLAMPNVEIREQCEVSGFTRE
GSRISGVQTPAGDITGDQVILAAGAWSGELLKTLGLDLPVEPVKGQMILYKCASDFLSSM
VLAKGRYAIPRRDGHILIGSTLEHEGFDKTTTQAALESLKASAVELLPPLAHAEPVAQWA
GLRPGSPEGIPFIGPLEGFDGLWLNCGHYRNGLVLAPASCQLITDLLLEREPIIDPAPYA
PTGRLAG
>tr|A0A0Q0CFG5|A0A0Q0CFG5_PSEAP Amidase OS=Pseudomonas syringae pv. aptata OX=83167 GN=ALO85_02087 PE=4 SV=1
MESALLGEPDARQHANNLRLLAALPRGAGIERRERIVDYEIVELAALLREGNPQRLTAEQ
VLVEYWSRITRFNGPQETYGDNGKYNAFVRLEDFSTLLEQARLADQWLTTPDDQRGPAPP
LCGIPFGIKDSFAIQGLESKNGTQAFSGNLALRDATCVARLRAQGALIIGHTICSELSTH
TVGQFAGNAWDPIRTPGGSSQGSGVAPIARLCAAALGEETAGSIIIPAAANGASAIKPSL
GLVSGAGVMPLRSGWDVVGPMARSIRDASLILSIIAGPDGENDPQSLSAPSIGEQLSITP
TPGSLPLQGLTIGIPQTDWMSSPGQPPAQSYDEDYSAAFIRFKDQLETLGARVIDFPWLD
AEILENIPYSSPEPIHDVLDSDGSILMRVNAQAVTSYANQLETRHWSAVRDFADVLENQD
DRDYLLRRYPELFDQVASLIPVGIRLEAENRRRLQQGHFEKALNDYQIDFMMVLPLGAHV
GERAGALVRTLPVQRMYFDMPNALAWPMLTLPIGHGATGVPKQLPVTAAFWGRRFSEPLL

VQAAIDFQTHYCEYHTKAPPDPDFTSPVAFHLWTLDEIPWQYSADPLVIAEGRRKKS
>tr|A0A0Q0C4Z1|A0A0Q0C4Z1_PSEAP M24/M37 family peptidase OS=Pseudomonas syringae pv. aptata OX=83167 GN=ALO85_04094 PE=4 SV=1
MLRFIAPLFALLLCLPAHADSFITRALDKPVPGGVAVIDLGTGAQAPTAIYQGKPVLVVK
EQGTRWLAIVGIPLTVKPGTQQVTSGGRTLNFTVGSKKYPEQHITLKNKRQVNPNPEDNK
RIEGELAEQLRAYRSFSPGTPSNLILDKPVNGPLSSKFGVRRFFNGEERNPHSGLDFAVP
AGTPIKSPAAGKVILTGNYFFNGNTVFVDHGQGFISMFCHMSKIDVKVGDLVPRGGVVGK
VGATGRATGPHMHWNVSLNDARVDPAIFIGAFQP
>tr|A0A0N8TAC4|A0A0N8TAC4_PSEAP Regulator of RNA polymerase sigma subunit, Rsd/AlgQ OS=Pseudomonas syringae pv. aptata OX=83167 GN=ALO85_01574 PE=3 SV=1
MPLKGREKTMLESCQNAQERWGGVNKLIDRWLQSRLELVEAYDALGDTPEALAADLEGLQ
NFCGILVDYVSAGHFEVYEQLGDEARAFNDERGLELADTIYPRLDVITKFALTFNDRCEK
GDCSDAAVVAREFNQLGQLLHERFELEDCLIEVLHNSHKEEIAAQA
>tr|A0A0Q0C3F2|A0A0Q0C3F2_PSEAP Putative translation initiation inhibitor OS=Pseudomonas syringae pv. aptata OX=83167 GN=ALO85_100116 PE=4 SV=1
MMETPMTQRDVVFPAGRQALYERNRYSPALRSNGFLFVSGQVGSREDGSPEPDLEKQVRL
AFNNLNAILLAAGCTFDDVVDVTVFIVDPEARFERIWKVIPEFWGDAPHPTLTGVGVTWL
YGFDFEIKVIARLPEAVEQ
>tr|A0A0Q0DXZ7|A0A0Q0DXZ7_PSEAP Phenylalanine--tRNA ligase alpha subunit OS=Pseudomonas syringae pv. aptata OX=83167 GN=pheS PE=3 SV=1
MENLDALVSQALEAVQSAEDINALEQIRVHYLGKKGELTQVMKTLGNLPAEERPQVGALI
NVAKERVTEVLNARKASFEQAELTARLAAECIDVTLPGRGQTSGGLHPITRTLERIEQFF
THIGYGIAEGPEVEDDYHNFEALNIPGHHPARSMHDTFYFNANMLLRTHTSPVQVRTMES
QQPPIRIVCPGRVYRSDSDITHSPMFHQIEGLLVDRDINFADLKGTIEEFLRVFFEKELA
VRFRPSYFPFTEPSAEVDMECVMCSGKGCRVCKQTGWLEVMGCGMVHPNVLRMSGIDPEE
FQGFAFGMGVERLAMLRYGVNDLRLFFDNDLRFLAQFR
>tr|A0A0Q0DV07|A0A0Q0DV07_PSEAP Binding-protein dependent transport system inner membrane protein OS=Pseudomonas syringae pv. aptata OX=83167 GN=ALO85_01466 PE=3 SV=1
MHSIFEFGKSLRTPRFVKPGARTPYWADGICLAILTLFIGLFIYGLGQMMQPLTVLNSSP
MSLDPARLPEYALRTTLRMFIALGASLIFTFVVATLAAKSRKAEQVIIPALDILQSVPVL
GFLTFTVTFFMGLFPGRQMGVECAAIFAIFTSQAWNLAFSFYQSLRTVPSDLNEVSRQFG
LSAVLRFTRLELPFAIPGLVWNMMMSMSGGWFFVVACEAISVGDTTVNLPGIGSWLALAI
EQKNLAAIAWAVGAMALVILAYDQLLFRPIVAWADKFRFEQTASSKRPRSWMYSLLGRSR
VAPMLGSVLAAPWKCLMLIDWRVFSRLWKVKLTPGVARVLDYAWLGLVMAACLAGSVYLF
RFIEASLGASDMVTAFGLGLATMVRVIVLIVIASLIWVPVGIWIGLRPVWAERLQPIAQF
MAAFPANVLFPFAVIAIVGLHLNPDIWLSPLMVLGTQWYILFNVIAGASALPTDLREAAT
MFNMRGWQWWRRVALPGVFPYYITGALTASGGSWNASIVAEAVSWGDQHLEAAGLGSYIA
NATQAGDFPRVALGIVVMSVFVIAFNRLLWRPLYGFAERRLSLV
>tr|A0A0Q0C8F9|A0A0Q0C8F9_PSEAP Alanine transaminase AlaT OS=Pseudomonas syringae pv. aptata OX=83167 GN=ALO85_05398 PE=4 SV=1
MQAGDSSSIFGIITMQFNKSNKLANVCYDIRGPVLKHAKRLEEEGHRILKLNIGNPAPFG
FEAPDEILQDVIRNLPTAQGYSDSKGLFSARKAVMQYYQQKQVEGVGIEDIYLGNGVSEL
IVMSMQALLNNGDEVLVPAPDYPLWTAAVALSGGSPVHYLCDEQANWWPDLEDIKAKITP
NTKAMVIINPNNPTGAVYSREVLLGMLELARQHNLVVFSDEIYDKILYDDAVHICTASLA
PDLLCLTFNGLSKSYRVAGFRSGWIAISGPKHNAQSYIEGIDILANMRLCANVPSQHAIQ
TALGGYQSINDLILPPGRLLEQRNRTWELLNDIPGVSCVKPMGALYAFPRIDPKVCPILN
DEKFVLDLLLSEKLLVVQGTAFNWPYPDHFRVVTLPRVDELEQAIGRIGNFLKGYRQ
>tr|A0A0Q0FDA1|A0A0Q0FDA1_PSEAP Lon protease OS=Pseudomonas syringae pv. aptata OX=83167 GN=lon PE=2 SV=1
MSRPIGRRRGEIMKTTIELPLLPLRDVVVYPHMVIPLFVGREKSIEALEAAMTGDKQILL
LAQRNPADDDPDEKALYSVGTIATVLQLLKLPDGTVKVLVEGEQRGSVERFIEVDGHYRA
DVALIEEVDAPDRESEVFVRSLLAQFEQYVQLGKKVPAEVLSSLNSIDEPGRLVDTMAAH
MALKIEQKQEILEIIDLSARVEHVLALLDAEIDLLQVEKRIRGRVKKQMERSQREYYLNE
QMKAIQKELGDGDEGHNEIEELKKRIDAAGLPKDALTKANAELNKLKQMSPMSAEATVVR
SYIDWLVQVPWKAQSKVRLDLARAEAILDADHYGLDEVKERILEYLAVQKRVKKIRGPVL
CLVGPPGVGKTSLAESIANATNRKFVRMALGGVRDEAEIRGHRRTYIGSMPGRLIQKMTK
VGVRNPLFLLDEIDKMGSDMRGDPASALLEVLDPEQNHNFNDHYLEVDYDLSDVMFLCTS
NSMNIPPALLDRMEVIRLPGYTEDEKINIAVKYLSPKQIQANGLKKGEISFDEEAIRDII

RYYTREAGVRGLERQIAKVCRKAVKEHAMEKRFAVQVTADMLEHFLGVRKFRYGLAEQQD
QIGQVTGLAWTQVGGELLTIEAAVVSGKGQLIKTGSLGDVMVESITAAQTVVRSRARSLG
IPADFHEKHDTHIHMPEGATPKDGPSAGVGMCTALVSALTQIPVRADVAMTGEITLRGQV
LAIGGLKEKLLAAHRGGIKTVIIPEENVRDLKEIPDNIKQDLQIKPVKWIDEVLQIALQY
APEPLPDVATDLVAKEEKRESDSKDRISTH
>tr|A0A0Q0DN10|A0A0Q0DN10_PSEAP Uncharacterized protein OS=Pseudomonas syringae pv. aptata OX=83167 GN=ALO85_03186 PE=4 SV=1
MTYNWDLIERLLHDVQNNGTPSTSTEFETLLNRSYIEPRPREEGGDGSTYMLTKRGASLL
ALIDSSIPGNDHPRQVLNEQAGDPLDPALFDTIAKKPQIA
>tr|A0A0Q0FTD7|A0A0Q0FTD7_PSEAP Uncharacterized protein OS=Pseudomonas syringae pv. aptata OX=83167 GN=ALO85_03653 PE=4 SV=1
MGTVLPAIAPQTLYVTIRRDELRTLKEEREQLQKKVAHLSSILQQSQLSAPGKMLQA
>tr|A0A0Q0BXR6|A0A0Q0BXR6_PSEAP Uncharacterized protein OS=Pseudomonas syringae pv. aptata OX=83167 GN=ALO85_101743 PE=4 SV=1
MPTLEHGHDSVRPDASRYTRSATYLLTIVPHAPVECLA
>tr|A0A0N8T7Z6|A0A0N8T7Z6_PSEAP TetR family transcriptional regulator OS=Pseudomonas syringae pv. aptata OX=83167 GN=ALO85_00534 PE=4 SV=1
MKTTDDSVAVASKGRKNNPEKNREDILQAAVAEFVAHGLSGARVDAIAERTKTSKRMIYY
YFGSKEQLYVEVLEKLYGAIRNTESQLNLSELEPVEAIHRVVEFTFDHHDSNVDFVRIVC
IENIHNGENVKQSESIHAKSQNIIRALDGILRRGEASGLFREGVHPVDLHLMISSFCFYR
ISNRHTFSEIFQIELWSEEVKQRHKAMICDAVLRYLKR
>tr|A0A0Q0CED9|A0A0Q0CED9_PSEAP Uncharacterized protein OS=Pseudomonas syringae pv. aptata OX=83167 GN=ALO85_02405 PE=4 SV=1
MSDTATDAVGLLARITQQSERVAMDSVLGTPVIRLGLITTLYFRNGHTVEMKRRVEACFL
RFYEAFKTRLKWQLFKRMRRLSGSGFASTRRQIVESPPDEQFIWSIASATQAEVATYSLF
VMNTSEGQADNDRSCLKMVLPWSYLTEPDGLKQYEEWIRYLSSELQAEHGFGGLACVLPC
DGHQYLPREFRLAQDFSGLMVDPGPHIESLRLLDRIKGVSWYTVLGDAFVRQLGGSDKMR
GEMSAHSEIVFHTYRNGLIIRAGEVPELGGRGLPPPQSYVTVNRMIKPIRLQDTGNLHPY
LVPGIGFTDETTAQWYARFDEKPLPPVNAGQACPRSGNWFSSAQSGSRRHFDEGELMPAF
AHIKSKKTQWFWAGMHS
>tr|A0A0Q0IA30|A0A0Q0IA30_PSEAP Uncharacterized protein OS=Pseudomonas syringae pv. aptata OX=83167 GN=ALO85_02332 PE=4 SV=1
MTSPDQTVLCTNRYDPLDRLAPRYFIQPFADCFVKRGNFIGWYWRCHFSGLNYKIKFVGP
IQC
>tr|A0A0Q0D8I7|A0A0Q0D8I7_PSEAP Putative permease OS=Pseudomonas syringae pv. aptata OX=83167 GN=ALO85_01929 PE=4 SV=1
MPTFSARQVLIASWILVFGGLLLVLPFKLLPSLLAGLLVFELVNMLTPKLQRLISGERAR
WLAVALLGTLVVSLLTLIFAGAISFVLHEAENPGASLDKFMTLVDRARGQLPPFIDAYLP
ASAAEFQVSLGQWLQKHVGELQLIGKGAAHMFVTMLIGMVLGAIIALQRISDVHARKPLA
AALFDRLHLVSRAFRNIVFAQIKISLLNTAFTSVFLAIVLPLCGIHLPLTKTLIIMTFLL
GLLPVVGNLMSNTLIFIVGMSISIWVALAALGYLIVIHKVEYFLNARIVGGQINAKSWEL
LLAMLLFEAAFGLPGVVAGPIYYAYLKSELQKEGLV
>tr|A0A0Q0D6G1|A0A0Q0D6G1_PSEAP Sensor histidine kinase OS=Pseudomonas syringae pv. aptata OX=83167 GN=ALO85_05120 PE=4 SV=1
MTLKHRYWRITLYVLLILAGAALCAGLAMRQAQRHAMSEDAARAEGQLALYANTLHTLIE
RYRALPSVLALDPEIRAALSGPVTGEVQNALNLKLEKINSAAHSSTLELLDRHGLAIGAS
NWRTPNSYVGHNYAFRPYFLQTRAKGTGRFYAVGVTTGIPGYFLSSAVLNDTGGFMGAMV
VKLEFPSLEQEWDQGEDLLLVSDEKGIVFIANQPGWRYRELLPISMEDRATLLRTRQYDK
QVLSPLRSRLIDSFSENSHLSRVDGPYGTTDYLWQSLPLTDENWTLHLLLRKPPPSSEDVR
NAGLAAAGIWLALVFLSLFLYQRWRLARLRERNRDELERLVQARTRDLQTAQDGLVQSAK
LAALGQMSAALAHEINQPLTAQRMQLATLRLLLDQGRIDEACKALVPVDQQLTRMAALTS
HLKTFARKSPSGLRERLDLATVVDQALLLLDPRLREEHVDCVLHLARPAWVRGDAIRLEQ
VMINLLRNALDAMRECRSRHLEVRIDAVDNYWRLSVRDNGSGIAAENLPSIFDPFFTTKP
VGDGLGLGLAVSYAIIHELGGQLTAQNYADGAEFWFSLPNDFLEA
>tr|A0A0N8T8W1|A0A0N8T8W1_PSEAP Chemotaxis protein OS=Pseudomonas syringae pv. aptata OX=83167 GN=ALO85_00442 PE=4 SV=1
MDPTDLGPGTATWLGGTGTILLGGFLWLRKFLSRDATDRAMDNADIGTVRRLNELLDSER
QARKEAEARADQFAKERNELAAAVGRMEGKIEALTSHIVQLTDKVTTQSAEIARLRSQLG
GANDAQMRN

>tr|A0A0Q0DJA9|A0A0Q0DJA9_PSEAP Alpha/beta hydrolase family protein OS=Pseudomonas syringae pv. aptata OX=83167 GN=ALO85_01879 PE=4 SV=1
MSDIIKRLRLLTLRGTFTLTSRILPGITGRYLARRFVTPQASGRAKAAEILSTATDVQTG
SLELDGIRIATYVWGDPSSQPYVLLAHGWSSYAMRFIGWVPLLRSMGYAVVGFDQPAHGL
SAGSISHMTQFVAVQQHVGRHFGKPSAVIAHSLGASSIVFAQEQSWRPDRFILIAPFLAP
TDSVLQLFDSVGVAHTVFKPFEGVLHSLTGRRFADYDASSRLPLLDRPALVIHDRRDRET
PWEKGARFAELWPGARLFTTEGLGHNRLIDHPSVITEAMKFLTPDHR
>tr|A0A0Q0DI65|A0A0Q0DI65_PSEAP Extracellular solute-binding protein OS=Pseudomonas syringae pv. aptata OX=83167 GN=ALO85_03039 PE=4 SV=1
MNKLFAASLLAAGLAFASAAQAAPTLLNVSYDVMRDFYKDYNSAFQKHWKDEKHEDVTVQ
MSFGGSSKQARSVIDGLPADVITMNMATDINALADNGKLVPEDWGSRLPNNSAPFTSATV
FIVRKGNPKAIKDWPDLIKDGVEVIVPNPKTSGNGRYTYLSAWGYTLKKGGDEAAAKKFV
GDLFSHVPVLDTGGRGATTTFMTNQIGDVLVTFENEAEMIAREFGRDQFEVVYPSVSAEA
EPPVSVVDKVVDRKQSRALAEAYLKYLWSPEGQEIAAKNYLRPRDPQVLAKYTDRFPKVD
FLSVEKTFGDWRTVQKTHFIDGGVFDQIYPGK
>tr|A0A0Q0D3Q4|A0A0Q0D3Q4_PSEAP Deoxyribodipyrimidine photolyase OS=Pseudomonas syringae pv. aptata OX=83167 GN=ALO85_01717 PE=3 SV=1
MQLIWLRSDLRAQDNTALTAAMERGPTLAVYLLSPAQWRSHDDADCKVDFWLRNLVELEK
ALGALNVPLLIREADSWDQAPEVLAKLCAQFKVEGLHLNQEYGINETRRDQAVQKAMEKA
GVHFNSYLDQLLFKPGSILTKTGNYFQVFTQFKKVCYTRLHEAMPHPVRTPKAQPAQSIK
SDAVPDQVKGFATPSKTLRDLWPAGEAEARRRMDAFADEQISYYKTERDFPAKPGTSQLS
AYLAAGVISPRQCLHAALASNQGEFETGDVGTVTWINELLWREFYKHTLVGYPRVSRHRA
FRPETEALKWRDAPEELRAWQEARTGLPIIDAAMRQLLETGWMHNRLRMVVAMFLTKNLL
IDWREGERFFMRHLIDGDLASNNGGWQWSSSTGTDSAPYFRIFNPLSQSERFDPEGVFIK
RWLPELADMNKKQIHDPSLIGDLFGAADYPKPIVDLSKSRARALAAFKALPARQPAAGVE
HG
>tr|A0A0Q0DQ57|A0A0Q0DQ57_PSEAP Ferric iron reductase protein FhuF OS=Pseudomonas syringae pv. aptata OX=83167 GN=ALO85_01972 PE=4 SV=1
MSLPLFAGPFARFGRTLLTADDARPLMTLPDLLRPERLDQLLLTVYGPQLMPDQLPVLVS
QWAKFYFMQLIPPVLVASLVHDWHWPLQLGQVALALDERGVPSGVRLAGEGRVWRGMPVD
PFQRFAGLLDDNLQPFITSLSAYGGLSAAVLWSSAGDYLEGCLAQLATCSDASLAAGRAL
LSEKKRPDGRANPLFQAVRYVPQAQGGEPRRQRRVCCLSHRVEWVGRCEHCPLPG
>tr|A0A0Q0BUE6|A0A0Q0BUE6_PSEAP Putative phosphate starvation-inducible ATPase PhoH OS=Pseudomonas syringae pv. aptata OX=83167 GN=ALO85_01324 PE=4 SV=1
MNAPIEPHRFTLEPFEAHRFANLCGQLDEHLRLIEQRLDIEIRNRGNQFELIGDPKQNHS
AENLLRRLYRETKGTELTPDMVHLFLQESGVDGLDNHPAAEVGVSLRTKKGMIRPRGLNQ
QRYVKEVLSNDINFGIGPAGTGKTYLAVACAVDALEREQIRRILLVRPAVEAGEKLGFLP
GDLAQKIDPYLRPLYDALYEMLGFEYVAKLIEKQVIEVAPLAYMRGRTLNNSFIILDESQ
NTTVEQMKMFLTRIGFGSTAVITGDITQVDLPKGTRSGLTHVIDVLKDVPGISFTHFKPK
DVVRHPLVQRIVEAYERFDDRLSDGPSGNNSYPRDKHRDA
>tr|A0A0Q0IHR8|A0A0Q0IHR8_PSEAP Uncharacterized protein OS=Pseudomonas syringae pv. aptata OX=83167 GN=ALO85_02224 PE=4 SV=1
MGMAVGKRVGHRFIVLEARLMPVISKWIERIRMFWPASRRKSGLLAPPIIAELLPDVPGG
EPNLLPSSAWLAPLRVDFSMWQNSGPSPGDPEYLRVYWNDMLLEEKIWTEPVQPADLFSM
IAQQYLIEGQHRLRYSVETANQVQTESETLVFVIDKTAPVFEDEGALIFPEEIISDGLTA
AWLDTHDDTLLAEVPAYFSPSPGDIITWYWSSTPTGSEHTGTLTLEASDIGSAINIAFGR
QLILESGDGIRYASYRLKDRSGNAGPRALAVSLLVCAQPVPRVLPPPRVQEATGSASASR
LDPVDVFQGATVSIPEDAVIFPGETVRVQWAEPGSVGSFLTEIADSRLFSIPPTQVAQHF
GKSIPVYYEVFEKSADSPHISDRHTLSIMGMTGFPVVQCDKVSGGRLSLHDIAEGGYARF
TLDSWSFMGTDQFVSVEVHGLSSADNALLVVSVLDEYPVPVVDDEIDAGHISKTDLNRFM
IGTQLDVRVRVSFDQTLSWQPFPSLRATLYA
>tr|A0A0Q0C3M5|A0A0Q0C3M5_PSEAP Sensor histidine kinase OS=Pseudomonas syringae pv. aptata OX=83167 GN=ALO85_01464 PE=4 SV=1
MMRMREPESYGKAAMAVRLIALLMLALVIAVADTLTDLEIAVGVFQIVVVLLAVRFLPAT
GVIAMALLCMVLTVISYEMTTSRGSEASGLINCIISLAAIAMTTWLALRMALAIRSVHEA
RSQLARIARVNQLGELTASIAHEVNQPLSAIVTSGNACQRWLATEPVNLEKARQAVDRMI
SDANRAGDIIVRVRALAKRSSTHKEWISVADTVAEIVALAHSEIEGQGVALLVDVPEGLP
PLLADRVQIQQVLLNLMLNGVDAMKKLEADQAQLEIRVGWHDGGDIGFAVSDSGIGVQPE
NIHRLFDAFYTTKEEGMGIGLAISRSIIEAHGGRIWVTPNPAIGATFHFTLPTGKMET

>tr|A0A0Q0FG01|A0A0Q0FG01_PSEAP Uncharacterized protein OS=Pseudomonas syringae pv. aptata OX=83167 GN=ALO85_01029 PE=4 SV=1
MHPLFINIKKAILDIIEDQLTNNEEAPDSEIWNILVDELDLTVEQADAAIAMRPRFRCEI
FIAGQSPLYQTNTVTFDPLEKKLVAAEPLSFDQILEIYTMLLKSRPGYRLKLGAHWAAGL
NSEGELYCTHLNPCDKNVMFEVYDFDRDAFVDGRWQYETEEQTRAAIDKPEFIR
>tr|A0A0Q0C7A5|A0A0Q0C7A5_PSEAP Acetolactate synthase OS=Pseudomonas syringae pv. aptata OX=83167 GN=ALO85_02993 PE=4 SV=1
MRHIISLLLENEPGALSRVVGLFSQRNYNIESLTVAPTEDPTLSRLTLTTVGHDEVIEQI
TKNLNKLIEVVKLVDLSESAHIERELMLVKVKATGAQRAEIKRTTDIFRGQIVDVTASVY
TVQLSGTSDKLDSFIQAIGTAAILETVRSGVTGIARGDKVLSI
>tr|A0A0Q0BUD1|A0A0Q0BUD1_PSEAP Putative inner membrane peptidase OS=Pseudomonas syringae pv. aptata OX=83167 GN=ALO85_00497 PE=4 SV=1
MDFVVDYASFLAKTVTLVIAIVVVLVAIASMRGKGRRKSSGQLQVTRLNDFYKGLRERLE
QSLLDKERLKTLRKEQGKALKKEKKQAEPKARVYVLDFDGDIKASATESMRHEITALLTL
ATDRDEVVLRLESGGGMVHSYGLASSQLARIRQAGIPLTVCIDKVAASGGYMMACIGNKI
ISAPFAILGSIGVVAQLPNVNRLLKKHDIDFEVLTAGEYKRTLTVFGENTEKGREKFQQD
LDITHDLFKNFVASYRPQLSIDEVATGEVWLGMAAVDKLLVDELKTSDEYLAERAKDADV
FHLHYVQRKSLQERMGMAAATSADQLATKWWGRLTQQRFW
>tr|A0A0Q0C5Z7|A0A0Q0C5Z7_PSEAP Uncharacterized protein OS=Pseudomonas syringae pv. aptata OX=83167 GN=ALO85_01933 PE=4 SV=1
MSVMTVTDSTFQKDVIEASNQKLVVVEFSTKGKVNKRGEENASARMDTIFNALEEKYAVK
VSLVRGEIELDSDLKDSLSPVTSTEYQINHGPTMVFFKGGARAREDLVGLQTKERMTQIL
DELLPAS
>tr|A0A0Q0C8N6|A0A0Q0C8N6_PSEAP Regulatory protein LysR OS=Pseudomonas syringae pv. aptata OX=83167 GN=ALO85_03388 PE=3 SV=1
MDLFQAMRVYVKVVEAGSLTAAAQACSISTTMVGNHLRALEERLGVSLLQRTTRRQRLTE
FGTAYYERCLEVLGLVAESEQLAEQAHGDPTGTLRITAPLTFGSEVLAPALVEFSRRFPE
VKLDLVLTNQRLDMIDNGFDVAIRLGNIPESSPLIARPLQDYTLTICAAPDYLARHGTPT
HPEDLRQHNCLAFAYPAGDEWRTAGTQWRMTGADGDVLVEVSGSMVANTSAGLYQAARAG
MGVVMLPDALVQRDLQEGRLVALFPDYHLPRRPTNLLYVQDRYRLPKLRSFVEYATGLWG
R
>tr|A0A0Q0C6W2|A0A0Q0C6W2_PSEAP Uncharacterized protein OS=Pseudomonas syringae pv. aptata OX=83167 GN=ALO85_00798 PE=4 SV=1
MPSSKREQAQTDRRLVSALTHACETAKAEMPGFCWLTHEVDYAAFPASLQVVWVFDTLAD
KNAALERGLDKRMIGLTVEALDDAQVSLSNAAAHVHVDCEEQCLRENAGDWQQRIKRKYA
RRG
>tr|A0A0Q0CXG3|A0A0Q0CXG3_PSEAP Uncharacterized protein OS=Pseudomonas syringae pv. aptata OX=83167 GN=ALO85_01263 PE=4 SV=1
MDLMVGDTRTLSARALGNEFILQELAQLRDELIQQVFDHPFLVQCRAASMPIDRLKSFLV
QQGKYSRYFTRYICQLMSHLEGDEDILAVFENLFEELGFGEVAEPTHSAIYREMLCSFDL
NLEAHATLPSTQHLIDTMMNFCKQPNGIYGLSALCLGAEAIVPHLYSDIVTGFSGQGVAA
ERLRFFTLHIECDDGHADTLLAILSRLVMEKPTRFEIVRHAAVMMIKARLEFLDKL
>tr|A0A0Q0D061|A0A0Q0D061_PSEAP Uncharacterized protein OS=Pseudomonas syringae pv. aptata OX=83167 GN=ALO85_03153 PE=4 SV=1
MNRLCCALLVMLPVTALAYPIEVEKQYQDVKIDYVAHDVYHDTGSITVNNYGEVDAKCSV
VFINGPEAPRTRRVQVGAGKSLDVSSRFNRSIIKLRIRLSCQPA
>tr|A0A0Q0DX43|A0A0Q0DX43_PSEAP Threonine efflux protein OS=Pseudomonas syringae pv. aptata OX=83167 GN=ALO85_03788 PE=4 SV=1
MYEITLLTALAGAFIVLIISPGPNFLVITQLSFSQSRQQGICAGLGVASGSILWALLAAT
GLGLMFQQLPWLQPTLQLLGGAYLTWLGSKSLRSAGSQPKPRDIAALGIGSLSRAYRFGL
LTNLTNPKALAFYTSVFTTVVTTPGMPMWVRTAGVSIIAVLAISWFVLLATLFSIPAVQAR
YQRMKKAIDIVTGLFMIAFGLRLLIGLLPAGFIH
>tr|A0A0Q0FW13|A0A0Q0FW13_PSEAP Type III effector HopAH1 OS=Pseudomonas syringae pv. aptata OX=83167 GN=ALO85_05347 PE=3 SV=1
MIEALTTLIQALLKRLLGDSFASNSSANNPSENPSPASSGPGRASASPGGAASSTPDSGL
SEQSNQAQPFNNTGLGSHGGANTVPDRIASPEAAQKMVEDVKNAGGETLRIQMTHDQIND
PAQMAKLKAMVDAGDKQGVKIQFTFRDNANGGGGNVLSGDKLKQAADDVKNVVSALGNHP
SFVLDTFNEGGKSATQGWADMQSTLIKSARDAGYKGDIVVEDSNWGGGLTAGGESGLVKY
ADQLKAANGSNNPGLIGSIHEYASGSDASSRLSSEIKALTSAGFKPQIGEVGNANWTGGG
NFEQRDGANQAVKDNMGALKAAGADVLPWMDQFENGKIKHDVGFSKGDQFS

>tr|A0A0Q0C9P3|A0A0Q0C9P3_PSEAP Putative DNA helicase OS=Pseudomonas syringae pv. aptata OX=83167 GN=ALO85_00383 PE=4 SV=1
MNIKIEALIARKIGFASHQNAVPLVRELSIWNQEESGFENLTLTLSTDPDFVESRTWRID
RIHAGDRLNISERDIKLNAGYLSGLTESLSAEVVMRLSQGDDVLVEQRFPTELLARTEWG
GLSAMPELLAAFCMPNDPAVDRVLKAASQVLRRAGKKDGIDGYESKSRTRTWELASAIWS
GVCSFQLSYALPPASFEQQGQKVRPPSVVLENGVATCLDTALLFAAALEQAGLNALLIMT
KGHAFAGVWLQPQEFSQLLTDEASAVRKRLDLKEMVVFETTLATQTPAPGFSQAITVAER
QLDDEQFIMAIDLHRARMQKIRPMALVSVTEGSDDTADAPPTIEGLEEAPALPGFDVEIN
TDSEGPAGKLTLWQRKLLDLTTRNRLLHLPDSAKGVRLICPDPAVLEDLLSSGQRIRILS
VPDLKSSGRDVALYEQQNNENLVDEVARQALARGEVLASLEKVKLEATLIDLFRKARSDL
EEGGANTLFLAIGFLKWKKTADDPKTYSAPLILLPVKLERKSALSGVTLSLLDEEPRFNL
TLLELLRHDFELVIPGLDGELPSDESGIDVTGIWNVVRRAVRDVPGFEVSTELVLGTFSF
AKYLMWKDLNANAAQLLQSPLVKHLLEPGGAREGFSGSDEFPRPERLDSSITPAELFTPL
PADSSQLSAVVASAGTHSFVMDGPPGTGKSQTIANMIAHNLALGRRVLFVAEKRAALDVV
YRRLAEKGLGEFCLELHSSKASKVEVLKQLDRAWDVSDALSPEAWEREATRLQTLRSRLN
ELVEVLHRRWSNGLSLHQAIGRVVRDHGPQTPRLTWPSDTQHDAAEYSQLLDLARRLGLN
GDAARNLSGKFGALAQTQWSNAWQAHIANAARELPQALDALSATSERLLGLTHLDLPVSE
PAQVQHLNELATLVVESYGLNLSFAFAPDAVARIEAARRACQLIESYRELEEGLSLDYAD
EACRRIPVEAMRSEWRDAEGKFWFLATIAKKKVAKKLASSGGAAGLPDTLGDLAIFERLY
AQLNELDTLAGSLQSIPGWQGLTSDVSQIFRAASMAENLRGLMSGLAQSPDHLISLRSAV
ARLVVEANEMLAPGGQIGVALSALQQALSRYEDAARLFAELSAAGTDTQPSVGALRATSL
SIQQHEEQLKAWCDWCRVREQAGEAGLASLARALEAGSLSPAASEETFVTAYAHWFATQG
IDGEPLLRNFVAAEHMSDIAAFVRLDDEMAKLTVRYIRAKLCGLIPSKNDIPKSSGFAIL
KHELQKSRRHKPVRQLAVEMGDALNRLAPCMLMSPLSIAQFLPADQPPFDLVIFDEASQI
APWDAIGSIARGRQVVIAGDPRQMPPTNFFNRGPNASDDDTAEDMESILDECLGAGVPSH
SLSWHYRSRHESLIAFSNHRYYDSNLITFPAAETRASAVEWRRVDGVYAKGKGRYNQAEA
EAIVSETVKRLTDPAFVAAGHSIGIITLNSDQQKLINDLLDNARKQYPEIEPFFQDTQTE
PVVVKNLETVQGDERDLIMLGIGYGPTEPGAQVMSMNFGPLNKDGGWRRLNVAITRSRQE
MLVFSSFDPSMIDLNRTNARAVRDLKHFIEFAQRGPKALAEAVQGSVGGYDSPFEEAVAQ
GLRRLGWQVVPQIGVSRFRIDLGIVHPDRPGDYLAGVECDGATYHSAATARDRDKVRGAI
LSGLGWNLVRLWSTEWWVDKEGALQRLNAALNDLLVQSREETADQQITEQPTAEPIVIDF
STVPRISGESPDERVASISELPAEMGESRIALSHGLTPAAAGQKGIYRIADLSSLAPLIQ
ADQFHLPGYTPVLRQLIEEVLRQEAPIRDSALIQRIARAHGFQRSGRLIRDRVLELAEQH
HYVQQAGFEEVFVWHSEGDVAGWTTYRIPASSEDTRAIEEVAAEELRIAATGVDAEDKAL
EVARLFGIKRLTGPARERMERIFST
>tr|A0A0Q0FDI4|A0A0Q0FDI4_PSEAP TPR repeat OS=Pseudomonas syringae pv. aptata OX=83167 GN=ALO85_100100 PE=4 SV=1
MLWPSGSVDPASLRLWRYVMQHWKRTIEQANRCFSLGEWVEARELYLQALALAQVLFERW
PDVDEAVAACVISHHNLADLHLSLGQPEESAEYLCAIHQHLLQTMQSQRLPPALREAALR
HSSKTYAELLSFISEHGEYPRTHRLLNSSSEHTRSALQRHSAAPSGLFYGAH
>tr|A0A0Q0DEZ5|A0A0Q0DEZ5_PSEAP Uncharacterized protein OS=Pseudomonas syringae pv. aptata OX=83167 GN=ALO85_100550 PE=4 SV=1
MDSNTNRLTLRLELALLISKAHTQDSIYQQVLNKDTPPDRFSSMYWEDVLDKLEDLALLD
HVENFTPDHSSYIEDAALLRSYWTLREWCGIDIYPDSPSALTERL
>tr|A0A0Q0BU40|A0A0Q0BU40_PSEAP Uncharacterized protein OS=Pseudomonas syringae pv. aptata OX=83167 GN=ALO85_01882 PE=4 SV=1
MMSNDVYGGRPFAPQPPLSSAQDAAERLQLTLDSGAVVGTWVWDIIADQLTGDERFARTF
GLCPKLCAKGLPLELVIASIHPDDSARVDKSIEEALQSSETYRCEYRVLHEDGIYRWIEA
SGKIERDSRGKPVRSPGVLLDIDSRRTAEEERDRLNELLRIFTAAVPGVVYAKDLEGRML
VANRGTADLIGKPPEFFIGKTDLDFLDDQQQARILMETDRRIMQNNVSEQIEEQVNLADG
SAAIWLSTKAPLLDENGEVIGLIGSSIDVTARKKAEEAVRELNQTLEQRIEQAVFEREQI
EDALRHSQKMDAVGQLTGGIAHDFNNLLAGISGSLELITKRLAQGRVGDVDRYVSVAQGA
VRRAASLTHRLLAFSRRQTLSPRVTDVNGLIHDMEELIARTVGPEIDIKVVAQNDLWPAL
IDHAQLESSLLNLCLNARDAMPNGGRIIIETANASLEECTDPDHGIPAGEHLSIRVTDTG
IGMSPDIAAKAFEPFFTTKAIGAGTGLGLSMVYGFVRQSGGQIRVESIEGQGTSVVMHLP
RHTAENAPRPVEPEVIEEPPHHTGETVLIVDDEPSVRMLVAEVISGLGYNCLEAADAQSG
LQILQSDIRIDLLISDIGLPGGMNGREMADAAGDCRPGLPTLFITGYAKTSVLDDCHLRP
CTQVLTKPFGLDALAGRVTGLIGADAVARGYS

>tr|A0A0Q0C681|A0A0Q0C681_PSEAP CDP-diacylglycerol--glycerol-3-phosphate 3-phosphatidyltransferase OS=Pseudomonas syringae pv. aptata OX=83167 GN=ALO85_03732 PE=3 SV=1
MPMNIPNLITVLRVLLIPIFILLFYMPYHWSYMAASAVFAFAAATDWLDGYLARRLEQST
PFGAFLDPVADKLMVAVALVLLVQAHANLWLTLPAAVIIGREIVISALREWMAEIGARAQ
VAVSNMGKWKTAAQMLALVILLGNPPAITVWVILGYALLLVAAGLTLWSMVQYLRAAWPH
LRTTAEKK
>tr|A0A0Q0DNC1|A0A0Q0DNC1_PSEAP Isocitrate lyase OS=Pseudomonas syringae pv. aptata OX=83167 GN=ALO85_00737 PE=4 SV=1
MALTREQQIAALEKDWAENPRWKNVTRTYSAADVVRLRGSVQPEHTFARLGADKLWNLVT
QGAKPSFRPDKDFVNCMGALTGGQAVQQVKAGIQAIYLSGWQVAADNNSAESMYPDQSLY
PVDSVPTVVKRINNSFRRADQIQWKAGKAPGDEGYIDYFAPIVADAEAGFGGVLNAYELM
KNMIEAGAAGVHFEDQLASVKKCGHMGGKVLVPTQEAIQKLVAARLAADVAGVPTIILAR
TDANAADLLTSDCDPYDKPFVTGERTHEGFYKVRAGLDQAISRGLAYAPYADLIWCETAR
PDLDEARRFAEAIKKEYPDQLLSYNCSPSFNWKKNLDDATIARFQRELSAMGYKHQFITL
AGIHNMWHSMFNLAHDYARNDMTAYVKLQEQEFADAAKGYTFVAHQQEVGTGYFDDMTTV
IQGGVSSVTALTGSTEEEQFH
>tr|A0A0Q0FNJ5|A0A0Q0FNJ5_PSEAP Uncharacterized protein OS=Pseudomonas syringae pv. aptata OX=83167 GN=ALO85_02733 PE=4 SV=1
MRKLVLLAALFSPLAMAQAIIVAPHSLMRLPGNSSVLQVERLEVSDYGTLLIPAGIDDVK
IGELVLGHEARIAIVPGVQSFNMVVLHGEFGTGSQITARGAPGTFEKPARPGRNLTLRLQ
SLDAEELSIDARGGAGSPGYAGLDGGSGEAPGCTWGQAGRGYNGDSGGNGHDGAAGAQVR
LELPQSYPAERIKVNVAGGAAGKGGEGGKAGKGGASKGCVVYRAEGGKSGRQGEPGLPGA
PGPAGALILQRL
>tr|A0A0Q0C1P3|A0A0Q0C1P3_PSEAP Uncharacterized protein OS=Pseudomonas syringae pv. aptata OX=83167 GN=ALO85_01712 PE=4 SV=1
MLKSIANALLFQIGWFACVLGGNSFWLLIAVGVLAVHFLWISSWAAEGRLILTVTLIGIV
LDSALMTLGVFDFGTGGYLLPLWLALLWAVLGTTLNHCLAWTAKPLWRAVVLGAIGGPMS
YYAGSQLAQVHLPLGVWPGMLVLGLVWAGVFPLLQWLAARAARHSGEPL
>tr|A0A0Q0ISM0|A0A0Q0ISM0_PSEAP Extracellular solute-binding family 5 protein OS=Pseudomonas syringae pv. aptata OX=83167 GN=ALO85_02589 PE=4 SV=1
MRLAALPFLLVPLLLPVAAQASNLVVCTEASPEGFDVVQYNSLTTTNASADVLMNRLVDF
DAKSGKLVPSLAESWTVSKDGLSYDFKLRSGVKFHTTDYFKPTRELSADDVLFSFQRMLD
PQNPWHKIAQSGFPHAQSMQLPALIKSIEAPDAHTVRFTLNHADSTFLATLSMGFASIYS
AEYTAQLLKAGTPEKLNSQPIGTGPFVFKRFQKDAAVRYEAFDDYFAGKSDIDTLVYAIT
PDANVRLQKIRQDECQIALSPKPLDIQAASEDKSLKVEKTEAFMTAFVAINSQHPPFDKP
EVRQAINLAFDKDNYLKAVFEGTAEKANGPYPPNTWSYAKDLPGYQHDVAKAKALLAKAG
VKEGLKTTIWTRPSGSLLNPNPSLGAQLLQADLKQIGIDAEIKIIEWGELIRRAKAGEHD
LLFMGWAGDNGDPDNFLTPQFSCAAVQSGTNFARYCDKTLDKLIADGKSVSDQTERSKLY
HQAQQQIQQQALWLPLAHPTAAALVRKDVTGYQVSPFGRQDFYKVQVK
>tr|A0A0Q0BT09|A0A0Q0BT09_PSEAP Biosynthetic peptidoglycan transglycosylase OS=Pseudomonas syringae pv. aptata OX=83167 GN=mtgA PE=3 SV=1
MKNRRTAHGLYNGPLNGFGQAISMLQSILRRLFKALLWFAAGSVLVVLVLRWVPPPGTAL
MVERKVESWVDGEPIDLQRDWEPLDKISDNLKIAVIAGEDQKFAEHWGFDVDAIQAAILH
NERGGSIRGASTLSQQVSKNLFLWSGRSYLRKGLEAWFTLLIELLWSKERILEVYLNSVE
WDEGVFGAQAAAQHHFRTNASALSAQQASYLAAVLPNPRQWSASHPSSYVSRRAGWIRQQ
MRQLGGDEYLQGLNSSRRW
>tr|A0A0Q0C585|A0A0Q0C585_PSEAP PIN_12 domain-containing protein OS=Pseudomonas syringae pv. aptata OX=83167 GN=ALO85_04145 PE=4 SV=1
MTLATKNVFIDTEFYVKAEMDFESQTFKSFGELCQKGELVHITSTVVEQEVKKKILESIK
EGLKNLDTFKKRAGFLRNDEALANEIFTTKTEDELKRKGYEDFQEFLELTSAEIVDMSKV
DPNEILEMFFKQIRPFGAKKQTEFRDAFSLLAIRSALKGNEKIYVISSDPDHKAFCKGTE
QFISIDTLSSMLDLCYKHIDARSQFVEKFIAEKMEAIRAEIKSQLEDADGYNSSTWEDAE
VDDFEVIEIDDFEPQITHLDNESCRLNFDISVKFRVEVSGPDMVNSHYDREDDRYFTFDE
TEREDEEEHNFSVEVNIYFEDDEGEFINEEFELTIIDLGKGIEFGVEETPFEDPRM
>tr|A0A0Q0CCL0|A0A0Q0CCL0_PSEAP MobC domain-containing protein OS=Pseudomonas syringae pv. aptata OX=83167 GN=ALO85_200009 PE=4 SV=1
MENSGRSINVHLGSDLKRRWVSYCEHVGRSPGAALKEAIEHQLLRPNKHEQTKKYQQIEN

GRERKVRFEILLTNSERAAIQERASFERCSSRRWIIDAIRVGLTHEPQFSMTEIDALGES
NYQLLALGRNLNQIARRLNEGHYEPVTVERICALNHLIEKHTDIVSSAIRASLERWQIMP
TT
>tr|A0A0N8T9Z8|A0A0N8T9Z8_PSEAP Alpha/beta hydrolase fold protein OS=Pseudomonas syringae pv. aptata OX=83167 GN=ALO85_200004 PE=4 SV=1
MKPTDESCAQGTCDYWMVSAAAAKLGAHANTLGIRHIKDGALRLQFTREIAYYATSITND
VSQGKKSPEQGLQELKNEQSSLLSQSLVIAQKSVGLVAGAFQFVAGAGICYASVGTLCVL
GGGLLMAHGANNVYENGRNLWDGSTNAEGPVREAYQGAAKFMGAAEAEGNIAYGVADLGL
SAFGLARTVLKPDAWRLFKYVRTDYVRGYTEASKKGLFLEATSDGFTINSIHDELKK
>tr|A0A0N8T9W3|A0A0N8T9W3_PSEAP Uncharacterized protein OS=Pseudomonas syringae pv. aptata OX=83167 GN=ALO85_101546 PE=4 SV=1
MSSGFMPDEQEVFDVGRGVVFEALFADDRQPVCVMAK
>tr|A0A0Q0BS83|A0A0Q0BS83_PSEAP Glycosyl transferase PslC OS=Pseudomonas syringae pv. aptata OX=83167 GN=ALO85_04559 PE=4 SV=1
MGNRTSPWSIVMAPLRVACVIPTYNGRKDLERLLDSLAAQTASFDTLIVDSSSSDGTLEL
AQSRCANVLRIDSKDFNHGGTRQMMVDLHPDYDVYVFMTQDAYVEDINAIANLLLPFADP
KVGAVCGRQLPHKDANLLAQHARLFNYPPTSQVKTLADAGTLGIKTPFMSNSFAAYRGEA
LRAIGGFPRHVILSEDMYVTAKMLIDGWKAAYEGSAVCRHSHNYSLREEFRRYFDIGVFQ
AREAWIYETFGGIGGEGMRYVKSELKFLGPRRILWWPVSFVRNALKLLAYKLSRQEKRLP
RGVKKKLGMYARYWDSPYA
>tr|A0A0Q0C446|A0A0Q0C446_PSEAP Acyltransferase super protein OS=Pseudomonas syringae pv. aptata OX=83167 GN=ALO85_02761 PE=4 SV=1
MPVHVIDSLATVTPAQWDALVPGNQPFLRHAFLSSLEDSGSLGPRSGWRAEHVLHYENDQ
LVAALPAYRKWHSYGEYVFDHAWADACRRAGIAYYPKLLVAVPFSPVGGSRILSATPQGG
IELLGELPAYLQREGLSSAHVNFTDPAADALLETQPGWLQRIGCQFHWQNRGYRDFQDFL
DALSSRKRKQMRKEREQVAGQGIEFEWLEGAQMSEVLWDFVYACYSNTYEVRGQAPYLTR
EFFSLLAERMPESIRVVIALQGSRPVAMAFSLIGDDSFYGRYWGCLTEFDRLHFETCFYQ
GMDYAIAHGLQRFDAGAQGEHKLIRGFEPQITRSWHYLMHPGLKDAVSEFLEQERVGVMA
YAEDARSALPYRQAE
>tr|A0A0Q0D0R0|A0A0Q0D0R0_PSEAP Cold-shock protein, DNA-binding OS=Pseudomonas syringae pv. aptata OX=83167 GN=ALO85_00726 PE=4 SV=1
MIEGKVKWFNNAKGFGFINAEGNNEDLFAHFSAIEMDGYKTLKAGQKVRFEVAQGPKGLH
AVKIKGLEDKKLDPAKETHPLHQQDHAHADA
>tr|A0A0N8T9J8|A0A0N8T9J8_PSEAP Protein translocase subunit SecY OS=Pseudomonas syringae pv. aptata OX=83167 GN=secY PE=3 SV=1
MAKQGALSALGKGGMSELWARLRFLFLAIIVYRIGAHIPVPGINPDRLAELFRQNEGTIL
SLFNMFSGGALERMSIFALGIMPYISASIIMQLMTAVSPQLEQLKKEGEAGRRKISQYTR
YGTVVLALVQAIGMSVGLASQGVAFSGDLGFHFVAVTTFVAGAMFMMWLGEQITERGVGN
GISMLIFAGIVAGLPRAIGQSFESARQGDINIFALVAIGLLAVAIIGFVVFIERGQRRIA
VHYAKRQQGRKVFAAQTSHLPLKVNMAGVIPAIFASSILLFPASLGSWFGQSEGLGWLQD
ISQSIAPGQPLNILLFSAGIIFFCFFYTALMFNPKDVAENLKKSGAFIPGIRPGEQSARY
IDGVLTRLTMFGALYMTAVCLLPQFLVVAANVPFYLGGTSLLIVVVVVMDFMSQVQSHLV
SHQYESLMKKANLKGYGGSGGMLR
>tr|A0A0Q0DXU9|A0A0Q0DXU9_PSEAP 50S ribosomal protein L35 OS=Pseudomonas syringae pv. aptata OX=83167 GN=rpmI PE=3 SV=1
MPKMKTKSGAAKRFLKTANGIKHKHAFKSHILTKMSTKRKRQLRGSSLLHPSDVAKVERM
LRLR
>tr|A0A0N8TA52|A0A0N8TA52_PSEAP Metal ion ABC-type transport system, periplasmic substrate-binding protein OS=Pseudomonas syringae pv. aptata OX=83167 GN=ALO85_03397 PE=3 SV=1
MKRLMMLSSLAALALSAFASLAQAKPLEAVASFTVIADMVSTVGGERVHVRSLIGPNGDP
HVYEPTPSDAQALKNADLAFVSGLHLEGWMDRLIKASGYKGQPVVLSEGIKTRSMDEDGK
RIVDPHAWNSAANGVIYYVRNIVAALKKADPEGASVYQANGDRYIVELQQLDTYARDQIRS
IPAARRKILTSHDAFGYFGDAYGVTFLSPLGLSTESEASAADVSKLIRQIKTEHVSAYFF
ENSSDPRLVKQIAEASGAQPGGELYVESLSPADGPAPTYAKMFRYNVDTLTAAMKRNQ
>tr|A0A0Q0C249|A0A0Q0C249_PSEAP Probable alpha-L-glutamate ligase OS=Pseudomonas syringae pv. aptata OX=83167 GN=rimK PE=3 SV=1
MKIAVLSRNPRLYSTRRLVEAGIERGHEMVVIDTLRAYMNIASHKPQIHYRGKPLEGFDA
VIPRIGASVTFYGCAVLRQFEMMGVFPLNESVAIARSRDKLRSLQLLSRRGIGLPVTGFA
HSPDDIPDLIQMVNGAPLVIKVLEGTQGIGVVLCETATAAESVIEAFMGLKQDIMVQEYI

KEAGGADIRCFVVGDKVIASMKRQAKPGEFRSNLHRGGSASLIKITPEERMTALRAAKVM
GLSVAGVDILRSNHGPLVMEVNSSPGLEGIEVTTSKDVAGMIIEYLEKNSGPHMTRTKGK
G
>tr|A0A0Q0D7J0|A0A0Q0D7J0_PSEAP Na+/proline, Na+/panthothenate symporter
OS=Pseudomonas syringae pv. aptata OX=83167 GN=ALO85_02919 PE=4 SV=1
MAWSLLWQNTSHLRNAAVTQTANEFTKARRASLIAAIFMMATSAIGPGFLTQTATFTATL
GAAFAFGILASILIDFVVQLNVWRIVTLTKMRAADLANAAIPGSGYLLAVLVIFGGLVFS
LGNIAGAGLGLNALTGLDPKWGGALSALIAIAIFSSHRAGIAMDRIMMVLGTLKVCLILF
AAFASHPPLGEALRQTILPDMVDFAAITTIVGGTVGGYITYAGAHRLLDRGTVGVENIDV
VSKAALTGILVTGVVRYILFLAILGVVASGVVIDVSGKGANPAAQAFTAAAGNFGLIMFG
LVLWAAGISSVIGASYTSMSFITVFSKRITERARNLATVGFILITVVVYVMWGKPPAALL
VFAGGFNGLILPLGLSIFMYVGWRRSDMMDGYHYPRWLLVLGVLTCLLSWYMAFKSVGPI
FAFINAA
>tr|A0A0Q0CFU0|A0A0Q0CFU0_PSEAP LysR family transcriptional regulator
OS=Pseudomonas syringae pv. aptata OX=83167 GN=ALO85_03601 PE=3 SV=1
MAFDLNDVALFVQVVRSGSFAQAARQLGMPSNTISRRVQQLEMQLDTRLLQRSTRKLVLT
QAGEEFYDRCVGAVDGVMEAAEQLASGRDEPSGLVRIAAMADFFDFFSMDWVAGFLARYP
RVQLEFVLSDDHADLIADRLDIAFRGGPLQDSGYVGRQLIDEGHDTMVASPAYIAAHGMP
DSLQSLHSHYCVSAVHRGGVTAWRLLGPDTKMHEVHIASRFNANTAQALRKATLAGLGIA
LLPATLIRSDLRDGRLVPVLPRYQRISHGLHVLYPSRQHLPPAVSAFIALVLDKLRDKAF
AAQ
>tr|A0A0Q0DJS4|A0A0Q0DJS4_PSEAP Toluene tolerance protein Ttg2D
OS=Pseudomonas syringae pv. aptata OX=83167 GN=ALO85_00175 PE=4 SV=1
MISILRRGLLVLLATLPMLANAAATPSAHDLVERTTKELLSDLATNREQYKANPSAFYDA
LNRIVGPVVDADGISKSIMTVKYSRKASPEQVKRFEENFKRSLMQFYGNALLEFNNQGIT
VAPAKDEGNDRTSVDMQVKGSNGAVYPVSYTLVKLNDEWKVRNVVINGINIGKLFRDQFA
DAMQRNGNNLDTTINNWAGEVAKTKSTVDATQKPVQ
>tr|A0A0N8TA36|A0A0N8TA36_PSEAP Sodium:proton antiporter OS=Pseudomonas
syringae pv. aptata OX=83167 GN=ALO85_200102 PE=4 SV=1
MKFIKKKIVVINYTGTVGKTTIAANLLWPRMGGAPLYAIESINETAENLGLDVEKLRGNA
FRELFKRLMLEDQAIIDVGASNVEDFMANLEEFDEAHEEVDYFVIPVTSGTKEQKETVSM
ISSLASLGVPPEKILVLFNRVKKDVKTEFPIIFAFHQRASAFTLNTECAVFESELFDALS
IHRISMQSVMDDDTDYKELLKDKEASAQERDRWSDMYGLKLLCKGVNRKLDGVFAALFDL
EVIK
>tr|A0A0Q0DYV8|A0A0Q0DYV8_PSEAP Putative Membrane protein TctB
OS=Pseudomonas syringae pv. aptata OX=83167 GN=ALO85_01988 PE=4 SV=1
MVIQRIFAGVLLLACIGLALMAWPYQAPFSYEPVGPRAFPLLMLGLMGVALLYLLIRPTP
IVHTEEDPELDRETLIKIGVCIVLLLIFAGLFEPLGFILSSILIGIPMARLYGGRWLPSV
VVVILMSVGLYLLFDKAMDVPLPLGLLSVLEN
>tr|A0A0Q0DKD8|A0A0Q0DKD8_PSEAP Repressor protein c2 OS=Pseudomonas
syringae pv. aptata OX=83167 GN=ALO85_03569 PE=4 SV=1
MLRALRAVAFLEFLVAAARARIVTTDIFKGIAHRFLVSVTAVWTVYMAVLVVVVIVVMVA
IGAMHMGLLVHRFYSAIKSGAHYPANHGQAVSAYWDSHSASF
>tr|A0A0Q0IDX7|A0A0Q0IDX7_PSEAP Glutamate synthase, beta subunit
OS=Pseudomonas syringae pv. aptata OX=83167 GN=ALO85_02815 PE=4 SV=1
MAERLSNDFQFIDVGRKDPKKKLLRQRKKEFVEIYEPFKPQQSADQAHRCLGCGNPYCEW
KCPVHNFIPNWLKLVAEGNILAAAELSHQTNTLPEVCGRVCPQDRLCEGACTLNDGFGAV
TIGSVEKYITDTAFAMGWRPDMSKVVPTGKRVAIIGAGPAGLGCADVLVRGGVTPVVFDK
NPEIGGLLTFGIPEFKLEKSVLSHRREVFTGMGIEFRLNTEVGKDVTIDQLLAEYDAVFM
GMGTYTYMKGGFPGEDLPGVHDALDFLIANVNRNLGFEKAPEDFVDMKGKRVVVLGGGDT
AMDCNRTSIRQGAKSVTCAYRRDEENMPGSRKEVKNAKEEGVRFLYNRQPIAIIGEGKVE
GVKVVETRLGEPDARGRRSPEPIPGSEEIIPAEAVVIAFGFRPSPAPWFEQFTISTDSQG
RVVAPEQAQYKHQTSNPKIFAGGDMVRGSDLVVTAIFEGRNAAEGILDYLGV
>tr|A0A0N8TA20|A0A0N8TA20_PSEAP L-aspartate oxidase OS=Pseudomonas
syringae pv. aptata OX=83167 GN=ALO85_05012 PE=3 SV=1
MCRYPNTYTSGRQGQNSQMSQHFQHDVLVIGSGAAGLSLALTLPDHLRIAVLSKGDLANG
STFWAQGGVAAVLDDTDTVQSHVEDTLNAGGGLCNEDAVRFTVEHSREAIQWLIDQGVPF
TRGDDAGTDESGFEFHLTREGGHSHRRIIHAADATGAAIFRTLLEQAIKRPNIELLEQRA
AIDLITERRLGLEGQRCLGAYVLNRHSGEVDTYSARFTILASGGAAKVYLYTSNPDGACG
DGIAMAWRSGCRVANLEFNQFHPTCLYHPQAKSFLITEALRGEGAYLKLPNGERFMQRFD

ERAELAPRDIVARAIDHEMKRLGIDCVYLDISHKPEAFIKTHFPTVYERCLEFSIDITRE
PIPVVPAAHYTCGGVMVDSKGHTDVPGLYAIGETSFTGLHGANRMASNSLLECFVYARSA
AADIEKQLADVQMPADLPAWDASQVTDSDEDVIIAHNWDELRRFMWDYVGIVRTNKRLQR
AQHRVRLLLDEIDEFYSNYKVSRDLIELRNLAQVAELMIRSAMQRKESRGLHYTLDYPEM
LPEALDTILVPPTYAG
>tr|A0A0Q0C6K3|A0A0Q0C6K3_PSEAP LuxR family transcriptional regulator OS=Pseudomonas syringae pv. aptata OX=83167 GN=ALO85_01209 PE=4 SV=1
MGSAMTKSATRDLLIQQMVSTITELDGQAAIENALEWLRRECNSERAMFFQFKGPVLLTF
ITSNVDDAWGDAYRQHDLITQDPVIRCYRNQLGFLNWQDAFRQYPVTAFYDALVDTCELL
PAISYGYTNHCRGADSVTSVCTLGEVARPISAEQKYLMSSLMPVLHSVARGLKFCSRGLT
HRELEVLQWARDGKTAWEISMIRDVSESTVKYHLKTIYSKLGVANRAQAVGEALCRGLIR
>tr|A0A0Q0CVH1|A0A0Q0CVH1_PSEAP YciI-like protein OS=Pseudomonas syringae pv. aptata OX=83167 GN=ALO85_03806 PE=4 SV=1
MLYAIIATDVENSLEKRLSVRPAHLERLNALKDEGRLVLAGPHPAVDSNDPGAAGFSGSL
IVAEFESQSVAKAWAEADPFVAAGVYANVVVKPFKQVLP
>tr|A0A0Q0IL40|A0A0Q0IL40_PSEAP Homogentisate 1,2-dioxygenase OS=Pseudomonas syringae pv. aptata OX=83167 GN=hmgA PE=3 SV=1
MAIHSSSDALVYQSGFGNQFSSEALSGALPAGQNSPQKHPLGLYAEQFSGTAFTVVRSEA
RRTWLYRIKPSAAHPRYQRMDRQIAGQEQGPINPNRLRWNAFDIPAAPTDFIDGLIPLAS
TSAAEQAEGVSVYLYTANTSMQRAFFSADGEWLVVPQQGRLRIVTELGLLDIEPLEIAVL
PRGLKFCVQLLDSSARGYLCENHGCALRLPELGPIGSNGLANSRDFLTPVAWFEDSRQPI
QLVQKFLGELWSTQLEHSPFDVVGWHGNNVPYKYDLRRFNTLGTVSYDHPDPSIFTVLTS
PGAIHGQANIDFVIFPPRWMVAENTFRPPWFHRNLMNEFMGLIDGAYDAKAEGFMPGGAS
LHNCMSAHGPDNVTAEKAIAAELKPHRIDNTMAFMFETGKVLRPSRHALDCPQLQTDYDA
CWKDMARTFTQEPR
>tr|A0A0Q0CXN1|A0A0Q0CXN1_PSEAP 5-hydroxyisourate hydrolase OS=Pseudomonas syringae pv. aptata OX=83167 GN=ALO85_01161 PE=3 SV=1
MGKLTTHVLDAAHGCPGSAIRIELYRVEDHRLLHIVSTLTNSDGRCDAPLLQGETYQSGV
YQLHFKAGDYYRTRGVVLNKPAFLDEVVLRFGIDAGQEHFHVPLLISPYSYSTYRGS
>tr|A0A0Q0FMX9|A0A0Q0FMX9_PSEAP 4-amino-4-deoxy-L-arabinose-phosphoundecaprenol flippase subunit ArnF OS=Pseudomonas syringae pv. aptata OX=83167 GN=ALO85_00890 PE=3 SV=1
MASVALVSAAQLGMRWSMSRLPSPAHSLELHNSTQLDLSALMVVCASITAYALSMLFWLL
ALRVLPLSRAYSLLSISYALVYTLAAALPFFHETFTVSKTVGVTLIVAGVLIINLRRVPR
PSPQDLPHENQRFR
>tr|A0A0Q0DID1|A0A0Q0DID1_PSEAP Type III effector HopC1 OS=Pseudomonas syringae pv. aptata OX=83167 GN=ALO85_100754 PE=4 SV=1
MTIVSGHIGKHPSLTTVQAGSSASVENQMPDPAQFSDGRWKKLPTQLSSITLARFDQDIC
TNNHGISQRAMCFGLSLSWINMIHAGKDHVTPYASAERMRFLGSFEGVVHARTVHNFYRT
EHKFLMEQASANPGVSSGAMAGTESLLQAAELKGLKLQPVLEDKSNSGLPFLIACKQSGR
QVSTDEAALSSLCDAIVENKRGVMVIYSQEIAHALGFSVSSDGKRATLFDPNLGEFHTHS
KALADTIENISSADGLPLIGVQVFASKIH
>tr|A0A0Q0BUG4|A0A0Q0BUG4_PSEAP Glycosyltransferase OS=Pseudomonas syringae pv. aptata OX=83167 GN=ALO85_100110 PE=4 SV=1
MTDIDAALNSTEKPVSTRLLIIGYVWPEPRSSAASGHMMQLIQCFLEQDWQITFASPATE
GEHRADLLSLGIREVHIALNDSSFDVFVSELQPDVVLFDQFMIEEQFGWRVEQQCPDALR
ILETSDFQSLRHARHQLLKDLLNSDCAEAGPCSADVSAPQLFRRIASSDLAQREIASLYR
CDLNLMTSDVEIALLSGSFGLPAALLHWCPLMIDGAPDSFRPFAERAHFLSIGNFRHAPN
WDAVLWMKTAIWPLIRQQLPDAQLHIYGAYTPPKASALHNASQGFHVKHWAEDALEVMSH
ARVCLAPLRFGAGIKGKLMDAMLCGTPSVTTPMGAEAMSGGLPWPGIIADNPAEIAGAAV
RLYQDQNLWLEAQQAGQALLQARFQHRQHCASLIERIDTLRNELAEHRLANFTGAMLRHH
HHKSTKYMAQWIEAKNRNKDEKPA
>tr|A0A0Q0I9Y1|A0A0Q0I9Y1_PSEAP Poly granule-associated protein PhaF OS=Pseudomonas syringae pv. aptata OX=83167 GN=ALO85_04065 PE=4 SV=1
MAGKKKTDKDGSSWMGEVEKYSRQIWLAGLGAYSKISNDGSKLFDTLVKDGEKAEKLTKV
EAQKQLDDVKNTSKSAKSRVDDVKDLAVGKWNELEEAFDKRLNGAISRLGVPSRSEVQAL
HSKVDQLTRQIELLVGAKTKPAASRTVAAKPAAAKSAAKPLVKAAAKPVAKAADKSVAAR
PAVKKAAARPVEAANKPAAASTGKTSTATKPAAAKKPAARKPAAKPATKPSTPAASEKPA
IQS

>tr|A0A0Q0DXM8|A0A0Q0DXM8_PSEAP Oxidoreductase, FAD/FMN-binding protein OS=Pseudomonas syringae pv. aptata OX=83167 GN=ALO85_03691 PE=4 SV=1
MSETNKHVTAPLDRGHLKLKNRFAVAPMTRVSADEQGSATQTMARYYERFAKGGFGLIIT
EGNYTDRSSSQGYRFQPGLTDDEQAQAWRATTDAVHRHGSVMIAQLMHAGALSQANRFVS
ESAGPSAIRPKGEQMAFYYGAGSYRIPRAMSDKDIAEAIDGFYHAAQLAIGTAGFDGVEI
HGANGYLLDQFLTDYSNQRTDQWGGEVGQRLRLTLEVIAAVKKVAGQAPVGVRLSQGKVN
DFQHKWAGGERDAEIIFAALADAGVDYIHVTEHEAWQPAFAQGQASSLVELARRYAPDVT
LIANGGLHDPQKADQVLREGADIIAVGKGALANPDLPELLRQGAALRDFDPAILGPIANI
KDSELV
>tr|A0A0Q0C7T3|A0A0Q0C7T3_PSEAP AMP nucleosidase OS=Pseudomonas syringae pv. aptata OX=83167 GN=amn PE=3 SV=1
MTQAFVVVDTAEQAVERLAELHERATSALSQALKRYLKDRVEPTAEERAQFSYPELRLVY
KCQGEVPVTTRAYAKVQLPGTYSVTVTQPKAFKKYLLEQLVPLMNDFTVTVEVGMSEQSI
PYPYVVEQGDELAGTGVTAAALARVFPSTDLSAATDGIADGLYDWANVDPLPLALFDAAR
VDFSLRRLVHYTGSDWRHVQPWILLTNYHRYVDQFILHGLEKLREDPRFVRMVLPGNVVV
DKSMGQDEAQAIVASVVWHRYQMPAYHLIAEDGHGVTLVNIGVGPSNAKNITDHLAVLRP
HCWLMIGHCGGLRQSQTIGDYVLAHAYMRRDGILDRVLPPHIPIPALAEVQLALQESAAQ
ITGERGEELKKRLRTGTVLTYDDRNWELRWAQERPLINLSRAVAVDMESGTIAAQGYRLR
VPYGTLLCVSDKPLHSEIKLPGSANAFYERAVSQHLKIGIAALDLLRTELNSLHSRKLRS
FDEPPFR
>tr|A0A0Q0IMI1|A0A0Q0IMI1_PSEAP Oxidoreductase, short chain dehydrogenase/reductase family OS=Pseudomonas syringae pv. aptata OX=83167 GN=ALO85_01018 PE=4 SV=1
MNLQKNSATGVIFMSEFWNKAFDLNGRCAVITGGAAGIGLACASLLVARGARVALLDRDP
AVAEVAAGLGSGHIGIAVDLRRVDQVNSTIDSVFEHFQRIDYLVNSAGVAMLDKAVDVSE
EAWDTTLEINLKASFFVAQACARHMLGQGSGRIVNLASQAAVIGLDRHVAYCASKAAVVG
MTKVLAMEWAPHINVNAISPTIVETALGKKAWAGEVGERAKTQIPAGRFAQPEEIAGLAL
YLLSDAASMITGENVVIDGGYSIQ
>tr|A0A0Q0CY33|A0A0Q0CY33_PSEAP Bacterioferritin comigratory protein OS=Pseudomonas syringae pv. aptata OX=83167 GN=ALO85_01045 PE=4 SV=1
MTVTVDHPVPDFQAPATSGQTVELSALKGQQVVIYFYPKDNTPGCTTQGQDFRDHIAQFQ
AANTVVLGVSRDSLKTHENFKAKQSFPFELISDKDEAVCQLFDVIKLKKLYGKEYLGVDR
STFLIDKEGVLRQEWRGVKVPGHVAEVLAQAEALNKG
>tr|A0A0Q0D1J1|A0A0Q0D1J1_PSEAP Glutamate-1-semialdehyde aminotransferase OS=Pseudomonas syringae pv. aptata OX=83167 GN=ALO85_00404 PE=3 SV=1
MNRPLDPANIPDIPASAVSQWPDVDSLYRRFDELVKQPIRPIKRDAMNKIMGYFDERCQG
SKRLSEAAKKVIPGGVQHNLAFNYPFPLAFAQAHGAHLTDVDGNRYTDFLQAGGPTLLGS
NHPAIREQVDRILDECGPVTGLLHEYEVKLAELVCESMPGVDMLRLLGSGTEAVMAAVRL
ARAYTRKKWVIKIGGAYHGWSDQLVYGMRLPGTGRMEATGIPRGATANTQESPPNNLDAL
RRRLQINRLRGGTAAIMVEPFGPESGTRPVHADFNRQLRALCNEFDALLIFDEVVTGFRA
GLGGAQAYFNVMPDITVLGKCLTGGYPMAGAIGGRRDVMQLLAGGIGTSSRRAFVGGTLS
ANPLSCVAGYYALVEARRLNAPALAGRAGDRMRAGLEEIINRRGLPYVAYNMGSIVHLQT
SGVLLLDTGNPLKLLRARNEAKTRKHMMEEMGAAYTAHGLITLAGSRLYTSLADTDDVID
DALERFDAVFQLV
>tr|A0A0Q0DAP9|A0A0Q0DAP9_PSEAP Major facilitator transporter OS=Pseudomonas syringae pv. aptata OX=83167 GN=ALO85_03345 PE=4 SV=1
MSTPSDKGSVFETDLPARLDRLPWGRFHTLLVVALGITWLLDGLEVTLAGSVAGALKASP
ALNLSNSDIGLAGAAYIAGAVLGALFFGWLADRLGRRKLFFITLLLYVGATAATAFSFSV
WSFMLFRFLTGMGIGGEYTAINSTIQEFTPARYRGWVDLTINGTFWLGAALGAIGSIVLL
DPLWIGAELGWRLCFGIGAVLGLLVLLMRLWLPESPRWLLIHGQAEQAKQIVEQIEADLQ
RQGHVLPAVSGSPLRLHARDHTPLGEVARTLLVTFRQRSLVGLTLLTAQAFFYNAIFFTY
ALVLTDFYGVPAERVGWYVLPLALGNFCGPLLLGRLFDVVGRRVMISLTYGVSGVLLAIS
GYLFQQGLLDVTQQAIAWMVIFFFASAAASSAYLTVAETFPLEIRALAIAVFYAFGTGLG
GMIGPTLFGELIETGERSNVLIGYLIGAGLMSLAAIVQSIWGTAAERKSLEDVARPLSQA
GD
>tr|A0A0Q0IBQ2|A0A0Q0IBQ2_PSEAP Pirin-related protein OS=Pseudomonas syringae pv. aptata OX=83167 GN=ALO85_03476 PE=3 SV=1
MLTLRKASDRGAADHGWLKSFHTFSFAGYRNPQEQGFSDLLVINDDRVSAGKGFGQHPHR
DMEIFSYVLEGALEHKDTLGTGSVIRPGDVQLMSAGTGVAHSEFNHSHTEGVHFLQIWIV
PNVAGAAPNYQEKHFSPEQKRGRLQLIISPEGENGALKVRQDARVYAGLFDGDETATLEL

AANRYAYVHVARGSVVLNGEQLGEGDGVRVRDEQTLSLSAGVDAEVLVFDMRPNELPQMP
>tr|A0A0Q0E0U9|A0A0Q0E0U9_PSEAP Uncharacterized protein OS=Pseudomonas syringae pv. aptata OX=83167 GN=ALO85_102128 PE=4 SV=1
MSLDQDSKRALSNNCIIEAQLDISQDRRIGLPFVTSI
>tr|A0A0Q0D799|A0A0Q0D799_PSEAP Elongation factor Tu OS=Pseudomonas syringae pv. aptata OX=83167 GN=tuf PE=3 SV=1
MAKEKFDRSLPHVNVGTIGHVDHGKTTLTAALTRVCSEVFGSAAVEFDKIDSAPEEKARG
ITINTAHVEYKSLIRHYAHVDCPGHADYVKNMITGAAQMDGAILVCSAADGPMPQTREHI
LLSRQVGVPYIVVFLNKADLVDDAELLELVEMEVRDLLSTYDFPGDDTPIIIGSARMALE
GKDDNEMGTTAVRKLVETLDSYIPEPVRVTDKPFLMPIEDVFSISGRGTVVTGRIERGIV
KVQDPLEIVGLRDTTVTTCTGVEMFRKLLDEGRAGENCGVLLRGTKRDDVERGQVLVKPG
SVKPHTQFEAEIYVLSKEEGGRHTPFFKGYRPQFYFRTTDVTGSCELPEGVEMVMPGDNV
KVSVTLIKPIAMEDGLRFAIREGGRTVGAGVVAKIIA
>tr|A0A0Q0CEC4|A0A0Q0CEC4_PSEAP S-adenosylmethionine-dependent methyltransferase OS=Pseudomonas syringae pv. aptata OX=83167 GN=ALO85_02352 PE=4 SV=1
MSDRIELEFSRKYDKTHARQYLEKHQDGIWRKLSHHRDEQLARKALHMAGDPGLVLDLPC
GAGRFWPLLAEKPNRSIIGADNSADMLSTACASQPADVVKRVQPLQTSAFAIDLPDNAVD
SIFCMRLLHHVGQSSDRMALLREFQRVTRDSVIVSLWVDGNFKAWKRARLENKRQQKNGR
ESYQNRFVLPVVTAETEFEQAGFRIQNRMDFLPMYAMWRVYVLRKR
>tr|A0A0Q0FV37|A0A0Q0FV37_PSEAP tRNA synthetase, s-II OS=Pseudomonas syringae pv. aptata OX=83167 GN=ALO85_03381 PE=4 SV=1
MTQKTVSGAIFIAAGAEEAIVPALWGQDTFIEKAGGSEIIGQMWAFEDKAGRPCCLIPEA
TALFQERSEALLEGRREALFFYVARCYRYERPQAGRYREFTQLGLEILGPSPQQALLRSQ
AICTGFLDSLGLDYELNTAVKRGLSYYLEGNGFEVRCSRLGAQQQVVGGGAYREGAGFGI
GLERLVLALECG
>tr|A0A0Q0FQ85|A0A0Q0FQ85_PSEAP 30S ribosomal protein S3 OS=Pseudomonas syringae pv. aptata OX=83167 GN=rpsC PE=3 SV=1
MGQKVHPIGIRLGIVKEHTSVWYADGRTYADYLFADLKVREYLQDKLKSASVSRIDIHRP
AQTARITIHTARPGIVIGKKGEDVEKLRQDLTKQMGVPVHINIEEIRKPELDGMLVAQSV
AQQLERRVMFRRAMKRAVQNAMRIGAKGIKIQVSGRLGGAEIARTEWYREGRVPLHTLRA
DIDYANYEAHTTYGVIGVKVWIFKGEVIGGRQEELKPQAPAPRKKAAK
>tr|A0A0Q0DT79|A0A0Q0DT79_PSEAP Catalase OS=Pseudomonas syringae pv. aptata OX=83167 GN=ALO85_02616 PE=3 SV=1
MPLLNWSRHMVCLTAAGLITIPTAYAADTLTRDNGAVVGDNQNSQTAGAEGPVLLQDVQL
LQKLQRFDRERIPERVVHARGTGVKGEFTASADISDLSKATVFKSGEKTPVFVRFSSVVH
GNHSPETLRDPHGFATKFYTADGNWDLVGNNFPTFFIRDAIKFPDMVHAFKPDPRTNLDN
DSRRFDFFSHVPEATRTLTLLYSNEGTPAGYRFMDGNGVHAYKLVNAKGEVHYVKFHWKS
LQGLKNLDPKEVAQVQSKDYSHLTNDLVGAIKKGDFPKWDLYVQVLKPEELAKFDFDPLD
ATKIWPDVPEKKIGQMVLNKNVDNYFQETEQVAMAPANLVPGIEPSEDRLLQGRVFSYAD
TQMYRLGANGLSLPVNQPKVAVNNGNQDGALNTGHTTSGVNYEPSRLEPRPADDKARYSQ
LPLSGTTQQAKITREQNFKQAGDLYRSYSAKEKTDLVQSFGESLADTDTESKNIMLSYLY
KADPDYGTRVAEVAKGDLSKVKSLAASLKD
>tr|A0A0Q0C012|A0A0Q0C012_PSEAP Dipeptide ABC transporter, permease protein OS=Pseudomonas syringae pv. aptata OX=83167 GN=ALO85_00012 PE=3 SV=1
MSTPTPAVAVDQSLLYPSPYKEFWQAFSKNKGAVAGLLFMILIVFCALFAPWVAPHDPSE
QYRDFLLTPPVWLEGGQWQFILGTDELGRDLLSRLIQGSRLSLLIGLASVVMSLIPGILL
GLLAGFFPRVLGPSVMRLMDIMLALPSLLLAVAIVAILGPGLINTVIAIAIVSLPSYVRL
TRAAVMGELNRDYVTAARLAGATLPRLMFITVLPNCMAPLIVQATLSFSSAILDAAALGF
LGLGVQPPTPEWGTMLASARDYIERAWWVVSLPGLTILLSVLAINLMGDGLRDALDPKLK
NAA
>tr|A0A0Q0CA11|A0A0Q0CA11_PSEAP Peptidyl-prolyl cis-trans isomerase OS=Pseudomonas syringae pv. aptata OX=83167 GN=ALO85_00027 PE=3 SV=1
MSEVNLSTDETRVSYGIGRQLGDQLRDNPPPGVDLEAIVAGLRDAFGGQPSRVGQEEMSA
SFKVIREIMQAEAAAKAEQAAGAGKAYLAENAKREGVTTLASGLQFEVVTAGTGPKPTRE
DQVRVHYHGTLIDGNVFDSSYDRGEPAEFPVSGVIAGWTEALQLMNAGSKWRLHVPSELA
YGAQGVGSIAPHSVLVFDVELLDVL
>tr|A0A0Q0BXU1|A0A0Q0BXU1_PSEAP Carbonic anhydrase OS=Pseudomonas syringae pv. aptata OX=83167 GN=ALO85_01086 PE=3 SV=1

MNELQDLIDNNARWADAIKQEDPEFFAKLARQQTPEFLWIGCSDARVPANEIVGMLPGDL
FVHRNVANVVLHTDLNCLSVIQYAVDVLKVKHILVTGHYGCGGVRASMQDRQFGLIDGWL
RTIRDLYYENRELLAKLPTEEERVDRLCELNVIQQVANVGHTSIVQNAWHRGQSLSVHGC
IYGIKDGRWKSLDVTISGFEQLPPQYRLRPQD
>tr|A0A0Q0FKU5|A0A0Q0FKU5_PSEAP SOS response-associated protein OS=Pseudomonas syringae pv. aptata OX=83167 GN=ALO85_01738 PE=3 SV=1
MCGRYALFRWTPAFAALPGFPADQQAQWNISPADWVLIMRAAENADGIELVRARWGLTPA
WLTDLSKTPAHARAETVAEQPMFRDAFTHRRCLLPANGFYEWRGTVRKRPFWLTPGEGAA
LFFAAIWEAYPVQGHTYLSVAVVTQAAATQRRPLILDAKGQKDWLAADTPLPTLQALLAA
PQTALRERPLANLVNDPKLNAPECLTPLS
>tr|A0A0N8TA79|A0A0N8TA79_PSEAP Response regulator receiver domain protein OS=Pseudomonas syringae pv. aptata OX=83167 GN=ALO85_03856 PE=4 SV=1
MIDHDILSDDERDALHEVMLTPDRSAPQRVLIVDDDSDARELLAEILSLNDISCMTAPGG
DTALQMIHSRQSIGLLITDLRMAPFDGLDLIRKVRESDRAELPIIIVSGDANVRDAIDAM
HLNVVDFLLKPLNTRQLVKLVKRELGIS
>tr|A0A0Q0BTM5|A0A0Q0BTM5_PSEAP S-transferase OS=Pseudomonas syringae pv. aptata OX=83167 GN=ALO85_03123 PE=4 SV=1
MTQTSYTPPKVWQQETKAGGTFASINRPVAGPTHDKVLPVGKHPLQLYSLATPNGVKVTI
MLEELLALGHSGAEYDAWLIRISEGDQFSSGFVEINPNSKIPALLDRSHETPVRVFESGS
ILLYLAEKFSSFLPADPAGRTETLNWLFWQMGAAPYLGGGFGHFYAYAPEKLEYPINRFA
MEAKRQLDVLNRRLGEHRYLAGDTYTIADIAVWPWYGALVQNKVYSAAEFLSVHEYPNLI
RWTEEIAARPAVIKGQKVNRTWGEEADQVPERHQASDLDK
>tr|A0A0Q0FGV5|A0A0Q0FGV5_PSEAP tRNA-2-methylthio-N(6)-dimethylallyladenosine synthase OS=Pseudomonas syringae pv. aptata OX=83167 GN=miaB PE=3 SV=1
MAKKLYIETHGCQMNEYDSSRMVDLLGEHQALEVTARAEDADVILLNTCSIRERAQDRVY
SQLGRWRELKLANPEMVIAVGGCVASQEGAAIRDRAPYVDVVFGPQTLHRLPEMIDAARV
TRLPQVDVSFPEIEKFDHLPEPRVDGPSAYVSVMEGCSKYCTFCVVPYTRGEEVSRPFDD
VLSEVIHLAENGVREVTLLGQNVNGYRGTTHDGRVADLADLIRVVAAVDGIDRIRYTTSH
PLEFSDSLIQAHAEVPELVKHLHLPVQSGSDRILAAMKRNHTTLEYKSRLRKLRAAVPGI
SISSDFIVGFPGETEKDFDNTMKLIEDVGFDFSFSFVYSPRPGTPAADLKDDTPEALKKE
RLAALQHRLNQQGFEISRQMVGSIQRILVTDYSKKDPGELQGRTENNRIVNFRCDNPKLI
GQFADVHIDDAQPHSLRGSLLQ
>tr|A0A0Q0CEW1|A0A0Q0CEW1_PSEAP Aldo/keto reductase OS=Pseudomonas syringae pv. aptata OX=83167 GN=ALO85_02877 PE=4 SV=1
MSLKTLHDLHRPLGSTGLNVSPLGLGTVKLGRDQGVKYPSGFKIPDDQQAQMLLRMARDM
GINLIDTAPAYGTSEERLGPLLRGQRQDWVIVSKVGEEFVNGQSSHDFSAAHTRTSVERS
LKRLETDFLDLVLVHSDGNDLAILNDAGVYQTLADLKREGKIRGFGFSGKTVEGGLLALR
DGDCAMVTYNLNEQDEKPVLDYAQAHGKGILVKKALASGHVCLTPGIDPVHASFQLLFEH
PAVASAIVGTINPLHLAHNVATAAAVICRQA
>tr|A0A0Q0DEI1|A0A0Q0DEI1_PSEAP Copper chaperone CopZ OS=Pseudomonas syringae pv. aptata OX=83167 GN=ALO85_03074 PE=4 SV=1
MQLFNVQGMTCGHCVRAVTEAIKHEDPSAEVQVELASKQVKVQSSLSPERIVSLISEEGY
QAQPA
>tr|A0A0Q0IBB6|A0A0Q0IBB6_PSEAP 30S ribosomal protein S9 OS=Pseudomonas syringae pv. aptata OX=83167 GN=rpsI PE=3 SV=1
MSATQNYGTGRRKTATARVFLRPGTGNISINNRSLDNFFGRETARMVVRQPLELTETVEK
FDIYVTVIGGGVSGQAGAIRHGITRALMQYDETLRGALRKAGFVTRDAREVERKKVGLRK
ARKRPQYSKR
>tr|A0A0N8T865|A0A0N8T865_PSEAP Uncharacterized protein OS=Pseudomonas syringae pv. aptata OX=83167 GN=ALO85_01283 PE=4 SV=1
MIARPSVRWLTASALCLLGLSGNALAHNPMCECKQIDTEQIRCTGGFSDGSGAPGVTLDV
IGYDETILVPGKLGEDSTVTFKRPASEFYVLFDAGPGHVVEIDQADIQAP
>tr|A0A0Q0IDY5|A0A0Q0IDY5_PSEAP Gluconate transporter OS=Pseudomonas syringae pv. aptata OX=83167 GN=ALO85_02822 PE=4 SV=1
MSPSPGISLLVYAAVAIIALIVLIARYRLNPFIVITLVSVGLALVAGMPASGVVASYEAG
VGKTLGHIALVVALGTMLGKMMAESGGAEQVARTLVDRFGERNAHWAMVCIAFLVGLPLF
FEVGFVLLVPIAFTVARRVGVSILMVGLPMVAGLSVVHALVPPHPAAMLAVQAYQASVGQ
TLFYAILIGIPTAIIAGPVYAKFIVPRIHLPAENPLAKQFLDREPRQKLPSFGLTMATIL

LPVVLMLLGGWANLISTPGSAFNSFLLFIGNSVIALLLATLLSFWTLGIAQGFNRDSILK
FTNECLAPTASITLLVGAGGGLNRILIDSGVTNEIVGLAQDFHLSPLLMGWLFAALMRVA
TGSATVAMTTASGIVAPVALGLGYPHPELLVLATGAGSVIFSHVNDGGFWLIKEYFNMTV
AQTFKTWTVLETLISVVAFALTLGLAQVI
>tr|A0A0Q0D552|A0A0Q0D552_PSEAP PepSY-associated TM helix family protein OS=Pseudomonas syringae pv. aptata OX=83167 GN=ALO85_00898 PE=4 SV=1
MSASFSSPEPRSSGAFVAFLKRLHFYIGVFVGPFMLVAALSGVVYALTPQIEDSLYAHAL
HTDSRGPAMSLQAQIQRAQASAGPGLSVAAVRPAPREGDTTRVMFSDPGFGPSEHRALFL
DPVSGEIRGDMKVYGTSGILPLRTWIDQFHRGLLLGDVGRIYSELAASWLWVAALGGLVL
WAVRRRRQSRERGNVRRWHATTGVWLLLGLLFFSATGLTWSQYAGDNIGVLRAYYGWSTP
SVATSLGKSAAMPMSMPMDEHAEHHMHMAPTTQAAELDPALFDSVLASARAAHIDAAKVE
IRPSASNDKAWVVSEIDRSWPTQVDAVSIDPQTLDVVDRVRFSDYSLPAKLTRWGIDAHM
GVLFGLANQLVLVVFASGLAAMVVMGYVMWWRRRPMLSQPRNQQATLLSLWRSLNPGAQW
ALILGALLTGFALPVLGVSLLGFIILDALLGYRRAARPLVEGKV
>tr|A0A0Q0CDZ2|A0A0Q0CDZ2_PSEAP Cold-shock protein OS=Pseudomonas syringae pv. aptata OX=83167 GN=ALO85_00248 PE=4 SV=1
MLKIVHLVTGAAALLLSFIPSLRSEAASLYLQNPDAICLAFLGLLNLILAPVIPYWNRGP
RHNLQNLVSALLVIAVIVQTLTLLVPLPDIAGQPAILASLAVAIVAVALHLGVSFYRSYT
PSSAPQSHDMGNRDTGTVKWFNTSKGFGFISRDSGDDIFVHFRAIRGEGHRVLVEGQRVE
FSVMNRDKGLQAEDVIAALPRR
>tr|A0A0Q0IEV4|A0A0Q0IEV4_PSEAP Cytochrome family protein OS=Pseudomonas syringae pv. aptata OX=83167 GN=ALO85_02114 PE=4 SV=1
MSRVSLRLWDPLVRLFHVSIAGVFVANYFFNEAGGDWHIWLGYYAVAWLAVRVVWGFIGP
RSARWSDFWPHPARLRAHIRSLIDRRPIHRLGHSPLGALVMVLMMVLIFGMGLTGFLMEE
VDALWGADWPQDIHEILANTLCALVVLHMAAAIFESVQVRDNLPLSMLTGKRRPLPEDRH
R
>tr|A0A0Q0DL31|A0A0Q0DL31_PSEAP Uncharacterized protein OS=Pseudomonas syringae pv. aptata OX=83167 GN=ALO85_02655 PE=4 SV=1
MPMALPLGLLFSLLFGVMIWMLPAPFVVAAAIGIVAVATIMRRPVRGLLLFCLIGTFLPY
STVQIGIRTTVSEALIMLTWASYLLHAVFQERQRVPHMLRPERLLVALMLFSAFPFLAGQ
LTVVAEGNGPINWIRWLFNLSILFLVPRLLTDLKTLENAVIALLGGTLLLLLLSIGVYVT
KGSATAIIPILGSLGYSGADILSQSLLSLASRMGSPWMHPNVAGGALAMLLPLAFCFGMT
RSGAARKLGLTVAALGAVGLLLTGSRGALLSLIAMMLLMARRRIPHLGRLLMGGLVAGIV
LLMFYPPLQDRLMGLFSDDDASTAIRFLEYSHFPDAVATFPFGIGFKTDPPVLGYTEFGI
SNLWLNYIYKIGIPGMLLFIAITVSWWKEVRPGSGRIVLTRDNGIALGCTTGVLAALFSG
LFDHYFSFTSVLVAFFWLFVGISLYETRRLRASAYTTPRHLEASHEPGASS
>tr|A0A0Q0DEC1|A0A0Q0DEC1_PSEAP ABC transporter, periplasmic substrate-binding protein OS=Pseudomonas syringae pv. aptata OX=83167 GN=ALO85_01278 PE=4 SV=1
MPTLRTLTLALIMSGLFSNSLRAADSKTDSSQPVRVLASLPITYGLSEILLKDSGVVLQR
AAAANLPGSRQTAYFTGRGADALRGLANNADAVIGLRSIWADDPLYPNARRSNIRIVEID
AARPVDGSLPGIAVQPGQGTDGLNSQPWLASNNMGRMADVLAADLVRLAPAAKPKIEANL
AAFKQQLLKLSASSEAALAGADNLSVVSLSDRFSYLISGLNLELIDTQALTDEQWTPEAL
SKLSATLKDNDVALVLDHRQPPAPVKTAIEAAGSKLLVLGIDGADPLAELQGDIQGIIGV
LAGME
>tr|A0A0N8T9W0|A0A0N8T9W0_PSEAP Phosphonate transport protein PhnE OS=Pseudomonas syringae pv. aptata OX=83167 GN=ALO85_00814 PE=3 SV=1
MTTHAAYTQVAGKPNGSRYLGWGLLLAALAWAWHGAEMNPMALVRDSSNMATFAADFFPP
DFREWRSYLKEMLVTIQIALWGTALAIVCSIPLGILCAENITPWWIHLPLRRCMDAFRSI
NEMVFAMLFVVAVGLGPFAGVLALWISTTGVLAKLFAEAVEAIEPGPVEGVRATGASALQ
EVIYGVIPQVMPLWISYALYRFESNVRSATVVGMVGAGGIGVILWENIRAFAFVQTSAVL
LVIIVVVSVIDIMSQRLRKQFI
>tr|A0A0Q0DP26|A0A0Q0DP26_PSEAP Phenol degradation meta-pathway protein OS=Pseudomonas syringae pv. aptata OX=83167 GN=ALO85_00038 PE=4 SV=1
MSLKNYFLSASGLAMAILWLSSTACATENAAPTTAAGVFDFGAGFMPPTTPNGTVGMRIS
NYRADVIKDSHGNDSPNDFQINVLAIGLAYLRMTEQEFLGARYGFAVVPIFFKMDADLGV
NAGGQRVFSDSASLFRQADLQVVPIILDWKLGPGLGINTQLMIQAPTGDYDKNRLVSPGT
HHWTVSPLLNATYISPGGFEVSSSFQIDINARNPDTDYRSGVEYRHEFAVGQHVGDWTVG
IGGYYYRQLSDDDAPGLTRGNRARVLAAGPAVSYFKPGAGLPPIWLHAYKEFDARNRAEG
YTVALRTGISF

>tr|A0A0Q0DQE3|A0A0Q0DQE3_PSEAP Amino acid ABC-type transport system, periplasmic substrate-binding protein OS=Pseudomonas syringae pv. aptata OX=83167 GN=ALO85_01730 PE=3 SV=1
MNYRALLGIGLVTLAASTQVLAGATLDRVQKNKELVNVLMESYPPFSFLNDKNELDGFDV
DVAKAVAQKLGVKLRLETPSWDVIAAGHWSGRYDICVCSMTPSKARSEVFNFPVEYYASP
AVIVVNATDDRIHSAKDLSGKKVGLTSASSYESYLNKNLVIDGAEDKPLQYPFEDVQIAP
YDNDNVAFQDLGLGAGKRLDAILTNLVTAKPRLDQDKRFKLAGEPLYEEPNSVAIEKDDP
EWDAKVRQVFAELKSDGTLSKLSQKWIGADISK
>tr|A0A0N8T9G4|A0A0N8T9G4_PSEAP 3',5'-cyclic adenosine monophosphate phosphodiesterase CpdA OS=Pseudomonas syringae pv. aptata OX=83167 GN=cpdA PE=3 SV=1
MLIAHISDTHVRPRGQLYQGVVDSNAMLAAAVDTINALDPPPDLIVFSGDLVDEGRPEEY
AMARELLQPLRQKLLMIPGNHDHRQNLRDAFPEHDYFINDQDCSFVYSGSAPVRIIGLDI
SVPDQHHGDMTDTATQWLDRTLALEPDKPTLIMMHQPPFSSGIPYIDAYRCERGERLAEV
VCRYPAIERIVCGHIHRFMQLRFGGTLMCTAPSTTTAIALRLHPEAADASYVEPPALLLH
HWKADTGLITHWVPIGRFEGPFDFA
>tr|A0A0Q0BT53|A0A0Q0BT53_PSEAP Formate/nitrite transporter OS=Pseudomonas syringae pv. aptata OX=83167 GN=ALO85_02488 PE=4 SV=1
MSLNTPQQIAEIAVESGVKKAHLGLPSLMILGFLAGAFIALGFLLDIHVSTMIPHEWASL
GNLLGAVVFPVGLILVVLAGGELLTGNMMSLPMALFAGRIGLGQLVRNWVLVTLANLIGA
LFVAYCFGHVVGLTEGAFMTKTVAIANAKVGASFEQAFISGIGCNWLVCLAVWLSYASKD
VIGKVVGMWFPVMAFVAIGFQHIVANMFVIPAAIFAGALSWAQFGDNFVPVFLGNAVGGA
VFVGLAYHLAFFNAATKPVEMSRTSGVQTPE
>tr|A0A0Q0BV11|A0A0Q0BV11_PSEAP Mechanosensitive ion channel family protein OS=Pseudomonas syringae pv. aptata OX=83167 GN=ALO85_100144 PE=4 SV=1
MTPPSSLNGPDAFMDIHSLWLKTQEIWDMLDQHPWIRTGLALILLLTAALVLGRVARFLV
LYAVRMLGRQPSLHWVNDFRHNKVFHRLAQMVPSLVIQFGLTLVPGLSTAGRNVIGNIAM
AFTILFMTLAIGALLNALLDIYARTEHARTRSIKGYVQLSKMILYVFAGIIIVATLIDRS
PLLLLSGLGAMSAVILLVYKDTLLSFVASVQLTSNDMLRVGDWIEMPQVGADGDVVDITL
HTVKVQNFDKTIVSIPTWRLMSESFRNWRGMQQSGGRRIKRSLFIDASGVRFVRDDEEQQ
LMQIHLLTDYIGRKQAELLAWNQAQGNVAQMSANRRRMTNIGTFRAYALAYLKSHVDINP
NMTCMVRQLEPTSQGIPLEIYCFTRTTVWVDYERIQGDIFDYLITVMPEFGLSLYQQPSG
ADMRVGLRGEPARVGATPLVEPLPTEHSG
>tr|A0A0Q0FFG2|A0A0Q0FFG2_PSEAP Uncharacterized protein OS=Pseudomonas syringae pv. aptata OX=83167 GN=ALO85_03116 PE=4 SV=1
MKTARLEDMVKGWFVGGFEPAVFSTESCEVGVKTYKAGEKEAAHYHKVATEITVLIAGSV
NMRDQVWHAGDIIVLEPGDITAFEALTDATTVVVKVPGALADKYVV
>tr|A0A0Q0DM98|A0A0Q0DM98_PSEAP Uncharacterized protein OS=Pseudomonas syringae pv. aptata OX=83167 GN=ALO85_01136 PE=4 SV=1
MVYAIAVLCVLGIAAGQIMFKLSAISLQRAGTFFDIKTIMILGSAFALYGLTTIAWVWVL
QKIELGKAYPLMAMAFVLVPIGSHFILGERFQVQYFVGVLFIMFGIVLAVKS
>tr|A0A0Q0DLJ3|A0A0Q0DLJ3_PSEAP Putative lipoprotein OS=Pseudomonas syringae pv. aptata OX=83167 GN=ALO85_00334 PE=4 SV=1
MAMKTTRKPHLKLLGIFTLVLALTGCSSMLFYPEQGVPFTPDKARLQYQDVNLTAADGTR
LHGWWLPAKEGVPVKGTVLHLHGNGGNLSWHLGGVWWLPEQGYQVLMLDYRGYGESQGEP
SLPAVYEDVQAAFDWLNTAPQVQGKPLVVLGQSIGGALAVHYLSEHPQERSRLKALVLDS
VPASYRSVARNSLSKSWLTWPLKAPLSWLIPEADSAVNGLPKLAGTPMLIFHSMDDTLVP
LANGIDLYKAAPPPRVLQLTRGEHVQTFADPLWRQVMLRYLDDPTHFNGLRRLAEVPNYP
TPATPPAP
>tr|A0A0Q0IE48|A0A0Q0IE48_PSEAP Gluconolactonase OS=Pseudomonas syringae pv. aptata OX=83167 GN=ALO85_01119 PE=4 SV=1
MDAELIVDAQNATGESPVWSVRDQALYWVDIPKGELHRWDFKQNSTRSWKAPQMLACIAA
DSRGGWVAGMENGIFHLQPCEDGSLISTLLASVEHAQAGMRFNDGRCDRQGRFWAGTMFM
DMAAGARVGALYRYSASQTTLDAQLKDFIVPNGLAFSPDGKIMYLSDSHPSVQQIWAFDY
DTDSGTPRNRRLFVDMNQHLGRPDGAAIDTDGCYWICGNDEGLVHRFTPAGKLDRSLVVP
VKKPAMCAFGGPNLDTLFVTSIRPGGDIGDQPLAGGVFALRPGVQGLEEPTFQG
>tr|A0A0Q0DBZ8|A0A0Q0DBZ8_PSEAP DNA-binding protein HU, alpha subunit OS=Pseudomonas syringae pv. aptata OX=83167 GN=ALO85_01406 PE=3 SV=1
MRKPEVAAAIAEKADLTKEQSNRVLNAILEEITGALNRKDSVTLVGFGTFLQRHRGARTG

KNPQTGEPVKIKASNTVAFKPGKALKDSVNP
>tr|A0A0N8T9U1|A0A0N8T9U1_PSEAP Glycogen synthase OS=Pseudomonas syringae pv. aptata OX=83167 GN=glgA PE=3 SV=1
MISAAVKTQKGESFNQPVGELTHLPVFSNKSVPVLQMPTSTVQPQPTMSQVNRRKVLFVT
SELADLVKTGGLGDVSAALPRAMRHLHDVRVLIPGYPQVINSGNPIHIISQLGGHAALPP
CKVGRMDMKDGLVIYVLICPELYEREGTPYADSTGRDWSDNHIRFARLGLAAAEFAAGEV
KSQWCPELVHAHDWPAGLAPAYMRWRGQSTPSIFTVHNLAYQGTVSTASSRELGIPDSAI
TPEGMEFYGQLSFIKAGMAFASHITTVSATYAREITTPEFGCGLEGFLQSKANKGQLSGI
PNGIDESWDAATDEHLICHFAPNEWTRKEINADYVRELFELDASTGPLFAVVSRLVYQKG
LDLTIGVAEHIVNNGGQIAIIGRGEPEEEDAMRELAARFPGRVGVRIGFNETDARRMFAG
SDFLLMPSRYEPCGLSQMYAQRFGSLPVARNTGGLADTIEDGVTGFLFNESTVESYTEAL
NRALQVFAHPELLNAMRCRAMAAPFNWHQAVEPYADLYRDLLKKNVSISSNY
>tr|A0A0Q0C5B2|A0A0Q0C5B2_PSEAP Uncharacterized protein OS=Pseudomonas syringae pv. aptata OX=83167 GN=ALO85_04973 PE=4 SV=1
MGFNNFSDELAVLEIVRSSNFVRPGKLTSGQIYLALIRLQSDVPAAGDFMAMIDELVKEG
LLVSAAVLEHDISFPYVQHIVLGVTETGAAVLEDRSFQADLFYMCSTVPFQLQRQERTDS
RNCQSRTDEPHLPCSLCRA
>tr|A0A0Q0DLY7|A0A0Q0DLY7_PSEAP Uncharacterized protein OS=Pseudomonas syringae pv. aptata OX=83167 GN=ALO85_01308 PE=4 SV=1
MPLNEQRLSMRLERVATHVPPGARLADIGSDHGYLPVALLNRGVIAAAVAGEVALTPFCA
AERTVRENDLEDQVSVRLADGLAAIEAEDAITAISLCGMGGETIRDILEAGKARLSGQER
LILQPNGGEQPLRVWLMEHDYRIVSEEVLRENRFDYEIIVAERTGPVKYTAEELFFGPLQ
MQARSPAFLLKWQRLLGKKHKTLRNFERAQQAVNQEKLQDVAQQVRWITALLA
>tr|A0A0Q0D2H4|A0A0Q0D2H4_PSEAP Uncharacterized protein OS=Pseudomonas syringae pv. aptata OX=83167 GN=ALO85_100444 PE=4 SV=1
MHRPTPSRQRCVHPVVLEDRFAASINTQPFRTSSLTGRLTLRSELEQAPAHWIWQVIADG
KVGSRRTTIGLFLDRDLEPGGHDLIDHEQIKVIYTETLHRKNTVYHSAHFQGGRLVLLEA
NPCTLRIRGEFSFNMSSINLDVTDGAFDLYCR
>tr|A0A0Q0FHR0|A0A0Q0FHR0_PSEAP DUF2059 domain-containing protein OS=Pseudomonas syringae pv. aptata OX=83167 GN=ALO85_03541 PE=4 SV=1
MTRLRAICTAVALVCASGQVFADTASHNASAETFLKLANADRLGAPVYAQVQQMFAQRFA
ETKAPASKKALLETYQAKANTALDQAIGWNKLKPEMVKLYTANFTESELKDLVAFYQSPL
GKKVMTKMPELAQQSAQLTQSKLESAVPVVNKLLADMTAELEPAKAAAPAPAKKP
>tr|A0A0Q0C5R0|A0A0Q0C5R0_PSEAP dTTP/UTP pyrophosphatase OS=Pseudomonas syringae pv. aptata OX=83167 GN=ALO85_00154 PE=3 SV=1
MPSLYLASGSPRRRELLTQIGVPFTVLSAQIDETPFDHETPAAYVERLALGKAQAGLSVL
SAEQQACVMGADTAVVLDGRILGKPVDEADALAMLMALTGREHDVLTAVALCDRQRSETR
IVTSRVRFRPIQTHEAQAYWTSGEPADKAGGYAIQGLAAIFVEGLQGSYSGVVGLPLCET
AELLGHFGIPCWQCLEGDKS
>tr|A0A0N8T9K9|A0A0N8T9K9_PSEAP Adenylosuccinate synthetase OS=Pseudomonas syringae pv. aptata OX=83167 GN=purA PE=3 SV=1
MGKNVVVLGTQWGDEGKGKIVDLLTEHATAVVRYQGGHNAGHTLVIDGEKTVLHLIPSGV
LREGVQCLIGNGVVVAPDALLREIIKLEEKGIPVRERLRISPSCPLILSYHVALDQAREK
ARGEFKIGTTGRGIGPAYEDKVARRGLRIGDLFHRERFAAKLGELLDYHNFVLVNYYKEP
AIDFQKTLDECMEYADMLKPLMLDVTAALHEMRRDGKDIMFEGAQGSLLDIDHGTYPYVT
SSNTTAGGIATGSGFGPMYLDYILGITKAYTTRVGSGPFPTELFDDVGAFLAKRGHEFGA
TTGRARRCGWFDAVILRRAIEINSISGLCLTKLDVLDGLETINICIGYENEEGAVIDAPT
DADSYLGLRPVYEEMPGWSESTLGAKTLEELPAAARAYIKRVEELVGAPIDIISTGPDRN
ETIVLRHPFG
>tr|A0A0Q0D343|A0A0Q0D343_PSEAP Prophage PSSSM-04, putative transcriptional regulator OS=Pseudomonas syringae pv. aptata OX=83167 GN=ALO85_00445 PE=4 SV=1
MQTTGSHGMNEILDQLRKEFATPCPSLSAVRERYFSHLSNDRNLLRKINAGRIDLKVSRT
GGSRQGHPFVYLHDLANYLSAIVTNRAA
>tr|A0A0Q0DIQ2|A0A0Q0DIQ2_PSEAP Bacteriophage Mu tail sheath OS=Pseudomonas syringae pv. aptata OX=83167 GN=ALO85_00438 PE=4 SV=1
MAISFNNIPSDVRVPLFYAEMDNSAANSASASMRRLIVAQVNDDVSGPELGSLVLVPSVA
LAKNIGGQGSMLAAMYETWRKADPTGEVWCLPLLNTEGAKAGAKVTLTGAATEAGLLNLY
VGGMRVQATVVNGATAAQAATALSVKINATPDLPITAAVEAGVLTLSCKWSGASGNDIQL
EFNRQGKTNGEVIPAGLTAAVTAMTGGVGTPDQLKALAALGDEPFEFICMPWTDTSTLDA

WKAAMDDSTGRWSWARQLYGHVYSAKRGTVGTLVAAGQLRNDQHITLQGVENGVPQPVWL
QAAALAARTAVFISADASRPTQSGTMPGIDPAPASQRFTLTERESLLRYGIATAYYEGGY
VRIQRSITTYQKNAYGQADNSYLDSETMHQSAFIIRRLQGIITSKYGRHKLANDGTRFGA
GQPIITPSTIRGELIAQYARLEEEGHVENAETFAQHLIVERDGNDPSRVNVMFPPDYING
LRVFALLNQFRLQYDEAA
>tr|A0A0Q0DQI0|A0A0Q0DQI0_PSEAP Uncharacterized protein OS=Pseudomonas syringae pv. aptata OX=83167 GN=ALO85_01798 PE=4 SV=1
MATPSAPTLPIPVVKGASAKNEISLSKGIVLELPAFKDPRCTFVILNLVNADNSNRPLLT
GSSPITSGDPTIITLENTGTDPSMIFQPTQKARITGSVQVTGMDTWPDTPESAIYSLVQ
>tr|A0A0Q0DFP1|A0A0Q0DFP1_PSEAP Sel1 repeat-containing protein OS=Pseudomonas syringae pv. aptata OX=83167 GN=ALO85_01321 PE=4 SV=1
MNRTGRTLVLGCLLLVYPLLASAGGNSLLVPAMGRCTLNTQPENLPAALEACQQAAKGGD
AQAQYELGEFYYEGKNAPRDLPQALNYFEQASLQGHAQAQYQLGLMFSRGEGVQANNIQA
YVVLKMAAVNGSEEALSRQRCRVTSSKWPLRYWGRFSASTCLNCRPPKGAVLSLRCPDQE
SFRAIAIHTCNAYFSGIGMGNGITLLVPRASLIFGVPMRSTSSMRTIECIGTKLRTTPSN
WALSFSSLGSTTTVVRSPKTNSSTSRKPHRSLW
>tr|A0A0Q0BXX6|A0A0Q0BXX6_PSEAP Uncharacterized protein OS=Pseudomonas syringae pv. aptata OX=83167 GN=ALO85_01094 PE=4 SV=1
MSASSIEPFTSLPSILAGPLLRRQEADRLVLWLVGSRPLRLTMRLRHAATDDPTAGFNDY
VLDDSQCQVIAIGRHAFIHLIDLQLDAELPRDTWIDYDLLVDGEGGIAEWAPHLLYEGHR
YPNFVVRSSIDHLLHGSCRKPHHSAAEGLLCVDRLLADIQEPLQRPALLMMSGDQIYADD
VAGPMLRAIHVLIERLGLFDERLDGAIVDDSPRLYAHPASYYQRADLLPALKSNDTLRER
FFGGTSKPVFTSSTADNHLVTLAEVMAMYLLVWSPTPWTLIEPGRPQLEDGEVERYAKEQ
HLVEAFAKGLGGVARAMAHLPCMMIFDDHDITDDWNLSANWEQTAYGHPFSKRIIGNALL
AYLLCQGWGNNPDALNGLVKQAHALSSEAVSNDNWLDSQAQDQLIDQLLKFQQWHYVLPT
SPALVVLDTRTRRWRSESNFNHPSGLLDWEALSELQQELIDHKSAIIVSPAPVFGVKLIE
AIQRIFSWCGYPLLVDAENWMAHRGAANVILNIFRHSRTPGNYVILSGDVHYSFVYEILI
RQRSGGPHIWQITSSGIKNEFPRRLLDTFDRLNRWLYSPRSPLNWFTKRRLMKVVPRTPE
HSKAGERLWNSAGVGQVLFNEQGQPTLINQLNANGDAPTCFVEPEEYRDQRSDEERGFLE
NNRETRDSHR
>tr|A0A0Q0C1H9|A0A0Q0C1H9_PSEAP Rhs element Vgr protein OS=Pseudomonas syringae pv. aptata OX=83167 GN=ALO85_101867 PE=4 SV=1
MRKDLQVPFMLAITDCQPDLHIISFAGHEALNETYRFDIWMIGSDPQLDVRSLLNREAFL
SFGESGSGRHGLIDHAARLHRGARVSLYHLILVPDLHRLVQHRQRRTYQDLAIPQLIVQL
LEAHSIGANAYRFEHVNGLYPARPLHIQYDESDLHLLQRLCEEEGMHFRFEHRQSGHRLV
FSDDAASFPAQPLPVRLKHPQTSGAPRETLEHLAERLGFPPVSRQPTDHPQPARAPEHPL
TIAANHAFQPPGFRGAISPQEALSRQRGSRRLERLRCERRDVRGRSTDTALQCGEVIQVL
DHPEPLLNDQWLVTEVVHAGRQLQVLKGAAAHDALAILRILVEAQRSSPEAEQLQGNGYC
NSFRVIPWATPFRPSINHPRPAATGEHFATLQRSDTQPGQPGYRSIRFDWHVQSPQDEPV
QRWPMAEVACPEVDRLAPGARVRVRFLDNHPERPVICAVLSPGHELDGMTAQPAGCIRVA
SGEHLHVNAHDGLLVRGQAAALHVDAQRILIVGHKDQQSLKSGWRLSIKARMPSF
>tr|A0A0Q0DJS7|A0A0Q0DJS7_PSEAP Bifunctional poly C5 epimerase / O-acetylhydrolase PsmE OS=Pseudomonas syringae pv. aptata OX=83167 GN=ALO85_100855 PE=4 SV=1
MGTDGSETLRAGAGRGTVEAGAGNDRLFGGAGGDTLSGGAGADTFVYTQLSDSYHNDASG
SYSPPTSMATAMT
>tr|A0A0N8T9C4|A0A0N8T9C4_PSEAP Acetyl-coenzyme A carboxylase carboxyl transferase subunit alpha OS=Pseudomonas syringae pv. aptata OX=83167 GN=accA PE=3 SV=1
MNPNFLDFEQPIADLQAKIEELRLVGNDNSLNIGDEISRLQDKSKTLTESIFGNLTSWQI
ARMARHPRRPYTLDYIENIFTEFDELHGDRHFSDDAAIVGGIARLDNQPVMVIGHQKGRE
VREKVRRNFGMPRPEGYRKACRLMEMAERFKMPILTFIDTPGAYPGIDAEERNQSEAIAW
NLRVMARLKTPIIATVIGEGGSGGALAIGVCDQLNMLQYSTYAVISPEGCASILWKTAEK
APDAAEAMGITADRLKGLGIVDKVIAEPLGGAHRDPVAAAALIREELSSQLAMLKEFDND
ELLARRYDRLMSYGL
>tr|A0A0Q0BRP0|A0A0Q0BRP0_PSEAP Inner membrane protein OS=Pseudomonas syringae pv. aptata OX=83167 GN=ALO85_04294 PE=4 SV=1
MKKRPVLLSWPGSPNLPVETMTYKPPGSRLSRLLYGILAYTALGIGIVAIFVPGLPTTEF
ILLAAWAATRSSPRLNAWLENHRVFGPILHNWRNGRLVARKAKISATISMLVCAVVMLSL
IGHAWWLYLALTGMALGNLWIWSRPEPDKIDR

>tr|A0A0Q0DQQ0|A0A0Q0DQQ0_PSEAP TetR family transcriptional regulator OS=Pseudomonas syringae pv. aptata OX=83167 GN=ALO85_03949 PE=4 SV=1
MPAMTAIKTKSKGGRPRQDQAGEAERRLLDAALQRFLACGFEGTSCEDIARAAGASKATL
YARYANKEALFEAVVRRHVATLLVPAETIPALPLADRLRHVGHGILEHALQADTLAMMRL
VIATSNRTPLLAAEVNRIGWEGGFMRVRSAILAGPDQPSDPDALARQFIDLVFAPHQLRA
LLGEDRGVLIQGIPERVEWGVRMVGGAGLLG
>tr|A0A0N8TAC0|A0A0N8TAC0_PSEAP Chromosome partitioning protein ParA OS=Pseudomonas syringae pv. aptata OX=83167 GN=ALO85_01498 PE=4 SV=1
MAKVFAIANQKGGVGKTTTCINLAASLVATKRRVLLIDLDPQGNATMGSGVDKHNLENSV
YDLLIGECDLGEAMQFSEHGGYQLLPANRDLTAGEVVLLEMQMKESRLRNALAPIRENYD
YILIDCPPSLSMLTLNALVAADGVIIPMQCEYFALEGLSDLVDNIKRIAELLNPQLKIEG
LLRTMYDPRLSLINDVSAQLKEHFGEQLYDTVIPRNIRLAEAPSFGMPVLAYDKSSRGAL
AYLALASELVRRQRRGAKTAQPT
>tr|A0A0Q0FT80|A0A0Q0FT80_PSEAP Regulatory protein, LysR:LysR, substrate-binding protein OS=Pseudomonas syringae pv. aptata OX=83167 GN=ALO85_05423 PE=3 SV=1
MESLGSIAVFVLVAETRSFTQTGKRLGISSSGIGKSIGRLEESLGVRLFHRSTRSIALTL
EGSIFLERCRRILSELEAAEMELSNARDLPKGRLRISLPLVSGLVMPVIIDFMRTYPDIE
LDIDFSDRMVDIIEEGFDIVLRTGDPGDSGLMSKRMGSFQLRIVGAPDYFARHAIPRHPG
DLSQHACLLHKFPSTGRFEHWPLRREAGVDDLELPQAMVCNTTEILVDVARAGLGIACLP
DFMISSAIRNGELIPVLDDYTVHTGTFRLLWPSSKHLSLRLRVFIDFMHAHLFAPPTV
>tr|A0A0Q0DWZ5|A0A0Q0DWZ5_PSEAP Urease subunit gamma OS=Pseudomonas syringae pv. aptata OX=83167 GN=ureA PE=3 SV=1
MDLTPREKDKLLIFTAGLVAERRLARGVKLNYPEAMAYISAALLEGARDGQTVAELMHYG
TTLLSRDQVMEGIAEMIPEIQVEATFPDGTKLVTVHQPIA
>tr|A0A0N8T7W9|A0A0N8T7W9_PSEAP Uncharacterized protein OS=Pseudomonas syringae pv. aptata OX=83167 GN=ALO85_04133 PE=4 SV=1
MTNAAMTSNEQIKLLIFSMLLAPTIPLFVGIIPALFLIFGVVMMKKNSDFSHIETSAKIY
KYYVYLLLTGLILFGFYCATTLGASNSWDRLDQQFYASIALSAVAVFYIVVMNFLFLKPL
RCHSAWVEKNGIFSSKAKVVENSNDVDIIKGEKLRTFSVADELIKWAKLKDDGHITEQEF
NDARKKLLQ
>tr|A0A0Q0BX32|A0A0Q0BX32_PSEAP 4-diphosphocytidyl-2-C-methyl-D-erythritol kinase OS=Pseudomonas syringae pv. aptata OX=83167 GN=ispE PE=3 SV=1
MTMSTPQLVLPAPAKLNLMLHILGRRPDGYHELQTLFQFLDYGDELGFTVREDGEIRLQT
DVPGVPHDSNLIVKAARALQQQSGCTLGMDIWLEKRLPMGGGIGGGSSDAATTLLALNHL
WQLGWDEDRLAQLGLTLGADVPVFVRGHAAFAEGVGEILTPVDPEEPWYLVLVPQVSVST
AEIFSDPLLTRDTPPIKVRPVPKGNSRNDCKAVVERRYPEVRNALNLLGKFTEAKLTGTG
SCVFGAFPNKAEADKVSALLTETLTGFVAKGSNISMLHRKLQIL
>tr|A0A0Q0C7V6|A0A0Q0C7V6_PSEAP Acyl carrier protein OS=Pseudomonas syringae pv. aptata OX=83167 GN=acpP PE=3 SV=1
MNMQTREDIFEILRTAMVELFELEPERVTLEANLYQDLEIDSIDAVDLIDHIKRKTGKKI
AAEEFKSVRTVNDVVEAVYKLVNSAE
>tr|A0A0Q0FL38|A0A0Q0FL38_PSEAP DUF2007 domain-containing protein OS=Pseudomonas syringae pv. aptata OX=83167 GN=ALO85_01737 PE=4 SV=1
MQKIYEPENLMEAELLLAMLASEGVNAHLAGRDLLGAMGELPGLGLLALKVEDAQVAHAR
ALITAYNSASPLSGDEPDDFPDVLVC
>tr|A0A0Q0FCH9|A0A0Q0FCH9_PSEAP Putative integral membrane protein OS=Pseudomonas syringae pv. aptata OX=83167 GN=ALO85_01872 PE=4 SV=1
MSTLLIYGATGYTGRMAAERASALGLNVEIAGRNQQRLASLAAQLDVKYRLFEADQAEGF
LSGISVLLNFAGPFVQTAEPLMRACIKAGVDYLDITAEINVYRLAERLGAEAAANQVMLL
PGVGWDVVPTDSLAVQVAGRVQGPTALNIALQVPGSMSRGSAMSVSEIIGAGVLARIDGE
LVATPDAEPRHFDFGQGPVLCVPLSFGDLVTAWHSTGIPNIAMFVHIAGDAFPQGDLSQL
PDGPTPEQREAHRARAVIEVTDAKGAIARSIIETVNGYTYTPLAAVEAARRVLDGERQTG
FATPAKVFGGGFAESIVGTLVTDM
>tr|A0A0Q0IES2|A0A0Q0IES2_PSEAP Flagellar biosynthesis anti-sigma factor FlgM OS=Pseudomonas syringae pv. aptata OX=83167 GN=ALO85_02133 PE=4 SV=1
MVIDFSRLNNSQSVAGASRTSPSKDTAKTAAPDPAVTAGVKSTGETFSLSSEAQQLQKIT
DKLTDLPTVNSARVAELKQAIADGSYTVDSNRVASKLLNFETQR

>tr|A0A0Q0FM13|A0A0Q0FM13_PSEAP Putative hemolysin OS=Pseudomonas syringae pv. aptata OX=83167 GN=ALO85_00229 PE=4 SV=1
MRFEYDESDGLSIFKNTRFRSALRKKLGGDYRTFPKTQFSENSTDVFGGAVVKVWYDAND
EVKGVQLYHPEAEFYYFGKQLLGSNVEELEFFFSSIGQSLIFDDDGTGFNAENNTIRFYV
PDFDEFQKKARVECVYIDIAKA
>tr|A0A0Q0ILC0|A0A0Q0ILC0_PSEAP Uncharacterized protein OS=Pseudomonas syringae pv. aptata OX=83167 GN=ALO85_100363 PE=4 SV=1
MKPRISAWRGVSLIMRGALAFYTVTGIIYSEGALAIIHFIFAAERWPYSCSASRQKPRQY
LSFVI
>tr|A0A0Q0INY1|A0A0Q0INY1_PSEAP Uncharacterized protein OS=Pseudomonas syringae pv. aptata OX=83167 GN=ALO85_03493 PE=4 SV=1
MTVYNLRMFTMTGVILITAAGMANAATNTSAQSTSREHQSALLDSQSQSSDEMLARDWNL
SPQEWQRYQTLMQSSRGVYSPGLDPLTALGIEARNDEERRRYAELQVKAEAQRTAKELAY
QRAYDDAFKRLYPNLMPIASSASQGATASPANQGNGRLAVFVKDDCPECSVRVKALQAQK
QPFDVYMVGSQNDDERIRNWAIVSGIAPANVRTRQITLNHDGGRWLGLSLGGELPAVVRE
VNGQWLRQ
>tr|A0A0Q0C4D5|A0A0Q0C4D5_PSEAP LysR family transcriptional regulator OS=Pseudomonas syringae pv. aptata OX=83167 GN=ALO85_00284 PE=3 SV=1
MMYTNRWEGLDEFVAVAECGQFTAAALRLGVSSSHVSRQIVRLEERLQTRLLYRSTRKVA
LTEAGQTFLHHCQRLQDGREEALRAVGDLASEPKGLLRMTCAVAYGERFITPLVTRFMDL
YPHLRVDIELSNETLDLVHEGMDLAIRLGRLQDSRLVATRLAPRQMYLCASPSYIERYGR
PHSLSELNRHNCLIGGSDTWQLQQNGREFSQRVQGNWRCNSGQAVLDAALHGVGLCQLPD
YYVQKHLNDGTLVSLLEAHQPPNTAVWGLYPQQRHLSPKVRKLVDYLKEGLAMRDEYRQ
>tr|A0A0Q0BX24|A0A0Q0BX24_PSEAP Uncharacterized protein OS=Pseudomonas syringae pv. aptata OX=83167 GN=ALO85_01601 PE=4 SV=1
MHTSFRTLAAALLMGGSAMVMAANDGQSRVNELLSSAPEYRTTWAKVVKKEERLPDWVIN
LSGASEQQMTAAREDDDNYLVGPLCETADTCKSKRLIVAFSLDKKDAYAMLVEVPAGLPA
DKSPTRHATYRYLGKPDEGMQKLLNEELRKDPNWY
>tr|A0A0Q0DMF0|A0A0Q0DMF0_PSEAP ABC transporter OS=Pseudomonas syringae pv. aptata OX=83167 GN=ALO85_01132 PE=4 SV=1
MSLLSVSNLGKAYRVYASEFQRIGRWFGIPTKPSEEHWVLKHISFSIEPGEAIGIVGQNG
AGKSTLLKMITGTLQPTEGHVQVNGRIAAILELGMGFTPDLTGRQNVYHAAGLMGFSADQ
INGVIDEIEAFAEIGEYFDEPVRMYSSGMQMRVAFAVATAIRPEILIVDEALSVGDSYFQ
HKSFERIRQFQAQGTTLLIVSHDRGSIQALCSRAILLEKGTVIKDGKPEEVMDFYNALIA
EKENATVQLRELEDGSVQTRSGSGEATIGAVSLHNAAGERIEYVSVGEPVSLRINAQVNS
AIPELVVGYLIKDRLGQPVYGTNTHHMGCKVVDLQAGESLDYSFNFPANLGVGSYSVAVA
LHTTDSHLSRNYEWVDLTLVFNVVNISQSEFVGLAWLPPVVEFSR
>tr|A0A0Q0DN85|A0A0Q0DN85_PSEAP Nitrite reductase, small subunit OS=Pseudomonas syringae pv. aptata OX=83167 GN=ALO85_00644 PE=4 SV=1
MNASQTILAGDNAPTAAKPHWQTLCGRADLVANSGVVAWLDGAQVALFYLPETAQGEQLF
AIDNRDPKSGANVIGRGLIGQIAGELVIASPLYKQHFRLHDGSCIEYPEQRLEVWPVRLK
GDQVEIAV
>tr|A0A0Q0IAR4|A0A0Q0IAR4_PSEAP Uncharacterized protein OS=Pseudomonas syringae pv. aptata OX=83167 GN=ALO85_04139 PE=4 SV=1
MIKQQSITVMNLYEEITKQGYRIEEMRSHSNKNRVYIINENNERIGYYSKMEGTKAKIIL
KKEPLFEDYCADNGFLESLRFPRNANGSAYKAINNLYKKFVAWKKTEEMNKCFDKVLNEL
ISTEKNN
>tr|A0A0Q0CFH7|A0A0Q0CFH7_PSEAP Ferric siderophore transport system protein, ExbD/TolR family OS=Pseudomonas syringae pv. aptata OX=83167 GN=ALO85_04359 PE=3 SV=1
MAFSTQDTDEVLSEINVTPLVDVMLVLLVVFIVTAPLLTNSIPINLPKTESVAPVEQKDP
LVVSIDGQGKLFINKDEIQPGLLESSLKAAKDKTPDVRVQLQADNGVNYGEVARAMASIE
RAGITKLSVITAK
>tr|A0A0Q0CD50|A0A0Q0CD50_PSEAP Type III effector HopBA1 OS=Pseudomonas syringae pv. aptata OX=83167 GN=ALO85_00915 PE=1 SV=1
MLNRISSSSPTSYVSSGSSSAGINPSINVRPPRGGPVDTLVGAASDNNLVYIGDEHGKLF
IPKLITESAAKLKNAGVDHLAVEFVKHSDGAAFREALSDGKSAVKHFLEASWGRHGDAWL
DKVSEALCSAHRAGIYVSGIDRKMAIDQPKTPMQKILYMKKRLALNVAWDAAATREASAV
CANKSIVWGGAGHFSNSKTDGPKDMRPGLVISFDLTGRGSSRINDADEHSHIVIAGEDN

>tr|A0A0Q0IIW8|A0A0Q0IIW8_PSEAP Uncharacterized protein OS=Pseudomonas syringae pv. aptata OX=83167 GN=ALO85_01720 PE=4 SV=1
MNQINPRKLLLSKWTAATPLNREKHFLVTELFKDEEGTVLEVELQAVLTQRSERLPWQVL
QQANTWRMGWK
>tr|A0A0Q0CEU0|A0A0Q0CEU0_PSEAP 50S ribosomal protein L22 OS=Pseudomonas syringae pv. aptata OX=83167 GN=rplV PE=3 SV=1
MEVAAKLSGARISAQKARLVADQIRGKKVGEALNLLAFSSKKAAEILKKVLESAVANAEH
NEGADVDDLKVSTVFVNEGRSLKRIMPRAKGRADRIVKRSCHITVKVADK
>tr|A0A0Q0IRD3|A0A0Q0IRD3_PSEAP Short-chain dehydrogenase/reductase SDR OS=Pseudomonas syringae pv. aptata OX=83167 GN=ALO85_02017 PE=4 SV=1
MTRKVAVITGAASGIGQALAVAFARQGVAVAGGFYPADPHDPDETRRLVAEAGGECLMLP
LDVTFTESVDDLAAQAVKLFGRIDYAVANAGLLRRAPLLEMTDERWNEMLDVDLTGVMRT
FRAAVRHMGEGGALVAISSIAGGVYGWQDHSHYAAAKAGVPGLCRSLAVELAPKGIRCNA
VIPGLIETPQSLDSKNSLGPEGLAQAAKAIPLGRVGRADEVAALVRFLCSDDASYLTGQS
IVIDGGLTVRWPE
>tr|A0A0Q0DLI6|A0A0Q0DLI6_PSEAP Aspartate--tRNA(Asp/Asn) ligase OS=Pseudomonas syringae pv. aptata OX=83167 GN=aspS PE=3 SV=1
MLAGVTLRGPGLALVLCRCLVPLSASQERNRHRIRITRSENTIMMRSHYCGQLNESLEGQ
EITLCGWVHRRRDHGGVIFLDIRDREGMAQVVFDPDRADSFAAADRVRSEYVVKVVGKVR
ARPAGAVNANMASGAIEVLGYELEVLNESETPPFPLNEYSDVGEETRLRYRFIDLRRPEM
AEKLRLRSRITTSIRRYLDDNGFLDVETPILTRATPEGARDYLVPSRTHPGSFFALPQSP
QLFKQLLMVAGFDRYYQIAKCFRDEDLRADRQPEFTQIDIETSFLNEEDIIGLTEKMVRQ
LFKEVLDLEFGDFPHMTFEEAMRRYGSDKPDLRNPLELVDVADQLTGVEFKVFSGPANDP
KGRVAALRVPGAASMARSQIDDYTKFVSIYGAKGLAYIKVNERAKGPEGLQSPIVKFIPE
DNLNVILDRVGAVDGDIVFFGADNFKIVSEALGALRIKIGNDLKLHTCEWAPMWVVDFPM
FEENDDGSFTALHHPFTAPKCTPEELEANPATALSRAYDMVLNGTELGGGSIRIHRKEMQ
QAVFRLLGIAEDEQQEKFGFLLDALKYGAPPHGGLAFGLDRLVMLMTGAQSIREVIAFPK
TQSAADVMTQAPGVVDAKALRELHIRLREQPKAE
>tr|A0A0Q0BV26|A0A0Q0BV26_PSEAP DSBA oxidoreductase OS=Pseudomonas syringae pv. aptata OX=83167 GN=ALO85_03192 PE=4 SV=1
MNTVGPLKIDVWSDYVCPFCYLQLAVLEQLQQTYGERLEFNWHAFELRPDPLALLDPSAD
YLRETWSRSVLPMADRRQVTMKMPSVQPRSRKVLEAAAFARNAGSFEAFHKEAYRAFFEK
GLDIGETHTLLELAATLGLDRQAMEQALNAGHFEKAVMDDQQLAQKLGLRAVPVLLLRRS
GEALEDARVFNGTLPFDRLSQEIDALATWA
>tr|A0A0Q0D9X0|A0A0Q0D9X0_PSEAP Putative signal transduction protein OS=Pseudomonas syringae pv. aptata OX=83167 GN=ALO85_03976 PE=4 SV=1
MTEVAHVNDPHHAPSVIRQLLEKLAINYNEVVDEKNLPPARKVQAVLVEDAVGALLILFP
QSQLLDLSRITELTGRKLTAVPHERLARMLTKHNLRVLPGLPALTSSPCLYDERLLQEPT
LLVGSGEPGVLLEISSDDFKGMLSKASAAHFGQPVATIRPNLDRPDDDPAEITQAMHAFT
ARRIQQRLEATLEIPPLAGTAQKIIKLRVDPDATIDDITGVVETDPALAAQVVSWAASPY
YASTGKIRSVEDAIVRVLGFDLVINLALGLALGKTLSLPKDQPQQTTPYWQQSIYTAAVI
EGLTRAIPRASRPEGGLSYLAGLLHNFGYLLLAHVFPPHFTLICRQLEVNPHLHHSFIEQ
HLLGISREQIGAWLMRYWDMPDELSIALRFQHDPHYKGEHHVYANLVYLAVRLLRANGIG
SGPEQDIPDELFDRLGLTRDKANESVKKVLEAEVLLRELASQFSK
>tr|A0A0Q0CZV3|A0A0Q0CZV3_PSEAP Prephenate dehydrogenase OS=Pseudomonas syringae pv. aptata OX=83167 GN=ALO85_03527 PE=4 SV=1
MVDVIAEQSVRPVIGRLVVVGLGLIGGSFAKGVRESGLCGEVVGVDLDPQSRQLAVELGV
VDRCEADLATACRGADVIQLAVPILAMEKLLALLAPIDLGHAILTDVGSAKGNVVRAARQ
AFGHMPSRFVPGHPIAGSEQSGVEASNAALFRRHKVILTPLTETDPQAVAIVDQLWSALG
ADVEHMQVERHDEVLAATSHLPHLLAFGLVDSLAKRNENLDIFRYAAGGFRDFTRIAGSD
PTMWHDIFMANRDAVLRTLDSFRTDLDALREAVDAGDGAQLMDVFTRARAAREHFGRILA
SRARVDTAPEEPRALHSGSAEGVSHRIKGPVDVTSAVLFMVATSISEGCDLLLEDVPVSS
SCEGAIDILRLMGGDIVLQNVRKLANETVADLHVRSARLKGADIPEALVPLALNAFPVLL
VAAACAEGRTILRGAQALQADESECVRLMADGLLALGIEAEPVLDGIIIEGGVPGGGEVD
ARGDQRIVMAFRVASLRASAPIRIQACADAAALFPHFLALCAQVGMRVAQEDKK
>tr|A0A0Q0DPY6|A0A0Q0DPY6_PSEAP Uncharacterized protein OS=Pseudomonas syringae pv. aptata OX=83167 GN=ALO85_00782 PE=4 SV=1
MTDKKDEKKPEPQSDVPAHSTPEEKERLKDFNKDGIPPGVC
>tr|A0A0Q0E0L8|A0A0Q0E0L8_PSEAP Type II secretion system protein OS=Pseudomonas syringae pv. aptata OX=83167 GN=ALO85_03296 PE=3 SV=1

MASKAVKVIVYTWEGVDKKGTKTSGELSGHNLALVKAQLRKQGINPTKVRKKSASIFGKG
KKIKPLDIAFFSRQMATMMKAGVPLLQSFDIISEGAENPNMRTLVNSLKQEVSAGNSFAT
ALRQKPEYFDDLFCNLVDAGEQAGALESLLDRVASYKEKTEKLKAKIKKAMTYPAAVLIV
AVIVSGILLIKVVPQFQSVFAGFGAELPTFTLMVIGLSVFVQEWWLAIIGIFFASIFMFK
KSYKKSQKFRDSLDRFLLKVPIIGPLIFKSSVARYARTLATTFAAGVPLVEALDSVAGAT
GNVIFKNAVNKVKQDVSTGMQLNFSMRSTGVFPSLAIQMTAIGEESGALDSMLDKVATYY
EDEVDNMVDSLTSLMEPMIMAILGVVVGGLVIAMYLPIFKLGNAV
>tr|A0A0Q0DA33|A0A0Q0DA33_PSEAP Ribonuclease T OS=Pseudomonas syringae pv. aptata OX=83167 GN=rnt PE=3 SV=1
MSEDNFDDEHEGHGGGGGSRHPMAARFRGYLPVVVDVETGGFNSATDALLEIAAVTIGMD
EKGFVFPEHTYFFRVEPFEGANIEAAALEFTGIKLDHPLRMAVSEETAMNDIFRGVRKAL
KANGCKRAVLVGHNASFDLGFVNAAVARMDMKRNPFHPFSSFDTATLAGLAYGQTVLAKA
CQAAGIDFDGREAHSARYDTEKTAELFCGIVNRWKEMGGWEDFDD
>tr|A0A0Q0FWN7|A0A0Q0FWN7_PSEAP Transglycosylase OS=Pseudomonas syringae pv. aptata OX=83167 GN=ALO85_01424 PE=4 SV=1
MGIIGTIFIGLIVGLLARFLKPGDDSMGWIMTIVLGIAGSLAATYGGQALGIYQAGEGAG
FLGALVGAIILLVIYGMIKKK
>tr|A0A0Q0DGU2|A0A0Q0DGU2_PSEAP Uncharacterized protein OS=Pseudomonas syringae pv. aptata OX=83167 GN=ALO85_03210 PE=4 SV=1
MTVCFVRGESGADTSSRLDPQRPDFNMTTDFQHLTTRQSALSVTQLAEQQAKLSGPVGLF
AKLCRQLRSHGRQSPLLEDIARLEGRKRQWLAERAVQFALGLHGRRSEAENPFKGLVRED
LCCIVFDDPSPHTLVERYAASEALRAQDSEYFVKLIATTRNTVERRIVFHGLLEHFDRLL
PIEKSIYPLNYRAVQQAHLDHEEALYGALIKQQPIGTLLERHTPEWLLNNLSSFELSIDQ
ACG
>tr|A0A0Q0BTF9|A0A0Q0BTF9_PSEAP FAD:protein FMN transferase OS=Pseudomonas syringae pv. aptata OX=83167 GN=ALO85_04783 PE=3 SV=1
MGLRRFGKVAGCLVLLVCSGCGQERKLESFGGPTMGSHYSVVYARASGQPEPAAVRPQVE
AILDEVDQQMSLWRTDSDIERFNALPANSCQVMPEPVLKMIGVGEQLAQESDGAFDLTVK
PLMDLWGLGAHTLFEQMPLVRQLARTQALVGYRNLRIVGDRLCKSAAVQVDLNSIAAGYA
VDRISERLDVLGLHDYLVQATGELKVSGLKPDGSPWRVTLDAPLDDGTVTQKVFPLEGYA
VSTSGDFRRYTDHEGWRISDTINALTGKPISHSLASVTVIDPSALMADGLSSLLLILGPQ
RGWDYAQEHKIPAFFVIRADKRFIIRSNASFDRLVVEQPR
>tr|A0A0Q0DXH6|A0A0Q0DXH6_PSEAP Alkyl hydroperoxide reductase C OS=Pseudomonas syringae pv. aptata OX=83167 GN=ALO85_03668 PE=3 SV=1
MPIINSQVKPFKATAFKNGAFVDVSDADFKGKWSVVFFYPADFTFVCPTELEDLADNYEE
FKKLGVEIYSVSTDTHFAHAAWHNTSPAIGKIQYTMIGDPTLTISRNFDVLIEEAGLADR
GTFVINPEGQIKIVELNDGGVGRDASELLRKIKAAQYVAAHPGEVCPAKWKEGEATLAPS
LDLVGKI
>tr|A0A0Q0FNB2|A0A0Q0FNB2_PSEAP Outer membrane lipoprotein SlyB OS=Pseudomonas syringae pv. aptata OX=83167 GN=ALO85_02741 PE=4 SV=1
MRKSALLVASFTAMALTLGGCTSNLTGDSYSRDEARTVQTVRMGTIESLRPVKIEGTKTP
IGGAAGAVVGGVGGSAIGGGRGSIVAAVIGAVAGGLLGSATEEGLTRTQGVEITVREDDG
SMRAYVQQVQENEIFRVGERVRIMSVNGTSRVTH
>tr|A0A0Q0IHL3|A0A0Q0IHL3_PSEAP Uncharacterized protein OS=Pseudomonas syringae pv. aptata OX=83167 GN=ALO85_05291 PE=4 SV=1
MRAHEARNFYTYKERPMYNATTEKKENVLTAPVIAAALNDGLLPVDSLNTPLEVLLNVWQ
GARPGYTYQLYFDGVLIGLKKEILPSQMPGDDLMLHIPPELLKEGRHSVAYAVENPINMV
VEFSAETAVIVDLTPPVDPLLAPIIFPAQTQNGLTSDELQTMGNVLSGTIASYNGMQEGD
VVRTYWNDLPGPMAVVSKDDVGLRRTMVDFTRSFLELIGDIEAPVYYTVTDLAGNLSMAS
EAVDVKLQLTHVTPLPTPTLKEAIGTTLDPANASSGATVVIDAVANLQAGDQVITQWQWP
NGNDTREKTLTGAEAGKTLEVVFAAALVTANAGQTVAISYVVNRVNGLVQVSGTLALQIL
AAQPELVLDTSPVTLGGKVYLLPGSPDLLPNFPADTTLLRQASGGQAPYQYASSDPLVAK
VDSNGLTSVRGKGTAIITATDALGASKQYTVTVTGVIHCIGLGSGSFSQISKNAGNNGAR
IPTIHELVDIHALYGNRWPMGKGNYWSSTVSSAGIGGWNWYYVKNMVTGGNFKLKSHNSS
LGVGIR
>tr|A0A0Q0CH22|A0A0Q0CH22_PSEAP Aspartate/glutamate ABC-type transport system OS=Pseudomonas syringae pv. aptata OX=83167 GN=ALO85_02038 PE=4 SV=1
MISIKNVNKWYGDFQVLTDCSTEVSKGEVVVVCGPSGSGKSTLIKCVNALEPFQKGDIVV
DGTSISDSKTNLPKLRSRVGMVFQHFELFPHLSIVENLTIAQIKVLGRSKAEATEKGLKL

LERVGLSEHAHKHPGQLSGGQQQRVAIARALAMDPVVMLFDEPTSALDPEMVNEVLDVMV
QLAQEGMTMMCVTHEMGFARKVANRVIFMDKGQIVEDCAKEEFFGDVSARSERAQQFLAK
ILQH
>tr|A0A0Q0DCH7|A0A0Q0DCH7_PSEAP tRNA uridine 5-carboxymethylaminomethyl modification enzyme MnmG OS=Pseudomonas syringae pv. aptata OX=83167 GN=mnmG PE=3 SV=1
MVDFPSRFEVIVIGGGHAGTEAALASARMGVKTLLLTHNVETLGQMSCNPAIGGIGKSHL
VKEIDALGGAMAMATDKGGIQFRVLNSRKGPAVRATRAQADRVLYKAAIRETLENQPNLW
IFQQACDDLIVEQDQVRGVVTQMGMRIFAESVVLTTGTFLGGLIHIGMQNYSGGRAGDPP
SIALAQRLRELPLRVGRLKTGTPPRIDGRSVDFSVMTEQPGDTPIPVMSFLGNKEQHPPQ
VSCWITHTNARTHEIIASNLDRSPMYSGVIEGIGPRYCPSIEDKIHRFADKESHQVFIEP
EGLTTHELYPNGISTSLPFDVQLQIVQSIRGMENAHIVRPGYAIEYDYFDPRDLKYSLET
KVIGGLFFAGQINGTTGYEEAGAQGLLAGTNAALRAQGRDSWCPRRDEAYIGVLVDDLIT
LGTQEPYRMFTSRAEYRLILREDNADLRLTEKGRELGLVDDARWAAFCTKRESIELEEQR
LKSTWVRPGTEQGDAIAAHFGTPLTHEYNLLNLLTRPEIDYASLISITGQGCIDPQVAEQ
VEIKTKYAGYIDRQQDEIARLRASEDTGLPEDIDYAAISGLSKEIQSKLGITRPQTLGQA
SRIPGVTPAAISLLMIHLKKRGAGRQLEQSA
>tr|A0A0Q0DQW4|A0A0Q0DQW4_PSEAP Major facilitator family transporter OS=Pseudomonas syringae pv. aptata OX=83167 GN=ALO85_100461 PE=4 SV=1
MNPAQFDDKTSALTTWMTLLERGIRVAVSIIDPFQIKTPRPAMTTTTVEQVDPATLKKVI
VAAAIGNFVEWFDFAVYGFLATTIALQFFPTGDSSAALLKTFAVFAVAFAFRPLGGIFFG
MLGDRIGRKKTLAMTILLMAGATTLIGLLPTYAAIGVMAPVLLTIIRCAQGFSAGGEYAG
ACAYLMEHAPRTQRAWYGSFVPVSTFSAFAAAAVVAYALESFLSTEAMGNWGWRLPFLIA
APLGLVGLYLRWKLDETPAFQAVAQEHAVAHSPLKDTLRHHAAAMCCLGAFVSLTALSFY
MFTTYFATYLQVAGGLSRATALLVSLIALAFAAALCPLAGAYSDRVGRRATMATACILLM
VVVFPSFLMASSGSFIASIIGVMLLAVGAVLCGVVTAALLSETFPTRTRYTASAITYNMA
YTLFGGTAPLMATWLISMTGSNLSPAFYLIAVAALAMAGGMALPETSKISLHDVPVASEE
PVVGSIA
>tr|A0A0N8T8H5|A0A0N8T8H5_PSEAP Uncharacterized protein OS=Pseudomonas syringae pv. aptata OX=83167 GN=ALO85_03496 PE=4 SV=1
MTPLMITLMVVAGIALLIAIGYLNHVAENGKLEKARTKVELGDRLRRCSEITEVFPGQLM
SPALKLLLARLELNVVQRMLNMEKGNAQLKHRIGELEEQIAKGEGIEIPNPVTPIQTEAK
AKDVRFLLEALHGQVTRAAHDGFLQTSEAKHWIREIRTILVNLHIEFFNNLGQTALSQDQ
PGQARLAFERGVQYLRNQPDPVTYQQQLLAMEKQLARANSVVLTNTAPTEDDDNELTEGL
KQDDTESEWKKKVIYD
>tr|A0A0Q0IN19|A0A0Q0IN19_PSEAP Uncharacterized protein OS=Pseudomonas syringae pv. aptata OX=83167 GN=ALO85_100744 PE=4 SV=1
MTLFQTLEQRLCHHHVTDPARPNDQNFHVLSKNDQKRSGRPIRLRDVPTQPVASLS
>tr|A0A0Q0D0T2|A0A0Q0D0T2_PSEAP NADH:ubiquinone oxidoreductase subunit M OS=Pseudomonas syringae pv. aptata OX=83167 GN=ALO85_00749 PE=4 SV=1
MILPWLILIPFIGGLLCWLGERFGSTLPRWIALLTMTLELVLGLWLWATGNFGYAPVPGA
DPTWTLEFHHPWITRFGIDVHLALDGLSLLMILLTGLLGVLSVLCSWKEIQRKVGFFHLN
LMWILGGVVGVFLAIDLFMFFFFWEMMLVPMYFLIALWGHSSSDGKKTRIYAATKFFIFT
QASGLIMLVAILGLVLVHFNQTGVITFAYADLLKTKLTPGTEYILMLGFFIAFAVKLPVV
PLHSWLPDAHAQAPTAGSVDLAGILLKTAAYGLLRFALPLFPNASAEFAPIAMTLGLVGI
FYGAFLAFAQTDIKRLIAFSSVSHMGFVLIGIYSGSQQALQGVLIQMIAHGLSAAALFIL
SGQLYERLHTRDMREMGGLWSRIAYIPAISLFFAAASLGLPGTGNFVGEFLILLGSFASS
PWITAIATSGLVFGSVYSLIMIHRAYFGPSKSDEVLKGMDGREMIMVLGLAVLLVVLGVF
PQPFLDTSAATMHGVQQWLGTAFTQLASAR
>tr|A0A0Q0ID62|A0A0Q0ID62_PSEAP Secretion protein HlyD OS=Pseudomonas syringae pv. aptata OX=83167 GN=ALO85_01669 PE=3 SV=1
MPVRISRLVTFGLAAALAALAGCGEEKPAAPELPRVYVQTVKSADFAASVALTGDVQARV
QTRLSFRVNGKIIQRNVDVGDRVTARQVLARLDPKDLQINVDSAAAAVAAEQARVNQASA
AFVRQQKLLPKGYTSRSEYDSAQAALRGSESSLKAAQAQLANAREQLGYTALVADAPGVI
TARQAEVGQVVQATMPIFDLARDGARDAVFNVYESLFVKPPTNQAVQVTLLDNPNIKVSG
KVREVTPAVSAQTGTLQVKIALDPLPEGMDLGSVVSVALSAPANASVELPWAALTKDLGE
QLGKPAVWVVDEQGKVNLRKVTVTRYLTAKVIIGDGLKSGEKVVVAGGQLLHPDMQVEIA
EQPKQPNAQVQP
>tr|A0A0Q0CV40|A0A0Q0CV40_PSEAP SIR2_2 domain-containing protein OS=Pseudomonas syringae pv. aptata OX=83167 GN=ALO85_04043 PE=4 SV=1

MPSLNFEKQAQDYYNKAPLIILGSGASAAHGMSGMWALANHLVANTEISGLSADEIEAWD
RFCKVLDAGVDLESALHQVAVTDELTSRIIKSTWSLINAEDIHIYYESLQKQAMFPLSRL
LRHMFKSSLPKLNILTTNYDRLAEYACDQEGIHHYSGFTHGFFRQLAAPNEINSARRVNI
WKVHGSLDWFKSPLDDIVALSNIQGIPVNYKPEIVTPGTQKYQTTHLEPYRSIINNADQA
ITAANSYLCIGYGFNDEHIQPKLMARCQRQNIPITIITYALSDAAKKLITDGKAQSYLAI
ERGETDDQSIVYSSWDKTPVTVERNIWSLEGYLSLIM
>tr|A0A0Q0C785|A0A0Q0C785_PSEAP Flagellar biosynthetic protein FliQ
OS=Pseudomonas syringae pv. aptata OX=83167 GN=ALO85_04033 PE=4 SV=1
MTPEVAVDLFREALWLTTVLVAILVVPSLLCGLLVAMFQAATQINEQTLSFLPRLLVMLV
TLIVIGPWLLKIFMEYMLSLYTSIPTLIG
>tr|A0A0Q0C128|A0A0Q0C128_PSEAP Tyrosine--tRNA ligase OS=Pseudomonas
syringae pv. aptata OX=83167 GN=tyrS PE=3 SV=1
MKSVEEQLALIKRGADELLVEAELVEKLKRGQPLRIKAGFDPTAPDLHLGHTVLINKLRQ
FQDLGHQIIFLIGDFTGMIGDPSGKSATRPPLTREQVLDYAETYKSQVFKILDPAKTEVA
FNSTWMDQLSPADFIRLSSQYTVARMLERDDFDKRYKSNQSIAIHEFLYPLVQGYDSVAL
KADVELGGTDQKFNLLMGRELQRAYGQEPQCILTMPLLEGLDGVKKMSKSLGNYVGIQEA
PGIMYSKLVSIPDSLMWRYFELLSFRSMEEIDGLKADCEAGANPRDIKIKLAEELVARFH
GEEAAANAHRSAGNRMKEGELPDDLPEISVAAAEDMPISAVLNKAGLVKNAAVARDLLAS
GGVRIDGEVVDRSFVFKLGATHVCQAGKKAFGRVMLVSEESSN
>tr|A0A0Q0BSK8|A0A0Q0BSK8_PSEAP Rod shape-determining protein MreD
OS=Pseudomonas syringae pv. aptata OX=83167 GN=ALO85_00153 PE=3 SV=1
MVSHVSGRNGWVVWLTFAIGLLLSVSPLPQFMEIFRPLWLALLLTFWVLALPHKYGMTTA
WILGLMEDVLYGTLLGQNALILSLITFLVMSLQQRLRMFPMWQQCLVLLVVFGLAQLAQL
WLSALTGNRQPTLALVLPALVSALLWPWISYGLRGLRKRLKIN
>tr|A0A0Q0DUA1|A0A0Q0DUA1_PSEAP Serine protease OS=Pseudomonas syringae
pv. aptata OX=83167 GN=ALO85_03300 PE=3 SV=1
MKLLAPGANTALANAHCTWNLESGKSSVFGEYAAVALLAVNDKRQPMGDPALLHQEQGWM
EWSGGPQDVGCTLRLDRLPTGSDRVLLMVYVYAAMGPIRDIGSLHLKVDGDIEHRLDLRD
NGEAAIIIGEFYQRNEQWKFRALSEGSAYGLSAFGRKIGLDVDDRHPRRPSAGSGGGPRH
ESATGTAFVVGPAHVMTCAHVIEDMGVFYITSLEGRYKAEPVVIDRRNDIALLRVQGAPL
LSPVTFRDGQGCEPGDTVAVLGYPLASISGGGLQVTQGGISGLFGLHNDASLFQFTAPIQ
PGSSGSPLFDNGGAVIGMVTSTVPDGQNMNFAVKSALLLAFLQACRIDAAHARPERSYTT
TEISRTAQSSLWLVEASRQ
>tr|A0A0N8T9G5|A0A0N8T9G5_PSEAP Uncharacterized protein OS=Pseudomonas
syringae pv. aptata OX=83167 GN=ALO85_00960 PE=4 SV=1
MTDHITIIKRFVMKNILLLAAAVGCSLGLSAQATTFAYISSPTDGLISQYRLDEKSGALS
LVEQTRAGDQVNPMAITPDGKALFAALRAKPYQVLSFSIEPGTGHLKPLSQAPLAESLAY
LSTDRSGRFLFGASYGADLLSVQPIDAQHRPSDSIQTYKTGMHAHSVRTDPSNRFVYAGN
LGVDRVLQYRLEPKDGKLVPIGEGYVAVPENTGPRHLAFSSDGKFLYVVGEMNGTVTAFA
INQKTGALKQTSQADGIPKRLNLAPGQARDARNNDLKDDPTPRIWAADIRIAPNGKWLFI
SERTSSSVSVFKVEPTNGKVAFVDNYPVQEKQPRNIAVSPNGRWLLVSGEKSDKVGSYAI
GEKGALQRVSEAPSGKGALWIEMLSQPSE
>tr|A0A0Q0C4P4|A0A0Q0C4P4_PSEAP Uncharacterized protein OS=Pseudomonas
syringae pv. aptata OX=83167 GN=ALO85_00977 PE=4 SV=1
MTKVTFPNACQLMRWHFHPVGFEASMDAPGSMIARLFDRASGETVVAIAGLPCSTVMNAA
DVERIIEAIEDELELFGTHTQRQIAGFI
>tr|A0A0Q0IP22|A0A0Q0IP22_PSEAP HupE/UreJ protein OS=Pseudomonas syringae
pv. aptata OX=83167 GN=ALO85_01920 PE=4 SV=1
MNYKKALGALALLLVPTLALAHPGHGDNGLIAGISHPIGGLDHLLAMLAVGLWAAQQQGS
ARWALPCTFVGTMLIGGMLGFEGLDLPALESGIAASVLALGLAVALAVRPPLALAVAATA
LFALFHGVAHGLELPEMSSPWAYAAGFVAATAALHALGYAMVRVLPQAAAPLVRIAGAAS
AAAGVWLLAA
>tr|A0A0Q0DE64|A0A0Q0DE64_PSEAP Thioesterase OS=Pseudomonas syringae pv.
aptata OX=83167 GN=ALO85_01274 PE=4 SV=1
MSTPVSLTLFCLPYSGASAMFYSPWRRKLPEWLNVRPLELPGRGMRMDEPLQTDIVQLAS
HLADEISADLDKPYALFGHSLGGLLAFELAHVLRERGLPAPLALFASATAGPVRRDVSEY
ATAKTDAQLLDRLRTLKGTSENVITNQELMQLMLPILRADFLLCGSFVYGKREPLSVPIH
VFGGKQDSISVEQLLDWQEETCTGFSLDMFEGHHFYLVDEQTQLLRLLRRYCEQHLTRWR
NSASRHMNRAAG

>tr|A0A0Q0C3R6|A0A0Q0C3R6_PSEAP Band 7 protein OS=Pseudomonas syringae pv. aptata OX=83167 GN=ALO85_01457 PE=4 SV=1
MSQPVDEAVAQRPPVNSPWLQASRLAFIGLYGVTLLAALAWATSNVRQIDPQNRAVVMHF
GAIERVQNAGLLVAWPQPFEQVVLLPSADRVIERRVETLLRSPAALKADEIATLSAPMSD
ALAGSGFLLTGDAGVVQLDVTVFYKVTDPTAFVLQGEHVLPALDRLVNRSAVALTAARDL
DTILVARPELIGADSQAAERRERLRGDLVRGINQRLAELKATGIGIGVEVARVDVQSSLP
TSAVNAFNAVLTASQQADQAVANARTDAEKLTQTANQQADRTLQVAHAQASERLAKAQAA
TATVVSLTQSAETRSDPGLMQRLYRERVPVILHQAGSVTTVDPKDDSRLIIQGAPQ
>tr|A0A0Q0ILS6|A0A0Q0ILS6_PSEAP RHH_4 domain-containing protein OS=Pseudomonas syringae pv. aptata OX=83167 GN=ALO85_00246 PE=4 SV=1
MEIRHDPFVEDFNMTLAQPHSKSVRLNGLATCLRLEKVYWNILSGIASSNECSVNAVLSY
IDREVHLRYGGVKNFSGLIRVVCVAHILKGDCLENSHV
>tr|A0A0Q0FP61|A0A0Q0FP61_PSEAP Uncharacterized protein OS=Pseudomonas syringae pv. aptata OX=83167 GN=ALO85_02427 PE=4 SV=1
MTTASPDTALCPLCGFSNQCSLADPRTVDQPCWCFSQSIDPALLAALPDNLRDKACLCPR
CAGIKDAALNPQARPATE
>tr|A0A0Q0DJI5|A0A0Q0DJI5_PSEAP Putative phosphoenolpyruvate synthase regulatory protein OS=Pseudomonas syringae pv. aptata OX=83167 GN=ALO85_00575 PE=3 SV=1
MKRSAFFISDGTGITAETLGQSLLAQFENITFNKFTRPYIDSVEKARAMVQQINNAADKD
DVRPIIFDTIVNQDIREILATSNGFMIDIFSTFLAPLEQELSSHSSYSVGKSHSIGHNSN
YMERIEAVNFALDNDDGARTHYYDKADIILVGVSRCGKTPTCLYMAMQFGIRAANYPLTE
DDMERLQLPAALKQHREKLFGLTIDPDRLTAIRHERKPNSRYSSYAQCEFEVREVESLFR
RENIAHINSTHFSVEEISAKVLVEKGVERRFK
>tr|A0A0Q0C568|A0A0Q0C568_PSEAP 3-deoxy-D-manno-octulosonic acid transferase OS=Pseudomonas syringae pv. aptata OX=83167 GN=ALO85_02874 PE=3 SV=1
MNRTLYTVLFHLGLPLVALRLWLRARKAPAYRQRIGERFARGLPVMQRGGIWVHAVSVGE
SIAAAPMIRSLLVQYPQLPITVTCMTPTGSERIKALFASEPRIQHCYLPYDLPWAAAVFL
DQVQPRLGIIMETELWPNHIHQCSLRGIPVVLANARLSERSARGYARFAGLTRPMLAEMA
WFAVQTEAEAQRFRDLGARPECVAVTGSIKFDLSIDPQLLQRAAQQREQWQIKQRPVWIA
ASTHAGEDESVLAAHRTLLTSHPDALLILVPRHPERFDSVHALCQQQGFATVRRSSAQAV
TADVSVLMGDTMGELLFLYALADIAFVGGSLVPNGGHNLLEPAALAMPVLSGPHLFNFLE
IAAMLRKAGALQEVNDAAALAAAVQGLFDQPQQARNMADAGLAVMKANQGALQRLLDGIG
RLMSRR
>tr|A0A0Q0CZS0|A0A0Q0CZS0_PSEAP Uncharacterized protein OS=Pseudomonas syringae pv. aptata OX=83167 GN=ALO85_05415 PE=4 SV=1
MKESMKEIAKFFLLLLAVCFVALAVSIVLPPPDGGVFAYSVLLSAIALMLIICGDLSSGR
INATGDIMRAIESRRSLRLTVTAYVLGCSMVASITVLIYRLAGHW
>tr|A0A0Q0BW32|A0A0Q0BW32_PSEAP Uncharacterized protein OS=Pseudomonas syringae pv. aptata OX=83167 GN=ALO85_01164 PE=4 SV=1
MNNWISALAELQARGEPGILVTIIEELGSTPRNAGSKMVVCAEHIHDTIGGGHLEYKAME
IAREMLASGTRQTRLERFNLGASLGQCCGGVNVLLFEPMGEPVAQIAVFGAGHVGRALVP
LLASLPCRVRWIDSREQEFPESMPEGVTKIINDEPVDEVAQLPVGTYCIVMTHNHQLDLE
LTAAILRRGDFGYFGLIGSKTKRVKFEHRLRERGFDASLLQRMRCPMGLTEVKGKLPIEI
AVSIAAEIIATYNVSFGQHTANAEPIARLLPASRRSQSQ
>tr|A0A0N8TAA3|A0A0N8TAA3_PSEAP Sugar ABC transporter permease OS=Pseudomonas syringae pv. aptata OX=83167 GN=ALO85_03260 PE=3 SV=1
MNLRTLKKALLRLAFWGLVGVVLLYAIFPFYYAVLTSLKPSSALFEVSYWITEADFSNYA
AVLSQASFLQAIANSLVVALSVVVLALFLGLTASYALGRVKFRGRGTVLMMVLGVSMFPQ
VAVLSGLFEVIRALGLYNTSWALILSYTIFTLPFTVWVLTTFMGQLPHELEEAAIMDGAS
PWVTLTRVLLPLLWPALVTTGLLAFIAAWNEFLFALTFTLTDAQRTVPVAIALISGGSSH
ELPWGLLMAASVLVTVPLVILVLIFQRRIVSGLTAGALKG
>tr|A0A0Q0DMW0|A0A0Q0DMW0_PSEAP Uncharacterized protein OS=Pseudomonas syringae pv. aptata OX=83167 GN=ALO85_02854 PE=4 SV=1
MLLMLYLIAITAEAMTGALSAGRRGMDWFGVVLIACVTALGGGSVRDVLIGHYPLTWVKH
PEYLVLTSVAALVTIFIAPLMRHLRSLFLVLDALGLVAFTLIGCMTALEAGHGLLIASVC
GVITGVFGGILRDIFCNDIPLIFRRELYASVSFLAAWCFLLCQYLQLPDEQAVLITLFGG
LLLRLLAIRFRWEMPKFVYKDEP

>tr|A0A0Q0IKK6|A0A0Q0IKK6_PSEAP GntR family transcriptional regulator OS=Pseudomonas syringae pv. aptata OX=83167 GN=ALO85_00905 PE=4 SV=1
MQRFDPDIPIDNSSKLALDALRQIVAQHAAFPQRALPTERDLAADFGVSRRAVRRALSVL
EAEGQVWRRQGKGTFVGPTPPSAAMSFARLSGRTNFTEVMEARLHLEPALASLAAVRANG
EQMAILRRLAERTTRQQVAEQTDAEGIELWDSALHRAIAEAAGNRLMLDIFEMLDAIRLD
PAWRDLRHRARNADRLDTYSHDHDDIVSAIESRDPIKAATAMRGHLRALQQALNTVINQD
LEASL
>tr|A0A0N8T8B1|A0A0N8T8B1_PSEAP Phosphoribosylaminoimidazole-succinocarboxamide synthase OS=Pseudomonas syringae pv. aptata OX=83167 GN=purC PE=3 SV=1
MEKREELYRGKAKSVYKTDDADRLILLFRNDTSAFDGKRIEQLDRKGMVNNKFNAFIMQK
LEEAGIPTQFDKLLGDNECLVKKLDMIPVECVVRNYAAGSLVKRLGIEEGTRLNPYTFEL
FLKDDAKGDPFINESHVVAFGWGTAEQLVRMKELSIKVNDVLTKLFDDAGLLLVDFKLEF
GVFHGEIVLGDEFSPDGCRLWDKDTRKKMDKDRFRQGLGDVIEAYEEVANRLGVPL
>tr|A0A0Q0FF64|A0A0Q0FF64_PSEAP Nucleotidyl transferase OS=Pseudomonas syringae pv. aptata OX=83167 GN=ALO85_03120 PE=4 SV=1
MLQIIVPMAGAGSRFAVAGYIDPKPLIPVHGVPMIKVVIDNLTPECPHRFIFICQAEQVA
RYGLREKLNAWAPGCEIVELGGLTGGAACSVYAARHLLDDSQQVMIANSDQYVDVDINAY
LQAINESDAEGLIMTMKASDPKWSFVGFSAAGLIDRVVEKEVISDEATVGIYNFRRAGQL
ISAIEAMVAKDLRVNGEFYVAPVYNELIGQGARVIHYSIGAEGHGMYGLGIPADLNQFLS
LPLSRQAASGKEA
>tr|A0A0Q0DIP8|A0A0Q0DIP8_PSEAP Segregation and condensation protein A OS=Pseudomonas syringae pv. aptata OX=83167 GN=ALO85_03810 PE=3 SV=1
MTAVEAVDSQANAQQELPFALVYGRAVTQMPLDLYIPPDALEVFLEAFEGPLDLLLYLIR
KQNIDILDIPVAEITKQYMGYVELMKTVRLELAAEYLVMAAMLAEIKSRMLLPRSAEVEE
EEGDPRAELIRRLQEYERFKAAAEGIDGLSRVGRDVIVPKLDAPEARARKLLPDVSLEEV
LMSMAEVLHRGDMFESHQVSREALSTRERMSDVLERLKGGGFVPFAELFTAEEGRLGVVV
TFMAVLELVKESLVELVQNEPFAAIHVRARAE
>tr|A0A0Q0C861|A0A0Q0C861_PSEAP Uncharacterized protein OS=Pseudomonas syringae pv. aptata OX=83167 GN=ALO85_04320 PE=4 SV=1
MPLSSDTLRRTLVIWLYAVAVAHVLGSLVFTWAGFSGLLDGYLTSLEQAFWTEAVPAAAR
AQQVWWMALFGATLQTYSVYMLALVHLGNRLKSAMPWGWLIAGLLLWAPQDILISVRGGV
WSHVWLDLAALLALLPPLIWLYRHDRRTSAVHSSKDPRHV
>tr|A0A0Q0C1P8|A0A0Q0C1P8_PSEAP HDIG domain protein OS=Pseudomonas syringae pv. aptata OX=83167 GN=ALO85_01747 PE=4 SV=1
MRLPAAWHAYARRWLSMLKKILVAELALGMHIHKICRRWTNDPFWIGLVEVMLDDVEVLK
RIQQSGTKEVWIETGKSRVVKPQTSPASPSASEDTTPASLETEVLRARLICGRARDAVMS
MFTEARMGRAMDVGNVQLLVEEISNSILRHPHALISLSRLKTSDEYTYMHSVAVCALMVA
LAKRMGMPDDQVREAGVAGLMHDVGKMMIASAVLNKPGRLTRDEFEIIKAHPELGVKILM
DTQPVAASVIDVCLHHHEKVDGSGYPHGLKGDQISIFARMGAVCDVYDAVTSDRPYKKGW
GVAHSIREMASWKGHFDDMVFQSFVKTVGIYPIGTLVRLESGRLGVVVEQGEASLLKPKV
KVFMSARTGKTFAAQVIDLDCPSDPDGILRIEKTTDWGLEEVDTLWAGIPA
>tr|A0A0Q0DHW9|A0A0Q0DHW9_PSEAP Uncharacterized protein OS=Pseudomonas syringae pv. aptata OX=83167 GN=ALO85_00028 PE=4 SV=1
MNYQEFIGHAQAGRVDELNLISIEGGIYLLDVRMNGTSVMLKDPAGKTLHLRSVEHARDL
LKDIPVVPFYLVHCVVHDELCGMPVNDRSEMRLPISFHSSWS
>tr|A0A0Q0DLG7|A0A0Q0DLG7_PSEAP UDP-3-O-acylglucosamine N-acyltransferase OS=Pseudomonas syringae pv. aptata OX=83167 GN=lpxD PE=3 SV=1
MSITIKLGQLAEFLGATLRGDKDIEITGLATLQEAGPGQVSFLANPKYRKLLVDTQATAV
LLKPADAEGYTGNALVVPDTYLAYARISHFFDPKPKSSAGVHPTAVIAADALIDPAASIG
AFAVIESGVRIAAGVTIGAHCFIGARCEIGEGGWLAPRVTLYHDVRIGKRVVIQSGAVLG
GEGFGFAQDKGIYHKVAQIGGVTLGDDVEVGVNTAIDRGALADTRIGNGVKLDNQIQIAH
NVQVGDHTAMAACVGISGSTKIGKHCMLAGGVGLVGHIDICDGVYITGMTMVTHSITEPG
SYSSGTAMQPADEWRKSAARLRKIDDMARRLQKLEKVVETVTSSGNRSSDG
>tr|A0A0Q0BSX2|A0A0Q0BSX2_PSEAP Peptidase, M23/M37 family OS=Pseudomonas syringae pv. aptata OX=83167 GN=ALO85_100309 PE=4 SV=1
MFQASKQWLRGVFFAAHGGHTRPDIFSHNSSVSPTHMLRALIALALICLLNPAFAEDERV
QTQKQIDAARQDVAELQKVLGKLQEERAGVQKDLRTTETEMGKLEKQVEVLQKELKKTET
ELQKLDSEKKKLNSARVEQQRLIAIQARAAYQSGRQEYLKLLLNQQHPEKFARTLTYYDY
LSQARLEQLRNFNETLRQLANVEKDIDLQQAQLLVQKSNLDSQSEELAKVRQERQQALAK

LNQDYKARDQKMQARQQDQADLAKVLKTIEETLARQAREAEEARQKALVAAREAEEKRQR
EAEAVARNNSSNSSSKSDDEPAPRRPVKAPGAVVSSAGVSYGGPFAQAKGKLPWPIDGRL
LARFGEARGDDERTKWDGVMISAAAGSQVRAVHGGRVVFADWLRGAGLLVILDHGNGYLT
LYGHNQSLLKSAGDIVKAGEAISTVGNSGGQDTAALYFAIRQQGRPSDPAQWCRTQG
>tr|A0A0Q0IQX2|A0A0Q0IQX2_PSEAP 50S ribosomal protein L4 OS=Pseudomonas syringae pv. aptata OX=83167 GN=ALO85_200040 PE=4 SV=1
MNGFESPTSVESTHAVFRLSWLAYANTVMTIIFFIVVSIAIGQWTAHNAQSDAAFRLGMT
ASAFILVVSLSVCIYNILFLKSARVYTDDVGVWLYRGILPWSKGVRGVKWRDIEDAMYYT
GFFSWVFRAYTVRVGHRFTKTSEIIVPHLAQGHKAVEHINHLHQTILKSEQAAELA
>tr|A0A0Q0FP48|A0A0Q0FP48_PSEAP Uncharacterized protein OS=Pseudomonas syringae pv. aptata OX=83167 GN=ALO85_100969 PE=4 SV=1
MVHRDNASTGQSRQKSGQWINRCEGIANQLTDTISKNFCYVNNRG
>tr|A0A0N8T7Z9|A0A0N8T7Z9_PSEAP Ribosomal RNA large subunit methyltransferase K/L OS=Pseudomonas syringae pv. aptata OX=83167 GN=rlmL PE=3 SV=1
MWQGNALPAADGSGPGIHMSDRYELFLTCPKGLEGLLAEEATALGLQETREHTSAIRGSA
DMETAYRLCLWSRLANRVLLVLKRFPMKDAEDLYHGVLDVEWQDHLESDGTIAVEFSGHG
SGIDNTHFGALKVKDAIVDKLRTPDGERPSVDKINPDLRVHLRLDRGEAILSLDLSGHSL
HQRGYRLQQGAAPLKENLAAAILIRAGWPRIAAEGGALADPMCGVGTFLVEAGMIAADIA
PNIKRERWGFSAWLGHVPALWRKLHDEALARAETGLAKTPSWIRGYEADPRLIQPGRNNI
ERAGLSDWIKVYQGEVATFEPRPDQNQKGLVICNPPYGERLGDEASLLYLYQNLGERLRQ
ACLNWEAAVFTGAPDLGKRMGIRSHKQYSFWNGALPCKLLLIKVTPDQFVTGERRTPEQR
QIERENPVEVEVVERKLNKNGNPIKPEPVVVEQARLSEGGQMFANRLQKNLKLMGKWVRR
EGIDCYRVYDADMPEYSLAIDLYHDWVHVQEYAAPKSIDPEKASARLFDALAAIPQALNI
DKNRVVIKRRERQSGTKQYERQSAQGQFLEVSEGGVKLLVNLTDYLDTGLFLDHRPMRMR
IQREAAGKRFLNLFAYTATASVHAAKGGARSTTSVDLSRTYLDWARRNLSLNGFSDKNRL
EQGDVMAWLQANRDEYDLIFIDPPTFSNSKRMEGIFDVQRDQVELVDLAMARLAPGGVLY
FSNNFRKFVLDENLSQRYAVEDITAQTIDQDFARNGKIHRAWKIMARS
>tr|A0A0Q0C504|A0A0Q0C504_PSEAP YD repeat-containing protein OS=Pseudomonas syringae pv. aptata OX=83167 GN=ALO85_04309 PE=4 SV=1
MDDTPETRRVLCRYRYDALDRIAVVDPEAQEAVSRFYQKNRLTVEIQGATRRRVFHGEDR
LLAEYKTEGSSVRADLLGTDMQGSVLEAVSGQERQSLAYSPYGHRAQGGPFSGFNGERAD
PVTGHYLLGKGYRAFNPVLMRFNSPDSLSPCGRGGLNAYAYCQGDPVNRSDPGGHISSPF
LARMRMHFKPRSSVTIENLPDEMIGKILERLSFTDVNELGKASTRLNSAIAMQAAWHLKK
RWAGGNNPSALSRLNDVVKNEPSHSVSAFMNDHFKTGFEEAVNNVDQIMSNQIQAAKRAE
QSFFEEFTGISNASHYEFGEQLAIKRQEHARSPDMLREINRDVGKRTRLTGAKFTSIGRK
ADMMRLVAEIRTALNSP
>tr|A0A0N8T9X4|A0A0N8T9X4_PSEAP Cysteine synthase OS=Pseudomonas syringae pv. aptata OX=83167 GN=ALO85_01957 PE=3 SV=1
MIGTPRSLDCEDAMTLQYQTIADCVGNTPLVRLQRMAGNTSNTLLLKLEGNNPAGSVKDR
PALSMITRAELRGQIHPGDTLIEATSGNTGIALAMAAAIKGYRMILIMPDNSSAERKAAM
TAYGAELISVSKDQGMEGARDLAERMQAEGRGKVLDQFANGDNPEAHYTSTGPEIWRQTG
GTVTHFVSSMGTTGTIMGTSRYLKEQNPDVQIVGLQPMEGASIPGIRRWPYEYLPKIYQS
DRVDRIIDMGQTEAEDTMRRLAREEGIFCGVSSGGSVAGALRISQEVENAVIVAIICDRG
DRYLSTGVYDSPN
>tr|A0A0Q0CG64|A0A0Q0CG64_PSEAP Translation initiation factor IF-3 OS=Pseudomonas syringae pv. aptata OX=83167 GN=infC PE=3 SV=1
MRQDKRAAPKAPINENISAREVRLIGADGEQIGIVSIDEALRIAEEAKLDLVEISADALP
PVCRVMDYGKSIFEKKKQVAAAKKNQKQIQVKEIKFRPGTEEGDYQVKLRNLVRFLSDGD
RAKVSLRFRGREMAHQELGMELLKRVEGDLLEYGSVEQHPKMEGRQLIMVIAPKKKK
>tr|A0A0Q0FWT0|A0A0Q0FWT0_PSEAP Transposase OS=Pseudomonas syringae pv. aptata OX=83167 GN=ALO85_01526 PE=4 SV=1
MAAVNEGLISSRRYRVGAALIGADGRSYVIGTINTKARQVVLIGQLGESHVYQDDDFRNT
VASGDLRSVVRIETPCGVEEVFEPRFLSPTEIKSRDERQEYIRVVEQLIEDFTWKDIYIE
IQRRYGSHRTVPSRRSIERYWRIYKESYSPNCLAAHFSDRGVHHSLSMEPEVQEIILNVI
ESKYCSSSRFSIQDIVHAINIRCSEKSAEIGVELGGVSRRTVGRFIARLNLKNIKGRLNP
RTFRLIMRNACSYLDVKEPYARVEGDSTVLDIFIVDASGNVIGCPTFYALIDTATMTIVG
IHLTIQAASQVGLMQSLQFAFSPKGEPFKTQWSCMHEWPAPADIRTLVLDNGSDCHGPMI
VKASQHLGMMLEYCVAGAPYQKPFIERFFGTLNTMLIKKLPGAKKSHDKRETHGLEDAQK
SATLTIEKLNEVIIRWITDSYHIKKSDRLSEKFGQEFTPLQALTRLSQQYVVFPAPSAEE

LMEACRHYLEVKLSITREGINYQRQFYQNEYVSALFKTNSTVKVDVCINPLDCRSVHIYD
LGLKEWVTVQNKNPNMPAISFEQAKEIRKAHYKSDFELSGEDYVINHINIIEDANAQKRP
KGKIRENQRAQRTVDKANAAAIAPEQQLPIAETVLAAAPTEIVVAPHRRKK
>tr|A0A0Q0DC86|A0A0Q0DC86_PSEAP Uncharacterized protein OS=Pseudomonas syringae pv. aptata OX=83167 GN=ALO85_101101 PE=4 SV=1
MLDIFSANKKRRPVSGLTRTPLSGPVLSGDAAFSVEGGVL
>tr|A0A0Q0BSQ5|A0A0Q0BSQ5_PSEAP Nucleotide-binding protein ALO85_00163 OS=Pseudomonas syringae pv. aptata OX=83167 GN=ALO85_00163 PE=3 SV=1
MRMIIVSGRSGSGKSTALDVLEDNGFYCVDNLPAGLLPELAERALINTELAEPLLAVSID
ARNLPSHLTRFPQMLDEVRSRNIQCDVLYLDADEATLLKRFSETRRRHPLSTADRSLAEA
IRDETTLLGPIIDLADLKINTTHLNLYQLRDALKLRLLNKPEPGTAFLIESFGFKRGMPV
DADLVFDVRCLPNPYWKPELRDHSGLEQPVIDYLSVQPDVEEMFQDIFAYLNKWLPRFAA
SNRSYVTIAIGCTGGHHRSVYLTERLGQVLQQSLKNVQVRHRDLS
>tr|A0A0N8T957|A0A0N8T957_PSEAP Uncharacterized protein OS=Pseudomonas syringae pv. aptata OX=83167 GN=ALO85_05214 PE=4 SV=1
MPGETTGDTGHILPDEDFFMMFDWWADKAPPRCIDITPKWGSTIDIYLDDIGRIDTAKTA
PEVIARLKQCPGRPDPFEP
>tr|A0A0Q0IMX3|A0A0Q0IMX3_PSEAP 30S ribosomal protein S19 OS=Pseudomonas syringae pv. aptata OX=83167 GN=rpsS PE=3 SV=1
MPRSLKKGPFIDLHLLKKIEVAAEKNDRKPVKTWSRRSMILPQMVGLTIAVHNGRQHVPV
LVNEDMVGHKLGEFAGTRTYRGHVADKKAKR
>tr|A0A0Q0DAP3|A0A0Q0DAP3_PSEAP Uncharacterized protein OS=Pseudomonas syringae pv. aptata OX=83167 GN=ALO85_03406 PE=4 SV=1
MKRTIDAAELNICFSEDLEKALFKWFVASFLMGKRIQADIACEAYRVIVEKHQRDTPRKL
AHCTHRELVAMLGQAHYVRYDESTAYRLSALCAKLNDDYAGKIGRIREVSEDQAHFEKRL
CEFAGVGPKTVEIFMREAGKALY
>tr|A0A0Q0D3Y5|A0A0Q0D3Y5_PSEAP BMPF super protein OS=Pseudomonas syringae pv. aptata OX=83167 GN=ALO85_03883 PE=4 SV=1
MLAPKAFLDALSGHASRLFNGETPVPRSEFETQFKALLQSGFSKLDLVSREEFDSQMAVL
ARTRARLEALEAKMAELEEKAGGAAGTEKAE
>tr|A0A0Q0BZX5|A0A0Q0BZX5_PSEAP LuxR response regulator receiver OS=Pseudomonas syringae pv. aptata OX=83167 GN=ALO85_04479 PE=4 SV=1
MSESADRPLRVIVADDHPIFLIGLRVVLEQDNLASVVAEACNPDELLQALERHACDVLVT
DFMMPVERQNDGLRLLQRIRRDFPTLPVIVVTTLSNAGLFQSMLDLDVRGLLSKASLAGE
LPAAIKGVRRGELFVADSVRSVPSEARQHGSDALLPADRLSPRELEVLRLLSAGHTVGRI
AAQLNRSKQTVSAQKVAAMRKLGLANDAALFIYLQESGMS
>tr|A0A0Q0C9C6|A0A0Q0C9C6_PSEAP Methyl-accepting chemotaxis protein OS=Pseudomonas syringae pv. aptata OX=83167 GN=ALO85_03851 PE=4 SV=1
MSLRNMNIAPRAFLGFAMIGTLMLILGVFALSQMSKIRSATEVLADSNVPSIKSLDRFAE
VSIRLRVLSYRLLVNRDPETQQKTIELMAMRNKQITDAQAVYEKLISGPDERDLYSQYVQ
LLGQYRQLEDRLKTLTRSNNIDELQGLLNNEMVNNSDQMNVVLRKLVDINTAQLNAVKEQ
ASVEYDSAFNMVIGLLIAATLLTIVFAWMLIKSITTPIATALLAAETIAQGNLTKPISID
GSDEAGRLLLAMKTMQDKLRDTLQGISGSATQLASAAEELNAVTDESARGLVQQNDEIEQ
AATAVNQMTSAVEEVARNATSTSQASRSAATSAGDGRDLVQETVSAIERMSGDVQGTAEL
IISLANESRDIGKVLDVIRGLADQTNLLALNAAIEAARAGEAGRGFAVVADEVRALAHRT
QQSTSEIERMISSIQTGTEQAVSSMRNSTERAESTLNIAKGAGQALNTINSAVEEINERN
MVIASAAEEQAQVAREVDRNLVNIRDLSAQSTNGANQTSAASTELSRLAIDLNGMVARFA
L
>tr|A0A0Q0C7R4|A0A0Q0C7R4_PSEAP Peptidoglycan D,D-transpeptidase MrdA OS=Pseudomonas syringae pv. aptata OX=83167 GN=mrdA PE=3 SV=1
MSQPIRLKDHEKDARLVRGRVVFGAVAVVLLVCVLIARLYYLQVIQYEYHSTLSENNRVH
VQPIPPSRGLIYDRNGVVVADNRPSFSLSMTRERAGEWSSVLDTIVEILQLTPDDRIIFE
KRMKQGRRPFEPVPILFELSEEQIALIAVNQFRLPGVEVVAQLVRHYPQGPHFAHSVGYM
GRINEKELKSLDPINYSGTHHIGKTGIERFYEPELHGQVGYEEVETNARGRVLRVLKRTD
PIPGKDIVLSLDIKLQEAAEAALGGRRGAVVALDPATGEVLAMVSAPSFDPNLFVTGISF
KAYAELRDSIDRPLFNRILRGLYPPGSTIKPAVAIAGLDTGAVTPGSRVFDPGYYQLPNY
DHKYRNWNRTGDGWVDLDTAIMRSNDTYFYDLAHKVGIDRLATYMNKFGIGQKVSLDMFE
ESPGLMPSREWKRATRRQAWFPGETLILGIGQGYMQATPLQLAQATALVANKGVWNRPHL
ARTIEGKAPVDENPMPDIVLRDPANWGRVNHGMQEVMHGARGTARKAAIGAQYRIAGKSG
TAQVVAIKQGEKYDRTKVQERHRDHALFVGFAPADNPKIVVAVMVENGESGSGVAAPVVR

QVLDAWLLDENGHLKPEYAGSLNLEAAAREE
>tr|A0A0Q0DMN3|A0A0Q0DMN3_PSEAP TonB-dependent siderophore receptor OS=Pseudomonas syringae pv. aptata OX=83167 GN=ALO85_200070 PE=3 SV=1
MFRKSSLYVRAGLGFAAFSLALPSFAADLPPAIDDSTLATLPQVTVKATKRKPALHQQTN
SGALGSHAVIDTPFSFKSVGSDEIQARQAGILSEAFKYDASVTSISSSYGTHPATLAVRG
LPLDDLNGYKVDGMANINRGVEMPLEMFERVDVLKGLSGFMYGFGSPGGIVNYVTKRPTD
KTTLSVDAGYQSDNVYKEHLDAGGRLDDPRFGYRVNVVHEEGDAASGDAKVNRTAVGVAL
NAQLSDDLSVDLDTLYQKRNTSGGTDIIVNTKNALPSPIDGSKRLYSNGSYTDVDYSLST
VSATYKFSPDWTGKLAYRYSDSTRRYVKDQYQISSDAGAYTDKVTSEYHAYDYNDTMGTL
EGKFTTGWFTHDVVLGASYSQLNSDKSVVTPKTTVGKGNLYNPTIFSVYNIDYSGGTYGD
DNVKESAVFASDTIGLGENWSLLAGLRNENYREQTRSSATSAVTPYKARPATPTVALMYK
PTKDVTLYASYVESLESGGTAPSSAVNSDQTLSPLLSKQYEVGVKAQQRNWSATAAAFRI
DRGAEYTNDANVYVQSGTIRYQGLELNASVDATPDLTVEGSVMSLDSAYHDAGDGVDGNR
AAGAAHYQAATQVTYRVPMVPGMLLHTGAQRIGEMAVDSGNVHTLPAYSLFDAGGGYRLR
LGDGHALTLGANVTNLANKKYWTYYQENYLQPGSPRTLSLNTRYDF
>tr|A0A0N8TA99|A0A0N8TA99_PSEAP Binding-protein dependent transport system inner membrane protein OS=Pseudomonas syringae pv. aptata OX=83167 GN=ALO85_03261 PE=3 SV=1
MPVSSTDYPGDEELVTRETPVQRRRLRAAWLFLTPMLVCLVLVAAWPLLRTFWFSLTDAD
LSDTGSGSFVGLSNYLLYDGVIWSGVLVDPQWWNAVRNTLHFTVVSVGLEMVLGLMVALL
LNVKFTGRALVRALILIPWAIPTIVSAKIWSWMLNDQFGIINHMMLGLGLIDAPLAWTAD
ADLSMWAVIIVDVWKTVPFVTLLMLAALQMLPSDCYEAARVDGIHPVRVFWRVTLPLLMP
ALLVAAIFRILDSLRVFDVIYVLTSNSSSTMSMSVYARQHLVEFQDVGYGSAASTLLFLV
VAVIAMLYLYLGRRQLEVRS
>tr|A0A0Q0DH72|A0A0Q0DH72_PSEAP GntR family transcriptional regulator OS=Pseudomonas syringae pv. aptata OX=83167 GN=ALO85_00677 PE=4 SV=1
MNKTMNKLSSDERLPLYQRLRDSLAEQIANNRWRPGEAIPTEAALSAEYGLSTGTVRKAI
DLLVSDNILERQQGRGTFIRRAQFQSSLFRFFRFQTEAGERRVPESRILSVEPVDAPSSV
AQALGMGAGAPVIRMVRLRLLDGQPVLAEEIWLPRARFQALLELDLDTQGPLLYPIYEDV
CGQVVACAQESLTAETVNDVYGRLLQIPVGSAVVVIERLARNYAGEPLEWRRSRGHASHF
RYSVDIR
>tr|A0A0N8T8H0|A0A0N8T8H0_PSEAP Phosphatidylcholine synthase OS=Pseudomonas syringae pv. aptata OX=83167 GN=ALO85_01093 PE=3 SV=1
MITNQRIARLNAWGAHGFTATGVVLAFLATIALFNNEPKACLLWLGAALIVDGVDGSLAR
KVNTTSVLPHFDGSVLDLVIDYLTYVFIPALFIYRYIPVPDYTLLLSISVILVSSLFCFC
NVDMKSKDNYFQGFPAAWNVVALCLYILSPAPWLTFITIIALALLTMTRMKFLHPFRVRR
FMPLNIAVTTVWLLSSALLIINHPHNGPVVMGLWLAMSAYFLGICIWRTSLEWLGRLKA
>tr|A0A0Q0CFR2|A0A0Q0CFR2_PSEAP Uncharacterized protein OS=Pseudomonas syringae pv. aptata OX=83167 GN=ALO85_03662 PE=4 SV=1
MMQNMAERWHLLPVQRSHLLIHWPYLEAKNMIRILSIIAVLGLTGCATASNTYLKNGKQG
LSIDCSGEAMSWASCYEKADASCAGTGYEIVGTDGTPQPAESDKTLGVDVGNYKSRSVLV
VCK
>tr|A0A0Q0DJT0|A0A0Q0DJT0_PSEAP Response regulator receiver OS=Pseudomonas syringae pv. aptata OX=83167 GN=ALO85_03961 PE=4 SV=1
MMTAVVLPAVPRVLIAESDPWVRETLSDLVLGVRADIELEMCTDGKQAVEWMKKHLPDLI
IAARELPGIDGLSLLRGVRNLRRQPAIPFILISNRNDSASVREVVPLAPTAYLTKPLNTE
GLRQRLEGLLLERSAPVAGAVPALTPGLTLTKFLDKRRDIADGAPLFVDVATAVTLSQTA
SGIDATLLEQELRNDPHITAVLIAAANTAAQHLGKPIQTMGGALAILGPVQVANIASGLG
KKRMAVLTYDALLAKAHELWTLSQRTADYARILGGMLELDAERCFCAGLLQSLGDLAVLG
CLQEWLLAGGHLDEQVIGQSLEQYSAAFGSALRTRWRLPLELRELIAAVYQYNTGIYTRE
VLTMNLAGQMARLGEEESVTTLIKTKSAKLLKLSVGDLQRLRKKLTGVTDPSLLRPLTSE
TEVAVDAVDDEPDLLDLAPEAPAEEPAALEDPAGAVQGPPEK
>tr|A0A0Q0BWC7|A0A0Q0BWC7_PSEAP Hydroxydechloroatrazine ethylaminohydrolase OS=Pseudomonas syringae pv. aptata OX=83167 GN=ALO85_03062 PE=4 SV=1
MPAIRIWLKNPLAVFTANDLDARGGLVIEGSVITEVLAAGQLPSKHCNQTFDAREHVLLP
GLINTHHHFYQTLTRAWAPVVNQPLFPWLKTLYPVWARLTPEKLAVASKVALAELLLSGC
TTAADHHYLFPDGLEHAIDVQVQSVRELGMRAMLTRGSMSLGEADGGLPPQQTVQQGEVI
LADSQRLIETYHQRGDGAQIQIALAPCSPFSVTPQIMAENAAMAEKLDVRLHTHLAETLD
EEDFCLQRFGLRTVDYLDSVGWLGPRTWLAHGIHFNPDEIARLGAAGTGVCHCPSSNMRL

ASGICPTLDLLAAGAPLGLGVDGSASNDASNMILETRQALYLQRLRYGAEKITPQLVLGW
ATKGSAQLLGRTDLGELAVGKQADLALFKLDELRFSGSHDPLSALLLCGADRADRVMIAG
QWRVIDGQVEGLDIKQLIADHSQAARELIHG
>tr|A0A0Q0CVE9|A0A0Q0CVE9_PSEAP Peptidase M48, Ste24p OS=Pseudomonas syringae pv. aptata OX=83167 GN=ALO85_02263 PE=4 SV=1
MKIYKLIAVLILLPLGLNLIGAWQLQRSVENTERLVEIQADLAHARPYFERLARSPDALT
RTMDIDGENITLKEALSRLHEAEQGLDFVVVLGSVTTWLARAVIALCLLAALVGGAGLAG
LNWAAARARQSRERLLHTFSRVRRILPFVLVGHIVAMGAAVSAILCFEALGLWHVGRMSA
GELKLIIVVLMLVAACLYSIWRMGKQLGVMLHMFEPTAMEVLGQQVTPEEAPVLWAYVRD
LAERLGALSPDHIVLGMTEGFYVTSSDVSLLPSDAVLKGRTLHVPILYLGLIDAAETSAV
IGHELSHFAGEDTEYSLRFLPIYDGIGRSLGVIAENMVVSGWLQRTILRPAFMLGVYFME
SFDQAVNHWSRVRELAADAAGASLAGNASAASALVRISAIDPLLQERIYTHITRATNPRR
GEVFTKDLPNSVLHELAGKTFTLPDEEMAVQLPHPSDTHPSNGERIAALQVAADEAIRSG
TRPVVAAQARAAMDHYFSNPQALRIRLTEDFIKHHVANDAEVVTELRARARTVSGDVVLH
EGARLRGLLLTLFLAPLLGLGIYLIAEALSFPQGLAEKRNSMLIAGGILTAFMLMLLPFA
LRMCLRADKTALLLTPDHFVFANLKSPVPIQHIADFELNVAYGTFLTLHLQDDAPLPERV
SRSFSAPNAKVFRKKRRVLLALARFSRDGKKLKPDELGELIADYVNAGTARHLLQERFEQ
GKR
>tr|A0A0Q0IMF1|A0A0Q0IMF1_PSEAP LysR family transcriptional regulator OS=Pseudomonas syringae pv. aptata OX=83167 GN=ALO85_00994 PE=3 SV=1
MRIEPLPPLPNLIAFEAAVRHGSFTRAATELNLTQSAISRQVAQLEEFLGRALFRREHKR
IHLTVAGQIYAEQIQRVLTLCAEATAQVITHSGDQQLTLACSSGVAALWLGPLLGRYRDA
FPAVDIRLRVVDGLDSLIRAEFDLALYFLREVSPAGLDGRLLFAESVNAYCSPDYLGGRL
LEPAQLLDHCLLMLEDGQRQWLSWGDWFGRQGVDASLIRKRMTVNLYPVLVDLAVAGHGI
VLGWQPMIDRHVQSGALVPACRKSVGAVGGYYLLTPSGKTPRRAARAFEKWLADEITTLA
PSP
>tr|A0A0Q0C6I1|A0A0Q0C6I1_PSEAP RND-type multidrug efflux system, membrane fusion protein MexE OS=Pseudomonas syringae pv. aptata OX=83167 GN=ALO85_03676 PE=3 SV=1
MHQTISHLRYPLTALALIVISACGRAPEATSAPGAAKVSVAKVLEQPVNEWDEFTGRLEA
PETVEVRPRVAGQIDQVAFTDGALVKKGDLLFQIDPRPFQSEVRRLEAQLQQARAVASRS
DSEAQRGERLRSNNAISAELAESRTTSAQEAKAGVAAIQAQLDLARLNLSFTRVTAPISG
RVSRAEITAGNIVTADVTALTSVVSTDKVYAYFDADERVFLKYTELARKGQRGQTTPVYL
GLSNEPGNPHLGQMNFVDNQVNPRTGTIRGRAVFDNADGAFTPGLYARLKLVGSGTYSAV
LINDEAVGTDLGKKFVLVMDKDNKPAYRAVELGPKIEGLRIVRNGLAKDDTIVVKGLQRV
RPGQPVDPEVTPMASADTLAALLKQRQALEASNLPQVAPKAAVKIASAAAPRG
>tr|A0A0Q0C7Y2|A0A0Q0C7Y2_PSEAP Glutamate synthase subunit alpha OS=Pseudomonas syringae pv. aptata OX=83167 GN=ALO85_02814 PE=4 SV=1
MKAGLYQPDEFKDNCGFGLIAHMQGEPSHHLLQTAVQALTCMTHRGGINADGKTGDGCGL
LIQKPDGFLRAIAKQHFDVDLPRQYAVGMVFLNQDDARADAARENMNREILAEGLDLVGW
RQVPIDTSVLGRLALERLPKIEQVFIGGEGLSDQEFAIKLFSARRRSSVSNAADTDHYIC
SFSHKTIIYKGLMMPADLTAFFPDLSDERLQTAICVFHQRFSTNTLPKWPLAQPFRFLAH
NGEINTITGNRNWAQARRTKFTNDLMDLDELGPLVNRVGSDSSSMDNMLELMVTGGIDLF
RGVRMIIPPAWQNVETMDPDLRAFYEYNSMHMEPWDGPAGVVMTEGRHAVCLLDRNGLRP
ARWVTTKNGYITLASEIGVWDYKPEDVIAKGRVGPGQIFALDTETGQILDTDAIDNRLKS
RHPYKQWLRKNALRIQATMEDNDHGSAFYDSEQLKQYMKMYQVTFEERDQVLRPLGEQGQ
EAVGSMGDDTPMAVLSRRVRSPYDYFRQQFAQVTNPPIDPLREAIVMSLEICLGAERNIF
QESPEHASRVILSSPVISPAKWRSLMNLERPGFERHIIDLNYDESLGLEAAVRSVADQAE
EAVRAGHTLIVLSDRHIAPGKLPVHASLAVGAVHHRLTERGLRCDSNILVETATARDPHH
FAVLIGFGASAVYPFLAYEVLGDLIRTGEVLGDLYEVFKNYRKGITKGLLKILSKMGIST
VASYRGAQLFEAIGLSEEVCDLSFRGVPSRLKGARFVDIEAEQKALAAEAWSPRKPIQQG
GLLKFVFGGEYHAYNPDVVSTLQAAVQQGDYSKFKEYTSLVDQRPVSMIRDLLQVRTIDQ
PLNIDEIEPLSEIFKRFDSAGISLGALSPEAHEALAEAMNRLGARSNSGEGGEDPARYGT
VRSSKIKQIATGRFGVTPEYLVNADVLQIKVAQGAKPGEGGQLPGGKVNGLIAKLRYAVP
GVTLISPPPHHDIYSIEDLSQLIFDLKQVNPAALVSVKLVAEAGVGTIAAGVAKAYADLI
TISGYDGGTGASPLTSIKYAGAPWELGLAETHQTLRGNDLRGKVRVQTDGGLKTGLDVIK
AAILGAESFGFGTAPMIALGCKYLRICHLNNCATGVATQNEKLRKDHYIGTVDMVINFFT
YVAEETREWLAKLGVRSLEELIGRTDLLDILPGETEKQQHLDLTPLLGSDHIPADKPQFS
QVDRNPPFDKGLLAEKMVEMAKPAIESLSGGDYELDICNCDRSIGARISGEIARLHGNQG
MNKAPVTFRFKGTAGQSFGVWNAGGLNMYLEGDANDYVGKGMTAGKLVIVPPKGSPFKTN

ESAIIGNTCLYGATGGKLFAAGTAGERFAVRNSGAHTVVEGTGDHCCEYMTGGFVCVLGK
TGYNFGSGMTGGFAYVLDLDNTFVDLVNHELVEIQRISGESMEAYRTHLQSVLNEYVAET
DSEWGRNIAENLDDYLRRFWLVKPKAANLKSLLSSTRANPQ
>tr|A0A0Q0FID3|A0A0Q0FID3_PSEAP Putative lipoprotein OS=Pseudomonas syringae pv. aptata OX=83167 GN=ALO85_00660 PE=4 SV=1
MIKFRLASLLSLLTLQACTSMAESEEPKQRFEDYRQQTLELIRNQRNFQTADHQAELGWN
APRQWTPSGTPKGGVLLVHGLGDSAWSFHDVAQTLAAQGYLVRTVLLPGHGTKPEDMLDV
RLEQWQQVVREQAQLLGREVPKVYLGGFSTGANLVLDYAYGHDEIAGLLLFSPAFRSNSG
YAWLTPWIGWARPWLAAPNEGLRPMQTPVRYMNMPTNGFAQFYRSSALAQDRLHQRRYDK
PVFIAIAEHDSVLDTDYVLDNFSQRFSNPASRLIWYGDLPARAANTPRVEARKDYLPEYR
ISRFSHMGLLFSADNPLYGLSGSQRICWNGQSTADTAKCMAGEPVWYSDWGYTEPGKIHA
RLTFNPYFEWQTHAMLGVLNATP
>tr|A0A0N8T877|A0A0N8T877_PSEAP Two-component system response regulator OS=Pseudomonas syringae pv. aptata OX=83167 GN=ALO85_01268 PE=4 SV=1
MTGDFRILIIDDQRPNLDLMEQLLAREGLTNVLSSTEPLRTLDLFNSFEPDLVVLDLHMP
EFDGFAVLEQLNRRIPTNDYLPIMVLTADATRDTRLRALALGARDFISKPLDALETMLRI
WNLLETRALYKSLRELVPAENIELLRQPRTLAQQ
>tr|A0A0Q0C5E0|A0A0Q0C5E0_PSEAP Fe2+/Zn2+ uptake regulation protein OS=Pseudomonas syringae pv. aptata OX=83167 GN=ALO85_04558 PE=4 SV=1
MFNRQGITTRTLLRQTAVAAQAQSPALETERPGNEHIRMLLKSFGLRTSLVRLKVLDALL
VSSEEGHPLGVRGVHSQLLRLDVPLSFLSVREVLKRLCDEGVIDLNDDKTYSLHPRAREW
LGEVGKDGAR
>tr|A0A0Q0FEZ8|A0A0Q0FEZ8_PSEAP Extracellular solute-binding protein OS=Pseudomonas syringae pv. aptata OX=83167 GN=ALO85_01184 PE=4 SV=1
MMSCFMKRLAVLIDGVARKLVTGVMLFIPLMAAPQVFAAQIVRVAAVHFPPYMVRPEKGE
DAGLLPKLVDALNGEQDEYEFVMIPTSVPRRFRDFTEGRFDLAVFENPDWGWKDIPHEQI
DMGLEDAEVYVARRVEGRGQDYFDTFADKRLALFSGYHYGFANFNTDQKYLSEHFSITST
YSHDSNLLMVVRGRADIAPVTRSYLIDFMAHNKDEAAQLMVSDRIDQTYRQYALLRPNGS
IDVAHFKQLLERLRADGQLAKIFEPLHIKVLAVNAEMPGK
>tr|A0A0Q0C055|A0A0Q0C055_PSEAP Phosphoenolpyruvate carboxylase OS=Pseudomonas syringae pv. aptata OX=83167 GN=ppc PE=3 SV=1
MADIDARLREDVHLLGELLGNTIREQRGAEFLDKIERIRKGAKAGRRGSAEGAEQLSSSV
DGLGDDELLPVARAFNQFLNLANIAEQYQLMHRRDDKQPLPFESRVLPELLDRLKVEGHS
PDALARQLSKLEIELVLTAHPTEVARRTLIQKYDAIAAQLAALDHRDLNSSERTQITSRL
QRLIAEAWHTEEIRRIRPTPVDEAKWGFAVIEHSLWHAIPNYLRKADHALHAATGLHLPL
EAAPIRFASWMGGDRDGNPNVTAAVTREVLLLARWMAADLYLRDVDNLAAELSMQQASDA
LRASVGDSAEPYRAELKRLRERLRATRNWANASLSETLPAPEAVLQDNRELLDPLMLCFQ
SLHECGMGVIADGPLLDCLRRAVTFGLFLVRLDVRQDSSRHCAAMTEITDYLGLGRYEEW
DEQTRIDFLLRELNNRRPLLPSYFKPAADTAEVLATCREVAAAPAASLGSYVISMAGSAS
DVLAVQLLLKESGLQRPMRVVPLFETLADLDNAGPVIETLLGLPGYRSRLHGPQEVMIGY
SDSAKDAGTTAAAWAQYRAQERLVEICREQQVELLLFHGRGGTVGRGGGPAHAAILSQPP
GSVAGRFRTTEQGEMIRFKFGLPDIAEQNLNLYLAAVLEATLLPPPPPQPAWRTMMDQMA
GDGVSAYRAVVRENPEFVEYFRQATPEQELGRLPLGSRPAKRREGGVESLRAIPWIFAWT
QTRLMLPAWLGWEAALSKALERGEGEVLAQMREQWPFFRTRIDMLEMVLAKADADIARLY
DERLVSAELQHLGAHLRDLLSQACNVVLGLTGQTQLLAHSPETLEFISLRNTYLDPLHLL
QAELLSRSRNREASLDSPLELALLVSVAGIAAGLRNTG
>tr|A0A0Q0DME4|A0A0Q0DME4_PSEAP Histidine kinase, HAMP region:Cache: chemotaxis sensory transducer OS=Pseudomonas syringae pv. aptata OX=83167 GN=ALO85_04742 PE=4 SV=1
MLPAVLLRCLVMNLKFRHKILLAAAGVVVLAFALFTFYNDYLQRKTINQNLESSVGQAGQ
LTASSVQNWLSGRILVLENLTQNVAFQGVNSDLSGLVSQSALVSTFQFTYVGDSKGAFTQ
RPNVDMPAGYDPRQRPWYKSTVDAGKTILSAPYLASVGGLVVTIASPVKAAGQVTGVVGG
DLSLDTLVKIINSVEFGGLGYAFLVSGDGQIIVSPDKEQVMKNLKDVYPGQTISLDARLR
EVTLNGQQRLLSFTPITGLPSADWYIGLSIDKEKAYAPLSQFRSSAMVAVLIAVAVIALL
LSLLIRALMRPLTDMGRAMQDIAQGEGDLTRRLSIQGKDEFAALGGSFNQFVERVHASIV
EVSSATLQVHDLSQRVVNSSNSSIVGFDEQSARTNNVAAAINELGAATQEIARNASDASI
QASEARNQAEDGQVVVEKTIHAMSALSAKISDSCTQIEQLNASTENIGHILDVIKGISQQ
TNLLALNAAIEAARAGEAGRGFAVVADEVRNLAHRTQTSADEIHKMIGGLQTGSQHAVLN
MNESQTSSQESVEVANQAGSRLREVTRRIGDIDGMNQSVAAATEEQTAVVETLNIDINQI
NTLNQQGVANLNETLKDCAALSQQAGRLKQLVDSFKI

>tr|A0A0Q0CHM4|A0A0Q0CHM4_PSEAP FAD linked oxidase OS=Pseudomonas syringae pv. aptata OX=83167 GN=ALO85_03342 PE=4 SV=1
MSGPLYSWGRYPQVAQQGHECDSIKRLPAHISQTVARHHTSLPFGNGRSYGDSCLASSDH
VLDMSGLDRFIAADWQRGVVQLEAGMTLSQLLAAAIPHGWFLPVTPGTQYVTIGGALAND
VHGKNHHRRGTFGCHVRRFELIRHDQPPMICSAVDNPRFFAASIGGLGLTGVISWVELQL
MPIRSAQIDSAAVRFANLAEFFTLSQELDAQHEYSVAWIDCLARGAHSGRGVYIVGNHAR
YGSLSVEARNRLSVPFTPPVSLVNSLSLRAFNSLYWHAHSDLPTPRRTTCEAFFYPLDRM
LHWNRLYGRKGFQQYQCVIPEHCAPQAMQLLLDAIAASGQGSFLAVLKRCGDIVSPGLLS
FPMQGTSLALDFAQTRDLAGSLFPRLDAIVRDAGGRLYPAKDAHMSGSDFRQAYPAWEQL
EALRAPSLMSRFWKRVMS
>tr|A0A0Q0C4Q0|A0A0Q0C4Q0_PSEAP Dihydroxyacetone kinase, L subunit OS=Pseudomonas syringae pv. aptata OX=83167 GN=ALO85_01015 PE=4 SV=1
MSEQFSTQHGTAIVADLVSVIVANREYLSEVDGAIGDGDHGINMAKGFAHCGRTIEGRQL
SLAEALDELTLSLMEGIGGSMGPLYGSLFIGMADQVRGCEQIDATAFASLLRGGLTSLQD
ITEAGVGDKCLMDTLIPAVEAFEQAHARGESFSAALDAMKNAASQGRDSTRDLVAKIGRA
SRLGERSLGVLDAGAVSCCLILTQLADSVQPRLKDR
>tr|A0A0Q0FUQ3|A0A0Q0FUQ3_PSEAP Tripartite tricarboxylate transporter protein TctA OS=Pseudomonas syringae pv. aptata OX=83167 GN=ALO85_05008 PE=4 SV=1
MKMDTFGYLGQGFGVALTPYNLITALCGTLIGTVVGLLPGLGPINGVALLIPIAFALGLP
PESALILLAAVYLGCEYGGRISSILLNIPGEASTVMTTLDGYPMARQGLAGVALSLSAWS
SFIGAFIATCGMVLFAPLLAKWAIAFGPAEYFVLMVFAIVCLGGMAGDRPLKTFIAALIG
LFLSAVGIDANSGVYRFTGDNIHLTDGIQFVVVLVLGLFSISEILLLLEKTHRGQEAVKAT
GRMMFNVKEAAAVFVVNIRCGLLGFILGVLPGAGATLASAVAYMTEKRIAGASGKFGQGD
MRGLAAPETAIGATACGALVPMLTLGVPGSGTTAVMIGALSLYNITPGPMLFQQQPDIVW
GLIASLFIANIMLVILNIPMIRIFTRILAVPNWALVPVIAIITGIGVYAVHATTFDLFLM
VGIGIFGYILRKLDFPLSPILLGFILGGLMEQNLRRALSISNGELGILWSSPITLGVWVV
TVFMLLFPLLRIWRKRAKQKAAAAHG
>tr|A0A0Q0BRH4|A0A0Q0BRH4_PSEAP Uncharacterized protein OS=Pseudomonas syringae pv. aptata OX=83167 GN=ALO85_04255 PE=4 SV=1
MLRQLSLEGSQTSMKPSKPITRAYCVELQQELSIAAARREYFSQEPPRARFEFLCSTEAC
RTMGAKVTGVRYDVKPQDNPTFVAAHFRANPHYDHNSGCEWVDERAEAEEELNDPVEETD
VQTRLRKAKRKLDDYIDIFDPVIKAPTKDASGPEKPDAGAQRPAATLPVTEPAKPPTARY
SETRTNNLERLVDSYRQAKAELSLEDFKLLKLKIPGQGEVSLRSYFRHIKYAQSGDNGRV
LFGGGLVEQRYGLGFKFRFFDRLDGKLVTLYVSPAQMQAYRSSRYISELLSHADDVKFFT
VYALGTVTQSPSGKSYGLEVDDLRHLAIVLGPAKEPLEEGKTPEA
>tr|A0A0Q0BXW0|A0A0Q0BXW0_PSEAP Sulfate transport system permease protein CysT OS=Pseudomonas syringae pv. aptata OX=83167 GN=ALO85_03967 PE=3 SV=1
MSRRISPVIPGFGLTLGYTLVYLSLIVLIPLAAMFVHAAQLTWDQFWTIITAPRVIAALK
LSFGTAFYAALINGVIGTLLAWVLVRYTFPGRKIIDAMIDLPFALPTAVAGIALTALYAP
NGWVGQFAADLGFKIAYTPLGITLALTFVTLPFVVRTVQPVLADIPREVEEAAACLGARP
LQVFRHILVPALLPAWLTGFALAFARGVGEYGSVIFIAGNMPMKTEILPLLIMVKLDQYD
YTGATAIGVMMLVVSFILLLLINLLQRRIETPS
>tr|A0A0Q0DDV9|A0A0Q0DDV9_PSEAP Ligand-gated channel OS=Pseudomonas syringae pv. aptata OX=83167 GN=ALO85_200071 PE=4 SV=1
MLRRVVQHIPEKHFRMIRYFGFLANRVCGKYLPKVYEALKMATPGPTPKLYFVQMAKAFL
NVDPFRCVLCGARMVYTAAISGLTVQGLVLNAQAIAQMRYVKP
>tr|A0A0Q0ILM4|A0A0Q0ILM4_PSEAP ThiF family protein OS=Pseudomonas syringae pv. aptata OX=83167 GN=ALO85_00316 PE=4 SV=1
MVMSAEDPRFAGIARLYGVEGLARLRAAHVAVVGIGGVGSWAAEAMARSGVGEISLFDMD
DVCVSNSNRQLHALDSTVGRPKVDVMAERIRAINPDCIVHAVSDFVTRDTMAECITPDMD
FVIDCIDSVNAKAALISWCKRRKIQMVTTGGAGGQIDPTLIQIADLNRTFNDPLASKVRS
TLRRDYGFSRTPNRHYSVPCVFSTEQLRYPKPDGSICLEKKFVGEGVKLDCAGGFGAVMM
VTATFGMVAATRAVDKLVAGTRRPSERVKPAAATPDQA
>tr|A0A0Q0FMC2|A0A0Q0FMC2_PSEAP Uncharacterized protein OS=Pseudomonas syringae pv. aptata OX=83167 GN=ALO85_200178 PE=4 SV=1
MTAEEYLLDRFGPLMSMAQVADLLDRSPDGFRVAMYTDSDLSRKLKPTVVRVGRRVFFRT
IQVNDALGLDAIAE
>tr|A0A0Q0FSW6|A0A0Q0FSW6_PSEAP Cobalamin biosynthesis protein CbiG OS=Pseudomonas syringae pv. aptata OX=83167 GN=ALO85_03627 PE=4 SV=1

MSTCIRPDPATLQPGAPLVAGLGCRRGCSAALLRDLLEQSLQALELDIGRVTALASIDLK
ETEPGLLELAGQLAVPLVCFNAAQLVAWELHLTHRSAQVFQNTGCHGVAEGSALALAAQL
GDGTATLLIERQKSAQATFALAISPAHSG
>tr|A0A0N8T8R2|A0A0N8T8R2_PSEAP Ribosomal RNA large subunit methyltransferase G OS=Pseudomonas syringae pv. aptata OX=83167 GN=rlmG PE=3 SV=1
MPLLISPFAELDLIRQPEQQDEPLQAFDAADEYLLNHVAQTGLSLPSRVLVLNDSFGALA
ASLAPHATVISSTDSFLAAQALEKNLARNGMSYDAVPHIPASEALQGPFDWVLIRVPKTL
ALLEEQLIRLQGQLAPGARVIAAAMVKHLPRSAGDLLEEYVGPVQASLAVKKARLLFATP
QPMEVRTSPYPTRYRLDEPAIELLNHANVFCRDGLDIGTRAFLPHLPKNLGTARVADLGC
GNGVLAITSALDNPQAHYTLVDESLMAVQSAAENWQATLGDRDVRIQAGDGLEMQEPDSL
DVVLCNPPFHQQQVVGDFLAWRMFLQARAALVTGGALYIVGNRHLGYHTKLSRLFRGVEQ
VAATPKFVILKARK
>tr|A0A0Q0C7C2|A0A0Q0C7C2_PSEAP Zinc-containing alcohol dehydrogenase superfamily protein OS=Pseudomonas syringae pv. aptata OX=83167 GN=ALO85_01644 PE=4 SV=1
MKALLCKAFGPASTLVLEDVLGPAIKKNEILLDVHAAGVNFPDTLIIEGKYQFKPPFPFS
PGGEAAGVIAAVGENVSHLKPGDRVMALTGWGSFAEQVAVPHYNVLPIPQGMDFTTAAAF
SMTYGTSMHALTQRARLQPGETLLVLGASGGVGLAAVEIGKALGARVIAAASSAEKLEVA
RNAGADELINYSETSLKDEIKRLTNGNGADVIYDPVGGDLFDQAIRSIAWNGRLLVVGFA
SGRIPELPVNLTLLKGASVVGVFWGSFAQRQPQDNAANFKQLFAWFEEGKLKPLVSTVYP
LERAGEAIDLLGGRRAVGKVVVLVGKT
>tr|A0A0Q0C1Q9|A0A0Q0C1Q9_PSEAP Phospho-2-dehydro-3-deoxyheptonate aldolase OS=Pseudomonas syringae pv. aptata OX=83167 GN=ALO85_03727 PE=3 SV=1
MNSSVAVTPSDLSSHAVNLADNSPASKRLPGTLELKMRLPLGAALTEQVAAHRRAVRAIL
EGQDSRLLVIVGPCSIHDPRSALEYAERLADLAAQVSDQMLLVMRAYVEKPRTTVGWKGL
AYDPRLDGSDDMAEGLTLSRQLMLEMLGLGLPIATEILQPMAAGYFDDLLSWVAIGARTT
ESQIHREMASGLPMAVGFKNGTDGGVTVASDAMRSAAHPHRHFGMDRHGHPAIIETQGNP
DTHIVLRGGHGGPNYDHQSVANVQASLSKNNVASRIMVDCSHANSGKDPLRQPGVFEDVL
EQRLSGNTSLIGMMIESHLFDGCQALGGTLQYGVSVTDGCLGWEGTERLLRRAVDRLRYR
>tr|A0A0Q0BTH2|A0A0Q0BTH2_PSEAP Pyoverdine biosynthesis-controling sigma factor PvdS OS=Pseudomonas syringae pv. aptata OX=83167 GN=ALO85_01273 PE=3 SV=1
MTEHVITSKCDSPLLQAFVDNYLLLVKIAARIVGCRSRAEDVVQDAFFRLRSAPQATLTF
KAQLSYLFQIVRNLAIDHYRKQALEQKYTGPEAEGLNVVIQGASPEISHINFSSLEKIAD
ALTELPPRTRYAFEMYRLHGVPQKDIAKELGVSPTLVNFMIRDALIHCRKVNGAQAEA
>tr|A0A0Q0DJC3|A0A0Q0DJC3_PSEAP Putative 3-hydroxyisobutyrate dehydrogenase OS=Pseudomonas syringae pv. aptata OX=83167 GN=ALO85_04977 PE=4 SV=1
MSILESSAHCRPKGLAASSSNSYKIFDRDADMTLKTGVIGLGNMGGGMAATLAGKGFDVS
GFDLSQAALAQAESKGVKPVADRTQLIQSVDILILSLPKAEHVESVCLGAGGINEVGRKG
LIVVDTTTSTPEMSRKVAAELAKNGIAFIDAPVSGGPKGAATGTMSMVIGAEDADLARAM
PVLEGMSGTRVHVGQCGAGNVAKIANNMLAACHLISTAEAVAMAARAGVDPEKLLQGLNA
GSGRSGATQVMFPTWVLNKAYDSGFTMGLMRKDVGLASDLADSLDMDLPLSRVVAQLWQA
SSETLADNEDFCAIVQRTDGKLYGHGE
>tr|A0A0Q0D9G5|A0A0Q0D9G5_PSEAP C4-dicarboxylate transport protein OS=Pseudomonas syringae pv. aptata OX=83167 GN=dctA PE=3 SV=1
MKMPTTPLRKTAMTTRQPIYKSLYFQVIVAIVIGILIGHFYPDTGKALKPLGDGFIKLIK
MVIAPIIFCTVVSGIAGMQSMKSVGKTGGYALLYFEVVSTIALLIGLIVVNVVQPGAGMN
IDVSTLDASKIAAYVTAGQDQSIVGFILNVIPNTIVGAFANGDILQVLMFSVIFGFALHR
LGAYGKPVLDFIDRFAHVMFNIINMIMKLAPLGAFGAMAFTIGAYGVSSLVQLGQLMICF
YITCVLFVVFVLGAIARAHGFSIFKLIRYIREELLIVLGTSSSESALPRMLIKMERLGAK
KSVVGLVIPTGYSFNLDGTSIYLTMAAVFIAQATNTHMDITHQITLLLVLLLSSKGAAGV
TGSGFIVLAATLSAVGHLPVAGLALILGIDRFMSEARALTNLVGNAVATVVVAKWVGELD
TDKLQSELTSGGSAILETRPEDDLGVAEGPTPAAAVNTTKTV
>tr|A0A0N8T8K7|A0A0N8T8K7_PSEAP Putative hemolysin-type calcium-binding region OS=Pseudomonas syringae pv. aptata OX=83167 GN=ALO85_05320 PE=4 SV=1
MSSTFVVAWSNTMIFNTKDFGALGDGVTDDTAAIQAAIDAAAAAGGGEVVMGAGTYVVSG

GEEPSDGCLMLKSNVTLSGAGMGETIIKLADGSDTKVTGIVRSAYGEETHDFGMKNLTLD
GNRDATTGKVDGWFNGYIPGSDGKDSNVTLDSVEIKDCSGYGFDPHEQTVNMVIKNSVSH
GNGLDGFVADYLSDSVFENNVAYDNDRHGFNVVTSTHDFTLSNNVAYGNGSTGIVVQRGS
ENIPSPANITITGGAVYGNAAEGVLIKLSSQVSVSGVDIHDNGNAGVRIYGSSGVDIFDN
TLSNNALGSAVPEIIIQSYDDTTGVSDKFFSGSDNLIRGNTITGSDNSTYGVAERNEDGT
DRNSIVGNTISHTSKGLTLVYGDGSFAGDAFPLVTVNGTEGNDVINGGAAHEQIFGLAGK
DTLNGGSGDDILVGGAGADKLTGGAGADTFRFDQLTDSYRTATTSSTDLVTDFDISQDRI
DLANLGFTGLGSGKGGTLNISYNAILDRTYVKSLDADASGNRFELGLSGNLKDTLNASHF
IFQHVVEGTAGGDTLTGTDGNDVLNGNAGTDRIDGGAGADTITGGADADTLTGGAGADLF
IYNSRLDSYRNYTASGTKQSDTITDFNPAEDRIDLSSIGLRGLGDGSANTIYLSVNADGS
KTYIKTDAVDSTGNRFEIALQGNLADKLSASNFIFSTAAAANQAPVLNTPLMDQNITELK
AFSYAVQPGSFSDPDSNTLTYSATLADNSALPDWLKFDSKTLTFSGTPGGTASGLYSVLL
TASDATGASVADSFAINVGNVTPGILTGTENAEALYGTEGNDTILGLGGDDTLRGDTGAD
IINGGAGRDALYGGADADTFVYSALTDSYRDYDAGGLTATDTIYDFTPGQDKIDVSALGF
LGLGNGEDHTLYMTLNETGDKTYIKSATADADGNRFEIALSGNLIDTLTDADFVFGQREA
QEILYLPTLGQSNARLLRMTEDDNQSGTSEMVKDLTRYTDYDVRSQFTDANGDGIDLAVG
GSTVVGYSTGTQDEQRVSWRLVDTDEPGPALLRATELLKSQLASLTAIDKVTTGIIWGQG
EEAAQEIARATDKQAAADLYKASTLKVFDYLHAQIGDFTVYIAETGHYQTDAAKARGYTE
EKINAIVEGTGYVRSVQEAIATERADVKLAVDYTDLPLRYEVNPLVYPDDVWHLHEESAE
IVGQRLADFIADDLGFQGNAADNNDPAAIFESGQNEGGHIFGTSDDDTLVGSSGNDVLDG
DQGADDMSGGDGNDIYVVDNAFDTVTESNDSPSQVDTVVSSVSWTLGANLENLLLTGVSA
INGTGNAVKNVIIGNASNNILDGAAGADMLTGGDGSDSYYVDDAADRVVETNADAKVGGV
DTVYSSLASYTLGANLENIVINSSGAANATGNALDNLIYAGAGDNVMDGRGGNDTVSYLF
ATAGVTVALNTSAQQATGGSGLDTLKGTENLIGSQFADTLTGNKNANTLSGGAGNDTLSG
GAGDDVLIGGSGADTLIGGTGADHYVFNGSSDTGLGGLRDIINGFKTVEGDKLDFSGLDA
RPLTDGHDAFVFIGNAAFSANNSGELRFADGVLYGNIDDNVGADFEIQLTGVQTLQAADI
IV
>tr|A0A0Q0IAI4|A0A0Q0IAI4_PSEAP Membrane-bound lytic murein transglycosylase F OS=Pseudomonas syringae pv. aptata OX=83167 GN=mltF PE=3 SV=1
MLGACVEKPTTLERVKEDGVLRVITRNSPATYFQDRNGETGFEYELVKRFAKDLGVELKI
ETADNLDDLFDQMNKPGGPVLGAAGLIETPKRKSQARFSHSYLEVTPQVVYRNGQSRPTD
PGDLVGKRIMVLKGSAHAEQLAALKAQNPRIEYEESDAVEVVDLLRMVDEGQLDLTLVDS
NELAMNQVYFQNVRVAFDLGEARAQRWAVAPGEDNSLLNEINAYLDKVEKNGTLQRLKDR
YYGHVDVLGYVGAYTFAQHLQERLPKYEKHFQTSAKKEQVDWRLLAAIGYQESMWQPTVT
SKTGVRGLMMLTQNTAQAMGVSNRLDARQSIQGGAKYFAYIKDQLDDSIKEPDRTWLALA
SYNIGSGHLEDARKLAQNEGLNPDKWLDVKKMLPRLAQKKWYSKTRYGYARGGEPVHFVA
NIRRYYDILTWVTQPQLEGSQVADGNLHVPGVDKTQPPVPPASPVPSSSNSTDESAL
>tr|A0A0N8TAA9|A0A0N8TAA9_PSEAP Uncharacterized protein OS=Pseudomonas syringae pv. aptata OX=83167 GN=ALO85_01571 PE=4 SV=1
MMDMLGLPVSAYWLEPLLVGAQILLILLAGYVLQRIVGGFLTGLGERYPLPPELLVPVRG
GLRWFIMGSAFVLVLGRLGVSATVLWTALSGFVAVAAVAFFAMWSVLSNLLCAILIFTIG
PFRIGDMVELVDTLDKPGVKGRVVAINLMFTTLIETPEAGGALVQVPNSQFFQKSVRRWR
GSDLFPQMVVAKQPDEQD
>tr|A0A0Q0C5R5|A0A0Q0C5R5_PSEAP UDP-N-acetylmuramoyl-tripeptide--D-alanyl-D-alanine ligase OS=Pseudomonas syringae pv. aptata OX=83167 GN=murF PE=3 SV=1
MLKPMTFSELVVPLDSRLVGGDCSFAGVSTDSRGIEPGQLFVALTGPRFDGHEYLDQVAA
KGAVGALVEREVQGAALPQLVVLDTRKALAQLGAMNRNAFVDRPVAAVTGSSGKTTVKEM
LASILRTRGPVLATRGNLNNELGVPLTLLELAPEYTSAVIELGASRVGEIAYTVSLAKPH
VAMITNAGTAHVGEFGGPDRIVEAKGEILEGLDAGGTAVLNLEDKAFPIWRKRAGNRNVL
TFALTDSAADVYASELSRDARGCPAFTLNSPLGTARVQLNLLGTHNVSNALAAAAASCAM
GVPLEGIVTGLNNVQPVKGRAVAQIASNGTRVIDDTYNANPASINAAVDILTGFTGRTVL
VLGDIGELGDWAEQGHREVGAYAAGKVSALYAVGPLMTHAVSAFGEHAYHFASQAELIAA
LGAEQDPDTTLLIKGSRSAAMEHVVAALCGSSLEKH
>tr|A0A0Q0IK43|A0A0Q0IK43_PSEAP RulB protein OS=Pseudomonas syringae pv. aptata OX=83167 GN=ALO85_200083 PE=4 SV=1
MDLFKCLARMGKQPLRIAAAFSSLMCAQQCFAQIPVTVTSQVTESPMTIAEFASNSTRWS
QQVQQMASQIDQMKQQYGAITGSRGLGRVFDDPQLREYLPQEWQAVYDSVKRGGYTGLDG
RAETIYADNKVFDACQSFAQDEQRTACQAQAVKPSQDKAFALDAYDQAKGRLRQLDQLMG

QIDKTQDPKGIAELQGRIAAEQAMIQNEQTKLQMFKMVAQAEDKVQEQRQRELNAQILDK
RGFETRTMLNLGGN
>tr|A0A0Q0C0R2|A0A0Q0C0R2_PSEAP Dienelactone hydrolase family protein
OS=Pseudomonas syringae pv. aptata OX=83167 GN=ALO85_02208 PE=4 SV=1
MSSNIEIRSVVYQIDGQPYESRLVYDTKATAAQPGLLMAPNWMGVSKGAEDIAKAVAAEG
YVVLLADLYGQQIRPGNNDEAGAAMTPLKNDRGLLRKRMQAGLDQLLGQGGASLDSSKIA
TFGFCFGGCCSLELARTGAELKAAISFHGTLDTPNPADAKNIKGSVLVLHGASDPLVPKE
QLPAFEDEMNAAGVDWQLYSYGGAFHSFTDPHANVPGMMMYDAKVSSRAFKSMHDLLKEV
FG
>tr|A0A0N8TA42|A0A0N8TA42_PSEAP Methyl-accepting chemotaxis protein I
OS=Pseudomonas syringae pv. aptata OX=83167 GN=ALO85_100376 PE=4 SV=1
MSATFAYPADSFSPEIVLMSKSLRLQILGLLGGSLALVLVIALACFNFLSDNVQAYRSLI
DGPVRASQLVDEANLQFKIQVQEWKNVLLRGKTPAERDKYWEQFEEQERKVQSTLGRLAE
MHGTDAALKSQVQTLQQAHQALGTAYRTGRDAFVAAGGDAGAGDMAVKGVDRPTSEQMAG
LVEQLHKSSDAQSVAISHEAQRTILVGAVVMLVSALLVGLLALWMVNRNLIAPIRMLIEY
VAQLSEGKLGARVEVTRQDELGRLAVAANTLRDFLADTFNRLKRSSTDLDTAGGELNAIA
ELMAQGTHEQFERTDQVATAMTEMSATAQDVARHAADAARAADEADQSAQDGERVMQLTI
ATITRMRNEIALTADVIERLETDSGRIGKVLEVIRSIADQTNLLALNAAIEAARAGDAGR
GFAVVADEVRTLAQRTAVSTSEINQIINSVQTGASDAVQAIQGGQARSEESVEQVANAGA
TLQRITMAVENIRDMNRQIATAAEEQTSVAEDISRNLTEITAIAVTNQDNVKRTQNASQE
LRGLSTGLGDVIARLGA
>tr|A0A0Q0DSX5|A0A0Q0DSX5_PSEAP Chemotaxis sensory transducer protein
OS=Pseudomonas syringae pv. aptata OX=83167 GN=ALO85_05137 PE=4 SV=1
MKNQVSLATRIGLGFAAVVSLLILITAVGIQRVGFIDSTLEDVGENAAKVQRYAINFRGS
VHNRAIALRDAVLVNNDQDLALHLAEMTRLEKDYVDSAVPMEQLFTKPNVSAEERQLLRG
IKEIEQQTLSSTTSVIALRRAGDIAGAQALLLSQTSGNYSEWLKRINALIDHEEASIRVQ
LDNVQATASQFRGLMLLATAFAVLLSIVLSAFIIRFVKKTLGAEPVDVAEAIRRLAAGDL
QQTITTAYPDSVMGVLRTALNRLSETITDVRMAAQEVSQSSTVLSAASSANNAQIMVQTR
EAEQVATAISEMAATVSEVSGYAAQAADAARLADSEVANGNTLVEGTTTAIEQLAATLTE
TTSTVEQVSRHGEEIEKVIEVINSIASQTNLLALNAAIEAARAGEHGRGFAVVADEVRSL
ANRTQLSTQEIQNMISTLQGGTETAAQNMRDSCELVNRAVEQTRNAQSALSRINQEVSAI
NHMNAQIASASVQQSAVAEDVAINITSIHDSTLKSANGSQQVATASEELAQLADRLTRKV
AFFKAG
>tr|A0A0Q0DLU5|A0A0Q0DLU5_PSEAP DUF4129 domain-containing protein
OS=Pseudomonas syringae pv. aptata OX=83167 GN=ALO85_02387 PE=4 SV=1
MRLTESTVAIRPRSPWEAIDLGVLLAGRHRGVLMSSWAIVTLPFFCVLSALFWDYPTAAI
LLFWWLKPAFERLPLLILSQSLFGATPTLRQALKAWPATLKEQLLPSLLWRRLSLSRSFQ
LPVQQLEHLAGTERALRIGLLSQKDLRAAQLLTIVGAHLEYALWIGLLVLLYALIPQQIQ
IDWHWMSLLDVENQWLWIDHLTNLFYALALILWEPVYVACGFTLYLNRRTVLEAWDIELI
FRRLRQRLIGTAYAVLMGVGLMLSAAPEPVWADQQSYSCPLPESAPAQSAPSVAAPDTPR
LTRQSLTSEAARSQIHNVLQQPPFRNPKTETGWRFPEKQKGQSAARDTRDQSWLMTWLNR
LLRAASALASLIEVLLWGVVIGLVGLVIFRYRDWFRTFVGRKTSKAKTTRPTPEQLFGLQ
VNAESLPDDVAGRAEQLWNSQPREALGLLYRALLSRLLSDYRLPLKNADTESQVLERIAA
LNQPLLSEFSRALTAHWQNLAYGHRLPPAHARQELCDGWRRLFNQAPQP
>tr|A0A0Q0C271|A0A0Q0C271_PSEAP Syringomycin synthetase E (Fragment)
OS=Pseudomonas syringae pv. aptata OX=83167 GN=ALO85_01288 PE=4 SV=1
MQQNTLNFDLSVEEIFPALLAGATLTPSREIFGSEGTEDYGIHPTVLHLTTAHWHTLVAE
WHKQPQVAEQRLQHVRLINVTGDALSAQKLKLWDEMRPAHTRLINTYGPTEATVSCTAAY
VSHDAAAGSEGSGNATIGKPMANTR
>tr|A0A0Q0C096|A0A0Q0C096_PSEAP Putative insecticidal toxin protein
OS=Pseudomonas syringae pv. aptata OX=83167 GN=ALO85_02377 PE=4 SV=1
MSSITHTNTPQLAVSDSRGLPVRSVQFYRGADGQPVDARVTQYYFDKAGRLIASRDPRFS
SRLKYGICAPVNLMQIVSLSGALLLSKSVDSGWRVSLNGEAGQLVDSCDGRENPRQIEYD
RLLRPLAINESGRMTERFTYGGPATAEHNQCNQLIRHDDTAGSRLLLDYGLSSRALREKR
YFLQSPDSPDWPLAEPDRDALLEPVGLQTRWAFNAQGEAQVQTDANSNVQRFSHGVDGQL
RAVELTLANTEQPQTLVSAIHYDAFNQVEQETAGNGVVSRYVYDQQDGRLIELSALSADG
SVLQKLNYSYDPAGNVLLINDASQPDRYCGNQRIEPINRYCYDTLYQLIEATGREVRNGA
SHGPALPGLQPLPTLDPCQVSNYTQRYSYDAAGNLLQMRHEGAHNFTRNMHVAPDSNRSL
PDDDGDVDFATSFDANGNLLQLVRGQVMGWDARNQLQHITTVQRKDAPNDDERYVYDGQG
QRCRKISTAQASGRTLTNEVRYLPGLEVRTTADGETLHVVTAQAGRNSVRVLHWEAGKPG

AIANDQVRYSLGDHLGSSTLELDQQGGLISQESYYPFGCTAWWAARSAVEAKYKTVRYSG
KERDASGLYYYGFRYYAPWLQRWINPDPAGVIGGNNRYGMVDNSPVSKVDPDGLMPKPYQ
GKGDEYEKKSEARNETILARGREQIRQMNQSNPQKMDQTLELMKLSYQGSISSLGASTAD
SKLLVGMVMGEESLHHLPALKKSYRSLDNIVNEYIGGERYNQFAITKGSIGHAYVTFTDP
HKRIFLSNELVDKHTMGNALAVSHELSHLMDERTLDFAYLSSPLVKEKRATLSKAQLTSH
FDGLAKASYRLSQGLENDYIFSRIKDVALRGQLKEAELMSLFEVSDAQDMKVERLSSPVV
RANILRRNADSVAALGMLVSHKSLTAKLTSWGQYTHG
>tr|A0A0Q0D449|A0A0Q0D449_PSEAP HAD family hydrolase OS=Pseudomonas syringae pv. aptata OX=83167 GN=ALO85_02507 PE=4 SV=1
MPLAIFDLDETLIGGDCATLWSEQMGRLGWVDSTSFMQRNDELMAAYSAGELAMEDYMAF
SLEPMAGRTPEEIDHLVGPWVEDVIEPIIYSDACTCIAKHRAKGDRILVISASGVHLVKP
IAERLGIDEVLGIELDVQHGVYSGATVGVLTYREGKITRLMEWLDAEGENLEGASFYSDS
RNDLPLLLNVDHPNVVNPDAVLLEHAQKAGWPVHDWS
>tr|A0A0Q0DLP1|A0A0Q0DLP1_PSEAP Ribonuclease HII OS=Pseudomonas syringae pv. aptata OX=83167 GN=rnhB PE=3 SV=1
MEPARTVAFTVRSPMMQTGLDFTLVEDLVAGVDEVGRGPLCGAVVTAAVILDPTRPILGL
NDSKKLTEAKREKLYVEIQEKALCWFIARAEVEEIDQLNILHATMLAMQRAVEGLSITPR
LALIDGNRCPQLSVPSAPVVKGDSKVPAIAAASILAKVSRDREMAAFELIYPGYGIGGHK
GYPTPVHLEALARLGPTPIHRRSFAPVRAAHEARATIMMGGSLAPSVGLLQD
>tr|A0A0Q0BWQ3|A0A0Q0BWQ3_PSEAP Uncharacterized protein OS=Pseudomonas syringae pv. aptata OX=83167 GN=ALO85_01040 PE=4 SV=1
MFEHLDLNALIAAYGYWVIFLGCLLEGETVLILGGMAAHQGALQLLQVIGLATLGGMLGD
QLLFWTGRYSGERLLPKLKRHQSAIKRVEGLIQRYPTTSVFAVRFLYGMRLVGPMVIGAS
RLSPWRFALINVLSAAVWATLFVCAGYWAGEALQHLLGDLKPYRLPIFLAVVALVTVAAL
VRYLRRRSRATHD
>tr|A0A0Q0D0U2|A0A0Q0D0U2_PSEAP Nitrite reductase, large subunit OS=Pseudomonas syringae pv. aptata OX=83167 GN=ALO85_04620 PE=3 SV=1
MLNNPTMKVVGPPRLIDVDRQSRKDQRATRVLPATWLDGTPEYPIRQQAKAPGTERFRAL
FFCFEKPDWPRPGLAMNTTVFPPNDVKTLIVVGNGMVGHHCVEQLIAGGALERYRIHVFG
EETQRAYDRVHLSEYFTGRDAESLAMSEMSVYEQPGLTLHLGVPVLEIDRDRHEIITAEG
CFSYDKLILATGSYPFVPPIEGARGNSRLVYRTLEDLDSIRYAASKARRGVVVGGGLLGL
EAANALKSLGLEAHVVEFAPRLMPVQLDELGGAALKARIEALGVGVHLSRATQSISDGKQ
YRYRMNFANDEFLETDLIVFSAGIRPQDALARQCELALGPRGGIAIDEHCRTSDADIYAI
GECAAWNGSVFGLVAPGYQMARGVAAQLCEQDTDAFFGADMSTKLKLLGVDVGSIGDAHG
ALAGAVSYRFIDEATASYRRLVVSADGKQVLGAVLVGDNSYYDTLLQYAQNGIPLPADPS
SLILPRGEGAPTLGADALPATATICSCHNVSKGAVCSAIDNGCTDLAAIKSCTKAATGCG
GCTALLKQVFEHELIARGVAVDKSLCEHFAYTRQELYSMVRVEGIETFDELLTRHGKGAH
GCDICKPAVGSILASCWNRPITEPSLVPLQDTNDTFMANMQKNGTYSVVPRIPGGEITPD
GLIAIGAVAKKYDLYTKITGGQRIDLFGAQLHELPDIWSELIEAGFETGHAYGKSTRTVK
SCVGSTWCRYGVQDSVAMALRIEDRYKGLRSPHKLKFAVSGCTRECAEAQSKDVGVIATE
NGWNLYLCGNGGMRPRHAELFATDLDDETLIRYIDRFLMLYIRTADKLQRTSVWRETLEG
GLEYLKAVIIDDSLGLAAELESQMQLVVDRYECEWANALKDPEKLKRFRTFVNDGRADPD
VQFVKERAQRRPAKPEELALIPLFKEVV
>tr|A0A0Q0DRL9|A0A0Q0DRL9_PSEAP ABC-type transporter, ATP-binding protein OS=Pseudomonas syringae pv. aptata OX=83167 GN=ALO85_02046 PE=3 SV=1
MNPLLLNLRNLACGYQNQRVVQDLNLHLNAGDIGCLLGSSGCGKTTTLRAIAGFEPIHAG
EITLAGEVISRPGWTLPPEKRRIGMVFQDYALFPHLSVAENIAFGIRNHPRLDQLIAELL
ELVNLGALGKRYPHELSGGQQQRVALARALAPEPQLLLLDEPFSNLDGELRRRLSHEVRD
ILKARGTSAILVTHDQEEAFAVSDHVGVFKEGRLEQWDTPYNLYHEPRTPFVASFIGQGY
FIRGQMMDPQSVHTELGVLRGNRAYPGVSGAAVDVLLRPDDIVYAPHSDLKARVIGRTFQ
GASTLYRLQLSTGSQLEAIFPSHADHGQGEEVGIRVAAEHLVLFPAPGSVAVHTLNS
>tr|A0A0Q0CFK6|A0A0Q0CFK6_PSEAP Urease accessory protein UreG OS=Pseudomonas syringae pv. aptata OX=83167 GN=ureG PE=3 SV=1
MENSMNSQPLRVGIGGPVGSGKTALTLALCLALRDRYNLAVVTNDIYTREDADFLVRNEA
LAPERIIGVETGGCPHTAIREDASINLEAVDQLNRRFEGLDLIIVESGGDNLSATFSPEL
SDLTIYVIDVSAGDKLPRKGGPGICKSDLLVINKIDLAPLVGASLEMMDSDTRRMRGEKP
FVFSNQKTGQGLEQIIAFIERQGLLNAAA
>tr|A0A0N8T8J1|A0A0N8T8J1_PSEAP UvrABC system protein B OS=Pseudomonas syringae pv. aptata OX=83167 GN=uvrB PE=3 SV=1
MSEFQLVTRFEPAGDQPEAIRQLVEGIDAGLAHQTLLGVTGSGKTFSIANVISQIKRPTL

VLAPNKTLAAQLYGEFKAFFPNNAVEYFVSYYDYYQPEAYVPSSDTFIEKDASINDHIEQ
MRLSATKALLERKDAIIVTTVSCIYGLGSPETYLRMVMHIDRGDKLDQRALLRRLADLQY
TRNDMDFARATFRVRGDVIDIYPAESDLEAIRIELFDDEVESLSAFDPLTGEVIRKLPRF
TFYPKSHYVTPRETLIEAMENIKVELQERLEYLRSQNKLVEAQRLEQRTRFDLEMMLELG
YCNGIENYSRYLSGRPSGAPPPTLFDYLPPDALLVIDESHVSVPQVGAMYKGDRSRKETL
VEYGFRLPSALDNRPMRFDEWEAISPQTIFVSATPGNYEAEHAGRIVEQVVRPTGLVDPQ
IEIRPALTQVDDLLSEIHKRAALEERVLVTTLTKRMSEDLTDYLSDHGVRVRYLHSDIDT
VERVEIIRDLRLGTFDVLVGINLLREGLDMPEVSLVAILDADKEGFLRSDRSLIQTIGRA
ARNLNGRAILYADRITGSMERAIGETERRREKQIAFNLEHGITPKGVFKDVADIMEGATV
PGSRSKKRKGMAKAAEENARYENELRSPSEINKRIRQLEEKMYQLARDLEFEAAAQMRDE
IGKLRERLLAV
>tr|A0A0Q0FHQ4|A0A0Q0FHQ4_PSEAP Competence protein ComEA-like protein
OS=Pseudomonas syringae pv. aptata OX=83167 GN=ALO85_05401 PE=4 SV=1
MMEETAPAVAHEQRKDRVNLNTADAQTLQKELAGIGKNKADAIVAYREANGDFTSIDELI
EVKGIGKAILERNREKLALD
>tr|A0A0Q0DX13|A0A0Q0DX13_PSEAP AsmA family protein OS=Pseudomonas
syringae pv. aptata OX=83167 GN=ALO85_01925 PE=4 SV=1
MTRSRKIFAWTGATLLVVLAILVIVIVTFDWNRIKPTLNAKVSEALHRPFAINGDLDVRW
TREPELGGWRAWVPSPHFVADDLSLGNPEWSKTPQMVTLKHVEFRLSLLPLLAQQVVIPR
IDLTGPEAKLERLADGRANWVFDLPKSDPNAEPSKWVMDIGAIKFDKGLVSFNDQKLNAN
LDVVIDLLGKPIPFSEIVGSKEAKKAQDKGAVPQDYAFGLAVTGQYHGQKLSGTGKVGGL
LALKDANQPFPVQADVKVGDTHAVIAGTLTDPQNLGALDLRLKLSGASLSNLYPLTGVTL
PDSPAYSTDGRLIAKLHDPAGANFRYEGFNGKIGNSDIHGDLGFVASQPRPKLSGVLVSN
QLLFSDLAPLIGADSNAAQKKRGGESKQPSGKVLPVEEFQTDRWRAMDADVEFTGKRIVQ
SPDLPFTDLYTHLVLDDGQLSLEPLRFGVAGGKLDADIRLNGQNKPMQGRAKLSARNFKL
KQLFPTFEPMKTSFGELNGDADISGRGNSIAALLGSANGEMKMLVNDGAISRGLMEIAGL
NVGNYVVGKLFGDKDVKINCAASNFGIKDGLATSRLFVFDTENAIIYINGTANLKTEQLD
LQINPESKGFRVFSLRSPLYVNGPFAKPNAGVQSGPLLLRGAGMVLLGATVGPVAGLLAL
VATGDSEPNQCGPLLEQMRTGKAPKTVK
>tr|A0A0Q0D9Y0|A0A0Q0D9Y0_PSEAP Glycerol uptake facilitator protein
OS=Pseudomonas syringae pv. aptata OX=83167 GN=ALO85_02044 PE=3 SV=1
MGFFLRRTNNEHPRLSQGAETKTKNEVSMSIALKQPTLTGQCVAEFLGTALMIFFGTGCV
AALKVAGATFGLWEICIIWGMAVSMGIYLSAGISGAHLNPAVSIALSLFAGFEKRKLPFY
ISAQIAGAFCGAGLVYLLYISLFFDFEHAHHIIRGSEQSLELASVFSTYPNPAISVGQAF
LVEVVITTILMGVIMALGDDSNGLPRGPLAPLLIGLLVAVIGSSMGPLTGFAMNPARDFG
PKLMTFFAGWGEMAFTGGRDIPYFLVPIFAPILGACLGAAGYRALIARHLPSAAPVEHEK
EAPVVRGKVQVSS
>tr|A0A0Q0DJ93|A0A0Q0DJ93_PSEAP AraC family transcriptional regulator
OS=Pseudomonas syringae pv. aptata OX=83167 GN=ALO85_04963 PE=4 SV=1
MVMPAIESIQVFQALNSSPNANLEQSAPLGDGLVAALWNNRHDSQEYHAPTHHTLSCYIE
DGTGTFRRGRPDQKGAPGKLCILPAGHESAWVVNGEIRLAHLYFSPEQFALGCVTLLDRE
PREMQLRENTFLEDPAQAARFRQLIRLNWAEPGERLLTSSLAHEMVSHALLNQAGLREGL
RLKGGLAPHLRRHLVDFIEMHLADPLSLGQLAGLCALSEYHFARMFRESFGLPPHQYLLA
RRIEQARQLLRSTGKPLGEIALACGFASASHFSNRFRQATGATPGEYRTALGQ
>tr|A0A0Q0BX48|A0A0Q0BX48_PSEAP Alginate biosynthesis protein AlgX
OS=Pseudomonas syringae pv. aptata OX=83167 GN=ALO85_01804 PE=4 SV=1
MRKGIVMHAHLIKLLSLSSLTAALMAAAGVARADDSQAPTFKAEPCCSLCPAAHDAKNYT
TRYQQNFTTLVQAQGDWLFRTQEDLRTEFDTTPAGYRRMKELHDAFKSKGVELVVVYQPT
RGLVDRNKLFPAERDKFDYDKALKNYQAMLGRFSKMGYWVPDLSPLTNEQQAHDFYFRGD
QHWTPYGAQRTAKIVAETVKKVPGYSDIPKREFESHISGRMGKTGTLHNMAGQLCGTSYA
IQYMDQFTTEPKGEAGDGDLFGDSGNPEITLVGTSHSGKNYNFAGFLQEYMGADVLNVAF
PGGGLEGSMLQYLGSEDFQKRPPKILIWEFSPLYRLDQETIYRQMMSLLDNGCEGKPAVM
SASTTLKPGTNEVLVNGKNGIKDIRNGSNQIDIKFDDTSVKVLQARLWYMNGRHEDLKIE
KPETSDTDGRFAFELREDEDWANQQLLALEIQGPEAGTAPQKVEAKVCKRNVFPSAATHT
AQAGL
>tr|A0A0Q0FWQ7|A0A0Q0FWQ7_PSEAP Alginate biosynthesis regulatory protein
AlgP OS=Pseudomonas syringae pv. aptata OX=83167 GN=ALO85_01572 PE=4 SV=1
MSAKKKPVNTPLHLLQQLSGSLLEHLEDACSQALADAEKLLAKLEKQRGKAQEKLHNSRI
KLQDAATAGKSKAQAKAKGAVSELEELLDALKDRQTETRTYILHLKRDAQESLKLAQGIG
RVKEAVGKILTTRDAKPVAPKAAAKAPVAKTPAAKAPAKTAAKAPVKAAAAKPVAKAATK

PSAVAKPAVKTAAAKAPVKAAAKPAARTTAAAKPVAARTTAAKPAAAKPAATKAPAAKPT
ASSAAKPAAAKAAAAKPAAKAPVKAPVKAPAKAPVKAPVKAAAKPVAKPAVKPAAKPAAA
KPATPAPAAAAKPTTPAPAAPAAAPATPANGATPTSAS
>tr|A0A0Q0C711|A0A0Q0C711_PSEAP Flavin prenyltransferase UbiX
OS=Pseudomonas syringae pv. aptata OX=83167 GN=ubiX PE=3 SV=1
MSGPERVTVAMTGASGAPYGLRLLDCLVREDREVHFLISKAAQLVMATETDVRLPPKAMM
MQAFLTEYTGASAGQIRVYGRDDWMSPVASGSGAPSAMVVVPCSTGSLSAIATGACNNLI
ERAADVTLKERRQLILVPREAPYSSIHLEHMLKLSNMGAVILPASPGFYHQPQTIDDLVD
FVVARILNLLNIPQDMLPRWGEHHIGSDE
>tr|A0A0Q0C215|A0A0Q0C215_PSEAP Amino acid adenylation (Fragment)
OS=Pseudomonas syringae pv. aptata OX=83167 GN=ALO85_05494 PE=4 SV=1
TLGLDEQVLDPADGDIAEQLLKRLDPRHTRLDIRQAPMLRIGYAHDEVNNRWLGMLLFHH
LVDDATSLRILRSEIEAHMLGQQASLPPSVPYRNYVAQAMLGVSREEHEVFFRDMLGDID
EPTLPFGLQDVQGDGSGIEEARLLVDVDLSRRLRVQARQLGVSSASLYHLAWARLLGAVS
GKEDVVFGTVLLGRLQGGAGSDRALGMFINTLPLRVTLGEQGVRSGLKATHARLSGLLAH
EHASLVLAQRCSGVPASTPLFSSLLNFRHTGDVAASDNAQVAWEGIQALHGEERTNYPLT
LCVDDIGEGFALTVMAEAQIGAKRVCAYMHCVLENLVQALEQTPDAALGALRILPGIERQ
QLLESWNTPHALQADDTLIHRTFEAWVVAQPNAVALHYEDRTLTYAELNTRANQVAHYLL
GLGVQPDDRVAICVERSLEMIVGLLGVLKAGAGYVPVDPAYPAERIAYQLQDSAPMAVLA
DAAGLALLGTFDGPRVDLHSPALQAQPGHNPQLTGLSPRHLAYVIYTSGSTGLPKGVMVE
HRNVARLFSATHSWFGFNEQDVWALFHSFAFDFSVWEIWGALLHGGRLLIVPQLISRSPQ
DCYALLCSAEVTVLNQTPSAFRQLLNAQGESDQRHSLRQVIFGGEALDTGMLKPWYARVI
NAGTQLVNMYGITETTVHVTYHPLVAADAQRAGVSPIGVRIPDLQLYVLDARREPVPVGV
VGELYVGGAGVARGYLNREALTAERFLANPFSKDPQARLYRTGDLGRWMDDGSLDYLGRN
DDQVKIRGFRIELGEIQAALAACDTVREALVLVREDQPGDKRLVAYVIAAPGHEIVATDL
RERLLLSLADYMVPSAFVALGSFPLTANGKLDQKALPAPDAQALAMREYAPPEGDVEIAI
AQIWQSLLQVPQVGRHDHFFELGGHSLLAVKLIERMRQIDLSADVRVLFSQPTLAALAAA
VGGHTDVQVPDNLIVPGCERITPDMLPLADLDQAAIDRVVASVPGGLANIQDIYALAPLQ
EGILYHHLAADEGDPYVLQMLFAFDDRECLASFAAALQNVVERHDILRTAVVWEGLEQPV
QVVWRNAKLSLEEVAIDPLDGDVLTRLRERFDPRHYRLDIGQAPLMRIAYAEDNTHQRLV
GMLLFHHLALDHTSLEVVVEEMQASLQGQIEQLPAPVPYRNHVAQARLGISQAEHEAFFR
DMLGDIDEPTLAYGIQDVQGDGSGIEEVHQVLDTQLSSRIRSIARQLGVSAASLAHLAWA
QVAGRVSGREEVVFGTVLMGRMQGGNGADRALGMFINTLPLRISVGSQSALAAVKLTHQR
LSTLLGHEHASLSLAQRCSGVPSSLPLFSTLLNYRHSNGGAASSETLSAWQGIQTLSMEE
RTNYPLCLNVDDLGDDFMLTIQAVQQISATRIGEYMQVALRSLVQALEHTPQAALNSLPI
LPDDERELLLAGFNDTAHPYPRDVLIHQLIEQQAAQRPDACAVRGDSGPLLTYAKLNQQA
NQLAHRLIELGVEPDARVAVSLRRGPEMVVALLGILKAGGAYVPIDPDLPSARQAYMLSD
SAPRALLTSQDLLASLPALSVTALVLDGHDESARLALQPINTPDAKALGLKPNHLAYVLY
TSGSTGQPKGVMNEHLGVVNRLLWARDAYHVDSNDRVLQKTPFGFDVSVWEFFLPLLAGA
ELVMARPGGHQEPDYLAQVMTDAGITLLHFVPSMLDVFLEHRSTRDF
>tr|A0A0Q0DYR9|A0A0Q0DYR9_PSEAP ABC transporter OS=Pseudomonas syringae
pv. aptata OX=83167 GN=ALO85_01980 PE=4 SV=1
MSFIRVRNVWQQYADQVVLEGLNLDVAEGEFCTLVGASGCGKSTFLRLLLGQESPSRGLI
TLDGTPLRNEPDASRGVVFQRYSVFPHLSVLDNVALGLELPRSSWLGRLFGQAKSEAREH
AAQLLGKVGLGHALDKYPTQLSGGMQQRLAIAQALIMKPRVLLLDEPFGALDPGIRKDMH
HLLLDLWRETKLTVFMVTHDLSEGFNLGTRLLVFDKVRHDPHAPGAYGARITYDIPLNSE
RRAERAAIDSLLNVSEEPVQ
>tr|A0A0Q0CF25|A0A0Q0CF25_PSEAP Uncharacterized protein OS=Pseudomonas
syringae pv. aptata OX=83167 GN=ALO85_02861 PE=4 SV=1
MSNFSLPLLLRAPTPTQSSLTFSEANPRDLKRWISRLPKANLGEMARQLYQGLTELNQLI
TPSENRLQLLELLRPEVYFVCKHLERHFLNQAIVLDERPRKVANLCQALQNHLATGYKQI
IQQVASRYTRDQAGLFSMALQRALHGLNGPLIRANQLYCPVQDGLWLELHQIYQIARQYQ
LHNTPVEEPLAHHSKVLSVEQTYVIALLMGCSRCNQMRQLNIGKLADALEAWSPLVKLQS
PATQSSLFALDPTVDKPPLYRTLFSDEQSPRLLGINTFALAEAINQYLALPDDQRTPSPL
LIPTGINTDLLQHLAVAWGDVAERTFQRTPGKGTLRLCIGMSALHFYVAGQKSFSELLQL
NSATSAASFSLKMANDDVDDPWASAFDTQRGKSSSVLLPYEEIEYPKNDGDSESLSNNQH
TFPTFDLNIVNHSPGGYCLAWQKDVPQQLQAGELVGIQDDAGQGWSIAMVRWIRQVRSGG
TQMGIELIAPFALPCGMQLIREQQNSQYLRTLMLPEVRAMGKPPTVLAPRLPFQEGNKVM
INVNGEERRASLSNRQVSSASYNQFEYQMYDAPKAAEIEKARPDQEFDSLWGTL

>tr|A0A0Q0C571|A0A0Q0C571_PSEAP Putative membrane protein OS=Pseudomonas syringae pv. aptata OX=83167 GN=ALO85_04104 PE=4 SV=1
MKAAHPEVVATNRVNPGETLRQARESRSWSLPDVALRLNLTVTSLTNLENGQFEKLPGHT
FARGYVRAYAKLLELDQAALVEQFDQFTGTDGKGSSVHALGRIEEPVRLSHNILRIVSLL
LLVILVGGGFFWWQDQATLRGKDQSGLNMEHVEVESADGTTQIHPLDEPDEPAVAETPAS
GQTSLPLNTGAPASEAPAAAPAPAAPASSVTGHATAQPQTPAAATPPASAPQAPVAAPNV
PSMPTAPAEQPAPVMAGAGQVTVQFVADCWTQVTDGNGKVLVSGLKRKGESLDVNGKPPL
TLRLGYARGAQVSYNGQPVDVAPFTSGETARLKLGQ
>tr|A0A0Q0IJ12|A0A0Q0IJ12_PSEAP Alginate biosynthesis protein Alg44 OS=Pseudomonas syringae pv. aptata OX=83167 GN=ALO85_01808 PE=4 SV=1
MNTAVNANVVHESEAQRQHARIKIPAKLRLLNDQPNAPLVRVEDLSAGGLSFVAPSGQKF
SEGQIVKGRLQFIIDNLGLAMDVDLQVRSIDNASNRIGCQFQNLEPQDIATLRHLITSHL
SGEVVTLGEVLATLQRDNFTKARKVKGKDSGMSAMGRLRAVTFSLGVFIVGLAAFGFIFK
TIYGLYFVSHASAGLVTIPSMDVTMPREGTVQSLVAQNGEAAKGAPLASFNTSMLEMLKG
SLTGEDLQPAKIEELYGKQMSGTMTSPCDCVVARQLVADGQFASKGQVIFQLVPRNAPAT
VEARFTYRQFNDVKPGTRVSFQIAGEDKLRTGQIVSSTNLSDADLSTDIRVQIKPDESLS
SSLAGRPVEVVSDRGPSVNWLIDKAMAAGL
>tr|A0A0Q0CFV1|A0A0Q0CFV1_PSEAP Filamentous hemagglutinin, intein-containing (Fragment) OS=Pseudomonas syringae pv. aptata OX=83167 GN=ALO85_101560 PE=4 SV=1
MDQVIAITPVAAAAAQTVTRVEGLPSSNFVSKPQKYLIETNPVLTELKQFMSS
>tr|A0A0Q0D5M0|A0A0Q0D5M0_PSEAP Amino acid adenylation (Fragment) OS=Pseudomonas syringae pv. aptata OX=83167 GN=ALO85_04453 PE=4 SV=1
LAEYMLPSAFVLLDELPLTPNRKLDRNALPAPDVDALISRGYEAPQGETETQIVAIWQDL
LGVEQVGRHDHFFELGGHSLLAVSLIERLRKQGLNLNVKTVFTAPSVREMALAISQDKHV
LFQVPANRIPAHCTQLTPDMLPLVELSGAQIELITSAVPGGAANIQDIYPLAPLQDGILF
HYLLNRERDAYLMRSMIEFDSRARLDAFLEGLQTVIDRHDILRSSVHWIGLPQAVQVVHR
QAQLPVHTLTLTPDEDALSQLDRLSDPGRMRLDLRQAPLLLAYIAGDPNSERWLLALIDH
HMISDHVTVELILEEIRLLMRGQSADLLPAQPYRDFVAQTLASPSSAHEAYFARRLADVD
SPTAPFDLLEVQGDGNDVEEAKLALSSDLCARIRTQARERGMSPAVLFHVAWAQVMARCT
DRDDVVFGTVVTGRLQGTVGAERAMGMFMNTLPVRVQLTTQGAQELVMATHRDLSELLSH
EQASLALAQRCSSVATGVPLFSSLLNYRHQGEDSRLQWPGMRLLDGTERTNYPLCLSVND
YGSELDLIIHSMQPADPQRL
>tr|A0A0Q0IA27|A0A0Q0IA27_PSEAP Amino acid adenylation (Fragment) OS=Pseudomonas syringae pv. aptata OX=83167 GN=ALO85_100843 PE=4 SV=1
MVVGLLGIFKAGGAYVPLDPAYPEQRLRYMLEDSTPVAVLVQAETRALLGELAVPTLDLQ
DAEWEVEAEHNPVVPAITPQHLAYVIYTSGSSGKPKGVGGPHQAMVNRLYWMHSTFGGQR
LEKHAQKTSISFLDSVTETLMPLLFGAQLHIVSPLASRDPLQLWQAVTEHQLTRLVLVPS
LLEELSRIENRQVSPEKRLIVCSGEVFSSSLLARTRAWLPSATILNFYGSSEAAGDSTFY
LCDSAVQAGHSLPIGRPIANTRIYLLDAHGQPVPIGVIGEIHIGGAGVARGYLNMPELTA
ERFLDDPFSAEPAARMYRSGDLGRWLADGNIEYLGRNDDQVKLRGFRIELGEIESKLSEY
PGVREAVVLVREHRSGDKRLVAYLTAQEGAVLSAAQLREQLSQGLAEYMIPSAFVTLARF
PQTPSGKLDRRALPAP
>tr|A0A0Q0FT44|A0A0Q0FT44_PSEAP Uncharacterized protein OS=Pseudomonas syringae pv. aptata OX=83167 GN=ALO85_101447 PE=4 SV=1
MVLSLWFRCFRLMKSGCCAVCCNAGDHSKGASIKAMLLGQKRLDAADKT
>tr|A0A0Q0C4W9|A0A0Q0C4W9_PSEAP Membrane-bound lytic murein transglycosylase D OS=Pseudomonas syringae pv. aptata OX=83167 GN=ALO85_101809 PE=4 SV=1
MPCRELERRACHVGGHVQDIPHMAAVALHVSSPRLLARSVQIAVLALGALCVGCQSVDDS
QSRLDRLPRLVAGLGHRSDDAQADPRAVTTVSDLPVYNGEDVWQRVAQRCRLVDGQGVNE
RIARQRDWLLNNRGFLTGASKRATPYLHFIVERLDERNMPLELALLPMIESSYNPLANSP
AAAAGLWQFIPSTGRQFNLNQSATYDARRDVVASSKAAMDYLTRLHDQFNNDWLLALAAY
NAGEGTVARAIDANRRRGMPVDYWHLNLPRETQDYVPRLLALSLVVRNPGAYGVQLTPVA
NMPYFDVVELNHTVDLTQLAATTGVDEAQLLRLNPAFLRKKTPDGPGRLLIPKTHTQVLT
AGIERITGESPTTAVIPASPGKRVIEPQPASQVAERAPVPGPLQAAGREQTAGLRQVLER
EPAPESVQPVRREPQPKVVQIARQEPLPGPAQPVQAAEQVTVSESIQPARQAPPQATSER
SLLDRRIQKRLYIDDRSSPRKRDEIAYRDEPRELPNGPRVVVYASDPRQ
>tr|A0A0Q0FLE9|A0A0Q0FLE9_PSEAP Uncharacterized protein OS=Pseudomonas syringae pv. aptata OX=83167 GN=ALO85_03957 PE=4 SV=1

MWTVYEMLEARLVRAFLFLVFSNSQDMSASTIREHSDSLLMVGEISMCDQRIVDSRRHNA
DLLDKKINRRETLVNSHSLMAGYIEAMNNWKADEQELNEKRQSLSTRLEQIKQQAVEDMA
KARPAETDAATAYAQAVAWGDTEGEKTASADAQKAAKNLATAAEHNRRQDLIISALEQEL
LTVDRYIAEAQEKHKGIERDALWLSQTVLEEKWNEAAKTLFDVGGRLWANYNLLGLDQVN
LLKLAVPQAGERGGNWTWHELSGRARNYGAQDLLQLNNISTPQQAALVSQLEELTDESSE
QHGADVVGVWLKK
>tr|A0A0Q0FBJ6|A0A0Q0FBJ6_PSEAP Carbohydrate ABC-type transport system, ATP-binding protein OS=Pseudomonas syringae pv. aptata OX=83167 GN=ALO85_04287 PE=3 SV=1
MATLELRNVNKTYGSNLPDTLKNIDLSIKDGEFLILVGPSGCGKSTLMNCIAGLESISGG
EILIDGQDVSGTSPKDRDIAMVFQSYALYPTMSVRENIAFGLKIRKMPQADIDAEVARVA
KLLQIEHLLNRKPGQMSGGQQQRVAMGRALARRPKIYLFDEPLSNLDAKLRVEMRTEMKL
MHQRLKTTTVYVTHDQIEAMTLGDKVAVMKDGIVQQFGTPKQIYTDPANLFVASFIGSPP
MNFIPLRLQRKDGQLIAALDSGQARCELPVGVSDAGLEDREIILGIRPEQIMLAGAESTG
MPTIRAEVQVTEPTGPDTLVFVTLNQSKVCCRLAPDVAPQVGESLTLQFDPARVLIFDAQ
SGERLGVLGARQAAERENNVSPVEPQVIN
>tr|A0A0Q0DV33|A0A0Q0DV33_PSEAP Sulfate transporter/antisigma-factor antagonist STAS OS=Pseudomonas syringae pv. aptata OX=83167 GN=ALO85_04894 PE=3 SV=1
MQLSCPRSACRSGRSASSTFNSPEGPMRWLNRHKFLPFLAWLPQQNRASVGRDALVGLSG
AILALPQSIAYALIAGLPPEYGLYAAIVPVLVACLWGSSWHLICGPTAAISIVLYASVSP
LAVPASQDYIMLILLLTFIAGVFQLLLGMMRFGALVNFVSHSVVLGFTLGAAVVIALGQM
PNLLGIDLPSQTTALKSLTTVLEHWREVDLSSLMLGLLTLALGIGLKLLVPRWPTLLIAL
VSSSLLVWLWPAMFGHVRVVSAFVGHLPPFSPLPLDVELILKLLPTAVAVGMLGLVNSLS
IARSLSARSQQMLNANQEVRAQGLSNMVGSLFSGYLSAGSFTRAALSYEAGARSPLAGVF
SALWVALFAVAGASLISHIPIPAMAASILLICWGLVDRRGIRALFRVSRAEFFVMALTCL
ATLLLELQTAIYAGVLASLFFYLKRTSQPRVQQWREGDEEVLRVGGSIFFGASHYLQTRL
QRTEGVRVVIDAQQINFIDYSGVEMLHQEARRLGRQGRVLVLRNARPQVIEELHKLEGPE
NCPILFED
>tr|A0A0Q0FE70|A0A0Q0FE70_PSEAP Uncharacterized protein OS=Pseudomonas syringae pv. aptata OX=83167 GN=ALO85_101010 PE=4 SV=1
MRGRGLSVGIKSSSGARICRTGKQRYGMFRRMANRMF
>tr|A0A0Q0CXL0|A0A0Q0CXL0_PSEAP Major facilitator superfamily transporter OS=Pseudomonas syringae pv. aptata OX=83167 GN=ALO85_01240 PE=4 SV=1
MSSSALDGSQHPLKSSFFLLFLTIFIPFGLGHFVSYLFRTVNAVIYVDLQTDLSLPASSL
GLLTGVYFLTFAAAQIPLGVMLDRYGPRSVQAPMLLFAVAGSVIFSISSTETGLLIGRGL
IGLGVAGSLMSAIKACAIWLPVERLPLSTACLLSIGGLGAMASTTPLHALLNWLTWREAF
LVLALLTFCVAVVIHLSVPKAYESKKTRYSDMFAAVGKLYSSWTFWRLALYSVFAHAIYM
SVLSLWMGPWLRDMAGLSDSGMANVLLFGAIAMVAGSLTFGAITDYLRRFGVQPIMICGT
GMLIFVGFQVLMASGLPVSPYLVAMGFSFFGTSTTMNYAIVAQSVSPELAGRVSSSFNLV
VFVLAFFLQWLMGVVLNLWPASQGAGHSLEAYQWSLGLMIALQLPGVLLWLSFRPWKRKV
>tr|A0A0Q0C9N9|A0A0Q0C9N9_PSEAP Acetyl-CoA acetyltransferase OS=Pseudomonas syringae pv. aptata OX=83167 GN=ALO85_05353 PE=3 SV=1
MSAYLCHHERSITMRISTMHDVVIVAATRTAVGSFQGSLASVAAVDLGAAVIRQLLARTG
VDGAQVDEVIMGQVLTAGAGQNPARQAAIKAGLPFSVPAMTLNKVCGSGLKALHLATQAI
RCGDAEIIIAGGQENMSLSNYVLPGARTGLRMGHASMVDTMITDGLWDAFNDYHMGITAE
NLAQQYDISREAQDEFAALSQQKALAAIEAGRFVDEITPILIPQRKGDPLSFATDEQPRA
GTTAETLAKLKPAFKKDGTVTAGNASSLNDGAAAVILMSAARAEQLGLPVLARIAAYANA
GVDPAIMGIGPVSATRRCLNKAGWSLADLDLIEANEAFAAQSLSVGKELGLDPQKLNVNG
GAIALGHPIGASGCRVLVTLLHEMIRRDVKKGLATLCIGGGQGVALALER
>tr|A0A0Q0CXT5|A0A0Q0CXT5_PSEAP Transglutaminase domain-containing protein OS=Pseudomonas syringae pv. aptata OX=83167 GN=ALO85_05314 PE=4 SV=1
MRGSITVQARRRHAVSIHIALHHVTHYRYDRAVELGPQIVRLRPAAHSRTRVLSYSLKVL
PENHFINWQQDPQGNYLARLVFPEKTDEFRVEVDLVAEMAVFNPFDFFLEPYAENIPFTY
ASEEQRELAPYLEKLPLTPRFQAYLDSISREPIPAIDFLVGLNQRLSQDVAYLIRMEPGV
QTPEFTLQNASGSCRDSAWLLVQLLRHLGMAARFVSGYLIQLKADVEALDGPSGTDVDFT
DLHAWCEVYLPGAGWVGLDATSGLFAGEGHIPLACSPEPSSAAPISGLVEPCETDFSHEM
SVERIWEAPRVTKPYTEAQWQDIQALGRQIDADLLRDDVRLTMGGEPTFVSIDDRDGAEW
NTAALGPRKRELSAELFQRMRGHYAPLGIVHFGQGKWYPGEQLPRWSLNCFWRKDGQPVW

RNNTLIADETRDYGATGELAGRFLASVAERLKLPARFVFPAYEDNFYYLWREGALPVNVT
AEDSRLGDELERARLRKVFAQGLDKMIGQVLPLARNADGDSWQSGRWYLRDEHCRLVPGD
SALGYRLPLASQPWVKAAEYPFIHPTDHNQDFPELADSDSLTSVLKSTDTDAERAPKIDE
SADWLTRTALCAEAREGRLYLFMPPLQKLEEYLELVAVIEAAAEELQCPVLLEGYEPPSD
PRLCNFRITPDPGVIEVNVQPSASWDELVERTEFLYEQARLTRLTTEKFMIDGRHTGTGG
GNHFVLGGATPADSPFLRRPDLLRSLISYWHNHPSLSYLFSGLFIGPTSQAPRIDEARND
SLYEMEIAFAQMPAPGEEVAPWVVDRLLRNLLIDVTGNTHRAEFCIDKLYSPDGATGRLG
LLELRAFEMPPHARMSLAQQLLLRALVARFWREPYAPAKLARWGTQLHDRFMLPHFIEQD
FADVVADLNAAGYPVQADWFSAHLEFRFPKVGDYAVNGIELELRQALEPWHVLGEEGSAG
GAVRYVDSSLERLQVHVRGLPPQRYVLTCNGVAVPLQPTGRAGEFVAGVRYRAWQPANCL
QPTIGVHAPLVFDLLDTWMHRSLGGCEYHVAHPGGRNYETLPVNANEAESRRLSRFFRIG
HTPGKLDVPSLNINDELPMTLDMRLQ
>tr|A0A0Q0III7|A0A0Q0III7_PSEAP Uncharacterized protein OS=Pseudomonas syringae pv. aptata OX=83167 GN=ALO85_00440 PE=4 SV=1
MKITPIVAHLQATCPSFAGRISAGIDWAAVALGDQLAHPSAYVIATGDQSTANDLQNVIR
QSITDTIDVVVVLDGGDKRGQEASEQLHALRAELWRALVGWNPDHDYDAMQYTGGALVQI
SGDRVTYRFGFAAQFQLGRNTSDQPAETWHEAYLDGLPGFTGATIEMDCVDPADPNLKSP
GPDGRIEAKFTAEVTP
>tr|A0A0Q0CF03|A0A0Q0CF03_PSEAP Lipopolysaccharide heptosyltransferase I OS=Pseudomonas syringae pv. aptata OX=83167 GN=ALO85_02895 PE=4 SV=1
MRVLLIKTSSLGDVIHALPALTDAARALPGIRFDWVVEEGFAEIPAWHPAVDRVIPVAIR
RWRKHLWQTFRSGEWRRFKQQVREQRYDLVIDAQGLFKSAWLTRYVDAPVAGLDRDSARE
PVASRFYDRAFPVARGQHAVERLRQLFAQALGYPQPSGMGDYGLKPLATLDDSLQAPFVL
FLHGTTWDTKHWPELYWRQLAELMAARGLQVQLPWGNPAEKARAERIAEGLSSAHVLPKL
NLAGVARVLASAQACVAVDTGIGHLAAALDVPTISLFGPTNPGLTGAYGKSQVHLASDYP
GCTPCLQKKCTYQPSADDQRRFDLKREWPMCFTRLNPERVASQLGALLLAKEPG
>tr|A0A0Q0DI39|A0A0Q0DI39_PSEAP Putative translation initiation inhibitor endoribonuclease, YjgF family OS=Pseudomonas syringae pv. aptata OX=83167 GN=ALO85_03023 PE=4 SV=1
MAGEMKPISTSLAAAPGGHYSQAMLHQGMLHVSGQLPVRADGSHSVGEPFEVQAAVALDN
LLAILGAAGCNTDDLLKVTIYIAGVQHWPAFDRLYAGYLGEHRPARAVVPVPELHHGYLI
EIEALARCSQST
>tr|A0A0Q0BWC0|A0A0Q0BWC0_PSEAP Copper-exporting ATPase OS=Pseudomonas syringae pv. aptata OX=83167 GN=ALO85_05303 PE=3 SV=1
MRQXXRVERALAKVPGVNSVTVNLASERAHVDTAAHTDPQTLIDAVSRAGYGATLSQDRH
AEADQKARHLHHERLALLLAILLALPLVLPMLLSPLGVHWMLPAWLQFALATPVQFILGA
RFYVAAWKAVRAGAGNMDLLVAIGTSAGYGLSVYQWLHAAPGTTPHLYFEASAVVIALVL
LGKYLESRAKRQTASAIRALEALRPERALRVVDGLEQDVAISALQLDDRVLVKPGERFPV
DGEVVEGRSHADEALITGESLPVVKQPGDKVTGGAINGEGQLLIRTTALGAETVLARIIR
LVEDAQAGKAPIQKLVDKVSQVFVPTVLVIALLTLAGWLLVGASLEVALINAVAVLVIAC
PCSLGLATPTAIMAGTGVAARYGILIKDAEALERAHEVDAVVFDKTGTLTSGTPRITNMS
AVLGNEEHLLQLAGALQRGSEHPLARAVLDVCHEWQLTPDAAQNSRVLSGRGIAGTVEGL
ELALGNRRLLEESGLPMGDLAESARIWEAEGRTLSWLIEQAPEPKVIGMFAFGDTLKPGT
DQAIRALNARGITSHLLTGDNRGSAQVVARALGIHDVHAEVLPADKAATVTQLKSHHVVA
MVGDGINDAPALAAADVGIAMGGGTDVAMHAAGITLMRGDPRLVPAALDISRRTYAKIRQ
NLFWAFVYNLIGIPLAALGYLNPVLAGAAMALSSVSVVSNALLLKTWKPRDLEDAP
>tr|A0A0N8T9D5|A0A0N8T9D5_PSEAP DNA mismatch repair protein MutS OS=Pseudomonas syringae pv. aptata OX=83167 GN=mutS PE=3 SV=1
MNKAISDLSAHTPMMQQYWKLKNQHLDQLMFYRMGDFYEIFYEDAKKAAKLLDITLTARG
QSAGQSIPMCGIPYHAAEGYLAKLVKLGESVVICEQIGDPATSKGPVDRQVVRIITPGTI
SDEALLDERRDNLIAAVLGDERLFGLAVLDITSGNFSVLEIKGWENLLAELERINPVELL
IPDDWPQGLPAEKRRGSRRRAPWDFERDSAHKSLCQQFATQDLKGFGCENLTLAIGAAGC
LLSYAKETQRTALPHLRSLRHERLDDTVILDAASRRNLELDTNLSGGRDNTLQSVMDRCQ
TAMGTRLLTRWLNRPLRDLTILQARQTSITCFLERYRFENLQPQLKEIGDIERILARIGL
RNARPRDLARLRDALSALPELQQAMTDLDAPHLQQLAQTASTYPELADLLQRAIIDNPPA
VIRDGGVLKTGYDAELDDLQSLSENAGQFLIDLEAREKARTGLGNLKVGYNRVHGYFIEL
PSKQAEQAPADYIRRQTLKGAERFITPELKEFEDKALSAKSRALAREKMLYETLLEDLIG
HLAPLQDTAAALAELDVLSNLAERALNLDLNCPRFVAEPCMRIEQGRHPVVEQVLSTPFV
ANDLALDDSTRMLVITGPNMGGKSTYMRQTALIVLLAHIGSFVPAASCELSLVDRIFTRI
GSSDDLAGGRSTFMVEMSETANILHNATDKSLVLMDEVGRGTSTFDGLSLAWAAAECLAQ

LRAYTLFATHYFELTVLPESEPLVTNVHLNATEHNERIVFLHRVLPGPASQSYGLAVAQL
AGVPGKVISRAKEHLQRLETTSLPHEQPRAKPGKPAVPQQSDMFASLPHPVLDELSKVKV
DDMTPRQALDLLYTLQTRL
>tr|A0A0Q0DQW5|A0A0Q0DQW5_PSEAP Acyl-CoA dehydrogenase OS=Pseudomonas syringae pv. aptata OX=83167 GN=ALO85_05152 PE=4 SV=1
MLLLWIVVLVVGIAWLAHRRTAPLPALGVVAVYLLAMGIFSHAPGWLLTVFWLLWLAVFI
PVMLPDLRRKHFTAPMFSWFQKVLPPMSQTERDAIDAGTVWWDGELFSGRPDWDKLLSYP
KVQLTEEEQAFIDGPTEELCAMVSDWEIGQAMDLPPEAWAHMKAHGFFALIIPKEFGGKG
FSAYAHSQVAMKLATRSGDLASTVMVPNSLGPAELLLHYGTDEQRNHYLPRLARGEDIPC
FALTGPLAGSDAGAMTDTGIICKGQWQGEEVIGLRLTWEKRYITLGPVATLLGLAFKAHD
PEHLLGEKEDLGISLALIPTETPGVEIGRRHLPLGAAFMNGPNSGKDVFIPLSYLIGGQP
MLGKGWMMLMNCLSVGRSISLPAVGTGAAKYTSLVTGQYAKVREQFNVPISAFEGIQEAL
ARIGGNAWLMDSARMLTANAVDLGEKPSVLSAILKYHLTERGRECIGHAMDVHGGKGIIM
GPNNYLGRNWQGAPIFITVEGANILSRNLMIFGQGAIRCHPFVLKEMALAGREDKQQALI
EFDSLLLKHIGFAVSNAASTFILNLGFGHFERAPGNGLSQGYFRALNRQAAAFAMLADLS
MMLLGGELKRRERLSARLGDVLSHMYLASAALKRYHDLGSPDHMSPLFRWAMEESLGHSE
RAMDEILSNFPNRILGGVLRAVVFPFGRRHKGPSDRLDAEVAQVLGRAKGDPTLEELLAG
CYRPQSADDPVGALQQAIDLLSAAYPLHKKLQVAIKSGQVKPDAGEPVIDAALRIGVLQA
DEAQTLRTAEAARRKVIDVDDFDKEELTLAAGKIR
>tr|A0A0Q0BSS0|A0A0Q0BSS0_PSEAP Sulfate adenylyltransferase subunit 1 OS=Pseudomonas syringae pv. aptata OX=83167 GN=cysN PE=3 SV=1
MSHQSDLISEDILAYLGQHERKEMLRFLTCGNVDDGKSTLIGRLLHDSKMIYEDHLEAIT
RDSKKSGTTGDDVDLALLVDGLQAEREQGITIDVAYRYFSTAKRKFIIADTPGHEQYTRN
MATGASTCDLAIILVDARYGVQTQTRRHSYIASLLGIKHIVVAINKMDLNGFDESVFESI
KADYLKFAEGIAFKPTTMAFVPMSALKGDNVVNKSERSPWYTGQSLMEILETVEIASDRN
YADLRFPVQYVNRPNLNFRGFAGTLASGIVHKGDEIVVLPSGKSSRVKSIVTFEGELEQA
GPGQAVTLTMEDEIDISRGDLLVHADNVPQVSDAFDAMLVWMAEEPMLPGKKYDIKRATS
YVPGSIASITHRVDVNTLEEGPASSLQLNEIGRVKVSLDAPIALDGYSSNRTTGSFIVID
RLTNGTVAAGMIIAKPVSGGGSQHHGELAHVSTEERAQRFGQQPATVLFSGLSGAGKSTL
AYAVERKLFDMGRAVYVLDGQNLRHDLNKGLPQDRAGRTENWSRAAHVARQFNEAGLLTL
AAFVAPDAEGRERAKALIGKDRLLTVYVQASPTVCRERDPQGLYAADGDNIPGDSFPYDV
PLNADLVIDTQTLNVEESVRQVLDLLRSRGAI
>tr|A0A0Q0C293|A0A0Q0C293_PSEAP MarR family transcriptional regulator OS=Pseudomonas syringae pv. aptata OX=83167 GN=ALO85_00579 PE=4 SV=1
MKHFSPDNFESSLIGLLVGRTNALKDRMLDKYLLPYDVTFAQFKVLIIIAQFSTDTPVEL
CRHLSLDSGSMTRMLDRLEQKGLVVRQRSATDRRQVRLALTEQGQALCDLLPQIGADAMN
DTFAALDSEEVESLKRILTKVLVAADDHITLTRLSFNPKAKR
>tr|A0A0Q0ILS0|A0A0Q0ILS0_PSEAP Chemotaxis protein methyltransferase WspC OS=Pseudomonas syringae pv. aptata OX=83167 GN=ALO85_00343 PE=4 SV=1
MKVDDSRFFSFLKERIGLDVTSVGEAIIERALRQRAAAAHCADSDAYWHLLVSSPQEQQA
LIEAVIVPETWFFRYPESFLSLGMLARERIASLAGARPLRILSLPCSTGEEPYSIAMALF
DAGVDARQFKVDAIDISPVSIAKAEHGVYGKNSFRGSDISYRERYFHPVADGFEIADSVR
ACVSFQPGNLLDPKLASHVAYDIVFCRNLVIYFDRQTQQHVFKVLKQLTREDGLLFIGPA
EGNLLAAIGMRSTGIAQSFAFRHAPVDAAVPAPAPKQVAAPVAPRPVVEAPARAVAPRPA
PRTSAAFAPIAKPVAAQANSEVSAVLDKIAGLANEGKTAEARAACERYLQQHEPVAQVFY
WLGLLSEVEGRVPQAQGFYRKALYLQPQHSESLAQLAALLAAQGDTAGARRLQDRAARGA
NKQGNH
>tr|A0A0Q0IP87|A0A0Q0IP87_PSEAP Endoribonuclease L-PSP OS=Pseudomonas syringae pv. aptata OX=83167 GN=ALO85_03738 PE=4 SV=1
MSDSIQRTSVGDFPISQSVTVPASTSLVFVSGTLPDLADPHAPAGTPAAYGNTEVQTVSV
FNKLRRILQQQDLDLGDIVQLRVFLVGAEETAGKLDFAGLQAGYTQFFGTPEQPLKPART
ALQVVALPLPGALIEVEAIAARPA
>tr|A0A0Q0FHC2|A0A0Q0FHC2_PSEAP Uncharacterized protein OS=Pseudomonas syringae pv. aptata OX=83167 GN=ALO85_02835 PE=4 SV=1
MSIDKSPKEHWASHQERGSFMLMKLTAWGMRVLGRRLLSPVLYGIVLYFFVFGRRARHSI
WQYQQHLAGWSGKPELRPSQRRVFGQFMAFADSLLDKLDVWNGRLGIDQIEIVDQALLRQ
NLRGQRGQMLVGAHLGNLEVCRALAELGEKVTMNVLVHTKHAERFNRLLGEAGATHLRLI
QVSELDPAIMLQLSQRLDEGEWLAIAGDRVALHGGRNVRVDFLGQPATFPQGPWLLAGLL
KCPVNLFFCLKGAKGYRVILEPFADAIEWRRSDRAQVIEQWATRYAERLGHYCLEAPQQW
FNFYPFWESADESGS

>tr|A0A0Q0CCK5|A0A0Q0CCK5_PSEAP VbhA domain-containing protein OS=Pseudomonas syringae pv. aptata OX=83167 GN=ALO85_200124 PE=4 SV=1
MNAPDSIDVLDAEMLRRQKAVDYARASLALEGVVISTDEEQRAQAFVNGKLTLAEFISVE
IAQ
>tr|A0A0Q0DNW0|A0A0Q0DNW0_PSEAP Uncharacterized protein OS=Pseudomonas syringae pv. aptata OX=83167 GN=ALO85_03494 PE=4 SV=1
MKLPLRPSLSRATVLQILALIVLIGMLAYQQWQILHLKTGFESAANLETVNAITQRINGV
DDRLDAASQLKSVTIDDFRAGQQALSNRINAVQALLKQVQEAAKDAAQQGASMQEVVVMN
ARIEELQVKLQDLRAAKPASVQISAAKPKTPPPSRKSTAQSKVPEAPPPFSVVGVEYRGG
ERFLSVAPPGSTQLSQLNLIRPGDMVAGSNWQLNSLDDSRALFSINGSTRILPLRP
>tr|A0A0Q0D8I0|A0A0Q0D8I0_PSEAP Riboflavin biosynthesis protein RibD OS=Pseudomonas syringae pv. aptata OX=83167 GN=ALO85_01912 PE=3 SV=1
MLTEQAALDVHYMARALEMARKGLYSTHPNPRVGCVIVREGRIVGEGWHARAGEPHAEVH
ALRQAGELARGATAYVTLEPCSHQGRTPPCADALIEAGLTRVVAAMQDPNPEVSGRGLLR
LMNAGIGVQCGVLESEARALNKGFLKRMETGLPYVRVKMAMSLDGRTAMASGESQWITGP
EARSAVQRLRAQSSVVLSGADTVLADKARLTVRPDELGLDAELTALAAARAPLRVLIDGR
LRVPLDAPFFQAGSALVVTCAAASARGRYQEEGHEMLALADSAGHVDLRRLMGELAARGV
NEVLVEAGPRLAGAFARLGLVDEFQLFIAGKFLGSSARPLLDLPLAQMSEALELNIVEMR
AVGNDWRVIALPVRTPGV
>tr|A0A0Q0FR78|A0A0Q0FR78_PSEAP Putative aspartate/tyrosine/aromatic aminotransferase OS=Pseudomonas syringae pv. aptata OX=83167 GN=ALO85_02945 PE=4 SV=1
MADQGSPRRFARIDRLPPYVFNITAELKMAARRRGEDIIDLSMGNPDGATPPHIVEKLIT
VAQREDTHGYSTSRGIPRLRRAISNWYKKRYEIDIDPESEAIVTIGSKEGLAHLMLATLD
QGDTVLVPNPSYPIHIYGAVIAGAQVRSVPLVPGVDFFDELEKAIRGSIPKPKMMILGFP
SNPTAQCVELDFFERVVALAKQYDVLVVHDLAYADIVYDGWKAPSIMQVPGAKDIAVEFF
TLSKSYNMAGWRIGFMVGNPELVNALARIKSYHDYGTFTPLQVAAIAALEGDQQCVLDIA
EQYRQRRNVLVKGLHELGWMVENPKASMYVWAKIPQAYAHLGSLEFAKKLLAEAKVCVSP
GIGFGEYGDDHVRFALIENQDRIRQAVRGIRSMFRADDKLKS
>tr|A0A0Q0IA37|A0A0Q0IA37_PSEAP PBPb domain-containing protein OS=Pseudomonas syringae pv. aptata OX=83167 GN=ALO85_02316 PE=4 SV=1
MRRVHGTLHASFTIECRNMFKRLLLALIGVGVLSSEGVRASESTPYSMVLLTENFPPFNM
AVDGKNFAQENNIDGSAAEVVREMFKRANIGYSMTLRFPWDRVYKLALEKPGYGVFSTTR
LPERENLFKWVGPIGSYDWIMLARGDSPITLTSLGQARDYRIGAYKGDAIGERLKAMGLN
PILMLRDRDNVKKLANGQIDLWAVGDPVGRYLAKLEGVTGLKTALRFNSAELYLAVNKST
PDEVVRRLQKALDQMRVEGWVDAAKARYQ
>tr|A0A0Q0D717|A0A0Q0D717_PSEAP Regulatory protein, LysR:LysR, substrate-binding protein OS=Pseudomonas syringae pv. aptata OX=83167 GN=ALO85_04704 PE=3 SV=1
MLPHRRCIDQGMLMDLRQLEAFAAVMSAGSVTAAGKMLGRSQPSVTRAIQELEQELGFAL
FERSGPKVTPTHKAFMMYGEVESALLGVRNIRQRAQHIAQEENHQIKLVAISALAAGLLP
AALSRLPEHLRPQQIQLQSMSPENVVHAVLSKTMDLGAVSLPLEHRGLEIHWIGEAPCVA
VLPADSALAAHEVLSMELLRQQTLITMANPYRFRRRIDKAFQEAGGQPPRMLDTNTSLIA
MQMARVGLGIALVDPFTAMGVPVQGVVVRPIACNIPFFFGLISAFASPLSDVASAMIDEI
ADSAKILLPEMIMHEASAHDALLQSIYAE
>tr|A0A0Q0CIS1|A0A0Q0CIS1_PSEAP Oxygen-dependent coproporphyrinogen-III oxidase OS=Pseudomonas syringae pv. aptata OX=83167 GN=hemF PE=3 SV=1
MLMSTRTEAVKAYLLDLQDRICTALEQEDGSAHFMEDAWTRPAGGGGRTRVIENGTVIEK
GGVNFSHVFGSNLPPSASAHRPELAGRGFEALGVSLVIHPHNPHVPTSHANVRFFIAEKE
GEEAVWWFGGGFDLTPYYGVEEDCVHWHRVAERACAPFGEDVYPRYKAWCDSYFHLKHRD
EPRGIGGLFFDDVNQWDFDTSFAFIRAIGDAFINAYLPIVRRRKAAAYTVQQREFQEFRR
GRYVEFNLVYDRGTLFGLQSGGRTESILMSLPPQVRWGYDWKAAPGSEEARLTDYFLTDR
DWLADN
>tr|A0A0Q0IA47|A0A0Q0IA47_PSEAP Adenine DNA glycosylase OS=Pseudomonas syringae pv. aptata OX=83167 GN=ALO85_02307 PE=3 SV=1
MQPEQFSSAVLDWYDRHGRHDLPWQQDITPYRVWVSEIMLQQTQVSTVLSYFDRFMEALP
SVQALADAPEDEVLHLWTGLGYYTRARNLQKTAKIVVADHGGEFPRNVEKLILLPGIGLS
TAGAIASLSMGIRAPILDGNVKRVLARFTAQEGYPGEPKVAKQLWATAERFTPHSRVNNY
TQAMMDLGATLCTRSKPSCLLCPLERSCEAHMLGLETRYPIPKPRKTVPQKRTLMPMLAN
EDGAILLYRRPSSGLWGGLWSLPELDDFDDLQHLATQHALQLGEHHELPGLIHTFSHFQL

SIEPWLVKVRESADHVAEADWLWYNLATPPRLGLAAPVKKLLKRAADVLNAGESS
>tr|A0A0Q0FI61|A0A0Q0FI61_PSEAP N-acetyltransferase GCN5 OS=Pseudomonas syringae pv. aptata OX=83167 GN=ALO85_03227 PE=4 SV=1
MKQNATTYSQRLLRGQSPSYERLQARLAEDGSQIDADPLALHCGWGRLLIGHTYPDPAAL
AQDLLNEKPGERDIALYVAAPQQVLAQSPQQLFLDPSDTLRLWFSDYRPAQRVFRGYRIR
RAHNENDWQAINNLYLARGMLPIDPELLTPRHEGGPVYWVAEDEGSKTIIGSVMGLNHQK
AFNDPEKGSSLWCLAVDPQCTRPGVGEVLVRHLIEHFMSRGLSYLDLSVLHDNDQAKALY
AKLNFRNLPTFAIKRKNGINESLFLGPGPQADFNPYARIIVEEAHRRGIDVQVDDADAGL
FTLSYGGRRIRCRESLSDLTSAVSMTLCQDKSLTHRALKAAGLRLPAQQRAGSADDNLAF
LEAHGQIVVKPLDGEQGQGVAVDLRTIDDVQSAIEQARQFDTRVILESFHEGLDLRIVVI
GFQVVAAAIRRPAEIIGDGRHTIKQLIEAQSRRRAAATDGESRIPMDQETERTVREAGFD
YADILPMDQRLAVRRAANLHTGGCLEDVTAILHPVLSDAAVRAARALDIPVVGLDLMVPA
ADQPEYVFIEANERVGLANHEPQPTAERFVDLLFPHSLPVHI
>tr|A0A0N8T9C3|A0A0N8T9C3_PSEAP Formate-dependent phosphoribosylglycinamide formyltransferase OS=Pseudomonas syringae pv. aptata OX=83167 GN=purT PE=3 SV=1
MTQIGTPLSPTATRVLFCGSGELGKEVVIELQRLGVEVIAVDRYENAPAMQVAHRSHVIN
MLDGAALRAVIEAEKPHFIVPEIEAIATSTLVELEAEGFTVIPTARAAQLTMNREGIRRL
AAEELKLPTSPYHFADTFEAYSKAVEDLGFPCVVKPVMSSSGKGQSLLKSTDDVQKAWDY
AQEGGRAGKGRVIVEGFIDFDYEITLLTVRHIGGTTFCAPVGHRQEKGDYQESWQPQAMS
PVALAESERVAKAVTEALGGRGMFGVELFIKGDQVWFSEVSPRPHDTGLVTLISQDLSQF
ALHARAILGLPIPLIRQFGPSASAVILVEGQSTQTAFANLGAALAEPDTALRLFGKPEVN
GQRRMGVALARDESIEAARAKATRASQAVNVEL
>tr|A0A0Q0DC22|A0A0Q0DC22_PSEAP Pyridoxal kinase PdxY OS=Pseudomonas syringae pv. aptata OX=83167 GN=pdxY PE=3 SV=1
MTRASAFSRRPWACHHQAAALANSISTKMFPRVSMTECSSVGVVSLTVLSDLQPYCATYI
SYYCICIVFRIPPPMKRTPHLLAIQSHVVFGHAGNSAAVFPMQRIGVNVWPLNTVQFSNH
TQYKQWTGEVLAPQQIPALIDGIAAIGELGNCDAVLSGYLGSAAQGRAILTGVARIKAAN
PKALYLCDPVMGHPEKGCIVAPEVSDFLLQEAAAMADFMCPNQLELDSFSGRKPESLHDC
LAMARALLARGPKAIVVKHLDYPGKAADGFEMLLVTAEASWHLRRPLLAFPRQPVGVGDL
TSGLFLSRVLLGDDLVAAFEFAAAAVHEVLLETQACGSYELELVRAQDRIAHPRVKFEAV
RL
>tr|A0A0Q0D8C6|A0A0Q0D8C6_PSEAP Efflux pump membrane transporter OS=Pseudomonas syringae pv. aptata OX=83167 GN=ALO85_04366 PE=3 SV=1
MSLFFIKRPNFAWVLALFILLAGLMALPSLPVAQYPDVAPPQITITATYPGASAKVLVDS
VTSVIEDELNGAKGMLYYESTSNSTGSAEINVTFVPGTNPDLAQVEVQNRIKKAEARLPQ
TVLSQGLQVEQASSGFLLIYTLNYKDGAANKDTVALADYAARNVNNEISRVNGVGRLQFF
AAEAAMRVWIDPQKLVGFGLSIDDVNAAIRAQNVQVPAGSFGSSPGSSLQELTATLAVKG
TLDNPEEFGRIVLRANEDGSAVHLSDVARVAVGSQDYSFESRLNGQRAVAGAVQLSPGAN
AIQTARAVEQRLTELSVNFPEGVGFSIPYDTSRFVDVAIDKVIYTLVEAMVLVFLVMFLF
LQNIRYTLIPTIVVPVCLAGTLAIMYLMGFSVNMMTMFGMVLAIGILVDDAIVVVENVER
IMAEEGLSPAAATVKAMQQVSGAIFGITLVLAAVFLPLAFMGGSVGVIYQQFSLSLAVSI
LFSGFLALTFTPALCATLLKPIPAGHHEKRGFFGGFNRLFGTFTDRYERVSFSMIKRAGR
YMLLYLGIVGLLGFFYLRLPESFVPVEDQGYLIIDVQLPPGATRSRTDLTAQLLENYMLS
REATGAVTMLLGFSFSGMGENAGLAFPTLKDWSERGKGQSAAEEAAAFNQHFAGLGDGTV
MAVTPPPIDGLGTSGGFSLRLQDRAGLGREALLAARDKLLGEANGNPKILYAMMEGLAEA
PQLRLNIDREKARALGVSFESISNALSTAFGSSVISDFANAGRQQRVVVQAEQSSRMTPE
SVLKLYVPNSSGTLVPLGAFVSTHWEQGPVQIARYNGYPAFRISGDAAPGVSTGEAMAEI
ERIVSELPQGIGYEWTGLSYQERLASGQAVGLFGLALLVVFLLLVALYESWAIPLVVMLI
VPVGALGSVLAVTAVGMPNDVYFKVGLITIIGLAAKNAILIVEFAKELWDQGHSLRDAAL
QAARLRFRPIVMTSLAFILGVVPLTLATGAGAASQRAIGTGVIGGMLSATLLGVVLVPIF
FVWVLSALRRKPHAQQIADDQQLSEK
>tr|A0A0Q0DY33|A0A0Q0DY33_PSEAP 4'-phosphopantetheinyl transferase OS=Pseudomonas syringae pv. aptata OX=83167 GN=ALO85_01971 PE=3 SV=1
MTRLSHLPICCPELIRHWPLPQALPGALLVSGHFDPLKLVDGDFQRCELQMPASIQRSVA
KRQTEFLAGRLCAREAMRQLDGRLHVPAVGEDRAPIWPADVCGSITHSTGWAAAVVAHKQ
QWRGLGLDTENLLSHDRASRLAGEILTAAELAEMAAGPEDQIALRVTLTFSIKEALFKAL
YPIVHKRFYFEDAQLLEWSADGHARLQLLIDLSSEWHAGKELDGQFSVQDDHLLSLVAVE
G

>tr|A0A0Q0C369|A0A0Q0C369_PSEAP Acyl-CoA dehydrogenase OS=Pseudomonas syringae pv. aptata OX=83167 GN=ALO85_03878 PE=4 SV=1
MGIHGNATCVMNFDGATGYLIGPANKGLNCMFTFMNTARLGTALQGLAHAEVAFQGGLQY
ARDRLQMRSLTGPKAPEKAADPIIVHPDVRRMLLTMKAFAEGTRAMVYFTAKQVDIVKYS
EDPEQKKAADALLAFMTPIAKAFMTEVGFEAANHGVQVYGGHGFIAEWGMEQNVRDSRIS
MLYEGTTGIQALDLLGRKVLMTQGEALKGFTKIVHKFCQSNEGNEVVQEFVTPLAALNKE
WGDLTLKVGMAAMKDREEVGAASVDYIMFSGYACLAYFWADMARVAAEKLAAGASDEAFY
RAKIQTARFYFQRILPRTRTHVAAILSGAANLMDMKEEDFGLAY
>tr|A0A0Q0BTQ5|A0A0Q0BTQ5_PSEAP Thymidylate synthase OS=Pseudomonas syringae pv. aptata OX=83167 GN=ALO85_03057 PE=4 SV=1
MPFTLVGPCEFREEIRKSRFITLATPIASPADAQAFIEQHSDLNATHNCWAWKLGGQYRS
SDDGEPGGTAGRPILAAIEAQAFDQVVVLVIRWYGGIQLGTGGLARAYGGGANKCLQQAE
RLPLIQRASFHLECSFSELALVKLRLGELNGLVESENFMANGVQMSIAVGPEHTDTLQRQ
LADLSRGRILLHAAPEQ
>tr|A0A0Q0DCB9|A0A0Q0DCB9_PSEAP 16S rRNA SAM-dependent methyltransferase B OS=Pseudomonas syringae pv. aptata OX=83167 GN=ALO85_01538 PE=3 SV=1
MNPRLAAAKALAAVLSGKASLNSSLPTQLDKVEVRDRGLTQDLAFGTARWQPRLSALAAK
LLQKPFKAADADVEALLLVGLYQLFYSRIPAHAAIGETVGCADKLKKPWAKGLLNAVLRN
AQRDGEALLIELEHDPVVRTAHPRWLQKALKAAWPEQWEAICAANNAHPPMILRVNRRHN
RRDQYLELLNTAGLQASACTFSQDGIVLAEPCDVRSLPGFAEGWISVQDEAAQLAADLLE
LAPGQRVLDACCAPGGKTCHLLEVQPQLSGVVAVDLEAKRLVRVRENLDRLGLDAELIAA
DARETAQWWDGKPFQRILLDAPCSATGVIRRHPDIKLTRQADDIAALAALQGELLDALWP
TLQVGGMLVYATCSTLPTENTDVIEAFLARTSGARELDIAGQAGQPPAGIKQAHGRQLLA
QQGGHDGFYYAKLIKIAAARE
>tr|A0A0Q0FT62|A0A0Q0FT62_PSEAP Uncharacterized protein OS=Pseudomonas syringae pv. aptata OX=83167 GN=ALO85_03623 PE=4 SV=1
MSNQLSSLLHLPVRLPEPQPTLQAIELGHRLGKLSRRTRQIFLLSRLDGLAYADIAAFMS
VDIARVERAMLRALGKAYVPGAADTTSAAIQATLQDRASRWYVHLQSPAATASERIEFRH
WLDADAAHLSAFQNSERLWRQLQAPASLLGASGWHRRKRRVYLVWCLLTAFICSLMVTAE
AMS
>tr|A0A0Q0CA24|A0A0Q0CA24_PSEAP Luciferase OS=Pseudomonas syringae pv. aptata OX=83167 GN=ALO85_01558 PE=4 SV=1
MKRLADVKISTLDLVPVRHDKGPAESLRNSLNLAQHVEKLGYNRFWVAEHHNMDGIASSA
TAVLLAYLAGGTSTIRVGSGGIMLPNHAPLVIAEQFGTLASLYPGRIDLGLGRAPGSDQM
TARALRRERSGSADDFPDDVTELMEYLGPRTPDQKVIAVPGSNTNVPIWLLGSSLFSAQL
AGMRGLPYAFASHFAPRYMHEAIRVYRNHFQPSEVLDKPYVMLGVPLSAADTDEHAEYLA
TSVYQRILALMRGHSLMQRPPVESMDGLWLPHEREAVASFLGLAMVGGPDKIRAKLDVLL
EQTDADELIFTCDMYEHEDRLRSYEILAQVAHG
>tr|A0A0Q0CGA3|A0A0Q0CGA3_PSEAP Amidase OS=Pseudomonas syringae pv. aptata OX=83167 GN=ALO85_04648 PE=4 SV=1
MLCNGLAAHLRRTRGASPVNCCRTHAQARCRALRNATASALITRTDTPQSFGKARMLVPE
LTRQYDEAFQQFDVLVMPTMPFVATTLTAADAPIEEYVHSALNMLANTAPFDLTGHPATS
VPAGLADGLPVAMMIVAPRFKDALALRVAQAYETARGAFPTPPGV
>tr|A0A0N8T8Q4|A0A0N8T8Q4_PSEAP Dimethylmenaquinone methyltransferase OS=Pseudomonas syringae pv. aptata OX=83167 GN=ALO85_00385 PE=4 SV=1
MSMNKQIAHRQQQLSDAVIEQYRDIAASTIGHLTDAGYLRGIRPLFEGARLVGNVVTVKL
QLPDGSILRDALLNSLPGDVLVIECVGDEHCACWGELRTLAGLIKGLAGVIVSGAVTDVA
ALREHRLPVFSRGVSAMTTRTLGESGELNGLIHIGGVEVRPGDIAVGDDDGVFILSPQQA
NELLPELLGKESADRKRREELIDRLNGRQPELGS
>tr|A0A0Q0C3J5|A0A0Q0C3J5_PSEAP Regulatory protein LysR OS=Pseudomonas syringae pv. aptata OX=83167 GN=ALO85_00874 PE=3 SV=1
MFELSQLRCFTTVATELNFRRAAERLNMTQPPLSRQIQILEHNLGVELFTRSTRSVALTA
AGRAFFIEAQTLLERAQQAAISARRFAEGDIGSVTISFVGSAVYEFLPRVIAEARLKQPQ
VKISLTEMNTYQQHEALRARRIDLGIVRAPLFQPGYESECLVREPFVLAVPSSHRLAHAD
SVAVADLDGAPFLMYSHSAYPPFNELLTGMFRSAQVAPEYVQWLGSSLTILALVNAGMGL
ALVPRCATNVVFKDVVFRDIDLGEGVQSELHLVWRTDNDNPACRMLLEAIRAAVRADEN
>tr|A0A0Q0DM87|A0A0Q0DM87_PSEAP Penicillin-binding protein 1A OS=Pseudomonas syringae pv. aptata OX=83167 GN=ALO85_05222 PE=4 SV=1
MIFSGNPKALIRLLKFFWWSLVAVFCSLLLGLSGAFLYLSPTLPSVDALRSIQLQIPLRV
FTSDGKLIAEFGEMRRTPIRFAEIPPNFINALLSAEDDNFANHYGVDPGSLMRAATQLVK

SGHIQSGGSTITMQVAKNFFLTSERSFSRKTTEILLALQIERQLTKDEILELYVNKIYLG
NRAYGIEAAAQVYYGKSIRDVSLAQMAMIAGLPKAPSRFNPLANPTRAKERRDWILGRMY
RLGKIDQTAYQAALSEPLNASYHVPTPEVNAPYIAEMARAEMVGRYGSEAYTEGFRVTTT
VPSDLQEHANKSIQQGLIDYDQRHGYRGPESRFPGMTRVNWVQELTKQRTINGLEPGIVT
QVDKTGLRVLTHNGEKEETVDWATMKWARPYLNTNSLGANPKQPGDVAKVGDLVRLLRQD
DGSLKFSQIPVAQSALVSLDPQNGAIRSLVGGFAFEQSNYNRAMQAKRQPGSSFKPFIYS
AALDNGYTASSLVNDAPIVFVDEYLDKVWRPKNDTNTFLGPIRLREALYKSRNLVSIRLL
QALGIDSTIDYITRFGFSKQDLPRNLSLALGTATLTPMEIATGWATFANGGYKVSPYLIQ
TIENRDGQTLFVANPPSVPVNDVNTAPNTSTFAVHDSPSVVAGPPGAPAQPQAPAVAERI
VDGRTTYILNSMLQDVIKRGTGRRALSMNRPDIAGKTGTTNDSKDAWFSGYNADYVTTVW
SGFDQPESLGRHEFGGTVSLPIWMDYMSAALKDKPPHQQAEPPGILSLRVDPLSGRAATP
STPNAFFELFKSEDTPPSVNELGGSSAPGSPLPADESAPIDLF
>tr|A0A0Q0CBH5|A0A0Q0CBH5_PSEAP Nucleoside ABC transporter membrane protein OS=Pseudomonas syringae pv. aptata OX=83167 GN=ALO85_01757 PE=3 SV=1
MELISHWLGSAPDFAVPFALAALGLILIERSGVLALGVEGLMLVGALAGIGMQLSLGSPG
LSLLASMAAAGLVSLLFALMVLVLRINQVIAGLALVFFCQGLTGLLGTLLGWTNRPVSGL
QAVELWPLSELPVVGRVFVQNPLVYLTVVIFIAVVWLLDRTRTGLRLRAVGENPQAADAA
GISVLGYRLAAIVAGSALMGLAGGFIAVLSTKMWIADMTGGRGWIAVSLVIFARWSPWRA
LAGALLFGGIEALIPQLAATGVRLPQYFVLMTPYAVTLLVMVWVASGKRQSSSQPGALGE
PYIREERR
>tr|A0A0Q0BTQ0|A0A0Q0BTQ0_PSEAP Uncharacterized protein OS=Pseudomonas syringae pv. aptata OX=83167 GN=ALO85_05661 PE=4 SV=1
MNMPEMNKITIITALSFLGVASQASAALVQYDTNSYGLDCATGFTTDPTGYKFGNSYRGD
GYFFRYFDRMGNPDIFFIKDKVTGEYSLAPDDIFSPNKTNQQMTDFIIQYGFEDPTAPLS
SCYPIVDAPVLTPPSNPTFSFNNTIMNACPAGQTVGGVQGGSTYRTGTIPFYSTYDYDNG
VSTTYVKSFNGLFSEVVMTDNGDGTSTFPQYGAADVDTYGTWDADSVQACSTPPVAPTNP
TGPTEPTTPSNPPDSGHVPISSVDFHEFCPAGFTVGGVSGASTVRVGTGPIFASYDAATN
TTSWSMKQNATGAYDNFYTAHSAVGLIPYPTGTQALVDKYGSMNPGSGPACKQVVTAPVL
PTYSFDLPYNEFCKAGESVGGAIGGSNVRTGKVPVYFRFDSVTFTSKYFFYDGTTYTPLQ
MVEQNGLTNFPTITEDMVKKYGALDTNSIQSCQTVPVTLPDPVPPQEGWMEDGYEDCAVG
STVGGVEGGSTKRHGTVWVWYGYDSYLQEQDFFKSAKTDELVRFNYNELAGYEAPEWNIG
KGIIDSYGTWNEDSMPACEPVRTFIDAVETKTENCASPLTGTLTLERPYKQWSDGVNDSY
GDWKTTVNACVVPPVVVEPTPPVTVPPVVTPVDPTPPVTVPPVVVPPVDPAPPVANFVTI
KDFELVREMCDEGQTGEKTFRFDWTYDLYVDGTIKNFTPKIRKALTDTCKTLTDNLLETT
PVANYEACPNGQKGIIIITGQNVTYSLSGTKFVEISRQNNCVAETTSLDPQYKTEDCPVG
QTGSLTKVRYVQTKDDGSINYPYGEGYEVSQNNCSSQSEADTANQGENSAAHGILANQSV
KASDSAQIKIMTDYVNNASSDFGNYKLNITVDSINVDSQKLGLLAKAWVAKTGGKINLGS
LPRSAISYVGKGNITKENVSKTVIGEVSFNSNTGKLTVHYKQTRSFLDSGSIQSVDVPLI
DAGLAGSALNSVKAY
>tr|A0A0Q0C8A8|A0A0Q0C8A8_PSEAP Uncharacterized protein OS=Pseudomonas syringae pv. aptata OX=83167 GN=ALO85_200132 PE=4 SV=1
MDVTKIAQTLFDFVDAALQSALVTGMAKVMLGLGALFGTMWLINFTLRNIQWLYRGMNVA
FEDVAKEIAKMAFITACAFNLEWYVNTIVPFVTGLPNWMGGAMGGAMSGQEGLQTNQIDS
LIATYIENLQALISAMNFNIITTSFANVWLGIQGVVFYVLGGIPFLLAAVATLFVLKVST
TAIVSVGPLFIAFLLFDQTKQYFWGWASAIGGFMLAQVLVSVVLAIEIGFINTAMIKDGS
LNTSLAGNFVILLVFGTFTALVIELPSQASSIMGGGPSGGGLVSKISGFSAARGMTRGVA
AGISKLRGRGGNNIK
>tr|A0A0Q0FI27|A0A0Q0FI27_PSEAP Uncharacterized protein (Fragment) OS=Pseudomonas syringae pv. aptata OX=83167 GN=ALO85_102009 PE=4 SV=1
MRQAALIIVPMLHVGMPVMVLCVTSVPQRNVHALHAAAAGVMLFNRDGTDDAECDV
>tr|A0A0Q0BXB7|A0A0Q0BXB7_PSEAP 50S ribosomal protein L25 OS=Pseudomonas syringae pv. aptata OX=83167 GN=rplY PE=3 SV=1
MNEFTLNAEQRSDLGKGASRRLRRLASLVPAVVYGGDKAPESISMLAKEVAKLLENDAAY
SHIIELNVGGKKQNVIIKALQRHPAKGHVMHADFVRVIAGQKLTAIVPIHFLNEEAPVKK
GGEISHTTTELEVTCLPKDLPEFIEVDLGALEVGDNVHLSELKAPKGVEFVALAHGTDLA
IANVHAPRIVKDEEEGEAEEGAAE
>tr|A0A0Q0C3X4|A0A0Q0C3X4_PSEAP Non-homologous end joining protein Ku OS=Pseudomonas syringae pv. aptata OX=83167 GN=ku PE=3 SV=1
MAFRVKNVSGWAGFPALVRLPKLFSDRPCHFSRTNRSQTMARAIWKGAISFGLVHIPVAL

VSATTSTSVDFDWLDKRSMDPVGYKRINKVTGKEVTSENIVKGVAYEKDRYVVLSEEEIR
SAHPKSTQTIDIFAFVDSQQIPLQNIDTPYFLTPDKRGEKVYALLRETLIDTKKVALANV
VLHTREHLAAVMPLESALVMVILRWPADVRELDALELSDAVTDAKLNKSERDMAKRLVKD
MSADWQPEHYRDTFQDKIMHLVKTKADEGQIEDVETDPAEEERKSADVIDLTDLLRRSLA
GKGGASKGKASKPAAKEKPADKDSPRPKRPAARKKTSKAS
>tr|A0A0Q0BRR0|A0A0Q0BRR0_PSEAP Glycine cleavage system transcriptional activator OS=Pseudomonas syringae pv. aptata OX=83167 GN=ALO85_05594 PE=3 SV=1
MNEIQIIGYSGASMRPDDEAPLSLPPLKALQAFEQTARFGNLARAAEVLDLTPSAVSHQL
ARLEALIGRQLFVRAARGVTLTPVGEQYLKDVSGVLHSLAVATERAASDVSLDCLRLHSA
PSFGLLWLMPRLEAFRVSHPDIQINLSCSYESLHFSRDKIDVDIRHGRPNWPSYEVRTLR
DEKFAVLASPQLLARRPVESAAGLLDRELILSEATLLKWPEWFARHGLARPEKPYALSFD
RSYMTLEAASHGLGFALESTLLAQKYLATGELIEVAPQALTAPVAAHHLVFPKAHSSFPR
VRRFLEWMEGELGQGFVF
>tr|A0A0Q0D6X2|A0A0Q0D6X2_PSEAP LysR family transcriptional regulator OS=Pseudomonas syringae pv. aptata OX=83167 GN=ALO85_00995 PE=3 SV=1
MNTRRLTPSMSLLLAFEAAARHGSFTKAADELALTQSAVSRQVQALEAQLEVELFKRDGR
RIELTTAGALYQHELAAALGRIRSATLQTIAHKAEGGTLHLAVLPTFGSKWLLPRMNDFY
TRHPGYVVHIHSRIVHADLTPAASEMNAIICAGNGNWPGYIAHPLVSEKLVVIASPAALA
GYQSMTPAQVAQHALLSVVSRPNAWSDWFDSNRLDHHIMRPGPSFELTSHLIQAVAAGIG
IALVPRILVQDEIHSGELVTLFEPLDSGRNYYLAYATRFQNLPSLCVFRDWLLSTPFPDP
L
>tr|A0A0Q0C4Z8|A0A0Q0C4Z8_PSEAP Uncharacterized protein OS=Pseudomonas syringae pv. aptata OX=83167 GN=ALO85_04173 PE=4 SV=1
MIETTNGNKKGVVLFRVNARAFSKTSLRLKDNDSRFCEELNAEMVGSDGKVIQAYIDTYR
YYEKNQEKVERHDGLPF
>tr|A0A0Q0CHK9|A0A0Q0CHK9_PSEAP Uncharacterized protein OS=Pseudomonas syringae pv. aptata OX=83167 GN=ALO85_03437 PE=4 SV=1
MLKGFVSKDYAVLVIIASLIVILLLGVGFTSRPSDWAGWMQAIGLIVGLMAAVAVPAIQR
KQEAQLAHKQIRDREVGYARRMQYLCGELSELQGRISLNLTHLRASDRHSLKYTLQDYLH
RLFESHKQDLNDDRVVLAYELRQVANDLIDELDSGRTDRVVFMALEKRLQKLTHRCQVNA
AMAERG
>tr|A0A0Q0FX03|A0A0Q0FX03_PSEAP Putative acyl-CoA dehydrogenase OS=Pseudomonas syringae pv. aptata OX=83167 GN=ALO85_05419 PE=3 SV=1
MLICVAGCIDRLPKAFRSVFTRFAMADYKAPLRDMRFVLNEVFEVSKLWAQLPELAETVD
AETVEAILEEAGKVTSKTIAPLSRNGDEEGCHWKDTVVTTPAGFPEAYKIYAEGGWVGVG
GNPEFGGMGMPKVVSAQVEEMLNSASLAFGLYAMLTSGACVSIDTHATEALKTKFLPNMY
SGVWAGSMCLTEAHAGTDLGIIRTRAEPQADGAYQITGSKIFITGGEHDLTENIIHLVLA
KLPDAPPGPKGISLFLVPKFMVGDDGSVGERNAVSCGSIEHKMGIQASATCVMNFDAATG
YLIGEPNKGLAAMFTMMNYERLGVGIQGLASGVRSYQAAVEYAQDRLQSRAPTGAQNKDK
AADPIIVHPDVRRMLLTMKAFNEGGRAFSSYVALQLDIAKFSEDDTARKRADDLAALLTP
VSKAFLSDIGLETTIHGQQVFGGHGYIREWGQEQLVRDVRITQIYEGTNGIQALDLVGRK
IVGSNGALYQLFSDEVRQFISGSSNALSEFTQPLSKALNMLDELTGWVLDRSRNNPNEIG
AASVEYLHLFGYTAYAYMWAMMAKAALGKEQQEEFYASKLGTARFYFARLLPRIHSLDAS
VRAGSESLYLLKASQF
>tr|A0A0Q0DNY7|A0A0Q0DNY7_PSEAP Uncharacterized protein OS=Pseudomonas syringae pv. aptata OX=83167 GN=ALO85_00386 PE=4 SV=1
MSLAQAFSESEWALLSEALRLRPAGEHDPRSLSARALLDDAVCEQLLGVLGPVIGSPTQA
ITASLLAKRISFLSTGACLYAMSVYDKGLTLSLDNSVIEYAHDDGLWTSSMPLLDTTPVG
YEPGAREAWRDQIATTLFRDLLQPLWETFNRVTGISRRILWENTAVRVYSLYDKRMDKVE
DPAIRQRCEADFDWLLHQADPTLFGLTYNPLKHFRRPPVVLEAEGKSIRFRRTCCFYYDA
SNPVEYCSTCPLLRPKKCR
>tr|A0A0Q0IDR4|A0A0Q0IDR4_PSEAP Flp pilus assembly protein CpaB OS=Pseudomonas syringae pv. aptata OX=83167 GN=ALO85_01363 PE=4 SV=1
MSSRVTMVLAVLFLLGALIAGYLGIVVGRPTEVPVETPPSAPPVTTEQTTPPAPTPHVEE
ALRHNVVVLAKDIPAYTAITADDLTIERLKVAPPGSFEAIDKIVGRSSWRTLTAGSWLTE
SNFEAGGTLARMIRPHERALALQVDEVTGASGQLSPGDYVDVLLYLPEDVSNPNRSSQVV
VPALRVLGVGALLGPDRNGKPVQDLNADERLKQEQQRINARTVTVAVPQALVARLMLASQ
TGVLRLAVRSADEGNLQDYWANEAGGPEAAERLDSANRQLVSFRQLSLGPASAPTAGATP
RTSRVVEVIRGNQITQQTP

>tr|A0A0Q0D4V0|A0A0Q0D4V0_PSEAP Methylmalonate-semialdehyde dehydrogenase OS=Pseudomonas syringae pv. aptata OX=83167 GN=ALO85_02768 PE=4 SV=1
MDAWFAEGRIAEMNASLNHTTTSIARVKLLINGEWTDSQSSEWQDIVNPATQEILAQVPF
ATASEVDAAVAAAHAAYRTWRETPIGVRMRIMLKLQALIREHSKRIAAVLSAEQGKTLAD
AEGDIFRGLEVVEHACSIGTLQMGEFAENVASGVDTYTLRQPIGVCAGITPFNFPAMIPL
WMFPMAIACGNTFVLKPSEQDPLSTLLLVELAIEAGVPPGVLNVVHGGKEVVDAICTHKD
IKAVSFVGSTAVGTHVYDLAGRHGKRVQAMMGAKNHAVVLPDANREQTLNALVGAGFGAA
GQRCMATSVVVLVGASRQWLPDLKARAQKLKVNAGQEPGTDIGPVISRRAKQRILDLIES
GVREGATLELDGRDVSVPGYEGGNFVGPTLFSGVTTDMQIYTQEIFGPVLVVLEVETLNE
AIALVNANPFGNGTGLFTQSGAAARKFQSEIDVGQVGINIPIPVPVPVFSFTGSRGSKLG
DLGPYGKQVVQFYTQTKTVTSRWFDDDSVNDGVNTTINLR
>tr|A0A0Q0BU06|A0A0Q0BU06_PSEAP Succinylglutamate desuccinylase OS=Pseudomonas syringae pv. aptata OX=83167 GN=astE PE=3 SV=1
MLALGKLLELTLAGREPAEKTQLTVEGVRMRWMGEGALEVRPPQARDNGTDLLLSAGIHG
NETAPIELLDELIRSIARGDLKPRARILFLFGNPDAMRRGARFVEQDVNRLFNGRHLQSG
GAEALRACELERLAASFFSLPERYRLHYDLHTAIRGSTIEQFALYPWKEGRQHSRLELAR
LRAAGMSAVLLQNKPSIVFSAYTYDQLGAEAFTLELGKARPFGQNQQVNLGPLRLCLEQL
IEGTEPERDDDLEGLQLFSVAREVIKRTDAFTFNLADDVENFSPLEKGYVLAEDAGGSRW
VVEEEGARIIFPNPKVKNGLRAGILIVPTDADSLG
>tr|A0A0Q0DKA8|A0A0Q0DKA8_PSEAP Uncharacterized protein OS=Pseudomonas syringae pv. aptata OX=83167 GN=ALO85_200212 PE=4 SV=1
MITSRWLVLFPVLLTGCGADEAVRSVDWYKAHNAERAIHISECERDPGRLALTPNCVNAK
QAENELRLAERGFRKRETLDLKEP
>tr|A0A0Q0FJF5|A0A0Q0FJF5_PSEAP NodT family outer membrane efflux lipoprotein OS=Pseudomonas syringae pv. aptata OX=83167 GN=ALO85_100854 PE=3 SV=1
MRQEQFCRNLSETLRRQAGYQRSTWAGQVAGMRRMNKPVSPWLFAVLSLSGCSLIPDYQR
PAAPVAAHYPQSFNSAPALSGPLAADMGWRDLFRDPTLQQLIESALLNNRDLRVAALNVE
AYQAQYRIQRADLFPAISATGSGTRQRQPASITQTGGPVTSGTYSATLGISSYELDLFGR
IRSLSGQASLTYLSSEEAQRSAQLSLVASVATAYLTWRADQELLALTQDTLMSYEKSLKL
TTRSQQVGASSSLDLAQSRTSVESARASLATFQRQVAQDLNNLTLLVGTTVADSAPSRPL
SSEWLAEVPAGLPSDLLQRRPDILEAEYQLQSANASIGAARAAFFPSITLTANAGTSSTE
LSGLFKAGSGGWVFSPQINIPIFNAGSLRASLDYSKIQKDISVSKYEKSIQTAFQEVSDG
LAARQTYKRQLKAQSDLVQASQEYYSLAERRYRIGVDSSLTFLDAQRSLFSAQQTLITDR
LSQLTAEVNLYKALGGGWTERTSKPL
>tr|A0A0Q0FH49|A0A0Q0FH49_PSEAP Precorrin-8X methylmutase OS=Pseudomonas syringae pv. aptata OX=83167 GN=ALO85_01389 PE=4 SV=1
MIDYIRDGQEIYRNSFSIIRAEARLDTIPADLEKLAVRVIHACGMVEVIEDLRFSPGAGA
AGRNALAAGAPILCDARMVSEGITRTRLPANNPIICTLHDEGVREMALELGNTRSAVALE
LWRPHLEGSVVVIGNAPTALFYLLEMLDAGAPKPALILGFPVGFVGAAESKAMLAADSRG
VPFVIMQGRRGGSAMAVAAVNALATEIE
>tr|A0A0Q0DIF8|A0A0Q0DIF8_PSEAP Rhs family protein (Fragment) OS=Pseudomonas syringae pv. aptata OX=83167 GN=ALO85_04432 PE=4 SV=1
MLSIQRTPTDGGRKLGVTAEKLEFAYDILGRLTQETSPQGALAYDYDPLSNLTTLTLPTG
QHLNHLYYGSGHLHQLNLDGQLISDMERDDLHREIYRTQGKLTSCFGYDALGRKTWQYAT
TLPAEKLSQIQNPLIKPERYVEHAYNPVHRRYEYDPAGELSRTLDKLRGEVTYEYEANGR
LLEHNPKKRFDGEEFRYDAAGNRLNFNTSRFDRVKDNRLKQWSNHEYKYDAWGNLVEKIV
GIVRWQTFTYDSENRLVK
>tr|A0A0N8T9E1|A0A0N8T9E1_PSEAP Cytochrome c assembly protein OS=Pseudomonas syringae pv. aptata OX=83167 GN=ALO85_04486 PE=4 SV=1
MPNSASFQARPSGSMSPLPPSLLPSLAAAIIYAAATVYQSLRLSQAVKPDKRLLCLLGTL
AVIAHAIGLFSQLTHSVGLGLDFFNSASLIAASVIVVTLIATSRIPVENLLVLLFPLGMV
TVLLAQFAPSGTVQPIMEEPGILSHILLSLLAYGMFTIAAFQALLLLLQDYRLKHKHPSG
LIKNFPPLQTMESLLFGFLWAGWSLLSLSLISGWLFVENLFAQHLVHKTLLACLAWIVFS
VLLWGRNRLGWRGHKAIRWTLGGFCLLMLAYFGSKLVREFILHI
>tr|A0A0Q0C9S5|A0A0Q0C9S5_PSEAP Uncharacterized protein OS=Pseudomonas syringae pv. aptata OX=83167 GN=ALO85_04504 PE=4 SV=1
MSRVNDNTVQVYAYSAIPLAHKKTRRSGSFFYQYHSDILSVAVPAMVIILDLRSASCATV
DGYRLLLREHVVQG

>tr|A0A0Q0BSM5|A0A0Q0BSM5_PSEAP GAF/GGDEF/EAL domain protein OS=Pseudomonas syringae pv. aptata OX=83167 GN=ALO85_04401 PE=4 SV=1
MIEYPLRIIMNASAPIPLNETQRLLRIRELCVLEDTSDDVFDEIVAMTAAFFETPIALIS
IVDEHRQWFRARVGLNARETPRNVSFCAYTILSDTLFEIPDATLDQRFVNNSLVTGHPDI
RYYAGAPLITDDGIALGSLCVIDTKPREPMSEYQTQMLKRFASLVMKRIVGLRLSCFIDQ
PTGLYNRSRLQEDISQAVASSVDYQLVVVDMITSAFLNDIVKALGYSFSQDLVTAIKSRL
ECLLPPTCSLYKVSPTRFGFLLAGDRTPEHLFRTILKDFETPVECRGIPVQMQVGLGVVT
LNRNPAEEQDWMRMVISAADDARDRDLGWAMYEPHFDAAQQRAFKLLSSLTGAVHADDQL
RLVYQPRIDLDSGLCTSVEALLRWNHPTLGAIGPAEFVPLAEKTALMRPLSLWVLTSAIE
QAARWQQQGFDFRIAINVTPEDLTGPAFTDRMIRLLGKHKIDPTRFELEFTEGALMHNPA
EVRHQLERMRQMGMDVAIDDFGTGYSNWNYLRQLPATTVKLDQSLIRNLASDKTDQRLVK
ALIGLAKKLGYWVVAEGIETDEIRRLVKQWGCDEGQGYLIAKPMEAEALLNWIGPNQRLQ
SAAQAAEAAIVRDKRL
>tr|A0A0Q0D3I4|A0A0Q0D3I4_PSEAP Peroxyureidoacrylate/ureidoacrylate amidohydrolase RutB OS=Pseudomonas syringae pv. aptata OX=83167 GN=rutB PE=3 SV=1
MTTSATVAGVDLPDVQPARELPARPEPLRMKPGETALVVVDMQNAYASLGGYLDLAGFDV
SSTGPVIANINKACAAARAAGIPVIFFQNGWDPAYVEAGGPGSPNWHKSNALKTMRKRPE
LEGQLLAKGGWDYQLVDELTPQPGDIVVPKIRYSGFFNSSFDSVLRSRGIRNLVFTGIAT
NVCVESTLRDGFHLEYFGVVLADATHQAGPEFAQQAALFNIETFFGWVSSVDDFCTTFSP
VGQLS
>tr|A0A0Q0C4G3|A0A0Q0C4G3_PSEAP Uncharacterized protein OS=Pseudomonas syringae pv. aptata OX=83167 GN=ALO85_05099 PE=4 SV=1
MPCTACTKASWPRPSAFNWVSTPTTAIDQEVHVMLRRQAMALGSLLRSACTLGSWTLFKQ
KNTRPLLLSARDDGDGKHYAVGFKLDGTQVFATQVGQRCHDIINHPDLPLAVFIARRPGT
ESYLIDLRDGRLLQTIVSQPDRHFYGHAVIHRDGEWLYATENDTRDPGRGLLGVYRFVDE
RLVHSAEIPTHGIGPHQVSWMPDGETLVVANGGIRTEAESRVEMNLDAMQPSLVIMQRDG
TLLSKEALSQQMNSVRHMGIASDGTIVAGQQFMGDSHELAELLAIKRPGQPFQAFPVAEE
QLRAMAHYTASVAVHSELRLVALTAPRGNRFFIWDLDSGALKLDAPLPDCAGVGAVADGF
VVTSGQGRCRFYDCRKAQLLAQPLDLPAGLWDNHLHLAQVG
>tr|A0A0N8T8Y8|A0A0N8T8Y8_PSEAP Polysaccharide deacetylase family protein OS=Pseudomonas syringae pv. aptata OX=83167 GN=ALO85_01812 PE=4 SV=1
MRIVLAVCAGLFWLQAQAAAPVIATIDRSVWPERLETPGLFDVASRAEILAFAHELYLSE
KLDEEGLKQRLGLRFVNVPSLNVVRHRLWLRLLENYQNAQKSCEQDANFCVLVEDMDQLR
QRAEEYQVSNDSFYANWARPGAAFHQRYLNELLFMAALFPQTSSEIERYNSDEMSGDEMP
DRTFMLNFESGPSPADGGTDWLADFMRQQKMTATFFVLGKSLQERLDSTSVAGVQALYRQ
QCVGIQGWEYRSHSQWLDWQDSVRRSAALVQQVLPDNFVPLFRPPYGHRRADSGDFFRAE
HLRVSLWNIDAEDDTGRLSAEQVGDRVLTLMLLWRKGTVVFHDVANKAQSALPLLLANTA
QSELIWDDCRNISEAPEEQGLQSSEEAAPEGE
>tr|A0A0Q0C1L9|A0A0Q0C1L9_PSEAP Major facilitator superfamily permease, Bcr/CflA subfamily OS=Pseudomonas syringae pv. aptata OX=83167 GN=ALO85_03621 PE=4 SV=1
MSLSSNDSTSPDVKSDGRAPNHLGFLAFTTLCFFAASSTPTPLYHLYQEAWGFSAGVLTL
IFAVYAFSLLAALLMGGSLSDYLGRRPVIFGALLLQSVSMLLFILASDVSWLVAARLLQG
FATGLAASALGAALLDNDSVKGPLINSISPMLGMAVGALGTALLAQFAPMPLMLGYCLLL
AAFLSQALYLGRVEETVSRQPGVWQSLKPSLHVPQQARRMLWLVLPVDIAAWALGGFFLS
LSPSLLVAATGSTSPLNGGLAVAALTLSGAAAILTLRQREPVLGLWVGASFLPLGVAVIL
LSINLGLLWLFFVGAVVAGVGFGSSFLGALRMLMPLAHAHERGGLMSAFLVLSYLAFCIP
ALVAGYFAHSAGLVMTSNVYGAVVITLALIALASLIVRRVANVNKNAHA
>tr|A0A0Q0IMI9|A0A0Q0IMI9_PSEAP Error-prone DNA polymerase OS=Pseudomonas syringae pv. aptata OX=83167 GN=dnaE2 PE=3 SV=1
MTSEYAELHCLSNFSFQRGASSARELFERAVRHGYKALAITDECTLAGIVRAWQASKSTG
LPLIVGSEMHIENGPKVVLLVENQAGYEALCTLITVARRRAKKGEYRVLREDFEPAPDGL
LALWLPDLDGDAQACLAGGRWLRERFTERLWLGVGLHRGPDDEQRLADLLALAQSLGIPA
VASGDVHMHARGRRALQDTMTAIRHHTTVAEAGHLLFANGERHLRPLDALSEHYPDWLLA
ESVRIARRCTFDLGDLKYEYPHELVPKGQTSTSWLRELTERGVRRRWPGGLTPATRAQVE
KELALIAEKKFDSYFLTVHDIVEFARSQHILCQGRGSAANSAVCYALGITELNPEQSNLL
FERFISRERNEPPDIDVDFEHDRREEVIQYIFRRYGRGRAALTAVASTYHGSGAMRDVAK
VLGLPPDQINALAEAFSRWSDSLPSPERLREYGFDADTPILKRVLALTGELIGFPRHLSQ
HPGGFVISEHPLETLVPVENAAMADRTIIQWDKDDLDLVGLLKVDILALGMLSALRRTFD

LVHLHRGQRWTLASLPGDDRKTYEMISRADTVGVFQIESRAQMAMLPRLRPEKFYDLVIE
VAIVRPGPIQGDMVHPYLRRRNGEEAVTYPPKLESVFKRTLGVPLFQEQVMEVAILAADY
TPGEADELRRAMAAWKRHGGLEPHRERLRTGMLKNGYEADFADRIFEQIKGFGSYGFPES
HAASFALLTYASCWLKCHEPAAFTCALINSWPMGFYSPDQLLQDARRHHIEIRPVDVRYS
DWDCSLEPLDHPDRTRNLAIRLGLRMVRSFREDDALRIEAARAKRPFVDATDLTLRAELD
SRAAEALADSGALRGLIGHRHRARWEVAGVEAQRPLFDDLPSEETQVILPLPTVAEDLVA
DYTTLGTTLGPHPLALLRRQLAAKRFRSSQDLLSLENDRTLSVVGLVIGRQRPGTASGVT
FVTLEDEFGMVNVVVWRDLAERQRKVLVGSQLLQVFGRLESKSGVRHLIAQRLYDLTPLL
TGLDVRSRDFQ
>tr|A0A0Q0C8F2|A0A0Q0C8F2_PSEAP Cobyrinic acid a,c-diamide reductase OS=Pseudomonas syringae pv. aptata OX=83167 GN=ALO85_03558 PE=4 SV=1
MTEQAFSPEERAAVYRAIAERRDMRHFIGGTVAPELLARVLEAAHQAPSVGLMQPWRFIR
ISDPLLRGRMQAQVEEERIRTAEALGERTDEFMKLKVEGINDCAEVLVAALMEGREQHIF
GRRTLPEMDMASLSCAIQNLWLASRAEGLGMGWVSLFDPEALAELLGMPDGAKPLAIICL
GPVKEFYPAPMLVMEGWAQARPLHELLYENQWGVSQ
>tr|A0A0Q0C5W5|A0A0Q0C5W5_PSEAP Ferric siderophore transport system protein, TonB family OS=Pseudomonas syringae pv. aptata OX=83167 GN=ALO85_04361 PE=4 SV=1
MGNVQTAASAHDVPWRQTPSGDLVDLGRPFRAPLALSRLQSTPKPILGRREAILLGALAL
VFHGAIIYWLSQNPTSALPVVPPEIPPMTIEFSQPAPPVVETPPPPPEPVVQPVVEPPPA
VEDELAIKPPPPKPIPKPKPQPPKPVVKPVVKPVAKPVEQPPTPPVPTAPVAAPAPPAPP
APKPVTPASASASAGYLRNPAPEYPSLAMRRGWEGTVLLRVHVLASGKPGEIQIQKSSGREQ
LDEAALAAVKRWSFVPAKQGDVAQDGWVSVPIDFKIN
>tr|A0A0Q0DFR8|A0A0Q0DFR8_PSEAP Hybrid sensor histidine kinase LadS OS=Pseudomonas syringae pv. aptata OX=83167 GN=ALO85_01317 PE=4 SV=1
MDGLNATTYLGASMRYLLIIILALWLPLQSHAVEFDENTRFLSLGRAIQVFEDPTGEATIE
SVSAASHAGAFQPVPAGTFNAGYSRSAFWLKVELTYRPTDASIHNDWLLELAYPPMDHID
FYGPDADGQPTRAWHTGDMLPFSSRQFAQNNYLFQLDLPPGQTRTLYMRIRSEGAVQAPL
YLWSTHAYLDAQPSKVYVFGLIYGVLLGMLVYNLFIYLSVREVDYLYYLLYVASFGLYQM
SINGVAIEYLWPDSPWWANASTPFLISLATLFACQFSRSFLGTAQLSRWLDRLLLAVIGA
AVLVMGMALFLSYGPALRGAAQLVMAGALTIYLAGIVAVIKGERVGRYFVLAWSVFMVSG
LIFGLMLLGYLPNTFLTMYASHIGTVLEMAFLSMALADRINHARRQQAQTLLAAGQDLER
LNQQLANSNRLKDEFLATLTHELRTPMNGVIGSLEVMQTLDMDDELEMYQQTAASSARDM
MSMINGILTLTELQAGVLYADCESFAPEDLAYRLEERFSGAAQAKGLILTLDLDDKLPPN
LRGDASKLYQCIECLVDNAIKFTRKGAVQVRFSGHPQDLERLYLRVEVMDSGIGFSRLDE
ARLYQHFFQVDGSMTREYGGLGIGLAICRKLVELQGGELSHRSEPGKGSCFTLSVPMLMV
VPGAVRRAPELKAQWGI
>tr|A0A0Q0CV97|A0A0Q0CV97_PSEAP Uncharacterized protein OS=Pseudomonas syringae pv. aptata OX=83167 GN=ALO85_02264 PE=4 SV=1
MDSIQQPEDDGIIRIQRLVPGQVDVVLGSHPRRKRVDAPRRSRPYGLWLACLIAAACVAG
LITSHARDPKALAVPPVVQSIPETRTVEAEAQPVAAADPAPTHIRKTAELVPIAATVAPP
APVQPLDDCMKNGNVIDEAVANCRFGQLHRPAQAEPATGMVSADYMADFKANASRNSARS
SKPYSVATASIREADGRNRYRAQWRVYGNTIDGDSVCENFAIRSFERRECRKAAAVNFRE
ECREWTKRAARNRDEDSKNAQQRYCEVTANFSP
>tr|A0A0Q0DYR6|A0A0Q0DYR6_PSEAP Uncharacterized protein OS=Pseudomonas syringae pv. aptata OX=83167 GN=ALO85_101578 PE=4 SV=1
MAPGAEGIIRGTPRNSLVGGRLPYTVRLGSYPVLKSYELLDEPGVIRIPSGYTNPGCELL
LLAVNV
>tr|A0A0Q0FTR2|A0A0Q0FTR2_PSEAP ABC transporter OS=Pseudomonas syringae pv. aptata OX=83167 GN=ALO85_00820 PE=4 SV=1
MSPMTTLIEVRDLSKTFTLHQHNGVVLNVLRGLNFSVRAGECLVLSGQSGAGKSTLLRTL
YGNYLPAAGSIRVQHEGEWTELVGATPRQVLEVRRKTLGYVSQFLRVIPRVSSLDVVMEP
ALARGWSREQAQERAQLLLGRLNIPQRLWPLAPSTFSGGEQQRINIARAFMVPWPVLLLD
EPTASLDDANRRVVLELVDEAKQAGAALIGIFHDRDAREAVANRQLDMTPVDLTAKELLQ
C
>tr|A0A0Q0C6R1|A0A0Q0C6R1_PSEAP Mn2+/Fe2+ transporter OS=Pseudomonas syringae pv. aptata OX=83167 GN=ALO85_100285 PE=4 SV=1
MHRMSHTPSLKETATPVAEPRFKKLARLLGPGIIAVLSWLGAGDLITSSVAGASYGYAMM
WVLAISLLLRFLIVNIIARFQLCNNQGMTILQGYAQLHPLFAWFMLGYALLMGHLINAYM
IKGAGEALAMLLHIDQPLLCSVAVVIAVWLLVGRNIYAMIEGVMKVLLAIMTLAFLTLAI

MSGPDVTGIIKGTIGFSIPPDEGVHGALLVAVSVIGAVAGSIANFVHPYVMREKGWTGPE
HKRIQRNDLLFAVIVGIIINLAIWVVGVEILRPNGIQVNTLADLGKALEIFFGPLGWYIF
FIGVFATLFASISGKTTAFPMLITDAFQHIQPKRRERYGKVFHNDPMHRWFMLFILVTPL
IWSLPGMPDFVTLTLGVNALNIIGLPVISLGLLIMSNQKSLLSKEYRNNWFENIALTFAT
GLALWVAFQLGTELLA
>tr|A0A0N8T889|A0A0N8T889_PSEAP Inner-membrane translocator
OS=Pseudomonas syringae pv. aptata OX=83167 GN=ALO85_03060 PE=3 SV=1
MDIDLLSNIFYAMVRCGTPLLLVALGELICEKSGVINLGQEGMMLFGAVIGFIVALNTGN
LWLGVLLAMLAGMLLSALFALVALVFNANQVATGLALTIFGVGLSSFVGAAWVGKPLSGF
EPVAIPLLSDIPLIGRMLFAQDLLVYLSFALFALVAWALLKSRVGLIIQAVGENPDAASA
MGLPVLRVRALAVLFGGAMAGLAGAYLSLAYTPMWAENMSAGRGWIALALVVFASWRVWR
LLLGAYLFGLASILHLVAQGIGLAIPSNLLAMLPYVATIIVLVLLSRDALRTRLYAPVSL
GQPWRAGH
>tr|A0A0Q0C258|A0A0Q0C258_PSEAP Cobyrinic acid a,c-diamide synthase
OS=Pseudomonas syringae pv. aptata OX=83167 GN=ALO85_04020 PE=4 SV=1
MRVWAVANQKGGVGKTTTTIALAGLLADSGKRVVVVDLDPHGSMTSYFGHNPDELEHSAF
DLFLHKGAVPDGLPGQLLLPTSDKRISLIPSSTALATLERQSPGQSGLGLVVAKSLAQLW
QDFDYALIDSPPLLGVLMVNALAASQQLAIPVQTEFLAVKGLERMVNTLTMINRSRKVPL
PYTIVPTLFDRRTQASMGTLKLLRDSFPEHVWQAYVPVDTRLRDASRLGLTPSQNDSKSR
GVIAYRALLKHMLAEQLNAQVA
>tr|A0A0Q0CX36|A0A0Q0CX36_PSEAP HSR1-like GTP-binding protein
OS=Pseudomonas syringae pv. aptata OX=83167 GN=ALO85_00453 PE=4 SV=1
MTEADSTRAIRLAVVGHTNVGKTSLLRTLTGDVSFGEVSHRPSTTRHVEGARLSVDGEAL
VELYDTPGLEDAIALLDYLERLDRPGERLDGPARLTRFLEGSEARQRFEQEAKVMRQLLD
SDAGLYVIDAREPVLAKYRDELAVLASCGKPLLPVLNFVSAQQHREPEWREALSRLGLHA
LVRFDSVAPPEDGGRRLYESLALLLESARPRLERLIDDQEKQRTAKRHSAARLIAELLID
CAACRRSVETTADQEQQAVEALRKAVRQREQQCVEALLKLYAFRKDDVSSSDLPLMDGRW
GDDLFNPETLKLMGVRVGGGVAAGAAAGAGVDLMVGGVTLGAAALVGAIAGGALSTARSY
GGRLLGKFKGRRELTVDDAVLRLLALRQRHLLLALGVRGHAALHSIELTTPQDKTWRKGK
IPEPLSRARAHPQWSTLNPQPKPNQAERQEAIDELAAVVLQD
>tr|A0A0Q0DIZ9|A0A0Q0DIZ9_PSEAP MFS transporter OS=Pseudomonas syringae
pv. aptata OX=83167 GN=ALO85_04214 PE=4 SV=1
MDGLANSLCLFWIDDTPGGCLAIYKNRDQTIMITTMTLDAVSTTRANAYRKTAWRLMPFL
MLCYLCAYLDRVNVGFAKLQMMNDLAFSETVYGLGAGMFFIGYFLCEVPSNLILHRVGAR
RWIARIMISWGIISALFAFVETAWQFYALRFLLGVAEAGLAPGLLLYLTYWFPSYRRARM
TVLWFIAIPLSGMIGGPLSGYIMTRFAGVHGWAGWQWMFVLEAIPTVIVGLLVLSYLKDG
VHQATWLSAEEKELVNKELQEDNSQKVVHASVGAFIRDKRLWLLACIYFCVVMGQYAITF
WLPTLIRNAGVSDPLHIGLMTSLPYLCAIVVMLLMGRSGDKHRERRWHLVGPMIAGALGL
SLAALFGGNLLLSVLSLCLAAAGILSASSMFWMLPTTLLGGVSAAAGIAGINSFANLAGF
CSPYLIGWITTTTGSSAIGMYLITGVLFIGAYLVLRIPAASVNR
>tr|A0A0Q0C8T5|A0A0Q0C8T5_PSEAP Taurine catabolism dioxygenase TauD/TfdA
OS=Pseudomonas syringae pv. aptata OX=83167 GN=ALO85_03384 PE=4 SV=1
MFTYEEGCTMLATSLVQLSQEPIKPGIGARVFNGKQDLLSGRFAGEIRELLEQRGVLVFP
QIHFSDAEQIAFTRTLGTFCPEPGDGQNITRISLDVKENPAGAEFLKGSLYWHIDGTSSD
SPILASLLSCKVPASWGGNTGFCNTYSAYEGLSSADRQHYDSLRVIHAPWASLLYYNPEP
GLAMLEAMQAIGEKELPLVWKHRSGRKSLILGCTAQQVVGLSLAQSARILVGLREWATSE
AFSYSHAWQVGDLVIWDNTGTMHRAEAYDPECGRMMHRTKLQGEEPFA
>tr|A0A0Q0IN67|A0A0Q0IN67_PSEAP Protein HflC OS=Pseudomonas syringae pv.
aptata OX=83167 GN=ALO85_05229 PE=3 SV=1
MMSNKSLIALIVGVILAVIAWNSFYIVSQTERAVLLQFGRVVQADVQPGLHVKVPYVNQV
RKFDGRLLTLDAPTQRFLTLEKKAVMVDAYAKWRVKDAERFYTATSGLKQIADERLSRRL
ESGLRDQFGKRTLHEVVSGERDALMADITGSLNRMAEKELGIEVVDVRVKAIDLPKEVNR
SVFERMSTEREREAREHRAKGNELAEGIRADADRQRRVLLAEAYRESEEARGDGDAQAAA
IYSKAYGQDQEFYAFYRSLRAYRESFANKSDVMVLDPNSEFFRYMEKAKP
>tr|A0A0Q0C592|A0A0Q0C592_PSEAP Non-ribosomal peptide synthase:amino acid
adenylation (Fragment) OS=Pseudomonas syringae pv. aptata OX=83167
GN=ALO85_05488 PE=4 SV=1
MKWRVSDMDKSAAERIAQRFVGLPVEQRRQILAKMYETGQSFKLLPIAVTRHAAARIPLS
YAQQRMLFLWQMEPGNAAYNVPMAVRLNGALNRQALSEALDLLVQRHETLRTRFVSEDGA
FYQEILEQATVALEFASVAPADIEQQVRAELQKPFDLLSGTLLRVKLFQLSESEHVLTVC

MHHVVSDGWSGEVLIREFVQLYQAQVSGQPVPLPALAIQYADYAIWQRAWLEAGEGERQL
HYWKQQLGSEHPLLSLPLDHPRPLQPSHRGASVRVDLPQQLSAQLKSLARNNGQTLFMVT
LAALSVVLSRFSGQSDLRIGAPNAGRTRSELEGLIGFFINTQVLRVQVDERQSFAELLDQ
VKQVVTGAQSHQELPFEHLVDALAPERNPGHNPLFQFKINQHVLAADGNGQRVSGLTVDE
FPMGSSDARFDLAFDFTDTPDGIRGYFTYATDLFEAQTIERIADALRRVLQALVSDTDRR
LADLPHAVSSLVAEQPQDFACTDFLSLWQQGLRAGRGKTALCVGQQVLSFDELETRSNQF
ARYLHAQDIKPGMTVALCLDRSVEWVISLLALLKLGAVYLPLDSAQPAERLQQLVRDSGT
VLLVHAPEDDKAARLGVCPVLAFDAALWAAVDNQSLDVRVLPGQAAYIIYTSGSTGQPKG
VVISHGALANYVQGVLERLALNDGASMAMVSTVAADLGHTLLFGALASGRTLHLLSHEQA
FDPDGFARYMAEHQVDVLKIVPSHLQGLLQAANPADVLPGQVLILGGEASAWALIEQVRA
LKPGCRVINHYGPTETTVGILTHEVAGPIDACRSVPVGQPLANGKARVLDAYLNPVAERV
TGELYLGGRGLAQGYLGRAAMTAERFVPDPDANGQRLYRAGDRARWVDGVLEYLGRADDQ
VKIRGYRVEPGEVGQLLQTLDNVAEAVVLAQPLDSDETRLQLVAYCVAVAGTSLNVDQLR
EHLAARLPEYLVPAQIMLLERLPVTANGKLDKRALPRPGVVKQRYTAPVGEIEEKLAAVW
ADVLKLEQVGSTDNFFELGGDSILSLQIIARAKRQGIKLSPKQLFEKQTIGQLASVAKLI
QKKPAAVAEHISGSLPLLPIQARFFELDIPERQHWNQALILKPLQTLEAAHLQSALTALI
EQHDALRLGFTQQDGQWQATFGVLNARELLWTHELDSIERLPELADEAQRSLDLKNGPLL
RGVLINLPQGEQR
>tr|A0A0Q0D1S9|A0A0Q0D1S9_PSEAP Oxidoreductase molybdopterin-binding subunit OS=Pseudomonas syringae pv. aptata OX=83167 GN=ALO85_00052 PE=4 SV=1
MNPFTYSKPTDITAAVNLSGPATRFIAGGTNLLDLMKENVATPEHLIDITGLPLKDVSET
ASGGVMIGALVSNADLAYHPLIEARYPLLSKAVLAGASPQLRNMASTGGNLLQRTRCYYF
YDTGVPCNKREPGSGCPARTGLNRIHAILGASEDCVATHPSDMCVALAALEAVVHVQGRA
GRRTIEFADFHRLPGDAPQRDNQLADDELIVAVELPANGFVSHNAYLKIRDRASYAFALI
SVAAAIDLDGDVIRDVRLALGGVAHKPWRDKAVEALLVGKPVTRENFAAAADAMLEDAQP
LEHNGFKVRLARRAIIRALSDAATGGSAQ
>tr|A0A0Q0DLV5|A0A0Q0DLV5_PSEAP DNA polymerase III, delta subunit OS=Pseudomonas syringae pv. aptata OX=83167 GN=ALO85_01332 PE=4 SV=1
MKLAPAQLGKHLQGTLAPVYVISGDDPLLCQEAADAIRAAARQQGFDERQVFSADASFDW
GTLLQASASMSLFAERRLLELRLPSGKPGDKGAAALMEYCARPAEDTLLLISLPKLDGSA
QKTKWGKALVEGAQTQFVQIWPVDIGQLPQWIRQRLSQAGLAATQDAVELIAARVEGNLL
AAAQEIEKLKLMAEEGQITVETVQAAVADSARFDVFGLTDAVLNGEAAHALRMLEGLRGE
GVETPVILWALTRELRALANMSQQFSQGVPLDKVFSSARPPIWDKRKPLMGKALQRHSAK
RWSQLLMDAQRIDAQIKGQAAGSPWSSLSRLALLMAGQRLALPAE
>tr|A0A0Q0D417|A0A0Q0D417_PSEAP Sensory box/diguanylate cye domain protein OS=Pseudomonas syringae pv. aptata OX=83167 GN=ALO85_05543 PE=4 SV=1
MVLMEKQPPTSLSGTPITDMPAAAETLLALMHAQAEVARLSEREQLFSSLLVSVNAVLWA
FDWQTQQMIYVSPAYERIFGRSAGLLLADYSEWRDSIYPDDLNYAERSLNEVIEKGSIED
REYRIIRADGEVRWLSDKCFVSQRADVSQRLIVVGIAEDITEKKHLEDELQRLATTDVLT
QSSNRRHFFECAQREFELARLNGTPLAFLLLDIDDFKLVNDTYGHQEGDTVLQRIAESGR
SALRRGDLFGRIGGEEFAAVFPGCAPDMAMQIAERLQREIQRQNFQSGGTTFSITASQGL
TNLGADDPSLETLYGRADAAMYQAKRQGKNQIVLV
>tr|A0A0Q0DIM4|A0A0Q0DIM4_PSEAP Uncharacterized protein OS=Pseudomonas syringae pv. aptata OX=83167 GN=ALO85_02244 PE=4 SV=1
MDIEKLKQKTQKLREVIEDFKKSDHVVETFRAEIEPLMELAEFGIITAKLQWEDIPGRYL
FTEEGLQQYSHLEHAFAEFRIELTGGETPLLRRLKREMGEE
>tr|A0A0Q0DVJ4|A0A0Q0DVJ4_PSEAP Bifunctional glutamine synthetase adenylyltransferase/adenylyl-removing enzyme OS=Pseudomonas syringae pv. aptata OX=83167 GN=glnE PE=3 SV=1
MSLPLLAEFPAILLPSISRARQMFQAALTALSEDALASFNAWPEARQAAFDRVCAASDFV
TEQVCRDPQMLLQLADSGELERSFSAGQLRGQLADALSSAASEDELGRNLRRQRARQQVR
IIWRDLTRQANLIETCRDLSDMADASIDLAYQWLYPRHCQQFGTPTGRHSGKPQHMVILG
MGKLGAVELNLSSDIDLIFGYPEGGETVGTKRALDNQEFFIRLGQRLIKALDPVTVDGFV
FRVDMRLRPYGSSGALVLSFNALEQYYQDQGRDWERYAMIKARVVGGDQAAGAELLAMLR
PFVYRRYLDFSAIEALRTMKQLIQQEVKRKGMAENIKLGAGGIREVEFIAQAFQLIHGGR
DLSLQQRPLFKVLKTLEGQGYLPSAVTEELREGYEFLRYTEHAIQAIADRQTQMLPDNEQ
DQARVALIMGFADWAAFHERLMYWRGRVAWHFRQVIADPDADPDDEQDEDDEVVVGGEWL
PLWEESQDEEAAGRQLLQAGFVDAERALKHLANLRSSPNLRSMQRLSRERLDAFIPRLLA

QAVEHEKPDLVLERVLPLVEAVARRSAYLVLLTENPDALRQLLTLCAASPWIAEQIARFP
LLLDELLNEGRLFNPPLAPELAAELRERLIRIPEDDLEQQMEALRHFKLAHSLRVAASEI
SGSLPLMKVSDYLTWLAEAILDQVLALAWRYSVARHGTPLRPDGTLCDPGFVIVGYGKVG
GIELGHGSDLDLVFIHDGDLEAETDGAKPIDTAQFFTRLGQRVIHLLTTQTNSGQLYDVD
MRLRPSGASGLLVSSLGAFARYQANEAWTWEHQALVRARVMTGSRDVGEQFEKVRADVLG
RERDLDKLRAEVSEMRAKMRDNLGTRATAAGRAANAFEAAVPFDLKQDAGGIVDIEFMVQ
YAALAWSREHPALLQYTDNIRILEGLEEAGLLPDTDAGLLREAYKAYRSAAHRQALQKQA
GVVSGDQFHAQRREVMRIWTQMGLS
>tr|A0A0Q0D652|A0A0Q0D652_PSEAP DUF2156 domain-containing protein
OS=Pseudomonas syringae pv. aptata OX=83167 GN=ALO85_00255 PE=4 SV=1
MMRDNMSDSNETIDAEQPVSPPRHNWRELLSKYRQPIGLAVMLLLFSLALIACRHMLVEM
DWYALHDSLFAVPLPALGGALLAAIVGYIILMGYELSASRYANVDLPIKTLLLGGFTSFA
IGNAVGLSMLSGGSVRYRLYSRHGVGAIEIARMTVFASLSLGCALPPLAALATLSNLSGA
SLALKLPVNVLAVVATAVLIGSLLLAVFVYRRRLPEQDIPHNILVRLGRRTLRLPDLKLT
LIQLVITALDVAAAAMVLYLLLPHAPPLGAFILIYLLALAAGVLSHVPGGVGVFEAILLA
AFANELGAAPLAAALLLYRLIYVILPLLLACLTLLFTEAQRLLPTRQAMRVASGLAAPIL
ALLVFLSGVVLLFSGATPEIDTRLENVGFMIPHRLIDASHFGASLVGVLCLLLAQGLRRR
LSAAWMLTSILLLVGAVLSILKGFDWEEASMLLLTACLLLTFRRSFYRPSRLLELPFSPV
YLIASICVVGASIWLLLFAYQDVPYSHQLWWQFTLDADAPRGLRSALGSAVLLVVVSLTW
LLRTARPVIKLPDAADLDKAAEILKASKQPDGGLVLTGDKAILFHPAGNAFLMYSHRGRS
LVALYDPIGPTQQRAELIWQFRDLCDVHHARPVFYQVRAENLPFYMDIGLTAIKLGEEAR
VDLRRFDIDAKGKEMKDLRYTWNRGGRDGLSLEIYEPGQAPIDELKVISDAWLTGKNVRE
KGFSLGRFSTEYLKYFRIAIVHFQGKPVAFSNLLETTSPELASLDLMRSHPDAPKLTMEF
MMVGLILHYKAQGYARFSLGMVPLSGLQPRRGAPITQRLGSMIFRRGEQLYNFQGLRRFK
DKFQPDWEPRYMAVPAGLDPLVALADTAALIAGGLTGLVKR
>tr|A0A0N8T9L2|A0A0N8T9L2_PSEAP Lipid A export ATP-binding/permease
protein MsbA OS=Pseudomonas syringae pv. aptata OX=83167 GN=msbA PE=3
SV=1
MTTSESSATSSVKIYFRLLSYVRPYVGIFLLSIIGFVIFASTQPMLAGILKYFVDGLTNP
EAVLFPNVPYLRELQLLQAVPLLIVLIAAWQGLGSFLGNYFLAKVSLGLVHDLRVELFNK
LLVLPNRYFDTTNSGHLISRITFNVTMVTGAATDAIKVVIREGLTVVFLFIYLLMMNWKL
TLVMLAILPLIAVMVGTASKKFRKQSKKIQVAMGDVTHVASETIQGYRVVRSFGGESYEQ
ARFAQASNSNTKKQLRMTKTGAIYTPMLQLVIYSAMAVLMFLVLFLRGEATAGDLVAYIT
AAGLLPKPIRQLSEVSSTIQKGVAGAESIFEQLDVEEEVDTGTIERDRVTGHLEVKNLSF
FYPQTARQVLNDISFSAAPGQMIALVGRSGSGKSTLANLIPRFYGHDMGNILLDGVEIND
YRLRNLRRHIAQVNQNVTLFNDTIANNIAYGDLAGAPRADIEAAAADAYAKEFIDQLPQG
FDTQVGENGVLLSGGQRQRLAIARALLKNAPLLILDEATSALDTESERHIQAALDHVMKG
RTTLVIAHRLSTIEKADLILVMDAGQIVERGTHSELLAQNGYYARLHAMGLDEPAPVGAV
>tr|A0A0Q0FE60|A0A0Q0FE60_PSEAP Penicillin amidase OS=Pseudomonas
syringae pv. aptata OX=83167 GN=ALO85_03570 PE=4 SV=1
MPMASPALRHFLPRFGAAAAAASFLSLAGCQLGGGDPETVLPVSGTFPLKGLAQNVSVRR
NTMGMPLIESSSYHDALFTLGYVHAGDRIGQMLGMRLLAQGRLSEMAGADALDVDRLMRS
VNLKQNASDLYNAASPRLKRFFDVYARGVNAYLFRYRDKLPAEIASSGYKPEYWKPEDSA
LIFSLLNFSLSVNLQEELSALVLAQKVSADKLAWLLPTYPDEELPFAEADKLKGLNLNNQ
VTGLSDLNRIALQLSDLNMLGVAASSNWAIAPQRSRNGKSLLASDMQLPAGLNSAWSFVQ
IRAPKYQVSGVTIAGMPLVLSGFNGKLAWSMSSVKGDNQDLFLEKIKREGNRVSYMADGK
WLPAAAHQETFLVKGGSPLRETVYETRHGALLNASATPPVNGLSLALQVPNFKDDKSLDA
FFDLSRAPNVEKAFDTSREIRAITLNMIFADASNIGWQVTGRFPNRREGQGLLPSPGWDG
KYDWDGFADSMLHPYDQDPRQGWLAAANQRTIPKGYGMQLSNSWGYPERAERIAELANGG
KQDLRSTIAMQYDQTTTFAAKLKTMFQAPGMSKPLKQAIDALPEADRNKAREAFTRLMAF
DGKLSATSADAALYELFLQESAKQIFLDELGPETSPAWQALVANASSSYSPQADHLLGRD
DSPYWDDVKTPQKEDKPAILARSLAAAITSGDSLLGSDHKAWQWGKLHRDNWVSTSPLAK
QLGGGEFNRSATPAGGDHTTLNVSGFEWGKGFDARMAPGLRMIVDFSLVEPMTGLISTGQ
SGNPASPYYANSIEPWQKGQYQSIPVQQQNYEKGYGKQRLTLTPGK
>tr|A0A0Q0DXM1|A0A0Q0DXM1_PSEAP Pyridoxal-phosphate dependent enzyme
family/ornithine cyclodeaminase family protein OS=Pseudomonas syringae
pv. aptata OX=83167 GN=ALO85_03785 PE=4 SV=1
MYNSLCEIVHDQTFLKVTGLGADFFLKMESLNPAGSIKLKTAVGLINDLQARGLLGPDTI
LIESSSGNLGVALAMICAERGVPFTCVVDPNSSSHNIRMMRSYGAEVIQVEIPDANGGFL
GTRIELIRQKVASDPRYVWLNQYENAANPRAHARTTARSISQHFGHVDYVFVGAGTTGTL

MGCIQHFQRHHPTTRIIAVDSVGSVTFDTPASRRFIPGLGTSQRPPIFNADGIHALEMVP
ESHTVAMCRILARSKGLLVGGSTATVIAAVHAWRERIEPGSVVVALSPDWGERYLDTLYD
DQWVEQRFGREVLSMTLADLSSSEAATSPGSVKTPSRASSLPQNRCVNVVRAHATPCGSE
LARXXXXXAKTAVQSQRLRVQARLRRSRVQPSGWPPSG
>tr|A0A0Q0DVB2|A0A0Q0DVB2_PSEAP Uncharacterized protein (Fragment) OS=Pseudomonas syringae pv. aptata OX=83167 GN=ALO85_100712 PE=4 SV=1
MHWVTLRVVLQRNVMLCLSSLHIKALFYRDVTDGANCDLSDRAKGPDEVGPET
>tr|A0A0Q0DSK4|A0A0Q0DSK4_PSEAP Regulatory protein, TetR OS=Pseudomonas syringae pv. aptata OX=83167 GN=ALO85_00851 PE=4 SV=1
MRVTKAQAQANRAHIVETASVQFREHGYDGVGVADLMAAAGFTHGGFYKHFGSKSDLMAE
SAACAFSQTVANSTGINPAQFIDYYLSREHRDNLGTGCPMAALSGDAARQSDEVKATFAD
GIESLLAALAPAQAAPGDEEWKQARANTIDRFAHALGALLLSRSCPNDSPLADEILEVCR
AKMRAQLESGQTD
>tr|A0A0N8T970|A0A0N8T970_PSEAP Malonate decarboxylase acyl carrier protein OS=Pseudomonas syringae pv. aptata OX=83167 GN=mdcC PE=3 SV=1
METLSFEFPAGQSPKGRALVGVVGSGDLEVLLEPGKPGTLSIQVVTSVNGAALRWQHLFE
RMFDGQTPPALSIDIHDFGATPGVVRLRLEQGFEEIGHD
>tr|A0A0Q0BZ12|A0A0Q0BZ12_PSEAP ISPpu14, transposase Orf1 OS=Pseudomonas syringae pv. aptata OX=83167 GN=ALO85_00868 PE=4 SV=1
MQPQRRSYSRSFKAQVIQECAQPGASIASIALSHSLNANLFHKWIRVQAQKNTAPQPAFN
GAEYGKKKPYR
>tr|A0A0Q0CHE1|A0A0Q0CHE1_PSEAP Ultraviolet light resistance protein A OS=Pseudomonas syringae pv. aptata OX=83167 GN=ALO85_200097 PE=4 SV=1
MILLLGGEKGGSGKSCLAQNLAVWLQARGGDVLLLDADPQGTTADWAAERSAQGDLPAIP
VVQAHGNIRQTLTDLRGRYRQIVVDAGGSDSEALRSAMTVATHMLIPFRPKRRDLKTLPK
VDNLARLATAVNPALNVRAVITQCPALPSQIQRILDAKEACRSFGIQPLDAITTARNVYD
DADEDGCSVLESNSDARAKEEIETLAAELWGAAKWD
>tr|A0A0Q0CG05|A0A0Q0CG05_PSEAP Precorrin-4 C11-methyltransferase OS=Pseudomonas syringae pv. aptata OX=83167 GN=ALO85_03626 PE=4 SV=1
MTVYFIGAGPGDPELITVKGQRLIRSCPVIIYAGSLVPLAVLEGHQAVQVTNSAELHLEQ
IIELIKRAHAQGQDVARVHSGDPSLYGAIGEQIRHLRELGIPFEIIPGVTATAACAALLG
VELTLPDVSQSVILTRYADKTSMPNGEELASLASHRATMAIHLGVNNLQRIVEELMPHYG
ADCPIAVVHRASWPDQDWAVGTLADIHEKVQAKGFRRTALILVGRVLADDTFSESSLYRA
GHAHLFRPV
>tr|A0A0Q0DD22|A0A0Q0DD22_PSEAP Enoyl-CoA hydratase/isomerase OS=Pseudomonas syringae pv. aptata OX=83167 GN=ALO85_00062 PE=4 SV=1
MSSQVSSARPVRPSASRDSIVIGVRNHIGHLTLNRPEGLNAIDLDMVRTLRQQLERWADD
PSVHAVVLRGAGGKAFCAGGDIRSLYESHQNGQDLHHTFFAEEYELDLAIHRYRKPVLAL
MDGLVLGGGMGLVQGADLRVVTERSRLGMPEVAIGYFPDVGGSYFLSRLPGELGTWLGVT
GSQIGAADALYCGLANWSMNSELLPRLDHMLDHLKWKSTPLKDLQGALAKLATQRLPTPP
LEALRHAIDHFFGLPDVPSMLEQLQQVVIGDTREWALDTVSRMKAHSPLGMAVTLEMLRR
GRHLSLPACFAMELHLDRQWFERGDLIEGIRALIIDKDKKPQWKHASAQDVSAAQVQSFF
SGLEN
>tr|A0A0Q0D5F1|A0A0Q0D5F1_PSEAP Mixed non-ribosomal peptide synthetase/polyketide synthetase OS=Pseudomonas syringae pv. aptata OX=83167 GN=ALO85_02446 PE=4 SV=1
MMNRHAENNARNPQTLLSRFAEQVRRDPQALAVIDRQVRLTYAQLASASKRIAKGLLAQG
TGPADPIALCMPRCWQWVATILAVLKVGAVVVPLDRASPARRRQLMLDDGGCVGLVTLGE
DADSLAAPQRGWYVSVEALLEFPDQPALPLPEDFAASSFLFYTSGTTGTPKAVDLGERGL
LRLARTDSCLDTRAGERVACLSNPAFDACNFELWAPLLNGGCCVIIAEADLQDAQQLARV
LETQQVDSLFMTVSLFNTLSADNPACFASLRQVLIGGEQVSAAAVRAWYQANPDSRCRIF
NAYGPTECTTFAVCYPIPRDFAGDAVPIGRPLPDTGVQVLDAQQRAVARGEAGELYLSGS
GVARGYRNRPTETEQRFLRLPTIDAGDILHYRTGDQVRVNADGLIEYLGRIDRQVKVRGF
RIEPGEVEQRILEHPQVAQVHVCTRRQAAEDHQLLAFIVPREALDYRDFDQHLRDNLAVW
MRPHQLFVLQRLPLTANGKIDQCALLEQPLKPWRPQAGSRADEQHSPTLDWLLTQARRLL
AQPELNGEDDWLGSGGDSLKAMRLRSAIRTHWRREITLGAVLSESFSALAKRLGDEQGAA
SAYPPAPLVNHSGRAPATAEQSRLWLMQQRTPQATAYNVPIILHLAADVQLPALADAVQR
LLTRHSGLRTAFVSAADGLHQEVHQRDAVCQTFAKGAFSEQTWRSFAALVFDTPFDLATS
ALCKAWLLPFADGSCRLLLNLHHVAIDGWSMNLLFDDLVQLYDDALQGRPSAEPSPGLGT
LEFALWQRQWRVDPRYRDQRRALAGLHRQHPASSPALLPVREPGPQALLYRQPLGATRSA

ALDRFCSRQRVTRYEVLFSVYAWSIYALTGCERPRIASPVSNRPLSEFEDAIGMFANTVL
IPTAFDGDKALGQQLHQQTATVREVLALQDVALADLVEDLRLSSSSALFDFMFVLENTDY
ARLAHTGLRATLEFNETVQAKCPLTLLVVDAGSQLECWWEYQCSYFDAGQMVAVNQLLQQ
GLDLLLEKPSATLDALLTPYRFNLPPASQGDSAEPPFNTAADWFAYQVSCTPDAPALVDN
QQCISYAELDALADTLAATLIEQCPLPEDNDAPLQVVLYLQASVEHIVALLALARLNLTA
VPVDPGYPLAVQRQVMQQAQPGCVLYSATTEVALEQLNADRQVCHRVDLSAAARPFERRR
HAGQRPLYTLFTSGSTGTPKGVQVPDRTLCNLLHWQRNEGQLPAKSVTLQFSMLSFDVSF
QEIFSTLCGGGCYHLINPRWRQDAQALLSYLVQTRIERLFLPCVALQHLAQTAVSQGVYP
QALREVITAGEQLLCTEALRNWFGGMPQARLFNHYGPTETHVVSAWRLPAAVQDWPLRAP
IGRAVSNARLLLVDEHDRPVPRGSQGYLLVAGPMISRCYLADPALNAARFVELPQPEGMT
LFYRTGDLARADANACLHYLGRDDQQIKISGQRIELGQIEAALLQVAQVSNAVVAMQAEP
PRLVAWLRSEGTLPDALQLDRQISLHLPAHVRIDEYRRVDAWPRTPSGKIDRRALGDLGE
VLQRQRTGLPAAPLSALEQQLSELFVAVIGRDIEPDQTFFEAGATSLGLMRLHARYNEVL
PQPVAMAALFEHVSVRRLAQHLSIPAGQLSGAGKGSERGAKAGQQPMAIIGMSVNVAGAS
NLAEFWAMVQGNGLGIERFDAAEGLVGARSQLTGLLDFDPDYFGISLQEARLMDPQQRHL
LMGCVQALQHAGLTPKADGPRIGLIASCGETTYFQQMLRETAEGDLPDGFQMALHHDKDF
LATKAAYHLDLGGPALSVQAACGSSLIAVHLAAAMLRQGDSDVMLAAGVLIDPTLTDGYR
YRPQHIFSRDGLCRPFSDDASGTIGASGYGVVVLKPLDRAQADGDRIYALVEASALNNDG
RAKMSYTAPSVSGQSAVISEALRKAGINGADMGYIEAHGTGTLLGDPIEVAALTKAFGAA
PAAGCALASVKSQVGHLGAAAGVVGLIRATLAVFHGVIPPNLGFTRINPQIDLQHSPFYI
PGTSRPWPEGRRRLAGVSSFGIGGTNAHVIVGAAPQQTARVEDSPACLLLSAHSRTALER
DMLAIEAWLNAFPEQQAALLRYLQNGRRALPWRFAMICQPGQTPRLQAASIKQVTLSDVR
VNASEHSPQAVLDAWYAGASIEGSTGSTPPPWDLPPSAFDLQTFRFKPAARAELQPSSAV
ERQPLADWFYQRQWQRVNRLRTQPTGEGRDVLVVCSHERLDATTLGSLQGVYRHVIEVQA
GNGYQQLGPNRYELDPLDPVAFGRLIATLDQNGQTASELDWLHALPLSVGGAVDEQSLAA
AQWACLDTLSALLQAWGQNAQKSVLRLWLLSWQACPVNGQVMRPELAALAGITEVAPQEY
PIRCHWLDLPTAQLSVQARHLAALLAEPASLPRRMAIRDGYLWQPQLLASPLANPMADSN
LLPANGTLLVLGGTGGIGRTLCEHLLQSSQRRVVLLSRGGECPKELHAYASRIDSIAADI
ADLARWPTVLEHLRERYGHFDGVIHAAGTGAGSLIRQRDAQVLSEAMASKTRGMLAVEAL
IEKMTPGFVLYCSSMSALFGGAGHLDYAAASAVLDGFTHYRPHAENGCVRLGINWDIWRD
VGMAAASGVGDDAHQRHLAVGMSAQEGCRIFDAAITAQLPQLLISTTDPETARQFYPVRH
SAGSTLADIAPVLTANIEAPDRLPGHLHDCLCKWLGVSALQPDASLYELGADSLTLLDLI
DELQAATGVVFQLSQFSHKVSLAEVLTLVQASGAVTVADENQWADAVRIDQWHAGAGRER
LYLIHPVGGDVQAYRELASALHPDLGVWVIADPALRLPELPNISVDERAQLYLEALQAHH
PDGTAWRLAGWSFGAWVTQAMCSLLTEDCEQPLLYLIDPPAPDAGHELAQIGEAQIQQVF
QREFSARRGVNTASEDASSYLQSLIVCCQNNMASMAGHQPEALLRTPVRLFIATQPNPYG
MGSAWRIADLQRAWQGLMPHMLSWQALDTDHYGIVAAPWAQVIAEMINADLSSIKG
>tr|A0A0N8TA16|A0A0N8TA16_PSEAP Pyridoxine 5'-phosphate synthase
OS=Pseudomonas syringae pv. aptata OX=83167 GN=pdxJ PE=3 SV=1
MTHSTRILLGVNIDHVATLRQARGTRYPDPVKAALDAEEAGADGITVHLREDRRHIQERD
VLLLKDVLQTRMNFEMGVTEEMLAFAERIRPAHICLVPETRQELTTEGGLDVAGQEARIK
AAVERLAKIGCEVSLFIDADERQIAASKRVGAPAIELHTGRYADAQTPTEVAEELQRVAD
GVAFGLAQGLIVNAGHGLHYHNVEAVAAIKGINELNIGHALVAHALFVGFKAAVAEMKAL
IVAAAR
>tr|A0A0N8T9H5|A0A0N8T9H5_PSEAP Nitrate/sulfonate/bicarbonate ABC-type
transport system, periplasmic substrate-binding protein OS=Pseudomonas
syringae pv. aptata OX=83167 GN=ALO85_00981 PE=4 SV=1
MPQPPVSFRLLSPRALIRWMASTCVLALSLNGAVEAAETAPAEIRLDYAYYSPVSLALKH
FGWLEKALPDAKVTWVLSQGSNRSLEYLNSGSVDFASSASLSAVLARANGSPIKSVYVYS
RAEWTALVVRKDSPLQSVAELKGKKIAATKGTDPYLFTLRALQKAGLKKDDVELLHLQHP
DGRTALEKGDVDAWAGLDPHMAASEVQSGSRLLYRNRDFNSYGVLSVTETYAKEHPQAIR
TVLSAYEQAREWAVKNPDELARLLATESGLPLEVARLQLTRTDLSNPQLSAQDAEASKAA
APILVAEDLVRKGVNVEQVIDQLITPEFSKAVIATP
>tr|A0A0Q0FEH0|A0A0Q0FEH0_PSEAP DNA polymerase III subunit gamma/tau
OS=Pseudomonas syringae pv. aptata OX=83167 GN=dnaX PE=3 SV=1
MSYQVLARKWRPRSFREMVGQAHVLKALINALDSQRLHHAYLFTGTRGVGKTTIARIIAK
CLNCETGITSTPCGTCSVCKEIDEGRFVDLIEIDAASRTKVEDTRELLDNVQYAPSRGRF
KVYLIDEVHMLSSHSFNALLKTLEEPPPYVKFILATTDPQKLPATILSRCLQFSLKNMTP
ERVVEHLTHVLGVENVPFEDDALWLLGRAADGSMRDAMSLTDQAIAFGEGKVMAADVRAM
LGTLDHGQVFDVLTALLEGDARGVLEAVRHLAEQGPDWNGVLSEILNVLHRVAIAQALPE

GVDNGHGDRDRVLALAQALPAEDVQFYYQMGLIGRRDLPLAPDPRGGFEMVLLRMLAFRP
ADSEDAPRQPLKPVGISQATVDSRKAVADATPVASAAPAVSTAPVMAAPDPAFEALPPAA
PAMPAAARPEPAVQPELKPEPQAAPVEDLDLPWNEPKSPAAEPAAETAPPTESKAEPVAE
PVLETVSEQPDLTPMPTPAPASPVPAAPEAEPAQPAAEQPVTPAMLEAIPDSAYLSMHAD
SMPSGSGPMGRDDEPPADDDYVEPDIDIDPASYSYLDELAHESVVELEAVEPEPAPAAKP
ATGLAAEWLDVFPKLPISGMTGSIAANCTLISVDGDNWLLHLDPAHSALFNSTQQRRLND
ALNQYHGRTINLSIELIKPEQETPAQAASRLRAERQRQAEASIQADPYIQQMLQQFGAVI
REDTIKPVDAPAVPAQ
>tr|A0A0Q0DIG8|A0A0Q0DIG8_PSEAP AsmA domain-containing protein
OS=Pseudomonas syringae pv. aptata OX=83167 GN=ALO85_02308 PE=4 SV=1
MKAFGKILGLFILGLLLIIVALGFALTHLFDPNDYKDEIRQLARDKANVELTLNGDIGWS
LFPWLGLELHDAHVATLTAPDQPFADVQMLGLSVRVLPLLRRQVQMSDIRVEGLNLTLHR
DENGHGNWEDIGKPAAASTEAPGETTPAPAPDAPKADRPSQPIRLDIDSLTVNNARVDYS
DARKGRTFMAESIQLSTGPIREASDIPIKLTAFLSTNQPVVRAKTELVGALRMDRVLKRY
QFEDMRLSGEVAGEPLQGKTVTFSAQGQLLVDLAANIAEWNSLKVSANQLRALGELRARD
LDKTPQISGGLSIAQLDLRAFFENIGQPLPAMSEGALSKVEMVTRLAGTPTSLALENLNL
KVDDSTFTGRIAVDDFAKQALRVQLKGDTFDADRYRPAESEESKGAKAARKSEVQSGEAA
AAAGDSPLPEQPTKAAWSTARLLPLERLRTLDVEADLSFGKLTLNKLPIDNAALKTQANA
GLIKVDTLSGDLYNGNFEVKGNLDARPDTPVATVQTRIVKVPVERILESQGQTPPVRGML
NLDSNLTGTGNSEKALIDSLNGTASFALNNGVLVNANLEQQLCKGISILNRKTLSGEPRA
KDTPFQQLNGNLVIRNGVASNPDLKVRTPGMAVNGNGDVDLRVLGMNYRVGIIVEGDKSD
MPDPACEINPRFVGIEWPVQCRGPLELGAKACRLDKEGVGQIAAKLAGDRISERLEDKLN
DKLGDKVSPELKDALKGLFKR
>tr|A0A0N8T8J2|A0A0N8T8J2_PSEAP S V aminotransferase OS=Pseudomonas
syringae pv. aptata OX=83167 GN=ALO85_100141 PE=3 SV=1
MSQTPLPDPQAQTLMTDRRTFLKQAGLLAAALPLGSGLATAAMTPATQPASTDKWTRLKQ
LFNQDPGYVHLSNFLVTSHPAPVRQAIEQHRAHIDRNPGLAMDWDLGETERREHEVRVWA
GKYLKAQPGQIALTGSTTEGLAIIYGGLHVRPDQEILTTEHEHSCVRDILKFRQQRDGTQ
VRKIRLFKDPATVSVDEIIGSIARSIQPKTRVLGMTWVQSGSGVKLPIGAIGDLVEEHNR
NRDDQDRILYVVDGVHGFGVENLDFPEMKCDFYVAGTHKWLFGPRGTGIVCARSEQVKDL
TPLIPTFSEASGFSTIMTPGGYHSFEHRWALNEAFKLHLQLGKADVQARIHQLNSYLKQR
LKAQPNVELVTPMDPALSAGFSFFRLKGQSCDDVAAWLMKQRMVVDAVSRDVGPVVRTAP
GLLNSEAEIDRFMMLLARRA
>tr|A0A0Q0ILI8|A0A0Q0ILI8_PSEAP Uncharacterized protein OS=Pseudomonas
syringae pv. aptata OX=83167 GN=ALO85_102166 PE=4 SV=1
MAGTRWVRKGAILSDHRCRVIIASFYRLSVKLQSRRKTST
>tr|A0A0Q0C7I4|A0A0Q0C7I4_PSEAP Thioredoxin OS=Pseudomonas syringae pv.
aptata OX=83167 GN=ALO85_01638 PE=3 SV=1
MSSDLIKHVTDATFEAEVLKAQGPVLVDYWAEWCGPCKMIAPVLDEIAGTYEGKLTVAKL
NIDQNPETPAKHGVRGIPTLMLFKGGNVEATKVGALSKSQLAAFLDANI
>tr|A0A0Q0BX78|A0A0Q0BX78_PSEAP Uncharacterized protein OS=Pseudomonas
syringae pv. aptata OX=83167 GN=ALO85_101911 PE=4 SV=1
MAVISFSVIRYVDPFQGGGILLENHREYKVQLNCIQPAPRRPAQKNRAHGPVSLFSRSPL
SE
>tr|A0A0Q0D028|A0A0Q0D028_PSEAP Cyclic peptide transporter OS=Pseudomonas
syringae pv. aptata OX=83167 GN=ALO85_05484 PE=4 SV=1
MNKPTPHSRTLTRHTMTRTTESLARETLRILKPFWWLVALSTLLGIVSGLSVTGLLATIN
NAMNMPGGPDTQTALLFAGLCVLTLACSTLSNLSTNYVGQRVVANLRRELAAKVLVAPIE
QLERYRAHRLIPVLLNDVNTISTFALSVAPMVISFTVTLGCLAYLAVLSWQILALTVLTV
VLGTGAQYLAHAFGMRSILAARNSEDELQKHYQALSAGAKELRIQRKRRQHMLDEKIHGA
TEHICRSNIRAANIFVSAETFGSMLFFAVIGMAIAFQALWPTTEKTVLGGFVLVMLYMKG
PLERLITSLPGISRAQIAMRRIAELSWKFSSPEPHLLVSDRPTSLASMHTLELHNLRYDY
PPVEGSDAFHLGPVDLSIKQGDIVFIVGENGCGKTTLIKLLLGLYTPQQGEIRLNGQAVT
AENLDDYRQLFTTIFADYYLFDEPLQGQAALPQDAGKYLERLDIAHKVRIENGAFTTTDL
STGQRKRLALINAWLDERQVLVFDEWAADQDPAFRRVFYTELLPELKQQGKTIIVISHDD
RYFYIADQLVRMQSGQIQVEQVHSEPRDKTITA
>tr|A0A0Q0E174|A0A0Q0E174_PSEAP Cyclopropane-fatty-acyl-phospholipid
synthase OS=Pseudomonas syringae pv. aptata OX=83167 GN=ALO85_01455 PE=4
SV=1
MLTQLPTALQNLHLPLRLKLWDGHQMDLGPSPSVTIVVKDPQLVSDFSHPSLGLLGSAFV

EGRLELEGSISEVVRVCDELTHALVDDDDSAGNPVRVEHDKDTDAASIAYHYDVSNDFYQ
LWLDREMVYSCAYFKTGDESLEQAQQDKFHHLCRKLRLQRGEYLLDVGCGWGGLARFAAR
EYGVKVFGITLSQQQLALARERVQAEGLQDKVDLQLLDYRDLPQDARFDKVVSVGMFEHV
GHANLPLYAQRLFGAVKEGGLVMNHGITARHTDGRPVGRGAGEFIDRYVFPNGELPHLAM
ISAQISEAGLEVVDVESLRPHYAKTLGHWSARLEARLDEAARIVPEQTLRIWRLYLAGCA
YGFTKGWINLHQILAVKPFADGKTGLPLTREDIYG
>tr|A0A0Q0CDU2|A0A0Q0CDU2_PSEAP LysR family transcriptional regulator YeiE OS=Pseudomonas syringae pv. aptata OX=83167 GN=ALO85_100259 PE=3 SV=1
MRINLRQLQIFCAITRCGSTTSAGSALALSQSATSAALNELESALGTRLFDRVGKRLLLN
DSGQALLPRAMKMLENAQQIEDSFLLPDAALNARLRIGSSSTIGNYVVPQIVGALRGSTP
QLRIDVDIGNSALIARKVAQFEIDMGLIEAPCHLPELIAEPWLVDEMLIVAGSAHPLAGQ
EHVSLGELQSAEWLLREQGSGTREEVEHLLLRHLHDLQDMRQVGSSEAIMRTLAQGIGIS
CLSRRVVADLLASGQLRTLASPLPPLNRRFFMIRHRDKFISPSLERFREACRQAADQADT
LRHGLSHNANPTSSSAP
>tr|A0A0Q0ITB8|A0A0Q0ITB8_PSEAP Uncharacterized protein OS=Pseudomonas syringae pv. aptata OX=83167 GN=ALO85_01521 PE=4 SV=1
MSAASTLVLLDTNAYLRLAKRVRPMLGVGFGQKNYVLTILKDVEEEVHRSGALKFKFPWF
DGEDLAAERVAKQVRLSADEKGQLEAAQSVLRGWVLTNPTVYTTAGRSPPSSTDCRVLAF
GQIRDAIVVTDDLGMHKLAQDFGIAVWHGHELLKKMLTAKLITNEQVKSIFEALELNGDL
TETWRQAKHIAFLKLFGKG
>tr|A0A0Q0E0I9|A0A0Q0E0I9_PSEAP Sucrose-6-phosphate hydrolase OS=Pseudomonas syringae pv. aptata OX=83167 GN=ALO85_03258 PE=3 SV=1
MSFSVNAMSNPMSPALERAHHALEEGLSQLVHDYRPGYHLAPPAGWMNDPNGVVFFRGEY
HVFYQHHPFDAKWGPMYWGHAKSADLVHWQHLPIALAPGDDFDRDGCFSGSAVVCGDTLA
LIYTGHTWLGEVGDERFIRQVQCLATSTDGISFVKHGAVIDTPPQDEMIHFRDPKVWRQD
GHWYLIAGARLGDKPLLPIYRSTDLHAWEFVSYVSTGAEGDGYMWECPDLFGLNGRDILL
YSPQGMPALRYERLNRFHTGYRVGQVDSQWQFTGGPFIELDSGHDFYAAQTLVAADGRRL
LWAWVDMWESPTPTEAHHWRGMLGLPRELEVRENRLCVQPARELTALRNESLPGTPWWSE
AGSQWLADIHGDMLEIHVHLDLLDCTEGHLGVALRCSQDGQEQTLLYYDAALKRLVLDRD
QSGTHVSGQRSVAIDPQQDRLELRIFLDRSSIEVFEQNGSFSFSSRLYPQTDSLGVKLIA
NGTGGRVCIADAWQLSSGYLSVNH
>tr|A0A0Q0BTM4|A0A0Q0BTM4_PSEAP Acriflavin resistance protein OS=Pseudomonas syringae pv. aptata OX=83167 GN=ALO85_04275 PE=3 SV=1
MNGRGSVSAWCIDHPVATLLLTFALVLLGVIAFPRLPIAPLPEAEFPTIQVTAQLPGASP
ETMASSVATPLEVQFSAIPGMTQMTSSSALGSTNLTLQFTLNKSIDTAAQEVQAAINTAA
GRLPADLPSLPTWRKVNPADSPVLILSVSSSLIPGTELSDVTETILARQLSQIEGVGQVF
ITGQQRPAIRVQAAPEKLAALGLTLADIRQAVQQTSLNLAKGALYGKDSISTLSSNDQLF
KPQDYAQLIVSYKNGAPVQLKDVARVVAGSENAYVKAWSGDQQGVNIAIFRQPGANIVDT
VDRIQGELPRLQEMLPASVDVSVLNDRTRTIRASLHEVELTLMIAVLLVVAVMALFLRQL
SATLIVSAVLGVSLIASFAMMYLFGFSLNNLTLVAIVVAVGFVVDDAIVVVENIHRHLEA
GQGMREAAIKGSGEIGFTVVSISFSLVAAFIPLLFMGGVVGRLFKEFALTATATILISVV
VSLTLAPTLAALFMRAPTHHLHQKPGFGERLLASYERGLRKALAHQRLMLGVFGLTLALA
VVGYILIPKGFFPVQDTAFALGTTEAAADISYPDMVEKHLELAKIVGADPAVLAFSHSVG
VSGSNQTIANGRFWISLKPRAERDVSVSEFIDRLRPKLAKVPGIVLYLRAGQDINLSSGP
SRSQYQYVLKSNDGELLNTWTQRLTEKLRSNPAFRDMSNDLQLGGSVTHIDIDRSAAARF
GLTTADVDQALYDAFGQRQISEYQTEVNQYKVILELDAQQRGKAESLAYFYLRSPLTSEM
VPLSALAKVSAPRMGPLSISHDGMFPAANLSFNLAPGVALGDAVRMLDQAKNEIGMPASI
IGSFQGAAQAFQSSLANQPWLILAALVAVYIILGVLYESFVHPLTIISTLPSAGIGALLL
LWMMGQDFSIMALIGVVLLIGIVKKNGILLVDFALQAQREQGLTPHEAIYEACITRFRPI
IMTTLAALLGALPLMLGFGVGSELRQPLGIAVVGGLLVSQMLTLFTTPVIYLQLERLFHR
RHPAPDAPALALHK
>tr|A0A0Q0FP17|A0A0Q0FP17_PSEAP Insecticidal toxin protein OS=Pseudomonas syringae pv. aptata OX=83167 GN=ALO85_05109 PE=4 SV=1
MSTNSSTTRWGVRPSQSPPKAGCADRPIGSGTPSVRTKTIQPRKCWPRERQPSVVNSAVH
SHTPELLVSEVRGLVVRQVLLHRTEVAEAAAMRVTKRRFDLAGRMIAATDPRLADANRST
VYSLGGNALATESVDAGWRVALFGEAGQVLNGWDARGNERQLEYDLLLRLRNIIEQNRCA
ERFTYGQTDAAGHNQCNQLVRHDDTAGSRLLQDYSLHGSVLSETRHFMLAAEAADWPSAE
PDRNELVEPAGLQTCRVFNAQDEVLTQTDASGNSQLSTHNLAGQLHSTDLILNDSMHART
LVSAIRYNAFNQVEQETAGNGVVSLYAYDQQDGRLIGLSAISADGTLLQQLNYSYDPVGN

ILLVNDTSQPDRYCDNQLIEPISHYRYDTVYQLIEATGREVRNGASHGPALPGLQPVPTL
DPCQVSNYTQSYSYDAAGNLLQMRHEGSHAFTRNMHVAPDSNRSLPDDDGDVDFATSFDA
NGNMLKLVRGQAMGWDARNQLALVTLLQRKDAASDEERYVYDGQGQRCRKISTAQASGRT
LINEVRYLPGLEIRTTADGEILHVIIAQAGRSSVRVLHWQAGKPYAINNDQVRYSLGDHL
GSNTLELDQQGGLISQESYYPFGGTAWWAARSAVEAKYKTVRYSGKERDASGLYYYGFRY
YAPWLQRWINPDPAGDVDSLNAYNFVSNNPVMLIDKDGRVGERIAAAYAPNEPALPSYFK
DTYLSEASNDQMTADARAGALWNDPPRFLGAGYAYAPAWYYNANLQQRIDLLNERSWMSH
GIVTTLFNHDHDPVKKPYEITSRHWNNVDEFTNTYTPEKWMIQSNFKASKSNDYHASDVI
RYQYETVSKSLGFFGVLPSRVENQYVMNTKTLSTTSGLESSTPHLLDLYLNETPIGKGTS
ISLTEFQMEAMWVQRQIDPVLDPISHFTLGVKPINHFKL
>tr|A0A0Q0DLZ1|A0A0Q0DLZ1_PSEAP Multi anti extrusion protein MatE
OS=Pseudomonas syringae pv. aptata OX=83167 GN=ALO85_01358 PE=4 SV=1
MSTVSGRIETLGHPAFDLATMPALPGVLMSTLLTDWRHRSTHRRVWALATPMILSNISVP
LVALVDSAVIGHLPHAHQLGAVAVGATLYTFLAWAMGFLRMGTTGFAAQAAGRSDSAALR
RILLQGLLLAVGLALLLGVIALPFSHLALTMMQPSADLQQMTLDFFHTRLFGLPAALASY
ALVGWFLGAQNARAPLAILLVTNAVNIVLNLWFVMGLDWGVVGSARASVLAEWTGVVLGL
LLARNTLRAWPGRIVWSALRLWSNWRPLLAVNRDIFLRTLALQSVMFLITVQGARLGDAT
VAANALLLNGLLLTAHALDGLAHAVEALCGHAIGARDRDTLRRSLVVAGGWSLISSVVFA
LFFLVAGHGFVSLQTDIPEVRATAMMYLPYLALMPLLAVWSYLLDGLFIGATRAREMRNA
MLFSAACIAPLAYLASSHGNHALWLTFLAFTLLRGLSLGTIAWRLTRSDGWFVH
>tr|A0A0N8T9U8|A0A0N8T9U8_PSEAP Adhesin/filamentous hemagglutinin,
ShlA/HecA/FhaA family OS=Pseudomonas syringae pv. aptata OX=83167
GN=ALO85_03579 PE=4 SV=1
MNNAIASKEKLNLAVGVSLSSQQVAALTHDIVWLEEHEVNGEKVLVPVLYLAQADNRLGP
TGALIAGNDVSLIAGQNLDNVGTLHAANNLSAVAGNNLSAVAGNNLINTGLIEAGNRLDL
LAGNDLINTAGGIIKGRDVSLTSINGDVINERSITSMDNSVRGQRHNEFADSAARIEAAN
DMSVSAGRDVINKGSVLESGRDMSIQAGRDVTIAPTEVTNSLFSDSKHNSSDITQLGSTA
SAGRDLTVQAGRDIAIIASQIDAKRDIAMAATENLTISSAADEEHSLSKSKKLTRQEDHV
SQVSSDLDAGGSVALQAGQNLSVISSRITAGKEAYLVAGENLDILAAQDSDYSLYDKKKR
GSFGAKNFKRDEVTDTRNISSEITTGGNLTLASGGDQHYQVAKLNSGNDLLINSGGAIDF
EGVKDLHDESHTKNKSSAAWFSTKGKGTTDETFRQSELVAKGEVAIQAVNGLRIDVKQVN
QQSVSQSIDAMVKADPQLAWIKDAEKRGDIDWRQVKEIHESFKYDNSGLGPAAQIVVAIL
MAAVMGPAGFGLSAGIGGAVATSVATTAVTSTINNKGDLGAVFKDVTSSSAIKGYAMAGV
MAGFVPTINPKNLGLDLASVQTVATKVITESVIKTAIMGGSFKDNLGSSIVSTGIATGGA
IAAGKIGDFTLFDEGKLTKVGMHAVLGGLMSEAMGGDFRTGALAAGANEMVVDYLAQRML
PSDLGKNSQAYQGGVSKLMTASELIGALTAAATGGDASAAAAVTANSTQYNYLAHNQLKE
AAEKLRKCAPAECDSIVAAYKKVSEDQTIEAIINCRFDVSLCKPSSRDVANTVADLSSVY
DALGDGSKYAKDSLQILINENLEFQETLAVATTGASAQAVAETLQAKLKLTPEETVELAE
AIGGGLAAAAGGGCIPQGACESVKAA
>tr|A0A0Q0ICZ3|A0A0Q0ICZ3_PSEAP HNHc domain-containing protein
OS=Pseudomonas syringae pv. aptata OX=83167 GN=ALO85_04722 PE=4 SV=1
MPLSMRTRTGDTMSTANPPSHTAKLDRILADAQRDREMGYRDKALRMYPHVCGRCAREFA
GKRLSELTVHHRDHNHDNNPQDGSNWELLCLYCHDNEHSRYTDQQYFADGSLGSPKTAKA
THNPFAALAGLMKKDE
>tr|A0A0Q0CIV6|A0A0Q0CIV6_PSEAP Oligopeptidase A OS=Pseudomonas syringae
pv. aptata OX=83167 GN=ALO85_04901 PE=3 SV=1
MRMATIGSFFGSDRRVERVWLLRSASIVIKMAECFFKARCKTVSANNPLLQSYDLPPFSA
IRAEHVKPAIEQILADNRAAIADILAKQGSTPTWAGLVLTMDELNDRLGAAWSPVSHLNA
VCNSAELREAYESCLPALSAYSTEMGQNRALFQAYEALANGPEAAGFDVGQKTILEQSLR
DFRLSGIDLPPEQQKRYAEVQSKLSELGSQFSNQLLDATQAWTKLVADESALAGLTDSAK
QQMAAAAKAKDLEGYLITLEFPSYYAVMTYAEDRALREEVYAAYATRASDQGPNAGKNDN
TPVMEQILDLRQELAQLLGYANYAELSLATKMAESSDQVLSFLRDLAKRSKPFAAQDLEQ
LKAYAAEQGCPDLQSWDSGFYGEKLREQRYSVSQEILRAYFPVDKVLGGLFTIVQRLYGI
EIAEQKGFDTWHPDVRLFEIKENGQHVGRFFFDLYARANKRGGAWMDGARDRRRTAQGTL
QSPVANLVCNFTPAVAGKPALLTHDEVTTLFHEFGHGLHHLLTRVEHAGVSGINGVAWDA
VELPSQFMENWCWEPEGLALISGHYETGEPLPQDLLEKMLAAKNFQSGLMMVRQLEFSMF
DFELHATHGDGRSVLEVLEGVRDEVSVMRPPAYNRFPNSFAHIFAGGYAAGYYSYKWAEV
LSADAFSKFEEDGVLNAETGRAFREAILARGGSQAPMVLFVDFRGREPSIDALLRHSGLS
EDAAA

>tr|A0A0N8T9S2|A0A0N8T9S2_PSEAP Methyl-accepting chemotaxis protein OS=Pseudomonas syringae pv. aptata OX=83167 GN=ALO85_03747 PE=4 SV=1
MKMLRQLKIAARTSACFALMVVLVFGLGIFSIQQLHGIREQSLEIENDALPGIALGDDIA
LAVEKTRTTVAKMLASHDPAQVTLAHTELLEKKAGFQKAVEAYDPLITEDDERALVEGLK
STYQGYIDRAEKVYTLINENQADAGRALVWGEMKGMAERIEAALGKLEKINDDSEAESSA
AATSVYENAVAVTQAVMFLTVLLTVLLAWRLTKSLAVPISQALHSSETIAAGDLRPSAIN
REGTDEAALLLQSMERMRGNLNQTLTQVGDAAHQLASATEEMSALMVNSNADLVVQNSEI
EMAATAVTEMSQAVDEVARNAVTTSVESKASSVSAREGQEELNQTVQSILELTRNVGTAS
TEAQALATRTLDITKVLDVIRAVSEQTNLLALNAAIEAARAGDAGRGFAVVADEVRALAH
RTSESTREIETMIGHIQQGTKSTLVALEVSTEQAQRTKQQAESANAVLASIASSVMVIDE
RNTVIASASEEQALVAREVDRNLVRIRDLSAQSAVRTSQTGSASQSLAELASDLTTTLGQ
FKLR
>tr|A0A0Q0DMD1|A0A0Q0DMD1_PSEAP Probable sugar efflux transporter OS=Pseudomonas syringae pv. aptata OX=83167 GN=sotB PE=3 SV=1
MITSPPNEQAGSWLGVFALALAAFIFNTTEFVPIGLLSNIGQSFEMTPAQVGLMLTIYAW
VVSLMSLPMMLATRNIERRKLLMFVFGLFVVSHVLSAIAPSFAILLVSRVGIAFAHAVFW
SVTASLAVRIAPPGKQVQALGLLATGTSLAMVLGIPLGRVLGEALGWRTTFLGIAGIAAL
VVFLLVRALPLLPSQNSGSLRSLPILFKRPRLMAIYLLTAIVVTAHFTAYSYIEPFTQTV
SQLSGEMTTILLLVFGGAGIMGSIIFSLFSDRFPNGLLITAIGTLTLCLLMLLPLSGDAT
TLGTLTIIWGMAIMCFGLTLQARVLSLAPDATDVAMALFSGIFNIGIGGGALLGSVVSSH
LGVANVGIVGGLLALGGLGLCCYTTHHFGKVRPAAEMQAESK
>tr|A0A0Q0BW61|A0A0Q0BW61_PSEAP Pyoverdine sidechain non-ribosomal peptide synthetase module I OS=Pseudomonas syringae pv. aptata OX=83167 GN=ALO85_01287 PE=4 SV=1
MNPEHAQKLARRFVELPLEKRRLFLDGMRKENMDFSLFPIPSCAGLAERDGLSYAQQRMW
FLWQLDPHSAAYNLPMSVCLNGPLELPLLERAFSALVERHESLRTTFGQEGDRAFQRVAP
PAPVSIRLTDLSALPPEQRWASARQAMAEQAAQTFDLQRGPLFTVQVLRLAEQEHLLLLN
LHHMITDGWSMNVLIDEWLRGYDALLAGKPLPFQPLLVQYRDYAVWQRCWLEAGEQARQL
DYWRSHLGEEHPLLELPTDRPYPALPSHDGARLELALEPELLRNLKSLAQRQGVTLFVVL
LATFKSLLHRYSGQTDIRVGGLIANRTRSETEGLIGCFINTQVLRSEVTAQTRFVDLLNI
VRDASTGAQAHQELPFDAIIDALQPERSQSHNPLFQVMFNHQPVVADLLDKQLSGGLRVA
NLPAEQQALAQRSHAAASDLMLATSGEGEQLHAAFTYATDIFDESTIARLAGHWCNLLAS
VCADPLQTIAELSMLSADERKQLLAVDSAANADSNTAAHRLFEAQVQRTPDAAALILASE
GQSPGLSYTELNQRSNRLAWQLREQGVGADVLVGVALDRSLDMPVALLAVLKAGGAYVPL
DLNAPSERLRHVLADSGVKLLLTHSDQLTRLPELTDMQCLCIDRMNSETASVQNLDGPID
PASLAYVIYTSGSTGRPKGVAISHAALAEFVTLGANYSDLREGDRVLQFATHSFDGFVEQ
FYPPLCRGAAVVLRDERLWDSATFHQAIVEHGVTLADLPAAYWLTLVQDFAASPPAHYGA
LRQVHVGGEAMAVEGLRLWHKAGLGHVRLLNTYGPTEATVVSSIHDCSTLTPQQVSWRGV
PIGQALAGRRLYVLDDQMNLLPQGAVGELYIGGPGLARGYHAQPGLSAERFVADPFVSGE
RLYRSGDRARLRADGAVEYIGRVDHQVKIRGFRIELGEVESRLQQCTGVREAVVLAVELA
GSTQLVAYAVPDVAASTEAEQLALRQSIRSQLQAFLPDYMVPTHMLLLPELPLTPSGKLD
RKALPAPDASQLQARYRAPHSEVEICLASIWADVLHAPQVGLDDHFFELGGHSLLATQVI
ARIKTQLGISLPLRTLFEKPLLSELAVEVAALTDNSMDNDWSDMDQFMDSLEEFGA
>tr|A0A0Q0BX07|A0A0Q0BX07_PSEAP Two-component system osmolarity sensory histidine kinase EnvZ OS=Pseudomonas syringae pv. aptata OX=83167 GN=ALO85_01600 PE=4 SV=1
MKTPLWFPQSFFSRTLWLVLIVVLFSKALTLVYLLMNEDVLVDRQYSHGVALTLRAYWAA
GENDREAIAEAAGLIRVVGGGVPEGEQHWPYSEIYQRQMQAELGADTEVRLRVHAPPALW
VRAPSLGDGWLKVPLYPHPLRGQKIWNVLGWFLAIGLLSTASAWIFVRQLSQPLKRLVFA
ARQLGQGRSVRLPVSDTPSEMTEVYRAFNQMAEDVEQAGQERELMLAGVSHDLRTPLTRL
RLSLELMNDNEFSEGMVRDIEDMDAILDQFLAFIRDGRDEEIEEVDLGHLVRDVVAPFND
DEDSIRLCLEPIPPFPLRRVSMKRLLTNLIGNAKHHAGNGIEVAAYVSGDRNAPYVVLSV
LDRGAGIDPSELDTIFNPFIRGDRARSGKGTGLGLAIVKRIAAMHGGNVELRNRSGGGLE
ARVRLPLGLMLPRDAI
>tr|A0A0Q0DKA7|A0A0Q0DKA7_PSEAP MotA/TolQ/ExbB proton channel OS=Pseudomonas syringae pv. aptata OX=83167 GN=ALO85_200002 PE=4 SV=1
MLFVVMLGGKHPKAKIEVHDVVFAAADSLEATYPQLRESWFGSSKGLHIDAWMAVDGIED
WKVELSPLAPRPGSPRLFFLNLGGYESGVFGEAHHYLLVVAGDRREAAAKGKLQILSHWT
QAHTDVVLDVDDCLPIDEVNGRYVHLAEGKHRGVAQRNDYIVLPR

>tr|A0A0Q0BTH9|A0A0Q0BTH9_PSEAP Pyoverdine biosynthesis regulatory protein OS=Pseudomonas syringae pv. aptata OX=83167 GN=ALO85_01286 PE=4 SV=1
MSHALSLDIRPLLAGAGSLPMLVQAPEPGLDLMEALGELKPLVAEHLYSAGGILFRGFEV
GGAEAFREFAAGFGDPLLNYEFGSTPRSNVTKGVYTSTEYPAHQSIPLHNEQAYTLEWPM
KIWFYSMIAAQTGGETPIADSREIYRRIPARIRERFVEKKLMYVRNYGNGLDVEWSQVFN
TDDESVVEAYCRAHNIECEWKDDGELRTRQICQAVSRHPVTRDTVWFNQAHLFHISNLQP
EVRETLLDVVDEEDLPRNVYYGDGSPLEETLLDEIRGVLDECTVSFPWLENDVLMLDNML
TAHSRAPFTGKRKVVVAMAQGHSDK
>tr|A0A0Q0C511|A0A0Q0C511_PSEAP Protein translocase subunit SecE OS=Pseudomonas syringae pv. aptata OX=83167 GN=secE PE=3 SV=1
MNPKAEASDSRFDMLKWLLVVVLVVVGVVGNQYYSAEPILYRVLALLVIAAAAAFVALQT
GKGKAFFVLAKEARAEIRKVVWPTRQETTQTTLIVVAVVLVMALLLWGLDSLLGWLVSLI
VG
>tr|A0A0Q0CXX7|A0A0Q0CXX7_PSEAP Uncharacterized protein OS=Pseudomonas syringae pv. aptata OX=83167 GN=ALO85_05302 PE=4 SV=1
MAAGLAFGVIVSQAGLSTPLSSATFTSGVSAMRWSAVSLFSILGFLAITPATQAAQDYGI
LIISRERLEVATSCEIGIYVQDRLAGRLFQEDSVSFNLPAGEVSVRLSTLRGNVVGCEPG
MEAPRSTKIQLRAGDILKYRIAADLNGMYLKKADLNY
>tr|A0A0Q0IP94|A0A0Q0IP94_PSEAP Lysine/ornithine N-monooxygenase family protein OS=Pseudomonas syringae pv. aptata OX=83167 GN=ALO85_03784 PE=4 SV=1
MGNSTPHTLHSDAVCIGFGPAGIALACAFADAREANDPLGAMSVRYLEAAQDSQWHRELL
LAGTDINHHVFRDLVTPRNPRSRFSFAMYLKEHRRMFDFGLLGRPASRHEWSDYLVWVAA
QVDSQTSFNTPVTGIDPVLRHGRLHELRVCTPDTSFTTRNVVLSSGSAPRIPRAFESLIG
PTLFHTSQFLSRLQAFGEQLPKRWLVLGSGQSASESVLELVSRDAGIEVHSVHRSAGFKL
TQLGQFPNRVFAPDHVDYFHSLDPAARQRFLDWSRSTNYAGIDPDESQKLFSLIYEDAIA
GRKRLQTYAYSVISAIEPVAEGYRVELTDTFSQRTQVLEVDALVLGTGYQQYLIPPLLSG
LQPWLQADVDGGLLIDRDYQVATQGECDVNIWVNGLSERSHGISDSQSFSLMALRAERIA
TALQQAVAPAAEPLLAQWK
>tr|A0A0Q0CE43|A0A0Q0CE43_PSEAP Uncharacterized protein (Fragment) OS=Pseudomonas syringae pv. aptata OX=83167 GN=ALO85_101968 PE=4 SV=1
MEGLGLCKRAAMIANADKSRAQAKTKALFLTHEMHRLHRPLREQV
>tr|A0A0N8T9S3|A0A0N8T9S3_PSEAP ABC transporter OS=Pseudomonas syringae pv. aptata OX=83167 GN=ALO85_05454 PE=3 SV=1
MTPSTRGPDMSENLIEIRDLSVAFNGQKVVSNLSLDVHRGECLALVGESGSGKSVTAHSI
LQLLPRSGTLTTGSITYRGQQLVGADDRTLRKLRGNRIAMIFQEPMTSLNPLHSVARQIG
ETLLLHRGISGREAQKRIVELLELVGIQQPEKRLKAYPHELSGGQRQRVMIAMALACEPE
LLIADEPTTALDVTVQRKILLLLKELQQRLNMSLLLISHDLNLVHSVAQRVCVMRAGEIV
EQSDCKSLFKSPQHPYSRLLLDAEPAGEPLPRDTRETVLQVDNLKVWFSLTGGILRRHKE
YLKAVDDISLSIERGKTLGIVGESGSGKSTLGQAILRLLESRGSIRFRGQALDGLSQKQM
RPWRKEMQVVFQDPYGSLSPRMSVAQIISEGLEVHSQYNPAKCDAEVIRALEEVGIDPQS
RHRYPHEFSGGQRQRIAIARALVLKPALILLDEPTSALDRTVQKQVVALLRELQEKHGLT
YLFISHDLAVVKALAHDVIVVKDGKVVERGASHDVFDAPQHPYTQELLAAAHPE
>tr|A0A0Q0DPD2|A0A0Q0DPD2_PSEAP Ribosomal RNA large subunit methyltransferase M OS=Pseudomonas syringae pv. aptata OX=83167 GN=rlmM PE=3 SV=1
MNTLFMHCRPGFEGEVCSEIADHAARLDVAGYAKARPDTACAEFICTEADGAERLMNGQR
FDKLIFPRQWARGLFLELPETDRISVILAQLAAFPTCGSLWLEVVDTNDGKELSNFCKKF
EAPLRKALTQAGKLVDDPRKPRLLLTFKSGREVFLGLADADNSAMWPMGIPRLKFPREAP
SRSTLKLEEAWHHFIPRDQWDERLSGDMTGVDLGAAPGGWTYQLVRRGMLVTAIDNGPMA
ESLMDTGLVQHLMADGFTYKPRQPVDWMVCDIVEKPARNAALLETWLGEGLCREAVVNLK
LPMKQRYAEVRRLLDRIEEGFQARGVRVSIGCKQLYHDREEVTCHLRRLDVAKAARK
>tr|A0A0Q0C1H5|A0A0Q0C1H5_PSEAP Outer membrane efflux protein OS=Pseudomonas syringae pv. aptata OX=83167 GN=ALO85_01792 PE=3 SV=1
MPRRIIRGLWPVGLLAFTLVLSGCIGTHGITPQGSVLHADSLATDQAIQSASLDAHWPAA
QWWRAYGDAQLDRWVDMATLGSPSLALAAARVRQAKAMAQVAESAESVQVQGNASLMRHD
WPEDQFYGPGALADTRTWDNNASLGLSYALDLWGRERNASEQAVDLAHVSAAEQRQAQLE
LQENVVRAYIRLSLDYARLDIAEAALAQQQQILDLAQRRLKGGIGTHFDVSQAETPLPEA
HRQIDALQEAIALSRNQLAALAGKGPGQGAALQRPALSLHTALTLPSALPAELLGQRPDV

VAARWQVAAQARGIDVAHAGFYPNVDLVGSLSYMATGGGMLEFLTGSKLGYKVGPAISLP
IFDAGRLRAELGRASAAYDQAVAHYNQTLIMALKNVSDQLIRRASMDKQQALAAESVASA
QKTYDIALIAWQRGLTDYLNVLNARTQLFRQQQVQQQVEAARLEAYAGLVVALGGYADGN
NNGNGHFAHTVAESEGGQ
>tr|A0A0Q0IMU1|A0A0Q0IMU1_PSEAP TonB_C domain-containing protein OS=Pseudomonas syringae pv. aptata OX=83167 GN=ALO85_200202 PE=4 SV=1
MSTLILSRRLPPRQLRHWPHQPVGNCNVAPSSLVVQQHSDNAPRTWPHAALAIALAIGLH
AALAAWLMADHPQAPEPTPPMPITVQWVPPPVAPPVAQPTPPEPTPQPPAPAPKPAPPEP
KPAELPKPKAQPRTATSKPVAKPVAPAPSVAAPTPAPAAAPAPRPAPAAPQTTTAPIGRA
GYLNNPPPVYPPAAARRHQEGTTMLRVHVLPNGRTDQVQVLQSSGVPALDEAAQAAVRQW
TFIPAKRGDTPVEGWVNVPMAFKLAP
>tr|A0A0Q0DPJ4|A0A0Q0DPJ4_PSEAP Electron transfer flavoprotein, beta subunit OS=Pseudomonas syringae pv. aptata OX=83167 GN=ALO85_02188 PE=4 SV=1
MLRTHIDRLVPPGDFFSPESEESMKVLVAVKRVVDYNVKVRVKADNSGVDLANVKMSMNP
FCEIAVEEAVRLKEKGVATEIVVVTIGPTTAQEQLRTALALGADRAVLVESAEELTSLAV
AKLLKAVVDKEQPQLVILGKQAIDSDNNQTGQMLAALSGYPQGTFASKVEVTGDKVAVTR
EIDGGLQTVSLSLPAIVTTDLRLNEPRYASLPNIMKAKKKPLEVLTPDALGVSTASTNKT
LKVEAPAARSAGIKVKSVAELVEKLKNEAKVI
>tr|A0A0Q0DMG9|A0A0Q0DMG9_PSEAP Uncharacterized protein OS=Pseudomonas syringae pv. aptata OX=83167 GN=ALO85_03513 PE=4 SV=1
MKASWDIFCSVVDNYGDAGVTWRLARQLVAEHGLQVRLWIDDLSAFVRLCPGADPQARQQ
WQEGVSVCQWPTEWLNTDIPDVVIEAFACRLPTRYTEAMLQRSPRPLWLNLDYLSAEDWV
SGCHGLPSPQSNGLKKFFFFPGFSDTTGGLLREKNLIEQRQVFQQNSAARQAFLSGLGIE
ALADARLISVFAYENPALGSWLDALVCDSRPTHLLVPEGRILGDLQRWLGTKDPLRAGFI
QACGGLTVQVLPFIRQELYDQLLWSCDFNIVRGEDSFVRAQWAGRPMLWHIYQQEDDAHL
PKLDAFLTLYLAGLSPAAAQALNQFWQRWNVGGDLGECWAALAEHWPQIERHAEHWSQQQ
AAQTDLATALVQFYVNSL
>tr|A0A0Q0FBN8|A0A0Q0FBN8_PSEAP Type III secretino system effector protein HopM1 OS=Pseudomonas syringae pv. aptata OX=83167 GN=ALO85_05592 PE=4 SV=1
MTGIRVGGSGSTEIVQANQPQPSAAVTQAHPHAVSPSSNPPLTASQSAAQAPESSAAGAA
RLPVAPRHLPTLEKFRAEQPTVKGTSTPTISANAALLIGSLLQSEKLPFEVMAARLSPER
YALQQFHGSDLQQALGRFAEPGHEPGKAETEQLIKGFAQSLADQLEHFQLMHDATADAFG
PGGLRDRNTLAVSQAALGEYAGRASKSIEAGLNHSLAVLDERIAALDSQLEGATEDSRPG
LLMDRQALETARAMLSDLHADFCKSPEAKRLNAVAAHTQMDALIDKLNVDRNSVGGWKGF
GPIVAAAVPQFMVSMLHLGYIRTATGDAMKDAVPEKSADASMKRALAVGLTAGVAHEGVT
NLLKPMVQAGFQKAGLNERLNMVPLKGIDTDSVIPDPFELKNDNGALVRKTPEEAAEDKA
FVESERAVLNQKKVQVSSTHPLGEMIPYGAFGGGQAVRQMLNDFNLLNGQTLSARAVTSG
IAGAISATTQTIAQLNSTYVDPRGRKIPVFTPDRANADLGKDLAKGLDLREPAVRTAFYS
KAVSGVQSAALNGALPPVAVQPQGASGTLSARNILRNMALAATGSVSYLSTLYANQSVTA
EAKALKEAGMGGAVPMLPRTESAWNTIRHPDRASLPHTFQPDTLGGVPRAVESAYHMGRG
ALQLPTQLVVDTLRGVEDGVSKGVSALRNSRNAPETPLPADNADPVDVELSSMEEGRRG
>tr|A0A0Q0C7K9|A0A0Q0C7K9_PSEAP Putative trans membrane protein OS=Pseudomonas syringae pv. aptata OX=83167 GN=ALO85_100173 PE=4 SV=1
MCDGVIEGVLMSPLDGYTAMPSRQRGAIGLMAALTMGLALLCTLTVIDSGRLYLEKRSLQ
RVADIAALEAAGRRGTCSGTAASAPDFANQSATRNGFVPNTDGRTLVTRCGTLTVDVAGP
RVFVADSTQALAIQVVAAHPVPRSIAAGIGALFEKTPSPPNVTISATAVAASAAPLAALT
IRSAGVTVDSTGAALLNPVVGSLLGGTLNLSVANWQGIADTNLSLLSYLNRLKTDLSLTA
VGYNDVLNTSVSVSQLLQSAINVISPNATLGGAAAIQGLQALKLASGATTVLLGDLLSVQ
SGADLAALNTNLRLMDLVQGIAQLANKKSGVLTATQVNIAGLAQVTTRIQVIEPPQLSAI
GDPSKIDPLDPRNGANRIYVRTAQMRALVSINLPVLGTMTSLVNTAGSVIGSLTPILNSA
LSLNLAGLGTSATCALGLTSCMVTDFKFLTDSSSSAGPRIDMSLSLASADSYVSGFTCTS
NASKTLNVKTDAALLSAKIGSIDSSSAFPASTDPAAITALPVPVIDIGTTTCQKIAGLLG
NCTARKPFGGGGIGLTFDNVSQSALGSSTVTSTTFSSPNLPEISSAPFYLTRVAHTKPST
LLNGTASSVKVNVYKPATSNVLGNVITGAAATLNSLTLALDAIVDDTLPRLLTSVVDPLF
ESLGVGMGATDVGTNLSCNLGQATLVI
>tr|A0A0Q0DWS8|A0A0Q0DWS8_PSEAP Putative superfamily II DNA/RNA helicase, SNF2 family (Fragment) OS=Pseudomonas syringae pv. aptata OX=83167 GN=ALO85_05396 PE=4 SV=1

WRIGQKKDVRVIFFGYIGSSQITCLQLMAKKIAVSQSTSGDVPESGLDSLNRDGDSVEMA
LARQLINA
>tr|A0A0N8T8E9|A0A0N8T8E9_PSEAP Znf/thioredoxin_put domain-containing protein OS=Pseudomonas syringae pv. aptata OX=83167 GN=ALO85_04855 PE=4 SV=1
MTDSFVTQCPHCQTSFRVSHAQLSVARGVVRCGACLQVFNAARQLLEQRASDDSEKEALP
ASPVIAALPEPEPLPTPVPEPEQKPEPQPEPQPEPEKAPEEDDPWQVSELDLDNLNLDEE
LARLEQRETRRPDTFARPVSDIDNDAVSLTAKRDSRQSDEAAWVDTLHNDDVEQLPELHA
EVIPDSDSAEAAEPPEDDKRTEPSLSLDKDLDDDEPPMPVIIQRKAAPAEKVERWSAVDD
DDDDDDHEPEPEPRGKRSRSEPAVRDQTLLDLTDDPLQLDWQRPKPRWGRRLAWGLLIVL
ALAGLAGQYVWYHFDQLARQDQYRPLFQQICPQVGCKVPSKVDISQLKSSNLVVRSHPEF
QGALVVDAIIYNRASFSQPFPLLELRFSDTGGQLIASRRFKPSEYLSGEMAGKEEMPPQT
PIHIALDILDPGAKAVNYSLNFRSPE
>tr|A0A0Q0C2V1|A0A0Q0C2V1_PSEAP Uncharacterized protein OS=Pseudomonas syringae pv. aptata OX=83167 GN=ALO85_200125 PE=4 SV=1
MLGYHLLKENTTQAIFYEYSFHFGRRRADLISAEEGMIIGYEIKSAFDRIDRLADQLNSY
TQLFDFVYVVCDRKNIGAIRKLTPERIGIYLCTPTGIRRIRKAKQIKNFDSITTLDAIPI
ECLRRAFKTNGKSKLEICEQISRTSKREEIKIFFREYIIQKYGAQTAHLQGELSSSIMLD
DVYSLSLAPNKLGT
>tr|A0A0Q0FHW6|A0A0Q0FHW6_PSEAP Cobyric acid synthase OS=Pseudomonas syringae pv. aptata OX=83167 GN=cobQ PE=3 SV=1
MSTLMVQGTTSDAGKSTLVTALCRWLTRQGVKVVPFKPQNMALNSAVTADGGEIGRAQAV
QAQACYLEPHTDMNPVLLKPNSDTGAQVIIHGRAVTTMNAVAYHGYKEIAMQAVLESHRR
LGESYPVIVVEGAGSPAEINLRANDIANMGFAEAVDCPVLLIADINRGGVFAHLVGTLEL
LSPSEQARVKGFIINRFRGDIALLQPGLDWLEARTGKPVVGVLPYVMDLHLEAEDGLDQR
QTDKVEHVLNVVVPVLPRISNHTDFDPLRLHPQVNLQFIGPGQAIPPADLIILPGSKSVR
SDLNYLRNNGWDTAIERHLRYGGKLMGICGGLQMLGEQVHDPLGLEGAAGSSPGFGLLAM
STVLEAEKQLRNVRGRLTLEDAEVSGYEIHAGVTTGPALEQAAVQLDDGRCDGAQSADGQ
VLGTYLHGLFESPAACSALLRWAGLTNVQSVDYHALRERDIERLADLVEKHLDGPLLREL
CGLEAN
>tr|A0A0Q0DS55|A0A0Q0DS55_PSEAP 50S ribosomal protein L10 OS=Pseudomonas syringae pv. aptata OX=83167 GN=ALO85_200081 PE=4 SV=1
MKSVRMLTIITASLFSAGLNIAIASPTSVHLYNWYAFIAPDTPKEFEHETGSKLLIDSFD
SAEIMQSKVMAGRTGYDVVVATSNVLPSLITAGVLQPLDRSQLGNWSHLDSEILAKVSVN
DPGNQYAVPYLWGTTGIGYDVDKIKSALGDDAPVDSWDLVFKEENISKLQQCGVAMLDSP
SEIISIALHYLGLPHNSKNPDDYQKAQALLLKIRPYVRYFDSSKIDADLADGNICMVVGW
ANGALAAQELNEKNKTGRRIAYSIPREGALVWSENMVLLKDAPHPQEGMAFINYMLRPEV
IAKTSNHTLYPSANKDAKQFVEPKLRNNASVYLDKDTISRLFPLEPLPLKLERIRTRAWV
KVKSGI
>tr|A0A0Q0BY48|A0A0Q0BY48_PSEAP 30S ribosomal protein S1 OS=Pseudomonas syringae pv. aptata OX=83167 GN=ALO85_03525 PE=3 SV=1
MSESFAELFEESLKTLNLQAGSIITAIIVDIDYQAGWVTVHAGLKSEGLIPLEQFHNDAG
ELTIKIGDEVHVALDAVEDGFGETKLSREKAKRAECWIVLEAAFAAEEVVKGVINGKVKG
GFTVDVNGIRAFLPGSLVDVRPVRDTTHLEGKELEFKVIKLDQKRNNVVVSRRSVLEAEN
SAEREALLESLQEGQQVKGIVKNLTDYGAFVDLGGVDGLLHITDMAWKRIKHPSEIVNVG
DEIDVKVLKYDRERNRVSLGLKQLGEDPWVAIKARYPESTRVTARVTNLTDYGCFAELEE
GVEGLVHVSEMDWTNKNIHPSKVVQVGDEVEVMVLDIDEERRRISLGIKQCKSNPWEDFS
GQFNKGDKISGTIKSITDFGIFIGLDGGIDGLVHLSDISWNEVGEEAVRRFKKGDELDTV
ILSVDPERERISLGIKQLESDPFSEYVTVNDKGAIVRGIVKEVDAKGAIITLADDIEATL
KASEISRDRVEDARNVLKEGEEIEAKIISVDRKSRVISLSIKSKDVEDEKEAIQSLRSKP
EVAESTGPTTIGDLLRAQMEKQN
>tr|A0A0Q0CXI5|A0A0Q0CXI5_PSEAP Alpha/beta hydrolase family protein OS=Pseudomonas syringae pv. aptata OX=83167 GN=ALO85_01203 PE=4 SV=1
MLSGISLLRVPNALRLLALPITLILSLAAATASAAAPSAVQRPISVDTENGKLYGTLLMP
RSDKPVPVVLIVAGSGPTDRDGNNPEGGRNDSMKRLAVILASNNIASVRYDKRGVAASKA
VTPDERNLSVERYVADVQLWARALKANPRLGQLILLGHSEGALVATLAAEKVGAAALISV
AGTGRPVDQVLREQFQERLPPDLLQRSDQLLDELKAGKTDDNVPAELEVVFRPSVQPYLI
SLFRQDPAAAFGALHIPALIVQGRNDIQVGVGDALLLQKAKPDAQLALIDGMNHVLRIVP
DDLQQQLLSYRNPTQPLASEVTERILSFIKALPHPEKRSDAR

>tr|A0A0Q0DQQ8|A0A0Q0DQQ8_PSEAP Putative FAD-dependent oxidoreductase OS=Pseudomonas syringae pv. aptata OX=83167 GN=ALO85_05540 PE=4 SV=1
MVHQAGFEPTTPAFGGQYSIQLSYWCDAGAMILTWVRGVQLRAVCDTNHPARPATAFHHP
TPFVVHKGTAHRYRHNRQLVHDAPHTTPSVRSHRGPLINQFKSIERSPCRVLITGASIAG
CAAAWWLTQRDCDVTVVEQAPAFRDGGQNVDVRGAAREVLRLMGLEQAVKDLNTGETGIA
WVDEDNRTAARIDLAGLDGDGPTAELEVLRGDLARLLYEASSADAFYRFGDRIVSVEHDA
EGVSVTFEGGGEERFDLLIIAEGVGSRTRELVFPGENQPRRMDLASAFFTVPRAPTDSQT
ARWYNAVGGRSAGVRPDNRSTTRAFFNVQGRSHLTVGNSVAEQKAFLQASFAGAGWEVPR
LLEGMEAAEDFWFDDLRQVKIDRWSNGPVVLLGDAAWCVTPLGGVGASLSLIGAYVLAGE
LTKTDTIAEALASYEYVLRPLVKKSQSVPKLVPRLVHPRSQTGVKILRAVQRLVATPFIS
KRAARALTPAVQSFTLPDYR
>tr|A0A0Q0FJC9|A0A0Q0FJC9_PSEAP Regulatory protein, LuxR OS=Pseudomonas syringae pv. aptata OX=83167 GN=ALO85_00410 PE=4 SV=1
MDDYMPNTLLPQIGRAIASIGSGNFSSMFHKLIDTQLAVDATHLSSLPQPWQTPPASAPT
VFNETVTSSRGTLLLSDSIHLYPDQASENFSCKITVFRALPAHEFSEKERRQLGDISPLL
FSILEKHVNALQSAMPGIEHKKPESIEKRFQERLRETGLTLSERETQVCLGLLAGHTALE
QAERLALKVNTVGSYQRRAAIKLGISGRNSLMRWMYGSSVGVFSTC
>tr|A0A0Q0DF30|A0A0Q0DF30_PSEAP CDP-diacylglycerol--serine O-phosphatidyltransferase OS=Pseudomonas syringae pv. aptata OX=83167 GN=ALO85_02995 PE=3 SV=1
MSERPEEPIKASDAESLLPIDEHVEEGHDAEGRQVRHRGIYLLPNLFTTANLFAGFYSII
SSMSAQSAMSAGDTAGASKYFAFAAIAIFVAMVLDGLDGRVARMTNTQSAFGAEYDSLSD
MVAFGVAPALLAFGWALGDMGKVGWMVAFIYVAGAALRLARFNTQVGKADKRYFIGLASP
AAAGVVAGTVWAFSDFGIQGSKLSFLVALLVAAAGMLMVSNIKYNSFKELDLKGRVPFVA
ILAVVLVFAVVFSDPPRILLLIFLAYALSGPIQYLLRRRKA
>tr|A0A0N8T8V8|A0A0N8T8V8_PSEAP Prophage PSSSM-04, tail protein OS=Pseudomonas syringae pv. aptata OX=83167 GN=ALO85_00431 PE=4 SV=1
MVVIRTAEHYAGQLQALLPPGPAWDPERVPELQQVITGLSREFARIDGRAFDLLNEMDPA
TVSELVPDWERVMNLPDPCLGLKPLFADRRLSVRQRLVATGGQNAAFYIDIAISQGYPDA
TVTEHRAPRMGRSRFGQAHFGTWSAQFMWTLNTGGRQRLGRRFGASYWGERFGVNPGLAI
ECLIRRAAPAHSVEFVNFN
>tr|A0A0Q0DBD0|A0A0Q0DBD0_PSEAP Phospholipase D/transphosphatidylase OS=Pseudomonas syringae pv. aptata OX=83167 GN=ALO85_02585 PE=4 SV=1
MLVALCVSGCAHQQVASEPSQAMPSSGSAFGRSIQAMAMPHEGRSGFRLLPDSSDAFKAR
AELIRNAKSSLDLQYYIVHDGLSTRALIDELLKAADRGVRVRILLDDTTSDGLDQAIATL
AAHPNIQIRLFNPQNLGRETGITRSLGRLMNLSRQHRRMHNKLWLADSSVAIVGGRNLGD
EYFDAEPNLNFTDIDMLSVGPVAEQLGHSFDQYWNSTLSKPIGEFIYFLPDGEDLAEARR
KLDDSLEQAHQQHKALYERLMAYKTQPRMKTWLNELVWAYNQALWDAPTKVLAQGEPDPH
LLLTTQLAPELLNTHKELMLISAYFVPGQEGLLYLTGRADAGVNVSLLTNSLEATDVPAV
HGGYAPYRKALLEHGVKLFELRRQPGDTETMRGSGPHLFKKSHSLVSSESSLHSKAMIFD
RQKVFVGSFNFDPRSVLWNTEVGVLVDSPQLAEQLRELTLQGMAPSLTYEARLEDGKVVW
VTEDNGQIHTLHTEPGDWWRRFNAWMSRAIGLERML
>tr|A0A0Q0IRN7|A0A0Q0IRN7_PSEAP Uncharacterized protein OS=Pseudomonas syringae pv. aptata OX=83167 GN=ALO85_200135 PE=4 SV=1
MTRTPVVCVSHKSITSGSGGRPVTEATRIRCLNWLGRREVARRIDAVIGGAVRIDDESRT
ILREFAAVS
>tr|A0A0Q0DPS7|A0A0Q0DPS7_PSEAP Uncharacterized protein OS=Pseudomonas syringae pv. aptata OX=83167 GN=ALO85_05469 PE=4 SV=1
MGSGKAWDVSSNYQARGGFDRRCFQKRVPLALSSRSGRPTVNPTNHKAMTPRTMAAVISS
TVWMLPSRTNDSTTAGMTLADSKANVMKIVMAFLNTPGKYLIVGTGTPRFGSKALAGQAL
TDAIGYKDWVLSYVSQFVPVVNIWDGFTEAITVEGLHPNILGADFISSRVVPIITANFEF
PGIPLPTDAGDVYSAIRPFGCLNANPLLAGTGGSLPAGVNAAAGSVLADGYKALGSGLAG
ITTRWFEEPAAYGEARCIELRGKMAAAGGYIYMQPTANVIQTNLAAGDVIEMVSAVEIMG
SSRGILAWEAELTITKTVSGASSTFYYRSMDKYQEPFTMPASFAGALETQRSTID
>tr|A0A0Q0IRF9|A0A0Q0IRF9_PSEAP Amino acid adenylation (Fragment) OS=Pseudomonas syringae pv. aptata OX=83167 GN=ALO85_05564 PE=4 SV=1
MSMIELLALLEEKDVQLAVKGDQLLVSGKRQSLMEPSVVAMLRENKAALIELINAGEYYS
GKADEVDVPAQAIVPGCERITPDMLPLIELDQAAIETIVARVPGGVPNVQDIYPLASLQE
GILYHHIAAEQGDPYVLQAQFTIASRERFDEFTAALQQVINRHDILRTSVVWEGLDEPVQ
VVWRKADLVLSETTIDPAAGPASEQLQQRFDPRHYRLDISQAPLLRLAFTHDEVNQCWVA

MLLFHHIAIDHAALDQVHHEIHAYLYGKAHTLGEPVPYRNYVAQARLGVTNKQHEGFFRE
MLGDVDEPTLPFGLQDVRGDGHGIEEAHQPLPAELSQRLRAQARLQGVSAASLHHLAWAR
VLGCLCGRNDVVFGTVLLGRMRGGEGVGRALGMFINTLPLRVDVGEQDVRAGVKATHARL
TALLGHEHASLALAQRCSGVSAPTPLFSALLNYRHSSAGAASDQEVTDQADQVWEGIEVR
GGEERTNYPLILSLDDLGEGFSLNVQAVAGIGAQRVCGYMQTALESLVHALEQTPQAPLN
SLLILPADEREQLLVDFNDTSLDYPQQQTIHGLFEAQAERTPEALAVVHGEQRLTYRELN
EQANRLAHALRKQGVQPDSRVGICVER
>tr|A0A0Q0CC79|A0A0Q0CC79_PSEAP Permease OS=Pseudomonas syringae pv. aptata OX=83167 GN=ALO85_02460 PE=4 SV=1
MARLPFARLLSLAVRQLMRDARAGELRVLFFALLVAVAASTAIGYFGARLNGAMLLRATE
FLGADLILEGSSPARAEQVEEGKRLQLKHAQIVVFSSVIATDAGIQLSSIKAVDDAYPLR
GELKSAADLYQPEQAGSGPQPGEAWVEARLLPAVDLKVGDDLDVGSKTLKLSRILTYEPD
RAGNFYSLTPRVMINLQDLAATGVVQPGSRVTYREMWSGPPQALAAYRKAIEPGLEPHQE
IKDARDGSQQIGGALGKAERYLNMASLVAVLLAGVAVALSASRFAARRFDASALLRCLGL
SRGETMTLFSLQLAIIGLLASLSGALLGWLAQLGLFALLQNLLPATVPPGGVLPALAGMG
TGLVALAGFALPPLAALGRVPPLRVLRRDMLPIPASSWLVYGAALLALGLIMWRLSLDLV
LTFALLGGGIVAALLLGSLLLLALNSLRRLLSGASLPDNRWLSA
>tr|A0A0Q0D6J2|A0A0Q0D6J2_PSEAP AmpG-related permease OS=Pseudomonas syringae pv. aptata OX=83167 GN=ALO85_05098 PE=4 SV=1
MLLPRLLGGYSGVVVEKYGYHNFFLITALLGIPTLVMIILQWNREIRTAKTEPPQEASVI
DKP
>tr|A0A0N8TA97|A0A0N8TA97_PSEAP Histidine kinase, HAMP region: chemotaxis sensory transducer OS=Pseudomonas syringae pv. aptata OX=83167 GN=ALO85_03284 PE=4 SV=1
MKWFYDLKISTKLITSFLVVLALTAAMGVFAILQLGQVNQAAQDIKENWMPSMRAAAGMR
FYAANYRLKENRHIAADAAPEKAQMELEAAEARKQFEARLAIYDKLISSDDDRQLFNSVS
ASWAAYLKVSNDLFDQSRQNLEAQARALLKGESKTHFDEVTTQLQKMVELNEAGATIAGN
KGTSLYETSRISIIVVLIAALLIGLGLALFIARIISRPLKEAATAAEQLAEGNLNAHIGH
GSKDETGMVLNAMRNMVGKLSHIIGEVRNAADNLASASEEVSATAQSMSQATSEQAASVE
ETSASVEQMSASINQNTENAKVTDGMASKAAKEATDGGESVQQTVVAMKKIAQRISIIDD
IAYQTNLLALNAAIEAARAGEHGKGFAVVAAEVRKLAERSQVAAQEIGELSSSSVDMAEK
AGKLLDEMVPSINKTSDLVQEISAASEEQAAGVAQINTAMTQLNQVTQQNASSSEELAAT
AEEMSSQAEQLQQAMSFFTLDASPRAVAQSATFDSRNSPNRQPPRPPQQAPRKAFAQSMA
GAPDESEFTRF
>tr|A0A0Q0DMC4|A0A0Q0DMC4_PSEAP Methyl-accepting chemotaxis protein OS=Pseudomonas syringae pv. aptata OX=83167 GN=ALO85_01087 PE=4 SV=1
MPRFLRRQATTTTLLRRFKITLRLVICFAITSLLMVALGAFCLLQMQAIRTQGEAVESGA
LPSIATADAIAIGLVKLRSETTRLIANADDPGAVINSKINVEQLRNEVEKGFSEYLARVQ
SGTEHDSIVALQDAYKAFMPGLQDQIALIEQNKLDEARMLANTMLSLQGDLMDMQVQLLR
ELNKQSASAAVEAAGASYEQTRIIALSAIGLVLALTLLLAWRLSVSIIHPVRQALHIAST
IADGDLSEHPIPDGKDETAQLLITLGRMRTNLHSTIDQIYAAATQLSQSVQEMGSIAEAS
ALNLQLQNTEIEQAAVAVNQMSQAAIEVAGNASNTVTESEASTQAAAQGQDKLSATILSI
KELTENVLESSHQAQGLAERTQSISSILDVIRAIANQTNLLALNAAIEAARAGEAGRGFA
VVADEVRSLAQRTSASTAEIEGLISGVQQSTQQTASSLRHTATQANLTMEQAASTGEALQ
VIIHSTATINDRNLLIASAAEQQAQVATEVDRNLSSIRELSSQTASGAQQTTVASNALSM
LANDLNLMVQRFVL
>tr|A0A0Q0C2W0|A0A0Q0C2W0_PSEAP Aldehyde oxidase and xanthine dehydrogenase OS=Pseudomonas syringae pv. aptata OX=83167 GN=ALO85_03414 PE=4 SV=1
MSENIIGVPQRRIDGGKKVSGQARYAADHPMGKMLYAYGVYSTIANGRVVAIKDQQAKAM
PGVVDIFHHDNFPSLHRTPNTKLSFAKMLSASKADEHRLPFEDDRVYYPGQFVALVVAES
FEQARAAAYRVTVEYDQRPAVKDLAEGMRVNGARDGGAGHNRGNPDSAFEGAKVKVDQTY
TTPVEVHNPMEMHASTAWWQDGKLFVYESTQGVVNHRNLLANVFDLSPDQVEVRAPFIGS
GFGGKLWPWPHSVAACAAAKVTGRPVQLVLPRAQMFTTVGHRPETHQRLRLATDASGKLV
SIRHESFNNTSMLDDYTENCGGVTKSLYACDNVLVSHKISPINRGTPTSMRAPGAAPGLF
ALESAIDEMALASGMDPLAFRKLNLSDKDQSAGLPWSSNHLPEAIDKAAERFGWHARKAE
VGSMREGDEIIGYGMGACNWEAYQVPTDARVILRSDGTALAQCGLQDIGTGTYTIVAQTV
SQLTGIPMERVEVELGSSSFPAGPVSGGSWVTASVMPAIAGATREALQKLRQYAVSKDAV
FAGQDAEAIKVENGQLVMGEQRASFVDVLNGQRLSRAEGTFQSGMPEAGKYSFRSFGVHF
VEVRWDPGISSLRVSRVVSAIDVGKVVNPLAARNQVEGAIVMGIGMALFEAGEYDPRSGM

PVNNNYAEYVVPVHADQPDIDVLLLDYPDYNLGEFGARGIGEIGVTGLAAAVANAVYHAT
GKRVRSLPISKEKLMAGL
>tr|A0A0N8T9B4|A0A0N8T9B4_PSEAP Trans membrane pair OS=Pseudomonas syringae pv. aptata OX=83167 GN=ALO85_02611 PE=4 SV=1
MIIPPSIITKGNVVMSNSQPHIDRQPGVASKTLRERAMHAALFEIGGVILVAPLLAWLMD
QSLAMMGAMTVMISTIAMLWNMTYNALFDRLRNRYGFTMSLTVRVLHATGFEAGLILAVV
PLAAWWLTISLMEAFWLDIGLLLMFLPYTLLFNWAYDTLRERLVQRRLARCEV
>tr|A0A0Q0C307|A0A0Q0C307_PSEAP Polyphenol oxidase OS=Pseudomonas syringae pv. aptata OX=83167 GN=ALO85_100639 PE=3 SV=1
MGIAIARRFRLVAVLAQAGSRGVHRVNDWLIPDWPAPAQIKSCVTTRSGGVSLAPFDSFN
LGDHVDDSPQAVATNRQRLTSSLNARPAWLKQVHGVDVVPADPSRVVEADASWTSTPGVA
CTIMTADCLPALFCRRDGTRVAAAHAGWRGLAAGVLEATANSLQSAPEDIMVWLGPSIGQ
PSFEVGPEVREAFTASHPQTAAAFVASSNPDRFMADIYALARLRLAAQGISAVYGGGLDT
FTDSRFFSYRRAARTGRFASLIWIEHA
>tr|A0A0Q0DYC3|A0A0Q0DYC3_PSEAP Amino acid adenylation (Fragment) OS=Pseudomonas syringae pv. aptata OX=83167 GN=ALO85_05282 PE=4 SV=1
GVKEAVVVAREDNPGDKRLVAYVVAQPQSQLTAAELRAELAPQLAEYMLPSAFVLLDELP
LTPNRKLDRKALPVPADDAFASREHVEPQGATEIALAQIWQNLLDLEQVGRHDQFFELGG
HSLLAMRLISQVRHQLGVELGLAALFAHPELSTLAAAIAQAGRSNLPDIVPVARDQAWPL
SFGQQRLWFLAQMEGASAAYHIPAGLSLHGKLDLKALQRALERIVARHEGLRTTFMQGDD
GQPVQRISPAHTGFNLQVHDLQGLADAEEKLQALASEESLQSFDLQQGPLIRGRLIRMAE
DHHVLLLTLHHIVSDGWSVGVLTRELSALYAAFSQDQDDPLAPLDLQYLDYAVWQRRWLS
GDLLQQQSNFWQQTLADAPALLMLPTDRARPALQDYAGAALPVVFDKDLTRGLKALSQRR
GSTLFMTVMAAWAGLLGRLAGQDDVVIGTPVANRTRSEVEGLVGLFVNTLAIRVDLSDKP
TAETLLARVKQQTLDAQAHQDLPFEQVVEVINPLRSLSHSPIFQAMLSWENNEASDLTLG
DMTLKSIELAADTAQFDLTLDMAEVDEQLVGTLEYATALFDESTMRRYLGYFQRVLEAMV
ADDQQVLEHVNLLGVDEREHLLVGLNATEAPYPQDFTIHQLFEERVQAQPNAIALAFEAQ
RLSYAELNRQANRLAHHLISLGIKPDDRVAICVERGVEMLIGVLGVLKAGAAYLPLDRAY
PAERLAYMIEDSAPSALLTQRDVQAHLPTLDLPLVLLDEDQRDTFNERDDNPVVENFGAH
NLAYVIYTSGSTGVPKGVMVEHRGLLAVSAAWEKLYALHAPLNHLQMAGFSFDVFSADLI
RSLGFGGTLVLCPRETLMDPPALYRLLSEESIGFADFVPAVLNALLGWVEETGHDLSFMR
TVVCGSDIWTAHSARQLRKLCGDHVQIVQAYGVTEASIDSTCFEFEANSQVDGVLPIGRA
LANTRIYLLDELGQPVPPGVAGELYIGGAGIARGYLNLPQLTAERFMDSPFVAGERLYRS
GDMARYRADGNIEFLGRNDSQAKLRGLRLELGEIEARLAEVAGVRESLVVIREDSGGTPK
LIAYFVEYATREESGPTLTPRALRQQLQLNLPEYMIPAAFVRMAALPLSANGKLDRRALP
EPDADAFDQQDFEAADGPLETAIAAIWADMLGVAQVGRNDDFFALGGHSLLVMRVLAQVR
QQLNLEVSPSAFFAAPVLRQFAELLANTQGNARLVITSAQRSGALPLSFAQQRLWFLAQL
EGGSAAYHIPAGLRMRGSLDQASLQRALDRIVARHEALRTTFVQEQGQPAEQRISAAETG
FRLQLQVLAGQNDAEDTLLAIAAQEASEHFDLVNGPLVRGRLVRMADDDHVLLVTMHHIV
S
>tr|A0A0Q0C7W0|A0A0Q0C7W0_PSEAP Arginine/lysine/ornithine ABC-type transport system, ATP-binding protein OS=Pseudomonas syringae pv. aptata OX=83167 GN=ALO85_02066 PE=4 SV=1
MYKLTIDGLHKSYGDNHVLKGVSLKASSGDVICLIGASGSGKSTFLRCINFLEQPSDGAM
SLDGKQVRMVSDKDGMRVADPDELQRIRTRLAMVFQHFNLWAHMTVLENITMAPRRVLGV
PKAEAEARARKYLEKVGLPERVADQYPAFLSGGQQQRVAIARALAMEPEIMLFDEPTSAL
DPELVGEVLKVIQGLAEEGRTMILVTHEMGFARKVASQVVFLHQGQIEESGHPDEVLNNP
KSERLQQFLSGNLK
>tr|A0A0Q0DSF8|A0A0Q0DSF8_PSEAP Succinate-semialdehyde dehydrogenase OS=Pseudomonas syringae pv. aptata OX=83167 GN=ALO85_03435 PE=4 SV=1
MEKLFMYPDVQLYIDGQWRASRDNRTLSVINPATGEALGTVAHAAIADLDEALAAAERGF
ATWRATSAYDRYKLMQKAANLIRERADSIARIMTQEQGKPLAEARMETLSAADIIDWLAE
EGRRSYGRLVPSRGVSIEQKVIKEPVGPVAAFTPWNFPINQVVRKLSSALAAGCSIIVKA
PEETPASPAELIRAFADAGVPAGVIGLVYGDPAEISGYLIPHPVIRKVTFTGSTPVGKQL
AALAGKHMKRATMELGGHAPALVFDDADIDLAARVLATAKFRNAGQVCVSPTRILVQRKV
LDAFTEKFVGLTREIKVGNGLEAGTTMGPVANDRRIPALVDLVDDAVRGGATLSAGGKAV
EGPGYFFEPTVLSGLKPHMRIMNEEPFGPVALLVPFDTLEEAIAESNRVPFGLASYAFTT
SMKTAQALSTYIEAGMLSINHQGIGLPEVPFGGIKDSGYGSEGGTEAIEAYLNTKLVTQY
N

>tr|A0A0Q0DHN7|A0A0Q0DHN7_PSEAP Pyoverdine sidechain non-ribosomal peptide synthetase module II OS=Pseudomonas syringae pv. aptata OX=83167 GN=ALO85_00371 PE=4 SV=1
MLVIHHLLVDGVSWRVLLEDLQHAYLALAQGQPVLLAAKTTSLQRWAEQLQQYAAGETLK
AERDYWLQALQGKDQPLPRDKPEGTMRNRDAAHASSWLSRDLTHKLLKVAPAAYRTQVND
LLLTALAQVICEWSQQPSVLIQLEGHGREDLFDDTDLSRTVGWFSSLFPLRLTPQTAPGA
SLCGIKEQLRAVPDKGIGYGVLRYMGEGPFAQQLAALPQARVTFNYLGQFDGSFNEHQGA
LFVPSADSAGTALCEDGPLGNWLSLNGQVFDGQLQLDWSFSREVYHASTIDTLARRYEQA
LTTLIEHCTAGHQGVTPSDFPLARLTQAQLDGLPIAAGQIEDIYPLSPMQQGMLFHSLFD
ERAGNYINQLQVNIRGLDVPRFRNAWQAAVDHHDVLRSCFVSQREQSLQVVQRQVEVPFV
ELDARGQPENWLDDWAQADRQQGFDLAQGPLLRLAVLRIADDAWHLVYTSHHILMDGWSS
SRLLGEVLQRYSGQMPAKQAGRYRDYIQWLQHQDAALSERFWTAELAKLDEPTRLLQAFK
SSAEGQGYGDYIQLIDSDGTRRLSEFAREQRVTLNTLLQSAWLLLLQRYTGQSSVTFGAT
VAGRPAELPGVEEQLGLFINTLPVIASPRAEQTVADWVQQVQAKNLALREHEHTPLYDIQ
RWARNSGEALFDNILVFENYPVSEALQRAPDGLVFSDLRNQEQAHYPLTLVVEATEVLSV
RFSYDRQHFSAEGILQLAAHFDHLLQSLCASATTPLGELALPTAWTQSVQRYPSEHCAHQ
RIETQAERNPQAIALSFGGEQLSYRQLNQRANQLAHKLREQGVGPDVRVGLAAERGLEMI
VGLLAILKAGGAYMPLDPDYPQDRLSFLMHDSGIELLLTQAPLLGKLPIPEQVQTLDLAD
ELDGYSTENPLNQTAPANLAYVIYTSGSTGKPKGTLLAHHNLMRLFAATDDWFHFNEQDV
WTLFHSFAFDFSVWEIFGALLHGGRLVIVPREVTRSPEEFHALLVEQQVTVLNQTPSAFK
QLMRVACDSSLPLSLQKIIFGGEALDVASLKPWFERFGDQTPQLINMYGITETTVHVSYR
PIALADTQNPASPIGEAIPDLSWYVLDADFNPVAQGCRGELHIGHAGLARGYHNRAALTA
ERFVPDPFSSEGGRLYRTGDLARYRAGGMIDYAGRIDHQVKIRGFRIELGEIEARLQAHP
AIREVSVLALDGQLAAYLVPNEPDQDQQSLRETLKNELRAHLPDYMVPTHFIVLDSMPLT
ANGKLDRKALPAPDASQLQATYSAPQGELEQQLAAIWADVLKVERVGRSDNFFELGGHSL
LAVQMLVRVREQLQHEVSLKDVFEQPLLADFCATLQEKNGESDHAQDELTKSLEALKRLS
AEEIDNLIA
>tr|A0A0Q0IFV3|A0A0Q0IFV3_PSEAP Secretion protein HlyD OS=Pseudomonas syringae pv. aptata OX=83167 GN=ALO85_101858 PE=3 SV=1
MRTQAELDLARRELDRTRLIAPFAGRVVARHAQPQSLLPAGQVLLDIESAAEQQVVAAVP
LALADSLEPGDLASASSSADGTVGFDLALEGISPRADDGLVRTAVFRVLRPAGRLPSGVT
LLVQMHPAADAQSLSVPVQALWMGTSSHVAEVFVHQPGGTVAVRSVTLGTVREGRAVVVS
GLVAGEQVVTAGAAFLQDGQPVTLFHSDTRLTGDAQ
>tr|A0A0Q0D577|A0A0Q0D577_PSEAP Diguanylate cye/phosphodiesterase domain signal transduction protein OS=Pseudomonas syringae pv. aptata OX=83167 GN=ALO85_00876 PE=4 SV=1
MLKKLPGALRALMSVPADNPRLLQAQYVALSRQLPLMYFVLLVNTWALAFTHWRTAPVWL
TLLVPALLTVICGVRARKWLRTVNRPPPAPAIILATLRGTHGLAGVIAAGFAAWSLSLYP
YGDAYSQAHVAFFMGITVIVCIFCMMHLRPAALTTALVVNTAFVIFFASTYNITFIATAV
NIVLVSIGMLIILHYQYRDFTSLVNVQVKTEQLSNENLLLANQDSLTGLPNRRQFFQTLD
AAMIEAVAQRSELAVGVLDLDGFKPVNDLYGHRIGDRLLMLVAERLTSASSDKVHVSRLG
GDEFALVIKGDMDSDALLAFGKHLCDLMHESFELSDMPIQIGATLGLATYPATADNATQL
FEFADYALYQGKNHNPGTTCLFSASHREQLHVDGVTEQALRRANLETEFHVLFQPIIESC
TRETVAFEALARWNSPELGSVSPAHFIPIAERIGMINKLTAPLLTRSLHMALSWPAPVRL
SFNLSAHDCASLESVSRIVEIIKNSGFDASRLDLEITETAVMQDIAQVQQAITQFRKLGC
GISLDDFGTGFSSLSQLHALALTKLKIDRSFVTGVHNNPASYKIVKSLVALSLDMSLGCV
IEGVETQDELNALTTLGCTMVQGYFYSPPISFAETLAWIEIPDEERTFGYAIS
>tr|A0A0N8T9D1|A0A0N8T9D1_PSEAP PAS protein OS=Pseudomonas syringae pv. aptata OX=83167 GN=ALO85_04481 PE=4 SV=1
MPNFAGFSNHRVESFVVKQASMQSVVREAGSTSGCSAWRKVWVLLALIFAGAANAAQSLP
FSLTAPFVVSADLALKDQDRAWLDQLKVLRVGIAIADYEPIDITSDRNRYQGISADYLGL
VSDQLNLPVQVTGYAKRDEAIEALRGGKIDLLTSANGFERGVKGLSFSTEYMPDRSVVVG
RGNDLSPPSNLAGKKVVLLDGYADSEVLHRVYPDSQIIIAPNLYSALEALSQGEVDVFIG
NEVIVRAYTALRPYLGLQIKFESRLPPVGFSFAVRGDEQRLLTFINRALDSIAPSTSREV
LGRWTMGLGADVEGQRIRLTSAERRWLLKHPSVTIATVQHPPYIYKDKNGHWVGLNADVL
SRISRMTGLQFVHQELPSTQLSIDMLRAGQADMNTTLAENAERRRFLDFTYSFGGNSWMF
VVRSDRSSHISLDSLTGKVLALPARHALEDLIRREHPLIQLRLVDTYDQARALVESGAAD
ATIQNEVGAYLFPSGQLKVARSVEGQWSPDRFSVIKTQPELLGILNKALEEFPVAELRSI
RLKWLGSALPQPSLWGRIPPWVFWVVSLALLIGLVSLTWSSRLKVQIRQRQRVQRQLNDQ
LAFKHALLDGIPNPIYVRDLKGRLISCNRSYEQSLGISFEQMNGRRLTDVNLIPRALAEQ

MHTDYLTLLENHQPVFSDRTIELPGKRMDVWQWTVPFFAADGQLQGLLGGWIDITERKQL
EQQLQEAMHLADQANEAKSAFLASMSHEIRTPMGAIIGLLELECEQALRLGKIPSQGLQV
AHRSATELVALIGESLDLARIEAGGMQLSLAVTSLQGLFDGVIELFSAQAGEKGVELRLE
FSGQARGDYWLDPFRLRQVLHNVLGNALKFTRQGAVVVTLDVTHDSPESTRVRIGIQDSG
EGIDPQRQQQVFQPFTQASDDTAAHYGGSGLGLSITRQLVELMKGDISLHSEPGKGTLVT
IDLPLTRVSEPVLPADDVTDVLVDTRSLHLLVVDDMSANRLVLTRQLEFLGHQVTAVEDG
KAALSSWCEGVFDAVITDCNMPGISGYALTQAIRQIEEKEQRQRCSVIGCTANAMSDEAA
RCEQAGMDGLLIKPLSLARLAHELADRVREPTFDIGTLQTMTQANPQQMQRLLSELWKNL
RHEHALLEPAVSANDWETLSACLHRLKGAASLVDAVPLAKACAALDDSVRLQSTASLAER
WQALEVAMTGLRADIELQLVAVPETAGPGN
>tr|A0A0Q0BZ90|A0A0Q0BZ90_PSEAP Uncharacterized protein OS=Pseudomonas syringae pv. aptata OX=83167 GN=ALO85_00682 PE=4 SV=1
MIGSLRPLEWALLAFAAVLAALIALIFSGAAHSPDWLPEQVPRNPVDQRAQVHSAPSVTL
DSLANTWKTPLFSPDRSPDVAVRKADAQATNLAGLTLTGVIMDGSLRVALLKQASGPALK
VRQGDRLPNGWTLDRLEPTQATFKLDDRSQVLRLPALRLPPPSNTPPITLTNDSTL
>tr|A0A0Q0FK97|A0A0Q0FK97_PSEAP Tyrosine-protein kinase OS=Pseudomonas syringae pv. aptata OX=83167 GN=ALO85_04344 PE=4 SV=1
MAGPARHNRPLPAARVSLRADLRAALALHRRLASPFSVRSNRINDRHEPSFPEFPSGLPD
RSGNSPADAVRSQDPDPVDRRRVFPDRPGLCHSGHTGVSGHRHDSDRASENRHRGHAGSQ
CQATLGVAELIKSRALLGKVVDDLQLNRLQTPDLFPVIGPYLYRTFKPARDGELAQPLFG
LTQYAWGGEKIEVFQLEVPEHLLGEKLTLTAGKPGQFSLYDSEHNLLLGGALNRVVEGHG
IKIQVATLQARPGTDFTISRQRTLSTALIYQNRLKIAEAGRDSGIIYLSIEDQDAQRANR
ILDEVSHLYVRQNVERSSAEAAQRLQFLRSQLPAVRKQLEESETALNTFQTSARSVDLSI
ETKGVLDQVVSLDSMLSELKLKRVELERLYTREHPTYRSLMSQMNQLEQQRLGLLKKIDA
LPMTQQELLRLTRDMQVISQTYTLMLNKSQEQDILRAGNIGNVRVIDNADTNVERPVKPM
KTLIVLIATLLGALVAMTMVFVRQAFYRGVESAEIIENLGMPVYASLPYSRQQEHLDKRD
HAGQESKLLSIAAPAELAIESLRSLRTSLHFAMLEARNNVLMISSPTPGAGKSFVSSNLA
TIIAQTGKRVLLIDADMRKGYLHRLFGLQPKHGLSDTLAARLRCTEVINTTRVRHLDFIS
CGFAAPNPSELLMHDNFHKMLAELSPLYDLILIDTPPILAVTDATLVGRQAGTCLLVARF
GMTTVQEIEASKRRLGQNGILIKGAIFNAVVRKATTSDYDCAAYGYNYHPAPR
>tr|A0A0Q0CCN9|A0A0Q0CCN9_PSEAP RecBCD enzyme subunit RecC OS=Pseudomonas syringae pv. aptata OX=83167 GN=recC PE=3 SV=1
MPLWFGIRFHAPVKEASMSVTTSLSAGFMVVQGNRPDELRSLVVSWMRRYPLAPLENEIA
LVQSNGIAQWLKLALAEDAQDEDNGGCGIAAAIDVQLPGSFMWTLYRMVLGADEIPQTSL
LDKTPLTWRLMRLLPSLINQPHFEPLQRFLTDDTDLRKRYQLSERLSDLFDQYQVYRADW
LEDWAAGRHQLRDVRGQSRPLSAANCWQAELWRALLDDVGAEGMSQSRAGVHQRFIERIG
NMTEAPPSLPARVIVFGISSLPAQALEALAGLARFSQVLLCVHNPCRHHWTDIVADKDLL
RHQYKRQARKSGMPMVLDPQALHQHAHPLLAAWGKQGRDYINLLDSHDDPRSYRSSFKDE
RIDLFSEAQPTTLLNQLQDDILELRPLDETRELWPAVDPLEDRSIRFHVAHSAQREVEVL
HDQLLARFSKDPGLRPRDVIVMVPDIDSYAPHIRAVFGQIEREDRRFIPFTLADQGQRGR
EPLLIAVEHLLKLPDSRFPVSEILDLLDVPALRARFRIQERDLPTLHRWIEGAGIRWGLN
AQQRAGLGLPDALEQNSWHFGLRRMLLGYAVGAGAAYDGIEPYDEIGGLDAALIGPLVAL
IDALQVAHKELSTPTTPEAWGARLQSLLQLFFLADSEHDDYLLAQLETLRENWLETCATV
RLTDELPLTVVREAWLAGLDQGRLSQRFLAGSVNFCTLMPMRAIPFKVVCLLGMNDGDYP
RAQPPLDFDLMGSDYRPGDRSRREDDRYLLLEALLSARDQLYVSWVGHSIRDNSERPASV
LIGQLRDHLASGWKLAGETDPDVKQDDERLLKALTVNHPLQPFSANYFHAGTGYFSFARE
WRLLHETDLQVPVPQELPAHEQEEPLSIAQLQDFLRNPVKHFFSQRLKIYFEVAEAPLAD
EEPFVLDALERYGLSESLLGAGMSSPDNMDAALQTQALKLQASGLLPLAGFGTLMQSELI
EPLPDVLRRYHDLLTLWPETLSSALPISFSHSGVSIDGWLGGLHRNARGELLLVTAIPNS
IGSKKTRKWHRLIRPWVNHLVACACDLPLSTALVASDETLMLEPLDKASAVTTLNHLLTA
WLHGMQEPLPVAVKTAFAWLGQPADKAEAAGRKAYEGDGQTTDGERRESTALARQFPDFD
ALMGSEEFAGWCETLYKPIYDAPWQSLSGGEGGA
>tr|A0A0N8T932|A0A0N8T932_PSEAP Putative Lipoprotein OS=Pseudomonas syringae pv. aptata OX=83167 GN=ALO85_04002 PE=4 SV=1
MDKEWSMVRALPWFLIAGLLAGCAGKPAAEEVESDSPTQPLQLSCYQAGWQAETVPVVYK
RGGPEVWDRYEFIPRTGNVGCL
>tr|A0A0Q0DAJ8|A0A0Q0DAJ8_PSEAP DUF1330 domain-containing protein OS=Pseudomonas syringae pv. aptata OX=83167 GN=ALO85_05377 PE=4 SV=1
MAMKAYWIAHVDVTDPQQYSEYTQRAPAAFELFGGRFLARGGRSEALEGRDTPQRTVIIE
FESYEKAVACYRSPEYQHAMSHRQGASKAEIVIVEGQF

>tr|A0A0Q0D586|A0A0Q0D586_PSEAP LysR family transcriptional regulator OS=Pseudomonas syringae pv. aptata OX=83167 GN=ALO85_04661 PE=3 SV=1
MLLLWLSGQRAIRLHAKHVKFSRPPWVDLLTDTLSSEKQIPRSVQTLSGVLMSLTLRQVR
YFVATAEIGQISQAAIHLNISQSAVTTAIKELESMLGTLLFQRSAQGMSLTEAGRHFLNR
AYVILRSVDDALNSPLPDIRASGVLRLAASYTVIGYFLPHHLQRLEHWHPDVAIEVHEQE
RQAIEQGLLQGRFDMAVVLTANITHPDIISETLFNSERRLWLSSHHPLCQRASVSLADVA
QEPYILLTVDEAEQSAMRYWELAGQQPNVRIRTSSVEAVRSMVANGSGVAILSDLVHRPW
SLEGKRIETITVTDTVTPMSVGLAWHRERELSPAMQAFRNYFHDAFLAPQQLSARR
>tr|A0A0Q0CGP6|A0A0Q0CGP6_PSEAP Syringopeptin synthetase A (Fragment) OS=Pseudomonas syringae pv. aptata OX=83167 GN=ALO85_05172 PE=4 SV=1
MGCRRLLRATRPTLCEKKCMAFVLTAAQRNIWLDQMTQGDSPLYNIGGYVEIEGPFDSGI
FQRALDLLIEKHDALRMVLLEERDEEGVPMQTFATSMPMLVTPFDLQGRDDPRQAAEHWM
QTQLEQPFKLEGGPLFRVHLLKLEAQRFYFVIHAHHIALDGWGIDRLCASVSELYSVLHD
QRVPDLAAPSYVDFIKDDRQYRESPRFALDQAYWLDKYRDIPDPLLAPRHQARIQHPATS
RSGHLSYGMDRGLEQQIEELALRLNASSFHVHLAVLYVYFTRACQRDELVIGLPILNRST
ASYKKTLGLFTQLSSVRLKFSPDLTFAELVQGICRAVKQDYRHQRFPVSDLNSLLELRRA
ERTQLFDLTFSYERQNQLLRFGQASARAFKCSNNHEQTPLAIHLRSNACDTESRLHYIYN
EAYFQHDEIVPLAERLQQVTEQGLANEQLPVHEFSLISKTDALQIQTWNAGQPHSAEYLT
IHQQFEARAAERPDAVALMFEGQTLSYGGLNARANKVAHRLLAQGVRPDDRVAICVERGP
AMIIGLLGILKSGAGYVPLDPAYPLERLAYTLGDSAPVALLSQQSVQQALPVSDVPLIYL
DDVDLQDERACNPQVAVKPSDLAYVIYTSGSTGLPKGVMVEHRNVARLFSATRDWFGFNE
QDIWALFHSFAFDFSVWEIWGALLHGGRLLIVPQLVSRSPEDFHDLLCSAGVTVLNQTPS
AFRQLIAAQGEKEQAHSLRQVIFGGEALDTAMLKPWYARSVNADTQLVNMYGITETTVHV
TYYPLQPEDAQRVGASPIGKRIPDLQLYLLDAYGHPLPPGAVGELYVGGAGVARGYLNRD
ELNATRFLDDPFASEPGARMYRSGDLGRWLPDGSLEYLGRNDEQVKIRGFRIELGEIEAQ
LSACQGVREAVVVVREDEPGDKRLVAYVIGTADLEPDATYLREQLRLSLAEHMLPSAFVS
LQAFPLTANGKLDRKALPLPSAEAYARRQYEAPQGLTETRLAGLWTELLGVEQVGRHDQF
FELGGHSMLAVKLIERMRDIGLNADVRVLFGQPTLASLAAASGKVTEVQAPTNKIPAYCR
HITPDMLPLAALTQAQIDRVVASVPGGVSNVQDIYGLVPLQEGILYHHLANEEGDPYLLQ
ALLRVDSFEQLLDFTEALQQVIDRHNILRTAVAWEELDEPVQVVWRQARLRVEAFLPHPD
QGDVAAQLQAQFDPRRIRMDLHQAPMMRLHYAEDPVDQSWVAVLLFHHLIDDATSLALLG
AEIEAFRQGRGEHLPASVPYRNHVAQARLSVSREEHEAFFRDMLADVDEPTLPFGLQDVQ
GDGSGVDEALSPVEPKLAGRLRAHARRLGVSNASLHHLAWAQVVGRLSGRQDVVFGTVLM
GRLLSGQGAERALGMFINTLPLRVAAGEQGIEAAVRTTHARLAALVSHEHAPLSLAQGCS
GVAAPTPLFSALMNYRHVAVGAQTPQEAGQTRLWGGVEVLGGEERTNYPLSLSVDDLGES
FGLTVQAVAGIDARRICGYMHTVLEQLADALDNRPDAALHSLDWLPAHERRQLLEDFNAF
DSAYPQDLLLHQLFEAQAAAQPDSVAVTYEGQRLSYAELNQWANQIAHRLIAAGVGADDR
VAICVERSLEMIAGLVGILKAGAGYVPLDPSYPEERLAYMLEDSAPKMLLTQRGLRERFA
QAAMPVLLLEAEARAESGIDRAPATNPQLAGLGAQHLAYLIYTSGSTGQPKGVAMPHAPL
VNLMHWQIAQSVEDRRPRQRTLQFAALGFDVAFQEIFSTLCAGGELSLIHADTRLNFRRL
FEHICEQRIERLYMPCIALQALAEAVVDEPEQPECLLQDVITAGEQLRITEPMRQLFARL
SDARLHNHYGPTESHVVTALTLDGDPQAWPTLPSIGQPVANTRIYLLDEHMRPVPVGVAG
ELYIGGAAVARGYLNRDDLSAERFIADPFASNDVGNPHARLYKTGDLACWQADGRIVYLG
RNDDQIKVRGFRIELGEIEARLGQYPGLRDTAVLAREDVPGEKRLVAYFSVQAGQVVPQA
DQMRLHLQALLPDYMVPAAYVHLEKLPVSPNGKLDRRALPQPSADAFVSRDYQAPLGDTE
TTLAALWCEVLSVERVGRQDHFFELGGHSLLAVKLIERMRQAGLSADVRVLFGQPTLAAL
AAAVGCGIEIEVPANGIPEGCRHITPDMLTLTALDQASIERIVAGVPGGAANVQDIYPLA
PLQEGILYHHIAAEQGDPYLLQATFALRDPAHVRRFAEGLQAMIDRHDILRTALVWEDLE
HPLQVALRQVELSMEQITLAPDGADIVAELQRRFDPRECRLDLSQAPLIHLAFAEDSENQ
RWVAILLFHHIALDHTALDVMSAEMLAHLQGTAAQLPPAMPYRNYVGQARLGVSREQHEA
FFREMLGDVDEPTLPFGLLEVQGDGQGVKEVRHDLPADLCQRLRTQARQLGVSAASLHHL
AWARVLGAASGREDVVFGTVLLGRMQSGLGADRALGMFINTLPLRVDVGTGGVRQAVRLT
HNRLTALLGHEHAPLVLAQRCSEVSATSPLFSALLNYRHSPAREASGGERWADAQVLGVR
ERTNYPLSLSVDDQGEGFLLSVQVAAGFDGQRICSYMQTALNHLVEALESAPDSAVRDLS
VVPETESQQLLVAFNDTARDYPQQQTVHGLFEAQVRAYPEACAAIHDGVAVSYAELNTRS
NRLARHLLGLGVQPGDSVAILLERSHDLLASQLAVLKCAAVYVPLDVNAPVERQAFMVED
SQARVLLTRSRISLTASTQRVDLDGLTLDGLKDTDLALPQSSESVAYIMYTSGSTGVPKG
VLVPHRAISRLVINNGYADFNAQDRVAFASNPAFDASTLDVWAPLLNGGCVVVIGQHDLL
SPLNFQRLLLEQAVTVLWMTAGLFHQYASGLGEAFSRLRYLIVGGDVLDPAVIGRVLANN
APQHLLNGYGPTEATTFSATYEIVSVGNGSIPIGKPVGNTCLYVLDSQGQPVPLGVPGEL

YIGGQGVARGYLNRDELTLEKFVADPFDSAPQARLYRTGDLVRWRADGNLEYLGRNDDQV
KIRGFRVELGEIEARLAEHSDIREAVVLCRQDEPGDKRLVAYVTAQQPETALDIEHLRNH
LQGLLPDYMVPAAYVQLDALPLTANGKLDRKALPAPDALSLISHGYEAPQGELETLLASI
WADVLKVEQVGRHDHFFELGGHSLLAVTLIERMRQVGLSADVRVLFSQPTVAALAAAVGG
GTEVVVPANLIPEHCEYITPELLPLISLSQMQIDQIAASVSGGMANVQDIYPLAPLQAGI
LYHHISTEGGDPYTLKALFEISDRTRLDAFSGALQGVINRHDILRTAVLWQGFDEPVQVV
LRRAELQVTELLLDPADGPVDEQLHERFDPRHYRLDVRHAPLMRIVFSHDPLNGRWLAML
LFHHMAIDHVALEVLKHEIQSGLLGEADALAASVPVPYRNYVAQARLGVSQAGHETFFRK
MLGDIEEPTLPFGLQDVQGDGRDIEGASLTLDARLDQRLRAQARQQGVSAASLVHLAWGQ
VLANVSSKCDVVFGTVLMGRMQSGEGADRAMGMFINTLPLRVDVGAITVVEGLKTTHERL
TALLGHEYAPLVLAQRCSGVAAPLPLFSALLNYRHSAPGSVSERTAQAWEGIYALNSEER
TNYPLTLNVDDLGEGFSLTALVTSSIGAKRVCNYMQMALETLVTALEQTPLASLQELSIL
PAEEREQLLVGFNNTALDYPHQQTIHGMFEAQVERTPDAVAVVHGEQRLSYRQLNEQANR
LAHALRKQGVRPDSRVGICVERGVEMVVGLLATLKAGGGYVPLDPAYPAERIAYMLQDSA
PAAVLAHTATQGLLADISMPVINFDLSDWQDESVQNPQVAGLTSAH
>tr|A0A0N8T871|A0A0N8T871_PSEAP Aspartate/tyrosine/aromatic amino acid aminotransferase OS=Pseudomonas syringae pv. aptata OX=83167 GN=ALO85_01192 PE=4 SV=1
MAFSERVSRLKSSLIREILAAAQRPEVMSFAGGLPAESMLPKVEWADMPVALGQYGMSEG
EPALREALAADARALGIDCDASQVMVVSGSQQTLDLAAKLYIGKGTEILLEAPTYLAALQ
IFQLFGAHCITVPLQAEGPDLLAMRQRLEQHTPAFAYLIPTFQNPSAVRYSQASRDAVAA
LLDEFGVTLIEDEPYRELSFDGGSARPIVSGLKTASWIYTGTVSKTLLPGLRVGYLIASP
DLFPHLLRLKQSADLHTNRLGQWQALQWIGTEHYREHLVTLCEFYRGRRDAFESALQTHF
SDLATWNSPQGGLFFWLTLKQPLDTRTLLDTALAHDVAFMPGEPFFADPDANHGHLRLNF
SHIDPARLDEGIKRLASVVRAAHLLQAA
>tr|A0A0Q0C4Q9|A0A0Q0C4Q9_PSEAP Putative transcriptional regulator AguR OS=Pseudomonas syringae pv. aptata OX=83167 GN=ALO85_05096 PE=4 SV=1
MPAAQPRATPMTRAATPRKPQARSQARIDSILDAARTLLAEQGVASLSIYSVAERAGIPP
SSVYHFFASVPALLEALTADIHAAFRASLQAPIDHDQLTTWRDLSRIVELRMLAIYNADA
AARQLILAQHGLTEINQADRQHDIELGQLMLEVFDRHFQLPALPYDVDVFALAMELGDRV
YARSVQLHDEITPRMAEEGMRVFDAYLGLYLPVFLVKRAN
>tr|A0A0Q0FJA4|A0A0Q0FJA4_PSEAP Aldolase OS=Pseudomonas syringae pv. aptata OX=83167 GN=ALO85_00405 PE=4 SV=1
MSNVQQQIVDLSRHLSRRGFFAATGGNLALRIDALHIAVTPSATDYFSMRPEDVCVLRLK
DLKQLSGERSPSVESELHAKVLRSRPDVNCSIHTHQPLASACTLFGKSLDVPYPPLWQSL
GRRIQLVGYAPSGSGWLAGKLEKTIRPDQNAYLMRNHGVLCCGPDIEATLKRLEDLETFC
RDHLLNQIKANSRGKPESRVAIARLIDALTSAGTHALSPQPFSETQS
>tr|A0A0Q0D6X7|A0A0Q0D6X7_PSEAP Dienelactone hydrolase family protein OS=Pseudomonas syringae pv. aptata OX=83167 GN=ALO85_04703 PE=4 SV=1
MARQPAKMQAALNDSEGSIPMERLTAKDFAPELLELYDYYAHGRINRREFLDRAALFTFG
GLTASALLASLSPNYALAEQVEFTDPDIIAEYVSYPSPKGHGQVRGYLVRPAKAAEKVPA
VVVAHENRGLNPYIEDVARRVAKAGFIALAPDGLSSVGGYPGNDDKGRELQQTVNPEKLM
NDFFAAIEWLMKHDATTGKVGITGFCYGGGVANAAAVAYPELGAAVSFYGRQPNAEDVVK
IKAPVMIHYGELDTRINEGWPAYEKALKAAGKTYETYIYPGANHGFHNDSTPRYDEAAAK
LAWDRTLGWFNKYLV
>tr|A0A0Q0C6X7|A0A0Q0C6X7_PSEAP Bcr/CflA family efflux transporter OS=Pseudomonas syringae pv. aptata OX=83167 GN=ALO85_100065 PE=3 SV=1
MNSPVRRSLQGKPGKLARCSASVNGHPMNLRTILILGSLSAFGPLAIDFYLPGFPAMASY
FGTDEKHVQLTLAAYFLGLSLGQLAYGPVADRFGRRIPLLVGVTLFMLASVACAFAPSLE
WLIAARFVQALGGCAGMVLSRAIVSDKCNAVESAKVFSQLMLVMGLAPILAPMLGGVLVS
TFGWQSIFVSLSLFSGACLVAVALGLPESMPATTPRQPLSGALGQYLNLFKDSVFIGHAL
TGGIAMAGMFAYVAGSPFVFIKLYGVPAEHYGWLFGMNAAGFILAAQINARILRKTGPAF
LLSRMVWVYLAAGLVLLAISLLRTEALWPLLVPLFICVASLGCIIPNASACAMNGQWARA
GSASALLGCLQFSVAAVAASLVGLLHDGTATPMALVISLCGVLTVGLSVLTRRAQARRDA
SLQTA
>tr|A0A0Q0IRH4|A0A0Q0IRH4_PSEAP Amino acid adenylation:thioester reductase OS=Pseudomonas syringae pv. aptata OX=83167 GN=ALO85_04329 PE=4 SV=1
MKRLEIVLIGHSLTLSELNAELLDHGHGVRHLSDQQALDAVTMPDGGVLIEDGSLYLSQE
HLSAFGHCTHLRLRVGFGNALEYGLPRLELLCWHGAAQARRLIVREWLPVEESGNGRVLR

DATVAAMVDLATLQISRLSREDDYFNGLTSVTSAQSDRQHGLQAIDQLLFEHRLNQTDQP
HLLKLAETPITERLEQALLTFAERPALTIRNQTLSYRQLHAHSLAIQRLLRPLLAHAKAD
APPVIGICLRKSAELYAGILAILGCGAVYLPLDPGQPVQRQQYILENSGAMLLLHDGTHP
LAAAEFPALDIAAVPVEDVASAHTCAAADQDAPCMALYTSGTTGQPKGVLLSQRNLSHFT
AWYAEHVSLNEHSRVLQFSTLSFDSSIIDIFPTWLSGAELVVPDEDQRRDPLQLVRVLQQ
GITHAFLPPALLSILPLDQPLGLEHVMTGGDVCEPHVIAQLTGQCHFHNLYGPTEATVLV
TACEFASSSSNRHLGRPIANSQAWILDEQLQPVAEQTQGELYIVGPGVCLGYVNNPELTA
ERYVWLTKPDGQLMRAYRTGDMAKWTADGIVLSGRRDNQVKIRGFRVEPEEIEHCLRDSG
LYRQVAVVIDRQRRILAFLAQPYLEHDEQALKAHVQRLLPDYMHPAVYTMLPGMPFASNG
KVDRKALLEIPLSFIGQHTRRQPETEQERALLELWGELLELPPDDISTDESFFNLGGHSI
LLSRLLLGVRERFGRSIPINRFIEAPTVLTLASLIDSDGTSTGVLSAQALRDANPDFQLA
TLPVSRMGDVHKVIVTGANGFLGVHIVEALLDWGATEITCLVRETSGQSAQQRFEHALRQ
NRLDHLDLSRVKVYPADLTKPRLGLSEAVYERLDLEFGALVHNAANVNHVQDYETLIKDN
VAPVFECLKLCEGRRKKVFNFVSTLSACSAIDAAGNVLEQPAAATPPIYIKNGYNLSKWV
AERILQCARDQGAWVNIYRPGNIAFNSVTGVCQPQKNRLMLMLKGSLQLGQVPAFAINFD
LMPVDFLARFIGFHASRYQPEHAVFNLHNPEPLSWSDYVYAFREAGRQFELVSVEQWQAQ
LRRVDSQNALFGVLGFYLDGFEEDIGDISMIEHRNTLNGIRRMGEQYPQKTPALLRRGCD
YLKEIDFI
>tr|A0A0Q0DAE9|A0A0Q0DAE9_PSEAP Uncharacterized protein OS=Pseudomonas syringae pv. aptata OX=83167 GN=ALO85_200185 PE=4 SV=1
MLNMKCITARPLLLRDLADPKYRYWIKQFAGEVAAPCVAESLMSFLSKHSLIT
>tr|A0A0Q0C4N1|A0A0Q0C4N1_PSEAP Replication protein A OS=Pseudomonas syringae pv. aptata OX=83167 GN=ALO85_200172 PE=4 SV=1
MSGGDNLYQYAPSAIGWIDPLGWSCSTALNVNKPKILNSPSLSVAERSFLERQFAKNQNA
LTRAAARGDLVWSPGTHDVRISSLQGSYRKAVTARFERMYGQPPNLSKINADHPIDLIVG
GSSTQRLQMLDESINKSVGSSLKNAGRKAGLKPGDKISSIIFN
>tr|A0A0Q0C1J2|A0A0Q0C1J2_PSEAP RND-type multidrug efflux system, outer membrane lipoprotein OprN OS=Pseudomonas syringae pv. aptata OX=83167 GN=ALO85_03674 PE=3 SV=1
MSAYKVFIPSLLVMALAACAVGPDYRAPVTAPAKLASAAQGSYDRSKVDRAWWQQFEDPT
LNKLVARSLDGNRDLRVAFARLKSARAIRDDVANDVMPVVTSRASSDIGKGQQPGITERR
VNSERYDLGLDMAWEVDLFGRIQRQLEASDADQEAAEANLYQLQVTLIAEVVDAYGQLRG
AQLREAIARDNLKNQQSSQDVTTQLRDAGVGNELDVVRAEARLAAVEATVPQLQAEQARQ
RNRIATLLGERPENLSVDLSPSKLPAIAKALPIGDPTQVLRNRPDIRAAERQLAASTARI
GVATADLFPRVSLSGFLGFTAGRGSQIGSSAARAWSLGPSITWAAFDLGSVRAQIRSADA
DAEGALANYEQQVLLALEESENAFSDYDKRQQRLVSLMRQSDASRSAARLASVQYREGTA
DFLVLLDAERERLAAEDAQAQAEIELYHGIVAIYKALGGGWQPQA
>tr|A0A0Q0BXV2|A0A0Q0BXV2_PSEAP Sulfate and thiosulfate ABC-type transport system, periplasmic substrate-binding protein CysP OS=Pseudomonas syringae pv. aptata OX=83167 GN=ALO85_03966 PE=4 SV=1
MSIRRFALAALASAVFAGSAVAKDYELLNVSYDPTRELYQDYNAEFVNFWKKSHPDDKVE
IKQSHGGSGKQARGVIDGLRADVVTLALAGDIDEIAKLGKTLPENWQTRLPDASTPYTST
IVFLVRKGNPKGIKDWGDLTKEGVSVITPNPKTSGGARWNFLAAWAYGLKAGGGDEAKAK
EYVQTLFKHVPILDTGARGSTITFVNNGQGDVLLAWENEAFLALKEDGGKDKFDIVVPSL
SILAEPPVAVVDKNAERKGNTEIATEYLKHLYSKEGQEIAAKNFYRPRDAEVAAKYEKQF
PKLDLVTIDKDFGGWKTAQPKFFNDGGVFDQIYQAQ
>tr|A0A0Q0CHC7|A0A0Q0CHC7_PSEAP Uncharacterized protein OS=Pseudomonas syringae pv. aptata OX=83167 GN=ALO85_200204 PE=4 SV=1
MAVKVNKSCPVSCRFTDEQFARFAEDIAQSGLKPARFFRDLVISRSPTFERSAIDKKRMQ
QVFEKSGHALNRVAYSANSAPYNGTVYQKQYLHWLNKLNAIQQLLLTVLSETDPPKPARP
VHNGNRGSPNVSGKKVHIIRFRLTQDEMTQFDDMIKRAGCSASTFFRELILNPTPVFREF
TGFRRRIVFIVNKAGNNISQLAYIAKSASDRGLITDSVRDKWYEALVVIETILLAGIEYA
D
>tr|A0A0Q0CXY4|A0A0Q0CXY4_PSEAP Iron dicitrate transport system, permease protein FecD OS=Pseudomonas syringae pv. aptata OX=83167 GN=ALO85_03066 PE=3 SV=1
MNHPRARLLLLMTLLALALLLSLSAGTVWLTPDAVLDHLIAHDSKDFEVWNHRLPRGLIA
IMTGCAFGLAGAIVQGVIRNPLASPEILGVTQGAGLALTIAIIGLPQLPVSALPFVACLG
GAAGALLLALYNTGVQFSGVRFALSGVAIAVTLSSITEFLILSNPLDINTALLALTGSLW
SRNWHHVLLATPLLLLIPLSVLLAKPLNLIGLGDEAAQSLGTGLTRTRWLAMGSAVLLTS

LGVGIIGPVSFVGLVAPHMARRLVGGHHQYLLPASMVLGALLLMLADTLGRTLIAPSEIP
AGILTAVIGAPYFLWLLARFKG
>tr|A0A0Q0BZY4|A0A0Q0BZY4_PSEAP CinA OS=Pseudomonas syringae pv. aptata OX=83167 GN=ALO85_00272 PE=3 SV=1
MNDYTVNEITGLADTLGRLLGAMNAQVTTAESCTGGGIAEAITRIAGSSAWFEAGYVTYS
NAQKTRQLGVPEMLFEQVGAVSQAVVEAMVRGAQRESGARFAVAVSGVAGPGGGSPDKPV
GTVWLCWGKDDALIARRCHFDGDRDQVRRQTVEAALHGLIQLARGEMPKQG
>tr|A0A0Q0C5X3|A0A0Q0C5X3_PSEAP Cysteine--tRNA ligase OS=Pseudomonas syringae pv. aptata OX=83167 GN=cysS PE=3 SV=1
MLSIYNTLTKSKEVFKPLDGNKVRMYVCGMTVYDYCHLGHGRSMVAFDLVTRWLRFSGYE
LTYVRNITDIDDKIINRARENGESFDALTARMIDAMHEDEARLNILKPDMEPRATDHIPG
MHAMIQTLIDKGYAYAPGNGDVYYRVGKFQGYGKLSRKKIEDLRIGARIEVDESKEDPLD
FVLWKGVKPGEPSWESPWGAGRPGWHIECSVMSTCCLGETFDIHGGGSDLEFPHHENEIA
QSEAATGKTYANAWLHCGMIRINGEKMSKSLNNFFTIRDVLEKYHTEVVRYLLVSSHYRS
AINYSEDSLRESKAALERFYHALKGLPVAEPAGGEAFVERFSTAMNDDFGTPEACAVLFE
MVREINRLRESDIAAAAGLAARLKQLASVLGVLQLEADDFLRAGAEGRVDAAEVEALIQA
RLAARAAKDWAESDRIRDQITAMGVLLEDGKGGTTWRLAD
>tr|A0A0Q0DNS3|A0A0Q0DNS3_PSEAP Outer membrane porin OS=Pseudomonas syringae pv. aptata OX=83167 GN=ALO85_05034 PE=4 SV=1
MFATSRWCPAGSSRTYKKSQVDTFKTICRSIGAHMTQLRTLSLALCSTLAGVTPITALAE
EQKDGFIEGSTLTVLNRNFYMNRDHRNGESSPTGNGYSEAWAQGIIGRFQSGFTQGTFGV
GVDAFAMIGIKLDTGDGRNGGRSAFDVLPVDEQGNARDEYTKVGGSLKARAFDTVVNVGD
VFPSTPVVAFGDSRLLPESFRGVTAVNNSISGLTVQGGRLHSMSQPVSSNLRDGFATFYG
GAVDAPWVAYFGGDYQATDALSLGLYASRLKDVWDQKYASASYVLPLSDDLALTGSLNYY
NATDDGKKLLGEFDNDIWSAKLALQYGAHTLSLSHQRNEGDDDFDYLRQSDSIYLANSIQ
YSDFNSPKERSWMVTYNLDMSTFGVRGLSFMTRYGKGTDADYSNANSTYMRRDAQGNPLT
DQKRWERDIEAKYIVQTGSLKDLSLRVRQATTRATAFESDLDEVRVIVEYPLSVL
>tr|A0A0N8T8P5|A0A0N8T8P5_PSEAP Alkaline metalloendoprotease OS=Pseudomonas syringae pv. aptata OX=83167 GN=ALO85_00703 PE=4 SV=1
MTKVKENAAIQLSAATSTSFDQINAFAHQYDRGGNLTINGKPSYSVDQAADYILRDDAAW
TDRDGNGTINLTYTFLTAKPAGFDNSLGTFSAFNAQQKAQAVLSMQSWADVAKVSFTQAA
SGGDGHMTFGNYSNGSAGGAAFAYLPSGNSRTDGQSWYLVDNSYKVNTTPDNGNYGRQTL
THEIGHTLGLSHPGDYNAGEGNPSYKDATYAEDTRGYSVMSYWSESNTDQNFVKGGAPSY
SSAPLLDDITAVQQLYGANMSTRAGDTVYGFNSTAGRDFYSATSASSKVVFSVWDGGGKD
TLDFSGFTQNQKINLNAASFSDVGGMVGNVSIAKGVVVENAVGGSGNDLLIGNAAANDLK
GGAGNDIIYGGGGADSLTGGAGADIFVFGASSDSNRAGQDTIRDFVSGQDKIDVSAISTL
SALQFVNAFSGHAGEAILNYNQSSNLGSLAIDFTGQGIGDFLVGTVGQAVAADIIV
>tr|A0A0Q0D2F0|A0A0Q0D2F0_PSEAP Alanine racemase OS=Pseudomonas syringae pv. aptata OX=83167 GN=ALO85_03024 PE=4 SV=1
MSASLSSLDTPVALLDVSRMQHNIQRMQQRMNELGVRLRPHVKTSKCLPVIQAQIAAGAS
GVTVSTLKEAEHCFAEGIDDVFYAVAIAPGKLEQALRLRRKGCRLSILTDSVTAARAIVA
FGQAHDERFDVWIEIDCDGHRSGLTVDDPSLVEVASTLVDGGMQLRGVMTHAGSSYDLDT
PAALQALAEQERRLCVSAAERIRQAGLPCADVSIGSTPTALSAQSLHGVTEVRAGVYVFF
DLVMHNIGVCQADELALSVLTTVIGHQQDKGWIIVDAGWMAMSRDRGTQRQSEDFGYGQV
CSESGEWIDGARVTGANQEHGIITLAAGSEADISERFPIGSRLRILPNHACATGAQFPDY
HACDSEGAIHTWSRLHGW
>tr|A0A0Q0C927|A0A0Q0C927_PSEAP JmjC domain-containing protein OS=Pseudomonas syringae pv. aptata OX=83167 GN=ALO85_00734 PE=4 SV=1
MHTPGCAGRFHFKGLHMNPDIPLQLLGGISARVFLRDYWQKKPLLIRQALPDFQSPIDAD
ELAGLALEEEVESRLVIENGERPWELRRGPFAEDEFSKLPERDWTLLVQAVDQFVPEVSE
LLENFRFLPSWRIDDVMISYAAPGGSVGPHFDNYDVFLLQGHGKRHWQIGQMCDAESPLL
QHADLRILAEFEKTEEWTLEPGDMLYLPPRLAHCGVAVDDCLTYSVGFRAPSAAEVLTHF
TDFLSQFIPDEERYTDADAQPVSDPHQIQHDALDRLKTLLAEHMSDERLLLTWFGQFMTE
PRYPELVTGPELEEQDLLDSLEQGAVLIRNPSARLAWSEVDDDLLLFASGQSRLLPGSLR
DLLKLICAADALHSENLGQWLADDEGRNLLCELVKQGSLGFADE
>tr|A0A0Q0DVM4|A0A0Q0DVM4_PSEAP Gluconate transporter OS=Pseudomonas syringae pv. aptata OX=83167 GN=ALO85_02950 PE=4 SV=1
MTPLMLMVVAGAGIALLLFLVLKYKFQPFVALMLVSIIVALVAGVKPADLVATIEGGMGK
TLGHIAIIIALGAMIGRIIELSGGAEALAKTLINRFGNRRTPLALTVAGFMVGIPVFFEV
GVIILMPLAYGVARAARKPLLIFALPMCAALLAVHAFLPPHPGAVAAASQLGADLGRVLM

LGIPIVAVLCMIGYFVAGRMTRKTYPMTDDIRAEVYGPHVTNEDLEAWARNDYSAVREAT
ETRTMGVEESASSLASKLPPAPAPGFGLIIALILLPIVLILMGTLATAMLPDTSTLRAVL
TVLGAPLVALLIDTLLCAWLLGSRRGWSRTQVSDVIGSALPGVAMVILIAGAGGVFGKVL
VDTGIGAVVSEALRNTGLPVLALGFLLTLLLRAVQGSTTVALVTTAGILSPLIATLDLSA
NHLALLCLAMGGGGLAMSHINDAGYWMFTKLAGLNVADGLRTWTVLVTLLGTLGFVITLL
LWPFV
>tr|A0A0Q0FLH6|A0A0Q0FLH6_PSEAP Uncharacterized protein OS=Pseudomonas syringae pv. aptata OX=83167 GN=ALO85_03954 PE=4 SV=1
MVPVNRSLVEKPASLTAPNGAGHREHLRADIHYQNPHTTAYGFSHYKEPDVVMALNTLFH
NKCAYCEGKITNTGPIDVEHFRPKGRVAGVLNHPGYWWLATEWTNLLASCIDCNRSRNQT
LLGSEDDQQLTQGERLSGKHDHFPVSGPRATCATDDHALEAPLLIDPTSVDPREHIYWKV
MVDQPLAVAVSQNGQASIDLLGLNRSKLAEARRELQLKLDEEFLCLKATISKIADEPTNE
GVRGWMPVLQMLIDKFFRHTEANNEYSAFARHIVESRYDKVSKMMDALLARVAADSPVES
AAR
>tr|A0A0Q0ICK8|A0A0Q0ICK8_PSEAP Uncharacterized protein OS=Pseudomonas syringae pv. aptata OX=83167 GN=ALO85_01155 PE=4 SV=1
MNRTISSALLASSLLAAAPAMAGDIFQWQSNSLTYLNGRDFAVNPENQQTFTFEHADSWK
YGDNFFFVDKIFYNGKKDATAGDNTYYGEFSPRLSLGKIFGQKFEFGPISDVLIAATYEF
GEGDNESYLIGPAFDLKIPGFDYFQLNFYQRQTEGNRPGDGVWQITPVWSYTIPVGKSDV
LIDGFMDWVVDNDKTSRGTYHSNLHFNPQIKYDLGKALNYPERQLYVGIEYDYWTNKYGI
KDTRSFDTDQNTASLLVKVFF
>tr|A0A0Q0D3X7|A0A0Q0D3X7_PSEAP Probable proton/glutamate-aspartate symporter OS=Pseudomonas syringae pv. aptata OX=83167 GN=gltP PE=3 SV=1
MSKGSSCLETAVKKHARRTGFGFAQATLNKGNNMKKAKLSLAWQILIGLVLGIAVGALLN
HFSAEKAWWITNILQPAGDIFIRLIKMIVIPIVVASLIVGIAGVGDAKKLGRIGLKTILY
FEVVTTIAILVGLVLANVFHPGTGIDMSTLGTVDISKYQQTTAEVQHDHAFIATILNLIP
SNIFAAMARGEMLPIIFFSVLFGLGLSSLQAEVREPLVKLFQGVSETMFKVTHMIMNYAP
IGVFALIAVTVANFGFASLLPLAKLVILVYVAIAFFAFVVLGLIARAFGFSVIRLMRIFK
DELILSYSTASSETVLPRVIQKMEAYGAPKAICSFVVPTGYSFNLDGSTLYQSIAAIFIA
QLYGIDLSIGAQLMLVLTLMVTSKGIAGVPGVSFVVLLATLGSAGIPLEGLAFIAGVDRI
MDMARTALNVIGNALAVLVISRWEGMYDDAKGERYWESLPHWRSKEPVPLRQPTAE
>tr|A0A0Q0DGA0|A0A0Q0DGA0_PSEAP Histidine kinase, HAMP region: chemotaxis sensory transducer OS=Pseudomonas syringae pv. aptata OX=83167 GN=ALO85_01128 PE=4 SV=1
MNSLLSPGMRLMGHFGFARKFQVLFLLFMLPLAGSAWVITKEYSNKLDVISGEQSGVRQL
LALDDLNVELSAQRDHAARWKAADILREPTPAAKAAMASVDAGIPRIAKALEGVQAVLVE
EKAPADTMNRFKALQAAVTGLDTASLRTVGWWPDGYDRFTTVLNLVQALREQIAMDSGLI
LDPWLETYMLMQLSTQQVPDLIERIGRLSSVGQTSVASGQFSLQSRLQMRDLRGRIDDSK
DQMQKAAGLLLSKLPEQLQPWADKYAAVMQVLEAQLKVIDDGVFGGSIKLKPEEFEKSID
TLTGSTTELRRQSLEALNGRLDYYHESSNTEFIPMALVFCVLVIMAIYLFACLQSSIRNS
ARGITTLAESLRDGNLCVEVAVQGRDELAAISRALNVAVVQLRTSLLGVNQETVRVGDAV
LTLSAQSSGTLSEVEDQQQQISQIAAAATELAATSQGVARSCEQASESARQTRQIAQQSS
QDSQRTTDSIQQLNQRLTETAAALGRVSEQGQQIQSVVDAIRGIAEQTNLLALNAAIEAA
RAGEQGRGFAVVADEVRSLSQRTQASTAQIAGTVDSLRSTVTQAVNLMEAACGQAVNDAQ
SVTGLGVRLGEIAIAVQGVTDTLAQISTAVEEQATTADEVSSNIQQVDQAAGRLLEGARA
VNQAADTLSQGSRALNDNTARFRLG
>tr|A0A0Q0FU01|A0A0Q0FU01_PSEAP Ribonucleoside-diphosphate reductase OS=Pseudomonas syringae pv. aptata OX=83167 GN=ALO85_01975 PE=3 SV=1
MQTDTTRENSPTGAPQASQTQQDLSATAPGQLRVIKRNGTVVPYTDDKITVAITKAFLAV
EGGNAAASSRIHDTVARLTEQVSATFKRRMPSGGTIHIEEIQDQVELALMRAGEQKVARD
YVIYRDSRAKERAVRAPEEQVQAHPSIRITRADGSFAPLDMGRLNTIVTEACEGLAEVDA
DLIQTETLKNLYDGVALKDVNTALVMTARTLVEREPNYSFVTARLLMDTLRAEGLGFLGV
ADSATHHEMADLYAKALPAYVTAGIKFELLNPVLAEFDLEKLGKAINHERDQQFTYLGLQ
TLYDRYFIHKDGVRFELPQIFFMRVAMGLAIEEKAREDRAIEFYNLLSSFDYMSSTPTLF
NAGTLRPQLSSCYLTTVPDDLSGIYHAIHDNAMLSKFAGGLGNDWTPVRALGSYIKGTNG
KSQGVVPFLKVVNDTAVAVNQGGKRKGAVCAYLETWHMDIEEFIELRKNTGDDRRRTHDM
NTANWIPDLFMKRVFDDGPWTLFSPSEVPDLHDLTGKAFQERYEYYEALTEYPGKIKLFK
TIQAKDLWRKMLSMLFETGHPWLTFKDPCNLRSPQQHVGVVHSSNLCTEITLNTNKDEIA
VCNLGSINLPNHIVDGKLDTDKLKRTVDVAVRMLDNVIDINYYSVPQAKNSNLRHRPVGL
GIMGFQDALYLQHIPYGSDAAVQFADTSMEAVSYYAIQASCDLADERGAYETFQGSLWSK

GILPLDSQQILIEQRGEKYISVDLKETLDWAPVRARVQKGIRNSNIMAIAPTATIANITG
VSQSIEPTYQNLYVKSNLSGEFTVINPYLVRDLKARDLWDSVMINDLKYYDGSVQQIERI
PQELKELYATAFEVDTKWIVDAASRRQKWIDQAQSLNLYIAGASGKKLDVTYRMAWYRGL
KTTYYLRALAATSTEKSTVNTGKLNAVSSGGHGPDDSAITAPRPTEAAPAGPAPVPKACA
IDEPDCEACQ
>tr|A0A0Q0DHT9|A0A0Q0DHT9_PSEAP Oxidoreductase FAD/NAD-binding:oxidoreductase FAD-binding region OS=Pseudomonas syringae pv. aptata OX=83167 GN=ALO85_00045 PE=4 SV=1
MTVSDDKFTRQTLQRVTPLTANLFTLRTTRDPGFRFRAGQFARLGVTKADGTVVWRAYSM
VSSPHDEFLEFFSIVVPDGEFTSELSRLREGDSLLVEKQAFGYLTLDRFVDGRDLWLLST
GTGVAPFLSILQDFEVWEKFERVILVYSVRESRELAYQQLIAELMQREYLAEYEDKFLFL
ATVTREQHPGALNGRITQLIENGELERAAGIALTPEHSRVMLCGNPQMIDDTRAILKQRE
MRLSLSRKPGQVAVETYW
>tr|A0A0N8T8T2|A0A0N8T8T2_PSEAP Aldehyde dehydrogenase OS=Pseudomonas syringae pv. aptata OX=83167 GN=ALO85_03020 PE=4 SV=1
MTSQSLNSHHVDPDTALKLYIDGGWSAPLDPVSIAVINPATEEVVAHVASGSAADVDRAV
AAARAAFASWSGTSPHARAQVIGRIYELIIERKEQLAQAISLEMGASISSARAMQVPLAA
EHVRVARDLLAIYRFHTIEGGTAIEREPIGVCGLITPWNWPLYQITAKVAPAIAAGCTVV
LKPSELSPLSALLFAQLVHDAGLPPGVFNLVNGSGPEVGGAMAAHPDIDMISITGSNRAG
ALVAQAAAPTVKRVTQELGGKSPNILLPDADFAKAVPPGVMAAFRNVGQSCSAPTRMIVP
RNRLAEVEALAAETAAAIVVGDPQLEGTVLGPIANEAQFHRVQAMIDAGLSEGAKLVCGG
PGRVQGHERGFYTRPTVFSEVDSSMRIAQEEIFGPVLCLIAYDTVEEAIAIANDTVYGLG
AHVQGQNLEQARAVASRIRAGQVHLNYPAWNPMAPFGGYKRSGNGREYGVQGFEEYLETK
AIVGFG
>tr|A0A0Q0DJU3|A0A0Q0DJU3_PSEAP Ribokinase OS=Pseudomonas syringae pv. aptata OX=83167 GN=rbsK PE=3 SV=1
MNPHRKCYLNNINKAVEASIGAGEVKITMSKIAVIGSNMVDLITYIERMPAQGETLEAPR
FAMGCGGKGANQAAAAALLGADVLMLSKVGDDAFADTTLANFQRLGIDTRFVERVPGVSS
GVAPIFVQENSHNSILIVKGANAHLSPADIDRAEAQLRDCALIVLQLEVELATVYHAIAF
AHRHAIPVLLNPAPALAGLSREHLAQLDFLIPNESELALISGQPVTCAEEAAQAARQLVA
LGVRHVIVTLGEQGALYVGAEGEWQVPGVKVQARDTTGAGDAFIGCFARHWSEDGNIRQA
MHQAVAYSACSVTGLGTQSSYPDAPTFARFLDASAN
>tr|A0A0N8T7V9|A0A0N8T7V9_PSEAP Type III secretion system helper protein HrpK1 OS=Pseudomonas syringae pv. aptata OX=83167 GN=ALO85_05591 PE=4 SV=1
MPSLTTWMATMRISSSPSPALGSIVNQPTSGELAAETPLAKASLTQGSAGGGQAFVQFGQ
ANDSPSSFSGTEQSGSSLMSLLTRSSSSSESTSSVDQDGDQVSPMTSVSATASSAPTAAS
SPANAPSATDAAFLDNSEYSSPEALKRWEPMVANLPPEEREQAAKELNRPIAAALMARED
GPNAEKAMAFINANPALKTAVDVGKDGGNADGKITNKDLKAFAKNMEKAADNADKDVAKY
MEDNPGADPQSLEMVRSAAVMRANMPLATAADPHHAVGAADKTDVDGNVSAEGLKALIKS
NPGLSGTLKQSCNMWSQAGFLSQVDEAGLTGRKKAAHSPDQVFDASNMSEWIRKSAPKNG
GQFASMLSDAATLNSVAGIDISKLNAQVFEKPEAYTGAQKAAVMIKLQQTQQSVIAGRDL
RNTEKTEAGLNERIARLQDDPDVQEYLNKSIPEQERSLVRSDSALQKAVTEQAQNVNSGK
ALQADMATADKAVDKHNPNPDYSGAITGLSAQLQLQKDLFPDAQVPTAQQVFNNQPDELQ
TKIADSYVRNFSEGGALKQLLGQKKSDAGESLQTADNQKAAYESVLPADFVNDERESYTA
STLSELQNSKKGRKLLEGKTDEEGGPSLAEQLAEQGIGGKAFNSVMGFASVSDRLASGDK
LGAAQGIIDSSRLGAEAIKGGIDTGAKMMGREASAGLGRLGGQMIGRAVGLVAGEATGLA
AGAALGAAIPVIGWAIDGAMALGFGISAIIDAVKKHKAQKAFDHNVDPVLDQFGIDRAH
>tr|A0A0Q0CIW8|A0A0Q0CIW8_PSEAP NAD-dependent epimerase/dehydratase OS=Pseudomonas syringae pv. aptata OX=83167 GN=ALO85_01480 PE=4 SV=1
MTVLVTGAAGFIGFHVARRLCELGVEVVGIDNLNDYYSVELKQSRLAILQRLPGFTFHRL
DITDTEGLSTLFSQNAFEQVIHLAAQAGVRYSLEQPNVYAQSNLVGFINVLEACRQYRPA
HLIYASSSSVYGANTRMPFQVEDAVDRPLSLYAATKRANELTAYSYCHLYGLRATGLRFF
TVYGPWGRPDMALFKFTKAMLAGQPVDIYNHGEMARDFTYIDDIVESILRLRLLPPDAVG
SEPPHQLFNIGRGQPVKLLEFVDCLEAALGLRAERRYLPLQAGDVLQTWADVSALSQWID
FQPQVSVDTGVRAFVDWYREHYQARICAPLQ
>tr|A0A0Q0DLJ1|A0A0Q0DLJ1_PSEAP Exopolyphosphatase OS=Pseudomonas syringae pv. aptata OX=83167 GN=ALO85_100092 PE=3 SV=1
MPEQLRVGGKHGSWHDGPVIEPDFLTMPHSTPKNFSLIAAIDLGSNSFHMVVAKANQGEI
RILERLGEKVQLAAGIDEERQLTEESMQRGLDCLKRFAQLINGLPLGAVRIVGTNALREA

RNRNDFIHRAEEILGHPVEVISGREEARLIYLGVSHTLADTPGKRLVADIGGGSTEFIIG
QRFEPLLRESLQMGCVSFTQRYFRDGKVTPARYAQAYTAARLEIMSIEHALHRLKWDEAI
GSSGTIRAIGVALKANGYGAGEVNAEGLAWLKRKLFKLGDVEKIDFDGIKPDRRAIFPAG
LAILEAIFDALELKRMDHCEGALREGVLYDLMGRHHHEDVRERTLSSLMERYHVDLEQAA
RVEAKALHALEQVAESWDLQHESYAELLSWAAKVHEIGLDIAHYHYHKHGAYLIEHSDLA
GFSREDQQMLALLVRGHRRNIPKDKFAEFGDEGIKLIRLCVLLRFAILFHHIRGTQEMPR
VTLRADGPNLDAEFPKGWLENNQLTQADFALEAEWLTRVGIVFSVR
>tr|A0A0Q0FMD3|A0A0Q0FMD3_PSEAP Phosphonate ABC-type transport system, permease protein OS=Pseudomonas syringae pv. aptata OX=83167 GN=ALO85_02772 PE=3 SV=1
MLSRDQRDPAATPRLLLTLLAVALLWPGIRLAELNPLVLLQADNARTMGSFLAGFWPVAH
SAEFLGLLLDATLQTLAIATAGIALALLLAVPASLLASQALSLSAASRGGRPGWLGQCLR
WPARGVLIFLRSVPEIVWALLFVRAVGLGPTAGVLAIAITYAGMLGKVYAEIFESVDQRP
AHALLQAGSGRLAALAYGILPNAAAELTSYTVYRWECAVRASVVMGFVGAGGLGQQIDLS
MRMFAGDEVASMLLTFLLLVLLADQLSRLLRRQFT
>tr|A0A0Q0DBG4|A0A0Q0DBG4_PSEAP Probable cytosol aminopeptidase OS=Pseudomonas syringae pv. aptata OX=83167 GN=pepA PE=3 SV=1
MELVVKSVSPETLKTATLVVTVGESRVLAGAARTVDILSGGAVSLILKRGDLAGKVGQSL
LLHNLPNIKAERVLLVGTGKEDELSDRQLKKIVGAALTCLKGLGGTDAAIALDDLSVKNR
DTYGMARLLVEALADGEYVFDRFKTQKADVRALKKITLLTDKIKAADVERASTHAQAIAT
GMALTRDLGNLPPNICHPTYLGEEAKALGKAHKNLKVEVHDEKKLADLGMGSFLAVAQGS
AQPPRLIVMNYQGGKKGDQPFVLVGKGITFDTGGISIKPASGMDEMKFDMCGAASVFGTL
RAVLELKLPINLVCILACAENMPSGTATRPGDIVTTMSGQTVEILNTDAEGRLVLCDALT
YAERFKPQAVIDIATLTGACVVALGGHTSGLLGNNDALINQLLDAGKQADDRAWQLPLFD
EYQEQLDSPFADIANIGGPKGGTITAACFLSRFTKAYHWAHLDIAGTAWLSGGKEKGATG
RPVPLLTQYLLDRAGV
>tr|A0A0Q0CFI2|A0A0Q0CFI2_PSEAP Transcriptional regulator NfxB OS=Pseudomonas syringae pv. aptata OX=83167 GN=ALO85_05623 PE=4 SV=1
MKTYPMELPADDKFLKALAVALVDHSNGTLKDIAQAAGVSKATLNRFCGTRANLVELLLN
HASDLMNQMVADADLQHAPPLEALQRLVDNHLMHREMLVFLVFQWRPDSLDESSGGRRWL
PYSDALDAFFLRGQREGLFRIDVGAAVLTEMFAALLSGMVDAERRGRVARVGMGALVTQF
FLQGAAAR
>tr|A0A0N8T9X6|A0A0N8T9X6_PSEAP Two-component sensor histidine kinase RstB OS=Pseudomonas syringae pv. aptata OX=83167 GN=ALO85_05003 PE=4 SV=1
MMNSIFLRIYGGVLGVLVLVALLGVLALHVLNQSRGEQYRERLAHGTFTIMADNLVPLDG
IERRRALAVWERLLGIPLSLQTLEQAHLDSSALGQLARGKVVVEQIGPHAARVYRQLSDK
EPLLLTGEVQQITEQLARATIYLLIDELVRFPVDEQPARLQALRSDKGFGFDLHLLALDQ
ADLDDDQRRRVYEGDTVMALGKGGDSIRVLAGIVDTNWVLEIGPLYQMNPYPLHWLLLIA
LMGLCLIGLVVYLLVRRLERRVLELEAAATQIAQGSLQTRVPTEGSDSVGRVAAAFNSMA
EHLQRSLMIQRELVRAVSHELRTPVARLRFGLEMVSDAATPEARRKYMIGMDSDIQDLDK
LVDEMLTYARLEQGAPTLNFQRVDLDALIDQVIAELAPLRADVRVSRGECLRAEEGEAAW
VEGEPRYLHRAVQNLVSNALRHADNEVRISYRLEAQQCRIDVDDDGPGVPEQAWEQIFTP
FMRIDDSRTRASGGHGLGLSIVRRIIHWHEGRALIGHSVSLGGACFSLSWPRTQVPR
>tr|A0A0N8T8Y6|A0A0N8T8Y6_PSEAP Putative iron-regulated membrane protein OS=Pseudomonas syringae pv. aptata OX=83167 GN=ALO85_01788 PE=4 SV=1
MKEGFRQSMAWLHTWVGLLFGWLLFAIFLTGTLSYFKDEITHWMQPEIPVRALDAGASVQ
QAQHYLQSHATGATRWFISLPDQRTPGLVVGWLPEGKQGQRDNFVRKLLDPQTGSEVQAR
DTRGGDFFYRFHYQLQMPYPWGRWLATSAAMVMLVALITGIIIHKKVFKEFFTFRLRKGQ
RSWLDGHNALGVLVLPFHLMITYSSLVIFMSMVMPASILSQYDTVQAFYSEAYPFPETVK
REGIATPLVALAPLVNAANAKWDSGQVGRVIVNNPGDSSASVLLTRDDKGSIVPDRGGAL
TFSGVSGELSNEVPERPLALLISSGMYGLHVGHFAAPLLRWLYFMFGVAGTAMIGTGLVM
WLGKRQLKHAKTGVLPLELRVVEVLNIASMSGLMLAVASFFWTNRLLPGGFAGRTNWEVS
VFFTFWALSFMHAALRKGRNAWREQLGLGALLFAGLPLLDLLICGRYLLDSLMSGNWVLA
AFDLTALVTGLFLGWAALKFKAVPIDKTARVSLPVAAQEAP
>tr|A0A0Q0FTS3|A0A0Q0FTS3_PSEAP Phosphonate metabolism transcriptional regulator PhnF OS=Pseudomonas syringae pv. aptata OX=83167 GN=ALO85_00815 PE=4 SV=1
MQLSRQVSPMYHELANILREELTTYQPGDFLPAEFQLAERFSVNRHTIRRAVDELVREGS
VLRRQGKGTQVLERPLIYPMQADSAYSKSWSAQGLGVEAILLRRQECQASREDAQHLGVD
EHAPLIELQTLRKLDGQAVSLIFHRYSARYSDLLADYRGGSVRQYLAERELPLSRAQSLI

GARLPSREEAAWLLMPRHMPALTVLTVSCDPAGQPVELSRSVSRADRFQYQVATPFKTT
>tr|A0A0Q0BSC0|A0A0Q0BSC0_PSEAP Uncharacterized protein OS=Pseudomonas syringae pv. aptata OX=83167 GN=ALO85_04562 PE=4 SV=1
MCDAHGQIWVESAFGHGVGLLKLEQGSAMSTASTPLSGVNQPLKGIGFILLATFLFASHD
TLSKYLSGFYPIILVVWARYVVHTLLMACIFMSSSGLRVLRTKRPGLQALRALALLGTSL
LFTSSLMFIPQAEATAVNFLAPLLVTALSVPLLHERVTRGQWTAVVVGFVGVMIIIHPGG
ELFTPAILLPLSSALCFALYQLLTRLVSPFDSPTTSNFFAGLFNTLLMSALVPFFWEVPA
LKHLPFLLALGACGMSAHLLLTQAFRFAAPAMLAPFSYCQIVFAGLLGYLVFDHTPSPTA
QAGIAIICLSGLAAAWQQRRKGG
>tr|A0A0Q0FL93|A0A0Q0FL93_PSEAP Fusaric acid resistance protein OS=Pseudomonas syringae pv. aptata OX=83167 GN=ALO85_01795 PE=3 SV=1
MKKFFSLFATLLVLAIALWIGRTLWVDYMDSPWTRDGRVRADIINVAADVSGTVVEVPVR
DNQWVKRGDLLMQIDPEHYRIAVKQAQALLASRKATWEMRKLNARRRADMDELVISAENR
DDASNVATSALADYQLAQAQLEAAELNLSRTRVLAAVDGYVTNLNVHRGDYARVGEAKMA
VVDMNSFWVYGFFEETKLPHLKVGDPADLQLMSGEVLKGHVESIARGIYDRDNPQSRELI
ADVNPTFNWVRLAQRVPVRIHLDQVPQDVLLAAGMTCTVIVRPVDG
>tr|A0A0Q0FEG2|A0A0Q0FEG2_PSEAP Uncharacterized protein OS=Pseudomonas syringae pv. aptata OX=83167 GN=ALO85_01151 PE=4 SV=1
MSMNPYLSIAPNVSRPAWHQAIPLPLLEAALILTWSSGFVGARFSLEHAPPLLVVFWRCV
MVTLILLPFVARQLRSIPAATLLKNAGIGLLAMTGYVAGVTQGIALGVPAGLAALFADLL
PMGMALLAAVVLGQRLAWQIWAGLFVGLIGVVLVTYSALAVGDAPLWAYGLPLLGMLSLA
IATLWQKQSGTAEPMALLPNLWLQCAVSSVAFAIIQGTQGSLAPVASTGFALSVLWTVGL
ATLGGYGLYWVCLRRATATRVASVLYLSPPVTMLWAWAMFDEPLSWQMASGMAVSGIGVW
MVVRAEQRQSAD
>tr|A0A0Q0CEI8|A0A0Q0CEI8_PSEAP Uncharacterized protein OS=Pseudomonas syringae pv. aptata OX=83167 GN=ALO85_101634 PE=4 SV=1
MLRNFLGAAFRQGLVPVGPMHGLAQTPGFAAPNQWGLALALPKYGIATSIFSAVAELDEA
MTRL
>tr|A0A0Q0FE37|A0A0Q0FE37_PSEAP Uncharacterized protein OS=Pseudomonas syringae pv. aptata OX=83167 GN=ALO85_00473 PE=4 SV=1
MLRLKDQAMSPFRILAATLVLFTGLQALPAVAADVTTGSGDPVYTIQNPPAFAMIGDLLI
ARPLLIAATVIGTGLFVIAAPFAAAGGNLGATGKALVVDPGKAAFVRCLGCTGDGYGKQQ
>tr|A0A0N8T9B6|A0A0N8T9B6_PSEAP Type III helper protein HopAK1 OS=Pseudomonas syringae pv. aptata OX=83167 GN=ALO85_02754 PE=3 SV=1
MNTINRNYYPVSGTSAQDAPAQTDQLQLQGQGIKPGHNSNLIDFGLTQQANGPHSSLNTL
GSRVQPTDTSTSSNMLGGNGEQVLNKLVQAIRNILNNLLSLLEGNLHNGSGPAQTQREQT
PTLAQSPSPSSSSSSPSTPQGNAEKPFVVQNDHPSEKPVSLQKNTEPTPAAPPQTAARTA
ERSSVTPDKTPARPDAVNPAVVNDPALPKTSTDTTKTDNTVKAAKTVTPAAHGQGADMSG
IVGFAKEANTTGGNGGEVVTVNTVADLKKYMEDDKARTVKLGANLSADSKVTINFGANKT
LLGTDKGNSLHNIYLASGKTASNDIFQNLNFDHDSRYRENGDMQMFISSGQKYWIDHNTY
SGTKDQNPKGLDKLLYVGGKADNVSLTNSKFQNNEYGVILGQPDDSAQAKAEYKGYPRMT
IANNVFSNLDVRAPGLMRHGLFHAYNNSIDNFHLGFTATGDATILSQANYFAKGVDVSDK
ASNSGVLDDYGDAHFKDIGSNVSFTQKSAVTAWTPSYQSNIKTAEAARAYDLAHAGANTV
N
>tr|A0A0Q0BZZ1|A0A0Q0BZZ1_PSEAP Superfamily protein (Fragment) OS=Pseudomonas syringae pv. aptata OX=83167 GN=ALO85_04429 PE=4 SV=1
ALVEINLATIASYKDGGVSAVVYAGTDGSLTVQMVRPPDDARKAKNSQNRGADPFTFGSP
TGGAPAE
>tr|A0A0Q0DD34|A0A0Q0DD34_PSEAP Catalase-peroxidase OS=Pseudomonas syringae pv. aptata OX=83167 GN=katG PE=3 SV=1
MSTESKCPFNHAAGGGTTNRDWWPKQLNLKILHQHSSLSDPMGESFDYAKEFKSLDIEAV
KQDLRNVMTQSQDWWPADFGHYGPLFIRMAWHSAGTYRTGDGRGGAGAGQQRFAPLNSWP
DNVSLDKARRLIWPIKQKYGRKISWADLIVLTGNVALESMGFKTFGFSGGRADVWEPEED
VYWGSETTWLGGEERYGVQKKMQQPGDGTLVAEPENHANEESRTASGERNLENPLAAVQM
GLIYVNPEGPEGVPDPVASARDIRETFGRMAMNDEETVALIAGGHAFGKTHGAGPADNVG
PEPEAAGLEEQGLGWRNKFGSGKGGDTITSGLEVTWTSTPTKWSNEYLENLFGFEWELTK
SPAGAHQWTPKNGAGAGKIPDAHDPSKRHAPSMLTSDLALRFDPAYEQISRRFLNNPEQL
ADAFARAWFKLTHRDMGPLARYLGPETPAEELLWQDPIPHVDHALVDDQDVAALKAKILA
SGLSVSQLVSTAWAAASTFRGSDKRGGANGGRLRLAPQKDWAVNQPAQLAGVLKTLEGIQ
SEFNAGQSNGKKVSIADLIVLAGNAGVEQAAKHAGQHVTVPFAPGRADASQEQTDVESFS

FLEPIADGFRNYQKGHYKVSAESLLVDKAQLLTLTAPEMTVLLGGLRVLNINVGQSKHGV
FTDQPGTLTNDFFKNLLDMGVEWKATAGGTDTFEARDRKTGAVKWTGTRVDLVFGSHAQL
RAIAEVYGSSDAHEKFVKDFVAVWTKVMNLDRFDLA
>tr|A0A0Q0FAU0|A0A0Q0FAU0_PSEAP Methyl-accepting chemotaxis protein OS=Pseudomonas syringae pv. aptata OX=83167 GN=ALO85_04075 PE=4 SV=1
MIMSRGLSKSLANASVSLKLAIGFGLVLLMTLMISATGWFSNQALIERGDRVTAIAEVNE
LTLQLRINRMRYEDLYNAETAAQVRSTLDQLDAALQTARNLLRSAENLQLLDVQIQATRD
YRQSFEDMSKAIESREVSRSQMGENADKAVDQADRIEAELLKEDNILAFNGIVGVSKLIQ
QARFQVRGYTYSGRPDFEKDANKAIDDAVTGINTLAGDISSTYSPMLQQAIAGLNGYRAA
VGRYRDAQAASKAALEKMTTLGVSMLATSNDLITRQNKSRDAESAKSVTMIAAATALALV
MSILAAWVITRQITTPLQETLEVVERVASGDLSRNLNVDRKDELGKLQATIQRMTVGLRE
LVGGIRDGVTQIASAAEELSAVTEQTSAGVNSQKVETDQVATAMHEMTATVQEVARNAEE
ASEAAVTADRQARDGERVVNEAIAQIERLASAVGNSSEAMGALKQESDKIGSVLDVIKSV
AEQTNLLALNAAIEAARAGEAGRGFAVVADEVRSLAQRTQKSTEEIEALIARLQSGTQQA
TTVMDSSRELSTSSVELTRRAGGSLESITKTVSAIQAMNQQIAAAAEEQSATAEEINRSI
INVRDVSEQTSAASEETAASSVELARLGNHLQVLVSRFTV
>tr|A0A0Q0CHB8|A0A0Q0CHB8_PSEAP Transposase OS=Pseudomonas syringae pv. aptata OX=83167 GN=ALO85_200210 PE=4 SV=1
MKNHVMAYALSALMLTTVLPAAHAAVPGVPVLDPSNLLALKANALAQAKQAMDALSTAKD
AITQTAQQYNHYKSIITGNDMLGGFLNDPALNKVMPLGDWADVYSTGRDIASLRDRYGLT
SDNASVQAKFDQMMSAADALERNYNASTERVKNAELLRARLNEVQTPQQKEDLQLRYQQE
LIEQQNQQMRLANMQMLQQQQEKMENEQRAQAFSDYMKGKTSVRPSYD
>tr|A0A0Q0C8B7|A0A0Q0C8B7_PSEAP Inorganic pyrophosphatase OS=Pseudomonas syringae pv. aptata OX=83167 GN=ppa PE=3 SV=1
MSYTDIPAGNAIPDDFFTVIEIPANHSPIKYEVDKPSGQIFVDRFLSTPMFYPANYGFIP
NTLSDDGDPLDVLVICPYPVSPGVVIRSRPVGVMYMTDEAGADAKVIAVPHEKLSSMYSN
VKECSDLPALLLAQIQHFFENYKALEPGKWVKMGRWGSADEAREDIRKSVAAYNLKKEAS
K
>tr|A0A0N8T851|A0A0N8T851_PSEAP Uncharacterized protein OS=Pseudomonas syringae pv. aptata OX=83167 GN=ALO85_00456 PE=4 SV=1
MKLVPKLLAAAVCFGLASQAIAATELKHWPEPAAKALDSMIAANANKGNYAVFDMDNTSY
RFDLEEALLPFMENKGLITRETLDPSLKLIPFKDTAEHKESLFSYYYRLCEIDDMVCYPW
VAQVFSGFTLQELKGYVDELMALKKPIPATYYEGDTVKQLNVEPPRVFTGQTELYNKLME
NGIEVYVMTAASEELVRMVAADPKYGYNVKPQNVIGVSTLLKDRKTGELTTARKQITAGK
YDPKSNMGLELTPYLWTPATWMAGKQAAILTYIDQWKKPVLVGGDTPTSDGYMLFHSVDV
SKGGVHLWINRKDKYMTQLNGMIKDNAAAQAKEKLPVTADKNWVIVKPDEIQ
>tr|A0A0Q0CVE0|A0A0Q0CVE0_PSEAP Filamentation induced by cAMP protein Fic OS=Pseudomonas syringae pv. aptata OX=83167 GN=ALO85_101251 PE=4 SV=1
MNPTAAFCNDRERYYAMLATADKGTKEGLEAWCTYVLEGIRDEVEKVDRLTDYAYLTKCI
LVPAVAFAREREWITETEESVLSAAIKLKIVKSADVAKVLPRQSSNQRTYLIRKLVDQGM
LLPLSAGAKQYTIGFSNNYLIRGVIKSLRDQGFIPEPLEKP
>tr|A0A0Q0DUV8|A0A0Q0DUV8_PSEAP Multidrug resistance protein NorM OS=Pseudomonas syringae pv. aptata OX=83167 GN=ALO85_04911 PE=4 SV=1
MQPSALKELWIILRLAGPLIASQMAHMLMVFTDTVMMGKIGPEALAGGGLGAATYSFVSF
FCVGVMAAVGTLVSIRHGAGDTEGATRLTQAGLWLAWGMALVAALLLWNLEPILLQFGQA
EANVHMAAQFLITLPFALPGLLSFMALRGFTSALGRAGPVMTISLAGAAANFVLNYALIH
QWLGLPNLGLMGIGLVTAIVTNCMALALALHIRRHPAYAAYPIRKGLSKLSRSHLGELWR
LGLPIGGTYAVEVGLFTFAAFCMGAMGSTQMAAHQIALQTVSMAFMVPVGMSYAVTMRIG
QHYGAGNLLMVRMAGRLGIGFGGCVMLMFGLLFWLAPHLVIGLFLDINDPAFAEIVVLAT
KLLAIAALFEFFDGTQTIAMGAIRGLKDARTTFLIGLGCYWLIAAPAAWLLGFSTDAGAS
GVWWGLALGLFCSAVALTYAFEWKTARLLRRDAAGVAVPG
>tr|A0A0Q0DB98|A0A0Q0DB98_PSEAP Ribosomal RNA large subunit methyltransferase F OS=Pseudomonas syringae pv. aptata OX=83167 GN=rlmF PE=3 SV=1
MTDTRKPPRKKPQRPAKPAAPREKATLHPRNRHQGQYDFAKLIKSSPQLAAFVILNPYGK
ESIDFANPQAVRVFNRALLKAFYGIAHWDIPADYLCPPIPGRADYLHFLADLLAEDNEGV
IPRGASIKALDIGTGANCIYPLLGHSDYGWQFVGSDIDSTAIAAATTIVKANGLSKAISV
RQQGNRKQILLGLLDSSERFHVSLCNPPFHASLEEAQRGSQRKWRALGKADPKRKLPVLN
FGGQSQELWCDGGEIGFVTQLIQESALLPSQVAWFSTLVSKASNLPPIQSALKKAGVLEV
KVVEMGQGQKQSRFVAWTFLDKAQRAQF

>tr|A0A0Q0CE55|A0A0Q0CE55_PSEAP Uncharacterized protein OS=Pseudomonas syringae pv. aptata OX=83167 GN=ALO85_00262 PE=4 SV=1
MMNNDVLRSVRYMLDISDSKIVEITALTGFEVSKSDVIAFMKKEEEEGYLDCSDEIMAHF
LDGLVYFKRGKDDSRPPQAIDLPITNNIVLKKLRVAFELKEDDMHAILKSVDFPVSKPEL
SALFRKVGHTNYRACGDQLLRNFLKGLTLRVRG
>tr|A0A0Q0C3L7|A0A0Q0C3L7_PSEAP Methyl-accepting chemotaxis protein OS=Pseudomonas syringae pv. aptata OX=83167 GN=ALO85_01467 PE=4 SV=1
MLSIKTNLGSFVAHKKAFSGDFMNSRFANISVNMKLALGFGTVLFFTAVLALVGWTSLDK
LIYRTDRIGDITRLSDNLTTLRIARLQYMLTDGDETAARNMQSKLDVFKAQQQSLLVSFN
NPVNLKPLRELADVTRDYEASLNSMRAAYQAGAKVRDEMTANGTAAMQAVESLNNAVMQI
DPSDPARFDLAQLANSARQDLVLVRYEVRGYTGNPNDKTETAAFQQLDSAISHLDRLKAA
FGTANREQIAQFESALRNYRSSVDAFKATTQTAASVRKDLTTQGATIVKLGEELYGLQMQ
MGKEDTAHARSLQMGCVVLVMLLGILAAVVITRQITRPLRDTLAIVERIASGDLTHTEAV
TRRDELGVLQQGIQRMGTTLRELISGIRDGVTQIASAAEELSAVTEQTSAGVNSQKVETD
QVATAMHQMSATVHEVARNAEQASVAASDADKQAREGDKVVGEAIQQIERLAAEVVRSSD
AMNVLEQESDKIGKVMDVIKAVAEQTNLLALNAAIEAARAGEAGRGFAVVADEVRGLAQR
TQQSTEEIEGLVSALQNGTRQVSGIMLGSRTLTDSSVELTRRAGTSLESITRTVSSIQAM
NQQIAAAAEQQSSVADEISRSIVNVRDVSEQTAEASEETAASSVELARLGGQLQMMVSHF
RV
>tr|A0A0Q0DYF4|A0A0Q0DYF4_PSEAP 1-aminocyclopropane-1-carboxylate deaminase OS=Pseudomonas syringae pv. aptata OX=83167 GN=ALO85_00623 PE=3 SV=1
MMLDALGWQPTAPLERLNLPWLQKAGVEVAILRLDLIDALISGNKWFKLSEHLSLAVDAG
AEGLISLGGAHSNHLHALAAAGKRFGFPTVGLLRGHAQQTPTVLDLQAFGMQLHWLGYAG
YRERHAAGFWSPWQARYPGLYPIAEGGGGLAGALGCGRLRAMLDAQLGHLGWGDYHGWWL
AAGTGTTLAGLLLAEAGARPVYGAMAVPEDHGVAPNIIAVLNDAAQAQADVKPCLPPGCV
LLDASRGGFARTDAALLDFIAGSEAQSGIPLEPLYTGKALLALHDEVLAGRFAPGTRLVF
VHTGGLQGRRAMCL
>tr|A0A0Q0D5Y2|A0A0Q0D5Y2_PSEAP Short-chain fatty acid transporter OS=Pseudomonas syringae pv. aptata OX=83167 GN=ALO85_02721 PE=4 SV=1
MAANIEESRSARFALRCAAWAERWFPDSWVFAALAVVIVTLATLAIGARPAEAAKAFGDG
FWSLIPFTMQMAFVVIGGYVVASSPPAVRLIDRLARVPRNGRSAVAWVALISMLASLLNW
GLSLVFGGLLVRALARRTDLRMDYRAAGAAAYLGLGAVWALGLSSSAAQLQANPASLPPS
ILAITGVIPFTETIFLWQSGVMLAALVVISLIVAYATAPGPNSARDAKACGVDPAFNLPP
LPPRTRPGEWLEYSPLLIIILMVLLGVGWLFSEFSSKPAITAISGLNTYNFLFIMLGALLH
WRPRSFLDAVARAVPTTTGVLIQFPLYGSIAALLTTVKGGDAQTLAHYISTFFTSIASHD
TYAILMGVYSAILGFFIPSGGGKWIIEAPYVMQVANDLQYHLGWAVQIYNAAEALPNLIN
PFYMLPLLGVLGLKARDLIGFSFVQLLVHTPLVLFLLWALGTTLAYVPPVVP
>tr|A0A0Q0DKR2|A0A0Q0DKR2_PSEAP Chorismate synthase OS=Pseudomonas syringae pv. aptata OX=83167 GN=aroC PE=3 SV=1
MVFWLIFRSARMSGNTFGKLFTVTTAGESHGPALVAIVDGCPPGLELDLQDLQRDLDRRK
PGTSRHTTQRQEADEVEILSGVFEGKTTGASIGLLIRNTDQKSKDYSAIKDLFRPAHADY
TYHHKYGIRDYRGGGRSSARETAMRVAAGAIAKKYLASQGIVIRGYMSQLGPIQIPFKTW
DSVEDNAFFCPDPDKVPELEAYMDQLRRDQDSVGAKITVVAEGVMPGLGEPIFDRLDAEL
AHALMSINAVKGVEIGAGFDCVAQRGTEHRDEMTPQGFLSNHAGGILGGISSGQPIIAHL
ALKPTSSITTPGRSIDVDGNAADVITKGRHDPCVGIRATPIAEAMMAIVLLDHLLRHRGQ
NANVSVNTPVLGQV
>tr|A0A0Q0CYQ1|A0A0Q0CYQ1_PSEAP Beta-ketoacyl synthase OS=Pseudomonas syringae pv. aptata OX=83167 GN=ALO85_04826 PE=4 SV=1
MSSTKTDLDSTQVAIVGVGLRLPESDSIDAFWSKLKAGKSLISEVNEKRWSKAELYGDPK
TGGDKTSSIWGGFVDDIECFDANFFGISPRESKFMDPQQRIAMEMAWHAFEDAGICPSSV
KGTNTGVFMGVCHWDYAELLALNHTPIDPYFPTGTAYSILSNRISYYFDLKGPSISIDTA
CSSSLVSLALAVNAIKSGECELALAGGVNLIWSPQHFIAFTKNGMLSKDGKSYTFDDRAN
GYVRGEGGGLLLLKALDKAIEDGDNIYAVVRGIANNHGGKTGSLTVTNPQAQADLIAELY
RRSNIDVRDVSYIETHGTGTPLGDPIEIHGLKKAFAQASHAVDDPQSRKNYCGIGSVKTN
IGHLEGAAGVAGIIKILAALKNKELPGNLNFKSINPKIELTDTPFYIVDKHQPWASPAEG
STGRIAGISSFGFGGSNAHAVIQEFIPETAQLSARAVADNHPVFAIPVSAKSSESLVAYA
QRLLAFLEDNPSDLPGIAHALQCQRDALTERVCFVVESIDELKLAIRSFIDTRAAAEHTF
VGNSYDAGEGKKLRSYAEENRASLTENWMTNHDMLSLAQYWVFGVDVDWNDLYLDASLIP
AKVRLPLYSFERTAYWFPKKNADVAKPKQPGTVEFTLSSKAFFLEDHTVNNQSILPGVHY

LEMILSTIPRADKMINVDFSNVFWLSPFAIELSDDDVDIRKSIYLDFDSKQADANAASGP
PGKGFRFYTHPKNIEGQAKDNVRVHVQGEYQPGSTVADRIDLPDMSSFKEIHRDQCYQYL
QSTGIYHGPRLQGVEQIHVGVDEVWVKVSIAGSQQSFSAHPAILDSVLQAGIATALSNNA
SRYPYVPFSLDSLSVYQIFPTTVWARIREKNRFAGTKASLTFDLNIFTEQGEQVASAKNF
TMFLLRSPVGEQMTSQNNSGSNEPSSQNLYFSKQWVVDSTDHQQPPISSTAPADRANHVL
ALYVGSTIQDDRWIANYRNQVSAQTSCTFQAMALSSPEAVSSAPTIEAYSHVFSYIKKLL
ETKPKTIQHLLVCASSTLPDVIRSSLTALLRVAHNENPRIQGRFVSIENSPLVDSGSIIA
REFLIADGAFSVRHDAQGLRRIERITAQPVRPVQGKIPVLNEQPVFWITGGLGGIGQLIS
RQLAADFPGCTLYLTGRKTAAEQQAVFSALKSEIQARGGKVDYQPLDITDVVKVENFVAA
LKNTHHSVDVIFHAAGHIADNFILRKEVKDSLPVLSPKLDGTLAIDQAIKRLPLGKFVLF
SSVASTFGNAGQSDYAAANAFLDAFAIDRNQRVREGEGTGQTIAINWPLWQEGGMKMDAA
TLASVHETTGMEPLPTHEAMHALRYALTTTDDSPILVTYGNQEKIHAYIAGLNQRSVVPE
GGSQESPDPSKSGDDVDNEALFQYAQDYLKGKLAIVIERDAKTIRGDQKIEEYGFDSIMA
VEMIRQLEGQFGQLSKTLFFEYFTINEVADYLMDEYSEKLKSLYLESRAPVVEVAAHTDT
PSKDKQVAEEATAAPAPSINKQIEAVPVASQVAADDRDQHDIAIIGLSGRYPKSRNMDEL
WELLKSGQHAFEKIPADRWKHDAIYYKERDVWGKSTIKTGGFLDDIDKFDPRYFQISQLE
AEKMSPEVRLFLEVGVEALEDSGYSKESLQQLYKGDVGVVAGTMSNHYNLYGFQNNLTRG
GSASGSYTGTIPNMLSYFYGFTGPSLFVDTMCSATSTCIHVAVQMLRSGESKIVVAGGMN
LLLHPYNLISSSQEHFTTKTSDVIRSYGIGADGTILGEGVGAVVLKTLADAKRDGDNIWG
IIKGTALSNAGVRNGFTVPQPHMQALAIEKAIEDAQIDPQTIGYFEGHGSGTELGDPVEI
KGATQAFRKYTDKKQFCPIGSIKSNVAHLLAAAGIAGISKILLQFKHQQIVPSLGADTLN
SNIRFEDSPFYVHKELQSWPAPRSESNLVLPRRASITSIGAGGMNSHMILEEYVDPRTTS
RSPRQTQQQLFVFSATNRDSLVRNLQGFSSYLKDHRDVSPVSLAYTLQVAKNQMKCRLAF
CADSVNEVIGSIDSWLGGLSSNRSVRFVDSVSGATKEFSRRELSQAIESAELETLAEYWT
NGSTVDWFALHGLNDQAALGAMIKVRIPAYQFEKKRCWFEVLEDAPSVIDPLGNRNKYHP
LVRDNTSDLSGIRYSADLIEIEILDYLYRVKKQPQLITFSLLDAAIAVMKQADPQSGYTS
VRWELLAPTPVVPSQLCYAVALNDQTGQFSDVYISSTADASTPHQLCARVEFAVQAGDGR
RVLQQPFPAFANATVRESLSGAQVSKALEEHKIVFGDYVNAISELQLIGEQRYRVHLKDT
DYRHNHFARNTTFLSELGSALQQAVLYIALRQGLDHWSDYALCACNVQLDLASVSKVSSL
LFSLESSGQALRGTIALADKNGTLLSLLDDVYIANSQAIEPRAAKTIETASFRSQPVFRH
ETAESPRPVAAAGTESDGLGKAVSAIVAKLLKFDESEVDARTSFYEFGFDSIGLTQLSKE
VNETLGARITPAVFFDCENIEALCFYLKDQGVSGTSEEAPQNSASAVNQAATPVSRVEKP
EAERNHRLPHPHESIAVIGMAGRFPGAASVEALWSNLLNNQDSIQDFPYQRYNQDTQAAF
AGVACVKKGGYLDKVDGFDAAFFNISRVEAEFMDPQQRQVLEVVWHAVENAAYDPSQLPS
DTGVFIGVSNNDYRALLQKDQELDGYIATGNSHAMLANRVSFFLDIHGPSESIDTACSSS
LVAIHNACESLRSGSASFAIAGGVNLLLDIQGFAEPDHAGMLSLDGRCNTFSRNANGYVR
GEGVGVVILKPLSLAQKDGDNILAVIESSAHQHGGRAKSLTAPNAKSQAELLRTTLKKID
LSQLQYVEAHGTGTELGDPVEVNALKQAVTETHGLPIYLGALKSNIGHLESAAGVAGLIK
AVKVLSERTVPANLHCSPLNPYIELHDSRFVINERQSSLKASDQGIFAAVSSFGFGGSIA
SVVLRRYESNRTTARAHPAFSGHEVVLVLSAKTRRGLVSQAINLRQHLSSHPEQIEEVAY
TLQVGRAELSYRLSIVAMDSADGLSKLTRFIESEAALSDADFSTGANELNHVSIFTGHIK
GSKTEKMSNEALPKLLTLDNANRVAERFVKGVKFNWNLLYGGARPNRVVLPGYPFEQQRY
WLPKDQVTTWYDADNFSNEQFVEHSFYENLLDRVLLDDLDTETVLQLLRKD
>tr|A0A0Q0ISW7|A0A0Q0ISW7_PSEAP Uncharacterized protein OS=Pseudomonas syringae pv. aptata OX=83167 GN=ALO85_03270 PE=4 SV=1
MALSGDEADDHEFRSRSYPVQEDMAQQRRVWRFERIGWAGLIVLIVLTLAGLFSKGPLSQ
VEPQTADGKLQVSYERFSRNGSQDDMIVTSKGASSEMRYLVVGSGLLEGVSIEKLNPQPA
PLHSEGRDLVIPMQADKDGLATLYLTVRSNGVGLYRGQMHMLGGETLPMPRFIYP
>tr|A0A0Q0C836|A0A0Q0C836_PSEAP Tripartite ATP-independent periplasmic transporter DctQ OS=Pseudomonas syringae pv. aptata OX=83167 GN=ALO85_01117 PE=4 SV=1
MSLMKNTLLRINDTLYRVCIGIAGLSVLIMTLIIPWGIFARYILGSGSSWPEPTAILLMV
VFTFFGAAASYRAGAHMAVSMAVDRMPQQVRRVAGVVVQILMAIVALFMIFKGFKLCATT
WNQFVGELPFLRVGISYLPIPLGGLVTLVFVLERLLLGDQSHRAVMRFDVIEESEGAA
>tr|A0A0Q0DST2|A0A0Q0DST2_PSEAP Outer membrane porin OS=Pseudomonas syringae pv. aptata OX=83167 GN=ALO85_00841 PE=4 SV=1
MSKLAHDAASAVSAPFFRGKRCGLALGLLGISAIPDPAQAQGFLDDSHGTLTLRNYYMDR
DYKDDGAKTATREWAQGFIMNAESGFTQGPVGFGLDARALVGVKLDSSPDRSGTELLPVS
ASDGRAADDYSRLALTGKLKFHETTVKTGDVSIFLPFAFASPSRLLPQTFRGTTLTSRDI
DDLTLNAGYIDRVNKRDSSNYQAISIASPNRRFNGAATSSHMAYAGGDYQVDKDLSLRAY

HAEVDDLYTQNTLALLHKLAIGDGVLSTDLRSFFSRDTGSAKAGEVDNQNLSALLGYKWG
GHSVSLGYMHSSGDTATPYVSGTELMGLSEMTMSSDFLNAKERTWQAIYDYDFTAVGLVG
LRSRLRYVRGDNIELAAFNADDRKEREFQMELGYVVQSGPLKNVGLLARKSIYRNDFPAG
AAFRDENQTRFIVQYSLPLW
>tr|A0A0Q0DLT1|A0A0Q0DLT1_PSEAP Exodeoxyribonuclease I OS=Pseudomonas syringae pv. aptata OX=83167 GN=ALO85_05113 PE=4 SV=1
MSRPTLRRSFVQDQPVTSSIFWYDYETTGINPRNDRALQMAGIRTDTDLNEIAPPVNLHC
QLSDDILPHPAACMITGITPATLAEKGLCEADFMTRVHAELSAPGTCGAGYNTLRFDDEV
TRYSFYRNFFDPYAREWQGGNSRWDLIDVVRAAYALRPDGIVWPEQDGRVTLKLERLTAA
NGIDHGQAHDALSDVRATIALARLIREKQPKLYDYLFALRSKQKVQEQVRLMQPLVHISG
RFSAARNYLGVVLPLAWHPHNRNALIVCDLHLDHSPLLHEGAETLKRRLYTRHDALGDGE
LPVPLKLLHINRCPVIAPLGVLRSEDQQRLQLDMAGYQARAAQLSERREVWHDKLAAVYG
KDDFVASEDPEQQLYDGFIGDRDRRLCEQVRQAEPEQLARDAWPFDDARLPELLFRYRAR
NFPDTLSGEEQTRWRDFCQQRLRSPEWGAPNTLHDFTTAWVECSLSAAPEQLEVLRQWQD
YANKLSNRLGV
>tr|A0A0Q0IP26|A0A0Q0IP26_PSEAP Putative methyltransferase OS=Pseudomonas syringae pv. aptata OX=83167 GN=ALO85_04988 PE=4 SV=1
MRAVDPAPAVDAYRSAGNSGSGAVAVRQGAMIAPQHLQRTLSELLGDARLTITALPGTEL
KLWLIDDANMDRAFSPDETRRILEDPPYWCFCWASGLALARFLAENPHWVAGKRVLDFGA
GSGVAGIAALRAGALEVVACDLDPLALAACRANAELNQVPLRYSTDFFAEADRFDLILVA
DVLYDRANLPLLDQFLSRGREALVADSRVRDFKHAAYQRLTMLHAHTLPDLAEPHEFRDV
SLYHAAR
>tr|A0A0Q0DPQ4|A0A0Q0DPQ4_PSEAP FAD dependent oxidoreductase OS=Pseudomonas syringae pv. aptata OX=83167 GN=ALO85_03782 PE=4 SV=1
MLIDYEIAVIGAGITGASIAAKLCSAGVSVVLIDKGAAGSLGASGYSGGLVRLYDSDPLL
MELAAYSIRLMDDGVFATTYASALRRTGVIYRAATDQLDNLHRAIDQYGSERYPMRLLSG
HELDGRRYPHCPTLERVNLFEPRACVGNVRQAVAALCQSVRQQGLLLEHREIKSIDCRAP
DLVHIDLGSATLRCRAVVVAAGAWSQHLVSHPGLEARSIPLARVLTEGDWSMPVIDAVTQ
SYGIPLTRHIVQTGCGARDSGLWPEHLAQPDARHREDACKRVAQLCASAGKPQVLDVLPG
FDSYSADGRPMLGFCAERSPVYLAAGMSGLGFKFAPGVAHIAVEQLRRHLAGKDRACSGW
SALSPGRSMPGFSAQDLALASVQS
>tr|A0A0Q0C9P9|A0A0Q0C9P9_PSEAP Uncharacterized protein OS=Pseudomonas syringae pv. aptata OX=83167 GN=ALO85_05645 PE=4 SV=1
MDGVSAQLHVFHRRNVTVVIFFLHERAXXXXXXXXXXXXXXXXXXXXXXHQAVRHDAVGIR
LERLLGRIAEHQAGSVHWPVRAEQMFADKTVDTVWSCNTIGIRKLVXXXXXXAVLKDIVR
ALGLIVHADGCHQGRKYFPLFTREIFCDRQADTTYLESGPSHVFMTWWHRTQFDTPYCVR
TAFTGDVAFKHHIQLTVIGINERAGVAKVPGRVLRFGLIIGAVGLEQXHALIGRHNTATY
FGDRCAGCDAGVIKINIAFVIQQYGGARPLSGGGRYTLGHYATTKDPVLAGTRVPVAHAC
TAMTIFPVTLDVITAIGVEVLRIIGVTVSRQGQDDRPAVDXXXXXXXXXXXXXXXXXXXX
XXXXXIFIDLQRIGAGPDSGSVFDVTC
>tr|A0A0Q0C569|A0A0Q0C569_PSEAP Uncharacterized protein OS=Pseudomonas syringae pv. aptata OX=83167 GN=ALO85_04206 PE=4 SV=1
MNTSQHEFSRFITALGAQLGTSLTWQNGVCALYDGQDNEAAVIELPEHSEMVIFHCRVGR
CPERSADLQHLLSLNFDVARLHGCWFAVDQGDVRLCAQRELVSLDEPAFCDVTRGFIAQA
REARAFLHA
>tr|A0A0Q0ICG7|A0A0Q0ICG7_PSEAP Guanine deaminase OS=Pseudomonas syringae pv. aptata OX=83167 GN=ALO85_01163 PE=3 SV=1
MSPNESAMSSVSRRKAYRAAIVHSLADPAEVALEASYEYFADGLLLVEDGKIVSVGHAAD
LLERLDDDVELIEYPDALITPGFIDTHIHLPQTGMIGSYGEQLLDWLNTYTFPCESQFAD
PAHSAQVADIFIKELLRNGTTTALVFGSVHKESVEAFFSAAQALDLRMIAGKVMMDRNAP
DYLVDTPETGYADSKALIERWHDKGRLSYAVTPRFAPTSSPEQLSLAGQLLTEYPGLYLQ
THISENLQEIEWVKALFPERKHYLDVYDHFNLLGERSVFAHGVHLCDEQCARLAQTGSAI
AFCPTSNLFLGSGLFNLPMTEKHKVNVGLGTDVGGGTSFSILQTLNEAYKVMQMQGARLN
PFKSLYLATLGGARALRLEDKVGSLKPGNEADFLVLDYNATPLLSYRLKQAKTIEEILFV
LMTIGDDRTVKQTWSGGRLVHDRG
>tr|A0A0N8T910|A0A0N8T910_PSEAP Deoxyribose-phosphate aldolase OS=Pseudomonas syringae pv. aptata OX=83167 GN=deoC PE=3 SV=1
MMNSLEPAALAQAIDHTLLAADASREQIATLCAEAREHGFYSVCVNSSQVPFAARQLAGS
AVKVCAVVGFPLGAGLSASKASEAALTIAAGAQEIDMVLNIGWLKDGLFDEVRDDIAAVL
QACGSVPLKVILETCLLDEAQKVRACEICRDLGVAFVKTSTGFSRSGATLEDVALMRRVV

GPDIGVKASGGVRDVATARAMIEAGATRLGTSSGIAIVTGAGTGAGY
>tr|A0A0Q0DB02|A0A0Q0DB02_PSEAP ABC transporter OS=Pseudomonas syringae pv. aptata OX=83167 GN=ALO85_04241 PE=4 SV=1
MLAPSLETIGASKHFGAFRALDEVSFKVRAGTVHALLGENGAGKSTLVKGIIGYSPLQSG
SILINNREHEIRTPRDSHQLQIGMVYQHFTVAPGLSVAENLVLSRGDLPWRIDWASEHQR
LEAFMAKMPFRLDIHRPVSSLAAGEKQKLEILKQLYLERRLVILDEPTSVLTPQEADEVL
GLMQAMAHRGELTVLMITHKFREVSTYADDVTVLRKGRWVGSAAVADTTTQQMAAWMMGE
AQVRGVSTERPAMAPRPPQLQVAGLDVPGDKGTLAVKNLSLAVHPGEIVGVAGISGNGQR
ELTEALLGQRAFSAGHIAVDGQPYHARREQMQRLRVFSLPEEPLRNACIGTFSVADNLAL
RNFDQPPLCRQGWRLDRRAAGVQARSLIEQFQVSPNDPNRPIGTLSGGNVQRAVLARELT
HDVAVLIVANPVFGLDFASVALIHQRLIDARNSGTAVLLLSEDLDELLSLADRIVVIHDG
ELVFETSAADADRTELGRHMAGAEHLAGEPA
>tr|A0A0Q0FB90|A0A0Q0FB90_PSEAP Uncharacterized protein OS=Pseudomonas syringae pv. aptata OX=83167 GN=ALO85_02305 PE=4 SV=1
MHQALLLSFEDITLAIAATHGAIIMPGYCVMDFLLNACLSTHGLEAVAESMGLALRRLKL
ANVT
>tr|A0A0Q0FV00|A0A0Q0FV00_PSEAP LPD7 domain-containing protein OS=Pseudomonas syringae pv. aptata OX=83167 GN=ALO85_200189 PE=4 SV=1
MLIRVSGYNTGAQEYLEQGNKSGREFTRDELDHRLIIEGQLSVTRAIYESIPDHGQDRYL
TFTLSFKEDTVSPELLKAVTTDFKNFFMHAYKPEEFNLYAEAHLPKMKTVTDRKTGEVID
RKPHIHIIIPRINLLSGNEANPVDVYKNHEKYFEAFQEHINQKYGLSSPRENVRADITDA
ASVLSRYKGDDFYGKNRQFKQQLVKQVIERGVTSRADFYALVAEHGETRIRNEGKGNEYI
SVKLPGDAKGTNLKDTIFQDAFIVRRELKKPPLEASVIQERLLAWPQRAREIKYVNKATP
KFRKQYSQASPEERVRLLAERENNFYRAHGDDYESVHTWQRQRDNQRSPAETAGRRTAAP
ADGLQDLSVSDVADHRQAGPTRSRDGALLLPSDAHVHVGQSQPGGDSGLRSSVPAGGRGR
RAGSTTERGRGGSQPAAVSQETKGTATPAGETGRRRAGAGKPRNTRVRAGRVVPPYAQNP
HRVATIADIEERGRRLFDPLKRPSDNALVFYRSSSHPRVAGQLPQARAATTTPVQSTAGR
RPRSSGKPRPPRQWRPGAVPPYAKNPHRVATVADIEQRARMLFDPLKRPADKALVFKRAS
IKALTVNKHASTVAAYFTRQAQHNQIAPAHRRAIRRIDQQYFALRRAVFSDQRLTRQDKA
QLVSVLTFERLKAREQFHNPKPNIEVNLMGSAAIRNLLDDEKEDPGFSISGARGPGPEGV
RERVKRVMDRFAKQVDPVAASERARDLSAKDLYTRKAKFSQNVHYLDKQTDKTLFVDTGT
TISMRRTGITEAGVSVALQLARERFGSTLTINGTAEFKKLVIEAVAKNGLDVHFTDKAMN
QSLADRRAELDIERHGQSIGPATDLPRHVDDATRDVRDQADRLGVTVPIEALYGQGKTAD
QVSQALATQLDTVPEPERIAFVETVAITLGIPERGQPKGDQAFAQWQAQRAQPAANSAAS
ATSEAQANVPTPSDAVSEPVSPEAAKPALANDPDLQSPSELVRLEAQWRRDFPMSEAEVR
ASDTVMGLRGEDHAVWIIATNDKTPEAAALLTAYMENDSYREAFKASIVAAYKQVENSPK
LVDDLDHLTAMAAQIVNEVEERLFPTPQAATGQTAPSRSKVIEGTLIEHGEAPYQHNDDN
QMSYFVTLKPEGGKPRTVWGVGLEEAMSDADLKQGDQVRLQDLGTQPVVVQVIEEDGTVT
DKTVNRREWSAQPVAPEREVAETTPKGQAAAAGTPELSSPDEDDGMSVD
>tr|A0A0Q0DSD7|A0A0Q0DSD7_PSEAP ABC transporter ATP-binding protein OS=Pseudomonas syringae pv. aptata OX=83167 GN=ALO85_03364 PE=3 SV=1
MEAGMAFLQLEGLSKRYGSIDAVVATNLAVDKGEFVSLLGPSGCGKTTTLQMIAGFVDVS
DGRIVLDGRDITHAKPSSRGLGVVFQSYALFPHMTVADNVAFGLRMRKVPAAELHPRVKR
VLELVRLGQHAERYPRELSGGQRQRVALARALVIEPPVLLLDEPLSNLDANLREEMQFEI
RRIQNEVGITTLMVTHDQAEALSISDRVVVMQSGRITQIDEPYKLYEHPRTRFISGFVGK
ANMLQGDLDSSGIPQIRQVPGDGSLTLSLRPEKIDLVAPGCGRLSGRIVTRYFLGSQWLY
RIQTGIGEVTVVRRNDGQVPLEQGAAVDMDWPTELLRVLDADEVRA
>tr|A0A0N8T9D0|A0A0N8T9D0_PSEAP Uncharacterized protein OS=Pseudomonas syringae pv. aptata OX=83167 GN=ALO85_101468 PE=4 SV=1
MRFPCCFHRHITAELVRDSDSRSHVPRALADRHSHELSENVPLLREQVRSYGLRPESKTG
WRTAKIAAYKIFE
>tr|A0A0Q0BW85|A0A0Q0BW85_PSEAP Methylmalonate-semialdehyde dehydrogenase OS=Pseudomonas syringae pv. aptata OX=83167 GN=ALO85_03052 PE=4 SV=1
MSSIQHLIHGEFVSAEGRSANVFNPSTGQVIHTVALADRETVQKAIDSAKAAFPAWRNTP
PAKRAQVMFRFKQLLEQNEARIAQLISEEHGKTLEDAAGELKRGIENVEYACAAPEVLKG
EYSRNVGPNIDAWSDFQPLGIVAGITPFNFPAMVPLWMYPLAIACGNCFILKPSERDPSS
TLLIAELLHEAGLPKGVLNVVHGDKVAVDALIEAPEVKALSFVGSTPIAEYIYKEGAARG
KRVQALGGAKNHAVLMPDADLDNAVSALMGAAYGSCGERCMAISVAVCVGDQIADALVAK
LVPQIQSLKIGAGTTCGLDMGPLVTGQHRDKVGGYIEDGVQAGATLVVDGRNLQVAGHEQ
GFFMGGCLFDRVTPEMRIYKEEIFGPVLCIVRVDSLEQAMQLINDHEYGNGTCIFTRDGE

AARLFCDEIEVGMVGVNVPLPVPVAYHSFGGWKRSLFGDLHAYGPDGVRFYTRRKAITQR
WPQRAGHEASQFAFPSL
>tr|A0A0Q0DEZ9|A0A0Q0DEZ9_PSEAP Glutamine amidotransferase OS=Pseudomonas syringae pv. aptata OX=83167 GN=ALO85_01070 PE=4 SV=1
MCELLGMSANVPTDIVFSFTGLMQRGGRTGPHRDGWGIAFYEGRGLRLFQDPAASSESEV
AQLVQRYPIKSEVVIGHIRQANVGKVCLSNTHPFVRELWGRNWCFAHNGQLADFTPGVTF
YRPVGDTDSEAAFCDLLNRVREAFPEPVDVEELLPTLIQACAEYRSKGVFNCLLSDGDWL
FCFCSTKLVQITRRAPFGPARLKDVDVIVDFQAETTPHDVVTVIATEPLTENENWNRYEP
GQWSLWRKGESVAQGSVDAVTETARS
>tr|A0A0Q0CWQ6|A0A0Q0CWQ6_PSEAP DNA repair protein RecN OS=Pseudomonas syringae pv. aptata OX=83167 GN=ALO85_00120 PE=3 SV=1
MLVHLSVHNYAIVEHLDLELDRGMSVITGETGAGKSIMLDALGLTLGDRADSGVVRPGAD
KADILATFDLGDIPEAQTWLKERDLDNDGPCILRRVITAEGRSRSYINGSPCPQGDLKAL
GELLIDIHSQHEHQSLLKTDTHRRLLDEYAGATDLARQVHLAAQRWRQTRQELERLSNSG
DEQRARHQLLSYQLEELESLSLGENELEQLEQEHKDLTNAESLLSICRQVVEQCSESDSG
NVLNALTASLHRLGSVDHSPSALSEATGLLSSAQIQVEEAVGELNRFLDHFDADPARLQQ
LEERLDAIYTLARKHRIQPGEVATLQQKLLDEIETLNANDESIERLEHEVQAFARHYQDK
ARELSDLRHNSATKLASAVEQEIHRLGMPGGRFQIDLKANASVEPSPHGLEQVELLVSAN
PGQPLKALAKVASGGELSRISLAIQVITAQTSRVPTLVFDEVDVGIGGPTAEIVGQLLRR
LGERGQVMTVTHLPQVAAQGHQHLFVHKVRDNDATRTAVSRLSKTERIEEVARMLGGIDL
TKESLAHAKKMVVTAKS
>tr|A0A0Q0D9Y8|A0A0Q0D9Y8_PSEAP Glycine cleavage T protein OS=Pseudomonas syringae pv. aptata OX=83167 GN=ALO85_05010 PE=4 SV=1
MGLDTIQDHANHALQFSIFGFGTLHEPAEHILILTGQQTAVQIAVHVIQMWVDLTDKRHQ
QHVQFKHAAAAVPVKAVEFNVFDHGALLKVGGKYTAVSRAREYRNGRSSFHVPVCITSTR
HYDVPPGLFSQSAMTMAHTAFFCTLTHEGVLAVRGVDASKFLQGQLTCNLNYLNEDKSSL
GARCTQKGRMQSSFRIVFEGDGCLLAMAGELIEAQLLDLRKYAVFSKSKLTDESADWVRF
GLQDGDAALVSLGLDLPQETDSVVRANDLIAIRVSPGRAELWVRSAQADSIKSRLAAQLS
EGPLNDWLLGQIRVGIGQVFGSTREEFIPQMINLQAVGGVSFKKGCYTGQEIVARMQYLG
KLKRRLYRLTLSDEEIPQPGTALFSPVHASAVGNVVMAAQDGQNIELLAVLQGDAAEDGR
INLGSPEGAALQMSELPYTLDSKLETQR
>tr|A0A0Q0DMQ1|A0A0Q0DMQ1_PSEAP Flagellar rod assembly protein/muramidase FlgJ OS=Pseudomonas syringae pv. aptata OX=83167 GN=ALO85_02150 PE=4 SV=1
MDMPKSGISSAVDSGAYTDVNRLASLKHGDKDSVENQKKVAREFESLFVSQMLKAMRSAN
EVLAKDNPMNTPATRQYQDMYDQQLAVTLSTRGNGIGLQDVLMRQLSKDKGINHAAPINT
TDAAKAATAAIDAAPAKTGLATSVYQRPLWATRSVAADQAAAAASASGEGRNDMAMLNSR
RLSLPAKLTDRLLAGIVPSATAAVSNSPVPARATAGSTGNNGDWTLDPNLAPAPEYVRSM
AQPPLAPAKRAFSNADQFVETMLPLAKEAAARIGVDPVMLVAQAALETGWGKSIMRQQDG
SSSHNLFGIKAAGSWKGAEARAITSEFRDGKMVKETADFRSYDSYADSFHDLVSLLQNNS
RYKEVVNSADKPEQFVKELQKAGYATDPDYASKISQIAKQMKSYQTYAAAAGSSTTL
>tr|A0A0Q0C8Q5|A0A0Q0C8Q5_PSEAP Xylulose kinase OS=Pseudomonas syringae pv. aptata OX=83167 GN=xylB PE=3 SV=1
MFLGIDCGTQGTKVLVLDTESGTVLGEGSAPHSLISDHNGRREQDVQQWLDALQQATRDA
LAAAGISGQQIQGIGVSGQQHGLVMLDAQGQVLRPAKLWCDTESAPENQRLLDYLGGAQG
SLRRLGLVIAPGYTVSKLLWTKEQYPDLFERIDKVLLPHDYLNYWLTGRCCTEFGDASGT
GYFNVRTREWDLPLLAHIDPSGRLGKALPQLLQAHESVGVLRPEVARLLGLNPDALVSSG
GGDNMMGAIGTGNIQSGLITMSLGSSGTVYAYAEEARVSEHESVATFCSSSGGWLPLICT
MNLTNATTAIRELFALDITGFNQAVARAPIGAEGVLMLPFLNGERVPALPDATGSIVGLD
STNLTQANLSRAVVEGTTFGLRYGLDLLRDSGIKSEKIRLIGGGSKSAVWRQIVADIMNT
PVICTDHSEAAALGAAIQAAWCWSRANGKAQDLQQLCERCVSLDQGSETHPVVHNVAAYQ
QVYQRYQAQLRKV
>tr|A0A0Q0C1K1|A0A0Q0C1K1_PSEAP Hypoxanthine phosphoribosyltransferase OS=Pseudomonas syringae pv. aptata OX=83167 GN=ALO85_04943 PE=4 SV=1
MTCSGLNGCVPLPAFLKLPYLESVMSADLEHIRQVMREADCLYTESEVDAAIARVGAQIN
AELAERNPVVFCVMNGGLIFSGKLLTHLNFPLEASYLHATRYRNETTGGDLFWKAKPEVS
FMDRDVLIIDDILDEGHTLGAIIDFCKHAGARAVHTAVLIDKDHDRKARPDLKADYVGLP
CIDRYIFGFGMDYKGYWRNAAGIYAVKGM
>tr|A0A0Q0IA41|A0A0Q0IA41_PSEAP Uncharacterized protein OS=Pseudomonas syringae pv. aptata OX=83167 GN=ALO85_05081 PE=4 SV=1
MKTLLRLLKSFWFLVPILWLISLAVCWFFAPRISWLQGYTLEAMAIISAFYLLLIVLRQY

RRIRTEHNLENLVQIEVDRSLKSTGEFRDQQVLRDRLKHAIAMLRTDRSAGGGGSSALYD
LPWYLVIGMSAAGKTSLLTRSGLSASIASSANDTESGTQHCDWYFSPEAVMIDTAGRYLR
DDQSASEFAAFLRMLKKQRSKAAVNGLVLVVSLPELLAASSAERNEMGARLVTRIEEYTD
CLDANPPIYLMLTKTDQLPGFNQAFDGMDLNERQQPLGMTFGLNEINTESLRTVLDRKLA
NLQAHVSRHVDAQIIALGADANSALLNFPNYFAELSTILEQFLQHFARSNVNGTQLLLRG
LYFTSALQTDKQLTPVYEDELSESFVLRPAKPYGAVEEEEEEEEHRKISDRSYFITDTFR
RVIFPDRDLTLYQSRHGRDRSIGPIVIAAALFAGLAFIGWQALSFQKNREWLANISQQLH
ELEQSPDRAQRLASGQGLELLRNQLATIEKYRTKGVPLQLSGGLYRGDDIYFATQTAYLQ
QLRTQALEPITLKLQLQMREFNEFAKTMDPQYGFGAAPAGSDSKTGKARAQGRKLLDRGA
RNVAAGRPTSLSSIPRSTGDVTNRLTGAARNTASQTRSEAIAALRNPATANDAAELSATT
GGLSLSEEMLGRLDERQVASIIESYNALKLYLILTQPTTHPETEFVNAALPVAWADISSE
ANPISDDVIEDNSPVYVQLLKNGDAPAMPRNEQLIDETRKSLKFFMISSSLVDREYLRLQ
LESSRQFPAIGLNDLVPMPGRQLLYGSEAVPAIFTRQGWEEFVKPELIKLVSGNLRNESD
WVLDGEGGDSVVQKANFVREFMSRYKRDYTKVWYTMIDSVGVRRFSDMANATQQLSLLSD
VRNSPVKLLLASVNDNTQWDVPAVREAQQTGVKRDDGFWGKVTGIFDNKEAAASVLVSPL
PAVDDGSLAKRFEPVSRVFATQNAEGADSTIMDRYLAALRKLKVRMNNIQRSQDVGKSSK
QLISETLEGQPSEVTNVRNYVETTVDTSQGGLSTSLQSLFSLPIQFAWETLRDPAGEQIA
KAWAKQVAKPWEQVMAHRYPIAADSRNEASVKDLQRFVDPESGLLPNFKRNEIGNLSDGE
GLGMGSGAKAAPLVNPKMVSNIDKASSLGEVIASLSDRDNGFEIMLEPSAYFTDIIFTLD
GQEQHYRNGKTSWSRFSWPGTTTAPGARLDVVTLSGERITVFDYTGRWGLLRMNDSARVA
DLDGIQQRFSWNTAKGPVSLVVRNYGGVKLTDLANVKALSALNATDGRTK
>tr|A0A0Q0FD53|A0A0Q0FD53_PSEAP Nudix hydrolase domain-containing protein OS=Pseudomonas syringae pv. aptata OX=83167 GN=ALO85_00218 PE=4 SV=1
MKRVHVAAAVIRGADGSVLIARRADTLHQGGLWEFPGGKVEEGETVQAALARELQEELGI
LVTAARPLIKVCHDYPDKQVLLDVWEVSAFTGQAHGAEGQPLVWASPRELANYDFPAANQ
PIVAAARLPGEYLITPDGLDNIELLRGMQKAIAGGSKLVQLRAPGGYDPKYRDLAVDAAG
LCAGKAQLMLKGPLEWLGDFPSAGWHLTAEQLRKYASRGRPFPENRWLAASCHNAEELAL
AEQMGVDFVTLSPVQPTLTHPDAQPLGWPQATQLIADFNRPVFLLGGVGPAERQQAWESG
AQGVAGIRAFWPDEIV
>tr|A0A0N8T977|A0A0N8T977_PSEAP AB hydrolase-1 domain-containing protein OS=Pseudomonas syringae pv. aptata OX=83167 GN=ALO85_00916 PE=3 SV=1
MHRFISHPAIGLLFSVALLASAVSAAPLPAATESDWIASQFTFHTGEKLANLKLHYITLG
NPKNPAILFLHGTYRPGSDMLSKDFGGELFGPGQPLDASKYYIISTDGIGVGQSSKPSDG
LRTRFPTYNYDDMVQAQYRLVTEGLGIKHLRLIIGNSMGGMQTWIWGQNWPQMMDALVPM
ASQPTEMSSRNWMMRRLLVESIKQDPAWNNGNYSAQPSSLRLANAMFSVATSGGTLAYQA
TAPTRAQADKLVDERLSAAQTADANDFIYQWQSSADFNAAPGLKKIQAPVLAINSADDER
IPPETGLMQASMKELKHARLLLIPASADTRGHGTTSMAKFYSKELKQFMRETEKVK
>tr|A0A0N8T8A7|A0A0N8T8A7_PSEAP Glycine cleavage system transcriptional regulator OS=Pseudomonas syringae pv. aptata OX=83167 GN=ALO85_04718 PE=4 SV=1
MSSTPTVREQFLVISALGANPMELTNVLCRASHENRCSVVTSRLTRHGECSALILQVSGS
WDALARLETGLSSLSKKHAFTTSVVRSATLENRPEALPYVAYVSSAYRPDIINELCQFFI
DHNVELENLICDTYQAPQTGGTMLNATFTVTLPAGTQISWLRDQFLDFADALNLDALIEP
WRPQAPM
>tr|A0A0Q0CH89|A0A0Q0CH89_PSEAP Uncharacterized protein OS=Pseudomonas syringae pv. aptata OX=83167 GN=ALO85_200181 PE=4 SV=1
MASNEVLKKLTFESYNAMSRPAMFWNIPIMPMVGLLMGGLVFGVAATFLLSWVWGLVAAS
PFLIALIALRVVGLIDPQYLRRIRFARRRLWLNLKYGKPLLLTPFNPQWSEFYGARFSHK
RYAPGKESAIDALSGPRTHGDPTRQSPGEPDPVKGGFQ
>tr|A0A0N8T984|A0A0N8T984_PSEAP Urea amidolyase-related protein OS=Pseudomonas syringae pv. aptata OX=83167 GN=ALO85_00850 PE=4 SV=1
MIKVLKPGLATSVQDLGREGYYHLGIPPSGALDQYALSAANQLVGNPPGAAALECTLLGP
ELEFQCDTLAAVCGAHMAARLDGVEMHHDTAFAVKAGQVLRFDFPKDGARTYLAVAGGID
VPLVLGSRSTYTLGALGGFQGRRLAADDLLPIGTPSGKGRAGASLPMALRQSLGGEVYLR
VVPGLYYERLTEFAATSFFSEPWSVGSEADRIGYRFKGGRALTFQPREQPFGAGSDPSNI
VDSCYPIGSIQVPAGLEPIVLHRDAVSGGGYAMIGTVISADLDLIGQMQPNQTARFVAVT
LEEALVARKSYKKKLACLSKLFPS
>tr|A0A0Q0IDL2|A0A0Q0IDL2_PSEAP Malonyl CoA-acyl carrier protein transacylase OS=Pseudomonas syringae pv. aptata OX=83167 GN=ALO85_01297 PE=4 SV=1

MNVRTDALARDTASPLNQGMVYPLHRVFMFGGQGCHYYQMASHLFLTHSTFREAMLRLDL
IGQRILGKSVIAEIYRKDKSRSDDFSSITFTHPSIVMVQLALVSTLAAEGITADSVIGTS
LGEFIAACVAGAISDTDLLAAICDQALLIEQFAGQGKLPKGGMLSALITPDERDALLAEF
PEVAVAGVHHSRHVTFSGDTWVIDALEKSCQQRAFLYQRIAVDYAFHSPAIRPLKAASLA
SIKKYTTGAFTLPFYSCATVARCRVIDAEHFWGIVESPINFADTIDLIEASATGATPVYV
DLSPSGSLHGFVKQHLGAFRAKQVPVSLTLMSLMSNDTTQYQAGVLTLKRLAKAYSESAI
AQPGPQDTTLVNHSMEKTRTSVVGNNPAKNTALVFPGQASQRVGMGRELFERFRRQTEIA
SDILGYSIEQLCLEDPQKQLNKTQYTQPALYTVCALQYLAYREDNEVVIEFLAGHSLGEY
AALFAADVFDFETGLRIVKHRGELMSSANAGSMAVILNYKAQDLQQLLNDNGITSIDIAN
INSPDQLVIAGPHQALEAFEGIINSLAGTYIPIPVGAPFHSRYMQPIAQDFERFLEPFTF
RPMNKTVISNVTGLPHQNEMLKNQLVKQLYSPVQWLDSVRYLMGLEVDEYVEIGPGDVLT
KLTTKIRNKCSPLVLGEKVIRQVPGSTESVESKVARQPAKDGLGMAFKSRWSRGDEFSSY
FGLAYPHVLTLSEDNLDYSFIERAQACGHLAFQAVPWTAEAIDSGSLDSILSRLGAKRSN
DSGCVGVAITHSENDKVLFSKVLAHQLNAVALTGVYGMTTELVYYRVISAYQGRFSSTPQ
KNKLFIRVDDPRCVQGFYQKLPQGVLERLHLQGRIDAAQFEFAKNIAACDAIILNNEEHD
LEALMGINRLRVEHPELDIKIGIEGALSAPNDFTQGTLSQIDFFVSSELNVLIGESFLPG
LIRQTLFSASDAFIVDLPDPKTIHFGGRAKTYITNDPAIRQRAGNAHGWLSDDQRPTLCR
LLPSNYLAISEWLKVQFVNKQPSLAELSSALLNGV
>tr|A0A0N8T8F8|A0A0N8T8F8_PSEAP N-acyl-D-amino-acid deacylase
OS=Pseudomonas syringae pv. aptata OX=83167 GN=ALO85_05223 PE=4 SV=1
MLYDLIIRDALVIDGSDTPGVRADVAISKGRIQRIGSLSNASAREEVDAAGRVLAPGFID
VHTHDDTVVIRKPEMLPKISQGVTTVIVGNCGISASPVSLTGNPPDPMNLLGDAAAFVYP
RFTDYRAAVENARPAVNVAALIGHTALRSNHMDDLLRSASPEEIAAMRVQLRDSLEAGAL
GLSTGLAYASAFSAETSEVKQLAEELSAFGAIYTTHLRSEFEPVLEAMAEAFDIGRHARS
PVIISHMKCAGAGNWGRSPQLLAALEKAAQDHPVGCDCYPYAASSSTLDLKQVTDAFRIT
ITWSTPHPDQGGRDLQEIAADWGLSLLDAARKLQPAGAVYYGMDEGDVERIMSHPLAMIG
SDGLPEDPFPHPRLWGAFPRVLGHFSRDKGLFALHTAVHKMTGLSAARFALHERGLIREG
YWADLVLFNPQTVRDVADFKDPQRAAQGIDGVWVNGRLSYADGKPHGERQGRFLPRSGSL
LEGFADAKAPT
>tr|A0A0Q0D8N9|A0A0Q0D8N9_PSEAP Molybdenum transport system permease
OS=Pseudomonas syringae pv. aptata OX=83167 GN=ALO85_03773 PE=3 SV=1
MPLGSADIAAIWLTLKLASLTTVILLIIGTPIALWLARTDSWLKGPIGAVVALPLVLPPT
VIGFYLLLLLGPNGAVGQLTQSLGLGTLTFSFAGLVIGSVLYSMPFVVQPLQNAFAAIGT
RPLEVAATLRAGPWDTFFHVILPLAKQGFITAAILGFAHTVGEFGVVLMIGGNIPEKTRV
VSVQIFDHVESMEYLQAHWLAGAMLVFSFLVLLALYSSGKSRTGWS
>tr|A0A0N8T7V2|A0A0N8T7V2_PSEAP Putative lipoprotein OS=Pseudomonas
syringae pv. aptata OX=83167 GN=ALO85_04218 PE=4 SV=1
MFKQCLLLATAISLSGCWSLMYHLDGERCVYPGTRHGWAWGTKDVASTWPWLIDVPFSLA
LDTLFLPYDLTAFLPENLGGDDRECHFNDGLNVLG
>tr|A0A0N8T8C0|A0A0N8T8C0_PSEAP PaaI-like thioesterase OS=Pseudomonas
syringae pv. aptata OX=83167 GN=ALO85_01597 PE=4 SV=1
MDIPEDLTHSAYFKMLGCELRRLDEGVAEVALPLEAHLRNRGNVMHGGAIFSLVDIAMGL
ACSSVHGFDQRSVTIECKINYVRGVSEGEVLCIAKVLHAGRRTLVVEAEVVQGDKLVAKA
QGTFAVI
>tr|A0A0Q0IM22|A0A0Q0IM22_PSEAP Beta-lactamase OS=Pseudomonas syringae
pv. aptata OX=83167 GN=ALO85_02370 PE=4 SV=1
MPRGFVRLCLVLILGFSAQGVVAQPWPAEQWPSQSVPLSAAIEELEAYAFPPRDDATRKG
IRTDALLVIRDGEVVYERYGGVTTAQTPHLTWSISKSVMATVLGVAFAEGRFQLNDPVAK
FYTPFKAHPEIAIKDLMHWASGLDWQEDYEYAPLNSSVVAMLYTRGRDDMARFTADHRAA
QPPGKTYLYSSGDSTVLAAALKKMVGDEAYPGYPWTALFDPLGIRSAVWETDGAGTFVGS
SYAYMTARDLARIGLLMQRGGQWQDRQLLNKEWMDFALMPFAGAGVDASVEVPGGQWWLN
RTADGGKGPWPDAPPDTFAALGHWGQALYVIPDQKLVIVRYADDRDSTYNHNEMLKRVQA
AFGKEGGQ
>tr|A0A0Q0BZ06|A0A0Q0BZ06_PSEAP Probable 4-deoxy-4-formamido-L-arabinose-phosphoundecaprenol deformylase ArnD OS=Pseudomonas syringae pv. aptata
OX=83167 GN=arnD PE=3 SV=1
MQAGLRIDVDTYRGTREGVPRLLDILDEAQVKATFFFSVGPDNMGRHLWRLVRPTFFWKM
LRSRAASLYGWDILLAGTAWPGKPIGRDLGPLMRRALDAGHEIGLHAWDHHGWQANAGHW
SDRQLTAQIHRGIDCLSDILGHPVVCSAAAGWRADQRIVQAKEAFGFRYNSDCRGASLFR
PLLADGSLGTAQVPVDLPTFDEVIGPHLQPNAFNDYILGRFAARRLNVYTLHAEVEGIIM

AEGFRQLLKRANTQGIHFAPLGHLLPDAIERLPSGHIVRGHLPGREGWLGVQQ
>tr|A0A0Q0CVU2|A0A0Q0CVU2_PSEAP Hva1_TUDOR domain-containing protein OS=Pseudomonas syringae pv. aptata OX=83167 GN=ALO85_05598 PE=4 SV=1
MLPPPNTPNSTERTPQISRSPLEDISMTTAFKVGDAVHWNSEAGQVHGKVTKVHVQDVEF
MGKHRPASKDDPQYEVKSDKTGHSAMHHGDALKHA
>tr|A0A0Q0C371|A0A0Q0C371_PSEAP Acyl-CoA dehydrogenase OS=Pseudomonas syringae pv. aptata OX=83167 GN=ALO85_03877 PE=4 SV=1
MPDYKAPLRDIRFVRDELLGYEAHYQSLPACHDATPDMVDAILEEGAKFCEQVLAPLNRV
GDTEGCTWSESGVKTPTGFKQAYKQFVEGGWPSLAHDVAHGGQGLPESLGLAVSEMVGEA
NWSWGMYPGLSHGAMNTISEHGTPEQQDAYLTKLVSGEWTGTMCLTESHCGTDLGMLRTR
AEPQADGTYKVTGTKIFISAGEHDMADNIVHIVLARLPDAPAGTKGISLFIVPXXXXXXX
XXXXXXXXSSTRWAFTAMPPV
>tr|A0A0Q0D9D7|A0A0Q0D9D7_PSEAP Amidase family protein OS=Pseudomonas syringae pv. aptata OX=83167 GN=ALO85_00824 PE=4 SV=1
MQKSSELLGKSATELRALIGNKQLSPVELLDACIERIESLNPKINAFAATCFERARDEAL
VAEQAVMQGKSLGLLHGLPIGIKDLEETAGVLTTYGSQLFRDNIPAQDNLFVARLRAAGA
IMVGKTNVPELGAGANTRNVVWGATGNPFNPELNAGGSSGGSAAALAVDMVPLCSGSDTG
GSLRIPAALCGIVGLRPSPGLVPSERKKLGWTPISVVGPMGRNVADTLLQLRASAGLGQS
DPLSYAIAADEFAPRPIDLSQLRVGYSEDFGACAVDDRIRAVFREKISALKSLFKSCEAI
DLNLTSAHRTFDVLRAEAFVAGLQDAHDRDPDALGPNTRANFDMGAAMSLQDCVKAHGEQ
SRIFRGFQKQFEHYDLILAPTTPVSPFPWSELYLREVNGVPLDNYYRWLALCYTITLTTN
PALSLPCGADHNGMPFGLQVIGGFRGDAKLLACADAIEQATLNDPRLSRPRPDLQKLLAS
AVDLTHIVTHPPIYGGSRTGKPEIGAM
>tr|A0A0N8TAB5|A0A0N8TAB5_PSEAP Putative outer membrane protein OS=Pseudomonas syringae pv. aptata OX=83167 GN=ALO85_101833 PE=4 SV=1
MVTHHIAANETLAAIAKSKHLEVSEDPMLMDEGKALILEIHCTA
>tr|A0A0Q0DG00|A0A0Q0DG00_PSEAP 3-oxoacyl-synthase II OS=Pseudomonas syringae pv. aptata OX=83167 GN=ALO85_02827 PE=3 SV=1
MKRVVVTGMSGITSVGSDWPTIDASFTANRSGIRRMDEWNRFAELNTRLAGPIDDFAVPG
HWTRKQLRSMGRVSRLAVAAAEQALIDAGLLNDPIIRDGRMGTACGSSTGSTDEIKAFGN
MLINSVAVGLNANSYVRMMPHTTAANISIFFGLTGRLIPTSSACTSGSQGIGYAYEAIKF
GRLPLMLAGGAEELCPTEAMVFDALYATSLKNDAPHTTPRPYDSGRDGLVIGEGGGMLVL
EELEHAQARGAHIYAEIVGFGSNADGAHSTRPEQATMRRAMELALEDANLSPDAIGYVNG
HGTATEQGDIAETLATSSLFGPRMPLSSQKSFLGHTLGACGALESWFSIEMLNSDRYMHT
LNLVDIDPQCGELDYIVSEPRHMSNEYVMNNNFAFGGVNTSLIFRRWG
>tr|A0A0Q0D6K0|A0A0Q0D6K0_PSEAP Major facilitator transporter OS=Pseudomonas syringae pv. aptata OX=83167 GN=ALO85_02366 PE=4 SV=1
MPLSLLILALSAFAIGTTEFVIMGLLPDVAADLGVSIPGAGWLVTGYALGVAVGAPFMAM
ATAKLPRKAALVTLMGIFIIGNLLCALASDYNVLMFARVVTALCHGAFFGIGSVVAAGLV
PANRRASAVALMFTGLTLANVLGVPLGTALGQYAGWRSTFWAVTVIGVIALIGLIRFLPT
NRNEEKLDMRAELGALRGAGIWLSLTMTALFSASMFTLFTYIAPLLGEVTGVPPNGVTWT
LLLIGLGLTAGNVIGGKMADRRLSSTLIGVFVSMAVISTVLSWTSTALIPTEITLFLWAV
AAFAAVPALQINVVTFGKAAPNLVSTLNIGAFNVGNALGAWVGGSVIAHGLGLTSVPLAA
ATLAVLALLITLITFRQTGNPDLAPATH
>tr|A0A0Q0IS00|A0A0Q0IS00_PSEAP Uncharacterized protein OS=Pseudomonas syringae pv. aptata OX=83167 GN=ALO85_05381 PE=4 SV=1
MASLSEEPMNKVLYAVLLSSSLLVAAGCDQLESSTKHVVNAAADSAKQVVDDTHKAATQA
LDEARQELSLQEPPPNSAETENTSDKEI
>tr|A0A0Q0C763|A0A0Q0C763_PSEAP Prepilin OS=Pseudomonas syringae pv. aptata OX=83167 GN=ALO85_04717 PE=4 SV=1
MEQEPLGMNVLKHSQTRKNGFVVIELLFGLIIFAIASAIGVSLMADRMDAQNYQIAAQQQ
QQIAEAASKYLKDNFATVYGSAGTTTPATITPQMLRNTNYLPASFSDTNAFGQSFVVLAR
RVNVNQLESIVITTGGQAIDEIGTRTIAENMGAPGGFIPFNNTGVIQGVRGGWQLALSNY
GINPGVGHTASALFLQDGTLSNDYLYRNAIPGKPELNRMNTALSMGGNNVNDVAALNASG
TVTVGGNVDTAGSVNAVGNVSASGSVTAQGNVSASGSVTAQSNVIAAGDVYAQSVNASAN
LTGAAARISGETVTGGWFRTQGDTGWYSEKWGGGWYMSDSDWVRVYGDKNLYTAGNIRGG
TVTSEGRATVGEYLQLNGVATAGTACAANGMVGRTSTGRSLSCDNQVWVVNGSSAPTCTA
KTIPGYDANDVTTYACPVGYTKVGWDTAGSGQRFSSTPGIVVGQNDYATIFCCQF

>tr|A0A0Q0DGU9|A0A0Q0DGU9_PSEAP PAS domain two component sensor histidine kinase OS=Pseudomonas syringae pv. aptata OX=83167 GN=ALO85_03203 PE=4 SV=1
MTVEQVSLPVTEDLYEHAPCGLLITLPNGTIERANLTFCRWLGLERDAVVGRRFQELLSL
AGKAFLQTHWAPTLLAQGSIAEVKVELIHADGRPIPMMLNAVRREYPSGYLHEVAVFLAE
ERNKYERELLAARKIAEELLQQQMTVQSELTSARNRLRLAHAEAEIRAIFAEQMIGIVSH
DLRNPLAAIKMAAGLLERTPLESRQERILGHINHSTDRADRMIVDLLDFTHARVGSGITV
VPQPVDFHAVVARGVEELRQVFPDRVLTHRSEGQGACSADPDRLLQMLGNLVSNAINYGT
DDGEVLITSSFDTWMIKLSVHNVGEPIPMEKVDDLYEPVVRVVSDSDETRTVGLGLFIVR
EIVRAHLGDVTVRSSAEEGTTFTVAFPRPPAKTEV
>tr|A0A0Q0DB86|A0A0Q0DB86_PSEAP Uncharacterized protein OS=Pseudomonas syringae pv. aptata OX=83167 GN=ALO85_04148 PE=4 SV=1
MEVIKQSITIMTEIIEPTVKQEKSNKDKLIDLDLELARAKESVKKTESKIKNATAEIGRL
SDLILDEKATDNQNTLWKKKKEYRATLEESKKVKDKIVNSLITEITKMSEVINKDTKVIA
ADEQEAILKLSVADVTKLILHLFKVTNTQNLKELTEDVTKKFTSFQ
>tr|A0A0Q0DIQ7|A0A0Q0DIQ7_PSEAP Prophage PSSSM-04, putative chitinase OS=Pseudomonas syringae pv. aptata OX=83167 GN=ALO85_00428 PE=4 SV=1
MPINQQQLLQILPNAGPKAEAGVFVPALNIAMARYAIDTRLRIAAFIAQIGHESGQLRYV
RELGSDSYLAKYDTGQLALRLGNTPEADGDGQLYRGRGLIQVTGRTNYEACGEALGLDLL
AQPQLLEQPDHAAMSAAWFWDRANLNTLADQGDFLMITRRINGGTNGLADRQALYQRALE
VLP
>tr|A0A0N8T7S5|A0A0N8T7S5_PSEAP Uncharacterized protein OS=Pseudomonas syringae pv. aptata OX=83167 GN=ALO85_04059 PE=4 SV=1
MPKISSYFGGGSSVQTYDATVQHNQSNGTVHNTFGASQLGLPAYNGYGVTVSSGTLAMLA
DATWANRVVKSAIPSGAGNQRADIINSGGESWARLHLADAKYPGGGSTGQLKRAQKFQGG
TCAVHASAAAAALQSRGVRYPVNRVRTSLPDGNTHEFLLLGDRRQSGDRNTVVVDPWATY
PSACTLDQAVLHDASRGTHHPVTQLLASYRNEIWQSSIASADVQRLSRIEVLGTAELEKK
LGKAGLPGLGSQQLVNHALSDDRFGRFDVRVATDPSTLYKDDSGAGWQSFDPLIR
>tr|A0A0Q0FV62|A0A0Q0FV62_PSEAP Precorrin 6A synthase OS=Pseudomonas syringae pv. aptata OX=83167 GN=ALO85_03462 PE=4 SV=1
MKQILLIGMGAGDPEQITLQAVAALNRAEVIFILDKGYVDDQLLRLRKELCQRFITHERY
RLVQVQDPRREDDPQAYERGIEGWHEQRAILFERLLTDELGVDQVGAFLVWGDPSLYDST
MRILDQVLARGKEVFEYQVIPGITSVQSLVARHRIPLNRIGESIHITTGRRMATAPLQQP
GNVVVMLDAHCTFERYVGQGLDIYWGAYLGTPDEILVAGRLDDVCEQIKRLRSEARERKG
WIMDTYLLRKPA
>tr|A0A0Q0C6M7|A0A0Q0C6M7_PSEAP Uncharacterized protein OS=Pseudomonas syringae pv. aptata OX=83167 GN=ALO85_101428 PE=4 SV=1
MDESGSSNDYGDQPTSRHKWIKARFALELIGLRDHFSCLSHKLVNLSLEFLKFQLVLLTR
HPTSYQTLACISQAAFMKQGYASHLLCKRLKKLRHISFQRSNQGNRNACRRAQAKTHAPC
IRFSATLKSTGL
>tr|A0A0Q0DKV7|A0A0Q0DKV7_PSEAP Histidine kinase, HAMP region: chemotaxis sensory transducer OS=Pseudomonas syringae pv. aptata OX=83167 GN=ALO85_02449 PE=4 SV=1
MSAKWLDNLNVSKKLGLGFAAILLGVLTVTVIGYSSTNLLIERMGKSSKVAEIKADVLNA
RIAAQAYATGPTAAGVQNYASALDTLSRSVDQGLQVFVISSNLAKLREIKEQVGGLKQTF
DQLVGINQKVDEALKPIIKVSDDVSATFENLLNKTIDDTLRSPNETGIRQVKIAGDLRNG
MTNFRLVFRRYLSVPNADNRQATYKAADALIAQVDAARSQLPVEANAAVDAALVALKQYK
VLMTSISDMLQQTEQIRNDLQQQSVATAARADDLAALQVISAKKEQGTAVVQLLSVALVV
LLVGIFAAFLITRQITVPLSSTVIAARRIADGDLTHDTSTTRQDELGLLQNTMQHMTVSL
RGLIGGIGNGVTQIATAAEQLSAVSEQTSAGVTLQKNEVDQVATAMNEMASTVQEVARNT
EDASQAAKQASERAAHGSSVVQHATREIGQLAGEVQQLGQAMQRLTEDSGKIGSVIDVIK
AVAEQTNLLALNAAIEAARAGEQGRGFAVVADEVRSLAQRTQNSTTEIEALIQALQKGTG
AASGLMDASLQRTEGTVVLARQAEQALVEINQSIGTIEQMSQQISAAAEQQSAVTEEINR
SVLSVRDIADQSASATEQSAASTVELARLGSDLQSMVARFKI
>tr|A0A0Q0D1F6|A0A0Q0D1F6_PSEAP Fanconi-associated nuclease OS=Pseudomonas syringae pv. aptata OX=83167 GN=ALO85_04625 PE=3 SV=1
MALNTSPLDNPFYYLENFRQVLDWIARRYDDLLDASERRFISEFAELPVPAQGLLVRMVM
RKGVLFRASKLGYVEIGDPHDAVLPLLAREWVDSAPPLGLSELFQLLRRDELSQCFKDHA
VKGPECKQEWLERLQPMYPSPQSLEQWHPTLPDAVFGLKIMPLCDRLRLLYFGNLYQEWS
EFVLADLGIYRYEKVEFSLESRGINQRADIDVCLQLHACREALETCLELHALAERVIAIE

CGNPWLLMRRGKLLFRIGQQAERMQDWPLAMAVYRQSSYPGARSRQIRVLERNAEYAAAL
ALAEQARLAPESDAEVQHLSRVLPRLQRKLGLTAARKRTVRAIARLDVQVTPVSGMSVER
LIRLHLEEEQGGEVHYVENALINSLFGLLCWRAIFAPLPGAFFHPFHSAPSDLYSPDFYQ
RRATLFDACLLQLESGEYLATIREHFESKYGLQSPFVFWGALTPELLEQALYCLPAEHLL
RWFRRLLQDIKANRTGMPDLIQFFPEQRRYRMIEVKGPGDRLQDNQLRWLDFCAEHGMPV
EVCYVQWAADDSVCVSSDLSDLQGALCPS
>tr|A0A0Q0D5T2|A0A0Q0D5T2_PSEAP Uncharacterized protein OS=Pseudomonas syringae pv. aptata OX=83167 GN=ALO85_02632 PE=4 SV=1
MKVREMQKKASQNVRALTHIVDPVGSGVPLDIAWLTGIIPLGFVPAEIAISPDETTLYVA
HESGREVSVVDIASASVTGAIKRAGAGAITLSPDGKRLYTIGQKRCYVVDTRMREVIRIL
PAANVNRILCSADGRFICLSFQNALGGLIRLIETENYTVENEFDLGALTNASLALGISPL
NDRFYATMLVDRTAPTDVLAISKVDYLQHDIPGFSDPRSMAFSSDGAWLYVGGIDEVYII
DAATNKTFYRERIGTQGYPVKVIGVTSDDRYVYAIYTCNYDIYRIDTVKGIATCIAYFPS
VGGAVLNKTGTYIYSSHPDMSWISIYRL
>tr|A0A0Q0D8B8|A0A0Q0D8B8_PSEAP Uncharacterized protein OS=Pseudomonas syringae pv. aptata OX=83167 GN=ALO85_101492 PE=4 SV=1
MPIRTIRARICSTVAREREGRSGSFMEDLTQDVRHVLRSRRCKHQKTIISFEGVSRTTTM
FYCAGAQQEHK
>tr|A0A0Q0C426|A0A0Q0C426_PSEAP Uncharacterized protein OS=Pseudomonas syringae pv. aptata OX=83167 GN=ALO85_02650 PE=4 SV=1
MPVKALTTREVYQQLRDAAMGTRSLKFTGTPSGFGLQQVDIDGWLLTLEVTEGSPTRCRA
CRCPLGREGSFESWLRTDPVSLLSGWEHAQIERLLGEAAAGQLGLDQVPGQSPESQ
>tr|A0A0Q0DGG3|A0A0Q0DGG3_PSEAP Phospho-2-dehydro-3-deoxyheptonate aldolase OS=Pseudomonas syringae pv. aptata OX=83167 GN=ALO85_03507 PE=3 SV=1
MSQPWSPDSWRALPIQQQPHYPDAEHLHRVEQTLASYPPLVFAGEARELRRQFAEVTQGR
AFLLQGGDCAESFMEFSAAKIRDTFKVLLQMAIVMTFAAGCPVVKVGRMAGQFAKPRSAN
DEIIDGVTLPAYRGDIVNGIGFDEKSRVPDPERLLQAYNQSTATLNLLRAFAQGGFADLH
QVHKWNLDFIANSALAEKYSQLAGRIDETLAFMRACGMDSSPQLRETSFFTAHEALLLNY
EEAFVRRDSLTNDYYDCSAHMLWIGDRTRQLDGAHVEFLRGVNNPIGVKVGPSMNTDDLI
RLIDILNPDNDPGRLNLIARMGANKVADHLPQLIRAVEREGRKVLWSSDPMHGNTIKASS
GYKTRDFAQILGEVKQFFQVHQAEGTYAGGIHIEMTGQNVTECIGGARPITEDGLSDRYH
THCDPRMNADQSLELAFLIAETLKQVRR
>tr|A0A0Q0DQR6|A0A0Q0DQR6_PSEAP Succinate-semialdehyde dehydrogenase family protein OS=Pseudomonas syringae pv. aptata OX=83167 GN=ALO85_03915 PE=3 SV=1
MIMLKNQLKDTSLLVDRAYVDGQWISADDGATLTINNPATGEALAQVPALQGVETLRAIE
AADRAWPAWRARPAAERAALLERWHQAMLDNVEDLALIMTFEQGKPLNESRGEIRYGASF
VKWFAEEARRSYGETIPAPSADRRLMTLKQPVGVCAAITPWNFPNAMITRKCAPALAAGC
PIIVKPSDLTPLSALALAVLAERVGIPAGVFNVITGMPTGIGEELTSNPVVRKISFTGST
PVGRLLMRQSAEHIKRLSLELGGNAPFIVFDDADLEQAVTGIMLSKFRNAGQTCVCANRI
LVQNGIYDRFAARLVEEVSKLKVGNGLEEGVTIGPLINPAAVSKVARHIDDALSQGAKLL
HGGSPDGTSQFVQPTVLGDTHAGMLLANEETFGPVAPLMRFTDEAEALALANATPYGLGA
YYFTQDLRRSWRFGEALEFGMVGLNTGIISMEVAPFGGIKQSGLGREGSSYGLDEYLEVK
AFHVGGL
>tr|A0A0Q0DJY6|A0A0Q0DJY6_PSEAP DNA polymerase III subunit epsilon OS=Pseudomonas syringae pv. aptata OX=83167 GN=dnaQ PE=4 SV=1
MYMRQQSMALQNLDNRSIVLDTETTGMPVTDGHRIVEIGCVELIGRRLTGRHFHVYLQPD
RESDEGAIGVHGITNEFLVGKPRFAEVADEFFEFIKGAQLIIHNAAFDVGFINNEFALMG
SQDRADITQHCSVLDTLMMARERHPGQRNSLDALCKRYGVDNSGRELHGALLDSEILADV
YLAMTGGQTSLSLAGNASDGNGSGEGSGNRASEIRRLPADRKPCRIIRASESELAEHEVR
MSTIAKACGAPPLWVQMLEAGAQASS
>tr|A0A0N8T8W2|A0A0N8T8W2_PSEAP Polyamine ABC-type transport system, permease protein I OS=Pseudomonas syringae pv. aptata OX=83167 GN=ALO85_00449 PE=3 SV=1
MTMLNPYTSPIERLWYYALRIICTLVLLFLVLPVLVIVPLSFNSGSFLVYPLQGFSLQWY
QDFFNSAEWMRALKNSMIVAPAATLLAMGFGTLAAIGLTRGNFPGKALVMALVISPMVVP
VVIVGVASYLFFAPLGLGNSYTSLILVHAVLGVPFVIITVSATLQGFNHNLVRAAASLGA
SPLTAFRRVTLPLIAPGVISGALFAFATSFDEVVVTLFLAGPEQATLPRQMFSGIRENLS
PTIAAAATLLIGFSVVLLLVLEWLRGRSEKLRTAQ

>tr|A0A0Q0DPM8|A0A0Q0DPM8_PSEAP Uncharacterized protein OS=Pseudomonas syringae pv. aptata OX=83167 GN=ALO85_03770 PE=4 SV=1
MERFELFELASAAFCHFTEEGNHEWRHQASDYLAYNKGGIVVGSLLNSRYVLHEADQSPY
HAAHFAFLDEKNDLIMRDYKKYGVLEGRYTYTETGQTLTLVEQPRQMNGVSYQRLSDEDQ
YRALVDASAIALDQGDFKAYVSLWERHSYSIFIRCLHCLDGFAVREDCTHCDGRGFTEDP
QCANKLPQSLNSQRSAPKK
>tr|A0A0Q0C5H9|A0A0Q0C5H9_PSEAP Levansucrase LscA OS=Pseudomonas syringae pv. aptata OX=83167 GN=ALO85_04570 PE=3 SV=1
MRGMSESIASVATCDAYSKEVTSLFNPVICSCSLPVNFDKCLLCKKFSATRSFIGFRLSS
TPECPTHSAPRHCTHACRVAFLSALLVQAPAKDIIFMSNINYAPTIWSRADALKVNENDP
TTTQPLVKPDFPVMSDKVFIWDTMPLRELDGTVVSVNGWSVIVTLTADRHPDDPQYLGAN
GRYDIKRDWEDRHGRARMCYWYSRTGKNWIFGGRVMAEGVSPTTREWAGTPVLLNDKGDI
DLYYTCVTPGATIAKVRGRIVTSDQGVELKDFTQVKKLFEADGTYYQTEAQNSTWNFRDP
SPFIDPNDGKLYMVFEGNVAGERGTHSVGAAELGPVPPGHEEVGGARFQVGCIGLAVAKD
LSGEEWEILPPLVTAVGVNDQTERPHYVFQDGKYYLFTISHKFTYADGVTGPDGVYGFVG
EHLFGPYRPMNASGLVLGNPPAQPFQTYSHCVMPNGLVTSFIDSVPTSGDDYRIGGTEAP
TVRILLKGDRSFVQEEYDYGYVPAMKDVQLS
>tr|A0A0Q0C8X1|A0A0Q0C8X1_PSEAP Aerotaxis receptor Aer OS=Pseudomonas syringae pv. aptata OX=83167 GN=ALO85_03241 PE=4 SV=1
MRVNMPITQTERTFPASERLISTTDLNSHITYCNDAFVTLSGFTREELVGQTHNLVRHPD
MPASVFAHMWETIKQGKPWMGVVKNRSKQGDYYWVSAYVTAVYENGRIVGYESVRSLPTR
DQVRRAEALYARLRAGKSAVSSSSSAAYHLIRQLPMILCALALAVGVYLLDDLPSIIMIP
IVMVVLGVFLEMRQRRSIRKTLEEHPKAFTSALIALTYSDNRGPQAQLDLAMLSEEARLQ
TALTRLADTGESVRQHAGRSAQLSLRQAESLDQQRSEADQSATAINQMAATIQEVTHNVQ
NTAHAAEEADKLAQQGRGLADESLLAIRHMATSVTDIGNAVGELADATQSIGSVVDVITS
IAQQTNLLALNAAIEAARAGEQGRGFAVVADEVRSLASRTQSSTEQIQQIITSLRDGADR
AVQTASKGEQISQESVASVEAVQKALDGISQSVTRITGMSQQMASASEEQSHVAETISQQ
ITRIAQLCDESASQAQQGSQISSELEEMAHYLHSLAERFSR
>tr|A0A0Q0C134|A0A0Q0C134_PSEAP Phosphoglycolate phosphatase OS=Pseudomonas syringae pv. aptata OX=83167 GN=ALO85_04533 PE=3 SV=1
MPRMSGFEQLFAGKLPKLIMFDLDGTLVDSVPDLAVAVDTMLAELGRPIAGLESVRAWVG
NGAPVLVRRALANDLDHSGVDDALAEQGLEIFMRAYAQKHEFTVVYPGVRETLKWLQKMG
VEMALITNKPERFVAPLLDEMKLGRFFRWIIGGDTMAQKKPDPAALFFVMKMAGAPASQS
LFVGDSRTDVQAAKAAGVACVALSYGYNHGRPIAEENPAMVIDDLRRLIPGCLDMDAEIL
LPDIKRPSSRESIVVVTRKLWMKVIKALARWRWRA
>tr|A0A0Q0C8N0|A0A0Q0C8N0_PSEAP Pyoverdine sidechain non-ribosomal peptide synthetase module IV OS=Pseudomonas syringae pv. aptata OX=83167 GN=ALO85_100320 PE=3 SV=1
MGICVERSLELVIGLLAIIKAGGAYVPLDPDYPEDRLAYMMQDSGIGLLLTQTSLQERLP
VPAQVHSLCLDQDGDWLEGYSTANPVSFSHPLNLAYVIYTSGSTGKPKGAGNSHRALVNR
LHWMQKAYKIDGSDTVLQKTPFSFDVSVWEFFWPLMTGARLAVALPGDHRDPERLVQTIR
EHQVTTLHFVPSMLQAFMTHPQVESCNTLRRVVCSGEALPAELAAQVLKRLPQAGLYNLY
GPTEAAIDVTHWTCTTDDVLSVPIGRPIDNLKTHILDDGLLPAAQGVAAELYLGGVGLAR
GYHNRAALTAERFVPDPFDEHGGGRLYRTGDLARYRDAGVIDYAGRIDHQVKIRGLRIEL
GEIEARLHEHAAVREVTVIDIDGPSGKQLVAYLVPTATAEAPDVLRERLQAHLKAQVPDY
MVPSYFVVIDSMPLTANGKLDRRALPKPDVARSQQGYVAPRSAFEQRLAALWEQVLHVER
VGLNDNFFALGGHSLLAVSLAGRIRETFDISIKLHDLLLLQTLGELADFMRADEARVKSA
VIAMNANGSAHAPLFCLPPGGGGTYSYYPLAGRLSDSRRVYGLVNKAYVVPGWFDTSWQD
MVDYYVEQIRMTQPHGPYNLLGWSMGGALAVEVAHVLERAGEVVSFLGLVDTQLPASVGM
QWVEEEPHVTTQQGENYYRSLIKSLQAFVPGLQEQTIVDLIETARQSVTGESEVIDQVIK
QIALQHAMNVDSLRSMFQDIAVQDEIETGYKLLEANAKLSQAFTLRTLNVQVDCWWAGQS
RKPGQIARAEAVLLEQCSVNGLRSSTTIDQRHDNLVIAEAFLQGLAERLV
>tr|A0A0Q0FJ68|A0A0Q0FJ68_PSEAP Diguanylate cye domain protein OS=Pseudomonas syringae pv. aptata OX=83167 GN=ALO85_00634 PE=4 SV=1
MSCASVLLADNQHLRSRLFMFRIIQPHRWKLAVLMVASNLGLLAFLAFGTVKPVSEWQWL
DIVGEGGSALLSLVWLFLVFKSRPAGRVTNYLSIGLSCVFFSWWIDALDEFIRLPTEIEW
DHWLESGPMPVGLILLTLGIFHWHREQLAISAQMEKREGGFREHRLYDKLTPLGSADYLK
RQLVISLEQSVCQQQPLSLLALDLDGFSAINNAFGHAEGDGVLSALNHLLVLNLRRQDLL
CRLAGDRFVVLLPNTAESQAKLLAQELQQAVASLAHKTRQQGERIHLSASVAVVMALNET
PDNLLRRLNLSMARARHPLTRTA

>tr|A0A0Q0BTT1|A0A0Q0BTT1_PSEAP Regulatory protein, LysR:LysR, substrate-binding protein OS=Pseudomonas syringae pv. aptata OX=83167 GN=ALO85_01052 PE=3 SV=1
MFSSERLKGIDVFVIVADMGSFTAAAERLNLTNSAISKSIARLEGRLRTRLFQRTTRRLS
LTEAGETFYRTCTTVLSDLEEVEIALTSTQTEPQGKVRIDLPASYGRLHVLPLILDFMKQ
HPRLQPHVSFSDRYVDPVHEGIDIVVRIGGSDAWPAAMAHQFFGTQKLIFCASPEYVNRY
GQPKTVSDLDDHQCIGYGQNDGMAIPWYFKGRQPGDMERRIMHPHIAVGDGEGEVMAVLA
GLGIAQLPTWLVQVHLNSGRLVEVLPDLATEGLPMNLVWLRSREALPKVSALVAYLGANL
TPAGRVERA
>tr|A0A0Q0DMW3|A0A0Q0DMW3_PSEAP AttF/G component of AttEFGH ABC-type antimicrobial peptide transport system OS=Pseudomonas syringae pv. aptata OX=83167 GN=ALO85_03231 PE=4 SV=1
MRVFCWTLRALLSHWRRHPVQFFSVVTGLWLATALLTGVQALNSQARESYQRASQLIGGE
PQTRITASSGGLFPQALFIELRREGWPVSPMLQGRIVLKGREDRRLQLMGIEPVTLPPGS
ALAGQTLNAEQVVDFLTPPGMTWIAPQTLQALGLEEGQQPLTETGVALPPLHAKPDMAPG
VLLTDIGFAQPLLGQPGQLSGMLLAKDFARQNPVLPAALNDLLVIKKSGEENNLERLTES
FHLNLNALGVLSFIVGLFIVHAAIGLALEQRRGLLRNLRACGVSARLLIAALGVELGALA
LLGGIFGVVSGYLLASLLLPDVAASLRGLYGAEVAGQLNLSLWWWLSGIGLSLLGALLAG
ANSLLRAARLPLLALADAQAWQQAHARWLQRQAWVAVLGAVVAVSALLFGTSLMLGFVMM
SALLLSAALALPVLLDAMLGGLLKRSRSVLGQWFLADCRQQLPALSLALMALLLAMAANI
GAGSMTSGFRQTFNSWLEQRLTAELYVSPQNPEQAGPLNTWLSQQQDVSAVLPNWQVPVQ
VQGWPADLFGVIDHGTYRQHWALLESVAGDPWNVLRDEDTVMLSEQLARRLQLGLEDTLS
IPVPTGKWTPRIVGIYADYGNPKGHLLVNARHLLAHWPQSMPVRFNLRVDQAAIPSLVTR
LQARFKLDDNHIIDQSQLKGWSSQVFERTFAATAALNSLTLGVAGVALFISLLTQSQSRL
GQLAPLWALGVTRRQLMLLNLGQTWLLAVLTLVLALPLGLLLAWCLDAVINVRAFGWRLP
LQVFPLQLLQLMALAMLATLLASAWPLLKLYRSRPADLLRTFANEQ
>tr|A0A0Q0DVT2|A0A0Q0DVT2_PSEAP N-acetylmuramoyl-L-alanine amidase OS=Pseudomonas syringae pv. aptata OX=83167 GN=ALO85_100009 PE=4 SV=1
MCHFGFGIQIDMGLGMRMRALVTVVGLLLMALAVEAVAATQVRSVRLWRAPDNTRLVFDL
TGPVQHSLFTLTSPDRLVIDINGATLGGPLNVPTANTPISSMRSAQRTPTDLRVVIDLKK
GVTPKSFTLAPNQQYGNRLVVDLYDNAADANPTPVIPDTAANTAPAVPVSPAKPEIKLTP
VPNGKRDIVVVIDAGHGGEDPGASGGAGQKEKNVVLSIAKELQRQVNAEKGYRAELTRTG
DYFIPLRKRTEIARSKGADLFVSIHADAAPSSAAFGASVFALSDRGATSETARWLADSEN
RSDLIGGAGAVSLDDKDRMLAGVLLDLSMTASLSSSLNVGQKVLSNIGRVTSLHKSRVEQ
AGFMVLKSPDIPSILVETGFISNANEANKLGSASHQQALARSITSGVKQFFQQNPPQGTY
IAWLRDNGKLAQGPRNHVVRSGETLAMLAARYDMNIATLRSANNLKSDELKIGQDLRIPS
SEVATQ
>tr|A0A0N8T8Z2|A0A0N8T8Z2_PSEAP FecR protein OS=Pseudomonas syringae pv. aptata OX=83167 GN=ALO85_100335 PE=4 SV=1
MNPNNESLNKSDISPAVAQQAVGWLLEMQEGTLDARRQHAWQLWLNGNAEHQRAWAHMQR
VNQRLSGLSSPLAHAALNAPKSASRRHALKLLLLLGAGSAAGWGLREQIALQPLLADYDS
GVGEQRKVALSDGSQVQLNTASAVDVRFDAQQRLIELLQGEILMTASADTRPLNLLSAEG
TVRASTGASRFNLRQLNGRTQLAVFAGALEISPAGKSGPGLMLQASQQVTFSRDAWDKVR
PLDAGSGAWVDGMLVASRMRLADFLAELSRYRRGRLNCDARVAGLLISGSYPLADSERIL
DMLELALPVRVQRFTRYWVNVQARV
>tr|A0A0Q0DQ08|A0A0Q0DQ08_PSEAP Extracellular solute-binding protein OS=Pseudomonas syringae pv. aptata OX=83167 GN=ALO85_04659 PE=4 SV=1
MTLEEHRIQLSLQFADLPTDRRRRDIEADRGFADRSAPRHFEEVAQGSGLYAVELHRCSC
VAFSAMKGSKYCACGIDTLCLESCRGATHQKKPIKRTLRPRCFPSSRICDPLPFDTSTLP
FGDDGMGIKGFARSIALLSLFSSFSVLAGKADDTLVYTSDSEPENISPYHNDLREGVILG
RLIWDNLVYRNPDNGEYQPMLATSWKQVDDTTIDFQLRQGVKFHNGAPFTADDVVFTLNY
VVSPESKVVTVQNVDWIKSAEKLGDYSVRLHLKKPFPPALEYLSNAVPMFPKKYFEEVGL
AGFSRKPIGTGPYKVTAIATGEGVKMDKNPDYFKDSPQGQPKIGHINFRVIADAETRLAE
LMTGGVDWTWRVAPDQAENLKAMPNLVVTSGATMRIGFLILDARGTSSADSPMKHLKVRQ
AINYAINRDGLASQLVGGESKPLQVACYPGQFGCDTTAATVYNYDPAKAKALLAEAGYPN
GFETEIFAYRDRDYVEAIIGNLRAVGINAKLRYLKYAALRDQQRGGKVPMSFQAWGSFSI
LDTSASAGTWFKGNPDDNIKDPQVQGWLQTADNALDPQVRKDNYRKALQRISEQAYWAPL
FNYSMNYAYVSDLNFKPYPDELPRFVLSSWK
>tr|A0A0Q0DAZ1|A0A0Q0DAZ1_PSEAP Uncharacterized protein OS=Pseudomonas syringae pv. aptata OX=83167 GN=ALO85_05608 PE=4 SV=1

MRRRQVCLVATGLVKSAGVLAHGYRSARSSVGMPFVTLRVMDSRRPAHSGEDAERPERHT
HAWAR
>tr|A0A0Q0BT59|A0A0Q0BT59_PSEAP DnaJ domain-containing protein OS=Pseudomonas syringae pv. aptata OX=83167 GN=ALO85_02293 PE=4 SV=1
MLYAAGWSFAEELLKDICRCAWPVGFAQGGAEDGCLNRMNVRLDADEGFIDVSRNARILW
IRAFSCGLVAVASDLHWSYRLFGRYSCIENGSQPWTASAMQRTPTHYELLSVARDASPEQ
IKKAYRKLAQKLHPDRNPDPYASDMMGVVNASHDVLADPSRRAAYDAQLAANEHKARMDA
ARRKQAHAARGQAVHVYAATSAATAAAPSQAARSGPAPKSSSYASASPSRDKRRRSAWRW
ALLFVVFCAGGAWMGYDPGAGKSFVPSEPVPVAQTWVKPAPVMPAAPVEEPVASPAKPVD
AAASECGVPALDPMGAPWPDKAGYVKDMPLLKDNGWSQITVDNSAGESAVYAKVTDAVGR
RAFRHAFVPAGAVFTFAKMDPGLYLLKYKMMSTGCAFASGRILLEETPMGSQIKSSAYKL
TLRKLQNRSVPFARLKDDQF
>tr|A0A0Q0C734|A0A0Q0C734_PSEAP Uncharacterized protein OS=Pseudomonas syringae pv. aptata OX=83167 GN=ALO85_03331 PE=4 SV=1
MSLDENFVRAIYDELFEENFNRYKEVLNQPIDDGKDSFARARNALALLDETERSHVINFF
KVVMFDSASVILGTLDGVHFPDDLDGDFLLLCDGKEIQGSLADIFIGKAQDAGVYE
>tr|A0A0Q0FBF1|A0A0Q0FBF1_PSEAP Uncharacterized protein OS=Pseudomonas syringae pv. aptata OX=83167 GN=ALO85_05085 PE=4 SV=1
MMLDDLLPYYEKELSHLRFLGQEFAAQYPKIASRLLIEGDNCEDPHTERLIEAFSFLSAR
VHKKLDDEFPEIVESFLEVLYPHYLRPTPSMSIVEFNMGKQEKVTESYRVARHTELHANP
VDGIVCKFRTCYPVELWPIAVQSASFIEMERSAFNGHSADLIARLRIGLTATSDVLFGKM
EMDSLRFFLDGESTLMHQLYELLFNNLAKATLSFEDEGRTREVVLPAGALKSVGYSLDEG
LVDYSERSFLGYRLLHEYFTFPDKFMFFDLSGFARILAGKEIGKVEINFYFSDYDLTDRL
ARLTQNVSRNNFKLNCTPIINLFRQQAEPIKLTHVQHEYAVTPDVRLQSSAEVVSIDRVR
RVKKINGNDQVGTCHPFFEPRGDQGPGQSFWIARRRPTQSRQSDGSNMFIRVVDRDLELI
DSNNDTLSIRLTCSNRDLPLMLPFGGERGDFNIPSNSVIKDIRCLRKPTATVRVPLGNGV
IWRLISHLSLNHMSLVSKGREVLLELLSLYNYRNVSAIRKQINGIVSVSSEPVVARIGHP
RPNFVRGVGITLKFDESQYTGSGVFLFGMVLDHFFGQYCSMNSFTQLTLRTLQREKRVVQ
WPPRTGDQPLV
>tr|A0A0Q0BZN3|A0A0Q0BZN3_PSEAP Peptidase_M75 domain-containing protein OS=Pseudomonas syringae pv. aptata OX=83167 GN=ALO85_02602 PE=4 SV=1
MVHSKRMTYPLLTRKTLMKKTPLALLLTLGLLQTPLAAFAATAPLDLVGPVSDYKIYVTE
NIEELVSHTQKFTDAVKKGDIATAKKLYAPTRVYYESVEPIAELFSDLDASIDSRVDDHE
QGVTAEDFTGFHRLEYALFSQNTTKDQGPIADKLLSDVKDLEKRVAELTFPPEKVVGGAA
ALLEEVAATKISGEEDRYSHTDLYDFQGNIDGAKKIVDLFRPQIEQQDKAFSSKVDKNFT
TVDKILAKYKTKDGGFETYDKVKENDRKALIGPVNTLAEDLSTLRGKLGLN
>tr|A0A0Q0ITS7|A0A0Q0ITS7_PSEAP LysM domain protein OS=Pseudomonas syringae pv. aptata OX=83167 GN=ALO85_04890 PE=4 SV=1
MRQHDKGIHRMRKSLLALLLLTASGLAQAQVQLREGYPQSYTVVAGDTLWDISGKFLREP
WKWREIWRANPQVHDPDLIYPGDTLALTWVDGQPRVTLNRGESRGTIKLSPRVRSTPMVE
AIPSIPLGAINAFLISNRIVDNAEQFEKAPYIVAGNAERVLSGNGDRAYARGALDPSHSV
YGIFRQGKTYIDPKTQEVLGINADDVGSAEVVATEGDVSTLILQRSTQEVRLGDRLLSSE
ERAISSTFLPSAPQAPIDGLILDVPRGVTQVSVLDVVTLDKGKRDGLTEGNVLAIYKTSE
TVRDRITGELVKIPDERAGLLMVFRTYDKLSYALVLQANRSLAIMDKVRNP
>tr|A0A0Q0CBT4|A0A0Q0CBT4_PSEAP Methyl-accepting chemotaxis protein OS=Pseudomonas syringae pv. aptata OX=83167 GN=ALO85_05528 PE=4 SV=1
MDFKRMCSMPVAPRFLDHYRKADRIMLGLIWLLFIYALGLAFWFDTFTQAVVVGGGTAVV
LTGLYRAIGGTRLMRCCFGVGLMVMTALHINQAHGQVEIHFGIFVLLAVLTFYRDWLPIL
VAAVTIALHHIGFHALQHSGFPVYVMHHGFGWSMVLVHAVYVVVESAILVYLAVQNQAEA
VENQDMLDRMLATTNQFSTDSQSSQQSGKHVSLAQRFEQFLAQITGLVDGVVRDTRGLGE
LGHDLAKASSTLETGAQHQLSEIARMTGAMQRMGDAMNDISSHVTQAVQRAGDASDQVAH
GRDSVDRAQSEITQLAARITTTDETVQALANQSEQIGKVLDVIGSIAEQTNLLALNAAIE
AARAGEQGRGFAVVADEVRNLAQRTASSTKEIQTIIEDLQKGSRQAATAMNDSLQGVGRC
VEDSQRASQSLRAVGEGIGHITRLNGLIATTTEQQSAVSREMADQLRSVQTIAEHTAANI
GVLATSSQSLSPLAIRLEALGQSFHA
>tr|A0A0Q0IAE9|A0A0Q0IAE9_PSEAP Type III secretion system-associated chaperone, CesT family OS=Pseudomonas syringae pv. aptata OX=83167 GN=ALO85_04176 PE=4 SV=1
MTIQDLLNALAIRLESGPLSLDANHLCCLKVNELDMTLERIEQQNTLFVYLCVGTLSTPA
SSTLLSDILAANLFHYGSSDGAAFGLDEKNNEVLLFQRFDPLRIDEDHFVSACVQMIEVA

KIWRAKLLHGHSAPLASSTRLTKAGLMLTMAGTIR
>tr|A0A0Q0D9R2|A0A0Q0D9R2_PSEAP Alpha/beta hydrolase fold protein OS=Pseudomonas syringae pv. aptata OX=83167 GN=ALO85_02601 PE=4 SV=1
MTISSDAAMRLCETVRMLRHLALNLPDPVCLSFLARDNDPLPEPACSSVRATERAINAAF
ARIGVRTLQYLHGGEAQLSSFEYFISRKLCEALDYSYEHFDDAEDVTAFNRLLKHFEFSG
AVPHIETLHLTTRDHAPLLVHASANRELPPVVLALPCGIPFDLCRDWFNALSERFFVVTW
ETRGLFGACEAFDQIAVDTDAQVADMISVMNHFRLSTAHLMGICGGAVIALSAAAAHADR
VNSLSLWHGDYNLGDNDLRTAHQQNFEWLMESAAQDRGEAADLQTMFLDQATLATTPEPI
AHVVLYPYVNPELFYRYARLNDALNKTELAPRLTRITAPTLVVAGDADSTTHIGGSRYIA
ASIKDATLHVERDGSHLAFFASSHQSKQTAFSFLEEVLQPAVA
>tr|A0A0N8T9U9|A0A0N8T9U9_PSEAP Acyl-CoA dehydrogenase/oxidase domain protein OS=Pseudomonas syringae pv. aptata OX=83167 GN=ALO85_100365 PE=4 SV=1
MPIRVRWASACRHWSTKVWISFPCLPAVRHLSAGGRWPALXXXDLGLCKLYEGHTDALAI
MAELGAPPPEQFSTWGMWAAEPPRARVNISGAGDALRLHGRKAWCSGASALSHALITAWD
AEHRQQLVAVRLDQPGVHMTGDGWRAVGMGATGSIDVVFDNAIGQAIGKPGGYLDRPGFW
QGGIGIAACWYGASRAIAQRLVTHVGKREDPHALAHLGAVDVAMHAAIDVLRAAAAHLDQ
APAENAEMLARRCRAYVEQAADLVIQHVGRAVGAGPYCKDPHFARLITDLPVFLRQSHAE
QDLAALGQLSGKALPAARTWSL
>tr|A0A0Q0C360|A0A0Q0C360_PSEAP 8-amino-7-oxononanoate synthase OS=Pseudomonas syringae pv. aptata OX=83167 GN=bioF PE=3 SV=1
MSFDLRTRLDARRAAHLYRQRPLLQSPQGPQVIVDGQPLLAFCSNDYMGLANHPEVIAAW
QAGAERWGVGGGASHLVIGHSAPHHELEEALAELTGRPRALLFSNGYMANLGAVTALVGQ
GDTVLEDRLNHASLLDAGLLSGARFSRYLHNDVSSLEARLEKSVGDTLVVTDGVFSMDGD
IADLPALARATRAKGAWLMVDDAHGFGPLGANGAGIIEHFGLSMDDVPVLVGTLGKSFGT
SGAFVAGSEELIETLIQFARPYIYTTSQPPALACATLKSLQLLRTEHWRREHLTRLIQQF
RRGAEQIGLQLMDSFTPIQPIMIGDASRALRLSQLLRERGLLVTAIRPPTVPAGSARLRV
TLSAAHSEADVQLLLNTLEQCYPLLDASHSSEPVHA
>tr|A0A0Q0IBT7|A0A0Q0IBT7_PSEAP Insulinase-like:peptidase M16 OS=Pseudomonas syringae pv. aptata OX=83167 GN=ALO85_00466 PE=4 SV=1
MITRNAPRHALLGLILAGSLGAFVALPALADDAPAEPSQAAPALGSNLQTLKELDGKAPA
RRALNIQTWNTAEGARVLFVESRELPMFDMRLTFAAGSSQDQKSPGIALLTNAMLNEGIK
GKDVNAIAQGFEGLGADFSNGSYRDMAVASLRSLSAADKRDPALKLFSEVVGKPTFPADS
LARIKNQLIASFETQKQNPGAIASKELFNRLYGDHPYAHPSEGDAKSVNAITLAQLKAFH
AKGYAAGNAVIALVGDLSRDEAQAIAAQVSASLPKGPALAKVADPVEPKAGTTHIEFASN
QTHLMLAQLGVDRNDPDYAALTVGNSVLGGGGFGSRLMTEVREKRGLTYGVSSGFTAMQV
AGPFMIGLQTRAEMSENTLKLVQDIIRDFLANGPTQKEVDDVKRELTGSFPLTAASNSAI
VGQLGAIGFYNLPLTYLEDYMAAAQNVTVEQVKAAMSKHLSADKMVIVTVGPTVEQKPLP
APTDKPVRQPAGVPEH
>tr|A0A0Q0IJ49|A0A0Q0IJ49_PSEAP Syringopeptin synthetase C (Fragment) OS=Pseudomonas syringae pv. aptata OX=83167 GN=ALO85_04440 PE=4 SV=1
RMQGGEGTERALGMFINTLPLRVSVGEQGVRDGVKATHKRLTALLGHEHASLALAQRCSG
VVAPTPLFSALLNYRHSGGGSASEQAMQAWQGIHALSSEERTNYPLTLNVDDLGEGFKLT
ALTTTAVGAQRICGYMHTALDQLIQALEQASTAPLDSLSILPADERERLLVGFNDTALDY
PQQQTIHGLFEAQVERTPEALAVVHGEQRLTYRELNEQANRLAHALRKQGVQPDSRVGIC
VERSAEMVVGLLAILKAGGGYVPLDPAYPVERIAYMLQDSAPAAVLAQTATQGLLADVSV
PVINLDLSDWQDQSVQNPQVPGLTSAHLAYLIYTSGSTGLPKGVMIEHRNTVNFLSWAHN
AFDASALEKTLFSTSLNFDLAVYECFAPLTSGGSIEIVKNVLEL
>tr|A0A0Q0DKP2|A0A0Q0DKP2_PSEAP ATP-binding region, ATPase-like:Histidine kinase OS=Pseudomonas syringae pv. aptata OX=83167 GN=ALO85_01271 PE=4 SV=1
MQSPPHDPASALAIRNQYRQSQSRAARLRLLVDTGQELTHLPPQAMRQCVLQRACAFVAM
DHGLLLEWSADNGVQTTASHGSAERLATLETAADPLAIGPQWLERPAADLPCMLLLPLRG
ADEGSFGTLLLANSVGINAPDNEDIESLQLLATLLAAHLENNRLLDALVVREQTMSELVR
QLFTAQEDERKRMAYDLHDGLAQTLAGLHQRLQGFAGRCPALPAPLDADLQAILKLAQHC
VGEGRQLISGLRPSVLDDFGLLQAVDKEADRLREAGVAVHWRSRSLTRLPSHLEIALFRI
AQEGINNVLKHASAGRVELALELSDEHISLLLEDNGSGFITQKPFNGKGVQKLGLVAMQE
RASLLGGRLTCVSRPGHGTRLRAIVPFTADKAMT
>tr|A0A0N8T9I7|A0A0N8T9I7_PSEAP Conjugative transfer region protein OS=Pseudomonas syringae pv. aptata OX=83167 GN=ALO85_100117 PE=4 SV=1

MTELQQDQHEDGTLRFLPSRLNNQPVVIGGLTADEMWATVFGCSGIGFVIGLPLAFIITP
SMPVVCALIGGVLGLLIASRVLRRLKRGRPETWIYRRVQLQTAMLGPTSFNKANLVLRSG
NWTCRRSEQQ
>tr|A0A0Q0CWG6|A0A0Q0CWG6_PSEAP Malate/L-lactate dehydrogenase
OS=Pseudomonas syringae pv. aptata OX=83167 GN=ALO85_04556 PE=3 SV=1
MKTSRRSLSATIRLLPLGVSLHNPQRYSRGLSFASSLSLAFSIYRHEQEFQMRANSADQS
TRTVSFEQLTDLLRRIFVAHGTSAEVAEALAENCASAQRDGSHSHGIFRIPGYLSSLASG
WVNGKAVPVVEDVGAAFVRVDAGGGFAQPALAAARALLIAKARSAGIAVLAIRNSHHFAA
LWPDVEPFAEQGLVALSMVNSMTCVVPHGARQPLFGTNPIAFAAPRAGSEPIVFDLATSA
IAHGDVQIAAREGRLLPVGMGVDCDGQPTEEPRAILEGGALLPFGGHKGSALSMMVELLA
AGLTGGNFSFEFDWSKHPGAQTPWTGQLLIVIDPDKGSGQSFAQRSEELVRQLHGAGQER
LPGDRRYSERAQSMVHGISITQADLERLQALAGH
>tr|A0A0Q0BYN5|A0A0Q0BYN5_PSEAP Probable lipid II flippase MurJ
OS=Pseudomonas syringae pv. aptata OX=83167 GN=murJ PE=3 SV=1
MNLLKSLAAVSSITMVSRVLGFVRDTIIARTFGAGMATDAFFIAFKLPNLLRRIFAEGAF
SQAFVPILAEYKSQQGEEATRTFISYVTGLLTLALALVTLLGVIFAPWVIWATAPGFVDT
PEKFALTSDLLRVTFPYILLISLSSMAGAILNTWNRFSVPAFVPTLLNVSMIFFALFLTP
YFDPPVMALGWAVLVGGLLQLLYQLPHLKKIGMLVLPRLNLRDTGVWRVMKQMLPAILGV
SVSQISLIINTIFASFLVAGSVSWMYYADRLMELPSGVLGVALGTILLPILSKTYAQRDR
QEYSRILDWGLRLCFVLVLPCTLALGLLAEPLTVSLFQYGKFDALDAAMTQRALVAYSVG
LLGIILIKVLAPGFYAQQNIRTPVKIAIFTLIVTQLLNLAFIVPLQHAGLALAISVGACI
NAGLLFWQLRKQDLFQPQPGWMKFLFKLVIAVAVMSAVLLGLMHVMPAWDEGHMLERFLS
LGALVAAGVVTYFAMLLLLGFRLRDFARKAIM
>tr|A0A0N8TAC2|A0A0N8TAC2_PSEAP Ribonuclease P protein component
OS=Pseudomonas syringae pv. aptata OX=83167 GN=rnpA PE=3 SV=1
MHVWPPKTAAQFFRVVVPKAVSVWQFDKSAQEVSRDFSREKRLLTPRHFKAVFDSPTGKV
PGKNLLLLARNNDLDHPRLGLVIGKKSVKLSVERNRLKRLMRESFRQHQDSLVGWDIVIV
ARKGLGDVENPELIQHFGKLWKRLARSRPIPEEKSEPAGVDSPDA
>tr|A0A0Q0DGF5|A0A0Q0DGF5_PSEAP Adenosylcobinamide-GDP
ribazoletransferase OS=Pseudomonas syringae pv. aptata OX=83167 GN=cobS
PE=3 SV=1
MPLSVCIRLMPVSACCWSVMVVSCACWWRRRVVCRVSNCCRWWSGMARCCRFRSLLIAYS
RRSEMLPFWIALQFLGSLPIRLPGMPRPEELGRSLLFYPLVGAVFGTLLLGFNTLLSGAP
LMLHAALVLTAWVLLSGGLHLDGLADSADAWLGGFGDRERTLKIMKDPRSGPIAVVTLVL
VLLLKFAAILALIESHASVWLLLAPVIGRAAMLGLFLGTPYVRSGGLGQALADHLPRGPG
RKVLLATAIACVLLAGWSGVMVLLVCAACFFWLRQLMMRRLGGCTGDTAGALLELLELAV
LLTLALL
>tr|A0A0N8T7U0|A0A0N8T7U0_PSEAP Phosphoglycerate mutase family protein
OS=Pseudomonas syringae pv. aptata OX=83167 GN=ALO85_02289 PE=3 SV=1
MKQVILIRHGQSAANAGEASVDHATIPLTLKGVEQAQSVARSFTHAPDLIVASPFSRAQS
TAMATAATFPATPLETWPIQEFTYLEPARCANTTVAQRRDWVEAYWARSDPAFTDGAGAE
SFSGFITRAQAFLARLAEHPAQRIAVFSHGQFINAVAWLIERKPQDICGRAMADWRDYEI
KNHVPNGGGYILFRHVGPDDWRICSQPSQNAESR
>tr|A0A0Q0DFT3|A0A0Q0DFT3_PSEAP Flp pilus assembly protein, ATPase
TadZ/CpaE OS=Pseudomonas syringae pv. aptata OX=83167 GN=ALO85_01365 PE=4
SV=1
MSQSLSQTFLAITRNNTDLEWLQSALGSLGQVVSAGTGSLDDLLALVDVTFASVVFVGLD
REHLMTQSALIESALEAKPMLAIVALGDGMDNQLVLNAMRAGARDFVAYGSRSSEVAGLV
RRLSKRLPAVTPNPNMSGLSVLYGVQSDDDGALIATHLAMVVHKSGQRTLLLDLGLPRGD
SLLMLGLESTFSFGDALRHLRRLDATLIDSAFTTSESGLRILAYSEADDHLEQSSAAELY
MLLSALRQHFQHVVVNLVGQPDSEALRSLVSHCDQLLWYTDQSVLGCRRNLTVLNNWREK
GMKMQHAGLLVDRYQRSVAPNSETVSKTFGLPVLAVLPLAPELRLNAKNQGVTLFELASR
DALCSGLRRLGEHLARHAEAREKPDQGWLARLWGNR
>tr|A0A0Q0C683|A0A0Q0C683_PSEAP Sulfurtransferase OS=Pseudomonas syringae
pv. aptata OX=83167 GN=ALO85_00469 PE=4 SV=1
MSIAQLINPEQLAERQKTPGLVILDCRYALEDSDYGQRSYAQGRIEGASFADLKRDLSGP
VIKGKTGRHPLPDPAALLQCFQAWGINADSDVVLYDDGPGMYAARAWWLLAWMGKREGVY
LLDGGLKAWHAAGLPLSLDAPNREPGHFSGEPDMSMVLSGSRLQGRLGRPEMTLIDARAE
ARFRGDVEPLDPVAGHIPGAQCAACTDNLGADGRFLPPEQLRQRFAEKLQGRPPESLVSY
CGSGVTACHNLFALCLAGYPLATLYAGSWSEWITDPEREIATGAD

>tr|A0A0Q0C0Q5|A0A0Q0C0Q5_PSEAP 3-isopropylmalate dehydrogenase OS=Pseudomonas syringae pv. aptata OX=83167 GN=leuB PE=3 SV=1
MSKQILILPGDGIGPEIMTEAVKVLELANEKYQLGFELTHDVIGGAAIDKHGVPLADETL
ERARAADAVLLGAVGGPKWDTIERDIRPERGLLKIRSQLGLFGNLRPAILYPQLADASSL
KPEIVAGLDILIVRELTGGIYFGAPRGTRVLDNGERQAYDTLPYSESEIRRIAKVGFDMA
MVRSKKLCSVDKANVLASSQLWREIVEQVAKDYPEVELSHMYVDNAAMQLVRAPKQFDVI
VTDNLFGDILSDQASMLTGSIGMLPSASLDTANKGMYEPCHGSAPDIAGKGIANPLATIL
SVSMMLRYSFNLTDAADAIEKAVSLVLDQGIRTGDIWSEGKVKVGTQEMGDAVVAALRNL
>tr|A0A0Q0C5L1|A0A0Q0C5L1_PSEAP Uncharacterized protein OS=Pseudomonas syringae pv. aptata OX=83167 GN=ALO85_04426 PE=4 SV=1
MFILLPRHPSMAFRPLNARPPAVLLREAKPLKAIFRHAERLSHLQRLLESQLQPAAREHC
HVASWREGTLLLIVTDGHWATRLRYQQKRLHRQMMAFDEFINLTRIVFKVQPPEAPRGAA
THTIDLSAVAAENIQATAEGITDPRLRAALERLASHAKDRK
>tr|A0A0Q0DU81|A0A0Q0DU81_PSEAP GYF_2 domain-containing protein OS=Pseudomonas syringae pv. aptata OX=83167 GN=ALO85_05352 PE=4 SV=1
MRSGVDALEQWFVMNAGKQLGPMDADAARLIARESPGAWCWKQGMPDWQPIYQVPQVRAM
KKDGVTPFPDPTPDMVQPKPSGLPAQAAPTPAPASVPARRPSFTPEPNASGGYGLAQTTD
GVDFKLYGTETQFIELELDPGESAVAEAGAMMYKTCDVQMETIFGDGSNQSSGLLGSLFG
AGKRMLTGESLFTTVFSQQGSGKGRVAFAAPYPGTILPLNLRDFGGKLICQKDSFLAGAK
GVSIGIQFQKKILTGLFGGEGFILQKLEGDGWVFVHMGGTVRKIELAAGEALDVDTGCLA
AMTQTVDYDIRMVGGGIKSMLFGGEGVFFARLTGPGTVWLQSLPFSRLAGRMLAAGPSGV
GRSER
>tr|A0A0Q0CFY2|A0A0Q0CFY2_PSEAP Amidase family protein OS=Pseudomonas syringae pv. aptata OX=83167 GN=ALO85_03595 PE=4 SV=1
MIESKPSSMPTVIEAATRVREGYLTPIHLTELCLTAIETHNSTLNAFGDVYAEAALEQAA
SMTAELQRGQVRGPLHGIPFGIKDLFSTAGLRTTRGSLTALESVPVQDAPIIRRLKNAGA
IILGKTATTEFGWTGASTSRVFGNGRNPWAPSLTSGGSSSGSAIAVAARMVPAALGSDGG
GSVRIPGSFCGAFALKGTLGRIPTWPWSATEMLSHAGPITRSVRDSALLFDILSGPDPLD
HQALPAPDESFLARCDQPLQPLRIGFCPTLFDTQVDAQVAAAVDAAVGNIARSLPVMVST
LKPDWQDPLATFETLWVAGRGIAYGKALAQKLDQLDPGFADLIRRSAQYSLSDYLQALQQ
RAAFANQVHAMFDDYDLLLMPTLPILPFAADDVAPVGYAGQDGAVPWARWTPFTYPFNIT
GNPAANLPCGRSEAGLPIGLQVVGPRFADAQVLQFCAAVEAIAPWDQHLPPVSGQ
>tr|A0A0N8T8L4|A0A0N8T8L4_PSEAP Membrane fusion protein (MFP) family protein OS=Pseudomonas syringae pv. aptata OX=83167 GN=ALO85_04634 PE=3 SV=1
MHRCARHPPYTVAEPDRSPAGAERRSHAGIRCHGSGVAGLVRTAIVGAGSASAPSIAISP
CTCRAFHEPSVRHAEQAVRRMNSPIDTHYAVPGKERGVQFFVRAGWILMLAGAGSFFLWA
SLAPLDQGIAVQGTVVVSGKRKAVQSLDGGVVSKILVSEGQRVKEGEPLFRLDQTQVEAD
VQSLRAQYRMAWASLARWQSERDNLDEVRFPAELIAAGQGQDPDPRLALVLEGQRQLFSS
RRQALAREQSGLQASIEGAGLQLAGMRRARSDLMAQADSLRKQLSNLEPLAQNGFIPGNR
LLEFQRQLSQVQQSLAQNAGETGRIEQGIVESRLRLQQQREEYQKEVRSQWADAQVKALT
LEQQLASAGFSLQHSAILAPADGIAVNLGVHTEGAVVRAGETLLEIVPQGTRLEVEGRLP
VQLIDKVASHLPVDILFTAFNQSRTPRVSGEVSLISADQMQDEKTGQPYYVLRTSVGDAA
LEKLNGLVIKPGMPAEMFVRTGERSLLNYLFKPLLDRAGSALTEE
>tr|A0A0Q0FQR0|A0A0Q0FQR0_PSEAP Pyrroline-5-carboxylate reductase OS=Pseudomonas syringae pv. aptata OX=83167 GN=proC PE=3 SV=1
MSKTRIAFVGAGNMAASLIGGLRAQGVEAALISASAPGAETRERIANDHGIKVFADNAEA
IQGADVVVLAVKPQMMKDVCQGLKSSLEPHQLIVSVAAGITCASMTQWLGEQPVVRCMPN
TPSLLRQGASGLYATDNVTSEQREQAETLLAAVGVAVWVEQEKHMDAVTAVSGSGPAYFF
LMMEAMTAAGVKLGLPEDIAKKLTLQTALGSALIATGSDVDAGELRRRVASPGGTTEAAI
KAFQAGGFEALVETALTAADHRAAELAEQLGK
>tr|A0A0Q0CXS9|A0A0Q0CXS9_PSEAP DNA-binding protein OS=Pseudomonas syringae pv. aptata OX=83167 GN=ALO85_05311 PE=4 SV=1
MKSPTLEKLDQLATRIGIHPLELLVSCYAKQASMSQQALLDMLDERLRNA
>tr|A0A0Q0C7A0|A0A0Q0C7A0_PSEAP Cell-wall associated Rhs family protein with YD-repeats and PAAR motif (Fragment) OS=Pseudomonas syringae pv. aptata OX=83167 GN=ALO85_04502 PE=4 SV=1
GDHYVIEIDDTGNMTGLTLPDGNQLAFEYDEFARLLKETDPLGRSIRYQYHHLTTLITQV
DYPDGSTWKA

>tr|A0A0Q0C0X1|A0A0Q0C0X1_PSEAP Xaa-Pro aminopeptidase OS=Pseudomonas syringae pv. aptata OX=83167 GN=ALO85_05619 PE=4 SV=1
MSGSAGESMSTQSNASSGVAERLAQTRALMSRERIDAYLVPSADPHLSEYLPGYWQGRQW
LSGFHGSVGTLIITQDFAGIWADSRYWEQATKELAGSGIELVKLMPGQQGPLEWLADEAK
AESVVAVDGAVLAVASSRTLASKLYERGARLRTDIDLLTELWQDRPALPSHPIYEHLPPQ
ASLDRSEKLARVRQIIVERKADWHFIATLDDIAWLFNLRGADVSYNPVFIAFALIGPQSV
TLFVDSKKVPDSVRARLEREAINLMEYTQIGAALRELPKDARLLVDPARVTCGLLDYLDS
EVTLVEGLNPSTLLKSRKTETDTAHIRQAMEQDGAALCEFFAWLDSALGREPVSELTIDE
KLTQARERRPGYVSPSFATIAGFNANGAMPHYRATEAEHARIEGDGLLLIDSGGQYLGGT
TDITRMVAIGTPSAEQKQDCTRVLKGVIALSRAHFPKGIQSPLLDAIARAPIWSEGVNYG
HGTGHGVGYFLNVHEGPQVIAYQAPATPQTAMLPGMITSIEPGTYRPGRWGVRIENLVIN
QEAGKTEFGEFLRFETLTLCPIDTRCLEVSMLNAEERAWLNDYHVQVLTRLSPFLQGTAL
LWLQARTIPV
>tr|A0A0N8T8L8|A0A0N8T8L8_PSEAP Glycos_trans_3N domain-containing protein OS=Pseudomonas syringae pv. aptata OX=83167 GN=ALO85_00714 PE=4 SV=1
MNDYAALVTETPEEHPFATFVRILGKGKRGARNLTREEAREAMGMLLDEKVEDTQLGAFL
MLLRHKEESPEELAGFTEAVRERLHAPDMQVDIDWPTYAGKKRHLPWYLLAAKCLAQNGV
RILLHGGGAHTAGRLYTEQLLDLLKIPLCRNWSAVKTSLEQDRLAFIPLADWAPQLQRMI
DLRNTLGLRSPIHSLARILNPLQARCGLQSIFHPGYQSVHRDASSLLGDNAIVVKGDGGE
IEINPDTISHLYGTTGGQPWDEEWPALSPRRHVKPATLDPQQLLALWRGEIEDSYPQLAL
ISTMALALRGLGVDRDEAFAQAQVFWDRRDINL
>tr|A0A0Q0DZ67|A0A0Q0DZ67_PSEAP Sodium/hydrogen exchanger family protein OS=Pseudomonas syringae pv. aptata OX=83167 GN=ALO85_05363 PE=4 SV=1
MAARKRPTSDRYPANYFNLLPCKGVPPLRPLLRAHFDGGSFPMSFIVCMGVLGVLLMLLA
LTSSYLRWMPVTTSAVCLGFGFGIGPMGLDIVDLDFEKSYEWLERLTEVAVLFSLFGTGI
KLRLPLRGKAWHSAYWLAGPVMLATIAGVTVAGHYIIGLDWGVSLLLGAMLSPTDPVLAG
MVQVNNAQDQDRLRFGLSGEAGLNDGTAFPFVIFALLYLSSGGAMSDWVQGWMIKDLIWA
VPAGLLMGYGMGRGVGHLMIFLRIKNADSTTSPNDFLALALIALAYVFAQSVGAFGFLSV
FAAGFGLRQAEVRVTRSELPSEHVAQPVLGHMTSSLEQGTVAQVDDMSDSQLAAGVMMGD
MLAFGSLVERAMEVLLITLLGAALAMYWDWRAIGLGIALFCVIRPASVWLLVSRRLLNVR
QKALVGWFGIRGIGSLYYLCFALSHGLAHDVGHVVIGMTLSVVALSILVHGISIQPLLER
YERSTAASPD
>tr|A0A0Q0DU66|A0A0Q0DU66_PSEAP Beta-ketoadipyl CoA thiolase OS=Pseudomonas syringae pv. aptata OX=83167 GN=ALO85_02398 PE=3 SV=1
MRDVFICDAIRTPIGRFGGGLSSVRADDLAAVPIRALMERNPSVNWEEIDEVFLGCANQA
GEDNRNVARMAALLAGLPQSVPGVTLNRLCASGMDAIGTAFRAIASGEIELAIAGGVESM
SRAPFVMGKADAAFSRNMKLEDTTIGWRFINPAMKAQYGVDSMPETGDNVATDFRISRAD
QDAFALRSQQRTAVAQAAGFFEEEIVPVRVAHKKGETLVDKDEHPRGDTSLETLSKLKPV
NGPDRTVTAGNASGVNDGAAALILASAEAVKKHGLTPRARVLGMASAAVAPRVMGIGPVP
AVRKLVERLGLAVTDFDVIELNEAFASQGIAVLRELGLADDAPQVNPNGGAIALGHPLGM
SGARLVLTALHHLEKTGGRRGLATMCVGVGQGLALAIER
>tr|A0A0Q0DDY4|A0A0Q0DDY4_PSEAP Membrane protein in purine degradation operon OS=Pseudomonas syringae pv. aptata OX=83167 GN=ALO85_01156 PE=4 SV=1
MLAHMMEWLNLGVRWIHMIVGVAWIGASFYFVWLENNLNRSNPREGLSGDLWAIHGGGIY
HLEKYKLAPPTMPENLHWFKWEAYSTWLSGVALLCVVFYANPTLYLLAPGSSLSGAEGVH
IGLGSLFVGWFIYSFLCDSPLGKRPALLGVVLFVLLVAAAYGFSKVFSGRGAYLHVGAIM
GTIMVGNVFRIIMPAQRALVAAIAENRTPDPSLPAKGLLRSRHNNYFTLPVLFIMISNHF
PSTYGSEYNWLILAGIALLAVLVRHYFNTRHDSHRFAWTLPVAALGMLCLAYVTGPAQAP
AKIVYQPLPGTAVGGHRADEQPAQPAAAPAQAAPAQPEPAKVAGADFNKVHDVIRQRCSV
CHSATPTSQLFSVAPAGVMFDTPEQIQQQAPRIKAQAITAPIMPLGNITQMTQQERDLVG
AWIDQGAHIN
>tr|A0A0Q0ID93|A0A0Q0ID93_PSEAP Peptidase M24 OS=Pseudomonas syringae pv. aptata OX=83167 GN=ALO85_04928 PE=4 SV=1
MTATPACACHSASRWTPSGRLLPMIQIPKSEYARRRKALMAQMEPDSIAILPAAAVAIRN
RDVEHVYRQDSDFQYLSGFPEPEAVVVLIPGREYGEYVLFCRERNPERELWEGLRAGQEG
AIREYGADDAFPINDIDEILPGLIEGRDRVYSAMGSNPEFDRHLMDWINVIRSKAHLGAQ
PPKEFVALDHLLHDMRLYKSAAEIKVMRCAADISARAHVRAMQACRAGLHEFSLEAELDY
EFRKGGSKMPAYGSIVASGRNGCILHYQQNDAVLRDGDLVLIDAGCEIDCYASDITRTFP
VNGRFSPEQKAIYELVLKAQHAAFEAIGPDKHWNQAHEATVKVITAGLVELGLLRGDVGE

LIESEAYKMFYMHRAGHWLGMDVHDVGEYKVGGEWRVLEVGMTLTVEPGIYISPDNLEVA
KKWRGIGVRIEDDVVVTRQGCEILSGGVPKTVAEIEALMAAAQGRVA
>tr|A0A0Q0C8Q1|A0A0Q0C8Q1_PSEAP CrtC domain-containing protein
OS=Pseudomonas syringae pv. aptata OX=83167 GN=ALO85_05334 PE=4 SV=1
MYSGHSPMNSKWLMPLVLVGLLAACDDKPQPESGFAGLGNTADSYAQVTPGKVFTFPQDH
GQHPGFRIEWWYITATLKDDAGQQFGVQWTLFRNALRPGQQTGSGWNDGTIWLGHAAATS
SSGHYVAERYARGGIGQADVTLAPFSAWIDDWAFTSSVHSGDPLTAMQLKASGKDFRYDL
NLTSSKPLVLQGEQGYSQKSEAGQASYYYSQPFFDAKGSVEIQGKSYQVTGHAWLDREWS
SQPMTANQSGWDWFSLQLADGDRLMLYRIRHKNGAPYLTGNWISADGTTRMLHANEISLE
PLKETLIGEHKVPTRWRVRVPDRNLDITTEALNPAAWMGVSIPYWEGPVKFSGSQEGVGY
LEMTGY
>tr|A0A0Q0FMQ6|A0A0Q0FMQ6_PSEAP Oligopeptide/dipeptide ABC transporter
OS=Pseudomonas syringae pv. aptata OX=83167 GN=ALO85_00904 PE=3 SV=1
MSATANLTENGDGTVLSISDLTVRFAGAPANVVDGVSFSVKRGKTLAIVGESGCGKSVTS
MGLMGLLPTTAKVDASDSLLIDEALLGMSEERLLDVRGNRMAMIFQEPMTSLNPVFTIGE
QIAESVMRHQGLSDKAARQRALDMLEKVRVPDARQRLDAYPHELSGGMRQRAMIAMALAN
DPALIIADEPTTALDVTIQAQILSLIANLQTETGTAMILITHDLGVVAEVADEVMVMYAG
RVVESGPVKTLFDDPQHPYTIGLMGSMPSIGPREGRLATINGRVPTPAEMPSGCRFAGRC
PFVIQQCRDERPPLLELSPGHFAACIRAPLEQHVGVSA
>tr|A0A0Q0C0X5|A0A0Q0C0X5_PSEAP Uncharacterized protein OS=Pseudomonas
syringae pv. aptata OX=83167 GN=ALO85_102032 PE=4 SV=1
MVDQHHLAPASGSGERTHQTGSAGANDYNVSNGHSVVPRSSGHDRSHAPRQAMHKPFFSG
RRPWEXXXNVFAKGPVYSPKIYRLNYRLREQVRSHALRAEA
>tr|A0A0Q0C8R5|A0A0Q0C8R5_PSEAP Putative lipoprotein OS=Pseudomonas
syringae pv. aptata OX=83167 GN=ALO85_100471 PE=4 SV=1
MNRASKREPDRSHGLNRRLCKSLMLSVGLLILAGCGTQRVQEPELTPEQARAQIMRLMPA
TATDRQGWATDIHAAFAAQKIPLTTENLCSVMAVTEQESTFQVDPAVPDMGHIARAEINR
RAARLHIPNALIATALRVRSPDGKTYGKRLDSARTEKDLSAIFDDFIGMVPLGQALFGNF
NPVKTGGPMQVSIAFAEKHAEDYPYTVDGSIRREVFTRRGGMYFGIAHLLGYPVNYTQSL
YRFADFNAGWYASRNAAFQNAVSRATGIELALDGDLIRFDSTSPGSTELAVRTLGDRLGM
NKSQIWSQLKQGDTLEFEETDLYSKVFALADRAAGKPLPRAILPGITLKSPKITRNLTTA
WFAERVDDRRERCVQRAPK
>tr|A0A0Q0C5U0|A0A0Q0C5U0_PSEAP Alkanesulfonate transporter substrate-
binding subunit OS=Pseudomonas syringae pv. aptata OX=83167
GN=ALO85_05040 PE=4 SV=1
MSCNAGSSKARRMASMCWCRTCRAVSKMSRGCWCRSCSGAGCFAPSMKARPCAKTWACNG
LLIDFDRCRMQVLMSAESGLPRPSARIKGRFLYRIKGSPMKSFTFIAAILAFSGLFGTAA
ADEPDTLRIGYQKGSITLVLAKEHSLLEKRFPQKKIKWVEFPAGPQMLEALNIGSLDIAS
TGDIPPIFAQVAGADLVYIGAEPPKPQVETLLVRNESPVHSVAELKGRKVALQKGSSSHN
VVLRLLNKAGLTFKDIQPIYLTPADARAAFENGSVDAWAIWDPYYSIAMSEGHTRLLANG
EGLGLSGPFYTARRAFAEENAALVQQVLDELTVADGLSRSQRAESIQILARSMGLSEAVM
TQYLDHRPVSPSLPITPEIIRAQQATADLFYDNHLIPKRVDIQQVVWQKP
>tr|A0A0Q0FBW7|A0A0Q0FBW7_PSEAP Type III secretion apparatus protein,
YscD/HrpQ family OS=Pseudomonas syringae pv. aptata OX=83167
GN=ALO85_04182 PE=4 SV=1
MFELRVLNGQHQGAALPLIGEQWSIGSAGQHDLALDDAGVESLHCRLQRVDDNWTLNAEQ
GAVCDEQGNARPSIDLTLNNAFMLGSVWLCVSSAGDEWPSVPAVIPKQPEAESGPARNDV
PLEKVKSRSQFLNRTTGIIAGLLVGVIGSAWSLTRPPAIAMDQSPAHLAAATTEASPDTP
KAPARAANPVTDKRIRLSSADDVRHQLSTMLSDRLLTDVSVEETPDGLVLNGDLKEESLL
VYQRMLQRFKTLYDSPVTVLDNVGSNRNTLPFVVVQIMTGPHAHLVTADGRRVYVGDEVD
GLRLTRIDNQRLQFDGNRHVEVNW
>tr|A0A0Q0FVM1|A0A0Q0FVM1_PSEAP Amino acid-binding ACT OS=Pseudomonas
syringae pv. aptata OX=83167 GN=ALO85_03858 PE=4 SV=1
MANGTRFTVSELPSGHAYTGMTDAVSKPSVQKEELTVDHLVVTLVAPDKPGQVERIARCI
AEHGGNWLESRMSRMAGQFAGILKVGVEAEKYDDLIKALNGLSAHGIRMLLAESVVETSD
TTRLISMELVGNDRPGIVRDITRLLAGQGVNVEHLITDVSPAPMSSELLFQAKAVLGVPQ
DLSLDDLQMALETLADDLMVELVLGAKE
>tr|A0A0Q0C269|A0A0Q0C269_PSEAP Lysine exporter protein LysE/YggA
OS=Pseudomonas syringae pv. aptata OX=83167 GN=ALO85_03958 PE=4 SV=1
MESLQQWLSFAMIALFVTLTPGPAVIMALSNSITHGPARAMVGSLGNALGLIVVATAVTA

GLGAVLVASASAFLVLKIAGAGYLVYLGIKQWRSARSAFDALADVPKAVSTGSLFIKGIS
VALTNPKAILFFIAFLPQFIQPGTFQVEQTGVLIVTFAGCSVVAHVFYVLLAQTLKRHLN
SARRRQNVNRVFGASFIGLGLSLFTLKGRAA
>tr|A0A0Q0C6S8|A0A0Q0C6S8_PSEAP Oligopeptide/dipeptide ABC transporter ATP-binding protein-like protein OS=Pseudomonas syringae pv. aptata OX=83167 GN=ALO85_00827 PE=3 SV=1
MALLHVENLRVEIPLGQDTLHAVRGLDFQVERGEMLCIVGESGCGKSLTSLALMDLLPRK
ARRTATTLSLDGIDMLTQSERQMCDLRGNRLAMIFQEPMTSLNPAYSIGDQLSEVLTRNR
KVSRKEALQRAAQMLEKVGISNAGERLRQYPHQLSGGLRQRVIIAMALMCEPDVIIADEP
TTALDVTIQAQILRLIRDIQKELGLAVIFITHDLGLVARIADRVAVMYAGQIVETAPAIE
LFENPQHPYTRGLLASIPIPGRTQPGQPLGSIPGLVPSLVGEQHGCAFRNRCPQAIPACA
DDIPPINSHNHMTRCLFAAAGAEPVFHREGVPS
>tr|A0A0Q0FU19|A0A0Q0FU19_PSEAP Nucleoside triphosphate pyrophosphohydrolase OS=Pseudomonas syringae pv. aptata OX=83167 GN=ALO85_04999 PE=4 SV=1
MQAKADCGGLKLNRCDRRKPMYTVQDLLNLMARLRDPQFGCPWDLKQTYASIVPHTLEEA
YEVADAIEQGDLDHLKGELGDLLFQVVFYAQLAKEERRFEFDDVIDGITRKLLRRHPHVF
PTGELYAPAETPRLTDEQVNLRWDEIKAEERAEKAGVPEQLSLLDDVPRALPALSRAAKL
QKRAAQVGFDWPAALPVVDKVREELDEILEAMVDNDAEGIAEEVGDLLFSVVNLARHLKV
DPETALRSANSKFDRRFRFIEQALRHLQRPIEACSLEEMDALWGEAKRQEKSTPGCG
>tr|A0A0Q0D3I8|A0A0Q0D3I8_PSEAP Bifunctional glyoxylate/hydroxypyruvate reductase B OS=Pseudomonas syringae pv. aptata OX=83167 GN=ALO85_01790 PE=3 SV=1
MKKTVLAFSRVSPEMAERLAQDFNVIVPNPKQGDINAQFAEALPESHGLIGAGRKLGREQ
LQNATKLEVVSSISVGYDNYDVEYLSERGILLTNTPDVLTESTADLGFSLIMSSARRVAE
LDAYTKAGQWTRSIEPPHFGTDVHGKTLGIVGMGNIGAAIARRGRLGFNMPILYSGNSRK
TELEQELGAQFRSLDQLLAEADFVCLVVPLSEKTQHLIGRRELSLMKPGAILVNIARGPI
VDEPALIEALQNGTIRGAGLDVYEKEPLSESPLFQLKNAVTLPHIGSATTETRQAMADRA
YGNLRSALLGQRPQDLVNPQVWKG
>tr|A0A0Q0CVS2|A0A0Q0CVS2_PSEAP Type III helper protein HrpW1 OS=Pseudomonas syringae pv. aptata OX=83167 GN=ALO85_04209 PE=4 SV=1
MSIGINSSTSYQPASTQLDFSALSGKSPQTNTFSEESTAQAVDPSALLFDTASQKDVSFG
KPDNTVQSPTDSSAATDPQSNVVKLLSALVTSLLQMLMNLNKKQDTGQDANEWQDPFQNN
GGLGTPSTEGGGGGTEAASSGDDGGGGTEATSGGDEGGGGTTAATGGGDGGGTSPTTEGD
GGGSYASTGADGSGTPSTEDGTGGGGGGGDGVTPQVTPQLANPGRSSGNGTVSDTTGSLE
QSGNVNVVKDTIKVGAGQVFDGHGATFTADKSMGTGDQDEHQKPLFELAEGAVLKNVNLG
ENEADGIHVNAKNSEQVTIDNVHAQNVGEDMITVKGEGGAKVTNLNITNSSANGADDKVI
QLNADTHLKVDGFKATDFGTLVRTNGGKQFDDMSVELNGVDATHGKFALVKSDSDDLKLA
TGDIAMTDVKHAYDKTKASTQHTEL
>tr|A0A0Q0CHL7|A0A0Q0CHL7_PSEAP Amino acid permease OS=Pseudomonas syringae pv. aptata OX=83167 GN=ALO85_03403 PE=4 SV=1
MTMATAETSSLRRAITAPMLTLFILGDVLGAGVYALAGTIAGRVGGAIWAPLLIALCFAL
LTAASYAELVTKYPRAGGAAVYAEKAFGKPLLSFLVGFSMVAAGVTSAAGLAVAFAGDYF
QALVDWPAQWVCVAFLVIVGLLNARGIKESLSANLVMTVIELSGLLLVIAAAVWFVSQGQ
GVPQRVFELDTASPATAILGASLLAFYSFVGFETSANLAEEIKDVRRVYPRALFGALLAA
GAVYMLVGVGAAMVLPVEQLKDSQAPLMEVVSASGLGIPAPWFAVIALIAVANGALLTMI
MASRLTFGMAREGLLPAVMGSVLPKRRTPGLAILATTLVAIALSFTSTLTILAETVVLLL
LFVFLSVNIAVLVLKRDKVEAGHFQVHWVIPLLGILSCLLLLTQQGPETWLRAGIMLAVG
AALYGLTRLGSSALASHSR
>tr|A0A0Q0FNN5|A0A0Q0FNN5_PSEAP Uncharacterized protein OS=Pseudomonas syringae pv. aptata OX=83167 GN=ALO85_100763 PE=4 SV=1
MPSMFPGIDASRLTVMAMLGMLGAAWKPIHLVWQCAGARYGFRAERSPASLGGRFFGPSL
>tr|A0A0Q0BZ68|A0A0Q0BZ68_PSEAP Uncharacterized protein OS=Pseudomonas syringae pv. aptata OX=83167 GN=ALO85_00854 PE=4 SV=1
MNSTHPDDASDQARPPPSVKPETRLLESEPSAPHAGVQATPHKAIHPAFMDMPAAPYPGL
ENQTGMPFSSFYLSRYIAFASIAIVAIGVCIAIFFLNLDGDWPQINRVLAAAALWFANLI
SAVLNTVYWFIRRAPKGLVILLAMQWVVALAGAVSLLLQN
>tr|A0A0Q0DQN7|A0A0Q0DQN7_PSEAP Cupin_3 domain-containing protein OS=Pseudomonas syringae pv. aptata OX=83167 GN=ALO85_03906 PE=4 SV=1
MPQPTVLLLARADLSPVDTEFTTGPIDAHDSFDNERRTAFIDDQGIAAGLVHFGTSLSVA

AYPYTEMLVMHRGSVTLTSGAESLTIKTGESAVIGRGTQVRIDAQPDSLWAFCASTQASG
PDKPGITALDRLAMLTPSSPPDPAIMISALPQCRSNNLFEDSASTLRIGVWDSTPYERIS
RPHKIHELMNLIEGSVELSLENGPSLTVNTGDTVFVAQGAPCKWTSTAYVRKFYAVT
>tr|A0A0Q0D436|A0A0Q0D436_PSEAP Major facilitator transporter
OS=Pseudomonas syringae pv. aptata OX=83167 GN=ALO85_05539 PE=4 SV=1
MPEADACAIQSVFQPHRGHSYGAPKRRATKRTGKLSDQSTPNTPLVRFCDKIGIPYPMFW
GFVGLLIFMIGDGVELGYLSPFLSERGMPGDEVAMIFTIYGVTVGISSWLAGALSNIWGP
KRVMFLGLIIWSVFEVLFLVYGLQELSYSMILLTYGLRGFGYPLFAYGFLVWIAASTPSR
KMGMAVGWFWVAYAAGLPMLGSLVASIAIPLIGALLTFWLSFALVVIGGVIGLVGMREAH
GMRRLAPEGERPLVSMAKNITIMWTRPKVSLAGIVRVINTSSMFGFLVVMPGFFMQTVGF
TLEEWLRLLTIVFVFNIIGNLGSSVISTKIGYRNTILWFGAVGSTISTPLFFFMPQAFPN
DFLIASIFGAFYGLTLACFVPLSALAPALAPNNKAAALSVLSLGAGASTWVGPAVVAIFN
ESYGIEGVVWAFSGLYALSAVLTVFLKDPEPVHDPILIKIKRRIKVFAPRLDYRWAHAED
PKQSQS
>tr|A0A0Q0C0L6|A0A0Q0C0L6_PSEAP Binding-protein-dependent transport
(System) inner membrane component OS=Pseudomonas syringae pv. aptata
OX=83167 GN=ALO85_01002 PE=3 SV=1
MNVIEKAPIGSTLLQAQSPWRRVLTEFFSSPTAVTGLVVLLLIVLVAALAPWIVVQNPYD
LMQLNVMDARMPPGSLNGDSGYTYWLGTDGQGRDLVSAIIYGLRISLWVGIGSALIAAVL
GTLLGLVSAYVGGWLDALLMRLVDLLLSFPVILMALMILAWLGKGVGNVMLTLILLEWAY
YARTARGQALTESRREYVDAARGQGVGPLRIVVGHILPNCLPPLIVIGALQIARAITLEA
TLSFLGLGVPVTEPSLGLLIANGFQYMLSNEYWISLFPGLALLITIVAINLVGDRLRDVL
NPRLQR
>tr|A0A0Q0C224|A0A0Q0C224_PSEAP Uncharacterized protein OS=Pseudomonas
syringae pv. aptata OX=83167 GN=ALO85_200184 PE=4 SV=1
MIFFGYIGSSQITCLQLMAKKIAVSQSTSGDVPESGLDSLNQDGDSVEMALARQLINS
>tr|A0A0Q0IBV4|A0A0Q0IBV4_PSEAP Signal recognition particle receptor FtsY
OS=Pseudomonas syringae pv. aptata OX=83167 GN=ftsY PE=3 SV=1
MFGSNDDKKTPAEAGEKKGLFGWLRKKPQQAEPKPAESSPTQPDPIADQVAATPQVEPHV
ASQSVPEVTVEAAAPASQLHVEPGAPSPQPWLTLPVTEEPVALSDEREPHVTPAIPEQNH
AQVVPEPLEPEPVVAESVADISPLAMPEPLLVQPVAEPQPQPVQPAAQELAVPAPAPTPE
PETGKVGFFARLKQGLSKTSASIGEGMASLFLGKKAIDDDLLEEIETRLLTADVGVEATS
VIIKSLTQKVARKQLTDAQALYTSLQGELAAMLKPVEQPLVIKSEHRPFVILVVGVNGAG
KTTTIGKLAKKLQLEGKKVMLAAGDTFRAAAVEQLQVWGERNHIPVIAQHTGADSASVIF
DAVQAAKARGIDVLIADTAGRLHTKDNLMEELKKVRRVIGKLDADAPHEVLLVLDAGTGQ
NAINQAKQFNQTVTLTGLALTKLDGTAKGGVIFALAKQFGLPIRYIGVGEGIDDLRTFEA
EPFVQALFAERERP
>tr|A0A0Q0CYL1|A0A0Q0CYL1_PSEAP Uncharacterized protein OS=Pseudomonas
syringae pv. aptata OX=83167 GN=ALO85_04936 PE=4 SV=1
MEHRGAARLHARSGRVVDSLDADGTGLDDRRSHALQPPAVAGRCSVDRGGGGQAVLRRAE
QSRRHGAHRLVHRRWHFAIGGGIFRTFTAETLCHRGRRKTRPRPDCRTGHAMRKPFLSHP
AMKIAVLGLALCTAITALAQDKPADFASQTPLSLSGEGPWYRIELPLAVQLNARQTDLSD
VRVFNAEGQPQAYSITPRQPAPEQEPTPIEVNWFALYSTQEAGDTAPVIRIERSSNGSVI
EVQPQSDIEAGEEVLRGWLLDTSAIKAPLEQLVIDWSTEREGFQNFSIEASDDLQHWRNW
GEGQVARLSFADEVVEQREVGLPGQSARYLRLLWRSPHSAPLLISAHLLSASSDTPATPL
TWSPPVKGSVESPNEYVWQLPTGLPIGRVKIDITQPNSLAPATLYGRLDAGKPWQPISSG
LLYRLSQNNSDVLQDQLQLSGRIVQQLKLVVDDRGGGLGSEAPQLSVAVPATEVVFLARG
NGPFTLAIGNPSVKAANLSLATLIPDLSAEKLATLGTASPATAAVANMSAAPIAVQQSTD
FKRLGLWAVLLLGVVFLGWMAFSTLRAAKR
>tr|A0A0Q0C8S2|A0A0Q0C8S2_PSEAP Putative long-chain-fatty-acid-CoA ligase
OS=Pseudomonas syringae pv. aptata OX=83167 GN=ALO85_03183 PE=4 SV=1
MSHEREAFWLRLQTLADEQPRAIALQGEDRTLDYAALVEEITQRREQLRVANARIVALML
DNSPDALLWDLAVLFEGLSCVALPPFFSLTQRRHCLQRSQADTVITGSLFAQELQASDYR
QHGAFWQRRAAEQVYMPVGTTKLTFTSGTTGTPKGVCLSADSILRVARGLHEASQSVRPV
HHLSLLPLAILLENIGCYAALYAGARISLPSQQELGIQGASGVDASRLLMFLQQRRPDSM
ILVPQLLLLLVTACEAGAFDASMLSFAAVGGARVSLDLLSRAQAAGLPVFEGYGLSECAS
VITLNRPDAQRAGSVGRPLPHIWLRLADDGEVLVAGSPMLGYLGDTTPAPAWWPTGDLGE
LDADGYLYLKGRKKHQFVTSFGRNVNPEWIEAELTQSHCIAQAFVYGEALPSNHALLWPS
RPGTTDLQIDEAVNKANQALPDYARVTSWTRLDEPFSPANGLSTANGRPRREAILERYQT
LFTPSNLTQGALS

>tr|A0A0Q0DSI8|A0A0Q0DSI8_PSEAP Amine oxidase A OS=Pseudomonas syringae pv. aptata OX=83167 GN=ALO85_100994 PE=4 SV=1
MDIAGGYLMARVSCAASRCVGLTCPIFPMFSGVRRMLSGWLRACALIVAGLVSVSTLADE
KQRTAIVVGGGLAGLTAAYELQAKGWQVTVLEAKPSLGGRSGLATSEWIGNTKAQPVLNR
YLDSFKLSTVPAPEFVRTPSYLIDGVYFTQADLAVKQPATAEAIKRYNDTLDNLARSVDD
PENPASNSTLFALDQINVANWLDRLNLPVTARQLINQQIRTRYDEPSRLSLLYLAQQSRV
YRSVDERDLRAARLPGGSAVLTQAFVKQLKTVKTNSPVTAVIQEKDKVTVKAGATSYTAD
YVVMAVPLRSLGKIQMTPALDAQHMGAIKSTNYGWRDQIMLKFKTPVWDSKARMSGEVFS
NTGLGMLWVEPALKGGANVVINLSGDNARIMQAFGDKQMVDQVLIRLHAFYPEARGAYTG
YEIRRYSVDPSTGGSYLAFGPGQISKYWRLWERPILRVAFAGEHTDTLYPGTLEGALRSG
QRAAGQVQDLAAGKSFEPVKVAPPKAPAPASQKSDGNFFSNLFGGSSDKPAEPAKPQAGK
PGFFSRMFGGGEEPAPAPAPVEKAVEPAPAPAPAAPPAPVVAPVVQPAKPPVKAEPAKKP
ATKAEPAKKVQSKEAARKEAAKKEAAKKEAAKKEAAKKAQSKPAPAKAPAEAAPADANN
>tr|A0A0Q0D7I0|A0A0Q0D7I0_PSEAP Heme chaperone HemW OS=Pseudomonas syringae pv. aptata OX=83167 GN=ALO85_02942 PE=3 SV=1
MTHDSPAQPLFLGESGFSSESPRPALPHLPPLSLYIHIPWCVRKCPYCDFNSHTASPVLP
EQEYVDALLADLDLDLPHVYGRELQSIFFGGGTPSLFSADALGRLLQGVEQRIRFASDIE
ITLEANPGTFEQAKFSAYRGLGINRLSIGIQSFEESKLKALGRIHNGDEAIRAADMARQA
GFDNFNLDLMHGLPDQSQDEALADLRQAIALAPTHLSWYQLTLEPNTVFWNQPPVLPEDD
ILWDIQETGQQLLATHGYAQYEVSAYAQPGKPARHNLNYWAFGDFIGIGAGAHGKLSHPD
GRIMRTWKTRLPKDYLNPDKPFQAGSKLLPLDELPFEFLMNALRLTNGVDAALFRERTGL
SLDSLAEARRQAEQKGLLHQDPTRLIATPQGQLFLNDLLQYFLI
>tr|A0A0Q0IT06|A0A0Q0IT06_PSEAP Transcriptional regulator, LysR family OS=Pseudomonas syringae pv. aptata OX=83167 GN=ALO85_100375 PE=3 SV=1
MILKKYQMIDLKLMRQYVVVAETLNFRKAAQRLNMSQPPLSVAIRRLEECLGVQLLLRST
RSTQLTTAGVVFLAEARQTLLQAERACEMARRSAAGMLGTIRLGFVDSVVDGLLPALLRR
YQAANPNVDIQLQEATPPEQLEGLRNDRLDVGILVLPVIDPGNIQIEPLFEDRMVAVLPQ
DHPLAEQTSIGLAALADQSWILFAPHHGPGMHSHILLACASAGFTPRVVQQPRQMQTTAA
LVAGGMGVALMPQRYARRQPGRLACRELTGPGTPVAYVLALAYRELSPCAQSLRDVIMEL
VQSPAFKLASAES
>tr|A0A0Q0FUD6|A0A0Q0FUD6_PSEAP Syringopeptin synthetase A (Fragment) OS=Pseudomonas syringae pv. aptata OX=83167 GN=ALO85_05503 PE=4 SV=1
VPSALKALLDVDGLPATVHTVNVAGEALKRSLVENLFEKTGVQRLCNLYGPSETTTYSSW
VAMDREDGFAPHIGKPVGNTQFYLLDEQQQPVPLGVAGEIYIGGAGVARGYLNRDDLTAE
RFLKDPFSQNPAARMYRTGDLGRYLPDGNIEYLGRNDDQVKIRGFRIELGEIDARLAKHP
AVHEAVVTAREDIPGDKRLVAYYTLAAGHTVVDIDSLRSHLQQRLPEYMVPAIYVMLDKL
PLTPNGKLDRKALPAPDSDSLISRGYEAPQGETETQIAVIWQELLGVEQVGRHDNFFELG
GHSLLAVSFIGHMRQSGLSVDVRALFGQPTLAALAAAIGGSTEIVVPANLINDDCQQITP
ELLPLINLTQVQIDQVVATVPGGVANVQDIYPLAPLQEGILYHHLSAETGDPYVLQTQFV
FDDHERMDAFVHALQTVIDRHDILRTSVVWQGLDSPVQVVWRQARLHLDALELDPANGGI
AEQLHSRFDPRHYRLDIGQAPLMRVAYAADPLNQRICAMLLFHHMALDHAALEVVKHEIQ
SCLLDEAEALAASVPVPYRNYVAQARLGVSQADHETFFRDMLGDIDEPTLPFGLGNVQGD
GRDIEEASLVLDAQLNLRLRAQARQQGVSAASLVHLAWAQVLGNLSDKRDVVFGTVLMGR
MQSSEVADRALGMFINTLPLRVDVGAITVVEGLKATHERLTALLGHEHAPLVLAQRCSGV
AAPLPLFNALLNYRHSAVGNVSERDTQAWQGIHALSSEERTNYPLIVNVDDLGTGFRLNV
QAVTGIDARRICTYMQTTLSHLVKALEFAADSVVCDLPVVPEAERQQLLVAFNDTTLDYP
QQQTVQSLFEAQVRACPKACAAIHDGVAMSYAELNTRANRLARHLLGLGVQPGDSVAILL
ERSLDLLASQLAVLKCSAVYVPLDINVPVERQTFMIEDSQARVLLTHSQMSLTTAAQRVD
LDNLTLDGLKDTDLALPQSSESVAYIMYTSGSTGTPKGVLVPHRAISRLVINNGYADFNA
QDRVAFASNPAFDASTLDVWAPLLNGGCAVVIGQNDLLSPRDFQRLLLEQSVNVLWMTAG
LFHQYASGLGEAFSRLRYLIVGGDVLDPAVIGRVLANSPPQHLLNGYGPTEATTFSATYE
ITSVGNGSIPIGKPVGNSRLYVLDGQGQPVPLGVPGELYIGGQGVAKGYLNRDELTDEKF
VADPFDSDPQARLYRTGDLVRWRADGNLEYLGRNDDQVKIRGFRVELGEIEVHLAEHADV
REAVVLCRQDVPGDKRLVAYITARQPETVLDIESLRSHLQGTLPEYMVPAAYVQLDALPL
TANGKLDRKALPVPDAQSLISRGYEAPQGEVEVALAEIWAEVLQVERIGRNDNFFDLGGH
SLLAMRMVSQVRLQLGVELPLGGLFARGELAAVAAALGGVARSELTAILPVKRNQPMPLS
FAQQRLWFLAQMDGGNQAYNIPMALSLEGTLDVTALTRALARIVERHETLRSRLVSREGA
AQVLFAPTCADATLSVEDIRHVPHRLQEAIRVEAVAPFNLADGPLIRAHLLHVADDRHVL
LLTVHHIVADGWSMGVLSQELLALYPAFCQGQPDPLPPLAIQYTDYAVWQRRWLSGERLQ
HQAAYWRQALEGAPTLLTLPTDRPRPAQQDFAGASLAVQLNARLTADLRQLAQRQGVTLY

MALMTAWATTLTRLSGQAEVVIGSPVAGRGRSELEDLVGLFVNTLAVRIDTSGNPSGEAL
LAQVKTRVLEAQEHQDLPFEQVVEVVRPTRSLAHPPLFQNTLNWMPGKHAAASLGDLRVV
PIEQVSQVSKFDLSLNLSEQGETLVGTLDYATALFDEATAQRYLGYFVQVLETLANDEQT
HLDRITLVGEEERRYLLETLNTTALAQVNDEQTVHAMIEARAASAPEGVAAQVGEQCLHY
GELNHRANALAHHLISLGVGVDDRVAVMARRGLDTLVAMLAVLKAGAGYVPVDPSHPDER
IAYLLTDSAPKVVLTQQALMSRVPETAAPVIAFDRPEWPQRLENPQVAGLDATHLAYVIY
TSGSTGQPKGVMVEHRTLGNLIAWHCQTFDLQAGSHTASVAGFGFDAMAWEVWPALCAGA
TLHLPPAEVSNEHIDALLDWWIAQPLQVAFLPTPVAEYAFSRNLSHPTLRTLLIGGDRLR
QFQRDPGFAVINNYGPTEATVVATSGRLLPDGSLDIGTPIANTRVYLLDEHQQLVPLGVA
GELYVAGEGVARGYLNRAEMTAERFLRDPFHANSRMYRTGDLARWNADGTLDYLGRNDDQ
VKIRGLRVELGEIEAQLSQLPGIEEALVLAREDEPGQPRLAAYFIERAGSLSMPVSELRA
ALLAVLPGYMVPSAFIRLDAWPLTANGKVDRKALPLPDREALPDREYEAPQGVVETAVAE
IWSSLLHVERVGRHDHFFELGGHSLLAVNLIAQMRRQGLDADIRTLFTQPTLAALAAAVG
GGNEITVPENAIPDNCTRITPEMLPLADLDQAAIDLIVASVPGGVANVQDIYPLAPLQEG
ILYHHLAADAGDPYVLQSVFEIADRARLNAFVGALQSVVDRHDILRTAVQWQGLETPVQV
VCRQARLPVEQVELDPLEGPIMAQLQQRFDPRHSRLDMSQAPLLRLVFAEDQPNQRWAAI
LLFHHMVLDHTALEVVLHDMQAHLLGQADQLEVAIPYRNYVAQARLGVSREDHEAFFREQ
LGDIDEPTLPFGLLDVQGDGRDIEESSLTLDRQLNLRLRAQARQLGVSAASLVHLAWAQV
LSQVSGRQSVVFGTVLIGRMQSGEGAERALGMFINTLPLRVDLAGQGARAGVRATHAQLT
ALLGHEHAPLVLAQRCSGVAAPLPLFSALLNYRHSSVGEPSAESIEAWRGIDVQSGEERT
NYPLTLNVDDQGDGFRLSVLVTGKVGAGRVCGYMQTALDNLLVALEQSPDTALESLPILP
AAEREQLLVGFNDTALDCPHEQTIHGLFEAQVERTPEALAVVHGEQRLTYRELNEQANRL
AHTLRKQGVQPDSRVGICVERSAEMVVGLLAILKAGGGYVPLDPAYPAERIAYMLQDSTP
AAVLAQSATEALLTDVSVPVINLDQNNWQEESVRNPQVAGLTSAHLAYLIYTSGSTGLPK
GVMIEHRNTVNFLSWA
>tr|A0A0Q0DE87|A0A0Q0DE87_PSEAP Uncharacterized protein OS=Pseudomonas syringae pv. aptata OX=83167 GN=ALO85_01261 PE=4 SV=1
MNGSTLPFDDATSKDFKGNVLLANSDIELALVKAVWQEVYSEELGWLDGDDTDPRNDRYH
ANSVYLLATDDEGSAVGVMRMIIDGDQGLPIDNFADISGIRATAQGALIECARLMVPARH
RDQIFSAYPYGIFAALVKAALQYCLKEKNYDVLANCFIDTPHTPIKALAHMGFRDLNIRF
RDELHETSECTVLHLDIKDMLSIFYGPEKSLGHYIMARA
>tr|A0A0Q0D7H3|A0A0Q0D7H3_PSEAP Uncharacterized protein OS=Pseudomonas syringae pv. aptata OX=83167 GN=ALO85_102162 PE=4 SV=1
MLTALDALRPTSSHAARRRFVTRSVTQGIPTLEREER
>tr|A0A0N8T8Y1|A0A0N8T8Y1_PSEAP FMN reductase (NADH) RutF OS=Pseudomonas syringae pv. aptata OX=83167 GN=rutF PE=3 SV=1
MPTSSAAPAILPIAAVSQLAFRDAMSGLAAAVNVITTDGPGGRAGFTATAVCSVTDQPPT
LLVCINRSASVYDAFIENGRLCVNTLGNGQQDLSNLFGGKSSQQERFACGQWEAGVTGAQ
ILDSAKLALDCKVSQSVSVGTHDILFCEVVDIRHQSGADALVYFGRRYHHLPSETPVA
>tr|A0A0Q0CAI3|A0A0Q0CAI3_PSEAP Galactonate dehydratase OS=Pseudomonas syringae pv. aptata OX=83167 GN=ALO85_02183 PE=4 SV=1
MKITKLTTFIVPPRWCFLKVETDEGVVGWGEPVVEGRAHTVAAAVEELSDYLIGKDPRNI
EDIWTVLYRGGFYRGGAIHMSALAGIDQALWDIKGKALGVSVSDLLGGQVRDKIRVYSWI
GGDRPADTARAAKEAVERGFTAVKMNGTEELQFLDTFDKVDLALANVAAVRDAVGPNVGI
GVDFHGRVHKPMAKVLMKELDPYKLMFIEEPVLSENYEALKELAPLTSTPIALGERLFSR
WDFKRVLSEGYVDIIQPDASHAGGITETRKIANMAEAYDVALALHCPLGPIALAACLQLD
AVCYNAFIQEQSLGIHYNESNDLLDYVKHPEVFDYDKGMVKIPNGPGLGIEINEEYVIER
AAIGHRWRNPIWRHADGSFAEW
>tr|A0A0N8T8L0|A0A0N8T8L0_PSEAP Putative D-galactonate transporter, major facilitator superfamily OS=Pseudomonas syringae pv. aptata OX=83167 GN=ALO85_00662 PE=4 SV=1
MSTSNSRMADGTGSVLNSAVSKVKRHVLPLFVIMFIVNYIDRVNIGFVRSHMEHDLGIGA
AAYGLGAGLFFIGYALFEVPSNILLQKVGARIWLTRIMLTWGIVAACMAFIQTETHFYIL
RFLLGVAEAGFFPGVIYYFTRWLPGVERGKAIAIFLSGSAIASLISGPLSGLLLQITGFG
LKGWQWMYFIEGMFSVGLCFFVWFWLDSKPHDAKWLTREEQDALVNAIDAEQAAREAATP
VKASIGKLLKDGQIILFCLIYFFIQLTIYAATFWLPSIIRKMGSLTDFEVGMFNSIPWLL
SIIGMYVFATLSAKWKRQQAWVAIALLIAAAGMFMSTTGSPIFAFVAICFAALGFKSASS
LFWPIPQAYLDARIAAAVIALINSVGNLGGFVAPTTFGLLEEHTGSIQGGLYGLTATSII
AAIIVFAARNNPKPGATPVAAIQTPATH

>tr|A0A0Q0DAE5|A0A0Q0DAE5_PSEAP Outer membrane porin OS=Pseudomonas syringae pv. aptata OX=83167 GN=ALO85_02281 PE=4 SV=1
MQNKEDKMLNTRMGMLALGIISAGQAVAQGQADSKGFVEDSSLNVNLRNAYINRDYKNDR
EDKSEWGQAFMGAFASGFTQGTVGVGVDAFALYGIRLDGGKGRSGNGGIDFFKQGDSGAA
ADDLSRAGAAVKARFSNTVIKYGDQMPQLPVLNYDNSRLLPESYTGTLITSKEIDGLEVV
AGRFTQEARKSAEGRDSGDLKSINVYGASYKFTDEFSAAFYASDNEDVFKKQYLNLNYVF
ALPQDQSLTFDFNGYKTRLDKDFTASDARDNKIWSLAATWAVGIHSFTLAHQRSTGDMGY
AYGGYRNDGGFGDGGNTIYLANSYWSDFNGKDERSWQAAYGVDLSGLVLPGLSYKAAYVR
GDNIDDGTAGSGDGTEREIFSQLTYVVQSGPAKDLSIRLRNSFLRVSDDAQAYNSEGNET
RIFVDYPINVF
>tr|A0A0Q0BXH0|A0A0Q0BXH0_PSEAP Biotin carboxylase OS=Pseudomonas syringae pv. aptata OX=83167 GN=ALO85_01374 PE=4 SV=1
MLEKVLIANRGEIALRILRACKELGIKTVAVHSTADRELMHLGLADETVCIGPAPANLSY
LNIPAIISAAEVTGATAIHPGYGFLAENADFAEQVEKSGFAFIGPKADTIRLMGDKVSAK
DAMKLANVPTVPGSDGPLPEDEDTALRIGREVGYPVIIKAAGGGGGRGMRVVHREEDLIE
AASQTRAEAAAWFSNPMVYLEKYLTNPRHVEVQVISDGQGQAIHLGDRDCSLQRRHQKVL
EEAPAPFIDEKAREDVLAACVKACIDIGYRGAGTFEFLYENGRFYFIEMNTRVQVEHPVS
EMVTGIDIVKEMLSIAAGNKLSYTQDDVVIKGHALECRINAEDPKTFVPSPGLVKHFHAP
GGNGVRVDSHLYSGYKVPSNYDSLIGKVITWGATREEAMARMRNALDEIVVDGIKTNIPL
HRDLTRDEGFCEGGVNIHYLEHKLANQ
>tr|A0A0Q0IH09|A0A0Q0IH09_PSEAP Glycine betaine/choline OpuC ABC transporter, ATP-binding protein OS=Pseudomonas syringae pv. aptata OX=83167 GN=ALO85_04374 PE=4 SV=1
MAATHADSKRITEMIELQNLSKTFQSNGKEVKAVDSVSLTVNEGEICVFLGPSGCGKSTT
LKMINRLIMPTSGKVLINGEDTTGLDEVTLRRNIGYVIQQIGLFPNMTIEENIVVVPKLL
GWDKQRCHDRARELMSMIKLEPKQYLHRYPRELSGGQQQRIGVIRALAADAPLLLMDEPF
GAVDPINREMIQNEFFEMQRALNKTVIMVSHDIDEAIKLGDKIAIFRGGKLLQIDHPDTL
LAHPADDFVSSFVGQDSTLKRLLLVKAEDAADNAPSVSPETPVADALEVMDENDRRYIVV
TDSENKAMGYVRRRDLHRQQGTCAQFLREFNATAAYDEHLRILLSRMYEFNRAWLPVLDA
ENVFLGEVTQESIAAYLSSGRSRGMKTSIVSPAEQVAS
>tr|A0A0Q0BZC1|A0A0Q0BZC1_PSEAP GNAT family acetyltransferase OS=Pseudomonas syringae pv. aptata OX=83167 GN=ALO85_05393 PE=4 SV=1
MSRDTGESMEPILKLDSARLRMRQWRDDDLPAFARMSADPRVMRYFPKPLSHLESAAMIG
RLRGHFAELGFGLWALERKDTGEFIGFTGLHVVGFKAPFTPAVEIGWRLAYDHWGLGFAS
EAAWTALGCGFERLELKEIVSFTAVNNQPSQKVMQAIGMQQDESGSFDHPNLEDGHPLKP
HVLYRISHEQWLKTLKP
>tr|A0A0Q0BRG3|A0A0Q0BRG3_PSEAP GMP synthase [glutamine-hydrolyzing] OS=Pseudomonas syringae pv. aptata OX=83167 GN=guaA PE=3 SV=1
MALDIHAHRILILDFGSQYTQLIARRVREIGVYCELHPFDMDDEAIREFAPKGVILAGGP
ESVHEADSPRCPQAVFDLGVPVFGICYGMQTMAEQLGGKVAGSELREFGYARVDVVGKSR
LLDGIEDHIDADGLFGLDVWMSHGDKVTKLPENFHILASTPSCPIAGMADDARGYYGVQF
HPEVTHTKQGGRILSRFILDICGCEALWTPSKIAEDAIAQVRAQVGTDNVLLGLSGGVDS
SVVAALLHKAIGDQLTCVFVDNGLLRLHEGEQVMAMFAENMGVKVIRANAEEQFLNNLAG
ESDPEKKRKIIGRTFIDVFDAESCKLDNIKYLAQGTIYPDVIESAGAKSGKAHVIKSHHN
VGGLPEEMNLKLVEPLRELFKDEVRRLGLELGLPYDMVYRHPFPGPGLGVRILGEVKKEY
ADILRRADHIFIEELRKADWYHKVSQAFVVFQPVKSVGVVGDGRRYAWVVALRAVETIDF
MTARWAHLPYELLETVSGRIINEIEGISRVTYDVSSKPPATIEWE
>tr|A0A0Q0DL20|A0A0Q0DL20_PSEAP Hybrid two-component sensor histidine kinase/response regulator CvgSY OS=Pseudomonas syringae pv. aptata OX=83167 GN=ALO85_02614 PE=4 SV=1
MTPTDLQHLQSRLAALEQENASLKESLSVTGREAEYALAQSQERYRFLFNAMDEGFCIIE
FFDGPHGPLSDYLHVEANPAYEYHAGISNVVGKKLREMVREEADGWVEFYGDVLRTGKPI
RFERELVATGRYLALTAFRIEPASRRQVAVLFQDITERKRAELALKQLNETLEARIVEAV
AERNVLADVVNGTNAIIHVVDCNFRWMAINGAALREFERLFGVRPQVGDSMLDLLDDRPQ
SKADLERRWTRALAGEEFAESVSFSDSNDPASHFEIRYSTLRNAQGQAIGAYLFAYDVSE
RLREQKRLSQAEEALRQSQKMEAVGQLTGGIAHDFNNLLTGITGSLELLKTRVSQGRFNE
LDRYIGAAQDASKRAASLTHRLLAFSRRQTLDPKPVDVNRLVIGMEELIRRTVGPHITVE
VVTSVGLWSTFIDAPQLENALLNLCINARDAMPRGGRITIETANRWIDERGSQSRELIPG
QYLSLCVSDTGSGMSPEVINRAFDPFFTTKPLGQGTGLGLSMVYGFVRQSGGQVRIYSEP
DQGTNMCLYLPRHYLGTPEEIESADASESTSVQTERTVMIVDDEPTIRMLVAEVLDDQGY

IPIEAGEGASALKVLESDARIDLLVTDVGLPGGMNGRQLADAARIIRPDLKVLFITGYAE
NAIIGNGHLDPGMWVLTKPFTMEAFASRIYEMIERED
>tr|A0A0Q0BWL6|A0A0Q0BWL6_PSEAP Glycosyl transferase, group 1
OS=Pseudomonas syringae pv. aptata OX=83167 GN=ALO85_04349 PE=4 SV=1
MRIAIVHDWLVSYAGAERVLASLINVWPAADLFAVIDFLSDQDRAHLHGKVARTTFIQKL
PGARKHYSRYLPLMPLAIEQLDLSGYDLIISSSHAVAKGVLCGPDQLHISYVHSPIRYAW
DLQHQYLQESGLSKGIKGGLARLILHYIRLWDQRTSTGVDAFIANSGFIGARISKAYRRD
STVIYPPVDTLGFTAQGTRGDFYLCASRMVPYKRMPMIVEAFAAMPDKRLIMIGDGPDLA
KAQAIASQVSNVTLLGFQPSNVLLEHMRSARAFVFAAEEDFGISPVEAQACGTPVIAFAK
GGVMETVRGLDHPQPTGVFYRQQTVASLIAAIGEFEAAQSRISPEACRANAERFSVARFE
QEIKAFVEDRLALSSRVHLARLPQSQWVVQASPGLGSELPNRVVPLKIV
>tr|A0A0Q0BY37|A0A0Q0BY37_PSEAP Nicotinate-nucleotide--
dimethylbenzimidazole phosphoribosyltransferase OS=Pseudomonas syringae
pv. aptata OX=83167 GN=cobT PE=3 SV=1
MSNSWWLKPAQAIDVPMREAALARQQQLTKPAGSLAQLERLAVQLAGLQGRELPAVDQLW
IAIFAADHGVVAEGVSAYPQEVTGQMLHNFVNGGAAISVLARQLSAQLDVVDLGTVSPMD
LPGVRHLRIGAGTANFAQGPAMSVEQGLAALQAGRDSVLRAKAVGTELFIGGEMGIGNTT
AASAVACSLLECAAPLLVGPGTGLNAEGIQHKTHVIERALALHAEQAGDPLSSLFCLGGF
EIAALTGAYLACAQEGIAVLVDGFICSVAALVAVRLNPSCRNWLLFGHRGAEPGHRHLLE
TLQAEPLLDLGLRLGEGSGAALAVPLVRLACELHNGMATFAEAAVADRPA
>tr|A0A0Q0IPD8|A0A0Q0IPD8_PSEAP 4-alpha-glucanotransferase OS=Pseudomonas
syringae pv. aptata OX=83167 GN=ALO85_05437 PE=3 SV=1
MMFWRNRMNNEQLERLATEAGLSVHWVDANARPQTVSPDVLRKVLEALGYPAESGEAIDA
SLLRLQKASHGTSAPPLLTVDVESNLDLSQWFAPQTPFTLHLEDGSSLDARLTSNAELPA
LAPPGYQQLEIAGQHLTIAVAPKTCFSMAMATETSKPHGWGLTAQLYSLRREGDGGFGDT
EALEKVLRSAGERGADALGISPIHAMFANDPHRYSPYSPSSRLFLNSLYASPGAIVGESA
WRQAIEDARVGDEMKRLETQTLIDWPAAANAKWKALHALYEGFSSGDHPLHEDFNSFRHS
GGEALENHCRFEALCAESETLNISNNWHEWPEQFKDPRGDAVAEFAKSHREQISFHAFAQ
WLIARGLERAQVAARSSGMRVGLIADLAVGADGAGSQAWSRQDELLSALTVGAPPDILNR
AGQSWGISAFSPDGLKRNGFRAFIEMLRANFAHAGGLRIDHVMGLQRLWVIPQGAPPSEG
AYLNFPLDDMLRLLSLESWRHKAIVLGEDLGTVPEGLSEKLSARAILGMRVLLFEQNNGQ
FKPILDWSDQALATTSTHDLPTLAGWLSELDIEWNARLGHIDDQHESQWREERTREYESL
RRALSQNIDSMPSDTEDPAQIIDASISYLGHTRAPLVLLPIEDALGVEEQANLPGTIDSH
PNWRRRLPGDAASLLDNPNAARRLELLSRSRLQATERDQ
>tr|A0A0Q0DN99|A0A0Q0DN99_PSEAP Adhesin/filamentous hemagglutinin,
ShlA/HecA/FhaA family (Fragment) OS=Pseudomonas syringae pv. aptata
OX=83167 GN=ALO85_04604 PE=4 SV=1
QQAVVARTGQAFIDGQTSNEAQFKYLMNNAIASKQQLNLAVGVSLNSQQVAALTHDIVWL
EEHEVNGEKVLVPVLYMAQADNRLGPTGALIAGNDVSLIAGQNLDNVGTLRAANNLSAAA
GNDLVNSGLIEAGNRLDLLAGNDLINKSGGIIAGRDVTLTALRGDVINERTVTSHQSASG
DATWRQDFADSAARIEAANDMSLQAGRDVKNTGSVLQAGRDLSIAAGRDVAIDSAQTENG
QTRGANSSNSSITQLGSTVSAGRDLTAQAGRDINVIASSIDAKRDIAMAATENLTLSSAA
DEQHSYGKSKKVTEQEDHVSQVSADLKAGGSVALQAGQNLDIIASRINAGSNIALDAAHD
LTIASAQDESSYFYAKKSKGSFGRSSSKQQESYESNNVASVINAGQDLTINTTKAADGSI
SINGGHDVSVIGSRLSAGNDLVLGATNDVTVLSGVDEQGAYSQKSKSGSFGVSKSGKSEL
KTSATQVASELSAGNDVVVASGKDITLRASNIDAGNDVDLRAGLVDKTGDINLLSANDEA
SRHSDEYKKKTGLSVSGGFLSISSAKEAGGQAQSSTSVGSQINAAGNVSLQTERDINIVG
SGVNAGANVSLNAGQDVNILAAQSSQSNQDWEKNRQSGIGVSSNANGVTFFAGVDRTKEK
NRLEQQSAAASQVRAGEDLTVNAKRDINQVGSDLQALNDINLTAGRNIKIDTARESQTVE
QQRENSRNGLSASIDHNYGSTKDAISGAGKGEDATSKGSSTLKAVDSTSQFLSGPTGDGK
FGNSKQGTSQQVVETTNRASTLDAGNNLNISASNDVQISGGQLQAGRDINIKGRDVTLDA
AKGSYSQETSEQKSWSGIHGSSSGGFKLGVGGSSGIAKGDQSQEASTVTTLQAGRDVTIK
ASNDLNLIGTQAQAERNINLNAGNTLNIKAAQNDSSTENTRKNGGGEAGIAIGPGGIGVY
ASVNIGKGDLEREGKQQQEAYLYAGNHLGFTSGQDTNIAGATLRGNDVVGRVGRDFNVTS
LP
>tr|A0A0Q0CIM9|A0A0Q0CIM9_PSEAP Uncharacterized protein OS=Pseudomonas
syringae pv. aptata OX=83167 GN=ALO85_01560 PE=4 SV=1
MVDAWFDYDQESSMKRFILAATCTLLATSALAAPKSCEELKAEIEAKIQANNVTSYTLEI
VSNEEATDPSMIVGSCDNGTKKIIYQLNGR

>tr|A0A0Q0C6J8|A0A0Q0C6J8_PSEAP Regulatory protein, MarR OS=Pseudomonas syringae pv. aptata OX=83167 GN=ALO85_01191 PE=4 SV=1
MLDLKKPANQQMAMEAFFFGYQAFTAKADEMLARRGFSRVHQRIVFFIARYPGLSVKELL
TVLGVSKQALNAPLRQLIAMNLVHSTAPENDKRKRLLGLTDEGALFEQGLRREQVKLLQR
VFAEAGQDAVDGWLTVNRALGQTLQSGACPTGADAE
>tr|A0A0Q0FGX3|A0A0Q0FGX3_PSEAP Penicillin-binding protein 1C OS=Pseudomonas syringae pv. aptata OX=83167 GN=ALO85_01361 PE=4 SV=1
MKPEFLLRLPRTFKWLAGAIMLLIALLWLADRLWPLPLPRDDLARVVLAEDGTPLWRFAD
ANGVWRYPISNEQVSPYYLEALLTYEDRWFYSHPGVNPLALLRATWQNLSGARVVSGGST
LSMQVARLLDPHSRTLSGKFRQLWRTLQLEWHLSKDQILSLYLNRAPFGGTLQGVAAASW
AYLGKSPQNLTRAEAALLAVLPQAPSRLRPDRHPQRAQQARDKVLRRLAEFQVWPQASVN
EALEEPLWLAPRQEPSLAPLLARRLNRPHSPPLIRTTLDASLQRRMEDLLMGWRARLPER
TSAAILVVEAESMAVRAYVGSVDINDAKRFGHVDMVTALRSPGSTLKPFLYGMAMDAGLI
HSESLMQDVPRRYGDYRPGNFSSGFGGPVAASSALSMSLNLPAVQLLEVYGPKRFAAELR
NGGVPLTLPPLAEPSLALILGGAGSRLEDLVTGYSAFARGGRSADVRLQPQDRLRERRMM
SPGAAWIIRRILSGQSRPDIDPRAELVQRPQLAWKTGTSYGFRDAWAIGVGPRFLVGVWI
GRPDGTPVPGQFGLASAAPLMLQVHDVLVNRDSQRGIAAPLQPVPLNVGVAAICWPLGQP
MSKSDPNCRRQRFAWTLDGTTPPTLQAADQPLGLGLQERVWVNAKGLRVAANCPDAQAQD
IALWPAPLEPWLPRAERRDARLPPADPDCPPQNLNLAPPLSIVGVREGDNLRLPAASRQA
LRLTLSALGGSGHRWWFIDGKPLADTDTRQDFTPTLSKPGRYQLSVLDESGQTARVEFSV
VE
>tr|A0A0Q0IP10|A0A0Q0IP10_PSEAP ISPsy19, transposase OS=Pseudomonas syringae pv. aptata OX=83167 GN=ALO85_00370 PE=4 SV=1
MPKTGRPRSIAAEHYPVLVKLAHAQPYSSQAELALVFFAETGITAHPDTFAKALKMAGIT
RVKQRAKGSFQSPEPNKSYGYNETHRRQLPEQLYPSCLTDTEWALVADLFESQGGRGVPP
LHSRRTLLEACCYVVRTGCSWRMLPRDFPHWDNVYKTFRRWSAQGKFEQMHDRLRAQWRE
REERADSPSAAILDSQSTRSSPQGGDSGYDAGKKVKGRKRSLIVDTLGLLLAVSISAASV
QDRDAADDAVAYSKEKYPSLSTLFVDSAYAGKWAQRTHQLHAIDVQVIRGPNNRRTGQWH
SEQGDLFSVEPVQTGFVVMPKRWVVERTHAWNERARRLIMHHDRLFAVSEAWVWLAEARI
LARRLTT
>tr|A0A0Q0DGX2|A0A0Q0DGX2_PSEAP Putative ribonucleoside transporter OS=Pseudomonas syringae pv. aptata OX=83167 GN=ALO85_03170 PE=4 SV=1
MQAFDSTLKGAETTAHPPAWGAVFSMALCVVVLIASEFMPVSLLTPIAQDLGISQGQAGQ
AISISGFFAVLTSLLNTPLTGHLDRKPVLLGFSLLLLVSGVIVTLASNGWLFMTGRALLG
IAIGGFWSMSTATVMKLVPKQSVAKGLALINGGNALAATVAAPLGSFMGQYIGWRGAFFL
VIPLAALAFAWQWLSLPAMRSQPNNRARNPFRLLRNPQVAVGMSAILLLFMGQFAVFTYL
RPFLEEITGVSVNTLSLMLLALGASGLVGTYLIGRLLHKGLYACLIVIPLLMAVLAVALT
GLGHSPQSVAIVLAVWGLIATPAPVAWGLWLSRTLPDDAEAGGGLMVATIQLAITAGAGI
GGALFDSLGWWSPFAFGGVLLACSAALAWTARDNASVQESNTGQGRADKRGSISQRGNTP
>tr|A0A0Q0CGV1|A0A0Q0CGV1_PSEAP NAD-dependent glutamate dehydrogenase OS=Pseudomonas syringae pv. aptata OX=83167 GN=ALO85_04579 PE=4 SV=1
MDCKGTLVSTPSSSLPNKSQSGVPQMAFFTAASKADFQHQLQAALAQHISEQALPQVALF
AEQFFGIISLDELTQRRLSDLAGCTLSAWRLLERFEHAHPQVRVYNPDYERHGWQSTHTA
VEVLHHDLPFLVDSVRTELNRRGYSIHTLQTTVLSVRRGAAGELLELLPKGTAGDDVLQE
SLMYLEIDRCANVSELNVLARELEQVLGEVRAVVEDFGPMKARLHELLASIDANESNTDV
EEKAEIKVFLQWLVDNHFTFLGYEEFEVRNDAEGGQLVYDESSFLGLTRLLRPGLTREEL
HIEDYAVKYLQEPVLLSFAKAAHPSRVHRPAYPDYVSIRQIDASGKVIKECRFMGLYTSS
VYGESVRQIPYIRRKVAEVERRSGFDAKAHLGKELAQVVEVLPRDDLFQTPVDELFTTVM
SIVQIQERNKIRVFLRKDPYGRFCYCLAYVPRDVYSTEVRQKIQQVLMDRLKASDCEFWT
FFSESVLARVQLILRVDPKINLDIDVAQLENEVIQACRSWKDDYASLVVESFGEAHGTNV
LADFPKGFPAGYRERFAAHSAVVDMQHVLSLSEANPLVMSFYQPLAGGRQQLHCKLYHAD
TPLALSDVLPILENLGLRVLGEFPYRLHHANGREFWIHDFAFTYGEGLNLDIQQLNDTLQ
DAFVHIVRGDAENDAFNRLVLTAGLPWRDVALLRAYARYLKQIRLGFDLGYIATTLNNHT
DIARELTRLFKTRFYLARKLGSDDLDDKQLRLEQAILTALDDVQVLNEDRILRRYLDLIK
ATLRTNFYQADANGQSKSYFSFKFNPRLIPELPKPVPKFEIFVYSPRVEGVHLRFGNVAR
GGLRWSDREEDFRTEVLGLVKAQQVKNSVIVPVGAKGGFVPRRLPTTGNRDEVQAEAIAC
YRIFISGLLDITDNLKEGALVPPVNVVRHDDDDPYLVVAADKGTATFSDIANGIAIDYGF
WLGDAFASGGSAGYDHKKMGITAKGAWVGVQRHFRERDINVQQDSISVIGIGDMAGDVFG
NGLLMSDKLQLVAAFNHLHIFIDPNPDPATSFAERQRLFNLPRSSWTDYDTSIMSAGGGI
FPRSLKSIAITEQMKARFDIKADKLTPTELLNALLKAPVDLLWNGGIGTYVKSSDESHAD

VGDKANDALRVDGNELRCKVVGEGGNLGMTQLGRVEFGLNGGATNTDFIDNAGGVDCSDH
EVNIKILLNEVVQAGDMTEKQRNQLLESMTDEVGHLVLGNNYKQTQALSLAARRAYERIA
EYKRLMSDLEARGKLDRAIEFLPAEEQIAERIAAKQGLSRAELSVLISYSKIDLKEALLE
SRVPDDDYLARDMETAFPPSLGAKFSTAMRGHRLKREIVSTQIANDLVNHMGITFVQRLK
ESTGMSAAAVAGAYVIVRDIFHLPHWFRQIEALDYKVSAEVQLALMDELMRLGRRATRWF
LRSRRNELDAGRDVAHFGPHLAALGLKLDELLEGPTREIWQTRYQAYVEAGVPELLARMV
AGTTHLYTLLPIIEASDVTGQNAADVAKAYFAVGSALDITWYLQQISSLPVENNWQALAR
EAFRDDVDWQQRAITVSVLQMADGPSEIDARLALWLEQHTLMVERWRAMLVELRAASGTD
YAMYAVANRELLDLAMSGQGITV
>tr|A0A0Q0FN94|A0A0Q0FN94_PSEAP TonB-dependent siderophore receptor
OS=Pseudomonas syringae pv. aptata OX=83167 GN=ALO85_02716 PE=3 SV=1
MNSPSFPMRLTLLAFCCSIGSPAWAAAEAATRQDGPVVLGETDISADKADPHALPPVYAG
GQVARGGQLGVLGNADIMDVPFTMSSYTEQLIEDQQAETVGDVLLNDASVRQSSGYANAS
HIFVIRGLKLTTDDISYNGLYGVLPRQIISTDALERVEVFKGPNAFINGVTPTGSGIGGG
VNLQPKRAQDTPTRRFTSDISSDGRVGEHLDLGQRFGEGNRFGARLNLSQREGETGVEDE
EHRSKLFALGLDYRGDNFRLSGDFAYQKQRINQSRNTVFVDSALTSVPHAPDSDTNYAPN
WTWTETEDTFGMSRAEYDLNEDWTLYAAVGAKHTREVGVYGSPTVHDAAGNTTAAPSFIP
HDEDNKTAMAGINGKLQTGAVSHKVNFGLSGIWTEQRSAYLFGGTSSNNLYDPVSQERPA
LDSFTAGDLNDPGIVGKSRMRSVAFSDTLGFFDDRLLLTAGLRRQQLRVQGYEYGSGPRN
ALYDEAITTPVYGVVFKPWHYVSFYANRIEGLAQGPTAPDTVTINPGEVFAPSRSKQVEA
GVKLDMGTFGASLGVYRIEQPADGYQVLEGTGSRYVRDGQQRNRGVEMNVFGQPVSGLRL
LAGLTLMDTEVSGTASGSNDGNRAVGVPTFQFNAGADWDIPGIEGAAINARMLRTGGQYV
DAANNLSIPTWNRFDLGARYTFNVAQKDVTLRANVENLMDKDYWASANGGYLTQGEPRTL
KLSGTIDF
>tr|A0A0Q0DSI6|A0A0Q0DSI6_PSEAP Methyl-accepting chemotaxis protein
OS=Pseudomonas syringae pv. aptata OX=83167 GN=ALO85_03354 PE=4 SV=1
MLRNAPLSMKLLLILIFPLVGFLAFAGLFVADKSENLGDMRRAVTATSAAQKVSDVVTTL
QRERGASGVFLGSGGKSMQDKLKTFRQETDKAISEMRAQSTDGIPGPDKVNRAFEELIAL
RLKVDSLGINNTESGTRFTDIIKTLIGFSYSMESSIEDPEILRGLSSLNQFVDMKERAGR
ERVLLGLAFNQNRFDAALLSRFSRNLGEFSGYFEAFQRWSPEAFKTKLNAVLQQPGSLEV
ARLQRLGFDTPLGDPLNVKPEDWFNLSTARIDMMANVEAELGQNVVGLATDARSSAQSSL
YVAVATVVLMLIVVLWLASVIIRNIKVAVVDVNRTLMALSTRDLTARTRYVGKDEFGEIS
RNLDNMAQQISDVIRDIGSATAQVATAAEQSSAVALQTNQNVAQQRQGTDQVATAISEMS
ATVKDVARSTTDAAEMSQRVNNSTLQGKTEIDNTIGLIQGLSVQAEETSRIIDELKGESN
SISSVLDVIRGVADQTNLLALNAAIEAARAGEQGRGFAVVADEVRNLAKKTQDSTVSIQK
MIANLQSGSERAAASMQETLGKAQEGASNVVRAGELLEEIAEGIATISDRNIQVASAAEE
QSLVAEEIHRNVDDINSLVIQVSAGAEQTAVTSRELARLAEQQQGLVGRFKVS
>tr|A0A0Q0IP56|A0A0Q0IP56_PSEAP 2-nitropropane dioxygenase, NPD
OS=Pseudomonas syringae pv. aptata OX=83167 GN=ALO85_03586 PE=4 SV=1
MTTINTALTSLLGIDHPLILAPMGGASGGRLAAAVSRAGGLGLVGASYGDPQWMARELEM
MREVQQPWGVGLVMFTVAKQFELLLLLALEYRPAVVALSFGDVRPFVRPIHQANAKVIVQV
HDVDQALEALDAGADALIVQGAEAGGHSLRRASLPLFPAVRDAVGEHVVLIGAGGIADGR
GMAAALALGMDGVMMGTRFLASQEALPSERVKQRLLQAVASDTVRTRIFDQVRGIDWPEG
YSGRVIGNDFSASWVGEEQAFAASAGQLRLDYEAALAADDVSIRAIWAGEVADLIKEVLP
AQLIVESTLRGYAASVERLRTTR
>tr|A0A0Q0DT96|A0A0Q0DT96_PSEAP Exopolysaccharide biosynthesis protein
PslE OS=Pseudomonas syringae pv. aptata OX=83167 GN=ALO85_05195 PE=4 SV=1
MVRETDMINIRSFRDLLRLLFIFQREVRITVLATFVIILLGAFLLPNRYESTALLLVKPG
RDSSTVPIELSDRQAIVIPSGLRDPLLDEERMLSGRPIMRAVSEKYLAELSMVPPETGAL
ASVKNGIKTVVGALVNGVRSVLQFIGLVEHRSQAERLAEDLEKNFKVSHEPGSSVMELRF
TWSDPEVAQTVLKTWITEYQTQRTKTLGRVSLYAFYEGEVKATGANIIEYKKQIQNYLNQ
LSAVSISQRLADTSQALNDLRTERNNTTRSIASTKAGLDLLKKQLAEQPKTVSAGRELAL
NPNRQDLQNRINGKEVERQEMLRSFKDEAPPIRAINEEVSNLKKLLKEQDATVQRSESIT
PNPIYNRMQNVYADQQTSYARLQTQFIQQNEQIAQLERDRQQALNLEPELSRLQNELDAA
EKSYALYKNSLEKSRIDRELDNSQISNIATIEEATFNPSRVFPKSLMMLFLAFPLSVVVG
AVALYSFYLLDQRIHDGDKIESSFGVPVWTSLPDLERAQERSAALTSNLHRVYGILPLDQ
VEERGLTIGFTSVKYGAGVSFVIARLSTLLIEQGHRVRTEGRSPAIPGEIVLINASALSA
NQDAFVLLRKADLIVLVVRAQDTTVPMLEDTLNNLNTAFKKVDGIIINRRRFEVPEKVLS
FLKRIGSRG

>tr|A0A0Q0IA16|A0A0Q0IA16_PSEAP Uncharacterized protein (Fragment) OS=Pseudomonas syringae pv. aptata OX=83167 GN=ALO85_04452 PE=4 SV=1
MFGPTGAGKSATLNNLLNQLIAVYAPRLFIVEAGNSFGLLGDFAKKLGLTVNRIKLAPNS
GVSLAPFADAIRLVNTPSQVKTLDADDLESSDEHINAKADEDNDERDVLGEMEIVARLMI
TGGEEKEEARLTRADRSVIRHCILAAARVCSDADRTVLTEDVRNALRTAGEDTSIPEGRR
NRMLEMAEAMDMFCMGADGEMFNRTGTPWPEADLTIVDLATYAREGYNAQLSIAYISLIN
TVNNIAERDQYKGRPLVNVTDQLFNVNRFSGHYRRNEMNSNQ
>tr|A0A0Q0IPL2|A0A0Q0IPL2_PSEAP Putative glutathione S-transferase OS=Pseudomonas syringae pv. aptata OX=83167 GN=ALO85_100413 PE=4 SV=1
MRGASLQHLARSTAMYTLFGTQGSGSAAIEIALRRCSVPFTLSDACGWEEGPGKEALRRV
NPMMQVPTLVLADGAVLTESAAILIHLGLEHPDSGLLPGKSAQRAQALRALVYIAANCYA
AIGIIDYPQRWLAGSDDGLERLLEAGARTRLHEHWETFADLFGDAGIWCCDAPGAVEILA
SVVTQWSGAREHLRNVRPDFHALLLLVDAEPVVNAVIRRHWP
>tr|A0A0Q0DR40|A0A0Q0DR40_PSEAP Amino acid adenylation (Fragment) OS=Pseudomonas syringae pv. aptata OX=83167 GN=ALO85_05497 PE=4 SV=1
VNFLSWAHSAFDASALEKTLFSTSLNFDLAVYECFAPLTSGGSIEVVKNVLELQHGEHDI
GLINTVPSALKALLDVDGLPATVHTVNVAGEALKRSLVESLFEKTGVQRLCNLYGPSETT
TYSSWVAMDREDGFAPHIGKPVGNTQFYLLDQQQQPVPLGVVGEIYIGGAGVARGYLNRD
DLTAERFLKDPFSQNPAARMYRTGDLGRYLPDGNIEYLGRNDDQVKIRGFRIELGEIDAR
LAKHPAVHEAVVNAREDVPGDKRLVAYYTLSAGQASVDIDSLRGWLQEQLPAYMIPVAYV
LLDALPLTPNGKLDRKALPAPDVDALIRRGYEAPQGETETLLAGIWSDLLKVERISRHDQ
FFELGGHSLLAMRLISQIRQQLGVELSLAALFAHPELSALALAIAQAGRSILPDIVPVAR
DQVWPLSFGQQRLWFLAQMEGASAAYHMPVGLSLRGELDRRAMQRALERIVARHEGLRTT
FIQGDDEQPLQRISPADAGFNLQLHDLQGLVDAKEKLQALASEESLQSFDLEQGPLIRGR
LIRMAEDQHVLLLTMHHIVSDGWSIGVLTRELAALYAAFSQGQDDPLAPLALHYLDYAVW
QRRWLSGDLLQQQSDYWQHTLADAPALLMLPTDRVRPAQQNYDGAVLPIVFDQDLTRGLK
ALSQRHGSTLFMTVMAAWAALLGRLAGQDDVVIGTPVANRTRSEVEGLVGLFVNTLAIRV
DLSDKPTAETLLAQVKTNTLGAQDHQDLPFEQVVEVIKPVRSLSHSPVFQAMLSWQDMGD
GDFALGDLQLESLSAGHTLSKFDLSLDIGEAQGQLFGSLEYATALFDESTIARYLGYLQR
LLHAMVADDRQVLEQVPLLDAVERKHLLVDLNANDVPYPQSCTIHQLFEEKVQAQPDAIA
VAFQAQRLSYAELNRQANRLAHHLIGMGIGPDDRVAICVERGVKMIVGLLGVLKAGAAYV
PLDPAYPAERLAYMINDSQPSALLTQRGLQERLPALSMPLVLLDDEHCQGFTECDDNPVV
PTLSVRNLAYVIYTSGSTGNPKGVMIEHRGLVNYSVDAARLFGLSQSDTVLQQNTLNFDL
SVEEIFPALLAGATLAPSREIFGSEGTENHGIYPTVLHLTAAHWHTLVAEWHNQPQAAEQ
RLAEVRLINVTGDALSAQKLKLWDEVRPAHTRLINTYGPTEATVSCTAAYVSHDAVAGSE
GSGNATIGKPMANTRIYLLDAHQQPVPYGVAGEI
>tr|A0A0Q0FMP5|A0A0Q0FMP5_PSEAP Putative 3-hydroxyphenylpropionic transporter MhpT OS=Pseudomonas syringae pv. aptata OX=83167 GN=ALO85_00840 PE=4 SV=1
MRLAMNSPLRRSTLTIVLCFIVALIEGFDLQAAGTAAAGLRQTFALEPKMMGWVFSAGII
GLLPGAFFGGWIADRIGRKKILVSAVLLFGVFSLSTAYVESFSGLLLVRFMTGLGLGAAL
PNLIALCAEAVSERHRGVAISVMYAGVPLGGALAAVVAMFTSEHWQTTFIIGGLVPLLVA
PLMILLLPESSAFKQQTTADSVRHSSVTALFGEGRARTTLALWLGYFFTLTVMYMLLNWL
PSLLIGQGFSKPQAGMVQMLFNIGGALGSLLGGLLLDRCNGIKVVLFVYAGLLTALAGVG
LSVGIVPMAIAGFAAGVFVIAAQLVLYALAPPSYPTSVRATGVGAAVAIGRLGSVAGPLA
AGQLLGAGAGTAGVLLATSPGLVIAALAAITVLSRSVAIEKRELKAAESS
>tr|A0A0Q0C2Z1|A0A0Q0C2Z1_PSEAP Cinnamyl alcohol dehydrogenase-like protein OS=Pseudomonas syringae pv. aptata OX=83167 GN=ALO85_03453 PE=3 SV=1
MLSYGVTLIPIEQVTVMVKTYSYAAQSAQDTLKPHQFERRSPGADDVQIDILYCGVCHSD
LHTARNEWHNTLYPSVPGHEIVGRVTAVGSDVKQFKVGDLAGVGCMVDSCQQCTSCTEGD
EQYCESGFTGTYNGPVFGGENTFGGYSDKIVVKEKFVLRISHDADLAAVAPLLCAGITTY
SPLRHWKVGPGKKVGIVGLGGLGHMGVKIAHAMGAHVVLFTTSPNKREDGLRLGADEVVV
SKDAAQMAAQANSLDFILNTVAAPHDLDAFLSLLKRDGTMTLVGAPAEPHPSPAVFNLIF
KRRSLAGSLIGGIQETQEMLDFCAEHGIVSDIEKINMQDINEAYERMLKGDVKYRFVIDM
DSLKQEAAADSNAA
>tr|A0A0Q0D602|A0A0Q0D602_PSEAP MOSC:MOSC, N-terminal beta barrel protein OS=Pseudomonas syringae pv. aptata OX=83167 GN=ALO85_02667 PE=4 SV=1
MLRLSSLYRFPLKSCKAESMQRASFDALGLAGDRRWMLVDASNGRFFTQRALPHMSQLSV
LWNASGGVTLSAPGFEPQDVAVPLNIDLNLRGVTVWRDSLQVPDAGDEAAEWVSRFIGKP

TRMVYLPAERARWIPGGYQTVNDRVSFADGFPLLLIGQGSLDDLSARMGRPMEMLRFRPN
LVIEGAEAFAEDSWKRIRIGDIEFQLLTPCARCILTTVDPATGERSADREPFATLKTYRE
VEGNVLFGQNVANEGVGELEVGMSVEVLE
>tr|A0A0N8T9Q7|A0A0N8T9Q7_PSEAP Uncharacterized protein OS=Pseudomonas syringae pv. aptata OX=83167 GN=ALO85_101231 PE=4 SV=1
MCNHAGKSVWAQRGNDAMDASVENFDEQEFYLEIDLNVLNHLGIGLYSSTPAVVTEIVAN
AWDADAHLVDVDIQNDKIIVQDDGHGMGPGELQARFLRVGYARRDQPKGNKSDTLERPVM
GRKGIGKLAMFSLADQIDIWTKKKGLPAVSARINVEQLRLDIQSAKKYVLEKQDAEYEWG
DKTGTRIVLSKLTAGTDKTESFLRPRIARRFSVLGDLHKFKVVIGDSEVTTQDRGYHSDV
QFWWDLEDDTRSMQKPLLKNLATDEEGNECVKRVNDTVVVDKHAYNLRGFIATVAKPKSL
KKTDDNINQISLFANGRVFQEDMLKDIGNAKVFNSYIVGEIHADFLDADGIDRATANRES
VKTGDPLVSAVRTWLKKTLDEIANQWDEWRRQQNVESDDNRTQIALERWYATLTDKRDRK
LAQKLITPILSAEHSNDDVKNQDIKRDLIRSAIVGFEKLRIRKQLDKLEKVTDVLSVEFQ
RLFINLDSVEATHYHEITRSRLQVIEKFEKEIADTDALEKVAQNYLFDHLWLLDPTWGPV
GESRVMEKTLTKELKDIAPDAPTGARIDIAYRTSTGRHVIVELKKPDKKSVDVDDLTKQG
RKYRGAVNEYLRKHNDIGGLNGRKPPIDVVFVTAELPRTTDGDALEILRKNEMQSFTYKG
MIVNARRAYQEYLDASPTVSVIDDIISNIT
>tr|A0A0Q0E0Z8|A0A0Q0E0Z8_PSEAP YeeE/YedE OS=Pseudomonas syringae pv. aptata OX=83167 GN=ALO85_04863 PE=4 SV=1
MRFLLPVLEIPMNNSLTATPGRKFGAPLAALFLLLMGAQFLLLSVGTRQVMLWIVGAALG
VTLYHAAFGFTSAWRVFIRERRGAGLRAQMVMLAVAVVLFFPALGAGTLFGQPVTGLVAP
VGISVVVGAFIFGIGMQLGGGCASGTLFTAGGGNARMLVTLLFFILGSLIATHHVDWWFA
LPSFPAVSVVKTFGVLPALLVNLALFGLIALVTVKLEKRRHGQLEAPPVTDHRGLSRVLR
GPWVLVWGAVALALLNYATLALAGRPWGITSAFALWGAKAASGLGVDVGSWVFWQSAANA
KALAAPVWEDITSVMDIGIMLGALLAAGLAGRFAPSLDIPTRSLVAAVIGGLLLGYGSRL
AYGCNIGAYFSGIASGSLHGWLWLVAAYAGNVVGVRLRPIFFTGERPQVALNGC
>tr|A0A0Q0E008|A0A0Q0E008_PSEAP Amine/polyamine ABC-type transport system, permease protein OS=Pseudomonas syringae pv. aptata OX=83167 GN=ALO85_03866 PE=3 SV=1
MKRSPLFVAQLAFTLLVCAFMLVPVVLSLLAGLTRNYFLGLSSGLTFDWLVQVWQAYSPT
VWLSLQLAVACAICVCLVGVPAAYALVRMNNRFSRAFEELMVLPVAMPGLASALALLLTY
GQFGGFRSSWLFILVGHVLFTLPFLVRPVMAVMQRQQLPVLEEAAASLGAGPIKRFFSVV
VPNCRAGILAGVLMVVTLSLGEFNLTWMLHTPMTKTLPVGLADSYASARLEVASAYTLLF
LLLIVPLLIALQAISARLARGESR
>tr|A0A0N8T9C1|A0A0N8T9C1_PSEAP Methylated-DNA--cysteine S-methyltransferase ADA OS=Pseudomonas syringae pv. aptata OX=83167 GN=ALO85_05187 PE=4 SV=1
MALTGAVLSKLKAGSTMDSTERTQPARALRTEDDPRWDILVNRRSDAGAAFVYGVLTTGI
YCNPCSPTRLPRPENVVFFDSAHDAEAAGFRPSLRNAGDSHALQLKHAQAVAQACRLIDA
AESMPTLTELAEQVGMSGFHFHRIFKRLTGLTPKAYSVASLRSRVKVQLSQDVSITRALY
EAGYNANSRFYEASQNMLGMKPSEYRAGGTNVDIRFALGESSLGSILVATSSKGICAISL
GDDPHALIEAFQDQFPNANLIGADAAFEQLVAEVVGFVESPATGLALPLDIRGTVFQERV
WQALRDIPAGSTATYTQIATQIGLPSAVRAVANACGANKLAVAIPCHRVVRSDGSLSGYR
WGVERKRKLLEIEAAEGTGLFSGR
>tr|A0A0Q0IL74|A0A0Q0IL74_PSEAP Beta-lactamase OS=Pseudomonas syringae pv. aptata OX=83167 GN=ALO85_02738 PE=4 SV=1
MLPVRSQPLQIQGHYELQFEAVREAFAALFDDPQERGAALCIQVGGQTVIDLWAGTADKD
GAEAWHTDTILNLFSCTKTFTSVAVLQLVEEGKLKLDEPVARLWPEFAAAGKASITLRQL
LCHQAGLPAVREPLPAEALYQWDTMTAALAAEEPWWTPGQGHGYAAITYGWLVGEMLRRA
DGRGPGESIAARIARPLGLDFHVGLADDQFHRVAHIARGKGNAGDDAAQRVLQATMREPA
SITAKAFTNPPSIMTSTNKPEWRRMQQPAANGHGNARSLAGFYNGLLDGSLLEADMLNEL
TREHSLGQDKTLLTSTRLGLGCMLDQPGVVNATYGLGPKAFGHPGAGGSVGFADPDHEVA
FGFVTNTLGPYILMDPRAQKLVGVLRECLQ
>tr|A0A0Q0FI95|A0A0Q0FI95_PSEAP LysR family transcriptional regulator OS=Pseudomonas syringae pv. aptata OX=83167 GN=ALO85_03169 PE=3 SV=1
MSTPKLNDLQAFLQVARDQSFTKAATKLGVTPSALSHTLRGLEERMGLRLLARTTRNVAP
TEAGERLMRSIGPLLDQIAAEVEVLGELRDKPAGTIRITCSDDAAEGIIRPMLAGFLAQY
PDIQIEICIDYGFTNIVSERFDAGIRLGESISKDMIAVRLGPDWRLSVVGSPAYFERHPV
PLQPQDLTQHTCINIRHSPNGSCYAWEFEKGSRKLNIRVNGQFTSNSMIHVLNAALDGVG
LAYVPDSMAEPHIASGRLKEVLVDWSPYFEGFHLYYPNRRQASPAFSAFVEAVRYRG

>tr|A0A0Q0DTD3|A0A0Q0DTD3_PSEAP Glycolate oxidase, subunit GlcD OS=Pseudomonas syringae pv. aptata OX=83167 GN=ALO85_02636 PE=4 SV=1
MMNILYDERIDGVLPAVDKQLLLQALQQQLPDLDILHRPEELRPYECDGLSAYRTTPMLV
ALPRHVEEVQTLLRLCHARRVPVVARGAGTGLSGGALPLEKGVLLVMARFNQILDIDPSA
RLARVQPGVRNLAISQAAAPHGLYYAPDPSSQIACSIGGNVAENAGGVHCLKYGLTVHNL
LKVEILTVEGEPMTLGSDTLDSPGFDLLALFTGSEGMLGVITEVTVKLLPRPQVAKVLLA
AFDSVEKAGRAVADIIAAGIIPGGLEMMDNLAIRAAEDFIHAGYPVDAEAILLCELDGVE
ADVHADCERVSEVLTLAGATEVRQAKDEAERVRFWAGRKNAFPAVGRLSPDYYCMDGTIP
RRELPGVLRSIRELSEQYGLRVANVFHAGDGNMHPLILFDANQPGELERAEALGGKILEL
CVKVGGSITGEHGVGREKINQMCAQFNSDELTFFHAIKAAFDASGLLNPGKNIPTLHRCA
EFGAMHIHAGQLPFPDLERF
>tr|A0A0Q0D086|A0A0Q0D086_PSEAP Cation/acetate symporter ActP OS=Pseudomonas syringae pv. aptata OX=83167 GN=ALO85_03219 PE=3 SV=1
MIRRLSAALGLAAFAPTLWAADALTGAVQKQPLNVAAIVMFVVFVAFTLYITYWASKKNK
TASDYYSAGGKITGFQNGLAIAGDYMSAASFLGISALVYTSGYDGLIYSIGFLVGWPIIL
FLIAERLRNLGKYTFADVASYRLKQKEIRTLSACGSLVVVAFYLIAQMVGAGKLIQLLFG
LDYAVAVVLVGILMCLYVLFGGMLATTWVQIIKAVMLLSGASFMALMVMKHVNFDFNMLF
SEAIKVHPKGEAIMSPGGLVKDPISAFSLGLALMFGTAGLPHILMRFFTVSDAKEARKSV
LYATGFIGYFYILTFTSVSARSCWSAPIRRSRMRQAPCWAVTTWRRFTSPMPSEAVCSWA
SSRQSRSPLFWQWLPV
>tr|A0A0N8T8B3|A0A0N8T8B3_PSEAP NAD-dependent malic enzyme OS=Pseudomonas syringae pv. aptata OX=83167 GN=maeA PE=3 SV=1
MTKTSRPLYISYAGPSLLEMPLLNKGSAFTPQERVEFNLIGLLPQNVETIEEQVTRVYSQ
YKQCASDLDKHIYLRSIQDNNETLFFRLLDSHLDEMLPIIYTPTVGQACQEFSKIYRTHR
GLFISYPERDRIDDILRSATKDRIKIIVVTDSERILGLGDQGIGGMGIPIGKLSLYTACG
GISPAYTLPIVLDVGTNNRELLDDPMYMGWRHERVSGKEYEDFIALFIDAVQRRWPDVLL
QFEDFAQSNAMPLLEKYRDELCCFNDDIQGTASVAVGTLLAACKAKNETLGQQKVVFVGA
GSAGCGIAEHIIAAMRIEGLSESEARKRIFMVDRFGLLTEGMDNLLDFQKRLAQKTADVS
GWTAGSEAFPQLLDVVTHAGATVMIGVSGQRGLFTEQVIRELHKHCAKPLVMPLSNPTSK
VEATPEEILRWTDGNALVATGSPFAPVEINGRTVHIAQCNNSYIFPGIGLGVVACKASRI
TDRMLMAASNALAECSPMVTGKGDAVLPPLKEIQQVSRKIALAVAREAQAEGLALETTEE
ALLEAIERNFWLPGYRAYRRRSV
>tr|A0A0Q0C506|A0A0Q0C506_PSEAP Uncharacterized protein OS=Pseudomonas syringae pv. aptata OX=83167 GN=ALO85_200106 PE=4 SV=1
MLPRLGGVPERPKGSDCKSDVYDFEGSNPSPSTRFSASCKLCGYSLVVEPQPSKLMMRVR
FPLPAPGLWFMRGVLLL
>tr|A0A0Q0C911|A0A0Q0C911_PSEAP Peptidase U32 OS=Pseudomonas syringae pv. aptata OX=83167 GN=ALO85_00641 PE=4 SV=1
MSLPKHHLELLSPARDVTIAREAILHGADAVYIGGPSFGARHNACNEVSDIAELVKFAHR
YHARIFTTINTILHDDELEPARKLIHHLYDAGVDALIVQDLGVMEMDIPPIELHASTQTD
IRTLSRAKFLDQAGFSQLVLARELNLQEIRAIADETDAAIEFFIHGALCVAFSGQCNISH
AQNGRSANRGDCSQACRLPYTLKDDQGRVVAFEKHLLSMKDNNQSANLRALVDAGVRSFK
IEGRYKDAGYVKNITAYYRQRLDEILEDRTDLARASSGRTAHFFVPDPEKTFHRGSTDYF
VSDRKIDIGAFDTPTFTGLAVGTVEKLGKRDLIAVTHEPLSNGDGLNVQIKREVVGFRAN
IAELKGEFEEDGQKRWRYRVEPNEMPAALSSLRPNHPLNRNLDHNWQQALLKTSAERRIG
VQWQVTLREDHLHLKATSEEGVSVTVGLDGPFGTANKPEQALDQLRDLLTQLGTTIYHAQ
DVRLDAPQAFFVPNSQLKTLRRDAIEALTEARIKAHPRGGRKAETTPPPVYPESHLSFLA
NVYNQKARDFYHRHGVQLIDAAYEAHEETGEVPVMITKHCLRFSFNLCPKQAKGVTGVRT
KVAPMQLVHGDEVLTLKFDCKPCEMHVIGKMKGHILDLPLPGSAAAKSVVGHITPEDLLK
TARQRSPH
>tr|A0A0Q0DJZ0|A0A0Q0DJZ0_PSEAP DNA internalization-related competence protein ComEC/Rec2 OS=Pseudomonas syringae pv. aptata OX=83167 GN=ALO85_02526 PE=4 SV=1
MRTGMIALALGLLALRFLPALPPTWLLLLMPTLALMLLPFRTYPLALFLLGFTWACVSAQ
WALSDRLAPRLDGQTLWVQGKVVGLPSVAEGVVRFELEGAWSRRAKLPARIRVAWYGGQP
VNSGERWRMAVKLKRPSGLVNPYAFDYEAWLLAQRIGATGTVVDGERLAPARAAWRDALR
QRLLAVDAQGREGGLAALVLGDGSGLSSSDWQVLQDTGTVHLLVISGQHIGLLAGVIYAL
VAGLARWGLWPRSLPWLPWACALAFSAALGYGLLAGFEVPVRRACVMVAMVLLWRLRFRH
LGVVWPLLLSFNVVLIFEPLVTLQPGFWLSFAAVGILILIFSGRLGAWRWWQSWTRAQWL
IAVGLLPILLALNLPISLSGPFANLLAVPWVSVIVLPPALLGTLLLPVPVVGEGLLWLAG

GALQWLFVFLDAVAAALPAWLPSAVPIWAWWLSLLGALLLLLPKGVPMRPLGWPLLLLCV
FPPLESVPEGQVDVLQLDVGQGLAILLRTRNHTLLYDAGPRFGEFDIGQRVVVPAMRKAG
VRHLDLMLISHSDADHAGGAAAVHQAFPVSRVLGGELARLAPQLDARLCESGARWEWDGV
VFSTWRWEQGPDGNPASCILSVDARGERLLLAGDIDVSAERAAIDSGFDLRAHWLQSPHH
GSRTSSSKAFLRAVAPVGVLISRGRNNAFGHPHPLVMARYRGLGIASYDSAELGAVRLQL
GTFGAPQAERAQRRFWRD
>tr|A0A0Q0DIC3|A0A0Q0DIC3_PSEAP Potassium-transporting ATPase KdpC subunit OS=Pseudomonas syringae pv. aptata OX=83167 GN=kdpC PE=3 SV=1
MQPHLIEDVQMSNVLRPALSLIVLMSLITGVAYPLVVTGVAQVAFPEQANGSLVYDASGK
VRGSALIAQSFTGDEWFQSRPSAGAFATVASGASNFAPSNPALATRVKEDAAKLANVSQE
PIPMALLTTSGSGLDPHLSPEAVAWQAGRVAAARQLPLDKVQALIDANTQRPLIGPPVVN
VLALNMSLNQLPSAPRNAQL
>tr|A0A0Q0CZI3|A0A0Q0CZI3_PSEAP Glycosyl transferase family protein OS=Pseudomonas syringae pv. aptata OX=83167 GN=ALO85_04757 PE=4 SV=1
MSRHLDRGRETLMDMFHRHRQADEHDLATVALECALQTPEYRPEALVWKGIEALPQDPKL
AFIYLLNAAHAFPLRADIHALLGRSIIAQGQPLLANRYMSNAWQKLPADASLRMMLWQAR
SLSETPEDLRRIILAHLPDITAANELALVLKLLAAQTGLPGTLGVVRYLPDVQEIHGWAI
DVHNVQTPVSLQLEANGHLISMVASAPHPLLTAAGLPATHGGIRIKVPNATPAVHVRFDN
GTALLGSPVSAMPVFVAPPAALKVGDKQPVDVLIPVYDGLEETLECINSALEARKRNRTP
HRLVVIEDATPVPALRKALKVLAGKGKITLVQNPINLGFIRSMNRAMALSPRQDVVWLNA
DTRVQGDWLDRLRTTAYADETIASVTPFTNNGELMSFPESRFSHSMPSAAEQVRLDDLAR
LTGSPAMELETGCGFCLYIKRSALDSVGYLDEVELLRGYGEETDWCLRARGLGWRHVGAP
NVFVAHQGGISFGAEKALRVAHNNAILKRRYPDASSRYENFCLRDPIRPARQALQRARLA
QLTEQLSTQALRQLHIANSTASQAPLSLTWRNKGQHAQATLQANVLPLAFSLDYELPADT
EQLLQDLRSLPLDELIYQNLANCPEALCALAEQLDRPYRIVCRDDALLTPDSRFDSADFA
RKAQSIELPWQALRERYAAALPQADILIRSAPQALPNNDSVPTTLLIADRLSDPDIAKQW
LELGRRITHENQSLVLLVAEDGPWVKPLLATGAIHALPIAQGLSQADCILLAGCTAALSL
ERSPGASWRAADLAVELGLPLYAMPGPLAHEAGALPINTLPISLSRT
>tr|A0A0Q0DPI5|A0A0Q0DPI5_PSEAP Beta-glucosidase-related glycosyl hydrolase OS=Pseudomonas syringae pv. aptata OX=83167 GN=ALO85_03613 PE=3 SV=1
MNNLNQPITSTGRTLALSAFKQTLGLSLLTFSVMQANLALAAVTPATGNEVEARVSSILD
NMSQSEKINFTRVNDGHMIPSLLKWGIKGTVAYDSSMGVHVNNATFGAQYPSQSALAATW
SINRAKEFGLAIAYETRISGGQQMLSPGANLYRTPYNGRSAEYVSGEDPFLGAVLAPAIV
NGIQAQGIQASGKHYLANEQEANRQAIDVKVDERTLRELYLPGFESMVKNANVASIMCGF
NKVNGDYACENHHLITEVLKGEWGYQGMVISDFNAIHDAFKGAWAGTDIDMPSGLQFTEA
NLLPYLWSGQLTQNVIDDKVKRNLRGIVSYDLQNNLNTAKTLEHPEYGMRAALNTARESI
VLLRNENTAAGKPLLPLDRSAKIAVIGNWAHDVPASPFGTANSPPNSYVTELSGLQQLAS
SSSDVTYLSEMSLNPASSVWYQPATGDNGISNAGVKAEYFSNTSFSGDPVLTRVEPGVNL
NWTTGSNVTNAGSTAVSGFSPTAGAFSARFTTTIKPTISGAQAFKVRADGPYKLWVNDEL
IVQSDGVPYSGDVVNALTTSGKTAALSAGKTYTVKLEYQRVQGNFIPALGGLTGVQMSWA
ALRPPKDLSKYDAVVVATGTTSENEGEGSDHGFDLPDQQAELISFVAKANPNTIVVMHGG
GVANMQPWANKVGAALQAWFPGQQGGQALAEILYGKINPSGKLPVTIDKNIEDNPSYASY
PDPAAYRGSNPLTEMTYSEGLYMGYRGYDKKHAKPLYPFGYGLSYTTFSYSDLKLSTNVL
TPGSTVDVKFTVTNTGDKAGFEVAQLYVQPVKPAVDRPEKELKGFTKVYLQPGESKTVSV
PIDSRSLAYYVDKTASWDVDAGKFKILVGADSENLTLNRTLITLYPEKLTTRDSNPLPLP
LRKAVQVSAAQTY
>tr|A0A0N8T822|A0A0N8T822_PSEAP Uncharacterized protein OS=Pseudomonas syringae pv. aptata OX=83167 GN=ALO85_102173 PE=4 SV=1
MPTPRGHTVLDAPRPVLDDVRPAERQTLNDPLLPQPLVQRVAADAQTFGQL
>tr|A0A0Q0IFU3|A0A0Q0IFU3_PSEAP D-isomer specific 2-hydroxyacid dehydrogenase, NAD-binding protein OS=Pseudomonas syringae pv. aptata OX=83167 GN=ALO85_00665 PE=3 SV=1
MTPLKIAVLDDWQNVASTVVDWSVLDSVGKVSFLHDYPADTSTMARRLRDFDIICVMRER
TLFDATLLSQLPALKLLVTGGMRNAAIDTAAAKRQGIVVCGTESYKHAAPELTWALIMGI
TRNLVAEANSLRAGNWQAGLGSDLHGKTLGILGLGSIGKWIARYGQAFGMKVIAWSQNLT
PEAAAESGVTYVSKQQLFEQADVLSVHLVLSDRSRDLVDAEALSWMKPGAYLINSSRGPI
IDQAALIETLQQRRIAGAALDVFDIEPLPADHPFRTLDNVLATPHIGYVTENNYRTFYGQ
MIEDIQAWHAGSPIRVFG

>tr|A0A0Q0C638|A0A0Q0C638_PSEAP Pilus retraction protein PilT OS=Pseudomonas syringae pv. aptata OX=83167 GN=ALO85_05453 PE=4 SV=1
MIMDFPALLKILASQDGSDLYLSTGAPPCAKFNGVLKPLGSETFKPGEVAVIAQALMDEE
QKLEFQRELEMNLAVSMAGIGRFRINIFMQRNEVSIVARNIKLDIPRFEDLFLPPVLLDV
IMEKRGLVLFVGATGSGKSTSLAALIDYRNRNASGHIITIEDPVEFIHRHKKSIVNQREV
GVDTRSFHAALKNTLRQAPDVILIGEIRDRETMEHALAFADTGHLAISTLHANNANQALD
RIINFFPEERRAQLLHDLGNNLKAFVSQRLVRTPDGKRRAAVEVMMGTPTIRDLIQRNEL
TELKGIMEKSGSLGMQTFDTALFNLAVEGAISEEEALKNADSQNNVRLRLKLHTEGGAGT
LTTPPPAPTGSSTASTAEWGLVDDDAPGPQA
>tr|A0A0N8T8B9|A0A0N8T8B9_PSEAP Sulfate transporter family protein OS=Pseudomonas syringae pv. aptata OX=83167 GN=ALO85_01626 PE=4 SV=1
MGITALKTALPRELLASVVVFLVALPLCMGIAIASGMPPAKGLITGIVGGLIVGWIAGSP
LQVSGPAAGLAVLVFEVVREHGMAMLGPILVLAGLLQLLAGRFKLGCWFRVTAPAVVYGM
LAGIGVLIVLSQAHVMFDSGPKPSGLDNLIAFPSTLIQAFGPGTGMQAGMLGLGTMLIMW
GWEKLRPQSLRFVPGALLGVGIATGISLFMALQVKRVEVPDNLADAIDWLRPADLMNLAD
PAILVAAIVVAFIASAETLLSASAVDRMHSGQRSDFDKELSAQGVGNMLCGLLGALPMTG
VIVRSSANVQAGATTRFSTIFHGLWLLAFVLLLSSVLQSIPVASLAGVLVYTGLKLVDLK
AFRGLGRYGRMPMFTYAATAIAIIFTDLLTGVLVGFGLTLVKLAFKASRLKINVVELAGE
KEFELRLVGAATFLKVPALTQALGTIPQGSTVHVPLGNLSYIDHSCLELLEEWGRSNAAH
GTRLLIESRGLKRRLEGRIYTTTGIGSGAA
>tr|A0A0N8T8K1|A0A0N8T8K1_PSEAP ATP-dependent RNA helicase SrmB OS=Pseudomonas syringae pv. aptata OX=83167 GN=ALO85_03185 PE=3 SV=1
MFFVEVAPVFSEFALHERLLKAVAELKFVEPTPVQAAAIPLALQGRDLRVTAQTGSGKTA
AFVLPILNRLIGPAKVRVDIRAVILLPTRELAQQTLKEVERFSQFTFVKAGLITGGEDFK
VQAAMLRKVPDILIGTPGRLLEQLNAGNLDLKHVEVLVLDEADRMLDMGFSEDVERLAGE
CAGREQTMLFSATTGGAGLREMIGKVLKDPQHLQVNSVSELASGTRHQIITADHNVHKEQ
VLNWLLANETYQKAIIFTNTKAMADRLYGRLVALEYKAFVLHGDKDQKDRKAAIDRLKQG
GAKIMVATDVAARGLDVEGLDMVINFDMPRSGDDYVHRVGRTGRAGSDGLAISLICHGDW
NLMSSVERYLKQSFERRVIKEVKGTYGGPKKVKASGKAVGVKKKKTDAKGDKKKVAAKGP
TKRKTVNRPKSDLVSQDGMAPLKKRSTPAPAAE
>tr|A0A0Q0BXL6|A0A0Q0BXL6_PSEAP RHS family protein (Fragment) OS=Pseudomonas syringae pv. aptata OX=83167 GN=ALO85_04434 PE=4 SV=1
YNPIHRRYEYDPAGELSRTLDKLRGETQYEYEANGQLLARNTGRVVDGEEFRYDAAANRL
NFNTSRFDHVKDNRLRQWANHEYKYDAWGNLIEKVVGIVRWQTFTYDCENRLVKTETMAD
TQVESTSSYQYDSLGRRVAKQSEIKGQTDHKRFLWQGLRMLREDSPGQSSLYL
>tr|A0A0Q0ILC7|A0A0Q0ILC7_PSEAP Putative pyridine nucleotide-disulfide oxidoreductase OS=Pseudomonas syringae pv. aptata OX=83167 GN=ALO85_02628 PE=4 SV=1
MFDTCMETACGCVVGGAGLAGMGFLFNALKSGAMPGLAAKGLIVIDASDRPGTGALGDYR
ITANSVGDVFIDCLRDPALREVFEPLENSAAYWRIKAQAQSAPQLSDVGLLMAEASRLVL
DHIVQRYGVEVWSGTRLDEVICEGDEFCLHVDSVYGERRVRCQALVLNLGGRQDPQHLID
DLARQGLTLPDHCTVQSADRLLRMNAVQLREVFAPALADNGRITIVGGSHSAFSTLENLA
DALEFAGLQELSLVHRSSIRLFYESVELAHAAGYVFDAQLDVCPVSGRVNRSGGLRYRAL
EIGRQALQNGRVGKTGVRVQMLQTLGGRPDEYEQARLALADSAVIVQCSGYQPVLPRLSD
ANGNLVSLREAKGGLDSDAGGCPFDQHGRRIKGMHLFGLGAGLGVDPQLGSEPAFDGRIY
GVWQFHHDASRVVLEAVTRRLAHQPVAAETFISEGFMQAALGLQAG
>tr|A0A0Q0BTV5|A0A0Q0BTV5_PSEAP Uncharacterized protein OS=Pseudomonas syringae pv. aptata OX=83167 GN=ALO85_04170 PE=4 SV=1
MEKAMCVFGIIGVLTNKLQMKTISQNEHVIFKAVGDTKDFYLRWKKGELEVSLYNFSSCE
LQQFYEASVQCAKYDRFVQLFVLSPSKKEYQWGSEFFLLKSLNNDFDRAFVIIPDEQDSL
EIHEFNALAQSGYTDVIIKGQRVSILRAYKYWSENVEGKNYKKHSQSFEDFMTKTKKFSS
LE
>tr|A0A0Q0C500|A0A0Q0C500_PSEAP S-adenosylmethionine:tRNA ribosyltransferase-isomerase OS=Pseudomonas syringae pv. aptata OX=83167 GN=queA PE=3 SV=1
MRVADFTFELPDSLIARHPLAERRSSRLLTLDGPTGALAHRQFTDLLEHLRPGDLMVFNN
TRVIPARLFGQKDSGGKLEILVERVLDSHRVLAHVRSSKSPKPGSSILIDGGGEAEMVAR
HDALFELRFAEEVLPLLERVGHMPLPPYIDRPDEGADRERYQTVYAQRAGAVAAPTAGLH
FDQPLLDAIAAKGVETAFVTLHVGAGTFQPVRVEQIEDHHMHSEWLEVGQDVVDAVAACR
ARGGRVIAVGTTSVRSLESAARDGELKPFSGDTDIFIYPGRPFHVVDALVTNFHLPESTL

LMLVSAFAGYPETMAAYAAAIEHGYRFFSYGDAMFITRNPAPTAPQESAPEDHA
>tr|A0A0Q0C0M2|A0A0Q0C0M2_PSEAP Nitrate/sulfonate/bicarbonate ABC-type transport system, permease protein OS=Pseudomonas syringae pv. aptata OX=83167 GN=ALO85_00980 PE=3 SV=1
MTSRNKSLPASAQATVPDKAKRTLWQRYRAPLKGLVVPAVIIVLLEMIVRIGWLPAYQMP
APSEIVLTLRDLADGALWKHISASLLRVLSGFAIGASLALVFAAWVGLSREAEAYLEPTF
AGLRSIPSLAWVPLLLLWLGIGETSKIVLIAIGAFFPVYLNGVAAIRGIDRKLVEVGQMY
GLSRYRLTRRILLPAALPGLFTGLRSGMSLSWMFLVAAELIAATKGLGYLLSDGRETSRP
DIVLAAIIILATLGKLSDGLLASLEKRFLAWRDTFNGQSREG
>tr|A0A0Q0C7I8|A0A0Q0C7I8_PSEAP Putative membrane protein OS=Pseudomonas syringae pv. aptata OX=83167 GN=ALO85_01694 PE=4 SV=1
MLWICLVVGGALLGYSLSYDEWAGTLMGAVMGWVFWAGIRVRLLDRQSADQLQQLLATRE
SLDVLQQRLSALERQSAPGVPPAQAPAPLPLDAVIVEAPTDGPELIWDLPDEAPQATTPA
EPAPQILQPTRTAKANPLDSAINAAKDWLLGGNTVLRVGVVVLFLGLAFLLRYATEGMVV
PLEARYAAVAASAMALLGLGWWLRMRNGPYALMLQGAGVGVLYLTVFAAMKLHTLLDPTL
GFALLVAITSFSAILALTQNSLALACAGALGGFAAPLLASTGQGSHISLFSYFALLNAGI
IAIAWFKAWRILNLIGFFGTFGIGFAWGLQAYTPALFWSTEPFLMVFFLMYLAIGLLFAR
RKLLELDSAPPDHTREAMLRWAARQGDYVDGTLLLGTPIAGFGLQYALVEHLEFGAAFSA
LALGLIYMALARWLAARAPSQTALLMETCLALGVVFATLAIPLGLGSQWTTSAWAVEGAA
IFWLGLRQQRRLAQAFGLLLQLGASAILISGGTDSSPQAFANDYWTPMILAVAAMVSAWC
VFRLNSLTLIDQRTARTLLIIWGAFWWMVSLTSAVMLHAPEAYLAAAFLLALALSVALWA
AIAIRLRWPDLAALCSVLTPLSVALLMISVDLHNHPAGRLGWLAWLLVFAVHLLTLRYLT
GMQSILARRVAHTVGCLLIITVLALELRFGLLQLSEHYNAWRWLGWAIVPSLFLLAMSST
RQWPWPVKACPEAYHLGAAAPLAVLMLAWFWLANTFSDGAADPLPYIPLLNPLEIGLLLS
LAGVCLWLRKHVMRLGNAALLVAGASLFALVTAMVMRTAHHWADVPWNTGALLDSMRVQA
GLSIVWTLMALALMIGGHMRSNRQLWLGGAALIGVVVVKLFFVELSNRGGMERIVSFIGV
GILLLVVGYFAPLPPKHSVTEVGEKPGPGPTAAPDTL
>tr|A0A0Q0CVX7|A0A0Q0CVX7_PSEAP Pili assembly chaperone OS=Pseudomonas syringae pv. aptata OX=83167 GN=ALO85_04270 PE=4 SV=1
MPMSAFAGSCKALLTAVLLTSGLAAQGAVSLSGTRLIFDGQYREVTLEVRNRGTSEALVQ
AWLSDAADDDDTPGELRKALSFVVTPPLSRLPVGGRQALRVLYQGAGMPQKYESLLHLYV
LEIPRRQDGAGQLNIAVRQRINVFYRPPGLDGDPADTPARLLWTLTHDPSHALVLTVSNP
TPYHAALQALRIDGVQLGDHLLLAPGVQISMVVPASVEPSATHRFSYQALTDYGGRRTHC
AQLTGQTATTARLPKNNSIQDEC
>tr|A0A0Q0C0I9|A0A0Q0C0I9_PSEAP Cytochrome c biogenesis ATP-binding export protein CcmA OS=Pseudomonas syringae pv. aptata OX=83167 GN=ccmA PE=3 SV=1
MIPATPFLQATALACERDGRMLFENLDLHLHAGDMLQISGPNGCGKTSLLRLLCGLMQPT
AGQVVLDAQPVDRGPAAPGSKLLWIGHAPALKDVLTPLENLSWLSALHQPADAGAIATAL
DAVGLAGFEDVPCHTLSAGQQRRVALARLYLPGPPLWLLDEPFTXXXXXXXRQGIEQLEK
HLAEHCEHGGMIVMTTHHSLSRLPAGYRDLDLGQWSA
>tr|A0A0N8T9K8|A0A0N8T9K8_PSEAP Type IV pilus bioproteinsis protein PilJ OS=Pseudomonas syringae pv. aptata OX=83167 GN=ALO85_02924 PE=4 SV=1
MSSTDRSREGARSRTQIILLFVALIVFIMLLFANFAYLNTQSNYDKQYIGHAGELRVLSQ
RIAKNATEAAAGKTQAFKLLADARNDFDVRWGYLRKGDPATGLPAAPDLIRDELRTVQRD
WEGLRKSTDVILASEQTVLSLHQVAATLAETIPQLQAEYEKVVENLLQSRAPAAQVVVAQ
RQALLAERILGSVNTVLAGDETAVQAADAFGRDASQFGRVLNGMLEGNATLRISQVEDRD
ARARLAEIAELFEFVSGSVDEILETSPELYQVREASGNIFNTSQTLLDETSVLANSLENL
AKRRTVNTVGGYVLGLLALASIILIGLVMVRETNRQLRETAQKSERNQTAIMRLLDEIEN
LADGDLTVTASVTEDFTGAIADSINYSIDQLRELVVTINLTAEQVAAAVTETQATAMQLS
AASEHQALQISAASTAINDMAASIDQVSSNASESSAVAERSVTIANKGNEVVQNTIHGMD
NIREQIQDTSKRIKRLGESSQEIGDIVSLIDDIADQTNILALNAAIQASMAGDAGRGFAV
VADEVQRLAERSSSATKQIETLVRAIQNDTNEAVISMEQTTSEVVRGARLAQDAGVALGE
IEGVSRVLAELIESITDAAHQQAESAGQISQIMTVIQQTTSQTTSGTSATAESIGNLAKM
ASEMRRSVSGFALPSSKKAG
>tr|A0A0Q0DFG1|A0A0Q0DFG1_PSEAP N-formimino-L-glutamate deiminase OS=Pseudomonas syringae pv. aptata OX=83167 GN=ALO85_04934 PE=4 SV=1
MKSMSAFFAERALLSDGWASNVRLEVGQDGILTRVEANAEQQGAEKVHGPLLPGMPNLHS
HAFQRAMAGLAEVAGNPNDSFWTWRDLMYRLVGRISPQQLGVIARQLYIEMLKGGYTSVA
EFHYVHQDSDGKPYADPAELALQISQAAGSVGIGLTLLPVLYSHSGFGGQAPNEGQRRFI

HSTDSYLDVQARLRPVLAAQPAQQLGLCFHSLRAVTPQQISEVLAASDRQCPVHIHIAEQ
QKEVDDCLSWSGRRPLQWLYENAPVDNRWCLVHATHAEADEVALMAQSGAVAGLCLTTEA
NLGDGIFPAVDYIAQGGRWGIGSDSHVSLSVVEELRWLEYGQRLRDQRRNRLYRSDQPMV
GRTLYDAALAGGAQALGQQMGTLQVGQRADWLVLDGNDPYIATASDDAILNRWLFAGSDR
QIRDVAVNGRWVIREGRHAAEEQSKREFAQVLRELLG
>tr|A0A0N8T9R5|A0A0N8T9R5_PSEAP YbbN-like thioredoxin domain protein
OS=Pseudomonas syringae pv. aptata OX=83167 GN=ALO85_04987 PE=4 SV=1
MPCGLYSWQHSDSSRRAMSQETAYIFDATAANFDQLVIDKSFDQPVLVDFWAEWCAPCKV
LMPLLQQITESYQGELLLAKVNCDIEQDIVARFGIRSLPTVVLFKDGQPVDGFAGAQPES
EIRKILDQHVVMPPPPAADPLKQAQALFAESRFAEAEAQLKVLLGEDNTNGAALILYARC
LAERGELSEAKAVLDAVTGDAHKAELAGAKAQLTFLAQAATLPDAAELKSRLAQNPQDDE
AALQLAIQQLSRQQYEAALEGLLKLFIRNRNYAEGLPHKTLLQVFDLLGNDHPLVTAYRR
KLFAALY
>tr|A0A0Q0IQ64|A0A0Q0IQ64_PSEAP Phosphonate metabolism protein PhnM
OS=Pseudomonas syringae pv. aptata OX=83167 GN=ALO85_00821 PE=4 SV=1
MLNEQVFSNARVVTAEQVFTGTVVVRDGLIAEVESGCSQLSQAQDMQGDYLLPGLVELHT
DNLEKHLSPRPGVDWPSMSAVISHDAQIVAAGITTVFDALSIGDINPKGKRMQQLPAMLQ
AISDANAAGLTRAEHVLHLRCEVSHPDTLSVFRDLVDQPLVQLVSVMDHAPGQRQFALES
KYREYYMGKYHMNNEEMDRFIVEQVANSNEYADRYRRAIVELCLARGLSIASHDDATMAH
VEESAGFGMNIAEFPTTLEAARGCRQLGMSVLMGAPNIVRGGSHSGNVAAASLARDGLLD
ILSSDYYPASLLQSAFMLAAQDNACDLPQAVNMVSRTPAVAAGLADRGEIRVGLRADLIQ
ARDHAGLPVIDKVWRQGKRVF
>tr|A0A0Q0DU77|A0A0Q0DU77_PSEAP PAP2 super family protein OS=Pseudomonas
syringae pv. aptata OX=83167 GN=ALO85_02350 PE=4 SV=1
MSKTVAPPYSRSINPWIYLGIPVAVALILMLLELTSLDMDIAKLAFDPVSGQFIGRHSYF
LEDVLHDRAKQLVMVFGAVAMIGFAASFKVQRLIPWRRELGCLVLSMALSTAFVTPVKVV
TSVQCPWSLKEFGGQEIYSELLSPRPATDKPGRCWPGGHAATGFTLFALFFAFRDRRPRM
AKMGLALAFGLGTVFSVGRMLQGAHFFSHNIWTAVFCWLICLGVYYFVLYRPEPKGLENS
KVQPTH
>tr|A0A0Q0IB41|A0A0Q0IB41_PSEAP Phosphoenolpyruvate synthase
OS=Pseudomonas syringae pv. aptata OX=83167 GN=ALO85_00574 PE=3 SV=1
MVEYVVSLDKLGVHDVEHVGGKNSSLGEMISNLAGAGVSVPGGFATTAQAYRDFLELSGL
NEQIHAALDALDVDDVNALARTGAQIRQWIMEAEFPEQLNAQIRTAFAELSAGNPNLAVA
VRSSATAEDLPDASFAGQQETFLNIRGVENVIRAAKEVFASLFNDRAISYRVHQGFDHKL
VALSAGVQRMVRSETGTAGVMFTLDTESGFRDVVFITGAYGLGETVVQGAVNPDEFYVHK
DTLAAGRPAILRRNLGSKAIKMIYGEEAKAGRSVKVIDVEPADRARFCLSDAEVSELAKQ
AMIIEKHYKCPMDIEWAKDGDDGKLYIVQARPETVKSRASANVMERYLLKETGKVLVEGR
AIGQRIGAGKVRIIKDVSEMDKVQPGDVLVSDMTDPDWEPVMKRASAIVTNRGGRTCHAA
IIARELGIPAVVGCGNATHTLQDGQGVTVSCAEGDTGYIFEGELGFDIKTNSVDAMPDLP
FKIMMNVGNPDRAFDFAQLPNAGVGLARLEFIINRMIGVHPKALLNYSVLPPEIRESVDK
RIAGYDDPVGFYVEKLVEGISTLAAAFTPKKVIVRLSDFKSNEYANLIGGKLYEPEEENP
MLGFRGASRYISENFRDCFELECRALKRVREEMGLTNVEIMVPFVRTLGEASQVIDLLAA
NGLKRGENGLRIIMMCELPSNAILAEEFLEYFDGFSIGSNDLTQLTLGLDRDSGVIAHLF
DERNPAVKKLLSNAIQACNKAGKYIGICGQGPSDHPDLARWLMDQGIESVSLNPDSVLET
WFFLAEAQAPV
>tr|A0A0Q0C9X0|A0A0Q0C9X0_PSEAP Methionyl-tRNA formyltransferase
OS=Pseudomonas syringae pv. aptata OX=83167 GN=fmt PE=3 SV=1
MTEPLRIVFAGTPEFAAEHLKALLDSPHQIVAVYTQPDRPAGRGQKLMPSPVKQLALQHD
VPVMQPPTLRDPAAQAELAALQPDLMVVVAYGLILPQVVLDIPRLGCINSHASLLPRWRG
AAPIQRAVQAGDAESGVTVMRMEAGLDTGPMLLKAVTPITAQDTGGTLHDRLAELGPPAV
LQAIAGLADGSLVGEVQDDSLANYAHKLNKDEARLDWTRPADELERLVRAFNPWPICHST
LNEETLKVLAADLAEGQGAPGTILGASKDGLIVACGQNALRLTRLQLPGGKPLNFTDLFN
SRREKFAIGTVLGQ
>tr|A0A0Q0DJU7|A0A0Q0DJU7_PSEAP Histidinol-phosphate aminotransferase
OS=Pseudomonas syringae pv. aptata OX=83167 GN=hisC PE=3 SV=1
MSKFWSPFVSDLVPYVPGEQPKLTRLVKLNTNENPYGPSPKAIDAMRAALTDDLRLYPDS
NSDLLKHAVADYYKVQPNQVFLGNGSDEVLAHIFHALFQHDAPLLFPDISYSFYPVYCGL
YGIDYETVPLDEQFQIRAEDYARPNAGIIFPNPNAPTGCLLGLDKVEQIIKASPDSVVVV
DEAYIDFGGETAITLVDRYPNLLVTQTLSKSRSLAGLRVGLAVGHPDLIEALERIKNSFN
SYPLDRMANVGGAAAFEDREHFETTRNKVIESREVLVEQLQGKGFEVLPSAANFIFARHP

QHDAAALAAKLREQGVIVRHFKQQRIAQFLRISIGTPEQHQALLEGLSDI
>tr|A0A0Q0BVQ5|A0A0Q0BVQ5_PSEAP Hemolysin activator protein, HlyB family OS=Pseudomonas syringae pv. aptata OX=83167 GN=ALO85_04614 PE=4 SV=1
MTHLFDINMLPQGRAVYPHALKLALLLLLAIAPATSLAATAPASLPGDQDLIRDRQDRLL
EEQRRRLEELKDLPGKQTAPAAPSSPADTRCFTINRIELKGADSLSAAERDALTKPYIGK
CLGVAQLNELLKVITDHYLEKGLVTTRAYLPQQDLSTGDLQVLVIEGKLEHLRGDPASGL
SASELAMTFPGREGQMVNLREIEQMVDQLNRLPSNQAQMELTPGKAVGGSDVVVKNTPQK
PWRANLSRTNEGQRSTGEQQWNAGLEWDNPSGLADQFSLRGGHDAISDRQKASRNASLAY
SLPWGWWTFSYLYNTSEYRSVAQASNVNFKQDGDSQNHQFKAERVVYRDALSKTSLNAGL
SYLRTNNYVEGSKLAVSSNRITEAQFGINHGRRVGSAFVNIDLGMQQGIGALDAQGNHDP
RPGEADARYRKYTGTLSYLHPFQLWGENLSFTSLATGQRSEDVLFSSQRTSLGGSASVRG
YKDEFLSGDSGGYWRNELRLTRPVTLDWLRPVFAEYGAAAGYDQGVIRNDRYNGNQHGRL
SSNSFELFTRGQHVAASVTFAHSLERPDVIEREAPIYFRMDFFL
>tr|A0A0Q0DDP4|A0A0Q0DDP4_PSEAP Putative membrane protein OS=Pseudomonas syringae pv. aptata OX=83167 GN=ALO85_02483 PE=4 SV=1
MTNSRSAFIPSWLRPVLRPLLDPYRRYRHARLIHAARIAVGLLVTILLTTGLNLPHGEWA
SVTMLIVIGGLQHHGNIGKKSVERAYGTLIGAGLGLIVVVQQGYLEMPLLTYAMMSVMCG
FFAYHAIGKGGYTALLSAITLFIVAGHGYNPISDGLWRTVDILIGIALALAFSFALPLYA
VFSWRYNLASGLRDCAKVYGRIVQGQPVTADEHLKLTARLNGTMLQLRSLLPSVSKEVKM
SMVELDAIQGHFRMCLSTLEILANIRPANLNQLADETLKASLDADYRQIRRQLIGMARAL
QTGATERLERTTETPETPATPAVKAVIPTELKGYHLMTQQLALNLDGLQARLAKTAKRWK
F
>tr|A0A0Q0DAQ4|A0A0Q0DAQ4_PSEAP Pyridine nucleotide-disulfide oxidoreductase OS=Pseudomonas syringae pv. aptata OX=83167 GN=ALO85_03361 PE=4 SV=1
MSDQAIAIVGAGPAGIRAAQTLLAHGIKACLIDEGLRGGGQIYRRQPDNFQRSAKALYGF
ESAKAVAVHEALDTLAAQIDYRPQTLVWNAEDHQLDTLQNGTAATVDFSHLIVATGATDR
ILPVPGWTLPGVYSLGAAQVALKYQGCAIGQRVVFCGSGPLLYLVAYQYAKAGAKVLAVL
DSAPFSAQCKALPALLGQPATLAKGIYYRAWLSAHGIQVHQGVQLTRIDGEKRVDGIQWQ
RNGKSGHLACDAVAFAHALRSETQLADLLGCEFAWSALNRAWLPTRDECGRSSVSGIYLA
GDGAGIMGADAAEMAGELAALGLLQDIGVVADTARIDTLKTALRRIERFRHGLETAFPFS
ENWAATVADDTLVCRCEEVSAGEIRSAVQDGHWEINRVKAMCRVGMGRCQGRMCGLAAAE
IIARESGRPVEHVGRLRGQAPIKPLPFGLGMQPMEKQSVETQP
>tr|A0A0Q0IQA4|A0A0Q0IQA4_PSEAP Formyltetrahydrofolate deformylase OS=Pseudomonas syringae pv. aptata OX=83167 GN=purU PE=3 SV=1
MQHEKNHFILKISCPATSGIVAAVTSYLAGNSCYIGEMAQFDDEYSGTFFMRAVFRFNDG
HEGDIQQLKAGFDAVANDFAMQWELHDTRRPMRVLLMVSKFDHCLTDLLYRYHKGEMDMT
ITAIVSNHLDLRPMAEREGIRFIYLPVTRETKAAQEAALMKVVDETGTELVVLARYMQIL
SDDLCQQLAGRAINIHHSFLPGFKGAKPYHQAYERGVKLIGATAHYVTSDLDEGPIIEQE
VQRVDHVYLPADLVAAGRNNETIALSRAVKYHLEHRVFLNTDRTVVFR
>tr|A0A0Q0CI66|A0A0Q0CI66_PSEAP Cupin_3 domain-containing protein OS=Pseudomonas syringae pv. aptata OX=83167 GN=ALO85_02587 PE=4 SV=1
MSIESIVDFSEASTAAEHYRPAPEKVFKGDPAQTLYNHYNSPCGQMSAGVWNGEPGQWQV
NYSEHEYCEIVQGVSVLRDEQGHAKTLRAGDRFVIPAGFKGTWEVLETCRKIYVVFEAAA
DK
>tr|A0A0Q0D419|A0A0Q0D419_PSEAP Putative NAD-dependent dehydrogenase OS=Pseudomonas syringae pv. aptata OX=83167 GN=ALO85_03971 PE=4 SV=1
MMNASTTTCQILIVGGGAAGIATAASLLARDATLQITLVDPADTHYYQPGWTMVGAGIFT
PQSTARSMASLIPTGVQWIKAAVATFEPQHNTLTLADGRVLGYQQLVVCPGLKLDWSAID
GLVETLGANGVTSNYRYDLAPYTWKLVQNLKQGRALFTQPPMPIKCAGAPQKAMYLSCDH
WLKSGRLAQINTQFYNAGGVLFGVADYVPALMKYVERYAIDLKFSHRLVAVDGPGKRATF
IRTQADGSSETVEQSFEMLHVVPPQIAPDFIRSSPLADAAGWVDVDPATLKHRQFANVHG
LGDVTNTSNAKTAAAARKQAPVVANNVLVALGRRDGAAVYDGYGSCPLTVEKGRIVLAEF
TYGGKVAPSFPRWLLEGRQPTRLAWWLKARVLPALYWHGMLKGHEWLAKPRKADSRHG
>tr|A0A0Q0CEK3|A0A0Q0CEK3_PSEAP Putative damage-induced mutaganesis involved protein ImuB OS=Pseudomonas syringae pv. aptata OX=83167 GN=ALO85_00986 PE=4 SV=1
MLWACVLLPQLALDGVMRRHGNPDEPLALISGSAQRRVLQSVNPAARALGLKAGQSLTAA
QALVPNFTTIEHDPADIEHLQQLLAAWAYGFSSNVSLKYPRVLLMEIESSLKLFGPWPVF
EARLREELTTQGFRHRIVVAPNPIAARMLANMHDGLSIDCPHELRRRLEQMPLERIGLSR

ETATALTRMGLRSVRQVLALPRDTLARRFPASVLQHLDTLLGERPVALECYTPPDFFDVR
IELNFDVESHQALLFPLKRLIADLVLFLAGRDSGVQRFSLHLEHVPGPETVVPETVVPVG
LLSAERDASMLFELARGRLEQVLVASPVRAVRLLARDLPDFVPAHRQLFDERVQQTLPWE
QLRERLRARLGDESVNGLRAQADYRPECAWQAHSSSKPVLPARGFTRPGWLLREPQPLPV
QATRIVAGPERIESGWWDGGDVRRDYYLVETASGQRAWAYRAVGEQGELLLHGWFA
>tr|A0A0Q0ISM9|A0A0Q0ISM9_PSEAP Coenzyme A biosynthesis bifunctional protein CoaBC OS=Pseudomonas syringae pv. aptata OX=83167 GN=ALO85_05165 PE=3 SV=1
MGTAVPVRSLCYLSPSFLHDDLAWGGVMAVVCSLDQKGRSMQRLYRKRIVLGVGGGIAAY
KSAELVRRLRDQGAEVRVVMTRGGAEFITPLTMQALSGHPVHLDLLDPAAEAAMGHIELA
KWADLILIAPATADLIARLAQGIANDLLTTIVLATDATVAIAPAMNQAMWRDASVQANTR
LLEERDFRVFGPASGSQACGDVGFGRMLEADDLAQCAADCFQRLTLTGKHVLITAGPTQE
NIDPVRYITNHSSGKMGFALAEAAVEAGARVTLITGPVNLPTPDRVSRIDVVSARDMLAA
CEAAIPCDLFIASAAVADYRPEVVAPHKLKKDPSNGDGLLLQMVRNPDILATIATRPDRP
FSVGFAAETEHLLDYAARKLKDKNLDLIVANDVANPSIGFNSEENACSVIDRALHATLFA
QTSKAKIARQLVTFIADRMNQV
>tr|A0A0Q0CIW2|A0A0Q0CIW2_PSEAP DUF4105 domain-containing protein OS=Pseudomonas syringae pv. aptata OX=83167 GN=ALO85_01403 PE=4 SV=1
MRRFRAWLLAGALSLVGTHAFASLKLELHTDGLDAPQQQASQALLDEALHALPPSFVEAL
DRTVEVNWSADMPQNAYGQAAGPYQLYLNSHLLASLTDGSAATAQTGRPHGTVRRELLAT
VLHELTHVYDRARLWSPSERAAIFRCTSRSSSLGKVGLPDNCRGQTERRFTLSDDPRLLD
LAGWQQYVGRRGDREEHNGQVARSPDIYETTSPLEFVAVNMEYFLLDPAYACRRPALYAY
YKERFGWAPAAHNECPKFYPYLNAGSDFGREPLGKLDPERVYAVDYLLAEANQNWASRWG
HSMLRLVICKPGRPRGPDCRLDLDQHLVLSYRAFVGDVQLSSWDGLMGAYPSRLFVLPLA
QVIDEYTKTELRSLASVPIKLSRPEIEGLVQHAAEMHWSYDGNYYFLSNNCAVENLKLLR
AGTHNPKLVGLDSILPNGLLEVLEGRGLADTSVLDDPKEALRLGYRFDSYRDRYQAMFEV
LKKHLPIKQNTVEDWMALPASERSQWFGKADLRTSAAILLLEQASLRRQLLLAQDEVKQS
YLGARELKDGKVSNATKTLQEILASSGFLSRPAELLGSDGYGLPQAGEWQRLEAESSQRQ
KQLKRLTGDLDKEVRALLEPERAKEIAANEANLKQVSEHLRALHKAAGGLQLP
>tr|A0A0Q0DKT8|A0A0Q0DKT8_PSEAP N-acetyltransferase GCN5 OS=Pseudomonas syringae pv. aptata OX=83167 GN=ALO85_04764 PE=4 SV=1
MVDYHDLSASQRQQLGYLEVTQEQIQFSGDIYTALNTLLVRPSPNICGFVLLGEEKPVGF
FLLKRDDCLPHWAQEDLTATLHALQIDHREQGKGLGKACLQALPAALRLKWPDITQLMLS
VDADNLAAIGLYTGQGWVDTGEAYRGRIGFERRLTLTL
>tr|A0A0Q0FVP2|A0A0Q0FVP2_PSEAP Iron ABC transporter periplasmic iron-binding protein OS=Pseudomonas syringae pv. aptata OX=83167 GN=ALO85_05160 PE=4 SV=1
MGFTTISPHLRTRDTPMQASKRLLAALTLTLLGSTAQAADEVVVYSSRIDELIKPVFDAY
TASTGVKIKFITDKEAPLMQRIKAEGQNATADLLLTVDAGNLWQAEQMGILQPFTSPVIE
ANIPPQYRSSTHGWTGLSLRARTIAYSTDRVKPAELSTYEALADKNWEGRLCLRTAKKVY
NQSLTATLIETHGAEASEKILKGWVNNLSTDVFSDDIAVLEAINAGQCDVGIVNSYYYGR
LHKEKPDLAVKLFWPNQADRGVHVNLSGIGLTKYAPHPEAAKALVEWMTGPEAQKIFSSV
NQEFPANPKVAPSEEVASWGQFKADTLPVEVAGKRQAEAIRMMDRAGWN
>tr|A0A0N8T9T1|A0A0N8T9T1_PSEAP Putative ABC-type transporter, periplasmic substrate-binding protein OS=Pseudomonas syringae pv. aptata OX=83167 GN=ALO85_03783 PE=4 SV=1
MMPSVDSRLPGLRVGVSALFDPDDTPHARTFMRALAVARNCIPGLERVQWHFLDDAADAV
RGADVARQMIDWKADLVIGHFSSDAAVAAAPLYRHAGIALLTPAATIDCLTLEHPNVFRF
CPSDRQLAADLVSWLATRQWPRVHVQADDSAHGRALCVAISAALASAGLQRVDELEQADV
EVFAGRLKPSREHWQARRQAGSTRPLVLTDDAASPYLGRAAAHDGNTFVIGFGAPDSAGH
RCQAQSLHRSLFAAEPQTYFRESLLMLYVLAEVARSGLRAGQLPDAFRHSTFNTPLGTVS
FDQGELRSATTCLWTPGPTGLSRITR
>tr|A0A0Q0C891|A0A0Q0C891_PSEAP Uncharacterized protein OS=Pseudomonas syringae pv. aptata OX=83167 GN=ALO85_200201 PE=4 SV=1
MSNVRNIVSSFRYEISAGTRKCDAKSSHTISKGEKHFAYEKIPGQRLNICMVCAPAIIEK
AQEELRKIADELKK
>tr|A0A0N8T890|A0A0N8T890_PSEAP Cu-responsive transcriptional regulator OS=Pseudomonas syringae pv. aptata OX=83167 GN=ALO85_03077 PE=4 SV=1
MNIGQAAKTSGLSAKMIRYYESIGLLQAAHRSDSGYRLYSADDLHTLAFIKRSRDLGFSL
EEVGKLLTLWQDRQRASADVKALARQHIAELNRKIDELASLRDTLQELVEHCQGDDRPDC

PILKDLASGGCC
>tr|A0A0Q0BXM2|A0A0Q0BXM2_PSEAP Uncharacterized protein OS=Pseudomonas syringae pv. aptata OX=83167 GN=ALO85_100689 PE=4 SV=1
MSLANHRRGAVSAAQTRVFEPERPEKGHKSAENRALPKAGAPRKVC
>tr|A0A0N8T9E5|A0A0N8T9E5_PSEAP Chaperone protein HscC OS=Pseudomonas syringae pv. aptata OX=83167 GN=ALO85_05116 PE=3 SV=1
MPALPTHKTKTTPVALIQNHAKHRNTLRAGSIFEMIVGIDLGTTNSLVAVWRDGSSELVT
NALGETLTPSVVGLDDDGQILVGKAARERLQTHPEKTTALFKRYMGSAQEIRLGSATYRP
EELSSLVLKSLKADVERAFGEPVTEAVISVPAYFSDAQRKATRIAGELAGLKVEKLINEP
TAAALAYGLHQKEGETSFLVFDLGGGTFDISILELFDGVMEVRASAGDNFLGGEDFDQVM
VEHFVNLHRDEPDFPSTELIAPALRREAERVRRALGQDGSADFVLRHADREWRKTITQEQ
MSDFYAPLLNRLRAPAERALRDARIRVADLDEILLVGGTTRMPLIRKLAASLFGRFPSIA
LNPDEIVAQGAAVQAALKHRDAALEEIVLTDVCPYTLGIETITQVGNRYESGHYLPIIER
NSVVPVSRVKTVQTVSDDQEHVMVRIFQGESRMVKDNIALGELIIPIPKAKAGEVALDVR
FTYDNNGLLEADVMTQLTGERHKLVIENNPGVMTPDEIQERLQVLEALKVHPREQQANTH
LIARLERLYQECLGSDRELIDHWTTQFQHVLETQDERQISEIRKQLKQEVDTFEQGAR
>tr|A0A0Q0BVS6|A0A0Q0BVS6_PSEAP Achromobactin biosynthesis protein AcsF OS=Pseudomonas syringae pv. aptata OX=83167 GN=ALO85_04510 PE=3 SV=1
MIIICIRKSSEDLQMLNNGLLHSTTSQKTNANYLDRQSKFESNVRSYPRKLPLAIAKAHG
VWVTDVEGTTYLDCLAGAGTLALGHNHEAIMASLDSFLTSGMPMHTLDLTTAVKDAFSET
LLSLLPGQGRDYCLQFCGPSGADAVEAAIKLAKTATGRHNIISFSGAYHGMTHGALALTG
NTGPKNAVANLMPGVQFLPYPHEYRCPLGIGGEAGTEALSYYFTQFIEDVESGVSLPAAV
ILEAVQGEGGVNPAPAAWLRQIREVTRKHGILLILDEVQAGFGRTGKMFAFEHAQIEPDV
IVMSKAVGGGLPLAVLGFKREFDAWSPGNHAGTFRGNQMAMATGLATLEVLQRQNLAGQA
AKRGDWLKEQLGLLQQRYPAIGNVRGRGLMLGIEIVDERKPADRLGSLPMDPDLAVAIQQ
HCFKQGLLLERGGRNGNVIRLLPPLIITEEQCQLVIQRFEQALKAALSQLRQ
>tr|A0A0Q0CWD1|A0A0Q0CWD1_PSEAP Periplasmic binding protein/LacI transcriptional regulator OS=Pseudomonas syringae pv. aptata OX=83167 GN=ALO85_00517 PE=4 SV=1
MRGDARHRFCAVRPNNNHKIGDELMKLPFAGRLLAVAMFAAVTAVMPLSTVYAQTPEKPK
VALVMKSLANEFFLTMEDGAKAYQKEHATEFDLISNGIKDESDTSAQIRIVEQMIASNVN
ALVIAPADSKALVPVLKKATDAGIKVVNIDNQLDVDVLKSKSLQIPFVGPDNRKGAKLVG
DYLAKKLTAGDEVGIIEGVSTTNNAQQRTAGFKDAMDEAKMKVVSTQSGNWEIDKGNAVA
SAMLNEYPNLKALLAGNDSMALGAVSAVRAAGKAGKVMVVGYDNINAIKPMLKDGRVLAT
ADQYAAKQAVFGIEAALKLVKGEKVDTNDKGVIETPVQLVTQP
>tr|A0A0N8T8Q2|A0A0N8T8Q2_PSEAP Aldehyde dehydrogenase OS=Pseudomonas syringae pv. aptata OX=83167 GN=ALO85_00409 PE=3 SV=1
MIQSARVSPDTTFHASGLTELFEAQQAAFRAQGAPSFDQRIADLNAVLRMVSDNQNRLLE
AVSADFGNRSFDETRLGELMPVVNGIKHIRSHLKAWMRPSRRKAGIVFRPATARVVYQPL
GVVGILAPWNYPLTLTLVPLIEALAAGNRVMIKPSELTPRTSELLKQLLGEAFPAEQVVV
VTGDATLAGQFSELPFDHLLFTGSTHVGRQVMAAAARNLTPVTLELGGKSPVVVCDDFSI
TKAARMIAIGKLFNAGQTCVAPDYVLVPREQVDSFASEWLAAAKKLYPTLDGNSDYTSII
SRRHYDRLMAMASQAVATGAKAWQHNAPATAGERKIAPMAFTQVSLQADVMGEEIFGPLL
PILAYESLDEAIAFINARPAPLALYCFSNDQVSVDRVLNRTQSGGVTINGTMLHATQDDL
PFGGVGDSGTGAYHGYEGFVRLSHARGVLKLGRFNMSDKVSAPYGRLTRLVTRFMIGK
>tr|A0A0Q0C9E2|A0A0Q0C9E2_PSEAP GntR family transcriptional regulator with aspartate aminotransferase domain OS=Pseudomonas syringae pv. aptata OX=83167 GN=ALO85_02584 PE=4 SV=1
MTLYVNLAELLGSRIENGFYRPGDRLPSVRALSVEHGVSLSTVVQQAYRVLEDNGLAAPKP
KSGYFVSASRKAPALPAVGRPVQRPVDISQWDQVLDLIRVAPLEAVTQLGRGMPDVTSPT
LKPLMRALSQLSRHQDMPGLYYDNIYGVRRLREQIARLMLDSGCNLTANDLIVTNGCHEA
LSASVRAICSPGDIVAVDSPSFFGAMQTLKGVGMKAMEIPTDPLTGISLEALELALEQWP
IKVIQLTPNCNNPLGYVMPEARKRALLTLAQRFDVAIIEDDVYGDLAYNYPRPRTIKSFD
EDGRVLLCSSFSKTLAPGLRIGWVAPGRYLEQVLHMKYIGTGSTAPQPQLAIADFIEGGH
YEPHVRRMRSQYQRSRDQMIDWVMRYFPEGTRASRPQGGFMLWVELAEGFDTLRLNRALL
GKGVQIAVGSIFSASGKYRNCMRMNFAAKPNREIENAVRIVGETIALLNAQTD
>tr|A0A0Q0CX30|A0A0Q0CX30_PSEAP 4Fe-4S ferredoxin OS=Pseudomonas syringae pv. aptata OX=83167 GN=ALO85_00472 PE=4 SV=1
MSLIITDDCINCDVCEPECPNEAISQGEEIYVINPNLCTECVGHYDEPQCQQVCPVDCIP
LDENHVESKEQLMDKYRIITSKA

>tr|A0A0Q0C4U0|A0A0Q0C4U0_PSEAP Polysaccharide deacetylase OS=Pseudomonas syringae pv. aptata OX=83167 GN=ALO85_00991 PE=4 SV=1
MIKFVLKAFCVLAIAASLSACISAPISLTAKTAAQLQQQPPVRFLLTFDDGPSASTFYNP
SITVLDSLADNPLQPGIKAVFFVQTGATGAGNSDQGRAIMHRQHEEGHLLGFHTATPHHT
NHRSLSDEELEQSLTQGCAIIASITGVPTTLLRPPFWNYDKRTFSAYQRRHLQVLLTDLS
ANDGKIWGYNFSLRRRSNMVSQLSQVRERIAAGELPAVDGVIPVVVTFHDLNRYTARHAR
EYLQILVDSAGQTGVRLADKPFYDDSAQLQRAALVRTVKAAGEPVSLPGAWSWIWDHNAH
>tr|A0A0Q0D1M6|A0A0Q0D1M6_PSEAP TonB-dependent siderophore receptor OS=Pseudomonas syringae pv. aptata OX=83167 GN=ALO85_04511 PE=3 SV=1
MSRWSFQVSAQKIRELPSMSAVSSYRLRPLLHFSLLLTLSSSPLFISTSWADTSGRRAYE
VPAGSLSAALTRFAGQAGVNLSVDPALVNGRNSSGLSGEFAVEEGFARLLQGSGLQLMPV
GEQAYTLIPAPESASAGLQLAPTSIVGDSGVTDGQEYAGGQVARKGSQGMLGSRDFMETP
FSMTTYTKDAVKNQQARTLGDLIASDPSVRATNPAGGRYEQFTIRGLSLFNSDVSYNGLY
GILPTYTIDMEMAERVDILKGPSQLINGISPRGSVGGGINVVPKRATDKPITELTTSYAS
KGQVGAAVDVARRFGEDDKFGIRFNGVKQSGDTEWDHQSVDRDMAVLGLDFRGDRLRLST
DIGHTERDTDAPQERVQVGANAKVPNANDVRDNYAQSWSKARTKDTFGAVNAEYDLSDSV
MLYGGVGARKSNHDFLRHAVSVTNDAGDFSVQPRDFTRDENVRTATAGARSWFTTGPVSH
EVNLAASYFYMDFENGGARYAAAPSNLYNPVDTPTPSNPTRTDSKVYTENRFSGVALTDT
LGFFEDRLLLTLGARWQRVKVDDWTDDVKGDTAYDEEKVSPSGGILYKVTDDFSVYANYM
EGLSQGKIAPSTSVNEDQIFPPFVSRQVEVGAKYDLGSFAFTASVFRIKQPAYDTNTTSR
VFGPNGKRRNDGVELTMFGEPMKGFRLLGGVMYIDSELTNTVNGTFDGNRAPATPKYNVN
LGAEWDVPGVNGLTLTGRGIYTGSQYLDQANSKSIDSSERFDVGARYAFKVDQKDITLRA
NVENVMNKYYWSSAGASDDSEPGLTLSTPRTYLLSATVGF
>tr|A0A0Q0IEH3|A0A0Q0IEH3_PSEAP Protease HtpX OS=Pseudomonas syringae pv. aptata OX=83167 GN=htpX PE=3 SV=1
MMRILLFLATNLAVVLIASITLSLFGFNGFMAANGVDLNLNQLLVFCAVFGFAGSLFSLF
ISKWMAKMSTGTQIISQPRTRHEQWLLQTVEQLSRDAGIKMPEVGIFPAYEANAFATGWN
KNDALVAVSQGLLERFSPDEVKAVLAHEIGHVANGDMVTLALVQGVVNTFVMFFARIIGN
FVDKVIFKTENGQGIAYYITTIFAELVLGFLASAIVMWFSRKREFRADDAGARLAGTSAM
IGALQRLRSEQGVPVNMPDSLTAFGINAGLKKGLAGLFMSHPPLEQRIEALRRRG
>tr|A0A0Q0DS79|A0A0Q0DS79_PSEAP Uncharacterized protein OS=Pseudomonas syringae pv. aptata OX=83167 GN=ALO85_200114 PE=4 SV=1
MFLQRFHLPTVEHIHFNPHDLSVKNRLITYSPNSSRVVIEGLPQIFWEDGLPWREANLWA
MERVTNGEALLKTVSSNLNGLLNYAKFLESRRLQWFEFPSRKADRCLVQYRGALIKARDA
GHISPATASEYMRNCISFYRWVRRRGLLNPLLPLWKDKRYFIKFFDQVGFERTLAGTTTD
LGIPNRKRIGVTLEGGLLPVSAVDRGAILDFAKQNASPELYLMLALGFFTGTPPTMCG
>tr|A0A0Q0DNS6|A0A0Q0DNS6_PSEAP Secretion protein HlyD OS=Pseudomonas syringae pv. aptata OX=83167 GN=ALO85_00672 PE=3 SV=1
MLASLIGQCYSLMTFPFATLLRSCCRVSGNIRPLRSLYTQPGVVLGFISLLSLSACSAGK
PAEKALVRVTAQPVALLNVASSVTLTGDIQARKVTEQSFRVSGKLIKRYVDVGDRVRVGQ
VLARLDAREQKTELASASAEVAVRQSRLHLAEQNYQRQQVLLPKGYTNLSEYQRARSGLD
SAQGDLAALQAQQANAHDQVGYTELFAAANGVVTARYAEEGQVVQAATAIFSVAHDGERE
AVFSAYESLLRADQTGAAVTVSPLGQPEIQLSGIIREITPIVSAANGTLRVRVALQGAAA
SLALGSVVSARLQTQSQSAFALPWSALSRTQGQPAVWRVDAQARVRLTPVKVLRYEQGQV
IVSEGLSEGDRVVFRGLQFLYPGQRVEVAEQAPAEQSVSLASSGAAR
>tr|A0A0Q0IGN0|A0A0Q0IGN0_PSEAP Proteinral secretion pathway protein M OS=Pseudomonas syringae pv. aptata OX=83167 GN=ALO85_00683 PE=4 SV=1
MRRPLTSRERRGAALMILALVLCLAYWLLIDSWFAGPLRDINEQTDQLREQQQRYAGLLQ
QGDSLREQLEQARRDPASSTSLLPGEDPSAVAADLMQRIADLISSQASTGGGCELTQRMP
ITPEQDSAEPYRQVKVSLTLNCAIEPLTAILHALEYQRPFLFIDEMSMRRDANAPASGAA
GKLVVHLLVRGYLQPASAGEGEQ
>tr|A0A0N8TA83|A0A0N8TA83_PSEAP Putative methyltransferase OS=Pseudomonas syringae pv. aptata OX=83167 GN=ALO85_02581 PE=4 SV=1
MVDSMSCNRQKIADLRRQIPSFECVPGCHDCCGPVTTSPEEMSRLPRKTAAEQDAALDEL
NCVHLGPQGCTVYDERPLICRLFGTTASLPCPNGRRPVELIHPRVEKQIHEYMASTRQVL
V
>tr|A0A0Q0DC81|A0A0Q0DC81_PSEAP N-acetylmuramoyl-L-alanine amidase OS=Pseudomonas syringae pv. aptata OX=83167 GN=ALO85_04873 PE=4 SV=1
MCSLRRSMHRRQVLLNMLLASAALTLPLGAFATQIRNARLWRTNDKLRLVLDLSGPVQYK
TFTLTAPDRLIIDVSGSRLTGDFSQLALDRTVIKSIRSGHYGQGDDTRIVLDLTAPVQLN

SFLLGPEGGQGHRLVLDMSSTAHAPVQMAELPPPVPVPIPTSKAHTGRDIMVVVDAGHGG
KDPGAVGSRGEREKDVVLSIAQLLARRLKREKGFDVRLVRNDDFFVPLRKRVEFAHKSNA
DMFISVHADAAPRLTASGASVFALSEGGATSATARFMAQRENGADLLGATSLLNLKDKDP
MLAGVILDMSMNATIAASLQLGHTVLGSLEGITTLHQKRVEQAGFAVLKSPDVPSILVET
GFISNSRDSQRLVTARHQQAVADGLFDGLQRYFQRNPPVDSHMAWLQAQKQEQPV
>tr|A0A0Q0BVS2|A0A0Q0BVS2_PSEAP Regulatory protein, LysR:LysR, substrate-binding protein OS=Pseudomonas syringae pv. aptata OX=83167 GN=ALO85_01265 PE=3 SV=1
MSRIFDVDTHLWRTFKSVSETRSITRSAVALCKTPPAISMQIKKLEALLELKLFVRGDDG
FRLTVIGEEVLSAAEKILAMHDNLFRLKNKNPDSELRLGMPDDYALFFLQGIVKALSRSN
PDLKIKLVCKTSALLIDDVDQGRLDMAVVAVPESRVMEHSEYIRREELHWIGDARLIRAG
HPIPLVHFPDGCICREVSVQSLQFAGMASEIRFTSESNFAVFNAISAGVGIGVGELSLIP
GDTPIITSSLLPALPAIRMSVFSNNDRISTAVLSRISTSVVRSVNAVCERNILGKTRPEN
AFAVGAF
>tr|A0A0Q0BT00|A0A0Q0BT00_PSEAP RNA polymerase sigma factor RpoH OS=Pseudomonas syringae pv. aptata OX=83167 GN=rpoH PE=3 SV=1
MLNCTLPVRWRFFRMTTSLQPAYALVPGANLEAYVHTVNSIPLLTPEQERELAESLYYEQ
DLDAARQMVLAHLRFVVHIARSYSGYGLAQADLIQEGNVGLMKAVKRFNPEMGVRLVSFA
VHWIKAEIHEFILRNWRIVKVATTKAQRKLFFNLRSQKKRLAWLNNDEVHRVAESLGVEP
REVREMESRLTGHDMAFDPAAEADDDSAFQSPANYLEDHRYDPARQLEDSDWTDNSTANL
HQALDVLDERSRDILYQRWLAEEKATLHDLAQKYNVSAERIRQLEKSAMNKLKTSIAA
>tr|A0A0Q0FKY2|A0A0Q0FKY2_PSEAP Cyanate transport system protein OS=Pseudomonas syringae pv. aptata OX=83167 GN=ALO85_01823 PE=4 SV=1
MTEKLMTAKAQPSPQPTTDQPHPALKRPWLLLLGLVLVALNLRPALSSIAPLLDTVSDSL
GMSAAEAGLLTTLPVLCLGLFAPLAPILARRFGSERVVMVVLLTLAAGLAVRSLFGQVGL
FAGSLLAGASIGIIGVLLPGIVKRDFPHKAGAMTGVYTMALCLGAAIAAGSTVPLSQYVG
GSWQIGLGFWLVPALIAAALWFPQAREVHGTHRDVYRVRGLLRDPLAWQVTLYMGLQSSL
SYIVFGWLPSILIGRGLTPTQAGLLLSGSIIAQVISALAVPYLATRGKDQRLAIMLVMSL
TLTGLFGCLYAPLGGLWGWAILLGVGQGGTFSLALALIVLRSRDSHVAANLSSMAQGVGY
TIASTGPFAVGVVHDMTGSWSAIGWIFAVLGLGAIIAGMGAGRALQVQVSSEKV
>tr|A0A0N8T928|A0A0N8T928_PSEAP Citrate synthase OS=Pseudomonas syringae pv. aptata OX=83167 GN=ALO85_02479 PE=3 SV=1
MEKTMAEAKVLSGAGLRGQVAGQTALSTVGMAGAGLTYRGYDVRDLAAEARFEEVAYLLL
YGELPNQAELSAYMDKLKRLRDLPQALKEVLERIPADTHPMDVMRTGASMLGTLEPERSF
DQQRDSTDRLLAAFPAIMCYWYRFSHDGKRIDCTSDEDSIGGHFLHLLLDRAPSELHRKV
MDVSLILYAEHEFNASTFTARVCASTLSDLYSCVTAAIGTLRGPLHGGANEAAMEMIQRF
ASAEEATKGTLGMLERKEKIMGFGHAIYKESDPRNEVIKGWSKKLAEEVGDTVLFPVSEA
IDKVMWEQKKLFPNADFYHASAYHFMGIPTKLFTPIFVCSRLTGWAAHVFEQRANNRIIR
PSAEYIGVEQRAFVPLEQR
>tr|A0A0Q0DNB6|A0A0Q0DNB6_PSEAP NADH-quinone oxidoreductase subunit N OS=Pseudomonas syringae pv. aptata OX=83167 GN=nuoN PE=3 SV=1
MDLTIQHFIALAPLLITSLTIVVVMLAIAWRRNHSQTFLLSVAGLNLALLSIYPALKVAP
LVVTPLLHIDNFACLYMAIILASTLACVTMAHAYLGDGKVGYPGNREELYLLILLAAAGG
LVLVSAQHLAGLFIGLELLSVPVYGLVAYAFFNKRSLEGGIKYMVLSAAGSAFLLFGMAL
LYAEAGSLSFDGIGKAIAATGMPSPIASLGLGMMVVGLAFKLSLVPFHLWTPDVYEGAPA
PVAAFLATASKVAVFAVLVRLFQVSPAASSGVLHDVLAVIAVASILVGNLLALTQTNLKR
LLGYSSIAHFGYLMIALVASKGMAVEAIGVYLATYVLTSLGSFGVITMMSSPYSGRDADA
MFEYRGLFWRRPYLTAVMTLMMLSLAGIPLTAGFIGKFYIIATGVESQLWWLVGALVIGS
AIGVFYYLRVMVTMYLVDNKLPRHDAAINWAQRTGGVMLLAVAILTFVLGVYPQPLLDLV
LNAGLHVAG
>tr|A0A0N8T7S0|A0A0N8T7S0_PSEAP Methyl-accepting chemotaxis protein OS=Pseudomonas syringae pv. aptata OX=83167 GN=ALO85_04076 PE=4 SV=1
MFRLINQALGNMNVRFKLSLGFGLVLLLTLIITLTGWHGLYTMIDRSESLSDIAQLNGLT
KDLRAERITDRVQKTPESTALVTEKLNEMKAQLTLLHTQSEEEATIKLLNGQFEMLNRLE
KTFADVRDNRQTRNQIRTRLEQTSEQALQAIAQVEVEVLKSVSQEQDSSERMEEFTNISQ
LRQQVQTARYQVQTYTFTTRDADESAAIAAIDEALKEIGQIAQDQGNESLQGLTPATAAL
QSYRERLGEFKQIQTKAEADQEIMAGLGETLLDSVAALNRLQTAQRDSEAANASAMLSSV
AGLALLIGLLAAWIMTRQITVPLQQTLGAAARIAQGDLSRDISVTRRDEMGQLQGSMQTM
TVSLRELVGGISEGVSQIASAAEQLSAVTKQTCIGVTSQKDETDQVATAMNEMAATVQEV
ARNAQQASQAAAQADQQARSGDEVVGQAIIQIEQLAREVLNSTQSMSALKQESNKIVGVL

DVIKSVSQQTNLLALNAAIEAARAGEAGRGFAVVADEVRGLAQRTQKSTEEIEELIAALQ
TGTQQVATTLDNSRTLTDNTVELSRRAGGALEQITRTVATIQHMNEQIAAASEEQSVVAE
QINRNVISVRDISEQTAAASEQTAASSIELARLGTHLQTLVGKFRVG
>tr|A0A0Q0DIZ4|A0A0Q0DIZ4_PSEAP Sugar transporter family protein
OS=Pseudomonas syringae pv. aptata OX=83167 GN=ALO85_04224 PE=3 SV=1
MSSSSTSAAAGQQGRQRAASNRLIFISVLVATMGALAFGYDTGIIAGALPFMTLPADQGG
LGLDAYSEGMVTASLIVGAAFGSLASGYISDRFGRRLTLRLLSVLFIAGALGTAIAPSIP
FMIAARFVLGIAVGGGSATVPVFIAEIAGPSRRARLVSRNELMIVSGQLLAYVLSAVMAA
LLHTPGIWRYMLAVAMVPGVLLLVGTFFVPASPRWLASKGRFDEAQDVLEQLRPSTQDAQ
REIDEMKAQDEEARHRPKARELLRQRWVIKLLLIGVGLGFTAQFTGVNAFMYYTPIILKN
TGMGTNAALTATIGNGVVSVIATLLGIWAIGRYGRRHLLMTGLVIVILMQAALGCVLQFM
PQNMTQSYTALACILVFLLFMQMCISPVYWLLMSELFPMQVRGLLTGTAVSMQWLFNASV
AFTFPIAVDTIGNPTFFIFAAINIGSLIFVFLCLPETKGKSLEQIEKHLKKEL
>tr|A0A0N8T987|A0A0N8T987_PSEAP Non-ribosomal peptide synthetase
OS=Pseudomonas syringae pv. aptata OX=83167 GN=ALO85_02445 PE=3 SV=1
MNAYQLLELFGRHAVVLEVDGDKLRCKAPRGFLNDEMLQALKQHKAELIALLSGTDPAAI
PRRAAGQTAVPLSFSQRQLWFLDQMEPGNAFYNVPTAVLLKGALDVPVLERALNELIMRH
EILRTTFASVDGEPRQLVHPAMPLVMPGVDLRDLSPTARDAHVSMAVEQEAKAPFDLASG
PLLRASLLRLADEEYLWLYSVHHIIADGWSMGVILQEVTTVYGDFLRGQASSLAPLAVQY
ADYACWQQQRLSDEALAGQLDFWKRTLADAPPLLDMPADRPRPTVQRYVGATFSSTVDGT
TLRALNALARQTQGTLFNVLIGALSVLLWRHSGQRDLCIGTPFANRSRPEIEPLIGHFVN
TQVIRQRLDPQQTFAELLREVRATLLDVHAHQDVPFDRVVEAVNPQRDTAYSPLFQVMMV
LQNTPGNAAQMQGLSMTPYGTGSATAKFDLAFEWVERDGVLSLLVEYNTDLFDQTSIERL
SGHYRQLLEQVALNPKQPVGALTLISDAEREQILHAWNSPAPLAQPVDCVHRLIEAQVAR
RQAECAVIFEGRSLSYSQLNSQANRLAHHLLTLGVGPDVRVAVCIERSLELPLAVLAVLK
AGGAYVPLDPDYPSGRLRHILDDTSPVVLLAQGPTRKILREALQGADCEVPILDVQADAV
LWAECPSDNPQPQRVGVNADHLAYVLYTSGTTGLPKGAMVTHRGLSNLLLWCQQFCGEHG
SMLHKIPFGFDASAWEIFWPLLTGGRLVIARPGGHFEPGYLAQVVREQSVTAMVFVPAML
QLFLEVEEVSACLSLKDVFSGGGELSPAVARLFQERLPHARLHNVYGPTETTVISSVWTL
EPGADVPPRQLPIGRPIANTRFYVLDERDAPVPAGVTGQLHIGGVGVARGYLGQDELTAE
RFIDNPFVAGDRLYRSGDLARYRPDGQLEFLGRNDFQVKLRGIRLELSEIEARLDLFPGI
RTSVALIVGDTAQNQRLVACCVTDSTVDESALRAHLATTLSSAVMPSAFLWLDALPLTVN
GKVDRAALAALADQDLADRQVNLGSPRDHIELTLYQIWKDLLLVPQIGIRDNFFNVGGTS
IAAIKMAYEIGRAFSVEVPVRVILGHPTIEALGGWLRSGASLAAAQDNLIEFRRGAGQRN
VVCIHPAGGTAFCYLSLAKELPESIGVYGVQSPGLNPGESTEPSVEAMADAYLRRIAALT
SQPLVLTGLSFGGLVAYEMARRLTAAGHRQVTVVLLDTQGSDDPGFRQQIGTVDMAEFRD
KLVRFNGMYPGIEDAQVERYFHLYNHNRLAMAAYECAPQAGRIVLIQAREGFTRPQLHEL
RSFWRRRAGNGYKARLVHGGHWDMLESAEVHRVSQTLRQELQRFDTQEAQ
>tr|A0A0Q0BXK5|A0A0Q0BXK5_PSEAP Putative cytoplasmic protein
OS=Pseudomonas syringae pv. aptata OX=83167 GN=ALO85_03939 PE=4 SV=1
MSSEALRLPLYPQLWDQTSRLLLESANFSVRAWTYPSGVKALSLENSRGKLVILPWQGQM
IWSAEFDGVDLTMLNMFTQPRPSASVIGTYGCFMFHSGLLRNGCPGPEDDHALHGEMPCA
PMDDAWLQAGEDEHGAYLRLGGTYEYVQGFGDHYRASPCVTLRPASGLFEIGMQVVNLAG
KPMDLMYMAHMNYAYVDGARLTQPLGCERTRVRASVPAHVRPTPAWNAYIAELSQDPTRL
EVLDSPALYDPEIVCFFDDVRGDAQGQAHFFLDHPDGAAFYTRYSPRQFEHAARWILHNT
DQQVAAFILPATCEPEGYRAELAKGNVRSLAPGASAEFSVTTGYLNAPERRALQP
>tr|A0A0Q0DV20|A0A0Q0DV20_PSEAP Integrating conjugative element membrane
protein, family OS=Pseudomonas syringae pv. aptata OX=83167
GN=ALO85_100130 PE=4 SV=1
MADVADRAKQQQDHEQSFVGSLFTLPFRFAAVMFLSLGGAIIVEWICMYFFWPEAGWQHA
RAMLDHELSWLSKGFLQSVVVQEPGRTATWLVQTTYDWLVVKTGLQDWVQHTSDYAATGQ
RSRGFDMRYMLGVGVSKFQDYGLAALYTTLVFCVRLVILTLAIPLFVMTAFVGFVDGLVR
RDLRRFGAGRESSYLYHKARATMVPLVIIPWSLYLALPFSVSPLLVLLPCAALLGLAVSI
TASSFKKYL
>tr|A0A0Q0FLE2|A0A0Q0FLE2_PSEAP Uncharacterized protein OS=Pseudomonas
syringae pv. aptata OX=83167 GN=ALO85_05538 PE=4 SV=1
MQPTFGCEVFCMTAFYLKLLITPALMLAISLAARRWGTGVAGLLSGLPMTSALVMLFLSL
EQGTQFASMAVPGALAGLAAIQATYLFYFLVTRRVSALTGCILALAVYGATAFLMNLLGL
LALSIIFTLLMVALIIVATSKQTPPDVASYVALPRWVIPMRMLTATLLLLAITASATWLG
PVVSGLLAPIPVIAWPLAVFVHVQGGRYELAAIIRGNAIGAVGVVSFYLTLRYSILQWGA

LLSIAAAVLLAVVVTAGLARLLAKR
>tr|A0A0Q0DLQ2|A0A0Q0DLQ2_PSEAP Zinc metalloprotease OS=Pseudomonas syringae pv. aptata OX=83167 GN=ALO85_00300 PE=3 SV=1
MSALYMILGTLIALGVLVTFHEFGHFWVARRCGVKVLRFSVGFGTPLLRWHDRQGTEYVV
AAIPLGGYVKMLDEREGNVPPELADQSFNRKTVGQRIAIVIAGPTANFLLAIAFFWVLAM
MGSEQVRPVIGAVESGSIAQQAGLTAGQEIVAVDGEPTSGWSGVNLQLVRRLGESGTIAF
KVRDQGSTVDTSRELVLNDWLRGAEEPDPIKSLGIRPWRPALLPVLAEIDPKGPAQSAGL
KSGDRLISMDGQPLEEWQQVVDRVRERPEAKISLRIERDGVQMDVPVTLAAKGEGKAAAG
YLGAGVKAVDWPPEMLREVSYGPFAAMTEGVKRTWNMSVLTLDSLKKMLFGELSVKNLSG
PITIAKVAGASAQSGIGDFLNFLAYLSISLGVLNLLPIPVLDGGHLLFYLIEWARGRPLS
EKVQGWGAQIGISLVVGVMLLALVNDLGRL
>tr|A0A0Q0DLE7|A0A0Q0DLE7_PSEAP Uncharacterized protein OS=Pseudomonas syringae pv. aptata OX=83167 GN=ALO85_04713 PE=4 SV=1
MPRGITAPQGGSPPVLEDLMPNRTQLFHIEECPDLYVDACVCDEQRNLIFLSAWGRDTAM
QEFLARLTLGTTENGLDQFHIVMNDQRIPVFPDTDLLEKRTTRQLRGTLFGSLLHLWLFD
QRCSQPDRANHSAYALINQAQDPFDRLWPLIVDTCPLPFLPHWREPVMEVLTAHNMLHPL
PGAIGSVTAWRLSLQLDVLEKALGELIRAGKLTTEVTA
>tr|A0A0Q0CGC6|A0A0Q0CGC6_PSEAP Phosphoribosylglycinamide formyltransferase OS=Pseudomonas syringae pv. aptata OX=83167 GN=purN PE=3 SV=1
MPAICDVVVLLSGTGGNLQAMIDSFKDGASPVRIRAVISNRADAFGLQRARDAGIETCVL
DHTAYEGRKAFDAALIERIDAFQPQLVVLAGFMRILSAGFVRHYHGRLLNIHPSLLPRYK
GLHTHKRALEAGDTEHGCSVHFVTEELDGGPLVVQAVISVQLHDTPATLAQRVHVQEHRI
YPLAIRWFAEGRLSLGEQGALLDSQLLPASGHLIRH
>tr|A0A0Q0BWZ6|A0A0Q0BWZ6_PSEAP Polyphosphate kinase OS=Pseudomonas syringae pv. aptata OX=83167 GN=ppk PE=3 SV=1
MNTEALIEAAVQVDVPEATPVVEPDIEVIPAIEAPAAAVPAIVAPNLDDSSLYIHRELSQ
LQFNIRVLEQALDESYPLLERLKFLLIFSSNLDEFFEIRVAGLKKQITFAREQAGADGLQ
PHQALARISELVHGHVDRQYAILNDILLPELEKHQVRFIRRRHWTAKLKAWVRRYFRDEI
APIITPIGLDPTHPFPLLVNKSLNFIVELEGIDAFGRDSGLAIIPAPRLLPRVIKVPEDV
CGPGDNFVFLSSMIHAHADDLFQGMKVKGCYQFRLTRNADLALDSEDVEDLARALRGELF
SRRYGDAVRLEVADTCPKHLSDYLLKQFNLNESELYQVNGPVNLTRLFSITGLDSHPELQ
YPPFTPAIPKLLQNSENVFSVVSKQDILLLHPFESFTPVVDLLRQAAKDPHVLAVRQTLY
RSGANSEIVDALVDAARNGKEVTAVIELRARFDEESNLQLASRLQAAGAVVIYGVVGFKT
HAKMMLILRREAGEIVRYAHLGTGNYHAGNARLYTDYSLLTSDDALCEDVGKLFSQLIGM
GKTLRMKKLLHAPFTLKKGMLDMIARETQFALDGKPAHIIAKFNSLTDPKIIRALYKASQ
SGVRIDLVVRGMCCLRPGIAGVSHNIHVRSIIGRFLEHTRVFYFLNGGDEQMFLSSADWM
ERNLDKRVETCFPVEGKKLILRVKKELESYLTDNTHSWLLQSDGRYVRSTPTGNQNPRSA
QATLLERLSNPVLSVR
>tr|A0A0Q0ICA9|A0A0Q0ICA9_PSEAP Chromosome partition protein Smc OS=Pseudomonas syringae pv. aptata OX=83167 GN=smc PE=3 SV=1
MRLKCIKLAGFKSFVDPTTVNFPSNMAAVVGPNGCGKSNIIDAVRWVMGESSAKNLRGES
MTDVIFNGSTSRKPVSQASIELVFDNSDGTLVGEYAAYAEISIRRKVTRDSQNSYYLNGT
KCRRRDITDIFLGTGLGPRSYSIIEQGMISKLIEAKPEDLRNFIEEAAGISKYKERRRET
ENRIRRTHENLARLTDLREELERQLERLHRQAQAAEKYQEYKAEERQLKAQLSALRWQAL
NDLVGQREAVIGNQEVGFEALVADQRSADASIERLRDGHHDLSERFNLVQGRFYSVGGDI
ARVEQSIQHGQQRLRQLQDDLREAERARQETESHLGHDTTLLATLGEELEMLEPEQEMTS
AAAEESAIALEDAEAAMQGWQEKWDVFNQQSAEPQRQAQVQQSRIQQLEQSIERLAERQR
RLAEERQLLAADPEDAAILQLSEDLATRDMTLEELHMGEEQAVERLEQLREALQHASQAQ
QQAQGELQRLNGRLASLEALQQAALDPDTGTAEWLRDQQLAERPRLAEGLSVEAGWELAV
ETVLGADLQAVLVDDFDGLDLAGFQQGDLRLLSAGADTVRAPGSLLEKVDSTVDLSAWLS
QVIPVENLDEALARRAQLSAGQSLISRDGYWVGRHFLRVRRASEAQSGVLARGQELQRLS
LERDEREATLATLEEQLLVLREQQSQEEDAREQLRRRVQDETRQQSELKAQLSAVRVKVE
QLTLRRTRLDEELAELGEQRAVEHEQLGESRLQLQGALDSMAQDTEQREVLQAQRDALRE
RLDRIRQDARQHKDHSHQLAVRLGSIKAQYDSTRQALERLRMQSERLTEKREQLSLNLEE
GEAPLEELRLKLEELLERRMVVDDEMRIAKNALEDADRELREAEKRRTQAEQQSQLLRGQ
LEQQRLEWQALTVRRKALQDQLHEDGYDLHGVLATLTPDASEQAAEQQLESIAGRIQRLG
AINLAAIDEYQQQSERKRYLDAQDADLVEALDTLENVIRKIDKETRNRFKDTFDQINSGI
QALFPKVFGGGSAYLELTGEDLLDTGVTIMARPPGKKNSTIHLLSGGEKALTALALVFAI
FKLNPAPFCMLDEVDAPLDDANVGRYARLVKEMSQTVQFIYITHNKIAMEMADQLMGVTM

HEPGCSRLVAVDVEQAMALVDA
>tr|A0A0Q0E149|A0A0Q0E149_PSEAP Two-component DNA-binding response regulator OS=Pseudomonas syringae pv. aptata OX=83167 GN=ALO85_01463 PE=4 SV=1
MSTAATASPTEEPIIYVLDDDLSVRSSLEDLLASVGLRSMLFGSTREFLDTPRPDAPGCL
ILDIRMPGMSGLDFQEHMARSGISLPVIFITGHGDIPMSVRAMKAGAVEFLTKPFRDQDL
LDAIQQGLAQDRSRRQSAAVEAELQRRHASLNLGEQQVMELVVSGLLNKQIAARLNVSEI
TVKVRRGSVMRKMEADSLADLVKFAERLKELH
>tr|A0A0Q0D8G8|A0A0Q0D8G8_PSEAP Uncharacterized protein OS=Pseudomonas syringae pv. aptata OX=83167 GN=ALO85_01907 PE=4 SV=1
MHIMLRIRGTVGQWPVDLTLELDEGDWAQLGAQLKGVAPEAPAAATENKPLNQDDRLWQV
AQELLHKAEQMSGPDLLGQLEGLAGSTAAGKHLLVRLRHSANVKVESGGDAPLYSWVEAT
>tr|A0A0Q0DDT3|A0A0Q0DDT3_PSEAP LPS-assembly protein LptD OS=Pseudomonas syringae pv. aptata OX=83167 GN=lptD PE=3 SV=1
MIRNLFQPIERRAAPPRADGAQEARTNKMALKSPAFRKKFPLLVTGSLLAMQPLATQFVV
AAEQYDCSVSASGAWNCAPKSNAAAVDLPPRPVHDTTSVSSNGTVTSQGTSSGEQSAGTQ
LVTEAKGKGLKSRSADYSHLDWVPREKLTAAQLAETGPYCSGAYVEPIRPGMDDKTPMKD
APMFVGAKASRYEQEAQVATLAGDVVMRQGSMQVQAQEAALHQAENRGELNGDVRLRDNG
ALIVGDKAELQLDTGEARVDNAEYVLHKSNIRGNALYAKRAENAIIRLKDGTYTTCEPNS
NAWTLKGNNITLNPATGFGTATNVTLRVKDIPVLYTPYIYFPIDDRRQSGFLPPTIAAGG
DNGFTLVTPYYFNLAPNYDATLYPRYMADRGLLMEGEFRYLTKGSEGQFGGAYLNDENDD
RKQQSDYDKTRWMINWQHKGGLDTRWLTQVDYTDISDPYYFQDLETDQIGVKRTDYLNQQ
GSLTYRGDSFAAVLNAQAYKLATVANVTPYNRLPQLTLNGTLPYNPGGLKFDYQTEAVRF
ERDLRSGAFIDEDGNSETRLDNNISGLARANGDRLNLAPSVSLPMNWTYGFLTPKLKYVY
TQYDLDLDSQGKNTLLAGEEYSSSQSRSVPIFSVDSGLYFDRNTNWFGKDYRQTLEPRLF
YLYVPEKDQTDIPVFDTSESTFNFSSLFRDNRFTGSDRIGDENKLSLGVTNRWIEDNGFE
RQRFSIGQALYFEDRKVQLPGVAFADRDDARSKVSPYALEYEYRFNRDWRFNSDFNWDPD
SRSTRSGSAMFHYQPEDNPNKIVNLGYRYRNDQIRYDESTGRWVVGGGDYGTPGSPNYVK
DYYKIQQHDFSVIWPIVPQWSLISRWQYDYNRERTIEAFGGFEYDNCCWKMRLVNRYWID
YDEFSQAAPQNEKGDRGIFLQIVLKGLGGVTGAKVDSFLDKGIQGYREREDQAF
>tr|A0A0Q0IFJ8|A0A0Q0IFJ8_PSEAP LysR family transcriptional regulator OS=Pseudomonas syringae pv. aptata OX=83167 GN=ALO85_00729 PE=3 SV=1
MDRIQEMTLFAALAEQTSFAGVARHFGLSTATVTRAVASLESRLGILLVVRTTRNMRLTE
AGQRFAADCRRLLADLDEAESAAGGLHALPGGLLTVTAPQMFGALHVVPVMTSFLERFPA
VDIRAILVDRVVSMLDEGVDVAVRIGTLPDSSLTAIPTGSVRRMVCASPAYLEKHGAPQH
PDDLRQHSTVSTTTAERSPHWLFRIDDKNYSVDVASRLSLTSYQAAISAALQGWGLTQVP
YYQIREHLQEGRLKCVLETFEIIPEPVHVVYLEGRRRSSKVRAFVDFCVSALRHDLQFEQ
Q
>tr|A0A0Q0FMV6|A0A0Q0FMV6_PSEAP Secretion protein HlyD OS=Pseudomonas syringae pv. aptata OX=83167 GN=ALO85_00926 PE=3 SV=1
MRKIHVYGLLALAALSVSGWILFGRAEPTPAAVAAPATSLTVEAVQPHREDWPQELVASG
ALAPWQEAVISAETGSLRIASLKADIGDQVKKGQVLATLADDSVLAEENKQKSAVAQATA
QLQEARSNARRAAVVGQSGALSEQQLEEYRVKVQTAEANLASANADLRSIRIKLKQTRIV
AVDDGIISGRKALLGDVVSAGSEMFRMIRDGRIEWQAELDAQQLPGVKPGQLARVMLPGG
IEVQGRVRLVSPVLDGKTSRALVYVSLPVGAVARAGMYASGRIELPSSPALTVPDTSVIL
RDGHSYVFVLGKDMRVSQHVVEIGRRRGSALEIVGGLAEQAQIVRSGGAFLNDGASVTLV
NAEARAQ
>tr|A0A0N8T8A4|A0A0N8T8A4_PSEAP Syringomycin synthetase E (Fragment) OS=Pseudomonas syringae pv. aptata OX=83167 GN=ALO85_04336 PE=4 SV=1
AQVRTQPGAIAVAFQGQRLSYAELNRQANRLAHHLIGLGIGPDDRVALCVERGVEMMVGL
LGVLKAGAAYVPLDPAYPAERLAYMIEDSTPSALLTQRDLQERLPALNLPLVLLDDDQRQ
AFTACDDNPVVPNLGVRNLAYVIYTSGSTGNPKGVMIEHRGLVNYSVDAARLFALSPTDT
VLQQNTL
>tr|A0A0Q0BUH9|A0A0Q0BUH9_PSEAP Histidine kinase, HAMP region: chemotaxis sensory transducer OS=Pseudomonas syringae pv. aptata OX=83167 GN=ALO85_04740 PE=4 SV=1
MLENLMSSWFANISVNMKLALGFGLVLVFTAILALTGWTSMGGLINRSNWMSDITSLNAQ
LTKLRVTRLQYMVADGDEKVAETVQTSLDSFKAYQEKLRASFKSPENLKMLDQLGIVIAD
YQKSLNNMRSGYKASTAARDELTTHASKSLAVFEQLVTEVRNMDPADANRFEQYRLVTDA
KDDLRVARYEVRGYTTNATPETEQAAVSKLDSAIKDLDALKTTFSGTQADQLRQLETSLM

AYRTTLQNFKAATGTIVQARKEMTTQGQDIVKISEDMYKLQLDRRDQESAQARTTQITCT
LLAMILGIIAAVIITRQITRPLQETLAVVDRIASGDLTQTLAVTRRDELGVLQQGIQRMG
TTLRELIGGIRDSVVQIASAAEELSAVTEQTSAGVNSQKVETDQVATAMHEMSATVAEVA
RNAEQASQAASNADREARDGDKVVGEAIAQIERLANEVGRSADAMTQLEQESDKIGKVMD
VIKAVAEQTNLLALNAAIEAARAGEAGRGFAVVADEVRGLAQRTQQSTVEIETLVAALQS
GTRQVSSIMLNSRELTVSSVTLSRKAGTSLGSITQTVSSIQAMNQQIAAAAEEQSAVAEE
ISRSIVNVRDVSEQTASASEETAASSVELARLGGQLQTMVSHFRI
>tr|A0A0Q0IL91|A0A0Q0IL91_PSEAP Virulence factor MVIN-like protein
OS=Pseudomonas syringae pv. aptata OX=83167 GN=ALO85_05194 PE=4 SV=1
MLGSTLWLTLATLTGLAAGFAREWLLVASWGAGSQSDAFLVSMFLPEALRMSLAAGLLSA
AALPLYQQRTAERQQRWLGGMAPRLLLTGLAVSVILLLSAGGLVRLIGPGLDADGYAQAA
SGLRWLAWCAPGFMLHALFCVPLQARSRFVLAGLGSLLFNLPPVIYLATFSHASTPTGLA
SACVLGSVLMPGVLLPALYRSGWRPWQWQSEAGAMRELLQRIGPLLSSNLASQGLALLER
MVASLLGEGAVTWVNLARKLINLPLIALMSLNQVLLGLMSGSIGDQRLSLLKRGLGSATL
LTLPAVAGLIGAAGALVTLLLPNQTQGGPLPELLAWFAVPLMFGAWNALLARYAYAAGDT
RLPLNCELVGSLCNALLLGALPFAFGLTGIALAALGGALVTGVLLMRRQSLLHVLPWRSH
WLLASLLMAIAALLLHPLHDTWLQLGLSCVYGALLLVGLALWLKPWRNVSA
>tr|A0A0Q0IT49|A0A0Q0IT49_PSEAP Periplasmic binding protein
OS=Pseudomonas syringae pv. aptata OX=83167 GN=ALO85_01477 PE=4 SV=1
MNCLSARLALAALLCSPMAHAAYPVTVQSCDRSVTFTAAPQRAVSNDVNLTKMMVALGLQ
SHMVGYSGITGWNKPDQALLRDLGKLPELSSKYPSLETLLNANADFYFAGWNYGMRVGGD
VTPQSLAPLGIQAYELTESCAQIMPRTEATLEDVYNDLLNLGRIFDVQTRAETLVAQMRR
RVSDVEKRVAGKSPPRVFLYDSGEDRPMTAGRLAIPQALISAAGGRNVMGDVAASWTHVN
WESVVQSNPEVIVIVDYGEVSAAQKQHFLESNPALQSVDAIRNRRYVVLPYVAVTPGIDN
VTAIETLAAAFHDVKR
>tr|A0A0Q0CAN0|A0A0Q0CAN0_PSEAP Camphor resistance protein CrcB
OS=Pseudomonas syringae pv. aptata OX=83167 GN=ALO85_200151 PE=4 SV=1
MRPTDAIRGKEGEIRRLIETYGFIEKVYYALCGPQLSS
>tr|A0A0N8T893|A0A0N8T893_PSEAP NAD-dependent aldehyde dehydrogenase
OS=Pseudomonas syringae pv. aptata OX=83167 GN=ALO85_03098 PE=3 SV=1
MRYAHPGTEGAIVNFKERYGNYIGGEFVAPVKGQYFTNTSPVTGKAIAEFPRSTAEDIDK
ALDAAHAAAAAWGSTSVQARSLALLKIADRIEANLEVLAITETWDNGKAIRETLNADIPL
AADQFRYFAGVLRAQEGSAAEIDGNTVAYHIHEPLGVVGQIIPWNFPILMAAWKLAPALA
AGNCIVLKPAEQTPLGISVLIELIGDLLPPGVLNIVQGYGREAGEALASSKRIAKIAFTG
STPVGSHIMKLAADNIIPSTVELGGKSPNIFFEDIMSAEPEFIDKAAEGLVLAFFNQGEV
CTCPSRALVQESIYPQFMEAVLRKVEKIKRGDPLDTDTMVGAQASEQQFDKILSYLDIAK
GEGAELLTGGKVEKLEGSLSTGYYIQPTLLKGNNKMRVFQEEIFGPVVSVTTFKDEAEAV
AIANDTEFGLGAGLWTRDINRAYRVGRAIKAGRVWTNCYHLYPAHAAFGGYKKSGVGRET
HKIALEHYQQTKNLLVSYDTNPLGFF
>tr|A0A0Q0C934|A0A0Q0C934_PSEAP Putative sugar diacid recognition
OS=Pseudomonas syringae pv. aptata OX=83167 GN=ALO85_00659 PE=4 SV=1
MFALDRALAQDIVDRAMAILPYNVNVMDYLGIIIGSGDAERLCTRHEGAQRVLANRQVVE
IDSLAASQLGGVKPGVNLPLMLDHQLVGVLGITGEPDEVRVYGELVKMTAEMLMEQRRQQ
ADLQWRAQRSEDLLARLLLPECPDSLVDEARQLGLQPHMARQVVLLQFAEEGLAASQAAK
WLGTRYADSWCVLREPILLYWCRATGKERDDALLLKQFDEHHWPVQRMATVAQSADLPTL
RQACAAARDLLDYASQARPAQRLLLRLEAYRLPVLFWRYRHDWLAEHVAEPLERLHTHTQL
LDTLCKWFEYSGESQACAEALGIHRNSLRYRLEKIGELTGCDPYKTDDLLRLYLGAQMIT
RQT
>tr|A0A0Q0DDV2|A0A0Q0DDV2_PSEAP Sensor y box histidine kinase
OS=Pseudomonas syringae pv. aptata OX=83167 GN=ALO85_02988 PE=4 SV=1
MIRLLRTGFIGCLALLLMAQMANTQADEPETPAVAQLSSAQRNWLDQHGPLRVGLVLRAP
YAQFDQRLQQLSGANVDLMNALAATLPVELLWRNFPDQSALESALAKGEIDVAPGLMQTP
AGLRLWLFSDPYLRVSQLLIGERDGSTAVDLEKLDSLSRVAVRMPSTTADYLRSNFAHLN
LQGVPLERQALQLLLSQQARYAVVDEAQLSRLLREPEFAGLAVVGDIGLPQLLRVASRRD
MPELAAIVSEALRAIPAKELEQLHARWMPLTPSHFIESTKLWKNLCILLLVMLLACFAIV
IWQRRQQQALEQELLAAREELARRVEEEEALRLAQFSIDQSTVGILWVNWDSRVRYANRA
AESMLGYASGQVVERPLIDFEPGLHMDRWLNLWKNARSSEDSPQVFETHCLRADGSLLPV
DVSLSFLRFRDAEYLVVFLTDVSERRRAHEQLRDLSAHLESVREEEKAHIAREVHDELGQ
MLTVLKLETSMCELAYADLDPGLSERLESMKRLIAQLFQLVRDVATALRPPILDAGIASA
IEWQARRFEARTQIPCLVQVPDNLPTLSDARATGMFRILQEALTNVMRHAKAHTVEISLT

LQDGMMCMSIADDGQGFVIESGRAVSFGLVGMRERVLMLGGRLELDSEVGEGTTLRAYIP
LDTLAQERKQ
>tr|A0A0Q0FAR1|A0A0Q0FAR1_PSEAP RelA/SpoT protein OS=Pseudomonas syringae pv. aptata OX=83167 GN=ALO85_05554 PE=3 SV=1
MARGSALPSIDALADRLSAYLGKDQVNLVRRAYFYAEQAHDGQRRRSGEAYVTHPLAVAN
ILADMHMDHQSLMAAMLHDVIEDTGIAKEALSAQFGETVAELVDGVSKLTQMNFETKAEA
QAENFQKMAMAMARDIRVILVKLADRLHNMRTLEVLSGEKRRRIAKETLEIYAPIANRLG
MHSVRIEFEDLGFKAMYPMRSSRINQAVKRARGNRKELVNKIEESLSHCLAVDGIEGDVS
GRQKHLYGIYKKMRGKRRAFNEIMDVYAFRIIVDKVDTCYRVLGAVHNLYKPLPGRFKDY
IAIPKANGYQSLHTTLFGMHGVPIEIQIRTREMEEMANNGIAAHWLYKSSDDEQPKGTHA
RARQWVKGVLEMQQRAGNSLEFIESVKIDLFPDEVYVFTPKGRIMELPKGSTAVDFAYAV
HTDVGNSCIACRINRRLAPLSEPLQSGSTVEIVSAPGARPNPAWLNFVVTGKARTHIRHA
LKLQRRSESINLGERLLNKVLNGFECNLDKIGQERIQLVLNEYRVEVLEDLLEDIGLGNR
MAYVVARRLLGEGDQLPSPEGPLAIRGTEGLVLSYAKCCTPIPGDPIVGHLSAGKGMVVH
LDNCRNISEIRHNPEKCIQLSWAKDVTGEFNVELRVELEHQRGLIALLASSVNAADGNIE
KISMDERDGRISVVQLVVSVHDRVHLARVIKKLRALTGVTRITRMRA
>tr|A0A0Q0BY07|A0A0Q0BY07_PSEAP tRNA pseudouridine synthase A OS=Pseudomonas syringae pv. aptata OX=83167 GN=truA PE=3 SV=1
MANIDNPAAEMAAEGFSRIALGVEYKGSRYCGWQRQASGVLTVQETLEDALSKVAASPVS
LMCAGRTDAGVHACGQVVHFDTQAERTLKAWVMGANINLPHDVSVTWARVMPATFHARFK
AIARRYRYVIYNDQIRPAHLNQEITWNHRPLDVERMAQAAEYLVGTHDFSAFRAGQCQAK
SPVKQLHHLRVTRHGKMIVIDVRANAFLHHMVRNIAGVLMTIGTGERPVEWAREVLESRI
RRTGGVTAHPYGLYLVGVEYRDEFPLPERYIGPHFLTGFAELDG
>tr|A0A0Q0DHR4|A0A0Q0DHR4_PSEAP Uncharacterized protein OS=Pseudomonas syringae pv. aptata OX=83167 GN=ALO85_00403 PE=4 SV=1
MSSMRDSLRQQWFDLRERLMGQVDSHSSVSLRLPGGQSMWLGKLDDLAPQVVDCNDSAAD
DGQTHAAIYRARADVGAVLLGGGAFAKSLVDFGGVLPILFDEQARHIGHMATPASSEQPL
ARLLKRGGNAAVIDSTPVVMGTTGARMVLNAELFEKCAKAYTLAKACGTHLTLLPWWVVL
VANGRLMKDEKRAAQCFAEGRIPPETRGY
>tr|A0A0Q0DTF8|A0A0Q0DTF8_PSEAP FAD dependent oxidoreductase OS=Pseudomonas syringae pv. aptata OX=83167 GN=ALO85_02582 PE=4 SV=1
MNARVQHPPRHDQHVASYYAASSHAQADHPALQGELKVDVCVVGGGFSGLNTAIELAERG
FSVALLEARKIGWGASGRNGGQLIRGVGHGLEQFANVIGSEGVRQLKLLGIEAVEIVRQR
IERHAIDCDLKWGYCDLANKPRDLHSLAEDAEALRSLGYRHALQLLQPEEMHSVVGSSRY
VGGLIDMGSGHLHPLNLALGEAAVAQSLGVRLFEHSAVTKIDYGPQVRVQTAAGSVQAQT
LVLGCNAYLYDLNPEISGKVLPAGSYIIATEPLSEAQAREVLPQDMAVCDQRVTVDYYRL
SADRRLLFGGACHYSGRDPQDIAAYMRPKMLDVFPQLAAVKIDYQWGGMIGIGANRLPQI
GRLKQHPNVFYAQAYSGHGLNATHLAGRLLAEAIAGQHSDGFELFAKVPHMTFPGGKHLR
SPLLALGMLWHRLKELR
>tr|A0A0Q0D6F6|A0A0Q0D6F6_PSEAP Putative type VI secretion system effector, VgrG family OS=Pseudomonas syringae pv. aptata OX=83167 GN=ALO85_05097 PE=4 SV=1
MSTLARNERLVLYNGATMLKDLTALFAPQNRRLIKLTTVARDEQELLLERFSGTESLSEL
FSFELSMISRDAGLELKSQIGQPAQLAIELANGESRYINGYISAFSLEGSDGGLARYSAT
LSPWLWMLSRRVDSRIFQEQTIEAVIRTVFAAYGALPDFEFQLSQPLKTHSYITQYRESD
LTFVLRLLEHEGLFFYFDHNQEKHTLIILDHSRDLSPLPQQPTIRYHSASVTETADSITE
WRSHRRLQSGRMSIQTFDYKQPRNSLPVGMPSLNEQGNVDSYEVYDVLDHYSHGTFNDGE
RLVRQRLEAIEVQGKTFTGNSNCRAMYPGHTFELTQHFDHDRGSAEDRSFLLITVKHEGS
NNYLSDEGAGYTNEFVCIRHKIPYRHPITVPRPSIAGPLSAIVVGPEGEEVFTDELARIQ
VRFHWQRGDSLPQGTTWLRVAMPSAGSGFGHQFMPRIGQEVLVTFLAGDIDRPLVTSVLY
NNINLPPRFSKASGLPGNRTLSGIRTQEHKGSGFNELLFDDTPGSLRARMGTTHQATALN
LGKLTDPRTDGTAQPRGNGAELRTDAAIALRAAQGMLLTTYARTDAKGSQLDREELLKLL
AECGELFKSLGETAAARGSQAVDVKGIEALRQSLDQWPAPDNNGLGDPVLAMTAAAGIAS
ATPRSQAHYAGENHDTTAQDNLQLTSGAAMHLQAGKGLSAFAQDAGISAIANRGKVLVQA
QEDDIALNAQKNLHLSAVEGEVVITAPTIRLVADDGSYIKIGGGVEIGSQGKVTVHASEH
DWIGPKTESAAIPSFGRDPAAQQVTFHYPGHSEQSPRAAADHSYEIKLEDGSLVKGMTNA
DGLTERVEREMMHQAQVSALRSGTPKGGAQ
>tr|A0A0Q0DGL3|A0A0Q0DGL3_PSEAP Flagellar hook-length control protein OS=Pseudomonas syringae pv. aptata OX=83167 GN=ALO85_02172 PE=4 SV=1
MPLATNALLQATTSVTARSGVANNAVKSADSSKDGASSFSNVYAKQAKDTAPAREDAPVK

PARDKTAPDKDKVAAGKDKPASDQANAADKSSVADSGNDLPAKPAAADDDAGHSDAKQDD
AQASADPALTEGQAVVDPALDPALQAMVAQAPGAPVAKVPPSSEVVTAPVVSALTTTQAA
PPAVVEDAFNPDADPLDGLDAVQLALENATAKTQLAAQNAQAANKATPSNAEADPNQNIV
NNLSVLSEQLPSEGSSTESSDKSFSGLIGEGLKDVKGAAGDTRVDNFADRLAALSQAAQP
VRAAAAPAAPLMNQPLAMHQSGWTEGIVDRVMYLSSQNLKTADIKLEPAELGRLDIRINM
APEQQTQVTFMSAHLGVRDALESQMSKLRESFVQQGLGNVDVNVSDQSQQQAQQQAQEQA
SRAQRSGRGGGRSSGDSSDEIAGVDAAIPVSQPAARVIGTSEIDYYA
>tr|A0A0N8TA32|A0A0N8TA32_PSEAP UmuC domain-containing protein
OS=Pseudomonas syringae pv. aptata OX=83167 GN=ALO85_200138 PE=4 SV=1
MSNKPTQVFGLVDCNSFYASCERVFRPDLAKVPIVVLSNNDGCVIARSYDAKPYVKMGAP
YFQIKDVLRRHGIQSFSSNFALYGDLSQRVMAIIESMVPAVEVYSIDKNKLHTVDVQS
>tr|A0A0Q0DAL7|A0A0Q0DAL7_PSEAP ABC transporter ATP-binding/permease
OS=Pseudomonas syringae pv. aptata OX=83167 GN=ALO85_03335 PE=4 SV=1
MPVFKRFISFMHQALRAMHLVWTTSRALSIGLIVATLIAGLLPALAAWLGQRIVDAVVAA
MQLHASAGEAPLWPVLRYVLFEAGVLALLAAAQRGLSVQQALLRVLLGQKVNTLILEKAQ
TLSLSQFEDSEFYDKLVRVRREASTRPLALVTKSLGLLQNLISLISFAVLLVHFSPWALL
ILVLGALPVFFAEAHFSGDAFRLFTRRAPETRRQNYIETLLSHEGYIKEVKLFGFAPLLL
KRYRDTFERLYAEDRRLTVRRDGWGFLLGLLGTAAFYLAYAWVVVDAVHGRITLGQMTMY
LVLFKQGQAAVSSSLSAISGLYEDGLYLADLYVYLGQPVTPSAGSLAVGAQPGDGMRFEN
VSFTYPGTSRPALENIDLHLAPGRSVALVGENGSGKTTLIKLLTRLYQPDQGRILLDGSD
LQEWDEDALRSRIGVIFQDFIRYQFQVGENLGVGDTQAFHDEGRWREAAAQGMAAQFIEQ
LDKGYATQLGRWFAGGQELSGGQWQKIALSRAYMRRDADILILDEPTAALDAGAEAAVFE
HFREYAKGRMTLLISHRFSSVRNAEHIVVLEHGRVLERGDHDSLIVAAGRYAALFDLQAQ
GYR
>tr|A0A0N8T8J6|A0A0N8T8J6_PSEAP Na+/solute symporter OS=Pseudomonas
syringae pv. aptata OX=83167 GN=ALO85_03220 PE=3 SV=1
MVAGLTLAGASAVSHDLYASVIKAGKANEKDEIRISKITTIALAVVAIFLGIAFESQNIA
FMVGLAFSVAASCNFPILLLSMYWKNLTTRGAMIGGWMGLISAVVLMVLGPTIWVQILHH
EKAIFPYEYPALFSIAIAFVGIWFFSITDKSKAAEGERALFYPQFVRSQTGLGASGAVSH
>tr|A0A0Q0C4U6|A0A0Q0C4U6_PSEAP Nitroreductase OS=Pseudomonas syringae
pv. aptata OX=83167 GN=ALO85_03802 PE=4 SV=1
MLDHFSRRQSMQALDVLLNRVSVPRLVDPAPDAAQRDILFGAALRAPDHGQLKPYRFLTV
EGAARERMGEMLAQALQESGVEVTPQALEKARLGPLRAPLVVVVIARLQDHFKVPHSEQR
ITAGCAAHGVLLAAYALGIGAVWRTGELSYAPQVAKGFGLEAGEEVLGFLYLGTPLNPPR
EAPKVDVGGFVSEWQG
>tr|A0A0Q0DBQ9|A0A0Q0DBQ9_PSEAP Uncharacterized protein OS=Pseudomonas
syringae pv. aptata OX=83167 GN=ALO85_100876 PE=4 SV=1
MLLKPTSRNASLKLPSTNAAPEGTQSNPVQLEYKECVMSKVYPIEDAVPLPLDEDEKKPE
KKVLTKDEKIEKEKRELLSSVAAADFSSLKPRVAYVLNLYPHTRNSDISLSLKYWEIFQP
DLYKESGILPKDLFKLERLHYIVRARAKIQNEYELFLADTEIRKSRRRNEEKMEGAVIDD
SPPVKKVHIFADETGKTHKFVMVASVWVLNGRAVFSTSKAISEWKAKSVWKNREIHFTKF
GKSDYETLKSYLGIILENREYLGFKYIAVERAKTNRPIEETILKLHELMLARGATHELES
GRIDLPREMEVTVDEEQSLDSFVISDLRDRVGETFKKRYDDKLQLTSLQTASSKNSALIQ
LSDVIAGAIGRILNHEGERNFKDDMADLVVQMLDLKIEEGDIDGLDSAARFNV
>tr|A0A0Q0DZA5|A0A0Q0DZA5_PSEAP Uncharacterized protein OS=Pseudomonas
syringae pv. aptata OX=83167 GN=ALO85_03339 PE=4 SV=1
MPLDSRTSMCIALFLLGAAITASFLASSGPVQWMDNGMFLANAHEGQYFSQSLGPLEHPF
YQLFNTVFFNLFGARSLSLLNSVLLLPLAWVVYQLAQSVGAGARQALLAAAATTLTHGVF
WVSTKAEVYLLHTLLVLLAYALSFNDSPVLTPLKKLFAIGVLTGLAASVHQLTFVVLLPL
YLHLLFQHKARVLITLPGFMLGFAAAAVAVINDLNAGMGLLDIAHRYMTGASATVAGPEW
EGSLLRFDKLLEEKSAVALLLLSLIGPQLAGLLFPRGSRLRLLWSAALLNLVFAISYNVT
DRFTFFLPGAAILTILGMIQLRRILPRTAARGTLLSLSALSAPVVILLTYSLYANGVITL
PAHKEQLPYRDDIRYFMVPYLRDRSAEHFVRAYEPVVPGGALIIADWTPMGAMRSAQAIG
LLAGRTLATCDDDVAIRAAMRSNGVYLPRLSYCATVAQDHRLEEQPVGYSLHAE
>tr|A0A0Q0IKM4|A0A0Q0IKM4_PSEAP Regulatory protein, LysR:LysR, substrate-
binding OS=Pseudomonas syringae pv. aptata OX=83167 GN=ALO85_00908 PE=3
SV=1
MLCELKYSVGHIRKNKMRNSIQHIRAFLSVAQTGSFSKAAAALNLSPSALTVQIQQLEDW
LGVALLERSPRHVCLTGAGQNALLPMEKLLLDLDNIVSAARDIAASRRGLITIAALPSLC
SGVLPAVLKTFRERFPGVEVRLRDVVAQRIDALVMEREVDFGLGVQARPTHGLEFNAILE

DRLCLFAPKDHPLANRRAVKLNEIAGFPIVLTGRESSVRDLVERVFADEQLPLSAGLEAN
YMSTVLALVRQGQGITLLPESADDGRGDLSKVPIDHPGVVRQIGLITRTEQTMAPAVVEF
VRLLEDLRTLGHHSRTEAIAHNETVMPRR
>tr|A0A0Q0C037|A0A0Q0C037_PSEAP Two-component system response regulator WspR OS=Pseudomonas syringae pv. aptata OX=83167 GN=ALO85_04476 PE=4 SV=1
MRRKIMNDLQTDELKTPDENSAMVLLVDDQAMIGEAVRRGLAGHESIDFHFCADPHQAIA
QAVQLKPTVILQDLVMPGLDGLTLVREYRSNPLTRDIPIIVLSTKEDPLIKSAAFTAGAN
DYLVKLPDNIELIARIRYHSRSYMTLLQRDEAYRALRVSQQQLLDTNLVLQRLMNSDGLT
GLSNRRHFDEYLELEWRRAIREQSQLSMLMIDVDYFKPYNDNFGHLEGDEALRQVAKAIR
NSCSRPSDLPARYGGEEFAMVLPNTSPGGARLLAEKLRQSVAGMNIPHIAPVPGSSLTVS
IGVATMTPQVGMQSRQLILDADKGLYQAKNNGRNQVAAG
>tr|A0A0Q0DMP1|A0A0Q0DMP1_PSEAP Uncharacterized protein OS=Pseudomonas syringae pv. aptata OX=83167 GN=ALO85_200134 PE=3 SV=1
MSFSTSNDDDAIGDINITPLVDVMLVLLVTFIVTAPLLNNAIPLDLPQTVATTSLDQADP
VVVSVDASGGVFIDSQAVALEQLPEALQTLHSKDPDVAVSLRADQATGYGRVAQVLADVQ
KAGISRLSVITESP
>tr|A0A0Q0BZM0|A0A0Q0BZM0_PSEAP OmpA/MotB OS=Pseudomonas syringae pv. aptata OX=83167 GN=ALO85_02739 PE=4 SV=1
MSSNKSLVLALCIAVTGCAQTPQSDSAATGGHWWQFGSGSESADAGTAAKAPAPSPAPAP
VAKAAATPAPAAGAENSAPWYWPFGSNDNAQAKPDAVADKPAAKPAAPAMVAKSETDTKW
WWPFGNDSSKDEPKALAMPDPKVTQAWLDEYEPRLRAAIKDSPFQLERREDLLAITAPVD
SSFNPDRPAMLLPNTLGPITRLAKVVEGDQKTAVLILGHADTSGPTEANQKISQERAQSV
AAIFRLSGLERNRLSQRGMGAVMPRAANDSLQGRALNRRVEILMTPQDTMRALMSRYALP
PVAPTMVATQDVKPVAPPPAAAPAKKAAAKKDTAKKAAPAKASTAKKAAPAKTTAAAKKP
AATKAKAPAKKDDSQASN
>tr|A0A0Q0CX51|A0A0Q0CX51_PSEAP Ribosomal RNA large subunit methyltransferase J OS=Pseudomonas syringae pv. aptata OX=83167 GN=rlmJ PE=3 SV=1
METGRHKGPGVSRKFPSSERDESRKFVTNSCSLLRFAKLRSLPGAAMNYRHAYHAGNHAD
VFKHLTLTRLIALMARKEQPFAYLDTHAGLGLYDLKGDQASRTGEWLEGIGRLWNATDLP
ALASDYLQVLHDMNPDGELRYYPGSPELARRLTRERERVLLNEKHPEDGRLLKENMKGDR
RVAVHLGEGWHVPRALLPVAEKRAVMLIDPPFEQLDEMKRCAVALKETIGRMRQTVAAIW
YPIKDTRLLRRFYQDLAETGAPKLLRVELFVHPLDTPASLNGSGLAIANPPWGLEEELRE
LMPYLAQKLGQTQGGWKMDWLIAE
>tr|A0A0Q0DBD8|A0A0Q0DBD8_PSEAP AcetylCoA_hyd_C domain-containing protein OS=Pseudomonas syringae pv. aptata OX=83167 GN=ALO85_05161 PE=4 SV=1
MPYSCSIEHAVDHVLAQLPEHIHLGMPLGLGKPNRFVNALYQRISELPERRLTIYTALTL
GRPTPGEGLQARFLEPFLQRVFADYPELDFLAALRRDKLPANIRVQQFFMQPGSLLNSQS
AQQDYVSSNYSHAARDINANGLNLVAQLVARDEQRPGTLSLSCNPDVTLDLLPMIAKRRA
AGETILMLGQVHADLPYMPGDSELDVDAFDVLIDEDERSTLFSTPNMPVGYQDHLIGLHA
STLVRDGGTLQIGIGSMGDALTGALLARQADNETWRGLLAELNMSNWQTLIDREGGVQPF
ASGLYGCSEMFVNGLLVLADAGIVRRKVYADAELQRLANMGTLDEDAHPGGVVVHGGFFL
GPQSFYARLRELPAERLAQFNMTAISYINELYGQEELKRLQRRDARFINSAFTVTLMGAA
VADQLEDGRVLSGVGGQYNFVAQAHALEGARSILMLRSWRESGGEVSSNIVWQYGHTTIP
RHLRDIVVTEYGIADLRGQTDATVIERILNITDSRFQSGLIEQAQKAGKLPKDFRLDPRF
TDNTPERLKDTASRYPSLFTEYPLGCDFTAEERDLLRALNWLKSKLKLTEILELGKATLD
APDPEAFLAHLQRMQLDAPQGLREELYQRLLLAGLQNSTGLAG
>tr|A0A0Q0CFM7|A0A0Q0CFM7_PSEAP DNA topoisomerase OS=Pseudomonas syringae pv. aptata OX=83167 GN=ALO85_03771 PE=4 SV=1
MPDSTAISQPASELHYVDDTQPGLTRKVLRGKFAYFDTQGQRIKDESEIKRINALAVPPA
YTDVWICADPLGHLQATGRDARGRKQYRYHPRWREIRDQDKYSRLIEFGHALPKVRKQIE
AQLAQPGMGREKVMATVISLLDATLIRIGNSQYAKENRSYGLTTLRNKHVEVKGGQILFE
FRGKSGVEHKVSVKDRRLANVIKSCMELPGQNLFQYLDEDGVRHAVTSSDINAYLQSLTG
SDFTAKDYRTWAASALALATLQKLHWEPEADAKRHIVDMVKAVSKQLGNTPAICRKCYIH
PAVLEGFLLGNLAKLPRSRQRKGLRLEEVALASYLRILADKVEAVVKDAVVKDSKA
>tr|A0A0N8TA13|A0A0N8TA13_PSEAP Oxidoreductase, molybdopterin-binding OS=Pseudomonas syringae pv. aptata OX=83167 GN=ALO85_100093 PE=3 SV=1
MARGMARQHSECSAINRIAMTRSEAVMTRTEHYRACHLCEAICGLVIETVVEADSAPRIT
SIKGDPLDTFSRGHICPKAVALQDIQNDPDRLRQPMLRSGDQWQPIAWQQAFDLVAERLY
AIQQRHGQNAVAVYQGNPSVHNYGLMTHSNYFLGLLKTRNRFSATSVDQLPHHLTSFLMY

GHGMLLPIPDIDHTDFMLILGGNPLASNGSIMTVPDVEKRLKAIQQRGGKLVVIDPRRSE
TAAIADQHLFVRPGGDAALLFGLLNTLFEEGLTRESHLPVDGLEQVRHAIAGFTAEAMSP
RCGIAAEQIRQLARDFAAADKAVCYGRMGVSTQTFGTLCHWLAQLINLVTGNLDRVGGAL
CTEPAVDLVSSTSGGHFNLWQSRVSGLPEYGGELPVSALAEEMLVDGEGQVRALVTVAGN
PVLSTPNGRQLEQALEGLEFMLSIDLYINETTRHADLILPSTSALENDHYDTTFNTLAVR
NVTRFNRAIFDKPEGTLHDWEIFVGLASAFAAKAERALKPTLPPAQMIDRGLRAGLYGDA
SPHKLSLETLDSHPHGLDLGALKANLAQRLKTANGRIQAAPDVIMADLARFAAEPAPQAG
ELLLIGRRHVRSNNSWMHNYHRLVKGKPRHQLLMHPDDLACRGLSDGQQVRVSSRVGMIE
VQVLGSLDMMPGVVSLPHGWGHSRAGVKMAIAQSLPGVSANDLTDERQLDVLSGNAALNG
VPVQVVAC
>tr|A0A0Q0DS94|A0A0Q0DS94_PSEAP Riboflavin biosynthesis protein
OS=Pseudomonas syringae pv. aptata OX=83167 GN=ALO85_02785 PE=3 SV=1
MQLVRGLHNLRPEHRGCVATIGNFDGVHRGHQAILARLRERAVELGVPSCVVIFEPQPRE
FFAPETAPARLARLRDKLELLSAEGVDRVLCLSFNQRLCKLSAASFVETVLIDGLGVQHL
EVGDDFRFGFDRVGDFDFLQQAGTTYGFTVEAAQTVEIEGIRVSSTKVRKALAASDFALA
ERMLGRPFQIAGRVLHGQKLARQLGTPTANVQLKRRRVPLSGVYLVSTEIDGKAWPGVAN
IGVRPTVTGDGSAHLEVHLLDFAGDLYDRRLTVAFHHKLRDEQRFASLEALKTAINADVA
AARAHWHG
>tr|A0A0Q0FDF7|A0A0Q0FDF7_PSEAP Dihydropteroate synthase OS=Pseudomonas
syringae pv. aptata OX=83167 GN=ALO85_00131 PE=3 SV=1
MTSALYPTRLPXNRVLDLAHTHVMGILNATPDSFSDGGRYSQLDAALRHAEAMVQAGATL
IDVGGESTRPGARPVSASEEVERVAPLVEVIARELDVIISVDTSTPEVMLATAGLGAGLI
NDVRALQRPGALEAAASTGLPVCLMHMLGEPGTMQNDPHYDDLVGEVCAFLAERMKHCVA
AGIGQQQIILDPGFGFAKTLEHNLSLFKHMEALHALGRPLLVGVSRKSMIGAVLGRPVDQ
RLSGGLALAVLAMAKGARILRVHDVAETADVVRMITAVEAAE
>tr|A0A0Q0IR41|A0A0Q0IR41_PSEAP GGDEF domain-containing protein
OS=Pseudomonas syringae pv. aptata OX=83167 GN=ALO85_02010 PE=4 SV=1
MKFKASFQARIAGVLIVLLLIVVSAVYIAVKVATEEAVRTQAQAQLEVGSRVFERLIDLR
GKRLRDTVQLVAADFGFRDAVASADSSTIRSVLLNHGKRINASDMFLLGMDGTVIASTVQ
KVPEGSRFVYDQALRNAKRAGQSVLIVPGGGDPHLLVEATVLAPLPIGRVVMGFTIDSDI
AEELRSLSGLEVSFLTVEDGKNGDLISTQPEALHAGLIELMRSSSEGQMLLTEQSNLNFL
SQTLMLANTNNGGDGQVIALLQSPLDKAYQAFAPLNQKIFWISMAALVASLIGTLALARS
VSLPVQVLATAAKRIGDGDYKTPVTLVRSDELGMLADAINTMQQGIAVREGQLAHNALHD
NLTGLPNRALVMERLGSSIAADRAVALLSLSVENLATISESVSAEGLDQLLRQVGQRLQS
NLRAGDTVARLGANEFLLLLDNTSSAGAVGMADAVQRLLSEPQRIDNHELELECCIGITV
YPEHGDSAQELLNRAVIARKDAAFLPGRLQIYQDGRDLAHQRQITLIRDLRKAAQNGELM
LHYQPKLDIRQGYVRQAEALLRWAHPQFGSVSPAEFIVLAERTGSIYLLTNWVIEEAMRQ
LAEWRKRGLVLQVSVNISADDLLGDDLVGYVVKLLKQYAVPAEQLLFEITESAVMSEPEK
ALIVLHRLRDCGISLSIDDFGTGYSSLAHLKRLPVQELKIDQSFVRNLDETSEDAVIVRS
TIEMSHNLGLKVVAEGVEYQHSLDLLRRWHCDTAQGYLISRPLTASAFEAWIATYQASPG
LMVN
>tr|A0A0Q0BVR2|A0A0Q0BVR2_PSEAP SNF2-related:helicase OS=Pseudomonas
syringae pv. aptata OX=83167 GN=ALO85_04774 PE=4 SV=1
MSSILEKVLAAAWTDLFHERTLERGRDYARQKRIVLEDSSPQIIRTACRGSGLEIYTQTL
LFKDPDRYKNYLTCKCTCPVRNDCKHCVAALLFLQDPHNRPLLQAALASHQQEPEKPVEQ
KPRLPERLIDDLQPRPRLILASIEFSAYEPRNGRMQRHIQHRAALAFAYGKHYAVGTTNV
SILVSSLGSDLVNQKSDIIEHTDTETLRIRRQGERERQLRQELADLGFKVATRQSKALPD
SAGDMYELPNDKAWLHFVLDDLPRLREQGWEIDIQEEFGFDVTPVEDWYAVIDEENERDW
FDLELGIIVNGERLSLLPILINLIRSHPELMSPSAVAKRGDEEQLLVQLNHFTQSGGSPI
QIALPYGRLKPVLATLGDFYWRQEGNNALRMSKADAARLTQLEDLPLTWQGGDRLRHFAE
RLINIKDAPVSLPDGLNATLRPYQLEGLSWMQSLRELEVGGVLADDMGLGKTLQTLAHLL
MEKQAGRLDRPALAVMPTSLIPNWLDESEHFTPDLKVLALYGANRHQDAGNLQDYDLILT
TYALLPRDLELLEKQPFHVLILDEAQYIKNPNSKAAQAARNLNARQRLCLSGTPLENHLG
ELWSLFHFLMPGWLGNSKEFNSNYRTAIEKHGNQDRLHHLNARIKPFLLRRTKEQVATEL
PPKTEIIHWVDLNDAQRDVYETVRLAMDKKVRDEITRKGVARSQIIIILEALLKLRQVCCD
LRLVNQDMPVNSKQGTSGKLNSLMEMFEELLAEGRKILLFSQFTSMLSLIEEELKQRGIA
YALLTGSTRDRRTPIHDFQSGKLPIFLISLKAGGTGLNLTAADTVIHYDPWWNPAAENQA
TDRAYRIGQDKPVFVYKMIARGTVEEKIQRLQREKSALASGVLDGRTSGDWKLRDEDLQA
LFAPLPAAPKKTRK

>tr|A0A0Q0DEE9|A0A0Q0DEE9_PSEAP Syringomycin synthetase E (Fragment) OS=Pseudomonas syringae pv. aptata OX=83167 GN=ALO85_00226 PE=4 SV=1
KPMANTRIYLLDAHQQPVPYGVAGEIYIGGDGVARGYLNLEEVNAERFLADPFSNSPDAR
MYKTGDLARYMVDGRIEYLGRNDFQVKVRGFRIELGEIEARLGNCKGVKEAVV
>tr|A0A0Q0C4T5|A0A0Q0C4T5_PSEAP Uncharacterized protein OS=Pseudomonas syringae pv. aptata OX=83167 GN=ALO85_01000 PE=4 SV=1
MSDVNAPAGLAALEARLQQDLAWLDLPAKPWVKPRISAGQAVLDVAIIGGGMAGLALSAE
LRNLGVAAVIFDQSPAGFEGPWATTARMQTLRSPKQLTGPALGLPALTFRAWYEAQFGVD
GWALLDKIPRLQWAEYLRWYRKVLALDVRNEHRISRVAPRADGLIELDVVTPVQTRFLLA
RHVVLATGRDGLGGPWVPDFAWQLPEHLWTHSAAGLQDSWFAGKRVAVIGGGASAMDSAA
TALEAGATRVDLLIRRAELPRINKGKGVGNPGMVHGYWRLPDIWKWRIRHYLNTQQVPPP
RGSTLRVSASGKARFMLDSPVLSVEESAAGGVWLHTPKARIEADFVVFATGFRTDFRQRP
EFAPFSAQIRVWQDRFEAPSGETDAELAVLPDLGNCFEFQEKTPGACPGLNHIHCFSYPA
ALSYGAVSGDIPAISEGSKRLAHALVGQLFNEDIDLHFDTMLDYAEPELLGDEWVVSQPS
AAELRQ
>tr|A0A0N8T7X2|A0A0N8T7X2_PSEAP Type III effector HopAA1 OS=Pseudomonas syringae pv. aptata OX=83167 GN=ALO85_05593 PE=4 SV=1
MSVLFADGLPDVCNCYHSRTDAPLPCLFAGQSSGQTRLVALLRNLRNREGVSMHINRTGS
PQPSIELELERFYSASQSLASSSVRELSPAEQTMLDSITDYLKDHVFAAHKLPLSESTVD
QDAVHAHNEQIDNIIDSRARRLLDEGETPATIADTFAKAEKFDRMATTASTALRATPFAA
ASVLQYMQPAINKGDWLPTPLKPLTPFVSGALSGIMDQVGTTVMNRATGNLHYLSTSPEK
LHDAMAASVKRHSPGVMRQAVDLGIAVQTYSARNAVRTVLSPALASRPAVQGAVDISVSA
AGGLVANAGFGNRMLSVQARDHLRGGAFVLGIKDKQPKADLSEETDWLDAYNAIKSASYA
GAALNAGKRLAGLPLDVATDGLKAVRSLVSATGLAQNGLALAGGFAGVGKLQEMATKNIT
DPATKAAVSQLTNMAGSAGVFAAWTTAAVATDPAVKKAESFLQDTVKSAASSSTSYVADQ
TVKLAKAGIDASGEAITATGASLRDTLRRRNAREPDIEEGGIAAGSPSAVPFQPGRS
>tr|A0A0Q0DD43|A0A0Q0DD43_PSEAP Methyltransferase domain protein OS=Pseudomonas syringae pv. aptata OX=83167 GN=ALO85_00068 PE=4 SV=1
MTDEAFAQADPEWLALIRAAREWLSGPLGQLLLEEERRILDEELGRYFGGYLVHYGPSAQ
SPPVAPQVQRNVRLGAPLPGVEIVCEEQAWPLSEHAADVVVLQHGLDFCLSPHGLLREAA
SSVRPGGHLLIIGINPWSTWGIRHVFARDALRKARCISSSRVADWLNLLGFALEKRRFGC
YRPPLASAAWQSRLAGLERLGEGRQSPGGGFYVLVARKLVVGLRPLRQVRRDPIGKLIPM
PMAKVSRNIQDQA
>tr|A0A0Q0D7C4|A0A0Q0D7C4_PSEAP Uncharacterized protein OS=Pseudomonas syringae pv. aptata OX=83167 GN=ALO85_200179 PE=4 SV=1
MNNRLSVAMQAVEKATGLAMAYFNDRHTLDITTKSAQDLVSRADFEVEQLLRAELSKHFP
DDTILGEEMGGEFITDGWVIDPIDGTGNYLRGTPLWGIAVAYMSAGEPEIGVVAYPALGY
TLAARTRDGLLRNGVPFVRPQPPEHLRIAGVGENTRWDAEEMGKLHLSLRRQGWGLAGYR
CATIGLAFAALGQTDGYMEKFTSLWDIAAGAVICREAGLLCTIEGEQKQGSMTVMVGREE
LMGIFGA
>tr|A0A0Q0DJ80|A0A0Q0DJ80_PSEAP Phosphate acetyltransferase OS=Pseudomonas syringae pv. aptata OX=83167 GN=ALO85_04953 PE=3 SV=1
MLRPLALLNREASMQTFFIAPTDFGVGLTSISLGLVRTLERAGLKVGFFKPIAQPHPGDL
GPERSTELMARTHGLKPPKPLGLAHVERMLGDGQLDELLEEIINLYQQAAVGKDVLVVEG
MVPTRSASYAARVNLHLAKSLDAEVILVSAPENEVLADLSGRVELQAQLFGGPKDPKVLG
VILNKVRTEESMEVFASRLKEHSPLLRSGDFRLLGCIPYRAELNAPRTRDVAELLGAQVL
NAGDYDQRRMTRIIICARTVLNTVPLLKPGVLVVTPGDRDDIILAVSLAAINGVPLAGLL
LTSDTVPDPRILELCRGALQAGLPVLSVSSGSYDTANRLNQLNKEIPIDDRERAENITDF
VASHLDANWLHQRCGTPRELRLSPAVFRYQLIQRAQAANKRIVLPEGNEPLTIQAAAICQ
ARGIARCVLLARPEEVHAVAQAHGIELPQGLEILDPDLIRERYVAPMVELRKSKSLNAPM
AEQQLEDPVVIGTVMLALDEVDGLVSGVIHSTANTIRPALQLIKTAPGCTLVSSVFFMLF
PEQVLMYGDCIMNPHPSASELSEIALQSADSATAFGISPRVAMISYSSGNSASGEEVEKV
REATQLARETRRDLLIDGPLQYDAAANEQVARQLAPDSAVAGRANVFVFPDLNTGNTTYK
AVQRSADCVSLGPMLQGLRKPVNDLPRGAQVDDIVYTIALTAIQADTLS
>tr|A0A0Q0CXF2|A0A0Q0CXF2_PSEAP Uncharacterized protein OS=Pseudomonas syringae pv. aptata OX=83167 GN=ALO85_01202 PE=4 SV=1
MTDANTMTETLAEPASAEVAVPVPPQPWEDVLPESFQMLRLSPLPTDRATGGRPLRFVQF
GRAERHSKALSLLRITVQLPGQRVRKEQNHLDIWADHEKRMVRFGPESGLQIEPWNRGIG
RFMIAHAVHWAQKRWSSYKIEGVALASKDGLNEDTRLRRDHFLRTLGFEVTYADAQHMKG
AIKDVHVGNLHSTWNNDKVQIIEILEASQMLEKAEKNLIEQEVTIRQHEDRVSKYKREDS

GLRFTIACLVTFAVFQAGLLIWIATHR
>tr|A0A0Q0FH65|A0A0Q0FH65_PSEAP Uncharacterized protein OS=Pseudomonas syringae pv. aptata OX=83167 GN=ALO85_01296 PE=4 SV=1
MPGNAGMTNLFISNVVFDFGESKSIDSLSSCISPDELKAFRAGGFEYYVNESRDVYAVCE
KVAAKVLDESHVVREEVTDIVYISSTKFIDKVVGLSVYLSKLAANIGIHKATLYALSAGE
CGNFVHALRFCGQILQERPDSKVLLVFAEKLMTDEERHYPGTAILSDGVAVCLVTCEKTR
NCYEVIARSEATDHRVTKAYAEQKNIDALRLALTAIHTLSRDIFLKVDFDKSAISHFITN
NFIMHLSKMYIMEVGGDASKLRLPTLPPKAHVFTIDSLLNLTGTPMDDGDLAFCLSFGMF
FWGACIVKYHA
>tr|A0A0N8T8G2|A0A0N8T8G2_PSEAP Phospholipase D/transphosphatidylase OS=Pseudomonas syringae pv. aptata OX=83167 GN=ALO85_01105 PE=4 SV=1
MPFYPSYRRLSLSYRAVIAALLLGSSCAQADFAIPGFELVHTVPVGSDLQTPDLRAPGEV
WRELFDGARQRIDIEQFYVADHAGSVMDKVLESLTAAGQRGVKIRFLLEEKGLKLSDPQT
LERLRAIPNLTLRVLPYAKLTGSGIIHAKFLVVDGRQAFIGSQNFDWRSLEHIHETGLRI
DEPTVVRQTQAIFDQDWLAQAAITEGKPVPVPRPVDSTLPNGNYLIASPQRYNPPGVVDS
QTELPRLLAQAKSEVRVQLLDYAPLSYGPDKTRPYYAVIDNALRSAAARGVSIKLMVSDW
NTGMPEVAYLKSLALVPNVQVRIVTLPMAAQGFIPYARVIHSKTMDIDDQVAWVGTSNWL
GGYLDNSRNLEVVMHDGSMAKRIGQLHEQLWDGPYAKPIDINRDYPEPHPGKPNAPDH
>tr|A0A0Q0D2N4|A0A0Q0D2N4_PSEAP DNA-binding response regulator OS=Pseudomonas syringae pv. aptata OX=83167 GN=ALO85_02221 PE=4 SV=1
MAAQAGDRRIMQALPDTSLDATSTDQRWSVRALIVDDDVAIRELLCDYLTRFNINARGVT
DGTQMRQALTVETFDVVVLDLMLPGEDGLSLCRWLRSTSDIPILMLTARCEPTDRIIGLE
LGADDYMAKPFEPRELVARIQTILRRVRDERSDQRTTIRFDAWRLNSVLRQLTAADGLVV
PLSNAEFRLLRVFLERPHRVLSREQLLDAARGRSIEAFDRSIDLLVSRLRQKLGDDPKNP
QLIKTVRGEGYLFDARELG
>tr|A0A0Q0IKR0|A0A0Q0IKR0_PSEAP Macrolide export ATP-binding/permease protein MacB OS=Pseudomonas syringae pv. aptata OX=83167 GN=macB PE=3 SV=1
MGHTSGSGGLTMSRALLELKGVTRRFVAGEKDFIALNDINLTINAGDLIAITGASGSGKS
TLMNVLGCLDHANSGSYKVDGRETGTLTDDELAELRRDHFGFIFQRYHLLPHLAAIQNVE
MPAIYAGTGKSMRVERAQKLLERLGLSGHLEHRPSQLSGGQQQRVSIARALMNGGEIILA
DEPTGALDSVSGKEVMNILLELNSAGHTVILVTHDEKVAAHAERIIEMRDGEIIADRVNT
DRPIINEKATERLPSKPRQGNRLMANIGLFQEAFVMAWVALISHRMRTLLTMLGIIIGIT
SVVSIVAIGEGAKRYVLKDIEAIGSNTIEVFPGSDFGDTKSMDIQTLALSDVAALSSQYY
IDSATPNIGRNMLLRYRNIDVSATVSGVSPSYFQVRGTKMGSGVGFNKDDARRQAQVVVI
DYNTRIRLFGPKVDPLGQVILVGNLPCTVIGVTENKKNIFDTSKNLNIWMPYETASGRLL
GQSYLDSITVRVKDGQPSKVVEDNVNKLMQKRHGTKDFFTYNLDSVMQTVQKTSQSLALL
LSLIAVISLAVGGIGVMNIMLVSVTERTREIGIRMAVGARQSDIRQQFLVEAVMVCLIGG
VIGIGLSFVIGYVFSLLVKEWQMVFSVGSIVTAFICSTLIGIVFGFVPARNAAQLDPIEA
LARD
>tr|A0A0Q0DKT2|A0A0Q0DKT2_PSEAP Glutamine ABC transporter permease OS=Pseudomonas syringae pv. aptata OX=83167 GN=ALO85_00864 PE=3 SV=1
MTSKFDLSAILQGEYAELIVKGIETTLQLALVAWCLAMAIALLLVSIRLTGNKYAERLVA
GYVSYQRNVPTLVQLMLWYFGIPTLLSESTQIWLANYSTEYLFSVIALGLCQAAYFSEDI
RSGLRAIPAGQVEASRALGLGYVRSMRYVILPQGVRNCLPSLINHTVLLFKNTSLVMAIG
VVELTYATREVENYTFRTFEAYLVATVVYLAISLMLMGLGALLARQFSKAMAR
>tr|A0A0Q0DJ90|A0A0Q0DJ90_PSEAP Magnesium transporter MgtE OS=Pseudomonas syringae pv. aptata OX=83167 GN=ALO85_01853 PE=3 SV=1
MTEREVKKTQESLQDRLAQVVDLLQRQRIVEDLTHRQEGQHQDRVENLVHRQNLVELQRK
LEDLHSADVAYILEALPLEDRLTVWQLVKADRDGDILLEVSDSVRETLIADMDDHELLAA
ARDMDADELADLAPELPRDVVHELMETLDSQQRERVRSALSYDEDQVGALMDFEMVTIRE
DVSLEVVLRYLRRLKELPGHTDKLFVVDSEGVLKGVLPIKRLLVNDPEKQVGEVMANDPV
SFHPEDDAYDAAQAFERYDLISTPVVDKNGKLIGRLTIDEMVDLIREESESEVLNMAGLR
EEEDIFASVWRSLRNRWAWLAVNLVTAFLASRVIGLFEGSIEKLVALAALMPIVAGIGGN
SGNQTITMIVRAMALDQVNTGNTARLLRKELAVGLINGLLWGGVIGVVAYMLYGSWSLGV
VMTAAMTLNLLLAALMGVSIPMALARLGRDPAMGASVMITAMTDSGGFFIFLGLATIFLL
>tr|A0A0Q0D0G1|A0A0Q0D0G1_PSEAP Regulatory protein, LysR:LysR, substrate-binding protein OS=Pseudomonas syringae pv. aptata OX=83167 GN=ALO85_03173 PE=3 SV=1
MFMNNTLRRIDLNLLVTLDALLSEQNVTRAAHRLNLTQPTVSLQLGRLRQVLEDPLLLPG

PRGMTPTARAEELREPLRQALAALESALLPGRDFDPATADHTWRVAASDYATIALIWPSL
GRLRSSAPGTRLALLNKHPVSLAADLESGRLDLALHTREEAPPKLRQRSLIHERYVLAGR
HDHPDLVQPVSLTRFCELEHAVMSPNGGGFAGSTDQALAALGLSRRVVLSAPHFGSLVSA
LTSSDLVAVVPERLVRGQPTLVVQEPPLSIPGFEMLMLWPERLHRDPAHRWLRELMVSAI
D
>tr|A0A0N8T8L6|A0A0N8T8L6_PSEAP Proteinral secretion pathway protein H
OS=Pseudomonas syringae pv. aptata OX=83167 GN=ALO85_00688 PE=4 SV=1
MRTPVASRGFTLMEMLVVLVLMSIAVGLVGFGLQQGLSTASERRAVGDMVEALRATRVRA
IVTGQPARTEFNLRKATFKAPGKREMHWPESLRVTMQTASDLGSAVEFYPDGGSSGGNVV
VADGDRRWRIDIGWLTGSVQVRTL
>tr|A0A0Q0C2P4|A0A0Q0C2P4_PSEAP Uncharacterized protein OS=Pseudomonas
syringae pv. aptata OX=83167 GN=ALO85_200140 PE=4 SV=1
MNVIKKTVTALTLGVALAASMSAWAAQDRWTFDGDIHNNSLAVSPDETTAVVSYSQRPDV
VVYDLKTGKVRQVLTGYVTPRNIVFSPTGDVFYLSDSSLGVVRKIDTKTLKVIADIPLGA
GAFGTTLNKDGSLLYVNNEAASTLSVIDLDHQRPVAVVPGFSQPRQGIRVSPDGKTVYVT
NFLGDKITLVDSKTNTIEGEITGFNKLRAISISADGNTLYAANSGSNSIAVVDTQKRAIT
TTVMVGKDPYGAALTPDGLHVYSGNLGDNSLSVIDTKTLKVTTTVTGLKAPRQAIVFTKD
KSKAYVLNEDLSISTVDLASNKVVSTLKAD
>tr|A0A0N8T9R3|A0A0N8T9R3_PSEAP Urease subunit alpha OS=Pseudomonas
syringae pv. aptata OX=83167 GN=ureC PE=3 SV=1
MKISRQAYADMFGPTVGDKVRLADTELWIEVEKDFTTYGEEVKFGGGKVIRDGMGQGQLL
AADVVDTLITNALIIDHWGIVKADVGIKNGRIAAIGKAGNPDIQPDVTIAVGAATEVIAG
EGMILTAGGVDTHIHFICPQQIEEALMSGVTTMIGGGTGPATGTNATTVTPGPWHMARML
QASDSFPMNIGFTGKGNVSLPGPLIEQVKAGAIGLKLHEDWGTTPAAIDNCLSVADEYDV
QVAIHTDTLNESGFVETTLAAFKNRTIHTYHTEGAGGGHAPDIIKACGSPNVLPSSTNPT
RPFTRNTIDEHLDMLMVCHHLDPSIAEDVAFAESRIRRETIAAEDILHDLGAFSMLSSDS
QAMGRVGEVIMRTWQTADKMKKQRGPLPQDGPGNDNFRAKRYIAKYTINPAITHGISHEV
GSIEVGKWADLVLWRPAFFGVKPTLILKGGAIAASLMGDANASIPTPQPVHYRPMFASFG
SSLHATSLTFISQAAFDAGVPESLGLKKQIGVVKGCRTVQKKDLIHNDYLPDIEVDPQTY
QVKADGVLLWCEPADVLPMAQRYFLF
>tr|A0A0Q0C264|A0A0Q0C264_PSEAP TonB system transport protein
OS=Pseudomonas syringae pv. aptata OX=83167 GN=ALO85_05532 PE=3 SV=1
MEGSGLLEKEEVELPLGTTASGTEGSQGHFNNVWIKSELTMLMNLPGCTLKPLALAVRRH
PRALLCALAMGMSGAQAADAVDADTSGSAVSTSAAVVPATTQLQRVEVTGSAIRRVDAET
AVPITILRADDLKKQGVTTTQEMVQRITGSQSINNSAGSVGAGTGGASFADMRGIGANKT
LVLLNGRRLANNALSGVGTPNGSAIDLNMIPFAAIDRVEVLRDGASALYGTDAIGGVINF
ITKKSLTDGSLTLGGETPTASGGGATKDMSASWGFGDLEEDRFNVMGVFNYNKQQNLDAN
DRSFAKDYVPGRGLNQTSGTAYPGNYFQGDNSANPLGRNCSGPSLIASNGICRFSTREYI
DLIPQTEKTSFFGRATGKLADDHNVSLEYFWARNNNHTDIGPAPLTGLSLDSSSPFYPGN
GSTPAPTDFTLDPTQPVSTAWRETAAGPRGSSDQNTSQRFLLNFDGLVGGWDYNVGASYN
QNKVLSSLTDGYISDAAMLNGLANGTLNPFGPQTAAGQALIDANQYHGQYSSAVGRVAGL
DARISREIGDWFGAGPAGIALGGEYRKEKMHQEYEAFVNDVSSLGADAAGSVEGDRSVKA
EYAELNVPVLDSLELTAAVRHDKYSDFGSTTNPKYSFRYQPFKQLVVRGAYSEGFRAPSL
YELYAPRSTTCTQGYYNDPVLCAGGVVQPGGNAGRDCQQQFLNNGGGNTELAPEKARNMT
LGFVYQPVNNLSMGLDFWWIHISNQIQAFPESTVFDDPNTYADRYVRAADGSIANIVTGN
ANLGIVKTSGVDVTLDYRFPNTPYGQFGLGMTGTYVTRYDFQNIIDGPYTDKVGDFQGDG
VISRWKHVLTGTWALGDTRASITNRFTSGYNDYDRTTNARVASYALWDMSVGHTFDKVLD
IDAGVRNMFDRNPPFSNQAYNFQSGYDPRYADPLGRTLFARATYHF
>tr|A0A0Q0DBV4|A0A0Q0DBV4_PSEAP Type III effector HopAG1 OS=Pseudomonas
syringae pv. aptata OX=83167 GN=ALO85_03278 PE=4 SV=1
MINPVKHNFSHLGFSNAQSTSALGPASNKVPNFVSRGRGKGVPVEEFNTAEECRLAGRQD
SVLDSIDGKEFMRLLQKYTASETTEEEFADLRASIPRYSIELAKSDHPKVLYRGISLDDE
AASLLLNTSKGYRSREIAHGLIHGLRVVKGVYTATGVASASTVSAVSQGFALVNANRKKE
TPVLFVLKAMPAVPALNHSGAKGVTLSESRLPLSVASKSEHEVILDITNRYEITQARRSG
EFIVVDMTVLGRSKRGGEFALVETDKWKQLFGAKGSNPGGLFQAPNGVKWYVKTNPSANR
LRNEVLASRLYRAAGIDVPDIELASRKGKPALISKLIVGNPKDLDTLAKNSQLKCGFAVD
AWLANWDVIGLTGDNVIFNHRNKPVRIDLGGALVFRAQGEHKGNQFGTTPMELVTMLSRE
DNSSSRAFRKIERNDIREGIAAIEKIPDARIAALCAEHGPGNHSERIELGKRLISRKKWL
VDMKQTLPYIHRQKNERGNVVTVKKPTSPSAADTWRDRYATAVFVPHSAVRGSMNNLPFR
SFIPPNTMDGWRRFTTRAVNFTEPEFKRSQHLAPASGAIIFEPDGRVWITEPTNHPFGAT

HAFPKGKQEAGLNLRTNALKEVYEETGLLVEFHGFIGDYDRTTSRTRYYLAKRIDGTPSD
MGFESQSVKLANITEAKRLLPNTLDIAILRDAERAYLKGPFK
>tr|A0A0Q0DP03|A0A0Q0DP03_PSEAP PAAR domain-containing protein OS=Pseudomonas syringae pv. aptata OX=83167 GN=ALO85_04430 PE=4 SV=1
MVSCQLAGTHTLNGKPPAVLGDKATCPLHAGEFAFIEGHPSRKLNGIPVVLHGHRLACGC
QGVASHAMNVRVV
>tr|A0A0Q0DIC4|A0A0Q0DIC4_PSEAP Uncharacterized protein OS=Pseudomonas syringae pv. aptata OX=83167 GN=ALO85_05574 PE=4 SV=1
MIENPMNLLAPALFVTNRVRFPMKFAILGFIVLIPLLLLGTRAMLSLNTSITGIKHEQVG
QQYLLDVTPILRLTMIQRALTHGMLSGDTNAVANAARNAEKLNDAYATLAAQDAKFSTQL
ATTDRVQTLRTASVQLVERAKAGEAPLVIFSAWNDQLTDLMNFVYYITATSGMILDEEAG
SLYLIDLSSIRLPRQINLVGQIRGLASGFSADRPLDDTTRIFAQTLLKQELLMRQELQQS
LSLLRREEPKLSGVVQPSVTTAITDLENLRKDLVDYLDPSKRSNVNANTLGERGNAVVAQ
FYKAQDQMQESLRIRIDERLNTLSNERTFGTELFIAIAILLLYAFVGIYKALRIGVDELL
AVTARIAQGDLSARVRINSHDEIGDVGEGLNRMVTAFSGSLSQVERSSHSVSDAAKRLEI
SIGQAKASMNSQQAETEQVATAINEMTASVADVAQNTEGAARAAESASSASNDGLKVMIE
TSAAIEALANEVELSASKVEALATHSKEIGGVIEVISTIADQTNLLALNAAIEAARAGEQ
GRGFAVVADEVRTLASRTQNSTEEIRRIIQQLQGATDAAVQQMKAGQTRARECIQAASQA
SGSLNQINEAVDGIVGMNTQIASAAVQQHSVSEDINRNVIGIRNSSLIVMEGVEDNAVTA
DELSLLANELRSVVGKFKLSSNA
>tr|A0A0Q0C6E1|A0A0Q0C6E1_PSEAP ABC_trans_aux domain-containing protein OS=Pseudomonas syringae pv. aptata OX=83167 GN=ALO85_03603 PE=4 SV=1
MTLRLKLMVLAAAVGLGACTSTQTHYYTLIAPMSATSTAASKPMPFQFEMLPVIMPVQVD
QPPLVVRQGNGSLAILDTERWGSPLGDEFHDALTPQLERRFGSRDMAGLPKNSDQPTLSI
RTDVRRFESMPGNYALIDVVWTLGLREAGATAGSKRQSLTCSSVIREEAGEGMENLIIAH
QKAVAQLADKIAVTAASWTAQRASRCL
>tr|A0A0Q0BVD8|A0A0Q0BVD8_PSEAP 4Fe-4S ferredoxin OS=Pseudomonas syringae pv. aptata OX=83167 GN=ALO85_00491 PE=4 SV=1
MQLAPEVNVMLDTLLPILLFSALALAALGAWRRVTLWRNGRSSKVDWLGGLLAMPKRYMV
DLHHVVARDKYIANTHVATAGGAVASIVLAILVHGFGLHNRLLGYALLLMTAVMFVGAVF
VYLRRLNPPARLSKGPWMRLPKSLMAFSASFFLVTLPVAGILPEHFGGWVLVVVLGLGVL
WGVSELFFGMTWGGPMKHAFAGALHLAWHRRAERFGGGRSTGLKPLDLNDRSAPLGVEKP
QDFTWNQLLGFDACVQCGKCEAACPAFAAGQPLNPKKLIQDMVVGLAGGTDAKFAGSPYP
SLDGKGKPLGEHGGSPHQPIVNGLVDAETLWSCTTCRACVEECPMMIEHVDAIVDMRRHL
TLEKGATPNKGAEVLDNLIATDNPGGFAPGGRMNWAADLNLTLLSEKKTVDVLFWVGDGA
FDMRNQRTLRAFVKVLKAAGVDFAVLGLEERDSGDVARRLGDEATFQTLARRNIQTLSQY
SFQRIVTCDPHSFHVLKNEYGAFGGDYQVQHHSTFMAELVRNGALTLGQHKGASVTYHDP
CYLGRYNGEYEAPREVLRALGIEIREMQRSGFRSRCCGGGGGAPITDIPGRQRIPDMRMD
DIRETAAELVAVGCPQCTAMLEGVVEPRPLIKDIAELVADALIEDVVPSTPTPAKREPAE
VL
>tr|A0A0Q0C5E5|A0A0Q0C5E5_PSEAP OprD family outer membrane porin OS=Pseudomonas syringae pv. aptata OX=83167 GN=ALO85_00537 PE=4 SV=1
MRVNSPCKLLCGLSAAVAASLTPISGHSAGFVEDAKVNLNLRNFYINRNFVDPANTQNYA
EEWTQNFILDARSGFTQGTVGFGVDALGLYSLKLDGGKGTGGTQLLPIHSDGRPADDFGR
LAVAGKARFSKTELKVGEWMPVLPILRSDDGRSLPQTFQGGQITSKEIDGLTLYGGQFRG
NSPRNDASMEDMSLNGRTAFTSDRFNFGGGEYVFNEKRTQVGVWYAELEDIYHQQYFNLL
HSQPLGSWTLGANLGYFQGKDDGQSLAGDLDNKTWSALLSARHGGNTFYLGLQKVSGDSA
WMRVNGTSGGTLANDSYNSSFDNAKEKSWQLRHDYDFVAMGVPGLTLMNRYISGDNVHTG
AITDGEEWARETELAYVFQSGAFRSLSVKWRNSTMRRDYNTNQFDENRLIVSYPLSLL
>tr|A0A0Q0DKH4|A0A0Q0DKH4_PSEAP DUF2059 domain-containing protein OS=Pseudomonas syringae pv. aptata OX=83167 GN=ALO85_04983 PE=4 SV=1
MTRMRFHSPKPLITRQIHANTGRIAACRQESPMRRLFVLLLMFCTSPAWADSYDQLYKSA
GWPEQRAHFNDALKAAQQRYSNNLPPAVYQALVNNSNQRFAPQAMDQRASKRLRESLKDP
APSLQFFQSPLGRKIVNAELTATRADQLAKHAHGLPHIEADATRQLLIGHLAQALPAREA
GAEVSLAIAGVAADSLSQMIPGLLGGGQAQGMLEGQRERLMAQMSADLNNTLLYVYRDLS
DPELEEFSTFAESPEGKAYYQAALAAIRAGLAVGQSASSLNP
>tr|A0A0Q0FDT8|A0A0Q0FDT8_PSEAP ABC transporter, substrate-binding protein, aliphatic sulfonate OS=Pseudomonas syringae pv. aptata OX=83167 GN=ALO85_03474 PE=4 SV=1
MPTTLVRWSQRLLKGLVLSLVASLNTQAVNLIVGDQSYSARTVMEAAGVLKDLPYELEWK

QFTAGSPVAEALNVGSLDIGLLGDSPALFMGALGAPIKVIGVSRQNLDGVAIVVRKDSPI
KTVADLAGKKVAIWKGSFSQQLMLTALDKAGVARDAVDYRYLSALDSSHALDGGSVDAIA
TWEPYVTQQERQGARIIATAEGLIPAQSFIVANDLAIKDKRAQISDFLQRLQAARAWSVS
DPQHNERYANAWAALTKADAEVARRWFSRAQVVVVPITAQVVAGAQQTIDFFNNAGLTKR
YPAASVFDESFSAALADQAQAVR
>tr|A0A0Q0DKU0|A0A0Q0DKU0_PSEAP Prophage PSPPH05, helix-destabilizing protein OS=Pseudomonas syringae pv. aptata OX=83167 GN=ALO85_100445 PE=4 SV=1
MGISQSEPKFSAAYTVRGSTMSMTLLIEVTGIQRSGVAAKSQKPYTMFQAFVHLPNIPYP
QKTDFYASTPSEVPQPGTYECDVIADVRDGRLEFTCDPRQGRRKNIPPLSAAMTKAG
>tr|A0A0N8T7Y7|A0A0N8T7Y7_PSEAP Alanine--tRNA ligase OS=Pseudomonas syringae pv. aptata OX=83167 GN=alaS PE=3 SV=1
MEEPMKSAEIREAFLGFFEEQGHTRVASSSLIPGNDPTLLFTNAGMNQFKDCFLGQEKRA
YTRAVTSQKCVRAGGKHNDLENVGYTARHHTFFEMLGNFSFGDYFKRDAITYAWTFLTSE
KWLNLPKEKLWVTVYATDDEAYDIWTKEIGVPAERMVRIGDNKGAPYASDNFWTMGDTGP
CGPCSEIFFDHGPEIWGGPPGSPEEDGDRYIEIWNNVFMQFNRTADGVLHPLPAPSVDTG
MGLERVSAVLQHVHSNYEIDLFQSLLAASAKAIGCSNDNQASLKVVADHIRSCGFLIADG
VLPSNEGRGYVLRRIIRRACRHGNKLGAKGSFFYQIVAALVAEMGSAFPELVQQQSHIER
VLKGEEEQFAKTLEQGLKILEQDLADLKGTVVPGEVVFKLYDTYGFPMDLTGDIARERNL
TLDEAGFEREMDAQRVRARSASSFGMDYNSLVKVDVATQFTGYSATTGSASVVALYKEGQ
SVSHLNEGEEGVVILDITPFYAESGGQIGDSGFLQAGDARFDVSDTTKTGGAFLHHGVVA
SGSLSVGAQVETQVADEVRDATKLNHSATHLLHAALRQVLGEHVQQKGSLVDSQRLRFDF
SHFEAIKPEQLRALEDIVNAEIRKNTEVMTEETDIDTAKKKGAMALFGEKYGDSVRVLSM
GGEFSVELCGGIHASRTGDIALFKIVSEGGVAAGVRRIEAVTGAAALAWLNSAEDQLKEA
ATLVKGNRDNLLDKLTAVIERNRLLEKQLEQLQAKAASAAGDDLSSAALDVKGVKVLATR
LDGQDGKALLALVDQLKNKLGRAVILLGSVHEDKVVLVAGVTKDLTGQLKAGDLMKQAAA
AVGGKGGGRPDMAQGGGVDAGALDSALALAVPFVEQGI
>tr|A0A0Q0FW04|A0A0Q0FW04_PSEAP DUF2236 domain-containing protein OS=Pseudomonas syringae pv. aptata OX=83167 GN=ALO85_05354 PE=4 SV=1
MEFIRTRIENQVISLTGLALGQLDLESPKGDPGLFGPQAVCWEVHGDFTSMLVGGISALM
LQTLHPLALAGIWDHSNFRQDMLGRLRRTGQFLSGTTYGSTRDANWLIDKVRNIHMNIVG
TAPDGRPYAASDPDLLTWVHVAEVSSFLAAHVRYLNPDMSLAEQDAYCAETALVAERLGA
RNVPRSRQAIADYLADIRGQLVCDQRSREVMRLLLDAPAPNWLAKPFGVLMMRAGIDLLP
DWASDMLDVTQSPCKRRMIRLGVNSTAPILRWAVRDGSAQRARRRMGV
>tr|A0A0Q0ID35|A0A0Q0ID35_PSEAP DEAD/DEAH box helicase (Fragment) OS=Pseudomonas syringae pv. aptata OX=83167 GN=ALO85_04711 PE=4 SV=1
MMNDLPLAAPAGHPCVPTLNIGLTEFIEEFGDELLESLNRSNPPVYAGIDNPARQWVLDG
LKRQPFPAQAQVVQAIAALLLDQNEQAGIINAEMGTGKTMMAIALAAVMHGAGYRRTMVI
APPHLVYKWRREILETIPDARVWVLNGPDTLVKLLKLRDQLGDTYDGRQEFFILGRVRMR
MGFHWRLAFWQRRAGGGRSLAACPDCGRLLQDQEGNLITAEEFQREERRRRCDHCDAALW
TLMRPGKSDGGSRRNTILKSMCRIPTIGPVRAERLLSDFGEDFLASMLLDNVSEFINLMD
AKGNFIFSDRQAKRMERTMANIEFGFGEGGYQPTEFIKRYLPDGCFDLLVVDEGHEYKNS
GSAQGQAMGVLAAKARKTVLLTGTLMGGYADDLFYLLFRILTRRMIEDGYRPNARGSMAP
AAMSFMRDHGVLKDIYTERDGSSHKTAKGKKLSVRTVKAPGFGPKGIHRFVLPFTVFLKL
KDIGGNVLPGYREEFIDVPMSPDQEAAHRKLAQTLTIELRQALARRDTTLLGVVLNVLLA
WPDCCFRPEVVKHPRSRDTLAFVPSIFEEDELMPKEQALLDLCLAEKARNRKVLAYSVYT
GTRDTTSRMKRVLEQSGLKVAVLRASVDTAKREDWILDQVDRGVDVLITNPELVKTGLDL
LDFPTIAFMQTGYNVYTVQQAARRSWRIGQK
>tr|A0A0Q0C1R6|A0A0Q0C1R6_PSEAP Uncharacterized protein OS=Pseudomonas syringae pv. aptata OX=83167 GN=ALO85_01744 PE=4 SV=1
MTSTSPFWRRARLPLAVSLASSLAGPAFAVTFNVGEIEGSFDSSLSVGASWSTANPNKNL
IGANNGGRGLSQTSDDGHLNFKRGETFSKIFKGIHDLELKYGDTGVFLRGKYWYDFELKD
ENREFKDISDSGRKEGAKSSGFQLLDAFVYHNYSIADQPGAVRFGKQVISWGESTFIQGG
INSVNPVDVSAFRRPGSEIKEGLIPVNMFYLSQSLTDNLSAEGFYQLEWDQTVVDNCGTF
FSQPDVIADGCNNNLAVLRSRSGLNSSLTAAGLPAGARGAVFNSLSQQGVVFGNPDEGAV
VRRGPDRDARDSGQFGFALRYNFEPLDTEFGAYMMNYHSRAPIFSGRGGSAASFSPQGLV
GSLVRNGIPASTAAALAPSLLPVVVAGNSSYYVEYPEDIRLYGLSFSTTLPTGTAWSGEI
SYRPNAPIQVNTTDILYSGISPLNPNVSLLNGQPNTDQAGYRRKEITQMQTTFTQFFDQV
MGAERLTVVGEVGWTHVGGLESTSKIRYGRDPVYGPGPLPGGQCATLNAGTLTGAEQNNL
TRYCEDDGFTTANSWGYRARAIWDYNSVFAGVNLKPSVAWSHDVKGYSPGPGGNFEEGRK

AVSLGLDAEYQNTYTANLSYTNFFDGKYTTVDDRDFVALSFGMNF
>tr|A0A0Q0DQ50|A0A0Q0DQ50_PSEAP Uncharacterized protein OS=Pseudomonas syringae pv. aptata OX=83167 GN=ALO85_102052 PE=4 SV=1
MRLSFRTLQRGNAVRDALRRKEDAERLERRIQARSDEPTAHPKHMKHPSFCHHCAVSLVK
QGLQKIDQFLTTGEPEQAIYLGS
>tr|A0A0Q0IH19|A0A0Q0IH19_PSEAP Dipeptide ABC-type transport system, ATP-binding protein OS=Pseudomonas syringae pv. aptata OX=83167 GN=ALO85_00015 PE=4 SV=1
MSIQAQVLNLFMDLQQEFNTAYVFISHNLSVVRHVADTVLVMYLGRPAEMGPKEEIYNRP
LHPYTQALLSATPTIHPDPLKPKIKIVGELPNPLNPPSGCAFHKRCPYATERCAAEVPEL
RLVDNRQVACHYAEQFVAA
>tr|A0A0Q0BVS0|A0A0Q0BVS0_PSEAP Rhs family protein (Fragment) OS=Pseudomonas syringae pv. aptata OX=83167 GN=ALO85_03463 PE=4 SV=1
RTLDKLRGEVTYEYEANGRLLEHNPEKRFDGEEFRYDAAGNRLNFNTSRFDRVKNNRLKK
WSNHEYKYDAWGNLVEKIVGIVRWQTFTYDSENRLVKTETMANSQVESTSSYQYDSLGRR
VGKQWEIKGKTDHRLFLWQGLRLLREESPGQSSLYLYEPGSYAPLARVDEKEGEVENKVY
YFHTDQIGTPLEMTDAEGQIVWQAKYRPWGAIEKLVVNEVEQNLRFQGQYFDVETGLHYN
TFR
>tr|A0A0Q0FQ09|A0A0Q0FQ09_PSEAP Type IV secretory pathway VirB4 component (Fragment) OS=Pseudomonas syringae pv. aptata OX=83167 GN=ALO85_05556 PE=4 SV=1
MMTLFQRLKSRASLNSAMQTDIAGDPLLSEPAAVELEQPEDDLVEDLKASDQNDVDAALE
RYLKRLEDQGIPAPGDWRNPKQKPATVGDVAALYDVKPSFVDLLPWIEYLPAEQAMLLED
GKSLAAFFELTPIGTEGRDPEWLRKVRDALENALQNSFDELDTSPWVVQFYAKDETSWDD
YLRNLREYIRPQAKGSPFSELYLDLFKHHLDAIAKPGGLFEDTTVSKLPWRGQQRRVRVV
VYRRVVGDAAFRGQTPAMHLDNICQRFTGGLANAGIKSKRMDGYDIRHWLLQWFNPHPDH
LGTTLKDFDRFFQLVNKRTEDAGEDLPLVTGDDFAQGLFYREPKSDSQKGLWYFDGQPHR
VIMLDRLREAPKTGNLTGETRKGGDALHALFDKLPEDTVLAITLVITPQDVLEAHLEKLA
RKSVGDNQASLLTREAVNDARKLIGREHKLYRGSIAFYLKGNDEAQLTARSMQLTNALLG
AGMEPVASDDEVAPLNSYLRWLPGNFDVNQKRSMDWYVQMMLAQHVANLCPVWGRSSGTG
HPGITLFNRGGAPLTFDPFNKLDRQMNAHMFLFGPTG
>tr|A0A0Q0ILW6|A0A0Q0ILW6_PSEAP Uncharacterized protein OS=Pseudomonas syringae pv. aptata OX=83167 GN=ALO85_02354 PE=4 SV=1
MKLEMARGLFIFGALAVATVAVAALEQPSTRILITQHGEGYCPLPRVAKNLVNVQPDHDL
LLFMYSLAQGTGIKN
>tr|A0A0Q0C640|A0A0Q0C640_PSEAP Binding-protein dependent transport system inner membrane protein OS=Pseudomonas syringae pv. aptata OX=83167 GN=ALO85_03468 PE=3 SV=1
MPMSDTTLNLADPGFLVVKKPEQSRVSQSVRPRWQVPGFVLRLLSPLVLLLLWELASQTG
LLPVRVIAAPSQIGGTLWSMIVSGELAHHLWVSLQRALLGLSIGVSIGVVAALVTGLSRR
GEIVLDSPMQMLRTIPSLALVPLFILWFGIGEFTKVALIVMGTTFPVYLNLFSGIRNIDP
KLIEAANTLGLSRRELIWHVILPGSLPAFFVGLRYSLGISWLALVFVEQINTTAGIGYLA
SDARDFMRTDVIVICLLIYSVLGLVIDAIIRTLERYALAWRPTFVRH
>tr|A0A0Q0D8S3|A0A0Q0D8S3_PSEAP Chitin-binding protein OS=Pseudomonas syringae pv. aptata OX=83167 GN=ALO85_03762 PE=4 SV=1
MSDATTTGVKHGRITDPATRGSYAMDQSWVLEWHLHGIESGKNFPDTIGGEFAGFKSDVP
SAEPPKDGLIVSGGWTDERECLNYTDREVADRLAAKGNAEGWPRQGVKGVSTFMVTWRYE
APHVTRGYRWFITKDGWDESTRLTRAHFQEKPFYEEISLLDPYSEHREELAPKTEISAVL
PEGKQGHHVILLLWIVAESPMAFYQTLDVDFDKPPVK
>tr|A0A0Q0CVK2|A0A0Q0CVK2_PSEAP Uncharacterized protein OS=Pseudomonas syringae pv. aptata OX=83167 GN=ALO85_04136 PE=4 SV=1
MDMKRAVSFAVVATILTLTGCADLQKQAGAYMPGSQPAQPTTASYEGGQQVQFIPGNAKA
TNTSYQANNTSTANGQQDSSFIGDMMKTATDSVKSEAGSTVRSTVRGLFSR
>tr|A0A0Q0CIF0|A0A0Q0CIF0_PSEAP Inner-membrane translocator OS=Pseudomonas syringae pv. aptata OX=83167 GN=ALO85_03274 PE=3 SV=1
MKTLDLTRQRILPQSLFKRVLHDFPGVVSIGLFFALCFVLFALVTDNFLSGANLLNVIRQ
NAPLLIVAVAMTLVVTTGGIDLSVGSTLALVGALAAMALNAWHLPWPLVLLGGLAVGAMI
GVLNGYFIAYAGIPAFIVTLATLTIIRGLAMLLTQGYSIPVPREETAFLALGRGWFLGVP
LTAWLALLVTLGGVLVLGKMRFGRYLTGIGANAESVRRAGVNHRGVLLRVYMLSGMAAAM
AGMIVTARLGSGSSNQGEGFELEVIAAVVLGSTSLFGGFGTIVGTLLGVLTMAAIKNGLI

LAHVSPFYTQIATGLIVLLAIWLNTRILRTGRPLGRAGA
>tr|A0A0Q0C7M6|A0A0Q0C7M6_PSEAP Two-component response regulator with GGDEF domain OS=Pseudomonas syringae pv. aptata OX=83167 GN=ALO85_01352 PE=4 SV=1
MTYPEDPSRDRLKHHFAQRVIHQARQILETWQRLQKAEWSVGDMAELKESTQRLSRFAER
FEQVEHSALAQEISQALDVVEANRGRLNSRVITDLNRLMQRLSRTGLRHGDRFEQTALPP
LRKPVYIVLQDHERAERLAKQLEFFGLAALSLHNVAEFQRAMAQRHPAAIVMDVDFSGPG
KGLELASQAQEGLEQKLPLLFFSVNETDTPTRLAAVRAGGEEFLTGALEASSLLEKIEVL
TCVAQYEPYKVLIIDDSRAQALHTERVLNAAGIVTRVLLDPIQAMSELAEFQPDLIILDM
YMPGCTGTELAKVIRHNDRYVSVPIIYLSAEDDQDKQLDAMSEGGDDFLTKPIKPRHLIT
TVRNRAARARNLKARMVRDSLTGLYNHTHILQLLEDCSFRSRREGKPLCFAMLDIDHFKR
VNDSHGHPMGDRVIKSLALFLKQRLRKTDFIGRYGGEEFAIVMPDTDIQSAHGVLDDIRH
RFAEIHYPAQPADLFCTFSAGVVALGAHDDSLSLASQADTALYCAKHAGRNRVHSVLVDV
PYHVVRTEIEA
>tr|A0A0Q0DLG0|A0A0Q0DLG0_PSEAP Signal recognition particle protein OS=Pseudomonas syringae pv. aptata OX=83167 GN=ffh PE=3 SV=1
MVSVRRSLRYNLRPAQAAGSPSAISALRAGPHQPGFDESLKGSRMFENLTDRLSQTLRHV
TGKAKLNEDNIKDTLREVRMALLEADVALPVVKDFVNNVKERAVGTEVSRSLTPGQAFVK
IVQAELESLMGAANEDLVLNVTPPAVILMAGLQGAGKTTTAGKLARFLKERKKKTVMVVS
VDVYRPAAIKQLETLANDIGVTFFASDISQKPVDIAQAAIKEARLKFIDVVIVDTAGRLH
VDVEMMGEIQALHAAVKPAETLFVVDAMTGQDAANTAKAFGDALPLTGVILTKVDGDARG
GAALSVRAITGKPIKFIGMGEKSDALEPFHPDRIASRILGMGDVLSLIEQAEQTLDKDKA
DKLAKKLKKGKGFDLEDFRDQLQQMKNMGGLGGLMDKLPSIGGVNLSQMGNAQGAAEKQF
KQMEAIINSMTPAERRDPDLVSGSRKRRIAMGSGTQVQDIGRLIKQHKQMQKMMKKFSAK
GGMAKMMRGMGGMFPGGGMPKM
>tr|A0A0Q0FV18|A0A0Q0FV18_PSEAP NAD(P)-bd_dom domain-containing protein OS=Pseudomonas syringae pv. aptata OX=83167 GN=ALO85_03350 PE=4 SV=1
MIVVTGATGQLGRLVIEQLLSRVPASQIIAAVRSPEKAADLSRQGIQVRQADYSQPATLD
SAFAGADKVLLISSSEVGQRLPQHKAVIDAAKRAGVQLLAYTSVLHADTSALSLAKEHRE
TEDYLRANGLPFALLRNGWYTENYTAGIPGALAHGAVMGCADEGRISSASRLDYAEAAAV
LLTSKTAQAGNVYELAGDESYTLSEFAAQLSTQSGKSLPYVNLPQAEFEAALIQAGLPDF
VARLLADSDAAAAKDALFDDSRQLSALIGRPTTPLSATIAETLKG
>tr|A0A0N8T8Q3|A0A0N8T8Q3_PSEAP Syringafactin synthetase SyfB (Fragment) OS=Pseudomonas syringae pv. aptata OX=83167 GN=ALO85_04517 PE=4 SV=1
LAREDEPGQPRLVGYFTERAGAASSTVEQLRTALLARLPGYMVPGALVRLDSWPLTANGK
VDRRALPVPDRTALSTGEYQAPQGDLESALALIWSELLQVERVGRYDRFFELGGHSLLAM
RMVSQVRQRLSLELALGDLFADSSLIAVAQCLSAAARSQLPVIDVQPRNGPVPLSSAQQR
IWFMAQMEDANSAYNVSLGLKLNGPLDSRALTRALERIVARHDSLRSRFSQEDDIAWVQA
APVTEVPRISWQDLRDQDPQALQAVIREEAAQPFDLQHDLPIRGRLLCLAEDRHVLLLTV
HHIVADGWSLGVLTRELTALYQAFSQGQEDPLPALALQYGDYAVWQRNWLDDERLSHQAD
YWQQTLTGAPVLLTLPTDRPRPAHQDYTGASVALDLDPRLSNDLRAFCQAQSVTPFMLFM
GAWAVLLARLSGQEEVVVGMPVANRRHAEIEGLIGLFVNTLAVRVDTSGEPDVVTLLERI
KARVVEAQDHQDLPFEQVVERLRPPRSLAHSPLFQASLAWDGSQGLELQLGDMQLEPLDE
QAAFAKFDLALSVSDGPDHFRCIVEYATALFDHGTVERHLGYLKAILRAMVADSQTVVSH
IRLLSEAERRQLTDGFNAPDAVYPQEQTLHNQFEAQAQRTPDAIAVSYEEESWSYATLNA
HANRIAHRLIGMGIGADDRVAICTPRGLQMIAGLLGILKAGAAYVPLDPAYPLERLAYTL
DDSAPVALLSQRSVQGTLPVSEVPVICLDDDLQDESVCNPQVPVTPGNLAYVIYTSGSTG
KPKGVMIEHRNVARLFSATEEWFGFNQQDVWALFHSFAFDFSVWEIWGALLHGGHLVVVP
QLVSRSPEDFYTLLCSTGVTVLNQTPSAFRQLIAAQGENGQAHSLRKVIFGGEALDTVIL
KPWYARNVNAGTQLVNMYGITETTVHVTYYPLQPEDAQRLGASPIGRRIPDLQLYVLDAR
GEPVPMGVVGELYVGGAGVARGYLHREALTAERFLDNPFSHAANARMYRTGDLGRWLADG
SLEYMGRNDEQVKIRGFRIELGEIAARLNDHPDVLDAVVVAREDVPGDKRLVGYYTSAQD
KAGLDIEQLRAWLSGLLPEYMVPAAYVRLASLPVTANGKLDRKSLPAPDRDSIASRAYEA
PQGPIEIALASLWAELLHVEQVGRQDNFFELGGHSLLAVTLIARMRRLDMRADIRVLFVQ
PTLAALAEAVGGDTEIDVPANLIDAHCQRITPELLPLVALDQPAIERIVARVPGGAANVQ
DIYPLGPLQTGILYHHLTAGDRDPYLLQPQFAFADASRLEAFCHALQRVIERNDILRTGL
FWEGLQAPVQVVWRQAPLRVEETALQDLFNAPRMDLTQAPLLQLVYAHDPANQRIAAVLR
YHHVIMDHIALDILSHELQAILLDDETQLAAPVPYRNYIAHVVRGPGEDAHEAFFREQLG
DIDEPTLPYGLAMASAEQIPGEARLTLDSDLCRQIRDQARQLSVSAATLMHLAWAKVLGQ
LSGRDSVVFGTVLLGRLRGGEGGERALGVFINTLPLRMDLVGHCARSAVLDLHGRLVGML

AHEHAQLALTQRCSALPAGAPLFNTLLNYRHSAVPEVDDPANNTAWQGIEVVHAEERSNY
PLTLSIDDFGDSFSLTAQAAPGIDPQRICAYVQQAVVHLVQALEQQSAAALIESSVVPEA
EREQMSVSFNDTRRDYPRGQPVHRLFERRAALHPHAVAAVHGRRSLTYGELNERANHLAH
YLLGQGVRPNEHVAILLPRSLELLISQLAVGKCAATYVPLDVNAPAERQHYMLDDCQAKC
VLTQSATSMGSTVQRIDLDQLNLDDQPAHDPGLPQASDTAAYVMYTSGSTGAPKGVRVAH
RGIARLVLNNGYADFNEQDSIAFASNPAFDASTMEVWGALLNGGQLLVIEHTTLIDPMRF
SAALRQGNVSVLFLTSALFNQYVQLIPEALSGLRLLLSGGERADPASFRTLLAQAPGLHL
LNAYGPTETTTFATASDVRTLADHAESVPIGTPIGNTTVYVLDAHQHLTPLGAIGELYIG
GDGVALGYLNRPDLTAEKFIADPFSDQPGAMMYRTGDLGRWLEDGQLECLGRNDDQVKIR
GFRIELGEIVNCLHQLPGISEAVVLAREDEPGHVRLVAYFTSRLDAEAPAPEQMRAHVQA
NLPEYMVPGAFVELTVLPLTANGKLDSRALPKPDHSSLLGLAYEPPQGEIEIALAQIWAE
VLQLERVGRHDHFFDLGGHSLLAMRMVSQVRQQMGMELPLGELFALGELAAVASALAGAG
RSELSLILPAPRDQSLPLSFAQQRLWFLAQMEGGNQAYNIPLALSLQGSLDVAALTAALG
RIVERHETLRSRFIAREEGAEVVFTAPAAMSFLHIEDLRQNPQTLAERVAAEATAAFDLT
RGPLIRGCLLQVEDARHVLLLTVHHIVSDGWSMGVLTRELLALYPALRRGETDPLPALGI
QYVDYAVWQQGWMSGERLQHQAAYWRQVLEGAPTLLTLPADRPRPAQQDFAGASLNVRLD
GQLTAGLRALAQRQGVTLYMTLLTAWGALLARLSGQAEVVVGSPIAGRGRAELEGLIGLF
VNTLAIRIDTASAPTGEVLLAQVRTRVLEAQDHQDLPFEQVVEIVRPTRSLAHGPLFQTT
LNWLAGETTLPEMDGLSLATVEQQSQVSKFDLSLNLGEQGDALFGTLDYATALFDEATVQ
RYCGYFEQLLRTLVSNQQAVLADIPLVGPQEREYLLDTLNASAVSLPQGQTIHALIEARA
EGMPEAVAARVGEQSLSYAQMNRQANSLAHHLISLGVRPDDRVAVVARRGLDTLVSLLAV
LKSGACYVPVDPAHPDERINYLLTDSAPVVVLAEQAFMARLPALTVPLLALDRPQWPEQP
ANPLVSGQTPGDLAYVIYTSGSTGQPKGVMVEHRTLANLVHWHCQAFALHAGSHTATVAG
FGFDAMAWEVWPALCAGATLHIPPAEISNEQLDVLLDWWLAQPLQVAFLPTPVAEYAFSR
ELYHPTLHTLLIGGDRLRQFHREPGFAVINNYGPTETTVVATSGRLLPDGSLDIGKPIAN
TRVYLLDEQQQLVPTGVAGELYIGGEGVARGYLNQPQLTAERFLSDPFCEHPQARMYRTG
DLARWNADGTLDYLGRNDDQVKIRGMRIELGEIEAQLTSLPGIEESLVVAREDEPGQSRL
VAYFIEQGPRPALDIARLRADMLDRLPGYMVPSAFVRLDAWPLTANGKVDRRALPVPDRD
AMPGREYEPPQGELEMALADIWSDLLQVEQVGRNDHFFELGGHSLLAVTLIARMRRRGMD
ADIRVLFAQPTLAALARAVGSGAQVKIPANLIGADCASITPDLLPLVKLDQAGIDRVVAS
VTGGASNIQDIYPLGPLQAGIFYHYLSAAEDDPYRLQARFAFADRSRLEAFSQALQQVIA
RNDVLRTSLCWEGLETPVQVVWRHADLPVIEVPLAALDAPEPLELVRAPLLRLVHADDPE
NGRIVAVLLFHHLIMDHVALDLLSQELQAVLLDQAAQLPAPVPYRNYIAQTLLGAGDDAH
ETFFREQLGDLDEPTLAYAQTWLPGPDVAGEARLRLDMDLSRRLRHQVRQLGVSPASLMH
LAWAQVLGRLSGRDSVVFGTVLLGRLNGSEGAERTLGVFINTLPLRIDLADQSARDALSQ
THRRLTGLLAHEQASLAIAQRCSALPAGAPLFSALLNYRHSAAPGEANEAAGKAWKGIEL
LQSGERSSYPLTLSVDDLGEAFDLTALTSKGIDARRVCAYLACAVEGLVDALEQAPAGPI
QALNILPGAERNELLDGFNADRLTAECPLPVHLRIEQQALEQPNALAAQAGDQHLSYGEL
NARANALAHHLIGLGVRPDDRVAVVARRGLETLAGLLAVLKAGACYVPVDPGHPDERISY
LLENSEPVVVLAQFDLLTRLPELQVPVIALDRPDWSQRTDNPSVPEMTTQHLAYVIYTSG
STGLPKGVMVEHRTLNNLVDWHCEAFNLRAGSHTASVAGFGFDAMAWEVWPALCAGAVLH
LPPAEIGNEQLDVLLDWWLAQPLQVAFLPTPVAEYAFSRELHHPTLQTLLIGGDRLRHFN
RDPGFAVVNNYGPTETTVVASSGRMQPGKVLHIGKPVTHARLYVLDSRGQPVPLGVPGEL
YIGGAGVARGYLNRADLTAERFLDDPFSDRSGARMYRSGDLVRWLSDGTLEYLGRNDDQV
KIRGVRIEPGEIEQHLAQCPGVGEAVVTTQRLDDGTLRLVGYFTRRDASLNSAALRAHLL
GQLPEYMVPAVFVGLDALPLTQNGKVDRKALPAPDLAALANLAYQAPSTALEERLADLWA
EVLEVGKIGRHDSFFELGGHSLSAIRLVSLLQKAGVSLTLAELFQHPSVAALAGLLDQRP
GSPDEAREVITVRAGGSESPLFLLHDFTGLDAYFPVLGQHLQGDFPIYGLPGIGLGQQQL
RTMECLAARMVERIRQVQPRGPYRLAGWSFGGVLAYEVATQLLGMDEAVTFLGLIDSYVP
RLTDQGKARWQGPDLLERQLLSHCTAHWQAQAGEGAAKLARLNSLSEQAPLPDFATLLQL
CRDEGLLYEELALASDQQLHHYLDREVAHGQALAHYQLESLSLPIHLFRAEQRPMAPIGS
SSTLGWGEILSADLLRCVDVPGDHMTMMQAPHVAVLGLSIVQALHSAPATPPPPPAHQSL
LAIQSGRDGHAPVFCVPGAGDSVTSFIGLAEALGPDWPIHGLQPRGLDGRSVPHSRVEAA
AQSHVQAIEALYPKGPLHLVGHSFGGWAAHAMAVKLQASGREVVSLTLIDSEAPGGEGLD
GKPYTATAVLERLIEALQLSSGRSLELDPLRFAGSDTVTQMHLLQQAMVRVGLLPPRLAA
QALQGIVRTFASALRTVYRPEPGGYSGRASLVLVDDPQLDALDNQLEHAACASQWQHLLP
QLSLWHGPGNHFSVLKAPDVYSLAAWWYDGLAVGVGETQ
>tr|A0A0N8T8Y5|A0A0N8T8Y5_PSEAP Alginate biosynthesis protein AlgG OS=Pseudomonas syringae pv. aptata OX=83167 GN=ALO85_04966 PE=4 SV=1
MALLMRTAKGVPEMNSHTSNGRSRHWPHALLESALLTSALLMASGVALANAPVVPDAQKA

LVKELHQAKTYTITSPPTAPLEMAKPVLPDLKGYTAEAALKKIVRNKPGKVTVSRMMEET
GLKEFIGGDNKMAEWVNRQRGIPQAIMISDGYVNLQDLAKKVPKQFLNEVSPGVYVARLP
ILIKETGTFEIDSKTKELRLSQEKGSFIVSEGKMLITHTQVNGWSETRNSLATYRKPDEF
RPFLLTWGGSETWIANTKMASLGYDQSKSYGISISQYTPNTAKVLKRPEPTGWIIDSEFS
DMWYGFYCYETRDFVVKGNTYRDNIIYGIDPHDRSHGLIIAENDVYGTKKKHGIIISREV
DNSFIFRNKSHNNKLSGVVLDRNSVGNIVAYNEIYQNHTDGITLYESGNNLLWGNRVIAN
RRHGIRVRNSVNIKLYENVAMGNGLMGVYGHIKDLSDTDRDIELDPFDAQVSLIMVGGEL
SSNGSGPLSIDSPLSVELYRVSMLMPTKEVGISLNGVLGERQDEILDLLVRQKKAVLIDP
VESQTELRE
>tr|A0A0Q0DPX2|A0A0Q0DPX2_PSEAP Methyl-accepting chemotaxis protein OS=Pseudomonas syringae pv. aptata OX=83167 GN=ALO85_00805 PE=4 SV=1
MSIKLRLFLLIGTSVLTVMIISLVNYLGNTRTEAAMLDSEVSMAALGNHLEADMMHDALR
ADVLSAMLFGLGRSNSSRDEVQVSLKEHAALFRKVVSDNLQLPLTDAIKAELSRIKPSLD
AYISAGERIVGLAIDSPDRAQQELGTFSSAFTQLEGQMSSLSDLIEDNSKASGERTRQAI
SSANVTLAVVLSISILLLLIQGHWVTRSIMIPLASASRIADGIAHGNLREPIAESGGRDE
ASMLIRSLAIMQRDLRSMIEVVRSNANDVSGMSRQLSSGCHEVADSSRQQSSAAGTMSAA
TSEMTASIEEITRHAGQALEMASQAESLAKNGGRVIHQVVSDMDSIARSAQQSAQVIRTL
DKDSEGIFNIIQVIKGIADQTNLLALNAAIEAARAGEQGRGFAVVADEVRSLAGRTSAST
QEITTMVARIQQSTREAVTSMEAGVAQVDKGMAVTAEVERAISDILDATLSTTQLVNDIT
RTIGEQSLASNEIAHQVEMIASMSEGNSRVIGQTASTTDELSVMAGQLSQSVDRFQL
>tr|A0A0Q0C6A2|A0A0Q0C6A2_PSEAP Type IV secretion system protein (Fragment) OS=Pseudomonas syringae pv. aptata OX=83167 GN=ALO85_05147 PE=4 SV=1
MRFLNRSTGRTDGPALTISNGEVTMDLFKCLARMGKQPLRIATAFSSLMCAQQCFAQIPV
TVTSQVTESPMTIAEFASNSTRWSQQVQQMASQIDQMKQQYGAITGSRGLGRVFDDPQLR
EYLPQEWQAVYDSVKRGGYSGLDGRAEMIYADNKVFDACQSFAQDEQRAACQAQAVKPSQ
DKAFALDAYDQAKGRLRQLDQLMGQIDKTQYPKGIAELQGRIAAEQAMIQNEQTKLQMFQ
MVAQAEDKIQEQRQR
>tr|A0A0N8T9T8|A0A0N8T9T8_PSEAP MvaT-like transcriptional regulator MvaV OS=Pseudomonas syringae pv. aptata OX=83167 GN=ALO85_03672 PE=4 SV=1
MSRLAEFRAAEKALQEQLAQLESLKNDAGLKKEIEFEQKLQDLMGTYGKSLRDIIAILDP
SSATSLVAPAGPKRRRARVVKVYQNPHTGELIETKGGNHRGLKAWKEQYGVDTVDSWLRA
>tr|A0A0Q0CZQ4|A0A0Q0CZQ4_PSEAP DNA gyrase subunit A OS=Pseudomonas syringae pv. aptata OX=83167 GN=gyrA PE=3 SV=1
MLAAASFVYPEWVCEVSPEKGIRLLMGELAKEILPVNIEDELKQSYLDYAMSVIVGRALP
DARDGLKPVHRRVLFAMSELGNDWNKPYKKSARVVGDVIGKYHPHGDTAVYDTIVRMAQP
FSLRYLLVDGQGNFGSVDGDNAAAMRYTEVRMTKLAHELLADLHKETVDWVPNYDGTEMI
PAVMPTRIPNLLVNGSSGIAVGMATNIPPHNLGEVIDGCLALIDNPELTVDELMQYIPGP
DFPTAAIINGRAGIIEAYRTGRGRIYMRARSIVEDIDKVGGRQQIVITELPYQLNKARLI
EKIAELVKEKKLEGITELRDESDKDGMRVVIELRRGEVPEVILNNLYAQTQLQSVFGINI
VALIDGRPRILNLKDLLEAFVRHRREVVTRRTVFELRKARERGHILEGQAVALSNIDPVI
ALIKASPTPAEAKEALIKTPWESSAVVEMVERAGADSCRPENLDPQYGLREGKYFLSPEQ
AQAILELRLHRLTGLEHEKLLGEYQEILNQIGELIRILNSATRLMEVIREELELIRSEYG
DARRTEILDARLDLTLGDLITEEERVVTISHGGYAKTQPLAVYQAQRRGGKGKSATGIKD
EDYIAHLLVANSHTTLLMFSSKGKVYWLKTYEIPEASRAARGRPLVNLLPLSDGEYITTM
LPVDLEAMRKRADEEGEALEGELDDAENSSETEEERKARIKAADKKKAPFIFMSTANGTV
KKTPLVAFSRQRSSGLIALELDEGDILISAAITDGEQEIMLFSDGGKVTRFKESDVRAMG
RTARGVRGMRLPEGQKLISMLIPEEGSQILTASERGYGKRTAISEFPEYKRGGQGVIAMV
SNERNGRLVGAVQVLDGEEIMLISDQGTLVRTRVGEVSSLGRNTQGVTLIKLASDEKLVG
LERVQEPSEVEGEELEGELVDGVIVDAAEVDDAGEGLQADAASDEDEPQN
>tr|A0A0Q0C277|A0A0Q0C277_PSEAP Oligopeptide/dipeptide/nickel ABC-type transport system, periplasmic substrate-binding protein OS=Pseudomonas syringae pv. aptata OX=83167 GN=ALO85_03910 PE=4 SV=1
MADKKTDSALITGQDSLRVFEGLNRGLSRRDALRMLGVAGVVAAGSTSLFGAAGKVFADD
ADAPVKGKPGGRIRVAGMSSSTADTLDPAKGALSTDYVRHFMFYNGLTRFDSHLVPHMEL
AEKIESDNATVWTVTLRQGVTFHNGKALTAGDVVFSLSRHKDPATGSKVMPLMAQFSEIK
ATGPLEVQITLSSPNAELPSILAVSHLLIVPEGTTDFSKGIGTGPFTVKEFNPGVRSVSA
RNPNYWKPGLPYLDEIEFIAIADEPSRVNALLSGDVHMINEVNPRSTTRIAASAKHRVVD
APSGNYTDLIIRQDQMPGKSPEFTEAMKYLLDREQVKSAVFRGYARVGNDHPISPGSRYF
NADLPQRAYDPEKAKFLLKKAGMESITMAVAASPAATGSVDIAVLLQQSAKQAGLTLNVN

RLPSDGYWSNHWMKHPLSFGNINPRPNADVIFSQFFQSTAPWNESGWKNDQFDQLLVLAR
GETDDAKRGKMYGDMQALVSQNCGVGIPVFISNIDGVDKRVQGYSSNPLGGFMGYMFSEQ
VWLDA
>tr|A0A0Q0IDG0|A0A0Q0IDG0_PSEAP RND-type cation/multidrug efflux system, inner membrane pump protein OS=Pseudomonas syringae pv. aptata OX=83167 GN=ALO85_01671 PE=3 SV=1
MKGNFNLSEWAIKHQSFVWYLMFVALLMGVFSYMKLGREEDPSFTIKTMIIQTRWPGATV
DETLEQVTDRIEKKLEELDSLDYVKSYTRPGESTVFVYLRDTTNAKAIPEIWYQVRKKVD
DIRGQFPQGLQGPSFNDEFGDVYGSIYAFTADGFSMRQLRDYVEKVRADIRDVPGLGKVE
MIGQQDEVVYLNFSTRKLAALGIDQSQVVQSLQSQNAVTPAGVIEAGPERISVRTSGQFA
SEKDLAAVNLRINDRFYRLSDIADITRGYTDPPKPLFRFDGKPAIGLAIAMQKGGNIQSF
GKALHAQMDATTAELPVGIGVHKVSDQAEVVNKAVGGFTSALFEAVIIVLLVSFVSLGFR
AGLVVACSIPLVLAMVFVFMEYSGITMQRISLGALIIALGLLVDDAMITVEMMVTRLEMG
ESKEQAATYAYTSTAFPMLTGTLVTVAGFVPIGLNNSSAGEYTFTLFAVIAVAMLVSWVV
AVLFAPVIGVHILSANIKPKSEEPGRIGRAFNGSMLWAMRHRWLAIAITVGLFAASLFSM
QFVQNQFFPSSDRPEILVDLNLPQNASINETRKVVDRFEASLKDDPDIERWSTYIGQGAL
RFYLPLDQQLENPFYAQLVIVSKGLEERGALTARLQKRLRDDFVGIGSYVQALEMGPPVG
RPLQYRVSGENIDKVRQHAIELATLLDQNPHVGEVIYDWNEPGKVLRIDINQDKARQLGL
SSEDVAKLMNSVVSGSTVTQVRDDIYLINVIGRAEDAERGTPETLQNLQIVTPTGTSIPL
LAFATVGYELEQPLVWRRDRKPTITVKGAVRDAIQPTDLVKQLQPEIDKFAAGLPVGYKV
ATGGTVEESSKAQGPIASVAPLMLFLMATFLMIQLHSIQKMFLVASVAPLGLIGVVLALI
PTGTPLGFVAILGVLALIGIIIRNSVILVTQIDAYEKSGYLPWDAVVEATEHRRRPILLT
AAAASLGMIPIAREVFWGPMAYAMIGGIIIATLLTLLFLPALYVAWYRIKEPTDEQRREA
EEKNEDENAQPAH
>tr|A0A0Q0FU53|A0A0Q0FU53_PSEAP Binding-protein dependent transport system inner membrane protein OS=Pseudomonas syringae pv. aptata OX=83167 GN=ALO85_200059 PE=4 SV=1
MFFSTPIGTTRQYNFKASCVVYFSLPYLNQIKPYPVVYTGFLLATL
>tr|A0A0Q0CDV3|A0A0Q0CDV3_PSEAP Histidine kinase, HAMP region: chemotaxis sensory transducer OS=Pseudomonas syringae pv. aptata OX=83167 GN=ALO85_02619 PE=4 SV=1
MKAGNYAAARTQLNSLSSESFTKVRSYMRTMIDSNNRQIKEGAAAAADLKANSVLMLEIG
VVIAFLVAIMLGVLITRMITRPLAVAVASAQRIAGGDLTQPIVSNSGDEAGQLLNALSDM
QNGLKNTIQQITSASDQLASAAEELSAVTDESTRGLTRQNDEIQQAATAVNQMTAAVEEV
ARNAVSTSEASKSATDDAVDGRGQVDHTVKGITTMVHEITASTGAVSELAGHVREISKVL
DVIRSIAEQTNLLALNAAIEAARAGEQGRGFAVVADEVRALAHRTQASTVEIEGMISTVQ
SGADGAVAAMGKSLSLATNTQELAQRAGAALEKITLGVATINERNLVIASASEEQAQVAR
EVDRNLINIQDLSTQSAAGANQTSASSQELSRLATSFNSLVANFKL
>tr|A0A0Q0FUH8|A0A0Q0FUH8_PSEAP Aminopeptidase N OS=Pseudomonas syringae pv. aptata OX=83167 GN=ALO85_04598 PE=4 SV=1
MRTEQPKMIYLKDYQAPEYLIDETNLTFELFDDHSLVHAQLVMRRNPERGAGLPALVLDG
QNLELVSVKLSDVELSAADYQLTDDHLTLHPKADTFTVDSTVRIHPESNTALEGLYKSSG
MFCTQCEAEGFRKITYYLDRPDVMSKFTTTVSADKQGFPILLSNGNPVASGEEEGGRHWA
TWEDPFMKPAYLFALVAGDLWCIEDTFTTMNERNVTLRIYVEPENVDKCQHAMTSLKKSM
RWDEETYGREYDLDIFMIVAVNDFNMGAMENKGLNIFNSSAVLARAETATDAAHQRVEAI
VAHEYFHNWSGNRVTCRDWFQLSLKEGFTVYRDAGFSADMNSATVKRIQDVAYLRTHQFA
EDAGPMAHAVRPDSFIEISNFYTLTVYEKGSEVVGMLHTLLGAEGFRQGSDLYFARHDGQ
AVTCDDFIKAMEDANGVDLTQFKRWYSQAGTPRLAVSESYDAAAKTYSLTFRQSCPTTPG
QPGDQKQPFVIPVELGLLDSKGSEIALRLANETTATGTTRVLSVTEAEQTFTFVDVAEKP
LPSLLRGFSAPVKLSFPYDRDQLMFLMQHDSDGFNRWDAGQQLSVQVLQELIGQHQQGQP
LVMDQRLITALGSVLADDGLDQAMVAEMLSLPGEAYLAEISEVADVDAIHAARDFARQQL
AEGLFDGLLARYQANREVSKVTPYVAEAAHFARRALQNIALSYLMLSGKPEVLAATIDQF
DTSDNMTERLTALAVLVNSPFTAERDKALAVFAENFKSNALVMDQWFSVQAASTLPGGLQ
RVQELMKHPAFSIRNPNKVRALIGAFAGQNLVNFHAADGSGYRFLADLVIELNALNPQIA
SRQLAPLTRWRKYDSARQALMKAELERILASGKLSADVYEVVSKSLAE
>tr|A0A0N8T9Q5|A0A0N8T9Q5_PSEAP Choline transporter OS=Pseudomonas syringae pv. aptata OX=83167 GN=ALO85_05043 PE=3 SV=1
MPAHNRRLTLAVSAFCSWFYQSGGTQPMSSSSSSSSGMVRMNAPVFYFAASFILIFGIVV
IAFPQASGEWLLAAQNWAANTVGWYYMMVMTLYLVFVVVTALSGFGKIKLGADHDEPEFS
YLSWAGMLFAAGISITLFFFCVSEPLTHLLQPPQGEGGTAEAARQGMQLLFLHWGLHGWG

VFAFVGMALAYFAYRHNLPLALRSALYPLIGKRINGPIGYAVDGFGIIATIFGLGADMGF
GVLHLNSGLDYLFGVAHTHWIQVGLITLMMGAAILVAIAGVDKGVRVMSDINMLLACALL
LFVLFAGPTQHLLNTLVQNIGDYLGALPSKSFDVYAYNEPSDWLGGWTVFYWAWWIAWAP
FVGLFIARISRGRTIREFVFGVLLIPLGFTLAWMSIFGNSAIDQVLNHGMTALGQSAIDD
PSMTLYLLLETYPWSKTVIAVTVFISFVFFVTSADSGTVVLSTLSAKGGNPDEDGPKWLR
VFWGVATALITSGLLFSGSIDALKSAVVLTSLPFSLILLLMMWGLHKAFVMESQRKIAQL
YSLAPVSGSRRGGWRQRLSQAVHYPSRDEVYRFLDQTVRPAIEEVTAVFVEKGLNVVNVP
DPSNDSVTLEIGHGEERPFIYQVQMKGFFTPSFARGGMGSKQLNNRRYYRAEVHLSEGSQ
DYDLVGYTKEQVINDVLDQYERHMQFLHLVR
>tr|A0A0Q0DYZ3|A0A0Q0DYZ3_PSEAP HAD superfamily hydrolase OS=Pseudomonas syringae pv. aptata OX=83167 GN=ALO85_04324 PE=4 SV=1
MSEQQKQRGPIKAVIFDMDGLLLDTEGVYTEVTHLIASRHGRTFDWSVKQHTIGRGARDF
SDYVIKALELPMSIDEFLEVREPMLEERFPRAAAMPGAEALVRHLAAHNIPIAVGTSSSV
HYFKAKTTLHRAWFELFDTVVTADDPEVGAAKPAPDIFLVAARRLGVSPADCLVFEDSPF
GVTAAKAAGMYAVAVPDSHMPVEQYEHADLLLGSLADFPLTAWGLPELA
>tr|A0A0Q0BRB7|A0A0Q0BRB7_PSEAP Uncharacterized protein OS=Pseudomonas syringae pv. aptata OX=83167 GN=ALO85_02301 PE=4 SV=1
MHPTPAKGPLMASPDKQQKRAKRAKIKAKQNRSGKAATSLVHPVFASALVNQPFDDVELD
LNTFDFSDIVENGFDPADYDDLFTAMKQAEAISQLALCVVFLQYPVLALVISEEDEDPAT
DFMMGLLIVYRAMYHDEDEDSAIEWIESPAFQKDYTLASDLLTKRDTRAAR
>tr|A0A0Q0IAX5|A0A0Q0IAX5_PSEAP N-succinylglutamate 5-semialdehyde dehydrogenase OS=Pseudomonas syringae pv. aptata OX=83167 GN=astD PE=3 SV=1
MNSLYIAGAWQDGQGEVVNSLNPVTQQVLWSGRGASAAQVEQAVQAARQAFPAWALLSLD
QRIAVLEAFTARLKHHADALAQCIGEETGKPLWESATEVTSMVNKIAISVQSYRERTGEK
SGPLGDATAVLRHKPHGVVAVFGPYNFPGHLPNGHIVPALLAGNVVLFKPSELTPKVAEL
TVKCWIEAGLPAGVLNLLQGGRETGIALAANPGIDGLFFTGSSRTGNALHQQFAGRPDKI
LALEMGGNNPLIVDQVQDIDAAVYTIIQSAFISAGQRCTCARRLLVPEGDWGDALLARLV
GVSATIEAGAFDQQPAPFMGSVISLEAARALLDAQRNLLANGALTLLEMRQPQPGSALLT
PGIIDVSAVPDRPDEELFGPLLQVIRYAGFDAAIAEANATRYGLAAGLLSDSAARYQQFW
LHSRAGIVNWNKPLTGAASSAPFGGVGASGNHRASAYYAADYCAYPVASLEAGSLTLPAT
LTPGIRLS
>tr|A0A0Q0IFB3|A0A0Q0IFB3_PSEAP Amino acid adenylation (Fragment) OS=Pseudomonas syringae pv. aptata OX=83167 GN=ALO85_05343 PE=4 SV=1
MTSSHQHVSNNSNVSYELSSVQQGIWLGQIANPDIPLYNIGMKLEIKGDLDIPMFEKAIN
LVVRHNDALRLALFQEGDIARQRVLPSVDITLELVDFSGYVDSVERAQEYLQHAFSRPFN
PLEGILWEAQLVRCSASRHYWLGRYHHLVMDGSGATLFCHAVAKAYNNLLVGIDELEEGP
SYRDFLVKDQAYLNSPRLERDRSFWRERYAQIPPSLLQWSGDHAVGSMCKSGHIRSTIKR
DLFNAVAAVATEHGLSPVTVFFAVVSAYFSRVGGAEEVIIGMPLHNRTTARQKQTIGMFS
SVIPIGVRVDPNRSLLELMGDVAAELRRCYRHEHFPIAELNRALNIGHSRRKQIFDITLS
FEKFDADFFFGGAPSKAIRMYSGFDQTPLSIAICDYYSDEDVVVDFNFNLACFRPDEVER
IRDRITLLLESVAAHESTPIGQLALMGEAERRQVLVEFNATHQALQQDLLVHQLFEQQAQ
QQPQALALVCGDERVTYAELNERSNQVAHALLSLGIAPDDRVAICVERSVEMVVGLLGIL
KAGAAYVPLDPGYPPERLRYMLEDSAPVAVLVQRTTRELLGALAMPVLDLQSVNRAAEAW
HDRVLPTVAPQHLAYVIYTSGSTGQPKGVMIEHRNLVNLVAWHCEAFGLTHRKRVSSVAG
VGFDACVWELWPALCVGASLSLLPGQALGNDVDALLGWWRRQDLDVSFLPTPIAEIAFAQ
GIEPASLQTLLIGGDRLRQFPNPDSRVALINNYGPTETTVVATSGLIDATQSVLHIGRPI
ANTQVYLLDAHGQPVPIGVSGEIYIGGAGVARGYLNRPELTAERFLDDPFSAETAARMYR
TGDLGRWLADGNIEYLGRNDDQVKLRGVRIELGEIEAQLRQIADIRDAVVIAQEDTPGEK
RLTAYYTMQEAAQAMTAQTLRAALQARLPEYMVPAAYVKVSEWPLTPNGKLDRRALPAPE
DDAYASRDYEAPAGEVERALAEIWQELLGVDRAGRNDHFFELGGHSLLAMRLISLVRQRL
NVELELAALFANPQLEALACVVAQAQNNTLPQIVPTSREAQLPLSFAQQRLWFLAQMEGA
SAAYHIPAGLRIVGALDEAALQRALNRIVARHEVLRTTFVQTGDQAVVLCIHPEETGCPL
RKYDLTTHADSSSELARLMDEEAIGRFDLQQGPLIRGSLVRLSDDEHVLLLTMHHIVSDG
WSMGVLTRELGALYASGCQAEADPLPQLSIQYADYAVWQRGWLSGEVLHKQSTYWQTALL
DAPALLMLPSDRVRPAQQDYTGDAVPVVLDATLSHELKALSRRHGTTLFMTLLAGWATVL
SRLSGQDEVVIGSPVANRMSAEVEGLIGFFVNTLALRVNVSDDPTVEALLSRVKACTLAA
YEHQDLPFEQVVELLKPVRSLSHSPLFQAMLSWQNTPPAELALEGLELTLLDSVARTTKY
DVSLDLAEVEGRIVGSLEYATALFDRETAQRYVAYLERALRAMVENERQVVGAIELLGKT
ERRQVLVEFNATHQAWPQDLLVHQLFEQQAQQQPQALALVCGDERVTYADLNERSNQVAD

ILLSLGIAPDDRVAICVERSVEMVVGLLGILKAGGAYVPLDPGYPPERLRYMLEDSVPVA
VLVQRTTRDLLGALAMPVLDLQGVNWAAETEHDRVLPTVTPQHLAYVIYTSGSTGQPKGV
MIEHRAIVNRLLWAQDQYRLSREDRVLQKTPFGFDVSVWEFFLPLLAGAQLVIARPGGHQ
DPEYLAELIADSGVTILHFVPSMLQSFLNQAGPLACSTLRQVFCSGEALPYSLLKHFEQR
FAHVQLHNLYGPTEAAVDVTYWHCVPDLHAGIVPIGRPVANTRMYLLDPHMQPVPVGVCG
EIYIAGVQLARGYLNRDELTAERFVKDRFSNDPTARMYRSGDLGRWLADGNIEYLGRNDD
QVKLRGFRIELGEIEAQLAGCPGVGEAVVIAREDTPGDKRLIAYYTTREAKEIIAVQTLR
AALQASLPEYMVPAAYVKLPGWPLTPNGKLNRRALPVPEADAYVSRGYEAPCGTVETALA
EIWQELLGVERVGRHDHFFELGGHSLLAVSLMGRMRQAGLSADVRLLFAQPTLSAFAEVL
GNGHEVEVPANGIVVGCTRITPSMLPLADLSEDAIERIVRAVPGGTANVQDIYALAPLQE
GVLFHHLASVEGDPYLLNTRFALPSEDHLKAFFRALQGVIARHDILRTAVLWEGLDEPVQ
TVWREAPLAVEALELKAADRDIEQQLQERFDPRHYRLDLTKAPLMKMAYAYDEANSRWVA
ILLFHHMVLDHTALEVVQHEVQADLLGQAAGLPDSVPYRNHVAQARLGVSQAAHEAFFQE
MLGQVTEPTLPFGLHDVQGDGRDIKQARQAVAAELAGRIRRQARSLGVSAASLHHLAWAQ
VLGRTSGQEDVVFGTVLLGRMQGGGGADRSLGMFINTLPLCLNVGEQGVREGVKATHARL
TALLGHEHASLALAQRCSAIAPPLPLFSALLNYRHSAEPQVSDQGDRAWGGIEVLGWNER
TNYPLTLAVDDLGEGFALTVQATAGIDPQRVCAYMETALESLVDALEHSPESLLRSLEML
PRSERQLLQEWNATAVDYPQGTCVHQLFEAQVEKTPEAIALVFEARTFTYAQLNARANQL
AHHLIGLGIGPDDRVAICVERSPEMVVGLLGILKAGAAYVPLDPAYPEQRLRYMLEDSAP
AAVLVQSATRALSGELAVPLLDLEGGCWEAEADHDPVARAVKPDHLAYVIYTSGSSGQPK
GVLIEQRGFLNLMHWYLAELKLASDDAVLLVSSYSFDLTQKNILGPLLVGGTLHLAREAF
IPEVLLEQIQRERITHINLSPSAFNTLIDANDSRQLDSIRRVVLGGEPIQVARLEMLPEP
RPEFINSYGPTECSDVVAWHRLVDDIDCYRSSVFPIGRPIANTRIYLLDTHGQPVPIGVI
GEIHIGGAGVARGYLNLPELTAERFLDDPFSAEPAARMYRSGDLGRWLADGNIEYLGRND
DQVKLRGFRIELGEIESKLSECPGVREAVVLVREHR
>tr|A0A0Q0DSY5|A0A0Q0DSY5_PSEAP Putative periplasmic ligand-binding protein OS=Pseudomonas syringae pv. aptata OX=83167 GN=ALO85_03486 PE=4 SV=1
MVMNSEEQTLIDGLFSKLKDAETASAPRDAAAEARIKEHLTRQPAAPYYMTQAILVQEAA
VNQLNQQVKQRDEQIQQLQAELQQAKGQASSAPASGGFLSSIFGSSAPRTSQPQPSQPSS
GGWRDGGGFNPAPAPAAAQGGYAAPAPAAGSGFLGGALKTAAGVAGGVLLAEGISSMFSH
HSQPQEIVEVIHDAPQAQDTGLSNDQGGWGQPDQQYTADDSFDDGGDSFSDDDSFV
>tr|A0A0Q0DB34|A0A0Q0DB34_PSEAP Pili assembly chaperone: pili assembly chaperone OS=Pseudomonas syringae pv. aptata OX=83167 GN=ALO85_04271 PE=4 SV=1
MNVSMFPDFFRYTMLAALLFCASTAQAGIVLNTTRVIYQGTDKEVSLGVHNSGGGEILLQ
SWLESPVAAAGTHESLPNLPFIVTPALTRMAGGGRQLLRIIHSGTDMPTDRESVLWLNVQ
EIPQTAAENTLQIAVRQRIKVFFRPDGLQGDPQQAPERLNWQWIDDTGVHVENPGPYHVS
MLRISIRQHDTELALLDSQMLAPHQRLNIPLHHTPASAPLLLSFVSLNDYGGQVPYQATL
IHKQPVNADKVSPR
>tr|A0A0N8T8L5|A0A0N8T8L5_PSEAP ABC transporter OS=Pseudomonas syringae pv. aptata OX=83167 GN=ALO85_00640 PE=4 SV=1
MNALPHPPSAPVLGCSGLGYSVRGAALLRDVDLSIAAGETLAVVGPNGSGKSTLLKLLAG
IQKPVSGSVSLGGQALEALSRRDVARLLAVVEQQAETSDAVTVLDAVELGRTPWLSALEP
WSAEDDAIVRQALQDVDMLHLQKRAWHTLSGGERQRVHIARALAQRPQILLLDEPTNHLD
IQHQLTILGLVRALPVTTVIALHDLNQALDCDRVAVMEKGRLVALGAPLEVLTPERLLST
FGVVAHWLTDPSDGAKILRLRSR
>tr|A0A0Q0DDX0|A0A0Q0DDX0_PSEAP Peptidase M20:Peptidase M20 OS=Pseudomonas syringae pv. aptata OX=83167 GN=ALO85_04787 PE=4 SV=1
MLITLSYILIHICRTSLACGVEQRIGVKMLFNSRSSEGTSMHSRRFPITAAIGFSLASMG
LFSMVASAAELSPDQLLKKAQAEQPAYLDTVRQLVDIDTGTGQAIGLKTVSAMLVERLKA
LGAEVSTTPAEPSAGDNIVGTFEGNGTRSFLLMVHYDTVFGPGTAAKRPFRLDSERAYGP
GVADAKGGVAMILHALQLLQNEQFKAFGTLTVLFNPDEETGSSGSKKVIAELARQHDYVF
SYEPPDKDAVTVATNGINGLILDVKGKSSHAGSAPEAGRNAAIELAHQLLQLKDLGDPDK
GTTVNWTLIKGGEKRNIIPSSASAEADMRYSDLSESDRVLADGQRMVKKTLVDGTEVTLR
MEKGRPPLARNPGSEQLAKTAQTLYQKTGRTLEPIAMRFGTDAGYAYVPGSAKPAVLETM
GVVGAGLHADDEYIELSSIAPRLYLTVALITQLSKGDATP
>tr|A0A0Q0CVA9|A0A0Q0CVA9_PSEAP APH domain-containing protein OS=Pseudomonas syringae pv. aptata OX=83167 GN=ALO85_02277 PE=4 SV=1
MPLATLIRRSSLPCPEVSVDQALQLLSEHYGLGGTLKALGSQQDRNFLLDTGTRRYVLKI

CHGAYSTTELNAQHAALHHLAGHSAFKVPGVIRANGTEQLLSVDIDGQAVHVRLLEFIDG
QSLGHVEHLGRDIVVELGELCAHVDIALADFDHPGLQRILQWDPRHAHALIKHLLPVIKD
ADARACLIEAGERAHRRLLPLIAALPIQAVHLDISEHNAVWRREAGHPWHLQGLIDFGDL
LSTWRVADLSVTCAALLHHAEGDPLYILPAIAAYHALNPLKIEELQALWPLIVARSAVLV
LSSEQQASVEPGNAYIQANLAGEWNIFDVATSVPMALMEAAILRALGLDLPPVEQPAYSP
LLPSLSGLQPVLVDLGVLSEHFVAGNWEQSGIDEYLLSQAAGDSGLAASRFGEYRLSRTL
PDCASEPETFALHVELHVPAATPLHAPFDGTLRLTADAAVLLVGERISLKLWGVLPEASL
AGQVAASTLIGQGGGSLLLQLCTEPDLSPPLFTTPAWADVWRAVCPSPSALLGFDCDAPA
LQDAAQLLARRDASFARSQKHYYQAPPQIERGWRNHLIDMQGRSYLDMLNNVAVLGHGHP
RMAHEAARQWSLLNTNSRFHYAAIAEFSERLLKLAPEGMDRVFLVNSGTEANDLAIRLAW
AYSGGRDMLSVLEAYHGWSVATDAISTSIADNPQALSTRPDWVHPVTAPNTYRGPFRGAD
SAPEYVRSVDQALATLAEQQRQVAGFICEPVYGNAGGISLPPGYLQQVYQKVRAVGGVCI
ADEVQVGYGRLGHYFWGFEEQGVVPDIISMAKGMGNGHPLGAVITRREIAEALEAEGYFF
SSSGGSPVSCRIGMAVLDVMEEEKLWDNARIVGDHFKARLQALADKHPLVGAVHGMGFYL
GMELVRDRHTLEPATEETARLCERLRELGIFMQPTGDHLNILKIKPPICTTRRSVDFFVD
NVSKVLRELE
>tr|A0A0Q0DXW3|A0A0Q0DXW3_PSEAP Nitrilase/cyanide hydratase and
apolipoprotein N-acyltransferase OS=Pseudomonas syringae pv. aptata
OX=83167 GN=ALO85_00795 PE=4 SV=1
MTFPILAAAQFCSTRGDIQHNLSGHLAFMQRAAELGASYLLFPELSLTGYEPDLARELVL
LPDDARLAPLVALAVKLQLTTTVGVPLRGPSDSVLIGALTFTAAGDVIAYAKQYLHPGED
AVFTAGNQDCFLLVDQHRIGLCVCADFAHAEHAQRMAQGGSWVYAASVLISPGGYEYDAG
LLSGHAGRHSLPVLMANHGGPTGGXXSRRGAVVCGTRQGAGLAGWKVEVAVW
>tr|A0A0Q0C3T7|A0A0Q0C3T7_PSEAP Uncharacterized protein OS=Pseudomonas
syringae pv. aptata OX=83167 GN=ALO85_01525 PE=4 SV=1
MPVRNKGVKPVRDVAKRTKRGRRAYIVPTDRNANGIVAGESFLERNHLLLLSLMPGFTRI
SDQSICVDLENENLLISRQDLPKKDGKKAVAYTADSEIERHDGQIFICESKPSAFLEKHA
DKHARAERILATYGKQFITFTEEKFSPVFISNLENLKKALSPAQKERAAGACEKVGIALK
NRKRWPTLELKESGALSNADIFFGLAYGVLSADLSENVFSGDGWVNAAHGSLDHLKLMDI
>tr|A0A0Q0CG59|A0A0Q0CG59_PSEAP Putative M20 peptidase family dipeptidase
OS=Pseudomonas syringae pv. aptata OX=83167 GN=ALO85_04658 PE=4 SV=1
MSSRSHAIENVTEQFDNGAFFALLGRSVAYPTQSQEAESLPELYRYLHEFITPHVERLGF
SVTVHDNPVAGRGPLMIATRIEAAELPTLLSYGHGDVVRGYDAQWQTGLSPWQVIERGER
WYGRGTADNKGQHLINLTALEQTLKARDGKLGFNVKLLLEMGEEDGSPGLSAFCQAHAEE
LAADIFIASDGPRLAAARPTLFLGSRGVFNFELRVNLREGAHHSGNWGGLLANPGIILAN
AIASMVDEHGRVKVAGLMPKAIPDAVKAALADIEVGGGPGDPDIDADWGNPALSLSEKVF
GWNTLDVLAFKTGNPDAPVHAIPGKAHALCHIRFVVDSDYNAFIPAVRAHLDAHGFDRVE
VRQTRMDVMHATRLSPDSPWVGWALDSLATTTGKKPALLPNLGGSLPNDVFAEVLGLPTV
WVPHSYPACSQHAPDEHLLAPVVKESLQIMAGLFWDLGTDGARLTREQRAQELSE
>tr|A0A0Q0C0M9|A0A0Q0C0M9_PSEAP DUF1989 domain-containing protein
OS=Pseudomonas syringae pv. aptata OX=83167 GN=ALO85_00987 PE=4 SV=1
MQYQPVHPATEKPSMYKDYPAAYQVSKGAALQVDIAFYERIRTNAQQRTLVEQFEVPIRT
GRAWKVKAGQVFRVTTPSGPQVGDFNVWNANDPRERLWAARTRQLQGAHVSTHDRLWSNL
PFLRPMVTITDDSLASYGTDEHGGRLHDLLGTRCDPYVNKMLTGEDFHHHCHSNLTRAVL
PHGLTEFDVHDVLNIFQCTGLNHDDLYFMKACPAQKGDYLEFFAEIDLLCALSTCPGGDL
SLPMWGPDAQDPLSVCRPLGIEIYDLDASLLDGWQSPERAAYNGLHGLQIAKADWER
>tr|A0A0Q0BZ16|A0A0Q0BZ16_PSEAP Diaminobutyrate--2-oxoglutarate
aminotransferase OS=Pseudomonas syringae pv. aptata OX=83167
GN=ALO85_00928 PE=3 SV=1
MEELSNLKKLESNARTYAATFQQLFVSGKGMRVKDANGQEYLDCLSNAGTLALGHNPPIV
RDAVIEFLNSDHLQQALDLATPAKHAFVEELFATLPASMRDNSKILFCGPSGSDAVEAAI
KLARHYTQRSPLMSFHGGYHGMTAGALSAMGNLNPKAGLLAQGTHFLPFPYRFRCPFGTD
GEHTDRLSIEYIRTVLSDPESGVPKPAAMVVEVIQGEGGCIAASAEWLRAVREITRELGI
LLIIDEVQTGLGRTGNLFAIEHSGITPDILVLSKAIGGGYPISVIVYAEHLDTWGPGMHA
GTFRGNQVAMVAGAATMRHIKEHDLVGNATRRGEQLHSGLEDIARQFPFIGDVRGRGLML
GVEIVKPASGSRPGAGDGARARAIKLECFKNGVIIETGGRNSAVLRFLPPLNITESEVGM
VLDRFEQSLKSSSAARVSSVSAVG
>tr|A0A0Q0BUA2|A0A0Q0BUA2_PSEAP Thiol:disulfide interchange protein DsbD
OS=Pseudomonas syringae pv. aptata OX=83167 GN=dsbD PE=3 SV=1
MRGLSIFETAMRRLLCLLLLILALPASAAGLLDSRPSSTLGGGSLDNSKDFLPVRQAFQL

SLIETTPESIKLRLVATDGYYLYRHRFQFRTEPADIGLGEAQLPKGEQKHDEYFGDVEVY
HGILDIDLPRKPGEQRPFTLAVTYQGCADKGLCYPPETERLSIGDVAASPANQASATPPA
ATAGWSWKELALFFLAGVGLTFTPCVLPMLPILSGVVLRGQVGGLRGLSLSLAYVLPMAA
CFALLGALMGVFGAGLNLQARLQSAWVLVPFSAFFVIFAIAMFGAFELRLPQSISSRLDR
IAGKTQGGSLWGAAVLGVVSSLLVSPCVSAPLAGALLYISASGDALGGALKLFALGLGMG
APLLLIATGGATWLPKSGPWLVTVKNAIGVLLLGLAIGLLSRVLPGQVTLLLVGLLSAGV
ALFLGALEFNVKTTRQKLAQLLGLALLVYALACWYGALSGQTDPMRPLGREYATANNGAV
AQAASQWKTITTSAELDRVMQEAQSEGKPLVLDWYADWCISCKVIEHEVLPDPGVVARLS
GYKQVRFDMTDSNAEQRALLDRYKLFGPPALLFFDKNGEERQTVRVVGEIDAAGLIERLN
SANDQN
>tr|A0A0Q0DB80|A0A0Q0DB80_PSEAP Biotin synthase OS=Pseudomonas syringae pv. aptata OX=83167 GN=bioB PE=3 SV=1
MSASTTATLRHDWTLAEVRALFVQPFNDLLFQAQTVHRAHFDANRVQVSTLLSIKTGACP
EDCKYCPQSGHYNTGLEKEKLLEVQKVLEEAARAKAIGSTRFCMGAAWKHPSAKDMPYVL
EMVKGVKAMGMETCMTLGRLDQEQTEALATAGLDYYNHNLDTSPEFYGSIITTRTYAERL
QTLAYVRDAGMKICSGGILGMGESLDDRAGLLIQLANLPEHPESVPINMLVKVAGTPLEN
AEDVDPFDFIRMLAVARILMPKSHVRLSAGREAMNEQMQALAFFAGANSIFYGDKLLTTA
NPQADKDMLLFSRLGIKPEAGEGHADEVHQAAIEQALVEQQSSSMFYDAASA
>tr|A0A0Q0FUY0|A0A0Q0FUY0_PSEAP Bacterioferritin OS=Pseudomonas syringae pv. aptata OX=83167 GN=ALO85_200053 PE=4 SV=1
MSELLDPILEKLDTLGSALKIAISTAAPLNVSSGNWSFPGITRGELIERVTYLRELVANA
HQPTAESEKYITSIVERLIFLIDHTVPQLASNAGVAVPSFLITLDAVERGLSALLQDTKA
LALKNSQAVKKTTVQVRSIETRLRELTPRTKSLEDMVSRIEKAYDAADQLPTDLETLAEA
QESVSELLNFAKTDKSSIEELLEAISDHEQGLRAKSKEADEVLEKCVSAYSSATSLGLAA
AFSERSKALDWSMWGWVGGLMISLIVGGFAGSIQLRNLAFELTKADANSLGIGVNLVLSI
LSVGGPVWFAWLSTKQIGQRFRLSEDYAFKASISRAYEGYRREAARFDDGLERQLLSSAL
ARLDEQPLRLVETNSFGSPWHELLASDLVKDAVKTVPGFAEKVTALASSALQGRKAAASS
ALAVNDVPPEHTEEKA
>tr|A0A0Q0FH11|A0A0Q0FH11_PSEAP Alpha-2-macroglobulin OS=Pseudomonas syringae pv. aptata OX=83167 GN=ALO85_01359 PE=3 SV=1
MLNKGLFLACALALLSACDSSSTDKPAPATAPTTQATVTDTKPKPAVDLAALSKRYAGRE
LTVIDVSEIQVDGASTLSVSFSVPLDPEQKFDEKLHLVDSKSGKVDGAWELSDNLMELRL
RHLEPQRKLVLTVDAGLLAVNKAKLAAEYISRLETRDLQASVGFASRGSLLPTRLAEGLP
VIALNVDKVDVEFFRIKPDSMPNFLAQWDGASSLSSYSSEELLPMADLVYSGRFDLKPAR
NTRETLLLPIAGIKPLQQPGVYLAVMRASGTYSYSQPATLLTLSDIGLSVHRYSNRLDVF
TQALEGGKALGDISVDVYDDNGKVVAQGKTDSDGHAQLPLPAKAAVVLAHKDEQTSMLRL
NSSALDLAEFDISGPQAHPLQFFIFGPRDLYRPGETVLLNGLLRDSDGNTVKPQPVTVEV
RRPDDQVSRKFVWEADSNGFYQYQLPLADEAPTGRWQLVFDLGDGKPQLYEFKVEDFLPE
RLALELKGSDTPVAPADDPQFDITGRYLYGAPASGNTLTGQVYVRPLREAVPKLPGYQFG
SITEEDLKHDLELEPVTLDAEGHSTIAVQSAWAQAKSPLQLILQASLQESGGRPITRRLV
QPIWPAEHLPGVRALFGSSTGSDDYDNEAKGEAQTNGDGPADFEIVMADAAGNKLAADNV
KVRLIRERRDYYWNYSADDGWSYHFNEKFLNLNEETVSISKDATAKVSFPVEWGPYRVEV
EDPSTGMVSSLRFWAGYRWQDNTDGGAVRPDQVKLALDKPAYNDGDTAKVTVTPPAAGKG
YLLVESSEGPLWWQEIDVPAEGKSYDIPLDKKWARHDLYVTALVIRPGERKANVTPKRAV
GVLHLPLDRAQRKLALTVTGPEKMRPRQPLTLKVAAKNADGSVPKQVHVLVSAVDVGILN
ITSYATPDPFASLFGRKQYGADQLDIYGQLIEAGQGRLASMAFGGDALAKGGKRPDTSVT
IVALQSAPVTLNDAGEGEVSVDIPDFNGELRIMAQAWTDDRYGMAEAKTVVAAPIIAELS
TPRFLAGGDQTSVALDVSNLSGKAQKLDVKISAEGQLSVPGGDQSKPLQLKEGQRVTLKV
PVLAQGGLGQGKIKVLVEGLDLPGENLPAFTREWTVGVRPAYPAMLKHYRATLNDQPWSL
PEGALEAFEPAGREALLSLSNRPPLNLGEQIRALKAYPYGCLEQTASGLFPSLYADAATL
KRLGLTGETDAERKRKVEIGIERLLGMQRYNGSFGLWGSDSDEEYWLTAYVTDFLLRARD
QGFAVPPDALKKASERLLRYLQDAGQIQVNYSQNAEHTRFAVQAYAGLVLSRSQQAPLAA
LRSLFDRRSDARSGLPLVQLAVALEKMGDKPRADQVLMAGLAASRKNEWLADYGSSLRDQ
ALILALLEENDLAKDKRDERLFALADEVAANRYLSTQESNSLFMAGRNLLGKPEKDWTAS
IASGTQTRELSNRQPGLKLDGSLLASPLSVHNQSSEALYQQLTVSGYPTQTPAAGGENLS
IRREYLNLSGAPLNIGALKTGQLVLVHLEIGAKQRVPDALVVDLLPAGLELENQNLAQSA
ASLEDASEDVKTIRESMENAGIKHQEYRGDRYVAALDIEGSSTVHLLYLARAVTPGTYRV
PPPQVESMYRPNWQALGEAPAQMVVKGK
>tr|A0A0Q0DJJ3|A0A0Q0DJJ3_PSEAP Probable membrane transporter protein OS=Pseudomonas syringae pv. aptata OX=83167 GN=ALO85_00503 PE=3 SV=1

MRYVRAAILPLCRPRHKPSATMPFELSVDLTTLAILAVVAFIAGFIDAIAGGGGLLTTPA
LMTAGLPPHLVLGTNKLSSTFGSATASFTFYRRKLFDPGQWLHAVAGTAVGAALGAVIAH
YLPAEWLNQMLPVIVFGCGLYLLFGGTPKAPLESNAPIKKKWQLPQGLGLGFYDGVAGPG
TGAFWTVSTLLLYPVDLVKASGVARSMNFVSNAVALSVFIFSGQVDYIIGLSMGLAVMLG
AYFGAGTAIKGGAKFIRPVFITVVLGLTVRLAWQHWFGGA
>tr|A0A0Q0CDD2|A0A0Q0CDD2_PSEAP Family flavoprotein OS=Pseudomonas syringae pv. aptata OX=83167 GN=ALO85_100034 PE=4 SV=1
MATARTARHSQGATVSSITVRNGSGTTWACDPARDFASVAAHDVRNTLKERSGRMPHPSA
IDISLTMTDLAPSTARTVAIIGGGPAGLMAAEVLSQAGLRVDLYDGMPSVGRKFLLAGVG
GMNITHSEPYPAFLARYAERSPMIAPLLRGFDADALCQWIYALGIETFVGSSGRVFPTDM
KAAPLLRAWLKRLRDAGVTIHTRHRWSGWNADGSLRITHADGELAIKPAATLLALGGASW
ARLGSDGAWLPWLQARQVEVAPLQAANCGFEVSAWSDLLRDKFAGAPLKNIAMGLHGHAL
RLGECVLTATGVEGSLVYALSAQIREQINLHGSAVVDIDLLHGKALTDIQKALSKPRGSR
SMSKHLHSQLGLDGAKAALLRELAPREAFADPQLLSEAIKALPLPLIKPRPIDEAISTAG
GVTFEAMDERLMLRQLPGVFCAGEMIDWEAPTGGYLLTACFASGRAAGLGMVEWLQSR
>tr|A0A0Q0DML1|A0A0Q0DML1_PSEAP Acyl carrier protein phosphodiesterase OS=Pseudomonas syringae pv. aptata OX=83167 GN=ALO85_200042 PE=4 SV=1
MPCPWFDLPIEGENCVASQLKILILSGLLLFAGSFNLQAADVPALPMGAAVPADDKAAEK
ADPKADPKAADKPAAKADDKAADKDKADAKPADGAEPAAETPELLVQGGLLGAISTSIDD
VQEKLNLDDNLFEAWQLRADRAADELETLVNKRTTRSPWSVAGDFLALSFVWIASFAVLT
TLGRFLAVRLCRTPFMRVRARSQALLKYVLPFTLPAIVCLPLTLYVSHFMPSSVGRALAL
CFAYATSSGIVSTSVLLCVIVMFNFGHKRAGVRMIRRYAPRPLFVVGFLAALSDALTSPQ
IARQLGSNVTTSVAVFTGLFASVVFCMLIIKVRRPVAHLIRNRSLSSRLQRPALQQSLKI
FSRLWHLPILLMILVSAINLIGAGEDSQQALRCALFTTILLIATVFLSTVFQHLFKPTET
LGRGGHVYKARLLSLVYAVLRIALAIGFIEILGRIWGFSMFEFAQRNTLGRVISDSLSSI
GLIFVVTWLLWVVLDTAIQEALKPPVNHRSGRQPSTRIKTILPLLRNAVKIILVVICAIT
TMANLGINVAPLLAGAGVVGLAIGFGSQQLVQDVITGLFIIIEDTFSVGDWVVLSTGHSG
SVESLTIRTVRLRDGKGFVHSVPFGQIKAVTNQSRQFAYAFFSVQFTYDSDIDDALALIR
ETGQSISEDILLKHNLQGPLEVFGVDRMDLNGVVLTAQFRTSSGGQYAVGRAFNERLKRL
VDKNPNVRFAQTYPQMVMGPGFNNPQAGVEAEGPPAPSDPAMATPSAPAPAPLSLSKGNS
TPGPAAQ
>tr|A0A0Q0IR27|A0A0Q0IR27_PSEAP Syringopeptin synthetase C (Fragment) OS=Pseudomonas syringae pv. aptata OX=83167 GN=ALO85_05501 PE=4 SV=1
GLTSAHLAYLIYTSGSTGLPKGVMIEHRNTVNFLSWAHTAFDGAALEKTLFSTSLNFDLA
VYECFAPLTSGASIEVVKNVLELQHGEHDIGLINTVPSALKALLEINALPESVHTVNVAG
EALKRSLVESLFEKTSVQRLCNLYGPSETTTYSSWVAMDREDGFAAHIGKPVGNTQFYLL
DEQQQPVPLGVAGEIYIGGAGVARGYLNRDDLTAERFLKDPFSQNPAARMYRTGDLGRYL
ADGNIQYLGRNDDQVKIRGFRIELGEIDARLARHPAVHEAVVTAREDVPGDKRLVAYYTL
SAAQASVDIDSLRGWLQEQLPAYLIPVAYVLLDALPLTPNGKLDRKALPAPDAGALISRG
YEAPQGETETQIAAIWQDLLGVAQVGRHDNFFELGGHSLLAVSLIGRMRQSGLSSDVRVL
FGQPTLAALAAAVGAGTEVIVPANLIDEHCEYITPDLLPLADLTQAQIDQVVAGVPGGVA
NVQDIYPLAPLQEGILYHHIAAEQGDPYVLQTRFAFDNRQRLDAFAEALQLVIDRHDILR
TSVVWQGLDSPVQVVWRQAQLHLEALELDPANGDIAEQLHSRFDPRHYRLDIGQAPLMRV
AYAEDPLNQRICAMLLFHHIALDHVALEVVKHEIQTCLMGQAAQLGSPVPYRNYVAQARL
GVSEADHEAFFRDMLGDIEEPTLPFGLMNVQGDGRDIEEHTLVLEPQLDLRLRAQARQLG
VSVASLAHLAWAQVLSKVSGRQDVVFGTVLMGRMQGGEGAERSLGMFINTLPLRVDIDTC
AVRQAVRQTHTRLTGLLGHEHAPLVLAQRCSAVAAPLPLFSALLNYRHSAVGSVSEQAVQ
SWQGIHALSSEERTNYPLILNVDDMGAGFGLNVQAVAGIDARRICDYMQTVLSHLAEALE
SAPDSAVCDLPVVPETERQQLLVAFNDTALDYPQQQTVHGLFEARVRACPDACAAIHDGV
AMSYAEFNTRANRLARHLLGLGVQPGDCVAILLERSHDLLASQLAVLKCAAVYVPLDINA
PVERQTFMIEDSQARVLLTHSQVSLTTAAQRVDLDGLKVENFKGTDLALPQSSESVAYIM
YTSGSTGTPKGVLVPHRAISRLVINNGYADFNAQDRVAFASNPAFDASTLDVWAPLLNGG
CVVVIGQSDLLSPRDFQRLLLEQSVTVLWMTAGLFHQYASGLGEAFSRLRYLIVGGDVLD
PAVIGRVLANSPPQHLLNGYGPTEATTFSATYEITSVGNGSIPIGKPVGNSRLYVLDGQG
QPLPLGVPGELYIGGQGVAKGYLNRAELSATQFVVDPFSASENALMYRTGDLVRWRADGN
LEYLGRNDDQVKIRGFRVELGEIEARLAEHPDVLEAVVLCRQDTPGDKRLVAYVTAQQPV
DIEHLRSHLQGLLPEYMVPAAYVQLDALPLTANGKLDRKALPMPDAQSLISRGYEATQGE
VETLLASIWADVLKVGQVGRHDHFFELGGHSLLAVKLIERMRQVGLSADVRVLFSQPTLA
ALAAAVGGGTEVVVPANLIPERCEHITPDLLPLISLTQAQIDRVVKHVPGGAANVQDIYP
LAPLQAGILYHHISAEQGDPYTLKALFSLRDRARLDDFSHALQGVINRHDILRTAVLWEG

LEEPLQVVLRQAEMHVTEVDLDPADGPLDEQLHERFDPRHYRLDVRQAPLMQIVFSHDPL
NDRWLAMLLFHHLVNDATSLYVVLRELQAHLLGQHAALGQSVPYRNYVAQARLGVSEAQH
EAFFRDMLSDIDEPTLPFGLQDVQDGGRDLEEASVILPAELDLRLRAQARQAGVSAASLM
HLAWARVLGSVSARDQVVFGTVLLGRMQAGEGADRALGMFINTLPLRVDVGATTVVEGLK
ATHRQLTALLGHEHAPLVLAQRCSGVAAPTPLFSALLNYRHSGVNDVSAETATAWEGIES
FGAKDNTNYPLTLNVDDMGNGFSLTAQVSSPVGAKRVCGYMQTVLEHLADALEQSPSLAL
DSLPTLPVDEREQLLVQFNDTALDYPHEQTIHGLFEAQAKRTPEALAVVHGEQRLTYREL
NEQANRLAHALRERGVQPDSRVGICVERGAEMVVGLLAILKAGGGYVPLDPAYPAERIAY
MLQDSAPAAVLVQTATQGLLGDVSVPVINLDLSDWQDESVQNPQVPGLTSAHLAYLIYT
>tr|A0A0Q0FN62|A0A0Q0FN62_PSEAP High-affinity branched-chain amino acid ABC transporter, periplasmic amino acid-binding protein OS=Pseudomonas syringae pv. aptata OX=83167 GN=ALO85_02748 PE=4 SV=1
MAIRIIKTTTQEWSIMTKGTNKLSRLFAALVMAGVASHSFAADTIKIGIAGPKTGPVAEY
GTMQFNGAKMAIEQINAKGGVDGKKLEAVEYDDACDPKQAVAVANKVVNDGVKYVVGHLC
SSSAQPASDVYEDEGIIMVTPAATSPEITARGYKLVFRTIGLDSAQGPAAGNYIADVAKP
KIVAVIHDKQQYGEGIATAVKQTLEKKGVKVALFEGINAGDKDFSSLIAKLKQANVDFVY
YGGYHPELGQILRQSKEKGLNAKFMGPEGVGNESISQIAGEASEGLLVTLPKSFDQDPAN
QALTEAFKAKSQDPSGPFVYPSYSAVQVIADGIAAAKSEDTTKVAAAIHAGTFKTPMGEL
SYDEKGDLKNFQFVVYEWHFGKPKTEAAK
>tr|A0A0N8T8T6|A0A0N8T8T6_PSEAP N-acetyltransferase domain-containing protein OS=Pseudomonas syringae pv. aptata OX=83167 GN=ALO85_02204 PE=4 SV=1
MPDITHLSAIPTPTLYTPRLVLRPLQLEDAAAMQHLFNQWEVVCYLTHHVPWPYPDGEAL
RYLREDALPAMQDGEEWHWSIRLCGEQDHLIGAACMMDEPDNNRGFWLSPPYQGLGLMSE
VCVAIDRFWFETLKRPVLRVAKSALNEASCRLSRREGMRLVTVQEAQFVCGTTQEQIWEL
TRQEWQMNNHR
>tr|A0A0Q0IRV4|A0A0Q0IRV4_PSEAP Arabinose import ATP-binding protein AraG OS=Pseudomonas syringae pv. aptata OX=83167 GN=araG PE=3 SV=1
MQQAIELQPIGGALRFNGIGKVFPGVKALSDISFEARPGSVHALMGENGAGKSTLLKVLG
GSYQPSSGTLQIGEQSYQFKSTAESIAAGVAVIHQELHLVPEMTVAENLLLGHMPNRFGL
INRGAMYRRAGELLKGLADEIDPRTRLGDLSLGQRQLVEIAKAMSRNAHVIAFDEPTSSL
SAREIDRLMAIIVRLRDEGRVILYVSHRMEEIFRVCDAVTVFKDGRFVKTFEQMADLDHD
RLVTCMVGRDIQNIYNYRPRQHQGPSLRVTGLLGPGLHEPVTFAVQKGEVLGFFGLVGAG
RTELFRLLSGLTRSSAGTLQLDGKPLTLKSPRDAIEAGILLCPEDRKKEGIVPLSSVAEN
INIGARPRHVNLGCLIQGRWERDNARSQIKSMNVKTPSPEQQIMYLSGGNQQKAILGRWL
SMPMKVLLLDEPTRGIDVGAKSEIYEIIHNLAADGIAVIVVSSDLMEVMGISDRILVMSE
GTITGELNRDEADESRLLQLALPRTRG
>tr|A0A0N8T860|A0A0N8T860_PSEAP Uncharacterized protein (Fragment) OS=Pseudomonas syringae pv. aptata OX=83167 GN=ALO85_05615 PE=4 SV=1
MCYMSLVVGQLISDMERDDLHREIYRAQGKLTSCFGYDSMGRKAWQYATTLPAEKLSQIQ
NPLIKP
>tr|A0A0Q0BVT2|A0A0Q0BVT2_PSEAP Transport system permease protein OS=Pseudomonas syringae pv. aptata OX=83167 GN=ALO85_00388 PE=3 SV=1
MDKHFTVRYRRFSRQLSLTALTRLLTGLALTLLVALSSLALGKINLTPATLLSVFTGQAD
ASLVFIVEQLRMPRLALAALVGAALAVSGLILQSIIRNPLASPDLLGITSGASAAAVLYL
SFFSAALGAQFLPLAAISGAGLAALVIYLLAWNQGASPLRMVLIGVGVSALLAAVTTFIL
VFSPLTTTLSAYVWLTGSVYGASWPEPRALAGWLLLTLPLLALLARQVRMQQLDDALAQG
IGVRVQWLRAGLLLVSVALAGLAVAWGGAIAFVGLIAPHIAKRLMPPGFVGQALMAALIG
ANLVMLADLAGRTLFLPLDLPAGIFVAVLGTPFFLYLLINQRH
>tr|A0A0Q0DS15|A0A0Q0DS15_PSEAP Uncharacterized protein OS=Pseudomonas syringae pv. aptata OX=83167 GN=ALO85_05612 PE=4 SV=1
MAMIVALRLSTRRRTLVMSDLPLLYLLAGNGSSAEWWDDALPHFQQHQVVPLELPGFGNN
PQPPCEDLAAYADALLAATVKGSAIVAVGVNALLVMHALQRQPGHFCRSVLLAPVGAFLW
QRRLPALMSPLPIRKTIHWLLANKPTLFAHKFSRQSWPAAHYQRMGSGYARCRAFVPYWD
LLRADTALPLLEWVQDPIELVWGDQDKVLGIEQAAAWSAILARADLTISLKPGWGHYPWI
DAPAEFAQWLESGERGFVAHTKGGRLRLAAIGGQPVPEALSLVQGDDSALPAFLARQPDA
IWAVRSSSFGEDQADAANAGLSTTFLREPSHNVPARVAELHNAGVEEVVVQRFITPVLSG
IAFVRHLSVELEWVEGHLESLADGQASPERAIISRLGAAWSSGDFKPSHGLTEEALWDFL
QGVLRVFHYVPGDVEWAWDGRQLWLLQYRPISDYGWRRHLTAANIAEILPPQPSRLVEYA
QRRAAGSIPAIMARWDSRVLQDNEPFTALFGAASYINNDLFLARLADWGIASSSYADEVG

GATPHLPWRPLRLLRSLPVFLRMQRIARGHLLTLEKQLHRFDRELHALTAQGADGQQLAD
WFTRFYVFVVQGNLCIATSLASSGGDLLGRPPTAYDDLEHCPHRLPWETDPATPRPAATD
LPLQAFPTWPCFIRIAHRAGLPGMRGYYLQVREWYRDNLMRLFFRLHHAMPGADREHWFA
PHPDIRSRAGSFWQDGREGTEQATGFMIYPGQVQGILGDDILLEDTLDPGRHAHYQNARA
VIARMGGRLSHGSTLLRELRKPSAVLPQVDLAWVGREVLYVDGELRLAEGQA
>tr|A0A0Q0DMU7|A0A0Q0DMU7_PSEAP Phosphomethylpyrimidine synthase OS=Pseudomonas syringae pv. aptata OX=83167 GN=thiC PE=3 SV=1
MMSIKAKNAAHLSESAQVDSGSVQPFTRSQKIYVQGSRPDIRVPMREITLDVTPTDFGGE
INAPVTVYDTSGPYTDPNVIIDVRKGLADVRSPWIDSRNDTERLPGLSSNFGQQRLSDAE
LTALRFAHVRNPRRAKAGANVSQMHYARQGIITAEMEYVAIRENMKLQEARAAGLLTQQH
SGHSFGASIPKEITAEFVREEIARGRAIIPANINHVELEPMIIGRNFLVKINGNIGNSAL
GSSIEEEVAKLTWGIRWGSDTVMDLSTGKHIHETREWIIRNSPVPIGTVPIYQALEKVGG
AAEDLTWELFRDTLIEQAEQGVDYFTIHAGVLLRYVPLTAKRVTGIVSRGGSIMAKWCLA
HHKENFLYTHFEDICEIMKAYDVSFSLGDGLRPGSIADANDAAQFGELETLGELTKIAWK
HDVQTMIEGPGHVPMQLIKENMDKQLECCDEAPFYTLGPLTTDIAPGYDHITSGIGAAMI
GWFGCAMLCYVTPKEHLGLPNKDDVKTGIITYKIAAHAADLAKGHPGAQIRDNALSKARF
EFRWEDQFNLGLDPDTARSYHDETLPKDSAKVAHFCSMCGPKFCSMKITQEVREYAANQR
IEAVDVDVAKGLAEQAERFKQEGSQLYKKV
>tr|A0A0N8T9U3|A0A0N8T9U3_PSEAP Pyridoxal-5'-phosphate-dependent enzyme, beta subunit:ornithine cyclodeaminase/mu-crystallin OS=Pseudomonas syringae pv. aptata OX=83167 GN=ALO85_03786 PE=4 SV=1
MVDGEVVARLLAADPLACIGDVQAAYLDHKAGRTINPDSYFLRFAQAPANRIIALPASLS
GEQPVSGIKWISSFPGNIDAGLQRASAVLILNDPLTGYAFACLEASRISAMRTAASAVLA
ARWMNRQQRHVGRMSFIGAGFIARTILDMFVSDGWTMDAVSVFDQHQDSALALVGHAASH
HGLHSEPSDLATCLQADVVVFATTAPSPYVLEPVFQPGQLVLNISLRDLGPEVIAQANNI
LDDVEHCLKAQTSPDLAVQQYQHRSFITGTLAQLMTGQVELSPAKASIFSPFGLGVLDLA
VGQRVYRQAVAEGSALPVPQFFFESARW
>tr|A0A0Q0BT07|A0A0Q0BT07_PSEAP Putrescine-binding periplasmic protein OS=Pseudomonas syringae pv. aptata OX=83167 GN=ALO85_02338 PE=4 SV=1
MSISILSKALLAAAGLTLSISAQAESTVHIYNWSDYIGKTTLADFEAATGIKPMYDVFDS
NETLEAKLLAGRTGYDVVVPSNHFLGKQIKAGAFQKLDKSLLPNYKNLDPALLKRLEKND
PGNQYAVPYLWGTNGIGYNVEKVKAALGVDKIDSWAVLFEPENIKKLSSCGVAFLDSADE
MLPAVLNYMGLDPNSTKEADYKKAEAKLLAIRPYVTYFHSSKYITDLANGDICVAAGFSG
DVFQAKARAEEAGKGVKLAYSIPKEGGNLWFDMLAIPADSKNIKAASAFINYMLDPKVIA
KVSDEVGYANPNPASGEFMDQSIRTDEAVYPPQEVLDRLFVNSELPPKVQRLMTRSWTKV
KSGK
>tr|A0A0Q0D536|A0A0Q0D536_PSEAP Probable 2-(5''-triphosphoribosyl)-3'-dephosphocoenzyme-A synthase OS=Pseudomonas syringae pv. aptata OX=83167 GN=mdcB PE=3 SV=1
MSALQWTPQSASLAVRLADLAVDALIDEADLSPKPALVDRRGSGAHSDLHLGLMHSSALS
LWPTFKWMADAATQFGAVGQPLREALGRLGREGEATMLRTTSGVNTHRGAIWALGLLVTA
AALDPQECAPEAICARAAALAQIKDRQVLTQNSHGDQVVRRYGVMGAREQAQQGFPAVRL
FALPQLRRSRAAGSGEQNARLDALLAIMTTLDDTCVLHRAGIEGLNAMQHGAQRVLDAGG
SASLAGRRALNALDQHLLALNASPGGAADLLAACLFIDGLEPALGPVSRSA
>tr|A0A0N8T8J4|A0A0N8T8J4_PSEAP Hydroxyneurosporene synthase ABC-type export system, ATP-binding protein OS=Pseudomonas syringae pv. aptata OX=83167 GN=ALO85_03232 PE=4 SV=1
MLQVRNVFKRYTTAQGPLDVLRGVDLHLAQGESLALMGESGSGKSTLLHLVAGLDQVDDG
SIEVAGQRLDLMNESQLANWRRTEIGLVFQQFNLIGSLKIADNLAFQARLAGRVDAAWQT
HLIERLGLAALLDRYPEQLSGGQQQRVAIGRALASRPGLLLADEPTGNLDEATSDEVLQL
LLDILADSPTSLLMVTHSPRVAQRLSRKVVLHLGRLADEGKR
>tr|A0A0N8T976|A0A0N8T976_PSEAP Feruloyl-CoA synthase OS=Pseudomonas syringae pv. aptata OX=83167 GN=ALO85_00845 PE=4 SV=1
MSSDIRSSTLQSEDNAPRYRQVSIGHPPIEVREEQGFLHMRSLEPLARLPDRLLDRLVHW
AQLRPQQTFIAARDSRGGWRKVSYAEMLTDVRAIAQSLLAYDLSADRPLALLSGNDIEHL
QLALGAMYAGIPYCPVSPAYSVMSQDFAKLRHVCDILRPGLVFVSDAAPFQRAIDAIIPT
DTPVITVRGQLNGRPHLGFSSLLEEPGGSEADAAFLASGPNTIAKFLFTSGSTKLPKAVV
TTQRMLCANQQMLLQTFPVFGEEPPVLVDWLPWNHTFGGSHNIGIVLYNGGTFYLDNGKP
TTQGFAETLRNLKEISPTAYLTVPKGWEELVNALEQDAELRERFFSRIKLFFFAAAGLSQ
SIWDRLDRVAEQHCGERIRMMAGLGMTEAAPSCTFTTGPLSMAGYIGLPAPGCEVCLVPV

DGKLEGRFRGPHIMPGYWRSPQQTAEVFDENGFYCSGDAIKLADPAAPELGLMFDGRLAE
DFKLSSGVFVSVGPMRNRAVLEGSPYVQDVVITAPDRECLGALVFPRVHECRQLAGLDAQ
ATDAQVLASAPVREWFADWLARLNQGANGNASRLEWIVLLDQPASIDRGEITDKGSINQR
AVLQWRADKVEALYRDQDPDKLSAAPKA
>tr|A0A0Q0FBZ2|A0A0Q0FBZ2_PSEAP Two component DNA-binding response regulator GltR OS=Pseudomonas syringae pv. aptata OX=83167 GN=ALO85_04290 PE=4 SV=1
MSPVSKSILLVDDDQEIRELLDTYLSRAGFQVRTVGDGADFRRAFNDEPSDLLILDVMLP
DEDGFSLCRWIRQHPRQPHVPIIMLTASSDEADRVIGLELGADDYLGKPFSPRELQARIK
ALLRRAQFGQERPGGDVILFDEWRLDMVSHRLFHSDGEEVILSGADFALLKLFLDNPQQI
LDRDTIGNATRGRELMPLERIVDMAVSRLRQRLRDTGKSPRLIRTIRGSGYLLAANVNSQ
AGNGY
>tr|A0A0Q0C2N5|A0A0Q0C2N5_PSEAP 30S ribosomal protein S7 OS=Pseudomonas syringae pv. aptata OX=83167 GN=ALO85_200031 PE=4 SV=1
MAQETENRREALAELAQDSKARSYTAQIRDLYDVIENALDSGVSRTVIHQKLVDTGLAIS
LRHFDQALYRIRKSHKRAGQVEHPLPEKSDSAAVGKPALLDGEKTTPRANIRQSMKQIRK
EVENTNWAEVISEANRTAKS
>tr|A0A0Q0C602|A0A0Q0C602_PSEAP Lipoprotein OS=Pseudomonas syringae pv. aptata OX=83167 GN=ALO85_100020 PE=4 SV=1
MDITRCHSRLRAVDGNRASLASLQAVTGRWTVDSFMRTFMMRRMLLTLLFSVTTQAWAAE
PRELTWNDMIPPDAPVVKPVTAPLHDMSKLSDALAMEAAPAAHQLAPHAPVVKALDGKLV
RLPGYIVPLEVSEEGRVTEFLLVPYFGACIHVPPPPANQIVHVTSELGVKVDELYQPYWI
EGPMQAKSSSSELAEAGYQMQADKILVYELPDS
>tr|A0A0Q0DN68|A0A0Q0DN68_PSEAP Sensor histidine kinase OS=Pseudomonas syringae pv. aptata OX=83167 GN=ALO85_00753 PE=4 SV=1
MKSIQRRLSLGLIAVMVVVGLVMAQTSLWLFEVGLQRYLESGLRNESENLLVALVRGPAG
LQLDDHRLSGAYQRPLSGHYFRMDLPQGHWRSRSLWDFELPQPGVGLHANLELGKTGQSL
LMLTSEYRKFGKQISITVAQDYTPVRASFRLMQQIGLGLGLAALILVLVLQRITVRRALR
PLETVREQIFQLQQGQRSQLDNQVPLELEPLVAQINHLLAHTEDSLKRSRNALGNLGHAL
KTPLAVLLSLALSGQLDANPALRRTLQEQLENIRLRLERELNRARLSGDALPGARFDCDA
ELPGLFSTLGMIHGAHLQLINDVEAGMYLPWDREDILELLGNLLDNACKWADSEVRLDIL
SQHQGFCLLVDDDGPGIPEAQRAEVFSRGARLDEQITGHGLGLGIVRDIVEAWGGQLQLQ
ESPAGGLRVRIDLPERTAP
>tr|A0A0Q0C8Q8|A0A0Q0C8Q8_PSEAP Transposase, family OS=Pseudomonas syringae pv. aptata OX=83167 GN=ALO85_05642 PE=4 SV=1
MRQQFAVRSGFGDLAFLQNHQAIHACDGRQAVRDGDHGLALHQRLQALLNRRFDFRVECR
GGFVHDQDRRVLEQHAGDRDTLALTAGQLDAALTDMGVEAGATFGVREQRDELVGARLVN
RIPELFISCVRIAIQQVLANRAVQQRGVLGDHADLRAQAFLGDFGNILAVDEDPPALHVV
HAQQQVDQRRLARAGRSDQADLLTRSDVQVEVVNDPVAIAVMEIDAVEPDAAVFDLQRQG
AVFVIDGDGLRDTFQAVLHGADVLEDAVDHPHDPAGHVDDADDQTGGQCDRTHRDHRLRP
QPKGQTGGRDDQYAVHGGDGGVHRGDHAASQLTFFGLIAHRFASVLLLVVGVGEQLERGD
VGVAVDDAAHQS
>tr|A0A0Q0BUC1|A0A0Q0BUC1_PSEAP Cobalt-precorrin-6x reductase OS=Pseudomonas syringae pv. aptata OX=83167 GN=ALO85_01393 PE=4 SV=1
MKRILLLGGITEALAIARRLGPEHIYSLAGVGRVPSDLACQLKVGGYGGADGMAQYMGEQ
GVDLLLDATHPYAAQISHNAARAAKLAGIPCWALRRTAWQAGPGDDWREVADWSELATAL
LPFKRPLFTLGREPLQHLHEIPPHQFWTLRALDDYPGNERCEVIGARGPFMLEDERQLFE
QRRIDVLISKNSGSSSTEPKLDVAREHGLPVLILKRPQLPDVDRLFWGVDEVLEALGLD
>tr|A0A0Q0CBZ6|A0A0Q0CBZ6_PSEAP Uncharacterized protein OS=Pseudomonas syringae pv. aptata OX=83167 GN=ALO85_03886 PE=4 SV=1
MRAEGAENGMWHQSRIALRARPRGFHLVTDEIVAGLPPLRGCRVGLLHVWLQHTSASLTV
NENADPAVRRDFERFFNRLVPQGVDGYEHDDEGPDDLPAHFKASLLGCQLLLPVTAGRLA
LGTWQGIYLGEHRDAGGSRNVLATLQGEWT
>tr|A0A0Q0FW19|A0A0Q0FW19_PSEAP Stress protein OS=Pseudomonas syringae pv. aptata OX=83167 GN=ALO85_03306 PE=4 SV=1
MTQLVPGANAPVAAGPLTVEIIYSPIADADIDVSAFLLTASGKVRGDQDMCFYGQKSVNG
GALQQTEASAGRAVFSLDPSRLDSVIEKVALTATIYENKASFGSVSRLALNITGGIEADI
PTSGMKETALILGEFYLRQGAWKFRCVAQGFAGGLEPLAKNFGVEVAAPQDEPAPAPAPA
PAPVPAPAAKSTVSLSKITLDKTRASISLEKSSAGFGEIRVNLNWNRRNDSKGGGFFSMK
KSNAIDLDVGCLFEMQDGLKGAVQALGNSFGSLNTEPFIKLMGDDRTGSISDGEWLHING

AHWSKIRRILVYAFIYEGAPNWKETDGVVTIHAPGQPPIEVRLNEEGGRQGMCAIALLEN
DNGAVKVTRRVDFHNGHSNMDKAYGWGMRWAAGSK
>tr|A0A0Q0CDI3|A0A0Q0CDI3_PSEAP DUF1653 domain-containing protein
OS=Pseudomonas syringae pv. aptata OX=83167 GN=ALO85_02674 PE=4 SV=1
MDVLQGDRNMQLQPGLYRHYKGPHYRVFSVAKHSETEEEVVFYQALYGDFGMWVRPLNMF
LESVEVDGEHVPRFALVEAEPSLFSRT
>tr|A0A0Q0FPC7|A0A0Q0FPC7_PSEAP GCN5-related N-acetyltransferase
OS=Pseudomonas syringae pv. aptata OX=83167 GN=ALO85_00964 PE=4 SV=1
MPGINRLMTNPSSMTFTLRDASSADALCLAALGMQVFLDTYATQGIRQSIAREAMEAFSP
ANFAQQLIEPTALIVVAELQGHLIGFAQVALRTDHAMLGVSNAAELQRLYVQERFTGRGA
GRLLLDAAEQRATTRHASLLWATVWVGNPRALAFYPRQGYTWKGTPHYVFQGETHENHLF
AKPLMTTD
>tr|A0A0Q0FKF9|A0A0Q0FKF9_PSEAP Glycoside hydrolase family protein
OS=Pseudomonas syringae pv. aptata OX=83167 GN=ALO85_101023 PE=4 SV=1
MQLSTQALTWQREREQAALAVVDWQNAEQAQRRALEVRLHTNDSTIHKELSDAQTAQARL
RDRLATADLRLSVLLANSPANRDGMPAGTDTGGVVHGSPRGELDPAAAGRIVAITDYGDQ
GLIALKACQAYVREIAH
>tr|A0A0Q0IB78|A0A0Q0IB78_PSEAP PmbA protein OS=Pseudomonas syringae pv.
aptata OX=83167 GN=ALO85_04415 PE=4 SV=1
MMEKVMSATQSVGPQALPELQEQVEQIIAEARRQGASACEVAVSVDQGLSTSVRQREVET
VEFNRDQGFGITLYVGQRKGSASTSATGADAIRETVAAALAIAEHTSEDESSGLADAALM
AKEVMDFDLYHTWDITPEQSIEKALLCEAAAFDADSRIKNADGTTLNTHQGCRVYGNSNG
FIGGYASTRHSLSCVMIAEGEGQMQRDYWYDVNRQGELLADAQSIGRKAALRAASRLGAR
PVPTCEVPVLFAAELAGGLFGSFLGAISGGTLYRKSSFLEGALGQRLFPEWMTIDERPHL
KHAMGSSAFDSDGLATFAKPFVEGGDLVSYVLSTYSGRKLGMPSTANAGGVHNLFVTHGS
DDQAALIRRMGRGLLVTELMGSGLNMVTGDYSRGAAGFWVENGEIQFPVQEVTIAGNMRD
MFKQIVAVGSDLELRSNIRTGSVLIERMTVAGS
>tr|A0A0Q0FB39|A0A0Q0FB39_PSEAP dTDP-glucose 4,6-dehydratase
OS=Pseudomonas syringae pv. aptata OX=83167 GN=ALO85_100005 PE=3 SV=1
MRTCTPARKLPGVLFDLLLGFNGLIMPYALKRYAALLQNTQEGQFMRILVTGGAGFIGSA
LIRHLINNTEHEVLNFDKLTYAGNLESLQSIATDTRYEFVQADICDQARVSAVLERFAPQ
AIMHLAAESHVDRSIDGPAEFIQTNIVGTYSLLEATRAWWLKLPEAQRQAFRFHHISTDE
VYGDLHGVDDLFTETTPYAPSSPYSASKAASDHLVRAWHRTYGLPVVVTNCSNNYGPFHF
PEKLIPLVILNALAGKPLPVYGNGLQVRDWLYVEDHARALLKVVTEGEVGETYNIGGHNE
QKNIDVVRGICALLDELAPQHPAGVAQYSDLITYVVDRPGHDQRYAIDASKIDKELDWTP
EETFESGLRKTVQWYLDNLDWCRRVQDGSYQGERLGFTEPKDLIA
>tr|A0A0N8T8Q0|A0A0N8T8Q0_PSEAP Regulatory protein LuxR OS=Pseudomonas
syringae pv. aptata OX=83167 GN=ALO85_100919 PE=4 SV=1
MLSVYRSPSAKAFTSQERARLKDLSWVVLPIVKQHIATVTPTLVSTNPLPVTKSLKNKDA
MEGLRQRFLGRLEASALSLSSRETEVCVGLLAGLTVPQLAERLDLKVSTVESYFKRAAVK
MGIAGRSALLRWLHAEQRMQHPIPVEMFQAAV
>tr|A0A0N8T978|A0A0N8T978_PSEAP Uncharacterized protein OS=Pseudomonas
syringae pv. aptata OX=83167 GN=ALO85_04679 PE=4 SV=1
MLKHSSHMKSQWNNPLQGFPMKTPRPNTRLKSLINLRNLKMARSAHAFVRGNTAQFYEWL
HSQSGRRLPSGPPVWICGDCHAGNLGPTGDSRGRIDMHIRDLDQAVIGNPAHDLVRLGLS
LATAARGSDLPGVTTARMLEEMMQGYEEAFMGDDDEEPDRPAQVKAGMRSAVQRTWKHLA
KERFEGTQPTIPLGKHFWQLSRAERDAIKELCETPEIHALVTSLKGRSQDDRVRLLDSAY
WVKGCSSLGLLRYAVLMGVGDEEDEEFCLLDVKEAVAAAAPRTARARMPRDNGKRVVEGA
RNLSPGLGSRMVAVRMLDHSFFIRELLPQDMKLELDELTEQEAMQAAAYLAKVVGNAHAR
QMDLATRAAWIRDLQANRSDTLDAPSWLWSSVVQLVGSHEQGYLEHCRRYAL
>tr|A0A0Q0DG17|A0A0Q0DG17_PSEAP Arginine--tRNA ligase OS=Pseudomonas
syringae pv. aptata OX=83167 GN=argS PE=3 SV=1
MKDTIRQLIQQALTRLVTEGVLPEGLTPAIQVENARDKTHGDFASNIAMMLAKPAGMKPR
DLAEKLIAALPADEQVSKVEIAGPGFLNFFQNTAALAARLDAALADAHLSVRKAGAAQRV
VVDLSAPNLAKEMHVGHLRSTIIGDGVANVLTFLGDTVIRQNHVGDWGTQFGMLLAYLQE
KPATSDELSDLENFYRAAKQRFDESEEFAERARGLVVKLQAGDAECLALWTRFKDISLSH
CQETYERLNVKLTPADVMGESAYNDDLANVVNDLKASGLLVESNGAQCVFLEEFRTADDT
PLPVIVQKAGGGYLYATTDLAAIRYRSKVLKADRVLYFVDQRQALHFQQVFEVARRAGFV
HDGMQLEHMGFGTMNGADGRPFKTRDGGTVKLIDLLDEAEERAYTLVKEKNPEVAEAELR
SIAKAVGISAVKYADLSKHRASDYSFNFDQMLSFEGNTAPYLLYAYTRVAGVFRKLGSAF

DASKGQIVLAAPQEQELAARLAQFTETLNNVAEKGTPHVLCAYLYDLAGLFSSFYENCPI
LGAENPDQQQSRLRLAALTGRTLKQGLDLLGLETLERM
>tr|A0A0N8T900|A0A0N8T900_PSEAP Uncharacterized protein OS=Pseudomonas syringae pv. aptata OX=83167 GN=ALO85_100338 PE=4 SV=1
MIQGVDSSPLALLNSRRRERSAAAVRLTCIDSLKRYSAMDKTITVDQLASTDLRSVNEIW
LGGTHTELCLWRGKQVPTHDMLSEGTHDQNARRLCLQLVDPDDKAAVANVEALVRDRLER
MGSQGEFYPAENADTHQ
>tr|A0A0Q0DH83|A0A0Q0DH83_PSEAP NADH-quinone oxidoreductase subunit F OS=Pseudomonas syringae pv. aptata OX=83167 GN=ALO85_100048 PE=3 SV=1
MTITSFGPANRITRTAETHPLTWRLRDDGEPVWLDEYQTKDGYAAARKALTQQAPDDIVQ
SVKDSGLKGRGGAGFPTGVKWGLMPKDESMNIRYLLCNADEMEPNTWKDRMLMEQLPHLL
VEGMLISARALKAYRGYIFLRGEYVTAAKHLNRAVAEAKAAGLLGKNILGTGFDFELFVH
TGAGRYICGEETALINSLEGRRANPRSKPPFPAAVGVWGKPTCVNNVETLCNVPAIVNNG
NDWYKSLAREGSEDHGTKLMGFSGKVKNPGLWELPFGVQARELFEDYAGGMRDGFKLKCW
QPGGAGTGFLLPEHLDAQMYAGGIAKVGTRMGTGLAMAVDDSVNMVSLLRNMEEFFAQES
CGFCTPCRDGLPWSVKMLRAIENGQGQPGDIETLLGLVNFLGPGKTFCAHAPGAVEPLGS
AIKYFRSEFEAGIAPASATTLVPGKSPTVVGA
>tr|A0A0Q0C939|A0A0Q0C939_PSEAP Proteinral secretion pathway protein E OS=Pseudomonas syringae pv. aptata OX=83167 GN=ALO85_00691 PE=4 SV=1
MSLRSSKMPSFMPSAPVEVSRLLIPDAQQVCAWLIENAGLKTADLERAQRLQQESEGTEL
LGLLTRLGLVSEFELARAWAALLQAPLLLADAVPPLLDPLPPLTERFMRHYQVVPVGWSD
QGLQVLSANPGTLYPFQAIAYACEVPVRLSIGPRNEVETLIERYYGQGRSAMGTLIENLD
EEGGSLEDIEHLKDLASEAPVIRLVNLILQRAVEQRASDIHIEPFESQLKVRYRIDGVLH
EAEAPPSSSSAAVISRVKIMARLDIAERRLPQDGRIMLRIQGKELDLRVSTVPTSFGESV
VMRLLDRQTINFDFPSLGFDGERLDEFLDVLERPHGILLVTGPTGSGKTTTLYTALSRLN
TAERKIITVEDPVEYQLEGINQIQVKPSIGLDFAGALRSIVRQDPDVIMIGEMRDLETCR
IAIQSSLTGHLVLSTLHTNSAAASITRLLDMGVESYLIASTVNGILAQRLVRRLDPATRE
AFDAPPELIAEHGLERFTDERPIRLYRPRADAPGGGYRGRSAITELLVMNEELRSLLMRH
ADASTLEEAARRGGLRTLHEEGLRQAVAGVTSLEEVLRVTRGEGT
>tr|A0A0Q0BVV7|A0A0Q0BVV7_PSEAP Uncharacterized protein (Fragment) OS=Pseudomonas syringae pv. aptata OX=83167 GN=ALO85_04431 PE=4 SV=1
MEAQESDMELRDIHSMTRWGIAAIFGTVLLTYSGHWWGKAVAHGNAELAQKSEQTATQQR
TYSLEIRGAGIAIFHDNQSEIWSFIKKKNNNSTSIYSNDAKDYDASLDSRKISRDIKIRA
AFQHLAGDAIAYWPIPVFALGPPNLFDKAYRAAGLINSGRNAATLGVALFLWQDDESTTH
AQAMIERLFNFFDDNSQVPQALIASRDGDITRDVYRKPGTPALQNAQVVPTIFESMTGLL
VARSDRVDRYIRPYATQEPEDNQNKNTDLGKLWAFYWEQPRKFRKIYEDAEKAKGVKAPL
APGTISTAYWQSQLPTLWKTISNRGPGNFEPSPWLPIRWGQHQVKEFDAAPVLGYLHRPI
KAPMQDENGKRLKPALQAKALQAAWVQALDTLPDGQKP
>tr|A0A0Q0C697|A0A0Q0C697_PSEAP Uncharacterized protein OS=Pseudomonas syringae pv. aptata OX=83167 GN=ALO85_05417 PE=4 SV=1
MTCISVSSSMNLYEIVFSAELVAGAQPEKVRANLGKLFNADAERLDLLFSGRRLVLKNNL
DAATAEKYRATLERAGALVRVLEMNTPLPMEEVELAPPPDAPNWLRKTPVSPGRLQIKPR
DAYMAAFVDVDAPDYSVAEAGVELLPDKPQPVAPLLDLSRLSLAPVGSDMGQVEKPESQE
APDTSHLKIIPE
>tr|A0A0Q0BZI5|A0A0Q0BZI5_PSEAP Phenazine biosynthesis PhzC/PhzF protein OS=Pseudomonas syringae pv. aptata OX=83167 GN=ALO85_02610 PE=4 SV=1
MTTEVLKLAAFSDGDRGGNPAGVWIGDALPEDAVMQQIAADIGFSETAFAAPVEGGWRVR
YFSPLAEVPFCGHATIALGAALAAQQGDGVFQLTLNQAQITVEGHAQRSLTSAALQSPPT
FSKPVSVPLLEEALALFGYNHADLHERITPAHINGGAGHLLLALNSREKLKAMRYDQQTG
HDLMVREGWATILLVWAETDQLFHTRNPFAFGGVYEDPATGAATAALGGYLRDIGWPHGG
LIDIIQGEDMGSPSRLRAEIPQQPGSSIRVSGMARRL
>tr|A0A0Q0DIE2|A0A0Q0DIE2_PSEAP Potassium-transporting ATPase ATP-binding subunit OS=Pseudomonas syringae pv. aptata OX=83167 GN=kdpB PE=3 SV=1
MNLPINPAKNAAAKSTESQATESAKTGIAALWRPALVQAFVKLDPRQLKRSPVMLVVELT
AILTTVLCVIPNPQVPTSVCVQIALWLWFTVLFANFAEALAEGRGKARADSLKAGSEGLK
ARIRDDNGSLRIINASELRKGDVVRVEIGEMIPGDGEVIEGIAAVNEAAITGESAPVIRE
SGGDRSAVTGNTRLVSDWLLIRISANPGESTLDRMIALVEGAKRQKTPNEVALDILLIGL
TLIFLLVVITLQPFAHFAGGSLPLVFLVALLVTLIPTTIGGLLSAIGIAGMDRLVRLNVI
AKSGRAVEAAGDVHVLLLDKTGTITFGNRRCAAVYAAPGVTPKELGEGALLASLADDTAE
GKSIVEFLRNLHPMNEPTLSSVTAVPFSADTRLSGVDYQGHVYRKGAVDSLLAFIDMQRS

ELPPVLAREVEKIAKTGGTPLLVCANGRLLGAIHLKDVVKPGIRERFEELRKLGIRTVMV
TGDNPLTAAAIAAEAGVDDVLAEATPEKKLERIRQEQGEGRLVAMCGDGANDAPALAQAD
VGMAMNDGTQAAREAANMVDLDSDPTKLLDVVQIGKELLVTRGALTTFSIANDVAKYFAV
LPALFASIYPQLGVLNVMKLASPQSAIVSAIVFNALIIVVLIPLALRGVRVQAASAAHLL
RRNLLIYGLGGIVVPFIGIKLIDMLLVALGLV
>tr|A0A0Q0FI06|A0A0Q0FI06_PSEAP Proteinral substrate transporter:Major facilitator superfamily OS=Pseudomonas syringae pv. aptata OX=83167 GN=ALO85_05053 PE=4 SV=1
MHPIAIGLYKNNKHRGTPAMTPVSTHEVGDAEGNLVYRRITLRLIPFIFICYLFNYLDRV
NVGFAKLQMLDALNFSETVYGLGAGIFFIGYVLCGLPSNLALNRFGPRRWIGLMMITWGI
FSTCLLFVTTPVEFYVLRFLTGMAEAGFFPGIVLYLSRWYPNQRRGRIMALFMSAIPVSG
LLGGPFSGWILNHFAAGQGGMAGWQWMFLIQGVPTVALGALAFVLLCDKVEDARWLTLEQ
RQRVKTDITNDELSRPVHGKSSVASLLSMPFIWILGFIYFCIQSGVYAINFWLPSIIKNL
GFSDALVIGWISAVPYLMAGVFMLLVGRSADLRNERRWHLVVPMLMGATGLIIAANFATL
PIVAIIGLTIATMGALTSLPMFWPLPTALLSASVAAGGLALINSIGQMAGFLSPYLVGWI
KDQTGSTTLALYALAALTIVGSLVALRVSRSSAVKVAGPA
>tr|A0A0Q0DMY2|A0A0Q0DMY2_PSEAP Membrane protein OS=Pseudomonas syringae pv. aptata OX=83167 GN=ALO85_100051 PE=4 SV=1
MTQPLWSRPSKMGAGMGYLLFVTLIQAFSFSLIGVYLAGHVDSYFAVLVRVVLAGLVFIP
LTRWRQVEPRFMRSMLLIGALQFGVTYVCLYLSFRVLTVPEVLLFTILTPLHVTLIEDAL
NRRFNPWALLAAVVAVLGAGVIRYDGLDGDFLGGFLLLQLANFTYAAGQVLYKHLVARYP
SDLPHYRRFGYFYLGALAVVLPAFLIFGNPQHLPEASTQWIVLLFLGLCSTALGMYWWNK
GACMVNGGTLAVMNNLHVPLGLLINLLIWNQHEDFTRLLIGGGVIVASVWISRLGVRRVP
ESVSGTR
>tr|A0A0Q0C6W9|A0A0Q0C6W9_PSEAP Phenylalanine--tRNA ligase beta subunit OS=Pseudomonas syringae pv. aptata OX=83167 GN=pheT PE=3 SV=1
MKFSEQWLRGWVSPQVSRDELVARLSMAGLEVDSVTLAAGVFSGVVVGEVLSTEQHPDAD
KLRVCQVSNGSETFQVVCGAPNVRPGLKIPFAMIGAELPGDFKIKKAKLRGVESNGMLCS
AAELQAGEGNDGLMELAADAPVGQDIRVYLSLDDASIEVDLTPNRGDCLSVAGLAREVGA
LYAAEVTRPQIAAVSAAHDEVRPVEVLAPAACPRYLGRVIRNVDLSRPTPLWMVERLRRS
DVRSIDAAVDITNYVMLELGQPLHAFDLAEINGGIRVRMAEEGEKLVLLDGQEVSLRADT
LVIADHQRALAIAGVMGGEHSGVTASTRDIFLESAFFDTISVAGKARSYGLHTDASHRYE
RGVDWQLAREAMERATGLLLEITGGEAGPVIEVVSEQHLPSIAPVTLRASRVEQMLGLVI
ENAEIERLLKGLGLAVTAEADGQWRVEVPSHRFDISLEVDLIEELARLYGYNRLPVRYPQ
ARLAPQAKAEAKGDLPELRRLLVARGYQEAITYSFIDPKWFELFSPGAKPLLLANPISND
MAAMRASLWPGLVKALQHNLNRQQDRVRMFESGLRFVGQLDGLKQEPMLAGVVCGSRLPE
GWAQGRDVVDFFDVKADVEAVLGFAGALGEFTFTPGQHPALHPGQTARIERDGREVGFLG
AIHPELSKTLGLDRPVFVFELVLAEVSTGRLPKFHELSRFPEVRRDLALLADRDVSASAV
LDVIRENAGEWLTDLRLFDVYQGKGIDPHRKSLAVGLTWQHPSRTLNDDEVNATTLAILT
SLEERLNATLRK
>tr|A0A0Q0CXH8|A0A0Q0CXH8_PSEAP Pyoverdine sidechain non-ribosomal peptidesynthetase PvdI (Fragment) OS=Pseudomonas syringae pv. aptata OX=83167 GN=ALO85_04810 PE=4 SV=1
MERHGPVHGLTGGIRRMTGTTAARIAKRFVGLSLEQRQQFLSRLRQEGKDFSLLPVPVSR
HDASAVPLSFAQQRLLFLWQLDPSSDAYKMTTGLRLRGTLNESALRRAFDHLIERHEVLR
TVFHTDGDQAQQVLLHDQTVALESIDLAGLADVELRDAELALQVATVTGQAFDLRTGPLL
RAHLFRLADDEHVLIVSMHHIVSDGWSMDVMIQEFVHCYQAYCEGREPALPELPLQYADY
AIWQRSWLEAGEGARQLEYWRNRLGDEQPLLDAAPDFPRPATQSYQGEHLRFDFGVDLSR
RLNAFARTQGMTLFMLVLAGFSLFLSRKAGQRDIRIGVPNANRGRAETEGLIGFFINTQV
LRCQVDERLSYLDLLAQIRDTSLGAQAHQDVPFEQLVDQLAPERSLGHNPLFQAKFNQNV
VLKQKTALRLAGLEVSEYAFDKQGAHFDLALDITDDGTLIHGDMAYASDLYRRTTVEGFI
PELLALFQTLLDTPHAPLFSLGALAVEPRRDARAPAPHMLQLWDRQVERQPNAQAARCLQ
RTLTTLQLEQAANQLAHHLIRMDISEGQPVAVLMERSLDWLTAVLAIFKAGGVYMPLDVK
APDARLQQMLVNAQARVLLCAEGDVRHTSLGVAGCQGLTWTPALWQDLPVSRPDTRPSGD
SAAYVIHTSGSTGQAKGVLVSQSALASYLRGLLEQLQLAPEASMALVSTIAADLGHTVLF
GALCSGRTLHVLTEALGFDPDAFAAYMAEHQVGVLKIVPGHLAALLQAGQPADVLPQHAL
IVGGEACSPALIEQVRQLKPGCRVINHYGPSETTVGVLTYEVLMSPGSLPQDVCVSTGVH
AGDPLRSVPVGTPLPGASAYMLDDVLNPVGTQVAGELYIGGGSVALGYIGQPALTAERFV
PDPFAQDGTRAYRSGDRMRRNHQGLLEFIGRADDQVKVRGYRVEPAEVARVLLGLASVAQ
VSVLALPVDDDQTRLQLVAYCVAASGANLNVDSLREQLAARLPDYMVPTQIMLLDSLPLT

ANGKLDKRALPRPGVVKQRYTAPVGEIEEKLAAVWADVLKLEQVGSTDNFFELGGDSILS
LQIIARAKRQGIKLSPKQLFEKQTIGQLASVAKLIQKKPLAVAEQISGSLP
>tr|A0A0Q0IJR7|A0A0Q0IJR7_PSEAP Regulatory protein, ArsR OS=Pseudomonas syringae pv. aptata OX=83167 GN=ALO85_04005 PE=4 SV=1
MNLRVPAIRHDACDDLSALCKAGGDPLRLNVLRVLINDSFGVLELAQIFAIGQSGMSHHL
KVLAQADLVATRREGNAIFYRRALPHTELLGGRLHAALLDEVDTLDLPDDVQSRIGLVHR
QRASASQDFFARTAEKFRAQQDLIAGLAQYSESLLILLDKLSFGAQATALEVGPGDGGFL
PELARRFAQVIAMDNSPVMLDLARGVCERKALANVELRLADALTDAKVSADCVVLNMVLH
HFAVPAEALKQLAEHVKPGGSLLVTELCSHDQSWAREACGDLWLGFEQEDLARWAIAAGL
VPGESLYVGLRNGFQIQVRHFQRPAGDTHHR
>tr|A0A0Q0BUS8|A0A0Q0BUS8_PSEAP Phosphocarrier protein HPr OS=Pseudomonas syringae pv. aptata OX=83167 GN=ALO85_00162 PE=4 SV=1
MPVRQITIINKLGLHARAAAKFVGVAGKFPCKIRVGRTPESMVDGKSIMAVMMLAAGKGT
EIHLHTEGEFEQEALDGLVELIDNKFDEGE
>tr|A0A0Q0IT19|A0A0Q0IT19_PSEAP ABC transporter:TOBE OS=Pseudomonas syringae pv. aptata OX=83167 GN=ALO85_03259 PE=3 SV=1
MMKLKLANINKQLGGTRILRDVSLEIAAGEFVVFVGPSGCGKSTLLRLIAGLDSICAGDL
LIDGRRVNDLEPRERGVGMVFQSYALYPHMSVYDNISFGLKLAKTEKSSLRERVLKTAQI
LQLDKLLQRKPRELSGGQRQRVAMGRAMAREPDILLFDEPLSNLDASLRVQMRNEIARLH
KRLGSTMIYVTHDQVEAMTLADKIVVLNGGQVEQVGSPRELYERPASLFVAGFLGSPRMN
FLPVSLQAPGRNSLIDIPALGMKSLPFDSSNLTEDAQLTLGVRPEHITLNAANNGVGVTV
SGVEYLGSEVYVHLATGASEPLICRCDAKAGWQVGDQVALQFDDSNLHLFDADGLALKRH
TDASQHLTPGTTPHLVQAGTL
>tr|A0A0Q0DLP7|A0A0Q0DLP7_PSEAP Uncharacterized protein OS=Pseudomonas syringae pv. aptata OX=83167 GN=ALO85_00273 PE=4 SV=1
MNLEAAHLPRNRAEHAALLVIFPAPGLFDRFHRGFSRPFSGFFQRDEAPADRIPCSFVLH
IKVLFYKLPPGDRVAAMKVYTANRDAWLSGNARSPIRGKPRCHCTTRSSVITLPPSHIPG
RRPLLQTRATRRVSRLPHSSQELA
>tr|A0A0Q0DMD7|A0A0Q0DMD7_PSEAP Glycosyl transferase, group 2 family protein OS=Pseudomonas syringae pv. aptata OX=83167 GN=ALO85_01135 PE=4 SV=1
MTIGQQHQVKPDVIPTVDVEGRSKIAVLIPSYKVVAHILGVIADIGPEVDRIYVVDDCCP
DHSGAYVEQHCTDPRVVVLRNPQNQGVGGAVMTGYKAAIEENMDVIVKIDGDGQMDPALI
MNFINPILAGEADYTKGNRFFDLEEIRAMPRVRLFGNAVLSFMTKFSSGYWDLFDPTNGY
TAIHRDVAKHLPLDKISRRYFFETDILFRLNTLRAVVVDIPMHAKYGDEVSNLKVSKVVG
EFFVKHVRNFGKRIFYNYYLRDMSLASIELPVGLTLLLSGSVFGISHWISSIYTGVPNSA
GTVMLSALPIILGIQLILAFLGQDIASVPRRPFHLAKTKVKSKAGAV
>tr|A0A0Q0C3G0|A0A0Q0C3G0_PSEAP Uncharacterized protein OS=Pseudomonas syringae pv. aptata OX=83167 GN=ALO85_101704 PE=4 SV=1
MQLNMTSVCLLNSISQWVEAGIFPERPRQVFRPWKQV
>tr|A0A0Q0IB98|A0A0Q0IB98_PSEAP UPF0102 protein ALO85_00195 OS=Pseudomonas syringae pv. aptata OX=83167 GN=ALO85_00195 PE=3 SV=1
MQRSTRQQAGREAEAFALQFLQQQGLRLIEQNWLCKRGELDLVMLDGDTVVFVEVRYRRH
SGWGGAMESVDFRKQEKLVTAAQLFLQHATAWANYPCRFDVIAIEGEPGKAAPLNWIKSA
FDS
>tr|A0A0Q0CFT2|A0A0Q0CFT2_PSEAP Peptidase M14, carboxypeptidase A OS=Pseudomonas syringae pv. aptata OX=83167 GN=ALO85_03622 PE=4 SV=1
MTLTIGSDFDSGNILVLDASDPAHIRLAIRPDTQSAHFQWFHFKADGLNVGQTYGFSLIN
ASESTFNNAWTGYNAVASYDHQNWFRVASDFDGKALNFSLAAEQPQVWFAYFEPYSRERH
DWLIEQAQEKAGAQLLAVGKSVEGRDIQLLRKGDGAEGKRKIWMIAQQHPGEHMAEWFME
GVIERLQQKDDAPLQQLLAEADVYLVPNANPDGAFHGHLRTNASGKDLNRAWQDSTEALS
PEVSFIRQQMEKYGVDMFLDVHGDEKIPYVFTAACEGNPGYTAQQRQLEERFRSRLCEVS
DNFQTVHGYPRGAPGQANMNLAANAVGERYKCLSLTLEMPFKDHDNAPDPVTGWSGKRSA
QLAGEILTVLSEMVKELR
>tr|A0A0Q0CGE0|A0A0Q0CGE0_PSEAP Amino acid adenylation (Fragment) OS=Pseudomonas syringae pv. aptata OX=83167 GN=ALO85_05004 PE=4 SV=1
MVVGLLGILKAGGAYVPLDPGYPSERLRYMLEDSAPVAVLVQGETRTLLGELAVPTLDLQ
DGEWEVEAEHNPAVPAITPQHLAYVIYTSGSTGKPKGVANQHDGVVNRLWWAKSEYRIGA
DDRVLQKTPFGFDVSVWEIFLPLLAGAQLVMARPGGHQDPHYLMEVIERRSISMLHFVPS
MLQAFVNQTPAGRCSTLKRVLCSGEALPHALLLQGQAHFPKSELHNLYGPTEAAIDVTAW

HYVAEQDIGIVPIGRPIANTQIYLLDAHGQPVPIGVSGEIHIGGIGVARGYLNRPELTAE
RFLEDPFSTEPAARMYRSGDLGRWLADGNIEYLGRNDDQVKLRGYRIELGEIESQLAGCP
GVREAVVLAREHRPGDKRLVAYLTAQEGAVLSATQLREQLSQGLAEYMIPSAFVTLARFP
LTPNGKLDRRALPAPEDDAYASRGYEAPVGEIEHALAEIWQALLGLERVGRHDHFFELGG
HSLMAVQLVSCLRQRFEVEVALGDLFMHPTICELAASCHSGLSKALHPNLTTIRKEGAQH
PLFLIHEGSGDIGYAQQLAKQIPSDIPVYGFSASGLQSGEEHLTTIEAMASRYIEGIRHI
QPQGPYRVAGWSAGGTIAYEIAHQLIGAGATVEFLGLIDTTSDYRHLFDAQEGGYAHLDE
RPDFDEIEALLRLLPPDVRQGPVRHLAAARDFEALLAYTQVQGLLPEGIESDLAKRYLAL
IRSIGLALYRYMPPALPVRVTLFSAMGENRSDISIGWEALMPKEQLQVISIMGTHHSIVK
EPDIRELGRAILESVTNKGRASQTRLPLETV
>tr|A0A0Q0DGR5|A0A0Q0DGR5_PSEAP Twin-arginine translocation pathway signal OS=Pseudomonas syringae pv. aptata OX=83167 GN=ALO85_03821 PE=4 SV=1
MTISRRGFIAGLALTGAAVPAAYYAHRELTRVDLPETPGEATAGPADTATQRLADKLRGV
WTLRFEGRDAGLSGAPLEGLEMFLDIAPRGRGLRGYIDTAEQLRGDALPRFRVIGDLQPA
NAARLYLRVMDGHAGNDPHSDSPDYEFSLTLDEVWGAFGNAGSGTLSGRIERLDRPLALP
ELDNRLIAIKQVFPEARERVGLSPPFLAWLVSREHRLFHQLWHASRDKWHKLPEGKRDAL
RGIGWQPGPRDHERDARGPHKDRNGSGEDFFYMHRHMLIQARRIQDLPSWPRFPLPQPEL
ERDRLGFARYFDNHDGCSLPPNWLAHGDEEYTQLVSDIKSHETFHTHFQVWESQYRDPRF
LSKLTLGQFGSQVELELHDWLHMRWASVARDPANGQPVPMARRSDDFAERWFAPENDFLA
DPFSSHVNPVFWMFHGWIDDRIEDWFRAHERFHPGEVKRLEVNGVPWFAPGRWVNVSDPW
LGPETHGCSTVPGQAAGTTMEMDPEVMKLALRITFAADDRLSNLLRRVPRRPWYARNLLP
DRWF
>tr|A0A0Q0C6V8|A0A0Q0C6V8_PSEAP Uncharacterized protein OS=Pseudomonas syringae pv. aptata OX=83167 GN=ALO85_03107 PE=4 SV=1
MAHLIETMAYASATPWHGLGNPLSPKQPIEVWQREAGMDWQIQESAVHFKSDAVGHLSAI
HSFPEQKVLYRSDTKAPLSVVSQRYHTVQPRDVLEFYRDLTEVSGYELETAGVLKGGRKF
WALARTGQGTALQDNDRVNGYLLLATSCDGTLATTATPTTVRVVCNNTLAIALDGNTHAI
KVPHNTRFDPDTVKKQLGIAVSGWDEFMYRMRHLAECKVQWHEALGYFMSVLCDTSPSDN
LPEQLPNERALRKVQELYEGRGRGSQLASARGTAWGLLNAVTEFVDHERRARSTEYRMDS
AWFGQGAQIKQRALDQAIQLAA
>tr|A0A0Q0BSK2|A0A0Q0BSK2_PSEAP Tat pathway signal sequence domain-containing protein OS=Pseudomonas syringae pv. aptata OX=83167 GN=ALO85_04413 PE=4 SV=1
MLSASGWNKAREFTSYRAPEVFEVCHELDLVRCHTAPIFPCYGTFLPVSEGITVSQFDLK
RRRVIQAVGTVGAGLLLPGVAPAVIASVQDRPKMMDGVQSGDVFGDKAIVWSRSDRPSRM
VVEWDTRSMFTQPRRVVSALADQRSDFTARVELSGLPVDQAIFYRVSFEDAQSGVVSEPW
FGHLRSTPQQRRDIRFVWSGDTVGQGFGINPDIGGMRIYEAMRLRLPDFFIHSGDTIYAD
SPVPEQLTTEGGRVWRNITTEAKSKVAETLDEYRGNYRYNLMDENLRRFNAEVPQIWQWD
DHEVTNNWSPSKQLDDRYKVKDIGLLSSRARQAYLEYAPLRLQEADNGGRIYRKIPYGPM
LDVFVLDMRSYRAGNDANLADKPGPTTAFMGREQLDWLKRELTASRAQWKVIAADMPIGL
GVPDGEVSPGVQRWEAIANGNDGPALGRELEVAELLAYLRAQKVRDCVWLTADVHYCAAH
HYQPDLAVFQDFDPFWEFVAGPLNAGSYGPNVLDKTFGPELVFQKAPPAQNTSPFAGFQF
FGEVNIDGQTGELTVALRDLDGVSVFERKLQPVKEVSRIV
>tr|A0A0Q0D2L7|A0A0Q0D2L7_PSEAP Uncharacterized protein OS=Pseudomonas syringae pv. aptata OX=83167 GN=ALO85_02192 PE=4 SV=1
MNVFRCLGLLVVVLLSGCDAPDTSVPKPKTEAVITAPVSPEVHLKTEAPAQAAATDAKQI
KAPDTMHQLMPGVPVVPVVVGKEKSEPAAPQSVKSATSEKASSRVAAAKAPASKRKNAKD
AKTASVKDVRIKAPKLDLSLPPELVKELDPPAKVITNKRKPILPQMFSDKGDSANPGPFE
LEGRLLTNEMQLQMRNDNRRDVEGAALDFKFKQ
>tr|A0A0Q0DCF5|A0A0Q0DCF5_PSEAP Argininosuccinate lyase OS=Pseudomonas syringae pv. aptata OX=83167 GN=argH PE=3 SV=1
MTAVHSSVRPTTKRQLSMPARPGVNRASQGRSPHSSAADSSTIHCTNSDINARRTQSKPL
RIGSTSTRTSTSSSASTTSTSNACGRHSSGRKKSLPGAGRASCFTGSIRMAPVSALASSA
ASCPGIKMHQPCYHRVRSCARYRARHFQSATSESMSTDKTNQSWGGRFSEPVDAFVARFT
ASVTFDQRLYRHDIMGSIAHATMLAKVGVLTDAERDTIVDGLNTIQAEIEAGQFDWRVDL
EDVHMNIEARLTDRIGITGKKLHTGRSRNDQVATDIRLWLRDEIDLILSEITRLQQGLLG
QAEREAETIMPGFTHLQTAQPVTFGHHMLAWFEMLSRDYERLVDCRKRLNRMPLGSAALA
GTTYPIDRELTCTLLGFDVVGGNSLDGVSDRDFAIEFCSAASIAMMHLSRFSEELVLWTS
AQFQFIDLPDRFCTGSSIMPQKKNPDVPELVRGKSGRVFGALMGLLTLMKGQPLAYNKDN

QEDKEPLFDAADTLRDSLRAFADMIPAIKPKHAMMREAALRGFSTATDLADYLVRRGLPF
RDCHEIVGHAVKYGVETGKDLAEMSLEELRQFSNQIEQDVFAVLTLEGSVNARNHIGGTA
PEQVRAAVVRGQELLAGR
>tr|A0A0Q0C6Q7|A0A0Q0C6Q7_PSEAP Uncharacterized protein OS=Pseudomonas syringae pv. aptata OX=83167 GN=ALO85_01154 PE=4 SV=1
MTLAHDSISAVFFFTCSRIFILAAGSLLTLLDDIAMLLDDVALATKKTAGVLGDDLALNA
QQVTGVSADRELPVVWAVAKGSLLNKAILVPTALLISAFAPWAILPLLMVGGAFLCFEGF
EKIAHKWLHPETGEEHEQRKQASADASVDMVAFEKERVKGAIRTDFILSAEIIVLALGVV
SARPFMDQVGVLVIVAVLITVGVYGLVAGIVKLDDLGLYLKKSAASAAQKIGAGLLWLAP
VLMKVLSIVGTAAMFMVGGGILVHGLPFAHHWVEGVTEAAAGAVGGLSMVVPTLIDAVAG
VIAGAVLVLVVTLIGKVWRAAKE
>tr|A0A0Q0IRK3|A0A0Q0IRK3_PSEAP Uncharacterized protein OS=Pseudomonas syringae pv. aptata OX=83167 GN=ALO85_200182 PE=4 SV=1
MTTVEVSTLQHGLSLVEPAERALNEALRILKAGAADHEGAIEAVDSARLAYHQACVGLFG
FVQGIVAQAVLADQQSAATPGDTVMLNIEVDSGVTIQRAREEIASSAAMIRDNTDELKRV
ADAMYINGYLMSLQEHGLVSLHVLEQLRVERDRAFAGSEEARD
>tr|A0A0Q0BXY6|A0A0Q0BXY6_PSEAP Outer membrane porin OS=Pseudomonas syringae pv. aptata OX=83167 GN=ALO85_04738 PE=4 SV=1
MTPRLLKTRTARHPSRFTTFAETQRTVLGRLQVEAMPGDRHHVILEGVGRRSRAARKGDG
FTKQGSTTIMTSHTTRSRILLGVGGMTLIFPMLGMAEGFVDDTKATLNLRNAYFNRNFTN
PAFPNSAAPQNKAEEWTQSFILDAKSGFTQGVVGFGVDVLGLYSLKLDGGKGTGGTQLLP
IHSDGRPADDFGRLGVAGKARISKTELKIGEWMPVLPILRSDDGRSLPQTFRGGQVTSKE
IDGLTLYGGQFRGNSPRNDASMEDMSLNGRGAFTSDRFNFGGGEYVFNDKRTQVGVWYSE
LQDIYQQQFFNLLHSQPLGDWTLGANLGYFIGKEDGNKLAGDLDNKTAYALLSARYGGST
FYVGLQKLTGDTAWMRVNGTSGGTLANDSYNSSYDNAKEKSWQLRHDYNFATLGVPGLTL
MNRYISGDNVHTGNITDGKEWGRESELAYTVQSGALKNLNVKWRNSSLRRDFSTNEFDEN
RVFVSYPISLL
>tr|A0A0Q0BXL0|A0A0Q0BXL0_PSEAP Putative integrating conjugative element protein, family OS=Pseudomonas syringae pv. aptata OX=83167 GN=ALO85_03998 PE=4 SV=1
MTLYSLFTQARAFLKRRPFSVLLTAATPGTSFAALPGAQAPTRGTGTSFLQTFQNYAFDG
FTLLGLCMCAFGIILVGRHALGVYHEIHMGKAKWADLGSTAVVGVCLIGVTIYLVTTATN
IL
>tr|A0A0Q0DSL2|A0A0Q0DSL2_PSEAP Glutamine ABC transporter ATP-binding protein OS=Pseudomonas syringae pv. aptata OX=83167 GN=ALO85_00866 PE=4 SV=1
MHPMIMFSRINKWYGEYQALTDITAEVQRGEVVVLCGPSGSGKSTLIRTVNRLEDVQQGQ
ILFDGRDVNGTDANVNRLRSRVGFVFQSFNLFPHLSVLDNIILTPTKVRGLKSSEARARA
MELLDRVGLAHKAGAYPAQLSGGQQQRVAIARALAMEPPVMLFDEPTSALDPEMVGEVLG
VMKGLAKEGMTMMCVTHEMNFAREVADTIWFMDAGQILEKSSPEVFFQQPSHPRAQRFIA
DLRSH
>tr|A0A0Q0C9L5|A0A0Q0C9L5_PSEAP Recombination factor protein RarA OS=Pseudomonas syringae pv. aptata OX=83167 GN=ALO85_00719 PE=4 SV=1
MDLFSREPIAQPLAARLRATNLDEYVGQEHVLAHGKPLREALEQGALHSMIFWGPPGVGK
TTLAKLLAKVSDAHFETVSAVLAGVKEIRQAVEVARQQAGQYGKRTILFVDEVHRFNKSQ
QDAFLPYVEDGTLIFIGATTENPSFELNNALLSRARVYVLKSLDEAALRKLVNRALTEER
GLGKRQLALSDEGFAMLMSAADGDGRRMLNLLENASDLAEDGSEIDVGLLQSLLGDSRRR
FDKGGEAFYDQISALHKSIRGSNPDAALYWFARMIDGGCDPLYLARRVVRMASEDIGNAD
PRALPLCMSAWDVQERLGSPEGELAVAQAIVYLACAPKSNAVYMAFKAAMREAGEHGSLE
VPLHLRNAPTKLMKQLGYGEEYRYAHDEPDAYAAGEDYFPDELEPRQYYQPVPRGLELKI
GEKLRHLNALDDASPRKRRK
>tr|A0A0N8T9F3|A0A0N8T9F3_PSEAP Allophanate hydrolase OS=Pseudomonas syringae pv. aptata OX=83167 GN=ALO85_02429 PE=4 SV=1
MNDIALPNDLRLDTVRALYQSGKISPRQLILALREKAARLNNDYHLFIHLLSPAELEPYL
AGLDARDPKDLPLFGIPFAIKDNIDLAAIPTTAACPDYAYTPEHSASVVAHLIALGAVPM
GKTNLDQFATGLNGSRSPYGACPNSVSNEYPSGGSSAGSSLAVALGVSSFSLGTDTAGSG
RVPAALNNLVGMKASKGLISTAGVVPACRTQDCVSTFTATAREASELLALIAAFDPRDEY
SRRNPLWNDASAFGTPRPFRFGVPRAEDLQFFGCTEGPRLFNAAIDHLAALGGEAVTVDL
SPFLEAARLLYEGPWVAERYSVAGQLMEERPDAVLPVIRDVLAKAPQVSGVDTFRAQYRL
QALKATCDRALEGLECMVTPSIGRPVTSAELAAEPVLRNSELGYYTNFVNLLDYAAVAVP

SAFMGNGLPWGVTLFGRAFTDQYLLSLADALQRQTALPLIGGEAPRLPVPQTTARNDRAR
LVVCGAHLDGLALNWQLRQRGARLLETTQSSTDYRLYALAGGPPFRPGMVRVTEHGVAID
VEVWELPSIELGSFLTGIPAPLGLGKVQLADGRWETGFICEAYGLEGARDISHLGSWRTH
VQPQ
>tr|A0A0Q0DMX0|A0A0Q0DMX0_PSEAP Autotransporter barrel protein with S8 family peptidase passenger domain OS=Pseudomonas syringae pv. aptata OX=83167 GN=ALO85_05337 PE=4 SV=1
MFMKDALSTRHTSVKPLRQAIRAVNRASCGALACYLATLGVAHAAPYVEAGLAGNAASWR
SAEFNADWGLGAINADQAYAAGYSGKDIKLGIFDQPVYAPHPEFDSPNKVVNLVTSGIRE
YTDPYIPVKAGDAFRYDGAPSLDSGGKLGNHGTHVGGIAGGNRDGGPMHGVAYNAQIISA
DNGDPGPEDGIVLGNDGAVYQAGWNALVNSGARVINNSWGIGITDRFDKGGRDPAFPHFT
VQDAQVQFDQIRQILGTRPGGAYQGAIDAARSGVVTIFAAGNDYNLNNPDAMAGLGYFVP
EIAPNWLTVAALQQNPDAAAAATTPYTLSTFSSRCGYTASFCVSAPGTRIYSSVLNGTSL
ADLTVGWANKNGTSMAAPHVAGSMAVLMERFPYMTGAQVADVLKTTATDLGAPGVDALYG
WGMINLGKAVNGPSMFVTEADIPAEFRIHGAYGDSQFVADLPGVGAIVDAGKPTQRTCSG
PQCGLDVWSNNISGHGGLTKQGIGTLVLTGANSYSGPTLVNQGRLAINGSLASAVTVNNG
GILAGNGSVTSLNANRGGTVAPGNSIGTLQVAGDLNLAPGSTYAVEFSPTASDRIVVGGT
ATVSGANMALSLENDNPALLSSNQTSSVLGQQFNVLQAAGGVQGQFGSVTDNYEFLDGNL
AYSGTGVTLALARNGDSFASVAQTSNQRSVAQAIEQLSAGNSVYEGIILSQDTASARRAF
TQLSGEVHPAIATQLINDSRQLRDAVGDRLRVEGLYDQPAPGTEDNSVWVKALGAWGKNS
GSSDSASSTSSLGGLLAGVDGLISEHTRLGAMAGYSDTSLSLGDDTHSRASADSYHLGAY
IGHEQGALRLTAGASHSWHRIDVKRDLQFGAFSDRQKVKRDAQSSQVFTEAAYRLNLQPL
ALEPFANLAYVHFDSDSFTEKGGATALKGSDDTRDTVLSTLGMRAGNRFNLNDTQKLDVS
ATLGWQHNLSDTSSEQHLAFASAGNSFNVQSVSMDRDAAVVGARASLALGRDARINLDYN
GLLGARDKTHGVGLSLDWQF
>tr|A0A0Q0IEI4|A0A0Q0IEI4_PSEAP TetR family transcriptional regulator OS=Pseudomonas syringae pv. aptata OX=83167 GN=ALO85_03519 PE=4 SV=1
MALLETQRMRYALDHKAQTHQRIVKEASMRFRRDGIGATGLQPLMKALGLTHGGFYAHFK
SKDDLVEQALSHALDNVKGITSDVFARQDSLSEFIDLYLSIASRDAEDGGCPLPTMCLEL
GQRDQPSMTTDKIILHLLEQFDKPLAENGLESKSLPVLSALVGGLALARSAFDEELSQRI
LDTTRDYLKKEIKKSTN
>tr|A0A0Q0C669|A0A0Q0C669_PSEAP Alpha/beta hydrolase fold protein OS=Pseudomonas syringae pv. aptata OX=83167 GN=ALO85_03639 PE=4 SV=1
MQLCLSPEFRMSAVQLADGQLNYLLEGPAGAPVLVLSNSLGTDLHMWDNQVPAFSLHFQV
LRYDTRGHGKSLVTEGSYSIEQNGRDVLALLDALDIDKAFFCGLSMGGLIGQWLAINAPQ
RLHKVVLCNTAAKIGNPDVWNPRIETVLRDGQSAMVALRDASIARWFTPSFAHAEPAAVD
TVVGMLARTSPQGYAANCAAVRDADFREQIASIELPVLVVCGTEDAVTTPADGRFMVERI
QGSQLIELHAAHLSSVEAGEAFTGPVLAFLTAE
>tr|A0A0Q0IDK4|A0A0Q0IDK4_PSEAP Uncharacterized protein OS=Pseudomonas syringae pv. aptata OX=83167 GN=ALO85_101039 PE=4 SV=1
MSSPGIERTTAPPLGDGEVVVRDGDEQLATSTARPVNARSLND
>tr|A0A0Q0DNC8|A0A0Q0DNC8_PSEAP ABC transporter ATP-binding protein OS=Pseudomonas syringae pv. aptata OX=83167 GN=ALO85_00701 PE=4 SV=1
MSMSSQGNPAPLYKALADYKSALIGVGCFTALINVLMLAPSIYMLQVYDRVLTSQNQTTL
AMLTLMVVGFFAFIGLLEMIRSFVVIRIGSELEKRFNLRVYQAAFESNLHAGQRQAGQAL
GDLTFIRQFLTGPALFAFFDAPWFPIYLAVIFLFDPWLGVLASVGTVLLVGLACLNEWLT
RKPLAEASGYSQQSAQLATRHLQQAEAIQAMGMLEVLRRRWFHVHGRFLALQNRASDTGA
VISSISKALRLCLQSLVLGLGALLVINGDMTAGMMIAGSILMGRVLSPIDQLIAVWKQWS
SARLAYGRLDQLLKTYAEPAPRMPLPAPRGQISAEQVSAAPPGKTSPTIQQVSFQLQAGE
VLGVLGASGSGKSTLARVLVGVWPALGGTVRLDGADMHRWDREALGPYIGYLPQDIELFG
GSVAENIARFREGDASAVVAAAQLAGVHELILRLPQGYDTRLGDDGAGLSGGQKQRVALA
RALYGDPRLVVLDEPNSNLDAAGEAALTQAIAELKTRGCTVVLVTHRTQSLNQTDRLLVL
SEGRMQAFGATAQVLQALSGQPSSAQAAQAHPQSQSRPAPAGLSMSRQYGTQNRQSDV
>tr|A0A0N8T9G9|A0A0N8T9G9_PSEAP Cation/multidrug RND-type efflux system, membrane fusion protein OS=Pseudomonas syringae pv. aptata OX=83167 GN=ALO85_00953 PE=3 SV=1
MVDLFMQSSGSRKSRRWLISLLVLLVIVALCWWFWPASTSGKGADSNKKPATSGMARPGF
GGSAVAVPVRVAPATEGDFPIYYKALGTVTAMNTINVRSRVAGELVKIYFQEGQMVKAGD
LLAEIDPRSYQVALQQAEGTLATNQALLKNAQLDVQRYRGLFAEDSIAKQTLDTAESLVN
QYKGTIKTNQAAVADAKLSLDFSRIRAPIAGRVGLKQLDVGNLVAANDTTALVVITQTQP

ISVAFTLPEKDLSKVISRYRTGDKLPVEAWDRGDTKMQATGVLASLDNQIDVATGTLKFK
ARFDNSDEVLFPNQFVNVRLRADTLKKVTLVPTAAVQFGTDGTFVYVLDGDKKVKLKLLK
TGPSDETSTVITEGLAAGERVVLEGTDRLRDGAEVEVVNDSKEVPAGPAQKLQGKPDSKT
DKSAALGKVEKPNV
>tr|A0A0Q0D4S0|A0A0Q0D4S0_PSEAP ABC transporter OS=Pseudomonas syringae pv. aptata OX=83167 GN=ALO85_200045 PE=4 SV=1
MFYARKLNSDTGQVEVWECEWSDPGTGLAKKNFVRKYCNEGEQEDNPEQYSTAAAICWAP
GRTIGNIAVNSEGVFGSFTAKAGDNAVLPCHIVPCGKFRNGADRWYCKTHQIHWGVKADI
AAVPSSGEVTCSNHLMGMSYVVDPLVVDFNDFEEIGVWCSLPPALSSEKIVRRPPKIHVH
KRFSGEDKKRLDRDFDAIVCSYNQNLGLFSSNEITQIQITPPAAFEFVKSLEDGREMSCV
TCKSCGYPHLDLGSFANTPHAKHFCGNCGSDSVWSDGKIVSTPLKPLHDQFNNSNQYVVP
DRSLNMDEYPGLEFEVWSSTPAVLWTANRPQEMGIHVHIYERGMRGRRLIDDTFGEVIYQ
GRVLDRKILWQRMAGNTIY
>tr|A0A0Q0CHK4|A0A0Q0CHK4_PSEAP Uncharacterized protein OS=Pseudomonas syringae pv. aptata OX=83167 GN=ALO85_05361 PE=4 SV=1
MGNTSRQSAEAPALSNITNHNHRGARIGNLCIGTATTSMAGWERDESDALTHSRNLYQQN
HFVQPIRPSPSRGRKPKSLFPIADREQASRHAMTCSGNLRVRLPTSLLPLTFQRLQRVLH
VGTIGSGLLANITLATTEHLQGCAQLRVRITKLVLLRERRHGVDANFTGLGNPDDMVIQR
VFARSKQTSQFINSRLCVDVHVDAPSLIDCSIIDTSSSCARTSATQRD
>tr|A0A0Q0DXN3|A0A0Q0DXN3_PSEAP Extracellular solute-binding protein OS=Pseudomonas syringae pv. aptata OX=83167 GN=ALO85_05455 PE=4 SV=1
MSSAMIVVTAVQPVAFNPDIRLRTSVRIRVMRFVFSSLLALTLTFVGAAQSLSAAPQHAL
TVYGEAPRYPADFKHFDYVNPDAPKGGSMRRSAIEIGQFDHLMPFTDKGMGVSQINGMLY
SPLATRSMDEPYTVYGLVAEKMERGADGLSLRFYLNPKARFADGTPITASDVKYTYELLM
TQGNLQYRTQFEAVKGVEVESPLQVRFDFKQSDNRTLPLDLASLPVFPEHWWKTRDFASG
AGFEIPPGSGPYRISHVDPGRTVTFERDPDWWGKDLPVSKGLYNFDRFSIEYFGDTDVAR
TVLKGGAYDYNREFSATGYSIRYDGPALSDGRLQKAQLARGAVQSSQGFVFNLSRPMFQD
RRVRQALSLLWDFEWTNRQMMRNMYIRQNSFFSNSELAASELPTPAELKILEPLRGKVPD
QVFDSVFETPKTDGTGFIRDKQLQALALMKEAGWTPKGDELVNAQGEPFSFTFLNAQTGF
ERMLLPYKRTLAQIGIHFDIRRIDAAQYLNRVMARDYDMIVTGYPVSTSPGAELYSSFGS
KVAMDPGSSNYMALQDPAVDALIAGLLKADSKQTMTDYAHCLDRVLQWNYYWIPNYYPPG
SSTVWWNRFGIPKIQASNNEAIETWWEISPTPLTNEQFAEKRGASATVSEMQ
>tr|A0A0Q0C5V7|A0A0Q0C5V7_PSEAP UDP-2,3-diacylglucosamine hydrolase OS=Pseudomonas syringae pv. aptata OX=83167 GN=lpxH PE=3 SV=1
MILLISDLHLEQERPDITRAFLDLLAGRAREAESLYILGDFFEVWIGDDAMSPFQLSICK
ALRELSDSGTRIFLMHGNRDFMLGKGFCKAAGCTLLSDPSVVQLNGEPVLLMHGDSLCTR
DEGYIRMRRYLRHPLTLFILRHLPLGTRHKLARKLRNESRAQTRMKANDIVDVTPDEVPR
IMQQFGVRTLVHGHTHRPAIHKLQIGDQAARRIVLGDWDRQGWVLQVDEQGFNLSSFDFV
PETLALLN
>tr|A0A0Q0FE01|A0A0Q0FE01_PSEAP Putative SAM-dependent methyltransferase OS=Pseudomonas syringae pv. aptata OX=83167 GN=ALO85_00476 PE=3 SV=1
MSLPSLRLKANADRRLRAGHLWVYSNEIDVAATPLHGFAAGDQAILEAAGGKPLGIVAMS
PNNLICARLLSRDIKLPLDKSLLVHRLNVALSLRERLFDKPFYRLVYGDSDLLPGLVVDR
FGDILVVQLASATMENHKEDIIAALVQVIKPSGILFKNDSAARDAEGLNRYVETVFGLVP
EWVALEENGVKFEAPVMAGQKTGWFYDHRMNRARIAPYVKGKRVLDLYSYIGGWGIQAAA
FGASDVTCVDASSFALDGVERNAALNGFAEKMTCIEGDVFEALKELKAAEERFDVIVADP
PAFIKRKKDMKNGEGAYRRLNEQAMRLLSKDGILVSASCSMHLPEDDLQNILLTSARHLD
RNIQLLERGGQGPDHPVHPAIAETRYIKSITCRLLPNS
>tr|A0A0Q0D0P1|A0A0Q0D0P1_PSEAP Proteinral secretion pathway protein D OS=Pseudomonas syringae pv. aptata OX=83167 GN=ALO85_04631 PE=3 SV=1
MMGTFSGVPAIATLRTPLLCLATAVALGGCASQSDTLDPDNGMLQEALQGTGSQRPPVDP
RSERPPVEPPSQQTTSSPQRQIIKGNQRFIRQPAAAPAARQAETGDIVFNFTNQPIQAVI
NSIMGDLLHENYSIAQGVKGDVSFSTSKPVNKQQALSILETLLSWTDNAMIKQGNRYVIL
PSSQAVAGKLVPEMRVAQPSAGMSARLFPLRYISATEMQKLLKPFARENAFLLVDPARNV
LSLAGTPEELANYQDTIDTFDVDWLKGMSVGVFGLQRASVGELMPELQKMFGPESGMPLA
GMVRFLPIERTNSVVAISSQPEYLHEVGEWIHTIDEGGGNEPQMYVYDVRNMKATDLAKY
LRQIYGTGAIKEDAAAKVAPGLRTTTLSSLNSSGGNGVGGMSSSNGLGGTGSGLSNGGGF
GNSQGMNNSQNNGDSESEGDDQSGGDSDSASQEGSESGGNNKSLDASTRITAQKSSNQLL
VRTRPAQWKEIESAIKRLDNPPLQVQIETRILEVQLTGELDMGVQWYLGRLAGNSGTTGN
VTNTAGSQGAIGTGGAALASTDAFFYSFVSNNLQVALRALETNGRTQVLSAPSLVVMNNQ

QAQIQVGDNIPISQTSINTNTNAGTTLSSVEYVQTGVILDVVPRINPGGLVYMDIQQQVS
SADTNANNSDANGNPRISTRSVATQVAAQSGQTVLLGGLIKQDNAETVSAVPYLGRIPGL
RWLFGNTSKSKGRTELIVLITPRVITSSSQARQVTDDYRQQMQLIKPEVSRTSMQN
>tr|A0A0Q0C5A9|A0A0Q0C5A9_PSEAP Acetyl-coenzyme A synthetase
OS=Pseudomonas syringae pv. aptata OX=83167 GN=acsA PE=3 SV=1
MSAASLYPVRPEVAATTLTDEATYKAMYQQSVVNPDGFWREQAQRLDWIKPFTVVKQTSF
DDHRVDIKWFADGTLNVAYNCLDRHLEERGDSIAIIWEGDDPSEHREITYRELHAEVCKF
ANALRGQDVHRGDVVTIYMPMIPEAVVAMLACARIGAIHSVVFGGFSPEALAGRIIDCSS
KVVITADEGLRGGKKTALKANVDRALTNPETSSVQKVIVCKRTGGEIEWNRHRDIWYHSL
LEVASSTCAPKEMGAEESLFILYTSGSTGKPKGVLHTTAGYLLYAALTHERVFDYKPGEV
YWCTADVGWVTGHSYIVYGPLANGATTLLFEGVPNYPDITRVSKIIDKHKVNILYTAPTA
IRAMMAEGTKSVEGADGSSLRLLGSVGEPINPEAWAWYYNTVGKQNCPIVDTWWQTETGG
ILISPLPGATALKPGSATRPFFGVIPALVDNLGNLIEGAAEGNLVILDSWPGQSRSLYGD
HDRFVDTYFKTFRGMYFTGDGARRDEDGYFWITGRVDDVLNVSGHRMGTAEIESAMVAHP
KVAEAAVVGVPHDLKGQGIYVYVTLNGGEEPSDALRTELRNWVRKEIGPIASPDFIQWAP
GLPKTRSGKIMRRILRKIATAEYDALGDISTLADPGVVQHLIDTHKAMNAA
>tr|A0A0Q0C4L0|A0A0Q0C4L0_PSEAP Uncharacterized protein OS=Pseudomonas
syringae pv. aptata OX=83167 GN=ALO85_04049 PE=4 SV=1
MARRRPTSERIWPGRRPAMNLRKPRAWAALALSISTWPAAAGTVTSATLVASTLSPDCLE
YKVVGICYWLLCTPFGCKVKTSTKVRHFVPDAVVSSYSNTGENPWIEVSSLSSSTSFAQD
GGDGTTNHNNEDSLAKFKNADVIGHPGGATFSKFASASGYACQGAATPYMPYLLSTLDTV
AWRYGVPESVYPEALIPGRREVGGLTSGNMWGSVYPRSGFIHQADDYKAASVIAQRAGDV
VTRSSQVHVYQPLLAQPQPGYWPAGELIETDATTGKWQELTPTLSQSCAVFPNSQPRVQA
TDGGYAWALWRPYSCCKREGQTFLGSTDFQ
>tr|A0A0Q0IAM4|A0A0Q0IAM4_PSEAP Isochorismatase family protein
OS=Pseudomonas syringae pv. aptata OX=83167 GN=ALO85_04240 PE=4 SV=1
MSERHIASAPYPWPWNGQLHAHNTALIVIDMQTDFCGVGGYVDSMGYDLALTRAPIEPIK
ALLAAMRPLGFTIIHTREGHRPDLSDLPANKRWRSQRIGAGIGDPGPCGKILVRGEPGWE
IIDELAPLPGEIVLDKPGKGSFCATDLELILRTRGIDNLILTGITTDVCVHTTLREANDR
GFECLLLEDCCGATDPGNHAAALSMVKMQGGVFGAVGHSSMLRDLLGA
>tr|A0A0Q0FE18|A0A0Q0FE18_PSEAP Formamidopyrimidine-DNA glycosylase
OS=Pseudomonas syringae pv. aptata OX=83167 GN=mutM PE=3 SV=1
MSMPELPEVETTRRGIAPHLEGQRVSRVIVRDSRLRWPIPEDLDVRLSGQRIVQVDRRAK
YLLIQAEVGTLISHLGMSGNLRLVEAGLPALKHEHVDIELESGLALRYTDPRRFGAMLWS
LDPHNHELLIRLGPEPLTDLFDGQRLYERSRGKSIAVKPFVMDNAVVVGVGNIYATEALF
AAGIDPRREAGSVSRARYLKLAIEIKRILAYAIERGGTTLRDFIGGDGKPGYFQQELFAY
GRGGQPCKVCGTTLREVKLGQRASVYCPKCQR
>tr|A0A0Q0FTP1|A0A0Q0FTP1_PSEAP Uncharacterized protein OS=Pseudomonas
syringae pv. aptata OX=83167 GN=ALO85_100656 PE=4 SV=1
MRGGWFTARDTSAGTRAACTWPFTGDQTDMTAITLDADIKAKWPQGHCSHSPGNPEELMI
IAVDLLIKELGTEGARSFISQVLSRYAAAGLPA
>tr|A0A0Q0BUQ1|A0A0Q0BUQ1_PSEAP Bifunctional adenosylcobalamin
biosynthesis protein OS=Pseudomonas syringae pv. aptata OX=83167
GN=ALO85_03554 PE=3 SV=1
MLQLILGGARSGKSRLAEKLAADSALRVTYIATSQPLDGEMNQRVASHRARRPEHWGLVE
EPIELARVLRENAAPDCCLLVDCLTLWLTNLLMLDNPERLLQERESLLDNLAGLPGEIIF
VSNETGLGVVPLGELTRRYVDEAGLLHQALAERCQRVVLTVAGLPLTLKGTAL
>tr|A0A0Q0E162|A0A0Q0E162_PSEAP Glycine--tRNA ligase alpha subunit
OS=Pseudomonas syringae pv. aptata OX=83167 GN=glyQ PE=3 SV=1
MSQPTPAVRTFQDLILALQQYWAEQGCVVLQPYDMEVGAGTFHTATFLRAVGPETWNAAY
VQPSRRPTDGRYGENPNRLQHYYQFQVILKPNPDNFQELYLGSLKHIGLNPLVHDVRFVE
DNWESPTLGAWGLGWEIWLNGMEVSQFTYFQQVGGLECYPVTGEITYGLERLAMYQQGVD
SVYDLVWADGPFGKVTYGDVFHQNEVEQSTYNFEHANVDKLFELFDFYESEAARLIELEL
PLPGYEMVLKASHTFNLLDARRAISVTARQQYILRVRTLARSVAQAYLQARARLGFPMAP
PDLRDEVLAKLEAAQ
>tr|A0A0Q0DGX7|A0A0Q0DGX7_PSEAP Peptidoglycan-binding LysM OS=Pseudomonas
syringae pv. aptata OX=83167 GN=ALO85_03239 PE=4 SV=1
MLKSSLAAVEGAGRRARGAFGRGVLAVSLLTCSTLASALGLGEITLHSALNQPLNAEIQL
LETGGLSNEDVVARLASPEAFAKAGMERVFFLNDLRFTPILRGDRGVIRVVSSKPVTEPY
LSFLVQLARPNGDLLHEYTLLLDPATSAQGLAATRSRNQQRSATSASESRMPVAPPAAVQ

GKRYTVASGDTLNGIASRLQGPGNKVSASQLADGIRSLNPQIFAAGAGSALKVGQDLLLP
DAAVLPTAAAPAASAAAPSPKPVELQRTAEQLSAAAIENQQLTQSLEALKAQTQELQEEM
SGKDKQIIALRSDLAAAQSAATPAAPATVTPAPVSPAAAPAAPAQPVAASGSDSFLSLPI
LLAAVLIVLLLVAFAYSKRRQQRKESIASAVSPDDQLIKPAQASMLPVFEVPAVAPQPVV
QSPSTPAPAKPSPAQRSPGAAPDALDGVSIYIAYGRFTEAMGILRSALETQPERDDIRIR
LLELLAEQGDGSGFVREEQAALEHGVDPQTIQDIRDRFPQLKAAEIAPVAAVVPAVAVAA
STLSFDKSEPEPELYLQPEAEPEPAAQLDESAAAALNPQHADEFQLNLDDLSMDADWDLV
DPFDSPPPRAKPAAAPAPAEVDPGFSSDLTQLPEVFELSDEQFLSDFSEPEVVEPVEVLA
PSAADGLSDDFLDSFMNDDADFDLLDLEEPPLSQINQAQVLIEDGDLESAREILQQIIDE
SDEEHRRMARELLANIS
>tr|A0A0Q0IJ42|A0A0Q0IJ42_PSEAP TPR domain-containing protein
OS=Pseudomonas syringae pv. aptata OX=83167 GN=ALO85_100024 PE=4 SV=1
MHQFFRRLWVLPTALCHDAPTAGKPPFLYMNRSSALFLALAFLGGCQSMTPASSQPVPAQ
KDAPPAPEAKPQVYGSFTQDTLVSLLSAELAGQRNRFDIALDNYVTQAIKTQDPGVSERA
YQIAEYMGADQSALDTALIWARNAPGNLEAQRAAAIQLARAGRYDDSLVYMERVLQGQGD
THFDFLALSAAETDPDTRNGLLKSFDRLLGKYPNNGQLIFGKALLLQQNGDSEQSLKLLE
DNPPKEGEIAPILLHARLLQSMNRGKEAVPLLEKSIKKYPDDKRLRLTYARMLVEQNRME
DAKVQFSALLQQYPDDDELRFSLALVCLEAKAWDEAAGYLEELIARGAHVDSAHLNLGRI
HEERDDPQSALNEYAQVGPGPDFLAAQLRQADILVSNGNGAEAAKRLSEARAEEPDYAVQ
LYLIEAETLTSNDQLDRGWQVLNQALKQYPDDANLLYTRAMLAEKRNDLAQMEKDLRTII
KREPENAMALNALGYTLSDRTTRYTEARELIEKAHKISPDDPAVLDSLGWVNYRLGNLDD
AERYLRQALERFPDHEVAAHLGEVLWAKGEQREAKKVWAKALEQQPDSTVLRSTLRRLTG
SETL
>tr|A0A0Q0BVD3|A0A0Q0BVD3_PSEAP DEAD/DEAH box helicase OS=Pseudomonas
syringae pv. aptata OX=83167 GN=ALO85_00707 PE=4 SV=1
MNSQASSSDSSAFERLHPDVQQWVWSQGWTSLRDAQEWAIPALIDADRDVIIAASTAAGK
TEAAFLPILTHLLNNTDAPGAVLYISPLKALINDQWDRLTRLCERLEIPVVGWHGDVSAS
RKHRFLKSPRGILLITPESLEALFVNRGSALAGLFQPLRYIVIDELHAFIGSERGKQLQS
LMHRVETVVGKKLPRVGLSATLGEMALAREFLRPGQGRRMDEIISRASGQDLQVQVRGYT
MLPMKKLTENLQMVVTKDDGNDDQDEEQEEDVSGTRHAIAAHMYKVLRGSNNLIFPNSRD
KVEWYADRLRHLCENDGLPNEFLPHHGSLSRDLREDTERALKAGSPPASAVCTTTLELGI
DIGNIKAVAQIGPPPSVASLRQRLGRSGRRAGKPATLRAYCQEDTLTPDSDLSDQLRQNL
VQTIAMIRLLIEKWFEPPRANGLHASTLVQQCLSTIAQQGGAHASHLWNELIASGTFAAV
DKTDFMALLKELGEKKLIVQDSSGLLLPGEIGEKLVNHYEFYSAFSSDEEFRLLRDGKPL
GSIPVSRPLTLGQRIIFAGKRWQVMDVDLEKKVIAVIRARGGEPPVFDGLGAKIHDRVRE
EMRAVLTEVTPCPFLDANAQALLAEARQTFHRLGLADQRLTGSTSSSYLLTWAGDYTNDA
LCLLLNQAGIMCTASGLVLEISASQESVRAALARIAELDATDVEPLLKDVKNLIREKWDW
ALPKSLLIKSFASSQLDIPNAIALAKTLTI
>tr|A0A0N8T8P8|A0A0N8T8P8_PSEAP Syringopeptin synthetase B (Fragment)
OS=Pseudomonas syringae pv. aptata OX=83167 GN=ALO85_05546 PE=4 SV=1
VPGLTSAHLAYLIYTSGSTGLPKGVMIEHRNTVNFLTWAHAAFDAGTNNSALDKMLFSTS
LNFDLAVYECFAPLTSGGSIEVVKNVLELQHGEHDIGLINTVPSALKALLEVDGLPTSVH
TVNVAGEALKRSLVESLFEKTSVQRLCNLYGPSETTTYSSWVAMDRKDGFAPHIGKPVGN
TQFYLLDEQQQPVPLGVAGEIYIGGAGVARGYLNRDDLTAERFLKDPFSQNPAARMYRTG
DLGRYLPDGNIEYLGRNDDQVKIRGFRIELGEIDARLARHPAVHEAVVTAREDVPGDKRL
VAYYSVQSAQTEPGIDSLRGWLQEQLPAYMIPVAYVRLDAMPLTPNGKLDRKALPAPDSD
SLISRGYEAPQGEIETQIAVIWQELLGVEQIGRHDNFFELGGHSLLAVSLIGRMRHIGLS
TDVKALFSQPTLAALASAVGGGDEPGSDEVTVATNLITPGCQRITPDLLPLIKLTQEQID
LVVACVPGGAANVQDIYPLAPLQEGILYHHIAAEQGDPYVLQSQFAFQSRAHLDTFAQAL
QTVINRHDILRTSMHWESLDEPLQVVWRQVELSVEEIQLNPRFGDISRQLQERLDPRQIR
MDIRRAPLMRVVCALDTVNQRWVATLMFHHMILDHTALDQVRYEMQVCLLGQADRLGDSI
PYRNYVAQARQGVNEQDHELFFQDMLGDIDEPTLPFGLHDVQGDGNAIDQARLTLDNALS
QRLRVQTRQLGVSAASLLHLAFAQMLGRLSGRDQVVFGTVLMGRMQSGEGAERALGMFIN
TLPLRVDLGGEGVRDGVKATHRRLTGLLGHEHASLALAQRCSGVVAPTPLFSALLNYRHS
SVAVTDEAMAAWDGMQSLRLDEEERTNYPLTVNVDDTGEGFQFTSLVAASIGAQRLCDYL
QLAVAGLVEALEQAPQTPLHSISILPLSERTQLLEHWNPSGKTYAHETPIHRQFEARAAE
RPDAVALMFEAQTLSYGELNARANQVAHHLLALGVRPDDRVAICVERGPAMIIGVLGILK
SGAGYVPLDPAYPLERLAYVLGDSTPVALLSQRSVQQALPVCEVPLIYLDDADLQDESVR
NLQVPVKPADLAYVIYTSGSTGLPKGVMVEHRNVARLFSATQDWFGFNEQDVGALFHSFA
FDFSVWEIWGALLHGGRLLIVPQLISRSPEDCYDLLCSAGVTVLNQTPSAFRQLIAAQGK

SERAHSLRQVIFGGEALETAMLKPWYARNVNTGTQLVNMYGITETTVHVTYYPLQAEDAQ
RMGASPIGEHIPDLQLYVLDAHREPVPVGVIGELYVGGAGVARGYLNREALTAER
>tr|A0A0Q0C4Y0|A0A0Q0C4Y0_PSEAP Lipase OS=Pseudomonas syringae pv. aptata OX=83167 GN=ALO85_04217 PE=4 SV=1
MNANELLDPAYRWFMDEPSSNWTLHTLADIRARVSATWATAATARGEQHWTSSDSDNGIR
LCLYRPDLSHPHTDLPVILYIHGGGFVLGSPEMTDDDLAKLAVELQAIIIAVDYRLAPEH
PFPIPLEDCYAALEWIFCNGAAQGMNTSNLVIMGHSAGGGLAAALALLARDRGLRTIAGL
VLIYPMLDHRTGTPDGPPANPTTGTFSWSRQANHFCWECLRGAYAVDDDRKALFSPALAT
DLSDLPPTFICVGALDLFLEEDLAFGLSLSRSGVPVELHVYPGVPHMFDQLPGEQTTQAT
QDIARAMRRMIAAGLCD
>tr|A0A0Q0DES9|A0A0Q0DES9_PSEAP 1-deoxy-D-xylulose-5-phosphate synthase OS=Pseudomonas syringae pv. aptata OX=83167 GN=dxs PE=3 SV=1
MPTTFKEIPRERPVTPLLDRADTPHGLRRLGEAELETLADELRLELLYSVGQTGGHFGAG
LGVIELTIALHYVFDTPDDRLVWDVGHQAYPHKILTGRRARMSTLRQKDGVAAFPRRSES
EYDTFGVGHSSTSISAALGMAIASRLQGSERKSIAVIGDGALTAGMAFEALNHAPEVAAD
MLVILNDNDMSISRNVGGLSNYLAKILSSRTYSSMREGSKKVLSRLPGAWEIARRTEEYA
KGMLVPGTLFEELGWNYIGPIDGHDLPTLIATLRNMRDLKGPQFLHVVTKKGKGFEPAEV
DPIGYHAITKLEPLNAPAAQKKISAPKYSGVFGQWICDMADADARLVGITPAMKEGSDLV
AFSERFPERYFDVAIAEQHAVTLAAGMACEGSKPVVAIYSTFLQRGYDQLIHDVAVQNLD
VLFAIDRAGLVGEDGPTHAGSFDLSYLRCIPGMLVMTPSDENELRKLLSTGYLHTGPAAV
RYPRGTGPNAVIEANLEPVEIGKGVVRRQGQGVAILAFGVQLAEALVVAEKLDATVIDMR
FVKPLDEALVSEAAANHELLVTLEENAVMGGAGAAVSEFLARANILKSVLHLGLPDVYVE
HAKPAQMLTECGLDAQGIEAAINERLALIG
>tr|A0A0Q0DBT7|A0A0Q0DBT7_PSEAP Iron-sulfur-dependent L-serine dehydratase single chain form OS=Pseudomonas syringae pv. aptata OX=83167 GN=ALO85_04979 PE=4 SV=1
MMAISVFDMFKIGIGPSSSHTVGPMRAGALFVTELRNHNRLHSVERIEVRLYGSLSATGI
GHGSDRATVMGLMGEWPDQIDPSQVNQRIDALRADNQLMLAGEQAITFVWERDMCLLDES
LPYHPNGMTLCAYGKNGELHEQTYYSVGGGFVIDAEQAASGVLDSDTTVLPYDFFSGAQL
LRLCKTHGMSISELMMANEKVWRSEEEIREKIMVIWAAMRACVDKGLVETGILPGGLNVR
RRAYRLHQNLQNLDNPNVIGSTLSAMEWVNLFALAVNEENAAGGRMVTAPTNGAAGIVPA
VLHYFMKFKPTANDDDVVRFFLSAAAVGILCKKNASISGAEVGCQGEVGSACAMAAAGLA
EILGATPEQVENAAEIGLEHNLGLTCDPVGGLVQIPCIERNAIAAVKAINAAQMALRGDG
EHHISLDHAIRTMRDTGADMHDKYKETSRGGLAVNLIEC
>tr|A0A0Q0BTC4|A0A0Q0BTC4_PSEAP OPT superfamily oligopeptide transporter OS=Pseudomonas syringae pv. aptata OX=83167 GN=ALO85_04220 PE=4 SV=1
MLSTSPSTPLASPVERELSLRAVITGVVLGILLTPSNVYAGLKIGWSFNMSIIALLIGYA
IWQGLSKRSSGQLPWTLHESNINQTVASAAASIISGGLVAPIPAYTLLTGQQLDAIPMIA
WVFSVSFLGIWIAWYLRPSLLNDTSLKFPEGMATLETLLHIYNHGREAATRLKVLLSTAL
LSALAKWVDTFVWAFPRWSPSAQLERLTFTADPSLLLVGFGAIIGIRVGLTLLIGALLAW
GGLAPWLLEQGLVTLPADSSGPQFAALVEWLLWPGVSLMVCSTLASLAIRLWTLHASTKA
SGGTPRSMPKPGPAAGFALAILLVVSLQALLFGINLWMALLTIPLAICLAAVAARVVGAT
GIPPIGAIGQLSQLSFGIVAPGQVPINLMSANTAGGSAGQCTDLMNDFKVGRAIGATPRK
QLIAQTLGIFVGSIVGVLAYMALIPDPQSMLLTEEWPAPAVATWKAVAQTLTHGLDSLSA
SIRWAIFIGGLAGLLLGILDSTLPAHRARYLPSAAALGLAFVLPASVSLMMALGAVLTWL
VNCRWPSLTERFAITAAAGLIAGESITGVGASLWQMVQNGG
>tr|A0A0Q0DHQ8|A0A0Q0DHQ8_PSEAP Transmembrane protein OS=Pseudomonas syringae pv. aptata OX=83167 GN=ALO85_00402 PE=4 SV=1
MQTSSAVTRRHLSAALALGSVAVLMVGLQPLLLGELLAQDHVSLEGVGLVAMGEIVALGL
GVVLGDLLRSMLNLRLLTVIAILCAAGMDLVTANVTGDGPLTAARALAGLAEGLLLWGAV
SLIVQGPAPDRVAGAFMVVQTLAQALMAAALAYLILPDRSVHAGFVFLGMLTAGSVLFIV
WLPGRLPQQPDRQPDTTGFKWNLKQVLLLTLVFMQLSAVGGLWAYLEPLGIHAGLRAHAA
QLLVSAMLFMQVLGGCLGILLVRRVPDYSMLAIAAFLLAGIALGMYITADQGINVFLGLC
ALFALIYLMLTPFQVRIALQIDPSGRIAGLIPGMQLLGCAFGPLLASQWVSGDNAQPVLL
VSVWLSVSALCLIALLHRRAVDRRVGVVGGV
>tr|A0A0Q0BVY4|A0A0Q0BVY4_PSEAP Carbon starvation protein A OS=Pseudomonas syringae pv. aptata OX=83167 GN=ALO85_100071 PE=4 SV=1
MPSSQLCWNKKMKNNNSLMRHVPWLIVAIVGACALGVVALRRGEAINALWIVVAAVAIYL
VAYRYYSLFIATHVMQIDPLRATPAVVNNDGLDYVPTNKHILFGHHFAAIAGAGPLVGPV
LAAQMGYLPGTLWLIAGVVLAGAVQDFMILFLSTRRDGRSLGDMVREEMGRIPGTIALFG

CFLIMIIILAVLALIVVKALAESPWGMFTVMATIPIAMFMGIYMRYIRPGRIGEISIVGV
ILLLGSIWVGGMVAADPTWGPMFTFTGVQITWMLVGYGFVAAMLPVWLLLAPRDYLSTFL
KIGTILALAIGILILAPELKMPALTQFTNGTGPVWKGALFPFLFITIACGAVSGFHALIS
SGTTPKLLDNEKNARYIGYGGMLMESFVAIMAMVAASVIEPGVYFAMNSPAAVVGSDVVT
VAQTVSSWGFAITPEQLTAVAKDIGENTVLARAGGAPTLAVGIAQILHQVLPGENTMAFW
YHFAILFEALFILTAVDAGTRAGRFMLQDLLGSFVPALKRTESWTANAIGTGGCVALWGY
LLYQGVIDPLGGINTLWPLFGISNQMLAGIALMLASVVLIKMKRQRYVWVTMLPAVWLLI
CTVTAGLIKLFDANPAVGFLALAKKYSAAADAGQILAPAKTMDQMQHVIFNAYTNAGLTI
LFLFVVVSILFYAIKVGRAAWSTKERTDKEAPFQAMPPASQV
>tr|A0A0N8T8H7|A0A0N8T8H7_PSEAP Putative UDP-glucose 4-epimerase OS=Pseudomonas syringae pv. aptata OX=83167 GN=ALO85_03522 PE=4 SV=1
MSGDVALVAITGATGFVGSAVVRRLIERTGFAVRVAVRGAYVASSPRIDVVSAQSLAPDN
QWASFVAGADVVIHCAARVHVLNETAEAPDQEYFRANVTATLNLAEQAAAAGVRRFIFIS
SIKANGESTPVGAPFTASDPCNPLDAYGVSKHRAEEGLRELSARTGMQVVIIRPVLVYGP
GVKANFRSMMRWLDKGLPLPLGSIDNRRSLVAVDNLADLVTVCVDHPAAADQTFLVSDGE
DLSTTRLLREMGKALGKPARLLPVPAVLLKAAAALLGKKAFSQRLCNSLQVDISKTCTML
DWHPPVSIEHAMQDTARYYLEHDKHD
>tr|A0A0Q0C3M9|A0A0Q0C3M9_PSEAP Uncharacterized protein OS=Pseudomonas syringae pv. aptata OX=83167 GN=ALO85_100605 PE=4 SV=1
MSSMSALEVAKAIRLSISSARISTYENAARAVGRGLDEALTLYAWNALVSAAFLTPLHLC
EVIVRNGVADAIASVYGPEWPWSPGFEQSLPNVTGPVFKPKQELARARQKCGTTGAVIAE
LKFVFWEKMFTKRFEGRIWAPYLYRFFPNLEKCFTVSAHRAKIAADLEQIRLLRNRIAHH
EPIFSRNLRSDFAVIQRLTETRCAVSADWMNGQQQVISTVATKPF
>tr|A0A0N8T9E9|A0A0N8T9E9_PSEAP PS-II repair superfamily protein OS=Pseudomonas syringae pv. aptata OX=83167 GN=ALO85_05122 PE=4 SV=1
MIRNEGTMALLNKDEQRQVAEAIDRVEQRTDAELVTVLAARADDYAYMPLIWAGLIGLLL
PGTINYCLQWLSADELMLAQMSTFIIVALVCHLSKVSALLVPLSVRRWRAGNLARRQFLE
QNLHKTHDGTGILVFISEAERYVEILVDHGIASRLHDDTWKAMVDVFTQQVRDGQILQGF
LGCIHACGELLADHVPVTHGRNELPNRLVVLG
>tr|A0A0N8T8L2|A0A0N8T8L2_PSEAP Putative ABC transporter, periplasmic substrate-binding protein OS=Pseudomonas syringae pv. aptata OX=83167 GN=ALO85_04635 PE=4 SV=1
MVDPPHRRQGCTCLLDDICCSLCAGCHIGALPAHLDPPATGCLIKEIIMKSLYKHQLAAL
FLVCIAVSAAAQAEQLKVMTSGGFTAAYKLLGPQYAASSGDSLDTILGPSMGKAPEAIPN
RLARGEHADVVIMVGYALDDLIKQGKVDPASRVELADSRIGMVVKEGAAKPAIGTDSELK
ATLLKARSVAYSDSASGVYIEKELFKKLGIEKELAPKGKMIERIPVASVVAKGDYEVGFQ
QVAELLPIPGVTFVAKIPEDVQSVTRYAAGIPVNAEHPKEAKALLDYLASPKAQATVQST
GLDSVPR
>tr|A0A0Q0DQZ4|A0A0Q0DQZ4_PSEAP Tetraacyldisaccharide 4'-kinase OS=Pseudomonas syringae pv. aptata OX=83167 GN=lpxK PE=3 SV=1
MAFTDRLLDAWYKGHPALALLRPLEGLYRRVVERKRARFLAGEGDIYRAPVPVIVVGNIT
VGGTGKTPLILWMIEHCRRRGLRVGVVSRGYGATPPSLPWRVLPEQSASEAGDEPLLIVQ
RCGVPLMIDPDRSRAVQALLAAEPLDVILSDDGLQHYRLARDLELVLIDAARGLGNRRCL
PAGPLREPVERLGSVDALLYNGATADRDDGYAFRLKPSALINLRSGERQPVDYFPAGQAL
HAVAGIGNPQRFFNTLEGLHWRPVTHAFADHAVYSAEALTFTPALPLVMTEKDAVKCRAF
AADDWWYLAVDALPSDAFVGWFDQQLLRLSP
>tr|A0A0Q0D443|A0A0Q0D443_PSEAP L-carnitine dehydratase/bile acid-inducible protein F OS=Pseudomonas syringae pv. aptata OX=83167 GN=ALO85_02497 PE=3 SV=1
MSLPLQGIKVVELGQLIAGPFAAKILAEFGAEVIKVEPPLTGDPLRKWRLLHNGTSVWWA
VQSRNKHSVTLDLRQPQAQEMARRLIRDADVLVENFRPGTLEGWGLSWEALHALNPKLIM
LRVSGYGQTGPYRDRPGFGVIGEAMGGLRHLSGEAGRTPVRVGVSIGDSLSALHGVIGVL
LALRHREQNGGEGQQIDVALYESVFNMMESLIPEYSVFDTVREPAGSSLPGIAPSNAYRC
NDGKYALIAGNGDSIFRRLMEKIGRMDLASDPELQHNDGRVRHVARIDAAISTWTAERSM
DEVLAALNDARIPAGKIYDAADIASDPHYQARDMLLQGTLDDGTPVTLPGIVPKLQDTPG
QVRSSAPTLGQHTDAVLEAHGIDAGTRAEWRRLGII
>tr|A0A0Q0C8I7|A0A0Q0C8I7_PSEAP N-acetyltransferase GCN5 OS=Pseudomonas syringae pv. aptata OX=83167 GN=ALO85_03382 PE=4 SV=1
MQFKERINMHAPAPQILIQRMADIHLEGVTALYNEPAVCRQVLQMPYQSIEVWRKRLAAS
TERHVKLVALHGNEVIGSIGLEQYSRSRQSHVGAIGMGVASAWQGKGIGSKLLATALDIA

DNWMNLHRVELTVYVDNEAAQGLYRKFGFETEGRLRNYAVRDGLFVDALSMARLR
>tr|A0A0N8T8E0|A0A0N8T8E0_PSEAP Flp pilus assembly protein, ATPase TadA/CpaF OS=Pseudomonas syringae pv. aptata OX=83167 GN=ALO85_01366 PE=4 SV=1
MAEKLFGVSSRTQGKPDDGQGLKLVLHRYIIDGMEESGKSLLEGSRPALAQFVIDKVAEY
VARLRLAISRYEMERLAEELVDELTGFGPLEVLLRDTSVTEILVNGPGKVFVERDGVLHH
TDLRFIDSHHVERVMQRILAPLGRRLDESSPMVDARLPDGSRVNAIIPPIALDGPCLSIR
KFRKDMLKSADLVAMQTIDQSIFEFFQEAVGKRCNILVSGGTGTGKTTMLNVLSQLIDSN
QRLVTIEDVAELQLSHPHVVRLETRPPNAEGHGEVRASDLIRNSLRMRPDRIILGEIRGV
EVLDVLTAMNTGHDGSMSTVHANNAQDALLRLETLVGLTGRLIPEKTLRQMVCAALDVII
QLTRLPDGRRCVSEVVEVVGIRDDIYVTNTLFRVDRRTGVGFMREAAHAAGDKLRPGY
>tr|A0A0Q0CDN9|A0A0Q0CDN9_PSEAP GCN5-related N-acetyltransferase OS=Pseudomonas syringae pv. aptata OX=83167 GN=ALO85_02626 PE=4 SV=1
MLYSRAFFNFFKRLPPMFTHRPLEEKDVPLICEMPQNADELFYMFPRATYPLTASQLIHA
LETRHDSTVIEMDGEVVGFANFSLCTFRGRCSLGNVIIAPKARSRGVGRFMITTMMGIAF
DKHEASEMIASCYNHNVPGLLFYPRMGFRPFHIEEKLDKRGERVALIHLRLSGTDAKESM
QDTQE
>tr|A0A0Q0CVD4|A0A0Q0CVD4_PSEAP Uncharacterized protein OS=Pseudomonas syringae pv. aptata OX=83167 GN=ALO85_101509 PE=4 SV=1
MLFWAIFKRFSAFRAVLHCSPCEQKGNSGVGHHGSQPIGQCANGSFVHVRGTDPSSSSDG
RVHQYGFLHASGAQIECASGKPEGWNVYCVIKDERRGTTTPLYSQITSGTRATCINIYNS
KSYEFQE
>tr|A0A0Q0DY62|A0A0Q0DY62_PSEAP RHS family protein (Fragment) OS=Pseudomonas syringae pv. aptata OX=83167 GN=ALO85_102110 PE=4 SV=1
MTRHSNALGLNCYYRWETIDDQPRVVEHWTSDGDHYVI
>tr|A0A0Q0FHU5|A0A0Q0FHU5_PSEAP N-ethylammeline chlorohydrolase OS=Pseudomonas syringae pv. aptata OX=83167 GN=ALO85_05407 PE=4 SV=1
MNVINDGNLLMPNATAPLDLLLLPAWLVPVEPAGVVLKDHGIGIRDGCIVYIGPRAEALR
QNAVQVQELPGMLLSPGLINAHGHAAMTLFRGLADDLPLMTWLQDHIWPAEGKWVDEDFV
RDGTDLAIAEQLKGGITCFSDMYFYPKVAAERVHASGMRAQITVPVLDFPIPGAHTTAEA
LHNGIELFNDLAHHPRIKIAFGPHAPYTVSDENLEKVRVIADELDATIQMHVHETAFEVE
QAVEQRQERPLARLNRLGMLGPRFQAVHMTQISDADLALLVESNTNVIHCPESNLKLASG
FCPVERLWQAGVNVAVGTDGAASNNDLDLLGETRTAALLAKAVAGSATALDAHRALRMAT
LNGARALGIQAETGSLEIGKAADMVAFDLSGLAQQPIYDPVSQLIYATGRDCVSHVWVAG
KQLLDARQLTRMDEHALRDTAIAWGQRISGKAE
>tr|A0A0Q0DQ64|A0A0Q0DQ64_PSEAP Phosphoribosylformylglycinamidine cyclo-ligase OS=Pseudomonas syringae pv. aptata OX=83167 GN=purM PE=3 SV=1
MSKQPSLSYKDAGVDIDAGEALVERIKSVAKRTKRPEVMGGLGGFGALCEIPAGYKQPVL
VSGTDGVGTKLRLALNLNKHDTIGIDLVAMCVNDLVVCGAEPLFFLDYYATGKLNVDTAA
QVVTGIGAGCELAGCSLVGGETAEMPGMYEGEDYDLAGFCVGVVEKAEIIDGSKVAAGDA
LLALPSSGPHSNGYSLIRKIIEVAGADIENIQLDGKPLTELLMAPTRIYVKPLLKLIKET
GAVKAMAHITGGGLLDNIPRVLPEGAQAVVDVASWQRPAVFDWLQQQGNVAENEMHRVLN
CGVGMVICVAQEHVEAALKVLREAGEQPWVIGQIATAAEGAAQVELKNLKAH
>tr|A0A0Q0DMU9|A0A0Q0DMU9_PSEAP TonB-dependent siderophore receptor OS=Pseudomonas syringae pv. aptata OX=83167 GN=ALO85_05483 PE=3 SV=1
MSLPILTGKSQFMHNPSRLTPLSKALMLSQMFRSRPSMAAMGLALCMPVAGQAQETVADD
SNTTGTLNLAPTAIDSHYLGTSTDGTGSYTTGAVSIGKGAPQSLRETPQSVSVVTRQVMD
DNNLTSLSDVLEDTPGITFQYRNFGGHVYTSRGFSLLAESFLVDGIGGQGYQITGWMQPD
MAIYDRVEVLRGASGLLVGAGQPGGAVNLVRKRPTAENKFSITTRAGSWDQYRVDLDGSG
KLNDSGTLRGRMVAAYDDSGSYLDGRDSRTPLLYGIVEADLSDDTTLTMSLRRQEQVING
YSIYGLPRYSNGQSLGLSRSTNLAQKWNRHESDMTEVFTEVAHRFNEDWTSRTSLTYSQG
GFDQNIAYARGAVNPVTLAGSNFQGTLFRKDEVDSVGLNSQLEGNFNAFGLEHQVTLGAD
WSKQDANTHQATVTRRTAVNVFDVNENAFAKPARPAWQNDIDTTEERAGLYANTRIHLSE
PLSVVLGTRLSWYDYKYDVKTGAANSYESKQTQEFTPFAGLIYDINDSWSWYASYADIFT
PQANYVTAGGSPLDPAIGSNYETGFKGELFDKRMNLSMALFYIKQKDVAAVDVNTTELCP
TSSDGSCYLQDGISRSKGVDVEASGEVLPGLQVFGGYTYNMLRNNGDSDVAYETPKHMLR
LNTSYNLPGAWNRLTLGAGVSADSGYEVPYDKALGVAGRAIWDARASWKLDENWKVSLNA
ENLFDRKYYTTSIATDRSNVYGEPRSYVLTLRGDF
>tr|A0A0Q0DFK4|A0A0Q0DFK4_PSEAP Cobalamin biosynthesis protein CbiG OS=Pseudomonas syringae pv. aptata OX=83167 GN=ALO85_01387 PE=4 SV=1

MTSMTPAIVILGNGSLATARRIQQLFPDALIHGLAERVQGADRTYSEFGATLRQLYQQNT
PIIALCAAGIVIRTLAAVLLEKGAEPPVLAVAEDGSAVVPLLGGLGGVNVLARSIASGLG
VAPSITTSGELRFGTCLLNPPSGYALGDLELGKRFVSDLLSGEPVRIEGQAPWLAQAQLP
EDPQAALAIHVGSALREPSPHELLIYPRNVLVALTDLTDQLVEHVRSALREARIAGQSLA
CLLAAEEQMANPHLHKAAAELDVAVRFVSAAAASEMAMKAVPQLLPPLCVDAHTAIAVAA
QPLEVQSIGRGRGRVAVIGLGPGAADLMVPAVKAELARANDVLGYETYVRMAGPFRADQV
LHCTDNREEMQRARHAFELAAQGRSVVVVSSGDPGVFAMASAVLEALHESDNPHWHAVEL
EILPGVSASLATAAQAGAPLGHDFCVLSLSDNLKPWEIIEKRLDLACQADLALAFYNPIS
RSRPWQLGRALEIVRQHRIPATPVTLGRDIGRPGQTLRVITLGELTPEQVDMRTMVLIGS
SLTCTFARHEGGNWVYTPRWYGNKPTA
>tr|A0A0N8T9E8|A0A0N8T9E8_PSEAP PAS protein OS=Pseudomonas syringae pv. aptata OX=83167 GN=ALO85_05118 PE=4 SV=1
MTSWPPGYPAHFDSPKDGAFSTVNTPLDLPLGGGEMGRFIRETDWSTSALGPKQAWPAAL
LSSMNLMLSCPDSMYLLWGHDYTLFFNDAYSQVLPIAARQAQGQPIASVWGDAWEAVRPL
AEQALAGQSRRSDNIQRTIVRNGKPEQTWWSLSYSPLYGDSGLIEGVFCRVNETTLQVMA
EVRQSENEAFTDRVLSSINDCIKVLDLDSRLTFMSEGGQRIMEVSDFNAIRGCPWPDFWQ
DQGNLDAIAAVDAARAGQNASFTGSAQTLAGTTKWWHVQVSPIFGKDGKPEKILCVSRDM
TQLRDAEEALLSLNETLEQRVVERTQDRDRIWRLSTDLMLVAQFDGIISAVNPAWTQALG
WSEQQLLDSQFLALVHPEDVDSTLAAMSGLESGRTIPHFKNRYRHQDGSYRTISWTAVPD
KQFIHAVGRDIQAEEEANEALRLSQDALHQAQKLEAIGQLTGGVAHDFNNLLTVIRSCSD
LLKSSNLEEHRRVKYVEAISSTVDRAARLTGQLLAFARRQALQPEVFDVCKSVARIGEMM
DTLTGARIQVSIDLPAGPCFIFADGSQFDTALVNMVVNARDAMSGAGKLIIKVECAAHCT
ADQPASHSAGDYVKVSLTDTGAGIAKEKLGLIFEPFYTTKSVGQGTGLGLSQVFGFAKQS
GGEVLVESELGKGSRFTLCLPSAQSHEVVQEDHHHTLTPLPGLCVLMVEDNQDIGTYTRP
MLEQLGFQVLWVSSAAEALKELSGNPENFHVVFSDIAMPGMSGLELYAEIEARYPWMPVV
LTTGYSTEFATIAQDETHRFDLLQKPYSRDDLAAILHKAVSRSGEH
>tr|A0A0Q0FI16|A0A0Q0FI16_PSEAP Flagellar hook-associated protein FlgK OS=Pseudomonas syringae pv. aptata OX=83167 GN=ALO85_02151 PE=3 SV=1
MSLISIGLSGINASSAAINTIGNNTANVDTAGYSRQQVLTTASAQIALGQGVGYIGTGTT
LSDVRRIYNGYLDTQLQSSTALSADAVAYSGQASKTDTLLSDSATGISVQLADFFTKMQG
IATSATQSAERSSFLTQAGALSARFNSVSSQLSTQNDNVNTQLDTFTKQVNELTTTLASL
NKQITQASAGNATPNTLLDSRSEAVRKLNELVGVKVVENNGNFDIYTGTGQSLVSGGTSY
KMSASPSPSDPLQYNVQVAYGQTQTDVTSVLTGGSIGGLLRYRNEVLVPATNELGRTAMV
LSDQVNSQMNQGIDSKGNFGSNLYSSINSADAITQRSIGKTTNSVGSGNLNVTIGDTSKL
TANDYEVTFSDSSNFSVRRLPNGESVGAGSLADNPPKQFEGFSVSLNGNTLAAGDSFKVI
PTRTGAAGISVALTDAKDIAAAAPLTATAGSSNSGTGGFTQPVLNTKSNIYDSTQTADLR
NALKSSTPMKLVMGAANSSGVQSYTLINASGGAVLDQNGNAVGGSIIQGQANTVKLNVGY
TDTTTTPSSKTAFELQMTISGSPVANDTFSIGMTGGGSSDNRNALAVAGLQTAKTVGVIN
GGVGTSLSGAYASTVSVVGTLASQSKNDVTATAAVVSQAKSSRDSVSGVSLDEEASNLIK
YQQYYTASSQIIKAAQTIFSTLINSL
>tr|A0A0Q0DUW9|A0A0Q0DUW9_PSEAP Uncharacterized protein OS=Pseudomonas syringae pv. aptata OX=83167 GN=ALO85_01451 PE=4 SV=1
MRRVSTGLMAMLISTHLIAAPPRPSADLATCTRSATLLACNDAHGNSYSVATAGSTTWLK
GYEVLDKRRWAQTNSRYGQLTFFTGLASDGEAWVGTVQRVGWTTITRVSSSSGTRSKITC
SRLNGCR
>tr|A0A0Q0C0X7|A0A0Q0C0X7_PSEAP Flagellar motor rotation protein MotB OS=Pseudomonas syringae pv. aptata OX=83167 GN=ALO85_05232 PE=4 SV=1
MENNQPIIIKRVKRFGGGHHGGAWKIAFADFATAMMAFFLVLWLMSSATPEQLLAVAGYF
KDPVGFSDSGSPYVIDLGGSPEMSPNQTLNPEVQTTPSPDTVPIEAENAETKAEAVEQER
LEMLLQELQNKVQENPQLQKFKDQILFEITQDGLRIQIMDAENRPMFDSGSARLKPYFED
ILLALADTIKSVPNKVSISGHTDATPFVGSGGFGNWELSANRANAARRALVAGTYPDSQV
ARVVGYASSALYDHQNPTNPINRRIDIVVLTKKAQHRIEGDQNSGGAPVTPAAPAPATPA
APAVPAAPGASTAAPPDPQAYAQPHEIRQKLNIFDDGKLRVEPAQN
>tr|A0A0Q0CG44|A0A0Q0CG44_PSEAP Pyoverdine sidechain peptide synthetase III, L-Thr-L-Ser component (Fragment) OS=Pseudomonas syringae pv. aptata OX=83167 GN=ALO85_04446 PE=4 SV=1
IVSDGWSMPIMVEELMQFYQAASLGREITLAPLPIQYADYALWQRNWLEMGEQERQLAYW
KQQLGEQQPILELPTDRPRPALRSYQGARLDVELDAALLDGLKVLARQQGITLFMLLLAS
LQTLLHRYSGQADVRVGVPVANRTRSETQGLIGFFVNTQVLKAEFTTQTTVAELLQQVKH
TAVQAQAHQDLPFEQLVEALQPQRDLSRSPLFQVAYNHQSEGHNQSRELAGLRLEYQVFD

KHTAQFDLTLDTCERPDGLTAALTYATDLFDASTIERMASHWRNLLYGMCRDVNQRIADL
PLLSAEERQNTLRDWNRDLAVYPSAYCAHQRIETQAERTPLAIALSFGVEQLSYQQLNRR
ANQLAHKLREQGIGPEVRVGLAAERGLEMIVGLLAILKAGGAYVPLDPDYPQDRLSFLMH
DSGIQLLLTQTSLLDKLPIPAQVQTLDLADALDGYSTENPLNQTTPDNLAYVIYTSGSTG
KPKGTLLAHHNLMRLFAATDEWFTFSEKDVWTLFHSFAFDFSVWEIFGALLHGGRLVIVP
REVTRSPEEFHALLVEQQVTVLNQTPSAFKQLMRVACDSSLPLSLQTVIFGGEALDVASL
KPWFERFGDQTPQLINMYGITETTVHVSYRPIKLADTQNPASPIGEAIPDLSWYVLDADF
NPVAQGCSGELHIGHAGLARGYHNRAALTAERFVPDPFSSDGGRLYRTGDLARYRAAGII
EYAGRIDHQVKIRGFRIELGEIEARLQAHPAVREVHVLALDGQLAAYLVPAEPDQDQPTL
REMLKNQLRAHLPDYMVPTHFIVLDSMPLTANGKLDRKALPAPDASQLQATYSTPQGELE
QQLAAIWAYVLKLERVGRSDNFFELGGHSLLATQVISRIRQQLDVELSLRDLFEARDLAA
FALAAGAGQGNGAPRFIKADRTQPLGLSYAQQRQWFLWQLDPENTAYVIPAALRLRGSLD
IAALEHSFSALIARHETLRTTFRQQGEHAVQVIHAPRALTLSVESVPSGQTLEACVQQEM
QRPFDLEQGPLLRVRLLNLNTDEHVLILTQHHIVSDGWSMPIMVDELVRLYEGYSQGREV
SLTALDLQYADYALWQRTWMEAGEQARQLDYWKQQLGDQQPILELPADYPRPVVQSHAGA
RLAVELEPALIDGLKQVARQQGVTLFMLLLASFQSLMHRHSGQSDIRVGVPIANRNRAET
EGLIGFFVNTQVLRAEFDVHTTFNELLQQVKHTALQAQAHQELPFEQLVEALQPHRSLSH
SPLFQVMFNHQSQASANVCALPGLQVEALLPDSYPAQFDLTLNTAEHDGGLSAGLTYATA
LFDPRTIERMAGHWLALLQAICANAAQRIAEVPMLDRAERQQILHDWNATAADFPSEDCL
HSLIEAQVRATPDAPALIFAAEQLSYAQLN
>tr|A0A0Q0CDK4|A0A0Q0CDK4_PSEAP Glycoside hydrolase family protein OS=Pseudomonas syringae pv. aptata OX=83167 GN=ALO85_02658 PE=3 SV=1
MQRPSRFGQLVTRVLLGAALSCAAQASTVLKAPREVVWADFLGVNVQFQYFAPDIYQQQM
SRLDELGLNWVRLTIHWPVIEPQKDRYALTELDAAMAAIKAHNYNTLAYLVGSAPFASSA
PAGAKARDQYPPTDFNVFAARMTALAQRYPQVNNWQVWNEPNIVWLPKEDPAAYSRLLTT
TANAIRAVLPSKTIVTAGMAYYSQMHSTSGYMLQTLLDNGLGQQNIVAAYHPYSEYPEGD
STPDRDFLVRANAMNQLLHSNGVKQVWATEWGWSSYAGPVEMQHLIGTSGQADYTLRRLA
LMSAMDFQRIFLFNLSDLDERASARDQGYGLLDLQASPKPVYTALQNFLKITGPRLLPAE
PPAVSSVPDDLYAVPWTRADGTHLLMFWSAAGTSLNFPAITDAVVHDPLTGSRTPLSGSQ
GVTLLLKPTLQILEWKP
>tr|A0A0Q0DYI2|A0A0Q0DYI2_PSEAP Aconitate hydratase B OS=Pseudomonas syringae pv. aptata OX=83167 GN=ALO85_00582 PE=3 SV=1
MRVNTVLEAYRKHIEERAAQGIVPQPLNAEQTAGLVELLKNPPAGEEAFLVDLITNRVPP
GVDEAAYVKAGFLSALAKGEATSPLIDRKRATELLGTMQGGYNIVTLVELLDDATLAPVA
AEQLKHTLLMFDAFHDVAEKAKSGNAHAKAVLQSWADGEWFKKRPTLADKISLRVFKVTG
ETNTDDLSPAPDAWSRPDIPLHALAMLKMARDGIVPDVQGAIGPMKQIEEMRGQGFPIAY
VGDVVGTGSSRKSATNSVLWFFGDDVPYVPNKRAGGFCFGSKIAPIFYNTMEDAGALPIE
FDVSNINMGDVIDVYPYAGKVCKHDSDEVITTFEMKTPVLLDEVRAGGRIPLIIGRGLTS
KARAELGLPEFDLFKTPDQPAESTKGYTLAQKMVGKACGVAGVRPGTYCEPKMTTVGSQD
TTGPMTRDELKDLACLGFSTDLVMQSFCHTAAYPKPIDVKTHHTLPDFIMTRGGVSLRPG
DGIIHSWLNRMLLPDTVGTGGDSHTRFPIGISFPAGSGLVAFAAATGVMPLDMPESILVR
FKGKMQPGITLRDLVHAIPYYAIQAGLLTVEKKGKKNAFSGRILEIEGLDNLSIEQAFEL
SDASAERSAAGCTIKLAKEPIIEYLNSNITLLRWMIEQGYGDPRTLERRAQAMEAWVANP
ELLEADKDAEYAEIIEIDLADVKEPVLCAPNDPDDARLLSSVQGEKIDEVFIGSCMTNIG
HFRAAGKLLDQVKGQLPTRLWLSPPTKMDAHQLTEEGYYGIYGKAGARMEMPGCSLCMGN
QARVEPNATVVSTSTRNFPNRLGDGANVYLASAELASVASILGRLPTVEEYMGYANQIDT
MAADVYRYLSFDQIAEFREAAANANIPAVQV
>tr|A0A0Q0C9S3|A0A0Q0C9S3_PSEAP Uncharacterized protein OS=Pseudomonas syringae pv. aptata OX=83167 GN=ALO85_03302 PE=4 SV=1
MSNDRPLIFVDLDDTLFQTARKTPADIEKHVATLDITGNANGYMTNVQKSFAHWLLAHSD
VVPVTARSVEAYSRVKLPFTAGAICSHGGVMLDVMGRLDHDWNEQMKKTLASYQSRLHEL
SATTLAIGQELGFSLRGWVVEEAQLFHYVVTKHNESDDSILGKVLAEVQARGLLDGMHIH
GNGNNLAFLPEGLAKRYAVQEWLRRDKATNGERPVLGFGDSITDLGFMDECHWWATPARS
QLAKMFVGAAHE
>tr|A0A0Q0C286|A0A0Q0C286_PSEAP Syringomycin/cyclic peptide export system, ATPase/permease fusion protein SyrD OS=Pseudomonas syringae pv. aptata OX=83167 GN=ALO85_05181 PE=4 SV=1
MQGQQLMKTKQEKKARPGSIMRLLWSSHPWLTFFTLLTGLISGVASIAVVNVINQAIHEK
TFQRQSLFWFVGLSVVALLFRNGASLFPAYASMRIMTRLRIALCRKILGTPLEEVDRRGA
PNVLTLLTSDIPQLNATLLIMPTILVESAVFLFGIAYLAYLSWVVFAITISLMMLGVAMY

LLFFMGGMKFTHKVRDEFTAFNEYTHALVFGLKELKLNGIRRRWFSRSAIQESSIRVAKY
NYIERLWFTAAENVGQLTLSLLVGCLLFAAPMFAVIDAKTMSASVLAVLYIMGPLVMLVS
AMPMLAQGRIACTRLADFGFSINEPHPEPETSDTDNVLLLDHKKSWGSIQLKNVHMNYKD
PQSSSGFALGPIDLTIHAGELVYIVGGNGCGKSTLAKVFCGLYIPQEGQLLLDGAAVTDD
SRGDYRDLFSAVFSDFHLFNRLIGPDEKEHPSTDQAQTYLSTLGLEDKVQIEGLGYSTTT
ALSYGQQKRLALVCAYLEDRPIYLLDEWAADQDPPFKRFFYEELLPDLKRRGKTVLIITH
DDQYFQLADRIIKLVDGCIVSDVKCAVQDKRA
>tr|A0A0Q0BWS7|A0A0Q0BWS7_PSEAP BPL/LPL catalytic domain-containing protein OS=Pseudomonas syringae pv. aptata OX=83167 GN=ALO85_04529 PE=4 SV=1
MELNTLTRSDVQDSIIDICVEQGLQAEQDLLASVCSGAADHGLLFWRPTDRALVMPRRMS
RLPGFAEASETLSDNAWPVLLRETGGEPVPQSSATLNIALVYAQPSTDVDRDRIETAYLR
LCQPILDLLTELGGQASLGEVAGAFCDGRFNVNLDGRKMVGTAQRWRQSQGGTRPVVLAH
GALLLENERVQMAVAVNRFNELCELEPRVQAESHIALYEAFPGADVLERLAQTYQKLLAA
L
>tr|A0A0Q0FII2|A0A0Q0FII2_PSEAP Fimbrial assembly OS=Pseudomonas syringae pv. aptata OX=83167 GN=ALO85_00684 PE=4 SV=1
MNSSVSARLAPVYALREQVARQWRGSPAQHAWQWWVTELRACLPPRVRRWLGRETVEQTL
VWPLVEPLPDASPQVRRVLLLPPSAVLLQTLQLPAAAARNLSTVVGYELDRFTPFDAGQL
YFVARQDSRSASFVQVTLVAILGERLDAILSECAERGLRPDAVDVGTHVGADNLAAGQRM
GIDLLPMPLRPQQSRSGHRLQRGLIWLCAGLLLSAMLLWLNDRQSLLEDMQASVKAQKAQ
VGEVQQLRQQLTNTLGAANYLLRRKAAQPPLSALLSELTACLPSDTWIEHLEISDSAEVA
FSGQSAKASALIGRVKDCRSLDNAQFQGVIQPDTKTGKDQYSLRAHLHQNQQQENANAPS
TDKP
>tr|A0A0Q0IBR2|A0A0Q0IBR2_PSEAP Aliphatic sulfonates import ATP-binding protein SsuB OS=Pseudomonas syringae pv. aptata OX=83167 GN=ssuB PE=3 SV=1
MATPDLRNTPFVAPQAGQAPVQLRDVVRQFGRQRVIDGLNLDIAPGEFVALLGASGSGKT
TLLRTLAGLDEIDSGELRVPVARAAVFQEPRLMPWKSAWKNVVLGLRINDAKARAEAALT
EVGLAHRLNAFPATLSGGEAQRVALARGLVREPKLLLLDEPFAALDALTRIRMHQLIIDL
WRKHTPAVLLVTHDVDEAILLADRVIVLADGKIADDIRVDLPRQRDSGQAGFQLIRSRLL
GLLGVKVNAAADTAPQAPEQDVTLSALRRFANAR
>tr|A0A0Q0IJJ3|A0A0Q0IJJ3_PSEAP Transport permease protein OS=Pseudomonas syringae pv. aptata OX=83167 GN=ALO85_02525 PE=3 SV=1
MTVPNGELRPNLIALKTIVYREVHRFMRIWPQTLLPPAITMVLYFVIFGNLIGRQIGDMG
GFTYMQYIVPGLIMMSVITNSYGNVVSSFFGAKFQHSIEELMVSPVSPHTILVGYTLGGV
LRGLMVGFIVTMLSMFFTDLQVHHLGVTIIVVVLTATIFSLLGFINAVFARNFDDISIIP
TFVLTPLTYLGGVFYSINLLPPFWQTVSLANPVLHMVNAFRYGILGVSDIKISIALAFMI
VATFVLYIGCARLLVSGRGMRQ
>tr|A0A0Q0BTI8|A0A0Q0BTI8_PSEAP Regulatory protein, GntR OS=Pseudomonas syringae pv. aptata OX=83167 GN=ALO85_01167 PE=4 SV=1
MTFKAPDSLAEQIAHHLAERIIRGELKPGERIQEQKVTQALNVSRGSVREALLILERRHL
VVILPRRGAQVTVLNAHNVTSLCCMMRELYILLANAVVERWTTEEDLAPFMQIQQRLQAS
FEQQDVNAFVEESFNVMRAAYPFANNPYLEETVENLQPSMSRSYFIALEQRKADMGDYLA
LFDELLGAVVARDIPKIRVVLVSYCQRSCQLVLAALAAG
>tr|A0A0N8T7T4|A0A0N8T7T4_PSEAP Uncharacterized protein OS=Pseudomonas syringae pv. aptata OX=83167 GN=ALO85_100600 PE=4 SV=1
MTMNAEYLKQKTQKLRDVIEDLRKSDPVVEKLRAEIEPLMKLSESGMIIVKLQWRDIPGR
YLFTEEGLQQYPHLEHAFAEFRIELTGGETPLLRKLKREMGEE
>tr|A0A0Q0IER5|A0A0Q0IER5_PSEAP Sulfatase OS=Pseudomonas syringae pv. aptata OX=83167 GN=ALO85_02117 PE=4 SV=1
MSWLQSRRLHYWLGAVMVVFLLSALLRGVFFFGFSGIEPGTLFTHGEVAQTLGIGLRFDL
RLALLILLPVAVLLWLPGWNLISVRALRWLARGYLIVALAILTMIYIVDFGHYAYLGVRL
NASVLRYLEDAQISRDMLWQTYPVLWITVGWLLTVAVLGWVLIRLERVTLDREARPIKRG
SVVWGSALMVCATFLGLLGRVENLNLENPVPLRWSDAFFSGNSQVAALGLNPALFLYDTL
KVSQSNFDEPEVRKHYERVARYLGVPQPDVQGLSFKRHQTVQPYRLPGERPPNVMFVMLE
SLGTSAVGAYGNPLNPTPNLDHLATQSWFFKHFYVPVTGTAKTVWASITGVPDVTRQETA
TRHPLLTRQNTIINDFKGYEKLYMIGGNSGWANMNALIRRSIDDVRLYDERDWKSPQVDV
WGISDLDLFKESDRLLRALPADKPFFAYVQTAGNHRPFTIPKDNDGFQVSDKTVEQVQAA
GSRSVEQYNAVRLLDFNIGRLMELAKAGGYYENTIFVLFGDHNTRISQIPHMAPAFEQLG

LESNNVPLLIHAPGLLGTRVVDEAVGLADLLPTVAGMAGMDFTSGTMGRDIQQAAPEGER
VVPLVLREGTFPLIGGVTRNYLLQMEHDGSSPTLHDLSSPTPLDNVAEQNPQEFERLRDL
TRGLHETSRMMLYQNVR
>tr|A0A0Q0BUW4|A0A0Q0BUW4_PSEAP Cyanate hydratase OS=Pseudomonas syringae pv. aptata OX=83167 GN=cynS PE=3 SV=1
MPHSNISRAPRQHLTERVLQAKTAKNLTWAGLAEGTGLSVVYVTAALLGQHPLPEAVAEV
VAERLGLDRDAVAELQTIPLRGNVEDVSSDPTIYRFHEMVQVYGTTLKALVHEQFGDGII
SAINFKLDIKKVDDPEGGERAVITLDGKFLPYKPF
>tr|A0A0Q0CXW0|A0A0Q0CXW0_PSEAP Replicative DNA helicase OS=Pseudomonas syringae pv. aptata OX=83167 GN=ALO85_03144 PE=3 SV=1
MAMNDISAPEQYDLQTAALKVPPHSIEAEQAVLGGLMLDNNAWERVLDQVSDGDFYRHDH
RLIFRAIARLADQNSPIDVVTLAEQLDKEGQTSQVGGLGYLSELAKNTPSVANIKAYAQI
VRARATLRQLIGISTEIADSAFNPEGRTAEEILDEAERQIFQIAEARPKTGGPVSVNDLL
TKAIDRIDTLFNSDNAITGLSTGYTDLDEKTSGLQPSDLIIVAGRPSMGKTTFAMNLVEN
AVLRSDKVVLVYSLEMPGESLIMRMLSSLGRIDQTKVRAGQLDDDDWPRLTSAVNLLNDR
KLFIDDTAGISPSEMRARTRRIVREHGDIAMIMIDYLQLMQIPGSSGDNRTNEISEISRS
LKALAKEFNCPVIALSQLNRSLEQRPNKRPINSDLRESGAIEQDADVIMFVYRDEVYHPE
TEHKGIAEIIIGKQRNGPIGTTRLAFIGKYTRFENLAPGSYNFDDD
>tr|A0A0Q0CZ49|A0A0Q0CZ49_PSEAP Uncharacterized protein OS=Pseudomonas syringae pv. aptata OX=83167 GN=ALO85_02823 PE=4 SV=1
MQSMLRLMTLVCALLVYSGQACADEAVREQQLLLPIHIDAHREKVEALIVRPARAGKFPV
ALIINGSAAQPSSVHADWLAHIAHDFAHRGWLAASIVWPGYGRSTGRFMDEAGNCTNPDV
ARFLDTHGEELGAALAALRKRPDVDPSLALGVGISIGGASMLDLAARPDHPLAAVINISG
GVYHYSTMGSPEADCSLYQADLVRNFTTFGTNNPTPTLWMYAENDPYFSPDLVTRMVAGY
RSQGGHADYVALPPFGQDGHTLYKNGANKLLKPHIEDFLKANRLPGMDDEALMPLLSKLS
PADRAEANAYLLSVTEKAMAKSAEADGLFWFYGARSIKTARQRALGNCRVATGKACRIVA
QNMQLVNGWQATVAPTKK
>tr|A0A0Q0DTP4|A0A0Q0DTP4_PSEAP Protein-glutamate methylesterase/protein-glutamine glutaminase OS=Pseudomonas syringae pv. aptata OX=83167 GN=cheB PE=3 SV=1
MKIAIVNDMPMAIEALRRALAFEPAHQIIWVASNGADAVQRCVEQTPDLILMDLIMPVMD
GVEATRRIMAETPCAIVIVTVDREQNMRRVFEAMGHGALDVVDTPAIGGPNPKEAAAPLL
RKILNIDWLIGQRVGVERGAAAPVSAPSRRDRLVAIGSSAGGPAALEILLKGLPVSFPAA
IVLVQHVDQVFAAGMADWLSSASGLPVRLAREGETPQPGVVLLAGTNHHIRLLKDGTLAY
TAEPVNEVYRPSIDVFFESVTRYWNGEAVGVLLTGMGRDGAQGLKAMRERGFLTIAQDQA
SSAVYGMPKAAAAIDAAVEICPLPAIAPRLIEVFTQ
>tr|A0A0Q0C1R9|A0A0Q0C1R9_PSEAP Enoyl-CoA hydratase/isomerase OS=Pseudomonas syringae pv. aptata OX=83167 GN=ALO85_03617 PE=3 SV=1
MLYETLLIEVVGSVGLIKLNRPNALNALNARLINELNHALDQFEADGAIGCIVLTGSEKA
FAAGADIKEMVDLSYPQIYLDDLFRESDRVAARRKPLVAAVAGFALGGGCELALMCDFIL
AAENARFGQPEINLGVLPGMGGTQRLTRAVGKAKAMEMCLTGRLIDAHEAERAGLVARVL
PLDQLLPEALAVAAVIASKSLPVAMMVKESVNRAFEVSLAEGIRFERRVFHAAFASQDQK
EGMRAFIDKRQPDFKDC
>tr|A0A0Q0CG22|A0A0Q0CG22_PSEAP Zinc-containing alcohol dehydrogenase superfamily protein OS=Pseudomonas syringae pv. aptata OX=83167 GN=ALO85_03715 PE=4 SV=1
MSRTIRFHQFGPAEVLKVEEHPVASPVTGEVQVRVQAIGVSWYDVLWRQNLASTQARLPA
GLGYEMAGVVTALGDGVDDLQVGDNVASFPAADANQHPVYGESIVMPRGALTRYPDALTP
VQASVHYTPYLVAYFAYVDLARIKSGQTVLVTDASHCSGPAFVQLGKALGARVIAATKTE
DAREYLLSLGAEKVVVTEEQDLLMAINKYTDNRGVDAVFDGLGGPQMSIMGDVLAPRGSL
VLYGLQGGNQTPFPACAAFQKNIQFYVHCLGNFTGKPELGITQDKEALQRALQDINQMTR
DKVLMPLQTRSFPFDQVVQAHRYMDVCPIGGRAVLEVEAV
>tr|A0A0Q0D0S2|A0A0Q0D0S2_PSEAP Uncharacterized protein OS=Pseudomonas syringae pv. aptata OX=83167 GN=ALO85_00706 PE=4 SV=1
MNKIRAKDRDAVIQSLRAGVVPRVGQHLIQVGRAGELAALIKDVDRLAEGGSAFRIVVGE
YGAGKTFFLNLVRGIAMERKLVTMHADLNPDRRLHASGGQARSLYAELAKNMSTRTKPDG
GALQGIVEKFISQARTHASSKGVDSETVIRQNLAELTEMVNGYDFAEVIAAYCRGFNEGN
EQLKADAIRWLRGEFTTRTDARAALGVRSIVDDASVYDQLKLLSRFVRLAGFGGLMVCLD
ELVNLYKLANTQARNANYEQILRILNDSLQGSTDGLGFVLGGTPEFLMDTRRGLYSYPAL
QSRLAENTFAKAGYVDLSGPVIRLTSLTPEDFYVLLLNLRNVYAYGDAEHYLLPEEAIPA

FIEHCGQRLGEAYFRTPRTTITAFINLLAVLEQNPEADWRDLLGAIDIARDDGGKSDFTV
EADDELTSFKL
>tr|A0A0N8T8T8|A0A0N8T8T8_PSEAP Potassium-transporting ATPase potassium-binding subunit OS=Pseudomonas syringae pv. aptata OX=83167 GN=kdpA PE=3 SV=1
MHRYDYLLILAFLLLLLAPAPWLGRFYYRVMEGERTWLSPVLGPVERACYRISGVDPKTE
QSWKQYAWALLAFNLAGFVVLFAILMLQGMLPLNPQQLPGMEWSLAFNTTMSFVANTNWQ
AYSGEASLSYLSQMVGLTVQNFVSAATGLAVLVALCRGISRRSSQSLGNFWADMTRATLY
GLLPISIVLAVFLVWQGVPQNFGHYIDALTLQGADQSLPMGPAASQISIKQLGTNGGGFF
GVNSAHPFENPTAWSNLFELVSILLIPAALVFTFGHYVKDMRQSRAILGCMLALLLIGGA
VSLWAEYQPNPALNIAGVEQTAPLEGKETRFGTTGTVLWSVATTAASNGSVNGMHDSLNP
LTGMVALVNMMVGEVIFGGVGVGLNGMLLNVLIAVFLAGLMIGRTPEYLGKKLQAQEVRL
LVATLLVMPVGVLVLGAIAASLPGPAGAISNPGPHGFSQLLYAYTSATANNGSAFGGFSA
NTVFHNLMLSLAIFIGRFGYILPVLALAGSLAMKKAAPQGQNSFPTHGLLFVTLLTVTIL
LVGGLTFLPTLALGPIAEHLSLGF
>tr|A0A0Q0C9D7|A0A0Q0C9D7_PSEAP Sarcosine oxidase, alpha subunit OS=Pseudomonas syringae pv. aptata OX=83167 GN=ALO85_05513 PE=3 SV=1
MDTRRRLPPVFQHDAQYRDLRDSRDLSDRYQAAVHGSGRQGMSQTHRLPNGGRINRSKVL
NFTFNGQTYQGFEGDSLASALLANGVDIIGRSFKYSRPRGIFAAGSEEPNAVLQIGATEA
TQIPNVRATQQALYAGLVATSTNGWPSVNTDVMGILGKVGGKLMPPGFYYKTFMYPQSFW
MTYEKYIRKAAGLGRSPTENDPDSYDAMNQHCDVLIVGAGPAGLAAALAAGRSGARVIIA
DEQEEFGGSLLDSRESLDGKPATEWVSTVVAELKSLRNVTLLPRATVNGYHDHNFLTIHE
RLTDHLGDRAPIGMVRQRMHRVRAKRVVLAAGTHERPLVYGNNDVPGNMLAGAVSTYVRR
YGVAPGKKLVLSTNNDHAYRVALDWLDASLHVVAIADARHNPRGPLVEEARAKGIRILTG
SAVIEARGSKRVTAARVAAIDLKTHSVTSPGEWLECDVVASSGGYSPIVHLASHLGGKPV
WREDILGFVPGEAPQKRICVGGVNGVYSLADSLADGFEGGVRAASEAGFKVVEGVMPKAL
SRAEEPTLALFQVPHEKGTARAPKQFVDFQNDVTAAAIELATREGFESVEHVKRYTALGF
GTDQGKLGNVNGLAIAARSLNVTIPEMGTTMFRPNYTPVTFGAIAGRHCKHIFEPVRFTA
LHAWHVKNGAEFEDVGQWKRPWYFPKNGEDLPAAVARECKAVRDSVGLLDASTLGKIDIQ
GPDAREFLNRIYTNAWTKLDVGKARYGLMCKEDGMVFDDGVTACLADNHFLMTTTTGGAA
RVLQWLEIYQQTEWPDLKVYFTSVTDHWATLTLSGPNSRKLLSEVTDIDLGREAFPFMTW
KEGLVAGVPARVFRISFTGELSYEVNIQADYAMGVLEKIAEAGKQYNLTPYGTETMHVLR
AEKGFIIVGQDTDGSMTPDDLNMGWCVGRTKPFSWIGWRGMNREDCVREQRKQLVGLKPV
DPAKWLPEGAQLVFDTKQTIPMSMVGHVTSSYAHNSLGYSFAMGVVKGGLARMGERVFAP
LADGSVIEAEIVSSVFFDPKGERQNIE
>tr|A0A0Q0IA90|A0A0Q0IA90_PSEAP Sulfur carrier protein FdhD OS=Pseudomonas syringae pv. aptata OX=83167 GN=fdhD PE=3 SV=1
MPVTRKVSSASFATPAPEASQSYSYSNLEGTASARNELAEEVALAIAYNGISQAVMLVSP
TDLEDFIVGFSLGSGIIASHDEIYDFKLSGCGSAMQAEVEIASRAFWELKQQRRQLAGTS
GCGLCGVEAVEQALPDLKALPGAPLPPIEWLEGLRQRIGEFQPLGRHCGAVHAAVFMDGN
GQLLLGREDIGRHNALDKLIGALIRQDIPLAGGVAIVTSRCSLELIQKVLRAGIHTLVSL
SSPTGLALQWARRHNLNLIHLPQHSAPRVYSPAMEIQA
>tr|A0A0Q0DUQ8|A0A0Q0DUQ8_PSEAP Acyltransferase family protein OS=Pseudomonas syringae pv. aptata OX=83167 GN=ALO85_00937 PE=4 SV=1
MLISVQALRAFAAWVVVCHHFMQIFFDFKASGPVGQFFVSKGAVGVDIFFVISGLVIFLS
TQNSDMPARRFILHRLIRIVPAYWLYTAAMALLLLMAAPMLPHQVIGWQNFLLSLVFIPS
ENPGGYGLYPTLNVGWTLNYEMFFYLLFSMVFLFKQRHRPLIIAAALFAVSEVLARAGLI
SRFYGNDIIYEFLLGIGIGILYRRGLIKQALWLPLAGIAVGLYAINNLTNDQRLLNWGLP
SALIVLSCISLEPYFQGNRLLKVLGDCSYSVYLLHVLVLYGGLLMAQRFGLNPYVVFAFC
VPIIAIGAWVSYEWVEKGLYRRMKAWLDARRAVSQAQALT
>tr|A0A0Q0IAW3|A0A0Q0IAW3_PSEAP Helix-turn-helix, AraC type:AraC-type transcriptional regulator OS=Pseudomonas syringae pv. aptata OX=83167 GN=ALO85_01873 PE=4 SV=1
MTCMEKSLAELRCIVMRAQDKWTETGLPRVSMVRAEACADQVYQPMLHLVLQGTKTLSIG
EDVSRYTTGHYFLVPVDVPATGQIHADTPDKPYLAVSLTLDPEVIATLLMAEGKAPEAPQ
NACFEGIIAPDEMIDAWLRMLRLMDRPHEVAVLAPMIEREILFRALQGPLGGILRDVARP
DGRLTQIRRVTQWIREHYTEPFRVEPLAQMADMSVAAFYRHFKSVTAMSPIQYQKRLRLL
KARWLLLFDTLDATSIAYTVGYESASQFNREYARLFGLPPARDAARFRTPADELTAVS
>tr|A0A0N8T9G6|A0A0N8T9G6_PSEAP Amino acid adenylation (Fragment) OS=Pseudomonas syringae pv. aptata OX=83167 GN=ALO85_04710 PE=4 SV=1

MERTARGRTGRPSRQLFRAGRSFAAGGEPDCAPAPGRHRGRCPGAFRTTYAGGLRSHHRE
HGDYPVSINELLATLKAHEIHLTVKDGQLVVQGNRRALTENGLLEHLREHKPALIELIEQ
GDYQNGKRGAPALPANGIEQGCERITPEMLTLVKLDQPAIDLLMDAIPGGAANVQDIYPL
APLQQGILYHHVTATQGDPYVMQVQFAFSDQARRDAFAEALQSVITRHDILRTSIHWKGL
ETPLQVVWRHAELKVDSSPLAADLTMDLGQAPLMRLVCHAPVAGQRVEATLLFHHIAMDH
SALEVVRHEIQACLSGQAEMLGVPVPFRNYVGQALLGVSEAQHEAFFREMLGDLDEPTLA
YGLQDLSGEGDAIEEHSVTLDQQLCLRLRAQARTLGISVASLFHLGWARVLAGLAGQSRV
VFGTVLMGRLLGAEATERALGIFINTLPLRLDLDEQGVQAAVRATHQRLTALMRHEHAPL
ALAQRCSGVKAPTPLFNALLNYRHSAPAQASGETWQGIEVLQAQERSNYPLVLSVDDLGE
AFGLTAQAAAGIDPQSICAYMQWTMESVVDALEQTPQMSVDQLDIVPASERAQLLLDFND
RRADYERMLTTHQRIELLAEQQPDAIAAQVGKQHLSYRELNARANALADHLISLGVQPDD
RVAVVARRSLETLVGLLAVLKAGAGYVPVDPAHPDERIAYLLGDSAPVAVLAQPVFVERL
QGLGLPGLNTPLIELDLANWPEQQDNPHVDTLDSTHLAYVIYTSGSTGQPKGVMVEHRSL
NNLIDWHREAFDLHAGSHTASVAGFGFDAMAWEVWPALCAGATLHLPPAEIGNEQLDALL
DWWIAQPLQVAFLPTPVAEYAFSRNLRHPTLRTLLIGGDRLRQFHRDPGFAVINNYGPTE
ATVVATSGRLLPDGSLDIGKPVANTSIYLLDERQQLVPLGVPGELYIGGDSVARGYLNQP
QLTAERFVHDPFAGQPQARMYRTGDLARWNADGTLEYLGRNDDQVK
>tr|A0A0Q0IP75|A0A0Q0IP75_PSEAP DUF2384 domain-containing protein
OS=Pseudomonas syringae pv. aptata OX=83167 GN=ALO85_03778 PE=4 SV=1
MIPSSASKTTGRREAAASFWLLTHQLSQRSEAERLTNIQAGFAPRWIRAVREALALGPRQ
MEALFNASISTLERRQRQQQSLDLVASERLDRVAMIASHALQVFTTPERAGQWLVARNTS
LGGRTPLRLCETGLGTTQVHRALVALECASADAP
>tr|A0A0Q0DAS7|A0A0Q0DAS7_PSEAP Probable intracellular septation protein
A OS=Pseudomonas syringae pv. aptata OX=83167 GN=ALO85_03807 PE=3 SV=1
MKQFIDFIPLLLFFIVYKTEPRAVDILGNTYMVGGIFSATAMLIISSVVVYGILYVKQRK
LEKSQWLTLVACLVFGSLTLAFHSETFLKWKAPVVNWLFAVAFAGSHFIGDRPLIQRIMG
HALTLPAAIWTRLNIAWIIFFLFCGAANLYVAFTYQEFWVDFKVFGSLGMTLIFLVGQGI
YLSRHLHDTTPNTTKSED
>tr|A0A0Q0DP44|A0A0Q0DP44_PSEAP Two-component sensor histidine kinase
RoxS OS=Pseudomonas syringae pv. aptata OX=83167 GN=ALO85_00004 PE=4 SV=1
MLAPVQMLSATRQNLWRLTFIRILVLAAQAGSVGMAWLFDFLPLPWLQLSITLGCSLVLC
GLTVIRLRTSLPLTELEYALQLALDLLIHSALLYYSGGSANPFVSYYLVPLTIAAATLPW
RYSLILSGFALSMYTLLMVRSYPLETDSIARENMQIYGMWLSFALAAAVITFFAAKMAEE
LRRQEQLRAERREEGLRDQQLLAVATQAAGAAHELGTPLATMSVLLKEMRQDHSDPALQD
DLSVLQEQVKQCKQTLQQLVRAAEANRRMAVEYQPATRWLDESLNRWHLMRPEVSYRFYQ
LGKGEAPMLAPPPDLTQALLNLLNNAADACPDGLEVNLDWDSAEICISIRDHGAGVPLAI
AEQIGKPFFTTKGKGFGLGLFLSKASVTRAGGSVKLYSHEEGGTLTELRLPRDTRGEEA
>tr|A0A0Q0DAT2|A0A0Q0DAT2_PSEAP 3,4-dihydroxy-2-butanone 4-phosphate
synthase OS=Pseudomonas syringae pv. aptata OX=83167 GN=ribB PE=3 SV=1
MCEIFCLMVYHDSFTHKTGKLTMAFDRIEDIIEDYRQGKMVLLVDDENRENEGDLLLAAD
CCSPQAISFMAREARGLICLTLTDEHCQRLGLEQMVPSNGSVFATAFTVSIEATTGVTTG
ISAADRARTVQAAVNPHAVAEDLVQPGHIFPLRARDGGVLTRAGHTEAGCDLARLAGFTP
ASVIVEVMNDDGSMARRPDLEVFAKKHGIRIGTIADLIHYRLSTEHTIVRIGERELPTVH
GTFRLFSYEDRIEGGVHMAMVMGDIRRDAATLVRVHVVDPLRDLVGAEYNGPANWTLWAA
LQRVAQEGHGVVVVLANHESSQALLERIPQLTQPPRQYTRSQSRIYSEVGTGAQILQDLG
VGKLRHLGPPLKYAGLTGYDLEVIESIPFPG
>tr|A0A0Q0FPH3|A0A0Q0FPH3_PSEAP Oligopeptide/dipeptide ABC transporter,
ATP-binding protein OS=Pseudomonas syringae pv. aptata OX=83167
GN=ALO85_01004 PE=3 SV=1
MSSDQIPLIELHQVSKTFGKPVDERSVQGLLQRLHLTRAKPMTHAVDRVDLRIARGEVVG
LVGESGCGKSTLGRMVAGLLPVSSGTTLVDGKPLESLTPQERKALRLKVQMIFQDPSSSL
NPRLRVDRIVGEGALLHGLTDRKGADDYVSAQLQRAGLSPQLRQRYPHQFSGGQRQRIGI
ARALAVQPELLVCDESVAALDVSIQAQILNLFMDLRDELGLTYLFISHDLGVVEHLCDRV
IVMYLGRVVETASVDDLFARANHPYTQALLAQIPRFDIRSTRYDAIQGEIPSPLDPPAGC
HFHPRCPHAMARCREEVPALREVSPNHQSACHLNDQR
>tr|A0A0Q0DCU9|A0A0Q0DCU9_PSEAP Ribosomal RNA small subunit
methyltransferase I OS=Pseudomonas syringae pv. aptata OX=83167 GN=rsmI
PE=3 SV=1
MTVPVVSNSTPGSLYVVATPIGNLDDMSVRALKVLRDVALIAAEDTRHSARLMQHFGIST
PLAACHEHNERDEGSRFITRLLAGDDVALISDAGTPLISDPGYHLVRQARAAGVQVVPVP

GACALIAALSAAGLPSDRFIFEGFLPAKSAGRKARLERVKEEPRTLIYYEAPHRILECLQ
DMELVFGADRQALLAREITKTFETLKGLPLGELRAFVESDSNQQRGECVVLVAGWTPPDD
EDVIGEEARRVLDLLLAEMPLKRAAALAAEITGVRKNLLYQVALEKQKE
>tr|A0A0Q0DYA4|A0A0Q0DYA4_PSEAP CheW-like protein OS=Pseudomonas syringae pv. aptata OX=83167 GN=ALO85_04019 PE=4 SV=1
MLHFSLGPGMNRPVEVATRPKLALQSYLDALLYDATEVLEEPVSSIDEFQAAVLEEQAFD
ARMQAQIKPLAVAVAAPAPVAVKVQVAAPAPVVEVAPVIVAEAVQVEEAVPTVALVEPVA
ELSTLAAQRTPTPPPTSDGRPAWAAEPFECLLFDVAGLTLAVPLVCLGSIYPLAGQELTP
LFGQPDWFLGILPCQAGNLKVLDTARWIMPDRYRDDFRQGLQYVISVQGYEWGLAVHQVS
RSLRLDPNEIKWRAQRGHRPWLAGTVIEHMCALLDVAELAELIASGAVKQLNKSK
>tr|A0A0Q0FE95|A0A0Q0FE95_PSEAP Uncharacterized protein (Fragment) OS=Pseudomonas syringae pv. aptata OX=83167 GN=ALO85_03827 PE=4 SV=1
SSLYLYEPGSYAPLARVDQREGEGENRIYYFHTDQIGTPLEMTDADGQIVWQAKYRPWGA
IEKLVVNEVEQNLRFQGQYFDVETGFHYNTFR
>tr|A0A0Q0CYW3|A0A0Q0CYW3_PSEAP Pseudouridine synthase OS=Pseudomonas syringae pv. aptata OX=83167 GN=ALO85_01314 PE=3 SV=1
MHQGPLKWPFLFFDCRILMSRTQRPNSRRPAPKPAGAARRVAKAPPAEPKLILFNKPFDV
LTQFSDEEGRATLKDFIKIPGIYPAGRLDRDSEGLLLLTNDGQLQARIADPKHKLAKTYW
VQVEGEPSDEQLQQLRDGVQLNDGPTLPAEARQLDEPQLWPRNPPVRFRKSVPTSWLELV
IKEGRNRQVRRMTAAVGLPTLRLVRVRIGDWTLEGLDQGQYREVAAKL
>tr|A0A0Q0BVD4|A0A0Q0BVD4_PSEAP Protein GbcA OS=Pseudomonas syringae pv. aptata OX=83167 GN=ALO85_00487 PE=4 SV=1
MDVTATLSLGDPLEPARKATAEMLQNRERTYSLPQPFYSDERLFEIDMQEIFHKEWLIAG
MTCEIPTKGNYLTLQIGKNPILVVRGPDGAVNAFHNVCRHRGSRLCTAEKGKVAKLVCHY
HQWTYELDGRLLYAGTEMGDDFDMKQFGLKPVHVKTAGGYIFISLAENPPAIDEFLSTLR
HYMEPYDMENTKVAVQTTLMEKANWKLVLENNRECYHCSGSHPELLKTLLEWDDVTDPRA
DQTFKDHVAASAAAWDAEKIPYAHASFGLRNRIVRMPLLKGTVSMTMDGKVGCKKLMGRI
QNPDLGSMRILHLPHSWNHCMGDHIIVFTVWPISAQETMVSTKWIVHKDAVEGVDYDVER
MRQVWDATNDQDRRLAEENQRGINSSAYQPGPYSKTYEFGVVNFVDWYSSRMLENLGAEP
APYLKGVAVNHE
>tr|A0A0Q0IDP7|A0A0Q0IDP7_PSEAP Aminotransferase OS=Pseudomonas syringae pv. aptata OX=83167 GN=ALO85_01290 PE=3 SV=1
MSQPYSARSRAIEPFHVMALLARANELQAAGHDVIHLEIGEPDFTTAQPIIKAGQAALAD
GKTRYTAARGLPQLREAIAGFYAQRYGVDIDPQRILVTPGGSGALLLTSSLLVDPGKHWL
LADPGYPCNRHFLRLIEGEAQLVPVGPQERYQLTPELVAKHWNQNSVGALVASPANPTGT
LLSRDELAALSQALKARNGHLVVDEIYHGLTYGVDASSVLEVDNDAFVLNSFSKYFGMTG
WRLGWLVAPEDAVADLEKLAQNLYISAPSMAQHAALACFEPQTLAILEQRRAEFGRRRDF
LLPALRELGFGIAVEPEGAFYLYADISAFGGDAFAFCRHFLETEHVAFTPGLDFGRFQAG
HHVRFAYTQSLPRLEQAVERIARGLRSWGV
>tr|A0A0Q0D1V5|A0A0Q0D1V5_PSEAP ABC transporter OS=Pseudomonas syringae pv. aptata OX=83167 GN=ALO85_04369 PE=4 SV=1
MCSGIVRSKRASLNHMNRNAEYSAVNDTVQGAFLPRVWKLITPYWRSEEKTTAWLLLVSV
IALSLFSVQVSVFFNSWYRDFYNALQNKDLAAFTHLILYFSGIAAIAILAAVYRLYLTQL
LTIRWRRWLTEQHFARWLADKNYYQLEQGGYTDNPDQRLSEDLNEFTTSTLSLGLGLMSS
VVSLVSFSVILWGVSGSIELFGVTIPGYMFWAAMLYALVGSLLTHWIGKRLIPLNNQQER
FEADLRFALVRVRENAESIALSEGERNENQRLSGRFMMIWNNFRASMKVQKRLTFFTSGY
SQIALIFPFIVAAPRYFAGKIELGDLMQINSAFGNVQENFSWFISAYSTLASWRATSDRL
LSFRQAMTDNEGRQPAIERIHANDTLDLRHLGLSIAGGRQLLSDASLQVGAGERVMLSGR
SGSGKSSLLRALGGLWPEGSGQIRLPTGTALFLPQRPYLPIGTLREAMSYPQPAEHYPAL
RFAEVLEQCRLEHLIPRLDESNHWQRFLSPGEQQRVAFARALLVAPNWLYVDEATSAMDE
DDEAAMHQAVIDGLPGMTLISVGHRSSLKRFHGRHVRVEGGKLIEQA
>tr|A0A0Q0CIP2|A0A0Q0CIP2_PSEAP Metabolite-proton symporter, MFS superfamily OS=Pseudomonas syringae pv. aptata OX=83167 GN=ALO85_01420 PE=4 SV=1
MSSVPPSSTQPARPLTRSDYKTLSLSALGGALEFYDFIIFVFFAAVVGKLFFPVDMPDWL
RMMQTFGIFAAGYLARPLGGIIMAHFGDLLGRKKMFTLSIFMMAVPTLIMGLLPTYAQIG
MWAPILLLLMRVIQGAAIGGEVPGAWVFVSEHVPARRVGYACGTLTAGLTAGILLGSLVA
TAINSIYTPVEVSDYAWRIPFLLGGVFGLMSVYLRRLLHETPVFAELQLRKALAEEVPLK
AVLRDHRGAVALSMLMTWLLSAGIIVVILMTPTILQTIYGFPAATALKANSLAIVFLSVG
CIAAGALADRFGAGWVFLLGSAGLLISSWTFYHILGSHPDVLFPLYAVTGLFVGTIGAVP

YVMVKAFPPVVRFSGLSFSYNLAYAIFGGLTPMVVTFMLKSSPMGPAWYVAILCGLGMAI
GVFLLSRKR
>tr|A0A0Q0D4Q1|A0A0Q0D4Q1_PSEAP Amino acid adenylation (Fragment) OS=Pseudomonas syringae pv. aptata OX=83167 GN=ALO85_04491 PE=4 SV=1
MHFSELMAVLSTHAIRLQQEEDDLVILGDDEGLDSATLESLSVHKAELLTLVARMGGDWL
SPAFRITADMLPLAKLSQEAIDRIVDAVPGGAGNVQDIYPLAPLQEGILYHHLTAGQGDP
YVLQDLFGAESRERLDDFAQALQAVIDRHDILRTAMVWEGLDEPVQVVLREATLTVDELT
LDAADGPIETQLRERFDTRHHRLDLRDAPLMRMVCVEDPANARWVAILLYHHIAIDHAAL
ELVKHEMQAFLLGEGHALPEAVPYRNYVAQVRLGVGADAHEGFFREMLADIDEPTLPFGV
LETPGSDSLIEDVRLPVDDALSARLRTLAKQLGVSAASLHHLAWARVVGALSGKSDVVFG
TVLMGRMQGGNGADRALGMFINTLPLRVKLEGHAVREGVRTTHARLTALLGHEHASLAQA
QRCSGVAAPAPLFNSLLNYRHSASEPVVSSEALQAWKGLQSLGSEERSTYPITLSVNDLG
QGFSLTVQAMAQVGAERIGNYMLTALGALVEALEQQPQTPLQRLQILSSTERQQVLHAFN
DTARDYSRNSSLQELFEQRVATQPDALAAVQDDQQLTYHELNSRANALARHLTDLGVQPG
ERVALLLERSLDLLAGQLAIVKCGAAYVPLDINAPAERQAFMLQDCTARYVLTLARHHLP
EGIRRIDLDLLELQNDAPDPLPSASAESVAYIMYTSGSTGMPKGVLVPHRAVSRLVLNNN
YADFNARDRVAFASNPAFDASTLDVWAPLLNGGCVVVVEQSVLLSLDEFRALLLSQSVSV
LWMTAGLFHQYADGLMEAFARLRYLIVGGDVLDPAVIARVLKDGAPRYLLNGYGPTEATT
FSTIHEITSVGNGGIPIGRPIGNSQVYVLDALRQPVAIGVVGELYIGGQGVAKGYLNRPE
LNATQFIANPFSEDAGALLYRTGDLGRWNADGVIEYLGRNDDQVKIRGFRIELGEIEARL
VDCPGVREAVVLARQDEPGHKRLVAYVVGEENSVLSAVELRRELAASLAEYMVPSAFMVL
DSFPLTANGKLDRRALPVPDADAYASREFQAPEGEVEITLARLWSELLNVERVGRQDHFF
ELGGHSLLAVSLIERMRQAGLSADVRVLFSQPTLAALAAAVGASHDIKVPANLIDKGCER
ITPELLPLANLTQVQIDQVVATVPGGVANVQDIYALAPLQEGILYHHMAAEAGDPYVLQA
QFAFDNRERLDAFVQALQMVIDRHDILRTGVVWDGLDSPVQVVWRQAQLHLEGLELDPAD
GEIGAQLHSRFDPRHYCLDMTQAPLMRLVYAEDPLNQRITAMLLFHHMALDHTAMDVVQH
EMQAWLLGESETLSAPVPYRNYVAQARLGVSQADHEVFFRDMLGDIDEPTLPFGLQDVQG
DGHDIEEAVLVVDSQLSLRLRAQARQQGVSAASLVHLAWAQVLGKVSDRRDVVFGTVLLG
RMQAGEGADRALGMFINTLPLRVAVGGQGVRAGVKATHAQLTALLAHEHASLALAQRCSG
VSALTPLFSALLNYRHSAVGSASERTAQAWQGIRALNSEERTNYPLTLNVDDFGEDFKLT
ALATITVGAQRVCGYMHTALEHLVDALELMPQASLQSLSILPAVEREQLLVTFNDNALDY
PQQQTIHGMFEAQVERTPQTLAVVHGEQRLTYRELNEQANRLAHALRKQGVQPDSRVGIC
VERGADMVVGLLAILKAGGGYVPLDPAYPAERIAYMLQDSAPAAVLTQSATEALLADVSV
PVINLDLSNWQDESVQNPQVAELTSAHLAYLIYTSGSTGLPKGVMIEHRNTVNFLTWAHA
AFDAGTNHSALEKTLFSTSLNFDLAVYECFAPLTSGGSIEVVKNVLELQHGEHGEHDIGL
INTVPSALKALLDVDGLPESVHTVNVAGEALKHSLVESLFEKTSVQRLCNLYGPSETTTY
SSWVAMDREDGFAAHIGKPVGNTQFYLLDEQLQPVPLGVAGEIYIGGAGVARGYLNRDDL
TAERFLKDPFSPQPAARMYRTGDLGRYLPDGNIEYLGRNDDQVKIRGFRIELGEIDARLA
KYPAIHEAVVTAREDVPGDKRLVAYYTLSTGHASVDIDSLRSHLQEKLPEYMVPAIYVML
EKLPLTPNGKLDRKALPPPDSEALISHGYEAPQGEIEEQIAVIWQDLLGVAQVGRHDHFF
ELGGHSLLAVSLIGRMRQLGLRADVRVLFSQPTLAALAAAVGGGTEIVVPANLISVDCQR
IT
>tr|A0A0Q0D0Q0|A0A0Q0D0Q0_PSEAP Uncharacterized protein OS=Pseudomonas syringae pv. aptata OX=83167 GN=ALO85_00679 PE=4 SV=1
MHSRNPNKAKTAMDAAPRTSKISAILVKISAGIVWMIGVIWSIFTYLFPDPTILGLSVDF
INWKTLIIIVSTVMLFSGLGIAILARKLPSLLKLGVWMALSLFLCAVFFKLGGEYAKPSV
EFARSQKLFTENDNANMFGKRSENVDNLSIELLDCDQNGQSPTCTLELINKSADRDFRFL
NPVSLFEETGGALGLTQMHVGDAKYDRWDSFQLIRNVPTRVTLIFEATKGRLKKSPALKL
TFRDRESKENVLKFKEVKVN
>tr|A0A0Q0CDS1|A0A0Q0CDS1_PSEAP GGDEF domain-containing protein OS=Pseudomonas syringae pv. aptata OX=83167 GN=ALO85_05209 PE=4 SV=1
MRIGQAMDWLGLNVFADPPADVQLLIDCSHNLFLVALAYGVACAACFATLDIADRVIQVD
ALKSRRLWKALGALCLAGGVWAMHFISMLAFQAPLKIAYDLSITLISLLIVLITALVAMR
ALTIPELTLKRCLISAVVMGIGISVMHYLGMSAMRSTATQYYEPRMFALSILVAVLSSIA
LLTLSRHLRQYSGMFHQMFRYAVSLLLGAGLLTMHLMGMKALRLVIPEGVALHGPTTENS
QQLGLTIAAIALLIIAGSISAAMADKKLQGKEHDLQRVNVLLSQLDQARVSLQQVAHYDP
LTNLINRRGFNQIFAEKLQEHTFNKGMLAVMFLDIDHFKRINDSLGHDAGDELLKVIAER
IRSATRAQDVVARFGGDEFCILLSIPDYEEARHLAHRVMHKMKETIALAGRRMVMTTSIG
IAVFPRDGSTCDELLKHADLALYQSKDKGRNGVNFFNPALKTKASLELQLEEELRNALRE
GKGLQVYYQPIVDMRTGHVAKLEALVRWNHPHHGLLVPARFISIAETNGLIAELDNWVLR

RACHDLRTLGQEGLEQLIISVNCSALTLGRNELVEEVERALADADAAPGQLELEVTENAL
MGNISNTIQMLKHIRSLGVSLSIDDFGTGYSSLAYLKRLPLDTLKIDRSFIIDIPQSPQD
MEIVQAILVMAHTLRLKVVTEGVETQDQLEFLRQFGSDYVQGYLFSRPQPLERILPLARQ
MNQREPTTRWSALPQQHSEESGSETPAIPDVFAELRDNDDFASPRPVRT
>tr|A0A0N8T884|A0A0N8T884_PSEAP Uncharacterized protein OS=Pseudomonas syringae pv. aptata OX=83167 GN=ALO85_01226 PE=4 SV=1
MRQNHMNTLEWNEAALAKYLATHPTLKDEISALSPKEQQQQVQWAFEDEAESQGIETWEL
ALELIAESPEQLQSMRLEAHRQVAEALGMDWEEYCGFNDIQP
>tr|A0A0N8T814|A0A0N8T814_PSEAP Uncharacterized protein OS=Pseudomonas syringae pv. aptata OX=83167 GN=ALO85_04387 PE=4 SV=1
MGAVRRGALQRKQYMSAAPYFSSDALRARFTQDIQDAVAQSRISRDDGAWLQLLAAESTE
PANDALPRVDRLLINDQLPVNAELAAALFVSDAANPSARVFLITLTFGIEQFESRSALFS
ALQQRFDDITALSIVESERIKGSLFDACTQTVMGQQAGHLQRLAARLEALPDLREAAGKA
LQDVLVERGIDVVAQTVQLVHTPPGAAPAASTVVGTQYLAEAAMQAFSYDALPQGQTRRY
LDTKGAALPEAQAIVFDQALADSAASVGTAYEQLLSDYWASQWQGGRTLRDLGADALAEC
FRQHLLSSRAHRTMTEAEYRWLLTLLPSQPGAPAARVPRVRRLSVVVAGEDPVKLVGVFL
IDFPGQASSAAFLYSSQPGFLRFDDQAQAIEHVLRGPSHAGLLFYSSLNDHSVIGLGGQL
ELRHDTLSEDFFSEFIDALIALQARDLRYVLDLPALHSEKNPARIDDALDIRKLLDGRLL
NLHDAGRWRQDRLSFDSVWGAAAQSSISERPNMISDPSYNWVGKLKKLDALLGHVDAMHA
GADGCMHRALNLYLALIGGPPLDARKLWVLADVAEAKPVPVLTLALDRVCGYTSAPLTDS
VVLAGQITPVMGQPVLRLSLALLEQILICVQADFAHRFESQIGEFYARTIRQRDTSMHPG
VISALVREYSLRLELLVERRTATLPVVFIDNIQQVIDRPLPALREGLGEARVDAFRVTVE
YDPQAPAIKVPNAFVVTGRRPDSQPVLWILEQGIICFETRQLLEVYVAGRLAGIELGSQV
SGVIAEPDRRMLLDQRTRRGTLELKVVLQPIEGHFIEALQRDEVERQQRTVADLYRQSIA
WQVPSELFVNLMSAAERDDRNRQALNSLGVAIQFIIYKALVPSWVSDASKRDQVILVEVL
RRFYVTCVGEKDFLFDIPSFYDYSLEQLKHRFDKDFAEPRPDPENVCVSLTHYVPAPVAP
GEVPQSVPAAAQTVSENLVEFAINRFLSRQDGVILLSSRDDTPLHAALTPAYLRELVTSL
DIAASYRRMLNSVLSETTPDYIERRKLFVEQVPSLDILRAFTFKLKNELSEQAYRIVENV
LTMPDAVARLPVNGCQIIISPLKLLPAKEGWEPTAVLNTYVMGPKENQPGPWVLYAPLHD
EFVFKEYPDQAALVLDIHTSAYFQAYILDRIDPDLRKIYDKGGFVEPHLPFSTESSFDLP
LERPAPVTVQIDPYEGNCLLLMFKGALDILKLQVKQYSVTNAEHRRAATQYLFTLGAEQF
MALMPGRLGALIGIIQGQTLLNLSLISATDQQWGQAISEFMAALSVMISSGQNPAEVASL
TARENSTLVDDATSPDPLVQLIEGADTFEFSWGNSSLTQQIRERLREFEVHDMALYTLQR
DELLSVYRDPVTGRSYAAIGGKTYELQSDEDGWFIVSGSKIGPPVSLDADQQWRLDIPGG
LRGGGGALTRMEGSLVDDLVDEIMVVSARGMPEIRHRHRDMAQSIEDACLQARSYLENAL
DNLTRRLPDNTLDPRAEKILSDFFAQKVPDDRLREVTKKAVTDLYQELIDPSMSPIDSKR
YVIGVNRMGNESASAFMFEADPARRIFLTEQFFRLPTYRLKLSAQRAGEFKFPQHYRAAI
LIHELSHMVLKTDDIAYVDSQAPFIDLLEDAPTYRLRIRNELIYQQQKTLSFQTDRDKLF
KQLEEDSWRDLRRTDGNGKQTILRISGKSTLEKARDVFYEDVHKRADIMLKNADSVALLV
TLLGRERFVKP
>tr|A0A0Q0FWE2|A0A0Q0FWE2_PSEAP Phospholipid/glycerol acyltransferase OS=Pseudomonas syringae pv. aptata OX=83167 GN=ALO85_04885 PE=4 SV=1
MNSQLFRFRTLKERAPARTVNVTMSIMQAIRTFFFYLLLGTSSLLWCTLSFFIAPFLPFK
ARYRFINVYWCRCAIWLTKVFLNIKVEVTGAENVPKTPCVIISNHQSTWETFFLSAYFEP
MSQVLKRELLYVPFFGWAMAMLRPIAIDRENPKAALREIAKKGDELLKDGVWVLIFPEGT
RVPYGQVGKFSRGGTALAVNADLPVLPVAHNAGKYWPRDGWAKKPGTIQLVIGEPMYAEG
TGPRAIAALNDRVQTWNEDTQRAMGSPVEPAATPEKVTA
>tr|A0A0N8T817|A0A0N8T817_PSEAP Phosphoglucosamine mutase OS=Pseudomonas syringae pv. aptata OX=83167 GN=glmM PE=3 SV=1
MTTRKYFGTDGIRGRVGQFPITPEFMLKLGWAAGMAFRKMGACRILVGKDTRISGYMFES
ALEAGLSAAGADVLLLGPMPTPAIAYLTRTFHAEAGIVISASHNPHYDNGIKFFSGQGTK
LPDEIEMMIEELLDAPMTVAESENLGKVSRINDAAGRYIEFCKSSVPTSTDFAGLKVVID
CAHGATYKVAPNVFRELGAQVVVLSAQPDGLNINKDCGSTHMEALQAAVLAEHADMGIGF
DGDGDRVLMVDHTGTIVDGDELLYIIARDLHERGRLQGGVVGTLMSNLGLELALAEQGIP
FVRANVGDRYVIAELLERNWQIGGENSGHIVCFQHATTGDAIIASLQVILALRRSGVSLA
EARLKLRKCPQILINVRFAGSGVDPVSHPSVQEACARVTEQMAGRGRVLLRKSGTEPLVR
VMVEGEDETQVRAYAEELAKLVTEVCA
>tr|A0A0Q0DMC9|A0A0Q0DMC9_PSEAP Extracellular solute-binding protein OS=Pseudomonas syringae pv. aptata OX=83167 GN=ALO85_01005 PE=4 SV=1
MKPLVRSLLASALLLAAGSVTAQDLRIGYADPVSSLDPQLNNYAGDRSVALHAFESLVSR

RDDKTLPGLAKSWKVVDDTTWEFALRDDVKWQDGTPLTADDLVFSFERARSVPGSVASYA
GAMRTVESIKAKDDHTLIIKTRSPNANLLPDIDSIYIVSRHAGAAASSADYNSGKAMIGT
GPYRFVSFVPGDRTIFARNENYWGGRPTWEKVDFRFIANAANRTAALLAGDVDVIDKVSP
TDVARLRKTPNVNVFAYQGLRALIIQPSFRAGPNEFIRDNAGKPLAENPLLDVRVRKALS
LAINRPAINERIMQGTVTEANQWMPANTFGYNPQIKNIPYDVKQAKDLLAQAGFPEGFQL
TVHVPGDRYPQAPEVMQAVAQFWTRIGVKVQLEVLPWAVYAGKANKNELAISVIAWGNGT
GEAAYALTNILTTVDSSKGQGASNWGHYSNPLVDKALADSTAEFDEAKRRKILQDSVQIV
SDDVGIIPLFHYQNIWAARKGLKVEPLVSDRTAATMVTEQP
>tr|A0A0Q0CAD9|A0A0Q0CAD9_PSEAP Autotransporter barrel protein with pertacin-like passenger domain OS=Pseudomonas syringae pv. aptata OX=83167 GN=ALO85_03014 PE=4 SV=1
MSCRENNVVKSRGGAFQLAVIAFGALANVQASAAASGDNNGLPLDSKIVSGVLPAEELPD
ETVRRREIITVGPDTPFDSYHIIDQSVLNVVGGRVDFVGVRNATVNIESGVVESGISLTD
ALGNLSNATVKNATGVGIVLQGVIGAIKPGSSVSAHSSEVSGSGYGIAVGMWGALNIFNT
DVRGFASQGKGGQGIFASGANVLIADNSHVTGDMNGINLVDGSGNGVVDGNKSVTVIDRS
IVEGLGGAAIKVNQRVAFDIEADIAVQNQSELWSGNGNLLEVEDHSTANFNVDNSTLNGN
LVADDTSTLNITLQNGAQLNGDIVNGNRLAITSGSHWQMQGDNAVRSLSLQAGRVSFAGE
GFHTLSLNELSGAGTFGLRVDLDNGVGDLIDVNGQASGQFGLRVRNTGEEIVSADMAPLK
VVHTEGGDAQFSLLGGRVDLGAYSYLLEQQGNDWFIVGKHKVISPSTQSALALYSAAPAI
WMSELSTLRSRMGEVRASGRAGGWMRAYGNRLNATTSDGVDYRQKQSGLSLGADAPVEVS
NGQLVVGVLGGYSTSGIDLSRGTTGKVDSYYAGAYATWLLDEGYYVDGVLKLNRFRNKAD
VAMSDASKAKGDYTNNGVGGWVEFGRHIKLADDYFLEPFAQLSSVVVQGQELRLDNGMKA
KNDQTQSVLGKVGTSLGRSVALKDGGVLQPYVRVAVAQEFSRRNEVKANDVKFDNSLFGS
RGELGAGVSVSLSERMKLHADFDYMKGRHIEQPWGANVGLRLAF
>tr|A0A0Q0FNW7|A0A0Q0FNW7_PSEAP Elongation factor Ts OS=Pseudomonas syringae pv. aptata OX=83167 GN=tsf PE=3 SV=1
MQEDFEMAEITAALVKELRERTGEGMMDCKKALTKAGGDIEKAIDDMRASGAIKAAKKAG
NVAAEGAIAIKDDGKAAVIIEVNSQTDFLALQDDFKAFVAASVEKAFADKLTDVAPLIEA
QEAARLVLVGKVGENVNIRRLKRIEGDVVGTYLHGNKIGVVVTLKGGNVELAKDIAMHVA
ASNPEFLFPSEVSAEAIEREKNVFLQLNEDKIKGKPAEIVEKMVGGRITKFLAEASLVEQ
AFVKNPEVKVGDLAKKAGAEIVSFTYFKVGEGIEKPVDNFADEVAAQLAAAKQ
>tr|A0A0Q0FBV9|A0A0Q0FBV9_PSEAP Exodeoxyribonuclease 7 large subunit OS=Pseudomonas syringae pv. aptata OX=83167 GN=xseA PE=3 SV=1
MRQTGIAIMMPDMIKDPFARLGLDREVLTVSQLNGRARVLLEDVFSSIWVEGEISNLSRP
ASGHVYFTLKDSGAQVRCALFRQSAARVRQALKDGLQVKVRGKVSLFEGRGDYQLILDTV
EPAGDGALRLAFDALKAKLSDEGLFSAERKVALPLHPQRIGIISSPTGAVIRDIISVFRR
RAPRVELTLIPTAVQGREAINQIVRALKRADSRGFDALILARGGGSLEDLWCFNEEAVAR
AIDACVTPIVSAVGHETDVSISDFVADVRAPTPSAAAELLAPDSSDLHRRVDNLHRRLVS
RMQDRLMRERLRLEGISRRLRHPGERLRQQSQRLDDLDMRLRRAFEQNMHQRQLRLAHMQ
SRLAAQHPGRTLAFLRQRLDALAERLPRAIREQIKARKLQLQSQVQTLNVVSPLATLGRG
YSILLDERGHAIRNAAQTQTGQRLTARLGEGELHVRVEDNHLTPVTLSLLD
>tr|A0A0Q0D357|A0A0Q0D357_PSEAP 3-demethoxyubiquinol 3-hydroxylase OS=Pseudomonas syringae pv. aptata OX=83167 GN=coq7 PE=3 SV=1
MPMATERQYSPLDRLLLQADSAMRTLLPSSAHSQRPSPAVVQPEHKMSEVDTRHVAGLMR
INHTGEVCAQALYQGQALTAKLPKVRKAMEHAAEEEIDHLVWCEQRIHQLGSHTSVLNPL
FYSLSFGMGAIAGVISDRVSLGFVAATEDQVCKHLAEHLEQLPTEDGKSRAILQQMLSDE
EHHAESALEAGGFRFPAPVKFGMGVLAKVMTKSTYRI
>tr|A0A0Q0ICV0|A0A0Q0ICV0_PSEAP Iron-dicitrate transporter permease subunit OS=Pseudomonas syringae pv. aptata OX=83167 GN=ALO85_03065 PE=3 SV=1
MRRWLMAGVIIAACLGLFWVSLFALSSFSIRQVDAWNGLITQGREGGNIAYIVAQLRVPR
ALCAALVGACLGVAGALMQGITRNRLASPSLFGVTAGAALGLALFSTGLVALPFPGGALL
MTCLGGALAWITVFSLGGAWSPATAQGRLVLAGVAVAALCAALTRLTVILVEAQAQSVLN
WLAGSLANVGAAQLQLLWPCTLIGVVLAVACAPRLNLINLGEDAARSLGVRIGALRLLVF
VVSLLLVGASVCAVGPIGFVGLIAPNIARQWLGNDYRWLIPISAGLGAAIVLASDLISRA
VAFPVETPAGVVTALIGAPFFLFLARRAL
>tr|A0A0Q0C231|A0A0Q0C231_PSEAP Uncharacterized protein OS=Pseudomonas syringae pv. aptata OX=83167 GN=ALO85_100581 PE=4 SV=1
MQSTLSPDASYASVRYVMGSKVCVFSTTFVRLAGAGGVKVPKWNRTATPEGGAICTATSR
VTDLSTHAWTAEFTMK

>tr|A0A0Q0FWC2|A0A0Q0FWC2_PSEAP Heavy metal--translocating P-type ATPase OS=Pseudomonas syringae pv. aptata OX=83167 GN=ALO85_01456 PE=3 SV=1
MSGEAVHTHDADQPQAPGSLLSSAEQRSAARQLTLAMLALGLLGLGLVWRFFAPEQEGVS
QLLLGAASLLVAIPVISAGWHSLRHPSLHGITDQLIALAMLGAWATGDLMTAALLPIIMI
FGHVLEERSVIGSQEAIEALGRLTRSQARLLGADGSVREVDNATLKAGDQVEVRAGDRVP
ADGRVLSGQASLDTSSITGESVPLEAIVGVEVFGGAINLDGLLRIEVTRIGDQSTLGKVI
ALMQSAERSKPPITRLLERYAGSYMVLVLLIAALTWFITQDAQAMLAVLVAACPCALVLS
APATAIAGIAVAARHGILIRSSAFLEELADLNSLVVDKTGTLTYGRLRLQSVQADGPQHP
QLLELAASLGSASSHPVSRALAGLVDNARLLPLTDTRERQGLGVVASTWQGDAALGRPEL
FAQMGIITPAVPAHDGPLAGLSLDGVFLAWLLLADSVKPEAQAALFELRGLGLGRQLLLT
GDRQSVADNLAHDIGIADVQAQALPEDKLNRVMGEIAKGFRPMVVGDGVNDSLALKAGVV
GVAMGAGGADIALASADIVLIGSDLRRLGTCVRLSRQCRQTLQVNVIIGLGWTLAIVALA
AFGLLGAAGAMVAAILHNLSTLLVLGNAGRLLRFQEPLLKLDDAGVQHQGSRLR
>tr|A0A0Q0D430|A0A0Q0D430_PSEAP Sulfate ABC transporter permease OS=Pseudomonas syringae pv. aptata OX=83167 GN=ALO85_03968 PE=4 SV=1
MSYSSVSAAASANAARRGSAVSRRILIGLCWLVFALFLGLPLFIVVSQGLKNGLGAFFTA
ILEPDALSALKLTVIAVLISVPLNLVFGVSAAWCVSKYSFRGKSILVTLIDLPFSVSPVI
AGLVYVLMFGAQGLFGPWLQDHDIQIVFALPGIVLATIFVTVPFVARELIPLMQEQGTQE
EEAARLLGANGWQMFWHVTVPNIKWGLIYGVVLCTARAMGEFGAVSVVSGHIRGVTNTLP
LHVEILYNEYNHVAAFAVASLLLILALFILLLKQWSESRINRLRHNAAEE
>tr|A0A0Q0CWH2|A0A0Q0CWH2_PSEAP Nitrate/nitrite transporter OS=Pseudomonas syringae pv. aptata OX=83167 GN=ALO85_00562 PE=4 SV=1
MHMDTSFWKAGHKPTLFAAFLYFDLSFMVWYLLGPLAVQIATDLHLTTQQRGLMVATPIL
AGAVLRFFMGLLADQLSPKTAGVIGQVIVIGALLVAWQLGIHTYGQVLVLGLFLGMAGAS
FAVALPLASQWYPAQHQGKAMGIAGAGNSGTVLAALIAPVLAASFGWGNVFGLALIPLVL
TLIAFTLMARNAPQRSKPKSVADYLKALGDRDSWWFMFFYSVTFGGFIGLASALPGYFND
QYGLSPITAGYYTAACVFGGSLMRPLGGALADRFGGIRTLTVMYAVAAIGIAAVGFNLPS
SWAALALFVAAMLGLGAGNGAVFQLVPQRFRKEIGVMTGLIGMAGGIGGFLLAAGLGTIK
QNTGDYQLGLWLFAGLAVLAWFGLLNVKRRWRTTWGSAAVTAARV
>tr|A0A0N8T8J8|A0A0N8T8J8_PSEAP Bcr/CflA family efflux transporter OS=Pseudomonas syringae pv. aptata OX=83167 GN=ALO85_03250 PE=3 SV=1
MDTPQHTVGFKAAASLGLITILGPSAIDMYLAAMPQMTKDLNTDYATMQLTLTVFLLAMG
AGQLVFGPVIDALGRRRPLLAGLAAFILASLWASLSESMGAMLLARFFQGLSVALVLVIA
MSSVRDLSSGIRATQLFALLVTIQGAAPILAPAFGGIINVHFGWRAVMLALAIVGVLALL
NSSVCLPETLAREKRTPFRPASVFGTYRRLVADKRFILPALALSSVFFFLFGYVGGASFV
YQSGYALRSDIFGFVFGGTGVAMMLGAMTGSRLASRCSASLMALLGVAMMSGGSALALLA
VYVGADLYGIVPALFIAVFGFGIAEPSLMSIAMASQERALGATAALLGASTHILGSLATP
LAGSLAQIGAHAWLALLLAAALVSLALVFISVRSVSNNVIPVH
>tr|A0A0Q0DB89|A0A0Q0DB89_PSEAP Glycine betaine/L-proline transport ATP-binding subunit OS=Pseudomonas syringae pv. aptata OX=83167 GN=ALO85_05515 PE=4 SV=1
MHHAVAVDGGHRGPGRCRRPGQTSGQRAEHGRHRARFRSRSGNRIAGHHARPHLQTTRSQ
GRVRRMSIIKFDKVDVIFSKDPREALKLLDEGLTRDQILKKTGQIVGVENASLDIEKGEI
CVLMGLSGSGKSSLLRCINGLNTVSRGSLFVEHEGSQINIANCSAAELKMMRTKRIAMVF
QKFALMPWLTVRENISFGLEMQGRPEKERRKLVDEKLELVGLTQWRNKKPDELSGGMQQR
VGLARALAMDADILLMDEPFSALDPLIRQGLQDELLALQSKLSKTIVFVSHDLDEALKLG
SRIAIMKDGRIVQYSVPEQIVLNPADEYVRTFVAHTNPLNVLCGRSLMRTVDECTHVNGS
VCLDPSGDSWLDLADGNVIKGARQGATALDLQNWAPGQDVATLDRRPTLVHSNIGMRDAL
QIRYQTGNKLVLQEGNKVVGILGDTELYHALLGKNHG
>tr|A0A0Q0C9V7|A0A0Q0C9V7_PSEAP Putative Dipeptide ABC transporter, periplasmic dipeptide-binding protein OS=Pseudomonas syringae pv. aptata OX=83167 GN=ALO85_00007 PE=4 SV=1
MTTTEATMLKHAVIPFLIGASLLASAPFAHAASNLVFCSEGSPAGFDPGQYTTGTDFDAS
AETIFNRLSQFERGGTAVVPGLATKWDISDDGLTYTFHLREGVKFHTTPYFKPTREFNAD
DVLFTFNRMIDKDMPYRKAYPTEFPYFTDMAMDTNITKIEKIDDHTVKFTLGKVDAAFIQ
NMAMSFASIQSAEYAAKLLKEGKPELINQQPIGTGPFVFKSYQKDSNIRYTGNKDYWKPE
DVKVDNLIFAITTDASVRMQKLKKNECQITAYPRPADLEPLKADKNLKMPDQAGFNLGYI
AYNVMPEIKGEDHPNPLAQLKVRQALDMAVNKQQIIDSVYQGAGQLAVNAMPPTQWSYDT
TIKDAKYDPEKARQLLKEAGIKEGTEITLWAMPVQRPYNPNAKLMAEMLQSDWKKIGINA
KIVSYEWGEYIKRAKNGENGAMLIGWSGDNGDPDNWLGTLFGCDALNGNNFAKWCDKPFD

TLIHQAKETSDQAKRTELYKQAQHLLKDAVPMTPIAHSTVYQPMRTSVQDFKISPFGLNS
FYGVSVSGK
>tr|A0A0Q0BVW1|A0A0Q0BVW1_PSEAP Transcription-repair-coupling factor OS=Pseudomonas syringae pv. aptata OX=83167 GN=mfd PE=3 SV=1
MHGCHIDTHYIIFARKYSVILRKTDSHHLPPLQSPPLLQRLEPDLPVPVLRLPHLSAAAG
KQHWGNLPGATLSLAIAEAASAAKRFTLLLTADSQSAERLEQELKFFAPTLPVLHFPDWE
TLPYDLFSPHQDIISQRIASLYRLPELEHGVLVVPITTALHRLAPTKFLLGSSLVLDVGQ
KLDVEAMRTRLEASGYRYVDTVYEHGEFTVRGALIDLFPMGSKLPFRIDLFDDEIETLRT
FDPDTQRSIDKVESVRLLPAREFPLQKEEVTRFKARFRERFDVDFRRSPIFQDLSSGITP
AGIEYYIPLFFEETSTLFDYLPQDTQVFSLPGIEQAAENFWNDVRNRYEERRVDPARPLL
PPAELFLPVEDCFARLKNWPRVVASQQDVDAGAGRERFPAQSLPDLSIEAKATQPLAALS
RFLDDFPGRVLFTAESAGRREVLLELLERLKLRPKTVDSWLDFVDGKDRLAITIAPLDEG
LLLEQPALALIAESPLFGQRVMQRRRREKRTDGGNNDAVIKNLTELREGAPVVHIDHGVG
RYLGLATLEVENQVAEFLMLAYAEDAKLYVPVANLHLIARYTGSDDETAPLHRLGSETWQ
KAKRKAAEQVRDVAAELLDIYARRAAREGYAFADPKADYATFSAGFPFEETPDQQTTIDA
VRADMLAPKPMDRLVCGDVGFGKTEVAMRAAFIAVHGGRQVAILVPTTLLAQQHYNSFRD
RFADWPVTVEVMSRFKSAKEVNAAVADLAEGKIDIVIGTHKLLQDDVKIKNLGLVIIDEE
HRFGVRQKEQLKALRSEVDILTLTATPIPRTLNMAVSGMRDLSIIATPPARRLSVRTFVM
EQNKPTIKEALLRELLRGGQVYYLHNDVKTIEKCAADLAELVPEARIGIGHGQMRERELE
QVMSDFYHKRFNVLIASTIIETGIDVPSANTIIIERADKFGLAQLHQLRGRVGRSHHQAY
AYLLTPPRKQITHDAEKRLEAIANTQDLGAGFVLATNDLEIRGAGELLGDGQSGQIQAVG
FTLYMEMLERAVKSIRKGEQPNLDQPLGGGPEINLRVPALIPEAYLPDVHTRLILYKRIA
NAADEEALKDLQVEMIDRFGLLPEPTKNLVRITLLKLHAEQLGIRKIDGGPQGGRIEFAA
ETPVDPLTLIKLIQSQPNRYKFEGATLFKFMVPMERPEERFNTIEALLERLTPKTA
>tr|A0A0Q0BVB9|A0A0Q0BVB9_PSEAP Electron transfer flavoprotein, beta subunit OS=Pseudomonas syringae pv. aptata OX=83167 GN=ALO85_00489 PE=4 SV=1
MQPEMSVDKHAVQIISLVSIGAHPTSGRARRAEQDARAVELGLQLAGDNLQVLHAGNAEE
PALRAYLGMGLNELHVLEQPEGADALVALTDYLRDSAAQVVLAGSQAETGEGSGMLPFLL
AERLGWPLVTGLAEVESLNNGTALVLQALPRGQRRRLKVRLPFLATVDNAAPKPRQSAFG
PAQRGTLDTEIVQVVADELLTHQALQPARPRPKRLKVIKAKSGADRMKAATAKASGGGGQ
VLKGLRPEEGAAAILKLLIDEGVVK
>tr|A0A0Q0C3G3|A0A0Q0C3G3_PSEAP Putative transposase OS=Pseudomonas syringae pv. aptata OX=83167 GN=ALO85_101550 PE=4 SV=1
MMDMVHAWMIAQRDLVLEGSAISRALDYSLKRWAALSRYLDDGAVPIDNNWAENQIRLWA
LGRKNWLFAWSLRSGKRAAAIMSLIQSARLKPRNP
>tr|A0A0Q0C1I7|A0A0Q0C1I7_PSEAP Uncharacterized protein OS=Pseudomonas syringae pv. aptata OX=83167 GN=ALO85_101247 PE=4 SV=1
MAGQVGRGYGNGRVHVLASWAGGTLPRRPTGGFDKGLLEPGVCACHLAETERRRQAVPNG
GG
>tr|A0A0Q0DUE2|A0A0Q0DUE2_PSEAP ABC transporter OS=Pseudomonas syringae pv. aptata OX=83167 GN=ALO85_03275 PE=4 SV=1
MQTPGEVPRVQMSAITKHYGSIASLRGVDLLLQPGEVLGLVGDNGAGKSTLTKILSGAEL
PTSGRIVIDGEEVSFSGPADAQRCHIQMVYQDLSLCDTVDVAGNLFMGREPMRRVLGVPL
LDETRMHHEARQILEALGITIPDTRAQVRHLSGGQRQAVAIARAAAFGPKVLIMDEPTAA
LAVAEVEAVLELIRTISRQGVSVILITHRLQDLFLVCDRLMVMYDGANLAERRIADTDLN
EVVELIVGHKFTARSASA
>tr|A0A0Q0E0Q9|A0A0Q0E0Q9_PSEAP CheW-like protein OS=Pseudomonas syringae pv. aptata OX=83167 GN=ALO85_05348 PE=4 SV=1
MKEVIVSINLDQAQQTFIVEARELLQAMEESLLQLESEPGDQDAIGAVFRAAHTIKGSAG
LFGLTPIVSFTHIVEDVLDRLREGSVSVNAELIAVLLKSGDHMLELIDVVASRGEQMQQP
ALEREAALRQALQVFQAPASAGAADSASASVVSDDEPSAEVLWHISLRFGVDVFRNGMDP
LSFLRYLNTLGRMVQVTTVTDSIPAVEAWDPESCHLGFEIDFRSAAGHAAINEVFDFVRE
DCAVEITPVNETPDPVEPTGTELVSQPEHSPMVASGELLGDQRAVPRTPATATATAVERP
SSAGEQKNKDGRYVRVNADKLDELINLVGELVIASAGASLLAKSCDNDPLQEASSTVSGL
VEQILDGALHLRMIPIGDTFNRFRRVVRDVSQELGKDIDLIINGAETELDKTVVEKIGDP
LMHLLRNSMDHGIESAEARRAAGKPAKGHLSLNAYHDSGSIVIEIADDGAGLNRERILDK
AQQRGLVAAGASLTDQEIYNLIFEPGFSTAEAVTNLSGRGVGMDVVKRNITLLRGTVDLD
SQPGQGTIVRIRLPLTLAIINGFLVGIDQSTYVIPLDMVQECIELDEHNRQLTRDSGYLD
LRGEVLPLVYLRDHFNHEGPAARRQNVVVVRYAEHKAGLVVDELLGEFQTVIKPLGKLFG

ALRGISGSTILGSGAVALILDIPALLNQIVNMEARSTQAPQSLLPTSR
>tr|A0A0Q0DUI8|A0A0Q0DUI8_PSEAP Auxin-responsive GH3-like protein OS=Pseudomonas syringae pv. aptata OX=83167 GN=ALO85_04708 PE=4 SV=1
MMLAGDDVKRALWRSFMDSAYVTLDHWQRLFEQPEESQQRLLLRMLSASRDCAFGQAHDF
AGIRDSEEFRKRVPIHTYAQLQPWIERAQHEQGPILTASPPLFFERSSGNSAVQKHIPYT
QEFLGQLQGSLTVWLADMYRQVPEISHGSGYWSMSPPLQQPAMTANDIPIGSASDLQYLQ
GSAIAGLAGTLLIPELASDVAHWRRQTLLALIADAGLSFISVWSPTFLTSVLQPLLDTES
HESRQIVAWLEERLPATRQKALRHALAHGVFTELWPRLAAVSCWMDGPSRVYAQQLAARF
PQARWLPKGLFATEGVVSLPFGEGAGCPLAIGSHYLEFIGDDGLPKEAHALRMGETAQVL
LTTGAGLYRYALGDRVRVVGKLAGTPRVEFVGRCASTCDLVGEKLDEQLVERALAQCMDA
ADSACLIPDSSSALPHYVVLLCTSTTTLASICRNALANSIEMALQRSFHYAHARTLGQLG
PVRMRFVCGGAQRLAELLQRAAESTGIRAGDVKPRALISRLDTADALLAITEEPECPQL
>tr|A0A0Q0IM38|A0A0Q0IM38_PSEAP Uncharacterized protein OS=Pseudomonas syringae pv. aptata OX=83167 GN=ALO85_02410 PE=4 SV=1
MTTHPTRIGDIIMKTNTLIFGLAISVLASSAFALPATNVQSDLSSTTLVAEGGTKHTNVG
SIRVSADGADHVGANRLAADGADRVGANRQAADGADHVGANRLAADGADRVGANRLG
>tr|A0A0Q0FI54|A0A0Q0FI54_PSEAP GSDH domain-containing protein OS=Pseudomonas syringae pv. aptata OX=83167 GN=ALO85_03206 PE=4 SV=1
MLRKTLIATLCTAAILSLPLAASAATESYKSELGTVTVTPVVEGLDHPWALAFLPDKQGM
LVTERSGNLRVVSADGKLSAPLSGVPQVWAQKQGGLLDVVLSPDFAKDRMVYLTYSEGSG
KTAAEGDTAGTAAGRGRLSKDMTRLEDFEVIFRQEPKLSVGNHFGARMVFDRDGYLFIAL
GENNQRPTAQDLDKLQGKIVRIYPDGSVPKDNPFVGQKGVRPEIWSYGHRNQQGAALDPW
TGTLWTNEHGPKGGDELNIIERGANYGWPIATHGIDYSGQPIPESKGKVVEGTKIPFQVW
EVSPGLSGMAFYDHSLFKAWDHSVFIGALATEELIRLQFENDKIVHEERLLKDMKQRIRD
VRQGPDGYLYLLTDDDKGSLLKVGLAQ
>tr|A0A0Q0CWM4|A0A0Q0CWM4_PSEAP Liposaccharide ABC-type transport system, ATP-binding protein LptB OS=Pseudomonas syringae pv. aptata OX=83167 GN=ALO85_00167 PE=4 SV=1
MATLKAQHLAKSYKSRQVVRDVSISIDSGQIVGLLGPNGAGKTTCFYMIVGLVQADQGRV
LIDDLDVSHQPMHGRARAGIGYLPQEASIFRKLSVSDNIMAILETRKELDAGARRKELES
LLQEFHITHIRDNLGMSLSGGERRRVEIARALATAPKFILLDEPFAGVDPISVGDIKQII
HHLKAKGIGVLITDHNVRETLDICEMAYIVNDGQLIAEGDSETILANQLVKEVYLGHEFR
L
>tr|A0A0Q0DIN0|A0A0Q0DIN0_PSEAP Glutamine synthetase family protein OS=Pseudomonas syringae pv. aptata OX=83167 GN=ALO85_02334 PE=3 SV=1
MSVPPRAVQLNEANAFLKEHPEVLYVDLLIADMNGVVRGKRIERTSLHKVYEKGINLPAS
LFALDINGSTVESTGLGLDIGDADRICYPIPDTLCNEPWQKRPTAQLLMTMHELEGDPFF
ADPREVLRQVVAKFDEMGLTICAAFELEFYLIDQENVNGRPQPPRSPISGKRPHSTQVYL
IDDLDEYVDCLQDILEGAKEQGIPADAIVKESAPAQFEVNLHHVADAIKACDYAVLLKRL
IKNIAYDHEMDTTFMAKPYPGQAGNGLHVHISILDRDGKNIFTSEDPEQNAALRHAIGGV
LETLPAQMAFLCPNVNSYRRFGAQFYVPNSPTWGLDNRTVAVRVPTGTADAVRIEHRVAG
ADANPYLVMASVLAGVHHGLVNKVEPGAPVEGNSYEQHEQSLPNNLRDALRELDDNPVMA
KYIDPKYIDIFVACKESELEEFEHSISDLEYNWYLHTV
>tr|A0A0Q0FSQ8|A0A0Q0FSQ8_PSEAP Urease subunit beta OS=Pseudomonas syringae pv. aptata OX=83167 GN=ureB PE=3 SV=1
MIPGQYQIQPGDIELNAGRRTLSLTVANSGDRPIQVGSHFHFFETNDALTFDRAASRGMR
LNIPAGTAVRFEPGQSREVELVDLAGLRKVYGFAGRVMGEL
>tr|A0A0Q0C2Q0|A0A0Q0C2Q0_PSEAP MotA/TolQ/ExbB proton channel OS=Pseudomonas syringae pv. aptata OX=83167 GN=ALO85_200006 PE=4 SV=1
MLKKMSSLFCLVLFAFALSGCGDDKGQEFVGRWTGENKTRMGKPTYVMDISKDGEVFHIN
LETTADIVGYGKMKKDLQRLEAKAESDSVLSMAGGLATMRLEGKVIHFDGTTYSRSK
>tr|A0A0Q0C4R3|A0A0Q0C4R3_PSEAP Alpha amylase, catalytic region OS=Pseudomonas syringae pv. aptata OX=83167 GN=ALO85_04695 PE=4 SV=1
MQLNRLESGVSRPRNNTPEESIMAKKPGDATFIKDPLWYKDAVIYQVHVKSFFDSNNDGI
GDFAGLIEKLDYIAALGVNTIWLLPFYPSPRRDDGYDISEYRDVHSDYGTMADAKRFIAQ
AHKRGLRVISELVINHTSDQHPWFQKARNAKPGSKARDFYVWSDTDQKYDGTRIIFLDTE
TSNWTWDPVAGQYFWHRFYSHQPDLNFDNPHVLDAVLEVMRFWLDLGIDGLRLDAIPYLI
ERDGTNNENLPETHQVLKRIRAEIDANYPDRMLLAEANQWPEDTQLYFGDSKGPDGDECH
MAFHFPLMPRMYMALAQEDRFPITDILRQTPEIPENCQWAIFLRNHDELTLEMVTDRERD
YLWNYYAADRRARINLGIRRRLAPLVDRDRRRVELLNSMLLSMPGTPTLYYGDEIGMGDN

IYLGDRDGVRTPMQWSIDRNGGFSRADPASLVLPPIMDPMYGFQSVNVEIQERDPHSLLN
WNRRMLAVRKQQKAFGRGTLKMLSPSNRRILAYTREYTAPDGHSEVVLCVANVSSAAQAA
ELELSAYAGTVPVEMLGGSAFPPIGQLSYLLTLPPYGFYWFLLASENQMPSWHVEPAQSM
PDFPTLVLKKRLEELLEEPLRSTMENTSLAVYLPKRRWFAGKDKAIEKVSIAYAVRFGDE
AHPVLLSEIEVTAGGQTDRYQLPFGLLAEDDISSALPQQLALARVRRNRDVGLITDAFTL
ETFIRAVIQGMQSDTVIPCADGELRFEQSSQLAPLGLNAESEVRYLSAEQSNSSVVVGSS
LVLKLIRKVSAGTHPELEMGAFLTQAGFKNISPLLGSLVRVGNDGQPNLLMIAQGYLSNQ
GDAWEWTQNNLERAVRDELAHGVSGQEQHYNALLELADFSRSLGQRLGEMHQILASPTDN
ADFAVEVTSSQDSKASGASVNAQLERALQLLEQRMGELEKDDQQLVKDLLAQRKQIRQRV
EGLAKRSAGGLRIRVHGDLHLGQVLVVKGDAYLIDFEGEPARALEERRAKHSPFKDVSGV
LRSFDYAAAMAVRSAQSVDTSPEAAEARKRVADTYLAQARETFIEGYRSATTGIAHAWKD
AKGEDAALELFTLEKAAYEVIYEAENRPAWLAVPLQGLRGLLQPSDGESI
>tr|A0A0Q0FP23|A0A0Q0FP23_PSEAP Beta-propeller domains of methanol dehydrogenase type OS=Pseudomonas syringae pv. aptata OX=83167 GN=ALO85_100221 PE=4 SV=1
MIFVQTLFDQIMTVSGVGDGLCRGTCVLAKARRFNGFKKTPEVWILKSVSQWLLYVVMLL
GGVLRAEAEPSALVFPPMTGSVVDAAQMLDSQTTVRLARLLSAHEKATGERVVVVTLADL
QGTSIEEFGSRLGDAWGLGLHGKDDSVLLVVYRDKRKVFMEVGDVLKDRLNDAQASLIID
MLMTPEFDDNRFAVGIERGTRAVIAALGGQIPDYPEPKRQMSEVDEQTNDDQVWLIGIVL
TLIVLGIVIISVRRGGATRPARERAADRSGGRFGGGGASGNW
>tr|A0A0Q0D8L2|A0A0Q0D8L2_PSEAP Uncharacterized protein OS=Pseudomonas syringae pv. aptata OX=83167 GN=ALO85_101434 PE=4 SV=1
MAGSCQTAIMMQPGFRQKKPRGLIGRGAGLPAFQVQA
>tr|A0A0Q0DYT5|A0A0Q0DYT5_PSEAP Uncharacterized protein OS=Pseudomonas syringae pv. aptata OX=83167 GN=ALO85_01986 PE=4 SV=1
MADSSLRQWWATPLIGLLGGYLASQVGWPLPWMVGSLLAIILVRCLTPWQLAQIPGGRKC
GQLIIGIGIGLHFTPVVIEQVLAHFGLIFIGALVTSLSCLVGVWLMLRTGEDRPTAFFSS
MPGGSGEMVNLGARNGATLSSVAAAQSLRVLAVVLCVPAIFKYLLGDGAPALHASVVDWR
WLAVLLPLGAALAWLWQRLKQPNPWLFGPLLLSAVASVVWDLKIGLPNGASQLGQLLIGS
GLGCHFNREFFRRAPSFLARTLLGTALTMLIAALAALALSALTHLDLRSLTLGMMPGGIA
EMSLTAEVLQLSVPLVTAMQVMRLLFVLFLAEPLYRRWNKRLAD
>tr|A0A0Q0FIF2|A0A0Q0FIF2_PSEAP LysR family transcriptional regulator OS=Pseudomonas syringae pv. aptata OX=83167 GN=ALO85_04633 PE=3 SV=1
MRPGQAAPARSWKSRAAHGQRRSPVQRACHRRGRHAGQRLKSCPGSVRMSVNGRNDDAHH
NIKKAMICHPQHKKYFVMRRLNLNHLHTFSLVIAHGSFSAAAERLHLTQPAVSLQVRQLE
DQLKLQLIERVGKRLKPTSAGNVLLEHIARIDAVVEDALQALSSHASGIAGKIAIGTGAT
ACIHLLPPLLQALRRRFPELDVRVSTGNTEGILKAVEENLVDLALVTLPASGRSLHITPL
LEDEFVAIFASSQRPMPAHMTPERLSAQPLVVFEAGSSTRLLIDEWFLHAGIRVKPVMEL
GSIEAIKEMVAAGLGYSIVPRMAVAALHHRRGLQVLPLATALTRTLGIALRQDKPVSKAL
RQVLDALQDLKRS
>tr|A0A0Q0BVT3|A0A0Q0BVT3_PSEAP Lipoprotein releasing system, transmembrane protein LolE OS=Pseudomonas syringae pv. aptata OX=83167 GN=ALO85_01247 PE=4 SV=1
MFRPLPIFIGMRYTRAKRRKSFISFISMTSMIGLALGVLAMIVVLSVMNGFQREMSSRIL
GMVPHAVIAGNTPVDDWKPLADAALKNPEVTAAVPFTDMEGMLSYKGSMQPIQISGVDPA
LEHEVSIVTQHITRGSLEDLKPGEFGVVLGDITARRFRLSIGDKLTLIVPEVSSAPGGIT
PRMQRLTVVGIFKVGAELDGSMGLINIADAAQMQRWQPNQVQGVRLALKDLYKAPQVSTA
IAAGLGEGYRADDWTHTQGSLFSAMKMEKTMIGLLLLMIVAVAAFNIIATLIMVVNDKGA
DIAILRTIGATPRQIMAIFVVQGTVIGIVGTLIGGILGIIAALNVSSLVGWLERVSGQHI
FSSDVYFISNLPSELQAGDVLLICSAGFILSFLATIYPAWRAAQIQPAHALRYE
>tr|A0A0Q0DWX6|A0A0Q0DWX6_PSEAP ABC transporter OS=Pseudomonas syringae pv. aptata OX=83167 GN=ALO85_01904 PE=4 SV=1
MTQALIELSDLRFNWPGHPQLLDIPEFRLQSGETLFLKGPSGSGKTTLLGLLGGVQKPVS
GSIRLLNQELSALSSAARDRFRVDHTGYIFQQFNLLPFLSVRENIELPCHFSKVRAERAK
QRHGSVEKATTTLLAHLGLKDPAMLTRRADSLSIGQQQRVAAARALIGQPELVIADEPTS
ALDADSREAFIRLLFAECREAGASLLFVSHDQSLAPLFDRNLSLSDLNRAAVAVEI
>tr|A0A0Q0FN79|A0A0Q0FN79_PSEAP Carbohydrate kinase, PfkB OS=Pseudomonas syringae pv. aptata OX=83167 GN=ALO85_02688 PE=4 SV=1
MGQTRFATGRQLDLICLGRLGVDLYAQQVGARLEDVSSFAKYLGGSSANIAFGTARLGLR
SAMLSRVGDDQMGRFLVESLAREGCDVSAVKRDPERLTAMVLLGLKDRETFPLVFYRENC

ADMALRAEDIDEQQIASSKALLITGTHFSTDQVFKASSQALDYAEKHDVKRVLDIDYRPV
LWGLAGKADGETRFVADQKVSQHVQRILPRFDLIVGTEEEFQIAGGSTDLLAALRKVREL
TAATLVVKLGPQGCTVIHGAIPARLEEGAIYPGVQVEVLNVLGAGDAFMSGFLSGWLKEA
SDERCCQLANACGGLVVSRHACAPAMPTPAELDYLFNSPEPITRPDQDVALQRLHQVSVP
RKQWRQLFIFAFDHRGQLVELAQQAGRDLRSISQLKQLFVQAVERVEADLRKRGIEADVG
LLADQRFGQDSLNAATGRGWWVARPVEVQGSRPLAFEHGRSIGSNLLAWPQEQIIKCLVQ
YHPDDEPMLRLEQEAQIKALYDASKVSGHELLLEIIPPKDHPSPHPDVMLRSLKRLYNLG
IYPAWWKIEAQSAQVWQQLDELIQQRDPYCRGVVLLGLNAPVEDLAAGFAEARHSRVCQG
FAVGRTIFREPSRAWMAGEIDDATLISRVQSTFNWLIESWRESRA
>tr|A0A0Q0CG75|A0A0Q0CG75_PSEAP Uncharacterized protein OS=Pseudomonas syringae pv. aptata OX=83167 GN=ALO85_00801 PE=4 SV=1
MSAQRGLVLLARGGYAARGVVYLIIGLFAVLAAQGSSQPADSHSSLEALLSQPFGGVLVG
VVIVGLLAFAAWRVLQATRDVDHHGREFKGLVIRGGLLVGGFTYGALAFFALGLLVSGLK
SSGGSSDGGQAKDLLAAILSWDHSNLLVYVVALVPLGLGIVHIIKGYKASFEKYFEADED
VMKYVRPVSRFGLIARGVAFIEIAVLLAVSGSSYQAMHPPGMKDALNGLQDLPAGGLVLL
IVALGLIAFSVYSFAQAAWRRINMDVPDAPEAVARHFR
>tr|A0A0Q0DL57|A0A0Q0DL57_PSEAP AFG1-like ATPase OS=Pseudomonas syringae pv. aptata OX=83167 GN=ALO85_02608 PE=4 SV=1
MCLMDAVSPLQAYERAVQRGFQPDQAQLQAARQLQACYEGLADARGRARGVYLWGPVGRG
KTWLMDRFFESLSVPARRQHFHHFMRWVHKRMFELMGTSQPLTVVARELARDVRVMCFDE
LFVTDIGDAVILGGLLQVMFEEGVVLVCTSNQPPEQLYSHGHNRERFLPAVAAILKYMDV
VAVDGGEDHRLHPGQLHQRYWIAEAGRPSALQGIFEALSADQPVHDSQVMLGYRSINVVQ
HSDTAVWCRYRDLCEQPLAAMDFIALCDRFSVILLSEVPLLGAAQREAKIARGTEDGVEQ
VTAGDRELPQLSPNDDGVRRFIALVDECYDRRIPLYVEAPVPMDELYTEGYLSFAFRRTL
SRLQEMQLERFTES
>tr|A0A0Q0CZ09|A0A0Q0CZ09_PSEAP Peptidoglycan glycosyltransferase MrdB OS=Pseudomonas syringae pv. aptata OX=83167 GN=mrdB PE=3 SV=1
MKSNFDRILSSEDVMRRRATFLQRIHIDGPLLILLLTLAAGSLFVLYSASGKNWDLLIKQ
ASSFGIGLVAMVVIAQLEPRFMARWVPVLYVIGVLLLVVVDVMGHNAMGATRWINIPGVI
RFQPSEFLKIIMPATIAWYLSKRTLPPHLKHVAVSLALIGVPFILIVRQPDLGTSLLILA
SGAFVLFMAGLRWRWIISVLAAAVPAAVAMWFFFMHDYQKQRVLTFLDPESDPLGTGWNI
IQSKAAIGSGGVFGKGWLLGTQSHLDFLPESHTDFIIAVLGEEFGLVGICALLIIYMLLI
GRGLVITAQAQTLFGKLLAGALTMTFFVYVFVNIGMVSGLLPVVGVPLPFISYGGTSLVT
LLSAFGVLMSIHTHRKWIAQV
>tr|A0A0Q0CFX0|A0A0Q0CFX0_PSEAP S C beta-lactamase OS=Pseudomonas syringae pv. aptata OX=83167 GN=ALO85_100299 PE=4 SV=1
MAALNEAGIQRLDQVWRSFVEQGRIVGGVLLLACQGRLAYASARGWADREQQIPVTRRTR
FRLASLTKLLTSVAVLRLCERGVLDLHASVTHWLPAFRPRLANGREPLITLQHLLSHTSG
LSYGFEQAPDSAYQRAGVSDGLDRVAFDLQENLTRLARVPLLFEPGSAWGYSLATDVLGA
VVEQATGLKLSEAIDKWVTVPLRMHATSFRHLPEHQLASAYKDGLSGPQRIGDDDGVLLD
SAQARLSAARAFETDAYESGGAGMLGTADDYLRLLECLRLGGAPLLGEASTARLLGNAIG
QTSIASRGPGWRFGLGPMILTDPVLAGQRQGAGTWSWCGLYGSHYWVDPHAALSMVVLTN
TAVAGAWGEFADAIVDALYPRLGVRPGQIACGNCSG
>tr|A0A0Q0C2W3|A0A0Q0C2W3_PSEAP Orf12 OS=Pseudomonas syringae pv. aptata OX=83167 GN=ALO85_200150 PE=4 SV=1
MPDDVQRYLSSFFETVDGTNVAILYHSRLLGCSPMHSKLSWDERVCFHVMASPPKRRSPG
LAACKQ
>tr|A0A0Q0IM32|A0A0Q0IM32_PSEAP Binary cytotoxin component OS=Pseudomonas syringae pv. aptata OX=83167 GN=ALO85_02419 PE=4 SV=1
MLTPAALLENEDIARVTLKPVEYLNVIFDDEKSGGRSAGLVLTKEDILSLKRYERHALNI
PTSLSRVEQQLGFTKSGIPGLEPEDMLKTYKAINSHGKSWSGIEDGIKRTGFNIDLFAAQ
FSVQGQQIIDRIEKMDFARQLDLTVADLIIEEVRNTPPVPLEKNDQRVCSTLAEFLKKTA
SQIKNHQHAAETLEQHIDTFSSVLSVTLIPGINDKVKLAARSDLDQQIKELEKDIEQLTT
DIEQKNKEHKTALNTIAWSGFGGPIGVAIAGGIFGAKAEKIRKEKNRMVASKAQKVQLLK
EKVPLTAAVRSLQLMFDDMYIRMLDAHKGATHLKDLWLLLATYIERSAHELAAIKDDQAL
LIFAMQFQGVVMPWLEIKGKTTELLKIFDSALDQFKREQQPAIGIGR
>tr|A0A0Q0DH86|A0A0Q0DH86_PSEAP Acriflavin resistance protein OS=Pseudomonas syringae pv. aptata OX=83167 GN=ALO85_00674 PE=3 SV=1
MKERFNLSAWGLRHRTLVWYMMFVSLLMGSWSFLNLGREEDPSFAIKTMVIQARWPGATL
PDTLQQVTDRLEKKLEEIDALDYVKSYTLAGESTLFVFLKSETRSADIPAAWYQVRKKIS

DVRSELPSGIQGPAFNDEFGDVFGSIYAFTADGLSFRQLRDYVEQVRADIRSVPNLGKIE
LLGAQREVIYLNFSIRKLAALGIDQRQVLQSLQAQNSVTPAGVMEAGPERIAVRASGQFS
NEQDLEAVNLRFGDRFFRLSDLATIERRYADPPSSLFRFNGQPAIGLAVAMKQGGNIQAF
GTQLQQRIDDLTTELPLGIDVHLVSSQADVVEKAIGGFTHALFEAILIVLVVSFISLGIR
AGLVVACSIPLVLALVFVFMEYSGITMQRISLGALIIALGLLVDDAMITVEMMVTRLESG
DSLPQAATFAYTSTAFPMLTGTLVTVAGFVPIGLNSSSAGEYVFTMFAVIAVALLLSWLV
AVLFAPLIGVHILKASAQHAAPGRWMRGFSRLLVKALEHRGWVIGITLLMFIASLFAGRL
LQNQFFPDSDRPEILVDIYMPQNGSIEGTRQTMDRFEAALKGDADVLRWSSYVGKGAVRF
YLPLDQQLSNPFYGQLVIVSQGGEARDRLIERLRQRFRDDYVGVGGYVQPLNMGPPVGWP
VQYRVSGPDIEQVRSQAMALAAILDANPNIGQVIYDWNEPGKVLKIDIAQDKVRQFGLSS
EDVAQILNSLVSGTTITQVRDSTYLIDLVGRADSDERSSVQTLANLQIPTPGGASVPLLA
FATLSYEQEQPLVWRRDRLPTITLKANVLGTLQPAALVRQLKPDVDAFSADLPLRYSVAT
GGAVEASARSQGPILKVVPLMLLLVVSFLMIQLHSVKKLLLVVSVVPLGLIGVVAALLIS
GYPLGFVAILGVLALIGIIIRNSVILVTQIDEFIAAGESAWTSVVKATEHRCRPIMLTAA
AASLGMIPIAREVFWGPMAIAMIGGIAIATLLTLFFLPALYMVSYRIKPPAV
>tr|A0A0Q0C9A7|A0A0Q0C9A7_PSEAP Putative cAMP-binding/CBS domain signal-transduction protein OS=Pseudomonas syringae pv. aptata OX=83167 GN=ALO85_03855 PE=4 SV=1
MSTNNKNAEQAMSKADAFTQAGKTAVLQNIHGTMQFLQKFPPFNQMENAHLAYLVEQCQL
RFYAAGESIIKPGDGPVEHFYIVKQGRIVGERPHSAKGGTETTFEITTGECFPLAALLGE
RATRTEHLAAEDTFCLQLNKQAFIKLFALSTTFRDFALRGVSSLLDQVNQQVQQRAVETL
GTQYSLNTRLGELAMRHPVMCSPETPMRDAVRLMHEQQVGSIVIVDERQSPLGIFTLRDL
REAVADVNADFSAPVRHTMSLSPFHLSPDATAFDAAIAMTGRHIAHVCLVRDGRLCGVVS
ERDLFSLQRVDLVHLARTIRNAPRIEALAGLRGDIVQLVDRMLAHGASSTQITQIITLLN
DHTVCRVIELTLAEKGDPGIAFSWLCFGSEGRREQTLHTDQDNGILFEARDAAEAEQIRN
VLLPIAERINQNLAQCGFTLCKGNIMAGNPELCLSRLEWSQRFSTFIREATPENLLASSI
YFDLRVVWGDESSGEQLRRSILDQVADNRLFQRMMADNALRQRPPVGRFKDFVLARKGSE
KDTLDLKVEGLTPFVDGARLLALANGIGVSNTQERLRQLVEREVIDPLDGAAYEEAYHFI
QQTRMQQHQLQSRQNLAYSNRIDPDTLNHLDRRILRESFRQAQRLQTSLTLRYQL
>tr|A0A0Q0DQ40|A0A0Q0DQ40_PSEAP Two component sensor histidine kinase PhoQ OS=Pseudomonas syringae pv. aptata OX=83167 GN=ALO85_01967 PE=4 SV=1
MIRSLRLRLMLGAATLAVIFMLLMLPALQGAFSLALRGAIEQRLASDVTTMISAARVEND
HLLMPSVLPGEQFNLPGSRLLGYIYNRQGQMVWRSLSTEGENIDYRPQYDGQGSEFTKIK
EINGDEYFVYDVEIRLLGGRNAAFSIVAVQPLRGYQETIDGLRRKLYLGFGAALAVLLGL
LWMGLTWGLRALRGLSQELDQVESGVRDSLSEEHPSELLRLTDSLNRLLRSEREQRIRYR
DSLDDLAHSLKTPLAVLQGVSENIAKRPEDVEQAWVLQSQIERMSQQIGYQLQRASLRKS
GLVRHHVMLRPVVESLCNTLDKVYRDKQVKATLDLPEHCQVHMEEGALLEMLGNLLENAY
RLCLSEVRVSFTRSTTGDELCVEDDGPGVPQSQRARILERGERLDRQNPGQGIGLAVVKD
IIESYGAQLTLGDSPMGGAAFRIHFLAQ
>tr|A0A0Q0DA30|A0A0Q0DA30_PSEAP Metallophos domain-containing protein OS=Pseudomonas syringae pv. aptata OX=83167 GN=ALO85_02064 PE=4 SV=1
MNPYLAVTLAGEELWLLADKAIYYPAERSLLIADAHFGKAAAYRKLGQPVPHGTTQANLR
RLDSLLNAYPCDHLIFLGDFLHAPESHAVSTLATLQLWRAERSTLRITLIRGNHDKRAGD
PPASLGIDVVPEPLALGPFALQHEPDPHPDLHVLAGHVHPVYRLHGRGRQSLRLACFYLG
RQVSLLPAFGEFTGGFQIRPAPDSTIYVTGGDAVWRVA
>tr|A0A0Q0BZW8|A0A0Q0BZW8_PSEAP Putative ACR, YhhQ family OS=Pseudomonas syringae pv. aptata OX=83167 GN=ALO85_00369 PE=4 SV=1
MLFLIAYISSVVLINFAFSSAPHLDMIWSAWGGLVFVLRDMVQIRFGHGAIIAMLVALVL
SYVTSDPTIALASATAFAVSECIDWLVFSITRRPLRDRLWISSALSIPLDTFIFFGMIDA
LTAPVVLTALASKFAGVTIVWMIMAWRARNSACPG
>tr|A0A0Q0IB29|A0A0Q0IB29_PSEAP Uroporphyrinogen-III C-methyltransferase OS=Pseudomonas syringae pv. aptata OX=83167 GN=ALO85_00566 PE=3 SV=1
MSAKVWLVGAGPGDPELLTLKAVRALAEADVVLIDDLVNPAVLEHCPGARVIAVGKRGGC
RSTPQAFIHRLMLRYVRQGKCVVRLKGGDPCIFGRGGEEAQWLQERGVAVELVNGITAGL
AGATQCGISLTLRGISRGVTLVTAHTQDDSTLNWQALAQGGTTLVVYMGVAKLAEIRESL
LAGGMSGEMPVAMIENASLPWQRECRSSLNDMQQDAAAFQLKSPAILVIGAVAAHSNELL
NQTLTDHAQLNAG
>tr|A0A0Q0IRA6|A0A0Q0IRA6_PSEAP Uncharacterized protein OS=Pseudomonas syringae pv. aptata OX=83167 GN=ALO85_02054 PE=4 SV=1
MNTKYARTRSSMPTASDLTTAVEAFIARGGVIAVIPEGETAELTAPSSPRTKRAAEQRLE

VAGKVQQLRYLAGKGAGFTALQYSLRMNKRDVRRLATEHGVTINLSQPLIVPRSEALDDV
TDIDDVVAGHAMHYSALGYTASEIARILDLSLRQVFSIGKAYRFEFRPRQESD
>tr|A0A0Q0CYT0|A0A0Q0CYT0_PSEAP ATP-dependent helicase HrpB
OS=Pseudomonas syringae pv. aptata OX=83167 GN=ALO85_04836 PE=4 SV=1
MRHAGGSVSGDCRSAKTRRLIPQPHPGILSRISPGAPMTSLPIDAVLPALRQALSTRHEA
VLEAPPGAGKTTRVPLALLEETWLAGQTILMLEPRRLAARAAAERLASELDEKVGDTVGY
RIRLESRVGPKTRIEVVTEGILTRRLQDDPALEGVGLLIFDEFHERSLDADLALALSLNG
RELFRDDQPLKILLMSATLEGERLAALLDDAPVVRSDGRMFPVSMQWGRPFQPGEFVEPR
VVQTVLDALGSESGSLLVFLPGQAEIRRVNQQLAEALGERADILLCPLHGELDLNAQRAA
IEPAPKGTRKVVLATNIAETSLTIDGVRVVIDAGLARVPRFDPGSGMTRLETQRISRASA
TQRAGRAGRLEPGVCYRLWSEAQHDQLAAYGAAEILQADLAGLALQLARWGVTPAQLVWL
DVPPAAAYAQAQDLLVRLEALNNQPGQPPALTPHGQAMAELPAHPRIAHLLLRGHALGLG
ELACDVAALLGERDILRGAGADLHSRLTLLAGTERAARGAQGGVQRARQLSRQYRGYLRD
TANSPVGDPDHSRWLGALLALAYPDRVAQQRRAGGAEYRLANGRAALFAEADALMKQPWL
VIADLGSRQGQREERIYLAAEFDPALFDSVLAEQVISVDQIDWDEREGVFRAERQRKVGE
LIISREPLTGLDETTRSHALLALVRRKGLELLPWTPELRQWQARVALLRGLDIEKSSASE
WPDLSDAQLLATLENWLMPYLGKVTRLSHFSQLDLSSILRNLLPWPLPQQLEVQAPQTLQ
VPSGSNIRIDYSEHPPILSVRLQELFGLSDTPRIANGRQVLKLHLLSPARRPVQVTQDLA
NFWRSTYIEVKKDLKGRYPKHYWPDDPLVAEATARVKPRGT
>tr|A0A0Q0C354|A0A0Q0C354_PSEAP Rhs family protein (Fragment)
OS=Pseudomonas syringae pv. aptata OX=83167 GN=ALO85_04501 PE=4 SV=1
LDKLRGEVTYEYEANGRLLEHNPEKRFDGEEFRYDAAGNRLNFNTSRFDRVKDNRLKKWS
NHEYKYDAWGNLVEKIVGIVRWQTFTYDS
>tr|A0A0Q0DGQ0|A0A0Q0DGQ0_PSEAP ATP-binding region, ATPase-like protein
OS=Pseudomonas syringae pv. aptata OX=83167 GN=ALO85_02126 PE=4 SV=1
MSEETEQGLDFSMVIASTVHDMKNSLATLTQAHSQWQAKLSVQQRDTSEHGIIDYEFANL
NGMLVQLLGLYKLGVNQMPLRPDYHELDDFIEAQLAHHQDVLASRGIAARHAIDVASPLG
FFDRELIGSVVGNIIVNAIGFAREQISVSVGDEGGQLKITINDDGPGYPAYLIERQTDYV
QGINQGSGSTGLGLYFAAHIARLHVRNGMRGRIEIANGGVLGGAMFSMTLP
>tr|A0A0Q0C3F7|A0A0Q0C3F7_PSEAP Urea amidolyase-related protein
OS=Pseudomonas syringae pv. aptata OX=83167 GN=ALO85_00861 PE=4 SV=1
MIEVISATALATVQDLGRHGSLGYGVGTSGAMDTLSLRLGNLLLGNDEDAAGIEIPLFPF
EVRFVDDCTFAITGAACAASLDGERLPGNWVAQARKNQVLRLNYPTMGSRVYLCLAGGVD
VPLVLGSRSTQLRGAFGGWQGRALQQGDVIPAGAANEPRASDFGLQSSVAALPLMLDGLP
AIRVLPAAEFDCFSAEAQATFFAGEWKITTQSNRYGYRMEGTPLIAREALEIRSHGIVPG
VIQVPHGGQPIVQMRDAQPSGGYPKFATVIEADLWRLGQAPIGSKVRFVQCSYDEAVEAL
DTNHAFIEDARRMLALRSLQGHR
>tr|A0A0Q0FLU4|A0A0Q0FLU4_PSEAP Pseudouridine synthase OS=Pseudomonas
syringae pv. aptata OX=83167 GN=ALO85_05156 PE=3 SV=1
MVIICGMTNIAPPTPGVQLVEVAPELAGQRIDNFLITYLKGVPKTLIYRILRKGEVRVNK
GRIKPEYKLQAGDIVRIPPVRVPERDEPVPVAQALLQRLEAAIVYEDKALIVLNKPAGIA
VHGGSGLSFGVIEAFRQLRPDAKELELVHRLDRDTSGLLMIAKKRSMLRHLHEALRGDGV
DKRYMALVRGNWATALKQVRAPLMKSNLRSGERMVEVNEEGKEALTIFKVLRRFGDFATM
VEAKPVTGRTHQIRVHTLHAGHAIAGDTKYGDENFSREIRDLGGKRLFLHAYMLTVPMPD
GSKLNVQAPVDDMWAKTVERLSAP
>tr|A0A0Q0CVS9|A0A0Q0CVS9_PSEAP Putative Glucose ABC transporter,
permease protein OS=Pseudomonas syringae pv. aptata OX=83167
GN=ALO85_100000 PE=3 SV=1
MRLRPGFDTPHGILPMSSVAANSKASPMDALQRWLPKLVLAPSMFIVLVGFYGYILWTFA
LSFTNSTFLPSYKWVGLAQYARLMDNDRWWVASKNLAVFGGMFIAISLVIGVLLAVLLDQ
RIRREGMIRTIYLYPMALSMIVTGTAWKWLLNPGLGLDKMLRDWGWEGFRFDWLIDQDRV
VYCLVIAAVWQSSGFVMALFLAGLRGVDQSIIRAAQMDGASLPMIYWRVVLPSLRPVFFS
AVMILAHIAIKSFDLVAAMTAGGPGYSSDLPAMFMYSFTFSRGQMGMGSASAILMLGAIL
AILVPYLYSELRTKRHD
>tr|A0A0Q0DZ11|A0A0Q0DZ11_PSEAP 30S ribosomal protein S3 OS=Pseudomonas
syringae pv. aptata OX=83167 GN=ALO85_200052 PE=4 SV=1
MVIWLLPVAGFAVGLAYHLIGKPVDAGNNLIIDEIHDPKKIVPLRMVPMVLIGTVVSHLF
GASVGREGTAVQMGGALADQLTHVFRLRREDRRVILMAGISAGFASVFGTPLAGALFGLE
VLAIGRMRYDALFPCVVAAIVADQVGQAWGVVHTHYVIGEVVPVQLWSVMAVVAAGIVFG
LTGLLFATATHKLGAFVKRLITYSPLRPFAGGLLIAVAVWALGSNHYIDVDKYIGLGIPS

IVQSFQMPMAPWDWLGKMVFTVVSLGTGFKGGEVTPLFYIGATLGNALAPLLHLPFGMLA
GIGFVAVFAGAANTPLATIVMAMELFGPEIAPLAAIACIASYLVSGHTGIYHAQRVGHSK
HHRPLPEEIRLSDIKQFHAQSESASERKVTLAGEEK
>tr|A0A0Q0FKH5|A0A0Q0FKH5_PSEAP Prophage PSSSM-04, putative lipoprotein OS=Pseudomonas syringae pv. aptata OX=83167 GN=ALO85_00441 PE=4 SV=1
MHRCAIDFIARHWWRRLEIWLISMLLIAGCLMLGFQAGQWSANAEHTQQLAEVRNAYDAA
LGKRDRRLDRLAETTTQVADRVESAASIANQAAHAASRAADKADEALCKANQ
>tr|A0A0Q0FK48|A0A0Q0FK48_PSEAP Glycerol-3-phosphate dehydrogenase [NAD(P)+] OS=Pseudomonas syringae pv. aptata OX=83167 GN=gpsA PE=3 SV=1
MTTQQPVAVLGGGSFGTAIANLLAENGHQVRQWMRDPEQAEAIRVNRENPRYLKGIKVRP
EVEPVTDLTAVLDASELIFVALPSSALRSVLSPHVERLNGKMLVSLTKGIEAQSFKLMSQ
ILEEIVPQARIGVLSGPNLAREIAEHALTATVVASEDEALCQEVQAALHGRTFRVYASND
RFGVELGGALKNVYAIIAGMAVALDMGENTKSMLITRALAEMTRFAVSQGANPMTFLGLA
GVGDLIVTCSSPKSRNYQVGFALGQGLTLEEAVTRLGEVAEGVNTLKVLKVKAQEVQVYM
PLVAGLHAILFEGRTLSQVIEALMRAEPKTDVDFISITGFN
>tr|A0A0Q0CED4|A0A0Q0CED4_PSEAP Serralysin OS=Pseudomonas syringae pv. aptata OX=83167 GN=ALO85_05124 PE=4 SV=1
MCSMCDALTQLWDEGATGQGAAKQTATNQAALGHLTLDQQANYLTNGYWHDAYGGSQHHF
DVHAGGSLTVNLASLSASAQVVARYALQTWTNVSGLNFVETTAAAAINFSEQKSGAYSNS
NYAGSIISDSSVNIASDWVKYGLYYQQTYIHEIGHALGLGHAGNYNGSATFPNNAFYQED
SWKYSVMSYFSQDENTYSNASFGYVATPMLADIVAIQSLYGTAVTRTGDNTYSFNKTSIN
TGTDFVPGLVATIYDSGGNDTINVSTYVGAQTVDLRSEAFSSLYGGLSNIAIARGTVIEN
AITGAGADTLIGNASDNFLNANAGNDQLQGGDGNDRLMGGAGSDVLNGGNGIDTALYTEA
GARYFATYDTALVRNATLSVHDAQTSDVDTLSSVERLSFSDRNASLDELLMAFHSRYGAF
NAESDATVSLSFSTDLHHIALTEDQADIARLYSLFGRTPDYQGLKNWLTQQAIGSSYAEI
RDGFLNSIEGMQHYSGLGDRDFVLDLYQTVLHRTGEESGVSSWNTLLQAGVSRAAVADGF
LNSRESRDLSEGETGFIRIVAHNAWNNLDMVVGKGVATGTAGDDQISEQEVRLDSNAVSH
LAGNAGIDTFIFNDAASAYTISALDTGTLSVSRSTGAAAKFELSGFNVLDFADRELFVLD
SAQANIGRLYTILDRAPDIEGLKFWLSHGAAGATDAQVAGGFVQSAEFSQSLPNGSSNTA
FVEHLYHNVLDRGSDANGLAYWVQSLDGGTSRGQVAFNIANSAESAALTQGDAGFIHLVG
HADWV
>tr|A0A0Q0IKA9|A0A0Q0IKA9_PSEAP Rhs family protein (Fragment) OS=Pseudomonas syringae pv. aptata OX=83167 GN=ALO85_04433 PE=4 SV=1
RYVEHAYNPLHRRYEYDPAGELSRTLDKQRGEVTYEYEANGRLLEHNPEKRFDGEEFRYD
AAGNRLNFNTSRFDRVKDNRLKKWSNHEYKYDAWGNLVEKIVGIVRWQTFTYDCENRLVK
>tr|A0A0Q0D5Q3|A0A0Q0D5Q3_PSEAP Glycosyl transferase, group 1 OS=Pseudomonas syringae pv. aptata OX=83167 GN=ALO85_02657 PE=4 SV=1
MRILWTLPYLPWPTTSGGKTREYHLLRNLAARGHRITLLVQSKNPLDDDARAALEPWLER
LIVIDRRPLLSLRTLLAVAFAAPPMLASVNGYAPQLEKVFEQLLEEDWDIIQLQHTYAFQ
PFERALKRSGKPFVLTEHNVESDLGAASYDRLPRWANAFAVYDRWRYRRWETRVFRQTGE
LIAVTEDDAKALSRLSGRATSYVVNSVDCDYYASVHADRHSHRLLFIGNYEYGPNVDAIE
WALDEIMPKVWALAPHVRFAIGGFGMPTAWRERWPDPRIEWLGFVPDLRALQATASMFFA
PLRQGGGSKLKTLEAMAAGLPVVTTAQGVSGLAVRHGEHYLGSEDASGLATLIAEYTDKP
LQLEQIGEAGRVYVRARHDWSVSAAQLEVIYTRFSFSKQGLRSCV
>tr|A0A0Q0FUP8|A0A0Q0FUP8_PSEAP Uracil-DNA glycosylase OS=Pseudomonas syringae pv. aptata OX=83167 GN=ung PE=3 SV=1
MFLPDVTMTSDDRIKLEPSWKNALRDEFEQPYMAQLREFLRQEHAAGKEIYPPGPLIFNA
LNSTPLDNVKVVILGQDPYHGPNQAHGLCFSVQPGVPTPPSLVNIYKELKRDLNIDIPNH
GCLQSWADQGVLLLNTTLTVERANAASHAGKGWQHFTDRIIQVVSEHQPHLVFLLWGAHA
QSKQKLVDATKHLVLTSVHPSPLSAYKGFLGNGHFGRANKYLEQNGIAPVDWRLPAL
>tr|A0A0Q0BXF4|A0A0Q0BXF4_PSEAP Lipoyl synthase OS=Pseudomonas syringae pv. aptata OX=83167 GN=lipA PE=3 SV=1
MMTDTVQTLIPTLDVSERVARPKVEAGVKLRGAEKVARIPVKIIPTVDLPKKPDWIRVRI
PVSPEVDRIKQLLRKHKLHSVCEEASCPNLGECFSGGTATFMIMGDICTRRCPFCDVGHG
RPKPLDVNEPKSLAIAIADLRLKYVVITSVDRDDLRDGGAQHFADCIREIRLLSPGVQLE
TLVPDYRGRMDVALEITAAEPPDVFNHNLETVPRLYKAARPGSDYQWSLTLLQRFKQMVP
HVPTKSGLMLGLGETDEEVIEVMQRMREHDIDMLTLGQYLQPSRNHLPVQRFVHPDTFAW
FAEEGYKMGFKNVASGPLVRSSYHADEQAKIAKAML
>tr|A0A0Q0C4H5|A0A0Q0C4H5_PSEAP Histidine kinase OS=Pseudomonas syringae pv. aptata OX=83167 GN=ALO85_100081 PE=4 SV=1

MANRCCRPCMAWAIAWPRAGMEFKQSLAQRIIIAFALMSALVAGSFAIGIISTVHLVEEK
LISAGLGGDLNRLMLMDSVSDWSHRPKPDQLFYFTNGPGDFDLPKDIRHLEPGFHEVFRG
PLSYHAMIEVVDGRHYALLQDQSDFEERERVLFAVVLVGFVLALALAVFLGWVLARRVMA
PVVRLARQVRHRDQLLGLAPPLAPDYAADEVGELAVAFDATLGRLRQALIRERMFTSDVS
HELRTPLMVLASSCELLLENPALDLRGRRQVERIGRACEEMRDLVQTFLMLARTQREDPA
MTPKATLVGVAEQLISQWRDPIESKGLQLTYTPPADDQLSDIRYNATFLHAVMGNLLRNA
LHYTDHGFIALTLHAESFTVEDSGVGIPEEKREAMFEPFVRGNEQRGEGLGLGLSLVQRI
CTNQGWSVDLTPMEPHGCRFKVTLNVIKP
>tr|A0A0Q0DI91|A0A0Q0DI91_PSEAP Uncharacterized protein OS=Pseudomonas syringae pv. aptata OX=83167 GN=ALO85_03992 PE=4 SV=1
MAVGGRSWGSVSDALSVFRALLAVVMLAPMLAQAADAPEPRLYRYVDSRGVTVIDTQGVP
PDYVAKGYEVLNSRGRVVQVVPPAPTAEQIRQSDADKRQAAADAQLLSLYSSVAEVDRMK
ARKLAELDALVGVAQGNIQGLAAQQRSLQGQAADLERAGRPVQQALVDQLNDLRDQQQKL
QADIAGYQAARVKAEADFAVDRARVQRLTQ
>tr|A0A0Q0DK97|A0A0Q0DK97_PSEAP NADH:flavin oxidoreductase/NADH oxidase OS=Pseudomonas syringae pv. aptata OX=83167 GN=ALO85_04547 PE=4 SV=1
MEDRVMAFEAMFQPIQIGKLTIRNRVLSTAHAEVYATDGGMTTDRYVKYYEEKAKGGIGL
AICGGSSSVAIDSPQSWWKSVNLATDRIIPHFQNLADAMHKHGAKIMIQITHMGRRSRWD
GDHWPTLLSPSGIREPVHRATCKTIEPEEIWRVIGNYASAAARAKAGGLDGVELSAVHQH
MIDQFWSPRVNKRTDEWGGSFENRMRFGLEVIKAVRKEVGPDFCVGLRLCGDEFHPDGLS
HEDMKQIAKYYDDTGMIDFIGVVGSGCDTHNTLANVIPNMSYPPEPFLHLAAGIKEVVKA
PVLHAQNIKDPNQATRILEGGYVDMVGMTRAHIADPHLIAKIKMGQIDQIKQCVGANYCI
DRQYQGLDVLCIQNAATSREYMGVPHIIEKSAGPRRKVVVVGAGPAGMEAARVSAERGHD
VTLFEKKDFIGGQITTASKAPQRDQIAGITRWFQLELARLKVDLRLGTTADAATILDLRP
DIVVLAVGGHPFLEQNEHWGAAEGLVVSSWDVLDGKVAPGKNVLVYDTICEFTGMSVADF
LADKGCQVEIVTDDIKPGVAIGGTSFPTYYRSMYPKEVIMTGDMMLEKVYREGDKLVAVL
ENEYTGAKEERVVDQVVVENGVRPDEAIYYALKEGSRNKGQIDIDALFAIQPQPSLSQPG
DGYLLFRIGDCVAQRNTHAAIYDGLRLCKDF
>tr|A0A0Q0C4I8|A0A0Q0C4I8_PSEAP Ferric siderophore uptake system, TonB family protein OS=Pseudomonas syringae pv. aptata OX=83167 GN=ALO85_03978 PE=4 SV=1
MISTRQKLTRYSGSLLLVLAVHAIAIIVAVRWPAPQPVELPPAAMMVELAPVPEPAPPPP
PKVVQPPPPPAPEEPTPVPEVVQAPKPEIVVPKKVEKPKPKPQPPKPQKKPEPPKEKPAD
EKPVDTPPTTAQPAKPVAPKPAPAPSPPSNALPNWQGDLLGHLSKYKKYPEDARRRGMQG
VARLRFVVDADGNVLSYSIANSSGSSALDRATMEMIRRAQPLPKPPKEILNNGTIEILAP
FVYSLDKR
>tr|A0A0Q0CFQ5|A0A0Q0CFQ5_PSEAP Zinc-containing alcohol dehydrogenase superfamily protein OS=Pseudomonas syringae pv. aptata OX=83167 GN=ALO85_03777 PE=4 SV=1
MSELFKDDQSKLEAHMFKGILIDKDEAGYHVNLTDIDEARLPSGDVTINVSHSTLNYKDA
LAITGQSPIVRSFPMVPGIDLAGTVEASDTPEFKVGDKVLLNGWGVGEGHWGGLAQKARL
KSEWLIPLPQAFTPAQAMAIGTAGYTAMLSVLALENHGLTPDHGDVLVTGANGGVGSFAI
AILARLGYRVIASTGRASESDYLQQLGAAQIIDRQTLSQPGRPLAKEQWAAAVDSVGSHT
LANVCAGIRYRGVVAACGLAQGMEFPGSVAPFILRGVTLAGIDSVTRPRADRLEAWSRLA
SDLDMSLLPLISREVGLSEVIDLAPQLIGGHVRGRIVVDTAR
>tr|A0A0Q0DQM4|A0A0Q0DQM4_PSEAP Uncharacterized protein OS=Pseudomonas syringae pv. aptata OX=83167 GN=ALO85_200090 PE=4 SV=1
MSFDAFMKVDGVEGESLDDGHKGWVELLSYHYNAMQSISQTASSSGGATAGGVSLGDFQI
SKYLDRATPKLFELCCRGSHIKNVTIRIHRAGTEKFKYLDIVLEEVLISLVSGQGADQSG
FPVEVVNLNYGRIKFEYSQQRRADGGSAGIVSGGWDRTSNRPYA
>tr|A0A0N8TA35|A0A0N8TA35_PSEAP 50S ribosomal protein L15 OS=Pseudomonas syringae pv. aptata OX=83167 GN=ALO85_200076 PE=4 SV=1
MSHDNALILSLATSLIANADPLASSTHLPPARFFEDGTALNRLLLEAPYMARCSDDKTAT
RVRPREYALRYPYMQVNRPGMVSWLVFDLDHANALAWDDAGLPAPNLMVRNRKSGHSQLF
YAVPSVCTTENARAKPIQYMKAVYAAFAARLDADVDYHGGPVAKTPGHPWWETTEFHSHV
YELGELASAVELTVKPWATGPKLDQVSHSRHCILFEQLRYFAYSIVNRERELGSFESFMR
SLDAYAYNHNSFLKQGFSENLPLSSIRATVKSVGRWTWDRYTGDRRCHRGAMQLDGSLSL
TERQSLAAKRTNELRHKATESKIRAACRQLQDQGKALVRSAIATLAGVSVRTVASYTHIL
TEVSQPATVSVLRGNRKAVSVLPPQDRSPSTPPVQPAVGQPADGSVSGVQSGVHQISAVP
RGAQAGSAREIQHEDDS

>tr|A0A0Q0ILA7|A0A0Q0ILA7_PSEAP Uncharacterized protein OS=Pseudomonas syringae pv. aptata OX=83167 GN=ALO85_02749 PE=4 SV=1
MKPMTQEPSTLYAKLLGETASITWTELQPFFAKGALLWVELPLDLIEVAEAVAENDAARV
SAWLAEGRVGKVSETKALELVETDPLLWAVVVAPWVLIQNRAQD
>tr|A0A0Q0BT01|A0A0Q0BT01_PSEAP Uncharacterized protein OS=Pseudomonas syringae pv. aptata OX=83167 GN=ALO85_02282 PE=4 SV=1
MGLSSTFYTSVTPVPRIITDETPAKVVDEQQVKLFGSPKERLDFYRREIHYETTNLSNRT
NAYLSAQSFLVIAYASSMANMNTAWGAMFTLVVPPMLALLGLLSTLFAWPGIRAACDIIQ
HWHHKQAQLLLSEPVIGLTYDDSPLFSDWESSETGPKKSLLFSKRSPWLFSFFWVFLGGF
AVFVQLVAD
>tr|A0A0Q0FW71|A0A0Q0FW71_PSEAP MOSC-like protein OS=Pseudomonas syringae pv. aptata OX=83167 GN=ALO85_05351 PE=4 SV=1
MAFVVAGGADLRSGIVRASRRSGFSAYQSGAQCRFRCWNQFMSPLQELLADVPQQGRVRW
IGVRPQSRVEMIELDAVEARREAGLTGDHSRPGPRNARQVTLIQWEHLAVVGSLLNRAAD
NPIVPQDLRRNLVISGINLFSLKNRRFRIGQAIFETTGWCQPCARLEQRLGHGTFQAVRG
HGGITARVLQSGIIRLDDTLQVEPLEITDPWPPARQHA
>tr|A0A0Q0FF59|A0A0Q0FF59_PSEAP ArsR family transcriptional regulator OS=Pseudomonas syringae pv. aptata OX=83167 GN=ALO85_03071 PE=4 SV=1
MEYGPCISQIATLLADPKRSAMIWALMDGTARPADELAILAGLSTSSAGAHLARLTSGGL
LKQEVRGRKRFFRLAAPEVGAAVEALACASLASAEQINRRFAQPTPPLPLRRARICSDHL
GGEMAAELYQRLLEAGWIEQQEKRVEVTSKGVARFAERGIYIPALAQRKRETVCVCPKWN
DRNPHLGGALGAGLLQLFIQMGWLRTTEESSTLQVSSSGQREISKIAAAA
>tr|A0A0Q0DGE1|A0A0Q0DGE1_PSEAP L-sorbosone dehydrogenase OS=Pseudomonas syringae pv. aptata OX=83167 GN=ALO85_03561 PE=4 SV=1
MFKLRPHYAVLLLVAGGLAACGESSSLQVSDGTGPSPKLPEPNKTLIPTVNIAPAVGWEK
GAKPVAAPGTQVAAFAENLDHPRWLYVLPNGDVLVAETNSPAKPDDSKGIRGWVMEKVMG
RAGAGVPSANRITLLRDVDKDGVAETRTVFLQNLNSPFGMTLVGNKLYVADTDRLISFPY
ESGQTSISAQATKVVDLPGGTLNHHWTKNVIASKDGSKLYVTVGSNSNVAENGMDKEEGR
AAIWEVDAATGNHRIFASGLRNPNGMDWEPKTGKLWTAVNERDEIGSDLVPDYVTSVQDG
AFYGWPYSYYGQHVDERVKPQNPALVAKAIAPDYAVGPHTASLGLVFADGKTLAAPFNEG
LFIGQHGSWNRKPHSGYKVVFIPFSGGKPNGTPVDVLTGFLNKDEKAMGRPVGVVNDQRG
GLLVADDVGNKIWRVTSAKTAQ
>tr|A0A0Q0BUT6|A0A0Q0BUT6_PSEAP Short chain acyl-CoA dehydrogenase OS=Pseudomonas syringae pv. aptata OX=83167 GN=ALO85_00063 PE=3 SV=1
MHDLELSEEQVMIRDMARDFARNEIAPYAQAWEKAGWIDDALVAKLGELGLLGMVVPEQW
GGTYIDYVAYALAVEEISAADAATGTLMSVHSSVGCGPVLNYGTDEQKHTWLEKLATGRA
IGCFCLTEPHAGSEAHNLRTRTELQGDEWVINGAKQFVSNGKRAQLAIVFAVTDPQLGKK
GLSAFLVPTENPGFVVDRSEHKMGIRASDTCAVTLNNCRIPAANLLGERGKGLSIALSNL
EGGRIGIAAQALGIARAAFEAALAYARERVQFDKPIIEHQSIANLLADMHTRINAARLLT
LHAARLRSAGQACLSEASQAKLFASEMAEWVCSKAIQIHGGYGYLEDYPVERYYRDARIT
QIYEGSSEIQRLLIARELRHYLV
>tr|A0A0N8T7W5|A0A0N8T7W5_PSEAP Ultraviolet light resistance protein B OS=Pseudomonas syringae pv. aptata OX=83167 GN=ALO85_04134 PE=4 SV=1
MGRDRIVGCRAVESSQSLTMNKVFCLVDCDCFYVSAERLFDPTLIGVPVVALSNSDGCVV
SRCPIAKSKDSANGGLGIKMGAPFFEIKHLMKSHGLRVVSSNYALYQEVSNRVMRVLETF
APRIEVYSIDESWLDFSGVPGDLEVLGREIQTAVKKQTGIGVGVGFGPTKTLAKAANFSA
KKWRSQTGGVVVLMDEIRRDKLLNWMPVDEIWGIGRKLSARLEAMGIKKAIDLQRYDRKS
LRKLFNVNVEKTSMELAGVSCYSLSDGPEPKQTIASTRSFGERVTTLQGLREAVATYASR
ACAKLRSEGQLSSCLQVFIQTSRFDDKPYSRAAICGLVTPSNDTRLFVAAALEGLQKIYV
DGYRYAKAGIVLSQFCSGEGYQPDMFAPEPRRNSDVLMTVVDGINARYGRGSIRLAVEPP
IPQWGMKRDFLSDSYCTDWAQLKRVKMT
>tr|A0A0Q0C203|A0A0Q0C203_PSEAP Uncharacterized protein OS=Pseudomonas syringae pv. aptata OX=83167 GN=ALO85_03890 PE=4 SV=1
MTDQPPASTGTPCESPVDSAAPDLEAEAVIAFLLEHPDFFAEHDELLVSMRIPHQRGDTV
SLVERQLKLLRERNIEMRHRLSQLMDVARDNDRLFEKTRRLNLALMDATSLEELVIAVED
SLRQDFQVPFVSLILFSDSPMPVGRWVSSAEAQKAIGGLIGEKIICGALREHELAFLFGA
EQGKEVGSTAIASLNHLGLHGVLAIGSRDPQHYKSSVGTLFLSYIADVLSRLLPRFAHSL
RSVR
>tr|A0A0Q0IQF5|A0A0Q0IQF5_PSEAP Protein SprT OS=Pseudomonas syringae pv. aptata OX=83167 GN=sprT PE=3 SV=1

MPEQLNSRVETCYQQAEAFFKRTFKRPVVSFQLRGQKAGVAHLHENLLRFNPQLYKENAE
DFLRQTVPHEVAHLIAHQLFGGSIQPHGEEWQLIMRGVYELPPNRCHTYAVKRRSVIRYI
YRCPCPDSDFPFTAQRHGMVRKGRRYLCRRCREPLVFSGETRTE
>tr|A0A0N8T9W6|A0A0N8T9W6_PSEAP Nickel transport system substrate-binding protein OS=Pseudomonas syringae pv. aptata OX=83167 GN=ALO85_00793 PE=4 SV=1
MLSSKPFLALGLLGALFASHVNAHGLWTEQRRGNIEVVYGHGAEDNAFKAQKVSGAWAYD
LQGKMIPVTVQRLEDHARLVPLKPPAVMSVALDNGMWTRNAEKQWINEGRSKVPNGTDSI
HTFKYSVAIYAEGAHLPTFNKLNFVIVPQTDPLKVGAGKPLPVRVLIDGKPAAGIKLIGD
YRSAPDVVSAETDAQGLASVIVRNEGLNIIAAEVTLPVKDNADIAERGLFTSLTFVGEAH
HE
>tr|A0A0Q0DZF0|A0A0Q0DZF0_PSEAP Uncharacterized protein OS=Pseudomonas syringae pv. aptata OX=83167 GN=ALO85_03439 PE=4 SV=1
MIRQIPKIALFVGALALAGQANADRGWGGPAVVGAIVGAAVVGAAVSSGRDRTVYVEREP
VYVQQRPVYYAPPPVYYQPAPVYQVVERYPPPPPPGYYHHYYGPPPGPGYYYGPPRGRW
>tr|A0A0Q0FUV2|A0A0Q0FUV2_PSEAP Uncharacterized protein OS=Pseudomonas syringae pv. aptata OX=83167 GN=ALO85_101577 PE=4 SV=1
MDHRLGGSRQGVGMVLEKPASAGFLLGAIKSSLSPGYLRSAFLLRRFSRCTSRSLRRRIS
>tr|A0A0N8T8S9|A0A0N8T8S9_PSEAP Putative D-amino acid dehydrogenase, small subunit OS=Pseudomonas syringae pv. aptata OX=83167 GN=ALO85_05297 PE=4 SV=1
MTPQKSDVLIIGGGFMGASSAFFLRQHGRSVTLLERDQIGQYASGVNFGNVRRQGRFLGQ
LELSNRSWALWKRLPELIGEDLEFIPSGHMRVCYREDEIAELEAYAAAPEARELDLQILS
GKALHDRFPFLGAQVKGGSYAPHDGHANPRLAAPAFARAAIRAGARIEERTEVTEVQKVG
GEFQVTTADGQLFVAEQLLITAGAWGARLAEQFGESVPLEPNGPQMSVTEPVPYALPTVI
GVFTRIKEEVIYFRQIPRGNIIGGGNRNRPDMLNRRAYFKPESLINQMKQMKRLLPGAE
KLNIIRVWSGIESYTPDSLPIMGRSGKVDGLFYAFGFCGHGFQLGPGVGDVMAELISTGS
TRTLISPFDIRRFTDPTAIEMPLMSSLMSTGKLV
>tr|A0A0Q0C753|A0A0Q0C753_PSEAP NADH:flavin oxidoreductase/NADH oxidase OS=Pseudomonas syringae pv. aptata OX=83167 GN=ALO85_01051 PE=4 SV=1
MTLFNEFKLGNTTLNNRVVMAPMTRSRAPEDIATEQIALHYTQRGTAGLIVSEGTPISRE
GQGYLFNPGIYTPEQIKGWKLVTDSVHSVGGRMFAQLWHVGRVSHTSIQIDGKAPVSATT
KQAQGAVAFGYGEDGEPGFVPTSVPRPLTTEEVAQVVEDFAQAAQNAIDAGFDGVEIHGA
NGYLLEQFLNPLVNDRTDQYGASNLQDRLRFVFEVVDAACARIGADRVGIRISPYGQLFD
MPLYPEIDETYSALCAGMRERGIAYVHVMDQTHFFMAGESSAAQEQALRKLLKHCKAELG
NTALILAGDMTRERADALLEENLIDLAAFGQPFIGNPDLVARLKNGWPLTTPDRDTYYGG
DAHGYIDYTPYRA
>tr|A0A0Q0FUI3|A0A0Q0FUI3_PSEAP Nuclease SbcCD subunit D OS=Pseudomonas syringae pv. aptata OX=83167 GN=sbcD PE=3 SV=1
MRLFHTSDWHLGQNLHGQERDFEHACFLTWLLARLADRKPDVLLIAGDIFDTVNPPVKAQ
ERLYDFIVNAHEQQPLLTIVMIAGNHDSGSRIELPAPLMRRLRTHALGRVMWLDDGVLDA
ERLLLPLPDANGDIKAWCLALPFLRPAEVTGPTLGDDYLRGIGRVHELLIAAADLKRQPG
QALIAISHAHMAGGSVSEDSERSLIIGNAEALPASLFGPSITYVALGHLHKPQRVNGEDR
IRYSGSPIPLSFSEISYQHQILEINCDGETLASVEPLLIPRAVNLQRLGPTPLADLLVQL
KALPDIDLLADPDRQPWLEVRVRLDEPQPDLRNQIENALQGKAVRLVRIGAEYAGKGSAD
GSEGNATLIELDQLTPQELFSRAWQDNFGSEVDEQTLTDFATLLREVQQESEQP
>tr|A0A0Q0CZC5|A0A0Q0CZC5_PSEAP Chromosome segregation ATPase-like protein OS=Pseudomonas syringae pv. aptata OX=83167 GN=ALO85_04753 PE=4 SV=1
MGGFNARVQCSQYLAKRVCWVGMAAACCGVQSMSVPFYRAFEDRYRGSRELIHERQQVYV
PFLKPLHQLYPDGQALDLGCGRGEWLEILIQNGFQALGIDLDVGMLEACTALGLPVENVD
ALEKLRSLPDESMTVISGFHIAEHIPFGDLKVLVAEALRVLKPAGLLILETPNSENLVVG
TQTFYLDPTHTCPIPHLLLSFLTEYSRFSRSQLLRLQEPVALSEGGPVDLFSVLHGVSPD
YAIVAQKAAAPEQLEIFDAVFGKEYGMSLESLSRRYDSQVAGWIEKSDATCAELREQLNQ
VQQRGQELEQIVRALEESGTATETELREQLNQVQQRGQELEQIARTLEESGAATHAELRD
QLSHVQEQGKHLESSVHALQNNDEIEARIREVTLRAELAESRVHSFQLGQEAAQLRMNES
EARLNEAFSYLKELQAQVTQLQQDTGVTKVTDAVSAQARVDELQARLKESLDNAHHWWLK
ANENQVEQAQMHEIQAQLDQSLNNAHHWWLTATAYEARIAQFENSRSRWRITRPLRTSASL
SSKASRVPSSLFKRVVRSVLARSIRFVLNRPRLRTRLVNKAQAYPNFLNSVRQFALRHGI
IQPRPHEVTPVTAATEATDDGFPTASPRVARVYSDLKLAFERKENR

>tr|A0A0Q0C0M3|A0A0Q0C0M3_PSEAP Fusaric acid resistance protein region OS=Pseudomonas syringae pv. aptata OX=83167 GN=ALO85_03045 PE=4 SV=1
MLALYIALLMEMPRPYWAMATVYIVSSPFVGPTSSKALYRAAGTLAGAAASVVLVPMFVQ
TPLLLAIVVGLWTGTLLFLSLHLRTANSYALMLAGYTMPLISLPVVDNPQAVFDIAVSRT
EEIFLGIICAAVVGAMFWPRRLAPVFQATTEKWFSDAATYSQRFISRTCQPEEIGTLRNS
MVGSFNSLEMMIGQLSHEGARKQTVRNANELRGRMIHLLPVIDALDDALWALERRTPELL
ASLKPLLHKACDWLESTADGPQVEQWQQLHRDLQSLQPGSAQLDDRNQLLLSNTLFRLGE
WVDLWLDCRTLQYAIKTDDQSQWRAVYRHWRLGRLTPFLDRGLMLYSVTSTVLAIIAASV
LWILLGWKDGASAVALAAVSCSFFAAMDDPAPQIYRFFFWTLLSVVFASLYLFVVLPNLH
DFPMLVLAFAVPFICVGTLTVQPRFFLGTLLTIVNTSSFISIQSAYDADFMNFLNANLAG
PAGLLFAFIWTLVFRPFGVELAVKRLTRFSWRDIASLSENASLAEHRRMGVQMLDRLMQQ
LPRLTLTAQDTGIALRELRVALNMLDLLAYTRRATPAAQVLLRQVIEEVSGYFKQCRKAG
ERLPAPRGLLMAMDRARRSLTDQEMGDNPARLHLLHALSGLRLALLPGVEIVTVGGELTE
QLPHNIDGAPL
>tr|A0A0Q0FGQ1|A0A0Q0FGQ1_PSEAP Rhs family protein with PAAR motif (Fragment) OS=Pseudomonas syringae pv. aptata OX=83167 GN=ALO85_05388 PE=4 SV=1
MFSTFPVTLPDDNWLALKYDHLSRLIEETDPLGRKITYKHHLATTLVTQTTFPDGSTWKA
RYDSRGNLLVETDALGNKTEYLNSEDGLPHTIIDATYKSKYLWWNSLAQVVHFQDCSGKD
TYYRYDDRGHLIAVTDALNNTTTLERKPDGEVLRINHPDGSVESFTYNALGQVLSHTDGK
GQITRLSRNARGLQTRREDAKGKAVVYQYDKAIRLTALTNENNATYNFVYDNADRLIEEK
RIDNLTRRFSYNLGGHLTQVEEIGYGDKGEQPRRSTHLERDPIGRLLAKLNDDARQDYAY
DDGDRLLSIERKPTDTGRKLGVAAEKLEFAYDLLGRLVKETTPQGALAYDYDPLSNLTTL
TLPTGQHLNHLYYGSGHLHQLNLDGQLISDMERDDLHREIYRTQGKL
>tr|A0A0Q0D6T6|A0A0Q0D6T6_PSEAP Acriflavin resistance protein OS=Pseudomonas syringae pv. aptata OX=83167 GN=ALO85_00954 PE=3 SV=1
MNMSRLFILRPVATTLSMLAIVLAGLIAYTLLPVSALPQVDYPTIRVMTLYPGASPQVMT
SSVTAPLERQFGQMPGLTQMASTSSGGASVITLRFSLEINMDVAEQQVQAAINAATNLLP
TDLPAPPVYNKVNPADTPVLTLAITSKTMLLPKLNDLVDTRMAQKISQISGVGMVSIAGG
QRQAVRIKVNPEALAANSLNLSDVRTLISASNVNQPKGNFDGPTRVSMLDANDQLKSPEE
YANLILAYKDGAPLRLKDVAEIVDGAENERLAAWANRSQAVLLNIQRQPGANVIEVVDRI
KALLPSITENLPAGLDVVVLTNRTQTIRASVTDVQHELLIAIILVVLVTFLFLRRFSATI
IPSIAVPLSLVGTFGVMYLAGFSVNNLTLMAMTIATGFVVDDAIVMLENISRHIEEGETP
LQAALKGAKQIGFTLISLTLSLIAVLIPLLFMADVVGRLFREFAITLAVAILISLVVSLT
LTPMMCARLLKREPKEEEQSRFYRASGAWIDWLIDIYAGRLRWVLKHQPLTLLVALATLA
LTVLLYIVVPKGFFPVQDTGVIQGISEAPQSVSFAAMSQRQQALADIILKDPAVVSLSSY
IGVDGDNATLNSGRLLINLKPHGARDLTASEVIQRLQPEVDKLSDIRLFMQPVQDLTIED
RVSRTQYQFSMSSPDAELLTLWSEKLVDALGKRPELRDVASDLQDKGLQVYLNIDRDAAS
RVGVTVANITDALYDAFGQRQISTIYTQASQYRVVLQAASGSELGPAALEQIHVKTTDGA
QVKLSSLARVEQRQAQLAIAHLGQFPAVMMSFNLAPDIALGKAVKVIEEVEQEIGMPIGV
QTQFQGAAEAFQASLSSTLLLILAAVVTMYIVLGVLYESYIHPITILSTLPSAAVGALLA
LLISGNDLGMIAIIGIILLIGIVKKNAIMMIDFALDAERNRGVAPEQAIYDAALLRFRPI
LMTTLAALFGAIPLMLASGSGAELRQPLGLVMVGGLLLSQVLTLFTTPVIYLYFDRLGRR
WSRKPADPDRLERADA
>tr|A0A0Q0D7F9|A0A0Q0D7F9_PSEAP Aspartate carbamoyltransferase OS=Pseudomonas syringae pv. aptata OX=83167 GN=pyrB PE=3 SV=1
MTPLDAKRPLQLNHLGQLRHFLSLDGLPRELLTEILDTADSFLEVGARAVKKVPLLRGKT
VCNVFFENSTRTRTTFELAAQRLSADVITLNVSTSSTSKGETLFDTLRNLEAMAADMFVV
RHADSGAAHFIAEHVCPDVAIINGGDGRHAHPTQGMLDMLTIRRHKGGFENLSVAIVGDI
LHSRVARSNMLALKALGCPDIRVIGPKTLLPVGVEQYGVKVYTDLNEGLKDVDVVIMLRL
QRERMTGGLLPSEGEFYRLFGLTTARLEAAKPDAIVMHPGPINRGVEIESAVADGAHSVI
LNQVTYGIAIRMAVLSMAMSGQNAQRQFEQENAQ
>tr|A0A0Q0ILE8|A0A0Q0ILE8_PSEAP Uncharacterized protein OS=Pseudomonas syringae pv. aptata OX=83167 GN=ALO85_100133 PE=4 SV=1
MKTPTSGVTAKCSATPSFWIRTLERFLTVVARGLCVRHKDAQLFCIPGATGIRLSR
>tr|A0A0Q0D8A8|A0A0Q0D8A8_PSEAP Aldo/keto reductase OS=Pseudomonas syringae pv. aptata OX=83167 GN=ALO85_05039 PE=4 SV=1
MPQTREDSMTYIAAENRYEDMPYRRTGRSGLVLPALSLGLWHNFGDSTPIDTQRAMLRTA
FDLGINHFDLANNYGPPYGSAEINFGRLLREDFKHYRDELIISSKAGWDMWPGPYGQGGG
SRKYVLASLDQSLQRMGLDYVDIFYSHRFDPDTPLEETASALATAVQQGKALYIGISSYS

GAKTREIAALLKEWKVPLLIHQPAYNLLNRWVEKDLLDATEELGAGVIAFTALAQGLLSD
KYLNGVPKDARVNRPGGGSLQASHLSEQNIAHVRALNEIAQRRGQSLAQMALAWTLRDPR
VTSALIGASRPEQIIENVGALKNLAFSNEELTEIDSFAVEGGINLWEKPSLAE
>tr|A0A0N8T8D5|A0A0N8T8D5_PSEAP Rhs family protein with PAAR motif (Fragment) OS=Pseudomonas syringae pv. aptata OX=83167 GN=ALO85_05389 PE=4 SV=1
IVWQAKYRAWGAVEKVVVNEVEQNLRFQGQYFDVETGLHYNTFRYYDPEIGRFTTQDPIG
LLGGENLYKYVPNPTTWVDPWGWECWSTARKNHWKAEAKNPTQTYSPANMARMTKGQAPR
MKVEVINRKTGKPEIKDVSMELHHRDIPQRVGGDGVHQAGNLDALTPWAHESVDTFRHTG
YRLVDMIKGLDTWVK
>tr|A0A0Q0DA13|A0A0Q0DA13_PSEAP Uncharacterized protein OS=Pseudomonas syringae pv. aptata OX=83167 GN=ALO85_02024 PE=4 SV=1
MMLSEQPPPAQGQRYYALVAACLGWSGLVIQLYLILIGRYADHASLLGGLVRFSSFFTVL
TNMLVAVALSCALTTQQSAGHRFFRHPVVCGGIAVSIALVGIAYNILLRHLWHPEGWQWV
ADELLHDIMPLAFVLYWWLYVPKGTLRLSHIPLWAIYPLVYFAYVLLRGHMFGDYLYPFI
DVGTLGFPKAFINALGVLLGFVLIALVLLGVDKRASRRSR
>tr|A0A0Q0DJM3|A0A0Q0DJM3_PSEAP Uncharacterized protein OS=Pseudomonas syringae pv. aptata OX=83167 GN=ALO85_101312 PE=4 SV=1
MILAGGRRSEPGDASLVCESCLNCHAWQSTSTAWLRASRFDPRKRAFYVRKPGCVNASLR
EQVRSYALRAEAKANTVCF
>tr|A0A0Q0FJM4|A0A0Q0FJM4_PSEAP Serine hydroxymethyltransferase OS=Pseudomonas syringae pv. aptata OX=83167 GN=glyA PE=3 SV=1
MFSRDLTIAKYDADLFAAMEQEALRQEEHIELIASENYTSPAVMEAQGSALTNKYAEGYP
GKRYYGGCEYVDIIEQLAIDRAKELFGADYANVQPHAGSQANSAVYLALLQGGDTILGMS
LAHGGHLTHGASVSSSGKLYNAVQYGIDANGMIDYDEVERLAVEHKPKMIVAGFSAYSQI
LDFPRFRAIADKVGAYLFVDMAHVAGLVAAGVYPNPVPFADVVTTTTHKTLRGPRGGLIL
ARANAEIEKKLNSAVFPGSQGGPLEHVIAAKAVCFKEALQPEFKTYQQQVVKNAKAMAGV
FIERGFDVVSGGTENHLFLLSLIKQDISGKDADAALGRAFITVNKNSVPNDPRSPFVTSG
LRFGTPAVTTRGFKEAECKELAGWICDILADLNNEAVIDAVREKVKAICAKLPVYGA
>tr|A0A0Q0IJJ6|A0A0Q0IJJ6_PSEAP Aspartate-semialdehyde dehydrogenase OS=Pseudomonas syringae pv. aptata OX=83167 GN=ALO85_05158 PE=3 SV=1
MSQSFDIAVVGATGTVGETVVQVLEERNFPVGNLYLLASIESAGHSVPFRGKNVRVREVD
EFDFSTVRMAFFAAGPAVSRSHAEKAVAAGCTVIDLSGAFTSAQAPNLVPEINATLLHSR
GIAPCLVASPSASATAVALALAPLRGLIDIQSVMVTACLAVSALGREGVSELARQTTELL
NVRPLEARFFDRQMAFNLLAQVGTPDASGHLPLERRLVEELRELLALPSLKVSATCIQVP
VFFGDSFTVALRTAGAVDVAAVNAALESAEGIELVDAGDYPTPVGDAVGQDVVYVGRVRA
GTDDSEQLNLWLTSDNVRKGAALNAVQVGELLIKDYV
>tr|A0A0Q0DYJ0|A0A0Q0DYJ0_PSEAP EmrB/QacA family drug resistance transporter OS=Pseudomonas syringae pv. aptata OX=83167 GN=ALO85_00577 PE=3 SV=1
MSNNAPASFTPPSLLLCTIGLSLATFMQVLDTTIANVALPTIAGNLGVSSEQSTWVITSF
AVSNAIALPLTGWLSRRFGEVKLFLWATILFVIASFLCGISQSMPELVGFRALQGMVAGP
LYPMSQTLLIAVYPPAKRGMALALLAMVTVVAPIAGPILGGWITDSYSWPWIFFINIPIG
LFAVLVVRSQMAKRPVSTARQPLDYIGLLALIIGVGALQIVLDKGNDLDWFESNFIIFGS
LISLVALTFFVIWEMTDKHPIVNLRLFAYRNFRIGTLVMIGGYSGFFGINLILPQWLQTQ
MGYTATWAGLAVAPIGILPVLMSPFVGKYAHKFDLRLLAGLAFLAMGLSCFMRAGFNTDV
DFEHVAMVQLFMGIGVALFFMPTLSILLSDLPPNQIADGSGLATFLRTLGGSFAASLTTW
IWIRRANQHHAYLSENISTFDPATRHALDSLGGASPQAYAQLERTLNAQAYMMSTVDYFY
LLGWIFAGLILLVWLAKPPFAAKAGPAAGGGH
>tr|A0A0N8T920|A0A0N8T920_PSEAP Uncharacterized protein OS=Pseudomonas syringae pv. aptata OX=83167 GN=ALO85_100088 PE=4 SV=1
MTAESRRSDTSVSKGMTMKNRFKLFIGTTALILSASALAKPVLHTATIDQHGSVVAQSSP
WIKSVSYSSQKDYFATYKVFFKEGQFKNSPGFCSVSSIDTSDHERLFYGHAKLGGAATTE
QVNVLGLMVGKRGASGDSSMSFQLACSN
>tr|A0A0Q0C4F0|A0A0Q0C4F0_PSEAP Putative shikimate transporter, MFS superfamily OS=Pseudomonas syringae pv. aptata OX=83167 GN=ALO85_00367 PE=4 SV=1
MTSTLLSREDQDAPAKPVNSTTRVATASFIGTAIEFYDFYVYATAAALVIGPVFFPQTSG
TAQMLSSFLTFGIAFLARPLGSALFGHFGDRIGRKSTLVASLLLMGICTTLIGVLPGYAT
IGAWAPILLCVLRFGQGLGLGGEWGGAALLATENAPKGKRAWFGMFPQLGPSIGFLAANG

LFLALAMTLDDEQFRSWGWRIPFLLSAVLVMVGLYVRLKLEETPVFAKAMARHQRVSLPI
VETFSQHWKPMLLGAASMVVCYALFYISTVFSLSYGVATLGYTRETFLGLLCFAVLFMAA
ATPLAALASDRFGRKPVLIAGGVLAILSGFTMEPLLTHGSTWAVALFLCIELFLMGITFA
PMGALLPELFPTQVRYTGASAAYNLGGIVGASVAPFFAQKLVAMGGLSWVGGYVSAAALI
SVLAVLSLKETRDREL
>tr|A0A0Q0DPI4|A0A0Q0DPI4_PSEAP tRNA-dihydrouridine(16) synthase
OS=Pseudomonas syringae pv. aptata OX=83167 GN=dusC PE=3 SV=1
MQIALAPMEGLVDDILRNVLTKVGGIDWCVTEFIRVSERLMPAHYFYKYASEFHQGAKTD
AGTPLRLQLLGSDPVCLAENAAFACELGAPVLDLNFGCPAKTVNRSRGGAILLKEPELLH
SIVSQVRRAVPKHIPVTAKMRLGYENTDGALDCARALADGGAEQIVVHARTKVDGYKPPA
HWEWIARIQEVVKVPVVANGEIWTVDDWRRCREICGARDIMIGRGLVARPDLARQIAAAQ
KGEEVVPITWDELQPMLRVFWQDCLAKMTLIQAPGRLKQWLVLLTKSYPEATLIFNTLRR
ETDCDRISVLLGCAPGCLPRS
>tr|A0A0Q0CW64|A0A0Q0CW64_PSEAP Arginine utilization pathway regulatory
protein ArgR OS=Pseudomonas syringae pv. aptata OX=83167 GN=ALO85_01840
PE=4 SV=1
MFQIELSSKARLLRQYVSDVPMTAHRIGFLVWPGTKALTLALAEEALRVAQRVHPEVVYE
LSFLQAEATEPSALASAWQLPGEPWAGRLEGFQKLFLLADEPPAGVAPALGSALKQLVRA
GCSIGGLSAGVYPLAQLGLLDGYRAAVHWRWQDDFAERFPKVIATSHLFDWDRDRLTACG
GMSVLDLLLAVLSRDHGAELAGAVSEELVVERIREGGERQRIPLQNRLGSSHPKLTQAVL
LMEANIEEPLTTDEIAQHVCVSRRQLERIFKQYLNRVPSQYYLELRLNKARQMLMQTSKS
IIQIGLSCGFSSGPHFSSAYRNFFGATPREDRNQRRSSSPFELSPVPAERG
>tr|A0A0Q0IN34|A0A0Q0IN34_PSEAP Uncharacterized protein OS=Pseudomonas
syringae pv. aptata OX=83167 GN=ALO85_05243 PE=4 SV=1
MMRLSELKTAGRTPELPMSLTLADAAGSAELQLLTLLRVLPGQRYVGAGVWRGRTVLAKL
LVGDKAARHFQRELAGVRLLAEQGLTTPLLLADGLQDGEGGWLLFEFLEQAPSLGDAWNA
VKHLPPLADEQQAVLGEALSAIARQHAKGLWQEDLHLDNLLRHKGQLYLIDGAGIRAEQA
GTPLSRQKVLENLGVFFAQLPRSFEPFTEELLVHYLLSNAEHGLPMEALQKQIDKVRSWR
LKDFMSKTVRDCSLFSVEDSASVFRAIRREEEPAMLPVLSQADALLDKGHLYKTGGAASV
GRVEINGRPLVIKRYNIKNFSHWLKRFWRPSRAWHSWREGNRLMFLGIATPKPLAVQEKR
FLGLRSKAWLVTEFINGPDIIERFAPYVENGDAPEVELLALDRLFAQLIQARISHGDFKG
HNLFWHVDRWAMIDLDAMQQHSSQSSFAAAYARDRARFMRNWPTHSALHQLLEQRLPKLA
TD
>tr|A0A0Q0FGS7|A0A0Q0FGS7_PSEAP Gamma-glutamyl phosphate reductase
OS=Pseudomonas syringae pv. aptata OX=83167 GN=proA PE=3 SV=1
MTESVLDYMTRLGRAAREASRVIGRASTAQKNRALQATAAALDEARAELSAANALDLANG
QANGLEPAMLERLALTPARIDGMIVGLRQVAALADPVGAIRDMSYRPSGIQVGKMRVPLG
VVGIIYESRPNVTIDAASLCLKSGNATILRGGSEAIHSNRAIAACIERGLAEAGLPAAVV
QVVETTDRAAVGALITMPEYVDVIVPRGGKGLIERVSRDARVPVIKHLDGICHVYVSAHA
DLAKAQKIAFNAKTYRYGICGAMETLLVDQTIAADFLPSMAAQFREKGVELRGCERTREL
IDVIPATEDDWHTEYLDAILSIRVVSGLDEAIEHINHYGSHHSDAIVSDHQSQVRRFMAE
VDSSSVMVNAPTCFADGFEYGLGAEIGISTDKLHARGPVGLEGLTCEKYIVIGDGQLRGQ
A
>tr|A0A0Q0DXX0|A0A0Q0DXX0_PSEAP TonB-dependent siderophore receptor
OS=Pseudomonas syringae pv. aptata OX=83167 GN=ALO85_00794 PE=3 SV=1
MSSRRIASLAGLAIGVLGNTGYAEEGPQTIELESVNVTSDYQYETATSPIQGYRATRSAT
ATKTDTAIADIPQSISVVPASVLKDLGSNNVERALDFAGGVSKQNNFGGLTLYEYSIRGF
TTSEFYKDGFSANRGYPATPDAANIERIEVLKGPAASLYGRGDPGGTVNIVTKKPQREAF
TTLQTSAGSWDRYRTALDVNTPLDEEGNLLSRVNLAVEDNNSFRDHVESKRVFVAPSFSW
QLNPDTSLLVETEFVRHTSTFDRGVVAPNNKWSGVSRSTFLGEPDDDIDNDNNMVQFALD
HQLNDVWSLRLASHYKQGEMSGFASEARPLNADGHTVNRRYRERDNNWHDSITQLELRGR
FDGMGVEHEVLIGTEYENYRKNERVTTIAGSPYAIDIYNPVYGQPKPAGARSGTDFYEHI
ESRALNLQDQIVFTDKLRGMIGARYEHFDQQVDDFTTNATSGQRQDAFTQRAGLLYQLTP
QVGLFVNASTSFKPNNGLDAAGKTFDPEEGVGYEVGIKSELFDDRLSTTLAAFHIEKENV
LALDPGTDTNRAVGKARSQGLDLQFTGQVSEAIRVIGAFAYIDAEVTKGDQSIPTGSRIL
GVAKHSGSLLGVYEFQEGALRGSDVGAAYTYVGDRSGQSGSDFELPAYQTVDLLAHYKAS
DNVTVGLNLNNIFDEKYYERSYSNYWVAPGEPRNVTVSLTLNL
>tr|A0A0N8T8F7|A0A0N8T8F7_PSEAP SPOR domain-containing protein
OS=Pseudomonas syringae pv. aptata OX=83167 GN=ALO85_02813 PE=4 SV=1
MTSLHADEAFLGHYQLSHDPFAARVPGFKFFPAQRKPVLGQLHHLARYSQLLLAVTGPQG

SGKTLLRQALVASTNKQSVQSVVISARGAGDAAGVLQQVAQALSVAQAEMQSILSQVVQL
GLTGQEVYLLVDDAEQLGDSALEALLGLAAGTPEGRPHVFLFGEPSLLDRLDQMCADLQG
DVEGERFHVIELQPYTEEETREYLAQRLEGAGQGISLFTADQITDIHEQSDGWPGAINQV
ARDSMIEAMIASRSAVKRPSVGFKMPKKHVLALGAVVIVAVAAAVLIPGRGDKTGTPANA
PASAQAQLPLGEGKPTAQQSNNGSPAIEFAGNSQPMPLPMNGNQPVMRGPLAEAAGSSDS
DEDAVPTGSPAQPPTVTTTAPPAGVPAGQAAAQTPRSSIPAPTPAPAAKPAPAQTQVATA
KPAPAPAAKPAEKPAAAAAKPAAGGSWYSSQAPGHYVVQILGTSSEATAQAYVAEQGGEY
RYFKKTLQGKPLYVVTYGNFPDRAAALAAIKVLPAKVQAGKPWPRTVASVQQELGATR
>tr|A0A0N8T933|A0A0N8T933_PSEAP Transketolase OS=Pseudomonas syringae pv. aptata OX=83167 GN=ALO85_05559 PE=3 SV=1
MPRRSAMPSRRERANAIRALSMDAVQKANSGHPGAPMGMADIAEVLWRDYMKHNPANPSF
ADRDRFIMSNGHGSMLVYSLLHLTGYDLSIDDLKNFRQLHSRTPGHPEYGYTPGVETTTG
PLGQGFANAVGFAAAEKTLAAQFNRPGHSIVDHHTYVFMGDGCMMEGISHEAASLAGTLQ
LNKLIAFYDDNGISIDGEVEGWFTDDTPKRFESYGWLVIRNVDGHDPEEIKTAIETARKS
DQPTLICCKTTIGFGSPNKQGKEESHGAPLGADEIALTRAALKWNHGPFEIPADIYAEWN
GKEKGLAAEAEWDQRFADYSAAFPELANDFIRRMSGQLPADFAEKSAAYVAEVNAKGETI
ASRKASQNALGALGPLLPEILGGSADLAGSNLTIWKGCKSITGEDPNGNYLHYGVREFGM
GAMMNGIALHGGFVPYGATFLIFMEYARNAVRMASLMKKRVIYVFTHDSIGLGEDGPTHQ
PIEQLTSLRTTPNLDTWRPADAVESAVAWKHAIERDDGPSALIFSRQNLTHQARDEKQLS
YIARGGYVLRDCAAEPELILIATGSEVGLAVQAYDKLTEQGRNVRVVSMPCTSIFEAQDA
AYKQAVLPLQVSARIAIEAAHADYWYKYVGLEGRVIGMTTFGESAPAPALFEEFGFTLEN
VLSTAEELLED
>tr|A0A0Q0IE55|A0A0Q0IE55_PSEAP Outer membrane channel protein TolC OS=Pseudomonas syringae pv. aptata OX=83167 GN=ALO85_01149 PE=4 SV=1
MLRFVPVFLSLGLASVQVHADPTQPAAPQVTSASAYAVDLMKLYREARLEDPRMLSAYLR
AQSAVDSERDAFGGLLPQVTANGSYNRILRKDDTQREIYNNSSYSLNLTQYLYNKQAWET
YQKAKSTTEQKGHQAEDAQAEATVDLAKRYFQALSADDELELIMAERRATQKSLDRVSAM
YEKKMAVVTDLLDLKARVDLLAAQEVDARNQIRLSRAALSEIVGRPITEPLSRIRNDIAL
QVPSKSMDTWVAQAIDSNPLLKARESSLDVANAAVREGKGGHYPTLTFTLGAQQSDVGYD
NTLSPRSDSYVATIGVRVPIYSGGSTSARVRGLYNEQLATEQDVESVRRQVVKETTNAYL
TAFSSVEKIRANKNALASSEQSSIAAQKAFSYGVVNAVDVLTSVQNEFKARRDLLKAQYD
FITNLFILNRWAGRPPQESVDSVNVWLGGTNPAVEKAADSALKKTP
>tr|A0A0Q0C7Q9|A0A0Q0C7Q9_PSEAP PAS protein OS=Pseudomonas syringae pv. aptata OX=83167 GN=ALO85_04848 PE=4 SV=1
MHKRVCNLSLEKNRLSRWLGRGEATQVPQPLPVSSEPGPIGADLYLLLDDEGRIQSISPH
LLARLDVAAPQPEAQRLMSLLLPNSALVIEGVPRDWLGHMLDLDFKGDDAHTLHARGWVQ
AHGKGWLLQLLDISDLMEEASAARSRQQCLRFAGQMAERVRACNVERLTTVVSEQLEELA
QLWRIPCVALVLPEAGGVGWRVYCQYSAHTAPELWQVGQRLGTALDRFNVDATQQLRFGG
TVDEGALQSAFGNADGLLIPHSRDNVAKAWLMFGFYNAQQQAPDLGEREWLNLCSALAGP
VLFRMSEQHNRHHVERLAVLQDLLGTGWWELLPDRDEVLLAPQLARSLRLGDRVESLSRQ
DWLALIHPADRQELSSRLAQLRDKGTPLLLCVRLAQNDPTQETLWFRIQGQALIRGQVQR
VLGFMLDVSDIKNQETEAAAAHARLDNLIASSPAVIYVQRYDHGNLEPTFFSDSLTPLLG
WTLADCVDGQLAGYIHPEDHDIWFERNRQLLREGFVSRRFRLRNRNGQYHWLLDEARLLR
DDLGMPVEAVGLWLDVTEATLAAEHIRRSEERYRILVEDSPAMICRYTPDLVLSFGNRPL
ANYMECTPEQLTGINLGDWLSDEQREAFIKRIGQLTPEAPVSTEEICIELPGREYAWWIW
ADRGVFDEHGTLVEVQAVGRDNTDVRRSRMQLNQSAKMATLGEMSTGLAHEINQPLNVMR
MAVVNVLKRLGRGDVDIEYLTEKLNRIDTQVQRAARVVDHMRVFGRRSEVEEQLFDPAQA
VEGTISMLAECMKGKGVQLRVGGMIDAVRVRGHVDQLEQVLINLMVNARDALLSRREKDR
DFEPWISVEAERDENIIRLAVQDNGGGIDPRLLERIFEPFFTTKPIGVGTGLGLSVSYGI
VDQMGGQLSVANVGEGARFQIELPIFNADQTGS
>tr|A0A0Q0DM76|A0A0Q0DM76_PSEAP Putative acetyltransferase and hydrolase with the alpha/beta hydrolase fold OS=Pseudomonas syringae pv. aptata OX=83167 GN=ALO85_100429 PE=4 SV=1
MKTIWTPDGFEEWQRHFPATAFVRHPAELDWTDLFLQRWQVPLPSLPKNTLVVLVAGLYS
EFILYCNRACARSLTSAGHYVLRMPVRSSRGVIAQGRHIATLLGSRLKPGQRFVVLAHSK
GGLDTLAALSQNKDLLDACDGIALVQPPAGPSPIIDELLGHGAASAGRGYRMDRFRQAMI
NSAPLAAGTRDISSNRDPQVAEMLSALPATLHCLHVVSWSAARRSHFDTHHQRLNALRPG
HAHDGQFYMEHLALPGVPQICLPDLDHGQPILGGTGFDPTRFWRTLLDVLHQTRTPERST
PDRFRSLGYSPHKPR

>tr|A0A0N8T8K4|A0A0N8T8K4_PSEAP Uncharacterized protein OS=Pseudomonas syringae pv. aptata OX=83167 GN=ALO85_03167 PE=4 SV=1
MSARISPIVSEFETEEQAASYDKWFRAQVQASINDPAPNISHDQVMAEMRALLESKQPPS
DAG
>tr|A0A0Q0D7E8|A0A0Q0D7E8_PSEAP K(+)/H(+) antiporter NhaP2 OS=Pseudomonas syringae pv. aptata OX=83167 GN=nhaP2 PE=3 SV=1
MDATTINSLFLIGALLVAASILVSSLSSRLGIPILVIILAVGMVAGVDGGGIIFDNYATA
YLVGNLALAVILLDGGLRTRVASFRVALWPALSLATVGVLITTVLTGMMAAWLFNLSVIQ
GLLIGAIVGSTDAAAVFSLLGGKGLNERVTASLEIESGSNDPMAVFLTVTLIGMLASGQT
GLHWGLLGHLIQEFGIGSFIGLGGGWILLQLVNRINLAAGLYPILVIAGGLAIFALTNAI
HGSGFLAVYLCGLVLGNRPIRSRHGILHMLDGMAWLAQIGMFLVLGLLVTPHDLLPIAIP
ALGLALWMILVARPLSVMVGLLPFKAFHDREKAFIAWVGLRGAVPIILAVFPLMAGLPNA
QLYFNLAFFIVLVSLLLQGTSLPWMAKLLKVTVPPDPAPISRAALEVHVTSEWELFVYRL
GAEKWCIGAALRELKMPEGTRIAALFRGQQLLHPSGSTTLEVGDLLCVIGHEHDLPALGK
LFSQAPQRGLDLRFFGDFVLEGDARLGEVAALYGLKLDGIDPGMPLSQFIVQKNRGEPVV
GDQIEWNGTIWTVAVMDGNKIQKVGVKFPEGTRPGPGLFL
>tr|A0A0Q0C7Z5|A0A0Q0C7Z5_PSEAP YD repeat-containing protein (Fragment) OS=Pseudomonas syringae pv. aptata OX=83167 GN=ALO85_04438 PE=4 SV=1
SLPGLMPIEWTRFYASDLDVDSALGKGWVLPWEQSLRRKGSFLYLSDNQGRSVPFVTLDY
NQRIYNAQEQLYLVRTEGGHYLLQTLDNVFYYFGEVPDDNQPVPLQRVENALGHFLHFVR
SEDGVLTDICSTGGHRVHLHYDEVTNRLSTVKRIVGDQAVETLVRYGYDNNGQLNSVYNR
NGDSVRNFSYTDGLMTRHANALGLSCEYRWEVLDGKPRVVEHWTSDGEHFHFDYDFEARQ
TRVTDVLGRRAEVTYNKDRRVIASTDFGGEQYRIALDNTGNITGLTLPDGNQLGFEYDDL
SRLTAETDPLGRTTRYQHHYKTTLVKQITYPDGSIWKARYDSKGNLVAEIDALGQKTEYL
NSEDGLPHTIIDATYKSKYLWWNNLAQVERFQDCSGKSTWYRYDERDQLVAVTDALNNTT
TLERKPGGEVLSINHPDGTRESFTYNAHGQVLTHTNGKDQTTHLARNARGLPIRRQDPKG
F
>tr|A0A0N8TA92|A0A0N8TA92_PSEAP Na+/H+ antiporter OS=Pseudomonas syringae pv. aptata OX=83167 GN=ALO85_200131 PE=4 SV=1
MKRQIFPDTCVIDPTQGDRDMDVWVPVKSPAVGVDGAENTHLHAPASARIEQVVDGQAAQ
GIEPVTVMKEQWPQGVRQCEHQVLPGTLG
>tr|A0A0Q0C8D3|A0A0Q0C8D3_PSEAP Phosphoglycerate/bisphosphoglycerate mutase OS=Pseudomonas syringae pv. aptata OX=83167 GN=ALO85_03552 PE=4 SV=1
MTLHLDLLRHGETELGGGLRGSIDDALNESGWQQMRAAVAGGGPWTRIVSSPLQRCAHFS
EELAQRLSLPLHLEPGLQELHFGDWEGHSPAQLMETDAEGLGLFWADPYAFTPPNGEPVL
DFSNRVLSAVERLHQAYAGERVLLVSHGGVMRLLVAQARGLPREQLLQVVVGHGALLSIQ
VAADRVLTEI
>tr|A0A0Q0D8Q3|A0A0Q0D8Q3_PSEAP Regulatory protein, TetR OS=Pseudomonas syringae pv. aptata OX=83167 GN=ALO85_03695 PE=4 SV=1
MLGIMKKPAQDMRQHIIDVARSLMTNKGYTAVGLAEVLSTAGVPKGSFYYYFKSKEEFGQ
ALLEEYFSEYLGRVDALMARPGSGAERLMAYFNYWIETQGTDLPEGKCLVVKLGAEVCDL
SEDMRGVLEVGTANIIKRLTACVDMGVADDSIHPEGDHEGFAESLYQLWLGASLLAKVNR
STEPFEKALTMTKRLLR
>tr|A0A0Q0CVA3|A0A0Q0CVA3_PSEAP Organosulfonate ABC-type transporter system, permease protein OS=Pseudomonas syringae pv. aptata OX=83167 GN=ALO85_02329 PE=3 SV=1
MSLSASQRIIHRLAPWALPVLLLAVWQLSVSAGWLSTRILPAPSAVIEAGINLVASGEIW
THLAISGWRAGIGFAIGGGIGLALGFITGLSKWGERLLDSSVQMIRNVPHLALIPLVILW
FGIDETAKIFLVALGTLFPIYLNTYHGIRNIDPALVEMSRSYGLSGFSLFRHVILPGAMP
SILVGVRFALGFMWLTLIVAETISASSGIGYLAMNAREFLQTDVVVLAIVLYAVLGKLAD
LAARGLERVCLRWHPAYQVSKGGAA
>tr|A0A0Q0CXF9|A0A0Q0CXF9_PSEAP Uncharacterized protein OS=Pseudomonas syringae pv. aptata OX=83167 GN=ALO85_101090 PE=4 SV=1
MFAWSIHVISDRNRNPVIGLENGVTINRSSFRLAWRTLRFGKAEQLATEMAGVENLPVST
TGLATVLSPAMRLFDGCADMGLWDDDTYDLLIIRLPPDGSRSDTYRHLIRVARQGVIILR
>tr|A0A0Q0BYR2|A0A0Q0BYR2_PSEAP Long-chain acyl-CoA synthetase OS=Pseudomonas syringae pv. aptata OX=83167 GN=ALO85_03182 PE=4 SV=1
MSFFQTLQDATQEQRETLFNLPIIRQAMAGQVSLESYRAFLTQAYYHVRHTVPLMMACGA
RLPSRMEWLRAAVCEYIEDEYGHEKWVLNDLRACGGPADAVENGQPGAPVELMVAYLYDL

IARGNPVGLFGMVNVLEGTSIALATHAADSMRKGLGLGPEAFSYLSSHGSLDIGHMETFR
TQMDKVDDPDDQQAVIHASRMVYRLYTDMFRELPGAALTAHQESAHAAA
>tr|A0A0Q0DA87|A0A0Q0DA87_PSEAP YiiD_C domain-containing protein OS=Pseudomonas syringae pv. aptata OX=83167 GN=ALO85_02273 PE=4 SV=1
MTTIEPEHDAAHALEQVLHHDIPLTREMGMRVIDWHNQTLRLHLPLAPNVNHKSTLFGGS
LYCGAVLAGWGWLHLRLREARITDGHIVIQDGQISYPLPVRSDAIARCDAPELAQWDKFI
ATYQRRGRARLTLHTCISEQDSEEQAVRFVGQFVLHR
>tr|A0A0Q0BYF3|A0A0Q0BYF3_PSEAP Flagellar protein FlaG OS=Pseudomonas syringae pv. aptata OX=83167 GN=ALO85_02157 PE=4 SV=1
MDMSVKLNVTYPAVLPVSQAPVPDASVDKPADKAPVERVAATAESKGSGLHKDDSKDDAK
VKAAAEDIQKFFHEVKRNLEFSIDEDSGKVIVKVIASDSGEVVRQIPNAEILKLADSLSD
ANSLLFRAKA
>tr|A0A0Q0DN29|A0A0Q0DN29_PSEAP Histidine kinase OS=Pseudomonas syringae pv. aptata OX=83167 GN=ALO85_03178 PE=4 SV=1
MKSIRRRTLTLIIGLLLSGLLVLTLFNVHDSNHEIAEIYDAQLAQNARLLEGVMRMPMER
KEHAQLYQAFNQALAQAEPRVDGHPYEGKIAFQVWDKDNNSLVHTASAPTFTHPPQSTGF
SDFNDMAGHQWRAFVLRDEQHGLLVWVGERDDVRSDLVNRIVRHTVLPNLIGSLVLMIAI
WLAIRWGLRPLADFANTLRGRNAGALHPLQLNALPTELEPMQAALNRLLGQIQDLLKRER
RFIADAAHEMRTPLAVLRVHAQNVLEAHNEEQRKQSLGYLIIGVDRTTRLVNQLLTIARL
EPGPSPAGKVAVDLVSAVRDSLVQLTPWVLSKHLELSFECSEASRDVTVDFPALDIALHN
LITNAVNFSPEGGQVTVELTFSEHCFRLNVDDQGPGIDEQERERLFEPFYSRGNDQGVGL
GLAIVEKVALLMGGNVSLDNRPEGGLRASLQAPLR
>tr|A0A0Q0BTE7|A0A0Q0BTE7_PSEAP Diaminobutyrate--2-oxoglutarate aminotransferase OS=Pseudomonas syringae pv. aptata OX=83167 GN=ALO85_01276 PE=3 SV=1
MSVATRFIDDQPTQVAPAAAETLYQFDESPLLARQSRQESNARSYPRRIPLALKRAKGIH
IEDVEGRRFIDCLAGAGTLALGHNHPVIIEAIQQVIADELPLHTLDLTTPVKDQFVQDLF
GLLPPALADDAKIQFCGPTGTDAVEAALKLVRTATGRSTVLSFQGGYHGMSQGALSLMGS
LGPKKPLGALLGTGVQFLPYPYDYRCPFGLGGEQGVRANLHYLENLLNDPEAGVQLPAAV
ILEVVQGEGGVIPADLDWLRGVRRITEKAGVALIVDEIQSGFARTGKMFAFEHAGIIPDV
VVMSKAIGGSLPLAVVVYRSWLDTWLPGAHAGTFRGNQMAMATGSAVMRYLQEHSICAHA
TAMGARLSGHLRALQRDFPQLGDIRGRGLMLGVELVDPSGARDAQGHPPLFARLAPLIQR
ECLKRGLILELGGRHASVVRFLPPLIITAEQIDEVAEIFGRALNAAVAQV
>tr|A0A0Q0DT40|A0A0Q0DT40_PSEAP MaoC-like domain protein OS=Pseudomonas syringae pv. aptata OX=83167 GN=ALO85_02755 PE=4 SV=1
MLIICLECDPGSASKRLNDLVRALAATGHQVLQIKARSGCTNRRASIHFYCQDSHMTQVT
NTPYEALEVGQTASYSKTVEERDIQLFAAMSGDHNPVHLDAEYASKTMFKERIAHGMFSG
ALISAAVACELPGPGTIYIGQQMTFLKPVKLGDTLTVRLEILEKLPKFRVRIATRVFNQN
DELVVDGEAEILAPRKQQTVDLTVLPEITFG
>tr|A0A0Q0C3H7|A0A0Q0C3H7_PSEAP Acriflavin resistance protein OS=Pseudomonas syringae pv. aptata OX=83167 GN=ALO85_00925 PE=3 SV=1
MNISALSIRYPVPAVMLFLLLTLFGFLGFERLGIQDFPDTDLPAVVISASLEGAAPEQLE
TEVARKLEDKLTSLRLLKHVTTKISEGSVLINVIFEIDKDGNEALNEVRNAVDSAAAELP
ANLDTPSVTRLTTNTTALLTYVVDAPRMDEEALSWFVDNELSKQLLTVRGVAKISRVGGV
DREVQVDLDPTLMAGLGLSVTDIANRLRAMQKDNSGGQGDLGSGQQALRVLGGIDDPAAL
GAIRIPVSDGRMLAVQQLATVRDTHAERNTLAYRDGKPVIGFQVIRSLGFSDVGVTKDLR
QAVSEFARQHPDVHIEEASNAVEPVMENYRGSMALLYEGMLLAVLVVWWFLRDWRATIIV
ATALPLSIIPTFGVMYFAGFSLNTVSLLALALVIGILVDDAIVEVENIARHLRMGKTPRQ
AAIEASDEIGLAVLATTVTLVAVFLPTAFMGGISGKLFRQFGVTASAALMFSLLVARLLT
PMMAAYLLKARPHGEHDSGLMRRYLGWIHTSLSRRKTTMAIVGALFIGSLALIPLLPTSF
LPAQDIASSTVSLELPPGTSLAQTGEIALQAEKRLRAIPEVAHVFIAAGSGDAGGAGDRD
AKLTVDLLPRDQRTLKQSQVEASMRERLRSLPGVRVTVGGDGSGERLDIVLASDDGDLLE
RTAAALEPQLRQIKGIGNVTSSAAVQRPEIQMRPDVFRAAEQGISSQDIADTLRMATYGE
YSSSLGKINLSQRQVNVRVRMQPQVRTDLQSLAQLRIKGRDGQIALASLGELSMGSGPAE
IDRIDRLRNITLSIELNGSNLGEVMEQARQLPAMQNLPAQVKLVEQGELQLMSELFGNFS
LAMAVGVFCIYAVLVLLFHDFMQPLTILSALPLSLGGALLALLIGGMSFSMASVIGLLML
MGIVTKNSILLVEYAIMARRAPTVSRYEALIDACHKRARPILMTTIAMGAGMLPTALGWG
GESGFRQPMAVVVIGGLLASTVLSLLVVPVIFTYVDDGHERLKSLFRRPL
>tr|A0A0Q0DCB2|A0A0Q0DCB2_PSEAP Hexapeptide repeat-containing transferase OS=Pseudomonas syringae pv. aptata OX=83167 GN=ALO85_04900 PE=4 SV=1

MLHTALRWLTGLQAAPRRSFSSSMVSTSPAQVGVEPCLARMSAIAARLSARICSIAGLTC
SARIAEKGGRSYDCRRGLFALTVLHLALKKHSAILITIDADRSNQTRSTRLSLPKKEPIV
AIRKFQEHTPALGERAFVDHSAVVIGDVEIGADSSVWPLTVVRGDMHRIRIGARTSVQDG
SVLHITHAGPFNPDGFPLLIGDEVTIGHKAMLHGCTIGNRILVGMGTTIMDGAVVQDEVI
IGAGSLVPPGKVLESGFLYVGRPVKQVRALTEKEIAFFPYSAANYVKLKDQHLAEGFDKG
>tr|A0A0N8T8Y9|A0A0N8T8Y9_PSEAP ABC transporter OS=Pseudomonas syringae pv. aptata OX=83167 GN=ALO85_01755 PE=4 SV=1
MSQVNALQLTGISKSFDGFKALIDADFTACWGEVHALLGENGAGKSSLMNIAAGLYAPET
GTLLLDDNPVQLQGPRDASRYRIGMVHQHFKLVKPFTVAQNILLALPEQPGEGSYRKRLR
ALEQEIVGKAEALGFAIDPARTVDSLSVAEQQRVEILKVLLAGARILILDEPTAVLTDQE
AERLLLTVQAFARQGAAVVLVTHKMADVKRYADRVTVMRGGRTVQTLDPQKVSVEQLVQL
TVGESVPVAEHQPATPGEVRLQVKDLRSFGSSALNGVNLSLHAGQIFGIAGVGGNGQAEL
ANALMGLPQATEGEIHMAGFGDLRGASADQRRQLRIASIPADRYGAALAGSLSVAENFAI
GNIHSGQYGSFWRLGYKRLKQDAQRAVADFDVQGVRSLDQKAALLSGGNAQKLVIAREFS
RDPQLVLVHSPSRGLDVRATQAVHARLRAARDAGAAVLVISEDLDEVLALADRVGVMNGG
RIVAEFAHPADRQAIGKAMVSHD
>tr|A0A0Q0CAZ2|A0A0Q0CAZ2_PSEAP Extracellular solute-binding protein OS=Pseudomonas syringae pv. aptata OX=83167 GN=ALO85_04535 PE=4 SV=1
MPIAYQHCSWRKEHMLKSLKFTAITLGMMCAAQAMAATDLTVISFGGANKAAQEKAFYAP
WEKAGNGKIIAGEYNGEMAKVKTMVDTKSVSWDLVEVESPELARGCDEDMFEELDPKQLG
NAADFVPGAITTCGAGFFVWSTVLAYNADKVSTAPSGWADFWDTKKFPGKRGLRKGAKYT
LEFALMADGVAPKDVYKVLASKDGQDRAFKKLDELKPNIQWWEAGAQPPQYLASGDVVMS
SAYNGRIAAVQKESNLKVVWTGGVYDFDAWAIPKGSKNADAAKKFIAYMLSPEQQKTYSQ
NIAYGPANTQAVKLLDKETLQNMPTTPENIKDQVQMDVAFWTDNGESLEQRFTAWAAK
>tr|A0A0Q0CIE5|A0A0Q0CIE5_PSEAP Aryldialkylphosphatase OS=Pseudomonas syringae pv. aptata OX=83167 GN=ALO85_03273 PE=3 SV=1
MSLFRHPSPLPLGVDSGHAMTVLGPVPVERLGVTLMHEHILLDASGKWVAPSCCGERHLA
ERPVSIELLGELHMNPLVSRDNCQLFDVDLACEELLKYRALGGETVVDPTNLGIGRDPQA
LQRISRMTGLNIIMGAGFYLEPSHPHYVHERSVEQLAEQIIHDVGGGAEKPEVIAGLIGE
IGVSAAFTADEEKSLRAAARASAATGVPLSVHLPGWERLGHRVLDIVEAEGADLRHTVLC
HMNPSHSDPAYQHALAARGAFLEYDMIGMGYYFADEHAQSPGDEENARAIVDLIEHGFGS
QVLLSQDVFLKTMLTRYGGHGYGYLLKHFVPRLRRHGVTGEQLENLLIDNPRRVFQRGFQ
APFSSQRNATS
>tr|A0A0Q0BRC3|A0A0Q0BRC3_PSEAP YD repeat protein (Fragment) OS=Pseudomonas syringae pv. aptata OX=83167 GN=ALO85_05075 PE=4 SV=1
MFEAARFGDEISHTGALGGFLIGAVLGIALIATVAIATFTCGFGVALLAGLAAGVGGSLL
TAAGEAIGSMFSSPSGTIITASPNVYINNRKAAHVEKSIGACDKHPGPVKIAEGSTNVFI
NSVAAARKGDKLTCGATISGGSDNVFIGGGRYRYLPVDDEIPGWLRTTVDVLMAVAGAAG
GIAQLIKAGTQAGMKAVLPCALKFTAGFVAGEVGSRYVVEPVMRSAIGGLSGNPVDTTTG
RKLIPDEIDFSLPGL
>tr|A0A0Q0BWF9|A0A0Q0BWF9_PSEAP Uncharacterized protein OS=Pseudomonas syringae pv. aptata OX=83167 GN=ALO85_05062 PE=4 SV=1
MPMRALMRLSSVPVVALCLSMFSTVLCAADVTGSSDLQILPRLADTEIVDYRPAAELERV
YPMGSIRKISGQLRFDGQVSARGNLTSVTYQLPAERTSDDAFTAARESLQQQGAELLFWC
QARDCGESSLWANEVFGNAKLYGADDRQAYLLLRLAEPRSDTLVALYSITRGNRRAYLHV
EQFESSAPLGELLPTSATLLRQLRSTGKLELPRLSGEPQDAWVTLVSRALNLDSSLRATV
SGPSAGAWLGALIAKGVRASRLEAGVTEGKGLKIEVIR
>tr|A0A0Q0FBF9|A0A0Q0FBF9_PSEAP Segregation and condensation protein B OS=Pseudomonas syringae pv. aptata OX=83167 GN=ALO85_03811 PE=3 SV=1
MNLNEPRELAPLLEAFLLASGKPQTLERLYELFEEGERPEPPVFKKALEVLRKSCEGRAF
ELKEVASGYRLQIRDRFSPWVGRLWEERPQRYSRAMLETLALIAYRQPITRGEIEDVRGV
AVNSHIVKTLLEREWIRVVGYRDVPGKPAMFATTKGFLDHFNLKSLDELPPLADLREMET
EPVLDFEEAPVPAHLQALADASLDPDAEPDAPRDETSFRSLLVELDSMEQGLKTDFDDLL
RAEPEPEPEDGKGDDEV
>tr|A0A0Q0CFQ1|A0A0Q0CFQ1_PSEAP His/Glu/Gln/Arg/opine family amino acid ABC-type transport system, ATP-binding component OS=Pseudomonas syringae pv. aptata OX=83167 GN=ALO85_03688 PE=4 SV=1
MSTLIELSGFSKNFGDTPVLRDIDLSVKEGEVVVILGPSGCGKSTLLRCLNGLELGHAGS
LRFAGKALPANADWRLVRQQIGMVFQSYHLFPHMTVLDNILLGPLRVQKRSAAEARQQAE
ALLQRVGLTEKRDAYPRELSGGQQQRIAIVRALCMNPKVMLFDEVTAALDPEMVKEVLEV

ILDLAQGGMTMLIVTHEMAFARAVADRILFMDRGRIAEQNDPETFFSAPQTARAQQFLER
FSYVENMPKRKAS
>tr|A0A0Q0IP33|A0A0Q0IP33_PSEAP DNA-binding transcriptional activator OsmE OS=Pseudomonas syringae pv. aptata OX=83167 GN=ALO85_04989 PE=4 SV=1
MSKTLNHAIRLTSAFRERARNIQSAFHARSSCRSGGSAPASGWMQGTASSPSFFRTLGLS
SAPRRTGPKPALTCGAITMYKKLFAATFVLATMAGCSNTTVQNPVDYVTYRNEPLVKQVE
NGMTRQQVLTIGGEPSSTIERRVNPGTCNNYVMNKDGHKQVYHVSFNSDGRVTNKGFMTC
EQREKNEKAM
>tr|A0A0Q0IQB7|A0A0Q0IQB7_PSEAP Carbon-phosphorus lyase complex accessory protein PhnP OS=Pseudomonas syringae pv. aptata OX=83167 GN=ALO85_00823 PE=4 SV=1
MRLTLLGTGDARQVPVYGCQCAACTAALTNHDLRRLPCSALIECADQRWLIDSGLTDLTE
RFPPRSLSGILQTHYHADHAQGLLHLRWGQGLVIPVHGPADPEGLADLYKHPGILDFSQP
FAAFERRALGALQVTALPLAHSKPTFGYLFEGDGQRFAYLTDTVGLPDDTREYLQRAPLD
LLVLDCSMPPQPQPPRNHNDLTLALQTIEWLRPGRAVLTHVGHTLDAWLMEHSGQLPGNV
VVGRDEMSVL
>tr|A0A0N8T8U9|A0A0N8T8U9_PSEAP Putative threonine/homoserine efflux transporter (Fragment) OS=Pseudomonas syringae pv. aptata OX=83167 GN=ALO85_04337 PE=4 SV=1
RASTVNFKIAEIDRFVITYIEEHTHLAQYIAHSLIIPHHGPLPVPGSHPGCTSEMSEKSR
NIASALFPLGLLMIAMISIQSGASLAKSLFPVVGAQGTTTLRLVFASLILVAVLRPWRAN
LTAKSLRTVVIYGIALGCMNLLFYMSLQTVPLGIAVALEFTGPLAVALLSSRKPIDFLWV
TLAVIGLLLLIPLGTSAAAIDLLGAGYALGAGVCWAAYIVFGHKAGADNGVQTAALGVVI
AALFIGPIGVIHAGSALLDISLIPAALGVAILSTALPYSLEMIALTRMSARTFGTLASLE
PVFAALSGLVFLHESLSLTQWLAIGAIIFASIGATLGSANSKPQLVPAD
>tr|A0A0Q0D2M5|A0A0Q0D2M5_PSEAP KDP operon regulating sensor histidine kinase KdpD OS=Pseudomonas syringae pv. aptata OX=83167 GN=ALO85_02238 PE=4 SV=1
MSDSGRADALLAQLPREGRGRLKVFLGAAPGVGKTYAMLQAAHAQLRQGVKVLAGVVETH
GRAETEALLNGLPQQPLLRTDYRGMTLEEMDLDALLKAAPSLVLVDELAHTNAPGSRHAK
RWQDIQELLAAGIDVYTTVNVQHLESLNDQVRGITGVQVRETLPDWVLQEAFDLVLIDLP
PRELLERLRDGKVYVPEQARAAIDAFFTQTNLTALREMAMQTAAAQVDNDLAQGYRQLGQ
SAPAVRGRMLVGIDGDIHAERLVRHASRVAQRRHLPWSAVHVDDGQTLDEQSRARLQSAQ
QLAERLGGEAVSLRAGEVARTLVQHAIERRASVVLVGQSRRHWRRKIFGGGVAARLLREG
HGLEISVLDDSEELPDQPPRARPAREVVWFDYGLAFVATLIASLVAWGVAGVLALPNISL
IFLAAVLLVAVRSSMGPALACAGLSFLAYDFLFIPPSFSLNIQREEDVLTLLFFLLMSAL
TGKLAARQRRQLQALRDTQEQTSELLDLSGKMTAATDGKAVFSAAEQHFSGWKELHLCLV
DRDGEGGWVVETGGPLTFSEAERAAADWAWKHDQPAGSGTGTLPSGRWWWWPITAEEGPL
ALLGVCAREGQSFTAQHRRLLAALTQPLAQALARAQLAQELEAARLHGETEQLRSALLAS
VSHDLRTPLTSMRGSIDSLLALGEAIPLEDRRELLEGTRDEAERLDRYIQNLLDMTRLGH
GALKLARDWVSPADIVGSALNRLRAVLAPLQVSTQVTGELPLLYVHAALIEQALVNVLEN
AARFSPVDGRLQVAAGVVDSELFFSVSDEGPGIPEDERAKIFDMFYTAARGDRGGQGTGL
GLAICQGMIGAHGGRLTVEEGIDGLGTRITLFLPLQAQPDVELEESA
>tr|A0A0Q0BRS6|A0A0Q0BRS6_PSEAP Uncharacterized protein OS=Pseudomonas syringae pv. aptata OX=83167 GN=ALO85_04146 PE=4 SV=1
MSFDKKEDNLQWIWIWMGFIILIAGCVLFTMYGVYLPYLSHEHAAWSSFGSLLSGFFTLT
GTVATIATLLFLAKQNKDQQKVTQAQLDSLTFERYINHRKLFTDQLNELVAIHKNTITFR
DPSHLYNSIFTMNSPHHCEFSVPPVYDEDGDGTNHVGLLYRKLEGIKSALDLVQPEASDV
EQLCHDLIDLSYDLLMIEPAGNRRVGDVMFRDSAYGFNIFALNEFIDPAVKIANMIFKFT
NNPVIEGQLIQGNSSFVKEALLRTFFPRRTKSSLNVHDNIMGLSTFVWVYLVAQEIYVGN
EQLLPDSVRKLRIKLFSIESVNGLAEKANFDDVLNSCIHELKRKLSGMTHEDEHFVQAME
FNSRLRGLINHNA
>tr|A0A0N8T9T4|A0A0N8T9T4_PSEAP Transcriptional regulator, GntR family OS=Pseudomonas syringae pv. aptata OX=83167 GN=ALO85_03708 PE=4 SV=1
MYMTRQVRFDKKTQVINELIRRIESGQMSSGALLPGENLLAQEFNVSRGTLREALSELKR
RNFIATQTGVGSIVTYDGFSLDQRAGWAQALADTGAGVRTEILRLVAVQRPDLEVRLGTS
SLVALDRRRIAADGSIVSLERALIPAVDGLENLPQNGLLEQSLTVTMAAHGFIGDKGDQW
IGAGPLNRDDAVLLAREPGTVFLKAVRTTFDRRERFMEHVESLLDPVHFRLHLAFGSST
>tr|A0A0Q0DM68|A0A0Q0DM68_PSEAP Glycosyl transferase family protein OS=Pseudomonas syringae pv. aptata OX=83167 GN=ALO85_02836 PE=4 SV=1

MHKPCVIIPVFDHERAVPNVVQAVRNAGLPCILVDDASSPACEAVLQQLAQGEAIYLLRL
PINQGKGGAVMAGFREAIRLGFSHALQVDADGQHDLSDIPVFLEQSRQHPDALICGYPQF
DESVPKGRLYARYLTHVWVWINTLSLHIRDSMCGFRLYPLAPVLALIDSVQIGRRMDFDS
EILVRLSWRGQPMRWLPTRVHYPQDGLSHFRLFHDNALISKMHAKLFFGMLLRLPMILWR
RWRP
>tr|A0A0Q0C2X2|A0A0Q0C2X2_PSEAP Uncharacterized protein OS=Pseudomonas syringae pv. aptata OX=83167 GN=ALO85_03340 PE=4 SV=1
MTGSLLLSGWRYRAVLLSIVFSAMGYLGFSLWGGWQAVSAATGKVGLLGIGVALLMSAIN
YALRFVRWQLYLSVLGHPLAWRKSLRIYLAGFALTTTPGKAGEALRGVLLRPLGVPYPQS
FAAFVSERLSDLLAIVLLTLFGLSWYPQAQPMILLGLALVLTGSLVLSRRHLLERLQVSL
PTKNTRASSLWQQLFNVLLAARQCLHGNVLLTASLLSLLAWSAEALAFYWMLGWMGAQIP
LTFAVFTYALAMLAGALSFMPGGLGSAEAVMVGLLMFKGMPGADAIAATLLIRLATLWFA
VAVGAVMLSRFTGAVPAPTPQP
>tr|A0A0N8TA49|A0A0N8TA49_PSEAP DNA polymerase OS=Pseudomonas syringae pv. aptata OX=83167 GN=ALO85_05369 PE=3 SV=1
MDVQQGFILSRHWQDTPAGTEVDFWLATDQGPRHIRLPCQPSVAFIPADQGERARSLLRS
ERGVELRPLELCDFRHRPVLGVYCQQHRQLMNIEKLLRRGNVDVYEADIRPPERYLMERF
ITAPVQFGGTPDADGVLCNAQIKPAPDYRPNLKLVSLDIETTARGELYSIALEGCGQRQV
YMLGPVNGGDEALDFQLDYCDTRAQLLERLNEWLALHDPDAIIGWNVIQFDLRVLHEHAQ
RLQVPLRLGRGGEAMGWREHGSRNNHFFADVSGRLIIDGIEALRSATWSFPSFSLEYVAQ
TLLGEGKAIDTPYQRMDEINRMFAEDKPALARYNIKDCELVTRIFAKTQLLTFLLERATV
TGLPADRSGGSVAAFCHLYIPLMHRQGFVAPNLGERLPEASPGGFVMDSQPGLYESVLVL
DYKSLYPSIIRTFLIDPVGLIEGLRHPADSESVPGFRGARFSRTRHSLPAIVERVWQGRE
AAKRDKNAPLSQALKIIMNAFYGVLGSSGCRFFDPRLASSITLRGHEIMLKTRDLIQAQG
YTVIYGDTDSTFVWLGRPHGQEEAASIGKALVQQVNQWWREHLHAEYGLHSALELQYETH
FRRFLMPTVRGAEEGSKKRYAGLVTRADGSEEMIYKGLETVRTDWSPLAQRFQQELYLRI
FKRQPYQDYVRDYVQRTLSGELDDLLIYRKRLRRKLDDYQRNVPPHVRAARLADEYNDQQ
GRPRQYQSGGWISYVMTAAGPEPLEIRHAPIDYDHYVTRQLQPLADAILPFVDDDFSTLI
GGQLGLF
>tr|A0A0Q0CWJ7|A0A0Q0CWJ7_PSEAP Helix-turn-helix, Fis-type OS=Pseudomonas syringae pv. aptata OX=83167 GN=ALO85_00091 PE=4 SV=1
MSQTSASLAHDIIVQDSWTRCRDFGLSHQTRPSFGQLPGAEVSRLLERHNGLVQTTHQEV
LPVYENILSNSSCLILLADTQGQLLKSWGAQRFVESSQAQGFMPGASWSERGTGTNAIGT
ALACEQAIHIEPDEHFLKANRFMTGSASPIFDADRRIIAVLDVSSDSFLPPSHTLGMVKM
MSQSVENRLILDQFRDSHFQLIFNTGLNNLDSQWAGLLIFDESGQILCANRRADTLLGTA
PAGASIETLFKCPILQLLSETEARPFALHAFGNNRFQCLLKRPTRKPLKLHCVQAAQMPA
APRNLDLQAISLGDAKVEKAVRQAQRLLEKDIPLLIHGETGVGKEVFVKALHQASARASQ
PLIAVNCAAIPAELVEAELFGYERGAFTGANQKGSIGLIRKADKGTLFLDEVGDMPMPVQ
ARLLRVLQERCVQPLGSSELYPVDIRLISATNRGLRDQVQAGLFRQDLYYRISGLSIELP
PLRERTDKHALVQRIWERHRDAHQRAGFSREVLELFERHPWPGNLRQLNSVIQVALALAD
EQPIGTDHLPEDFLLDAHADEECRAPVRTQESATRLHSSEDLGQLFQAAGGNISQLAKRL
GVSRNTLYKRLREQRIV
>tr|A0A0N8T9Z7|A0A0N8T9Z7_PSEAP Amino acid adenylation (Fragment) OS=Pseudomonas syringae pv. aptata OX=83167 GN=ALO85_05183 PE=4 SV=1
MSQLPMTDIPRWMLYFFFNVLNPPADWHSSSAMPRRPSTGDEPIDFANRCFEMNSLIDNL
CTQGQELLPLASVQQGIWLDQLAYPHLPYYNIGMTLEIKGDIDIALFEKAIQMVVDGHDA
LRLSFSHERGMGGQRVLSKAPFTLEVTDLPDVDGDAEQAIAHLRESFRQPFESMTGQLWE
SRLVRCGPQLYYWFNRLHHLVADGIGAVLIAHTVSDAYSGLLEGKQTLAEGPSYLSFLEE
DKAYMTSSRYERDRLFWEQAYAQLPPVLLQRRADFKAGVTNGPAPSDQVQAMLPRALYNA
LTQFAGECNLSVAHVLVGVISTYFCRTAGVDEIVVGMPVHNRTNARQKATIGMFSSISPI
RVGFDPHETLVDLMNNIGTQLRRTYRHQRFPIAELNRSLRLAQNGRHQLFDVSLSFESFD
GDVTFCCEDPARVLMLDNGYEQTPMAIFVRDYHPSEDIHLDFNFNTAYFTLEEAQRVQQR
IFAMLEAVLNQHDTPLAHFPLMSETEHQQLSAFNETALAYPRDVVIHQLIEQQA
>tr|A0A0Q0FSN8|A0A0Q0FSN8_PSEAP Amino acid adenylation (Fragment) OS=Pseudomonas syringae pv. aptata OX=83167 GN=ALO85_04500 PE=4 SV=1
MVSRLRQRFEVEVALRDVFAEPTLQGLARQVANARLSAQTPLTLVDRDLPLPLSWAQQRL
WFLDQLDRAAGAAYHIPAGLRLRGRLDSDALQATLDRIVAR
>tr|A0A0Q0DTN3|A0A0Q0DTN3_PSEAP Single-stranded-DNA-specific exonuclease RecJ OS=Pseudomonas syringae pv. aptata OX=83167 GN=ALO85_04478 PE=4 SV=1
MACGRARLTAAGSDRNHIHRHCKENQLTRMRIEPRLLPDTLPFLGDLPPLLTRLYAARGV

ACEAELDKSLARLIPYQQLKGIDAAVDLLVTALREGQRMLIVGDFDADGATASTVGVLGL
RLLGAAHVDYLVPNRFEYGYGLTPEIVEVALQREPQLLITVDNGISSVEGVAAAKAAGLQ
VLVTDHHLPGHELPAADAIVNPNQPGCTFPSKSLAGVGVIFYVLMALRARLRETGWFQSN
QRAQPNLGELLDLVALGSVADVVPLDANNRILVHQGLMRIRAGRARPGLRAILEVARREP
SRITSTDLGFILGPRLNAAGRLDDMSLGIECLLCDDPTLAREMAVQLDELNQDRKSIEQG
MQREALAQLKDLPLESMPFGLCLFDAEWHQGVIGILASRLKERYHRPTIAFASAGEGVLK
GSARSVPGFHIRDALDAVAASHPHLISKFGGHAMAAGLSLPEENFPAFAEAFDQEVRRQL
DEQDLTGRLLSDGSLAVEEFHLELARALRNAGPWGQHFPEPLFHGVFQLVEQRVVGERHL
KMVLKTECGSVKLDGIAFGVDRDIWPNPNIRWVELAYKLDLNEFRGNETVQLMVVHIAPR
>tr|A0A0N8T7X7|A0A0N8T7X7_PSEAP Formamidase OS=Pseudomonas syringae pv. aptata OX=83167 GN=amiF PE=3 SV=1
MASGLGGLNKSPNGVVIGLAQLALPDPHTREALWAQTQKVVGMVAKARRSNPGMDLIVFP
EYSLHGLSMSTAPEIMCSLDGPEVIALREACKAHRIWGCFSIMEANPHGNPFNSGLIVDD
LGEIRLYYRKLHPWVPVEPWEPGDLGIPVCDGPRGSKLALIICHDGMFPEMARECAYKGA
DIMLRTAGYTAPIRHSWKITNQSNAFTNLMQTASVCMCGSDGTFDSMGEAMFVDFDGTIM
AEGGGRADEIVCCELRPDLVREARVHWGVENNIYQFGHRGYVAVKGGARDCPYTYMHDLS
AGQYRLPWEDDVVHTDGSSCGFAAPERDFKPHPQQLEGVTP
>tr|A0A0Q0DQU1|A0A0Q0DQU1_PSEAP Putative HAD superfamily hydrolase OS=Pseudomonas syringae pv. aptata OX=83167 GN=ALO85_03918 PE=4 SV=1
METLPAMGLIDYRALLIDCDEVLVDRDSGVWTALQPLLDNGLNTPGKDQVLTEFNDVIRT
LYPRFDELGFSGLLCFAHRQLAERLGIKASWEEGMSFARSVPNWTLFEDAPGAMLYLRKF
YRLLVYADRDLQDRGLLCERLGLEADSLLSLTTHPLDDTRWLAKMDLDTRDTLLVSRPPA
SAPGLGGLCLIRRSREQHSQPCAADICINSMADLVAQHQLSLRR
>tr|A0A0Q0IAQ0|A0A0Q0IAQ0_PSEAP Sensor histidine kinase OS=Pseudomonas syringae pv. aptata OX=83167 GN=ALO85_04289 PE=4 SV=1
MLGRMLLLTLLAVSLAQALSSVIWLSQLRATQMEGLVTSARSLAYSMAASVSYFRSLPLA
YRPMVLDQLRSMGGTRFVVSLNDHPLDMQIMQPTPRKQAVLDVVGEVLRQRLGSGPDITV
WFARPEELRIFNSGLKLDELPRSWAHYALTLEPVNPPVLVTQIEMAPGEWFYIASLLPEP
YTSLEEQELPLQQVSFIVLTSAFLLLFIGLLVHWQSRPLKRLARAARDMSLGADVEPVVE
GGGSEVIEVSRAFNAMRTRISRYLTERGQLFSAISHDLRTPITRLRLRVELLEDEQLQRK
FSRDLDELELLVKGALQCVKDTDIHENIEPVDLNALLECLVEPWLLADGNGRVTQQGEAH
AAYSGKPLALKRCIGNLIDNALKYGHRAHLKVEDDAHAFVLHVDDEGPGVPEQRLEHVFE
PHFRLAGSQQQGYGLGLGIARNIAHSHGGEVSLQNLRDGGLRVTLYLPRTAE
>tr|A0A0Q0DMT1|A0A0Q0DMT1_PSEAP Flagellar basal body protein OS=Pseudomonas syringae pv. aptata OX=83167 GN=ALO85_02146 PE=3 SV=1
MDKLLYVAMSGASENAIAQKAHANNLANVSTNGFMRDLEQARSMPVFGEVMPSRAYAMSE
RPGTDFSAGAMVQTGRELDIAVKGDGWIAVQTPDGGEAYTRSSGMNIDALGVLRDGSGLP
VMGNGGPIAVPPQQQIEIGADGTISIRSLGESPQVMAQVDRIKLVRPDLKTMEKGPDGLI
HTKTGRPADADATVQVESGFLQASNVNAVEEMTSVLALSRQFELHVKMMKTAEEDDQSMA
RVLQLG
>tr|A0A0Q0CG51|A0A0Q0CG51_PSEAP RND-type cation/multidrug efflux system, membrane fusion protein OS=Pseudomonas syringae pv. aptata OX=83167 GN=ALO85_00775 PE=3 SV=1
MFLFRPKATALLLLPLFLTACSESSGGRDPRTQPPLVRAATVANTDQASRSFTGVVVARV
QSDLGFRVSGKVLERLVDTGQTVKRGEPLMRLDPVDLGLQSQAQQQAVAAAVARARQTAD
DEARNRDLVVAGAISASAYDRIKSLADTAKAELSAAQAQASVARNATGYAVLLADADGVV
VDTLVEPGQVVSAGQPVVRLAKAGPREAIVHLPETLRPTMGDRAEARLYGDGARVIPARL
RLLSDAADPLTRTFEARYVLEGSNISAPLGSTVSLDIAVGKAGRQSLAIPIAALHDPGRG
PGVWVIAGTPAKVTWRAVQLLGVSDDSANVSGGLEAGEQVVALGTHLLHDGEEVLVQQGG
ITAEARP
>tr|A0A0Q0DJ67|A0A0Q0DJ67_PSEAP Phospholipid/glycerol acyltransferase OS=Pseudomonas syringae pv. aptata OX=83167 GN=ALO85_100188 PE=4 SV=1
MLQKAVLTNPSSFYPIRFKQESMMGEFDAIRPYNDAEVPAVLARLLSDKAFLAILTRFRF
PRLAGAFGWLLQPTLARKLRREFAGIDSVATLQDKVEYYVDHTIERATDGVTYTGVEQLK
SGCAYLFLANHRDIVMDPAFVNYAVYHAGLPTPRIAIGDNLLQKPFVSDLMRLNKSFIVH
RSITGRREKMAAYQLLSAYINHSITQDCQSIWIAQAEGRAKDGDDRTESAILKMFHMSRK
DEPFGDVIRSLNLIPVSISYEYDPCDSAKARELYIRATTGAYTKTPGEDDVSIALGITGY
KGRVHVNFAPPITEHFEDTKQLAIEMDRQILGGYRLFPVHYLAYAQWSDADPALEVPKAE
ALFPADELERAREEWETRLAGTPIEHRPYMIQQYATPVRNQYRVKAGLAL

>tr|A0A0Q0IN53|A0A0Q0IN53_PSEAP Putative hydro-lyase ALO85_02917 OS=Pseudomonas syringae pv. aptata OX=83167 GN=ALO85_02917 PE=3 SV=1
MNSLQLAREAASAARARYRQGFVAPTAGQAPGLTQCNMITLPREWAYDFLLFAQRNPQAC
PVLDVTEPGSHTTLLAEQADLRTDLPLYRVWRDGQLAEELSDVSHTWAQHTDLVSFLIGC
SFTFETDLMHAGIDVRHISEGCNVPMYRSNRVCRPAGRLQGNMVVSMRPIAADRVAEAAR
ITGRYPGVHGAPVHVGEPGLLGINDLATPDFGDAVTIRPGEIPVFWACGVTPQAVVMASR
VPFAVSHAPGHMFITDIPDSYYHV
>tr|A0A0Q0BU21|A0A0Q0BU21_PSEAP Thiol:disulfide interchange protein OS=Pseudomonas syringae pv. aptata OX=83167 GN=ALO85_01610 PE=3 SV=1
MRNLIISAALVAASLFGMSAQAATPIEAGKQYVELASAVPVAEPGKIEVIELFWYGCPHC
YAFEPTINPWVEKLPSDVHFVRIPAMFGGPWDAHGQLFITLDTMGVEHKVHAAVFEAIQK
EGKRLTDKNDMADFVATRGVNKDEFLKTFDSFAVKGKIAQYKELAKKYEVTGVPTMIVNG
KYRFDLGSAGGPEKTLEVADQLIAKERAAAAAAK
>tr|A0A0Q0FC29|A0A0Q0FC29_PSEAP Phosphogluconate dehydratase OS=Pseudomonas syringae pv. aptata OX=83167 GN=edd PE=3 SV=1
MHPRVLEVTERLIARSRDTRQRYLQLIRGAASDGPMRGKLQCANFAHGVAACGPEDKQSL
RLMNAANVAIVSSYNEMLSAHQPYEHFPNQIKQALRDIGSVGQFAGGVPAMCDGVTQGEP
GMELAIASREVIAMSTAVALSHNMFDAAMMLGICDKIVPGLMMGALRFGHLPTIFVPGGP
MVSGISNKEKADVRQRYAEGKATREELLDSEMKSYHGPGTCTFYGTANTNQLVMEVMGMH
LPGASFVNPYTPLRDALTVEAARQVTRLTMQSGSFMPIGEIVDERSLVNSIVALHATGGS
TNHTLHMPAIAQAAGIQLTWQDMADLSEVVPTLSHVYPNGKADINHFQAAGGMSFLIREL
LAAGLLHENVNTVAGYGLSRYTKEPFLEDGKLVWREGPLESLDENILRPVARPFSPEGGL
RVMEGNLGRGVMKVSAVAPEHQIVEAPAKVFQDQKELADAFKAGELECDFVAVMRFQGPR
CNGMPELHKMTPFLGVLQDRGFKVALVTDGRMSGASGKIPAAIHVCPEAFDGGPLALVRD
GDVIRVDGVKGTLQVLVEASELAAREPAINQIDNSVGCGRELFGFMRMAFSSAEQGASAF
TSSLETLK
>tr|A0A0Q0FW36|A0A0Q0FW36_PSEAP GATase1_like domain-containing protein OS=Pseudomonas syringae pv. aptata OX=83167 GN=ALO85_03272 PE=4 SV=1
MKQSVLLAGESWVSTSTHIKGFDQFDSVTAHNGARRLLAEMADSDYQITQMCADQAQTDF
PATVEALSQYQAVILSDIGANTLLLHPDTWLRGKRTPNRLKALREYVLQGGALMMVGGYF
SFQGINGGARYRGTPVEEVLPVRCLPYDDRVEVPEGFLPQINSGHPILQGLGGEWPYLLG
YNEVELHPEAQLLATVEGSQHPLLAAREVGAGRSLAWTSDLSEHWLPEAFLDWEGYRRFW
INSLDWLTRRT
>tr|A0A0Q0FK31|A0A0Q0FK31_PSEAP Chaperone protein HtpG OS=Pseudomonas syringae pv. aptata OX=83167 GN=htpG PE=3 SV=1
MSVETQKETLGFQTEVKQLLHLMIHSLYSNKEIFLRELISNASDAVDKLRFEALSXXXEL
LEGGAELKIRVSFDKDAKTVTLEDNGIGMSREDVITHLGTIAKSGTADFMKNLSGDQKKD
SHLIGQFGVGFYSAFIVADQVEVFSRRAGTPASEGVYWSSKGEGEFEVATVDKADRGTRI
VLHLKNGEEEFADGYRLRNIIKKYSDHIALPIELPKEQPPAAEGEEAPALEWETVNRASA
LWTRPRTEVKDEEYQEFYKHVAHDYENPLSWGHNKVEGKLEYTSLLYVPARAPFDLYQRE
APRGLKLYVQRVFVMDQAESFLPLYMRFVKGVVDSNDLSLNVSREILQKDPIIDSMKSAL
TKRVLDMLEKLAKNEPEKYKGFWKNFGQVLKEGPAEDFANKEKIAGLLRFASTSDDSGEQ
SVSLAEYLARAKEGQDKIYYLTGESYAQVKNSPHLEVFRKKGIEVLLLTDRIDEWLMSYL
SDFDGKGFVDVARGDLDLGKLDSEEDKKAQEEIAKDKEGLIERLKAALGESVSEVRVSHR
LTDSPAILAIGEQXXXADASDSGSQRAEGAGFQANLRIQPCAPIDQQARCRAERRPLRRS
VAHPVRSGRAGRW
>tr|A0A0Q0C047|A0A0Q0C047_PSEAP S-glutathione dehydrogenase OS=Pseudomonas syringae pv. aptata OX=83167 GN=ALO85_00283 PE=4 SV=1
MMIKSRAAVAFEAKKPLEIVEVDVAMPKAGEVLLRVVASGVCHTDAYTLSGADPEGIFPS
ILGHEGGAIVEAVGEGVTSVMVGDHVIPLYTPECRQCKFCRSGKTNLCQAIRATQGKGLM
PDGTTRFSYKGEPIFHYMGTSTFSEYTVLPEISVAKIDKQAPLEKVCLLGCGVTTGIGAV
LNTAKVKPGDTVAIFGLGGIGLSALIGAVKAKAARIIAIDINPAKFEIAKQLGATDCVNP
KDFDRPIQEVIVDMTDGGVDFSFECIGNVQLMRAALESCHKGWGESVIIGVAGAGQEIAT
RPFQLVTGRVWRGSAFGGVRGRTELPSYVDMAQTGEIPLDTFITHTMGLEDINKAFDLMH
EGKSIRTVIHF
>tr|A0A0Q0ITT6|A0A0Q0ITT6_PSEAP Putative Type I restriction-modification system, M subunit OS=Pseudomonas syringae pv. aptata OX=83167 GN=ALO85_01508 PE=4 SV=1
MADKLSLETLESWLWESANILRGSIDSSDFKNYIFGLLFLKRYNDVFDERVTKLREEENL
SYGEAQEEIEDKWGKYPISARWFDLISRTENIGEALDKAFATIEANNPELQHVLTATQYG

DKRVLADATLQRLLRHFNQYKLGNDDLYKADMLGDAYEYLIKQFADDAGKKGGEFYTPKA
VVQLVVELIDPRPGHSVYDPTCGSGGMLVESAHHVSGLPDGTLMGKPNVLLYGQEKNLGT
WAIAKLNLYLHNMHASIERGDTLVEPKHLDGDYLKTFDRVIANPPFSAKSWWTPLELSNE
NEQENGKKVKAPNYKQVSDPYGRLVYGVPPRGYADLAFAQHMLASLKADGRMGVILPHGV
LFRSGEEGKIREGLLFGTGAASGNQPGDVIEAIVGLPSALFYNTGIPACVLILNKQKPSA
LKDKVIIIDGSRDYLEGKAQNSLHAEDILRIVSTHKAAFEQQVEVENYCRLVTLDEIRSN
DGNLNIVRYIDNGESDEVVNVAATLAHMATLAKEEAQIDQRLNRYLMELGLLETGV
>tr|A0A0N8T846|A0A0N8T846_PSEAP Peptide methionine sulfoxide reductase MsrA OS=Pseudomonas syringae pv. aptata OX=83167 GN=msrA PE=3 SV=1
MTLRSEILVNKNVLPTAEQALPGRETPMALPETHFVNDNPLLGPFSSDVEFAIFGMGCFW
GAERRLWQQEGVVSTVAGYAGGFTPNPTYEEVCSGLTGHTEVVLVVYEPAKISYEALLKV
FWEVHNPTQGMRQGNDIGTQYRSVIYCTTPEQLDAAKASAETFQAELTKAGKGTITTEIE
QAPTVYFAEAYHQQYLAKNPQGYCGLGGTGVCMPA
>tr|A0A0Q0DXC8|A0A0Q0DXC8_PSEAP Multidrug resistance ABC-type transport system, ATP-binding and permease component fusion OS=Pseudomonas syringae pv. aptata OX=83167 GN=ALO85_03700 PE=4 SV=1
MENTMLRAFEKWLDPFPPDEVPPPPDGLARFLWACTRGARGYILAFALLCAGVSIYEAWL
FSFLGQVVDLLSTWKAGGDTAVQESSVLWGIGLVLLSSIVLVALRTMVQHQVLAINLPLR
LRWDFHRLMLRQSLSFFSDEFSGRVTTKVMQTALSVREVLFTIVEIVLGIGVYFIAIIAL
AGGFDLKLMLPFLGWLMLFGLAMLYFVPRLGKVGQEQANARSSMTGRVSDAYTNITTVKL
FSHSKREAHFARAAMEDFKLTGLAQMRLVSQFEIVNQALVVALIMGAGGYALWLWHQGQV
GTGAVAAITAMALRINGMSHWIMWQMTSLFENIGTVQDGMETLTRGPKVEDAPDAKALVT
RGGAVTFDNVSFNYNGERQVLDALSLTIRPGEKIGLVGRSGAGKSTLINLLLRFYDVDKG
SISIDGQDIAHVTQDSLRSAIGMVTQDTSLLHRSIRDNIAYGRPDATDEQIRSAAASAQA
DGFISQLSDKQGHSGYDTLVGERGIKLSGGQRQRVAIARVMLKNAPILLLDEATSALDSE
VEVAIQESLDEMMQGKTVIAIAHRLSTIAAMDRLIVMDDGRIIEQGTHAELLGKNGTYAR
LWQHQSGGFLGEDQGVAEDMG
>tr|A0A0N8T8Q6|A0A0N8T8Q6_PSEAP Amino acid adenylation (Fragment) OS=Pseudomonas syringae pv. aptata OX=83167 GN=ALO85_04451 PE=4 SV=1
IQPDDRVAICAQRSLEMVVGLLGILKAGGAYVPLDPGYPSERLRYMLEDSAPVAVLVQET
LQECLPAVNVPQVVLDSVDLSSGTGGLPCGNPDLDALGLTSRMPAYVMYTSGSSGVPKGV
LIEHRSVLRLVINNPYARITTEDCVAHGANTAFDASTWEIWSALLNGAKLLVVSQATLLN
AARLNHVLIEGGVTALWLTAGLFNEYVDVLAAAFSKLRYLLVGGDVLDPVSVAKVLSRAH
RPRHLINGYGPTETTTFASTYEIHALSDNSRSIPIGRPIGNTRIYLLDAHGQPVPIGVSG
EIHIGGAGVARGYLNLPELTAERFL
>tr|A0A0Q0BTS8|A0A0Q0BTS8_PSEAP Syringopeptin synthetase B (Fragment) OS=Pseudomonas syringae pv. aptata OX=83167 GN=ALO85_04488 PE=4 SV=1
MQDIYALAPLQEGILYHHLAAAEGDPYLQHALFAFDSRELLHNFAQALQDVIARHDILRT
AVFWERLDAPVQVVWREATLGLDE
>tr|A0A0Q0IQT5|A0A0Q0IQT5_PSEAP Protein-glutamate methylesterase/protein-glutamine glutaminase OS=Pseudomonas syringae pv. aptata OX=83167 GN=cheB PE=3 SV=1
MAVKVLVVDDSGFFRRRVTEILSSDPNIVVVGTATNGKEAIEQALALKPDVITMDYEMPM
MDGITAVRHIMQRIPTPVLMFSSLTHEGARVTLDALDAGAVDFLPKNFEDISRNPQKVKQ
LLCEKINSISRSNRRSSGFGSASATPAAPAPSTLTSRASAPAPAPARAVPSRTATPAAAS
AAPAHHAPAHPTTSGTAKRKAYKLVAIGTSTGGPVALQRVLTQLPANFPAPLVLIQHMPA
AFTKAFAERLDKLCKISVKEAEDGDVLRPGLALLAPGGKQMMVDGRGTVKILPGDERLNY
KPCVDITFGSAAKSYGDKVLSVVLTGMGADGREGARLLKQAGSTVWAQDEASCVIYGMPM
AIVKAELADAIYSLDDIGRHLVEACL
>tr|A0A0Q0D2W4|A0A0Q0D2W4_PSEAP DegT/DnrJ/EryC1/StrS aminotransferase OS=Pseudomonas syringae pv. aptata OX=83167 GN=ALO85_05621 PE=3 SV=1
MDRHDSAVACPAVNARLRKMPGHPLGNALIQRNMEASMINVTKTYLGDIDQFKRYVEGIY
ARGWLTNHGPLARDLTDRLKDYLGVRHIILTNNGTLALQVAYRALGLSGSAVTTPFSFVA
TSSSLQWEGIRPIFADIDAHTWNISPKHIENSIAEDTTAIVATHVFGNPCDVQAIEQIAR
EHKLKVVYDGAHAFAVRHAGQSVLNHGDISTLSFHATKLFHTIEGGAIITNDDELARQAH
LMCNFGISGVDRIDGIGINAKLNEFSAAMGLCILDNISNILEERAEIADHYTSSLQDYLD
LQTPQADSQLNHSYYPVALRDEQQLLSVRTALNQHKINPRRYFYPSLDTLEYLQPQPGQP
VSRALSERVLCLPIYPGLLKSEQDLVIRTLIEACNGTETDYRACTVARVC
>tr|A0A0Q0BX86|A0A0Q0BX86_PSEAP Peptidyl-tRNA hydrolase OS=Pseudomonas syringae pv. aptata OX=83167 GN=pth PE=3 SV=1

MAAVVSGSTLNSKGRAPVVTAIQLIVGLGNPGAEYEQTRHNAGAFFVERIAAAQRVNLVP
ERKFFGLTGRFTHQGQDVRLLIPTTYMNRSGQAVAALAGFYRIPVESILVAHDELDLPPG
VAKLKVGGGHGGHNGLRDIIAQLGNQNTFHRLRLGIGHPGDASKVSGFVLGRAPRAEQEK
LDASIDFALGVLPDIFAGEWNRAMKNLHSQKA
>tr|A0A0Q0DBQ1|A0A0Q0DBQ1_PSEAP Methionine aminotransferase
OS=Pseudomonas syringae pv. aptata OX=83167 GN=ALO85_04098 PE=4 SV=1
MIDSKLPNVGTTIFTVMSQLAAETGAINLSQGFPDFDGPQALRDAVCRHVTQGHNQYSPM
TGLPALRQQVAAKIARSYGRTVNPDSEITITPGATQAIFCAIHSVIRAGDEVIIFDPCYD
SYEPAVELAGGRCVHVQLGLDDFAIDWQKLSDALSPRTRMIVINSPHNPSGALISRAELD
RLAALIADRDIYLLSDEVYEHLVFDGARHVSVLDHEALYQRAFVVSSFGKTYHVTGWKTG
YVVAPPALTSELRKVHQYVSFCGVTPLQYALADFMAEHPEHVDELPGFYQAKRDFFCGHL
AESRFSFRPVGGTYFQLVDYSQIRPDLNDVDMALWMTREHGVASIPISVFYQSPPAGQRL
IRLCFAKQEETLRLAAEKLCAI
>tr|A0A0Q0C1H2|A0A0Q0C1H2_PSEAP Transcriptional regulator, AraC family
with amidase-like domain protein OS=Pseudomonas syringae pv. aptata
OX=83167 GN=ALO85_05449 PE=4 SV=1
MALVAITSVQFTTPMRTFWHEAASAAITLLLKRALALSASTLQLISRFMVGPSADRYCGV
SASLCTVETNYPAWRAAKRTAATRFCTAATCPLNAPMNGSINVPRCRSDESAEMAHDFYF
LLMPGFSAIGFISALEPLRVANRFGGDLYRWHVLSADGGAVQSSNGMSVNADAALESLKK
GATLLVVAGFEPLQFATPALQHWLRRLDHEGVTLGAIDTGACVLAETGLLDGHRLTLHWE
AIEAFKESYPQLTVTQELFEIDRRRITCAGGTASIDLMLDLIAQAHGPELAIQVSEQFVL
GRIRTRKDHQRMQIATRYGIANKKLVHVIGEMEQQTERPLSTLSLAEAINVTRHQLERLF
RLHLNDTPSNFYLGLRLEKARQLLRQSGLSVLEVSIACGFESPSYFTRSYRARFTKCPRE
DRRREGV
>tr|A0A0Q0DKA4|A0A0Q0DKA4_PSEAP Peptidase M19, renal dipeptidase family
OS=Pseudomonas syringae pv. aptata OX=83167 GN=ALO85_00494 PE=4 SV=1
MSPAELHADSIVIDGLIIAKWNRELFEDMRKGGLTMANCTVSVWEGFQATINSICTSQKL
MRENSDLVMPVHTSADIRKAKELGKTGILFGFQNAHAYEDQIGYVEIFKKLGVGIVQMCY
NTQNLVGTGCYERDGGLSGFGREIVAEMNRVGVMCDLSHVGSKTSEEVILESKKPVCYSH
CLPSGLKEHPRNKSDVELKFIADHGGFVGVTMFAPFLAKGIESTIDDYAEAIEYVMNIVG
EDAIGIGTDFTQGHGQEFFEYLTHDKGYARRLTNFGKIINPLGIRTVGEFPNLTETLLKR
GHSERVVRKIMGENWVNVLADVWGE
>tr|A0A0N8T858|A0A0N8T858_PSEAP SdiA-regulated OS=Pseudomonas syringae
pv. aptata OX=83167 GN=ALO85_01900 PE=4 SV=1
MAWYQSRPLNLSESPFMRRSTRSLVLLLALVPLIVVFVIANQRYRYVESALFDWQVFWQS
DSLQSIGLDQYRVVTEAQVIDGLQDDVSGLSYDPDRKSLFTVTNQNPELVELSLDGRVLR
RIPLVGFGDAEAVEYISPGTYVISDERRLRLIQIHVDDNTKSLDAAQAEQLTLGITASGN
KGYEGLAYDSVGKRLFVARERDPVQIIEVRGFPKTNTEAPGNLQVISNSKRDGRLSVRDL
SSLQFDETSGHLLALSDESKRILELDTSGHPIGSGSLAKGAMGLSKDVPQAEGMAMDAEG
ALYLVSEPNLFYVFRKP
>tr|A0A0Q0BTE5|A0A0Q0BTE5_PSEAP Glutaredoxin domain-containing protein
OS=Pseudomonas syringae pv. aptata OX=83167 GN=ALO85_01195 PE=4 SV=1
MTLFMKALRVGLGQLVIGIDFLTRPSRKKRDPQAQAAVAEAAQDLTLYQFHACPFCVKTR
RALRRLNVPVALRDAKNNELDRQTLLNEGGRIKVPCLRIEEGDKTVWMYESKVIIDYLNQ
RFGAI
>tr|A0A0Q0DTQ8|A0A0Q0DTQ8_PSEAP Proline--tRNA ligase OS=Pseudomonas
syringae pv. aptata OX=83167 GN=proS PE=3 SV=1
MSCLSNRQRPTMRTSQFLLATQKETPSDAVVVSHQLMLRAGMIRKLASGLYTWLPMGLRV
LRKVEAIVREEMDAAGALEILMPGIQPAELWQESGRWEQYGPELMRLVDRHNREFCLGPT
HEEVITDLARNELNSYKQLPINLYQIQTKFRDEIRPRFGLMRGREFVMKDAYSFHADHES
LQVTYDRMHLAYSNIFTRLGLKFRPVEADNGSIGGAGSHEFHVLAESGEDDIVFSDGSDY
AANIEKAEAIPREKTRPAATEELRLIDTPNTKTIAQLVEGFGLPIEKTVKTLVVHAAEEG
KLIALIIRGDHELNEIKASQLEQVANPLVMASEAELRDAIGAGAGSLGPLNLPLPCIIDR
SVELMSDFSVGANIDDKHYFGVNWERDLPVPTVADLRNVVAGDPSPDGKGTLEIKRGIEV
GHIFQLGTKYSEAMKCQVLGENGKPVNLAMGCYGIGVSRVVAAAIEQNSDENGIIWNDTL
APFQIALVPLRYETDAVREATDKLYADLTAAGFEVLLDDRDKKTSPGIKFADMELIGIPH
RIVVSDRGLAEGNLEYKSRTESQPQAIAVADVLSFIQGKVNR
>tr|A0A0Q0FSY6|A0A0Q0FSY6_PSEAP Uncharacterized protein OS=Pseudomonas
syringae pv. aptata OX=83167 GN=ALO85_101910 PE=4 SV=1
MVPVMLIAMRLPASEGTSLSSAYAVATRLVASRVSTRVRPIACRRLVFGVGTASGIRVAP

LAQENNPDQQCTGHAQNRALRLAMARGPAAGDFTPLRWMVWVRSSQVLPAQTPDTLSIRC
CTDNNESMR
>tr|A0A0Q0DYQ2|A0A0Q0DYQ2_PSEAP Regulatory protein, GntR OS=Pseudomonas syringae pv. aptata OX=83167 GN=ALO85_02013 PE=4 SV=1
MNEHKDVCQGDNNASVRESSVNRQKCMKPMSEEDPRTASRYAMIREVLRNAIVSGAAANG
LVLLEAPLAELFGTSRVPVRKALNLLHEDGLISRFDGRGYLVNPDAREVEPLRLSLSHKQ
FGLDSSEELVDTRPLGERIHDEIGAALSACIAFGHYRLDEQSAAQHYGVSRAVVREALMR
LRDRGLVEKEPYSQWLAGPLTAREITEDYELRACLEPEALRQTAQQLSRETLEAMLARVI
EAQDAEQCSLEEIERLEDDLHQQCLGGLQNRKIATLISQAQSPMIINRIFYRLLSIGADD
AMLAEHRLIIELLLHGAFDAAALNLKEHLLRARQRMLQRLKVLSVLPEQEVPGYLTRLS
>tr|A0A0Q0BUA7|A0A0Q0BUA7_PSEAP Thiamine-phosphate synthase OS=Pseudomonas syringae pv. aptata OX=83167 GN=thiE PE=3 SV=1
MKLRGLYAITDSQLLSGKFLSYVEAALDGGVTLLQYRDKNSDESRRLREATELLKLCERY
KTRLIINDDAELAARLGVGVHLGQSDGSLPDARALLGHKAIVGATCHGRVELAEQAKADG
ATYVAFGRFFNSQTKPGAPAVPLDLIAQVRARVHLPIAVIGGITLENAPQLVEHGADLLA
VVHGLFGAENTQEVTRRAKAFTALL
>tr|A0A0Q0DLR5|A0A0Q0DLR5_PSEAP Putative periplasmic or secreted lipoprotein OS=Pseudomonas syringae pv. aptata OX=83167 GN=ALO85_01604 PE=4 SV=1
MFSLKKIALITAAAALLGSGPVLAQPDLPTQLTEARQEGSIWTAFALNKHLSPFKIDVGV
EQGTATLKGKVENEVDRELAERIALDTKGIEKVDNQLEVDPAVATEPGTRTNMAQRFEDA
TLVATVKSKLLWSSVTEALNIEVDSKDGVITLKGRAQSPEAKELAGSLASNTDGVVSVNN
LISLSAADSIAAKTQPQSLTPTEEMSDAWITSKVKASLIYSRTLDGLNIKVDTKAGVVSL
NGVVANFAEKELAVEIAKNIRGVKGVNGDALKVMARSAG
>tr|A0A0Q0FVK5|A0A0Q0FVK5_PSEAP Uncharacterized protein OS=Pseudomonas syringae pv. aptata OX=83167 GN=ALO85_03839 PE=4 SV=1
MTDTYTASLPSISAAGRESLQHMLTTEGSEQLAAILQRPRPEGFTLLDEIQVLGAASAFG
RWADDIELLNTLKSVLFDDELNALGDSTERVTRSVDSPVSDAGPEHELAKAAFDIAQSHQ
RESHSPSVRTERIDRARGDYPRLLNTLRARLRDVKTGTCMTNSVFMAVADDSPLFAGVAF
GSSILQQFKTLLRVIARVDPAGDLDEDLLPGVRRKDLRYVLDLVDKTQMDFRQPFTCVLH
VRMNEGRPVHSLQLQGVSLGRQPAYATLSDLHLRVIRFTDSLTPLAPHLESADARCILAI
RDDQAVSLHGLLDYYGVDIPLAATRDDLLQVITALLPGEREAHLAMLARALPFTLAEHQR
FMLDTSQVYTFRQHKVQAHGTDDYGVIDLELEKEMRWHLDRCRTLLNSYRHFGASLQDNE
NALLAMFQQAGGITLKLVVYDSDGHHDVYREQHTVNPWRAKDLAGYFDAARQELSSYMIN
TIRYGEAEGAINIERLIVEVIVPNPVQRRHSLSDIDIEAFDIRSKEPPFLYVDPLALGAY
DVIRSRFVELTEELREVQWDVGHSVTLQRAVRYLLAHYQEDLPALPEAQRQAAYARRLND
AVNTYYRVIAQNLEDQQRLQVVLQPTQPEFVARELCRYLGLNESCWPVIARSSFDISYQA
GWINTGTFFKHEESAGYRTLLSVVYDEDLRRELALADNRDERLGRLAQALADKTRYTASL
DALQAKLTGLFDANIAFVNNGHLPARLTAPQAAFRLDAENYAIQATETYQTLEAERLVLV
SGTAAQRLQTLEGAALSDSDRPVHCIELLSADEKVRLEASALAFRQMLSARANDDDIQNM
STSAQHVLQLVHQVGGLIPVLGNFINLGFDIYDGNDEGIAMDALNIIAELVLCKMRTVPP
RVLGVLMLQGTNIWTMSQQVQALGESIKANDYEESVKNFGFMLVGLHSALHCGISAVKGL
RSARPHDQQTVSQRAVPDRSDLTETVRHPEHSDAIEGEHLARRRPSTSSSHWRVSEDGQI
SERQTGRKVTRIDWNGNAMVEGGARAVLLGGISEKIYMLNGFACRAVSGVAGTELRPLHA
LDLEAGQWRRRPQSTVDAPQLSRFAHTEAFPVGFSGKPERFLAPANTVSWYDNCVATLET
VPAQIIDSDSNAKLQTTALGVIEHKYVTERNGHVEVLEHAGTEVETTAFIRADGSRMEVA
RMLPAIPAYKPEIQAEIIAAQGMFVTVRISDTLQGLVGRKTVAGVLATLSDGSGQELVIE
ADRGVHYRGTVPIENFLALAPGETLHDQPPVALTLQKISPKADPKRALPHLWRDQSDYRN
GIAHDDFALELFYGSKAANEAYARNPQWVARNIDAVAKIKSRLPSSITTLENPFYVLDTP
AELAILFAARNQAALANTLLGHTIEWGAASRSRELSLGENILKQLRLNEPLLAAQENIPP
LASERSAFVSQLQEKLARNNLMMAEVSLKSGEKAWFAHSPARHLGMLAGHLHDEFYLSHS
GSVIDDIEAYFTDPSRIESIAVLSLDDVPETDARRIFLSSQRGYTYESVTSLEQPAEHAH
LSGAQLQLHIDDALYPERTRGVGIASAAVDLTGAEAISSGPKRGSYSIEGRDYVKLDNGR
IYRADWDERSGVFLLVPPQGRSRAETLALPWVRHIGGHEFTLINRPALKGGNVIDQMICS
IRTRRPAGSLATHESHSAHDAASGTLQVHHEDLHIMNDDVLVPGKLNVPSSLFHSIAADR
DGYVRLEAKAAVPESCISSTEFLVSKYAGGQVAFDYRGESLGDPTPLVPNVEVVALQDGQ
PVSLNITTTALDDWISARDHSDYFVGEQSFPEIGEGIGFIERGGLEGSPSSDYPFHFMFV
AGKVLDQNGQITGVLASDLSEPAREPDQNGVTPQLPARRNWNLGLFPSIDSMRGSPEPDS
NAYVQEHYYSVWVRAVRE

>tr|A0A0Q0FT02|A0A0Q0FT02_PSEAP Uncharacterized protein OS=Pseudomonas syringae pv. aptata OX=83167 GN=ALO85_100907 PE=4 SV=1
MNGLKVICKINDLKDISDEVVLGVLKDKIFRPDGKIDISLGSEYVVYGIEFWENSPWYYL
CVDVDDEYPKPYAADLFFVSNNKLSTHWQLGFCTNYNGSQVASLVFEEWAVDRRFYERLI
DGGEIEVGVFKKYKSLMDDEAK
>tr|A0A0Q0IC24|A0A0Q0IC24_PSEAP Penicillin amidase OS=Pseudomonas syringae pv. aptata OX=83167 GN=ALO85_04982 PE=4 SV=1
MCRVSSSQLRHNDSLHSCLRHLQRNRMKRSLFILAIIIALLAGVAGWYVNSKLPVRDGEI
AMSRLQAPVTLSYDERGVPHIRAENEADLYRALGYAHAQDRLFQMEMLRRLARGELAEVL
GPNLVETDKLFRTLRIREHADAYVARQDKNTPTWKALEAYIDGINQYQDTHARPMEFDAL
GIPRRRFTTEDTVSIAGYLAYSFAAAFRTEPLLTYVRDQLGPEYLKVFDLDWHPDGMLDS
KPALADADWKGLNQLAQLSHKALEDAGLPQFEGSNAWVISGSRTQSGKPILAGDPHIRFS
VPSIWYEAQLSAPGFELYGHFPALNPFALLGHNMDFGWSLTMFQNDDVDLIAEKTNPQNP
NQVWYHGQWTDMKRSEQQIAVKGQAPVTLTLLESPHGPIVNNVMGKNAGQTPIAMWWSFL
ASANPVLDGFYEANRADTLEKMRSAAEKIQSPGLNIVWANAKGDIGWWAAAQLPIRPQGV
NPIFILDGSTAQADKLGFYPFSANPHEENPARGYIVSANFQPLSPTGMQIPGYYNPAERG
QALNRQLSDSSIKWDLAASKALQLSTQTDYAPSILRPLIPVLRSVVSDPDERALVERLAS
WKGDYPVDSVGATLFNQFLFDLTEETFHDELGDAMFETLLSTRVLDAALPRLAADADSPW
WNNRNSPHEESRANTVKVAWRASVSHLRSLYGTNPDEWVWGKAHTLTQGHPLGSQKPLDM
IFNVGPYAAPGTHEVPNNLSSSIRPAPWPVGYGPSTRRLIDFADPAHSLGINPVGQSGAP
FDKHYSDQAKAFVNGEYLPQRFSEKDIAEHTEGVLRLVPGA
>tr|A0A0Q0IPJ0|A0A0Q0IPJ0_PSEAP TPR repeat-containing protein OS=Pseudomonas syringae pv. aptata OX=83167 GN=ALO85_05440 PE=4 SV=1
MPFDSCQPLPEAATRQALEIDMPRLSTTGLSPQPTSRRRWLIGAAAAALLVLSLVFWWWR
MPIQTAPVKLSHTYAQALEQAHDGKPGAARVLYQQLARTDLSDTQRISLLAELNDYPSPQ
ALKLLDAALSNPSAQVREAAIDVSVRLVSNSQRSLLLGPLLDDAEQSVRFRATSALLGLS
PDELGLYFAVLQQSAEAFQESLKSQPQSAQNQLQLARLYLQTGDLEPALTALQRATALDP
GNIEAALAHIELLDRKGQAEQARGLFAQLLEHNPGSSMLQHALGMWLLNHGQAEFAVLSL
AKATELAPDNNDYRYDLAVALHSLNELEAAQRQLTQIVQNQPANRKARVLLIQYWKENGQ
LQNVQILLAELEQQNPDDPVLQQGL
>tr|A0A0Q0DUI3|A0A0Q0DUI3_PSEAP Shikimate dehydrogenase (NADP(+)) OS=Pseudomonas syringae pv. aptata OX=83167 GN=aroE PE=3 SV=1
MMDQYVVFGNPIGHSKSPLIHRLFAEQTGQALNYQTALAPLDDFTAFAQAFFQTGRGANV
TVPFKEEAFRLADSLTERGQRAGAVNTLSKLDDGTLLGDNTDGAGLVRDLTVNCARSLRG
QRVLLLGAGGAVRGALEPLLAQQPAALVIANRTVEKAERLAQEFADLGPVFASSFDWLEE
SVDVIINATSASLAGELPPIAPSLIQPGVTFCYDMMYSNEPTAFCRWATEHGAAQAVDGL
GMLVEQAAEAFLLWRGVRPDSAPVLAELRRLLAAG
>tr|A0A0Q0IQW5|A0A0Q0IQW5_PSEAP Syringomycin biosynthesis enzyme 2 OS=Pseudomonas syringae pv. aptata OX=83167 GN=ALO85_02600 PE=4 SV=1
MSKKFALTAEQRASFEKNGFIGPFDAYSPEEMKETWKRTRLRLLDRSAAAYQDLDAISGG
TNIANYDRHLDDDFLASHICRPEICDRVESILGPNVLCWRTEFFPKYPGDEGTDWHQADT
FANASGKPQIIWPENEEFGGTITVWTAFTDANIANGCLQFIPGTQNSMNYDETKRMSYEP
DANNSVVKDGVRRGFFGYDYRQLQIDENWKPDEASAVPMQMKAGQFIIFWSTLMHASYPH
SGESQEMRMGFASRYVPSFVHVYPDSDHIEEYGGRISLEKYGAVQVIGDETPEYNRLVTH
TTRGKKFEAV
>tr|A0A0N8T915|A0A0N8T915_PSEAP Polyamine ABC-type transport system, ATP-binding protein OS=Pseudomonas syringae pv. aptata OX=83167 GN=ALO85_02508 PE=3 SV=1
MSFVSIENLQKSYASTSVFSDINCSIAKGEFVTLLGPSGCGKSTLLRCIAGLTPVNSGRI
LLDGNDLVPLTPQKRNIGMVFQSYALFPNMTVEQNVAFGLRIQKVSADESARRVAEILRM
VELHDFATRYPHQLSGGQCQRVALARSLVTRPRLLLLDEPLSALDARIRKNLREQIRAIQ
RELSLTTIFVTHDQEEALTMSDRIFLMNQGRIVQSGDAETLYTAPVDVFAAGFIGNYNLL
EADDATRLMQRPINSRVAIRPESIQLSLTGELEGEVRSHSLLGNVIRYRVQARGVDLVVD
VLNRSADDLHPDGRRVTLNIEPSALCALD
>tr|A0A0Q0C9W5|A0A0Q0C9W5_PSEAP Putative dodecin OS=Pseudomonas syringae pv. aptata OX=83167 GN=ALO85_04898 PE=4 SV=1
MTEAGSALYIASFSDLRRTAMSDHHTYKKVEIVGSSTTTIEDAINNALAEASKSLDLMEW
FEVTETRGHIENGKVAHFQVTLKVGFRITNS
>tr|A0A0Q0BY01|A0A0Q0BY01_PSEAP Ribonuclease E OS=Pseudomonas syringae pv. aptata OX=83167 GN=rne PE=3 SV=1

MCAERRFRQPGNRRYYMKRMLINATQPEELRVALVDGQRLYDLDIESGAREQKKANIYKG
RITRIEPSLEAAFVDFGSERHGFLPLKEISREYFKKAPEGRVNIKDVLSEGQEVIVQVEK
EERGNKGAALTTFISLAGRYLVLMPNNPRAGGISRRIEGEERNELREALNGLIAPADMGL
IVRTAGLGRSSEEMQWDLDYLLQLWTAIKEASLDRSAPFLIYQESNVIIRAIRDYLRQDI
GEVLIDSIEAQEEALTFIRQVMPQYASKIKLYEDSVPLFNRFQIESQIETAFQRVVELPS
GGSIVIDPTEALVSIDINSARATKGSDIEETALQTNLEAAEEIARQLRLRDIGGLIVIDF
IDMTPAKNQRAVEEKVRESLEADRARVQIGRISRFGLLEMSRQRLRPSLGESSGIVCPRC
NGTGIIRDVESLSLAILRLIEEEALKDRTAEVRAQVPIPVAAFLLNEKRNSITKIELRTR
ARIIILPNDQLETPHFEVQRLRDDSPEAHSSQTSYEIAAAAAEVEEIAPLAAATRTLVRQ
EAAVKTAPARANAPVPVEAAAPAPTPAPAPVAHEPSLFKGLVKSLVSLFATKEEPVAPVV
VEKPVAEQRPARNEERRNGRQQSRGRNNRRDEERKPREERAPREERAERAPREERAPREE
RAPREERTVREPREAREESAPREERPARTTRERKPREAREDRPVRELREPLDAAPAVNLA
REERPERAPREERQPRAPREERQPRAEQAAAAVSEEEEVLLNEEQTHDENQEGNDGSEGD
RPRRRSRGQRRRSNRRERQRDANGNVIEGSEEEEGSATDLSAGLGFTAAASAASSVISAP
AEADAHQQAERANSNAAAADAEPVHQETAAEAPVTETPVAETSATEAPVAETTVEAPAVE
APVADDAPVAQPAPEVEVQPAAVEAPAIAAQQSELFEAPHAERVVPFKPTPEPAPQAPVE
AAAHEEVPATESSELPTPAPAAVVEPVALKEEPAPYVAPQPVVEEQAPAPQEQAPAAEEA
APAPVVSSTGRAPNDPREVRRRKREEEARRQQEAAAASAPVVEAAPAATEAESAQPSPAA
EEKTEEAATEQPAVKPQHEAEKENEPKPLV
>tr|A0A0Q0CCQ4|A0A0Q0CCQ4_PSEAP 3-hydroxyisobutyrate dehydrogenase OS=Pseudomonas syringae pv. aptata OX=83167 GN=ALO85_02769 PE=3 SV=1
MNIAFIGLGNMGTPMARNLIKAGHALQVFDLNKSVLAEFAELGAQVSDSPAQAAQGTELV
ITMLPAAAHVRSVYLNDDGVLKGIASGVPAVDCSTIDPQTIRDIAAVAAQQGVVLGDAPV
SGGTGGAQAGTLTFMVGGSAGHFAVLKPVLEQMGRNIVHCGDVGTGQIAKICNNMLLAIS
MIGVAESMALGNALGIDTQVLAGIINTSTGRCWSSEAYNPWPGIVETAPASRGYSGGFGA
ELMLKDLGLATEAARAAHQPVVLGAVAQQLYQAMSLRGDGGKDFSAIIEGYRPKA
>tr|A0A0Q0DFF4|A0A0Q0DFF4_PSEAP Amino acid ABC transporter ATP-binding protein OS=Pseudomonas syringae pv. aptata OX=83167 GN=ALO85_04925 PE=4 SV=1
MASLLWATSSCATPCRCARAIWKGDCMPLLRISALHKYYGDHHVLKGIDLSVEEGQVVAI
IGRSGSGKSTLLRTLNGLESINDGVIEVDGEYLDAARADLRTLRQKVGMVFQQFNLFPHL
SVGENVMLAPQVVQKVPKAEAAKLARKMLERVGLGEKFDAFPDRLSGGQQQRVAIARALA
MSPKVLLCDEITSALDPELVNEVLSVVRQLAADGMTLIMVTHEMRFAREVGDKLVFMHQG
KVHEVGDPKVLFANPKTPELANFIGTVEHA
>tr|A0A0Q0C3E7|A0A0Q0C3E7_PSEAP Aminotransferase OS=Pseudomonas syringae pv. aptata OX=83167 GN=ALO85_100125 PE=3 SV=1
MPGADPPATIARPFLVRRWFICGHKRCLSTQNELVLWRAPRWLVTYSSSRPSTRNANVTA
SRIAARVLRIKPSPSSAAADRANELRRAGKSIINLVVGEPDFDTPAHIRQAACTAIENGE
TRYTLNAGTTALREAIAAKLQRENRLAYAPGQIVVTSGAKAAIFNAFAATLGVDDEVLIP
APYWVSYPDMVLACDGRPVTLACPEEDQFKLTATQLRNAITPRTRWLLLNSPSNPTGATY
SASEWRALADVLLDYPHVLVMTDDIYEHIRYDDAPLSHLLTVEPALRKRVLIINGVSKTY
AMTGWRIGYAAGPDDIIKAMATLQSQSTSNACSVSQAAAVAAFNGDQSFVSDSVKVYKQR
RDRCLELINAIDGLSCIKPEGAFYLYVNCGALIGKTTPSGSVLEADTDVVLYLLESQGVA
VVAGTAYGLAPFFRMSTATALETLEDGCARMATAVAALR
>tr|A0A0Q0CUY8|A0A0Q0CUY8_PSEAP YicC-like protein OS=Pseudomonas syringae pv. aptata OX=83167 GN=ALO85_03987 PE=4 SV=1
MVHSMTAFARVERAGAQGTLSWELRSVNHRYLEPHLRLPESFRDLEGAIREALRQGLSRG
KVECTLRFVEETAGKPLQVDLERAGQLIAAAESVAGLIKQPAALNPLEVLAWPGVLVADA
NDPQVLNTEALKLFNDALTELKSGRGREGADLARLLDERLSSIVEEVATLRTLVPQMLAT
QRQKVLDRFADMKAELDPQRLEQEMVLLAQKSDVAEELDRLSTHVTEVRRVLKAGGQAGR
RLDFLMQELNREANTLGSKAFDPRSTQAAVNLKVLIEQMREQVQNIE
>tr|A0A0Q0CXH4|A0A0Q0CXH4_PSEAP Acid phosphatase OS=Pseudomonas syringae pv. aptata OX=83167 GN=ALO85_04765 PE=4 SV=1
MNQREYLAMSDRPDDEDTNENPGRRRFLGGMAALGAGVTLSGYVSAREKPPGDNERHHHL
AGPALDRALRENVKTIVVIYAENRSFNNLFGDFPGVEKPLASLRPSDYEQRDRDGTRLSK
LPPAWGGVLQVGPQTVDGVTYPVGEQFQQDLPNAPFPLKGPNGEDLPLSLVTRDLWHVYY
QNQMQINDGKNDRFVAWADAGGLTMGNYAQTQYSLRLWDVAEEFVLCDNFFQGAFGGSFL
NHQYLISATAPVYPNAAESPAKSQIATLQSFNPLDPRLKPLDKSPASAMDGPPQFGPSAI
TPDNYAVNTMAPPYWPTWLRDPQNPDYSKPDLPNVLVPQSHEHIGDKLSKRNVDWAWYAG
AWQVTLDEFKDSTGIPKIPNFQYHHQPFNYFKQQGPQHPEERKKRLRDGGLGDESSTNRF

LADAEAGKLPAVTFYKPQGNLNMHAGYADVAAGDRHIDRVIKVLRKSPQWDNMVIVVTVD
ENGGWWDHVAPPKGDRFGPGTRVPALVISPFARKGKVDHTVYDTASILRLITRVHGLEKL
DGLKRRDEAMIARGQAPMGDLTNALQFPA
>tr|A0A0Q0IGZ9|A0A0Q0IGZ9_PSEAP Dipeptide ABC-type transport system, ATP-binding protein OS=Pseudomonas syringae pv. aptata OX=83167 GN=ALO85_00014 PE=4 SV=1
MSVVLTARDLTRHYEVSRGLFKGTALVRALNGVSFELEAGKTLAVVGESGCGKSTLARAL
TLIEEPSSGSLKIAGQEVAGASKAERKQLRKDVQMVFQSPYASLNPRQKVGDQLGEPLLI
NTSLSAAERREKVQAMMAQVGLRPEHYQRYPHMFSGGQRQRIALARAMMLQPKGW
>tr|A0A0Q0BWE9|A0A0Q0BWE9_PSEAP Iron-dicitrate transporter substrate-binding subunit OS=Pseudomonas syringae pv. aptata OX=83167 GN=ALO85_03064 PE=4 SV=1
MRAFRLPHLLACGLLTLIASVSQAAPIDIDDGQHKVHLPDTPKRVVVLEFSFLDGLASVG
VTPVGAADDGDANRVLPKVRKAVGEWQSVGLRSQPNIEVIARLKPDLIIADLGRHQALYN
DLASLAPTLMLPSRGEDYQGSLKSAELIGVALGKGPQMQARIEENRQHLKTIAEQIPANS
NVLFGVAREDSFSVHGPHSYAGSVLQAIGLKVPEVRKNAAPTEFVSLEQLLALDPGWLLV
GHYRRPSIVDSWSKQPLWQVLGAVRNKQVAEVDGDSWARNRGIMASEQIADDALAVLKGG
KAVLNP
>tr|A0A0Q0C1X9|A0A0Q0C1X9_PSEAP DUF1624 domain-containing protein OS=Pseudomonas syringae pv. aptata OX=83167 GN=ALO85_00834 PE=4 SV=1
MTTAVHSNRAPASRLRSIDALRGLIILFMLLDHVRETFFMHHQVIDPMDVTTTAPELFFS
RTLAHLCAPLFVLLTGLSAFLYGEKHQGKTDVAAFLLKRGLFLIALEFTLVSFAWTFQFP
PTIIYLQVIWAIGLSMVALSLMVFLPRWALVVIGVVIIAGHNLLDTVHFGVESAMHVPWA
ILHDRGWIEASDNLRLRTSYPLLPWIGIIALGYAAGPWFSSSYDGAKRRANLWKCGAGAL
LGFVVLRLINGYGEKPWSVGDSGIQTLMSFFNITKYPPSLLFICLTLGIGLLILIAFERS
QEKSWLRPLVVLGSAPMFFYLLHLYVLKILYLIALAIWGTNQGTYFGFDHMWGVWLTSVV
LAVLLFPAVRWFAALKARRRDISWLKYF
>tr|A0A0Q0DCC8|A0A0Q0DCC8_PSEAP GGDEF/EAL domain protein OS=Pseudomonas syringae pv. aptata OX=83167 GN=ALO85_01591 PE=4 SV=1
MPTPVEPLRLLLLAHEPFWQALLSGCLTAPGGNVILLHGANWDVFSARYANERNTVLLTT
PDLQPRTGACLLPTVVLLETEPLVGPTGVSDWLVRDALTGDTLRRCLRYVHERSRLEDSL
RRLVGQDPLTGIANRQGFQALLSARLSEGGGVALGHLDLDNFRRANDSLGHQAGDRLILQ
VVARLKNQLDAGDQIARLGGDEFALLIDTRTAPERALQMAERITDVLAEPYWVDGESLLL
GCSLGIAHSQAQPEADPLMWHAHIAMRQAKSMQGCTYHLFDERINRNARSLADLEGELRR
ALRRDELELHYQPRLCLDSGRIVGLEALVRWRHQERGLLPPSEFVPLAEQSGLIVPLGYW
VIFRALRDMQALREQGFAPLHMAINLSFRQFQDSQLLPTLNRLIAEHNVDPRWLEFELTE
TAVMRRSDQVRQTMDALRQLGVRFSLDDFGTGYSSFMHLNSLPIALLKIDMGFVGQMELR
EENRKLVNAMINLAHNLNLEVVAEGVETAGQLELLRSFGCNQVQGYLISRPLPVEELVTY
LRSQAAHAGTSRSG
>tr|A0A0Q0DNX7|A0A0Q0DNX7_PSEAP Regulatory protein, LuxR OS=Pseudomonas syringae pv. aptata OX=83167 GN=ALO85_00377 PE=4 SV=1
MGDAGFASNMYQIIAASVPIDCLDLSEWTIDENEGSVISIQPLGQETADHKPMILTVSQN
QETLLARMLSVDDFLLVHLKLPSSVGQASSGPTGTYHCNLVSRKSNRRCVISLHRDHHQR
DFSLHELSFLKNFSEALLPLLDRHAQTTRTLCTAHADNEAVTQGGQQLQREFNDKLSQAE
VRLSLREKEICLGILAGATVPELADKLHVKNSSIETYLKRAGAKLGVKGRHGLVKWMTGV
SQTA
>tr|A0A0Q0DKB3|A0A0Q0DKB3_PSEAP Uncharacterized protein OS=Pseudomonas syringae pv. aptata OX=83167 GN=ALO85_200199 PE=4 SV=1
MQVTAAVIQVIQVLAGGKGQCGQVAEWIVVVVQRALWRRFLCQAAQIVLPQVFGFVGCIN
ETEGRLTRC
>tr|A0A0Q0FKA4|A0A0Q0FKA4_PSEAP Low molecular weight phosphotyrosine protein phosphatase OS=Pseudomonas syringae pv. aptata OX=83167 GN=ALO85_04343 PE=3 SV=1
MFSSVLVLCVGNVCRSPMAVAMLHERLQATQVRIQSAGIAALSGSSIDPTAHAVLQSHRV
QPRRHAARQMSRELLRQADLILLMEQAHFSDVLELAPEVRGKAFLIGKWQDPLDITDPYR
RPASAFEQTYAQLSRCIDDWLPHFQSGATG
>tr|A0A0N8T7T2|A0A0N8T7T2_PSEAP Uncharacterized protein OS=Pseudomonas syringae pv. aptata OX=83167 GN=ALO85_02313 PE=4 SV=1
MSKAKGKPPILTLSPEHEQQAIDKLKRLFAERFELDLGSFEVAEVLELFTREIAPHYYNR
AIFDVQQQLKERFESIESDLWALEKN

>tr|A0A0Q0ID75|A0A0Q0ID75_PSEAP Autotransporter barrel protein with phosphatase-like passenger domain OS=Pseudomonas syringae pv. aptata OX=83167 GN=ALO85_01661 PE=4 SV=1
MCHDSYIEVSERLARVGALFDLSEAPAMRSNKPVVHVMVSSLILSLLAVSVQAASRANDN
RINGVDLLSGFNTLWTTGATWDTGTPTALGQSLLRRNLQIVVDRANSRTLAQETAAYFDD
RRDQSYSAISGLGSLSDAYKAGAGAFTTITQFDDSNKTVKYDDKGNGAGSSSSALGKVVD
LVGAVRNDASTTPAKSHYLYPRPWRQSLDGQSLAFVVAPSLRPAESTTPASDSGFPSGHT
NAAYLSAYALAYAIPERFSELMLRASEIGDNRIEAGMHSPFDVMGGRITATYFAIDNLSN
SANTQLRADARAQALAYFTAQCGGNVNNCMTAIDPATDRTSQHAQDKALYTARMTYGFDP
VGPTNLAAVVPTNAEVLLETRFPYLDASQRREILATTELSSGYAVIDQSGGYGRLNLYAA
GDGYGAFNSNVTVNMNASLGGYNAIDAWRNDISGSGALIKNGTGNLILTGNNTYSGGTLI
NGGTLTGHAQAFGSGTITDNATLVVDQSTNDTLANTLTGTGALIKRGIGSLNLTGNSSLS
GATTVQAGRLAVNGNLGNSIVSVQQGATLGGNGTVGGINVAQGGVVAPGNSVGQLNVNDD
VNLAQGSVYQVESDASGKADRIVASGRATINNSTLSLVEGGNWLAASRYSILSAAGGVSG
AFAAVQTNFAFLTPTLNYTATDVGLTLDRNAQTFASLATTRNAKAVAQGLDSAGAGNALW
RQVVQDDASTAQATFNALSNELHASTQSALIEDSRLVRNAMNARMQQAQSAQAFGSTTQT
LAGDASRGVVWTQAIGATGQTDSSRDASGLETRTSGLLFGADVPLDDTWRVGALAGFSNS
SFDLRHASGSTDSDNYHLGVYGGAKWGQLGLRLGAVRTWHELTAKRTLDLPGSSEHFKED
YKAATNQVFGELGYTLEMGNAQLEPFANLAHVRLDTDAFDENSNAISLQNKSQDNHITFS
TLGLRAATRLNAGSVVIKPNATLGWRRAYGDVTPESRSAFSGGDTFELSGAPIARSAAVL
GAGVDLGLSDTLSVGLSYDGQVSNDASDQSLNARVTLAF
>tr|A0A0Q0IIV8|A0A0Q0IIV8_PSEAP Homocysteine S-methyltransferase OS=Pseudomonas syringae pv. aptata OX=83167 GN=ALO85_01731 PE=4 SV=1
MKQGTTVILDGGMGRELQRRGAPFRQPEWSALALSEAPEAVSAVHAAYIESGAQVITSNS
YAVVPFHIGEERFAREGQALAALAGQLARESADASGGRARVAGSIPPLFGSYRPDLYQPQ
LAADVLKPLVAGLSPYVDLWLAETQSCILEAQTIRAGLPNDGKPFWLSFTLQDEDTDEVP
RLRSGEPVADAARAAAAMGVATLLFNCSQPEVIGGAIDAAREVFTSLNVDIAIGAYANAF
PPQPKDATANDGLDELREDLDPQGYQQWAADWVKRGATHIGGCCGIGPEHIAVLSRSL
>tr|A0A0Q0CVL8|A0A0Q0CVL8_PSEAP Type III secretion protein HrcQa OS=Pseudomonas syringae pv. aptata OX=83167 GN=ALO85_04186 PE=4 SV=1
MSALRLRKVDALLAQATRALGAGRRLGFSARGQHAELSVLPQHEDARIPADGVWLNTAVG
PLLLSDAEALLSLLGEVPLTLGGEYQAWYWQLFNQRLSTVIADLLAPVAPFSDAPTEPAI
GCRVLVRLGSERLDAHLHAAPATLLRLLGSADWQVLNRNVDESWSVATPLIVGELSLTRE
QIALLRPGDVVLPARCRFDSAGQGSVALAGRQWAARTDQQAQHLFLQLGHEEHSHHEY
>tr|A0A0Q0C9N1|A0A0Q0C9N1_PSEAP Phosphoenolpyruvate-protein phosphotransferase OS=Pseudomonas syringae pv. aptata OX=83167 GN=ALO85_03318 PE=3 SV=1
MLELTLEQISMAQSAVDKTAALKLIADHLVADGLVAEGYLTGLMNREQQGSTFLGQGIAI
PHGTPETRDLVFTTGVRLMQFPEGVDWGDGQMVYLAIGIAAKSDEHLRLLQLLTRALGEE
DLGQALREAKTPEDLLKLLQGAPQELALDAQMISLGVSADDFEELVWRGARLLRKADCVS
NGFAAVLQQVEALSLGDGLWWLHSEQTVNRPGLAFVTPDKPMRYLGQPLTGLFCLASLGE
AHQALLERLCLLLIEGRGHELGHATNSRAVLEALGGELPAEWPTRQIQLANAHGLHARPA
KILAQLAKEFDGEIRMRVLETGEAAVSAKSLSKLLSLGARRGQTLEFIAEPTIADDALPA
LIAAVEQGLGEEVEPLPAVSQAEAAPAPTPAAAKPLSAPPAGSVLQAVSASPGIAMGPAH
IQVLQSFDYPQRGESVAAERERLHKAIGEVRSDIENLIQRSKSKAIREIFITHQEMLEDP
ELTNEVEARLNNDESAAAAWAAVIETAAVQQEQLQDALLAERAADLRDVGRRVLAQICGV
ETVAAPDEPYILVMDEVGPSDVARLDPAQVAGILTARGGATAHSAIVARALGIPALVGAG
DEVLLLKPGTVLLIDSQRGRLTVAPDEETLQRAVQDRDAREQRLKAAAEARMEPAVTRDG
HAVEVFANIGDSTGTPAAVEQGAEGVGLLRTELLFMAHSQAPDEATQEAEYRRVLTDLGG
RPLVVRTLDVGGDKPLPYWPIAKEENPFLGVRGIRLTLQRPDVMESQLRALLRSADSGPL
RIMFPMIGTLEEWRQARDMTQRLREEIPVSDLQLGIMIEVPSAALIAPVLAKEVDFFSIG
TNDLTQYTMAIDRGHPTLSAQADGLHPSVLQLIDMTVRAAHANGKWVGVCGELAADPLAV
PILVGLGVDELSVSARSIGEVKACVRELTLSSAQQLAQNALTAGSAAEVRALVEAV
>tr|A0A0Q0C1A6|A0A0Q0C1A6_PSEAP Substrate-binding region of ABC-type glycine betaine transport system OS=Pseudomonas syringae pv. aptata OX=83167 GN=ALO85_02094 PE=4 SV=1
MNMKKALLTTLVSAGLLASAGASQAAGWCESGKPVKFAGLNWESAMLLTDILQVVLDKGY
GCTVDALPGNSIAMENALGTNDIQIFTEEWVGRSEVWNKAAAAGKVVGVGAPIVGAVEGW
YVPRYVIEGDAKRKLEAKAPNLKSISDLAQYSAVFKDQEEPGKGRFYNCPAGWTCELENS
EMLKSYGLESKYTNFRPGTGPALDAAILSSYKRGEPILTYYWSPTPLMGQVDLVRLEEKA

GVNKTIDIKVGVSKVFHDEAPELVAILEKVNLPIDLLNQNLGRMAKERIESPKLAKIFLK
EHPEVWHKWVSEDAAKKVDASL
>tr|A0A0Q0DLF1|A0A0Q0DLF1_PSEAP Ribonuclease D OS=Pseudomonas syringae pv. aptata OX=83167 GN=rnd PE=3 SV=1
MAIDIHWIRDDDSLARHCAQWQSLPFVALDTEFMRVDTFYPIAALLQIGDGQSAWLIDPL
LINDWRPLSALLENPDVIKVVHACSEDLEVLLRLTGSLPVPLFDTQLAAAYLNLGFSMGY
SRLVQEVLDIDLPKGETRSDWLQRPLSETQISYAAEDAVHLAELFTILRPRLSDDKYAWL
LDDGAELVANLRREVDPYEVYRDAKLAWKLSRAQLAVLRELCAWREREARARNLPRNRIV
REHSLWPLAKTQPDNLGALARIEDMHPRTVRHDGEFLLELIKTASVLPPEEWPPALPEPL
PIDAAGSIKRLRVIGQQYAERLDMAPELMLRKKTLEALLKSGYPDGPYQLPDSLRGWRRE
LMGQALLDSLATPGEPS
>tr|A0A0Q0BRL9|A0A0Q0BRL9_PSEAP Glyceraldehyde-3-phosphate dehydrogenase OS=Pseudomonas syringae pv. aptata OX=83167 GN=ALO85_05607 PE=3 SV=1
MQKDGCMTLRIAINGFGRIGRNVLRALYTQGYRQDLQVVAINDLGDSEMNAHLLRFDTVH
GPFSGTVECDKDSLTVNGDRISVSAIRNPAELPWKAQDIDVVFECTGLFTSRDKAAAHLT
AGARKVIISAPASGADATIVYGVNHDTLRQSHQIISSASCTTNCLAPVAQVLHRELGIEN
GLMTTIHAYTNDQNLIDVYHTDPYRARSATQSMIPSKTGAAEAVGLVLPELAGKLTGMAV
RVPVINVSLVDLTVTLKRETTVDEVNALMKEASQHSKVLGYNTLPLVSHDFNHNPLSSIF
DANHTKVSGKLLKVLSWYDNEWAFSNRMLDNCLALHNAQ
>tr|A0A0Q0D5G5|A0A0Q0D5G5_PSEAP Putative membrane protein OS=Pseudomonas syringae pv. aptata OX=83167 GN=ALO85_02439 PE=4 SV=1
MTVTTHPVYRYTPGPLHATLLAGTVPLFLGGLLSDIAYYQTFQIQWSNFAAWLIAGGLLF
CGLALLFALVNLVRADHKAGRPVVYFLLLLVTWVLGLVNAFEHAKDAWAVMPSGLILSVI
VTVLACVATWTGLTSLRSGGAE
>tr|A0A0Q0BS10|A0A0Q0BS10_PSEAP Amino acid adenylation (Fragment) OS=Pseudomonas syringae pv. aptata OX=83167 GN=ALO85_04526 PE=4 SV=1
MDRIVARHETLRTHFALHEGQAIQVIAPATQGFALITHDLRALDSAAQHKTQDEMQHEAV
ERLAGEEALAPFDLSRGPLIRGRLVQLSETEHILLVT
>tr|A0A0Q0D7M0|A0A0Q0D7M0_PSEAP Uncharacterized protein (Fragment) OS=Pseudomonas syringae pv. aptata OX=83167 GN=ALO85_05639 PE=4 SV=1
MEVVDDTIDLNKISSKGAVKYVTISVDYKDMRDGDQVRVIMEGLDEKQQAVTQSDLRPVS
SGNLSSNPNFRPMTFIWREEKIKVFNGGVDIYYQAYREGIWYNSIKRSLYVGSRLVGLAP
FLQDDDHQNIVGPLDPDKVFGYVNVRIPFSGTLVNDKITMFWHDSSLQNRLTETLTVTPG
NVDQDLNIEVYLDRTINSYRGKFVTVYYQIERTLPGGQNLRFNSPAYKFFVGTQQEQDAA
DSRVLKGAVVGGVKDGSMDVSLVRTGTTLTVPFAETEEGDKVTAYWQLAEGGAPTVLGTQ
AVVAANVNNDLVFNISAATVNTALNKKATAYYVIERQGLDGKPRKFRSQEASFSVGPQLA
DLSLPTPEVPAVVDAVLNPMVALNGTKVVIRPSAAFAVGDTVRLFFSGTGGAGTPDIATQ
TVSSLSQALEFIIPASAIGANMGSVVWVYYNVTRNGLARPIESQENLFKIERLGLDNFSA
PVIAQAPGGVLDLAAVNTDIVIVLKVWLFMAAGQLVWLSLQGVAKDGSFLEINVWTAKPL
TGTEAQQDLAITVARAEFDKLADGSEFEVFAKVSVDQDIEDLYAEEFPVLTVAIKSQVNP
SGGGSTGGASGTGSTGGAS
>tr|A0A0Q0C6Z0|A0A0Q0C6Z0_PSEAP Acetyl-CoA acetyltransferase OS=Pseudomonas syringae pv. aptata OX=83167 GN=ALO85_05310 PE=3 SV=1
MFLRSTRLIHSLEEREFSMTHPRRVAIVGGNRIPFARSNGPYATASNQDMLTAALEGLIE
RFNLHGLRMGEVVTGAVLKHSRDFNLTRECVLGSRLSAQTPAYDIQQACGTGLEAALLVA
NKIALGQIECGIAGGVDTTSDAPIGVNEGLRKILLQVNRSKTPGDKLKALMQLRPHHLKP
ELPRNGEPRTRLSMGQHCELMAQTWGIERGPQDQLALESHLKMAAAYAEGWQDDLMTPYL
GLIRDNNLRPDLTLEKLASLKPAFERSAKGTLSAGNSTPLTDGASVVLLASEEWARERGL
PVLAYWRDGEAAAVDFVNGAEGLLMAPVYAVPRLLARNGLTLQDFDYYEIHEAFAAQVLC
TLKAWEDADYCKTRLGLDAPLGSIDRSKLNVKGSSLAAGHPFAATGGRIVANLAKLLDAA
GSGRGLISICAAGGQGVTAILER
>tr|A0A0Q0CD84|A0A0Q0CD84_PSEAP Inosine-uridine preferring nucleoside hydrolase OS=Pseudomonas syringae pv. aptata OX=83167 GN=ALO85_101744 PE=4 SV=1
MNDACTPFKRPLRTKGTNVPIFHLQATMLMAGGIEAAKVDEQFLSVNSLSGLTQSLHQFA
ATCARSACMTRALSEASNCFKAFTCKSRC
>tr|A0A0Q0CFP7|A0A0Q0CFP7_PSEAP ThiJ/PfpI family protein OS=Pseudomonas syringae pv. aptata OX=83167 GN=ALO85_05475 PE=4 SV=1
MPCRPWSQLVPLNIGIYVYDDVEVLDFAGPYEVFTTATRMHARNSRDDRQLFNVFTIGRS
TAPVRARAGLKVDPDFSINDHPPVDCLIVPGGVVTAELEKPDVIHWISDQSEPGRVVAAV

CTGAFLLARTGKLAGKQVTTHWEDIDDLKEMFPSVDVLSTLRWVDEGSFVTSAGISAGID
MSLHLVERLHSRELAERTALQMDFDWTEND
>tr|A0A0Q0ICF8|A0A0Q0ICF8_PSEAP Putative alpha-helical domain protein with a conserved ER motif OS=Pseudomonas syringae pv. aptata OX=83167 GN=ALO85_01219 PE=4 SV=1
MLSRTASDLFWMSRYLERAENLARMLDVSYSLSLMPQDGRGDGLDEIAMPLLITGTLETY
IERHGELHAERMLHFFALDATNPASIYSCLGAARASAHAVRGRITADMWENINSTWLEIR
GIAQQGLTRYGMSRFCEWVKERSHLFRGATYGTIMRNDAFRFIRLGTFIERADNTMRMLD
ARYEMLELRGPQANAAADNSAAGYYQWSALLRALSSFEAYTEVYRDAPGARQVAELLLLR
ADIPRSLRACLEEMDVILASLPGVNGRPAQRMAAEMDARLRYTSIDEILSEGLHEWLNEF
IPMLAQLGDAIHTSYLEAA
>tr|A0A0Q0DMU2|A0A0Q0DMU2_PSEAP 3-oxo-C12-homoserine lactone acylase PvdQ OS=Pseudomonas syringae pv. aptata OX=83167 GN=ALO85_03823 PE=4 SV=1
MSRPLCGFLFAGLSFAVVLPAQALVEPGNQAARAEIRRTGFGVPHIVAANERGLGYGIGY
AYAQDNLCLLANEVVTVNGQRSRYFGPDKATLEQRNNMASDLLFQWLNTPQALADFWKAQ
PLEIRQLMQGYVAGYNRSLAEQTTQGLPQPCAAEWGRPISTDDLVRLTRRLLVEGGVGQF
AEALAGATPPALQKPLQVNAQQVQALQLAATRNQRFALERGSNAVAIGRELSANGRGMLL
ANPHFPWAGGMRFYQMHLTIPGKLDVMGAALPGLPLINIGFNQHLAWSHTVDTSKHFTLH
RLQLDPKDSTRYLLDGQSVAMGKQQVSVDVKQADGSLKSVPRIVYSSIFGPVVQWPGKLD
WDSKFAFSLRDANLQNDRVLQQWYAMDKADSLKAFEDSVHKIQGIPWVNTLAVDAKGQAL
YMNLSVVPNVDAARLARCSDPRIGTELIVLDGSRSECNWKVSAEAAQAGIYPSSRQPQLL
RTDFVQHSNDSAWMVNPAAPLKDFSPLISQDGQPLGQRARFALDRLGSLKKAGKISVENL
QAMVMDNEVYQAGQVLPDLLSFCASELGDDAARLTPLCTALKAWDGRADLNSGIGFVYFQ
RIVTSMQAVASRWRVEFDPRDPVHTPSGLAIENPQVATALRAAMLAAVDEVTKAGLSAGS
KWGDIQVSSISGKQIPIHGGPAGLGVYNAMQTIAGKGGKREVVSGTSYLQVVSFDEQGPR
AQGLLAFSESSNPQSAHSSDQTEAFSKKQWRALPFTDQQIKADPAYEVQVISEGANR
>tr|A0A0Q0IJ36|A0A0Q0IJ36_PSEAP Uncharacterized protein OS=Pseudomonas syringae pv. aptata OX=83167 GN=ALO85_01745 PE=4 SV=1
MKITKNLLQAGVLGMSLLATGVMAAVSESEAAKLGTSLTPMGAEKAGNAANTIPAWSPMP
KNAGAVDSKGFLANPYASEKPLFIITAANVEQYKDKLAPGQYAMFKRYPDSYRIPVYPTH
RGATVPDDVFAAIKKNATTTKLVGGGSGLENFDTAIPFPIPASGVEVIWNHITRYRGGSV
KRLVTQATPQPNGSYSLVYFSDQFVFRDKMRDFDPKNPGNVLFYFKQEVTAPARLAGGVL
LVHETLDQVKEPRSAWLYNAGQRRVRRAPQVSYDGPGTASDGLRTSDNLDMYNGAPDRYD
WKLIGKQEMYIASNSHKLDDPTLKYADIIKAGHINQDLTRYELRRVWHVTATLKEGQRHI
YAKRDFFIDEDTWQAAVIDHYDGRGQLWRVAEAHSENYYDKQVPWYAVETLYDLQSGRYV
ALGMKNEEKKAYEFGFTAGTSDFTPNALRQEGVR
>tr|A0A0Q0C1U3|A0A0Q0C1U3_PSEAP Cyclic di-GMP-binding protein OS=Pseudomonas syringae pv. aptata OX=83167 GN=ALO85_04960 PE=3 SV=1
MCTMKSSVAVNMGAFGVLACGISLLALMPAFALAADSPLTQAINRITADNRQERVVELRE
LGIDRPIILNATDARRELYLPVPANVPLTEATLNFDASYLNGEAGRNTLLLSLDGYPVRA
LGLNEEQGNASATLGVDKAARENGSVRLGIAWSSVISKVLCEDERAIGNVLRIDPHTRLT
YSYDASQLQDVGAVWNALPGKPGLLVAPGTLSNASYDAAWRLGVALERIGKQARILPFPA
VQDSVDLSGLTIPAELKQIPAFAGLEGKGQYTLRDPAEIGALLMLGQTPALQADLAISDP
QLLKAIDDAMDALQAQVQGLDASAASALGQWRERHIKKPLANSTGDDVSLALLGNRALLM
IKPESADKAIGLFSSAWNKLARNRQLTIGEEAAMPLSEDGRVALTRLGGKPGTVDVLAKT
DWSASFPLGSVAYDGRVPVTAMIDIAAAPGASGTPPVATLFLNDYLIGAMQLTADGKKER
IEARIPQYALAAQNVLRVSFQRQPVSNQCLETPQAFPISVLPTSHIVLDKVTPDENFSGM
AARFATDTQIMVPKGYLEHPASSLPQVIRIASASGVSPLRAQLSVSDDASVAVTPAKSFL
AFELPIKDGAESVKASNDGHLLINHKEQTLLDLKSLNHLASLQVIEAGGQHGMIYRTLGG
QTPVFERPVLLERGNAAVLADSGPIATFDAKDPTGSQMIEEEESTGIDAWRKPSLLWLIP
GGIVLFLILLLAGRSARRNRS
>tr|A0A0Q0FDX3|A0A0Q0FDX3_PSEAP Putative signal transduction protein OS=Pseudomonas syringae pv. aptata OX=83167 GN=ALO85_04540 PE=4 SV=1
MSEELTSEQIQLALQGISVPPQPQIMVDLQMEQYMPDPDLGAIARLIAQDPGLSGALLKI
VNSAHYGLSSKIASIDKAVNLLGSRTVINLINAQSIRGEMTDEAIVTLNRFWDTAQDVAM
TSLTLAKRIGSQTVDESYALGLFHDCGIPLMLKRFPDYMTVLEEAYANAGPDCRVVDTEN
HAFDTNHSVVGYYTAKSWRLPEHVTNAIANHHNALAIFQDDSSRDPTLKNLLAPLKMAEH
ICQSYRVLGSQDVDHEWESVGPLVLDYVALSEYDFEYLRESIRELGAR

>tr|A0A0N8T7V4|A0A0N8T7V4_PSEAP Carbohydrate ABC-type transport system, periplasmic substrate-binding protein OS=Pseudomonas syringae pv. aptata OX=83167 GN=ALO85_04288 PE=4 SV=1
MNSMSRLAVVISLASLFPLSANAADSKGTVEVVHWWTSGGEKAAVDVLKAQVEKDGFTWK
DGAVAGGGGSTAMTVLKSRAVAGNPPGVAQIKGPDIQEWASTGLLDSDALKEVAKSEKWD
SLLDKKVSDTVKYDGDYVAVPVNIHRVNWLWINPEVFKKAGIDKAPTTLEEFYAAGDKLK
KAGFIPLAHGGQPWQDSTVFEAVVLSVMGADGYKKALVDLDNGALTGPEMVKSLTELKKV
ATYMDADGKGQDWNQEAGKVINGKAGMQIMGDWAKSEWTAAKKVAGKDYQCVAFPGTEKA
FTYNIDSLAVFKQKDKGTTAAQQDLAKVAMGEDFQKVFSINKGSIPVREDMLSDMSKYGF
DSCAQTAAKDFLADSKTGGLQPSMAHNMATTLAVQGQFFDVVTNYINDPKADPAETAKKL
GAAIKSAK
>tr|A0A0Q0DTG6|A0A0Q0DTG6_PSEAP Major facilitator superfamily transporter OS=Pseudomonas syringae pv. aptata OX=83167 GN=ALO85_100171 PE=4 SV=1
MEMRWGTYFAVLASVLGVGLAMGVSMPLVSLRLAGWGYGSFAIGIMAAMPAVGVLLGAAL
ASRLAARFGTANLMRLCLWAGAVSVGALALLPYYWIWLVLRLTLGVILTIVFVLGESWIN
QLVIERLRGRLVALYGSTYALSQLAGPLLLGVIGTQADYGFWIGVALLVGSPFLLLGRTG
APSTESCNVTFADLSGFCRGMPAIAWAVALFAAFEALILTLLPLYCIRHGFTPEIALIMV
SVVVVGDALLQLPIGMLADRISRRAMFTGCAALLLASSLLVPLLIDTVLIWPLWTVFGAS
AGGLFTLSLILIGERYRDDSLVRANAHVAQLWGIGCLLGPLIAGAGSQWVSGEALPLMMA
AGALGLLILIRRRGAFGDAQAAPA
>tr|A0A0Q0ILU5|A0A0Q0ILU5_PSEAP Bifunctional uridylyltransferase/uridylyl-removing enzyme OS=Pseudomonas syringae pv. aptata OX=83167 GN=glnD PE=3 SV=1
MPQVDPDLFDRGQFQAELALKASPIAAFKKAIRRARDVLDERFRSGRDIRRLIEDRAWFV
DNILQKAWDQFEWSEDADIALLAVGGYGRGELHPYSDIDLLILLDSDDHEVFREPIERFL
TLLWDIGLEVGQSVRSVNECAQEGRADLTVITNLMESRTIAGPERLRQRMLEVTSTEHMW
PSKEFFLAKHAEQKKRHHKYNDTEYNLEPNVKGSPGGLRDIQTILWVARRQYGTLNLHAL
AGEGFLLGSENALLASSQEFLWKVRYALHMLAGRSEDRLLFDYQVRIAGLLGYEDNDAKL
AIERFMQKYYRVVMSIAELSDLIIQHFEEVILSDDSGTPQPINSRFQLHDGYIEATHPHV
FKRTPFAMIEIFVLMAQHPEIKGVRADTIRLLREHRHLINDEFRNDIRNTSLFIELFKCE
IGIHRNLRRMNRYGILGLYLPEFGHIVGQMQHDLFHIYTVDAHTLNLIKHLRKLQYTEVS
EKFPLASKIMGRLPKPELIYLAGLYHDIGKGRGGDHSELGAVDAQAFGTRHHLPAWDNRL
IVWLVSNHLVMSTTAQRKDLSDPQVIHDFAQFVGDEVHLDYLYVLTVADINATNPTLWNS
WRASLLRQLYTETKRALRRGLENPVDREEQIRRTQTAALDILVRNGTDPDDVEQLWSALG
DDYFLRHTAGDVAWHSDAILQQPADGGPLVLIKETTQREFEGGTQIFIYAPDQHDFFAVT
VAAMDQLNLNIHDARIITSSSKFTLDTYIVLDHEGGSIGNNPERIQDIREGLTEALRNPD
DYPTIIKRRVPRQLKHFAFAPQVTIHNDAQRPVTVLELLAPDRPGLLARVGKIFLEFDLS
LQNAKIATLGERVEDVFFITDANNHPLSDPQLCRQLQDAIVKQLSVNSEQGGDLRISI
>tr|A0A0Q0DQV9|A0A0Q0DQV9_PSEAP Pirin OS=Pseudomonas syringae pv. aptata OX=83167 GN=ALO85_05151 PE=3 SV=1
MRINLSNKQVYHDTSYHLIRSALSPMTQFRKVLSIQAGQPASDGAGVKLTRVFGGRGVEQ
FDPFLMLDEFGSDKPDDYIAGFPPHPHRGFETVTYMLEGRMRHEDHMGNVGLLQGGGVQW
MTAARGIIHSEMPEQEEGTMRGFQLWLNLPGKNKLSDASYQDIQPENVPSVSTQAGVQVV
VLAGHFDDGQVRQAGAVQRPDTEPLYFDFDMPAGSCISPHVPEGHLVLLYVYEGSVELEG
STQPIGKRQMARLSDSGEVQISSAAGARLLLIAGKPLREAIVQYGPFVMNTKEEIEQAIR
DFREDRLTA
>tr|A0A0Q0BU44|A0A0Q0BU44_PSEAP 2-octaprenyl-3-methyl-6-methoxy-1,4-benzoquinol hydroxylase OS=Pseudomonas syringae pv. aptata OX=83167 GN=ALO85_01653 PE=4 SV=1
MTMETRADVLIVGAGMVGSALALALQGSGLDVLVVDGGPLSVEPFDQQSTFEPRVSALSA
ASQRILQRLGVWEGIAARRISPYAHMQVWDGSGTGQIHFSASSVHADVLGHIIENRVVQD
ALLDRLHASDIGLLANARLEQMRHSGDDWLLTLADGRQLRAPLVVAADGANSAVRRLTET
PTREWDYLHHAIVTSVRTADPHRKTAWQRFTDDGPLAFLPLEREGEHWCSIVWSVTPAEA
ERLMVLEDDLFCRELEQAFEGRLGQVLSADSRLCVPLRQRHAKRYVARGLALIGDAAHTI
HPLAGQGVNLGFLDAAVLAEVLTHAARRGERWSDLRVLGRYERRRMPHNLALMAAMEGFE
RLFQADPLPLRWLRNTGLKMVNRMPEAKALFVREALGLSGDLPELARIEAR
>tr|A0A0Q0CEH7|A0A0Q0CEH7_PSEAP FAD linked oxidase OS=Pseudomonas syringae pv. aptata OX=83167 GN=ALO85_00996 PE=4 SV=1
MIALLKPTSEQVGHYDRFLNALAEAGFRGEIARDHGSRTVLATDNSIYQRLPQAAVFPMD
SHDVAIVARLAARPEHQQVVLTPRGGGTGTNGQSLTDGVVVDLSRHMNAILEINVEERWV

RVQTGVVKDQLNAALKPHGLFFAPELSTSNRATIGGMINTDASGQGSCTYGKTRDHVLEL
STVLLGGSYVNSHALAAEQLASEQARIDRAGDVYRCAQQIADTQAQLIKDVFPPLNRCLT
GYDLAHLREEDGRFNLNSVLCGSEGSLGFIVEARLNVLPIPKHSILVNVRYAGFMDALRD
AKALMAHKPLSIETVDSKVLMLAMQDIVWHGVAEYFPQDPATPTLGINLIEFSGDELSEV
EQRVHAFVAHLQTDTSVVRLGHTLAAGSAAVNRVYTMRKRSVGLLGNVKGEARPQPFVED
TAVPPENLADYIQEFRALLDSYGLQYGMFGHVDAGVLHVRPILDMKDPAQAALIRPISDA
VAALTKRYGGLLWGEHGKGLRSQYVPEYFGELYPALQALKAACDPFNQLNPGKIATPATV
PESRLTLVDEVMLRGDLDRTIDERVWQSYDSALHCNGNGACYNYDTDDAMCPSWKATRDR
IHSPKGRASLVREWLRLQGEQDVNVLTASARARSANVLKSLAERTVNSVARVAGQKDFSH
EVYDAMAGCLACKSCAGQCPVKVNVPEFRSRFLELYHSRYLRPIKDYLIGSLEFSIPHLA
RFPKLYNAIMGAKPVRYLLEHAAGMVDSPLLSLTDFRAACARWNVGIATPQRLAALSEQE
RKRTVVLVQDAFTRYFETPLAADWLELISRLGYQVYLAPFAPNGKPLQVQGFLQAFEKAA
AFNSQSLGKLAGYGIPLIGLDPAMTLVYRQEYSKTLGSENVPEVMLPQEWLASTVEEGRD
AGSAETYYFLPHCTEKTNEPASVGLWQKVFARAGLKLEVLASGCCGMSGTYGHETRNAKT
SDTIYRQSWQPLVARHGAEGRLVADGYSCRSQVKRQDGKVVKHPLQVLLERVRAL
>tr|A0A0Q0C5C4|A0A0Q0C5C4_PSEAP Threonine aldolase OS=Pseudomonas syringae pv. aptata OX=83167 GN=ALO85_01849 PE=4 SV=1
MTVGPGITPGLLTLQIAANATTPKRSRARRIARHYRRWGITPRPENVAVTLRGRREIFII
VRWIMHGHFRTVYLPFSYKESEYRERRRLIIMYRVVSNALLQAKGCMHYRSSTSGSFFVT
VIDLRSDTVTLPTAGMLDAMARAPVGDDVYGEDPTVNLLEATLAGRLGFDAALFVPSGTM
SNLLALMAHCERGDEYVVGQQAHTYKYEGGGAAVLGSIQPQPLEVQADGSLDLQQVLDAI
KPDDFHFARTRLLALENTMQGKVLPLDYLAAARTLTLEKGLALHLDGARLYNASVRLGVD
VHEITRHFDSVSVCLSKGLGAPIGSVLCGSHPLIAKARRLRKMLGGGMRQAGGLAAAGLY
ALDHQVERLADDHANAAFLAEGLSGLGYAVEPVQTNMVYVQIGERAASLKAFCAERGIVL
TASSRLRMVTHLNVSRAQVEQVIAAFAAFEHP
>tr|A0A0Q0IB90|A0A0Q0IB90_PSEAP Secretion protein HlyD OS=Pseudomonas syringae pv. aptata OX=83167 GN=ALO85_00061 PE=4 SV=1
MNESKTPDTEAPRNSPPPAPLTSPVTSKPRSTRKRVVSSVIFGVVALAGVLVVLYAWQLP
PFASPIESTENAQVKGQTTLIGPQLSGYVYEVPVQDFQFVKAGDLLVRLDDRIYRQRLDQ
AMAQLAVQKASLANNLQQRRSAEATIGQRQAELQNSIAQSRKSAADLRRNQALVTDGSVS
KSELDVTRAADAQANAAVAEARAVLQIAREDLQTVIVNRGSLEASVANAQAAIELARIDL
DNTRIVAPRDGQLGQIGVRLGAYVNSGAQLMALVPEQRWIVANMKETQMANVRLGQPVSF
TVDALDGHEMHGHVQRLSPAAGSEFSLLPADNATGNFVKISQRIPVRIVVDADQPMLEHL
RPGMSVVVSIDTSADGDVPPDPAVR
>tr|A0A0Q0C562|A0A0Q0C562_PSEAP 2-nitropropane dioxygenase NPD OS=Pseudomonas syringae pv. aptata OX=83167 GN=ALO85_04087 PE=4 SV=1
MNTTHFWSSIGVRLPIIQAPMVGVSTPILAAEVSNAGALGSIGLGASTPDQARALIKQTR
ELTDQPFNVNMFCHRPARFNQTQNDTWLAYIAKWFSEFDAAPPQEIREIYKSFLSDRVML
EVLLAERPAVVSFHFGTPDSAWVAELREAGIVTLGCATTLKEAQMLQKAGVDAIVAQGYE
AGGHRGVFDDAPNQDHELGLFPLTRMLTTHIRLPVIAAGGIMDGASINAALALGAAGVQM
GTAFILCPESSATPEHRAALISARAHETRVTSYVSGRPARGLANRLYLESTGQQIPVPAE
YPLAYDVTKALHAAAVLKGCHDFAAQWAGQGAPLARALPAAELIKTLVEEMRSASKSTTF
L
>tr|A0A0Q0CDL4|A0A0Q0CDL4_PSEAP Probable lipid kinase YegS-like OS=Pseudomonas syringae pv. aptata OX=83167 GN=ALO85_05196 PE=3 SV=1
MTQRRAMLILHGKQALNEDVRDAVADKRKQGWELDVRLTWEAGDAQRLVDEALAAGHRHI
VAGGGDGTLRDIAEALALAETKASLTILPLGTANDFARAAGVPLEVSKALQLMDVAPRAV
DLGEVGGKLFLNMATGGFGSQVTANTSEDLKKVLGGAAYLFTGLTRFSELHAAHGELTGP
DFHWRGDLLALGIGNGRQAGGGHELCPTAMADDGLLDISILPAPQEVVGTLRSLLEGGLG
IDNMFIRARLPWVELKSAQGLDINLDGEPLSGEDLRFEARPGALHVHLPADSPVLGGAPK
LSRPD
>tr|A0A0Q0DS70|A0A0Q0DS70_PSEAP Uncharacterized protein OS=Pseudomonas syringae pv. aptata OX=83167 GN=ALO85_200163 PE=4 SV=1
MGLKDLIRKPEQVSPSLEANDEAALAFISAAPVSATPEPKRKRKKASTFVRTTFSLSKDV
NRQIDKISLLPRTFRISRSDVIRAGIMALQELDKADLIALLENASNAEPITDSMEDE
>tr|A0A0Q0C1W7|A0A0Q0C1W7_PSEAP Histidine kinase, HAMP region: chemotaxis sensory transducer OS=Pseudomonas syringae pv. aptata OX=83167 GN=ALO85_04653 PE=4 SV=1
MTILQRVIGGFAVLVVLLLAMAGISYQSTHSISDRISVITGQSAPLSRAASELYVHVLRA
NQALLGVLVSTDPKQIDDGKQPFNESMTRFNQLLDSTPAYIGDHAELRDNLNQQRQLSAA

YAEQAQTLIASHRQHVLQALQSRVLQGYSSSQGAQLTGYLRDYIARERNAGSAETVAAGE
KLLLEVGKSYDGLAAHAATPNIQTLQRVLNLQDEVISTRTRELIAADPRAGRIAAVMVNR
LLNDLTGSDGVYQAYRQEAALAEQVDKQRQAAETRLQATLSKIGEFGNQSVAVANEAKAG
ADSTIATSLSLLLIACLLAVMAAAIIGTWVAFSLRRPLAAFREVLKTLTSGDMRVRFDVS
RRDEFGELGGYLNEFTQSLQQTFRQLIGSADNLALTASQNAQISEQTTRVVDEQKDRLNS
AASAMTEMESTVEEVARRAQDTRGAVDSTSELTGKVQKRVAETIVNIRQQAEQVNKASAV
TDELQKYGQNIDGIVDAIRTIAEQTNLLALNAAIEAARAGEQGRGFAVVADEVRSLASRT
QTSTSEIQEMIGQMQSKIHSAVEVMNESQIQSDHCVTLASGADQLLVEMGEAVDVIRDMN
IQIAAATEQQSATVQETSRMVTHINDSAQQAADGAEQSAISSQDLSKMARDQRELLHHFS
V
>tr|A0A0Q0FBA0|A0A0Q0FBA0_PSEAP PAS-domain protein OS=Pseudomonas syringae pv. aptata OX=83167 GN=ALO85_05092 PE=4 SV=1
MFGNKNKAELTAMKAQVDGLNGLTSALEKSMAVIELSLDGKILRANDNFLAAMGYRAEEL
TSKTHRDFCEPEMLRSREYADLWASLKAGKFISGTFKRVGKNGHSVWLEASYNPVTDAQG
NVVKVVKYALDVTTRVMSENETNSKLAAVDRAMAICEFEPNGNVITANNNFLNVMGYALT
EIKGKHHRTFCEPTLVNSPEYTEFWRKLNHGEFIGDQFKRIGKQGRVVWLEATYNPVFDV
DGKLYKIVKFASDITQRVEKQEADAHGASRAYHISAETEKVAEQGTLVVQDTAREMRQIS
ENIGASAKLVSQLGDRSEQITAIVNTIRGIADQTNLLALNAAIEAARAGDQGRGFAVVAD
EVRQLAGRTSGSTTEIAEMIGKILAETREAVASMDATQEGAIRGVTLADQAGQVIVQIRD
GASDAVEAVSMFANRMDEAEAFAKPGKR
>tr|A0A0Q0DRG3|A0A0Q0DRG3_PSEAP Lon protease OS=Pseudomonas syringae pv. aptata OX=83167 GN=lon PE=2 SV=1
MRATAPSKSHVQLLRCPMNDQQTDSPDLPNDADEHDVSLSSDSTSLALPGQNLPDKVYII
PIHNRPFFPAQVLPVIVNEEPWAETLELVSKSEHHSLALFFMDTPQEDPRHFKTDALPEY
GTLVKVHHASRENGRLQFVAQGLSRVRIRTWLKHHRPPYLVEVEYPQQPNEPTDEVKAYG
MALINAIKELLPLNPLYSEELKNYLNRFSPNDPSPLTDFAAALTSATGVELQEVLDCVPM
LRRMEKVLPMLRKEVEVARLQKEISAEVNRKIGEHQRQFFLKEQLKVIQQELGLSKDDRS
ADIEQFEQRLEGKTLPPQARKKFDEEIGKLKVLETGSPEYAVTRNYLDWTSSLPWGIYGA
DKLDLKHARKVLDQHHAGLDDIKARILEFLAVGAYKGEISGSIVLLVGPPGVGKTSVGRS
IAESLGRPFYRLSVGGMRDEAEIKGHRRTYIGAQPGKLVQALKDVEVMNPVIMLDEIDKM
GQSYQGDPASALLETLDPEQNVEFLDHYLDLRLDLSKVLFVCTANTLDSIPGPLLDRMEV
IRLSGYITEEKLAIAKRHLWPKQLAKAGVAKNKLSISDSALRAVIEGYAREAGVRHLEKQ
LGKLVRKAVVKLLDAPDSVIKIGPKDLEASLGMPVFRSEQVLSGTGVITGLAWTSMGGAT
LPIEATRIHTLNRGFKLTGQLGDVMKESAEIAYSYVSANLSKFGGDAKFFDEAFVHVHVP
EGATPKDGPSAGVTMASALLSLARNQPPKKGVAMTGELTLTGHVLPIGGVREKVIAARRQ
KIHELILPEPNRGNFEELPDYLKEGITVHFAKRFADVVKVLF
>tr|A0A0Q0C2F3|A0A0Q0C2F3_PSEAP ABC transporter OS=Pseudomonas syringae pv. aptata OX=83167 GN=ALO85_02461 PE=4 SV=1
MLAGLDLPSAGSVILSGRNLSELDEDQRARVRAEHVGFVFQSFQLLDSLNALENVMLPME
LEGRKDAREKARDLLERVGLGQRLTHTPRQLSGGEQQRVAIARAFAADPDVLFADEPTGN
LDSHTGERISDLLFELNKERNTTLVLVTHDERLAHRCRRLIRLEGGRLVAPLEP
>tr|A0A0Q0FST6|A0A0Q0FST6_PSEAP Glycoside hydrolase, family alpha amylase catalytic subunit OS=Pseudomonas syringae pv. aptata OX=83167 GN=ALO85_05434 PE=3 SV=1
MLHPLRLSSVSKPSASVAALAAQKIIRRQEVTMNTKSTTPAEDNSAPLDNPGSTPSRIRE
GLPFPLGASWDGLGVNFALFSANATKVELCLFDSTGEIELERIELPEYTDEIYHGYLPDA
HPGQIYGYRVYGPYDPKNGHRFNHNKLLIDPYAKQLVGELKWSEALFGYTIGHPDGDLSF
DERDSAPFVPKSKVIDEAYTWGRDQRVGTPWDKTIFYETHVRGITMRHPEVAEELRGTFA
GLGSAPVVEHIRKLGVTSVELLPIHAFVNDQHLLQKGMTNYWGYNSIAFFAPDPRYLASG
KIAEFKEMVAHLHHAGLEVILDVVYNHTAEGNELGPTLSMRGIDNASYYRLMPDDKRYYI
NDSGTGNTLDLSHPCVLQMVTDSLRYWASEMHVDGFRFDLATILGRYHDGFDERHSFLVA
CRQDPVLRQVKLVAEPWDCGPGGYQVGGFPPGWMEWNDKFRDTVRAFWKGDEAQLADFAA
RMTASGNMFNQRGRRPQASVNFITAHDGFTLHDLVSYNDKHNEANDENNQDGSNNNLSWN
HGVEGPTEDPEINSLRLRQMRNLFATLLLAQGTPMIVAGDEFARTQHGNNNAYCQDSEIG
WINWDLDEDGEALLKFVTRVIKLRQTYPILRRSRFLVGDYNEEIGVKDVTWLAPDGNEMS
VEQWHDANGRCLGMLMDGRAQETGIRRAGADATLLLVVNAHHDGVNFTLPEVPEGTHYEC
LIDTNQDDARSKEHFAFGSQYTVTPRSLLLFELKREDQQ
>tr|A0A0Q0DJB2|A0A0Q0DJB2_PSEAP Uncharacterized protein OS=Pseudomonas syringae pv. aptata OX=83167 GN=ALO85_01862 PE=4 SV=1
MSTHELVVKKEMTPAGIASDLLNVIKDIERSISESTDKTGAIEQRGMIKSMFASSKTDLI

DISKSQNKINELMLGLIQEIITLNTMSYSFLAAVIGELEQRAKSGWTDSEGRFQELSETG
QQFAGKAHDIFCKIAEGSKSTQTRIDLNKQDIDNLKNDFIAKAEVVQQSRQDIDNIKSVL
THKTAVVAKQSKDIGQIHSVLESKTERLASIDKLLETKSERLSSIDKLLASKADRLASID
KLLEEKSTVDQSQDMAIKQILQALEDGQRLDQQRVLEIHSLSQALEAQTERTSAAINVLQ
AGQAKAVRGVYAASTVAVISAGVTGLRLLGVL
>tr|A0A0Q0D595|A0A0Q0D595_PSEAP 3-ketoacyl-CoA thiolase OS=Pseudomonas syringae pv. aptata OX=83167 GN=ALO85_100690 PE=3 SV=1
MSGGGQYSAFSDVAIFDAVRTPWVDLGGALAAVSPIDLGIKVGRAVLARAAIEPDQVDSV
LAGSMAQASFDAYLLPRHIGLYSGVAQSVPALGVQRICATGFELLRQAALHVGEGGQMAL
CVAAESMSRNPIAAYTHRNGFSLGAAVQFKDFLWEALYDPAPAMDMIATADHLAKRHGLS
REAVDRYALLSHERALQSQVSGKWTEEIVPVSSERFALEGYQPCGIELPRGVEVVTQDSH
PRATHLAALSRLRAIHANGVQTAGNSCAVVDGAAAALVGSASQSTSPVLAHLLASAVTGV
APEFMGIGPAPAIRLLLQRSGLALRDIGRLEINEAQAAQVLAVAQTLELDHERLNCLGGS
LGLGHPLAATGLRLVMTLARQLRDTGLRYGIAAACVGGGQGMALLIENPHYASERV
>tr|A0A0Q0DPY0|A0A0Q0DPY0_PSEAP Sarcosine oxidase, subunit alpha family protein OS=Pseudomonas syringae pv. aptata OX=83167 GN=ALO85_04650 PE=3 SV=1
MVDAQSLQLLVPGRAPHRQRRDHPHLRSTRTIYRTYRLRPSRLQGDRRMTTPARLPAPMG
LLIDRTRPLTFSFDGKTYQGLQGDSIASALLANGRFLLSRSFKYHRPRGPLTMAGQDANS
LIQLPHEPNVMADTVALQEGLQATGQNFSGSLDNDRDAYLGKFSKFMPVGFYYRSFYKPK
GMWKLWEPIIRKKAGLGVLDLTFQPEYYDKAYLFTDLAVIGAGPAGLQAALSAANAGAKV
LLIEQQPILGGSLAYARFDVEGLRAEQLRRELVAAVEAHDNIEVLKQATCNAWFTDNYLP
VIQGKRLYKVRATHCLVCSGSFDQPVIFRNNDLPGVMLTSAVQRLIKLYAVKPGKRAVVL
TGNDDGYLAALDLHDQGVDVVAVADMRSSPSDRTLRLALEKRGIVCHVNTTVYEALHEKG
MRHVSGAELRKITGQGQVANGGSHVDCDLLCMSGGYMPVYQLLCQAGGKLAYDDQQAEFA
ISSLPQNLSIAGSVNGYHALDNVLADAAHAAASVVSALGLASADTPAALRSEAQVNFAWP
IFPHPDGKDFVDFDEDLQVRDIVNATRIGYRDIQLVKRYSTVGMGPSQGRHSALPTARLV
AASTQRSISETGVTTARPPFEAEKLAHVAGRAFDPHRQTPMHQRHVQAGAKMMPAGIWQR
PAFYGKASQRDACMQQEALHVRNKVGIIDVSTLGGLDVRGPDAAELLNRLYTFAFLKQPV
GRSRYALMTNEQGVVIDDGVCARFAEQHFYVTATTSGVDRIYQQMLKWNAQWRLNVDITN
VTAAIAAVNVAGPDSRKVLEQVCTDLDLSGAGFPYLGVRLGTVAGIKARLLRVGFVGELG
YEIHVPARHALRLWDALVEAGKAFDMRPFGVETQRLLRLEKGHVIISQDTDGMTHPAEID
MGWAVSRSKPFFVGRRSVDILEARPQKRKLVGFTLPKASPLPLEGHLVLKGPDISGNVTS
CEYSSTLGMIIGMAYAAFDQSTPGQQIPIRVEDGVVVQATVVKLPFFDPENQRQEL
>tr|A0A0Q0FMZ4|A0A0Q0FMZ4_PSEAP Amino acid adenylation OS=Pseudomonas syringae pv. aptata OX=83167 GN=ALO85_05141 PE=4 SV=1
MMVDKSSSRGTGFAHPDTEQPFDQTQCVHGLFEAQVQLNPDAIAARLGLDTLDYATLNTQ
ANRLAHYLRSLGVGPDVRVGICLERSLDMLVGVLAVLKAGGAYVPLDPTYPKARLAHMLA
DSTPRVLLSHAPARAVLLAALEQGEVATQVLDLADTWLWAAQPTHNPDPHAVGLTSRHLA
YVIYTSGSTGTPKGVMVEHRGLIAVSAAWERLYALHKPLNHLQMAGFSFDVFSADLIRAL
GFGGTLVLCPRDTLMDPPALYRLLSEARIDFADFVPAVLNPLLVWAKEAGRDLSFMSTVV
CGSDIWTAHSARQLRGLCGEHVQIVQAYGVTEASIDSTCFEFEPDSHVDAVLPIGRALAN
TRIYLLDAADERVPQGVTGELYIGGAGVARGYLNLPELTRERFIDSPFVAGERLYRTGDL
ARYRADGHLEFLGRNDSQAKLRGLRLELGEIEARLAEVAGVRDNVVVLREDNAGVPRLIA
YFREQSGAALTPKGLREHLQRSLPDYMIPAAFVRMDALPLTANGKLDRIALPEPGDDAFD
RHVFEAAQGALETALAAIWAEVLGVERVGRQDHFFALGGHSLLVMRVLAKVRQALHLEVS
PSSLFTAPVLQQFAERLSTAQQGNARPPITAVERSGAHTLSSAQQRLWFLAQMEGGNAAY
HMPLNLRLRGPLQVAALERSFNQLVARHEALRTTFFAVEGEGRQRVCAAETIIPLPVTDL
RGEHDAQARLLALIDAEGSKPFDLAVGPLMRVSLVRLADDDHVLLLTQHHIVSDGWSMGV
LTRELGLLYEAAIQQPAAHGQSLPLPPLAVQYVDYAAWQRQWLSGEVLDAQRRYWRETLS
GAPLLLELPTDHKRPAAQDYRGGFVPLVFDRALTARLRMLATQQGTTLFMNLLAAWSLLL
MRLSGQDDVVIGVPSANRSQQELEGLIGFFVNTLALRMTRAGSPTVASWLQQARSVALAA
QEHQDLPFDQVVELLNPPRSLAYSPVFQVLFAWEQHQDSDLELSGLEVSVLQSVQPVAKF
DLQLALSERDGQIVGGLEYASGLFEPETVVQMGEYLRRILMQMVEDSEQPLATLDLLNAA
QHRRIVHDWNRTERDTSAQPDCVKRFEAVAQRTPDAVALLADERALTYAELNQSANRLAN
YLIEQGVRPEQRVGLCLERSPQMVIGLLAILKTGAAYVPFDPAYPVERLAFMFADAAPSL
LLTQTALLAGLPPLPDTLPICCHDVEAQRWSEYSEANPQVPVSPGNLAYVLYTSGSTGRP
KGVAHSRAALDNLIAWQLEQTPVAQRVLQFASLNFDVSFQEICSTLCQGGSLVLMSEAGR
KDLAALRPTLVAEDVQRAFLPFAVLQQLASFTEAEASVPMLPKGGCEIITAGEALLVND
ELRAFVCGLGGAQLHNQYGPTETHVVSQFSLNCDQAGQWPDAPPIGRPIANARLYVLDGD

LNPVPVGVAGELYIAGACLARGYLNRPDLSAERFMPDPFNSQPGARMYRSGDLARFAADG
NVHYLGRIDQQVKLRGFRVELGEIDSLLHQQPGVREAVVLLREDVPGDKRLVAYVVGPAT
AETLRAELHRHLPEHMVPTAWVALAQLPLTRNGKLDRQALPVPERQAASAYVAPRDETEQ
QMVCIWAEVLKCQQVGIHDNFFELGGHSLLATRMIYMINQRMGAQLSLSSLFKTPVLMDL
AEQVRLGRSDGPSLDTPFAPIEADRSARYAPFPLTDIQQAYWFGREASVSLGGVSAHGYE
ELRIPGLDVPRFEQALNRMILRHDMLRVVFLGDGTQQVLDSVPTYHMPRNDLRGLSAAAA
QQALQVTRERQSHQVLDASRWPLFEFSLSLLDEGISHLHISLDALIVDAASTQILARELM
AFYADPQLQLPEPGLTFRDYVLAEQRLRNDSRYAQALDYWREKVATLAPAPDLPLVCQPE
SISQPHFTRRDRELSASQWSRLKELARQFAVTPSVMLLTAFSEVLALWSRQPRFTLSLPL
FNRMPLHPDVDEIIGDFTSLVLLEVSLDGAASFIDKARAVQARLWQDIDHSVVSGVRVLR
ELSQARGVQQTAMPIVFNSTLSEAAPELAEFNLADALNAEHMHSITQTPQVWLDHTLLEL
EGRLLFNWDSIDELFPQGLIEQMFVAYNALLDRLLDADAWNAGTVELIPLARLPVPEASP
VDSALMHELFDRQALAAPDALAVIGTQRQLSYRQLRAEARQLAALLQRRGVVPNQLVAVV
MERGWEQVVATLAIQYAGGAYLPLDPALPTERLEHILQRAEASLALTQPALLQRVEWPAQ
VQVISVSDPIVADDGLSDQSELHAVHLTPDDLAYVIYTSGSTGLPKGVVIDHRGAVNTLL
DINRRFAVGAADRVLAVSSLSFDLSVYDFFGTLAAGAAVVLLEPQQALDPAHWLTLIERH
QVSLWNSVPALFGMLLEYVQSERSALPSCLRVAMLSGDWIPLTLPERAWALQPTLQLVSL
GGATEASIWSIQYPLQQVDPAWRSIPYGKALDHQRFYVLDDALQVRPTWVAGQLYIGGVG
LAKGYWRDEALSAGSFFAHPLTGERLYRTGDLGRWLPDGNIEFLGREDTQVKVQGHRIEL
GEIEAALNRHPGVQSAVVRVLGEALGEKRLAGYVLKADPSLQASDFTQYLADKLPAYMVP
TSFTFVQEWPLSANGKVDKKRLPEPAQVQDAGPALEVEGPQEQQLVMIVQGVLKRPSIAA
DANLLNLGATSIDIVRISNALSGELQFRPNVAQLLAQPTLLNLLGMYRRNLANGSVVDSV
RQRAGSPEEVIEDPQQRARFKADQRGRRTFTSEVAALNLARPDDPAFARRFSDYRSVRQF
AEGPVPTEAFAGLLASLAQGQLHGEVKYQFPSAGGLYPIQCYLYVKPQRVTGVAAGAYYY
DPVEHRLLMLDSEPLDPDTYDYFVNRPVYENAAFSLFFIADMAAIRPLYGERSRDFCHIE
AGGMAQLLTMTAVEQGLGLCGMGSLEEQQLSALFDLGPNHQLIYSMVGGLRTADEQRRAQ
VEAFASAADQTDDASDMEEIEI
>tr|A0A0Q0DS30|A0A0Q0DS30_PSEAP Putative fluoride ion transporter CrcB OS=Pseudomonas syringae pv. aptata OX=83167 GN=ALO85_200196 PE=3 SV=1
MVGGYIIGLAIAFLAASPTMSPEWRLLIIMGFCGGRTAFSTFCAETIALIQEGRLLRALG
SVSLHVVGSVVMTAAGLLSYQIISTR
>tr|A0A0Q0D8J5|A0A0Q0D8J5_PSEAP Urea ABC transporter, ATPase protein UrtE OS=Pseudomonas syringae pv. aptata OX=83167 GN=ALO85_100004 PE=4 SV=1
MLTVSQLHQYYGGSHILRGLSFDVKVGEVTCLLGRNGVGKTTLLRVLMGLLPSKEGSVQW
EGKTITQFKTHQRVHAGIAYVPQGREIFGRLTVEENLLMGLSRFPGSEAKEVPAFIYELF
PVLLQMKHRRGGDLSGGQQQQLAIGRALASRPRLLILDEPTEGIQPSVIKEIGAVIKKLA
ERGDMAILLVEQFYDFAAELADQYLVMSRGEIVQQGRGENMESDGVRGLVTI
>tr|A0A0Q0ICD0|A0A0Q0ICD0_PSEAP Glyceraldehyde-3-phosphate dehydrogenase, type I OS=Pseudomonas syringae pv. aptata OX=83167 GN=ALO85_04780 PE=3 SV=1
MAHKQRRGHNVAPFFSPYMWKVPVTQKPDQCLGEWIDREALAEAMIPLIGQLYRNNNVVS
SIYGRSLINRSVIAILKAHRFARHRQSDAVELSVHETFPLIKAMSELKLGAASVDLGKLA
VKFKTEGNGRTPEQFVREELASVVGQQGTSPRKGTDVVLYGFGRIGRLLARILIEKTGGG
DGLRLRAIVVRKGAENDLVKRASLLRRDSVHGPFDGTITIDEANSTLTANGNLIQVIYAK
NPSEVDYTQYGIKDALLVDNTGVWRDAEGLGQHLACPGIDRVVLTAPGKGKLKNIVHGIN
HGEITADDKIVSAASCTTNAIVPVLKAVNDKFGITNGHVETVHSYTNDQNLIDNFHKGDR
RGRSAALNMVITETGAATAAKALPELAGKLTGNAIRVPTPNVSMAILNLNLEKQTTREE
MNEYLRYMALHSDLHKQIDFVNSQEVVSTDFVGSRHAGVVDAEATIVNDNRVVLYVWYDN
EFGYSHQVVRVMEDMADVNPPAFPQ
>tr|A0A0Q0DFQ0|A0A0Q0DFQ0_PSEAP Beta-ketoacyl synthase OS=Pseudomonas syringae pv. aptata OX=83167 GN=ALO85_04824 PE=4 SV=1
MKGVVMNNSNEIANFQRYIIDQLKAGRITKEQAVQALAKTDATGGRMREEQSGPPGSTRY
VPVWRDAPLGRALDVAQAHEQHCVVVVTEHGHERLVDTLKARLDQRLLARMTVVSAPEEA
DAKGTLADQMSGLASAMILDVFVSLKAQVTAILQHGKGRKSSADTLLQVFFLRPDSYAED
KVTHESVYVGVEAAISAMLKTLNKEHPSITTQLVALDEASLGQLSQNSLAEEQHSHAAYV
RYLKNVRYVQVFETLSERSDGASLQSFIKTDASYLIIGGVGGIGLHLADYILRQGQQARV
VLTGRRSEADLQSNTPYLQLVNTYGKSRVSYLTANAASYSDLKSLLIKVVASHHLEGIFH
CAGTLNDSFLVNKTVEQFENTLEAKVSGSIHLIQAANDVSQQTETGFDFILFCSSCAGQL
GFVGQIDYAAGNGFIDGFSDARNKVGRNGFDRLLSVNWPLWENAGMGVDQQALKELHVEY
GLLPLPVDVALNHVNTLLLAADTHRVLVTYGDPVKIDSQYVNAKPQRNLQTPALSQQTAQ

AASGKGAVTSVETAYDPLVAHLKQLISSVLKLETSKLDTFEPLQSYGLDSFSIMRINQAL
SEHFSRLSKTLLYEVESIHDLATTLSQLQPDAVQAWLGAVAPAASQQAPQASARQDAQPR
PAVPRAVDAQAGDAVAIIGINAQFPGAANIEAFWELLRNGVNAVTEIPESRWSLEDFYAT
DKIEAIRDGKSYCKQGGFLEQAYDFDEQFFKLSPREILAMDPQERLFMQSCWRALEDSGY
TKDKIKQQYDGDVGVYVGITKTGFNLYGPALWNQDERTIPTTSFSSVASRISYLFDLNGP
SMPIDTMCSSSLVAIHEAVHSLKAGRCQMAIVGGVNLYLHPASYVQLCASSMLSQKGQCH
SFGDQADGFVPGEGVATLILKPLRQAQADGDRIYATIKGSAVNHGGAAKGYTVPNPKAQT
AVIEKALNSANITAGELSYIEAHGTGTELGDPIELNALKACFSGNDLPESSCAIGSVKSN
IGHLEAAAGLAGVIKVVLQMQHRVLVPSLHAQTINPNLEFDQSPFYLQRELIEWPSRNNR
NRIAGVSSFGAAGVNAHVILEEYTAPQEQVVGAPVGQEHVQALVFPFSSNTEQGLKRNCR
RFLDFLQAQAPEASTAMDIATFIRATLRGLDNNLSLNQVDDSTPLVDAGLKPFDFYRLFE
AVNTRYGLNLSMLNWQGSVSIGRLVAVIQGGASSSTVPALRDVAFTLQTCRNAHQHRVAI
CARDLAGFIERLQRFLGEEDLQVNGSGLYAGVKTHRFEELSKQRALAHDRVQIAFAHHES
DALAQLWVNGSFEQWEGLYSDGLQPKKCRIPEYAFAANTHSFLDKVGSNAKAVRHLDQPS
EAGKVSAEIISHVPVRLEVPAVAGFIRERLANVLKMDEASINNEVSFVDYGLDSILGVDL
IHSLNEQYRLGLKTTCIYDYPNVEDLSRHAHGLITPVTIVDSSTQFARPAPQLLSESPAT
GQERNQKPIAIIGMSGRFGGADDTEAFWNNIRDGKDSTESVNRWDLADYLNQEKYRDGVC
QRGGFLNEIDKFDAIFFNISGVEATYMDPQQRLFLEEAWHALEDSGYAGSRLTPEVKDQF
GIYVGGWTGDYNEVFEASEAPAQALWGNMGSMIASRIAYYLDFKGPALTIDTACSSSLVA
IHQACKDLRLGITNTAIAGGVFVQTTPRLYVQATQAGMLSPTGTCSPFDENADGFVPGEA
VGAIILKRLDDALADGDNIRAVIYGDGINQDGRTNGITAPSAVSQEKLLKRVYKDFGITA
NDISMVEAHGTGTNLGDPIEFNALCAAFTTDSAHKNYCALGSVKANIGHTQFAAGISSVI
KSVLALEHSTIPPLIHHTKGNSRIDLAGSPFYVAKSSQPWQARNGRRLATISAFGASGTN
AHVVLGDLPQPVRHTAEKFGPQLILLSANSDPQLVKLLQKLREHLESDATVSLTNIAYTL
ACGRRHFGKRVAILCRSVSELANALGSLLSAAKPSMQIFTPSDLGQGNAQALLKQAHVYI
ENGQVNDASFQGSICSLPASPFNSMSFWASSKDKAPAKQASVARPSPPASAPRATAARSK
AASKYCEFTLTVDDFFIADHLVMGQKVLPGVMYIELMKRSVCAVYGDKFKNAYQIALNDI
AWVQPMVFTDATPRLPIIVEVTERGDNADLKVLSRDKKQLYAQAICELSSGQQVLAEDQS
FSHLKAQGIRFDEIDLSAAGLAQYRKVPAAQCYDLLRRHGITHKTRLQGIKALYFSPSTV
IAQLQLPSILANKQSPFSVHPCLADSALQAISILAVARDKAKQTRDQGGVQTRVPFAIDR
FQQYREIPLNAWSVGHVLNDGKKEIWTIVIYDAQSNVCAVLDGYRQRILSGVPRLSEA
>tr|A0A0Q0C0N9|A0A0Q0C0N9_PSEAP Fimbrial protein OS=Pseudomonas syringae pv. aptata OX=83167 GN=ALO85_05065 PE=4 SV=1
MVNKMLSVLGLALATTLSATVVSANPGTGTIRFEGKITATTCPIEVVNPGDGSIGNLVNM
GSVEASRFTGIGQELSGKSFALRVNDDGSCGLTGGGPNAATVTFIGNADSSGDYFAVTPT
EDGAKGVAIVIKDQTSTSIKPGGQSAPYTLVEGGVTDMVFNAYYRSTASTVQAGAASADV
QFVVAIN
>tr|A0A0Q0C0N2|A0A0Q0C0N2_PSEAP Uncharacterized protein (Fragment) OS=Pseudomonas syringae pv. aptata OX=83167 GN=ALO85_04331 PE=4 SV=1
MLSIQRTPTDGGRKLGVTAEKLEFAYDILGRLTQESSPQGTLAYEYDPLSNLTTLTLPTG
QHLNHLYYGSGHLHQLNLDGQLISDMERDDLHREIYRTQGKLTSCFGYDALGRKTWQYAT
TLPAEKLSQIQNPLIKPERYVEHA
>tr|A0A0Q0CI09|A0A0Q0CI09_PSEAP Sarcosine oxidase, delta subunit OS=Pseudomonas syringae pv. aptata OX=83167 GN=ALO85_03843 PE=4 SV=1
MLHIFCPHCGELRSEEEFHPSGQAHIPRPLDPGACTDEQWGDYMFFRDNPRGLHHELWIH
AAGCRQYFNMTRNTVTYEILETYPIGTRPQFTDQGDKV
>tr|A0A0N8T8Q9|A0A0N8T8Q9_PSEAP Putative xanthine/uracil/ascorbate permease OS=Pseudomonas syringae pv. aptata OX=83167 GN=ALO85_00054 PE=4 SV=1
MLERLFQLKAHNTNVRTEILAGVTTFLAMAYILFVNPSILGETGMDKGAVFVATCLAAAI
GSTVMGLIANYPIALAPGMGLNAFFTYTVVLHMGHSWQVALGAVFISAVMFFILSIFRIR
EWIINSIPLPLRSAIAAGIGLFLALIALHNAGIVVSNPATMVGMGDLKQPAPVLATLGFF
LIVALDYLKVRGAVLIGILAVTLVSIALGFSPFGGVVSMPPSLAPTFMQLDIMGALDVGL
VSIIFAFLFVDIFDNSGTLIGVAKRAGLMGKDGHMPKMGRALIADSTAAMAGSLLGTSTT
TSYIESAAGVSAGGRTGLTAVVVAVLFLLALFFAPLAGSVPAFATAPALLFVAVLMASGM
AEIDWDDITVAAPVVITALAMPFTYSIANGIAFGFISWTAIKLLSGRWRELNSALVILSI
LFVIKLGWFNA
>tr|A0A0Q0BR50|A0A0Q0BR50_PSEAP Uncharacterized protein OS=Pseudomonas syringae pv. aptata OX=83167 GN=ALO85_04058 PE=4 SV=1
MDTIASSDGVRKVCCPDGYRVSPWAWNRSAISATQLRLFIQGNIMQADPFQSFARFIIKQ

YPDTPQIHEAEKHPVYLLTFGADLDIHLIGSQSGYLNLVATFPAPKAWLELDGLRALLAE
NIFSLQHPQIILGLDAASGKLVLSLRQPLAELSDESVLSLFEAFLRQAIDLHSGRILPPS
TTSAESSREPLSKTRLQVTNNPSSLKEKSRA
>tr|A0A0Q0DLQ9|A0A0Q0DLQ9_PSEAP N-acetylmuramoyl-L-alanine amidase OS=Pseudomonas syringae pv. aptata OX=83167 GN=ALO85_01607 PE=4 SV=1
MKLYFSALLMLLLTACASGPRLDTSHPSVNFDGRAQYVVMHYTSTSLERSLQLLTHGEVS
AHYLIGDDSKATIYKLVDENARAWHAGESEWEGRTWLNSSSIGIEIVNPGFKETPTGRLW
YPYTEAQTQSIIVLLKDIVKRNRIDPKHIIGHSDIAPLRKQDPGPLFPWKRLAAEGLGNW
PDERQVAQRQALLAVNLPSITWFQQQLARLGYSTPQTGELDVATRQVLAAFQMHYRPARF
DGEPDAQSAAILQVLNHSN
>tr|A0A0Q0IKU6|A0A0Q0IKU6_PSEAP Putative zink transporter OS=Pseudomonas syringae pv. aptata OX=83167 GN=ALO85_02431 PE=3 SV=1
MNHSIAHHHQDSDLFGLLYGFRFRHGESGQEIGSAAALDYLQNPPQENDFIWLHLNLSHA
ACERWMKTQLDLPEEFFEALHEGSRSTRIEHVDATLMAVVNDVVFDFNRLSTDISTLWVC
ARQRLLVTARLQPLRSVDKLRSAVKRGEQFDSPLELLAHLLSDQGEVLTQFLRKTSVNVD
RIEDQLLSQRLSNNRSELGAMRRVLVRLQRLLALEPGSLMRLLHKPPQWLREQDVQALRQ
SIEESSLVINDLTALGERIKLLQEEIAANLNEQSSRTLFTLTVVTVLALPINIVAGFFGM
NVGGVPLASDPEGFWILVALVATFTLVAGRWAFRKRGDY
>tr|A0A0Q0DMT4|A0A0Q0DMT4_PSEAP ATP phosphoribosyltransferase regulatory subunit OS=Pseudomonas syringae pv. aptata OX=83167 GN=hisZ PE=3 SV=1
MCIMRQPGNSRLFCVCTIDWPYLNRNAARFFEESGLQGLSRAFVVPVIRRTTFCFTQGRP
VGWPPGSQGNGVMATVDRWLLPDGIEEVLPPEAARIEVARRQVLDLFQSWGYEFVVTPHI
EYLESLLTGAGSDLDLRTFKVIDPQSGRQMGFRADITPQVARIDAHTLKREGPSRLCYAG
SVLHAQPRALSSSRSPIQLGAELYGDASPSSDVEVISLMLAMLQLADVPDVHMDLGHVGI
YRGLARAAGLSGEVEQQLFDALQRKAIDEVIALTADLPPELATMLRALVDLCGGREVLDA
ARDRLAGAPAPVLAALDDLLSIAERLTARFPQLPLYFDLGELRGYHYHTGVVFAVFVPGV
GQSIAQGGRYDDIGADFGRARPATGFSTDLKTLVTLGQAEIVLPSGGIWVPDSTDAALWQ
MVCQLRSEGQRVVQALPGQQASAAREADCDRQLIQHGEHWQVMPLAS
>tr|A0A0N8T9S5|A0A0N8T9S5_PSEAP Phosphate ABC transporter ATP-binding protein OS=Pseudomonas syringae pv. aptata OX=83167 GN=ALO85_03735 PE=4 SV=1
MGEASTPVRAGRIQIQGVGKRFGSQQVLKDIDLSIEPGKVTVILGPSGSGKSTLLRTINH
LEKIDSGYITIDGDYVGYRRKGDLLYELKEREILKRRIDVGFVFQNFNLFAHLTAWENIA
EAPLAHKRWSKSEARFRANELLARVGLADKTDAYPRQLSGGQQQRVAIARALALDPKVLL
FDEPTSALDPELVGEVLDVIKGLTQLGVTLVVVTHEIGFAREVADHVVFLCEGRLIEQGP
PEQVFRQPRHPRTAAFLGKVL
>tr|A0A0N8TA51|A0A0N8TA51_PSEAP Spermidine/putrescine ABC-type transport system, ATP-binding protein OS=Pseudomonas syringae pv. aptata OX=83167 GN=ALO85_03420 PE=3 SV=1
MSAVIKDPAHQHQNTLVSLCNLNKHYGNFAAVDDISLDIQDGEFLTFLGSSGSGKSTTLS
MLAGFETPSSGEILVGGKSLVNVPPHKRDIGMVFQRYSLFPHLSVRDNIAFPLAIRKLPA
AEREKKVDAMLKLVQLEAFAHRRPSQLSGGQQQRVAIARALVYEPRILLMDEPLGALDKK
LREDLQDELRQLHRRLGITIVYVTHDQEEAMRLSQRIAIFSHGKIVGLGSGYDLYQDPPN
AFVASFLGNSNFLRLKAQGNALATFEGSTLAIRLTAGLKTDQDVLLMVRPEKAQALSVSQ
AAAIPLEAGWNEVNATVTEVLFLGESQTCSVVTAGGTAMTVKALSANGMSLKTGDPVRVR
WAAADACIYTEWTDSDLNKAAGTH
>tr|A0A0Q0DJT1|A0A0Q0DJT1_PSEAP Transporter OS=Pseudomonas syringae pv. aptata OX=83167 GN=ALO85_100297 PE=4 SV=1
MSDKYAPREWAAHERPTMPGSPSTPLHSTARRWAFAVVGVIVALTGGLGNALVVANLPYL
QGALGATSQDIAWLPAAYIMTNVSMNLLLVKFRQQFGLRAFTELFLVLYALVTLAHLFVN
DIDSAIAVRAAHGMVGAALSTLGLYYMIQAFPQKWRLKALVLGIGTAQLATPLARLVSED
LLQIAQWRGLYLFELGMALLSLGCVLMLKLPPGDRFKAFEKLDFLTFGLLATGFALFCAV
LSLGRIEWWLEAQWIGIASAASIALICAGLAIEHNRSNPLLITRWLGSGAILRLGLAVIL
FRVVLSEQSVGAVGFLQALNMGSQQLHTLYLVMLLGSVSGLAVSALTINPAHLIKPLLIS
LAMMAIGAWMDSHSTNLTRQSNMYLSQFLLAFGGTFFLGPTLVLGISGVLANPRNMVSFS
VLFGITQNIGGLIGSAALGTFQVVREKFHSSHVVEHLTLIDPLVQARLQSYSASYSSVIA
DPSLRNSQGIRAMATAATREANVLAYNDVFMLIALIAVLTMIWISARVLWLKMTTQPLAL
APVAPAASPTISNSGVKSS
>tr|A0A0N8T9T6|A0A0N8T9T6_PSEAP Transcriptional regulator FtrA OS=Pseudomonas syringae pv. aptata OX=83167 GN=ALO85_03790 PE=4 SV=1

MKNHLVVALIYNQLCTFEFGCTVEVFALKRPELGVSWYEFATFPVDDGPITAAGGITIVP
TPGEVLLENADTIIVPGWRGVESEVPQRLIEQLQAAHARGARICSICTGAFVLAAAGLLD
GHKVTTHWRYTDLLATMYPGLTIQPNELYVDQGSIVTAAGSAAGLDMMLHLVRNDHGSRV
ANMVAQRMVIPPHREGGQSQYASRQLVSASDGPISNLMDWIRSDLQRPHTIKEMASRAAL
STRTLHRSFMESTGLTPYDWLLGERIAYAKELLESSKLRLNEVVDRAGFGSEESFRRHFR
NLVGISPSSYRKQFARA
>tr|A0A0Q0BV24|A0A0Q0BV24_PSEAP tRNA 5-methylaminomethyl-2-thiouridine biosynthesis bifunctional protein MnmC OS=Pseudomonas syringae pv. aptata OX=83167 GN=mnmC PE=3 SV=1
MTITRHAQIDWDEHGNPRSRDFSDVYFSTESGLDETRHVFLVQNDLRRRFTELPDEGRLI
IGETGFGTGLNFLCAWQLFDECAPANARLQFVSVEKYPLSHADLQRALALWPELSPFAGQ
LLNQYVAVHEGFQRLVFAGGRVTLTLLIGDALQMLPQLDGQVDAWFLDGFAPAKNPEMWT
PELFAELARLSTSSTTIGTFTSTGWVRRSLNAAGFKMKRVPGIGHKWEVLRGAFIAWPED
VALPAAAKPWFARPAPIGGERKALVIGAGLAGCATAQSLAQRGWQVSLLERHAAPAQEAS
GNPQGVLYLKLSAHGTALSQLILSGFGHTRRLLERLQRGVDWDACGVLQLTFDDKEALRQ
KQLADAFPESLLHLLDQPAAEAQSGVALNSGGLFYPEGGWVHPPALCHAQAAHANISLIT
HQQALELRRVDDQWQVWSEEQLIDSAPVVVLAGAADIQQFSQSADLPLKRIRGQITRLPQ
TQASTALRSVVCAEGYVAPARLGEHTLGASFDFNSTDLTPNLADHLDNLGLLREISEDLT
SRLEAAALSPEQLQGRAAFRCTSPDYLPIVGPLADRKAFMQAYAALGKDARQVPDSACPW
LDGLYVNSGHGSRGLITAPLCGELIAAWLDNEPLPLPRSVAEACHPNRFALRGLIRGGGK
SPEKQDQRGASHAS
>tr|A0A0Q0DK20|A0A0Q0DK20_PSEAP Aminodeoxychorismate synthase, subunit I OS=Pseudomonas syringae pv. aptata OX=83167 GN=ALO85_05144 PE=4 SV=1
MPHWQGYNRQPRFQDRYSTMPICSIHPLPYSADPIAFFARIREAPGAVLLDSGRPTAVRG
RHDLLSAWPLQELSVADDEDGAAYLQRLRDSLKALGAAQLPDGCELPFAGGLIGYLSYEF
GRRLEPLPDQAIDDLQLPEARLGLYAWALISDHQEQTSQLIFHPSLAASERKRLIELFSV
DIPEPHTEFRLSQPFQASISADNYRHAFERIQAYILAGDCYQVNFAQRFQAQCVGDPWAA
YCALRAACPTPFAGYLSLPGADAILSLSPERFVRVSARQVETRPIKGTRPRSADPAQDAA
FAAELLASDKDRAENLMIVDLLRNDLGRSCRIGSVKVPELFSLESYPNVHHLVSSVTGEL
AAGNDALDLIGGSFPGGSITGAPKIRAMQIIDELEPTRRSLYCGSLMYLDVRGEMDSSIA
IRSLLVKDGQISCWGGGGIVADSDCEAEYQETFTKVRVLLETLEAL
>tr|A0A0Q0FLR9|A0A0Q0FLR9_PSEAP Putative type IV pilus bioproteinsis protein, FimV family OS=Pseudomonas syringae pv. aptata OX=83167 GN=ALO85_02555 PE=4 SV=1
MVQVRKLVLAIAAASALSSGMAQALGLGELSVKSTLNQPLVAEIELTEAQGLNATQVVPS
LATTADFAQAGVTRQAFLNDLTFTPVINATGKSVLRITSSKPVREPYVKFLVQVLWPNGR
LLREYSLLLDPPKFSPEAAAAAAAPAPAAAPAAPAQLATVAPSAEAPAAEAPAPAPSANP
AAVPPPAADKQSQYVTANNDTLWEIAAKVRTGGTVQQTMLAIQALNPDAFIGGNINRLKK
GQVLRLPTPQQTTALPQSQAVAEVSRQYSAWREGRRLPAGTRQVDATRRDRAGAAPSKVD
TSDNLSLVSASGKSAAKGAAGDSDVGNKLAVAQEALDTTRRDNAELKSRMNDLQSQLDKL
QRLVELKNGQLAKMQAAGAAVPPPAATPEAATPANAAVPASLVDANGAPVKPAGEIAPED
ALPAGAAEVATPAPEQPLAVEPVAAADEDEDALQKIINNPVLLGLIGGAVLLILALLLLF
LARRRAAKAEAEKHKRMARALAEESDFVSDMDMNAPPASFDGLDVPPPNVRMGGAAAAAA
GRERPADPLVQAEIHIAYGRMNQAIELLEEAVNEDPKRDDIRLKLMEIYAEQGNNKAYAA
HERKLVAAGKLEAEVEQREELNSTLKPVAPVAAPLASETSTASAAPVVAAAAAAATTAAL
VAELDAKYLEELLADDAAEQEAEPEPEPEIVATPEPEPEVTAPLAAEPVEEADPFDNDFD
LSLDEFEEPSVPEVSTVNDLDDLMLDEPKVSAVADDEELSFESVMQQQEEARAATTPQDL
ADFDLDLSEEDPALKSEDDFLLGLGEDPLDLGETSAVPPVSDDLELPEDFDLSLADEIET
DQASQAFATEIDDVNAELERLSQNLEHPPLDEPRFTAEDAAALDDEPDFDFMSGTDEAAT
KLDLARAYIDMGDADGARDILDEVVTEGDDGQKSEAREMLSRLA
>tr|A0A0Q0IEB7|A0A0Q0IEB7_PSEAP GAF domain-containing protein OS=Pseudomonas syringae pv. aptata OX=83167 GN=ALO85_101804 PE=4 SV=1
MVGATHMIDLQNTGAGLDGYGLLAAQLEALLADERDFIANAAQFSAFLYTQLDDLNWAGF
YLNRNEELVLGPFQGQIACVRIPFGRGVCGTAAQSRQTQRVQDVHEFPGHIACDSASNSE
LVVPLIKEGRLIGVLDLDSPSVGRFNEEDQAGIERLAAIFLASTDC
>tr|A0A0Q0DCQ0|A0A0Q0DCQ0_PSEAP Ferric uptake regulation protein OS=Pseudomonas syringae pv. aptata OX=83167 GN=ALO85_04408 PE=3 SV=1
MGDFHYLGWDNVNSDHTCIKRSCEAASARKLTMVENNELRKVGLKVTLPRVKILQMLDSA
EQRHMSAEDVYKALMEASEDVGLATVYRVLTQFESAGLVVRHNFDGGHAVFELADGGHHD
HMVNVESGEVIEFFDEEIEKLQKAIVERHGFDLVDHNLVLYVRNKKA

>tr|A0A0Q0DNZ6|A0A0Q0DNZ6_PSEAP EmrB/QacA family drug resistance transporter OS=Pseudomonas syringae pv. aptata OX=83167 GN=ALO85_00393 PE=3 SV=1
MSFNVPRRWVVFNVLLGTLTVSLSNSSLNPALPTFMEAFRIGPLLATWIVAGFMTSMGMT
MPLTSFLSQRIGRKRLYLWGVALFIGGSLLGALADSIALVITARVVQGIASGLMIPLSLA
IIFSVYEKHERGRVTGLWSAAVMLAPALGPLCGSLMLEWFSWRSLFLMNVPIGLLALLLG
VGVLPDSEPAERKPFDLIGYLLVASGIGLLMIAISRMHHAQALLDPVNQAMVLVAVACLV
AFVRVELSRQAPLLNLRLFNLRGYRLSVIIAVVQSVGMFECLVLLPLLVQSVLGYNPIWT
GLALLCTASFASLFGQWGGKALDRHGPRTVVAVGLLLTGVSTLALGMLKADAAIGVVFVL
MMIRGAGLGLSYMPVTTAGLNALPEPMVTQGAAMNNISRRLVASLAIVIASLWLEFRLNT
AGPAATPSAISEVFIATGLLILLALPCAWRFPLNDERAEAQPDAVEPR
>tr|A0A0Q0DLC8|A0A0Q0DLC8_PSEAP Uncharacterized protein OS=Pseudomonas syringae pv. aptata OX=83167 GN=ALO85_02671 PE=4 SV=1
MTVWIVLSIIGVMLSPLVWLRPSRQQSGRMALRMQARRLGMGMQLTPQEWPHWLSKEPPS
PCAQYHRPRLGSHADAWVYWQSEPGVWRNRWREPCEDAKLLPYLQTLPVDAYKVEADGQL
VAVYWAERGEAEVLQRIDALLKALA
>tr|A0A0Q0BX38|A0A0Q0BX38_PSEAP Alginate biosynthesis protein AlgE OS=Pseudomonas syringae pv. aptata OX=83167 GN=ALO85_01806 PE=4 SV=1
MKLNPLMAAGMGLGFTLLWACPTLAALTEQPQNFGIEIKATAQAEDDRDLGTRSGGDVNG
VGLDLRPWVYGERGDWSGYAMGQVVTATDIIQTDPLEQSNSDGSGTQTSRGTASERETDK
TYAALREFWIGYSGFTPYPGEILKIGRQRLRNDDGQWHDTNIEAINWSFDTTLLRAELGA
AQRFSEYRTDLTELAPDDEDRKHLFGSVSYQWTPGNWAGIRAHHSADDGKLKSQGQVLDD
LDKTSNGDLTWVGLQADSDAYNYRSNTNQLNYWGSLTWLNGTRDEIGVTSAPNDQFIAGE
KNSRDMNGWATDLGLRLRLDPQWQVGAAYSRASKDYVQNGLESNRSNWTGTRSRIHRFGE
AFQGEMANVETGSLFASWQMNEEYDASLIYHKFRRVDGNTGIGGSGINAVNDNQDGTFSS
LPLEDGSKDLGQEMDLVVTKYFKQGLLPASLSQSFDEPSALVRLRAGVFKPGDAYQSNVD
SYMHRAVVDVIWRF
>tr|A0A0Q0C4R2|A0A0Q0C4R2_PSEAP C4 dicarboxylate transport two-component response regulator OS=Pseudomonas syringae pv. aptata OX=83167 GN=ALO85_02274 PE=4 SV=1
MSSDTAIDSQIQVVLIDDDSHLRQALRQTLDLAGLNVLSLPEATGLAERIGRDWPGVVVS
DIRMQGMDGLELLDQLHAQDPDLPVLLITGHGDVPLAVKAMRAGAYDFLEKPFASDAMLD
SVRRALALRRLVLDNRSLRLALSDRQQLSTRLIGQSPPILRLREQIGALAGTRADVLILG
ETGAGKEVVARALHDLSNRRSGPFVAINAGALAESVVESELFGHEPGAFTGAQKRRIGKF
EFANGGTLFLDEIESMSLDVQVKLLRLLQERVVERMGSNQQIPLDIRIIAATKEDLRHAA
DQGRFRADLYYRLNVASLRIPPLRERGEDALMLFEHYADAASMRHGIPKHELKPGQRALL
LRHNWPGNVRELQNAAERFALGLELELEGADAEQAPPPGGGLSDQVESFERALIAAELAR
PHSSVRSLAEALGVPRKTLHDKLRKHGLTFGDSSGASDDND
>tr|A0A0N8T7W2|A0A0N8T7W2_PSEAP Ubiquinol oxidase subunit 2 OS=Pseudomonas syringae pv. aptata OX=83167 GN=ALO85_100217 PE=3 SV=1
MFNSKKLAGTGRAKCWRSKPNCTAQAAFRGREMSKKRYPRFFGFLALFCMLLLSGCDGVP
LLDPKGQVGIEQRNLIIIATLLMLIVVIPVILMTLIFAWKYRASNKAAKYTPDWSHSTKI
EIAVWGVPMLLLVFLGYITYVSTHSLDPYRPLDSDVKPITIQAISVDWKWVFVYPDYGIA
TVNKIVFPAKTPINFQVTSDSVMNSFFIPGLGGQIYAMAGMHTKLHLIANENHEFNGISA
NYSGAGFTGMKFKAIATSQADFDSWVSEVKSSPKKLGTAEYAELIKPSERNPVELFSSVT
PNLFQIVIDKYEGMNPGKKVVAGEKEVAGEAGAEKGKNSAAGAEE
>tr|A0A0Q0FUB3|A0A0Q0FUB3_PSEAP Chemotaxis sensor histidine kinase CheA OS=Pseudomonas syringae pv. aptata OX=83167 GN=ALO85_05565 PE=4 SV=1
MLTVNFREHPMSFGADEEILQDFLVEAGEILEQLSEQLVELESRPDDADLLNAIFRGFHT
VKGGAGFLQLHELVECCHIAENVFDILRKGERRVDSELMDVVLEALDTVNSMFGQVRERT
DVTPATPELLAALARLAEPQSADEVAAPEPEPVVEVQAAAAVAEPAAGDEITDDEFEQLL
DSLHGSNPVSAAPATAPAAVAAAPAGDEITDQEFESLLDQLHGKGKFAADAAPAPARAPV
AQGSAAPAGDEITDDEFEALLDQLHGKGSFDGAVAAPVAAAPVAVAAKAPAAASDEITDH
EFESLLDELHGKGKFEPEAIVANAPAPAAAAAPPPPVAKPAPAPAAKAEPAKPASAPAPA
RTPAPTGEKPVATEAETTVRVDTARLDEIMNMVGELVLVRNRLVRLGLNNGDEAMSKAVS
NLDVVTADLQTAVMKTRMQPIKKVFGRFPRLVRDLARQLKKEINLELVGEETDLDKNLVE
ALADPLVHLVRNAVDHGIETPEEREATGKSRGGRVILSAEQEGDHILLSISDDGKGMDPN
VLRSIAVKRGVMDKDAADRLSDTDCYNLIFAPGFSTKTEISDVSGRGVGMDVVKTKISQL
NGSINIYSTKGQGSKIVIKVPLTLAIMPTLMVMLGNQAFAFPLVNVNEIFHLNLSTTNVV
DGQEVVIVRDKALPLFYLKRWLVSSAAHEEQREGHVVILTVGTQRIGFVVDQLVGQEEVV

IKPLGKMLQGTPGMSGATITGDGRIALILDVPSMLKRYAARRI
>tr|A0A0Q0D7L0|A0A0Q0D7L0_PSEAP Membrane protein OS=Pseudomonas syringae pv. aptata OX=83167 GN=ALO85_100140 PE=4 SV=1
MASKSLRHTFRRLWALDKFSYSVRVFIALTGSMALCWYQNEMALLIPLFLGIIACALAET
DDSWQGRLNALAVTLVCFSIAALAVELLFPYPILFVCSLALASFCLTMLGALGERYGAIA
YGTLILSVYTMIGVDQRGGEVLDFWHEPMLLVAGAAWYGLLSVLWQMLFSNQPVQQALAR
LFRELGQYLKLKSTLFEPIRTLNVEARRLELAQQNGKVVAALNSTKEIILHRVGSGRPGS
KVSRYLKLYFIAQDIHERASSSHYPYNSLADAFFHSDVLFRCQRLLRQQGVACQALSESI
QLRQPFVYDPSFAEAMEDLHASLEHLRIQSNPAWRGLLRSLRALAANLGTLDRLISDASN
PDAVADATDSSLLDRSPRNLKDVWTRLRLQMTPTSLLFRHALRLPLALTIGYAMVHLIHP
SQGYWIILTTVFVCQPSYGATRLKLGQRIIGTAIGLTVGWVLFDLVTSPILQSMCAVLAG
VVFFVNRTTRYTLSTAAITVMVLFCFNQVGDGYGLFLPRLFDTLLGSLIAGLAVFLFLPD
WQGRRLNKVLANTLTCNSIYLRQIMQQYAKGKSDDLAYRLARRNAHNADAALSTTLANML
MEPGHFRKEADVGFRFLVLSHTLLSYLSGLGAHRDTQLPSDVREHLIDHVGATLAASIDE
IAAGLAEKKPVAVQSDVEEGLAAQLEQLPEDMDESQRLVQAQLALICRQLAPLRTLAAHL
IKAPEATATHAV
>tr|A0A0N8TA34|A0A0N8TA34_PSEAP Uncharacterized protein OS=Pseudomonas syringae pv. aptata OX=83167 GN=ALO85_200084 PE=4 SV=1
MPNALEKARRYIKSIKTAVDIESTEGAHNTAWGYQLALGDFDLITQAEKAMLDDEASVAK
KERLAELKAGRR
>tr|A0A0Q0BUK6|A0A0Q0BUK6_PSEAP Uncharacterized protein OS=Pseudomonas syringae pv. aptata OX=83167 GN=ALO85_04744 PE=4 SV=1
MDKGVKHDADEVDKGAKHDDKERHKADKHIDKEVKKDL
>tr|A0A0Q0FFC2|A0A0Q0FFC2_PSEAP ATPgrasp and Alpha-E domain-containing protein OS=Pseudomonas syringae pv. aptata OX=83167 GN=ALO85_03141 PE=4 SV=1
MPAEPSMPDLLDHYPLSEGTYHEMLDAEGAVRPHWRRLYEHMRRSSAAQLIQRQALLTRQ
IQENGVTYNVYADPKGADRPWELDLLPHIIAADEWQHVAAGIAQRARLLNAVLADLYGPQ
TLIANGLLPAELVFGHNNFLWPCQGVKPPEDTFLHMYAVDLARTPDGRWWVTADRTQAPS
GAGYALENRQSVARALPETYRDLQVRHLSGFFDALQQTLARQAPTSNEAPLIVLLTPGRF
NESYFEHLYLARQLGYPLVEGGDLTVRDATVYLKTLSGLRRVHAIMRRLDDDFCDPLELR
TDSALGVPGLLEAVRQGNVLVANALGSGVLESPGLLGFLPKISQYLFGEDLILPSVATWW
CGEPPVLAQALEKLPDLLIKPAFPSQHFAPVFGRDLNEEQRLALAQRMQSRPYAYVAQEL
AQLSHAPVLQADGTGLQPRAIGMRVYAVASLDGYRVLPGGLTRVAAEADADVVSMQRGGA
SKDTWVLGERSHLGEPWKGQRKLGVYDLIRRDPYLPSRVVENLFWFGRYCERCDDSARLL
RIMLTRYVDDDDPLALQAAVALAESLGMLPEAQEGEELKDRLLVALLGDEWPFSLRANLQ
RLQWAASQVRGKLSKENWQALVELQREALSLESEEPDFGELLDFLNRLVMSLAALSGFAL
DDMTRDEGWNFLMIGRRIERLKFLSTSLSAFLRGLVVFERAGLEWLLELGNSSITYRSRY
MAVPHLIPVLDLLLLDDQNPHAVLFQLKLVQRSLRRLNDEFDAPRDSSLSVLAQRLAAFD
LGSLENPLFGQSSIDAVLDGLADLLQSIGDSSGLASDRLALRHFAHVDDVSQRTVSV
>tr|A0A0Q0IHM1|A0A0Q0IHM1_PSEAP Sensor protein OS=Pseudomonas syringae pv. aptata OX=83167 GN=ALO85_03048 PE=4 SV=1
MRRQPSLTLRSTLAFALVAMLTVSGAGLYLYQSIEETVMQRSDHAVLARLDHFRKLLRYD
LTMDNLKGSPQLFENMLDSEEDIFIIGEPGKSPVISVNPQHAPLPELPTVAQDQPLRVXX
RSGMSLQGVPLRAAAVQVMSNGVEVRLQAAHLMVKEMAMLASFRQRIYIAVALAFLITAA
LGYVLLRRGLRPLRRMAAHAAAITPASLHKRLDSRDTPVELQQLSDAFNAMLDRLDDGYR
RLMQFSADLAHEIRTPVGSLMGHCQVALRQNRSADEYQALLASNLEELERISRLVESILF
LARADEAQAALERQSLDLHDELQRVAGYFEGLAEERNLTLSTHGQGTLLADPILLRRALS
NLVANAIRYADEDSEILMRVTAVDRHWKIDVENQGPVLPQATLARLFDRFYRGDASRHER
SDSNGLGLAIVTAIMQLHGGRVEVDQPAVGRICFSLIFPAA
>tr|A0A0Q0FLM3|A0A0Q0FLM3_PSEAP Oligopeptide/dipeptide ABC-type transport system, ATP-binding protein OS=Pseudomonas syringae pv. aptata OX=83167 GN=ALO85_03912 PE=3 SV=1
MSQLIRVQDLRVVADGDHGELEIVKGVSFALEKGEVLALIGESGSGKTTIALALLGYARR
GCKLAGGVVQVGEHDMLSLPESQLQGLRGNRVSYIAQSAAAAFNPAKKLIDQVIEGALIH
GLGSRAELEAKAVELFRDLALPNPESIGRRYPHQVSGGQLQRVMAAMALISDPLLVILDE
PTTALDVTTQIDVLRAFKRVVRDRGATAVYVSHDLAVVAQMADQIVVLNGGQIIETSSTA
ALLKGPVDAYSRSLLAAARPDKVLSPASEVAKDSTLLTITGLTAGYGKKNLQGMPMIRVL
EDIDLTIRRGQAIGVIGESGSGKSTLARVVAGLLDPAHGSLTFDGAELSGTLAGRTEEQF
RRIQMVFQNADTALNPMHSISAILARPLKMYFGLKGRALRERIDELMDLVRLPRELAERR

PNELSGGQKQRVNLARALAAKPDLILCDEVTSALDTVVGACILELLGELRRKLGVSYLFI
SHDISTVRALCDDIVVMYSGHKVEEGSREAFSRMPFHPYTDLLVHSVPELRQGWLETCGV
TSGNLPPISAPANNPELCTFLNRCPARIEGLCNKTAPGRRVIAGGSEILCHRDSDELQAV
QENLNPEIVGAYA
>tr|A0A0Q0D676|A0A0Q0D676_PSEAP Protein RecA OS=Pseudomonas syringae pv. aptata OX=83167 GN=recA PE=3 SV=1
MSTYTGVVAIKALIEFDYVRTSMDDNKKKALAAALGQIERQFGKGAVMRMGDHDRQAIPA
ISTGSLGLDIALGIGGLPKGRIVEIYGPESSGKTTLTLSVIAQAQKMGATCAFVDAEHAL
DPEYAGKLGVNVDDLLVSQPDTGEQALEITDMLVRSNAIDVIVVDSVAALVPKAEIEGEM
GDMHVGLQARLMSQALRKITGNIKNANCLVIFINQIRMKIGVMFGSPETTTGGNALKFYA
SVRLDIRRTGAVKEGDEVVGSETRVKVVKNKVAPPFRQAEFQILYGKGIYLNGEIVDLAV
LHGFVEKSGAWYSYQGSKIGQGKANSAKFLADNPEICKALEKQIRDKLLTPGVDTKAVGS
REAVAADDMSEADVDI
>tr|A0A0N8T8C6|A0A0N8T8C6_PSEAP 3-octaprenyl-4-hydroxybenzoate carboxy-lyase OS=Pseudomonas syringae pv. aptata OX=83167 GN=ubiD PE=3 SV=1
MAGLMKFKDLRDFVQQLEQRGELKRIQMPISPVLEMTEICDRTLRAKGPALLFEKPVGFD
IPVLGNLFGTPERVAMGMGAEAVSELREIGKLLAFLKEPEPPKGLKDAWSKLPIFRKVIA
MAPKVVKDAPCQEVVIEGDDVDLGMLPVQTCWPGDVAPLITWGLTVTKGPNKERQNLGIY
RQQVIGRNKIIMRWLSHRGGALDFRDWCVKHPGEPYPVAVALGADPATILGAVTPVPDSL
SEYAFAGLLRGSRTELIRCRGSNLQVPASAEIVLEGVIHPGEMADEGPYGDHTGYYNEVD
SFPVLTVERITHRIKPIYHSTYTGRPPDEPAILGVALNEVFVPILQKQFPEIVDFYLPPE
GCSYRMAVVTIKKQYPGHAKRVMLGVWSFLRQFMYTKFVIVTDDDINARDWNDVIWAITT
RMDPKRDTVMIDNTPIDYLDFASPVSGLGSKMGLDATNKWPGETSREWGRAIVKDEATTR
RVDEIWTQLGID
>tr|A0A0Q0DKM2|A0A0Q0DKM2_PSEAP Multidrug transporter OS=Pseudomonas syringae pv. aptata OX=83167 GN=ALO85_04664 PE=4 SV=1
MSQAPPMAWHRFSGCLRLQHWKPLKTAARAWRLLSLHCAEHLDMTTPSHPALTFKQALLA
MLGISLVLGLSALDQTVIGNALPSIVAELKGFELYAWVATGYLLASIVTIPIFGRLGDFY
GRKPFVLAATIIFTLASLLCALADSMLVLVIGRALQGVGGGMLIGTAFACVPELFPDTRQ
RLRWQVLLSAMFSVVNAIGPGLGGYLSGEFGWRSVFWLNLPLGVVALFFAWRFLPWYRPQ
GHRAHSAGAIRLDWLGALLIVLTLGSLQLFVEWLGQRSLSVSLTCGAVTLAAMAGLWFHE
RRCAFALLPAGLFADRSIRLLFIMSLLAGAIMFTLLFYLPLLLQGSYGYSPKDAGLLLTP
LALSITLGAIVNSRIVTRIANPNRLPVAGFAALSVACTALALVGLHAGFSTLLGLILLAG
LGLGFILLNLTVFTQTLAERQFLGIATALTQSLRLVGGLLGTAAMGVLVKLLYVTNLQSA
LNAAGQASALPRLADPQVLLQHTPANVDAALLIARTALAQAVGTGLLTCAGLGVLALLVL
HRLPRVELHRVPGATTPATDSCVTPAPEKQKET
>tr|A0A0Q0IPS4|A0A0Q0IPS4_PSEAP Malto-oligosyltrehalose trehalohydrolase OS=Pseudomonas syringae pv. aptata OX=83167 GN=ALO85_03650 PE=3 SV=1
MPLRTDDNWRHGAVLLDPEHTRFALWAPDASFVNVELQDGQSLAMLPQSDGWFVLEARCK
AGTRYRFRIDHELDVPDPASRAQVDDVHSESLVVDPDAYSWQNTAWNGRPWHEAVIYELH
VGAMGGYAGVEKHLPRLAKLGVTAIELMPLAQFPGDRNWGYDGVLHYAPQSSYGTPEQLK
HLIDTAHGLGLMVILDVVYNHFGPDGNYLGSYAKEFFRTDLHTPWGDAIDFRRPQVRDFF
VDNALMWLLEYRFDGLRMDAVHAIRDETFLPEFAERVRGRIEPGRHVWLNLENEFNQAGL
LKKGFDAQWDDDGHNTLHVLLTGETDAYYSDFAKEPTQKLARLLSEGFVYQGEPTRHGHT
RGEPSAHLSPSAFVLFLQNHDQIGNRALGERLPQLCAPAALKAATALLLLSPMIPLMFMG
DEWAASEPFLFFTSHHGELADAVREGRRKEFADFAAFADPEKREHIPDPNDIKTFEASRP
AFSAVDLDTDIGLDHRSWLEFYTQLLALRHQEIMPRLQGARFLKTDILGDKAVSARWTLG
DGSELRIDLNLSEHAVDAPPQAGAREIFSSAEKSSELSPRGILNAYTAIVSLKASDVLEE
QDEQ
>tr|A0A0N8T7Y9|A0A0N8T7Y9_PSEAP Aldehyde dehydrogenase OS=Pseudomonas syringae pv. aptata OX=83167 GN=ALO85_01888 PE=3 SV=1
MSTAKILELMRPYWGDRSVIASYVGGQFIDGHGAPVEVRNAHDDSLLLSFPDADESLVEV
ADKAARAAQQQWWALTAQARGRAMYQVGNLIRDELENLAQIESLTANKPIRDARVEVLKV
AEMFEYYAGWADKLHGEIIPVPTTHLNYVTYEPLGTVLQITPWNAPIFTCGWQIAPAIAA
GNAVILKPSELTPLSSLMVGVLIERAGVPKGLVNVIAGFGHSIGQAFIAKADIRKVVFVG
SPATGRHIAVAAAQRCIPAVLELGGKSANIVFDDADLEVALRGAQAAIFSGAGQSCVSGS
RLLVQESIFEKFTNALAVAATQFKVGDPSDPETQIGPINNAKQYNHVKNMVEQALKDGAK
[truncated: 683,981 more chars]
